# Asymmetric Decarboxylative Protonation and Deuteration of Cyanoacetic Acids Using an Organometallic Proton Shuttle

**DOI:** 10.1021/jacs.6c05281

**Published:** 2026-05-12

**Authors:** Wei-Feng Zheng, Guozhen Wu, Min Hou, Ke Li, Xiaotian Qi, Zhongxing Huang

**Affiliations:** † State Key Laboratory of Synthetic Chemistry, Shanghai Hong Kong Joint Laboratory in Chemical Synthesis, Department of Chemistry, 25809 The University of Hong Kong, Hong Kong 999077, China; ‡ College of Chemistry and Molecular Sciences, 12390 Wuhan University, Wuhan, Hubei 430072, China

## Abstract

The trajectory of proton is hard to control. Chiral catalysts known as “proton shuttles” are key to reactions involving proton transfers by hosting a proton and enabling its stereoselective addition. Although organic acids and bases are commonly used for this purpose, we demonstrate here that a rhodium–phosphoramidite complex can serve as an organometallic proton shuttle to intercept the deprotonation/CO_2_ extrusion/protonation sequence in the decarboxylation of cyanoacetic acids and dictate its stereochemistry. While the amino motif in the ligand accommodates the proton, the nearby rhodium helps fix the orientation of the ketenimine intermediate for protonation via a dative bond. Furthermore, the decarboxylation pathway is compatible with H/D exchange using deuterated solvent, thus allowing an asymmetric decarboxylative deuteration. This method bridges the bulk production of cyanoacetates and their facile substitution with postdecarboxylation transformations of nitrile to access structurally diverse stereocenters and deuterated entities.

## Graphical abstract

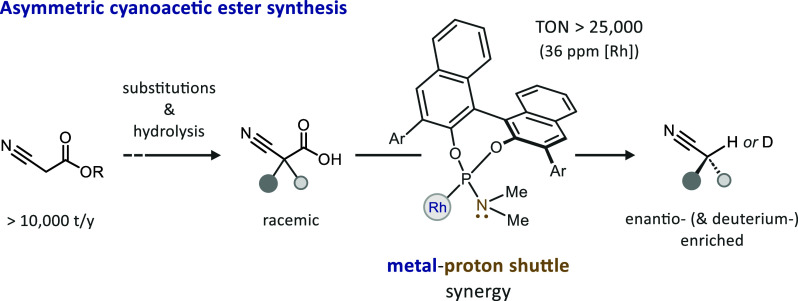

## Introduction

As the smallest charged species in organic transformations, the transfer of proton is fast and difficult to control. Particularly, asymmetric transformations with protonation as the stereodetermining step pose great challenges for catalyst design and development.^1^ Early attempts to modulate stereocontrol often employ chiral organobases with naturally occurring motifs, such as cinchona alkaloids and ephedrine derivatives (Scheme 1A).^2^ The Brønsted basic amines in these catalysts often serve as a shuttle to seize and store a proton, forming a chiral ion pair with a negatively charged reaction intermediate. During the subsequent protonation to yield product, the chiral skeleton of the base dictates the stereochemistry of the intermolecular transfer. Strong acids equipped with privileged chiral scaffolds are also capable of controlling trajectories of intermolecular proton exchange, largely facilitated by multiple weak interactions.^3^

**Scheme 1:**
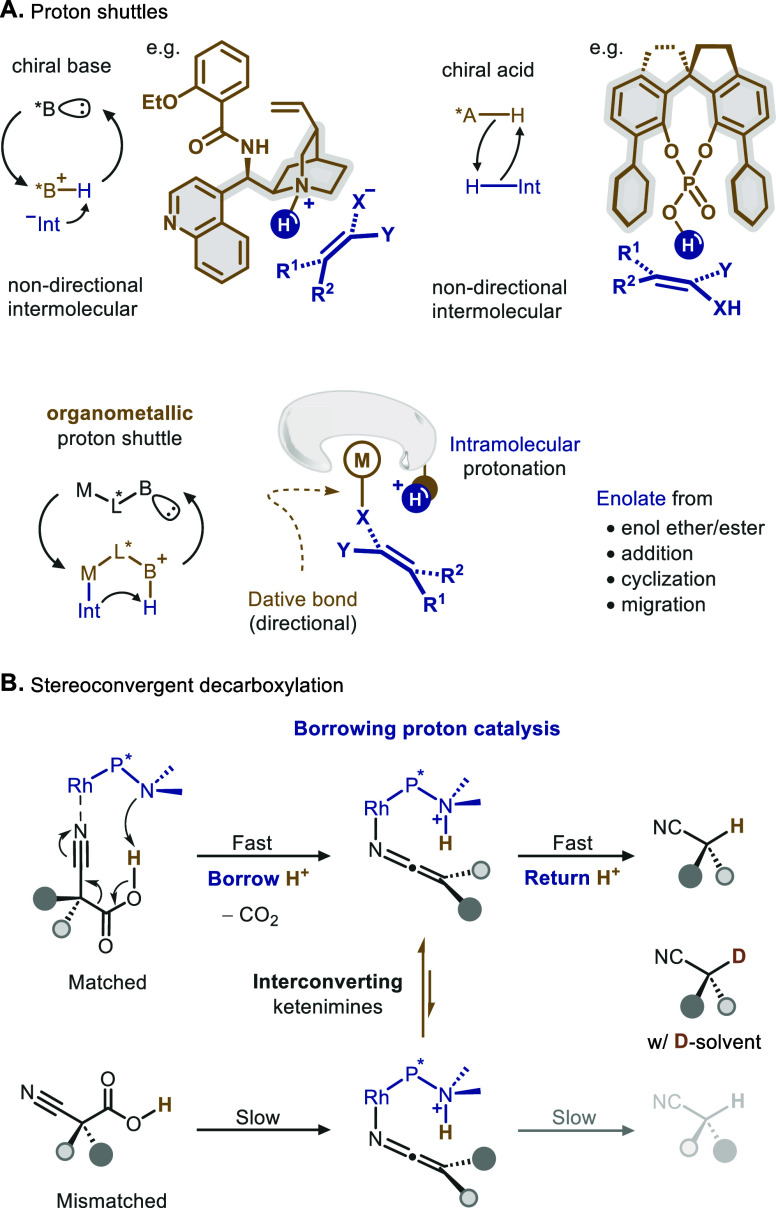
Organometallic Proton Shuttle for Asymmetric Decarboxylation

When a metal center is integrated into the scaffold of the proton host, the resulting organometallic shuttle confers unique advantages.^4^ The coordination between the metal and the proton-accepting intermediate turns the stereodetermining protonation into an intramolecular event. Compared with its intermolecular counterparts, where the donor and acceptor are loosely associated, the enforced spatial proximity of the proton promotes a rapid transfer that can outcompete undesired substitution with external proton sources. Besides the kinetic advantage, the directional nature of a dative bond imposes conformational constraints on the nucleophilic intermediate during the proton transfer, thus enhancing the stereochemical discrimination between competing diastereomeric complexes.

This type of metal–ligand cooperative catalysis has shown promise in protonating preformed enolate derivatives^5^ as well as those generated from conjugate addition^6^ and proton migration.^7^ Here, we aim to devise an organometallic shuttle catalyst for asymmetric decarboxylation, a reaction that is among the most challenging for imposing enantiocontrol (Scheme 1B).^1b,8^ The challenge largely stems from the spontaneous, uncatalyzed decarboxylative protonation as well as the racemic or achiral acid reactants acting as proton donors, both of which undermine stereocontrol.

Tailored bifunctional metal complexes can alleviate these issues by serving multiple roles. A synergistic activation can be envisioned when the Lewis acidic metal center complexes with the substrate, while the Brønsted basic shuttle seizes the acid proton to yield the anion and initiate CO_2_ extrusion. This ‘push-and-pull’ mode can provide an accelerated decarboxylation pathway that outcompetes uncatalyzed ones. By mediating dynamic isomerization of the resulting diastereomeric complexes, the metal center could also drive enantioconvergence from racemic acids. Consequently, rapid proton transfer from the nearby host to the kinetically favored complex within the chiral pocket yields the desired enantioenriched product while preventing interference in enantiocontrol caused by external acids.

In this work, rhodium–phosphoramidite complexes are found to be suitable for this dynamic kinetic manifold^9^ and unlock the elusive asymmetric decarboxylation of cyanoacetic acids. The borrowing proton catalysis is also compatible with facile H/D exchange between acid reactants and deuterated solvents. Thus, decarboxylation products can be obtained both enantio- and deuterium-enriched.

## Results and Discussion

### Development of Rhodium-Catalyzed Decarboxylation

We used disubstituted cyanoacetic acids as the model substrate because these racemates can be readily obtained from high-volume cyanoacetates and the nitrile group retained after decarboxylation offers diverse reactivity. Nevertheless, their asymmetric decarboxylation is rarely studied compared with related compounds such as malonic and β-keto acids.^8^ The combination of a cationic rhodium salt and a chiral phosphoramidite ligand^10^ (**L1**) was identified as the catalyst (Scheme 2A and Figures S1–S5). An almost quantitative yield and excellent enantiopurity of α-chiral nitrile (**1**) were obtained, confirming a high enantioconvergence. The ligand features a pair of electron-deficient aryl side arms on the binaphthyl scaffold and a dimethylamino motif as the proposed proton host (vide supra, Scheme 1B). We also obtained a crystallographic structure of the reactive rhodium/**L1** complex with an unexpected molecule of water ligated, presumably from adventitious moisture during the preparation (Scheme 2B). However, control experiments under anhydrous conditions, with added water, or with added molecular sieves, gave an almost identical yield and enantioselectivity of the decarboxylation, indicating a negligible role of trace water (Section 6 of Supporting Information for details). It is worth noting that besides a kinetic competitiveness (i.e., TOF_ini_) in catalyzing the decarboxylation, the robustness of the rhodium catalyst is also exceptional; a turnover number (TON) exceeding 25,000 was achieved at a ppm-level catalyst loading (Scheme 2C).

**Scheme 2:**
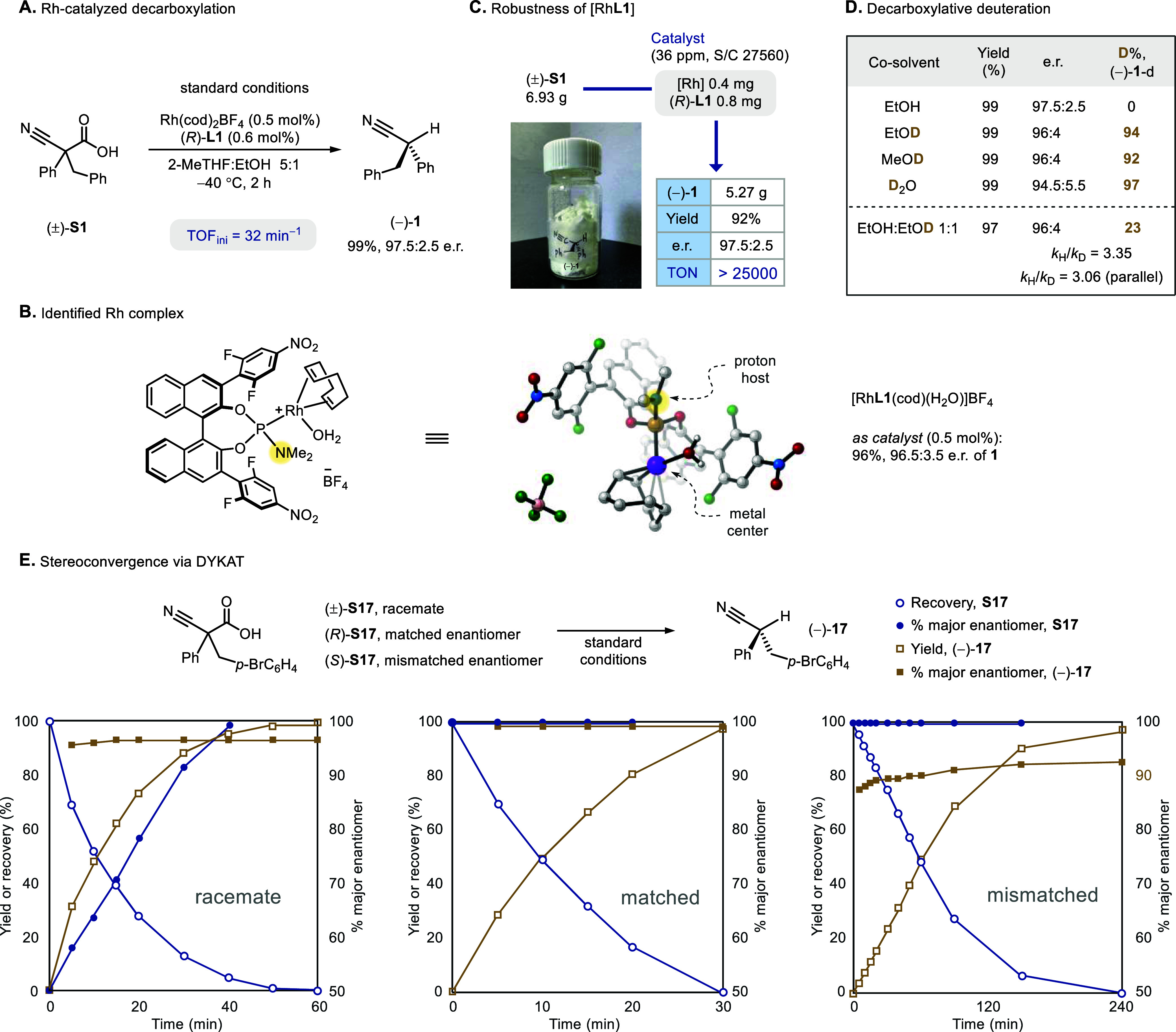
Identification of the Organometallic Proton Shuttle for Stereoconvergent Decarboxylation

The rhodium proton shuttle can also catalyze decarboxylative deuteration,^11^ offering a convenient access to deuterated stereocenters with higher configurational stability relative to their unlabeled analogs (Scheme 2D).^12–15^ Replacement of ethanol with deuterated protic solvents immediately led to a high level of deuteration, presumably benefiting from a fast H/D exchange between acid substrate and solvent (Figure S17). While heavy water caused a slight erosion in enantiocontrol, both deuterated methanol and ethanol afforded the labeled stereocenter in excellent yield and enantioselectivity. A high site selectivity of the deuteration was also observed, with no or negligible deuteration detected at the benzylic and aromatic positions. Meanwhile, both a mixed-solvent competition and parallel measurement (Figures S15 and 16) demonstrated a strong kinetic isotope effect, as expected from a reaction with extensive solute–solvent H/D exchange and deuterium transfer.^16^ It is worth noting that the amount of deuterated solvent can be largely reduced without a significant decrease in the deuteration rate (Figure S6). When run on a large scale, the high yield, minimal byproduct generation, and low catalyst loading of the decarboxylation can reduce contamination of the deuterated solvent, thereby facilitating its recovery and re-enrichment.

The stereoconvergence of the decarboxylation was also investigated with kinetic measurements (Scheme 2E). As a racemic mixture of **S17** proceeded through the decarboxylation, the enantiopurity of the product remained stable, while the acid substrate was enantioenriched over time. This observation suggests that the chiral catalyst is indeed involved in the CO_2_ extrusion and can differentiate between the two enantiomeric acids, despite the stereoselectivity of decarboxylation being determined by the protonation step later.^17^ We also examined the matched and mismatched cyanoacetic acids in their enantiomerically pure forms separately. As expected, the pair of decarboxylation reactions displayed contrasting rates. On the other hand, the enantioselectivity was marginally higher and lower compared with the reaction of racemic acid, respectively, suggesting an incomplete pre-equilibrium between the pair of rhodium-ketenimines from the matched and mismatched acids before the asymmetric protonation. Altogether, these kinetic data are consistent with a dynamic kinetic asymmetric transformation (DYKAT) of configurationally stable enantiomers with a pair of interconverting, catalyst-associated intermediates (vide supra, Scheme 1B).^9^

### DFT Calculation

The pathway of decarboxylative protonation was also studied using density functional theory (DFT) calculations under the ωB97XD/6-311+G­(d,p)-SDD/SMD­(THF)//ωB97XD/6-31G­(d)-LANL2DZ level of theory since this combination of functional and basis sets is classic and reliable for a rhodium-catalyzed reaction system (Scheme 3).^18^ The solvated rhodium complex (**Int-1**) was used as the starting point of the free-energy profile, partly due to the ease of water/protic solvent association (vide supra, Scheme 2B).

**Scheme 3:**
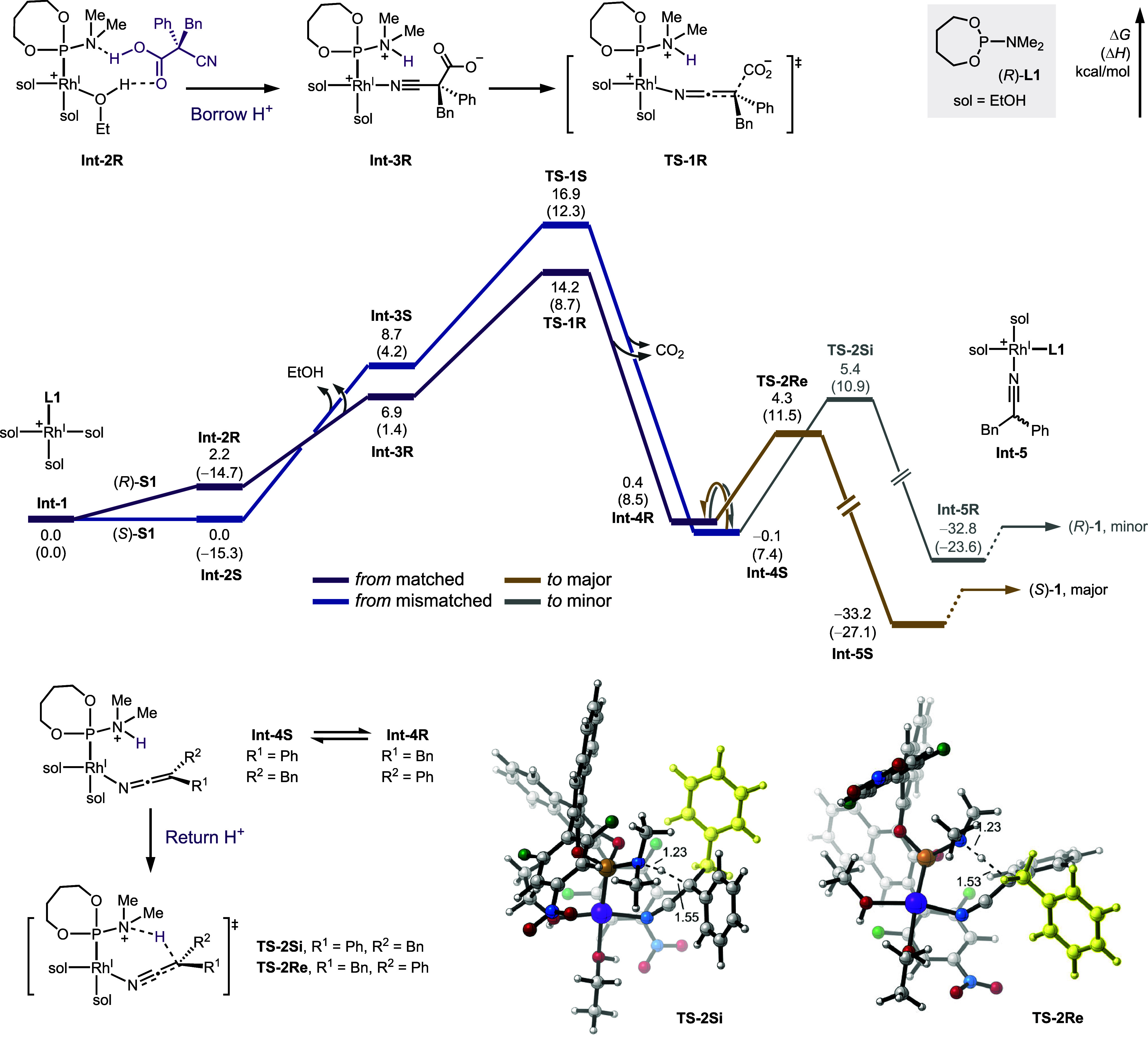
DFT Studies of the Decarboxylative Protonation Pathway

When matched cyanoacetic acid (*R*)-**S1** approaches, a hydrogen-bond complex (**Int-2R**) with the amine of the phosphoramidite ligand and an alcohol ligand was generated and served as the gateway to proton transfer.^19^ An alternative ligand dissociation and substrate association pathway was also calculated. However, the loss of an ethanol from **Int-1** is highly endergonic (Figure S18). The following, rate-determining step through **TS-1R** consists of the deprotonation, ethanol dissociation, as well as the complexation of nitrile to trigger the CO_2_ departure. The high barrier of the internal proton transfer (i.e., **Int-2R** to **Int-3R**) is proposed to be the origin of strong kinetic isotope effect (vide supra, Scheme 2D), as the deuterated cyanoacetic acid from the H/D exchange with solvent (Figure S17) has a stronger O–D bond that can slow down the deprotonation and thus the overall decarboxylation. Meanwhile, **Int-4R** after the CO_2_ extrusion validated our hypothesis of the organometallic proton shuttle. With ‘borrowed’ proton presiding on the amine, the ketenimine coordinates to the rhodium center.

We also calculated the pathway of mismatched (*S*)-**S1** to **Int-4S**, and the energy barrier of transition state **TS-1S** is higher than that of the matched transition state **TS-1R**, as expected from the slower reaction rate observed (vide supra, Scheme 2E). Our calculations indicate that the rhodium-ketenimine intermediate generated from the matched acid (**Int-4R**) is 0.5 kcal/mol higher in energy than its counterpart from the mismatched acid (**Int-4S**). Their facile interconversion, together with the kinetic advantage of the protonation transition state **TS-2Re** over **TS-2Si**, is central to the enantioconvergent and DYKAT nature of the decarboxylation. Structural analysis and quantitative steric-electronic effect dissection of the transition state (Figure S21) reveal that the greater steric repulsion by the benzyl group largely contributes to the enantioenrichment.

### Scope of Decarboxylation of Cyanoacetic Acids

The decarboxylation unlocks a synthetic sequence that begins with abundant cyanoacetates (**2**), harnesses the facile substitution of the active methylene (**3**), and leads to both deuterated (**6**) and nondeuterated (**5**) stereocenters (Table 1). Meanwhile, both ethyl and *tert*-butyl cyanoacetates can be used for the sequence, and their orthogonal hydrolysis conditions (i.e., basic and acidic) contribute to a good functional group tolerance. Overall, this synthetic protocol can be considered an asymmetric, nitrile variant of the textbook malonic and acetoacetic ester synthesis.

**Table 1:**
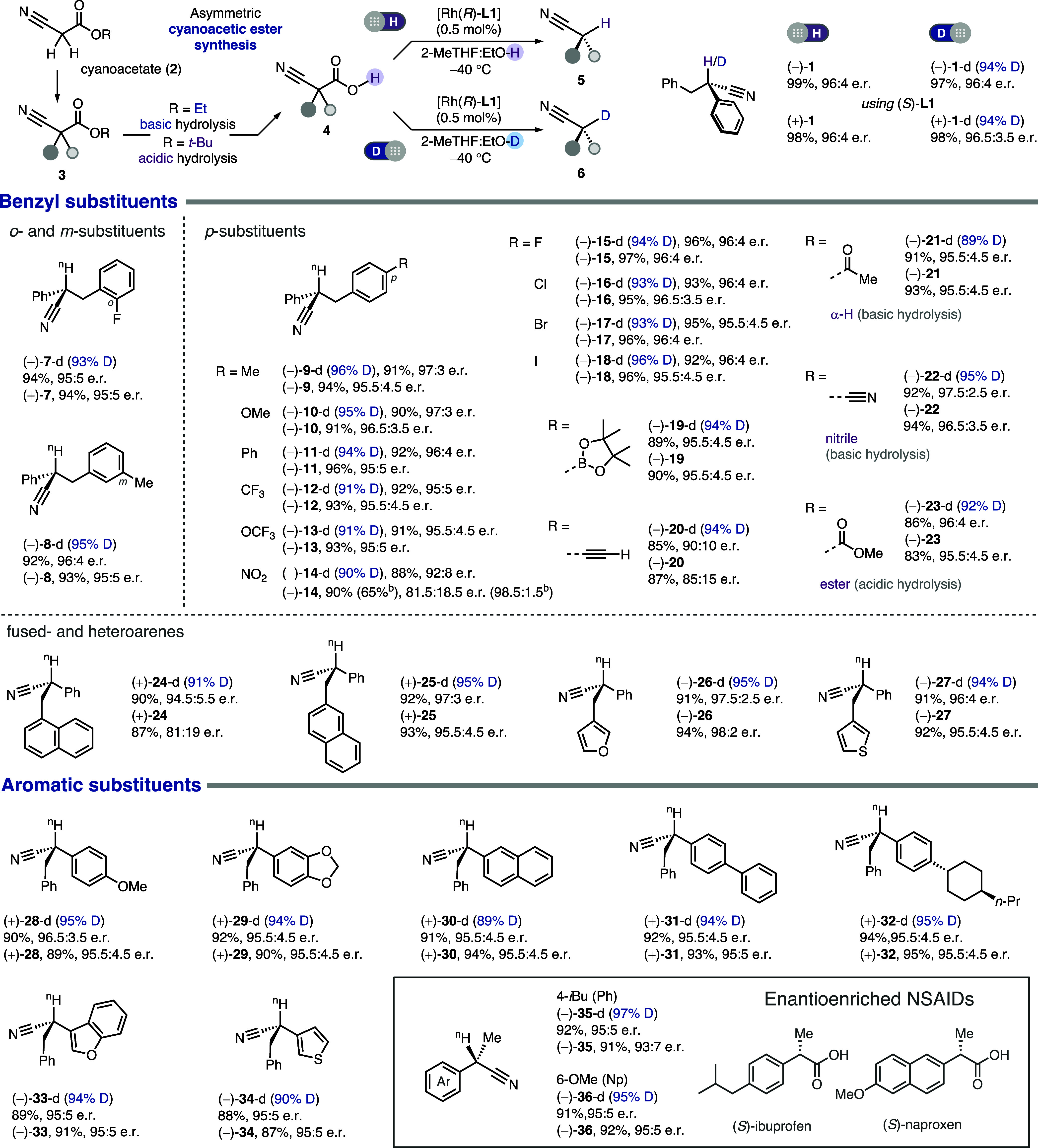
Scope of Benzyl and Aryl Substituents of Asymmetric Decarboxylation^a^

a Unless noted otherwise, the decarboxylative protonation was run with cyanoacetic acid (0.5 mmol), Rh­(cod)­BF_4_ (0.0025 mmol, 0.5 mol %), and **L1** (0.0030 mmol, 0.6 mol %) in a mixed solvent of 2-methyltetrahydrofuran (10 mL) and ethanol (2 mL) at −40 °C for 1–48 h. The decarboxylative deuteration was run with cyanoacetic acid (0.2 mmol), Rh­(cod)­BF_4_ (0.0010 mmol, 0.5 mol %), and **L1** (0.0012 mmol, 0.6 mol %) in a mixed solvent of 2-methyltetrahydrofuran (20 mL) and deuterated ethanol (EtOD, 4 mL) at −40 °C for 1–48 h. For detailed reaction conditions, see Supporting Information Section 6 and 7.

b The yield and enantioselectivity refer to the product after recrystallization.

With the pair of enantiomeric phosphoramidite ligands, deuterated and nondeuterated benzyl-phenyl stereocenters (**1**) of opposite configuration can be obtained with excellent yield, enantioselectivity, and level of deuteration. Different substitution patterns of the benzyl group and substituents of distinct electronic properties (**7**–**18**) are all compatible. However, the nitrobenzyl motif gave an inferior enantioselectivity (**14**), probably owing to the enhanced acidity of the aromatic C–H bonds that may create or interfere with weak interactions (e.g., hydrogen bonds). It is worth noting that the erosion of enantiocontrol is less significant for deuteration (**14**-d), and similar contrasts were observed in multiple cases (e.g., **24**, **46**, and **54**). Enhanced enantioselectivity with deuterated solvents or reagents has been sporadically observed in prior studies of assorted reactions.^20^ While the exact origin is unknown, it may result from the slower and more selective deuteron transfer (vide supra, Scheme 3, **Int-4** to **TS-2**) due to the stronger N–D bond and a solvent kinetic isotope effect can play a role as well. Versatile yet labile functional groups, including boronate (**19**) and terminal alkyne (**20**), were also compatible and enhanced the versatility of the decarboxylation products. The facile synthesis of disubstituted cyanoacetic acids, particularly the mild and flexible hydrolysis under basic or acidic conditions, was further showcased by the tolerance of methyl ketone (**21**), nitrile (**22**), and even methyl ester (**23**). Besides substituted aryls, naphthyl (**24** and **25**) and heterocyclic (**26** and **27**) rings are also suitable substituents at the benzylic position.

Substituents on the arene directly attached to the protonation/deuteration site are accommodated equally well (**28**, **29**). The catalyst is also compatible with distinctly shaped aryl (**30**–**32**) and heteroaryl (**33**, **34**) moieties. The diversity of tolerated aryl substituents allowed an easy, decarboxylative access to the enantioenriched nitrile analogues of naproxen (**35**) and ibuprofen (**36**), two nonsteroidal anti-inflammatory drugs (NSAIDs) famous for their chiral switching.^21^

The high compatibility of functional groups on both arenes aided the preparation of enantioenriched 1,2-diarylethanes (Scheme 4). These motifs are ubiquitous in bioactive compounds, drugs, and functional molecules, including a TRPM8 antagonist (**37**), inhibitors of HtrA1 (**38**), TRPC channels (**39**–**41**), and factor Xa (**42**), as well as a photoaffinity probe (**43**). The decarboxylative preparation enjoys a high regio- and enantioselectivity owing to the modular and sequential substitution of cyanoacetate that can introduce diverse combinations of aryl and benzyl substituents as well as the excellent stereocontrol of the rhodium catalyst. Meanwhile, selective deuterium labeling was enabled by the decarboxylative deuteration to offer equally efficient access toward enantioenriched isotopologues of these structures.

**Scheme 4:**
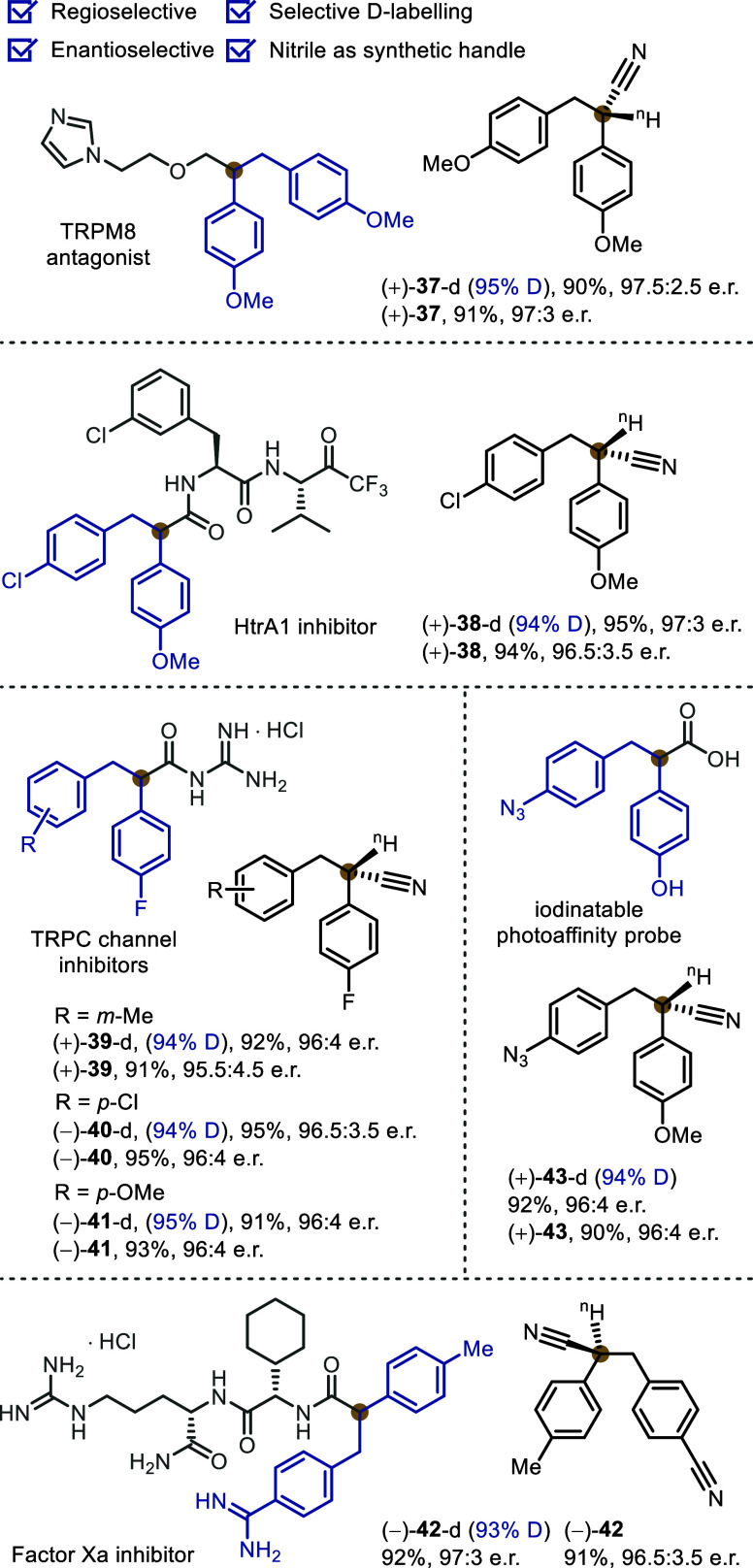
Motifs for Bioactive 1,2-Diarylethanes

Furthermore, the versatility of alkyl substituents extends beyond simple benzyl groups (Table 2). Alkyl chains of different lengths and shapes are well tolerated (**44**–**47**). However, the presence of a pendent and remote phenyl group (**46**) can adversely affect stereocontrol, likely due to disruptive π–π interactions with the ligand or metal–π complexation. In contrast, the interference of olefins (**48**–**50**) and alkynes (**51**) was less detrimental to the enantioselectivity.

**Table 2:**
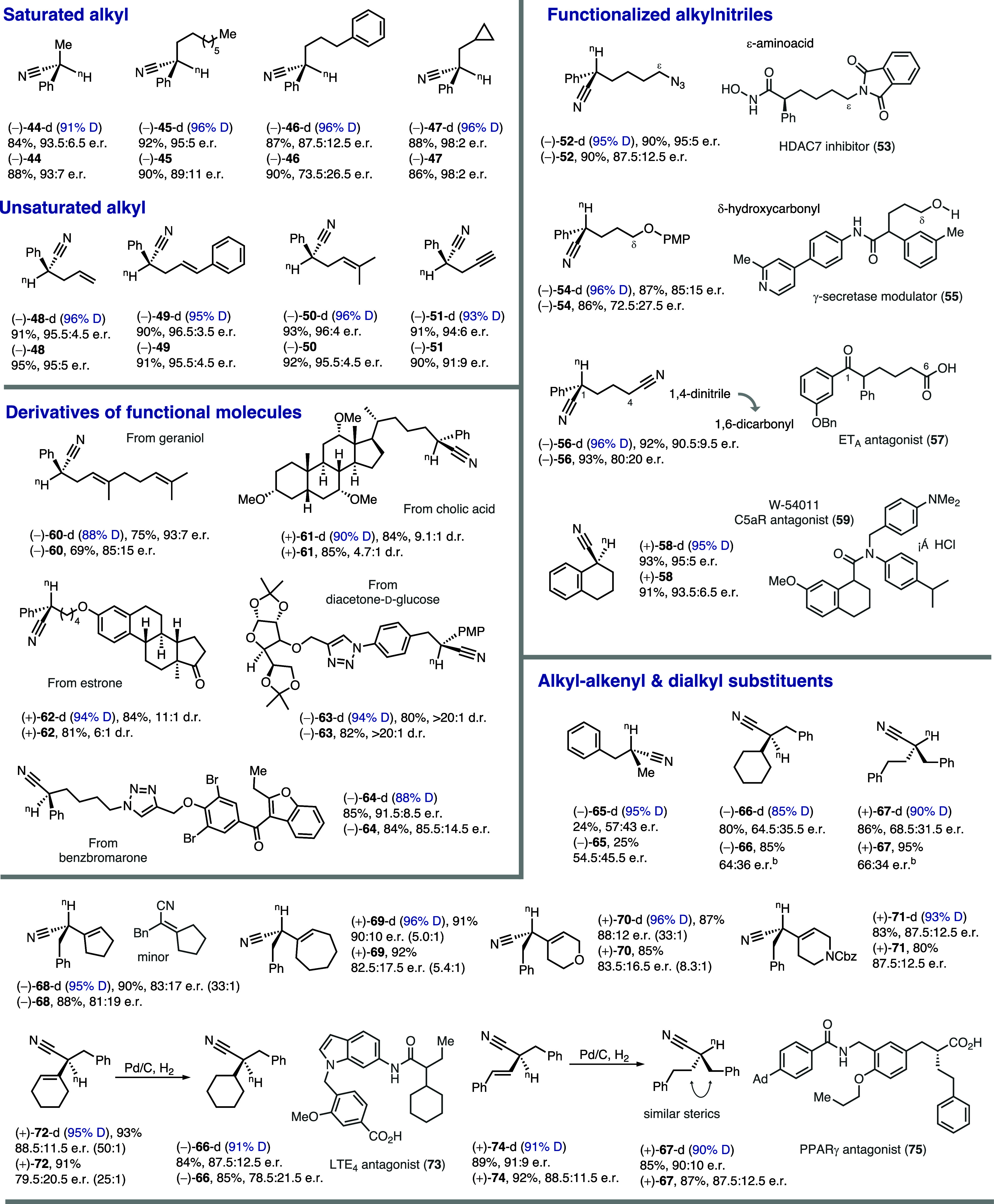
Scope of Alkyl and Alkenyl Substituents of Asymmetric Decarboxylation^a^

a Unless noted otherwise, the decarboxylative protonation was run with cyanoacetic acid (0.5 mmol), Rh­(cod)­BF_4_ (0.0025 mmol, 0.5 mol %), and **L1** (0.0030 mmol, 0.6 mol %) in a mixed solvent of 2-methyltetrahydrofuran (10 mL) and ethanol (2 mL) at −40 °C for 1–48 h. The decarboxylative deuteration was run with cyanoacetic acid (0.2 mmol), Rh­(cod)­BF_4_ (0.0010 mmol, 0.5 mol %), and **L1** (0.0012 mmol, 0.6 mol %) in a mixed solvent of 2-methyltetrahydrofuran (20 mL) and deuterated ethanol (EtOD, 4 mL) at −40 °C for 1–48 h. For detailed reaction conditions, see Supporting Information Section 6 and 7.

b These decarboxylation reactions were run using 5 mol % of rhodium catalyst and 6 mol % of **L1.**

Besides unsaturated bonds, heteroatom-embedded alkyl chains are also compatible and enhance the functional diversity of decarboxylation products. For example, an attached azide can not only participate in click reactions to connect an additional fragment (e.g., **64**) but could also, in principle, be converted to an amine to provide an ε-amino acid equivalent (**52**) frequently found in bioactive compounds, including an HDAC7 inhibitor (**53**). A protected alcohol was also tolerated (**54**), allowing the decarboxylation to access α-chiral carbonyl building blocks with a remote oxygen-containing motif (e.g., **55**). Meanwhile, the 1,4-dinitrile product **56** can serve as a precursor to enantioenriched 1,6-dicarbonyl molecules that are found in many bioactive molecules (e.g., **57**). The rhodium catalyst is equally effective in transforming cyclic cyanoacetic acids (**58**) into carbocycles with a deuterated stereocenter (e.g., **59**). Notably, adjusting the chain length of the alkyl substituent in the decarboxylation products is facile due to the modular substitution during cyanoacetic acid synthesis. Therefore, the potential to access diverse derivatization products is immense.

The easy substitution also allowed the attachment of naturally occurring fragments, such as geraniol (**60**), cholic acid (**61**), and estrone (**62**). The decarboxylation performed equally well for these complex acids, further showcasing its good compatibility with shape and functionality of substrate. On the other hand, connection of bioactive motifs, including protected glucose (**63**) and benzbromarone (**64**), is also feasible using click chemistry, and the Lewis/Brønsted basic triazole junction is tolerated under decarboxylation conditions.

While the current rhodium catalyst can decarboxylate cyanoacetic acids without an aromatic substituent (**65**–**67**), the enantioselectivity is low. Nevertheless, alkenyl groups can facilitate the decarboxylation. Cyanoacetic acids with these olefinic substituents can be easily synthesized via condensation with ketones, followed by a migratory alkylation. Both cyclic (**68**–**72**) and linear (**74**) alkenyl substituents are compatible with the decarboxylation, with only a small amount of migratory protonation product generated. We also demonstrated that the decarboxylation can be effectively followed by chemoselective hydrogenation of olefins in the presence of nitrile. This sequence presents a valuable approach for synthesizing enantioenriched α,α-dialkyl nitriles (**66** and **67**) currently hard to access through direct decarboxylation. Particularly, the synthesis of nitriles with sterically similar alkyl groups (**67**), often challenging to prepare, was made feasible and could provide important precursors for bioactive carbonyl compounds, such as antagonists **73** and **75**.

### Synthetic Application

The decarboxylation using organometallic proton shuttle can be applied to synthesize fluorinated stereocenters (Scheme 5A). Fluorodeuterated stereocenters can be prepared as well (**76**–**78**). These motifs are increasingly sought after in drug discovery (e.g., **79**–**81**) largely due to the unique physiochemical properties conferred by fluorine and the stabilizing isotope effects provided by deuterium. The rhodium–phosphoramidite complex also proved effective in decarboxylating malonic acids (**82**) to α-fluorinated acids. Although the enantioselectivity and deuteration rate were lower, the catalyst’s compatibility with acid substrates beyond cyanoacetic acids hinted a broad applicability of organometallic proton shuttles in controlling the trajectory of proton/deuteron transfer.

**Scheme 5:**
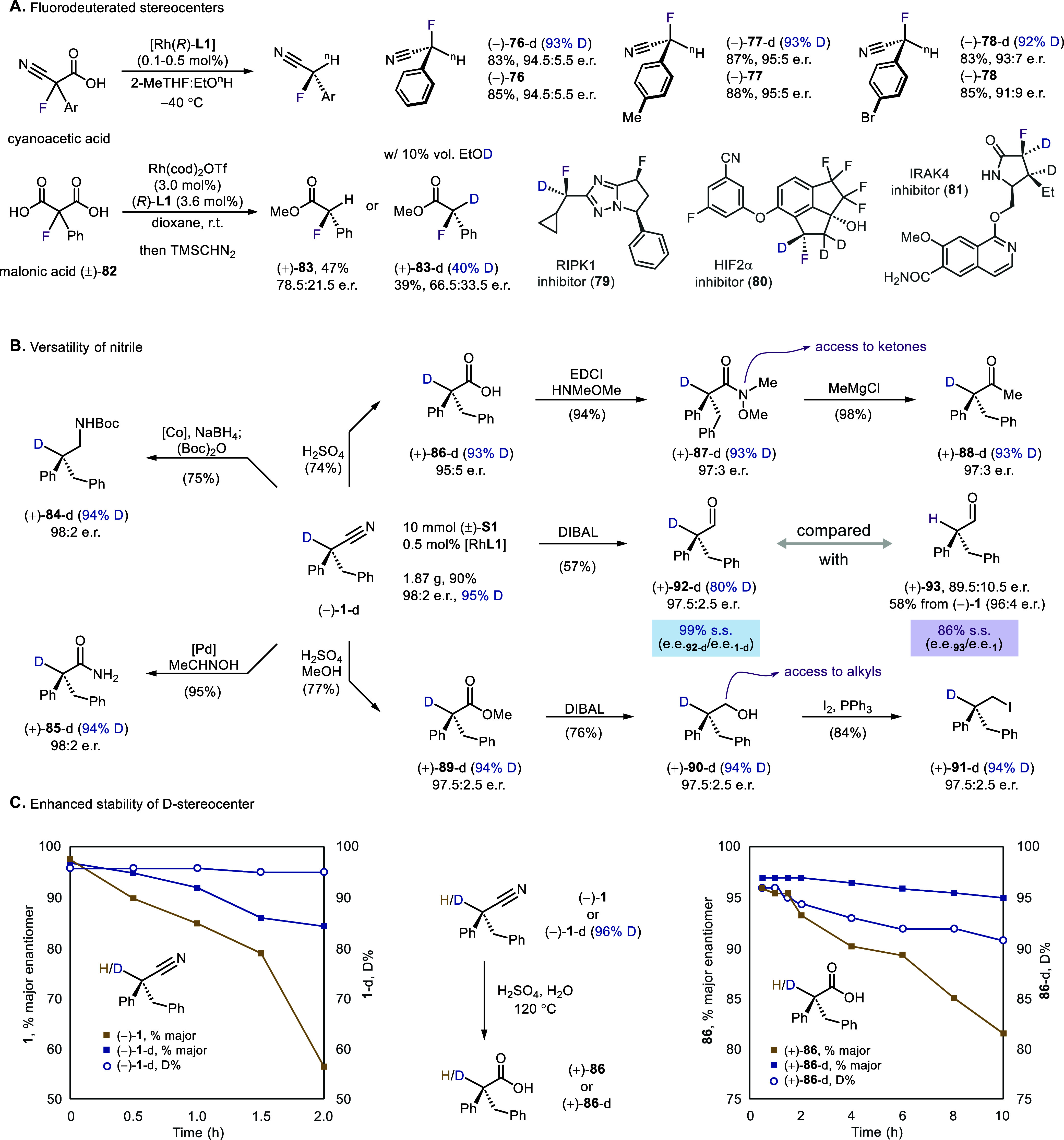
Access to Diverse Deuterated Stereocenters

The rich chemistry of nitriles provides a versatile platform for postdecarboxylation derivatization, particularly enabling efficient transfer of the deuterated stereocenter into valuable labeled and enantioenriched molecules of interest (Scheme 5B). Common reduction, hydrolysis, and alcoholysis conditions can be applied to generate deuterated amine (**84**), amide (**85**), acid (**86**), and ester (**89**). Importantly, the erosion in the deuteration rate and enantiopurity is minimal, even for transformations further downstream. As such, methyl ketone **88**, regio- and enantioselectively deuterated at the more congested α-position, was obtained from acid **86** via a Weinreb amide (**87**). On the other hand, the reduction to primary alcohol **90** and its further iodination (**91**) opens access to an alkyl moiety.

Meanwhile, the DIBAL reduction of the decarboxylation products is worth an additional discussion. Compared with the reduction of nondeuterated nitrile **1**, where the enantiopurity decreased largely (**93**), an almost complete stereoretention was observed for **1**-d (**92**). It is also interesting to note that the deuteration level of the aldehyde product is slightly diminished, suggesting a stereoretentive H/D exchange^14^ in the presence of an aluminum species.

The stabilizing effect of deuterium was also manifested by the hydrolysis of the nitrile (Scheme 5C). Monitoring of the reaction of nondeuterated decarboxylation product (**1**) displayed a considerable decline in enantiopurity for both the starting nitrile (**1**) and generated acid (**86**). In comparison, the deuteration rate and enantiomeric excess of their heavier isotopologues (i.e., **1-d** and **86-d**) experienced smaller decreases throughout hydrolysis. Taken together, these results are consistent with the enhanced resistance of deuterated stereocenters toward racemization due to the kinetic isotope effect.

The broad scope of decarboxylation and the robustness of the resulting deuterated stereocenters have facilitated the multistep synthesis of pharmaceuticals that carries along the labeled motif (Scheme 6A). For instance, a sequence of nitrile reduction, sulfonylation, and palladium-catalyzed cross-coupling from deuterated product **94** yielded AMPA receptor potentiator **96**. Similarly, a pathway concluding with copper-catalyzed C–N bond formation produced its analog **97** featuring a pyrazole motif, with equally good enantioselectivity and deuteration rate. Additionally, a Friedel–Crafts-type cyclization was orchestrated between the aryl substituent of the stereocenter and the alkylamine derived from nitrile (**100**), resulting in an unsaturated benzazepine that was subsequently hydrogenated to deuterated and protected lorcaserin (**101**).

**Scheme 6:**
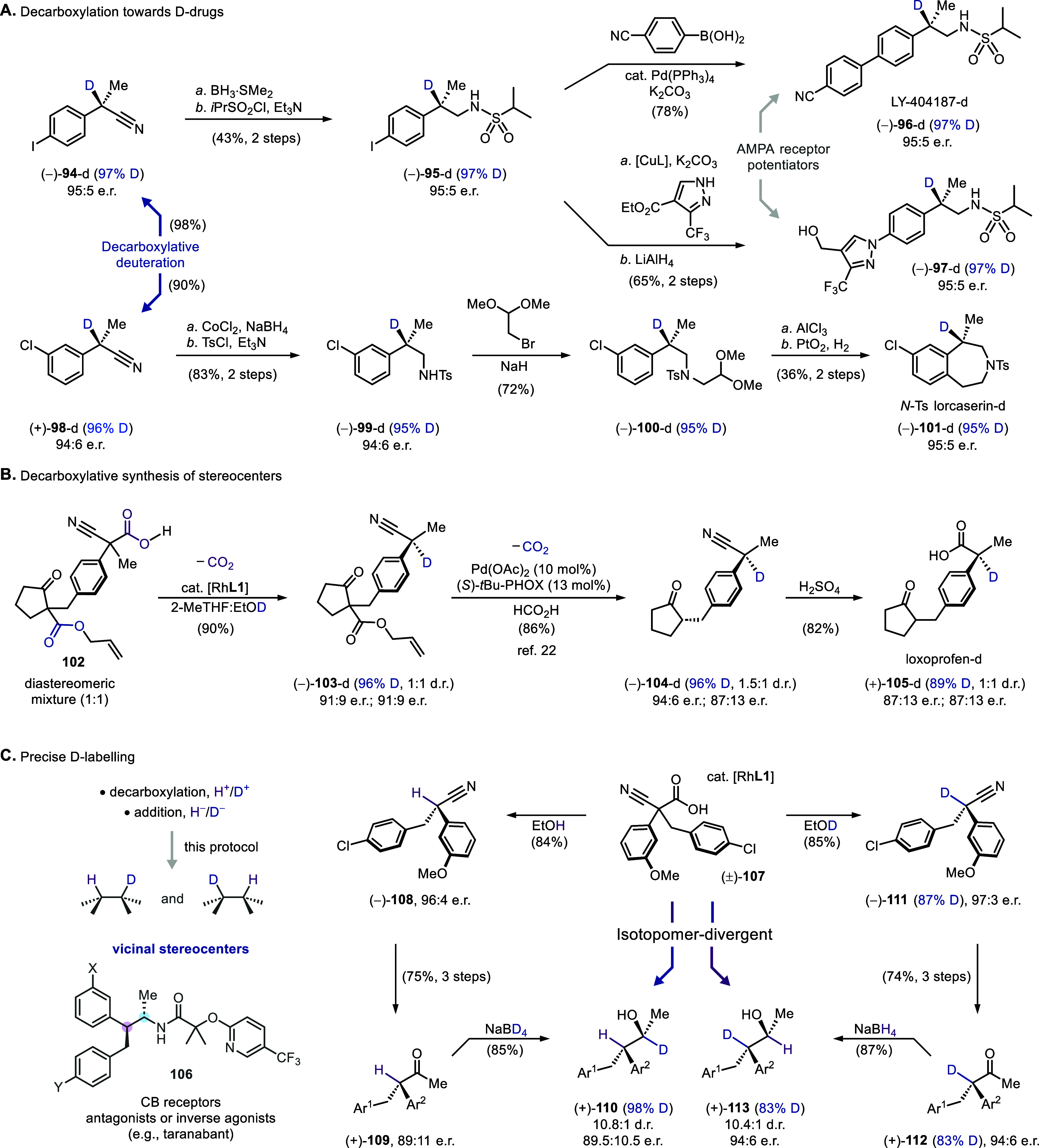
Synthetic Application of Asymmetric Decarboxylation

With racemic acid and ester starting materials readily available, decarboxylation is increasingly employed to create structurally diverse stereocenters. In this study, we demonstrate that two distinct decarboxylation reactions can be joined to synthesize the pair of stereocenters in loxoprofen (Scheme 6B). Starting from polyfunctionalized precursor **102**, easily obtained through straightforward substitutions, the rhodium-catalyzed decarboxylation of the cyanoacetic acid fragment yielded a deuterated acyclic stereocenter with excellent enantioselectivity (**103**). Subsequently, a Tsuji-Trost-type deallylative decarboxylation, a method proven to be effective for synthesizing multiple natural products,^22^ was used to construct the chiral cyclopentanone motif (**104**) across the arene. The final hydrolysis of nitrile afforded deuterated loxoprofen as a pair of enantiomeric diastereomers (**105**).

The prevalence of vicinal stereocenters in bioactive entities (e.g., **106**) and the high value of their deuterated variants motivated us to enable precise, site-selective deuterium labeling using the asymmetric decarboxylation and subsequent nitrile conversion (Scheme 6C). When the asymmetric decarboxylative protonation was followed by the conversion of nitrile to ketone (vide supra, Scheme 5B, **1** to **88**) and diastereoselective borodeuteride addition, alcohol **110** with monodeuterated vicinal stereocenters was obtained. Its isotopomer (**113**) is equally accessible by switching to decarboxylative deuteration and borohydride addition. Importantly, both reaction sequences exhibited good enantio- and diastereoselectivity, along with complete site selectivity for deuteration. Overall, these synthetic applications showcase the potential of the asymmetric decarboxylative deuteration in preparing labeled bioactive molecules, especially enantioenriched drug candidates with deuterated stereocenters.

## Conclusions

A ligand-assisted pathway of a rhodium–phosphoramidite complex was discovered to modulate proton transfer in asymmetric decarboxylation. While conventional acid- or base-mediated protonation hinges on multiple weak interactions, the organometallic proton shuttle operates in a synergistic fashion between the Lewis acidic rhodium center and the Brønsted basic amino motif in the ligand scaffold. In particular, the dative bond to the metal helps fix the orientation of the intermediate to the nearby amino proton host in a more directional manner, thus enhancing the enantiocontrol. This discovery unlocks a wealth of new possibilities for chiral metal catalysts as proton shuttle in asymmetric decarboxylation of accessible acid feedstocks and reactions involving proton transfer in general.

## Supplementary Material



## Notes

Supplementary figures, experimental procedures, NMR spectra for all new compounds, HPLC traces for chiral compounds, and crystallographic data (PDF)
