# Supplementary material for: Asymmetric Decarboxylative Protonation and Deuteration of Cyanoacetic Acids Using an Organometallic Proton Shuttle
Source: J Am Chem Soc. 2026 May 12;148(20):20985–96. doi: 10.1021/jacs.6c05281 (PMC13220274; doi:10.1021/jacs.6c05281)
Supplement: Supplementary file 1 [file ja6c05281_si_001.pdf]

Supplementary Information for

Asymmetric decarboxylative protonation and deuteration of cyanoacetic acids using an organometallic proton shuttle

Wei-Feng Zheng,[1] Guozhen Wu,[2] Min Hou,[1] Ke Li,[1] Xiaotian Qi,[2,*] Zhongxing Huang[1,*]

[1]State Key Laboratory of Synthetic Chemistry, Shanghai Hong Kong Joint Laboratory in Chemical Synthesis, Department of Chemistry, The University of Hong Kong, Hong Kong 000000, China.
[2]College of Chemistry and Molecular Sciences, Wuhan University, Wuhan, Hubei 430072, China
Correspondence to: qi7xiaotian@whu.edu.cn, huangzx@hku.hk

Table of Contents

Section		
1	Materials and methods	S4 [link]
2	Optimization of the asymmetric decarboxylative protonation reaction	S5 [link]
3	Preparation of rhodium catalyst and chiral ligand	S11 [link]
4	General procedures for the synthesis of substrates	S15 [link]
5	Characterization data of substrates	S19 [link]
6	General procedure for the asymmetric decarboxylative protonation/ deuteration reactions	S55 [link]
7	Characterization data of decarboxylation products	S57 [link]
8	Synthetic applications	S142 [link]
9	Crystal structures and crystallographic data	S166 [link]
10	Mechanistic studies	S170 [link]
11	Density functional theory (DFT) studies	S183 [link]
12	Supplementary references	S252 [link]
13	Copies of spectra and traces	S253 [link]

List of Supplementary Figures

Figure S1 [link] Screening of chiral ligands.

Figure S2 [link] Screening of rhodium catalysts

Figure S3 [link] Solvent effects

Figure S4 [link] Detail investigation of ligand structure and other reaction parameters

Figure S5 [link] Effects of catalyst/ligand loading and their concentration

Figure S6 [link] Deuteration using deuterated alcohol and heavy water

Figure S7 [link] Crystal structure for [Rh**L1**(COD)(H_2O)]BF_4 (CCDC 2302076)

Figure S8 [link] Crystal structure for (−)-**17** (CCDC 2298854)

Figure S9 [link] Crystal structure for (−)-**17-1** (CCDC 2323058)

Figure S10 [link] Crystal structure for (−)-**18** (CCDC 2321980)

Figure S11 [link] Investigation of background decarboxylation

Figure S12 [link] Full kinetic profile of the decarboxylation from racemic **S17**

Figure S13 [link] Full kinetic profile of the decarboxylation from enantioenriched (*R*)-**S17**

Figure S14 [link] Full kinetic profile of the decarboxylation from enantioenriched (*S*)-**S17**

Figure S15 [link] ^{1}H NMR spectrum of (–)-**1** with mixed H/D solvent experiment

Figure S16 [link] Initial reaction rate of competitive experiment

Figure S17 [link] ^{1}H NMR spectrum of H/D exchange experiments

Figure S18 [link] Calculated relative free energies of various Rh(I) complexes and their pathways

Figure S19 [link] Free energy profile of the protonation with ethanol that coordinates with the metal center

Figure S20 [link] Calculated activation free energy for the H/D exchange between **S1** and deuterated ethanol

Figure S21 [link] Quantitative steric-electronic effect dissection of protonation transition states **TS-2Si** and **TS-2Re**.

Figure S22 [link] Calculation results using alternative functions

Section 1. Materials and method

All reactions were run under ambient atmosphere. 2-Methyltetrahydrofuran (2-MeTHF) was purchased from Energy Chemical Co., Ltd. and used as received. Thin layer chromatography (TLC) was run on silica gel plates purchased from Yantai Huanghai Silica gel Development Co., Ltd. ^{1}H NMR, ^{13}C NMR, and ^{19}F NMR spectra were obtained on a Bruker 400, 500 or 600 spectrometer (400/500/600 MHz for ^{1}H, 100/125/150 MHz for ^{13}C, 376/471 MHz for ^{19}F). ^{11}B NMR and ^{31}P NMR spectra were obtained on a Bruker 400 spectrometer (128 MHz for ^{11}B, 162 MHz for ^{31}P). All ^{1}H NMR experiments were measured with tetramethylsilane (0 ppm) in $CDCl_3$ as the internal reference; ^{13}C NMR experiments were measured in relative to the signal of $CDCl_3$ (77.0 ppm). Data for ^{1}H NMR, ^{13}C NMR, and ^{19}F NMR were presented as following: chemical shifts (δ, ppm), multiplicity (br = broad, s = singlet, d = doublet, t = triplet, q = quartet, hept = heptet, dd = doublet of doublets, tt = triplet of triplets, td = triplet of doublets, m = multiplet), coupling constant (Hz), and integration. Chiral HPLC spectra were measured on an Agilent 1260 Infinity II. The $[\alpha]_D$ were recorded using INESA SGW-531 Automatic Polarimeter. Infrared absorption spectra were recorded on a PerkinElmer UATR two FT-IR and the data presented as per centimeter (cm^{-1}). High-resolution EI mass spectra were recorded on a Thermo Scientific DFS high resolution magnetic sector MS. High-resolution ESI-MS measurements were performed on a Bruker impact II high-resolution LC-QTOF mass spectrometer. Accurate masses from high-resolution mass spectra were reported for the molecular ion $[M]^+$, $[M+H]^+$, $[M+Na]^+$, or $[M–CO_2+H]^+$.

Section 2. Optimization of the asymmetric decarboxylative protonation reaction

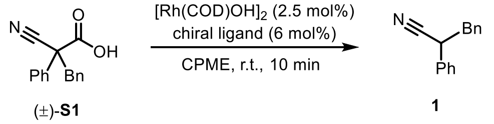

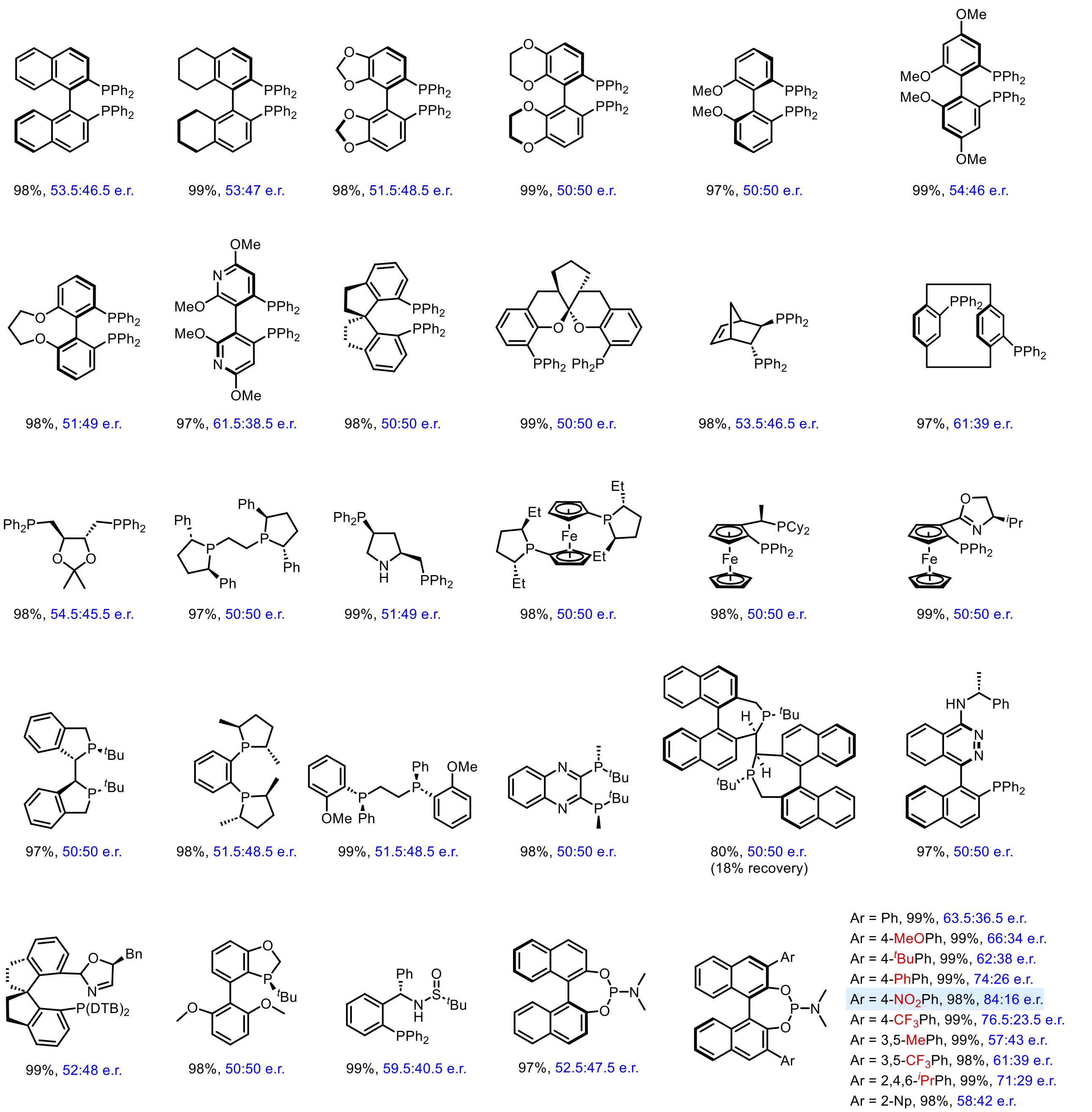

Reaction conditions unless noted otherwise: (±)-**S1** (0.1 mmol), $[Rh(COD)OH]_2$ (2.5 mol%), and chiral ligand (6 mol%) in CPME (2 mL) at r.t. for 10 min. The yield was determined by crude ^1H NMR analysis using 1,3,5-trimethoxybenzene as the internal standard and e.e. was determined by HPLC analysis of isolated product.

Supplementary Figure S1. Screening of chiral ligands.

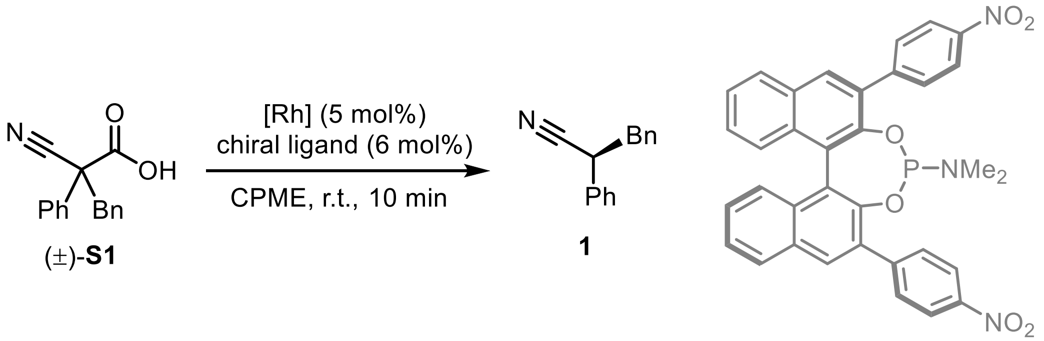

entry	[Rh]	Yield (%)	e.r.
1	$[Rh(COD)OH]_2$	98	84:16
2	$[Rh(COD)OMe]_2$	97	74:26
3	$[Rh(COD)Cl]_2$	99	78.5:21.5
4	$Rh(COD)_2BF_4$	98	87:13
5	$Rh(COD)_2OTf$	97	86.5:13.5
6	$Rh(COD)_2B(3,5\text{-diCF}_3C_6H_3)_4$	98	86:14
7	$Rh(COD)(CH_3CN)_2BF_4$	98	86:14
8	Rh(COD)(acac)	99	85.5:14.5
9	$Rh(C_2H_4)_2(acac)$	98	55:45
10	$Rh(NBD)_2BF_4$	97	78:22
11[a]	$Rh_2(OAc)_4$	0	/
12	$[Cp^*RhCl_2]_2$	99	52:48

Reaction conditions unless noted otherwise: (±)-**S1** (0.1 mmol), [Rh] (5 mol%), and chiral ligand (6 mol%) in CPME (2 mL) at r.t. for 10 min. The yield was determined by crude ^{1}H NMR analysis using 1,3,5-trimethoxybenzene as the internal standard and e.e. was determined by HPLC analysis of isolated product. [a] 99% Recovery.

Supplementary Figure S2. Screening of rhodium catalysts.

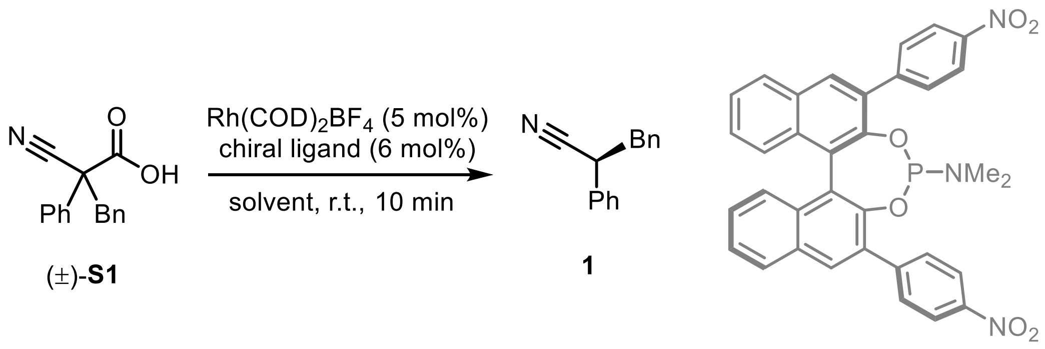

entry	solvent	Yield (%)	e.r.
1	Toluene	97	67:33
2	Acetone	96	84:16
3	Ethyl acetate	97	85.5:14.5
4	CH_3CN	98	77.5:22.5
5	$CHCl_3$	98	77:23
6	Cyclohexane	97	59:41
7	Et_2O	99	85.5:14.5
8	MTBE	99	86.5:13.5
9	THF	98	87:13
10	2-MeTHF	99	87.5:12.5
11	1,4-Dioxane	98	85.5:14.5
12	$^{i}Pr_2O$	98	83.5:16.5
13	DME	99	86:14
14	CPME	98	87:13

Reaction conditions unless noted otherwise: (±)-**S1** (0.1 mmol), $Rh(COD)_2BF_4$ (5 mol%), and chiral ligand (6 mol%) in solvent (2 mL) at r.t. for 10 min. The yield was determined by crude ^1H NMR analysis using 1,3,5-trimethoxybenzene as the internal standard and e.e. was determined by HPLC analysis of isolated product.

Supplementary Figure S3. Solvent effects.

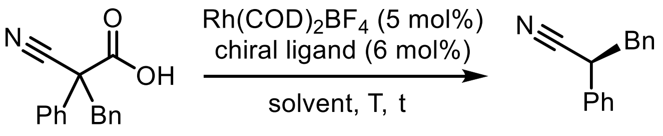

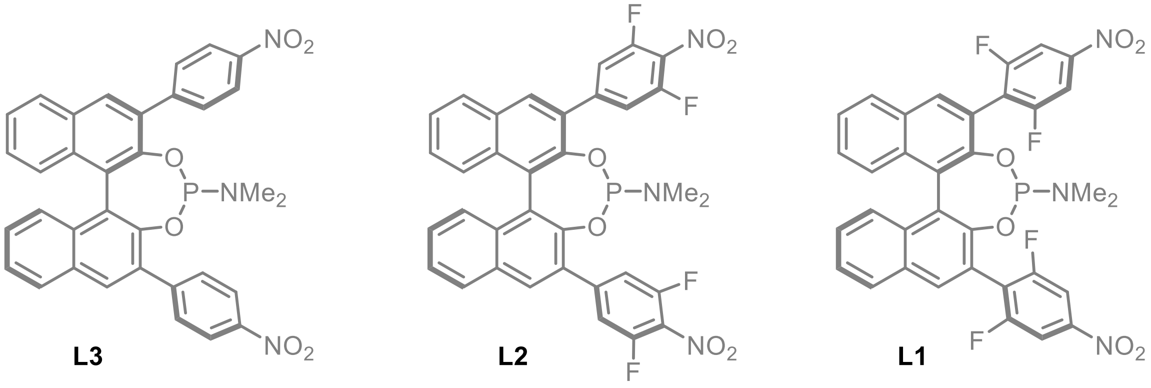

entry	chiral ligand	solvent	T (^{o}C)	t	Yield (%)	e.r.
1	**L3**	2-MeTHF	r.t.	10 min	99	87.5:12.5
2	**L2**	2-MeTHF	r.t.	10 min	98	91:9
3	**L1**	2-MeTHF	r.t.	10 min	99	92:8
4	**L1**	2-MeTHF:EtOH = 5:1[a]	r.t.	10 min	99	92.5:7.5
5	**L1**	2-MeTHF:EtOH = 5:1	0	1 h	99	94.5:5.5
6	**L1**	2-MeTHF:EtOH = 5:1	–20	1 h	98	96.5:3.5
7	**L1**	2-MeTHF:EtOH = 5:1	–40	1 h	99	97.5:2.5

Reaction conditions unless noted otherwise: (±)-**S1** (0.1 mmol), $Rh(COD)_2BF_4$ (5 mol%), and chiral ligand (6 mol%) in 2-MeTHF (2 mL) at desiganated temperature for t min. The yield was determined by crude ^{1}H NMR analysis using 1,3,5-trimethoxybenzene as the internal standard and e.e. was determined by HPLC analysis of isolated product. [a]2-MeTHF:EtOH = 5:1 (v/v).

Supplementary Figure S4. Detail investigation of ligand structure and other reaction parameters.

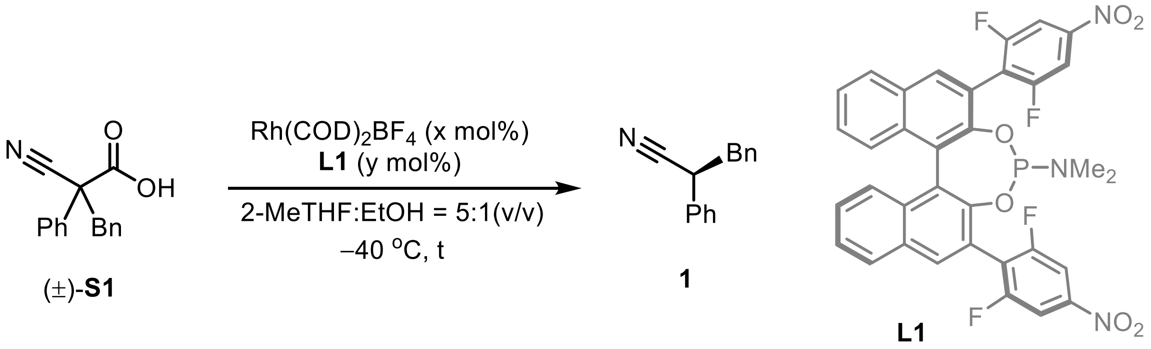

entry	x, y (mol%)	c (M)[a]	t (h)	Yield (%)	e.r.
1	5, 6	0.05	1	99	97.5:2.5
2	4, 4.8	0.05	2	99	97:3
3	2, 2.4	0.05	2	99	97:3
4	1, 1.2	0.05	2	99	96.5:3.5
5	0.5, 0.6[b]	0.05	2	99	96:4
6	0.5, 0.6 (w/o EtOH)[b]	0.05	10	98	95:5
7	0.1, 0.12	0.05	6	99	95.5:4.5
8	0.5, 0.6	0.1	2	99	95:5
9	0.5, 0.6	0.2	2	99	92.5:7.5

Reaction conditions unless noted otherwise: (±)-**S1** (0.1 mmol), $Rh(COD)_2BF_4$ (x mol%), and chiral ligand (y mol%) in 2-MeTHF:EtOH (5:1) at –40 °C for t h. The yield was determined by crude ^{1}H NMR analysis using 1,3,5-trimethoxybenzene as the internal standard and e.e. was determined by HPLC analysis of isolated product. [a]Concentrations refer to those of **S1** in 2-MeTHF. [b]The decarboxylation in 2-MeTHF/EtOH was complete at around 40 min via TLC monitoring, while the one without EtOH was much slower and required 10 h to finish.

Supplementary Figure S5. Effects of catalyst/ligand loading and their concentration.

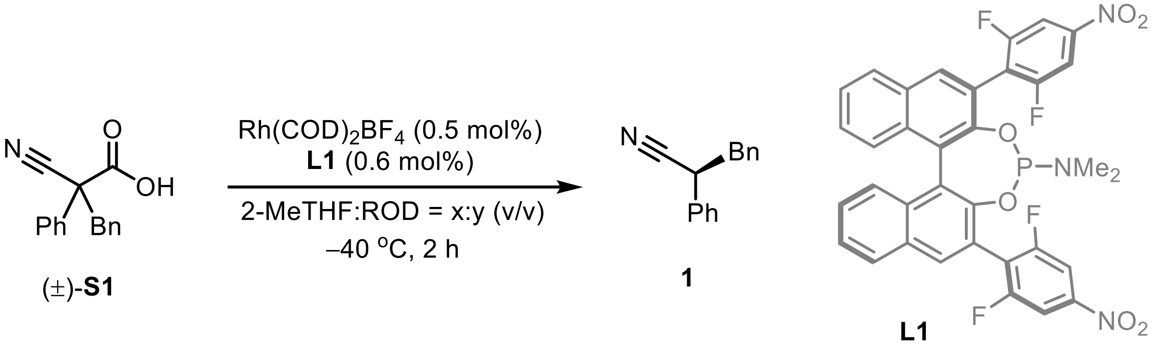

entry	ROD	c (M)[a]	x:y	v/v (mL/mL)	Yield (%)	e.r.	D (%)
1	MeOD	0.05	5:1	2/0.4	99	96:4	89
2	MeOD	0.025	5:1	4/0.8	99	96:4	91
3	MeOD	0.01	5:1	10/2	99	96:4	92
4	MeOD	0.01	10:1	10/1	99	96:4	90
5	D_2O	0.01	5:1	10/2	99	94.5:5.5	97
6	D_2O	0.01	10:1	10/1	99	94.5:5.5	96
7	EtOD	0.01	5:1	10/2	99	96:4	94
8	EtOD	0.01	10:1	10/1	99	96:4	92
9[b]	EtOD	0.01	5:1	20/4	97	96:4	94
10[b]	EtOD	0.05	5:1	4/0.8	99	96.5:3.5	88
11[b]	EtOD	0.05	2:1	4/2	99	96.5:3.5	91
12[b]	EtOD	0.05	1:1	4/4	99	96.5:3.5	94
13[b]	EtOD	0.1	2.5:1	2/0.8	99	96:4	85
14[b]	EtOD	0.1	1:1	2/2	98	96.5:3.5	93

Reaction conditions unless noted otherwise: (±)-**S1** (0.1 mmol), $Rh(COD)_2BF_4$ (0.5 mol%), and chiral ligand (0.6 mol%) in 2-MeTHF:ROD (x:y) at –40 °C for 2 h. The yield was determined by crude ^1H NMR analysis using 1,3,5-trimethoxybenzene as the internal standard, deuterium content and e.e. were determined by HPLC analysis and ^1H NMR analysis of isolated product. [a] Concentrations refer to those of **S1** in 2-MeTHF. [b] (±)-**S1** (0.2 mmol scale).

Supplementary Figure S6. Deuteration using deuterated alcohol and heavy water.

Section 3. Preparation of rhodium catalyst and chiral ligand

Preparation of rhodium catalyst $Rh(COD)_2BF_4$[1,2]

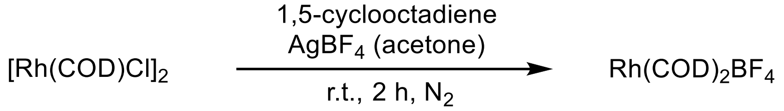

To a solution of $[Rh(COD)Cl]_2$ (0.4395 g, 1.0 mmol, 100 mol%) in dry dichloromethane (7 mL, DCM) under nitrogen atmosphere was added 1,5-cyclooctadiene (400 μL, 3.3 mmol, 330 mol%). After the mixture was stirred for 5 min, $AgBF_4$ (0.4485 g, 2.3 mmol, 230 mol%, dissolved in 3.5 mL acetone) was dropwise added to the reaction and stirred at room temperature for 2 h. The white precipitation was filtered through Celite to remove AgCl, the filtrate was diluted with 20 mL of tetrahydrofuran (THF) and the solvent was reduced to around 3 mL under vacuum. The precipitate was collected by filtration and washed with diethyl ether (5 mL × 3), then the $Rh(COD)_2BF_4$ was obtained as a red crystal (0.6678 g, 82%), which should be stored in fridge (2-8 °C). (**zwf-7-077**)

^{1}H NMR (400 MHz, $CDCl_3$) δ 5.35 (s, 8H), 2.72-2.56 (m, 8H), 2.55-2.43 (m, 8H).

^{19}F NMR (376 MHz, $CDCl_3$) δ −153.2 (s).

Preparation of (*R*)-**L1**[3,4]

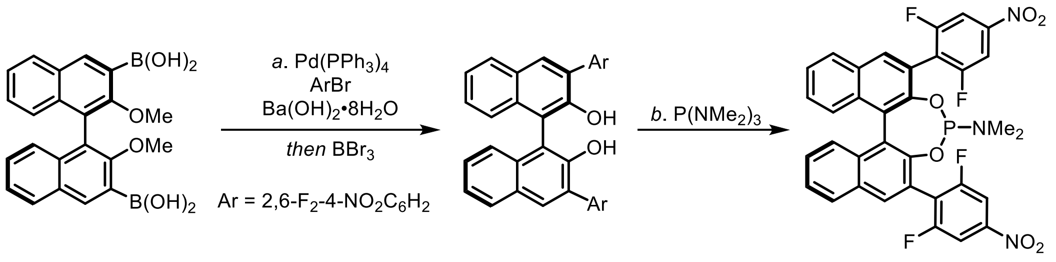

(*R*)-**I** (*R*)-**II** (*R*)-**L1**

Step a: To a solution of (*R*)-**I**[3,4] (4.0204 g, 10.0 mmol) in degassed 1,4-dioxane/water (80 mL, 3:1) were added 2-bromo-1,3-difluoro-5-nitrobenzene (7.1403 g, 30.0 mmol), $Ba(OH)_2·8H_2O$ (5.6837 g, 30.0 mmol), and $Pd(PPh_3)_4$ (0.5779 g, 0.5 mmol). The reaction mixture was stirred at reflux for 24 h and cooled to room temperature. The 1,4-dioxane was removed under vacuum, and to the resulting residue was added 1 N HCl (50 mL). The aqueous phase was extracted with CH_2Cl_2 (100 mL × 3). The organics were combined, washed with brine, dried with Na_2SO_4, filtered, and concentrated, the residue was purified by chromatography to give coupling product as yellow solid. R_f = 0.7 (Hexane/EtOAc 5:1). To a solution of the coupling product in CH_2Cl_2 (50 mL) was added 1.0 M solution of BBr_3 in CH_2Cl_2 (8.4 mL, 8.4 mmol, 300 mol%) at 0 °C. The reaction mixture was warmed to room temperature and stirred for 24 h. As the starting material was fully consumed as indicated by TLC, 1 mL water was added slowly to the reaction mixture at 0 °C. The organic phase (DCM) was separated, and the aqueous phase was extracted with DCM

(50 mL × 3). The organics were combined, washed with brine, dried with Na_2SO_4, filtered, and concentrated, the residue was purified by chromatography to obtain (*R*)-**II** (2.5813 g, 43% yield) as yellow foam solid. R_f = 0.3 (Hexane/EtOAc 5:1). (**zwf-3-037/041**)

^{1}H NMR (400 MHz, $CDCl_3$) δ 8.06 (s, 2H), 8.00-7.87 (m, 6H), 7.51-7.41 (m, 4H), 7.27 (d, *J* = 10.4 Hz, 2H), 5.31 (s, 2H).

^{13}C NMR (100 MHz, $CDCl_3$) δ 160.3 (ddd, J_1 = 251.3, J_2 = 12.3, J_3 = 7.2 Hz), 150.2, 148.0 (dd, J_1 = 10.9, J_2 = 10.8 Hz), 133.7, 133.6, 128.9, 125.0, 124.0, 121.5 (dd, J_1 = 20.6, J_2 = 20.2 Hz), 116.6, 111.3, 107.6 (dd, J_1 = 27.9, J_2 = 3.3 Hz).

^{19}F NMR (376 MHz, $CDCl_3$) δ –106.1 (t, *J* = 6.8 Hz), –106.5 (t, *J* = 7.0 Hz).

IR (neat, cm^{-1}) 3503, 3088, 1529, 1346, 1037.

HRMS (ESI) calcd $C_{32}H_{16}F_4N_2NaO_6^+$ $[M+Na]^+$: 623.0837. Found: 623.0839.

$[\alpha]_D^{18}$ = +49.0 (*c* = 1.05, $CHCl_3$).

<u>Step b</u>: To a solution of (*R*)-**II** (0.7375 g, 1.23 mmol, 100 mol%) in 30 mL dry toluene, $P(NMe_2)_3$ (335 μL, 1.84 mmol, 150 mol%) was added dropwise under N_2 at room temperature. The mixture was stirred at 100 ºC for 0.5 h, and then cooled to room temperature. The toluene was removed under vacuum, and the residue was purified by chromatography to obtain (*R*)-chiral ligand (0.5951 g, 72% yield) as yellow solid. R_f = 0.3 (Hexane/EtOAc 10:1). (**zwf-3-052**)

^{1}H NMR (400 MHz, $CDCl_3$) δ 8.06-7.85 (m, 8H), 7.53-7.44 (m, 3H), 7.41-7.30 (m, 3H), 2.14 (s, 3H), 2.11 (s, 3H).

^{13}C NMR (100 MHz, $CDCl_3$) δ 161.84, 161.78, 161.66, 161.60, 161.50, 161.42, 160.83, 160.75, 159.29, 159.22, 159.14, 159.07, 158.98, 158.90, 158.33, 158.25, 148.24, 148.13, 148.02, 147.86, 147.75, 147.64, 146.96, 146.94, 146.74, 146.71, 133.43, 133.25, 132.41, 132.20, 130.55, 129.99, 128.76, 128.61, 127.46, 127.32, 126.84, 126.76, 125.64, 125.51, 124.28, 124.23, 123.55, 123.54, 122.26, 122.07, 121.88, 121.68, 120.49, 120.18, 107.99, 107.95, 107.91, 107.70, 107.67, 107.62, 107.32, 34.96, 34.75.

^{19}F NMR (376 MHz, $CDCl_3$) δ –101.3 (t, *J* = 7.7 Hz), –104.3 (t, *J* = 7.5 Hz), –106.2 (dt, J_1 = 34.6 Hz, J_2 = 7.2 Hz), –109.2 (t, *J* = 7.0 Hz).

^{31}P NMR (162 MHz, $CDCl_3$) δ 150.8-150.3 (m).

IR (neat, cm^{-1}) 3088, 2895, 1748, 1531, 1347, 1038.

HRMS (ESI) calcd $C_{34}H_{20}F_4N_3NaO_6P^+$ $[M+Na]^+$: 696.0918. Found: 696.0915.

$[\alpha]_D^{20}$ = −207.2 (*c* = 1.06, $CHCl_3$).

The enantiomeric (*S*)-**L1** was prepared using the same procedure from (*S*)-**I**.

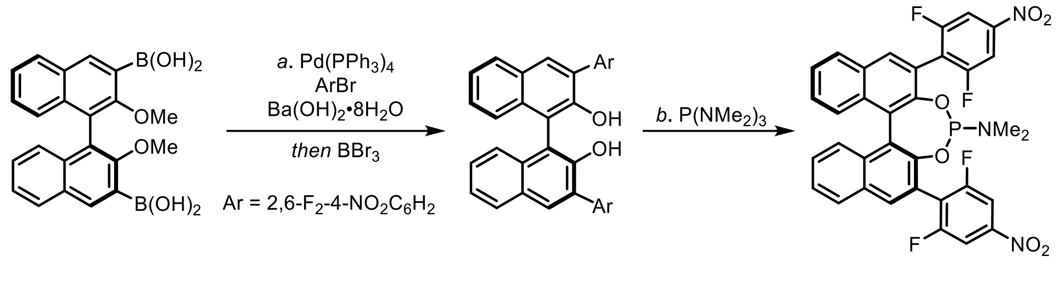

(*S*)-I (*S*)-II (*S*)-L1

(*S*)-**II** (5.0 mmol scale, 1.2052 g, 40% Yield) yellow foam solid. R_f = 0.3 (Hexane/EtOAc 5:1). (**zwf-7-040/043**)

¹H NMR (400 MHz, $CDCl_3$) δ 8.05 (s, 2H), 7.98-7.92 (m, 2H), 7.92-7.85 (m, 4H), 7.48-7.39 (m, 4H), 7.27 (d, *J* = 7.2 Hz, 2H), 5.38 (s, 2H).

¹³C NMR (100 MHz, $CDCl_3$) δ 160.3 (ddd, J_1 = 251.4, J_2 = 12.3, J_3 = 7.2 Hz), 150.2, 148.0 (dd, J_1 = 10.9, J_2 = 10.8 Hz), 133.7, 133.6, 128.9, 125.0, 124.0, 121.5 (dd, J_1 = 21.0, J_2 = 20.2 Hz), 116.6, 111.3, 107.6 (dd, J_1 = 27.9, J_2 = 3.3 Hz).

¹⁹F NMR (376 MHz, $CDCl_3$) δ −106.0 (t, *J* = 7.0 Hz), −106.6 (t, *J* = 7.0 Hz).

IR (neat, cm^{-1}) 3502, 3107, 1529, 1347, 1036.

HRMS (ESI) calcd $C_{32}H_{16}F_4N_2NaO_6^+$ $[M+Na]^+$: 623.0837. Found: 623.0832.

$[\alpha]_D^{18}$ = −47.7 (*c* = 1.00, $CHCl_3$).

(*S*)-**L1** (0.50 mmol scale, 0.2491 g, 74% Yield) light-yellow solid. R_f = 0.3 (Hexane/EtOAc 10:1). (**zwf-7-054**)

¹H NMR (400 MHz, $CDCl_3$) δ 8.06-7.86 (m, 8H), 7.56-7.46 (m, 3H), 7.45-7.34 (m, 3H), 2.13 (s, 3H), 2.11 (s, 3H).

¹³C NMR (100 MHz, $CDCl_3$) δ 161.85, 161.79, 161.67, 161.60, 161.50, 161.42, 160.83, 160.75, 159.30, 159.24, 159.14, 159.08, 158.99, 158.91, 158.34, 158.26, 148.25, 148.14, 148.03, 147.88, 147.77, 147.65, 146.97, 146.95, 146.75, 146.71, 133.44, 133.26, 132.40, 132.20, 130.56, 129.99, 128.76, 128.61, 127.46,

127.32, 126.85, 126.77, 125.63, 125.51, 124.28, 124.23, 123.55, 123.54, 122.26, 122.07, 121.88, 121.68, 120.51, 120.19, 107.98, 107.95, 107.91, 107.71, 107.66, 107.63, 107.59, 107.32, 34.96, 34.75.

^{19}F NMR (376 MHz, $CDCl_3$) δ −101.4 (t, *J* = 7.0 Hz), −104.3 (t, *J* = 7.5 Hz), −106.2 (dt, J_1 = 34.6 Hz, J_2 = 6.9 Hz), −109.3 (t, *J* = 7.0 Hz).

^{31}P NMR (162 MHz, $CDCl_3$) δ 150.7-150.2 (m).

IR (neat, cm^{-1}) 3098, 2894, 1748, 1530, 1347, 1038.

HRMS (ESI) calcd $C_{34}H_{20}F_4N_3NaO_6P^+$ $[M+Na]^+$: 696.0918. Found: 696.0921.

$[\alpha]_D^{20}$ = +207.1 (*c* = 1.02, $CHCl_3$).

Section 4. General procedures for the synthesis of substrates

Preparation of ethyl 2-cyano-2-arylacetate or *tert*-butyl 2-cyano-2-arylacetate intermediate

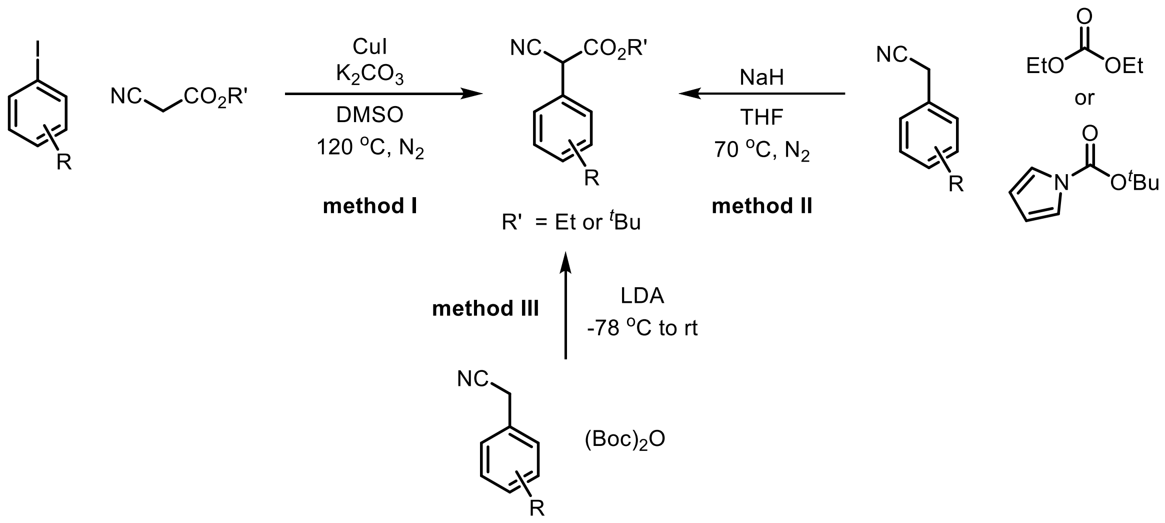

Method I: To a solution of CuI (10 mmol, 10 mol%) in DMSO (200 mL) were added iodobenzene (100 mmol, 100 mol%), ethyl 2-cyanoacetate or *tert*-butyl 2-cyanoacetate (200 mmol, 200 mol%), and K_2CO_3 (300 mmol, 300 mol%) at room temperature. The resulting mixture was stirred at 120 °C for 24 h monitored by TLC. When the iodobenzene was fully consumed, the reaction mixture was cooled down to room temperature, and 1 N HCl (100 mL) was added carefully to the reaction mixture. The aqueous phase was extracted with diethyl ether (100 mL × 3). The organics were combined, washed with water and brine, dried with Na_2SO_4, filtered, and concentrated. The residue was submitted to flash column chromatography (hexane/ethyl acetate) to afford the ethyl 2-cyano-2-arylacetate or *tert*-butyl 2-cyano-2-arylacetate.

Method II: To a suspension of NaH (60 mmol, 300 mol%, 60% in mineral oil) in THF (50 mL) were added substituted 2-arylacetonitrile (20 mmol, 100 mol%) and diethyl carbonate or *tert*-butyl 1*H*-pyrrole-1-carboxylate (48 mmol, 240 mol%) at room temperature. The resulting mixture was stirred at 70 °C for 2-6 h monitored by TLC. When the 2-arylacetonitrile was fully consumed, the reaction mixture was cooled down to room temperature, 1 N HCl (50 mL) was added carefully to the reaction mixture. The organic phase was separated, and the aqueous phase was extracted with EtOAc (50 mL × 3). The organics were combined, washed with water and brine, dried with Na_2SO_4, filtered, and concentrated. The residue was submitted to flash column chromatography (hexane/ethyl acetate) to afford the ethyl 2-cyano-2-arylacetate or *tert*-butyl 2-cyano-2-arylacetate.

Method III: To a suspension of lithium diisopropylamide (100 mmol, 200 mol%, 2.0 M in THF) in THF (50 mL) was dropwise added substituted 2-arylacetonitrile (50 mmol, 100 mol% dissolved in 50 mL THF) at -78 °C. After stirred at -78 °C for 1 hour, the reaction mixture was warmed to room temperature stirred

for 1 hour and cooled down to -78 °C again before addition of the di-*tert*-butyl decarbonate (60 mmol, 120 mol%, dissolved in 60 mL THF). The resulting mixture was stirred at room temperature for 4 h monitored by TLC. When the 2-arylacetonitrile was fully consumed, 1 N HCl (50 mL) was added carefully to the reaction mixture. The organic phase was separated, and the aqueous phase was extracted with EtOAc (50 mL × 3). The organics were combined, washed with water and brine, dried with Na_2SO_4, filtered, and concentrated. The residue was submitted to flash column chromatography (hexane/ethyl acetate) to afford the *tert*-butyl 2-cyano-2-arylacetate.

Representative procedure for substitution

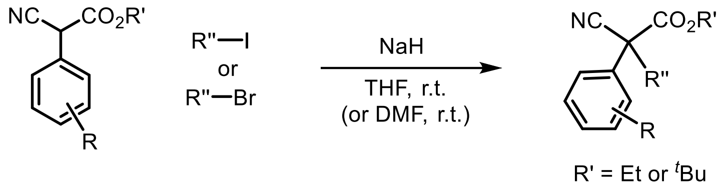

To a solution of ethyl 2-cyano-2-arylacetate or *tert*-butyl 2-cyano-2-arylacetate (10 mmol, 100 mol%) in THF (50 mL) was added NaH (15 mmol, 150 mol%, 60% in mineral oil). After the mixture was stirred for 1 h at room temperature, alkyl iodide or alkyl bromide (120 mol%) (for the unactive alkyl bromide, DMF was used as solvent) was added. The resulting mixture was stirred at room temperature until 2-cyano-2-arylacetate was fully consumed, as monitored by TLC. The reaction mixture was quenched with 1 N HCl (20 mL). The organic phase was separated, and the aqueous phase was extracted with EtOAc (50 mL × 3). The organics were combined, washed with water and brine, dried with Na_2SO_4, filtered, and concentrated. The residue was submitted to flash column chromatography (hexane/ethyl acetate) to afford the disubstituted ethyl 2-cyanoacetate or disubstituted *tert*-butyl 2-cyanoacetate. These substitution reactions were not optimized.

Preparation of alkenyl substituted ethyl 2-cyanoacetate intermediate

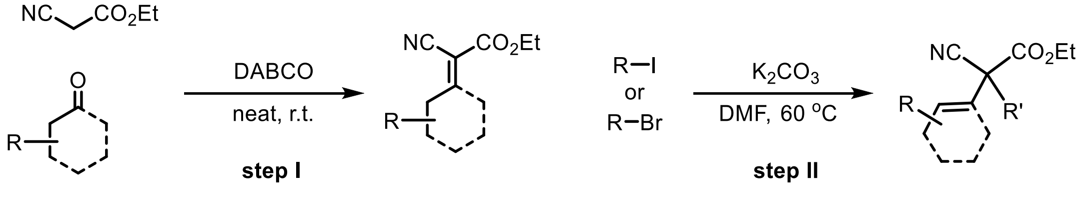

Step I: To a 100 mL flask were charged with ethyl 2-cyanoacetate (60 mmol, 200 mol%) and ketone or aldehyde (30 mmol, 100 mol%), then the 1,4-diazabicyclo[2.2.2]octane (3 mmol, 10 mol%, DABCO) was added. The resulting mixture was stirred at rt for 12 h, monitored by TLC. When the ketone or aldehyde was fully consumed, 1 N HCl (50 mL) was added to the reaction mixture. The aqueous phase was extracted with ethyl acetate (50 mL × 3). The organics were combined, washed with water and brine, dried with Na_2SO_4, filtered, and concentrated. The residue was submitted to flash column chromatography

(hexane/ethyl acetate) to afford the ethyl 2-cyano-2-alkenylacetate.

Step II: To a solution of ethyl 2-cyano-2-alkenylacetate (15 mmol, 100 mol%) in DMF (50 mL) were added K_2CO_3 (30 mmol, 200 mol%) and alkyl iodide or alkyl bromide (150 mol%). The resulting mixture was stirred at 60 °C until ethyl 2-cyano-2-alkenylacetate was fully consumed, as monitored by TLC. The reaction mixture was quenched with 1 N HCl (30 mL). The organic phase was separated, and the aqueous phase was extracted with EtOAc (50 mL × 3). The organics were combined, washed with water and brine, dried with Na_2SO_4, filtered, and concentrated. The residue was submitted to flash column chromatography (hexane/ethyl acetate) to afford alkenyl substituted ethyl 2-cyanoacetate. These substitution reactions were not optimized.

<u>Preparation of ethyl 2-fluoro-2-cyanoacetate intermediate or diethyl 2-fluoro-2-arylmalonate</u>

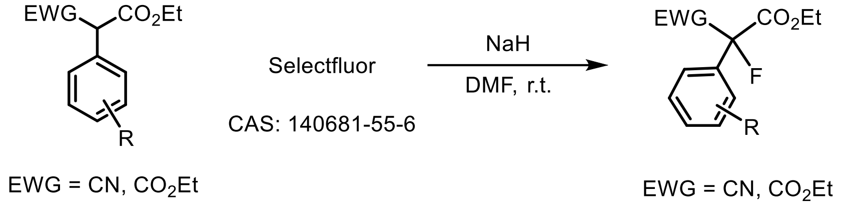

To a solution of ethyl 2-cyano-2-arylacetate or diethyl 2-phenylmalonate (20 mmol, 100 mol%) in DMF (80 mL) was added NaH (30 mmol, 150 mol%, 60% in mineral oil). After the mixture was stirred for 1 h at room temperature, 1-chloromethyl-4-fluoro-1,4-diazoniabicyclo[2.2.2]octane bis(tetrafluoroborate), (30 mmol, 150 mol%, Selectfluor, CAS: 140681-55-6) was added. The resulting mixture was stirred at room temperature until ethyl 2-cyano-2-arylacetate or diethyl 2-phenylmalonate was fully consumed, as monitored by TLC. The reaction mixture was quenched with 1 N HCl (40 mL). The organic phase was separated, and the aqueous phase was extracted with EtOAc (50 mL × 3). The organics were combined, washed with water and brine, dried with Na_2SO_4, filtered, and concentrated. The residue was submitted to flash column chromatography (hexane/ethyl acetate) to afford the fluorinated ethyl 2-cyanoacetate or ethyl malonate. These substitution reactions were not optimized.

<u>Representative procedures for hydrolysis</u>
Basic hydrolysis:

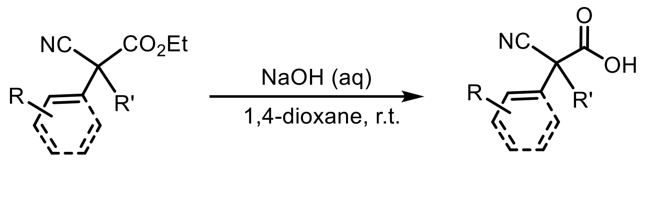

To a solution of disubstituted ethyl 2-cyanoacetate (100 mol%) in 1,4-dioxane (0.5 M) was added 0.5 M NaOH aqueous solution (120 mol%) at room temperature. The resulting mixture was stirred at room temperature and monitored by TLC. After the full consumption of cyanoacetate, 1,4-dioxane was removed under vacuum, and the aqueous layer was washed with diethyl ether (50 mL × 5) to remove the unreacted

starting material and decarboxylative byproduct (α-tertiary nitrile) until only the salt of disubstituted 2-cyanoacetic acid remained in the aqueous phase. Then the aqueous phase was acidified by 1 N HCl aqueous to pH = 2-3 and extracted with diethyl ether for three times. The organic phase was washed with brine, dried over $MgSO_4$, and filtered, and evaporated under vacuum without further purification to afford disubstituted 2-cyanoacetic acid.

Acidic hydrolysis:

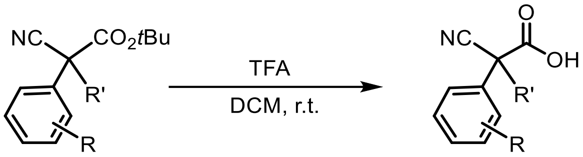

To a solution of disubstituted *tert*-butyl 2-cyanoacetate (100 mol%) in dichloromethane (0.1 M) was added trifluoroacetic acid (2000 mol%) at room temperature. The resulting mixture was stirred at room temperature and monitored by TLC. After the full consumption of cyanoacetate, dichloromethane and trifluoroacetic acid were removed under vacuum, and the residue was redissolved in diethyl ether (100 mL). 0.05 M NaOH aqueous solution was slowly added to the reaction mixture to adjust the pH to 8 ~ 9 under vigorously stirred. The aqueous phase was separated and washed with diethyl ether (50 mL × 3) to remove the unreacted starting material and decarboxylative byproduct (α-tertiary nitrile) until only the salt of disubstituted 2-cyanoacetic acid remained in the aqueous phase. Then the aqueous phase was acidified by 1 N HCl aqueous to pH = 2-3 and extracted with diethyl ether for three times. The organic phase was washed with brine, dried over $MgSO_4$, and filtered, and evaporated under vacuum without further purification to afford disubstituted 2-cyanoacetic acid.

Section 5. Characterization data of substrates

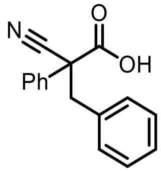

S1

zwf-7-039

S1 (8.0 mmol scale, hydrolysis time: 5 min, 1.6243 g, 77% Yield) solid. R_f = 0.4 (DCM/MeOH 5:1).

^{1}H NMR (400 MHz, $CDCl_3$) δ 8.38 (br, 1H), 7.64-7.52 (m, 2H), 7.47-7.36 (m, 3H), 7.30-7.19 (m, 3H), 7.19-7.11 (m, 2H), 3.70 (d, *J* = 13.6 Hz, 1H), 3.35 (d, *J* = 13.6 Hz, 1H).

^{13}C NMR (100 MHz, $CDCl_3$) δ 172.0, 133.5, 133.3, 130.3, 129.3, 129.2, 128.4, 127.9, 126.4, 117.3, 55.8, 43.5.

IR (neat, cm^{-1}) 3073, 2931, 2263, 1747, 1496, 1195.

HRMS (ESI) calcd $C_{15}H_{14}N^+$ $[M–CO_2+H]^+$: 208.1121. Found: 208.1124.

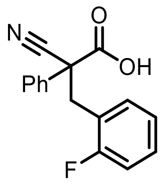

S7

zwf-7-109-1

S7 (7.1 mmol scale, hydrolysis time: 10 min, 1.4010 g, 73% Yield) solid. R_f = 0.4 (DCM/MeOH 5:1).

^{1}H NMR (400 MHz, $CDCl_3$) δ 9.47 (br, 1H), 7.62-7.51 (m, 2H), 7.46-7.35 (m, 3H), 7.28-7.20 (m, 1H), 7.17-7.09 (m, 1H), 7.06-6.94 (m, 2H), 3.76 (d, *J* = 14.0 Hz, 1H), 3.52 (d, *J* = 14.0 Hz, 1H).

^{13}C NMR (100 MHz, $CDCl_3$) δ 172.3, 161.3 (d, *J* = 246.2 Hz), 133.1, 131.7 (d, *J* = 3.6 Hz), 129.9 (d, *J* = 8.7 Hz), 129.4, 129.2, 126.4, 124.1 (d, *J* = 3.6 Hz), 120.9 (d, *J* = 15.2 Hz), 117.0, 115.6 (d, *J* = 22.4 Hz), 54.8, 36.2 (d, *J* = 2.2 Hz).

^{19}F NMR (376 MHz, $CDCl_3$) δ −115.0 to −115.2 (m).

IR (neat, cm^{-1}) 3075, 2939, 2267, 1746, 1492, 1179.

HRMS (ESI) calcd $C_{16}H_{12}FNNaO_2^+$ $[M+Na]^+$: 292.0744. Found: 292.0742.

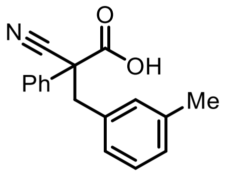

S8

zwf-7-086-9

S8 (8.3 mmol scale, hydrolysis time: 10 min, 1.7878 g, 81% Yield) solid. R_f = 0.4 (DCM/MeOH 5:1).

^{1}H NMR (400 MHz, $CDCl_3$) δ 7.61-7.53 (m, 2H), 7.46-7.36 (m, 3H), 7.15 0-7.03 (m, 2H), 6.98-6.90 (m, 2H), 6.73 (br, 1H), 3.66 (d, *J* = 13.6 Hz, 1H), 3.30 (d, *J* = 13.6 Hz, 1H), 2.25 (s, 3H).

^{13}C NMR (100 MHz, $CDCl_3$) δ 171.5, 138.0, 133.6, 133.5, 131.1, 129.2, 129.1, 128.6, 128.2, 127.2, 126.4, 117.5, 55.8, 43.5, 21.3.

IR (neat, cm^{-1}) 3478, 3033, 2929, 2252, 1727, 1450, 1064.

HRMS (ESI) calcd $C_{17}H_{15}NNaO_2^+$ $[M+Na]^+$: 288.0995. Found: 288.0992.

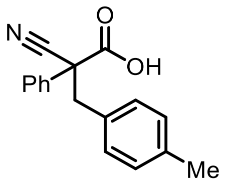

S9

zwf-7-086-1

S9 (6.8 mmol scale, hydrolysis time: 10 min, 1.3783 g, 76% Yield) solid. R_f = 0.4 (DCM/MeOH 5:1).

^{1}H NMR (400 MHz, $CDCl_3$) δ 8.03 (br, 1H), 7.62-7.53 (m, 2H), 7.46-7.35 (m, 3H), 7.10-6.98 (m, 4H), 3.66 (d, *J* = 14.0 Hz, 1H), 3.30 (d, *J* = 13.6 Hz, 1H), 2.29 (s, 3H).

^{13}C NMR (100 MHz, $CDCl_3$) δ 171.9, 137.6, 133.5, 130.5, 130.1, 129.20, 129.15, 129.09, 126.4, 117.5, 56.0, 43.2, 21.0.

IR (neat, cm^{-1}) 2978, 2852, 2242, 1711, 1492, 1280.

HRMS (ESI) calcd $C_{17}H_{16}NO_2^+$ $[M+H]^+$: 266.1176. Found: 266.1175.

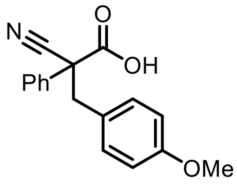

S10

zwf-7-055-12

S10 (4.0 mmol scale, hydrolysis time: 10 min, 0.5733 g, 51% Yield) solid. R_f = 0.4 (DCM/MeOH 5:1).

^{1}H NMR (400 MHz, $CDCl_3$) δ 8.60 (br, 1H), 7.61-7.52 (m, 2H), 7.45-7.36 (m, 3H), 7.07 (d, *J* = 8.4 Hz, 2H), 6.77 (d, *J* = 8.8 Hz, 2H), 3.76 (s, 3H), 3.64 (d, *J* = 14.0 Hz, 1H), 3.29 (d, *J* = 14.0 Hz, 1H).

^{13}C NMR (100 MHz, $CDCl_3$) δ 172.0, 159.1, 133.4, 131.4, 129.24, 129.18, 126.4, 125.6, 117.4, 113.8, 56.1, 55.2, 42.9.

IR (neat, cm^{-1}) 3009, 2933, 2252, 1711, 1496, 1250.

HRMS (ESI) calcd $C_{16}H_{16}NO^+$ $[M{-}CO_2{+}H]^+$: 238.1226. Found: 226.1228.

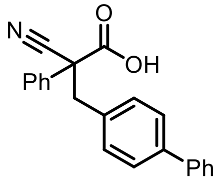

S11

zwf-7-055-3

S11 (8.5 mmol scale, hydrolysis time: 20 min, 2.1023 g, 76% Yield) solid. R_f = 0.4 (DCM/MeOH 5:1).

^{1}H NMR (400 MHz, $CDCl_3$) δ 9.63 (br, 1H), 7.64-7.56 (m, 2H), 7.54-7.49 (m, 2H), 7.49-7.36 (m, 7H), 7.36-7.30 (m, 1H), 7.25-7.19 (m, 2H), 3.73 (d, *J* = 13.6 Hz, 1H), 3.39 (d, *J* = 13.6 Hz, 1H).

^{13}C NMR (100 MHz, $CDCl_3$) δ 172.4, 140.7, 140.4, 133.3, 132.5, 130.7, 129.4, 129.3, 128.7, 127.4, 127.1, 127.0, 126.5, 117.3, 55.8, 43.2.

IR (neat, cm^{-1}) 2997, 2771, 2267, 1746, 1488, 1205.

HRMS (ESI) calcd $C_{22}H_{17}NNaO_2^+$ $[M{+}Na]^+$: 350.1151. Found: 350.1150.

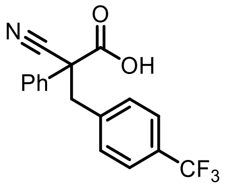

S12

zwf-7-055-7

S12 (6.4 mmol scale, hydrolysis time: 25 min, 1.3203 g, 65% Yield) solid. R_f = 0.4 (DCM/MeOH 5:1).

^{1}H NMR (400 MHz, $CDCl_3$) δ 7.59-7.52 (m, 2H), 7.49 (d, *J* = 8.4 Hz, 2H), 7.46-7.38 (m, 3H), 7.25 (d, *J* = 8.0 Hz, 2H), 6.21 (br, 1H), 3.73 (d, *J* = 13.6 Hz, 1H), 3.41 (d, *J* = 13.6 Hz, 1H).

^{13}C NMR (100 MHz, $CDCl_3$) δ 170.9, 137.6, 133.0, 130.7, 130.1 (q, *J* = 32.3 Hz), 129.5, 129.4, 126.3, 125.3 (q, *J* = 3.6 Hz), 124.0 (q, *J* = 270.4 Hz), 117.1, 55.3, 43.1.

^{19}F NMR (376 MHz, $CDCl_3$) δ –62.1 (s).

IR (neat, cm^{-1}) 2982, 2901, 2247, 1731, 1323, 1113.

HRMS (ESI) calcd $C_{16}H_{13}F_3N^+$ $[M–CO_2+H]^+$: 276.0995. Found: 276.0993.

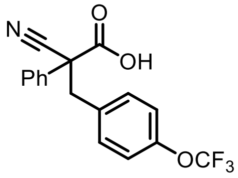

S13

zwf-7-109-12

S13 (7.4 mmol scale, hydrolysis time: 15 min, 1.7843 g, 72% Yield) solid. R_f = 0.4 (DCM/MeOH 5:1).

^{1}H NMR (500 MHz, $CDCl_3$) δ 7.59-7.49 (m, 2H), 7.47-7.37 (m, 3H), 7.16 (d, *J* = 8.5 Hz, 2H), 7.07 (d, *J* = 8.0 Hz, 2H), 6.28 (br, 1H), 3.68 (d, *J* = 13.5 Hz, 1H), 3.35 (d, *J* = 14.0 Hz, 1H).

^{13}C NMR (125 MHz, $CDCl_3$) δ 170.8, 148.9 (q, *J* = 1.8 Hz), 133.2, 132.3, 131.8, 129.4, 129.3, 126.3, 120.7, 120.4 (q, *J* = 255.8 Hz), 117.3, 55.5, 42.8.

^{19}F NMR (376 MHz, $CDCl_3$) δ –57.8 (s).

IR (neat, cm^{-1}) 3107, 2984, 2252, 1718, 1249, 1157.

HRMS (ESI) calcd $C_{17}H_{12}F_3NNaO_3^+$ $[M+Na]^+$: 358.0661. Found: 358.0665.

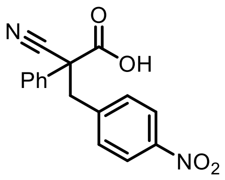

S14

zwf-7-055-8

S14 (3.3 mmol scale, hydrolysis time: 30 min, 0.6091 g, 62% Yield) solid. R_f = 0.4 (DCM/MeOH 5:1).

^{1}H NMR (400 MHz, $CDCl_3$) δ 8.08 (d, *J* = 8.8 Hz, 2H), 7.57-7.49 (m, 2H), 7.48-7.39 (m, 3H), 7.29 (d, *J* = 8.8 Hz, 2H), 5.88 (br, 1H), 3.77 (d, *J* = 13.6 Hz, 1H), 3.47 (d, *J* = 13.6 Hz, 1H).

^{13}C NMR (100 MHz, $CDCl_3$) δ 169.9, 147.6, 141.1, 132.7, 131.3, 129.6, 129.5, 126.2, 123.5, 117.0, 55.0, 43.1.

IR (neat, cm^{-1}) 3449, 2981, 2247, 1722, 1512, 1254.

HRMS (ESI) calcd $C_{15}H_{13}N_2O_2^+$ $[M–CO_2+H]^+$: 253.0972. Found: 253.0971.

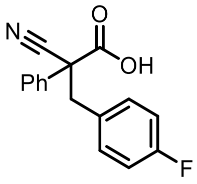

S15

zwf-7-055-5

S15 (9.0 mmol scale, hydrolysis time: 8 min, 2.0227 g, 83% Yield) solid. R_f = 0.4 (DCM/MeOH 5:1).

^{1}H NMR (400 MHz, $CDCl_3$) δ 8.10 (br, 1H), 7.63-7.50 (m, 2H), 7.48-7.35 (m, 3H), 7.16-7.03 (m, 2H), 6.99-6.82 (m, 2H), 3.66 (d, *J* = 13.6 Hz, 1H), 3.33 (d, *J* = 13.6 Hz, 1H).

^{13}C NMR (100 MHz, $CDCl_3$) δ 171.6, 162.5 (d, *J* = 244.9 Hz), 133.1, 131.9 (d, *J* = 7.9 Hz), 129.4, 129.3, 126.4, 117.2, 115.3 (d, *J* = 20.9 Hz), 55.8, 42.7.

^{19}F NMR (376 MHz, $CDCl_3$) δ −114.1 to −114.3 (m).

IR (neat, cm^{-1}) 3177, 2932, 2277, 1747, 1506, 1221.

HRMS (ESI) calcd $C_{16}H_{12}FNNaO_2^+$ $[M+Na]^+$: 292.0744. Found: 292.0746.

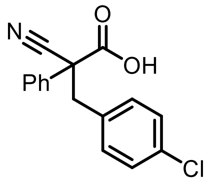

S16

zwf-7-055-1

S16 (8.7 mmol scale, hydrolysis time: 8 min, 2.1037 g, 85% Yield) solid. R_f = 0.4 (DCM/MeOH 5:1).

^{1}H NMR (400 MHz, $CDCl_3$) δ 7.47-7.35 (m, 2H), 7.48-7.35 (m, 3H), 7.19 (d, *J* = 8.4 Hz, 2H), 7.06 (d, *J* = 8.4 Hz, 2H), 6.40 (br, 1H), 3.64 (d, *J* = 14.0 Hz, 1H), 3.32 (d, *J* = 13.6 Hz, 1H).

^{13}C NMR (100 MHz, $CDCl_3$) δ 171.4, 134.0, 133.1, 132.0, 131.6, 129.4, 129.3, 128.6, 126.3, 117.2, 55.6, 42.9.

IR (neat, cm^{-1}) 3513, 2984, 2257, 1713, 1492, 1279.

HRMS (ESI) calcd $C_{16}H_{12}ClNNaO_2^+$ $[M+Na]^+$: 308.0449. Found: 308.0447.

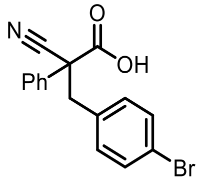

S17

zwf-7-055-6

S17 (7.0 mmol scale, hydrolysis time: 10 min, 1.6040 g, 69% Yield) solid. R_f = 0.4 (DCM/MeOH 5:1).

^{1}H NMR (400 MHz, $CDCl_3$) δ 7.58-7.49 (m, 2H), 7.47-7.39 (m, 3H), 7.36 (d, *J* = 8.4 Hz, 2H), 7.01 (d, *J* = 8.0 Hz, 2H), 6.13 (br, 1H), 3.63 (d, *J* = 14.0 Hz, 1H), 3.31 (d, *J* = 13.6 Hz, 1H).

^{13}C NMR (100 MHz, $CDCl_3$) δ 170.9, 133.1, 132.6, 132.0, 131.5, 129.4, 129.3, 126.3, 122.1, 117.3, 55.5, 42.9.

IR (neat, cm^{-1}) 3443, 2979, 2247, 1713, 1489, 1072.

HRMS (ESI) calcd $C_{15}H_{13}BrN^+$ $[M–CO_2+H]^+$: 286.0226. Found: 286.0224.

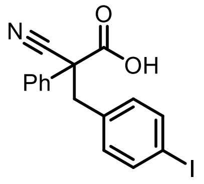

S18

zwf-7-086-10

S18 (8.0 mmol scale, hydrolysis time: 10 min, 2.0611 g, 68% Yield) solid. R_f = 0.4 (DCM/MeOH 5:1).

^{1}H NMR (400 MHz, $CDCl_3$) δ 7.65-7.58 (m, 4H), 7.47-7.35 (m, 3H), 6.93-6.84 (m, 3H), 3.62 (d, *J* = 13.6 Hz, 1H), 3.29 (d, *J* = 14.0 Hz, 1H).

^{13}C NMR (100 MHz, $CDCl_3$) δ 171.3, 137.5, 133.2, 133.1, 132.2, 129.4, 129.3, 126.3, 117.2, 93.9, 55.4, 43.0.

IR (neat, cm^{-1}) 2975, 2901, 2252, 1705, 1289, 1058.

HRMS (ESI) calcd $C_{16}H_{12}INNaO_2^+$ $[M+Na]^+$: 399.9805. Found: 399.9804.

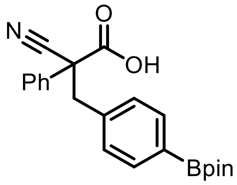

S19

zwf-7-109-10

S19 (6.9 mmol scale, hydrolysis time: 8 min, 2.1004 g, 81% Yield) solid. R_f = 0.4 (DCM/MeOH 5:1).

^{1}H NMR (400 MHz, $CDCl_3$) δ 7.67 (d, *J* = 8.0 Hz, 2H), 7.62-7.52 (m, 2H), 7.48-7.32 (m, 3H), 7.16 (d, *J* = 8.0 Hz, 2H), 6.47 (br, 1H), 3.72 (d, *J* = 13.6 Hz, 1H), 3.35 (d, *J* = 13.6 Hz, 1H), 1.32 (s, 12H).

^{13}C NMR (100 MHz, $CDCl_3$) δ 170.3, 137.0, 134.8, 133.7, 129.7, 129.2, 126.4, 117.5, 84.1, 55.5, 43.7, 24.74, 24.72.

^{11}B NMR (128 MHz, $CDCl_3$) δ 28.7 (br).

IR (neat, cm^{-1}) 3276, 2977, 2257, 1746, 1360, 1080.

HRMS (ESI) calcd $C_{21}H_{25}BNO_2^+$ $[M-CO_2+H]^+$: 334.1973. Found: 334.1970.

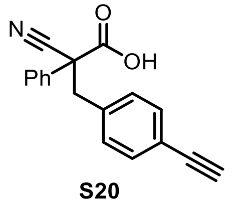

S20

zwf-7-086-4

S20 (6.2 mmol scale, hydrolysis time: 5 min, 1.0325 g, 61% Yield) solid. R_f = 0.4 (DCM/MeOH 5:1).

^{1}H NMR (400 MHz, $CDCl_3$) δ 7.58-7.49 (m, 2H), 7.46-7.38 (m, 3H), 7.35 (d, *J* = 8.0 Hz, 2H), 7.09 (d, *J* = 8.4 Hz, 2H), 6.08 (br, 1H), 3.67 (d, *J* = 13.6 Hz, 1H), 3.35 (d, *J* = 13.6 Hz, 1H), 3.07 (s, 1H).

^{13}C NMR (100 MHz, $CDCl_3$) δ 170.7, 134.4, 133.2, 132.1, 130.3, 129.34, 129.26, 126.4, 121.7, 117.3, 83.2, 77.8, 55.4, 43.4.

IR (neat, cm^{-1}) 3305, 2980, 2247, 2109, 1711, 1268, 1066.

HRMS (ESI) calcd $C_{18}H_{13}NNaO_2^+$ $[M+Na]^+$: 298.0838. Found: 298.0835.

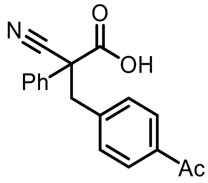

S21

zwf-7-055-4

S21 (5.8 mmol scale, hydrolysis time: 20 min, 1.1687 g, 69% Yield) solid. R_f = 0.4 (DCM/MeOH 5:1).

^{1}H NMR (400 MHz, $CDCl_3$) δ 7.81 (d, *J* = 8.0 Hz, 2H), 7.61-7.51 (m, 2H), 7.46-7.35 (m, 3H), 7.25 (d, *J* = 8.0 Hz, 2H), 6.56 (br, 1H), 3.75 (d, *J* = 13.2 Hz, 1H), 3.40 (d, *J* = 13.6 Hz, 1H), 2.56 (s, 3H).

^{13}C NMR (100 MHz, $CDCl_3$) δ 199.4, 169.3, 139.8, 136.1, 133.5, 130.7, 129.25, 129.22, 128.5, 126.3, 117.6, 55.3, 43.4, 26.5.

IR (neat, cm^{-1}) 3459, 2919, 2252, 1745, 1362, 1189.

HRMS (ESI) calcd $C_{18}H_{15}NNaO_3^+$ $[M+Na]^+$: 316.0944. Found: 316.0940.

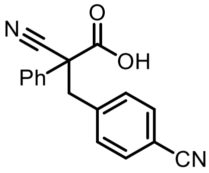

S22

zwf-7-055-9

S22 (4.5 mmol scale, hydrolysis time: 30 min, 0.8027 g, 65% Yield) solid. R_f = 0.4 (DCM/MeOH 5:1).

^{1}H NMR (400 MHz, $CDCl_3$) δ 7.56-7.48 (m, 4H), 7.47-7.38 (m, 3H), 7.24 (d, *J* = 8.4 Hz, 2H), 6.59 (br, 1H), 3.73 (d, *J* = 13.6 Hz, 1H), 3.42 (d, *J* = 13.6 Hz, 1H).

^{13}C NMR (100 MHz, $CDCl_3$) δ 170.0, 139.2, 132.8, 132.1, 131.2, 129.6, 129.4, 126.2, 118.3, 117.0, 111.7, 55.0, 43.4.

IR (neat, cm^{-1}) 3187, 2977, 2272, 2230, 1753, 1450, 1192.

HRMS (ESI) calcd $C_{16}H_{13}N_2^+$ $[M{-}CO_2{+}H]^+$: 233.1073. Found: 233.1075.

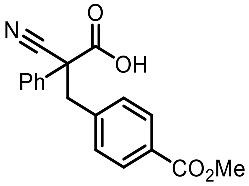

S23

zwf-10-036

S23 (8.0 mmol scale, hydrolysis time (TFA conditions): 6 h, 1.7954 g, 73% Yield) solid. R_f = 0.4 (DCM/MeOH 5:1).

^{1}H NMR (500 MHz, $CDCl_3$) δ 7.88 (d, *J* = 8.5 Hz, 2H), 7.58-7.48 (m, 2H), 7.44-7.35 (m, 3H), 7.21 (d, *J* = 8.0 Hz, 2H), 6.55 (br, 1H), 3.87 (s, 3H), 3.73 (d, *J* = 13.5 Hz, 1H), 3.39 (d, *J* = 13.5 Hz, 1H).

^{13}C NMR (125 MHz, $CDCl_3$) δ 169.5, 167.3, 139.3, 133.5, 130.4, 129.6, 129.3, 129.22, 129.20, 126.3, 117.5, 55.3, 52.3, 43.4.

IR (neat, cm^{-1}) 3429, 2957, 2265, 1709, 1437, 1297.

HRMS (ESI) calcd $C_{17}H_{15}NNaO_2^+$ $[M{-}CO_2{+}Na]^+$: 288.0995. Found: 288.0997.

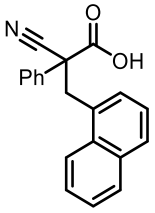

S24

zwf-7-109-13

S24 (4.3 mmol scale, hydrolysis time: 15 min, 0.4739 g, 36% Yield) solid. R_f = 0.4 (DCM/MeOH 5:1).

^{1}H NMR (400 MHz, $CDCl_3$) δ 7.91 (d, *J* = 8.0 Hz, 1H), 7.84-7.73 (m, 2H), 7.62-7.53 (m, 2H), 7.45-7.31 (m, 7H), 5.69 (br, 1H), 4.20 (d, *J* = 14.4 Hz, 1H), 3.90 (d, *J* = 14.8 Hz, 1H).

^{13}C NMR (100 MHz, $CDCl_3$) δ 171.4, 134.0, 133.7, 132.3, 130.0, 129.21, 129.18, 128.7, 128.6, 128.3, 126.5, 125.9, 125.7, 125.0, 123.5, 117.8, 55.0, 39.2.

IR (neat, cm^{-1}) 3488, 2981, 2252, 1717, 1277, 1177.

HRMS (ESI) calcd $C_{19}H_{16}N^+$ $[M–CO_2+H]^+$: 258.1277. Found: 258.1278.

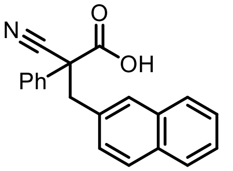

S25

zwf-7-086-8

S25 (4.7 mmol scale, hydrolysis time: 20 min, 0.9766 g, 69% Yield) solid. R_f = 0.4 (DCM/MeOH 5:1).

^{1}H NMR (400 MHz, $CDCl_3$) δ 7.79-7.74 (m, 1H), 7.74-7.61 (m, 3H), 7.62-7.53 (m, 2H), 7.47-7.34 (m, 5H), 7.20 (dd, J_1 = 8.4 Hz, J_2 = 1.6 Hz, 1H), 6.77 (br, 1H), 3.83 (d, *J* = 13.6 Hz, 1H), 3.49 (d, *J* = 13.6 Hz, 1H).

^{13}C NMR (100 MHz, $CDCl_3$) δ 171.3, 133.5, 133.1, 132.7, 131.1, 129.6, 129.23, 129.20, 127.94, 127.90, 127.5, 126.4, 126.1, 117.5, 55.8, 43.7.

IR (neat, cm^{-1}) 3192, 2982, 2277, 1751, 1450, 1066.

HRMS (ESI) calcd $C_{20}H_{15}NNaO_2^+$ $[M+Na]^+$: 324.0995. Found: 324.0993.

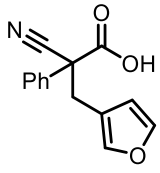

S26

zwf-7-133-1

S26 (8.5 mmol scale, hydrolysis time: 7 min, 1.4521 g, 71% Yield) oil. R_f = 0.4 (DCM/MeOH 5:1).

^{1}H NMR (400 MHz, $CDCl_3$) δ 7.64-7.50 (m, 2H), 7.47-7.36 (m, 3H), 7.32-7.25 (m, 2H), 6.20 (s, 1H), 6.02 (br, 1H), 3.54 (d, *J* = 14.4 Hz, 1H), 3.22 (d, *J* = 14.4 Hz, 1H).

^{13}C NMR (100 MHz, $CDCl_3$) δ 172.0, 143.0, 141.7, 133.1, 129.4, 129.3, 126.3, 117.6, 117.4, 111.5, 55.3, 33.7.

IR (neat, cm^{-1}) 3483, 2979, 2252, 1717, 1450, 1205.

HRMS (ESI) calcd $C_{14}H_{11}NNaO_3^+$ $[M+Na]^+$: 264.0631. Found: 264.0633.

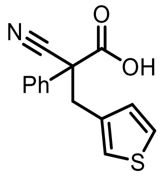

S27

zwf-7-086-6

S27 (8.5 mmol scale, hydrolysis time: 15 min, 1.4542 g, 66% Yield) solid. R_f = 0.4 (DCM/MeOH 5:1).

^{1}H NMR (400 MHz, $CDCl_3$) δ 7.63-7.52 (m, 2H), 7.48-7.35 (m, 3H), 7.19 (dd, J_1 = 5.0 Hz, J_2 = 3.0 Hz, 1H), 7.09 (d, *J* = 1.6 Hz, 1H), 6.87 (dd, J_1 = 9.2 Hz, J_2 = 1.2 Hz, 1H), 6.04 (br, 1H), 3.74 (d, *J* = 14.0 Hz, 1H), 3.40 (d, *J* = 14.4 Hz, 1H).

^{13}C NMR (100 MHz, $CDCl_3$) δ 171.0, 133.9, 133.5, 129.3, 129.2, 128.9, 126.3, 125.6, 124.9, 117.8, 55.5, 38.2.

IR (neat, cm^{-1}) 3491, 2981, 2262, 1715, 1247, 1066.

HRMS (ESI) calcd $C_{14}H_{12}NO_2S^+$ $[M+H]^+$: 258.0583. Found: 258.0580.

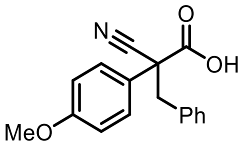

S28

zwf-7-055-13

S28 (9.8 mmol scale, hydrolysis time: 20 min, 2.1459 g, 78% Yield) solid. R_f = 0.4 (DCM/MeOH 5:1).

^{1}H NMR (400 MHz, $CDCl_3$) δ 7.48 (d, J = 9.2 Hz, 2H), 7.27-7.21 (m, 3H), 7.18-7.14 (m, 3H), 6.91 (d, J = 8.8 Hz, 2H), 3.81 (s, 3H), 3.66 (d, J = 13.6 Hz, 1H), 3.32 (d, J = 13.6 Hz, 1H).

^{13}C NMR (100 MHz, $CDCl_3$) δ 171.7, 160.1, 133.8, 130.3, 128.4, 127.8, 127.7, 125.4, 117.7, 114.4, 55.4, 55.1, 43.5.

IR (neat, cm^{-1}) 3489, 2974, 2247, 1710, 1511, 1254.

HRMS (ESI) calcd $C_{16}H_{16}NO^+$ $[M–CO_2+H]^+$: 238.1226. Found: 226.1225.

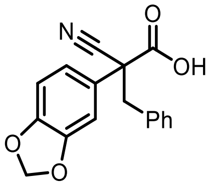

S29

zwf-7-109-5

S29 (11.2 mmol scale, hydrolysis time: 5 min, 3.0219 g, 91% Yield) solid. R_f = 0.4 (DCM/MeOH 5:1).

^{1}H NMR (400 MHz, $CDCl_3$) δ 9.37 (br, 1H), 7.29-7.23 (m, 3H), 7.21-7.14 (m, 2H), 7.08-7.03 (m, 2H), 6.85-6.77 (m, 1H), 6.01 (dd, J_1 = 5.2 Hz, J_2 = 1.2 Hz, 2H), 3.64 (d, J = 13.6 Hz, 1H), 3.32 (d, J = 14.0 Hz, 1H).

^{13}C NMR (100 MHz, $CDCl_3$) δ 172.3, 148.4, 133.5, 130.3, 128.4, 127.9, 126.8, 120.5, 117.3, 108.5, 106.9, 101.8, 55.4, 43.5.

IR (neat, cm^{-1}) 3508, 2981, 2261, 1720, 1493, 1039.

HRMS (ESI) calcd $C_{16}H_{14}NO_2^+$ $[M–CO_2+H]^+$: 252.1019. Found: 252.1021.

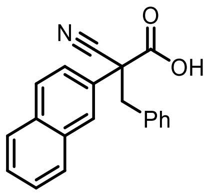

S30

zwf-7-055-11

S30 (5.0 mmol scale, hydrolysis time: 20 min, 1.0851 g, 72% Yield) solid. R_f = 0.4 (DCM/MeOH 5:1).

^{1}H NMR (400 MHz, $CDCl_3$) δ 8.03 (d, J = 1.6 Hz, 1H), 7.91-7.79 (m, 3H), 7.65 (dd, J_1 = 8.4 Hz, J_2 = 2.0 Hz, 1H), 7.57-7.48 (m, 2H), 7.24-7.10 (m, 5H), 6.15 (br, 1H), 3.78 (d, J = 13.6 Hz, 1H), 3.45 (d, J = 14.0 Hz, 1H).

^{13}C NMR (100 MHz, $CDCl_3$) δ 171.1, 133.6, 133.1, 132.9, 130.7, 130.3, 129.2, 128.4, 127.9, 127.6, 127.2, 127.0, 126.4, 123.1, 117.6, 55.9, 43.4.

IR (neat, cm^{-1}) 3233, 2973, 2262, 1740, 1257, 1082.

HRMS (ESI) calcd $C_{19}H_{16}N^+$ $[M–CO_2+H]^+$: 258.1277. Found: 258.1278.

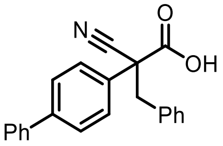

S31

zwf-7-109-2

S31 (5.0 mmol scale, hydrolysis time: 15 min, 1.1226 g, 68% Yield) solid. R_f = 0.4 (DCM/MeOH 5:1).

^{1}H NMR (400 MHz, $CDCl_3$) δ7.69-7.61 (m, 4H), 7.61-7.54 (m, 2H), 7.45 (t, J = 7.4 Hz, 2H), 7.41-7.34 (m, 1H), 7.27-7.17 (m, 5H), 6.87 (br, 1H), 3.74 (d, J = 13.6 Hz, 1H), 3.38 (d, J = 14.0 Hz, 1H).

^{13}C NMR (100 MHz, $CDCl_3$) δ 171.6, 142.2, 139.7, 133.6, 132.3, 130.3, 128.9, 128.5, 127.93, 127.91, 127.8, 127.1, 126.9, 117.4, 55.6, 43.5.

IR (neat, cm^{-1}) 3188, 2984, 2267, 1749, 1407, 1067.

HRMS (ESI) calcd $C_{21}H_{18}N^+$ $[M–CO_2+H]^+$: 284.1434. Found: 284.1433.

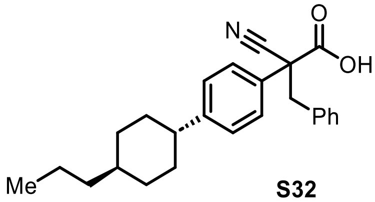

S32

zwf-7-109-7

S32 (6.4 mmol scale, hydrolysis time: 30 min, 1.8165 g, 76% Yield) solid. R_f = 0.4 (DCM/MeOH 5:1).

^{1}H NMR (400 MHz, $CDCl_3$) δ 8.03 (br, 1H), 7.49 (d, *J* = 8.4 Hz, 2H), 7.29-7.14 (m, 7H), 3.70 (d, *J* = 13.6 Hz, 1H), 3.30 (d, *J* = 13.6 Hz, 1H), 2.54-2.43 (m, 1H), 1.93-1.82 (m, 4H), 1.50-1.18 (m, 7H), 1.12-0.99 (m, 2H), 0.91 (t, *J* = 7.2 Hz, 3H).

^{13}C NMR (100 MHz, $CDCl_3$) δ 172.2, 149.3, 133.8, 130.7, 130.3, 128.4, 127.9, 127.7, 126.3, 117.5, 55.6, 44.1, 43.5, 39.6, 36.9, 34.1, 33.4, 20.0, 14.4.

IR (neat, cm^{-1}) 3203, 2917, 2277, 1742, 1412, 1067.

HRMS (ESI) calcd $C_{25}H_{29}NNaO_2^+$ $[M+Na]^+$: 398.2090. Found: 398.2091.

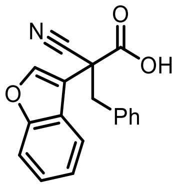

S33

zwf-7-109-4

S33 (7.5 mmol scale, hydrolysis time: 5 min, 1.6647 g, 76% Yield) solid. R_f = 0.4 (DCM/MeOH 5:1).

^{1}H NMR (400 MHz, $CDCl_3$) δ 7.83 (d, *J* = 7.6 Hz, 1H), 7.78 (br, 1H), 7.74 (s, 1H), 7.54 (d, *J* = 8.0 Hz, 1H), 7.41-7.15 (m, 7H), 3.74 (d, *J* = 14.0 Hz, 1H), 3.57 (d, *J* = 13.2 Hz, 1H).

^{13}C NMR (100 MHz, $CDCl_3$) δ 170.9, 155.9, 143.7, 133.1, 130.1, 128.6, 128.2, 125.4, 124.1, 123.6, 120.7, 116.5, 114.7, 112.2, 49.2, 41.3.

IR (neat, cm^{-1}) 2980, 2901, 2242, 1715, 1456, 1057.

HRMS (ESI) calcd $C_{18}H_{13}NNaO_3^+$ $[M+Na]^+$: 314.0788. Found: 314.0790.

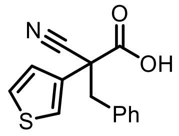

S34

zwf-7-109-3

S34 (12.2 mmol scale, hydrolysis time: 8 min, 2.5664 g, 82% Yield) solid. R_f = 0.4 (DCM/MeOH 5:1).

^{1}H NMR (400 MHz, $CDCl_3$) δ 9.36 (br, 1H), 7.49-7.43 (m, 1H), 7.43-7.37 (m, 1H), 7.31-7.22 (m, 4H), 7.21-7.15 (m, 2H), 3.64 (d, *J* = 13.6 Hz, 1H), 3.36 (d, *J* = 13.6 Hz, 1H).

^{13}C NMR (100 MHz, $CDCl_3$) δ 172.0, 133.6, 133.3, 130.1, 128.5, 128.1, 127.5, 125.6, 124.3, 117.3, 52.9, 43.9.

IR (neat, cm^{-1}) 2981, 2902, 2252, 1718, 1411, 1066.

HRMS (ESI) calcd $C_{14}H_{11}NNaO_2S^+$ $[M+Na]^+$: 280.0403. Found: 280.0405.

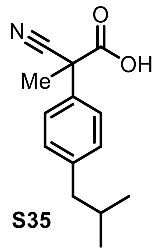

S35

zwf-7-129-14

S35 (14.9 mmol scale, hydrolysis time: 10 min, 2.8402 g, 82% Yield) solid. R_f = 0.4 (DCM/MeOH 5:1).

^{1}H NMR (400 MHz, $CDCl_3$) δ 8.55 (br, 1H), 7.45 (d, *J* = 8.4 Hz, 2H), 7.19 (d, *J* = 8.4 Hz, 2H), 2.48 (d, *J* = 6.8 Hz, 2H), 1.97 (s, 3H), 1.86 (hept, *J* = 6.8 Hz, 1H), 0.90 (d, *J* = 6.4 Hz, 6H).

^{13}C NMR (100 MHz, $CDCl_3$) δ 173.2, 143.0, 131.9, 129.9, 125.6, 119.1, 47.9, 44.8, 30.1, 24.1, 22.3.

IR (neat, cm^{-1}) 3175, 2963, 2257, 1701, 1273, 1057.

HRMS (ESI) calcd $C_{14}H_{17}NNaO_2^+$ $[M+Na]^+$: 254.1151. Found: 254.1148.

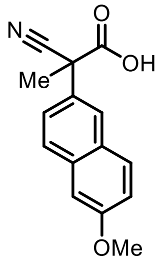

S36

zwf-7-129-13

S36 (11.4 mmol scale, hydrolysis time: 20 min, 2.5118 g, 86% Yield) solid. R_f = 0.4 (DCM/MeOH 5:1).

^{1}H NMR (400 MHz, $CDCl_3$) δ 9.10 (br, 1H), 7.96 (d, *J* = 2.0 Hz, 1H), 7.75 (dd, J_1 = 9.0 Hz, J_2 = 3.4 Hz, 2H), 7.55 (dd, J_1 = 8.6 Hz, J_2 = 2.2 Hz, 1H), 7.18 (dd, J_1 = 8.0 Hz, J_2 = 2.6 Hz, 1H), 7.11 (d, *J* = 2.4 Hz, 1H), 3.91 (s, 3H), 2.05 (s, 3H).

^{13}C NMR (100 MHz, $CDCl_3$) δ 173.1, 158.5, 134.5, 129.8, 129.5, 128.4, 128.1, 125.3, 123.3, 119.9, 119.0, 105.5, 55.3, 48.2, 24.1.

IR (neat, cm^{-1}) 3493, 2973, 2257, 1712, 1269, 1066.

HRMS (ESI) calcd $C_{15}H_{13}NNaO_3^+$ $[M+Na]^+$: 278.0788. Found: 278.0791.

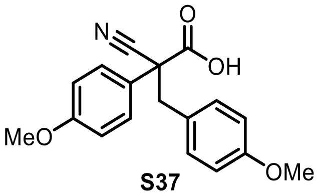

S37

zwf-7-129-1

S37 (10.2 mmol scale, hydrolysis time: 10 min, 2.7410 g, 86% Yield) solid. R_f = 0.2 (DCM/MeOH 5:1).

^{1}H NMR (400 MHz, $CDCl_3$) δ 7.48 (d, *J* = 8.4 Hz, 2H), 7.08 (d, *J* = 8.0 Hz, 2H), 7.00-6.86 (m, 3H), 6.77 (d, *J* = 8.0 Hz, 2H), 3.82 (s, 3H), 3.76 (s, 3H), 3.61 (d, *J* = 13.6 Hz, 1H), 3.26 (d, *J* = 14.0 Hz, 1H).

^{13}C NMR (100 MHz, $CDCl_3$) δ 171.8, 160.0, 159.1, 131.4, 127.8, 125.8, 125.4, 117.8, 114.4, 113.8, 55.4, 55.2, 42.8.

IR (neat, cm^{-1}) 3088, 2971, 2257, 1712, 1512, 1259.

HRMS (ESI) calcd $C_{18}H_{17}NNaO_4^+$ $[M+Na]^+$: 334.1050. Found: 334.1048.

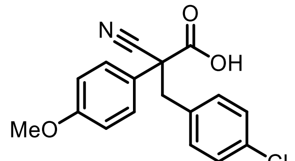

S38

zwf-7-129-2

S38 (6.6 mmol scale, hydrolysis time: 15 min, 1.4843 g, 71% Yield) solid. R_f = 0.4 (DCM/MeOH 5:1).

^{1}H NMR (400 MHz, $CDCl_3$) δ 7.45 (d, J = 8.8 Hz, 2H), 7.21 (d, J = 8.4 Hz, 2H), 7.07 (d, J = 8.4 Hz, 2H), 6.92 (d, J = 8.8 Hz, 2H), 6.37 (br, 1H), 3.82 (s, 3H), 3.61 (d, J = 14.0 Hz, 1H), 3.29 (d, J = 14.0 Hz, 1H).

^{13}C NMR (100 MHz, $CDCl_3$) δ 171.1, 160.1, 133.9, 132.2, 131.6, 128.5, 127.7, 125.0, 117.5, 114.5, 55.4, 54.9, 42.8.

IR (neat, cm^{-1}) 3473, 2983, 2262, 1749, 1513, 1057.

HRMS (ESI) calcd $C_{17}H_{14}ClNNaO_3^+$ $[M+Na]^+$: 338.0554. Found: 338.0556.

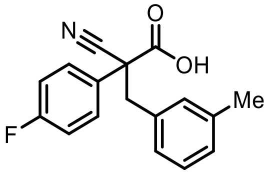

S39

zwf-7-129-9

S39 (4.8 mmol scale, hydrolysis time: 10 min, 0.9206 g, 68% Yield) solid. R_f = 0.4 (DCM/MeOH 5:1).

^{1}H NMR (400 MHz, $CDCl_3$) δ 7.55 (dd, J_1 = 8.8 Hz, J_2 = 4.8 Hz, 2H), 7.17-7.03 (m, 4H), 6.98-6.88 (m, 2H), 6.51 (br, 1H), 3.62 (d, J = 13.6 Hz, 1H), 3.28 (d, J = 13.6 Hz, 1H), 2.26 (s, 3H).

^{13}C NMR (100 MHz, $CDCl_3$) δ 171.1, 163.0 (d, J = 248.4 Hz), 138.1, 133.3, 131.0, 129.4 (d, J = 3.6 Hz), 128.7, 128.5 (d, J = 8.7 Hz), 128.3, 127.2, 117.4, 116.1 (d, J = 21.7 Hz), 55.2, 43.7, 21.3.

^{19}F NMR (376 MHz, $CDCl_3$) δ −111.9 to −112.0 (m).

IR (neat, cm^{-1}) 3078, 2988, 2247, 1718, 1507, 1066.

HRMS (ESI) calcd $C_{17}H_{14}FNNaO_2^+$ $[M+Na]^+$: 306.0901. Found: 306.0905.

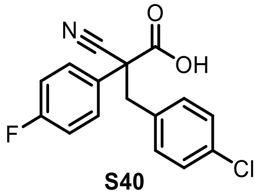

S40

zwf-7-129-11

S40 (12.5 mmol scale, hydrolysis time: 5 min, 2.7456 g, 72% Yield) solid. R_f = 0.4 (DCM/MeOH 5:1).

^{1}H NMR (400 MHz, $CDCl_3$) δ 7.52 (dd, J_1 = 8.8 Hz, J_2 = 4.8 Hz, 2H), 7.21 (d, J = 8.4 Hz, 2H), 7.16-6.98 (m, 4H), 6.03 (br, 1H), 3.62 (d, J = 13.6 Hz, 1H), 3.29 (d, J = 13.6 Hz, 1H).

^{13}C NMR (100 MHz, $CDCl_3$) δ 170.3, 163.0 (d, J = 248.4 Hz), 134.1, 131.8, 131.6, 129.0 (d, J = 2.9 Hz), 128.7, 128.4 (d, J = 8.6 Hz), 117.3, 116.3 (d, J = 22.4 Hz), 54.9, 43.1.

^{19}F NMR (376 MHz, $CDCl_3$) δ −111.5 to −111.6 (m).

IR (neat, cm^{-1}) 3092, 2981, 2252, 1719, 1406, 1076.

HRMS (ESI) calcd $C_{16}H_{11}ClFNNaO_2^+$ $[M+Na]^+$: 326.0355. Found: 326.0352.

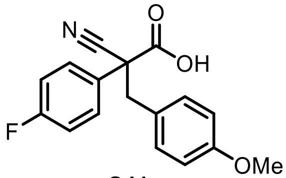

S41

zwf-7-129-8

S41 (6.1 mmol scale, hydrolysis time: 5 min, 1.3543 g, 74% Yield) solid. R_f = 0.4 (DCM/MeOH 5:1).

^{1}H NMR (400 MHz, $CDCl_3$) δ 7.54 (dd, J_1 = 8.8 Hz, J_2 = 4.8 Hz, 2H), 7.24 (br, 1H), 7.14-7.02 (m, 4H), 6.77 (d, J = 8.8 Hz, 2H), 3.76 (s, 3H), 3.61 (d, J = 13.6 Hz, 1H), 3.27 (d, J = 13.6 Hz, 1H).

^{13}C NMR (100 MHz, $CDCl_3$) δ 171.2, 162.9 (d, J = 248.4 Hz), 159.2, 131.4, 129.3 (d, J = 2.9 Hz), 128.5 (d, J = 8.6 Hz), 125.4, 117.5, 116.1 (d, J = 21.6 Hz), 113.8, 55.4, 55.2, 43.1.

^{19}F NMR (376 MHz, $CDCl_3$) δ −111.8 to −112.0 (m).

IR (neat, cm^{-1}) 3089, 2980, 2252, 1715, 1511, 1253.

HRMS (ESI) calcd $C_{17}H_{14}FNNaO_3^+$ $[M+Na]^+$: 322.0850. Found: 322.0848.

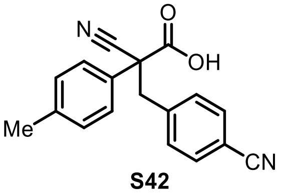

S42

zwf-7-129-12

S42 (12.3 mmol scale, hydrolysis time: 5 min, 3.0517 g, 85% Yield) solid. R_f = 0.4 (DCM/MeOH 5:1).

^{1}H NMR (400 MHz, $CDCl_3$) δ 7.78 (br, 1H), 7.52 (d, *J* = 8.0 Hz, 2H), 7.40 (d, *J* = 8.0 Hz, 2H), 7.28-7.18 (m, 4H), 3.70 (d, *J* = 13.6 Hz, 1H), 3.40 (d, *J* = 13.6 Hz, 1H), 2.38 (s, 3H).

^{13}C NMR (100 MHz, $CDCl_3$) δ 170.5, 139.7, 139.3, 132.1, 131.2, 130.0, 129.8, 126.1, 118.3, 117.1, 111.6, 54.7, 43.2, 21.0.

IR (neat, cm^{-1}) 30473, 2981, 2247, 2227, 1723, 1244, 1066.

HRMS (ESI) calcd $C_{18}H_{14}N_2NaO_2^+$ $[M+Na]^+$: 313.0947. Found: 313.0943.

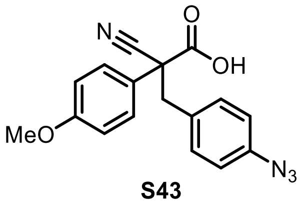

S43

zwf-7-129-4

S43 (8.2 mmol scale, hydrolysis time: 10 min, 2.1714 g, 82% Yield) solid. R_f = 0.4 (DCM/MeOH 5:1).

^{1}H NMR (400 MHz, $CDCl_3$) δ 8.70 (br, 1H), 7.46 (d, *J* = 8.8 Hz, 2H), 7.13 (d, *J* = 8.0 Hz, 2H), 6.98-6.84 (m, 4H), 3.83 (s, 3H), 3.62 (d, *J* = 13.6 Hz, 1H), 3.31 (d, *J* = 13.6 Hz, 1H).

^{13}C NMR (100 MHz, $CDCl_3$) δ 172.1, 160.2, 139.7, 131.7, 130.3, 127.7, 124.9, 119.0, 117.4, 114.6, 55.4, 55.1, 42.9.

IR (neat, cm^{-1}) 3204, 2988, 2267, 2117, 1748, 1509, 1182.

HRMS (ESI) calcd $C_{17}H_{14}N_4NaO_3^+$ $[M+Na]^+$: 345.0958. Found: 345.0955.

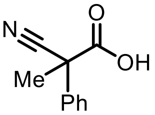

S44

zwf-7-055-14

S44 (5.0 mmol scale, hydrolysis time: 20 min, 0.6856 g, 79% Yield) solid. R_f = 0.4 (DCM/MeOH 5:1).

^{1}H NMR (400 MHz, $CDCl_3$) δ 7.61-7.49 (m, 2H), 7.46-7.35 (m, 3H), 7.21 (br, 1H), 1.98 (s, 3H).

^{13}C NMR (100 MHz, $CDCl_3$) δ 172.3, 134.8, 129.2, 129.1, 125.9, 119.0, 48.2, 24.2.

IR (neat, cm^{-1}) 3464, 2981, 2267, 1692, 1270, 1057.

HRMS (ESI) calcd $C_{10}H_{10}NO_2^+$ $[M+H]^+$: 176.0706. Found: 176.0708.

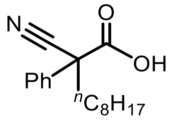

S45

zwf-7-133-4

S45 (7.0 mmol scale, hydrolysis time: 10 min, 1.6287 g, 85% Yield) oil. R_f = 0.4 (DCM/MeOH 5:1).

^{1}H NMR (400 MHz, $CDCl_3$) δ 7.62-7.52 (m, 2H), 7.46-7.36 (m, 3H), 7.23 (br, 1H), 2.45-2.29 (m, 1H), 2.22-2.02 (m, 1H), 1.55-1.18 (m, 12H), 0.87 (t, J = 6.6 Hz, 3H).

^{13}C NMR (100 MHz, $CDCl_3$) δ 172.4, 133.8, 129.2, 129.1, 126.2, 118.0, 54.3, 37.5, 31.7, 29.2, 29.07, 29.06, 25.4, 22.5, 14.0.

IR (neat, cm^{-1}) 3454, 2926, 2257, 1721, 1450, 1066.

HRMS (ESI) calcd $C_{17}H_{23}NNaO_2^+$ $[M+Na]^+$: 296.1621. Found: 296.1624.

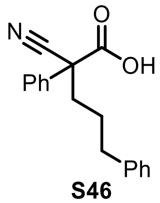

S46

zwf-7-129-15

S46 (4.9 mmol scale, hydrolysis time: 10 min, 0.8737 g, 64% Yield) solid. R_f = 0.4 (DCM/MeOH 5:1).

^{1}H NMR (400 MHz, $CDCl_3$) δ 8.38 (br, 1H), 7.57-7.47 (m, 2H), 7.45-7.33 (m, 3H), 7.32-7.05 (m, 5H), 2.77-2.53 (m, 2H), 2.39 (dt, J_1 = 12.8 Hz, J_2 = 4.7 Hz, 1H), 2.17 (dt, J_1 = 13.0 Hz, J_2 = 4.4 Hz, 1H), 1.94-1.64 (m, 2H).

^{13}**C NMR** (100 MHz, $CDCl_3$) δ 172.6, 140.7, 133.4, 129.24, 129.18, 128.4, 128.3, 126.2, 126.1, 117.7, 54.1, 36.8, 35.1, 26.8.

IR (neat, cm^{-1}) 3097, 2988, 2267, 1749, 1206, 1075.

HRMS (ESI) calcd $C_{18}H_{17}NNaO_2^+$ $[M+Na]^+$: 302.1151. Found: 302.1154.

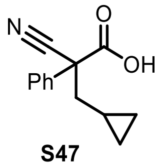

S47

zwf-7-133-5

S47 (9.2 mmol scale, hydrolysis time: 10 min, 1.7263 g, 87% Yield) oil. R_f = 0.4 (DCM/MeOH 5:1).

^{1}**H NMR** (400 MHz, $CDCl_3$) δ 7.59 (d, J = 6.8 Hz, 2H), 7.48-7.31 (m, 4H), 2.27 (dd, J_1 = 14.2 Hz, J_2 = 7.4 Hz, 1H), 2.18 (dd, J_1 = 14.0 Hz, J_2 = 6.0 Hz, 1H), 0.93-0.76 (m, 1H), 0.60-0.46 (m, 2H), 0.35-0.25 (m, 1H), 0.24-0.13 (m, 1H).

^{13}**C NMR** (100 MHz, $CDCl_3$) δ 172.6, 134.0, 129.2, 129.1, 126.3, 118.0, 54.4, 42.3, 7.2, 4.4.

IR (neat, cm^{-1}) 3478, 2983, 2247, 1725, 1450, 1067.

HRMS (ESI) calcd $C_{13}H_{13}NNaO_2^+$ $[M+Na]^+$: 238.0838. Found: 238.0839.

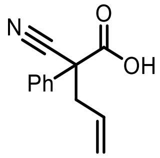

S48

zwf-7-086-2

S48 (9.3 mmol scale, hydrolysis time: 10 min, 1.6741 g, 89% Yield) oil. R_f = 0.4 (DCM/MeOH 5:1).

^{1}**H NMR** (400 MHz, $CDCl_3$) δ 7.63-7.51 (m, 2H), 7.48-7.34 (m, 3H), 6.50 (br, 1H), 5.90-5.60 (m, 1H), 5.35-5.14 (m, 2H), 3.12 (dd, J_1 = 14.0 Hz, J_2 = 7.6 Hz, 1H), 2.87 (dd, J_1 = 14.0 Hz, J_2 = 7.2 Hz, 1H).

^{13}**C NMR** (100 MHz, $CDCl_3$) δ 171.0, 133.3, 130.3, 129.22, 129.18, 126.3, 121.6, 117.6, 54.2, 41.7.

IR (neat, cm^{-1}) 3488, 2985, 2257, 1725, 1230, 1057.

HRMS (ESI) calcd $C_{12}H_{12}NO_2^+$ $[M+H]^+$: 202.0863. Found: 202.0862.

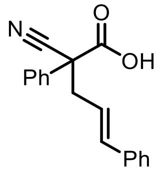

S49

zwf-7-055-2

S49 (9.5 mmol scale, hydrolysis time: 20 min, 1.7709 g, 67% Yield) solid. R_f = 0.4 (DCM/MeOH 5:1).

^{1}H NMR (400 MHz, $CDCl_3$) δ 7.64-7.55 (m, 2H), 7.45-7.35 (m, 3H), 7.33-7.19 (m, 5H), 7.06 (br, 1H), 6.58 (d, J = 15.6 Hz, 1H), 6.10 (dt, J_1 = 15.6 Hz, J_2 = 7.8 Hz, 1H), 3.26 (ddd, J_1 = 14.0 Hz, J_2 = 7.6 Hz, J_3 = 0.8 Hz, 1H), 3.08 (ddd, J_1 = 14.0 Hz, J_2 = 7.2 Hz, J_3 = 1.0 Hz, 1H).

^{13}C NMR (100 MHz, $CDCl_3$) δ 171.3, 136.33, 136.30, 133.3, 129.3, 129.2, 128.5, 127.9, 126.5, 126.3, 121.3, 117.6, 54.4, 41.1.

IR (neat, cm^{-1}) 3257, 2981, 2247, 1714, 1394, 1066.

HRMS (ESI) calcd $C_{18}H_{16}NO_2^+$ $[M+H]^+$: 278.1176. Found: 278.1174.

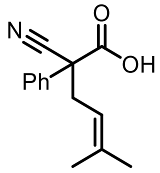

S50

zwf-7-109-11

S50 (10.0 mmol scale, hydrolysis time: 8 min, 2.1162 g, 92% Yield) oil. R_f = 0.4 (DCM/MeOH 5:1).

^{1}H NMR (400 MHz, $CDCl_3$) δ 7.63-7.53 (m, 2H), 7.47-7.34 (m, 3H), 7.02 (br, 1H), 5.14 (t, J = 7.2 Hz, 1H), 3.12 (dd, J_1 = 14.2 Hz, J_2 = 7.4 Hz, 1H), 2.83 (dd, J_1 = 14.4 Hz, J_2 = 7.2 Hz, 1H), 1.71 (s, 3H), 1.63 (s, 3H).

^{13}C NMR (100 MHz, $CDCl_3$) δ 172.0, 138.6, 133.6, 129.12, 129.06, 126.3, 118.0, 116.1, 54.3, 36.5, 25.9, 18.1.

IR (neat, cm^{-1}) 3498, 2988, 2257, 1726, 1450, 1068.

HRMS (ESI) calcd $C_{14}H_{15}NNaO_2^+$ $[M+Na]^+$: 252.0995. Found: 252.0997.

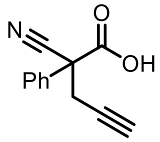

S51

zwf-7-086-3

S51 (4.5 mmol scale, hydrolysis time: 8 min, 0.7440 g, 83% Yield) oil. R_f = 0.5 (DCM/MeOH 5:1).

^{1}H NMR (400 MHz, $CDCl_3$) δ 7.61-7.52 (m, 2H), 7.48-7.37 (m, 3H), 5.81 (br, 1H), 3.27 (dd, J_1 = 16.8 Hz, J_2 = 2.8 Hz, 1H), 3.01 (dd, J_1 = 17.0 Hz, J_2 = 2.6 Hz, 1H), 2.16 (t, J = 2.4 Hz, 1H).

^{13}C NMR (100 MHz, $CDCl_3$) δ 169.3, 132.6, 129.5, 129.3, 126.1, 117.4, 77.1, 73.1, 53.6, 28.4.

IR (neat, cm^{-1}) 3483, 2988, 2257, 1729, 1233, 1056.

HRMS (ESI) calcd $C_{12}H_9NNaO_2^+$ $[M+Na]^+$: 222.0525. Found: 222.0524.

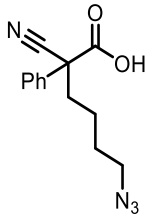

S52

zwf-9-010-4

S52 (1.9 mmol scale, hydrolysis time: 10 min, 0.3865 g, 78% Yield) oil. R_f = 0.4 (DCM/MeOH 5:1).

^{1}H NMR (400 MHz, $CDCl_3$) δ 7.62-7.52 (m, 2H), 7.46-7.37 (m, 3H), 5.81 (br, 1H), 3.28 (t, J = 6.6 Hz, 2H), 2.46-2.33 (m, 1H), 2.23-2.10 (m, 1H), 1.74-1.42 (m, 4H).

^{13}C NMR (100 MHz, $CDCl_3$) δ 170.8, 133.6, 129.2, 129.1, 126.0, 117.9, 54.0, 50.8, 36.9, 28.3, 22.6.

IR (neat, cm^{-1}) 2941, 2871, 2248, 2095, 1725, 1450, 1254.

HRMS (ESI) calcd $C_{13}H_{14}N_4NaO_2^+$ $[M+Na]^+$: 281.1009. Found: 281.1007.

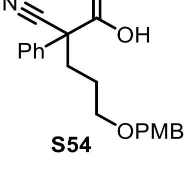

S54

zwf-7-133-2

S54 (8.1 mmol scale, hydrolysis time: 10 min, 2.1134 g, 77% Yield) solid. R_f = 0.4 (DCM/MeOH 5:1).

^{1}H NMR (500 MHz, $CDCl_3$) δ 7.55 (d, *J* = 6.5 Hz, 2H), 7.42-7.32 (m, 3H), 7.20 (d, *J* = 8.5 Hz, 2H), 6.83 (d, *J* = 9.0 Hz, 2H), 6.63 (br, 1H), 4.47-4.33 (m, 2H), 3.78 (s, 3H), 3.56-3.40 (m, 2H), 2.54-2.39 (m, 1H), 2.26-2.10 (m, 1H), 1.84-1.66 (m, 2H).

^{13}C NMR (125 MHz, $CDCl_3$) δ 170.3, 159.2, 133.9, 129.55, 129.50, 129.1, 129.0, 126.2, 118.2, 113.8, 72.4, 68.7, 55.2, 53.9, 34.5, 25.6.

IR (neat, cm^{-1}) 3093, 2919, 2272, 1741, 1450, 1233.

HRMS (ESI) calcd $C_{20}H_{22}NO_4^+$ $[M+H]^+$: 340.1543. Found: 340.1546.

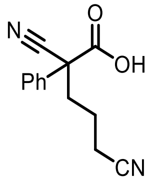

S56

zwf-9-010-1

S56 (4.5 mmol scale, hydrolysis time: 10 min, 0.8288 g, 81% Yield) solid. R_f = 0.4 (DCM/MeOH 5:1).

^{1}H NMR (400 MHz, $CDCl_3$) δ 9.06 (br, 1H), 7.65-7.50 (m, 2H), 7.49-7.36 (m, 3H), 2.62-2.20 (m, 4H), 1.97-1.65 (m, 2H).

^{13}C NMR (100 MHz, $CDCl_3$) δ 171.0, 132.7, 129.6, 129.5, 126.0, 118.4, 117.2, 53.4, 36.2, 21.4, 16.9.

IR (neat, cm^{-1}) 2950, 2838, 2245, 1716, 1455, 1278.

HRMS (ESI) calcd $C_{13}H_{13}N_2O_2^+$ $[M+H]^+$: 229.0972. Found: 229.0974.

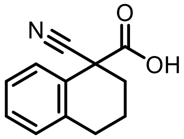

S58

zwf-8-115

S58 (10.0 mmol scale, hydrolysis time: 10 min, 1.8176 g, 90% Yield) solid. R_f = 0.4 (DCM/MeOH 5:1).

^{1}H NMR (400 MHz, $CDCl_3$) δ 8.86 (br, 1H), 7.42 (d, *J* = 8.0 Hz, 1H), 7.32-7.20 (m, 2H), 7.16 (d, *J* = 7.6

Hz, 1H), 2.86 (t, J = 6.4 Hz, 2H), 2.57-2.38 (m, 2H), 2.13-1.91 (m, 2H).

^{13}C NMR (100 MHz, $CDCl_3$) δ 174.1, 136.5, 130.1, 129.2, 128.8, 128.7, 127.0, 119.5, 47.7, 32.7, 28.3, 19.1.

IR (neat, cm^{-1}) 2938, 2870, 2243, 1703, 1262.

HRMS (ESI) calcd $C_{12}H_{12}NO_2^+$ $[M+H]^+$: 202.0863. Found: 202.0864.

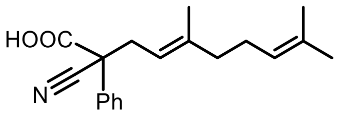

S60

zwf-7-086-12

S60 (5.2 mmol scale, hydrolysis time: 10 min, 1.1501 g, 74% Yield) oil. R_f = 0.4 (DCM/MeOH 5:1).

^{1}H NMR (400 MHz, $CDCl_3$) δ 7.62-7.55 (m, 2H), 7.44-7.37 (m, 3H), 7.19 (br, 1H), 5.14 (t, J = 7.2 Hz, 1H), 5.02 (t, J = 6.4 Hz, 1H), 3.12 (dd, J_1 = 14.4 Hz, J_2 = 7.6 Hz, 1H), 2.86 (dd, J_1 = 14.4 Hz, J_2 = 7.6 Hz, 1H), 2.11-1.92 (m, 4H), 1.65 (s, 3H), 1.62 (s, 3H), 1.56 (s, 3H).

^{13}C NMR (100 MHz, $CDCl_3$) δ 172.2, 142.1, 133.6, 131.6, 129.1, 129.0, 126.4, 123.7, 117.9, 116.1, 54.3, 39.7, 36.4, 26.3, 25.6, 17.6, 16.4.

IR (neat, cm^{-1}) 3483, 2974, 2252, 1715, 1450, 1066.

HRMS (ESI) calcd $C_{19}H_{23}NNaO_2^+$ $[M+Na]^+$: 320.1621. Found: 320.1623.

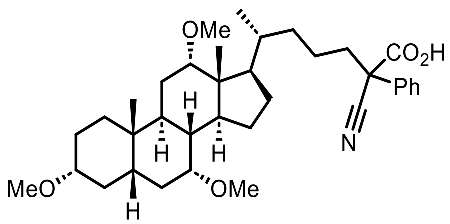

S61

zwf-9-010-5

S61 (0.8 mmol scale, hydrolysis time (with 2 M NaOH): 20 min, 0.3461 g, 75% Yield) oil. R_f = 0.4 (DCM/MeOH 5:1).

^{1}H NMR (400 MHz, $CDCl_3$) δ 7.64-7.55 (m, 2H), 7.44-7.34 (m, 3H), 5.93 (br, 1H), 3.41-3.30 (m, 4H), 3.27-3.17 (m, 6H), 3.16-3.12 (m, 1H), 3.10-3.01 (m, 1H), 2.40-0.96 (m, 27H), 0.90-0.86 (m, 5H), 0.62 (d, J = 4.0 Hz, 3H).

^{13}C NMR (100 MHz, $CDCl_3$) δ (diastereomer 1) 170.2, 134.4, 129.0, 128.7, 126.3, 118.6, 82.1, 81.0, 77.1, 66.9, 55.8, 55.7, 55.1, 46.9, 46.0, 42.5, 41.8, 39.5, 38.0, 35.4, 35.3, 35.2, 34.8, 34.1, 27.9, 27.6, 27.5, 26.4, 23.1, 22.7, 22.6, 21.9, 17.6, 12.4; (diastereomer 2) 170.0, 134.7, 129.0, 128.7, 126.3, 118.7, 82.1, 81.1, 77.1, 66.9, 55.8, 55.7, 55.1, 46.8, 46.0, 42.5, 41.8, 39.5, 38.2, 35.6, 35.3, 35.1, 34.8, 34.1, 27.9, 27.6, 27.5, 26.4, 23.1, 22.7, 22.5, 21.9, 17.5, 12.4.

IR (neat, cm^{-1}) 2933, 2865, 2257, 1738, 1450, 1084.

HRMS (ESI) calcd $C_{36}H_{54}NO_5^+$ $[M+H]^+$: 580.3997. Found: 580.3994.

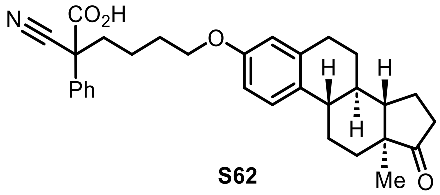

S62

zwf-7-133-3

S62 (7.4 mmol scale, hydrolysis time: 20 min, 2.8037 g, 78% Yield) oil. R_f = 0.4 (DCM/MeOH 5:1).

^{1}H NMR (400 MHz, $CDCl_3$) δ 7.81 (br, 1H), 7.62-7.54 (m, 2H), 7.45-7.35 (m, 3H), 7.16 (d, J = 8.4 Hz, 1H), 6.67 (dd, J_1 = 8.6 Hz, J_2 = 2.6 Hz, 1H), 6.60 (d, J = 2.4 Hz, 1H), 3.92 (t, J = 6.2 Hz, 2H), 2.96-2.78 (m, 2H), 2.57-2.32 (m, 3H), 2.23-2.17 (m, 8H), 1.74-1.35 (m, 8H), 0.90 (s, 3H).

^{13}C NMR (100 MHz, $CDCl_3$) δ 222.7, 171.0, 156.8, 137.7, 133.9, 132.0, 129.2, 129.0, 126.3, 126.2, 118.0, 114.6, 112.1, 67.2, 54.1, 50.3, 48.2, 43.9, 38.3, 37.3, 35.9, 31.4, 29.6, 28.8, 26.5, 25.8, 22.2, 21.5, 13.8.

IR (neat, cm^{-1}) 3454, 2970, 2924, 2252, 1734, 1497, 1233, 1056.

HRMS (ESI) calcd $C_{31}H_{35}NNaO_4^+$ $[M+Na]^+$: 508.2458. Found: 508.2459.

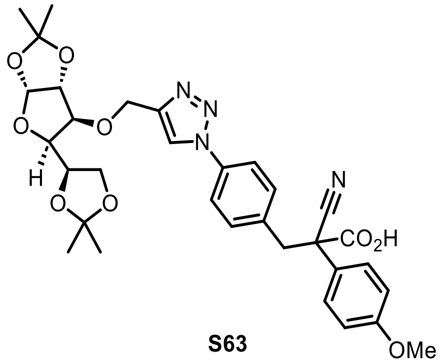

S63

zwf-9-010-3

S63 (2.2 mmol scale, hydrolysis time (with 2 M NaOH): 20 min, 0.8675 g, 64% Yield) solid. R_f = 0.4

(DCM/MeOH 5:1).

^{1}H NMR (400 MHz, $CDCl_3$) δ 8.12 (s, 1H), 7.61 (d, *J* = 8.4 Hz, 2H), 7.51 (d, *J* = 8.4 Hz, 2H), 7.36 (d, *J* = 7.2 Hz, 2H), 7.04-6.90 (m, 3H), 5.87 (d, *J* = 3.6 Hz, 1H), 4.90 (d, *J* = 12.8 Hz, 1H), 4.81 (d, *J* = 12.8 Hz, 1H), 4.64 (d, *J* = 4.0 Hz, 1H), 4.40-4.30 (m, 1H), 4.14-3.97 (m, 4H), 3.81 (s, 3H), 3.75 (d, *J* = 13.6 Hz, 1H), 3.38 (d, *J* = 13.6 Hz, 1H), 1.49 (s, 3H), 1.38 (s, 3H), 1.31 (s, 6H).

^{13}C NMR (100 MHz, $CDCl_3$) δ 168.8, 160.0, 145.2, 136.0, 135.7, 131.8, 127.6, 125.8, 121.1, 121.0, 120.23, 120.20, 118.1, 114.4, 112.0, 109.3, 105.1, 82.3, 81.6, 80.9, 72.2, 67.3, 63.2, 55.3, 54.9, 43.2, 26.8, 26.7, 26.1, 25.3.

IR (neat, cm^{-1}) 2984, 2935, 2247, 1729, 1511, 1254.

HRMS (ESI) calcd $C_{32}H_{37}N_4O_9^+$ $[M+H]^+$: 621.2555. Found: 621.2554.

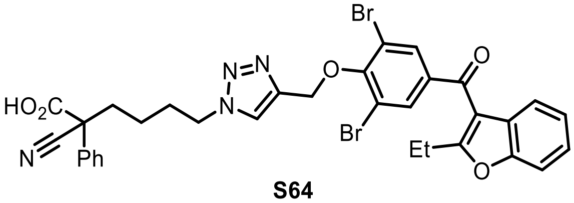

S64

zwf-9-010-6

S64 (0.8 mmol scale, hydrolysis time (with 2 M NaOH): 20 min, 0.3871 g, 65% Yield) solid. R_f = 0.4 (DCM/MeOH 5:1).

^{1}H NMR (400 MHz, $CDCl_3$) δ 7.96 (s, 2H), 7.80 (s, 1H), 7.58 (d, *J* = 7.2 Hz, 2H), 7.50 (d, *J* = 8.0 Hz, 1H), 7.43-7.28 (m, 5H), 7.26-7.22 (m, 1H), 6.63 (br, 1H), 5.30 (s, 2H), 4.40 (t, *J* = 7.0 Hz, 2H), 2.88 (q, *J* = 7.6 Hz, 2H), 2.55-2.40 (m, 1H), 2.19-1.94 (m, 3H), 1.65-1.51 (m, 2H), 1.35 (t, *J* = 7.4 Hz, 3H).

^{13}C NMR (100 MHz, $CDCl_3$) δ 188.4, 169.0, 167.4, 155.7, 153.7, 142.7, 137.5, 134.4, 133.6, 129.2, 128.9, 126.2, 126.1, 124.8, 124.03, 123.96, 121.0, 118.8, 118.6, 115.2, 111.1, 65.9, 54.1, 50.2, 37.1, 29.7, 22.6, 22.1, 12.1.

IR (neat, cm^{-1}) 2935, 2867, 2247, 1736, 1451, 1237.

HRMS (ESI) calcd $C_{33}H_{29}Br_2N_4O_5^+$ $[M+H]^+$: 719.0499. Found: 719.0495.

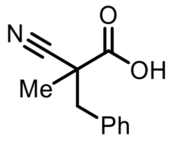

S65

zwf-7-055-15

S65 (12.5 mmol scale, hydrolysis time: 20 min, 2.0609 g, 87% Yield) solid. R_f = 0.4 (DCM/MeOH 5:1).

^{1}H NMR (400 MHz, $CDCl_3$) δ 7.42-7.27 (m, 5H), 6.14 (br, 1H), 3.27 (d, *J* = 13.6 Hz, 1H), 3.05 (d, *J* = 13.2 Hz, 1H), 1.63 (s, 3H).

^{13}C NMR (100 MHz, $CDCl_3$) δ 173.0, 133.8, 130.0, 128.6, 128.0, 119.2, 45.5, 43.2, 22.8.

IR (neat, cm^{-1}) 3108, 2988, 2262, 1744, 1452, 1199.

HRMS (ESI) calcd $C_{11}H_{12}NO_2^+$ $[M+H]^+$: 190.0863. Found: 190.0862.

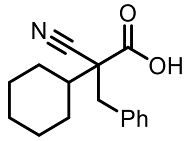

S66

lk-4-9

S66 (2.0 mmol scale, hydrolysis time: 6 h in EtOH with 2N NaOH aq. at 40 ºC, 261.9 mg, 51% Yield) solid. R_f = 0.6 (DCM/MeOH 5:1).

^{1}H NMR (600 MHz, $CDCl_3$) δ 7.31-7.27 (m, 5H), 3.25 (d, *J* = 13.8 Hz, 1H), 3.05 (d, *J* = 13.8 Hz, 1H), 2.07 (d, *J* = 12.0 Hz, 1H), 2.02-1.90 (m, 2H), 1.84 (d, *J* = 12.6 Hz, 1H), 1.74 (d, *J* = 12.6 Hz, 1H), 1.66 (d, *J* = 12.6 Hz, 1H), 1.45-1.23 (m, 5H).

^{13}C NMR (150 MHz, $CDCl_3$) δ 172.9, 134.3, 129.8, 128.6, 127.8, 117.6, 57.5, 44.6, 40.5, 29.4, 27.7, 25.94, 25.93, 25.7.

HRMS (ESI) calcd $C_{16}H_{19}NO_2Na^+$ $[M+Na]^+$: 280.1308. Found: 280.1310.

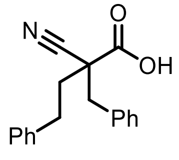

S67

lk-3-147

S67 (8.5 mmol scale, hydrolysis time: 30 min, 402.8 mg, 81% Yield) solid. R_f = 0.4 (DCM/MeOH 5:1).

^{1}H NMR (400 MHz, $CDCl_3$) δ 8.17 (br, 1H), 7.36-7.25 (m, 7H), 7.23-7.17 (m, 3H), 3.25 (d, J = 13.6 Hz, 1H), 3.13 (d, J = 13.6 Hz, 1H), 2.94 (dt, J_1 = 12.8 Hz, J_2 = 4.8 Hz, 1H), 2.72 (dt, J_1 = 12.8 Hz, J_2 = 5.2 Hz, 1H), 2.33 (dt, J_1 = 12.8 Hz, J_2 = 5.2 Hz, 1H), 2.14 (dt, J_1 = 13.0 Hz, J_2 = 4.8 Hz, 1H),

^{13}C NMR (100 MHz, $CDCl_3$) δ 173.5, 139.3, 133.5, 129.9, 128.7, 128.6, 128.4, 128.1, 126.6, 118.0, 51.7, 43.0, 38.7, 31.8.

HRMS (ESI) calcd $C_{18}H_{18}NO_2^+$ $[M+H]^+$: 280.1333. Found: 280.1333.

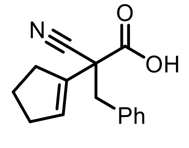

S68

zwf-10-085-3

S68 (8.5 mmol scale, hydrolysis time: 5 min, 1.6993 g, 83% Yield) solid. R_f = 0.4 (DCM/MeOH 5:1).

^{1}H NMR (500 MHz, $CDCl_3$) δ 8.33 (br, 1H), 7.35-7.25 (m, 5H), 6.01 (t, J = 2.0 Hz, 1H), 3.44 (d, J = 13.5 Hz, 1H), 3.17 (d, J = 13.5 Hz, 1H), 2.53-2.37 (m, 4H), 1.98 (pent, J = 7.5 Hz, 2H).

^{13}C NMR (125 MHz, $CDCl_3$) δ 171.6, 136.1, 133.9, 132.4, 130.0, 128.5, 127.9, 117.3, 53.2, 40.6, 32.4, 32.2, 23.2.

IR (neat, cm^{-1}) 3065, 2931, 2263, 1742, 1440, 1196.

HRMS (ESI) calcd $C_{15}H_{16}NO_2^+$ $[M+H]^+$: 242.1176. Found: 242.1177.

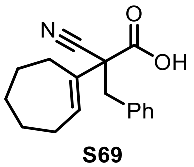

S69

zwf-10-064-1

S69 (8.1 mmol scale, hydrolysis time: 20 min, 1.7874 g, 86% Yield) solid. R_f = 0.4 (DCM/MeOH 5:1).

^{1}H NMR (500 MHz, $CDCl_3$) δ 7.33-7.26 (m, 5H), 6.53 (br, 1H), 6.19 (t, J = 6.5 Hz, 1H), 3.38 (d, J = 13.5 Hz, 1H), 3.12 (d, J = 13.5 Hz, 1H), 2.39-2.28 (m, 2H), 2.27-2.12 (m, 2H), 1.88-1.78 (m, 1H), 1.78-1.67 (m, 1H), 1.63-1.41 (m, 4H).

^{13}C NMR (125 MHz, $CDCl_3$) δ 171.5, 136.4, 134.1, 133.1, 130.2, 128.4, 127.7, 117.6, 57.8, 39.6, 32.2, 29.7, 28.2, 26.3, 25.7.

IR (neat, cm^{-1}) 3185, 2930, 2250, 1742, 1450, 1184.

HRMS (ESI) calcd $C_{17}H_{19}NNaO_2^+$ $[M+Na]^+$: 292.1308. Found: 292.1307.

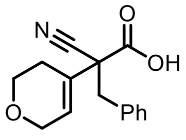

S70

zwf-10-064-3

S70 (10.2 mmol scale, hydrolysis time: 20 min, 2.2290 g, 85% Yield) solid. R_f = 0.4 (DCM/MeOH 5:1).

^{1}H NMR (500 MHz, $CDCl_3$) δ 7.34-7.26 (m, 5H), 6.47 (br, 1H), 6.04 (s, 1H), 4.30-4.14 (m, 2H), 3.83 (t, J = 5.5 Hz, 2H), 3.43 (d, J = 13.5 Hz, 1H), 3.15 (d, J = 14.0 Hz, 1H), 2.37-2.14 (m, 2H).

^{13}C NMR (125 MHz, $CDCl_3$) δ 168.7, 133.8, 130.1, 128.7, 128.5, 127.9, 126.3, 117.2, 65.1, 63.8, 56.2, 39.7, 25.1.

IR (neat, cm^{-1}) 3524, 3345, 2242, 1724, 1456, 1261.

HRMS (ESI) calcd $C_{15}H_{16}NO_3^+$ $[M+H]^+$: 258.1125. Found: 258.1126.

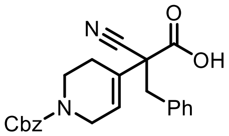

S71

zwf-10-064-4

S71 (10.5 mmol scale, hydrolysis time: 20 min, 3.3233 g, 81% Yield) oil. R_f = 0.4 (DCM/MeOH 5:1).

^{1}H NMR (500 MHz, $CDCl_3$) δ 7.39-7.25 (m, 10H), 6.46 (br, 1H), 6.05-5.87 (m, 1H), 5.14 (dd, J_1 = 12.5 Hz, J_2 = 13.5 Hz, 2H), 4.10-3.92 (m, 2H), 3.64-3.57 (m, 2H), 3.41 (d, J = 13.5 Hz, 1H), 3.13 (d, J = 13.5Hz, 1H), 2.34-2.14 (m, 2H).

^{13}C NMR (125 MHz, $CDCl_3$) δ 168.1, 155.8, 155.5, 136.0, 133.9, 130.1, 129.9, 129.8, 128.5, 128.4, 128.2, 128.0, 127.8, 124.4, 124.1, 117.4, 67.8, 66.9, 56.1, 43.3, 40.2, 39.7, 25.6, 24.9.

IR (neat, cm^{-1}) 3385, 2956, 2240, 1696, 1428, 1235.

HRMS (ESI) calcd $C_{23}H_{23}N_2O_4^+$ $[M+H]^+$: 391.1652. Found: 391.1653.

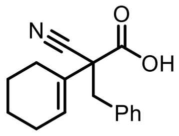

S72

zwf-10-018-1

S72 (8.1 mmol scale, hydrolysis time: 20 min, 1.7874 g, 86% Yield) solid. R_f = 0.4 (DCM/MeOH 5:1).

^{1}H NMR (500 MHz, $CDCl_3$) δ 8.43 (br, 1H), 7.38-7.25 (m, 5H), 6.05 (s, 1H), 3.39 (d, *J* = 13.5 Hz, 1H), 3.17 (d, *J* = 14.0 Hz, 1H), 2.23-2.00 (m, 4H), 1.76-1.65 (m, 2H), 1.65-1.53 (m, 2H).

^{13}C NMR (125 MHz, $CDCl_3$) δ 172.1, 134.1, 130.2, 130.1, 128.7, 128.4, 127.8, 117.5, 56.9, 39.9, 25.4, 25.3, 22.5, 21.4.

IR (neat, cm^{-1}) 3089, 2928, 2260, 1745, 1456, 1200.

HRMS (ESI) calcd $C_{16}H_{17}NNaO_2^+$ $[M+Na]^+$: 278.1151. Found: 278.1152.

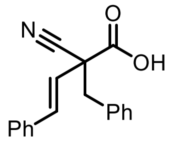

S74

zwf-10-085-4

S74 (4.4 mmol scale, hydrolysis time: 5 min, 0.9977 g, 90% Yield) solid. R_f = 0.4 (DCM/MeOH 5:1).

^{1}H NMR (500 MHz, $CDCl_3$) δ 7.41-7.26 (m, 10H), 6.86 (d, *J* = 15.5 Hz, 1H), 6.52 (br, 1H), 6.23 (d, *J* = 16.0 Hz, 1H), 3.44 (d, *J* = 13.5 Hz, 1H), 3.22 (d, *J* = 13.5Hz, 1H).

^{13}C NMR (125 MHz, $CDCl_3$) δ 171.3, 134.9, 134.4, 133.3, 130.2, 128.81, 128.76, 128.6, 128.1, 126.9, 122.3, 116.8, 54.2, 43.7.

IR (neat, cm^{-1}) 3060, 2935, 2186, 1722, 1455, 1268.

HRMS (ESI) calcd $C_{17}H_{15}NNa^+$ $[M+Na]^+$: 256.1097. Found: 256.1095.

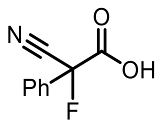

S76

zwf-10-018-10

S76 (25.0 mmol scale, hydrolysis time: 10 min, 4.2588 g, 95% Yield) solid. R_f = 0.4 (DCM/MeOH 5:1).

^{1}H NMR (500 MHz, $CDCl_3$) δ 7.64 (d, *J* = 7.5 Hz, 2H), 7.57-7.41 (m, 3H), 7.16 (br, 1H).

^{13}C NMR (125 MHz, $CDCl_3$) δ 165.7 (d, *J* = 29.9 Hz), 131.4 (d, *J* = 1.9 Hz), 130.9 (d, *J* = 23.5 Hz), 129.3, 125.5 (d, *J* = 5.4 Hz), 113.7 (d, *J* = 32.5 Hz), 86.9 (d, *J* = 196.9 Hz).

^{19}F NMR (470 MHz, $CDCl_3$) δ −146.9 (s).

IR (neat, cm^{-1}) 3486, 3434, 2177, 1882, 1704, 1304, 1063.

MS (70 eV, EI) *m/z* (%): 179 (M^+, 10.66), 134 (100); **HRMS** calcd for $C_9H_6O_2NF$ [M^+]: 179.0377. Found: 179.0381.

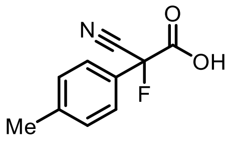

S77

zwf-10-085-6

S77 (11.9 mmol scale, hydrolysis time: 5 min, 2.0312 g, 87% Yield) oil. R_f = 0.4 (DCM/MeOH 5:1).

^{1}H NMR (500 MHz, $CDCl_3$) δ 7.50 (d, *J* = 8.0 Hz, 2H), 7.24 (d, *J* = 7.5 Hz, 2H), 5.87 (br, 1H), 2.37 (s, 3H).

^{13}C NMR (125 MHz, $CDCl_3$) δ 165.4 (d, *J* = 28.9 Hz), 141.7 (d, *J* = 1.8 Hz), 129.9, 128.3 (d, *J* = 23.5 Hz), 125.5 (d, *J* = 4.5 Hz), 114.2 (d, *J* = 33.4 Hz), 87.1 (d, *J* = 195.0 Hz), 21.2.

^{19}F NMR (470 MHz, $CDCl_3$) δ −144.2 (s).

IR (neat, cm^{-1}) 3492, 2926, 2277, 1746, 1614, 1257, 1053.

MS (70 eV, EI) *m/z* (%): 193 (M^+, 8.70), 148 (100); **HRMS** calcd for $C_{10}H_8O_2NF$ [M^+]: 193.0534. Found: 193.0536.

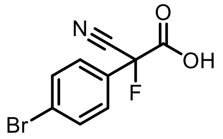

S78

zwf-10-085-2

S78 (12.0 mmol scale, hydrolysis time: 5 min, 2.6207 g, 90% Yield) oil. R_f = 0.4 (DCM/MeOH 5:1).

^{1}H NMR (500 MHz, $CDCl_3$) δ 7.60 (d, *J* = 8.5 Hz, 2H), 7.50 (d, *J* = 8.5 Hz, 2H), 5.68 (br, 1H).

^{13}C NMR (125 MHz, $CDCl_3$) δ 164.7 (d, *J* = 29.8 Hz), 132.5, 130.3 (d, *J* = 23.5 Hz), 127.1 (d, *J* = 5.4 Hz), 125.9 (d, *J* = 2.8 Hz), 113.8 (d, *J* = 32.5 Hz), 86.6 (d, *J* = 196.9 Hz).

^{19}F NMR (470 MHz, $CDCl_3$) δ −147.1 (s).

IR (neat, cm^{-1}) 3480, 3223, 2265, 1747, 1488, 1094.

MS (70 eV, EI) *m/z* (%): 256 (M^+, 2.65), 134 (100); **HRMS** calcd for $C_9H_5O_2BrNF$ [M^+]: 256.9482. Found: 256.9480.

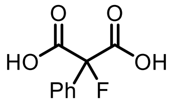

S82

zwf-11-030

S82 (10.0 mmol scale, hydrolysis time (200 mol% 2 N NaOH, EtOH as solvent): 2 h, 1.7606 g, 89% Yield) solid. R_f = 0.1 (DCM/MeOH 5:1).

^{1}H NMR (600 MHz, $CDCl_3$) δ 7.62-7.53 (m, 2H), 7.50-7.41 (m, 3H), 6.57 (br, 1H).

^{13}C NMR (150 MHz, $CDCl_3$) δ 167.2 (d, *J* = 27.0 Hz), 134.6 (d, *J* = 21.6 Hz), 130.6, 125.9, 126.8 (d, *J* = 7.7 Hz), 95.2 (d, *J* = 196.2 Hz).

^{19}F NMR (564 MHz, $CDCl_3$) δ −156.9 to −157.1 (m).

IR (neat, cm^{-1}) 3451, 2924, 2615, 1613, 1235, 1126.

HRMS (ESI) calcd $C_9H_8FO_4^+$ $[M+H]^+$: 199.0401. Found: 199.0400.

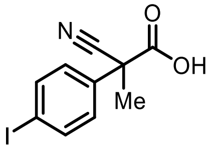

S94

zwf-10-085-8

S94 (10.5 mmol scale, hydrolysis time (0.5 N NaOH): 5 min, 2.3007 g, 73% Yield) solid. R_f = 0.4 (DCM/MeOH 5:1).

^{1}H NMR (400 MHz, $CDCl_3$) δ 7.77 (d, *J* = 7.6 Hz, 2H), 7.32 (d, *J* = 7.6 Hz, 2H), 6.29 (br, 1H), 1.97 (s, 3H).

^{13}C NMR (100 MHz, $CDCl_3$) δ 172.1, 138.4, 134.4, 127.8, 118.4, 95.3, 47.9, 24.0.

IR (neat, cm^{-1}) 2989, 2818, 2252, 1716, 1289, 1003.

HRMS (ESI) calcd $C_9H_9IN^+$ $[M-CO_2+H]^+$: 257.9774. Found: 257.9776.

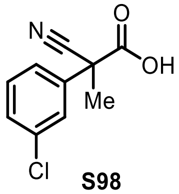

S98

zwf-10-085-7

S98 (11.6 mmol scale, hydrolysis time (0.5 N NaOH): 5 min, 1.7961 g, 84% Yield) solid. R_f = 0.4 (DCM/MeOH 5:1).

^{1}H NMR (500 MHz, $CDCl_3$) δ 7.58 (br, 1H), 7.55-7.53 (m, 1H), 7.49-7.44 (m, 1H), 7.42-7.33 (m, 2H), 1.98 (s, 3H).

^{13}C NMR (125 MHz, $CDCl_3$) δ 171.6, 136.6, 135.3, 130.5, 129.5, 126.2, 124.3, 118.5, 48.0, 24.2.

IR (neat, cm^{-1}) 3030, 2932, 2261, 1716, 1456, 1289.

HRMS (ESI) calcd $C_9H_9ClN^+$ $[M-CO_2+H]^+$: 166.0418. Found: 166.0419.

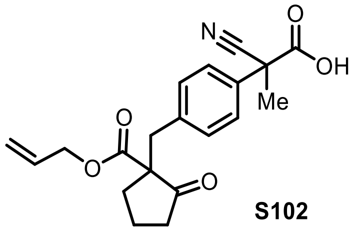

S102

zwf-11-055

S102[9-11] (5.0 mmol scale, hydrolysis time (TFA conditions): 9 h, 1.4998 g, 84% Yield) oil. R_f = 0.4 (DCM/MeOH 5:1).

^{1}H NMR (600 MHz, $CDCl_3$) δ 7.45 (d, *J* = 8.4 Hz, 2H), 7.19 (d, *J* = 8.4 Hz, 2H), 6.01 (br, 1H), 5.92-5.80 (m, 1H), 5.37-5.15 (m, 2H), 4.65-4.55 (m, 2H), 3.23 (dd, J_1 = 13.8 Hz, J_2 = 2.4 Hz, 1H), 3.08 (dd, J_1 = 14.1 Hz, J_2 = 3.9 Hz, 1H), 2.48-2.36 (m, 2H), 2.15-2.04 (m, 1H), 1.99-1.88 (m, 5H), 1.75-1.64 (m, 1H).

^{13}C NMR (150 MHz, $CDCl_3$) δ (diastereomer 1) 214.9, 170.9, 170.43, 137.6, 133.77, 131.3, 131.0, 125.9, 119.2, 118.8, 66.2, 61.40, 47.8, 38.3, 38.12, 31.7, 24.17, 19.4; (diastereomer 2) 214.9, 170.9, 170.41, 137.6, 133.79, 131.3, 131.0, 125.9, 119.2, 118.8, 66.2, 61.42, 47.8, 38.3, 38.14, 31.7, 24.23, 19.4.

IR (neat, cm^{-1}) 3524, 2961, 2246, 1722, 1219, 1145.

HRMS (ESI) calcd $C_{20}H_{22}NO_5^+$ $[M+H]^+$: 356.1492. Found: 356.1493.

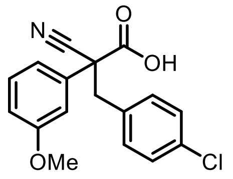

S107

zwf-11-087

S107 (12.2 mmol scale, hydrolysis time (0.1 N NaOH): 8 min, 2.5419 g, 66% Yield) oil. R_f = 0.4 (DCM/MeOH 5:1).

^{1}H NMR (600 MHz, $CDCl_3$) δ 7.32 (t, *J* = 8.1 Hz, 1H), 7.20 (d, *J* = 9.0 Hz, 2H), 7.15-7.04 (m, 4H), 6.93 (dd, J_1 = 8.1 Hz, J_2 = 2.1 Hz, 1H), 6.11 (br, 1H), 3.80 (s, 3H), 3.62 (d, *J* = 13.8 Hz, 1H), 3.31 (d, *J* = 13.8 Hz, 1H).

^{13}C NMR (150 MHz, $CDCl_3$) δ 170.6, 160.0, 134.6, 133.9, 132.1, 131.6, 130.3, 128.6, 118.6, 117.3, 114.5, 112.5, 55.5, 55.4, 42.8.

IR (neat, cm^{-1}) 3453, 2977, 2249, 1723, 1489, 1256, 1047.

HRMS (ESI) calcd $C_{16}H_{15}ClNO^+$ [M-CO_2+H]$^+$: 272.0837. Found: 272.0836.

Section 6. General procedure for the asymmetric decarboxylative protonation/deuteration reactions

General procedure for asymmetric decarboxylative protonation

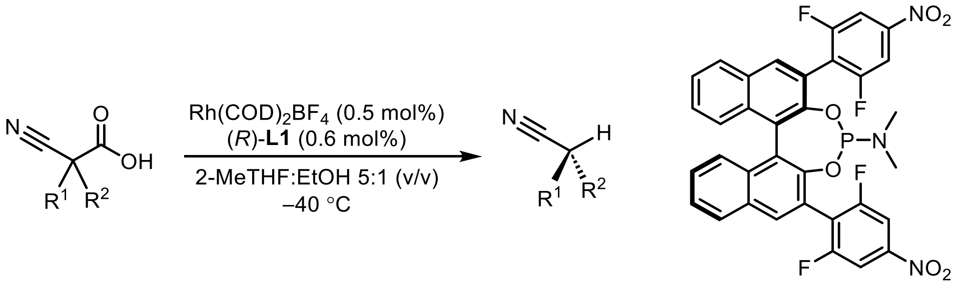

(*R*)-**L1**

To an oven-dried 100 mL Schlenk tube were added (*R*)-**L1** (2.0 mg, 0.003 mmol, 0.6 mol%), Rh(COD)2BF4 (1.0 mg, 0.0025 mmol, 0.5 mol%), and 2-methyltetrahydrofuran (4 mL, 2-MeTHF). After the reaction mixture was stirred at room temperature for 1 h, EtOH (2 mL) was added to the reaction and stirred for an additional 10 min at room temperature. Then the catalyst solution was cooled to −40 ºC and stirred for another 30 min. The disubstituted 2-cyanoacetic acid (0.5 mmol, 100 mol%) was dissolved in 2-MeTHF (5 mL) in another tube and cooled to −40 ºC for 10 min, which was added to the catalyst tube dropwise via syringe, and the tube was washed with 2-MeTHF (1 mL) to transfer the residue to the reaction. The reaction mixture was stirred at −40 ºC for 1-48 h. After the reaction mixture was warmed to room temperature, the solvent was removed under vacuum and the filtrate was purified by flash column chromatography (hexanes/ethyl acetate) to obtain the decarboxylative protonation product.

Control experiments on the influence of trace water

As an active rhodium catalyst was isolated with a ligated water (i.e., Scheme 2B in the manuscript), a set of control experiments were run to examine the role of trace water on the reactivity and enantioselectivity of the decarboxylation. These reactions were set up in a glove box using strictly anhydrous 2-methyl tetrahydrofuran and ethanol. Compared with the standard conditions (entry 1), adding either molecular sieves (entry 2) or water (entry 3 and 4) has negligible effects on both the yield and enantioselectivity of the reaction.

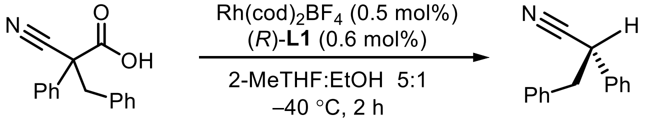

(±)-**S1** (−)-**1**

entry	Additive	Yield (%)	e.r.
1	none	97	94.5:5.5
2	4Å molecular sieves	98	94.5:5.5
3	H_2O (50 μL)	99	94.5:5.5
4	H_2O (100 μL)	99	94.5:5.5

General procedure for asymmetric decarboxylative deuteration

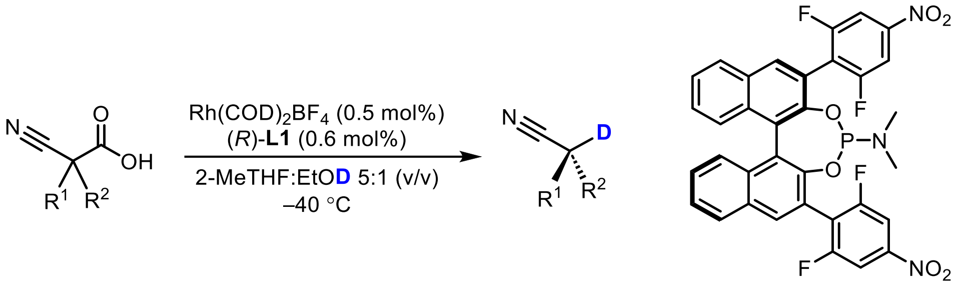

(*R*)-**L1**

To an oven-dried 100 mL Schlenk tube were added (*R*)-**L1** (0.8 mg, 0.0012 mmol, 0.6 mol%), $Rh(COD)_2BF_4$ (0.4 mg, 0.001 mmol, 0.5 mol%), and 2-methyltetrahydrofuran (8 mL, 2-MeTHF). After the reaction mixture was stirred at room temperature for 1 h, EtOD (4 mL, 99.5% D) was added to the reaction and stirred for an additional 10 min at room temperature. Then the catalyst solution was cooled to –40 ºC and stirred for another 30 min. The disubstituted 2-cyanoacetic acid (0.2 mmol, 100 mol%) was dissolved in 2-MeTHF (10 mL) in another tube and cooled to –40 ºC for 10 min, which was added to the catalyst tube dropwise via syringe, and the substrate tube was washed with 2-MeTHF (2 mL) to transfer the residue to the reaction. The reaction mixture was stirred at –40 ºC for 1-48 h. After the reaction mixture was warmed to room temperature, the solvent was removed under vacuum and the filtrate was purified by flash column chromatography (hexanes/ethyl acetate) to obtain the decarboxylation product.

An alternative procedure utilizing less solvent was applied for both protonation and deuteration:

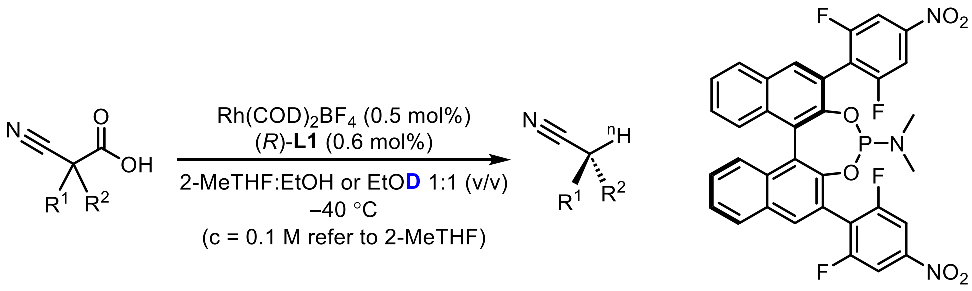

(*R*)-**L1**

To an oven-dried 100 mL Schlenk tube were added (*R*)-**L1** (0.8 mg, 0.0012 mmol, 0.6 mol%), $Rh(COD)_2BF_4$ (0.4 mg, 0.001 mmol, 0.5 mol%), and 2-methyltetrahydrofuran (0.8 mL, 2-MeTHF). After the reaction mixture was stirred at room temperature for 1 h, EtOH (2 mL) or EtOD (2 mL, 99.5% D) was added to the reaction and stirred for an additional 10 min at room temperature. Then the catalyst solution was cooled to –40 ºC and stirred for another 30 min. The disubstituted 2-cyanoacetic acid (0.2 mmol, 100 mol%) was dissolved in 2-MeTHF (1 mL) in another tube and cooled to –40 ºC for 10 min, which was added to the catalyst tube dropwise via syringe, and the substrate tube was washed with 2-MeTHF (0.2 mL) to transfer the residue to the reaction. The reaction mixture was stirred at –40 ºC for 1-48 h. After the reaction mixture was warmed to room temperature, the solvent was removed under vacuum and the filtrate

was purified by flash column chromatography (hexanes/ethyl acetate) to obtain the decarboxylation product.

Section 7. Characterization data of decarboxylation products

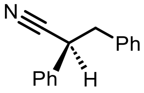

(–)-**1**

zwf-7-058

(−)-**1** was obtained as a white solid from the asymmetric decarboxylative protonation reaction of 2-cyanoacetic acid **S1** (125.7 mg, 0.5 mmol) for 2 h using the general procedure (102.2 mg, 99% Yield). R_f = 0.4 (Hexane/EtOAc 10:1).

HPLC analysis (Chiralpak IE-3, hexane/^{i}PrOH = 99/1, 0.8 mL/min, 205 nm; t_r (minor) = 17.06 min, t_r (major) = 18.85 min) gave the isomeric composition of the product: 96:4 e.r., $[\alpha]_D^{25}$ = –5.5 (c = 0.78, $CHCl_3$).

^{1}H NMR (400 MHz, $CDCl_3$) δ 7.42-7.18 (m, 8H), 7.12 (d, J = 6.8 Hz, 2H), 3.97 (t, J = 7.2 Hz, 1H), 3.24-3.05 (m, 2H).

^{13}C NMR (100 MHz, $CDCl_3$) δ 136.2, 135.1, 129.1, 128.9, 128.5, 128.1, 127.4, 127.3, 120.3, 42.0, 39.6.

IR (neat, cm^{-1}) 3030, 2932, 2242, 1604, 1454.

HRMS (ESI) calcd $C_{15}H_{14}N^+$ $[M+H]^+$: 208.1121. Found: 208.1122.

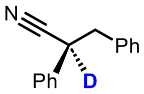

(–)-**1**-d

zwf-7-151

(−)-**1**-d was obtained as a white solid from the asymmetric decarboxylative deuteration reaction of 2-cyanoacetic acid **S1** (50.3 mg, 0.2 mmol) for 2 h using the general procedure (40.6 mg, 97% Yield, 94% D). R_f = 0.4 (Hexane/EtOAc 10:1).

HPLC analysis (Chiralpak IE-3, hexane/^{i}PrOH = 99/1, 0.8 mL/min, 205 nm; t_r (minor) = 18.93 min, t_r (major) = 21.03 min) gave the isomeric composition of the product: 96:4 e.r., $[\alpha]_D^{20}$ = –5.6 (c = 0.69, $CHCl_3$).

^{1}H NMR (400 MHz, $CDCl_3$) δ 7.41-7.19 (m, 8H), 7.17-7.08 (m, 2H), 3.99 (t, J = 7.4 Hz, 0.06H), 3.22-3.08 (m, 2H).

^{13}C NMR (100 MHz, $CDCl_3$) δ 136.2, 135.1, 129.2, 129.0, 128.6, 128.1, 127.4, 127.3, 120.3, 42.0, 39.4 (t, J = 21.0 Hz).

IR (neat, cm^{-1}) 3062, 2934, 2244, 1598, 1452, 1076.

HRMS (ESI) calcd $C_{15}H_{13}DN^+$ $[M+H]^+$: 209.1184. Found: 209.1185.

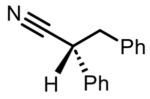

(+)-**1**

zwf-7-060

(+)-**1** was obtained as white solid from the asymmetric decarboxylative protonation reaction of 2-cyanoacetic acid **S1** (125.7 mg, 0.5 mmol) for 2 h using the general procedure (102.1 mg, 98% Yield). R_f = 0.4 (Hexane/EtOAc 10:1).

HPLC analysis (Chiralpak IE-3, hexane/^{i}PrOH = 99/1, 0.8 mL/min, 205 nm; t_r (major) = 17.21 min, t_r (minor) = 19.21 min) gave the isomeric composition of the product: 96:4 e.r., $[\alpha]_D^{25}$ = +4.2 (c = 0.71, $CHCl_3$).

^{1}H NMR (400 MHz, $CDCl_3$) δ 7.41-7.18 (m, 8H), 7.12 (d, J = 6.4 Hz, 2H), 3.98 (t, J = 7.2 Hz, 1H), 3.24-3.04 (m, 2H).

^{13}C NMR (100 MHz, $CDCl_3$) δ 136.2, 135.1, 129.1, 128.9, 128.5, 128.1, 127.4, 127.3, 120.3, 42.1, 39.7.

IR (neat, cm^{-1}) 3064, 2923, 2243, 1598, 1454, 1073.

HRMS (ESI) calcd $C_{15}H_{14}N^+$ $[M+H]^+$: 208.1121. Found: 208.1120.

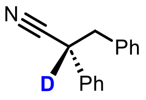

(+)-**1**-d

zwf-8-046

(+)-**1**-d was obtained as white solid from the asymmetric decarboxylative deuteration reaction of 2-cyanoacetic acid **S1** (50.2 mg, 0.2 mmol) for 2 h using the general procedure (40.9 mg, 98% Yield, 94% D). R_f = 0.4 (Hexane/EtOAc 10:1).

HPLC analysis (Chiralpak IE-3, hexane/^{i}PrOH = 99/1, 0.8 mL/min, 205 nm; t_r (major) = 20.66 min, t_r (minor) = 23.35 min) gave the isomeric composition of the product: 96:4 e.r., $[\alpha]_D^{19}$ = +5.3 (c = 0.55, $CHCl_3$).

^{1}H NMR (400 MHz, $CDCl_3$) δ 7.48-7.18 (m, 8H), 7.18-06 (m, 2H), 3.98 (t, J = 7.4 Hz, 0.06H), 3.26-3.00 (m, 2H).

^{13}C NMR (100 MHz, $CDCl_3$) δ 136.2, 135.1, 129.2, 129.0, 128.6, 128.2, 127.4, 127.3, 120.3, 42.0, 39.4 (t, J = 20.6 Hz).

IR (neat, cm^{-1}) 3061, 2928, 2243, 1594, 1446, 1082.

HRMS (ESI) calcd $C_{15}H_{13}DN^+$ $[M+H]^+$: 209.1184. Found: 209.1182.

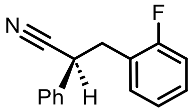

(+)-**7**

zwf-7-111

(+)-**7** was obtained as a white solid from the asymmetric decarboxylative protonation reaction of 2-cyanoacetic acid **S7** (134.6 mg, 0.5 mmol) for 6 h using the general procedure (c = 0.005, referring to the concentration of **S7** in 2-MeTHF) (105.4 mg, 94% Yield). R_f = 0.4 (Hexane/EtOAc 10:1).

HPLC analysis (Chiralpak IE-3, hexane/^{i}PrOH = 99/1, 0.8 mL/min, 205 nm; t_r (minor) = 16.14 min, t_r (major) = 17.76 min) gave the isomeric composition of the product: 95:5 e.r., $[\alpha]_D^{25}$ = +11.0 (c = 0.50, $CHCl_3$).

^{1}H NMR (400 MHz, $CDCl_3$) δ 7.41-7.21 (m, 6H), 7.15 (t, J = 6.8 Hz, 1H), 7.11-6.99 (m, 2H), 4.07 (t, J = 7.8 Hz, 1H), 3.30-3.09 (m, 2H).

^{13}C NMR (100 MHz, $CDCl_3$) δ 161.1 (d, J = 244.8 Hz), 135.1, 131.5 (d, J = 4.3 Hz), 129.3 (d, J = 8.0 Hz), 129.0, 128.2, 127.3, 124.3 (d, J = 3.6 Hz), 123.5 (d, J = 15.2 Hz), 120.1, 115.4 (d, J = 21.0 Hz), 38.2 (d, J = 1.4 Hz), 35.9 (d, J = 1.4 Hz).

^{19}F NMR (376 MHz, $CDCl_3$) δ −118.1 to −118.3 (m).

IR (neat, cm^{-1}) 3054, 2986, 2277, 1421, 1264, 1062.

HRMS (ESI) calcd $C_{15}H_{13}FN^+$ $[M+H]^+$: 226.1027. Found: 226.1025.

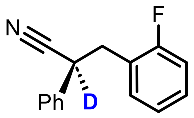

(+)-**7**-d

zwf-8-038

(+)-**7**-d was obtained as white solid from the asymmetric decarboxylative deuteration reaction of 2-cyanoacetic acid **S7** (54.0 mg, 0.2 mmol) for 6 h using the general procedure (c = 0.005, referring to the concentration of **S7** in 2-MeTHF) (42.5 mg, 94% Yield, 93% D). R_f = 0.4 (Hexane/EtOAc 10:1).

HPLC analysis (Chiralpak IE-3, hexane/^{i}PrOH = 99/1, 0.8 mL/min, 205 nm; t_r (minor) = 18.36 min, t_r (major) = 20.24 min) gave the isomeric composition of the product: 95:5 e.r., $[\alpha]_D^{20}$ = +14.9 (*c* = 0.76, $CHCl_3$).

^{1}H NMR (400 MHz, $CDCl_3$) δ 7.44-7.20 (m, 6H), 7.18-7.10 (m, 1H), 7.10-6.99 (m, 2H), 4.07 (t, *J* = 7.8 Hz, 0.07H), 3.28-3.10 (m, 2H).

^{13}C NMR (100 MHz, $CDCl_3$) δ 161.1 (d, *J* = 244.1 Hz), 135.0, 131.5 (d, *J* = 4.3 Hz), 129.4 (d, *J* = 7.8 Hz), 129.0, 128.3, 127.3, 124.3 (d, *J* = 3.6 Hz), 123.4 (d, *J* = 15.2 Hz), 120.1, 115.4 (d, *J* = 21.7 Hz), 37.9 (t, *J* = 21.3 Hz), 35.8 (d, *J* = 1.5 Hz).

^{19}F NMR (376 MHz, $CDCl_3$) δ −118.1 to −118.3 (m).

IR (neat, cm^{-1}) 3055, 2987, 2268, 1493, 1264, 1063.

HRMS (ESI) calcd $C_{15}H_{12}DFN^+$ $[M+H]^+$: 227.1089. Found: 227.1087.

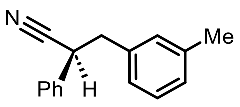

(−)-**8**

zwf-7-103

(−)-**8** was obtained as oil from the asymmetric decarboxylative protonation reaction of 2-cyanoacetic acid **S8** (132.5 mg, 0.5 mmol) for 2 h using the general procedure (102.6 mg, 93% Yield). R_f = 0.4 (Hexane/EtOAc 10:1).

HPLC analysis (Chiralpak IE-3, hexane/^{i}PrOH = 99/1, 0.8 mL/min, 205 nm; t_r (minor) = 16.15 min, t_r (major) = 17.77 min) gave the isomeric composition of the product: 95:5 e.r., $[\alpha]_D^{23}$ = –2.6 (*c* = 0.66,

$CHCl_3$).

^{1}H NMR (400 MHz, $CDCl_3$) δ 7.40-7.23 (m, 5H), 7.17 (t, J = 7.6 Hz, 1H), 7.06 (d, J = 7.6 Hz, 1H), 6.99-6.86 (m, 2H), 3.97 (t, J = 7.4 Hz, 1H), 3.18-3.02 (m, 2H), 2.30 (s, 3H).

^{13}C NMR (100 MHz, $CDCl_3$) δ 138.2, 136.2, 135.3, 129.9, 128.9, 128.4, 128.1, 128.0, 127.4, 126.1, 120.4, 42.1, 39.7, 21.3.

IR (neat, cm^{-1}) 3038, 2925, 2242, 1609, 1454, 1078.

HRMS (ESI) calcd $C_{16}H_{16}N^{+}$ $[M+H]^{+}$: 222.1277. Found: 222.1276.

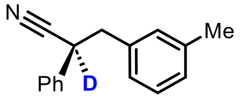

(–)-**8**-d

zwf-8-018

(–)-**8**-d was obtained as oil from the asymmetric decarboxylative deuteration reaction of 2-cyanoacetic acid **S8** (53.3 mg, 0.2 mmol) for 2 h using the general procedure (41.0 mg, 92% Yield, 95% D). R_f = 0.4 (Hexane/EtOAc 10:1).

HPLC analysis (Chiralpak IE-3, hexane/^{i}PrOH = 99/1, 0.8 mL/min, 205 nm; t_r (minor) = 17.35 min, t_r (major) = 19.15 min) gave the isomeric composition of the product: 96:4 e.r., $[\alpha]_D^{20}$ = –2.8 (c = 0.62, $CHCl_3$).

^{1}H NMR (400 MHz, $CDCl_3$) δ 7.44-7.23 (m, 5H), 7.18 (t, J = 7.6 Hz, 1H), 7.07 (d, J = 7.6 Hz, 1H), 7.00-6.87 (m, 2H), 3.97 (t, J = 7.4 Hz, 0.05H), 3.18-3.02 (m, 2H), 2.31 (s, 3H).

^{13}C NMR (100 MHz, $CDCl_3$) δ 138.2, 136.2, 135.3, 129.9, 128.9, 128.4, 128.12, 128.06, 127.4, 126.1, 120.4, 42.0, 39.5 (t, J = 20.6 Hz), 21.3.

IR (neat, cm^{-1}) 3030, 2923, 2247, 1609, 1454, 1077.

HRMS (ESI) calcd $C_{16}H_{15}DN^{+}$ $[M+H]^{+}$: 223.1340. Found: 223.1338.

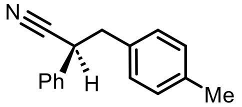

(–)-**9**

zwf-7-088

(−)-**9** was obtained as oil from the asymmetric decarboxylative protonation reaction of 2-cyanoacetic acid **S9** (132.7 mg, 0.5 mmol) for 2 h using the general procedure (104.4 mg, 94% Yield). R_f = 0.4 (Hexane/EtOAc 10:1).

HPLC analysis (Chiralpak IE-3, hexane/^{i}PrOH = 99/1, 0.8 mL/min, 205 nm; t_r (minor) = 15.59 min, t_r (major) = 16.71 min) gave the isomeric composition of the product: 95.5:4.5 e.r., $[\alpha]_D^{25}$ = −7.0 (*c* = 0.55, $CHCl_3$).

^{1}H NMR (400 MHz, $CDCl_3$) δ 7.40-7.21 (m, 5H), 7.09 (d, *J* = 7.6 Hz, 2H), 7.02 (d, *J* = 8.0 Hz, 2H), 3.96 (t, *J* = 7.4 Hz, 1H), 3.18-3.03 (m, 2H), 2.31 (s, 3H).

^{13}C NMR (100 MHz, $CDCl_3$) δ 136.9, 135.3, 133.2, 129.2, 129.0, 128.9, 128.1, 127.4, 120.4, 41.8, 39.9, 21.0.

IR (neat, cm^{-1}) 3033, 2920, 2242, 1609, 1454, 1076.

HRMS (ESI) calcd $C_{16}H_{16}N^+$ $[M+H]^+$: 222.1277. Found: 222.1279.

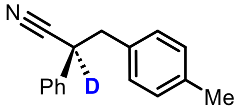

(−)-**9**-d

zwf-8-019

(−)-**9**-d was obtained as oil from the asymmetric decarboxylative deuteration reaction of 2-cyanoacetic acid **S9** (53.1 mg, 0.2 mmol) for 2 h using the general procedure (40.7 mg, 91% Yield, 96% D). R_f = 0.4 (Hexane/EtOAc 10:1).

HPLC analysis (Chiralpak IE-3, hexane/^{i}PrOH = 99/1, 0.8 mL/min, 205 nm; t_r (minor) = 19.07 min, t_r (major) = 20.58 min) gave the isomeric composition of the product: 97:3 e.r., $[\alpha]_D^{20}$ = −5.5 (*c* = 0.40, $CHCl_3$).

^{1}H NMR (400 MHz, $CDCl_3$) δ 7.44-7.18 (m, 5H), 7.09 (d, *J* = 7.6 Hz, 2H), 7.02 (d, *J* = 7.6 Hz, 2H), 3.96 (t, *J* = 7.0 Hz, 0.04H), 3.21-3.00 (m, 2H), 2.31 (s, 3H).

^{13}C NMR (100 MHz, $CDCl_3$) δ 136.9, 135.3, 133.2, 129.2, 129.0, 128.9, 128.1, 127.4, 120.4, 41.7, 39.5 (t, *J* = 21.0 Hz), 21.0.

IR (neat, cm^{-1}) 3033, 2923, 2242, 1608, 1454, 1077.

HRMS (ESI) calcd $C_{16}H_{15}DN^+$ $[M+H]^+$: 223.1340. Found: 223.1337.

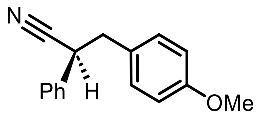

(−)-**10**

zwf-7-064

(−)-**10** was obtained as white solid from the asymmetric decarboxylative protonation reaction of 2-cyanoacetic acid **S10** (140.5 mg, 0.5 mmol) for 2 h using the general procedure (c = 0.025, referring to the concentration of **S10** in 2-MeTHF) (108.3 mg, 91% Yield). R_f = 0.3 (Hexane/EtOAc 10:1).

HPLC analysis (Chiralpak IE-3, hexane/^{i}PrOH = 99/1, 0.8 mL/min, 205 nm; t_r (minor) = 30.51 min, t_r (major) = 34.72 min) gave the isomeric composition of the product: 96.5:3.5 e.r., $[\alpha]_D^{25}$ = −7.2 (*c* = 0.53, $CHCl_3$).

^{1}H NMR (400 MHz, $CDCl_3$) δ 7.40-7.27 (m, 3H), 7.26-7.22 (m, 2H), 7.04 (d, *J* = 8.8 Hz, 2H), 6.82 (d, *J* = 8.4 Hz, 2H), 3.95 (t, *J* = 7.4 Hz, 1H), 3.77 (s, 3H), 3.17-3.02 (m, 2H).

^{13}C NMR (100 MHz, $CDCl_3$) δ 158.8, 135.2, 130.2, 128.9, 128.2, 128.1, 127.5, 120.4, 113.9, 55.2, 41.3, 40.0.

IR (neat, cm^{-1}) 3055, 2987, 2257, 1421, 1264.

HRMS (ESI) calcd $C_{16}H_{16}NO^+$ $[M+H]^+$: 238.1226. Found: 238.1227.

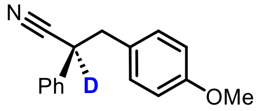

(−)-**10**-d

zwf-8-020

(−)-**10**-d was obtained as white solid from the asymmetric decarboxylative deuteration reaction of 2-cyanoacetic acid **S10** (56.3 mg, 0.2 mmol) for 5 h using the general procedure (43.1 mg, 90% Yield, 95% D). R_f = 0.3 (Hexane/EtOAc 10:1).

HPLC analysis (Chiralpak IE-3, hexane/^{i}PrOH = 99/1, 0.8 mL/min, 205 nm; t_r (minor) = 37.02 min, t_r (major) = 41.95 min) gave the isomeric composition of the product: 97:3 e.r., $[\alpha]_D^{20}$ = −7.6 (*c* = 0.86, $CHCl_3$).

^{1}H NMR (400 MHz, $CDCl_3$) δ 7.44-7.27 (m, 3H), 7.27-7.18 (m, 2H), 7.03 (d, *J* = 7.6 Hz, 2H), 6.81 (d, *J* = 7.6 Hz, 2H), 3.95 (t, *J* = 7.0 Hz, 0.05H), 3.77 (s, 3H), 3.19-2.98 (m, 2H).

^{13}C NMR (100 MHz, $CDCl_3$) δ 158.8, 135.2, 130.2, 128.9, 128.2, 128.1, 127.4, 120.4, 113.9, 55.2, 41.2, 39.7 (t, *J* = 20.6 Hz).

IR (neat, cm^{-1}) 3058, 2987, 2258, 1422, 1265.

HRMS (ESI) calcd $C_{16}H_{15}DNO^+$ $[M+H]^+$: 239.1289. Found: 239.1292.

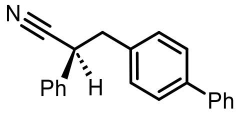

(–)-**11**

zwf-7-065

(–)-**11** was obtained as white solid from the asymmetric decarboxylative protonation reaction of 2-cyanoacetic acid **S11** (163.6 mg, 0.5 mmol) for 2 h using the general procedure (c = 0.025, referring to the concentration of **S11** in 2-MeTHF) (136.2 mg, 96% Yield). R_f = 0.3 (Hexane/EtOAc 10:1).

HPLC analysis (Chiralpak IE-3, hexane/^{i}PrOH = 99/1, 0.8 mL/min, 205 nm; t_r (minor) = 26.71 min, t_r (major) = 35.02 min) gave the isomeric composition of the product: 95:5 e.r., $[\alpha]_D^{25}$ = –6.2 (*c* = 0.30, $CHCl_3$).

^{1}H NMR (400 MHz, $CDCl_3$) δ 7.56 (d, *J* = 7.2 Hz, 2H), 7.51 (d, *J* = 8.0 Hz, 2H), 7.42 (t, *J* = 7.6 Hz, 2H), 7.38-7.23 (m, 6H), 7.19 (d, *J* = 8.0 Hz, 2H), 4.01 (t, *J* = 7.4 Hz, 1H), 3.27-3.09 (m, 2H).

^{13}C NMR (100 MHz, $CDCl_3$) δ 140.5, 140.2, 135.2, 135.1, 129.6, 129.0, 128.7, 128.2, 127.4, 127.3, 127.2, 127.0, 120.3, 41.7, 39.7.

IR (neat, cm^{-1}) 3055, 2987, 2244, 1421, 1264.

HRMS (ESI) calcd $C_{21}H_{18}N^+$ $[M+H]^+$: 284.1434. Found: 284.1430.

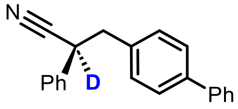

(–)-**11**-d

zwf-8-021

(–)-**11**-d was obtained as a white solid from the asymmetric decarboxylative deuteration reaction of 2-cyanoacetic acid **S11** (65.5 mg, 0.2 mmol) for 2 h using the general procedure (52.2 mg, 92% Yield, 94% D). R_f = 0.3 (Hexane/EtOAc 10:1).

HPLC analysis (Chiralpak IE-3, hexane/^{i}PrOH = 99/1, 0.8 mL/min, 205 nm; t_r (minor) = 35.77 min, t_r (major) = 47.82 min) gave the isomeric composition of the product: 96:4 e.r., $[\alpha]_D^{20}$ = –5.9 (*c* = 0.52, $CHCl_3$).

^{1}H NMR (400 MHz, $CDCl_3$) δ 7.57 (d, J = 7.2 Hz, 2H), 7.52 (d, J = 7.6 Hz, 2H), 7.43 (t, J = 7.2 Hz, 2H), 7.39-7.23 (m, 6H), 7.20 (d, J = 7.2 Hz, 2H), 4.03 (t, J = 7.4 Hz, 0.06H), 3.29-3.09 (m, 2H).

^{13}C NMR (100 MHz, $CDCl_3$) δ 140.6, 140.2, 135.2, 135.1, 129.6, 129.0, 128.8, 128.2, 127.5, 127.32, 127.28, 127.0, 120.3, 41.7, 39.4 (t, J = 20.6 Hz).

IR (neat, cm^{-1}) 3038, 2976, 2247, 1487, 1247.

HRMS (ESI) calcd $C_{21}H_{17}DN^{+}$ $[M+H]^{+}$: 285.1497. Found: 285.1495.

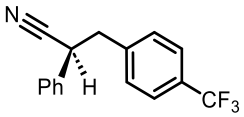

(–)-**12**

zwf-7-070

(–)-**12** was obtained as a white solid from the asymmetric decarboxylative protonation reaction of 2-cyanoacetic acid **S12** (159.8 mg, 0.5 mmol) for 2 h using the general procedure (127.6 mg, 93% Yield). R_f = 0.4 (Hexane/EtOAc 10:1).

HPLC analysis (Chiralpak IE-3, hexane/^{i}PrOH = 99/1, 0.8 mL/min, 205 nm; t_r (minor) = 12.54 min, t_r (major) = 13.55 min) gave the isomeric composition of the product: 95.5:4.5 e.r., $[\alpha]_D^{25}$ = –10.7 (c = 0.33, $CHCl_3$).

^{1}H NMR (400 MHz, $CDCl_3$) δ 7.54 (d, J = 8.0 Hz, 2H), 7.42-7.28 (m, 3H), 7.29-7.17 (m, 4H), 4.03 (t, J = 7.0 Hz, 1H), 3.31-3.12 (m, 2H).

^{13}C NMR (100 MHz, $CDCl_3$) δ 140.1, 134.5, 129.7 (q, J = 32.3 Hz), 129.6, 129.1, 128.4, 127.4, 125.5 (q, J = 3.6 Hz), 124.0 (q, J = 270.3 Hz), 119.9, 41.7, 39.3.

^{19}F NMR (376 MHz, $CDCl_3$) δ –62.5 (s).

IR (neat, cm^{-1}) 3068, 2944, 2241, 1456, 1321, 1107.

HRMS (ESI) calcd $C_{16}H_{13}F_3N^{+}$ $[M+H]^{+}$: 276.0995. Found: 276.0998.

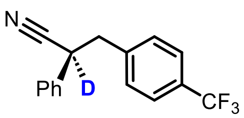

(–)-**12**-d

zwf-8-027

(–)-**12**-d was obtained as a white solid from the asymmetric decarboxylative deuteration reaction of 2-cyanoacetic acid **S12** (63.9 mg, 0.2 mmol) for 2 h using the general procedure (50.8 mg, 92% Yield, 91% D). R_f = 0.4 (Hexane/EtOAc 10:1).

HPLC analysis (Chiralpak IE-3, hexane/^{i}PrOH = 99/1, 0.8 mL/min, 205 nm; t_r (minor) = 15.57 min, t_r (major) = 16.96 min) gave the isomeric composition of the product: 95:5 e.r., $[\alpha]_D^{20}$ = –11.1 (*c* = 0.48, $CHCl_3$).

^{1}H NMR (400 MHz, $CDCl_3$) δ 7.55 (d, *J* = 7.6 Hz, 2H), 7.45-7.30 (m, 3H), 7.30-7.10 (m, 4H), 4.03 (t, *J* = 6.6 Hz, 0.09H), 3.30-3.09 (m, 2H).

^{13}C NMR (100 MHz, $CDCl_3$) δ 140.1, 134.5, 129.7 (q, *J* = 32.3 Hz), 129.6, 129.1, 128.4, 127.4, 125.5 (q, *J* = 3.6 Hz), 124.0 (q, *J* = 270.3 Hz), 119.9, 41.6, 39.0 (t, *J* = 20.9 Hz).

^{19}F NMR (376 MHz, $CDCl_3$) δ –62.5 (s).

IR (neat, cm^{-1}) 3067, 2944, 2242, 1420, 1320, 1119.

HRMS (ESI) calcd $C_{16}H_{12}DF_3N^+$ $[M+H]^+$: 277.1057. Found: 277.1055.

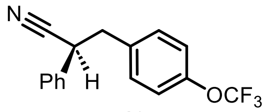

(–)-**13**

zwf-7-122

(–)-**13** was obtained as a white solid from the asymmetric decarboxylative protonation reaction of 2-cyanoacetic acid **S13** (167.7 mg, 0.5 mmol) for 3 h using the general procedure (135.1 mg, 93% Yield). R_f = 0.3 (Hexane/EtOAc 10:1).

HPLC analysis (Chiralpak IE-3, hexane/^{i}PrOH = 99/1, 0.8 mL/min, 205 nm; t_r (minor) = 12.25 min, t_r (major) = 12.62 min) gave the isomeric composition of the product: 95:5 e.r., $[\alpha]_D^{21}$ = –9.2 (*c* = 0.44, $CHCl_3$).

^{1}H NMR (400 MHz, $CDCl_3$) δ 7.41-7.29 (m, 3H), 7.27-7.20 (m, 2H), 7.18-7.08 (m, 4H), 4.00 (t, *J* = 7.2 Hz, 1H), 3.24-3.09 (m, 2H).

^{13}C NMR (100 MHz, $CDCl_3$) δ 148.5 (q, *J* = 1.4 Hz), 134.8, 134.7, 130.6, 129.1, 128.4, 127.4, 121.0, 120.4 (q, *J* = 255.7 Hz), 120.0, 41.3, 39.5.

^{19}F NMR (376 MHz, $CDCl_3$) δ –57.9 (s).

IR (neat, cm^{-1}) 3038, 2929, 2242, 1270, 1147.

HRMS (ESI) calcd $C_{16}H_{13}F_3NO^+$ $[M+H]^+$: 292.0944. Found: 292.0944.

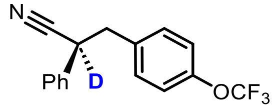

(−)-**13**-d

zwf-8-029

(−)-**13**-d was obtained as a white solid from the asymmetric decarboxylative deuteration reaction of 2-cyanoacetic acid **S13** (67.2 mg, 0.2 mmol) for 3 h using the general procedure (53.1 mg, 91% Yield, 91% D). R_f = 0.3 (Hexane/EtOAc 10:1).

HPLC analysis (Chiralpak IE-3, hexane/^{i}PrOH = 99/1, 0.8 mL/min, 205 nm; t_r (minor) = 13.99 min, t_r (major) = 14.41 min) gave the isomeric composition of the product: 95.5:4.5 e.r., $[\alpha]_D^{20}$ = –9.2 (c = 0.51, $CHCl_3$).

^{1}H NMR (400 MHz, $CDCl_3$) δ 7.45-7.29 (m, 3H), 7.28-7.20 (m, 2H), 7.18-7.04 (m, 4H), 4.00 (t, J = 7.2 Hz, 0.09H), 3.26-3.06 (m, 2H).

^{13}C NMR (100 MHz, $CDCl_3$) δ 148.5 (q, J = 1.5 Hz), 134.8, 134.7, 130.6, 129.1, 128.4, 127.4, 121.0, 120.4 (q, J = 255.7 Hz), 120.0, 41.2, 39.2 (t, J = 21.0 Hz).

^{19}F NMR (376 MHz, $CDCl_3$) δ –57.9 (s).

IR (neat, cm^{-1}) 3038, 2925, 2242, 1269, 1144.

HRMS (ESI) calcd $C_{16}H_{12}DF_3NO^+$ $[M+H]^+$: 293.1007. Found: 293.1010.

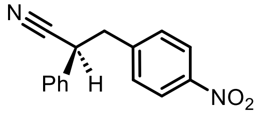

(−)-**14**

zwf-7-072

(−)-**14** was obtained as a white solid from the asymmetric decarboxylative protonation reaction of 2-cyanoacetic acid **S14** (148.2 mg, 0.5 mmol) for 6 h using the general procedure (113.9 mg, 90% Yield). R_f = 0.3 (Hexane/EtOAc 5:1).

HPLC analysis (Chiralpak IE-3, hexane/^{i}PrOH = 90/10, 0.8 mL/min, 205 nm; t_r (minor) = 27.33 min, t_r (major) = 29.33 min) gave the isomeric composition of the product: 81.5:18.5 e.r., $[\alpha]_D^{23}$ = –13.0 (c = 0.40, $CHCl_3$).

^{1}H NMR (400 MHz, $CDCl_3$) δ 8.15 (d, J = 8.8 Hz, 2H), 7.44-7.33 (m, 3H), 7.30-7.22 (m, 4H), 4.08 (t, J = 7.0 Hz, 1H), 3.35-3.24 (m, 2H).

^{13}C NMR (100 MHz, $CDCl_3$) δ 147.3, 143.4, 134.0, 130.2, 129.2, 128.6, 127.3, 123.7, 119.6, 41.5, 38.9.

IR (neat, cm^{-1}) 3072, 2929, 2242, 1272, 1197.

HRMS (ESI) calcd $C_{15}H_{13}N_2O_2^+$ $[M+H]^+$: 253.0972. Found: 253.0970.

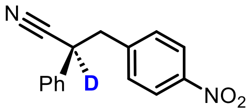

(–)-**14**-d

zwf-8-030

(–)-**14**-d was obtained as a white solid from the asymmetric decarboxylative deuteration reaction of 2-cyanoacetic acid **S14** (59.5 mg, 0.2 mmol) for 6 h using the general procedure (44.7 mg, 88% Yield, 90% D). R_f = 0.3 (Hexane/EtOAc 5:1).

HPLC analysis (Chiralpak IE-3, hexane/^{i}PrOH = 90/10, 0.8 mL/min, 205 nm; t_r (minor) = 31.97 min, t_r (major) = 33.80 min) gave the isomeric composition of the product: 92:8 e.r., $[\alpha]_D^{20}$ = –16.3 (c = 0.40, $CHCl_3$).

^{1}H NMR (400 MHz, $CDCl_3$) δ 8.15 (d, J = 8.4 Hz, 2H), 7.50-7.31 (m, 3H), 7.31-7.14 (m, 4H), 4.09 (t, J = 7.2 Hz, 0.10H), 3.37-3.18 (m, 2H).

^{13}C NMR (100 MHz, $CDCl_3$) δ 147.3, 143.3, 134.0, 130.3, 129.2, 128.6, 127.4, 123.8, 119.6, 41.5, 38.6 (t, J = 20.9 Hz).

IR (neat, cm^{-1}) 3078, 2932, 2242, 1273, 1191.

HRMS (ESI) calcd $C_{15}H_{12}DN_2O_2^+$ $[M+H]^+$: 254.1034. Found: 254.1038.

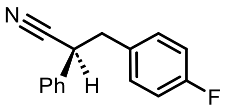

(–)-**15**

zwf-7-067

(–)-**15** was obtained as a white solid from the asymmetric decarboxylative protonation reaction of 2-cyanoacetic acid **S15** (134.8 mg, 0.5 mmol) for 2 h using the general procedure (109.7 mg, 97% Yield). R_f = 0.3 (Hexane/EtOAc 10:1).

HPLC analysis (Chiralpak IE-3, hexane/^{i}PrOH = 99/1, 0.8 mL/min, 205 nm; t_r (minor) = 17.50 min, t_r (major) = 18.72 min) gave the isomeric composition of the product: 96:4 e.r., $[\alpha]_D^{25}$ = –12.1 (*c* = 0.46, $CHCl_3$).

^{1}H NMR (400 MHz, $CDCl_3$) δ 7.41-7.28 (m, 3H), 7.27-7.19 (m, 2H), 7.11-7.03 (m, 2H), 7.02-6.90 (m, 2H), 3.98 (t, *J* = 7.0 Hz, 1H), 3.22-3.04 (m, 2H).

^{13}C NMR (100 MHz, $CDCl_3$) δ 162.1 (d, *J* = 244.1 Hz), 134.8, 131.8 (d, *J* = 2.9 Hz), 130.8 (d, *J* = 7.9 Hz), 129.0, 128.3, 127.4, 120.1, 115.4 (d, *J* = 20.9 Hz), 41.2, 39.7.

^{19}F NMR (376 MHz, $CDCl_3$) δ –115.0 to –115.1 (m).

IR (neat, cm^{-1}) 3055, 2980, 2255, 1511, 1263.

HRMS (ESI) calcd $C_{15}H_{13}FN^+$ $[M+H]^+$: 226.1027. Found: 226.1028.

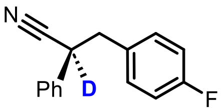

(–)-**15**-d

zwf-8-023

(–)-**15**-d was obtained as a white solid from the asymmetric decarboxylative deuteration reaction of 2-cyanoacetic acid **S15** (54.0 mg, 0.2 mmol) for 2 h using the general procedure (43.7 mg, 96% Yield, 94% D). R_f = 0.3 (Hexane/EtOAc 10:1).

HPLC analysis (Chiralpak IE-3, hexane/^{i}PrOH = 99/1, 0.8 mL/min, 205 nm; t_r (minor) = 19.39 min, t_r (major) = 20.83 min) gave the isomeric composition of the product: 96:4 e.r., $[\alpha]_D^{20}$ = –12.0 (*c* = 0.60, $CHCl_3$).

^{1}H NMR (400 MHz, $CDCl_3$) δ 7.43-7.28 (m, 3H), 7.28-7.18 (m, 2H), 7.12-7.02 (m, 2H), 7.01-6.90 (m, 2H), 3.98 (t, *J* = 7.4 Hz, 0.06H), 3.22-3.02 (m, 2H).

^{13}C NMR (100 MHz, $CDCl_3$) δ 162.1 (d, *J* = 244.1 Hz), 134.8, 131.8 (d, *J* = 2.9 Hz), 130.8 (d, *J* = 7.9 Hz), 129.0, 128.2, 127.4, 120.1, 115.4 (d, *J* = 20.9 Hz), 41.1, 39.4 (t, *J* = 20.6 Hz).

^{19}F NMR (376 MHz, $CDCl_3$) δ –115.0 to –115.1 (m).

IR (neat, cm^{-1}) 3058, 2989, 2247, 1510, 1265.

HRMS (ESI) calcd $C_{15}H_{12}DFN^+$ $[M+H]^+$: 227.1089. Found: 227.1091.

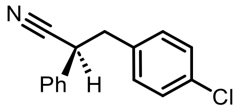

(−)-**16**

zwf-7-068

(−)-**16** was obtained as a white solid from the asymmetric decarboxylative protonation reaction of 2-cyanoacetic acid **S16** (142.9 mg, 0.5 mmol) for 2 h using the general procedure (115.1 mg, 95% Yield). R_f = 0.4 (Hexane/EtOAc 10:1).

HPLC analysis (Chiralpak IE-3, hexane/^{i}PrOH = 99/1, 0.8 mL/min, 205 nm; t_r (minor) = 17.46 min, t_r (major) = 18.97 min) gave the isomeric composition of the product: 96.5:3.5 e.r., $[\alpha]_D^{25}$ = –9.0 (*c* = 0.32, $CHCl_3$).

^{1}H NMR (400 MHz, $CDCl_3$) δ 7.41-7.29 (m, 3H), 7.28-7.18 (m, 4H), 7.03 (d, *J* = 8.4 Hz, 2H), 3.98 (t, *J* = 7.2 Hz, 1H), 3.21-3.04 (m, 2H).

^{13}C NMR (100 MHz, $CDCl_3$) δ 134.7, 134.5, 133.3, 130.6, 129.0, 128.7, 128.3, 127.4, 120.0, 41.3, 39.5.

IR (neat, cm^{-1}) 3058, 2988, 2242, 1492, 1265.

HRMS (ESI) calcd $C_{15}H_{13}ClN^+$ $[M+H]^+$: 242.0731. Found: 242.0734.

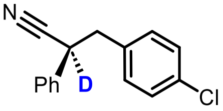

(−)-**16**-d

zwf-8-024

(−)-**16**-d was obtained as a white solid from the asymmetric decarboxylative deuteration reaction of 2-cyanoacetic acid **S16** (57.0 mg, 0.2 mmol) for 2 h using the general procedure (45.2 mg, 93% Yield, 93% D). R_f = 0.4 (Hexane/EtOAc 10:1).

HPLC analysis (Chiralpak IE-3, hexane/^{i}PrOH = 99/1, 0.8 mL/min, 205 nm; t_r (minor) = 20.13 min, t_r (major) = 21.90 min) gave the isomeric composition of the product: 96:4 e.r., $[\alpha]_D^{20}$ = –9.0 (*c* = 0.40, $CHCl_3$).

^{1}H NMR (400 MHz, $CDCl_3$) δ 7.48-7.28 (m, 3H), 7.28-7.15 (m, 4H), 7.03 (d, *J* = 7.6 Hz, 2H), 3.98 (t, *J* = 6.8 Hz, 0.07H), 3.24-3.01 (m, 2H).

^{13}C NMR (100 MHz, $CDCl_3$) δ 134.7, 134.5, 133.3, 130.6, 129.0, 128.7, 128.3, 127.4, 120.0, 41.3, 39.2 (t, *J* = 20.6 Hz).

IR (neat, cm^{-1}) 3031, 2923, 2244, 1490, 1249.

HRMS (ESI) calcd $C_{15}H_{12}DClN^+$ $[M+H]^+$: 243.0794. Found: 243.0798.

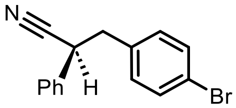

(–)-**17**

zwf-7-069

(–)-**17** was obtained as a white solid from the asymmetric decarboxylative protonation reaction of 2-cyanoacetic acid **S17** (165.0 mg, 0.5 mmol) for 2 h using the general procedure (137.2 mg, 96% Yield). R_f = 0.4 (Hexane/EtOAc 10:1).

HPLC analysis (Chiralpak IE-3, hexane/^{i}PrOH = 99/1, 0.8 mL/min, 205 nm; t_r (minor) = 18.83 min, t_r (major) = 20.61 min) gave the isomeric composition of the product: 96:4 e.r., $[\alpha]_D^{25}$ = –7.9 (c = 0.36, $CHCl_3$).

^{1}H NMR (400 MHz, $CDCl_3$) δ 7.45-7.27 (m, 5H), 7.26-7.19 (m, 2H), 6.98 (d, J = 8.4 Hz, 2H), 3.98 (t, J = 7.4 Hz, 1H), 3.20-3.04 (m, 2H).

^{13}C NMR (100 MHz, $CDCl_3$) δ 135.0, 134.7, 131.7, 130.9, 129.0, 128.3, 127.4, 121.4, 120.0, 41.4, 39.4.

IR (neat, cm^{-1}) 3029, 2922, 2244, 1488, 1011.

HRMS (ESI) calcd $C_{15}H_{13}BrN^+$ $[M+H]^+$: 286.0226. Found: 286.0228.

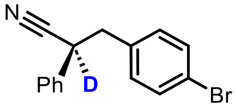

(–)-**17**-d

zwf-8-025

(–)-**17**-d was obtained as a white solid from the asymmetric decarboxylative deuteration reaction of 2-cyanoacetic acid **S17** (66.0 mg, 0.2 mmol) for 2 h using the general procedure (54.7 mg, 95% Yield, 93% D). R_f = 0.4 (Hexane/EtOAc 10:1).

HPLC analysis (Chiralpak IE-3, hexane/^{i}PrOH = 99/1, 0.8 mL/min, 205 nm; t_r (minor) = 21.99 min, t_r (major) = 24.12 min) gave the isomeric composition of the product: 95.5:4.5 e.r., $[\alpha]_D^{20}$ = –7.6 (c = 0.55, $CHCl_3$).

^{1}H NMR (400 MHz, $CDCl_3$) δ 7.48-7.26 (m, 5H), 7.27-7.18 (m, 2H), 6.98 (d, J = 7.6 Hz, 2H), 3.98 (t, J

= 6.8 Hz, 0.07H), 3.21-3.00 (m, 2H).

^{13}C NMR (100 MHz, $CDCl_3$) δ 135.0, 134.6, 131.7, 130.9, 129.1, 128.3, 127.4, 121.4, 120.0, 41.3, 39.1 (t, J = 20.6 Hz).

IR (neat, cm^{-1}) 3030, 2922, 2243, 14887, 1010.

HRMS (ESI) calcd $C_{15}H_{12}DBrN^+$ $[M+H]^+$: 287.0289. Found: 287.0290.

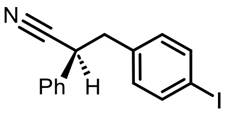

(−)-**18**

zwf-7-104

(−)-**18** was obtained as a white solid from the asymmetric decarboxylative protonation reaction of 2-cyanoacetic acid **S18** (188.6 mg, 0.5 mmol) for 2 h using the general procedure (c = 0.025 refer to 2-MeTHF) (160.2 mg, 96% Yield). R_f = 0.4 (Hexane/EtOAc 10:1).

HPLC analysis (Chiralpak IE-3, hexane/^{i}PrOH = 99/1, 0.8 mL/min, 205 nm; t_r (minor) = 21.37 min, t_r (major) = 23.96 min) gave the isomeric composition of the product: 95.5:4.5 e.r., $[\alpha]_D^{22}$ = −5.0 (c = 0.78, $CHCl_3$).

^{1}H NMR (400 MHz, $CDCl_3$) δ 7.61 (d, J = 8.4 Hz, 2H), 7.43-7.29 (m, 3H), 7.28-7.20 (m, 2H), 6.86 (d, J = 8.4 Hz, 2H), 3.98 (t, J = 7.2 Hz, 1H), 3.18-3.02 (m, 2H).

^{13}C NMR (100 MHz, $CDCl_3$) δ 137.7, 135.7, 134.7, 131.2, 129.1, 128.3, 127.4, 120.0, 93.0, 41.5, 39.4.

IR (neat, cm^{-1}) 3026, 2927, 2243, 1485, 1006.

HRMS (ESI) calcd $C_{15}H_{13}IN^+$ $[M+H]^+$: 334.0087. Found: 334.0083.

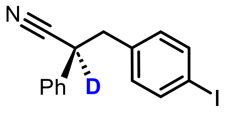

(−)-**18**-d

zwf-8-026

(−)-**18**-d was obtained as a white solid from the asymmetric decarboxylative deuteration reaction of 2-cyanoacetic acid **S18** (75.5 mg, 0.2 mmol) for 2 h using the general procedure (61.7 mg, 92% Yield, 96% D). R_f = 0.4 (Hexane/EtOAc 10:1).

HPLC analysis (Chiralpak IE-3, hexane/^{i}PrOH = 99/1, 0.8 mL/min, 205 nm; t_r (minor) = 26.46 min, t_r

(major) = 29.77 min) gave the isomeric composition of the product: 96:4 e.r., $[\alpha]_D^{20}$ = –7.0 (c = 0.79, $CHCl_3$).

^{1}H NMR (400 MHz, $CDCl_3$) δ 7.60 (d, J = 8.0 Hz, 2H), 7.41-7.28 (m, 3H), 7.27-7.18 (m, 2H), 6.85 (d, J = 8.4 Hz, 2H), 3.98 (t, J = 7.2 Hz, 0.06H), 3.17-3.00 (m, 2H).

^{13}C NMR (100 MHz, $CDCl_3$) δ 137.6, 135.7, 134.6, 131.2, 129.0, 128.3, 127.4, 120.0, 93.0, 41.4, 39.1 (t, J = 21.0 Hz).

IR (neat, cm^{-1}) 3026, 2925, 2243, 1484, 1002.

HRMS (ESI) calcd $C_{15}H_{12}DIN^+$ $[M+H]^+$: 335.0150. Found: 335.0151.

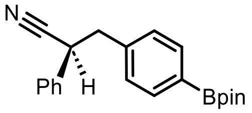

(–)-**19**

zwf-7-120

(–)-**19** was obtained as a white solid from the asymmetric decarboxylative protonation reaction of 2-cyanoacetic acid **S19** (188.6 mg, 0.5 mmol) for 3 h using the general procedure (149.8 mg, 90% Yield). R_f = 0.2 (Hexane/EtOAc 10:1).

HPLC analysis (Chiralpak IE-3, hexane/^{i}PrOH = 99/1, 0.8 mL/min, 205 nm; t_r (minor) = 23.37 min, t_r (major) = 29.51 min) gave the isomeric composition of the product: 95.5:4.5 e.r., $[\alpha]_D^{22}$ = –2.4 (c = 0.51, $CHCl_3$).

^{1}H NMR (400 MHz, $CDCl_3$) δ 7.73 (d, J = 8.0 Hz, 2H), 7.38-7.28 (m, 3H), 7.27-7.22 (m, 2H), 7.13 (d, J = 8.0 Hz, 2H), 4.00 (t, J = 7.4 Hz, 1H), 3.29-3.05 (m, 2H), 1.34 (s, 12H).

^{13}C NMR (100 MHz, $CDCl_3$) δ 139.3, 135.0, 129.0, 128.6, 128.2, 127.4, 120.2, 83.8, 42.2, 39.5, 24.8.

^{11}B NMR (128 MHz, $CDCl_3$) δ 30.6 (br).

IR (neat, cm^{-1}) 2979, 2929, 2242, 1613, 1357, 1142.

HRMS (ESI) calcd $C_{21}H_{25}BNO_2^+$ $[M+H]^+$: 334.1973. Found: 334.1971.

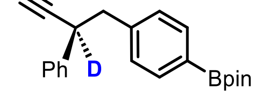

(–)-**19**-d

zwf-8-031

(−)-**19**-d was obtained as a white solid from the asymmetric decarboxylative deuteration reaction of 2-cyanoacetic acid **S19** (75.4 mg, 0.2 mmol) for 3 h using the general procedure (59.7 mg, 89% Yield, 94% D). R_f = 0.2 (Hexane/EtOAc 10:1).

HPLC analysis (Chiralpak IE-3, hexane/^{i}PrOH = 99/1, 0.8 mL/min, 205 nm; t_r (minor) = 24.23 min, t_r (major) = 31.42 min) gave the isomeric composition of the product: 95.5:4.5 e.r., $[\alpha]_D^{19}$ = −2.1 (*c* = 0.52, $CHCl_3$).

^{1}H NMR (400 MHz, $CDCl_3$) δ 7.73 (d, *J* = 8.0 Hz, 2H), 7.38-7.28 (m, 3H), 7.27-7.20 (m, 2H), 7.13 (d, *J* = 7.6 Hz, 2H), 4.00 (t, *J* = 7.2 Hz, 0.06H), 3.29-3.04 (m, 2H), 1.34 (s, 12H).

^{13}C NMR (100 MHz, $CDCl_3$) δ 139.3, 135.0, 129.0, 128.6, 128.2, 127.4, 120.2, 83.8, 42.2, 39.2 (t, *J* = 20.6 Hz), 24.8.

^{11}B NMR (128 MHz, $CDCl_3$) δ 30.5 (br).

IR (neat, cm^{-1}) 2978, 2934, 2242, 1612, 1358, 1145.

HRMS (ESI) calcd $C_{21}H_{24}DBNO_2^+$ $[M+H]^+$: 335.2036. Found: 335.2034.

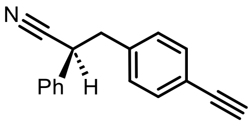

(−)-**20**

zwf-7-091

(−)-**20** was obtained as oil from the asymmetric decarboxylative protonation reaction of 2-cyanoacetic acid **S20** (137.5 mg, 0.5 mmol) for 7 h using the general procedure (101.0 mg, 87% Yield). R_f = 0.4 (Hexane/EtOAc 10:1).

HPLC analysis (Chiralpak IE-3, hexane/^{i}PrOH = 99/1, 0.8 mL/min, 205 nm; t_r (minor) = 20.73 min, t_r (major) = 22.60 min) gave the isomeric composition of the product: 85:15 e.r., $[\alpha]_D^{25}$ = −1.3 (*c* = 0.81, $CHCl_3$).

^{1}H NMR (500 MHz, $CDCl_3$) δ 7.47-7.28 (m, 5H), 7.25-7.18 (m, 2H), 7.07 (d, *J* = 7.5 Hz, 2H), 3.99 (t, *J* = 6.8 Hz, 1H), 3.25-3.09 (m, 2H), 3.07 (s, 1H).

^{13}C NMR (125 MHz, $CDCl_3$) δ 136.9, 134.7, 132.3, 129.2, 129.0, 128.3, 127.4, 121.2, 120.1, 83.2, 77.5, 41.9, 39.4.

IR (neat, cm^{-1}) 3038, 2929, 2242, 2104, 1494, 1078.

HRMS (ESI) calcd $C_{17}H_{14}N^{+}$ $[M+H]^{+}$: 232.1121. Found: 232.1120.

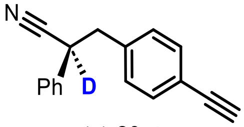

(−)-**20**-d

zwf-8-039

(−)-**20**-d was obtained as oil from the asymmetric decarboxylative deuteration reaction of 2-cyanoacetic acid **S20** (55.2 mg, 0.2 mmol) for 7 h using the general procedure (39.4 mg, 85% Yield, 94% D). R_f = 0.4 (Hexane/EtOAc 10:1).

HPLC analysis (Chiralpak IE-3, hexane/^{i}PrOH = 99/1, 0.8 mL/min, 205 nm; t_r (minor) = 27.26 min, t_r (major) = 30.20 min) gave the isomeric composition of the product: 90:10 e.r., $[\alpha]_D^{20}$ = −1.3 (c = 0.62, $CHCl_3$).

^{1}H NMR (500 MHz, $CDCl_3$) δ 7.56-7.28 (m, 5H), 7.27-7.20 (m, 2H), 7.07 (d, J = 7.2 Hz, 2H), 3.99 (t, J = 6.4 Hz, 0.06H), 3.25-3.09 (m, 2H), 3.07 (s, 1H).

^{13}C NMR (125 MHz, $CDCl_3$) δ 136.9, 134.7, 132.3, 129.3, 129.1, 128.3, 127.4, 121.2, 120.1, 83.2, 77.5, 41.8, 39.1 (t, J = 21.0 Hz).

IR (neat, cm^{-1}) 3033, 2934, 2242, 2109, 1494, 1020.

HRMS (ESI) calcd $C_{17}H_{13}DN^{+}$ $[M+H]^{+}$: 233.1184. Found: 233.1181.

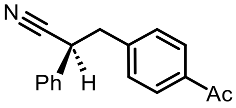

(−)-**21**

zwf-7-066

(−)-**21** was obtained as a white solid from the asymmetric decarboxylative protonation reaction of 2-cyanoacetic acid **S21** (146.7 mg, 0.5 mmol) for 2 h using the general procedure (116.4 mg, 93% Yield). R_f = 0.3 (Hexane/EtOAc 5:1).

HPLC analysis (Chiralpak IE-3, hexane/^{i}PrOH = 90/10, 0.8 mL/min, 205 nm; t_r (minor) = 40.67 min, t_r (major) = 47.57 min) gave the isomeric composition of the product: 95.5:4.5 e.r., $[\alpha]_D^{25}$ = −8.8 (c = 0.44, $CHCl_3$).

^{1}H NMR (400 MHz, $CDCl_3$) δ 7.88 (d, J = 8.4 Hz, 2H), 7.41-7.29 (m, 3H), 7.28-7.17 (m, 4H), 4.05 (t, J = 7.2 Hz, 1H), 3.29-3.16 (m, 2H), 2.58 (s, 3H).

^{13}C NMR (100 MHz, $CDCl_3$) δ 197.6, 141.4, 136.1, 134.6, 129.5, 129.0, 128.6, 128.3, 127.3, 119.9, 41.8, 39.1, 26.5.

IR (neat, cm^{-1}) 3055, 2987, 2242, 1743, 1422, 1264.

HRMS (ESI) calcd $C_{17}H_{16}NO^+$ $[M+H]^+$: 250.1226. Found: 250.1228.

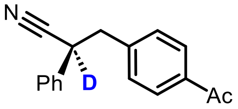

(−)-**21**-d

zwf-8-022

(−)-**21**-d was obtained as a white solid from the asymmetric decarboxylative deuteration reaction of 2-cyanoacetic acid **S21** (58.8 mg, 0.2 mmol) for 2 h using the general procedure (45.8 mg, 91% Yield, 89% D). R_f = 0.3 (Hexane/EtOAc 5:1).

HPLC analysis (Chiralpak IE-3, hexane/^{i}PrOH = 90/10, 0.8 mL/min, 205 nm; t_r (minor) = 45.77 min, t_r (major) = 54.37 min) gave the isomeric composition of the product: 95.5:4.5 e.r., $[\alpha]_D^{20}$ = −8.6 (c = 0.42, $CHCl_3$).

^{1}H NMR (400 MHz, $CDCl_3$) δ 7.89 (d, J = 8.4 Hz, 2H), 7.44-7.29 (m, 3H), 7.29-7.13 (m, 4H), 4.05 (t, J = 7.4 Hz, 0.11H), 3.32-3.12 (m, 2H), 2.59 (s, 3H).

^{13}C NMR (100 MHz, $CDCl_3$) δ 197.6, 141.5, 136.2, 134.6, 129.5, 129.1, 128.6, 128.4, 127.4, 119.9, 41.8, 38.9 (t, J = 21.0 Hz), 26.6.

IR (neat, cm^{-1}) 3055, 2985, 2244, 1745, 1419, 1265.

HRMS (ESI) calcd $C_{17}H_{15}DNO^+$ $[M+H]^+$: 251.1289. Found: 251.1290.

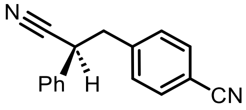

(−)-**22**

zwf-7-071

(−)-**22** was obtained as a white solid from the asymmetric decarboxylative protonation reaction of 2-cyanoacetic acid **S22** (138.3 mg, 0.5 mmol) for 2 h using the general procedure (109.2 mg, 94% Yield). R_f = 0.3 (Hexane/EtOAc 5:1).

HPLC analysis (Chiralpak IE-3, hexane/^{i}PrOH = 90/10, 0.8 mL/min, 205 nm; t_r (minor) = 29.05 min, t_r

(major) = 30.77 min) gave the isomeric composition of the product: 96.5:3.5 e.r., $[\alpha]_D^{25}$ = −19.4 (c = 0.39, $CHCl_3$).

^{1}H NMR (400 MHz, $CDCl_3$) δ 7.57 (d, J = 8.4 Hz, 2H), 7.43-7.30 (m, 3H), 7.26-7.17 (m, 4H), 4.06 (t, J = 7.2 Hz, 1H), 3.30-3.15 (m, 2H).

^{13}C NMR (100 MHz, $CDCl_3$) δ 141.3, 134.1, 132.3, 130.1, 129.1, 128.5, 127.3, 119.6, 118.5, 111.4, 41.8, 38.9.

IR (neat, cm^{-1}) 3038, 2929, 2242, 2222, 1493, 1107.

HRMS (ESI) calcd $C_{16}H_{13}N_2^+$ $[M+H]^+$: 233.1073. Found: 233.1072.

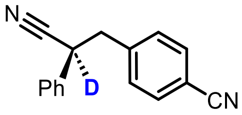

(−)-**22**-d

zwf-8-028

(−)-**22**-d was obtained as a white solid from the asymmetric decarboxylative deuteration reaction of 2-cyanoacetic acid **S22** (55.5 mg, 0.2 mmol) for 2 h using the general procedure (43.1 mg, 92% Yield, 95% D). R_f = 0.3 (Hexane/EtOAc 5:1).

HPLC analysis (Chiralpak IE-3, hexane/^{i}PrOH = 90/10, 0.8 mL/min, 205 nm; t_r (minor) = 31.59 min, t_r (major) = 33.33 min) gave the isomeric composition of the product: 97.5:2.5 e.r., $[\alpha]_D^{20}$ = −16.9 (c = 0.30, $CHCl_3$).

^{1}H NMR (400 MHz, $CDCl_3$) δ 7.57 (d, J = 7.6 Hz, 2H), 7.46-7.29 (m, 3H), 7.28-7.13 (m, 4H), 4.06 (t, J = 7.0 Hz, 0.05H), 3.32-3.11 (m, 2H).

^{13}C NMR (100 MHz, $CDCl_3$) δ 141.3, 134.1, 132.3, 130.1, 129.2, 128.5, 127.3, 119.6, 118.5, 111.4, 41.7, 38.7 (t, J = 20.6 Hz).

IR (neat, cm^{-1}) 3043, 2934, 2242, 2222, 1454, 1110.

HRMS (ESI) calcd $C_{16}H_{12}DN_2^+$ $[M+H]^+$: 234.1136. Found: 234.1139.

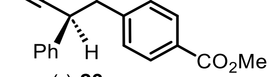

(-)-**23**

zwf-10-048

(−)-**23** was obtained as a solid from the asymmetric decarboxylative protonation reaction of 2-cyanoacetic acid **S23** (154.6 mg, 0.5 mmol) for 5 h using the general procedure (110.5 mg, 83% Yield). R_f = 0.3 (Hexane/EtOAc 10:1).

HPLC analysis (Chiralpak IE-3, hexane/^{i}PrOH = 80/20, 0.8 mL/min, 205 nm; t_r (minor) = 16.57 min, t_r (major) = 19.60 min) gave the isomeric composition of the product: 95.5:4.5 e.r., $[\alpha]_D^{19}$ = –0.7 (c = 0.51, $CHCl_3$).

^{1}H NMR (500 MHz, $CDCl_3$) δ 7.94 (d, J = 8.5 Hz, 2H), 7.38-7.29 (m, 3H), 7.25-7.21 (m, 2H), 7.18 (d, J = 8.0 Hz, 2H), 4.04 (t, J = 7.5 Hz, 1H), 3.90 (s, 3H), 3.28-3.15 (m, 2H).

^{13}C NMR (125 MHz, $CDCl_3$) δ 166.7, 141.2, 134.6, 129.8, 129.28, 129.26, 129.0, 128.3, 127.4, 119.9, 52.1, 41.9, 39.2.

IR (neat, cm^{-1}) 2954, 2926, 2245, 1715, 1276, 1112.

HRMS (ESI) calcd $C_{17}H_{16}NO_2^+$ $[M+H]^+$: 266.1176. Found: 266.1177.

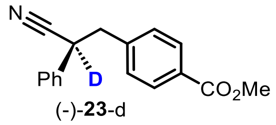

(-)-**23**-d

zwf-10-049

(−)-**23**-d was obtained as a solid from the asymmetric decarboxylative deuteration reaction of 2-cyanoacetic acid **S23** (62.0 mg, 0.2 mmol) for 5 h using the general procedure (45.7 mg, 86% Yield, 92% D). R_f = 0.3 (Hexane/EtOAc 10:1).

HPLC analysis (Chiralpak IE-3, hexane/^{i}PrOH = 80/20, 0.8 mL/min, 205 nm; t_r (minor) = 16.57 min, t_r (major) = 19.60 min) gave the isomeric composition of the product: 96:4 e.r., $[\alpha]_D^{19}$ = –0.9 (c = 0.49, $CHCl_3$).

^{1}H NMR (500 MHz, $CDCl_3$) δ 7.95 (d, J = 8.0 Hz, 2H), 7.40-7.29 (m, 3H), 7.25-7.20 (m, 2H), 7.18 (d, J = 8.5 Hz, 2H), 4.04 (t, J = 7.5 Hz, 0.08H), 3.90 (s, 3H), 3.26-3.16 (m, 2H).

^{13}C NMR (125 MHz, $CDCl_3$) δ 166.7, 141.2, 134.6, 129.8, 129.27, 129.24, 129.0, 128.3, 127.4, 119.9, 52.0, 41.7, 38.8 (t, J = 20.8 Hz).

IR (neat, cm^{-1}) 2958, 2929, 2246, 1714, 1275, 1112.

HRMS (ESI) calcd $C_{17}H_{15}DNO_2^+$ $[M+H]^+$: 267.1238. Found: 267.1236.

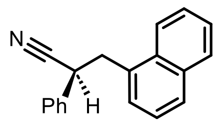

(+)-**24**

zwf-7-123

(+)-**24** was obtained as oil from the asymmetric decarboxylative protonation reaction of 2-cyanoacetic acid **S24** (150.9 mg, 0.5 mmol) for 3 h using the general procedure (112.4 mg, 87% Yield). R_f = 0.4 (Hexane/EtOAc 10:1).

HPLC analysis (Chiralpak IE-3, hexane/^{i}PrOH = 99/1, 0.8 mL/min, 205 nm; t_r (minor) = 22.70 min, t_r (major) = 26.21 min) gave the isomeric composition of the product: 81:19 e.r., $[\alpha]_D^{21}$ = +35.1 (*c* = 0.80, $CHCl_3$).

^{1}H NMR (400 MHz, $CDCl_3$) δ 7.97-7.84 (m, 2H), 7.78 (d, *J* = 8.4 Hz, 1H), 7.58-7.46 (m, 2H), 7.45-7.24 (m, 7H), 4.15 (t, *J* = 7.8 Hz, 1H), 3.69-3.53 (m, 2H).

^{13}C NMR (100 MHz, $CDCl_3$) δ 135.6, 133.9, 132.2, 131.2, 129.2, 129.1, 128.3, 128.2, 128.1, 127.3, 126.5, 125.7, 125.4, 122.6, 120.4, 39.5, 38.7.

IR (neat, cm^{-1}) 3067, 2944, 2242, 1454, 1078.

HRMS (ESI) calcd $C_{19}H_{16}N^+$ $[M+H]^+$: 258.1277. Found: 258.1278.

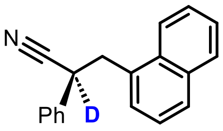

(+)-**24**-d

zwf-8-032

(+)-**24**-d was obtained as oil from the asymmetric decarboxylative deuteration reaction of 2-cyanoacetic acid **S24** (60.3 mg, 0.2 mmol) for 3 h using the general procedure (46.7 mg, 90% Yield, 91% D). R_f = 0.4 (Hexane/EtOAc 10:1).

HPLC analysis (Chiralpak IE-3, hexane/^{i}PrOH = 99/1, 0.8 mL/min, 205 nm; t_r (minor) = 24.43 min, t_r (major) = 28.16 min) gave the isomeric composition of the product: 94.5:5.5 e.r., $[\alpha]_D^{19}$ = +43.9 (*c* = 0.66, $CHCl_3$).

^{1}H NMR (400 MHz, $CDCl_3$) δ 7.97-7.84 (m, 2H), 7.79 (d, *J* = 8.0 Hz, 1H), 7.59-7.45 (m, 2H), 7.44-7.24 (m, 7H), 4.16 (t, *J* = 7.6 Hz, 0.09H), 3.69-3.51 (m, 2H).

^{13}C NMR (100 MHz, $CDCl_3$) δ 135.5, 133.9, 132.2, 131.3, 129.2, 129.1, 128.28, 128.25, 128.1, 127.3,

126.5, 125.7, 125.4, 122.6, 120.4, 39.5, 38.5 (t, J = 20.6 Hz).

IR (neat, cm^{-1}) 3063, 2942, 2242, 1454, 1083.

HRMS (ESI) calcd $C_{19}H_{15}DN^+$ $[M+H]^+$: 259.1340. Found: 259.1336.

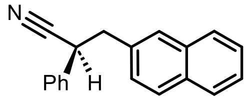

(+)-**25**

zwf-7-094

(+)-**25** was obtained as white solid from the asymmetric decarboxylative protonation reaction of 2-cyanoacetic acid **S25** (150.7 mg, 0.5 mmol) for 2 h using the general procedure (120.2 mg, 93% Yield). R_f = 0.3 (Hexane/EtOAc 10:1).

HPLC analysis (Chiralpak IE-3, hexane/^{i}PrOH = 99/1, 0.8 mL/min, 205 nm; t_r (minor) = 23.67 min, t_r (major) = 26.17 min) gave the isomeric composition of the product: 95.5:4.5 e.r., $[\alpha]_D^{21}$ = +5.4 (c = 0.50, $CHCl_3$).

^{1}H NMR (400 MHz, $CDCl_3$) δ 7.84-7.71 (m, 3H), 7.58 (s, 1H), 7.49-7.40 (m, 2H), 7.37-7.29 (m, 3H), 7.29-7.23 (m, 2H), 7.23-7.20 (m, 1H), 4.07 (t, J = 7.4 Hz, 1H), 3.40-3.21 (m, 2H).

^{13}C NMR (100 MHz, $CDCl_3$) δ 135.1, 133.7, 133.3, 132.5, 129.0, 128.3, 128.2, 128.1, 127.7, 127.6, 127.5, 127.1, 126.2, 125.9, 120.3, 42.3, 39.6.

IR (neat, cm^{-1}) 3058, 2928, 2242, 1495, 1075.

HRMS (ESI) calcd $C_{19}H_{16}N^+$ $[M+H]^+$: 258.1277. Found: 258.1279.

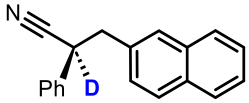

(+)-**25**-d

zwf-8-033

(+)-**25**-d was obtained as a white solid from the asymmetric decarboxylative deuteration reaction of 2-cyanoacetic acid **S25** (60.3 mg, 0.2 mmol) for 2 h using the general procedure (47.5 mg, 92% Yield, 95% D). R_f = 0.3 (Hexane/EtOAc 10:1).

HPLC analysis (Chiralpak IE-3, hexane/^{i}PrOH = 99/1, 0.8 mL/min, 205 nm; t_r (minor) = 30.43 min, t_r (major) = 33.86 min) gave the isomeric composition of the product: 97:3 e.r., $[\alpha]_D^{19}$ = +5.3 (c = 0.80, $CHCl_3$).

^{1}H NMR (400 MHz, $CDCl_3$) δ 7.85-7.68 (m, 3H), 7.58 (s, 1H), 7.51-7.39 (m, 2H), 7.39-7.14 (m, 6H), 4.07 (t, J = 7.4 Hz, 0.05H), 3.40-3.17 (m, 2H).

^{13}C NMR (100 MHz, $CDCl_3$) δ 135.1, 133.7, 133.3, 132.5, 129.0, 128.3, 128.2, 128.1, 127.7, 127.6, 127.4, 127.1, 126.2, 125.9, 120.3, 42.2, 39.3 (t, J = 21.0 Hz).

IR (neat, cm^{-1}) 3063, 2927, 2245, 1494, 1070.

HRMS (ESI) calcd $C_{19}H_{15}DN^+$ $[M+H]^+$: 259.1340. Found: 259.1343.

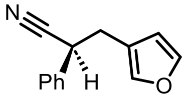

(–)-**26**

zwf-7-145

(–)-**26** was obtained as oil from the asymmetric decarboxylative protonation reaction of 2-cyanoacetic acid **S26** (120.7 mg, 0.5 mmol) for 8 h using the general procedure (92.5 mg, 94% Yield). R_f = 0.3 (Hexane/EtOAc 10:1).

HPLC analysis (Chiralpak IE-3, hexane/^{i}PrOH = 99/1, 0.8 mL/min, 205 nm; t_r (minor) = 23.10 min, t_r (major) = 26.47 min) gave the isomeric composition of the product: 98:2 e.r., $[\alpha]_D^{19}$ = –38.5 (c = 0.53, $CHCl_3$).

^{1}H NMR (400 MHz, $CDCl_3$) δ 7.44-7.24 (m, 6H), 7.23 (s, 1H), 6.22 (s, 1H), 3.95 (t, J = 7.2 Hz, 1H), 3.11-2.93 (m, 2H).

^{13}C NMR (100 MHz, $CDCl_3$) δ 143.1, 140.5, 135.1, 129.0, 128.2, 127.4, 120.4, 119.6, 110.8, 38.7, 31.5.

IR (neat, cm^{-1}) 3033, 2934, 2242, 1767, 1497, 1023.

HRMS (ESI) calcd $C_{13}H_{12}NO^+$ $[M+H]^+$: 198.0913. Found: 198.0912.

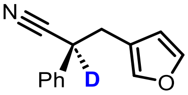

(–)-**26**-d

zwf-8-005

(–)-**26**-d was obtained as oil from the asymmetric decarboxylative deuteration reaction of 2-cyanoacetic acid **S26** (48.3 mg, 0.2 mmol) for 8 h using the general procedure (36.3 mg, 91% Yield, 95% D). R_f = 0.3 (Hexane/EtOAc 10:1).

HPLC analysis (Chiralpak IE-3, hexane/^{i}PrOH = 99/1, 0.8 mL/min, 205 nm; t_r (minor) = 25.00 min, t_r (major) = 28.78 min) gave the isomeric composition of the product: 97.5:2.5 e.r., $[\alpha]_D^{20}$ = –37.6 (*c* = 0.43, $CHCl_3$).

^{1}H NMR (400 MHz, $CDCl_3$) δ 7.47-7.27 (m, 6H), 7.25-7.19 (m, 1H), 6.23 (s, 1H), 3.96 (t, *J* = 7.0 Hz, 0.05H), 3.15-2.90 (m, 2H).

^{13}C NMR (100 MHz, $CDCl_3$) δ 143.1, 140.5, 135.0, 129.0, 128.2, 127.4, 120.4, 119.6, 110.9, 38.4 (t, *J* = 20.6 Hz), 31.4.

IR (neat, cm^{-1}) 3038, 2934, 2242, 1767, 1497, 1072.

HRMS (ESI) calcd $C_{13}H_{11}DNO^+$ $[M+H]^+$: 199.0976. Found: 199.0979.

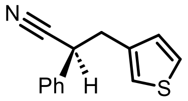

(–)-**27**

zwf-7-093

(–)-**27** was obtained as a white solid from the asymmetric decarboxylative protonation reaction of 2-cyanoacetic acid **S27** (128.6 mg, 0.5 mmol) for 2 h using the general procedure (98.3 mg, 92% Yield). R_f = 0.4 (Hexane/EtOAc 10:1).

HPLC analysis (Chiralpak IE-3, hexane/^{i}PrOH = 99/1, 0.8 mL/min, 205 nm; t_r (minor) = 18.88 min, t_r (major) = 20.60 min) gave the isomeric composition of the product: 95.5:4.5 e.r., $[\alpha]_D^{25}$ = –21.5 (*c* = 0.41, $CHCl_3$).

^{1}H NMR (400 MHz, $CDCl_3$) δ 7.39-7.28 (m, 3H), 7.28-7.21 (m, 3H), 7.03-6.94 (m, 1H), 6.87 (dd, J_1 = 4.8 Hz, J_1 = 1.2 Hz, 1H), 3.99 (t, *J* = 7.2 Hz, 1H), 3.30-3.11 (m, 2H).

^{13}C NMR (100 MHz, $CDCl_3$) δ 136.4, 135.1, 129.0, 128.2, 128.1, 127.4, 125.9, 123.2, 120.4, 39.0, 36.4.

IR (neat, cm^{-1}) 3073, 2929, 2242, 1455, 1077.

HRMS (ESI) calcd $C_{13}H_{12}NS^+$ $[M+H]^+$: 214.0685. Found: 214.0683.

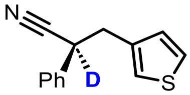

(–)-**27**-d

zwf-8-034

(–)-**27**-d was obtained as a white solid from the asymmetric decarboxylative deuteration reaction of 2-cyanoacetic acid **S27** (51.5 mg, 0.2 mmol) for 2 h using the general procedure (39.2 mg, 91% Yield, 94% D). R_f = 0.4 (Hexane/EtOAc 10:1).

HPLC analysis (Chiralpak IE-3, hexane/^{i}PrOH = 99/1, 0.8 mL/min, 205 nm; t_r (minor) = 23.65 min, t_r (major) = 29.49 min) gave the isomeric composition of the product: 96:4 e.r., $[\alpha]_D^{20}$ = –23.5 (c = 0.81, $CHCl_3$).

^{1}H NMR (400 MHz, $CDCl_3$) δ 7.43-7.29 (m, 3H), 7.29-7.20 (m, 3H), 7.06-6.93 (m, 1H), 6.88 (dd, J_1 = 4.8 Hz, J_1 = 1.2 Hz, 1H), 4.00 (t, J = 7.2 Hz, 0.06H), 3.28-3.11 (m, 2H).

^{13}C NMR (100 MHz, $CDCl_3$) δ 136.4, 135.1, 129.0, 128.2, 128.1, 127.4, 125.9, 123.2, 120.4, 38.7 (t, J = 20.6 Hz), 36.3.

IR (neat, cm^{-1}) 3093, 2929, 2242, 1452, 1080.

HRMS (ESI) calcd $C_{13}H_{10}DNNaS^+$ $[M+Na]^+$: 237.0567. Found: 237.0568.

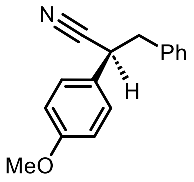

(+)-**28**

zwf-7-073

(+)-**28** was obtained as a white solid from the asymmetric decarboxylative protonation reaction of 2-cyanoacetic acid **S28** (140.9 mg, 0.5 mmol) for 2 h using the general procedure (105.4 mg, 89% Yield). R_f = 0.3 (Hexane/EtOAc 10:1).

HPLC analysis (Chiralpak IE-3, hexane/^{i}PrOH = 99/1, 0.8 mL/min, 205 nm; t_r (minor) = 32.95 min, t_r (major) = 35.94 min) gave the isomeric composition of the product: 95.5:4.5 e.r., $[\alpha]_D^{25}$ = +11.0 (c = 0.62, $CHCl_3$).

^{1}H NMR (400 MHz, $CDCl_3$) δ 7.35-7.21 (m, 3H), 7.20-7.05 (m, 4H), 6.86 (d, J = 7.6 Hz, 2H), 3.94 (t, J = 7.2 Hz, 1H), 3.79 (s, 3H), 3.22-3.03 (m, 2H).

^{13}C NMR (100 MHz, $CDCl_3$) δ 159.4, 136.3, 129.2, 128.59, 128.55, 127.3, 127.1, 120.6, 114.3, 55.3, 42.2, 38.9.

IR (neat, cm^{-1}) 3028, 2920, 2243, 1511, 1030.

HRMS (ESI) calcd $C_{16}H_{16}NO^+$ $[M+H]^+$: 238.1126. Found: 238.1124.

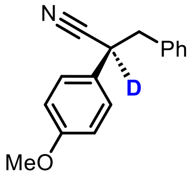

(+)-**28**-d

zwf-8-035

(+)-**28**-d was obtained as white solid from the asymmetric decarboxylative deuteration reaction of 2-cyanoacetic acid **S28** (56.4 mg, 0.2 mmol) for 2 h using the general procedure (43.0 mg, 90% Yield, 95% D). R_f = 0.3 (Hexane/EtOAc 10:1).

HPLC analysis (Chiralpak IE-3, hexane/^{i}PrOH = 99/1, 0.8 mL/min, 205 nm; t_r (minor) = 39.65 min, t_r (major) = 44.03 min) gave the isomeric composition of the product: 96.5:3.5 e.r., $[\alpha]_D^{20}$ = +11.4 (c = 0.60, $CHCl_3$).

^{1}H NMR (400 MHz, $CDCl_3$) δ 7.37-7.20 (m, 3H), 7.20-7.03 (m, 4H), 6.86 (d, J = 8.0 Hz, 2H), 3.94 (t, J = 7.2 Hz, 0.05H), 3.79 (s, 3H), 3.26-2.98 (m, 2H).

^{13}C NMR (100 MHz, $CDCl_3$) δ 159.3, 136.3, 129.2, 128.6, 128.5, 127.3, 127.1, 120.6, 114.3, 55.3, 42.1, 38.6 (t, J = 21.0 Hz).

IR (neat, cm^{-1}) 3028, 2919, 2242, 1512, 1027.

HRMS (ESI) calcd $C_{16}H_{15}DNO^+$ $[M+H]^+$: 239.1289. Found: 239.1290.

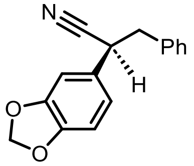

(+)-**29**

zwf-7-116

(+)-**29** was obtained as oil from the asymmetric decarboxylative protonation reaction of 2-cyanoacetic acid **S29** (147.7 mg, 0.5 mmol) for 3 h using the general procedure (112.7 mg, 90% Yield). R_f = 0.3 (Hexane/EtOAc 10:1).

HPLC analysis (Chiralpak IE-3, hexane/^{i}PrOH = 90/10, 0.8 mL/min, 205 nm; t_r (minor) = 16.69 min, t_r (major) = 21.46 min) gave the isomeric composition of the product: 95.5:4.5 e.r., $[\alpha]_D^{22}$ = +13.3 (c = 0.48, $CHCl_3$).

^{1}H NMR (400 MHz, $CDCl_3$) δ 7.36-7.21 (m, 3H), 7.19-7.08 (m, 2H), 6.83-6.71 (m, 2H), 6.68 (dd, J_1 = 8.0 Hz, J_2 = 1.6 Hz, 1H), 5.96 (s, 2H), 3.89 (t, J = 7.4 Hz, 1H), 3.20-3.03 (m, 2H).

^{13}C NMR (100 MHz, $CDCl_3$) δ 148.1, 147.4, 136.2, 129.1, 128.8, 128.6, 127.3, 121.0, 120.4, 108.5, 107.8, 101.3, 42.2, 39.3.

IR (neat, cm^{-1}) 3033, 2905, 2242, 1487, 1246, 1036.

HRMS (ESI) calcd $C_{16}H_{14}NO_2^+$ $[M+H]^+$: 252.1019. Found: 252.1016.

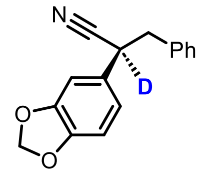

(+)-**29**-d

zwf-8-036

(+)-**29**-d was obtained as oil from the asymmetric decarboxylative deuteration reaction of 2-cyanoacetic acid **S29** (59.2 mg, 0.2 mmol) for 3 h using the general procedure (46.4 mg, 92% Yield, 94% D). R_f = 0.3 (Hexane/EtOAc 10:1).

HPLC analysis (Chiralpak IE-3, hexane/^{i}PrOH = 90/10, 0.8 mL/min, 205 nm; t_r (minor) = 17.89 min, t_r (major) = 22.76 min) gave the isomeric composition of the product: 95.5:4.5 e.r., $[\alpha]_D^{20}$ = +13.4 (c = 0.56, $CHCl_3$).

^{1}H NMR (400 MHz, $CDCl_3$) δ 7.40-7.20 (m, 3H), 7.19-7.06 (m, 2H), 6.85-6.71 (m, 2H), 6.68 (d, J = 7.6 Hz, 1H), 5.96 (s, 2H), 3.90 (t, J = 7.2 Hz, 0.06H), 3.23-3.00 (m, 2H).

^{13}C NMR (100 MHz, $CDCl_3$) δ 148.1, 147.5, 136.2, 129.1, 128.8, 128.6, 127.3, 120.9, 120.4, 108.5, 107.8, 101.4, 42.1, 39.1 (t, J = 20.6 Hz).

IR (neat, cm^{-1}) 3028, 2904, 2242, 1485, 1241, 1031.

HRMS (ESI) calcd $C_{16}H_{13}DNO_2^+$ $[M+H]^+$: 253.1082. Found: 253.1081.

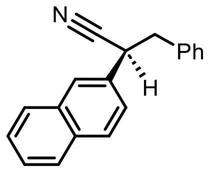

(+)-**30**

zwf-7-114

(+)-**30** was obtained as a white solid from the asymmetric decarboxylative protonation reaction of 2-cyanoacetic acid **S30** (150.5 mg, 0.5 mmol) for 4 h using the general procedure (120.4 mg, 94% Yield). R_f = 0.4 (Hexane/EtOAc 10:1).

HPLC analysis (Chiralpak IE-3, hexane/^{i}PrOH = 99/1, 0.8 mL/min, 205 nm; t_r (minor) = 28.70 min, t_r (major) = 34.47 min) gave the isomeric composition of the product: 95.5:4.5 e.r., $[\alpha]_D^{25}$ = +3.9 (*c* = 0.28, $CHCl_3$).

^{1}H NMR (400 MHz, $CDCl_3$) δ 7.90-7.74 (m, 3H), 7.71 (s, 1H), 7.54-7.44 (m, 2H), 7.33 (dd, J_1 = 8.8 Hz, J_2 = 1.6 Hz, 1H), 7.30-7.21 (m, 3H), 7.19-7.08 (m, 2H), 4.14 (t, *J* = 7.4 Hz, 1H), 3.31-3.11 (m, 2H).

^{13}C NMR (100 MHz, $CDCl_3$) δ 136.2, 133.2, 132.8, 132.4, 129.2, 128.9, 128.6, 127.8, 127.7, 127.3, 126.63, 126.56, 126.5, 124.9, 120.3, 42.0, 39.9.

IR (neat, cm^{-1}) 3026, 2929, 2241, 1496, 1200.

HRMS (ESI) calcd $C_{19}H_{16}N^+$ $[M+H]^+$: 258.1277. Found: 258.1274.

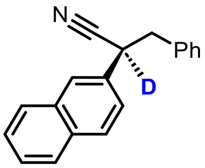

(+)-**30**-d

zwf-8-017

(+)-**30**-d was obtained as a white solid from the asymmetric decarboxylative deuteration reaction of 2-cyanoacetic acid **S30** (60.5 mg, 0.2 mmol) for 4 h using the general procedure (47.1 mg, 91% Yield, 89% D). R_f = 0.4 (Hexane/EtOAc 10:1).

HPLC analysis (Chiralpak IE-3, hexane/^{i}PrOH = 99/1, 0.8 mL/min, 205 nm; t_r (minor) = 33.84 min, t_r (major) = 41.08 min) gave the isomeric composition of the product: 95.5:4.5 e.r., $[\alpha]_D^{20}$ = +4.4 (*c* = 0.62, $CHCl_3$).

^{1}H NMR (400 MHz, $CDCl_3$) δ 7.90-7.75 (m, 3H), 7.72 (s, 1H), 7.57-7.42 (m, 2H), 7.38-7.21 (m, 4H), 7.21-7.07 (m, 2H), 4.16 (t, *J* = 7.4 Hz, 0.11H), 3.33-3.14 (m, 2H).

^{13}C NMR (100 MHz, $CDCl_3$) δ 136.2, 133.2, 132.8, 132.4, 129.2, 128.9, 128.6, 127.9, 127.7, 127.4, 126.7, 126.6, 126.5, 124.9, 120.3, 42.0, 39.6 (t, *J* = 20.6 Hz).

IR (neat, cm^{-1}) 3058, 2928, 2243, 1407, 1199.

HRMS (ESI) calcd $C_{19}H_{15}DN^+$ $[M+H]^+$: 259.1340. Found: 259.1342.

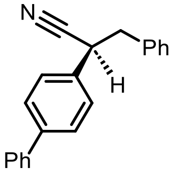

(+)-**31**

zwf-7-112

(+)-**31** was obtained as a white solid from the asymmetric decarboxylative protonation reaction of 2-cyanoacetic acid **S31** (163.7 mg, 0.5 mmol) for 4 h using the general procedure (c = 0.01, referring to the concentration of **S31** in 2-MeTHF) (132.2 mg, 93% Yield). R_f = 0.4 (Hexane/EtOAc 10:1).

HPLC analysis (Chiralpak IE-3, hexane/^{i}PrOH = 99/1, 0.8 mL/min, 205 nm; t_r (minor) = 36.83 min, t_r (major) = 47.66 min) gave the isomeric composition of the product: 95:5 e.r., $[\alpha]_D^{22}$ = +22.7 (*c* = 0.36, $CHCl_3$).

^{1}H NMR (400 MHz, $CDCl_3$) δ 7.57 (d, *J* = 8.4 Hz, 4H), 7.44 (t, *J* = 7.6 Hz, 2H), 7.39-7.23 (m, 6H), 7.16 (d, *J* = 8.8 Hz, 2H), 4.03 (t, *J* = 7.4 Hz, 1H), 3.27-3.08 (m, 2H).

^{13}C NMR (100 MHz, $CDCl_3$) δ 141.1, 140.1, 136.2, 134.1, 129.2, 128.8, 128.6, 127.9, 127.6, 127.4, 127.0, 120.3, 42.1, 39.4.

IR (neat, cm^{-1}) 3031, 2920, 2242, 1453, 1025.

HRMS (ESI) calcd $C_{21}H_{18}N^+$ $[M+H]^+$: 284.1434. Found: 284.1430.

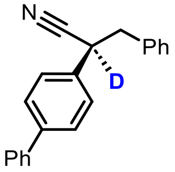

(+)-**31**-d

zwf-8-016

(+)-**31**-d was obtained as a white solid from the asymmetric decarboxylative deuteration reaction of 2-cyanoacetic acid **S31** (65.5 mg, 0.2 mmol) for 4 h using the general procedure (52.2 mg, 92% Yield, 94% D). R_f = 0.4 (Hexane/EtOAc 10:1).

HPLC analysis (Chiralpak IE-3, hexane/^{i}PrOH = 99/1, 0.8 mL/min, 205 nm; t_r (minor) = 43.76 min, t_r (major) = 56.61 min) gave the isomeric composition of the product: 95.5:4.5 e.r., $[\alpha]_D^{20}$ = +20.7 (*c* = 0.51, $CHCl_3$).

^{1}H NMR (400 MHz, $CDCl_3$) δ 7.64-7.52 (m, 4H), 7.45 (t, *J* = 7.4 Hz, 2H), 7.39-7.24 (m, 6H), 7.22-7.12 (m, 2H), 4.04 (t, *J* = 7.4 Hz, 0.06H), 3.28-3.09 (m, 2H).

^{13}C NMR (100 MHz, $CDCl_3$) δ 141.1, 140.1, 136.2, 134.1, 129.2, 128.8, 128.6, 127.9, 127.65, 127.62, 127.4, 127.0, 120.3, 42.0, 39.2 (t, *J* = 21.0 Hz).

IR (neat, cm^{-1}) 3030, 2919, 2242, 1486, 1023.

HRMS (ESI) calcd $C_{21}H_{17}DN^+$ $[M+H]^+$: 285.1497. Found: 285.1494.

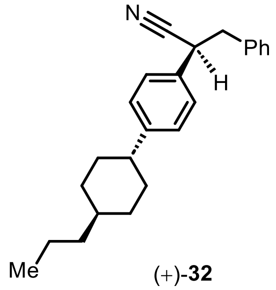

(+)-**32**

zwf-7-118

(+)-**32** was obtained as a white solid from the asymmetric decarboxylative protonation reaction of 2-cyanoacetic acid **S32** (187.9 mg, 0.5 mmol) for 6 h using the general procedure (157.7 mg, 95% Yield). R_f = 0.6 (Hexane/EtOAc 10:1).

HPLC analysis (Chiralpak IE-3, hexane/^{i}PrOH = 99/1, 0.8 mL/min, 205 nm; t_r (minor) = 16.39 min, t_r (major) = 17.76 min) gave the isomeric composition of the product: 95.5:4.5 e.r., $[\alpha]_D^{22}$ = +6.0 (*c* = 0.42, $CHCl_3$).

^{1}H NMR (400 MHz, $CDCl_3$) δ 7.34-7.24 (m, 3H), 7.22-7.13 (m, 6H), 3.95 (t, *J* = 7.4 Hz, 1H), 3.21-3.06 (m, 2H), 2.52-2.41 (m, 1H), 1.94-1.83 (m, 4H), 1.49-1.17 (m, 7H), 1.12-0.98 (m, 2H), 0.90 (t, *J* = 7.2 Hz, 3H).

^{13}C NMR (100 MHz, $CDCl_3$) δ 148.0, 136.5, 132.6, 129.1, 128.6, 127.4, 127.3, 120.5, 44.2, 42.2, 39.6, 39.5, 36.9, 34.2, 33.4, 20.0, 14.4.

IR (neat, cm^{-1}) 2919, 2847, 2240, 1496, 1077.

HRMS (ESI) calcd $C_{24}H_{30}N^+$ $[M+H]^+$: 332.2373. Found: 332.2375.

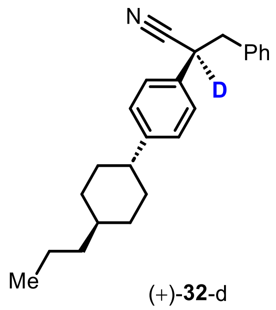

(+)-**32**-d

zwf-8-037

(+)-**32**-d was obtained as a white solid from the asymmetric decarboxylative deuteration reaction of 2-cyanoacetic acid **S32** (75.0 mg, 0.2 mmol) for 6 h using the general procedure (62.3 mg, 94% Yield, 95% D). R_f = 0.6 (Hexane/EtOAc 10:1).

HPLC analysis (Chiralpak IE-3, hexane/^{i}PrOH = 99/1, 0.8 mL/min, 205 nm; t_r (minor) = 18.18 min, t_r (major) = 19.56 min) gave the isomeric composition of the product: 95.5:4.5 e.r., $[\alpha]_D^{20}$ = +5.5 (*c* = 0.41, $CHCl_3$).

^{1}H NMR (400 MHz, $CDCl_3$) δ 7.34-7.23 (m, 3H), 7.22-7.08 (m, 6H), 3.94 (t, *J* = 7.4 Hz, 0.05H), 3.23-3.01 (m, 2H), 2.56-2.34 (m, 1H), 1.94-1.75 (m, 4H), 1.52-1.14 (m, 7H), 1.12-0.98 (m, 2H), 0.90 (t, *J* = 7.2 Hz, 3H).

^{13}C NMR (100 MHz, $CDCl_3$) δ 148.0, 136.5, 132.5, 129.1, 128.6, 127.4, 127.3, 120.5, 44.2, 42.1, 39.6, 39.2 (t, *J* = 20.3 Hz), 36.9, 34.2, 33.4, 20.0, 14.4.

IR (neat, cm^{-1}) 2920, 2846, 2241, 1495, 1075.

HRMS (ESI) calcd $C_{24}H_{29}DN^+$ $[M+H]^+$: 333.2436. Found: 333.2438.

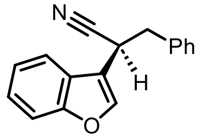

(–)-**33**

zwf-7-117

(–)-**33** was obtained as white solid from the asymmetric decarboxylation reaction of 2-cyanoacetic acid **S33** (145.6 mg, 0.5 mmol) for 3 h using the general procedure (c = 0.01, referring to the concentration of **S33** in 2-MeTHF) (112.3 mg, 91% Yield). R_f = 0.3 (Hexane/EtOAc 10:1).

HPLC analysis (Chiralpak IE-3, hexane/^{i}PrOH = 99/1, 0.8 mL/min, 205 nm; t_r (minor) = 23.69 min, t_r (major) = 27.42 min) gave the isomeric composition of the product: 95:5 e.r., $[\alpha]_D^{22}$ = –20.9 (*c* = 0.49, $CHCl_3$).

^{1}H NMR (400 MHz, $CDCl_3$) δ 7.60 (d, *J* = 7.4 Hz, 1H), 7.54-7.46 (m, 2H), 7.39-7.24 (m, 5H), 7.20-7.13 (m, 2H), 4.18 (t, *J* = 7.2 Hz, 1H), 3.34-3.22 (m, 2H).

^{13}C NMR (100 MHz, $CDCl_3$) δ 155.5, 142.5, 136.0, 129.1, 128.7, 127.5, 125.3, 125.1, 123.1, 119.2, 115.0, 112.0, 39.2, 30.0.

IR (neat, cm^{-1}) 3023, 2944, 2247, 1452, 1103.

HRMS (ESI) calcd $C_{17}H_{14}NO^+$ $[M+H]^+$: 248.1070. Found: 248.1071.

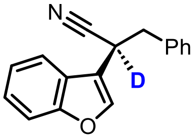

(–)-**33**-d

zwf-8-006

(–)-**33**-d was obtained as a white solid from the asymmetric decarboxylative deuteration reaction of 2-cyanoacetic acid **S33** (58.4 mg, 0.2 mmol) for 3 h using the general procedure (44.5 mg, 89% Yield, 94% D). R_f = 0.3 (Hexane/EtOAc 10:1).

HPLC analysis (Chiralpak IE-3, hexane/^{i}PrOH = 99/1, 0.8 mL/min, 205 nm; t_r (minor) = 27.73 min, t_r (major) = 32.07 min) gave the isomeric composition of the product: 95:5 e.r., $[\alpha]_D^{20}$ = –23.7 (*c* = 0.71, $CHCl_3$).

^{1}H NMR (400 MHz, $CDCl_3$) δ 7.59 (d, *J* = 7.6 Hz, 1H), 7.56-7.44 (m, 2H), 7.41-7.23 (m, 5H), 7.22-7.11 (m, 2H), 4.18 (t, *J* = 7.2 Hz, 0.06H), 3.34-3.20 (m, 2H).

^{13}C NMR (100 MHz, $CDCl_3$) δ 155.5, 142.5, 136.0, 129.1, 128.7, 127.5, 125.3, 125.1, 123.1, 119.2, 114.9, 112.0, 39.1, 29.7 (t, *J* = 21.0 Hz).

IR (neat, cm^{-1}) 3028, 2939, 2245, 1456, 1101.

HRMS (ESI) calcd $C_{17}H_{13}DNO^+$ $[M+H]^+$: 249.1133. Found: 249.1134.

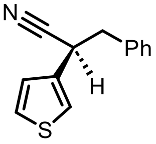

(–)-**34**

zwf-7-113

(−)-**34** was obtained as oil from the asymmetric decarboxylative protonation reaction of 2-cyanoacetic acid **S34** (128.6 mg, 0.5 mmol) for 4 h using the general procedure (93.2 mg, 87% Yield). R_f = 0.5 (Hexane/EtOAc 10:1).

HPLC analysis (Chiralpak IE-3, hexane/^{i}PrOH = 99/1, 0.8 mL/min, 205 nm; t_r (minor) = 23.16 min, t_r (major) = 24.43 min) gave the isomeric composition of the product: 95:5 e.r., $[\alpha]_D^{22}$ = –3.9 (*c* = 0.88, $CHCl_3$).

^{1}H NMR (400 MHz, $CDCl_3$) δ 7.38-7.22 (m, 4H), 7.19-7.07 (m, 3H), 6.98 (dd, J_1 = 4.8 Hz, J_1 = 1.2 Hz, 1H), 4.11 (t, *J* = 7.2 Hz, 1H), 3.22-3.10 (m, 2H).

^{13}C NMR (100 MHz, $CDCl_3$) δ 136.2, 135.0, 129.1, 128.6, 127.4, 127.0, 126.3, 122.8, 120.1, 41.0, 34.9.

IR (neat, cm^{-1}) 3107, 3033, 2924, 2242, 1454, 1078.

HRMS (ESI) calcd $C_{13}H_{12}NS^+$ $[M+H]^+$: 214.0685. Found: 214.0686.

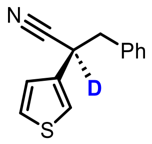

(–)-**34**-d

zwf-8-007

(−)-**34**-d was obtained as oil from the asymmetric decarboxylative deuteration reaction of 2-cyanoacetic acid **S34** (51.5 mg, 0.2 mmol) for 4 h using the general procedure (37.6 mg, 88% Yield, 90% D). R_f = 0.5 (Hexane/EtOAc 10:1).

HPLC analysis (Chiralpak IE-3, hexane/^{i}PrOH = 99/1, 0.8 mL/min, 205 nm; t_r (minor) = 27.93 min, t_r (major) = 29.32 min) gave the isomeric composition of the product: 95:5 e.r., $[\alpha]_D^{20}$ = –4.1 (*c* = 0.55, $CHCl_3$).

^{1}H NMR (400 MHz, $CDCl_3$) δ 7.49-7.22 (m, 4H), 7.22-7.05 (m, 3H), 6.98 (dd, J_1 = 5.0 Hz, J_1 = 1.4 Hz, 1H), 4.11 (t, *J* = 7.2 Hz, 0.10H), 3.27-3.05 (m, 2H).

^{13}C NMR (100 MHz, $CDCl_3$) δ 136.1, 135.0, 129.1, 128.6, 127.4, 127.0, 126.3, 122.8, 120.1, 40.9, 34.6 (t, *J* = 20.6 Hz).

IR (neat, cm^{-1}) 3104, 3038, 2929, 2242, 1455, 1081.

HRMS (ESI) calcd $C_{13}H_{10}DNNaS^+$ $[M+Na]^+$: 237.0567. Found: 237.0563.

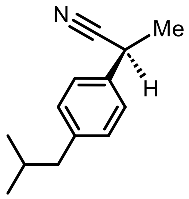

(−)-**35**

zwf-7-143

(−)-**35** was obtained as oil from the asymmetric decarboxylative protonation reaction of 2-cyanoacetic acid **S35** (115.8 mg, 0.5 mmol) for 24 h using the general procedure (c = 0.01, referring to the concentration of **S35** in 2-MeTHF) (85.8 mg, 91% Yield). R_f = 0.5 (Hexane/EtOAc 10:1).

HPLC analysis (Chiralpak IG-3, hexane/^{i}PrOH = 99/1, 0.8 mL/min, 205 nm; t_r (major) = 12.51 min, t_r (minor) = 17.37 min) gave the isomeric composition of the product: 93:7 e.r., $[\alpha]_D^{18}$ = −13.2 (*c* = 0.88, $CHCl_3$).

^{1}H NMR (400 MHz, $CDCl_3$) δ 7.25 (d, *J* = 8.0 Hz, 2H), 7.15 (d, *J* = 8.0 Hz, 2H), 3.87 (q, *J* = 7.3 Hz, 1H), 2.47 (d, *J* = 7.2 Hz, 2H), 1.85 (hept, *J* = 6.8 Hz, 1H), 1.63 (d, *J* = 7.2 Hz, 3H), 0.90 (d, *J* = 6.8 Hz, 6H).

^{13}C NMR (100 MHz, $CDCl_3$) δ 141.6, 134.3, 129.8, 126.4, 121.8, 44.9, 30.8, 30.1, 22.3, 21.4.

IR (neat, cm^{-1}) 2957, 2869, 2241, 1513, 1167.

HRMS (ESI) calcd $C_{13}H_{18}N^+$ $[M+H]^+$: 188.1434. Found: 188.1435.

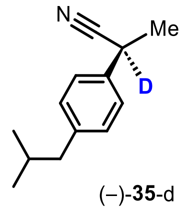

(−)-**35**-d

zwf-8-004

(−)-**35**-d was obtained as oil from the asymmetric decarboxylation reaction of 2-cyanoacetic acid **S35** (46.2 mg, 0.2 mmol) for 24 h using the general procedure (34.7 mg, 92% Yield, 97% D). R_f = 0.5 (Hexane/EtOAc 10:1).

HPLC analysis (Chiralpak IG-3, hexane/^{i}PrOH = 99/1, 0.8 mL/min, 205 nm; t_r (major) = 12.24 min, t_r (minor) = 16.83 min) gave the isomeric composition of the product: 95:5 e.r., $[\alpha]_D^{20}$ = −13.9 (*c* = 0.60, $CHCl_3$).

^{1}H NMR (400 MHz, $CDCl_3$) δ 7.25 (d, *J* = 8.4 Hz, 2H), 7.15 (d, *J* = 8.0 Hz, 2H), 3.87 (q, *J* = 7.3 Hz, 0.03H), 2.47 (d, *J* = 7.2 Hz, 2H), 1.85 (hept, *J* = 6.8 Hz, 1H), 1.62 (s, 3H), 0.90 (d, *J* = 6.4 Hz, 6H).

^{13}**C NMR** (100 MHz, $CDCl_3$) δ 141.6, 134.2, 129.8, 126.4, 121.8, 44.9, 30.6 (t, J = 20.3 Hz), 30.1, 22.3, 21.3.

IR (neat, cm^{-1}) 2955, 2871, 2241, 1515, 1164.

HRMS (ESI) calcd $C_{13}H_{17}DN^+$ $[M+H]^+$: 189.1497. Found: 189.1499.

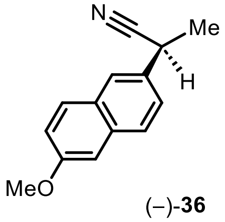

(–)-**36**

zwf-7-142

(–)-**36** was obtained as a white solid from the asymmetric decarboxylative protonation reaction of 2-cyanoacetic acid **S36** (127.8 mg, 0.5 mmol) for 24 h using the general procedure (c = 0.01, referring to the concentration of **S36** in 2-MeTHF) (97.4 mg, 92% Yield). R_f = 0.3 (Hexane/EtOAc 10:1).

HPLC analysis (Chiralpak IE-3, hexane/^{i}PrOH = 90/10, 1.1 mL/min, 205 nm; t_r (minor) = 10.20 min, t_r (major) = 10.59 min) gave the isomeric composition of the product: 95:5 e.r., $[\alpha]_D^{19}$ = –21.0 (c = 0.77, $CHCl_3$).

^{1}**H NMR** (400 MHz, $CDCl_3$) δ 7.80-7.68 (m, 3H), 7.38 (d, J = 8.4 Hz, 1H), 7.18 (dd, J_1 = 8.8 Hz, J_2 = 2.4 Hz, 1H), 7.13 (s, 1H), 4.02 (q, J = 7.2 Hz, 1H), 3.91 (s, 3H), 1.70 (d, J = 7.2 Hz, 3H).

^{13}**C NMR** (100 MHz, $CDCl_3$) δ 158.1, 134.0, 132.0, 129.3, 128.7, 127.8, 125.3, 124.9, 121.7, 119.5, 105.6, 55.3, 31.2, 21.4.

IR (neat, cm^{-1}) 2991, 2964, 2242, 1601, 1187.

HRMS (ESI) calcd $C_{14}H_{14}NO^+$ $[M+H]^+$: 212.1070. Found: 212.1071.

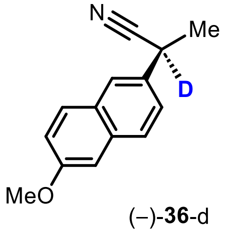

(–)-**36**-d

zwf-8-003

(−)-**36**-d was obtained as a white solid from the asymmetric decarboxylative deuteration reaction of 2-cyanoacetic acid **S36** (51.0 mg, 0.2 mmol) for 24 h using the general procedure (38.5 mg, 91% Yield, 95% D). R_f = 0.3 (Hexane/EtOAc 10:1).

HPLC analysis (Chiralpak IE-3, hexane/^{i}PrOH = 90/10, 1.1 mL/min, 205 nm; t_r (minor) = 10.31 min, t_r (major) = 10.65 min) gave the isomeric composition of the product: 95:5 e.r., $[\alpha]_D^{19}$ = –20.2 (*c* = 0.52, $CHCl_3$).

^{1}H NMR (400 MHz, $CDCl_3$) δ 7.80-7.65 (m, 3H), 7.38 (dd, J_1 = 8.4 Hz, J_2 = 2.0 Hz, 1H), 7.18 (dd, J_1 = 9.0 Hz, J_2 = 2.6 Hz, 1H), 7.12 (d, *J* = 2.4 Hz, 1H), 4.01 (q, *J* = 7.3 Hz, 0.05H), 3.91 (s, 3H), 1.69 (s, 3H).

^{13}C NMR (100 MHz, $CDCl_3$) δ 158.0, 134.0, 131.9, 129.3, 128.7, 127.8, 125.3, 124.9, 121.7, 119.5, 105.6, 55.3, 30.9 (t, *J* = 20.6 Hz), 21.3.

IR (neat, cm^{-1}) 2991, 2960, 2242, 1601, 1164.

HRMS (ESI) calcd $C_{14}H_{13}DNO^+$ $[M+H]^+$: 213.1133. Found: 213.1137.

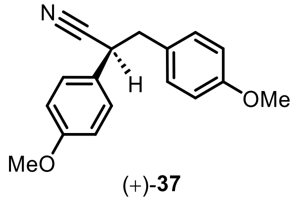

(+)-**37**

zwf-7-134

(+)-**37** was obtained as a white solid from the asymmetric decarboxylative protonation reaction of 2-cyanoacetic acid **S37** (155.6 mg, 0.5 mmol) for 24 h using the general procedure (c = 0.025, referring to the concentration of **S37** in 2-MeTHF) (121.8 mg, 91% Yield). R_f = 0.2 (Hexane/EtOAc 10:1).

HPLC analysis (Chiralpak IE-3, hexane/^{i}PrOH = 90/10, 0.8 mL/min, 205 nm; t_r (minor) = 19.51 min, t_r (major) = 21.51 min) gave the isomeric composition of the product: 97:3 e.r., $[\alpha]_D^{18}$ = +3.2 (*c* = 0.55, $CHCl_3$).

^{1}H NMR (400 MHz, $CDCl_3$) δ 7.14 (d, *J* = 8.4 Hz, 2H), 7.03 (d, *J* = 8.4 Hz, 2H), 6.86 (d, *J* = 8.8 Hz, 2H), 6.81 (d, *J* = 8.4 Hz, 2H), 3.90 (t, *J* = 7.6 Hz, 1H), 3.79 (s, 3H), 3.78 (s, 3H), 3.14-3.00 (m, 2H).

^{13}C NMR (100 MHz, $CDCl_3$) δ 159.3, 158.7, 130.3, 128.6, 128.3, 127.2, 120.7, 114.2, 113.9, 55.25, 55.15, 41.4, 39.1.

IR (neat, cm^{-1}) 2964, 2922, 2242, 1509, 1031.

HRMS (ESI) calcd $C_{17}H_{18}NO_2^+$ $[M+H]^+$: 268.1332. Found: 268.1330.

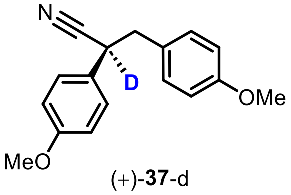
(+)-**37**-d

zwf-7-153

(+)-**37**-d was obtained as a white solid from the asymmetric decarboxylative deuteration reaction of 2-cyanoacetic acid **S37** (62.3 mg, 0.2 mmol) for 24 h using the general procedure (48.5 mg, 90% Yield, 95% D). R_f = 0.2 (Hexane/EtOAc 10:1).

HPLC analysis (Chiralpak IE-3, hexane/^{i}PrOH = 90/10, 0.8 mL/min, 205 nm; t_r (minor) = 20.49 min, t_r (major) = 22.69 min) gave the isomeric composition of the product: 97.5:2.5 e.r., $[\alpha]_D^{19}$ = +4.9 (*c* = 0.67, $CHCl_3$).

^{1}H NMR (400 MHz, $CDCl_3$) δ 7.14 (d, *J* = 8.8 Hz, 2H), 7.02 (d, *J* = 8.4 Hz, 2H), 6.86 (d, *J* = 8.8 Hz, 2H), 6.81 (d, *J* = 8.4 Hz, 2H), 3.90 (t, *J* = 7.2 Hz, 0.05H), 3.79 (s, 3H), 3.77 (s, 3H), 3.13-2.97 (m, 2H).

^{13}C NMR (100 MHz, $CDCl_3$) δ 159.3, 158.7, 130.3, 128.6, 128.3, 127.1, 120.7, 114.2, 113.9, 55.2, 55.1, 41.3, 38.8 (t, *J* = 20.6 Hz).

IR (neat, cm^{-1}) 2969, 2923, 2242, 1513, 1033.

HRMS (ESI) calcd $C_{17}H_{17}DNO_2^+$ $[M+H]^+$: 269.1395. Found: 269.1398.

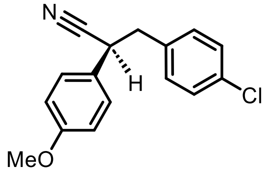
(+)-**38**

zwf-7-135

(+)-**38** was obtained as a white solid from the asymmetric decarboxylative protonation reaction of 2-cyanoacetic acid **S38** (158.1 mg, 0.5 mmol) for 2 h using the general procedure (c = 0.025, referring to the concentration of **S38** in 2-MeTHF) (127.3 mg, 94% Yield). R_f = 0.3 (Hexane/EtOAc 10:1).

HPLC analysis (Chiralpak IE-3, hexane/^{i}PrOH = 99/1, 0.8 mL/min, 205 nm; t_r (minor) = 38.11 min, t_r (major) = 39.39 min) gave the isomeric composition of the product: 96.5:3.5 e.r., $[\alpha]_D^{18}$ = +2.8 (*c* = 0.53, $CHCl_3$).

^{1}H NMR (400 MHz, $CDCl_3$) δ 7.24 (d, *J* = 8.4 Hz, 2H), 7.13 (d, *J* = 8.4 Hz, 2H), 7.02 (d, *J* = 8.4 Hz, 2H),

6.86 (d, J = 8.8 Hz, 2H), 3.93 (t, J = 7.2 Hz, 1H), 3.80 (s, 3H), 3.17-3.02 (m, 2H).

^{13}C NMR (100 MHz, $CDCl_3$) δ 159.4, 134.6, 133.2, 130.6, 128.7, 128.6, 126.6, 120.3, 114.3, 55.3, 41.4, 38.6.

IR (neat, cm^{-1}) 2974, 2939, 2242, 1510, 1030.

HRMS (ESI) calcd $C_{16}H_{15}ClNO^+$ $[M+H]^+$: 272.0837. Found: 272.0834.

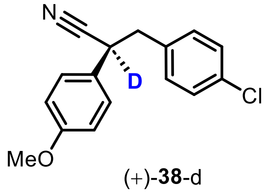

(+)-**38**-d

zwf-7-154

(+)-**38**-d was obtained as a white solid from the asymmetric decarboxylative protonation reaction of 2-cyanoacetic acid **S38** (63.2 mg, 0.2 mmol) for 2 h using the general procedure (51.7 mg, 95% Yield, 94% D). R_f = 0.3 (Hexane/EtOAc 10:1).

HPLC analysis (Chiralpak IE-3, hexane/^{i}PrOH = 99/1, 0.8 mL/min, 205 nm; t_r (minor) = 36.29 min, t_r (major) = 37.73 min) gave the isomeric composition of the product: 97:3 e.r., $[\alpha]_D^{18}$ = +2.3 (c = 0.62, $CHCl_3$).

^{1}H NMR (400 MHz, $CDCl_3$) δ 7.25 (d, J = 6.4 Hz, 2H), 7.13 (d, J = 8.4 Hz, 2H), 7.03 (d, J = 8.0 Hz, 2H), 6.87 (d, J = 8.4 Hz, 2H), 3.93 (t, J = 7.2 Hz, 0.06H), 3.81 (s, 3H), 3.19-3.01 (m, 2H).

^{13}C NMR (100 MHz, $CDCl_3$) δ 159.4, 134.6, 133.2, 130.6, 128.7, 128.6, 126.6, 120.3, 114.4, 55.3, 41.3, 38.3 (t, J = 20.6 Hz).

IR (neat, cm^{-1}) 2969, 2939, 2242, 1511, 1031.

HRMS (ESI) calcd $C_{16}H_{14}DClNO^+$ $[M+H]^+$: 273.0899. Found: 273.0897.

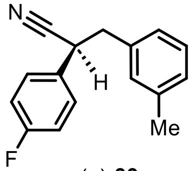

(+)-**39**

zwf-7-138

(+)-**39** was obtained as oil from the asymmetric decarboxylative protonation reaction of 2-cyanoacetic acid **S39** (141.6 mg, 0.5 mmol) for 2 h using the general procedure (c = 0.025, referring to the concentration of **S39** in 2-MeTHF) (109.2 mg, 91% Yield). R_f = 0.4 (Hexane/EtOAc 10:1).

HPLC analysis (Chiralpak IE-3, hexane/^{i}PrOH = 99/1, 0.8 mL/min, 205 nm; t_r (minor) = 15.58 min, t_r (major) = 16.46 min) gave the isomeric composition of the product: 95.5:4.5 e.r., $[\alpha]_D^{18}$ = +2.0 (*c* = 0.44, $CHCl_3$).

^{1}H NMR (400 MHz, $CDCl_3$) δ 7.26-7.14 (m, 3H), 7.12-6.98 (m, 3H), 6.97-6.84 (m, 2H), 3.97 (t, *J* = 7.4 Hz, 1H), 3.19-3.01 (m, 2H), 2.31 (s, 3H).

^{13}C NMR (100 MHz, $CDCl_3$) δ 162.4 (d, *J* = 246.2 Hz), 138.3, 135.9, 131.1 (d, *J* = 2.9 Hz), 129.9, 129.2 (d, *J* = 8.0 Hz), 128.5, 128.2, 126.2, 120.2, 115.9 (d, *J* = 21.7 Hz), 42.1, 39.0, 21.3.

^{19}F NMR (376 MHz, $CDCl_3$) δ −113.5 to −113.7 (m).

IR (neat, cm^{-1}) 3043, 2939, 2242, 1505, 1225, 1017.

HRMS (ESI) calcd $C_{16}H_{15}FN^+$ $[M+H]^+$: 240.1183. Found: 240.1184.

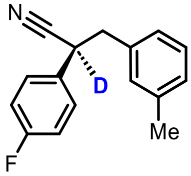

(+)-**39**-d

zwf-7-157

(+)-**39**-d was obtained as oil from the asymmetric decarboxylative deuteration reaction of 2-cyanoacetic acid **S39** (56.7 mg, 0.2 mmol) for 2 h using the general procedure (44.2 mg, 92% Yield, 94% D). R_f = 0.4 (Hexane/EtOAc 10:1).

HPLC analysis (Chiralpak IE-3, hexane/^{i}PrOH = 99/1, 0.8 mL/min, 205 nm; t_r (minor) = 15.36 min, t_r (major) = 16.26 min) gave the isomeric composition of the product: 96:4 e.r., $[\alpha]_D^{18}$ = +2.2 (*c* = 0.58, $CHCl_3$).

^{1}H NMR (400 MHz, $CDCl_3$) δ 7.27-7.13 (m, 3H), 7.12-6.97 (m, 3H), 6.96-6.84 (m, 2H), 3.97 (t, *J* = 7.4 Hz, 0.06H), 3.21-2.95 (m, 2H), 2.31 (s, 3H).

^{13}C NMR (100 MHz, $CDCl_3$) δ 162.4 (d, *J* = 246.3 Hz), 138.3, 135.8, 131.0 (d, *J* = 3.6 Hz), 129.9, 129.2 (d, *J* = 8.7 Hz), 128.5, 128.2, 126.2, 120.3, 115.9 (d, *J* = 21.7 Hz), 42.0, 38.7 (t, *J* = 20.6 Hz), 21.3.

^{19}**F NMR** (376 MHz, $CDCl_3$) δ −113.5 to −113.7 (m).

IR (neat, cm^{-1}) 3041, 2920, 2242, 1500, 1224, 1019.

HRMS (ESI) calcd $C_{16}H_{14}DFN^+$ $[M+H]^+$: 241.1246. Found: 241.1249.

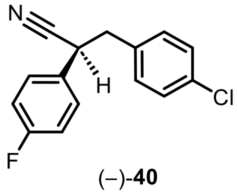

(−)-**40**

zwf-7-139

(−)-**40** was obtained as a white solid from the asymmetric decarboxylative protonation reaction of 2-cyanoacetic acid **S40** (151.9 mg, 0.5 mmol) for 2 h using the general procedure (c = 0.025, referring to the concentration of **S40** in 2-MeTHF) (123.7 mg, 95% Yield). R_f = 0.4 (Hexane/EtOAc 10:1).

HPLC analysis (Chiralpak IE-3, hexane/^{i}PrOH = 99/1, 0.8 mL/min, 205 nm; t_r (minor) = 18.98 min, t_r (major) = 19.78 min) gave the isomeric composition of the product: 96:4 e.r., $[\alpha]_D^{19}$ = –5.6 (c = 0.65, $CHCl_3$).

^{1}**H NMR** (400 MHz, $CDCl_3$) δ 7.25 (d, J = 8.4 Hz, 2H), 7.22-7.12 (m, 2H), 7.10-6.95 (m, 4H), 3.98 (t, J = 7.2 Hz, 1H), 3.22-3.03 (m, 2H).

^{13}**C NMR** (100 MHz, $CDCl_3$) δ 162.5 (d, J = 246.2 Hz), 134.2, 133.4, 130.6, 130.5 (d, J = 2.9 Hz), 129.2 (d, J = 8.0 Hz), 128.8, 119.9, 116.0 (d, J = 21.7 Hz), 41.3, 38.7.

^{19}**F NMR** (376 MHz, $CDCl_3$) δ −113.1 to −113.2 (m).

IR (neat, cm^{-1}) 3043, 2939, 2242, 1507, 1222, 1018.

HRMS (ESI) calcd $C_{15}H_{12}ClFN^+$ $[M+H]^+$: 260.0637. Found: 260.0634.

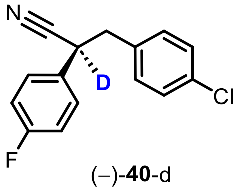

(−)-**40**-d

zwf-8-001

(−)-**40**-d was obtained as a white solid from the asymmetric decarboxylative deuteration reaction of 2-cyanoacetic acid **S40** (60.8 mg, 0.2 mmol) for 2 h using the general procedure (49.5 mg, 95% Yield, 94% D). R_f = 0.4 (Hexane/EtOAc 10:1).

HPLC analysis (Chiralpak IE-3, hexane/^{i}PrOH = 99/1, 0.8 mL/min, 205 nm; t_r (minor) = 18.92 min, t_r (major) = 19.75 min) gave the isomeric composition of the product: 96.5:3.5 e.r., $[\alpha]_D^{19}$ = –5.2 (*c* = 0.75, $CHCl_3$).

^{1}H NMR (400 MHz, $CDCl_3$) δ 7.26 (d, *J* = 8.4 Hz, 2H), 7.23-7.13 (m, 2H), 7.12-6.93 (m, 4H), 3.98 (t, *J* = 7.0 Hz, 0.06H), 3.23-3.00 (m, 2H).

^{13}C NMR (100 MHz, $CDCl_3$) δ 162.4 (d, *J* = 246.3 Hz), 134.2, 133.4, 130.6, 130.4 (d, *J* = 2.9 Hz), 129.2 (d, *J* = 8.7 Hz), 128.8, 119.9, 116.0 (d, *J* = 22.4 Hz), 41.2, 38.4 (t, *J* = 20.6 Hz).

^{19}F NMR (376 MHz, $CDCl_3$) δ –113.1 to –113.3 (m).

IR (neat, cm^{-1}) 3028, 2934, 2242, 1505, 1221, 1013.

HRMS (ESI) calcd $C_{15}H_{11}DClFN^+$ $[M+H]^+$: 261.0700. Found: 261.0702.

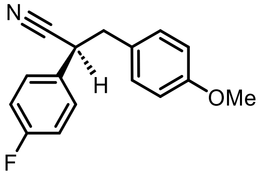

(–)-**41**

zwf-7-137

(–)-**41** was obtained as a white solid from the asymmetric decarboxylative protonation reaction of 2-cyanoacetic acid **S41** (149.8 mg, 0.5 mmol) for 2 h using the general procedure (c = 0.025, referring to the concentration of **S41** in 2-MeTHF) (119.2 mg, 93% Yield). R_f = 0.3 (Hexane/EtOAc 10:1).

HPLC analysis (Chiralpak IE-3, hexane/^{i}PrOH = 99/1, 0.8 mL/min, 205 nm; t_r (minor) = 31.28 min, t_r (major) = 33.89 min) gave the isomeric composition of the product: 96:4 e.r., $[\alpha]_D^{18}$ = –1.6 (*c* = 0.60, $CHCl_3$).

^{1}H NMR (400 MHz, $CDCl_3$) δ 7.23-7.14 (m, 2H), 7.07-6.96 (m, 4H), 6.81 (d, *J* = 8.8 Hz, 2H), 3.95 (t, *J* = 7.2 Hz, 1H), 3.78 (s, 3H), 3.18-3.00 (m, 2H).

^{13}C NMR (100 MHz, $CDCl_3$) δ 162.3 (d, *J* = 246.3 Hz), 158.9, 130.9 (d, *J* = 3.7 Hz), 130.3, 129.2 (d, *J* = 8.0 Hz), 127.9, 120.3, 115.9 (d, *J* = 21.7 Hz), 114.0, 55.2, 41.3, 39.2.

^{19}F NMR (376 MHz, $CDCl_3$) δ –113.5 to –113.7 (m).

IR (neat, cm^{-1}) 2964, 2929, 2242, 1507, 1216, 1025.

HRMS (ESI) calcd $C_{16}H_{15}FNO^+$ $[M+H]^+$: 256.1132. Found: 256.1130.

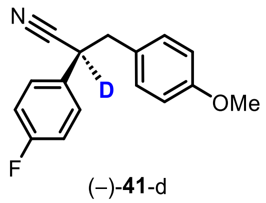

(–)-**41**-d

zwf-7-156

(−)-**41**-d was obtained as a white solid from the asymmetric decarboxylative deuteration reaction of 2-cyanoacetic acid **S41** (60.0 mg, 0.2 mmol) for 2 h using the general procedure (46.9 mg, 91% Yield, 95% D). R_f = 0.3 (Hexane/EtOAc 10:1).

HPLC analysis (Chiralpak IE-3, hexane/^{i}PrOH = 99/1, 0.8 mL/min, 205 nm; t_r (minor) = 31.21 min, t_r (major) = 33.78 min) gave the isomeric composition of the product: 96:4 e.r., $[\alpha]_D^{18}$ = −1.6 (*c* = 0.53, $CHCl_3$).

^{1}H NMR (400 MHz, $CDCl_3$) δ 7.24-7.11 (m, 2H), 7.10-6.93 (m, 4H), 6.81 (d, *J* = 8.4 Hz, 2H), 3.95 (t, *J* = 7.0 Hz, 0.05H), 3.78 (s, 3H), 3.18-2.97 (m, 2H).

^{13}C NMR (100 MHz, $CDCl_3$) δ 162.3 (d, *J* = 246.3 Hz), 158.9, 130.9 (d, *J* = 3.6 Hz), 130.3, 129.2 (d, *J* = 8.0 Hz), 127.8, 120.3, 115.9 (d, *J* = 21.7 Hz), 113.9, 55.2, 41.2, 38.8 (t, *J* = 21.0 Hz).

^{19}F NMR (376 MHz, $CDCl_3$) δ −113.5 to −113.7 (m).

IR (neat, cm^{-1}) 2959, 2925, 2242, 1506, 1215, 1025.

HRMS (ESI) calcd $C_{16}H_{14}DFNO^+$ $[M+H]^+$: 257.1195. Found: 257.1192.

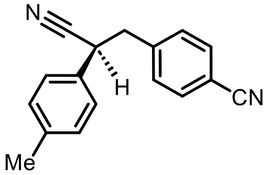

(–)-**42**

zwf-7-141

(−)-**42** was obtained as a white solid from the asymmetric decarboxylative protonation reaction of 2-cyanoacetic acid **S42** (145.2 mg, 0.5 mmol) for 2 h using the general procedure (c = 0.025, referring to the concentration of **S42** in 2-MeTHF) (112.7 mg, 91% Yield). R_f = 0.2 (Hexane/EtOAc 10:1).

HPLC analysis (Chiralpak IE-3, hexane/^{i}PrOH = 90/10, 1.1 mL/min, 205 nm; t_r (minor) = 22.36 min, t_r (major) = 23.56 min) gave the isomeric composition of the product: 96.5:3.5 e.r., $[\alpha]_D^{19}$ = −12.7 (*c* = 0.44,

$CHCl_3$).

^{1}H NMR (400 MHz, $CDCl_3$) δ 7.57 (d, J = 8.0 Hz, 2H), 7.22 (d, J = 8.0 Hz, 2H), 7.16 (d, J = 8.0 Hz, 2H), 7.10 (d, J = 8.0 Hz, 2H), 4.01 (t, J = 7.2 Hz, 1H), 3.28-3.14 (m, 2H), 2.35 (s, 3H).

^{13}C NMR (100 MHz, $CDCl_3$) δ 141.5, 138.4, 132.3, 131.1, 130.1, 129.8, 127.2, 119.8, 118.5, 111.3, 41.8, 38.6, 21.0.

IR (neat, cm^{-1}) 3023, 2924, 2242, 2225, 1505, 1215, 1078.

HRMS (ESI) calcd $C_{17}H_{15}N_2^+$ $[M+H]^+$: 247.1230. Found: 247.1233.

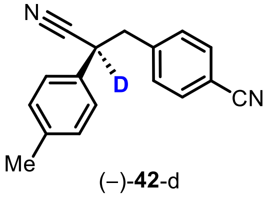

(−)-**42**-d

zwf-8-002

(−)-**42**-d was obtained as a white solid from the asymmetric decarboxylative deuteration reaction of 2-cyanoacetic acid **S42** (58.2 mg, 0.2 mmol) for 2 h using the general procedure (45.7 mg, 92% Yield, 93% D). R$_f$ = 0.2 (Hexane/EtOAc 10:1).

HPLC analysis (Chiralpak IE-3, hexane/^{i}PrOH = 90/10, 1.1 mL/min, 205 nm; t_r (minor) = 24.06 min, t_r (major) = 25.38 min) gave the isomeric composition of the product: 97:3 e.r., $[\alpha]_D^{19}$ = −14.1 (c = 0.68, $CHCl_3$).

^{1}H NMR (400 MHz, $CDCl_3$) δ 7.57 (d, J = 7.2 Hz, 2H), 7.22 (d, J = 7.2 Hz, 2H), 7.16 (d, J = 7.2 Hz, 2H), 7.10 (d, J = 7.6 Hz, 2H), 4.01 (t, J = 6.6 Hz, 0.07H), 3.30-3.10 (m, 2H), 2.34 (s, 3H).

^{13}C NMR (100 MHz, $CDCl_3$) δ 141.5, 138.4, 132.3, 131.1, 130.1, 129.8, 127.2, 119.8, 118.5, 111.3, 41.7, 38.3 (t, J = 19.9 Hz), 21.0.

IR (neat, cm^{-1}) 3018, 2924, 2247, 2225, 1507, 1217, 1021.

HRMS (ESI) calcd $C_{17}H_{14}DN_2^+$ $[M+H]^+$: 248.1293. Found: 248.1291.

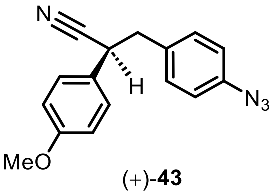

(+)-**43**

zwf-7-136

(+)-**43** was obtained as a white solid from the asymmetric decarboxylative protonation reaction of 2-cyanoacetic acid **S43** (161.2 mg, 0.5 mmol) for 2 h using the general procedure (c = 0.025, referring to the concentration of **S43** in 2-MeTHF) (125.6 mg, 90% Yield). R_f = 0.3 (Hexane/EtOAc 10:1).

HPLC analysis (Chiralpak IE-3, hexane/^{i}PrOH = 99/1, 0.8 mL/min, 205 nm; t_r (minor) = 58.37 min, t_r (major) = 61.04 min) gave the isomeric composition of the product: 96:4 e.r., $[\alpha]_D^{18}$ = +3.2 (*c* = 0.58, $CHCl_3$).

^{1}H NMR (400 MHz, $CDCl_3$) δ 7.13 (d, *J* = 8.4 Hz, 2H), 7.08 (d, *J* = 8.4 Hz, 2H), 6.94 (d, *J* = 8.4 Hz, 2H), 6.86 (d, *J* = 8.4 Hz, 2H), 3.93 (t, *J* = 7.2 Hz, 1H), 3.80 (s, 3H), 3.18-3.03 (m, 2H).

^{13}C NMR (100 MHz, $CDCl_3$) δ 159.4, 139.1, 132.9, 130.7, 128.6, 126.7, 120.4, 119.1, 114.3, 55.3, 41.4, 38.8.

IR (neat, cm^{-1}) 2969, 2934, 2242, 2111, 1506, 1250.

HRMS (ESI) calcd $C_{16}H_{14}N_4NaO^+$ $[M+Na]^+$: 301.1060. Found: 301.1065.

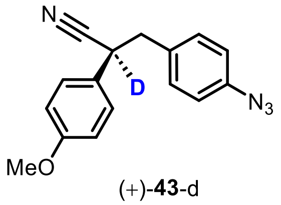

(+)-**43**-d

zwf-7-155

(+)-**43**-d was obtained as white solid from the asymmetric decarboxylative deuteration reaction of 2-cyanoacetic acid **S43** (64.5 mg, 0.2 mmol) for 2 h using the general procedure (51.3 mg, 92% Yield, 94% D). R_f = 0.3 (Hexane/EtOAc 10:1).

HPLC analysis (Chiralpak IE-3, hexane/^{i}PrOH = 99/1, 0.8 mL/min, 205 nm; t_r (minor) = 55.81 min, t_r (major) = 58.89 min) gave the isomeric composition of the product: 96:4 e.r., $[\alpha]_D^{18}$ = +4.0 (*c* = 0.72, $CHCl_3$).

^{1}H NMR (400 MHz, $CDCl_3$) δ 7.13 (d, *J* = 8.4 Hz, 2H), 7.08 (d, *J* = 8.4 Hz, 2H), 6.94 (d, *J* = 8.4 Hz, 2H), 6.86 (d, *J* = 8.4 Hz, 2H), 3.93 (t, *J* = 7.2 Hz, 0.06H), 3.80 (s, 3H), 3.20-3.00 (m, 2H).

^{13}C NMR (100 MHz, $CDCl_3$) δ 159.4, 139.1, 132.9, 130.7, 128.6, 126.7, 120.4, 119.1, 114.3, 55.3, 41.3, 38.5 (t, *J* = 21.0 Hz).

IR (neat, cm^{-1}) 2968, 2931, 2242, 2110, 1503, 1249.

HRMS (ESI) calcd $C_{16}H_{13}DN_4NaO^+$ $[M+Na]^+$: 302.1123. Found: 302.1124.

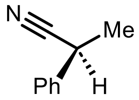

(–)-**44**

zwf-7-062

(–)-**44** was obtained as oil from the asymmetric decarboxylative protonation reaction of 2-cyanoacetic acid **S44** (87.6 mg, 0.5 mmol) for 12 h using the general procedure (c = 0.01, referring to the concentration of **S44** in 2-MeTHF) (57.4 mg, 88% Yield). R_f = 0.4 (Hexane/EtOAc 10:1).

HPLC analysis (Chiralpak IE-3, hexane/^{i}PrOH = 99/1, 0.8 mL/min, 205 nm; t_r (minor) = 12.81 min, t_r (major) = 13.39 min) gave the isomeric composition of the product: 93:7 e.r., $[\alpha]_D^{25}$ = –20.1 (*c* = 0.36, $CHCl_3$).

^{1}H NMR (400 MHz, $CDCl_3$) δ 7.44-7.28 (m, 5H), 3.90 (q, *J* = 7.2 Hz, 1H), 1.64 (d, *J* = 7.6 Hz, 3H).

^{13}C NMR (100 MHz, $CDCl_3$) δ 137.0, 129.1, 128.0, 126.7, 121.6, 31.2, 21.4.

IR (neat, cm^{-1}) 3032, 2937, 2241, 1452, 1077.

HRMS (ESI) calcd $C_9H_9NNa^+$ $[M+Na]^+$: 154.0627. Found: 154.0629.

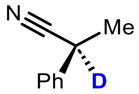

(–)-**44**-d

zwf-8-009

(–)-**44**-d was obtained as oil from the asymmetric decarboxylative deuteration reaction of 2-cyanoacetic acid **S44** (35.0 mg, 0.2 mmol) for 2 h using the general procedure (22.3 mg, 84% Yield, 91% D). R_f = 0.4 (Hexane/EtOAc 10:1).

HPLC analysis (Chiralpak IE-3, hexane/^{i}PrOH = 99/1, 0.8 mL/min, 205 nm; t_r (minor) = 14.45 min, t_r (major) = 15.06 min) gave the isomeric composition of the product: 93.5:6.5 e.r., $[\alpha]_D^{20}$ = –22.0 (*c* = 0.48, $CHCl_3$).

^{1}H NMR (400 MHz, $CDCl_3$) δ 7.52-7.20 (m, 5H), 3.90 (q, *J* = 7.2 Hz, 0.09H), 1.63 (s, 3H).

^{13}C NMR (100 MHz, $CDCl_3$) δ 137.0, 129.1, 128.0, 126.6, 121.5, 30.9 (t, *J* = 20.6 Hz), 21.3.

IR (neat, cm^{-1}) 3030, 2936, 2242, 1451, 1078.

HRMS (ESI) calcd $C_9H_8DNNa^+$ $[M+Na]^+$: 155.0690. Found: 155.0689.

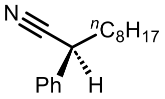

(–)-**45**

zwf-7-149

(–)-**45** was obtained as oil from the asymmetric decarboxylative protonation reaction of 2-cyanoacetic acid **S45** (136.5 mg, 0.5 mmol) for 10 h using the general procedure (c = 0.005, referring to the concentration of **Sx** in 2-MeTHF) (102.6 mg, 90% Yield). R_f = 0.8 (Hexane/EtOAc 10:1).

HPLC analysis (Chiralpak IE-3, hexane/^{i}PrOH = 99/1, 0.8 mL/min, 205 nm; t_r (minor) = 10.32 min, t_r (major) = 10.77 min) gave the isomeric composition of the product: 89:11 e.r., $[\alpha]_D^{18}$ = –19.3 (*c* = 0.83, $CHCl_3$).

^{1}H NMR (400 MHz, $CDCl_3$) δ 7.44-7.26 (m, 5H), 3.76 (t, *J* = 7.4 Hz, 1H), 2.00-1.76 (m, 2H), 1.56-1.18 (m, 12H), 0.87 (t, *J* = 7.0 Hz, 3H).

^{13}C NMR (100 MHz, $CDCl_3$) δ 136.1, 129.0, 127.9, 127.2, 120.9, 37.4, 35.9, 31.7, 29.2, 29.1, 28.9, 27.0, 22.6, 14.0.

IR (neat, cm^{-1}) 2925, 2856, 2242, 1454, 1077.

HRMS (ESI) calcd $C_{16}H_{24}N^+$ $[M+H]^+$: 230.1903. Found: 230.1901.

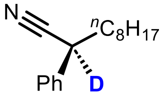

(–)-**45**-d

zwf-8-010

(–)-**45**-d was obtained as oil from the asymmetric decarboxylative deuteration reaction of 2-cyanoacetic acid **S45** (54.7 mg, 0.2 mmol) for 12 h using the general procedure (42.3 mg, 92% Yield, 96% D). R_f = 0.8 (Hexane/EtOAc 10:1).

HPLC analysis (Chiralpak IE-3, hexane/^{i}PrOH = 99/1, 0.8 mL/min, 205 nm; t_r (minor) = 10.26 min, t_r (major) = 10.67 min) gave the isomeric composition of the product: 95:5 e.r., $[\alpha]_D^{20}$ = –23.2 (*c* = 0.63, $CHCl_3$).

^{1}H NMR (400 MHz, $CDCl_3$) δ 7.47-7.26 (m, 5H), 3.76 (t, *J* = 7.4 Hz, 0.04H), 1.97-1.77 (m, 2H), 1.56-

1.17 (m, 12H), 0.87 (t, J = 7.0 Hz, 3H).

^{13}C NMR (100 MHz, $CDCl_3$) δ 136.0, 129.0, 127.9, 127.2, 120.9, 37.0 (t, J = 20.3 Hz), 35.8, 31.7, 29.2, 29.1, 28.9, 26.9, 22.6, 14.0.

IR (neat, cm^{-1}) 2924, 2852, 2241, 1456, 1077.

HRMS (ESI) calcd $C_{16}H_{23}DN^+$ $[M+H]^+$: 231.1966. Found: 231.1968.

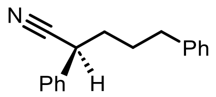

(−)-**46**

zwf-7-144

(−)-**46** was obtained as oil from the asymmetric decarboxylative protonation reaction of 2-cyanoacetic acid **S46** (139.9 mg, 0.5 mmol) for 48 h using the general procedure (c = 0.01, referring to the concentration of **S46** in 2-MeTHF) (106.2 mg, 90% Yield). R_f = 0.5 (Hexane/EtOAc 10:1).

HPLC analysis (Chiralpak IE-3, hexane/^{i}PrOH = 99/1, 0.8 mL/min, 205 nm; t_r (minor) = 19.76 min, t_r (major) = 21.31 min) gave the isomeric composition of the product: 73.5:26.5 e.r., $[\alpha]_D^{20}$ = –9.2 (c = 0.64, $CHCl_3$).

^{1}H NMR (400 MHz, $CDCl_3$) δ 7.50-7.05 (m, 10H), 3.75 (t, J = 7.0 Hz, 1H), 2.64 (t, J = 7.2 Hz, 2H), 2.00-1.71 (m, 4H).

^{13}C NMR (100 MHz, $CDCl_3$) δ 141.1, 135.7, 129.0, 128.4, 128.3, 128.0, 127.2, 126.0, 120.7, 37.2, 35.1, 35.0, 28.5.

IR (neat, cm^{-1}) 3027, 2935, 2240, 1495, 1450.

HRMS (ESI) calcd $C_{17}H_{18}N^+$ $[M+H]^+$: 236.1434. Found: 236.1436.

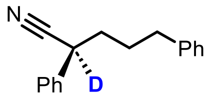

(−)-**46**-d

zwf-8-043

(−)-**46**-d was obtained as oil from the asymmetric decarboxylative deuteration reaction of 2-cyanoacetic acid **S46** (55.8 mg, 0.2 mmol) for 48 h using the general procedure (41.2 mg, 87% Yield, 96% D). R_f = 0.5 (Hexane/EtOAc 10:1).

HPLC analysis (Chiralpak IE-3, hexane/^{i}PrOH = 99/1, 0.8 mL/min, 205 nm; t_r (minor) = 19.78 min, t_r (major) = 21.21 min) gave the isomeric composition of the product: 87.5:12.5 e.r., $[\alpha]_D^{20}$ = –16.8 (*c* = 0.51, $CHCl_3$).

^{1}H NMR (400 MHz, $CDCl_3$) δ 7.50-7.00 (m, 10H), 3.75 (t, *J* = 6.4 Hz, 0.04H), 2.64 (t, *J* = 6.6 Hz, 2H), 2.14-1.62 (m, 4H).

^{13}C NMR (100 MHz, $CDCl_3$) δ 141.1, 135.7, 129.0, 128.4, 128.3, 128.0, 127.2, 126.0, 120.7, 36.9 (t, *J* = 20.2 Hz), 35.05, 35.03, 28.5.

IR (neat, cm^{-1}) 3027, 2932, 2240, 1494, 1451.

HRMS (ESI) calcd $C_{17}H_{17}DN^+$ $[M+H]^+$: 237.1497. Found: 237.1496.

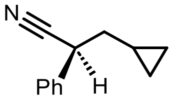

(–)-**47**

zwf-7-150

(–)-**47** was obtained as oil from the asymmetric decarboxylative protonation reaction of 2-cyanoacetic acid **S47** (107.9 mg, 0.5 mmol) for 10 h using the general procedure (c = 0.005, referring to the concentration of **S47** in 2-MeTHF) (74.1 mg, 86% Yield). R_f = 0.6 (Hexane/EtOAc 10:1).

HPLC analysis (Chiralpak IE-3, hexane/^{i}PrOH = 99/1, 0.8 mL/min, 205 nm; t_r (minor) = 14.31 min, t_r (major) = 15.33 min) gave the isomeric composition of the product: 98:2 e.r., $[\alpha]_D^{20}$ = –27.8 (*c* = 0.53, $CHCl_3$).

^{1}H NMR (400 MHz, $CDCl_3$) δ 7.42-7.28 (m, 5H), 3.86 (t, *J* = 7.2 Hz, 1H), 1.95-1.83 (m, 1H), 1.77-1.67 (m, 1H), 0.92-0.78 (m, 1H), 0.60-0.46 (m, 2H), 0.23-0.09 (m, 2H).

^{13}C NMR (100 MHz, $CDCl_3$) δ 135.9, 129.0, 127.9, 127.3, 121.0, 41.0, 37.8, 8.8, 4.7, 4.4.

IR (neat, cm^{-1}) 2925, 2856, 2242, 1450, 1030.

HRMS (ESI) calcd $C_{12}H_{14}N^+$ $[M+H]^+$: 172.1121. Found: 172.1123.

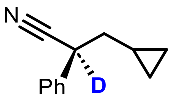

(–)-**47**-d

zwf-8-011

(−)-**47**-d was obtained as oil from the asymmetric decarboxylative deuteration reaction of 2-cyanoacetic acid **S47** (43.0 mg, 0.2 mmol) for 12 h using the general procedure (30.4 mg, 88% Yield, 96% D). R_f = 0.6 (Hexane/EtOAc 10:1).

HPLC analysis (Chiralpak IE-3, hexane/^{i}PrOH = 99/1, 0.8 mL/min, 205 nm; t_r (minor) = 14.69 min, t_r (major) = 15.68 min) gave the isomeric composition of the product: 98:2 e.r., $[\alpha]_D^{19}$ = –27.4 (*c* = 0.68, $CHCl_3$).

^{1}H NMR (400 MHz, $CDCl_3$) δ 7.55-7.20 (m, 5H), 3.86 (t, *J* = 7.4 Hz, 0.04H), 1.96-1.80 (m, 1H), 1.78-1.65 (m, 1H), 0.94-0.74 (m, 1H), 0.67-0.39 (m, 2H), 0.25-0.05 (m, 2H).

^{13}C NMR (100 MHz, $CDCl_3$) δ 135.9, 128.9, 127.9, 127.2, 121.0, 40.9, 37.5 (t, *J* = 20.6 Hz), 8.7, 4.7, 4.4.

IR (neat, cm^{-1}) 3007, 2928, 2238, 1493, 1019.

HRMS (ESI) calcd $C_{12}H_{13}DN^+$ $[M+H]^+$: 173.1184. Found: 173.1187.

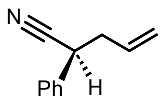

(–)-**48**

zwf-7-089

(−)-**48** was obtained as oil from the asymmetric decarboxylative protonation reaction of 2-cyanoacetic acid **S48** (100.9 mg, 0.5 mmol) for 5 h using the general procedure (c = 0.01, referring to the concentration of **S48** in 2-MeTHF) (73.3 mg, 95% Yield). R_f = 0.7 (Hexane/EtOAc 10:1).

HPLC analysis (Chiralpak IE-3, hexane/^{i}PrOH = 99/1, 0.8 mL/min, 205 nm; t_r (minor) = 11.75 min, t_r (major) = 12.35 min) gave the isomeric composition of the product: 95:5 e.r., $[\alpha]_D^{25}$ = –41.5 (*c* = 0.73, $CHCl_3$).

^{1}H NMR (400 MHz, $CDCl_3$) δ 7.47-7.26 (m, 5H), 5.89-5.70 (m, 1H), 5.24-5.10 (m, 2H), 3.85 (t, *J* = 7.4 Hz, 1H), 2.71-2.56 (m, 2H).

^{13}C NMR (100 MHz, $CDCl_3$) δ 135.2, 132.5, 129.0, 128.1, 127.3, 120.3, 119.3, 39.8, 37.5.

IR (neat, cm^{-1}) 3087, 2983, 2242, 1600, 1450.

HRMS (ESI) calcd $C_{11}H_{12}N^+$ $[M+H]^+$: 158.0964. Found: 158.0965.

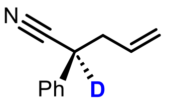

(–)-**48**-d

zwf-8-012

(–)-**48**-d was obtained as oil from the asymmetric decarboxylative deuteration reaction of 2-cyanoacetic acid **S48** (40.2 mg, 0.2 mmol) for 6 h using the general procedure (28.7 mg, 91% Yield, 96% D). R_f = 0.7 (Hexane/EtOAc 10:1).

HPLC analysis (Chiralpak IE-3, hexane/^{i}PrOH = 99/1, 0.8 mL/min, 205 nm; t_r (minor) = 14.11 min, t_r (major) = 14.98 min) gave the isomeric composition of the product: 95.5:4.5 e.r., $[\alpha]_D^{20}$ = –42.1 (*c* = 0.65, $CHCl_3$).

^{1}H NMR (400 MHz, $CDCl_3$) δ 7.50-7.27 (m, 5H), 5.91-5.65 (m, 1H), 5.28-5.10 (m, 2H), 3.86 (t, *J* = 7.2 Hz, 0.04H), 2.68-2.56 (m, 2H).

^{13}C NMR (100 MHz, $CDCl_3$) δ 135.1, 132.5, 129.0, 128.1, 127.3, 120.3, 119.3, 39.7, 37.2 (t, *J* = 20.6 Hz).

IR (neat, cm^{-1}) 3088, 2979, 2241, 1643, 1450.

HRMS (ESI) calcd $C_{11}H_{11}DN^+$ $[M+H]^+$: 159.1027. Found: 159.1029.

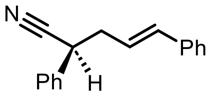

(–)-**49**

zwf-7-075

(–)-**49** was obtained as a white solid from the asymmetric decarboxylative protonation reaction of 2-cyanoacetic acid **S49** (138.7 mg, 0.5 mmol) for 2 h using the general procedure (c = 0.02, referring to the concentration of **S49** in 2-MeTHF) (106.7 mg, 91% Yield). R_f = 0.4 (Hexane/EtOAc 10:1).

HPLC analysis (Chiralpak IE-3, hexane/^{i}PrOH = 99/1, 0.8 mL/min, 205 nm; t_r (minor) = 19.27 min, t_r (major) = 21.31 min) gave the isomeric composition of the product: 95.5:4.5 e.r., $[\alpha]_D^{25}$ = –38.9 (*c* = 0.41, $CHCl_3$).

^{1}H NMR (400 MHz, $CDCl_3$) δ 7.43-7.26 (m, 9H), 7.25-7.22 (m, 1H), 6.49 (d, *J* = 15.6 Hz, 1H), 6.15 (dt, J_1 = 15.6 Hz, J_2 = 7.2 Hz, 1H), 3.90 (t, *J* = 7.2 Hz, 1H), 2.89-2.66 (m, 2H).

^{13}C NMR (100 MHz, $CDCl_3$) δ 136.6, 135.1, 134.3, 129.1, 128.5, 128.2, 127.7, 127.3, 126.3, 123.8, 120.3, 39.2, 37.9.

IR (neat, cm^{-1}) 3028, 2919, 2244, 1491, 1073.

HRMS (ESI) calcd $C_{17}H_{16}N^+$ [M+H]$^+$: 234.1277. Found: 234.1273.

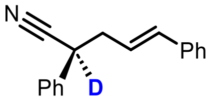

(−)-**49**-d

zwf-8-014

(−)-**49**-d was obtained as a white solid from the asymmetric decarboxylative deuteration reaction of 2-cyanoacetic acid **S49** (55.4 mg, 0.2 mmol) for 2 h using the general procedure (42.3 mg, 90% Yield, 95% D). R$_f$ = 0.4 (Hexane/EtOAc 10:1).

HPLC analysis (Chiralpak IE-3, hexane/^{i}PrOH = 99/1, 0.8 mL/min, 205 nm; t$_r$ (minor) = 26.84 min, t$_r$ (major) = 30.24 min) gave the isomeric composition of the product: 96.5:3.5 e.r., $[\alpha]_D^{19}$ = −38.6 (c = 0.57, $CHCl_3$).

^{1}H NMR (400 MHz, $CDCl_3$) δ 7.47-7.25 (m, 9H), 7.26-7.19 (m, 1H), 6.49 (d, J = 15.6 Hz, 1H), 6.15 (dt, J_1 = 15.6 Hz, J_2 = 7.3 Hz, 1H), 3.90 (t, J = 7.0 Hz, 0.05H), 2.90-2.65 (m, 2H).

^{13}C NMR (100 MHz, $CDCl_3$) δ 136.6, 135.1, 134.3, 129.1, 128.5, 128.2, 127.7, 127.3, 126.3, 123.8, 120.3, 39.1, 37.6 (t, J = 20.6 Hz).

IR (neat, cm^{-1}) 3030, 2920, 2244, 1490, 1078.

HRMS (ESI) calcd $C_{17}H_{15}DN^+$ [M+H]$^+$: 235.1340. Found: 235.1342.

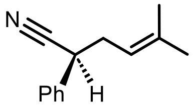

(−)-**50**

zwf-7-121

(−)-**50** was obtained as oil from the asymmetric decarboxylative protonation reaction of 2-cyanoacetic acid **S50** (114.8 mg, 0.5 mmol) for 3 h using the general procedure (c = 0.01, referring to the concentration of **S50** in 2-MeTHF) (85.5 mg, 92% Yield). R$_f$ = 0.5 (Hexane/EtOAc 10:1).

HPLC analysis (Chiralpak IE-3, hexane/^{i}PrOH = 99/1, 0.8 mL/min, 205 nm; t$_r$ (minor) = 12.13 min, t$_r$ (major) = 12.99 min) gave the isomeric composition of the product: 95.5:4.5 e.r., $[\alpha]_D^{22}$ = −34.7 (c = 0.75, $CHCl_3$).

^{1}H NMR (400 MHz, $CDCl_3$) δ 7.44-7.27 (m, 5H), 5.26-5.09 (m, 1H), 3.77 (t, J = 7.2 Hz, 1H), 2.68-2.48

(m, 2H), 1.72 (s, 3H), 1.54 (s, 3H).

^{13}C NMR (100 MHz, $CDCl_3$) δ 136.6, 135.6, 128.9, 127.9, 127.3, 120.8, 118.5, 37.8, 34.5, 25.7, 17.8.

IR (neat, cm^{-1}) 2969, 2917, 2242, 1454, 1078.

HRMS (ESI) calcd $C_{13}H_{16}N^+$ $[M+H]^+$: 186.1277. Found: 186.1273.

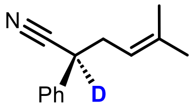

(−)-**50**-d

zwf-8-013

(−)-**50**-d was obtained as oil from the asymmetric decarboxylative deuteration reaction of 2-cyanoacetic acid **S50** (46.0 mg, 0.2 mmol) for 3 h using the general procedure (34.7 mg, 93% Yield, 96% D). R_f = 0.5 (Hexane/EtOAc 10:1).

HPLC analysis (Chiralpak IE-3, hexane/^{i}PrOH = 99/1, 0.8 mL/min, 205 nm; t_r (minor) = 13.57 min, t_r (major) = 14.62 min) gave the isomeric composition of the product: 96:4 e.r., $[\alpha]_D^{20}$ = –33.8 (*c* = 0.50, $CHCl_3$).

^{1}H NMR (400 MHz, $CDCl_3$) δ 7.45-7.27 (m, 5H), 5.30-5.07 (m, 1H), 3.77 (t, *J* = 7.2 Hz, 0.04H), 2.67-2.48 (m, 2H), 1.72 (s, 3H), 1.54 (s, 3H).

^{13}C NMR (100 MHz, $CDCl_3$) δ 136.6, 135.6, 128.9, 127.9, 127.3, 120.8, 118.4, 37.5 (t, *J* = 20.6 Hz), 34.4, 25.7, 17.8.

IR (neat, cm^{-1}) 2973, 2916, 2241, 1454, 1027.

HRMS (ESI) calcd $C_{13}H_{15}DN^+$ $[M+H]^+$: 187.1340. Found: 187.1337.

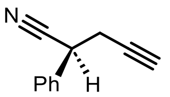

(−)-**51**

zwf-7-090

(−)-**51** was obtained as oil from the asymmetric decarboxylative protonation reaction of 2-cyanoacetic acid **S51** (99.6 mg, 0.5 mmol) for 7 h using the general procedure (69.5 mg, 90% Yield). R_f = 0.4 (Hexane/EtOAc 10:1).

HPLC analysis (Chiralpak IE-3, hexane/^{i}PrOH = 99/1, 0.8 mL/min, 205 nm; t_r (minor) = 18.04 min, t_r

(major) = 18.64 min) gave the isomeric composition of the product: 91:9 e.r., $[\alpha]_D^{25}$ = –42.6 (*c* = 0.27, $CHCl_3$).

^{1}H NMR (400 MHz, $CDCl_3$) δ 7.46-7.32 (m, 5H), 4.01 (t, *J* = 7.0 Hz, 1H), 2.86-2.72 (m, 2H), 2.16 (t, *J* = 2.6 Hz, 1H).

^{13}C NMR (100 MHz, $CDCl_3$) δ 134.1, 129.1, 128.6, 127.3, 119.4, 78.5, 72.4, 36.9, 25.9.

IR (neat, cm^{-1}) 3038, 2245, 2123, 1491, 1074.

HRMS (ESI) calcd $C_{11}H_{10}N^+$ $[M+H]^+$: 156.0808. Found: 156.0806.

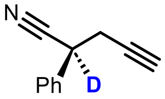

(–)-**51**-d

zwf-8-015

(–)-**51**-d was obtained as oil from the asymmetric decarboxylative deuteration reaction of 2-cyanoacetic acid **S51** (39.9 mg, 0.2 mmol) for 8 h using the general procedure (28.6 mg, 91% Yield, 93% D). R_f = 0.4 (Hexane/EtOAc 10:1).

HPLC analysis (Chiralpak IE-3, hexane/^{i}PrOH = 99/1, 0.8 mL/min, 205 nm; t_r (minor) = 22.38 min, t_r (major) = 23.00 min) gave the isomeric composition of the product: 94:6 e.r., $[\alpha]_D^{19}$ = –52.4 (*c* = 0.54, $CHCl_3$).

^{1}H NMR (400 MHz, $CDCl_3$) δ 7.55-7.28 (m, 5H), 4.01 (t, *J* = 7.0 Hz, 0.07H), 2.89-2.70 (m, 2H), 2.16 (t, *J* = 2.6 Hz, 1H).

^{13}C NMR (100 MHz, $CDCl_3$) δ 134.1, 129.1, 128.6, 127.3, 119.4, 78.5, 72.4, 36.6 (t, *J* = 21.0 Hz), 25.8.

IR (neat, cm^{-1}) 3035, 2243, 2121, 1498, 1075.

HRMS (ESI) calcd $C_{11}H_9DN^+$ $[M+H]^+$: 157.0871. Found: 157.0870.

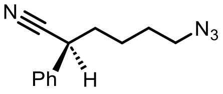

(–)-**52**

zwf-10-131

(–)-**52** was obtained as an oil from the asymmetric decarboxylative protonation reaction of 2-cyanoacetic acid **S52** (129.4 mg, 0.5 mmol) for 4 h using the general procedure (96.2 mg, 90% Yield). R_f = 0.4 (Hexane/EtOAc 10:1).

HPLC analysis (Chiralpak IE-3, hexane/^{i}PrOH = 99/1, 0.8 mL/min, 205 nm; t_r (minor) = 30.83 min, t_r (major) = 39.15 min) gave the isomeric composition of the product: 87.5:12.5 e.r., $[\alpha]_D^{20}$ = –19.1 (*c* = 0.49, $CHCl_3$).

^{1}H NMR (500 MHz, $CDCl_3$) δ 7.42-7.36 (m, 2H), 7.36-7.28 (m, 3H), 3.79 (t, *J* = 7.3 Hz, 1H), 3.27 (t, *J* = 6.5 Hz, 2H), 2.02-1.84 (m, 2H), 1.67-1.48 (m, 4H).

^{13}C NMR (125 MHz, $CDCl_3$) δ 135.5, 129.1, 128.1, 127.1, 120.5, 50.9, 37.2, 35.3, 28.2, 24.2.

IR (neat, cm^{-1}) 2941, 2866, 2240, 2092, 1454, 1259.

HRMS (ESI) calcd $C_{12}H_{14}N_4Na^+$ $[M+Na]^+$: 237.1111. Found: 237.1113.

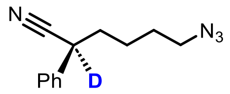

(–)-**52**-d

zwf-10-133

(–)-**52**-d was obtained as an oil from the asymmetric decarboxylative deuteration reaction of 2-cyanoacetic acid **S52** (51.6 mg, 0.2 mmol) for 4 h using the general procedure (38.9 mg, 90% Yield, 95% D). R_f = 0.4 (Hexane/EtOAc 10:1).

HPLC analysis (Chiralpak IE-3, hexane/^{i}PrOH = 99/1, 0.8 mL/min, 205 nm; t_r (minor) = 30.68 min, t_r (major) = 38.77 min) gave the isomeric composition of the product: 95:5 e.r., $[\alpha]_D^{20}$ = –30.9 (*c* = 0.23, $CHCl_3$).

^{1}H NMR (500 MHz, $CDCl_3$) δ 7.43-7.36 (m, 2H), 7.36-7.27 (m, 3H), 3.79 (t, *J* = 7.5 Hz, 0.05H), 3.27 (t, *J* = 6.5 Hz, 2H), 2.01-1.84 (m, 2H), 1.68-1.47 (m, 4H).

^{13}C NMR (125 MHz, $CDCl_3$) δ 135.5, 129.1, 128.1, 127.1, 120.5, 50.9, 36.9 (t, *J* = 20.3 Hz), 35.2, 28.2, 24.2.

IR (neat, cm^{-1}) 2940, 2866, 2240, 2091, 1455, 1260.

HRMS (ESI) calcd $C_{12}H_{13}DN_4Na^+$ $[M+Na]^+$: 238.1173. Found: 238.1172.

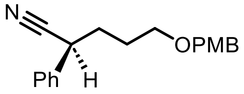

(–)-**54**

zwf-7-146

(–)-**54** was obtained as oil from the asymmetric decarboxylative protonation reaction of 2-cyanoacetic acid **S54** (169.4 mg, 0.5 mmol) for 24 h using the general procedure (c = 0.01, referring to the concentration of **S54** in 2-MeTHF) (126.7 mg, 86% Yield). R_f = 0.2 (Hexane/EtOAc 10:1).

HPLC analysis (Chiralpak IE-3, hexane/^{i}PrOH = 90/10, 0.8 mL/min, 205 nm; t_r (minor) = 16.68 min, t_r (major) = 18.95 min) gave the isomeric composition of the product: 72.5:27.5 e.r., $[\alpha]_D^{20}$ = –3.8 (c = 0.57, $CHCl_3$).

^{1}H NMR (400 MHz, $CDCl_3$) δ 7.42-7.27 (m, 5H), 7.23 (d, J = 8.8 Hz, 2H), 6.87 (d, J = 8.4 Hz, 2H), 4.40 (s, 2H), 3.88-3.73 (m, 4H), 3.55-3.40 (m, 2H), 2.03-1.96 (m, 2H), 1.83-1.70 (m, 2H).

^{13}C NMR (100 MHz, $CDCl_3$) δ 159.1, 135.8, 130.2, 129.2, 129.0, 128.0, 127.2, 120.8, 113.7, 72.6, 68.7, 55.2, 37.0, 32.8, 27.0.

IR (neat, cm^{-1}) 2936, 2859, 2240, 1512, 1245.

HRMS (ESI) calcd $C_{19}H_{22}NO_2^+$ $[M+H]^+$: 296.1645. Found: 296.1647.

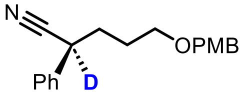

(–)-**54**-d

zwf-8-044

(–)-**54**-d was obtained as oil from the asymmetric decarboxylative deutertion reaction of 2-cyanoacetic acid **S54** (67.9 mg, 0.2 mmol) for 24 h using the general procedure (51.8 mg, 87% Yield, 96% D). R_f = 0.2 (Hexane/EtOAc 10:1).

HPLC analysis (Chiralpak IE-3, hexane/^{i}PrOH = 90/10, 0.8 mL/min, 205 nm; t_r (minor) = 17.26 min, t_r (major) = 19.76 min) gave the isomeric composition of the product: 85:15 e.r., $[\alpha]_D^{20}$ = –6.6 (c = 0.55, $CHCl_3$).

^{1}H NMR (400 MHz, $CDCl_3$) δ 7.42-7.26 (m, 5H), 7.23 (d, J = 8.4 Hz, 2H), 6.87 (d, J = 8.8 Hz, 2H), 4.41 (s, 2H), 3.84-3.74 (m, 3.04H), 3.52-4.41 (m, 2H), 2.03-1.95 (m, 2H), 1.81-1.70 (m, 2H).

^{13}C NMR (100 MHz, $CDCl_3$) δ 159.2, 135.7, 130.2, 129.2, 129.0, 128.0, 127.2, 120.8, 113.7, 72.6, 68.7, 55.2, 36.7 (t, J = 20.6 Hz), 32.8, 27.0.

IR (neat, cm^{-1}) 2936, 2858, 2239, 1511, 1245.

HRMS (ESI) calcd $C_{19}H_{21}DNO_2^+$ $[M+H]^+$: 297.1708. Found: 297.1710.

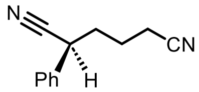

(–)-**56**

zwf-10-125

(–)-**56** was obtained as an oil from the asymmetric decarboxylative protonation reaction of 2-cyanoacetic acid **S56** (114.1 mg, 0.5 mmol) for 12 h using the general procedure (85.4 mg, 93% Yield). R_f = 0.2 (Hexane/EtOAc 5:1).

HPLC analysis (Chiralpak IE-3, hexane/^{i}PrOH = 99/1, 0.8 mL/min, 205 nm; t_r (minor) = 28.19 min, t_r (major) = 31.08 min) gave the isomeric composition of the product: 80:20 e.r., $[\alpha]_D^{20}$ = –13.9 (*c* = 0.50, $CHCl_3$).

^{1}H NMR (500 MHz, $CDCl_3$) δ 7.43-7.37 (m, 2H), 7.37-7.30 (m, 3H), 3.87 (t, *J* = 7.3 Hz, 1H), 2.43-2.34 (m, 2H), 2.10-2.00 (m, 2H), 1.90-1.74 (m, 2H).

^{13}C NMR (125 MHz, $CDCl_3$) δ 134.6, 129.1, 128.3, 127.0, 119.9, 118.6, 36.5, 34.2, 22.5, 16.5.

IR (neat, cm^{-1}) 2935, 2244, 1769, 1445, 1056.

HRMS (ESI) calcd $C_{12}H_{12}N_2Na^+$ $[M+Na]^+$: 207.0893. Found: 207.0894.

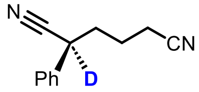

(–)-**56**-d

zwf-10-127

(–)-**56**-d was obtained as an oil from the asymmetric decarboxylative deuteration reaction of 2-cyanoacetic acid **S56** (45.7 mg, 0.2 mmol) for 12 h using the general procedure (34.2 mg, 92% Yield, 96% D). R_f = 0.2 (Hexane/EtOAc 5:1).

HPLC analysis (Chiralpak IE-3, hexane/^{i}PrOH = 99/1, 0.8 mL/min, 205 nm; t_r (minor) = 28.16 min, t_r (major) = 30.95 min) gave the isomeric composition of the product: 90.5:9.5 e.r., $[\alpha]_D^{20}$ = –21.1 (*c* = 0.56, $CHCl_3$).

^{1}H NMR (500 MHz, $CDCl_3$) δ 7.44-7.37 (m, 2H), 7.37-7.28 (m, 3H), 3.87 (t, *J* = 7.3 Hz, 0.04H), 2.44-

2.31 (m, 2H), 2.12-1.97 (m, 2H), 1.88-1.74 (m, 2H).

^{13}C NMR (125 MHz, $CDCl_3$) δ 134.6, 129.1, 128.3, 127.0, 119.9, 118.6, 36.2 (t, J = 20.3 Hz), 34.1, 22.5, 16.5.

IR (neat, cm^{-1}) 2933, 2243, 1769, 1444, 1056.

HRMS (ESI) calcd $C_{12}H_{12}N_2Na^+$ $[M+Na]^+$: 207.0893. Found: 207.0894.

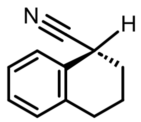

(+)-**58**

zwf-9-069

(+)-**58** was obtained as oil from the asymmetric decarboxylative protonation reaction of 2-cyanoacetic acid **S58** (100.7 mg, 0.5 mmol) for 2 h using the general procedure (71.8 mg, 91% Yield). R_f = 0.4 (Hexane/EtOAc 10:1).

HPLC analysis (Chiralpak IG-3, hexane/^{i}PrOH = 99/1, 0.8 mL/min, 205 nm; t_r (major) = 24.77 min, t_r (minor) = 29.54 min) gave the isomeric composition of the product: 93.5:6.5 e.r., $[\alpha]_D^{24}$ = +31.1 (c = 0.75, $CHCl_3$).

^{1}H NMR (500 MHz, $CDCl_3$) δ 7.40-7.29 (m, 1H), 7.25-7.16 (m, 2H), 7.14-7.05 (m, 1H), 3.97 (t, J = 6.3 Hz, 1H), 2.91-2.72 (m, 2H), 2.20-2.07 (m, 2H), 2.07-1.96 (m, 1H), 1.89-1.77 (m, 1H).

^{13}C NMR (125 MHz, $CDCl_3$) δ 136.3, 129.8, 129.7, 128.7, 127.9, 126.5, 121.7, 30.7, 28.3, 27.2, 20.7.

IR (neat, cm^{-1}) 2938, 2866, 2236, 1492, 1450.

HRMS (ESI) calcd $C_{11}H_{12}N^+$ $[M+H]^+$: 158.0964. Found: 158.0960.

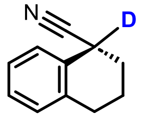

(+)-**58**-d

zwf-9-070

(+)-**58**-d was obtained as oil from the asymmetric decarboxylative deuteration reaction of 2-cyanoacetic acid **S58** (40.2 mg, 0.2 mmol) for 2 h using the general procedure (29.3 mg, 93% Yield, 95% D). R_f = 0.4 (Hexane/EtOAc 10:1).

HPLC analysis (Chiralpak IG-3, hexane/^{i}PrOH = 99/1, 0.8 mL/min, 205 nm; t_r (major) = 24.88 min, t_r (minor) = 29.68 min) gave the isomeric composition of the product: 95:5 e.r., $[\alpha]_D^{24}$ = +33.0 (c = 0.68, $CHCl_3$).

^{1}H NMR (400 MHz, $CDCl_3$) δ 7.41-7.30 (m, 1H), 7.28-7.15 (m, 2H), 7.15-7.06 (m, 1H), 3.96 (t, J = 6.4 Hz, 0.05H), 2.93-2.70 (m, 2H), 2.20-1.94 (m, 3H), 1.91-1.75 (m, 1H).

^{13}C NMR (100 MHz, $CDCl_3$) δ 136.4, 129.7, 128.7, 127.9, 126.4, 121.7, 30.4 (t, J = 20.6 Hz), 28.3, 27.1, 20.7.

IR (neat, cm^{-1}) 2937, 2866, 2236, 1492, 1450.

HRMS (ESI) calcd $C_{11}H_{12}N^+$ $[M+H]^+$: 159.1027. Found: 159.1029.

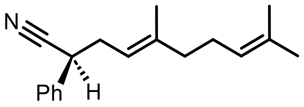

(–)-**60**

zwf-7-106

(–)-**60** was obtained as oil from the asymmetric decarboxylative protonation reaction of 2-cyanoacetic acid **S60** (148.5 mg, 0.5 mmol) for 24 h using the general procedure (87.8 mg, 69% Yield). R_f = 0.3 (Hexane/EtOAc 10:1).

HPLC analysis (Chiralpak IE-3, hexane/^{i}PrOH = 99/1, 0.8 mL/min, 205 nm; t_r (minor) = 10.14 min, t_r (major) = 10.50 min) gave the isomeric composition of the product: 85:15 e.r., $[\alpha]_D^{23}$ = –7.7 (c = 0.59, $CHCl_3$).

^{1}H NMR (400 MHz, $CDCl_3$) δ 7.42-7.27 (m, 5H), 5.23-5.14 (m, 1H), 5.13-5.02 (m, 1H), 3.77 (t, J = 7.2 Hz, 1H), 2.72-2.48 (m, 2H), 2.11-1.96 (m, 4H), 1.69 (s, 3H), 1.60 (s, 3H), 1.53 (s, 3H).

^{13}C NMR (100 MHz, $CDCl_3$) δ 140.2, 135.6, 131.6, 128.9, 127.9, 127.4, 123.9, 120.7, 118.3, 39.6, 37.7, 34.3, 26.4, 25.6, 17.6, 16.1.

IR (neat, cm^{-1}) 2918, 2854, 2242, 1453, 1378.

HRMS (ESI) calcd $C_{18}H_{24}N^+$ $[M+H]^+$: 254.1903. Found: 254.1901.

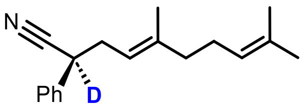

(–)-**60**-d

zwf-8-040

(–)-**60**-d was obtained as oil from the asymmetric decarboxylative deuteration reaction of 2-cyanoacetic acid **S60** (59.5 mg, 0.2 mmol) for 24 h using the general procedure (38.1 mg, 75% Yield, 88% D). R_f = 0.3 (Hexane/EtOAc 10:1).

HPLC analysis (Chiralpak IE-3, hexane/^{i}PrOH = 99/1, 0.8 mL/min, 205 nm; t_r (minor) = 11.20 min, t_r (major) = 11.62 min) gave the isomeric composition of the product: 93:7 e.r., $[\alpha]_D^{20}$ = –9.3 (*c* = 0.47, $CHCl_3$).

^{1}H NMR (400 MHz, $CDCl_3$) δ 7.50-7.28 (m, 5H), 5.27-5.13 (m, 1H), 5.13-5.00 (m, 1H), 3.77 (t, *J* = 7.2 Hz, 0.12H), 2.70-2.48 (m, 2H), 2.14-1.92 (m, 4H), 1.69 (s, 3H), 1.60 (s, 3H), 1.53 (s, 3H).

^{13}C NMR (100 MHz, $CDCl_3$) δ 140.2, 135.6, 131.6, 128.9, 127.9, 127.3, 123.9, 120.7, 118.3, 39.6, 37.4 (t, *J* = 21.0 Hz), 34.3, 26.4, 25.6, 17.6, 16.1.

IR (neat, cm^{-1}) 2919, 2855, 2243, 1451, 1377.

HRMS (ESI) calcd $C_{18}H_{23}DN^+$ $[M+H]^+$: 255.1966. Found: 255.1968.

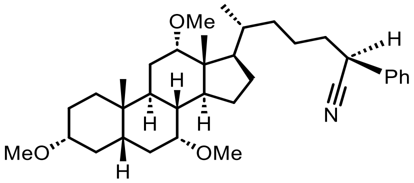

(+)-**61**

zwf-10-136

(+)-**61** was obtained as an oil from the asymmetric decarboxylative protonation reaction of 2-cyanoacetic acid **S61** (58.0 mg, 0.1 mmol) for 12 h using the general procedure (45.3 mg, 85% Yield). R_f = 0.5 (Hexane/EtOAc 5:1).

HPLC analysis (Chiralpak IE-3, hexane/^{i}PrOH = 90/10, 0.8 mL/min, 205 nm; t_r (minor) = 24.80 min, t_r (major) = 28.04 min) gave the isomeric composition of the product: 4.7:1 d.r., $[\alpha]_D^{19}$ = +34.4 (*c* = 0.36, $CHCl_3$).

^{1}H NMR (500 MHz, $CDCl_3$) δ 7.42-7.35 (m, 2H), 7.35-7.28 (m, 3H), 3.80-3.72 (m, 1H), 3.38-3.34 (m, 1H), 3.33 (s, 3H), 3.25 (s, 3H), 3.21 (s, 3H), 3.16-3.11 (m, 1H), 3.03-2.96 (m, 1H), 2.24-2.14 (m, 1H), 2.12-2.00 (m, 2H), 1.96-1.72 (m, 9H), 1.72-1.64 (m, 1H), 1.62-0.84 (m, 19H), 0.64 (s, 3H).

^{13}C NMR (125 MHz, $CDCl_3$) δ 136.1, 129.0, 127.9, 127.2, 121.0, 82.0, 80.7, 76.9, 55.8, 55.6, 55.3, 46.6, 46.0, 42.6, 41.9, 39.6, 37.3, 36.3, 35.5, 35.2, 35.1, 34.8, 34.4, 27.9, 27.7, 27.5, 26.7, 23.8, 23.1, 22.8, 21.9, 17.6, 12.4.

IR (neat, cm^{-1}) 2930, 2866, 2241, 1455, 1099.

HRMS (ESI) calcd $C_{35}H_{54}NO_3^+$ $[M+H]^+$: 536.4098. Found: 536.4095.

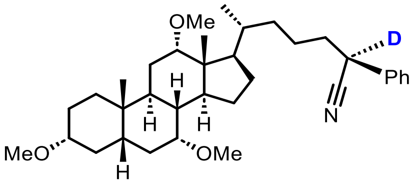

(+)-**61**-d

zwf-10-138

(−)-**61**-d was obtained as an oil from the asymmetric decarboxylative deuteration reaction of 2-cyanoacetic acid **S61** (58.1 mg, 0.1 mmol) for 12 h using the general procedure (45.1 mg, 84% Yield, 90% D). R_f = 0.5 (Hexane/EtOAc 5:1).

HPLC analysis (Chiralpak IE-3, hexane/^{i}PrOH = 90/10, 0.8 mL/min, 205 nm; t_r (minor) = 24.80 min, t_r (major) = 28.04 min) gave the isomeric composition of the product: 9.1:1 d.r., $[\alpha]_D^{19}$ = +41.1 (c = 0.41, $CHCl_3$).

^{1}H NMR (500 MHz, $CDCl_3$) δ 7.41-7.35 (m, 2H), 7.35-7.28 (m, 3H), 3.78-3.74 (m, 0.1H), 3.38-3.34 (m, 1H), 3.33 (s, 3H), 3.25 (s, 3H), 3.21 (s, 3H), 3.16-3.11 (m, 1H), 3.03-2.95 (m, 1H), 2.24-2.15 (m, 1H), 2.11-2.00 (m, 2H), 1.95-1.72 (m, 9H), 1.72-1.64 (m, 1H), 1.62-0.84 (m, 19H), 0.64 (s, 3H).

^{13}C NMR (125 MHz, $CDCl_3$) δ 136.0, 129.0, 127.9, 127.2, 121.0, 81.9, 80.7, 76.9, 55.8, 55.6, 55.3, 46.6, 46.0, 42.6, 41.9, 39.6, 37.0 (t, J = 21.2 Hz), 36.2, 35.5, 35.2, 35.1, 34.8, 34.4, 27.9, 27.7, 27.5, 26.7, 23.7, 23.1, 22.8, 21.9, 17.6, 12.4.

IR (neat, cm^{-1}) 2928, 2865, 2242, 1454, 1098.

HRMS (ESI) calcd $C_{35}H_{53}DNO_3^+$ $[M+H]^+$: 537.4161. Found: 537.4160.

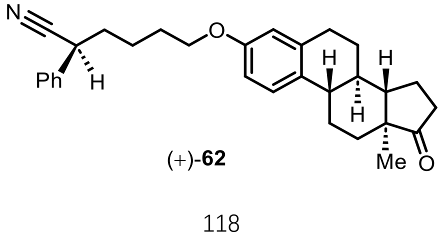

(+)-**62**

zwf-7-147

(+)-**62** was obtained as white solid from the asymmetric decarboxylative protonation reaction of 2-cyanoacetic acid **S62** (242.9 mg, 0.5 mmol) for 12 h using the general procedure (178.0 mg, 81% Yield). R_f = 0.2 (Hexane/EtOAc 5:1).

HPLC analysis (Chiralpak IE-3, hexane/^{i}PrOH = 80/20, 0.8 mL/min, 205 nm; t_r (minor) = 46.63 min, t_r (major) = 60.41 min) gave the isomeric composition of the product: 6:1 d.r., $[\alpha]_D^{20}$ = +96.7 (*c* = 0.42, $CHCl_3$).

^{1}H NMR (400 MHz, $CDCl_3$) δ 7.45-7.27 (m, 5H), 7.19 (d, *J* = 8.4 Hz, 1H), 6.68 (dd, J_1 = 8.8 Hz, J_2 = 2.4 Hz, 1H), 6.62 (s, 1H), 3.93 (t, *J* = 6.2 Hz, 2H), 3.81 (t, *J* = 7.4 Hz, 1H), 2.96-2.79 (m, 2H), 2.55-2.45 (m, 1H), 2.43-2.34 (m, 1H), 2.30-1.89 (m, 7H), 1.86-1.76 (m, 2H), 1.71-1.42 (m, 8H), 0.90 (s, 3H).

^{13}C NMR (100 MHz, $CDCl_3$) δ 221.0, 156.8, 137.7, 135.7, 132.1, 129.1, 128.0, 127.2, 126.3, 120.7, 114.5, 112.0, 67.2, 50.4, 48.0, 43.9, 38.3, 37.3, 35.8, 35.5, 31.5, 29.6, 28.6, 26.5, 25.9, 23.8, 21.5, 13.8.

IR (neat, cm^{-1}) 2938, 2875, 2242, 1733, 1455, 1058.

HRMS (ESI) calcd $C_{30}H_{36}NO_2^+$ $[M+H]^+$: 442.2741. Found: 442.2744.

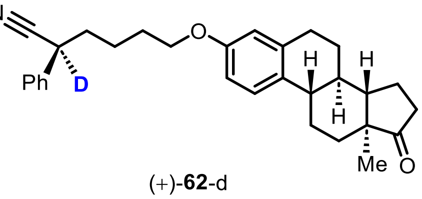

(+)-**62**-d

zwf-8-008

(+)-**62**-d was obtained as white solid from the asymmetric decarboxylation reaction of 2-cyanoacetic acid **S62** (97.1 mg, 0.2 mmol) for 12 h using the general procedure (74.1 mg, 84% Yield, 94% D). R_f = 0.2 (Hexane/EtOAc 5:1).

HPLC analysis (Chiralpak IE-3, hexane/^{i}PrOH = 80/20, 0.8 mL/min, 205 nm; t_r (minor) = 52.47 min, t_r (major) = 68.82 min) gave the isomeric composition of the product: 11:1 d.r., $[\alpha]_D^{19}$ = +111.6 (*c* = 0.45, $CHCl_3$).

^{1}H NMR (400 MHz, $CDCl_3$) δ 7.43-7.28 (m, 5H), 7.19 (d, *J* = 8.4 Hz, 1H), 6.69 (dd, J_1 = 8.8 Hz, J_2 = 2.8 Hz, 1H), 6.62 (s, 1H), 3.92 (t, *J* = 6.2 Hz, 2H), 3.81 (t, *J* = 7.2 Hz, 0.06H), 2.97-2.80 (m, 2H), 2.56-2.45 (m, 1H), 2.44-2.34 (m, 1H), 2.30-1.89 (m, 7H), 1.85-1.76 (m, 2H), 1.72-1.40 (m, 8H), 0.90 (s, 3H).

^{13}C NMR (100 MHz, $CDCl_3$) δ 220.9, 156.8, 137.7, 135.7, 132.0, 129.0, 128.0, 127.2, 126.3, 120.7, 114.4, 112.0, 67.2, 50.3, 47.9, 43.9, 38.3, 37.0 (t, *J* = 20.6 Hz), 35.8, 35.4, 31.5, 29.6, 28.6, 26.5, 25.8, 23.7, 21.5, 13.8.

IR (neat, cm^{-1}) 2935, 2876, 2241, 1733, 1452, 1053.

HRMS (ESI) calcd $C_{30}H_{35}DNO_2^+$ $[M+H]^+$: 443.2803. Found: 443.2805.

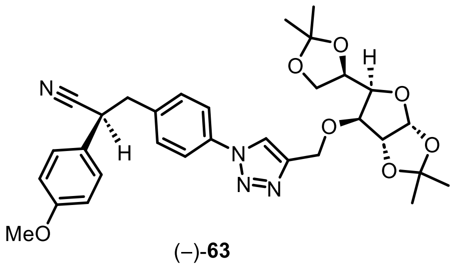

(–)-**63**

zwf-10-132

(–)-**63** was obtained as an oil from the asymmetric decarboxylative protonation reaction of 2-cyanoacetic acid **S63** (62.1 mg, 0.1 mmol) for 12 h using the general procedure (47.8 mg, 82% Yield). R_f = 0.3 (Hexane/EtOAc 1:1).

HPLC analysis (Chiralpak IE-3, hexane/^{i}PrOH = 70/30, 0.8 mL/min, 205 nm; t_r (minor) = 87.25 min, t_r (major) = 104.53 min) gave the isomeric composition of the product: > 20:1 d.r., $[\alpha]_D^{20}$ = –46.5 (c = 0.51, $CHCl_3$).

^{1}H NMR (500 MHz, $CDCl_3$) δ 8.11 (s, 1H), 7.66 (d, J = 8.5 Hz, 2H), 7.25 (d, J = 8.5 Hz, 2H), 7.15 (d, J = 8.5 Hz, 2H), 6.88 (d, J = 8.5 Hz, 2H), 5.90 (d, J = 3.5 Hz, 1H), 4.93 (d, J = 13.0 Hz, 1H), 4.87 (d, J = 13.0 Hz, 1H), 4.65 (d, J = 3.5 Hz, 1H), 4.41-4.34 (m, 1H), 4.15-4.07 (m, 3H), 4.06-3.99 (m, 2H), 3.81 (s, 3H), 3.27-3.16 (m, 2H), 1.50 (s, 3H), 1.41 (s, 3H), 1.35 (s, 3H), 1.33 (s, 3H).

^{13}C NMR (125 MHz, $CDCl_3$) δ 159.5, 145.7, 137.0, 136.2, 130.7, 128.6, 126.4, 120.6, 120.5, 120.2, 114.5, 111.9, 109.2,105.2, 82.6, 81.5, 81.1, 72.3, 67.5, 63.9, 55.3, 41.5, 38.6, 26.9, 26.8, 26.2, 25.5.

IR (neat, cm^{-1}) 2987, 2935, 2242, 1513, 1251, 1022.

HRMS (ESI) calcd $C_{31}H_{37}N_4O_7^+$ $[M+H]^+$: 577.2657. Found: 577.2655.

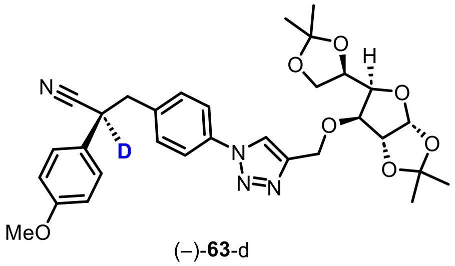

(–)-**63**-d

zwf-10-134

(−)-**63**-d was obtained as an oil from the asymmetric decarboxylative deuteration reaction of 2-cyanoacetic acid **S63** (62.0 mg, 0.1 mmol) for 12 h using the general procedure (46.3 mg, 80% Yield, 94% D). R_f = 0.3 (Hexane/EtOAc 1:1).

HPLC analysis (Chiralpak IE-3, hexane/^{i}PrOH = 70/30, 0.8 mL/min, 205 nm; t_r (minor) = 91.05 min, t_r (major) = 108.43 min) gave the isomeric composition of the product: > 20:1 d.r., $[\alpha]_D^{20}$ = –48.3 (*c* = 0.50, $CHCl_3$).

^{1}H NMR (500 MHz, $CDCl_3$) δ 8.11 (s, 1H), 7.66 (d, *J* = 8.5 Hz, 2H), 7.25 (d, *J* = 8.5 Hz, 2H), 7.15 (d, *J* = 8.5 Hz, 2H), 6.88 (d, *J* = 8.5 Hz, 2H), 5.90 (d, *J* = 3.5 Hz, 1H), 4.93 (d, *J* = 13.0 Hz, 1H), 4.87 (d, *J* = 13.0 Hz, 1H), 4.65 (d, *J* = 3.5 Hz, 1H), 4.42-4.32 (m, 1H), 4.18-4.07 (m, 3H), 4.06-4.00 (m, 1.06H), 3.81 (s, 3H), 3.27-3.16 (m, 2H), 1.50 (s, 3H), 1.41 (s, 3H), 1.35 (s, 3H), 1.33 (s, 3H).

^{13}C NMR (125 MHz, $CDCl_3$) δ 159.5, 145.7, 137.0, 136.2, 130.7, 128.6, 126.4, 120.6, 120.5, 120.2, 114.5, 111.9, 109.2,105.2, 82.6, 81.5, 81.1, 72.3, 67.5, 63.9, 55.3, 41.5, 38.3 (t, *J* = 20.3 Hz), 26.9, 26.8, 26.2, 25.5.

IR (neat, cm^{-1}) 2986, 2936, 2242, 1513, 1250, 1021.

HRMS (ESI) calcd $C_{31}H_{36}DN_4O_7^+$ $[M+H]^+$: 578.2720. Found: 578.2722.

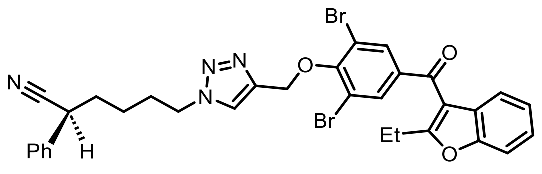

(–)-**64**

zwf-10-137

(−)-**64** was obtained as an oil from the asymmetric decarboxylative protonation reaction of 2-cyanoacetic acid **S64** (72.0 mg, 0.1 mmol) for 12 h using the general procedure (57.0 mg, 84% Yield). R_f = 0.3 (Hexane/EtOAc 2:1).

HPLC analysis (Chiralpak IE-3, hexane/^{i}PrOH = 70/30, 0.8 mL/min, 205 nm; t_r (minor) = 85.92 min, t_r (major) = 89.24 min) gave the isomeric composition of the product: 85.5:14.5 e.r., $[\alpha]_D^{20}$ = –3.1 (*c* = 0.27, $CHCl_3$).

^{1}H NMR (500 MHz, $CDCl_3$) δ 8.00 (s, 2H), 7.78 (s, 1H), 7.50 (d, *J* = 8.0 Hz, 1H), 7.44-7.35 (m, 3H), 7.35-7.28 (m, 4H), 7.26-7.23 (m, 1H), 5.32 (s, 2H), 4.39 (t, *J* = 7.0 Hz, 2H), 3.78 (t, *J* = 7.3 Hz, 1H), 2.90 (q, *J* = 7.5 Hz, 2H), 2.05-1.87 (m, 4H), 1.64-1.47 (m, 2H), 1.36 (t, *J* = 7.5 Hz, 3H).

^{13}C NMR (125 MHz, $CDCl_3$) δ 187.9, 167.0, 155.8, 153.6, 143.1, 137.5, 135.3, 133.6, 129.1, 128.2, 127.1,

126.3, 124.7, 123.9, 123.4, 120.9, 120.4, 118.8, 115.2, 111.1, 66.6, 49.9, 37.1, 35.0, 29.6, 23.9, 22.0, 12.1.

IR (neat, cm^{-1}) 2940, 2867, 2241, 1645, 1453, 1253.

HRMS (ESI) calcd $C_{32}H_{29}Br_2N_4O_3^+$ $[M+H]^+$: 675.0601. Found: 675.0600.

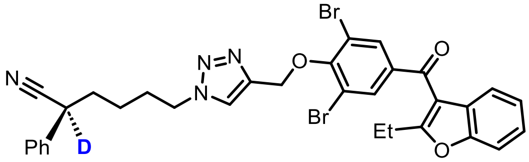

(–)-**64**-d

zwf-10-139

(–)-**64**-d was obtained as an oil from the asymmetric decarboxylative deuteration reaction of 2-cyanoacetic acid **S64** (72.1 mg, 0.1 mmol) for 12 h using the general procedure (57.8 mg, 85% Yield, 88% D). R_f = 0.3 (Hexane/EtOAc 2:1).

HPLC analysis (Chiralpak IE-3, hexane/^{i}PrOH = 70/30, 0.8 mL/min, 205 nm; t_r (minor) = 84.89 min, t_r (major) = 87.76 min) gave the isomeric composition of the product: 91.5:8.5 e.r., $[\alpha]_D^{20}$ = –4.3 (*c* = 0.37, $CHCl_3$).

^{1}H NMR (500 MHz, $CDCl_3$) δ 8.00 (s, 2H), 7.78 (s, 1H), 7.50 (d, *J* = 8.5 Hz, 1H), 7.44-7.35 (m, 3H), 7.35-7.28 (m, 4H), 7.26-7.22 (m, 1H), 5.32 (s, 2H), 4.39 (t, *J* = 7.0 Hz, 2H), 3.79 (t, *J* = 7.3 Hz, 0.12H), 2.90 (q, *J* = 7.5 Hz, 2H), 2.07-1.85 (m, 4H), 1.64-1.45 (m, 2H), 1.36 (t, *J* = 7.5 Hz, 3H).

^{13}C NMR (125 MHz, $CDCl_3$) δ 187.9, 167.0, 155.8, 153.6, 143.1, 137.5, 135.2, 133.5, 129.1, 128.2, 127.1, 126.3, 124.7, 123.9, 123.4, 120.9, 120.4, 118.8, 115.2, 111.1, 66.6, 49.9, 36.8 (t, *J* = 20.7 Hz), 34.9, 29.6, 23.9, 22.0, 12.1.

IR (neat, cm^{-1}) 2941, 2864, 2240, 1645, 1455, 1252.

HRMS (ESI) calcd $C_{32}H_{28}DBr_2N_4O_3^+$ $[M+H]^+$: 676.0664. Found: 676.0668.

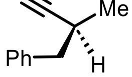

(–)-**65**

zwf-7-063

(–)-**65** was obtained as oil from the asymmetric decarboxylative protonation reaction of 2-cyanoacetic acid **S65** (94.6 mg, 0.5 mmol) for 7 days at room temperature using the general procedure (18.5 mg, 25% Yield). R_f = 0.3 (Hexane/EtOAc 10:1).

HPLC analysis (Chiralpak IG-3, hexane/^{i}PrOH = 99/1, 0.8 mL/min, 205 nm; t_r (major) = 14.74 min, t_r (minor) = 15.62 min) gave the isomeric composition of the product: 54.5:45.5 e.r., $[\alpha]_D^{23}$ = –3.0 (*c* = 0.80, $CHCl_3$).

^{1}H NMR (400 MHz, $CDCl_3$) δ 7.46-7.14 (m, 5H), 3.01-2.73 (m, 3H), 1.32 (d, *J* = 6.8 Hz, 3H).

^{13}C NMR (100 MHz, $CDCl_3$) δ 136.8, 129.0, 128.7, 127.2, 122.5, 40.0, 27.5, 17.6.

IR (neat, cm^{-1}) 3032, 2987, 2241, 1451, 1078.

HRMS (ESI) calcd $C_{10}H_{12}N^+$ [M+H]$^+$: 146.0964. Found: 146.0963.

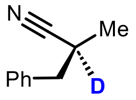

(–)-**65**-d

zwf-8-041

(–)-**65**-d was obtained as oil from the asymmetric decarboxylative deuteration reaction of 2-cyanoacetic acid **S65** (113.6 mg, 0.6 mmol) for 7 days at room temperature using the general procedure (21.2 mg, 24% Yield, 95% D). R_f = 0.3 (Hexane/EtOAc 10:1).

HPLC analysis (Chiralpak IG-3, hexane/^{i}PrOH = 99/1, 0.8 mL/min, 205 nm; t_r (major) = 15.82 min, t_r (minor) = 16.50 min) gave the isomeric composition of the product: 57:43 e.r., $[\alpha]_D^{19}$ = –4.8 (*c* = 0.61, $CHCl_3$).

^{1}H NMR (400 MHz, $CDCl_3$) δ 7.40-7.18 (m, 5H), 2.97-2.79 (m, 2.05H), 1.32 (s, 3H).

^{13}C NMR (100 MHz, $CDCl_3$) δ 136.8, 129.0, 128.7, 127.2, 122.5, 39.8, 27.2 (t, *J* = 21.0 Hz), 17.5.

IR (neat, cm^{-1}) 3029, 2982, 2239, 1454, 1082.

HRMS (ESI) calcd $C_{10}H_{11}DN^+$ [M+H]$^+$: 147.1027. Found: 147.1026.

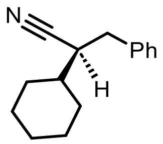

(–)-**66**

lk-4-10

(−)-**66** was obtained as oil from the asymmetric decarboxylative protonation reaction of 2-cyanoacetic acid **S66** for 12 h and room temperature using the general procedure: (8.8 mg, 41% Yield (0.5 mol% catalyst loading, 0.25 mmol **S66** was used), 18.1 mg, 85% Yield (5 mol% catalyst loading, 0.1 mmol **S66** was used)). R_f = 0.9 (Hexane/EtOAc 10:1).

HPLC analysis (Chiralpak IE-3, hexane/^{i}PrOH = 99/1, 0.8 mL/min, 205 nm; t_r (minor) = 16.64 min, t_r (major) = 17.69 min) gave the isomeric composition of the product: 54.0:46.0 e.r. (0.5 mol% catalyst loading), 64:36 e.r. (5 mol% catalyst loading), $[\alpha]_D^{20}$ = –0.019 (*c* = 0.90, $CHCl_3$).

^{1}H NMR (400 MHz, $CDCl_3$) δ 7.33 (t, *J* = 7.6 Hz, 2H), 7.29-7.20 (m, 3H), 2.96-2.82 (m, 2H), 2.68-2.60 (m, 1H), 2.00-1.90 (m, 1H), 1.87-1.64 (m, 4H), 1.59-1.46 (m, 1H), 1.32-1.15 (m, 5H).

^{13}C NMR (100 MHz, $CDCl_3$) δ 137.6, 128.8, 128.7, 127.0, 120.8, 40.7, 38.7, 35.8, 31.5, 28.9, 25.91, 25.85, 25.7.

IR (neat, cm^{-1}) 2926, 2854, 2236, 1451, 1054.

HRMS (ESI) calcd $C_{15}H_{20}N^+$ [M+H]$^+$: 214.1590. Found: 214.1591.

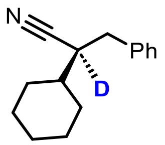

(–)-**66**-d

lk-4-13-1

(−)-**66**-d was obtained as oil from the asymmetric decarboxylative deuteration reaction of 2-cyanoacetic acid **S66** (25.7 mg, 0.1 mmol) for 12 h and room temperature using the general procedure (17.2 mg, 80% Yield, 85% D (5 mol% catalyst loading)). R_f = 0.9 (Hexane/EtOAc 10:1).

HPLC analysis (Chiralpak IE-3, hexane/^{i}PrOH = 99/1, 0.8 mL/min, 205 nm; t_r (minor) = 17.30 min, t_r (major) = 18.37 min) gave the isomeric composition of the product: 64.5:35.5 e.r., $[\alpha]_D^{20}$ = –0.025 (*c* = 0.85, $CHCl_3$).

^{1}H NMR (600 MHz, $CDCl_3$) δ 7.33 (t, *J* = 7.2 Hz, 2H), 7.30-7.21 (m, 3H), 2.94-2.79 (m, 2H), 2.67-2.62 (m, 0.15H), 2.02-1.90 (m, 1H), 1.87-1.65 (m, 4H), 1.57-1.46 (m, 1H), 1.32-1.14 (m, 5H).

^{13}C NMR (100 MHz, $CDCl_3$) δ 137.5, 128.8, 128.7, 127.0, 120.8, 40.4 (t, *J* = 20.8 Hz), 38.7, 35.7, 31.5, 28.9, 25.90, 25.86, 25.7.

IR (neat, cm^{-1}) 2921, 2852, 2239, 1450, 1057.

HRMS (ESI) calcd $C_{15}H_{19}DN^+$ $[M+H]^+$: 215.1653. Found: 215.1650.

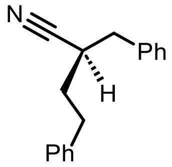

(+)-**67**

lk-4-6

(+)-**67** was obtained as oil from the asymmetric decarboxylative protonation reaction of 2-cyanoacetic acid **S67** for 12 h and room temperature using the general procedure (29.0 mg, 25% Yield (0.5 mol% catalyst loading, 0.5 mmol **S67** was used), 55.9 mg, 95% Yield (5 mol% catalyst loading, 0.25 mmol **S67** was used)). R_f = 0.7 (Hexane/EtOAc 10:1).

HPLC analysis (Chiampa IG-3, hexane/^{i}PrOH = 99/1, 1.0 mL/min, 205 nm; t_r (minor) = 32.60 min, t_r (major) = 37.36 min) gave the isomeric composition of the product: 66:34 e.r. (0.5 mol% catalyst loading), 66:34 e.r. (5 mol% catalyst loading), $[\alpha]_D^{20}$ = +0.113 (c = 0.98, $CHCl_3$).

^{1}H NMR (500 MHz, $CDCl_3$) δ 7.43-7.09 (m, 10H), 2.98-2.82 (m, 3H), 2.78-2.68 (m, 2H), 2.00-1.81 (m, 2H).

^{13}C NMR (125 MHz, $CDCl_3$) δ 139.9, 136.7, 128.9, 128.7, 128.6, 128.3, 127.2, 126.4, 121.4, 38.2, 33.3, 33.1, 32.9.

IR (neat, cm^{-1}) 3028, 2927, 2237, 1455, 1030.

HRMS (ESI) calcd $C_{17}H_{18}N^+$ $[M+H]^+$: 236.1434. Found: 236.1433.

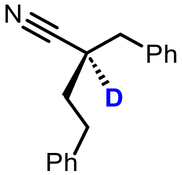

(+)-**67**-d

lk-4-3

(−)-**67**-d was obtained as oil from the asymmetric decarboxylative deuteration reaction of 2-cyanoacetic acid **S67** for 12 h and room temperature using the general procedure (23.3 mg, 20% Yield, 90% D (0.5 mol% catalyst loading, 0.5 mmol **S67** was used), 20.2 mg, 86% Yield, 90% D (5 mol% catalyst loading, 0.1 mmol **S67** was used)). R_f = 0.7 (Hexane/EtOAc 10:1).

HPLC analysis (Chiralpak IG-3, hexane/^{i}PrOH = 99/1, 0.8 mL/min, 205 nm; t_r (minor) = 42.38 min, t_r

(major) = 48.70 min) gave the isomeric composition of the product: 62.5:37.5 e.r. (0.5 mol% catalyst loading), 68.5:31.5 e.r. (5 mol% catalyst loading), $[\alpha]_D^{20}$ = +0.143 (*c* = 0.75, $CHCl_3$).

^{1}H NMR (500 MHz, $CDCl_3$) δ 7.40-7.09 (m, 10H), 2.96-2.82 (m, 3H), 2.77-2.69 (m, 1.10H), 1.99-1.81 (m, 2H).

^{13}C NMR (125 MHz, $CDCl_3$) δ 139.9, 136.7, 128.9, 128.64, 128.57, 128.3, 127.2, 126.4, 121.4, 38.1, 33.2, 33.1, 32.6 (t, *J* = 20.8 Hz).

IR (neat, cm^{-1}) 3032, 2917, 2239, 1454, 1027.

HRMS (ESI) calcd $C_{17}H_{17}DN^+$ $[M+H]^+$: 237.1497. Found: 237.1495.

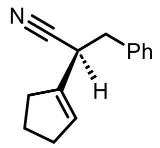

(−)-**68**

zwf-10-102

(−)-**68** was obtained as an oil from the asymmetric decarboxylative protonation reaction of 2-cyanoacetic acid **S68** (120.8 mg, 0.5 mmol) for 12 h using the general procedure (86.8 mg, 88% Yield). R_f = 0.6 (Hexane/EtOAc 10:1).

HPLC analysis (Chiralpak IE-3, hexane/^{i}PrOH = 99/1, 0.8 mL/min, 205 nm; t_r (minor) = 15.67 min, t_r (major) = 16.81 min) gave the isomeric composition of the product: 81:19 e.r., $[\alpha]_D^{20}$ = –4.8 (*c* = 0.67, $CHCl_3$).

^{1}H NMR (500 MHz, $CDCl_3$) δ 7.36-7.28 (m, 2H), 7.28-7.19 (m, 3H), 5.73-5.65 (m, 1H), 3.52 (t, *J* = 7.5 Hz, 1H), 3.04-2.90 (m, 2H), 2.49-2.25 (m, 4H), 2.00-1.87 (m, 2H).

^{13}C NMR (125 MHz, $CDCl_3$) δ 137.0, 136.8, 129.6, 128.9, 128.5, 127.1, 119.9, 37.7, 35.6, 33.0, 32.2, 23.3.

IR (neat, cm^{-1}) 2930, 2848, 2240, 1455, 1042.

HRMS (ESI) calcd $C_{14}H_{16}N^+$ $[M+H]^+$: 198.1277. Found: 198.1279.

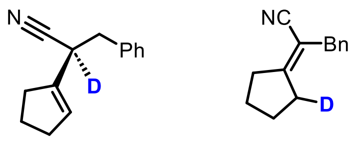

(–)-**68**-d **68'**-d

zwf-10-104

(–)-**68**-d was obtained as an oil from the asymmetric decarboxylative deuteration reaction of 2-cyanoacetic acid **S68** (48.4 mg, 0.2 mmol) for 12 h using the general procedure (35.9 mg, 90% Yield, 95% D) ((+)-**68**-d : **68'**-d = 33 :1 based on the ^{1}H NMR analysis of the crude product). R_f = 0.6 (Hexane/EtOAc 10:1).

HPLC analysis (Chiralpak IE-3, hexane/^{i}PrOH = 99/1, 0.8 mL/min, 205 nm; t_r (minor) = 15.82 min, t_r (major) = 16.99 min) gave the isomeric composition of the product: 83:17 e.r., $[\alpha]_D^{20}$ = –5.9 (*c* = 0.61, $CHCl_3$).

^{1}H NMR (500 MHz, $CDCl_3$) δ 7.40-7.15 (m, 5H), 5.72-5.65 (m, 1H), 3.52 (t, *J* = 7.3 Hz, 0.05H), 3.05-2.89 (m, 2H), 2.47-2.25 (m, 4H), 2.00-1.87 (m, 2H).

^{13}C NMR (125 MHz, $CDCl_3$) δ 137.0, 136.8, 129.6, 128.9, 128.5, 127.1, 119.9, 37.7, 35.3 (t, *J* = 20.8 Hz), 33.0, 32.3, 23.3.

IR (neat, cm^{-1}) 2925, 2849, 2239, 1453, 1044.

HRMS (ESI) calcd $C_{14}H_{15}DN^+$ $[M+H]^+$: 199.1340. Found: 199.1338.

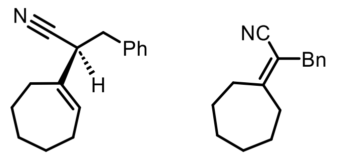

(+)-**69** **69'**

zwf-10-070

(+)-**69** was obtained as an oil from the asymmetric decarboxylative protonation reaction of 2-cyanoacetic acid **S69** (134.5 mg, 0.5 mmol) for 12 h using the general procedure (103.5 mg, 92% Yield) ((+)-**69** : **69'** = 5.4 :1 based on the ^{1}H NMR analysis of the crude product). R_f = 0.7 (Hexane/EtOAc 10:1).

HPLC analysis (Chiralpak IE-3, hexane/^{i}PrOH = 99/1, 0.8 mL/min, 205 nm; t_r (minor) = 15.57 min, t_r (major) = 16.77 min) gave the isomeric composition of the product: 82.5:17.5 e.r., $[\alpha]_D^{20}$ = +29.9 (*c* = 0.58, $CHCl_3$).

^{1}H NMR (500 MHz, $CDCl_3$) (+)-**69**: δ 7.36-7.18 (m, 5H), 5.80 (t, *J* = 6.5 Hz, 1H), 3.30 (t, *J* = 7.5 Hz, 1H), 2.99-2.85 (m, 2H), 2.33-2.18 (m, 2H), 2.15-2.08 (m, 2H), 1.83-1.68 (m, 2H), 1.62-1.41 (m, 4H). **69'**: δ 7.36-7.18 (m, 5H), 3.55 (s, 2H), 2.70-2.64 (m, 2H), 2.50-2.45 (m, 2H), 1.83-1.68 (m, 2H), 1.62-1.41 (m,

6H).

¹³C NMR (125 MHz, $CDCl_3$) (+)-**69**: δ 137.2, 136.9, 131.8, 129.0, 128.5, 127.1, 120.3, 43.4, 38.2, 32.1, 30.9, 28.1, 26.6, 26.4. **69'**: δ 162.7, 137.5, 128.6, 128.2, 126.7, 119.4, 107.9, 35.9, 35.4, 31.9, 29.02, 28.97, 27.4, 26.2.

IR (neat, cm^{-1}) 2922, 2850, 2237, 2207, 1455, 1030.

HRMS (ESI) calcd $C_{16}H_{20}N^+$ $[M+H]^+$: 226.1590. Found: 226.1591.

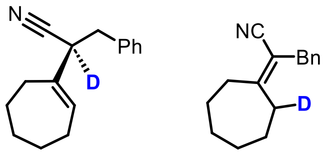

(+)-**69**-d **69'**-d

zwf-10-066

(+)-**69**-d was obtained as an oil from the asymmetric decarboxylative deuteration reaction of 2-cyanoacetic acid **S69** (53.5 mg, 0.2 mmol) for 12 h using the general procedure (41.1 mg, 91% Yield, 96% D) ((+)-**69**-d : **69'**-d = 5.0 :1 based on the ¹H NMR analysis of the crude product). R_f = 0.7 (Hexane/EtOAc 10:1).

HPLC analysis (Chiralpak IE-3, hexane/^{i}PrOH = 99/1, 0.8 mL/min, 205 nm; t_r (minor) = 15.69 min, t_r (major) = 16.93 min) gave the isomeric composition of the product: 90:10 e.r., $[\alpha]_D^{19}$ = +38.9 (c = 0.33, $CHCl_3$).

¹H NMR (500 MHz, $CDCl_3$) (+)-**69-d**: δ 7.36-7.18 (m, 5H), 5.80 (t, J = 6.5 Hz, 1H), 3.30 (t, J = 7.5 Hz, 1H), 2.99-2.85 (m, 2H), 2.33-2.18 (m, 2H), 2.15-2.08 (m, 2H), 1.83-1.68 (m, 2H), 1.62-1.41 (m, 4H). **69'-d**: δ 7.36-7.18 (m, 5H), 3.55 (s, 2H), 2.70-2.64 (m, 2H), 2.50-2.45 (m, 2H), 1.83-1.68 (m, 2H), 1.62-1.41 (m, 6H).

¹³C NMR (125 MHz, $CDCl_3$) (+)-**69-**d: δ 137.2, 136.9, 131.8, 129.0, 128.5, 127.1, 120.3, 43.0 (t, J = 20.8 Hz), 38.2, 32.1, 30.9, 28.1, 26.6, 26.4. **69'-**d: δ 162.6, 137.5, 128.6, 128.2, 126.7, 119.3, 107.9, 35.9, 35.4, 31.6 (t, J = 19.4 Hz), 28.99, 28.93, 27.3, 26.1.

IR (neat, cm^{-1}) 2921, 2851, 2236, 2208, 1454, 1030.

HRMS (ESI) calcd $C_{16}H_{19}DN^+$ $[M+H]^+$: 227.1653. Found: 227.1655.

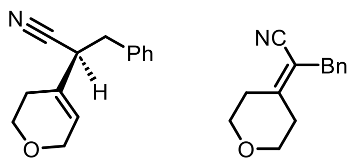

(+)-**70** **70'**

zwf-10-071

(+)-**70** was obtained as an oil from the asymmetric decarboxylative protonation reaction of 2-cyanoacetic acid **S70** (128.8 mg, 0.5 mmol) for 24 h using the general procedure (90.7 mg, 85% Yield) ((+)-**70** : **70'** = 8.3 :1 based on the ^{1}H NMR analysis of the crude product). R_f = 0.2 (Hexane/EtOAc 5:1).

HPLC analysis (Chiralpak IE-3, hexane/^{i}PrOH = 98/2, 0.8 mL/min, 205 nm; t_r (minor) = 31.82 min, t_r (major) = 34.94 min) gave the isomeric composition of the product: 83.5:16.5 e.r., $[\alpha]_D^{20}$ = +6.7 (*c* = 0.47, $CHCl_3$).

^{1}H NMR (500 MHz, $CDCl_3$) (+)-**70**: δ 7.36-7.19 (m, 5H), 5.74-5.68 (m, 1H), 4.16-4.08 (m, 2H), 3.83-3.78 (m, 2H), 3.37 (t, *J* = 7.3 Hz, 1H), 3.04-2.93 (m, 2H), 2.29-2.20 (m, 1H), 2.18-2.09 (m, 1H). **70'**: δ 7.36-7.19 (m, 5H), 3.79-3.75 (m, 2H), 3.71 (t, *J* = 5.5 Hz, 2H), 3.58 (s, 2H), 2.68 (t, *J* = 5.5 Hz, 2H), 2.51 (t, *J* = 5.5 Hz, 2H).

^{13}C NMR (125 MHz, $CDCl_3$) (+)-**70**: δ 136.4, 129.2, 129.0, 128.6, 127.3, 125.3, 119.4, 65.0, 63.8, 40.8, 37.6, 26.4. **70'**: δ 154.1, 137.2, 128.8, 128.1, 127.0, 118.4, 107.1, 68.3, 67.7, 35.2, 34.9, 31.2.

IR (neat, cm^{-1}) 2926, 2857, 2242, 2212, 2208, 1724, 1455, 1124.

HRMS (ESI) calcd $C_{14}H_{16}NO^+$ $[M+H]^+$: 214.1126. Found: 214.1127.

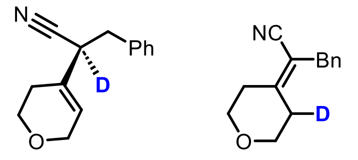

(+)-**70**-d **70'**-d

zwf-10-067

(+)-**70**-d was obtained as an oil from the asymmetric decarboxylative deuteration reaction of 2-cyanoacetic acid **S70** (51.6 mg, 0.2 mmol) for 24 h using the general procedure (37.3 mg, 87% Yield, 96% D) ((+)-**70-**d : **70'**-d = 33 :1 based on the ^{1}H NMR analysis of the crude product). R_f = 0.2 (Hexane/EtOAc 5:1).

HPLC analysis (Chiralpak IE-3, hexane/^{i}PrOH = 98/2, 0.8 mL/min, 205 nm; t_r (minor) = 32.00 min, t_r (major) = 35.12 min) gave the isomeric composition of the product: 88:12 e.r., $[\alpha]_D^{20}$ = +10.6 (*c* = 0.42, $CHCl_3$).

^{1}H NMR (500 MHz, $CDCl_3$) δ 7.36-7.20 (m, 5H), 5.74-5.68 (m, 1H), 4.11 (q, *J* = 2.7 Hz, 2H), 3.84-3.76

(m, 2H), 3.37 (t, J = 7.3 Hz, 0.04H), 3.02-2.93 (m, 2H), 2.30-2.19 (m, 1H), 2.18-2.08 (m, 1H).

^{13}C NMR (125 MHz, $CDCl_3$) δ 136.4, 129.1, 128.9, 128.6, 127.3, 125.3, 119.4, 65.0, 63.7, 40.4 (t, J = 20.8 Hz), 37.5, 26.3.

IR (neat, cm^{-1}) 2921, 2850, 2237, 2208, 1455, 1076.

HRMS (ESI) calcd $C_{14}H_{15}DNO^+$ $[M+H]^+$: 215.1289. Found: 215.1291.

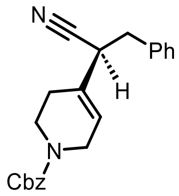

(+)-**71**

zwf-10-072

(+)-**71** was obtained as an oil from the asymmetric decarboxylative protonation reaction of 2-cyanoacetic acid **S71** (195.5 mg, 0.5 mmol) for 48 h using the general procedure (138.3 mg, 80% Yield). R_f = 0.2 (Hexane/EtOAc 5:1).

HPLC analysis (Chiralpak IE-3, hexane/^{i}PrOH = 80/20, 0.8 mL/min, 205 nm; t_r (minor) = 29.14 min, t_r (major) = 34.88 min) gave the isomeric composition of the product: 87.5:12.5 e.r., $[\alpha]_D^{20}$ = +8.4 (c = 0.46, $CHCl_3$).

^{1}H NMR (500 MHz, $CDCl_3$) δ 7.39-7.28 (m, 7H), 7.28-7.25 (m, 1H), 7.22-7.16 (m, 2H), 5.75-5.55 (m, 1H), 5.15 (s, 2H), 4.05-3.90 (m, 2H), 3.65-3.55 (m, 2H), 3.37 (t, J = 7.5 Hz, 1H), 2.95 (d, J = 7.5 Hz, 2H), 2.33-2.05 (m, 2H).

^{13}C NMR (125 MHz, $CDCl_3$) δ 155.2, 136.5, 136.2, 130.4, 130.1, 128.9, 128.6, 128.4, 128.0, 127.9, 127.3, 123.6, 123.0, 119.3, 67.1, 43.2, 43.0, 40.7, 40.3, 40.0, 37.7, 26.4, 26.2.

IR (neat, cm^{-1}) 3030, 2931, 2240, 2208, 1698, 1427, 1234, 1078.

HRMS (ESI) calcd $C_{22}H_{23}N_2O_2^+$ $[M+H]^+$: 347.1754. Found: 347.1753.

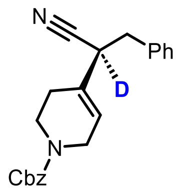

(+)-**71**-d

zwf-10-068

(+)-**71**-d was obtained as an oil from the asymmetric decarboxylative deuteration reaction of 2-cyanoacetic acid **S71** (78.3 mg, 0.2 mmol) for 48 h using the general procedure (57.8 mg, 83% Yield, 93% D). R_f = 0.2 (Hexane/EtOAc 5:1).

HPLC analysis (Chiralpak IE-3, hexane/^{i}PrOH = 80/20, 0.8 mL/min, 205 nm; t_r (minor) = 28.92 min, t_r (major) = 34.68 min) gave the isomeric composition of the product: 87.5:12.5 e.r., $[\alpha]_D^{20}$ = +8.5 (*c* = 0.42, $CHCl_3$).

^{1}H NMR (500 MHz, $CDCl_3$) δ 7.38-7.27 (m, 7H), 7.27-7.23 (m, 1H), 7.22-7.15 (m, 2H), 5.76-5.55 (m, 1H), 5.15 (s, 2H), 4.04-3.88 (m, 2H), 3.65-3.53 (m, 2H), 3.37 (t, *J* = 7.0 Hz, 0.07H), 2.94 (d, *J* = 7.5 Hz, 2H), 2.31-2.07 (m, 2H).

^{13}C NMR (125 MHz, $CDCl_3$) δ 155.2, 136.4, 136.2, 130.3, 130.0, 128.8, 128.6, 128.4, 128.0, 127.8, 127.3, 123.5, 122.9, 119.3, 67.1, 43.1, 43.0, 40.4 (t, *J* = 20.9 Hz), 40.2, 39.9, 37.6, 26.3, 26.1.

IR (neat, cm^{-1}) 3031, 2932, 2238, 2211, 1695, 1425, 1234, 1079.

HRMS (ESI) calcd $C_{22}H_{22}DN_2O_2^+$ $[M+H]^+$: 348.1817. Found: 348.1815.

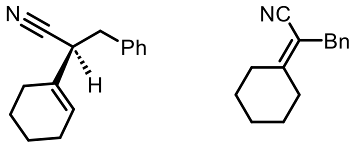

(+)-**72** **72'**

zwf-10-022

(+)-**72** was obtained as an oil from the asymmetric decarboxylative protonation reaction of 2-cyanoacetic acid **S72** (127.5 mg, 0.5 mmol) for 12 h using the general procedure (96.2 mg, 91% Yield) ((+)-**72** : **72'** = 25 :1 based on the ^{1}H NMR analysis of the crude product). R_f = 0.6 (Hexane/EtOAc 10:1).

HPLC analysis (Chiralpak IE-3, hexane/^{i}PrOH = 99/1, 0.8 mL/min, 205 nm; t_r (minor) = 16.03 min, t_r (major) = 17.88 min) gave the isomeric composition of the product: 79.5:20.5 e.r., $[\alpha]_D^{24}$ = +11.9 (*c* = 0.57, $CHCl_3$).

^{1}H NMR (400 MHz, $CDCl_3$) δ 7.35-7.19 (m, 5H), 5.72-5.64 (m, 1H), 3.27 (t, *J* = 7.6 Hz, 1H), 2.99-2.89 (m, 2H), 2.19-2.07 (m, 1H), 2.06-1.96 (m, 3H), 1.70-1.63 (m, 2H), 1.60-1.50 (m, 2H).

^{13}C NMR (100 MHz, $CDCl_3$) δ 137.0, 131.1, 129.0, 128.5, 127.1, 126.9, 120.2, 41.5, 38.0, 26.2, 25.0, 22.4, 21.8.

IR (neat, cm^{-1}) 2930, 2860, 2238, 2210, 1455, 1075.

HRMS (ESI) calcd $C_{15}H_{18}N^+$ $[M+H]^+$: 212.1434. Found: 212.1432.

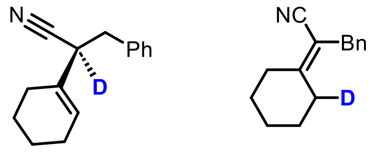

(+)-**72**-d **72'**-d

zwf-10-023

(+)-**72**-d was obtained as an oil from the asymmetric decarboxylative deuteration reaction of 2-cyanoacetic acid **S72** (51.2 mg, 0.2 mmol) for 12 h using the general procedure (39.4 mg, 93% Yield, 95% D) ((+)-**72**-d : **72'**-d = 50 :1 based on the ^{1}H NMR analysis of the crude product). R_f = 0.6 (Hexane/EtOAc 10:1).

HPLC analysis (Chiralpak IE-3, hexane/^{i}PrOH = 99/1, 0.8 mL/min, 205 nm; t_r (minor) = 16.02 min, t_r (major) = 17.80 min) gave the isomeric composition of the product: 88.5:11.5 e.r., $[\alpha]_D^{24}$ = +16.5 (*c* = 0.59, $CHCl_3$).

^{1}H NMR (500 MHz, $CDCl_3$) δ 7.37-7.17 (m, 5H), 5.71-5.64 (m, 1H), 3.27 (t, *J* = 7.8 Hz, 0.05H), 2.98-2.88 (m, 2H), 2.17-2.08 (m, 1H), 2.06-1.94 (m, 3H), 1.72-1.63 (m, 2H), 1.60-1.50 (m, 2H).

^{13}C NMR (125 MHz, $CDCl_3$) δ 137.0, 131.1, 129.0, 128.5, 127.1, 126.9, 120.2, 41.2 (t, *J* = 20.8 Hz), 38.0, 26.1, 25.0, 22.4, 21.8.

IR (neat, cm^{-1}) 2929, 2857, 2239, 2211, 1453, 1074.

HRMS (ESI) calcd $C_{15}H_{17}DN^+$ $[M+H]^+$: 213.1497. Found: 213.1496.

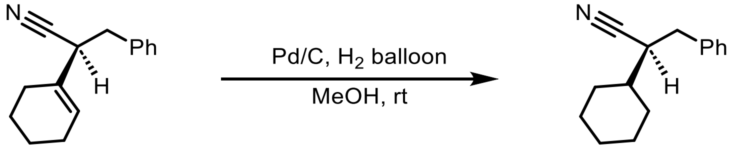

(+)-**72** (-)-**66**

zwf-10-144

To an oven-dried 25 mL flask was added (+)-**72** (63.4 mg, 0.3 mmol, 79.5:20.5 e.r.), Pd/C powder (10%, 50.1 mg), and sealed with a rubber septum and evacuated/refilled with nitrogen for three times. 10 mL of MeOH was added to the flask via syringe under nitrogen atmosphere. The reaction mixture was purged by H_2 for 20 min with a H_2 balloon, then sealed and stirred under H_2 atmosphere with a H_2 balloon. After stirred at room temperature for 12 h, the reaction mixture was filtrated with Celite, and the filtrate was concentrated under vacuum and purified by flash column chromatography to afford the (−)-**66** (54.7 mg, 85% Yield) as an oil. R_f = 0.9 (Hexane/EtOAc 10:1).

HPLC analysis (Chiralpak IE-3, hexane/^{i}PrOH = 99/1, 0.8 mL/min, 205 nm; t_r (minor) = 17.09 min, t_r (major) = 18.09 min) gave the isomeric composition of the product: 78.5:21.5 e.r., $[\alpha]_D^{20}$ = –1.7 (*c* = 0.43, $CHCl_3$).

^{1}H NMR (400 MHz, $CDCl_3$) δ 7.32 (t, *J* = 7.3 Hz, 2H), 7.29-7.18 (m, 3H), 2.95-2.80 (m, 2H), 2.70-2.59 (m, 1H), 2.01-1.90 (m, 1H), 1.89-1.62 (m, 4H), 1.59-1.45 (m, 1H), 1.32-1.14 (m, 5H).

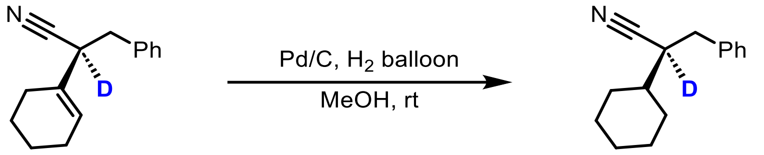

(+)-**72**-d (-)-**66**-d

zwf-10-145

To an oven-dried 25 mL flask was added (+)-**72**-d (63.2 mg, 0.3 mmol, 88.5:11.5 e.r., 95% D), Pd/C powder (10%, 50.2 mg), and sealed with a rubber septum and evacuated/refilled with nitrogen for three times. 10 mL of MeOH was added to the flask via syringe under nitrogen atmosphere. The reaction mixture was purged by H_2 for 20 min with a H_2 balloon, then sealed and stirred under H_2 atmosphere with a H_2 balloon. After stirred at room temperature for 12 h, the reaction mixture was filtrated with Celite, and the filtrate was concentrated under vacuum and purified by flash column chromatography to afford the (–)-**66**-d (54.1 mg, 84% Yield, 91% D) as an oil. R_f = 0.9 (Hexane/EtOAc 10:1).

HPLC analysis (Chiralpak IE-3, hexane/^{i}PrOH = 99/1, 0.8 mL/min, 205 nm; t_r (minor) = 16.70 min, t_r (major) = 17.65 min) gave the isomeric composition of the product: 87.5:12.5 e.r., $[\alpha]_D^{20}$ = –3.2 (*c* = 0.20, $CHCl_3$).

^{1}H NMR (400 MHz, $CDCl_3$) δ 7.32 (t, *J* = 7.3 Hz, 2H), 7.29-7.18 (m, 3H), 2.94-2.78 (m, 2H), 2.67-2.61 (m, 0.09H), 2.02-1.89 (m, 1H), 1.89-1.64 (m, 4H), 1.58-1.46 (m, 1H), 1.33-1.15 (m, 5H).

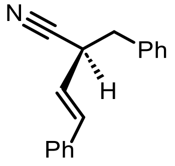

(+)-**74**

zwf-11-062

(+)-**74** was obtained as a solid from the asymmetric decarboxylative protonation reaction of 2-cyanoacetic acid **S74** (138.8 mg, 0.5 mmol) for 12 h using the general procedure (107.2 mg, 92% Yield). R_f = 0.4 (Hexane/EtOAc 10:1).

HPLC analysis (Chiralpak IE-3, hexane/^{i}PrOH = 99/1, 0.8 mL/min, 205 nm; t_r (minor) = 25.64 min, t_r (major) = 28.21 min) gave the isomeric composition of the product: 88.5:11.5 e.r., $[\alpha]_D^{23}$ = +35.9 (*c* = 0.46,

$CHCl_3$).

^{1}H NMR (500 MHz, $CDCl_3$) δ 7.37-7.23 (m, 10H), 6.69 (dd, J_1 = 16.0 Hz, J_2 = 1.0 Hz, 1H), 6.04 (dd, J_1 = 15.8 Hz, J_2 = 1.3 Hz, 1H), 3.67-3.60 (m, 1H), 3.11-2.97 (m, 2H).

^{13}C NMR (125 MHz, $CDCl_3$) δ 136.2, 135.6, 133.6, 129.1, 128.7, 128.6, 128.3, 127.4, 126.5, 122.3, 119.6, 39.6, 36.5.

IR (neat, cm^{-1}) 3031, 2897, 2243, 1496, 1077.

HRMS (ESI) calcd $C_{17}H_{16}N^+$ $[M+H]^+$: 234.1277. Found: 234.1276.

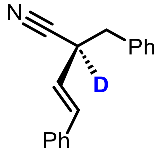

(+)-**74**-d

zwf-11-063

(+)-**74**-d was obtained as a solid from the asymmetric decarboxylative deuteration reaction of 2-cyanoacetic acid **S74** (55.6 mg, 0.2 mmol) for 12 h using the general procedure (41.9 mg, 89% Yield, 91% D). R_f = 0.4 (Hexane/EtOAc 10:1).

HPLC analysis (Chiralpak IE-3, hexane/^{i}PrOH = 99/1, 0.8 mL/min, 205 nm; t_r (minor) = 26.06 min, t_r (major) = 28.67 min) gave the isomeric composition of the product: 91:9 e.r., $[\alpha]_D^{23}$ = +41.8 (c = 0.44, $CHCl_3$).

^{1}H NMR (500 MHz, $CDCl_3$) δ 7.54-7.10 (m, 10H), 6.69 (d, J = 16.0 Hz, 1H), 6.04 (d, J = 15.5 Hz, 1H), 3.67-3.60 (m, 0.09H), 3.18-2.88 (m, 2H).

^{13}C NMR (125 MHz, $CDCl_3$) δ 136.2, 135.6, 133.7, 129.2, 128.70, 128.65, 128.3, 127.4, 126.5, 122.3, 119.6, 39.6, 36.2 (t, J = 20.3 Hz).

IR (neat, cm^{-1}) 3027, 2895, 2241, 1494, 1067.

HRMS (ESI) calcd $C_{17}H_{15}DN^+$ $[M+H]^+$: 235.1340. Found: 235.1343.

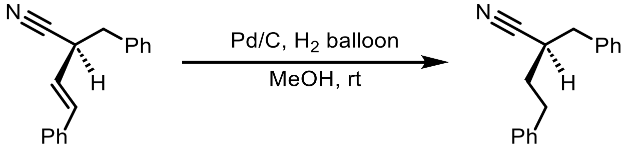

(+)-**74** (+)-**67**

zwf-10-146

To an oven-dried 25 mL flask was added (+)-**74** (70.2 mg, 0.3 mmol, 88.5:11.5 e.r.), Pd/C powder (10%, 50.2 mg), and sealed with a rubber septum and evacuated/refilled with nitrogen for three times. 10 mL of MeOH was added to the flask via syringe under nitrogen atmosphere. The reaction mixture was purged by H_2 for 20 min with a H_2 balloon, then sealed and stirred under H_2 atmosphere with a H_2 balloon. After stirred at room temperature for 12 h, the reaction mixture was filtrated with Celite, and the filtrate was concentrated under vacuum and purified by flash column chromatography to afford the (+)-**67** (61.7 mg, 87% Yield) as an oil. R_f = 0.7 (Hexane/EtOAc 10:1).

HPLC analysis (Chiralpak IG-3, hexane/^{i}PrOH = 99/1, 0.8 mL/min, 205 nm; t_r (minor) = 38.51 min, t_r (major) = 44.08 min) gave the isomeric composition of the product: 87.5:12.5 e.r., $[\alpha]_D^{20}$ = +41.0 (c = 0.45, $CHCl_3$).

^{1}H NMR (500 MHz, $CDCl_3$) δ 7.42-7.10 (m, 10H), 2.98-2.81 (m, 3H), 2.79-2.65 (m, 2H), 2.00-1.80 (m, 2H).

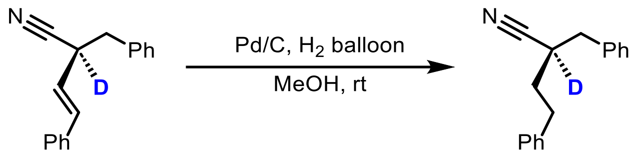

(+)-**74**-d (+)-**67**-d

zwf-10-147

To an oven-dried 25 mL flask was added (+)-**74-**d (70.0 mg, 0.3 mmol, 91:9 e.r., 91% D), Pd/C powder (10%, 50.2 mg), and sealed with a rubber septum and evacuated/refilled with nitrogen for three times. 10 mL of MeOH was added to the flask via syringe under nitrogen atmosphere. The reaction mixture was purged by H_2 for 20 min with a H_2 balloon, then sealed and stirred under H_2 atmosphere with a H_2 balloon. After stirred at room temperature for 12 h, the reaction mixture was filtrated with Celite, and the filtrate was concentrated under vacuum and purified by flash column chromatography to afford (+)-**67-**d (60.1 mg, 85% Yield, 90% D) as an oil. R_f = 0.7 (Hexane/EtOAc 10:1).

HPLC analysis (Chiralpak IG-3, hexane/^{i}PrOH = 99/1, 0.8 mL/min, 205 nm; t_r (minor) = 38.68 min, t_r (major) = 43.68 min) gave the isomeric composition of the product: 90:10 e.r., $[\alpha]_D^{20}$ = +46.6 (c = 0.49, $CHCl_3$).

^{1}H NMR (500 MHz, $CDCl_3$) δ 7.40-7.08 (m, 10H), 2.96-2.80 (m, 3H), 2.77-2.68 (m, 1.10H), 1.97-1.82 (m, 2H).

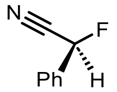

(–)-**76**

zwf-10-024

(–)-**76** was obtained as an oil from the asymmetric decarboxylative protonation reaction of 2-cyanoacetic acid **S76** (89.8 mg, 0.5 mmol) for 48 h using the general procedure (58.5 mg, 85% Yield). R_f = 0.7 (Hexane/EtOAc 10:1).

HPLC analysis (Chiralpak IG-3, hexane/^{i}PrOH = 99/1, 0.8 mL/min, 205 nm; t_r (minor) = 9.65 min, t_r (major) = 10.24 min) gave the isomeric composition of the product: 94.5:4.5 e.r., $[\alpha]_D^{19}$ = –49.0 (*c* = 0.53, $CHCl_3$).

^{1}**H NMR** (400 MHz, $CDCl_3$) δ 7.62-7.44 (m, 5H), 6.05 (d, *J* = 47.2 Hz, 1H).

^{13}**C NMR** (100 MHz, $CDCl_3$) δ 131.4 (d, *J* = 20.2 Hz), 131.2 (d, *J* = 3.7 Hz), 129.3 (d, *J* = 1.4 Hz), 127.5 (d, *J* = 4.3 Hz), 115.2 (d, *J* = 33.9 Hz), 80.1 (d, *J* = 180.6 Hz).

^{19}**F NMR** (376 MHz, $CDCl_3$) δ –167.4 (d, *J* = 47.4 Hz).

IR (neat, cm^{-1}) 3040, 2934, 2250, 1458, 1009.

MS (70 eV, EI) *m/z* (%): 135 (M^+, 100.00), 135 (100); **HRMS** calcd for C_8H_6NF [M^+]: 135.0479. Found: 135.0478.

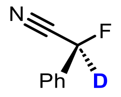

(–)-**76**-d

zwf-10-025

(–)-**76**-d was obtained as an oil from the asymmetric decarboxylative deuteration reaction of 2-cyanoacetic acid **S76** (35.9 mg, 0.2 mmol) for 48 h using the general procedure (23.0 mg, 83% Yield, 93% D). R_f = 0.7 (Hexane/EtOAc 10:1).

HPLC analysis (Chiralpak IG-3, hexane/^{i}PrOH = 99/1, 0.8 mL/min, 205 nm; t_r (minor) = 9.72 min, t_r (major) = 10.34 min) gave the isomeric composition of the product: 94.5:4.5 e.r., $[\alpha]_D^{19}$ = –48.0 (*c* = 0.54, $CHCl_3$).

^{1}**H NMR** (500 MHz, $CDCl_3$) δ 7.63-7.45 (m, 5H), 6.05 (d, *J* = 47.0 Hz, 0.07H).

^{13}**C NMR** (125 MHz, $CDCl_3$) δ 131.3 (d, *J* = 18.0 Hz), 131.2 (d, *J* = 3.6 Hz), 129.3 (d, *J* = 1.8 Hz), 127.5 (d, *J* = 4.5 Hz), 115.2 (d, *J* = 33.4 Hz), 79.9 (dt, J_1 = 179.8 Hz, J_2 = 24.4 Hz).

^{19}**F NMR** (470 MHz, $CDCl_3$) δ –167.4 (d, *J* = 46.5 Hz), –167.8 to –168.0 (m).

IR (neat, cm^{-1}) 3040, 2933, 2249, 1457, 1000.

MS (70 eV, EI) *m/z* (%): 136 (M^+, 100.00), 136 (100); **HRMS** calcd for C_8H_5DNF [M^+]: 136.0542. Found: 136.0538.

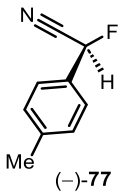

(–)-**77**

zwf-10-116

(–)-**77** was obtained as an oil from the asymmetric decarboxylative protonation reaction of 2-cyanoacetic acid **S77** (96.5 mg, 0.5 mmol) for 48 h using the general procedure (66.3 mg, 88% Yield). R_f = 0.6 (Hexane/EtOAc 10:1).

HPLC analysis (Chiralpak IE-3, hexane/^{i}PrOH = 99/1, 0.8 mL/min, 205 nm; t_r (minor) = 9.19 min, t_r (major) = 9.88 min) gave the isomeric composition of the product: 95:5 e.r., $[\alpha]_D^{20}$ = –50.9 (*c* = 0.49, $CHCl_3$).

^{1}H NMR (500 MHz, $CDCl_3$) δ 7.43 (dd, J_1 = 8.5 Hz, J_2 = 2.0 Hz, 2H), 7.28 (d, *J* = 8.0 Hz, 2H), 5.99 (d, *J* = 47.5 Hz, 1H), 2.40 (d, *J* = 2.5 Hz, 3H).

^{13}C NMR (125 MHz, $CDCl_3$) δ 141.6 (d, *J* = 3.6 Hz), 129.9 (d, *J* = 2.6 Hz), 128.5 (d, *J* = 20.8 Hz), 127.6 (d, *J* = 3.5 Hz), 115.4 (d, *J* = 34.3 Hz), 80.0 (d, *J* = 179.6 Hz), 21.3.

^{19}F NMR (470 MHz, $CDCl_3$) δ –164.5 (d, *J* = 46.5 Hz).

IR (neat, cm^{-1}) 2960, 2927, 2250, 1615, 1277, 1000.

MS (70 eV, EI) *m/z* (%): 149 (M^+, 62.11), 134 (100); **HRMS** calcd for C_9H_8NF [M^+]: 149.0635. Found: 149.0632.

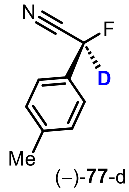

(–)-**77**-d

zwf-10-118

(–)-**77**-d was obtained as an oil from the asymmetric decarboxylative deuteration reaction of 2-cyanoacetic acid **S77** (38.7 mg, 0.2 mmol) for 48 h using the general procedure (26.5 mg, 87% Yield, 93% D). R_f = 0.6 (Hexane/EtOAc 10:1).

HPLC analysis (Chiralpak IE-3, hexane/^{i}PrOH = 99/1, 0.8 mL/min, 205 nm; t_r (minor) = 9.18 min, t_r (major) = 9.86 min) gave the isomeric composition of the product: 95:5 e.r., $[\alpha]_D^{20}$ = –49.3 (*c* = 0.50, $CHCl_3$).

^{1}H NMR (500 MHz, $CDCl_3$) δ 7.43 (dd, J_1 = 8.5 Hz, J_2 = 2.0 Hz, 2H), 7.27 (d, *J* = 8.0 Hz, 2H), 5.99 (d, *J* = 47.0 Hz, 0.06H), 2.39 (d, *J* = 2.5 Hz, 3H).

^{13}C NMR (125 MHz, $CDCl_3$) δ 141.7 (d, *J* = 3.6 Hz), 129.9 (d, *J* = 1.8 Hz), 128.4 (d, *J* = 20.9 Hz), 127.6 (d, *J* = 3.5 Hz), 115.4 (d, *J* = 34.3 Hz), 80.0 (dt, J_1 = 178.8 Hz, J_2 = 24.4 Hz), 21.3.

^{19}F NMR (470 MHz, $CDCl_3$) δ –164.4 (d, *J* = 46.5 Hz), –164.9 to –165.1 (m).

IR (neat, cm^{-1}) 29590, 2925, 2248, 1616, 1277, 1002.

MS (70 eV, EI) *m/z* (%): 150 (M^+, 68.07), 135 (100); **HRMS** calcd for C_9H_7DNF [M^+]: 150.0698. Found: 150.0694.

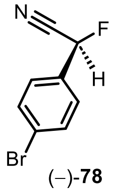

(–)-**78**

zwf-10-090

(–)-**78** was obtained as an oil from the asymmetric decarboxylative protonation reaction of 2-cyanoacetic acid **S78** (129.1 mg, 0.5 mmol) for 48 h using the general procedure (90.1 mg, 85% Yield). R_f = 0.5 (Hexane/EtOAc 10:1).

HPLC analysis (Chiralpak IG-3, hexane/^{i}PrOH = 99/1, 0.8 mL/min, 205 nm; t_r (minor) = 12.27 min, t_r (major) = 13.51 min) gave the isomeric composition of the product: 91:9 e.r., $[\alpha]_D^{19}$ = –32.3 (*c* = 0.83, $CHCl_3$).

^{1}H NMR (400 MHz, $CDCl_3$) δ 7.63 (dd, J_1 = 8.6 Hz, J_2 = 1.0 Hz, 2H), 7.42 (dd, J_1 = 8.4 Hz, J_2 = 1.6 Hz, 2H), 6.02 (d, *J* = 46.8 Hz, 1H).

^{13}C NMR (100 MHz, $CDCl_3$) δ 132.6 (d, *J* = 1.4 Hz), 130.3 (d, *J* = 21.0 Hz), 129.0 (d, *J* = 4.3 Hz), 125.7 (d, *J* = 3.6 Hz), 114.8 (d, *J* = 33.3 Hz), 79.4 (d, *J* = 182.0 Hz).

^{19}F NMR (376 MHz, $CDCl_3$) δ –168.4 (d, *J* = 47.0 Hz).

IR (neat, cm^{-1}) 3093, 2959, 2251, 1490, 1196, 1011.

MS (70 eV, EI) *m/z* (%): 212 (M^+, 48.96), 134 (100); **HRMS** calcd for C_8H_5BrNF [M^+]: 212.9584. Found: 212.9582.

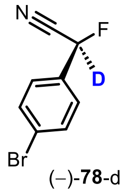

(−)-**78**-d

zwf-10-092

(−)-**78**-d was obtained as an oil from the asymmetric decarboxylative deuteration reaction of 2-cyanoacetic acid **S78** (51.7 mg, 0.2 mmol) for 48 h using the general procedure (36.7 mg, 83% Yield, 92% D). R_f = 0.5 (Hexane/EtOAc 10:1).

HPLC analysis (Chiralpak IG-3, hexane/^{i}PrOH = 99/1, 0.8 mL/min, 205 nm; t_r (minor) = 12.25 min, t_r (major) = 13.52 min) gave the isomeric composition of the product: 93:7 e.r., $[\alpha]_D^{19}$ = –34.8 (*c* = 0.78, $CHCl_3$).

^{1}H NMR (400 MHz, $CDCl_3$) δ 7.62 (dd, J_1 = 8.6 Hz, J_2 = 1.0 Hz, 2H), 7.41 (dd, J_1 = 8.6 Hz, J_2 = 1.8 Hz, 2H), 6.02 (d, J = 46.8 Hz, 0.07H).

^{13}C NMR (100 MHz, $CDCl_3$) δ 132.6 (d, J = 1.5 Hz), 130.2 (d, J = 20.9 Hz), 129.0 (d, J = 4.3 Hz), 125.7 (d, J = 4.3 Hz), 114.8 (d, J = 33.2 Hz), 79.1 (dt, J_1 = 180.6 Hz, J_2 = 24.6 Hz).

^{19}F NMR (376 MHz, $CDCl_3$) δ −168.4 (d, J = 47.0 Hz), −168.8 to −169.0 (m).

IR (neat, cm^{-1}) 3094, 2963, 2250, 1489, 1192, 1010.

MS (70 eV, EI) *m/z* (%): 213 (M^+, 24.73), 135 (100); **HRMS** calcd for C_8H_4DBrNF [M^+]: 213.9647. Found: 213.9643.

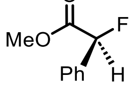

(+)-**83**

HM-1-096-3

(+)-**83** was obtained as an oil from the asymmetric decarboxylative protonation reaction of 2-cyanoacetic acid **S82** (60.3 mg, 0.3 mmol) by using $Rh(COD)_2OTf$ (4.2 mg, 0.009 mmol) as catalyst in 1,4-dioxane and stirred at room temperature for 14 days using the general procedure. Then 0.8 mL MeOH and 1.2 mL 2 M $TMSCHN_2$ (800 mol%) were added to the reaction mixture, which was stirred for an additional hour. (+)-**83** (24.2 mg, 47% Yield). R_f = 0.6 (Hexane/EtOAc 5:1).

HPLC analysis (Chiralpak IJ-3, hexane/^{i}PrOH = 90/10, 0.8 mL/min, 230 nm; t_r (minor) = 14.93 min, t_r (major) = 17.06 min) gave the isomeric composition of the product: 78.5:21.5 e.r., $[\alpha]_D^{20}$ = +46.1 (*c* = 1.00, $CHCl_3$).

^{1}H NMR (500 MHz, $CDCl_3$) δ 7.49-7.40 (m, 2H), 7.40-7.34 (m, 3H), 5.75 (d, *J* = 48.0 Hz, 1H), 3.74 (s, 3H).

^{13}C NMR (125 MHz, $CDCl_3$) δ 169.0 (d, *J* = 27.1 Hz), 134.1 (d, *J* = 19.9 Hz), 129.7 (d, *J* = 2.8 Hz), 128.8, 126.6 (d, *J* = 6.4 Hz), 89.3 (d, *J* = 185.1 Hz), 52.6.

^{19}F NMR (470 MHz, $CDCl_3$) δ −179.9 (s).

IR (neat, cm^{-1}) 3065, 2960, 1757, 1268, 1097.

MS (70 eV, EI) *m/z* (%): 168 (M^+, 23.63), 109 (100); **HRMS** calcd for $C_9H_9FO_2$ [M^+]: 168.0587. Found: 168.0582.

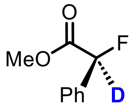

(+)-**83**-d

HM-1-096-2

(+)-**83-**d was obtained as an oil from the asymmetric decarboxylative protonation reaction of 2-cyanoacetic acid **S82** (60.2 mg, 0.3 mmol) by using $Rh(COD)_2OTf$ (4.2 mg, 0.009 mmol) as catalyst in 1,4-dioxane and stirred at room temperature for 7 days using the general procedure. Then 0.8 mL MeOH and 1.2 mL 2 M $TMSCHN_2$ (800 mol%) were added to the reaction mixture, which was stirred for an additional hour. (+)-**83-**d (20.1 mg, 39% Yield, 40% D). R_f = 0.6 (Hexane/EtOAc 5:1).

HPLC analysis (Chiralpak IJ-3, hexane/^{i}PrOH = 90/10, 0.8 mL/min, 230 nm; t_r (minor) = 14.80 min, t_r (major) = 16.84 min) gave the isomeric composition of the product: 66.5:33.5 e.r., $[\alpha]_D^{17}$ = +33.4 (*c* = 1.20, $CHCl_3$).

^{1}H NMR (500 MHz, $CDCl_3$) δ 7.48-7.40 (m, 2H), 7.40-7.35 (m, 3H), 5.75 (d, *J* = 47.5 Hz, 0.60H), 3.74 (m, 3H).

^{13}C NMR (125 MHz, $CDCl_3$) δ 169.0 (d, *J* = 27.5 Hz), 134.1 (d, *J* = 21.3 Hz), 129.7 (d, *J* = 1.3 Hz), 128.8, 126.6 (d, *J* = 6.3 Hz), 89.3 (d, *J* = 183.8 Hz), 52.6.

^{19}F NMR (470 MHz, $CDCl_3$) δ −179.9 (s).

IR (neat, cm^{-1}) 3062, 2959, 1753, 1265, 1095.

MS (70 eV, EI) *m/z* (%): 169 (M^+, 16.21), 110 (100); **HRMS** calcd for $C_9H_8DFO_2$ [M^+]: 169.0649. Found: 169.0645.

Section 8. Synthetic applications

Gram-scale asymmetric decarboxylative deuteration (**zwf-8-056**)

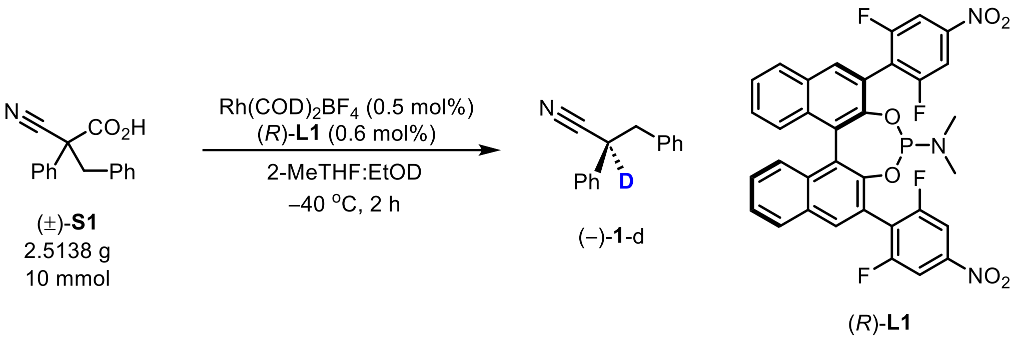

To an oven-dried 500 mL round bottom flask were added (*R*)-**L1** (40.6 mg, 0.06 mmol), $Rh(COD)_2BF_4$ (20.4 mg, 0.05 mmol), and 2-methyltetrahydrofuran (2-MeTHF, 160 mL). After the reaction mixture was stirred at room temperature for 1 h, EtOD (40 mL) was added to the reaction and stirred for additional 10 min at room temperature. Then the catalyst solution was cooled to –40 °C and stirred for another 30 min. The **S1** (10 mmol) was dissolved in 2-MeTHF (35 mL) in another flask and cooled to –40 °C for 10 min, which was added to the catalyst flask dropwise over 20 min via syringe, and the acid flask was rinsed with 2-MeTHF (5 mL) to transfer the residue to the reaction. The reaction mixture was stirred at –40 °C for 2 h. After the reaction mixture was warmed to room temperature, the solvent was removed under vacuum and the filtrate was purified by flash column chromatography to obtain (–)-**1**-d (1.8738 g, 90% Yield, 95% D) as a white solid, R_f = 0.4 (Hexane/EtOAc 10:1).

HPLC analysis (Chiralpak IE-3, hexane/^{i}PrOH = 99/1, 0.8 mL/min, 205 nm; t_r (minor) = 20.85 min, t_r (major) = 23.20 min) gave the isomeric composition of the product: 98:2 e.r.

^{1}H NMR (400 MHz, $CDCl_3$) δ 7.47-7.23 (m, 8H), 7.14 (d, *J* = 8.0 Hz, 2H), 4.00 (t, *J* = 7.4 Hz, 0.05H), 3.27-3.06 (m, 2H).

Derivatization of the deuterated product

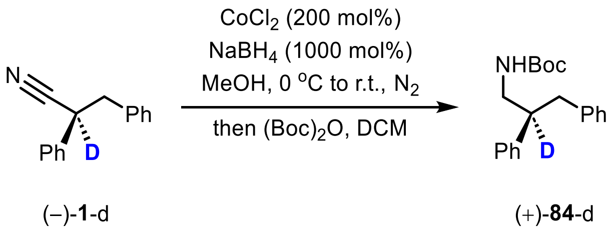

zwf-8-060

To a solution of (–)-**1**-d (62.5 mg, 0.3 mmol, 100 mol%, 95% D, 98:2 e.r.) and $CoCl_2$ (78.1 mg, 0.6 mmol, 200 mol%) in MeOH (3 mL) was added $NaBH_4$ (113.5 mg, 3 mmol, 1000 mol%) in portions at 0 °C under N_2, then the reaction mixture was stirred at room temperature for 3 h before quenching with saturated

$NaHCO_3$ at 0 °C. The reaction mixture was extracted with ethyl acetate (5 mL × 5). The combined organic phase was washed with brine, dried over $MgSO_4$, filtered, and evaporated under vacuum. The crude product was redissolved in DCM (5 mL) and $(Boc)_2O$ (103 μL, 0.45 mmol, 150 mol%) was added. The reaction mixture was stirred at room temperature for additional 1 hour and evaporated under vacuum. The residue was purified by flash column chromatography to obtain (+)-**84**-d (70.4 mg, 75% Yield, 94% D) as oil, R_f = 0.4 (Hexane/EtOAc 10:1).

HPLC analysis (Chiralpak IA-3, hexane/^{i}PrOH = 98/2, 1.0 mL/min, 205 nm; t_r (minor) = 7.92 min, t_r (major) = 9.02 min) gave the isomeric composition of the product: 98:2 e.r., $[\alpha]_D^{16}$ = +15.1 (c = 0.80, $CHCl_3$).

^{1}H NMR (400 MHz, $CDCl_3$) δ 7.33-7.24 (m, 2H), 7.23-7.15 (m, 3H), 7.15-7.08 (m, 3H), 7.08-6.94 (m, 2H), 4.36 (br, 1H), 3.65-3.40 (m, 1H), 3.33-3.12 (m, 1H), 3.06 (t, J = 6.8 Hz, 0.06H), 3.00-2.77 (m, 2H), 1.38 (s, 9H).

^{13}C NMR (100 MHz, $CDCl_3$) δ 155.8, 142.0, 139.5, 129.0, 128.5, 128.2, 127.9, 126.7, 126.0, 79.1, 47.2 (t, J = 20.2 Hz), 45.2, 40.5, 28.3.

IR (neat, cm^{-1}) 3026, 2929, 1698, 1496, 1164.

HRMS (ESI) calcd $C_{20}H_{25}DNO_2^+$ $[M+H]^+$: 313.2021. Found: 313.2023.

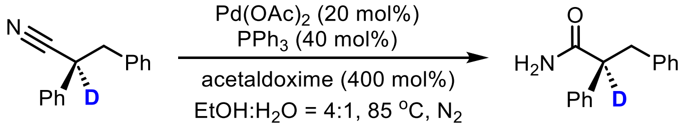

(–)-**1**-d (+)-**85**-d

zwf-8-059

To a solution of (–)-**1**-d (62.4 mg, 0.3 mmol, 100 mol%, 95% D, 98:2 e.r.), $Pd(OAc)_2$ (13.7 mg, 0.06 mmol, 20 mol%), and PPh_3 (31.5 mg, 0.12 mmol, 40 mol%) in $EtOH/H_2O$ (4.5 mL, 4:1) was added acetaldoxime (72 μL, 1.2 mmol, 400 mol%) at room temperature under N_2, then the reaction mixture was stirred at 85 °C for 3 h and cooled to room temperature. The reaction mixture was filtered through a Celite pad and washed with $EtOH/CH_2Cl_2$ (20 mL/20 mL). The combined organic phase was evaporated under vacuum, and the residue was purified by flash column chromatography to obtain (+)-**85**-d (64.5 mg, 95% Yield, 94% D) as white solid, R_f = 0.2 (Hexane/EtOAc 1:1).

HPLC analysis (Chiralpak IA-3, hexane/^{i}PrOH = 98/2, 1.0 mL/min, 205 nm; t_r (minor) = 35.22 min, t_r (major) = 38.12 min) gave the isomeric composition of the product: 98:2 e.r., $[\alpha]_D^{16}$ = +140.3 (c = 0.70, $CHCl_3$).

^{1}H NMR (400 MHz, $CDCl_3$) δ 7.42-7.06 (m, 10H), 5.87 (br, 1H), 5.49 (br, 1H), 3.62 (t, J = 7.4 Hz, 0.06H), 3.52-3.41 (m, 1H), 3.01-2.90 (m, 1H).

^{13}C NMR (100 MHz, $CDCl_3$) δ 175.4, 139.4, 139.3, 128.9, 128.7, 128.2, 127.9, 127.3, 126.1, 54.2 (t, J = 19.2 Hz), 39.2.

IR (neat, cm^{-1}) 3392, 3020, 2951, 1650 1394, 1133.

HRMS (ESI) calcd $C_{15}H_{15}DNO^+$ $[M+H]^+$: 227.1289. Found: 227.1286.

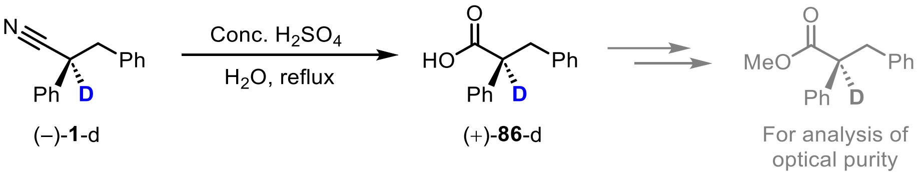

zwf-8-057

To a solution of (−)-**1**-d (62.5 mg, 0.3 mmol, 100 mol%, 95% D, 98:2 e.r.) in H_2O (0.75 mL) was added concentrated sulfuric acid (0.6 mL) at room temperature, then the reaction mixture was stirred under reflux for 10 h and cooled to room temperature. The reaction mixture was extracted with diethyl ether (5 mL × 5). The combined organic phase was washed with brine, dried over $MgSO_4$, filtered, and evaporated under vacuum. The residue was purified by flash column chromatography to obtain (+)-**86**-d (50.3 mg, 74% Yield, 93% D) as a white solid, R_f = 0.3 (Hexane/EtOAc 1:1).

HPLC analysis (Chiralpak IE-3, hexane/^{i}PrOH = 99/1, 0.8 mL/min, 205 nm; t_r (minor) = 9.01 min, t_r (major) = 9.53 min) gave the isomeric composition of the product: 95:5 e.r., $[\alpha]_D^{17}$ = +98.0 (c = 0.52, $CHCl_3$).

^{1}H NMR (400 MHz, $CDCl_3$) δ 7.37-7.13 (m, 8H), 7.13-7.04 (m, 2H), 3.84 (t, J = 7.8 Hz, 0.1H), 3.47-3.32 (m, 1H), 3.14-2.91 (m, 1H).

^{13}C NMR (100 MHz, $CDCl_3$) δ 179.2, 138.6, 137.8, 128.9, 128.7, 128.4, 128.1, 127.6, 126.4, 53.0 (t, J = 20.6 Hz), 39.1.

IR (neat, cm^{-1}) 3019, 2916, 1692, 1495, 1263.

HRMS (ESI) calcd $C_{15}H_{14}DO_2^+$ $[M+H]^+$: 228.1129. Found: 228.1127.

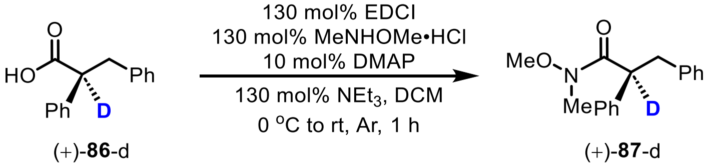

zwf-11-079

To a flask (25 mL) were added (+)-**x-**d (272.7 mg, 1.2 mmol, 95% D, 97:3 e.r.), EDCI (1-(3-dimethylaminopropyl)-3-ethylcarbodiimide hydrochloride) (299.3 mg, 1.56 mmol, 130 mol%), *N*,*O*-dimethylhydroxylamine hydrochloride (153.0 mg, 1.56 mmol, 130 mol%), DMAP (14.0 mg, 0.12 mmol, 10 mol%), NEt_3 (217 μL, 1.56 mmol, 130 mol%), and DCM (12 mL) at 0 °C under argon. The resulting mixture was stirred at rt for 1 h as monitored by TLC, diluted with DCM (10 mL), and quenched with H_2O (10 mL). After extraction with DCM (15 mL x 3), the combined organic layer was washed with brine (15 mL) and dried over anhydrous Na_2SO_4. After filtration and concentration under reduced pressure, the crude product was purified by column chromatography on silica gel to afford (+)-**87-**d (303.6 mg, 94% Yield, 93% D) as an oil. R_f = 0.5 (Hexane/EtOAc 10:1).

HPLC analysis (Chiralpak IC-3, hexane/^{i}PrOH = 95/5, 1.0 mL/min, 205 nm; t_r (major) = 16.76 min, t_r (minor) = 20.64 min) gave the isomeric composition of the product: 97:3 e.r., $[\alpha]_D^{23}$ = +112.3 (*c* = 0.38, $CHCl_3$).

^{1}H NMR (600 MHz, $CDCl_3$) δ 7.37-7.26 (m, 4H), 7.24-7.19 (m, 3H), 7.18-7.09 (m, 3H), 4.29 (s, 0.07H), 3.46 (d, *J* = 13.2 Hz, 1H), 3.26 (s, 3H), 3.09 (s, 3H), 2.95 (d, *J* = 13.2 Hz, 1H).

^{13}C NMR (150 MHz, $CDCl_3$) δ 173.8, 139.9, 139.7, 129.1, 128.5, 128.2, 128.1, 127.0, 126.1, 61.1, 49.3 (t, *J* = 20.7 Hz), 40.0, 32.1.

IR (neat, cm^{-1}) 3061, 2924, 1652, 1374, 1177.

HRMS (ESI) calcd $C_{17}H_{19}DNO_2^+$ $[M+H]^+$: 271.1551. Found: 271.1550.

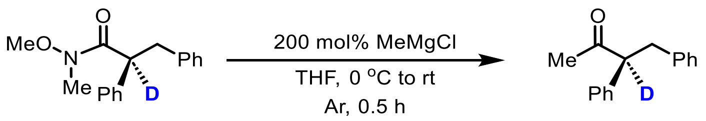

(+)-**87**-d (+)-**88**-d

zwf-11-080

To a flask (25 mL) were added (+)-**87-**d (54.2 mg, 0.2 mmol, 93% D, 97:3 e.r.) and THF (1 mL) at 0 °C under argon. Then MeMgCl (0.13 mL, 3.0 M in THF, 0.4 mmol, 200 mol%) was added to the reaction dropwise. After that the resulting mixture was stirred at room temperature for 0.5 h as monitored by TLC, quenched with saturated NH_4Cl (1 mL) at 0 °C and extracted with ethyl acetate (5 mL x 3), the combined organic layer was washed with brine (3 mL) and dried over anhydrous Na_2SO_4. After filtration and concentration under reduced pressure, the crude product was purified by column chromatography on silica gel to afford (+)-**88-**d (44.2 mg, 98% Yield, 93% D) as an oil. R_f = 0.8 (Hexane/EtOAc 5:1).

HPLC analysis (Chiralpak IC-3, hexane/^{i}PrOH = 99/1, 0.8 mL/min, 205 nm; t_r (major) = 11.46 min, t_r (minor) = 12.50 min) gave the isomeric composition of the product: 97:3 e.r., $[\alpha]_D^{23}$ = +328.1 (*c* = 0.31, $CHCl_3$).

^{1}H NMR (600 MHz, $CDCl_3$) δ 7.33-7.26 (m, 2H), 7.26-7.22 (m, 1H), 7.21-7.15 (m, 4H), 7.15-7.11 (m, 1H), 7.04 (d, J = 6.6 Hz, 2H), 3.91 (t, J = 7.2 Hz, 0.07H), 3.41 (d, J = 13.8 Hz, 1H), 2.89 (d, J = 13.8 Hz, 1H), 2.01 (s, 3H).

^{13}C NMR (150 MHz, $CDCl_3$) δ 207.7, 139.6, 138.3, 128.9, 128.8, 128.3, 128.2, 127.3, 126.1, 61.0 (t, J = 19.3 Hz), 38.2, 29.5.

IR (neat, cm^{-1}) 3063, 2921, 1652, 1374, 1177.

HRMS (ESI) calcd $C_{16}H_{16}DO^+$ $[M+H]^+$: 226.1337. Found: 226.1339.

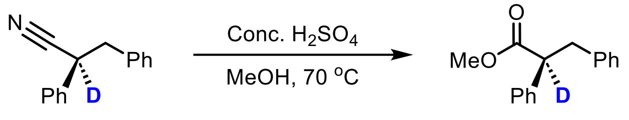

(–)-**1**-d (+)-**89**-d

zwf-8-058

To a solution of (–)-**1**-d (62.5 mg, 0.3 mmol, 100 mol%, 95% D, 98:2 e.r.) in MeOH (3 mL) was slowly added concentrated sulfuric acid (1.8 mL) at room temperature, then the reaction mixture was stirred at 70 °C for 24 h and cooled to room temperature. The reaction mixture was extracted with ethyl acetate (10 mL × 5). The combined organic phase was washed with brine, dried over $MgSO_4$, filtered, and evaporated under vacuum. The residue was purified by flash column chromatography to obtain (+)-**89**-d (55.4 mg, 77% Yield, 94% D) as oil, R_f = 0.6 (Hexane/EtOAc 10:1).

HPLC analysis (Chiralpak IE-3, hexane/^{i}PrOH = 99/1, 0.8 mL/min, 205 nm; t_r (minor) = 8.99 min, t_r (major) = 9.51 min) gave the isomeric composition of the product: 97.5:2.5 e.r., $[\alpha]_D^{17}$ = +117.0 (c = 0.62, $CHCl_3$).

^{1}H NMR (400 MHz, $CDCl_3$) δ 7.41-7.03 (m, 10H), 3.88 (t, J = 7.8 Hz, 0.06H), 3.58 (s, 3H), 3.46-3.36 (m, 1H), 3.09-2.91 (m, 1H).

^{13}C NMR (100 MHz, $CDCl_3$) δ 173.8, 139.0, 138.5, 128.9, 128.6, 128.3, 127.9, 127.3, 126.3, 53.1 (t, J = 20.8 Hz), 51.9, 39.7.

IR (neat, cm^{-1}) 3058, 3033, 2959, 1731, 1495, 1257.

HRMS (ESI) calcd $C_{16}H_{16}DO_2^+$ $[M+H]^+$: 242.1286. Found: 242.1283.

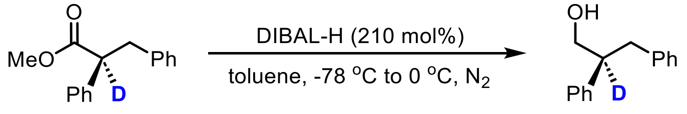

(+)-**89**-d (+)-**90**-d

zwf-8-072

To a solution of (+)-**89**-d (72.5 mg, 0.3 mmol, 100 mol%, 94% D, 97.5:2.5 e.r.) in toluene (6 mL) was added DIBAL-H (diisobutylaluminium hydride, 0.42 mL, 0.63 mmol, 210 mol%, 1.5 M in toluene) dropwise at –78 ºC under N_2, and the reaction mixture was stirred at the -78 ºC for 2 h and cooled to 0 ºC. The mixture was stirred for another 2 h at the same temperature before quenching with EtOD (1 mL, 99.5% D) and 1 N HCl (5 mL) at –78 °C. The reaction mixture was extracted with ethyl acetate (10 mL × 5). The combined organic phase was washed with brine, dried over $MgSO_4$, filtered, and evaporated under vacuum. The residue was purified by flash column chromatography to obtain (+)-**90**-d (48.4 mg, 76% Yield, 94% D) as oil, R_f = 0.1 (Hexane/EtOAc 10:1).

HPLC analysis (Chiralpak IE-3, hexane/^{i}PrOH = 99/1, 0.8 mL/min, 205 nm; t_r (minor) = 37.65 min, t_r (major) = 45.04 min) gave the isomeric composition of the product: 97.5:2.5 e.r., $[\alpha]_D^{17}$ = +76.4 (*c* = 0.97, $CHCl_3$).

^{1}H NMR (400 MHz, $CDCl_3$) δ 7.37-7.25 (m, 2H), 7.25-7.10 (m, 6H), 7.10-7.00 (m, 2H), 3.87-3.62 (m, 2H), 3.07 (t, *J* = 6.8 Hz, 0.06H), 3.05-2.96 (m, 1H), 2.94-2.80 (m, 1H), 1.44 (br, 1H).

^{13}C NMR (100 MHz, $CDCl_3$) δ 141.8, 139.9, 129.0, 128.6, 128.2, 128.0, 126.8, 126.0, 66.2, 49.6 (t, *J* = 19.9 Hz), 38.6.

IR (neat, cm^{-1}) 3026, 2922, 2859, 1495, 1028.

HRMS (ESI) calcd $C_{15}H_{15}DNaO^+$ $[M+Na]^+$: 236.1156. Found: 236.1155.

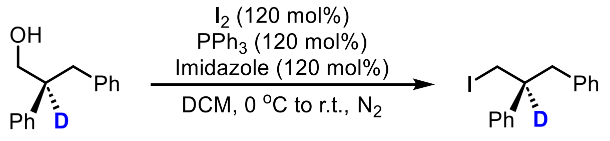

(+)-**90**-d (+)-**91**-d

zwf-8-076

To a solution of (+)-**90**-d (42.5 mg, 0.2 mmol, 100 mol%, 94% D, 97.5:2.5 e.r.), PPh_3 (63.3 mg, 0.24 mmol, 120 mol%), and imidazole (16.5 mg, 0.24 mmol, 120 mol%) in DCM (2 mL) was added I_2 (62.4 mg, 0.24 mmol, 120 mol%) at 0 ºC under N_2, and the reaction mixture was stirred at the room temperature for 6 h before quenching with saturated $Na_2S_2O_3$ (1 mL). The reaction mixture was extracted with DCM (5 mL × 5). The combined organic phase was washed with brine, dried over $MgSO_4$, filtered, and evaporated under vacuum. The filtrate was purified by flash column chromatography to obtain (+)-**91**-d (54.2 mg, 84% Yield, 94% D) as oil, R_f = 0.3 (Hexane).

HPLC analysis (Chiralpak IE-3, hexane/^{i}PrOH = 100/0, 0.8 mL/min, 205 nm; t_r (minor) = 18.14 min, t_r (major) = 28.41 min) gave the isomeric composition of the product: 97.5:2.5 e.r., $[\alpha]_D^{16}$ = +7.9 (*c* = 0.82, $CHCl_3$).

^{1}H NMR (400 MHz, $CDCl_3$) δ 7.37-7.14 (m, 6H), 7.14-7.08 (m, 2H), 7.08-7.00 (m, 2H), 3.51-3.40 (m, 1H), 3.40-3.31 (m, 1H), 3.20-3.14 (m, 0.06H), 3.14-3.07 (m, 1H), 3.02-2.92 (m, 1H).

^{13}C NMR (100 MHz, $CDCl_3$) δ 142.5, 139.2, 129.0, 128.4, 128.3, 127.4, 127.0, 126.3, 49.3 (t, J = 19.9 Hz), 41.9, 13.0.

IR (neat, cm^{-1}) 3025, 2920, 1601, 1494, 1189.

HRMS (FI) calcd $C_{15}H_{14}DI^{+}$ $[M]^{+}$: 323.0276. Found: 323.0274.

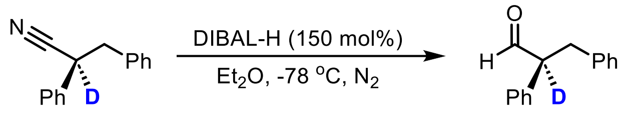

(–)-**1**-d (+)-**92**-d

zwf-8-064-1

To a solution of (–)-**1**-d (62.4 mg, 0.3 mmol, 100 mol%, 95% D, 98:2 e.r.) in diethyl ether (6 mL) was added DIBAL-H (diisobutylaluminium hydride, 0.45 mL, 0.45 mmol, 150 mol%, 1.0 M in hexane) dropwise at –78 °C under N_2, and the reaction mixture was stirred at the same temperature for 5 h before quenching with 10% w/w H_2SO_4 aqueous solution (3 mL) at -78 °C.[12] The reaction mixture was extracted with diethyl ether (10 mL × 5). The combined organic phase was washed with brine, dried over $MgSO_4$, filtered, and evaporated under vacuum. The residue was purified by flash column chromatography to obtain (+)-**92**-d (36.3 mg, 57% Yield, 80% D) as oil, R_f = 0.6 (Hexane/EtOAc 10:1).

HPLC analysis (Chiralpak IE-3, hexane/^{i}PrOH = 99/1, 0.8 mL/min, 205 nm; t_r (minor) = 10.30 min, t_r (major) = 10.94 min) gave the isomeric composition of the product: 97.5:2.5 e.r., $[\alpha]_D^{16}$ = +199.5 (c = 0.71, $CHCl_3$).

^{1}H NMR (400 MHz, $CDCl_3$) δ 9.73 (s, 1H), 7.37-7.25 (m, 3H), 7.24-7.09 (m, 5H), 7.08-7.00 (m, 2H), 3.83 (t, J = 7.2 Hz, 0.2H), 3.54-3.37 (m, 1H), 3.03-2.88 (m, 1H).

^{13}C NMR (100 MHz, $CDCl_3$) δ 199.92 (major), 199.87 (minor), 138.7, 135.6, 129.00, 128.95, 128.3, 127.7, 126.3, 60.9 (minor), 60.4 (t, J = 19.5 Hz, major), 36.1 (minor), 36.0 (major).

IR (neat, cm^{-1}) 3058, 3030, 2925, 1715, 1454, 1180.

HRMS (ESI) calcd $C_{15}H_{14}DO^{+}$ $[M+H]^{+}$: 212.1180. Found: 212.1181.

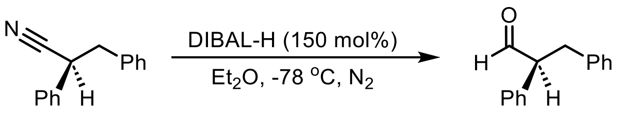

(–)-**1** (+)-**93**

zwf-8-067

Reduction using the decarboxylative *protonation* product for comparison: To a solution of (–)-**1** (41.3 mg, 0.2 mmol, 100 mol%, 96:4 e.r.) in diethyl ether (4 mL) was added DIBAL-H (diisobutylaluminium hydride, 0.3 mL, 0.3 mmol, 150 mol%, 1.0 M in hexane) dropwise at –78 °C under N_2, and the reaction mixture was stirred at the same temperature for 5 h before quenching with 10% w/w H_2SO_4 aqueous (2 mL) at -78 °C.[5] The reaction mixture was extracted with diethyl ether (10 mL × 5). The combined organic phase was washed with brine, dried over $MgSO_4$, filtered, and evaporated under vacuum. The residue was purified by flash column chromatography to obtain (+)-**93** (24.5 mg, 58% Yield) as oil, R_f = 0.6 (Hexane/EtOAc 10:1).

HPLC analysis (Chiralpak IE-3, hexane/^{i}PrOH = 99/1, 0.8 mL/min, 205 nm; t_r (minor) = 10.40 min, t_r (major) = 11.06 min) gave the isomeric composition of the product: 89.5:10.5 e.r., $[\alpha]_D^{17}$ = +160.0 (c = 0.65, $CHCl_3$).

^{1}H NMR (400 MHz, $CDCl_3$) δ 9.73 (d, J = 1.2 Hz, 1H), 7.39-7.09 (m, 8H), 7.09-7.00 (m, 2H), 3.83 (dt, J_1 = 7.4 Hz, J_2 = 0.9 Hz, 1H), 3.47 (dd, J_1 = 14.0 Hz, J_2 = 6.8 Hz, 1H), 2.97 (dd, J_1 = 14.0 Hz, J_2 = 7.6 Hz, 1H).

^{13}C NMR (100 MHz, $CDCl_3$) δ 199.9, 138.8, 135.7, 129.0, 128.3, 127.7, 126.3, 60.9, 36.1.

IR (neat, cm^{-1}) 3063, 3030, 2924, 1715, 1453, 1160.

HRMS (ESI) calcd $C_{15}H_{15}O^+$ $[M+H]^+$: 211.1117. Found: 211.1112.

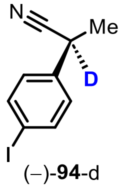

(–)-**94**-d

zwf-11-058

(–)-**94**-d was obtained as an oil from the asymmetric decarboxylative deuteration reaction of 2-cyanoacetic acid **S94** (1.5083 g, 5 mmol) for 4 h using the general procedure (1.2648 g, 98% Yield, 97% D). R_f = 0.6 (Hexane/EtOAc 10:1).

HPLC analysis (Chiralpak IE-3, hexane/^{i}PrOH = 99/1, 0.8 mL/min, 205 nm; t_r (minor) = 17.20 min, t_r (major) = 18.56 min) gave the isomeric composition of the product: 95:5 e.r., $[\alpha]_D^{21}$ = –24.6 (c = 0.52, $CHCl_3$).

^{1}**H NMR** (600 MHz, $CDCl_3$) δ 7.72 (d, *J* = 8.4 Hz, 2H), 7.11 (d, *J* = 8.4 Hz, 2H), 3.85 (q, *J* = 7.4 Hz, 0.03H), 1.62 (s, 3H).

^{13}**C NMR** (150 MHz, $CDCl_3$) δ 138.3, 136.7, 128.6, 121.0, 93.6, 30.6 (t, *J* = 20.9 Hz), 21.2.

IR (neat, cm^{-1}) 2985, 2937, 2240, 1485, 1396, 1006.

HRMS (ESI) calcd $C_9H_8DIN^+$ $[M+H]^+$: 258.9837. Found: 258.9838.

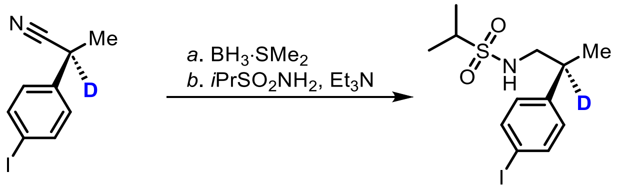

(−)-**94**-d (−)-**95**-d

zwf-11-060

To a solution of (−)-**94**-d (1.1618 g, 4.5 mmol, 100 mol%, 97% D, 95:5 e.r.) in THF (45 mL) was dropwise added $BH_3{\cdot}SMe_2$ (9.0 mL, 18 mmol, 2.0 M in THF) at room temperature. The reaction mixture was stirred at room temperature for 24 h before quenching with MeOH. The organic phase was evaporated under vacuum and the residue redissolved in the diethyl ether (50 mL). Then 1 M HCl aqueous (2400 mol%) was added to the reaction mixture, which was stirred at room temperature for 4 h. The aqueous was separated and adjusted pH to 9 ~ 10 with 0.1 N NaOH aqueous, the reaction mixture was extracted with diethyl ether (10 mL × 5). The combined organic phase was washed with brine, dried over $MgSO_4$, filtered, and evaporated under vacuum. The crude product (0.8398 g, 3.2 mmol) was redissolved in DCM (32 mL), DMAP (39.7 mg, 0.32 mmol, 10 mol%), NEt_3 (2.23 mL, 16 mmol, 500 mol%) were added. The reaction mixture was cold down to 0 ºC, 2-propanesulfonyl chloride (1.01 mL, 9.6 mmol, 300 mol%) was added to the reaction dropwise. The reaction mixture was stirred at room temperature for an additional 12 hours and evaporated under vacuum. The residue was purified by flash column chromatography to obtain (−)-**95**-d (0.7164 g, 43% Yield in two steps, 97% D) as a solid, R_f = 0.3 (Hexane/EtOAc 5:1).

HPLC analysis (Chiralpak IE-3, hexane/^{i}PrOH = 90/10, 0.8 mL/min, 205 nm; t_r (minor) = 24.38 min, t_r (major) = 28.13 min) gave the isomeric composition of the product: 95:5 e.r., $[\alpha]_D^{21}$ = –26.4 (*c* = 0.24, $CHCl_3$).

^{1}**H NMR** (600 MHz, $CDCl_3$) δ 7.65 (d, *J* = 8.4 Hz, 2H), 6.98 (d, *J* = 8.4 Hz, 2H), 4.15-4.05 (m, 1H), 3.30 (dd, J_1 = 12.9 Hz, J_2 = 7.5 Hz, 1H), 3.18 (dd, J_1 = 12.9 Hz, J_2 = 5.1 Hz, 1H), 3.09-3.01 (m, 1H), 2.93-2.90 (m, 0.03H), 1.32-1.24 (m, 9H).

^{13}**C NMR** (150 MHz, $CDCl_3$) δ 142.8, 137.8, 129.3, 92.1, 53.4, 50.0, 40.0 (t, *J* = 19.3 Hz), 18.7, 16.5, 16.4.

IR (neat, cm^{-1}) 3298, 2971, 2933, 1431, 1312, 1125.

HRMS (ESI) calcd $C_{12}H_{18}DINO_2S^+$ [M+H]$^+$: 369.0238. Found: 369.0236.

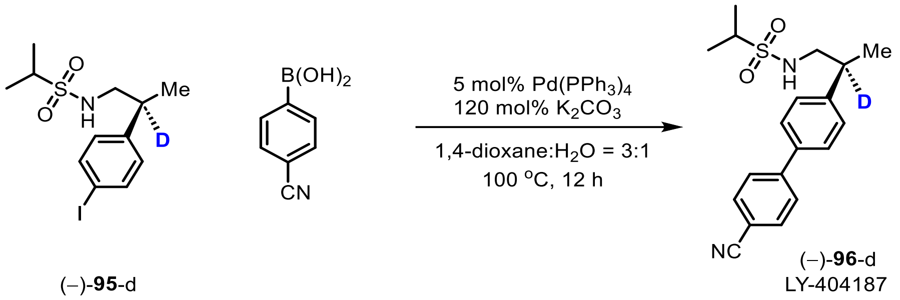

zwf-11-066

To a solution of (–)-**95**-d (110.4 mg, 0.3 mmol, 97% D, 95:5 e.r.) in degassed 1,4-dioxane/water (3 mL, 3:1) were added 4-cyanophenylboronic acid (53.3 mg, 0.36 mmol), K_2CO_3 (50.5 mg, 0.36 mmol), and $Pd(PPh_3)_4$ (17.5 mg, 0.015 mmol). The reaction mixture was stirred at reflux for 24 h and cooled to room temperature. Then 1,4-dioxane was removed under vacuum, and to the resulting residue was added 1 N HCl (5 mL). The aqueous phase was extracted with CH_2Cl_2 (10 mL × 3). The organics were combined, washed with brine, dried with Na_2SO_4, filtered, and concentrated, the residue was purified by chromatography to give (–)-**96**-d (LY-404187) (80.7 mg, 78% Yield, 97% D) as solid. R_f = 0.2 (Hexane/EtOAc 2:1).

HPLC analysis (Chiralpak IB-3, hexane/^{i}PrOH = 70/30, 0.8 mL/min, 254 nm; t_r (major) = 11.22 min, t_r (minor) = 14.57 min) gave the isomeric composition of the product: 95:5 e.r., $[\alpha]_D^{21}$ = –40.2 (c = 0.30, $CHCl_3$).

^{1}H NMR (600 MHz, $CDCl_3$) δ 7.72 (d, J = 8.4 Hz, 2H), 7.67 (d, J = 8.4 Hz, 2H), 7.57 (d, J = 8.4 Hz, 2H), 7.35 (d, J = 8.4 Hz, 2H), 4.21-4.12 (m, 1H), 3.37 (dd, J_1 = 12.9 Hz, J_2 = 7.5 Hz, 1H), 3.27 (dd, J_1 = 13.2 Hz, J_2 = 4.8 Hz, 1H), 3.15-3.03 (m, 1.03H), 1.40-1.22 (m, 9H).

^{13}C NMR (150 MHz, $CDCl_3$) δ 145.1, 143.8, 137.8, 132.6, 128.0, 127.6, 127.5, 118.9, 110.8, 53.4, 50.1, 40.1 (t, J = 19.3 Hz), 18.8, 16.5, 16.4.

IR (neat, cm^{-1}) 3288, 2973, 2937, 2228, 1604, 1316, 1062.

HRMS (ESI) calcd $C_{19}H_{22}DN_2O_2S^+$ [M+H]$^+$: 344.1538. Found: 344.1539.

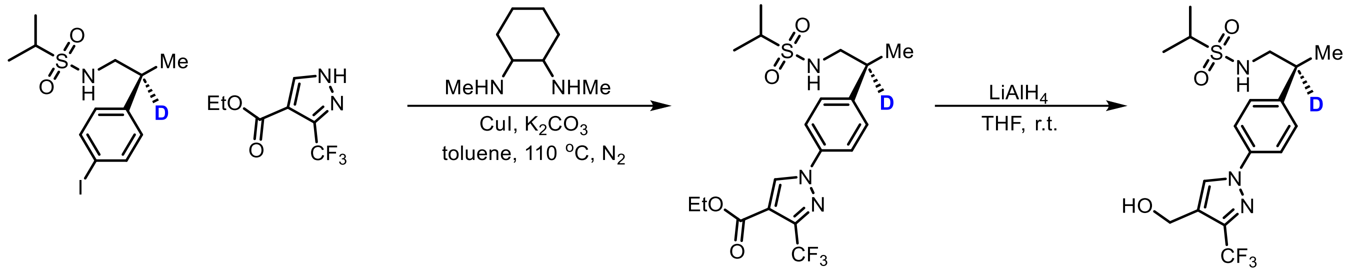

(–)-**95**-d **97'**-d (–)-**97**-d

zwf-11-067/068

To a solution of (–)-**95**-d (110.5 mg, 0.3 mmol, 97% D, 95:5 e.r.), ethyl 3-(trifluoromethyl)-1H-pyrazole-4-carboxylate (68.8 mg, 0.33 mmol), CuI (7.2 mg, 0.0375 mmol), and K_2CO_3 (207.4 mg, 1.5 mmol) in toluene (3 mL) was added *N,N'*-dimethyl-1,2-cyclohexanediamine (7.1 μL, 0.045 mmol) under N_2. The reaction mixture was stirred 110 ºC for 24 h and cooled to room temperature, filtered through Celite (3 cm), washed with ethyl acetate (5 mL × 5), and concentrated. The residue was purified by chromatography to give **97'**-d, which directly used for next step. R_f = 0.3 (Hexane/EtOAc 2:1).

To a solution of **97'**-d in THF (3 mL) was added $LiAlH_4$ (17.7 mg, 0.46 mmol). The reaction mixture was stirred room temperature for 12 h, filtered through Celite (3 cm), washed with ethyl acetate (10 mL × 5), and concentrated. The residue was purified by chromatography to give (–)-**97**-d (78.9 mg, 65% Yield (two steps), 97% D) as solid. R_f = 0.1 (Hexane/EtOAc 1:1).

HPLC analysis (Chiralpak IB-3, hexane/^{i}PrOH = 80/20, 0.8 mL/min, 205 nm; t_r (major) = 14.36 min, t_r (minor) = 16.76 min) gave the isomeric composition of the product: 95:5 e.r., $[\alpha]_D^{21}$ = –21.9 (*c* = 0.30, $CHCl_3$).

^{1}H NMR (600 MHz, $CDCl_3$) δ 7.97 (s, 1H), 7.62 (d, *J* = 8.4 Hz, 2H), 7.31 (d, *J* = 8.4 Hz, 2H), 4.74 (s, 2H), 4.26 (t, *J* = 6.0 Hz, 1H), 3.33 (dd, J_1 = 13.2 Hz, J_2 = 7.2 Hz, 1H), 3.22 (dd, J_1 = 13.2 Hz, J_2 = 5.4 Hz, 1H), 3.13-3.00 (m, 1.03H), 2.37 (br, 1H), 1.32-1.25 (m, 9H).

^{13}C NMR (150 MHz, $CDCl_3$) δ 142.9, 140.5 (q, *J* = 37.3 Hz), 138.0, 128.4, 127.9, 122.1, 121.5 (q, *J* = 267.6 Hz), 120.0, 54.8, 53.5, 50.0, 40.0 (t, *J* = 19.3 Hz), 18.8, 16.5, 16.4.

^{19}F NMR (564 MHz, $CDCl_3$) δ -61.0 (s).

IR (neat, cm^{-1}) 3291, 2975, 2937, 1498, 1288, 1126.

HRMS (ESI) calcd $C_{17}H_{22}DF_3N_3O_3S^+$ $[M+H]^+$: 407.1470. Found: 407.1467.

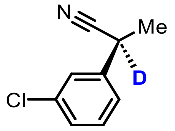

(+)-**98**-d

zwf-10-121

(+)-**98**-d was obtained as an oil from the asymmetric decarboxylative deuteration reaction of 2-cyanoacetic acid **S98** (209.6 mg, 1 mmol) for 4 h using the general procedure (150.0 mg, 90% Yield, 96% D). R_f = 0.5 (Hexane/EtOAc 10:1).

HPLC analysis (Chiralpak IE-3, hexane/^{i}PrOH = 99/1, 0.8 mL/min, 205 nm; t_r (minor) = 13.84 min, t_r (major) = 15.05 min) gave the isomeric composition of the product: 94:6 e.r., $[\alpha]_D^{23}$ = +1.3 (*c* = 0.76, $CHCl_3$).

^{1}H NMR (500 MHz, $CDCl_3$) δ 7.38-7.22 (m, 4H), 3.88 (d, *J* = 7.3 Hz, 0.04H), 1.62 (s, 3H).

^{13}C NMR (125 MHz, $CDCl_3$) δ 138.8, 134.9, 130.3, 128.2, 126.8, 124.8, 120.8, 30.4 (t, *J* = 20.8 Hz), 21.0.

IR (neat, cm^{-1}) 2988, 2940, 2243, 1598, 1478, 1080.

HRMS (ESI) calcd $C_9H_8DClN^+$ $[M+H]^+$: 167.0481. Found: 167.0484.

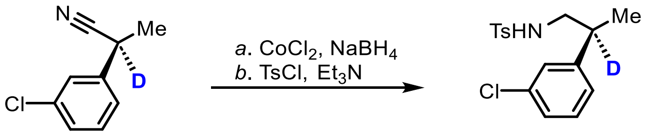

(+)-**98**-d (–)-**99**-d

zwf-10-152

To a solution of (+)-**98**-d (83.4 mg, 0.5 mmol, 100 mol%, 96% D, 94:6 e.r.) and $CoCl_2$ (130.0 mg, 1 mmol, 200 mol%) in MeOH (5 mL) was added $NaBH_4$ (190.1 mg, 5 mmol, 1000 mol%) in portions at 0 ºC under N_2, then the reaction mixture was stirred at room temperature for 3 h before quenching with saturated $NaHCO_3$ at 0 °C. The reaction mixture was extracted with ethyl acetate (10 mL × 5). The combined organic phase was washed with brine, dried over $MgSO_4$, filtered, and evaporated under vacuum. The crude product was redissolved in DCM (5 mL). TsCl (143.5 mg, 0.75 mmol, 150 mol%) and NEt_3 (140.0 µL, 1.0 mmol, 200 mol%) were added. The reaction mixture was stirred at room temperature for an additional hour and evaporated under vacuum. The residue was purified by flash column chromatography to obtain (–)-**99**-d (134.8 mg, 83% Yield, 95% D) as a solid, R_f = 0.5 (Hexane/EtOAc 2:1).

HPLC analysis (Chiralpak IB-3, hexane/^{i}PrOH = 98/2, 1.0 mL/min, 205 nm; t_r (minor) = 24.57 min, t_r (major) = 25.92 min) gave the isomeric composition of the product: 94:6 e.r., $[\alpha]_D^{20}$ = –12.5 (*c* = 0.22, $CHCl_3$).

^{1}H NMR (500 MHz, $CDCl_3$) δ 7.65 (d, J = 8.0 Hz, 2H), 7.27 (d, J = 8.5 Hz, 2H), 7.61-7.14 (m, 2H), 6.99 (s, 1H), 6.96 (d, J = 6.5 Hz, 1H), 4.73 (t, J = 6.5 Hz, 1H), 3.13 (dd, J_1 = 13.0 Hz, J_2 = 7.5 Hz, 1H), 2.98 (dd, J_1 = 12.8 Hz, J_2 = 5.3 Hz, 1H), 2.88-2.79 (m, 0.05H), 2.42 (s, 3H), 1.19 (s, 3H).

^{13}C NMR (125 MHz, $CDCl_3$) δ 145.1, 143.4, 136.7, 134.4, 129.9, 129.7, 127.2, 127.0, 126.9, 125.4, 49.3, 39.1 (t, J = 19.4 Hz), 21.4, 18.8.

IR (neat, cm^{-1}) 3278, 3225, 2971, 2874, 1571, 1321, 1154.

HRMS (ESI) calcd $C_{16}H_{18}DClNO_2S^+$ $[M+H]^+$: 325.0882. Found: 325.0883.

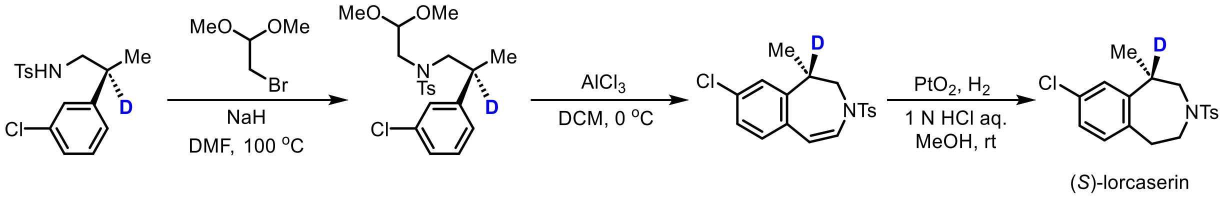

(–)-**99**-d (–)-**100**-d (+)-**101'**-d (*S*)-lorcaserin (–)-**101**-d

HM-1-012/016/019

To a solution of (–)-**99**-d (100.0 mg, 0.3 mmol, 100 mol%, 95% D, 94:6 e.r.) and 2-bromo-1,1-dimethoxyethane (0.1 mL, 0.84 mmol, 280 mol%) in DMF (1.5 mL) was added NaH (60.1 mg, 1.5 mmol, 500 mol%) under N_2, then the reaction mixture was stirred at 100 °C for 36 h. The mixture was cooled down to room temperature before quenching with saturated NH_4Cl. The reaction mixture was extracted with ethyl acetate (10 mL × 5). The combined organic phase was washed with brine, dried over $MgSO_4$, filtered, and evaporated under vacuum. The residue was purified by flash column chromatography to obtain (–)-**100**-d (92.1 mg, 72% Yield, 95% D) as an oil, which directly used for the next step. R_f = 0.4 (Hexane/EtOAc 5:1). $[\alpha]_D^{21}$ = –17.9 (c = 2.01, $CHCl_3$).

^{1}H NMR (600 MHz, $CDCl_3$) δ 7.64 (d, J = 8.4 Hz, 2H), 7.27 (d, J = 7.8 Hz, 2H), 7.22-7.14 (m, 2H), 7.07-7.03 (m, 2H), 4.42 (t, J = 5.1 Hz, 1H), 3.43 (d, J = 13.8 Hz, 1H), 3.36 (s, 3H), 3.34 (s, 3H), 3.19 (d, J = 13.8 Hz, 1H), 3.13 (dd, J_1 = 15.0 Hz, J_2 = 5.4 Hz, 1H), 3.05 (dd, J_1 = 15.3 Hz, J_2 = 5.1 Hz, 1H), 2.45-2.38 (m, 3.05H), 1.24 (s, 3H).

^{13}C NMR (150 MHz, $CDCl_3$) δ 146.1, 143.4, 136.6, 134.2, 129.74, 129.65, 127.6, 127.2, 126.7, 125.5, 104.6, 56.4, 55.3, 55.0, 50.8, 37.8 (t, J = 19.6 Hz), 21.5, 18.5.

To a solution of $AlCl_3$ (50.1 mg, 0.48 mmol, 400 mol%) in dry DCM (1.5 mL) was added (–)-**100**-d (50.2 mg, 0.12 mmol, 100 mol%, 95% D, dissolved in 1.5 mL DCM) at 0 °C under N_2. After stirred at the same temperature for 2 h, the reaction mixture was quenched with 1 M NaOH aqueous, extracted with DCM (10 mL × 5). The combined organic phase was washed with brine, dried over $MgSO_4$, filtered, and evaporated under vacuum. The residue was purified by flash column chromatography to obtain (+)-**101'**-

d (27.2 mg, 65% Yield, 95% D) as oil, which was directly used for the next step. R_f = 0.6 (Hexane/EtOAc 4:1). $[\alpha]_D^{21}$ = +236.6 (c = 1.00, $CHCl_3$).

^{1}H NMR (600 MHz, $CDCl_3$) δ 7.71 (d, J = 8.4 Hz, 2H), 7.31 (d, J = 7.8 Hz, 2H), 7.09 (dd, J_1 = 8.4 Hz, J_2 = 2.4 Hz, 1H), 7.06-7.01 (m, 2H), 6.88 (dd, J_1 = 10.5 Hz, J_2 = 1.8 Hz, 1H), 5.54 (d, J = 10.2 Hz, 1H), 4.05 (dd, J_1 = 13.2 Hz, J_2 = 0.6 Hz, 1H), 3.16-3.07 (m, 1.05H), 2.41 (s, 3H), 1.15 (s, 3H).

^{13}C NMR (150 MHz, $CDCl_3$) δ 145.2, 144.1, 135.8, 132.1, 131.8, 131.5, 129.9, 128.0, 126.9, 126.6, 126.1, 107.4, 50.2, 39.7 (t, J = 19.3 Hz), 21.5, 17.1.

To an oven-dried 25 mL flask were added (+)-**101'**-d (20.0 mg, 0.057 mmol, 95% D), PtO_2 (8.2 mg, 60 mol%), and 3 drops of 1 M HCl aqueous. The flask was sealed with a rubber septum and evacuated/refilled with nitrogen for three times. 2 mL of MeOH was added to the flask via syringe under nitrogen atmosphere. The reaction mixture was purged by H_2 for 10 min with a H_2 balloon, then sealed and stirred under H_2 atmosphere with a H_2 balloon. After stirred at room temperature for 12 h, the reaction mixture was filtrated with Celite, and the filtrate was concentrated under vacuum and purified by flash column chromatography to afford the (–)-**101**-d (11.1 mg, 55% Yield, 95% D) as a solid. R_f = 0.5 (Hexane/EtOAc 6:1).

HPLC analysis (Chiralpak IF-3, hexane/^{i}PrOH = 95/5, 1.0 mL/min, 210 nm; t_r (major) = 23.74 min, t_r (minor) = 25.74 min) gave the isomeric composition of the product: 95:5 e.r., $[\alpha]_D^{21}$ = –2.6 (c = 1.00, $CHCl_3$).

^{1}H NMR (600 MHz, $CDCl_3$) δ 7.61 (d, J = 8.4 Hz, 2H), 7.26 (d, J = 7.8 Hz, 2H), 7.10-7.04 (m, 2H), 6.96 (d, J = 7.8 Hz, 1H), 3.40-3.12 (m, 4H), 3.12-3.06 (m, 1.05H), 2.93 (dd, J_1 = 15.0 Hz, J_2 = 9.0 Hz, 1H), 2.39 (s, 3H), 1.38 (s, 3H).

^{13}C NMR (150 MHz, $CDCl_3$) δ 146.0, 143.3, 137.8, 135.3, 132.4, 131.3, 129.7, 127.5, 127.1, 126.3, 53.4, 48.1, 39.6 (t, J = 19.0 Hz), 35.9, 21.4, 17.4.

IR (neat, cm^{-1}) 2923, 2852, 1597, 1336, 1163.

HRMS (ESI) calcd $C_{18}H_{20}DClNO_2S^+$ $[M+H]^+$: 351.1039. Found: 351.1038.

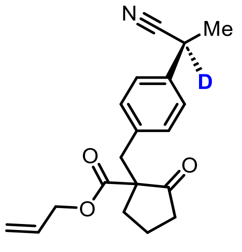

(–)-**103**-d

zwf-11-064

(−)-**103**-d was obtained as an oil from the asymmetric decarboxylative deuteration reaction of 2-cyanoacetic acid **S102** (533.2 mg, 1.5 mmol) for 4 h using the general procedure (0.4240 g, 90% Yield, 96% D). R_f = 0.3 (Hexane/EtOAc 5:1).

HPLC analysis (Chiralpak IH-3, hexane/^{i}PrOH = 98/2, 1.1 mL/min, 205 nm; t_r (minor1) = 35.91 min, t_r (minor2) = 38.22 min, t_r (major1) = 39.35 min, t_r (major2) = 40.96 min) gave the isomeric composition of the product: 1:1 d.r., 91:9 e.r., 91:9 e.r., $[\alpha]_D^{23}$ = −3.0 (*c* = 0.34, $CHCl_3$).

^{1}H NMR (600 MHz, $CDCl_3$) δ 7.25 (d, *J* = 8.4 Hz, 2H), 7.16 (d, *J* = 7.8 Hz, 2H), 5.95-5.80 (m, 1H), 5.35-5.21 (m, 2H), 4.67-4.55 (m, 2H), 3.87 (q, *J* = 7.2 Hz, 0.04H), 3.22 (dd, J_1 = 13.8 Hz, J_2 = 3.0 Hz, 1H), 3.11 (dd, J_1 = 13.8 Hz, J_2 = 4.8 Hz, 1H), 2.46-2.35 (m, 2H), 2.11-2.02 (m, 1H), 1.99-1.88 (m, 2H), 1.68-1.60 (m, 4H).

^{13}C NMR (150 MHz, $CDCl_3$) δ (diastereomer 1) 214.40, 170.41, 136.5, 135.55, 131.4, 130.95, 126.7, 121.5, 118.7, 66.0, 61.3, 38.4, 38.20, 31.7, 30.5 (t, *J* = 20.7 Hz), 21.13, 19.4; (diastereomer 2) 214.35, 170.43, 136.5, 135.57, 131.4, 130.96, 126.7, 121.5, 118.7, 66.0, 61.3, 38.4, 38.17, 31.7, 30.5 (t, *J* = 20.7 Hz), 21.16, 19.4.

IR (neat, cm^{-1}) 2981, 2889, 2240, 1723, 1514, 1141.

HRMS (ESI) calcd $C_{19}H_{21}DIO_3^+$ $[M+H]^+$: 313.1657. Found: 313.1655.

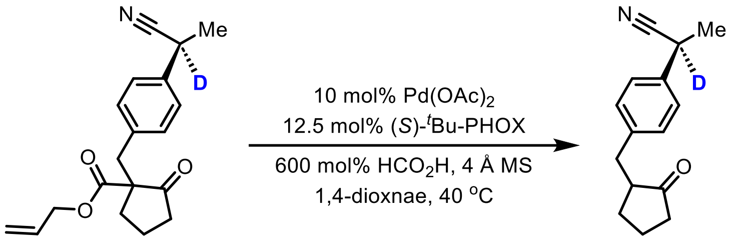

(−)-**103**-d (−)-**104**-d

zwf-11-070

To a 25 mL Schlenk tube equipped with a magnetic stir bar was charged with powdered 4Å molecular sieves (1.1250 g) and then thoroughly flame dried under vacuum (3 times, backfilled with N_2). After cooled to ambient temperature under N_2, $Pd(OAc)_2$ (11.2 mg, 0.05 mmol, 10 mol%), (*S*)-^{t}Bu-PHOX (24.2 mg, 0.0625 mmol, 12.5 mol%), and freshly distilled 1,4-dioxane (8 mL) were added, and the resulting slurry was stirred vigorously at 40 °C for 30 min. At this point, neat HCO_2H (113 μL, 3 mmol, 600 mol%) was added to the reaction mixture, followed immediately by the addition of a solution of (−)-**103**-d (156.3 mg, 0.5 mmol, 100 mol%, 96% D, 1:1 d.r., 91:9 e.r., 91:9 e.r.) in 1,4-dioxane (7 mL). When the reaction was completed as indicated by TLC, the reaction mixture was cooled to ambient temperature and then filtrated through a short column of silica gel eluted with ethyl acetate (10 mL × 5). The reaction mixture was concentrated, and the residue was submitted to flash column chromatography (hexane/ethyl acetate) to afford (−)-**104**-d (97.7 mg, 86% Yield, 96% D) as oil. R_f = 0.3 (Hexane/EtOAc 5:1).

HPLC analysis (Chiralpak IH-3, hexane/^{i}PrOH = 98/2, 1.1 mL/min, 205 nm; t_r (major1) = 42.21 min, t_r (minor1) = 45.06 min, t_r (minor2) = 51.37 min, t_r (major2) = 52.62 min) gave the isomeric composition of the product: 1.5:1 d.r., 94:6 e.r., 87:13 e.r., $[\alpha]_D^{23}$ = −37.7 (c = 0.21, $CHCl_3$).

^{1}H NMR (600 MHz, $CDCl_3$) δ 7.27 (d, J = 7.8 Hz, 2H), 7.18 (d, J = 7.8 Hz, 2H), 3.88 (q, J = 7.4 Hz, 0.04H), 3.13 (dd, J_1 = 13.8 Hz, J_2 = 4.2 Hz, 1H), 2.60-2.52 (m, 1H), 2.39-2.30 (m, 2H), 2.15-2.05 (m, 2H), 2.01-1.93 (m, 1H), 1.79-1.70 (m, 1H), 1.62 (s, 3H), 1.58-1.50 (m, 1H).

^{13}C NMR (150 MHz, $CDCl_3$) δ (diastereomer 1) 219.9, 139.9, 134.8, 129.5, 126.7, 121.6, 50.7, 38.0, 34.99, 30.5 (t, J = 20.7 Hz), 28.99, 21.2, 20.4; (diastereomer 2) 219.9, 139.9, 134.8, 129.5, 126.7, 121.6, 50.7, 38.0, 34.97, 30.5 (t, J = 20.7 Hz), 29.00, 21.2, 20.4.

IR (neat, cm^{-1}) 2964, 2938, 2877, 2239, 1733, 1514, 1153.

HRMS (ESI) calcd $C_{15}H_{17}DNO^+$ $[M+H]^+$: 229.1446. Found: 229.1447.

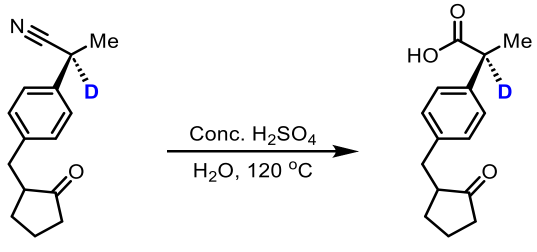

zwf-11-075

To a solution of (−)-**104**-d (68.5 mg, 0.3 mmol, 100 mol%, 96% D, 1.6:1 d.r., 91:9 e.r., 91:9 e.r.) in H_2O (0.75 mL) was added concentrated sulfuric acid (0.6 mL) at room temperature, then the reaction mixture was stirred under reflux for 6 h and cooled to room temperature. The reaction mixture was extracted with diethyl ether (5 mL × 5). The combined organic phase was washed with brine, dried over $MgSO_4$, filtered, and evaporated under vacuum. The residue was purified by flash column chromatography to obtain (+)-**105**-d (60.8 mg, 82% Yield, 89% D) as a white solid, R_f = 0.1 (Hexane/EtOAc 5:1).

HPLC analysis (Chiralpak IG-3, hexane/^{i}PrOH = 98.5/1.5, 1.3 mL/min, 205 nm; t_r (major1) = 33.05 min, t_r (major2) = 35.48 min, t_r (minor1) = 38.44 min, t_r (minor2) = 39.93 min) gave the isomeric composition of the product: 1:1 d.r., 87:13 e.r., 87:13 e.r., $[\alpha]_D^{23}$ = +41.3 (c = 0.23, $CHCl_3$).

^{1}H NMR (600 MHz, $CDCl_3$) δ 7.23 (d, J = 7.8 Hz, 2H), 7.12 (d, J = 7.8 Hz, 2H), 3.70 (q, J = 7.2 Hz, 0.11H), 3.11 (dd, J_1 = 13.8 Hz, J_2 = 4.2 Hz, 1H), 2.50 (dd, J_1 = 13.8 Hz, J_2 = 9.6 Hz, 1H), 2.40-2.27 (m, 2H), 2.16-2.04 (m, 2H), 2.00-1.90 (m, 1H), 1.79-1.66 (m, 1H), 1.59-1.44 (m, 4H).

^{13}C NMR (150 MHz, $CDCl_3$) δ 220.5, 180.4, 139.1, 137.6, 129.1, 127.6, 50.9, 44.5 (t, J = 19.3 Hz), 38.1, 35.1, 29.1, 20.4, 17.9.

IR (neat, cm^{-1}) 2970, 2937, 1704, 1221, 1154.

HRMS (ESI) calcd $C_{15}H_{18}DO_3^+$ $[M+H]^+$: 248.1391. Found: 248.1393.

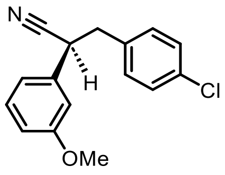

(–)-**108**

zwf-11-088

(–)-**108** was obtained as an oil from the asymmetric decarboxylative protonation reaction of 2-cyanoacetic acid **S107** (473.8 mg, 1.5 mmol) for 4 h using the general procedure (342.2 mg, 84% Yield). R_f = 0.3 (Hexane/EtOAc 10:1).

HPLC analysis (Chiralpak IE-3, hexane/^{i}PrOH = 99/1, 0.8 mL/min, 205 nm; t_r (minor) = 35.82 min, t_r (major) = 41.98 min) gave the isomeric composition of the product: 96:4 e.r., $[\alpha]_D^{23}$ = –2.7 (c = 0.24, $CHCl_3$).

^{1}H NMR (600 MHz, $CDCl_3$) δ 7.29-7.23 (m, 3H), 7.06 (d, J = 8.4 Hz, 2H), 6.86 (dd, J_1 = 8.4 Hz, J_2 = 2.4 Hz, 1H), 6.81 (d, J = 7.8 Hz, 1H), 6.76 (t, J = 2.1 Hz, 1H), 3.95 (t, J = 7.2 Hz, 1H), 3.78 (s, 3H), 3.17-3.08 (m, 2H).

^{13}C NMR (150 MHz, $CDCl_3$) δ 160.0, 136.1, 134.6, 133.4, 130.6, 130.1, 128.7, 120.0, 119.7, 113.7, 113.2, 55.3, 41.2, 39.5.

IR (neat, cm^{-1}) 2937, 2837, 2241, 1490, 1264, 1092.

HRMS (ESI) calcd $C_{16}H_{15}ClNO^+$ $[M+H]^+$: 272.0837. Found: 272.0839.

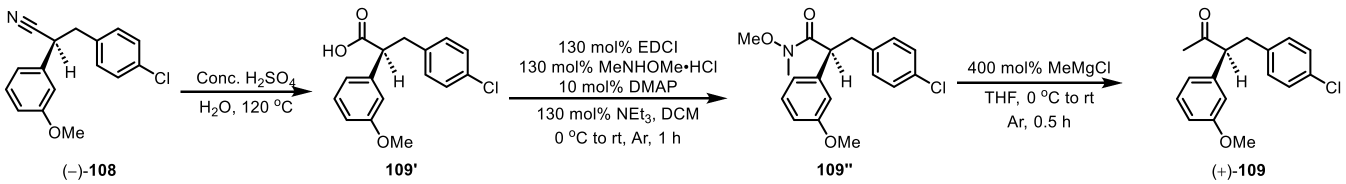

zwf-11-091/100/102

To a solution of (–)-**108** (271.6 mg, 1.0 mmol, 100 mol%, 96:4 e.r.) in H_2O (2.5 mL) was added concentrated sulfuric acid (2 mL) at room temperature, then the reaction mixture was stirred under reflux

for 12 h and cooled to room temperature. The reaction mixture was extracted with diethyl ether (10 mL × 5). The combined organic phase was washed with brine, dried over $MgSO_4$, filtered, and evaporated under vacuum. The residue was purified by flash column chromatography to obtain **109’** (238.1 mg, 82% Yield) as a white solid, which directly used for the next step. R_f = 0.1 (Hexane/EtOAc 5:1).

To a flask (25 mL) were added **109’** (145.5 mg, 0.5 mmol), EDCI (1-(3-dimethylaminopropyl)-3-ethylcarbodiimide hydrochloride) (124.7 mg, 0.65 mmol, 130 mol%), *N*,*O*-dimethylhydroxylamine hydrochloride (64.1 mg, 0.65 mmol, 130 mol%), DMAP (6.4 mg, 0.05 mmol, 10 mol%), NEt_3 (90 µL, 0.65 mmol, 130 mol%), and DCM (5 mL) at 0 ºC under argon. The resulting mixture was stirred at rt for 1 h as monitored by TLC, diluted with DCM (5 mL), and quenched with H_2O (5 mL). After extraction with DCM (10 mL x 3), the combined organic layer was washed with brine (10 mL) and dried over anhydrous Na_2SO_4. After filtration and concentration under reduced pressure, the crude product was purified by column chromatography on silica gel to afford **109’’** (160.1 mg, 96% Yield) as an oil, which directly used for the next step. R_f = 0.7 (Hexane/EtOAc 2:1).

To a flask (25 mL) were added **109’’** (133.5 mg, 0.4 mmol) and THF (4 mL) at 0 ºC under argon. Then MeMgCl (0.53 mL, 3.0 M in THF, 1.6 mmol, 400 mol%) was added to the reaction dropwise. After that the resulting mixture was stirred at room temperature for 0.5 h as monitored by TLC, quenched with saturated NH_4Cl (1 mL) at 0 ºC and extracted with ethyl acetate (5 mL x 3), the combined organic layer was washed with brine (5 mL) and dried over anhydrous Na_2SO_4. After filtration and concentration under reduced pressure, the crude product was purified by column chromatography on silica gel to afford (+)-**109** (109.4 mg, 95% Yield) as an oil. R_f = 0.6 (Hexane/EtOAc 5:1).

HPLC analysis (Chiralpak IG-3, hexane/^{i}PrOH = 90/10, 0.8 mL/min, 205 nm; t_r (minor) = 8.71 min, t_r (major) = 9.76 min) gave the isomeric composition of the product: 89:11 e.r., $[\alpha]_D^{23}$ = +272.1 (*c* = 0.21, $CHCl_3$).

^{1}H NMR (600 MHz, $CDCl_3$) δ 7.22 (t, *J* = 8.1 Hz, 1H), 7.15 (d, *J* = 7.8 Hz, 2H), 6.97 (d, *J* = 7.8 Hz, 2H), 6.80 (dd, J_1 = 8.4 Hz, J_2 = 2.4 Hz, 1H), 6.73 (d, *J* = 7.8 Hz, 1H), 6.68 (s, 1H), 3.82 (t, *J* = 7.2 Hz, 1H), 3.77 (s, 3H), 3.36 (dd, J_1 = 13.8 Hz, J_2 = 7.2 Hz, 1H), 2.86 (dd, J_1 = 13.8 Hz, J_2 = 7.8 Hz, 1H), 2.03 (s, 3H),

^{13}C NMR (150 MHz, $CDCl_3$) δ 207.1, 159.9, 139.5, 138.1, 131.8, 130.3, 129.9, 128.3, 120.7, 113.9, 112.8, 61.3, 55.2, 37.4, 29.4.

IR (neat, cm^{-1}) 2983, 2906, 1731, 1495, 1056.

HRMS (ESI) calcd $C_{17}H_{18}ClO_2^+$ $[M+H]^+$: 289.0990. Found: 289.0989.

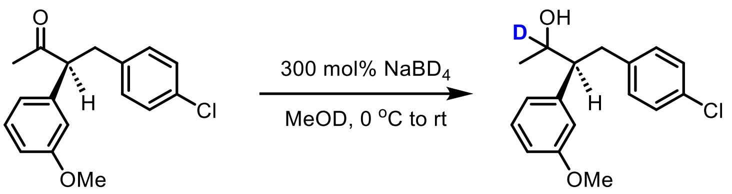

(+)-**109** (+)-**110**

zwf-11-104

To a solution of (–)-**109** (57.6 mg, 0.2 mmol, 100 mol%, 89:11 e.r.) in MeOD (1 Ml, 99% D) was added $NaBD_4$ (25.1 mg, 0.6 mmol, 300 mol%, 99% D) at 0 °C, then the reaction mixture was stirred at room temperature for 2 h. The reaction mixture was quenched with $NaHCO_3$ (0.5 mL) at 0 °C, extracted with diethyl ether (5 mL × 5). The combined organic phase was washed with brine, dried over $MgSO_4$, filtered, and evaporated under vacuum. The residue was purified by flash column chromatography to obtain (+)-**110**-d (49.3 mg, 85% Yield, 98% D) as oil. R_f = 0.3 (Hexane/EtOAc 5:1).

HPLC analysis (Chiralpak IG-3, hexane/^{i}PrOH = 90/10, 0.8 mL/min, 205 nm; t_r (minor1) = 9.11 min, t_r (major1) = 11.78 min, t_r (major2) = 12.16 min, t_r (minor2) = 14.24 min) gave the isomeric composition of the product: 10.8:1 d.r. (Based on the ^{1}H NMR analysis of the crude product), 89.5:10.5 e.r. (major). $[\alpha]_D^{24}$ = +108.7 (c = 0.22, $CHCl_3$).

^{1}H NMR (600 MHz, $CDCl_3$) δ (major diastereomer) 7.19 (t, J = 7.8 Hz, 1H), 7.14 (d, J = 8.4 Hz, 2H), 6.98 (d, J = 8.4 Hz, 2H), 6.81-6.68 (m, 3H), 3.98-3.95 (m, 0.02H), 3.76 (s, 3H), 3.10 (dd, J_1 = 13.5 Hz, J_2 = 6.3 Hz, 1H), 2.87 (dd, J_1 = 13.5 Hz, J_2 = 9.3 Hz, 1H), 2.78-2.70 (m, 1 H), 1.40 (br, 1H), 1.16 (s, 3H); (minor diastereomer) 7.19 (t, J = 7.8 Hz, 1H), 7.09 (d, J = 8.4 Hz, 2H), 6.90 (d, J = 7.8 Hz, 2H), 6.81-6.68 (m, 1H), 6.64 (d, J = 7.8 Hz, 1H), 6.59 (s, 1H), 3.98-3.95 (m, 0.02H), 3.73 (s, 3H), 3.26 (dd, J_1 = 13.5 Hz, J_2 = 4.5 Hz, 1H), 2.87 (dd, J_1 = 13.5 Hz, J_2 = 9.3 Hz, 1H), 2.78-2.70 (m, 1 H), 1.71 (br, 1H), 1.07 (s, 3H).

^{13}C NMR (150 MHz, $CDCl_3$) δ (major diastereomer) 159.5, 141.8, 138.7, 131.5, 130.4, 129.3, 128.2, 121.4, 115.1, 111.8, 69.0 (t, J = 22.1 Hz), 55.08, 55.06, 37.7, 21.4; (minor diastereomer) 159.4, 142.8, 138.9, 131.4, 130.4, 129.2, 128.1, 121.0, 114.7, 111.4, 69.0 (t, J = 22.1 Hz), 55.7, 55.1, 37.2, 21.5.

IR (neat, cm^{-1}) 3503, 2985, 2913, 1577, 1488, 1055.

HRMS (ESI) calcd $C_{17}H_{19}DClO_2^+$ $[M+H]^+$: 292.1209. Found: 292.1208.

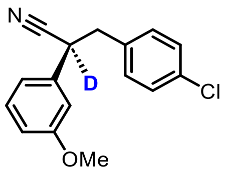

(–)-**111**-d

zwf-11-089

(–)-**111**-d was obtained as an oil from the asymmetric decarboxylative deuteration reaction of 2-cyanoacetic acid **107** (473.5 mg, 1.5 mmol) for 4 h using the general procedure (347.2 mg, 85% Yield, 87% D). R_f = 0.3 (Hexane/EtOAc 10:1).

HPLC analysis (Chiralpak IE-3, hexane/^{i}PrOH = 99/1, 0.8 mL/min, 205 nm; t_r (minor) = 35.80 min, t_r (major) = 42.07 min) gave the isomeric composition of the product: 97:3 e.r., $[\alpha]_D^{23}$ = –3.3 (c = 0.22, $CHCl_3$).

^{1}H NMR (600 MHz, $CDCl_3$) δ 7.29-7.22 (m, 3H), 7.05 (d, J = 8.4 Hz, 2H), 6.85 (dd, J_1 = 8.4 Hz, J_2 = 2.4 Hz, 1H), 6.81 (d, J = 7.8 Hz, 1H), 6.76 (t, J = 2.1 Hz, 1H), 3.95 (t, J = 7.2 Hz, 0.13H), 3.77 (s, 3H), 3.16-3.06 (m, 2H).

^{13}C NMR (150 MHz, $CDCl_3$) δ 159.9, 136.1, 134.6, 133.3, 130.5, 130.1, 128.7, 120.0, 119.6, 113.7, 113.1, 55.2, 41.1, 39.1 (t, J = 20.7 Hz).

IR (neat, cm^{-1}) 2938, 2832, 2241, 1493, 1261, 1090.

HRMS (ESI) calcd $C_{16}H_{14}DClNO^+$ $[M+H]^+$: 273.0899. Found: 273.0897.

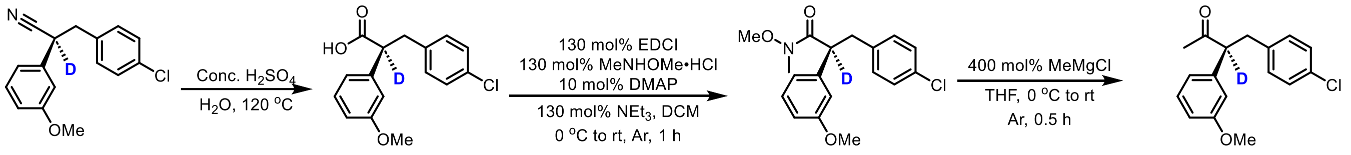

(–)-**111**-d **112'**-d **112''**-d (+)-**112**-d

zwf-11-092/101/103

To a solution of (–)-**111**-d (272.5 mg, 1.0 mmol, 100 mol%, 87% D, 97:3 e.r.) in H_2O (2.5 mL) was added concentrated sulfuric acid (2 mL) at room temperature, then the reaction mixture was stirred under reflux for 12 h and cooled to room temperature. The reaction mixture was extracted with diethyl ether (10 mL × 5). The combined organic phase was washed with brine, dried over $MgSO_4$, filtered, and evaporated under vacuum. The residue was purified by flash column chromatography to obtain **112'**-d (236.8 mg, 81% Yield) as a white solid, which directly used for the next step. R_f = 0.1 (Hexane/EtOAc 5:1).

To a flask (25 mL) were added **112'**-d (145.9 mg, 0.5 mmol), EDCI (1-(3-dimethylaminopropyl)-3-ethylcarbodiimide hydrochloride) (124.5 mg, 0.65 mmol, 130 mol%), *N*,*O*-dimethylhydroxylamine hydrochloride (64.0 mg, 0.65 mmol, 130 mol%), DMAP (6.3 mg, 0.05 mmol, 10 mol%), NEt_3 (90 μL, 0.65 mmol, 130 mol%), and DCM (5 mL) at 0 °C under argon. The resulting mixture was stirred at rt for 1 h as monitored by TLC, diluted with DCM (5 mL), and quenched with H_2O (5 mL). After extraction with DCM (10 mL x 3), the combined organic layer was washed with brine (10 mL) and dried over anhydrous Na_2SO_4. After filtration and concentration under reduced pressure, the crude product was purified by column chromatography on silica gel to afford **112''-d** (162.4 mg, 97% Yield) as an oil, which directly used for the next step. R_f = 0.7 (Hexane/EtOAc 2:1).

To a flask (25 mL) were added **112''-**d (133.9 mg, 0.4 mmol) and THF (4 mL) at 0 ºC under argon. Then MeMgCl (0.53 mL, 3.0 M in THF, 1.6 mmol, 400 mol%) was added to the reaction dropwise. After that the resulting mixture was stirred at room temperature for 0.5 h as monitored by TLC, quenched with saturated NH_4Cl (1 mL) at 0 ºC and extracted with ethyl acetate (5 mL x 3), the combined organic layer was washed with brine (5 mL) and dried over anhydrous Na_2SO_4. After filtration and concentration under reduced pressure, the crude product was purified by column chromatography on silica gel to afford (+)-**112-**d (108.7 mg, 94% Yield, 83% D) as an oil. R_f = 0.6 (Hexane/EtOAc 5:1).

HPLC analysis (Chiralpak IG-3, hexane/^{i}PrOH = 90/10, 0.8 mL/min, 205 nm; t_r (minor) = 8.70 min, t_r (major) = 9.77 min) gave the isomeric composition of the product: 94:6 e.r., $[\alpha]_D^{24}$ = +304.1 (*c* = 0.29, $CHCl_3$).

^{1}H NMR (600 MHz, $CDCl_3$) δ 7.22 (t, *J* = 7.8 Hz, 1H), 7.16 (d, *J* = 8.4 Hz, 2H), 6.97 (d, *J* = 8.4 Hz, 2H), 6.80 (dd, J_1 = 8.4 Hz, J_2 = 2.4 Hz, 1H), 6.74 (d, *J* = 7.2 Hz, 1H), 6.68 (t, *J* = 2.1 Hz, 1H), 3.85-3.75 (m, 3.17H), 3.35 (d, *J* = 13.8 Hz, 1H), 2.85 (d, *J* = 13.8 Hz, 1H), 2.03 (s, 3H),

^{13}C NMR (150 MHz, $CDCl_3$) δ 207.2, 160.0, 139.5, 138.1, 131.9, 130.3, 130.0, 128.3, 120.7, 113.9, 112.8, 60.9 (t, *J* = 19.4 Hz), 55.2, 37.4, 29.4.

IR (neat, cm^{-1}) 2988, 2901, 1734, 1493, 1057.

HRMS (ESI) calcd $C_{17}H_{17}DClO_2^+$ $[M+H]^+$: 290.1053. Found: 290.1050.

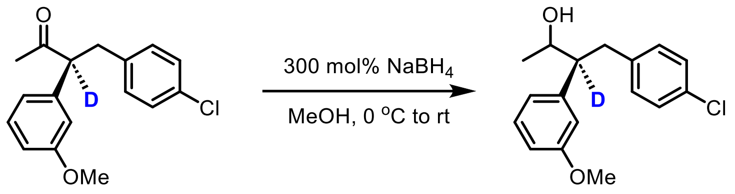

(+)-**112**-d (+)-**113**-d

zwf-11-105

To a solution of (−)-**112**-d (58.0 mg, 0.2 mmol, 100 mol%, 83% D, 94:6 e.r.) in MeOH (1 mL) was added $NaBH_4$ (23.1 mg, 0.6 mmol, 300 mol%) at 0 ºC, then the reaction mixture was stirred at room temperature for 2 h. The reaction mixture was quenched with $NaHCO_3$ (0.5 mL) at 0 °C, extracted with diethyl ether (5 mL × 5). The combined organic phase was washed with brine, dried over $MgSO_4$, filtered, and evaporated under vacuum. The residue was purified by flash column chromatography to obtain (+)-**113**-d (50.9 mg, 87% Yield, 83% D) as oil. R_f = 0.3 (Hexane/EtOAc 5:1).

HPLC analysis (Chiralpak IG-3, hexane/^{i}PrOH = 90/10, 0.8 mL/min, 205 nm; t_r (minor1) = 9.07 min, t_r (major1) = 11.71 min, t_r (major2) = 12.13 min, t_r (minor2) = 14.08 min) gave the isomeric composition of the product: 10.4:1 d.r. (Based on the ^{1}H NMR analysis of the crude product), 94:6 e.r. (major). $[\alpha]_D^{24}$ =

+117.7 (c = 0.29, $CHCl_3$).

^{1}H NMR (600 MHz, $CDCl_3$) δ (major diastereomer) 7.18 (t, J = 8.1 Hz, 1H), 7.13 (d, J = 8.4 Hz, 2H), 6.97 (d, J = 8.4 Hz, 2H), 6.80-6.68 (m, 3H), 3.99-3.90 (m, 1H), 3.75 (s, 3H), 3.09 (d, J = 13.8 Hz, 1H), 2.82 (d, J = 13.8 Hz, 1H), 2.77-2.71 (m, 0.17H), 1.47 (br, 1H), 1.16 (d, J = 6.0 Hz, 3H); (minor diastereomer) 7.18 (t, J = 8.1 Hz, 1H), 7.09 (d, J = 8.4 Hz, 2H), 6.90 (d, J = 8.4 Hz, 2H), 6.80-6.68 (m, 1H), 6.63 (d, J = 7.8 Hz, 1H), 6.58 (d, J = 1.8 Hz, 1H), 3.99-3.90 (m, 1H), 3.73 (s, 3H), 3.26 (d, J = 13.8 Hz, 1H), 2.82 (d, J = 13.8 Hz, 1H), 2.77-2.71 (m, 0.17H), 1.77 (br, 1H), 1.06 (d, J = 6.6 Hz, 3H).

^{13}C NMR (150 MHz, $CDCl_3$) δ (major diastereomer) 159.5, 141.7, 138.7, 131.5, 130.3, 129.3, 128.2, 121.3, 115.0, 111.8, 69.4, 55.0, 54.7 (t, J = 19.3 Hz), 37.6, 21.5; (minor diastereomer) 159.4, 141.8, 138.9, 131.3, 130.3, 129.2, 128.0, 120.9, 114.6, 111.4, 70.9, 55.2, 54.7 (t, J = 19.3 Hz), 37.1, 21.5.

IR (neat, cm^{-1}) 3499, 2981, 2932, 1570, 1481, 1048.

HRMS (ESI) calcd $C_{17}H_{19}DClO_2^+$ $[M+H]^+$: 292.1209. Found: 292.1212.

<u>Procedure for measurement of turnover numbers with **S1** at S/C = 27560</u> (**zwf-8-086**)

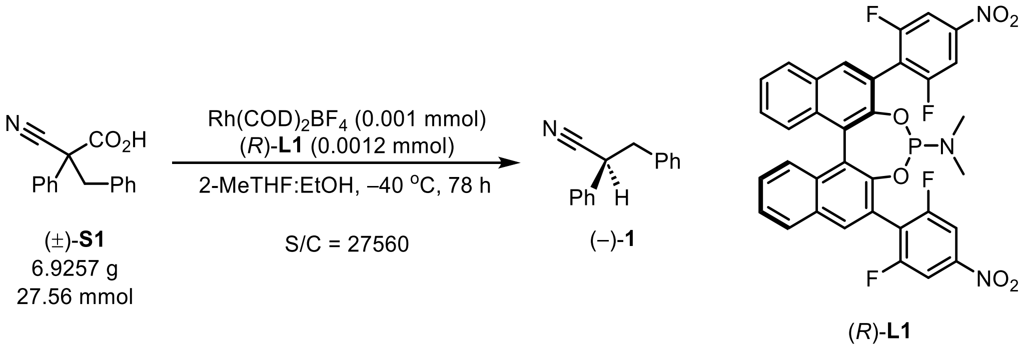

To an oven-dried 500 mL round bottom flask were added (R)-**L1** (0.8 mg, 0.0012 mmol), $Rh(COD)_2BF_4$ (0.4 mg, 0.001 mmol), and 2-methyltetrahydrofuran (20 mL, 2-MeTHF). After the reaction mixture was stirred at room temperature for 1 h, EtOH (20 mL) was added to the reaction and stirred for additional 10 min at room temperature. Then the catalyst solution was cooled to –40 °C and stirred for another 30 min. The **S1** (1.0 mmol) was dissolved in 2-MeTHF (4 mL) in another tube and cooled to –40 °C for 10 min, which was added to the catalyst bottle dropwise over 5 min via syringe, and the acid tube was rinsed with 2-MeTHF (1 mL) to transfer the residue to the reaction. The reaction mixture was stirred at –40 °C. After the full consumption of the acid starting material as monitored by TLC, the next portion of the **S1** (1.0 mmol) was added to the reaction using the same procedure (the total amount of **S1** was 6.9257 g, 27.56 mmol, and the total reaction time was 78 h). After the reaction mixture was warmed to room temperature, the solvent was removed under vacuum. The crude product was analyzed by ^{1}H NMR with CH_2Br_2 (193 μL) as the internal standard, 100% conversion of starting material with 97% NMR yield of (–)-**1** was

obtained. The crude product was purified by flash column chromatography to obtain (−)-**1** (5.2748 g, 92% Yield) as a white solid, R_f = 0.4 (Hexane/EtOAc 10:1). The turnover number was calculated to be 25447. *Note that this turnover number does not represent the limit of the catalyst, as we ran out of **S1** during the measurement and the cooling bath capacity became too small for the increasing amount of solvent.

HPLC analysis (Chiralpak IE-3, hexane/^{i}PrOH = 99/1, 0.8 mL/min, 205 nm; t_r (minor) = 19.58 min, t_r (major) = 21.74 min) gave the isomeric composition of the product: 97.5:2.5 e.r.

^{1}H NMR (400 MHz, $CDCl_3$) δ 7.41-7.20 (m, 8H), 7.13 (d, *J* = 7.2 Hz, 2H), 4.00 (t, *J* = 7.4 Hz, 1H), 3.24-3.07 (m, 2H).

<u>Measurement of initial turnover frequency (TOF_{ini})</u> (**zwf-8-081**)

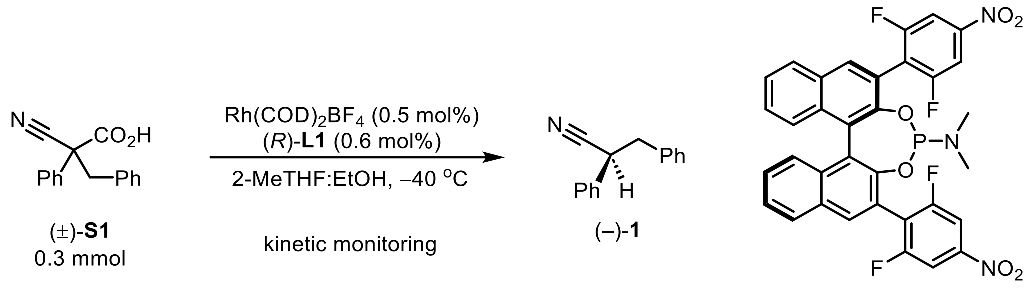

To an oven-dried 100 mL Schlenk tube were added (*R*)-**L1** (1.2 mg, 0.0018 mmol, 0.6 mol%), $Rh(COD)_2BF_4$ (0.6 mg, 0.0015 mmol, 0.5 mol%), and 2-methyltetrahydrofuran (2-MeTHF, 2.4 mL). After the reaction mixture was stirred at room temperature for 1 h, EtOH (1.2 mL) was added to the reaction and stirred for an additional 10 min at room temperature. Then the catalyst solution was cooled to −40 °C and stirred for another 30 min. **S1** (150.8 mg, 0.6 mmol) and 1,3,5-trimethoxybenzene (16.8 mg, 0.1 mmol) were dissolved in 2-MeTHF (7.2 mL) in another tube and cooled to −40 °C for 10 min. Then 3.6 mL substrate solution (0.3 mmol) was added to the catalyst tube one-pot via syringe to mark the start of the decarboxylation. A 0.2 mL aliquot was taken from the reaction mixture with a syringe after 0.5 min, 1 min, 2 min, and 3 min, and transferred to a tube containing MeOH (0.1 mL) frozen at −40 °C. Subsequently, $TMSCHN_2$ (0.1 mL, 2 M in hexane) was added to the tube and stirred vigorously to quench the reaction. The resulting reaction mixture was concentrated and analyzed by ^{1}H NMR spectra with 1,3,5-trimethoxybenzene as the internal standard to determine the yield of (−)-**1** and the recovery of **S1**.

entry	t (min)	NMR yield (%)	recovery (%)
1	0.5	8	91
2	1	13	87
3	2	18	81
4	3	25	75

using entry 1:

$$TOF_{ini} = \frac{8\%\ \text{(yield)}}{0.5\%\ \text{(cat loading)} \times 0.5\ \text{min}} = 32\ \text{min}^{-1}$$

Section 9. Crystal structures and crystallographic data

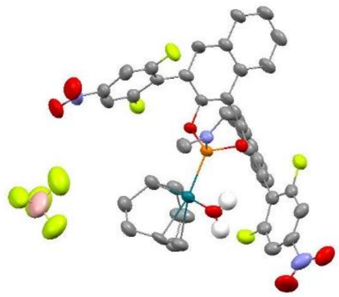

Supplementary Figure S7. Crystal structure for [Rh**L1**(COD)(H_2O)]BF_4 (CCDC 2302076).

Table S1. Crystal data and structure refinement for cu_231017zwf_0m.

Identification code	cu_231017zwf_0m
Empirical formula	$C_{42}H_{34}BF_8N_3O_7PRh$
Formula weight	989.41
Temperature/K	296
Crystal system	monoclinic
Space group	$P2_1$
a/Å, b/Å, c/Å	11.0582(5), 18.6301(8), 12.4381(5)
α/°, β/°, γ/°	90, 111.617(2), 90
Volume/$Å^3$	2382.22(18)
Z	2
$\rho_{calc}g/cm^3$	1.379
μ/mm^{-1}	3.917
F(000)	1000.0
Crystal size/mm^3	0.07 × 0.07 × 0.05
Radiation	CuKα (λ = 1.54178)
2Θ range for data collection/°	7.646 to 140.756
Index ranges	-13 ≤ h ≤ 13, -22 ≤ k ≤ 22, -13 ≤ l ≤ 14
Reflections collected	45503
Independent reflections	8875 [R_{int} = 0.1090, R_{sigma} = 0.0729]
Data/restraints/parameters	8875/68/574
Goodness-of-fit on F^2	0.986
Final R indexes [I>=2σ (I)]	R_1 = 0.0705, wR_2 = 0.1916
Final R indexes [all data]	R_1 = 0.0822, wR_2 = 0.2087
Largest diff. peak/hole / e $Å^{-3}$	0.72/-1.16
Flack parameter	0.232(18)

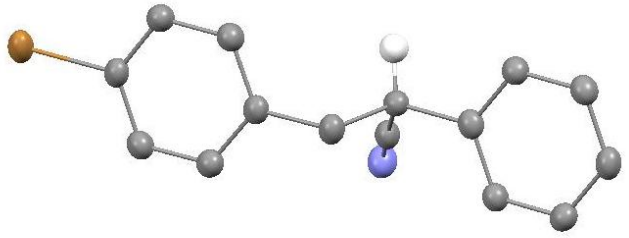

Supplementary Figure S8. Crystal structure for (−)-**17** (CCDC 2298854).

Table S2. Crystal data and structure refinement for cu_0927_1_0m.

Identification code	cu_0927_1_0m
Empirical formula	$C_{15}H_{12}BrN$
Formula weight	286.17
Temperature/K	150.00
Crystal system	monoclinic
Space group	C2
a/Å, b/Å, c/Å	20.9675(11), 5.7426(3), 10.8179(6)
α/°, β/°, γ/°	90, 99.780(2), 90
Volume/Å^3	1283.63(12)
Z	4
ρ_{calc}g/cm^3	1.481
μ/mm^{-1}	4.154
F(000)	576.0
Crystal size/mm^3	0.2 × 0.15 × 0.1
Radiation	CuKα (λ = 1.54178)
2Θ range for data collection/°	8.294 to 155.128
Index ranges	$-26 \le h \le 26$, $-7 \le k \le 7$, $-13 \le l \le 13$
Reflections collected	10497
Independent reflections	2629 [R_{int} = 0.0319, R_{sigma} = 0.0379]
Data/restraints/parameters	2629/1/154
Goodness-of-fit on F^2	1.206
Final R indexes [I>=2σ (I)]	R_1 = 0.0554, wR_2 = 0.1170
Final R indexes [all data]	R_1 = 0.0556, wR_2 = 0.1173
Largest diff. peak/hole / e Å^{-3}	1.39/-0.39
Flack parameter	0.072(9)

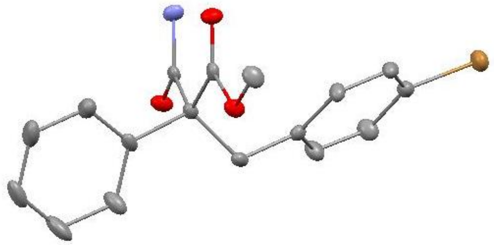

Supplementary Figure S9. Crystal structure for (−)-**17-1** (CCDC 2323058).

Table S3. Crystal data and structure refinement for 240102zwf_8_078.

Identification code	240102zwf_8_078
Empirical formula	$C_{17}H_{16}BrNO_3$
Formula weight	362.22
Temperature/K	173.00
Crystal system	orthorhombic
Space group	$P2_12_12_1$
a/Å, b/Å, c/Å	9.1514(2), 17.3526(3), 19.8107(4)
α/°, β/°, γ/°	90, 90, 90
Volume/Å^3	3145.95(11)
Z	8
ρ_{calc}g/cm^3	1.530
μ/mm^{-1}	2.419
F(000)	1472.0
Crystal size/mm^3	0.17 × 0.17 × 0.05
Radiation	GaKα (λ = 1.34139)
2Θ range for data collection/°	5.89 to 109.726
Index ranges	$-11 \leq h \leq 11$, $-21 \leq k \leq 20$, $-24 \leq l \leq 23$
Reflections collected	28916
Independent reflections	5951 [R_{int} = 0.0548, R_{sigma} = 0.0421]
Data/restraints/parameters	5951/0/399
Goodness-of-fit on F^2	1.039
Final R indexes [I>=2σ (I)]	R_1 = 0.0336, wR_2 = 0.0762
Final R indexes [all data]	R_1 = 0.0430, wR_2 = 0.0802
Largest diff. peak/hole / e Å^{-3}	0.24/-0.73
Flack parameter	-0.004(11)

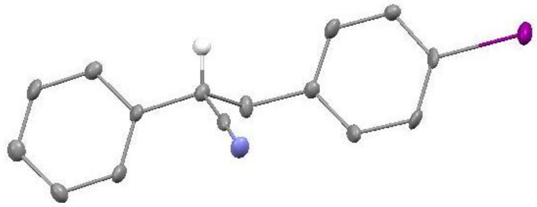

Supplementary Figure S10. Crystal structure for (–)-**18** (CCDC 2321980).

Table S4. Crystal data and structure refinement for 231030zwf.

Identification code	231030zwf
Empirical formula	$C_{15}H_{12}IN$
Formula weight	333.16
Temperature/K	173.00
Crystal system	monoclinic
Space group	$P2_1$
a/Å, b/Å, c/Å	5.7662(2), 15.2340(4), 15.4966(4)
α/°, β/°, γ/°	90, 100.5200(10), 90
Volume/Å^3	1338.38(7)
Z	4
ρ_{calc}g/cm^3	1.653
μ/mm^{-1}	12.771
F(000)	648.0
Crystal size/mm^3	0.17 × 0.17 × 0.15
Radiation	GaKα (λ = 1.34139)
2Θ range for data collection/°	7.138 to 110.036
Index ranges	$-7 \le h \le 6, -18 \le k \le 18, -18 \le l \le 18$
Reflections collected	26251
Independent reflections	5041 [R_{int} = 0.1285, R_{sigma} = 0.0925]
Data/restraints/parameters	5041/1/307
Goodness-of-fit on F^2	1.026
Final R indexes [I>=2σ (I)]	R_1 = 0.0568, wR_2 = 0.1313
Final R indexes [all data]	R_1 = 0.0643, wR_2 = 0.1349
Largest diff. peak/hole / e Å^{-3}	1.41/-1.96
Flack parameter	0.055(7)

Section 10. Mechanistic studies

10.1 Control experiments (zwf-8-065 and zwf-8-066)

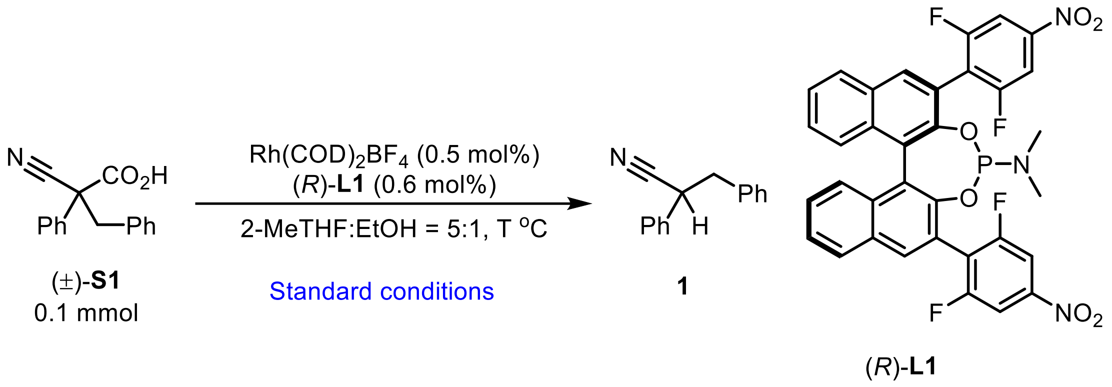

entry	T (°C)	Variation from standard conditions	Yield of **1** (%)	Recovery of **S1** (%)
1	–40	w/o (*R*)-**L1**	20	79
2	–40	w/o Rh(COD)$_2$BF$_4$	0	99
3	–40	w/o Rh(COD)$_2$BF$_4$ and (*R*)-**L1**	0	99
4	–40	w/o Rh(COD)$_2$BF$_4$, (*R*)-**L1**, and EtOH	0	99
5	r.t. (15 °C)	w/o (*R*)-**L1**	99	0
6	r.t. (15 °C)	w/o Rh(COD)$_2$BF$_4$	12	88
7	r.t. (15 °C)	w/o Rh(COD)$_2$BF$_4$ and (*R*)-**L1**	12	87
8	r.t. (15 °C)	w/o Rh(COD)$_2$BF$_4$, (*R*)-**L1**, and EtOH	12	88

Reaction conditions unless noted otherwise: (±)-**S1** (0.1 mmol), [Rh] (0.5 mol%), and (*R*)-**L1** (0.6 mol%) in 2-MeTHF/EtOH for 2 h. The yield and recovery was determined by crude ^{1}H NMR analysis using 1,3,5-trimethoxybenzene as the internal standard.

Supplementary Figure S11. Investigation of background decarboxylation.

10.2 The isolation and examination of a rhodium complex (zwf-7-050 and zwf-7-148)

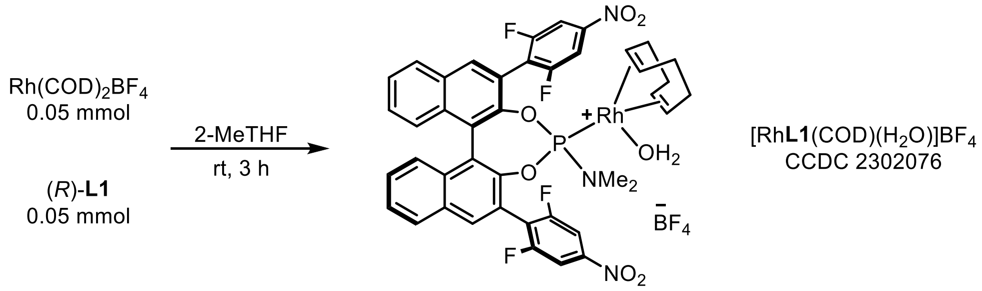

To an oven-dried 4 mL vial were added (*R*)-**L1** (33.7 mg, 0.05 mmol), Rh(COD)$_2$BF$_4$ (20.3 mg, 0.05 mmol), and 2-methyltetrahydrofuran (2-MeTHF, 2 mL). After the reaction mixture was stirred at room temperature for 3 h, the solvent was removed under vacuum. The residue was redissolved in dichloromethane (1 mL, DCM), and hexane (1 mL) was added to crystalize the [Rh**L1**(COD)(H$_2$O)]BF$_4$ over 5 days.

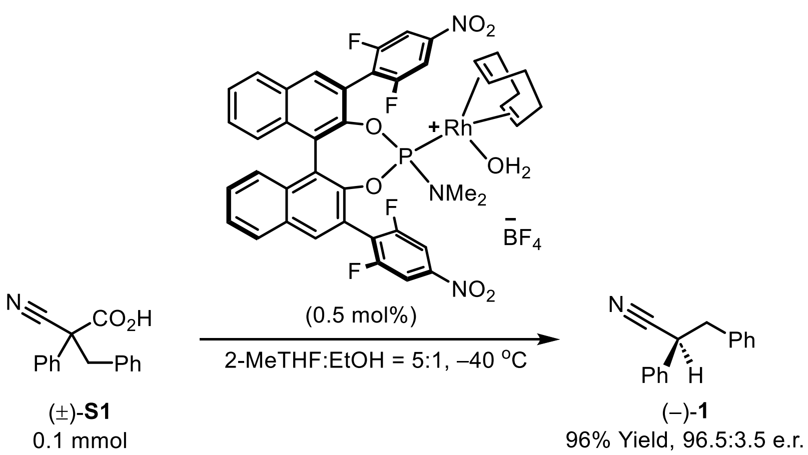

To an oven-dried 25 mL Schlenk tube were added [Rh**L1**(COD)(H$_2$O)]BF$_4$ (0.5 mg, 0.0005 mmol, 0.5 mol%), 2-methyltetrahydrofuran (0.8 mL, 2-MeTHF), and EtOH (0.4 mL), the reaction mixture was stirred at room temperature for 10 min. Then the catalyst solution was cooled to –40 °C and stirred for another 30 min. The **S1** (50.2 mg, 0.2 mmol) was dissolved in 2-MeTHF (2.4 mL) in another tube and cooled to –40 °C for 10 min. Then 1.2 mL of substrate solution (0.1 mmol) was added to the catalyst tube dropwsie via syringe. The reaction mixture was stirred at –40 °C for 2 h. After the reaction mixture was warmed to room temperature, the solvent was removed under vacuum and the residue was purified by flash column chromatography to obtain (–)-**1** (19.8 mg, 96% Yield) as white solid, R_f = 0.4 (Hexane/EtOAc 10:1).

HPLC analysis (Chiralpak IE-3, hexane/^{i}PrOH = 99/1, 0.8 mL/min, 205 nm; t_r (minor) = 19.41 min, t_r (major) = 21.54 min) gave the isomeric composition of the product: 96.5:3.5 e.r.

^{1}H NMR (400 MHz, CDCl$_3$) δ 7.40-7.22 (m, 8H), 7.14 (d, *J* = 6.4 Hz, 2H), 4.00 (t, *J* = 7.4 Hz, 1H), 3.26-3.08 (m, 2H).

10.3 Measurement of full kinetic profiles

10.3.1 From racemic S17

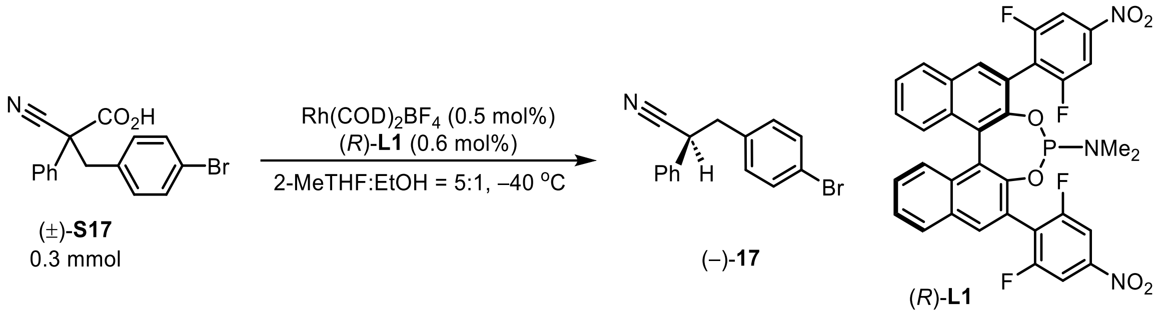

zwf-8-049

To an oven-dried 100 mL Schlenk tube were added (*R*)-**L1** (1.2 mg, 0.0018 mmol), Rh(COD)$_2$BF$_4$ (0.6 mg, 0.0015 mmol), and 2-methyltetrahydrofuran (2-MeTHF, 2.4 mL). After the reaction mixture was stirred at room temperature for 1 h, EtOH (1.2 mL) was added to the reaction and stirred for additional 10 min at room temperature. Then the catalyst solution was cooled to −40 ºC and stirred for another 30 min. racemic **S17** (198.1 mg, 0.6 mmol) and 1,3,5-trimethoxybenzene (16.8 mg, 0.1 mmol) were dissolved in 2-MeTHF (7.2 mL) in another tube and cooled to −40 ºC for 10 min. Then 3.6 mL of substrate solution (0.3 mmol) was added to the catalyst tube one-pot via syringe. A 0.2 mL aliquot was taken from the reaction mixture with a syringe after designated time and transferred to a tube containing MeOH (0.1 mL) frozen at −40 ºC. Subsequently, TMSCHN$_2$ (0.1 mL, 2 M in hexane) was added to the tube and stirred vigorously to quench the reaction. After concentration, the resulting reaction mixture was analyzed by ^{1}H NMR spectra with 1,3,5-trimethoxybenzene as the internal standard to determine the yield of (−)-**17** and the recoveries of **S17**. The residues were purification by the preparative TLC (Hexane/EtOAc 10:1) and the e.r. value were determined by HPLC analysis.

entry	t (min)	Yield of (–)-**17** (%)	e.r. of (–)-**17**	Recovery of **S17** (%)	e.r. of **S17**
1	5	31	95.5:4.5	69	58:42
2	10	48	96:4	52	63.5:36.5
3	15	62	96.5:3.5	39	70.5:29.5
4	20	73	96.5:3.5	28	78:22
5	30	88	96.5:3.5	13	91.5:8.5
6	40	95	96.5:3.5	5	99:1
7	50	98	96.5:3.5	1	/
8	60	99	96.5:3.5	0	/

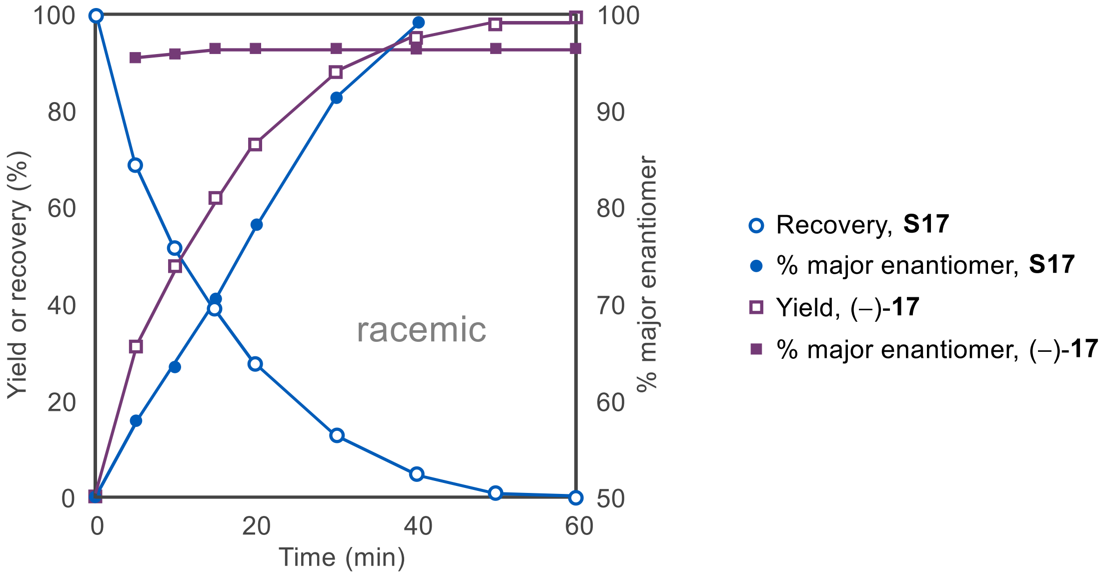

Supplementary Figure S12. Full kinetic profile of the decarboxylation from racemic **S17**.

10.3.2 From enantiopure (*R*)-S17 (matched enantiomer with catalyst)

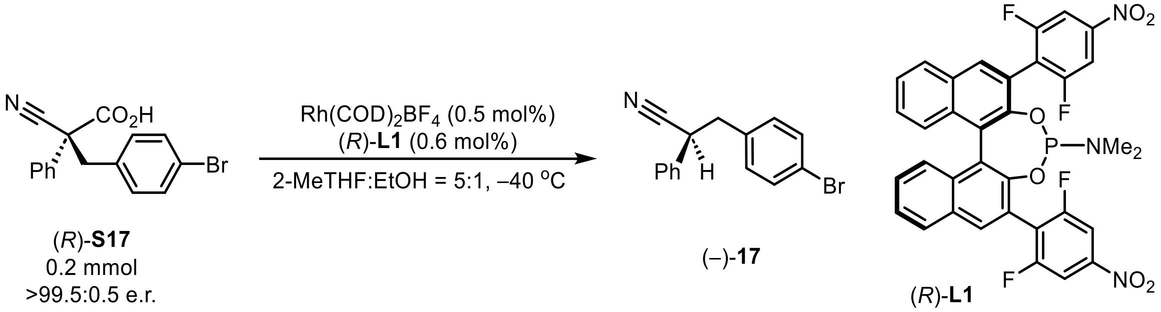

zwf-8-082

Enantioenriched (*R*)-**S17** and (*S*)-**S17** were obtained from the resolution service of Daicel Chiral Technologies (Shanghai) via the chromatographic separation of racemic **S17**.

To an oven-dried 100 mL Schlenk tube were added (*R*)-**L1** (0.8 mg, 0.0012 mmol), $Rh(COD)_2BF_4$ (0.4 mg, 0.001 mmol), and 2-methyltetrahydrofuran (2-MeTHF, 1.6 mL). After the reaction mixture was stirred at room temperature for 1 h, EtOH (0.8 mL) was added to the reaction and stirred for additional 10 min at room temperature. Then the catalyst solution was transferred to –40 ºC and stirred for another 30 min. (*R*)-**S17** (66.1 mg, 0.2 mmol, >99.5:0.5 e.r.) and 1,3,5-trimethoxybenzene (5.6 mg, 0.03 mmol) were dissolved in 2-MeTHF (2.4 mL) in another tube and cooled to –40 ºC for 10 min. Then the substrate solution was added to the catalyst tube one-pot via syringe. A 0.2 mL aliquot was taken from the reaction mixture with a syringe after designated time and transferred to a tube containing MeOH (0.1 mL) frozen at –40 ºC. Subsequently, $TMSCHN_2$ (0.1 mL, 2 M in hexane) was added to the tube and stirred vigorously to quench the reaction. After concentration, the resulting reaction mixture was analyzed by ^1H NMR spectra with 1,3,5-trimethoxybenzene as the internal standard to determine the yield of (–)-**17** and the

recoveries of **S17**. The residues were purification by the preparative TLC (Hexane/EtOAc 10:1) and the e.r. value were determined by HPLC analysis.

entry	t (min)	Yield of (–)-**17** (%)	e.r. of (–)-**17**	Recovery of (*R*)-**S17** (%)	e.r. of (*R*)-**S17**
1	5	29	99.5:0.5	70	>99.5:0.5
2	10	50	99.5:0.5	49	>99.5:0.5
3	15	67	99.5:0.5	32	>99.5:0.5
4	20	81	99.5:0.5	17	>99.5:0.5
5	30	98	99.5:0.5	0	/

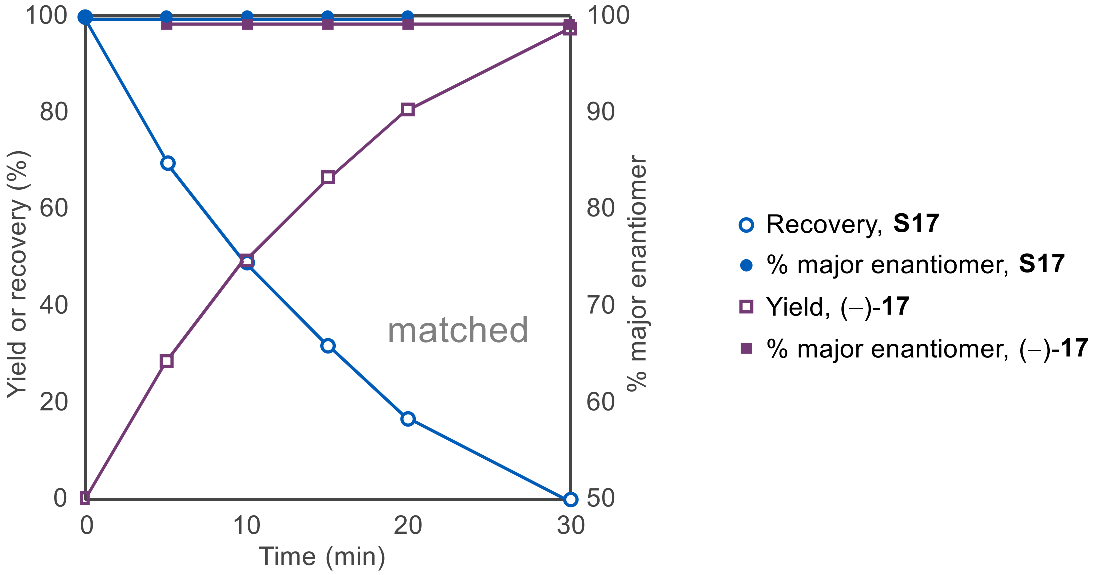

Supplementary Figure S13. Full kinetic profile of the decarboxylation from enantioenriched (*R*)-**S17**.

10.3.3 From enantiopure (*S*)-S17 (mismatched enantiomer with catalyst)

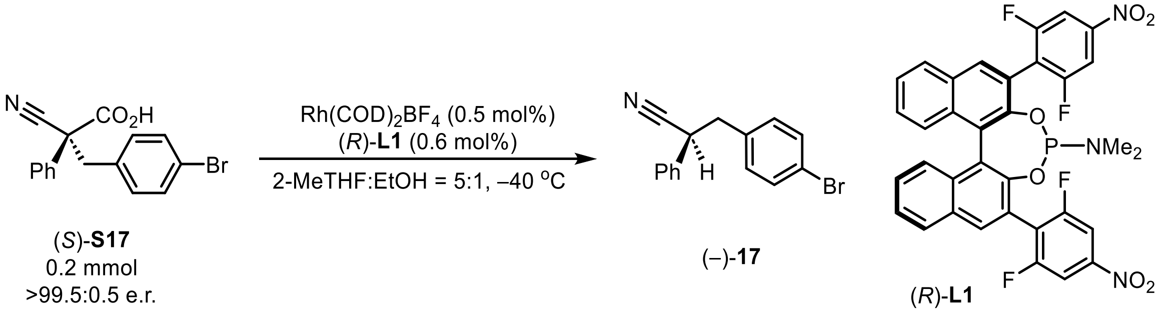

zwf-8-082

To an oven-dried 100 mL Schlenk tube were added (*R*)-**L1** (0.8 mg, 0.0012 mmol), $Rh(COD)_2BF_4$ (0.4 mg, 0.001 mmol), and 2-methyltetrahydrofuran (2-MeTHF, 1.6 mL). After the reaction mixture was stirred at room temperature for 1 h, EtOH (0.8 mL) was added to the reaction and stirred for additional 10 min at room temperature. Then the catalyst solution was transferred to –40 ºC and stirred for another 30 min. (*S*)-**S17** (66.2 mg, 0.2 mmol, >99.5:0.5 e.r.) and 1,3,5-trimethoxybenzene (5.6 mg, 0.03 mmol) were dissolved in 2-MeTHF (2.4 mL) in another tube and cooled to –40 ºC for 10 min. Then the substrate solution was added to the catalyst tube one-pot via syringe. A 0.2 mL aliquot was taken from the reaction mixture with a syringe after designated time and transferred to a tube containing MeOH (0.1 mL) frozen at –40 ºC. Subsequently, $TMSCHN_2$ (0.1 mL, 2 M in hexane) was added to the tube and stirred vigorously to quench the reaction. After concentration, the resulting reaction mixture was analyzed by ^{1}H NMR spectra with 1,3,5-trimethoxybenzene as the internal standard to determine the yield of (–)-**17** and the recoveries of **S17**. The residues were purification by the preparative TLC (Hexane/EtOAc 10:1) and the e.r. value were determined by HPLC analysis.

entry	t (min)	Yield of (–)-**17** (%)	e.r. of (–)-**17**	Recovery of (*S*)-**S17** (%)	e.r. of (*S*)-**S17**
1	5	3	88:12	97	>99.5:0.5
2	10	8	88.5:11.5	92	>99.5:0.5
3	15	12	89.5:10.5	88	>99.5:0.5
4	20	16	90:10	84	>99.5:0.5
5	30	24	90:10	76	>99.5:0.5
6	40	32	90:10	67	>99.5:0.5
7	50	41	90.5:9.5	58	>99.5:0.5
8	60	50	90.5:9.5	49	>99.5:0.5
9	90	70	91.5:8.5	28	>99.5:0.5
10	150	91	92.5:7.5	7	>99.5:0.5
11	240	98	93:7	0	/

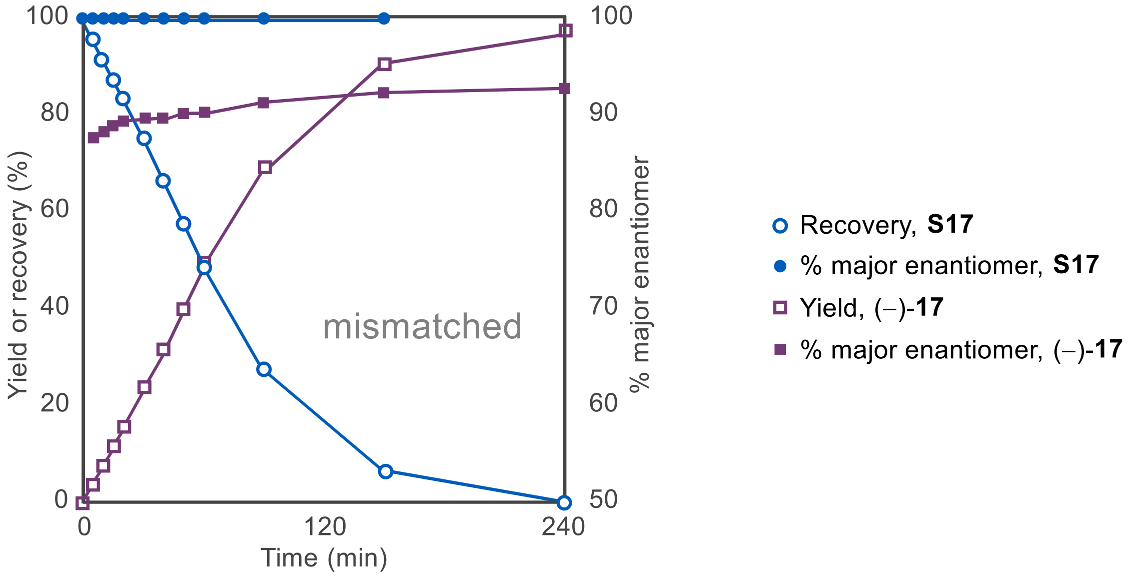

Supplementary Figure S14. Full kinetic profile of the decarboxylation from enantioenriched (*S*)-**S17**.

10.4 Determination of the absolute configuration of mismatched reactant (*S*)-S17

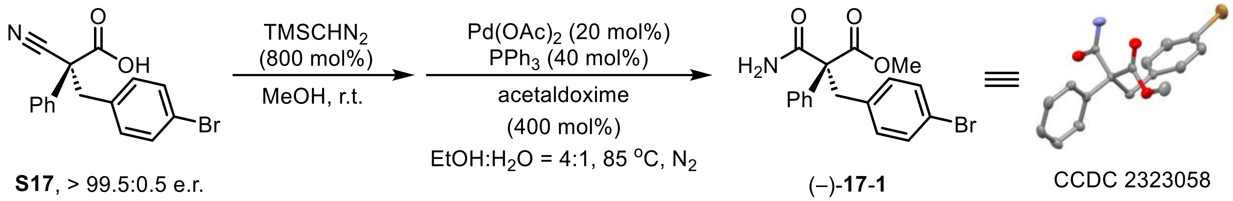

zwf-8-078

To a solution of **S17** (51.6 mg, 0.15 mmol, 100 mol%, >99.5:0.5 e.r.) in MeOH (2 mL) was added $TMSCHN_2$ (0.6 mL, 1.2 mmol, 800 mol%, 2 M in hexane), then the reaction mixture was stirred at room temperature for 1 h. The solvent was evaporated under vacuum, and the crude product was directly used for the next step.

To a solution of the crude ester (0.15 mmol) from above, $Pd(OAc)_2$ (6.8 mg, 0.03 mmol, 20 mol%), and PPh_3 (16.1 mg, 0.06 mmol, 40 mol%) in EtOH/H_2O (2 mL, 4:1) was added acetaldoxime (36 µL, 0.6 mmol, 400 mol%) at room temperature under N_2, then the reaction mixture was stirred at 85 ºC for 3 h and cooled to room temperature. The reaction mixture was filtered through a Celite pad and washed with EtOH/CH_2Cl_2 (10 mL/10 mL). The combined organic phase was evaporated under vacuum, and the residue was purified by flash column chromatography to obtain (–)-**17-1** (47.8 mg, 88% Yield) as a white solid, R_f = 0.5 (Hexane/EtOAc 1:1).

HPLC analysis (Chiralpak IB-3, hexane/^{i}PrOH = 90/10, 0.8 mL/min, 205 nm; t_r (minor) = 18.25 min, t_r (major) = 26.64 min) gave the isomeric composition of the product: >99.5:0.5 e.r., $[\alpha]_D^{16}$ = –38.9 (c = 0.79, $CHCl_3$).

^{1}H NMR (400 MHz, $CDCl_3$) δ 7.95 (br, 1H), 7.40-7.14 (m, 7H), 7.01 (d, J = 8.0 Hz, 2H), 5.78 (br, 1H), 3.99 (d, J = 12.8 Hz, 1H), 3.71 (s, 3H), 3.47 (d, J = 12.8 Hz, 1H).

^{13}C NMR (100 MHz, $CDCl_3$) δ 173.5, 171.8, 138.8, 135.6, 131.6, 131.3, 128.7, 128.0, 126.5, 121.0, 63.3, 52.7, 40.0.

IR (neat, cm^{-1}) 3419, 3177, 2949, 1724, 1676, 1485, 1199.

HRMS (ESI) calcd $C_{17}H_{17}BrNO_3^+$ $[M+H]^+$: 362.0386. Found: 362.0385.

10.5 KIE experiments

10.5.1 Experiment using a mixture of EtOH/EtOD

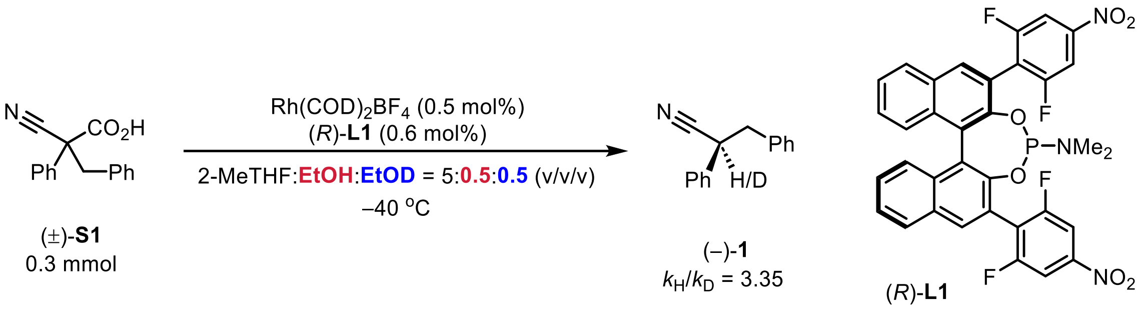

zwf-8-042

Following the general procedure, the reaction of **S1** (50.2 mg, 0.2 mmol), $Rh(COD)_2BF_4$ (0.4 mg, 0.001 mmol, 0.5 mol%), (R)-**L1** (0.8 mg, 0.0012 mmol, 0.6 mol%), 2-methyltetrahydrofuran (2-MeTHF, 20 mL), and EtOH:EtOD = 1:1 (2 mL/2 mL) for 2 h. The asymmetric decarboxylative protonation products were purified by flash column chromatography and analysis by ^{1}H NMR. The ratio of (–)-**1** to (–)-**1**-d is 3.35 (0.77:0.23).

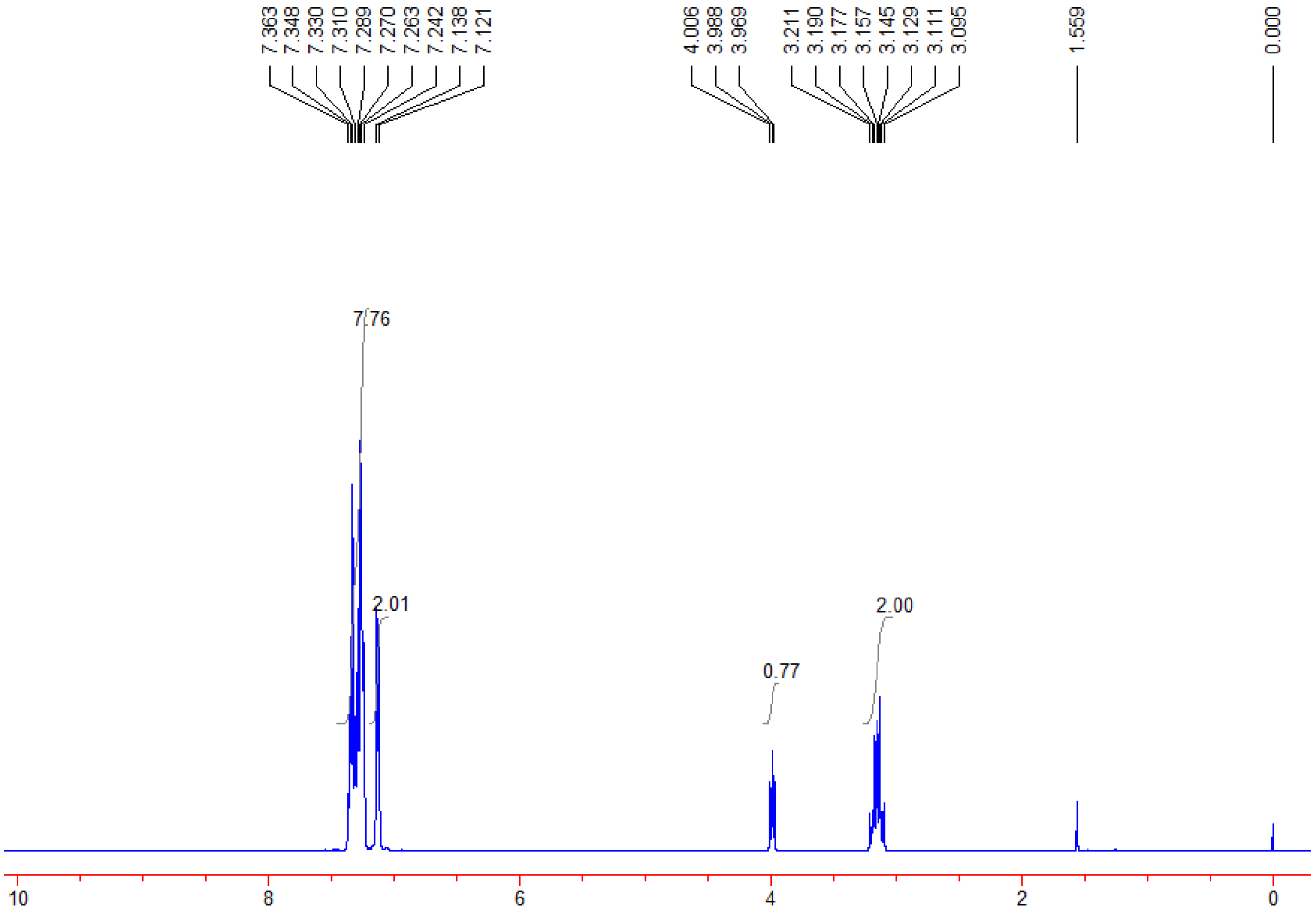

Supplementary Figure S15. ^{1}H NMR spectrum of (–)-**1** with mixed H/D solvent experiment.

10.5.2 Parallelled experiments

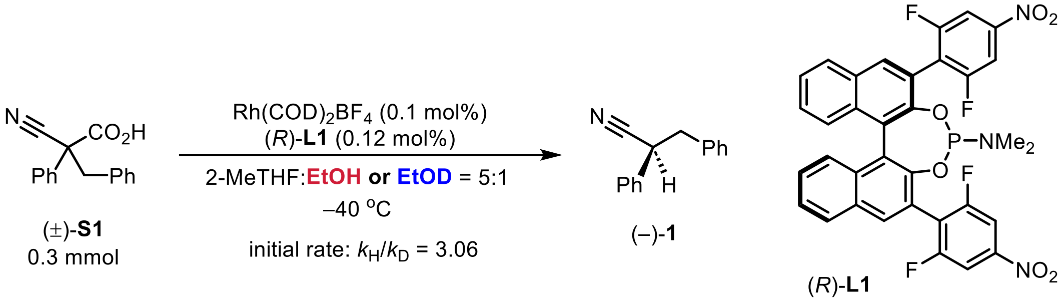

zwf-8-051 and zwf-8-080

To two oven-dried 100 mL Schlenk tubes were each added a catalyst solution of [$Rh(COD)_2BF_4$/(*R*)-**L1**] (0.0003 mmol/mL in 2-MeTHF, 1.0 mL, 0.0003 mmol, 0.1 mol%) and 2-methyltetrahydrofuran (1.4 mL, 2-MeTHF). After the reaction mixture was stirred at room temperature for 1 h, EtOH or EtOD (1.2 mL) was added to the reaction respectively and stirred for additional 10 min at room temperature. Then the pair of catalyst solutions were cooled to –40 °C and stirred for another 30 min. **S1** (150.8 mg, 0.6 mmol)

and 1,3,5-trimethoxybenzene (16.8 mg, 0.1 mmol) were dissolved in 2-MeTHF (7.2 mL) in another tube and cooled to −40 ºC for 10 min. Then 3.6 mL of substrate solution (0.3 mmol) was added to each catalyst tube via syringe. A 0.2 mL aliquot was taken from the reaction mixture with a syringe after 1min, 2 min, 3 min, 4 min, 5 min, and 6 min, and transferred to a tube containing MeOH (0.1 mL) frozen at −40 ºC. Subsequently, $TMSCHN_2$ (0.1 mL, 2 M in hexane) was added to the tube and stirred vigorously to quench the reaction. After concentration, the resulting reaction mixture was analyzed by ^1H NMR spectra with 1,3,5-trimethoxybenzene as the internal standard to determine the yield of (−)-**1** and the recoveries of **S1**. The KIE value (k_H/k_D) was determined to be 3.06 after kinetic analysis.

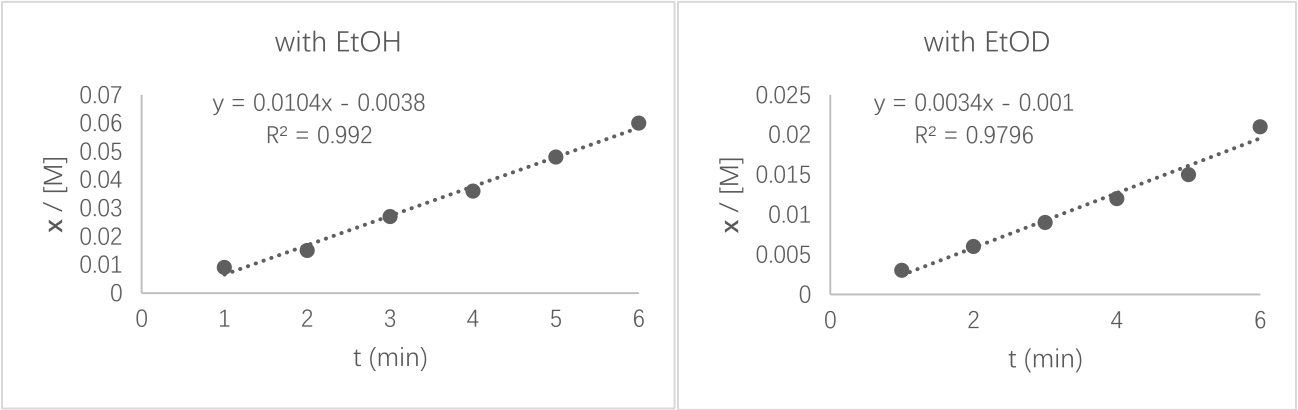

Supplementary Figure S16. Initial reaction rate of competitive experiment.

10.6 H/D exchange experiments of S1 with EtOD

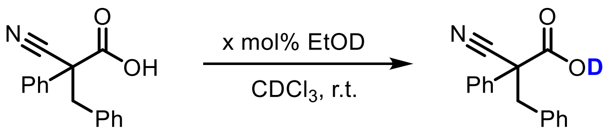

To four NMR tubes were added **S1** (25.1 mg, 0.1 mmol) and $CDCl_3$ (0.5 mL), respectively. After fully dissolved of **S1** in the solvent, 0 mol%, 500 mol%, 1000 mol%, 5000 mol% of EtOD were added to each tube, which were directly used for ^1H NMR experiments to investigate the H/D exchange. The exchange proceeds swiftly in all cases with added EtOD.

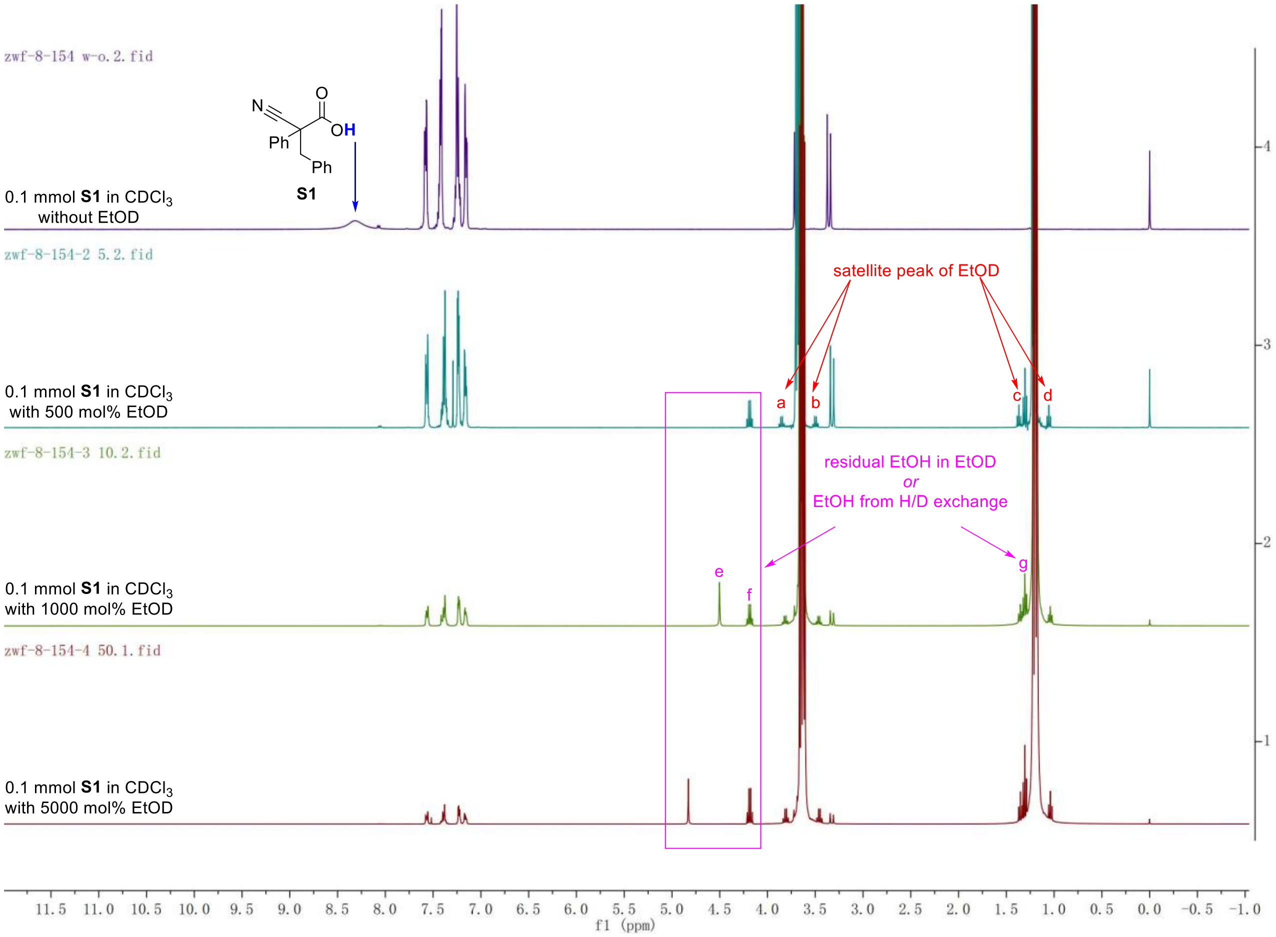

Supplementary Figure S17. ^{1}H NMR spectrum of H/D exchange experiments.

10.7 Monitoring of nitrile hydrolysis

10.7.1 From non-deuterated decarboxylation product (–)-1

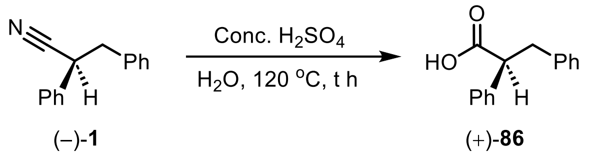

zwf-11-093

To a solution of (–)-**1** (41.5 mg, 0.2 mmol, 100 mol%, 97.5:2.5 e.r.) and 1,3,5-trimethoxybenzene (11.2 mg, 0.067 mmol) in H_2O (0.5 mL) was added concentrated sulfuric acid (0.4 mL) at room temperature, then the reaction mixture was stirred at 120 °C. Eight parallel experiments of the same scale were conducted and stopped at the designated time. The reaction mixture was extracted with diethyl ether (2 mL × 5). The combined organic phase was washed with brine, dried over $MgSO_4$, filtered, and evaporated under vacuum. The resulting reaction mixture was analyzed by ^{1}H NMR spectra with 1,3,5-trimethoxybenzene as the internal standard to determine the yield of (+)-**86** and the recoveries of (–)-**1**. The residues were purification by the preparative TLC (Hexane/EtOAc 10:1) and the e.r. value were

determined by HPLC analysis.

entry	t (h)	Yield, (+)-**86** (%)	e.r., (+)-**86**	Recovery, (–)-**1** (%)	e.r., (–)-**1**
1	0.5	45	96:4	54	90:10
2	1	65	95.5:4.5	34	85:15
3	1.5	78	95.5:4.5	21	79:21
4	2	88	93:7	11	56.5:43.5
5	4	97	90:10	0	
6	6	97	88:12		
7	8	95	85:15		
8	10	95	81.5:18.5		

10.7.2 From deuterated decarboxylation product (–)-1-d

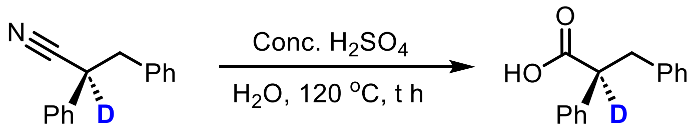

(–)-**1**-d (+)-**86**-d

zwf-11-107

To a solution of (–)-**1**-d (41.7 mg, 0.2 mmol, 100 mol%, 96% D, 97:3 e.r.) and 1,3,5-trimethoxybenzene (11.2 mg, 0.067 mmol) in H_2O (0.5 mL) was added concentrated sulfuric acid (0.4 mL) at room temperature, then the reaction mixture was stirred at 120 °C. Eight parallel experiments of the same scale were conducted and stopped at the designated time. The reaction mixture was extracted with diethyl ether (2 mL × 5). The combined organic phase was washed with brine, dried over $MgSO_4$, filtered, and evaporated under vacuum. The resulting reaction mixture was analyzed by ^{1}H NMR spectra with 1,3,5-trimethoxybenzene as the internal standard to determine the yield of (–)-**86**-d and the recoveries of (–)-**1**-d. The residues were purification by the preparative TLC (Hexane/EtOAc 10:1) and the e.r. value were determined by HPLC analysis.

entry	t (h)	Yield, (+)-**86** (%)	e.r., (+)-**86**	D%, (+)-**86**-d	Recovery, (−)-**1** (%)	e.r., (−)-**1**	D%, (−)-**1**-d
1	0.5	47	97:3	96	53	95:5	96
2	1	73	97:3	96	25	92:8	96
3	1.5	85	97:3	95	14	86:14	95
4	2	90	97:3	94	7	84.5:15.5	95
5	4	97	96.5:3.5	93	0		
6	6	96	96:4	92			
7	8	96	95.5:4.5	92			
8	10	95	95:5	91			

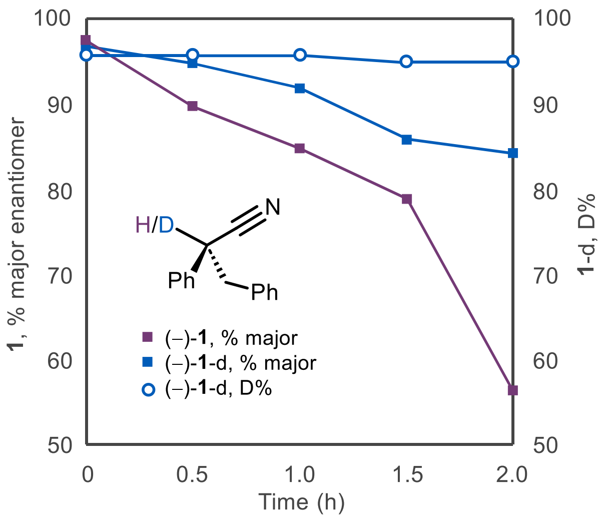

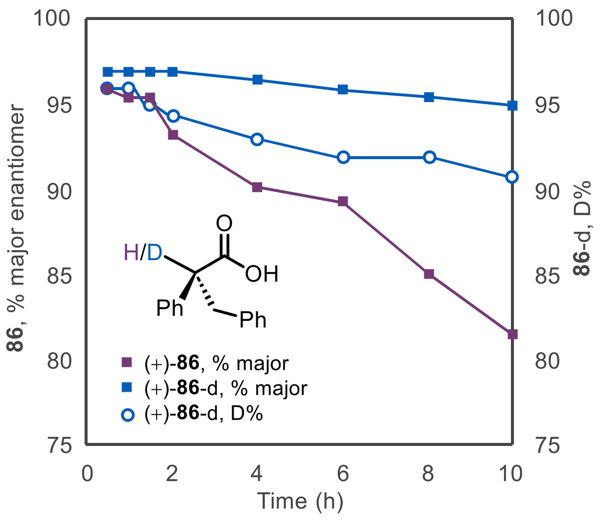

Section 11. Density functional theory (DFT) studies

11.1 Computational methods

All density functional theory (DFT) calculations were performed using the Gaussian 16 software package.[13] Geometries were optimized using the ωB97XD[14] functional with a mixed basis set of LANL2DZ for Rh and 6-31G(d) for other atoms. Vibrational frequencies were calculated for all the stationary points to confirm if each optimized structure is a local minimum on the respective potential energy surface or a transition state structure with only one imaginary frequency. Solvation energy corrections were calculated in tetrahydrofuran solvent with the SMD continuum solvation model[15] based on the gas phase optimized geometries. The ωB97XD functional with a mixed basis set of SDD for Rh and 6-311+G(d, p) for other atoms were used for single-point energy calculations. Three dimensional structures of optimized transition states were plotted using CYLview[16] and POV-Ray[17]. The intrinsic reaction coordinate (IRC) calculations were carried out to verify the located transition states connect the correct intermediates.

11.2 Comparison of two pathways prior to CO_2 extrusion with different Rh(I) complexes

Two pathways prior to CO_2 extrusion were considered. When the cyanoacetic acid substrate approaches the rhodium catalyst at its ground state (**Int-1**), a hydrogen-bonded complex (**Int-2S**) can be formed between the rhodium complex and the acid via a solvent bridge. Subsequently, proton transfer occurs between the acid and the amine moiety of the ligand, affording **Int-3S**. Alternatively, a pathway involving solvent dissociation and acid association is also possible. Although the catalyst–acid complex (**Int-2S'**) is lower in energy than the hydrogen-bonded complex, the dissociation of one ethanol ligand from **Int-1** (to form **Int-A**) is highly exergonic and therefore disfavored.

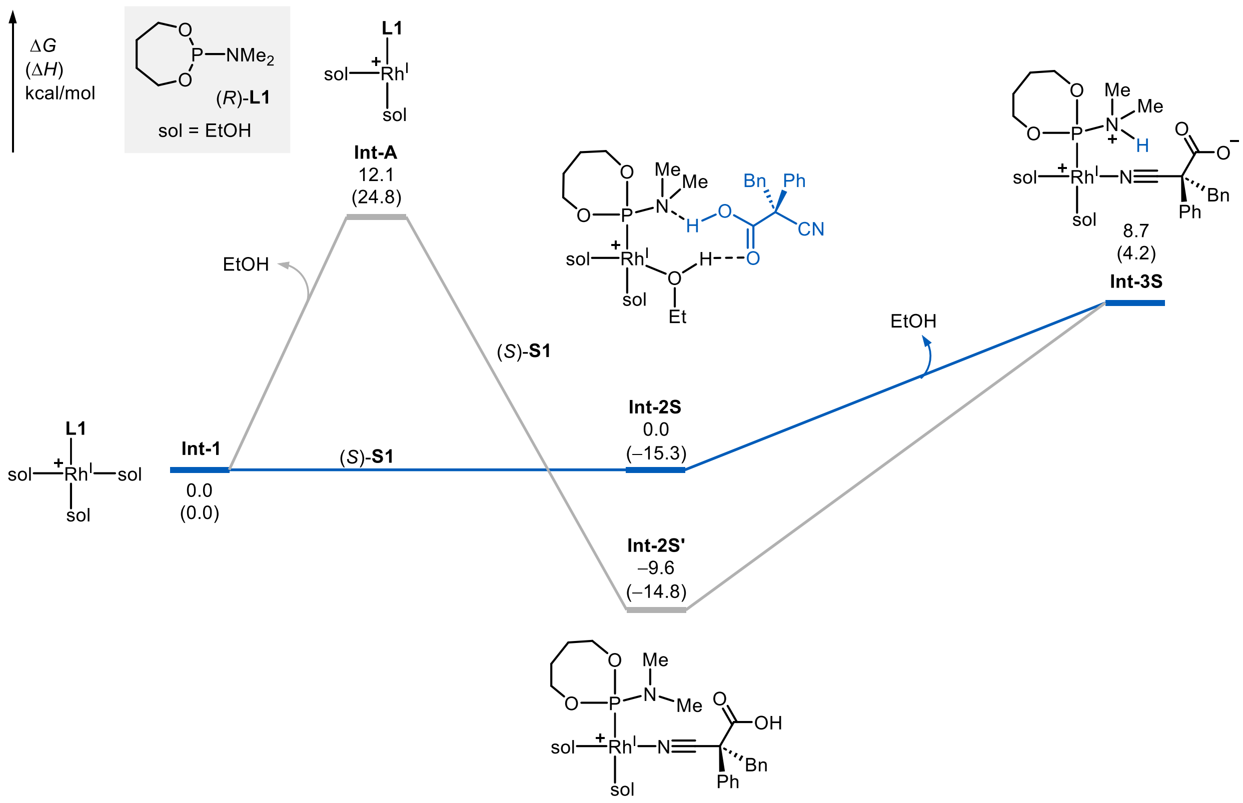

Supplementary Figure S18. Calculated relative free energies of Rh(I) complexes and their pathways. All energies were calculated at ωB97XD/6-311+G(d,p)–SDD/SMD(THF)//ωB97XD/6-31G(d)–LANL2DZ level of theory and relative to Rh(I) complex **Int-1**.

Since the experiments showed that the deuterium source of deuteration is EtOD, we also investigated the pathway of protonation with ligated ethanol (Figure S19). All the energy data were calculated at the same level of theory as provided in the manuscript. The higher activation free energy of **TS-4R** ($\Delta G^{\ddagger}$ = 9.9 kcal/mol) compared to **TS-2Re** ($\Delta G^{\ddagger}$ = 4.3 kcal/mol, Figure 3 in the manuscript) indicates this reaction pathway is kinetically disfavored. Moreover, the higher relative free energy of **TS-4R** than **TS-4S** suggests that the favored enantiomer calculated here is opposite to that observed in the experiment. Therefore, this protonation pathway with ethanol that coordinates with the metal center can be ruled out. Though this pathway is ruled out, the deuteration of the cyanoacetic acid is reasonable as described in manuscript, given that the amount of deuterated ethanol is 342.5 times greater than that of the cyanoacetic **S1** (Equation 1). The experimental data are collected from the general procedure for asymmetric decarboxylative deuteration in Section 6.

$$Ratio = \frac{amount\ of\ \boldsymbol{EtOD}}{amount\ of\ \boldsymbol{S1}} = \frac{\frac{4\ ml\ \times 0.806\ g/ml}{47.07\ g/mol}}{0.2\ mmol} = 342.5 \qquad (1)$$

11.3 Free energy profile of protonation with ethanol

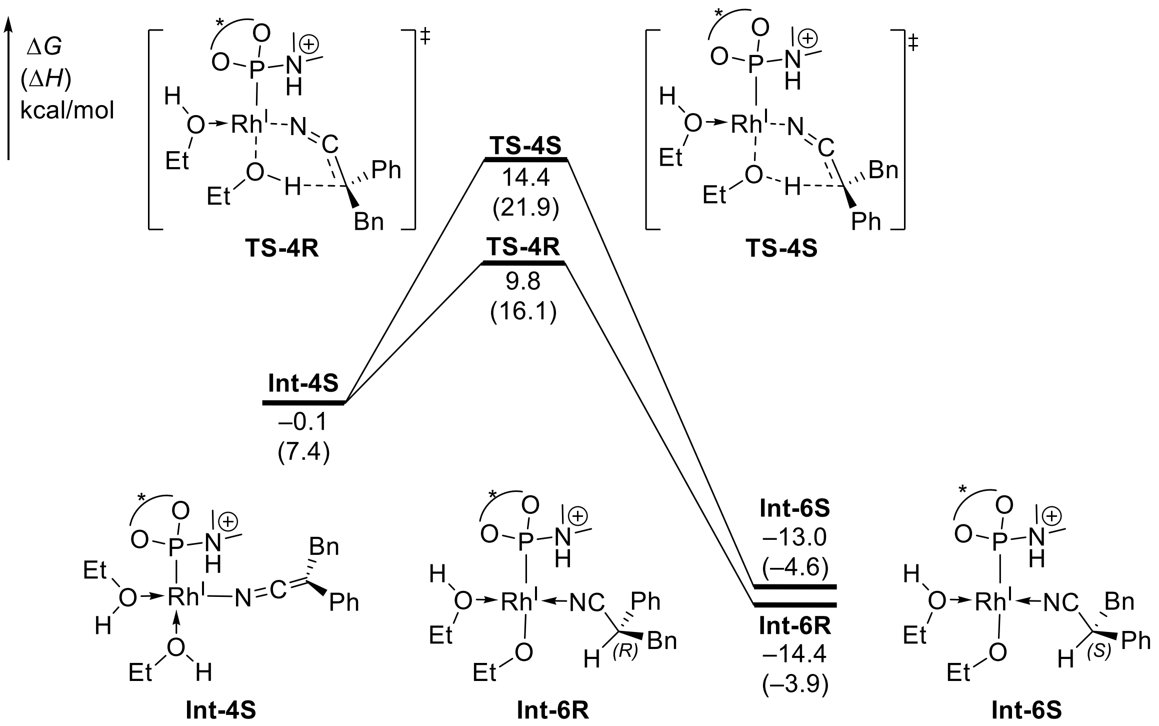

Supplementary Figure S19. Free energy profile of protonation with ethanol that coordinates with the metal center. All energies were calculated at ωB97XD/6-311+G(d,p)–SDD/SMD(THF)//ωB97XD/6-31G(d)–LANL2DZ level of theory. The chiral ligand (*R*)-**L1** is used in DFT calculation.

The simulation of H/D exchange between cyanoacetic acid **S1** and EtOD (via **TS-3**) indicates that the H/D exchange is a facile process as the activation free energy is only 12.4 kcal/mol (Supplementary Figure S20). Consistent with the NMR experiment (Supplementary Figure S17), these data indicated that under a large difference of concentration (H/D = 1/342.5), the deuterated cyanoacetic acid **S1-D** can form rapidly once it is added to the reaction system, which paves the way for following decarboxylative deuteration.

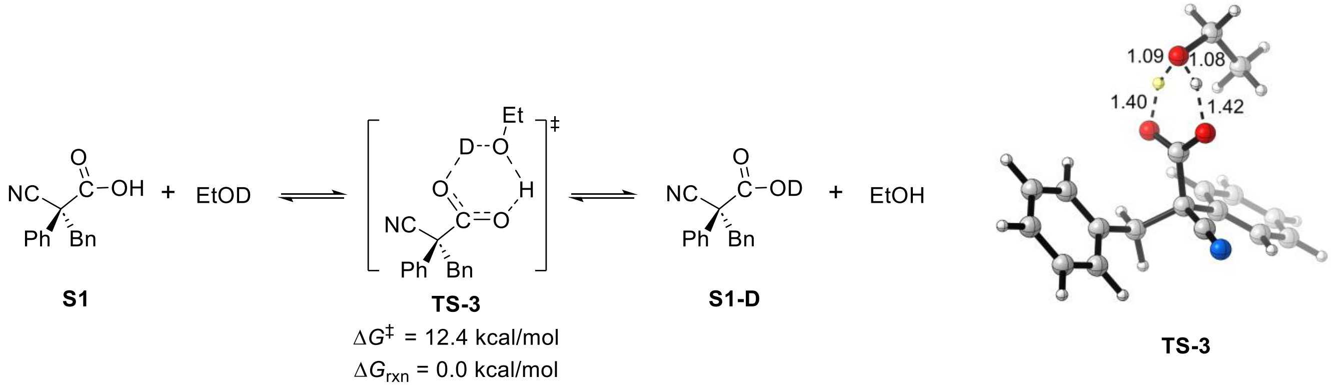

Supplementary Figure S20. Calculated activation free energy for H/D exchange between **S1** and deuterated ethanol. All energies were calculated at ωB97XD/6-311+G(d,p)–SDD/SMD(THF)//ωB97XD/6-31G(d)–LANL2DZ level of theory.

11.4 Quantitative steric-electronic effect dissection of the asymmetric protonation step

To further clarify the origin of the enantioselectivity in the protonation step, quantitative steric-electronic effect dissection[18] of protonation transition states **TS-2Re** and **TS-2Si** was performed using distortion interaction analysis and energy decomposition analysis based on absolutely localized molecular orbitals (ALMO-EDA).[19-22] The EDA calculation was performed using with Q-Chem[23] software package. In the EDA calculation, the ketenimine moiety is separated as an individual fragment and the rest part of the transition state structure is separated as the other fragment. The comparison results between **TS-2Re** and **TS-2Si** were summarized in Supplementary Figure S21. As shown in the pie chart, the steric effect is the dominant factor that promotes transition state **TS-2Re**. Combined with the structural analysis, the greater steric repulsion in **TS-2Si** can be attributed to the relatively larger size of the benzyl group compared to the phenyl group.

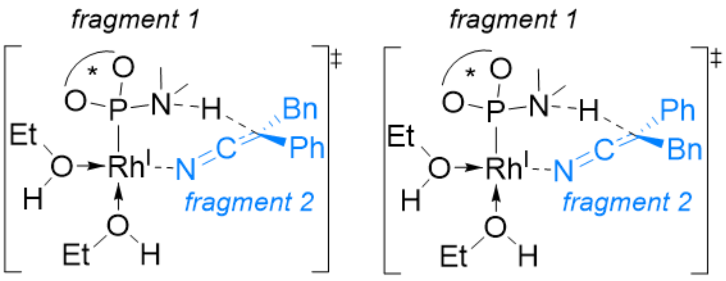

TS-2Si **TS-2Re**

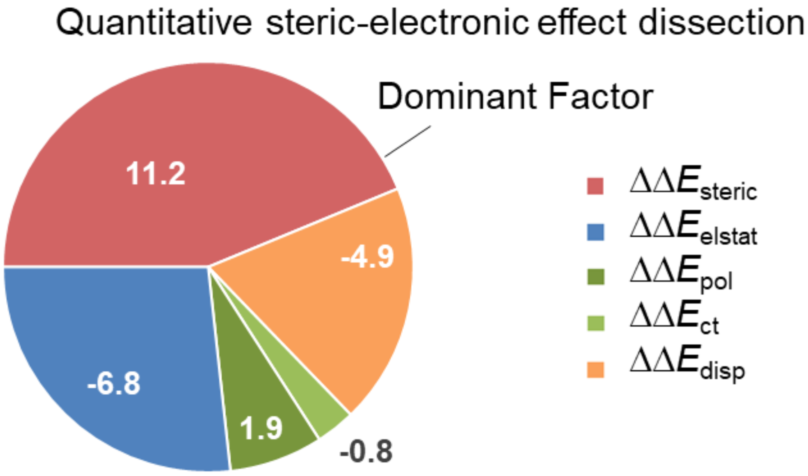

$\Delta\Delta E = \Delta E(\textbf{TS-2Si}) - \Delta E(\textbf{TS-2Re})$.

Supplementary Figure S21. Quantitative steric-electronic effect dissection of protonation transition states **TS-2Re** and **TS-2Si**. All energies were calculated at ωB97XD/6-311+G(d,p) –SDD/SMD(THF) level of theory. The energies are scaled in kcal/mol.

11.5 Calculation results using alternative functionals

Calculation of the reaction pathways using alternative functionals has been carried out. Results using M06/6-311+G(d,p)–SDD/SMD(THF)//ωB97XD/6-31G(d)–LANL2DZ and M06L/6-311+G(d,p)–SDD/SMD(THF)//ωB97XD/6-31G(d)–LANL2DZ level of theory have been obtained. These data and those calculated using the selected ωB97XD/6-311+G(d,p)–SDD/SMD(THF)//ωB97XD/6-31G(d)–LANL2DZ all demonstrated similar trends of energies for the asymmetric decarboxylation.

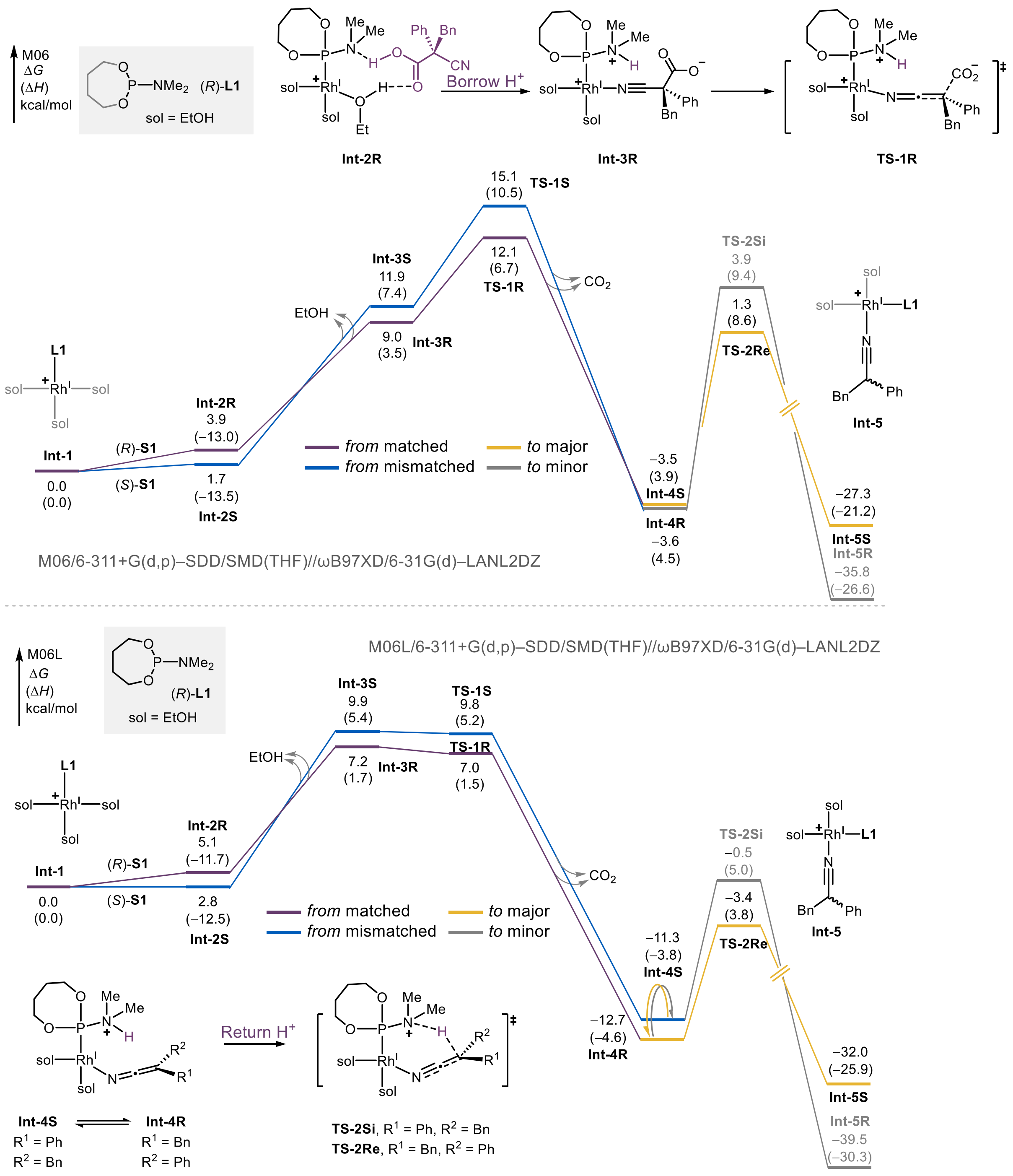

Supplementary Figure S22. DFT studies of the decarboxylative protonation pathway using alternative functionals. Energies were calculated at M06/6-311+G(d,p)–SDD/SMD(THF)//ωB97XD/6-31G(d)–LANL2DZ and M06L/6-311+G(d,p)–SDD/SMD(THF)//ωB97XD/6-31G(d)–LANL2DZ level of theory, respectively. The energies are scaled in kcal/mol.

Cartesian coordinates (Å) and energies of optimized structures

S1

ωB97XD SCF energy: -822.46579797 a.u.
ωB97XD enthalpy: -822.193764 a.u.
ωB97XD free energy: -822.255362 a.u.
ωB97XD SCF energy in solution: -822.46579797 a.u.
ωB97XD enthalpy in solution: -822.193764 a.u.
ωB97XD free energy in solution: -822.255362 a.u.

Cartesian coordinates

ATOM	X	Y	Z
C	-0.939028	-0.850987	0.042151
C	-1.080399	0.661672	0.206520
C	-0.666269	1.317630	1.364137
C	-1.555233	1.418014	-0.870585
C	-0.729300	2.707733	1.449350
H	-0.280993	0.753855	2.208200
C	-1.618742	2.803608	-0.784032
H	-1.876105	0.926521	-1.783084
C	-1.204874	3.454550	0.377600
H	-0.399681	3.201884	2.358349
H	-1.991695	3.376339	-1.628006
H	-1.252338	4.537544	0.443242
C	0.142401	-1.197708	-1.049873
H	0.177272	-2.284247	-1.161577
H	-0.218707	-0.777669	-1.993477
C	1.514551	-0.664490	-0.717996
C	1.899629	0.625136	-1.097579
C	2.419776	-1.454941	-0.003832
C	3.158914	1.115928	-0.764351
H	1.207414	1.250393	-1.654858
C	3.679887	-0.965711	0.331663
H	2.135618	-2.461945	0.291079

C	4.051872	0.322174	-0.046723
H	3.442824	2.119890	-1.066756
H	4.371451	-1.593316	0.886420
H	5.034803	0.704746	0.213069
C	-0.534163	-1.494045	1.303892
N	-0.200172	-2.010329	2.284024
C	-2.274826	-1.512758	-0.376938
O	-3.299369	-0.932196	-0.625761
O	-2.141909	-2.840988	-0.445531
H	-3.003624	-3.211056	-0.710854

S1-D

ωB97XD SCF energy: -822.44324473 a.u.

ωB97XD enthalpy: -822.174117 a.u.

ωB97XD free energy: -822.236665 a.u.

ωB97XD SCF energy in solution: -822.68812051 a.u.

ωB97XD enthalpy in solution: -822.418993 a.u.

ωB97XD free energy in solution: -822.481541 a.u.

Cartesian coordinates

ATOM	X	Y	Z
C	-1.003083	-0.803122	0.053755
C	-1.037751	0.714763	0.226982
C	-0.498867	1.325738	1.357393
C	-1.514037	1.516300	-0.815434
C	-0.441416	2.714268	1.450260
H	-0.108310	0.721359	2.169684
C	-1.456278	2.901357	-0.720800
H	-1.942035	1.062090	-1.701826
C	-0.918296	3.506344	0.412961
H	-0.015776	3.172146	2.337780
H	-1.835628	3.509433	-1.536470
H	-0.872147	4.588821	0.484660
C	0.047301	-1.202221	-1.051878
H	0.017751	-2.288899	-1.170098
H	-0.298357	-0.755694	-1.990027
C	1.448460	-0.742703	-0.733865
C	1.895633	0.521140	-1.127810
C	2.312884	-1.570645	-0.014189

C	3.179633	0.948900	-0.807538
H	1.228376	1.179091	-1.677924
C	3.597645	-1.143875	0.308606
H	1.974653	-2.553374	0.303266
C	4.033870	0.117098	-0.087813
H	3.512399	1.934241	-1.119953
H	4.257210	-1.798656	0.870045
H	5.036708	0.450532	0.162281
C	-0.628413	-1.479516	1.307601
N	-0.317798	-2.017324	2.283822
C	-2.369655	-1.379937	-0.379484
O	-3.334013	-0.746259	-0.718477
O	-2.340594	-2.721418	-0.373175
H(iso=2)	-3.219175	-3.021742	-0.657433

CO2

ωB97XD SCF energy: -188.51999993 a.u.

ωB97XD enthalpy: -188.504600 a.u.

ωB97XD free energy: -188.528881 a.u.

ωB97XD SCF energy in solution: -188.58249216 a.u.

ωB97XD enthalpy in solution: -188.567092 a.u.

ωB97XD free energy in solution: -188.591373 a.u.

Cartesian coordinates

ATOM	X	Y	Z
C	0.000000	0.000000	0.000000
O	0.000000	0.000000	1.164690
O	0.000000	0.000000	-1.164690

EtOD

ωB97XD SCF energy: -154.98233653 a.u.

ωB97XD enthalpy: -154.899127 a.u.

ωB97XD free energy: -154.930052 a.u.

ωB97XD SCF energy in solution: -154.98233653 a.u.

ωB97XD enthalpy in solution: -154.899127 a.u.

ωB97XD free energy in solution: -154.930052 a.u.

Cartesian coordinates

ATOM	X	Y	Z
C	-1.219238	-0.220235	-0.000050
H	-2.069802	0.469436	0.000041
H	-1.284707	-0.857886	-0.886965
H	-1.284703	-0.858073	0.886732
C	0.089951	0.545776	0.000046
H	0.141388	1.196625	-0.887547
H	0.141280	1.196586	0.887680
O	1.144221	-0.398016	0.000104
H(iso=2)	1.978498	0.084190	-0.000751

EtOH

ωB97XD SCF energy: -154.98233653 a.u.

ωB97XD enthalpy: -154.895938 a.u.

ωB97XD free energy: -154.926463 a.u.

ωB97XD SCF energy in solution: -154.98233653 a.u.

ωB97XD enthalpy in solution: -154.895938 a.u.

ωB97XD free energy in solution: -154.926463 a.u.

Cartesian coordinates

ATOM	X	Y	Z
C	-1.219238	-0.220235	-0.000050
H	-2.069802	0.469436	0.000041
H	-1.284707	-0.857886	-0.886965
H	-1.284703	-0.858073	0.886732
C	0.089951	0.545776	0.000046
H	0.141388	1.196625	-0.887547
H	0.141280	1.196586	0.887680
O	1.144221	-0.398016	0.000104
H	1.978498	0.084190	-0.000751

Int-1

ωB97XD SCF energy: -3237.44324405 a.u.

ωB97XD enthalpy: -3236.642535 a.u.

ωB97XD free energy: -3236.792327 a.u.

ωB97XD SCF energy in solution: -3239.39940546 a.u.

ωB97XD enthalpy in solution: -3238.598696 a.u.
ωB97XD free energy in solution: -3238.748488 a.u.

Cartesian coordinates

ATOM	X	Y	Z
C	2.306331	0.874899	-0.167434
F	4.598024	-1.334292	-2.494890
N	4.916085	-5.093469	0.460563
O	1.426970	-0.018727	0.411032
P	0.106584	-0.596519	-0.415283
Rh	-1.327997	-1.337499	1.061766
C	3.603021	0.410459	-0.510676
F	3.481281	-0.801566	2.035588
N	-6.220802	-0.733075	-2.406430
C	3.979150	-0.997840	-0.251458
F	-2.297307	1.984387	-2.845664
N	0.591161	-1.492185	-1.717024
O	4.739569	-5.515036	1.590301
C	4.462143	-1.834558	-1.260825
F	-3.869051	0.890566	1.438447
O	-0.330709	0.778853	-1.226813
C	4.780537	-3.167604	-1.061368
H	5.142661	-3.788162	-1.870820
O	-6.254209	-0.811847	-3.621606
C	4.612320	-3.672663	0.218196
O	-6.999454	-1.274372	-1.639400
C	4.165573	-2.900390	1.279303
H	4.068642	-3.314343	2.274472
C	3.873845	-1.574097	1.017222
C	4.472412	1.295313	-1.098887
H	5.472214	0.968146	-1.367986
C	4.093023	2.629985	-1.381409
C	2.790761	3.082204	-1.027109
C	1.903762	2.175106	-0.364092
C	0.539859	2.570435	0.077131
C	-0.544841	1.873149	-0.408910
C	-1.884334	2.211596	-0.077095
C	-3.012162	1.463270	-0.676471
C	-3.179826	1.372066	-2.061163
C	-4.221262	0.676622	-2.654591
H	-4.320288	0.623614	-3.731045

C	-5.131632	0.058391	-1.811749
C	-5.041534	0.119051	-0.429708
H	-5.774833	-0.366668	0.200262
C	-3.978503	0.826197	0.103250
C	-2.098036	3.240764	0.806595
H	-3.113485	3.520165	1.071313
C	-1.019295	3.938916	1.402845
C	0.317414	3.607136	1.041857
C	1.382642	4.279737	1.698286
H	2.406917	4.021595	1.453972
C	1.126339	5.241668	2.641770
H	1.952566	5.742031	3.136660
C	-0.202216	5.589761	2.978289
H	-0.386181	6.359285	3.720808
C	-1.251008	4.950341	2.372163
H	-2.275861	5.203143	2.628960
C	2.415418	4.405749	-1.377031
H	1.415644	4.756762	-1.146443
C	3.300027	5.238195	-2.014320
H	2.994394	6.245553	-2.278672
C	4.603731	4.797773	-2.337927
H	5.291892	5.471272	-2.838036
C	4.988426	3.519387	-2.031227
H	5.982322	3.164328	-2.288678
C	1.180024	-0.928061	-2.933367
H	2.270734	-1.024674	-2.925433
H	0.914081	0.123423	-3.028710
H	0.785757	-1.469121	-3.800381
C	0.882427	-2.906156	-1.511021
H	1.963280	-3.086184	-1.461188
H	0.473866	-3.494643	-2.341338
H	0.439370	-3.251994	-0.572891
O	5.312253	-5.747316	-0.487921
O	-2.731513	-1.901871	2.650690
C	-4.106753	-2.274301	2.433723
C	-4.683199	-2.963477	3.653922
H	-4.084344	-2.936944	1.567994
H	-4.677430	-1.379176	2.167586
H	-5.714449	-3.271329	3.454671
H	-4.098256	-3.851296	3.909356
H	-4.696928	-2.293297	4.520423

H	-2.697427	-1.187764	3.302887
O	-2.291643	-2.751612	-0.204164
C	-2.669911	-2.458224	-1.571603
C	-3.932755	-3.201290	-1.947766
H	-1.831535	-2.695700	-2.233278
H	-2.814246	-1.379251	-1.588753
H	-4.235618	-2.922190	-2.962388
H	-3.784340	-4.286214	-1.932209
H	-4.748871	-2.951504	-1.263185
H	-1.924503	-3.643951	-0.150421
O	-0.673645	0.057692	2.511764
C	0.505009	-0.239472	3.317277
C	1.186670	1.043755	3.737566
H	1.176603	-0.861072	2.726495
H	0.130908	-0.810070	4.169429
H	2.032085	0.812515	4.391585
H	1.579335	1.574289	2.863142
H	0.502624	1.705257	4.278831
H	-0.540367	0.919820	2.087529

Int-2S

ωB97XD SCF energy: -4059.93118069 a.u.

ωB97XD enthalpy: -4058.855504 a.u.

ωB97XD free energy: -4059.042589 a.u.

ωB97XD SCF energy in solution: -4062.11505264 a.u.

ωB97XD enthalpy in solution: -4061.039376 a.u.

ωB97XD free energy in solution: -4061.226461 a.u.

Cartesian coordinates

ATOM	X	Y	Z
C	-3.323253	0.833530	-1.089668
F	-2.539597	4.538688	-2.147733
N	-2.490196	6.370341	2.282334
O	-2.592261	0.669869	0.069447
P	-1.021670	0.167778	0.123750
Rh	-0.703559	-0.736961	2.083397
C	-3.798825	2.141628	-1.371848
F	-4.468321	2.102856	1.374113
N	4.172500	-3.628324	0.537834

C	-3.500823	3.249216	-0.435296
F	0.666220	-2.609506	-2.567384
N	-0.049427	1.402592	-0.503909
O	-2.764080	6.192910	3.456503
C	-2.869936	4.422742	-0.855874
F	-0.444643	-3.998314	1.774033
O	-0.975618	-0.801256	-1.212859
C	-2.532544	5.458001	-0.000332
H	-2.033303	6.348038	-0.360565
O	4.967937	-3.447196	-0.370578
C	-2.855005	5.299697	1.338065
O	4.459091	-3.887723	1.690463
C	-3.500595	4.175764	1.829871
H	-3.758040	4.089352	2.877341
C	-3.819123	3.179687	0.924423
C	-4.513111	2.337397	-2.526803
H	-4.888752	3.327823	-2.765468
C	-4.764603	1.275432	-3.429688
C	-4.307644	-0.035572	-3.117791
C	-3.615586	-0.252702	-1.882154
C	-3.198126	-1.610132	-1.439534
C	-1.874710	-1.850556	-1.152893
C	-1.393377	-3.116527	-0.723369
C	0.043218	-3.287477	-0.414201
C	1.037219	-3.001045	-1.350463
C	2.388469	-3.112602	-1.072835
H	3.129907	-2.902911	-1.831659
C	2.743258	-3.520056	0.203309
C	1.812827	-3.832547	1.183359
H	2.119004	-4.149356	2.171647
C	0.479555	-3.710604	0.842059
C	-2.299081	-4.136993	-0.560864
H	-1.955263	-5.115557	-0.239138
C	-3.686214	-3.931300	-0.772452
C	-4.151261	-2.656075	-1.203239
C	-5.552528	-2.450720	-1.311524
H	-5.927030	-1.477707	-1.607749
C	-6.432965	-3.466197	-1.036391
H	-7.500329	-3.288342	-1.119612
C	-5.967122	-4.742360	-0.645045
H	-6.677260	-5.537231	-0.442121

C	-4.621891	-4.968000	-0.514408
H	-4.251688	-5.940565	-0.202537
C	-4.532660	-1.072569	-4.060342
H	-4.163069	-2.071206	-3.854505
C	-5.198133	-0.819800	-5.233170
H	-5.355104	-1.623513	-5.945526
C	-5.675648	0.477160	-5.529006
H	-6.203681	0.658351	-6.459391
C	-5.459205	1.502293	-4.646290
H	-5.809098	2.506441	-4.868562
C	-0.233576	1.853476	-1.897989
H	-1.080325	2.539540	-1.995402
H	-0.382382	0.996467	-2.553654
H	0.674523	2.381115	-2.205913
C	0.153137	2.538201	0.414496
H	-0.664306	3.262203	0.321144
H	1.100380	3.027969	0.177298
H	0.192814	2.195183	1.450483
O	-1.934269	7.351828	1.822734
O	-0.619724	-1.694887	4.070495
C	-0.679135	-0.833648	5.227270
C	-1.951532	-0.009229	5.265907
H	0.202582	-0.196354	5.142895
H	-0.571916	-1.459069	6.119052
H	-1.955006	0.633728	6.151292
H	-2.023025	0.624384	4.374629
H	-2.839213	-0.651389	5.316236
H	-1.392358	-2.279951	4.067882
O	1.185247	0.046128	2.545901
C	2.211809	-0.866739	3.021222
C	3.189266	-0.135550	3.915404
H	2.720684	-1.305081	2.157112
H	1.682866	-1.658767	3.551820
H	3.970750	-0.828717	4.239987
H	3.674883	0.678876	3.371719
H	2.685345	0.271896	4.797554
H	1.634035	0.626330	1.893458
O	-2.551682	-1.813928	2.056210
C	-3.887704	-1.251968	2.097099
C	-4.891126	-2.322178	2.468751
H	-4.113057	-0.799221	1.130811

H	-3.831872	-0.456801	2.841026
H	-5.892476	-1.883529	2.513814
H	-4.914600	-3.121597	1.719444
H	-4.661406	-2.760346	3.444932
H	-2.525119	-2.530969	1.401324
C	4.812745	0.571783	-0.442579
C	5.411400	1.980128	-0.548103
C	6.565637	2.337688	0.141500
C	4.820882	2.890012	-1.427709
C	7.124461	3.598968	-0.046632
H	7.041000	1.630744	0.814241
C	5.378788	4.149896	-1.611004
H	3.929474	2.612192	-1.986593
C	6.534303	4.506685	-0.919770
H	8.028493	3.867559	0.490271
H	4.914372	4.851120	-2.297380
H	6.973763	5.488441	-1.064560
C	5.125183	-0.259394	-1.725599
H	4.709146	-1.262151	-1.595802
H	4.597414	0.217090	-2.556218
C	6.605906	-0.338619	-2.013323
C	7.212672	0.602312	-2.849118
C	7.393504	-1.328266	-1.420447
C	8.582309	0.560233	-3.085604
H	6.609617	1.380630	-3.309840
C	8.765097	-1.369484	-1.655786
H	6.933947	-2.071388	-0.775058
C	9.362333	-0.425980	-2.486791
H	9.040183	1.297546	-3.737954
H	9.366083	-2.144555	-1.190234
H	10.431764	-0.461126	-2.671118
C	5.313265	-0.152952	0.738573
N	5.650568	-0.768146	1.658807
C	3.311433	0.742449	-0.164860
O	2.911763	1.198677	0.893613
O	2.508221	0.393146	-1.148492
H	1.577975	0.612418	-0.863256

Int-2R

ωB97XD SCF energy: -4059.93491465 a.u.
ωB97XD enthalpy: -4058.858753 a.u.
ωB97XD free energy: -4059.043278 a.u.
ωB97XD SCF energy in solution: -4062.11461816 a.u.
ωB97XD enthalpy in solution: -4061.038457 a.u.
ωB97XD free energy in solution: -4061.222982 a.u.

Cartesian coordinates

ATOM	X	Y	Z
C	3.072878	0.385637	0.989272
F	3.075536	4.299286	1.414437
N	3.764535	5.343640	-3.212840
O	2.415622	0.187738	-0.209608
P	0.779263	0.070802	-0.343388
Rh	0.286161	-1.007819	-2.168958
C	3.806938	1.592287	1.132175
F	4.653361	0.962741	-1.491078
N	-5.117236	-2.871667	-0.884178
C	3.839132	2.568850	0.019457
F	-1.813844	-1.831697	2.443170
N	0.093848	1.570593	0.018738
O	4.080013	4.923084	-4.312197
C	3.463077	3.902921	0.195682
F	-0.533435	-4.122736	-1.448566
O	0.405010	-0.644676	1.096770
C	3.432767	4.831048	-0.831400
H	3.124858	5.854040	-0.658744
O	-5.996447	-2.633368	-0.076658
C	3.808132	4.390137	-2.090462
O	-5.278887	-3.088592	-2.072719
C	4.216829	3.089723	-2.342197
H	4.525030	2.779285	-3.331948
C	4.233500	2.211178	-1.273629
C	4.453263	1.819581	2.321106
H	5.023524	2.733456	2.457429
C	4.385236	0.891272	3.388707
C	3.666145	-0.325202	3.219324
C	3.040303	-0.589963	1.958240
C	2.350131	-1.876787	1.671828
C	1.029901	-1.864136	1.282373
C	0.297760	-3.046276	0.992555

C	-1.108373	-2.979474	0.529748
C	-2.126577	-2.376847	1.270517
C	-3.447312	-2.337020	0.847583
H	-4.216060	-1.879959	1.459270
C	-3.733593	-2.904831	-0.383156
C	-2.773452	-3.508623	-1.182632
H	-3.026653	-3.932546	-2.145328
C	-1.484129	-3.536402	-0.694680
C	0.952025	-4.251955	1.090174
H	0.413843	-5.172000	0.882015
C	2.328989	-4.324319	1.416457
C	3.048543	-3.128220	1.695295
C	4.448555	-3.218650	1.917648
H	5.018419	-2.315067	2.101218
C	5.084465	-4.433784	1.891231
H	6.155953	-4.482617	2.056768
C	4.361170	-5.624686	1.650465
H	4.878817	-6.578280	1.644010
C	3.012150	-5.569515	1.417381
H	2.447223	-6.476009	1.218917
C	3.575711	-1.216861	4.319914
H	3.006054	-2.134141	4.218836
C	4.189476	-0.922463	5.510981
H	4.104838	-1.613236	6.343892
C	4.925971	0.273552	5.669248
H	5.408892	0.488188	6.616894
C	5.017580	1.161703	4.630262
H	5.569710	2.090359	4.743817
C	0.255781	2.188884	1.350173
H	1.197854	2.739829	1.425270
H	0.219157	1.423260	2.124235
H	-0.573488	2.888199	1.501758
C	0.149733	2.566655	-1.063186
H	1.084447	3.137137	-1.016359
H	-0.697428	3.248470	-0.959556
H	0.091318	2.080546	-2.039854
O	3.407989	6.481268	-2.962655
O	-0.027429	-2.212421	-3.993030
C	0.242404	-1.572444	-5.258432
C	1.678081	-1.095061	-5.365632
H	-0.458021	-0.736471	-5.299843

H	-0.012402	-2.278732	-6.054770
H	1.840782	-0.609434	-6.332591
H	1.901422	-0.374148	-4.571013
H	2.381207	-1.933274	-5.290187
H	0.581556	-2.957222	-3.875912
O	-1.399446	0.111471	-2.777998
C	-2.593928	-0.636421	-3.140233
C	-3.431693	0.149129	-4.124293
H	-3.156839	-0.859777	-2.229263
H	-2.238719	-1.575586	-3.563463
H	-4.334437	-0.421186	-4.361607
H	-3.747318	1.102147	-3.691441
H	-2.876248	0.340231	-5.047890
H	-1.718934	0.859996	-2.229486
O	1.819656	-2.473775	-1.902014
C	3.254526	-2.265078	-1.882624
C	3.974652	-3.594407	-1.954131
H	3.520327	-1.706150	-0.984808
H	3.454515	-1.635356	-2.750397
H	5.055253	-3.423256	-1.963630
H	3.747907	-4.215304	-1.079661
H	3.703640	-4.145929	-2.859526
H	1.573300	-3.068771	-1.174524
C	-4.786838	1.392380	0.180235
C	-5.159779	0.717386	1.504288
C	-4.408701	0.913881	2.669696
C	-6.317944	-0.060733	1.575745
C	-4.794204	0.315355	3.863785
H	-3.517457	1.526044	2.652382
C	-6.700789	-0.659204	2.773118
H	-6.921086	-0.231168	0.690174
C	-5.937106	-0.478226	3.920517
H	-4.193299	0.471636	4.754220
H	-7.595014	-1.273185	2.797875
H	-6.230835	-0.948627	4.853662
C	-5.224794	2.908730	0.145288
H	-5.134156	3.253242	-0.888424
H	-6.283712	2.932240	0.416652
C	-4.409668	3.788050	1.062031
C	-4.807085	4.016438	2.381497
C	-3.224579	4.375571	0.605512

C	-4.024091	4.788258	3.235192
H	-5.732687	3.579246	2.744975
C	-2.439488	5.147971	1.457515
H	-2.930078	4.243169	-0.432265
C	-2.833752	5.348291	2.778670
H	-4.348383	4.956927	4.257335
H	-1.530991	5.611858	1.083343
H	-2.227095	5.955761	3.443129
C	-5.452436	0.715504	-0.949878
N	-5.937627	0.189875	-1.859555
C	-3.299106	1.350045	-0.211188
O	-2.925076	1.788474	-1.290770
O	-2.483509	0.832259	0.670479
H	-1.552174	0.987892	0.345919

Int-3S

ωB97XD SCF energy: -3904.88799617 a.u.
ωB97XD enthalpy: -3903.901052 a.u.
ωB97XD free energy: -3904.074649 a.u.
ωB97XD SCF energy in solution: -3907.03495739 a.u.
ωB97XD enthalpy in solution: -3906.048013 a.u.
ωB97XD free energy in solution: -3906.221610 a.u.

Cartesian coordinates

ATOM	X	Y	Z
C	3.207382	0.690453	0.107749
F	3.195446	4.219504	1.811659
N	0.683024	6.491124	-1.586053
O	1.931546	0.639474	-0.444389
P	0.618274	-0.080958	0.178518
Rh	-0.825163	-0.399198	-1.352153
C	3.768669	1.989165	0.209872
F	2.707041	2.223469	-2.418689
N	-4.218503	-3.624767	1.152463
C	3.003314	3.161944	-0.275521
F	0.284526	-3.344906	2.760489
N	0.130399	1.049878	1.557913
O	0.268630	6.457758	-2.730009
C	2.737011	4.255054	0.555077

F	-0.510768	-3.753143	-1.848951
O	1.272316	-1.172526	1.197428
C	1.988174	5.350302	0.159385
H	1.793318	6.176025	0.831757
O	-4.592542	-3.222867	2.244828
C	1.488280	5.340475	-1.132328
O	-4.950577	-3.932741	0.229573
C	1.711042	4.298559	-2.019445
H	1.311764	4.323178	-3.025266
C	2.471704	3.238141	-1.566421
C	5.003845	2.108693	0.795838
H	5.463114	3.087966	0.888501
C	5.688150	0.979247	1.308208
C	5.119769	-0.317662	1.160887
C	3.868267	-0.458671	0.473378
C	3.304928	-1.792292	0.124320
C	2.025563	-2.126168	0.501071
C	1.396949	-3.348535	0.154506
C	-0.039332	-3.549458	0.447825
C	-0.562583	-3.494748	1.739850
C	-1.917769	-3.560402	2.007952
H	-2.291941	-3.509608	3.022269
C	-2.770983	-3.686676	0.922389
C	-2.325797	-3.794349	-0.383655
H	-3.018626	-3.889709	-1.209089
C	-0.961207	-3.716384	-0.587886
C	2.123243	-4.259036	-0.569748
H	1.668823	-5.204025	-0.851642
C	3.434683	-3.969325	-1.018945
C	4.031703	-2.716291	-0.700751
C	5.297080	-2.410862	-1.268930
H	5.755226	-1.448646	-1.074196
C	5.940636	-3.315076	-2.075163
H	6.904276	-3.059678	-2.504124
C	5.363040	-4.573940	-2.357197
H	5.890448	-5.280523	-2.989474
C	4.134349	-4.890860	-1.841590
H	3.670901	-5.847601	-2.064538
C	5.802482	-1.424377	1.728776
H	5.368803	-2.415472	1.655026
C	6.995944	-1.249002	2.382344

H	7.500006	-2.105552	2.818247
C	7.574667	0.034975	2.500480
H	8.522350	0.153970	3.015052
C	6.931622	1.125333	1.976647
H	7.359146	2.119097	2.073928
C	1.195347	1.324461	2.573262
H	1.994390	1.895497	2.105083
H	1.574889	0.383754	2.966819
H	0.740132	1.911292	3.369360
C	-0.444830	2.329288	1.015654
H	-0.839769	2.895547	1.860241
H	-1.259538	2.133117	0.309702
H	0.360154	2.880376	0.528921
O	0.498047	7.385846	-0.781991
O	-2.243440	-0.380032	-2.996827
C	-3.595934	0.137486	-2.843449
C	-4.190488	0.488590	-4.188716
H	-3.486658	1.017297	-2.205739
H	-4.204126	-0.606840	-2.317613
H	-5.195446	0.892299	-4.031044
H	-3.586046	1.243667	-4.698842
H	-4.283832	-0.391093	-4.837067
H	-2.258871	-1.153516	-3.574971
O	0.687444	-0.232744	-2.853806
C	1.520678	-1.387427	-3.142003
C	2.892979	-0.960958	-3.615627
H	0.982547	-1.951006	-3.905976
H	1.588543	-2.004747	-2.243815
H	3.496590	-1.848044	-3.829620
H	2.830348	-0.353580	-4.523540
H	3.413251	-0.388642	-2.839211
H	1.266938	0.515797	-2.637868
C	-4.397664	0.890468	0.845430
C	-5.617481	0.561485	-0.022261
C	-6.201208	1.521036	-0.850203
C	-6.135158	-0.736986	-0.014802
C	-7.276584	1.177205	-1.666874
H	-5.822019	2.537059	-0.862515
C	-7.202692	-1.081486	-0.836500
H	-5.715297	-1.491079	0.645317
C	-7.773513	-0.123151	-1.670747

H	-7.727277	1.934552	-2.301148
H	-7.585233	-2.097120	-0.818263
H	-8.608865	-0.386156	-2.312302
C	-4.665523	0.715317	2.355462
H	-5.327625	1.541645	2.621925
H	-5.195971	-0.226138	2.532868
C	-3.408013	0.747882	3.191965
C	-2.663768	-0.424611	3.365770
C	-2.959098	1.923224	3.801139
C	-1.508340	-0.438927	4.147370
H	-3.024867	-1.342340	2.911324
C	-1.821451	1.908902	4.607388
H	-3.512252	2.842371	3.637344
C	-1.099015	0.729738	4.791260
H	-0.951202	-1.362732	4.278466
H	-1.509492	2.820210	5.109874
H	-0.228936	0.716818	5.441766
C	-3.297397	0.071782	0.386151
N	-2.361703	-0.444559	-0.063953
C	-3.852994	2.416665	0.529482
O	-3.102248	2.486047	-0.466568
O	-4.274730	3.260670	1.311951
H	-0.634201	0.554195	2.049733

Int-3R

ωB97XD SCF energy: -3904.88895654 a.u.

ωB97XD enthalpy: -3903.901483 a.u.

ωB97XD free energy: -3904.073614 a.u.

ωB97XD SCF energy in solution: -3907.03986433 a.u.

ωB97XD enthalpy in solution: -3906.052391 a.u.

ωB97XD free energy in solution: -3906.224522 a.u.

Cartesian coordinates

ATOM	X	Y	Z
C	-3.386120	0.074916	-0.435276
F	-3.818797	3.214296	-2.702779
N	-2.910746	6.530321	0.645093
O	-2.256507	0.419057	0.308286
P	-0.740229	0.138368	-0.235166

Rh	0.666128	0.312081	1.349545
C	-4.215873	1.143622	-0.855501
F	-3.855158	1.978109	1.826469
N	5.180096	-2.782718	-0.388385
C	-3.894815	2.534958	-0.459913
F	0.813946	-3.195445	-2.313626
N	-0.791986	1.272930	-1.694016
O	-2.741502	6.746831	1.831476
C	-3.729044	3.546274	-1.408857
F	1.252765	-2.892847	2.350794
O	-0.926756	-1.206356	-1.134072
C	-3.419362	4.855776	-1.083461
H	-3.294093	5.615555	-1.844580
O	5.623271	-2.695400	-1.518064
C	-3.257188	5.148072	0.260635
O	5.845222	-2.752751	0.636607
C	-3.393670	4.199080	1.263149
H	-3.266876	4.462416	2.305455
C	-3.716982	2.912473	0.874318
C	-5.310056	0.837751	-1.628921
H	-5.970469	1.632432	-1.962501
C	-5.593475	-0.495201	-2.011396
C	-4.755263	-1.555029	-1.559183
C	-3.645979	-1.247152	-0.708451
C	-2.773868	-2.296911	-0.115335
C	-1.419023	-2.266527	-0.356178
C	-0.505983	-3.195348	0.201697
C	0.959975	-3.075123	0.022885
C	1.582441	-3.109396	-1.223629
C	2.956176	-3.048778	-1.385080
H	3.412509	-3.078719	-2.365422
C	3.722899	-2.912665	-0.240384
C	3.179478	-2.862301	1.033937
H	3.800801	-2.752067	1.912593
C	1.804558	-2.951265	1.131389
C	-1.021795	-4.183182	1.006401
H	-0.349628	-4.913158	1.447095
C	-2.397639	-4.244144	1.330574
C	-3.292573	-3.281941	0.784872
C	-4.649561	-3.303801	1.204137
H	-5.341502	-2.561153	0.823937

C	-5.089575	-4.249065	2.095043
H	-6.128377	-4.248152	2.409246
C	-4.205795	-5.224958	2.611505
H	-4.574053	-5.970323	3.308675
C	-2.888207	-5.220524	2.237488
H	-2.196823	-5.956820	2.637093
C	-5.035675	-2.876381	-1.994643
H	-4.390516	-3.690261	-1.683052
C	-6.106630	-3.127555	-2.813707
H	-6.304513	-4.142515	-3.142899
C	-6.954078	-2.078968	-3.240334
H	-7.799191	-2.298222	-3.884330
C	-6.700312	-0.790951	-2.850017
H	-7.337439	0.023498	-3.182292
C	0.065305	0.883932	-2.869705
H	-0.280182	1.466567	-3.725616
H	-0.054490	-0.180758	-3.063234
H	1.095921	1.174766	-2.643256
C	-0.484645	2.680122	-1.257688
H	0.596006	2.762002	-1.109278
H	-1.047140	2.904979	-0.350643
H	-0.786331	3.346891	-2.066610
O	-2.816986	7.346209	-0.252665
O	2.035645	0.398253	3.012048
C	1.761439	1.188488	4.188366
C	2.275004	0.492400	5.432626
H	0.676638	1.299239	4.217302
H	2.210834	2.177455	4.057157
H	2.027072	1.084498	6.319429
H	1.820147	-0.497348	5.536788
H	3.361632	0.370587	5.399144
H	2.976924	0.498728	2.773916
O	-0.952635	0.304254	2.765408
C	-1.341185	-1.016248	3.244153
C	-2.791766	-1.024785	3.673897
H	-0.659255	-1.231795	4.067883
H	-1.161577	-1.743169	2.449204
H	-3.062204	-2.027231	4.019335
H	-2.975131	-0.313870	4.484953
H	-3.448808	-0.782857	2.830206
H	-1.735311	0.705217	2.353079

C	4.146012	1.010989	-1.475493
C	4.239553	0.264744	-2.794861
C	5.356528	0.461337	-3.610805
C	3.209239	-0.551897	-3.261118
C	5.444471	-0.167199	-4.848217
H	6.154935	1.120929	-3.289538
C	3.293377	-1.179132	-4.500611
H	2.334066	-0.721386	-2.642282
C	4.417133	-0.992358	-5.298631
H	6.322175	-0.005893	-5.465966
H	2.481126	-1.819289	-4.834628
H	4.491626	-1.481454	-6.264619
C	5.471873	1.032844	-0.673800
H	5.966945	0.059701	-0.760235
H	6.092430	1.784693	-1.168778
C	3.074430	0.541233	-0.630584
N	2.208159	0.311660	0.101679
C	3.639208	2.545948	-1.874654
O	2.403886	2.610304	-2.045030
O	4.546335	3.356875	-1.973467
C	5.298126	1.404921	0.781086
C	5.392758	0.428114	1.776425
C	5.024116	2.725404	1.155482
C	5.234099	0.763523	3.122195
H	5.628376	-0.597058	1.502678
C	4.855316	3.060285	2.496230
H	4.955831	3.486749	0.383829
C	4.965736	2.084133	3.485380
H	5.355405	-0.001363	3.884937
H	4.660203	4.092589	2.770875
H	4.867223	2.352973	4.533571
H	-1.769186	1.237354	-2.007790

Int-4S

ωB97XD SCF energy: -3716.37622443 a.u.

ωB97XD enthalpy: -3715.406696 a.u.

ωB97XD free energy: -3715.575143 a.u.

ωB97XD SCF energy in solution: -3718.44534151 a.u.

ωB97XD enthalpy in solution: -3717.475813 a.u.

ωB97XD free energy in solution: -3717.644260 a.u.

Cartesian coordinates

ATOM	X	Y	Z
C	-2.677803	-0.289549	-0.191773
F	-3.645101	3.279607	-1.538646
N	-3.305688	5.592134	2.647496
O	-1.634909	0.008565	0.684616
P	-0.044927	-0.031758	0.309554
Rh	1.407652	-0.030677	1.858448
C	-3.662430	0.720564	-0.366627
F	-3.576220	0.809136	2.452219
N	5.779785	-1.365191	0.123687
C	-3.604614	1.976342	0.415631
F	1.942571	-2.905789	-2.310885
N	0.078809	1.393379	-0.880775
O	-3.217099	5.490127	3.858043
C	-3.605694	3.230508	-0.201228
F	1.990808	-3.267389	2.358087
O	-0.021932	-1.168221	-0.875515
C	-3.515342	4.423308	0.494808
H	-3.502770	5.376832	-0.017002
O	6.278430	-1.033733	-0.938631
C	-3.428294	4.341902	1.875453
O	6.265836	-1.167166	1.223785
C	-3.441420	3.138089	2.563362
H	-3.389965	3.107042	3.643911
C	-3.540964	1.980968	1.811891
C	-4.662326	0.503509	-1.281776
H	-5.427051	1.260014	-1.428836
C	-4.713080	-0.679658	-2.056030
C	-3.752329	-1.702581	-1.828784
C	-2.756135	-1.515478	-0.814514
C	-1.841724	-2.616255	-0.413317
C	-0.484429	-2.414943	-0.441420
C	0.453123	-3.387810	-0.003419
C	1.892461	-3.051179	0.015905
C	2.578958	-2.728440	-1.152770
C	3.859353	-2.205941	-1.157445
H	4.348333	-1.929323	-2.082445
C	4.471089	-2.034792	0.075863

C	3.888759	-2.418807	1.272969
H	4.386607	-2.270709	2.221287
C	2.603299	-2.921259	1.206100
C	-0.026870	-4.587786	0.452306
H	0.671427	-5.348867	0.788086
C	-1.416798	-4.849358	0.528684
C	-2.344903	-3.852000	0.117301
C	-3.729175	-4.099330	0.312997
H	-4.452492	-3.337361	0.048366
C	-4.160275	-5.288654	0.843810
H	-5.222606	-5.457378	0.988306
C	-3.238379	-6.295292	1.209093
H	-3.597648	-7.233669	1.618558
C	-1.894592	-6.075755	1.059267
H	-1.173918	-6.833369	1.353735
C	-3.795666	-2.864640	-2.643094
H	-3.053787	-3.643025	-2.502841
C	-4.757625	-3.003702	-3.611083
H	-4.771156	-3.894985	-4.230204
C	-5.729774	-1.997876	-3.815570
H	-6.487317	-2.128167	-4.581297
C	-5.705552	-0.859162	-3.054516
H	-6.439464	-0.073582	-3.210005
C	-0.724602	1.367218	-2.141527
H	-1.747432	1.668569	-1.922545
H	-0.700055	0.368406	-2.569562
H	-0.264651	2.076910	-2.831728
C	-0.127188	2.670086	-0.125383
H	-0.033036	3.502761	-0.823871
H	0.634475	2.748915	0.649142
H	-1.119287	2.651174	0.322455
O	-3.293939	6.634234	2.017329
O	0.329391	-1.120967	3.379656
C	-1.049737	-1.040286	3.795959
C	-1.875437	-2.180452	3.242046
H	-1.410380	-0.079526	3.429975
H	-1.061143	-1.028896	4.890545
H	-2.917215	-2.073055	3.557544
H	-1.849784	-2.179155	2.151271
H	-1.516305	-3.152053	3.600846
H	0.609524	-2.046459	3.316860

H	1.081053	1.392343	-1.165739
C	3.061760	1.424799	-0.205346
N	2.866039	0.963723	0.892976
C	3.217510	2.037269	-1.388478
C	2.710302	3.429247	-1.503971
C	2.126242	3.883573	-2.696613
C	2.741277	4.302094	-0.406286
C	1.558912	5.153620	-2.771207
H	2.106274	3.236982	-3.568882
C	2.168181	5.566334	-0.481289
H	3.218784	3.978347	0.514993
C	1.564576	5.996291	-1.661910
H	1.110927	5.485012	-3.703524
H	2.204574	6.224691	0.381620
H	1.121876	6.985453	-1.722480
C	3.964478	1.377639	-2.534701
H	4.662035	2.110995	-2.955426
H	4.585576	0.568498	-2.132567
C	3.083252	0.832018	-3.643588
C	3.267091	1.214890	-4.973546
C	2.071419	-0.082231	-3.350322
C	2.450814	0.704335	-5.980666
H	4.053094	1.923433	-5.222774
C	1.260889	-0.606538	-4.352197
H	1.926600	-0.402032	-2.322234
C	1.443764	-0.207340	-5.673669
H	2.605724	1.017337	-7.008720
H	0.497346	-1.336490	-4.098284
H	0.813765	-0.610815	-6.460217
O	3.070687	-0.131692	3.305804
C	2.952336	0.481390	4.603724
C	4.225312	0.296398	5.406012
H	2.103890	-0.018847	5.073105
H	2.702575	1.542027	4.481071
H	4.106835	0.735709	6.401498
H	4.458434	-0.766090	5.520244
H	5.074514	0.788527	4.919994
H	3.717928	0.361337	2.768252

Int-4R
ωB97XD SCF energy: -3716.37112230 a.u.
ωB97XD enthalpy: -3715.401619 a.u.
ωB97XD free energy: -3715.571010 a.u.
ωB97XD SCF energy in solution: -3718.44354489 a.u.
ωB97XD enthalpy in solution: -3717.474042 a.u.
ωB97XD free energy in solution: -3717.643433 a.u.

Cartesian coordinates

ATOM	X	Y	Z
C	-2.866783	0.914027	-0.335967
F	-2.034623	4.671349	-1.152770
N	-1.148590	6.075729	3.346223
O	-1.871859	0.611210	0.593184
P	-0.426159	-0.046558	0.227733
Rh	0.854627	-0.786636	1.757753
C	-3.291780	2.269278	-0.378843
F	-3.449462	1.972792	2.416265
N	4.096798	-3.635190	0.660105
C	-2.755471	3.259858	0.582741
F	0.642794	-3.000666	-2.563434
N	0.335659	1.266548	-0.865647
O	-1.240129	5.804574	4.530057
C	-2.150063	4.447354	0.163053
F	-0.472170	-4.234054	1.827469
O	-0.788726	-0.965494	-1.079933
C	-1.621763	5.383953	1.034944
H	-1.149194	6.288588	0.674685
O	4.963826	-3.723274	-0.181892
C	-1.719419	5.109331	2.389933
O	4.290894	-3.371149	1.846451
C	-2.322940	3.964394	2.886722
H	-2.401168	3.785971	3.951311
C	-2.837910	3.071169	1.964746
C	-4.201936	2.634875	-1.338817
H	-4.544702	3.663913	-1.388482
C	-4.691742	1.703676	-2.285153
C	-4.292153	0.341799	-2.195905
C	-3.407874	-0.062691	-1.140747
C	-3.094248	-1.496836	-0.902731
C	-1.786927	-1.917946	-0.875362

C	-1.408929	-3.260268	-0.611336
C	0.014664	-3.593562	-0.386600
C	1.011432	-3.385943	-1.338872
C	2.362204	-3.492252	-1.056320
H	3.115427	-3.259667	-1.798351
C	2.704050	-3.803984	0.248751
C	1.774094	-4.120221	1.223404
H	2.063345	-4.374361	2.234541
C	0.442284	-4.008002	0.876325
C	-2.405205	-4.181407	-0.412601
H	-2.143272	-5.214895	-0.206634
C	-3.770528	-3.808105	-0.403854
C	-4.129202	-2.446509	-0.609800
C	-5.494409	-2.081666	-0.469377
H	-5.786803	-1.044150	-0.579428
C	-6.446579	-3.027370	-0.185466
H	-7.484193	-2.727809	-0.077904
C	-6.093215	-4.386610	-0.026642
H	-6.861056	-5.122431	0.188494
C	-4.781201	-4.765823	-0.127909
H	-4.492325	-5.803346	0.013762
C	-4.759161	-0.565260	-3.183074
H	-4.441299	-1.601260	-3.149433
C	-5.595488	-0.139799	-4.183681
H	-5.936049	-0.845463	-4.934615
C	-6.016264	1.207978	-4.251787
H	-6.684000	1.524954	-5.046070
C	-5.570805	2.110378	-3.322483
H	-5.876149	3.151701	-3.371081
C	-0.373641	1.715833	-2.105759
H	-1.141046	2.440901	-1.843054
H	-0.809562	0.850894	-2.600319
H	0.373803	2.173990	-2.757715
C	0.817105	2.419941	-0.053759
H	1.434283	3.050103	-0.696436
H	1.408038	2.044288	0.782126
H	-0.045792	2.974191	0.314152
O	-0.621002	7.070149	2.881285
O	-0.506992	-1.070038	3.427045
C	-1.934064	-0.872166	3.506678
C	-2.710774	-1.963139	2.799485

H	-2.108086	0.100765	3.051386
H	-2.198263	-0.812324	4.567662
H	-3.780576	-1.734011	2.825094
H	-2.403806	-2.041948	1.754121
H	-2.561992	-2.938295	3.274808
H	-0.287656	-1.948671	3.769707
H	1.182272	0.792864	-1.216870
C	3.063544	0.020590	-0.068657
N	2.489478	-0.811806	0.586884
C	3.639155	0.903009	-0.897515
C	3.183891	0.921292	-2.299150
C	3.438526	2.024688	-3.132067
C	2.402313	-0.125584	-2.828539
C	2.915212	2.081814	-4.420201
H	4.069781	2.833869	-2.780266
C	1.874966	-0.060394	-4.113371
H	2.214456	-1.008596	-2.224906
C	2.122345	1.048793	-4.918193
H	3.135418	2.943494	-5.043428
H	1.280255	-0.890111	-4.483382
H	1.721486	1.100176	-5.925236
C	4.663590	1.887052	-0.375179
H	4.296786	2.918062	-0.487229
H	4.779118	1.728419	0.704141
C	6.020621	1.771141	-1.047910
C	6.720907	2.914776	-1.431330
C	6.596637	0.519594	-1.275257
C	7.975923	2.814677	-2.027435
H	6.282659	3.896262	-1.260861
C	7.849865	0.415943	-1.869151
H	6.055981	-0.379872	-0.990840
C	8.543519	1.563663	-2.247704
H	8.507676	3.714683	-2.321317
H	8.286061	-0.563923	-2.038719
H	9.520506	1.481928	-2.713986
O	2.227452	-1.723229	3.215480
C	2.930082	-0.820113	4.097232
C	3.852011	-1.583207	5.027351
H	2.147589	-0.291994	4.646042
H	3.483079	-0.092421	3.492157
H	4.352615	-0.887111	5.707531

H	3.289063	-2.306515	5.624531
H	4.623302	-2.117568	4.463158
H	2.897364	-2.202138	2.686962

Int-5S

ωB97XD SCF energy: -3716.44009956 a.u.

ωB97XD enthalpy: -3715.470459 a.u.

ωB97XD free energy: -3715.636734 a.u.

ωB97XD SCF energy in solution: -3716.44009956 a.u.

ωB97XD enthalpy in solution: -3715.470459 a.u.

ωB97XD free energy in solution: -3715.636734 a.u.

Cartesian coordinates

ATOM	X	Y	Z
C	-3.104149	0.726990	-0.289409
F	-2.539430	3.777875	-2.723364
N	-0.771770	6.765557	0.578608
O	-1.957109	0.763943	0.483324
P	-0.544422	0.207784	-0.185844
Rh	1.140799	0.199233	1.235467
C	-3.575174	1.956676	-0.820206
F	-3.195344	2.825338	1.828537
N	4.318003	-2.797543	0.576013
C	-2.887604	3.218764	-0.465193
F	0.129620	-3.771948	-1.486127
N	-0.318946	0.893273	-1.677667
O	-0.614994	6.991252	1.765998
C	-2.386323	4.093716	-1.429896
F	0.282044	-2.778258	3.098000
O	-1.068729	-1.270146	-0.708848
C	-1.700272	5.256822	-1.124159
H	-1.314438	5.904384	-1.900626
O	4.959964	-3.281951	-0.333561
C	-1.521452	5.550766	0.217673
O	4.773895	-2.002588	1.400920
C	-2.010461	4.746609	1.235932
H	-1.875366	5.011231	2.276470
C	-2.693036	3.602986	0.864430
C	-4.658236	1.930908	-1.662486

H	-5.039929	2.858620	-2.078281
C	-5.282576	0.711552	-2.022501
C	-4.816337	-0.510038	-1.460497
C	-3.729470	-0.476068	-0.529047
C	-3.237749	-1.685285	0.183583
C	-1.904601	-2.013617	0.099780
C	-1.336238	-3.091991	0.828838
C	0.132467	-3.264490	0.794954
C	0.831605	-3.509269	-0.388553
C	2.212366	-3.445247	-0.479346
H	2.719914	-3.596453	-1.424535
C	2.898579	-3.115582	0.680273
C	2.283148	-2.944210	1.910305
H	2.835697	-2.689448	2.804590
C	0.905570	-3.027044	1.929075
C	-2.151835	-3.854213	1.623551
H	-1.733697	-4.682032	2.189083
C	-3.530032	-3.558515	1.756932
C	-4.080901	-2.444808	1.061224
C	-5.436672	-2.102347	1.310323
H	-5.866372	-1.233795	0.825389
C	-6.205434	-2.853379	2.162596
H	-7.238884	-2.573021	2.340125
C	-5.667509	-3.984674	2.817124
H	-6.292380	-4.572102	3.481890
C	-4.355205	-4.324013	2.621771
H	-3.922694	-5.178840	3.134236
C	-5.423736	-1.723253	-1.879004
H	-5.059168	-2.664824	-1.483059
C	-6.455476	-1.714071	-2.782669
H	-6.904689	-2.651479	-3.095141
C	-6.938330	-0.497098	-3.316243
H	-7.759750	-0.507393	-4.025212
C	-6.360960	0.688488	-2.945362
H	-6.714817	1.628843	-3.358650
C	-1.117611	0.588494	-2.866090
H	-1.825189	1.397235	-3.075997
H	-1.664818	-0.342639	-2.729819
H	-0.447190	0.478796	-3.727255
C	0.509491	2.084937	-1.820018
H	1.288319	1.911568	-2.574296

H	0.990099	2.340718	-0.873268
H	-0.098050	2.934835	-2.147449
O	-0.353200	7.454516	-0.335293
O	0.075803	0.151228	3.081471
C	-1.195413	0.702887	3.495604
C	-2.276234	-0.355500	3.533914
H	-1.430352	1.480670	2.773004
H	-1.041482	1.159680	4.478421
H	-3.230835	0.101199	3.811184
H	-2.394783	-0.818490	2.552678
H	-2.051910	-1.135392	4.270709
H	0.155311	-0.751866	3.423606
H	3.859775	0.715842	-2.949333
C	3.157955	0.035468	-1.143094
N	2.408377	0.135617	-0.271562
C	4.014635	-0.169468	-2.316754
C	3.487135	-1.400077	-3.057505
C	4.322513	-2.446318	-3.440911
C	2.118244	-1.478514	-3.336095
C	3.792341	-3.558333	-4.096233
H	5.384948	-2.417075	-3.226579
C	1.591759	-2.584971	-3.988811
H	1.451524	-0.683857	-3.007839
C	2.430661	-3.631264	-4.370353
H	4.451366	-4.369907	-4.387439
H	0.525992	-2.637442	-4.184416
H	2.022550	-4.500421	-4.876074
C	5.500342	-0.192949	-1.891223
H	5.704762	-1.112425	-1.333479
H	6.100094	-0.225682	-2.807259
C	5.863385	1.015363	-1.058403
C	6.184559	0.873522	0.292794
C	5.854107	2.295204	-1.620959
C	6.494021	1.991352	1.065648
H	6.207703	-0.119053	0.735785
C	6.149111	3.412846	-0.847401
H	5.628488	2.420567	-2.678210
C	6.469018	3.262958	0.500623
H	6.765582	1.866159	2.109751
H	6.142740	4.399992	-1.299002
H	6.711817	4.133169	1.102343

O	2.884817	-0.013945	2.567302
C	3.505599	1.205983	3.022629
C	4.625290	0.899872	3.997005
H	2.702992	1.772298	3.498178
H	3.870724	1.769592	2.156689
H	5.083824	1.831219	4.344057
H	4.247456	0.354002	4.866270
H	5.406184	0.296647	3.519703
H	3.564777	-0.559077	2.130061

Int-5R

ωB97XD SCF energy: -3716.42983243 a.u.

ωB97XD enthalpy: -3715.460644 a.u.

ωB97XD free energy: -3715.631882 a.u.

ωB97XD SCF energy in solution: -3718.49440677 a.u.

ωB97XD enthalpy in solution: -3717.525218 a.u.

ωB97XD free energy in solution: -3717.696456 a.u.

Cartesian coordinates

ATOM	X	Y	Z
C	3.344539	0.853821	0.529621
F	2.269015	4.322373	2.064849
N	0.973561	6.353858	-2.076261
O	2.334996	0.629303	-0.389677
P	0.856053	0.152240	0.195451
Rh	-0.605717	-0.279244	-1.394734
C	3.675699	2.202639	0.823117
F	3.709120	2.420444	-1.980425
N	-3.696906	-3.318843	-0.759390
C	3.010359	3.297541	0.081732
F	0.040914	-3.399225	2.196093
N	0.405601	1.145812	1.436351
O	1.008286	6.294431	-3.293085
C	2.323528	4.327302	0.725616
F	0.744581	-3.574249	-2.444990
O	1.344818	-1.138264	1.103544
C	1.656888	5.338003	0.054095
H	1.123604	6.113417	0.588303
O	-4.479554	-3.608188	0.121616

C	1.694702	5.309092	-1.330351
O	-4.020845	-2.815449	-1.838130
C	2.375251	4.334366	-2.044211
H	2.407143	4.346716	-3.125753
C	3.026484	3.357068	-1.314483
C	4.607086	2.447864	1.800669
H	4.880145	3.471188	2.040443
C	5.210248	1.390844	2.525241
C	4.888864	0.042843	2.200855
C	3.967875	-0.214261	1.135353
C	3.641659	-1.585022	0.659766
C	2.324355	-1.978702	0.617011
C	1.918163	-3.225896	0.072467
C	0.472069	-3.480448	-0.103959
C	-0.434442	-3.471048	0.958914
C	-1.807854	-3.486453	0.778986
H	-2.483495	-3.436381	1.623292
C	-2.271974	-3.516235	-0.527317
C	-1.437211	-3.603979	-1.629896
H	-1.815740	-3.631972	-2.643321
C	-0.079485	-3.582881	-1.379802
C	2.877756	-4.088959	-0.389575
H	2.585425	-5.047150	-0.809486
C	4.248837	-3.737963	-0.372344
C	4.641206	-2.460333	0.119301
C	6.007267	-2.088641	0.004871
H	6.322646	-1.104706	0.331101
C	6.929559	-2.957277	-0.520656
H	7.968217	-2.652451	-0.600366
C	6.544002	-4.242444	-0.965147
H	7.288533	-4.919756	-1.370561
C	5.229359	-4.619787	-0.897784
H	4.915048	-5.596297	-1.255604
C	5.464298	-0.996495	2.978457
H	5.204114	-2.027396	2.764389
C	6.332679	-0.707501	3.999964
H	6.758724	-1.514399	4.587767
C	6.675762	0.630793	4.300221
H	7.369373	0.841391	5.107704
C	6.123071	1.656267	3.579487
H	6.368715	2.689132	3.809988

C	0.992254	1.135203	2.776901
H	1.609759	2.026339	2.931037
H	1.602948	0.245991	2.920715
H	0.185490	1.128673	3.519008
C	-0.482604	2.275324	1.193792
H	-1.382440	2.188919	1.816550
H	-0.783106	2.317558	0.144526
H	0.021385	3.212057	1.451961
O	0.385252	7.198895	-1.424339
O	0.746794	-0.680663	-2.992798
C	2.025728	-0.117397	-3.364050
C	3.165867	-1.055458	-3.030892
H	2.109964	0.815527	-2.812338
H	1.981218	0.101047	-4.435807
H	4.118400	-0.583351	-3.288853
H	3.177984	-1.282144	-1.963064
H	3.094585	-1.993363	-3.593642
H	0.768400	-1.636998	-3.142729
H	-4.133682	1.288099	1.821245
C	-3.089005	0.154824	0.455393
N	-2.116172	-0.030478	-0.139977
C	-4.343368	0.436738	1.161363
C	-5.402734	0.846206	0.146593
C	-6.083103	2.051590	0.301342
C	-5.730853	-0.000998	-0.914151
C	-7.080534	2.412822	-0.600796
H	-5.846690	2.704216	1.137276
C	-6.725415	0.362372	-1.815909
H	-5.226940	-0.956779	-1.037033
C	-7.400370	1.571666	-1.662090
H	-7.609217	3.351499	-0.470016
H	-6.979101	-0.304652	-2.634065
H	-8.178578	1.853466	-2.364190
C	-4.770609	-0.763524	2.052649
H	-4.993607	-1.620976	1.410102
H	-3.926596	-1.039554	2.695654
C	-5.972052	-0.394645	2.887034
C	-7.264331	-0.657757	2.429451
C	-5.804598	0.269216	4.104081
C	-8.370159	-0.265895	3.177687
H	-7.404429	-1.167478	1.479880

C	-6.908847	0.662520	4.853981
H	-4.801376	0.471537	4.474634
C	-8.194655	0.395489	4.390363
H	-9.370227	-0.479205	2.813342
H	-6.765745	1.170807	5.802571
H	-9.057503	0.698126	4.975389
O	-2.146739	-0.811813	-2.896273
C	-2.920224	0.291381	-3.414275
C	-3.963206	-0.196800	-4.399174
H	-2.193062	0.952153	-3.889357
H	-3.388610	0.828047	-2.580404
H	-4.529818	0.653721	-4.790450
H	-3.494861	-0.720775	-5.237174
H	-4.672859	-0.875114	-3.912277
H	-2.773649	-1.460871	-2.527858

TS-1S

ωB97XD SCF energy: -3904.87668866 a.u.
ωB97XD enthalpy: -3903.891274 a.u.
ωB97XD free energy: -3904.064832 a.u.
ωB97XD SCF energy in solution: -3907.02042934 a.u.
ωB97XD enthalpy in solution: -3906.035015 a.u.
ωB97XD free energy in solution: -3906.208573 a.u.
Imaginary frequency: -290.8834 cm^{-1}

Cartesian coordinates

ATOM	X	Y	Z
C	3.339346	0.094708	0.231000
F	4.310215	3.649645	1.594718
N	3.060280	6.116311	-2.325802
O	2.160167	0.398844	-0.440261
P	0.640590	0.151650	0.074776
Rh	-0.707789	-0.100544	-1.526381
C	4.282564	1.151638	0.296943
F	3.623694	1.355157	-2.449733
N	-5.288396	-2.432672	1.070885
C	3.990914	2.434055	-0.387701
F	-0.846797	-2.662653	2.861067
N	0.387288	1.468244	1.350681

O	2.775507	6.060869	-3.508169
C	4.018676	3.656923	0.288394
F	-1.522820	-3.366176	-1.739897
O	0.823395	-0.991561	1.227207
C	3.721934	4.868979	-0.311594
H	3.747446	5.796431	0.245977
O	-5.669158	-2.115196	2.186159
C	3.388331	4.843299	-1.655786
O	-5.994034	-2.563079	0.089209
C	3.344050	3.673883	-2.399861
H	3.088984	3.686612	-3.451838
C	3.653006	2.498129	-1.743171
C	5.434486	0.938512	1.010544
H	6.179275	1.725334	1.080266
C	5.668038	-0.289500	1.678248
C	4.728703	-1.352368	1.552526
C	3.560620	-1.162234	0.740488
C	2.639021	-2.286242	0.409679
C	1.292393	-2.190102	0.675464
C	0.355895	-3.207664	0.353194
C	-1.101081	-3.028204	0.560071
C	-1.663040	-2.765589	1.809008
C	-3.023147	-2.596370	2.009420
H	-3.432317	-2.417589	2.995574
C	-3.846117	-2.669474	0.898859
C	-3.367692	-2.945525	-0.371173
H	-4.037243	-3.005683	-1.219746
C	-2.005162	-3.121930	-0.500139
C	0.837227	-4.350823	-0.235440
H	0.148774	-5.152509	-0.485493
C	2.202582	-4.495582	-0.579947
C	3.119621	-3.449447	-0.281329
C	4.459027	-3.578683	-0.735743
H	5.167582	-2.779607	-0.553586
C	4.862786	-4.698355	-1.417699
H	5.889100	-4.775074	-1.762071
C	3.958279	-5.752295	-1.680445
H	4.297335	-6.634643	-2.213075
C	2.654981	-5.649837	-1.271526
H	1.945372	-6.445297	-1.480358
C	4.969975	-2.556927	2.262171

H	4.250367	-3.365863	2.201450
C	6.094855	-2.699948	3.034588
H	6.259040	-3.625050	3.577720
C	7.040512	-1.654696	3.136141
H	7.927560	-1.787581	3.746524
C	6.828875	-0.474386	2.473351
H	7.542498	0.340538	2.553886
C	1.521456	1.655000	2.305132
H	2.362804	2.093848	1.775612
H	1.797250	0.692841	2.734084
H	1.184461	2.331589	3.088890
C	-0.014418	2.762793	0.713199
H	-0.238174	3.469210	1.514067
H	-0.905168	2.618445	0.098525
H	0.821708	3.122817	0.109326
O	3.096263	7.124604	-1.644561
O	-2.010291	-0.868179	-3.126688
C	-3.397353	-0.460292	-3.203344
C	-4.135766	-1.239461	-4.269939
H	-3.362071	0.605264	-3.432705
H	-3.866089	-0.582119	-2.222775
H	-5.176895	-0.904398	-4.303896
H	-3.684449	-1.091446	-5.254844
H	-4.143738	-2.313247	-4.045030
H	-1.986920	-1.826800	-2.983840
O	0.905248	-0.403362	-2.906483
C	1.331178	-1.772818	-3.129152
C	2.800693	-1.832872	-3.486313
H	0.699880	-2.143515	-3.938492
H	1.123032	-2.354342	-2.227009
H	3.098393	-2.874864	-3.636985
H	3.011109	-1.272283	-4.401871
H	3.415985	-1.431785	-2.672561
H	1.677337	0.112159	-2.623754
C	-4.242497	1.312038	0.843287
C	-5.505889	1.104751	0.056565
C	-5.525665	1.388575	-1.317656
C	-6.680864	0.637032	0.651939
C	-6.669964	1.178196	-2.076085
H	-4.638811	1.811950	-1.781619
C	-7.829711	0.432263	-0.109758

H	-6.710296	0.411475	1.712418
C	-7.830027	0.692116	-1.475123
H	-6.664068	1.419174	-3.135842
H	-8.727405	0.059347	0.373539
H	-8.727703	0.531519	-2.063789
C	-4.340612	1.270031	2.364486
H	-5.094952	2.012458	2.644244
H	-4.710114	0.291960	2.703909
C	-3.055010	1.574150	3.101583
C	-2.153899	0.546707	3.408121
C	-2.762573	2.863205	3.558871
C	-1.027028	0.778471	4.197435
H	-2.365757	-0.459197	3.067198
C	-1.632937	3.104776	4.339537
H	-3.441833	3.675046	3.319352
C	-0.772411	2.062487	4.679639
H	-0.373490	-0.050922	4.454291
H	-1.445203	4.107285	4.712540
H	0.078550	2.244369	5.330332
C	-3.154729	0.673454	0.268964
N	-2.244656	0.205525	-0.304010
C	-3.756840	3.341280	0.405134
O	-2.900739	3.342555	-0.436435
O	-4.490354	3.942765	1.124781
H	-0.421260	1.137209	1.910845

TS-1R

ωB97XD SCF energy: -3904.88281156 a.u.

ωB97XD enthalpy: -3903.896867 a.u.

ωB97XD free energy: -3904.069004 a.u.

ωB97XD SCF energy in solution: -3907.02668498 a.u.

ωB97XD enthalpy in solution: -3906.040740 a.u.

ωB97XD free energy in solution: -3906.212877 a.u.

Imaginary frequency: -287.8725 cm^{-1}

Cartesian coordinates

ATOM	X	Y	Z
C	-3.454029	0.004591	-0.277918
F	-4.052988	3.053424	-2.647702

N	-3.010662	6.520986	0.497428
O	-2.318711	0.387748	0.432505
P	-0.808546	0.127190	-0.128592
Rh	0.667781	0.704713	1.278361
C	-4.308160	1.052665	-0.707531
F	-3.806606	2.015210	1.930663
N	5.139974	-2.713067	-0.088501
C	-3.992151	2.462945	-0.376972
F	0.817209	-3.363619	-2.054720
N	-0.971681	1.140859	-1.694373
O	-2.789094	6.794414	1.663068
C	-3.898149	3.437803	-1.374789
F	1.157313	-2.686759	2.573231
O	-0.969026	-1.273240	-0.940378
C	-3.592375	4.764415	-1.124206
H	-3.524427	5.489932	-1.924861
O	5.609017	-2.677278	-1.211322
C	-3.357840	5.119115	0.193734
O	5.781866	-2.633089	0.948341
C	-3.421080	4.211940	1.240903
H	-3.239099	4.520574	2.262508
C	-3.745208	2.905412	0.926189
C	-5.414789	0.715371	-1.450217
H	-6.093826	1.492829	-1.786668
C	-5.682314	-0.627421	-1.806186
C	-4.815094	-1.664174	-1.356316
C	-3.700501	-1.327403	-0.521882
C	-2.817536	-2.360969	0.084202
C	-1.461442	-2.313919	-0.141342
C	-0.539488	-3.220199	0.432653
C	0.924264	-3.063036	0.264919
C	1.568054	-3.173633	-0.965499
C	2.942671	-3.088617	-1.109951
H	3.415415	-3.176913	-2.079038
C	3.682891	-2.850600	0.035724
C	3.115058	-2.711825	1.293046
H	3.720064	-2.517294	2.168137
C	1.741754	-2.825638	1.372035
C	-1.044210	-4.215054	1.233716
H	-0.363002	-4.929977	1.685547
C	-2.424120	-4.304755	1.532940

C	-3.328589	-3.354249	0.981256
C	-4.688866	-3.402374	1.387354
H	-5.388976	-2.665176	1.012100
C	-5.124864	-4.367618	2.258606
H	-6.167228	-4.386409	2.560336
C	-4.232771	-5.336527	2.772485
H	-4.597659	-6.098003	3.453838
C	-2.910022	-5.301021	2.420074
H	-2.210214	-6.027868	2.822306
C	-5.072510	-2.994382	-1.779637
H	-4.404277	-3.791171	-1.473210
C	-6.149908	-3.275442	-2.580222
H	-6.328787	-4.296877	-2.900153
C	-7.028735	-2.249886	-2.999407
H	-7.879381	-2.493004	-3.627381
C	-6.796915	-0.953749	-2.623165
H	-7.456473	-0.155705	-2.951579
C	-0.184531	0.652062	-2.873719
H	-0.496885	1.232880	-3.743555
H	-0.386889	-0.406207	-3.025880
H	0.870592	0.843008	-2.685162
C	-0.660288	2.577752	-1.406650
H	0.417695	2.672335	-1.275162
H	-1.190817	2.889432	-0.506654
H	-0.983094	3.170849	-2.263793
O	-2.967326	7.294764	-0.440874
O	2.135523	1.667501	2.557559
C	2.358704	1.333897	3.936611
C	2.844395	-0.091833	4.102207
H	1.401456	1.507681	4.432303
H	3.081007	2.050099	4.337823
H	3.001381	-0.319596	5.161612
H	2.119581	-0.804472	3.695300
H	3.795838	-0.227817	3.577568
H	2.995575	1.779162	2.111225
O	-0.825703	1.095347	2.791886
C	-1.087655	0.120978	3.833464
C	-1.860573	-1.084748	3.341067
H	-1.620829	0.647537	4.631197
H	-0.105251	-0.168320	4.207978
H	-2.063620	-1.754728	4.182121

H	-2.821163	-0.795630	2.902362
H	-1.280389	-1.639912	2.600524
H	-1.679469	1.336291	2.399147
C	4.144467	0.608888	-1.563541
C	4.084082	-0.135370	-2.848441
C	5.175682	-0.120345	-3.728106
C	2.935672	-0.832256	-3.251064
C	5.114336	-0.776549	-4.952696
H	6.081698	0.412021	-3.460528
C	2.873757	-1.487527	-4.475415
H	2.089698	-0.898887	-2.574758
C	3.965941	-1.463220	-5.337960
H	5.976843	-0.750096	-5.611668
H	1.973303	-2.033236	-4.745137
H	3.925862	-1.976013	-6.293558
C	5.519458	0.850889	-0.940264
H	6.113089	-0.070126	-1.000448
H	6.028317	1.603498	-1.554824
C	3.091594	0.423642	-0.681965
N	2.160782	0.348707	0.027408
C	3.490302	2.523912	-2.344603
O	2.293811	2.468775	-2.424064
O	4.507976	3.112354	-2.506462
C	5.497781	1.339345	0.490932
C	6.039063	0.559734	1.515467
C	4.974880	2.598219	0.817425
C	6.077842	1.026322	2.828903
H	6.456253	-0.415173	1.281002
C	5.002534	3.064113	2.131604
H	4.587684	3.237117	0.029691
C	5.561196	2.280198	3.142803
H	6.527208	0.411929	3.603636
H	4.613767	4.052573	2.359535
H	5.612458	2.655792	4.160729
H	-1.964879	1.068847	-1.944855

TS-2Re

ωB97XD SCF energy: -3716.36266994 a.u.

ωB97XD enthalpy: -3715.398763 a.u.

ωB97XD free energy: -3715.566919 a.u.
ωB97XD SCF energy in solution: -3718.43303991 a.u.
ωB97XD enthalpy in solution: -3717.469133 a.u.
ωB97XD free energy in solution: -3717.637289 a.u.
Imaginary frequency: -1201.2577 cm^{-1}

Cartesian coordinates

ATOM	X	Y	Z
C	-2.840358	0.618206	-0.492559
F	-2.446159	4.339873	-1.747868
N	-2.149632	6.404159	2.570136
O	-1.863055	0.545517	0.499378
P	-0.324378	0.050986	0.209884
Rh	0.985727	-0.359243	1.878143
C	-3.418418	1.892659	-0.742673
F	-3.735337	1.908740	2.053171
N	4.515404	-2.999075	1.228576
C	-3.099341	3.059577	0.110139
F	1.009561	-3.504315	-1.986668
N	0.294495	1.281316	-0.943744
O	-2.291752	6.265964	3.772039
C	-2.639994	4.265924	-0.425493
F	0.007045	-3.660284	2.586568
O	-0.551355	-1.134891	-0.901410
C	-2.327712	5.371993	0.345738
H	-1.962689	6.287773	-0.100906
O	5.386286	-3.191288	0.408147
C	-2.492953	5.252148	1.716583
O	4.687601	-2.472381	2.327860
C	-2.958616	4.095663	2.322983
H	-3.094419	4.038328	3.395144
C	-3.261761	3.028200	1.497304
C	-4.301814	2.013028	-1.787269
H	-4.758894	2.976254	-1.992657
C	-4.625442	0.912786	-2.615677
C	-4.093391	-0.368694	-2.307468
C	-3.234503	-0.513557	-1.169761
C	-2.803451	-1.851000	-0.687940
C	-1.466190	-2.128055	-0.544801
C	-0.991595	-3.359460	-0.017598
C	0.447070	-3.530294	0.275504

C	1.422705	-3.500535	-0.717848
C	2.777218	-3.401271	-0.449518
H	3.502609	-3.285323	-1.245011
C	3.141467	-3.367770	0.887502
C	2.240707	-3.531130	1.926815
H	2.550690	-3.524258	2.963098
C	0.903208	-3.598899	1.590867
C	-1.910976	-4.315461	0.328353
H	-1.567258	-5.262284	0.734433
C	-3.302331	-4.081795	0.220172
C	-3.765938	-2.825240	-0.259179
C	-5.163984	-2.575055	-0.244568
H	-5.540916	-1.611256	-0.564972
C	-6.043581	-3.538111	0.180148
H	-7.108018	-3.326201	0.186425
C	-5.581800	-4.801374	0.614213
H	-6.292935	-5.553989	0.938515
C	-4.237892	-5.063443	0.639038
H	-3.866955	-6.022341	0.989641
C	-4.409253	-1.457365	-3.161949
H	-3.991446	-2.436417	-2.954716
C	-5.224370	-1.276373	-4.250137
H	-5.449082	-2.117108	-4.898608
C	-5.772857	-0.005949	-4.540553
H	-6.420786	0.116934	-5.402156
C	-5.477946	1.065771	-3.739967
H	-5.885308	2.048825	-3.958224
C	-0.331932	1.412642	-2.288172
H	-1.237975	2.017640	-2.235004
H	-0.553543	0.423506	-2.684320
H	0.398811	1.898374	-2.940798
C	0.410663	2.592377	-0.247731
H	0.849984	3.307978	-0.945527
H	1.066115	2.476833	0.618526
H	-0.566178	2.946796	0.078349
O	-1.742224	7.406840	2.011574
O	-0.377937	-0.548274	3.522987
C	-1.809865	-0.359870	3.576398
C	-2.568592	-1.553050	3.036095
H	-2.000112	0.533071	2.985511
H	-2.069736	-0.153872	4.619760

H	-3.640767	-1.335016	3.027233
H	-2.259203	-1.777634	2.013343
H	-2.409897	-2.445822	3.650305
H	-0.159804	-1.405531	3.917376
H	1.482736	1.092011	-1.198087
C	2.925712	0.231854	-0.345863
N	2.630565	-0.298270	0.669183
C	2.982415	0.988902	-1.501063
C	2.980853	0.295558	-2.825513
C	3.664732	0.817355	-3.927968
C	2.220539	-0.866819	-3.000897
C	3.591038	0.184404	-5.166626
H	4.278035	1.705860	-3.823885
C	2.154985	-1.502012	-4.234172
H	1.658118	-1.268364	-2.164527
C	2.841623	-0.976486	-5.326713
H	4.134966	0.601053	-6.008832
H	1.563213	-2.405500	-4.339822
H	2.793134	-1.468408	-6.292942
C	3.676350	2.340372	-1.357465
H	3.260223	3.022649	-2.109761
H	3.412935	2.766809	-0.380780
C	5.192970	2.332209	-1.488113
C	5.841864	3.483819	-1.940302
C	5.962716	1.221681	-1.141746
C	7.229067	3.529461	-2.036988
H	5.254808	4.355911	-2.220774
C	7.351147	1.263435	-1.238544
H	5.484634	0.305239	-0.806497
C	7.988925	2.416998	-1.684775
H	7.715753	4.432747	-2.392110
H	7.933826	0.387955	-0.968639
H	9.071134	2.447529	-1.763247
O	2.426349	-0.895415	3.441091
C	2.933685	0.196934	4.238998
C	3.917259	-0.308653	5.275497
H	2.051603	0.643910	4.701580
H	3.393458	0.938127	3.575471
H	4.270807	0.525240	5.889730
H	3.445362	-1.046337	5.931082
H	4.788668	-0.769157	4.798381

H	3.186785	-1.320429	2.996589

TS-2Si

ωB97XD SCF energy: -3716.367810 a.u.

ωB97XD enthalpy: -3715.403160 a.u.

ωB97XD free energy: -3715.568428 a.u.

ωB97XD SCF energy in solution: -3718.43484024 a.u.

ωB97XD enthalpy in solution: -3717.470192 a.u.

ωB97XD free energy in solution: -3717.635484 a.u.

Imaginary frequency: -1211.1336 cm^{-1}

Cartesian coordinates

ATOM	X	Y	Z
C	2.600286	-0.027498	-0.349716
F	3.352708	3.708303	0.678449
N	2.188817	5.687115	-3.530617
O	1.403287	0.123123	-1.045890
P	-0.066541	0.043509	-0.326423
Rh	-1.829662	-0.042461	-1.574346
C	3.489212	1.080852	-0.360734
F	3.031204	0.983889	-3.129285
N	-5.656003	-1.899948	-0.089038
C	3.182931	2.276885	-1.178638
F	-1.700760	-2.955261	2.364948
N	-0.055271	1.352157	0.894583
O	1.949734	5.496487	-4.709881
C	3.126222	3.561883	-0.633445
F	-1.767998	-3.530949	-2.293154
O	0.156199	-1.153720	0.774805
C	2.808585	4.689923	-1.370649
H	2.759868	5.668802	-0.911616
O	-6.374069	-1.962926	0.884267
C	2.550345	4.510105	-2.720085
O	-5.937420	-1.320337	-1.138470
C	2.611451	3.272130	-3.341515
H	2.425644	3.164648	-4.402187
C	2.939747	2.186021	-2.551401
C	4.619276	1.014875	0.414963
H	5.313949	1.849234	0.423429

C	4.889389	-0.109686	1.231714
C	4.019831	-1.233763	1.182019
C	2.886919	-1.203772	0.304781
C	2.044060	-2.407016	0.074274
C	0.688113	-2.344845	0.287190
C	-0.186001	-3.426685	0.001373
C	-1.653073	-3.233441	0.041611
C	-2.360285	-2.938238	1.205288
C	-3.698743	-2.582945	1.210122
H	-4.211702	-2.316589	2.125548
C	-4.330730	-2.513593	-0.020223
C	-3.722886	-2.881218	-1.207944
H	-4.239511	-2.834725	-2.157484
C	-2.389609	-3.236014	-1.143924
C	0.361284	-4.595726	-0.461343
H	-0.288718	-5.435507	-0.689188
C	1.749291	-4.720469	-0.706421
C	2.604593	-3.606544	-0.478122
C	3.970596	-3.720941	-0.849775
H	4.633814	-2.873494	-0.724055
C	4.458333	-4.890855	-1.374045
H	5.504776	-4.957226	-1.654269
C	3.614955	-6.009921	-1.558786
H	4.020255	-6.929912	-1.967039
C	2.286764	-5.922179	-1.237163
H	1.622192	-6.767146	-1.393402
C	4.284072	-2.334295	2.039207
H	3.616171	-3.188743	2.031106
C	5.366077	-2.318347	2.882503
H	5.549061	-3.164085	3.537650
C	6.245915	-1.212010	2.909496
H	7.101398	-1.220001	3.576831
C	6.010965	-0.131204	2.101080
H	6.673682	0.729248	2.121237
C	1.055346	1.433563	1.884171
H	1.891003	1.996857	1.472426
H	1.372994	0.436466	2.176387
H	0.668599	1.947437	2.766530
C	-0.282504	2.671238	0.236807
H	-0.321910	3.435064	1.014795
H	-1.233665	2.653044	-0.296746

H	0.530110	2.887230	-0.461314
O	2.148526	6.762685	-2.960688
O	-1.025365	-0.543210	-3.500420
C	0.341312	-0.735300	-3.927150
C	0.898985	-2.078598	-3.507059
H	0.894811	0.083135	-3.474060
H	0.360351	-0.612311	-5.014943
H	1.957516	-2.136890	-3.778113
H	0.816903	-2.211716	-2.426184
H	0.377378	-2.906098	-3.999002
H	-1.557782	-1.296284	-3.795262
H	-1.053673	1.193881	1.597653
C	-2.874844	0.770676	1.141544
N	-3.018645	0.405295	0.026009
C	-2.407034	1.249905	2.352678
C	-2.586227	2.727207	2.565898
C	-1.751226	3.425389	3.449355
C	-3.538698	3.455948	1.843188
C	-1.863751	4.804616	3.597433
H	-1.007116	2.888915	4.028272
C	-3.642057	4.836616	1.982346
H	-4.212712	2.936233	1.166862
C	-2.804143	5.518773	2.859529
H	-1.207750	5.322688	4.290652
H	-4.389801	5.378173	1.410910
H	-2.887831	6.594877	2.972853
C	-2.506139	0.288504	3.554500
H	-3.382959	0.568336	4.150736
H	-2.694283	-0.717512	3.168249
C	-1.285018	0.220672	4.448536
C	-1.214581	0.947732	5.638858
C	-0.206405	-0.595923	4.099995
C	-0.076519	0.893353	6.440225
H	-2.055957	1.568065	5.937629
C	0.932385	-0.654961	4.897819
H	-0.263191	-1.192821	3.195220
C	1.003909	0.097532	6.067741
H	-0.038205	1.467512	7.360948
H	1.762526	-1.293608	4.607700
H	1.889356	0.052253	6.694261
O	-3.693088	-0.223024	-2.714960

C	-4.130266	0.979796	-3.385600
C	-5.408000	0.727883	-4.161219
H	-3.304213	1.253264	-4.044928
H	-4.259674	1.773732	-2.641446
H	-5.709048	1.639614	-4.686382
H	-5.263486	-0.065477	-4.900403
H	-6.224717	0.439049	-3.491466
H	-4.407102	-0.504836	-2.109462

TS-3

ωB97XD SCF energy: -977.43604404 a.u.

ωB97XD enthalpy: -977.082564 a.u.

ωB97XD free energy: -977.154886 a.u.

ωB97XD SCF energy in solution: -977.73393717 a.u.

ωB97XD enthalpy in solution: -977.380457 a.u.

ωB97XD free energy in solution: -977.452779 a.u.

Imaginary frequency: -737.7309 cm^{-1}

Cartesian coordinates

ATOM	X	Y	Z
C	-0.007749	-0.699450	0.210925
C	1.381859	-1.289706	-0.105629
C	2.012799	-2.192010	0.750480
C	2.027946	-0.912786	-1.288199
C	3.264496	-2.711884	0.428555
H	1.530702	-2.493610	1.674642
C	3.277919	-1.432305	-1.605675
H	1.558234	-0.196041	-1.953575
C	3.901602	-2.335551	-0.748465
H	3.739687	-3.414197	1.106723
H	3.764764	-1.129318	-2.528027
H	4.877270	-2.742004	-0.997330
C	-1.029151	-1.091678	-0.900288
H	-0.648823	-0.687942	-1.841004
H	-1.011130	-2.183571	-0.979020
C	-2.439060	-0.604146	-0.658506
C	-3.344477	-1.383345	0.066295
C	-2.860565	0.633825	-1.151396
C	-4.640141	-0.933754	0.300238

H	-3.029201	-2.346504	0.458200
C	-4.156803	1.084285	-0.920071
H	-2.161902	1.246823	-1.712651
C	-5.049852	0.302123	-0.192642
H	-5.329664	-1.550906	0.868454
H	-4.469967	2.048046	-1.311175
H	-6.061604	0.653305	-0.012024
C	-0.496578	-1.202861	1.503924
N	-0.898007	-1.665035	2.485577
C	0.176154	0.828216	0.306897
O	0.205305	1.477401	-0.783209
O	0.368809	1.332297	1.443786
H	0.702554	2.680239	1.144092
O	0.908636	3.454447	0.412729
C	2.329160	3.737933	0.343855
C	3.176525	2.484530	0.197833
H	2.567013	4.283315	1.259398
H	2.455390	4.414832	-0.503625
H	4.233502	2.765686	0.163230
H	3.027585	1.804525	1.041417
H	2.933680	1.944310	-0.721673
H(iso=2)	0.601456	2.756481	-0.370327

Int-1a

ωB97XD SCF energy: -2927.36254204 a.u.
ωB97XD enthalpy: -2926.741537 a.u.
ωB97XD free energy: -2926.869699 a.u.
ωB97XD SCF energy in solution: -2929.22594502 a.u.
ωB97XD enthalpy in solution: -2928.604940 a.u.
ωB97XD free energy in solution: -2928.733102 a.u.

Cartesian coordinates

ATOM	X	Y	Z
C	1.843498	-0.959707	0.180648
F	1.411029	-4.391821	-1.670408
N	-2.196859	-6.008155	1.039612
O	0.522095	-0.614474	0.456851
P	-0.442684	0.028231	-0.708213
Rh	-1.962727	0.889704	0.675889

C	2.133785	-2.347026	0.137724
F	0.596888	-2.402802	2.504643
N	-4.430372	1.526580	-2.313121
C	1.052215	-3.327184	0.394936
F	-0.034013	3.166892	-3.188452
N	-0.829522	-1.001903	-1.915546
O	-2.843238	-5.859370	2.062214
C	0.711341	-4.315318	-0.531299
F	-1.759113	3.645987	1.117874
O	0.638039	0.976988	-1.462416
C	-0.335931	-5.204030	-0.351543
H	-0.575659	-5.951741	-1.096495
O	-4.580873	1.353226	-3.507110
C	-1.060144	-5.095525	0.824844
O	-5.219457	1.204699	-1.437238
C	-0.767661	-4.159895	1.805369
H	-1.334212	-4.113880	2.726288
C	0.291762	-3.302040	1.568382
C	3.414107	-2.730522	-0.173563
H	3.666547	-3.785997	-0.208435
C	4.414518	-1.776551	-0.480116
C	4.103792	-0.387432	-0.437594
C	2.788018	0.020944	-0.043810
C	2.385772	1.450595	0.082206
C	1.283086	1.872856	-0.610965
C	0.671752	3.146586	-0.442194
C	-0.714948	3.193937	-0.967759
C	-0.994765	2.999093	-2.292351
C	-2.230005	2.488214	-2.777277
H	-2.368182	2.306987	-3.836338
C	-3.178392	2.150393	-1.868117
C	-3.026022	2.383245	-0.463865
H	-3.929077	2.535352	0.116715
C	-1.791959	2.945778	-0.041463
C	1.283753	4.054512	0.375801
H	0.839274	5.031660	0.538017
C	2.473012	3.709256	1.073118
C	3.003873	2.387441	0.978438
C	4.098887	2.040376	1.810695
H	4.488257	1.029300	1.785395
C	4.667869	2.968302	2.646459

H	5.505748	2.682506	3.274241
C	4.172620	4.289618	2.702755
H	4.639618	5.013721	3.362204
C	3.092913	4.647227	1.938301
H	2.688839	5.654239	1.991384
C	5.102926	0.540434	-0.833588
H	4.872223	1.599817	-0.848825
C	6.349713	0.109467	-1.208983
H	7.098824	0.833400	-1.513404
C	6.668779	-1.267684	-1.211707
H	7.662143	-1.590561	-1.505331
C	5.718735	-2.189306	-0.859763
H	5.945911	-3.251426	-0.876324
C	0.141759	-1.487567	-2.901978
H	0.423144	-2.523194	-2.691968
H	1.035992	-0.865104	-2.889829
H	-0.309900	-1.432677	-3.897270
C	-2.085058	-1.748531	-1.859111
H	-2.537753	-1.766942	-2.855212
H	-2.788504	-1.262905	-1.175109
H	-1.915312	-2.779215	-1.524658
O	-2.415576	-6.837360	0.174876
O	-3.149595	1.498470	2.377522
C	-4.536442	1.107840	2.544365
C	-5.027879	1.453004	3.933637
H	-4.543790	0.029944	2.372119
H	-5.142869	1.583482	1.765448
H	-6.061232	1.113907	4.050726
H	-4.413866	0.966617	4.696257
H	-5.014226	2.535583	4.103377
H	-3.031646	2.424788	2.634304

Int-1b

ωB97XD SCF energy: -3082.39964995 a.u.

ωB97XD enthalpy: -3081.688200 a.u.

ωB97XD free energy: -3081.827638 a.u.

ωB97XD SCF energy in solution: -3084.31017061 a.u.

ωB97XD enthalpy in solution: -3083.598721 a.u.

ωB97XD free energy in solution: -3083.738159 a.u.

Cartesian coordinates

ATOM	X	Y	Z
C	2.161049	0.733207	-0.092847
F	4.584110	-1.618855	-2.138583
N	4.149207	-5.434887	0.728848
O	1.162279	-0.118389	0.347746
P	-0.066679	-0.597903	-0.662268
Rh	-1.637918	-1.375160	0.668068
C	3.450028	0.175336	-0.283246
F	2.817266	-1.068231	2.185295
N	-5.846194	-1.381351	-2.343105
C	3.672283	-1.261347	-0.001352
F	-2.378106	1.855681	-3.044903
N	0.502859	-1.454336	-1.938692
O	3.770217	-5.855591	1.807665
C	4.226888	-2.123332	-0.951620
F	-3.839025	0.933642	1.330011
O	-0.372257	0.828555	-1.409168
C	4.398789	-3.481684	-0.744092
H	4.825369	-4.118807	-1.508108
O	-6.000497	-1.448149	-3.546726
C	3.999704	-3.989770	0.481699
O	-6.391443	-2.091903	-1.512158
C	3.459329	-3.198784	1.483821
H	3.173284	-3.617825	2.439801
C	3.319516	-1.849533	1.217062
C	4.442712	0.999372	-0.752727
H	5.442974	0.606179	-0.905993
C	4.187437	2.357456	-1.064630
C	2.885900	2.899491	-0.861517
C	1.864883	2.057897	-0.314401
C	0.493048	2.544298	-0.001066
C	-0.587880	1.907124	-0.564242
C	-1.933111	2.267921	-0.286666
C	-3.026132	1.433883	-0.836814
C	-3.173485	1.201006	-2.207411
C	-4.093274	0.305135	-2.732193
H	-4.169363	0.139172	-3.799305
C	-4.902258	-0.372265	-1.835741
C	-4.849362	-0.160195	-0.466187

H	-5.524711	-0.675785	0.204469
C	-3.909402	0.742551	-0.003824
C	-2.163276	3.310844	0.574258
H	-3.182517	3.608648	0.801991
C	-1.093706	3.980408	1.218506
C	0.249085	3.585466	0.953980
C	1.291887	4.202041	1.695149
H	2.317968	3.893767	1.530504
C	1.013156	5.174901	2.621159
H	1.823444	5.631206	3.180753
C	-0.317589	5.589964	2.856733
H	-0.519101	6.367489	3.586235
C	-1.347487	5.002638	2.170544
H	-2.375383	5.303597	2.352486
C	2.644923	4.245402	-1.241820
H	1.650834	4.663063	-1.126210
C	3.652663	5.015076	-1.764919
H	3.449578	6.040842	-2.055614
C	4.952239	4.485928	-1.936801
H	5.738514	5.110664	-2.347524
C	5.211010	3.184296	-1.597495
H	6.201936	2.762626	-1.739811
C	1.287712	-0.875862	-3.032776
H	2.347282	-1.129085	-2.923651
H	1.176996	0.207593	-3.046252
H	0.921478	-1.277582	-3.982725
C	0.568044	-2.911651	-1.843735
H	0.196451	-3.355685	-2.772565
H	-0.048742	-3.268554	-1.013202
H	1.599996	-3.245236	-1.678571
O	4.632668	-6.104211	-0.166488
O	-3.265075	-1.916998	2.012991
C	-4.150766	-2.991754	1.619161
C	-5.346266	-3.075777	2.544700
H	-3.537299	-3.892496	1.673670
H	-4.468595	-2.841213	0.578913
H	-5.982076	-3.915561	2.250625
H	-5.026946	-3.224337	3.579786
H	-5.957302	-2.166588	2.489648
H	-3.787528	-1.117556	2.178008
O	-0.495607	-0.762035	2.339133

C	-0.839896	0.526820	2.919695
C	0.348964	1.106713	3.655602
H	-1.677937	0.323549	3.588355
H	-1.173571	1.198827	2.126056
H	0.073454	2.075142	4.084176
H	0.680050	0.445409	4.461230
H	1.184317	1.278880	2.967488
H	0.427877	-0.695272	2.032886

Int-6R

ωB97XD SCF energy: -3716.38341491 a.u.

ωB97XD enthalpy: -3715.414662 a.u.

ωB97XD free energy: -3715.587963 a.u.

ωB97XD SCF energy in solution: -3718.46254507 a.u.

ωB97XD enthalpy in solution: -3717.493792 a.u.

ωB97XD free energy in solution: -3717.667093 a.u.

Cartesian coordinates

ATOM	X	Y	Z
C	3.257680	0.358987	0.585973
F	3.421073	4.011652	1.947313
N	3.071678	6.231884	-2.288808
O	2.295246	0.487999	-0.407056
P	0.722654	0.104742	-0.290446
Rh	-0.392807	-0.381899	-2.052409
C	4.052452	1.511519	0.823837
F	4.246825	1.593290	-1.987016
N	-5.211502	-2.744079	0.653015
C	3.840772	2.736991	0.019393
F	-0.972922	-2.202840	2.805135
N	0.069572	1.629673	0.663570
O	3.165021	6.132058	-3.498458
C	3.530091	3.962811	0.612541
F	-1.133543	-3.898568	-1.566891
O	0.685008	-0.788255	1.090017
C	3.280676	5.117646	-0.108244
H	3.033266	6.049523	0.383169
O	-5.720881	-2.241281	1.646673
C	3.356586	5.025762	-1.489620

O	-5.820314	-3.089522	-0.342789
C	3.676055	3.851035	-2.153076
H	3.739528	3.811966	-3.232863
C	3.921543	2.730957	-1.376973
C	5.001141	1.454299	1.812486
H	5.624121	2.321598	2.009544
C	5.180824	0.288378	2.594668
C	4.395878	-0.866878	2.322708
C	3.450784	-0.834333	1.241646
C	2.682860	-2.049938	0.857872
C	1.308878	-2.018529	0.866988
C	0.500805	-3.152742	0.603818
C	-0.974317	-3.052545	0.618055
C	-1.670543	-2.578510	1.731749
C	-3.049127	-2.484951	1.778992
H	-3.556036	-2.128124	2.665539
C	-3.750247	-2.891875	0.651519
C	-3.132612	-3.379293	-0.488388
H	-3.699834	-3.678763	-1.360027
C	-1.748873	-3.447381	-0.476134
C	1.131698	-4.334875	0.307261
H	0.540686	-5.223290	0.107067
C	2.540786	-4.413854	0.195816
C	3.334170	-3.260667	0.450645
C	4.735120	-3.341955	0.239057
H	5.351682	-2.464180	0.395822
C	5.311632	-4.514666	-0.178822
H	6.383201	-4.556375	-0.345874
C	4.526112	-5.669024	-0.399019
H	4.999892	-6.589880	-0.722823
C	3.169303	-5.617585	-0.216035
H	2.552759	-6.492841	-0.399548
C	4.558051	-2.001836	3.160052
H	3.946053	-2.881037	2.995820
C	5.470594	-1.992744	4.184393
H	5.574860	-2.868572	4.816865
C	6.272586	-0.855625	4.430545
H	6.994175	-0.868751	5.240716
C	6.125677	0.262555	3.653287
H	6.724552	1.149348	3.840402
C	0.606786	1.899795	2.027714

H	1.636748	2.245403	1.948052
H	0.564788	0.983460	2.613329
H	-0.010825	2.674433	2.488609
C	0.167826	2.817581	-0.231577
H	-0.285970	3.674091	0.267977
H	-0.349314	2.595988	-1.167287
H	1.220230	3.014080	-0.434243
O	2.753952	7.238298	-1.679204
O	1.219312	-0.553461	-3.426420
C	2.354166	-1.421229	-3.255291
C	1.959041	-2.856989	-2.975671
H	2.918182	-0.989224	-2.427040
H	2.963737	-1.339408	-4.160993
H	2.852684	-3.464256	-2.796865
H	1.315998	-2.911820	-2.092974
H	1.409974	-3.287612	-3.819588
H	0.612940	-0.893250	-4.125394
H	-4.625628	-0.263594	0.836092
C	-3.097425	0.135385	-0.515371
N	-2.123002	-0.120555	-1.081487
C	-4.253941	0.590005	0.262053
C	-3.757780	1.639349	1.248631
C	-3.943246	1.447419	2.616719
C	-3.137484	2.807822	0.795446
C	-3.510247	2.409607	3.525761
H	-4.450726	0.553665	2.969239
C	-2.713849	3.774456	1.705237
H	-3.012203	2.977951	-0.272515
C	-2.894471	3.573625	3.073286
H	-3.667773	2.254858	4.588222
H	-2.268300	4.697862	1.345463
H	-2.574692	4.331569	3.781416
C	-5.406925	1.073825	-0.659309
H	-5.064284	1.929345	-1.250594
H	-5.645832	0.265987	-1.359591
C	-6.613302	1.435401	0.171678
C	-6.858881	2.760595	0.533862
C	-7.466363	0.431353	0.635874
C	-7.943527	3.080578	1.345136
H	-6.198857	3.547860	0.177670
C	-8.548392	0.749898	1.450248

H	-7.284774	-0.606231	0.365440
C	-8.789317	2.075054	1.805441
H	-8.129778	4.115663	1.614487
H	-9.206940	-0.038094	1.801789
H	-9.637414	2.323763	2.435788
O	-1.203159	-0.951827	-3.753952
C	-2.536073	-1.322374	-3.937825
C	-2.717495	-1.894998	-5.336854
H	-3.209794	-0.457522	-3.807649
H	-2.844516	-2.079628	-3.195189
H	-3.759327	-2.188280	-5.507903
H	-2.437517	-1.151113	-6.089218
H	-2.081037	-2.775899	-5.470175
H	-0.932580	1.427587	0.770464

Int-6S

ωB97XD SCF energy: -3716.38814494 a.u.

ωB97XD enthalpy: -3715.418973 a.u.

ωB97XD free energy: -3715.588828 a.u.

ωB97XD SCF energy in solution: -3718.46409621 a.u.

ωB97XD enthalpy in solution: -3717.494924 a.u.

ωB97XD free energy in solution: -3717.664779 a.u.

Cartesian coordinates

ATOM	X	Y	Z
C	3.196832	0.412687	0.260805
F	3.584107	3.841659	2.082275
N	1.976217	6.567589	-1.520100
O	2.000445	0.625403	-0.405406
P	0.524548	0.102056	0.021447
Rh	-0.896709	-0.127237	-1.556849
C	3.991581	1.572903	0.462009
F	3.428666	2.058531	-2.254028
N	-4.946076	-3.465502	1.403626
C	3.521480	2.878296	-0.059261
F	-0.418897	-2.888523	2.847889
N	0.106942	1.355646	1.393245
O	1.735008	6.638088	-2.711450
C	3.323587	3.980777	0.775081

F	-1.308882	-3.683817	-1.692388
O	0.867639	-1.028672	1.165774
C	2.828563	5.194777	0.331010
H	2.677806	6.026726	1.006552
O	-5.286625	-3.417605	2.574761
C	2.529683	5.296385	-1.019009
O	-5.710107	-3.512477	0.452659
C	2.716864	4.255519	-1.915451
H	2.485481	4.367411	-2.966814
C	3.220533	3.068565	-1.411868
C	5.166664	1.442155	1.155581
H	5.793378	2.313210	1.321829
C	5.577411	0.190388	1.675881
C	4.786359	-0.967139	1.431062
C	3.591695	-0.847810	0.642332
C	2.804326	-2.047397	0.243381
C	1.470227	-2.136847	0.565497
C	0.666805	-3.263354	0.257591
C	-0.781556	-3.289737	0.563727
C	-1.281428	-3.120676	1.856054
C	-2.628928	-3.194235	2.163255
H	-2.979360	-3.104182	3.183441
C	-3.506736	-3.425551	1.113694
C	-3.089796	-3.592142	-0.196588
H	-3.793446	-3.758873	-1.001750
C	-1.728208	-3.525161	-0.438751
C	1.263039	-4.316959	-0.389424
H	0.676815	-5.197756	-0.632874
C	2.612459	-4.260441	-0.811690
C	3.393474	-3.106946	-0.523225
C	4.707460	-3.030445	-1.054844
H	5.306173	-2.144492	-0.877685
C	5.219368	-4.058996	-1.804566
H	6.222391	-3.978798	-2.211330
C	4.454987	-5.220677	-2.057996
H	4.878853	-6.027502	-2.646766
C	3.177721	-5.316712	-1.572400
H	2.572435	-6.195093	-1.777223
C	5.197235	-2.197320	2.007698
H	4.592536	-3.084970	1.860987
C	6.343018	-2.271565	2.758819

H	6.636571	-3.220376	3.196494
C	7.143193	-1.127098	2.974129
H	8.049828	-1.205390	3.564990
C	6.764140	0.078684	2.445871
H	7.362511	0.969428	2.615075
C	1.097525	1.501281	2.496441
H	2.012239	1.939872	2.105644
H	1.306434	0.520198	2.919450
H	0.675777	2.164220	3.252950
C	-0.237110	2.667229	0.774166
H	-0.621975	3.329177	1.551578
H	-0.992232	2.504612	0.003342
H	0.663982	3.086014	0.322945
O	1.791125	7.452426	-0.702625
O	0.451989	0.150474	-3.182648
C	1.605396	-0.672351	-3.436783
C	1.287653	-2.153641	-3.421539
H	2.321977	-0.407166	-2.658949
H	2.018813	-0.357911	-4.400532
H	2.203096	-2.733938	-3.578814
H	0.851411	-2.441879	-2.461222
H	0.571210	-2.413119	-4.207864
H	-0.272999	-0.055728	-3.820162
H	-0.758428	0.987855	1.815395
C	-3.561210	-0.225735	0.068101
N	-2.494471	-0.266391	-0.370020
C	-4.918601	-0.038135	0.576974
C	-5.554882	1.197293	-0.059590
C	-4.822189	2.159556	-0.752912
C	-6.932600	1.366979	0.098399
C	-5.459641	3.284651	-1.272078
H	-3.754156	2.035316	-0.909058
C	-7.565846	2.492074	-0.416208
H	-7.516242	0.613129	0.621625
C	-6.828798	3.456525	-1.100972
H	-4.880791	4.023914	-1.816702
H	-8.637122	2.611195	-0.289864
H	-7.323608	4.332664	-1.507437
C	-4.900983	0.005891	2.133807
H	-5.910113	0.284343	2.451522
H	-4.725070	-1.003761	2.513488

C	-3.885551	0.950579	2.728937
C	-4.056698	2.337692	2.675329
C	-2.763247	0.437631	3.388389
C	-3.135664	3.190176	3.278991
H	-4.928229	2.753189	2.178632
C	-1.854132	1.287518	4.017233
H	-2.620612	-0.638013	3.450756
C	-2.037928	2.669025	3.961709
H	-3.294314	4.263563	3.243372
H	-1.024274	0.865697	4.577900
H	-1.348737	3.334523	4.474393
O	-1.962826	-0.369059	-3.202255
C	-3.297462	-0.765712	-3.288444
C	-3.660513	-1.020501	-4.744869
H	-3.971402	0.006461	-2.877016
H	-3.476440	-1.687604	-2.705532
H	-4.707457	-1.329715	-4.838772
H	-3.512304	-0.111277	-5.335933
H	-3.025024	-1.809102	-5.160917
H	-5.495536	-0.921658	0.274954

TS-4R

ωB97XD SCF energy: -3716.354353 a.u.

ωB97XD enthalpy: -3715.390767 a.u.

ωB97XD free energy: -3715.557431 a.u.

ωB97XD SCF energy in solution: -3718.42543400 a.u.

ωB97XD enthalpy in solution: -3717.461848 a.u.

ωB97XD free energy in solution: -3717.628512 a.u.

Imaginary frequency: -1211.57 cm^{-1}

Cartesian coordinates

ATOM	X	Y	Z
C	-3.350486	0.040853	-0.105922
F	-4.304968	3.073530	-2.327697
N	-1.966908	6.281614	0.354761
O	-2.096167	0.451891	0.323755
P	-0.667757	0.012022	-0.292368
Rh	1.029172	0.156284	0.974880
C	-4.278804	1.093224	-0.323743

F	-3.316841	2.053832	2.141908
N	4.568337	-2.515908	-3.008710
C	-3.826889	2.487968	-0.106195
F	-0.200080	-2.968270	-3.123599
N	-0.447255	1.135690	-1.773845
O	-1.426579	6.507278	1.424376
C	-3.813065	3.430757	-1.135747
F	2.058189	-3.379781	0.975630
O	-1.100928	-1.218453	-1.279748
C	-3.249332	4.690012	-1.008377
H	-3.223676	5.383751	-1.838599
O	4.519819	-2.171115	-4.178084
C	-2.681199	5.003033	0.216981
O	5.584925	-2.654074	-2.352883
C	-2.699777	4.140311	1.302341
H	-2.258993	4.416776	2.250985
C	-3.289729	2.902946	1.116974
C	-5.538065	0.769191	-0.755086
H	-6.268333	1.554002	-0.928057
C	-5.903898	-0.578105	-0.996501
C	-4.963495	-1.620966	-0.757682
C	-3.663318	-1.292243	-0.240339
C	-2.668559	-2.354643	0.088058
C	-1.432613	-2.332795	-0.512436
C	-0.442899	-3.333196	-0.346163
C	0.857747	-3.186979	-1.038470
C	0.935276	-2.970787	-2.417465
C	2.124191	-2.750117	-3.086614
H	2.147580	-2.575728	-4.154209
C	3.286879	-2.775277	-2.332458
C	3.296772	-3.012517	-0.969163
H	4.221782	-3.014571	-0.407697
C	2.072756	-3.200369	-0.351786
C	-0.734195	-4.376637	0.494314
H	-0.004746	-5.166183	0.646536
C	-1.952580	-4.419699	1.219023
C	-2.928748	-3.398002	1.037658
C	-4.093015	-3.420217	1.848746
H	-4.831359	-2.633037	1.743785
C	-4.284687	-4.418155	2.770943
H	-5.176931	-4.414591	3.388867

C	-3.332252	-5.450618	2.926262
H	-3.503652	-6.237571	3.653401
C	-2.191971	-5.449368	2.165848
H	-1.446372	-6.230008	2.287216
C	-5.340585	-2.951216	-1.078717
H	-4.628008	-3.756529	-0.943743
C	-6.590534	-3.227197	-1.572611
H	-6.855813	-4.250667	-1.817590
C	-7.533879	-2.195017	-1.773670
H	-8.520029	-2.431848	-2.159162
C	-7.193226	-0.897672	-1.495727
H	-7.902424	-0.091917	-1.662590
C	-1.443616	1.027293	-2.875475
H	-2.415533	1.364494	-2.514992
H	-1.505896	-0.008462	-3.202535
H	-1.118335	1.668112	-3.697566
C	-0.234634	2.542615	-1.321964
H	-0.002177	3.161052	-2.189434
H	0.596438	2.568864	-0.616070
H	-1.144924	2.894705	-0.838740
O	-1.930644	7.009541	-0.622193
O	-0.072971	0.205663	2.819729
C	-0.964743	-0.860233	3.224877
C	-0.245360	-2.179254	3.404472
H	-1.713594	-0.913297	2.435260
H	-1.461738	-0.532666	4.143414
H	-0.967493	-2.961433	3.660902
H	0.265121	-2.467143	2.481086
H	0.494559	-2.123290	4.211232
H	0.554428	0.372315	3.537190
H	0.472693	0.797528	-2.100442
C	3.222390	0.599387	-0.625502
N	2.128685	0.242870	-0.914697
C	4.368113	1.030154	0.037424
C	5.501752	0.034390	0.128856
C	5.362309	-1.094280	0.945327
C	6.700446	0.220722	-0.561676
C	6.396281	-2.017760	1.060760
H	4.435580	-1.241671	1.493781
C	7.735558	-0.702726	-0.443129
H	6.835115	1.084365	-1.205518

C	7.587236	-1.824379	0.365833
H	6.273652	-2.886402	1.701516
H	8.658186	-0.546622	-0.993000
H	8.395130	-2.543515	0.454603
C	4.717256	2.515605	-0.142280
H	5.359931	2.794343	0.701537
H	5.322070	2.653767	-1.045286
C	3.526009	3.449869	-0.212989
C	2.715967	3.688298	0.903711
C	3.241956	4.136774	-1.395714
C	1.676510	4.614182	0.849037
H	2.913559	3.158698	1.830776
C	2.206202	5.067976	-1.454315
H	3.861269	3.967888	-2.273358
C	1.426329	5.318693	-0.328452
H	1.074031	4.808147	1.731596
H	2.027444	5.617557	-2.373999
H	0.651835	6.078984	-0.360504
O	2.795829	0.515587	2.018411
C	2.966024	0.808279	3.384662
C	4.410707	1.178614	3.690502
H	2.685726	-0.074592	3.981568
H	2.309570	1.646296	3.679902
H	4.539127	1.370665	4.760050
H	5.084852	0.369190	3.395844
H	4.702027	2.083336	3.145445
H	3.667897	0.875276	1.199436

TS-4S

ωB97XD SCF energy: -3716.34502459 a.u.

ωB97XD enthalpy: -3715.381250 a.u.

ωB97XD free energy: -3715.549864 a.u.

ωB97XD SCF energy in solution: -3718.41636469 a.u.

ωB97XD enthalpy in solution: -3717.452590 a.u.

ωB97XD free energy in solution: -3717.621204 a.u.

Imaginary frequency: -1076.5285 cm^{-1}

Cartesian coordinates

ATOM X Y Z

C	-3.424379	0.352300	-0.451552
F	-3.706562	3.558013	-2.696996
N	-2.171751	6.705657	0.578526
O	-2.229743	0.586029	0.228184
P	-0.765056	0.035982	-0.220911
Rh	0.949165	-0.006501	1.059252
C	-4.160210	1.507392	-0.827775
F	-3.605654	2.305085	1.826455
N	4.628506	-3.660424	-0.628057
C	-3.679247	2.857459	-0.454728
F	0.147318	-3.505809	-2.314768
N	-0.406272	1.051306	-1.759092
O	-1.912500	6.902151	1.752312
C	-3.473393	3.854834	-1.411992
F	0.876267	-3.117040	2.308959
O	-1.174509	-1.271090	-1.123734
C	-2.992683	5.117075	-1.108654
H	-2.835286	5.862941	-1.877072
O	5.001567	-3.933092	-1.752757
C	-2.711610	5.380379	0.222748
O	5.358824	-3.513889	0.342064
C	-2.903307	4.450757	1.233293
H	-2.689725	4.691011	2.266739
C	-3.395451	3.210044	0.868413
C	-5.314916	1.336406	-1.550188
H	-5.897019	2.204446	-1.844382
C	-5.759839	0.050494	-1.938132
C	-5.030313	-1.097004	-1.520846
C	-3.867403	-0.928304	-0.698708
C	-3.152423	-2.093271	-0.112818
C	-1.805759	-2.238653	-0.341888
C	-1.020635	-3.268604	0.232704
C	0.440299	-3.329715	0.001874
C	0.984256	-3.457252	-1.277302
C	2.343377	-3.555627	-1.513159
H	2.734363	-3.688097	-2.513441
C	3.182849	-3.502168	-0.410883
C	2.722660	-3.338024	0.886370
H	3.400890	-3.268879	1.725787
C	1.353270	-3.261583	1.054229
C	-1.654297	-4.179083	1.040464

H	-1.082004	-4.986284	1.488288
C	-3.035597	-4.076097	1.335224
C	-3.800146	-3.012001	0.778517
C	-5.153284	-2.870187	1.184186
H	-5.742256	-2.042585	0.806966
C	-5.719982	-3.764323	2.056659
H	-6.755152	-3.637592	2.357124
C	-4.971652	-4.846430	2.572822
H	-5.438949	-5.549312	3.254619
C	-3.655789	-4.994125	2.222597
H	-3.064007	-5.810443	2.626934
C	-5.465238	-2.372920	-1.966197
H	-4.902721	-3.256642	-1.686578
C	-6.579402	-2.494968	-2.756913
H	-6.892930	-3.477680	-3.094278
C	-7.321670	-1.355555	-3.143240
H	-8.204268	-1.471869	-3.763482
C	-6.917089	-0.109259	-2.744269
H	-7.470318	0.775445	-3.046247
C	-1.309445	0.916706	-2.936704
H	-2.237259	1.454375	-2.743077
H	-1.515656	-0.136826	-3.113704
H	-0.808389	1.355341	-3.802385
C	-0.165861	2.476148	-1.384649
H	0.647264	2.517628	-0.659097
H	-1.076848	2.887403	-0.951965
H	0.106604	3.031372	-2.283884
O	-2.016047	7.502678	-0.329075
O	0.040598	-0.365300	2.947830
C	-1.233889	0.100409	3.440577
C	-2.314052	-0.948056	3.290529
H	-1.465479	0.993815	2.860826
H	-1.089188	0.386379	4.486978
H	-3.270188	-0.552062	3.644943
H	-2.433718	-1.232034	2.243609
H	-2.087954	-1.847795	3.874164
H	0.139179	-1.313868	3.121016
C	3.196281	0.567111	-0.481767
N	2.085855	0.314393	-0.802076
C	4.363270	0.817654	0.246824
C	4.612556	2.280630	0.524160

C	5.844360	2.882843	0.262854
C	3.597097	3.042960	1.116391
C	6.051181	4.224777	0.574898
H	6.644710	2.311774	-0.197859
C	3.803928	4.382886	1.421596
H	2.646089	2.571548	1.359929
C	5.033721	4.979402	1.149778
H	7.013722	4.679896	0.363234
H	3.009602	4.960255	1.885644
H	5.198875	6.024590	1.391405
C	5.532828	-0.131879	-0.000317
H	6.341789	0.177616	0.672958
H	5.242192	-1.130240	0.342043
C	6.050546	-0.230415	-1.425101
C	6.848209	-1.325203	-1.771749
C	5.768242	0.726453	-2.401718
C	7.348897	-1.460844	-3.061320
H	7.059677	-2.090700	-1.029592
C	6.273519	0.594777	-3.694023
H	5.159486	1.593724	-2.158558
C	7.065180	-0.498518	-4.028094
H	7.956353	-2.324779	-3.312408
H	6.048104	1.352727	-4.438560
H	7.457096	-0.602957	-5.035135
O	2.751702	-0.116674	2.051727
C	2.923382	0.087111	3.440081
C	4.405371	0.208632	3.766175
H	2.491685	-0.760911	3.989321
H	2.391199	0.992724	3.763461
H	4.553538	0.309615	4.846004
H	4.946775	-0.680147	3.424299
H	4.837380	1.090176	3.279632
H	3.714501	0.363116	1.283534
H	0.527969	0.667871	-1.986423

Section 12. Supplementary references

1. Ensign, S. C.; Vanable, E. P.; Kortman, G. D.; Weir, L. J.; Hull, K. Anti-markovnikov hydroamination of homoallylic amines. *J. Am. Chem. Soc.* **2015**, *137*, 13748−13751.
2. Lee, B. J.; Ickes, A. R.; Gupta, A. K.; Ensign, S. C.; Ho, T. D.; Tarasewicz, A.; Vanable, E. P.; Kortman, G. D.; Hull, K. L. Synthesis of unsymmetrical vicinal diamines via directed hydroamination. *Org. Lett.* **2022**, *24*, 5513−5518.
3. Simonsen, K. B.; Gothelf, K. V.; Jorgensen, K. A. A simple synthetic approach to 3,3'-diaryl BINOLs. *J. Org. Chem.* **1998**, *63*, 7536−7538.
4. Wipf, P.; Jung J.-K. Formal total synthesis of (+)-diepoxin σ. *J. Org. Chem.* **2000**, *65*, 6319−6337.
5. Xu, P.; Huang, Z. Catalytic reductive desymmetrization of malonic esters. *Nat. Chem.* **2021**, *13*, 634−642.
6. Wang, H.; Qiu, S.; Wang, S.; Zhai, H. Pd-catalyzed umpolung of π−allylpalladium intermediates: assembly of all-carbon α-vinyl quaternary aldehydes through $C(sp^3)-C(sp^3)$ coupling. *ACS Catal.* **2018**, *8*, 11960−11965.
7. Zhang, Q., Zhang, J., Qian, H. & Ma, S. Aerobic oxidation of PMB ethers to carboxylic acids. *Chem. -Eur. J.* **2024**, *30*, e202401815.
8. Shibatomi, K. et al. Enantioselective decarboxylative chlorination of β-ketocarboxylic acids. *Nat. Commun.* **2017**, *8*, 15600−15606.
9. Chen, D.; Shen, A.; Li, J.; Shi, F.; Chen, W.; Ren, J.; Liu, H.; Xu, Y.; Wang, X.; Yang, X.; Sun, Y.; Ynag, M.; He, J.; Wang, Y.; Zhang, L.; Huang, M.; Geng, M.; Xiong, B.; Shen, J. Discovery of potent *N*-(isoxazol-5-yl)amides as HSP90 inhibitors. *Eur. J. Med. Chem.* **2014**, *87*, 765−781.
10. Doran, R.; Guiry, P. J. Catalytic asymmetric synthesis of sterically hindered tertiary α-aryl ketones. *J. Org. Chem.* **2014**, *79*, 9112−9124.
11. Craig, II, R. A.; Loskot, S. A.; Mohr, J. T.; Behenna, D. C.; Harned, A. M.; Stoltz, B. M. Palladium-catalyzed enantioselective decarboxylative allylic alkylation of cyclopentanones. *Org. Lett.* **2015**, *17*, 5160−5163.
12. Marcus, J. van Meurs, P. J.; van den Nieuwendijk, A, M. C. H.; Porchet, M.; Brussee, J.; van der Gen, A. Synthesis of γ-hydroxy-α,β-unsaturated esters and nitriles from chiral cyanohydrins. *Tetrahedron*, **2000**, *56*, 2491−2495.
13. Gaussian 16, Revision C.01, Frisch, M. J. et al. Gaussian, Inc., Wallingford CT (2016)
14. Chai, J.-D.; Head-Gordon, M. Long-range corrected hybrid density functionals with damped atom-atom dispersion corrections, *Phys. Chem. Chem. Phys*. **2008**, *10*, 6615−6620.
15. Marenich, A. V.; Cramer, C. J.; Truhlar, D. G. Universal solvation model based on solute electron density and on a continuum model of the solvent defined by the bulk dielectric constant and atomic surface tensions. *J. Phys. Chem. B* **2009**, *113*, 6378−6396.
16. Legault, C. Y. CYLview 1.0b, Visualization and analysis software for computational chemistry. Université de Sherbrooke (2009). (http://www.cylview.org).
17. Persistence of Vision Raytracer (Version 3.6), Persistence of Vision Pty. Ltd. (2004). (http://www.povray.org)
18. Zheng, W.-F.; Chen, J.; Qi, X.; Huang, Z. Modular and diverse synthesis of amino acids via

asymmetric decarboxylative protonation of aminomalonic acids. *Nat. Chem.* **2023**, *15*, 1672–1682.
19. Khaliullin, R. Z.; Cobar, E. A.; Lochan, R. C.; Bell, A. T.; Head-Gordon, M. Unravelling the origin of intermolecular interactions using absolutely localized molecular orbitals. *J. Phys.Chem. A* **2007**, *111*, 8753−8765.
20. Horn, P. R.; Mao, Y.; Head-Gordon, M. Defining the contributions of permanent electrostatics, Pauli repulsion, and dispersion in density functional theory calculations of intermolecular interaction energies. *J. Chem. Phys.* **2016**, *144*, 114107.
21. Horn, P. R.; Mao, Y.; Head-Gordon, M. Probing non-covalent interactions with a second generation energy decomposition analysis using absolutely localized molecular orbitals. *Phys. Chem. Chem. Phys.* **2016**, *18*, 23067−23079.
22. Mao, Y.; Demerdash, O.; Head-Gordon, M.; Head-Gordon, T. Assessing ion-water interactions in the AMOEBA force field using energy decomposition analysis of electronic structure calculations. *J. Chem. Theory Comput.* **2016**, *12*, 5422−5437.
23. Shao, Y. Gan, Z.; Epifanovsky, E.; Gilbert, A. T. B.; Gilbert, M. W.; Kussmann, J.; Head-Gordon, M. Advances in molecular quantum chemistry contained in the Q-Chem 4 program package. *Mol. Phys.* **2015**, *113*, 184–215.

Section 13. Copies of spectra and traces

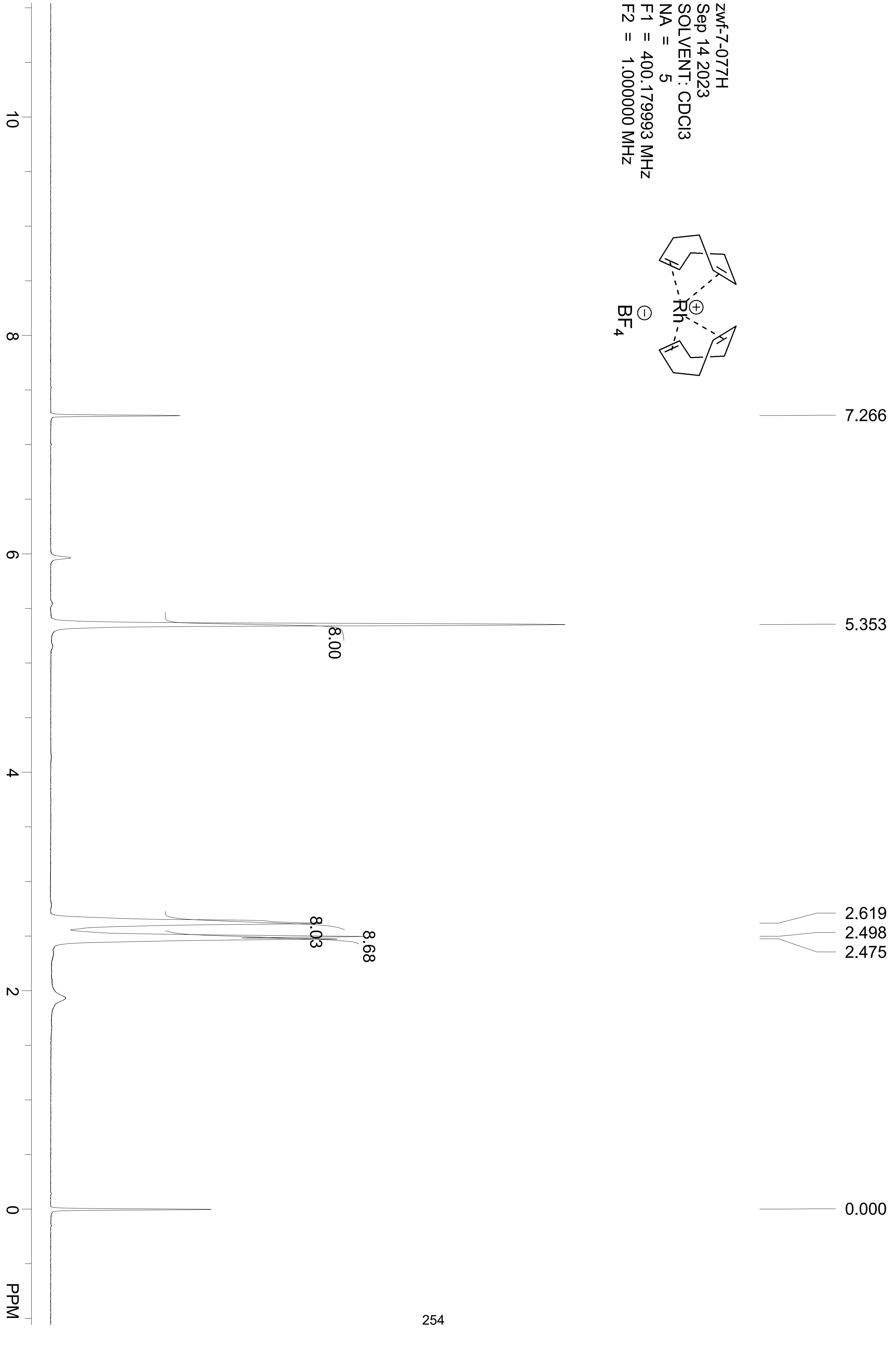
zwf-7-077H
Sep 14 2023
SOLVENT: CDCl3
NA = 5
F1 = 400.179993 MHz
F2 = 1.000000 MHz
Rh
BF4
7.266
5.353
2.619
2.498
2.475
0.000
8.00
8.03
8.68
10
8
6
4
2
0
PPM

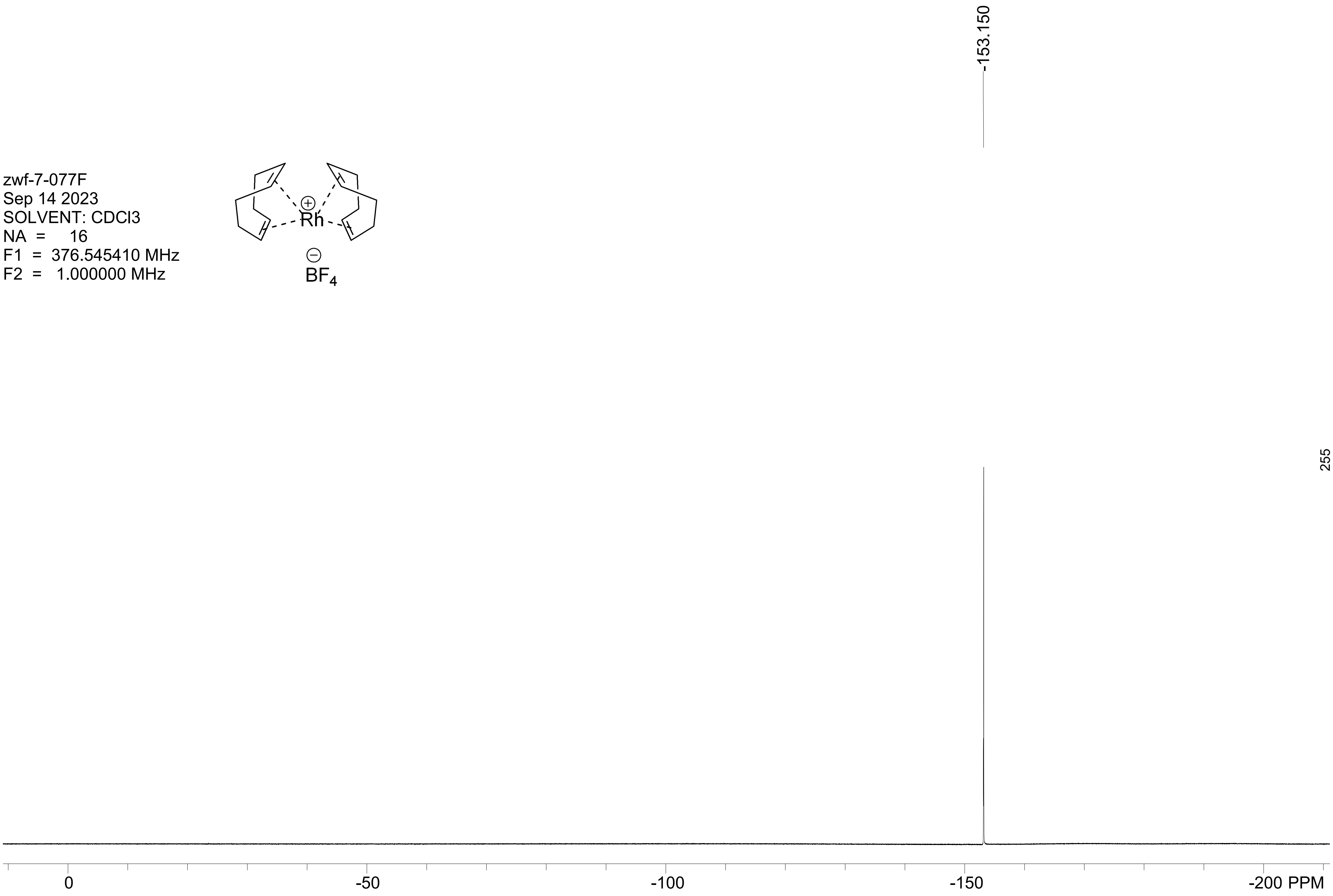
zwf-7-077F
Sep 14 2023
SOLVENT: CDCl3
NA = 16
F1 = 376.545410 MHz
F2 = 1.000000 MHz
Rh
BF4
-153.150
0
-50
-100
-150
-200 PPM

zwf-7-041H
Sep 01 2023
SOLVENT: CDCl3
NA = 16
F1 = 400.179993 MHz
F2 = 1.000000 MHz

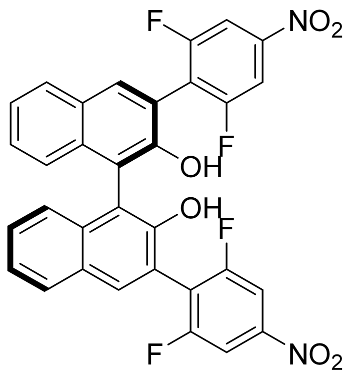

(*R*)-diol

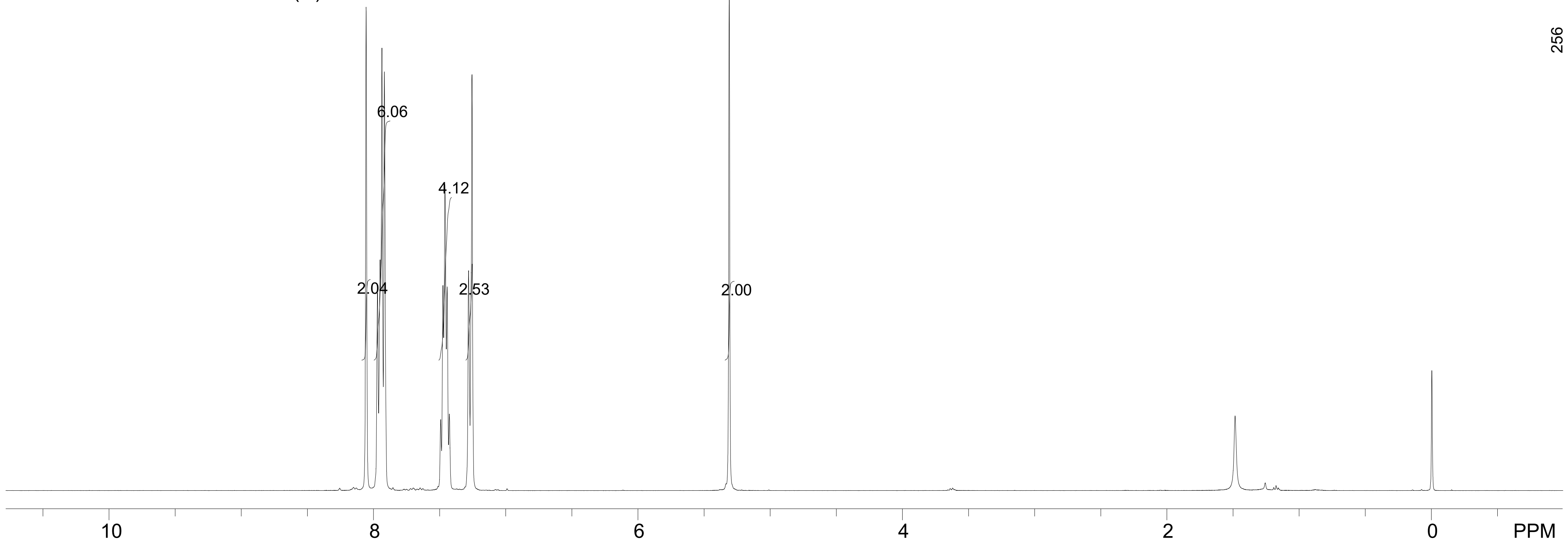

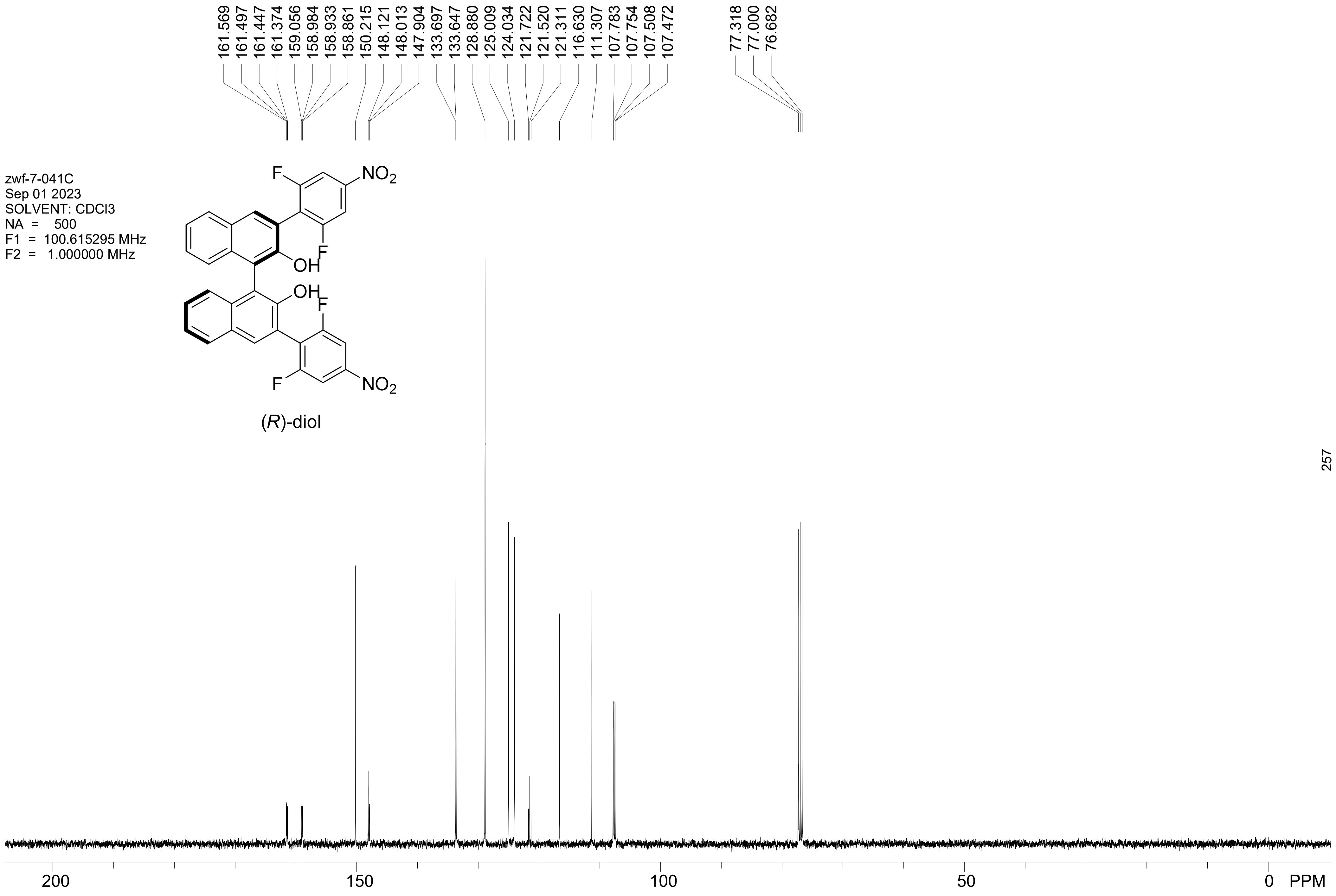
zwf-7-041C
Sep 01 2023
SOLVENT: CDCl3
NA = 500
F1 = 100.615295 MHz
F2 = 1.000000 MHz
(R)-diol
161.569
161.497
161.447
161.374
159.056
158.984
158.933
158.861
150.215
148.121
148.013
147.904
133.697
133.647
128.880
125.009
124.034
121.722
121.520
121.311
116.630
111.307
107.783
107.754
107.508
107.472
77.318
77.000
76.682
200
150
100
50
0 PPM

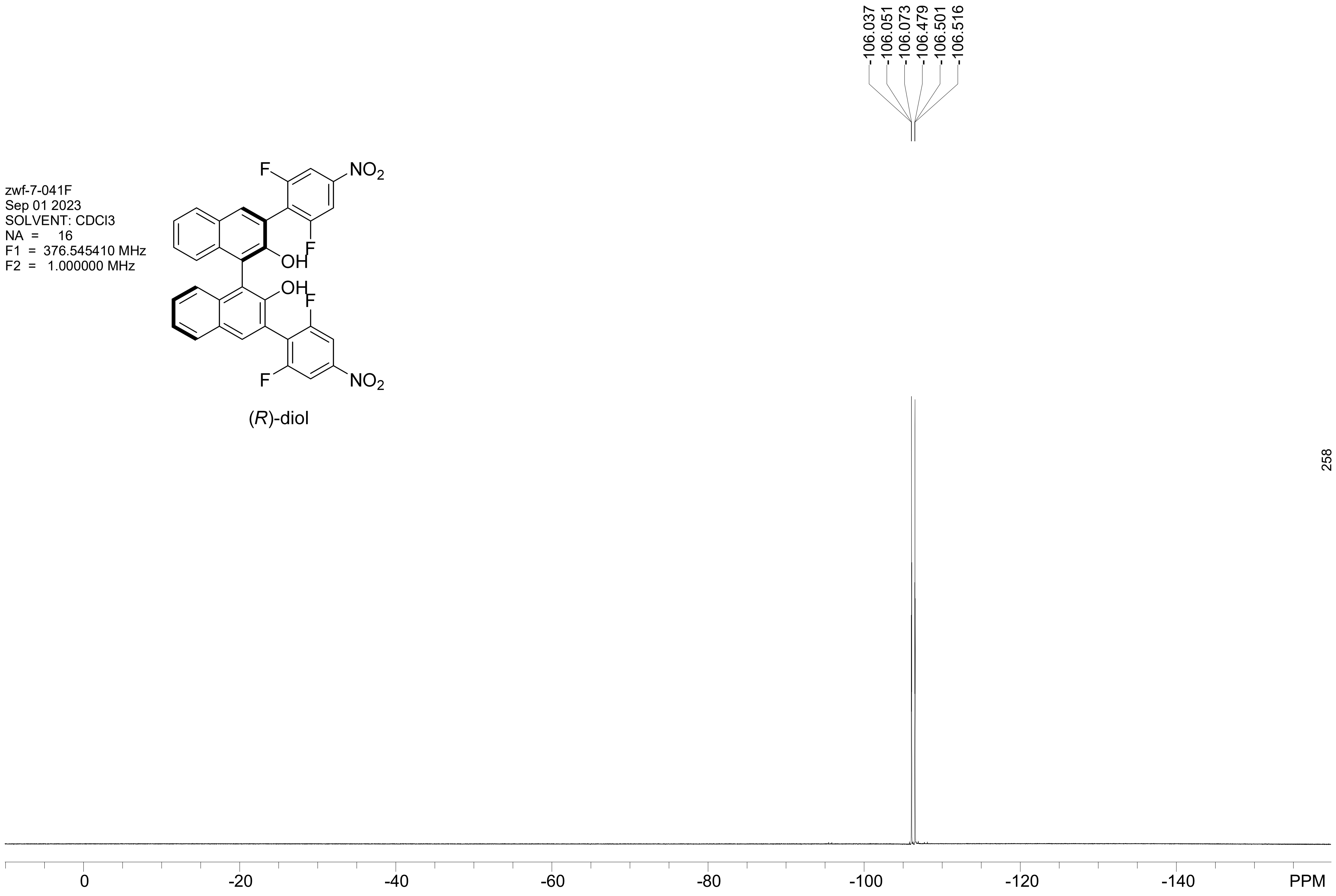
zwf-7-041F
Sep 01 2023
SOLVENT: CDCl3
NA = 16
F1 = 376.545410 MHz
F2 = 1.000000 MHz
F
NO2
OH
OH
(R)-diol
-106.037
-106.051
-106.073
-106.479
-106.501
-106.516
0
-20
-40
-60
-80
-100
-120
-140
PPM

zwf-7-052H
Sep 07 2023
SOLVENT: CDCl3
NA = 4
F1 = 400.140015 MHz
F2 = 1.000000 MHz

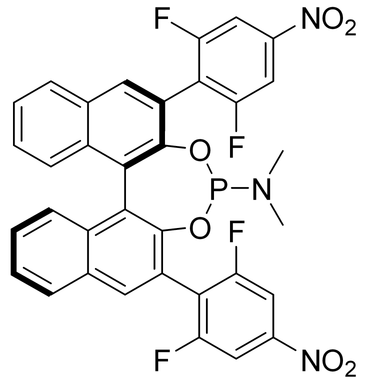

(*R*)-chiral ligand

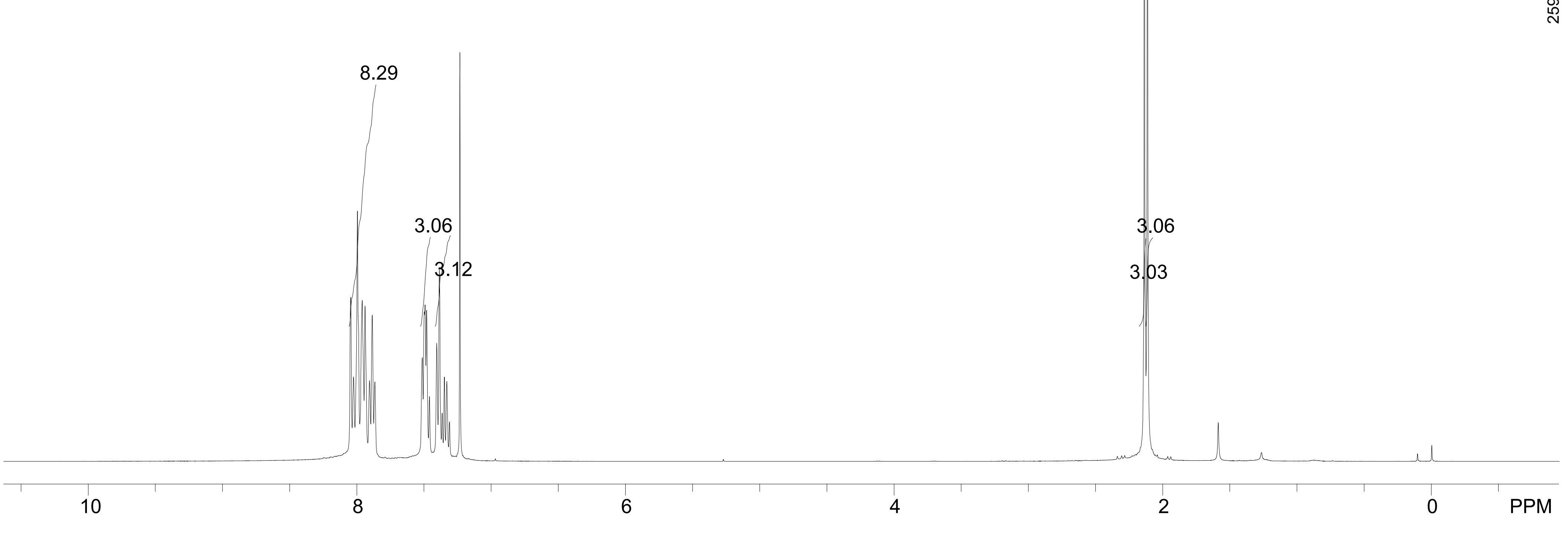

zwf-7-052C
Sep 08 2023
SOLVENT: CDCl3
NA = 400
F1 = 100.615280 MHz
F2 = 1.000000 MHz

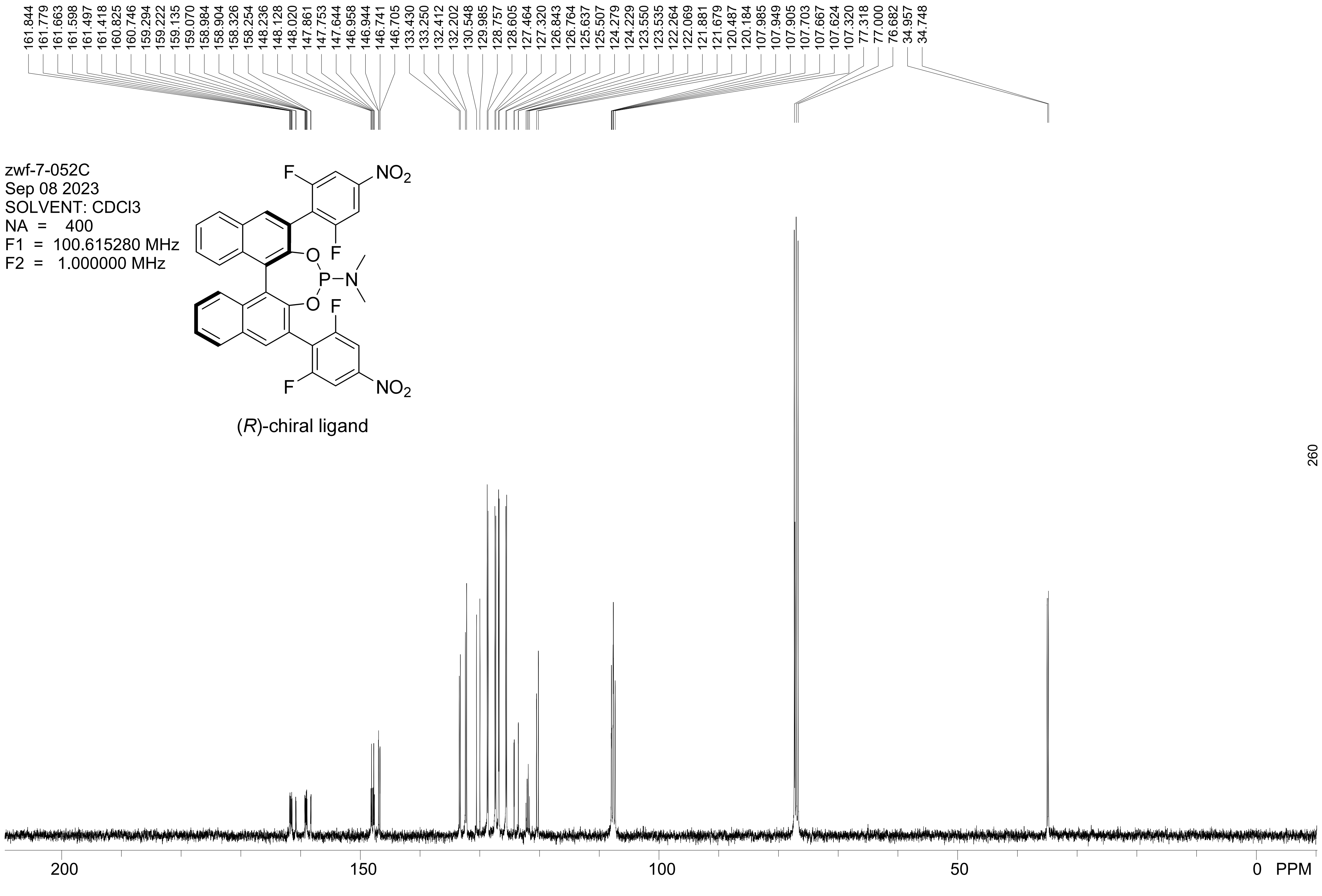

zwf-7-052F
Sep 07 2023
SOLVENT: CDCl3
NA = 16
F1 = 376.507782 MHz
F2 = 1.000000 MHz

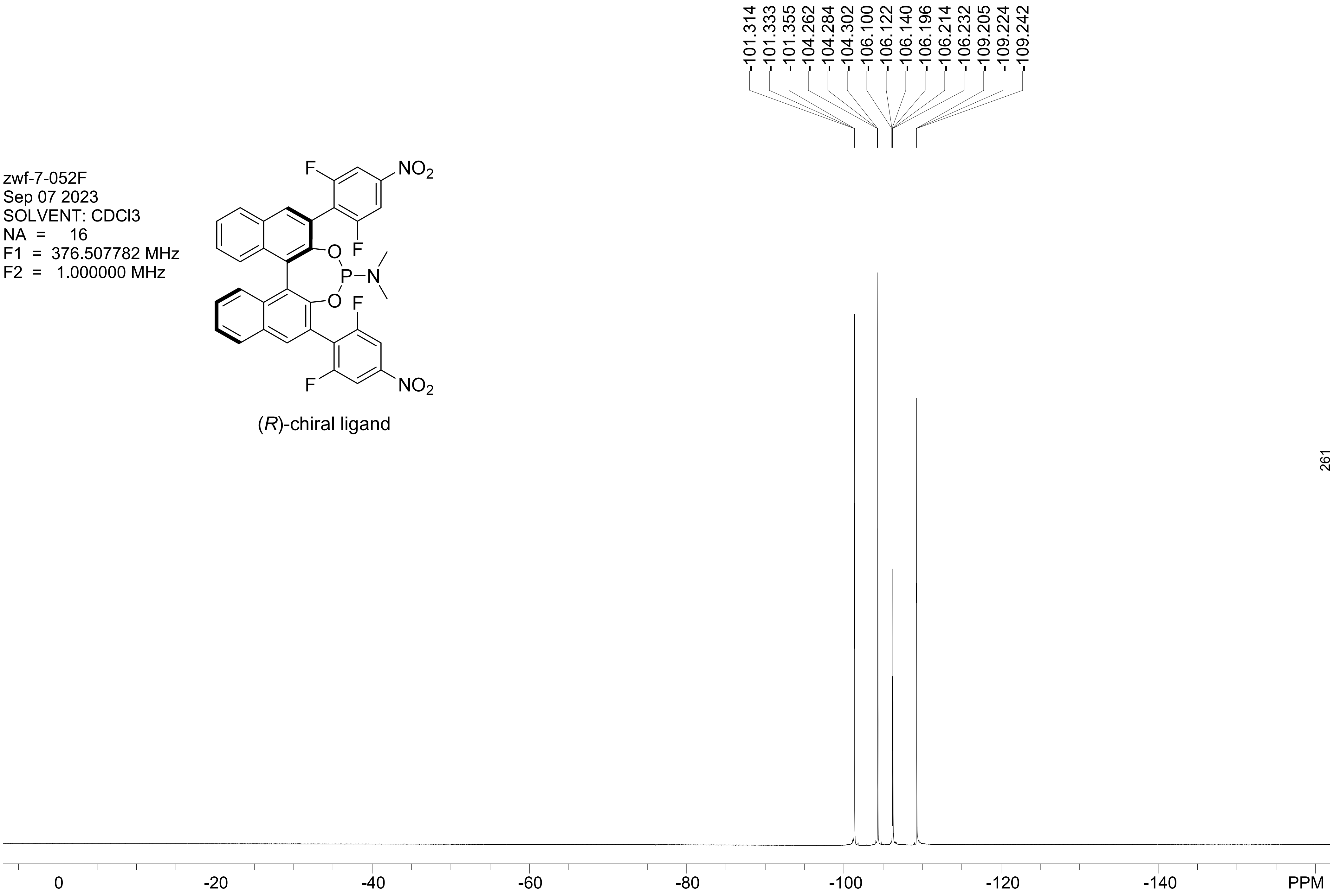

(*R*)-chiral ligand

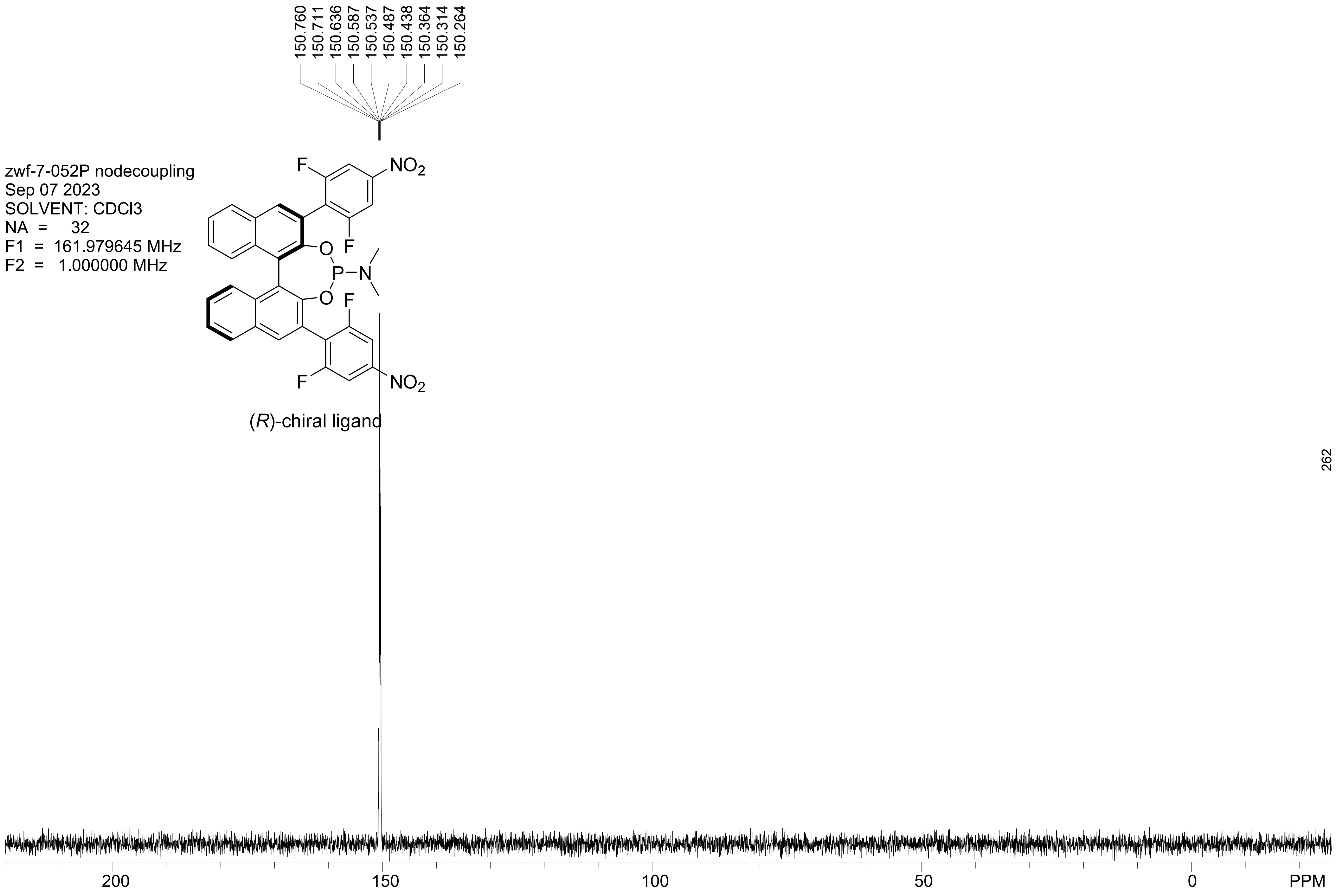
zwf-7-052P nodecoupling
Sep 07 2023
SOLVENT: CDCl3
NA = 32
F1 = 161.979645 MHz
F2 = 1.000000 MHz
150.760
150.711
150.636
150.587
150.537
150.487
150.438
150.364
150.314
150.264
(R)-chiral ligand
200
150
100
50
0
PPM

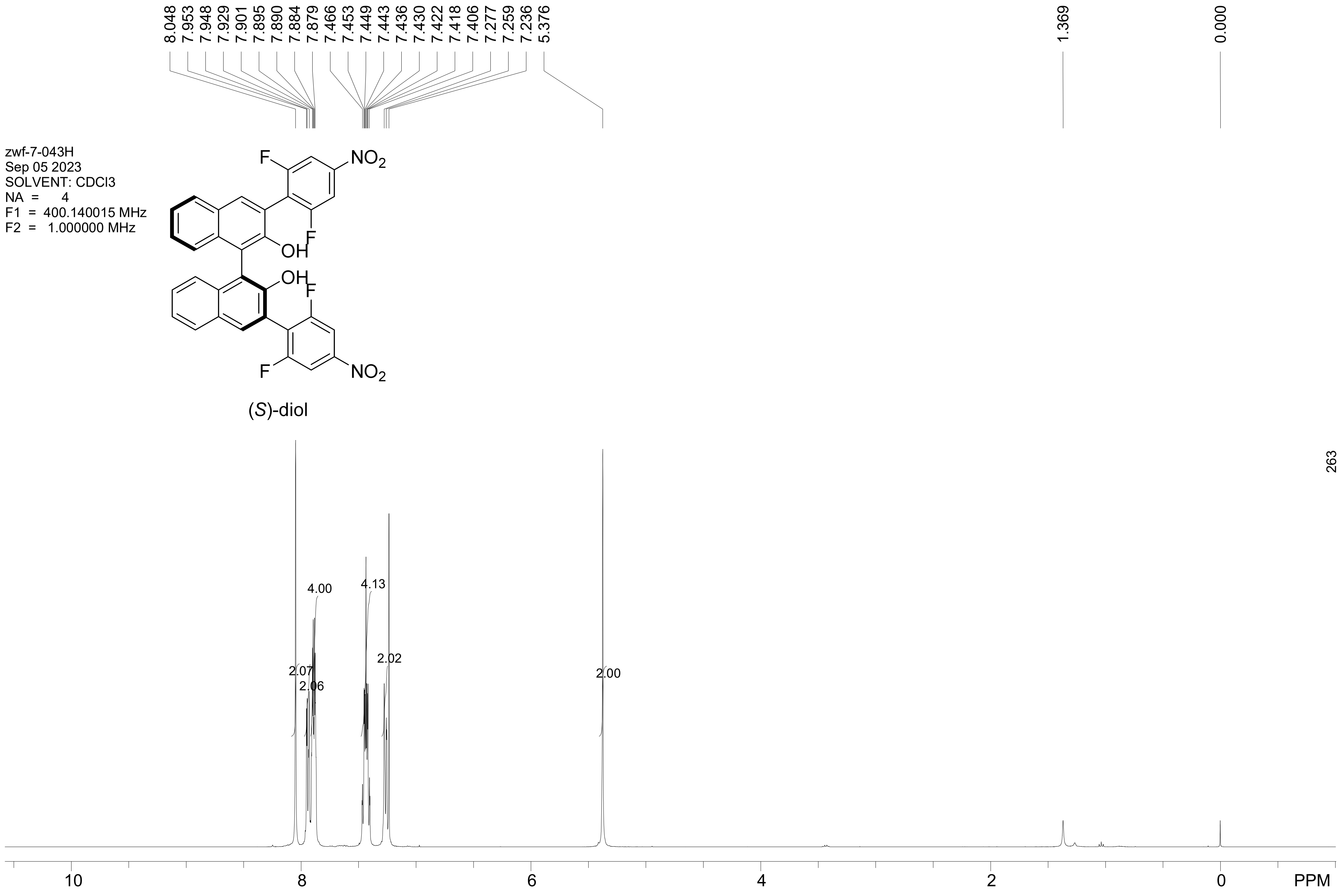
zwf-7-043H
Sep 05 2023
SOLVENT: CDCl3
NA = 4
F1 = 400.140015 MHz
F2 = 1.000000 MHz
(S)-diol
8.048
7.953
7.948
7.929
7.901
7.895
7.890
7.884
7.879
7.466
7.453
7.449
7.443
7.436
7.430
7.422
7.418
7.406
7.277
7.259
7.236
5.376
1.369
0.000
4.00
2.07
2.06
4.13
2.02
2.00
10
8
6
4
2
0
PPM

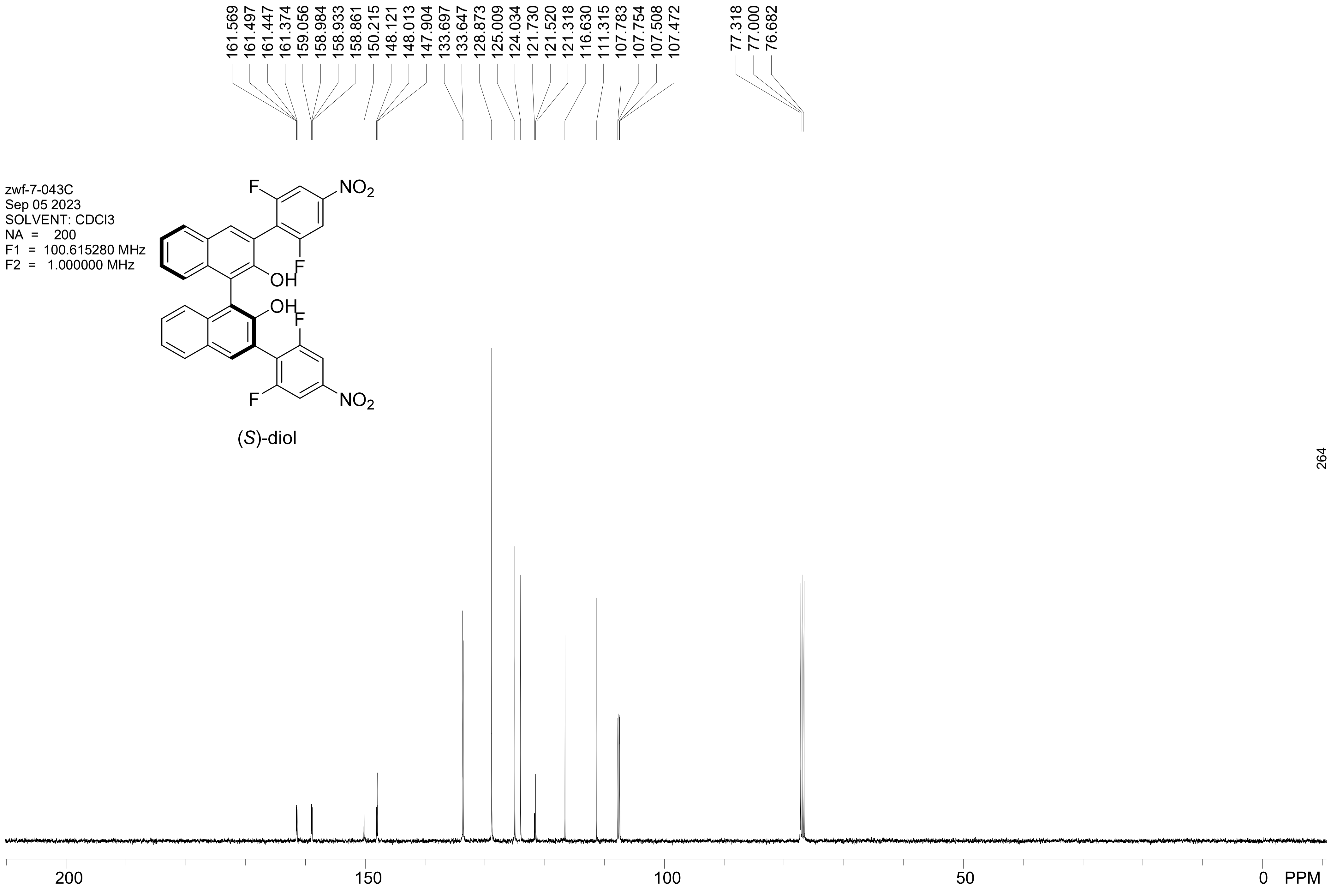
zwf-7-043C
Sep 05 2023
SOLVENT: CDCl3
NA = 200
F1 = 100.615280 MHz
F2 = 1.000000 MHz
(S)-diol
161.569
161.497
161.447
161.374
159.056
158.984
158.933
158.861
150.215
148.121
148.013
147.904
133.697
133.647
128.873
125.009
124.034
121.730
121.520
121.318
116.630
111.315
107.783
107.754
107.508
107.472
77.318
77.000
76.682
200
150
100
50
0 PPM

zwf-7-043F
Sep 05 2023
SOLVENT: CDCl3
NA = 16
F1 = 376.507782 MHz
F2 = 1.000000 MHz

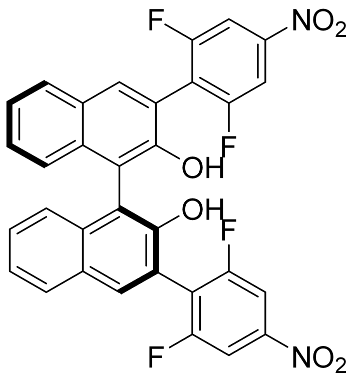

(*S*)-diol

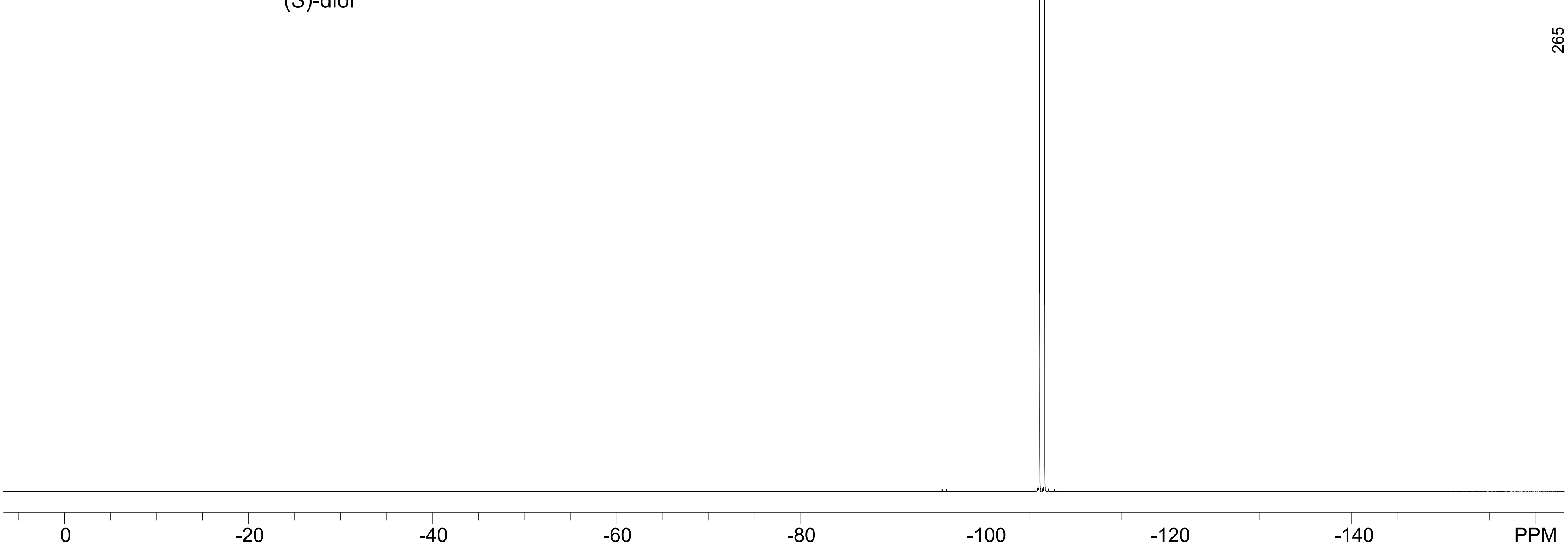

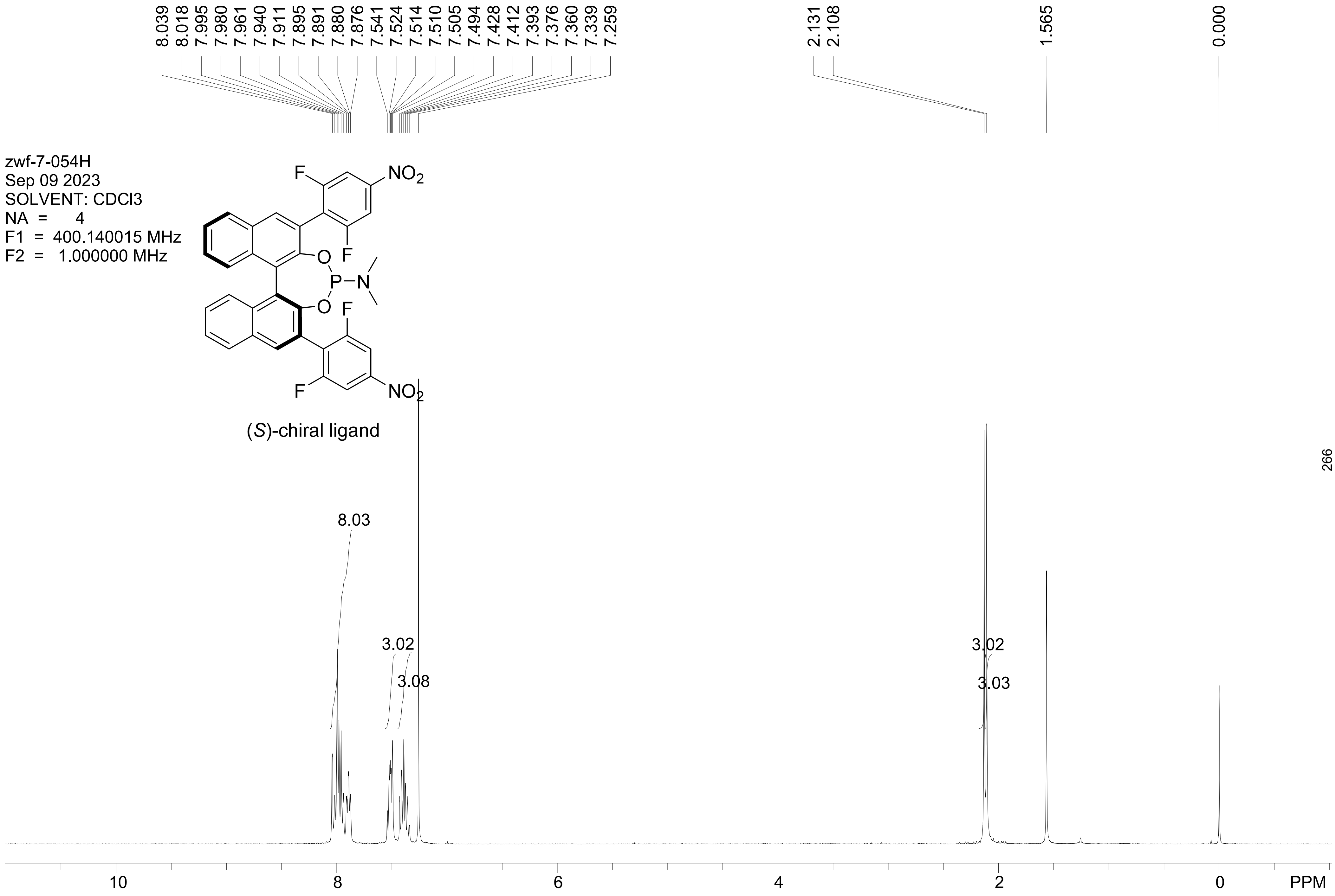
8.039 8.018 7.995 7.980 7.961 7.940 7.911 7.895 7.891 7.880 7.876 7.541 7.524 7.514 7.510 7.505 7.494 7.428 7.412 7.393 7.376 7.360 7.339 7.259
2.131 2.108
1.565
0.000
zwf-7-054H
Sep 09 2023
SOLVENT: CDCl3
NA = 4
F1 = 400.140015 MHz
F2 = 1.000000 MHz
(S)-chiral ligand
8.03
3.02
3.08
3.02
3.03
10
8
6
4
2
0
PPM

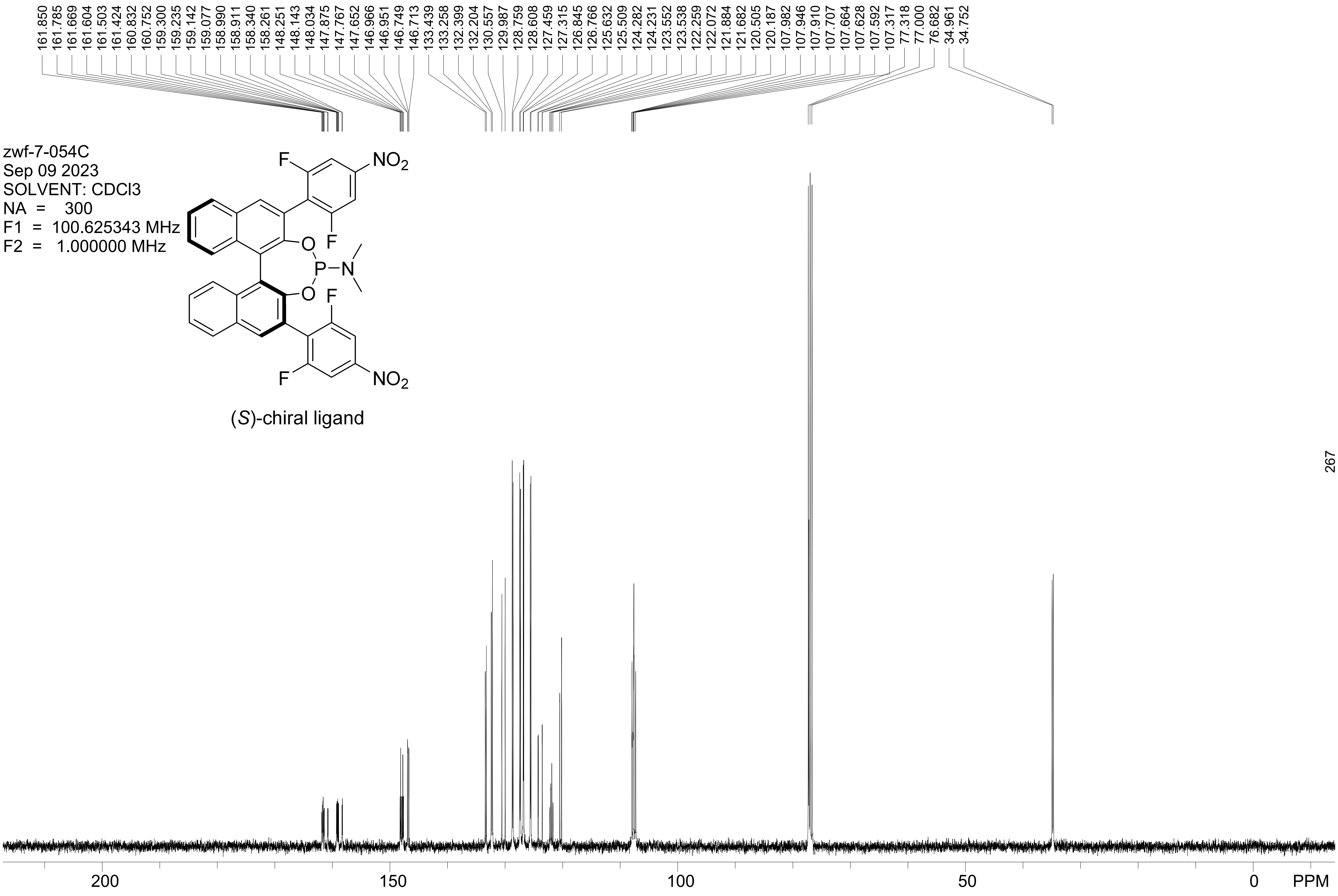

(*S*)-chiral ligand

zwf-7-054F
Sep 09 2023
SOLVENT: CDCl3
NA = 16
F1 = 376.507782 MHz
F2 = 1.000000 MHz

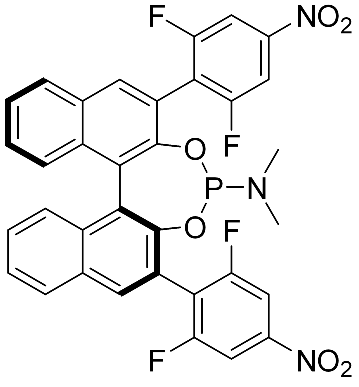

(*S*)-chiral ligand

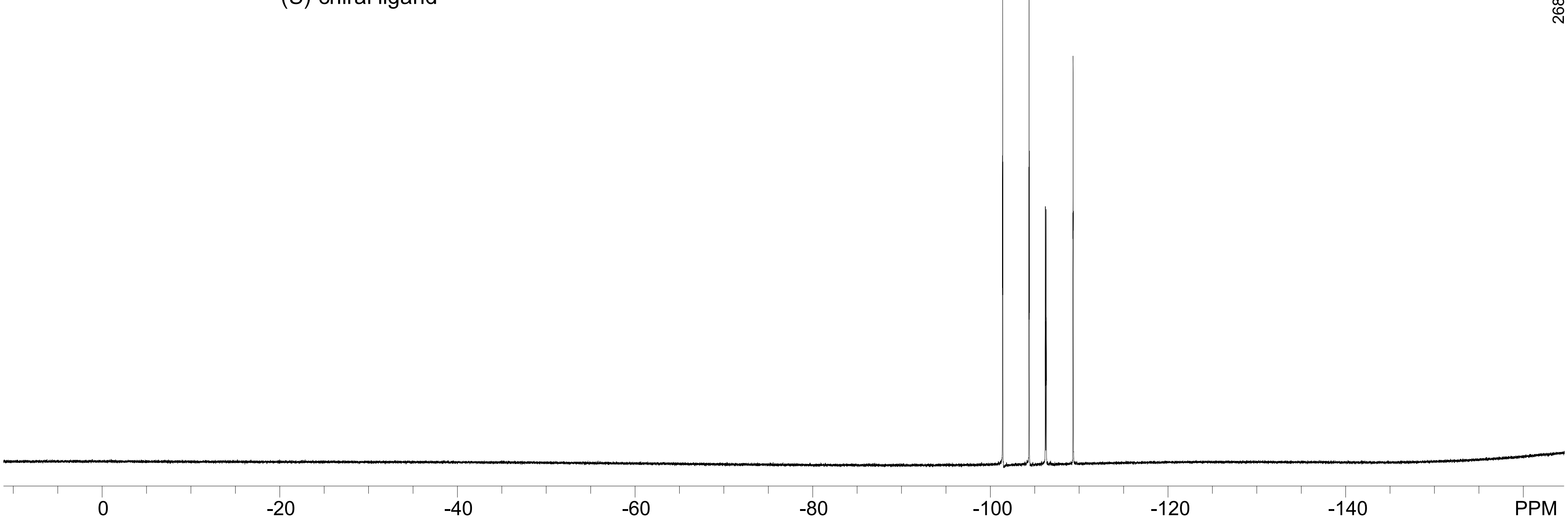

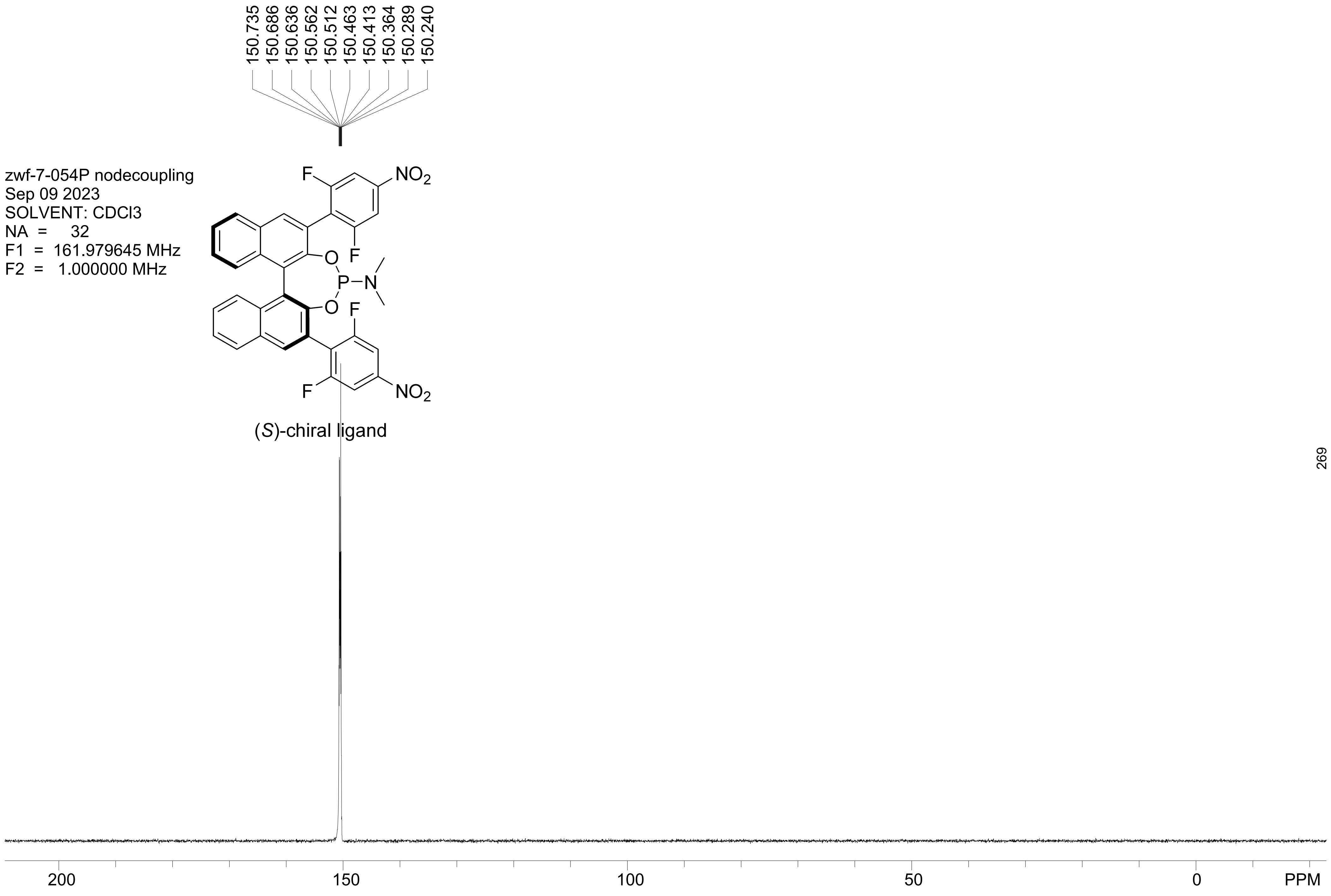

150.735
150.686
150.636
150.562
150.512
150.463
150.413
150.364
150.289
150.240
zwf-7-054P nodecoupling
Sep 09 2023
SOLVENT: CDCl3
NA = 32
F1 = 161.979645 MHz
F2 = 1.000000 MHz
F
NO2
O
P
N
(S)-chiral ligand
200
150
100
50
0
PPM

zwf-7-039H
Aug 28 2023
SOLVENT: CDCl3
NA = 4
F1 = 400.140015 MHz
F2 = 1.000000 MHz

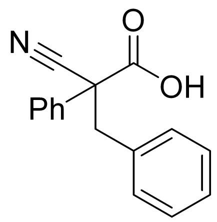

S1

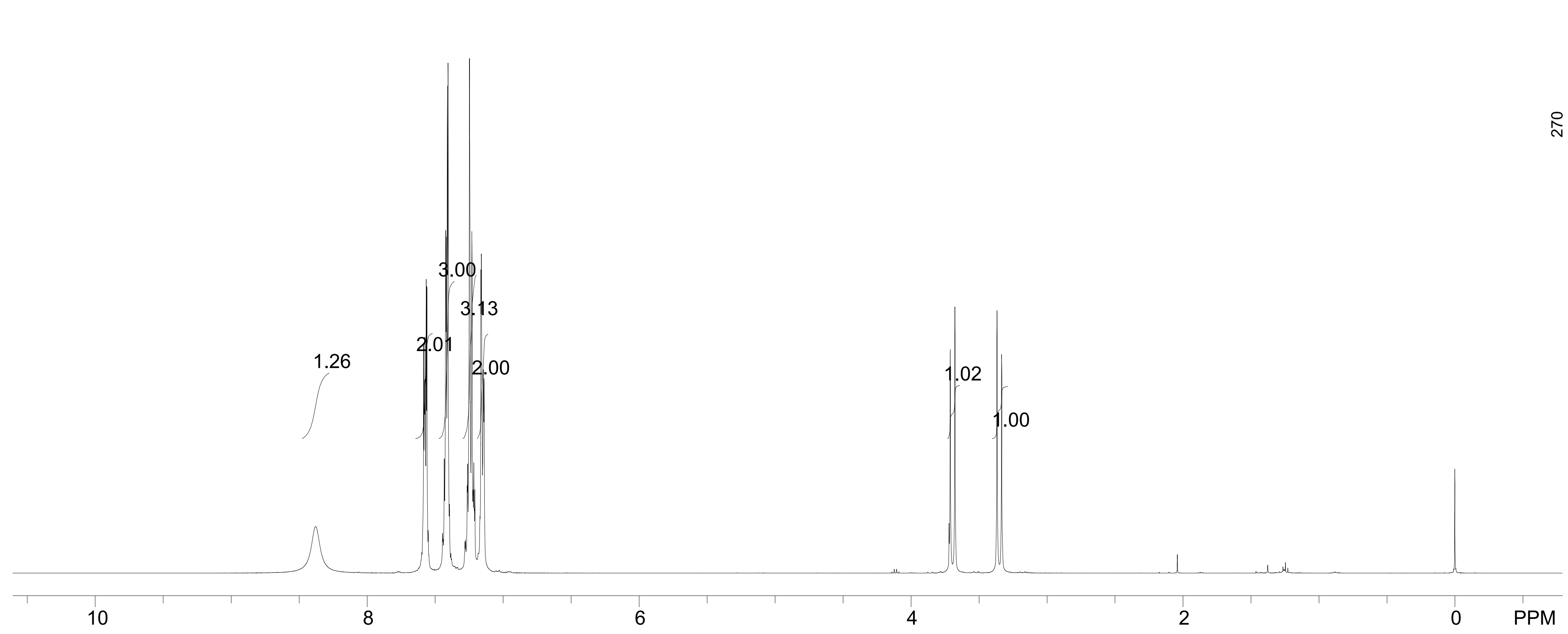

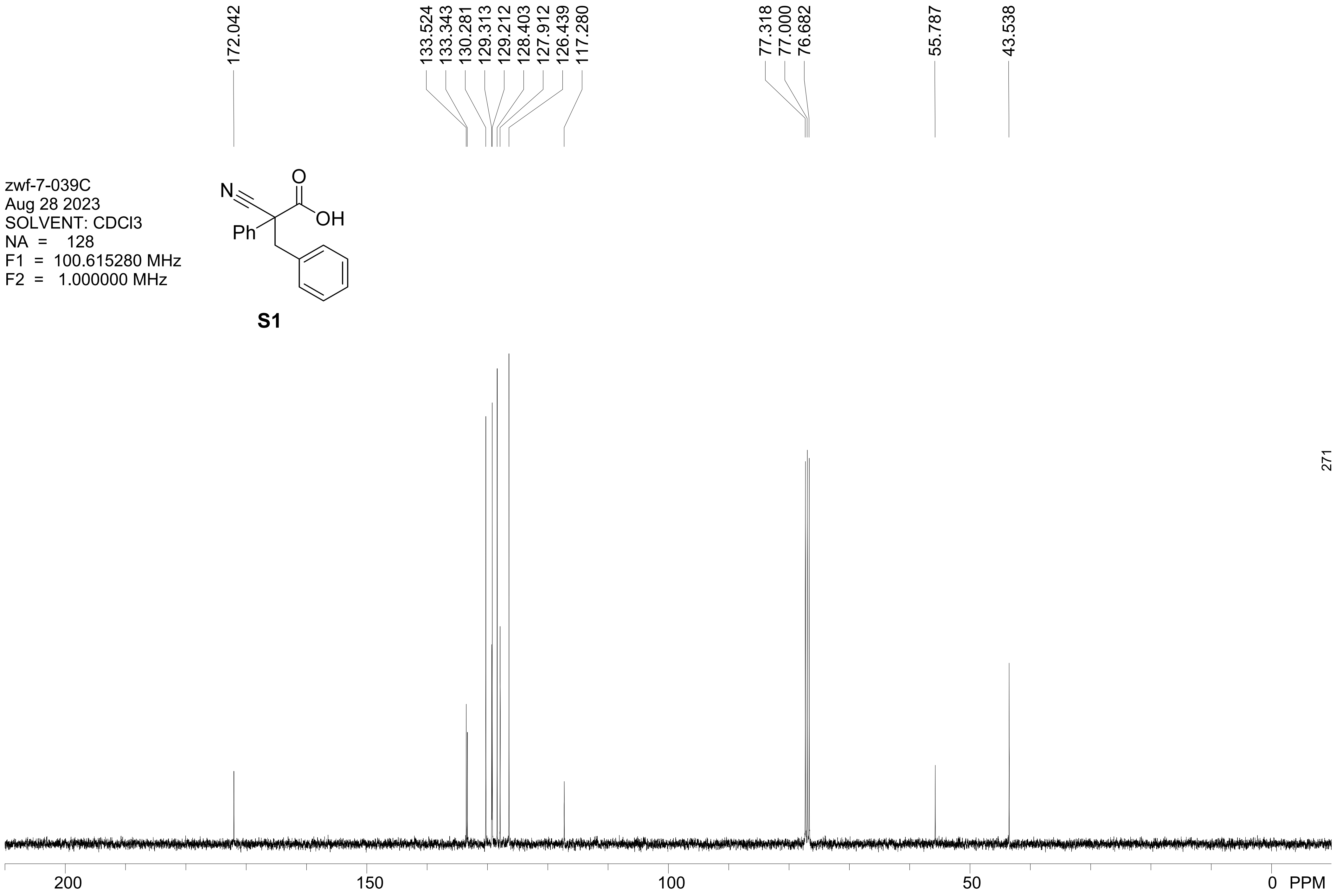
zwf-7-039C
Aug 28 2023
SOLVENT: CDCl3
NA = 128
F1 = 100.615280 MHz
F2 = 1.000000 MHz
N
Ph
O
OH
172.042
133.524
133.343
130.281
129.313
129.212
128.403
127.912
126.439
117.280
77.318
77.000
76.682
55.787
43.538
200
150
100
50
0 PPM

S1

zwf-7-109-1H
Oct 13 2023
SOLVENT: CDCl3
NA = 4
F1 = 400.140015 MHz
F2 = 1.000000 MHz

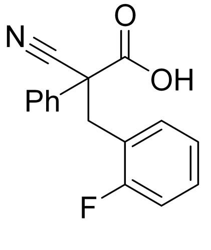

S7

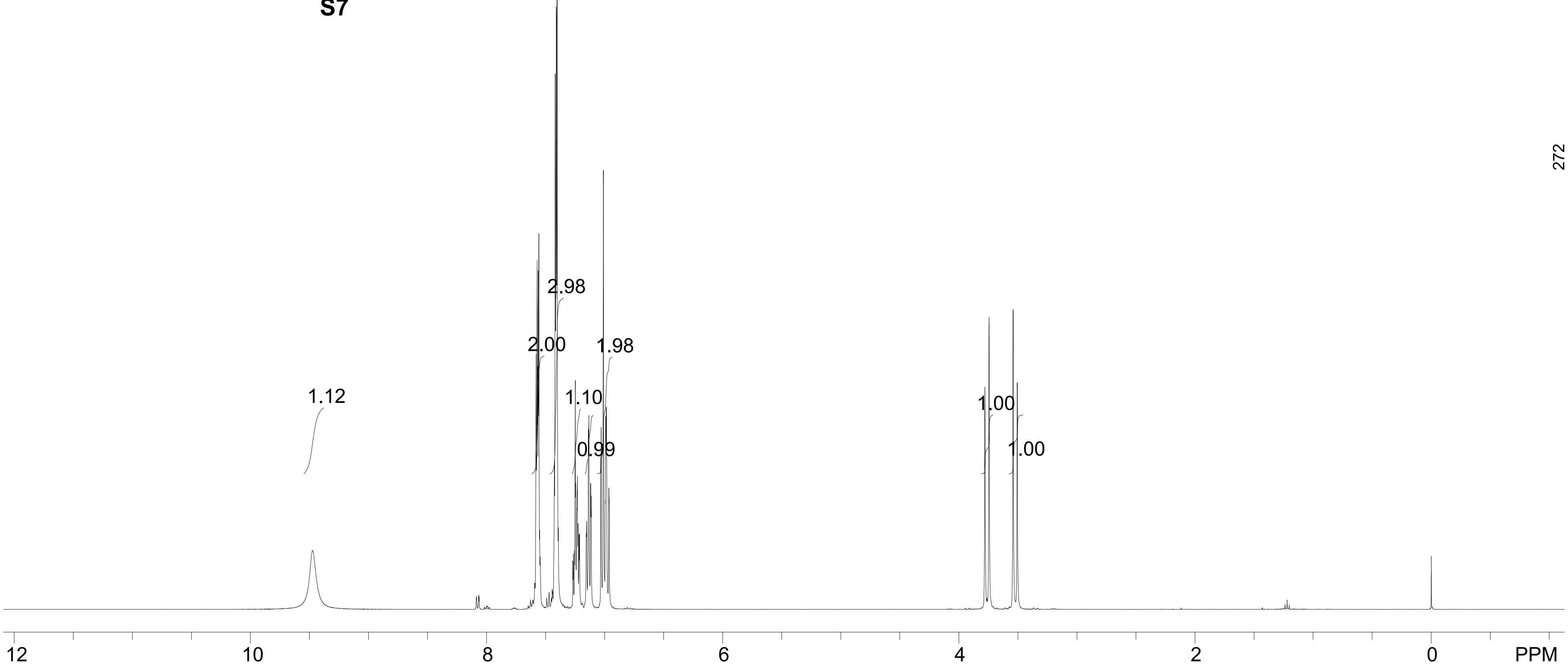

zwf-7-109-1C
Oct 13 2023
SOLVENT: CDCl3
NA = 100
F1 = 100.615280 MHz
F2 = 1.000000 MHz

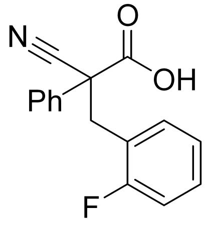

S7

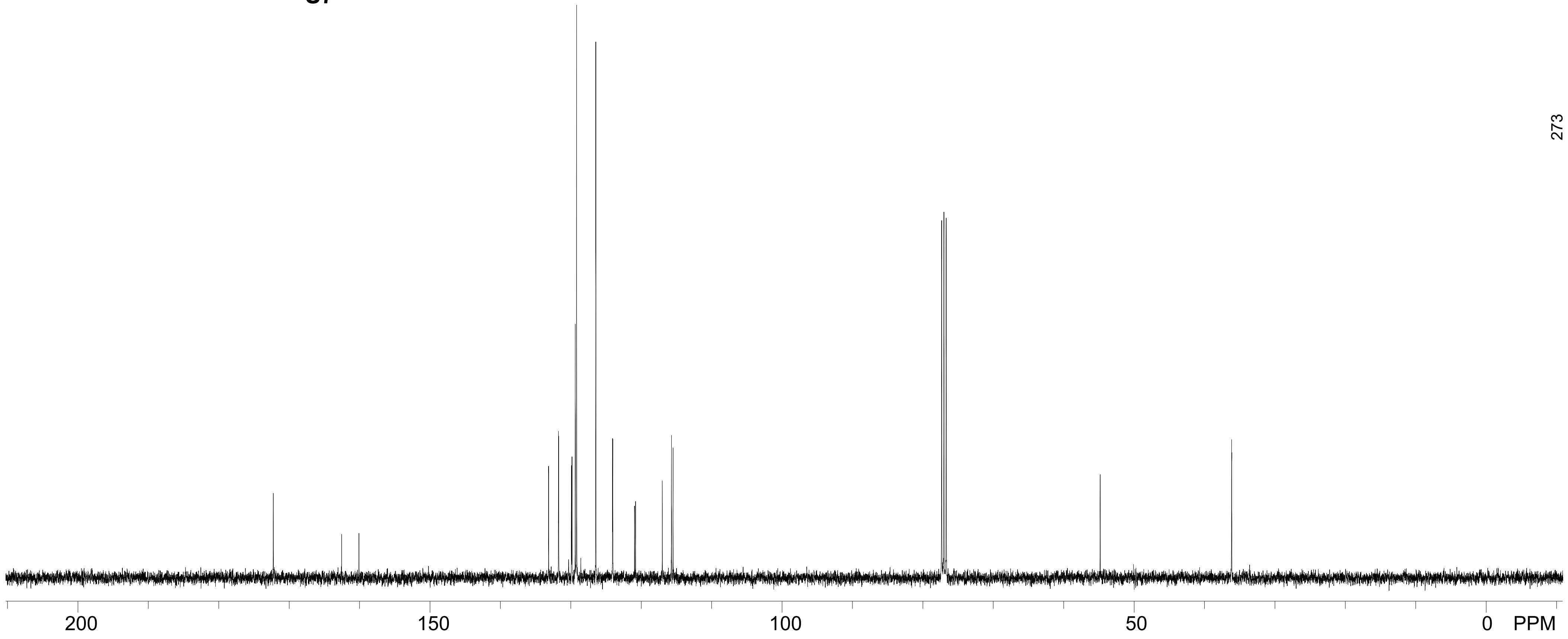

zwf-7-109-1F
Oct 13 2023
SOLVENT: CDCl3
NA =   16
F1 = 376.507782 MHz
F2 =   1.000000 MHz

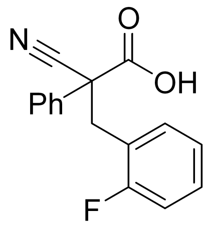

S7

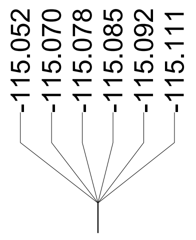

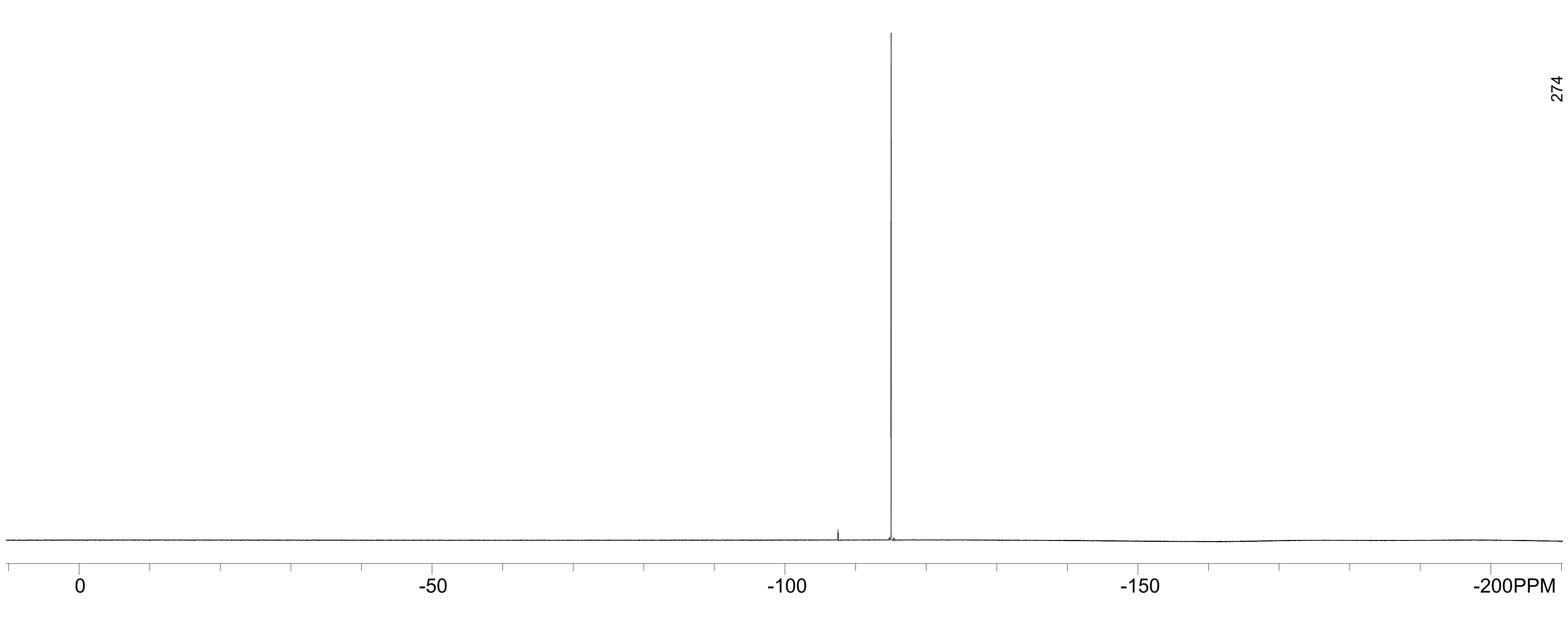

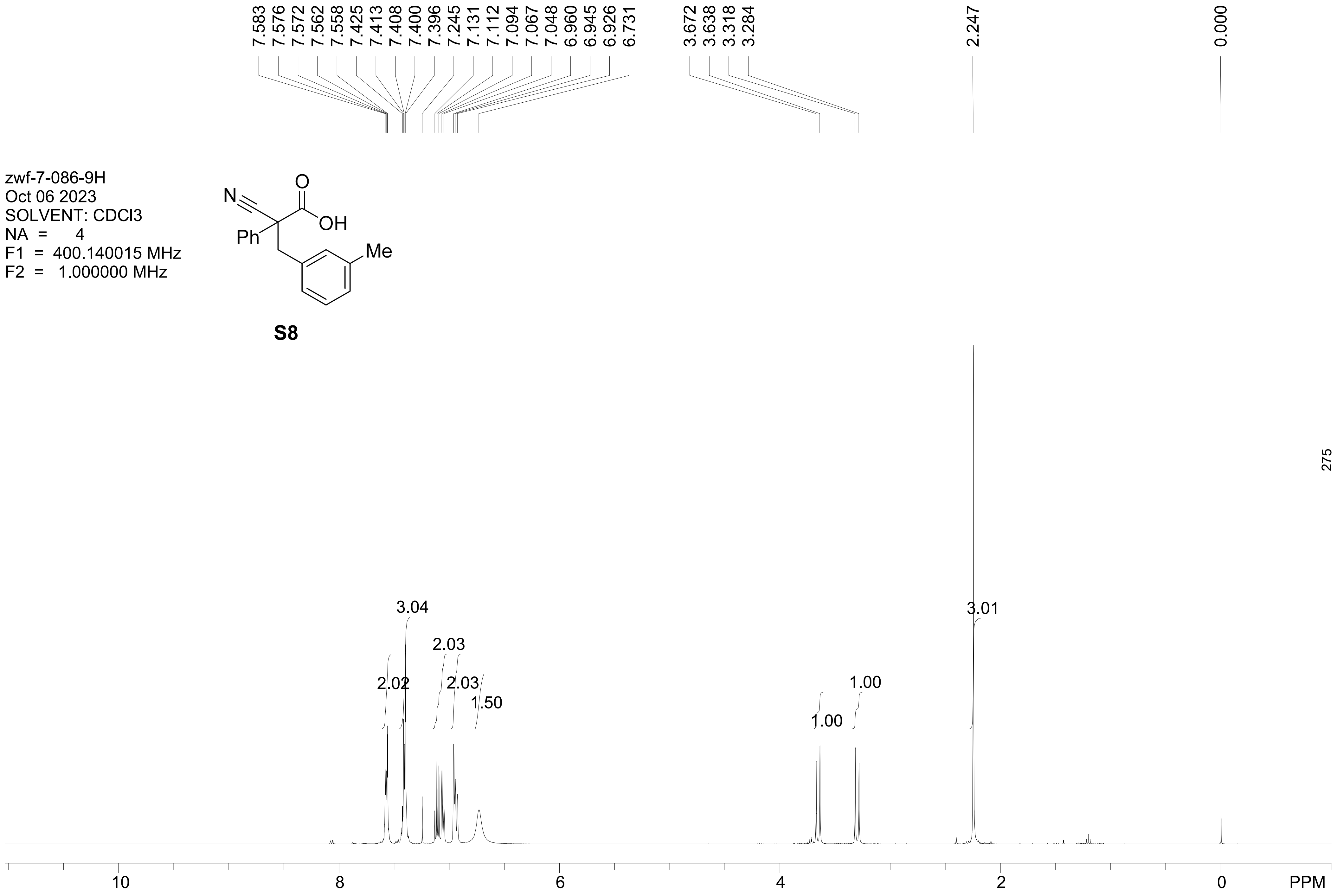
zwf-7-086-9H
Oct 06 2023
SOLVENT: CDCl3
NA = 4
F1 = 400.140015 MHz
F2 = 1.000000 MHz
N
O
OH
Ph
Me
S8
7.583
7.576
7.572
7.562
7.558
7.425
7.413
7.408
7.400
7.396
7.245
7.131
7.112
7.094
7.067
7.048
6.960
6.945
6.926
6.731
3.672
3.638
3.318
3.284
2.247
0.000
2.02
3.04
2.03
2.03
1.50
1.00
1.00
3.01
10
8
6
4
2
0
PPM

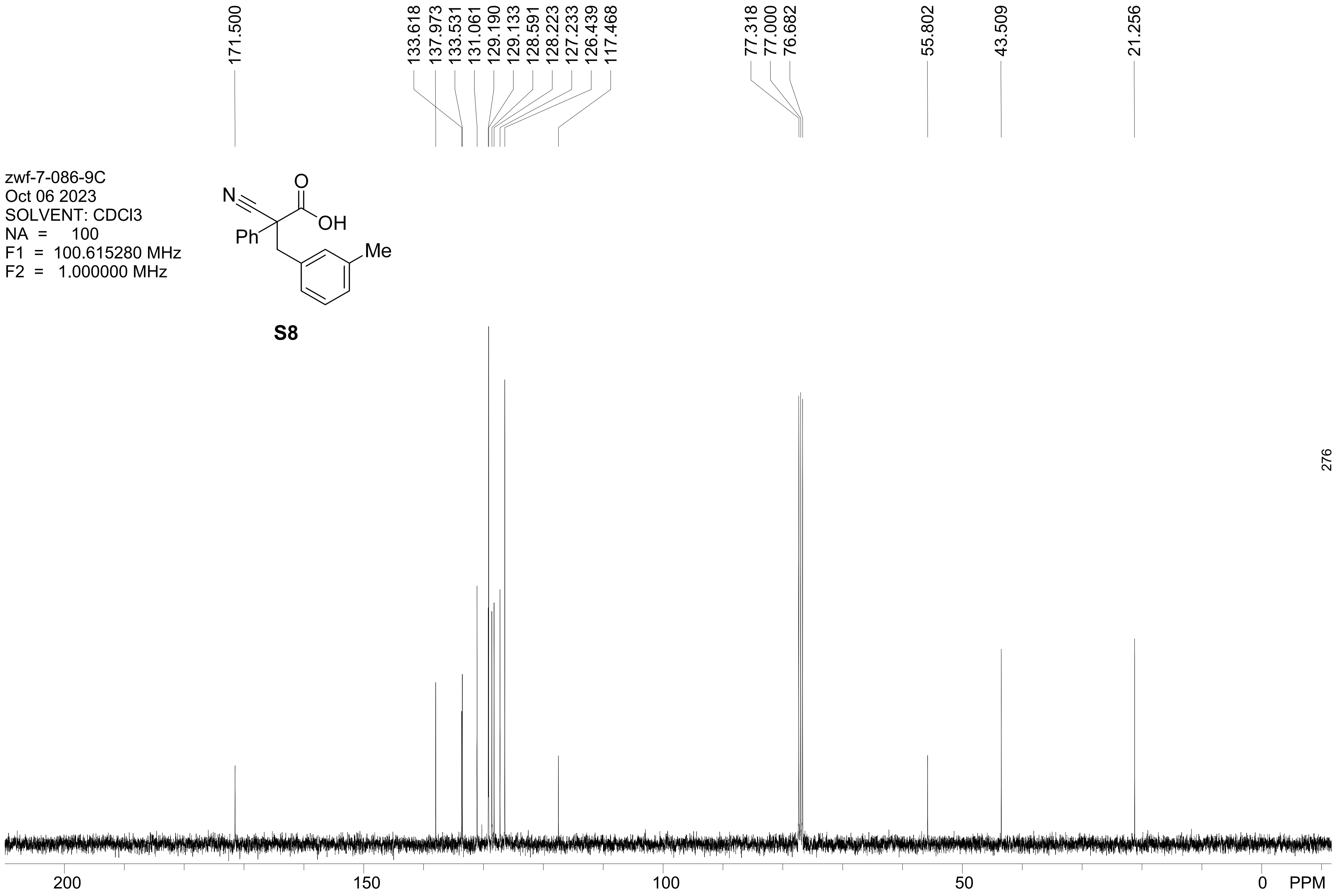
zwf-7-086-9C
Oct 06 2023
SOLVENT: CDCl3
NA = 100
F1 = 100.615280 MHz
F2 = 1.000000 MHz
N
O
OH
Ph
Me
S8
171.500
133.618
137.973
133.531
131.061
129.190
129.133
128.591
128.223
127.233
126.439
117.468
77.318
77.000
76.682
55.802
43.509
21.256
200
150
100
50
0
PPM

zwf-7-086-1H
Sep 22 2023
SOLVENT: CDCl3
NA = 4
F1 = 400.140015 MHz
F2 = 1.000000 MHz

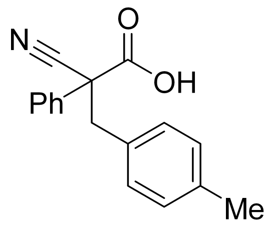

S9

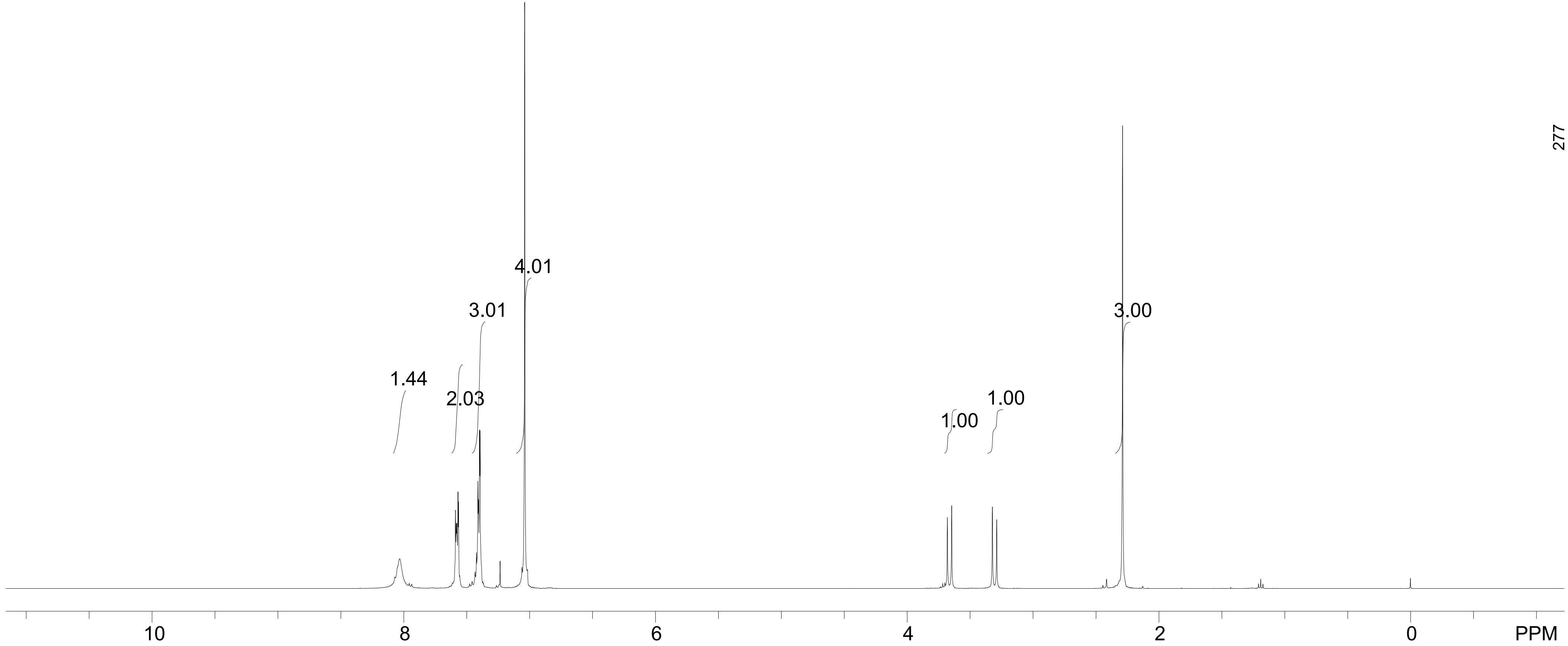

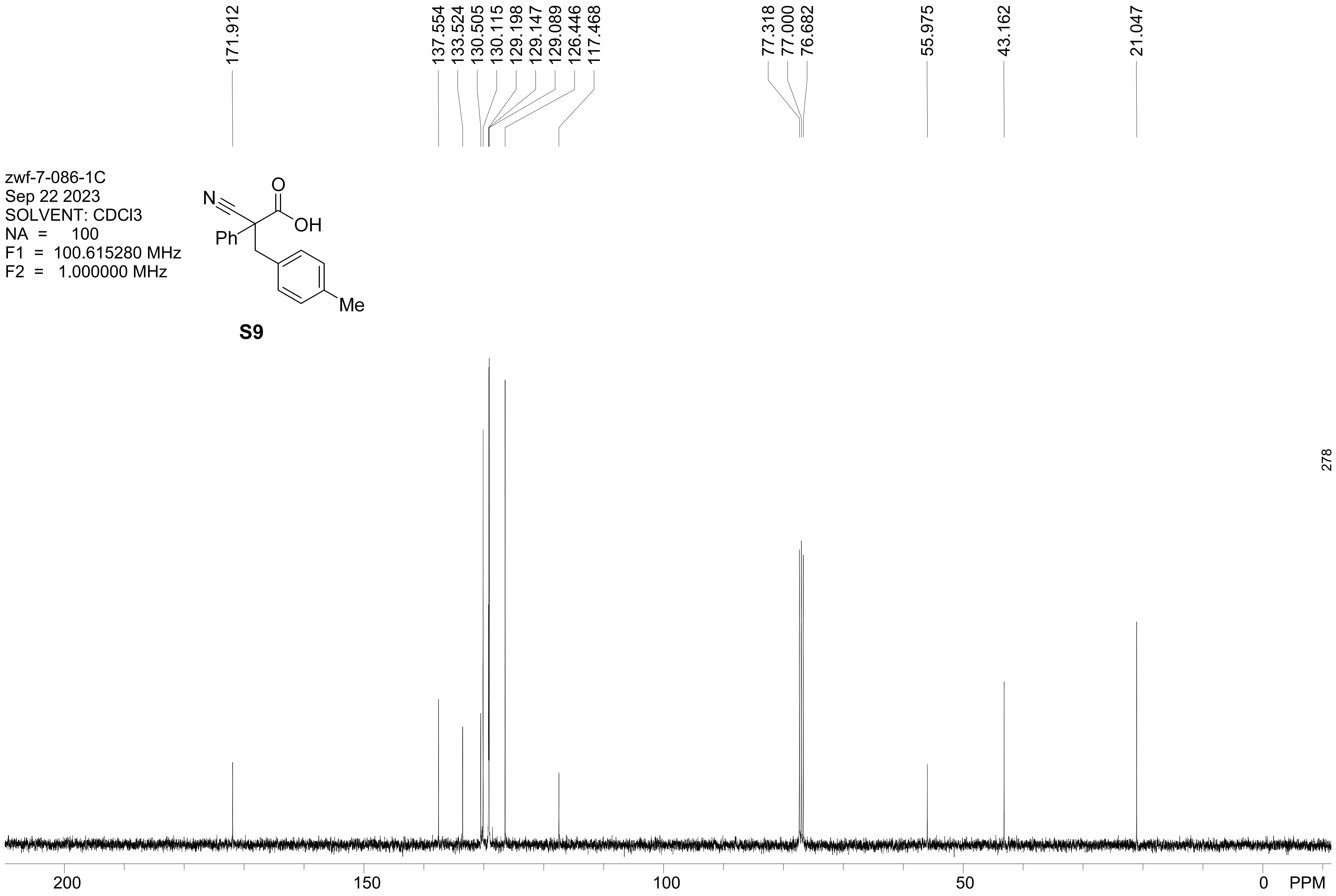
zwf-7-086-1C
Sep 22 2023
SOLVENT: CDCl3
NA = 100
F1 = 100.615280 MHz
F2 = 1.000000 MHz
N
O
OH
Ph
Me
S9
171.912
137.554
133.524
130.505
130.115
129.198
129.147
129.089
126.446
117.468
77.318
77.000
76.682
55.975
43.162
21.047
200
150
100
50
0
PPM

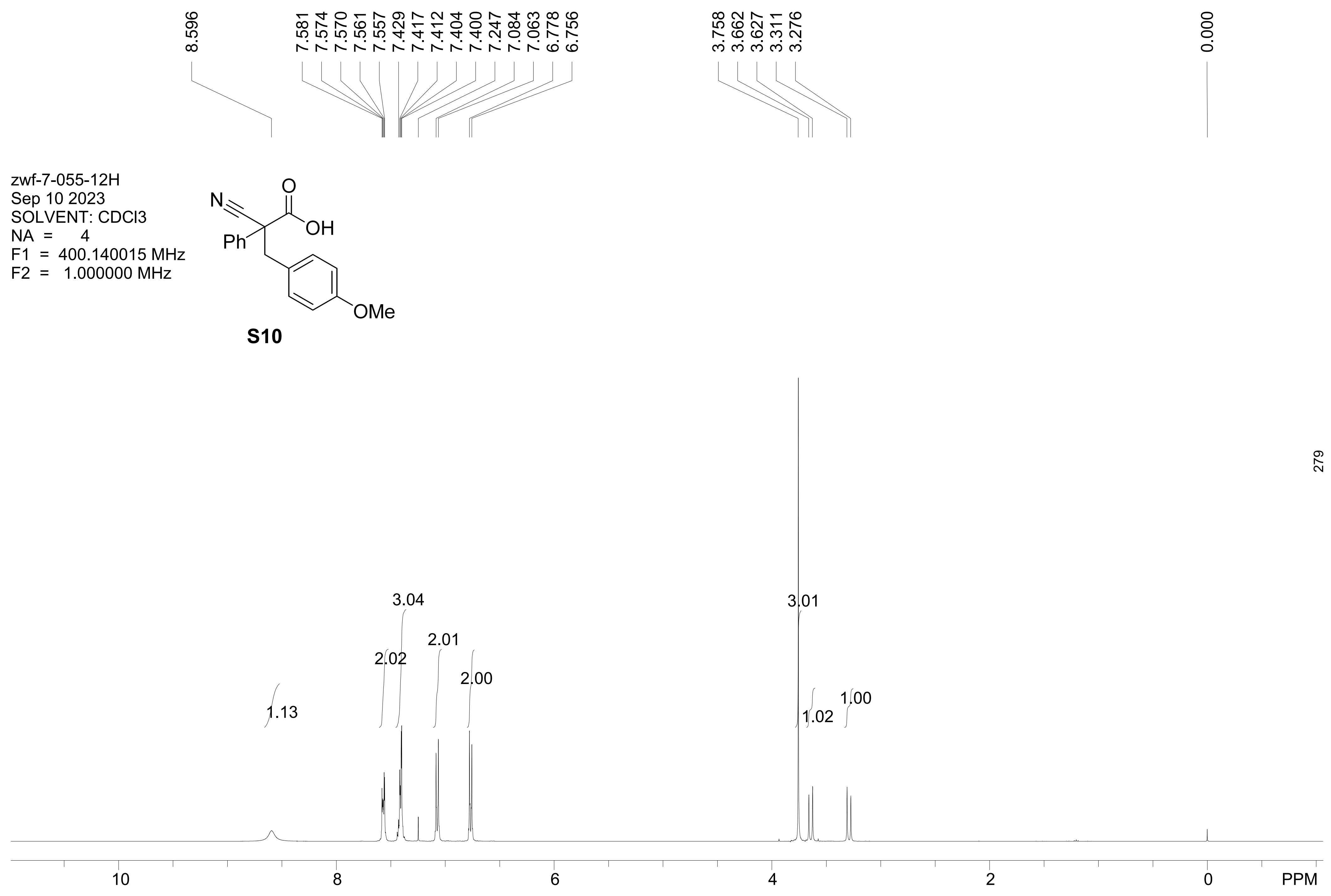
zwf-7-055-12H
Sep 10 2023
SOLVENT: CDCl3
NA = 4
F1 = 400.140015 MHz
F2 = 1.000000 MHz
N
O
OH
Ph
OMe
S10
8.596
7.581
7.574
7.570
7.561
7.557
7.429
7.417
7.412
7.404
7.400
7.247
7.084
7.063
6.778
6.756
3.758
3.662
3.627
3.311
3.276
0.000
1.13
2.02
3.04
2.01
2.00
3.01
1.02
1.00
10
8
6
4
2
0
PPM

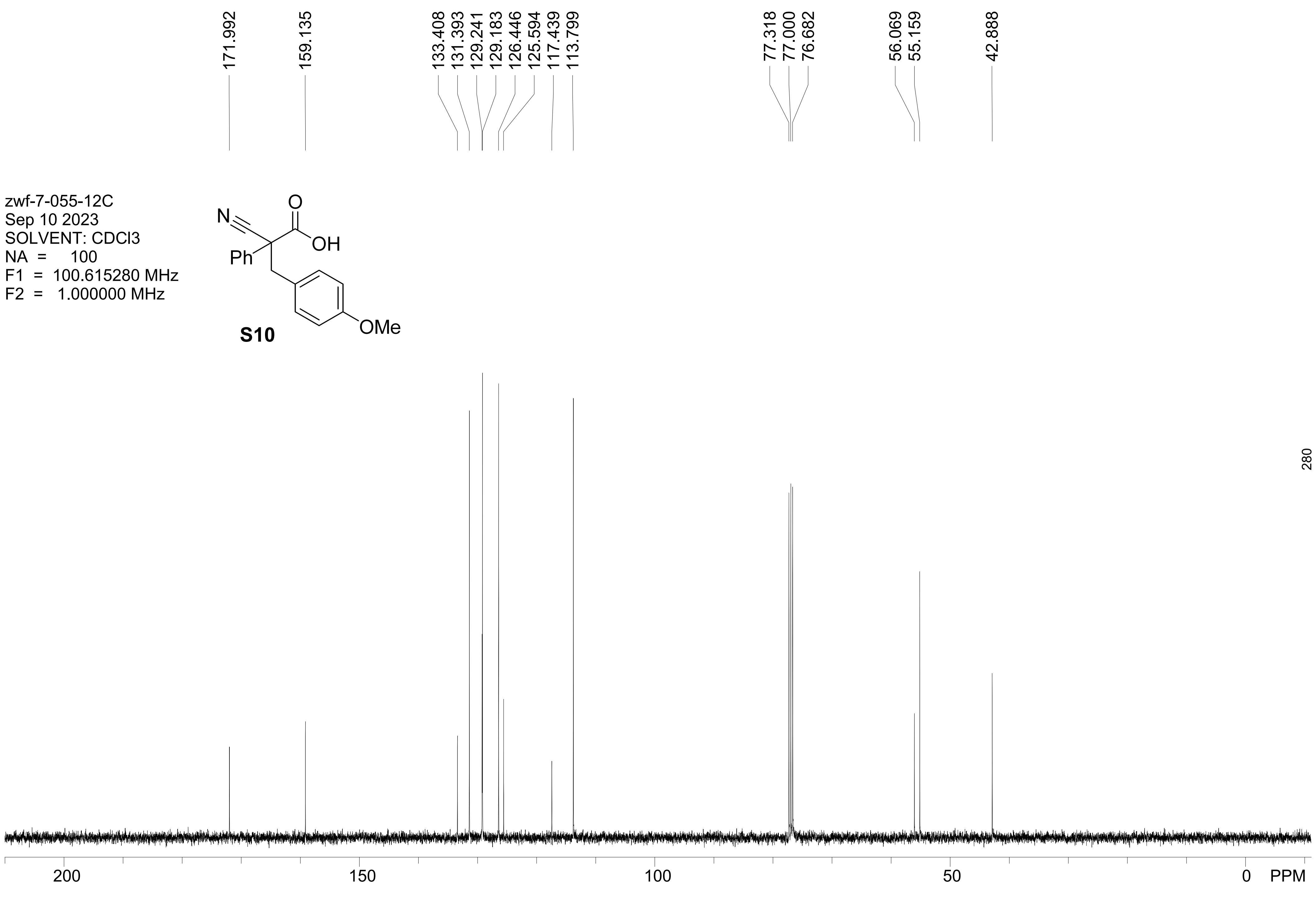
zwf-7-055-12C
Sep 10 2023
SOLVENT: CDCl3
NA = 100
F1 = 100.615280 MHz
F2 = 1.000000 MHz
N
O
OH
Ph
OMe
S10
171.992
159.135
133.408
131.393
129.241
129.183
126.446
125.594
117.439
113.799
77.318
77.000
76.682
56.069
55.159
42.888
200
150
100
50
0
PPM

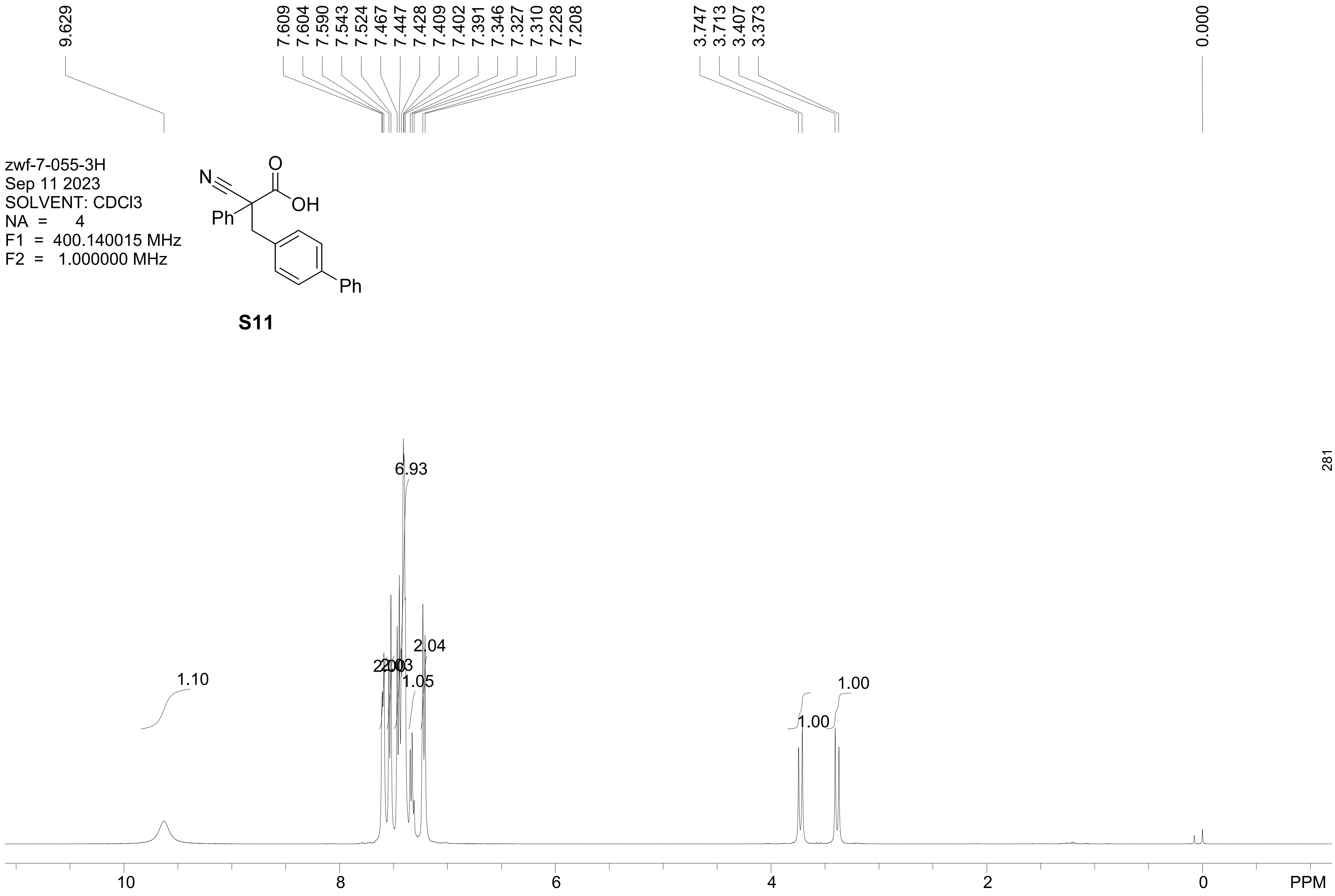
zwf-7-055-3H
Sep 11 2023
SOLVENT: CDCl3
NA = 4
F1 = 400.140015 MHz
F2 = 1.000000 MHz
N
O
OH
Ph
Ph
S11
9.629
7.609
7.604
7.590
7.543
7.524
7.467
7.447
7.428
7.409
7.402
7.391
7.346
7.327
7.310
7.228
7.208
3.747
3.713
3.407
3.373
0.000
1.10
6.93
2.04
1.05
1.00
1.00
10
8
6
4
2
0
PPM

zwf-7-055-3C
Sep 11 2023
SOLVENT: CDCl3
NA = 100
F1 = 100.615280 MHz
F2 = 1.000000 MHz

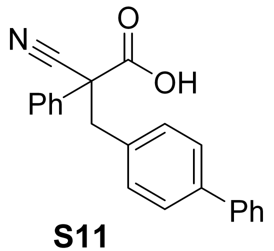

S11

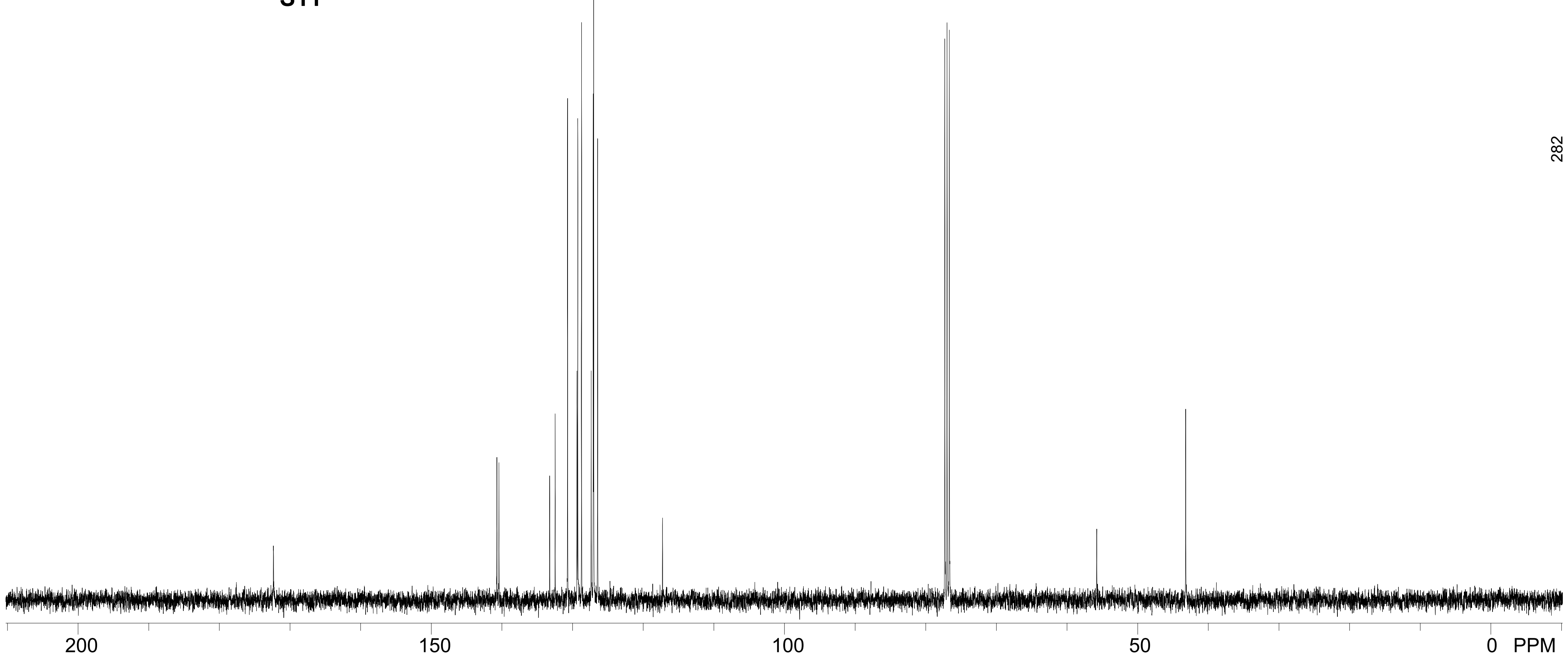

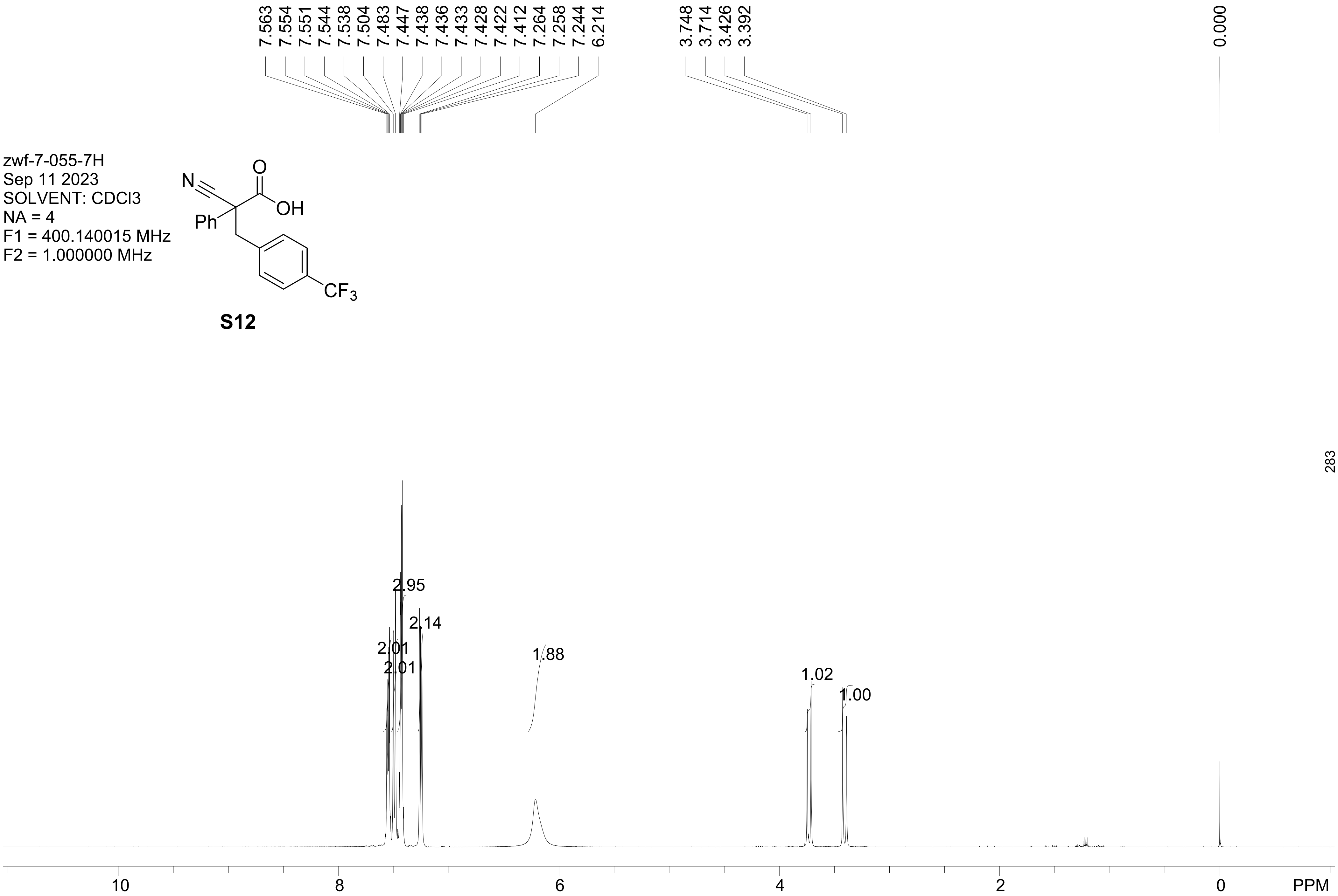
zwf-7-055-7H
Sep 11 2023
SOLVENT: CDCl3
NA = 4
F1 = 400.140015 MHz
F2 = 1.000000 MHz
S12
7.563
7.554
7.551
7.544
7.538
7.504
7.483
7.447
7.438
7.436
7.433
7.428
7.422
7.412
7.264
7.258
7.244
6.214
3.748
3.714
3.426
3.392
0.000
2.95
2.14
2.01
2.01
1.88
1.02
1.00
10
8
6
4
2
0
PPM

zwf-7-055-7C
Sep 11 2023
SOLVENT: CDCl3
NA = 200
F1 = 100.615280 MHz
F2 = 1.000000 MHz

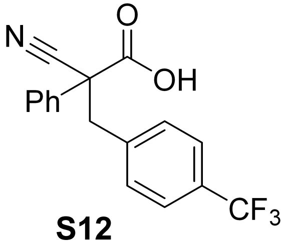

S12

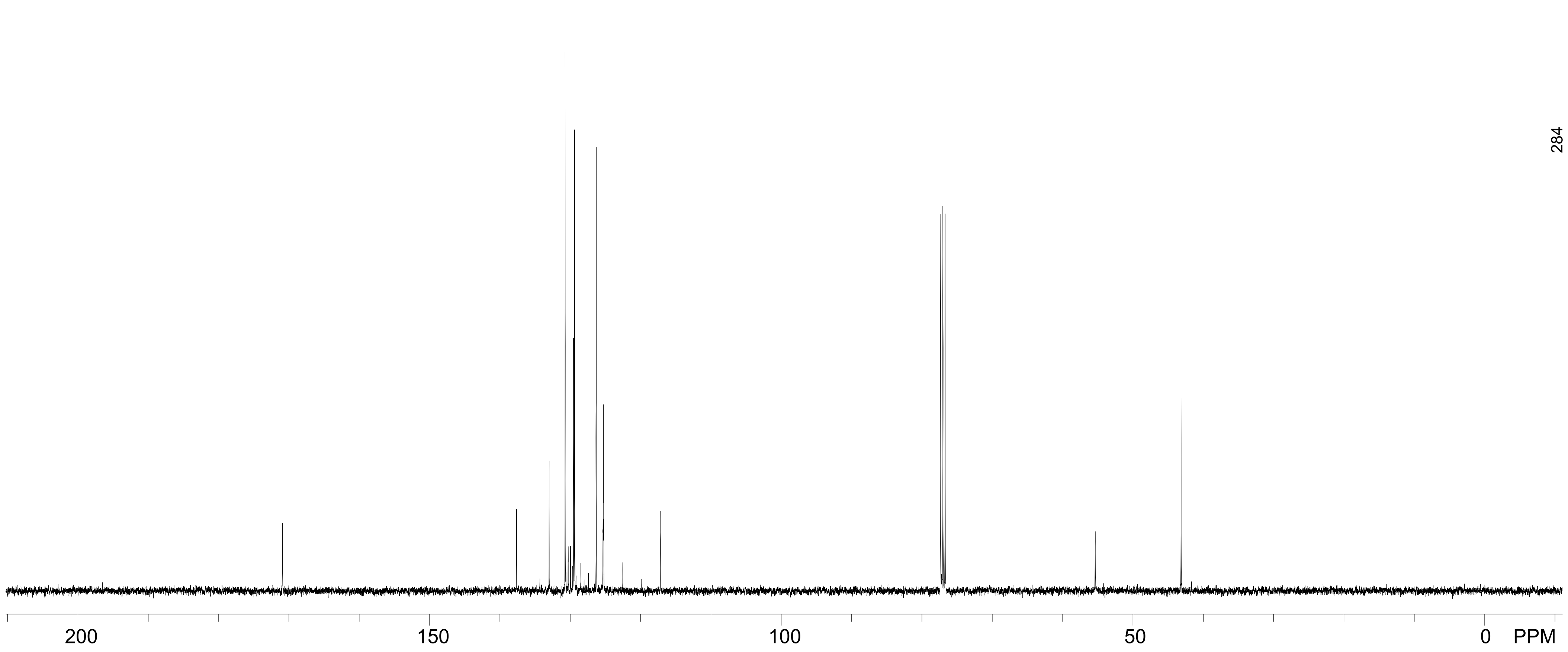

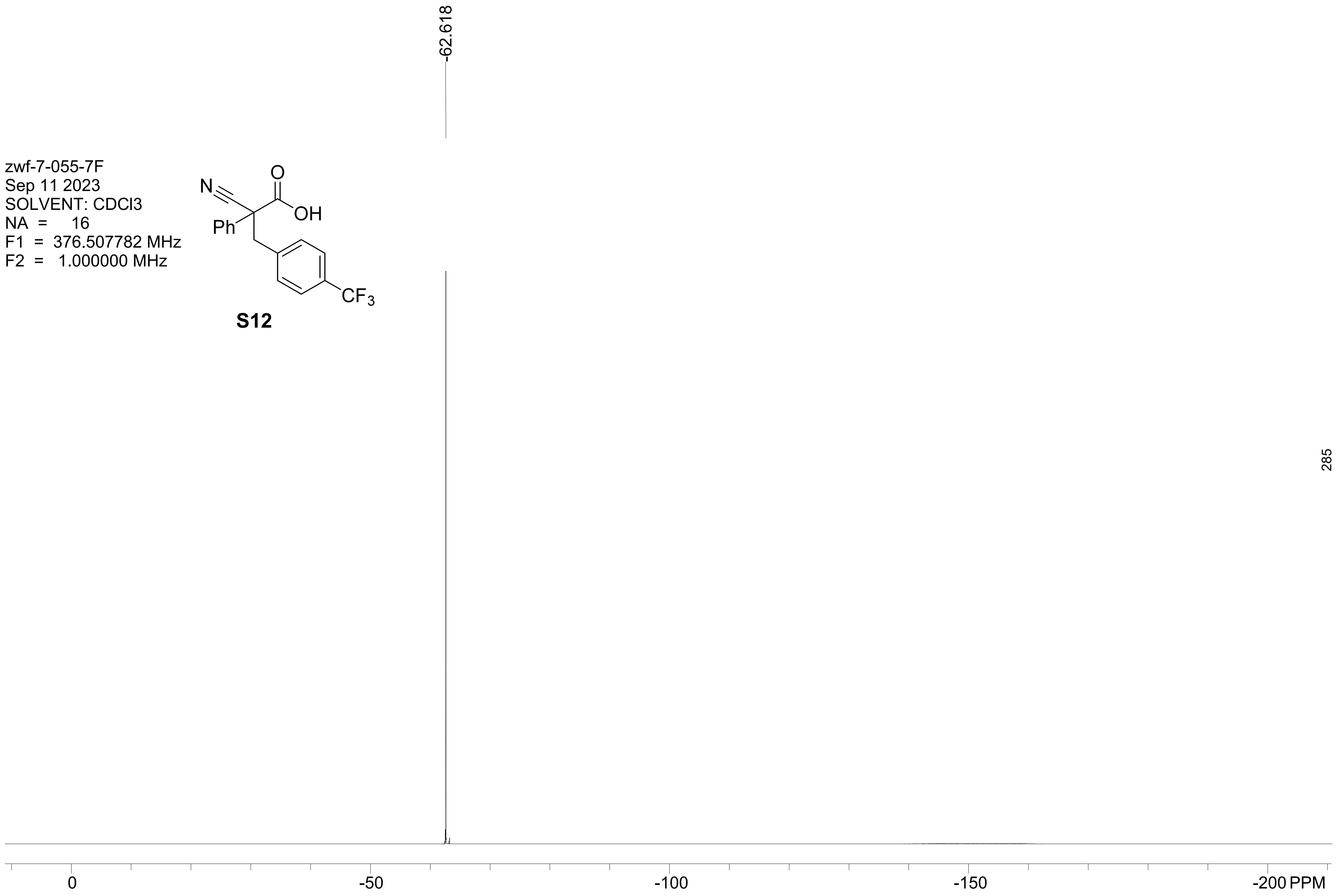
zwf-7-055-7F
Sep 11 2023
SOLVENT: CDCl3
NA = 16
F1 = 376.507782 MHz
F2 = 1.000000 MHz
N
O
OH
Ph
CF3
-62.618
0
-50
-100
-150
-200 PPM

S12

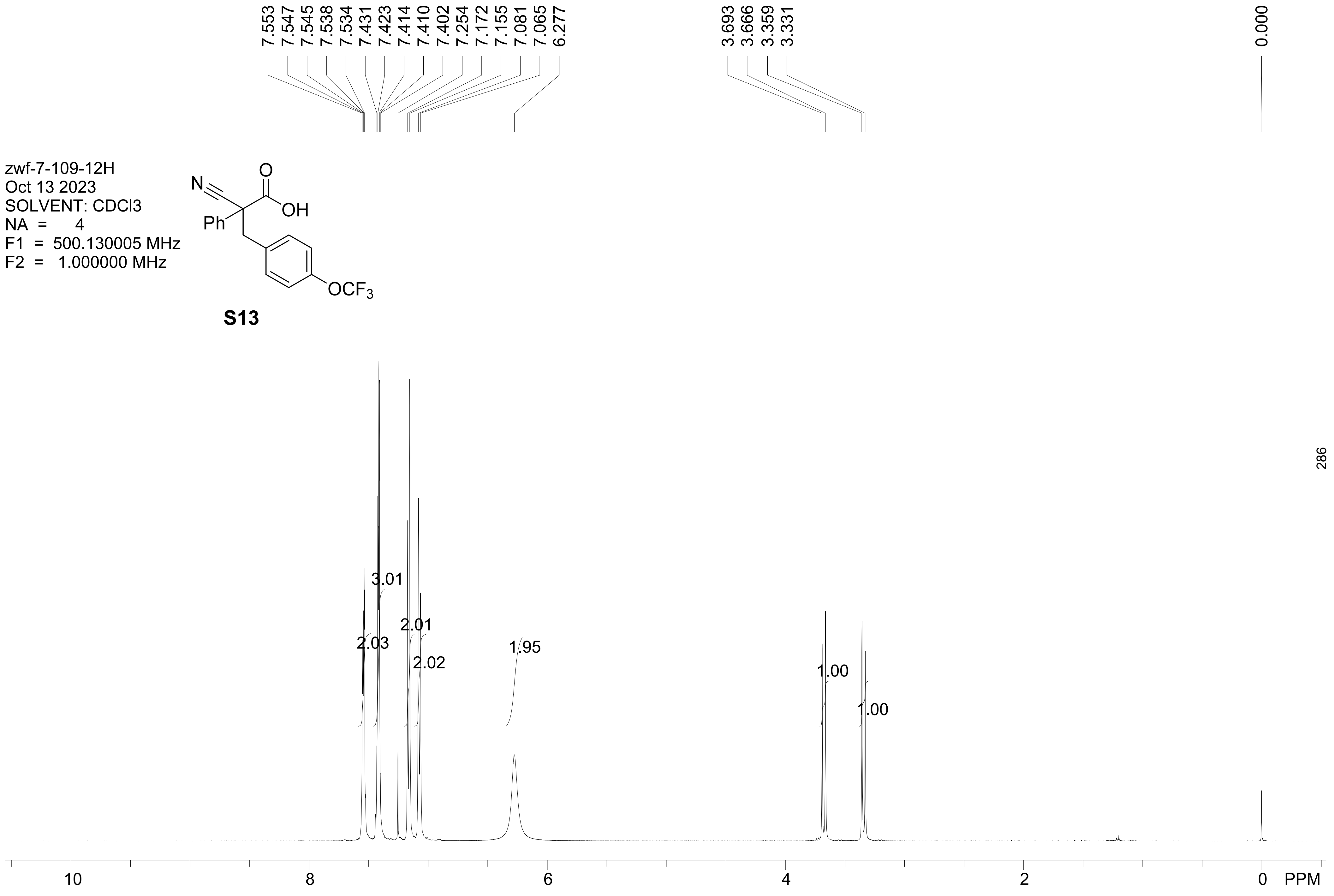
7.553
7.547
7.545
7.538
7.534
7.431
7.423
7.414
7.410
7.402
7.254
7.172
7.155
7.081
7.065
6.277
3.693
3.666
3.359
3.331
0.000
zwf-7-109-12H
Oct 13 2023
SOLVENT: CDCl3
NA = 4
F1 = 500.130005 MHz
F2 = 1.000000 MHz
N
O
OH
Ph
OCF3
S13
3.01
2.03
2.01
2.02
1.95
1.00
1.00
10
8
6
4
2
0 PPM

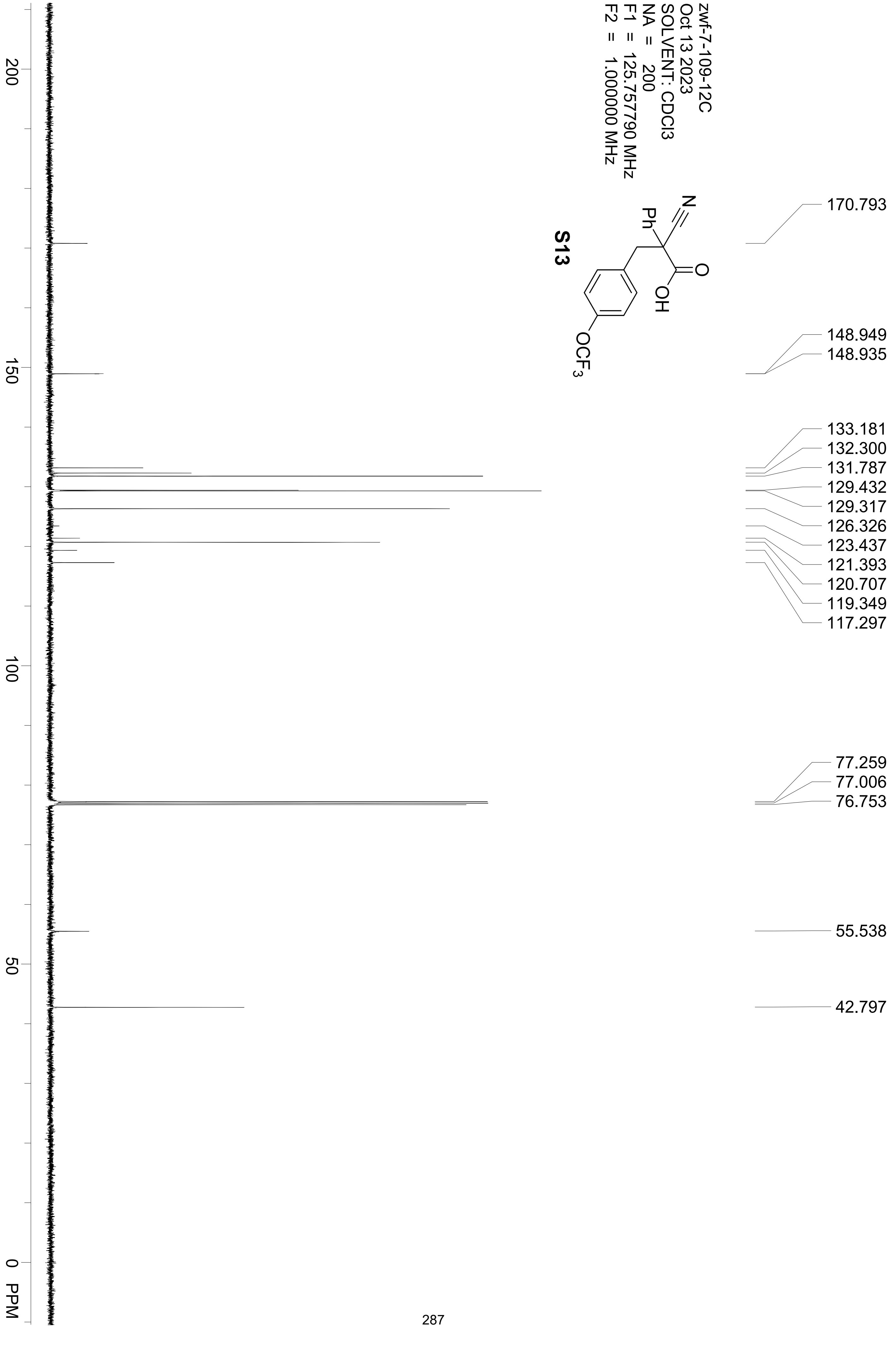
zwf-7-109-12C
Oct 13 2023
SOLVENT: CDCl3
NA = 200
F1 = 125.757790 MHz
F2 = 1.000000 MHz
S13
N
Ph
O
OH
OCF3
170.793
148.949
148.935
133.181
132.300
131.787
129.432
129.317
126.326
123.437
121.393
120.707
119.349
117.297
77.259
77.006
76.753
55.538
42.797
200
150
100
50
0 PPM

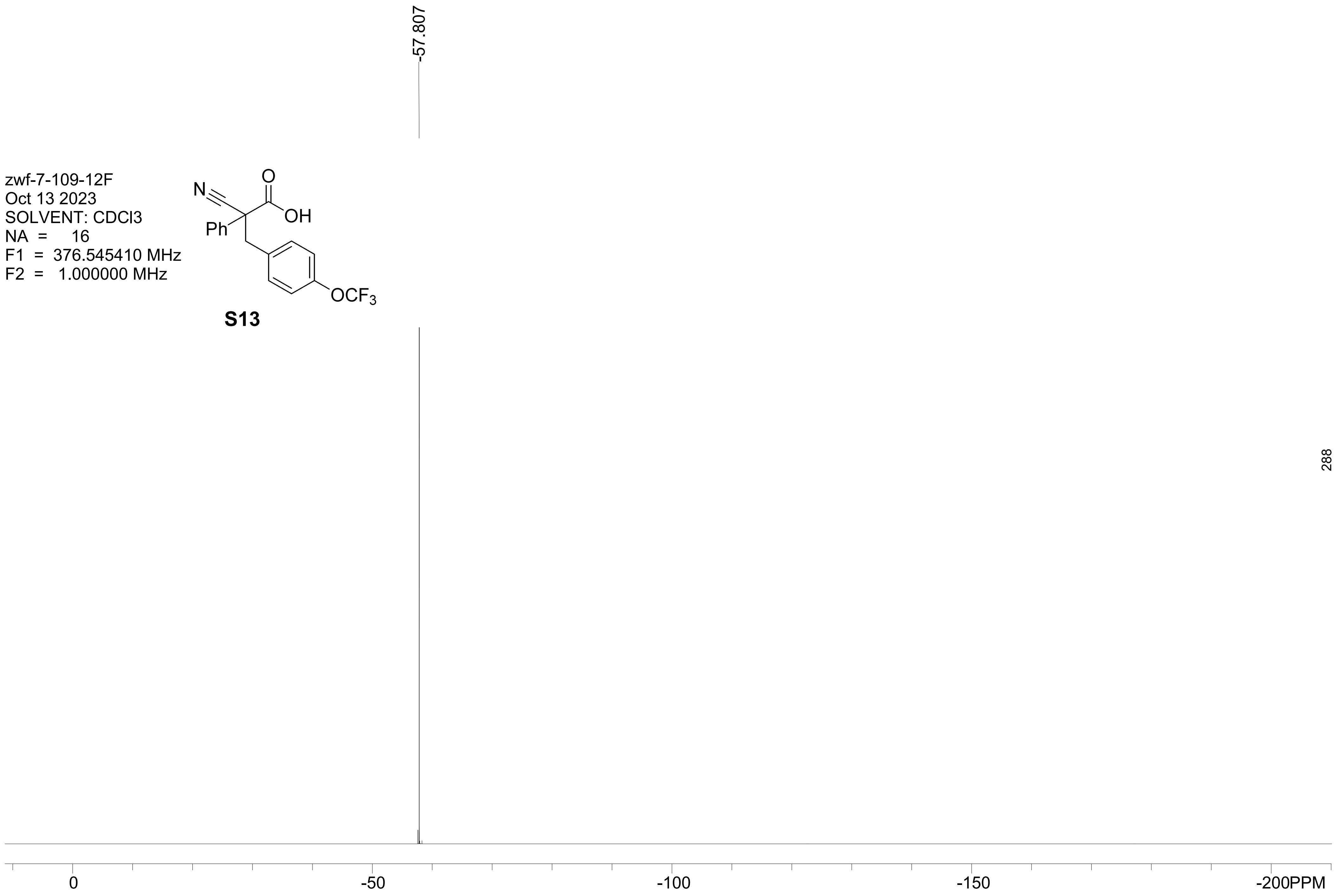
-57.807
zwf-7-109-12F
Oct 13 2023
SOLVENT: CDCl3
NA = 16
F1 = 376.545410 MHz
F2 = 1.000000 MHz
N
O
OH
Ph
OCF3
S13
0
-50
-100
-150
-200PPM

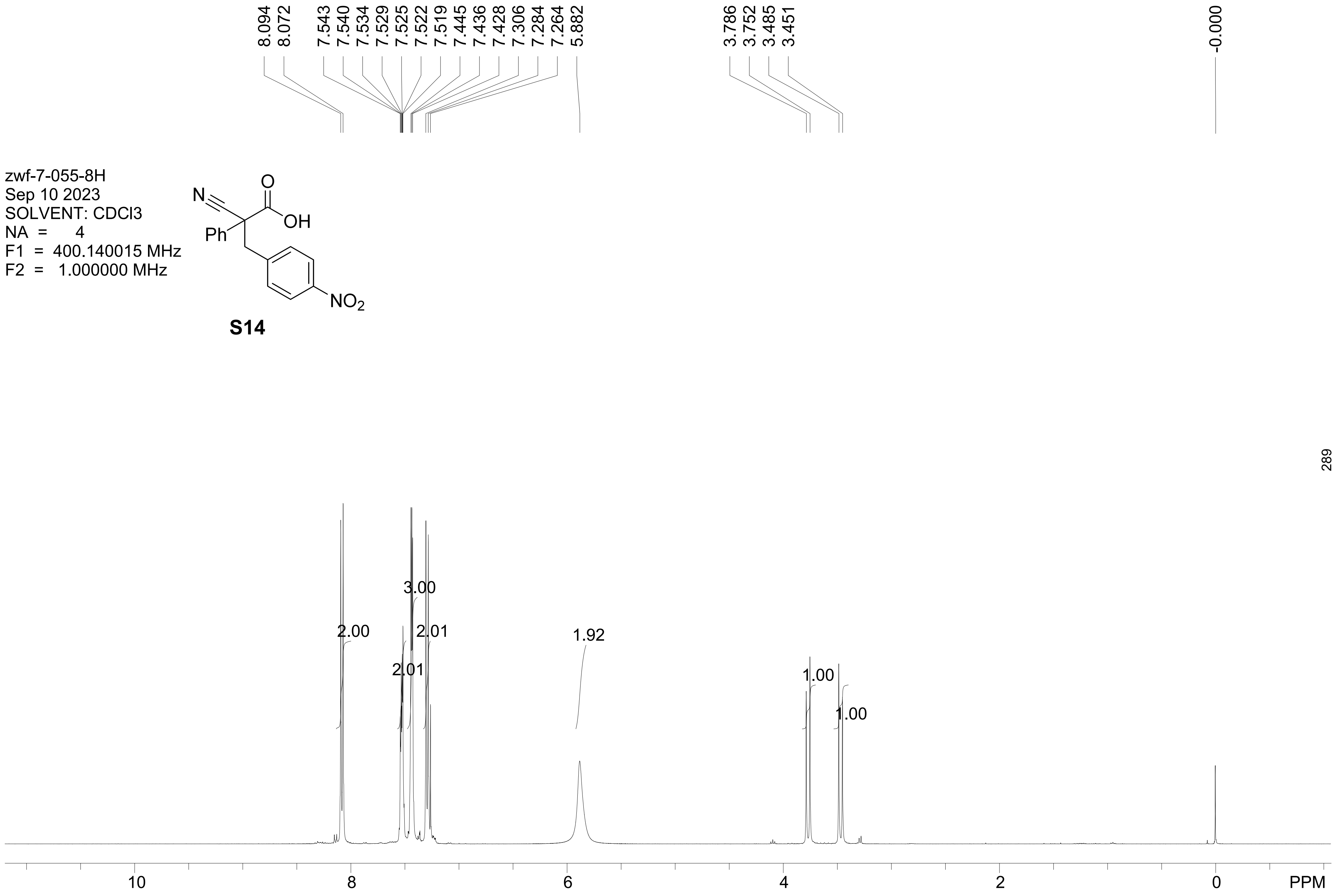
zwf-7-055-8H
Sep 10 2023
SOLVENT: CDCl3
NA = 4
F1 = 400.140015 MHz
F2 = 1.000000 MHz
N
O
OH
Ph
NO2
S14
8.094
8.072
7.543
7.540
7.534
7.529
7.525
7.522
7.519
7.445
7.436
7.428
7.306
7.284
7.264
5.882
3.786
3.752
3.485
3.451
-0.000
2.00
3.00
2.01
2.01
1.92
1.00
1.00
10
8
6
4
2
0
PPM

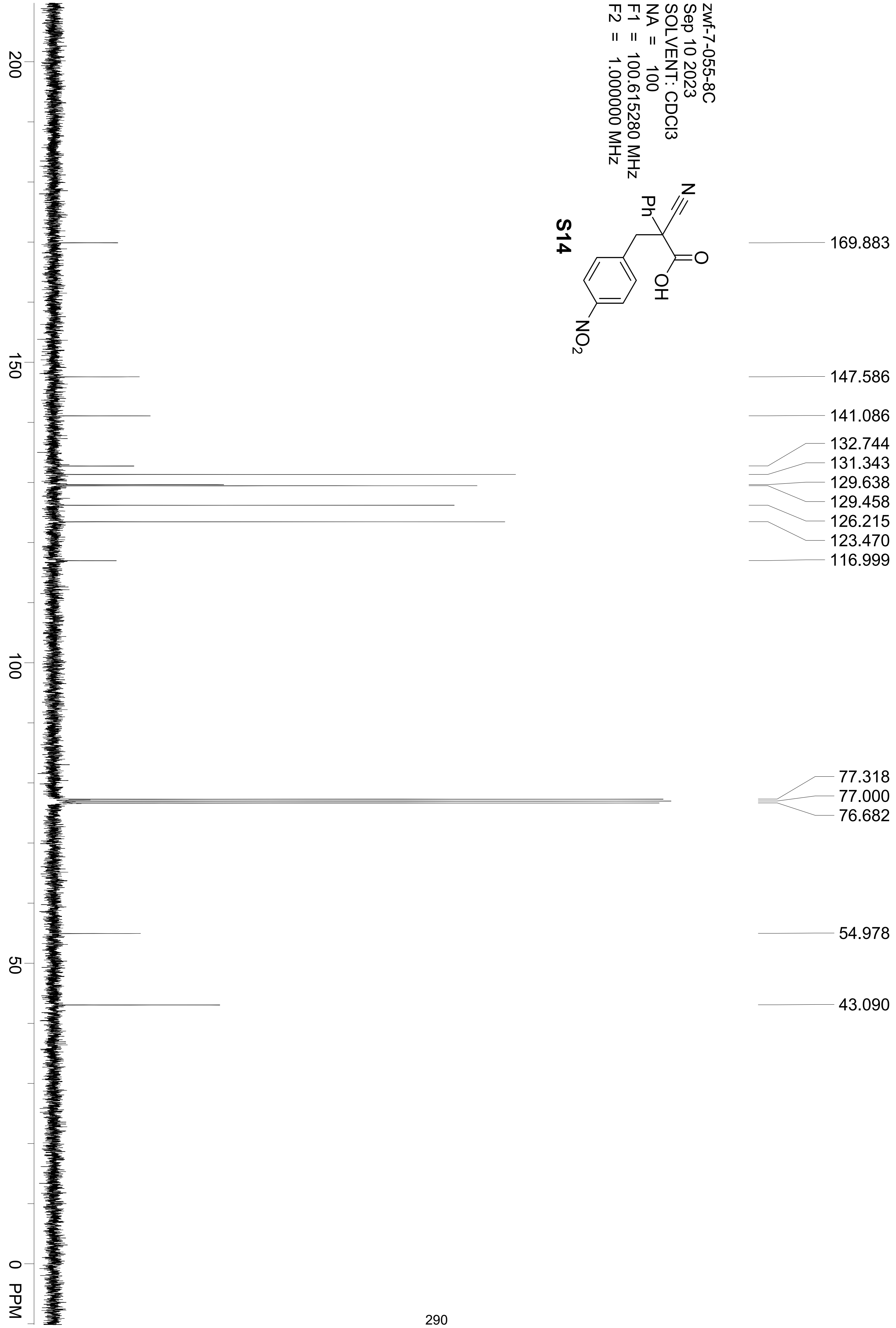
zwf-7-055-8C
Sep 10 2023
SOLVENT: CDCl3
NA = 100
F1 = 100.615280 MHz
F2 = 1.000000 MHz
S14
N
Ph
O
OH
NO2
169.883
147.586
141.086
132.744
131.343
129.638
129.458
126.215
123.470
116.999
77.318
77.000
76.682
54.978
43.090
200
150
100
50
0 PPM

zwf-7-055-5H
Sep 10 2023
SOLVENT: CDCl3
NA = 4
F1 = 400.140015 MHz
F2 = 1.000000 MHz

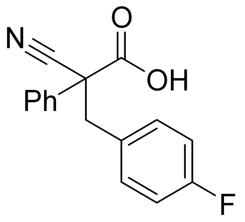

S15

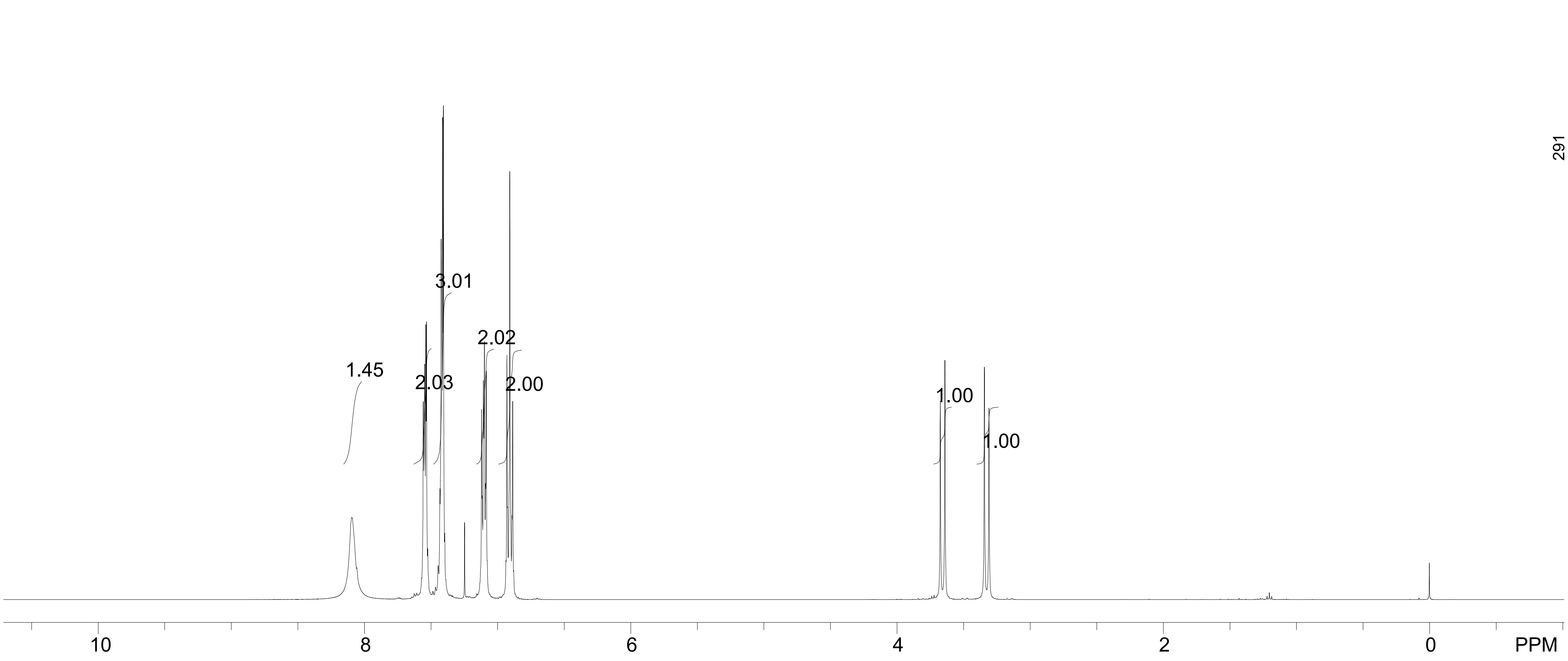

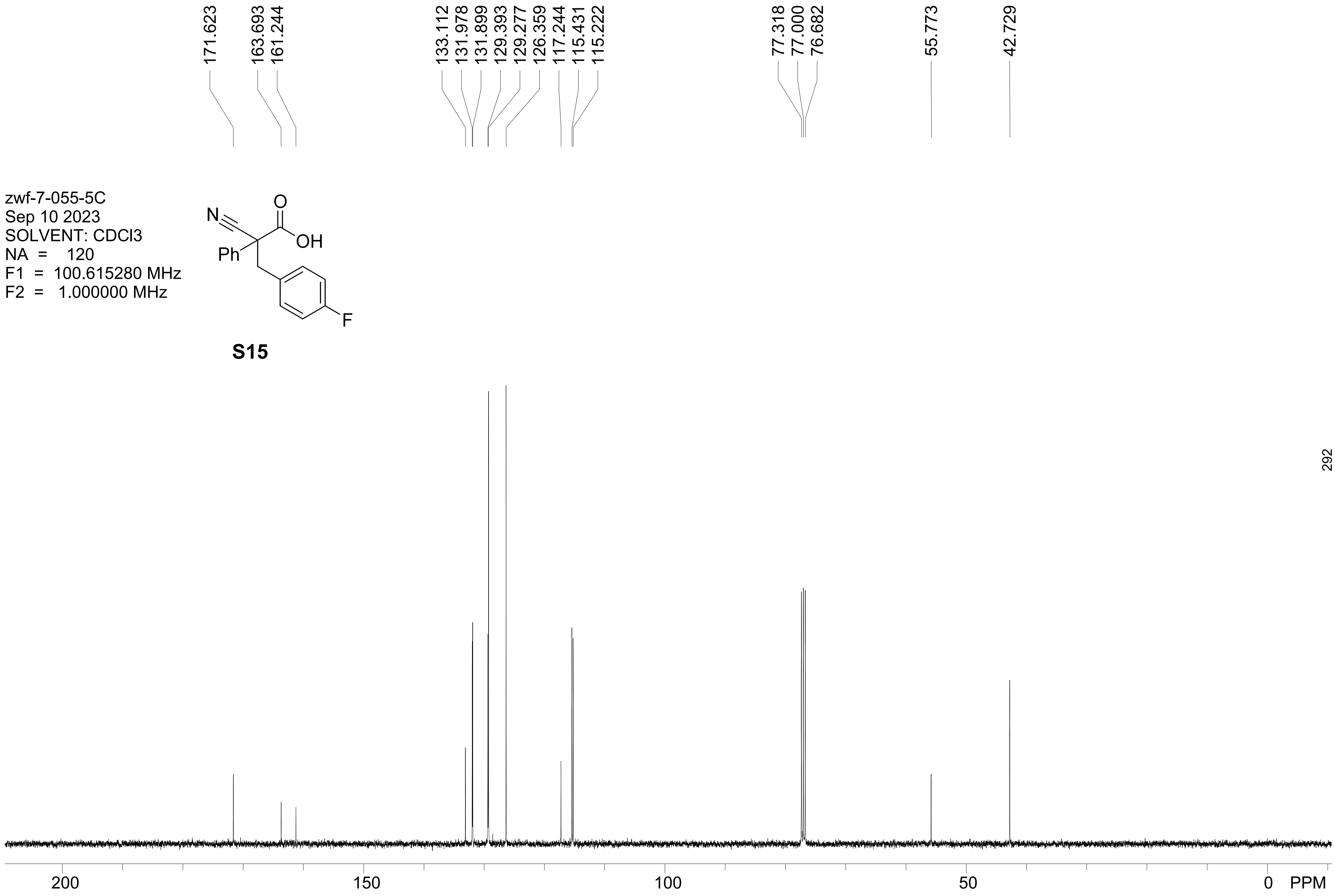
zwf-7-055-5C
Sep 10 2023
SOLVENT: CDCl3
NA = 120
F1 = 100.615280 MHz
F2 = 1.000000 MHz
N
O
OH
Ph
F
S15
171.623
163.693
161.244
133.112
131.978
131.899
129.393
129.277
126.359
117.244
115.431
115.222
77.318
77.000
76.682
55.773
42.729
200
150
100
50
0 PPM

zwf-7-055-5F
Sep 10 2023
SOLVENT: CDCl3
NA = 16
F1 = 376.507782 MHz
F2 = 1.000000 MHz

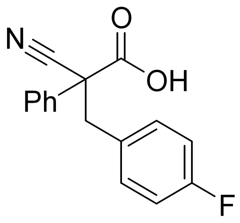

S15

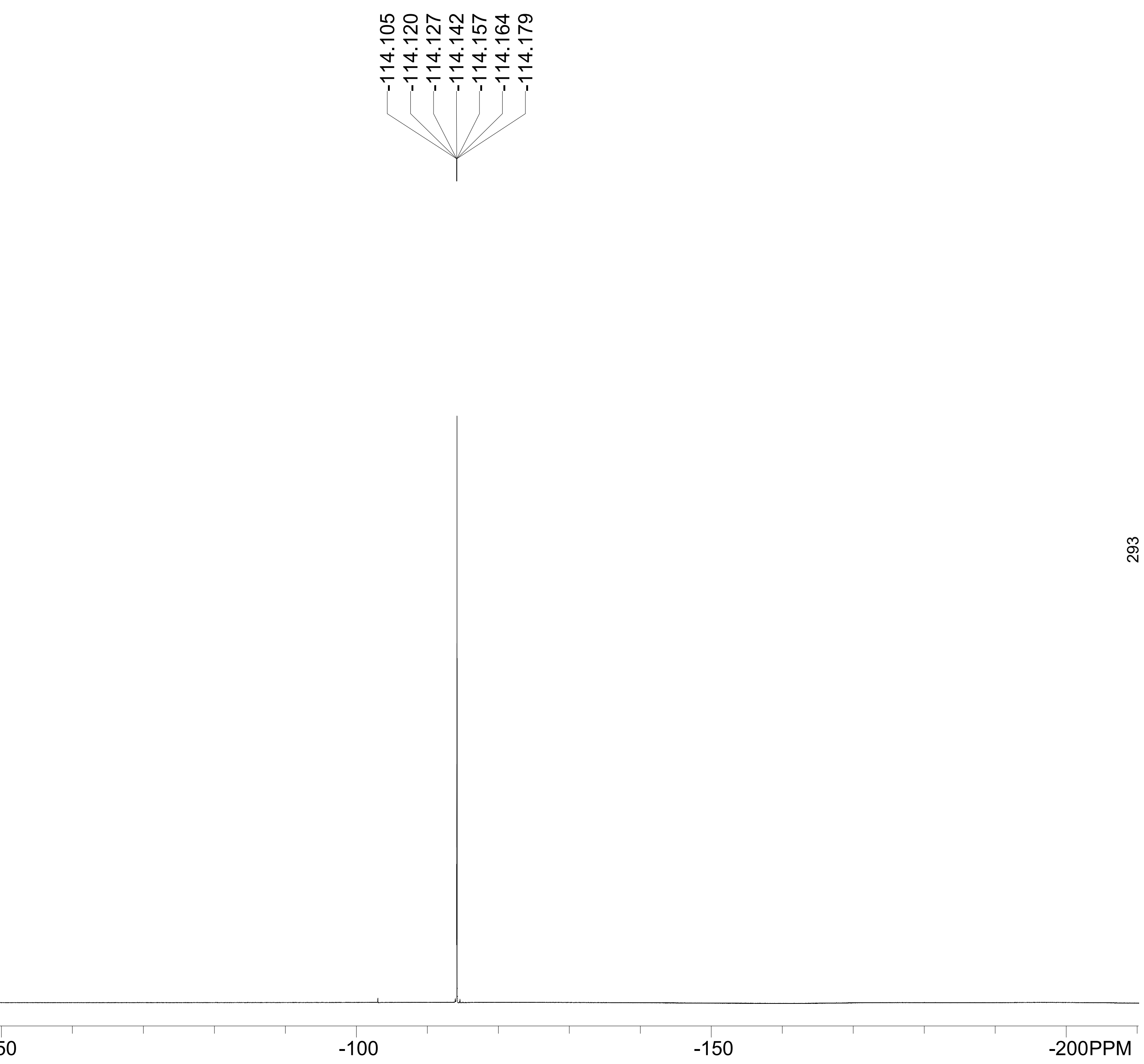

zwf-7-055-1H
Sep 11 2023
SOLVENT: CDCl3
NA = 4
F1 = 400.140015 MHz
F2 = 1.000000 MHz

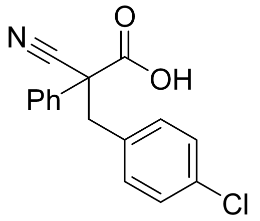

S16

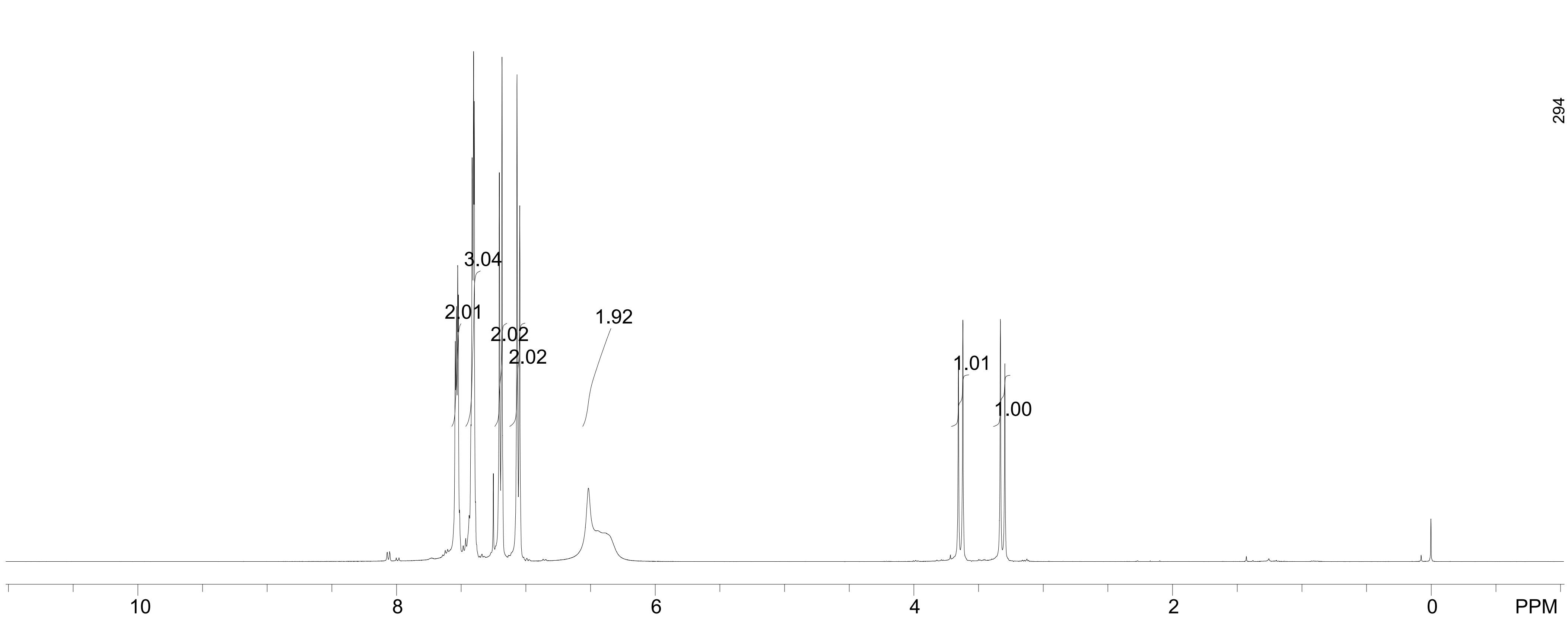

zwf-7-055-1C
Sep 11 2023
SOLVENT: CDCl3
NA = 100
F1 = 100.625343 MHz
F2 = 1.000000 MHz

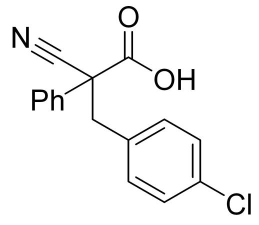

S16

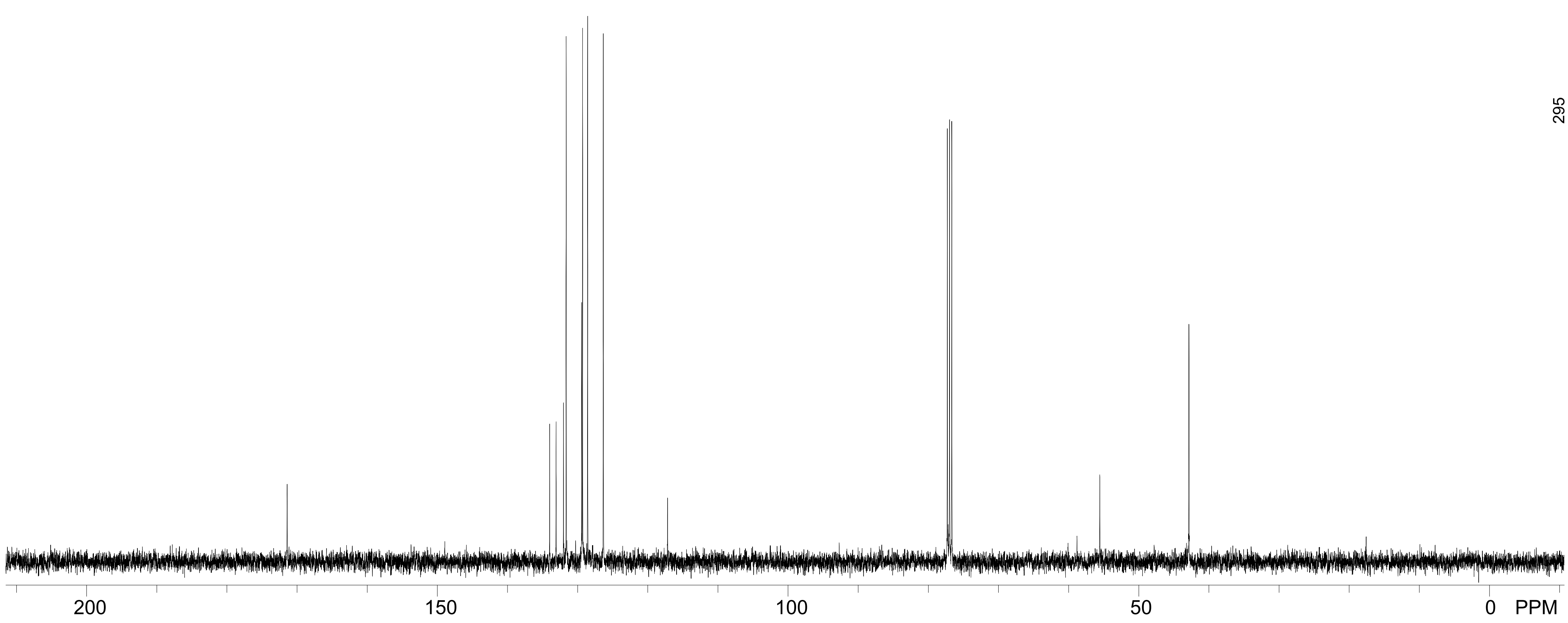

zwf-7-055-6H
Sep 10 2023
SOLVENT: CDCl3
NA = 4
F1 = 400.140015 MHz
F2 = 1.000000 MHz

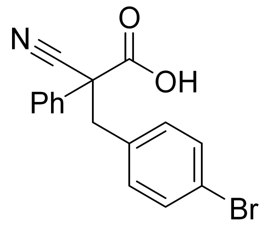

S17

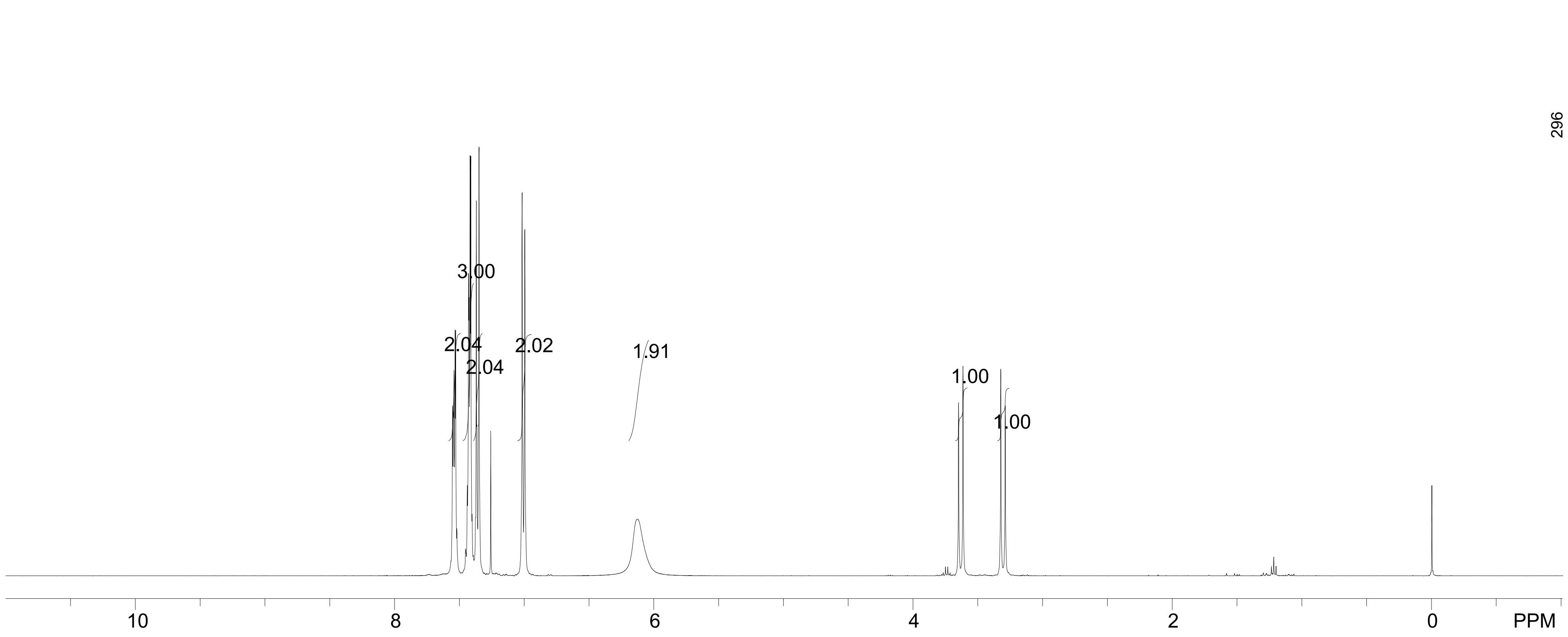

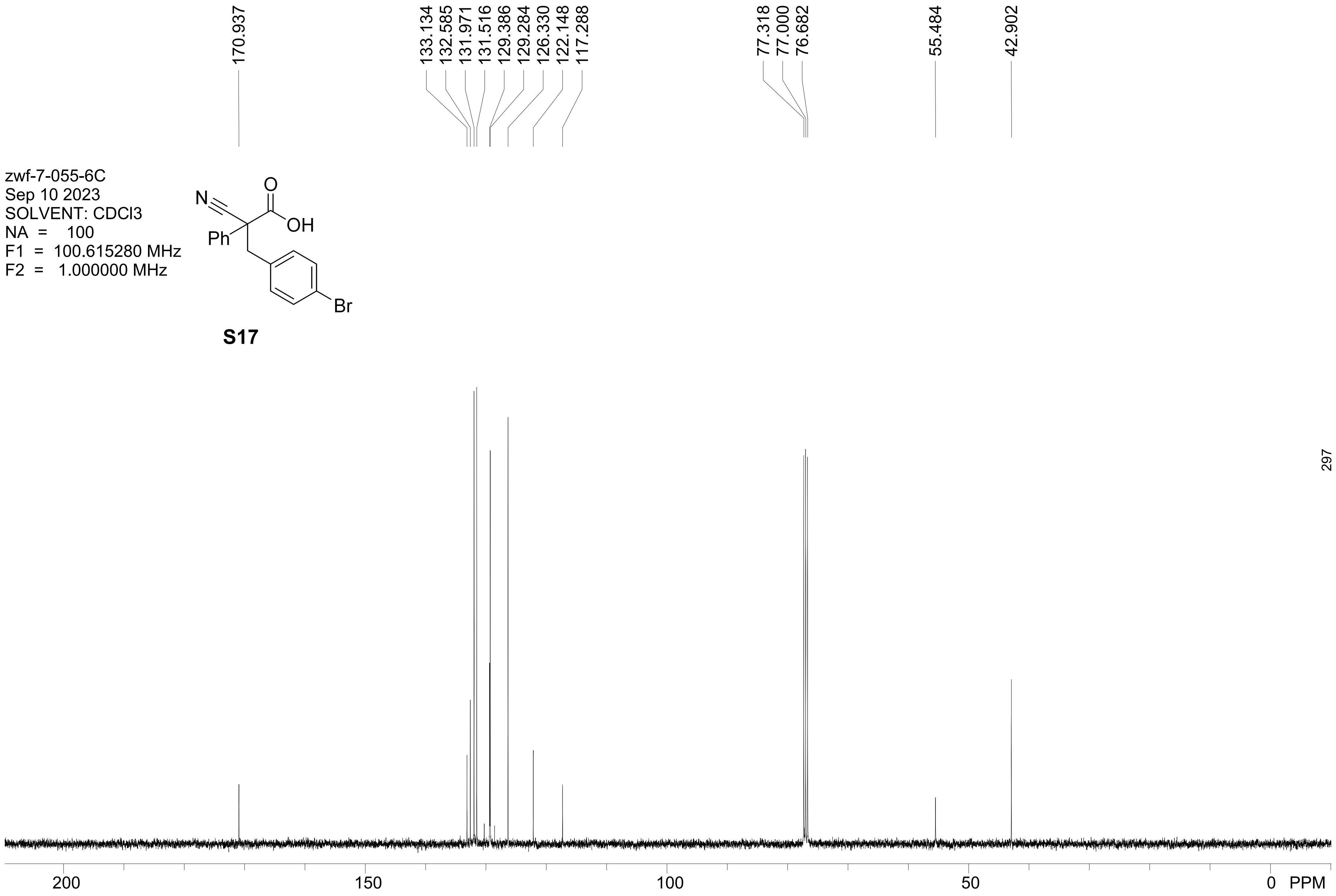

zwf-7-055-6C
Sep 10 2023
SOLVENT: CDCl3
NA = 100
F1 = 100.615280 MHz
F2 = 1.000000 MHz
N
O
OH
Ph
Br
S17
170.937
133.134
132.585
131.971
131.516
129.386
129.284
126.330
122.148
117.288
77.318
77.000
76.682
55.484
42.902
200
150
100
50
0 PPM

zwf-7-086-10H
Oct 06 2023
SOLVENT: CDCl3
NA = 4
F1 = 400.140015 MHz
F2 = 1.000000 MHz

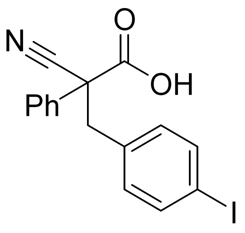

S18

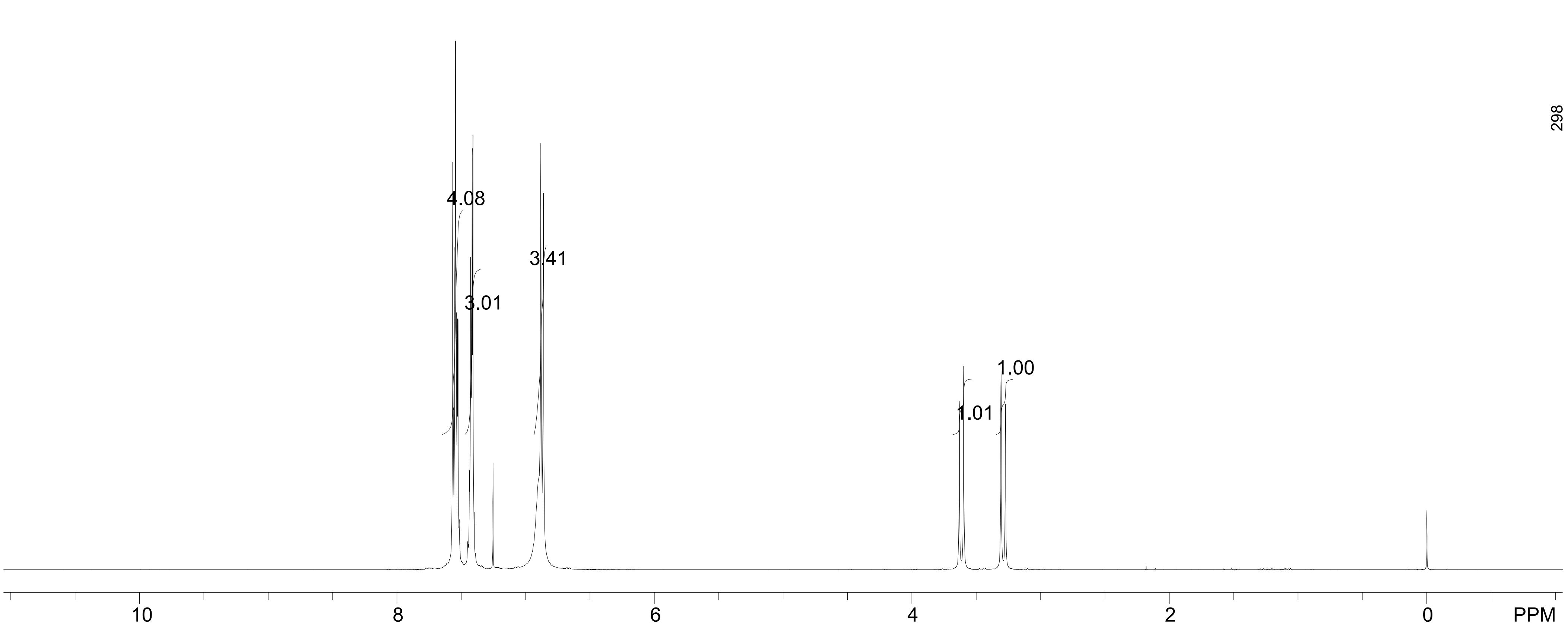

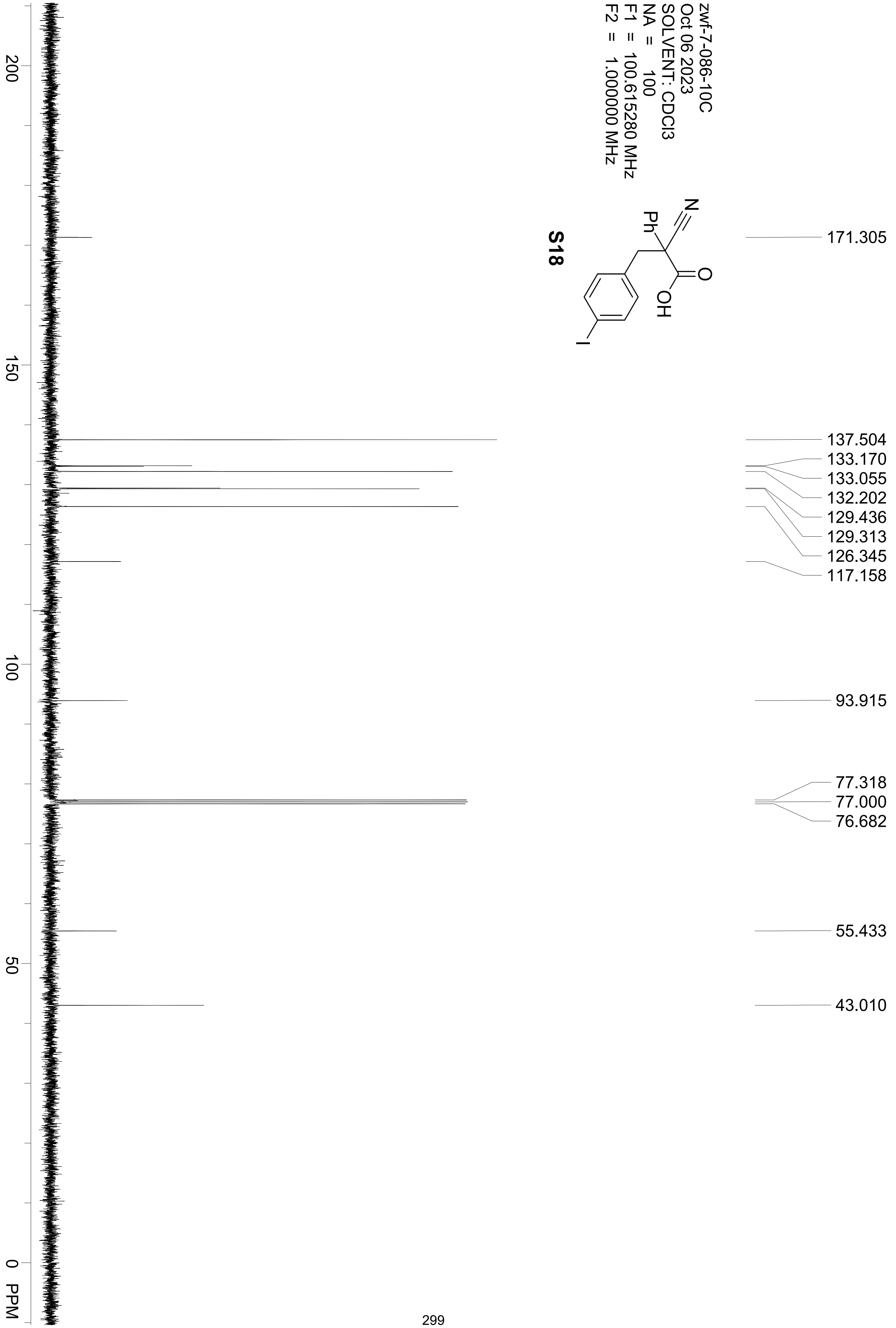

zwf-7-086-10C
Oct 06 2023
SOLVENT: CDCl3
NA = 100
F1 = 100.615280 MHz
F2 = 1.000000 MHz
S18
Ph
N
O
OH
I
171.305
137.504
133.170
133.055
132.202
129.436
129.313
126.345
117.158
93.915
77.318
77.000
76.682
55.433
43.010
200
150
100
50
0 PPM

zwf-7-109-10H
Oct 20 2023
SOLVENT: CDCl3
NA = 4
F1 = 400.140015 MHz
F2 = 1.000000 MHz

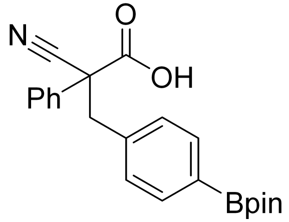

S19

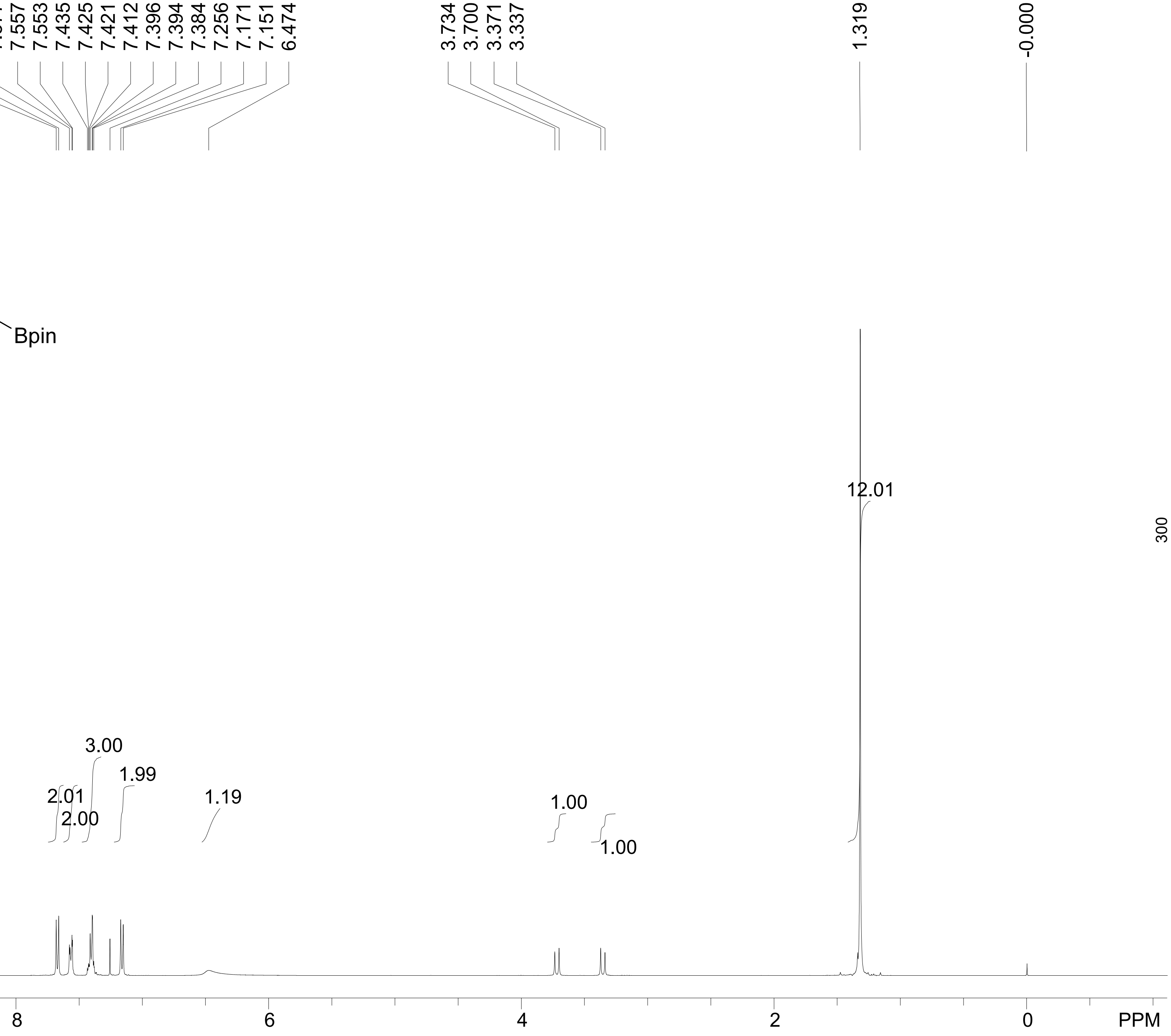

zwf-7-109-10C
Oct 20 2023
SOLVENT: CDCl3
NA = 1000
F1 = 100.615280 MHz
F2 = 1.000000 MHz

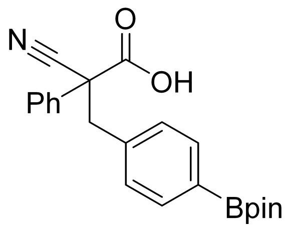

S19

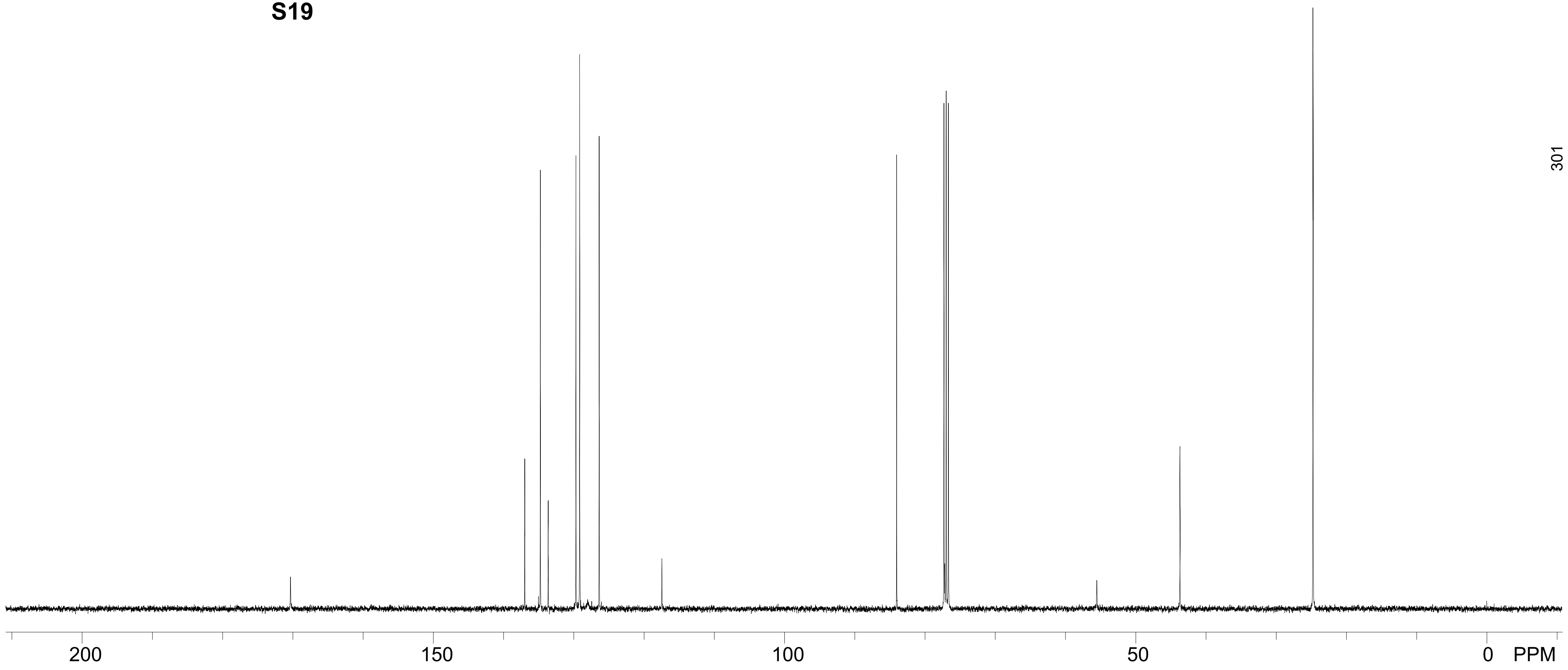

zwf-7-109-10B
Oct 20 2023
SOLVENT: CDCl3
NA = 700
F1 = 128.380814 MHz
F2 = 1.000000 MHz

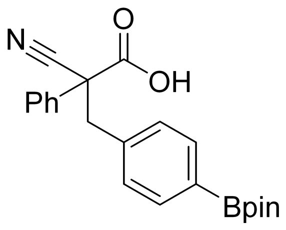

S19

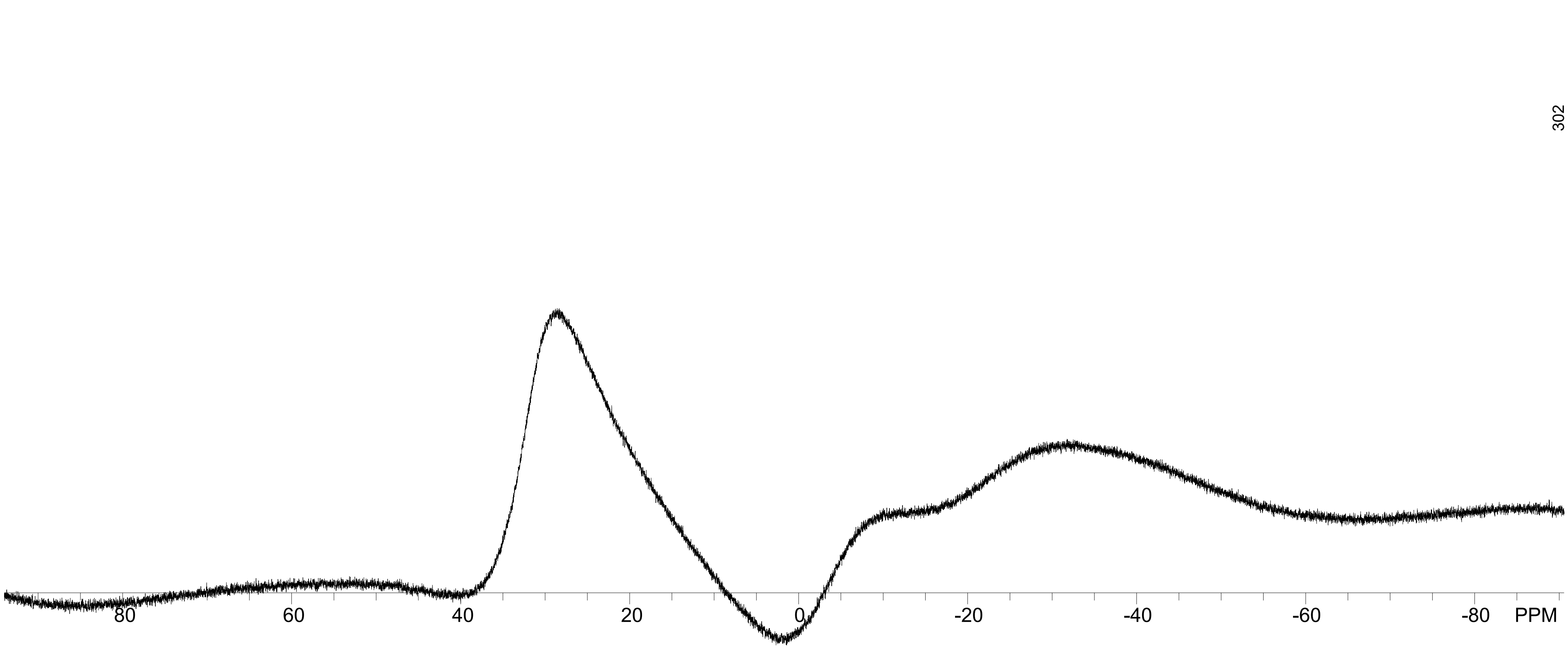

zwf-7-086-4H
Sep 23 2023
SOLVENT: CDCl3
NA = 4
F1 = 400.140015 MHz
F2 = 1.000000 MHz

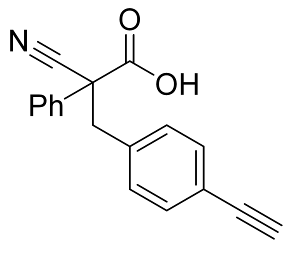

S20

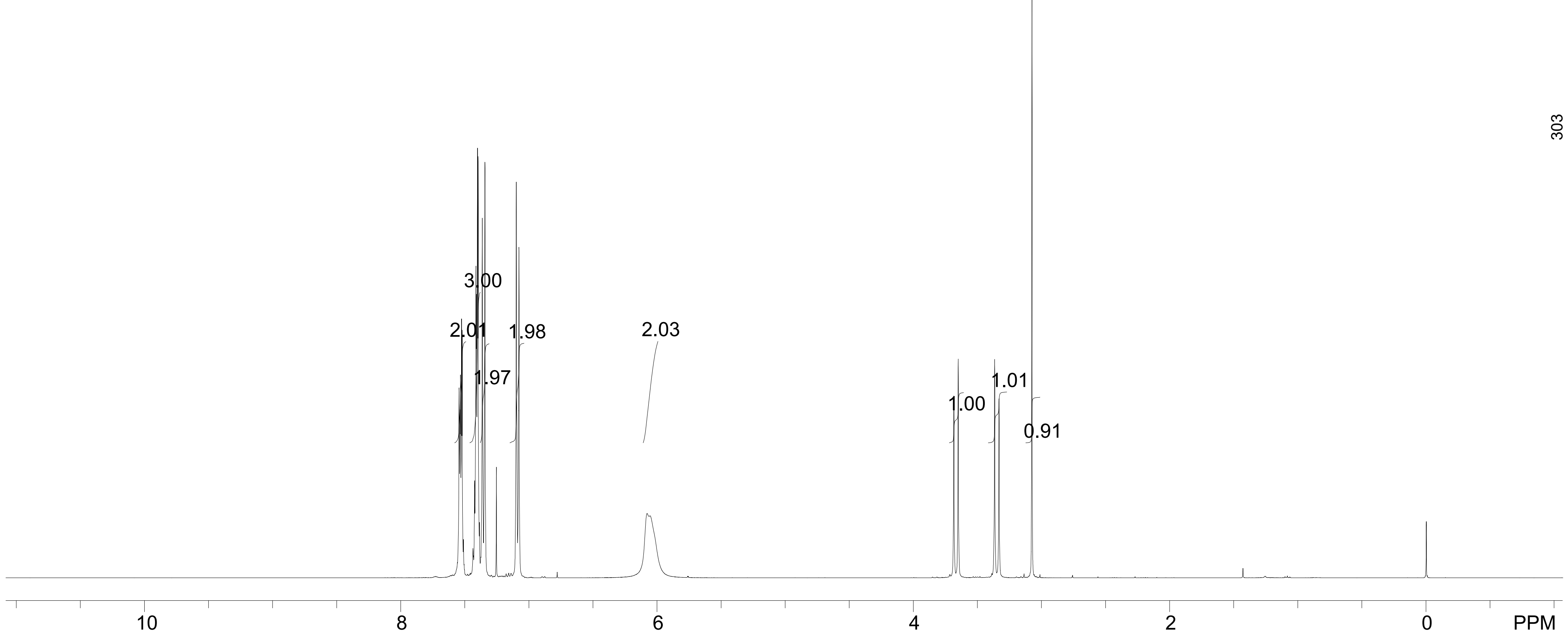

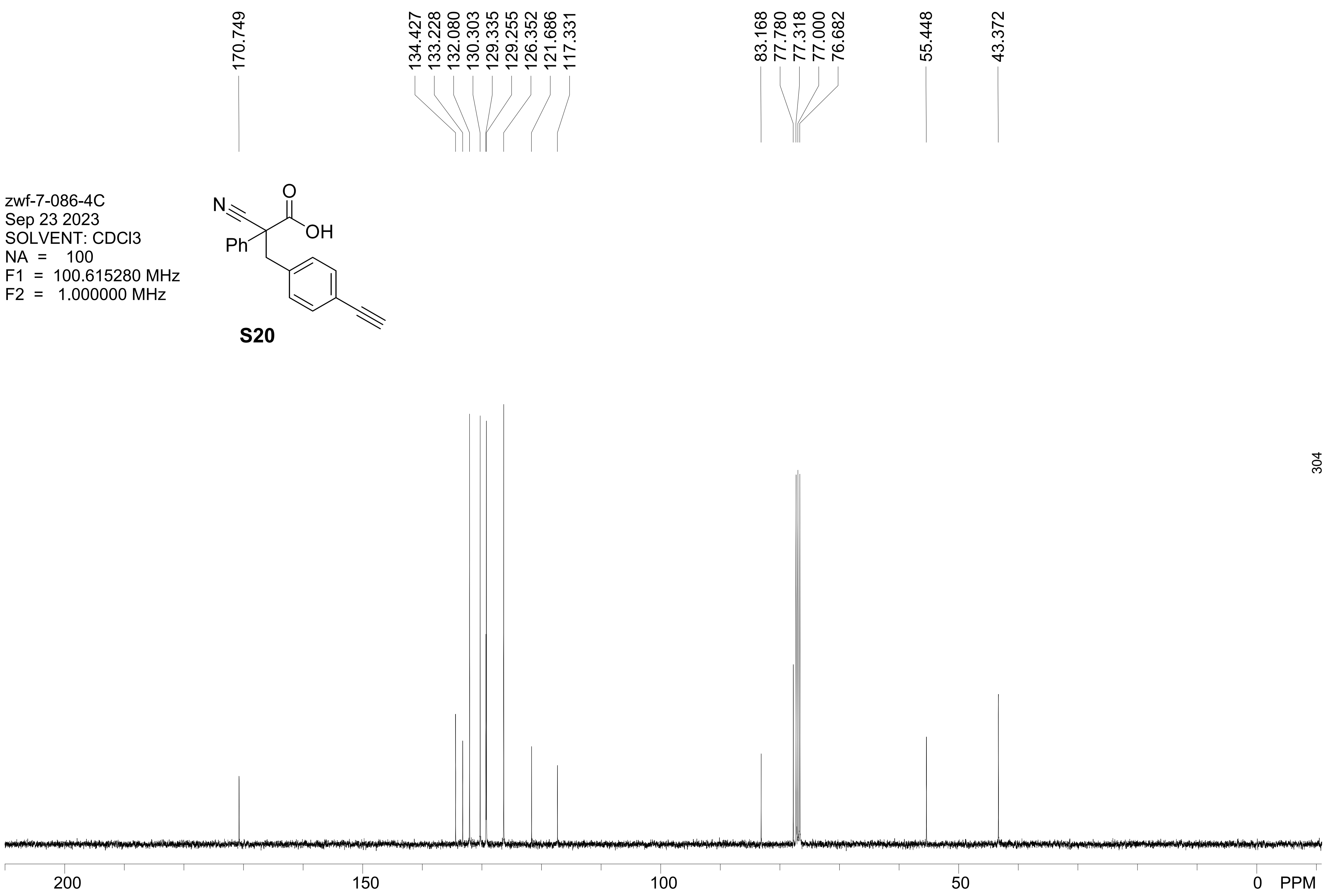
zwf-7-086-4C
Sep 23 2023
SOLVENT: CDCl3
NA = 100
F1 = 100.615280 MHz
F2 = 1.000000 MHz
N
O
OH
Ph
S20
170.749
134.427
133.228
132.080
130.303
129.335
129.255
126.352
121.686
117.331
83.168
77.780
77.318
77.000
76.682
55.448
43.372
200
150
100
50
0
PPM

zwf-7-055-4H
Sep 10 2023
SOLVENT: CDCl3
NA = 4
F1 = 400.140015 MHz
F2 = 1.000000 MHz

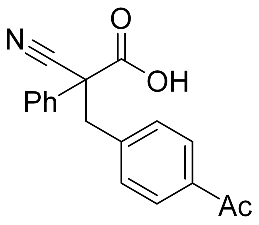

S21

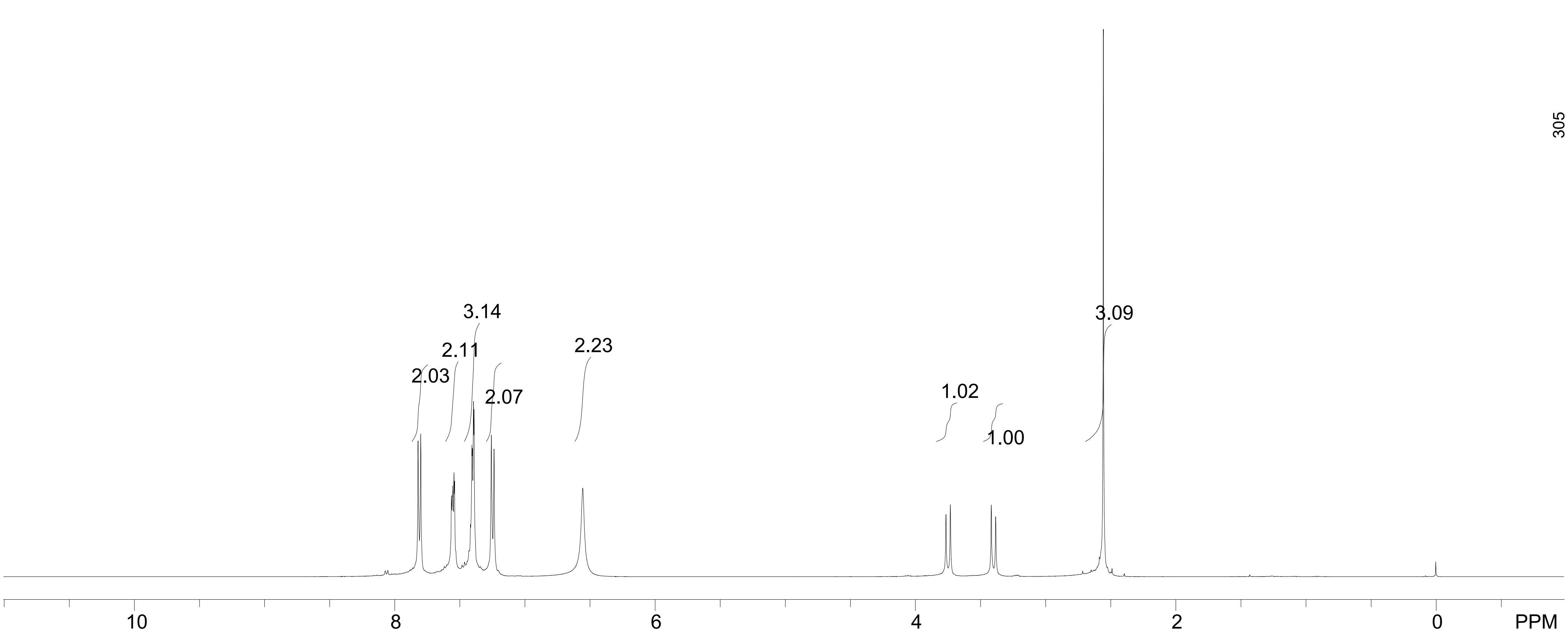

zwf-7-055-4C
Sep 10 2023
SOLVENT: CDCl3
NA = 100
F1 = 100.615280 MHz
F2 = 1.000000 MHz

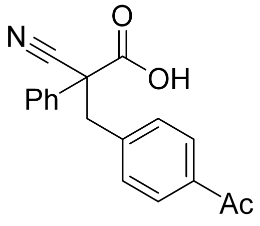

S21

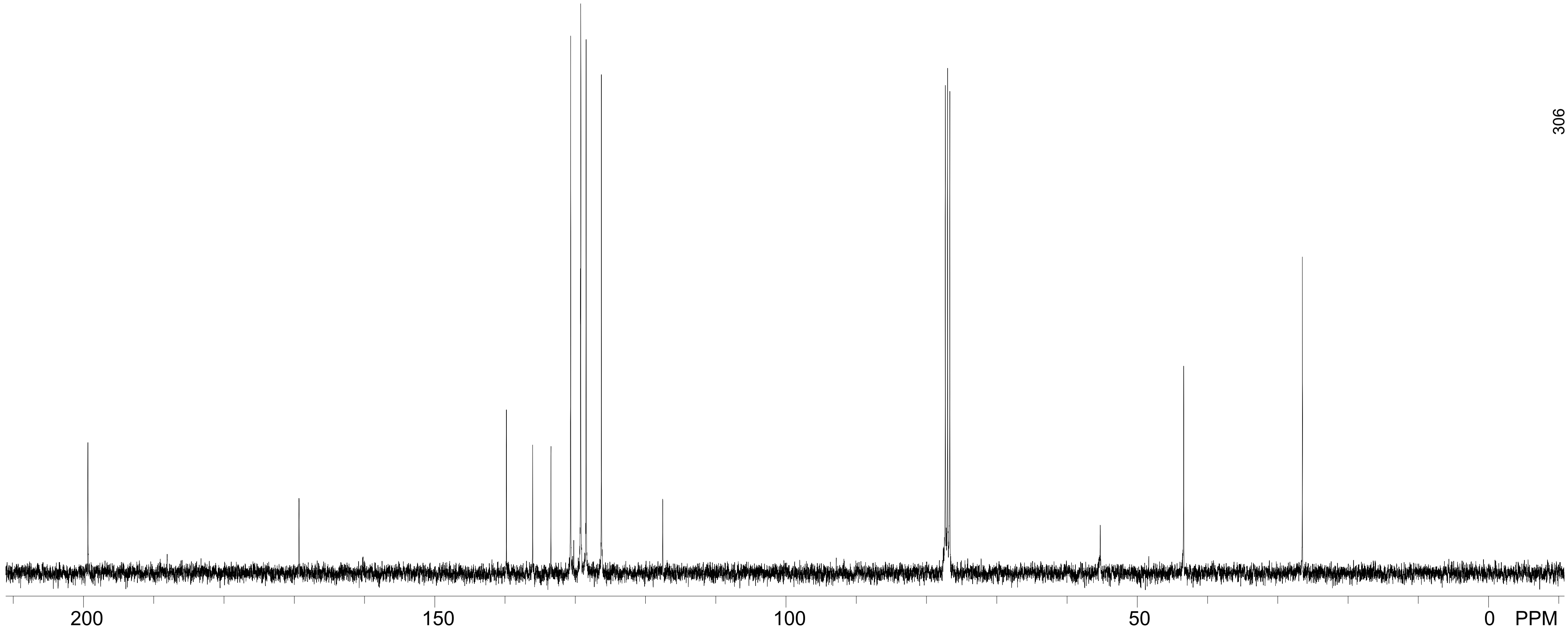

zwf-7-055-9H
Sep 12 2023
SOLVENT: CDCl3
NA = 4
F1 = 400.140015 MHz
F2 = 1.000000 MHz

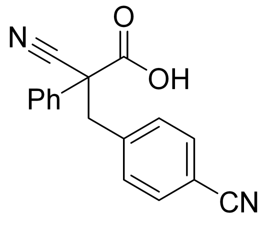

S22

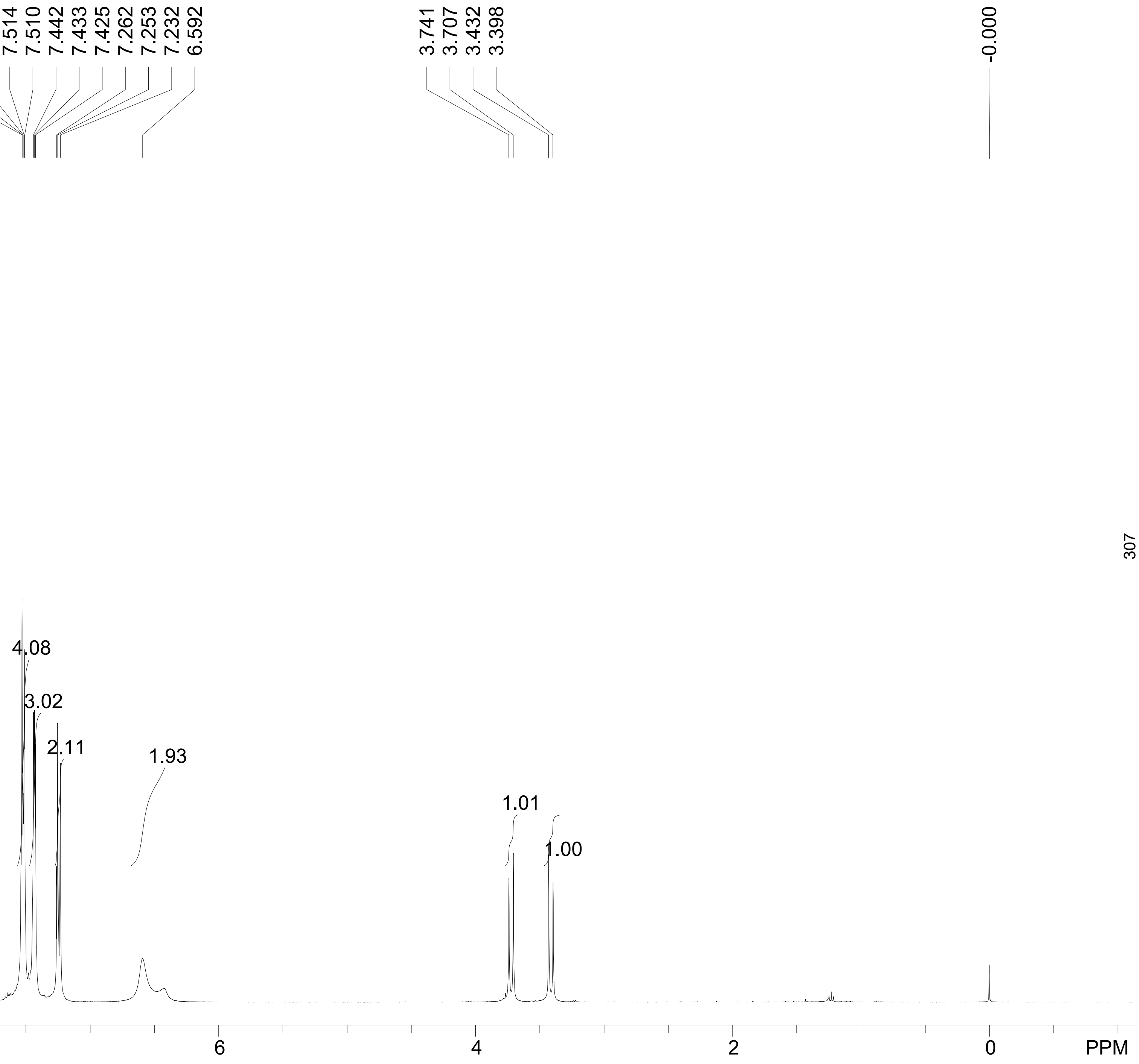

zwf-7-055-9C
Sep 12 2023
SOLVENT: CDCl3
NA = 100
F1 = 100.625343 MHz
F2 = 1.000000 MHz

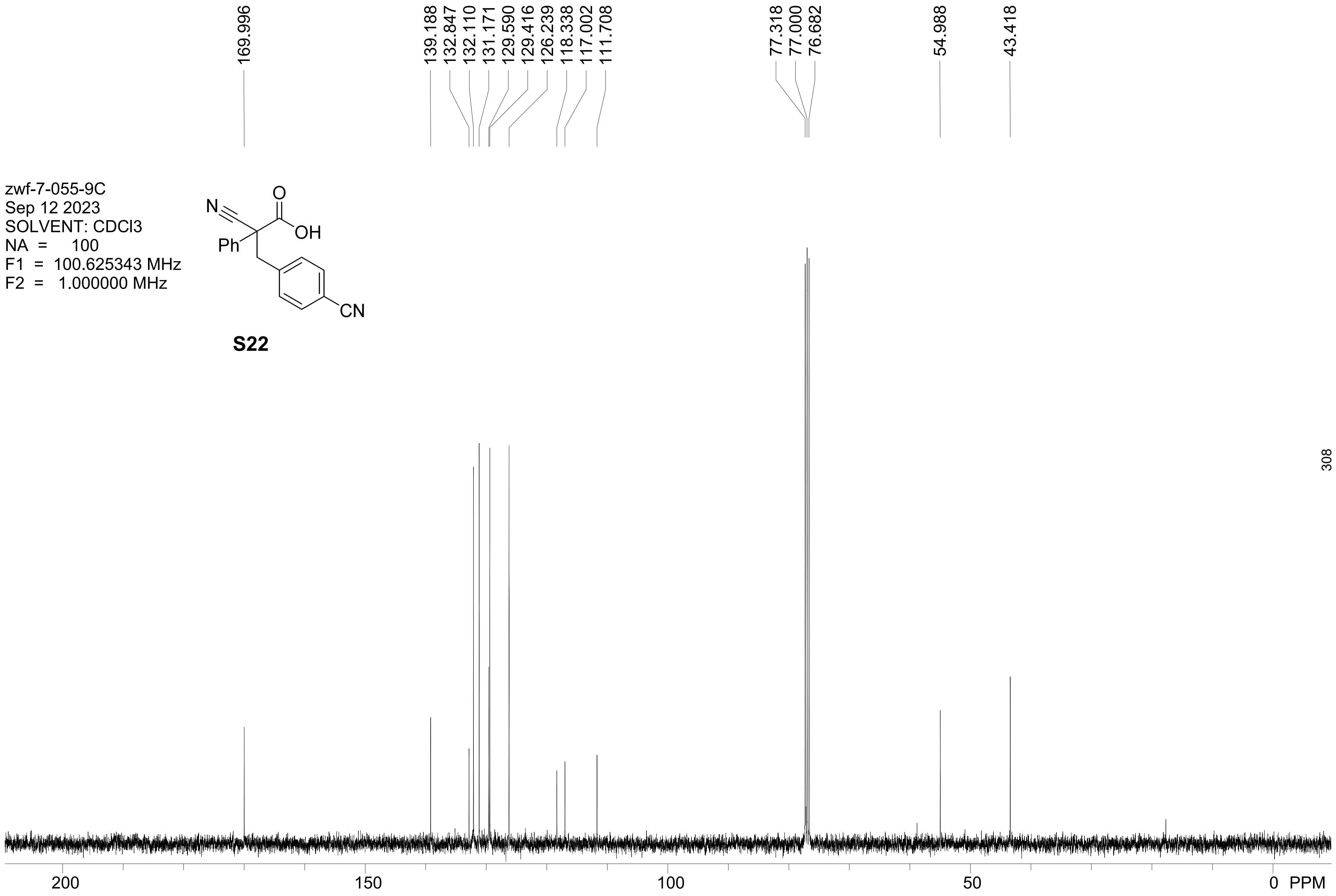

S22

zwf-10-036H
Jun 18 2024
SOLVENT: CDCl3
NA = 4
F1 = 500.130005 MHz
F2 = 1.000000 MHz

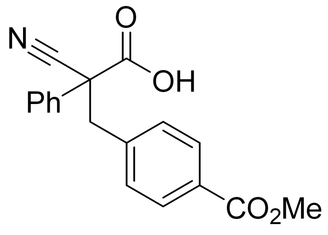

S23

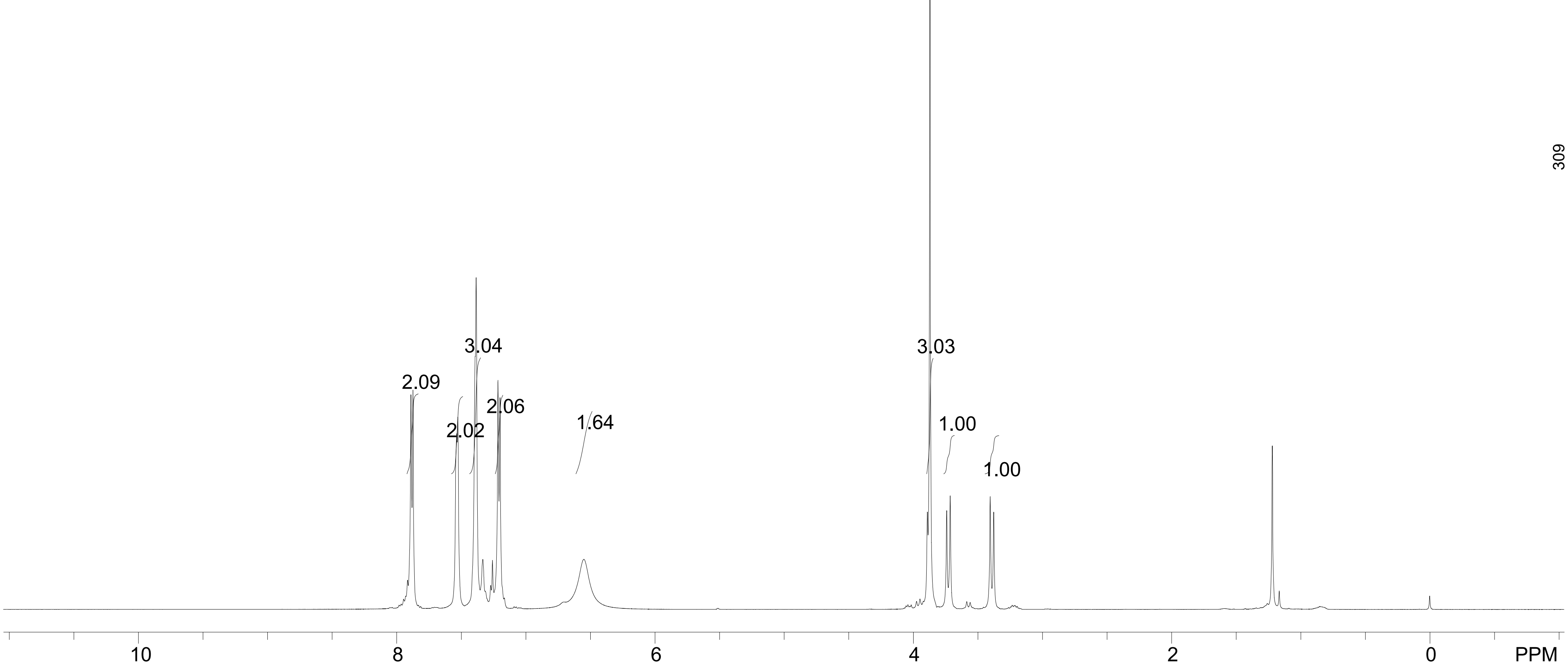

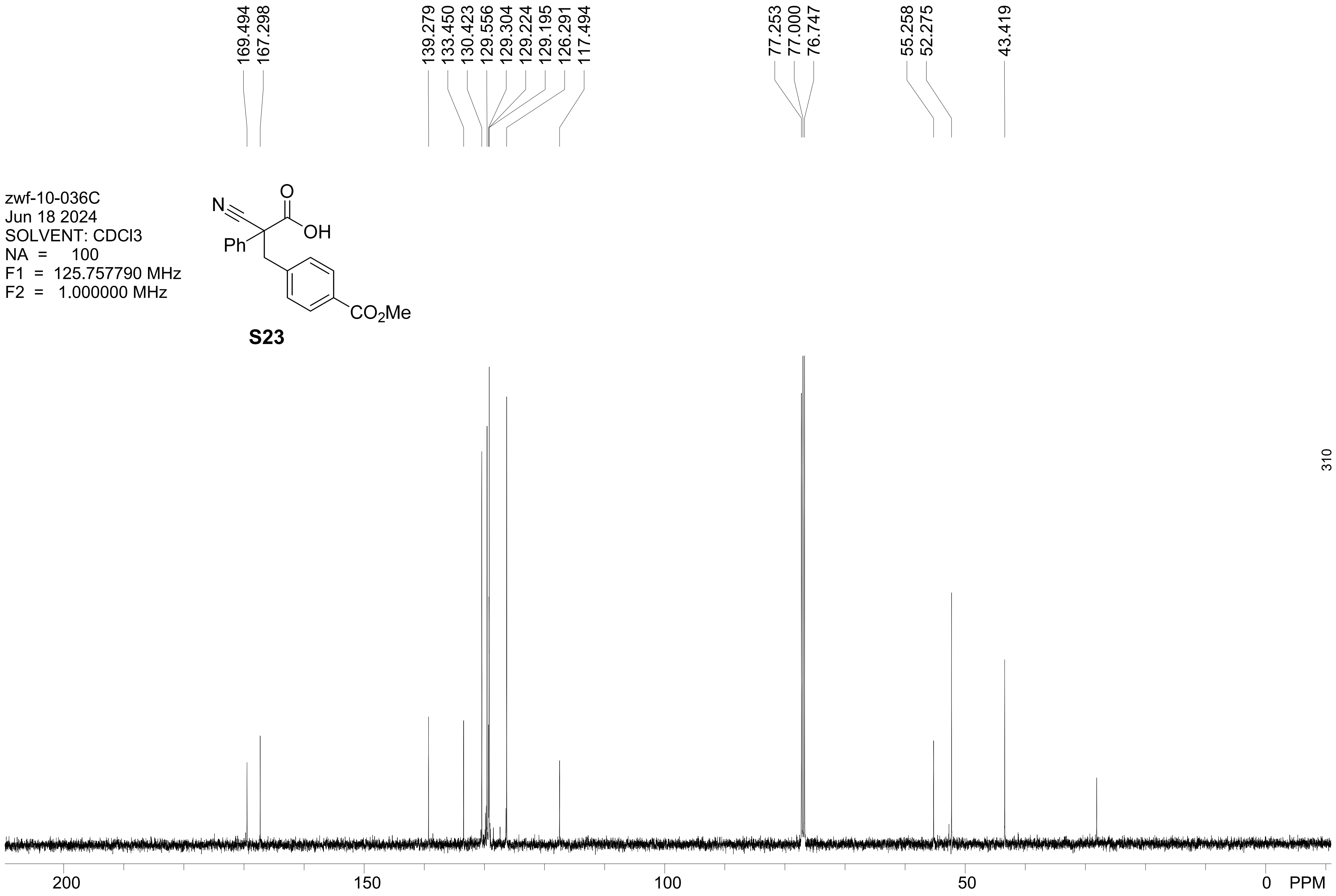
169.494
167.298
139.279
133.450
130.423
129.556
129.304
129.224
129.195
126.291
117.494
77.253
77.000
76.747
55.258
52.275
43.419
zwf-10-036C
Jun 18 2024
SOLVENT: CDCl3
NA = 100
F1 = 125.757790 MHz
F2 = 1.000000 MHz
N
O
OH
Ph
CO_2Me
S23
200
150
100
50
0 PPM

zwf-7-109-13H
Oct 18 2023
SOLVENT: CDCl3
NA = 4
F1 = 400.179993 MHz
F2 = 1.000000 MHz

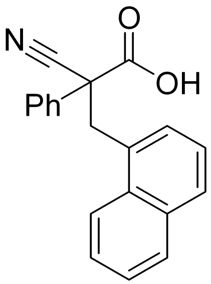

S24

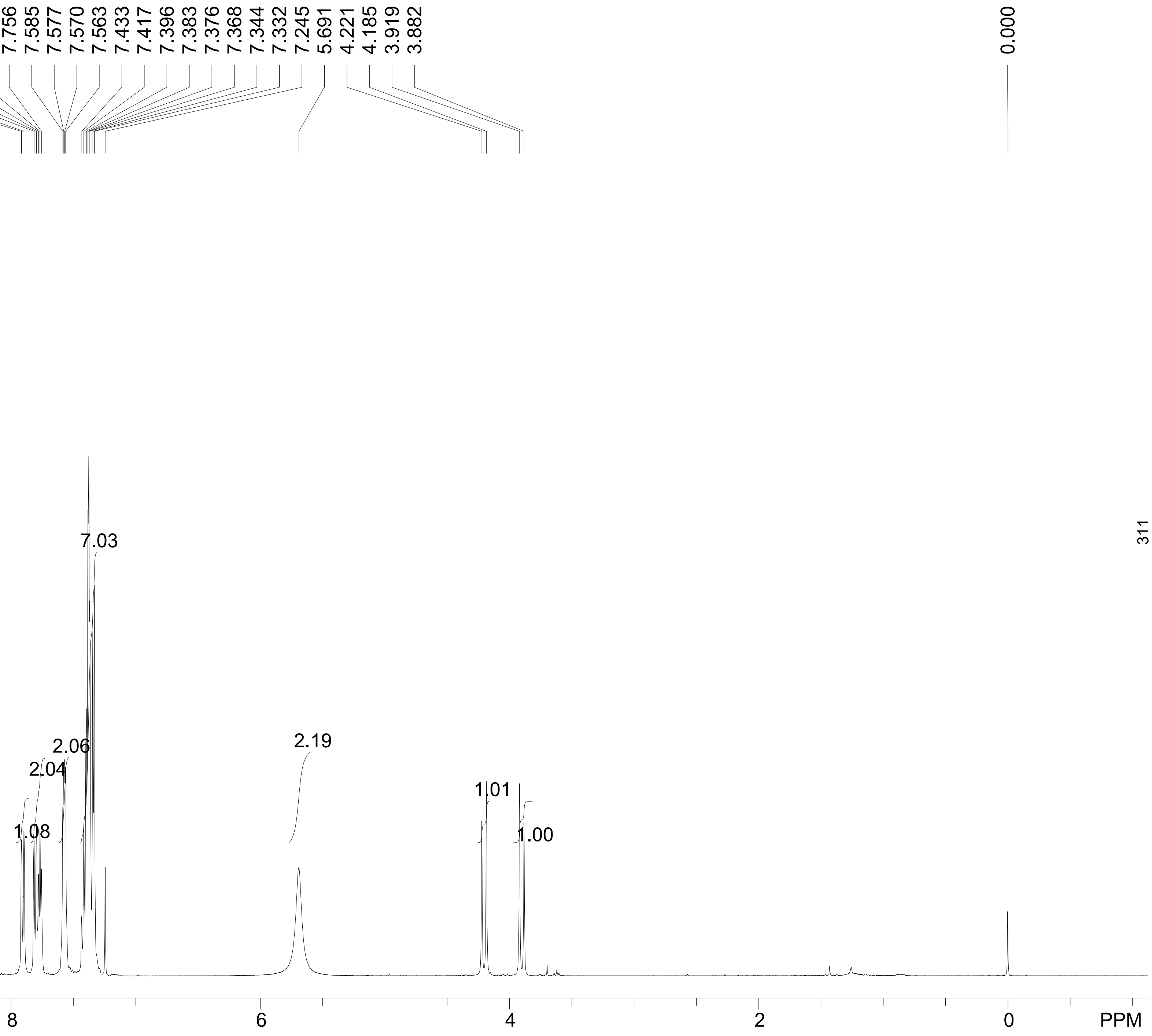

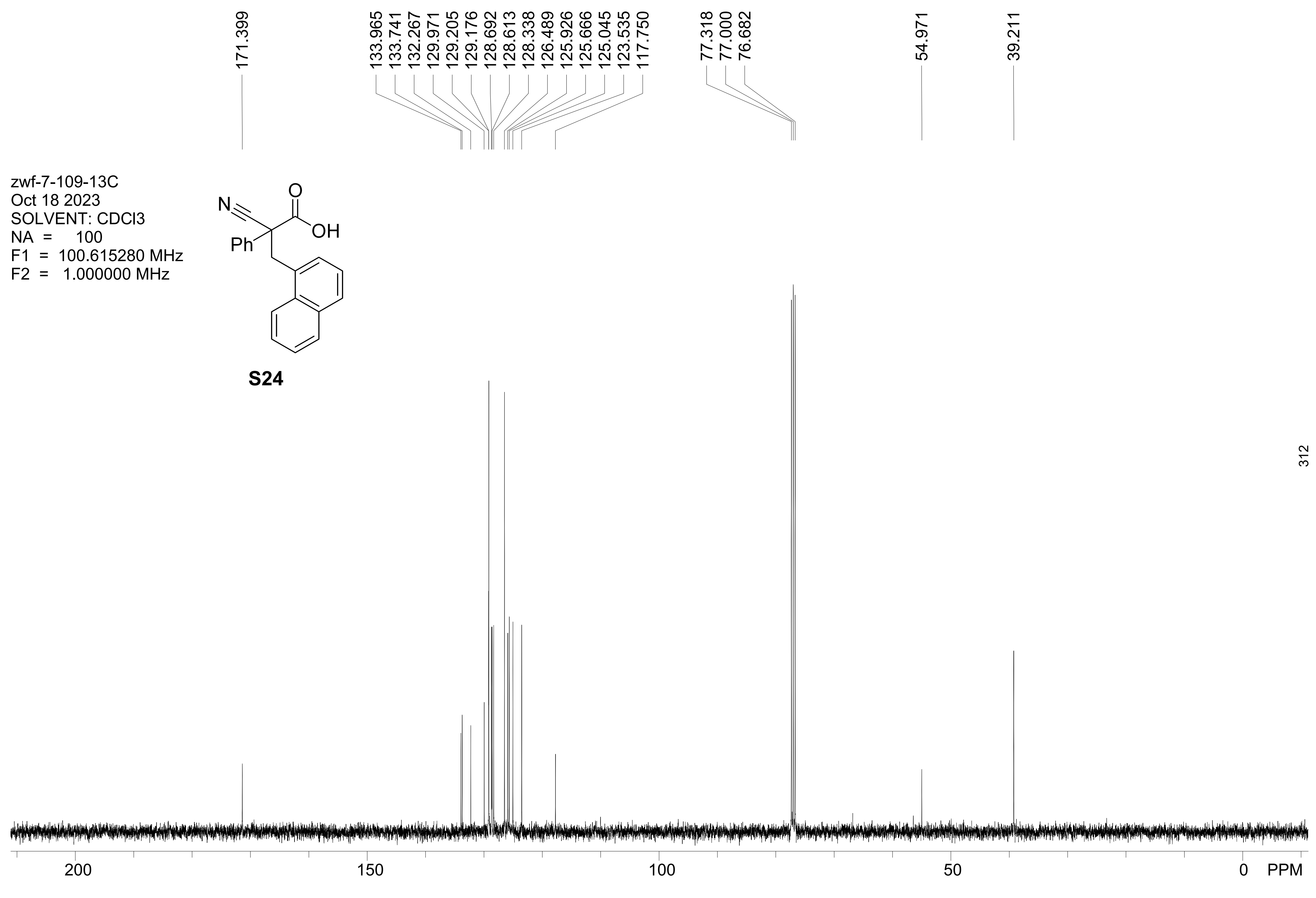
zwf-7-109-13C
Oct 18 2023
SOLVENT: CDCl3
NA = 100
F1 = 100.615280 MHz
F2 = 1.000000 MHz
N
O
OH
Ph
S24
171.399
133.965
133.741
132.267
129.971
129.205
129.176
128.692
128.613
128.338
126.489
125.926
125.666
125.045
123.535
117.750
77.318
77.000
76.682
54.971
39.211
200
150
100
50
0
PPM

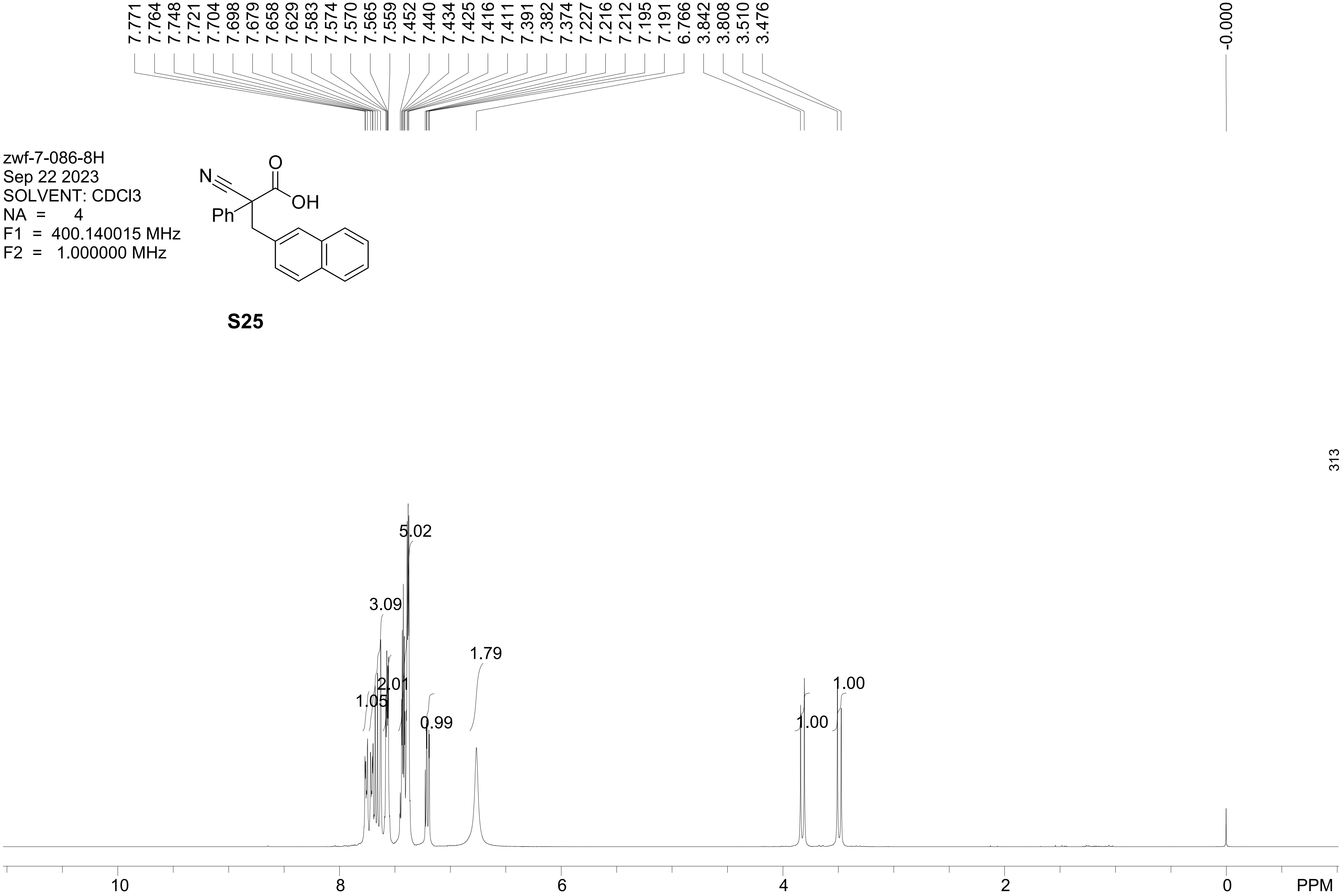
zwf-7-086-8H
Sep 22 2023
SOLVENT: CDCl3
NA = 4
F1 = 400.140015 MHz
F2 = 1.000000 MHz
N
O
OH
Ph
S25
7.771
7.764
7.748
7.721
7.704
7.698
7.679
7.658
7.629
7.583
7.574
7.570
7.565
7.559
7.452
7.440
7.434
7.425
7.416
7.411
7.391
7.382
7.374
7.227
7.216
7.212
7.195
7.191
6.766
3.842
3.808
3.510
3.476
-0.000
5.02
3.09
1.79
2.01
1.05
0.99
1.00
1.00
10
8
6
4
2
0
PPM

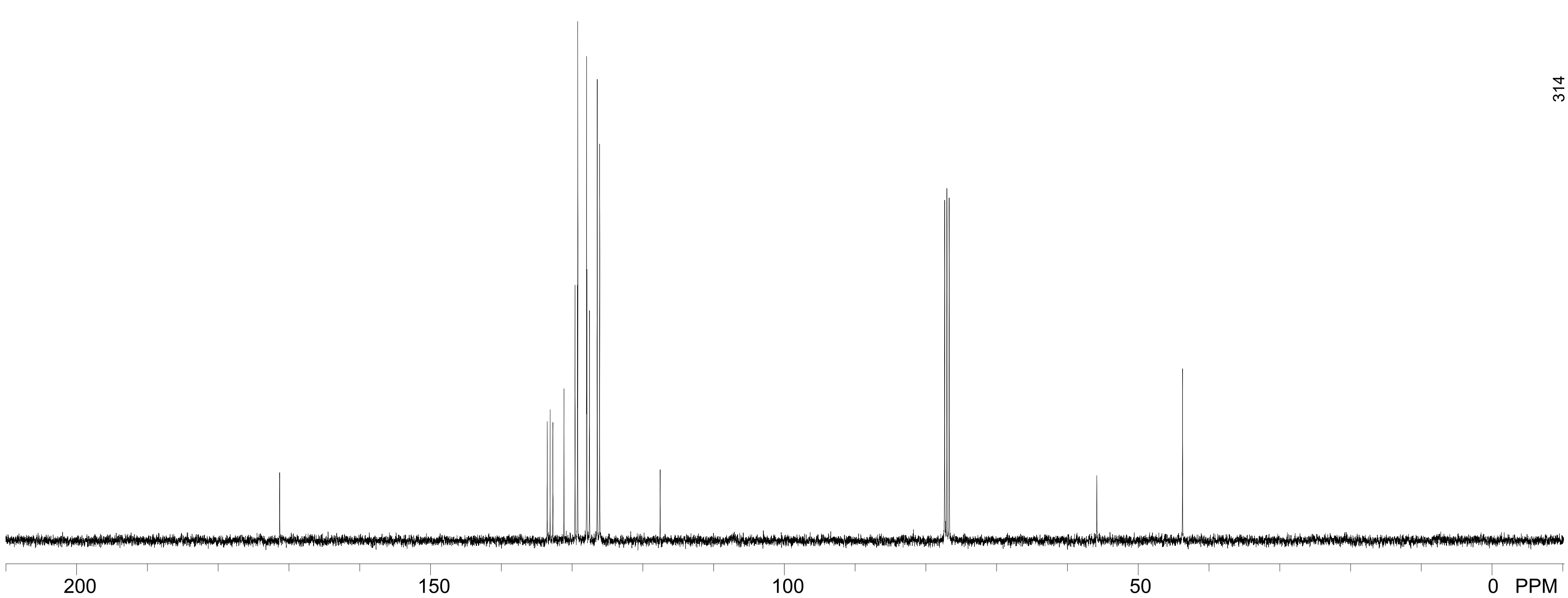
171.313
133.510
133.098
132.708
131.141
129.573
129.234
129.198
127.941
127.898
127.529
126.439
126.099
117.540
77.318
77.000
76.682
55.809
43.682
zwf-7-086-8C
Sep 22 2023
SOLVENT: CDCl3
NA = 100
F1 = 100.615280 MHz
F2 = 1.000000 MHz
200
150
100
50
0 PPM

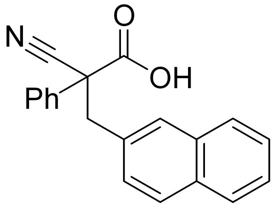
N
O
OH
Ph

S25

zwf-7-133-1H
Nov 03 2023
SOLVENT: CDCl3
NA = 4
F1 = 400.179993 MHz
F2 = 1.000000 MHz

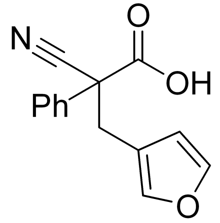

S26

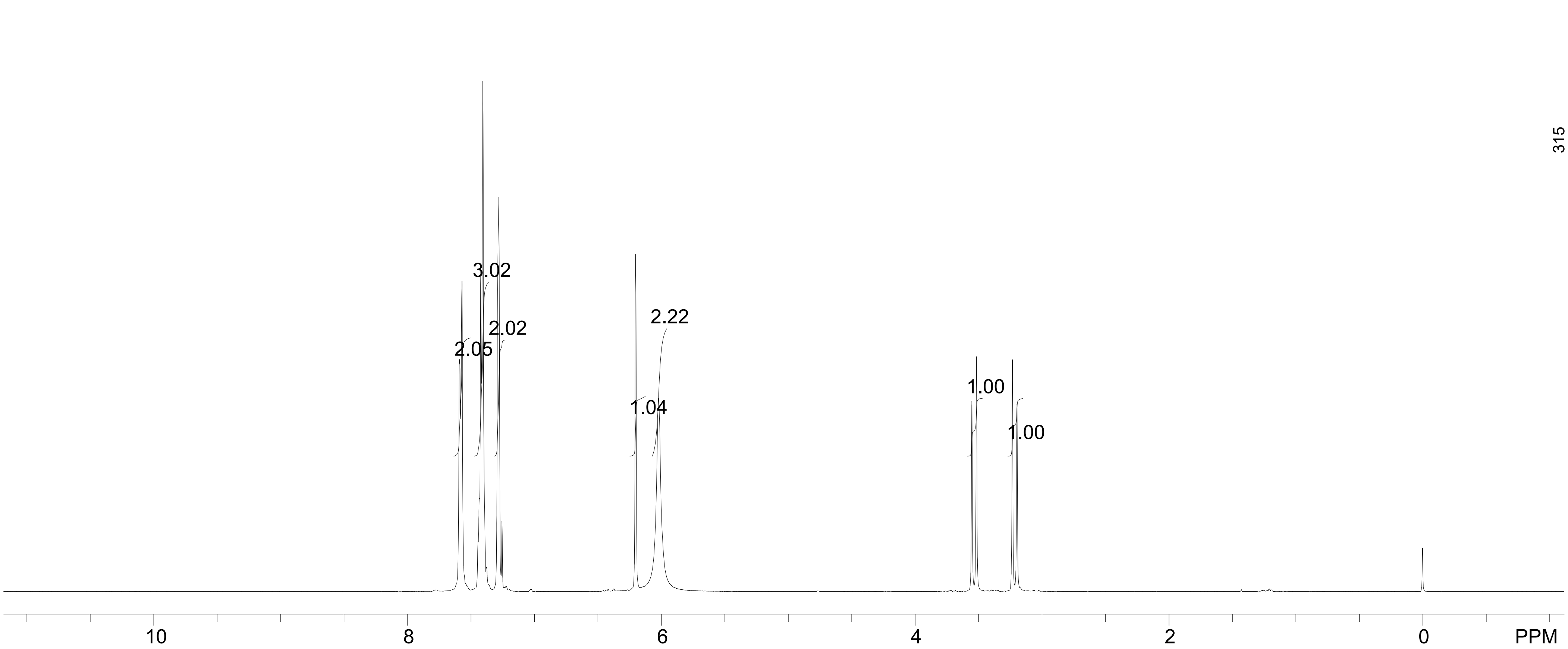

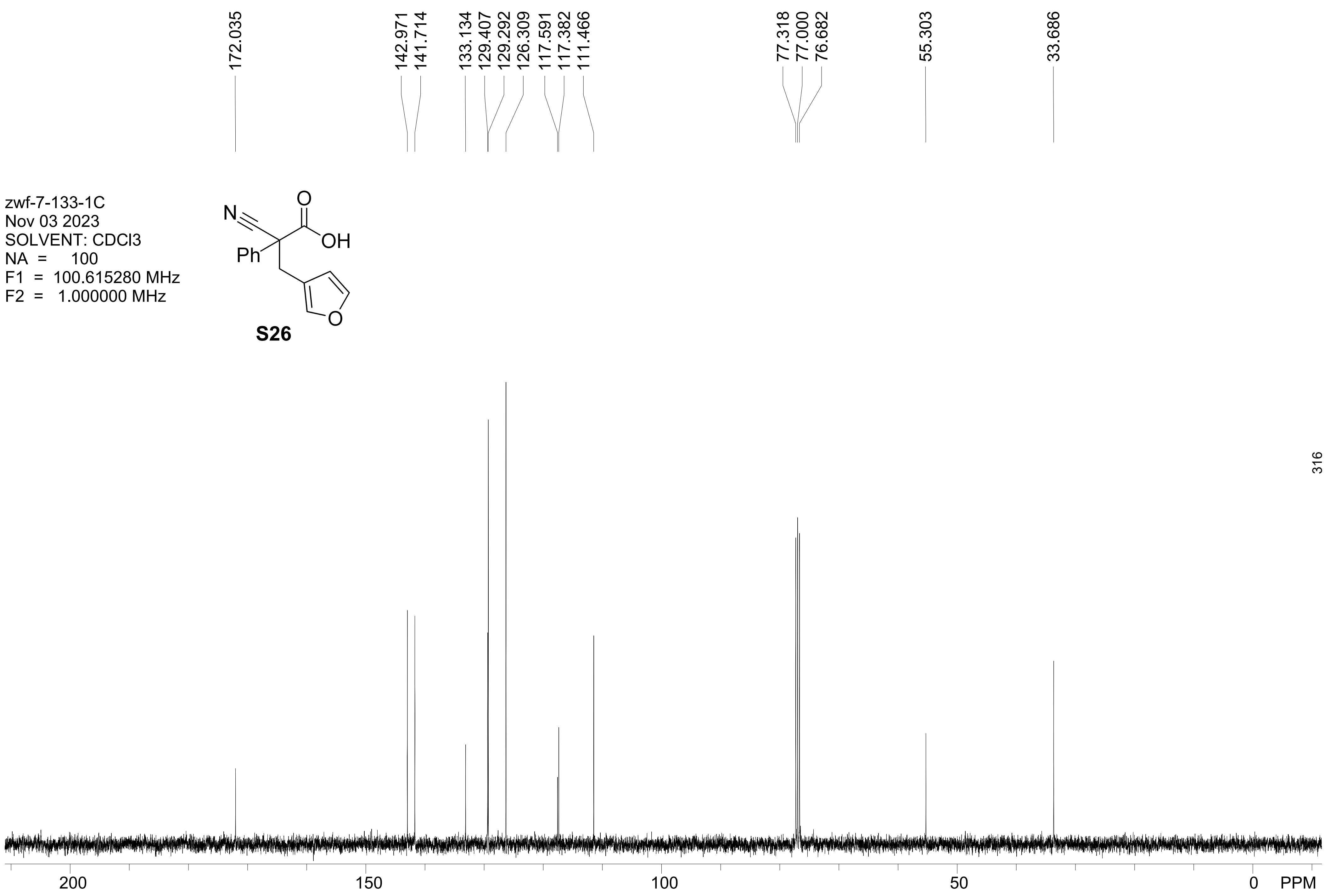
zwf-7-133-1C
Nov 03 2023
SOLVENT: CDCl3
NA = 100
F1 = 100.615280 MHz
F2 = 1.000000 MHz
N
O
OH
Ph
O
S26
172.035
142.971
141.714
133.134
129.407
129.292
126.309
117.591
117.382
111.466
77.318
77.000
76.682
55.303
33.686
200
150
100
50
0
PPM

zwf-7-086-6H
Sep 23 2023
SOLVENT: CDCl3
NA = 4
F1 = 400.140015 MHz
F2 = 1.000000 MHz

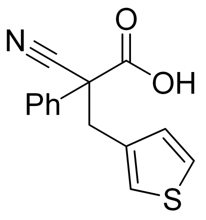

S27

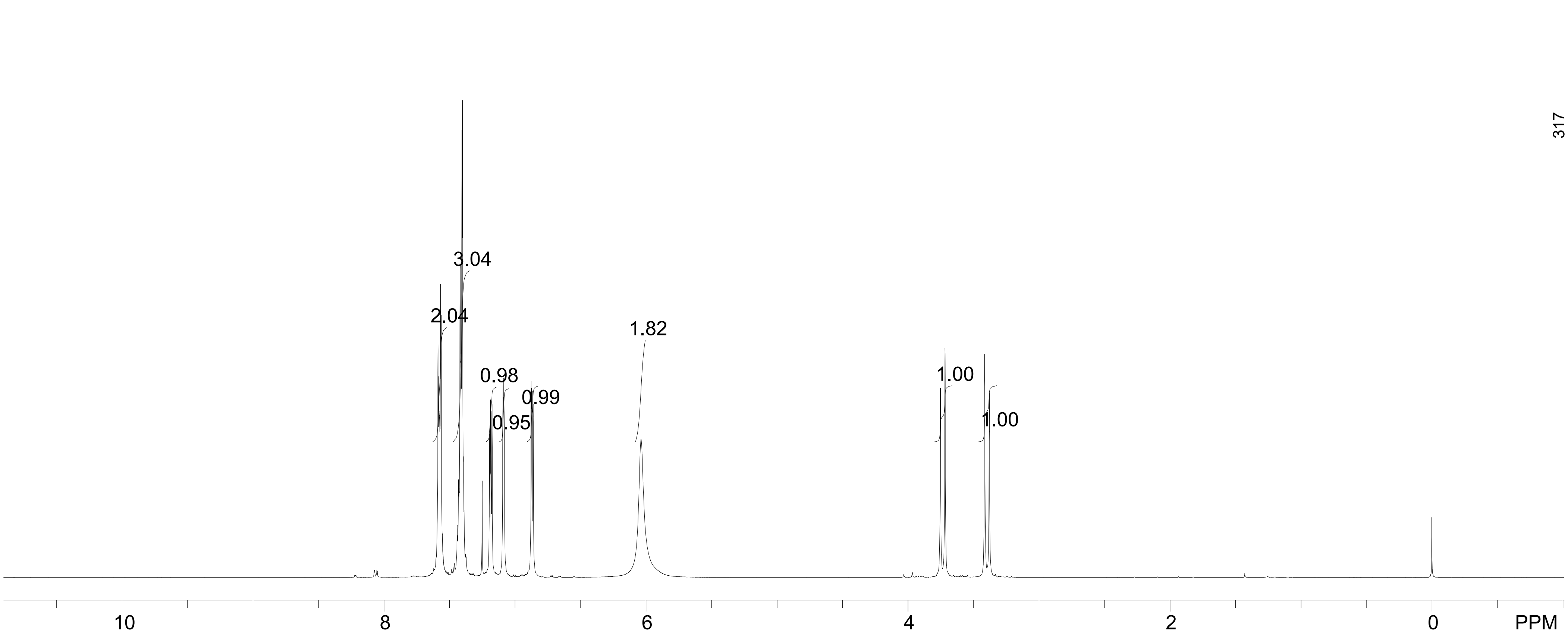

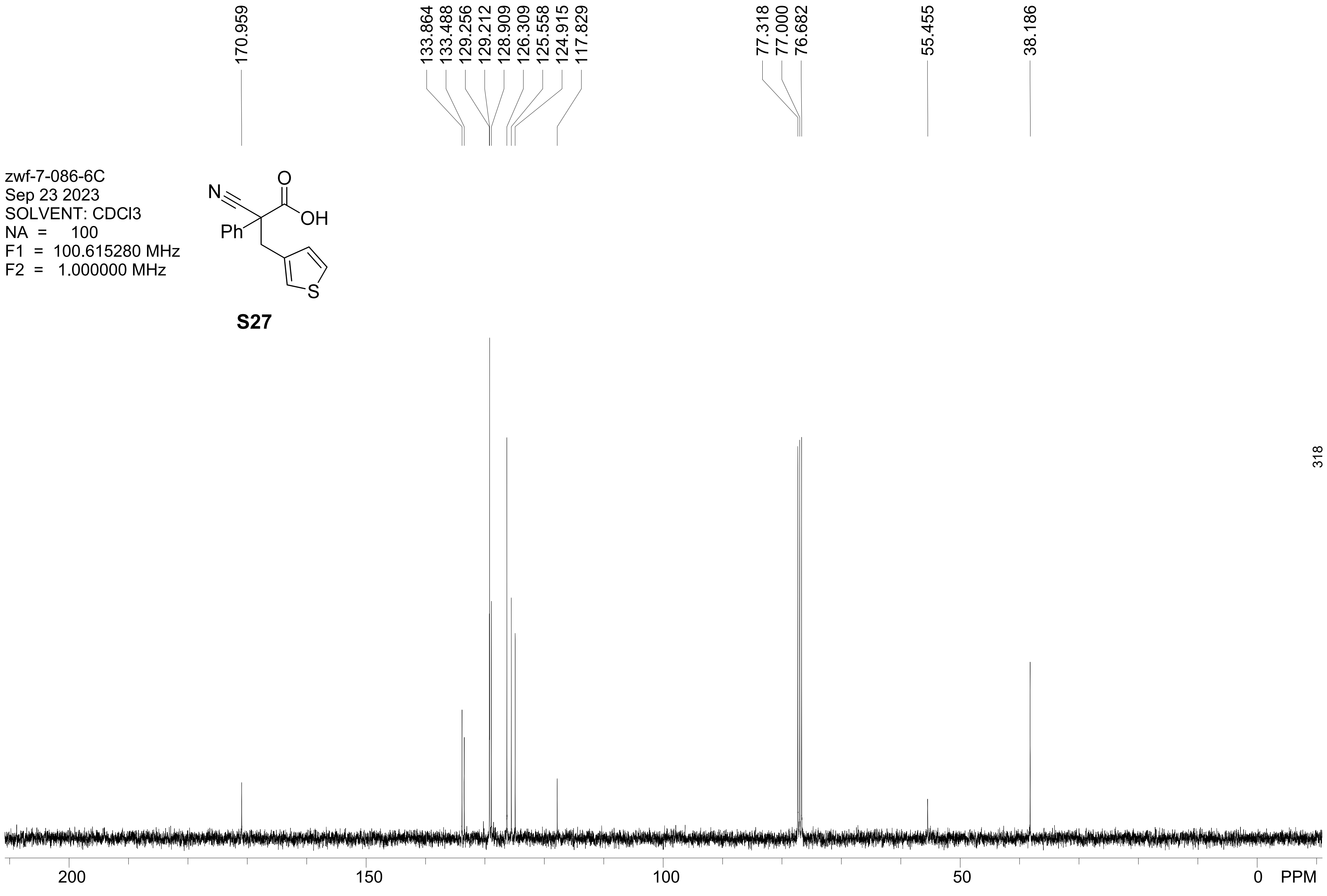
zwf-7-086-6C
Sep 23 2023
SOLVENT: CDCl3
NA = 100
F1 = 100.615280 MHz
F2 = 1.000000 MHz
N
O
OH
Ph
S
170.959
133.864
133.488
129.256
129.212
128.909
126.309
125.558
124.915
117.829
77.318
77.000
76.682
55.455
38.186
200
150
100
50
0 PPM

S27

zwf-7-055-13H
Sep 12 2023
SOLVENT: CDCl3
NA = 4
F1 = 400.140015 MHz
F2 = 1.000000 MHz

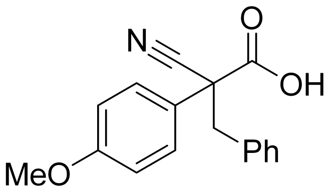

S28

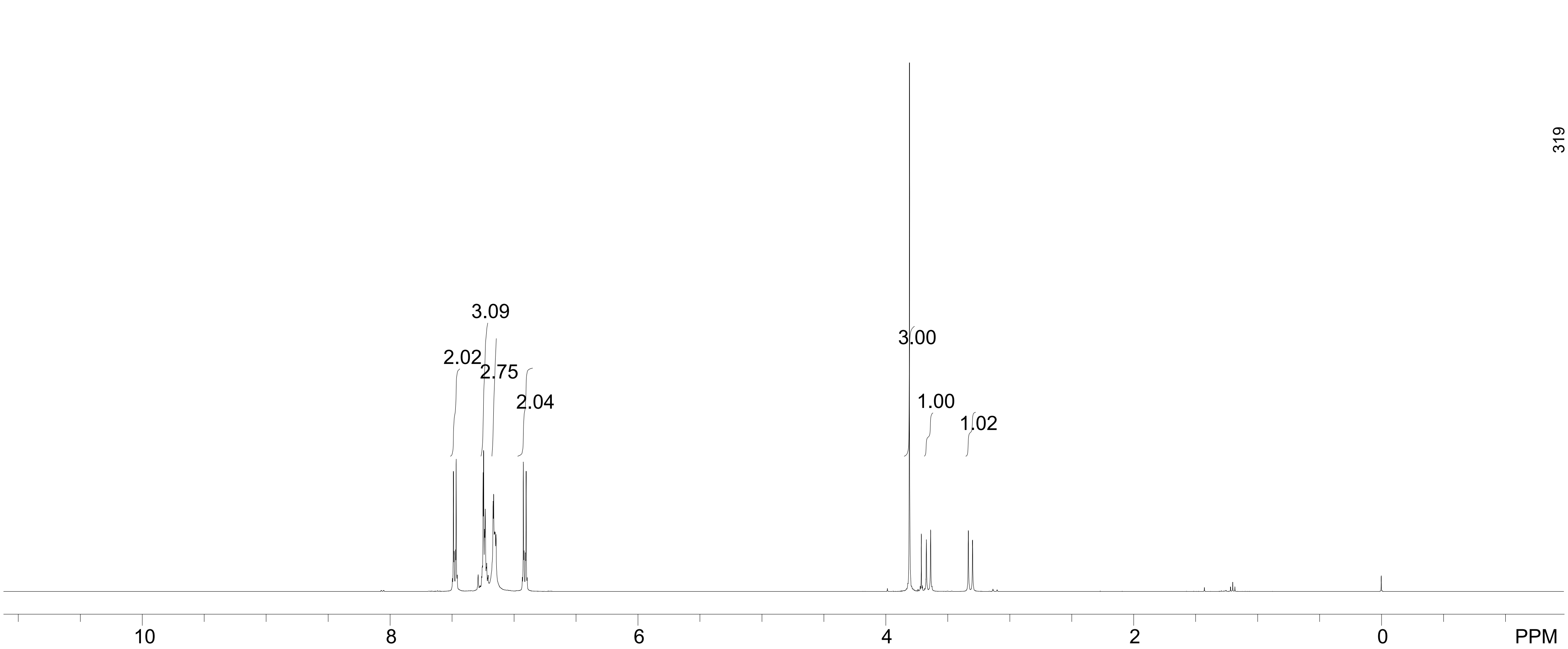

zwf-7-055-13C
Sep 12 2023
SOLVENT: CDCl3
NA = 100
F1 = 100.615280 MHz
F2 = 1.000000 MHz

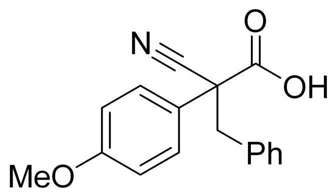

S28

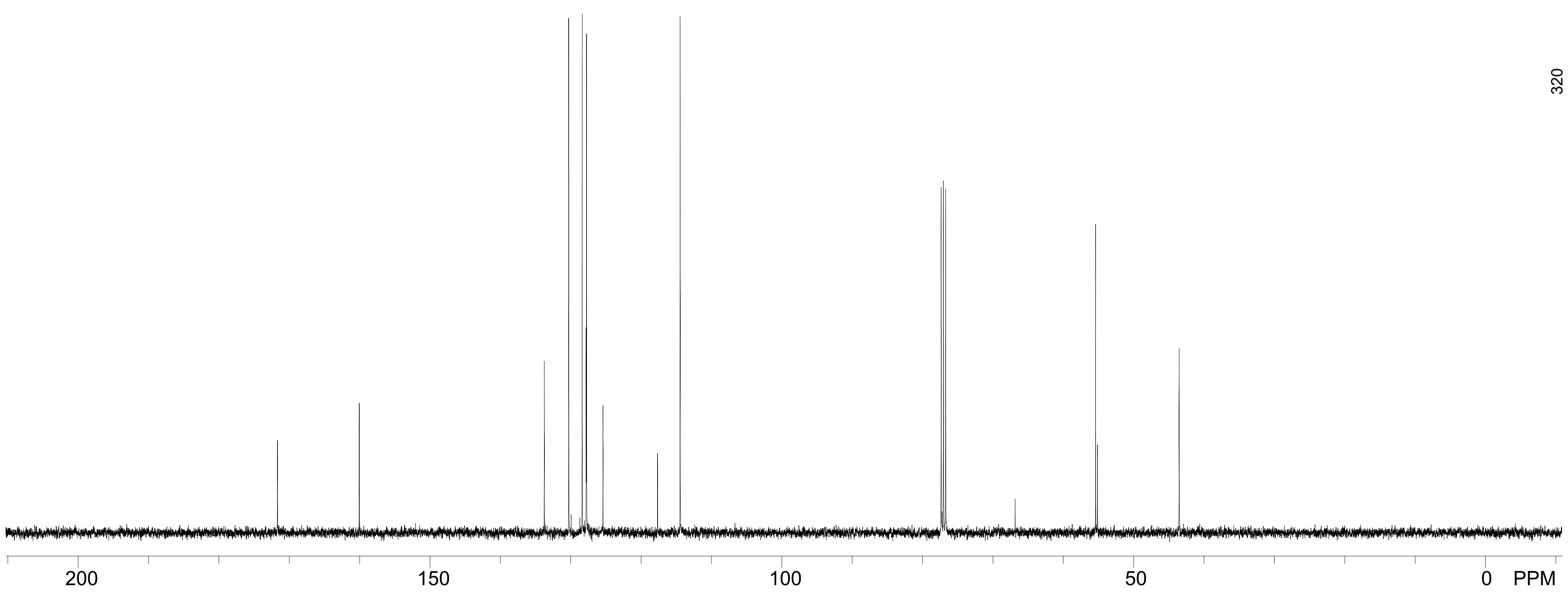

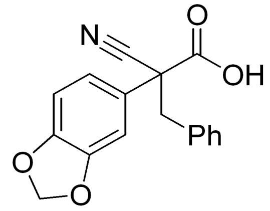

S29

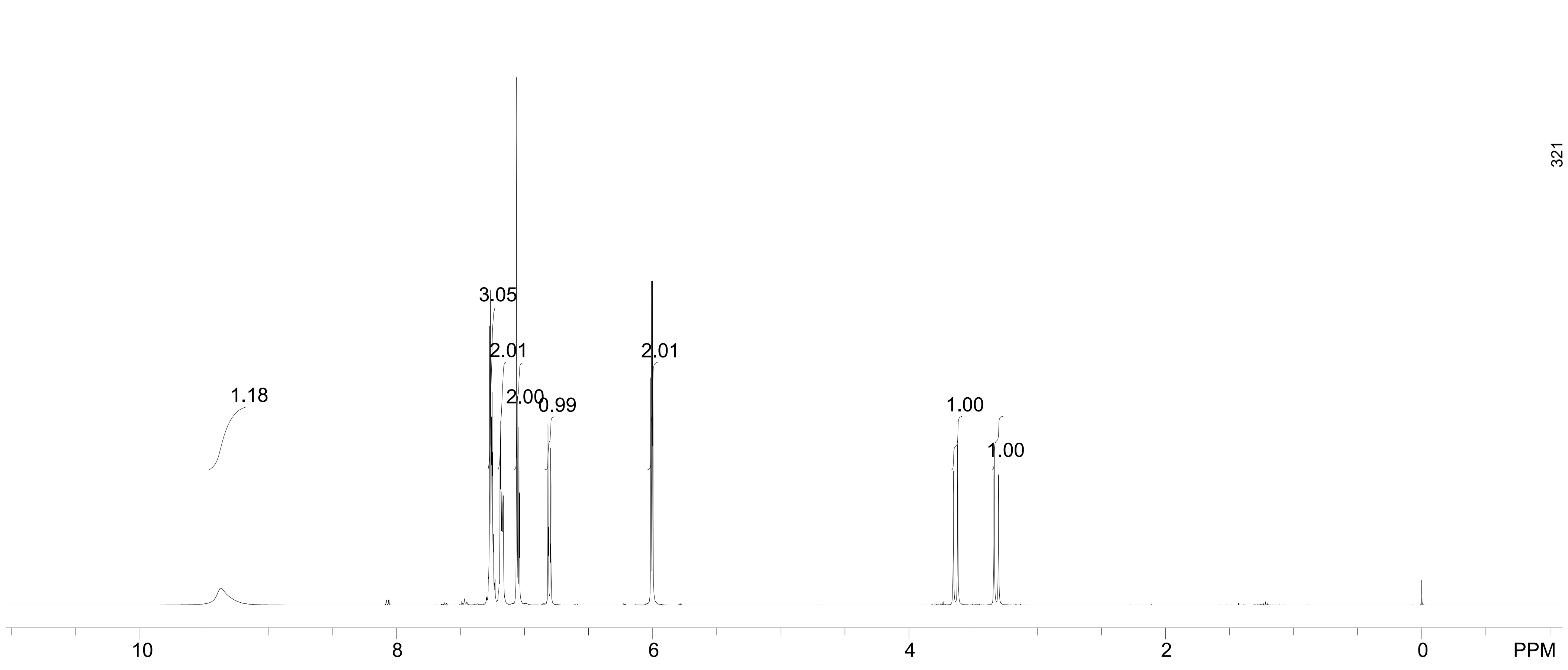
zwf-7-109-5H
Oct 14 2023
SOLVENT: CDCl3
NA = 4
F1 = 400.140015 MHz
F2 = 1.000000 MHz
9.369
7.271
7.266
7.259
7.253
7.249
7.243
7.190
7.186
7.177
7.173
7.166
7.061
7.057
7.044
7.039
6.817
6.812
6.799
6.795
6.014
6.011
6.001
5.998
3.652
3.618
3.335
3.300
-0.000
1.18
3.05
2.01
2.00
0.99
2.01
1.00
1.00
10
8
6
4
2
0
PPM

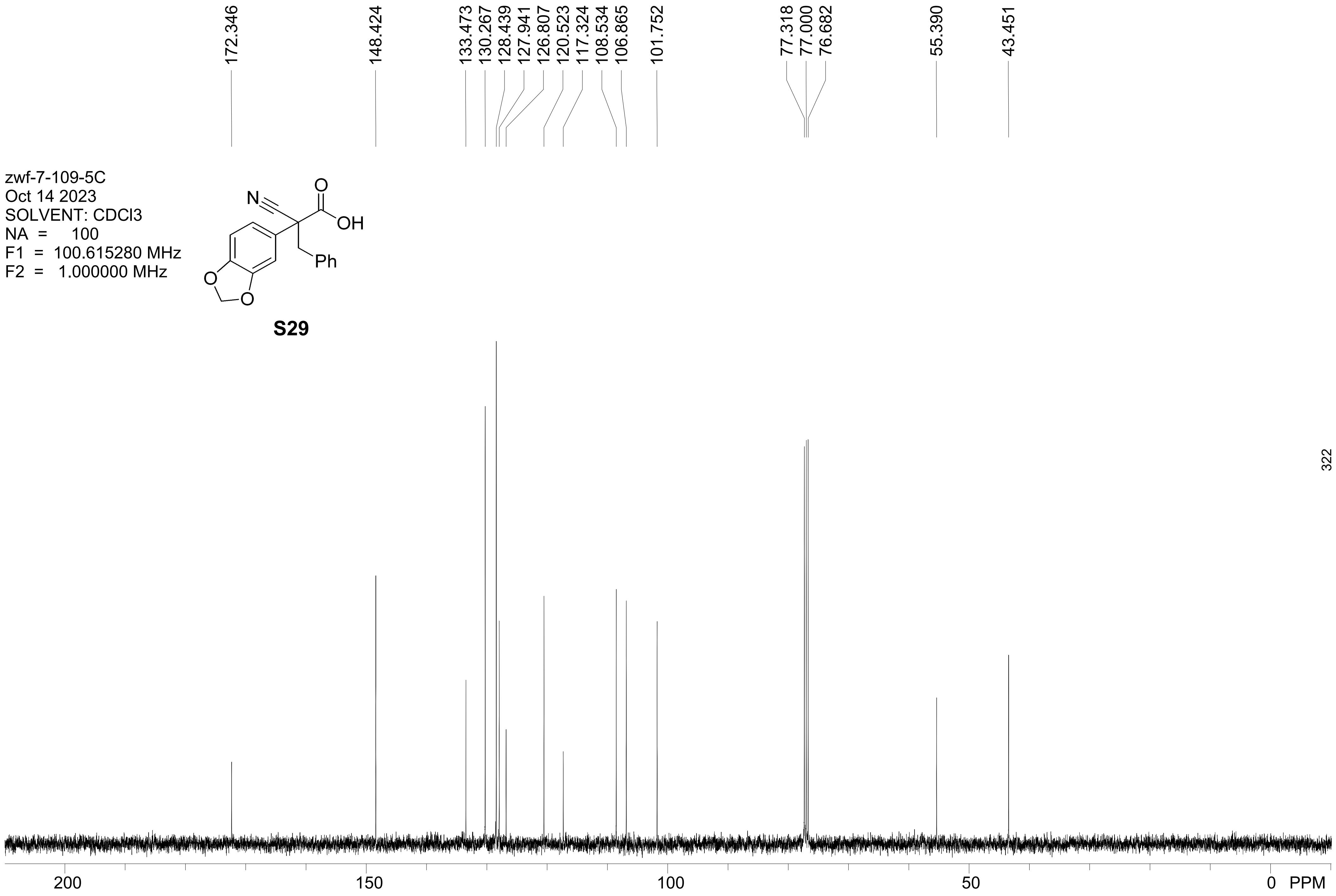
zwf-7-109-5C
Oct 14 2023
SOLVENT: CDCl3
NA = 100
F1 = 100.615280 MHz
F2 = 1.000000 MHz
N
O
OH
Ph
O
O
S29
172.346
148.424
133.473
130.267
128.439
127.941
126.807
120.523
117.324
108.534
106.865
101.752
77.318
77.000
76.682
55.390
43.451
200
150
100
50
0 PPM

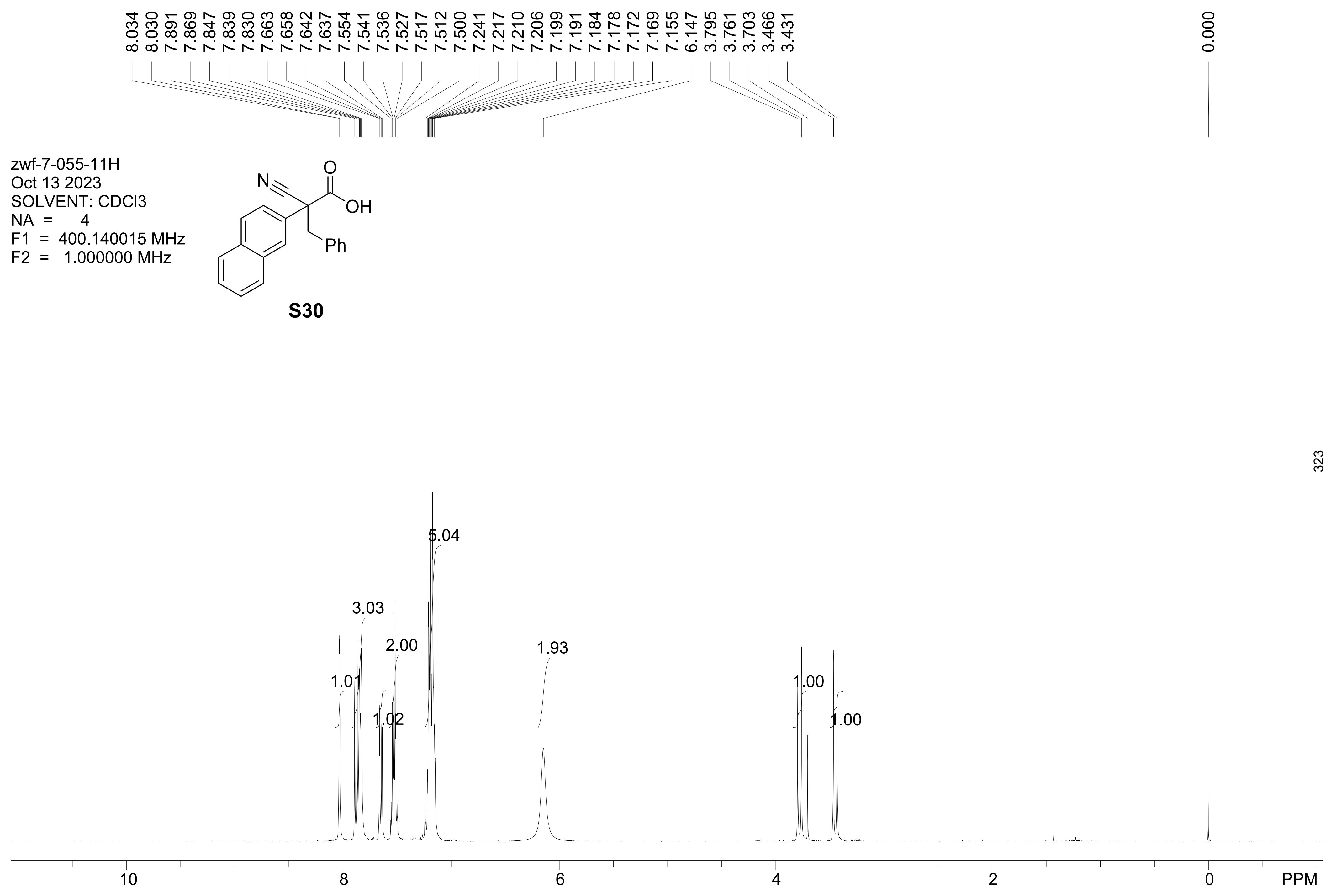
8.034
8.030
7.891
7.869
7.847
7.839
7.830
7.663
7.658
7.642
7.637
7.554
7.541
7.536
7.527
7.517
7.512
7.500
7.241
7.217
7.210
7.206
7.199
7.191
7.184
7.178
7.172
7.169
7.155
6.147
3.795
3.761
3.703
3.466
3.431
0.000
zwf-7-055-11H
Oct 13 2023
SOLVENT: CDCl3
NA = 4
F1 = 400.140015 MHz
F2 = 1.000000 MHz
N
O
OH
Ph
S30
5.04
3.03
2.00
1.93
1.01
1.02
1.00
1.00
10
8
6
4
2
0
PPM

zwf-7-055-11C
Oct 13 2023
SOLVENT: CDCl3
NA = 100
F1 = 100.615280 MHz
F2 = 1.000000 MHz

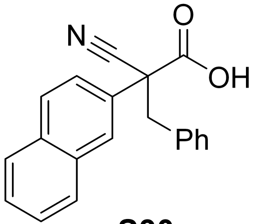

S30

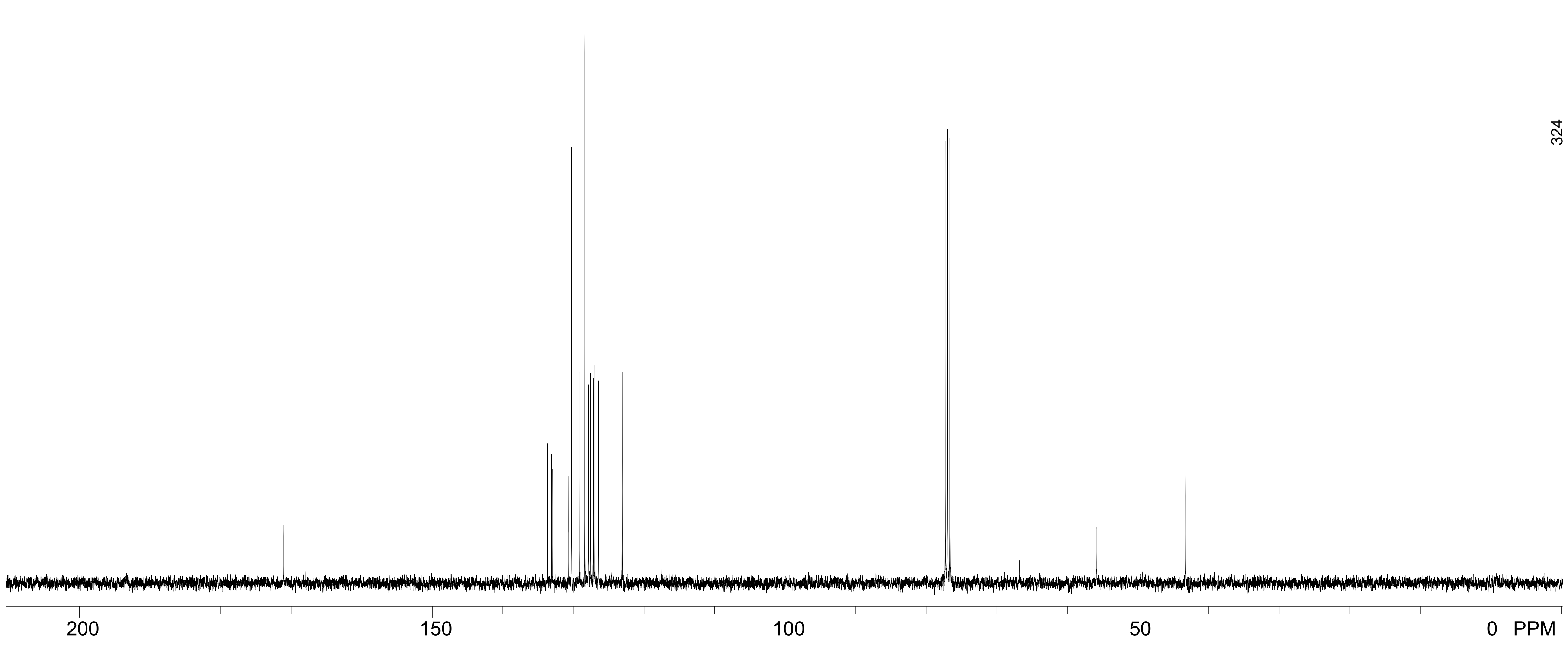

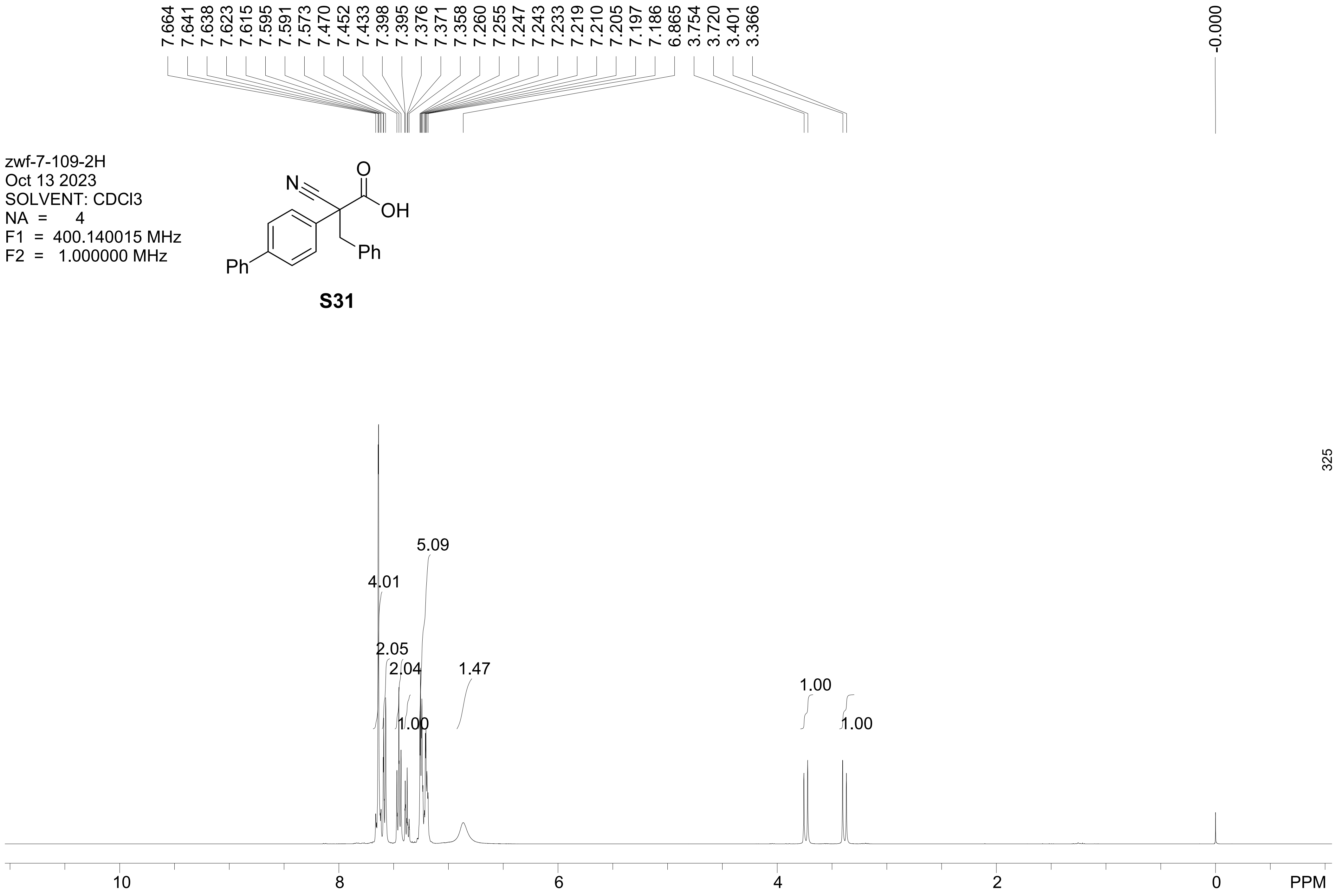
7.664
7.641
7.638
7.623
7.615
7.595
7.591
7.573
7.470
7.452
7.433
7.398
7.395
7.376
7.371
7.358
7.260
7.255
7.247
7.243
7.233
7.219
7.210
7.205
7.197
7.186
6.865
3.754
3.720
3.401
3.366
-0.000
zwf-7-109-2H
Oct 13 2023
SOLVENT: CDCl3
NA = 4
F1 = 400.140015 MHz
F2 = 1.000000 MHz
N
O
OH
Ph
Ph
S31
4.01
5.09
2.05
2.04
1.47
1.00
1.00
1.00
10
8
6
4
2
0
PPM

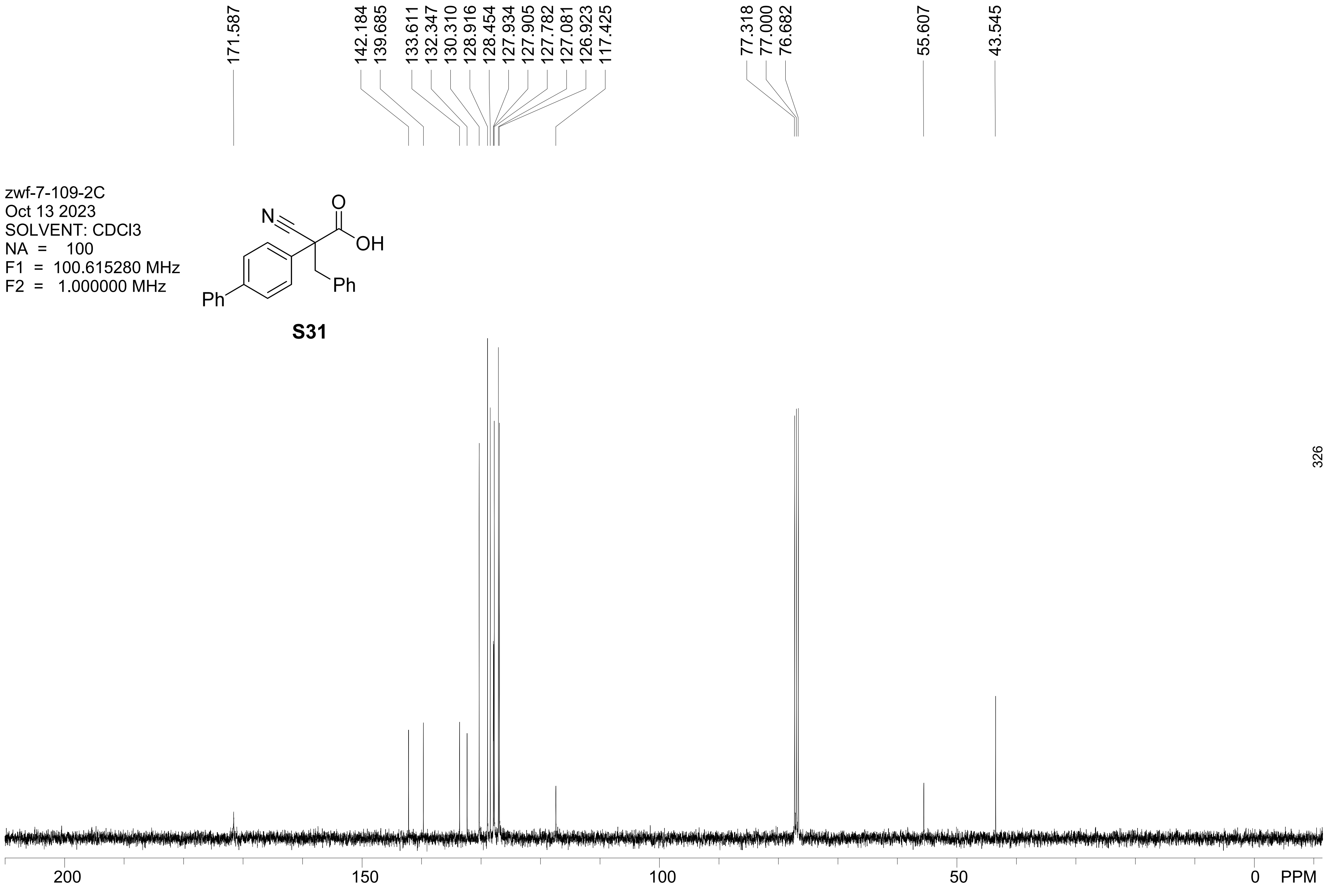
zwf-7-109-2C
Oct 13 2023
SOLVENT: CDCl3
NA = 100
F1 = 100.615280 MHz
F2 = 1.000000 MHz
N
O
OH
Ph
Ph
S31
171.587
142.184
139.685
133.611
132.347
130.310
128.916
128.454
127.934
127.905
127.782
127.081
126.923
117.425
77.318
77.000
76.682
55.607
43.545
200
150
100
50
0
PPM

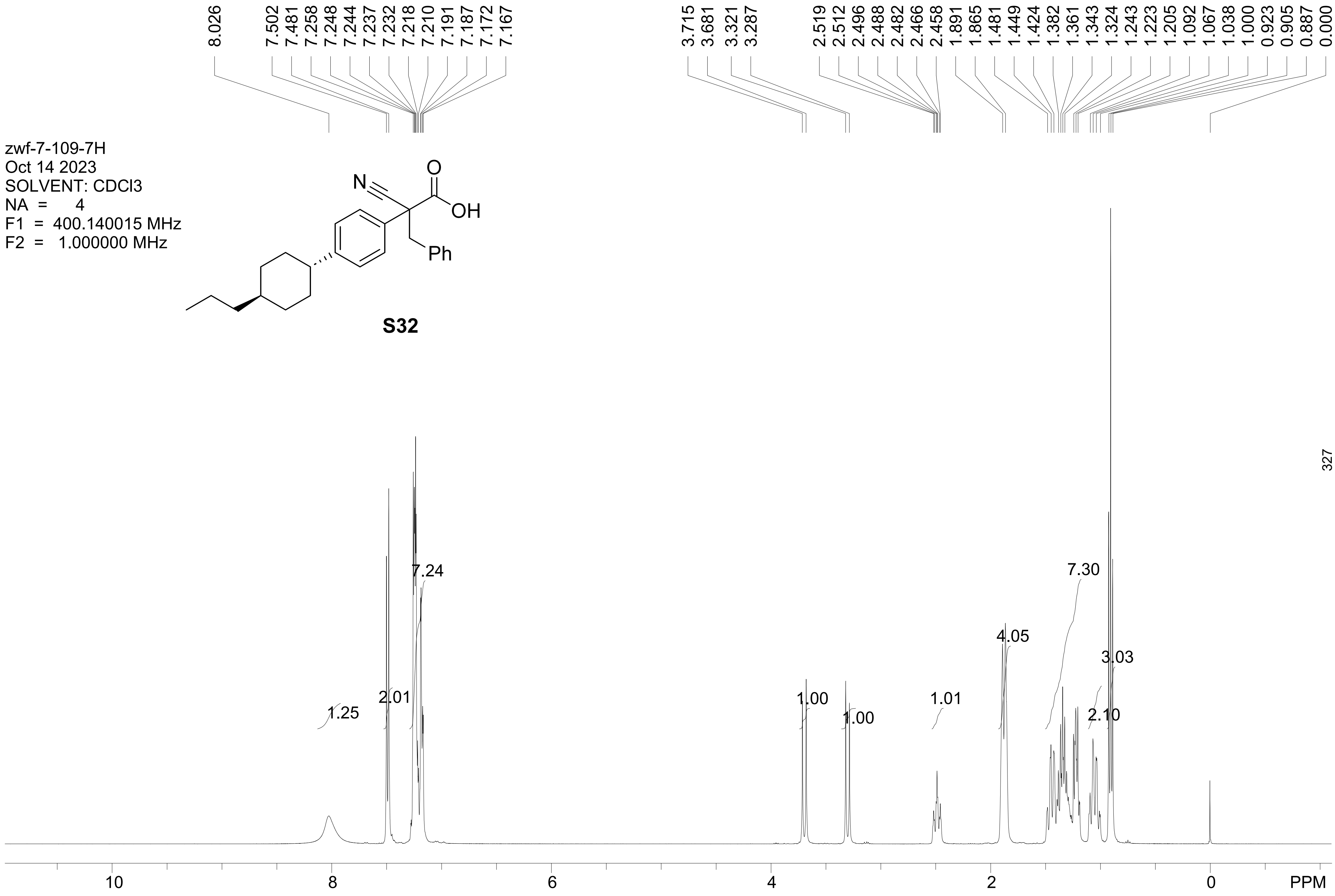
zwf-7-109-7H
Oct 14 2023
SOLVENT: CDCl3
NA = 4
F1 = 400.140015 MHz
F2 = 1.000000 MHz
S32
8.026
7.502
7.481
7.258
7.248
7.244
7.237
7.232
7.218
7.210
7.191
7.187
7.172
7.167
3.715
3.681
3.321
3.287
2.519
2.512
2.496
2.488
2.482
2.466
2.458
1.891
1.865
1.481
1.449
1.424
1.382
1.361
1.343
1.324
1.243
1.223
1.205
1.092
1.067
1.038
1.000
0.923
0.905
0.887
0.000
1.25
2.01
7.24
1.00
1.00
1.01
4.05
7.30
2.10
3.03
10
8
6
4
2
0
PPM

zwf-7-109-7C
Oct 14 2023
SOLVENT: CDCl3
NA = 100
F1 = 100.615280 MHz
F2 = 1.000000 MHz

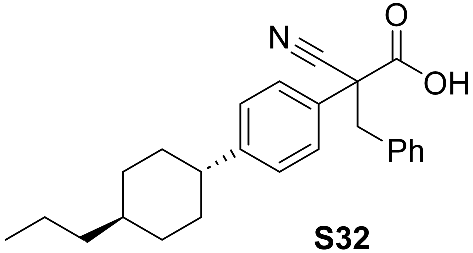

S32

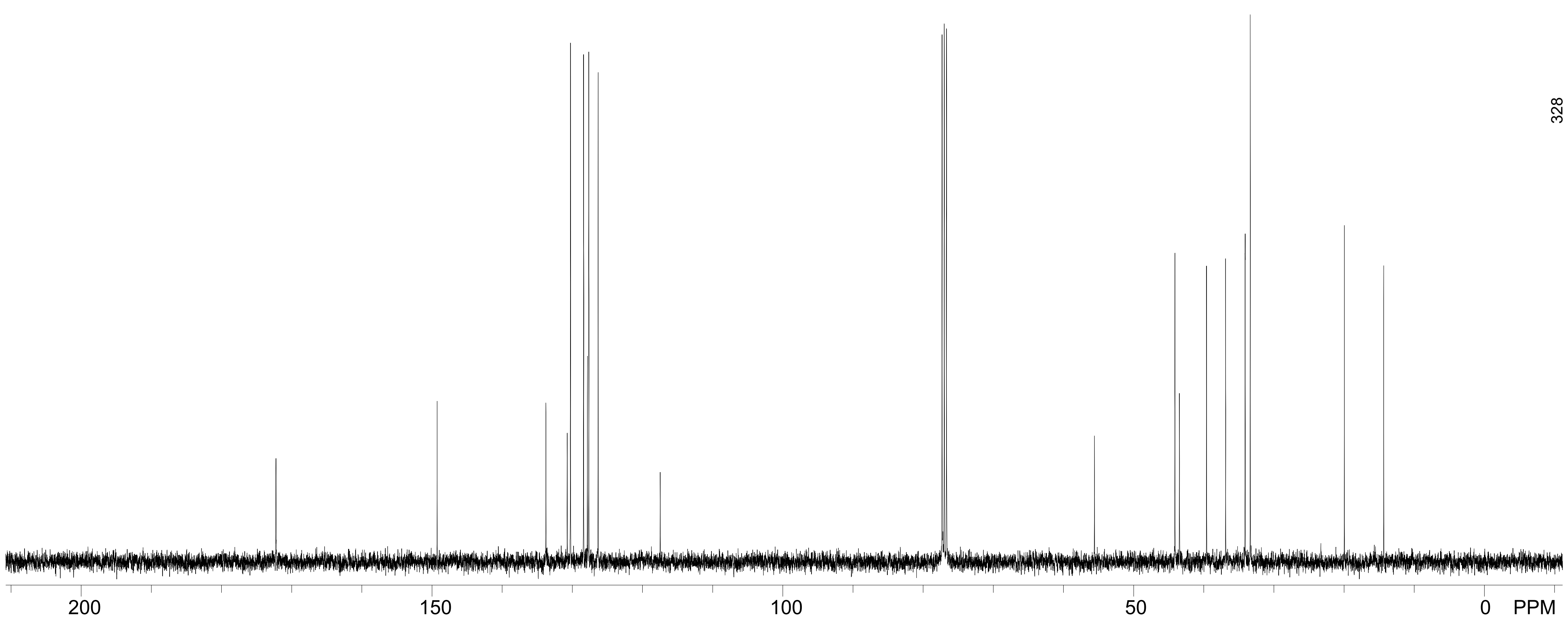

zwf-7-109-4H
Oct 14 2023
SOLVENT: CDCl3
NA = 4
F1 = 400.140015 MHz
F2 = 1.000000 MHz

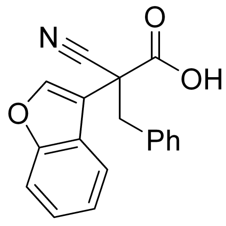

S33

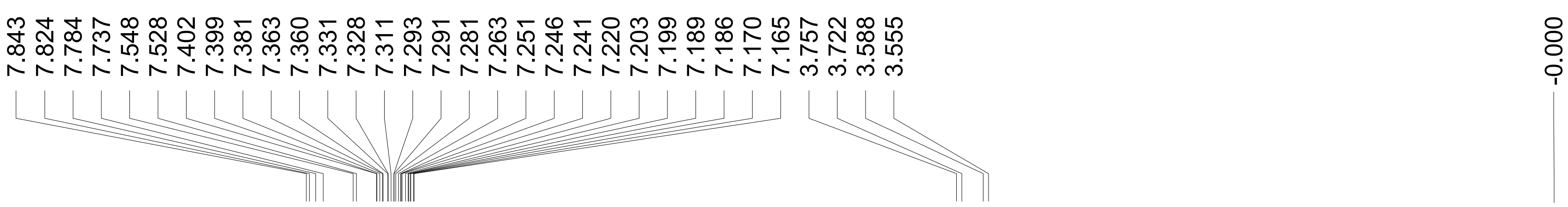

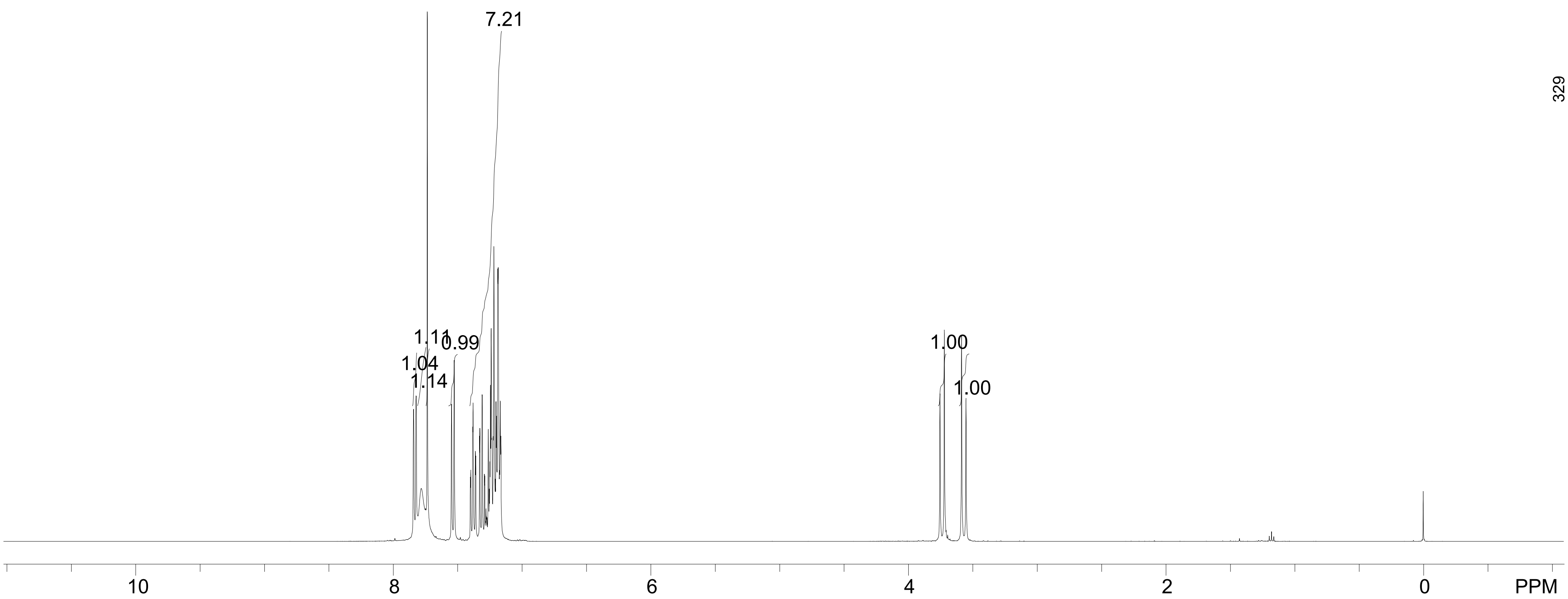

zwf-7-109-4C
Oct 16 2023
SOLVENT: CDCl3
NA = 100
F1 = 100.615280 MHz
F2 = 1.000000 MHz

S33

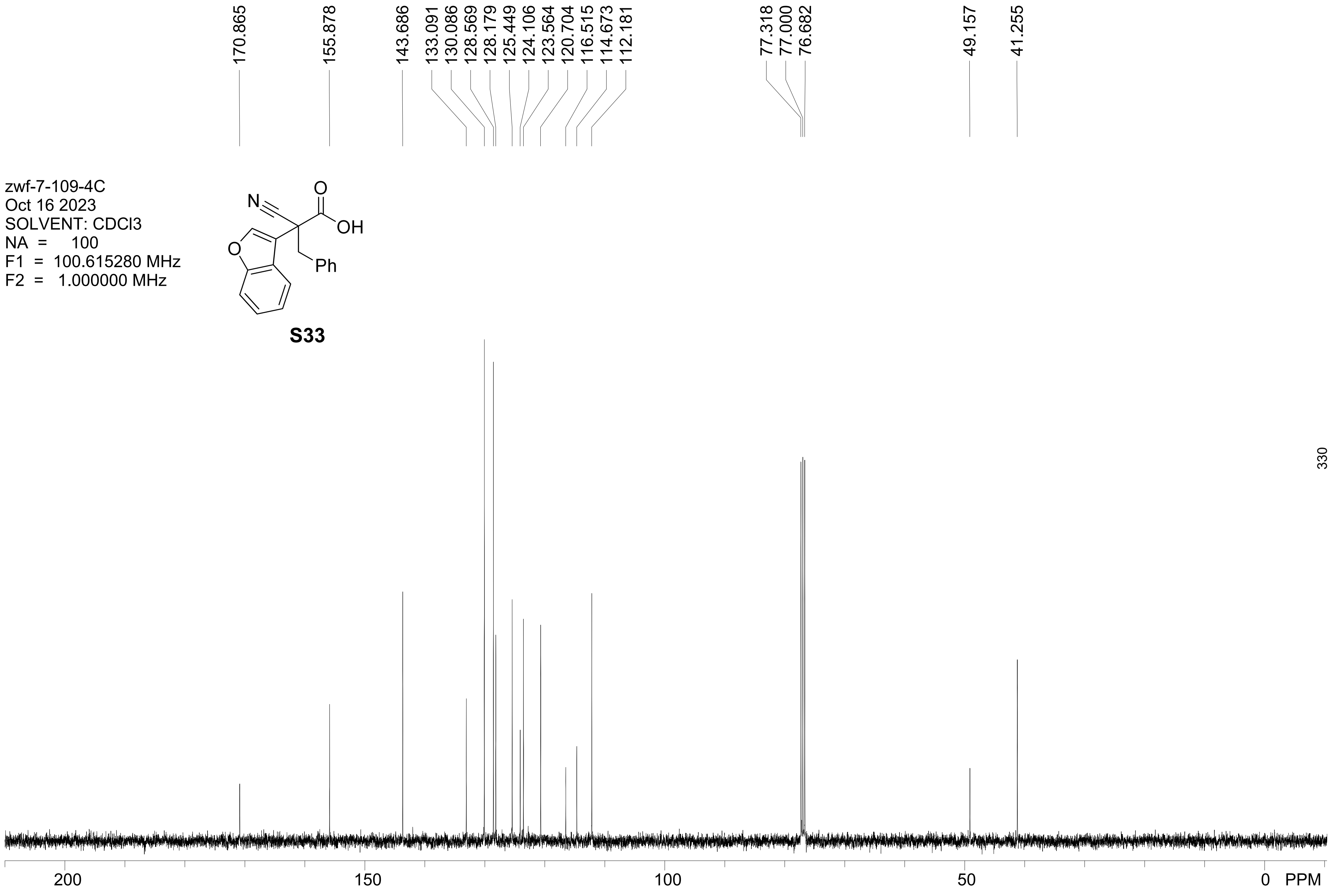

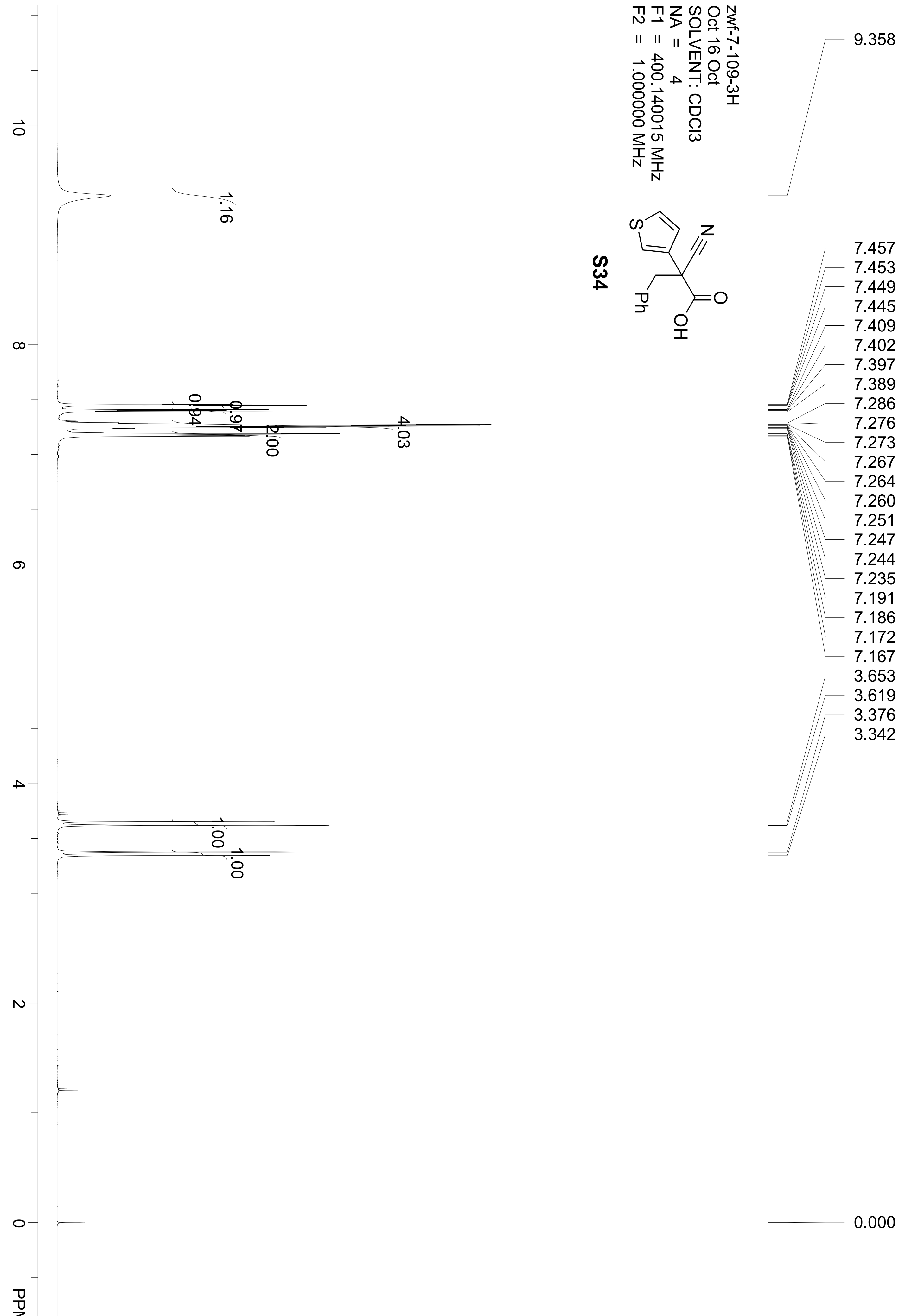
zwf-7-109-3H
Oct 16 Oct
SOLVENT: CDCl3
NA = 4
F1 = 400.140015 MHz
F2 = 1.000000 MHz
S34
N
O
OH
S
Ph
9.358
7.457
7.453
7.449
7.445
7.409
7.402
7.397
7.389
7.286
7.276
7.273
7.267
7.264
7.260
7.251
7.247
7.244
7.235
7.191
7.186
7.172
7.167
3.653
3.619
3.376
3.342
0.000
1.16
0.94
0.97
2.00
4.03
1.00
1.00
10
8
6
4
2
0
PPM

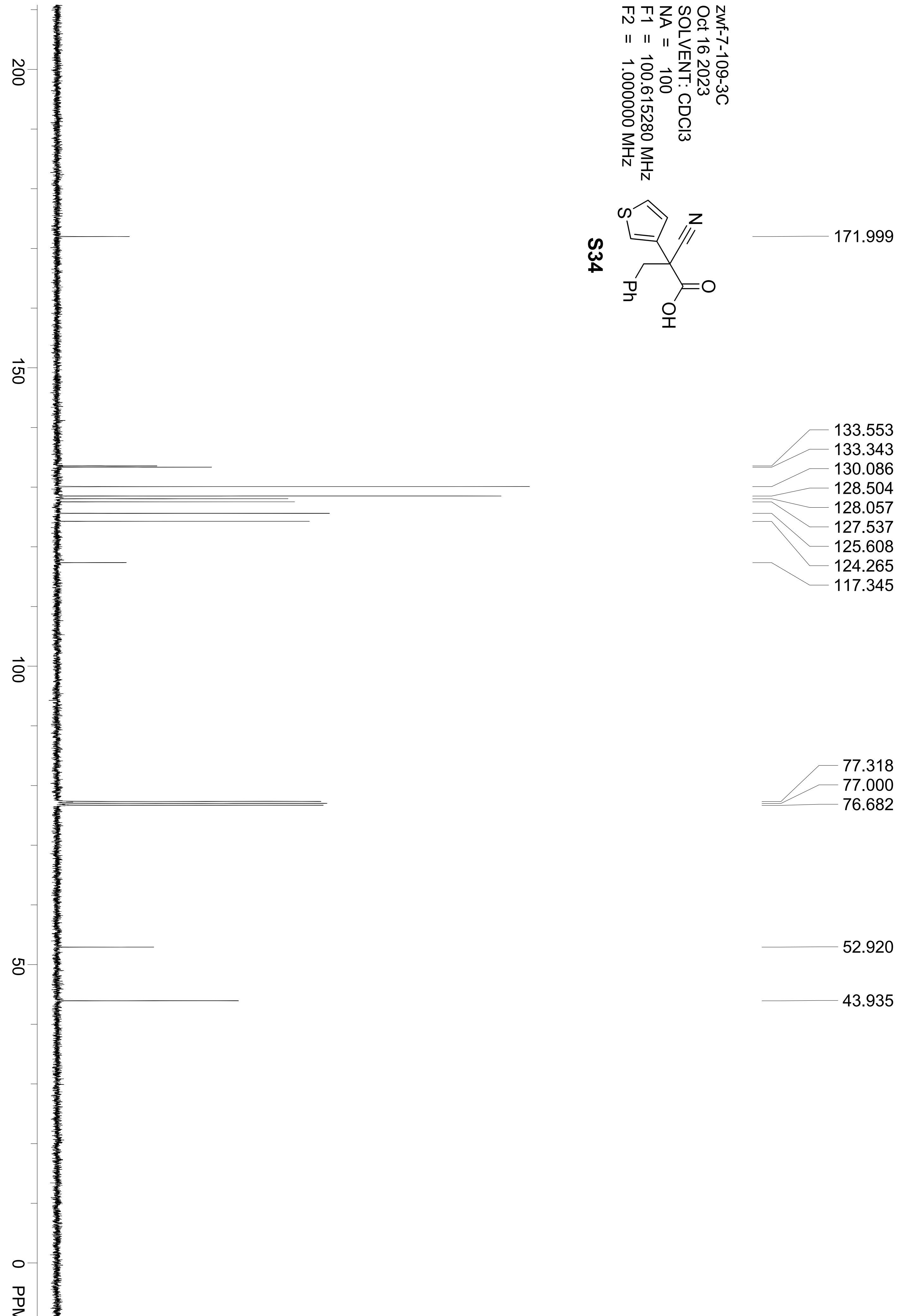
zwf-7-109-3C
Oct 16 2023
SOLVENT: CDCl3
NA = 100
F1 = 100.615280 MHz
F2 = 1.000000 MHz
N
O
OH
S
Ph
S34
171.999
133.553
133.343
130.086
128.504
128.057
127.537
125.608
124.265
117.345
77.318
77.000
76.682
52.920
43.935
200
150
100
50
0 PPM

zwf-7-129-14H
Nov 01 2023
SOLVENT: CDCl3
NA = 4
F1 = 400.140015 MHz
F2 = 1.000000 MHz

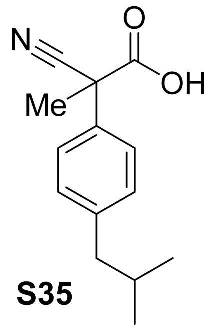

S35

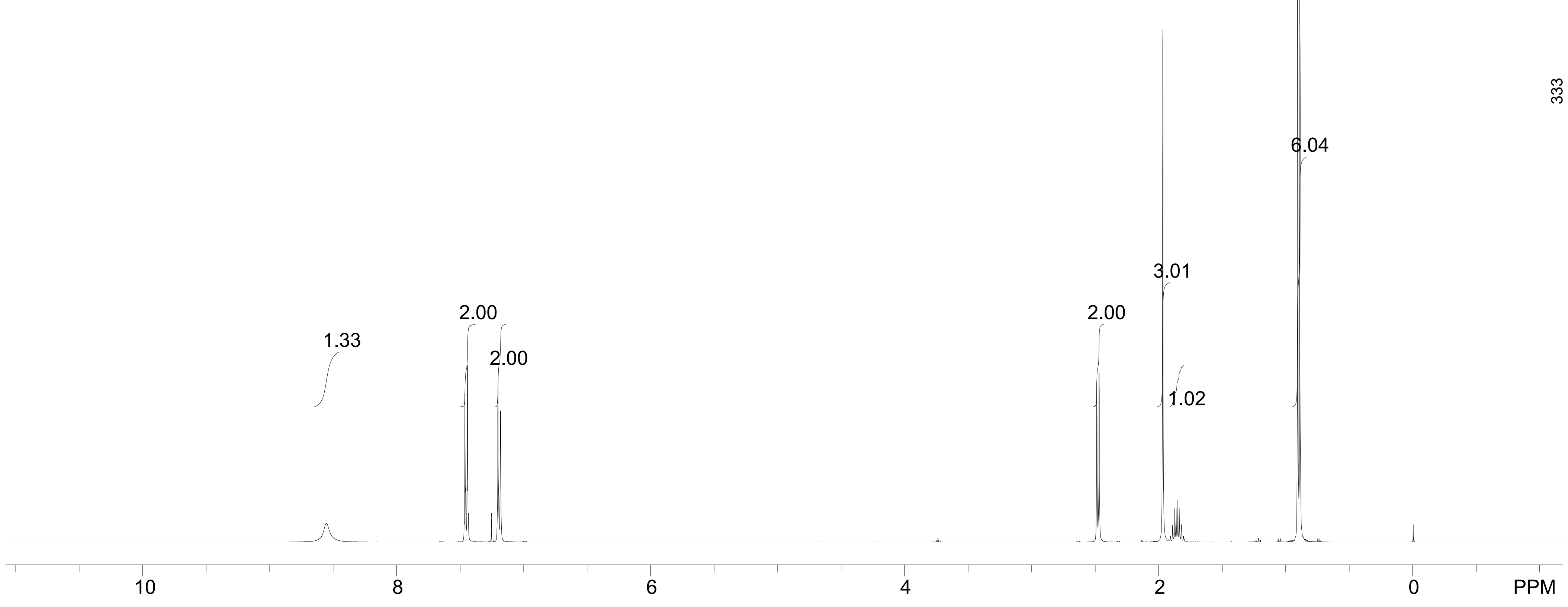

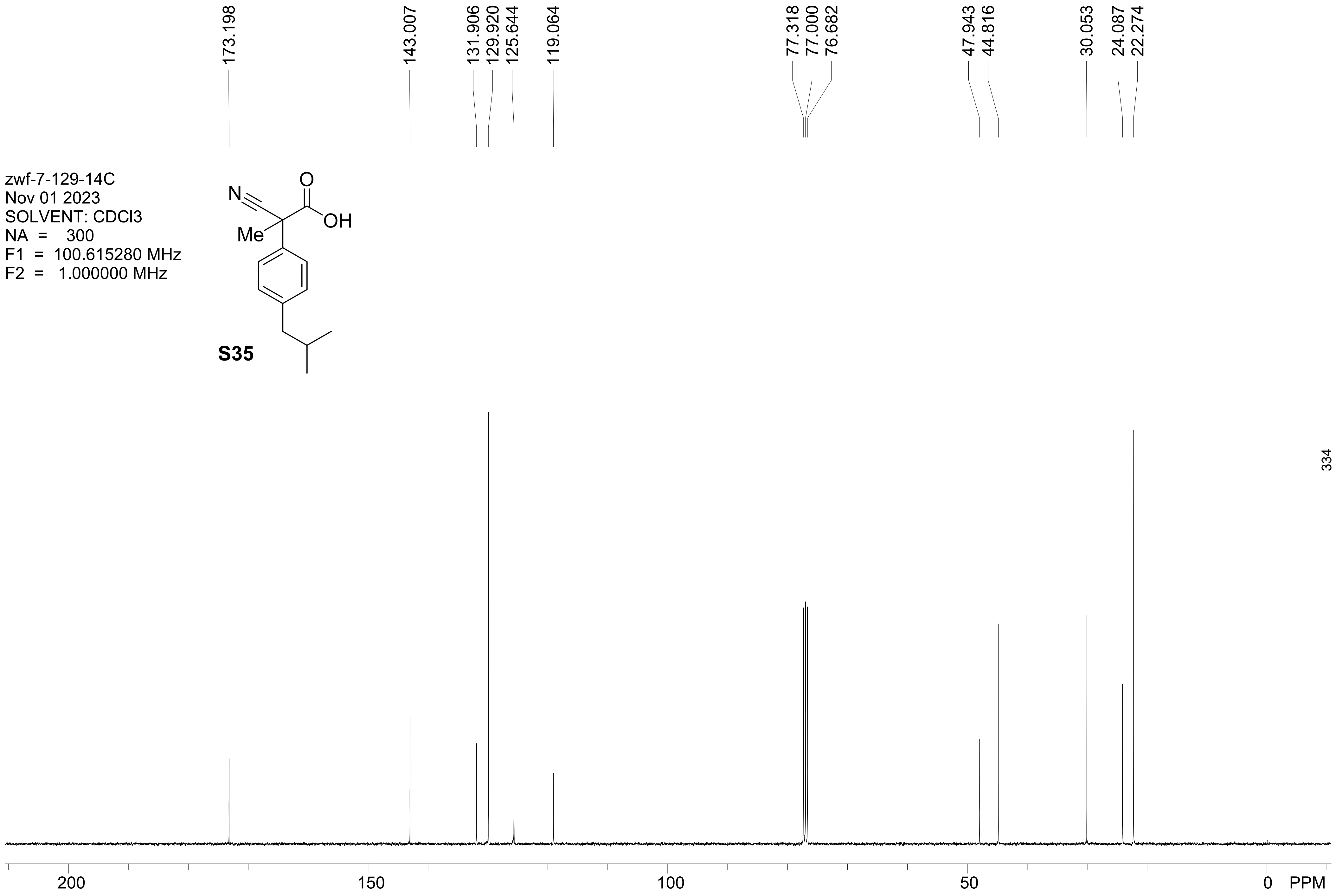
zwf-7-129-14C
Nov 01 2023
SOLVENT: CDCl3
NA = 300
F1 = 100.615280 MHz
F2 = 1.000000 MHz
N
O
OH
Me
S35
173.198
143.007
131.906
129.920
125.644
119.064
77.318
77.000
76.682
47.943
44.816
30.053
24.087
22.274
200
150
100
50
0 PPM

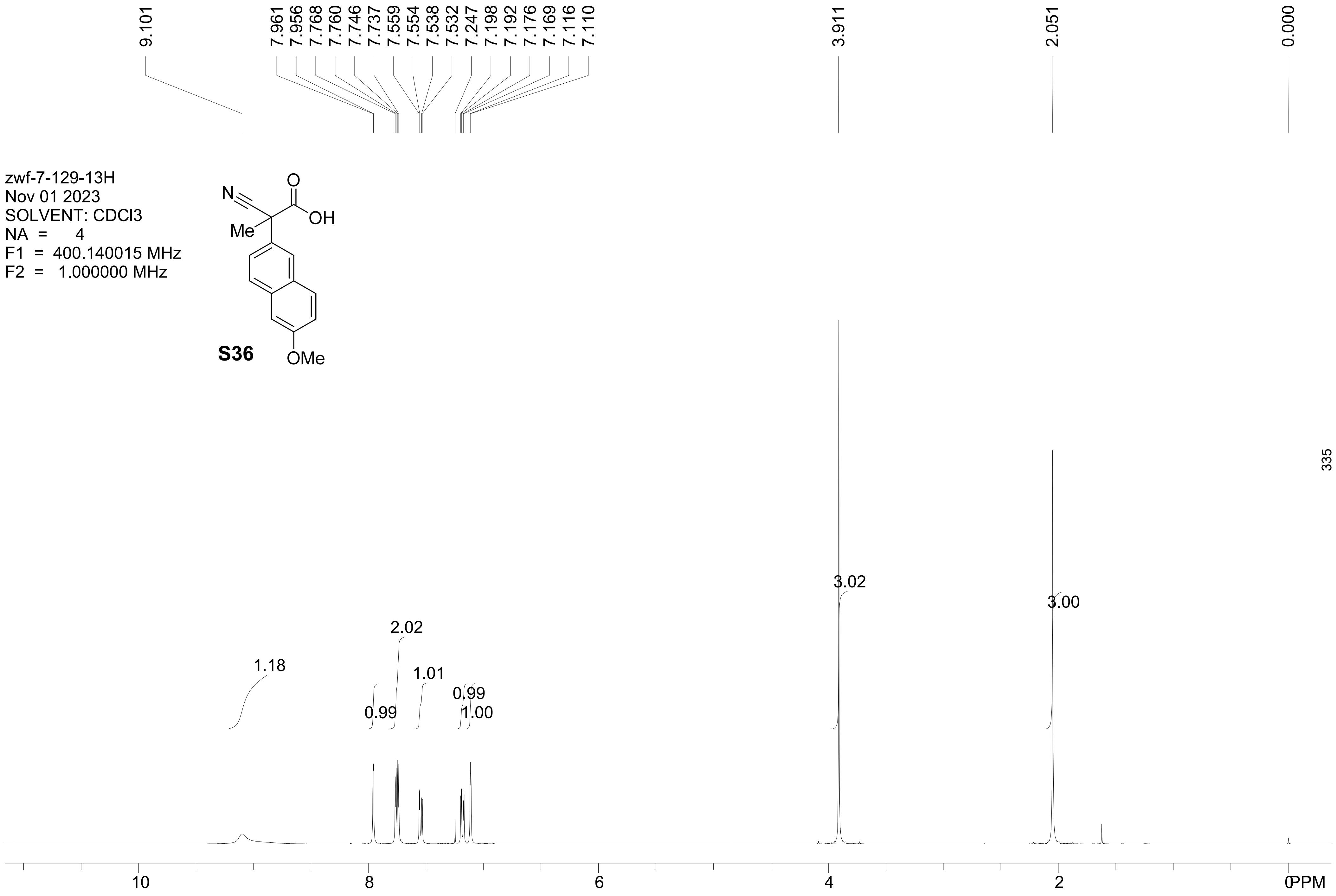
9.101
7.961
7.956
7.768
7.760
7.746
7.737
7.559
7.554
7.538
7.532
7.247
7.198
7.192
7.176
7.169
7.116
7.110
3.911
2.051
0.000
zwf-7-129-13H
Nov 01 2023
SOLVENT: CDCl3
NA = 4
F1 = 400.140015 MHz
F2 = 1.000000 MHz
N
O
OH
Me
S36
OMe
1.18
0.99
2.02
1.01
0.99
1.00
3.02
3.00
10
8
6
4
2
0
PPM

zwf-7-129-13C
Nov 01 2023
SOLVENT: CDCl3
NA = 100
F1 = 100.615280 MHz
F2 = 1.000000 MHz

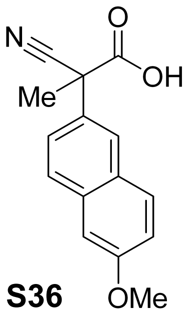

S36

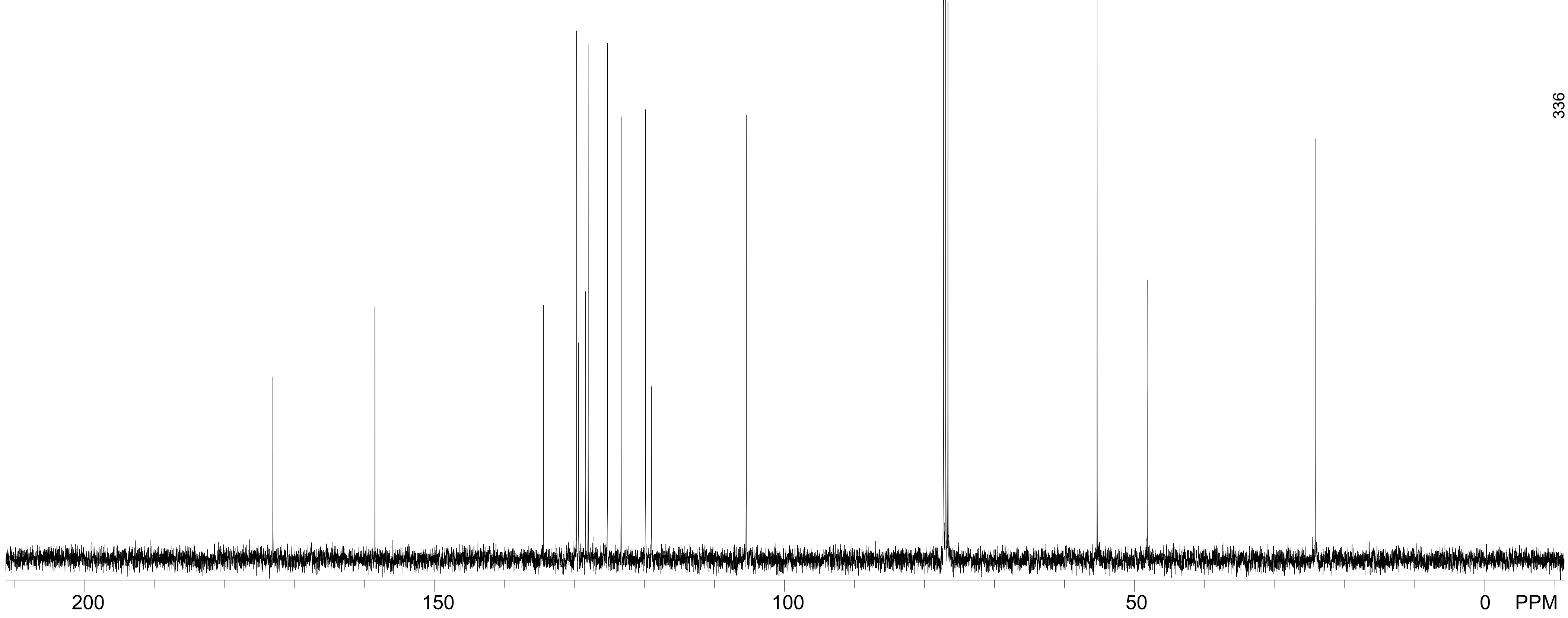

zwf-7-129-1H
Nov 01 2023
SOLVENT: CDCl3
NA = 4
F1 = 400.140015 MHz
F2 = 1.000000 MHz

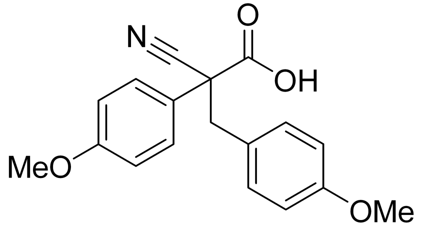

S37

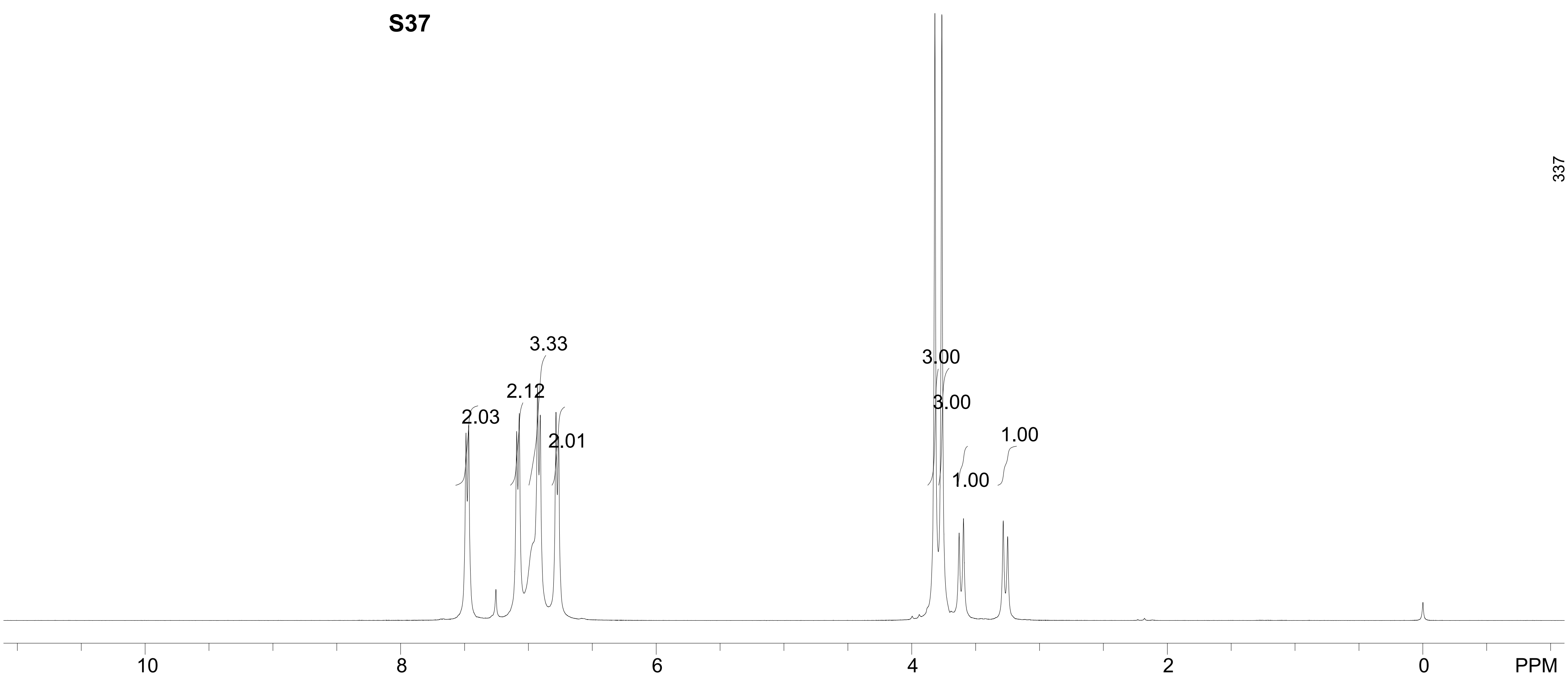

zwf-7-129-1C
Nov 01 2023
SOLVENT: CDCl3
NA = 100
F1 = 100.615280 MHz
F2 = 1.000000 MHz

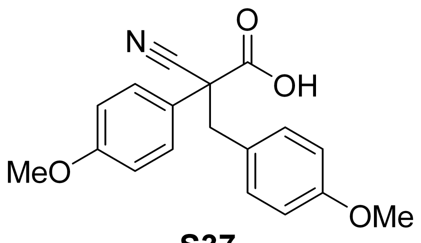

S37

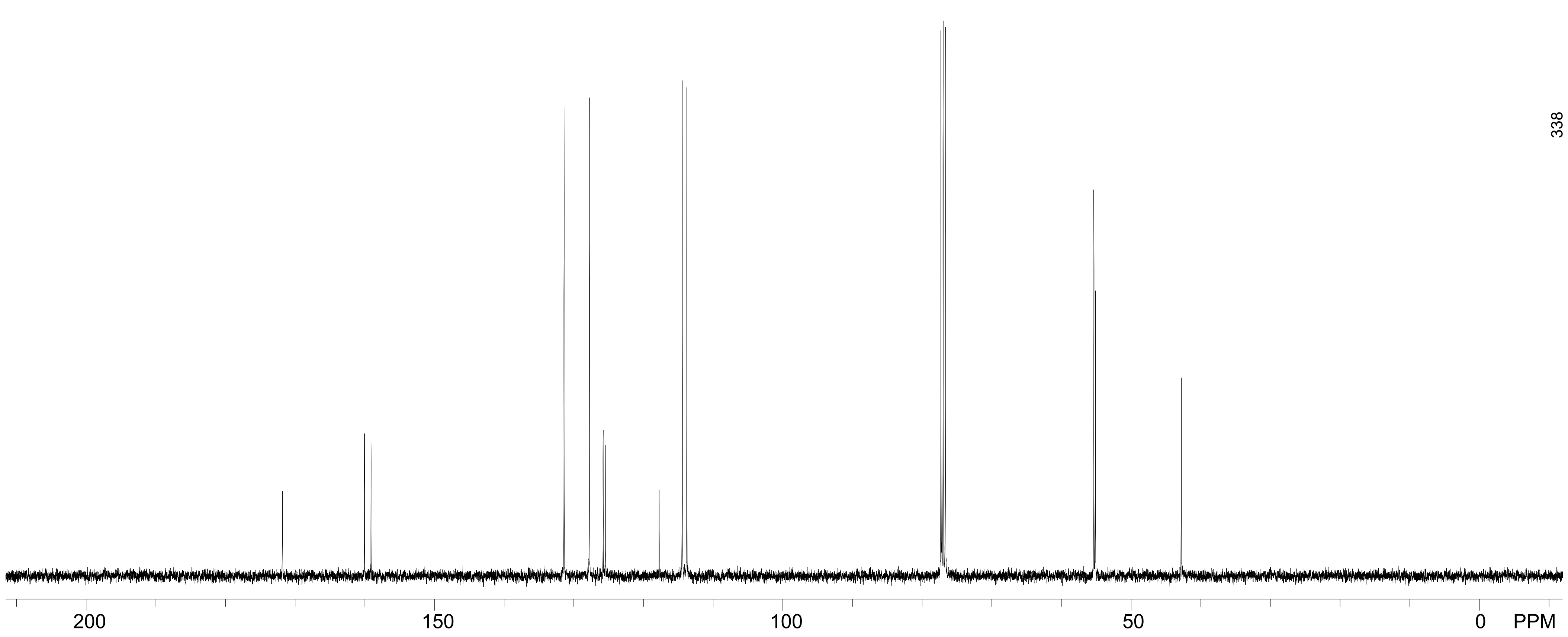

zwf-7-129-2H
Nov 01 2023
SOLVENT: CDCl3
NA = 4
F1 = 400.140015 MHz
F2 = 1.000000 MHz

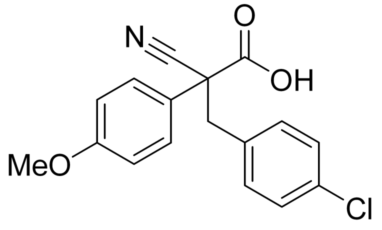

S38

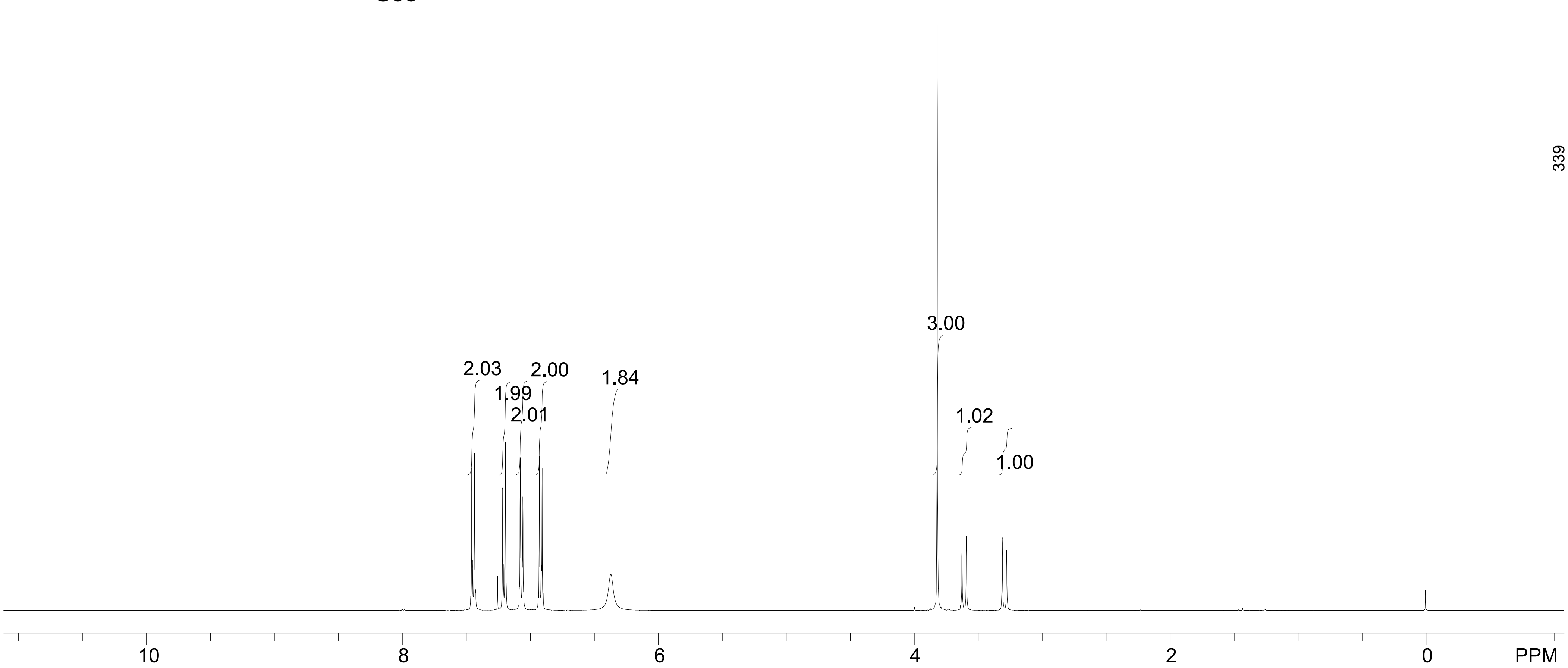

zwf-7-129-2C
Nov 01 2023
SOLVENT: CDCl3
NA = 100
F1 = 100.615280 MHz
F2 = 1.000000 MHz

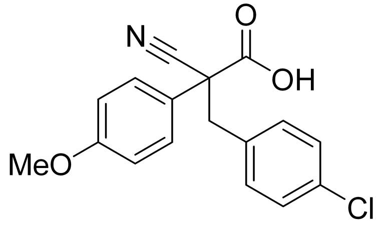

S38

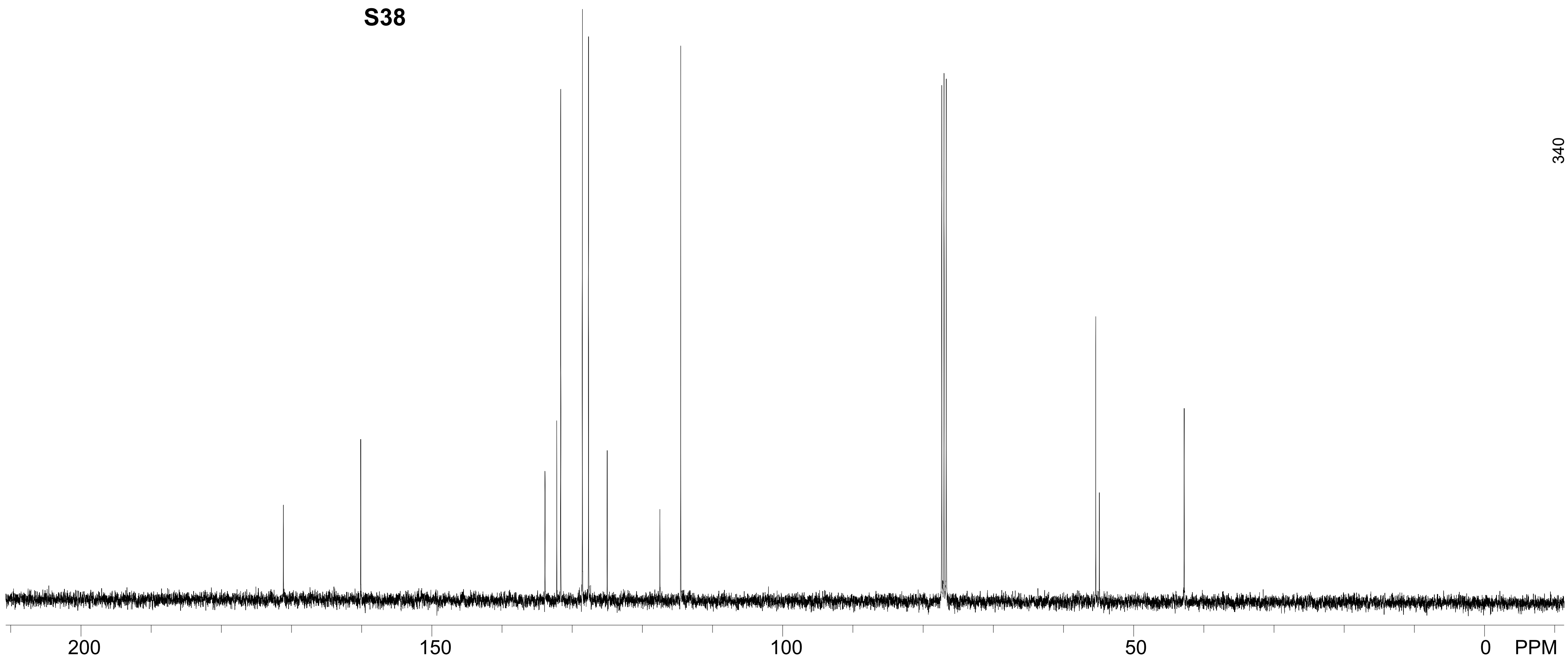

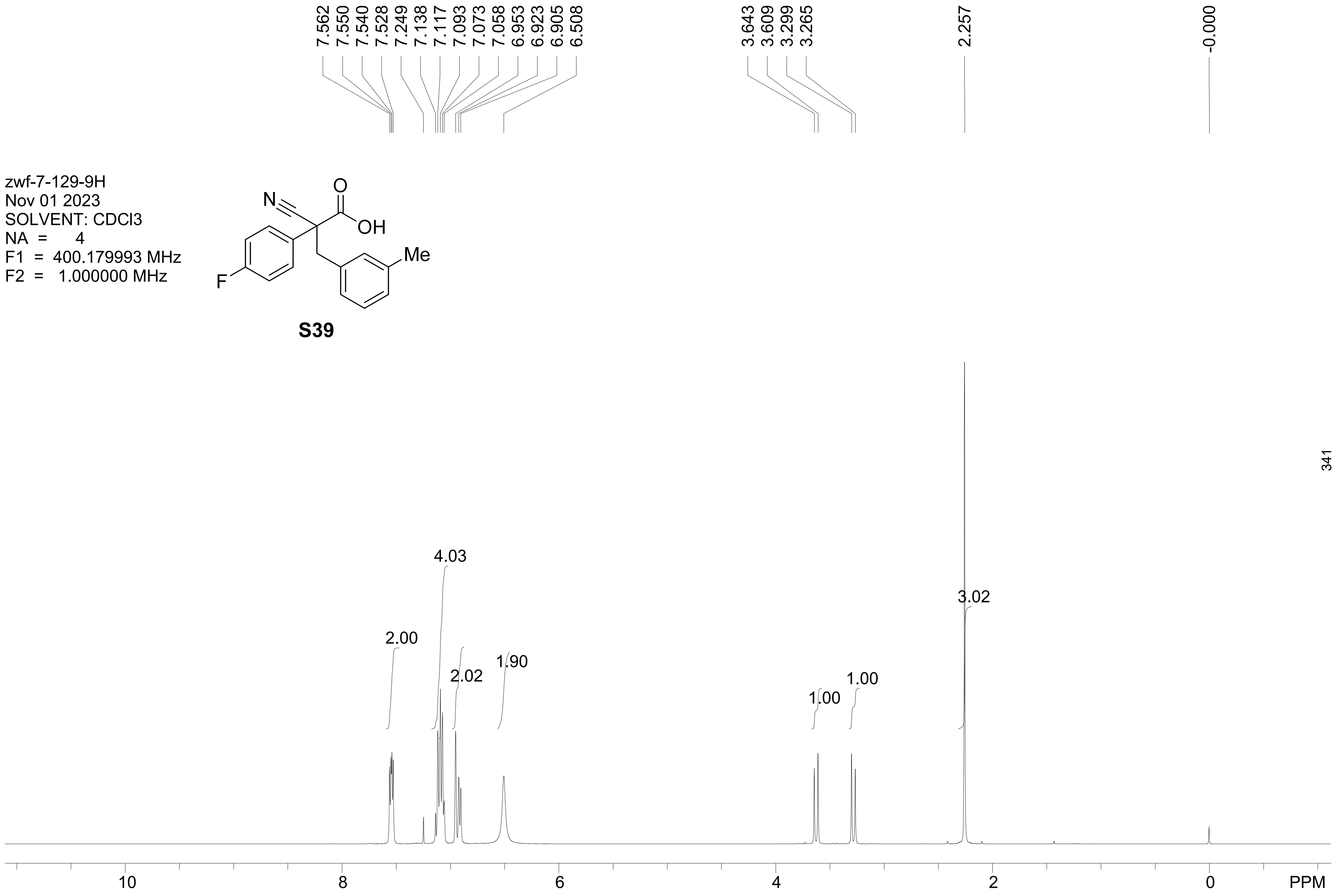

zwf-7-129-9H
Nov 01 2023
SOLVENT: CDCl3
NA = 4
F1 = 400.179993 MHz
F2 = 1.000000 MHz
N
O
OH
Me
F
S39
7.562 7.550 7.540 7.528 7.249 7.138 7.117 7.093 7.073 7.058 6.953 6.923 6.905 6.508
3.643 3.609 3.299 3.265
2.257
-0.000
2.00
4.03
2.02
1.90
1.00
1.00
3.02
10
8
6
4
2
0
PPM

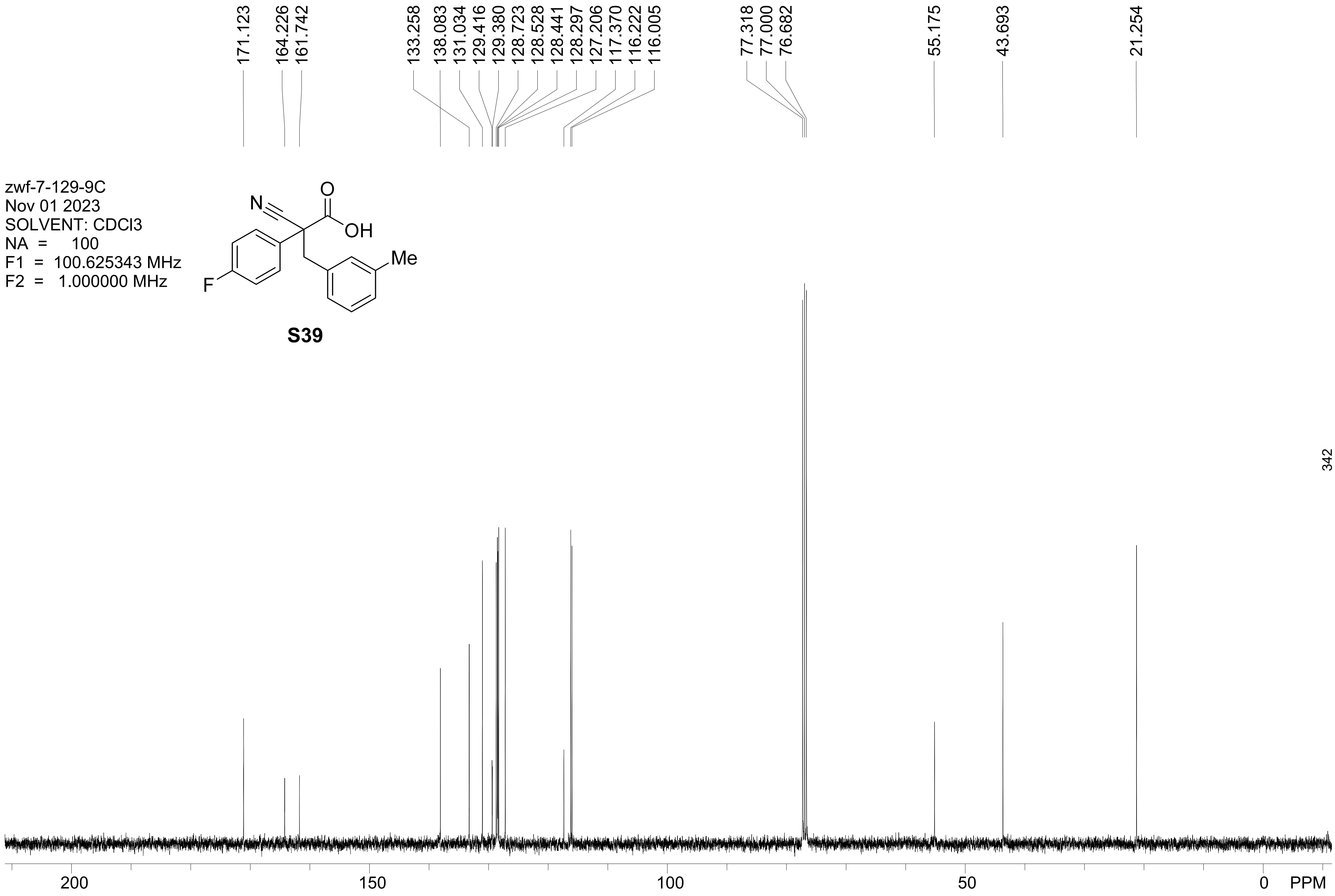
zwf-7-129-9C
Nov 01 2023
SOLVENT: CDCl3
NA = 100
F1 = 100.625343 MHz
F2 = 1.000000 MHz
N
O
OH
F
Me
S39
171.123
164.226
161.742
133.258
138.083
131.034
129.416
129.380
128.723
128.528
128.441
128.297
127.206
117.370
116.222
116.005
77.318
77.000
76.682
55.175
43.693
21.254
200
150
100
50
0
PPM

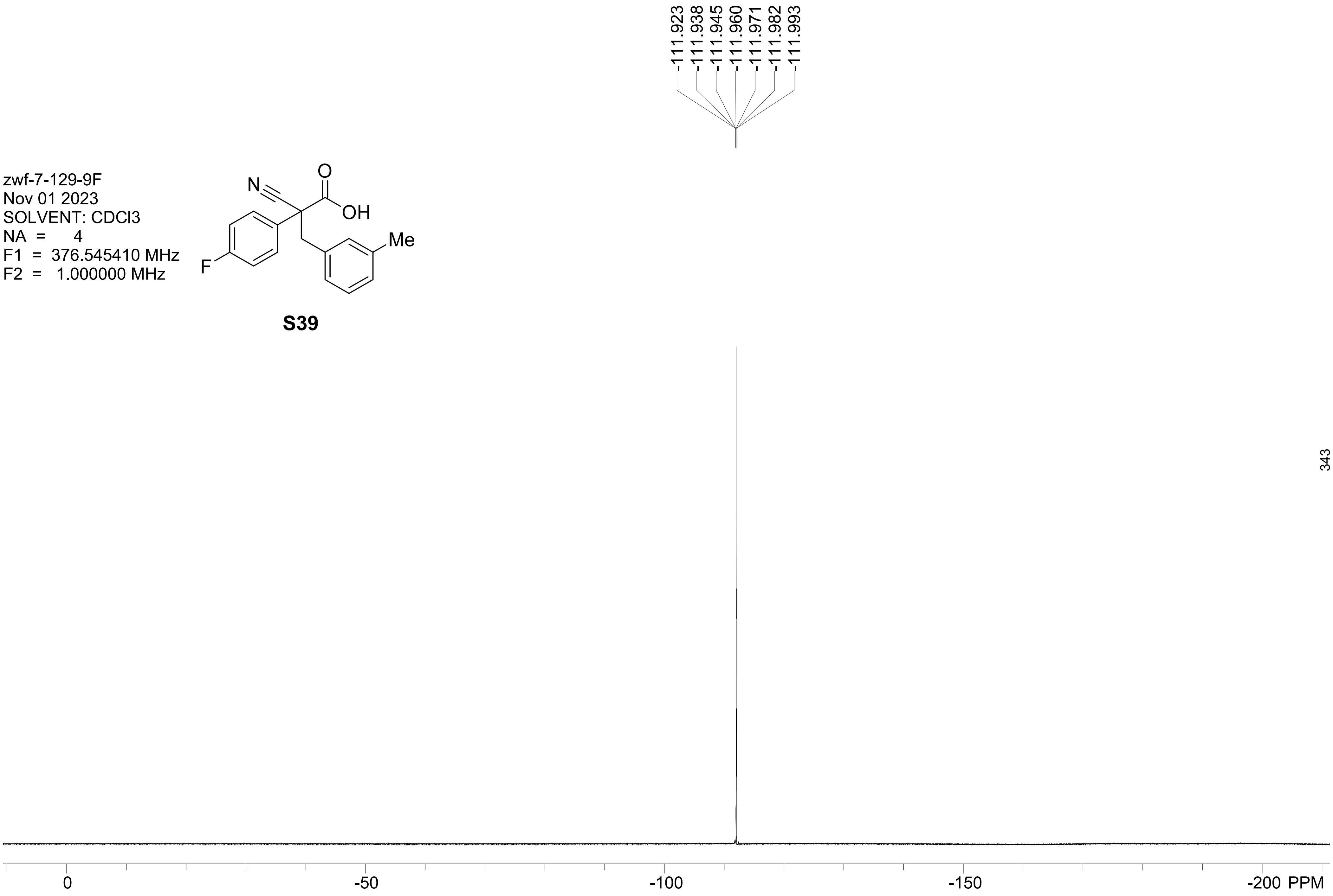
zwf-7-129-9F
Nov 01 2023
SOLVENT: CDCl3
NA = 4
F1 = 376.545410 MHz
F2 = 1.000000 MHz
S39
-111.923
-111.938
-111.945
-111.960
-111.971
-111.982
-111.993
0
-50
-100
-150
-200 PPM

zwf-7-129-11H
Nov 02 2023
SOLVENT: CDCl3
NA = 4
F1 = 400.179993 MHz
F2 = 1.000000 MHz

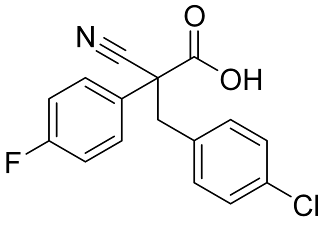

S40

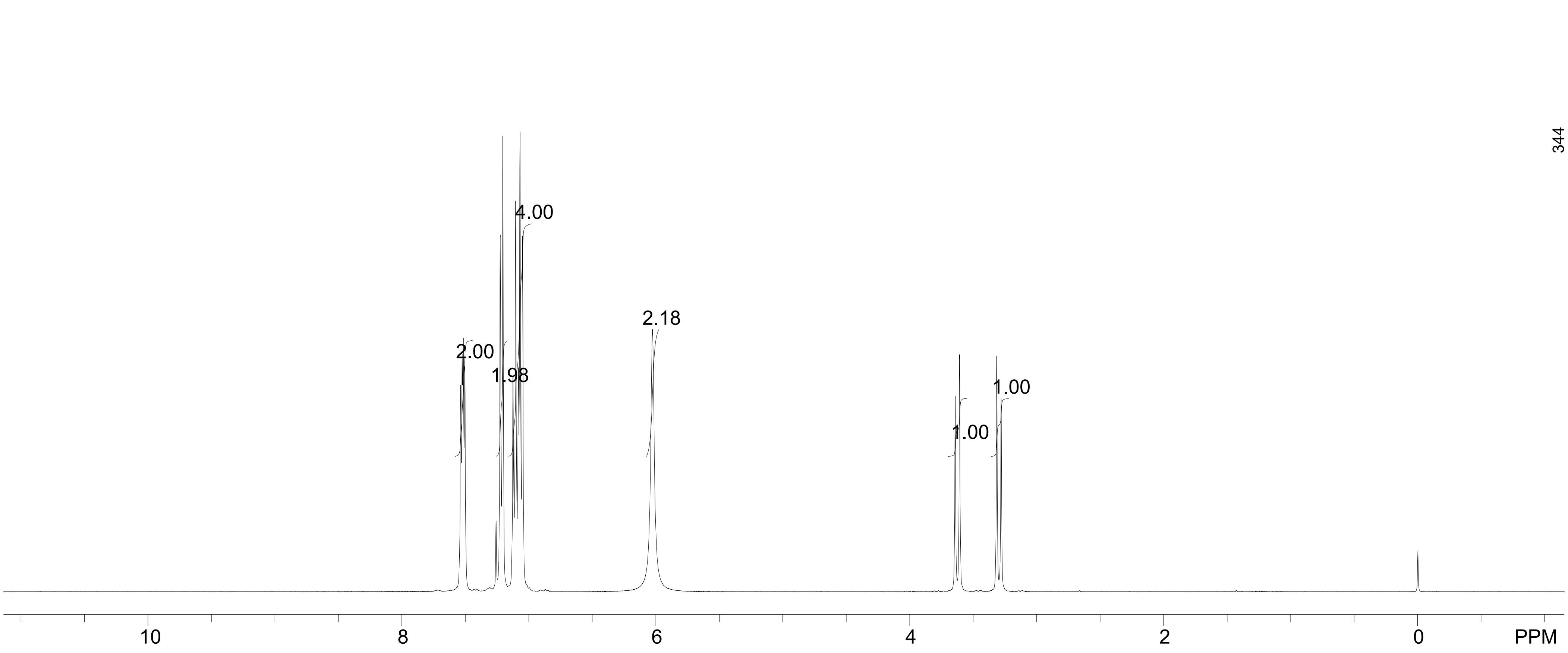

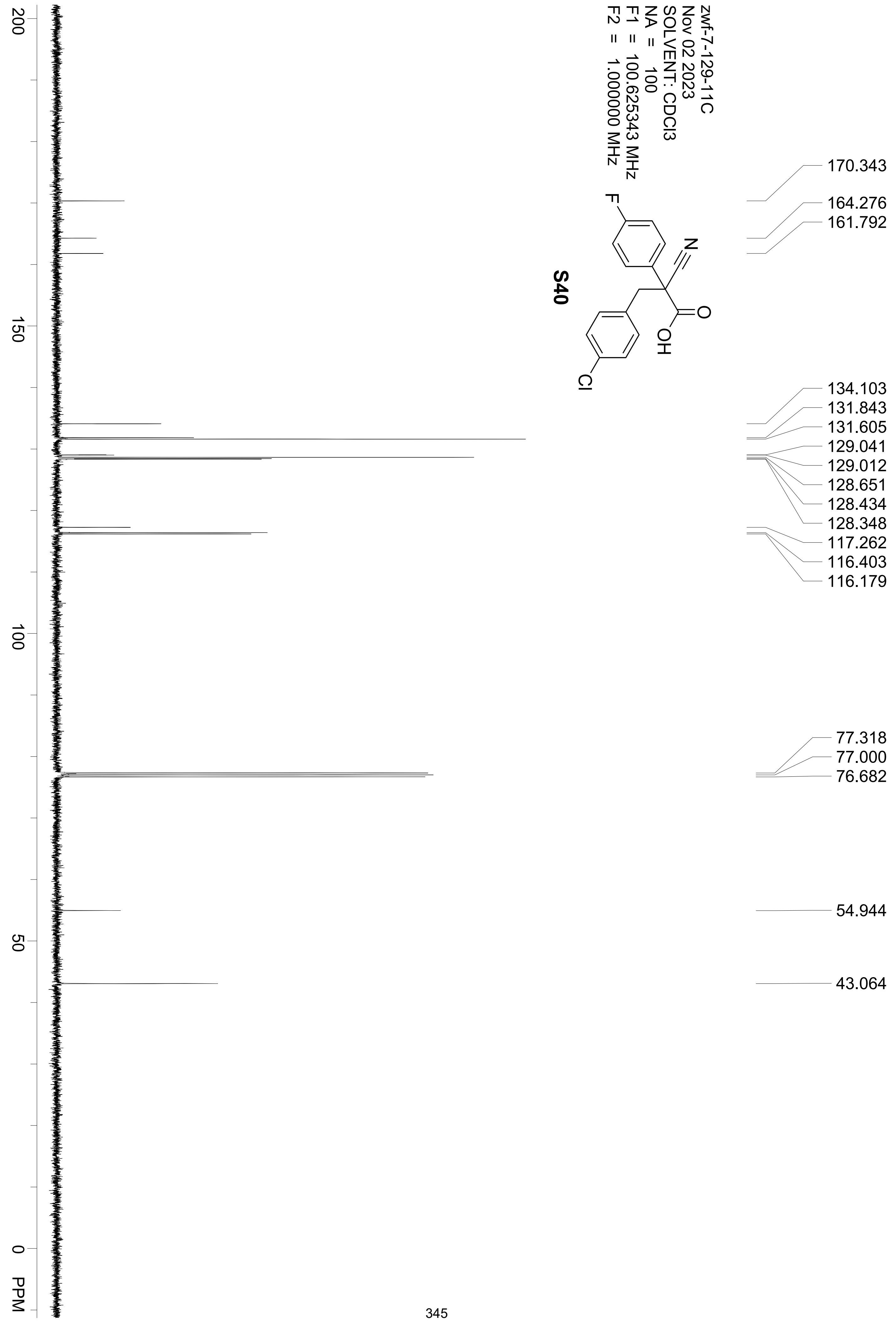
zwf-7-129-11C
Nov 02 2023
SOLVENT: CDCl3
NA = 100
F1 = 100.625343 MHz
F2 = 1.000000 MHz
S40
F
N
O
OH
Cl
170.343
164.276
161.792
134.103
131.843
131.605
129.041
129.012
128.651
128.434
128.348
117.262
116.403
116.179
77.318
77.000
76.682
54.944
43.064
200
150
100
50
0
PPM

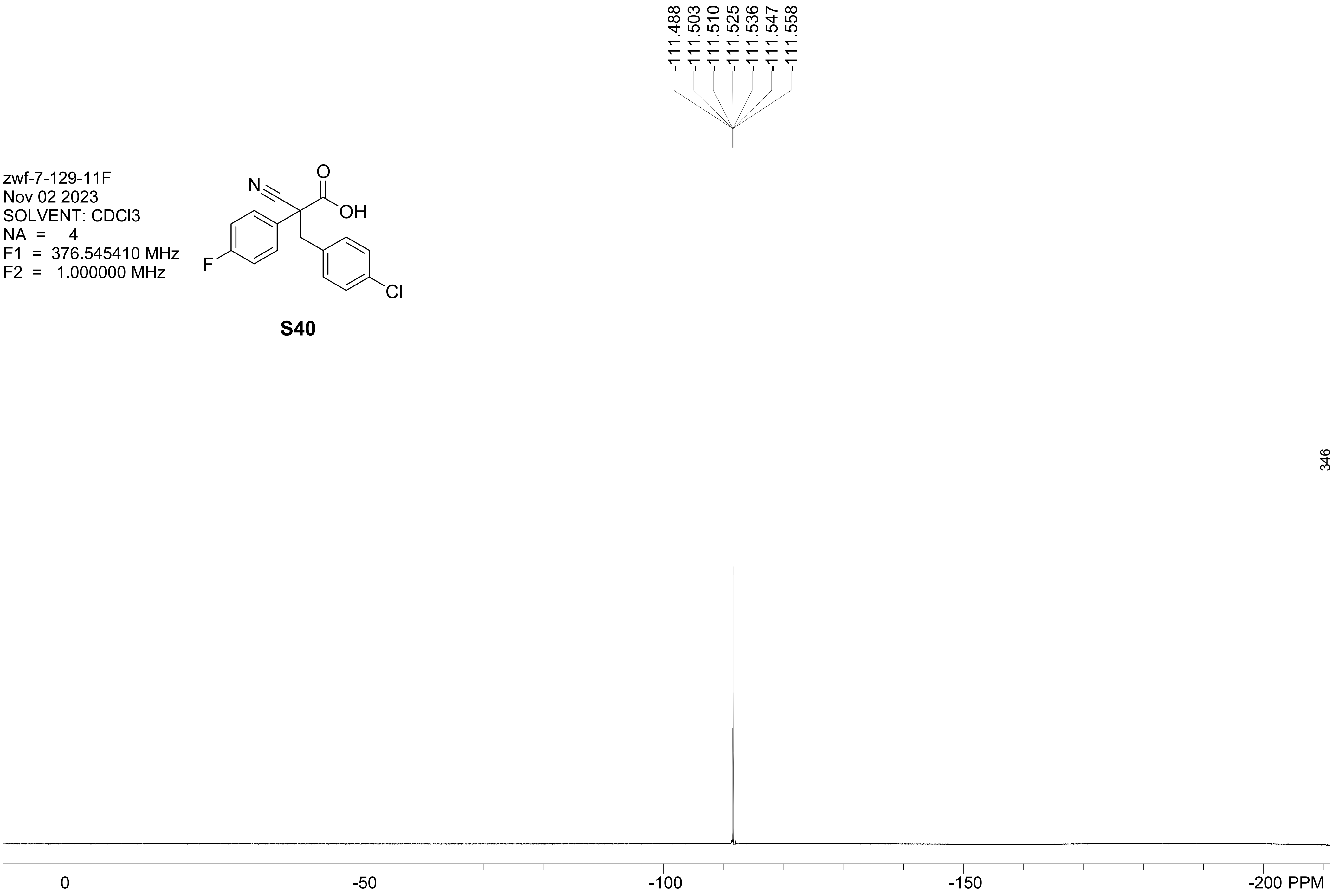
zwf-7-129-11F
Nov 02 2023
SOLVENT: CDCl3
NA = 4
F1 = 376.545410 MHz
F2 = 1.000000 MHz
-111.488
-111.503
-111.510
-111.525
-111.536
-111.547
-111.558
0
-50
-100
-150
-200 PPM

S40

zwf-7-129-8H
Oct 30 2023
SOLVENT: CDCl3
NA = 4
F1 = 400.140015 MHz
F2 = 1.000000 MHz

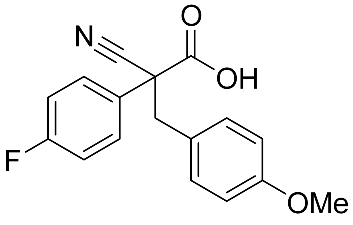

S41

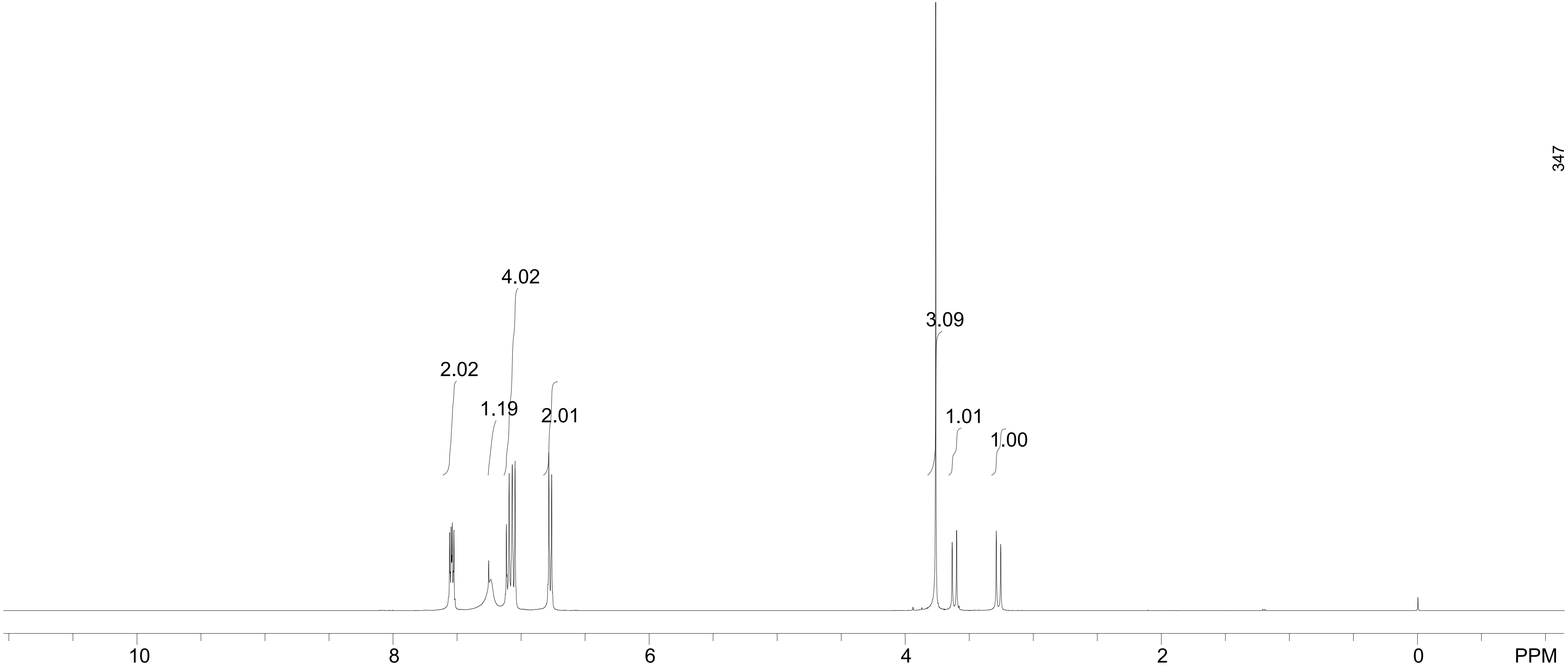

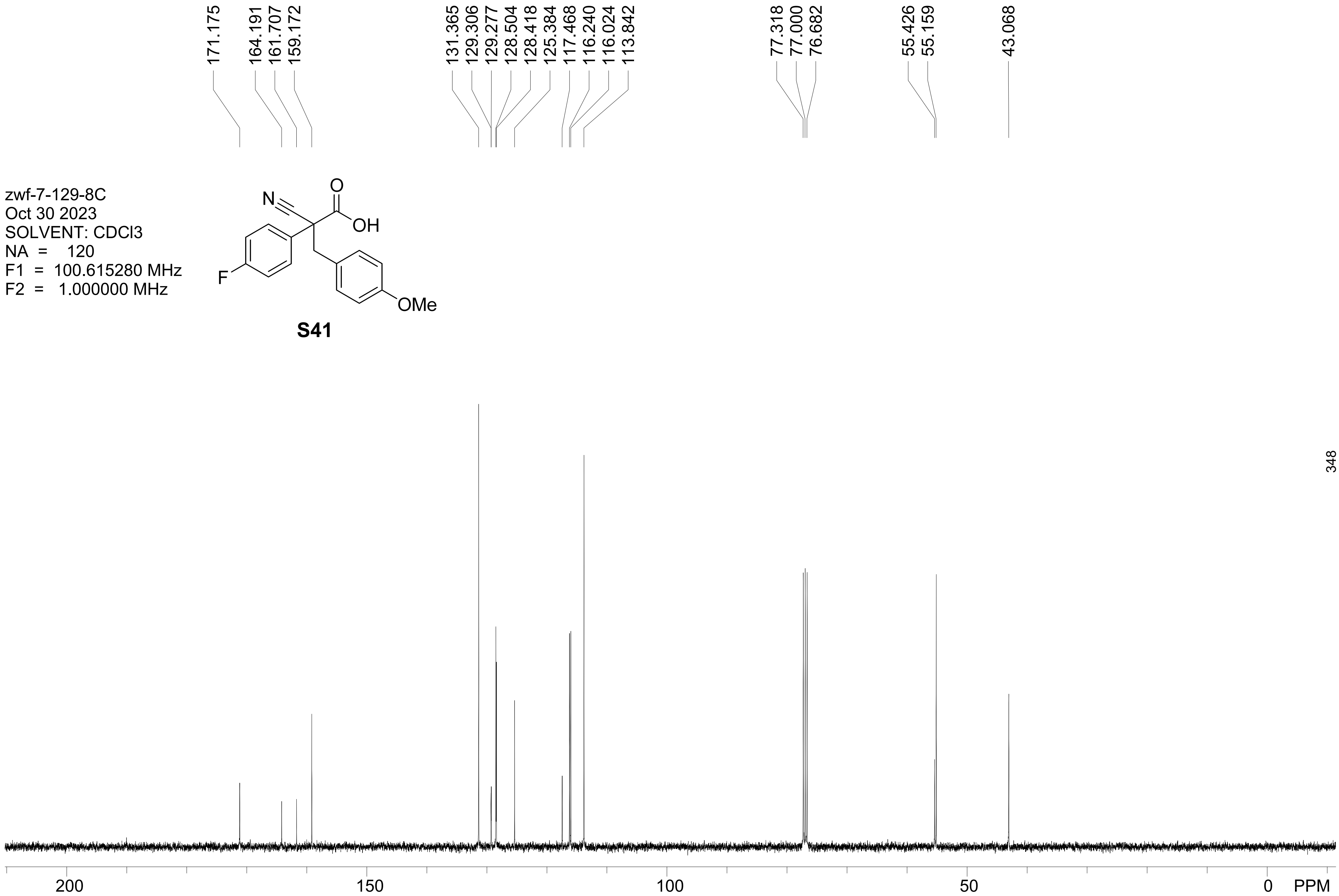
zwf-7-129-8C
Oct 30 2023
SOLVENT: CDCl3
NA = 120
F1 = 100.615280 MHz
F2 = 1.000000 MHz
N
O
OH
F
OMe
S41
171.175
164.191
161.707
159.172
131.365
129.306
129.277
128.504
128.418
125.384
117.468
116.240
116.024
113.842
77.318
77.000
76.682
55.426
55.159
43.068
200
150
100
50
0
PPM

zwf-7-129-8F
Oct 30 2023
SOLVENT: CDCl3
NA = 4
F1 = 376.507782 MHz
F2 = 1.000000 MHz

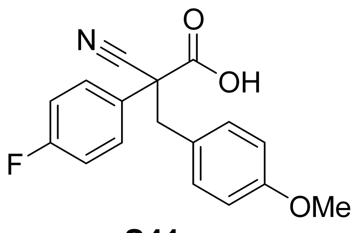

S41

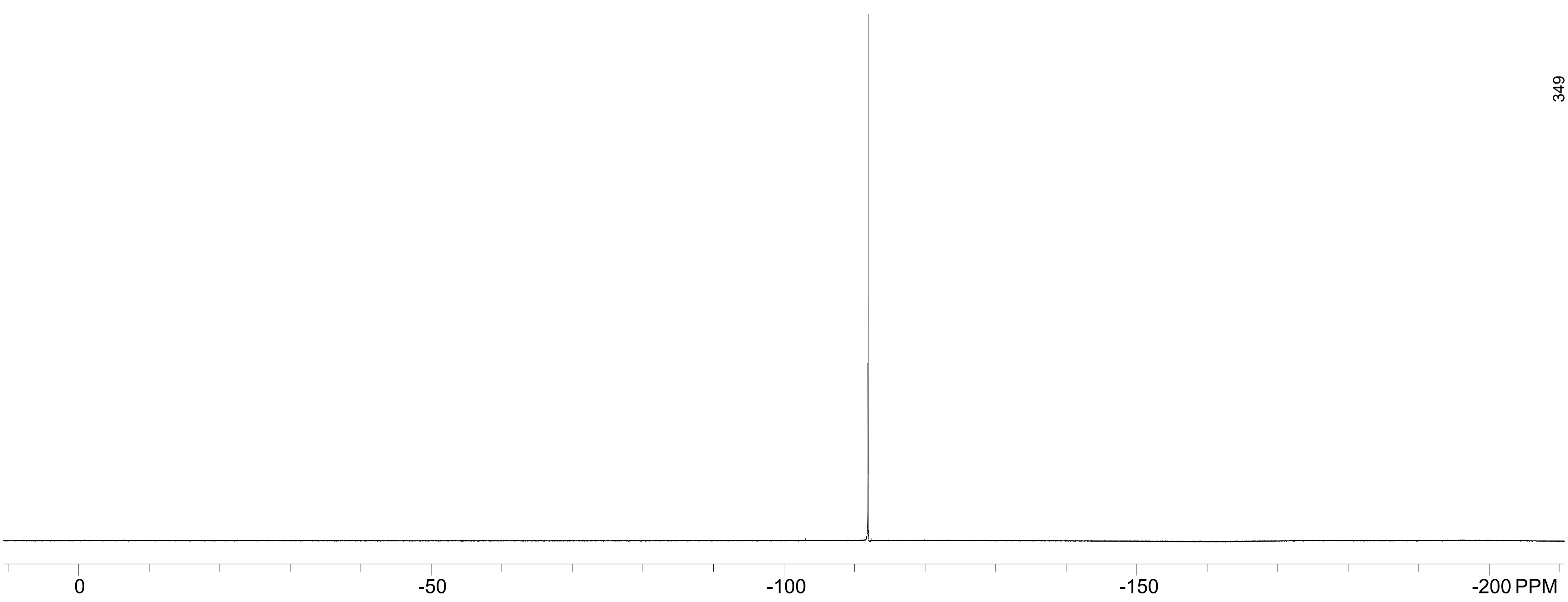

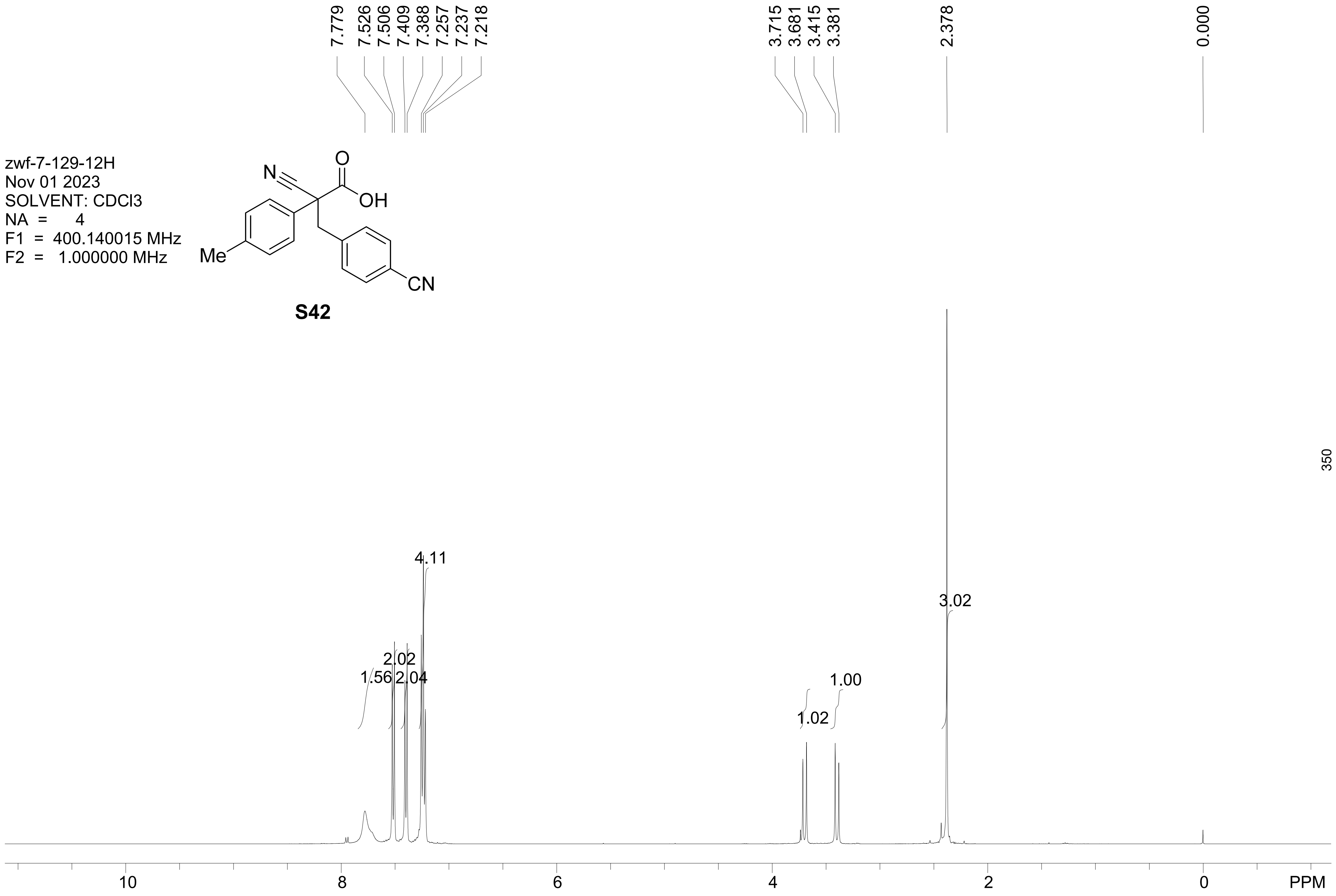
zwf-7-129-12H
Nov 01 2023
SOLVENT: CDCl3
NA = 4
F1 = 400.140015 MHz
F2 = 1.000000 MHz
S42
7.779
7.526
7.506
7.409
7.388
7.257
7.237
7.218
3.715
3.681
3.415
3.381
2.378
0.000
1.56
2.02
2.04
4.11
1.02
1.00
3.02
10
8
6
4
2
0
PPM

zwf-7-129-12C
Nov 01 2023
SOLVENT: CDCl3
NA = 100
F1 = 100.615280 MHz
F2 = 1.000000 MHz

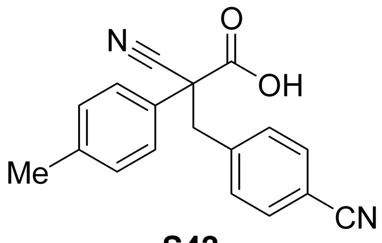

S42

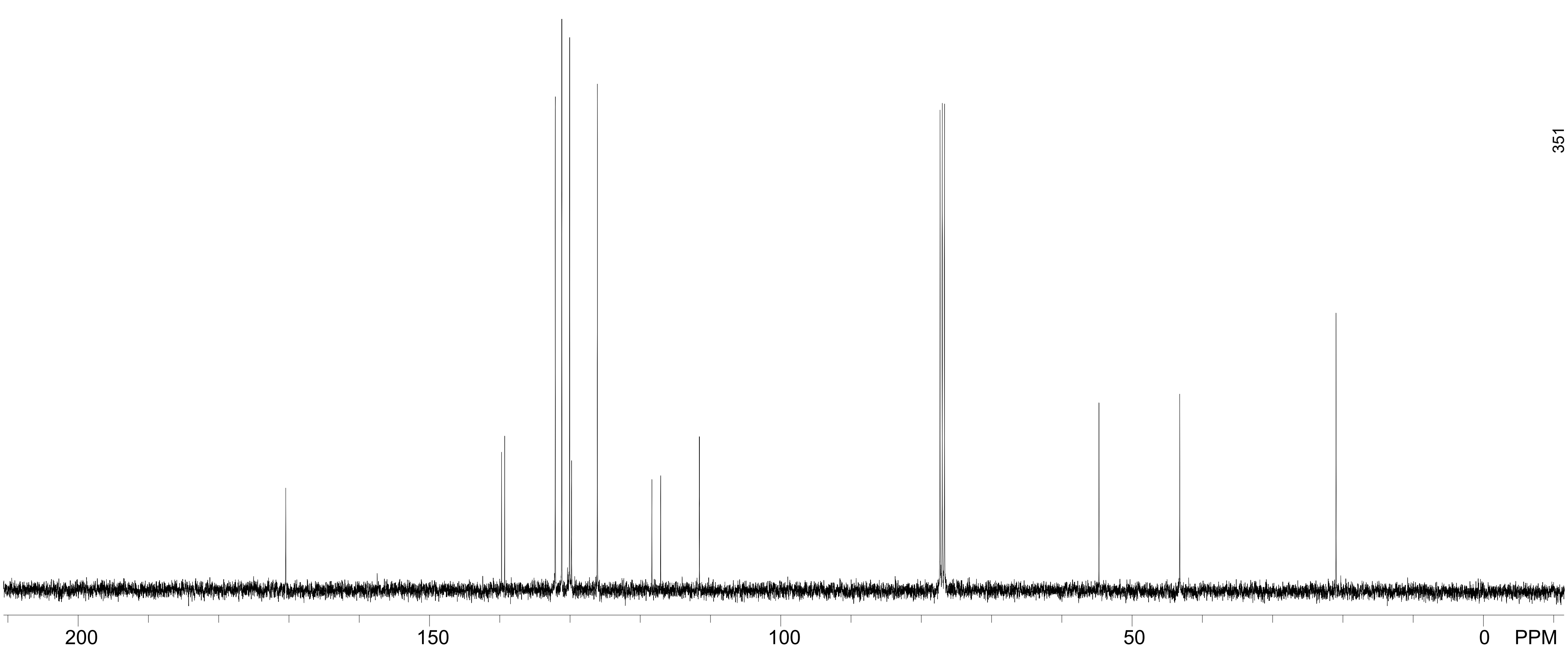

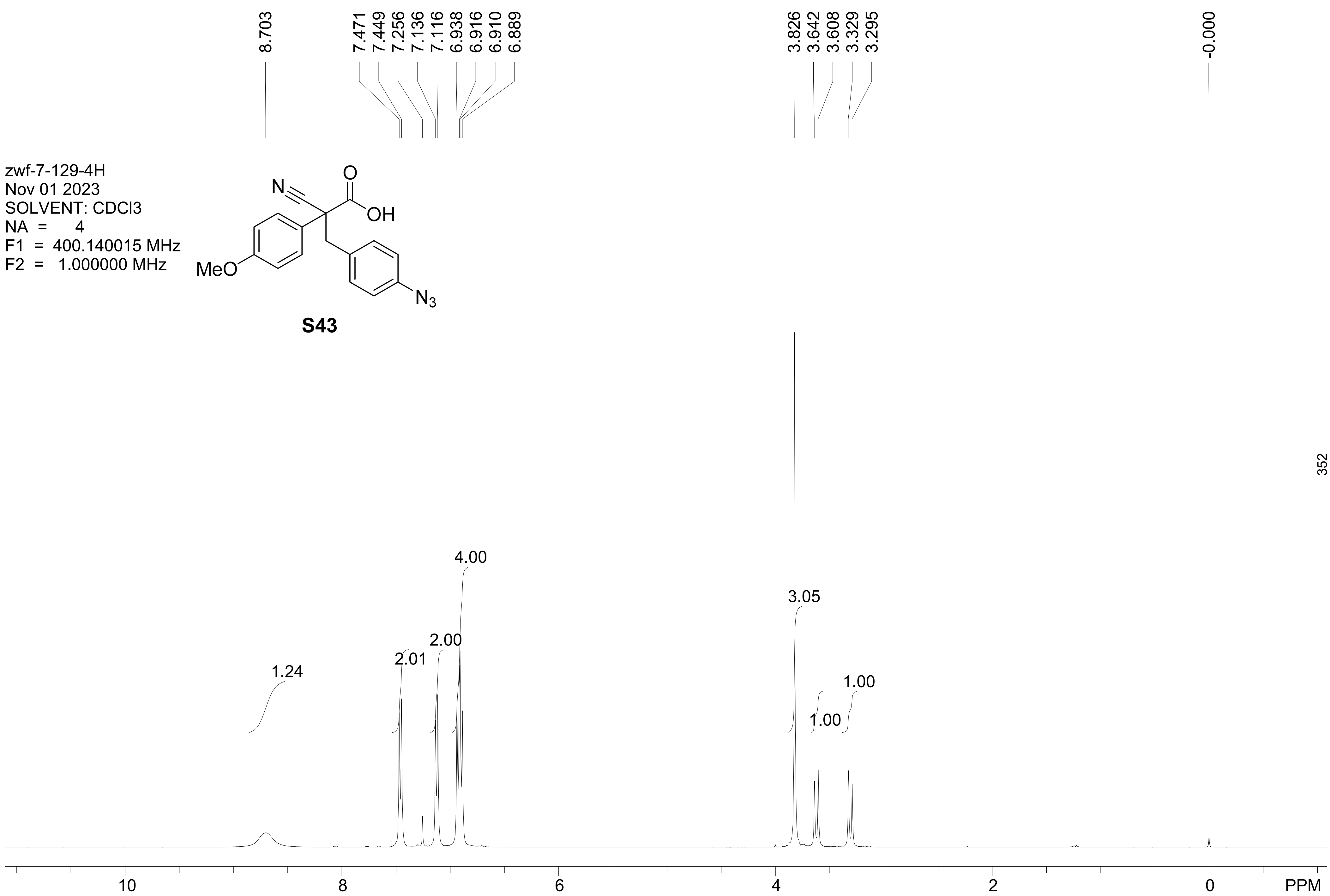
zwf-7-129-4H
Nov 01 2023
SOLVENT: CDCl3
NA = 4
F1 = 400.140015 MHz
F2 = 1.000000 MHz
N
O
OH
MeO
N3
S43
8.703
7.471
7.449
7.256
7.136
7.116
6.938
6.916
6.910
6.889
3.826
3.642
3.608
3.329
3.295
-0.000
1.24
2.01
2.00
4.00
3.05
1.00
1.00
10
8
6
4
2
0
PPM

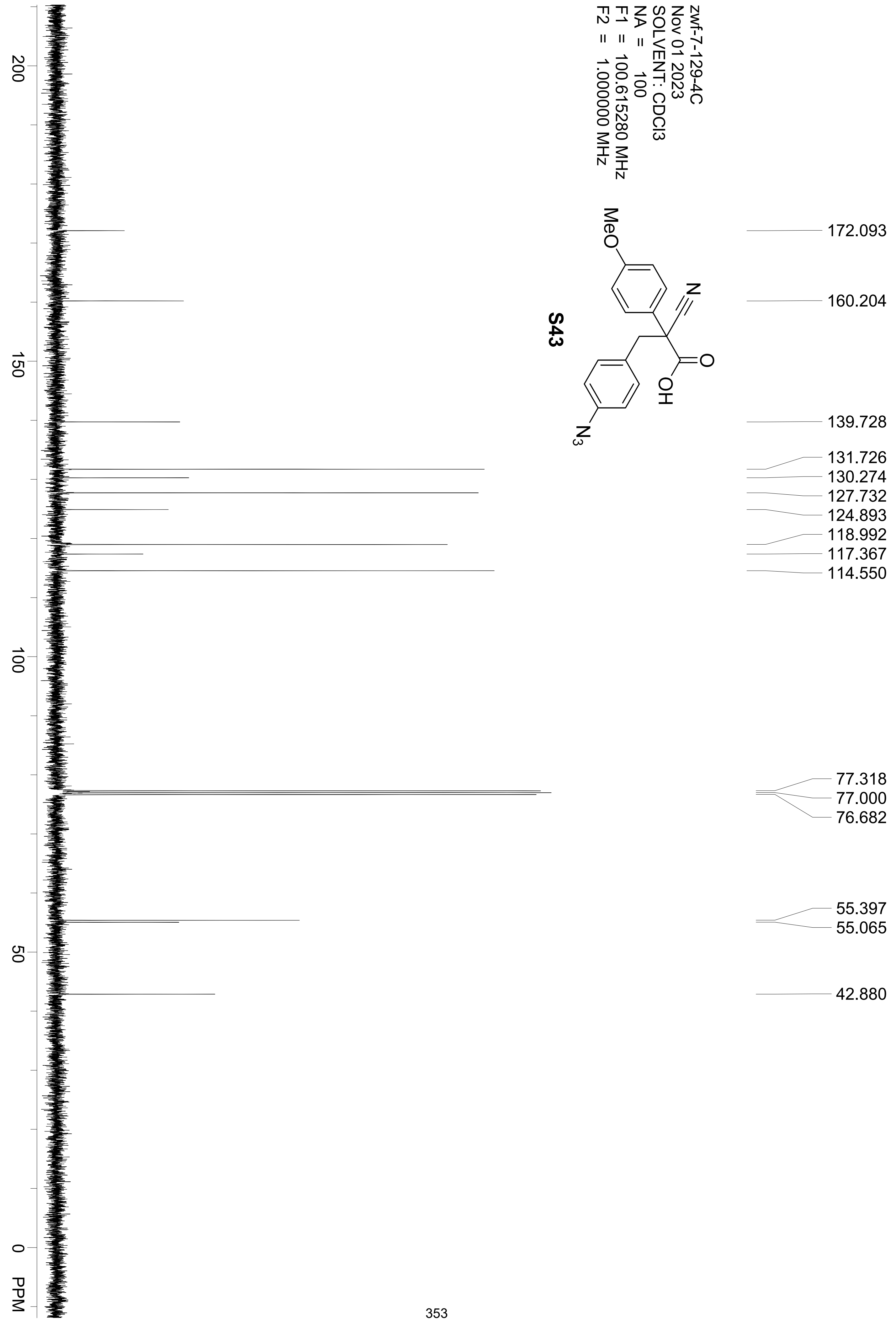
zwf-7-129-4C
Nov 01 2023
SOLVENT: CDCl3
NA = 100
F1 = 100.615280 MHz
F2 = 1.000000 MHz
MeO
N
O
OH
N3
S43
172.093
160.204
139.728
131.726
130.274
127.732
124.893
118.992
117.367
114.550
77.318
77.000
76.682
55.397
55.065
42.880
200
150
100
50
0
PPM

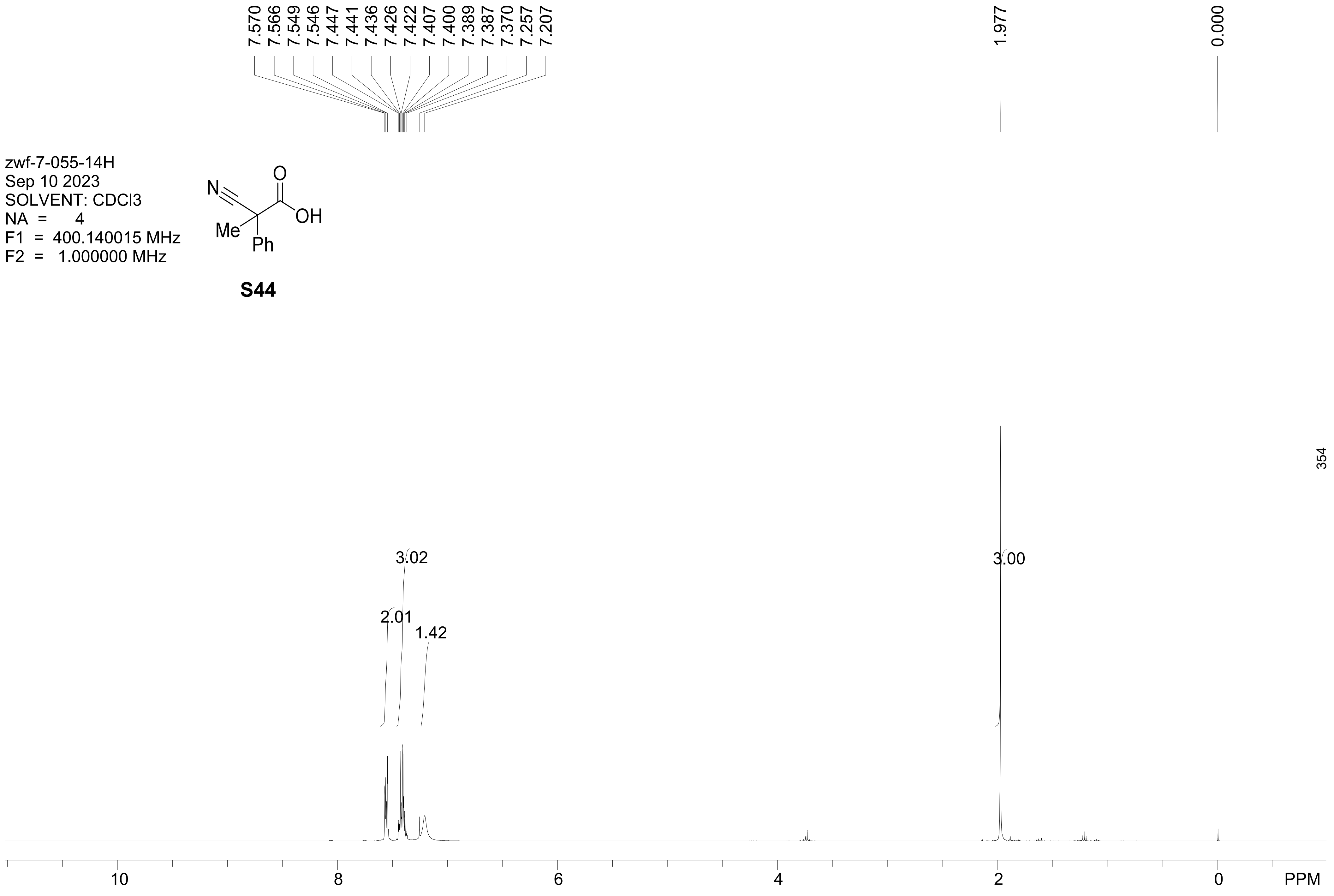
7.570
7.566
7.549
7.546
7.447
7.441
7.436
7.426
7.422
7.407
7.400
7.389
7.387
7.370
7.257
7.207
1.977
0.000
zwf-7-055-14H
Sep 10 2023
SOLVENT: CDCl3
NA = 4
F1 = 400.140015 MHz
F2 = 1.000000 MHz
N
O
OH
Me
Ph
S44
3.02
2.01
1.42
3.00
10
8
6
4
2
0
PPM

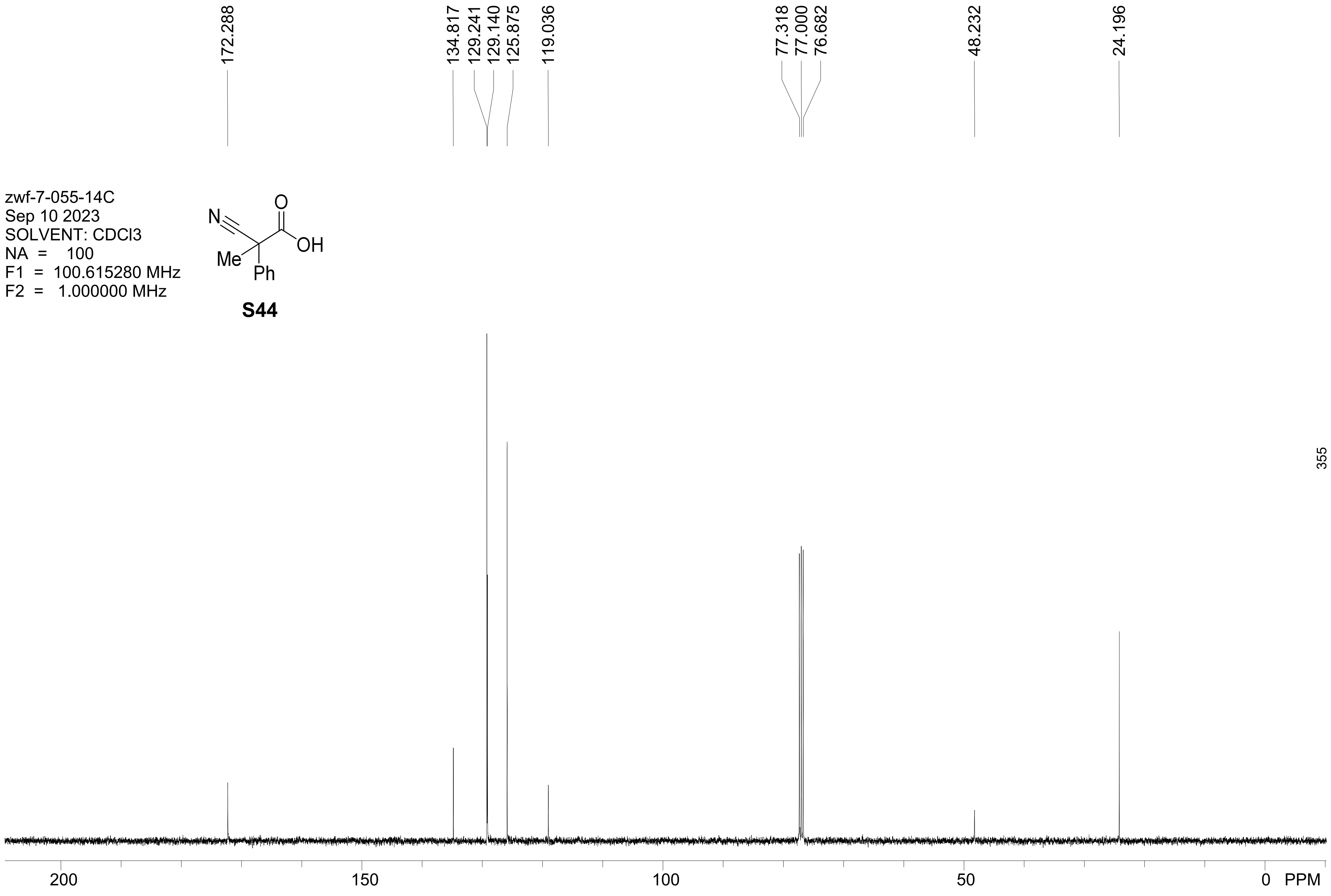
zwf-7-055-14C
Sep 10 2023
SOLVENT: CDCl3
NA = 100
F1 = 100.615280 MHz
F2 = 1.000000 MHz
N
O
OH
Me
Ph
S44
172.288
134.817
129.241
129.140
125.875
119.036
77.318
77.000
76.682
48.232
24.196
200
150
100
50
0 PPM

zwf-7-133-4H
Nov 05 2023
SOLVENT: CDCl3
NA = 4
F1 = 400.179993 MHz
F2 = 1.000000 MHz

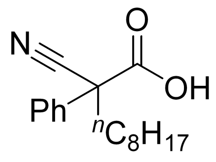

S45

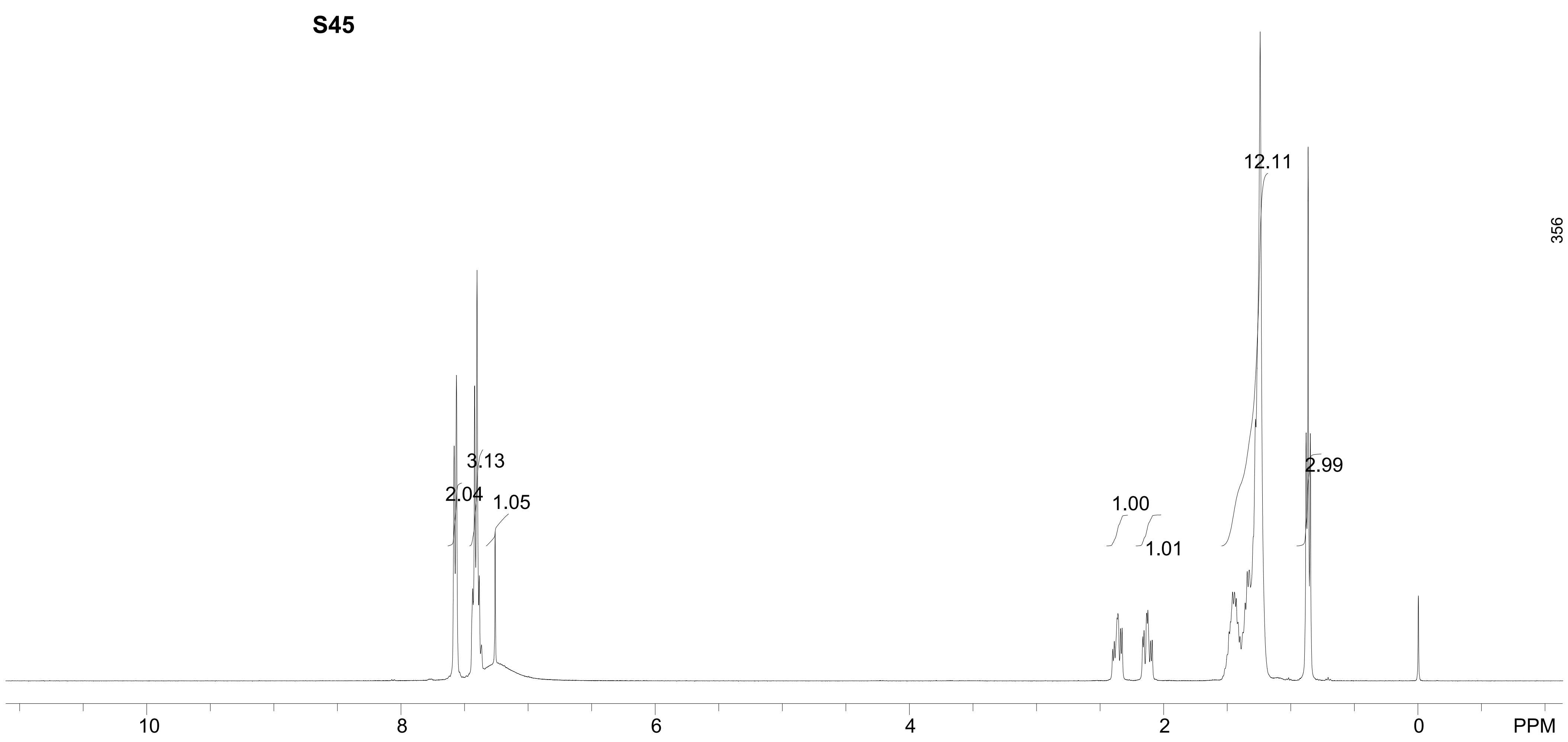

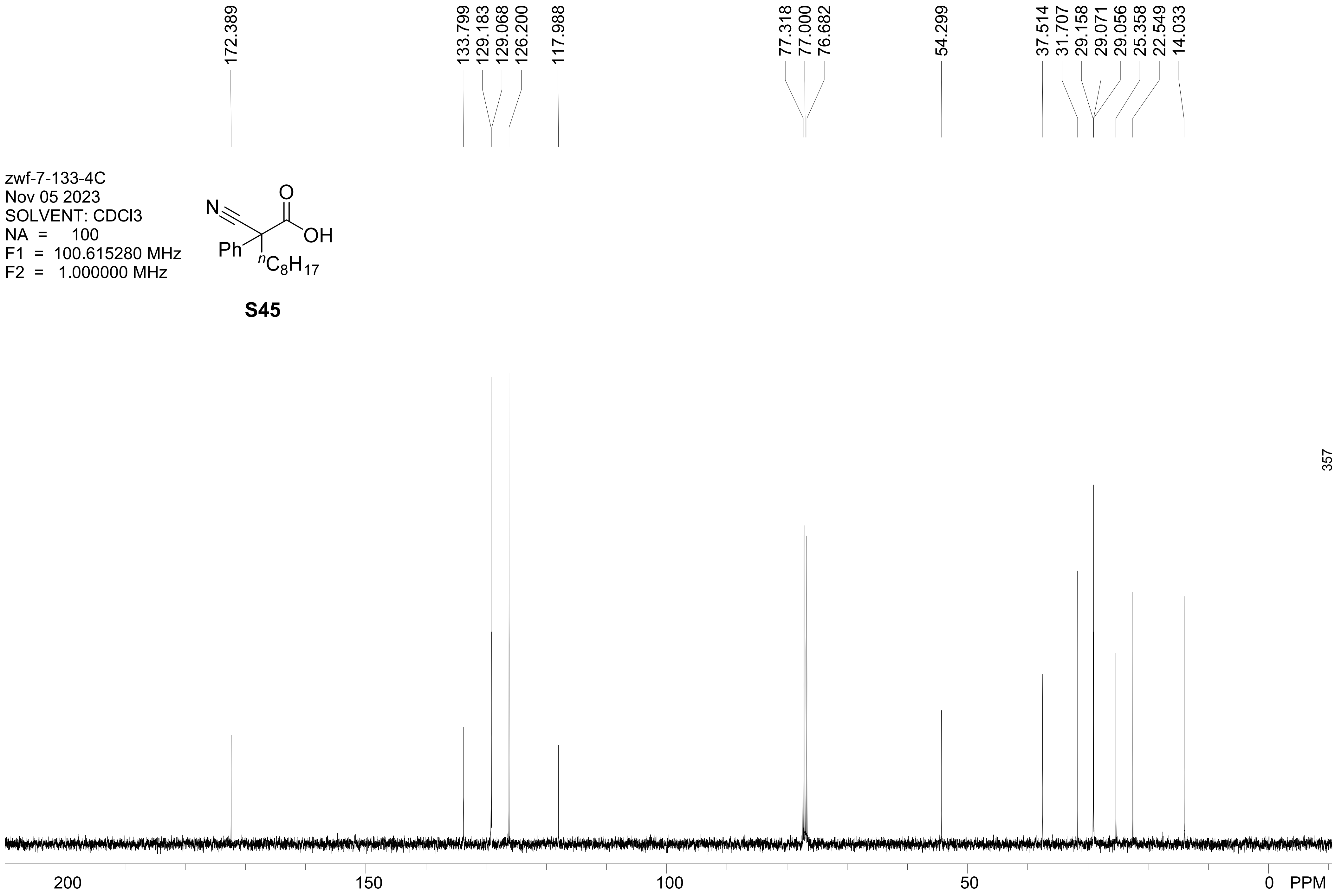
zwf-7-133-4C
Nov 05 2023
SOLVENT: CDCl3
NA = 100
F1 = 100.615280 MHz
F2 = 1.000000 MHz
172.389
133.799
129.183
129.068
126.200
117.988
77.318
77.000
76.682
54.299
37.514
31.707
29.158
29.071
29.056
25.358
22.549
14.033
200
150
100
50
0 PPM
N
O
OH
Ph
$^{n}C_8H_{17}$

S45

zwf-7-129-15H
Nov 02 2023
SOLVENT: CDCl3
NA = 4
F1 = 400.140015 MHz
F2 = 1.000000 MHz

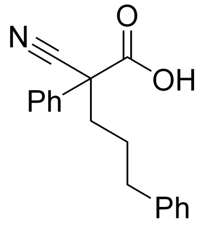

S46

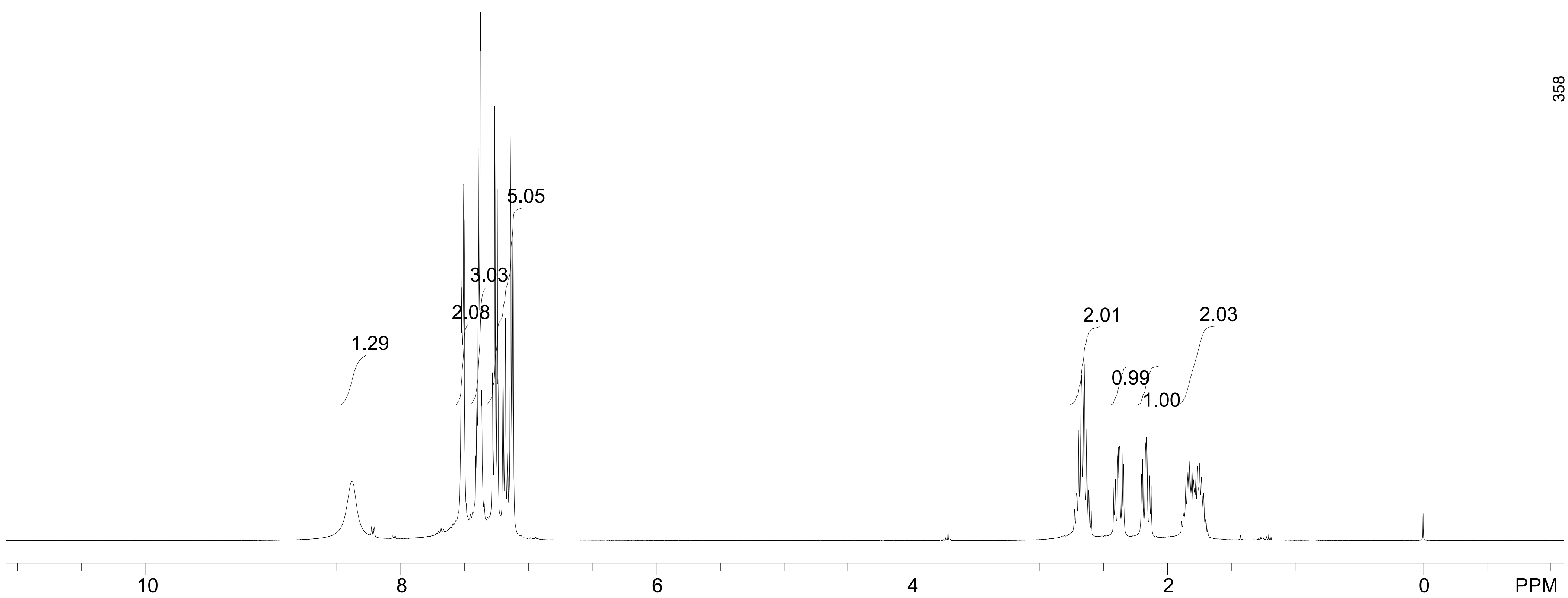

zwf-7-129-15C
Nov 02 2023
SOLVENT: CDCl3
NA = 100
F1 = 100.615280 MHz
F2 = 1.000000 MHz

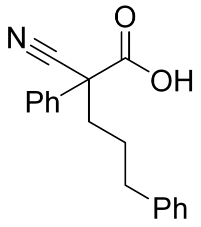

S46

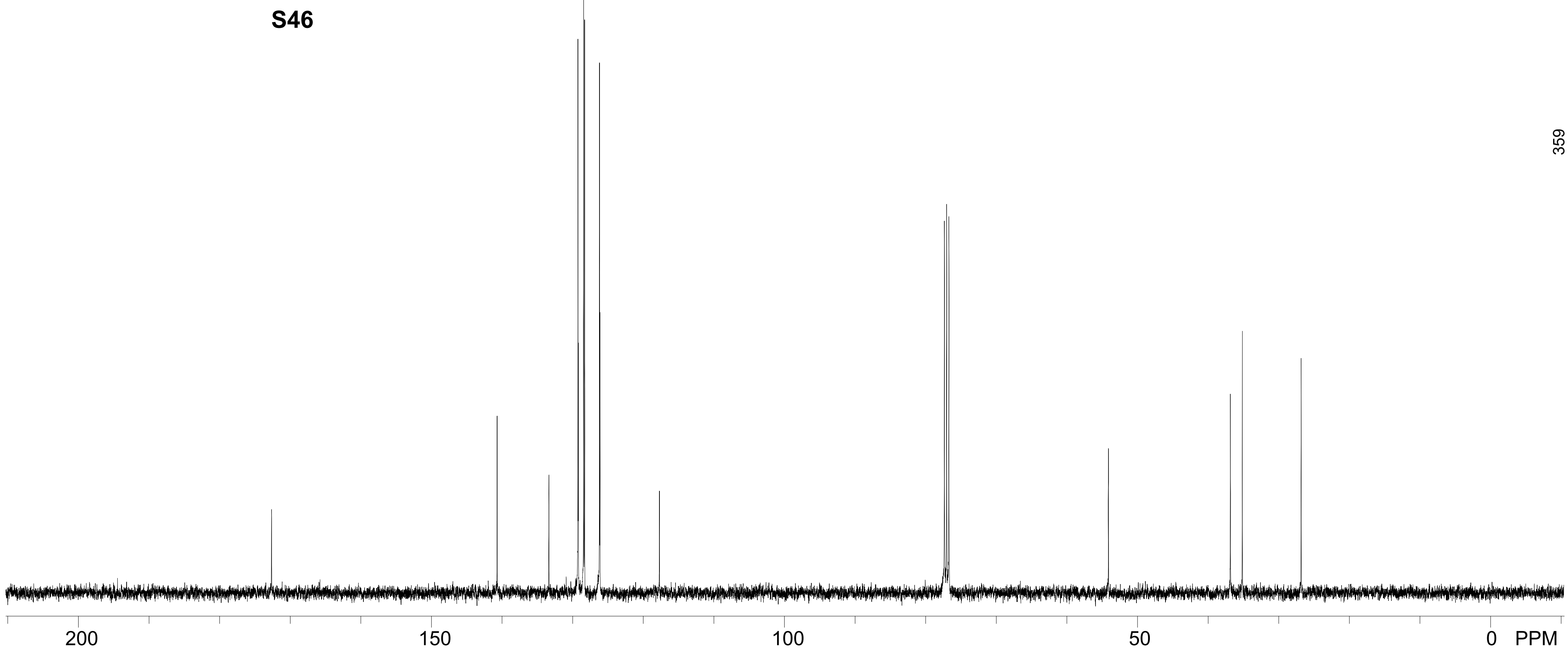

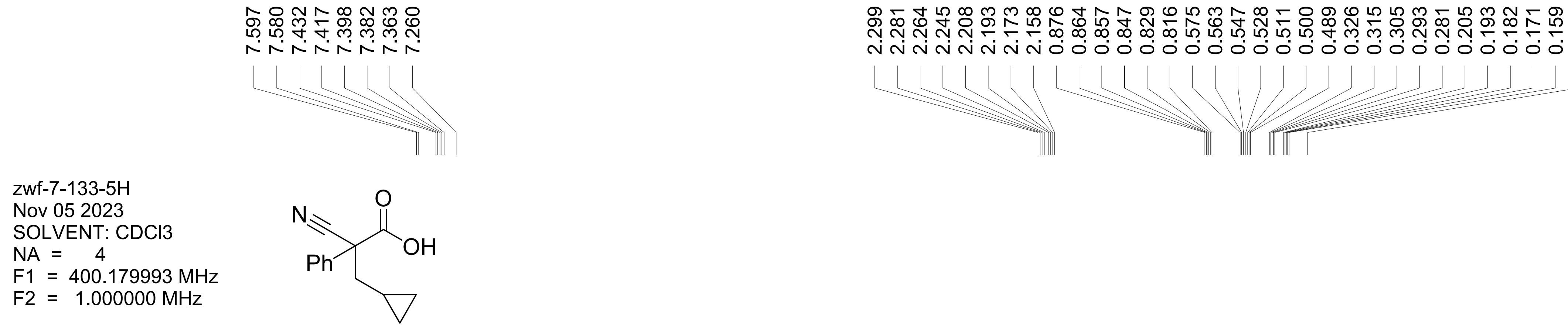
zwf-7-133-5H
Nov 05 2023
SOLVENT: CDCl3
NA = 4
F1 = 400.179993 MHz
F2 = 1.000000 MHz
N
O
OH
Ph
7.597
7.580
7.432
7.417
7.398
7.382
7.363
7.260
2.299
2.281
2.264
2.245
2.208
2.193
2.173
2.158
0.876
0.864
0.857
0.847
0.829
0.816
0.575
0.563
0.547
0.528
0.511
0.500
0.489
0.326
0.315
0.305
0.293
0.281
0.205
0.193
0.182
0.171
0.159
0.000
4.05
2.09
2.02
1.00
1.03
1.00
1.01
1.00
10
8
6
4
2
0
PPM

S47

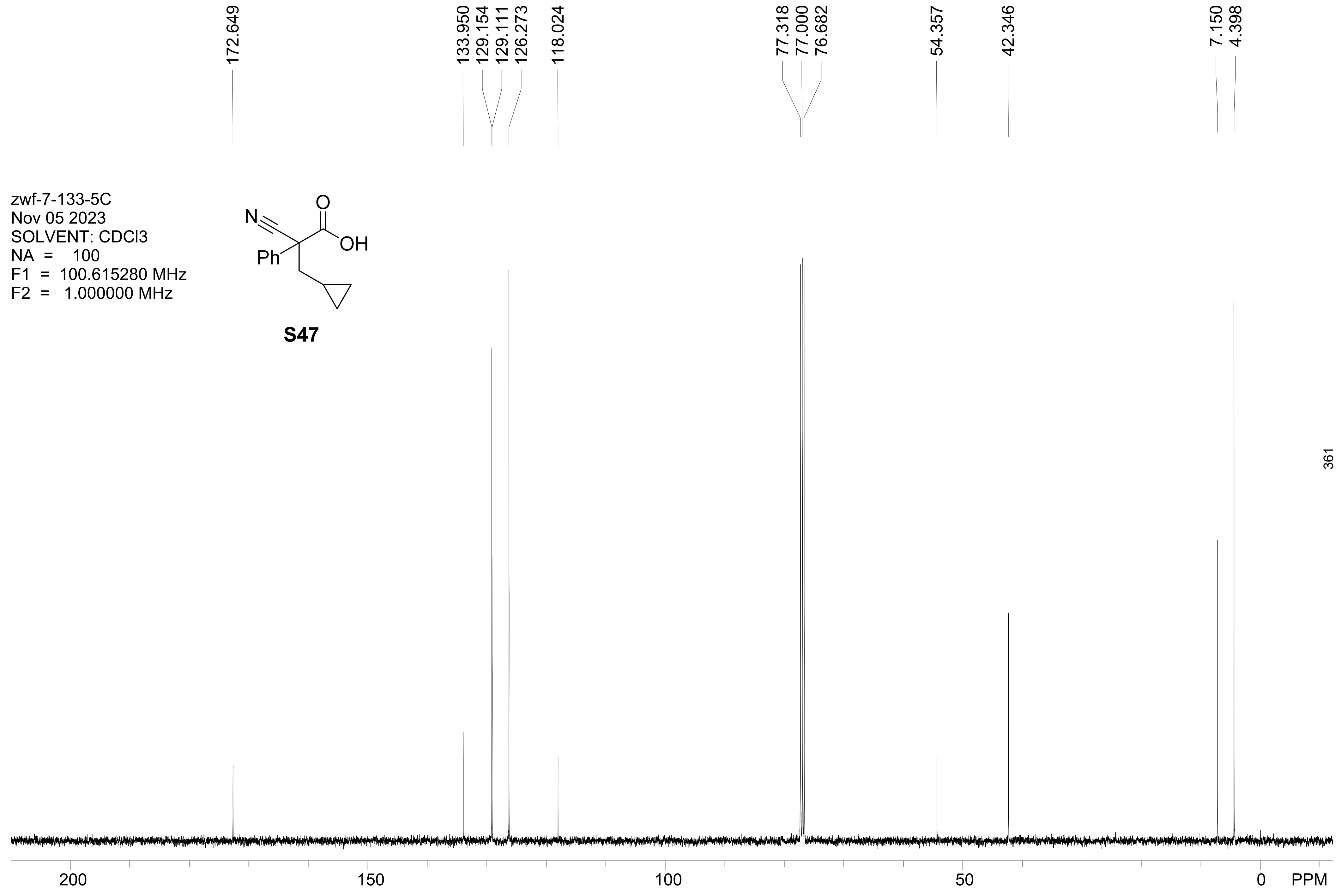
zwf-7-133-5C
Nov 05 2023
SOLVENT: CDCl3
NA = 100
F1 = 100.615280 MHz
F2 = 1.000000 MHz
N
O
OH
Ph
S47
172.649
133.950
129.154
129.111
126.273
118.024
77.318
77.000
76.682
54.357
42.346
7.150
4.398
200
150
100
50
0
PPM

zwf-7-086-2H
Sep 22 2023
SOLVENT: CDCl3
NA = 4
F1 = 400.140015 MHz
F2 = 1.000000 MHz

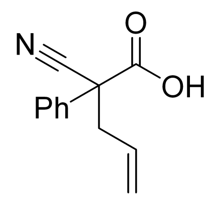

S48

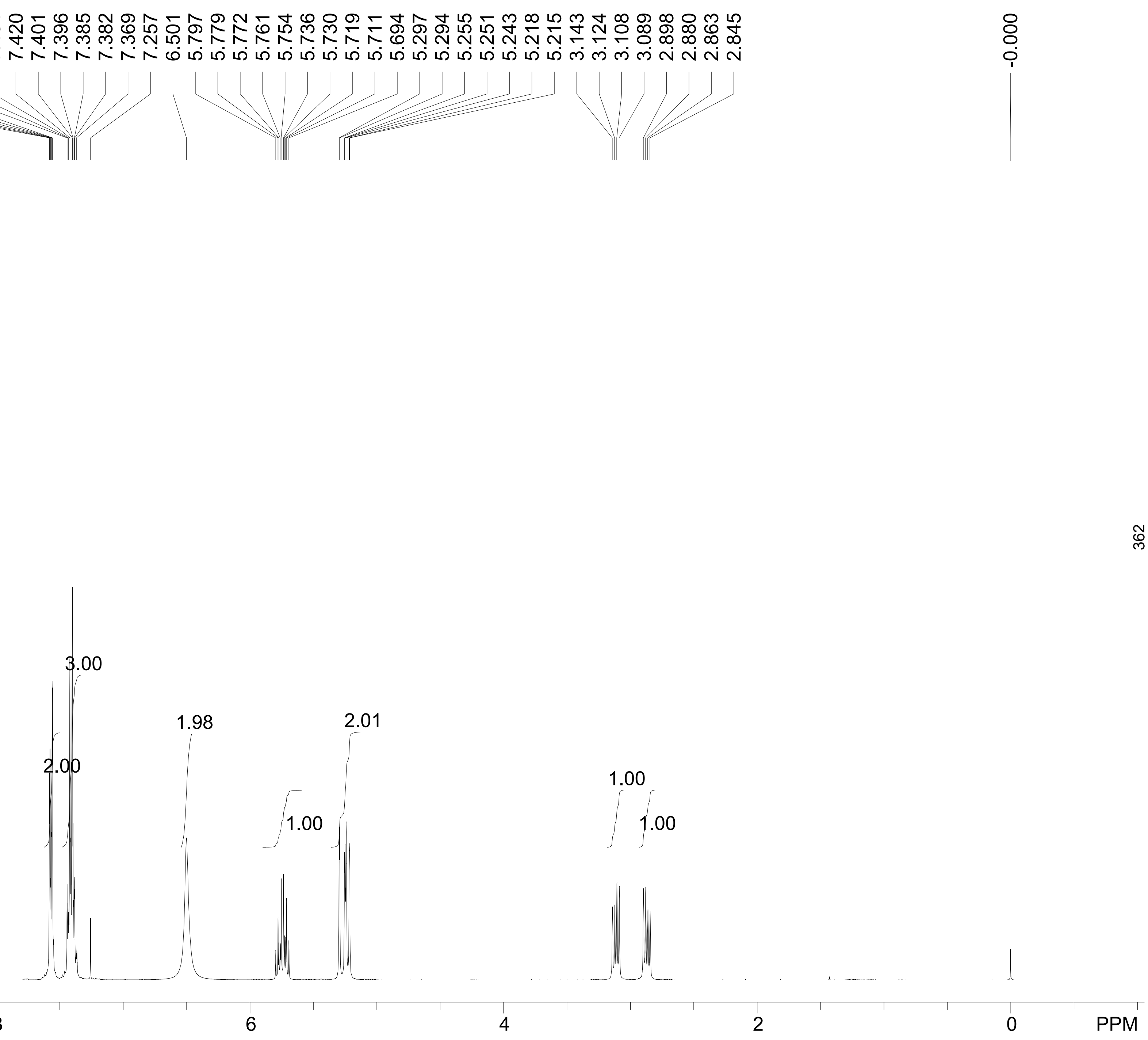

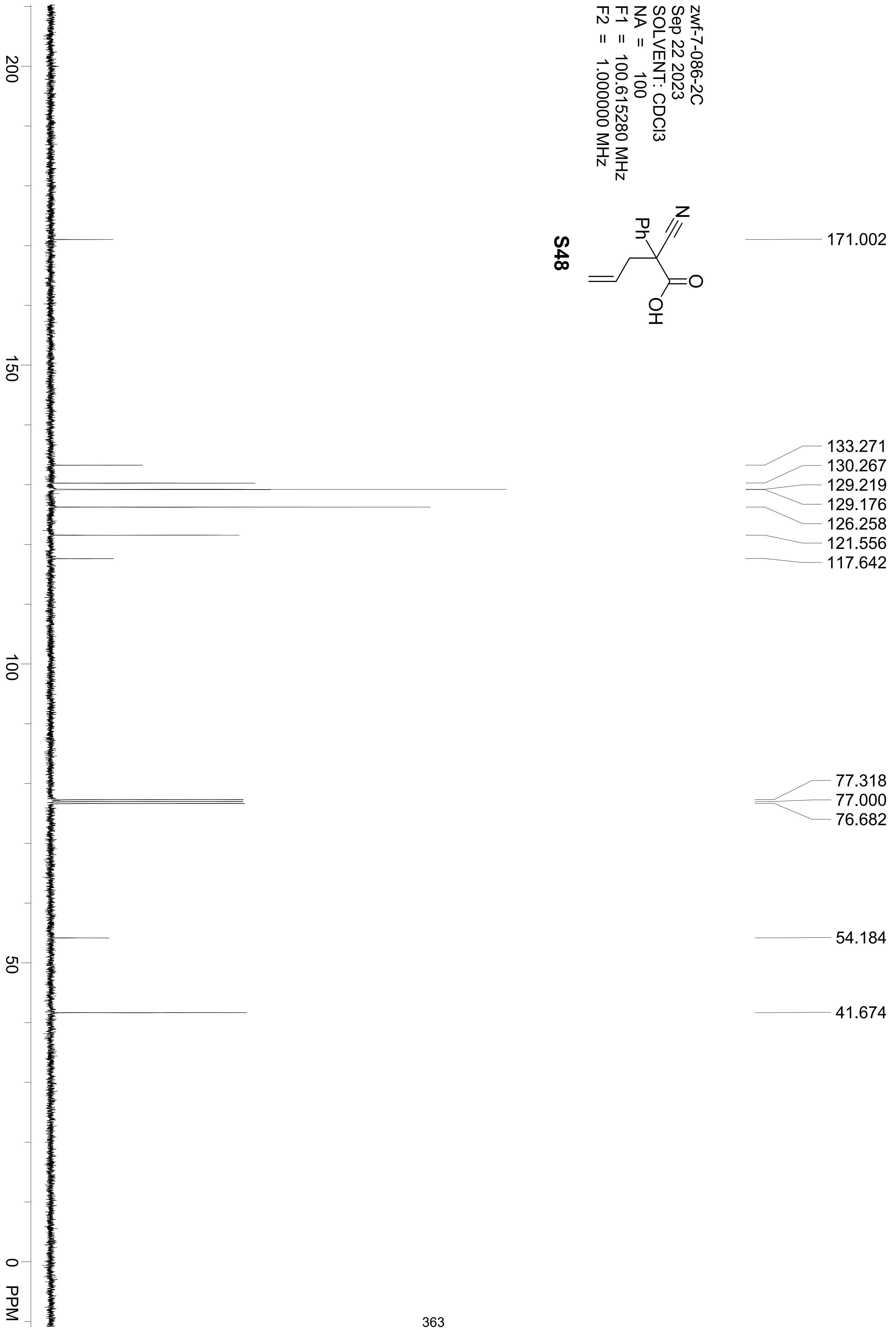
zwf-7-086-2C
Sep 22 2023
SOLVENT: CDCl3
NA = 100
F1 = 100.615280 MHz
F2 = 1.000000 MHz
N
Ph
O
OH
S48
171.002
133.271
130.267
129.219
129.176
126.258
121.556
117.642
77.318
77.000
76.682
54.184
41.674
200
150
100
50
0
PPM

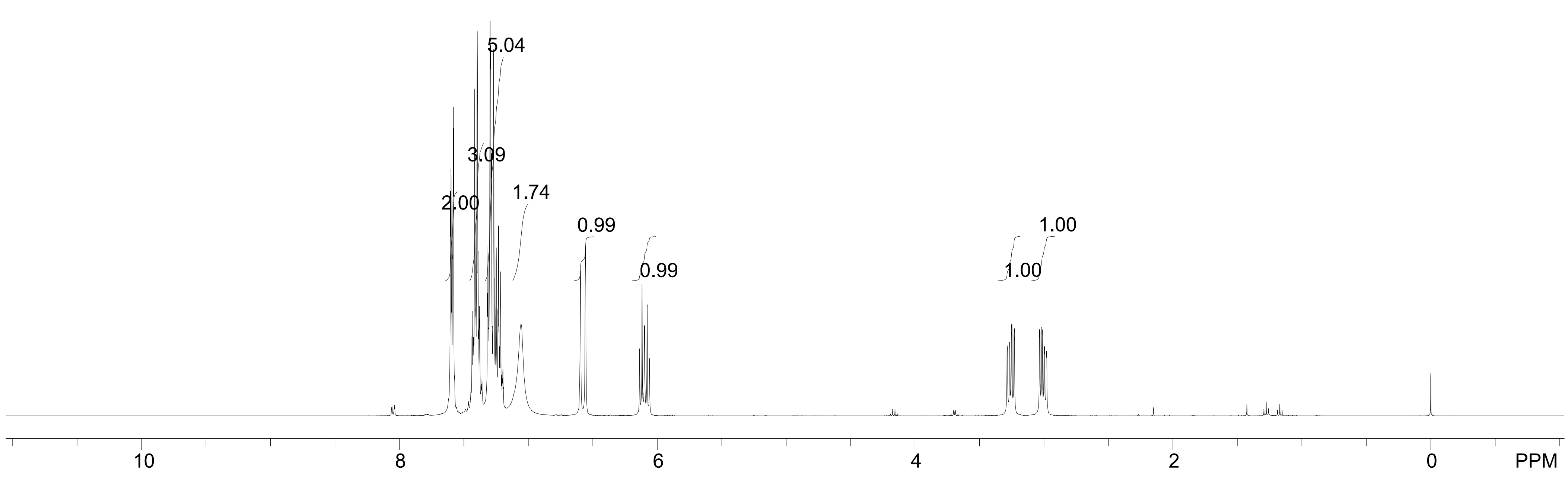
zwf-7-055-2H
Sep 11 2023
SOLVENT: CDCl3
NA = 4
F1 = 400.140015 MHz
F2 = 1.000000 MHz
7.604
7.599
7.583
7.580
7.437
7.430
7.425
7.415
7.411
7.396
7.379
7.376
7.318
7.313
7.296
7.293
7.285
7.268
7.249
7.231
7.214
7.197
7.056
6.596
6.557
6.137
6.118
6.100
6.079
6.060
3.288
3.286
3.269
3.267
3.253
3.251
3.234
3.232
3.036
3.033
3.018
3.016
3.001
2.998
2.983
2.981
-0.000
5.04
3.09
2.00
1.74
0.99
0.99
1.00
1.00
10
8
6
4
2
0
PPM

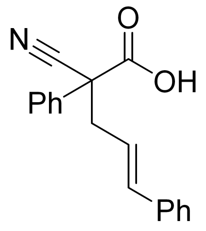
N
O
OH
Ph
Ph

S49

zwf-7-055-2C
Sep 11 2023
SOLVENT: CDCl3
NA = 100
F1 = 100.615280 MHz
F2 = 1.000000 MHz

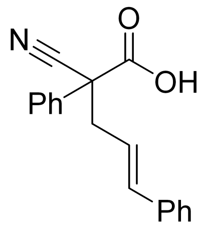

S49

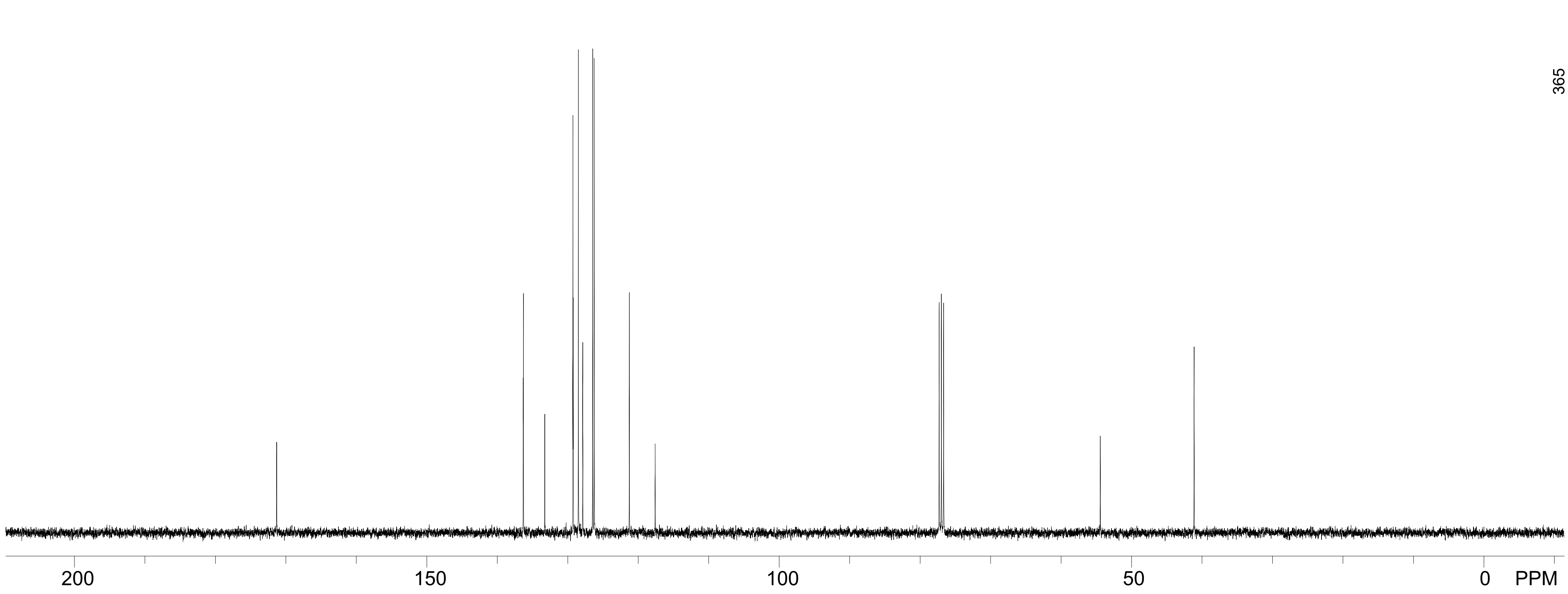

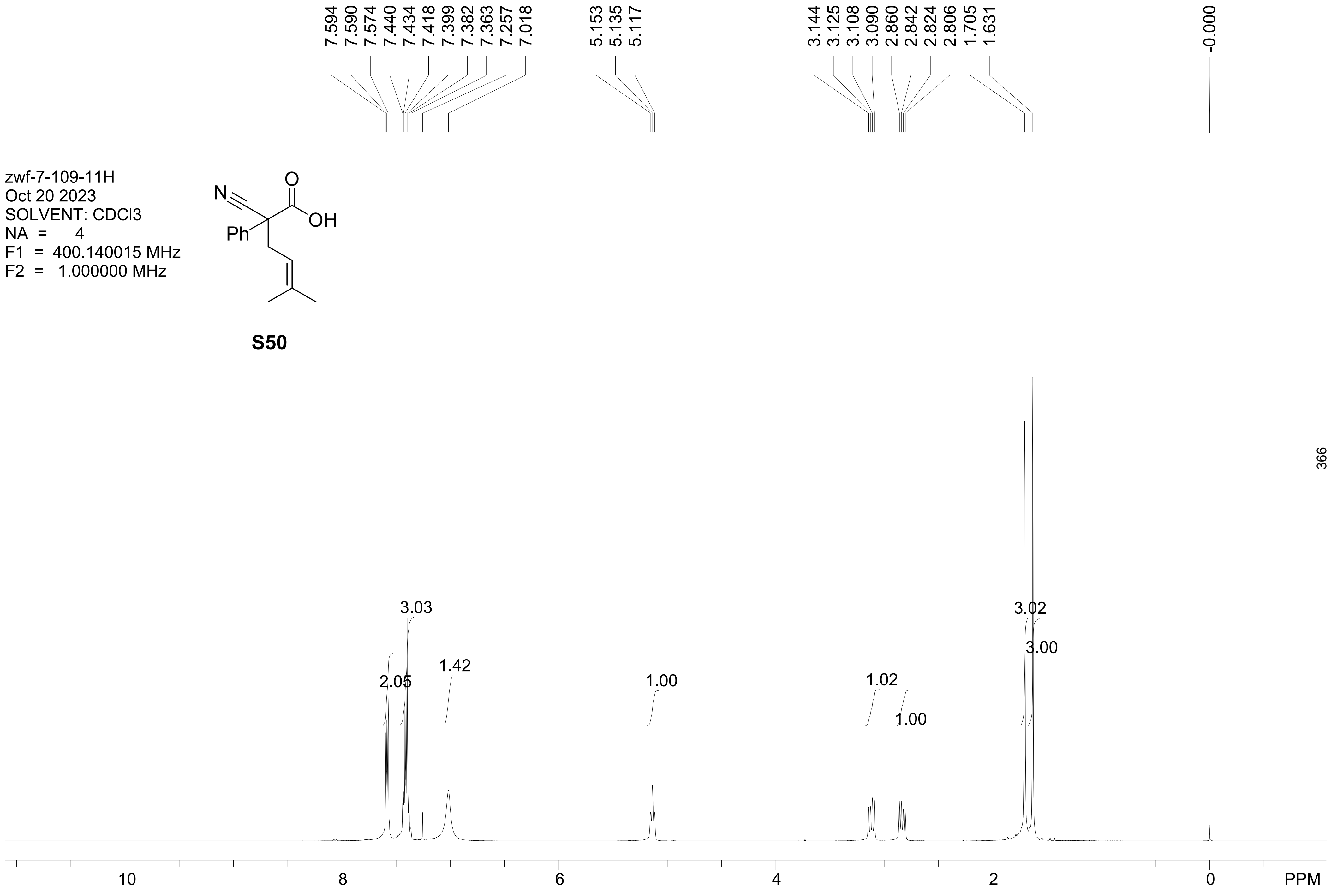
zwf-7-109-11H
Oct 20 2023
SOLVENT: CDCl3
NA = 4
F1 = 400.140015 MHz
F2 = 1.000000 MHz
N
O
OH
Ph
S50
7.594
7.590
7.574
7.440
7.434
7.418
7.399
7.382
7.363
7.257
7.018
5.153
5.135
5.117
3.144
3.125
3.108
3.090
2.860
2.842
2.824
2.806
1.705
1.631
-0.000
3.03
2.05
1.42
1.00
1.02
1.00
3.02
3.00
10
8
6
4
2
0
PPM

zwf-7-109-11C
Oct 20 2023
SOLVENT: CDCl3
NA = 100
F1 = 100.615280 MHz
F2 = 1.000000 MHz

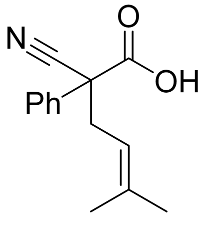

S50

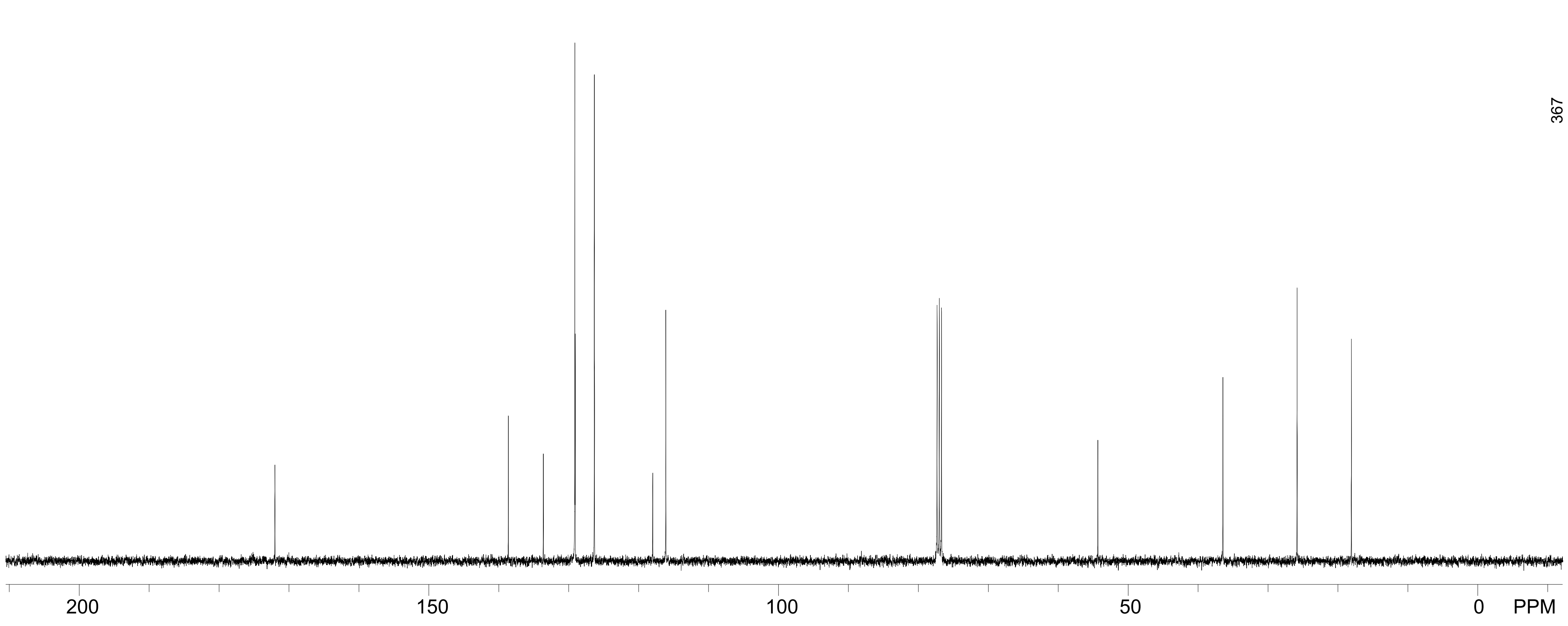

zwf-7-086-3H
Sep 23 2023
SOLVENT: CDCl3
NA = 4
F1 = 400.140015 MHz
F2 = 1.000000 MHz

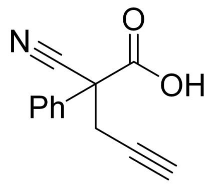

S51

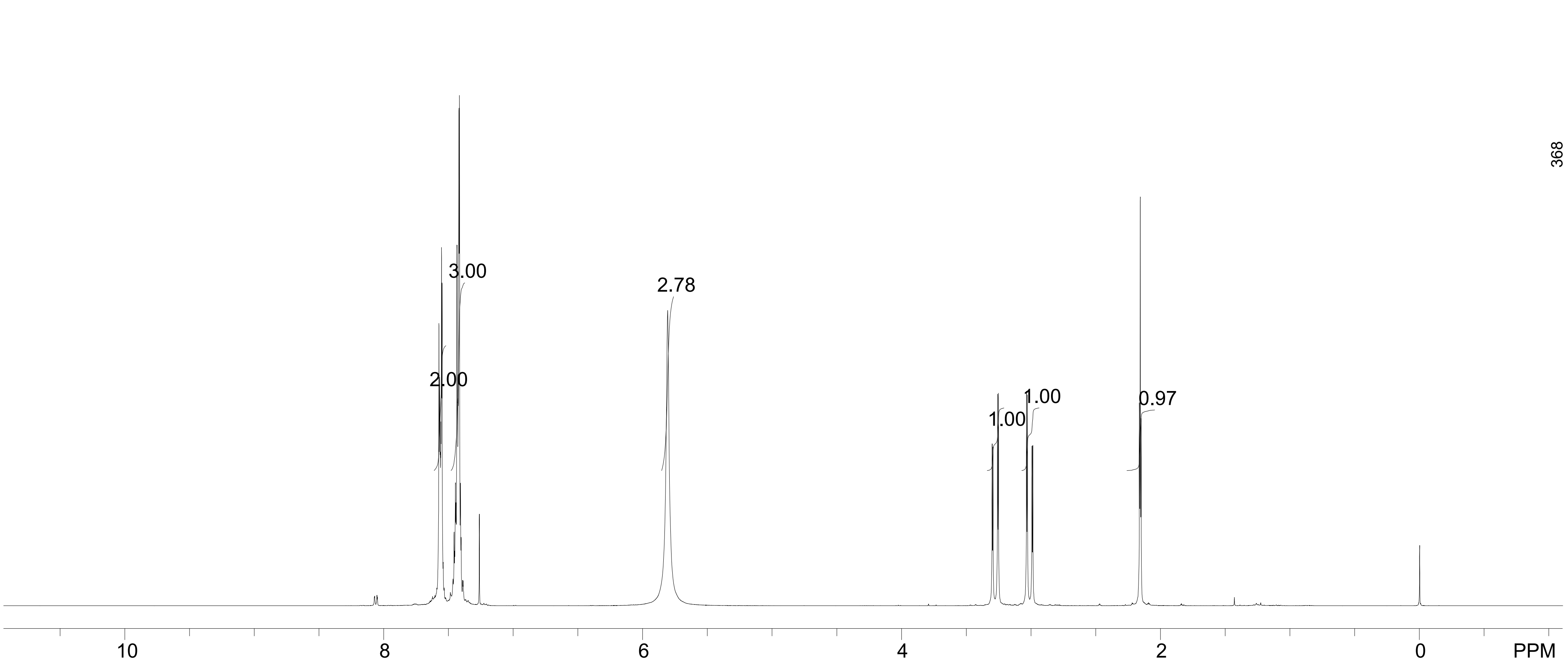

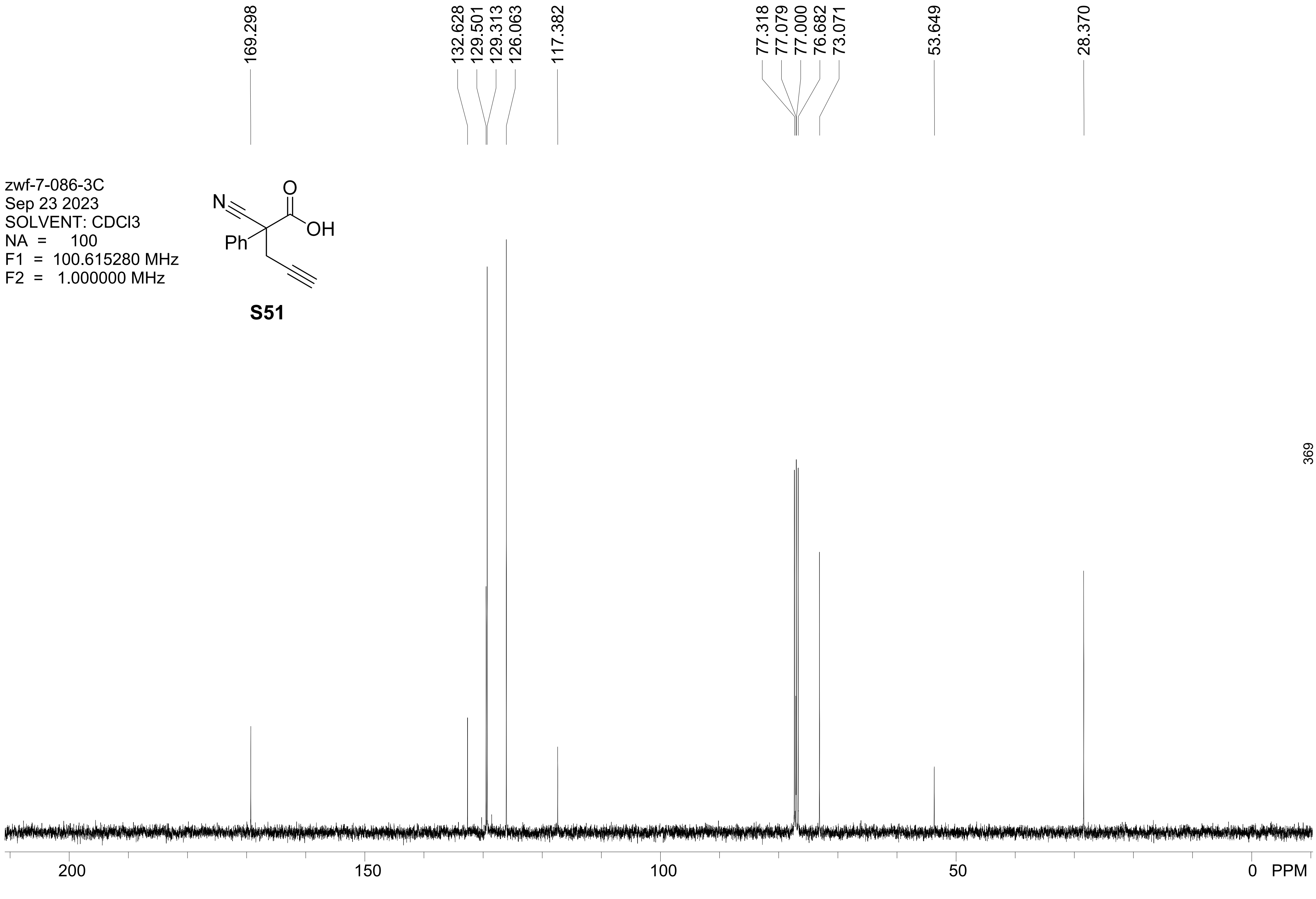
zwf-7-086-3C
Sep 23 2023
SOLVENT: CDCl3
NA = 100
F1 = 100.615280 MHz
F2 = 1.000000 MHz
N
O
OH
Ph
S51
169.298
132.628
129.501
129.313
126.063
117.382
77.318
77.079
77.000
76.682
73.071
53.649
28.370
200
150
100
50
0 PPM

zwf-9-010-4H
Mar 08 2024
SOLVENT: CDCl3
NA = 4
F1 = 400.140015 MHz
F2 = 1.000000 MHz

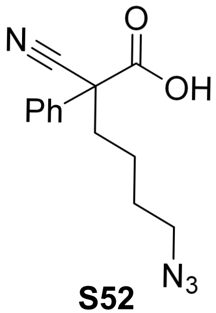

S52

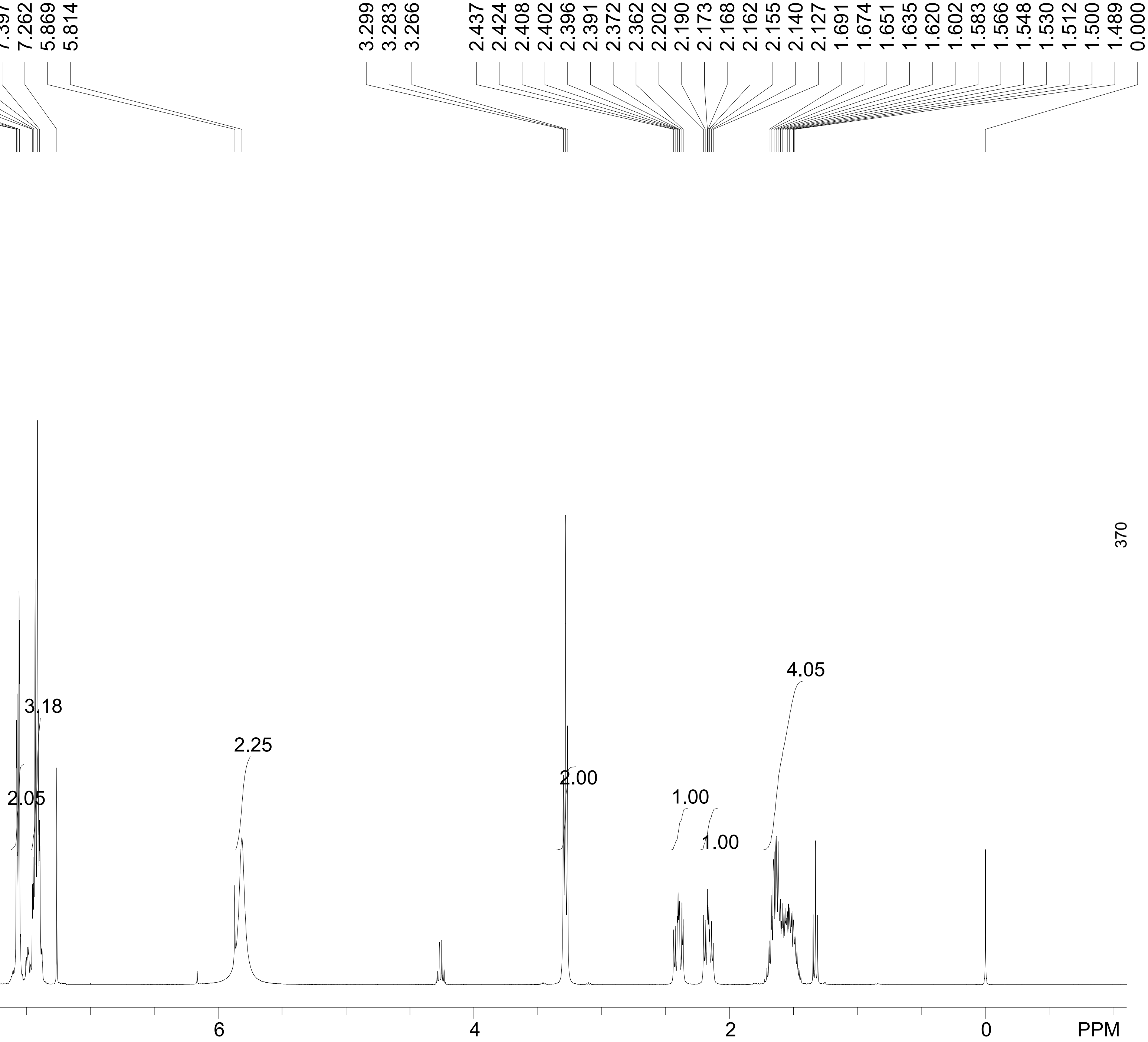

zwf-9-010-4C
Mar 08 2024
SOLVENT: CDCl3
NA = 100
F1 = 100.615280 MHz
F2 = 1.000000 MHz

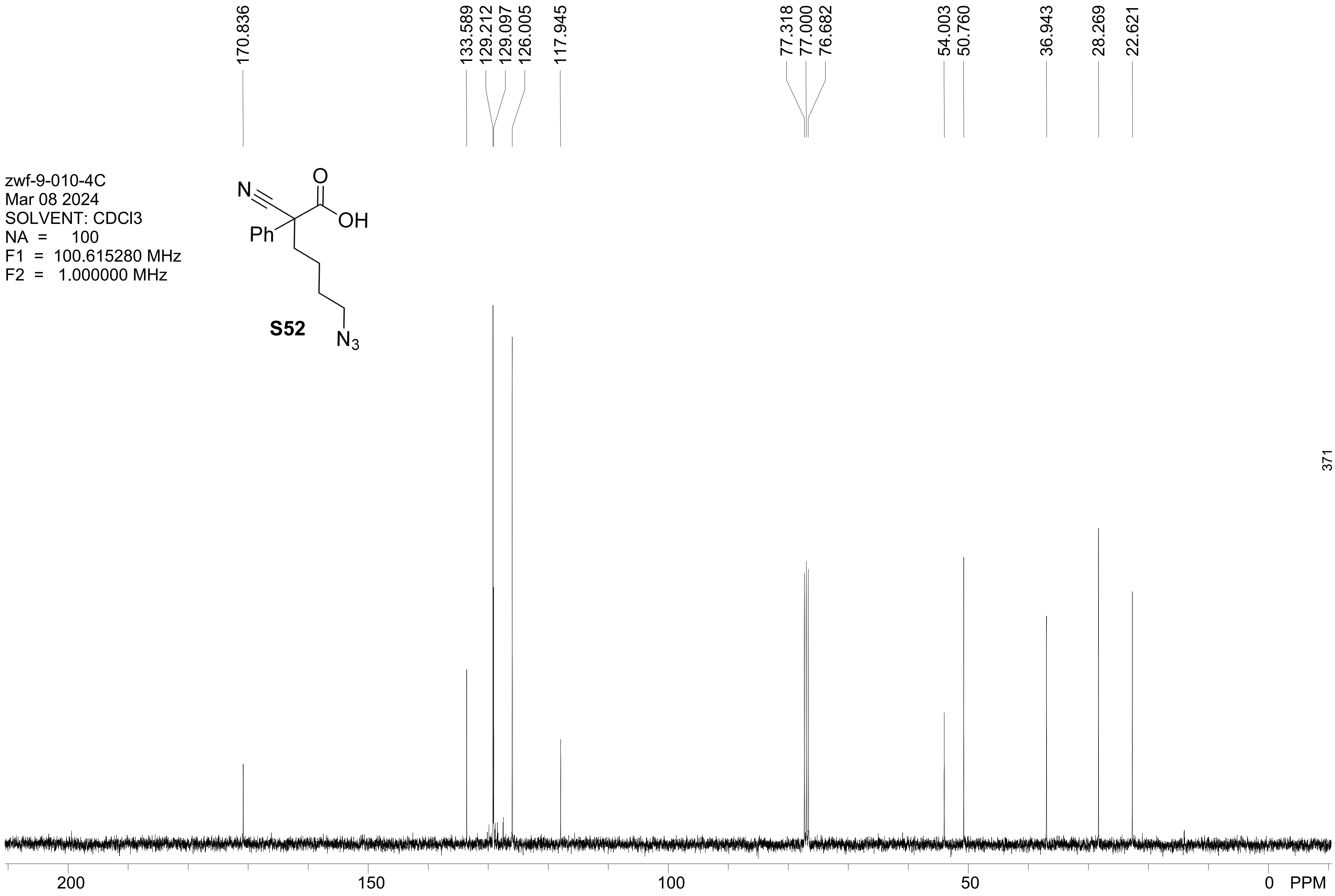

zwf-7-133-2H
Nov 03 2023
SOLVENT: CDCl3
NA = 4
F1 = 500.130005 MHz
F2 = 1.000000 MHz

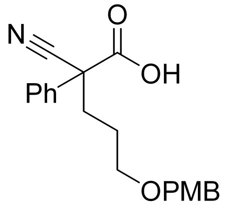

S54

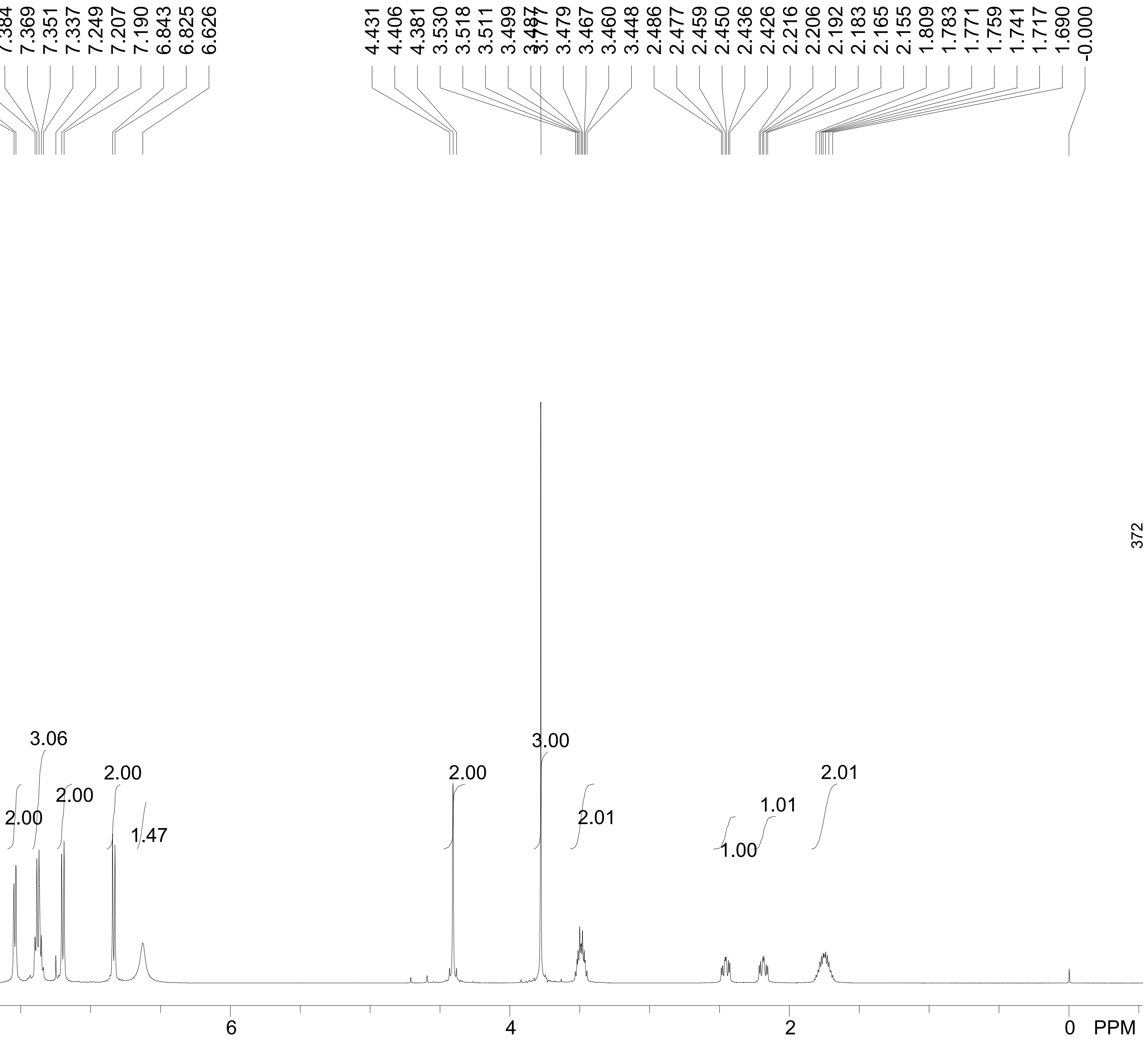

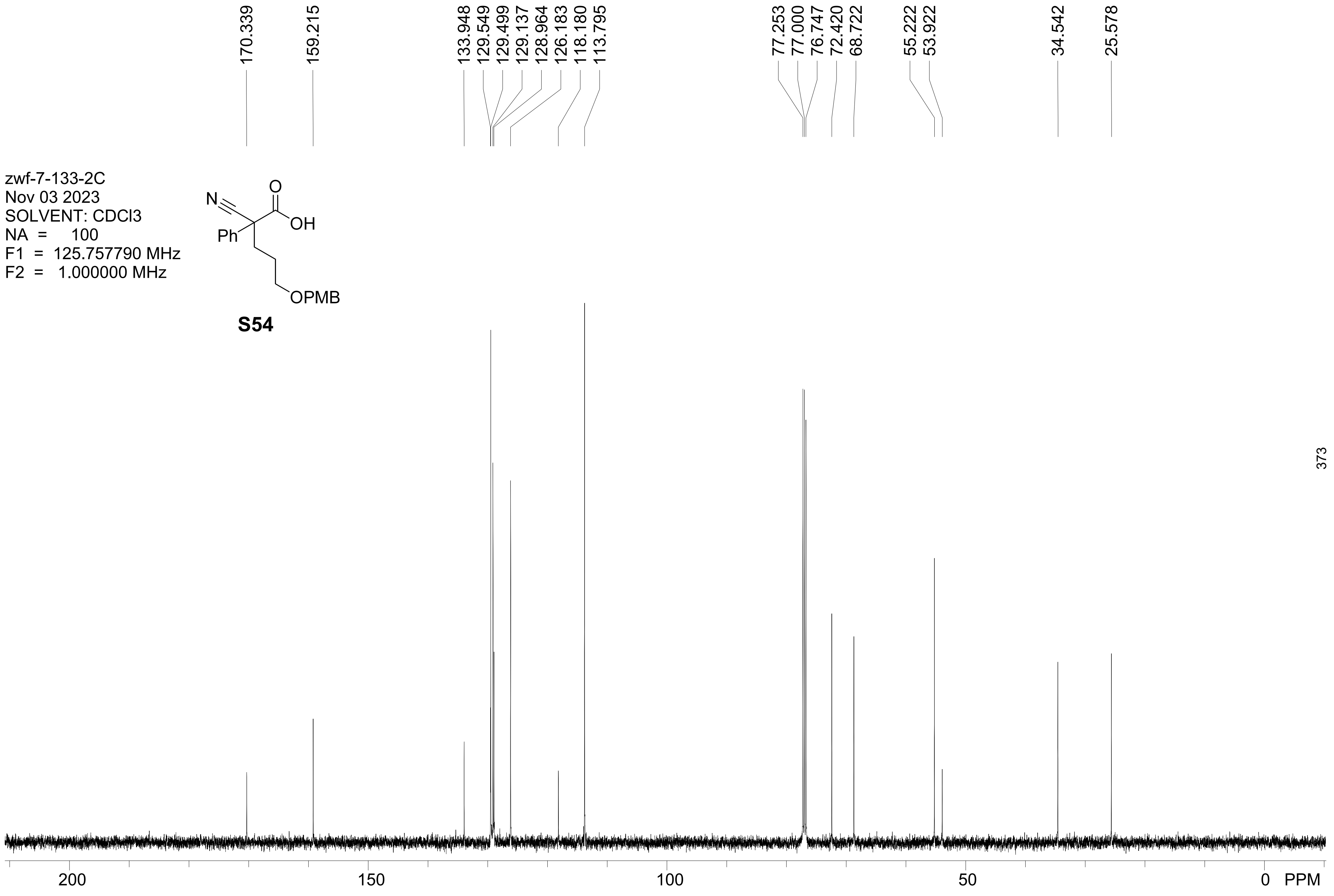
zwf-7-133-2C
Nov 03 2023
SOLVENT: CDCl3
NA = 100
F1 = 125.757790 MHz
F2 = 1.000000 MHz
N
O
OH
Ph
OPMB
S54
170.339
159.215
133.948
129.549
129.499
129.137
128.964
126.183
118.180
113.795
77.253
77.000
76.747
72.420
68.722
55.222
53.922
34.542
25.578
200
150
100
50
0 PPM

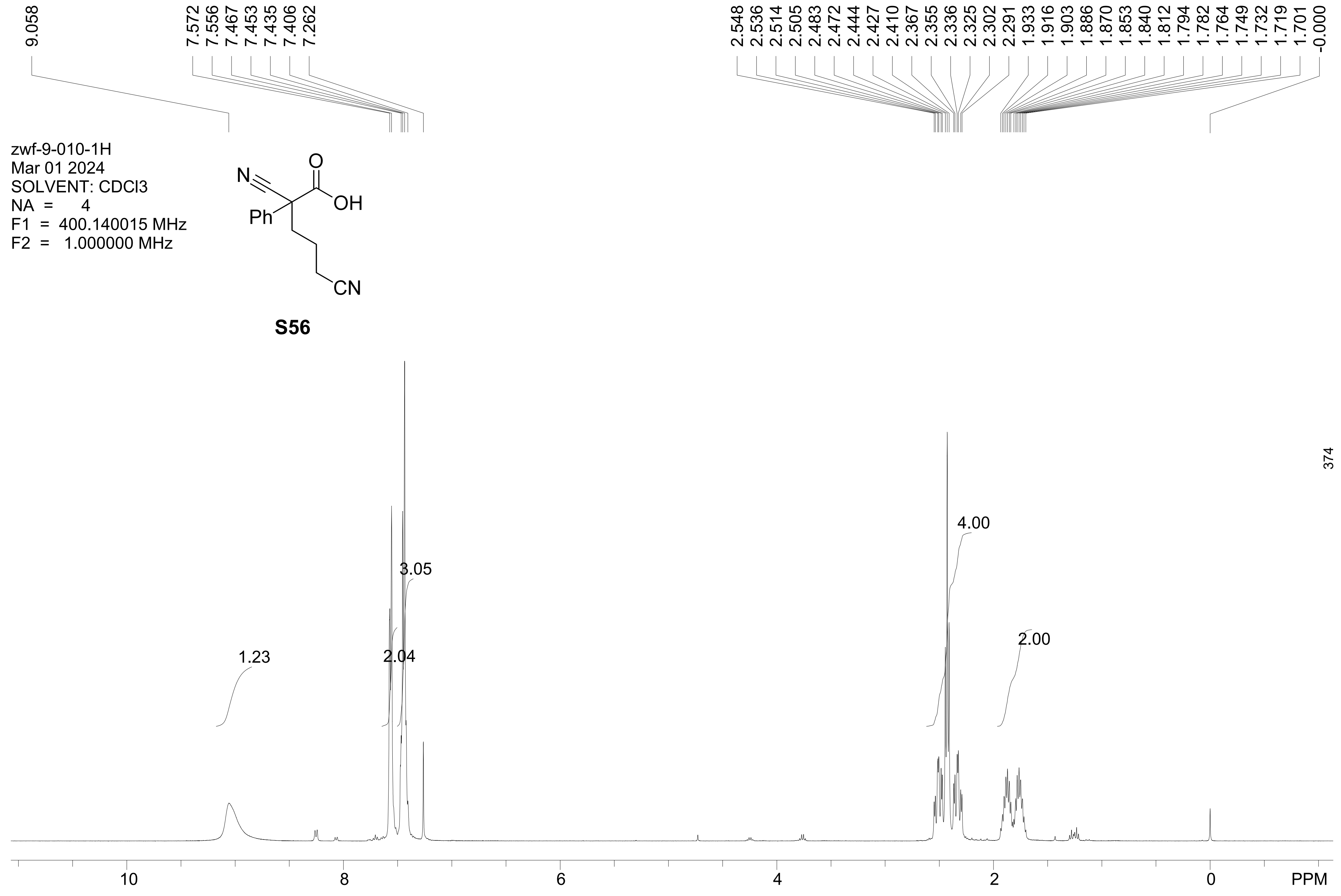
9.058
7.572
7.556
7.467
7.453
7.435
7.406
7.262
2.548
2.536
2.514
2.505
2.483
2.472
2.444
2.427
2.410
2.367
2.355
2.336
2.325
2.302
2.291
1.933
1.916
1.903
1.886
1.870
1.853
1.840
1.812
1.794
1.782
1.764
1.749
1.732
1.719
1.701
-0.000
zwf-9-010-1H
Mar 01 2024
SOLVENT: CDCl3
NA = 4
F1 = 400.140015 MHz
F2 = 1.000000 MHz
N
O
OH
Ph
CN
S56
1.23
2.04
3.05
4.00
2.00
10
8
6
4
2
0
PPM

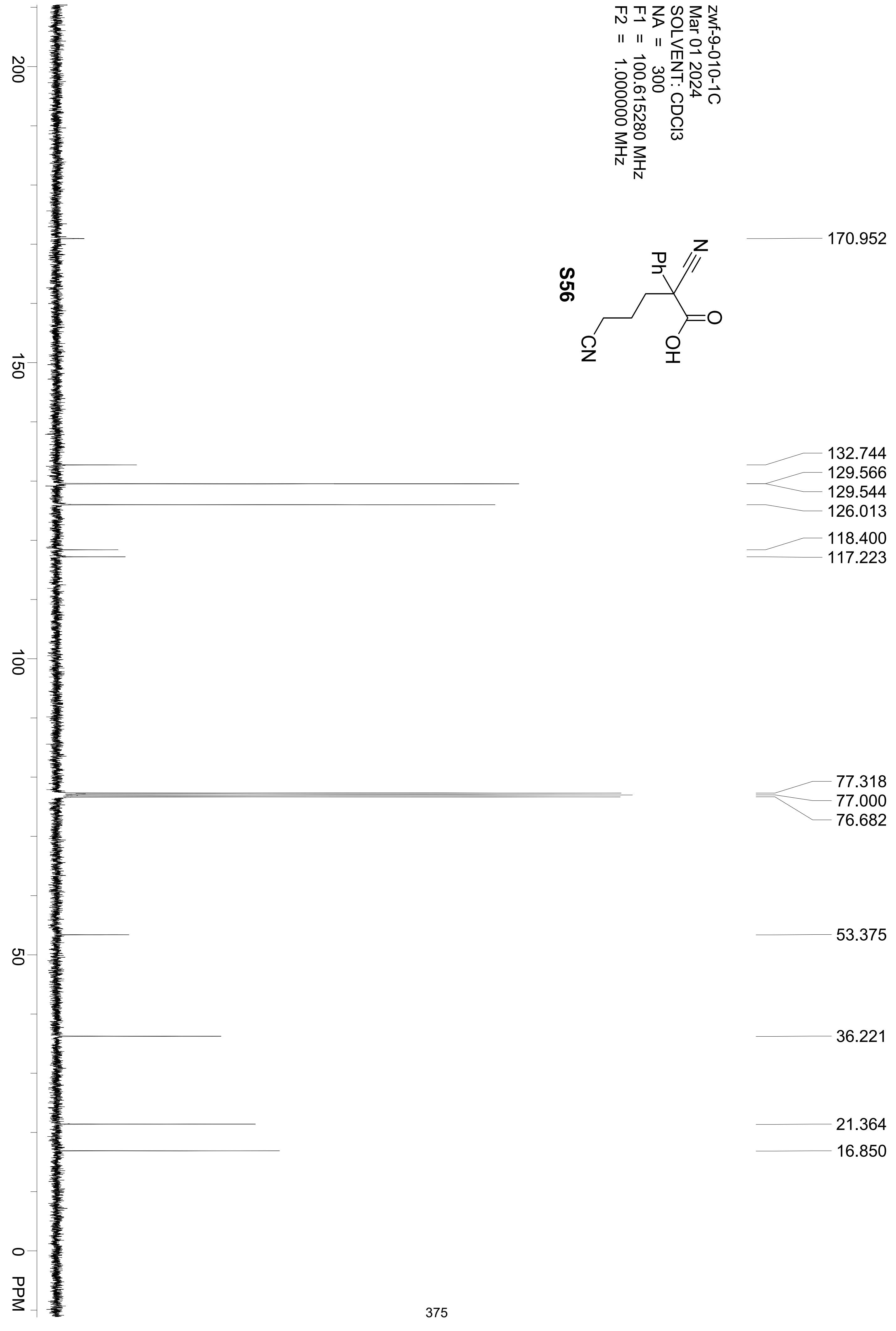
zwf-9-010-1C
Mar 01 2024
SOLVENT: CDCl3
NA = 300
F1 = 100.615280 MHz
F2 = 1.000000 MHz
S56
Ph
N
O
OH
CN
170.952
132.744
129.566
129.544
126.013
118.400
117.223
77.318
77.000
76.682
53.375
36.221
21.364
16.850
200
150
100
50
0
PPM

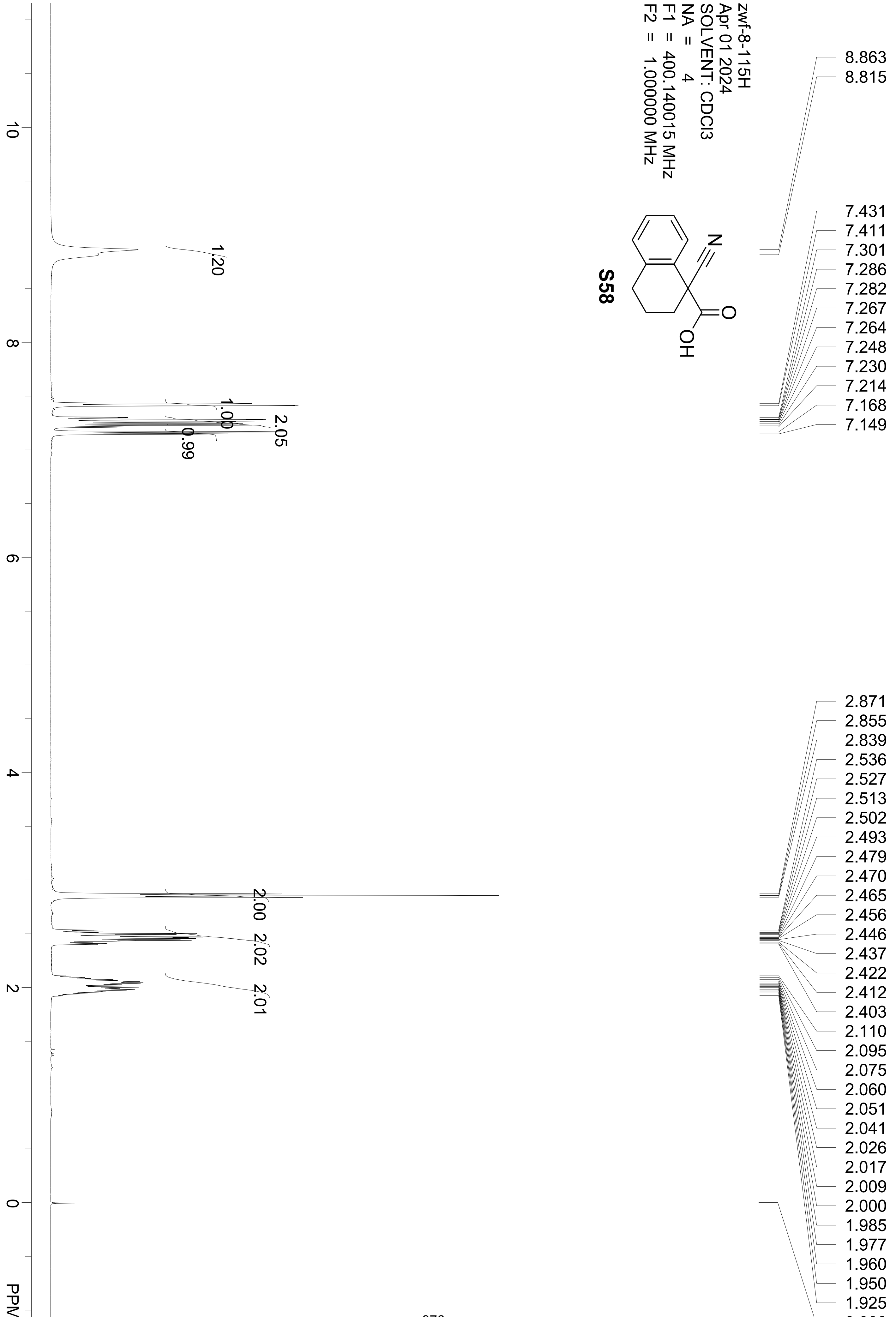
zwf-8-115H
Apr 01 2024
SOLVENT: CDCl3
NA = 4
F1 = 400.140015 MHz
F2 = 1.000000 MHz
N
O
OH
S58
8.863
8.815
7.431
7.411
7.301
7.286
7.282
7.267
7.264
7.248
7.230
7.214
7.168
7.149
2.871
2.855
2.839
2.536
2.527
2.513
2.502
2.493
2.479
2.470
2.465
2.456
2.446
2.437
2.422
2.412
2.403
2.110
2.095
2.075
2.060
2.051
2.041
2.026
2.017
2.009
2.000
1.985
1.977
1.960
1.950
1.925
-0.000
1.20
1.00
2.05
0.99
2.00
2.02
2.01
10
8
6
4
2
0
PPM

zwf-8-115C
Apr 01 2024
SOLVENT: CDCl3
NA = 200
F1 = 100.615280 MHz
F2 = 1.000000 MHz

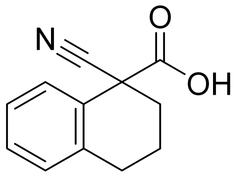

S58

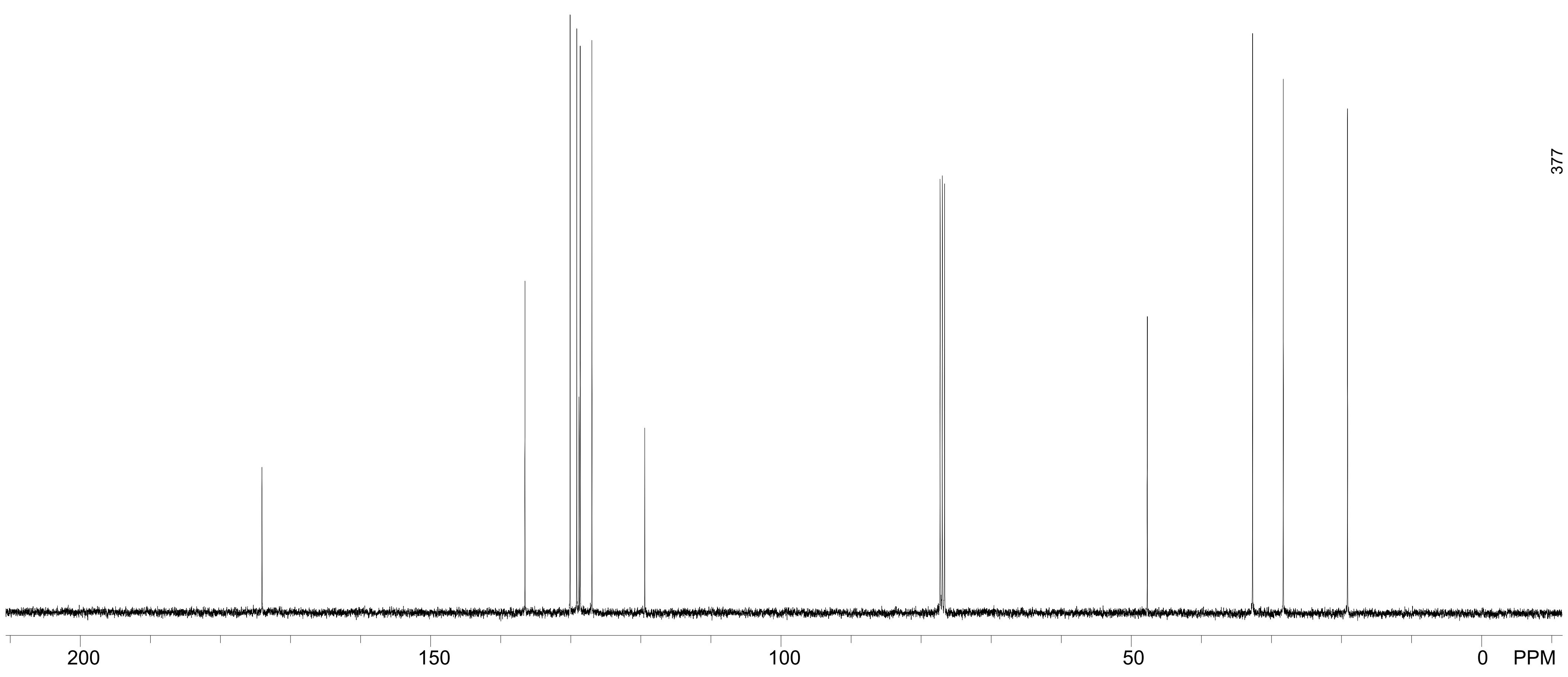

zwf-7-086-12H
Oct 06 2023
SOLVENT: CDCl3
NA = 4
F1 = 400.140015 MHz
F2 = 1.000000 MHz

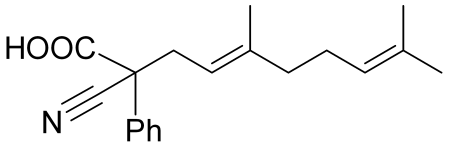

S60

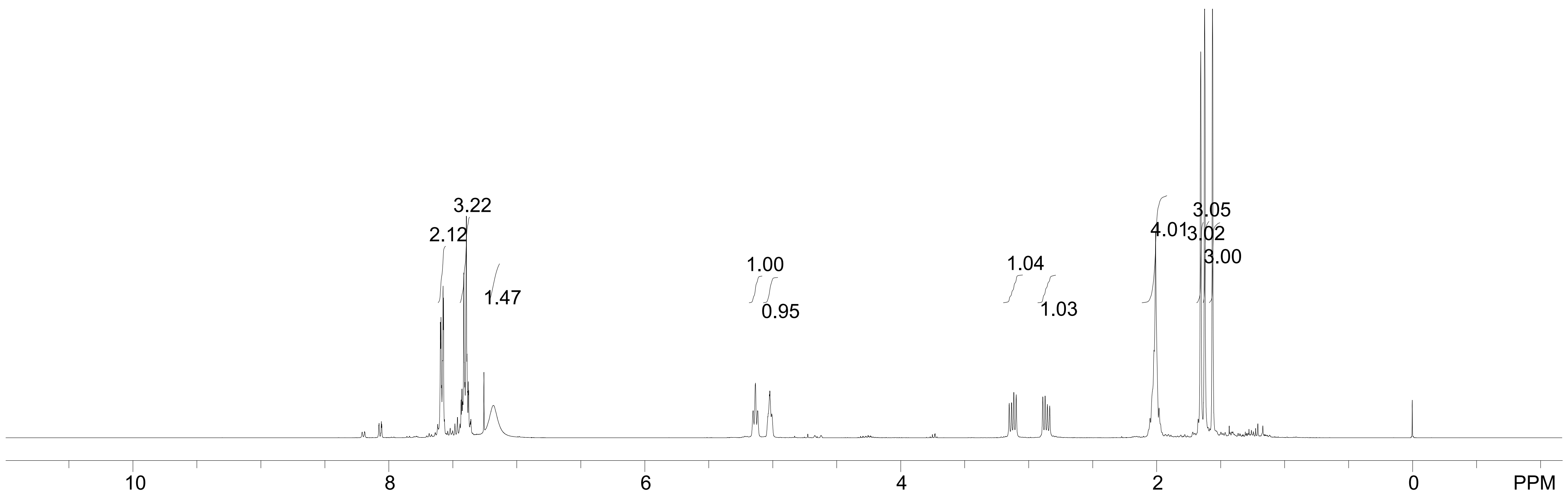

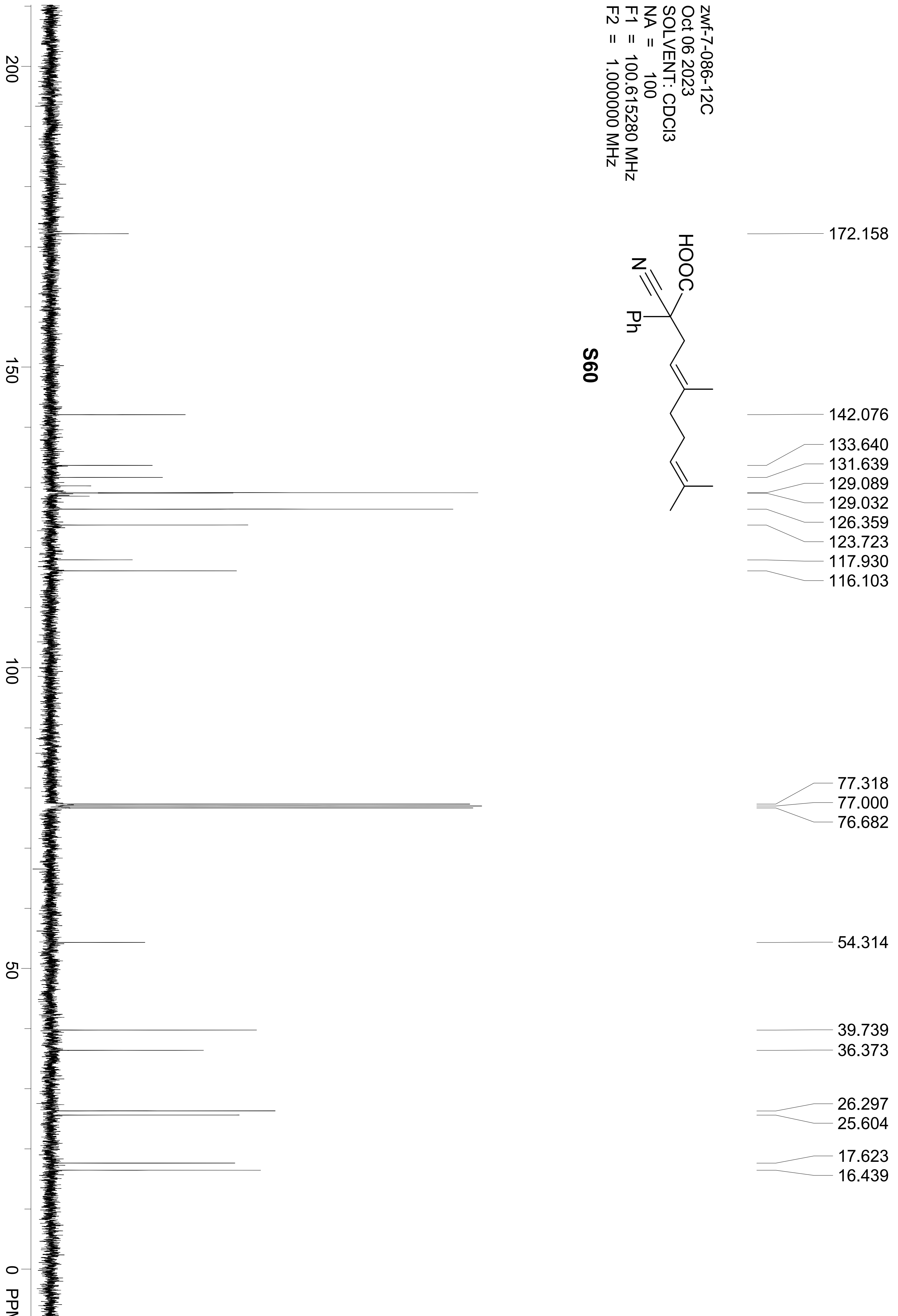
zwf-7-086-12C
Oct 06 2023
SOLVENT: CDCl3
NA = 100
F1 = 100.615280 MHz
F2 = 1.000000 MHz
HOOC
N
Ph
S60
172.158
142.076
133.640
131.639
129.089
129.032
126.359
123.723
117.930
116.103
77.318
77.000
76.682
54.314
39.739
36.373
26.297
25.604
17.623
16.439
200
150
100
50
0 PPM

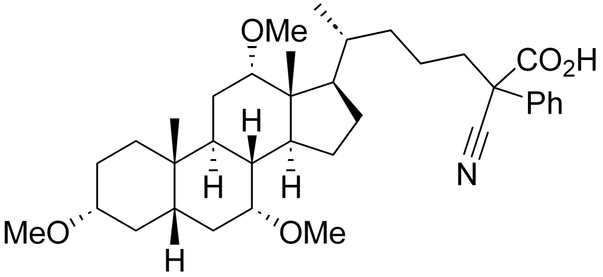
OMe
CO2H
Ph
N
H
H
H
MeO
OMe
H

S61

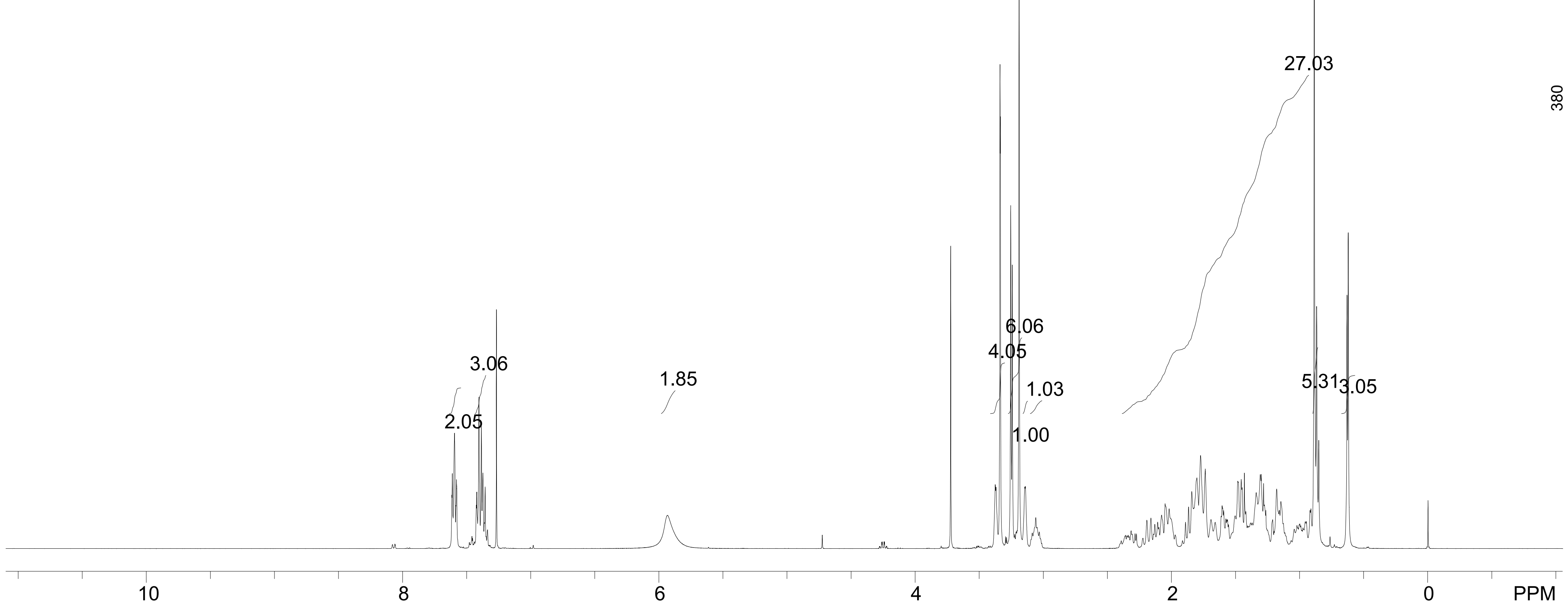
zwf-9-010-5H
Mar 22 2024
SOLVENT: CDCl3
NA = 4
F1 = 400.140015 MHz
F2 = 1.000000 MHz
7.615
7.611
7.594
7.579
7.421
7.404
7.385
7.372
7.355
7.337
7.267
5.931
3.374
3.367
3.337
3.334
3.253
3.721
3.241
3.188
3.144
3.138
3.085
3.057
3.047
3.030
2.314
2.192
2.050
1.842
1.773
1.605
1.483
1.432
1.308
1.180
1.000
0.913
0.887
0.868
0.852
0.632
0.622
0.000
3.06
2.05
1.85
6.06
4.05
1.03
1.00
27.03
5.31
3.05
10
8
6
4
2
0
PPM

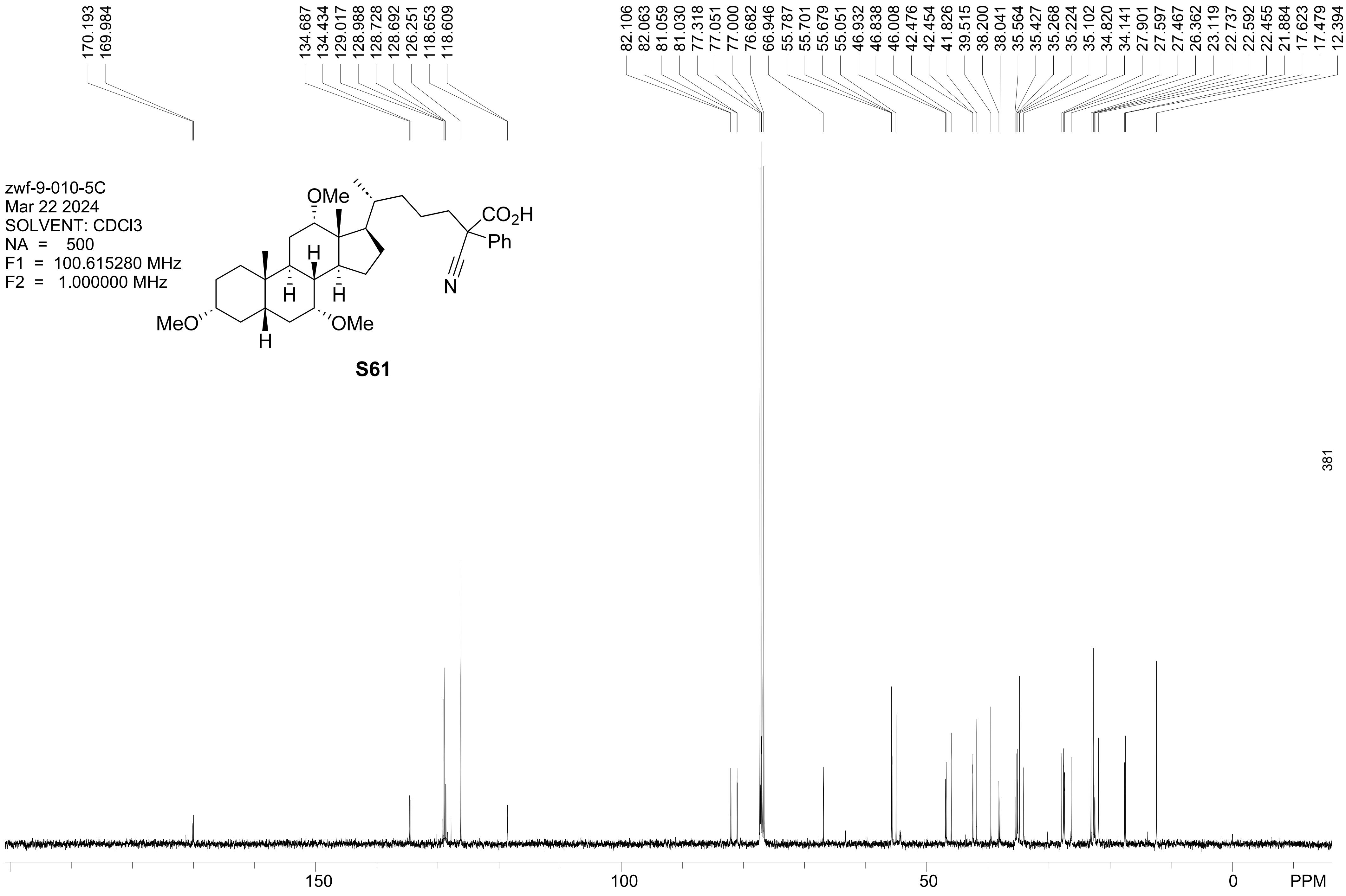
zwf-9-010-5C
Mar 22 2024
SOLVENT: CDCl3
NA = 500
F1 = 100.615280 MHz
F2 = 1.000000 MHz
OMe
MeO
OMe
CO_2H
Ph
N
H
S61
170.193
169.984
134.687
134.434
129.017
128.988
128.728
128.692
126.251
118.653
118.609
82.106
82.063
81.059
81.030
77.318
77.051
77.000
76.682
66.946
55.787
55.701
55.679
55.051
46.932
46.838
46.008
42.476
42.454
41.826
39.515
38.200
38.041
35.564
35.427
35.268
35.224
35.102
34.820
34.141
27.901
27.597
27.467
26.362
23.119
22.737
22.592
22.455
21.884
17.623
17.479
12.394
150
100
50
0
PPM

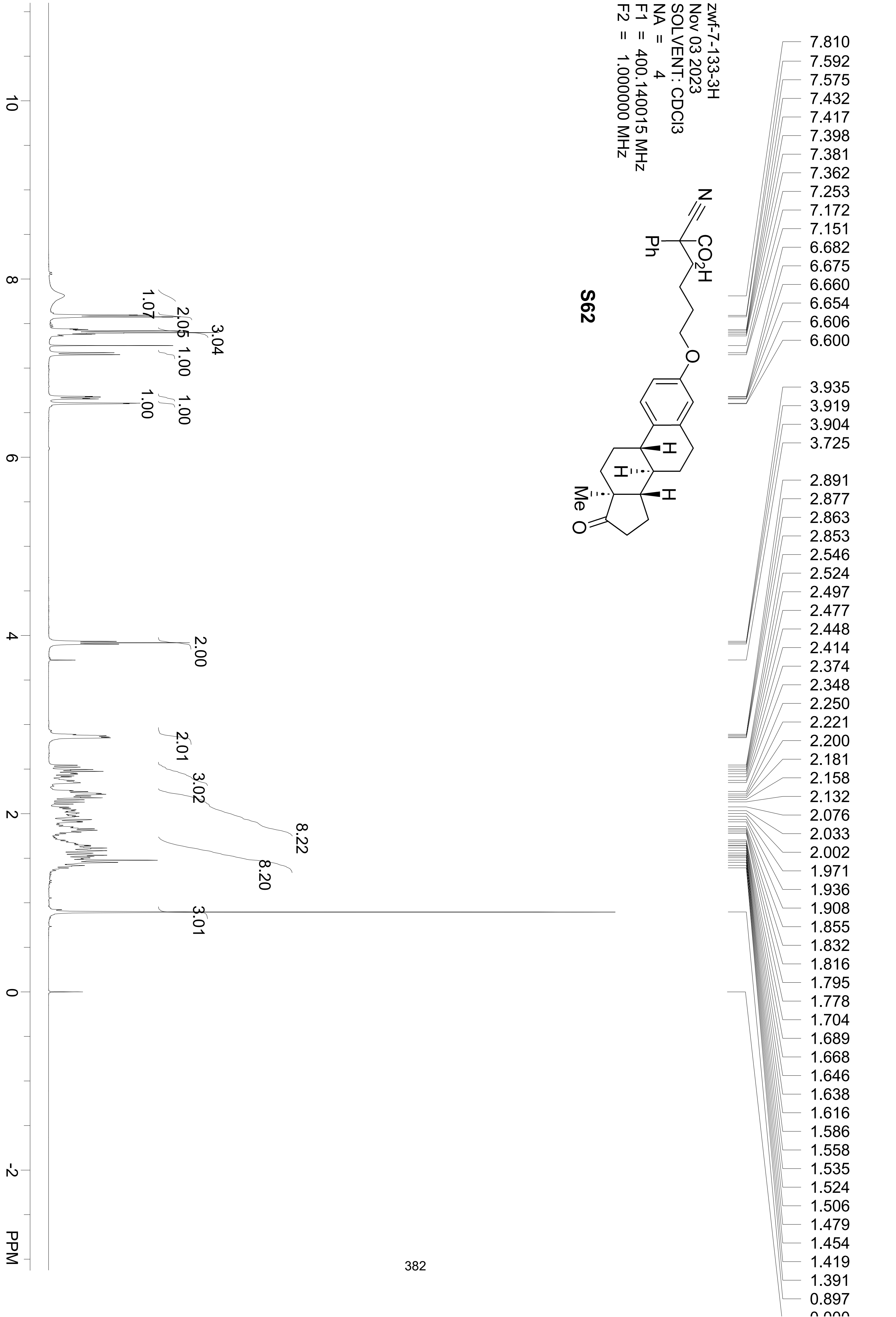
zwf-7-133-3H
Nov 03 2023
SOLVENT: CDCl3
NA = 4
F1 = 400.140015 MHz
F2 = 1.000000 MHz
N
CO2H
Ph
O
H
H
H
Me
O
S62
7.810
7.592
7.575
7.432
7.417
7.398
7.381
7.362
7.253
7.172
7.151
6.682
6.675
6.660
6.654
6.606
6.600
3.935
3.919
3.904
3.725
2.891
2.877
2.863
2.853
2.546
2.524
2.497
2.477
2.448
2.414
2.374
2.348
2.250
2.221
2.200
2.181
2.158
2.132
2.076
2.033
2.002
1.971
1.936
1.908
1.855
1.832
1.816
1.795
1.778
1.704
1.689
1.668
1.646
1.638
1.616
1.586
1.558
1.535
1.524
1.506
1.479
1.454
1.419
1.391
0.897
1.07
2.05
3.04
1.00
1.00
1.00
2.00
2.01
3.02
8.22
8.20
3.01
10
8
6
4
2
0
-2
PPM

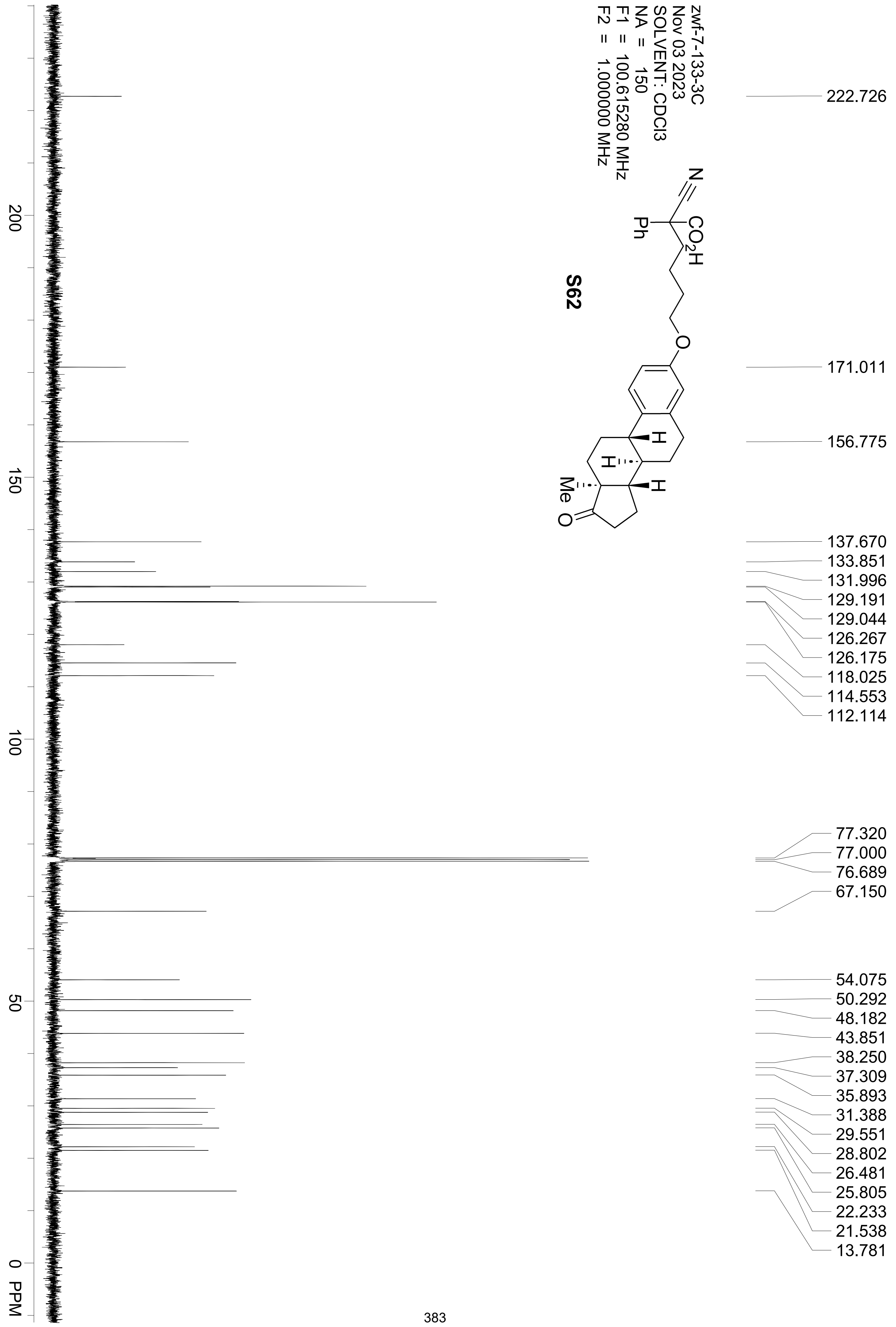

zwf-7-133-3C
Nov 03 2023
SOLVENT: CDCl3
NA = 150
F1 = 100.615280 MHz
F2 = 1.000000 MHz
CO2H
Ph
O
H
H
H
Me
O
S62
222.726
171.011
156.775
137.670
133.851
131.996
129.191
129.044
126.267
126.175
118.025
114.553
112.114
77.320
77.000
76.689
67.150
54.075
50.292
48.182
43.851
38.250
37.309
35.893
31.388
29.551
28.802
26.481
25.805
22.233
21.538
13.781
200
150
100
50
0 PPM

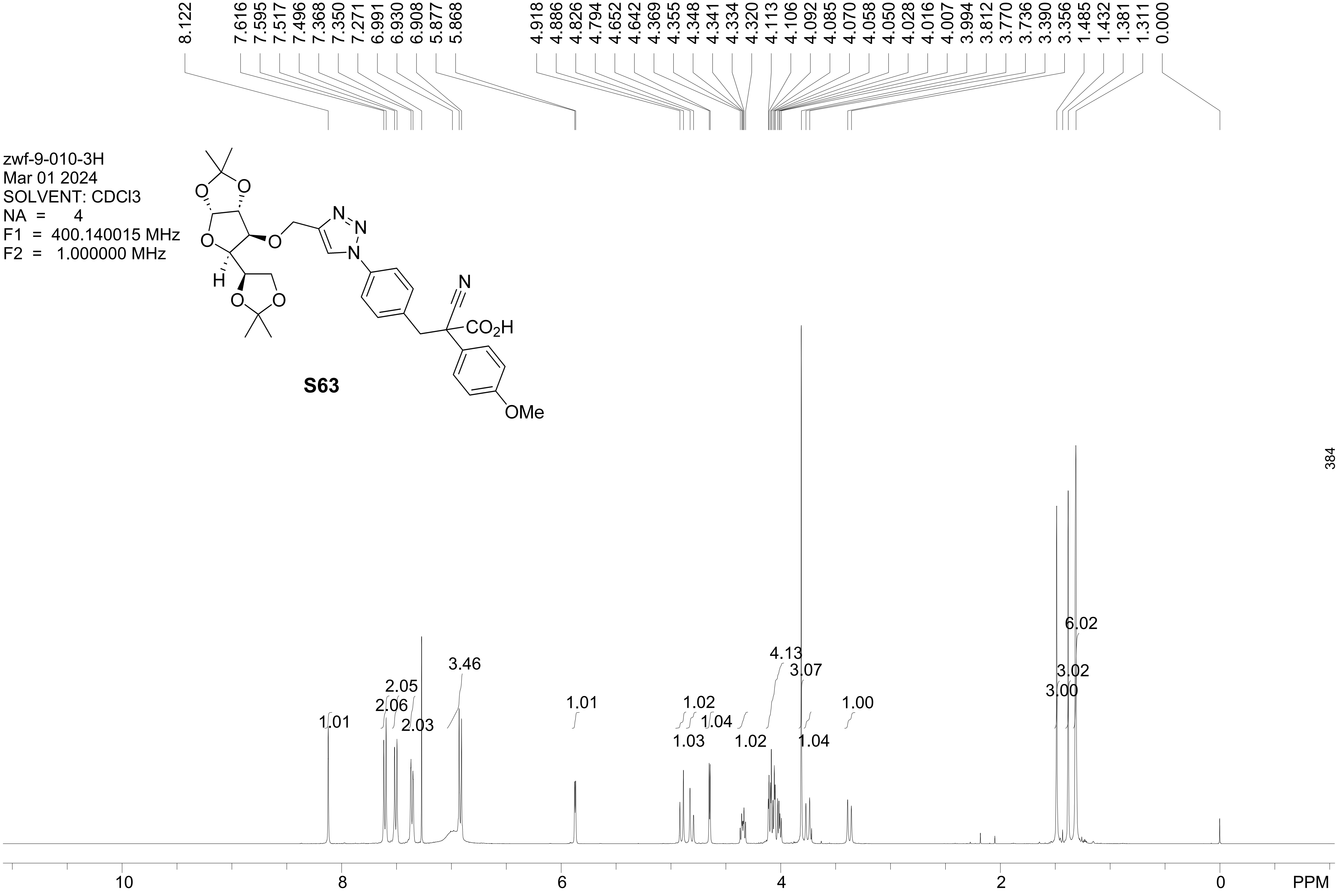
zwf-9-010-3H
Mar 01 2024
SOLVENT: CDCl3
NA = 4
F1 = 400.140015 MHz
F2 = 1.000000 MHz
N
CO2H
OMe
PPM

S63

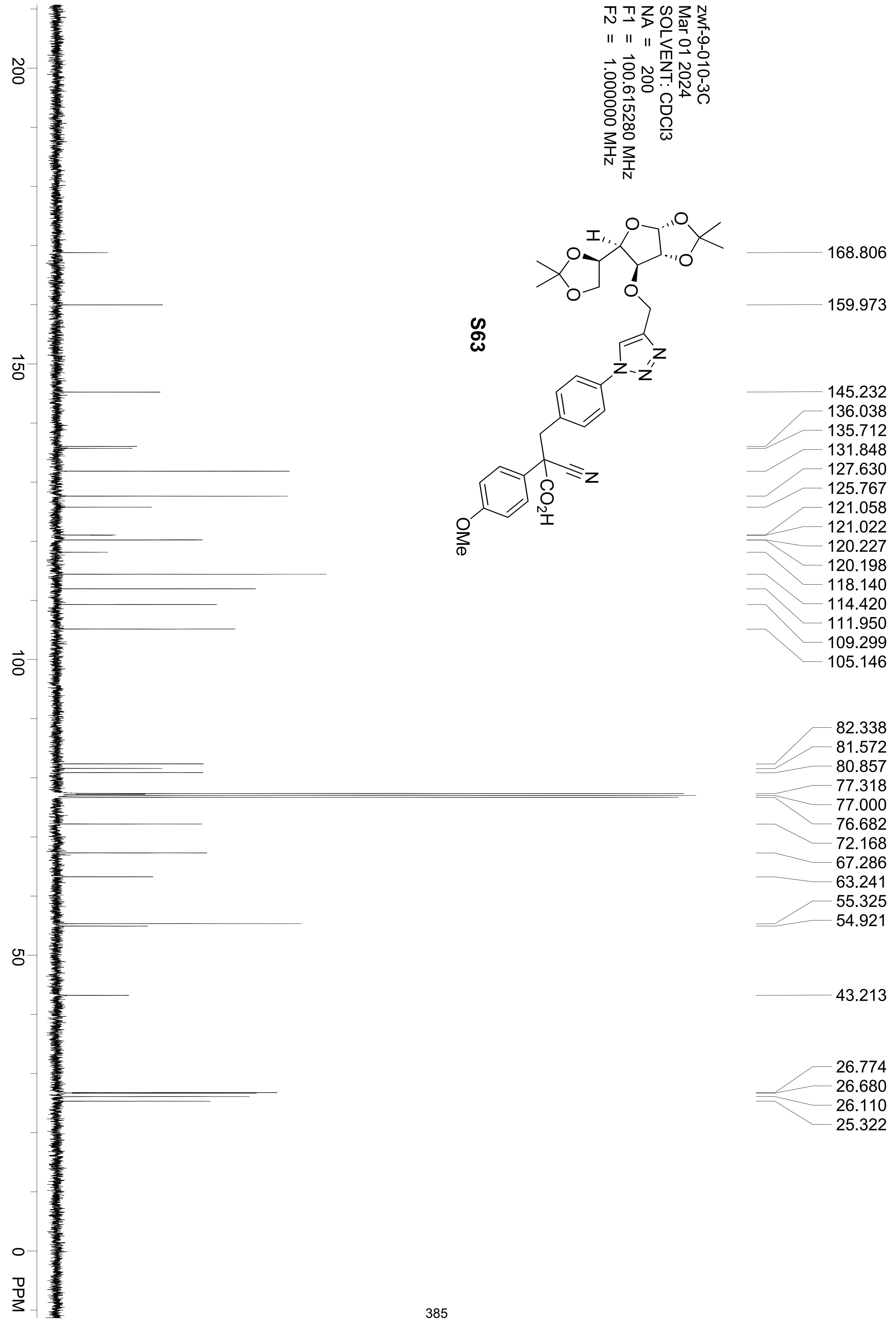
zwf-9-010-3C
Mar 01 2024
SOLVENT: CDCl3
NA = 200
F1 = 100.615280 MHz
F2 = 1.000000 MHz
S63
CO2H
N
OMe
H
O
N
168.806
159.973
145.232
136.038
135.712
131.848
127.630
125.767
121.058
121.022
120.227
120.198
118.140
114.420
111.950
109.299
105.146
82.338
81.572
80.857
77.318
77.000
76.682
72.168
67.286
63.241
55.325
54.921
43.213
26.774
26.680
26.110
25.322
200
150
100
50
0
PPM

zwf-9-010-6H
Mar 22 2024
SOLVENT: CDCl3
NA = 4
F1 = 400.140015 MHz
F2 = 1.000000 MHz

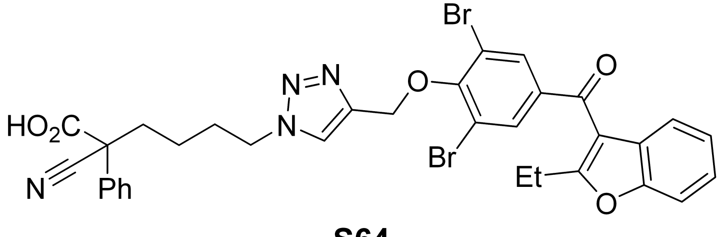

S64

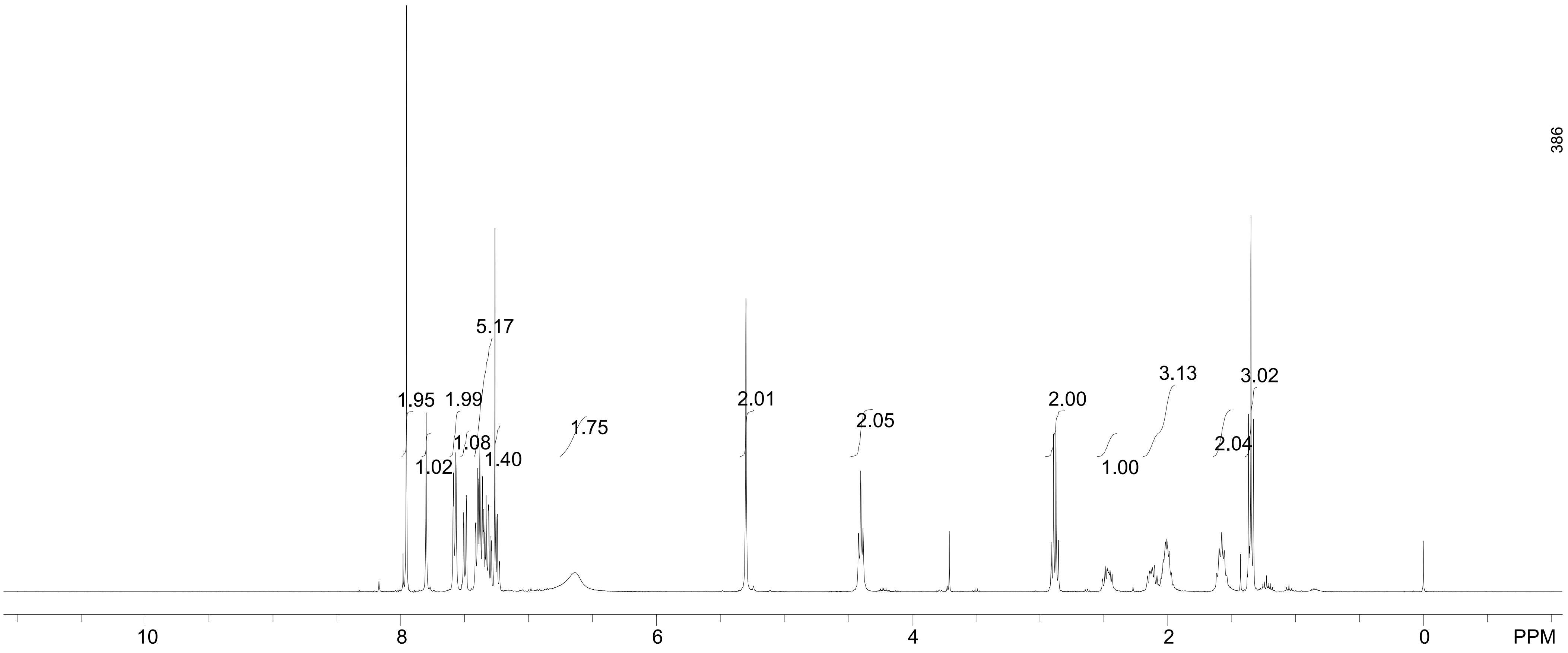

zwf-9-010-6C
Mar 22 2024
SOLVENT: CDCl3
NA = 200
F1 = 100.615280 MHz
F2 = 1.000000 MHz

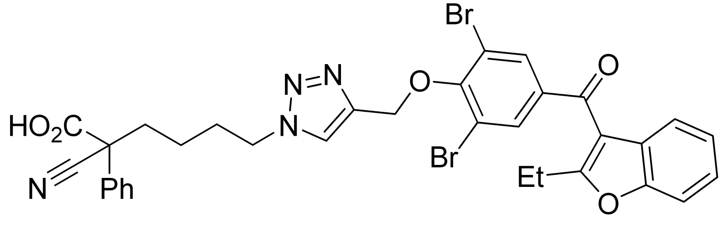

S64

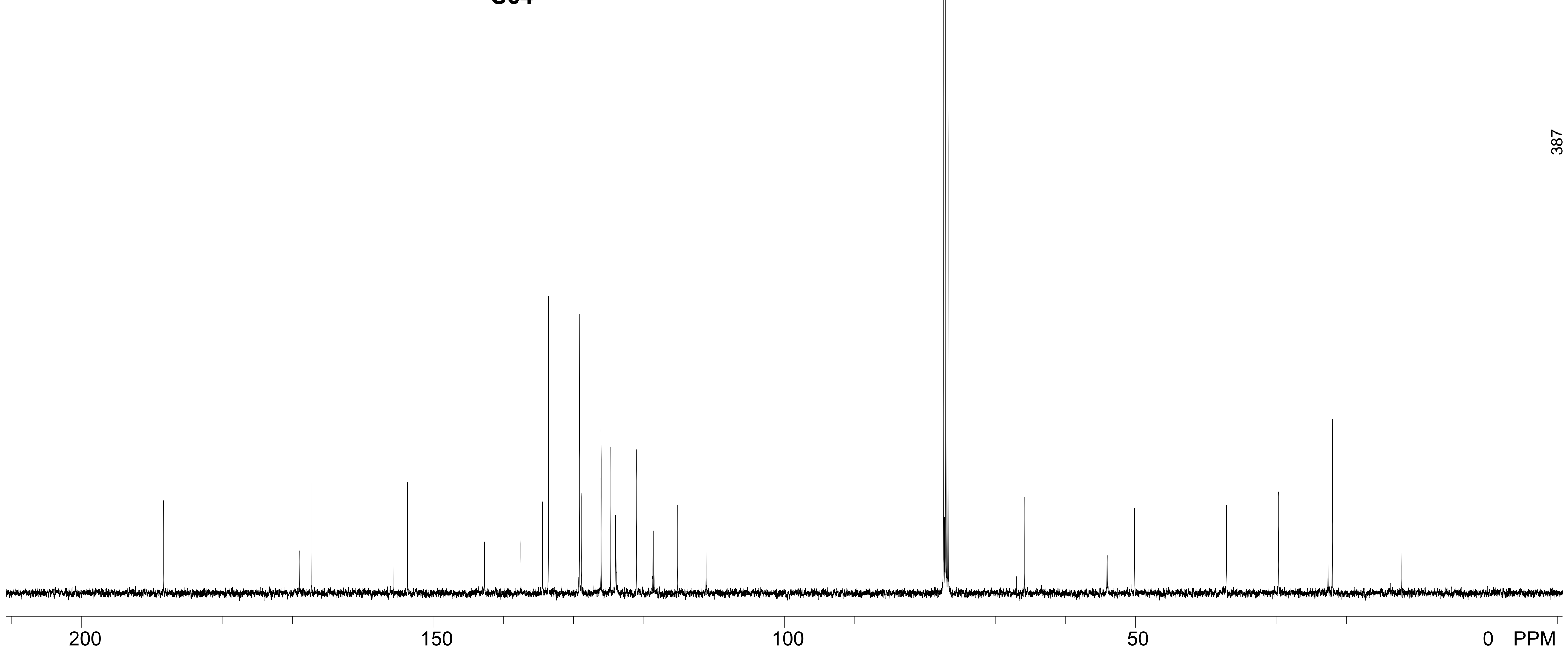

zwf-7-055-15H
Sep 12 2023
SOLVENT: CDCl3
NA = 4
F1 = 400.140015 MHz
F2 = 1.000000 MHz

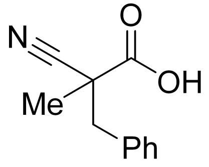

S65

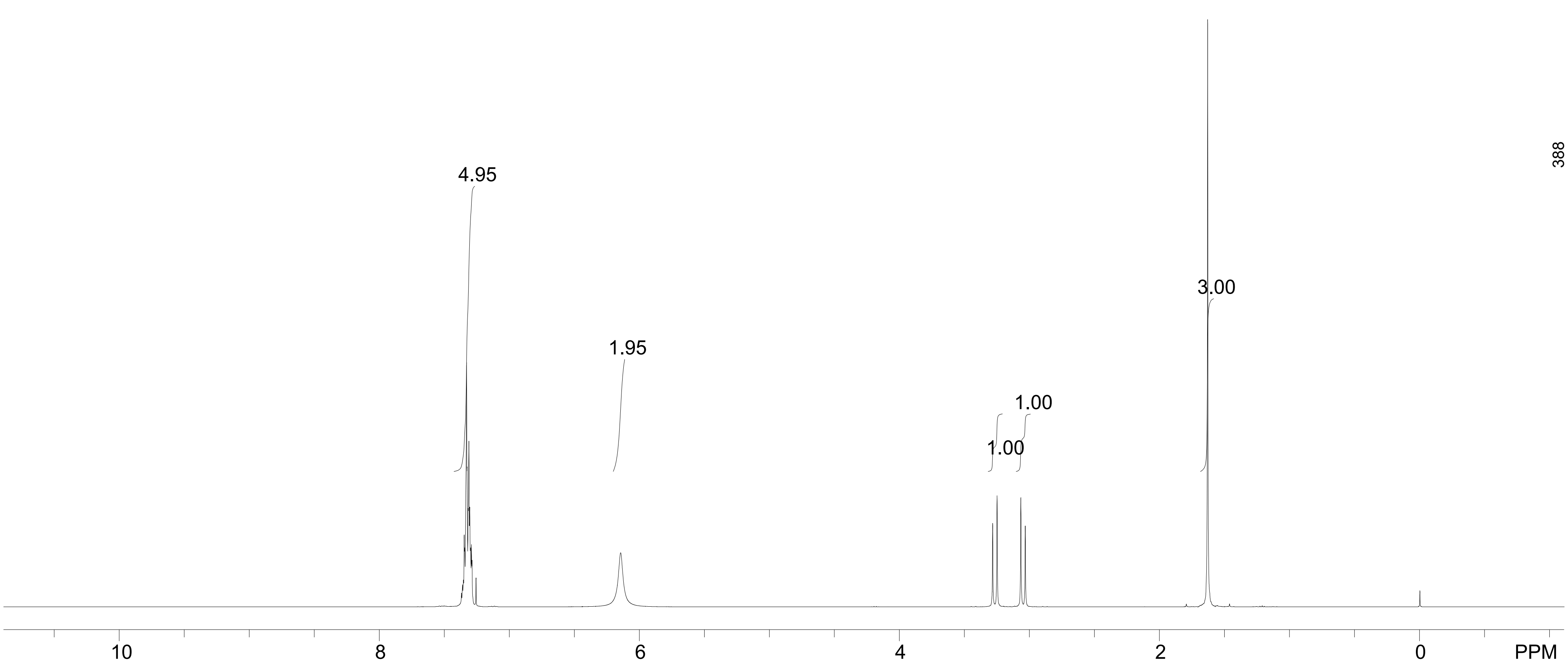

zwf-7-055-15C
Sep 12 2023
SOLVENT: CDCl3
NA = 100
F1 = 100.615280 MHz
F2 = 1.000000 MHz

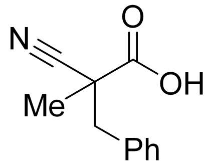

S65

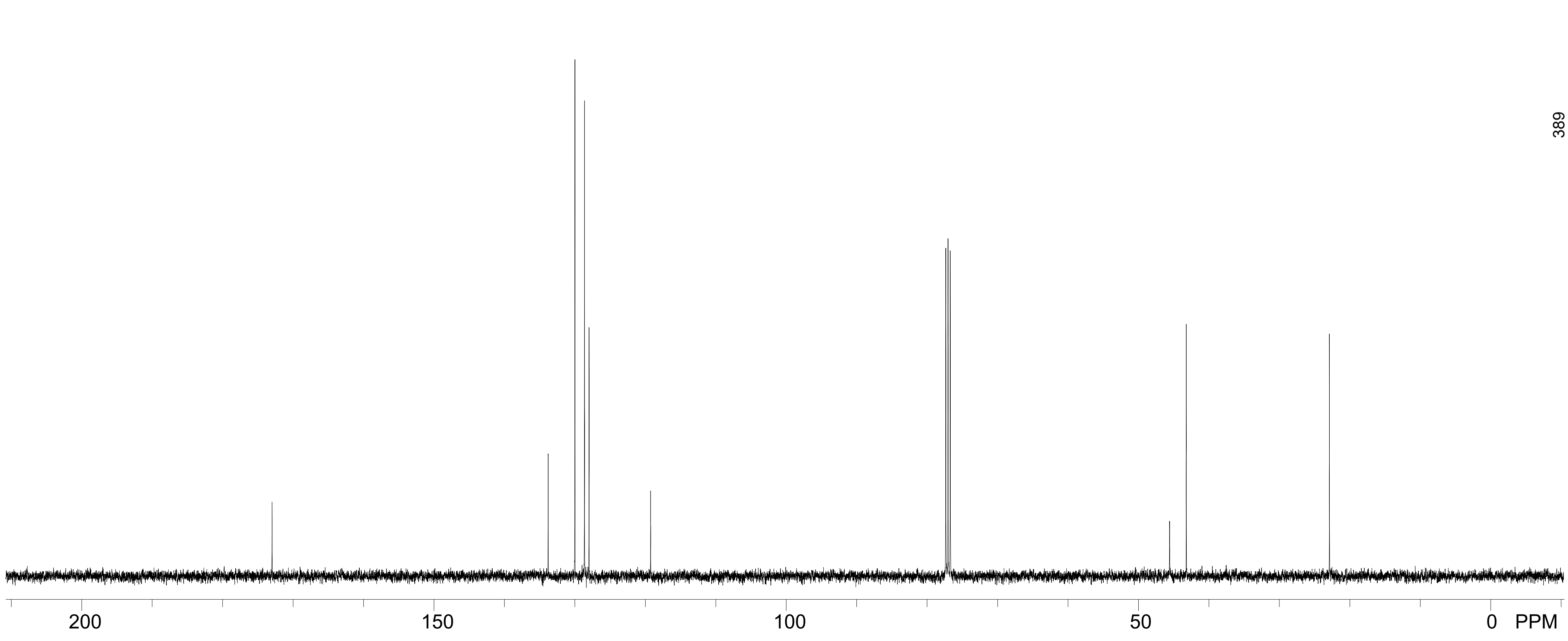

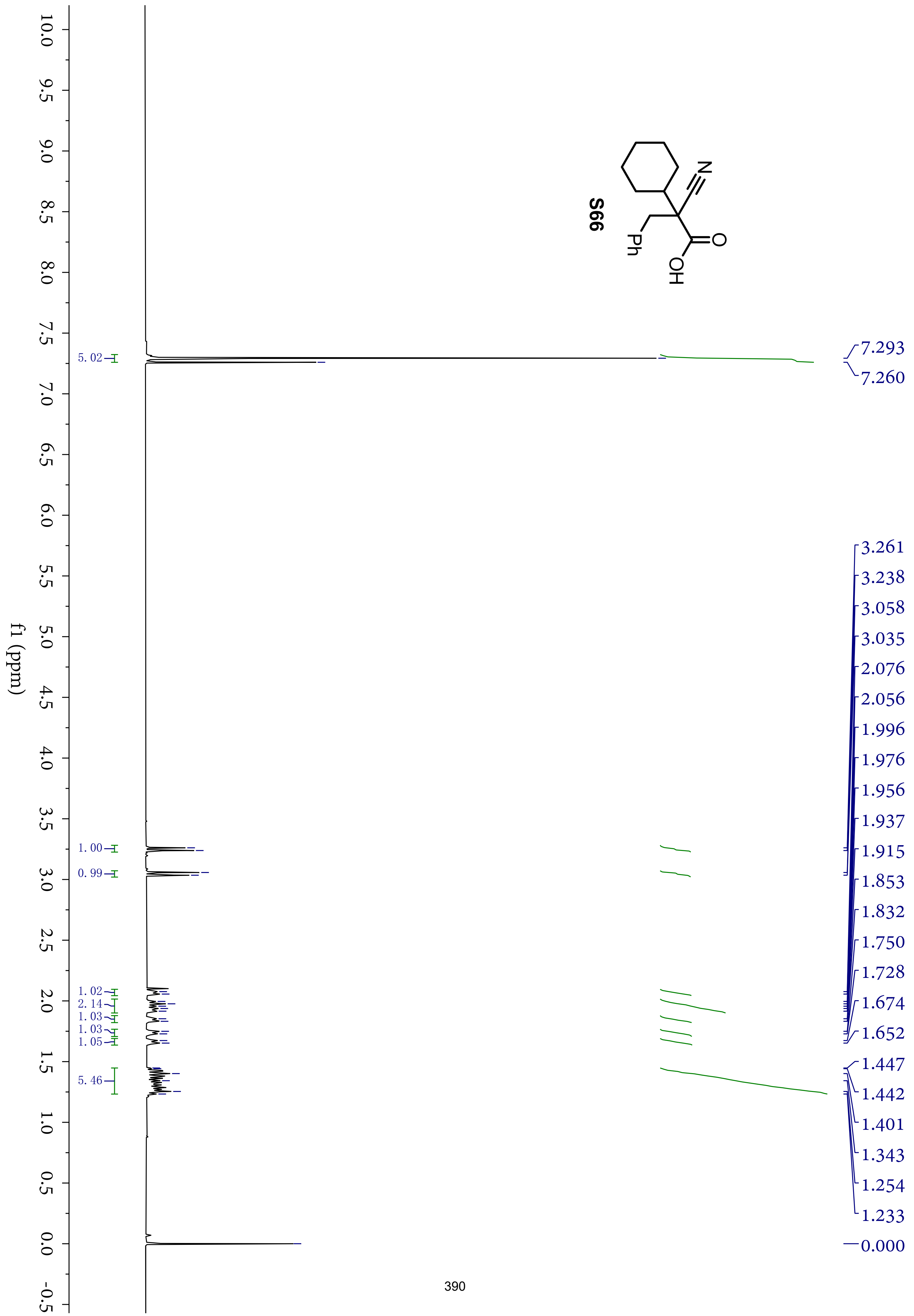
S66
Ph
OH
O
N
f1 (ppm)
10.0
9.5
9.0
8.5
8.0
7.5
7.0
6.5
6.0
5.5
5.0
4.5
4.0
3.5
3.0
2.5
2.0
1.5
1.0
0.5
0.0
-0.5
5.02
1.00
0.99
1.02
2.14
1.03
1.03
1.05
5.46
7.293
7.260
3.261
3.238
3.058
3.035
2.076
2.056
1.996
1.976
1.956
1.937
1.915
1.853
1.832
1.750
1.728
1.674
1.652
1.447
1.442
1.401
1.343
1.254
1.233
0.000

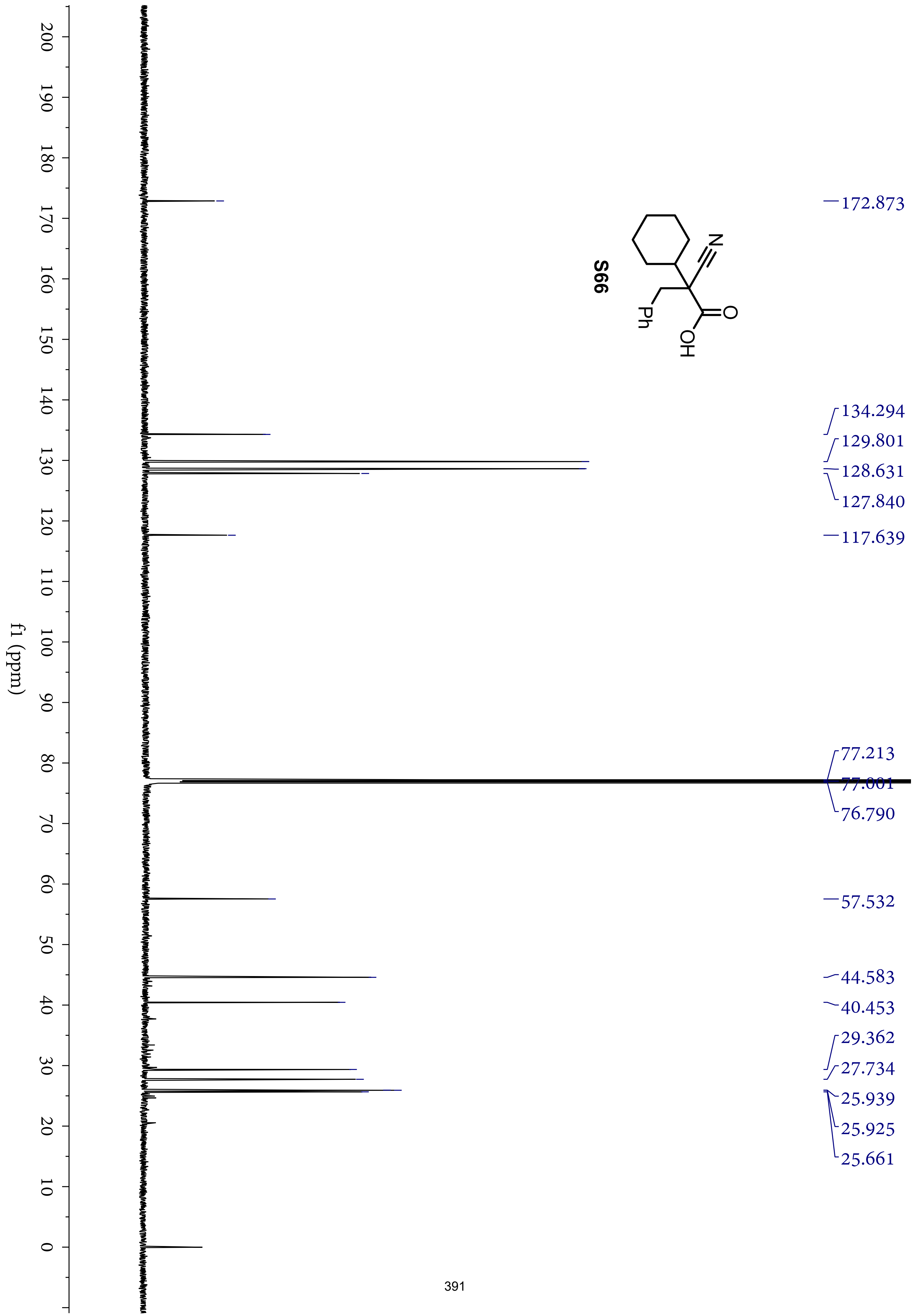
S66
N
O
OH
Ph
172.873
134.294
129.801
128.631
127.840
117.639
77.213
77.001
76.790
57.532
44.583
40.453
29.362
27.734
25.939
25.925
25.661
f1 (ppm)
200
190
180
170
160
150
140
130
120
110
100
90
80
70
60
50
40
30
20
10
0

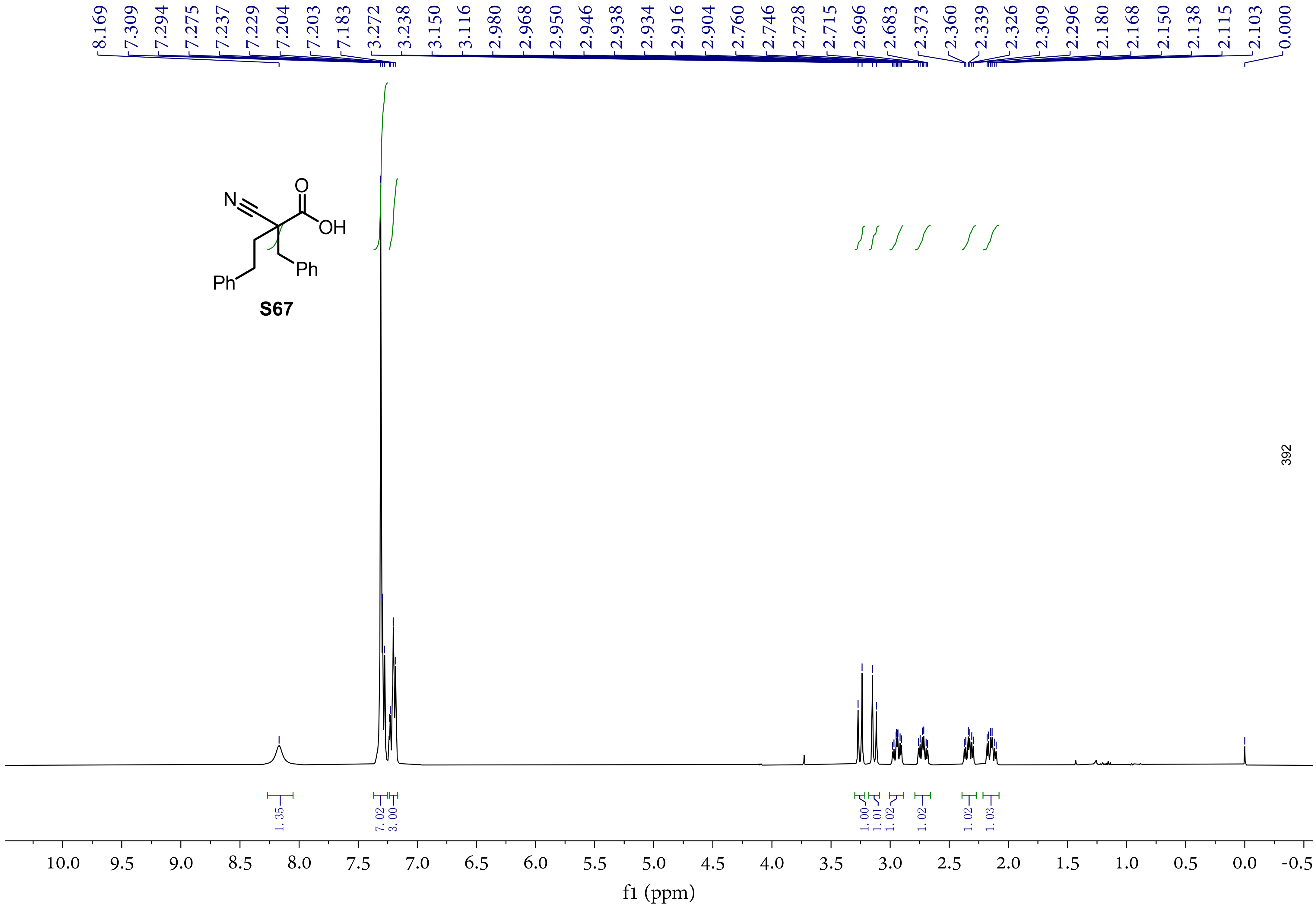
8.169
7.309
7.294
7.275
7.237
7.229
7.204
7.203
7.183
3.272
3.238
3.150
3.116
2.980
2.968
2.950
2.946
2.938
2.934
2.916
2.904
2.760
2.746
2.728
2.715
2.696
2.683
2.373
2.360
2.339
2.326
2.309
2.296
2.180
2.168
2.150
2.138
2.115
2.103
0.000
N
O
OH
Ph
Ph
S67
1.35
7.02
3.00
1.00
1.01
1.02
1.02
1.02
1.03
10.0
9.5
9.0
8.5
8.0
7.5
7.0
6.5
6.0
5.5
5.0
4.5
4.0
3.5
3.0
2.5
2.0
1.5
1.0
0.5
0.0
-0.5
f1 (ppm)

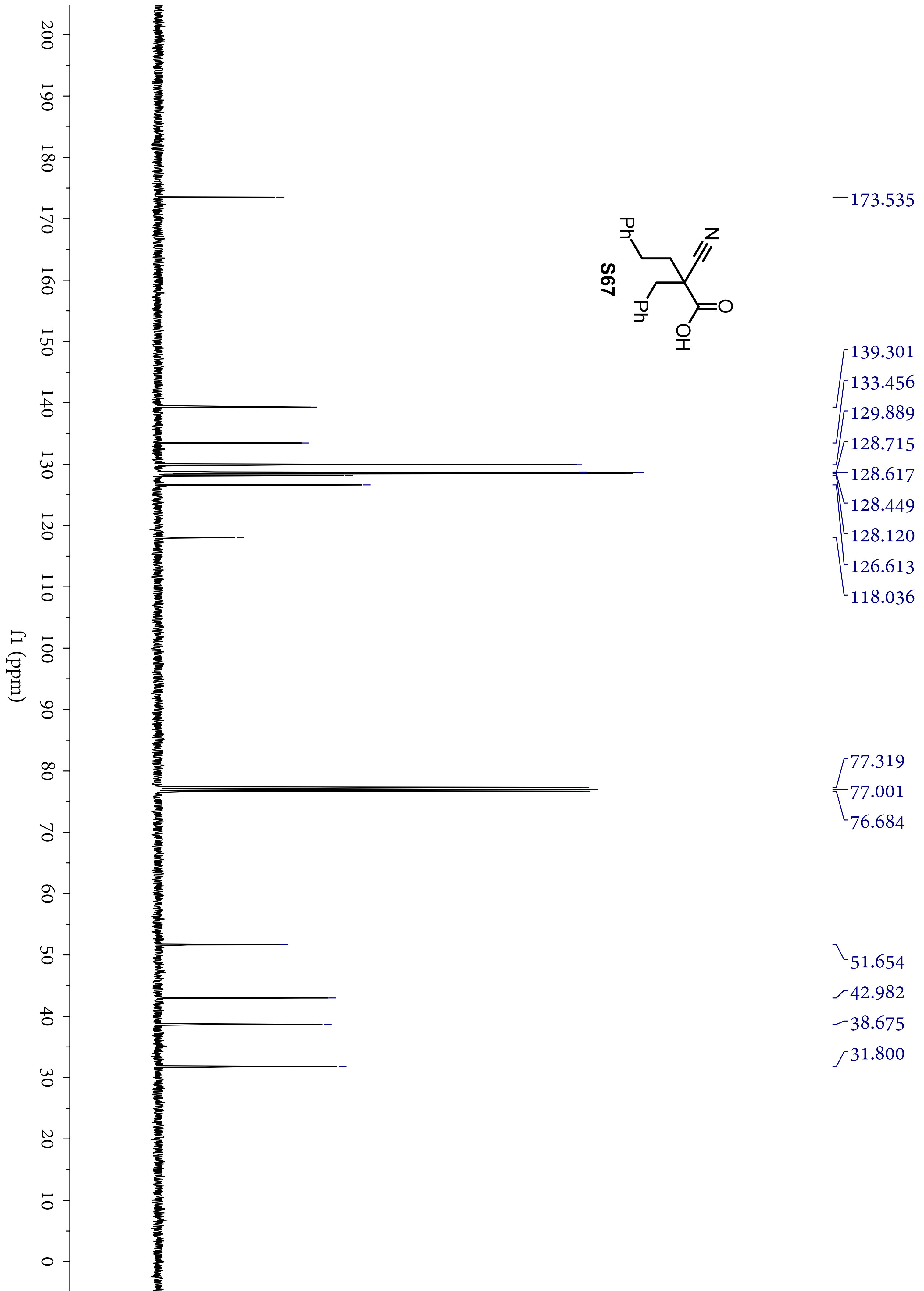

S67
Ph
Ph
N
O
OH
173.535
139.301
133.456
129.889
128.715
128.617
128.449
128.120
126.613
118.036
77.319
77.001
76.684
51.654
42.982
38.675
31.800
f1 (ppm)
200
190
180
170
160
150
140
130
120
110
100
90
80
70
60
50
40
30
20
10
0

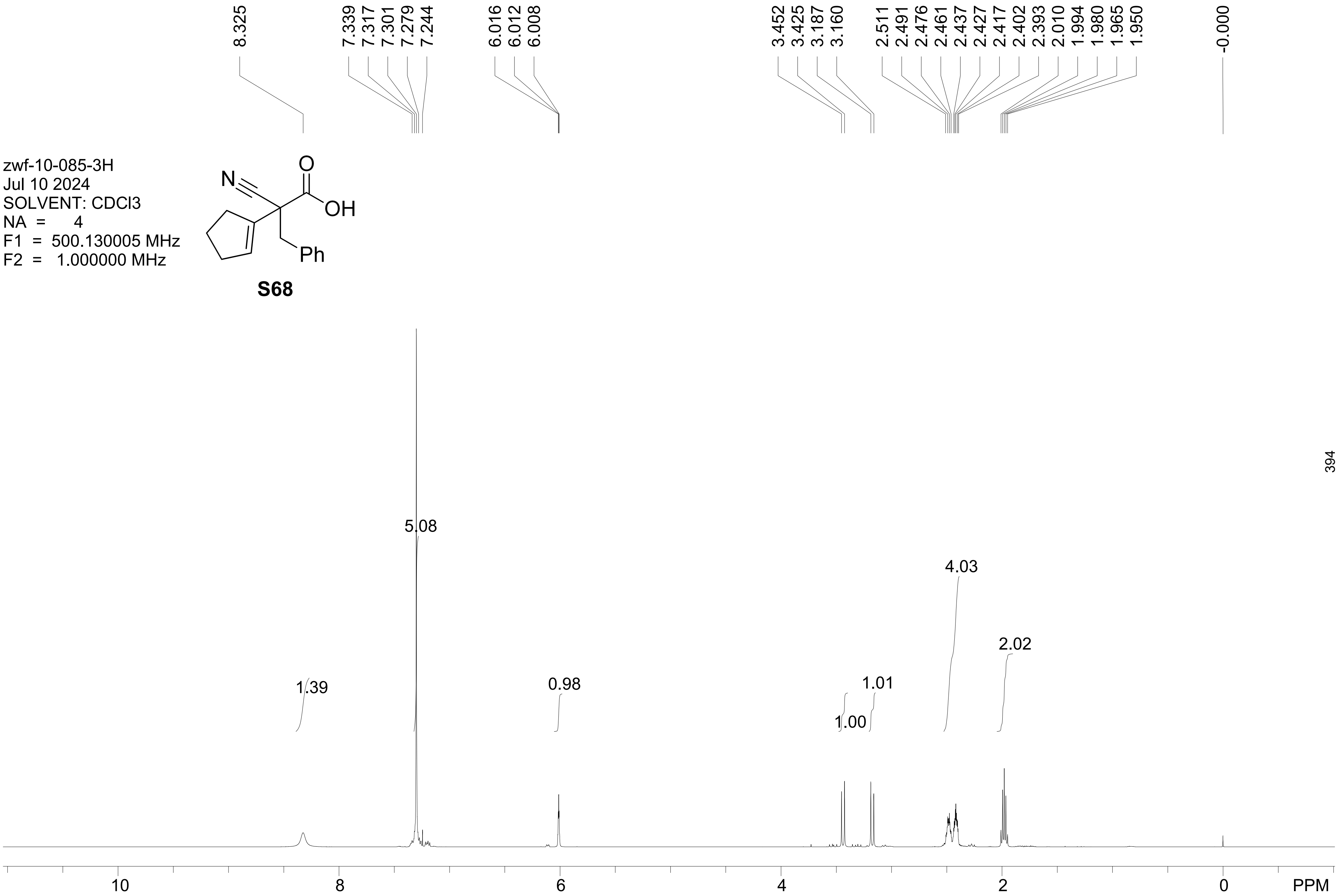
zwf-10-085-3H
Jul 10 2024
SOLVENT: CDCl3
NA = 4
F1 = 500.130005 MHz
F2 = 1.000000 MHz
N
O
OH
Ph
S68
8.325
7.339
7.317
7.301
7.279
7.244
6.016
6.012
6.008
3.452
3.425
3.187
3.160
2.511
2.491
2.476
2.461
2.437
2.427
2.417
2.402
2.393
2.010
1.994
1.980
1.965
1.950
-0.000
1.39
5.08
0.98
1.00
1.01
4.03
2.02
10
8
6
4
2
0
PPM

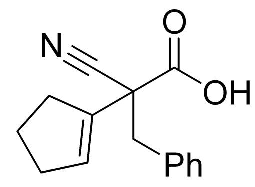

S68

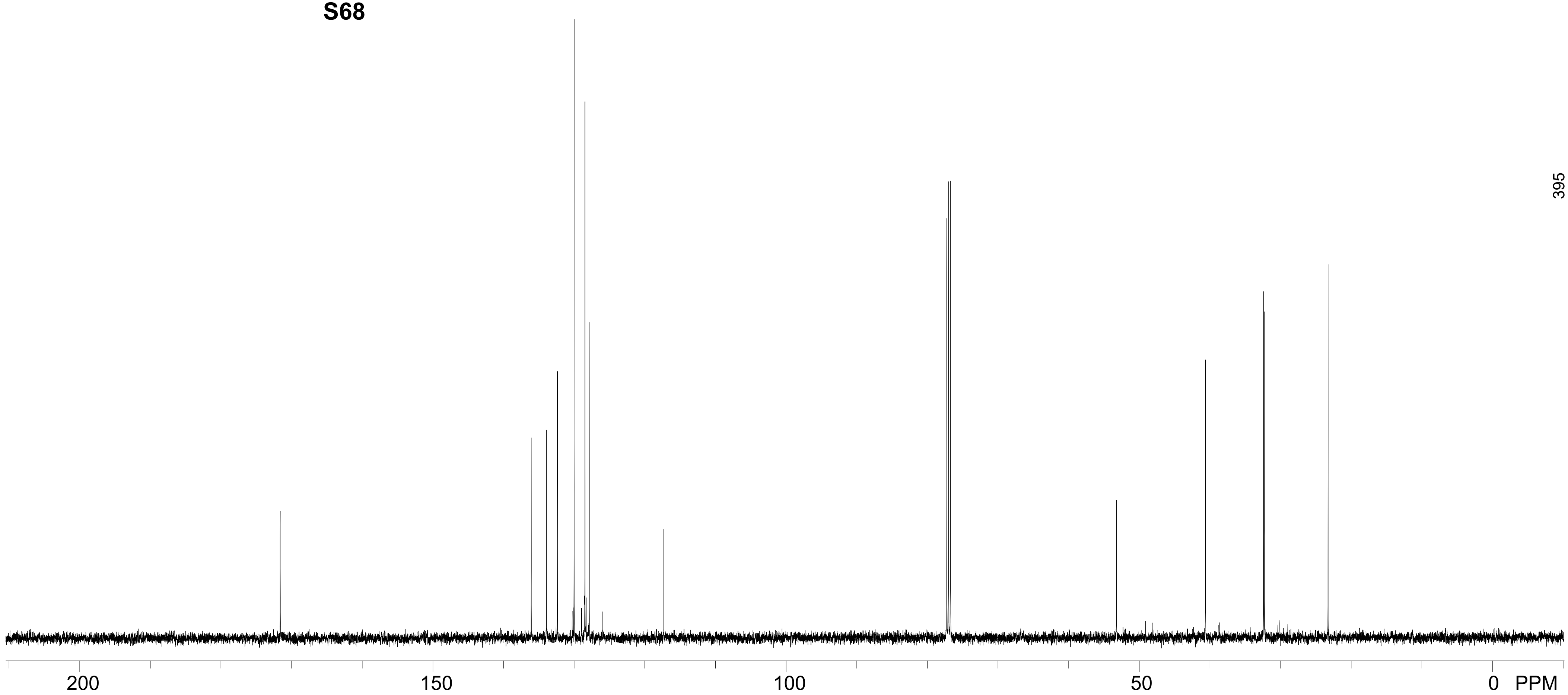
zwf-10-085-3C
Jul 10 2024
SOLVENT: CDCl3
NA = 100
F1 = 125.757790 MHz
F2 = 1.000000 MHz
171.610
136.079
133.926
132.381
130.019
128.480
127.866
117.313
77.253
77.000
76.747
53.207
40.609
32.375
32.216
23.230
200
150
100
50
0 PPM

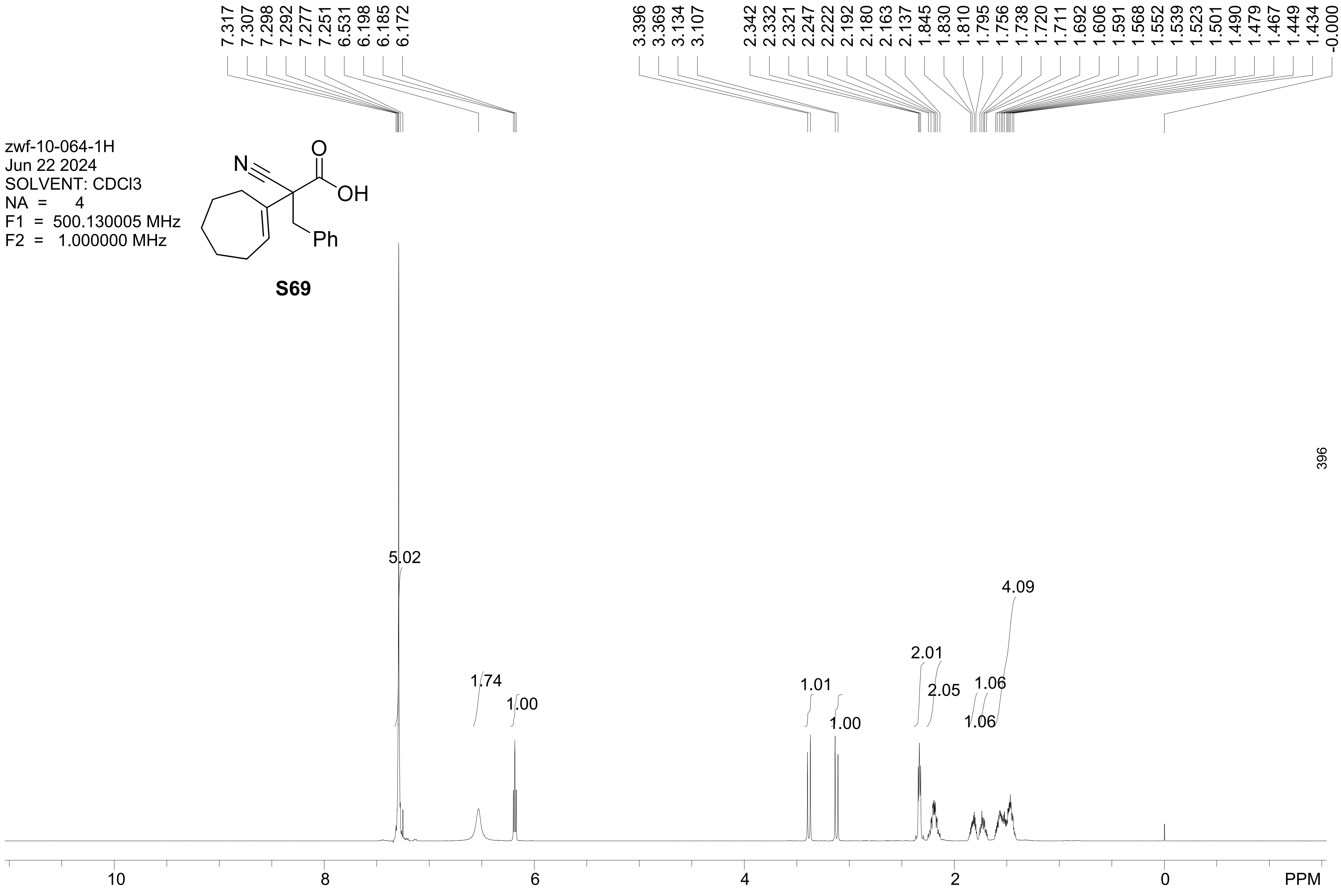
zwf-10-064-1H
Jun 22 2024
SOLVENT: CDCl3
NA = 4
F1 = 500.130005 MHz
F2 = 1.000000 MHz
N
O
OH
Ph
S69
7.317 7.307 7.298 7.292 7.277 7.251 6.531 6.198 6.185 6.172
3.396 3.369 3.134 3.107
2.342 2.332 2.321 2.247 2.222 2.192 2.180 2.163 2.137 1.845 1.830 1.810 1.795 1.756 1.738 1.720 1.711 1.692 1.606 1.591 1.568 1.552 1.539 1.523 1.501 1.490 1.479 1.467 1.449 1.434 -0.000
5.02
1.74
1.00
1.01
1.00
2.01
2.05
1.06
1.06
4.09
10
8
6
4
2
0
PPM

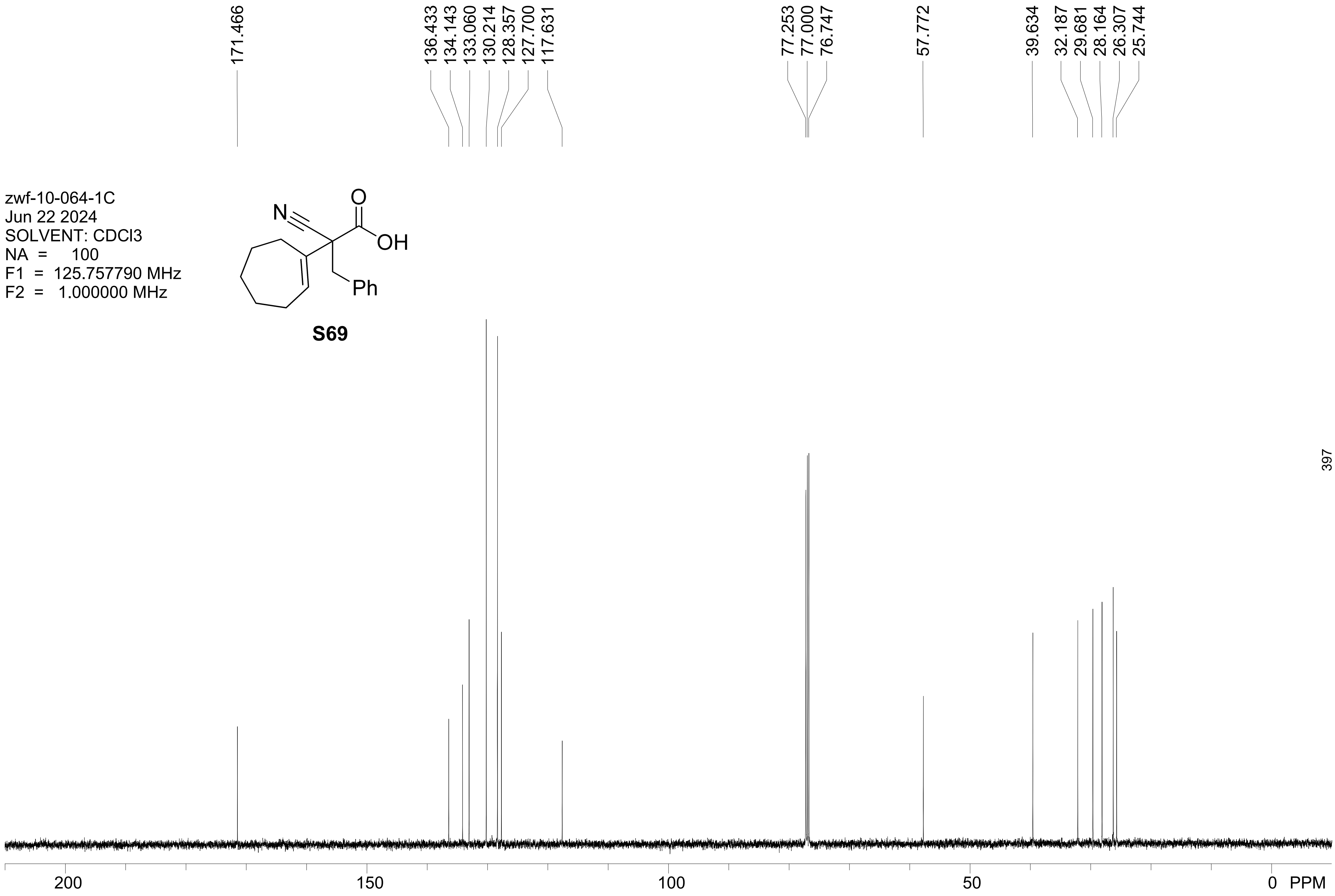
171.466
136.433
134.143
133.060
130.214
128.357
127.700
117.631
77.253
77.000
76.747
57.772
39.634
32.187
29.681
28.164
26.307
25.744
zwf-10-064-1C
Jun 22 2024
SOLVENT: CDCl3
NA = 100
F1 = 125.757790 MHz
F2 = 1.000000 MHz
N
O
OH
Ph
S69
200
150
100
50
0 PPM

zwf-10-064-3H
Jun 24 2024
SOLVENT: CDCl3
NA = 4
F1 = 500.130005 MHz
F2 = 1.000000 MHz

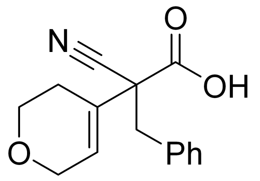

S70

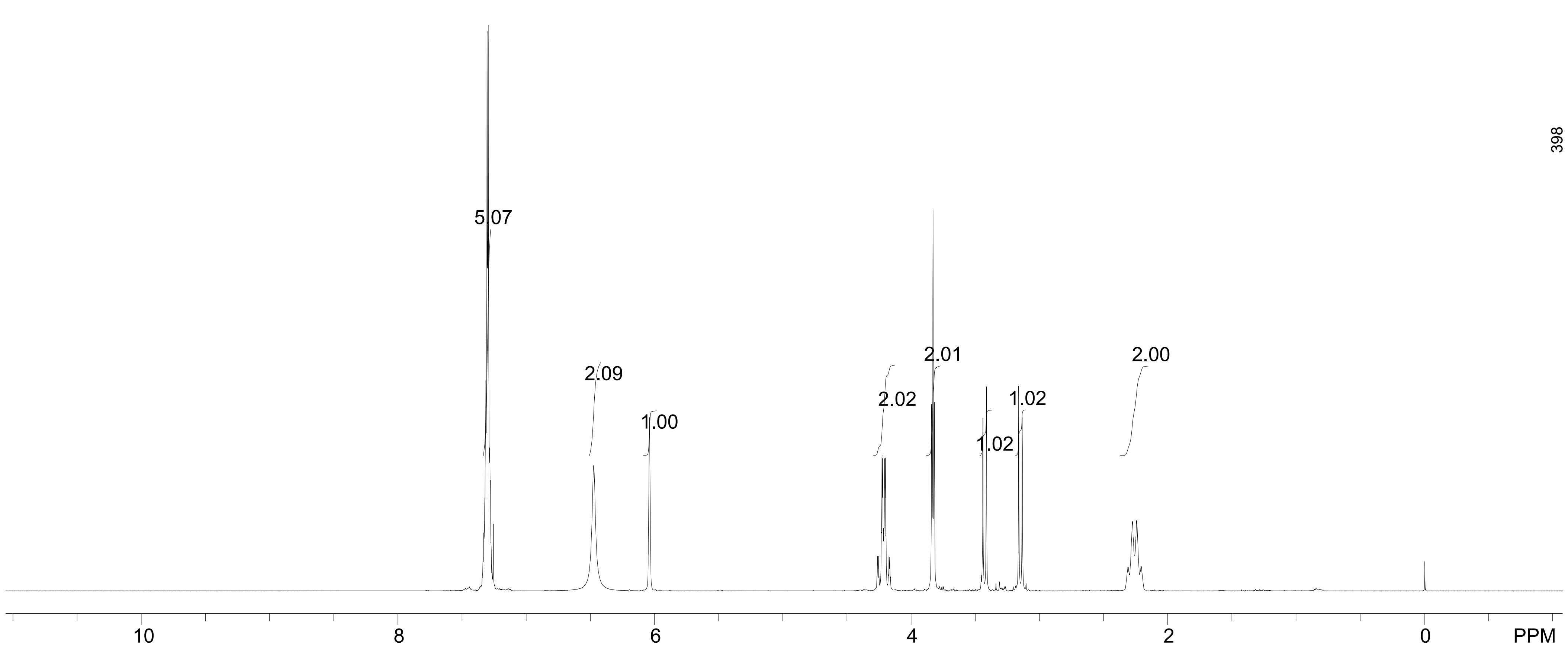

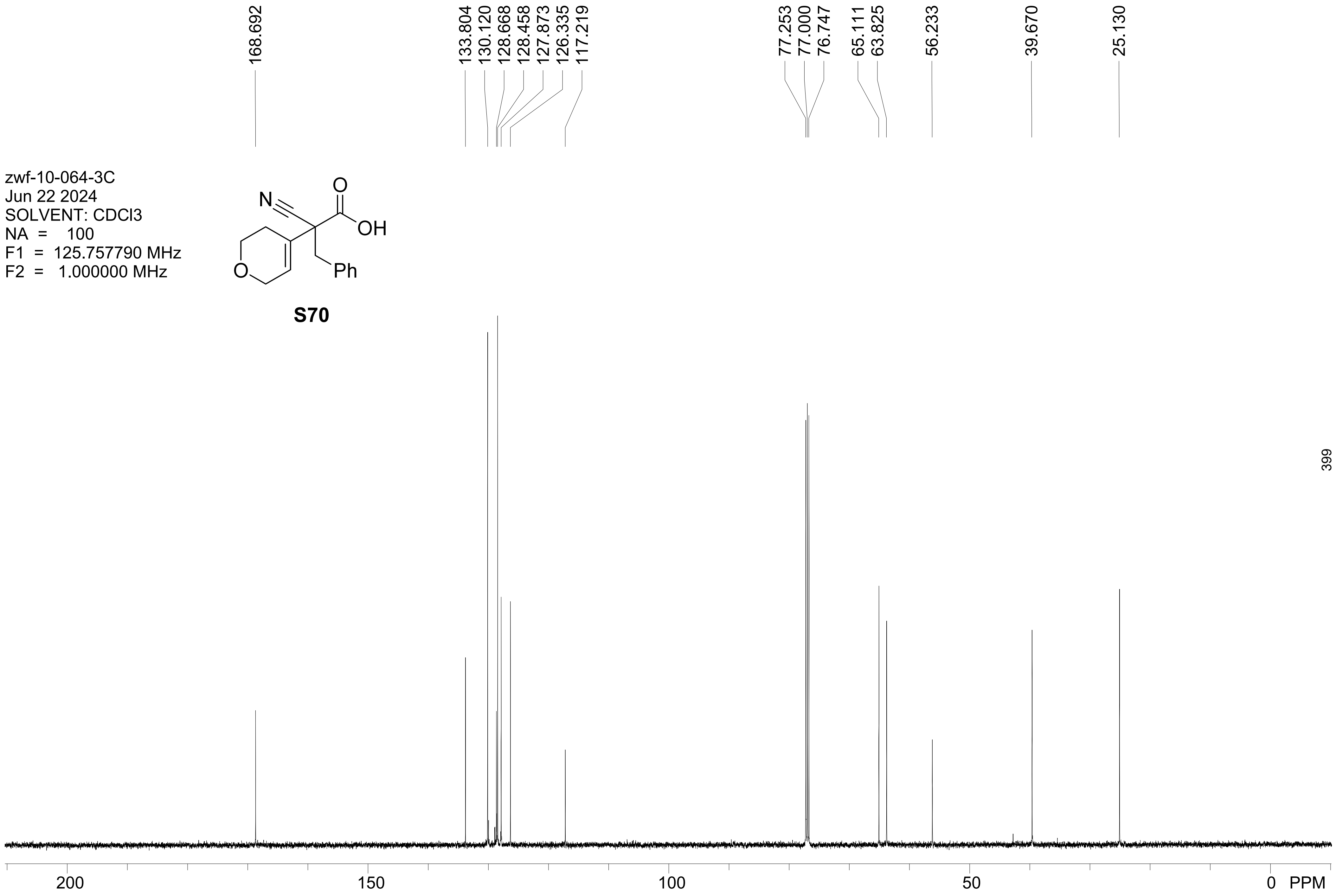
zwf-10-064-3C
Jun 22 2024
SOLVENT: CDCl3
NA = 100
F1 = 125.757790 MHz
F2 = 1.000000 MHz
N
O
OH
O
Ph
S70
168.692
133.804
130.120
128.668
128.458
127.873
126.335
117.219
77.253
77.000
76.747
65.111
63.825
56.233
39.670
25.130
200
150
100
50
0 PPM

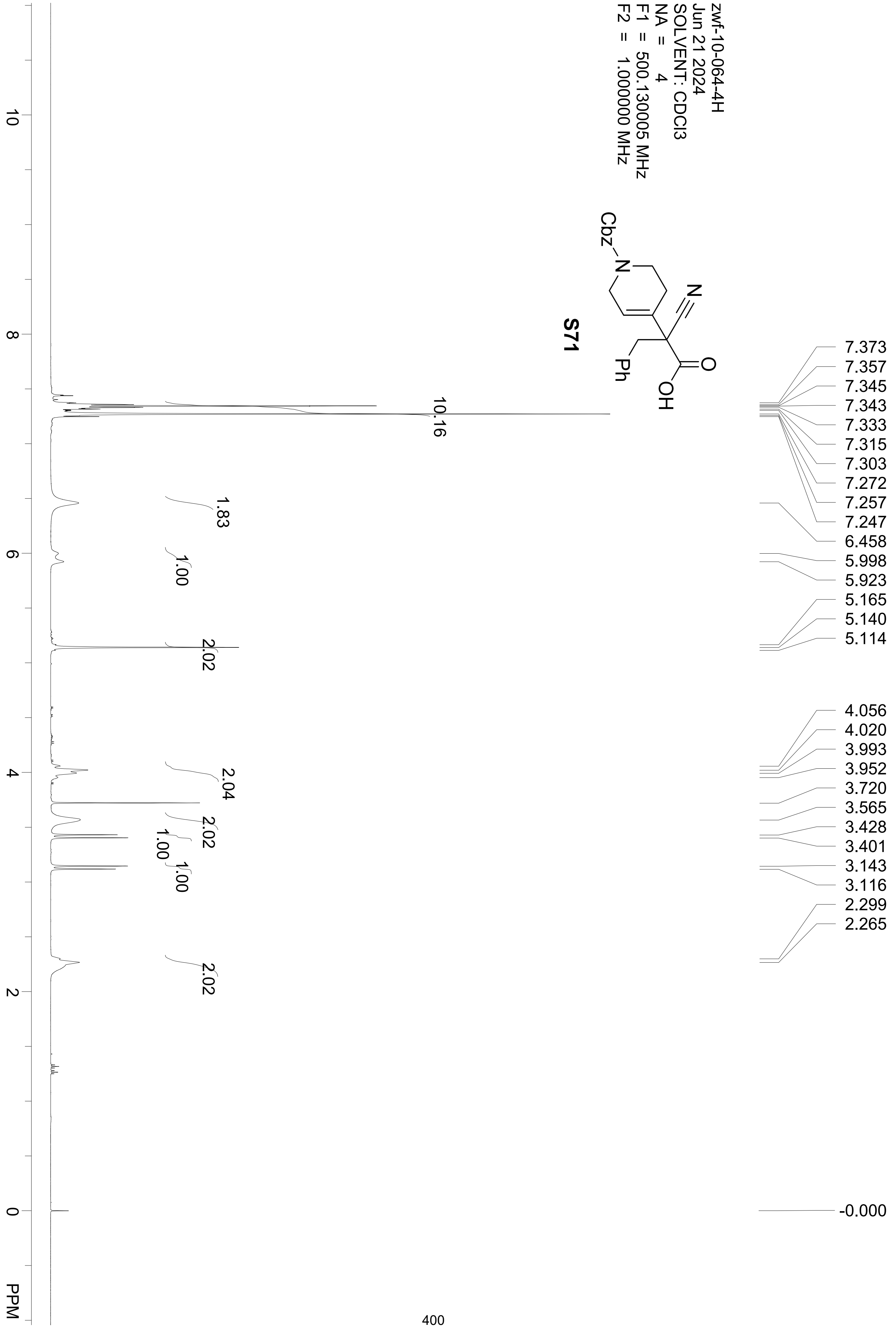
zwf-10-064-4H
Jun 21 2024
SOLVENT: CDCl3
NA = 4
F1 = 500.130005 MHz
F2 = 1.000000 MHz
S71
7.373
7.357
7.345
7.343
7.333
7.315
7.303
7.272
7.257
7.247
6.458
5.998
5.923
5.165
5.140
5.114
4.056
4.020
3.993
3.952
3.720
3.565
3.428
3.401
3.143
3.116
2.299
2.265
-0.000
10.16
1.83
1.00
2.02
2.04
2.02
1.00
1.00
2.02
PPM

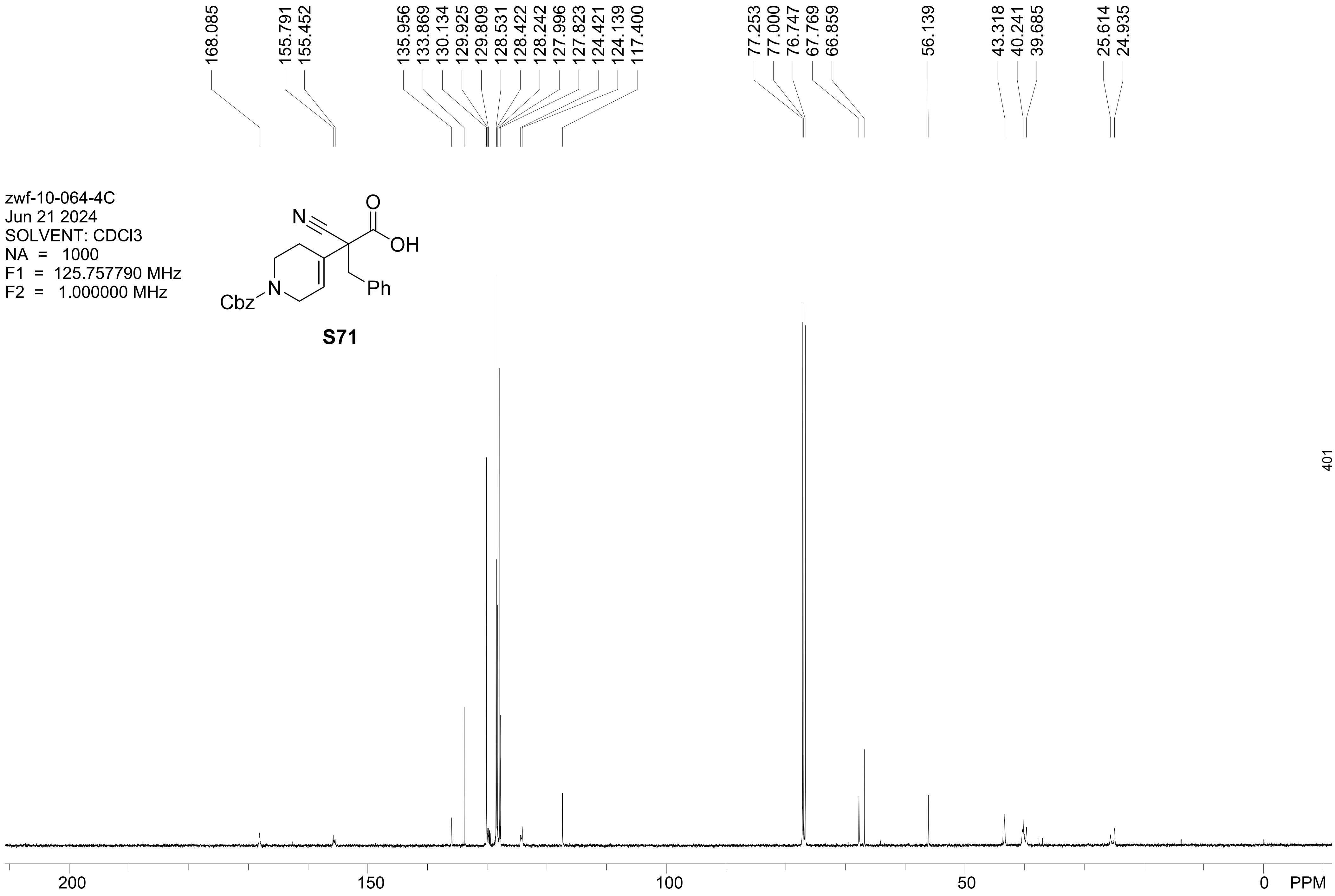
zwf-10-064-4C
Jun 21 2024
SOLVENT: CDCl3
NA = 1000
F1 = 125.757790 MHz
F2 = 1.000000 MHz
168.085
155.791
155.452
135.956
133.869
130.134
129.925
129.809
128.531
128.422
128.242
127.996
127.823
124.421
124.139
117.400
77.253
77.000
76.747
67.769
66.859
56.139
43.318
40.241
39.685
25.614
24.935
200
150
100
50
0
PPM
N
O
OH
Ph
Cbz
N

S71

zwf-10-018-1H
Jun 10 2024
SOLVENT: CDCl3
NA =   4
F1 = 500.130005 MHz
F2 = 1.000000 MHz

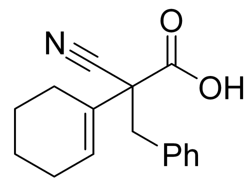

S72

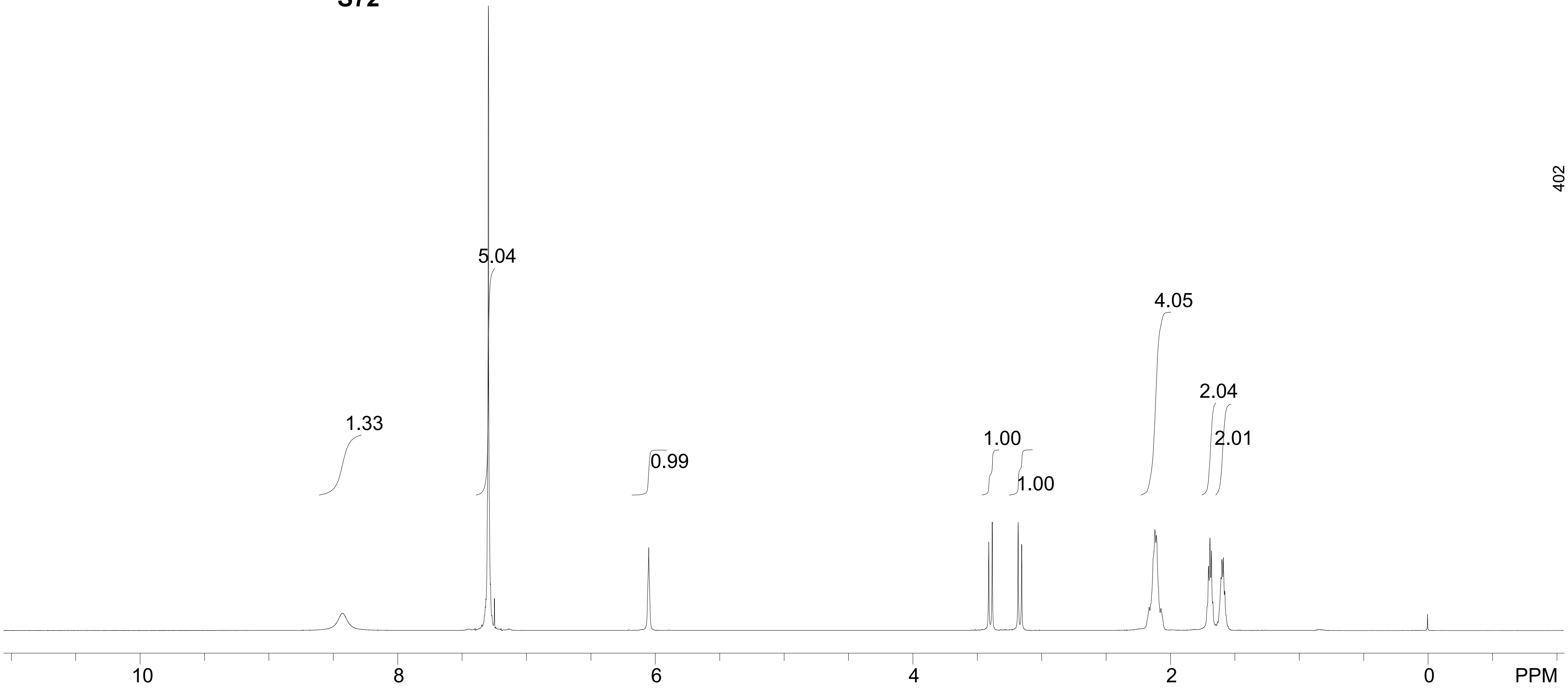

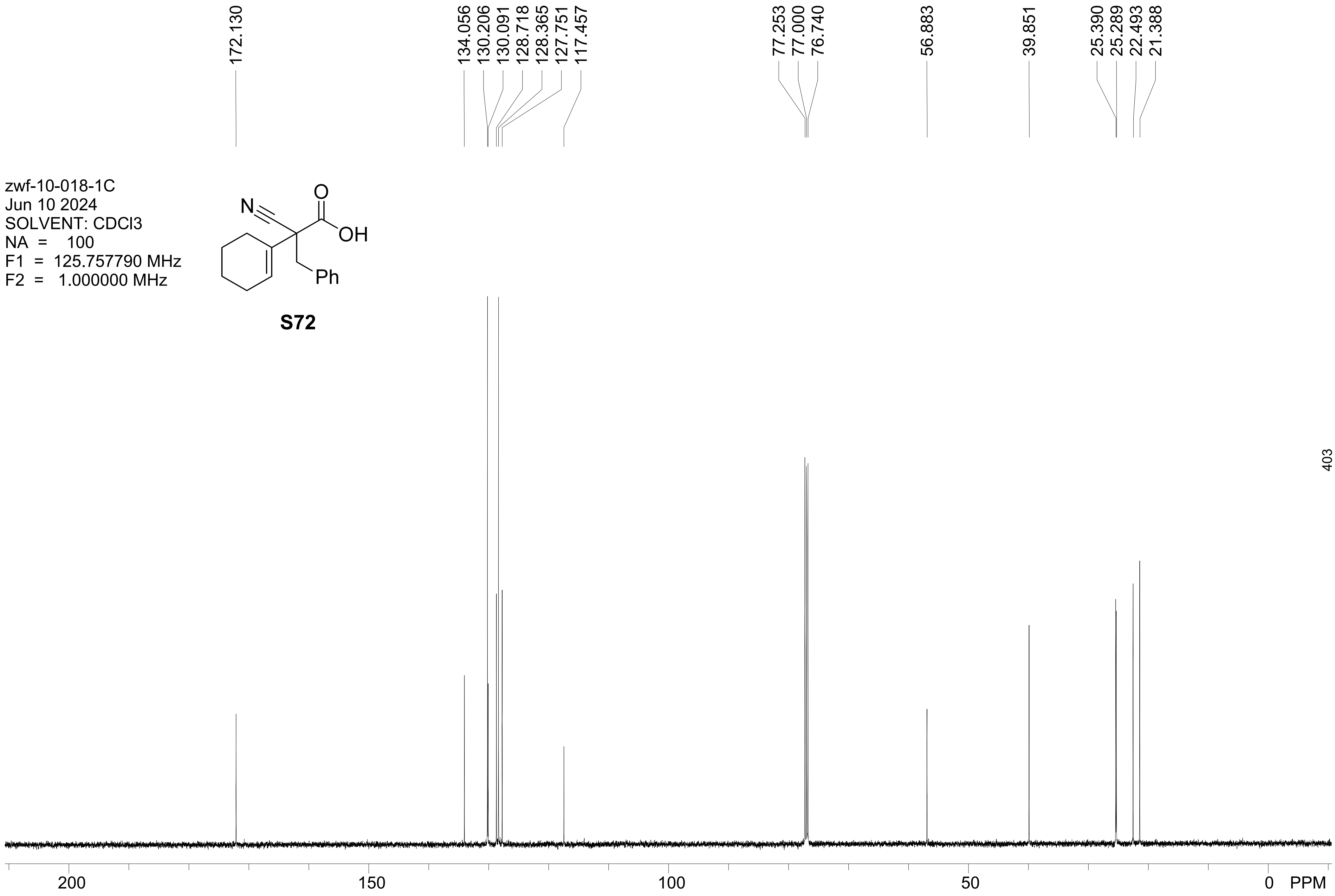
172.130
134.056
130.206
130.091
128.718
128.365
127.751
117.457
77.253
77.000
76.740
56.883
39.851
25.390
25.289
22.493
21.388
zwf-10-018-1C
Jun 10 2024
SOLVENT: CDCl3
NA = 100
F1 = 125.757790 MHz
F2 = 1.000000 MHz
N
O
OH
Ph
S72
200
150
100
50
0 PPM

zwf-10-085-4H
Jul 10 2024
SOLVENT: CDCl3
NA = 4
F1 = 500.130005 MHz
F2 = 1.000000 MHz

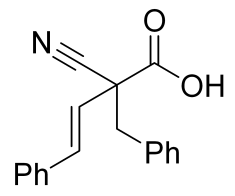

S74

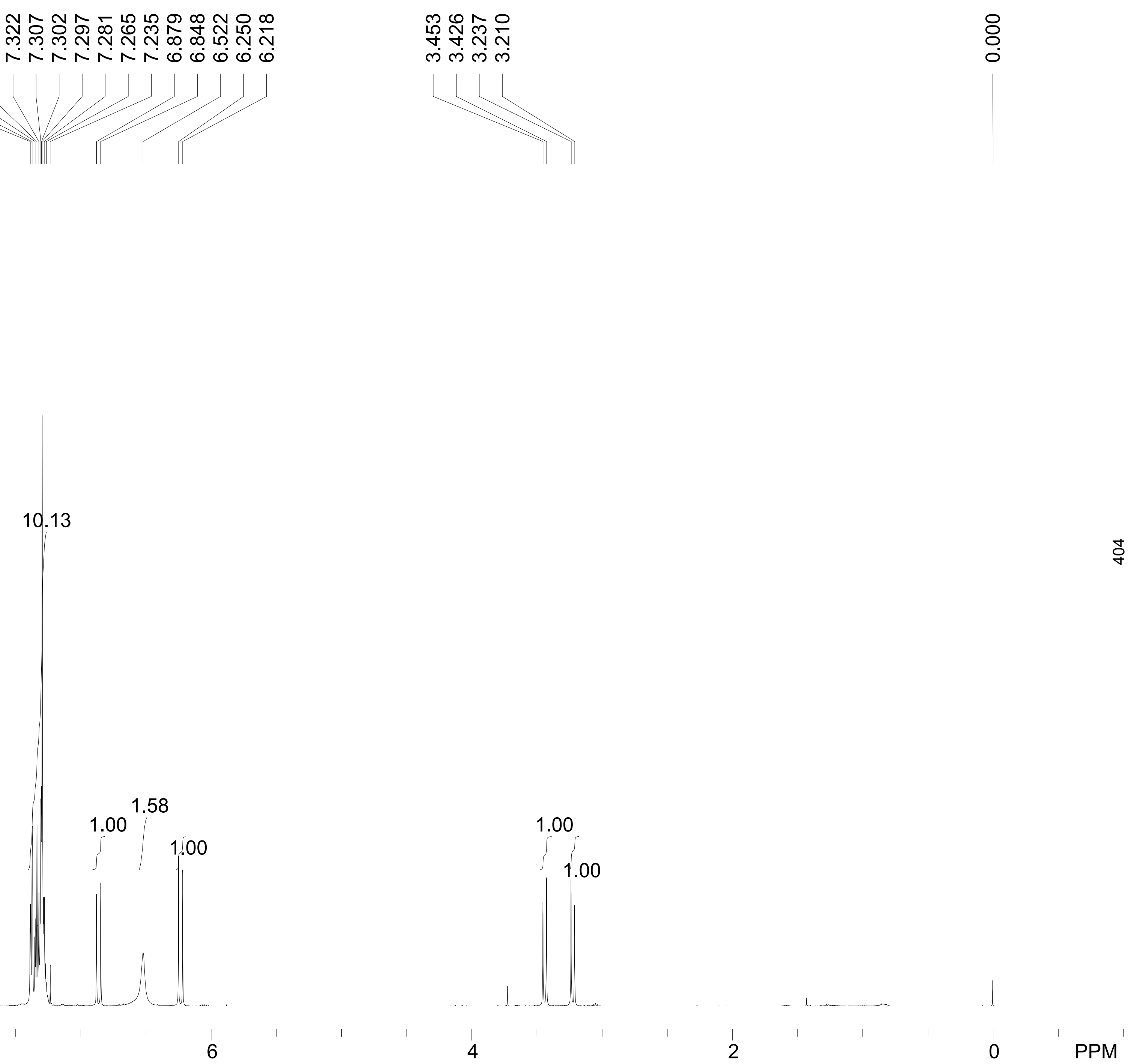

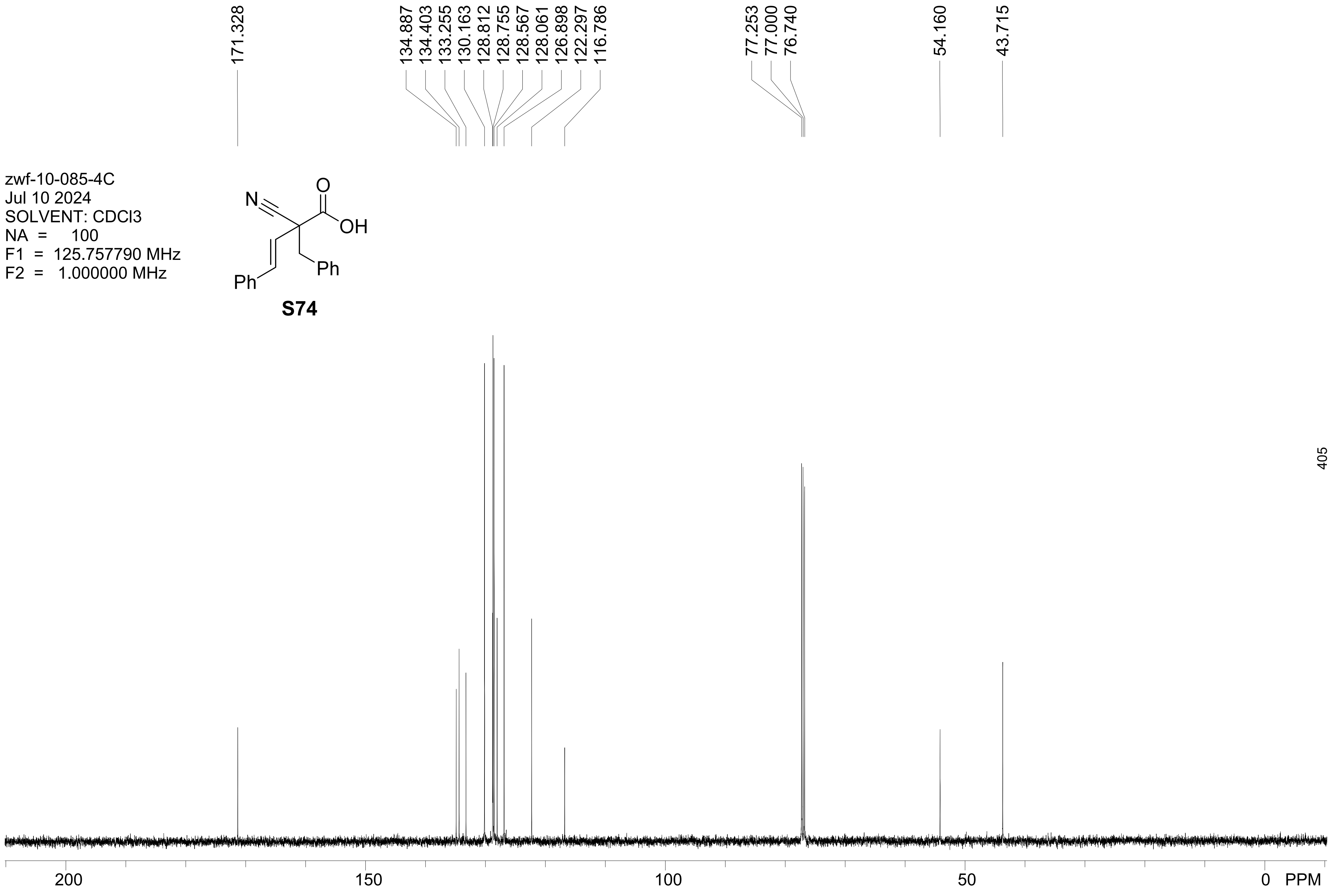
171.328
134.887
134.403
133.255
130.163
128.812
128.755
128.567
128.061
126.898
122.297
116.786
77.253
77.000
76.740
54.160
43.715
zwf-10-085-4C
Jul 10 2024
SOLVENT: CDCl3
NA = 100
F1 = 125.757790 MHz
F2 = 1.000000 MHz
200
150
100
50
0 PPM
N
O
OH
Ph
Ph

S74

zwf-10-018-10H
Jun 12 2024
SOLVENT: CDCl3
NA = 4
F1 = 500.130005 MHz
F2 = 1.000000 MHz

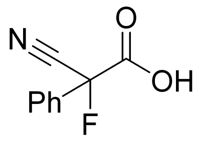

S76

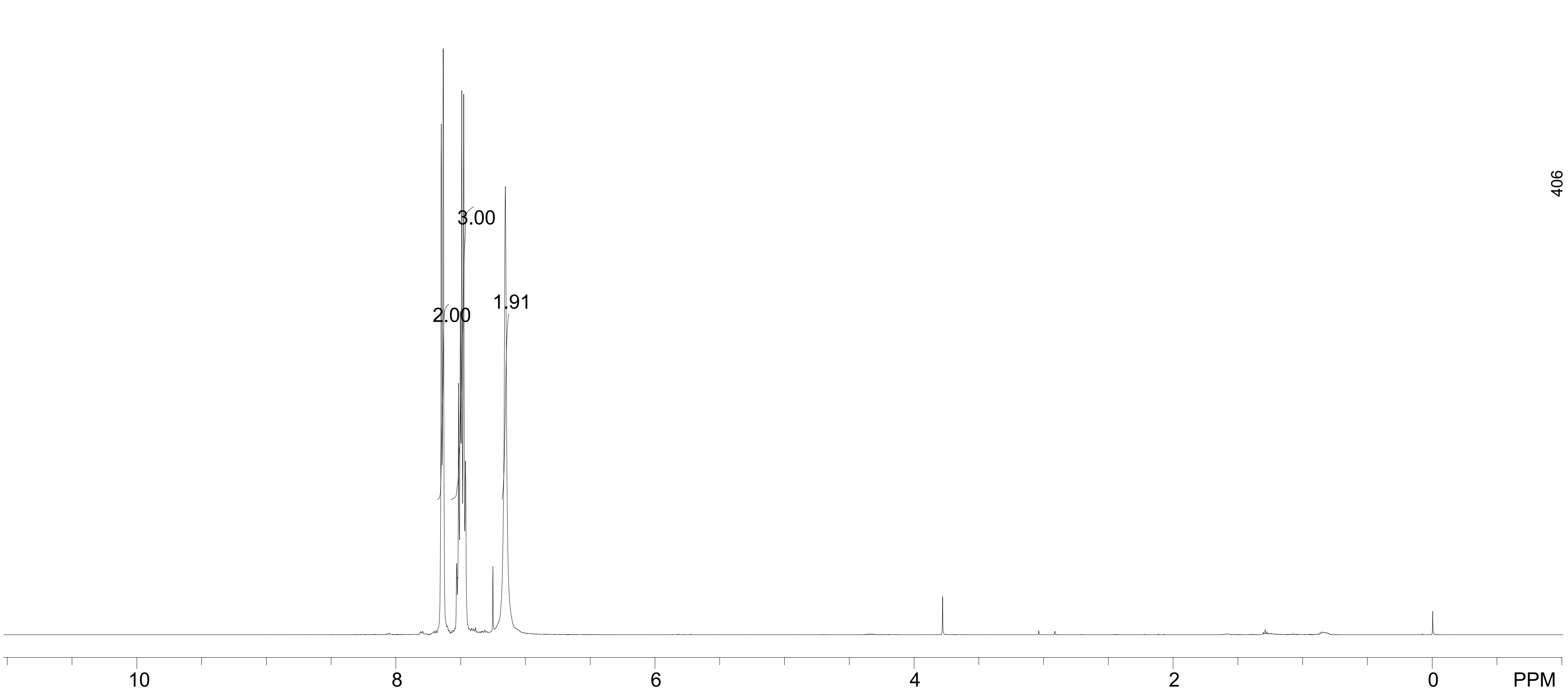

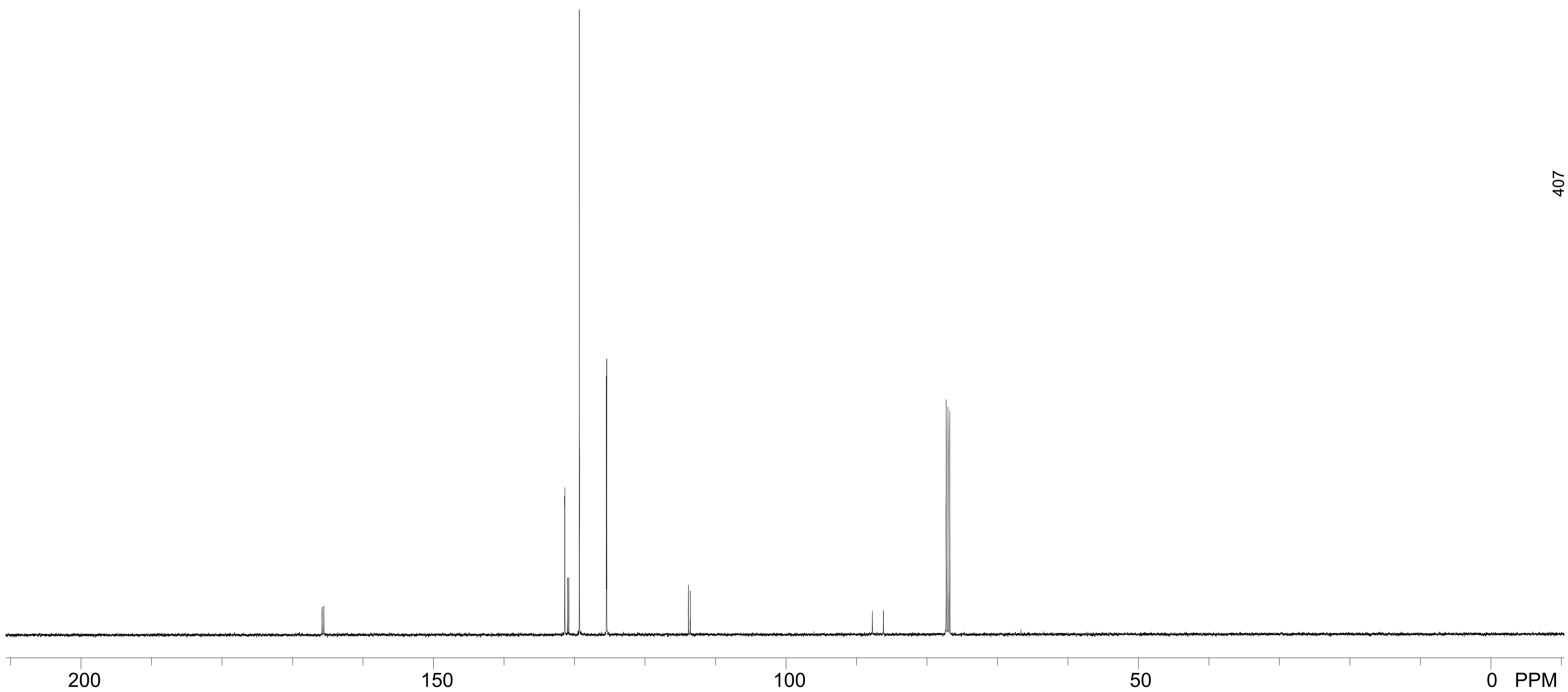

zwf-10-018-10C
Jun 12 2024
SOLVENT: CDCl3
NA = 100
F1 = 125.757790 MHz
F2 = 1.000000 MHz

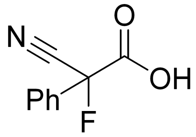

S76

zwf-10-018-10F
Jun 12 2024
SOLVENT: CDCl3
NA = 16
F1 = 470.592377 MHz
F2 = 1.000000 MHz

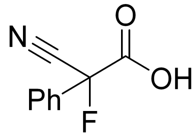

S76

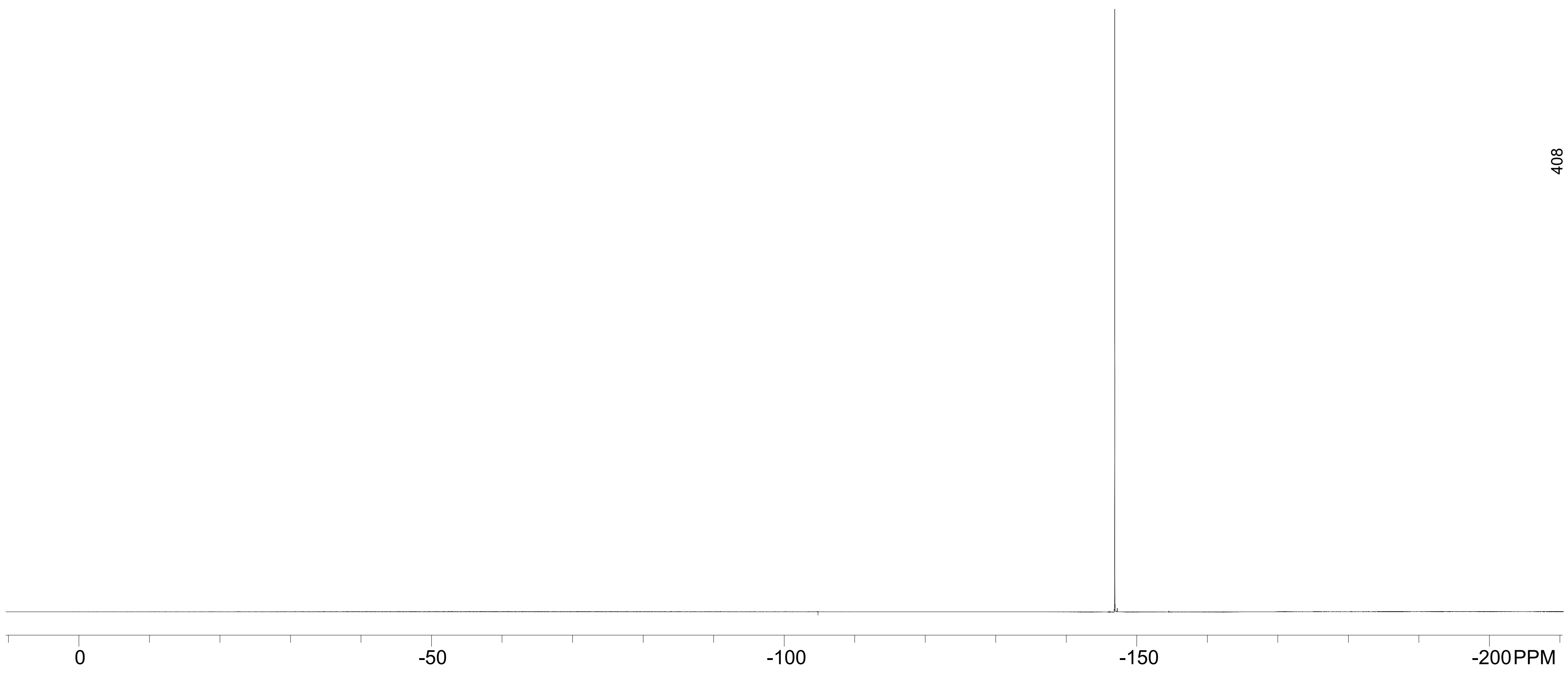

zwf-10-085-6H
Jul 12 2024
SOLVENT: CDCl3
NA = 4
F1 = 500.130005 MHz
F2 = 1.000000 MHz

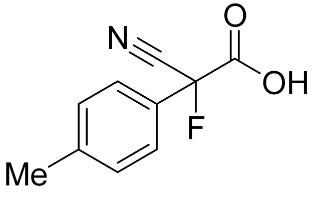

S77

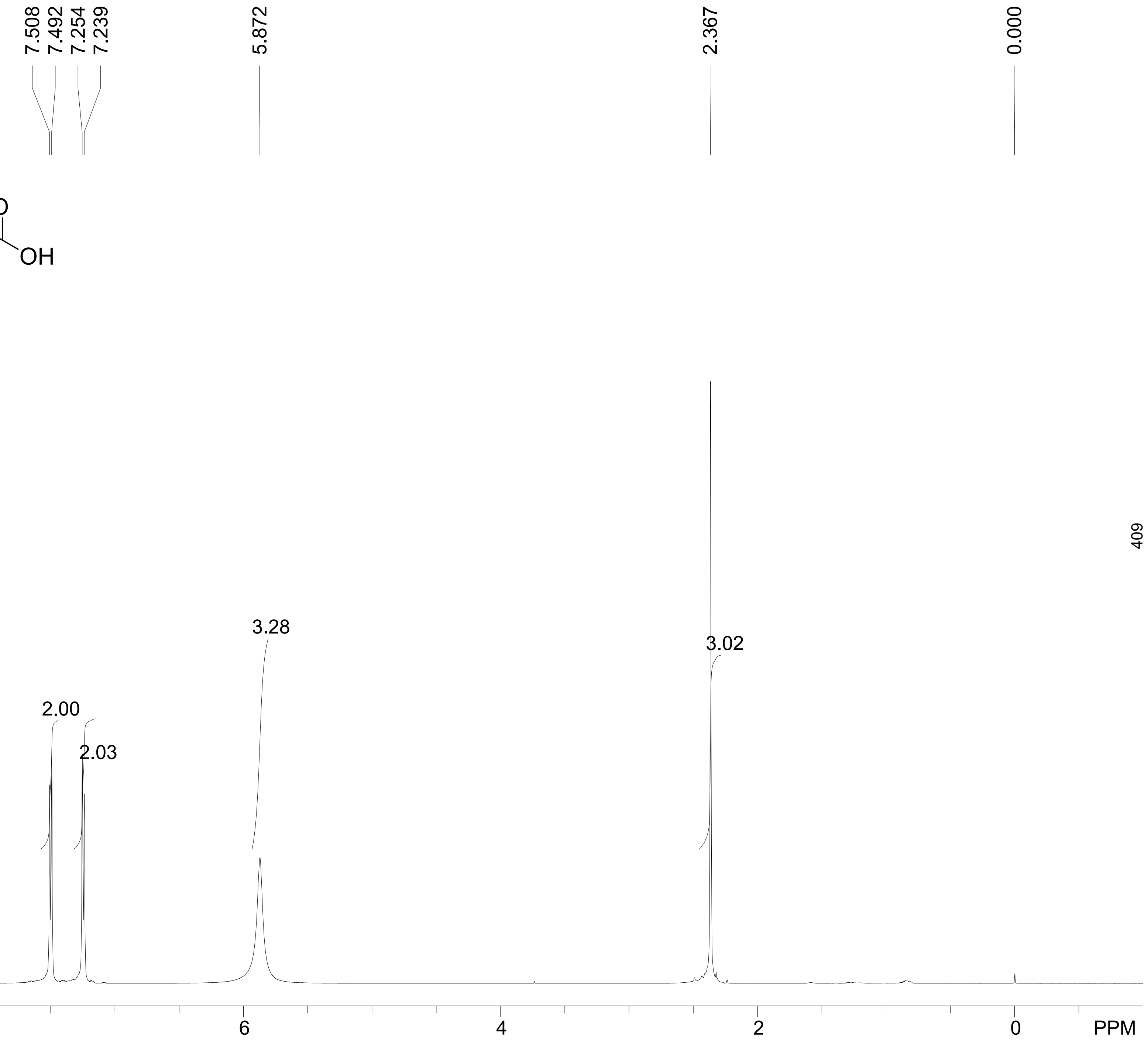

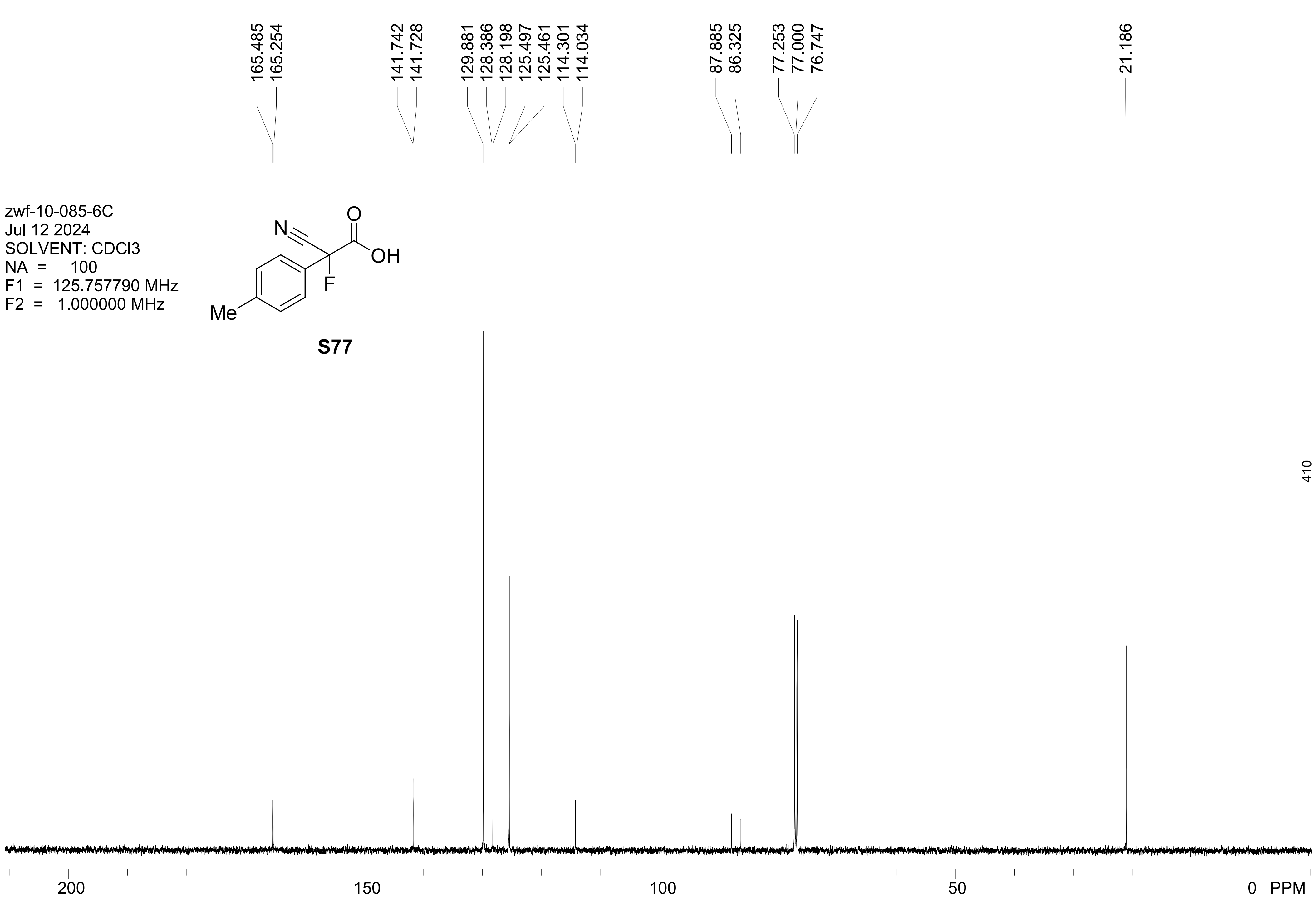
165.485
165.254
141.742
141.728
129.881
128.386
128.198
125.497
125.461
114.301
114.034
87.885
86.325
77.253
77.000
76.747
21.186
zwf-10-085-6C
Jul 12 2024
SOLVENT: CDCl3
NA = 100
F1 = 125.757790 MHz
F2 = 1.000000 MHz
N
O
OH
F
Me
S77
200
150
100
50
0 PPM

zwf-10-085-6F
Jul 12 2024
SOLVENT: CDCl3
NA = 16
F1 = 470.592377 MHz
F2 = 1.000000 MHz

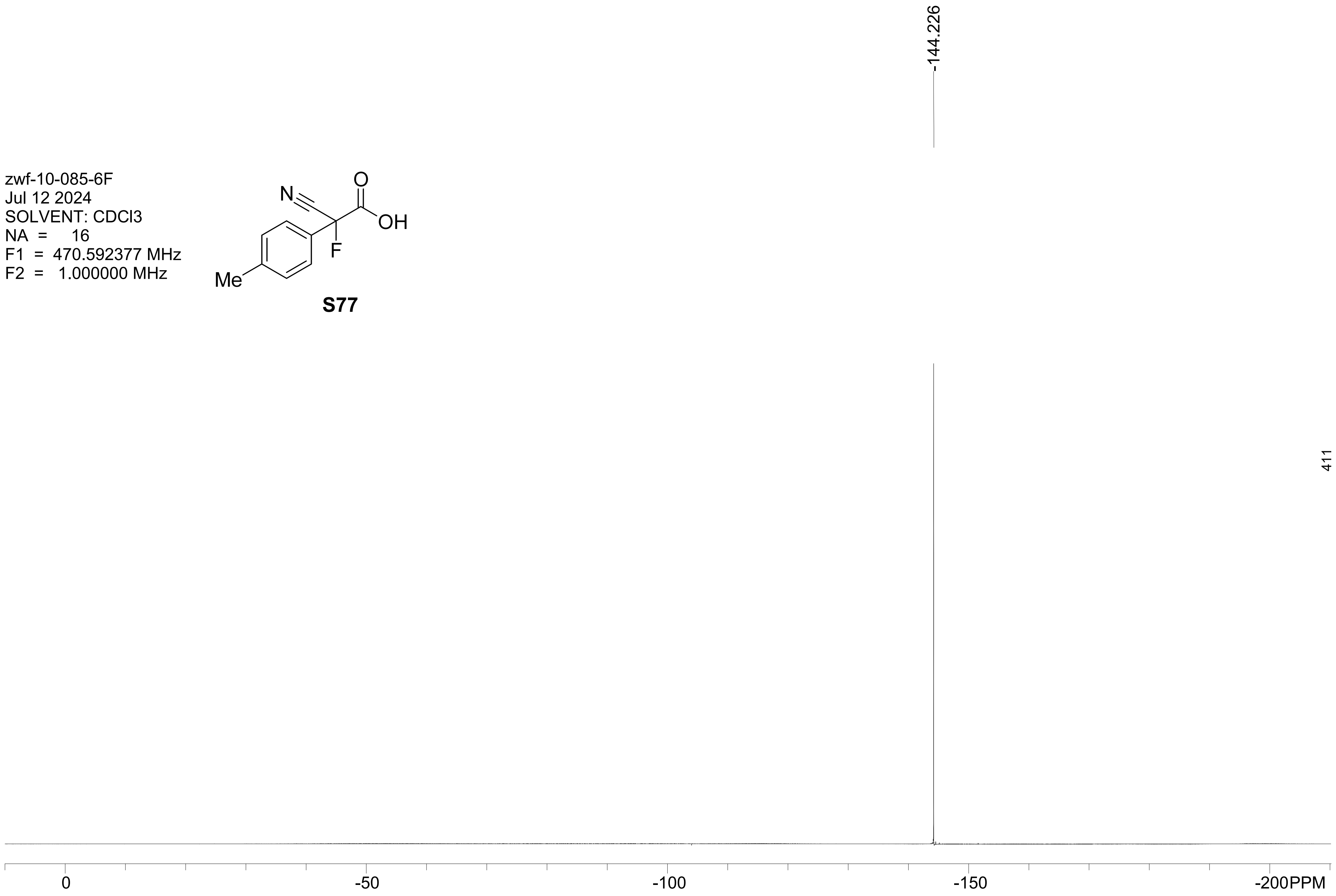

S77

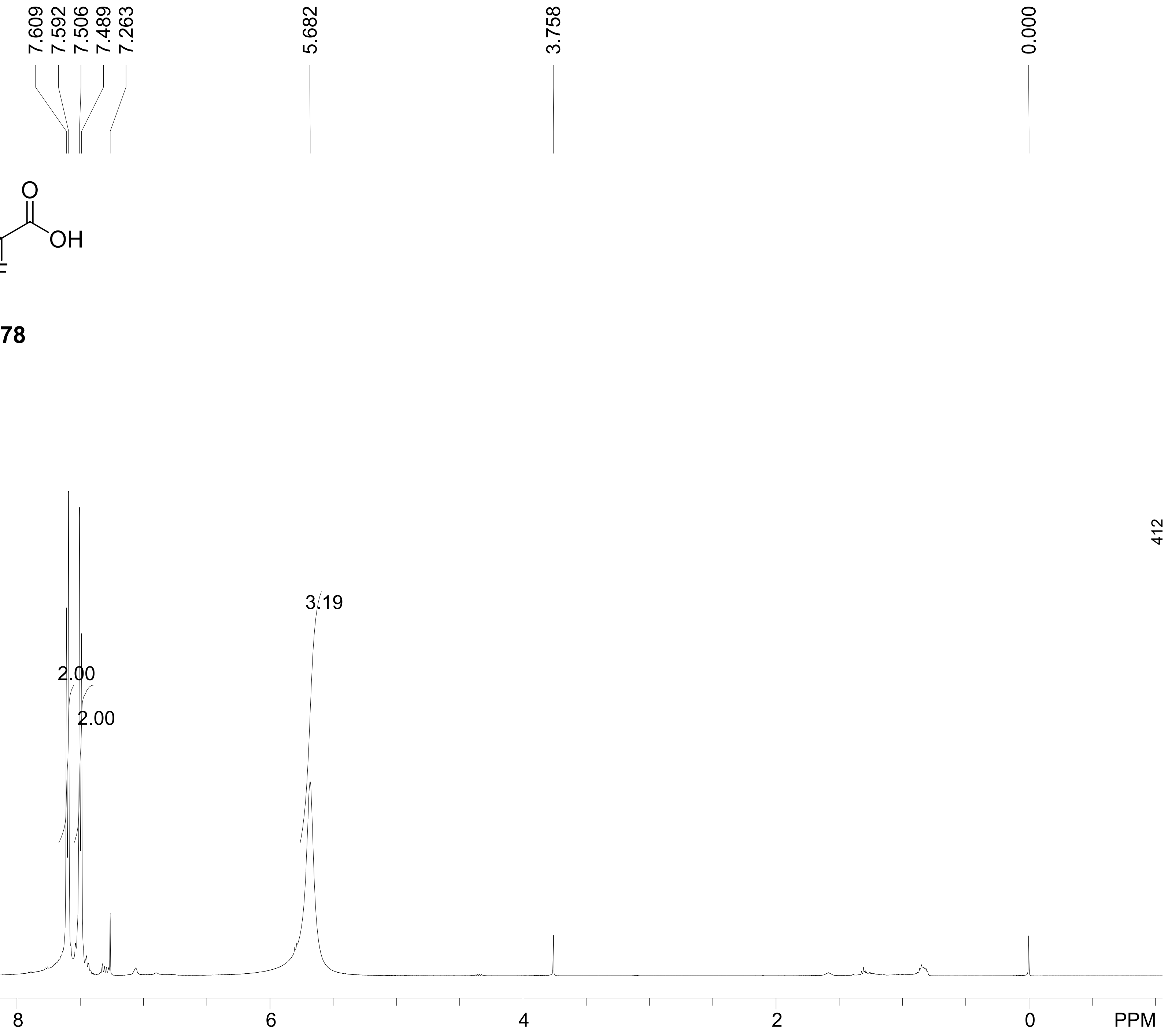
zwf-10-085-2H
Jul 07 2024
SOLVENT: CDCl3
NA = 4
F1 = 500.130005 MHz
F2 = 1.000000 MHz
7.609
7.592
7.506
7.489
7.263
5.682
3.758
0.000
2.00
2.00
3.19
10
8
6
4
2
0
PPM

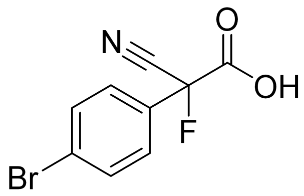
N
O
OH
F
Br

S78

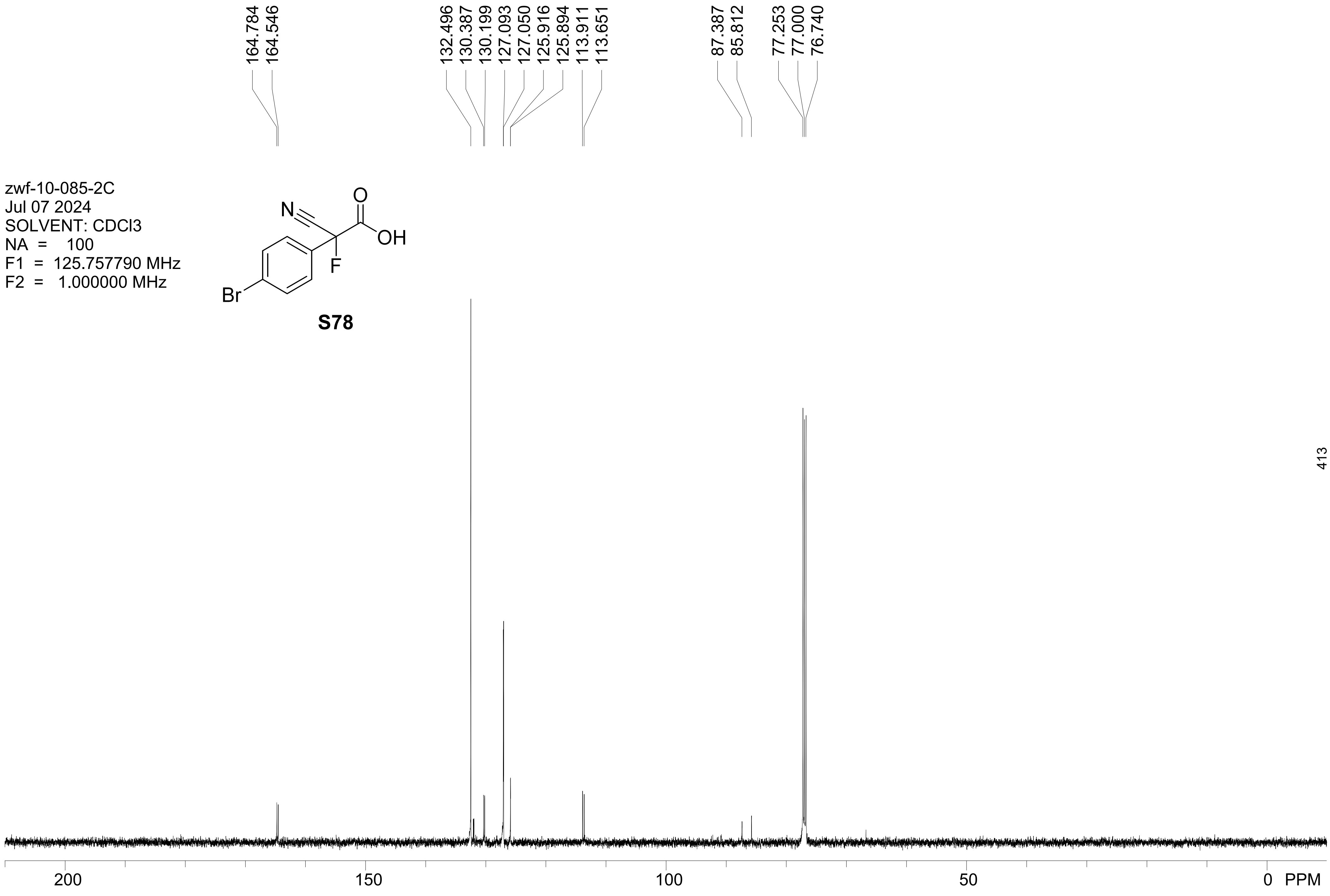
164.784
164.546
132.496
130.387
130.199
127.093
127.050
125.916
125.894
113.911
113.651
87.387
85.812
77.253
77.000
76.740
zwf-10-085-2C
Jul 07 2024
SOLVENT: CDCl3
NA = 100
F1 = 125.757790 MHz
F2 = 1.000000 MHz
N
O
OH
F
Br
S78
200
150
100
50
0 PPM

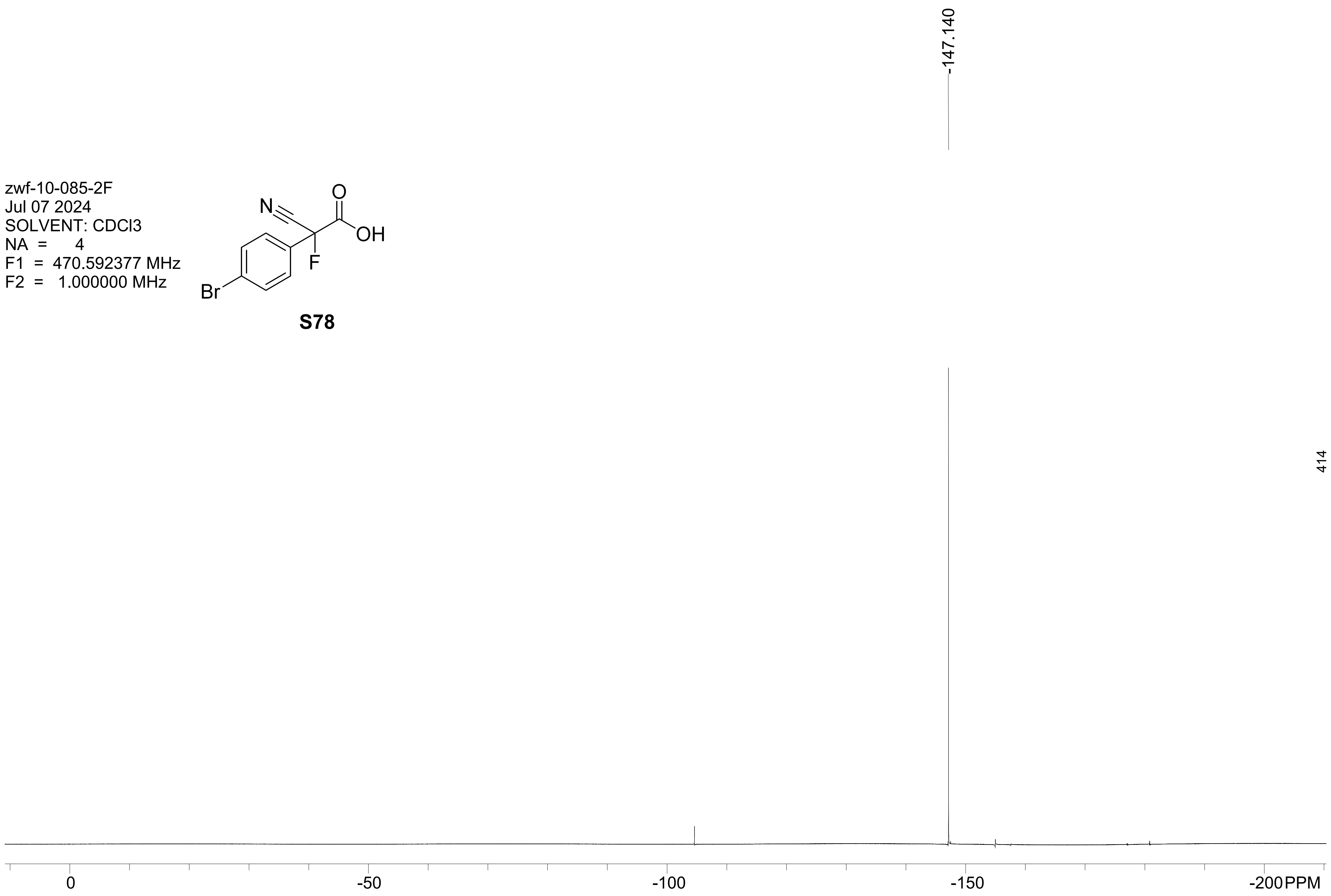
zwf-10-085-2F
Jul 07 2024
SOLVENT: CDCl3
NA =   4
F1 = 470.592377 MHz
F2 = 1.000000 MHz
N
O
OH
F
Br
S78
-147.140
0
-50
-100
-150
-200PPM

zwf-11-030H
Aug 10 2024
SOLVENT: CD3CN
NA = 4
F1 = 600.169983 MHz
F2 = 1.000000 MHz

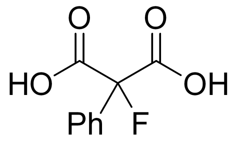

S82

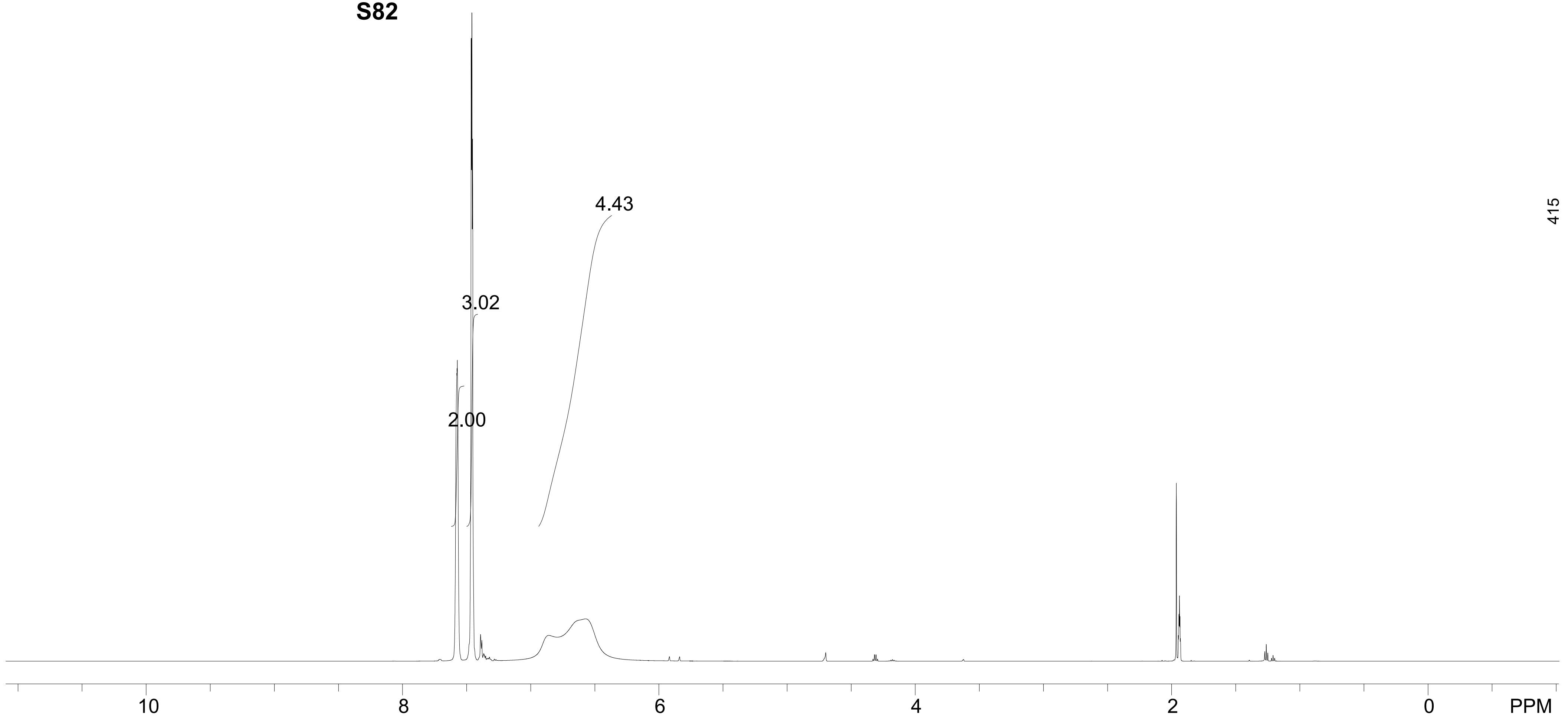

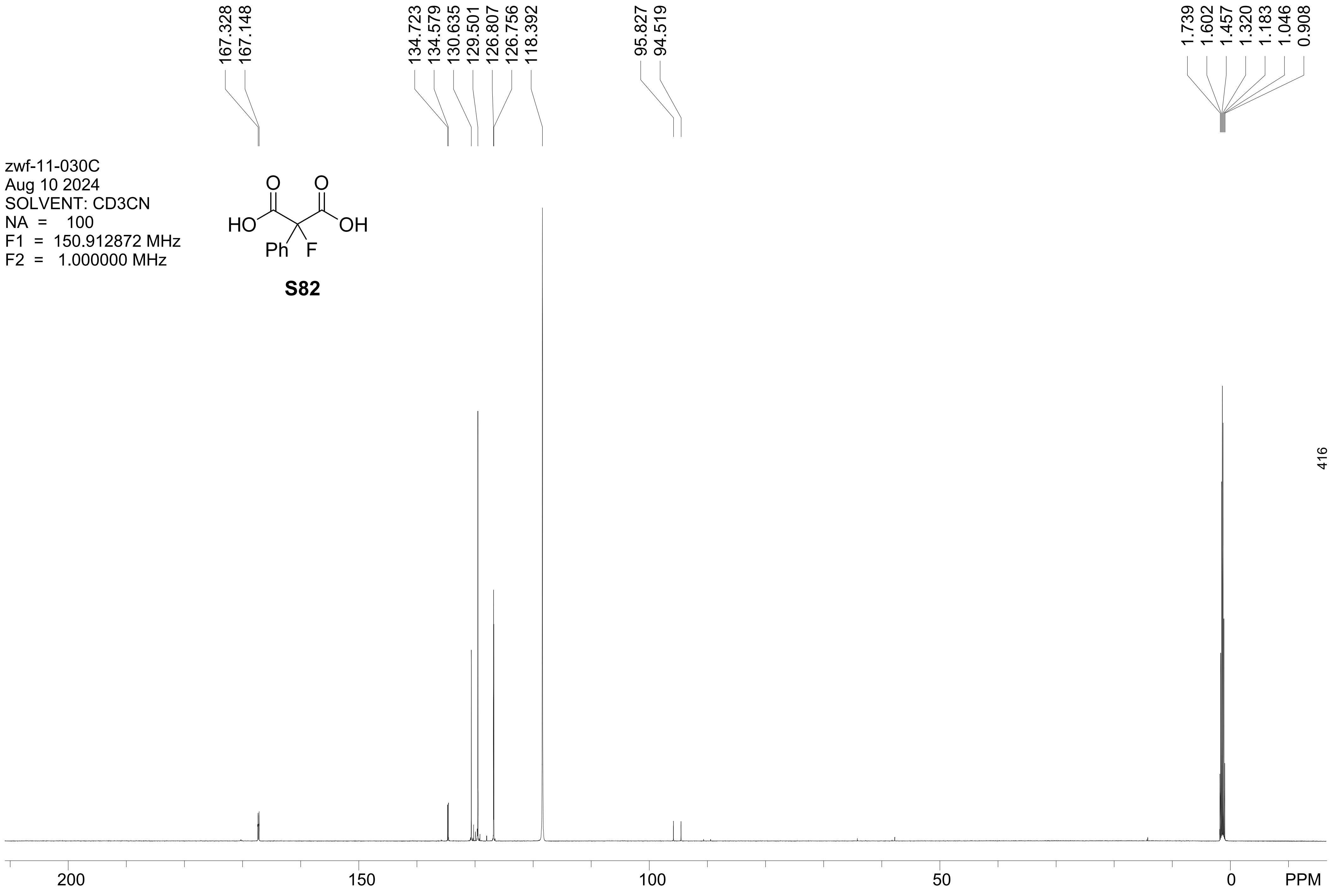
zwf-11-030C
Aug 10 2024
SOLVENT: CD3CN
NA = 100
F1 = 150.912872 MHz
F2 = 1.000000 MHz
HO
O
O
OH
Ph
F
S82
167.328
167.148
134.723
134.579
130.635
129.501
126.807
126.756
118.392
95.827
94.519
1.739
1.602
1.457
1.320
1.183
1.046
0.908
200
150
100
50
0
PPM

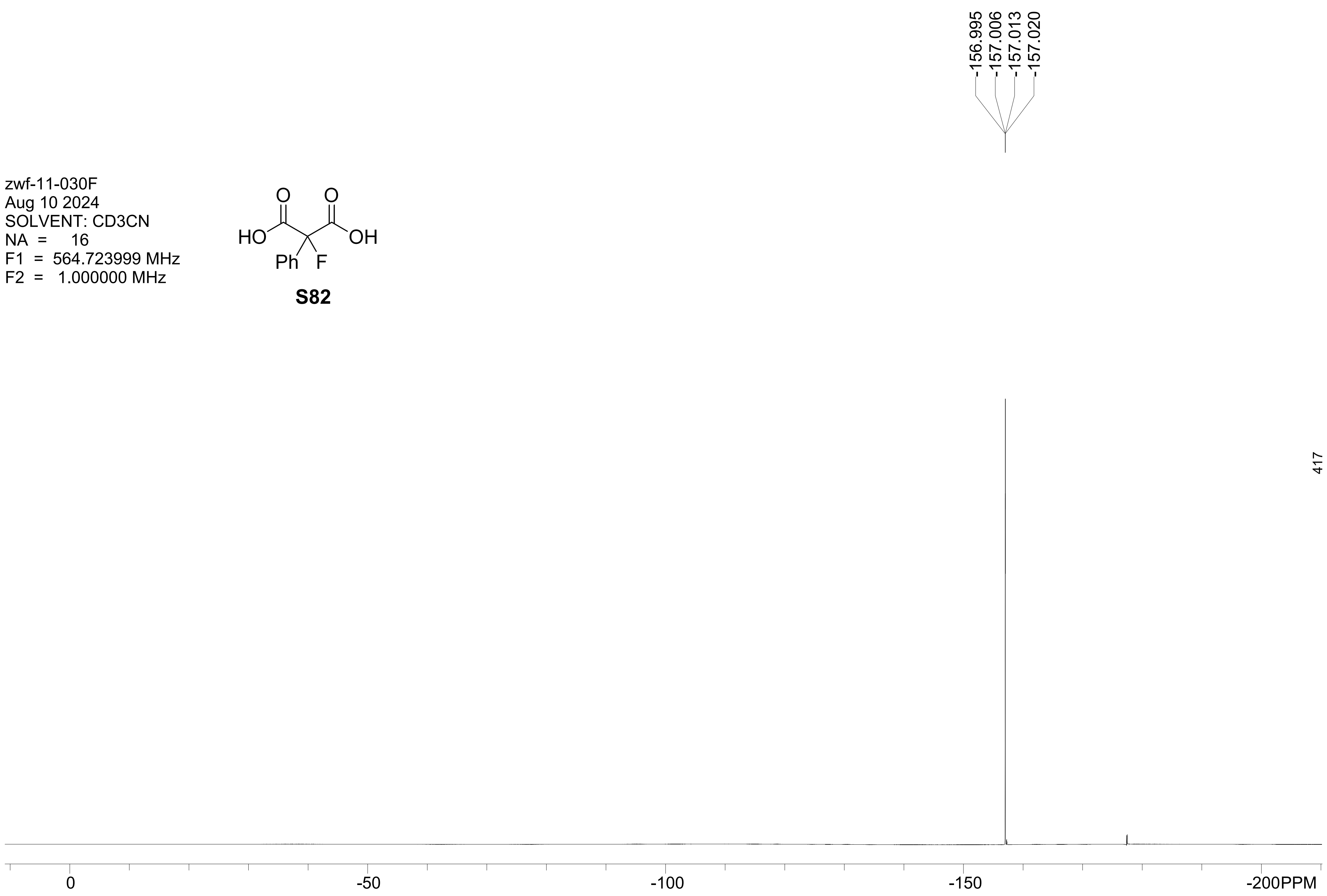
zwf-11-030F
Aug 10 2024
SOLVENT: CD3CN
NA = 16
F1 = 564.723999 MHz
F2 = 1.000000 MHz
HO
O
O
OH
Ph
F
S82
-156.995
-157.006
-157.013
-157.020
0
-50
-100
-150
-200PPM

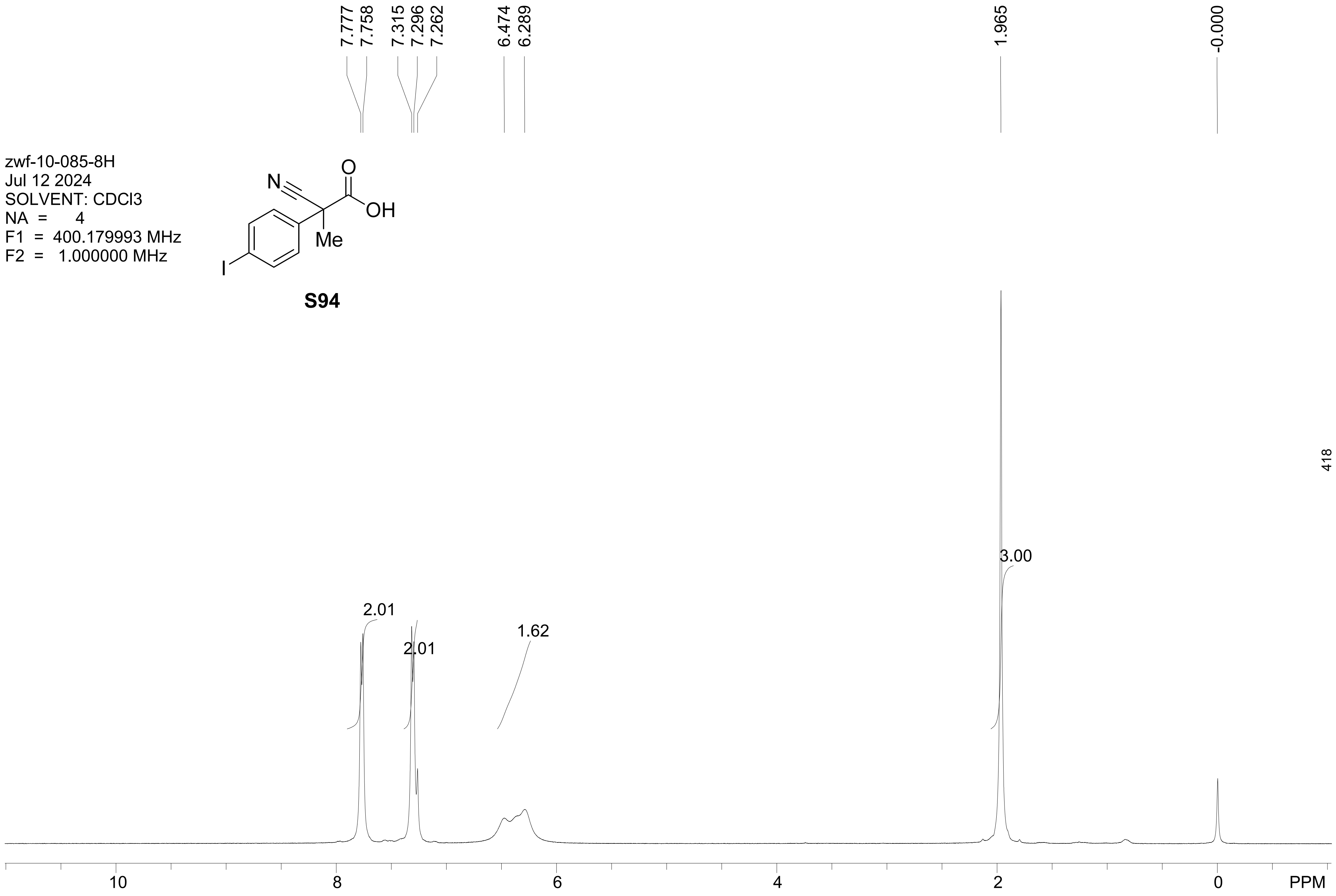

7.777
7.758
7.315
7.296
7.262
6.474
6.289
1.965
-0.000
zwf-10-085-8H
Jul 12 2024
SOLVENT: CDCl3
NA = 4
F1 = 400.179993 MHz
F2 = 1.000000 MHz
2.01
2.01
1.62
3.00
10
8
6
4
2
0
PPM
N
O
OH
Me
I

S94

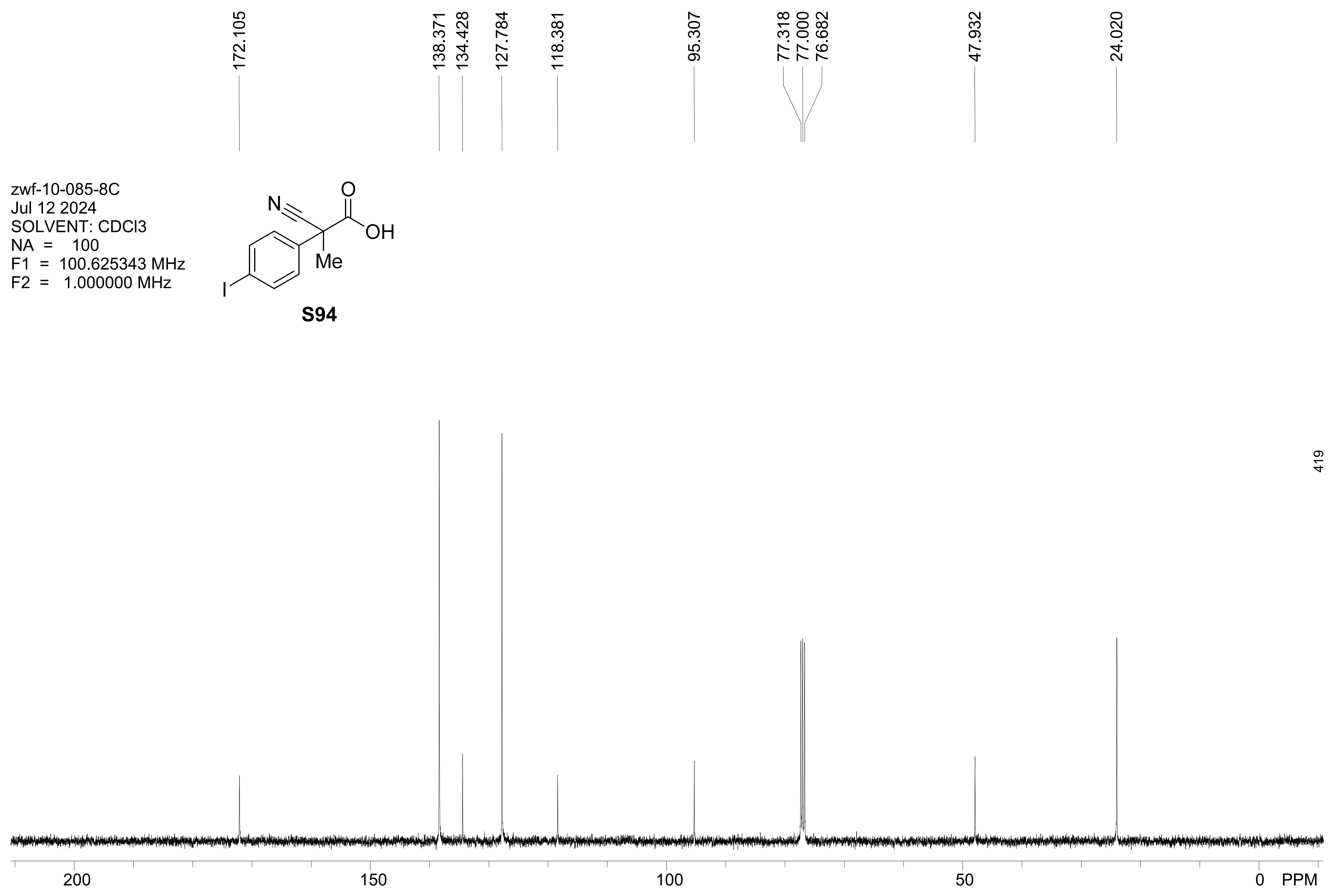
172.105
138.371
134.428
127.784
118.381
95.307
77.318
77.000
76.682
47.932
24.020
zwf-10-085-8C
Jul 12 2024
SOLVENT: CDCl3
NA = 100
F1 = 100.625343 MHz
F2 = 1.000000 MHz
N
O
OH
Me
I
S94
200
150
100
50
0 PPM

zwf-10-085-7H
Jul 13 2024
SOLVENT: CDCl3
NA = 4
F1 = 500.130005 MHz
F2 = 1.000000 MHz

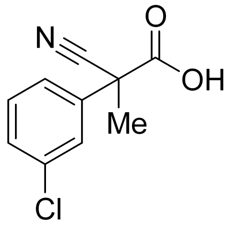

S98

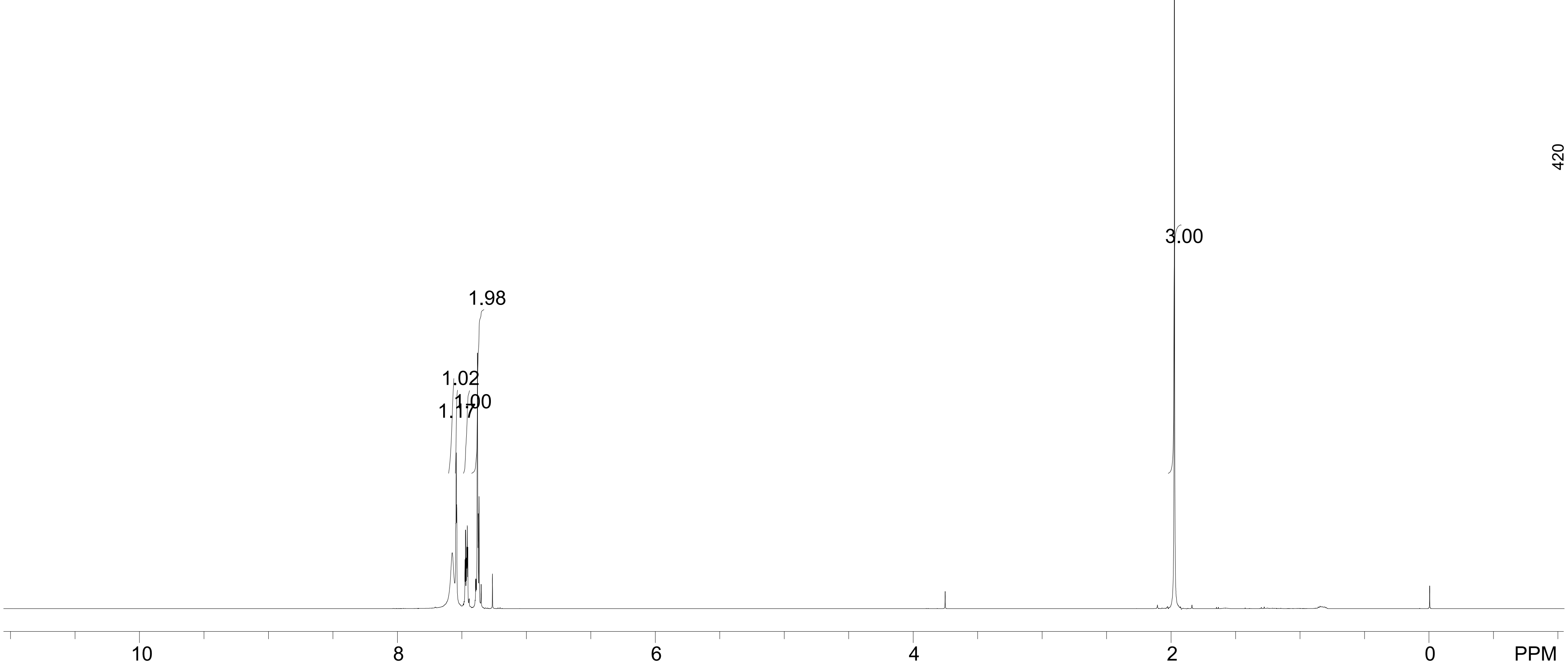

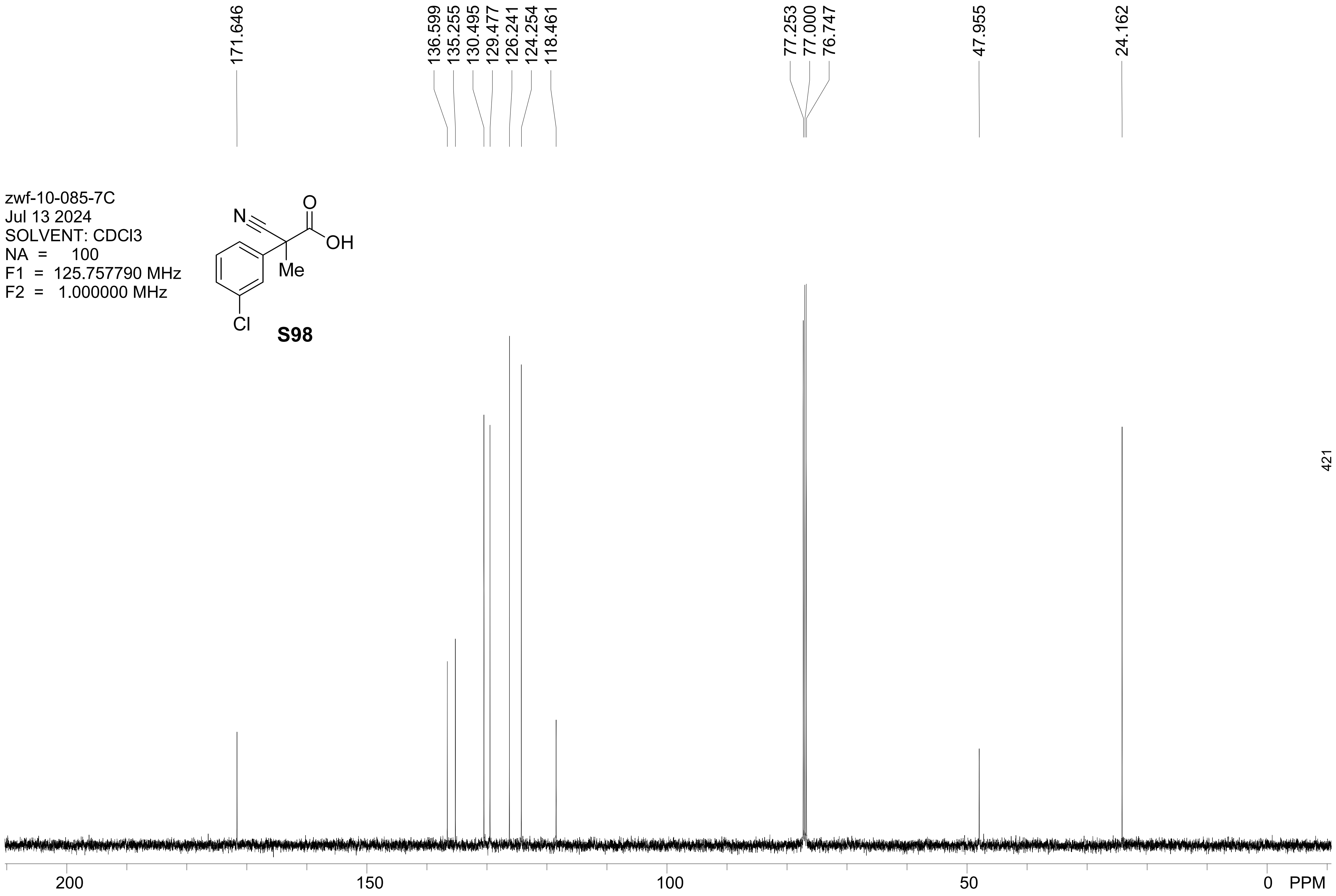
N
O
OH
Me
Cl
S98
zwf-10-085-7C
Jul 13 2024
SOLVENT: CDCl3
NA = 100
F1 = 125.757790 MHz
F2 = 1.000000 MHz
171.646
136.599
135.255
130.495
129.477
126.241
124.254
118.461
77.253
77.000
76.747
47.955
24.162
200
150
100
50
0 PPM

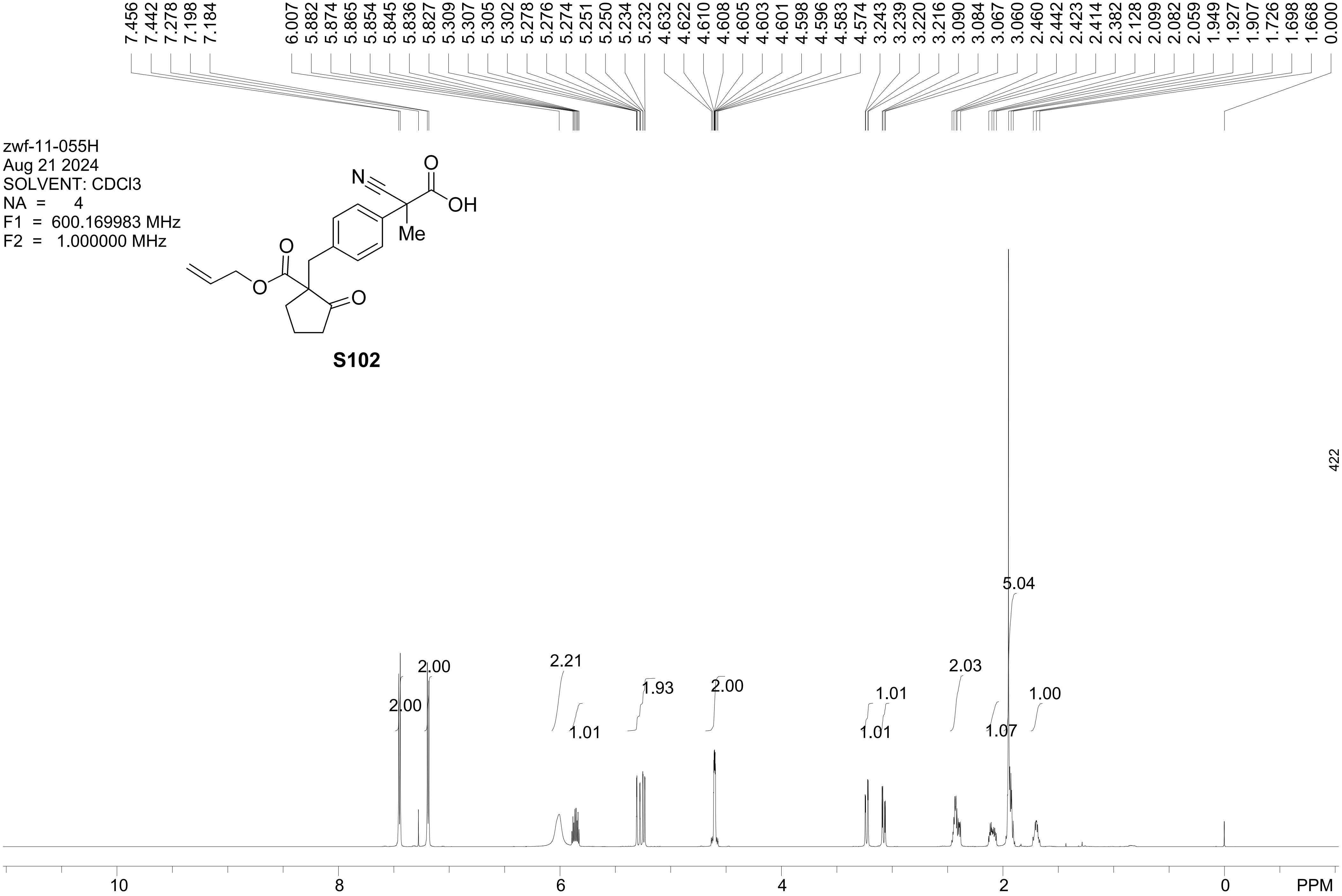
zwf-11-055H
Aug 21 2024
SOLVENT: CDCl3
NA = 4
F1 = 600.169983 MHz
F2 = 1.000000 MHz
7.456
7.442
7.278
7.198
7.184
6.007
5.882
5.874
5.865
5.854
5.845
5.836
5.827
5.309
5.307
5.305
5.302
5.278
5.276
5.274
5.251
5.250
5.234
5.232
4.632
4.622
4.610
4.608
4.605
4.603
4.601
4.598
4.596
4.583
4.574
3.243
3.239
3.220
3.216
3.090
3.084
3.067
3.060
2.460
2.442
2.423
2.414
2.382
2.128
2.099
2.082
2.059
1.949
1.927
1.907
1.726
1.698
1.668
0.000
N
O
OH
Me
O
O
O
2.00
2.00
2.21
1.01
1.93
2.00
1.01
1.01
2.03
1.07
5.04
1.00
10
8
6
4
2
0
PPM

S102

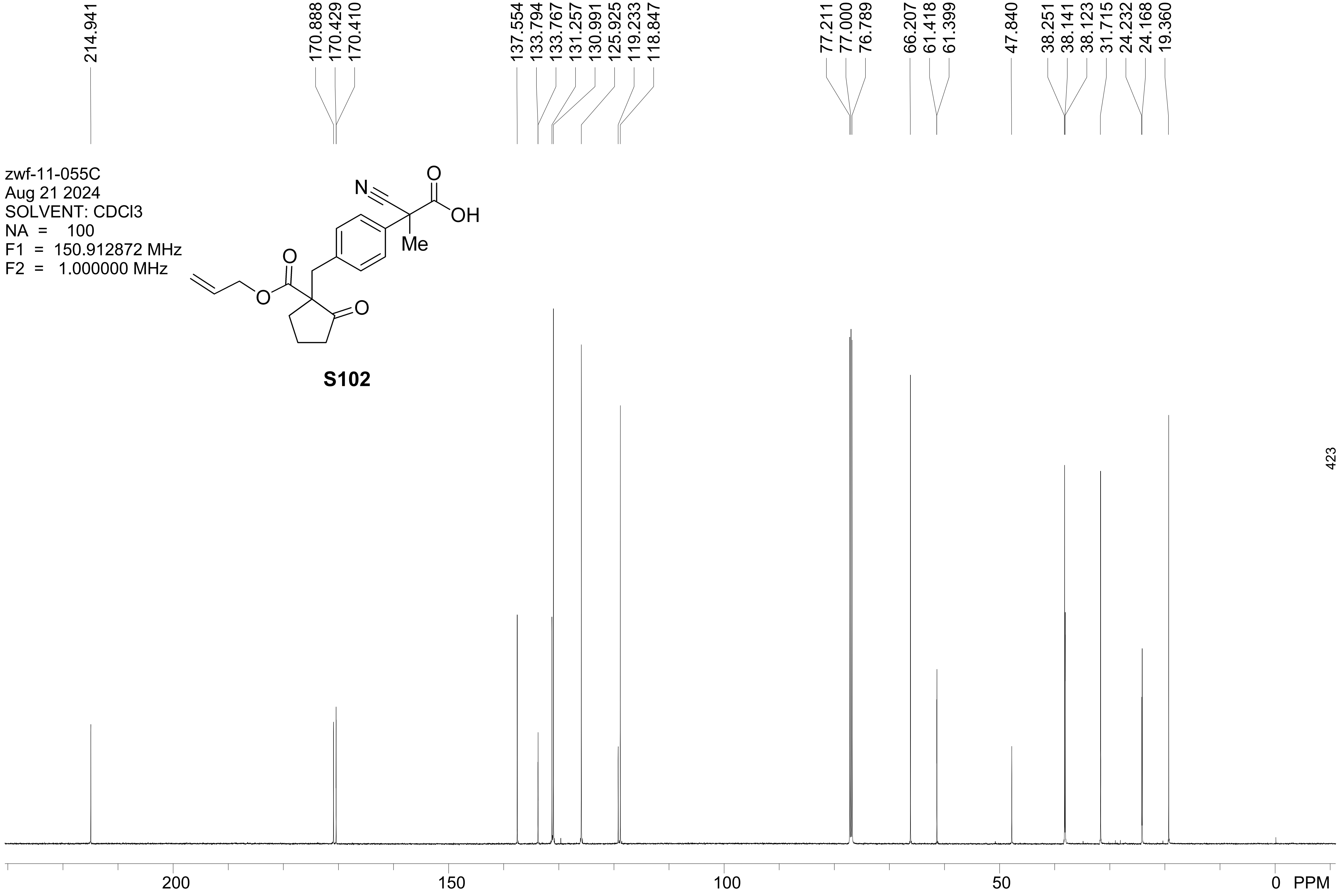
214.941
170.888
170.429
170.410
137.554
133.794
133.767
131.257
130.991
125.925
119.233
118.847
77.211
77.000
76.789
66.207
61.418
61.399
47.840
38.251
38.141
38.123
31.715
24.232
24.168
19.360
zwf-11-055C
Aug 21 2024
SOLVENT: CDCl3
NA =   100
F1 = 150.912872 MHz
F2 =   1.000000 MHz
N
O
OH
Me
O
O
O
200
150
100
50
0 PPM

S102

zwf-11-087H
Aug 29 2024
SOLVENT: CDCl3
NA = 4
F1 = 600.169983 MHz
F2 = 1.000000 MHz

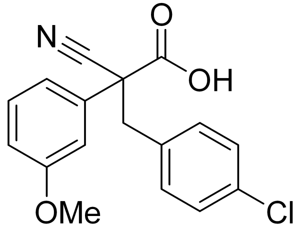

S107

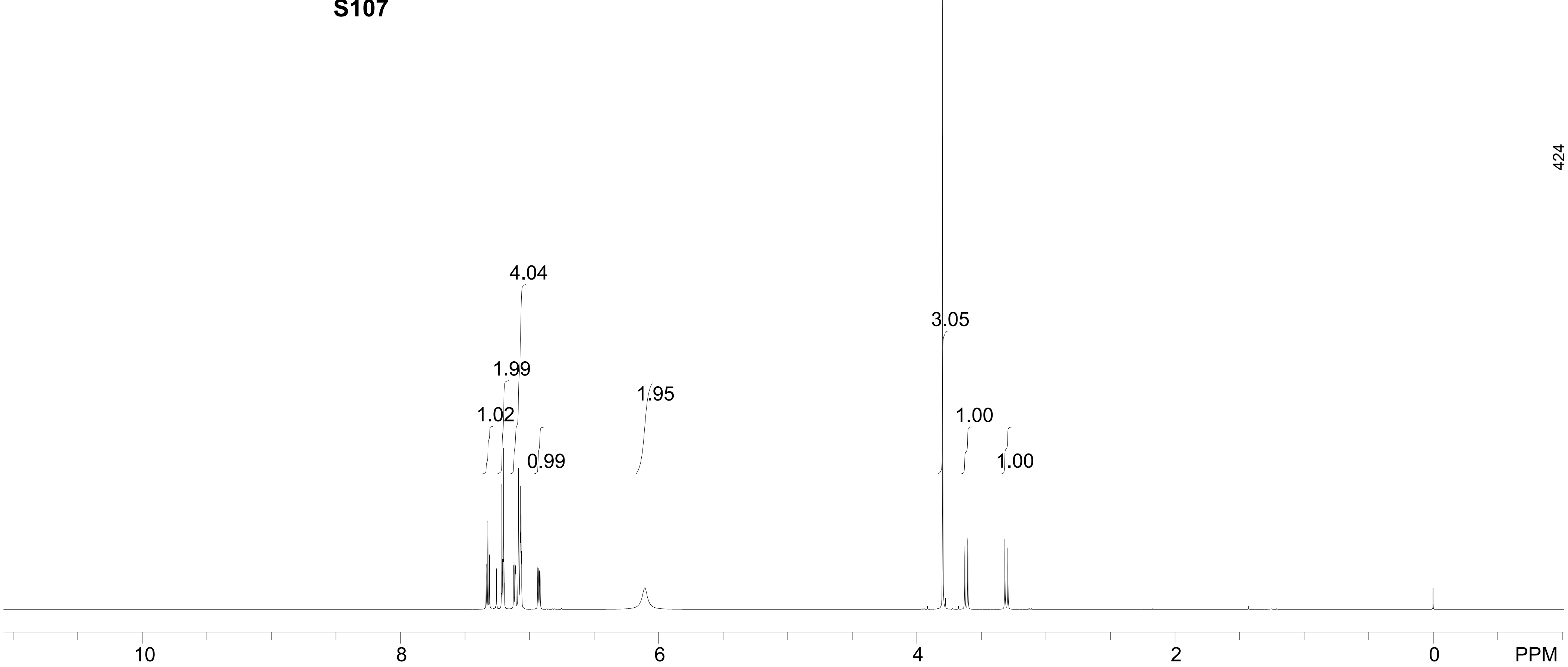

zwf-11-087C
Aug 29 2024
SOLVENT: CDCl3
NA = 100
F1 = 150.912872 MHz
F2 = 1.000000 MHz

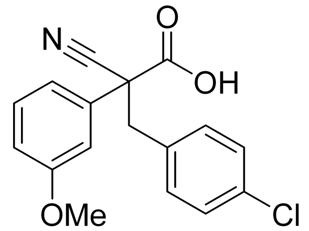

S107

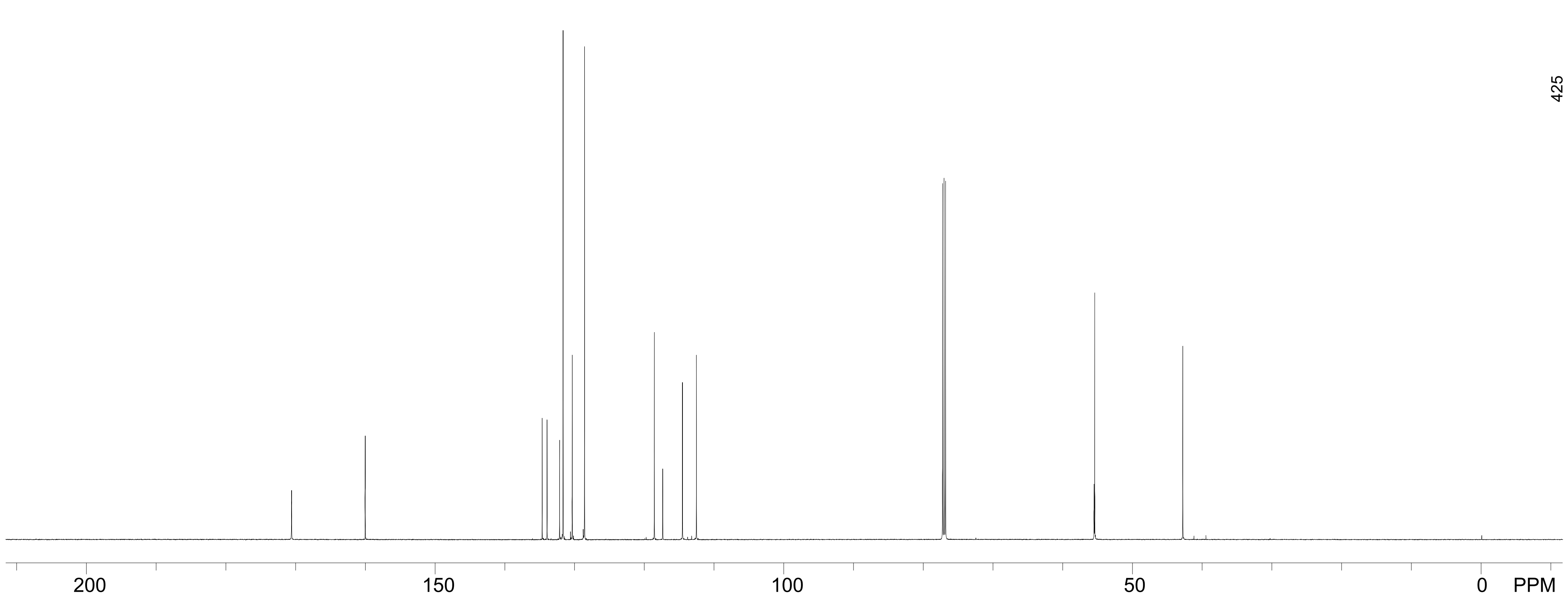

zwf-7-058H
Sep 11 2023
SOLVENT: CDCl3
NA = 4
F1 = 400.179993 MHz
F2 = 1.000000 MHz

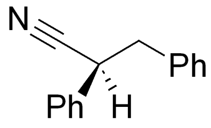

1

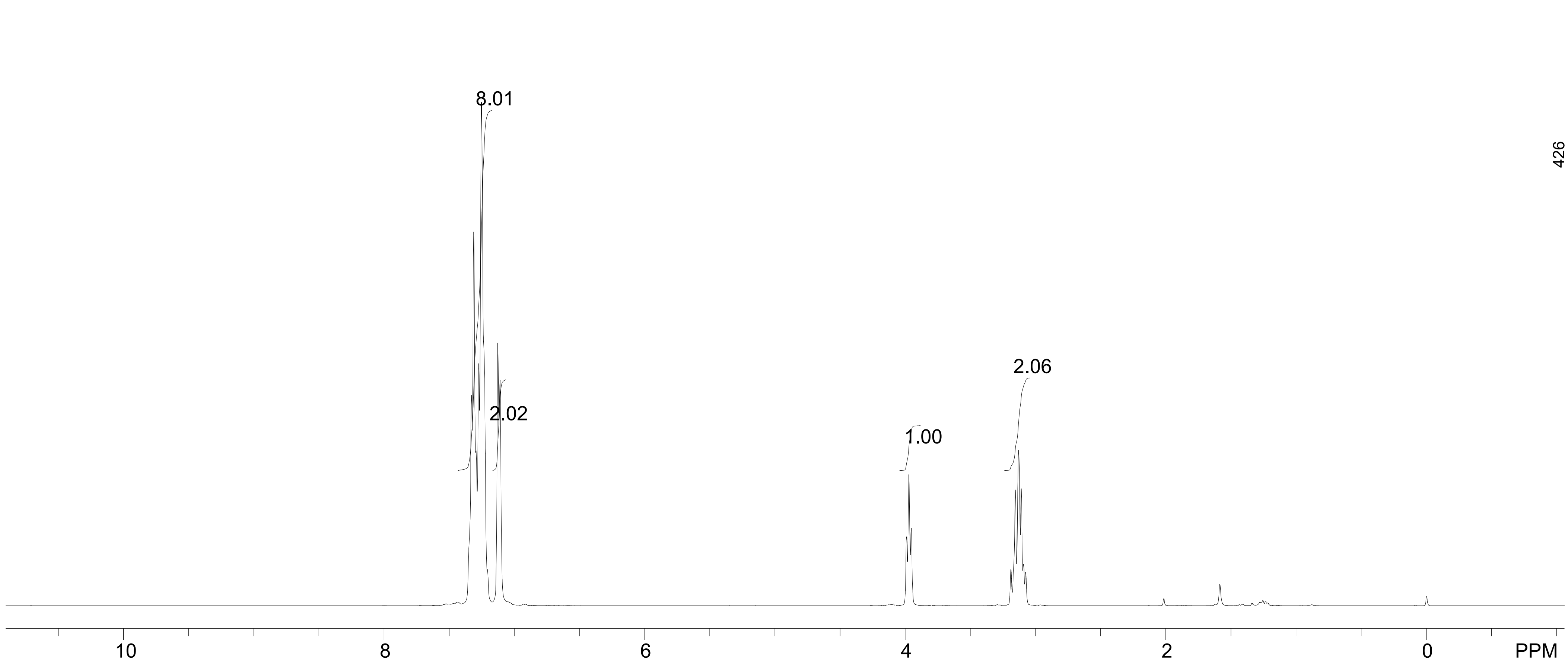

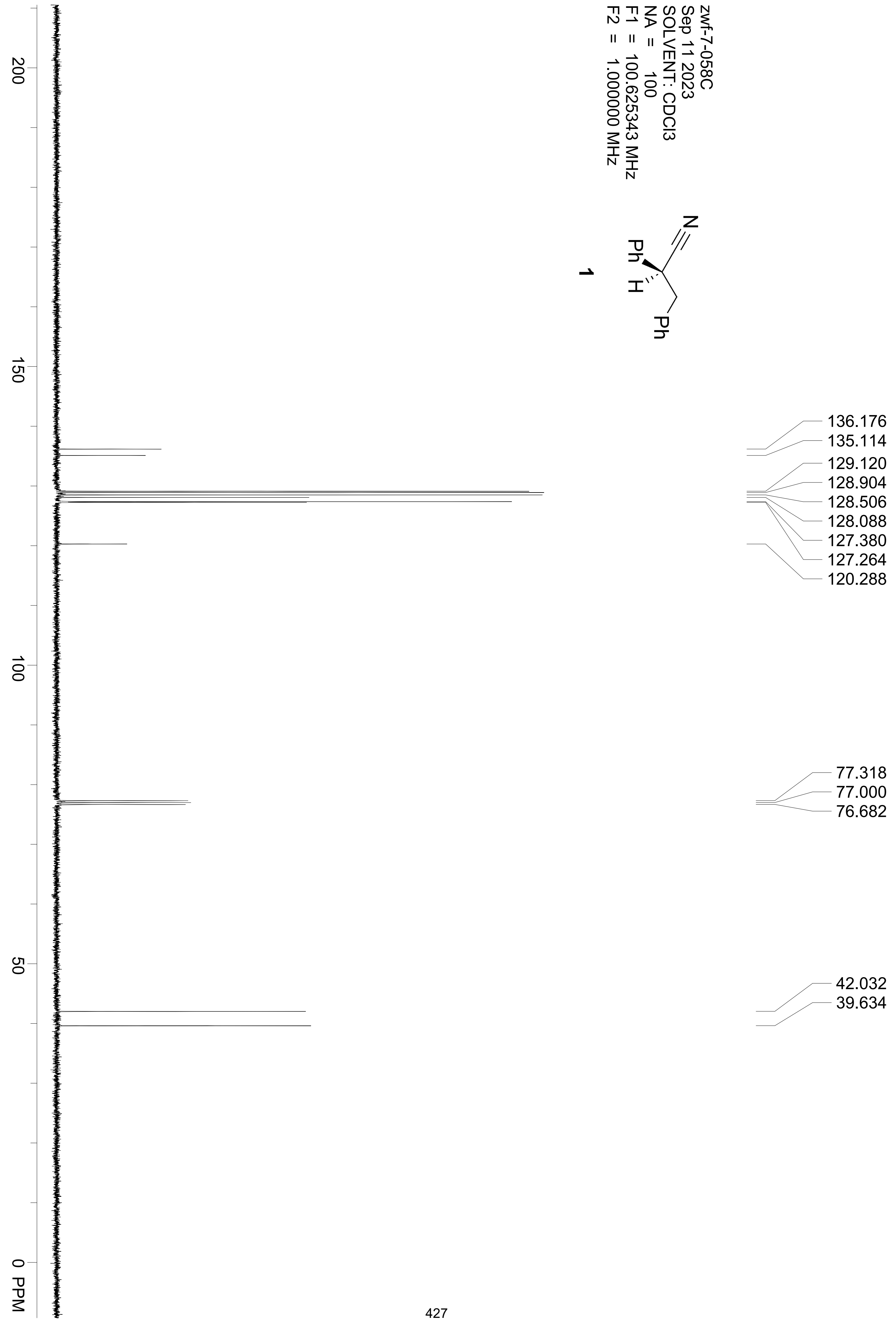
zwf-7-058C
Sep 11 2023
SOLVENT: CDCl3
NA = 100
F1 = 100.625343 MHz
F2 = 1.000000 MHz
N
Ph
H
Ph
1
136.176
135.114
129.120
128.904
128.506
128.088
127.380
127.264
120.288
77.318
77.000
76.682
42.032
39.634
200
150
100
50
0 PPM

Area Percent Report

Sample name: zwf-7-058-IE-99-1-0.8-205

Data file: C:\Users\Public\Documents\ChemStation\1\Data\zwf\zwf-S 2023-09-11 18-06-28\004-P1-C2-zwf-7-058.D

Injection Acquired Date 09/11/2023 19:25:44

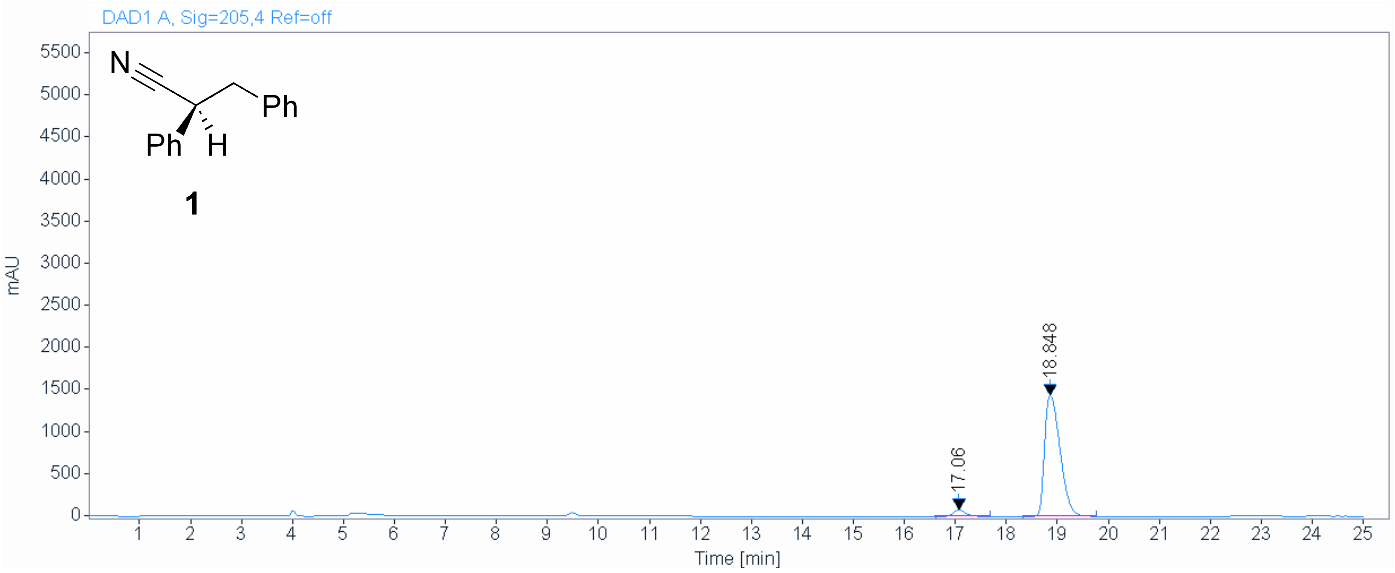

Signal: DAD1 A, Sig=205,4 Ref=off

RT [min]	Width [min]	Height	Area	Area%
17.060	0.2511	77.9977	1291.1536	4.0888
18.848	0.3299	1436.7190	30287.0117	95.9112
		Sum	31578.1653	100.0000

Area Percent Report

Agilent Technologies

Sample name: zwf-7-058-rac-IE-99-1-0.8-205

Data file: C:\Users\Public\Documents\ChemStation\1\Data\zwf\zwf-S 2023-09-11 18-06-28\003-P1-C3-zwf-7-058-rac.D

Injection Acquired Date 09/11/2023 18:59:37

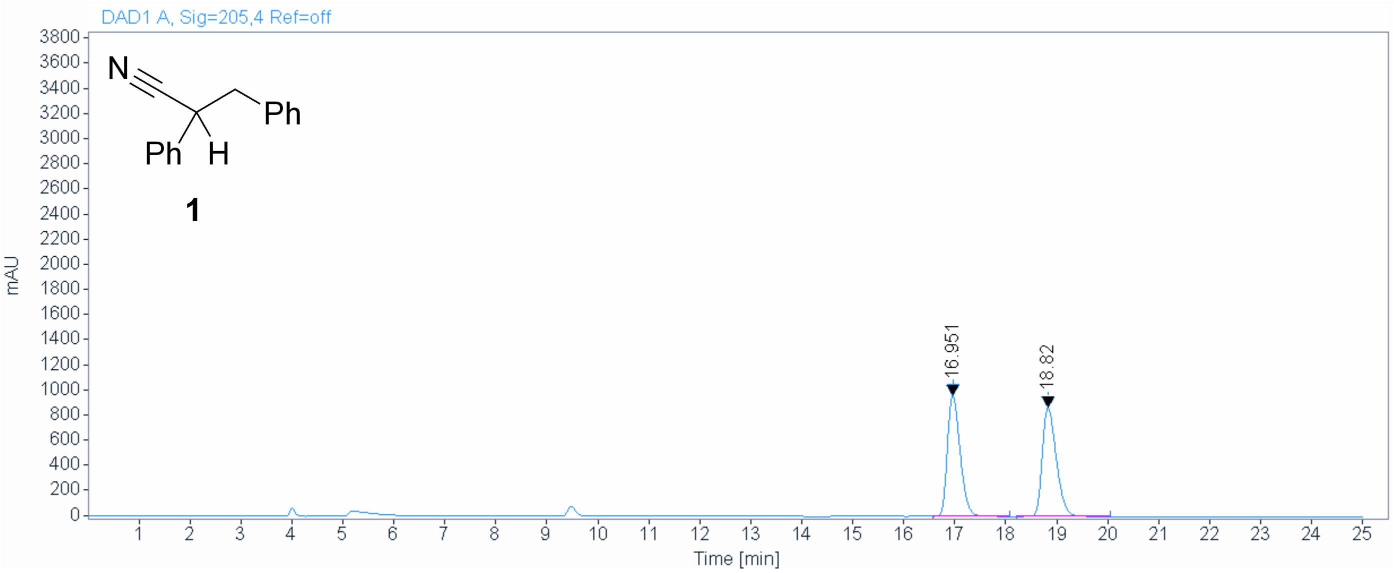

Signal: DAD1 A, Sig=205,4 Ref=off

RT [min]	Width [min]	Height	Area	Area%
16.951	0.2729	962.4082	16740.2598	50.0786
18.820	0.2983	867.9600	16687.6836	49.9214
		Sum	33427.9434	100.0000

zwf-7-151H
Nov 09 2023
SOLVENT: CDCl3
NA = 4
F1 = 400.179993 MHz
F2 = 1.000000 MHz

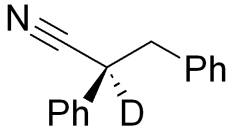

1-d

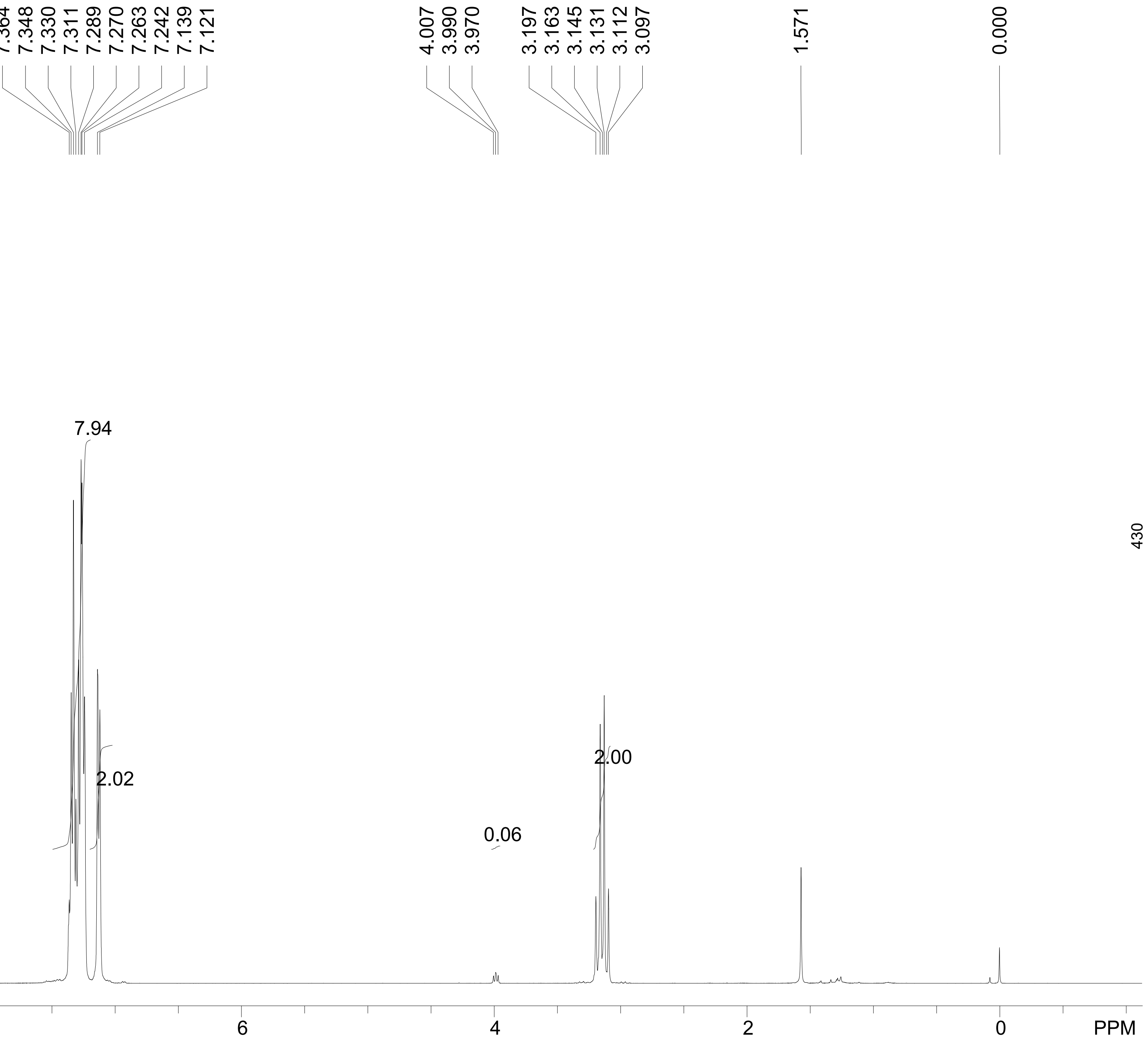

zwf-7-151C
Nov 10 2023
SOLVENT: CDCl3
NA = 160
F1 = 100.625343 MHz
F2 = 1.000000 MHz

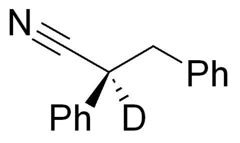

1-d

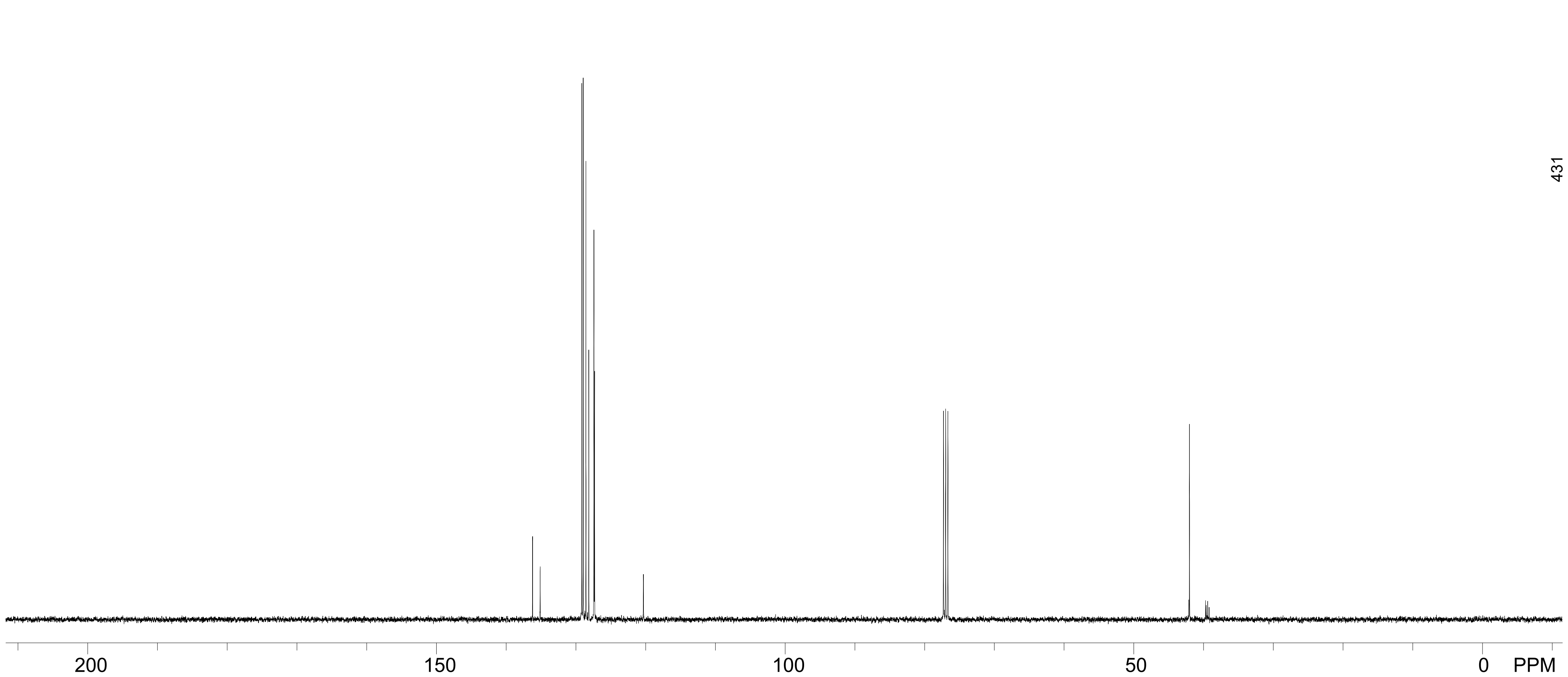

Area Percent Report

Agilent Technologies

Sample name: zwf-7-151-IE-99-1-0.8-205

Data file: C:\Users\Public\Documents\ChemStation\1\Data\zwf\zwf-S 2023-11-10 07-52-15\006-P1-C1-zwf-7-151.D

Injection Acquired Date 11/10/2023 09:54:34

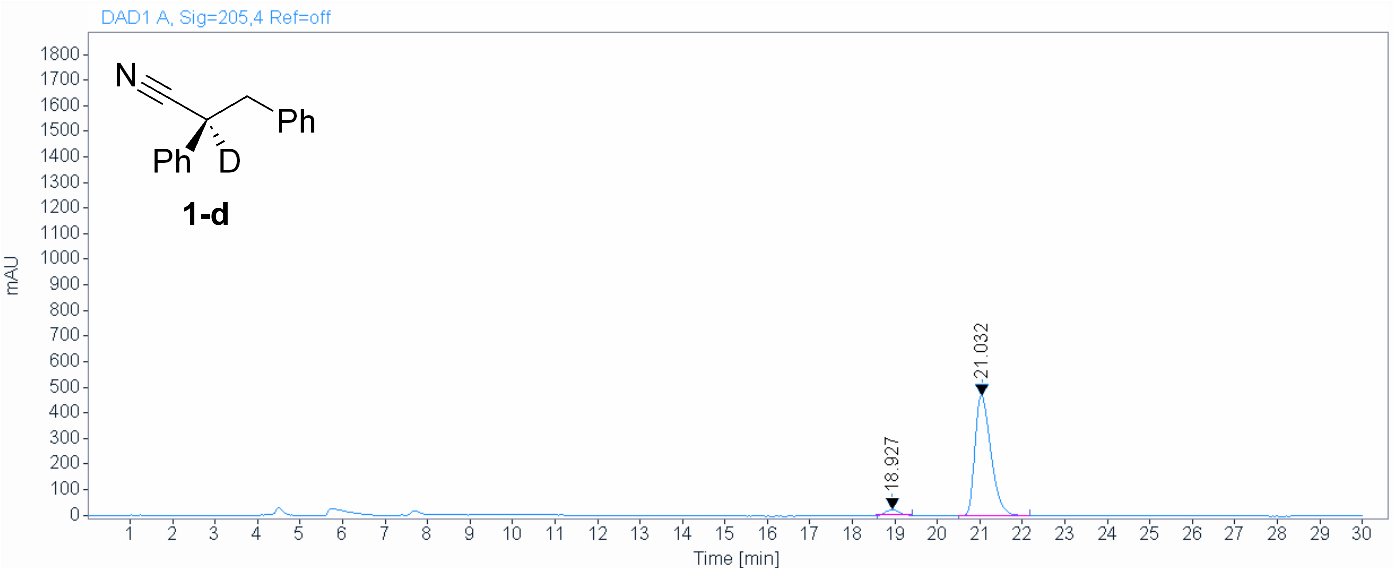

Signal: DAD1 A, Sig=205,4 Ref=off

RT [min]	Width [min]	Height	Area	Area%
18.927	0.3470	23.7956	495.4225	3.8493
21.032	0.3945	470.7209	12375.1504	96.1507
		Sum	12870.5729	100.0000

Area Percent Report

Agilent Technologies

Sample name: zwf-7-151-rac-IE-99-1-0.8-205

Data file: C:\Users\Public\Documents\ChemStation\1\Data\zwf\zwf-S 2023-11-10 07-52-15\007-P1-C2-zwf-7-151-rac.D

Injection Acquired Date 11/10/2023 10:25:40

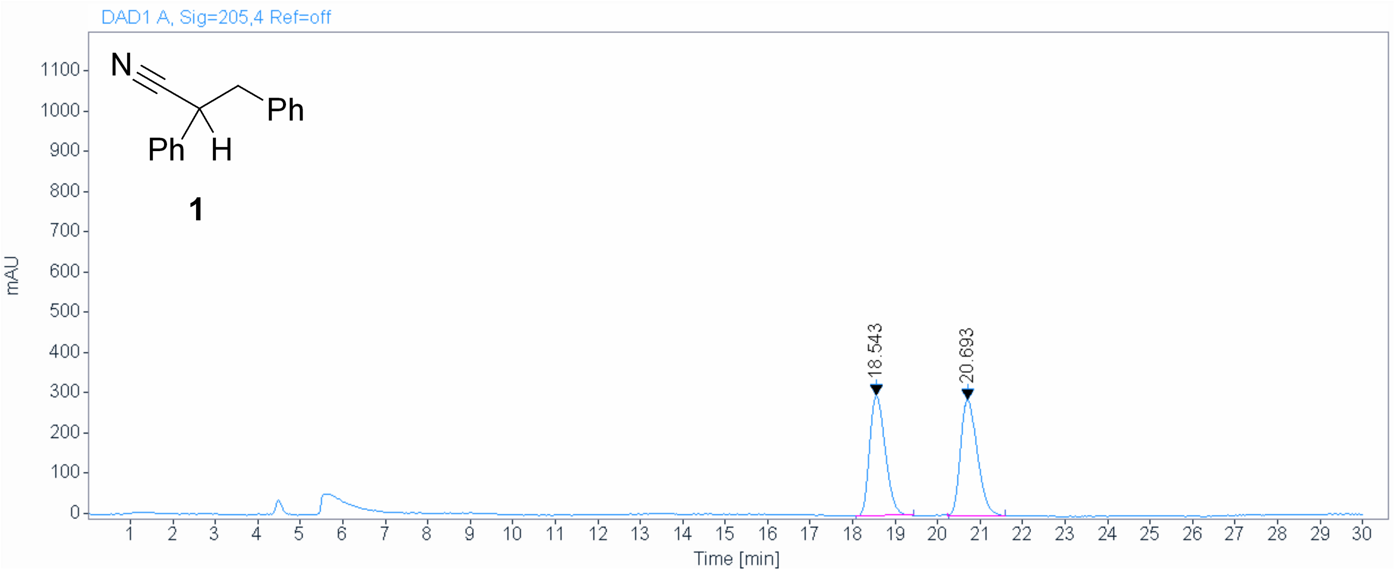

Signal: DAD1 A, Sig=205,4 Ref=off

RT [min]	Width [min]	Height	Area	Area%
18.543	0.3843	299.7573	7767.7627	49.4283
20.693	0.4587	288.7425	7947.4644	50.5717
		Sum	15715.2271	100.0000

zwf-7-060H
Sep 11 2023
SOLVENT: CDCl3
NA = 4
F1 = 400.179993 MHz
F2 = 1.000000 MHz

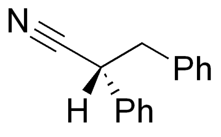

1

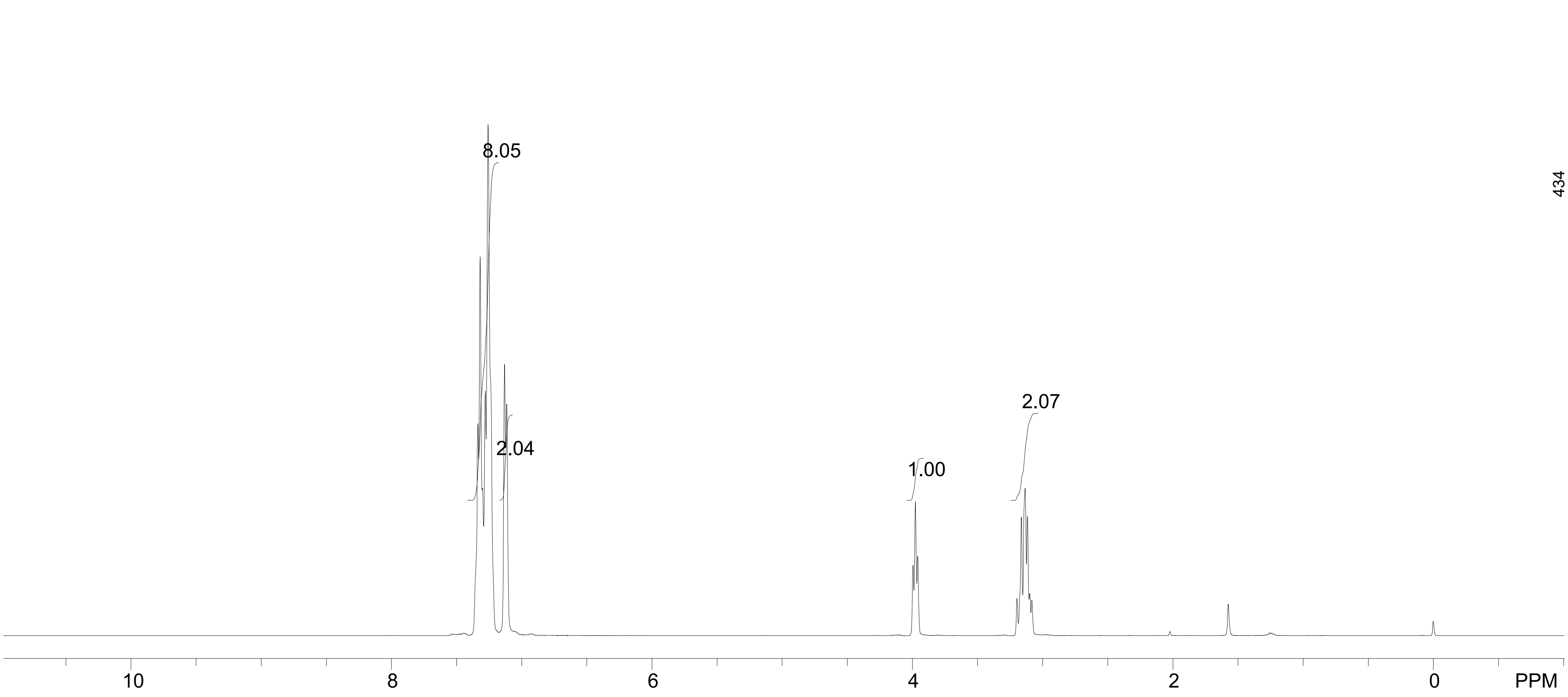

zwf-7-060C
Sep 11 2023
SOLVENT: CDCl3
NA = 100
F1 = 100.625343 MHz
F2 = 1.000000 MHz

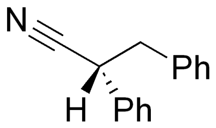

1

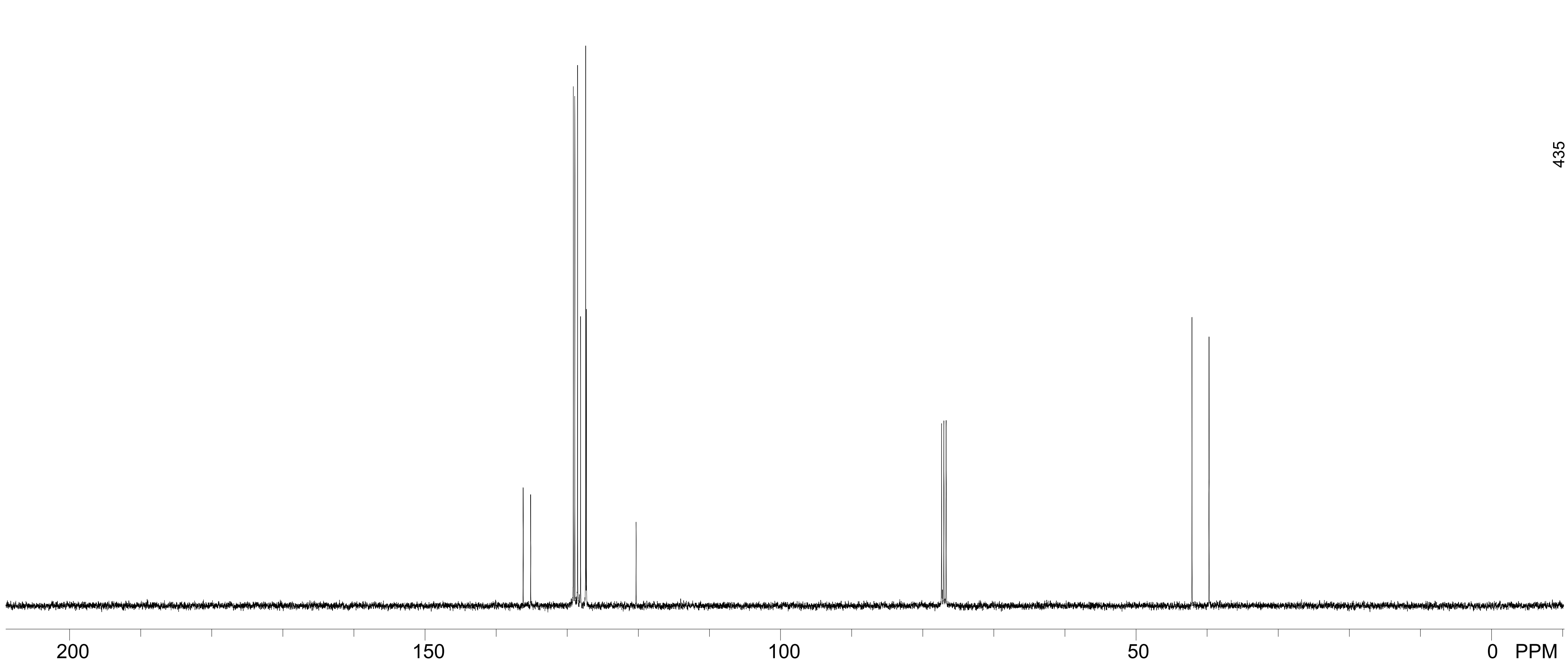

Area Percent Report

Sample name: zwf-7-060-IE-99-1-0.8-205

Data file: C:\Users\Public\Documents\ChemStation\1\Data\zwf\zwf-S 2023-09-11 18-06-28\002-P1-C1-zwf-7-060.D

Injection Acquired Date 09/11/2023 18:33:30

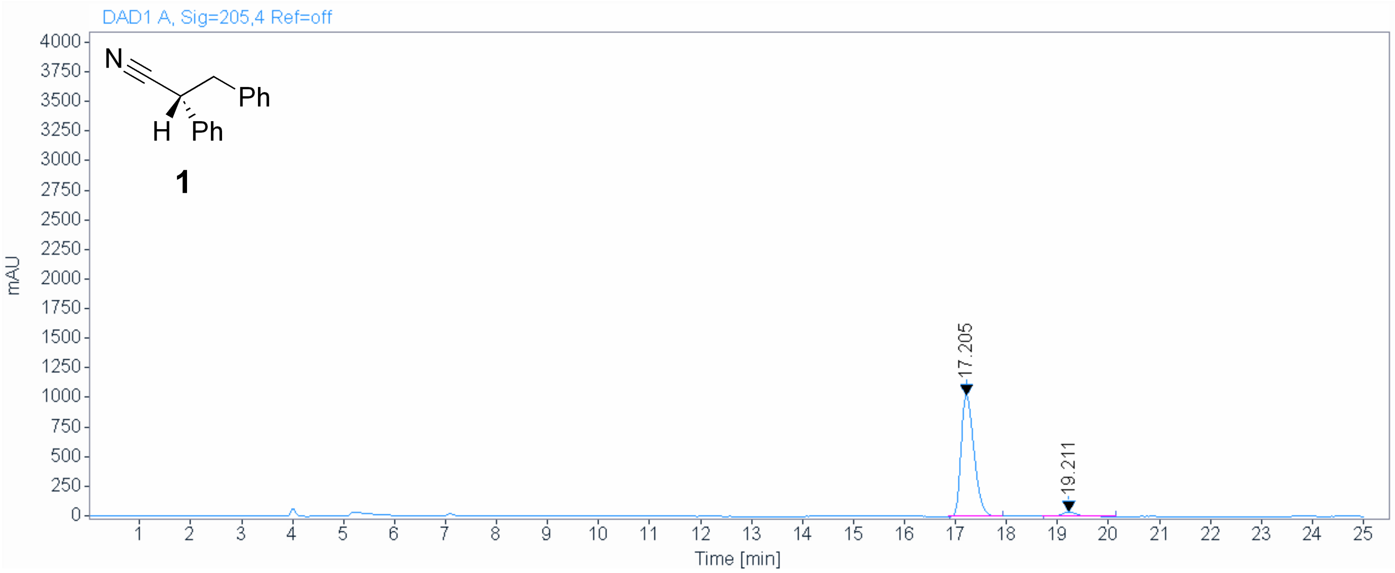

Signal: DAD1 A, Sig=205,4 Ref=off

RT [min]	Width [min]	Height	Area	Area%
17.205	0.2768	1021.3159	18107.0371	96.0170
19.211	0.2895	36.8966	751.1160	3.9830
		Sum	18858.1531	100.0000

Area Percent Report

Agilent Technologies

Sample name: zwf-7-058-rac-IE-99-1-0.8-205

Data file: C:\Users\Public\Documents\ChemStation\1\Data\zwf\zwf-S 2023-09-11 18-06-28\003-P1-C3-zwf-7-058-rac.D

Injection Acquired Date 09/11/2023 18:59:37

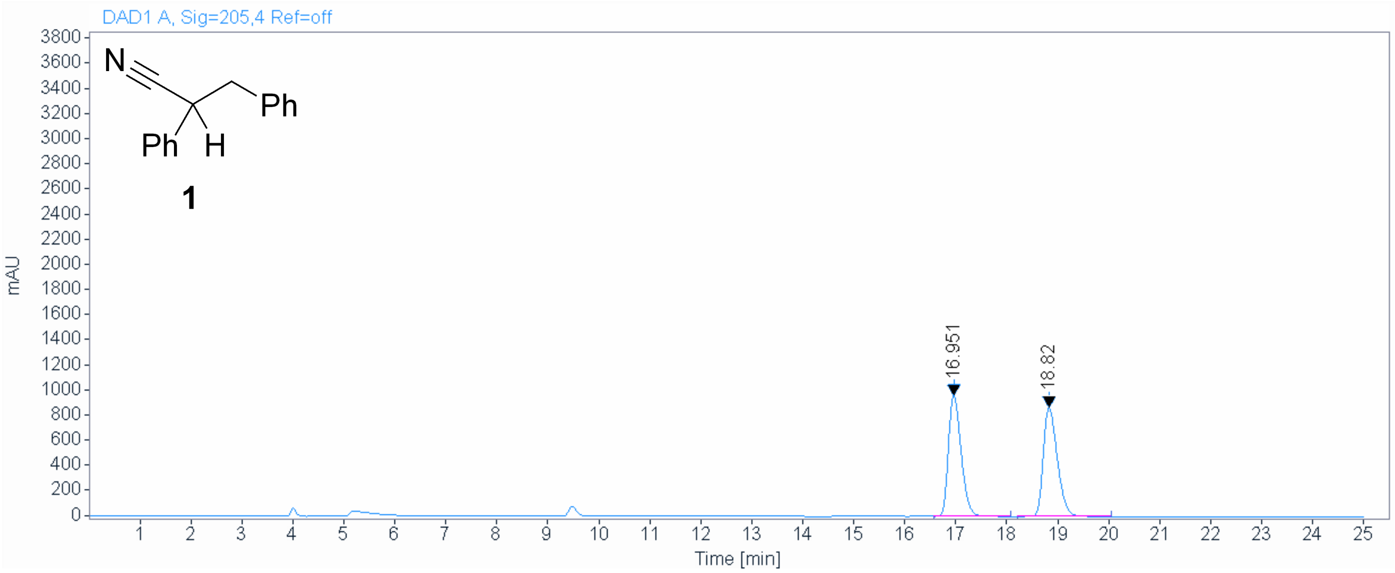

Signal: DAD1 A, Sig=205,4 Ref=off

RT [min]	Width [min]	Height	Area	Area%
16.951	0.2729	962.4082	16740.2598	50.0786
18.820	0.2983	867.9600	16687.6836	49.9214
		Sum	33427.9434	100.0000

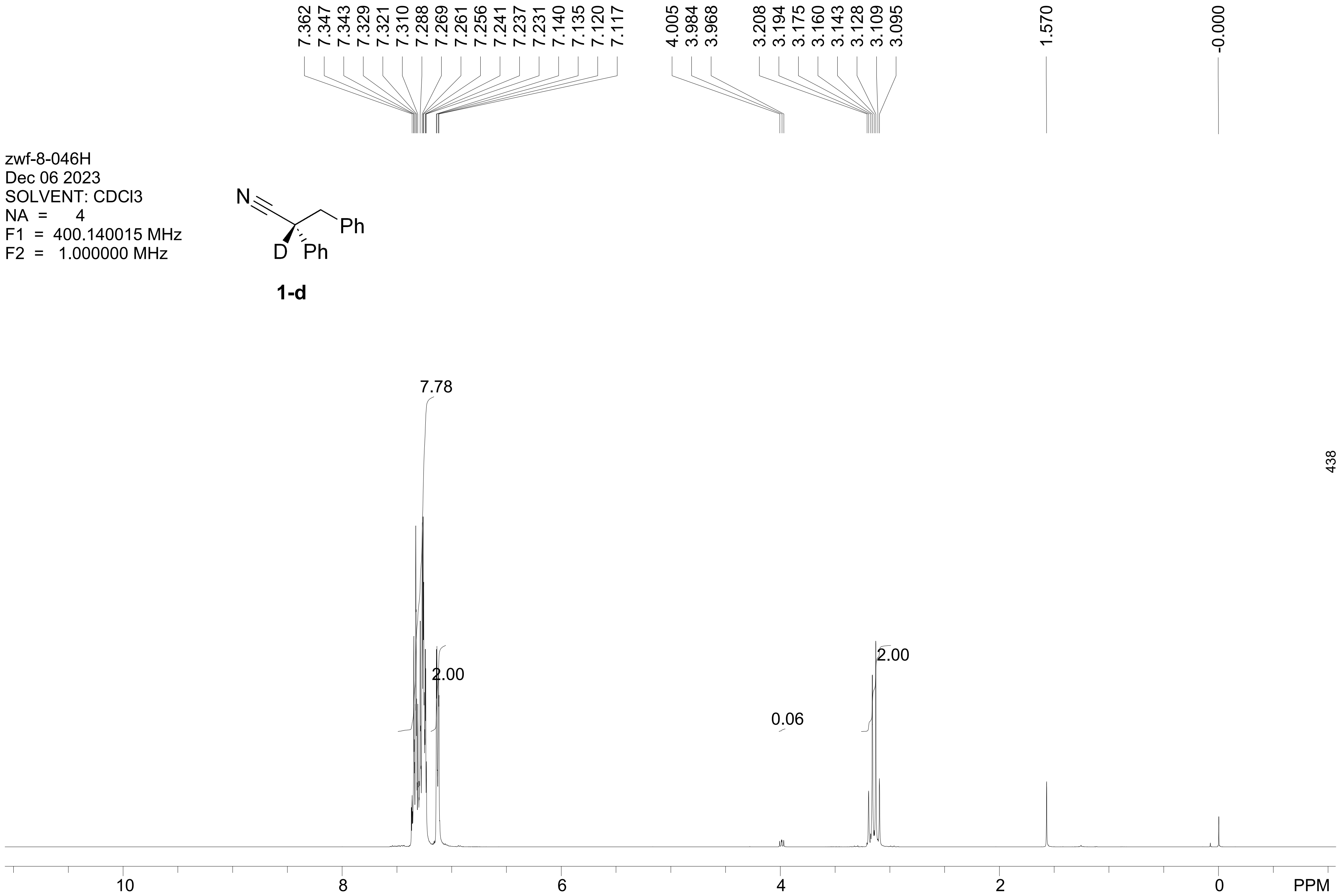
7.362
7.347
7.343
7.329
7.321
7.310
7.288
7.269
7.261
7.256
7.241
7.237
7.231
7.140
7.135
7.120
7.117
4.005
3.984
3.968
3.208
3.194
3.175
3.160
3.143
3.128
3.109
3.095
1.570
-0.000
zwf-8-046H
Dec 06 2023
SOLVENT: CDCl3
NA = 4
F1 = 400.140015 MHz
F2 = 1.000000 MHz
N
Ph
D
Ph
1-d
7.78
2.00
0.06
2.00
10
8
6
4
2
0
PPM

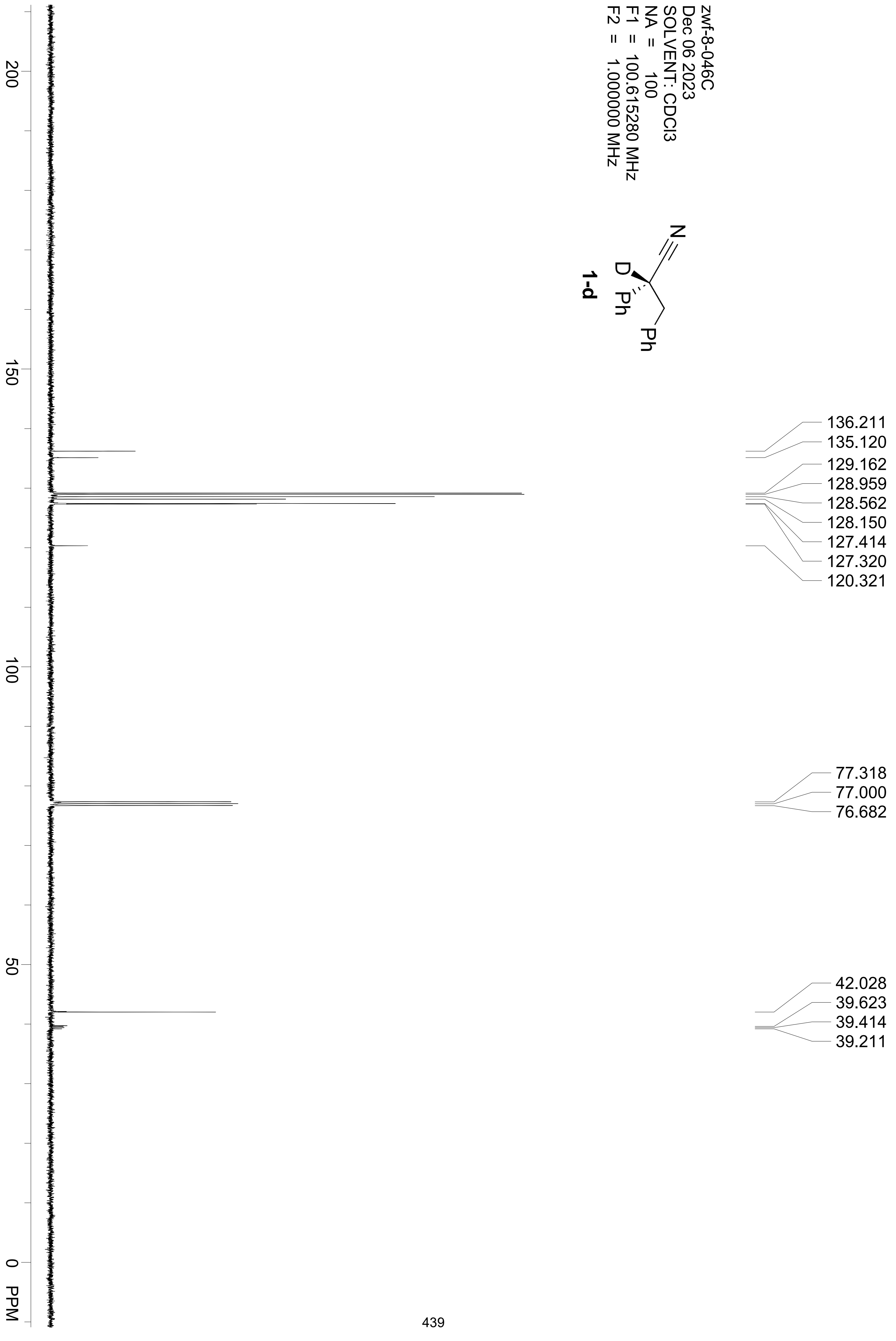
zwf-8-046C
Dec 06 2023
SOLVENT: CDCl3
NA = 100
F1 = 100.615280 MHz
F2 = 1.000000 MHz
1-d
136.211
135.120
129.162
128.959
128.562
128.150
127.414
127.320
120.321
77.318
77.000
76.682
42.028
39.623
39.414
39.211
200
150
100
50
0 PPM

Area Percent Report

Agilent Technologies

Sample name: zwf-8-046-IE-99-1-0.8-205

Data file: C:\Users\Public\Documents\ChemStation\1\Data\zwf\zwf-S 2023-12-06 20-07-35\020-P1-C6-zwf-8-046.D

Injection Acquired Date 12/07/2023 11:51:05

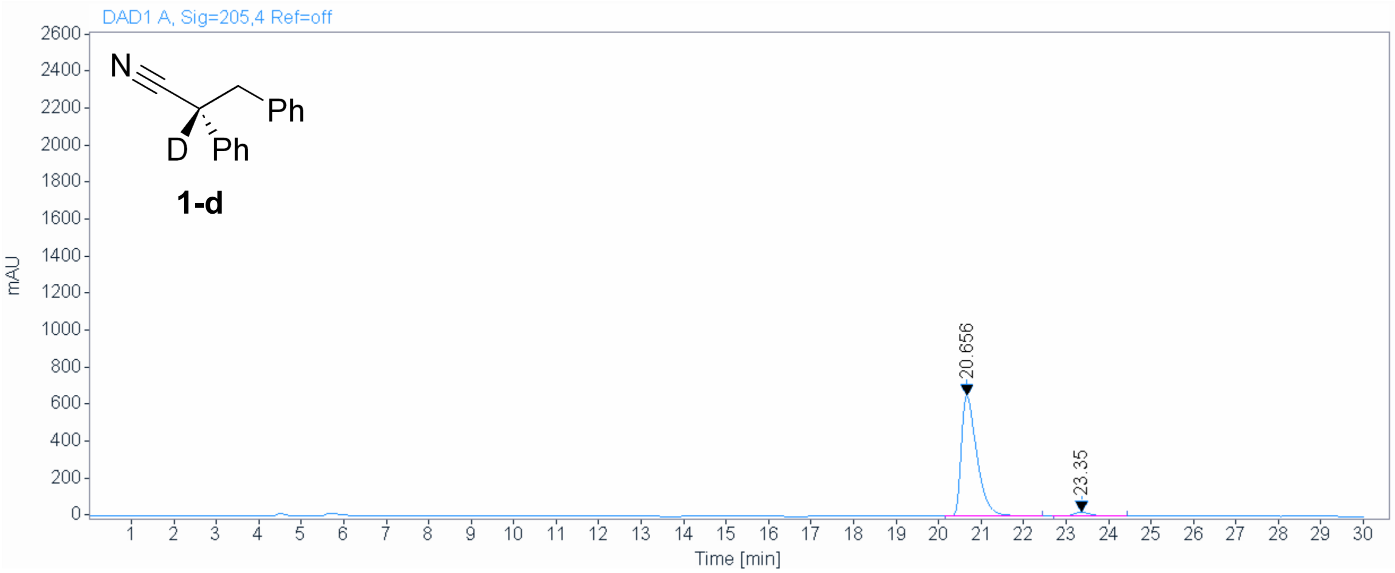

Signal: DAD1 A, Sig=205,4 Ref=off

RT [min]	Width [min]	Height	Area	Area%
20.656	0.3838	650.1743	16375.4590	96.5948
23.350	0.4477	21.4885	577.2736	3.4052
		Sum	16952.7325	100.0000

Area Percent Report

Agilent Technologies

Sample name: zwf-8-046-rac-IE-99-1-0.8-205

Data file: C:\Users\Public\Documents\ChemStation\1\Data\zwf\zwf-S 2023-12-06 20-07-35\010-P1-C7-zwf-8-046-rac.D

Injection Acquired Date 12/07/2023 02:55:08

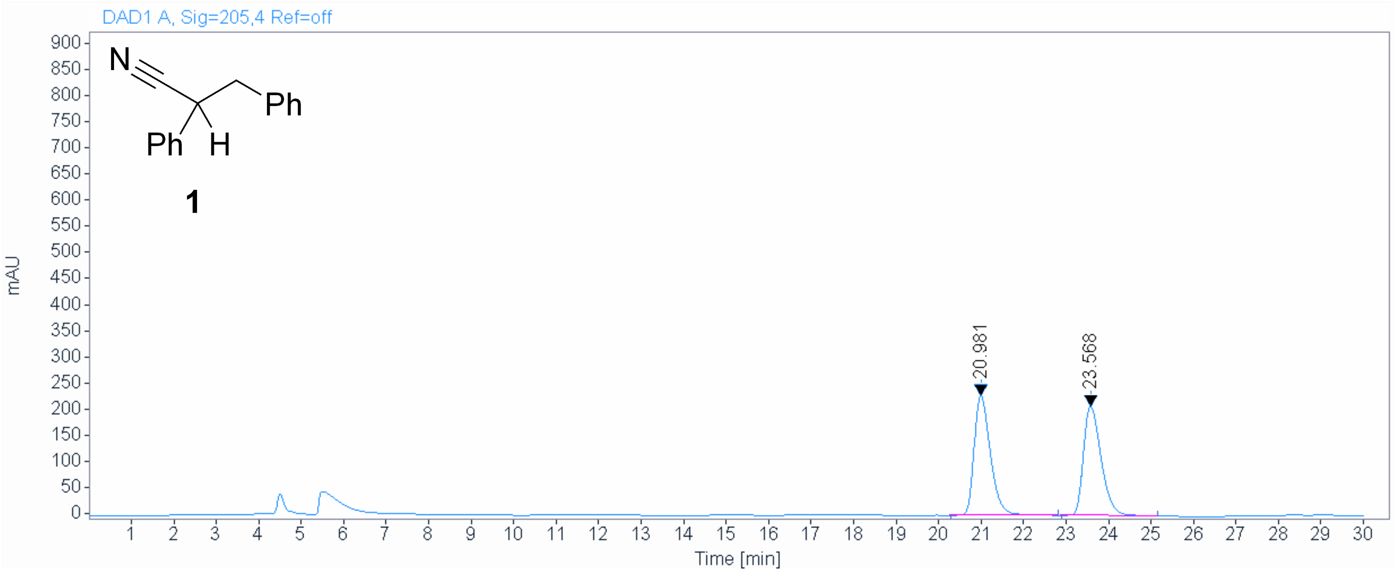

Signal: DAD1 A, Sig=205,4 Ref=off

RT [min]	Width [min]	Height	Area	Area%
20.981	0.4082	228.8855	6085.1563	50.2622
23.568	0.4424	208.8490	6021.6675	49.7378
		Sum	12106.8237	100.0000

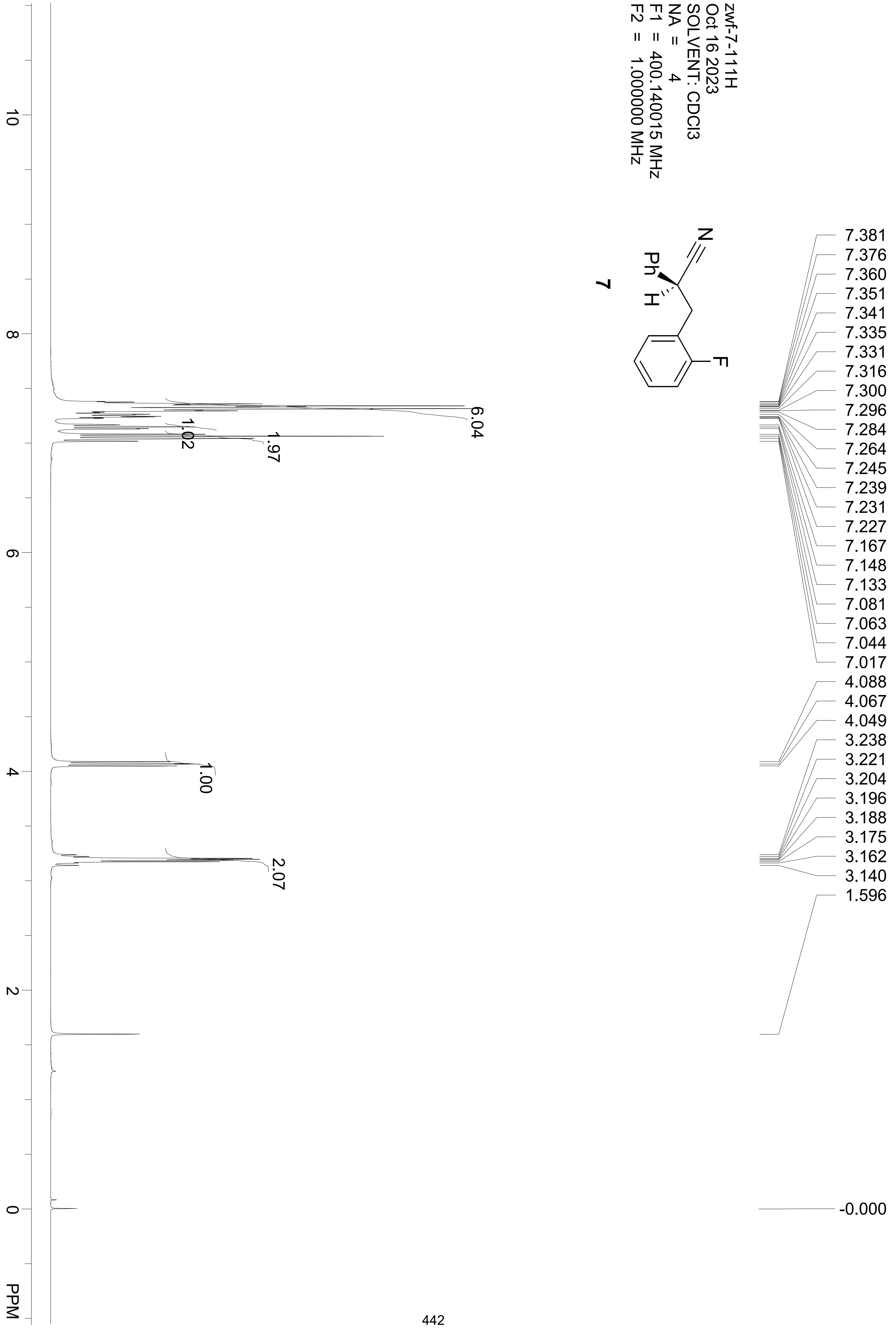
zwf-7-111H
Oct 16 2023
SOLVENT: CDCl3
NA = 4
F1 = 400.140015 MHz
F2 = 1.000000 MHz
N
Ph
H
F
7
6.04
1.02
1.97
1.00
2.07
7.381
7.376
7.360
7.351
7.341
7.335
7.331
7.316
7.300
7.296
7.284
7.264
7.245
7.239
7.231
7.227
7.167
7.148
7.133
7.081
7.063
7.044
7.017
4.088
4.067
4.049
3.238
3.221
3.204
3.196
3.188
3.175
3.162
3.140
1.596
-0.000
10
8
6
4
2
0
PPM

zwf-7-111C
Oct 16 2023
SOLVENT: CDCl3
NA = 100
F1 = 100.615280 MHz
F2 = 1.000000 MHz

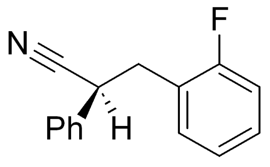

7

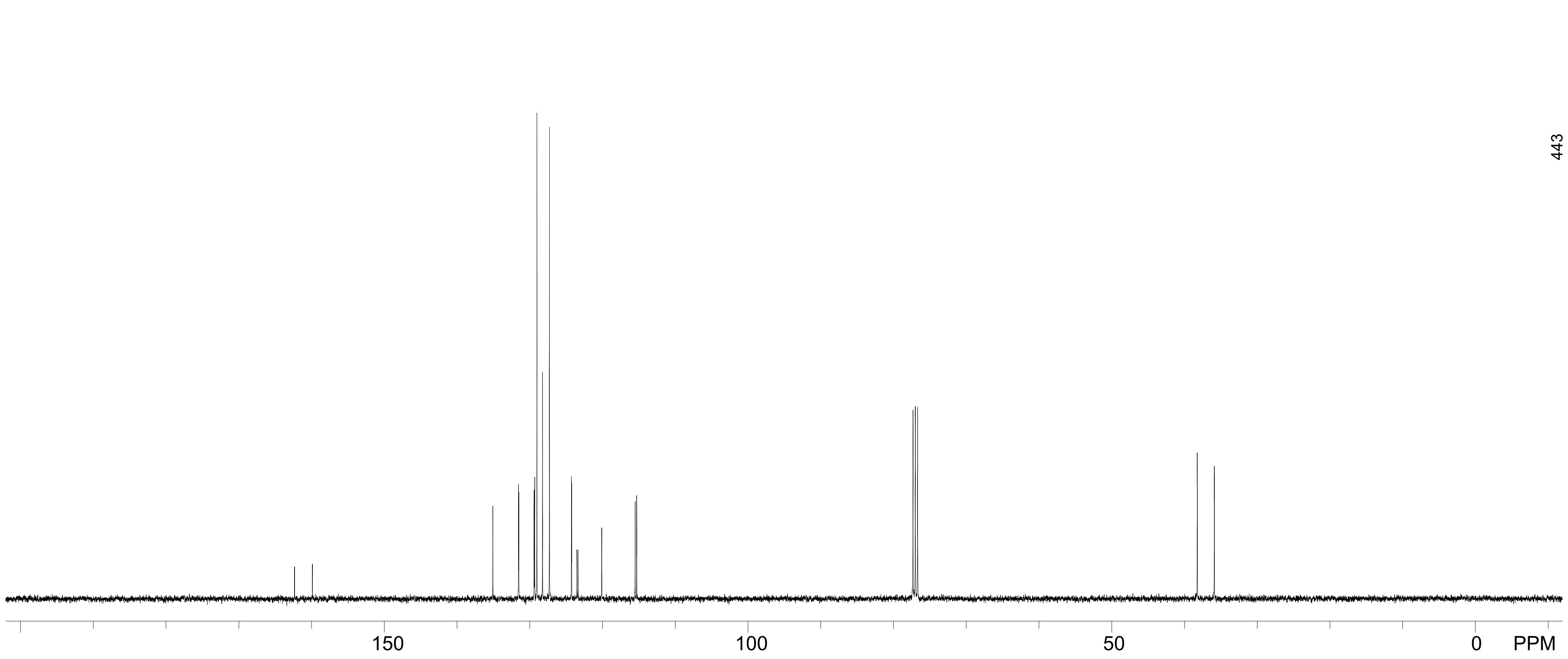

zwf-7-111F
Oct 16 2023
SOLVENT: CDCl3
NA = 16
F1 = 376.507782 MHz
F2 = 1.000000 MHz

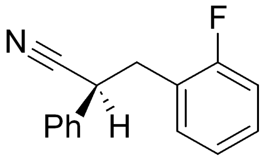

7

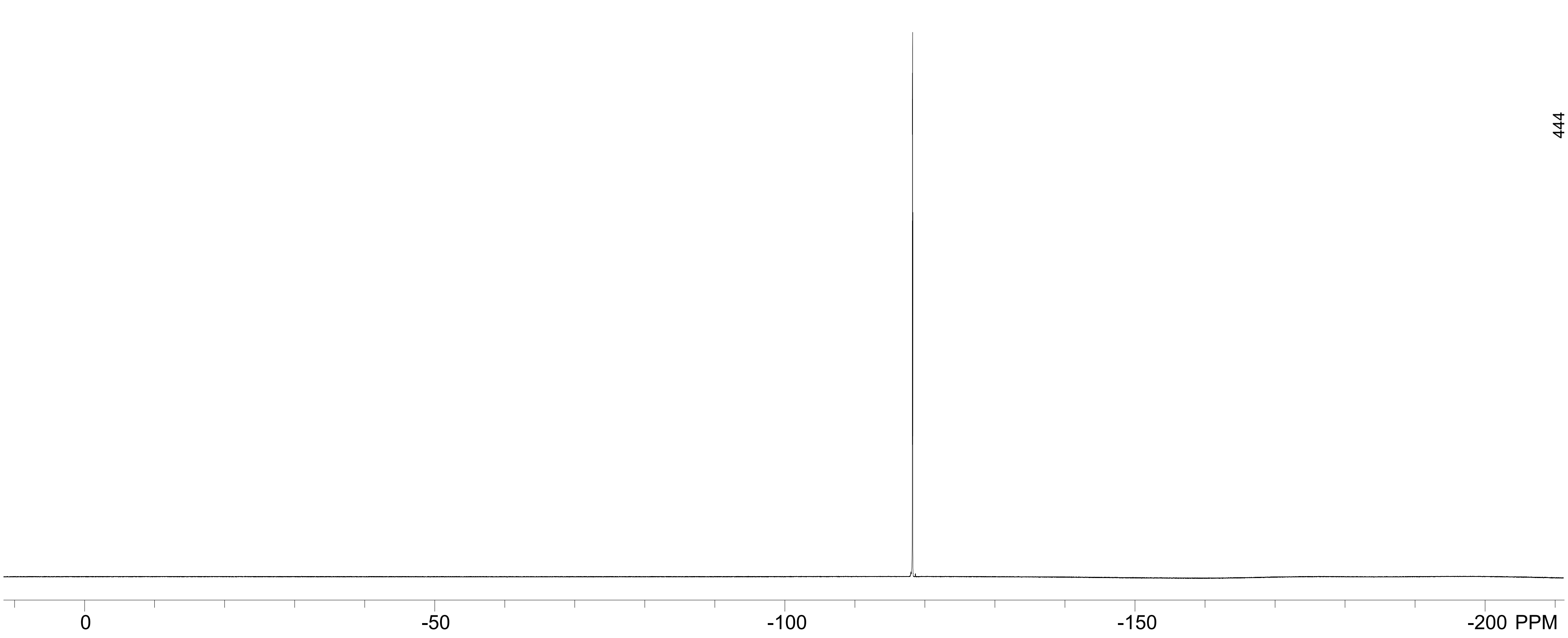

Area Percent Report

Sample name: zwf-7-111-IE-99-1-0.8-205

Data file: C:\Users\Public\Documents\ChemStation\1\Data\zwf\zwf-S 2023-10-17 08-55-39\023-P1-C1-zwf-7-111.D

Injection Acquired Date 10/17/2023 20:21:11

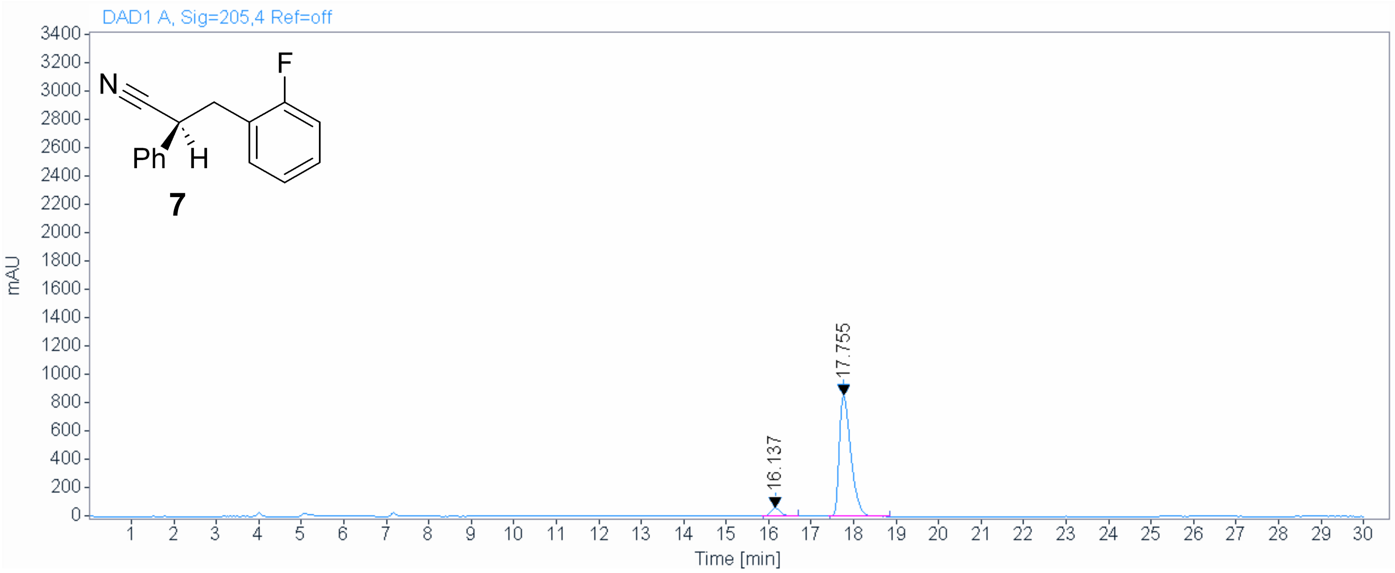

Signal: DAD1 A, Sig=205,4 Ref=off

RT [min]	Width [min]	Height	Area	Area%
16.137	0.2509	54.6702	823.0448	4.8295
17.755	0.2915	853.8515	16219.1572	95.1705
		Sum	17042.2020	100.0000

Area Percent Report

Sample name: zwf-7-111-rac-IE-99-1-0.8-205

Data file: C:\Users\Public\Documents\ChemStation\1\Data\zwf\zwf-S 2023-10-17 08-55-39\021-P1-C3-zwf-7-111-rac.D

Injection Acquired Date 10/17/2023 19:19:05

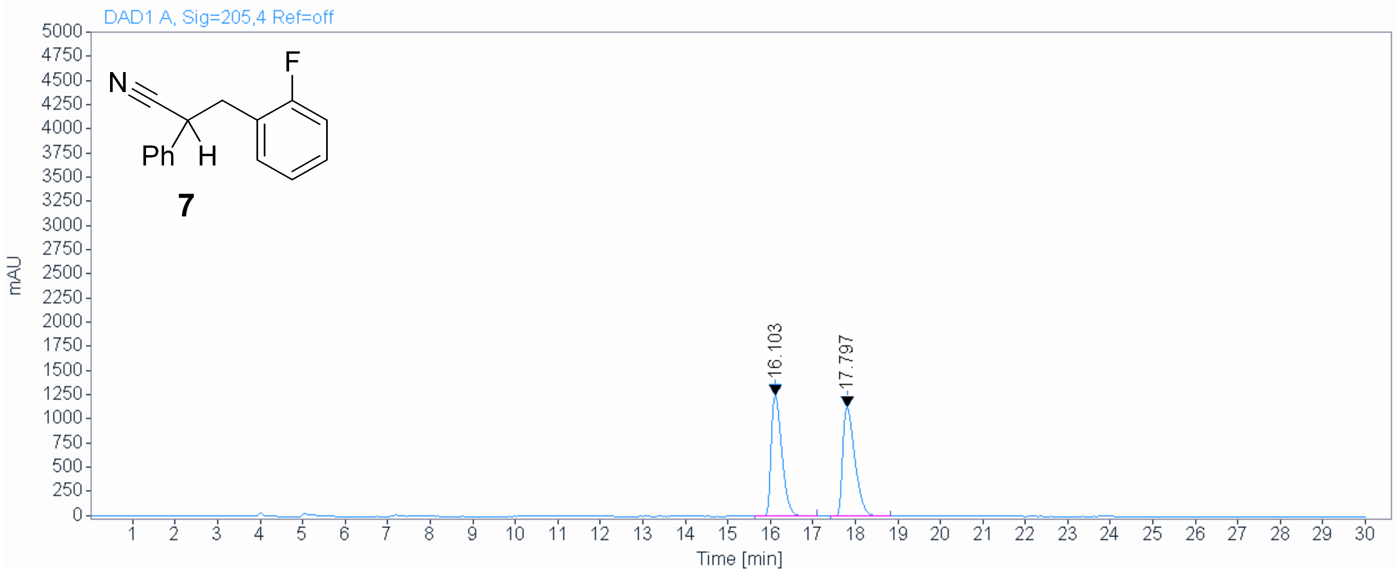

Signal: DAD1 A, Sig=205,4 Ref=off

RT [min]	Width [min]	Height	Area	Area%
16.103	0.2792	1248.4637	21968.4961	49.5588
17.797	0.3054	1127.3196	22359.6836	50.4412
		Sum	44328.1797	100.0000

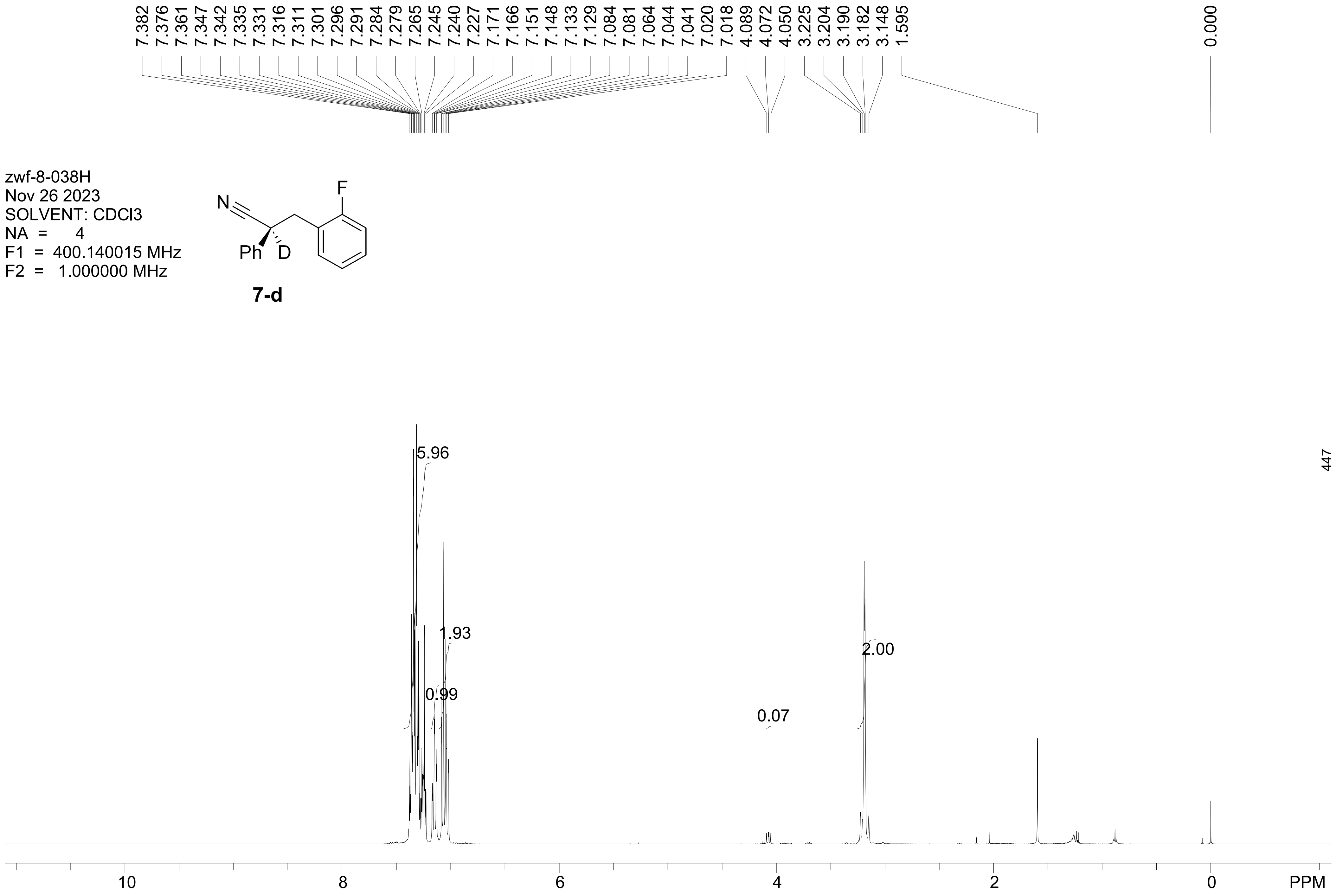
7.382
7.376
7.361
7.347
7.342
7.335
7.331
7.316
7.311
7.301
7.296
7.291
7.284
7.279
7.265
7.245
7.240
7.227
7.171
7.166
7.151
7.148
7.133
7.129
7.084
7.081
7.064
7.044
7.041
7.020
7.018
4.089
4.072
4.050
3.225
3.204
3.190
3.182
3.148
1.595
0.000
zwf-8-038H
Nov 26 2023
SOLVENT: CDCl3
NA = 4
F1 = 400.140015 MHz
F2 = 1.000000 MHz
N
F
Ph
D
7-d
5.96
1.93
0.99
0.07
2.00
10
8
6
4
2
0
PPM

zwf-8-038C
Nov 26 2023
SOLVENT: CDCl3
NA = 200
F1 = 100.615280 MHz
F2 = 1.000000 MHz

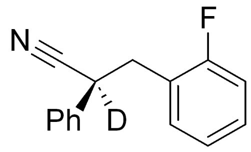

7-d

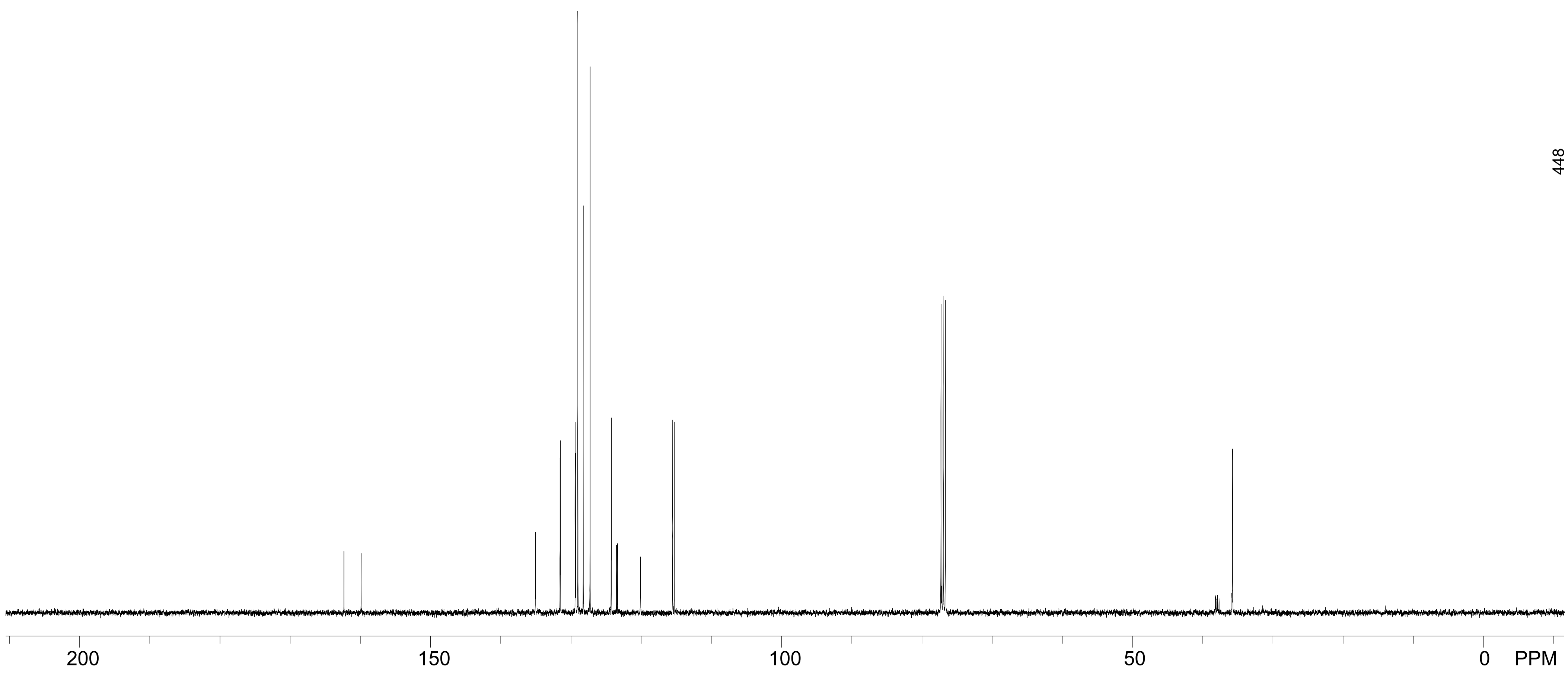

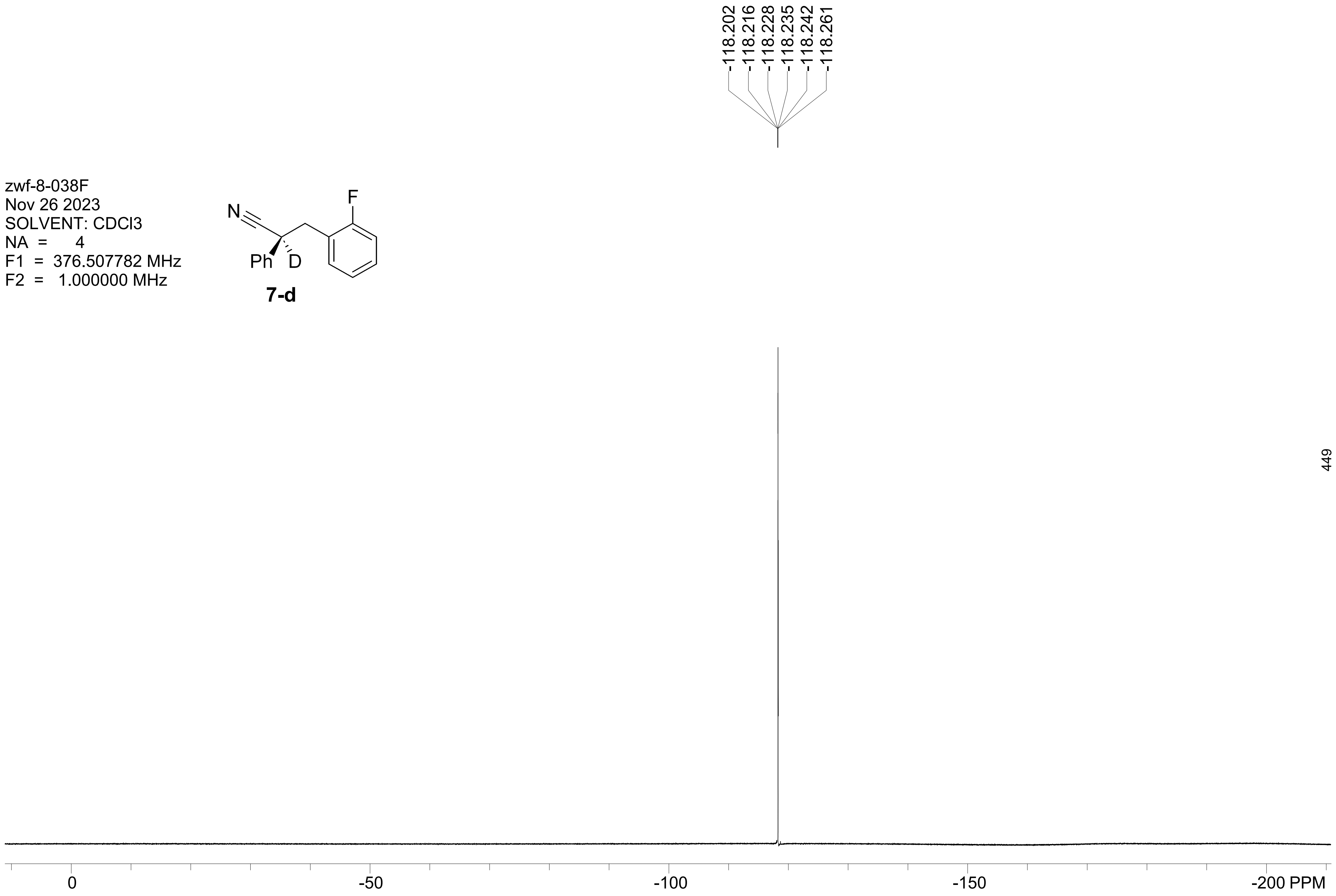
zwf-8-038F
Nov 26 2023
SOLVENT: CDCl3
NA = 4
F1 = 376.507782 MHz
F2 = 1.000000 MHz
N
F
Ph
D
7-d
-118.202
-118.216
-118.228
-118.235
-118.242
-118.261
0
-50
-100
-150
-200 PPM

Area Percent Report

Agilent Technologies

Sample name: zwf-8-038-IE-99-1-0.8-205

Data file: C:\Users\Public\Documents\ChemStation\1\Data\zwf\zwf-S 2023-11-29 11-54-07\004-P1-C1-zwf-8-038.D

Injection Acquired Date 11/29/2023 13:18:50

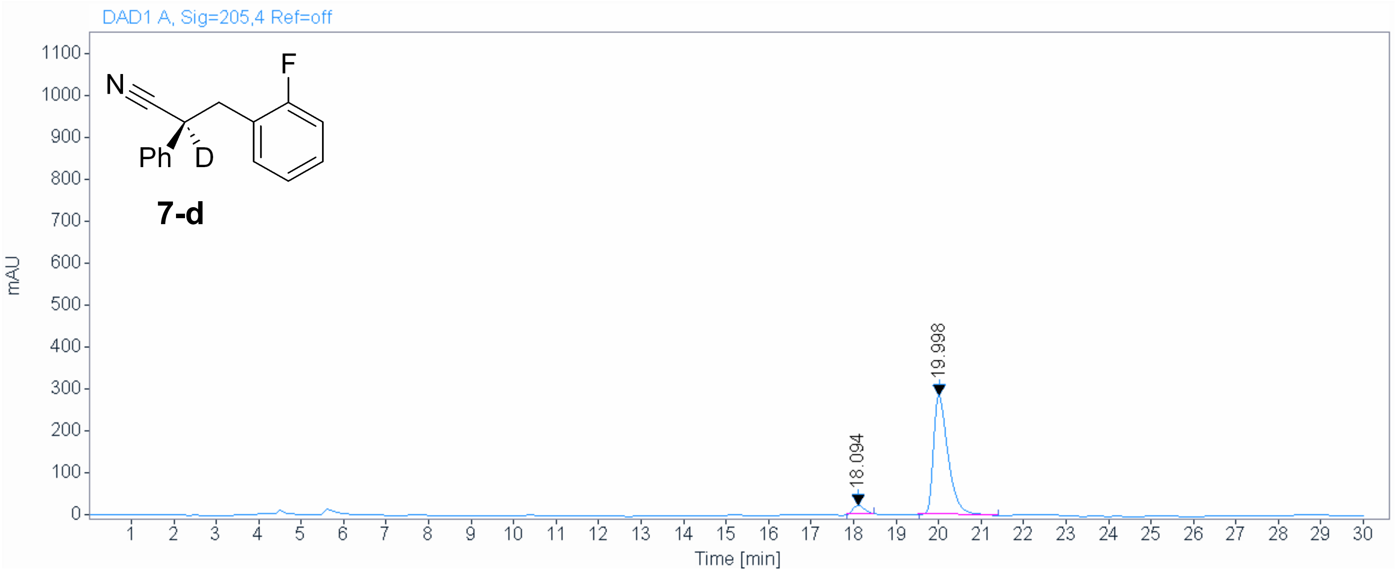

Signal: DAD1 A, Sig=205,4 Ref=off

RT [min]	Width [min]	Height	Area	Area%
18.094	0.3074	20.4883	377.9202	5.2404
19.998	0.3645	284.2604	6833.7051	94.7596
		Sum	7211.6252	100.0000

Area Percent Report

Sample name: zwf-8-038-rac-IE-99-1-0.8-205

Data file: C:\Users\Public\Documents\ChemStation\1\Data\zwf\zwf-S 2023-11-26 13-46-16\014-P1-B4-zwf-8-038-arc.D

Injection Acquired Date 11/26/2023 20:21:13

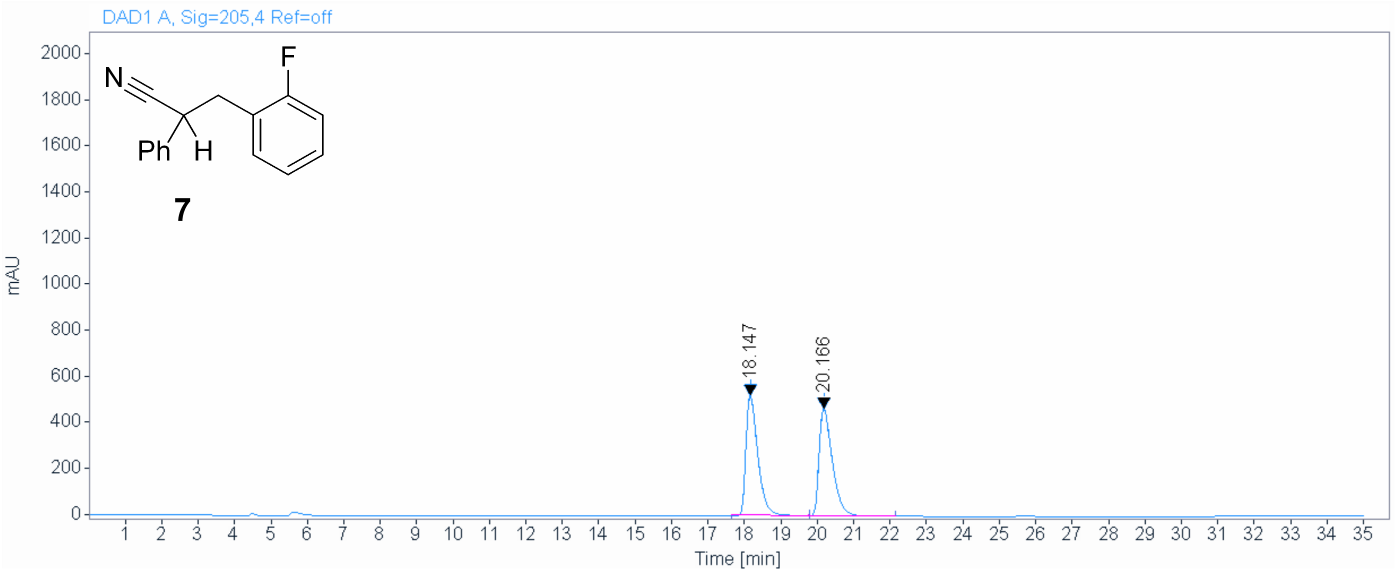

Signal: DAD1 A, Sig=205,4 Ref=off

RT [min]	Width [min]	Height	Area	Area%
18.147	0.3470	521.0298	11925.7910	50.2271
20.166	0.3863	465.2557	11817.9414	49.7729
		Sum	23743.7324	100.0000

zwf-7-103H
Oct 08 2023
SOLVENT: CDCl3
NA = 4
F1 = 400.140015 MHz
F2 = 1.000000 MHz

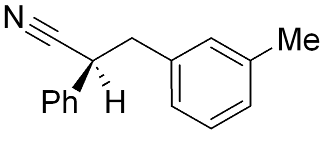

8

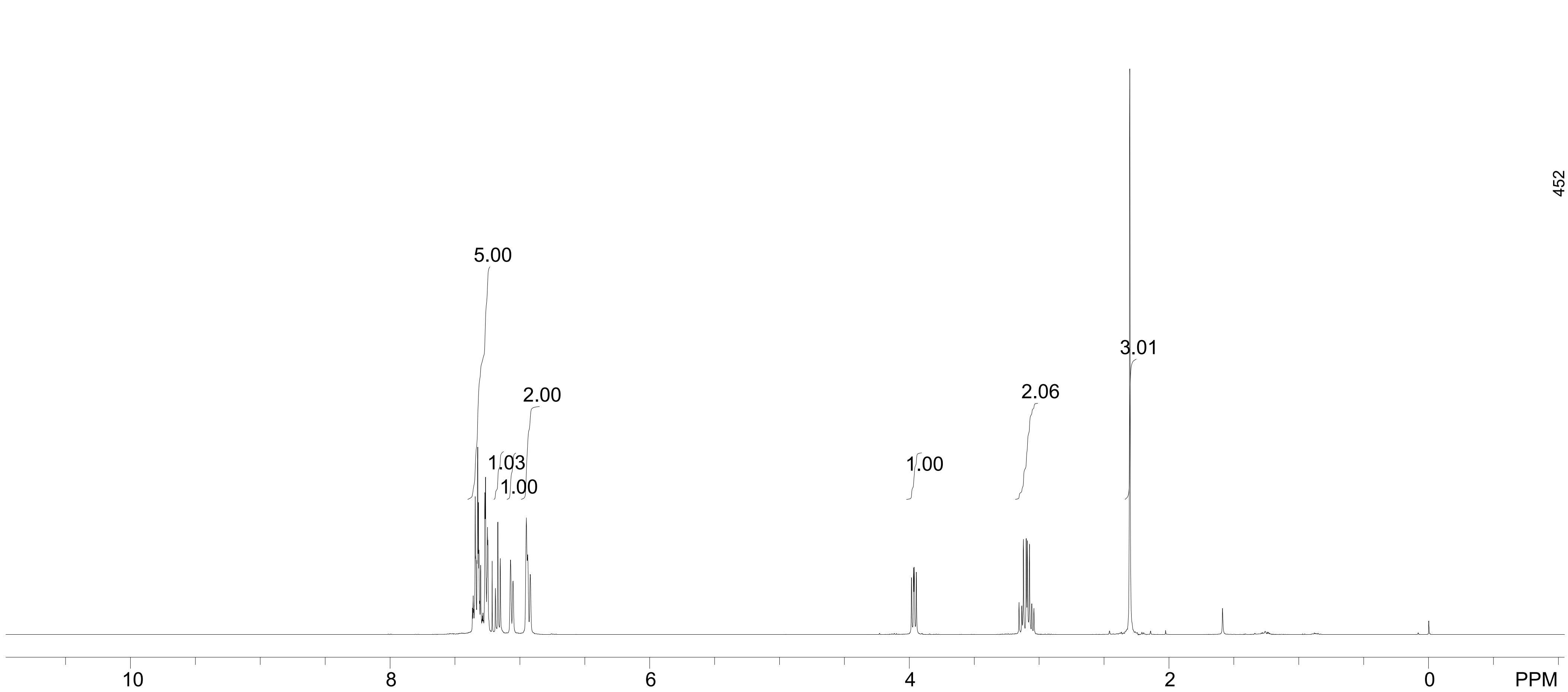

zwf-7-103C
Oct 08 2023
SOLVENT: CDCl3
NA = 100
F1 = 100.615280 MHz
F2 = 1.000000 MHz

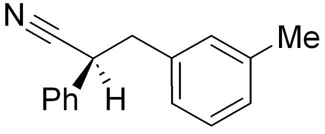

8

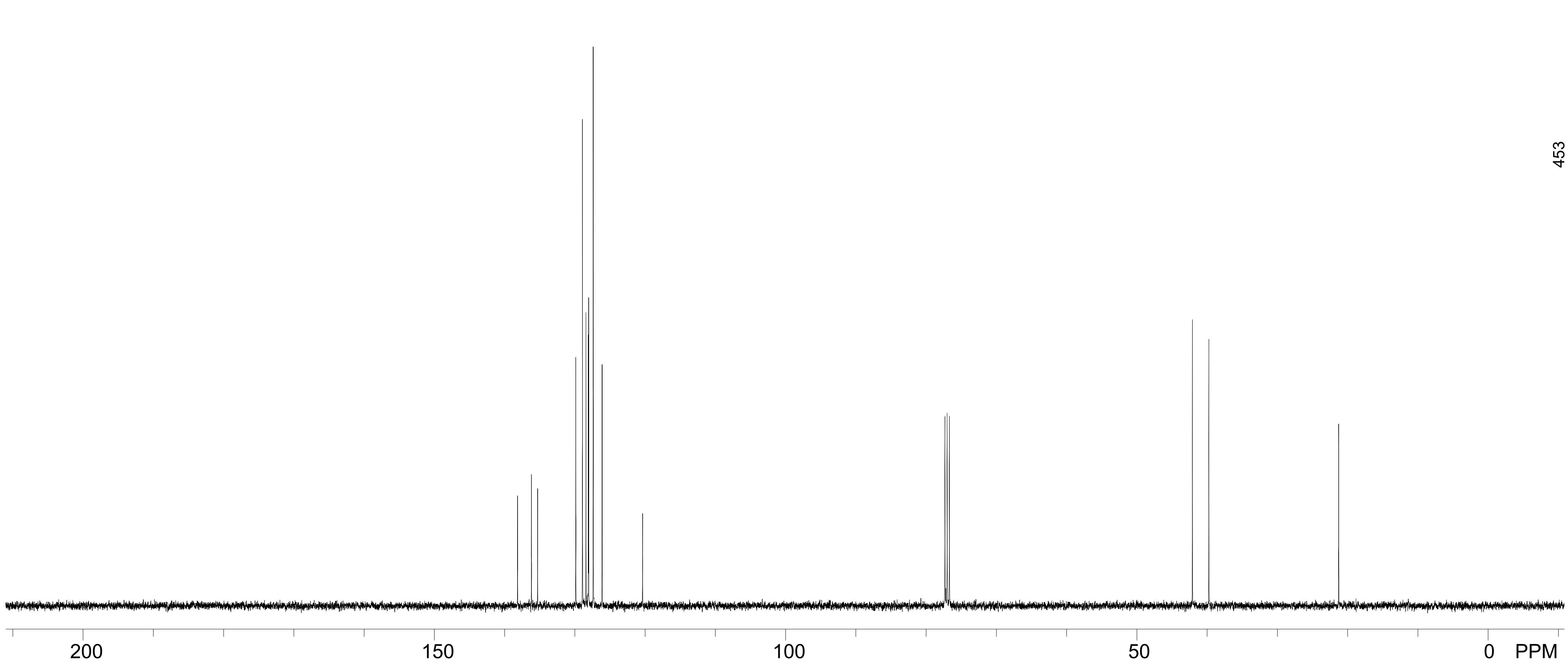

Area Percent Report

Agilent Technologies

Sample name: zwf-7-103-IE-99-1-0.8-205

Data file: C:\Users\Public\Documents\ChemStation\1\Data\zwf\zwf-S 2023-10-08 16-50-37\002-P1-C1-zwf-7-103.D

Injection Acquired Date 10/08/2023 17:17:43

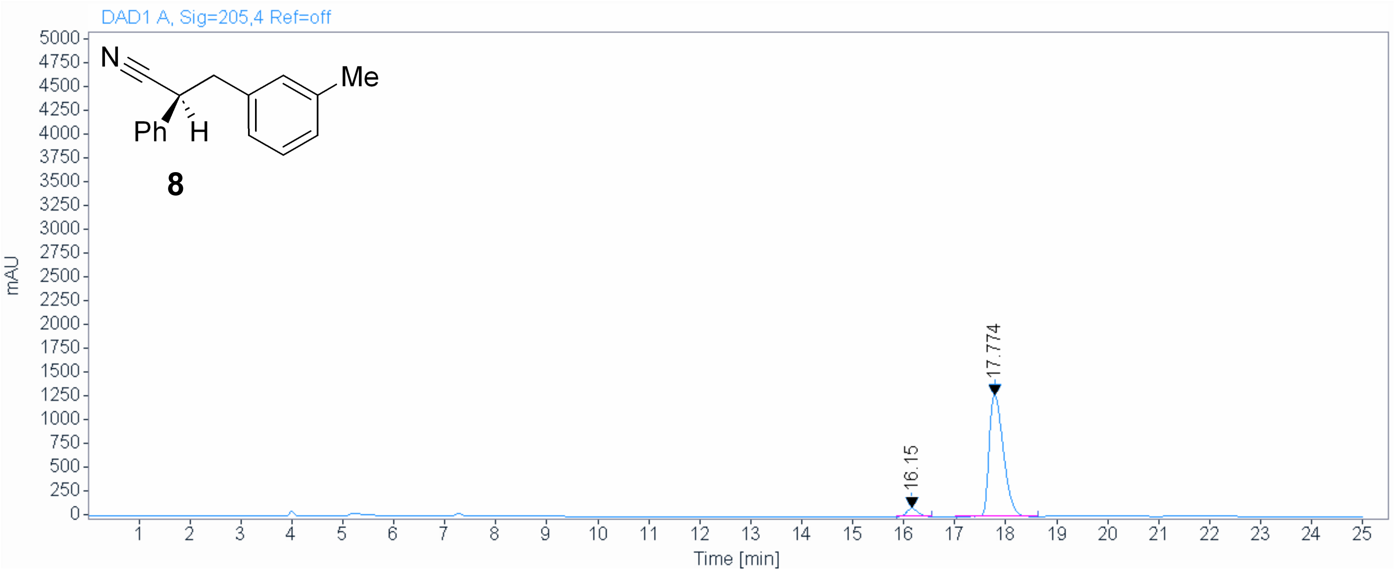

Signal: DAD1 A, Sig=205,4 Ref=off

RT [min]	Width [min]	Height	Area	Area%
16.150	0.2528	86.2848	1308.7836	5.0098
17.774	0.3059	1270.3320	24815.4453	94.9902
		Sum	26124.2289	100.0000

Area Percent Report

Agilent Technologies

Sample name: zwf-7-103-rac-IE-99-1-0.8-205

Data file: C:\Users\Public\Documents\ChemStation\1\Data\zwf\zwf-S 2023-10-08 16-50-37\003-P1-C2-zwf-7-103-rac.D

Injection Acquired Date 10/08/2023 17:43:45

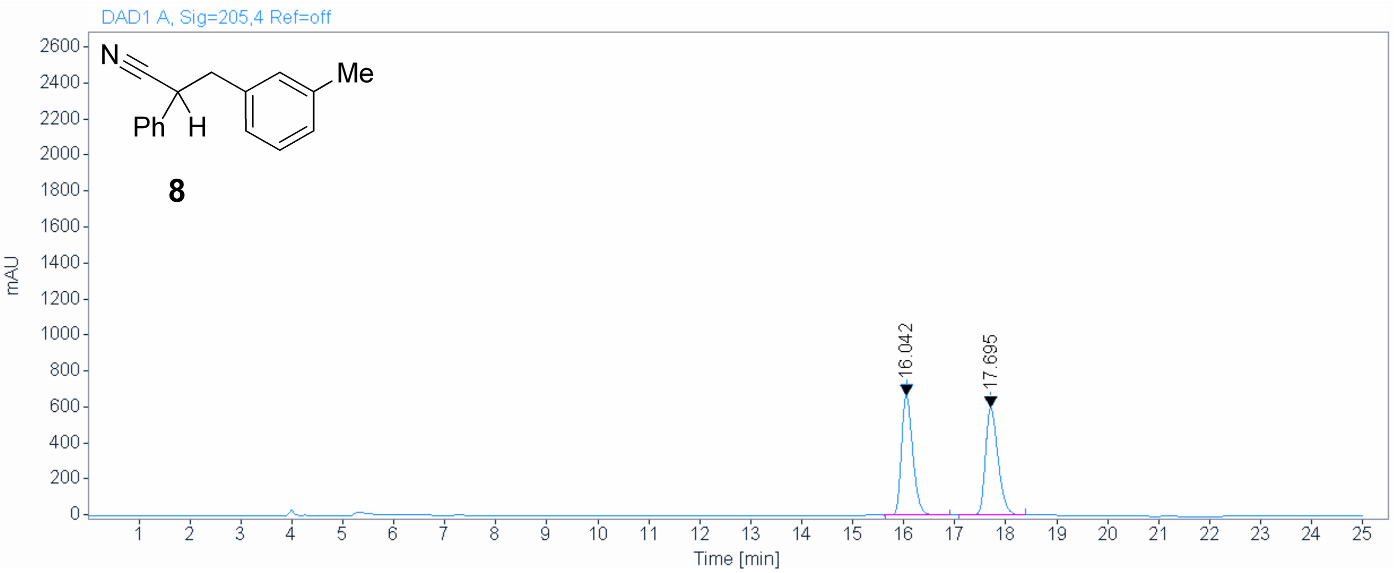

Signal: DAD1 A, Sig=205,4 Ref=off

RT [min]	Width [min]	Height	Area	Area%
16.042	0.2373	664.9059	10232.3555	49.9999
17.695	0.2592	598.0742	10232.3916	50.0001
		Sum	20464.7471	100.0000

zwf-8-018H
Nov 12 2023
SOLVENT: CDCl3
NA = 4
F1 = 400.140015 MHz
F2 = 1.000000 MHz

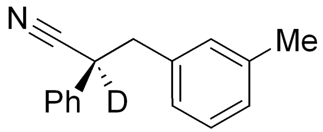

8-d

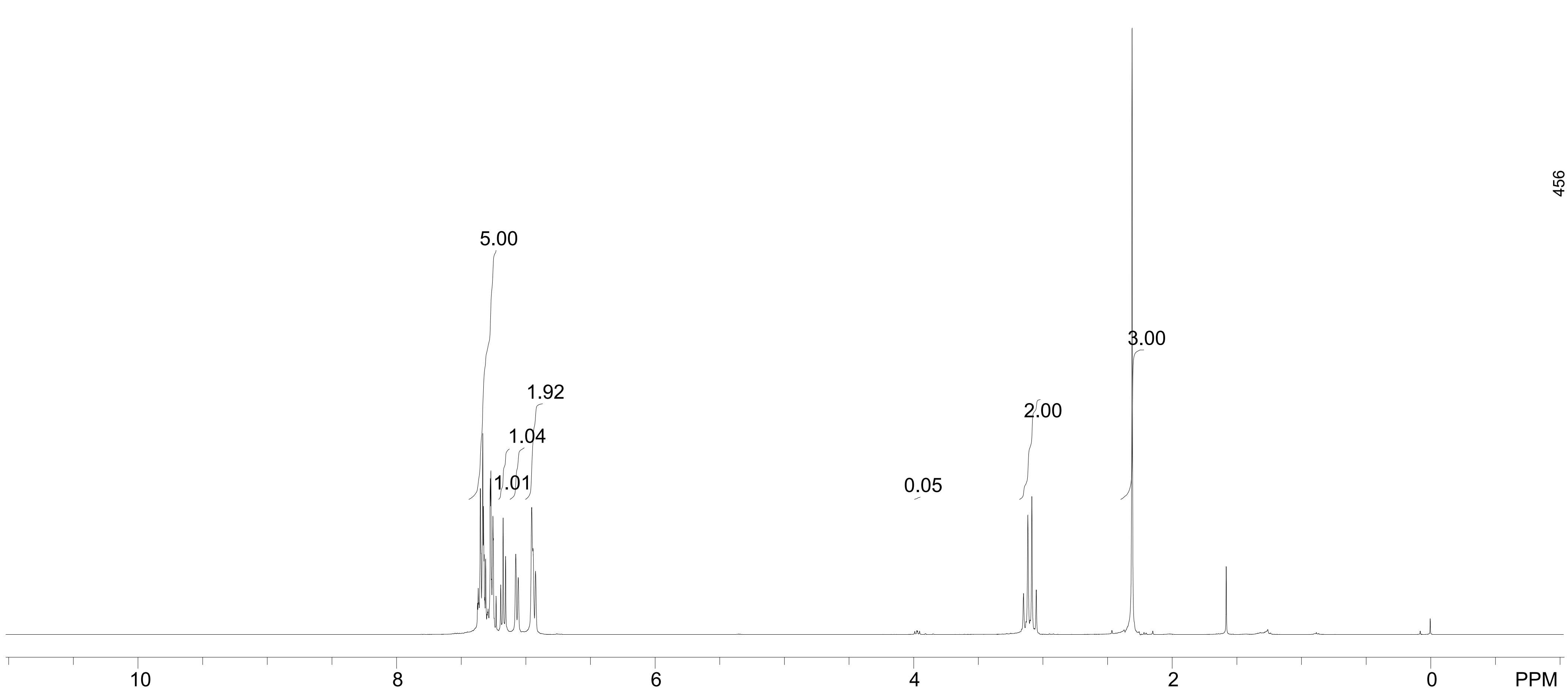

zwf-8-018C
Nov 12 2023
SOLVENT: CDCl3
NA = 150
F1 = 100.615280 MHz
F2 = 1.000000 MHz

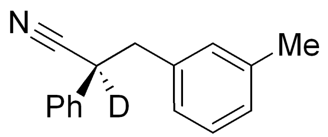

8-d

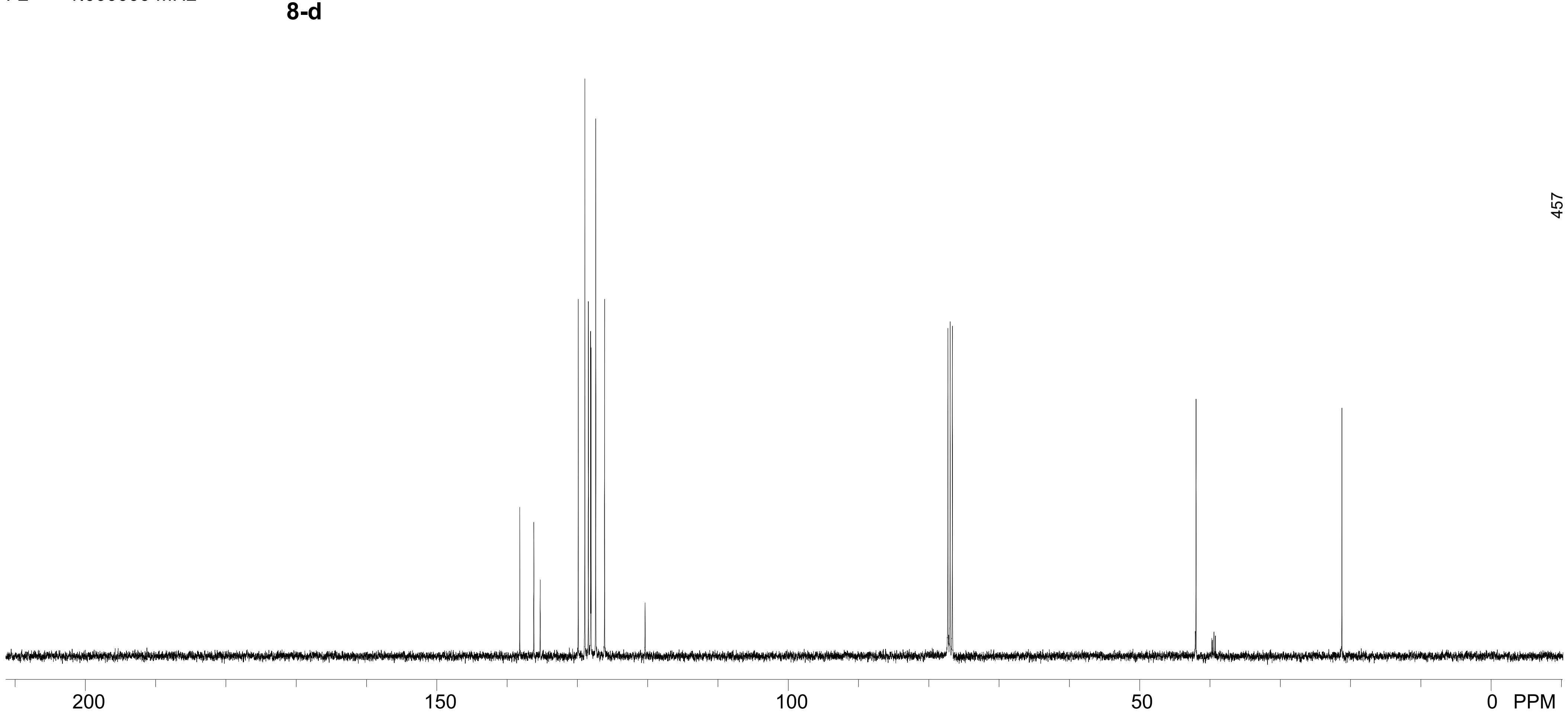

Area Percent Report

Sample name: zwf-8-018-IE-99-1-0.8-205

Data file: C:\Users\Public\Documents\ChemStation\1\Data\zwf\zwf-S 2023-11-12 11-02-17\028-P1-A1-zwf-8-018.D

Injection Acquired Date 11/13/2023 09:20:39

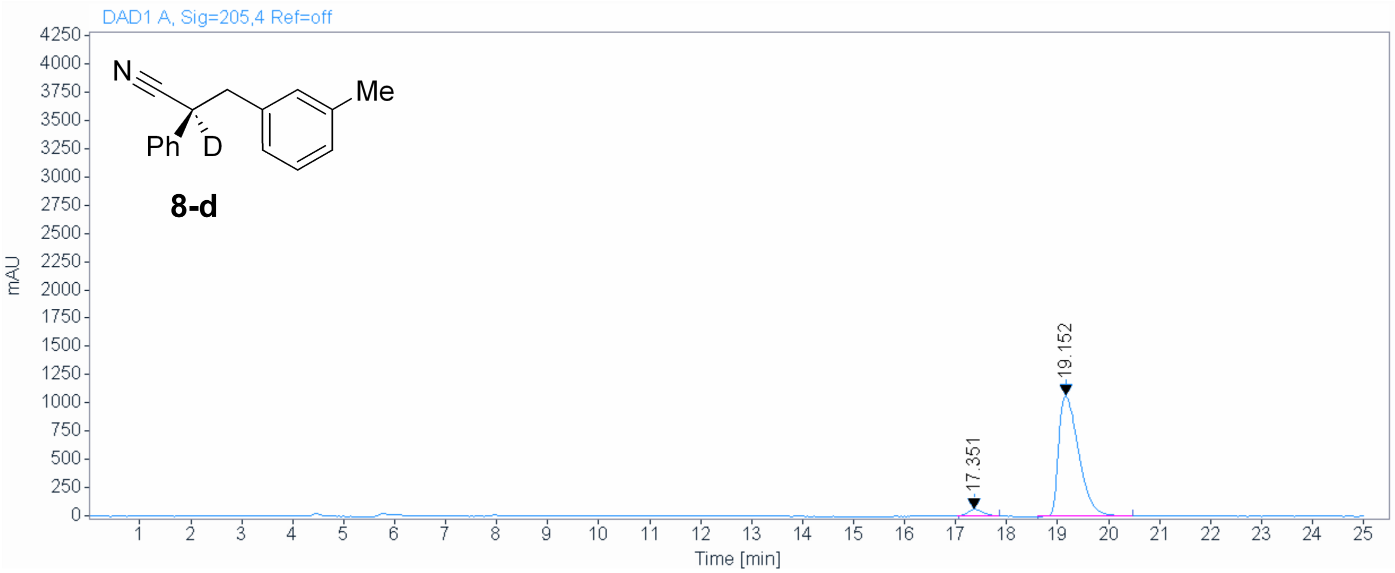

8-d

Signal: DAD1 A, Sig=205,4 Ref=off

RT [min]	Width [min]	Height	Area	Area%
17.351	0.3488	58.1227	1216.3157	4.0032
19.152	0.3314	1070.9240	29167.3867	95.9968
		Sum	30383.7024	100.0000

Area Percent Report

Agilent Technologies

Sample name: zwf-8-018-rac-IE-99-1-0.8-205

Data file: C:\Users\Public\Documents\ChemStation\1\Data\zwf\zwf-S 2023-11-12 11-02-17\029-P1-A2-zwf-8-018-rac.D

Injection Acquired Date 11/13/2023 09:46:43

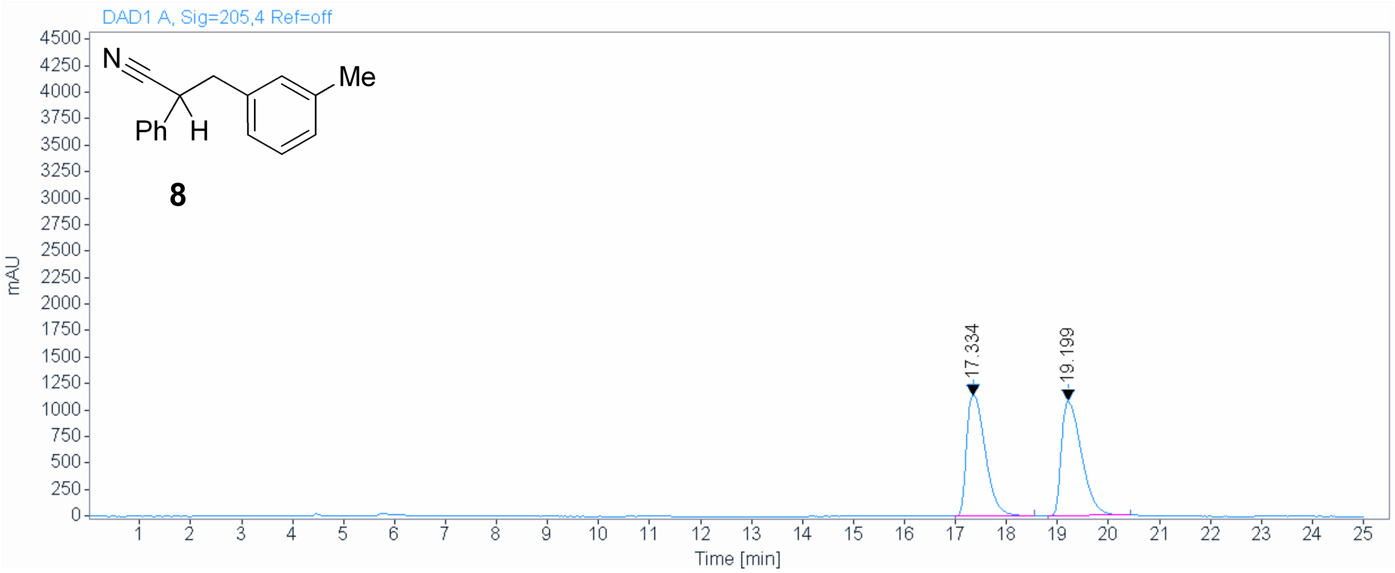

Signal: DAD1 A, Sig=205,4 Ref=off

RT [min]	Width [min]	Height	Area	Area%
17.334	0.4267	1139.0125	29157.6621	49.5072
19.199	0.3720	1088.4813	29738.0879	50.4928
		Sum	58895.7500	100.0000

zwf-7-088H
Sep 26 2023
SOLVENT: CDCl3
NA = 4
F1 = 400.140015 MHz
F2 = 1.000000 MHz

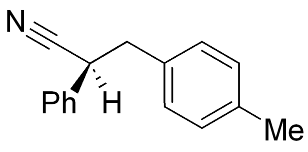

9

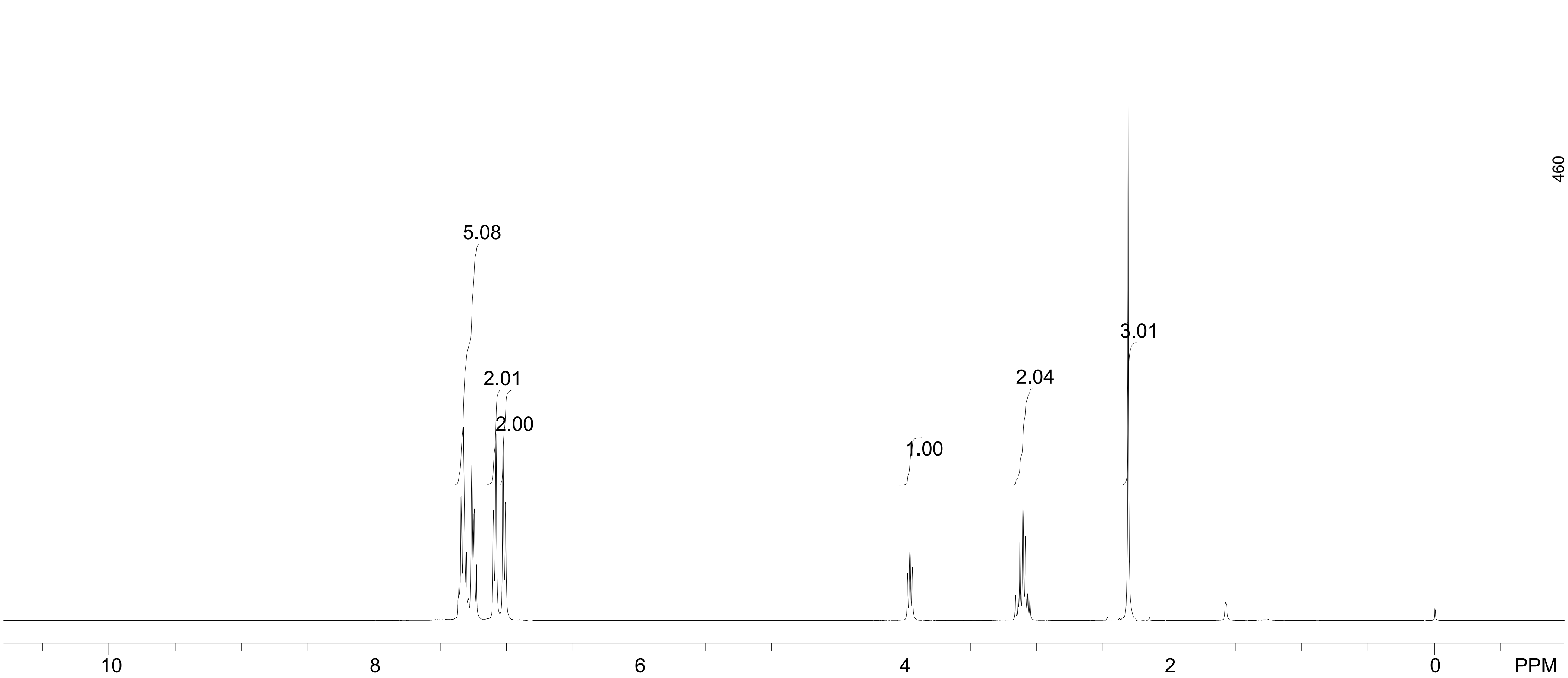

zwf-7-088C
Sep 26 2023
SOLVENT: CDCl3
NA = 100
F1 = 100.615280 MHz
F2 = 1.000000 MHz

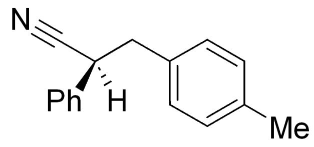

9

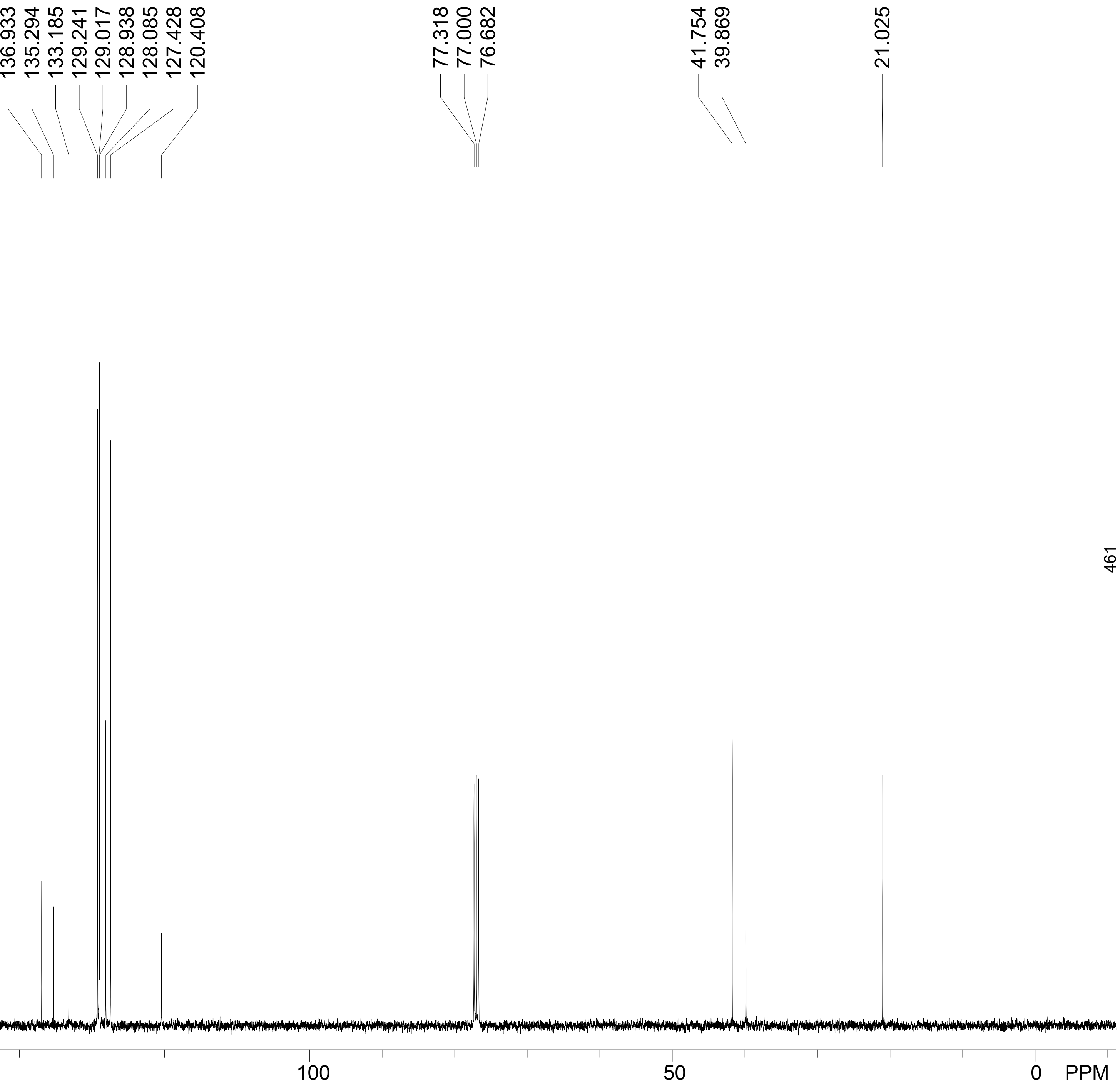

Area Percent Report

Agilent Technologies

Sample name: zwf-7-088-IE-99-1-0.8-205

Data file: C:\Users\Public\Documents\ChemStation\1\Data\zwf\zwf-S 2023-09-26 14-33-49\006-P1-C1-zwf-7-088.D

Injection Acquired Date 09/26/2023 16:33:01

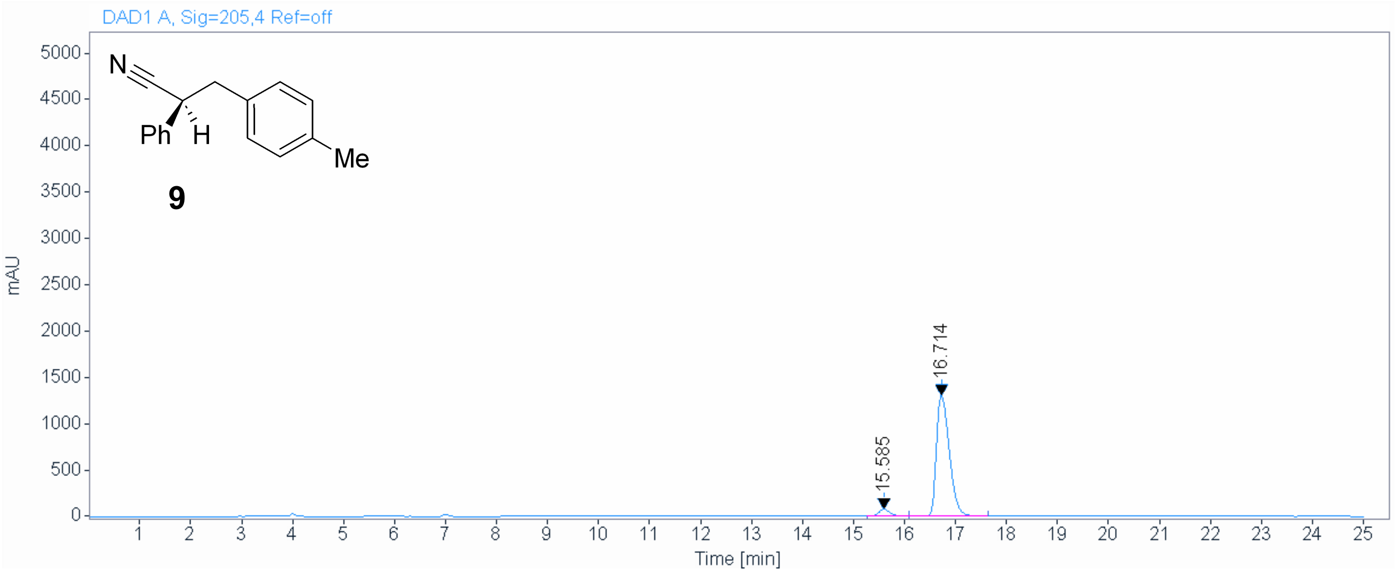

Signal: DAD1 A, Sig=205,4 Ref=off

RT [min]	Width [min]	Height	Area	Area%
15.585	0.2225	71.7066	1035.5859	4.3453
16.714	0.2767	1299.1819	22796.8086	95.6547
		Sum	23832.3945	100.0000

Area Percent Report

Agilent Technologies

Sample name: zwf-7-088-rac-IE-99-1-0.8-205

Data file: C:\Users\Public\Documents\ChemStation\1\Data\zwf\zwf-S 2023-09-26 14-33-49\007-P1-C2-zwf-7-088-rac.D

Injection Acquired Date 09/26/2023 16:59:04

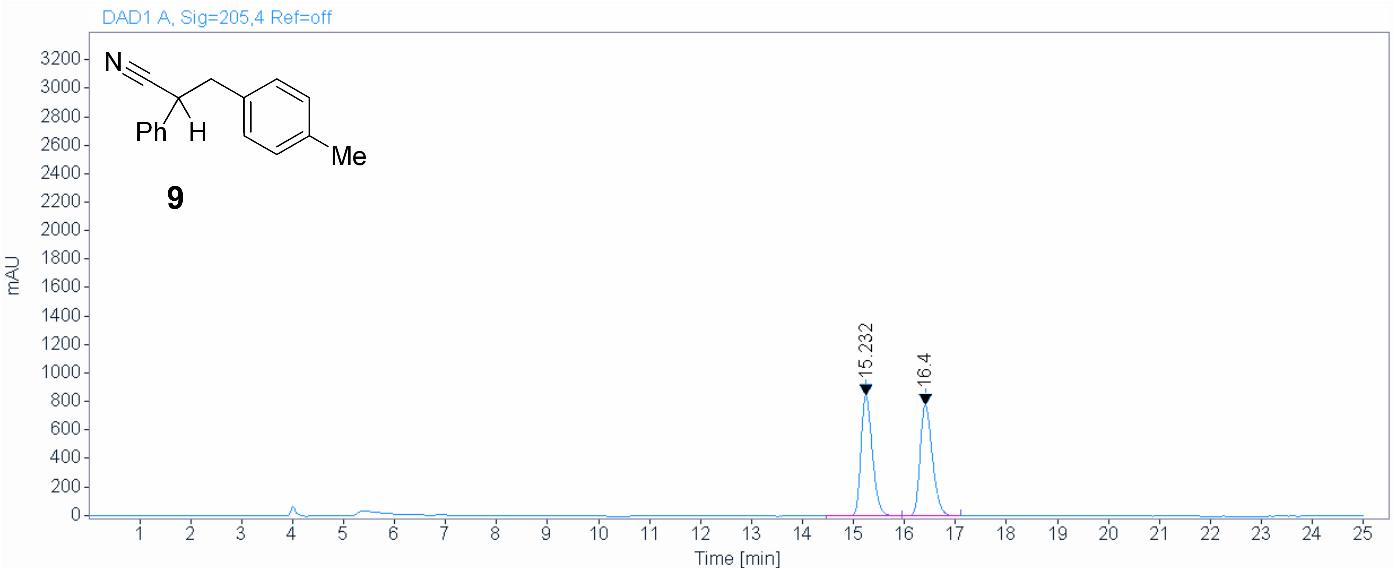

Signal: DAD1 A, Sig=205,4 Ref=off

RT [min]	Width [min]	Height	Area	Area%
15.232	0.2451	846.8530	13181.9814	50.1673
16.400	0.2577	780.5638	13094.0684	49.8327
		Sum	26276.0498	100.0000

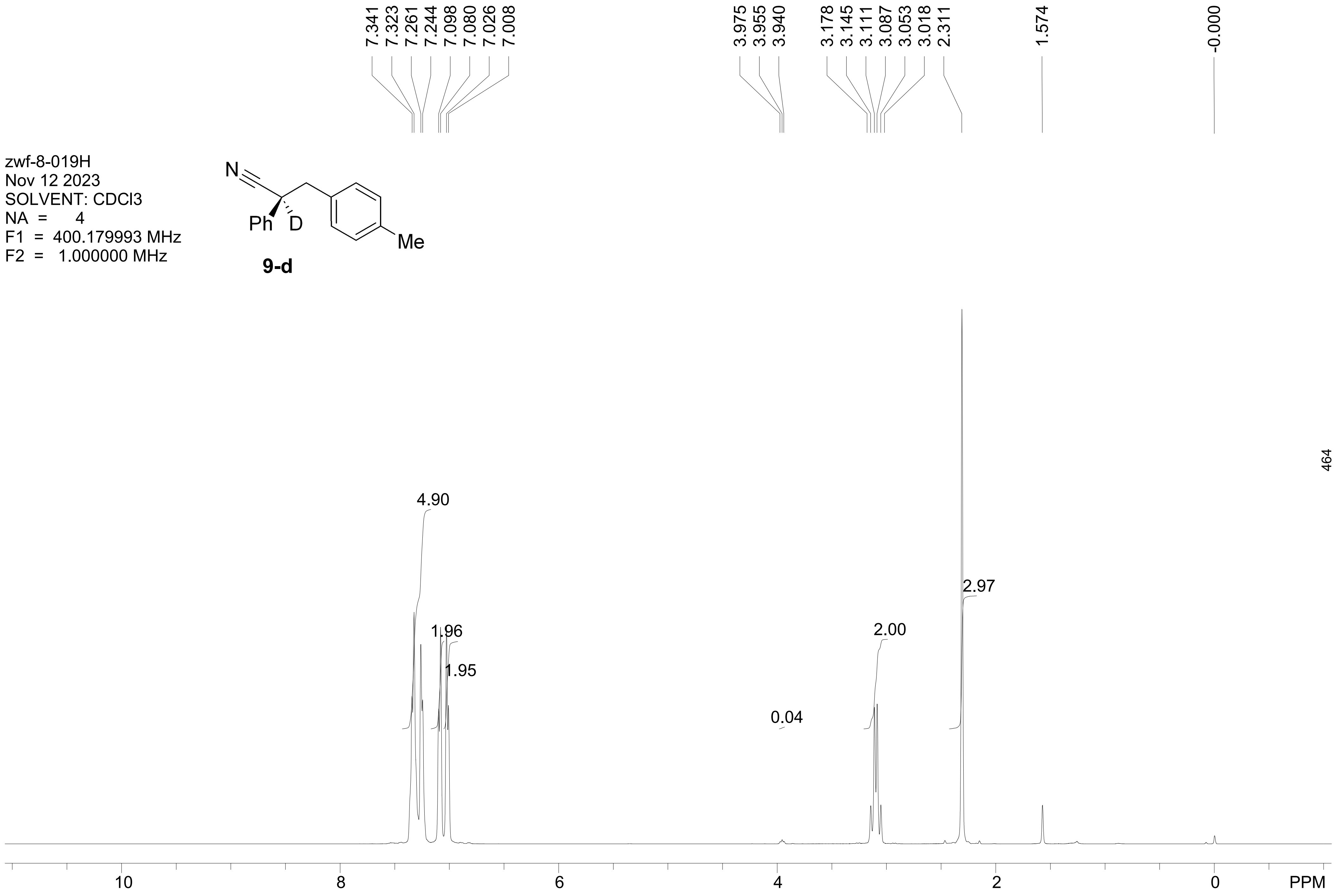
7.341
7.323
7.261
7.244
7.098
7.080
7.026
7.008
3.975
3.955
3.940
3.178
3.145
3.111
3.087
3.053
3.018
2.311
1.574
-0.000
zwf-8-019H
Nov 12 2023
SOLVENT: CDCl3
NA = 4
F1 = 400.179993 MHz
F2 = 1.000000 MHz
N
Ph
D
Me
9-d
4.90
1.96
1.95
0.04
2.00
2.97
10
8
6
4
2
0
PPM

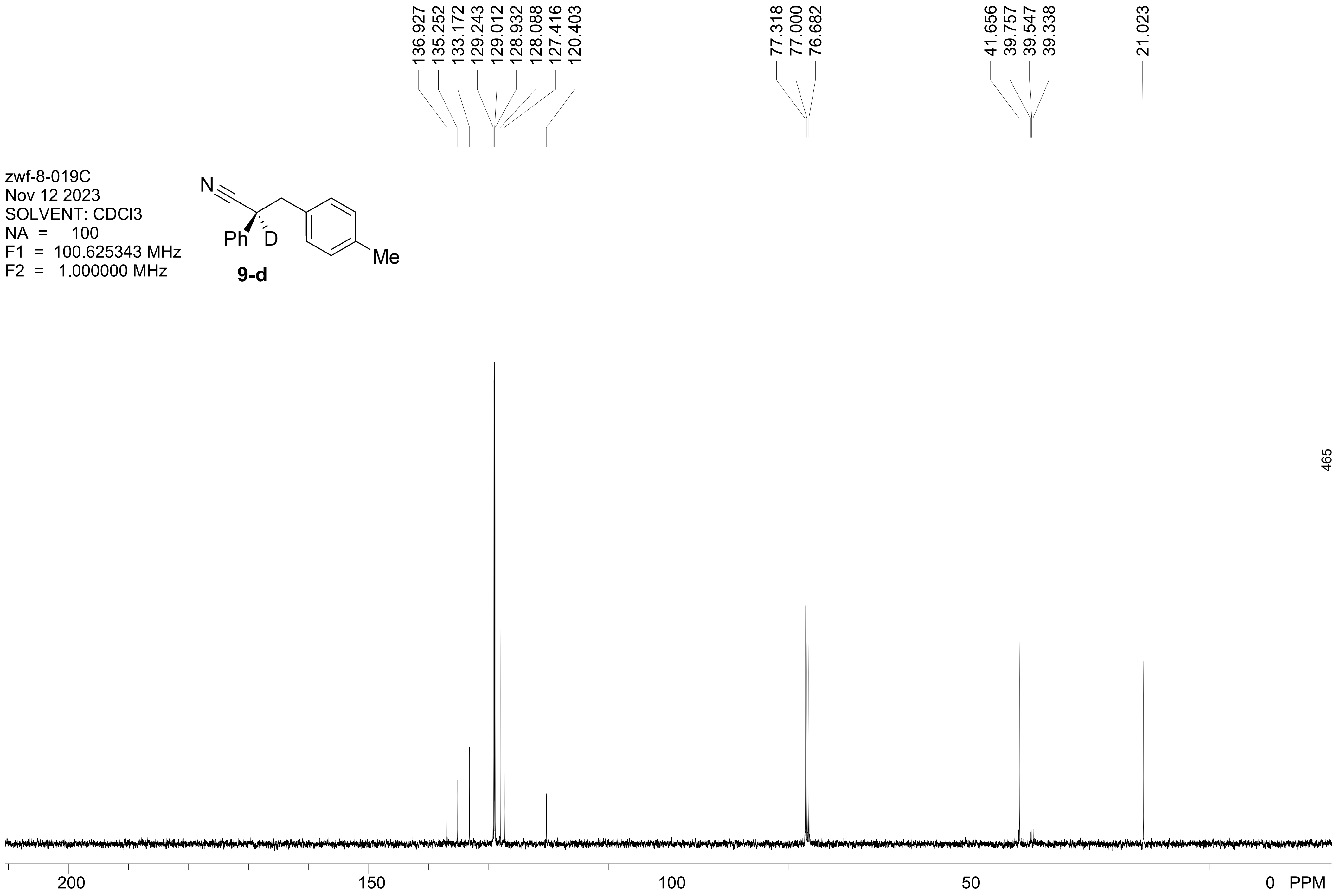
zwf-8-019C
Nov 12 2023
SOLVENT: CDCl3
NA = 100
F1 = 100.625343 MHz
F2 = 1.000000 MHz
N
Ph
D
Me
9-d
136.927
135.252
133.172
129.243
129.012
128.932
128.088
127.416
120.403
77.318
77.000
76.682
41.656
39.757
39.547
39.338
21.023
200
150
100
50
0 PPM

Area Percent Report

Agilent Technologies

Sample name: zwf-8-019-IE-99-1-0.8-205

Data file: C:\Users\Public\Documents\ChemStation\1\Data\zwf\zwf-S 2023-11-12 11-02-17\030-P1-A3-zwf-8-019.D

Injection Acquired Date 11/13/2023 10:12:45

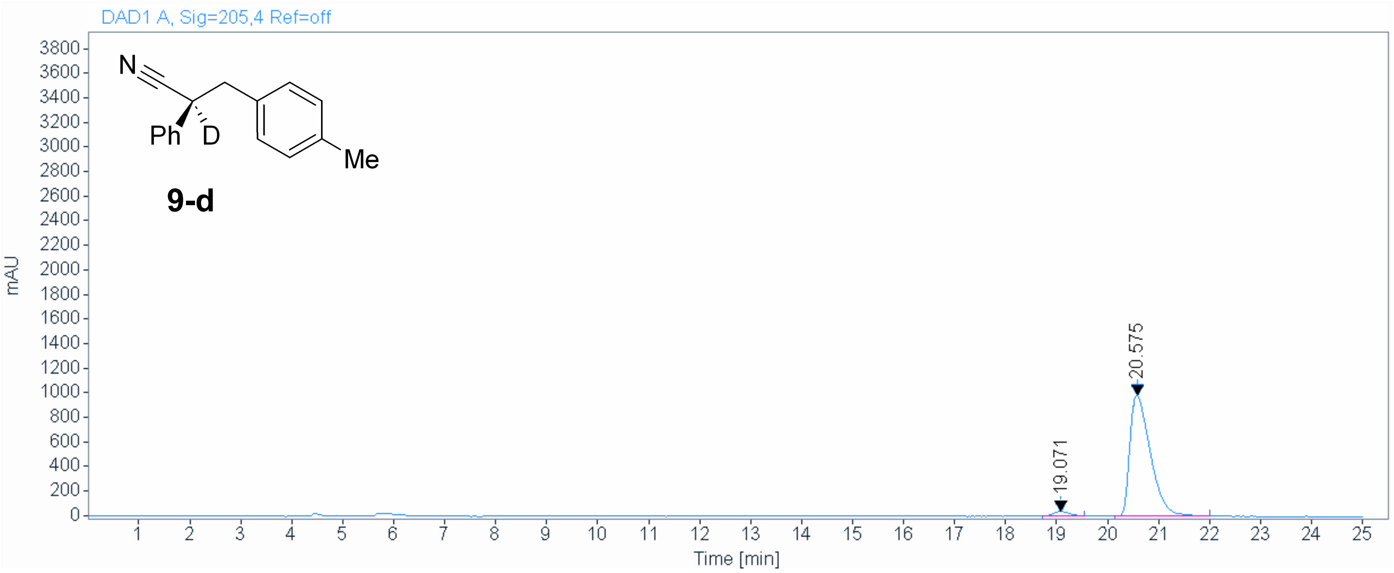

Signal: DAD1 A, Sig=205,4 Ref=off

RT [min]	Width [min]	Height	Area	Area%
19.071	0.3644	35.8821	784.4817	2.7745
20.575	0.4667	981.6821	27490.6074	97.2255
		Sum	28275.0891	100.0000

Area Percent Report

Agilent Technologies

Sample name: zwf-8-019-rac-IE-99-1-0.8-205

Data file: C:\Users\Public\Documents\ChemStation\1\Data\zwf\zwf-S 2023-11-12 11-02-17\031-P1-A4-zwf-8-019-rac.D

Injection Acquired Date 11/13/2023 10:38:46

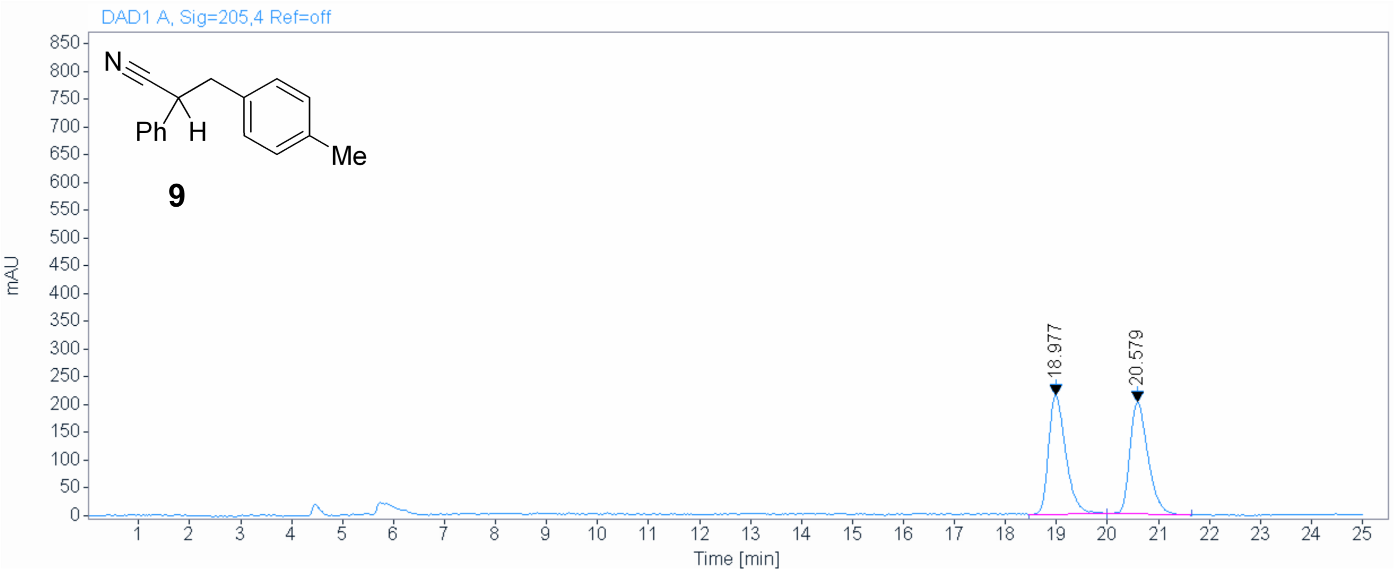

Signal: DAD1 A, Sig=205,4 Ref=off

RT [min]	Width [min]	Height	Area	Area%
18.977	0.3684	214.6501	5119.8584	50.3138
20.579	0.3220	202.4300	5055.9980	49.6862
		Sum	10175.8564	100.0000

7.369
7.362
7.347
7.329
7.320
7.309
7.307
7.254
7.249
7.242
7.234
7.230
7.047
7.025
6.825
6.804
3.968
3.952
3.931
3.774
3.149
3.115
3.094
3.077
3.059
-0.000

zwf-7-064H
Sep 18 2023
SOLVENT: CDCl3
NA = 4
F1 = 400.140015 MHz
F2 = 1.000000 MHz

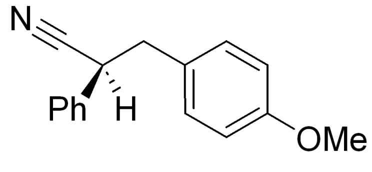

10

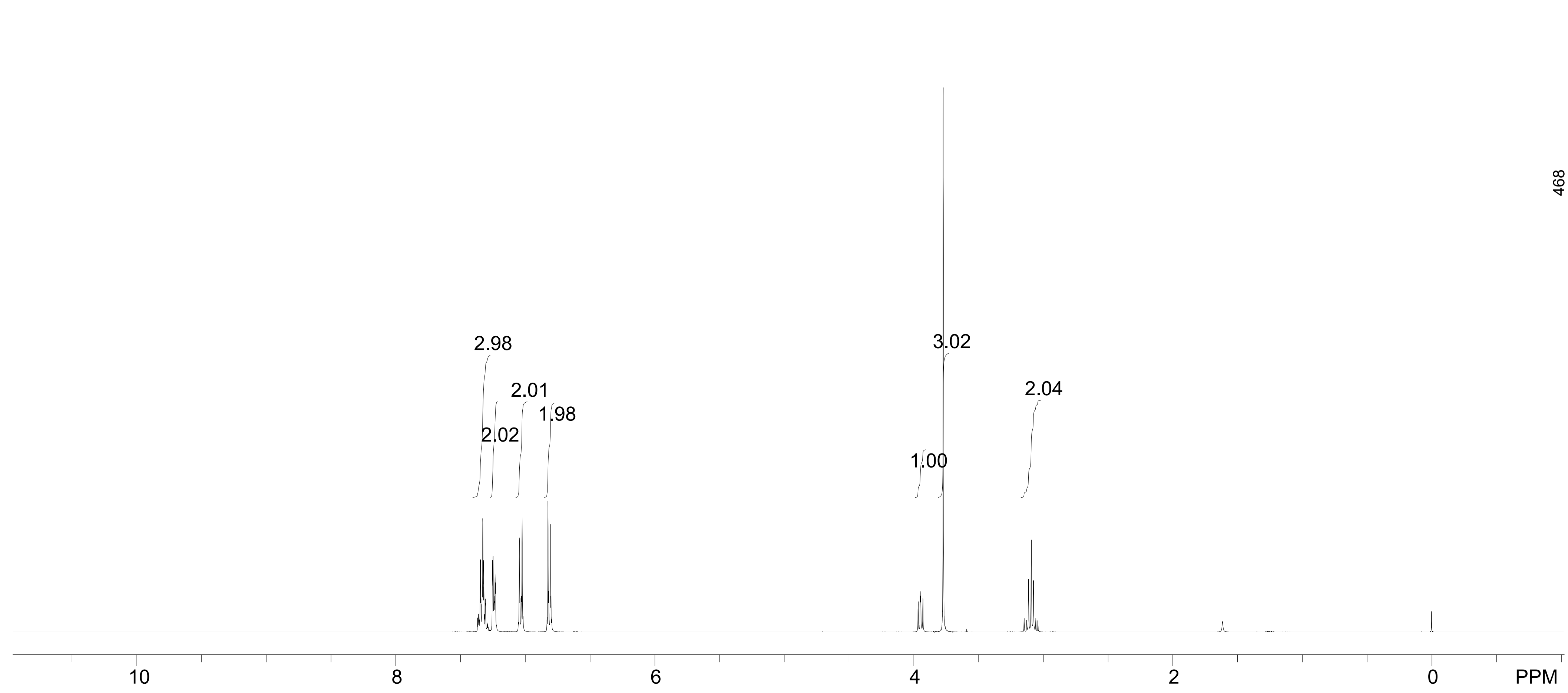

zwf-7-064C
Sep 18 2023
SOLVENT: CDCl3
NA = 100
F1 = 100.615280 MHz
F2 = 1.000000 MHz

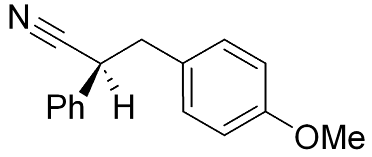

10

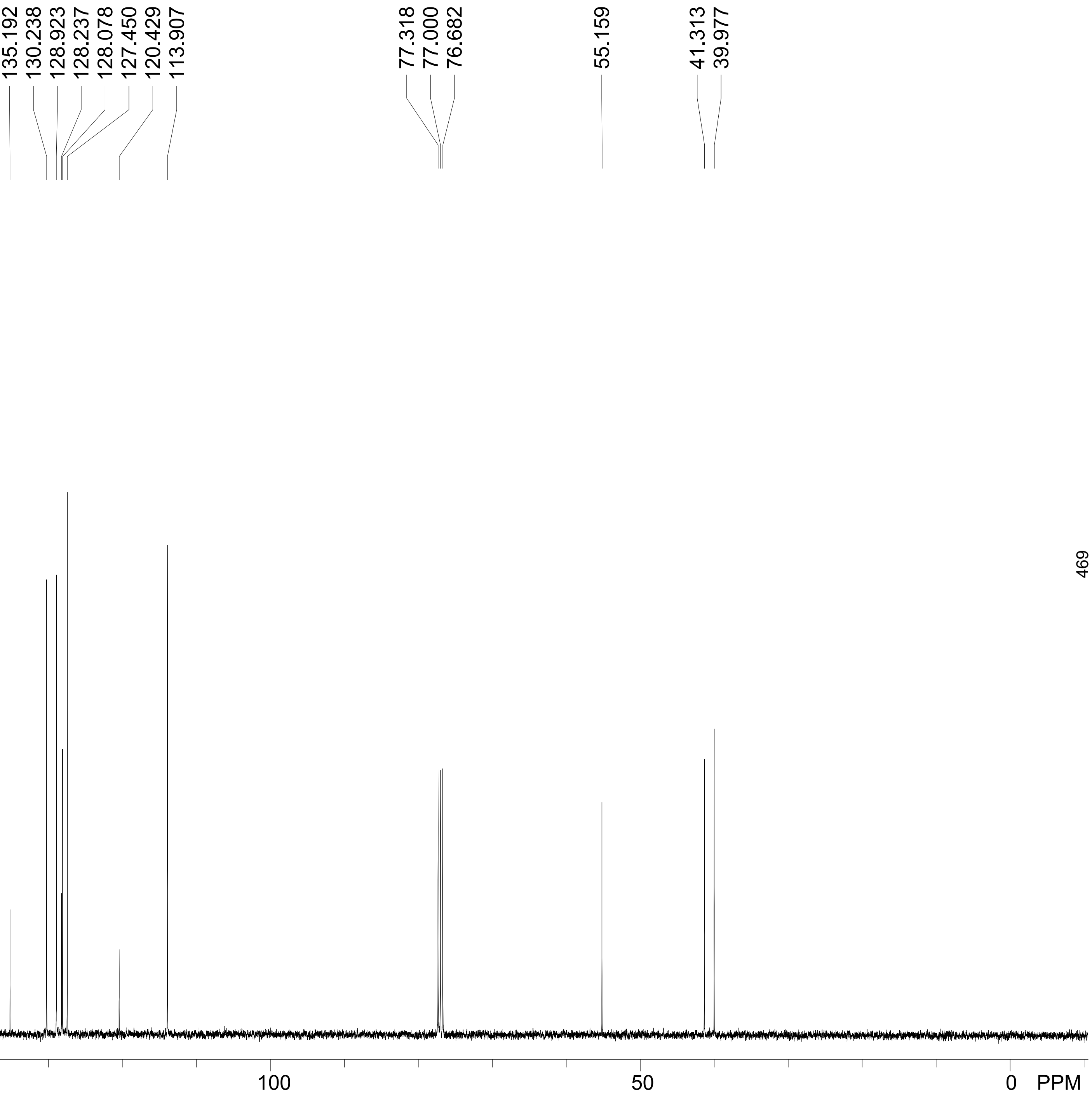

Area Percent Report

Agilent Technologies

Sample name: zwf-7-064-IE-99-1-0.8-205

Data file: C:\Users\Public\Documents\ChemStation\1\Data\zwf\zwf-S 2023-09-24 18-53-06\005-P1-C3-zwf-7-064.D

Injection Acquired Date 09/24/2023 20:19:09

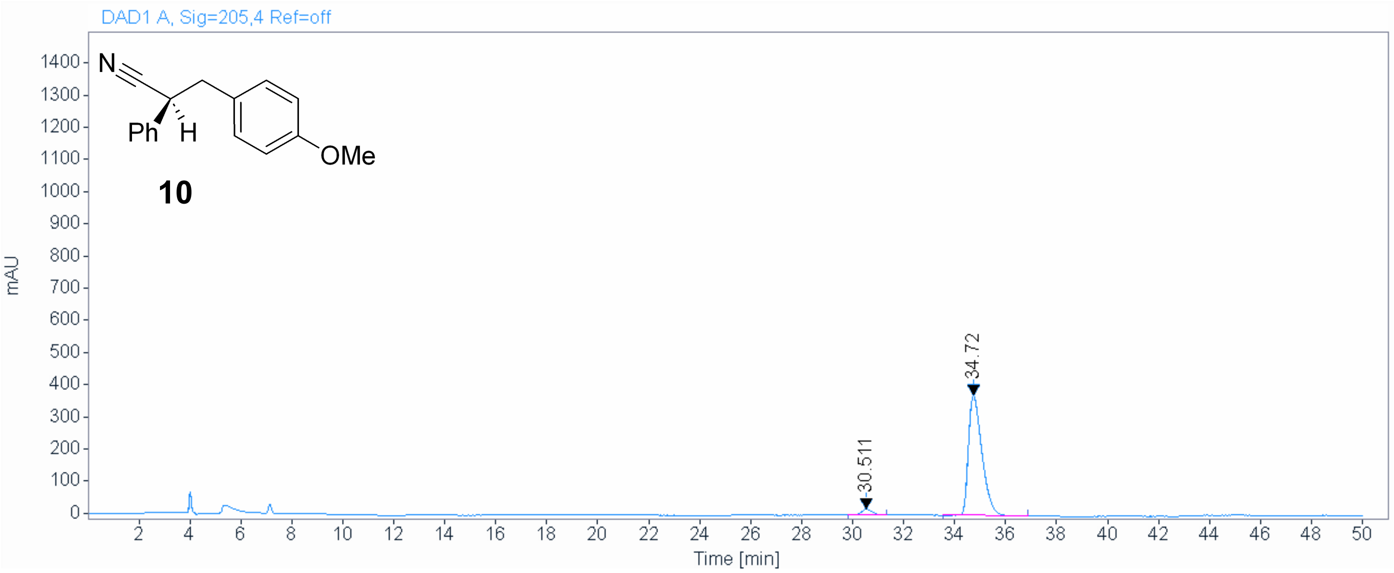

Signal: DAD1 A, Sig=205,4 Ref=off

RT [min]	Width [min]	Height	Area	Area%
30.511	0.3572	18.1396	536.6495	3.7375
34.720	0.6175	373.0719	13821.8652	96.2625
		Sum	14358.5147	100.0000

Area Percent Report

Sample name: zwf-7-064-rac-IE-99-1-0.8-205

Data file: C:\Users\Public\Documents\ChemStation\1\Data\zwf\zwf-S 2023-09-24 18-53-06\006-P1-C4-zwf-7-064-rac.D

Injection Acquired Date 09/24/2023 21:10:15

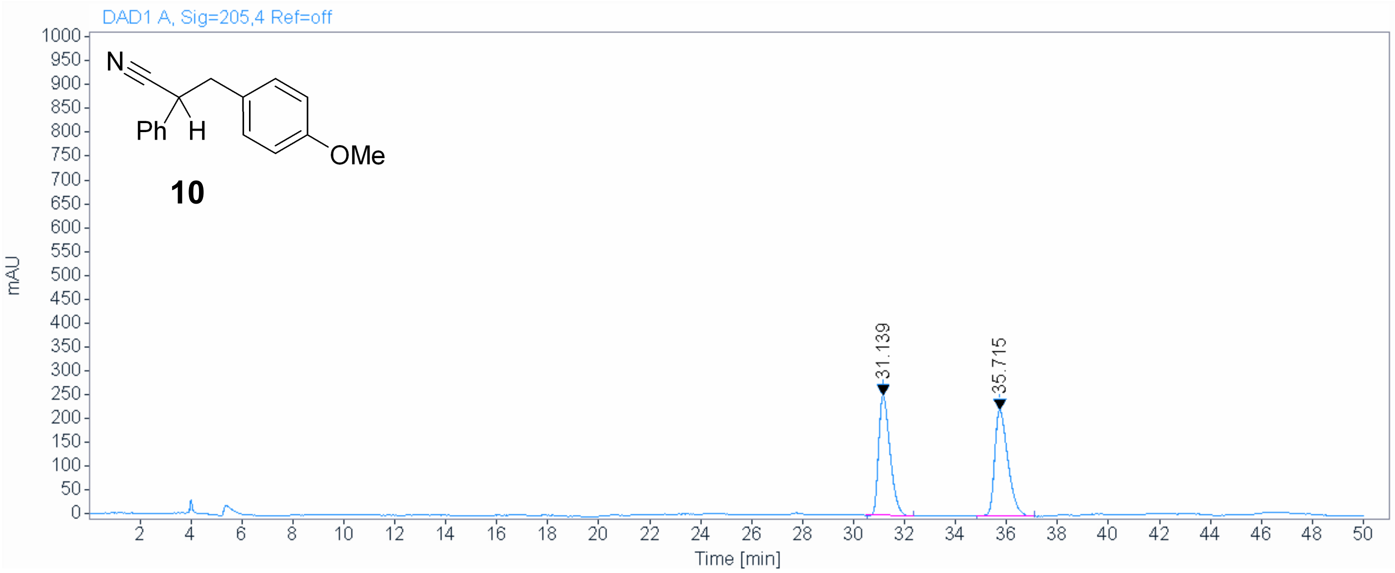

Signal: DAD1 A, Sig=205,4 Ref=off

RT [min]	Width [min]	Height	Area	Area%
31.139	0.4536	252.4328	7909.9263	49.3048
35.715	0.5368	223.3384	8132.9854	50.6952
		Sum	16042.9116	100.0000

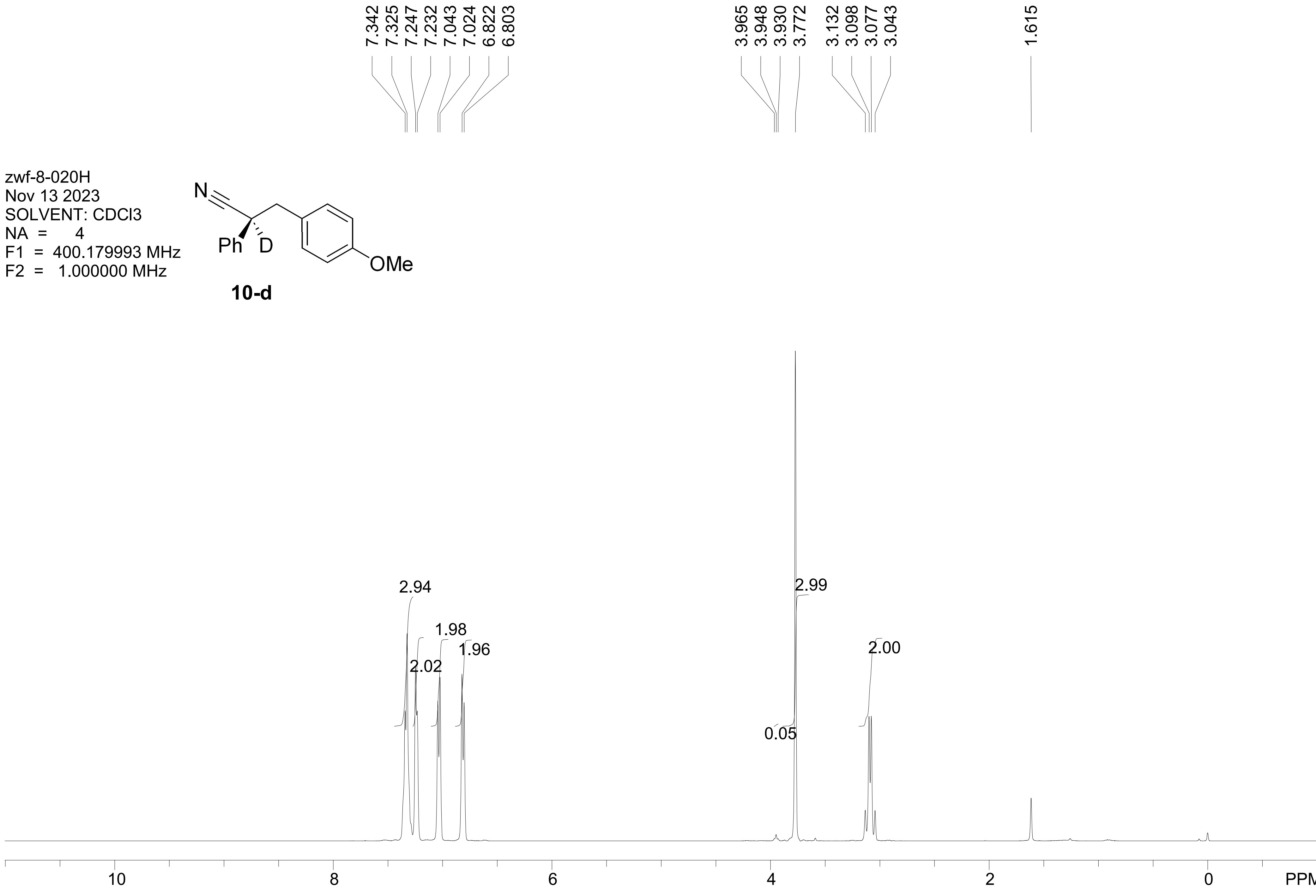
zwf-8-020H
Nov 13 2023
SOLVENT: CDCl3
NA = 4
F1 = 400.179993 MHz
F2 = 1.000000 MHz
N
Ph
D
OMe
10-d
7.342
7.325
7.247
7.232
7.043
7.024
6.822
6.803
3.965
3.948
3.930
3.772
3.132
3.098
3.077
3.043
1.615
2.94
2.02
1.98
1.96
0.05
2.99
2.00
10
8
6
4
2
0
PPM

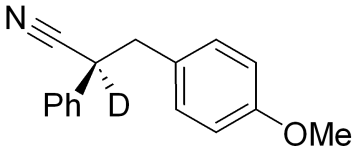
N
Ph
D
OMe

10-d

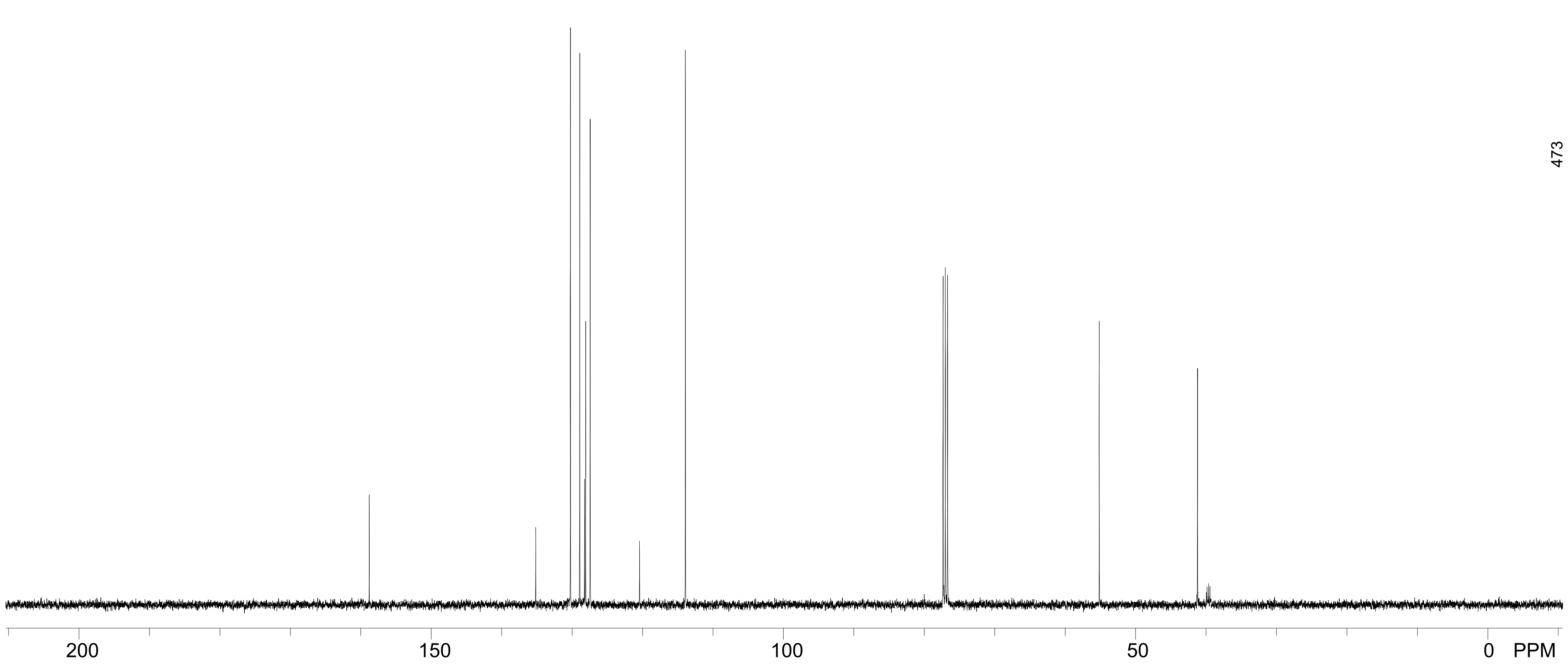
zwf-8-020C
Nov 13 2023
SOLVENT: CDCl3
NA = 100
F1 = 100.625343 MHz
F2 = 1.000000 MHz
158.802
135.165
130.240
128.918
128.239
128.080
127.438
120.425
113.918
77.318
77.000
76.682
55.154
41.216
39.865
39.656
39.453
200
150
100
50
0
PPM

Area Percent Report

Sample name: zwf-8-020-IE-99-1-0.8-205

Data file: C:\Users\Public\Documents\ChemStation\1\Data\zwf\zwf-S 2023-11-12 11-02-17\049-P1-B7-zwf-8-020.D

Injection Acquired Date 11/13/2023 20:07:27

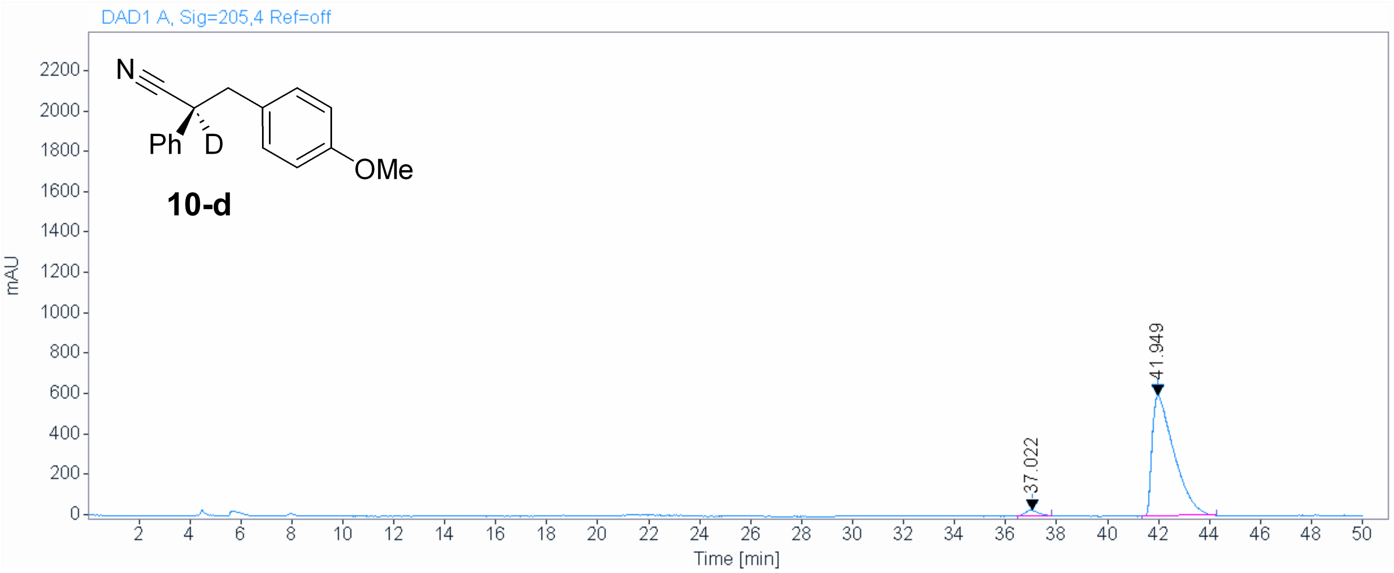

Signal: DAD1 A, Sig=205,4 Ref=off

RT [min]	Width [min]	Height	Area	Area%
37.022	0.6181	27.5834	1023.0328	2.7701
41.949	0.7170	597.5098	35908.7031	97.2299
		Sum	36931.7359	100.0000

Area Percent Report

Agilent Technologies

Sample name: zwf-8-020-rac-IE-99-1-0.8-205

Data file: C:\Users\Public\Documents\ChemStation\1\Data\zwf\zwf-S 2023-11-12 11-02-17\050-P1-B8-zwf-8-020-rac.D

Injection Acquired Date 11/13/2023 20:58:30

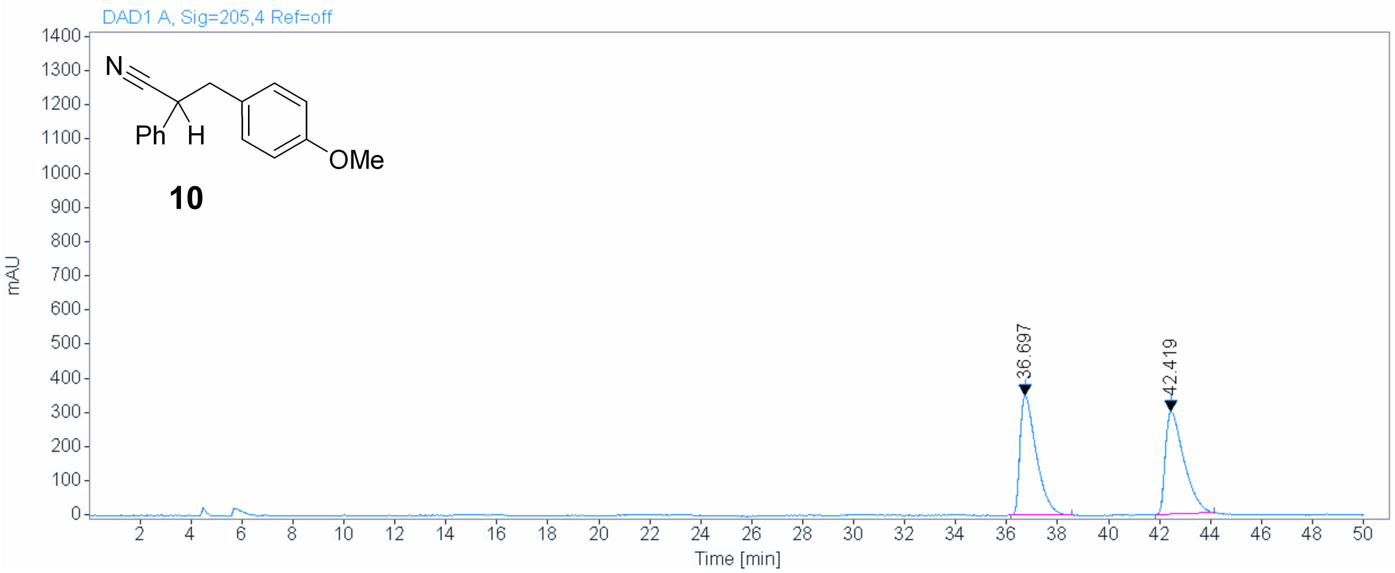

Signal: DAD1 A, Sig=205,4 Ref=off

RT [min]	Width [min]	Height	Area	Area%
36.697	0.7395	349.8299	15522.5615	50.1756
42.419	0.6113	301.0809	15413.9219	49.8244
		Sum	30936.4834	100.0000

zwf-7-065H
Sep 20 2023
SOLVENT: CDCl3
NA = 4
F1 = 400.140015 MHz
F2 = 1.000000 MHz

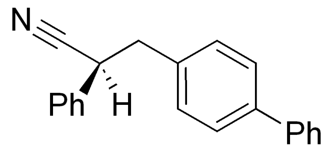

11

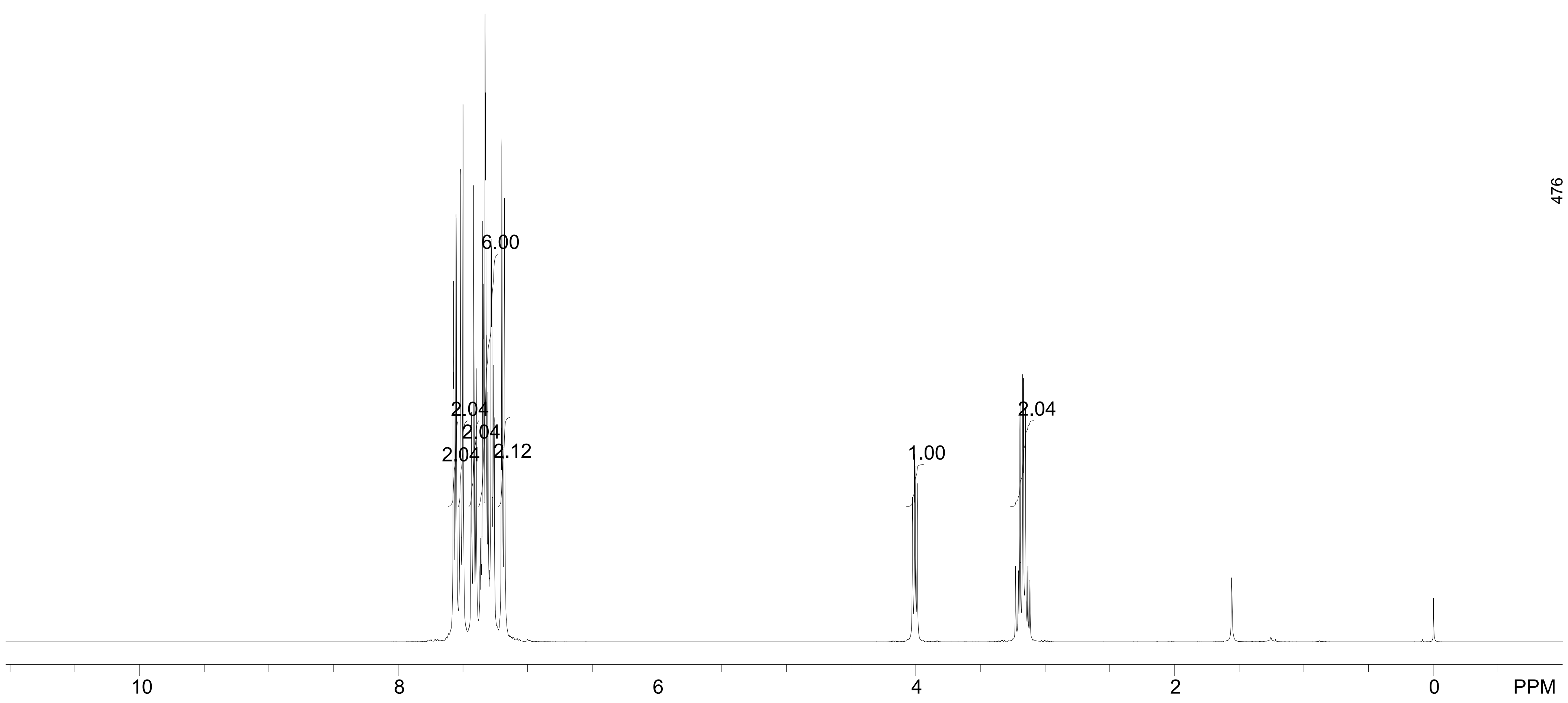

zwf-7-065C
Sep 20 2023
SOLVENT: CDCl3
NA = 100
F1 = 100.615280 MHz
F2 = 1.000000 MHz

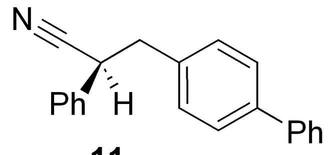

11

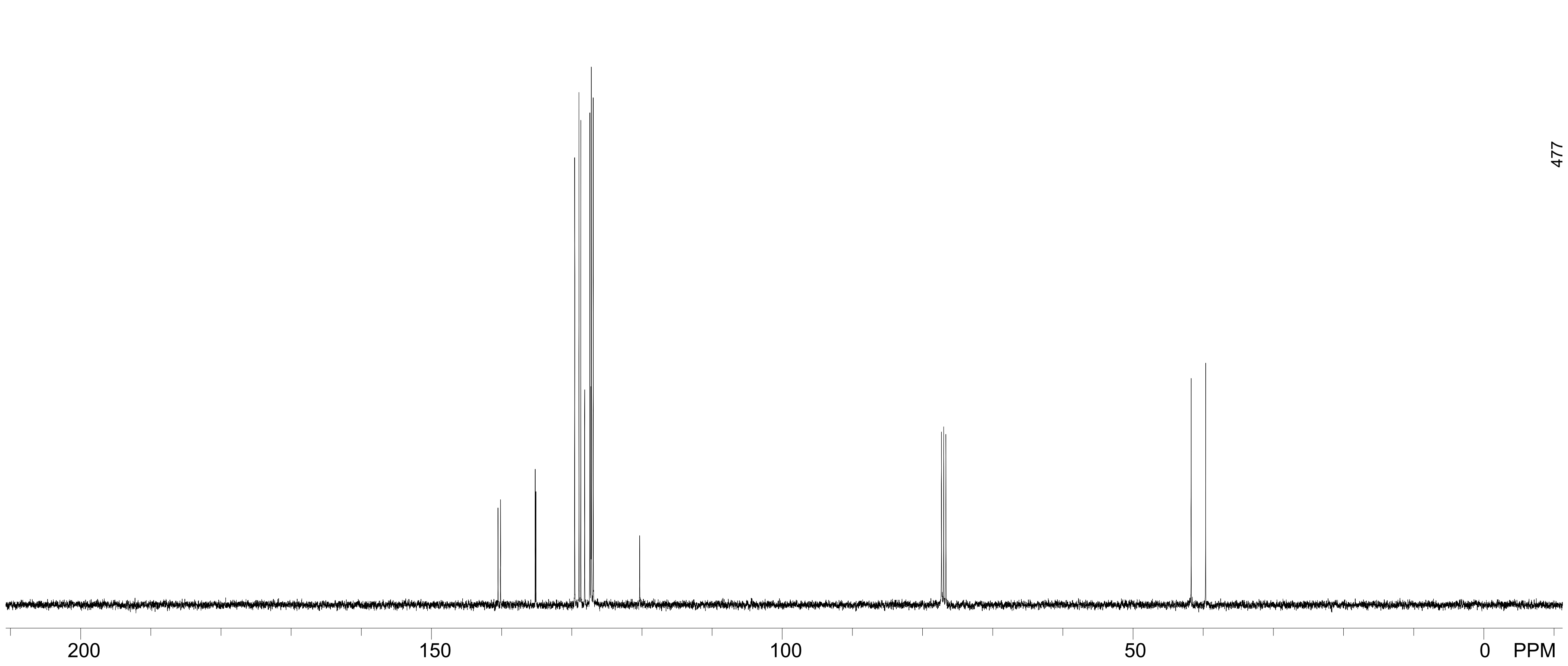

Area Percent Report

Agilent Technologies

Sample name: zwf-7-065-IE-99-1-0.8-205

Data file: C:\Users\Public\Documents\ChemStation\1\Data\zwf\zwf-S 2023-09-21 02-21-00\005-P1-C4-zwf-7-065.D

Injection Acquired Date 09/21/2023 03:51:16

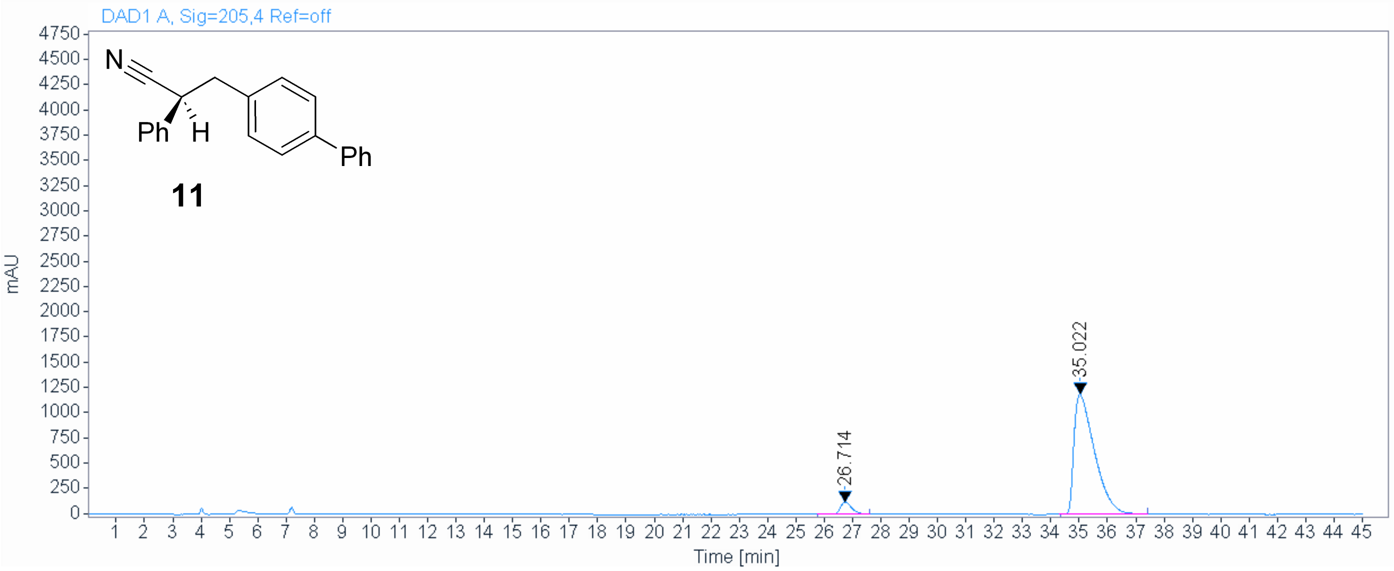

Signal: DAD1 A, Sig=205,4 Ref=off

RT [min]	Width [min]	Height	Area	Area%
26.714	0.4023	118.5383	3214.0437	5.1062
35.022	0.6200	1193.1547	59730.2852	94.8938
		Sum	62944.3289	100.0000

Area Percent Report

Agilent Technologies

Sample name: zwf-7-065-rac-IE-99-1-0.8-205

Data file: C:\Users\Public\Documents\ChemStation\1\Data\zwf\zwf-S 2023-09-21 02-21-00\007-P1-C6-zwf-7-065-rac.D

Injection Acquired Date 09/21/2023 05:23:24

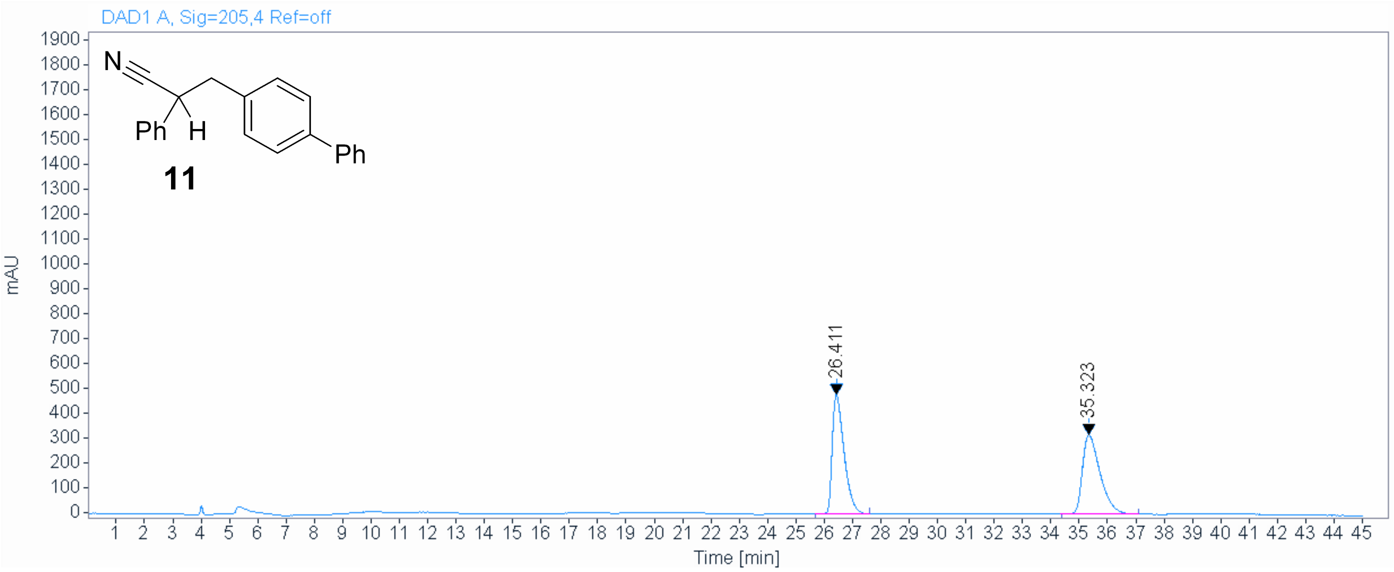

Signal: DAD1 A, Sig=205,4 Ref=off

RT [min]	Width [min]	Height	Area	Area%
26.411	0.4329	477.7765	13798.8262	50.1049
35.323	0.5878	318.1900	13741.0332	49.8951
		Sum	27539.8594	100.0000

zwf-8-021H
Nov 26 2023
SOLVENT: CDCl3
NA = 4
F1 = 400.179993 MHz
F2 = 1.000000 MHz

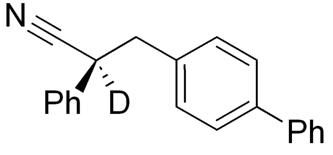

11-d

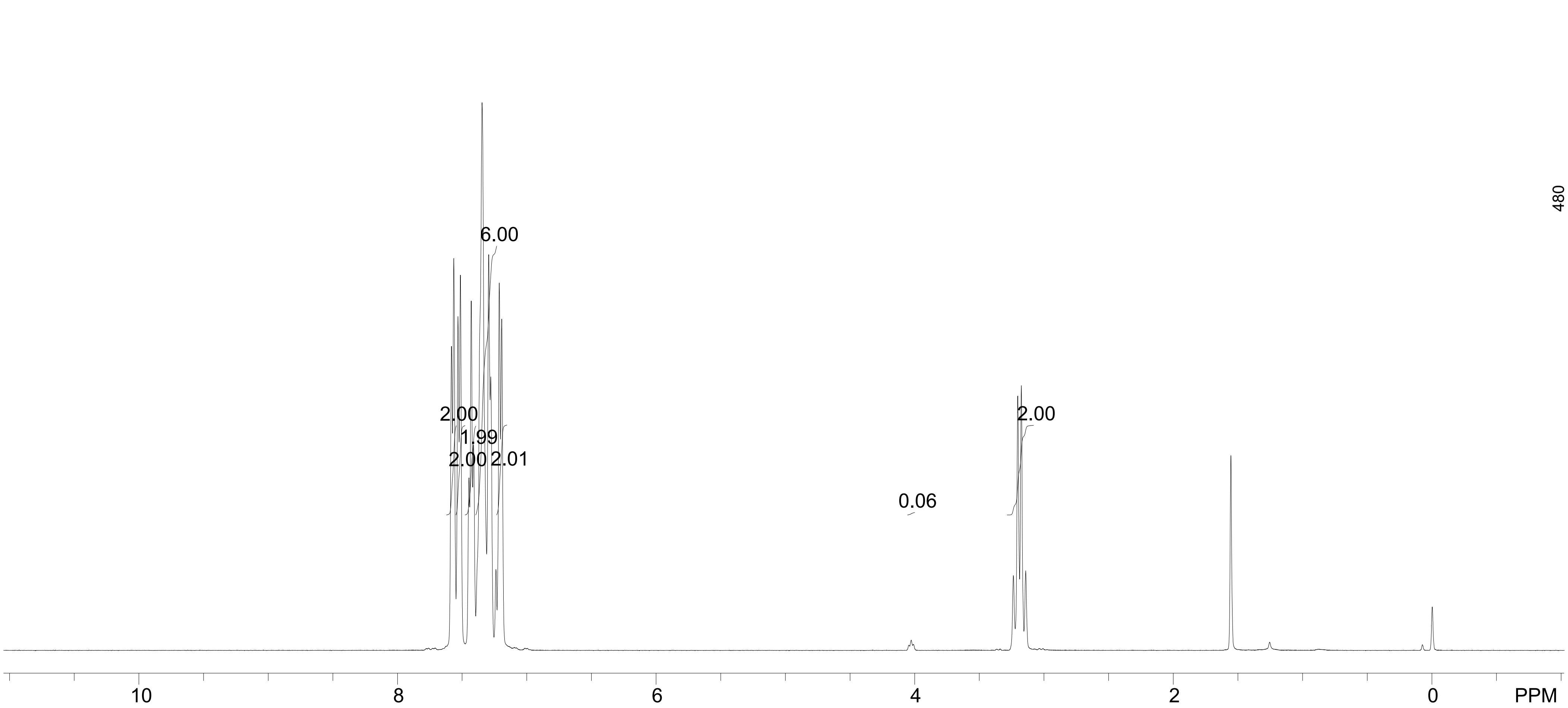

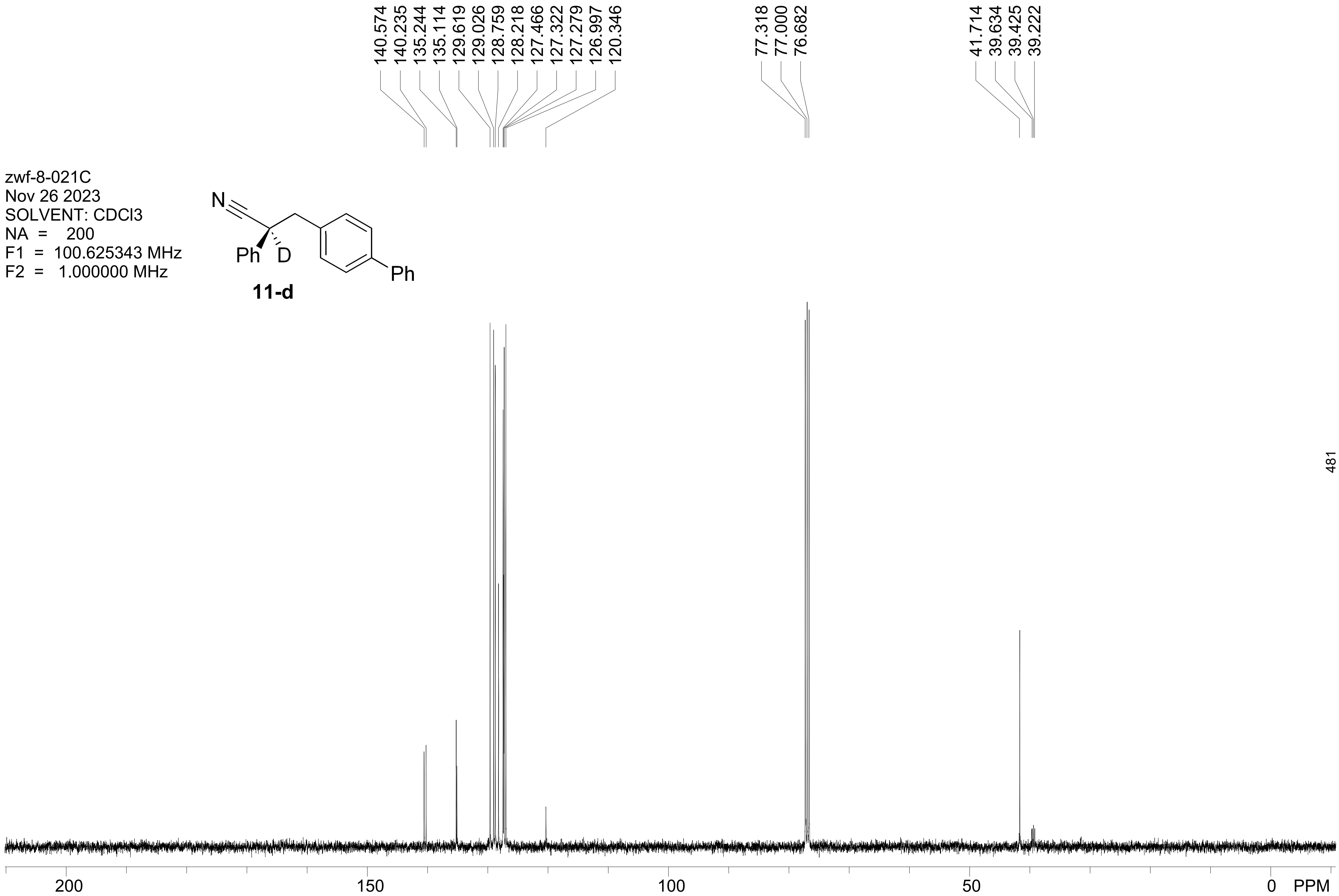
zwf-8-021C
Nov 26 2023
SOLVENT: CDCl3
NA = 200
F1 = 100.625343 MHz
F2 = 1.000000 MHz
N
Ph
D
Ph
11-d
140.574
140.235
135.244
135.114
129.619
129.026
128.759
128.218
127.466
127.322
127.279
126.997
120.346
77.318
77.000
76.682
41.714
39.634
39.425
39.222
200
150
100
50
0 PPM

Area Percent Report

Agilent Technologies

Sample name: zwf-8-021-IE-99-1-0.8-205

Data file: C:\Users\Public\Documents\ChemStation\1\Data\zwf\zwf-S 2023-11-26 13-46-16\019-P1-C8-zwf-8-021.D

Injection Acquired Date 11/27/2023 01:21:27

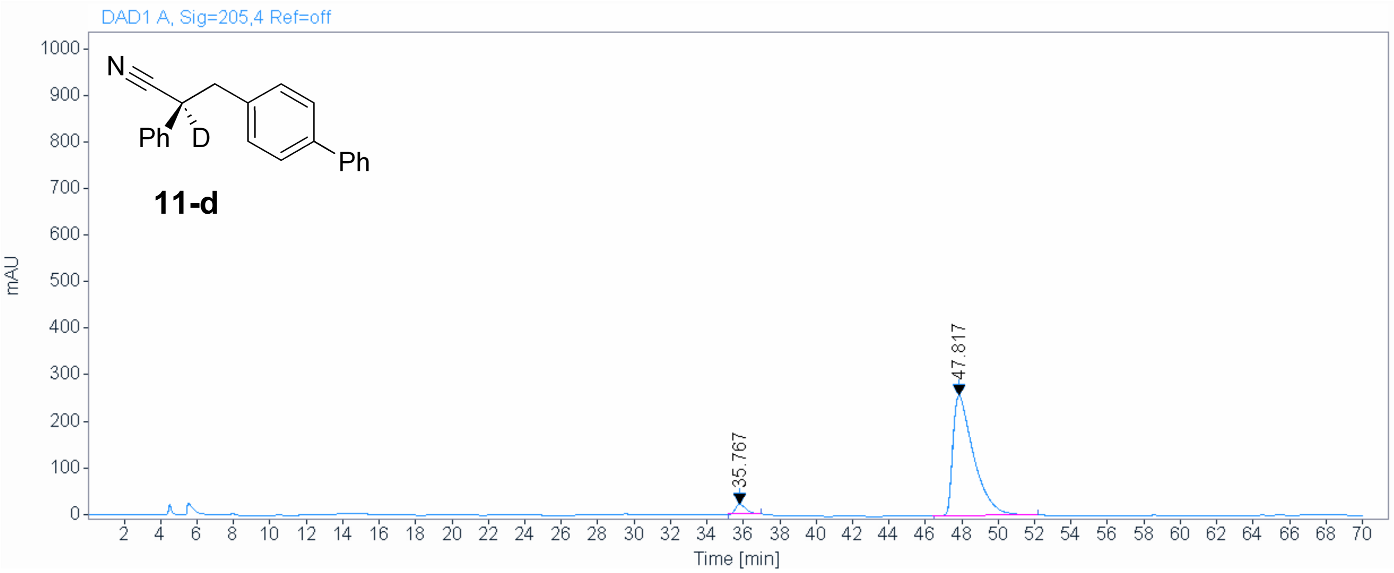

Signal: DAD1 A, Sig=205,4 Ref=off

RT [min]	Width [min]	Height	Area	Area%
35.767	0.6676	21.4454	859.0587	3.9890
47.817	1.1293	258.4738	20676.4922	96.0110
		Sum	21535.5509	100.0000

Area Percent Report

Sample name: zwf-8-021-rac-IE-99-1-0.8-205

Data file: C:\Users\Public\Documents\ChemStation\1\Data\zwf\zwf-S 2023-11-26 13-46-16\020-P1-C9-zwf-8-021-arc.D

Injection Acquired Date 11/27/2023 02:32:29

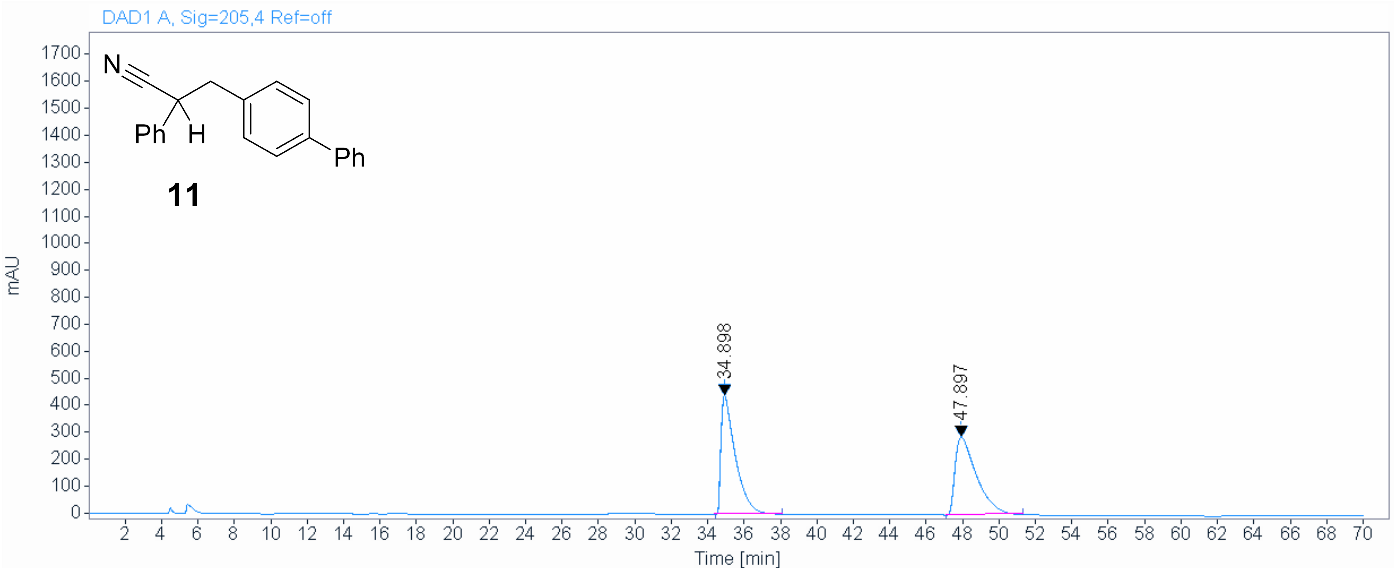

Signal: DAD1 A, Sig=205,4 Ref=off

RT [min]	Width [min]	Height	Area	Area%
34.898	0.7889	440.3772	24190.4727	50.6127
47.897	1.1813	289.4761	23604.8125	49.3873
		Sum	47795.2852	100.0000

zwf-7-070H
Sep 16 2023
SOLVENT: CDCl3
NA = 4
F1 = 400.179993 MHz
F2 = 1.000000 MHz

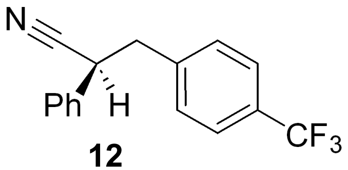

12

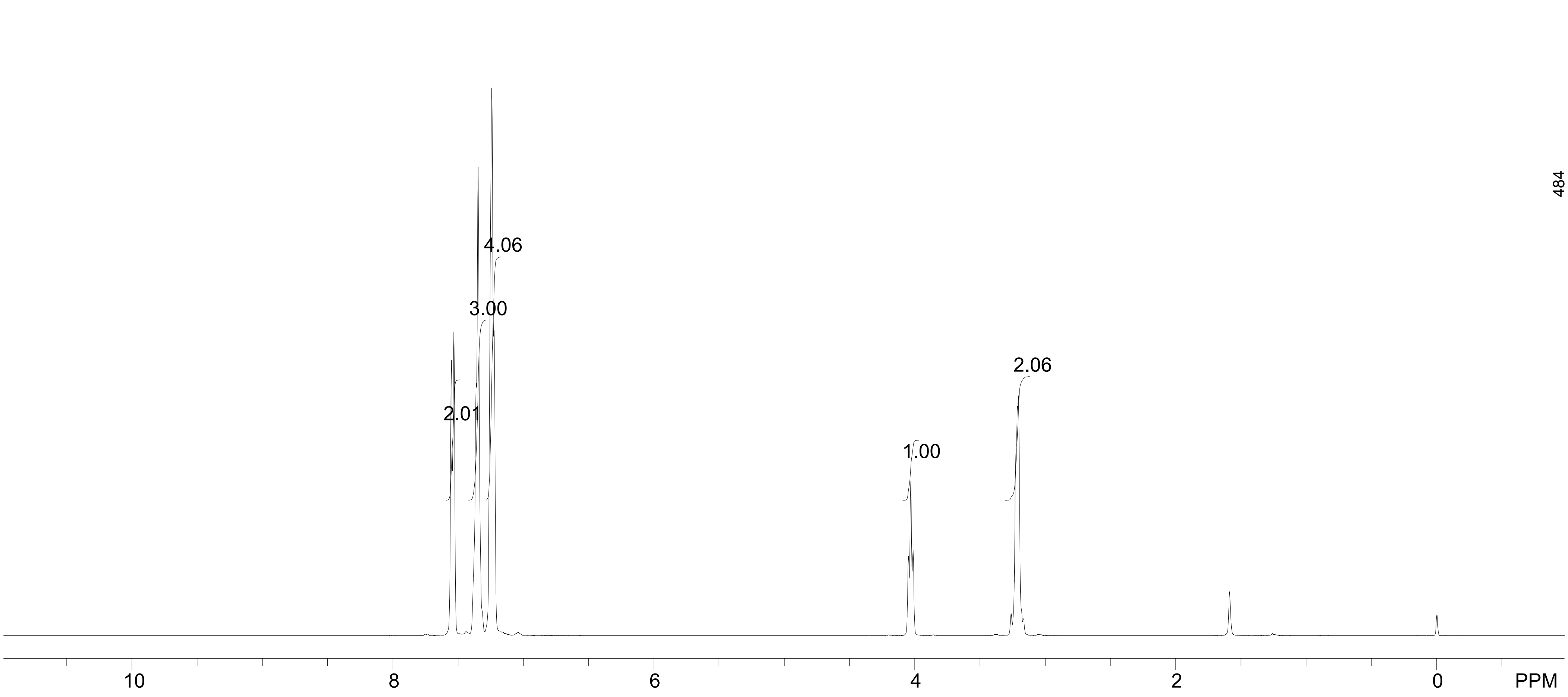

zwf-7-070C
Sep 16 2023
SOLVENT: CDCl3
NA =   150
F1 = 100.625343 MHz
F2 =   1.000000 MHz

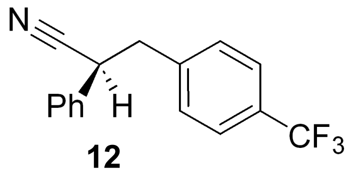

12

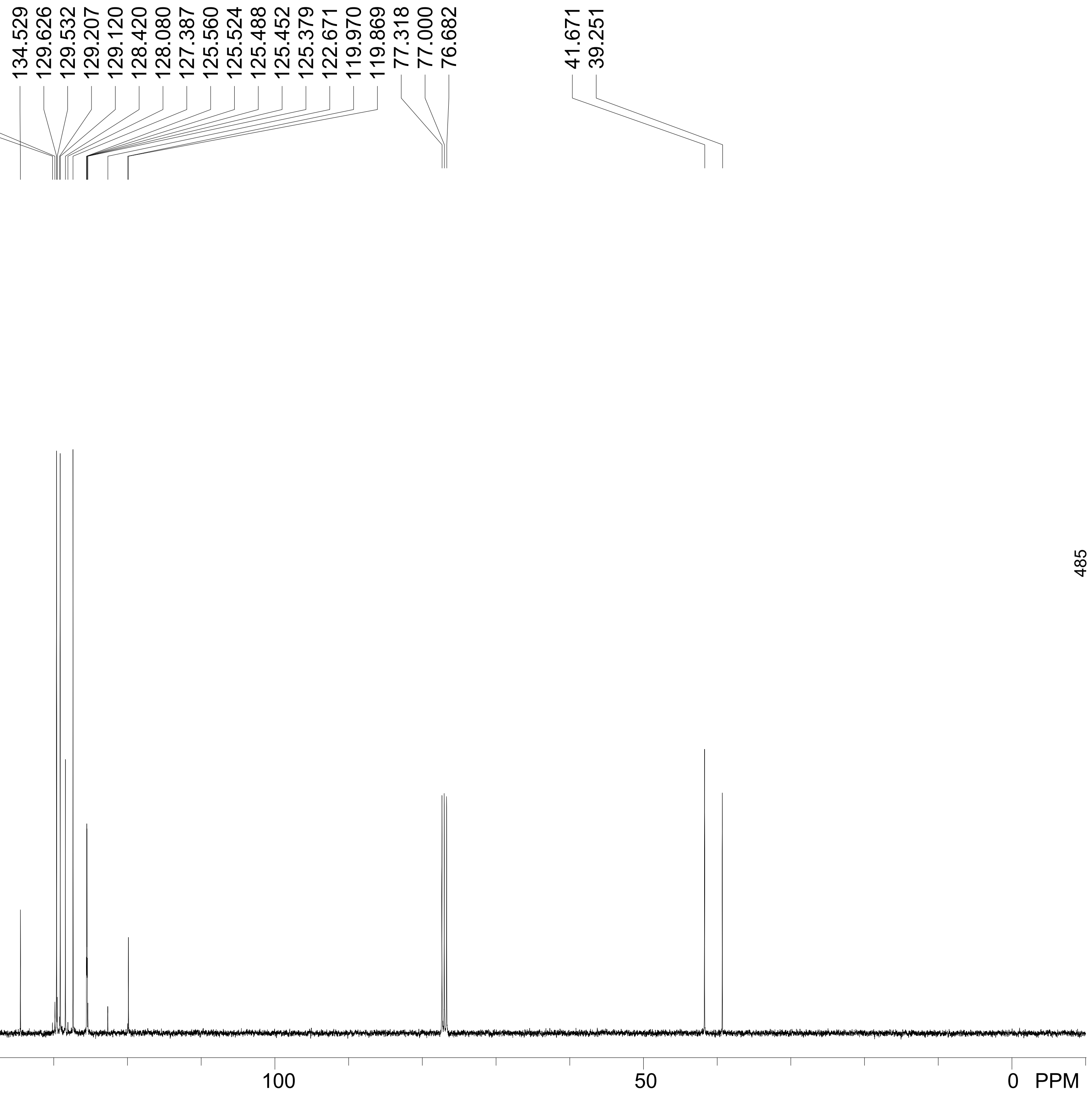

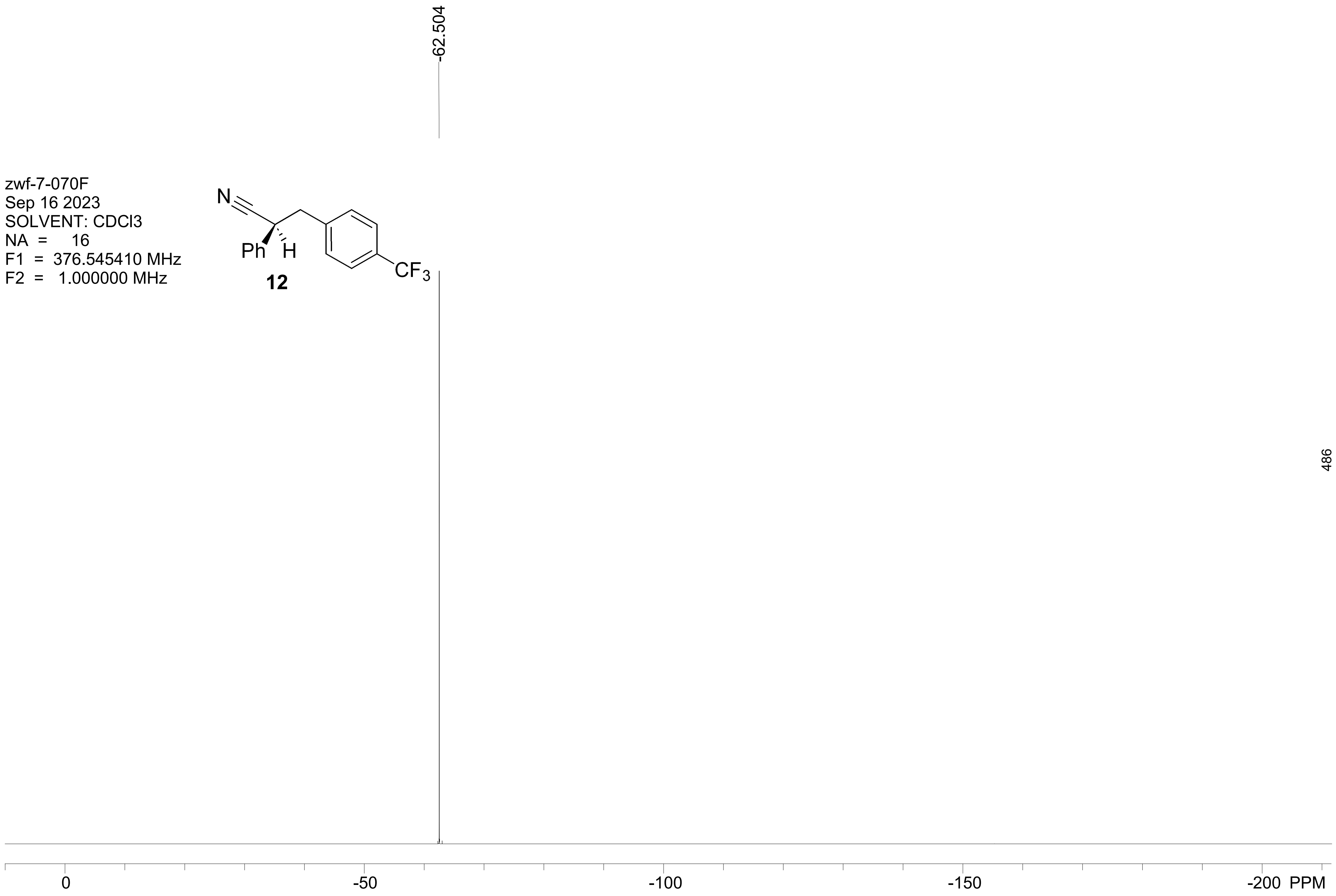

zwf-7-070F
Sep 16 2023
SOLVENT: CDCl3
NA = 16
F1 = 376.545410 MHz
F2 = 1.000000 MHz
N
Ph
H
12
CF3
-62.504
0
-50
-100
-150
-200 PPM

Area Percent Report

Agilent Technologies

Sample name: zwf-7-070-IE-99-1-0.8-205

Data file: C:\Users\Public\Documents\ChemStation\1\Data\zwf\zwf 2023-09-16 20-24-54\008-P1-C7-zwf-7-070.D

Injection Acquired Date 09/16/2023 23:24:07

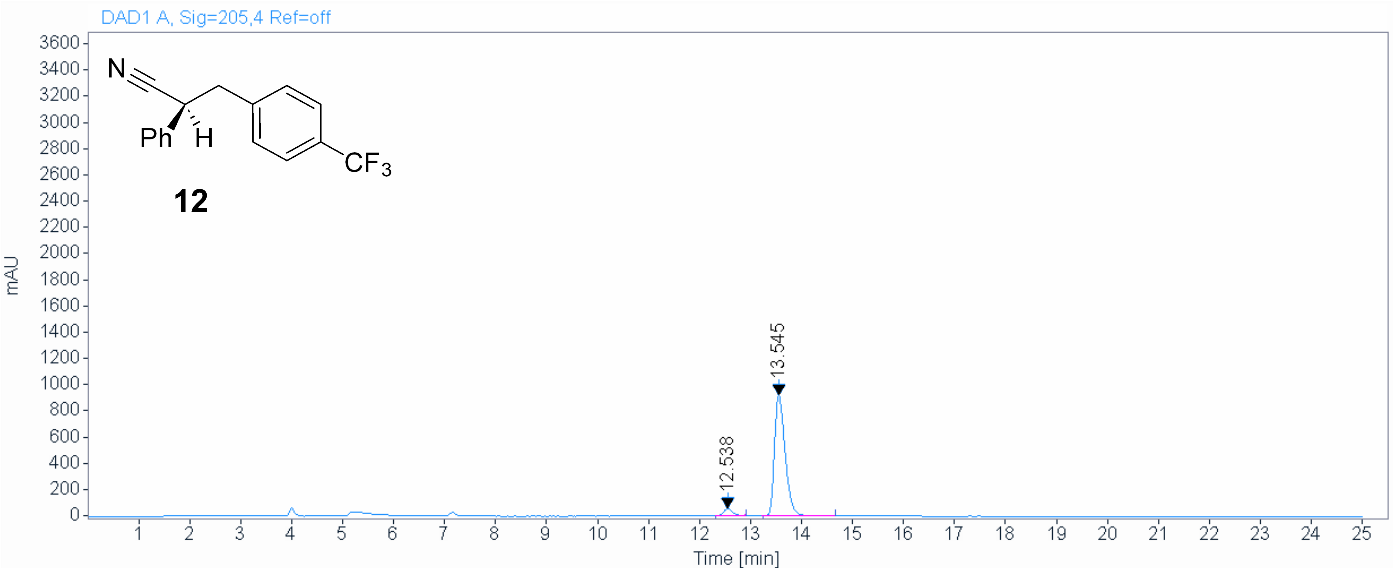

Signal: DAD1 A, Sig=205,4 Ref=off

RT [min]	Width [min]	Height	Area	Area%
12.538	0.2087	53.2565	666.7289	4.7481
13.545	0.2239	918.1798	13375.1719	95.2519
		Sum	14041.9008	100.0000

Area Percent Report

Sample name: zwf-7-070-rac-IE-99-1-0.8-205

Data file: C:\Users\Public\Documents\ChemStation\1\Data\zwf\zwf 2023-09-16 20-24-54\009-P1-C8-zwf-7-070-rac.D

Injection Acquired Date 09/16/2023 23:50:10

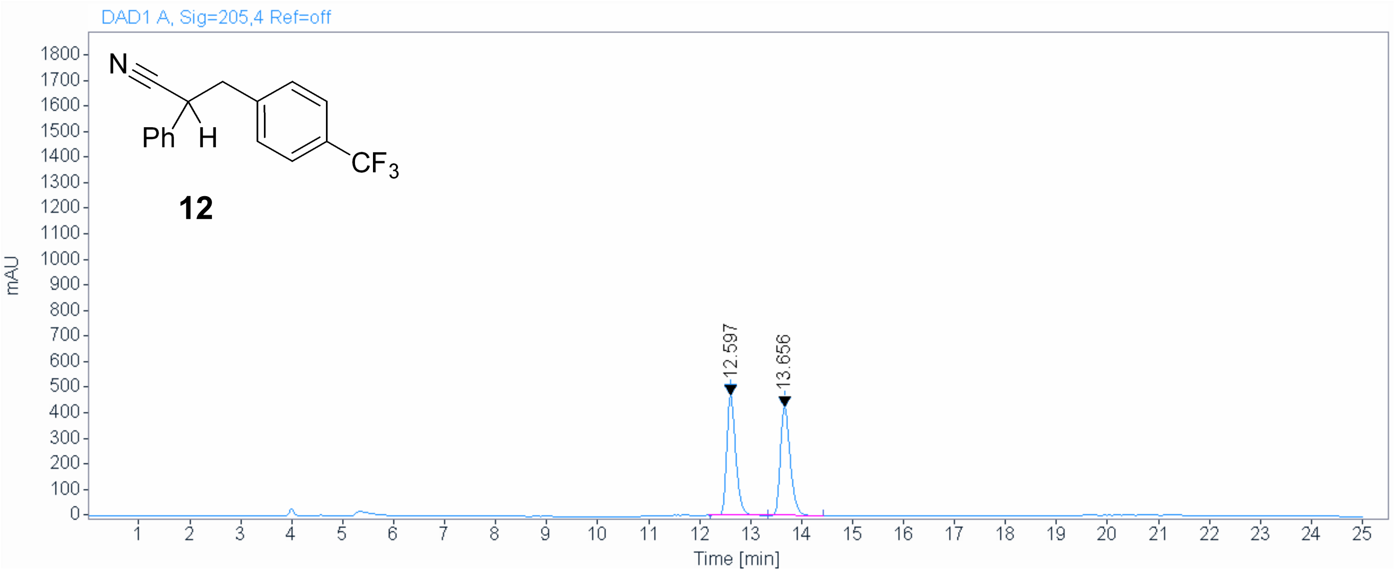

Signal: DAD1 A, Sig=205,4 Ref=off

RT [min]	Width [min]	Height	Area	Area%
12.597	0.1910	468.7794	5827.4150	50.2101
13.656	0.2087	424.7688	5778.6519	49.7899
		Sum	11606.0669	100.0000

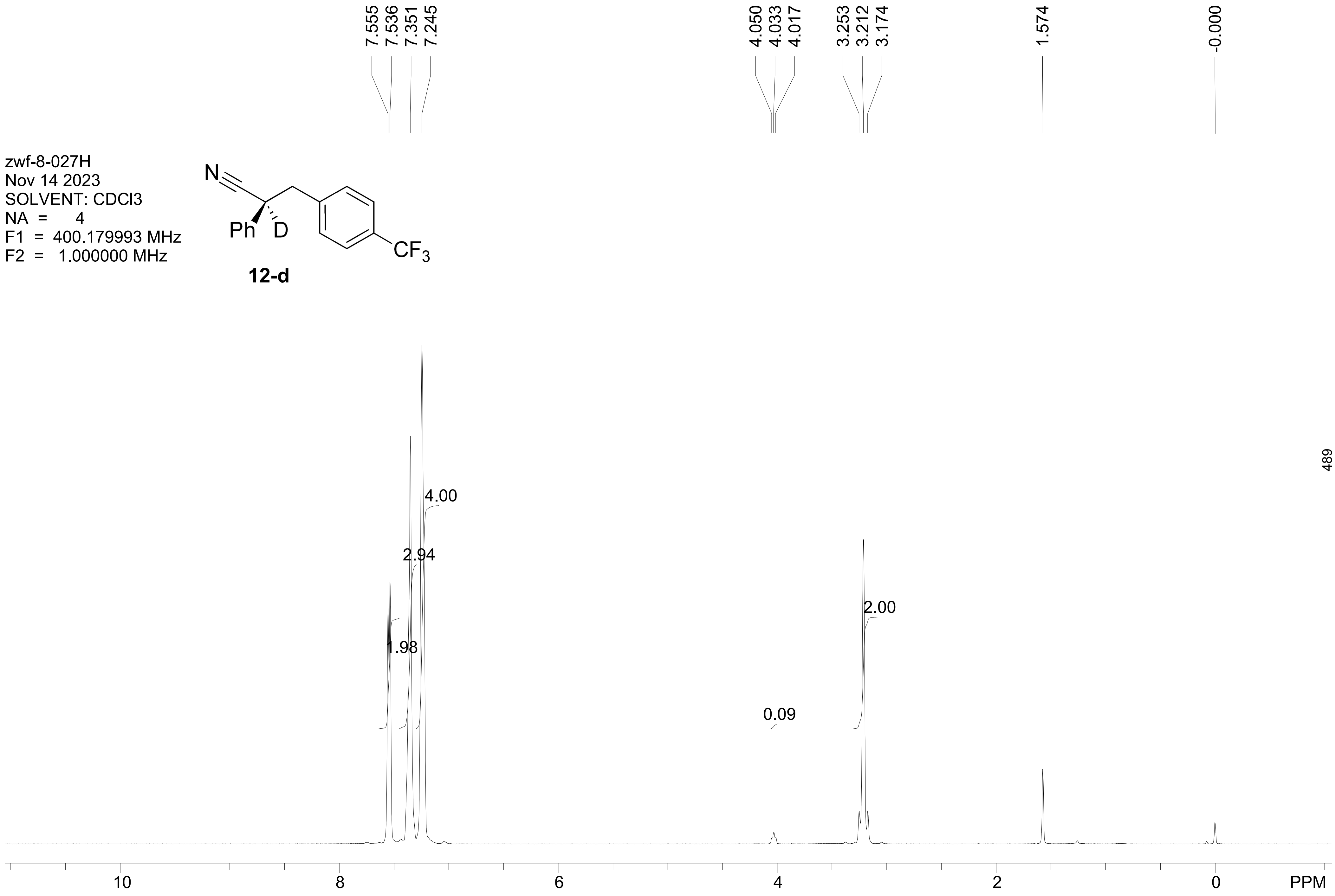
7.555
7.536
7.351
7.245
4.050
4.033
4.017
3.253
3.212
3.174
1.574
-0.000
zwf-8-027H
Nov 14 2023
SOLVENT: CDCl3
NA = 4
F1 = 400.179993 MHz
F2 = 1.000000 MHz
N
Ph
D
CF3
12-d
4.00
2.94
1.98
0.09
2.00
10
8
6
4
2
0
PPM

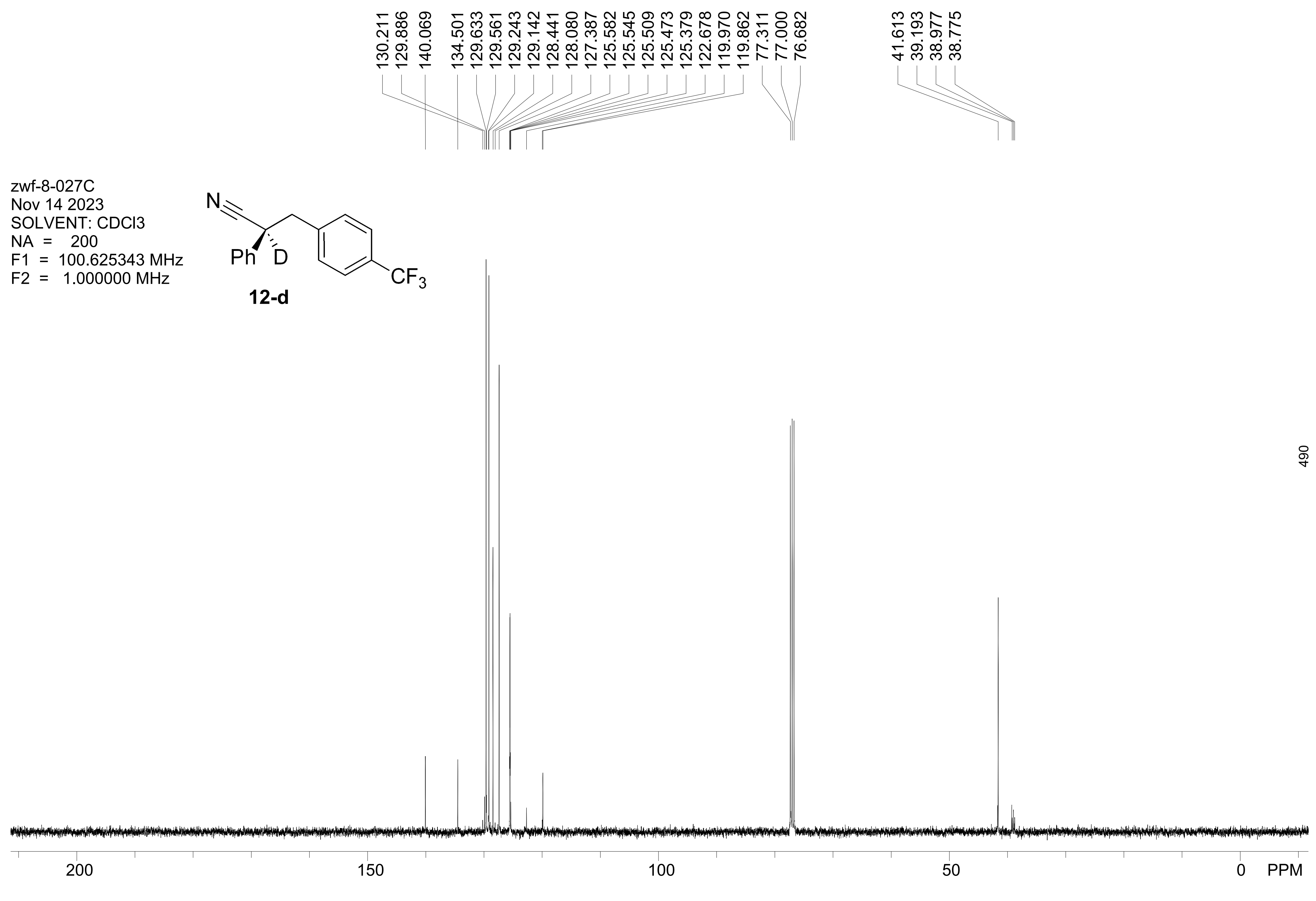

zwf-8-027C
Nov 14 2023
SOLVENT: CDCl3
NA = 200
F1 = 100.625343 MHz
F2 = 1.000000 MHz
N
Ph
D
CF3
12-d
130.211
129.886
140.069
134.501
129.633
129.561
129.243
129.142
128.441
128.080
127.387
125.582
125.545
125.509
125.473
125.379
122.678
119.970
119.862
77.311
77.000
76.682
41.613
39.193
38.977
38.775
200
150
100
50
0
PPM

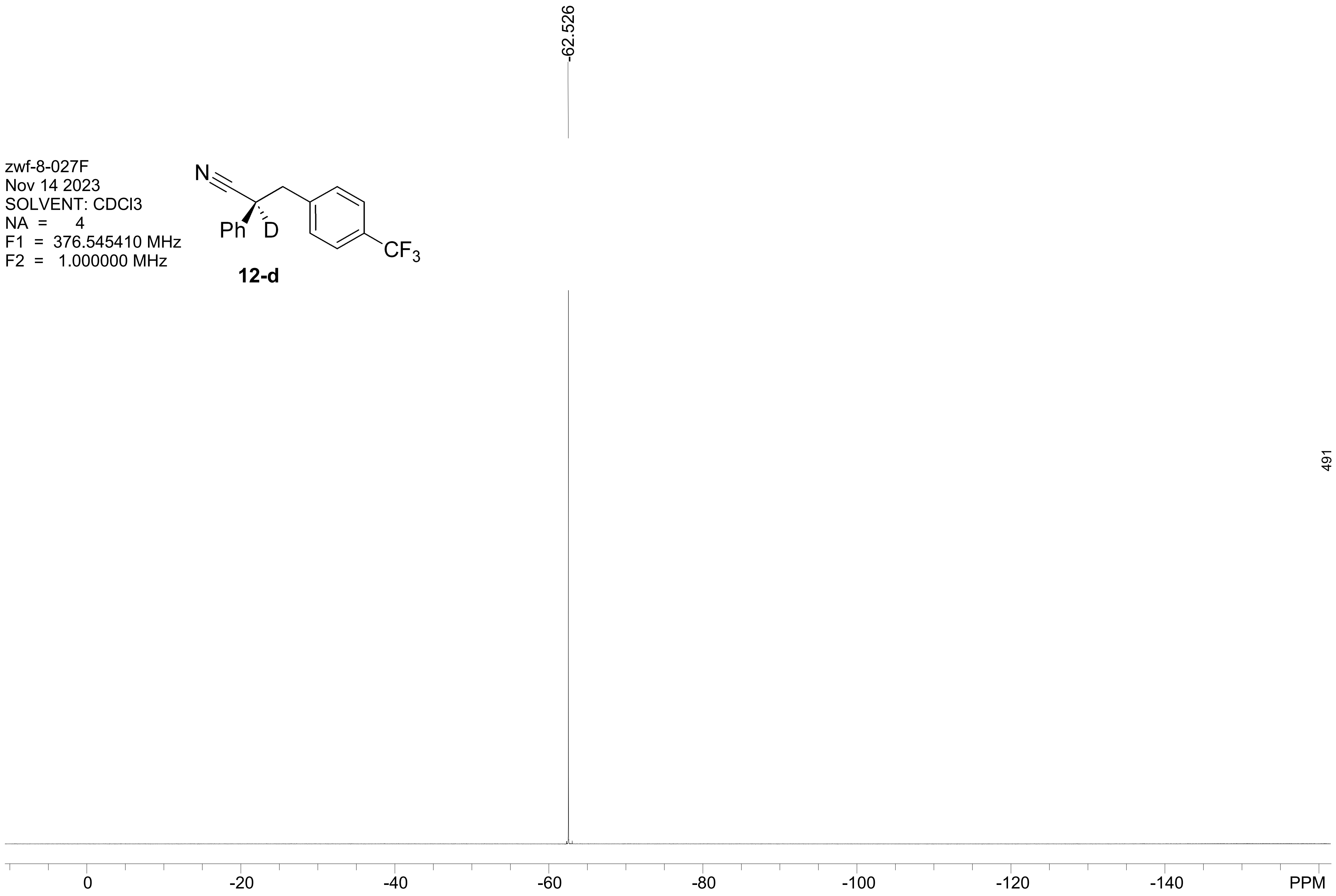
zwf-8-027F
Nov 14 2023
SOLVENT: CDCl3
NA = 4
F1 = 376.545410 MHz
F2 = 1.000000 MHz
N
Ph
D
CF3
12-d
-62.526
0
-20
-40
-60
-80
-100
-120
-140
PPM

Area Percent Report

Sample name: zwf-8-027-IE-99-1-0.8-205

Data file: C:\Users\Public\Documents\ChemStation\1\Data\zwf\zwf-S 2023-11-14 20-02-58\016-P1-C7-zwf-8-027.D

Injection Acquired Date 11/15/2023 04:39:57

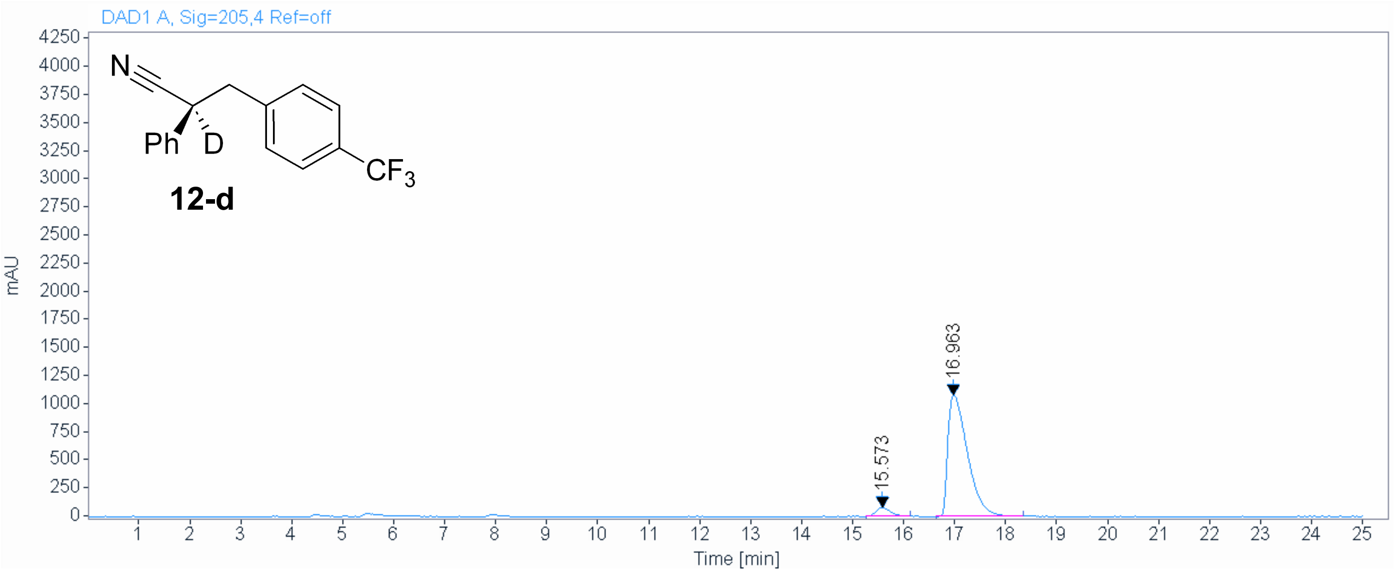

Signal: DAD1 A, Sig=205,4 Ref=off

RT [min]	Width [min]	Height	Area	Area%
15.573	0.3300	75.1619	1488.1492	4.9054
16.963	0.3524	1075.9642	28848.6074	95.0946
		Sum	30336.7566	100.0000

Area Percent Report

Sample name: zwf-8-027-rac-IE-99-1-0.8-205

Data file: C:\Users\Public\Documents\ChemStation\1\Data\zwf\zwf-S 2023-11-14 20-02-58\017-P1-C8-zwf-8-027-rac.D

Injection Acquired Date 11/15/2023 05:05:58

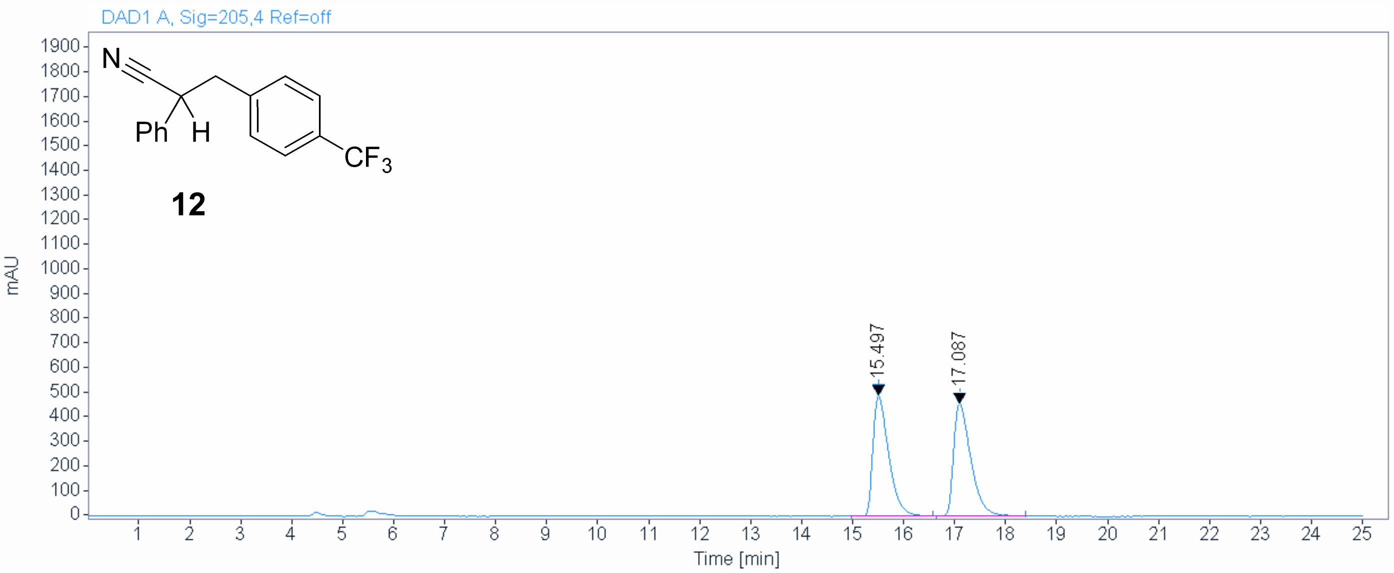

Signal: DAD1 A, Sig=205,4 Ref=off

RT [min]	Width [min]	Height	Area	Area%
15.497	0.3228	490.3266	10563.0547	49.4778
17.087	0.3422	456.6707	10786.0342	50.5222
		Sum	21349.0889	100.0000

zwf-7-122H
Oct 20 2023
SOLVENT: CDCl3
NA = 4
F1 = 400.179993 MHz
F2 = 1.000000 MHz

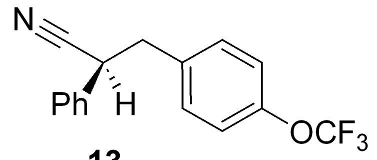

13

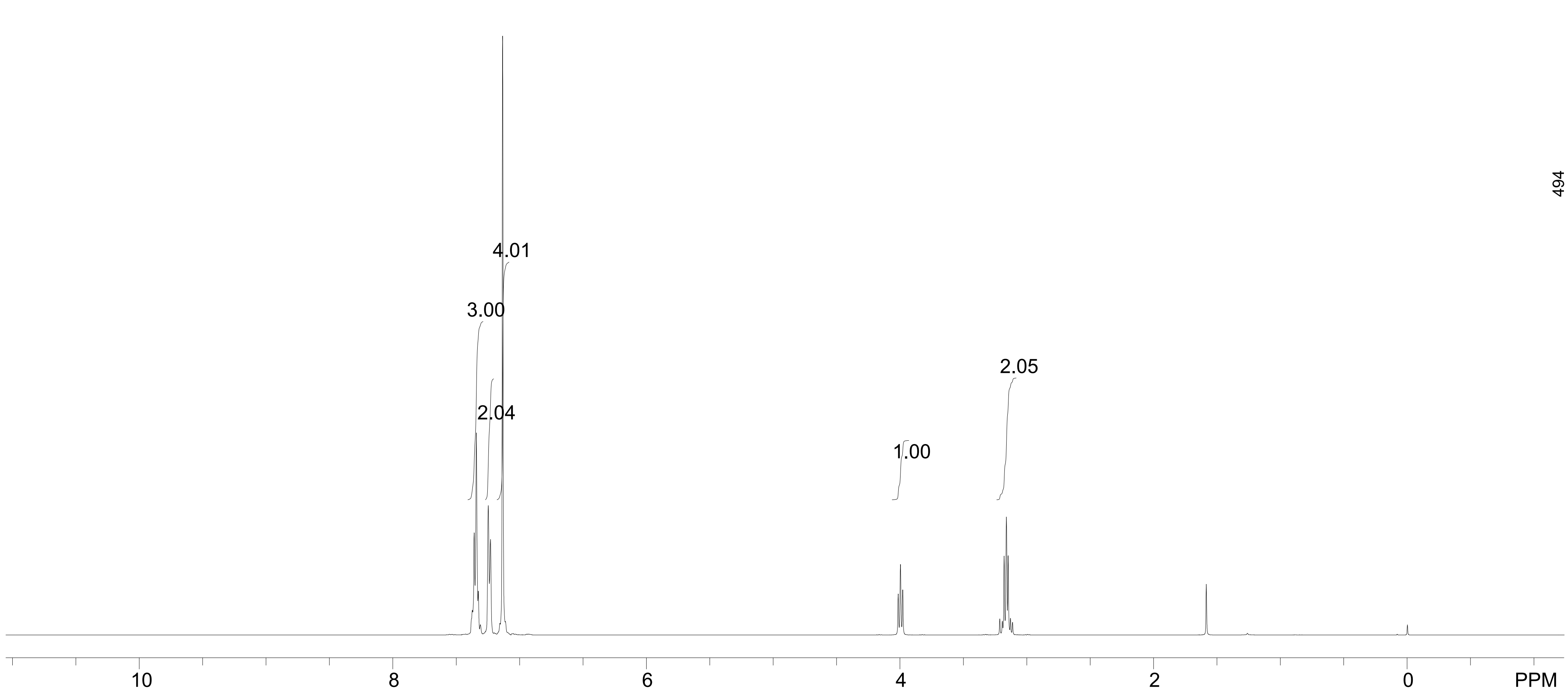

zwf-7-122C
Oct 20 2023
SOLVENT: CDCl3
NA = 256
F1 = 100.625343 MHz
F2 = 1.000000 MHz

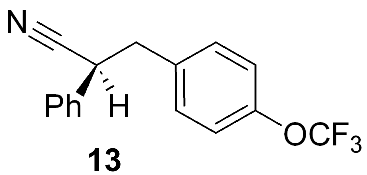

13

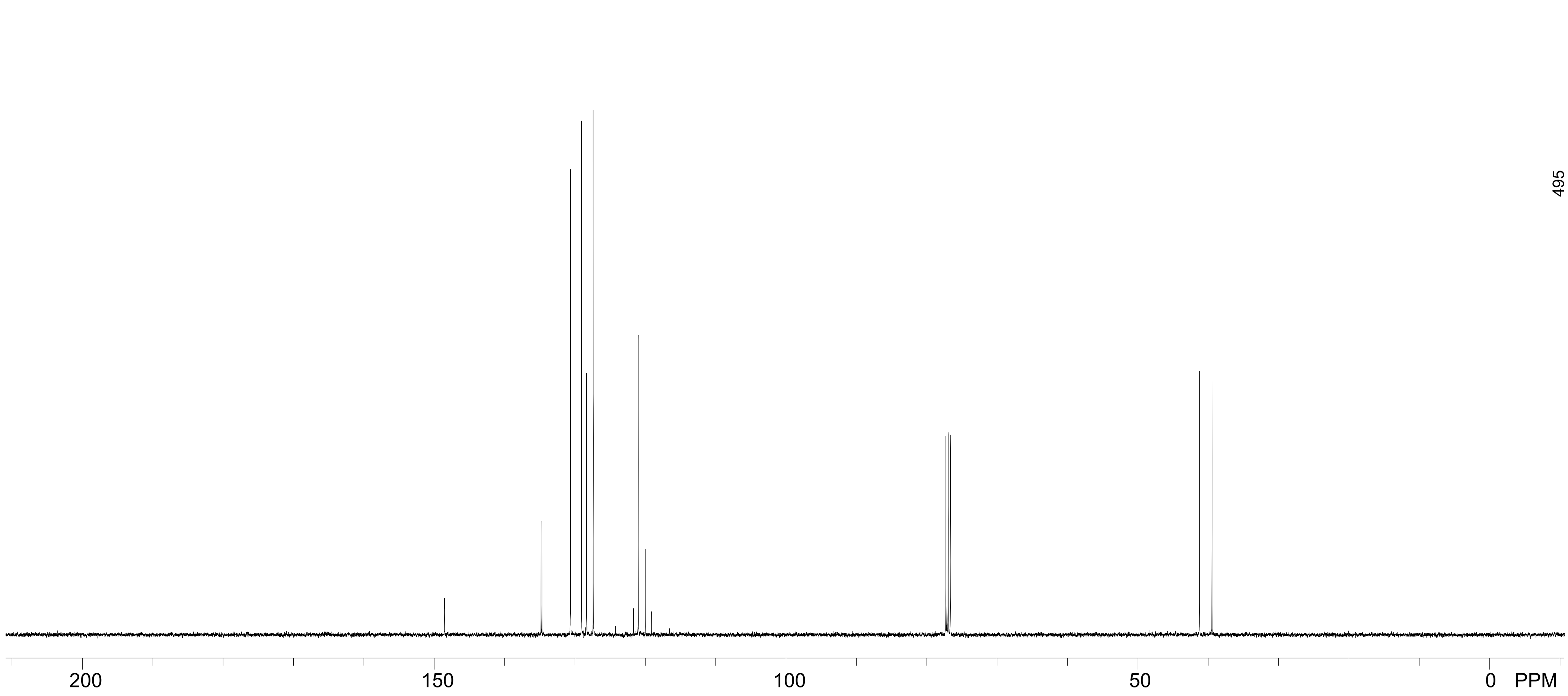

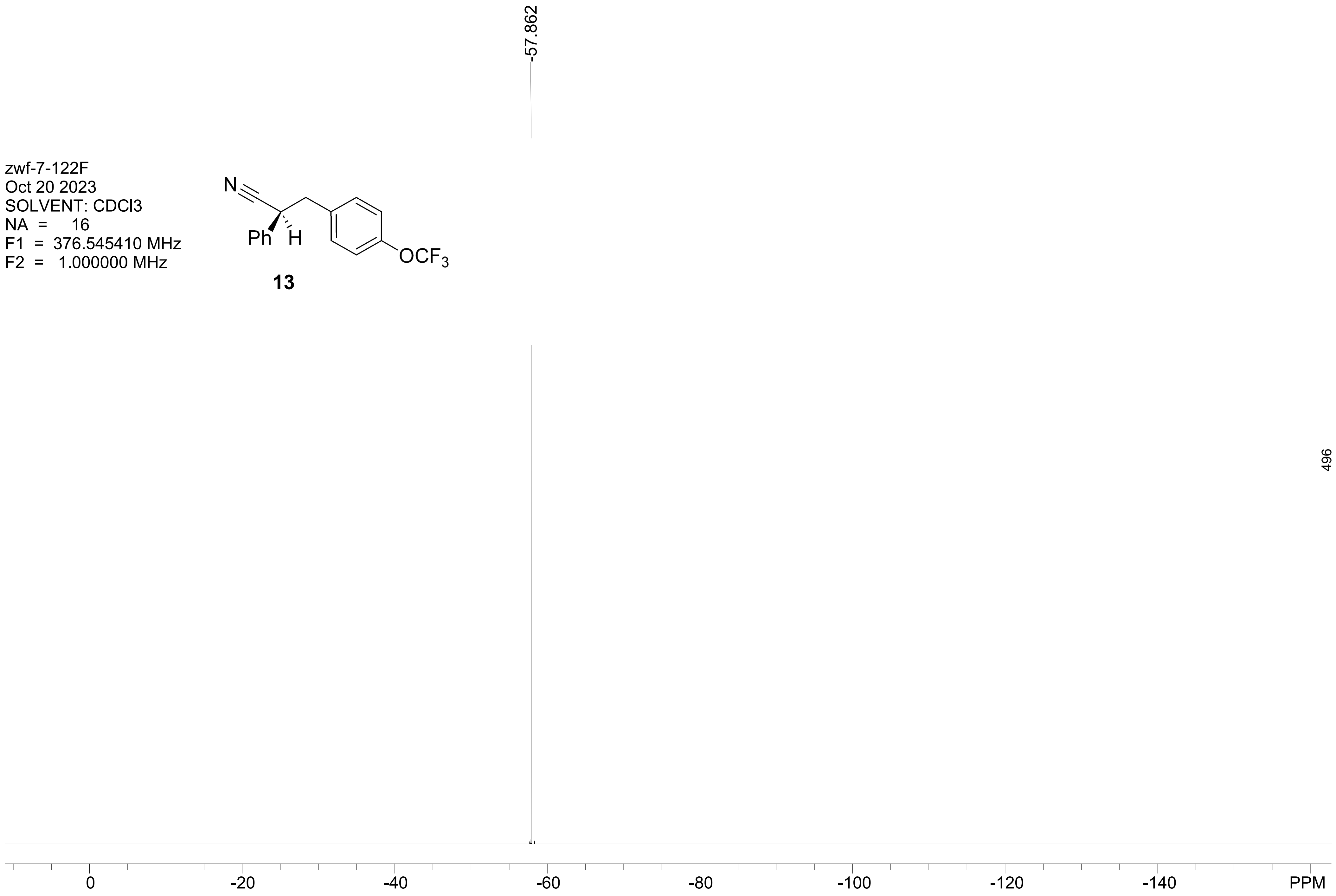
zwf-7-122F
Oct 20 2023
SOLVENT: CDCl3
NA = 16
F1 = 376.545410 MHz
F2 = 1.000000 MHz
N
Ph
H
OCF3
13
-57.862
0
-20
-40
-60
-80
-100
-120
-140
PPM

Area Percent Report

Agilent Technologies

Sample name: zwf-7-122-IE-99-1-0.8-205

Data file: C:\Users\Public\Documents\ChemStation\1\Data\zwf\zwf-S 2023-10-21 17-25-18\003-P1-C5-zwf-7-122.D

Injection Acquired Date 10/21/2023 18:13:30

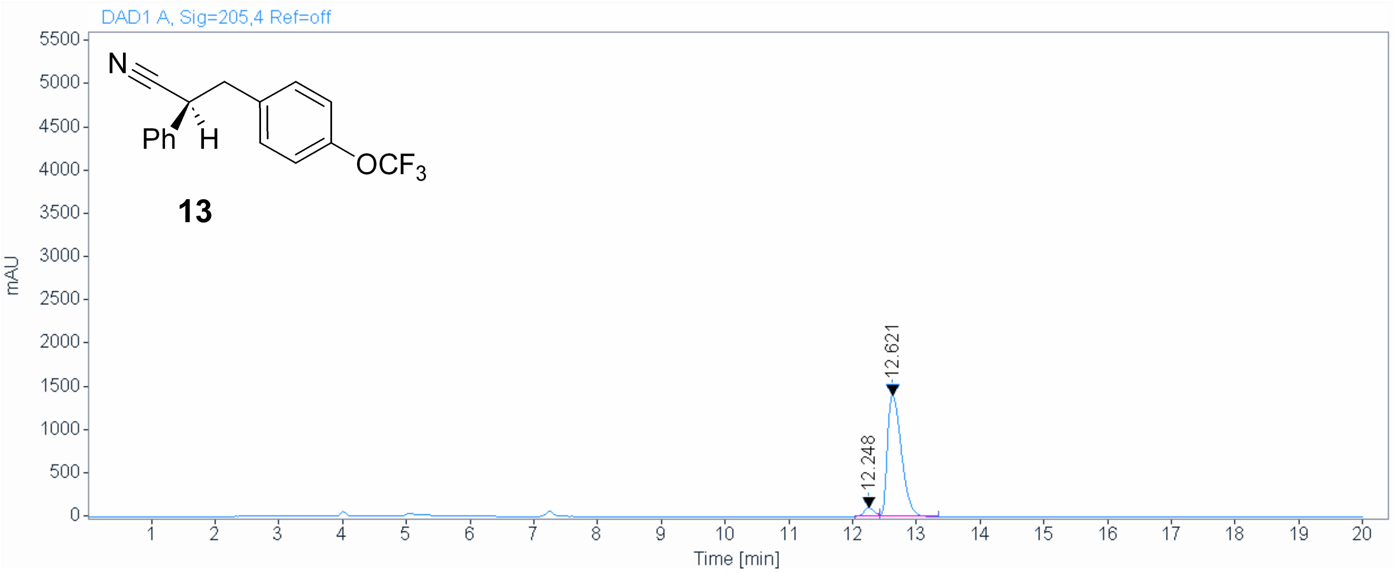

Signal: DAD1 A, Sig=205,4 Ref=off

RT [min]	Width [min]	Height	Area	Area%
12.248	0.1875	98.6460	1109.5765	4.9234
12.621	0.2551	1399.8564	21427.1523	95.0766
		Sum	22536.7289	100.0000

Area Percent Report

Agilent Technologies

Sample name: zwf-7-122-rac-IE-99-1-0.8-205

Data file: C:\Users\Public\Documents\ChemStation\1\Data\zwf\zwf-S 2023-10-21 12-48-40\003-P1-C2-zwf-7-122-rac.D

Injection Acquired Date 10/21/2023 13:36:43

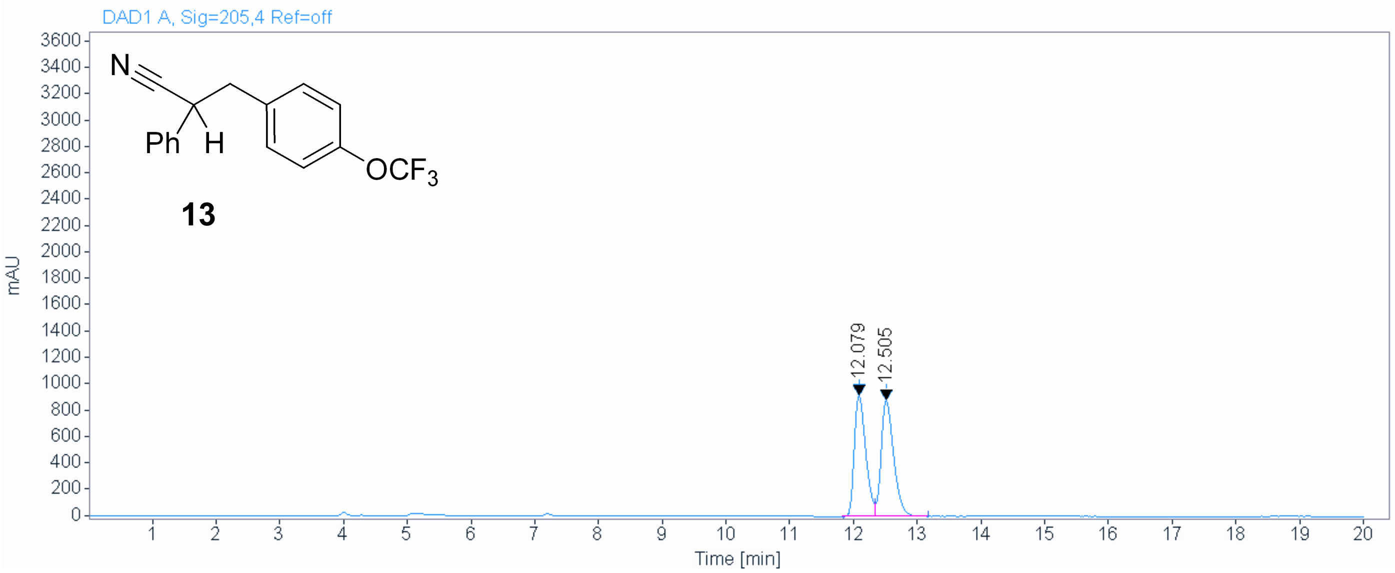

Signal: DAD1 A, Sig=205,4 Ref=off

RT [min]	Width [min]	Height	Area	Area%
12.079	0.1944	917.2980	11510.0879	48.9115
12.505	0.2052	881.0627	12022.3857	51.0885
		Sum	23532.4736	100.0000

zwf-8-029H
Nov 14 2023
SOLVENT: CDCl3
NA = 4
F1 = 400.179993 MHz
F2 = 1.000000 MHz

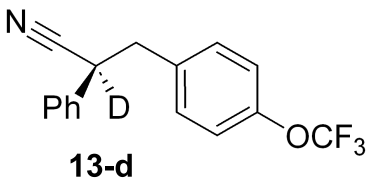

13-d

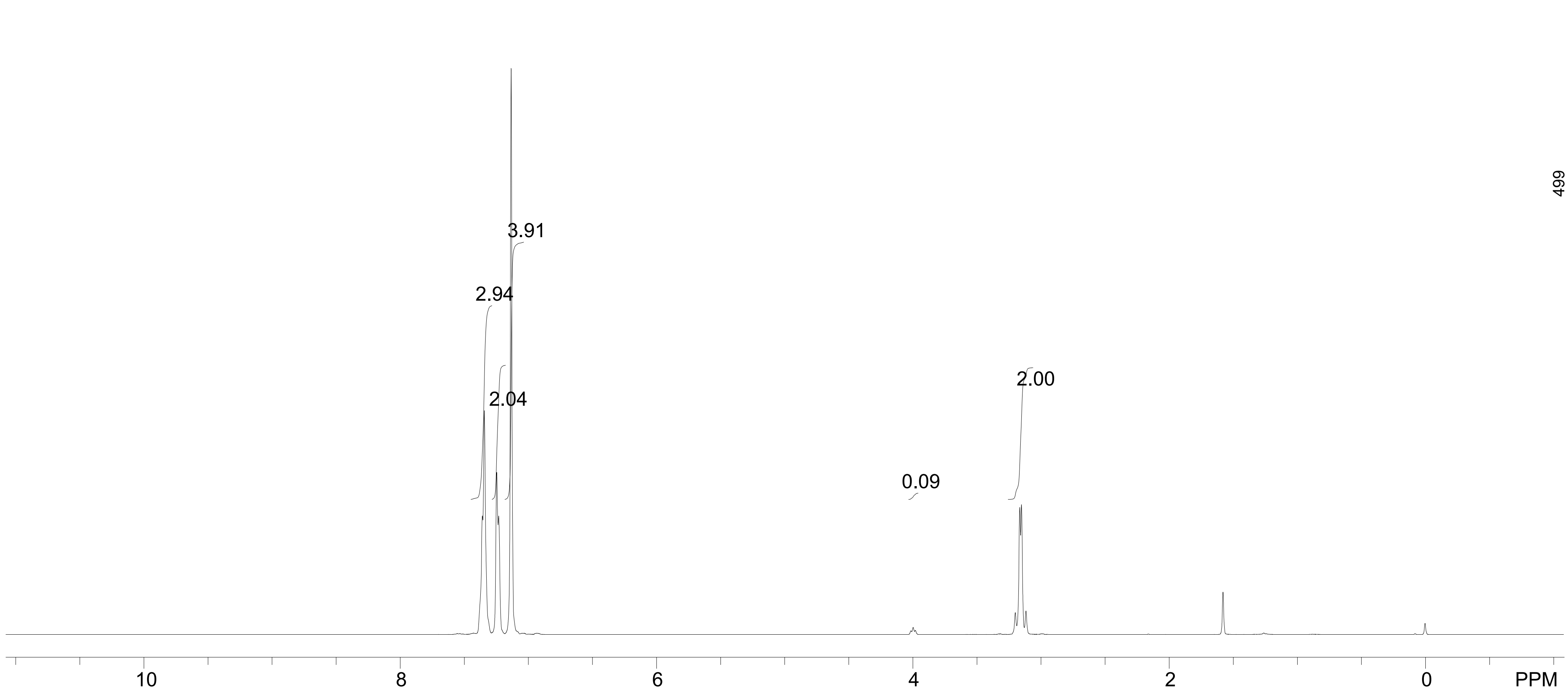

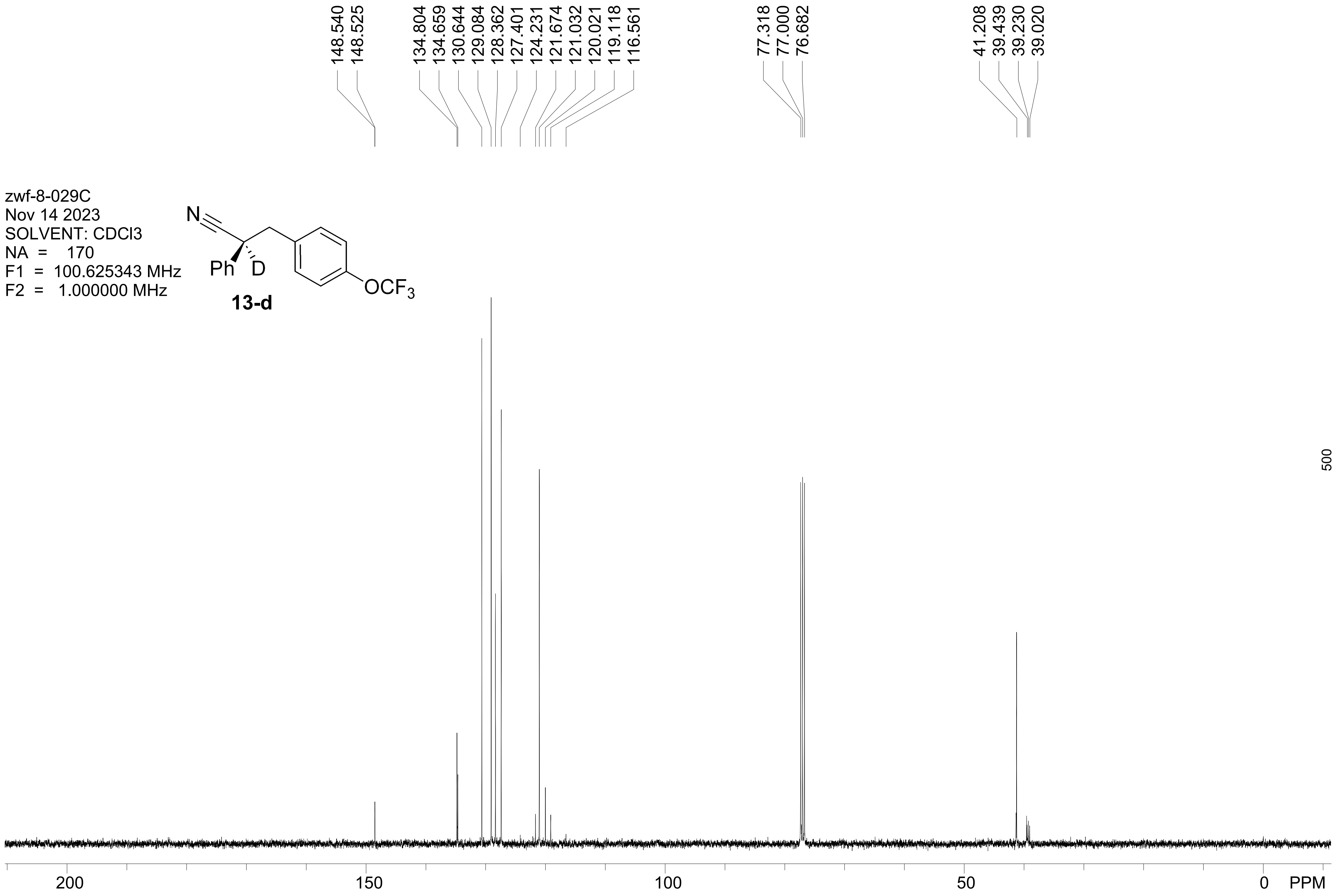
zwf-8-029C
Nov 14 2023
SOLVENT: CDCl3
NA = 170
F1 = 100.625343 MHz
F2 = 1.000000 MHz
N
Ph
D
OCF3
148.540
148.525
134.804
134.659
130.644
129.084
128.362
127.401
124.231
121.674
121.032
120.021
119.118
116.561
77.318
77.000
76.682
41.208
39.439
39.230
39.020
200
150
100
50
0
PPM

13-d

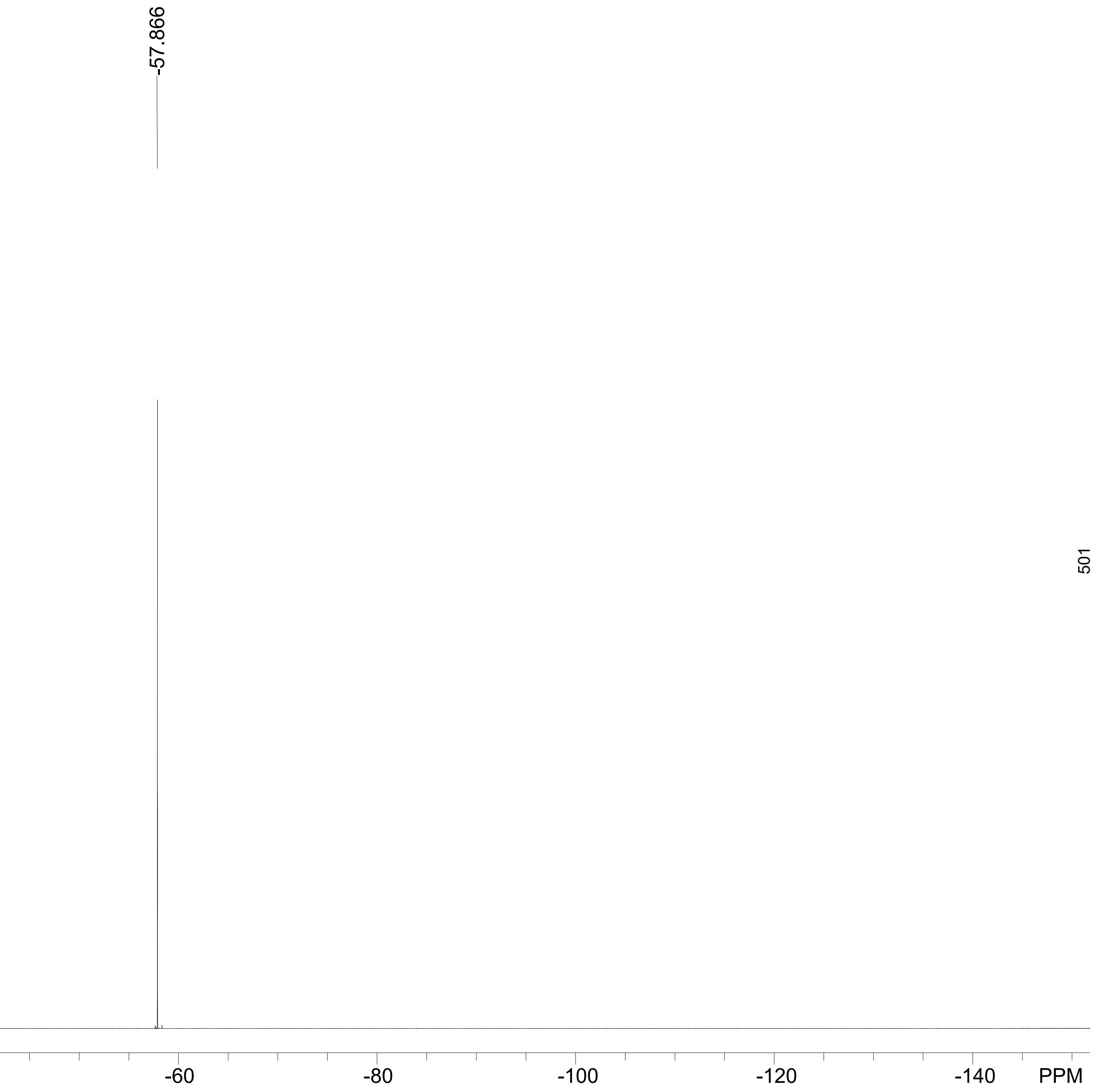
zwf-8-029F
Nov 14 2023
SOLVENT: CDCl3
NA = 4
F1 = 376.545410 MHz
F2 = 1.000000 MHz
-57.866
0
-20
-40
-60
-80
-100
-120
-140
PPM

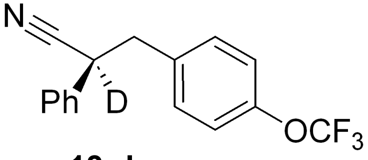
N
Ph
D
OCF3

13-d

Area Percent Report

Agilent Technologies

Sample name: zwf-8-029-IE-99-1-0.8-205

Data file: C:\Users\Public\Documents\ChemStation\1\Data\zwf\zwf-S 2023-11-14 20-02-58\018-P1-B1-zwf-8-029.D

Injection Acquired Date 11/15/2023 05:32:00

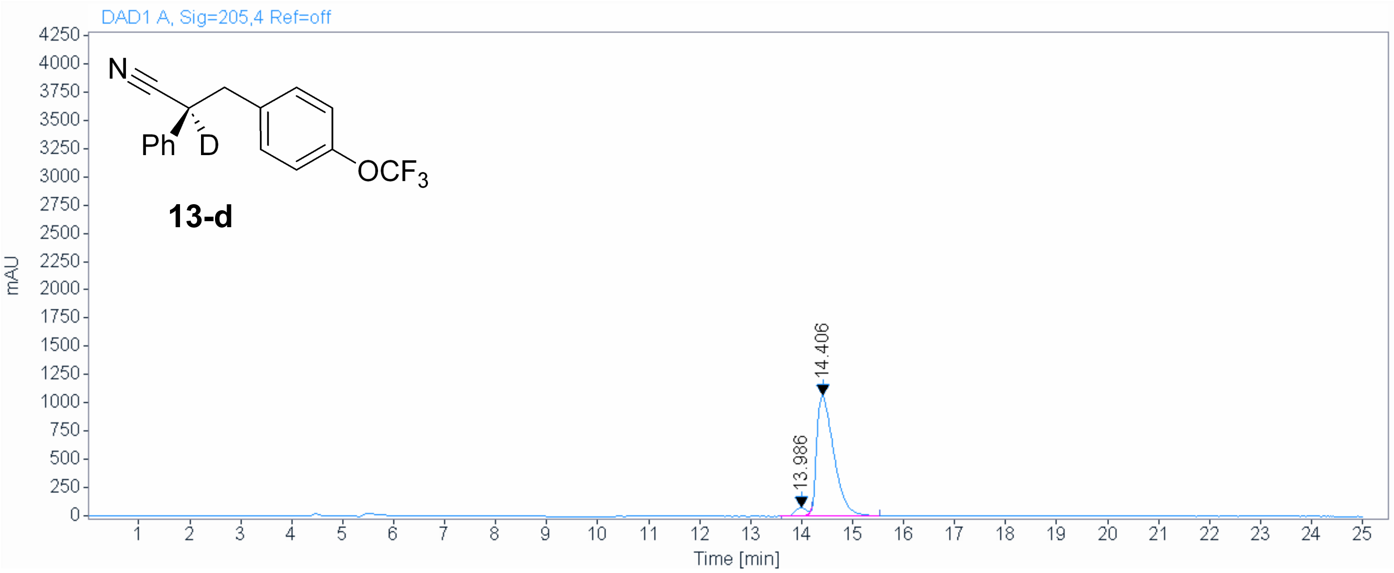

Signal: DAD1 A, Sig=205,4 Ref=off

RT [min]	Width [min]	Height	Area	Area%
13.986	0.2263	74.6075	1178.3378	4.5129
14.406	0.2883	1068.3981	24932.3535	95.4871
		Sum	26110.6913	100.0000

Area Percent Report

Sample name: zwf-8-029-rac-IE-99-1-0.8-205

Data file: C:\Users\Public\Documents\ChemStation\1\Data\zwf\zwf-S 2023-11-14 20-02-58\019-P1-B2-zwf-8-029-rac.D

Injection Acquired Date 11/15/2023 05:58:00

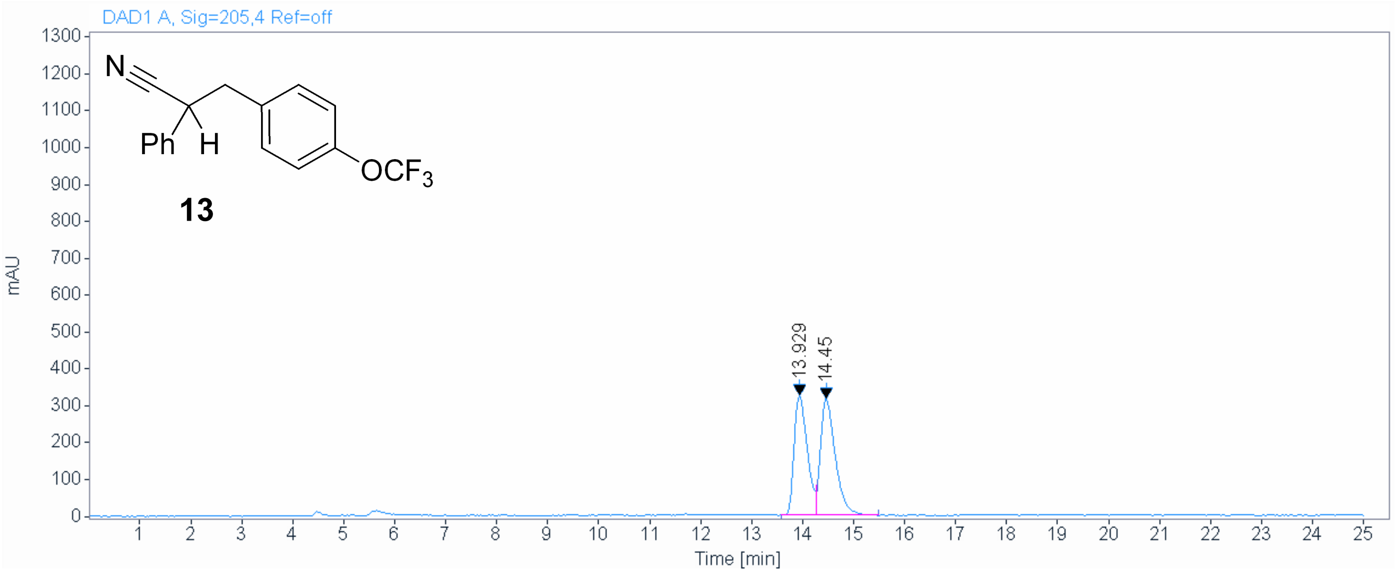

Signal: DAD1 A, Sig=205,4 Ref=off

RT [min]	Width [min]	Height	Area	Area%
13.929	0.3047	324.3994	5930.7920	48.4069
14.450	0.3339	315.4993	6321.1699	51.5931
		Sum	12251.9619	100.0000

zwf-7-072H
Sep 21 2023
SOLVENT: CDCl3
NA = 4
F1 = 400.140015 MHz
F2 = 1.000000 MHz

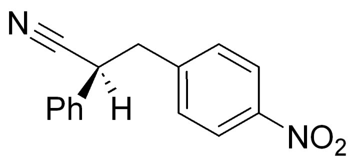

14

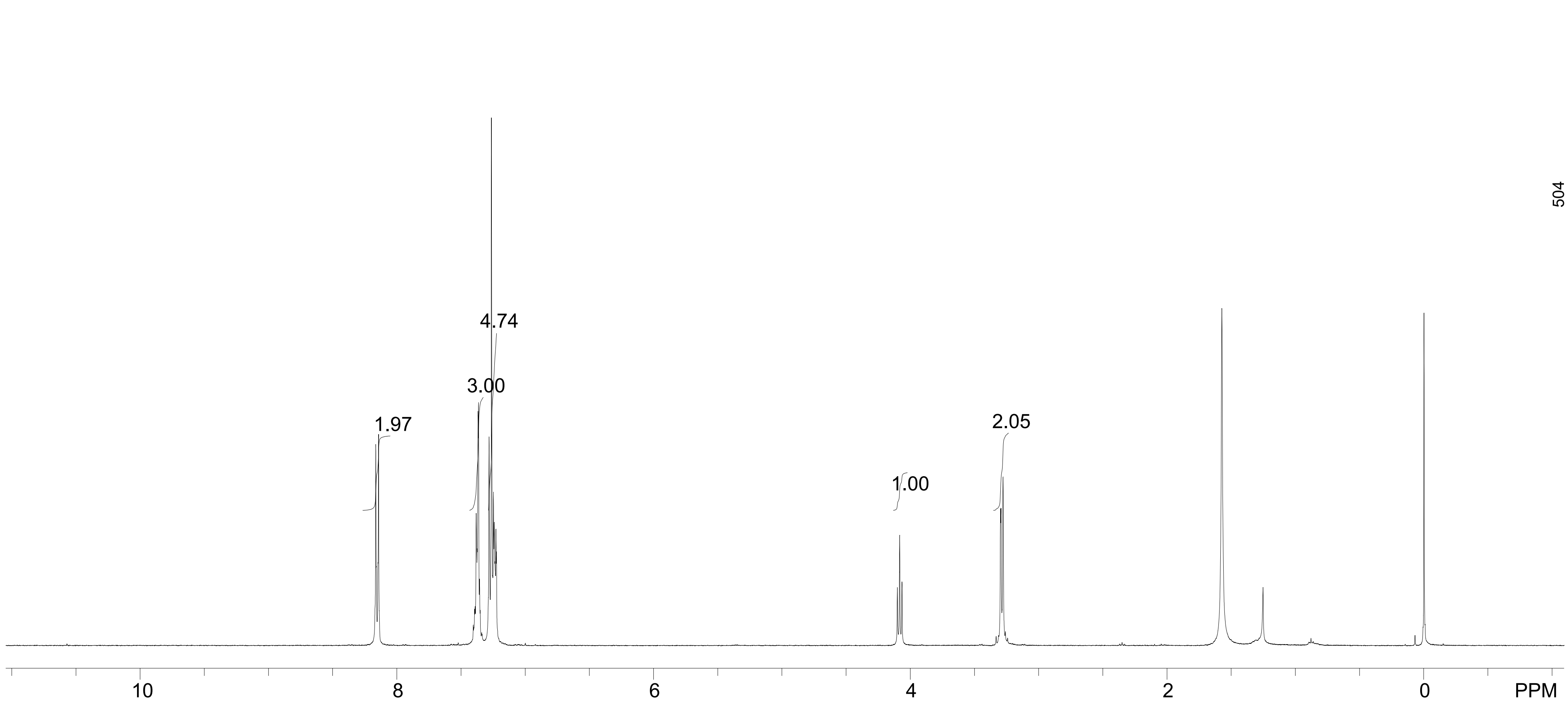

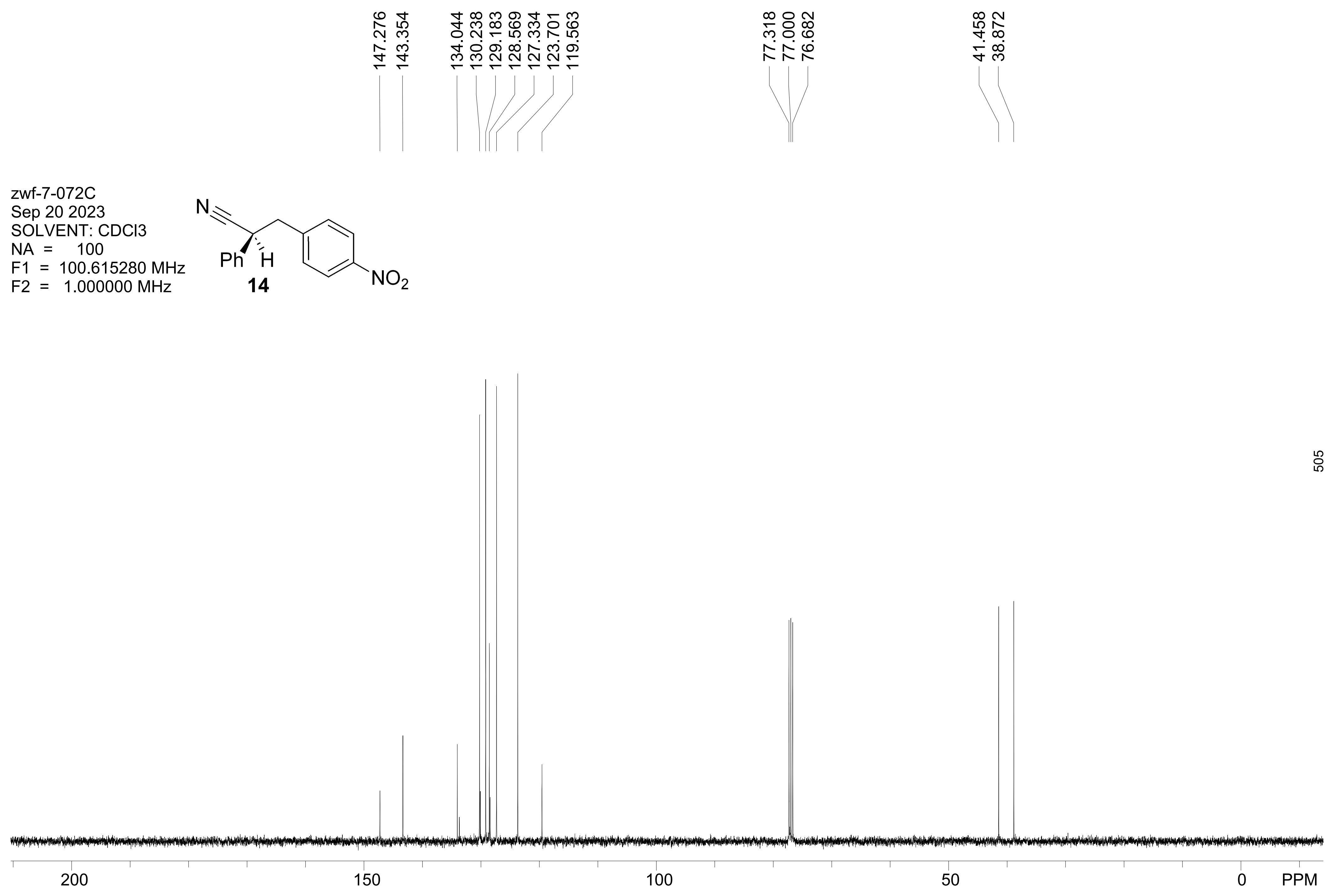
147.276
143.354
134.044
130.238
129.183
128.569
127.334
123.701
119.563
77.318
77.000
76.682
41.458
38.872
zwf-7-072C
Sep 20 2023
SOLVENT: CDCl3
NA = 100
F1 = 100.615280 MHz
F2 = 1.000000 MHz
N
Ph
H
NO2
14
200
150
100
50
0
PPM

Area Percent Report

Agilent Technologies

Sample name: zwf-7-072-IE-90-10-0.8-205

Data file: C:\Users\Public\Documents\ChemStation\1\Data\zwf\zwf-S 2023-09-17 19-52-41\017-P1-B3-zwf-7-072.D

Injection Acquired Date 09/18/2023 05:26:29

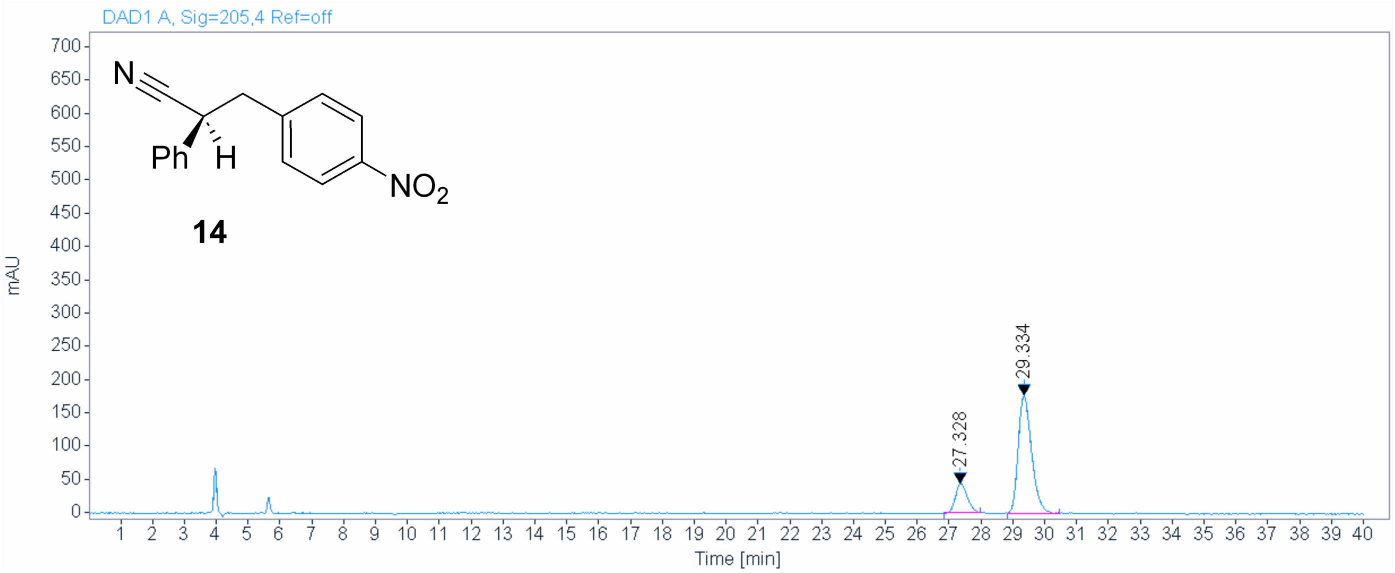

Signal: DAD1 A, Sig=205,4 Ref=off

RT [min]	Width [min]	Height	Area	Area%
27.328	0.4390	44.3927	1169.4260	18.3374
29.334	0.4108	177.2541	5207.8501	81.6626
		Sum	6377.2761	100.0000

Area Percent Report

Agilent Technologies

Sample name: zwf-7-072-rac-IE-90-10-0.8-205

Data file: C:\Users\Public\Documents\ChemStation\1\Data\zwf\zwf-S 2023-09-17 19-52-41\019-P1-B5-zwf-7-072-rac.D

Injection Acquired Date 09/18/2023 06:48:42

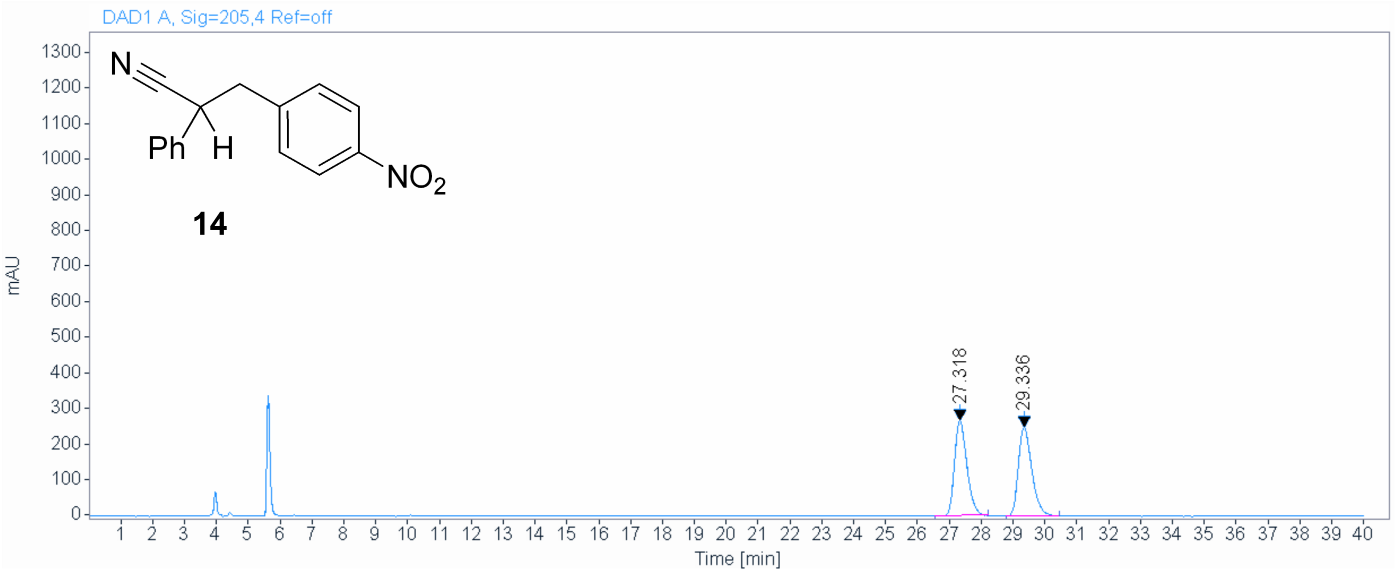

Signal: DAD1 A, Sig=205,4 Ref=off

RT [min]	Width [min]	Height	Area	Area%
27.318	0.3975	267.9370	7303.3149	49.9600
29.336	0.4166	249.0821	7314.9951	50.0400
		Sum	14618.3101	100.0000

Area Percent Report

Agilent Technologies

Sample name: zwf-7-072-single crystalized-IE-90-10-0.8-205

Data file: C:\Users\Public\Documents\ChemStation\1\Data\zwf\zwf-S 2023-10-17 08-55-39\011-P1-B7-zwf-7-072-recrystal.D

Injection Acquired Date 10/17/2023 14:28:26

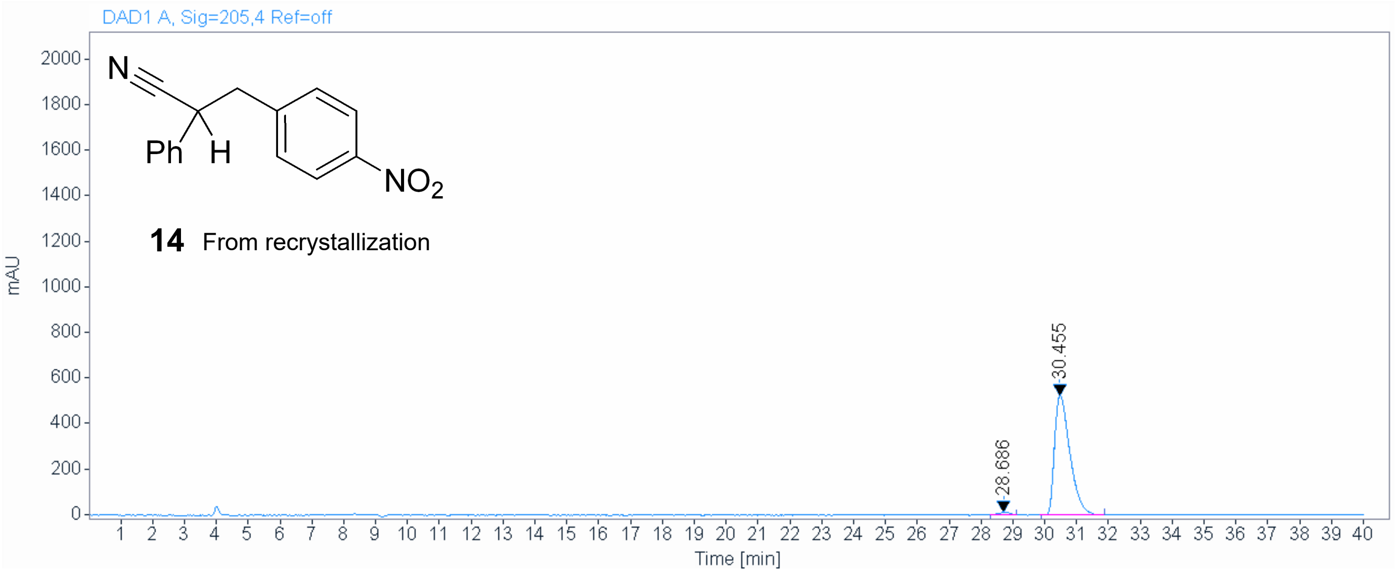

Signal: DAD1 A, Sig=205,4 Ref=off

RT [min]	Width [min]	Height	Area	Area%
28.686	0.3896	12.3240	288.0806	1.6037
30.455	0.4506	525.2230	17675.7344	98.3963
		Sum	17963.8149	100.0000

Area Percent Report

Sample name: zwf-7-072-rac-IE-90-10-0.8-205

Data file: C:\Users\Public\Documents\ChemStation\1\Data\zwf\zwf-S 2023-10-17 08-55-39\013-P1-B9-zwf-7-072-rac.D

Injection Acquired Date 10/17/2023 15:50:35

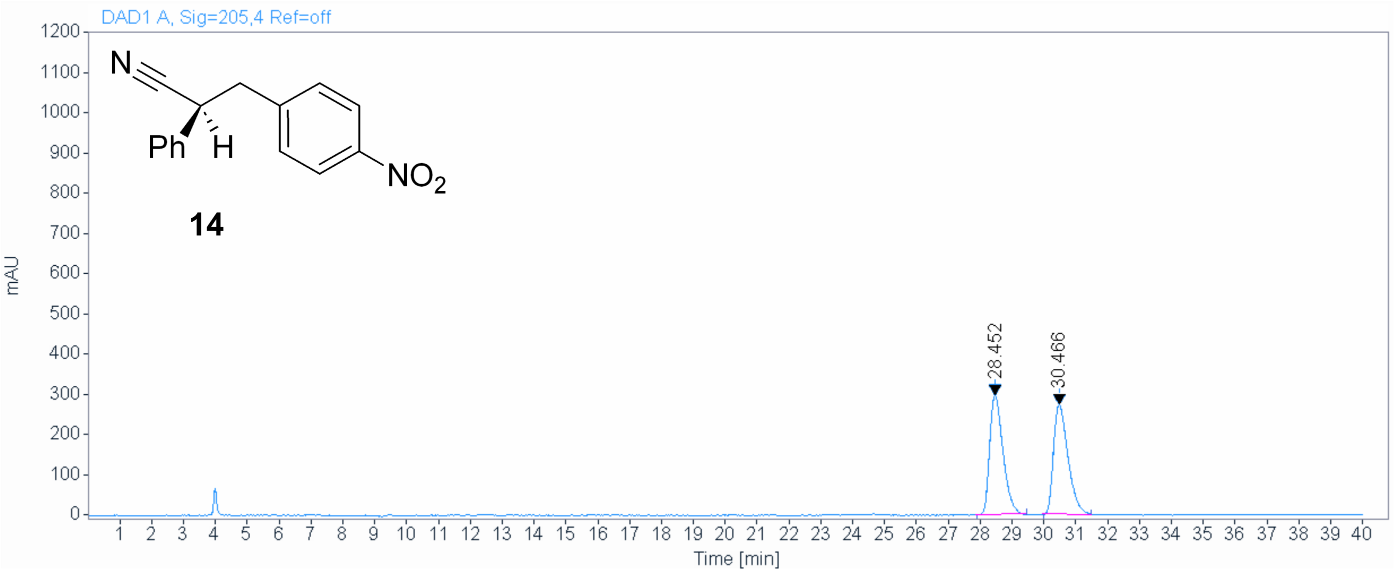

Signal: DAD1 A, Sig=205,4 Ref=off

RT [min]	Width [min]	Height	Area	Area%
28.452	0.4289	297.0482	8729.5137	50.0740
30.466	0.4058	273.1212	8703.7295	49.9260
		Sum	17433.2432	100.0000

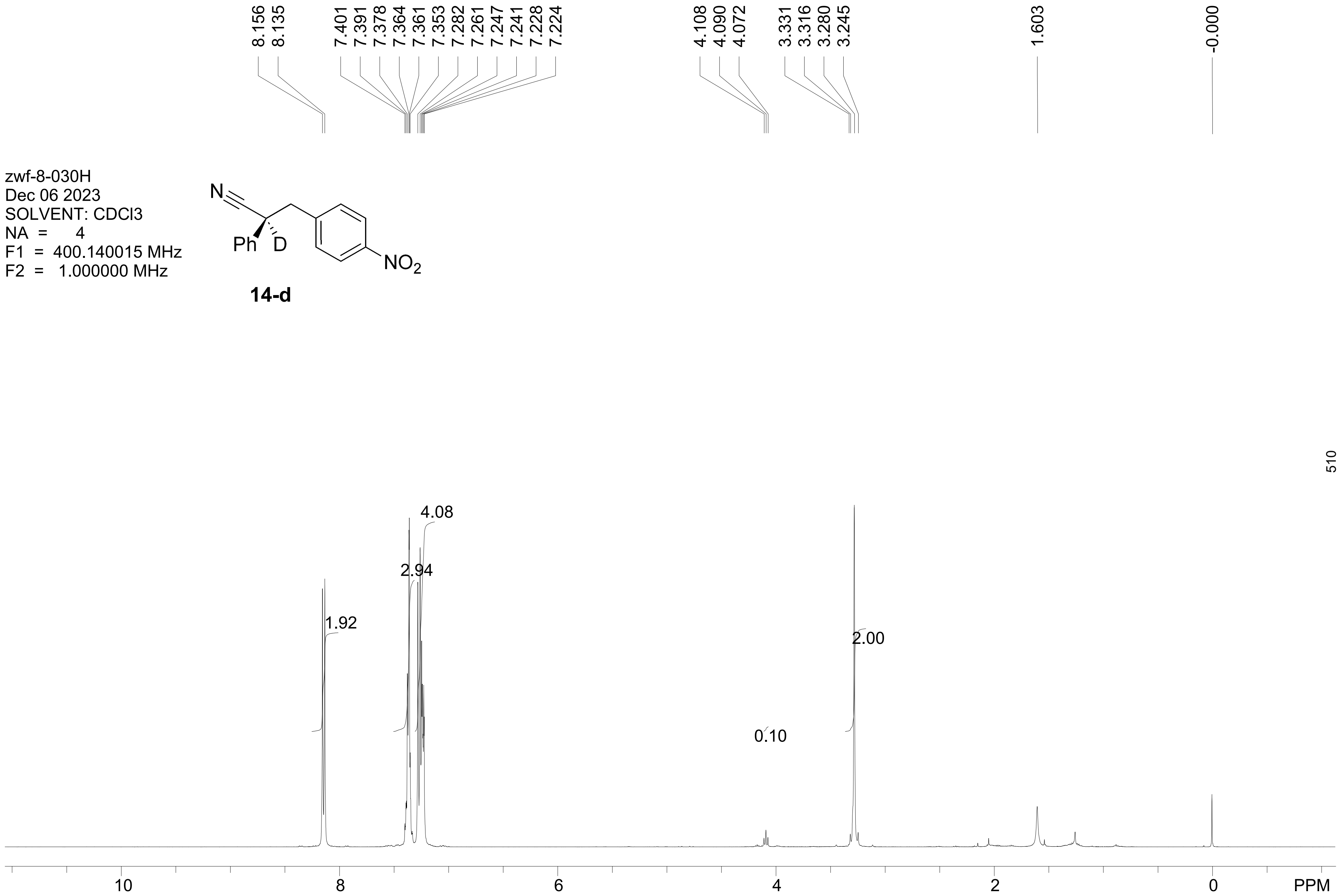
zwf-8-030H
Dec 06 2023
SOLVENT: CDCl3
NA = 4
F1 = 400.140015 MHz
F2 = 1.000000 MHz
N
Ph
D
NO2
14-d
8.156
8.135
7.401
7.391
7.378
7.364
7.361
7.353
7.282
7.261
7.247
7.241
7.228
7.224
4.108
4.090
4.072
3.331
3.316
3.280
3.245
1.603
-0.000
1.92
2.94
4.08
0.10
2.00
10
8
6
4
2
0
PPM

zwf-8-030C
Dec 06 2023
SOLVENT: CDCl3
NA = 150
F1 = 100.615280 MHz
F2 = 1.000000 MHz

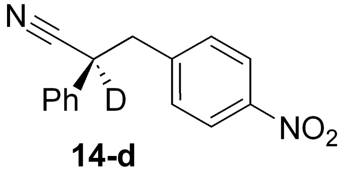

14-d

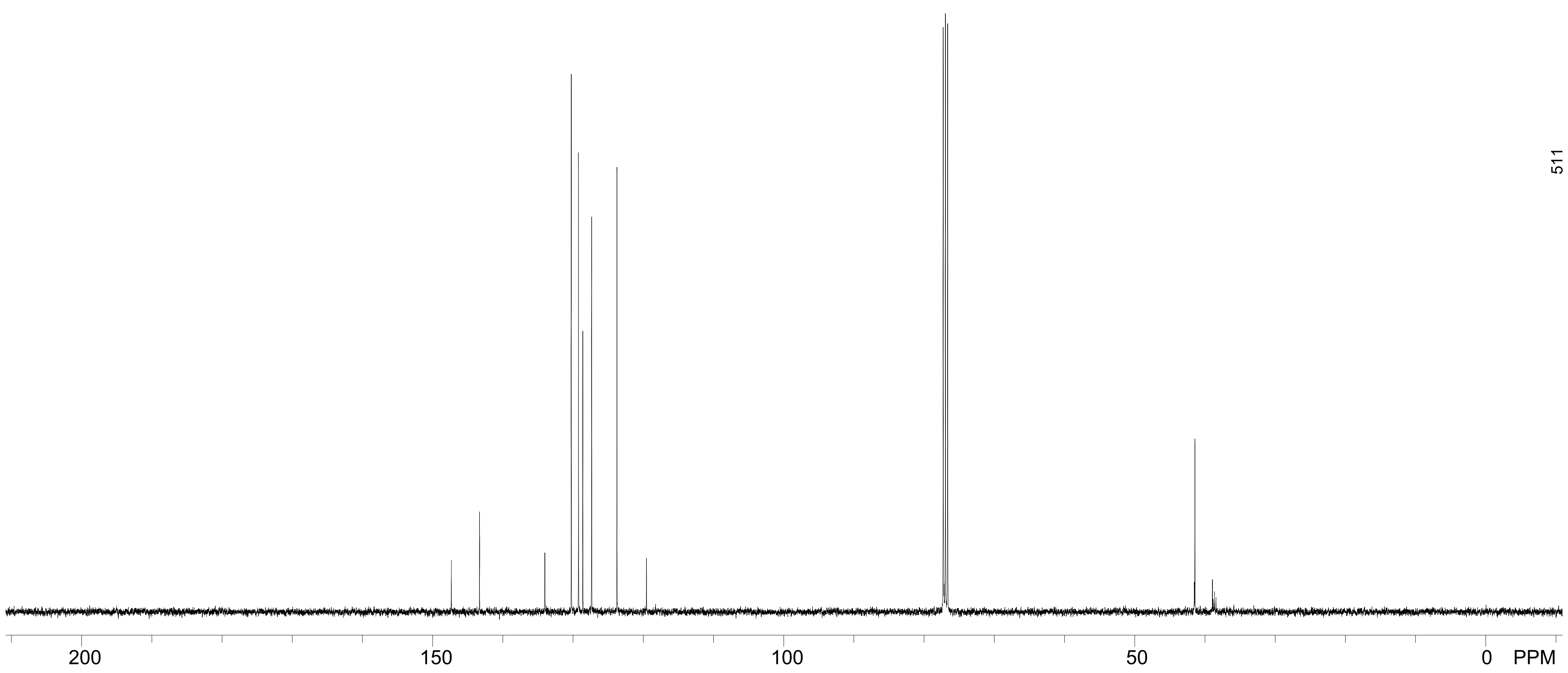

Area Percent Report

Agilent Technologies

Sample name: zwf-8-030-IE-90-10-0.8-205

Data file: C:\Users\Public\Documents\ChemStation\1\Data\zwf\zwf-S 2023-12-06 12-05-52\009-P1-B1-zwf-8-030.D

Injection Acquired Date 12/06/2023 18:45:41

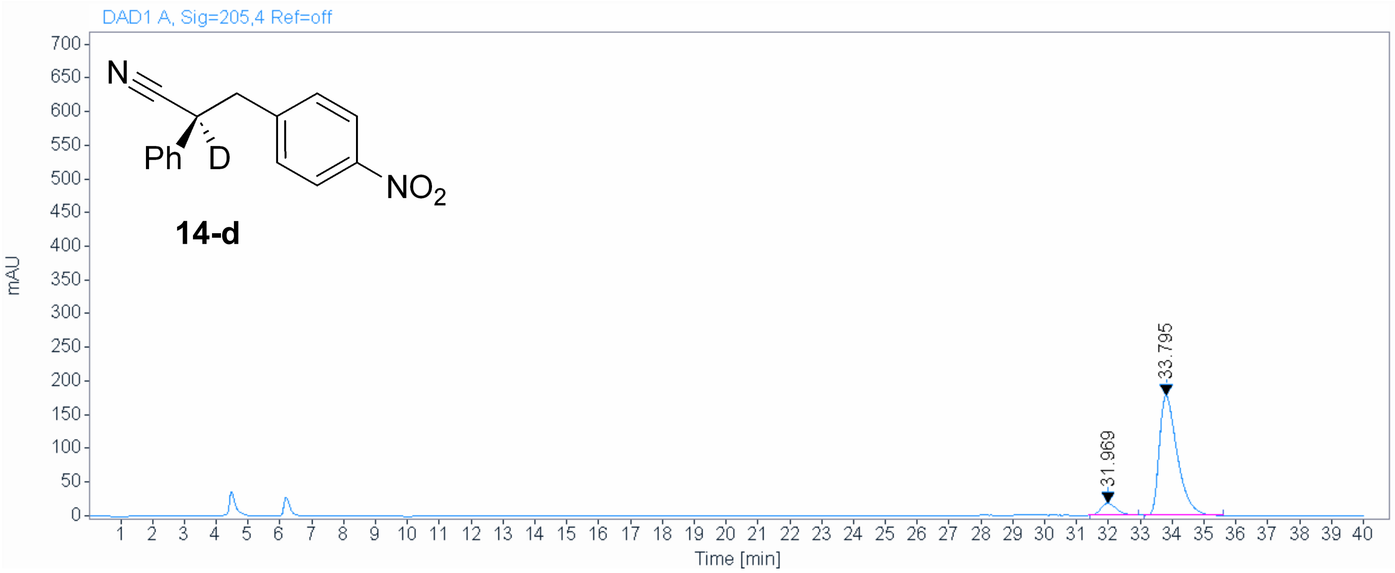

Signal: DAD1 A, Sig=205,4 Ref=off

RT [min]	Width [min]	Height	Area	Area%
31.969	0.5014	17.6685	604.9202	7.9584
33.795	0.5972	177.7435	6996.0957	92.0416
		Sum	7601.0159	100.0000

Area Percent Report

Agilent Technologies

Sample name: zwf-8-030-rac-IE-90-10-0.8-205

Data file: C:\Users\Public\Documents\ChemStation\1\Data\zwf\zwf-S 2023-12-06 20-07-35\007-P1-C5-zwf-8-030-rac.D

Injection Acquired Date 12/07/2023 01:11:52

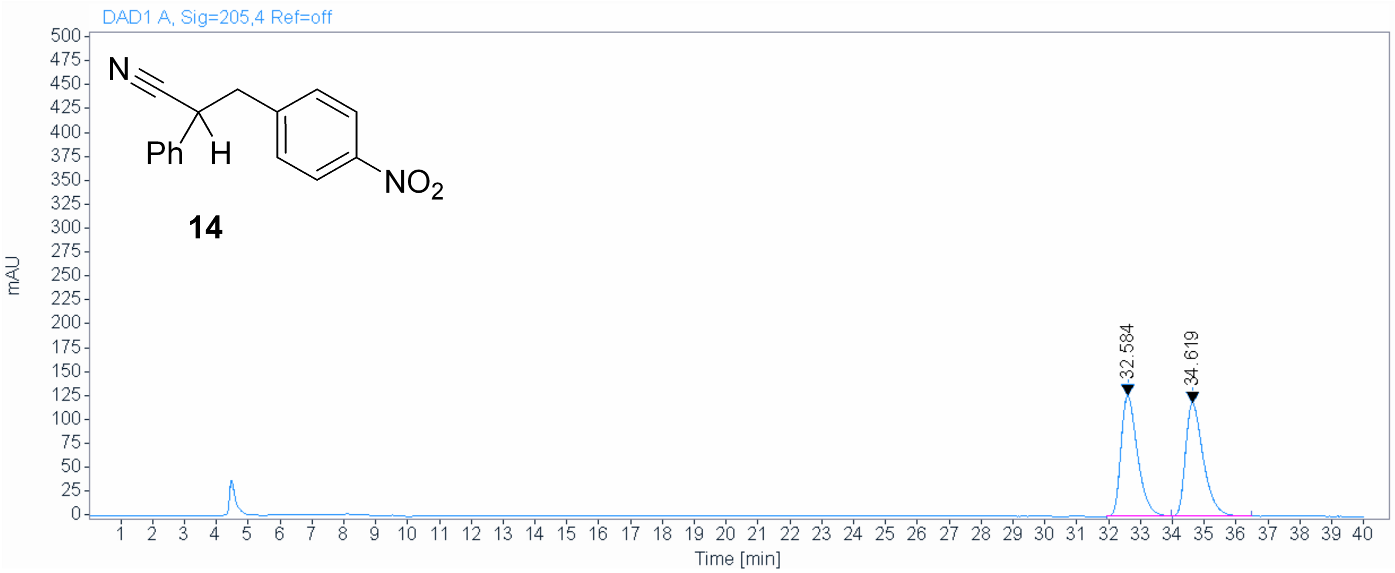

Signal: DAD1 A, Sig=205,4 Ref=off

RT [min]	Width [min]	Height	Area	Area%
32.584	0.5594	126.1576	4556.3672	49.8698
34.619	0.5946	118.0569	4580.1523	50.1302
		Sum	9136.5195	100.0000

zwf-7-067H
Sep 16 2023
SOLVENT: CDCl3
NA = 4
F1 = 400.179993 MHz
F2 = 1.000000 MHz

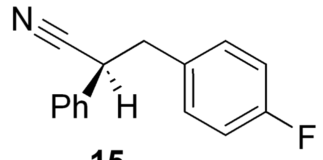

15

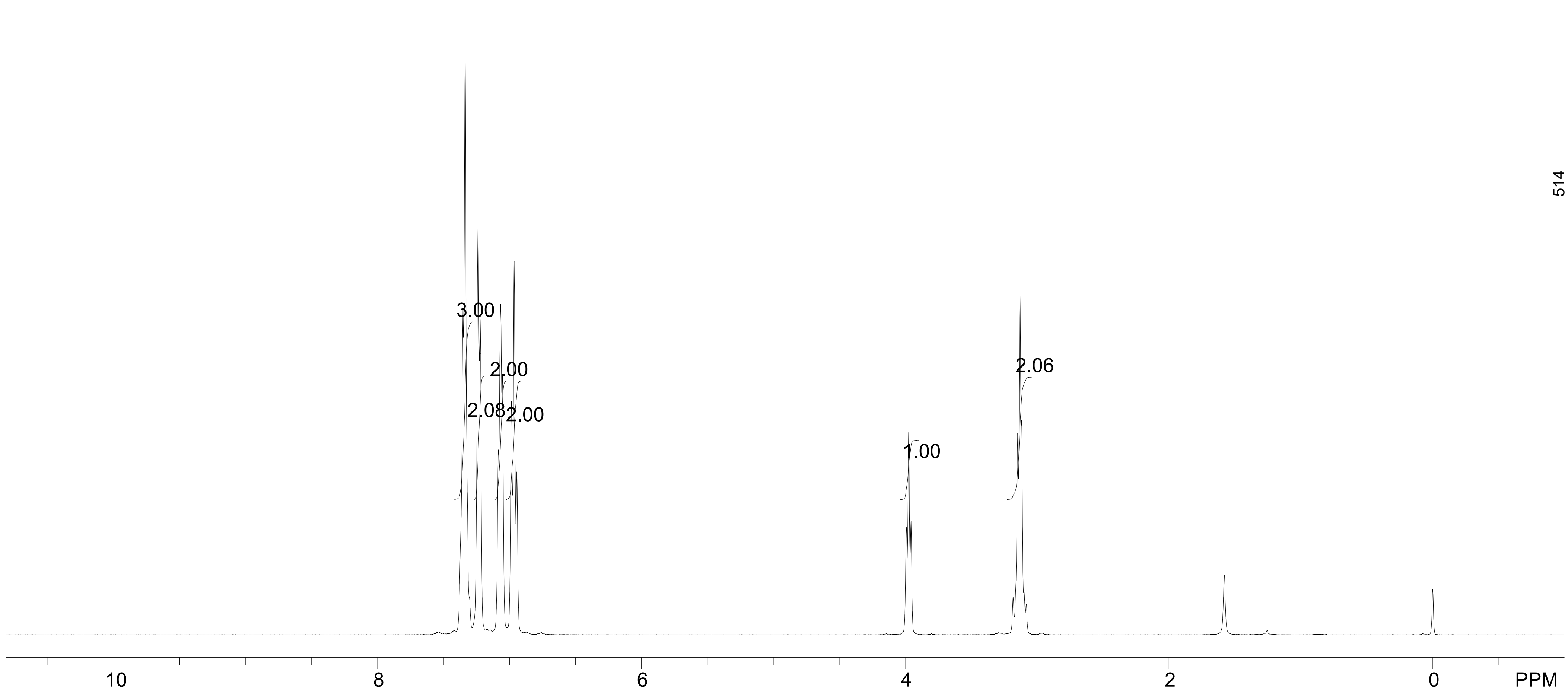

zwf-7-067C
Sep 16 2023
SOLVENT: CDCl3
NA = 100
F1 = 100.625343 MHz
F2 = 1.000000 MHz

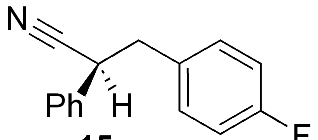

15

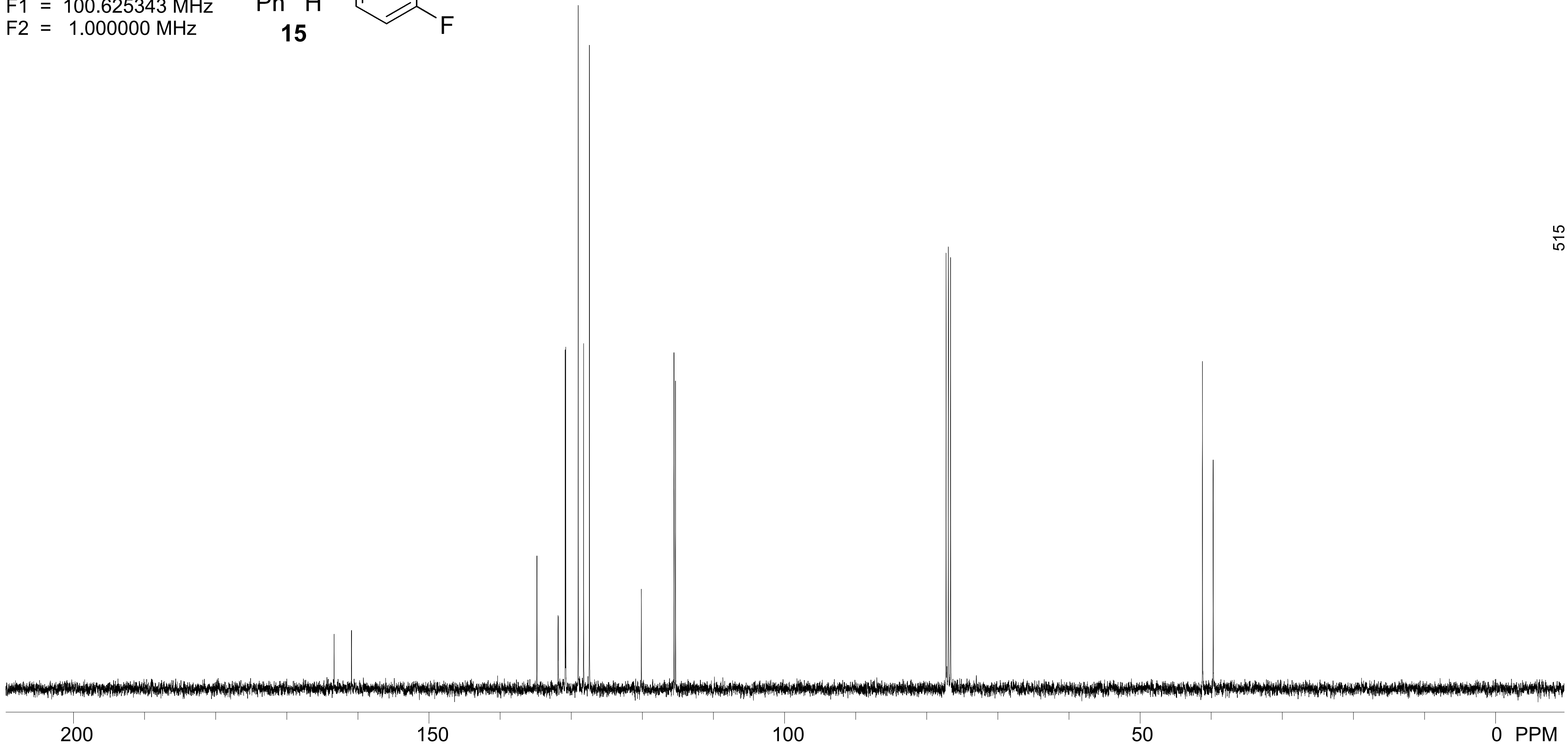

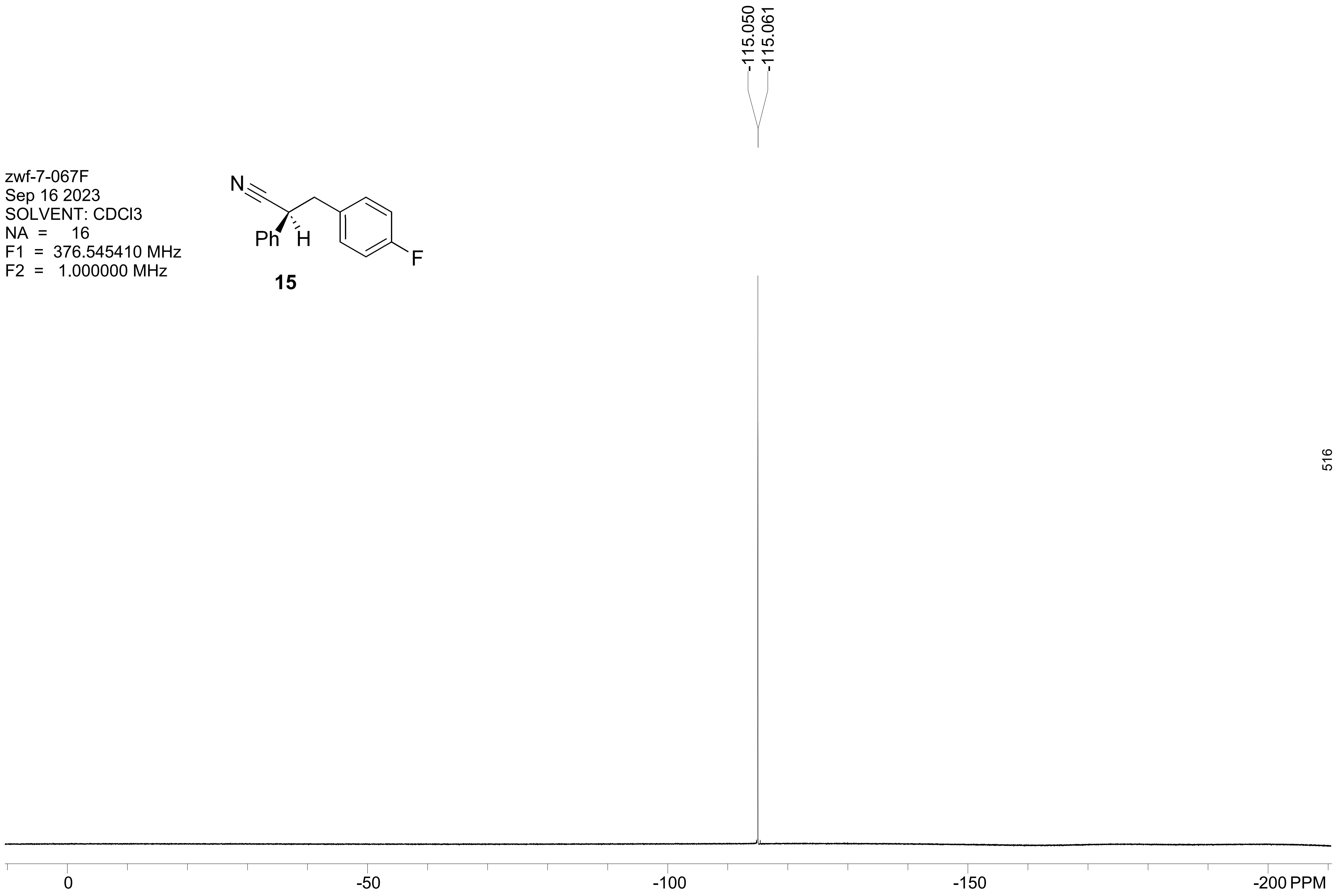
zwf-7-067F
Sep 16 2023
SOLVENT: CDCl3
NA = 16
F1 = 376.545410 MHz
F2 = 1.000000 MHz
N
Ph
H
F
15
-115.050
-115.061
0
-50
-100
-150
-200 PPM

Area Percent Report

Agilent Technologies

Sample name: zwf-7-067-IE-99-1-0.8-205

Data file: C:\Users\Public\Documents\ChemStation\1\Data\zwf\zwf 2023-09-16 20-24-54\002-P1-C1-zwf-7-067.D

Injection Acquired Date 09/16/2023 20:47:46

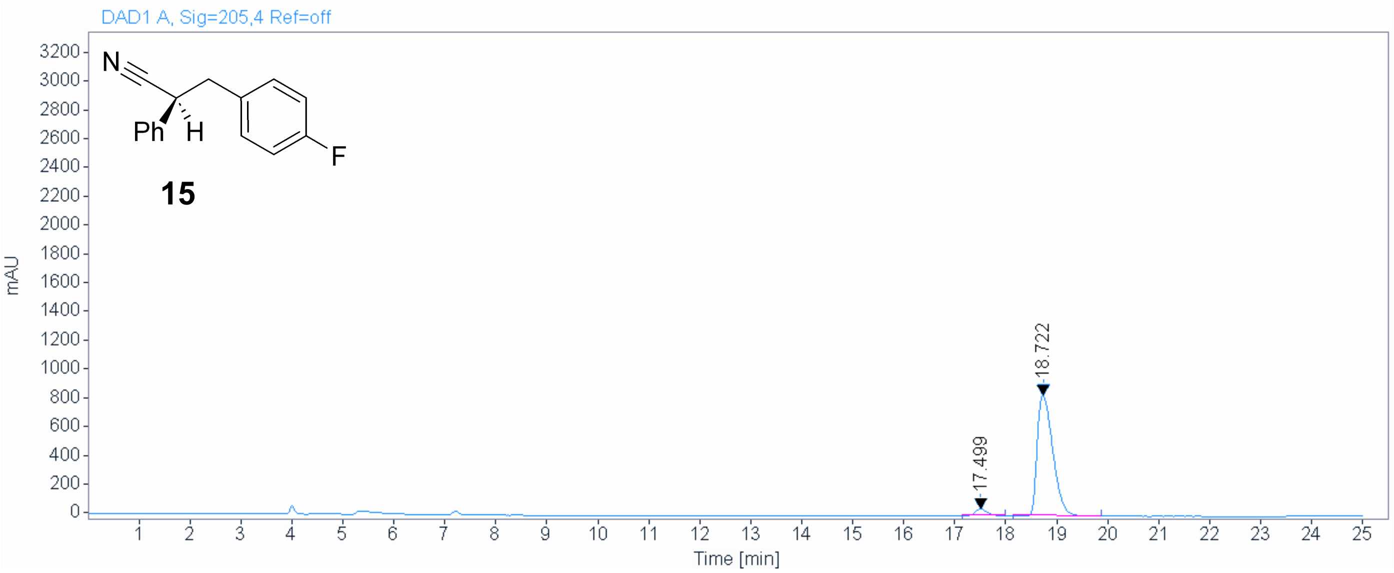

Signal: DAD1 A, Sig=205,4 Ref=off

RT [min]	Width [min]	Height	Area	Area%
17.499	0.2557	46.1909	758.9520	4.0845
18.722	0.3389	835.3256	17822.0957	95.9155
		Sum	18581.0477	100.0000

Area Percent Report

Agilent Technologies

Sample name: zwf-7-067-rac-IE-99-1-0.8-205

Data file: C:\Users\Public\Documents\ChemStation\1\Data\zwf\zwf 2023-09-16 20-24-54\003-P1-C2-zwf-7-067-rac.D

Injection Acquired Date 09/16/2023 21:13:49

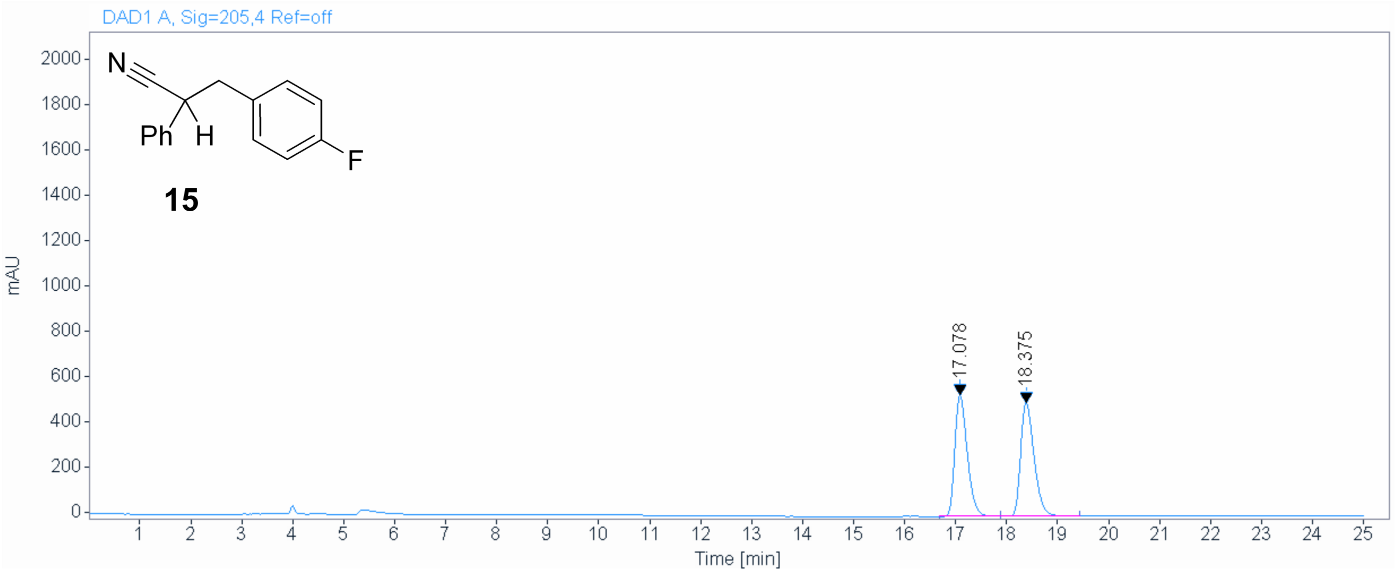

Signal: DAD1 A, Sig=205,4 Ref=off

RT [min]	Width [min]	Height	Area	Area%
17.078	0.2625	533.3809	9075.7422	49.7407
18.375	0.2827	498.3705	9170.3789	50.2593
		Sum	18246.1211	100.0000

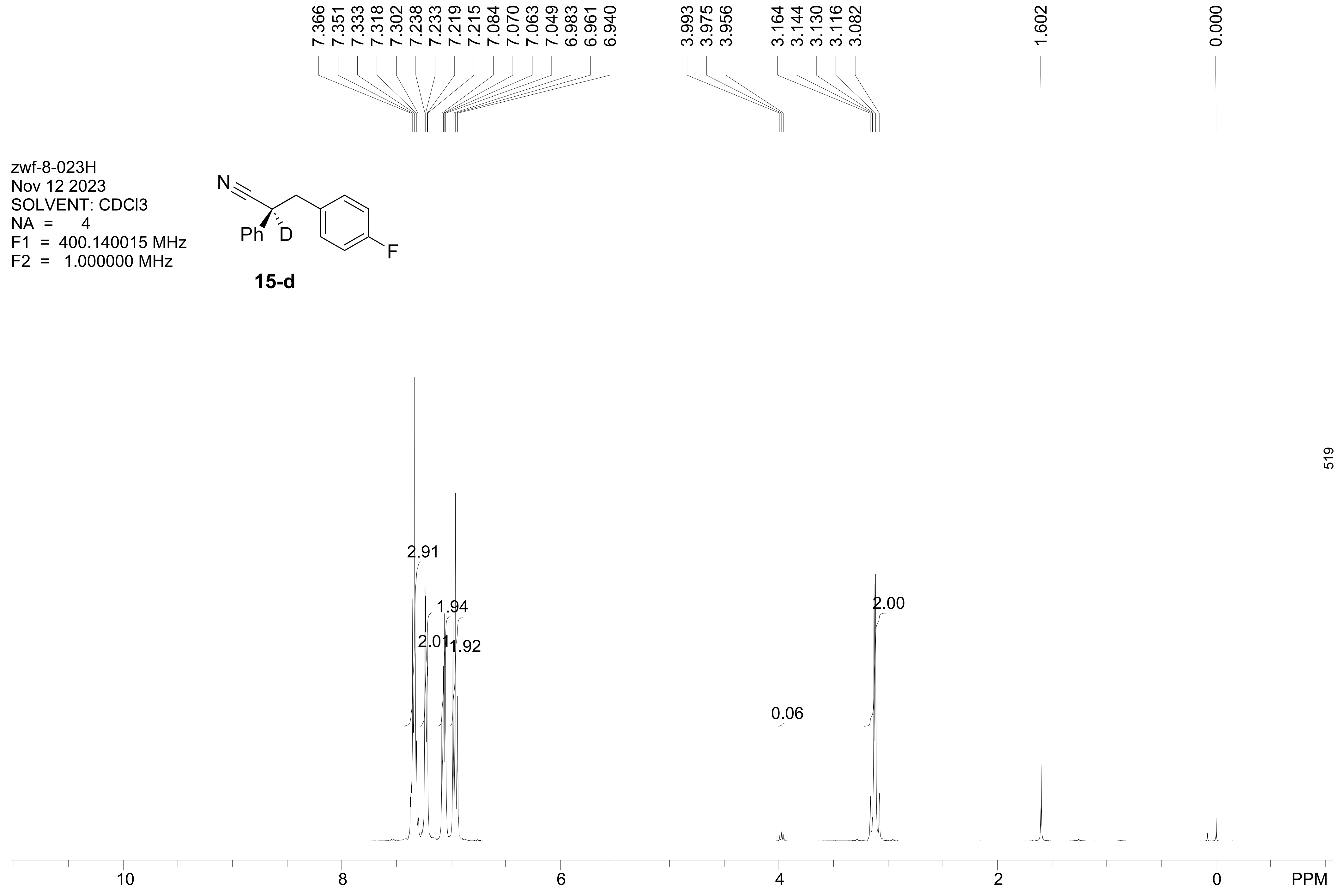
7.366
7.351
7.333
7.318
7.302
7.238
7.233
7.219
7.215
7.084
7.070
7.063
7.049
6.983
6.961
6.940
3.993
3.975
3.956
3.164
3.144
3.130
3.116
3.082
1.602
0.000
zwf-8-023H
Nov 12 2023
SOLVENT: CDCl3
NA = 4
F1 = 400.140015 MHz
F2 = 1.000000 MHz
N
Ph
D
F
15-d
2.91
1.94
2.01
1.92
0.06
2.00
10
8
6
4
2
0
PPM

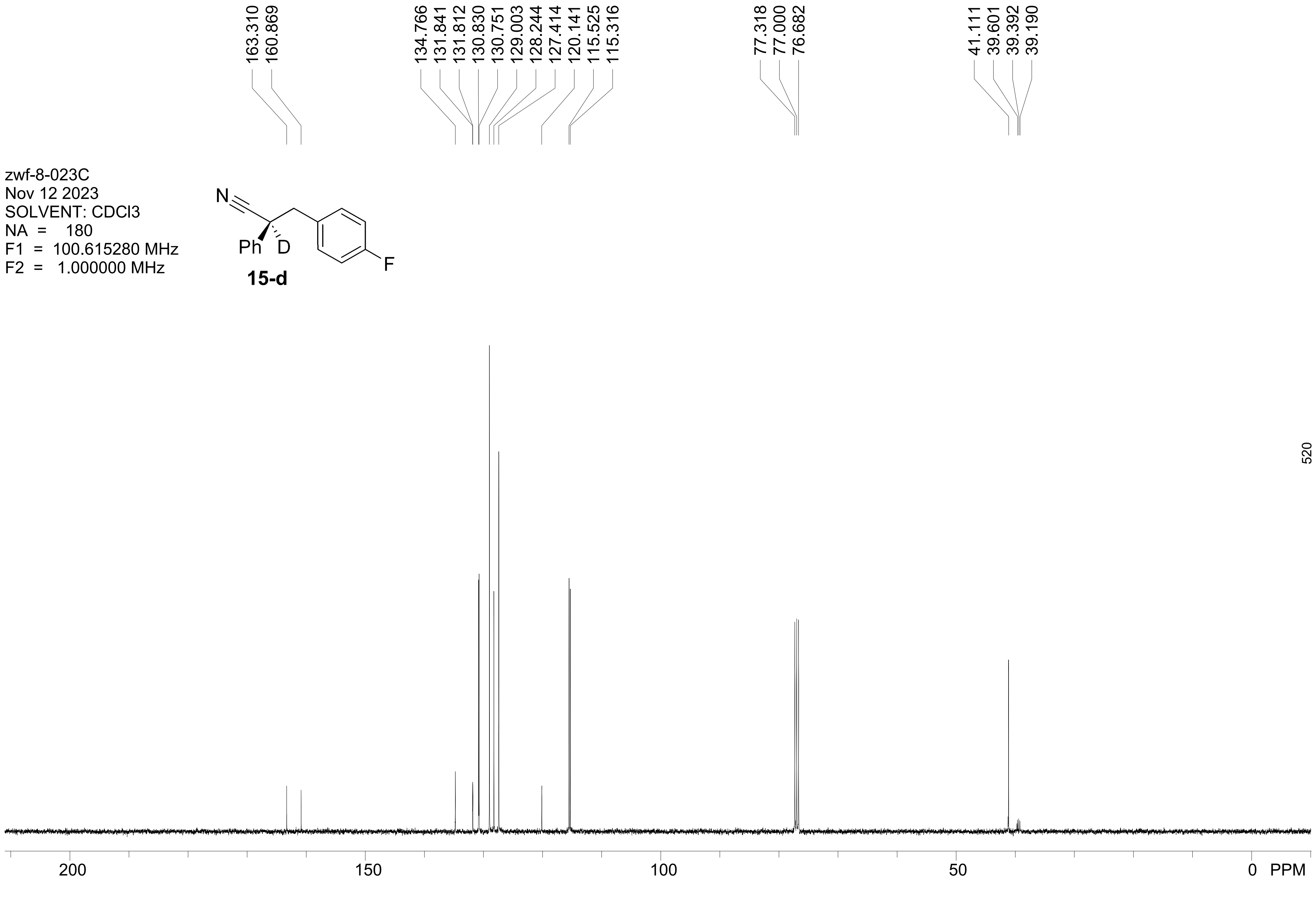
zwf-8-023C
Nov 12 2023
SOLVENT: CDCl3
NA = 180
F1 = 100.615280 MHz
F2 = 1.000000 MHz
N
Ph
D
F
15-d
163.310
160.869
134.766
131.841
131.812
130.830
130.751
129.003
128.244
127.414
120.141
115.525
115.316
77.318
77.000
76.682
41.111
39.601
39.392
39.190
200
150
100
50
0 PPM

zwf-8-023F
Nov 12 2023
SOLVENT: CDCl3
NA = 4
F1 = 376.507782 MHz
F2 = 1.000000 MHz

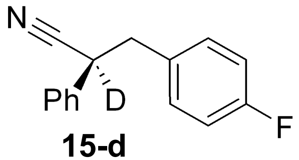

15-d

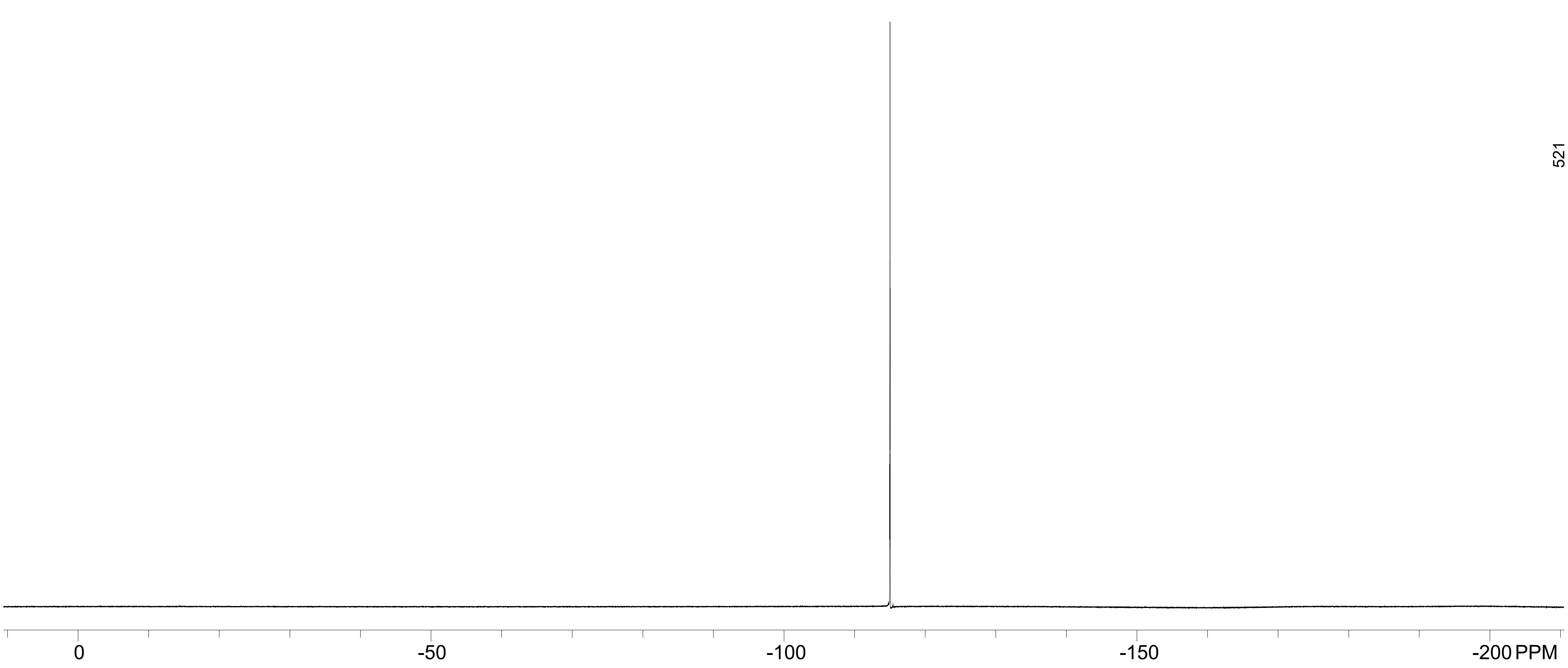

Area Percent Report

Agilent Technologies

Sample name: zwf-8-023-IE-99-1-0.8-205

Data file: C:\Users\Public\Documents\ChemStation\1\Data\zwf\zwf-S 2023-11-12 11-02-17\034-P1-A7-zwf-8-023.D

Injection Acquired Date 11/13/2023 12:56:53

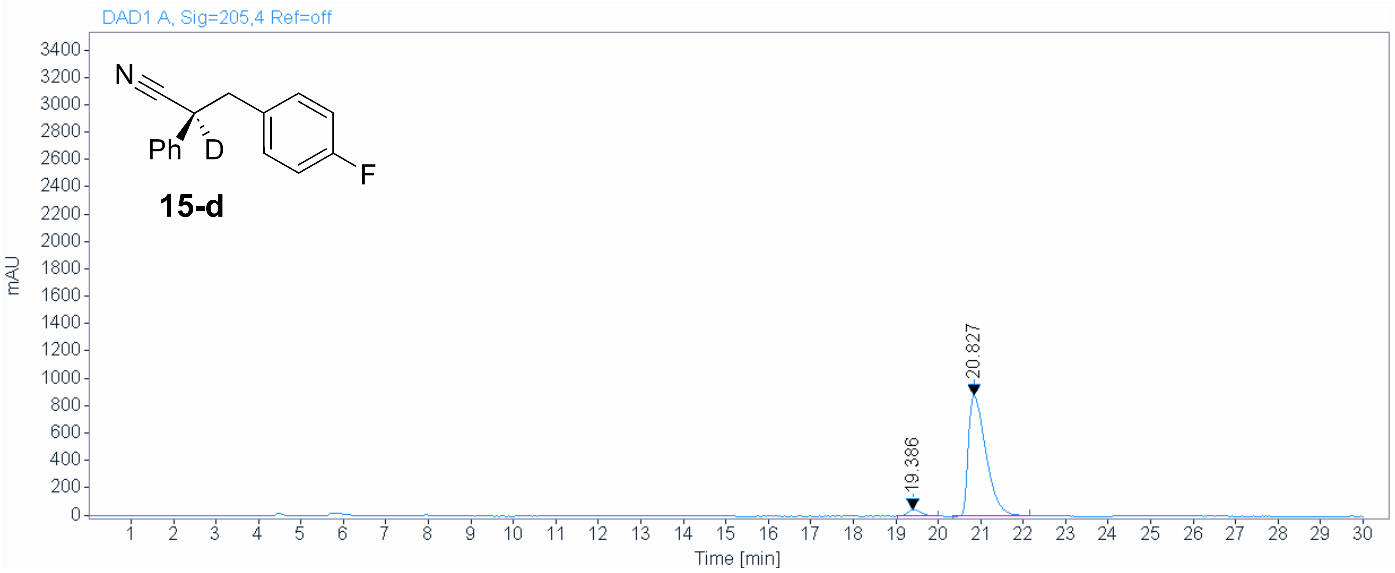

Signal: DAD1 A, Sig=205,4 Ref=off

RT [min]	Width [min]	Height	Area	Area%
19.386	0.3816	46.7313	1069.9821	4.0913
20.827	0.3795	882.8723	25082.9492	95.9087
		Sum	26152.9313	100.0000

Area Percent Report

Agilent Technologies

Sample name: zwf-8-023-rac-IE-99-1-0.8-205

Data file: C:\Users\Public\Documents\ChemStation\1\Data\zwf\zwf-S 2023-11-12 11-02-17\035-P1-A8-zwf-8-023-rac.D

Injection Acquired Date 11/13/2023 13:27:53

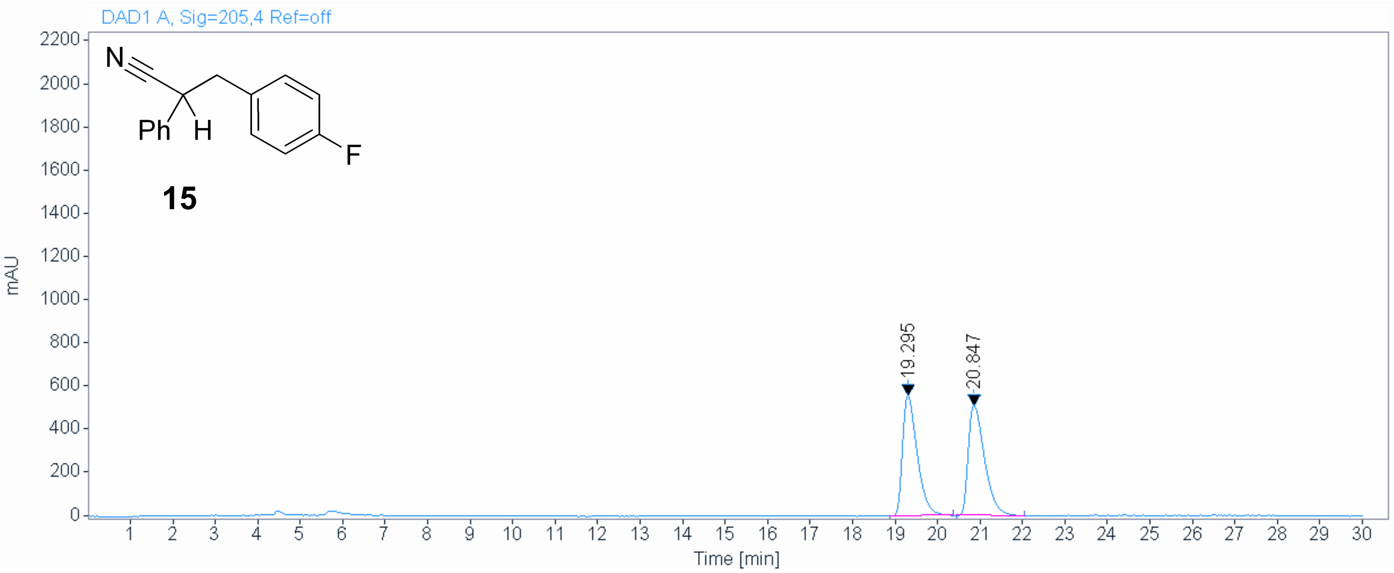

Signal: DAD1 A, Sig=205,4 Ref=off

RT [min]	Width [min]	Height	Area	Area%
19.295	0.3545	557.2631	13503.0205	49.9964
20.847	0.3765	510.1685	13504.9883	50.0036
		Sum	27008.0088	100.0000

zwf-7-068H
Sep 16 2023
SOLVENT: CDCl3
NA = 4
F1 = 400.140015 MHz
F2 = 1.000000 MHz

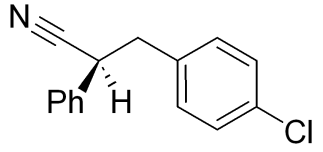

16

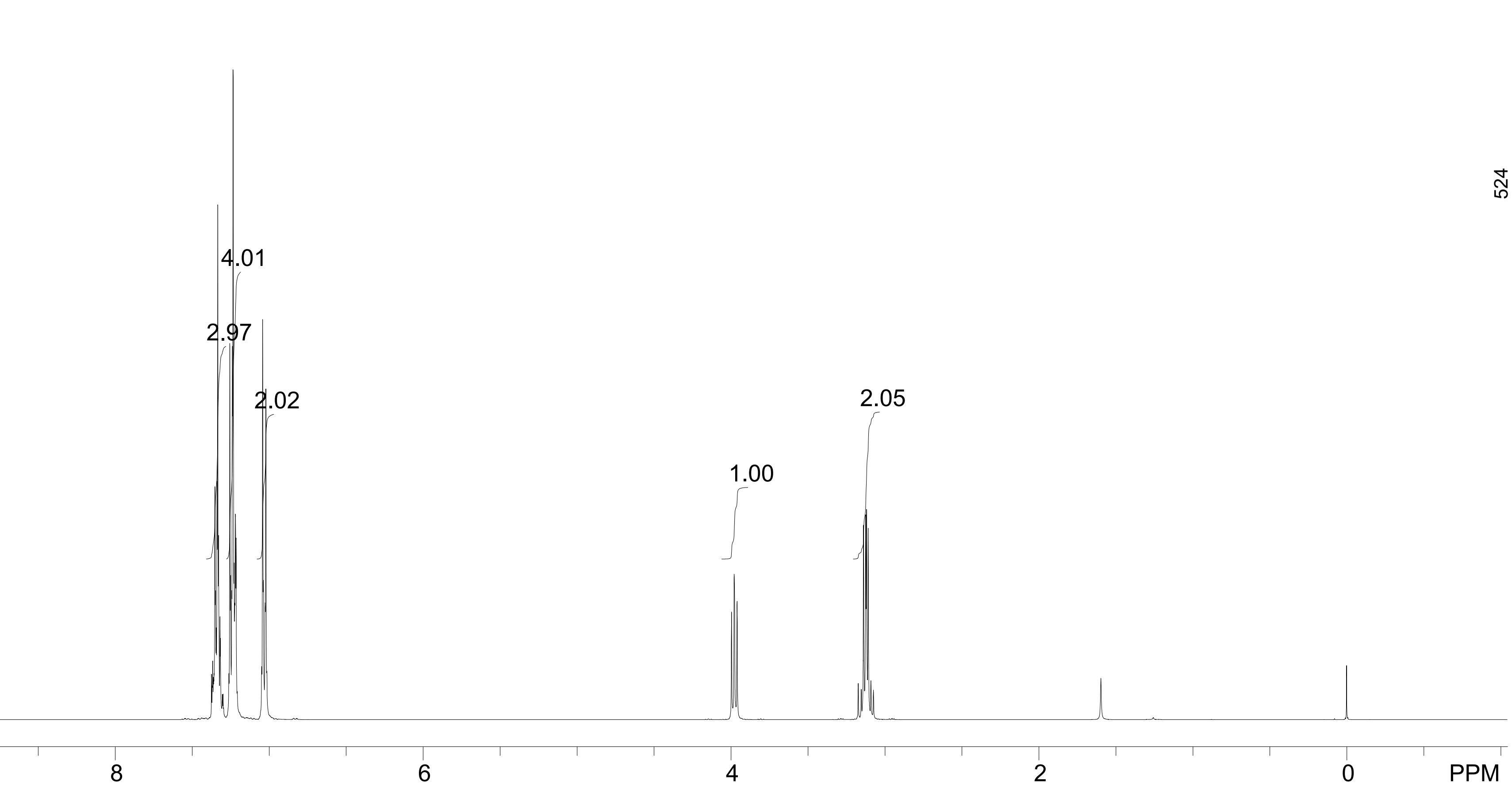

zwf-7-068C
Sep 16 2023
SOLVENT: CDCl3
NA = 100
F1 = 100.615280 MHz
F2 = 1.000000 MHz

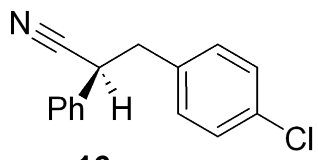

16

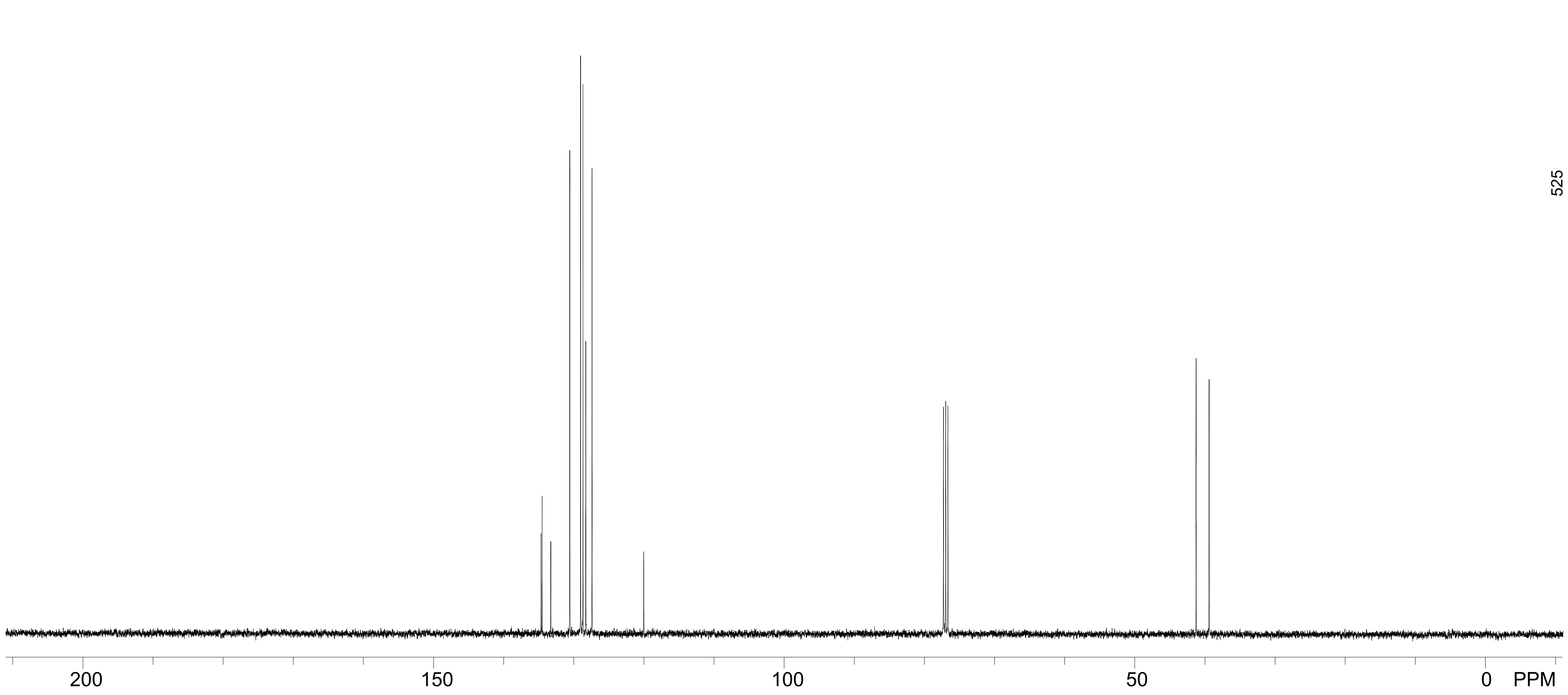

Area Percent Report

Agilent Technologies

Sample name: zwf-7-068-IE-99-1-0.8-205

Data file: C:\Users\Public\Documents\ChemStation\1\Data\zwf\zwf 2023-09-16 20-24-54\004-P1-C3-zwf-7-068.D

Injection Acquired Date 09/16/2023 21:39:53

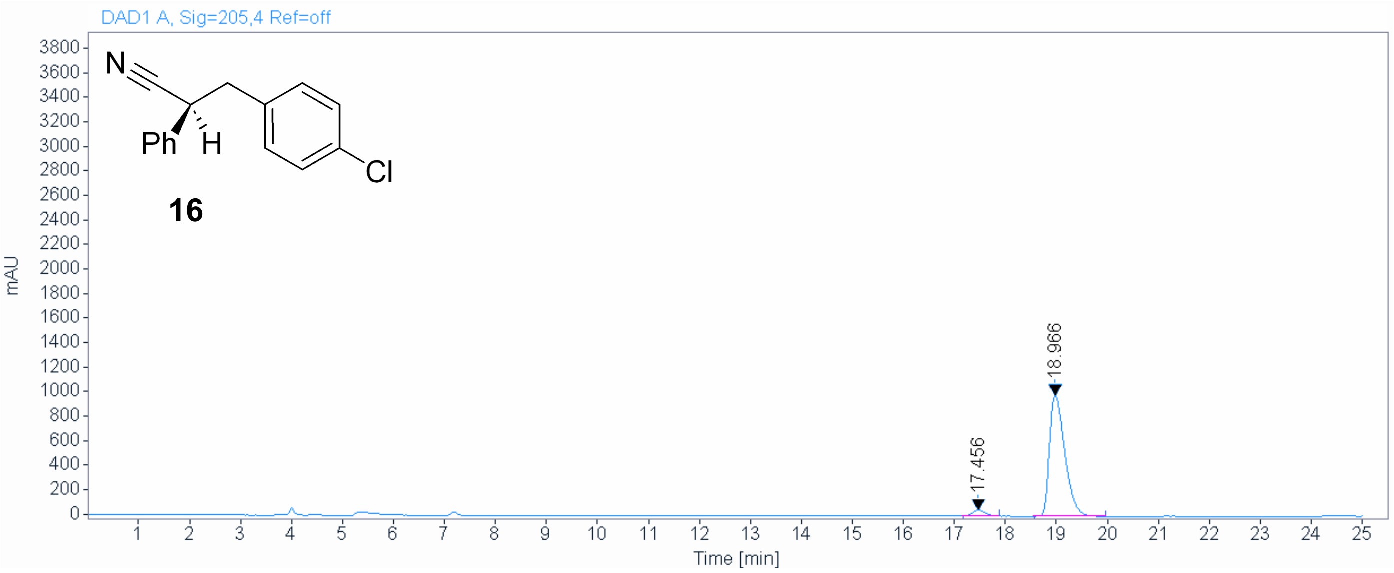

Signal: DAD1 A, Sig=205,4 Ref=off

RT [min]	Width [min]	Height	Area	Area%
17.456	0.2724	45.8798	749.8124	3.4963
18.966	0.3252	984.6678	20695.8125	96.5037
		Sum	21445.6249	100.0000

Area Percent Report

Sample name: zwf-7-068-rac-IE-99-1-0.8-205

Data file: C:\Users\Public\Documents\ChemStation\1\Data\zwf\zwf 2023-09-16 20-24-54\005-P1-C4-zwf-7-068-rac.D

Injection Acquired Date 09/16/2023 22:05:56

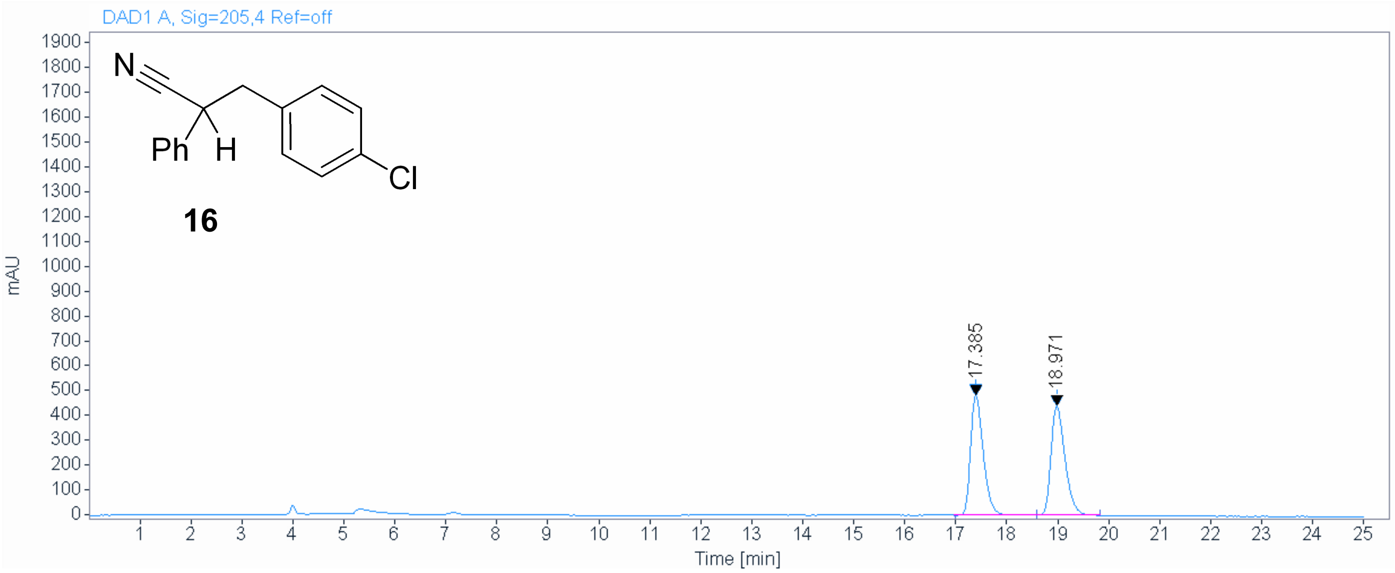

Signal: DAD1 A, Sig=205,4 Ref=off

RT [min]	Width [min]	Height	Area	Area%
17.385	0.2687	481.8952	8386.0176	49.9671
18.971	0.2927	439.9092	8397.0645	50.0329
		Sum	16783.0820	100.0000

zwf-8-024H
Nov 12 2023
SOLVENT: CDCl3
NA = 4
F1 = 400.179993 MHz
F2 = 1.000000 MHz

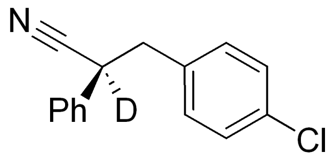

16-d

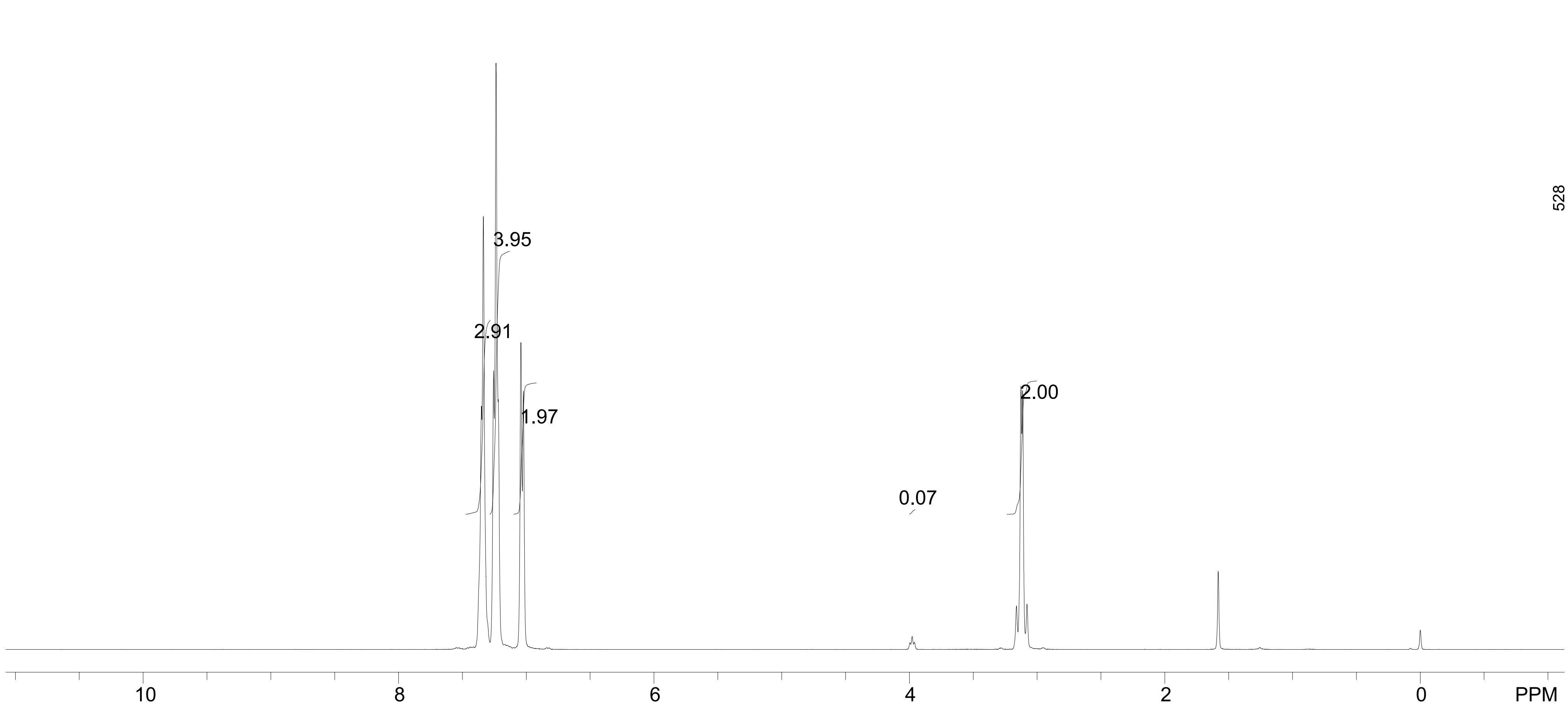

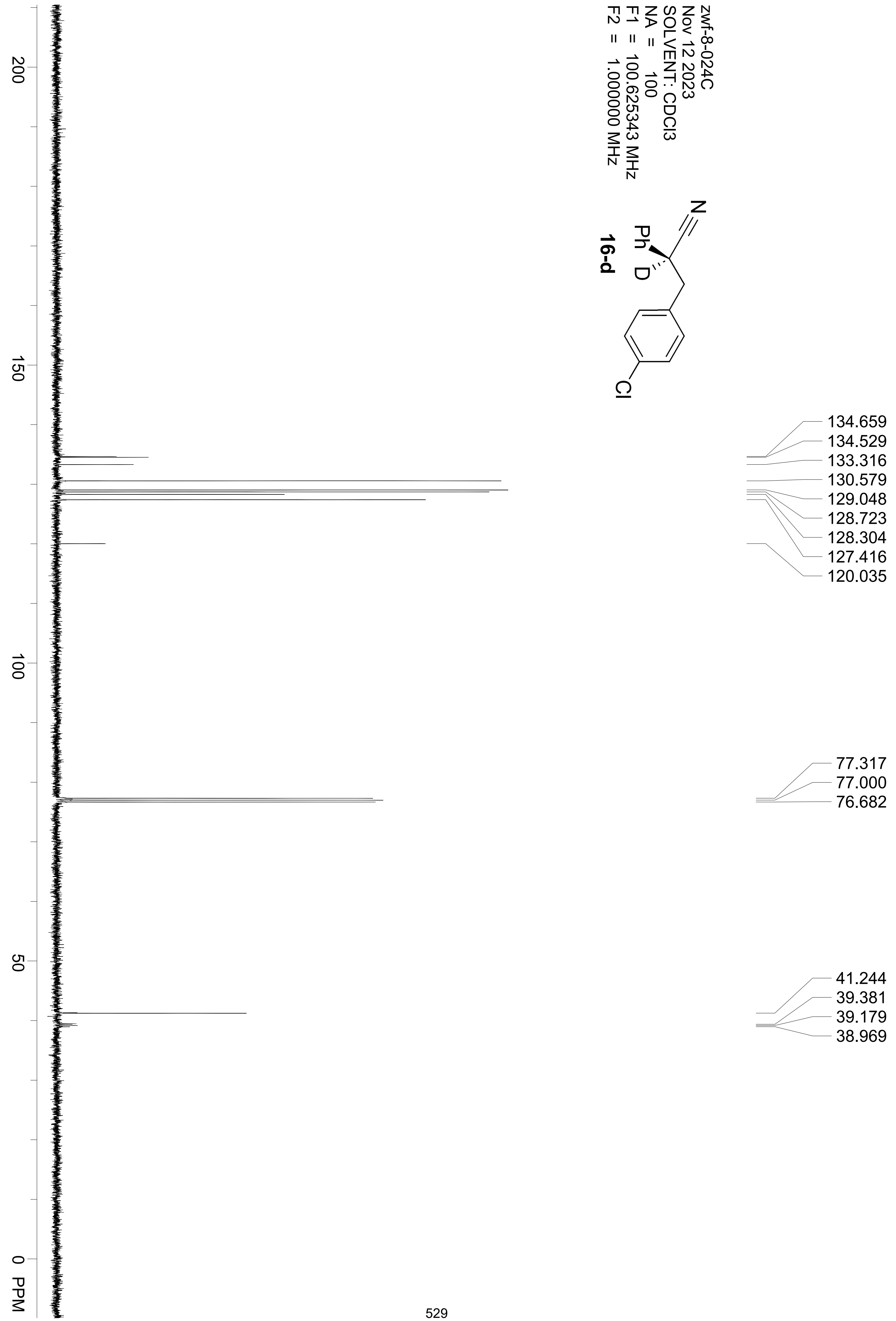
zwf-8-024C
Nov 12 2023
SOLVENT: CDCl3
NA = 100
F1 = 100.625343 MHz
F2 = 1.000000 MHz
N
Ph
D
Cl
16-d
134.659
134.529
133.316
130.579
129.048
128.723
128.304
127.416
120.035
77.317
77.000
76.682
41.244
39.381
39.179
38.969
200
150
100
50
0 PPM

Area Percent Report

Agilent Technologies

Sample name: zwf-8-024-IE-99-1-0.8-205

Data file: C:\Users\Public\Documents\ChemStation\1\Data\zwf\zwf-S 2023-11-12 11-02-17\036-P1-A9-zwf-8-024.D

Injection Acquired Date 11/13/2023 13:58:55

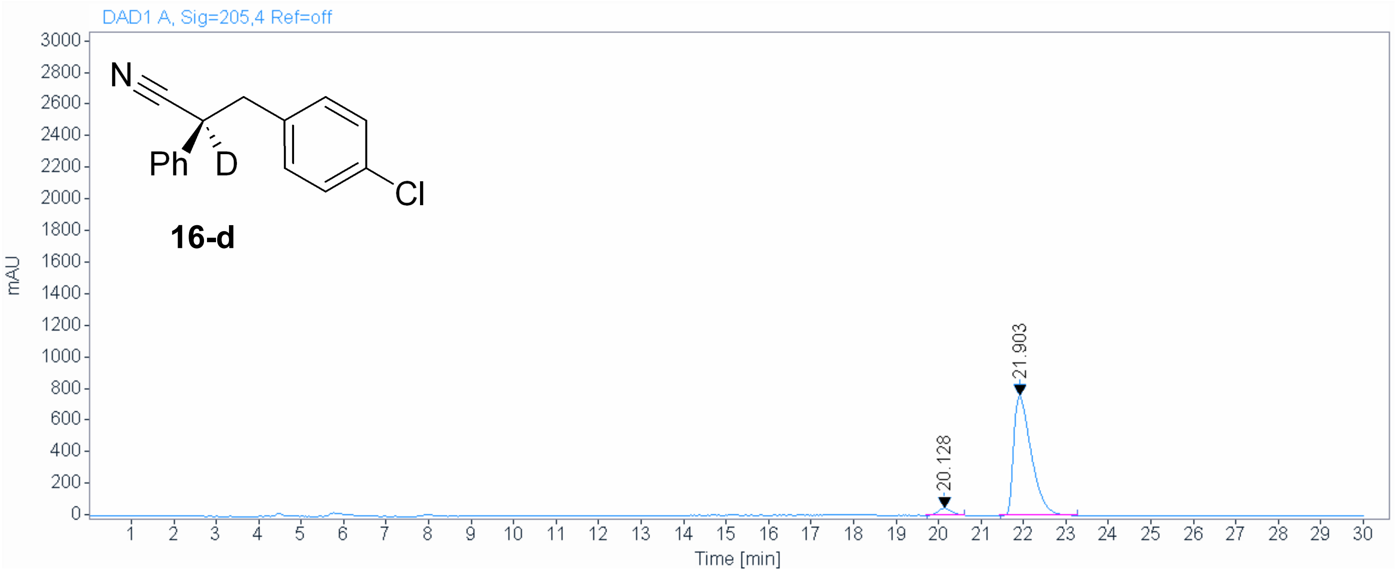

Signal: DAD1 A, Sig=205,4 Ref=off

RT [min]	Width [min]	Height	Area	Area%
20.128	0.3554	43.6034	929.8210	4.0473
21.903	0.4023	760.0301	22044.2383	95.9527
		Sum	22974.0593	100.0000

Area Percent Report

Agilent Technologies

Sample name: zwf-8-024-rac-IE-99-1-0.8-205

Data file: C:\Users\Public\Documents\ChemStation\1\Data\zwf\zwf-S 2023-11-12 11-02-17\037-P1-A10-zwf-8-024-rac.D

Injection Acquired Date 11/13/2023 14:29:58

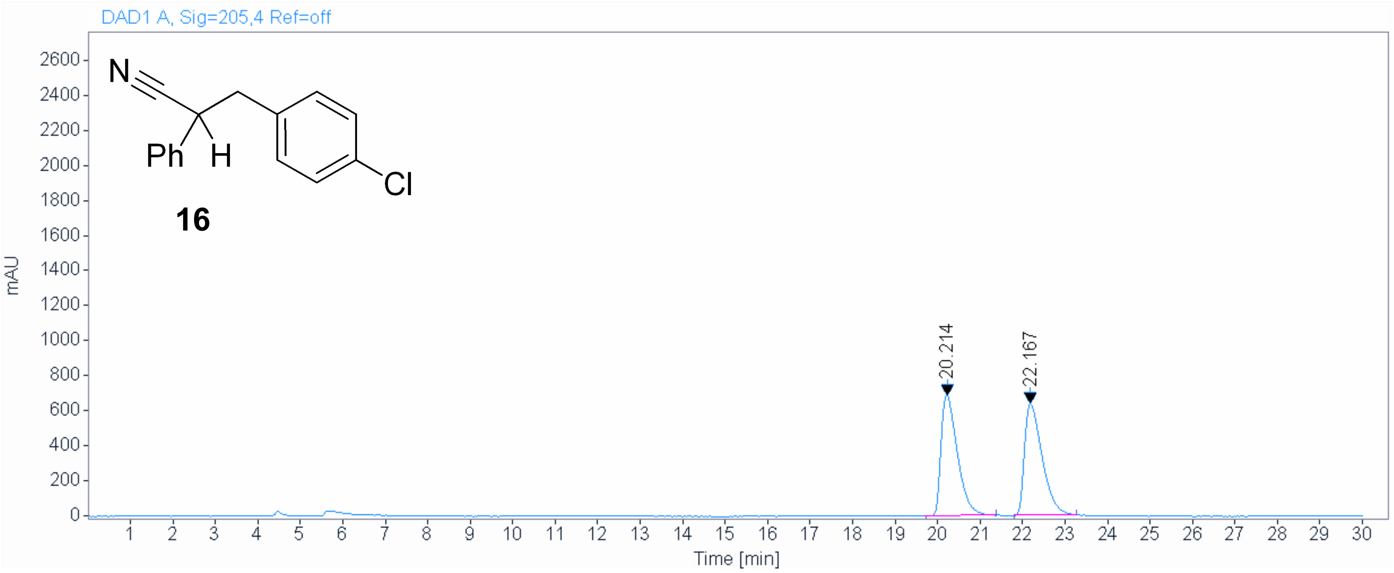

Signal: DAD1 A, Sig=205,4 Ref=off

RT [min]	Width [min]	Height	Area	Area%
20.214	0.3739	686.8068	18499.2207	49.9786
22.167	0.4219	639.2946	18515.0938	50.0214
		Sum	37014.3145	100.0000

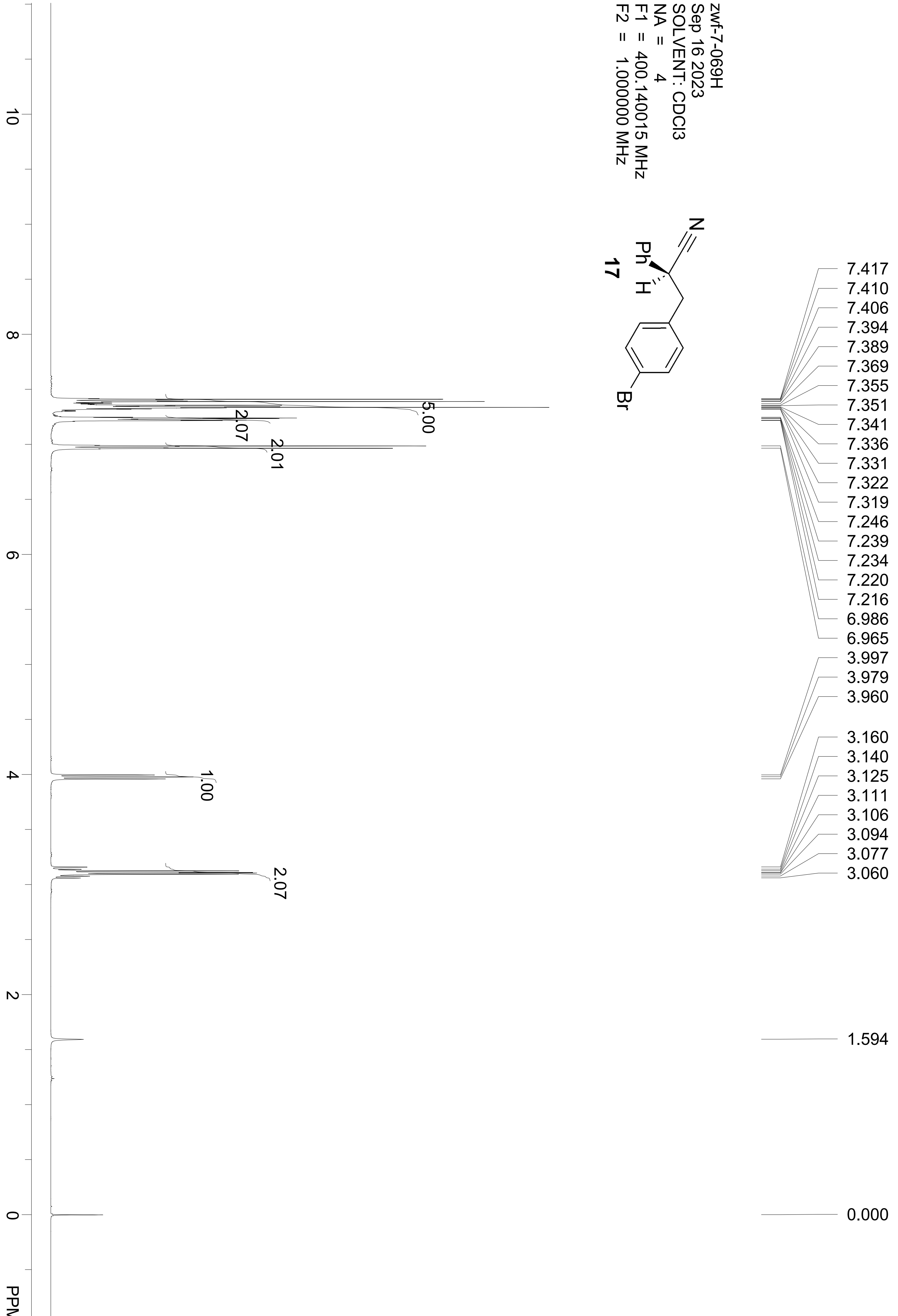

zwf-7-069H
Sep 16 2023
SOLVENT: CDCl3
NA = 4
F1 = 400.140015 MHz
F2 = 1.000000 MHz
N
Ph
H
Br
17
7.417
7.410
7.406
7.394
7.389
7.369
7.355
7.351
7.341
7.336
7.331
7.322
7.319
7.246
7.239
7.234
7.220
7.216
6.986
6.965
3.997
3.979
3.960
3.160
3.140
3.125
3.111
3.106
3.094
3.077
3.060
1.594
0.000
5.00
2.07
2.01
1.00
2.07
10
8
6
4
2
0
PPM

zwf-7-069C
Sep 16 2023
SOLVENT: CDCl3
NA = 60
F1 = 100.615280 MHz
F2 = 1.000000 MHz

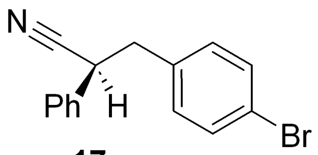

17

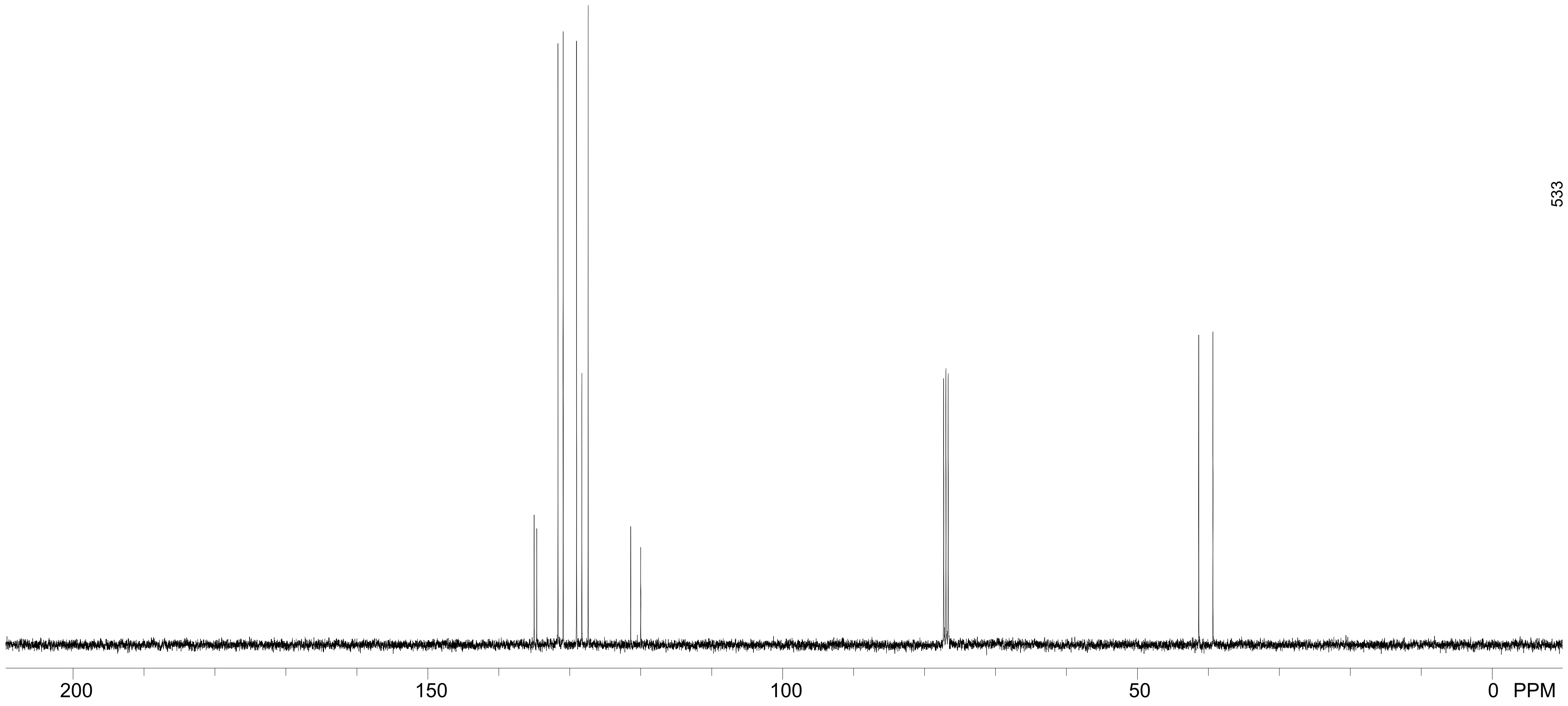

Area Percent Report

Agilent Technologies

Sample name: zwf-7-069-IE-99-1-0.8-205

Data file: C:\Users\Public\Documents\ChemStation\1\Data\zwf\zwf 2023-09-16 20-24-54\006-P1-C5-zwf-7-069.D

Injection Acquired Date 09/16/2023 22:32:00

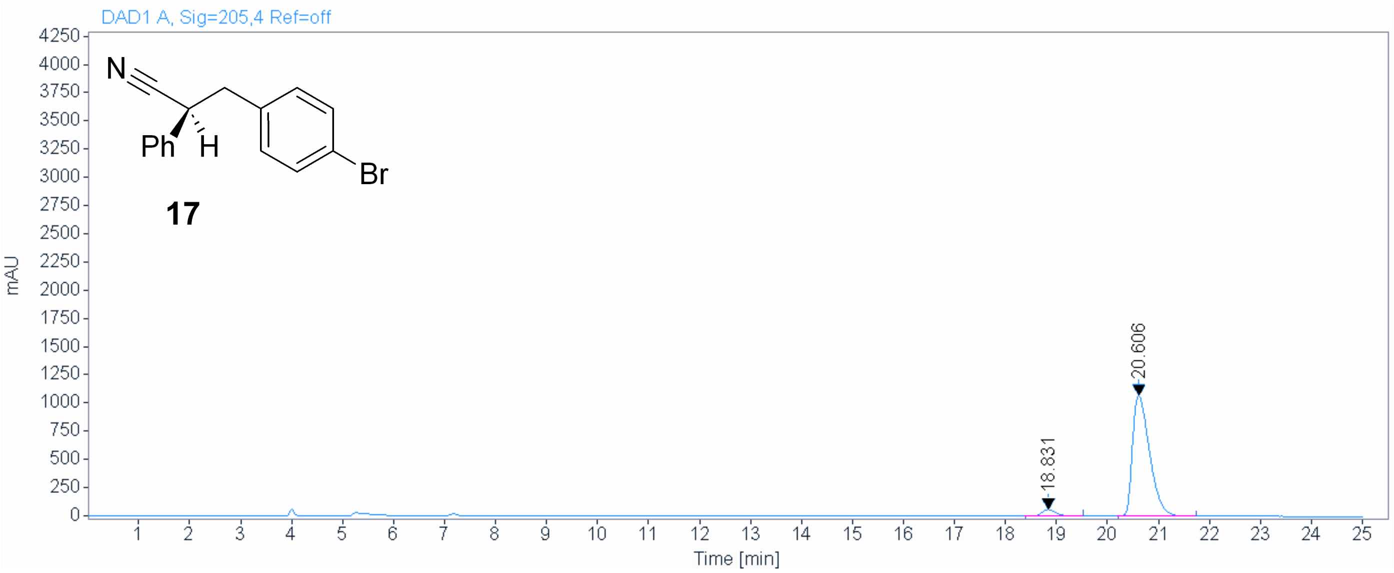

Signal: DAD1 A, Sig=205,4 Ref=off

RT [min]	Width [min]	Height	Area	Area%
18.831	0.2928	57.5878	1079.9946	4.1767
20.606	0.3589	1067.0901	24777.6426	95.8233
		Sum	25857.6372	100.0000

Area Percent Report

Agilent Technologies

Sample name: zwf-7-069-rac-IE-99-1-0.8-205

Data file: C:\Users\Public\Documents\ChemStation\1\Data\zwf\zwf 2023-09-16 20-24-54\007-P1-C6-zwf-7-069-rac.D

Injection Acquired Date 09/16/2023 22:58:04

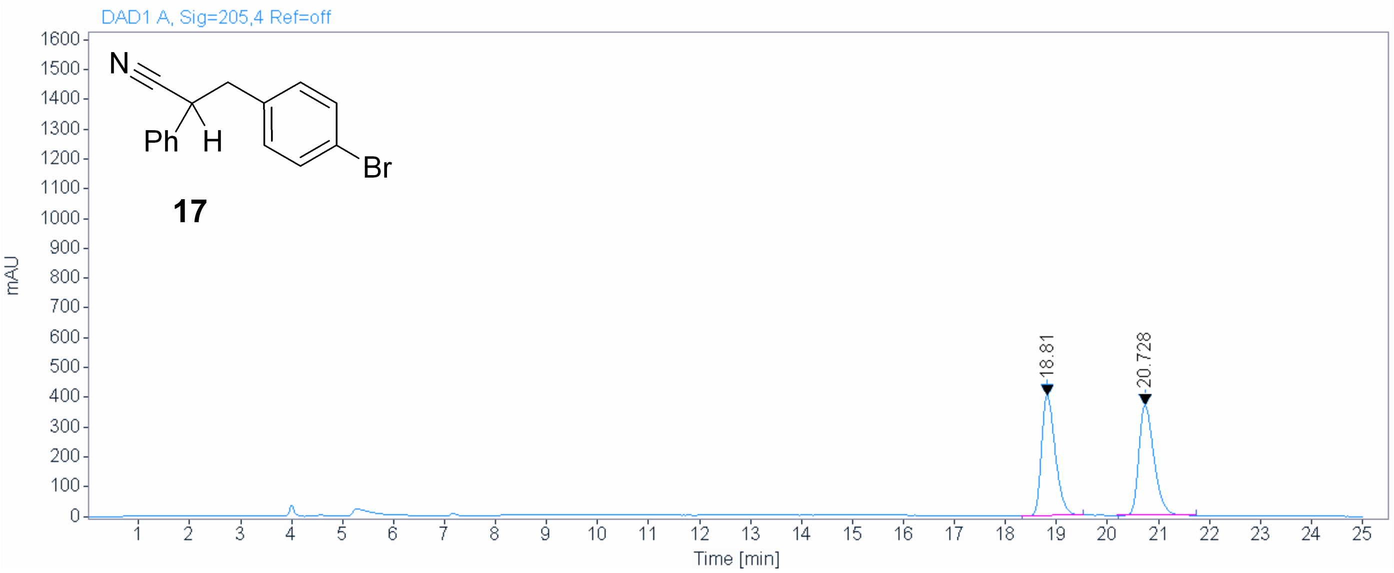

Signal: DAD1 A, Sig=205,4 Ref=off

RT [min]	Width [min]	Height	Area	Area%
18.810	0.2875	403.2875	7586.0684	49.6825
20.728	0.3195	370.8258	7683.0376	50.3175
		Sum	15269.1060	100.0000

zwf-8-025H
Nov 12 2023
SOLVENT: CDCl3
NA = 4
F1 = 400.179993 MHz
F2 = 1.000000 MHz

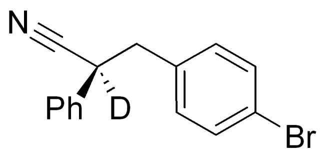

17-d

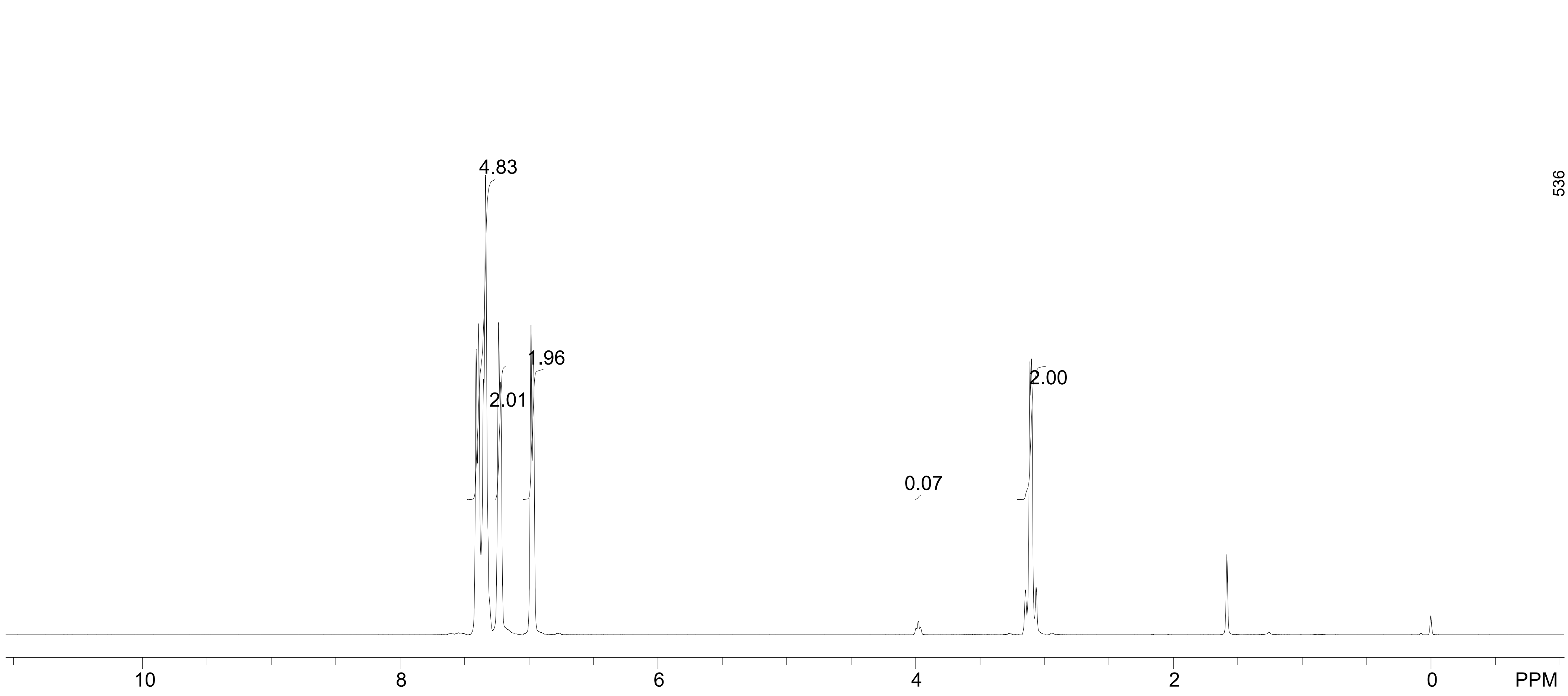

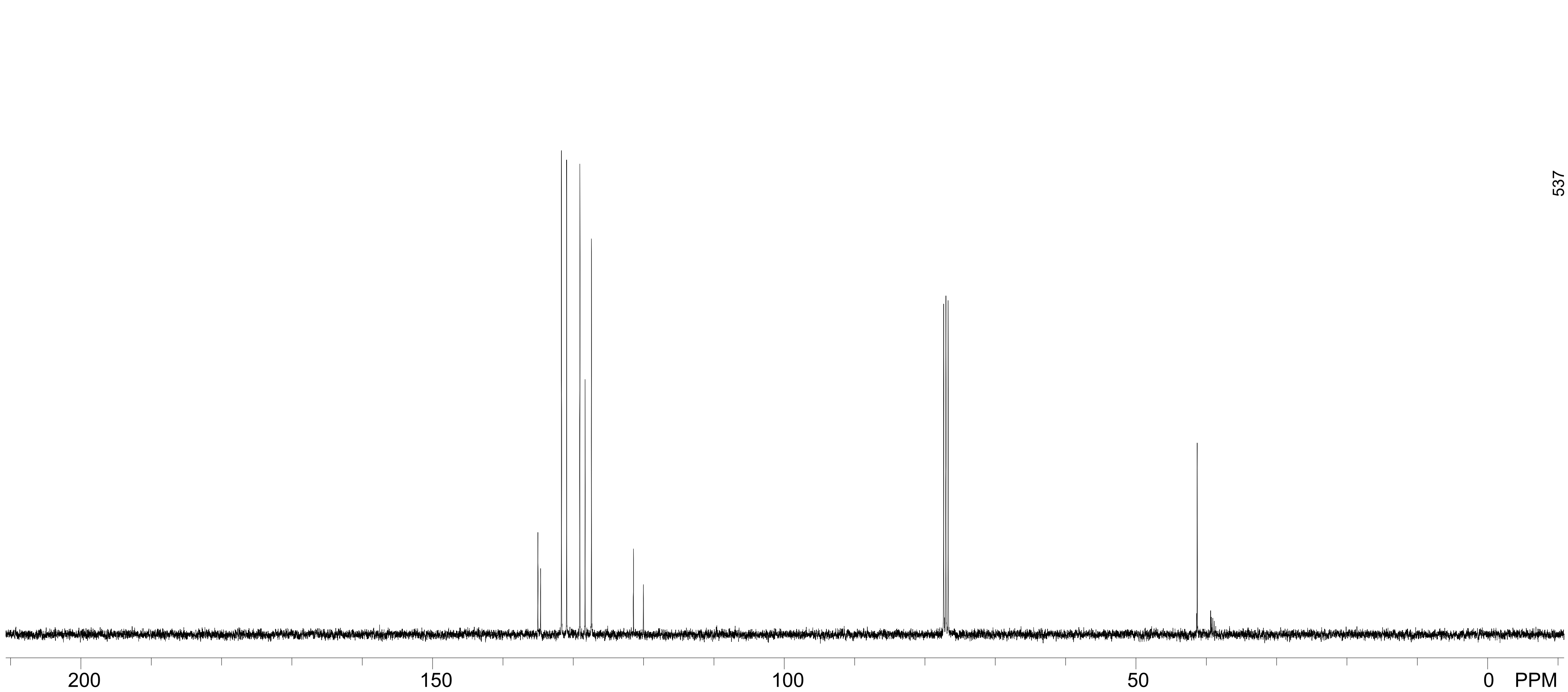
zwf-8-025C
Nov 12 2023
SOLVENT: CDCl3
NA =   100
F1 = 100.625343 MHz
F2 =   1.000000 MHz
135.028
134.631
131.670
130.933
129.055
128.311
127.409
121.429
120.013
77.318
77.000
76.682
41.295
39.287
39.085
38.876
200
150
100
50
0
PPM

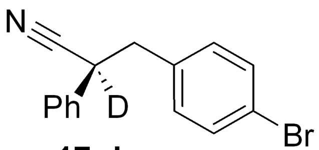
N
Ph
D
Br

17-d

Area Percent Report

Agilent Technologies

Sample name: zwf-8-025-IE-99-1-0.8-205

Data file: C:\Users\Public\Documents\ChemStation\1\Data\zwf\zwf-S 2023-11-12 11-02-17\038-P1-B3-zwf-8-025.D

Injection Acquired Date 11/13/2023 15:01:00

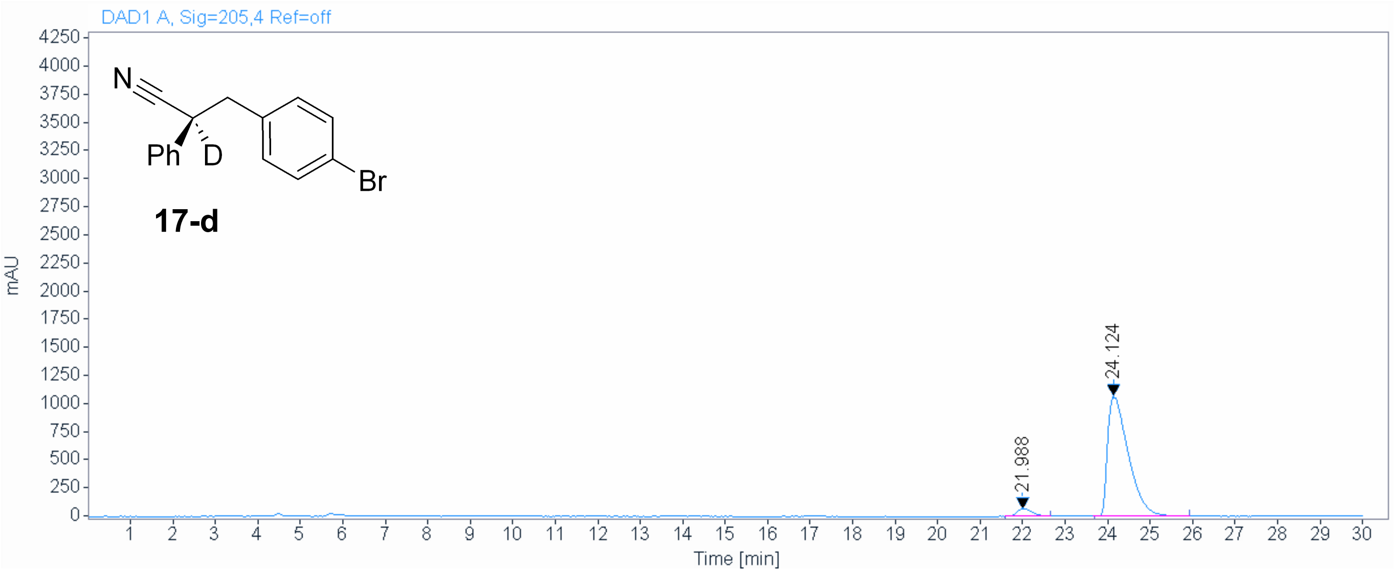

Signal: DAD1 A, Sig=205,4 Ref=off

RT [min]	Width [min]	Height	Area	Area%
21.988	0.4111	68.3855	1686.9910	4.4330
24.124	0.5638	1075.0680	36368.5430	95.5670
		Sum	38055.5339	100.0000

Area Percent Report

Agilent Technologies

Sample name: zwf-8-025-rac-IE-99-1-0.8-205

Data file: C:\Users\Public\Documents\ChemStation\1\Data\zwf\zwf-S 2023-11-12 11-02-17\039-P1-B4-zwf-8-025-rac.D

Injection Acquired Date 11/13/2023 15:32:02

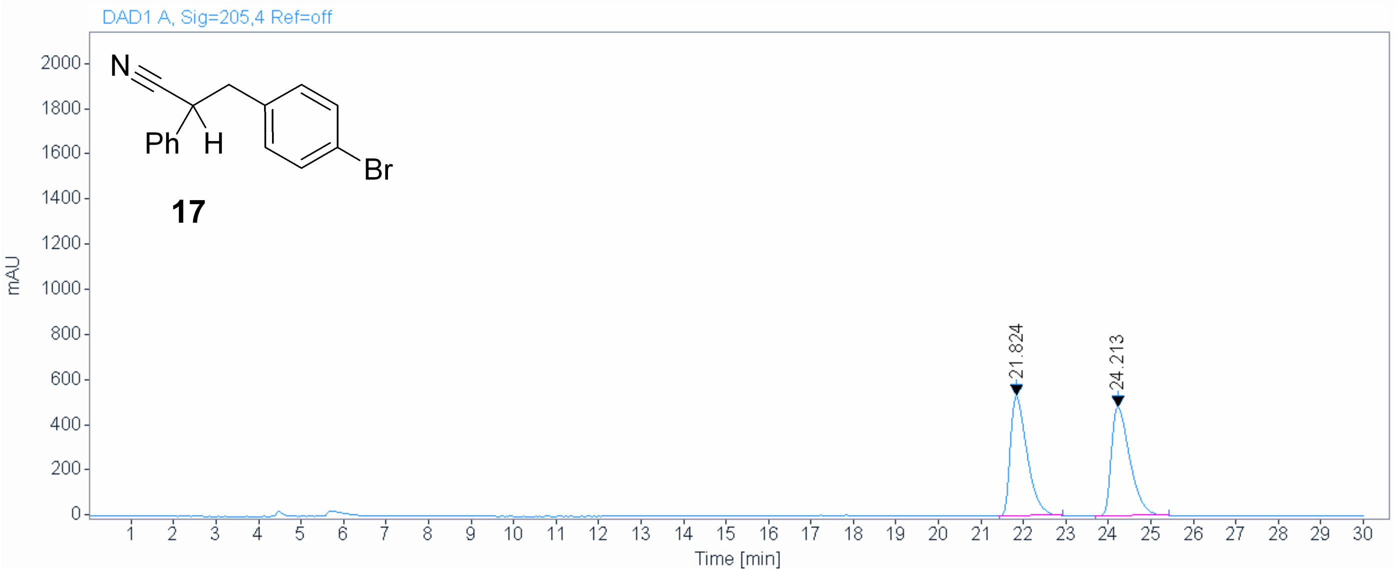

Signal: DAD1 A, Sig=205,4 Ref=off

RT [min]	Width [min]	Height	Area	Area%
21.824	0.3912	532.8704	14608.4473	49.9827
24.213	0.3754	482.2840	14618.5469	50.0173
		Sum	29226.9941	100.0000

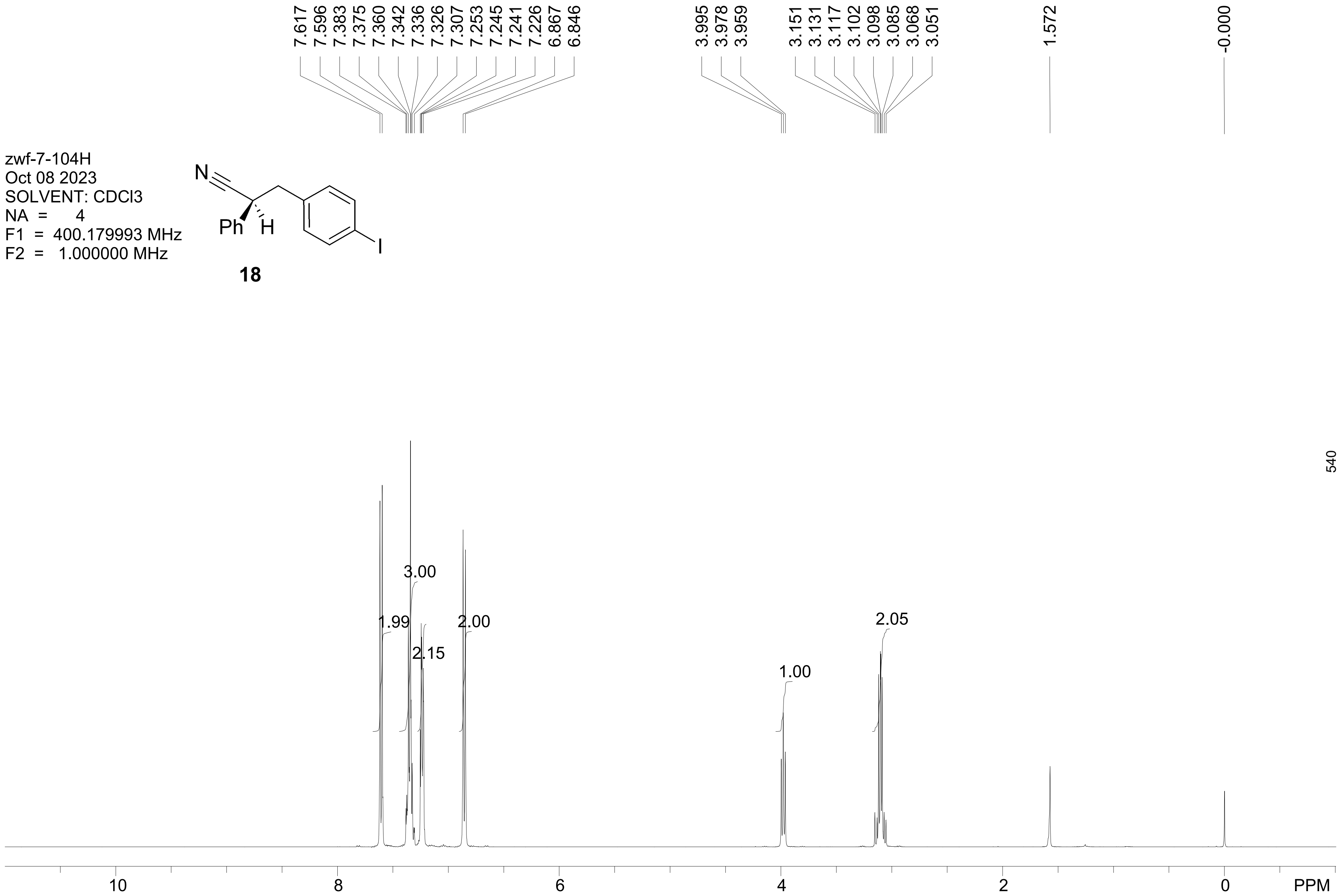
zwf-7-104H
Oct 08 2023
SOLVENT: CDCl3
NA = 4
F1 = 400.179993 MHz
F2 = 1.000000 MHz
N
Ph
H
I
18
7.617
7.596
7.383
7.375
7.360
7.342
7.336
7.326
7.307
7.253
7.245
7.241
7.226
6.867
6.846
3.995
3.978
3.959
3.151
3.131
3.117
3.102
3.098
3.085
3.068
3.051
1.572
-0.000
1.99
3.00
2.15
2.00
1.00
2.05
10
8
6
4
2
0
PPM

zwf-7-104C
Oct 08 2023
SOLVENT: CDCl3
NA = 100
F1 = 100.625343 MHz
F2 = 1.000000 MHz

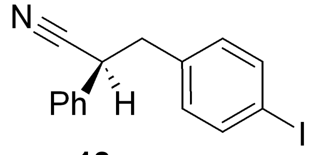

18

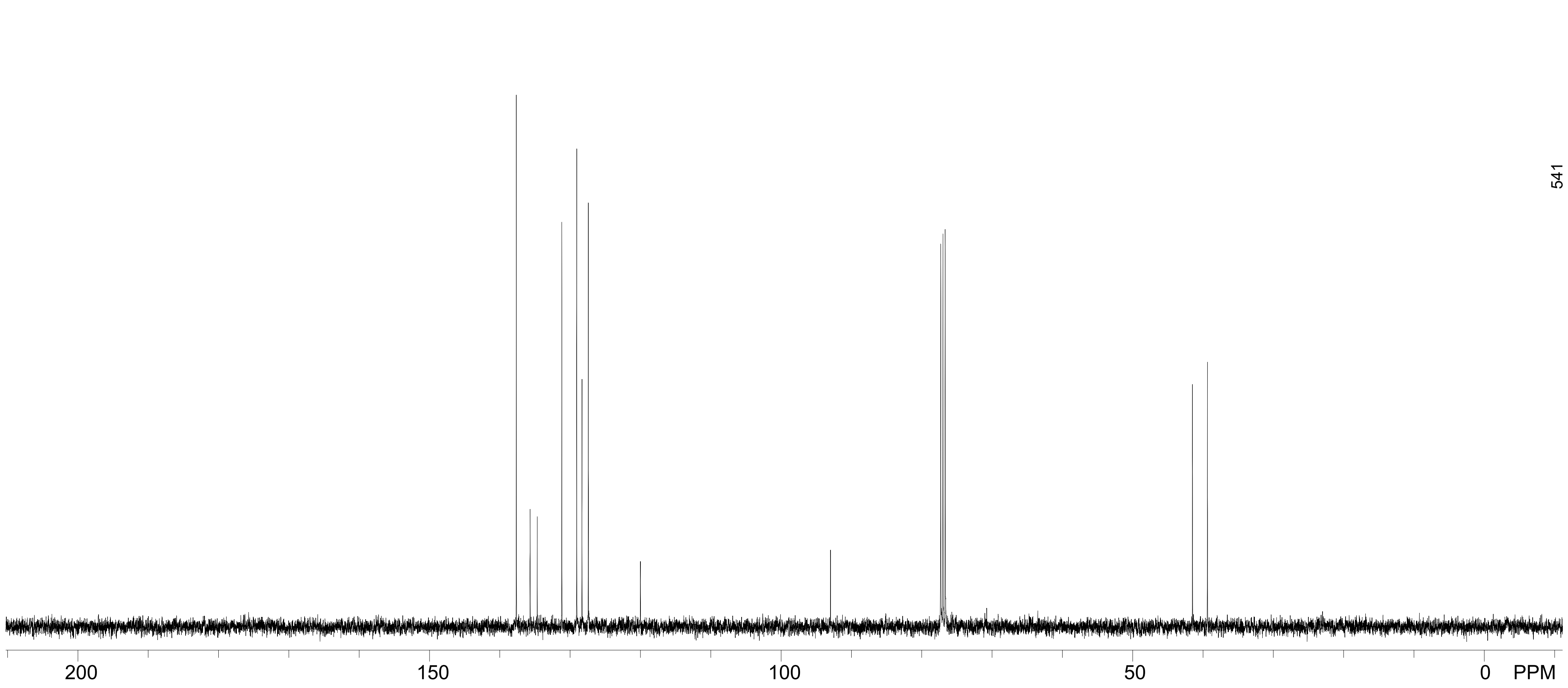

Area Percent Report

Agilent Technologies

Sample name: zwf-7-104-IE-99-1-0.8-205

Data file: C:\Users\Public\Documents\ChemStation\1\Data\zwf\zwf-S 2023-10-09 16-10-18\002-P1-C1-zwf-7-104.D

Injection Acquired Date 10/09/2023 16:38:08

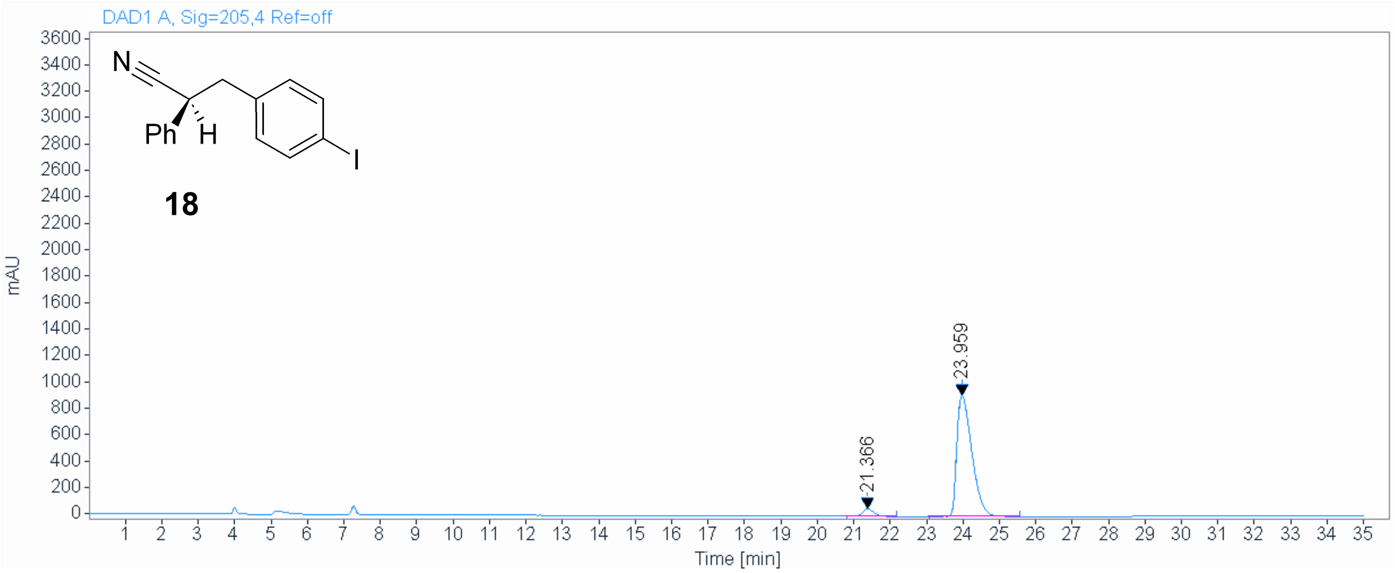

Signal: DAD1 A, Sig=205,4 Ref=off

RT [min]	Width [min]	Height	Area	Area%
21.366	0.3247	57.6275	1247.9913	4.5322
23.959	0.4308	915.9709	26288.3066	95.4678
		Sum	27536.2980	100.0000

Area Percent Report

Agilent Technologies

Sample name: zwf-7-104-rac-IE-99-1-0.8-205

Data file: C:\Users\Public\Documents\ChemStation\1\Data\zwf\zwf-S 2023-10-09 16-10-18\003-P1-C2-zwf-7-104-rac.D

Injection Acquired Date 10/09/2023 17:14:10

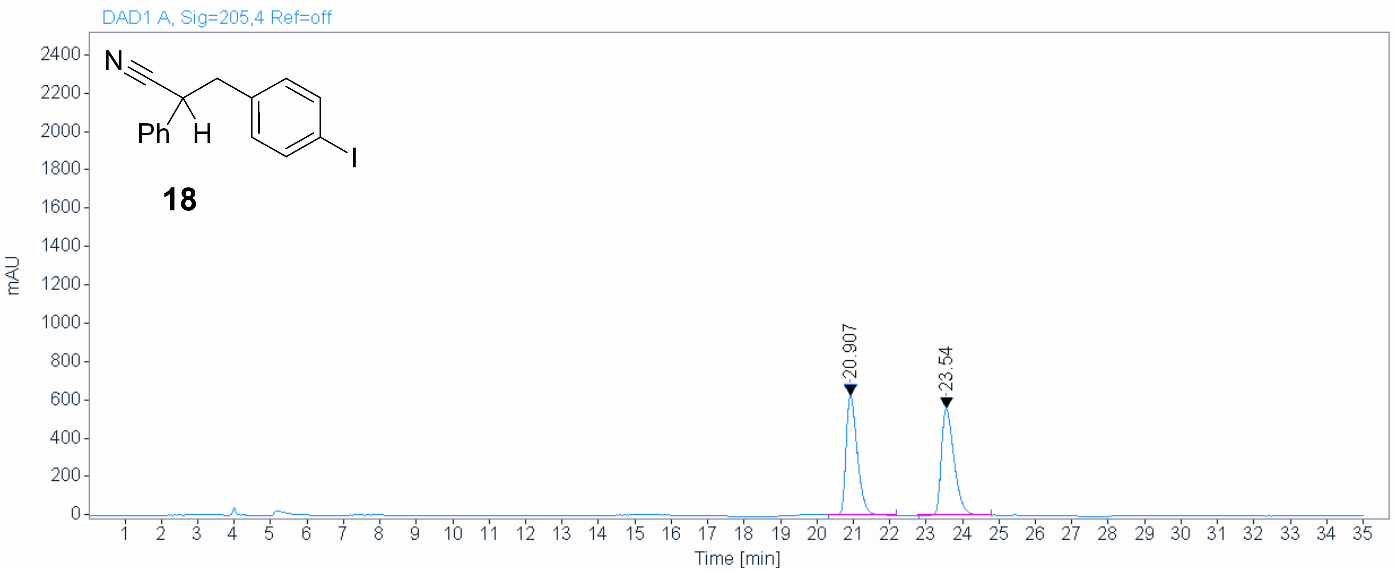

Signal: DAD1 A, Sig=205,4 Ref=off

RT [min]	Width [min]	Height	Area	Area%
20.907	0.3299	624.3407	13374.6377	49.7104
23.540	0.3787	558.4077	13530.4854	50.2896
		Sum	26905.1230	100.0000

zwf-8-026H
Nov 15 2023
SOLVENT: CDCl3
NA = 4
F1 = 400.140015 MHz
F2 = 1.000000 MHz

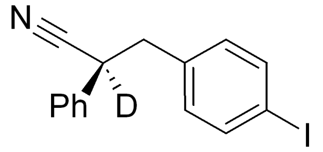

18-d

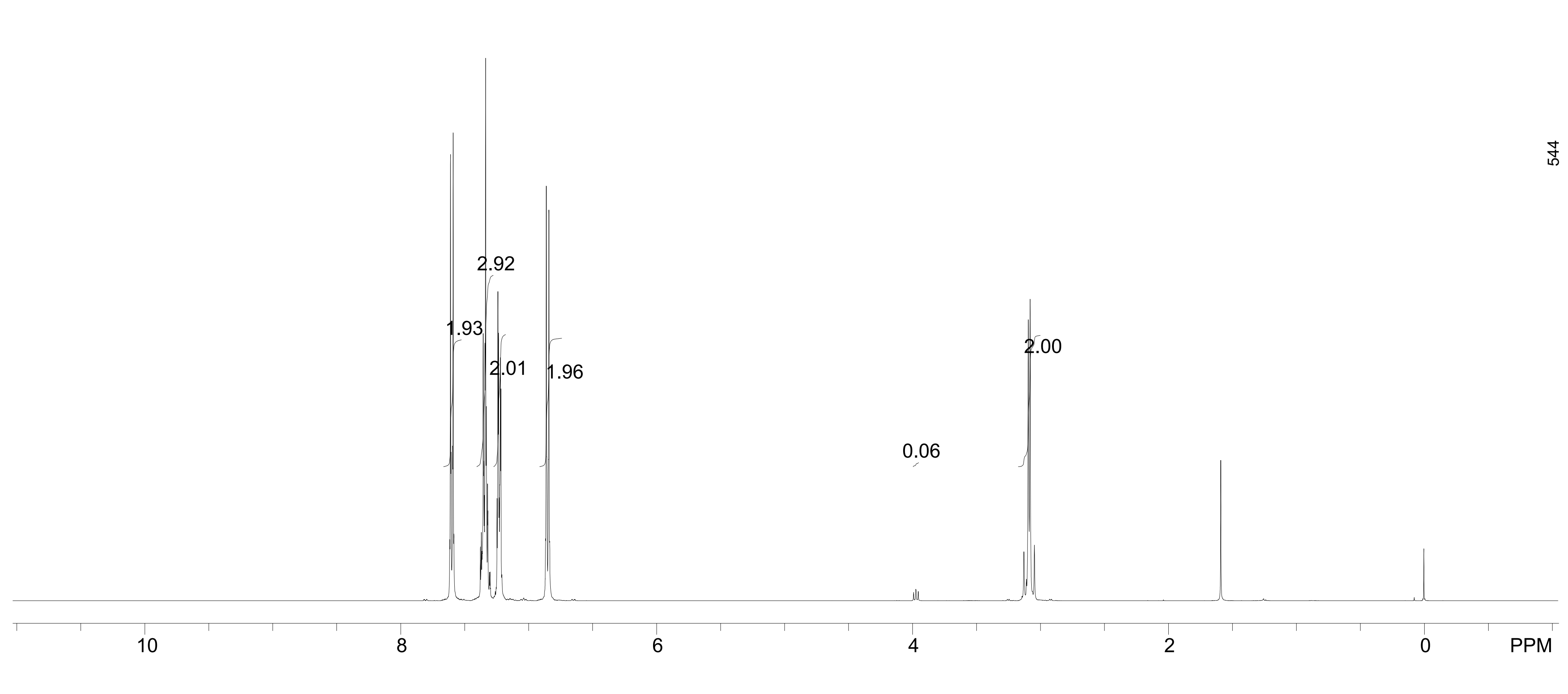

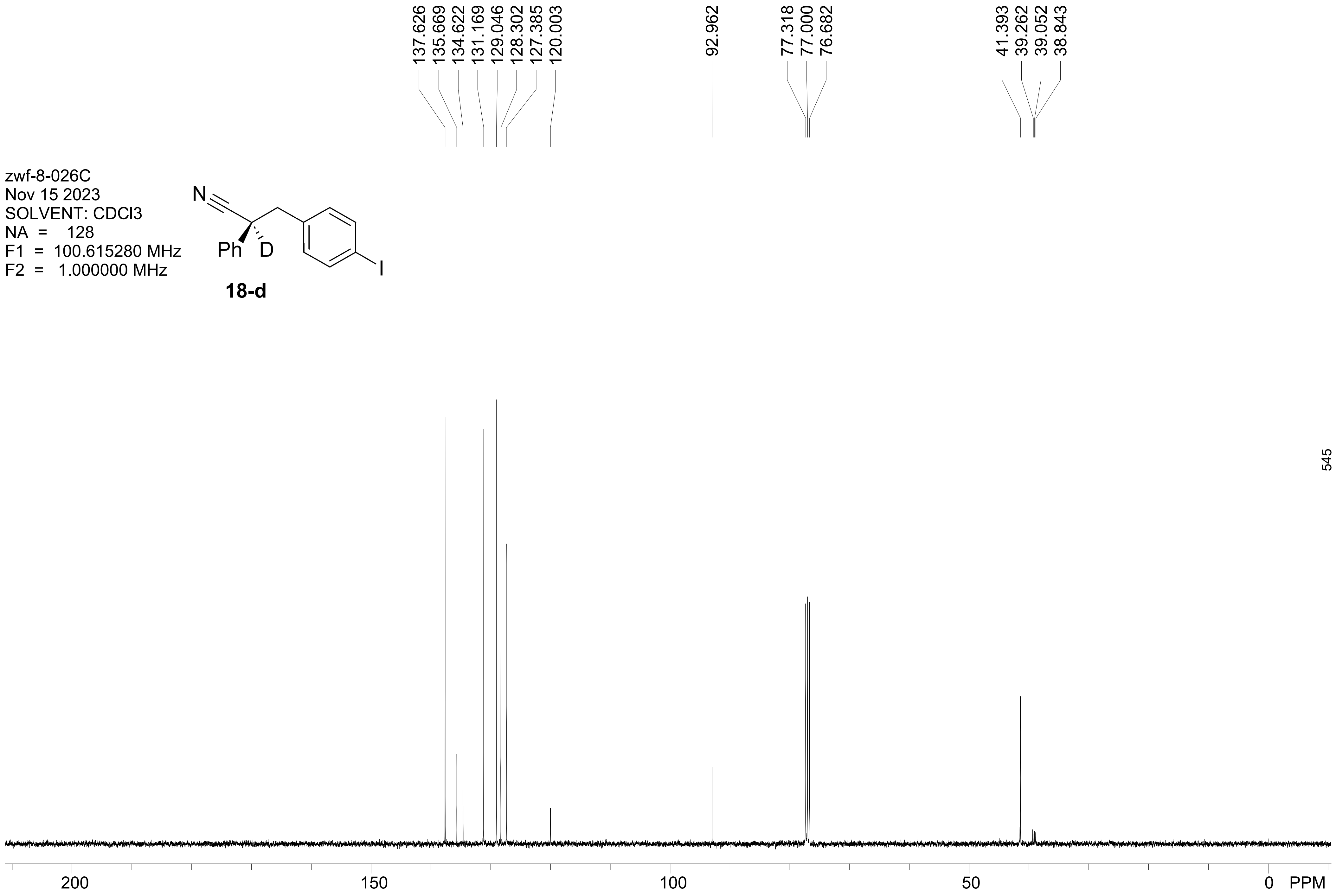

zwf-8-026C
Nov 15 2023
SOLVENT: CDCl3
NA = 128
F1 = 100.615280 MHz
F2 = 1.000000 MHz
N
Ph
D
I
18-d
137.626
135.669
134.622
131.169
129.046
128.302
127.385
120.003
92.962
77.318
77.000
76.682
41.393
39.262
39.052
38.843
200
150
100
50
0 PPM

Area Percent Report

Agilent Technologies

Sample name: zwf-8-026-IE-99-1-0.8-205

Data file: C:\Users\Public\Documents\ChemStation\1\Data\zwf\zwf-S 2023-11-17 07-38-48\002-P1-C1-zwf-8-026.D

Injection Acquired Date 11/17/2023 08:01:36

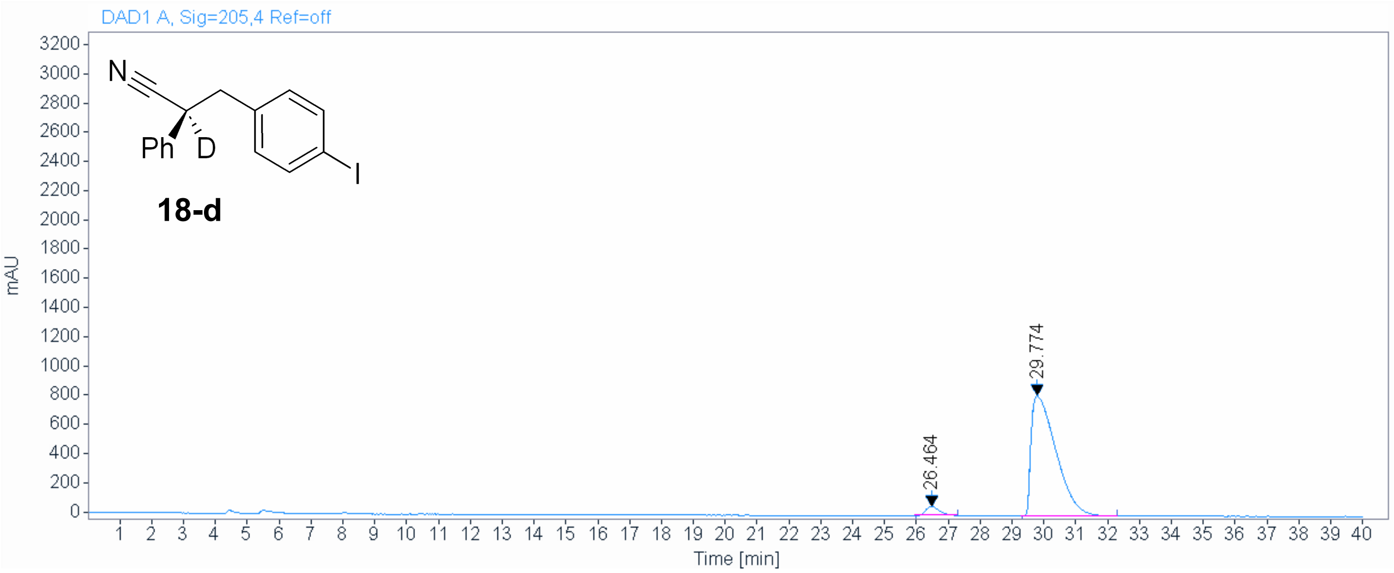

Signal: DAD1 A, Sig=205,4 Ref=off

RT [min]	Width [min]	Height	Area	Area%
26.464	0.4916	59.6212	1758.5437	3.7871
29.774	0.9016	825.8864	44676.4961	96.2129
		Sum	46435.0398	100.0000

Area Percent Report

Sample name: zwf-8-026-rac-IE-99-1-0.8-205

Data file: C:\Users\Public\Documents\ChemStation\1\Data\zwf\zwf-S 2023-11-17 07-38-48\003-P1-C2-zwf-8-026-rac.D

Injection Acquired Date 11/17/2023 08:42:36

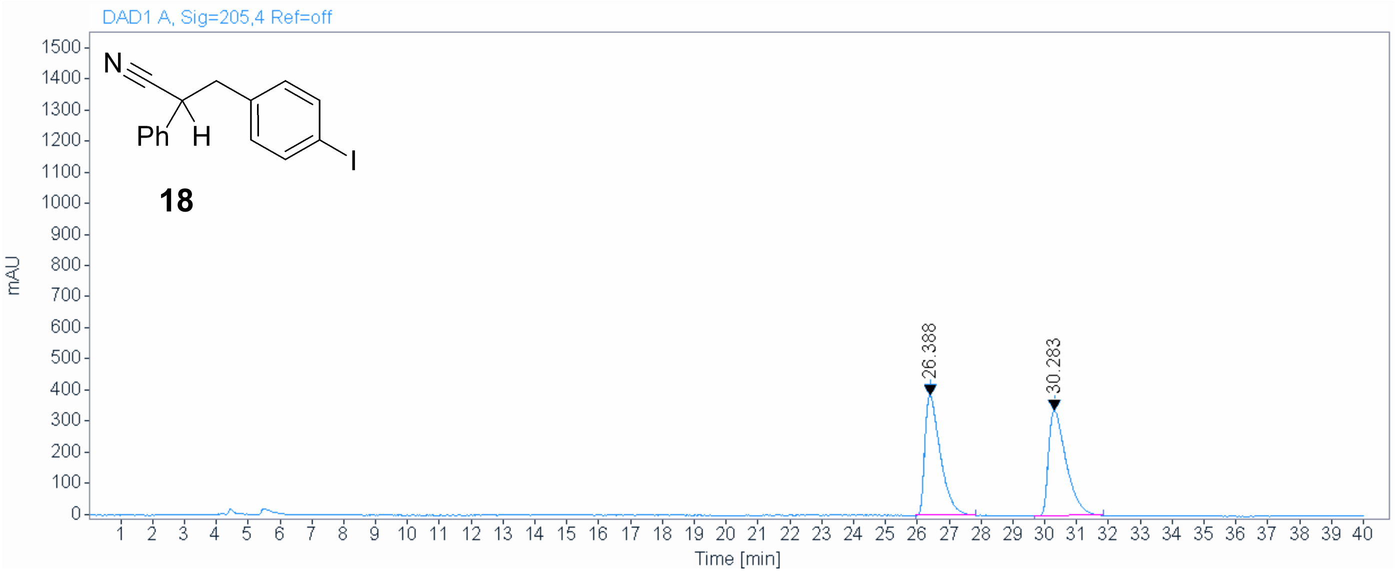

Signal: DAD1 A, Sig=205,4 Ref=off

RT [min]	Width [min]	Height	Area	Area%
26.388	0.4877	386.1003	13167.2549	50.0255
30.283	0.5220	338.8327	13153.8545	49.9745
		Sum	26321.1094	100.0000

zwf-7-120H
Oct 16 2203
SOLVENT: CDCl3
NA = 4
F1 = 400.140015 MHz
F2 = 1.000000 MHz

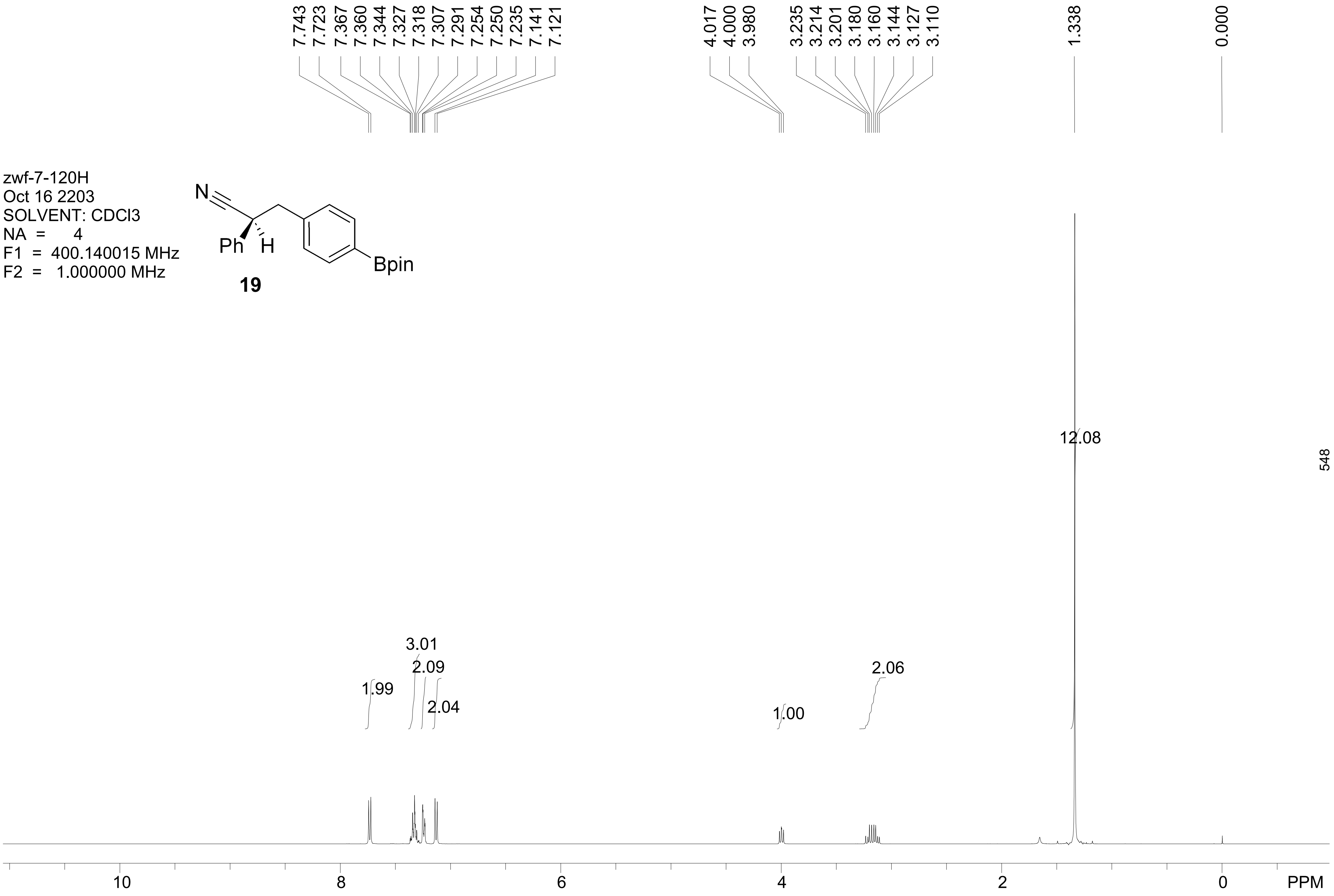

zwf-7-120C
Oct 16 2023
SOLVENT: CDCl3
NA = 100
F1 = 100.615280 MHz
F2 = 1.000000 MHz

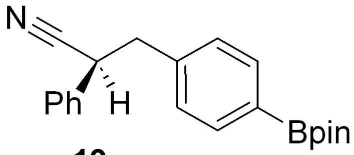

19

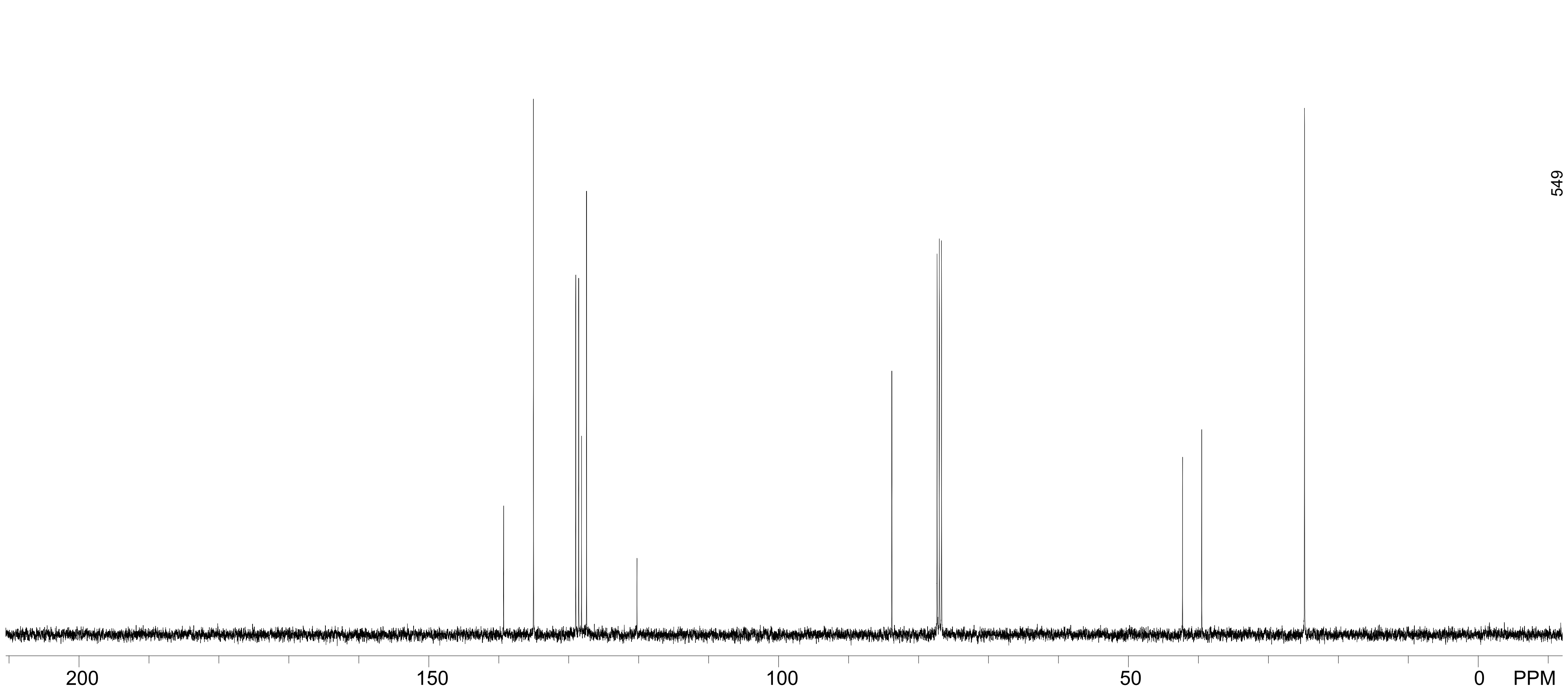

zwf-7-120B
Oct 16 2023
SOLVENT: CDCl3
NA = 256
F1 = 128.380814 MHz
F2 = 1.000000 MHz

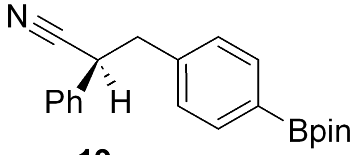

19

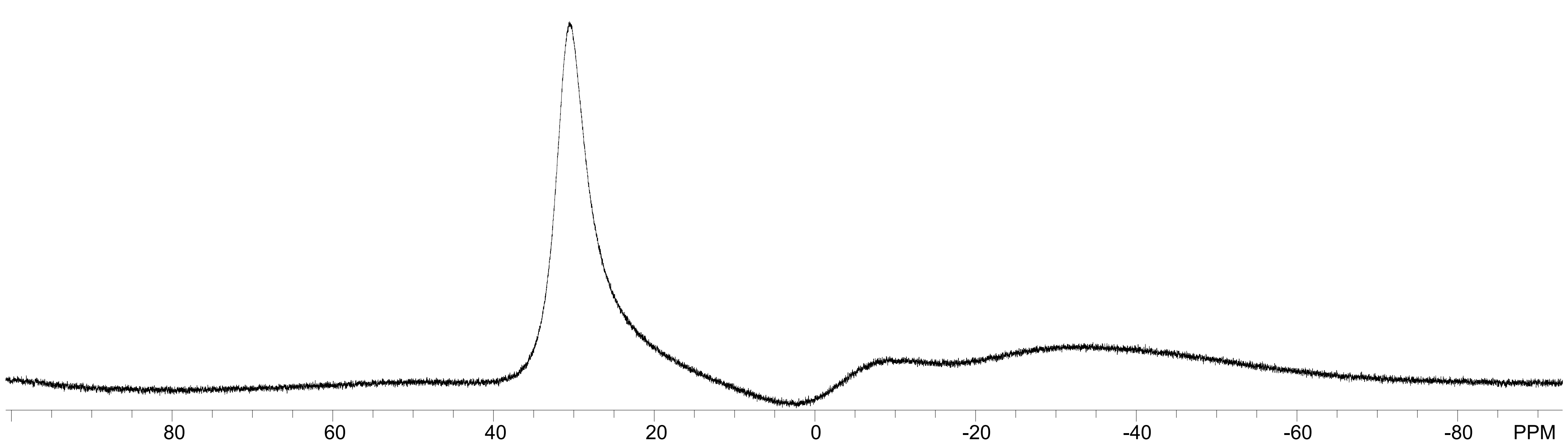

Area Percent Report

Agilent Technologies

Sample name: zwf-7-120-IE-99-1-0.8-205

Data file: C:\Users\Public\Documents\ChemStation\1\Data\zwf\zwf-S 2023-10-17 08-55-39\004-P1-C7-zwf-7-120.D

Injection Acquired Date 10/17/2023 10:30:58

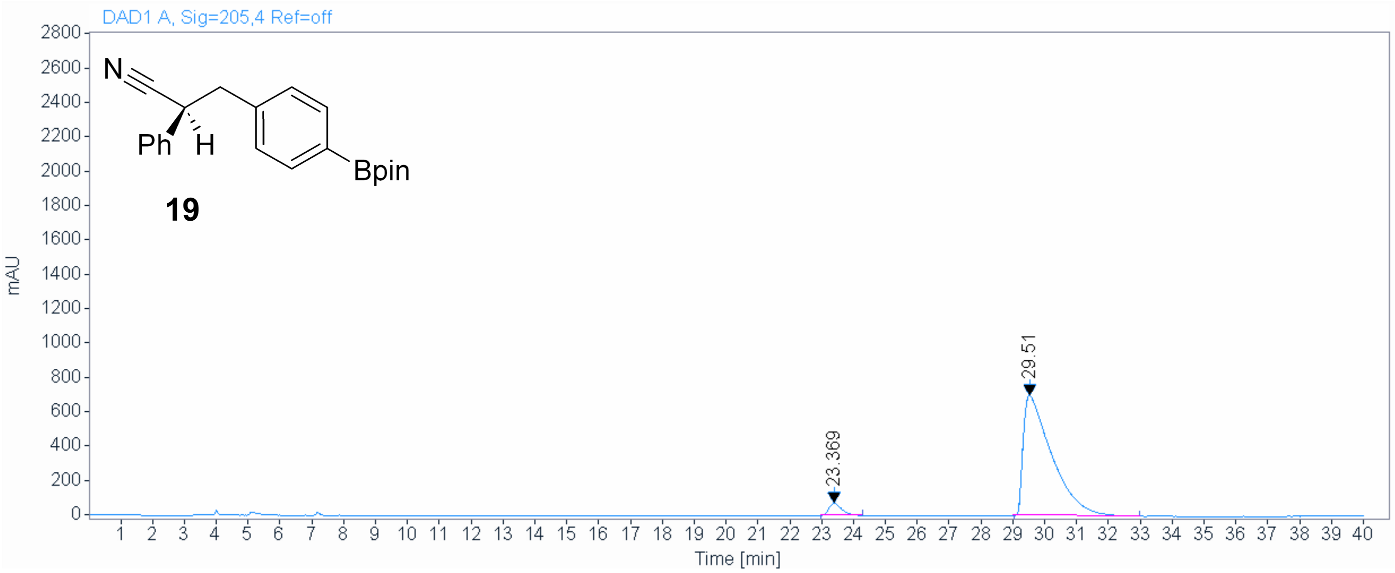

Signal: DAD1 A, Sig=205,4 Ref=off

RT [min]	Width [min]	Height	Area	Area%
23.369	0.4662	71.6346	2003.6951	4.3737
29.510	0.8287	699.4676	43809.1055	95.6263
		Sum	45812.8005	100.0000

Area Percent Report

Sample name: zwf-7-120-rac-IE-99-1-0.8-205

Data file: C:\Users\Public\Documents\ChemStation\1\Data\zwf\zwf-S 2023-10-16 21-38-05\005-P1-B4-zwf-7-120-rac.D

Injection Acquired Date 10/16/2023 23:59:57

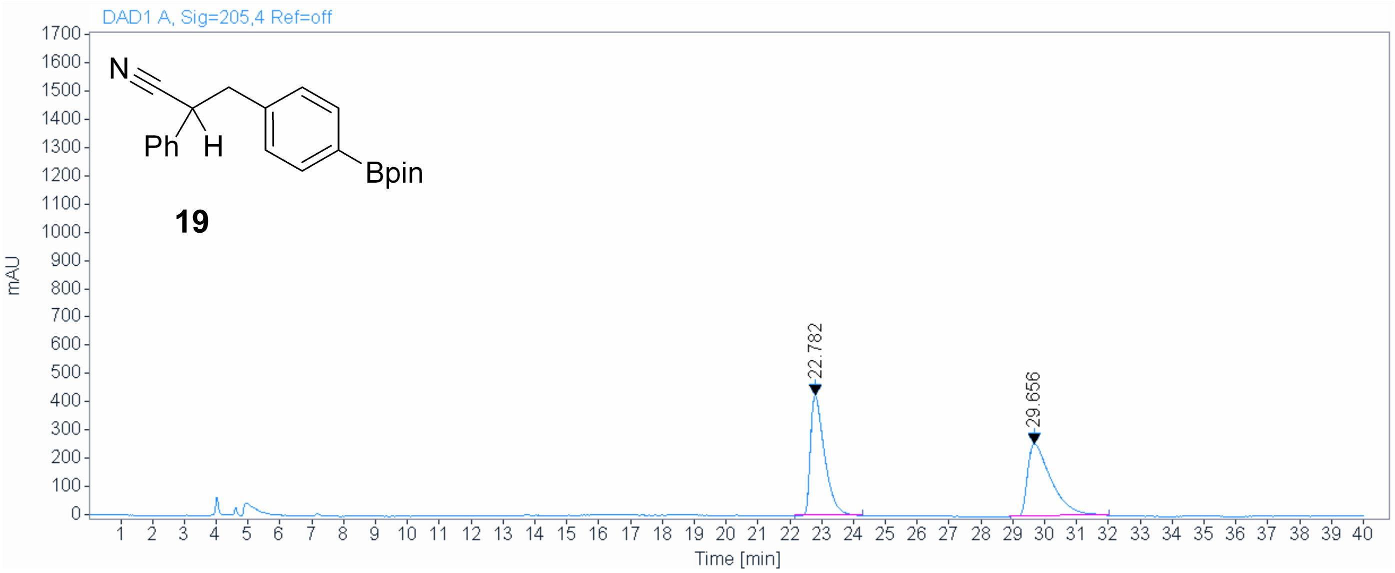

Signal: DAD1 A, Sig=205,4 Ref=off

RT [min]	Width [min]	Height	Area	Area%
22.782	0.4358	425.9754	13153.1904	49.6407
29.656	0.6816	255.6593	13343.5928	50.3593
		Sum	26496.7832	100.0000

zwf-8-031H
Nov 13 2023
SOLVENT: CDCl3
NA = 4
F1 = 400.179993 MHz
F2 = 1.000000 MHz

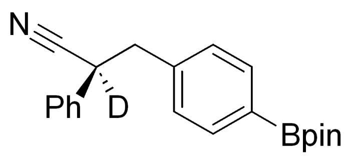

19-d

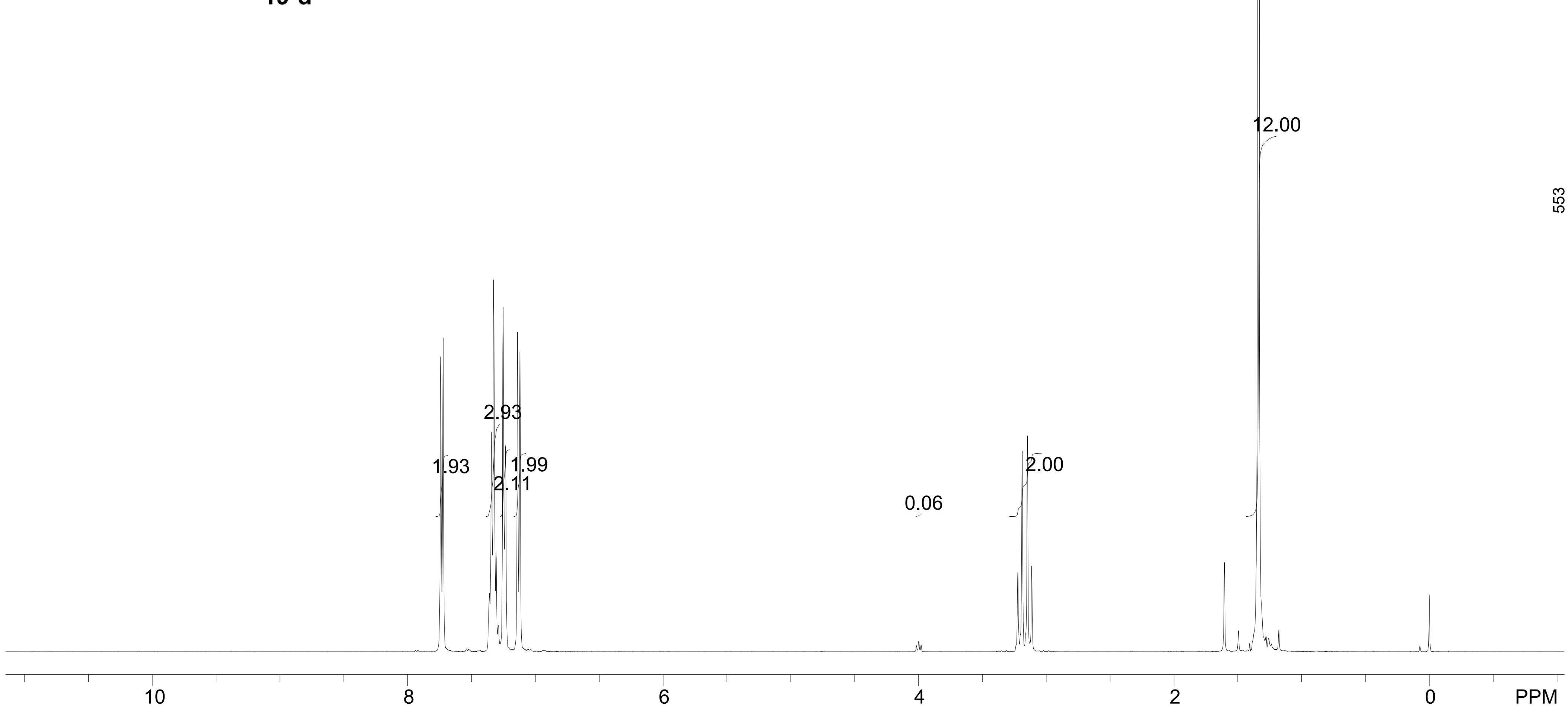

zwf-8-031C
Nov 13 2023
SOLVENT: CDCl3
NA = 150
F1 = 100.625343 MHz
F2 = 1.000000 MHz

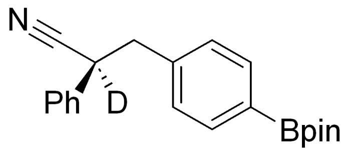

19-d

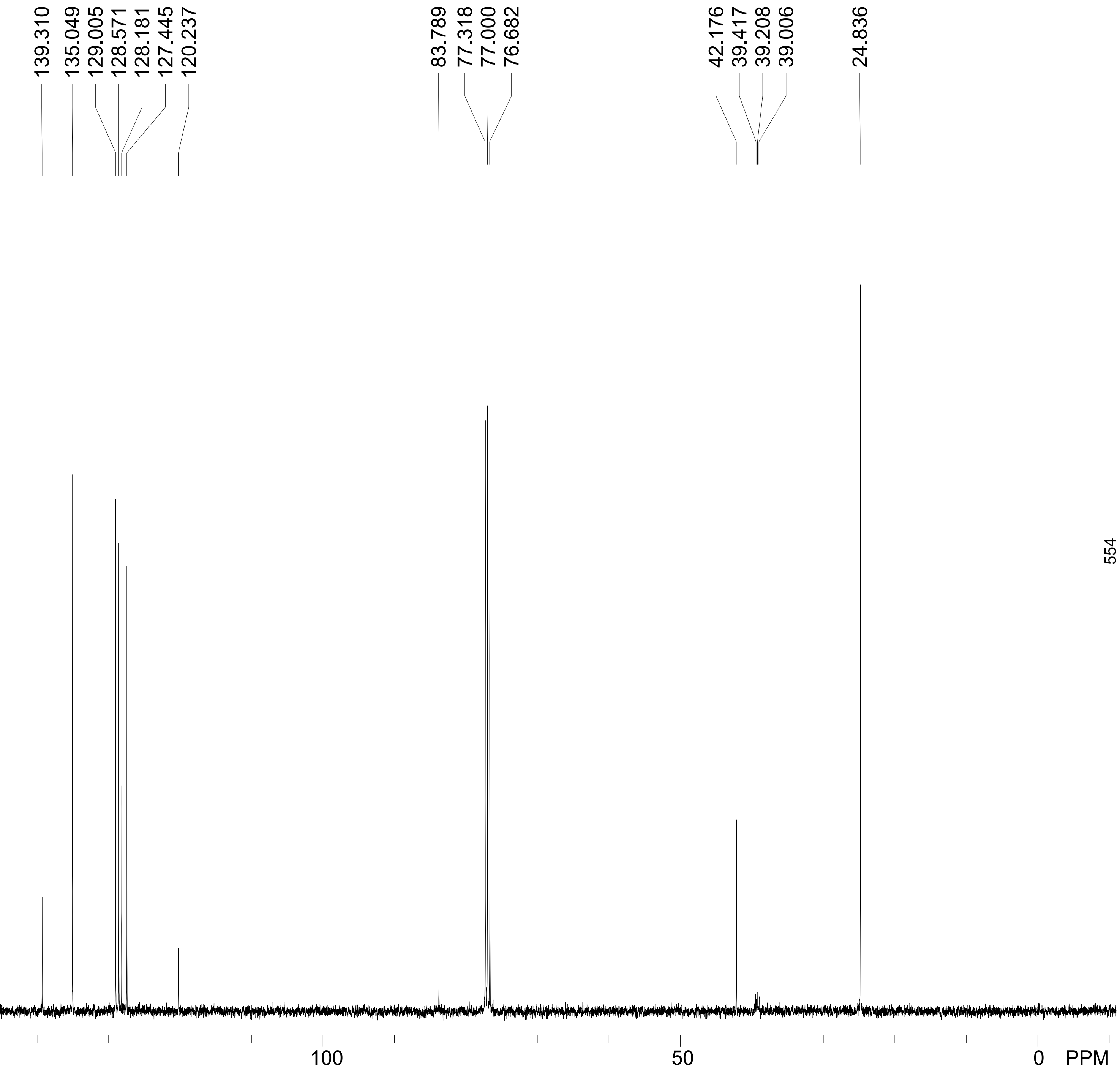

zwf-8-031B
Nov 13 2023
SOLVENT: CDCl3
NA = 256
F1 = 128.393646 MHz
F2 = 1.000000 MHz

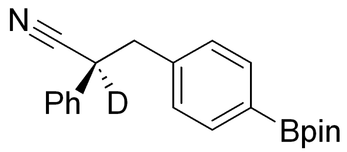

19-d

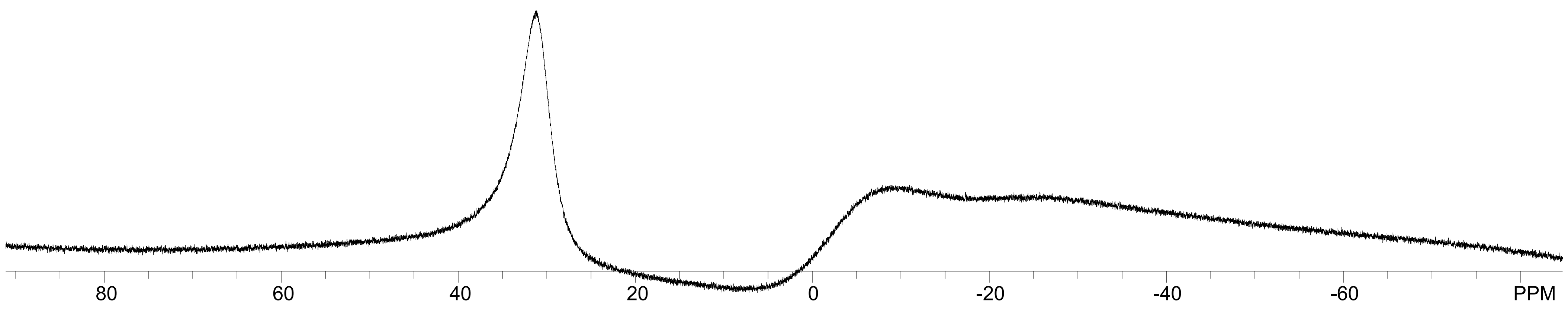

Area Percent Report

Sample name: zwf-8-031-IE-99-1-0.8-205

Data file: C:\Users\Public\Documents\ChemStation\1\Data\zwf\zwf-S 2023-11-15 14-21-28\006-P1-C1-zwf-8-031.D

Injection Acquired Date 11/15/2023 16:22:43

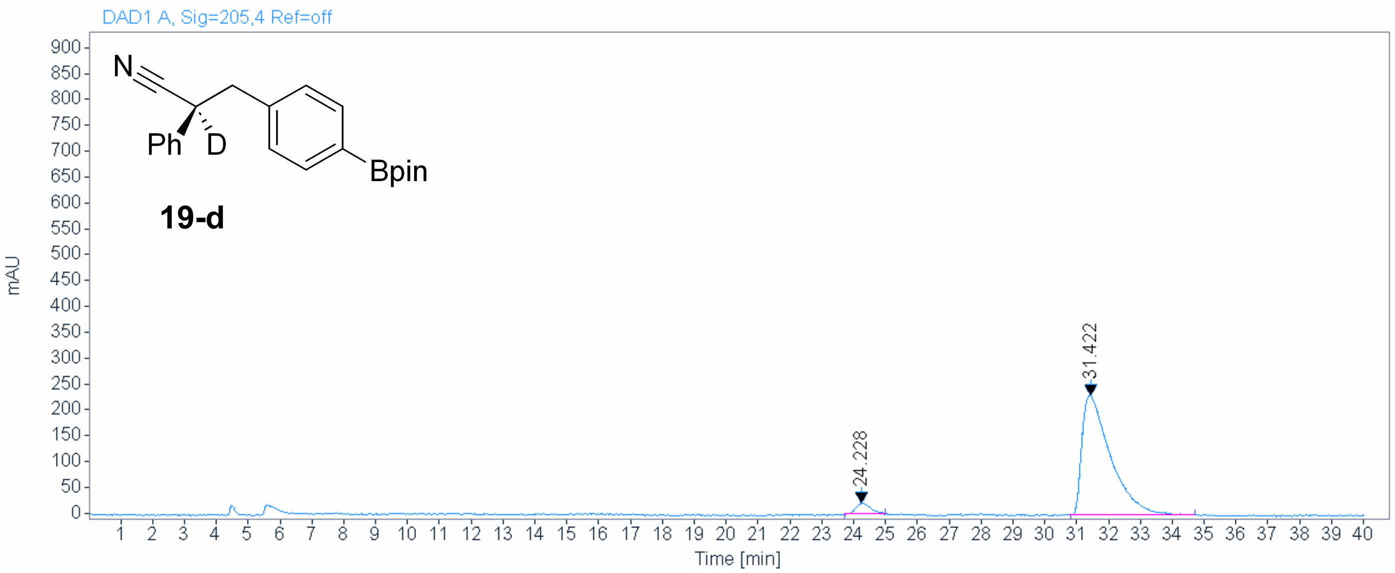

Signal: DAD1 A, Sig=205,4 Ref=off

RT [min]	Width [min]	Height	Area	Area%
24.228	0.5318	21.3667	681.7698	4.4817
31.422	1.0460	231.5218	14530.5762	95.5183
		Sum	15212.3460	100.0000

Area Percent Report

Agilent Technologies

Sample name: zwf-8-031-rac-IE-99-1-0.8-205

Data file: C:\Users\Public\Documents\ChemStation\1\Data\zwf\zwf-S 2023-11-15 14-21-28\007-P1-C2-zwf-8-031-rac.D

Injection Acquired Date 11/15/2023 17:03:48

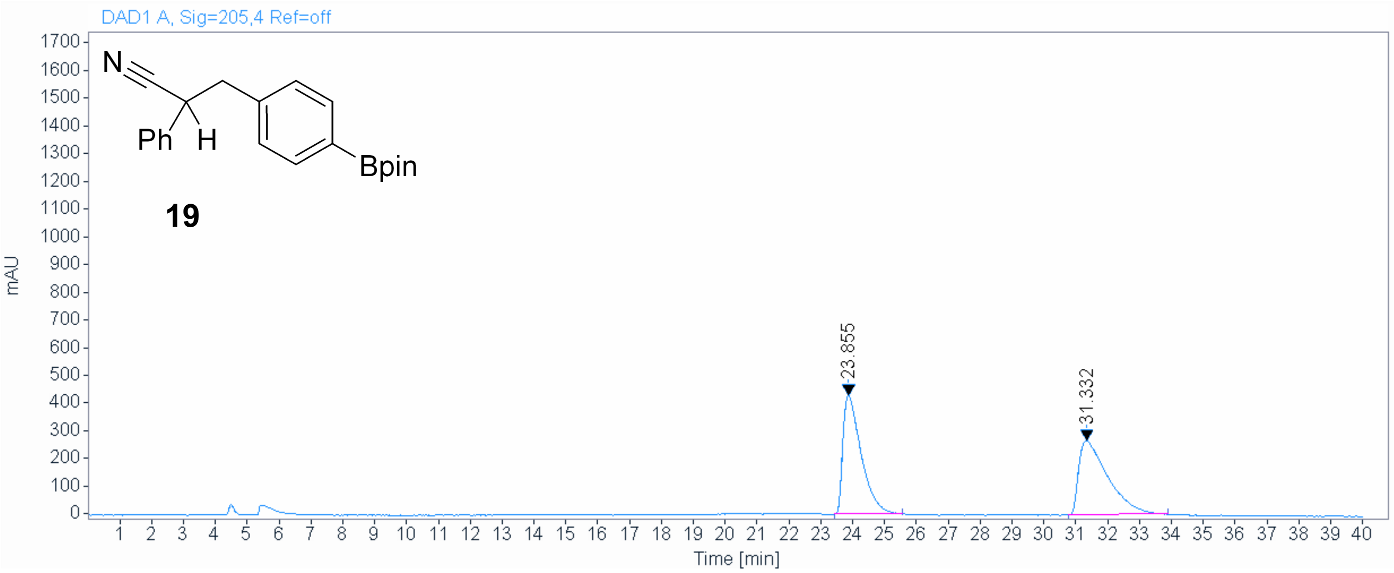

Signal: DAD1 A, Sig=205,4 Ref=off

RT [min]	Width [min]	Height	Area	Area%
23.855	0.4963	428.8006	17253.9063	50.2966
31.332	1.0624	267.4932	17050.4043	49.7034
		Sum	34304.3105	100.0000

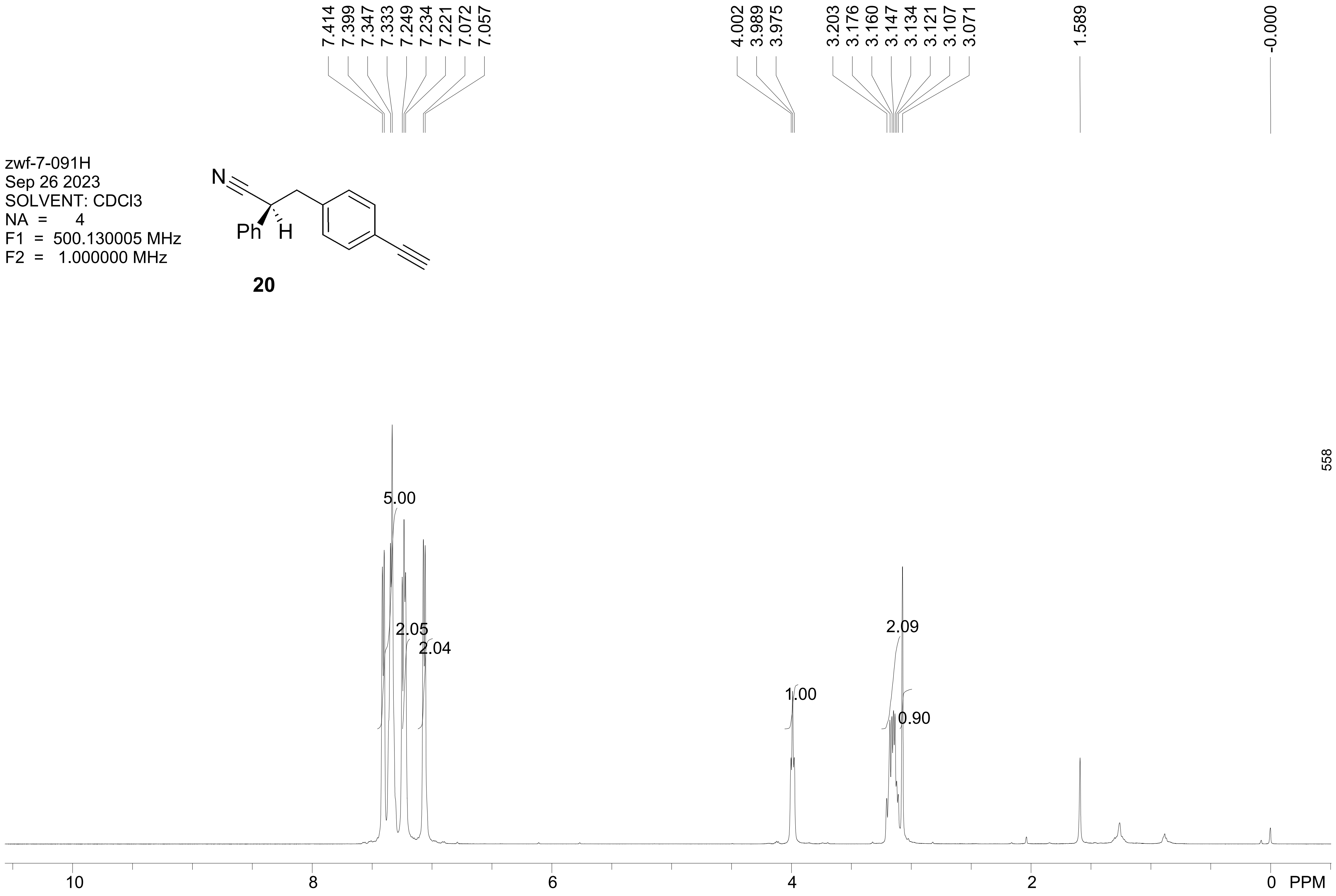
zwf-7-091H
Sep 26 2023
SOLVENT: CDCl3
NA = 4
F1 = 500.130005 MHz
F2 = 1.000000 MHz
N
Ph
H
20
7.414
7.399
7.347
7.333
7.249
7.234
7.221
7.072
7.057
4.002
3.989
3.975
3.203
3.176
3.160
3.147
3.134
3.121
3.107
3.071
1.589
-0.000
5.00
2.05
2.04
1.00
2.09
0.90
10
8
6
4
2
0 PPM

zwf-7-091C
Sep 26 2023
SOLVENT: CDCl3
NA = 100
F1 = 125.757790 MHz
F2 = 1.000000 MHz

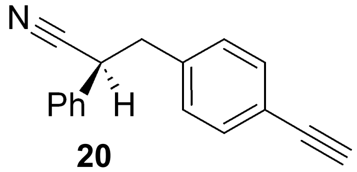

20

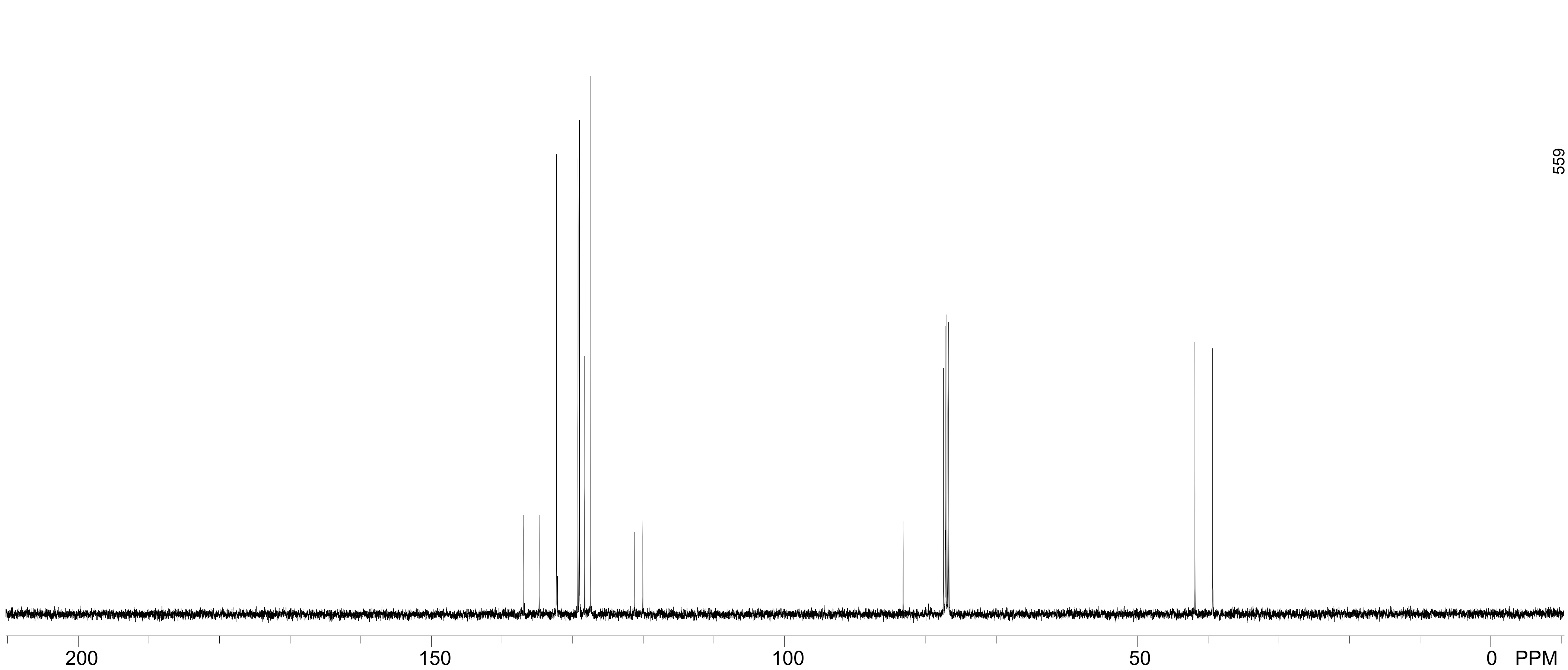

Area Percent Report

Agilent Technologies

Sample name: zwf-7-091-IE-99-1-0.8-205

Data file: C:\Users\Public\Documents\ChemStation\1\Data\zwf\zwf-S 2023-09-26 14-33-49\009-P1-A4-zwf-7-091.D

Injection Acquired Date 09/26/2023 17:51:14

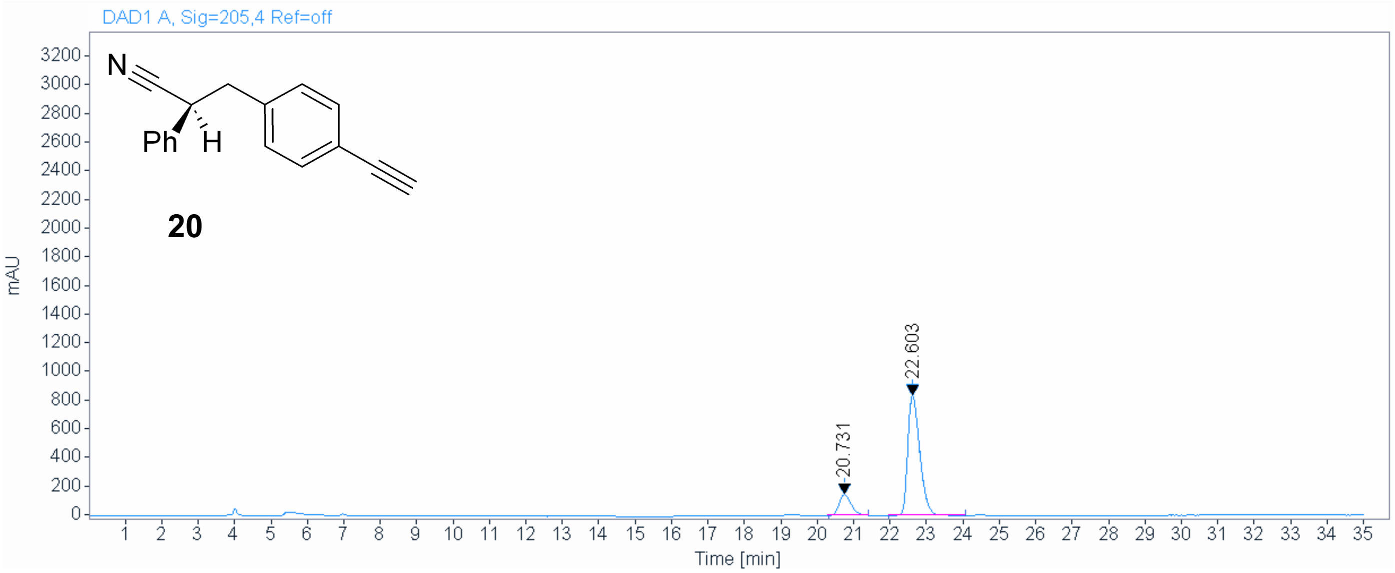

Signal: DAD1 A, Sig=205,4 Ref=off

RT [min]	Width [min]	Height	Area	Area%
20.731	0.3498	147.4239	3358.6411	14.8983
22.603	0.3498	835.2434	19185.1738	85.1017
		Sum	22543.8149	100.0000

Area Percent Report

Agilent Technologies

Sample name: zwf-7-091-rac-IE-99-1-0.8-205

Data file: C:\Users\Public\Documents\ChemStation\1\Data\zwf\zwf-S 2023-09-26 14-33-49\011-P1-A6-zwf-7-091-rac.D

Injection Acquired Date 09/26/2023 18:58:21

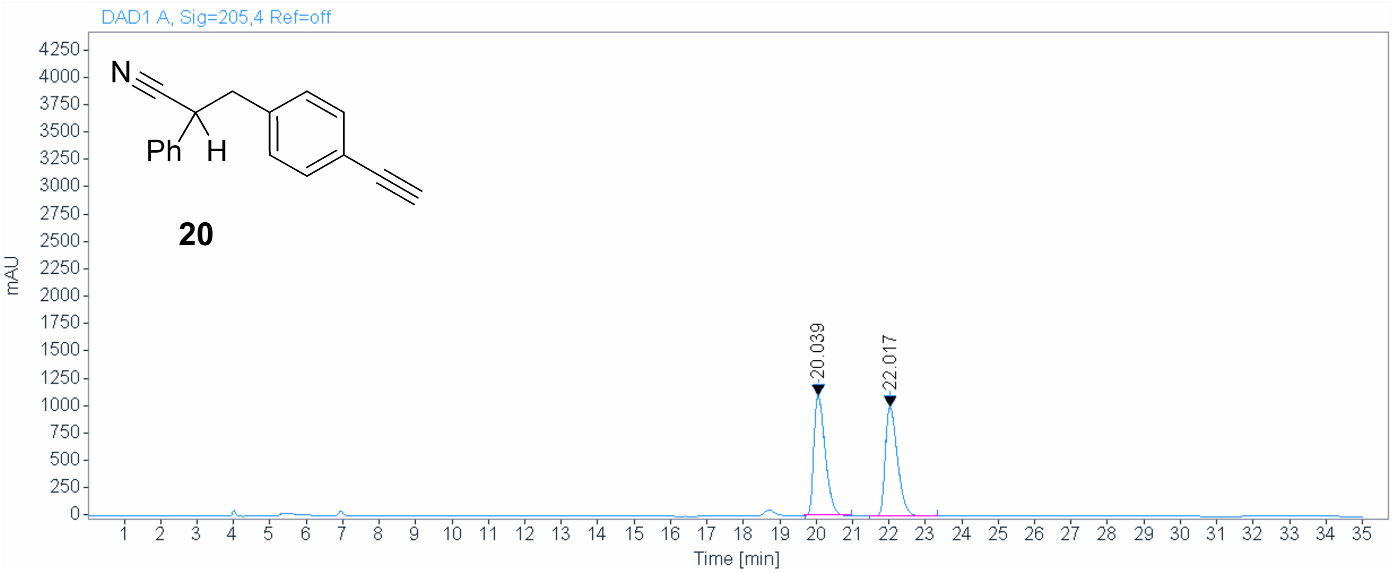

Signal: DAD1 A, Sig=205,4 Ref=off

RT [min]	Width [min]	Height	Area	Area%
20.039	0.3613	1096.4700	23766.0547	50.5629
22.017	0.3886	996.6828	23236.9316	49.4371
		Sum	47002.9863	100.0000

zwf-8-039H
Nov 26 2023
SOLVENT: CDCl3
NA = 4
F1 = 400.179993 MHz
F2 = 1.000000 MHz

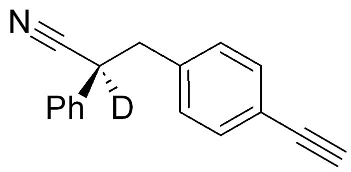

20-d

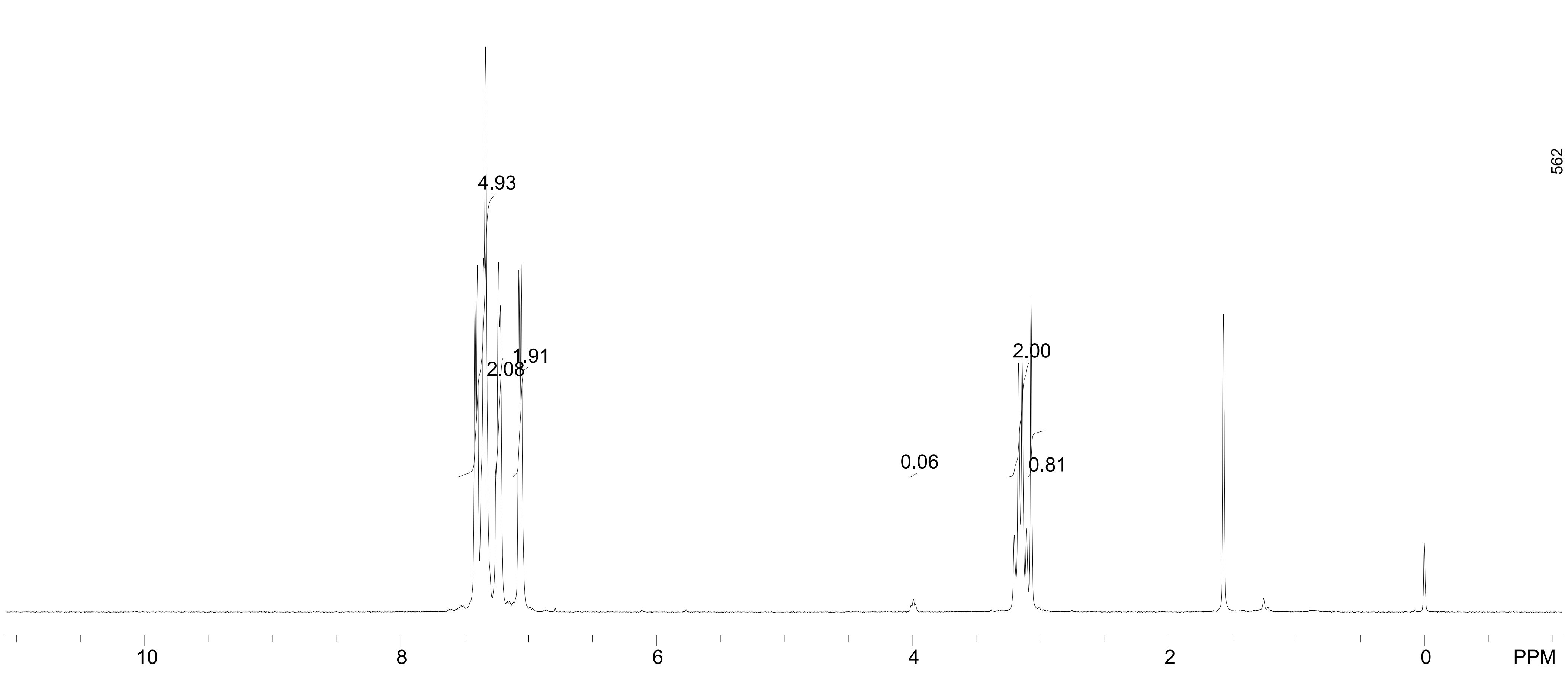

zwf-8-039C
Nov 26 2023
SOLVENT: CDCl3
NA = 300
F1 = 100.625343 MHz
F2 = 1.000000 MHz

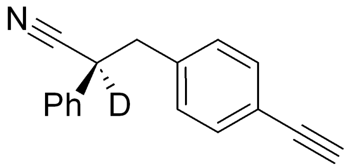

20-d

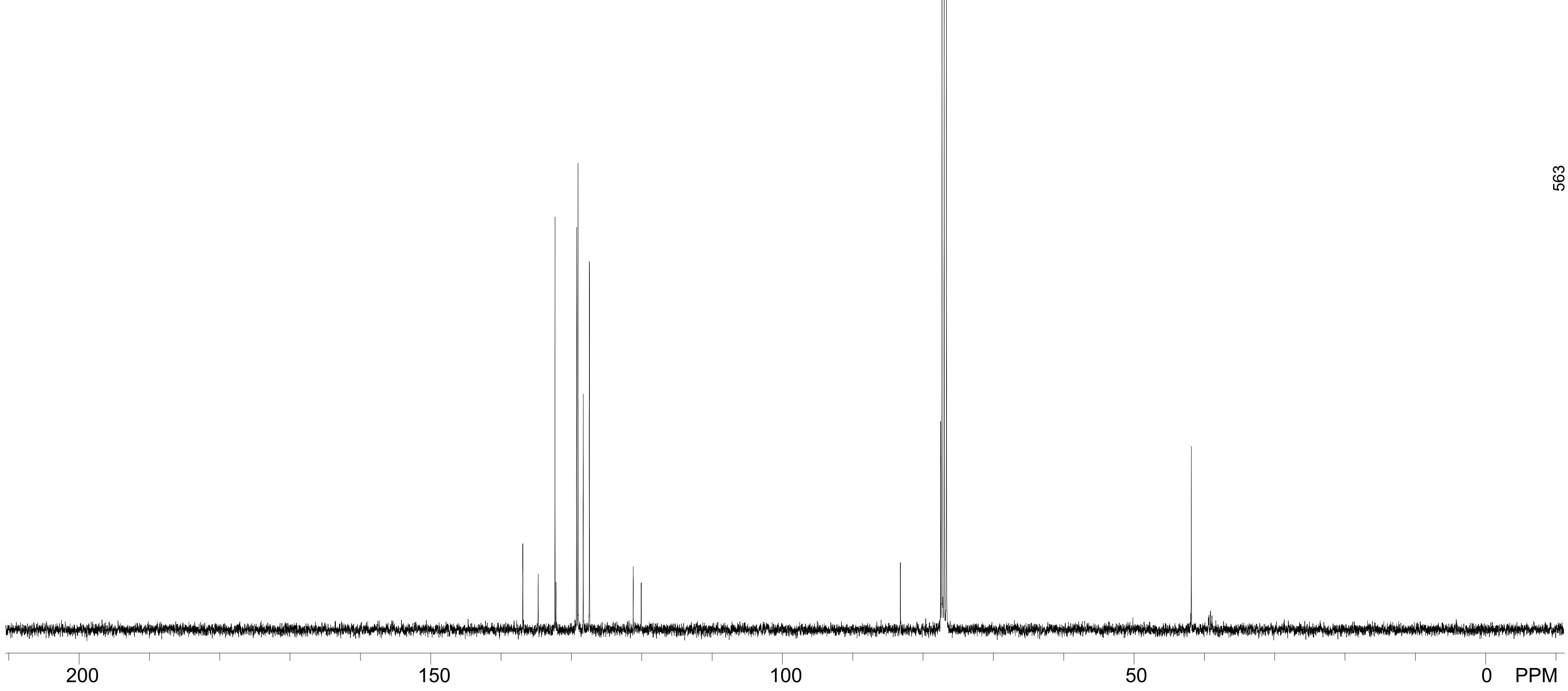

Area Percent Report

Agilent Technologies

Sample name: zwf-8-039-IE-99-1-0.8-205

Data file: C:\Users\Public\Documents\ChemStation\1\Data\zwf\zwf-S 2023-11-26 13-46-16\009-P1-B5-zwf-8-039.D

Injection Acquired Date 11/26/2023 18:01:04

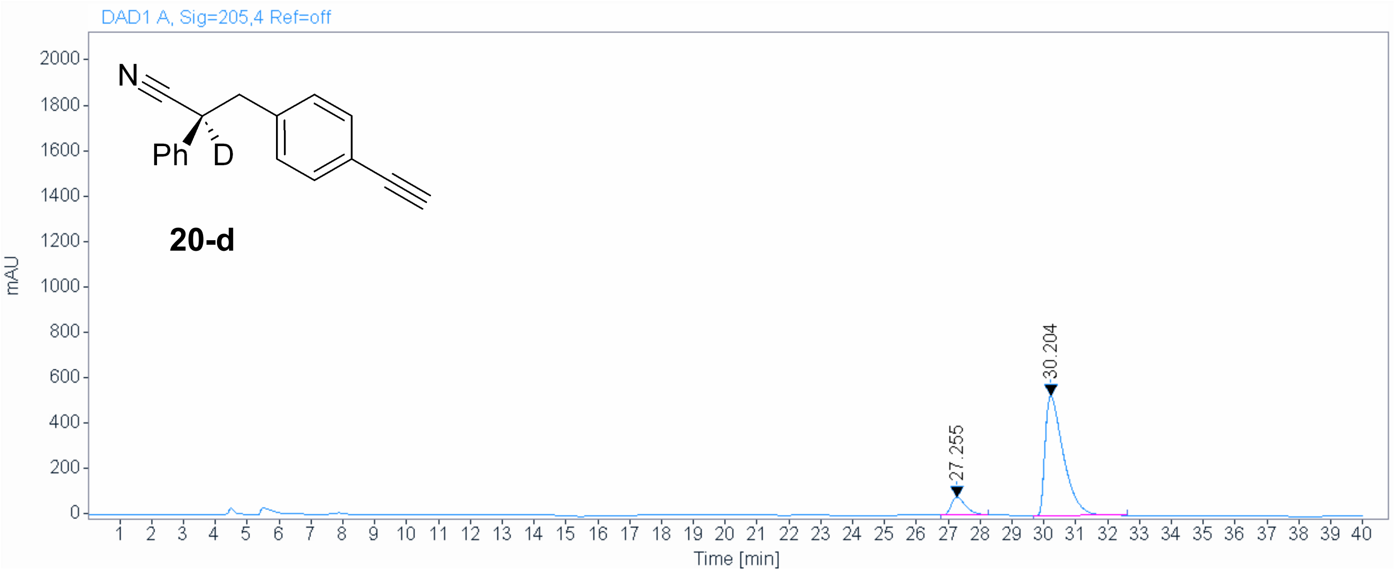

Signal: DAD1 A, Sig=205,4 Ref=off

RT [min]	Width [min]	Height	Area	Area%
27.255	0.5204	76.7181	2395.5830	10.1495
30.204	0.6031	529.7488	21207.4063	89.8505
		Sum	23602.9893	100.0000

Area Percent Report

Sample name: zwf-8-039-rac-IE-99-1-0.8-205

Data file: C:\Users\Public\Documents\ChemStation\1\Data\zwf\zwf-S 2023-11-26 13-46-16\008-P1-B9-zwf-8-039-arc.D

Injection Acquired Date 11/26/2023 17:19:59

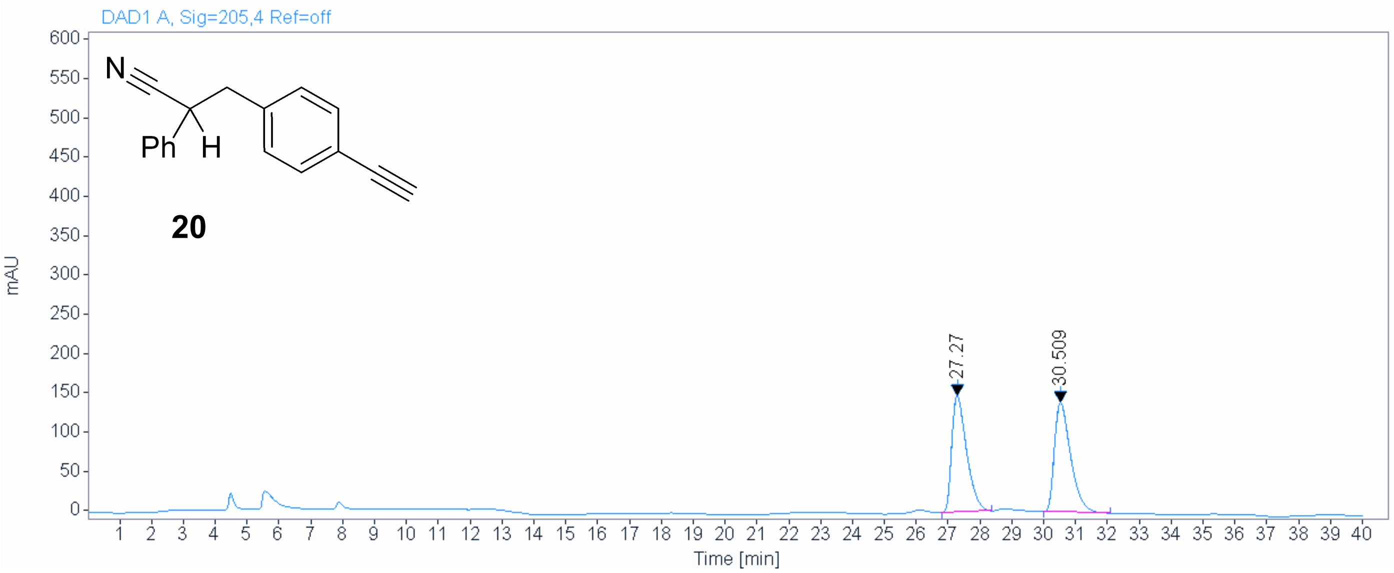

Signal: DAD1 A, Sig=205,4 Ref=off

RT [min]	Width [min]	Height	Area	Area%
27.270	0.4886	148.3653	4814.7417	49.2149
30.509	0.5920	139.8714	4968.3525	50.7851
		Sum	9783.0942	100.0000

zwf-7-066H
Sep 17 2023
SOLVENT: CDCl3
NA = 4
F1 = 400.140015 MHz
F2 = 1.000000 MHz

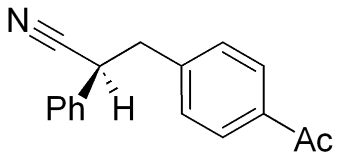

21

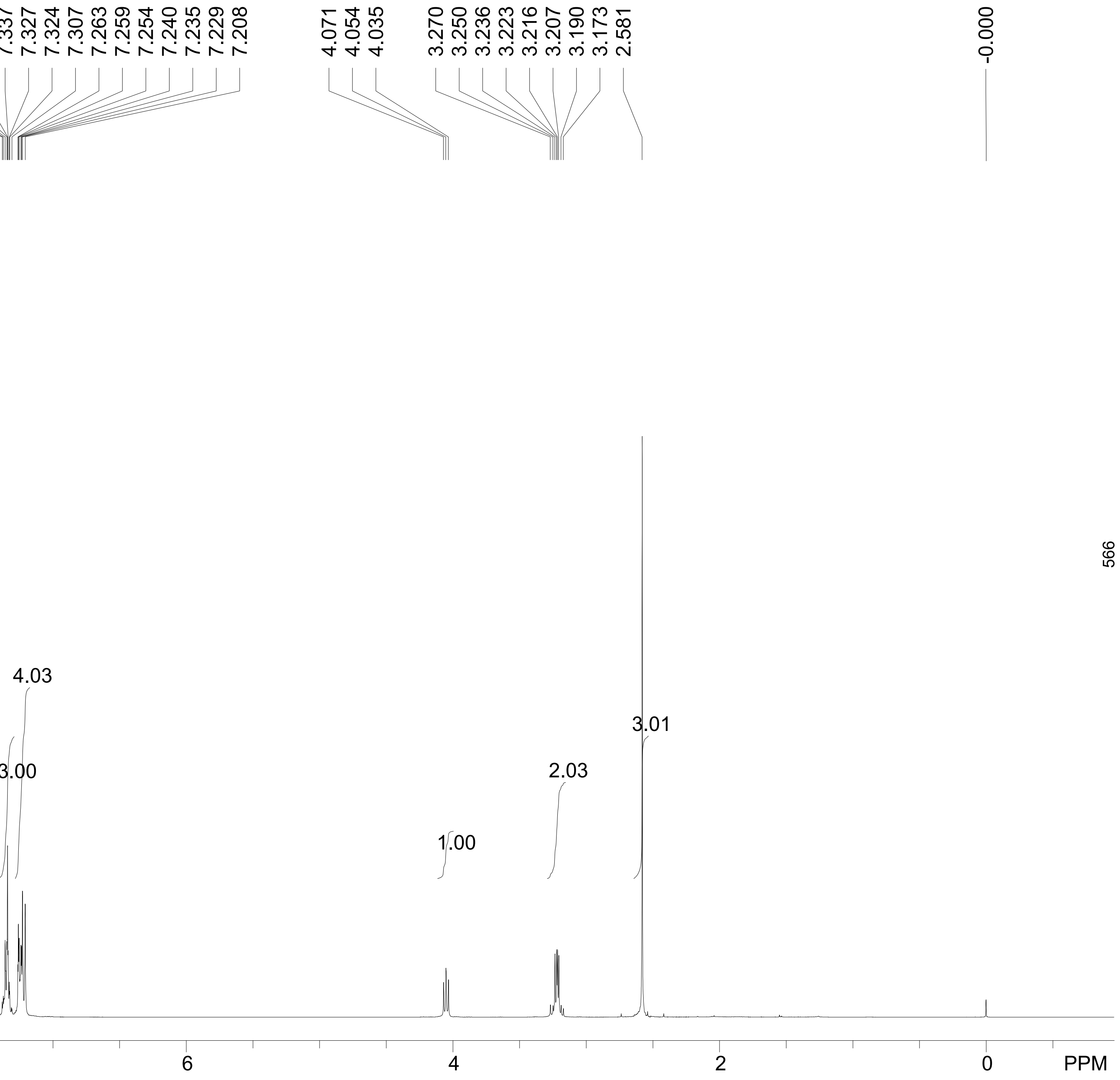

zwf-7-066C
Sep 17 2023
SOLVENT: CDCl3
NA = 100
F1 = 100.615280 MHz
F2 = 1.000000 MHz

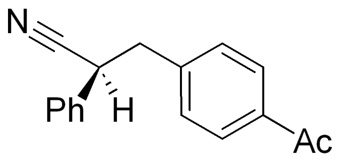

21

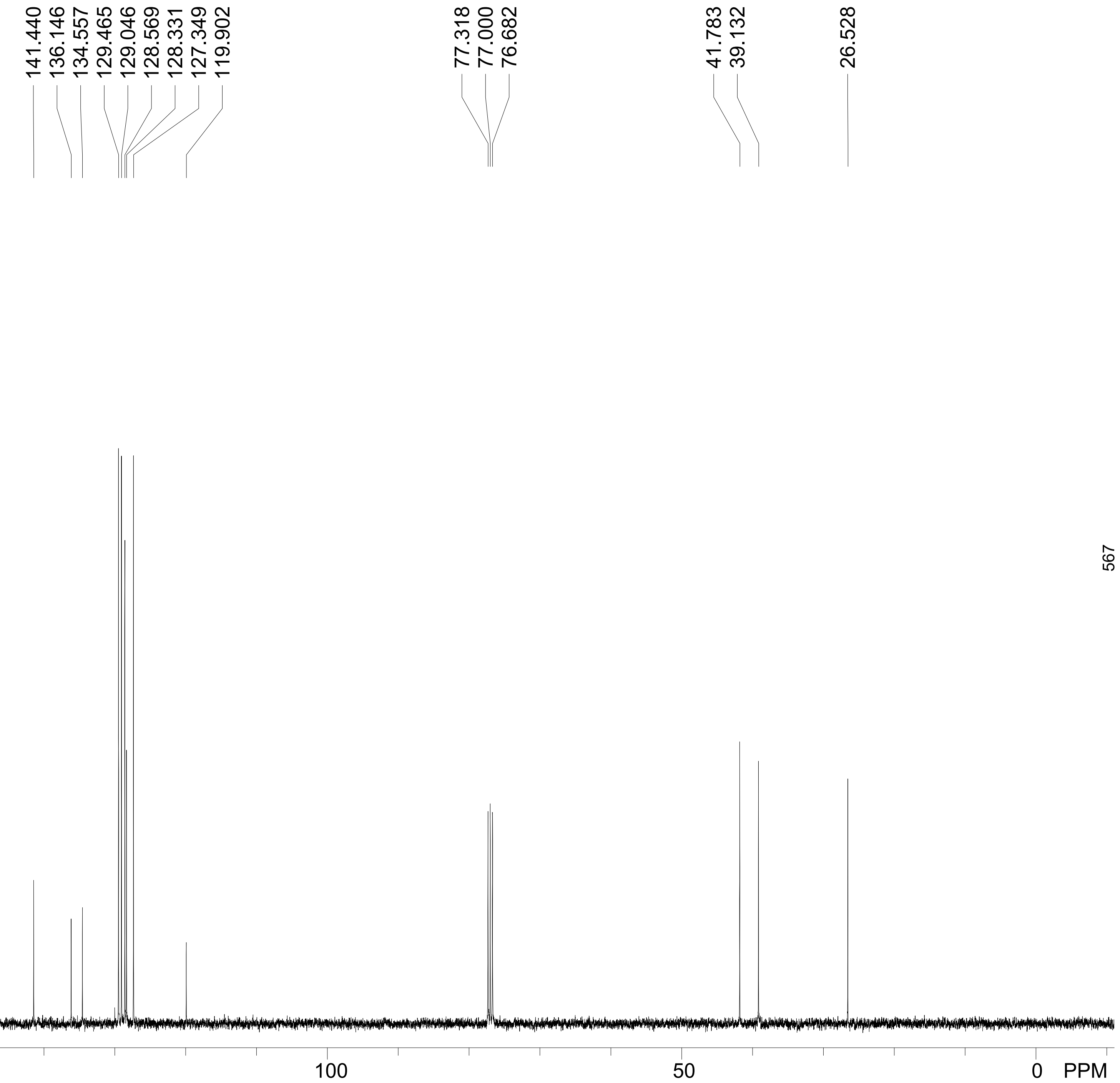

Area Percent Report

Agilent Technologies

Sample name: zwf-7-066-IE-90-10-0.8-205

Data file: C:\Users\Public\Documents\ChemStation\1\Data\zwf\zwf-S 2023-09-17 19-52-41\015-P1-B1-zwf-7-066.D

Injection Acquired Date 09/18/2023 03:24:18

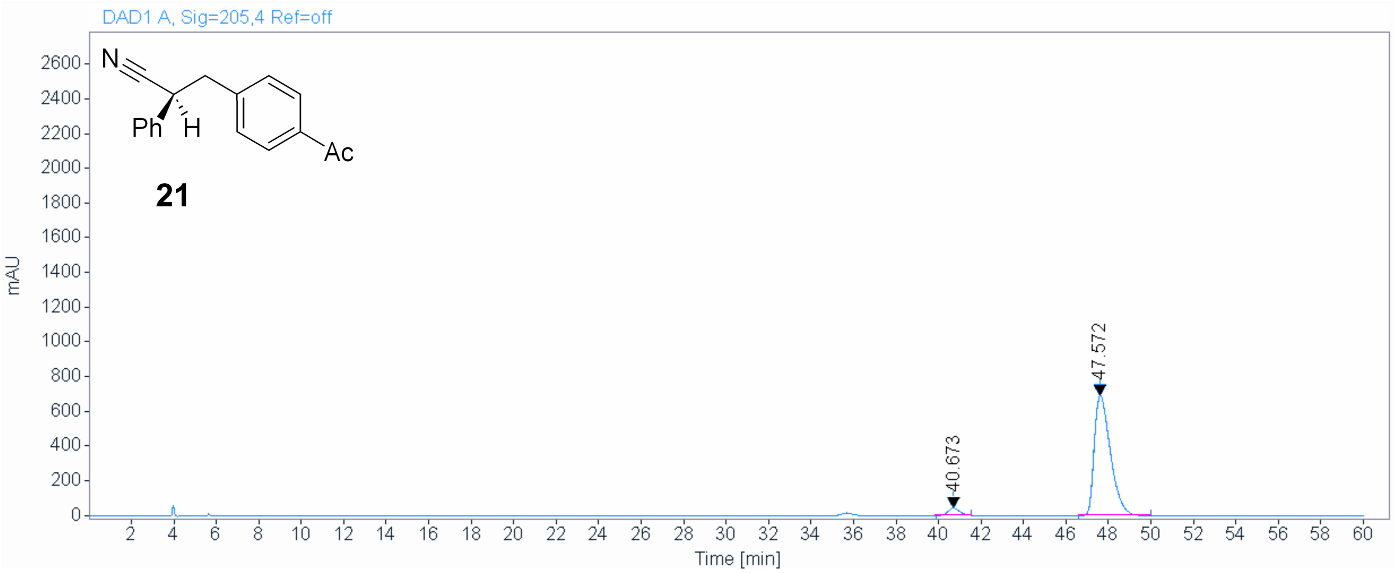

Signal: DAD1 A, Sig=205,4 Ref=off

RT [min]	Width [min]	Height	Area	Area%
40.673	0.4524	43.4888	1663.8723	4.3285
47.572	0.8856	692.0976	36776.2344	95.6715
		Sum	38440.1067	100.0000

Area Percent Report

Sample name: zwf-7-066-rac-IE-90-10-0.8-205

Data file: C:\Users\Public\Documents\ChemStation\1\Data\zwf\zwf-S 2023-09-17 19-52-41\016-P1-B2-zwf-7-066-rac.D

Injection Acquired Date 09/18/2023 04:25:24

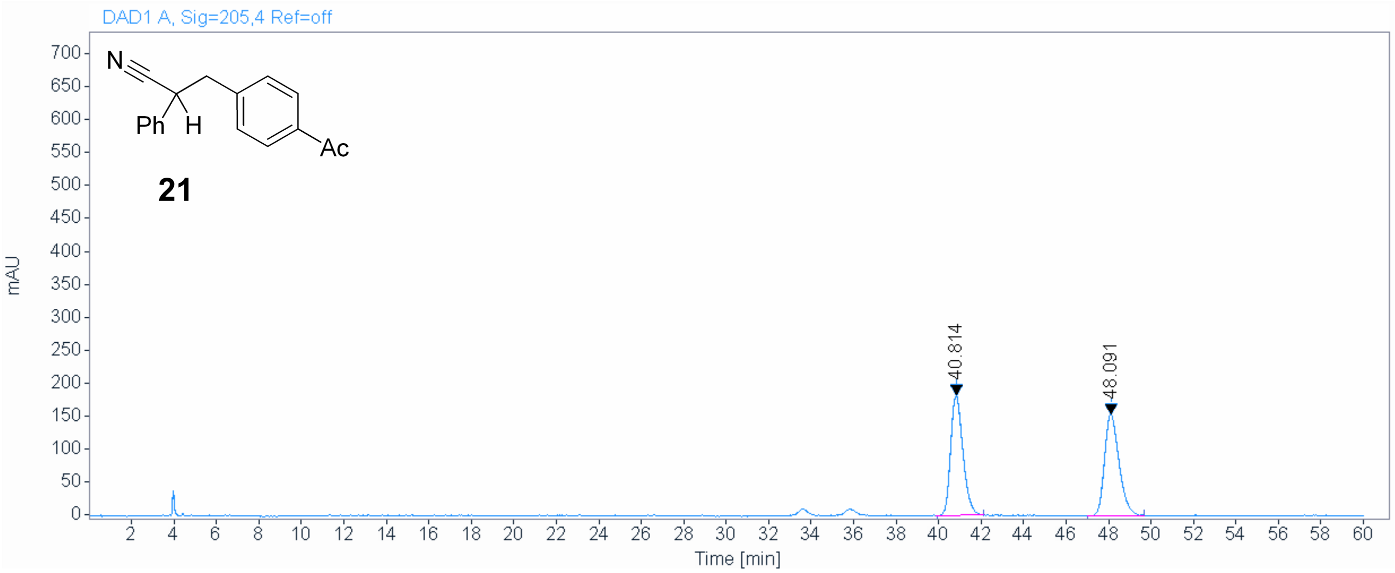

Signal: DAD1 A, Sig=205,4 Ref=off

RT [min]	Width [min]	Height	Area	Area%
40.814	0.5064	182.0716	7340.6147	49.8490
48.091	0.6163	153.5379	7385.1001	50.1510
		Sum	14725.7148	100.0000

zwf-8-022H
Dec 08 2023
SOLVENT: CDCl3
NA = 4
F1 = 400.140015 MHz
F2 = 1.000000 MHz

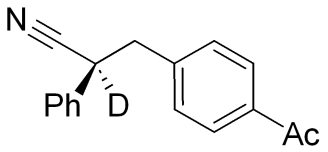

21-d

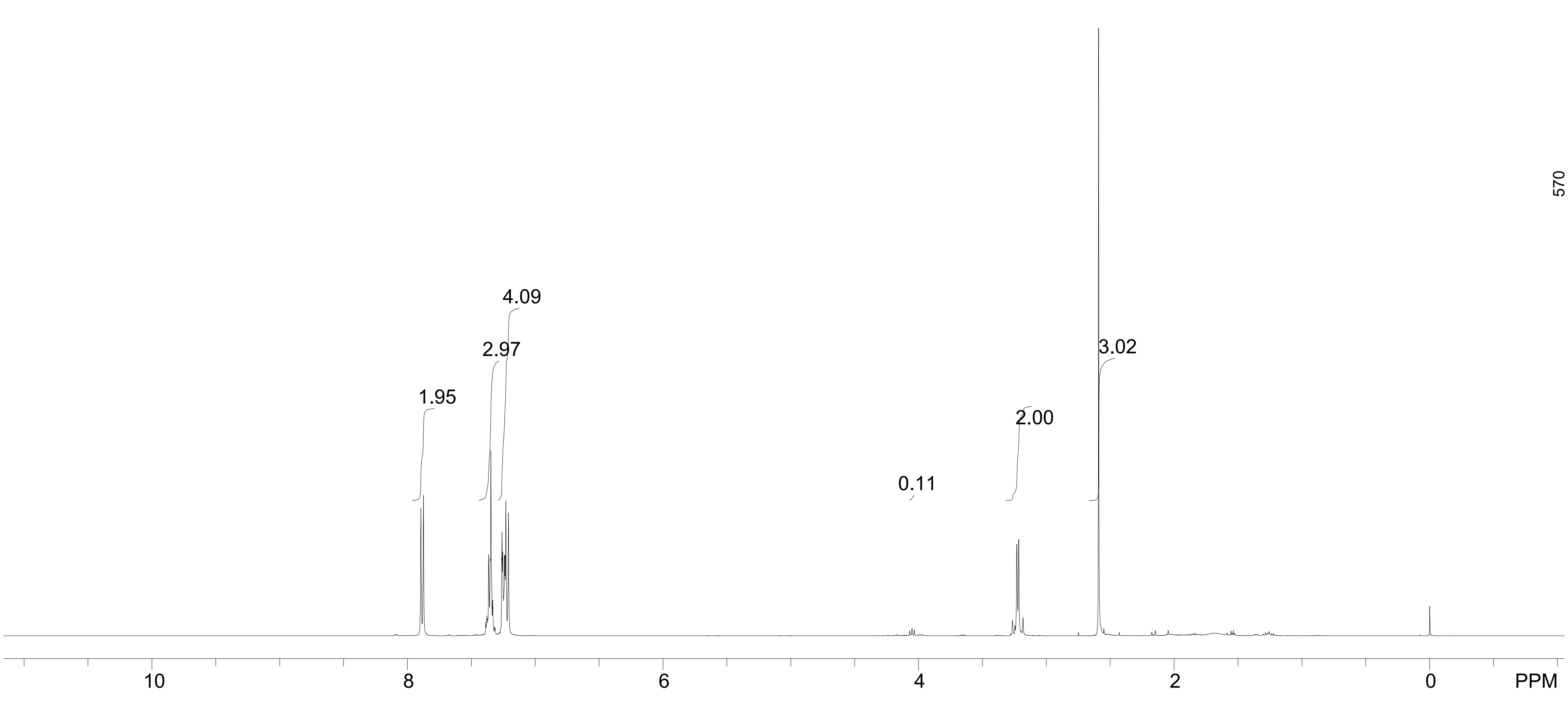

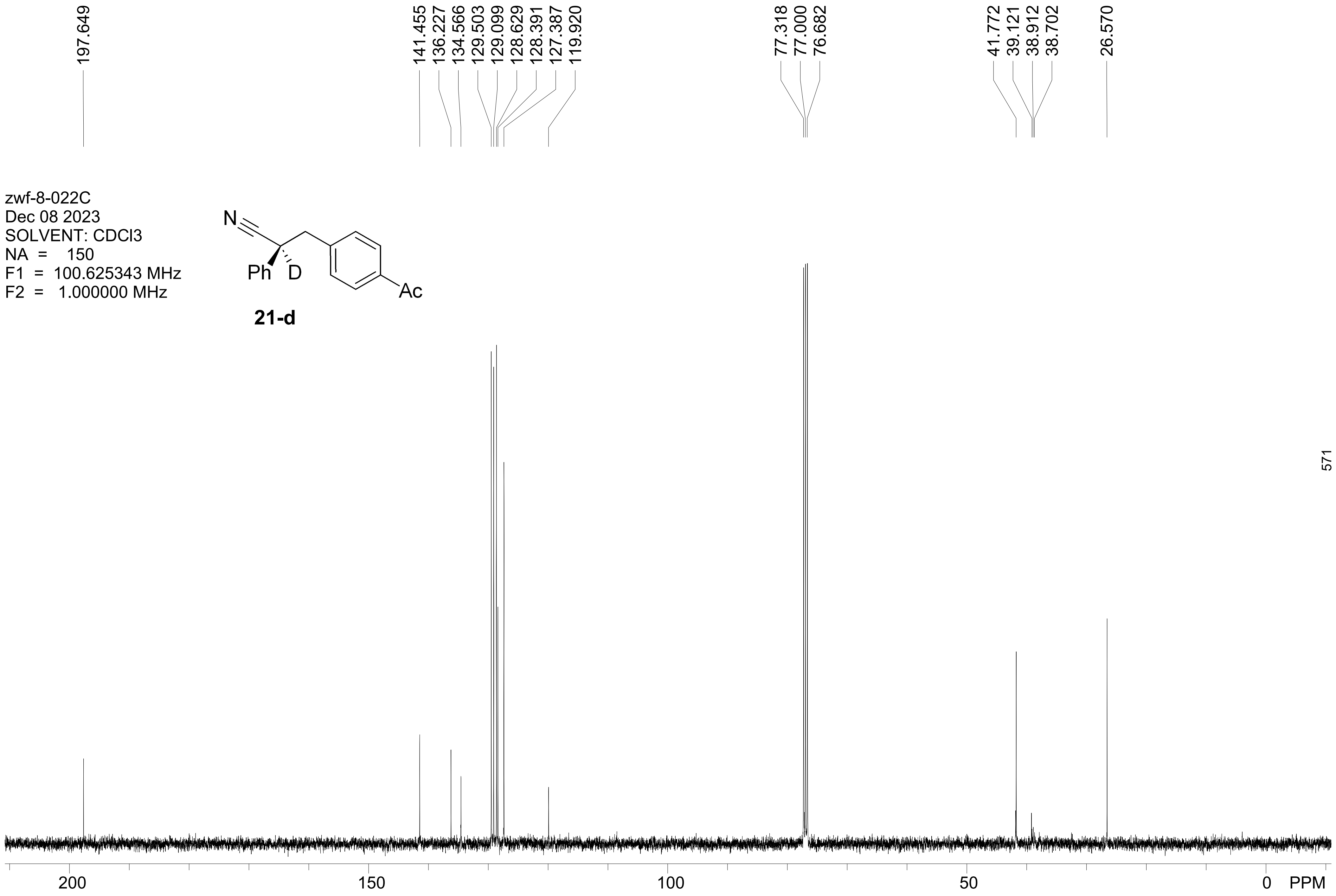

zwf-8-022C
Dec 08 2023
SOLVENT: CDCl3
NA = 150
F1 = 100.625343 MHz
F2 = 1.000000 MHz
N
Ph
D
Ac
21-d
197.649
141.455
136.227
134.566
129.503
129.099
128.629
128.391
127.387
119.920
77.318
77.000
76.682
41.772
39.121
38.912
38.702
26.570
200
150
100
50
0
PPM

Area Percent Report

Sample name: zwf-8-022-IE-90-10-0.8-205

Data file: C:\Users\Public\Documents\ChemStation\1\Data\zwf\zwf-S 2023-12-08 15-34-41\011-P1-C10-zwf-8-022.D

Injection Acquired Date 12/08/2023 22:37:04

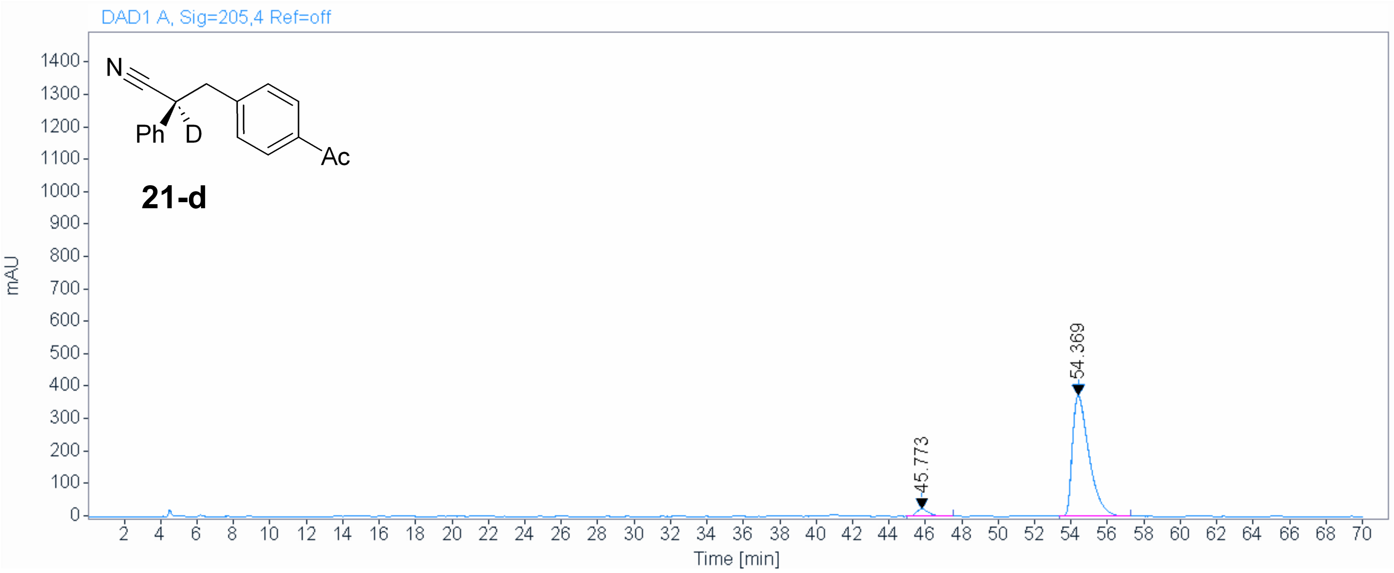

Signal: DAD1 A, Sig=205,4 Ref=off

RT [min]	Width [min]	Height	Area	Area%
45.773	0.6603	22.4385	1069.9451	4.3370
54.369	0.9516	372.2840	23600.0410	95.6630
		Sum	24669.9861	100.0000

Area Percent Report

Agilent Technologies

Sample name: zwf-8-022-rac-IE-90-10-0.8-205

Data file: C:\Users\Public\Documents\ChemStation\1\Data\zwf\zwf-S 2023-12-08 15-34-41\012-P1-C11-zwf-8-022-rac.D

Injection Acquired Date 12/08/2023 23:48:09

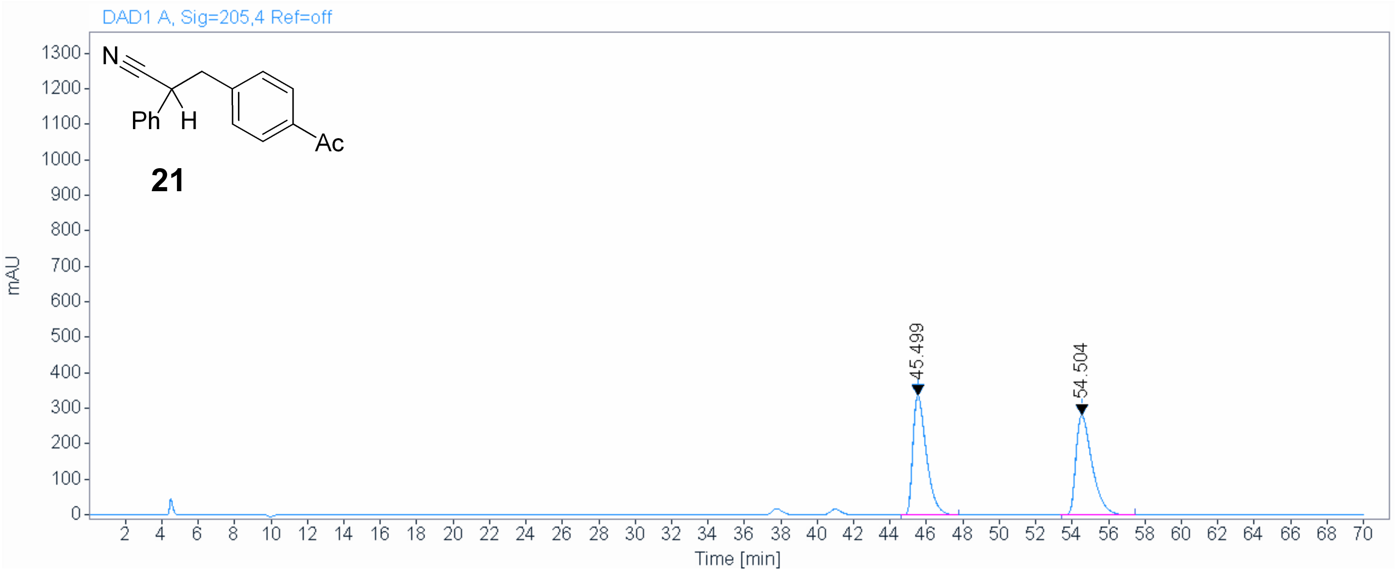

Signal: DAD1 A, Sig=205,4 Ref=off

RT [min]	Width [min]	Height	Area	Area%
45.499	0.7812	336.6529	17348.7305	49.9218
54.504	0.9215	281.4249	17403.0684	50.0782
		Sum	34751.7988	100.0000

zwf-7-071H
Sep 17 2023
SOLVENT: CDCl3
NA = 4
F1 = 400.140015 MHz
F2 = 1.000000 MHz

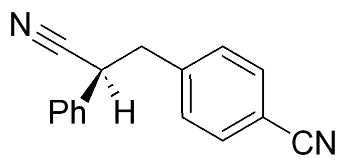

22

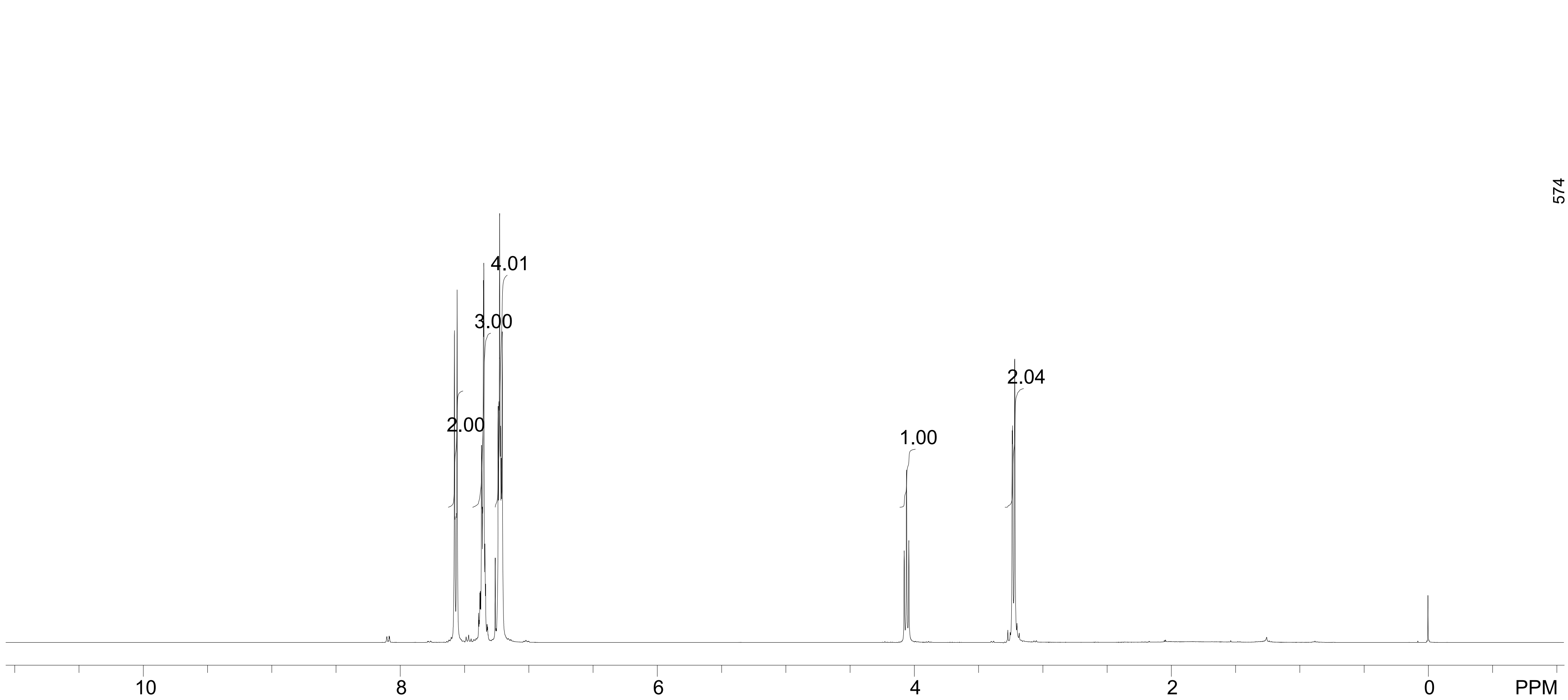

zwf-7-071C
Sep 17 2023
SOLVENT: CDCl3
NA = 100
F1 = 100.615280 MHz
F2 = 1.000000 MHz

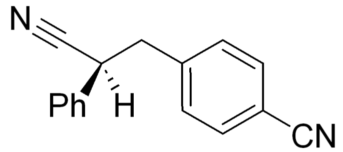

22

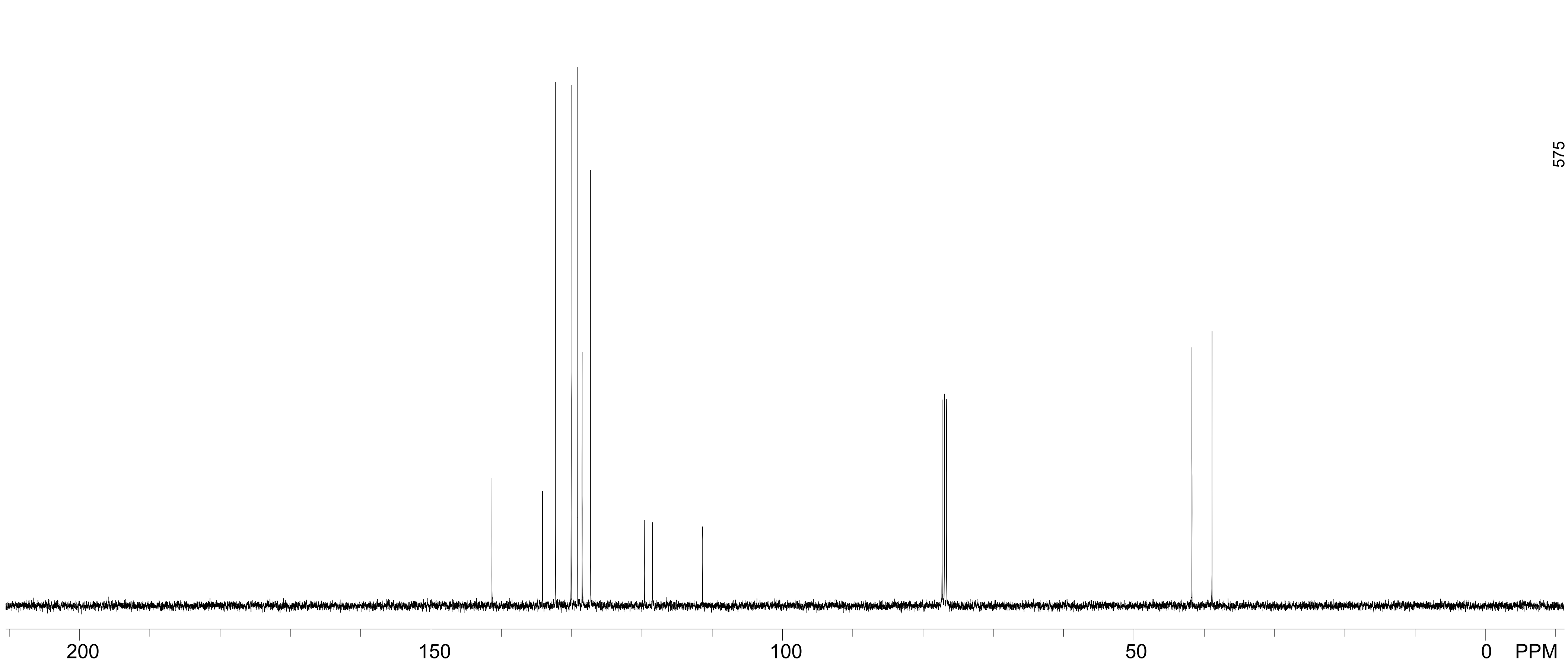

Area Percent Report

Agilent Technologies

Sample name: zwf-7-071-IE-90-10-0.8-205

Data file: C:\Users\Public\Documents\ChemStation\1\Data\zwf\zwf-S 2023-09-17 19-52-41\020-P1-B6-zwf-7-071.D

Injection Acquired Date 09/18/2023 07:29:49

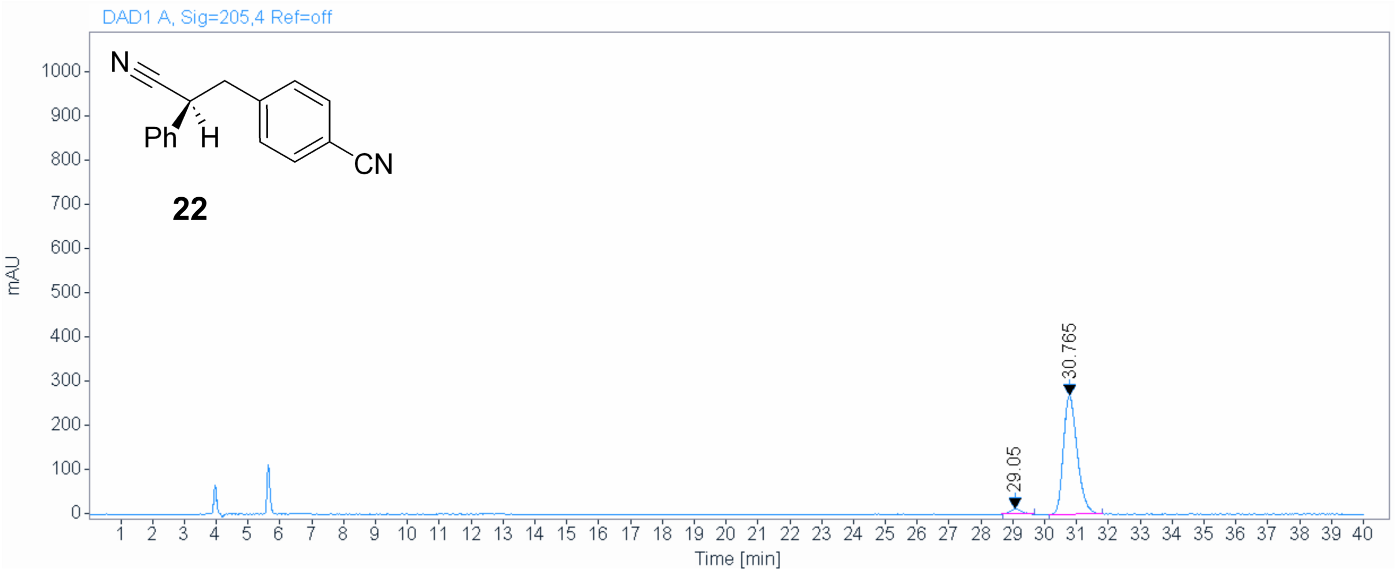

Signal: DAD1 A, Sig=205,4 Ref=off

RT [min]	Width [min]	Height	Area	Area%
29.050	0.4322	12.1360	314.7173	3.7162
30.765	0.4416	268.8335	8154.1777	96.2838
		Sum	8468.8951	100.0000

Area Percent Report

Agilent Technologies

Sample name: zwf-7-071-rac-IE-90-10-0.8-205

Data file: C:\Users\Public\Documents\ChemStation\1\Data\zwf\zwf-S 2023-09-17 19-52-41\022-P1-B8-zwf-7-071-rac.D

Injection Acquired Date 09/18/2023 08:52:04

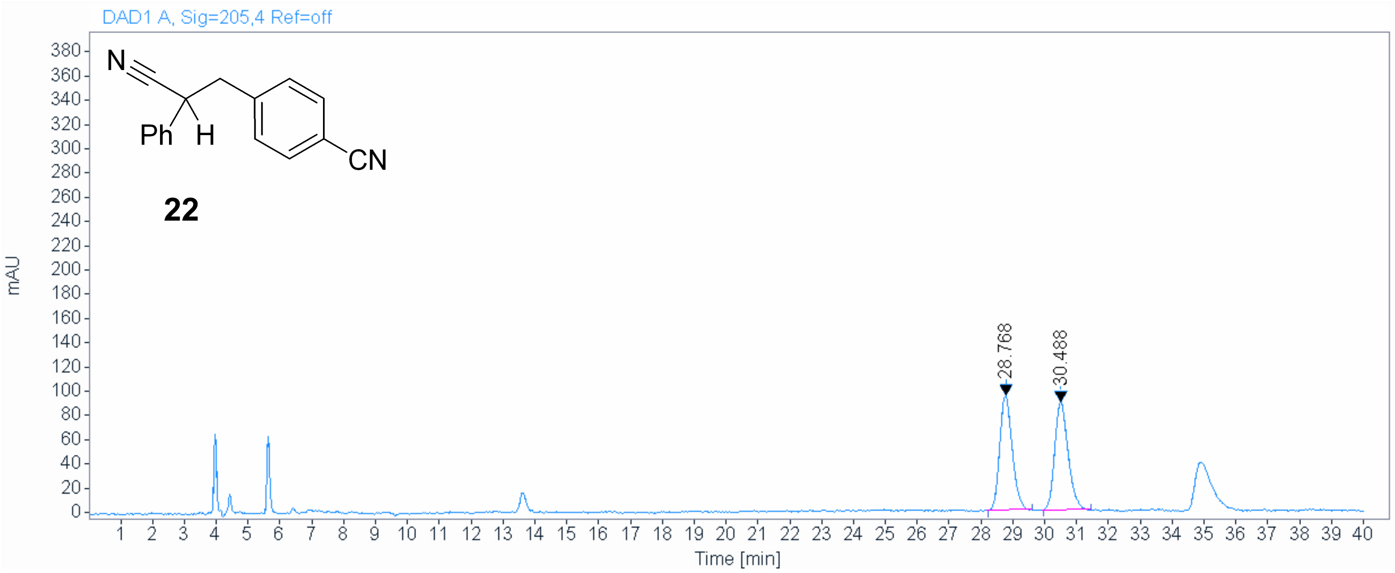

Signal: DAD1 A, Sig=205,4 Ref=off

RT [min]	Width [min]	Height	Area	Area%
28.768	0.3377	94.8185	2614.8325	49.7336
30.488	0.3796	89.4047	2642.8467	50.2664
		Sum	5257.6792	100.0000

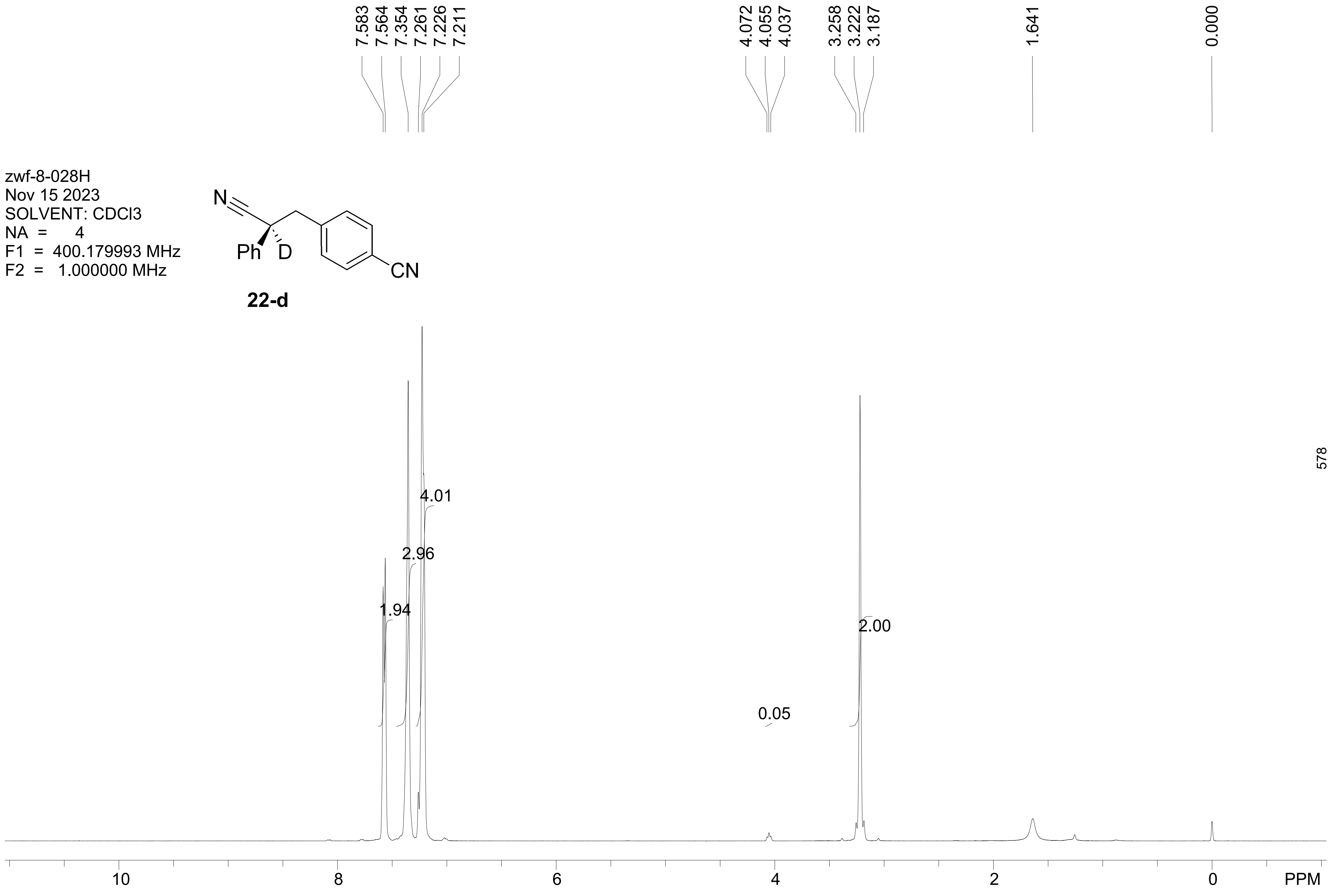

zwf-8-028H
Nov 15 2023
SOLVENT: CDCl3
NA = 4
F1 = 400.179993 MHz
F2 = 1.000000 MHz
N
Ph
D
CN
7.583
7.564
7.354
7.261
7.226
7.211
4.072
4.055
4.037
3.258
3.222
3.187
1.641
0.000
1.94
2.96
4.01
0.05
2.00
10
8
6
4
2
0
PPM

22-d

zwf-8-028C
Nov 15 2023
SOLVENT: CDCl3
NA = 150
F1 = 100.625343 MHz
F2 = 1.000000 MHz

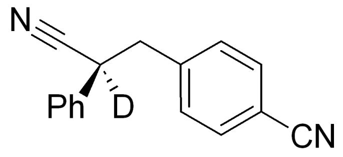

22-d

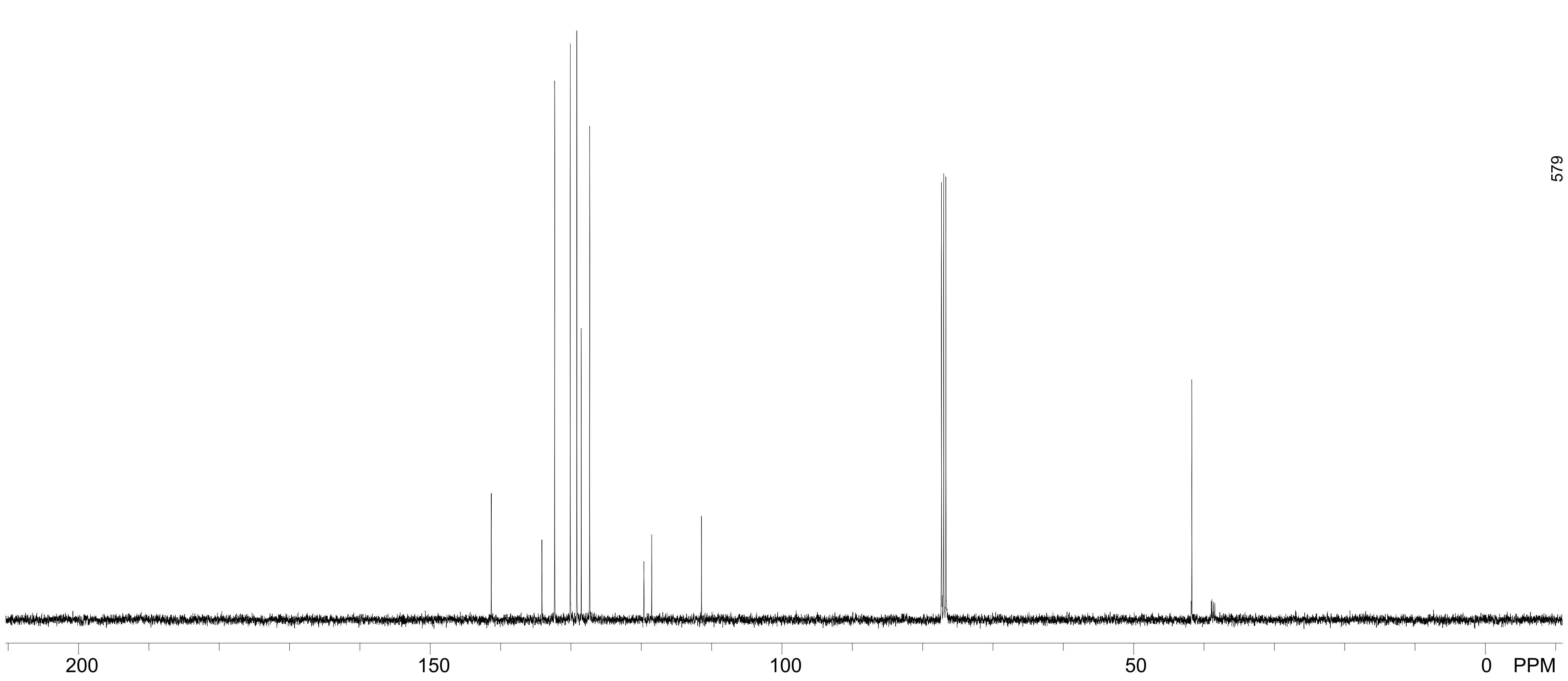

Area Percent Report

Agilent Technologies

Sample name: zwf-8-028-IE-90-10-0.8-205

Data file: C:\Users\Public\Documents\ChemStation\1\Data\zwf\zwf-S 2023-11-15 14-21-28\025-P1-A3-zwf-8-028.D

Injection Acquired Date 11/16/2023 05:03:08

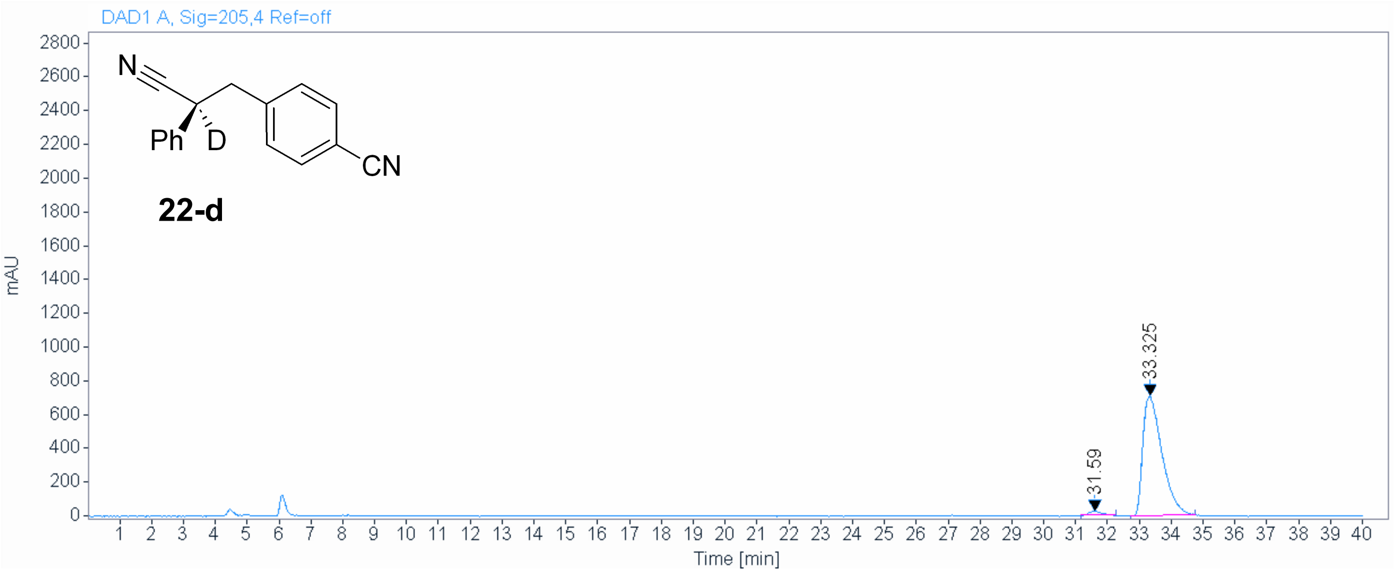

Signal: DAD1 A, Sig=205,4 Ref=off

RT [min]	Width [min]	Height	Area	Area%
31.590	0.4896	27.3130	802.4205	2.6414
33.325	0.4931	710.7025	29575.6250	97.3586
		Sum	30378.0455	100.0000

Area Percent Report

Agilent Technologies

Sample name: zwf-8-028-rac-IE-90-10-0.8-205

Data file: C:\Users\Public\Documents\ChemStation\1\Data\zwf\zwf-S 2023-11-15 14-21-28\027-P1-A6-zwf-8-028-rac.D

Injection Acquired Date 11/16/2023 06:25:21

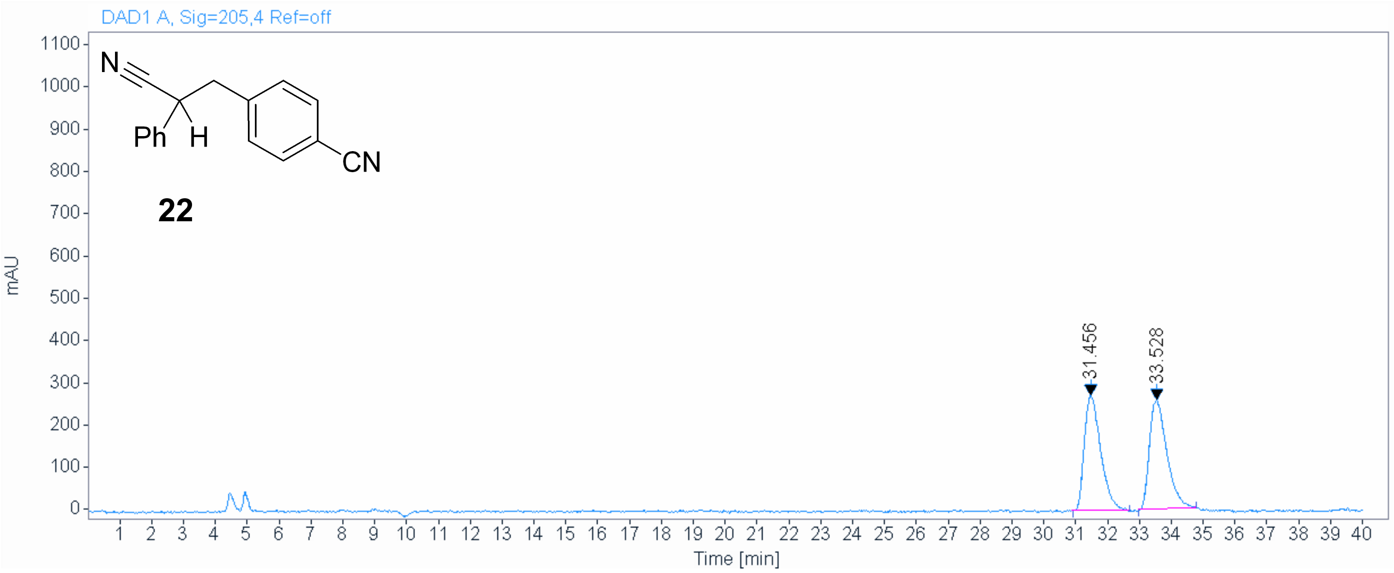

Signal: DAD1 A, Sig=205,4 Ref=off

RT [min]	Width [min]	Height	Area	Area%
31.456	0.4495	273.5796	9941.0527	49.8774
33.528	0.6420	259.3452	9989.9121	50.1226
		Sum	19930.9648	100.0000

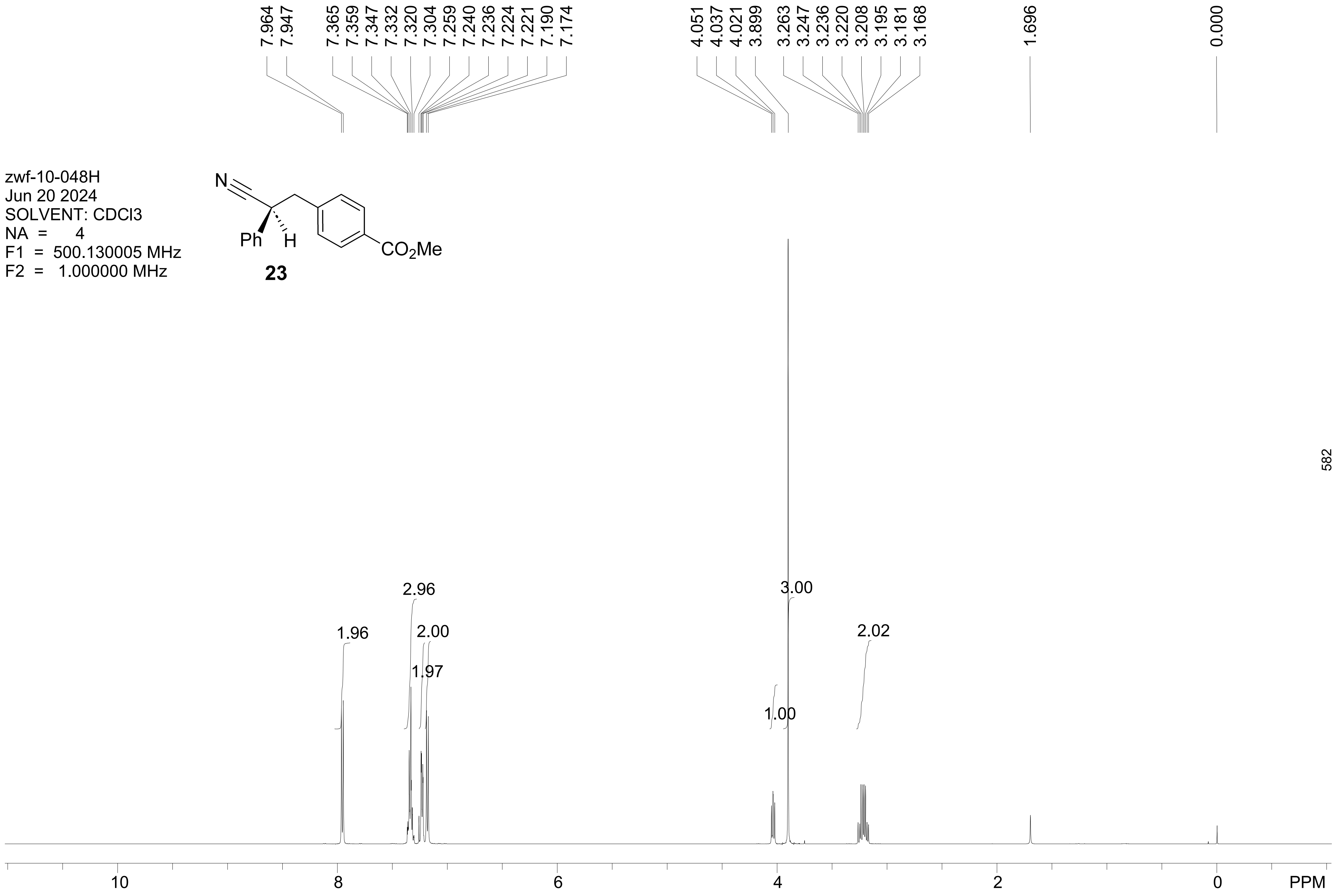

zwf-10-048H
Jun 20 2024
SOLVENT: CDCl3
NA = 4
F1 = 500.130005 MHz
F2 = 1.000000 MHz
N
Ph
H
CO_2Me
23
7.964
7.947
7.365
7.359
7.347
7.332
7.320
7.304
7.259
7.240
7.236
7.224
7.221
7.190
7.174
4.051
4.037
4.021
3.899
3.263
3.247
3.236
3.220
3.208
3.195
3.181
3.168
1.696
0.000
1.96
2.96
2.00
1.97
3.00
1.00
2.02
10
8
6
4
2
0
PPM

zwf-10-048C
Jun 20 2024
SOLVENT: CDCl3
NA = 100
F1 = 125.757790 MHz
F2 = 1.000000 MHz

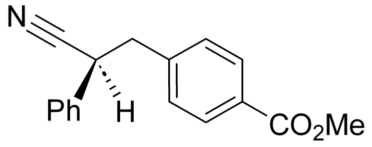

23

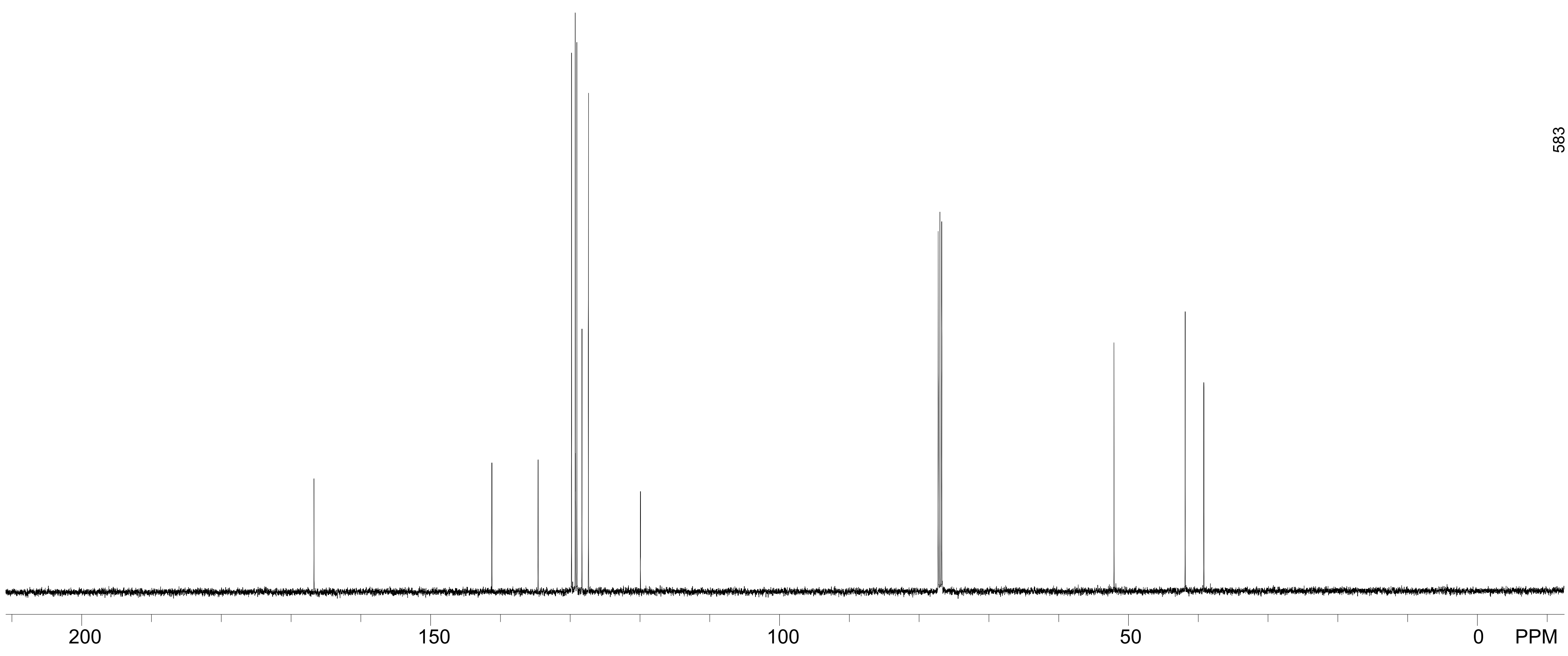

Area Percent Report

Sample name: zwf-10-048-IE-80-20-0.8-205

Data file: C:\Users\Public\Documents\ChemStation\1\Data\zwf\zwf-S 2024-06-20 20-48-43\002-P1-C1-zwf-10-048.D

Injection Acquired Date 06/20/2024 21:11:47

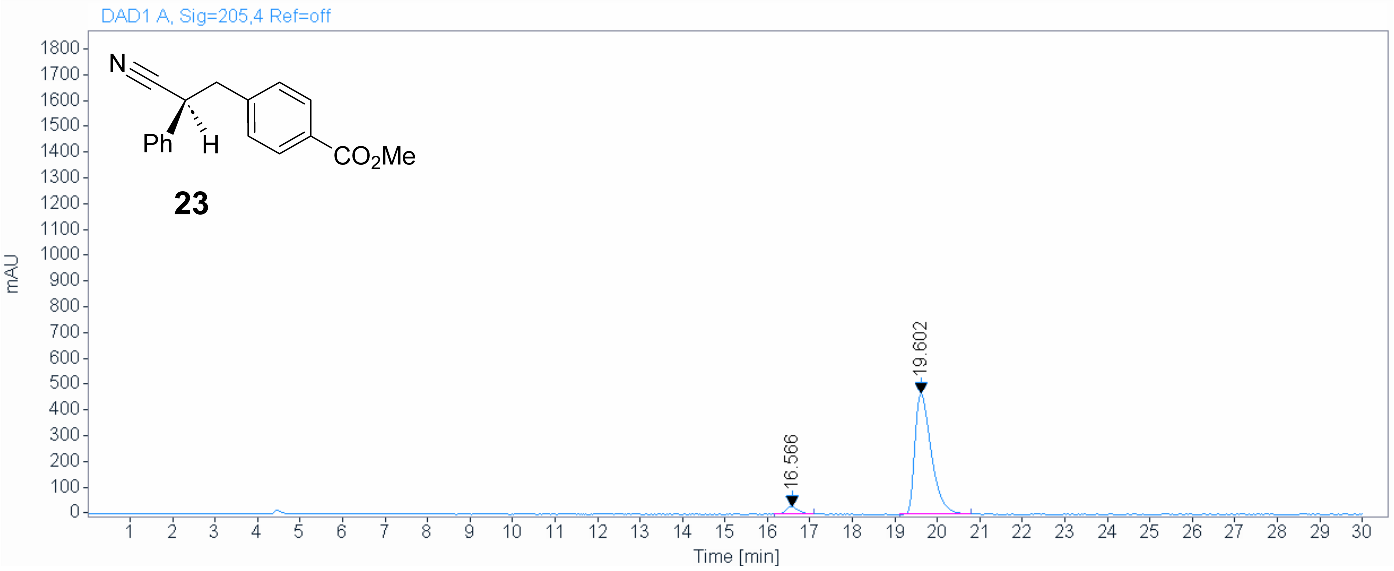

Signal: DAD1 A, Sig=205,4 Ref=off

RT [min]	Width [min]	Height	Area	Area%
16.566	0.2600	28.2013	589.7225	4.4757
19.602	0.3877	467.7768	12586.3828	95.5243
		Sum	13176.1053	100.0000

Area Percent Report

Agilent Technologies

Sample name: zwf-10-048-rac-IE-80-20-0.8-205

Data file: C:\Users\Public\Documents\ChemStation\1\Data\zwf\zwf-S 2024-06-20 20-48-43\005-P1-C3-zwf-10-048-rac.D

Injection Acquired Date 06/20/2024 22:44:43

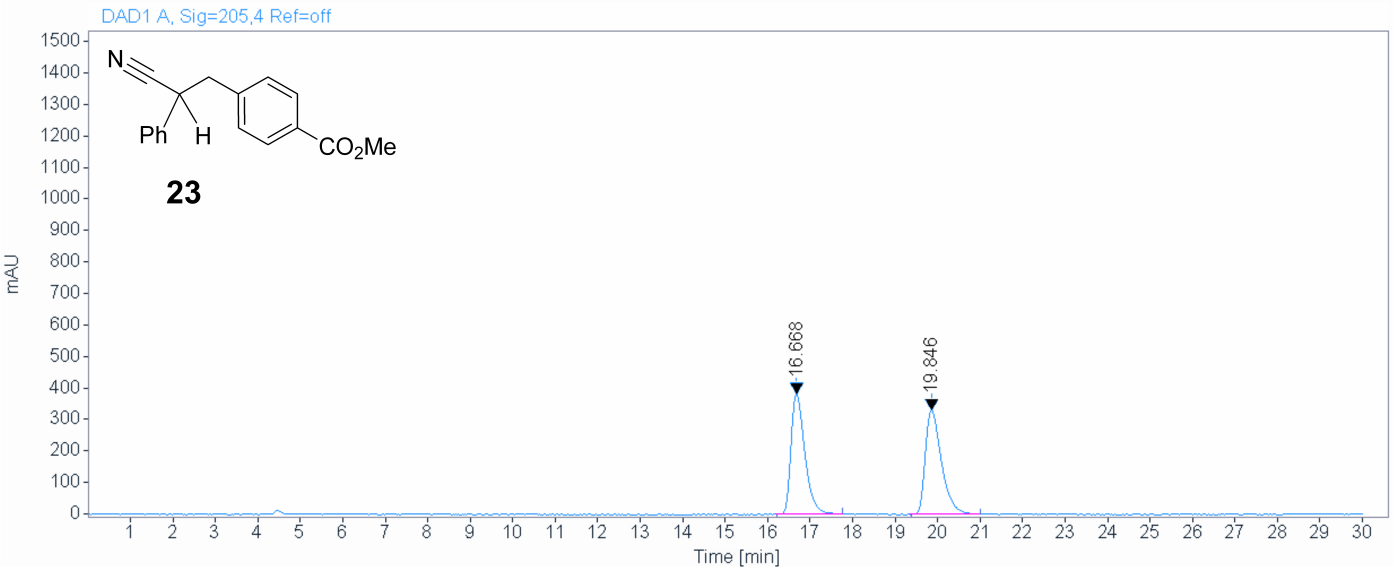

Signal: DAD1 A, Sig=205,4 Ref=off

RT [min]	Width [min]	Height	Area	Area%
16.668	0.3419	382.4466	8717.3643	49.7363
19.846	0.3745	330.9854	8809.7900	50.2637
		Sum	17527.1543	100.0000

zwf-10-049H
Jun 20 2024
SOLVENT: CDCl3
NA = 4
F1 = 500.130005 MHz
F2 = 1.000000 MHz

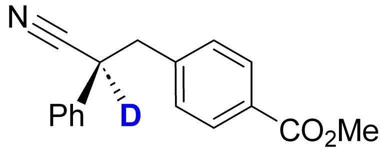

23-d

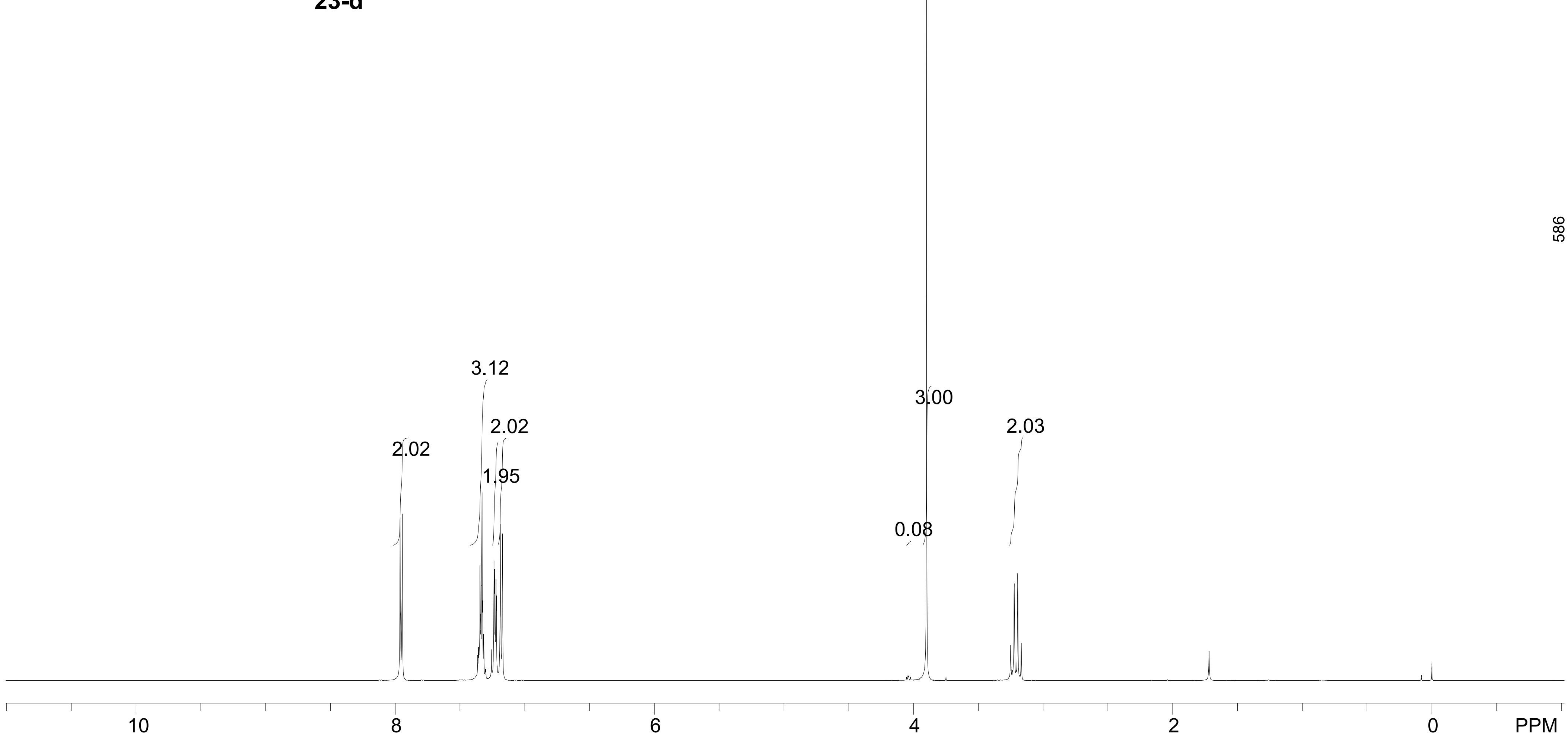

zwf-10-049C
Jun 20 2024
SOLVENT: CDCl3
NA = 100
F1 = 125.757790 MHz
F2 = 1.000000 MHz

23-d

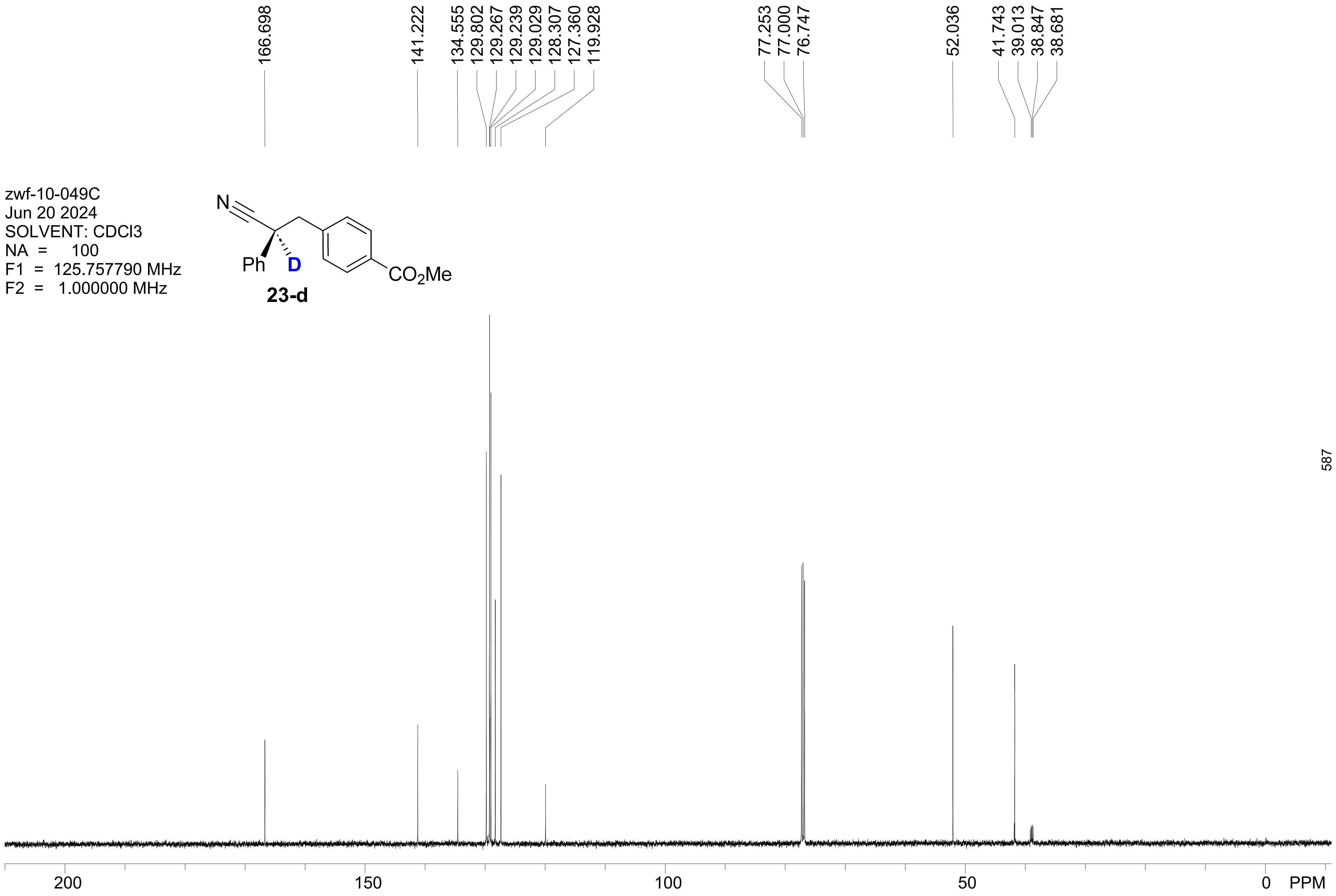

Area Percent Report

Agilent Technologies

Sample name: zwf-10-049-IE-80-20-0.8-205

Data file: C:\Users\Public\Documents\ChemStation\1\Data\zwf\zwf-S 2024-06-20 20-48-43\003-P1-C2-zwf-10-049.D

Injection Acquired Date 06/20/2024 21:42:46

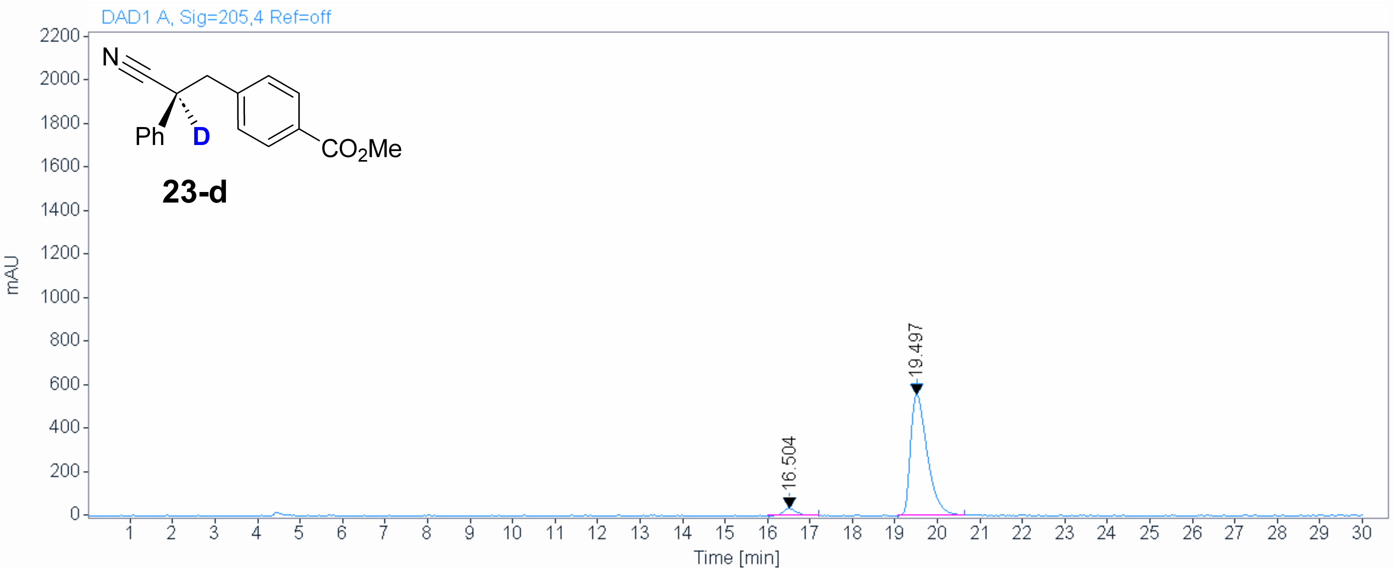

Signal: DAD1 A, Sig=205,4 Ref=off

RT [min]	Width [min]	Height	Area	Area%
16.504	0.2816	32.3931	743.1395	4.6548
19.497	0.3962	554.6559	15221.9814	95.3452
		Sum	15965.1209	100.0000

Area Percent Report

Agilent Technologies

Sample name: zwf-10-048-rac-IE-80-20-0.8-205

Data file: C:\Users\Public\Documents\ChemStation\1\Data\zwf\zwf-S 2024-06-20 20-48-43\005-P1-C3-zwf-10-048-rac.D

Injection Acquired Date 06/20/2024 22:44:43

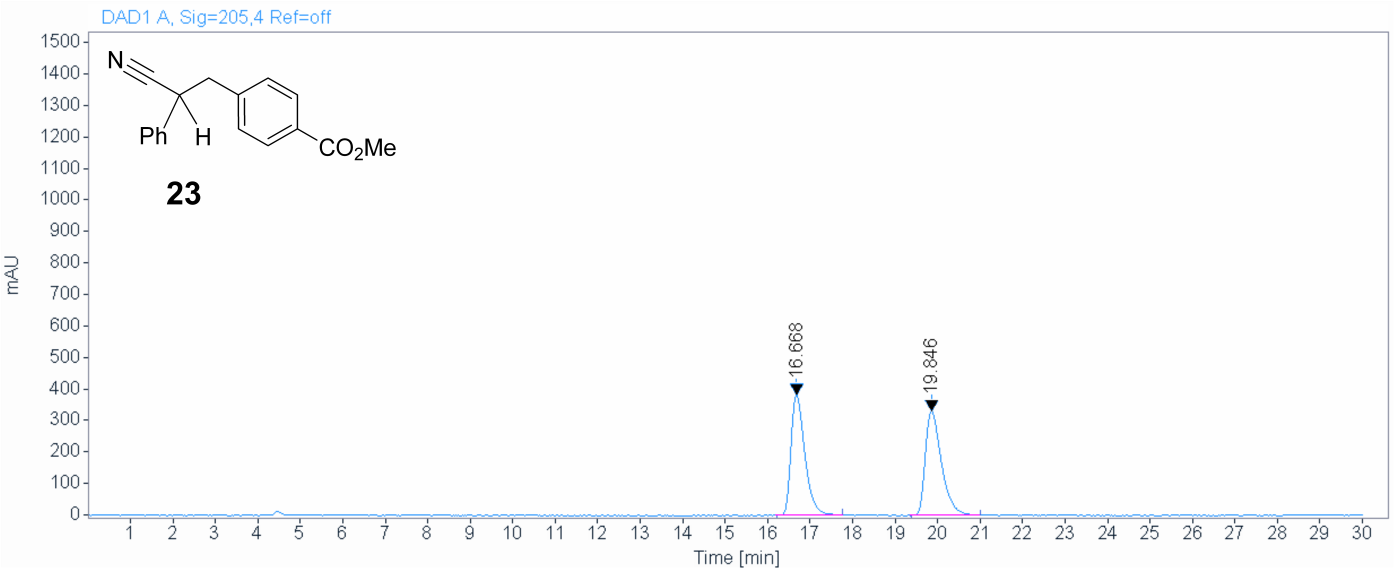

Signal: DAD1 A, Sig=205,4 Ref=off

RT [min]	Width [min]	Height	Area	Area%
16.668	0.3419	382.4466	8717.3643	49.7363
19.846	0.3745	330.9854	8809.7900	50.2637
		Sum	17527.1543	100.0000

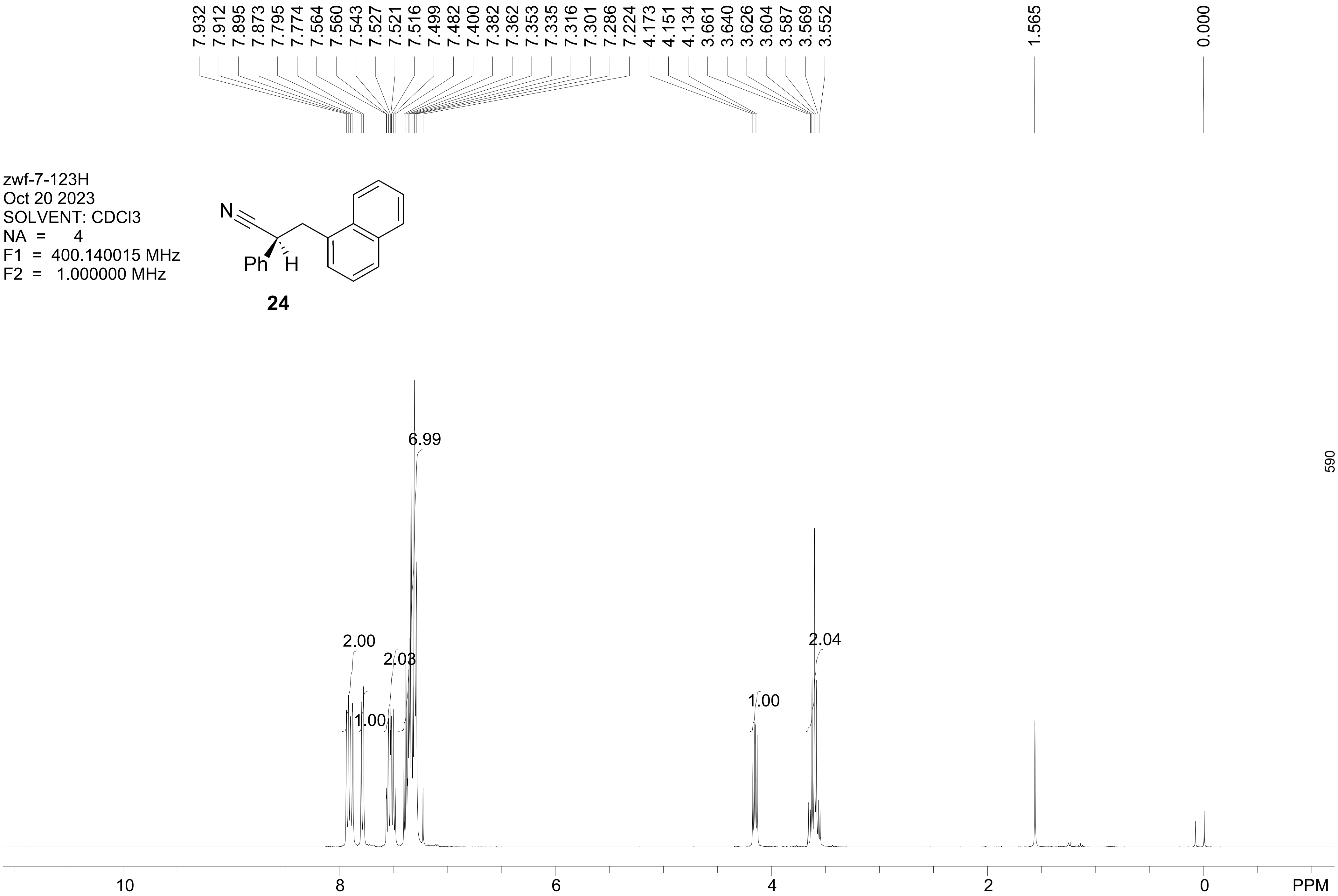
zwf-7-123H
Oct 20 2023
SOLVENT: CDCl3
NA = 4
F1 = 400.140015 MHz
F2 = 1.000000 MHz
N
Ph
H
24
7.932
7.912
7.895
7.873
7.795
7.774
7.564
7.560
7.543
7.527
7.521
7.516
7.499
7.482
7.400
7.382
7.362
7.353
7.335
7.316
7.301
7.286
7.224
4.173
4.151
4.134
3.661
3.640
3.626
3.604
3.587
3.569
3.552
1.565
0.000
6.99
2.00
2.03
1.00
1.00
2.04
10
8
6
4
2
0
PPM

zwf-7-123C
Oct 20 2023
SOLVENT: CDCl3
NA = 100
F1 = 100.615280 MHz
F2 = 1.000000 MHz

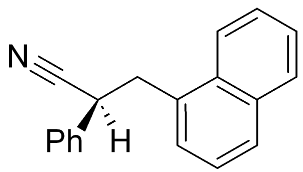

24

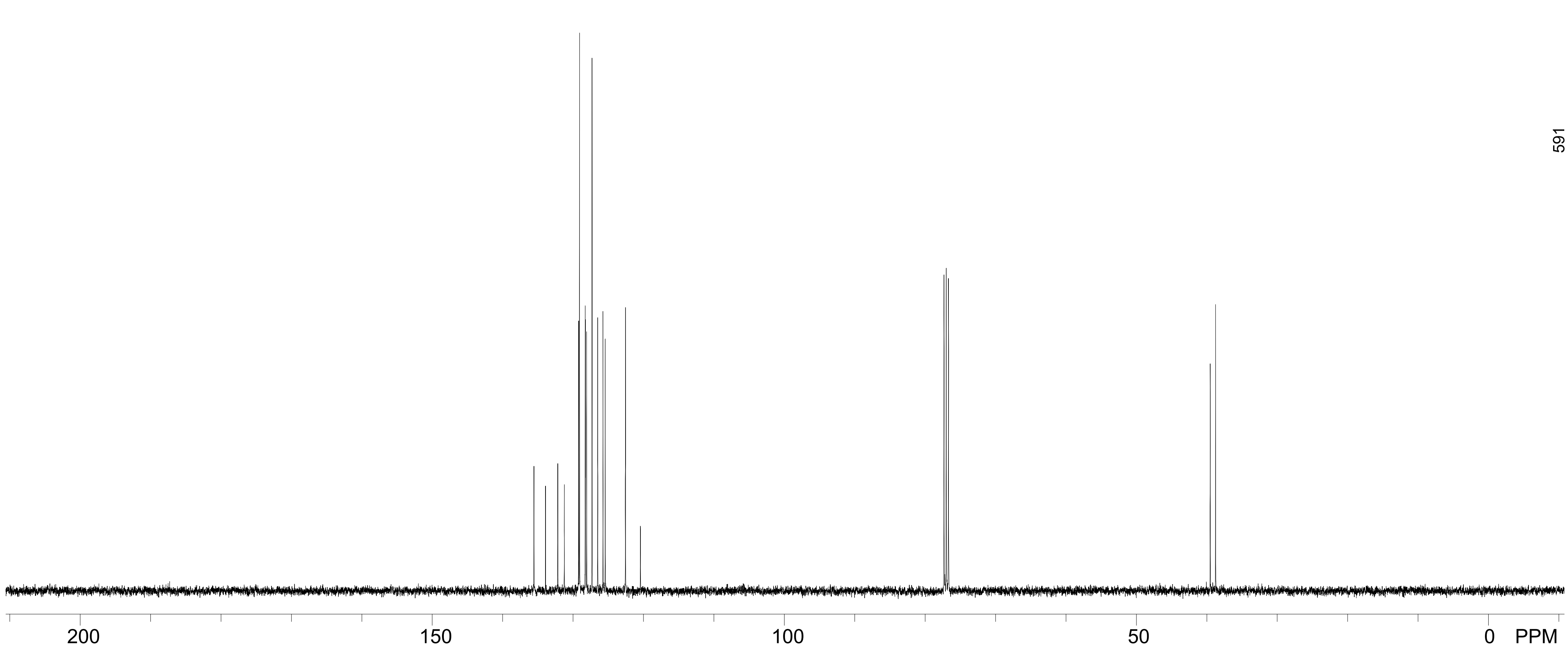

Area Percent Report

Sample name: zwf-7-123-IE-99-1-0.8-205

Data file: C:\Users\Public\Documents\ChemStation\1\Data\zwf\zwf-S 2023-10-21 17-25-18\004-P1-C6-zwf-7-123.D

Injection Acquired Date 10/21/2023 18:34:37

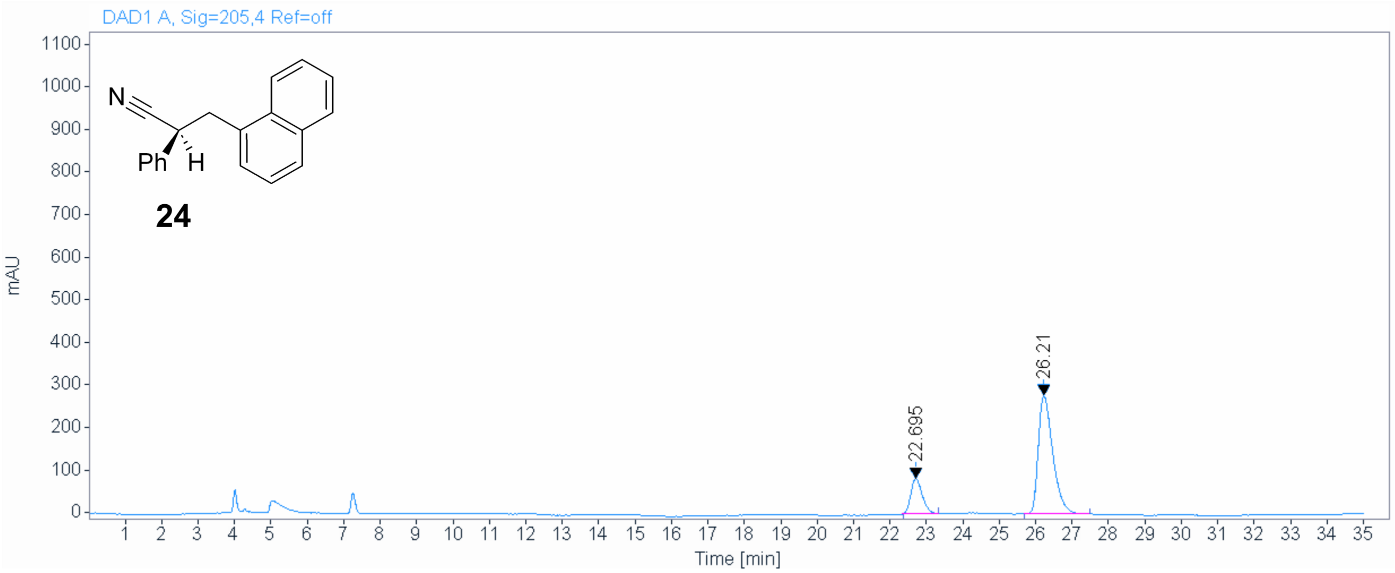

Signal: DAD1 A, Sig=205,4 Ref=off

RT [min]	Width [min]	Height	Area	Area%
22.695	0.3644	82.6107	1806.1863	18.9748
26.210	0.4623	278.0449	7712.7056	81.0252
		Sum	9518.8918	100.0000

Area Percent Report

Agilent Technologies

Sample name: zwf-7-123-rac-IE-99-1-0.8-205

Data file: C:\Users\Public\Documents\ChemStation\1\Data\zwf\zwf-S 2023-10-21 12-48-40\004-P1-C3-zwf-7-123-rac.D

Injection Acquired Date 10/21/2023 13:57:42

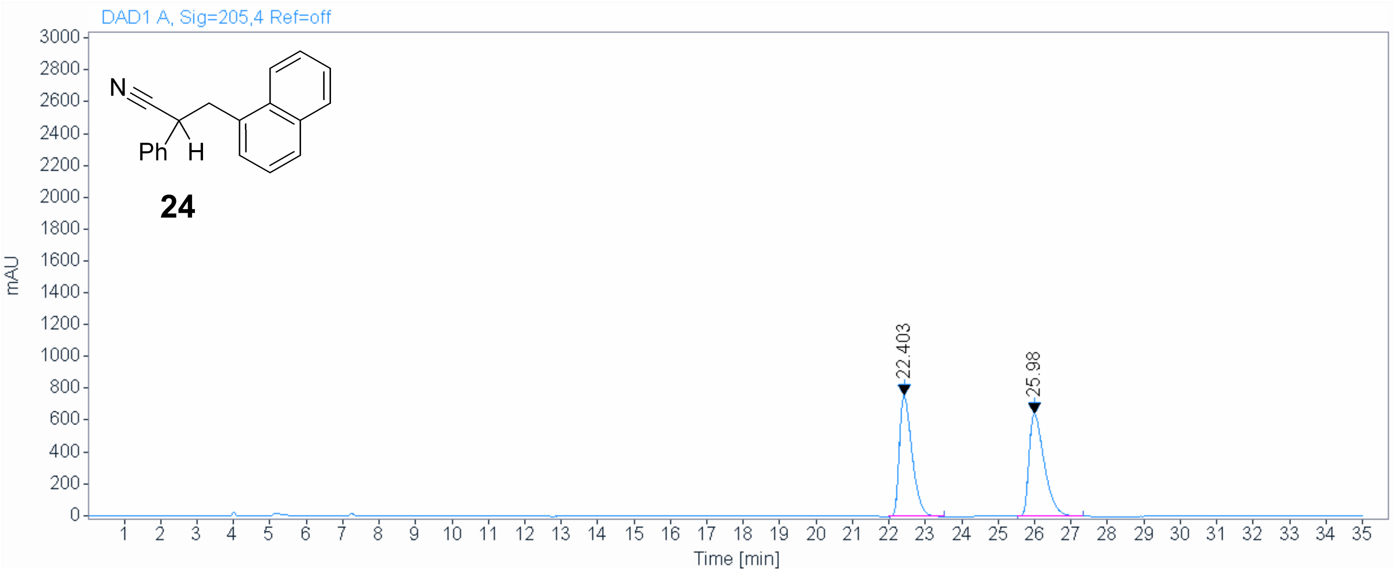

Signal: DAD1 A, Sig=205,4 Ref=off

RT [min]	Width [min]	Height	Area	Area%
22.403	0.3675	759.8231	18460.7461	49.7709
25.980	0.4464	642.2979	18630.6836	50.2291
		Sum	37091.4297	100.0000

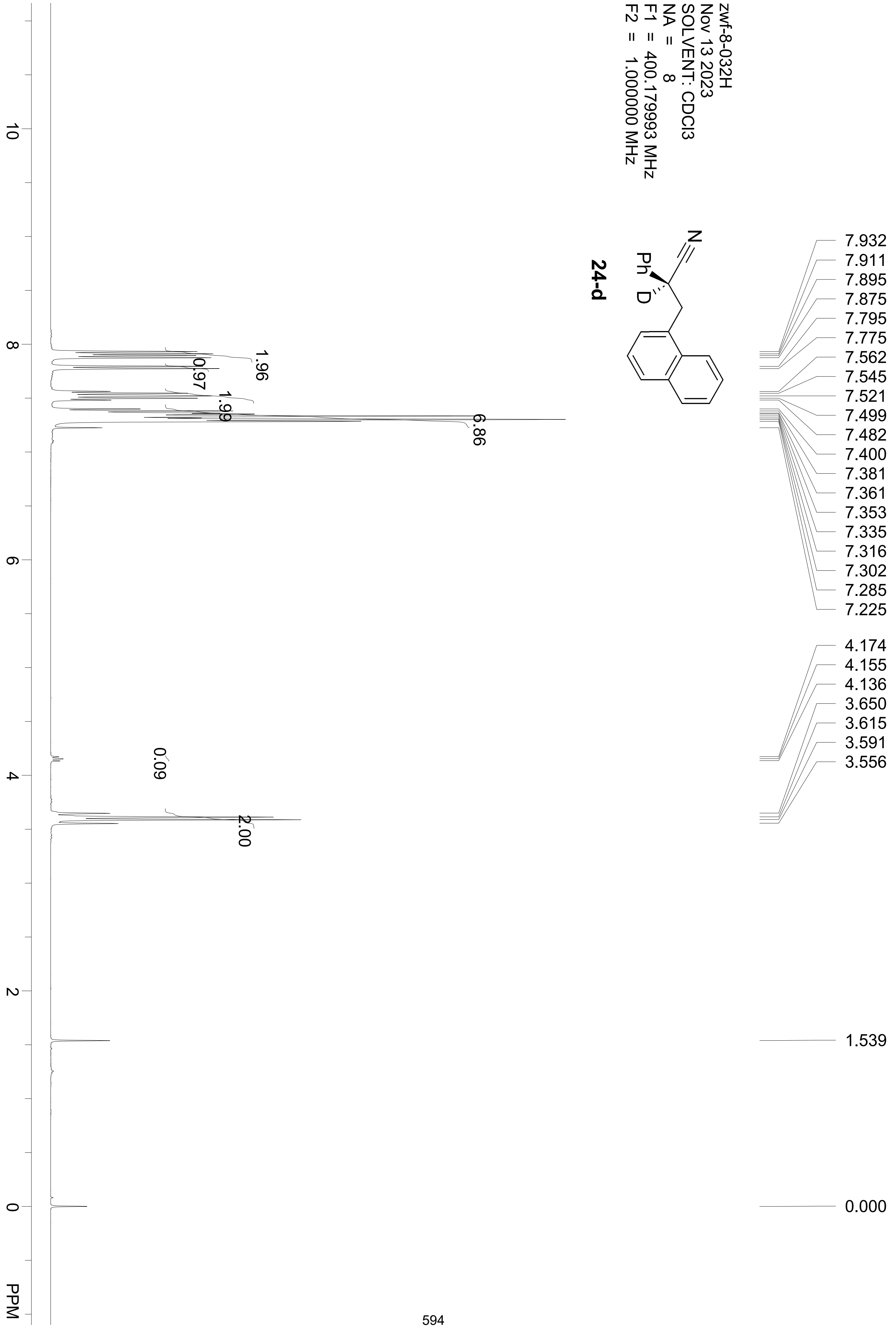

zwf-8-032H
Nov 13 2023
SOLVENT: CDCl3
NA = 8
F1 = 400.179993 MHz
F2 = 1.000000 MHz
N
Ph
D
24-d
7.932
7.911
7.895
7.875
7.795
7.775
7.562
7.545
7.521
7.499
7.482
7.400
7.381
7.361
7.353
7.335
7.316
7.302
7.285
7.225
4.174
4.155
4.136
3.650
3.615
3.591
3.556
1.539
0.000
1.96
0.97
1.99
6.86
0.09
2.00
10
8
6
4
2
0
PPM

zwf-8-032C
Nov 13 2023
SOLVENT: CDCl3
NA = 100
F1 = 100.625343 MHz
F2 = 1.000000 MHz

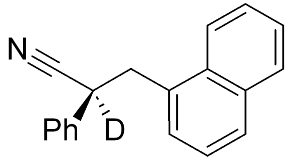

24-d

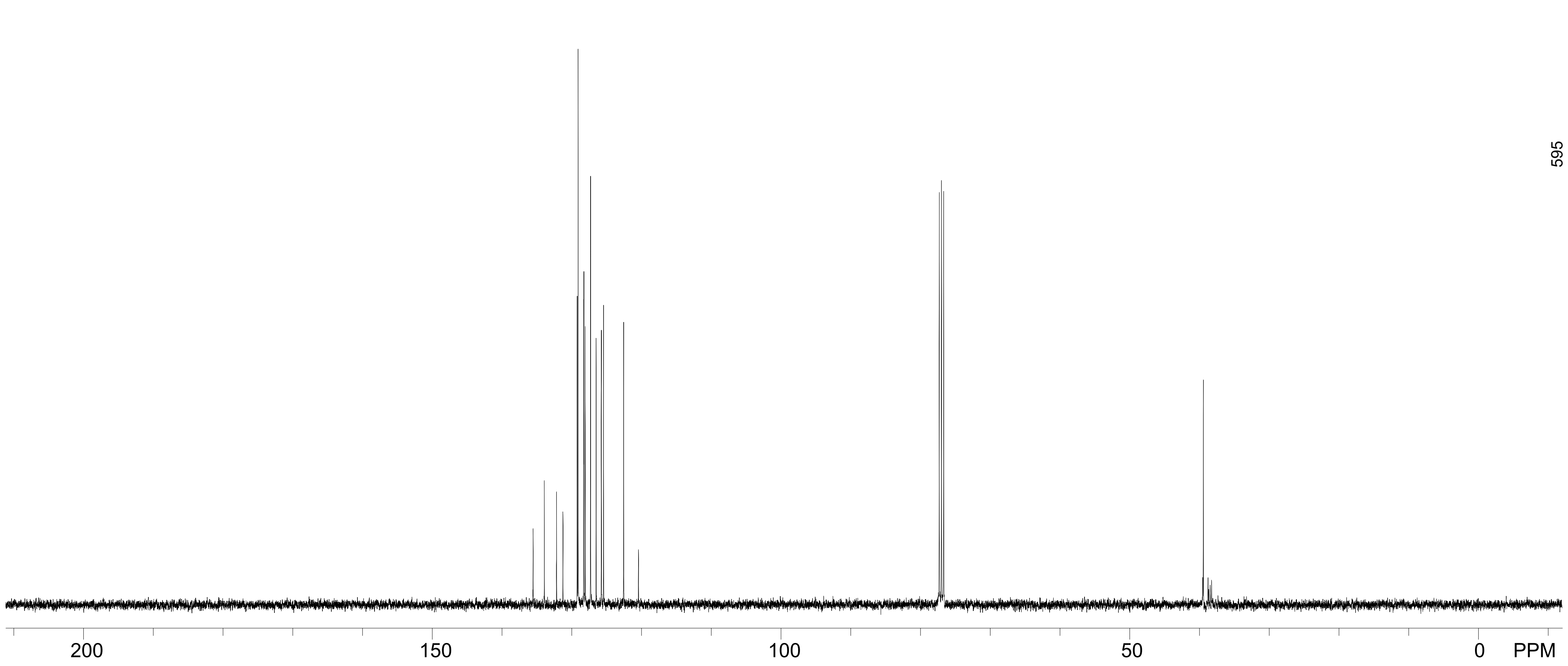

Area Percent Report

Agilent Technologies

Sample name: zwf-8-032-IE-99-1-0.8-205

Data file: C:\Users\Public\Documents\ChemStation\1\Data\zwf\zwf-S 2023-11-15 14-21-28\008-P1-C3-zwf-8-032.D

Injection Acquired Date 11/15/2023 17:44:57

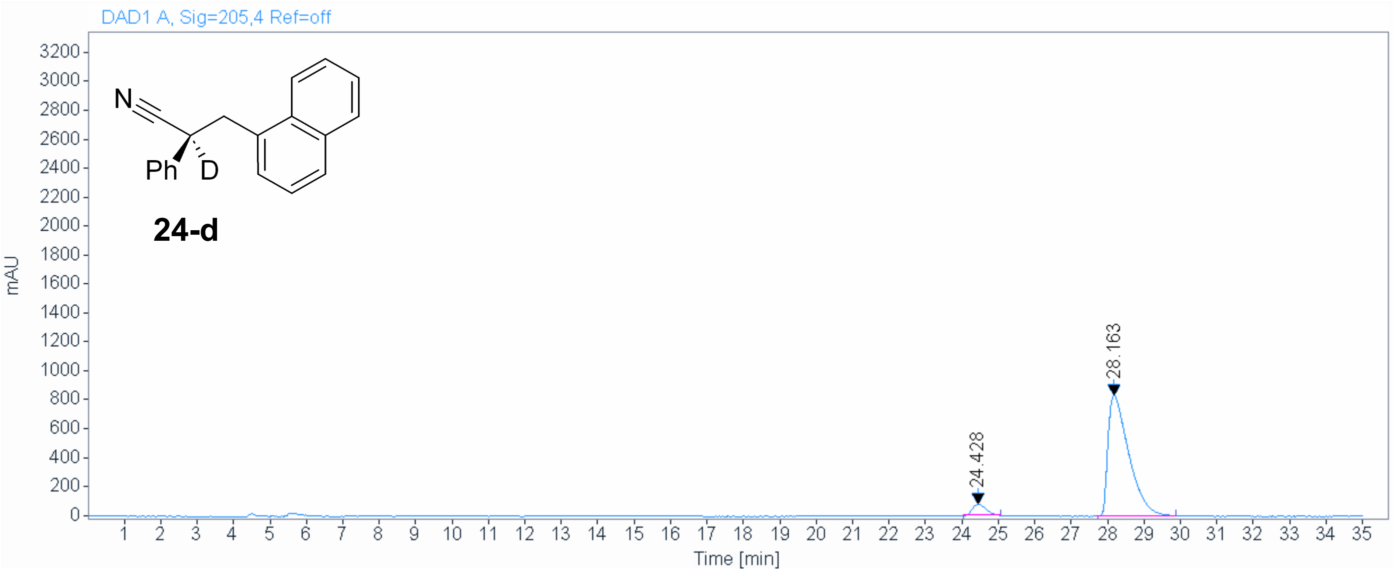

Signal: DAD1 A, Sig=205,4 Ref=off

RT [min]	Width [min]	Height	Area	Area%
24.428	0.4347	77.0568	2009.6523	5.7038
28.163	0.5244	834.0882	33224.0742	94.2962
		Sum	35233.7266	100.0000

Area Percent Report

Sample name: zwf-8-032-rac-IE-99-1-0.8-205

Data file: C:\Users\Public\Documents\ChemStation\1\Data\zwf\zwf-S 2023-11-15 14-21-28\009-P1-C4-zwf-8-032-rac.D

Injection Acquired Date 11/15/2023 18:20:58

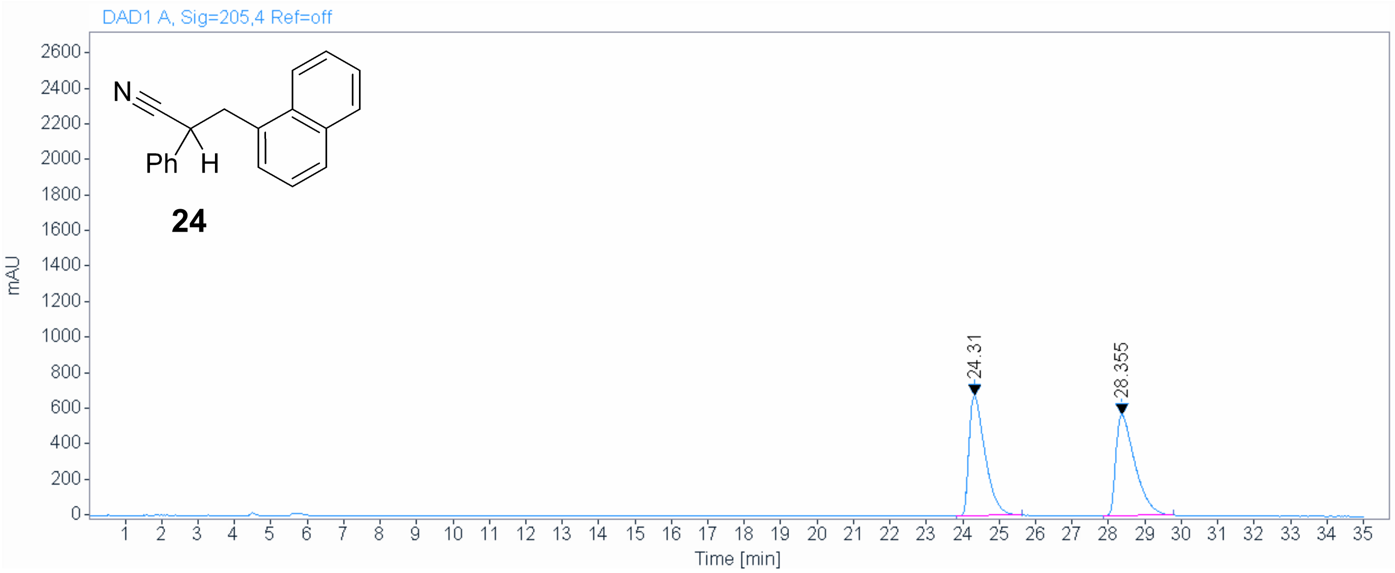

Signal: DAD1 A, Sig=205,4 Ref=off

RT [min]	Width [min]	Height	Area	Area%
24.310	0.4229	677.6952	20862.2852	50.0616
28.355	0.5003	569.3921	20810.9160	49.9384
		Sum	41673.2012	100.0000

7.808 7.793 7.784 7.767 7.745 7.738 7.579 7.472 7.460 7.455 7.450 7.447 7.442 7.436 7.425 7.352 7.344 7.331 7.313 7.309 7.301 7.286 7.280 7.269 7.263 7.255 7.250 7.245 7.229 7.225 7.216 7.208 7.204 4.087 4.066 4.050 3.361 3.341 3.327 3.307 3.289 3.272 3.255 3.238 1.556 -0.000

zwf-7-094H
Sep 26 2023
SOLVENT: CDCl3
NA = 4
F1 = 400.140015 MHz
F2 = 1.000000 MHz

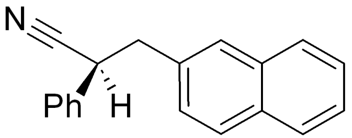

25

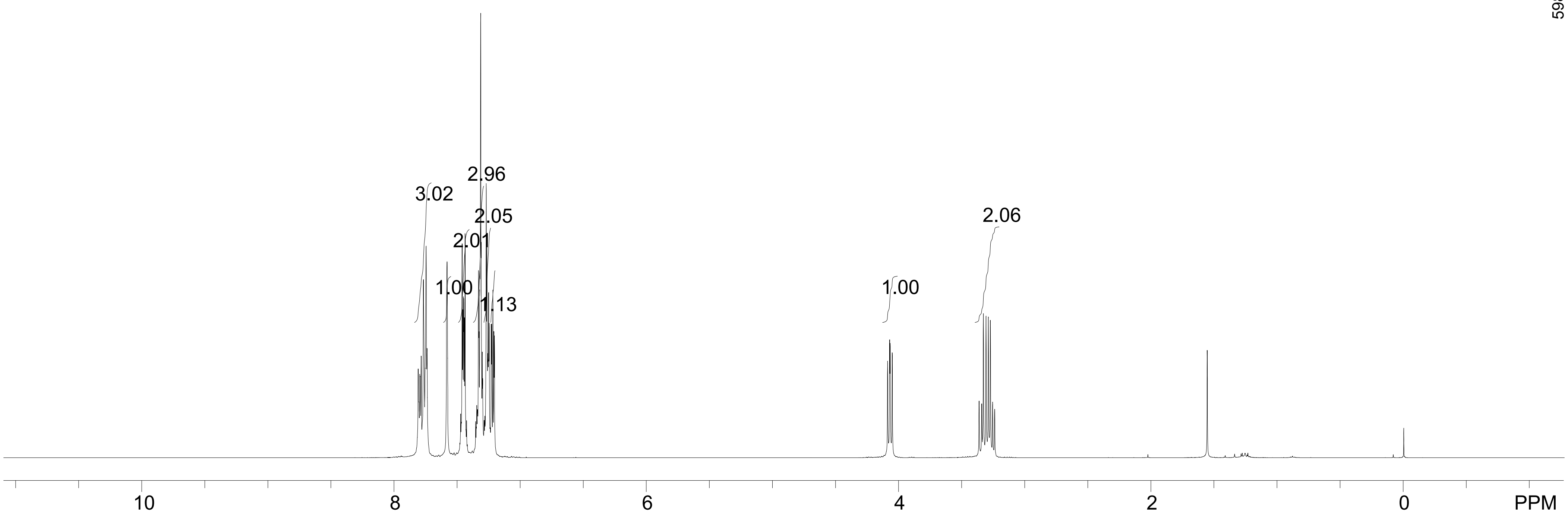

zwf-7-094C
Sep 26 2023
SOLVENT: CDCl3
NA = 150
F1 = 100.615280 MHz
F2 = 1.000000 MHz

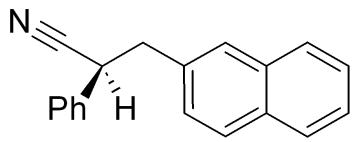

25

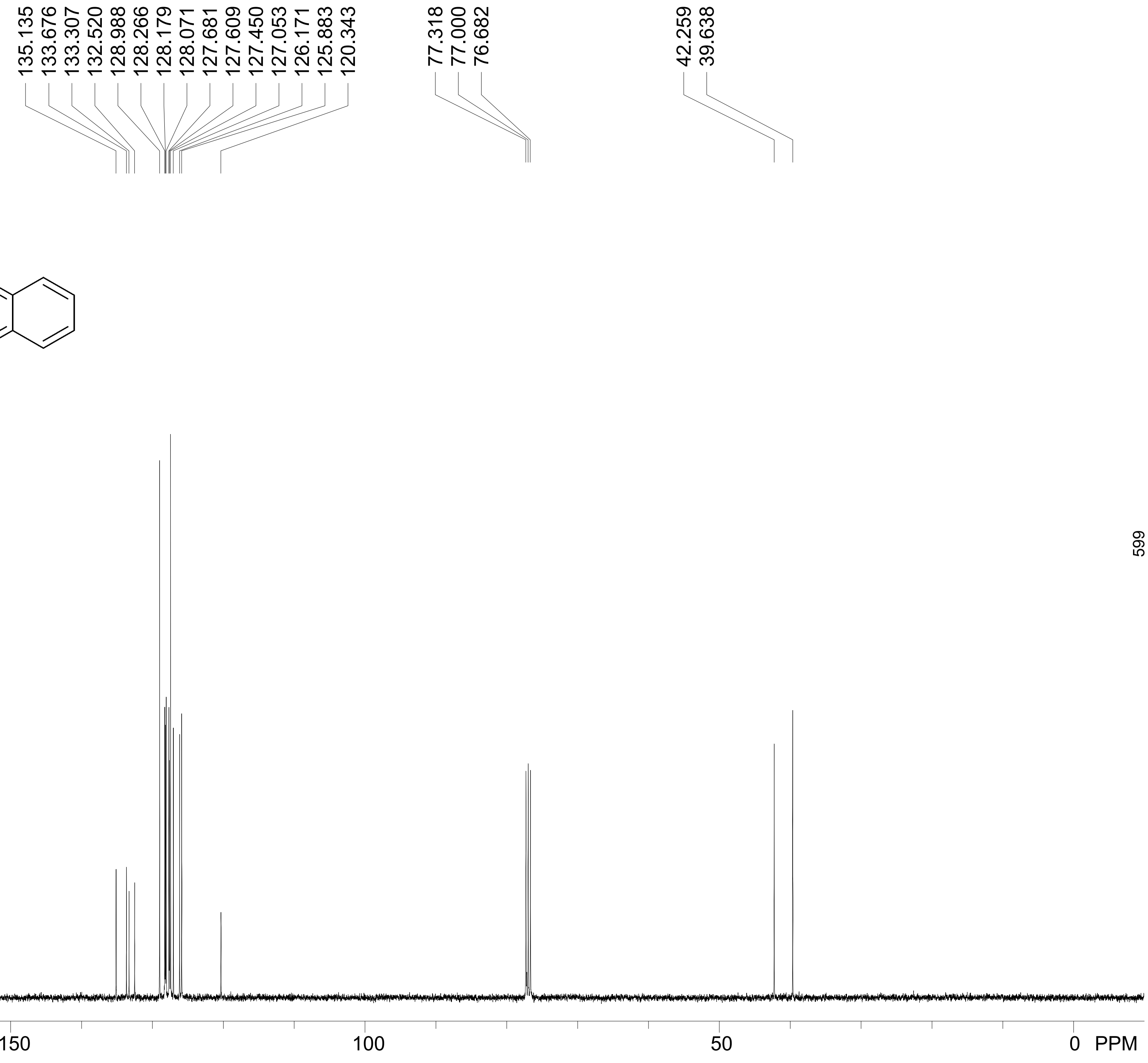

Area Percent Report

Agilent Technologies

Sample name: zwf-7-094-IE-99-1-0.8-205

Data file: C:\Users\Public\Documents\ChemStation\1\Data\zwf\zwf-S 2023-09-27 08-41-33\002-P1-C1-zwf-7-094.D

Injection Acquired Date 09/27/2023 09:09:23

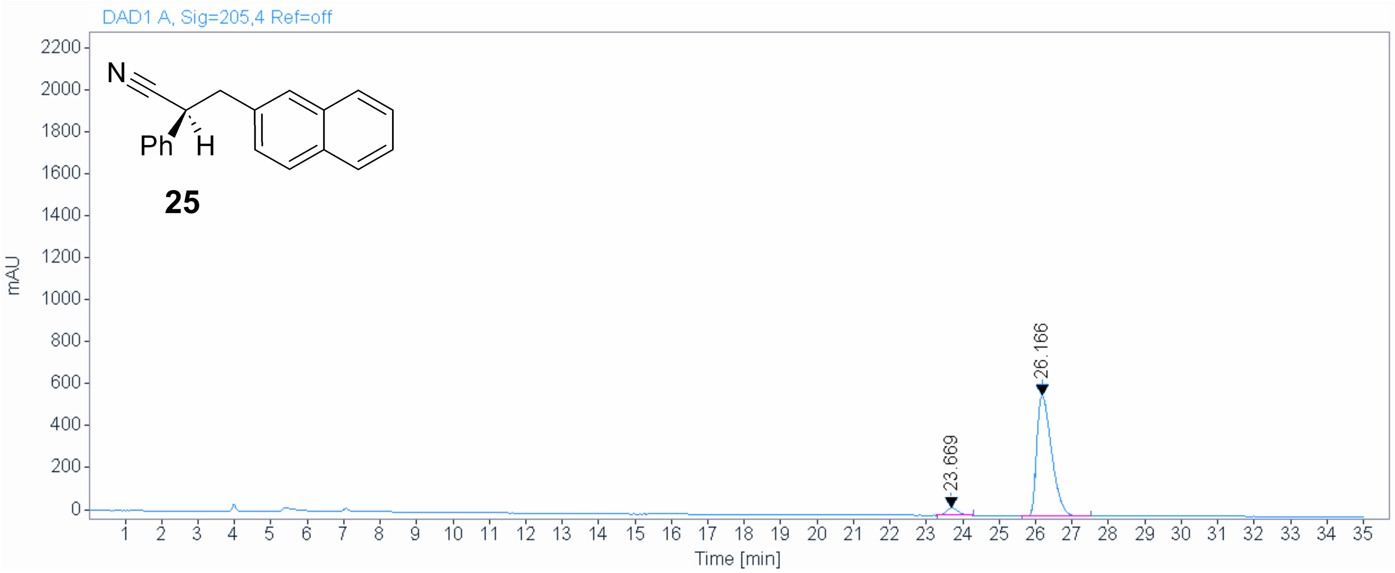

Signal: DAD1 A, Sig=205,4 Ref=off

RT [min]	Width [min]	Height	Area	Area%
23.669	0.3724	34.6284	773.7237	4.5400
26.166	0.4344	574.4220	16268.5566	95.4600
		Sum	17042.2803	100.0000

Area Percent Report

Sample name: zwf-7-094-rac-IE-99-1-0.8-205

Data file: C:\Users\Public\Documents\ChemStation\1\Data\zwf\zwf-S 2023-09-27 08-41-33\003-P1-C2-zwf-7-094-rac.D

Injection Acquired Date 09/27/2023 09:45:24

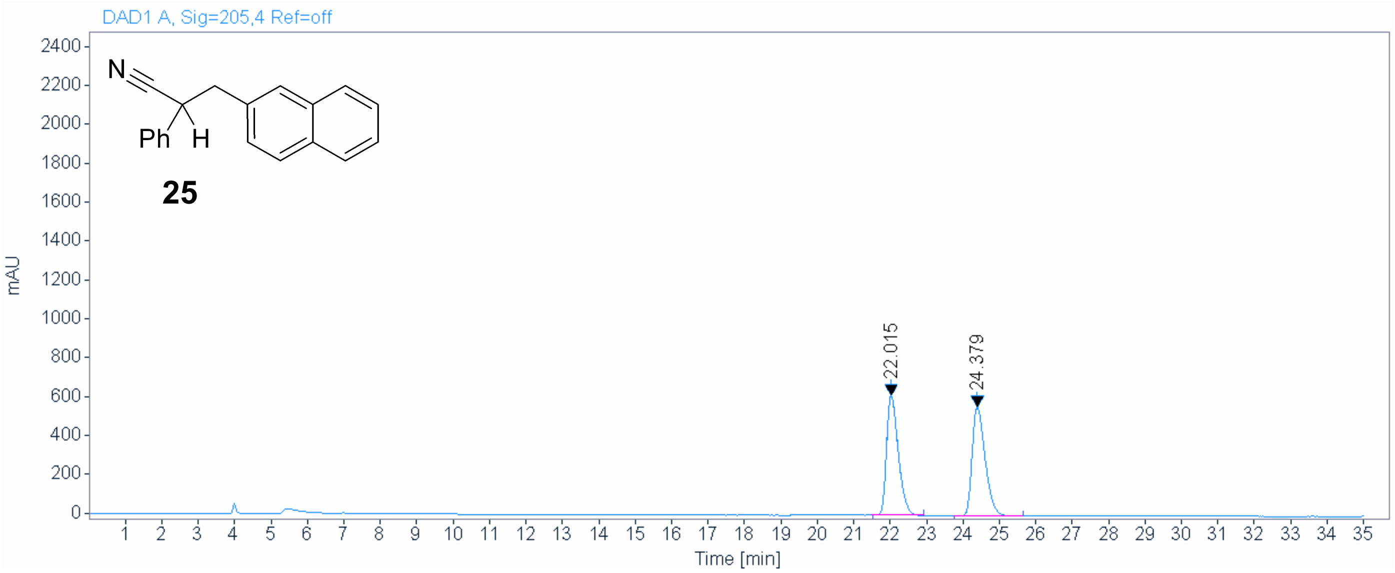

Signal: DAD1 A, Sig=205,4 Ref=off

RT [min]	Width [min]	Height	Area	Area%
22.015	0.3463	615.8998	13849.2949	49.7207
24.379	0.3875	560.5504	14004.9063	50.2793
		Sum	27854.2012	100.0000

zwf-8-033H
Nov 13 2023
SOLVENT: CDCl3
NA = 4
F1 = 400.179993 MHz
F2 = 1.000000 MHz

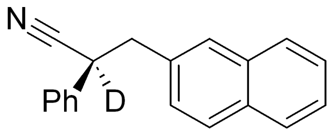

25-d

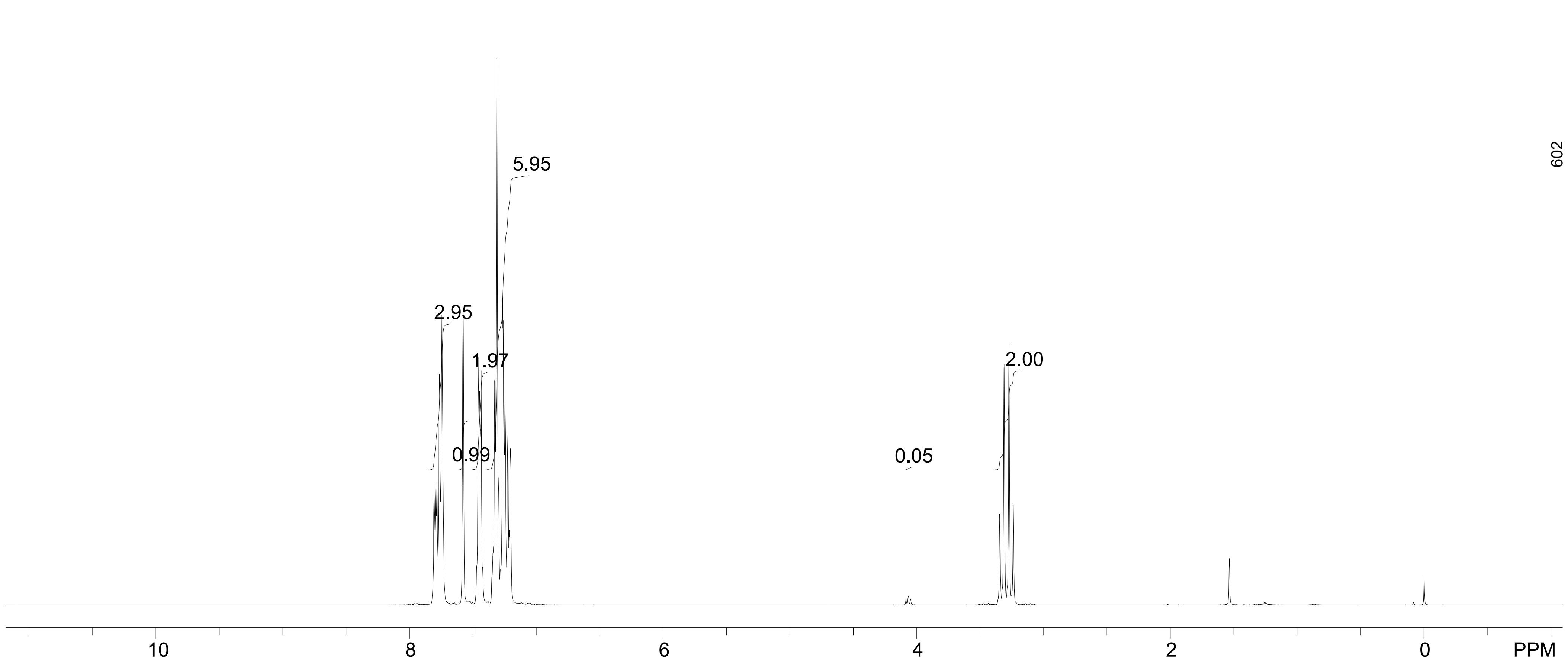

zwf-8-033C
Nov 13 2023
SOLVENT: CDCl3
NA = 100
F1 = 100.625343 MHz
F2 = 1.000000 MHz

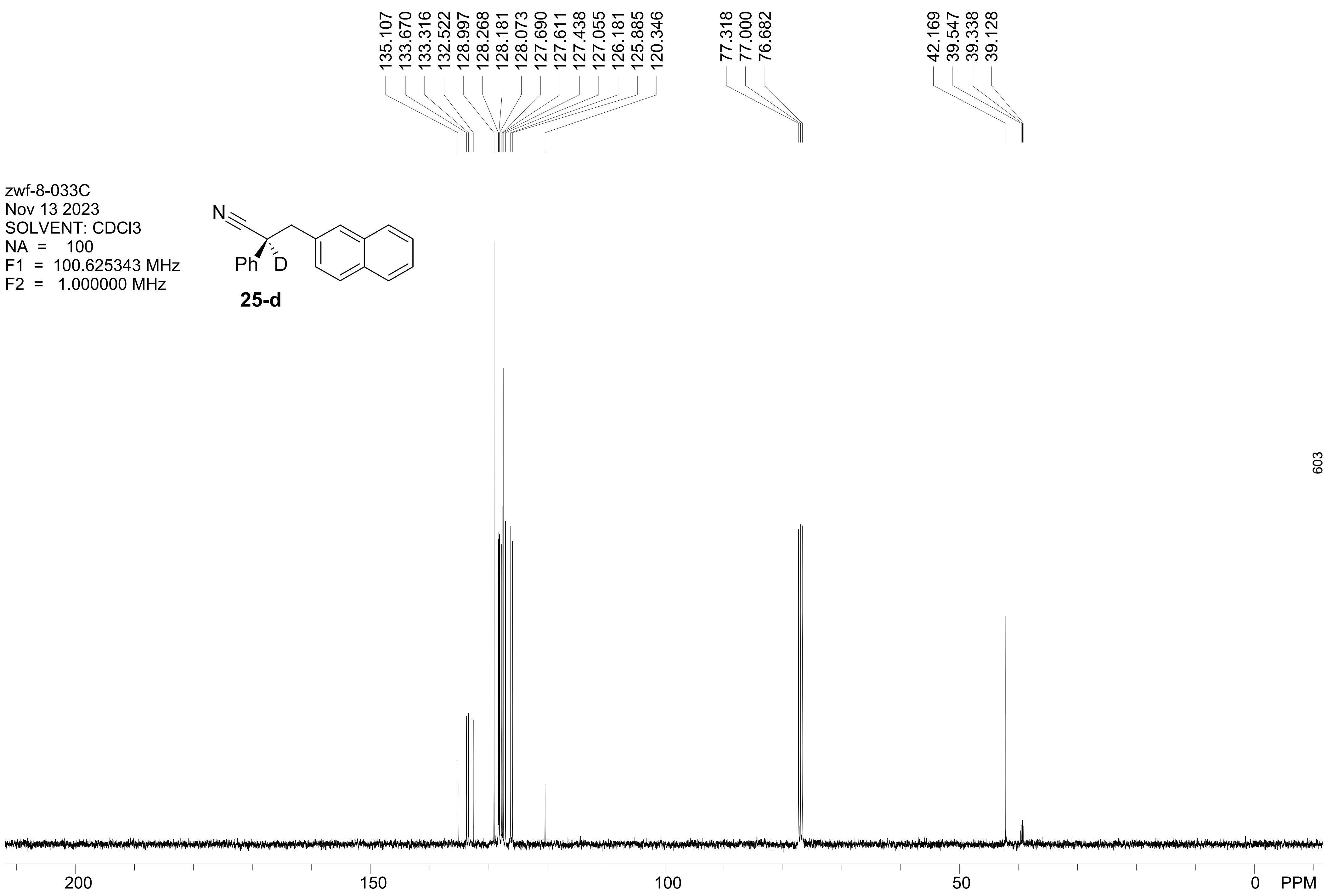

25-d

Area Percent Report

Agilent Technologies

Sample name: zwf-8-033-IE-99-1-0.8-205

Data file: C:\Users\Public\Documents\ChemStation\1\Data\zwf\zwf-S 2023-11-15 14-21-28\012-P1-C5-zwf-8-033.D

Injection Acquired Date 11/15/2023 20:19:11

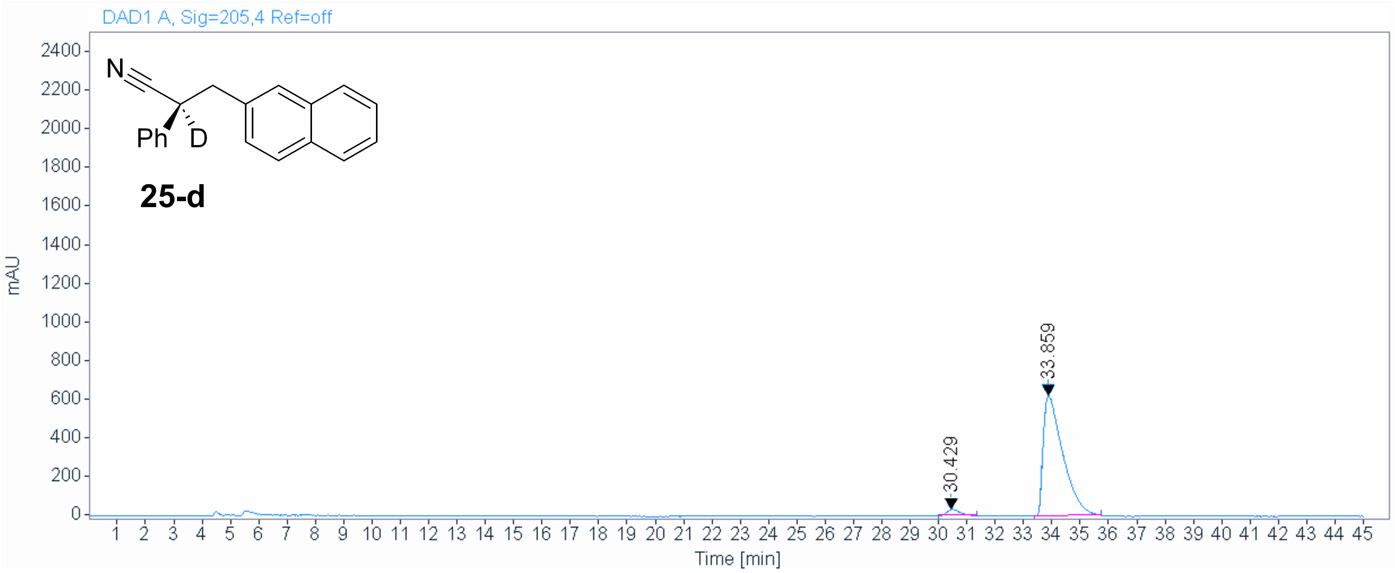

Signal: DAD1 A, Sig=205,4 Ref=off

RT [min]	Width [min]	Height	Area	Area%
30.429	0.5241	32.0426	1007.5408	3.2385
33.859	0.6307	623.9166	30103.9121	96.7615
		Sum	31111.4529	100.0000

Area Percent Report

Sample name: zwf-8-033-rac-IE-99-1-0.8-205

Data file: C:\Users\Public\Documents\ChemStation\1\Data\zwf\zwf-S 2023-11-15 14-21-28\011-P1-C6-zwf-8-033-rac.D

Injection Acquired Date 11/15/2023 19:33:03

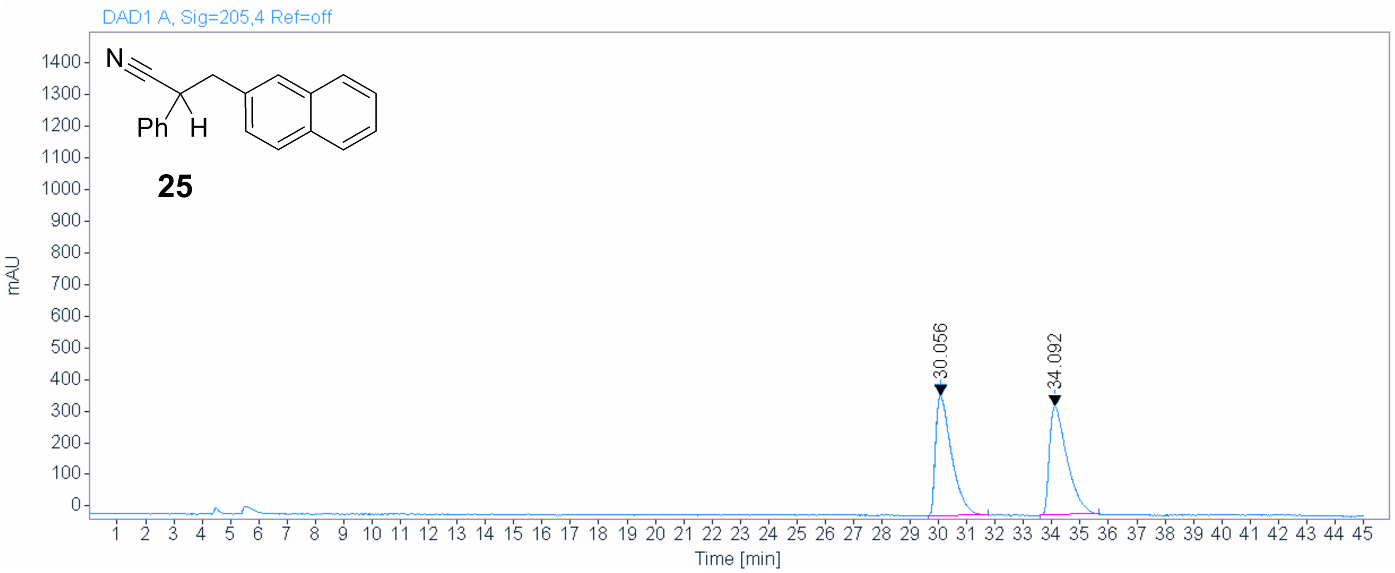

Signal: DAD1 A, Sig=205,4 Ref=off

RT [min]	Width [min]	Height	Area	Area%
30.056	0.5280	379.3584	15030.7217	49.9092
34.092	0.5386	344.4671	15085.4170	50.0908
		Sum	30116.1387	100.0000

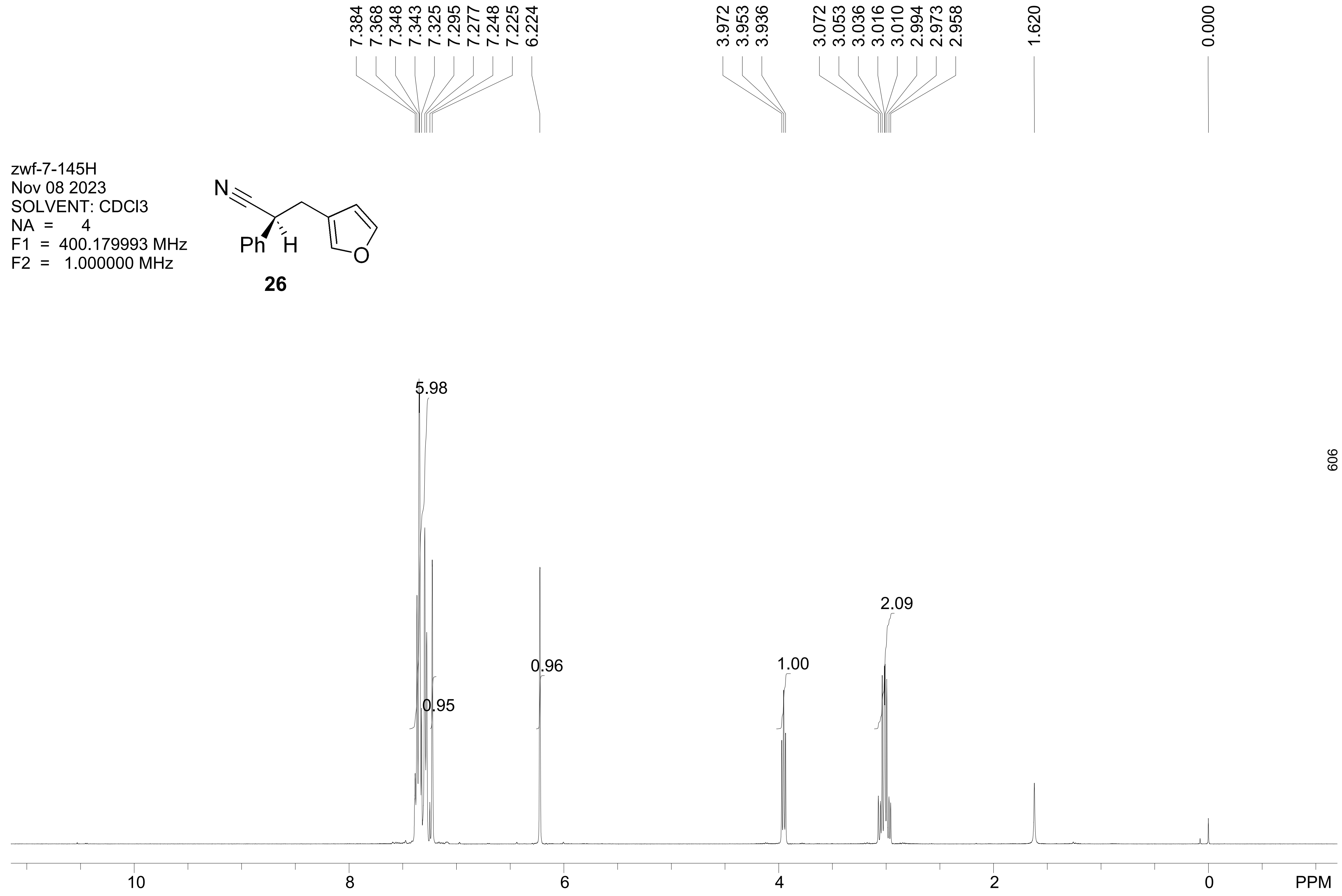
zwf-7-145H
Nov 08 2023
SOLVENT: CDCl3
NA = 4
F1 = 400.179993 MHz
F2 = 1.000000 MHz
7.384
7.368
7.348
7.343
7.325
7.295
7.277
7.248
7.225
6.224
3.972
3.953
3.936
3.072
3.053
3.036
3.016
3.010
2.994
2.973
2.958
1.620
0.000
5.98
0.95
0.96
1.00
2.09
10
8
6
4
2
0
PPM
N
Ph
H
O

26

zwf-7-145C
Nov 08 2023
SOLVENT: CDCl3
NA = 100
F1 = 100.625343 MHz
F2 = 1.000000 MHz

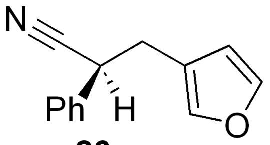

26

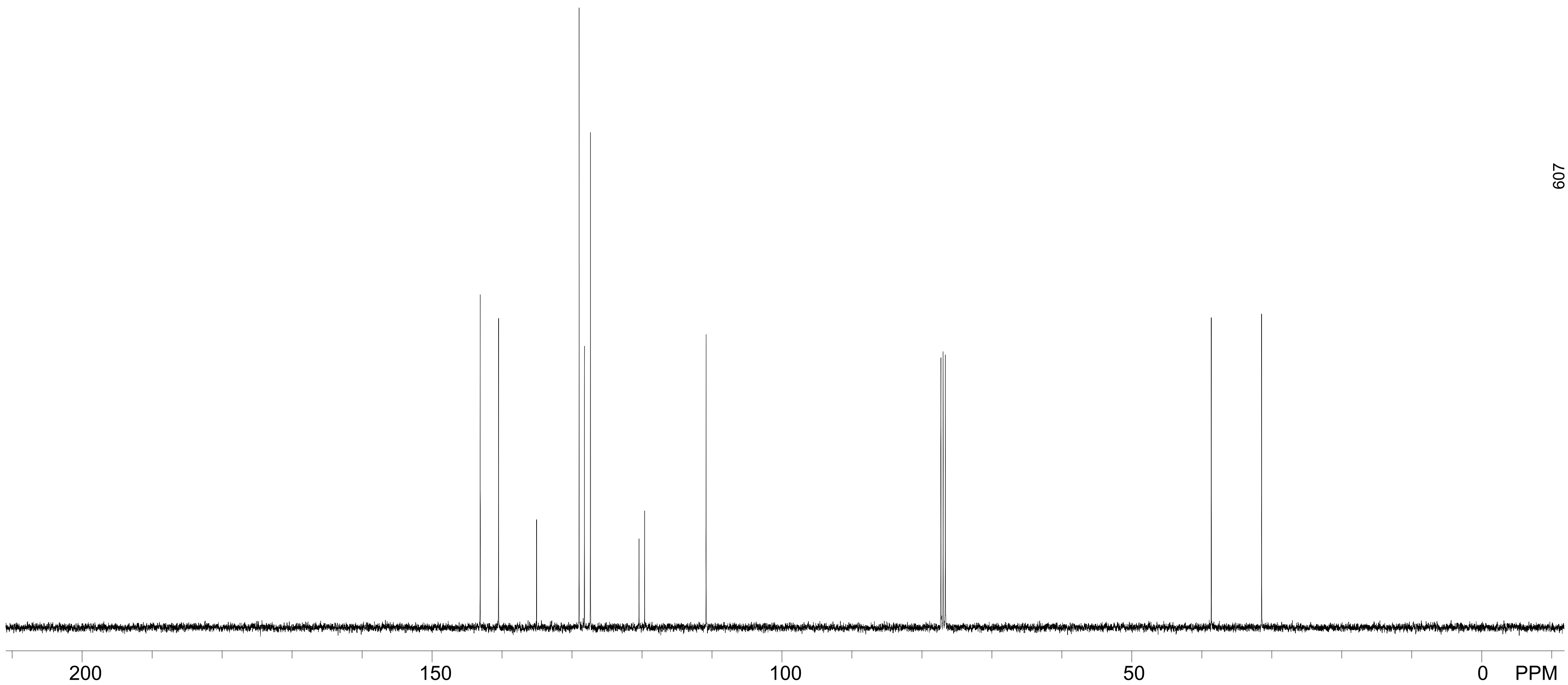

Area Percent Report

Agilent Technologies

Sample name: zwf-7-145-IE-99-1-0.8-205

Data file: C:\Users\Public\Documents\ChemStation\1\Data\zwf\zwf-S 2023-11-08 08-15-08\007-P1-C3-zwf-7-145.D

Injection Acquired Date 11/08/2023 10:30:46

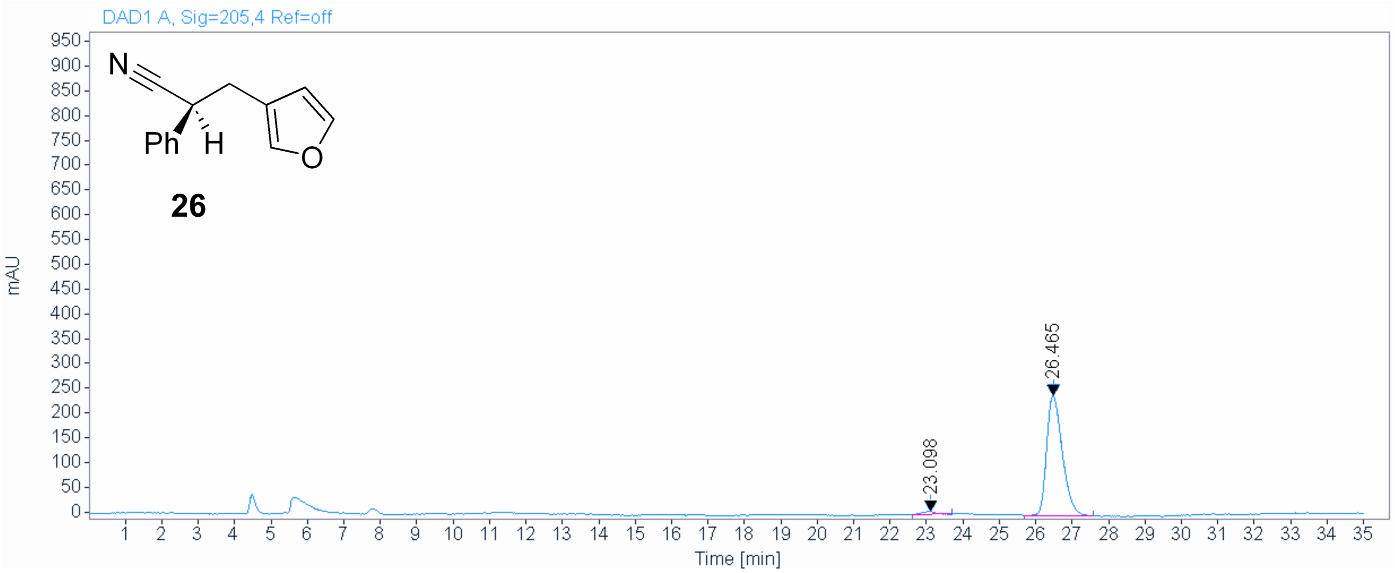

Signal: DAD1 A, Sig=205,4 Ref=off

RT [min]	Width [min]	Height	Area	Area%
23.098	0.4206	6.3264	159.6672	2.1380
26.465	0.4250	242.7398	7308.4956	97.8620
		Sum	7468.1628	100.0000

Area Percent Report

Sample name: zwf-7-145-rac-IE-98-1-0.8-205

Data file: C:\Users\Public\Documents\ChemStation\1\Data\zwf\zwf-S 2023-11-08 08-15-08\008-P1-C4-zwf-7-145-rac.D

Injection Acquired Date 11/08/2023 11:06:50

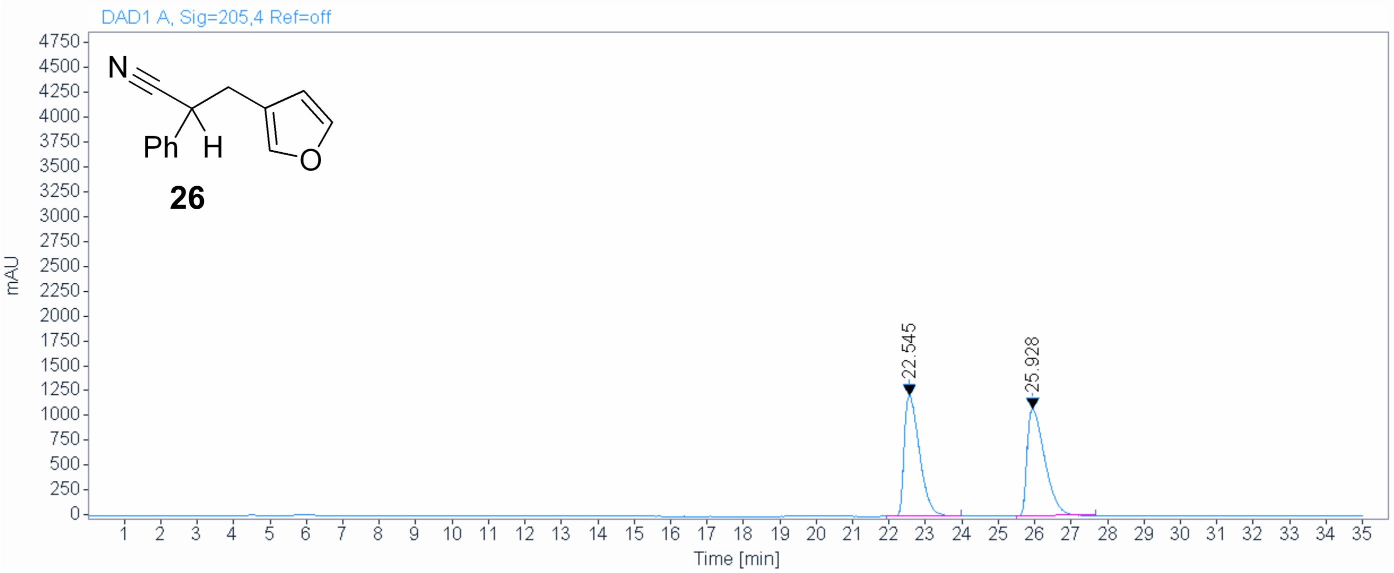

Signal: DAD1 A, Sig=205,4 Ref=off

RT [min]	Width [min]	Height	Area	Area%
22.545	0.3647	1212.9807	35420.4258	49.3123
25.928	0.4625	1075.9082	36408.2891	50.6877
		Sum	71828.7148	100.0000

zwf-8-005H
Nov 14 2023
SOLVENT: CDCl3
NA = 4
F1 = 400.140015 MHz
F2 = 1.000000 MHz

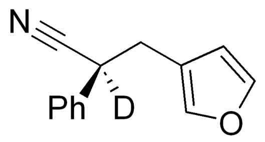

26-d

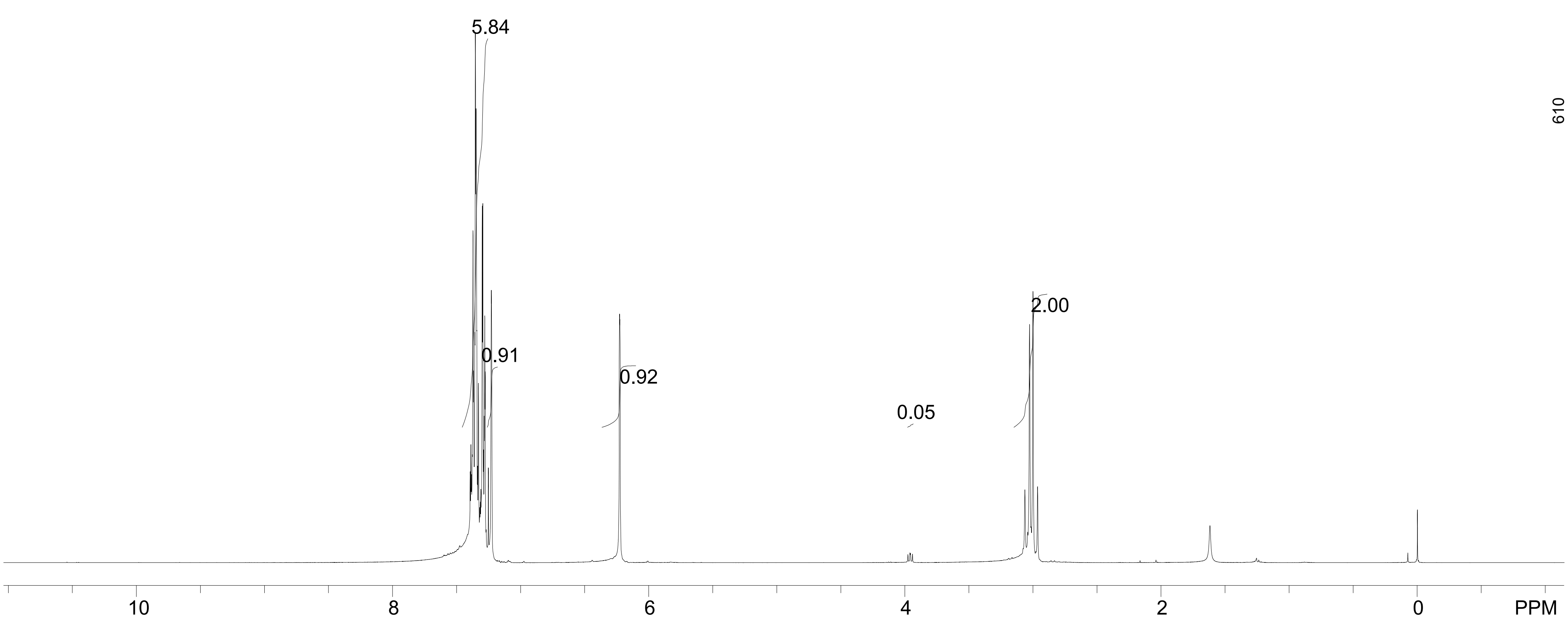

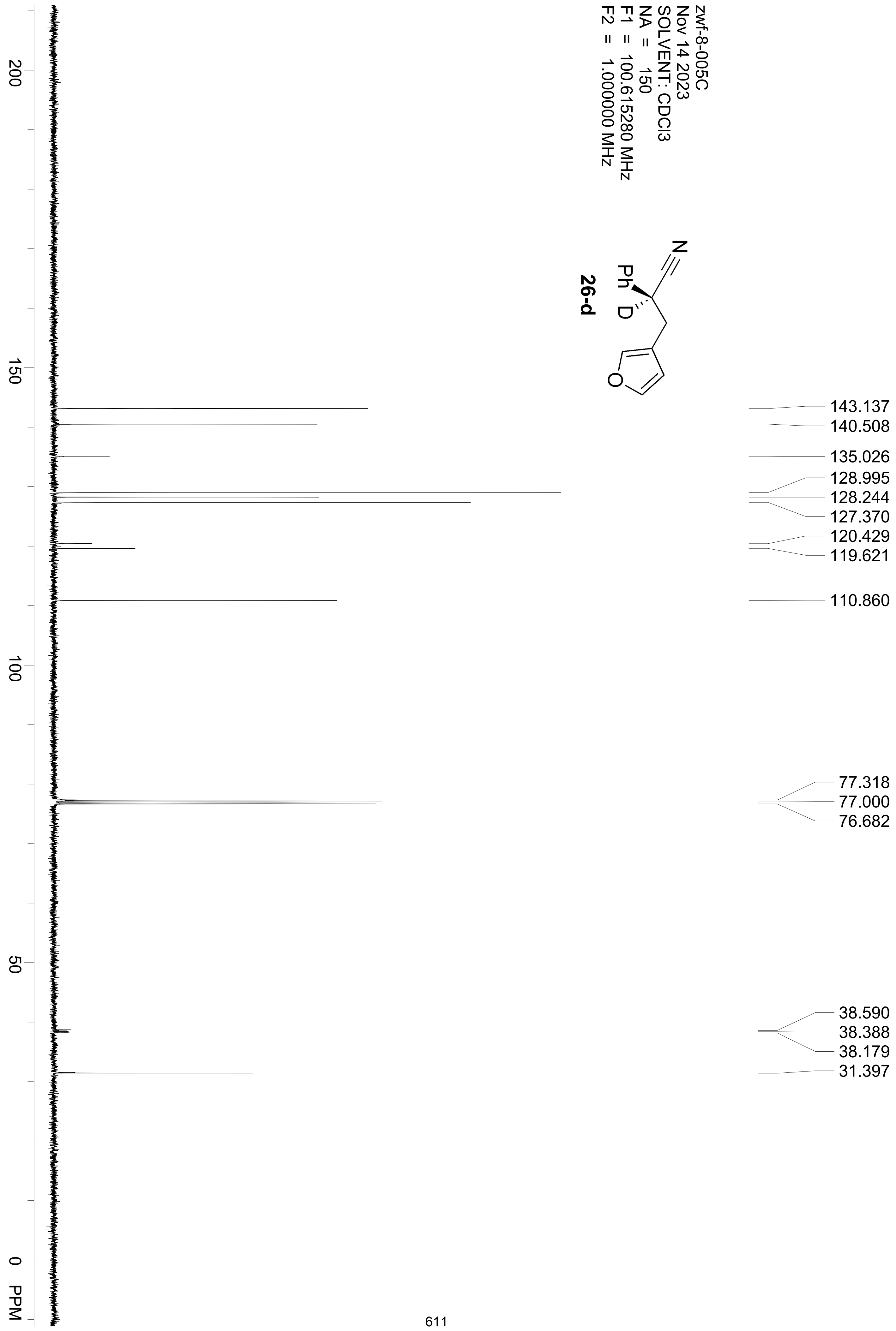
zwf-8-005C
Nov 14 2023
SOLVENT: CDCl3
NA = 150
F1 = 100.615280 MHz
F2 = 1.000000 MHz
26-d
143.137
140.508
135.026
128.995
128.244
127.370
120.429
119.621
110.860
77.318
77.000
76.682
38.590
38.388
38.179
31.397
200
150
100
50
0
PPM

Area Percent Report

Sample name: zwf-8-005-IE-99-1-0.8-205

Data file: C:\Users\Public\Documents\ChemStation\1\Data\zwf\zwf-S 2023-11-14 20-02-58\012-P1-C3-zwf-8-005.D

Injection Acquired Date 11/15/2023 02:05:44

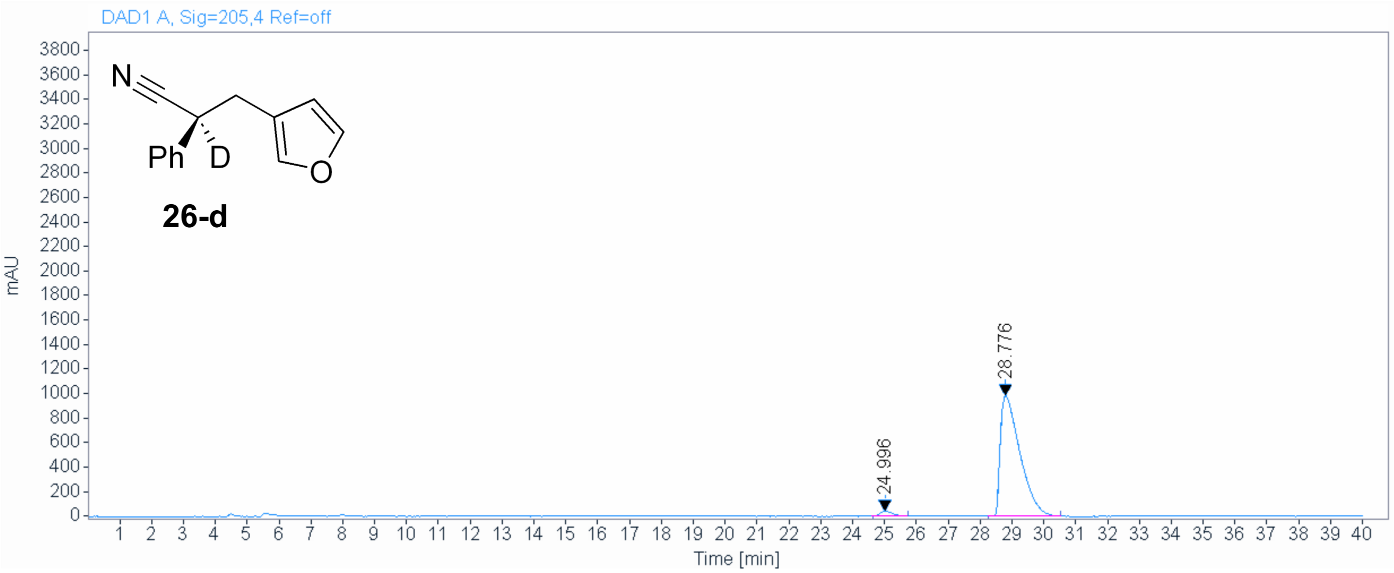

Signal: DAD1 A, Sig=205,4 Ref=off

RT [min]	Width [min]	Height	Area	Area%
24.996	0.3390	43.0947	1193.6187	2.7421
28.776	0.5151	985.5175	42335.9414	97.2579
		Sum	43529.5601	100.0000

Area Percent Report

Sample name: zwf-8-005-rac-IE-99-1-0.8-205

Data file: C:\Users\Public\Documents\ChemStation\1\Data\zwf\zwf-S 2023-11-14 20-02-58\013-P1-C4-zwf-8-005-rac.D

Injection Acquired Date 11/15/2023 02:46:47

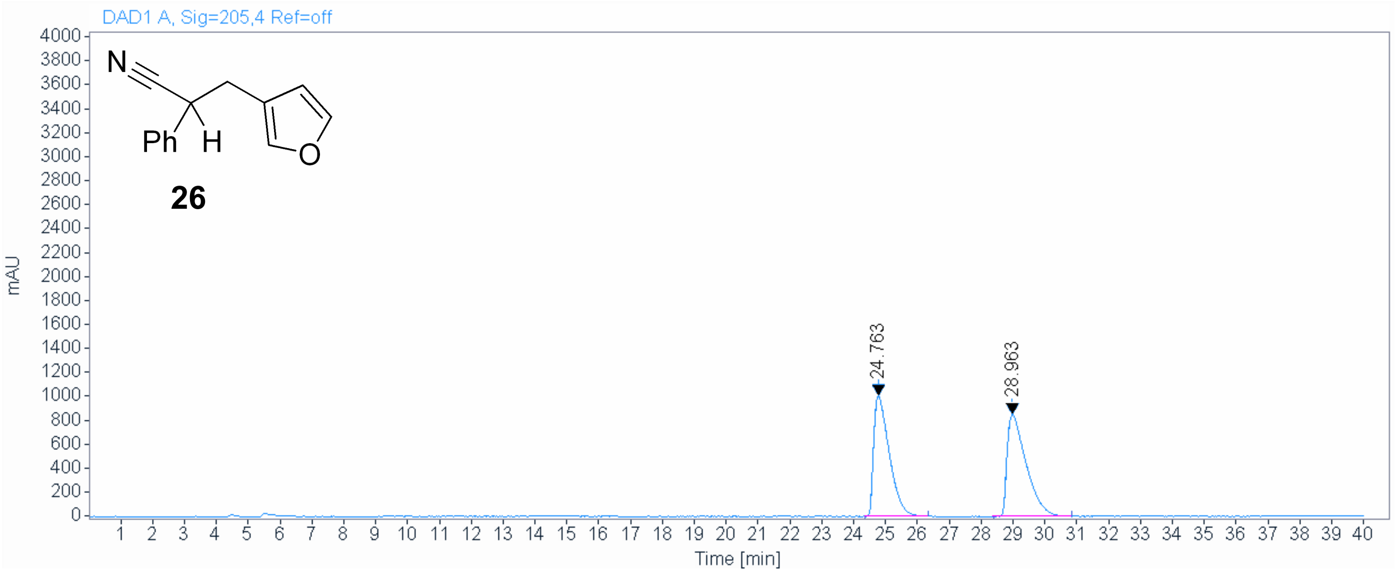

Signal: DAD1 A, Sig=205,4 Ref=off

RT [min]	Width [min]	Height	Area	Area%
24.763	0.4099	1008.9810	34285.8672	49.1938
28.963	0.4958	852.8981	35409.6602	50.8062
		Sum	69695.5273	100.0000

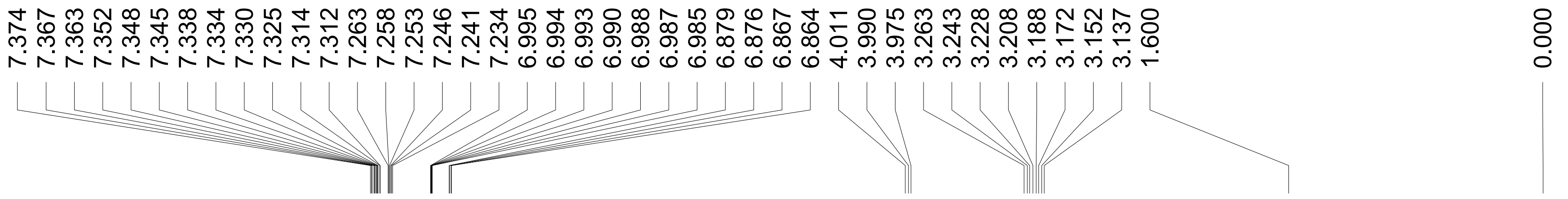

zwf-7-093H
Sep 26 2023
SOLVENT: CDCl3
NA = 4
F1 = 400.140015 MHz
F2 = 1.000000 MHz

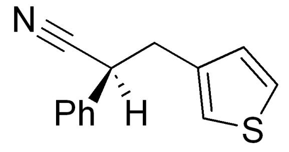

27

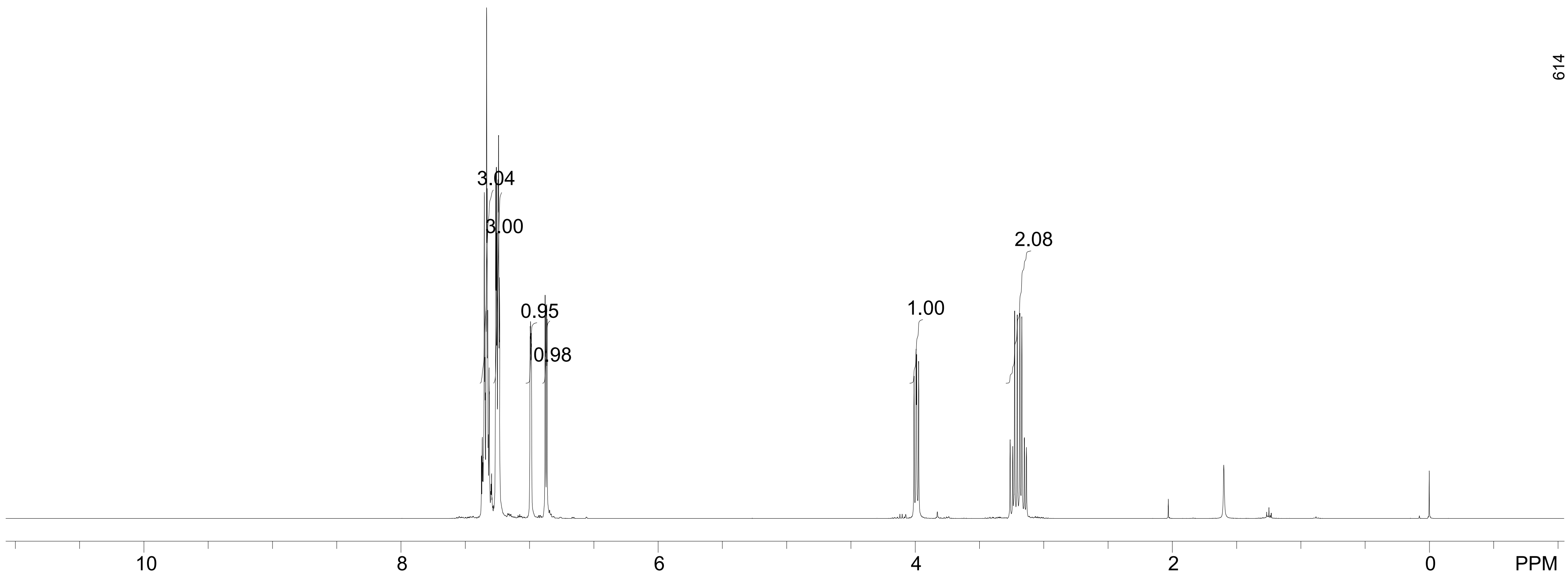

zwf-7-093C
Sep 26 2023
SOLVENT: CDCl3
NA = 100
F1 = 100.615280 MHz
F2 = 1.000000 MHz

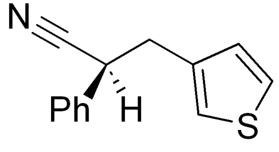

27

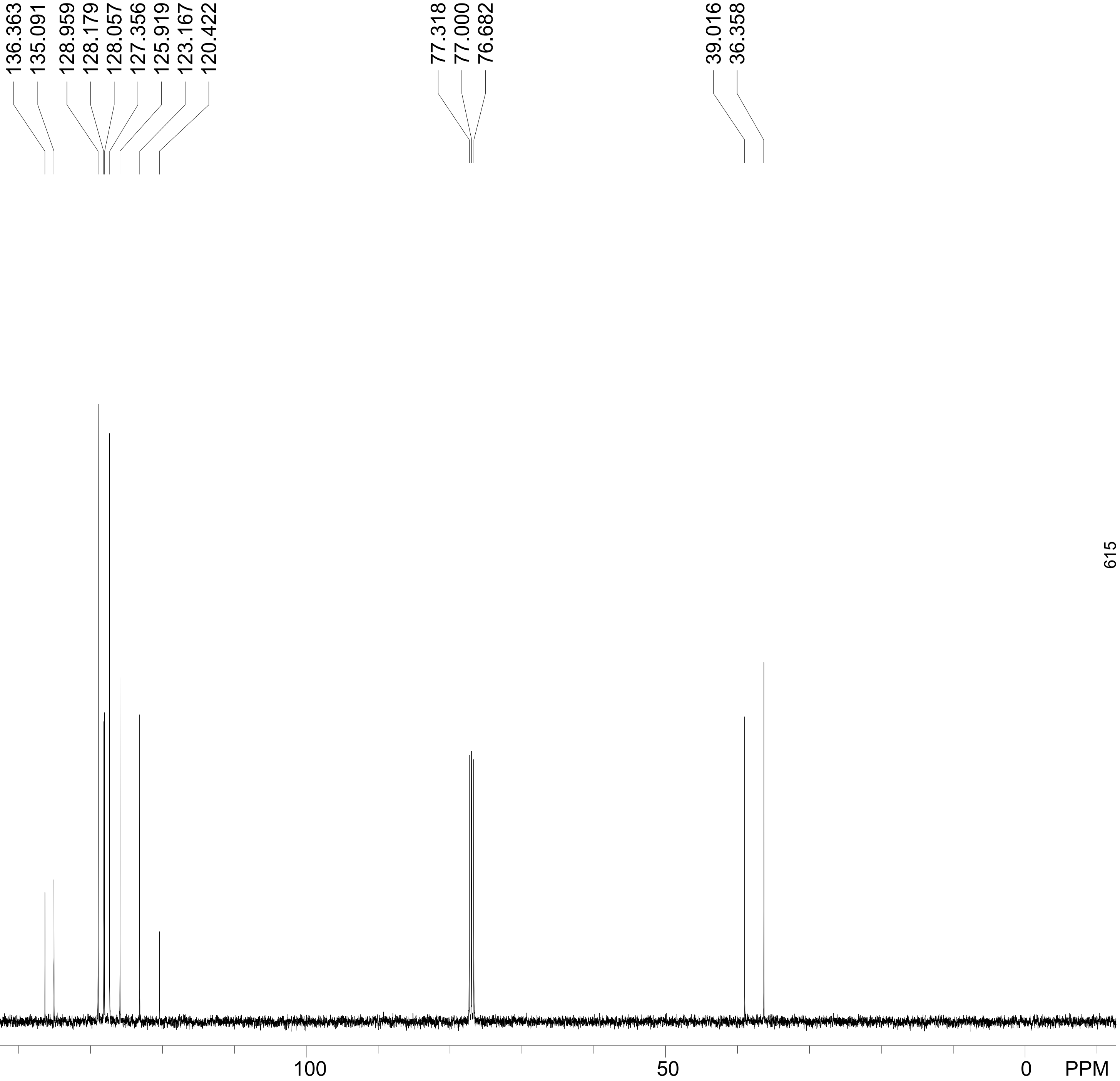

Area Percent Report

Agilent Technologies

Sample name: zwf-7-093-IE-99-1-0.8-205

Data file: C:\Users\Public\Documents\ChemStation\1\Data\zwf\zwf-S 2023-09-26 14-33-49\018-P1-B3-zwf-7-093.D

Injection Acquired Date 09/26/2023 22:11:12

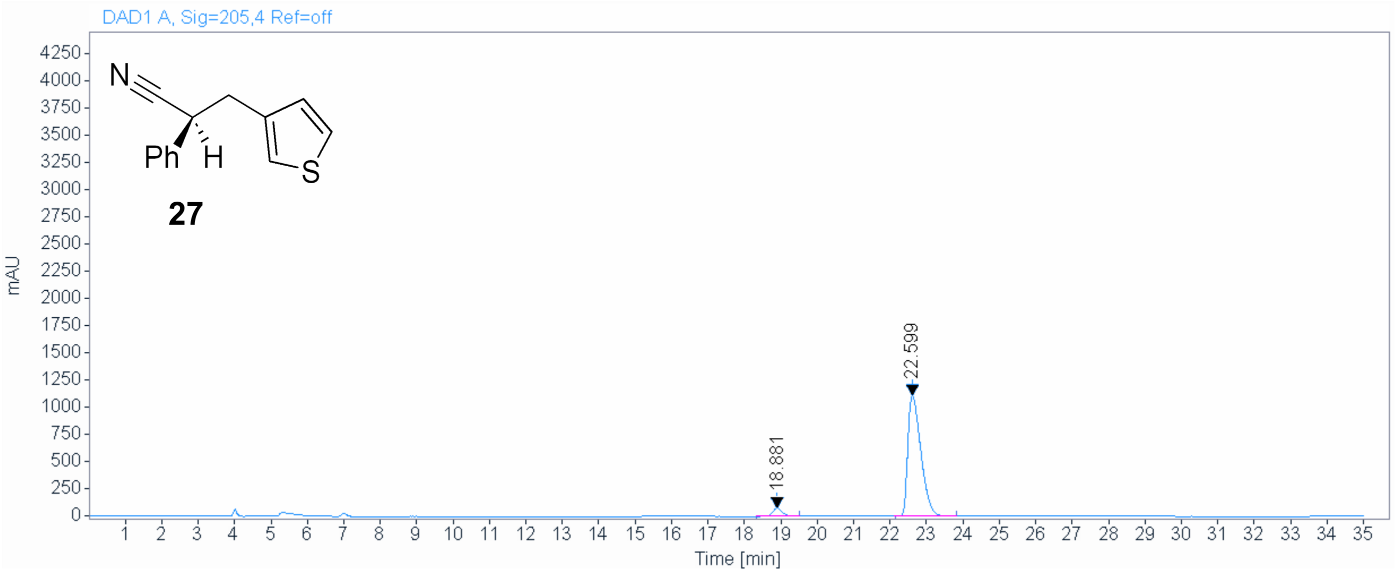

Signal: DAD1 A, Sig=205,4 Ref=off

RT [min]	Width [min]	Height	Area	Area%
18.881	0.2764	75.1079	1367.2754	4.7040
22.599	0.3713	1109.4366	27698.6445	95.2960
		Sum	29065.9199	100.0000

Area Percent Report

Agilent Technologies

Sample name: zwf-7-093-rac-IE-99-1-0.8-205

Data file: C:\Users\Public\Documents\ChemStation\1\Data\zwf\zwf-S 2023-09-26 14-33-49\019-P1-B4-zwf-7-093-rac.D

Injection Acquired Date 09/26/2023 22:47:19

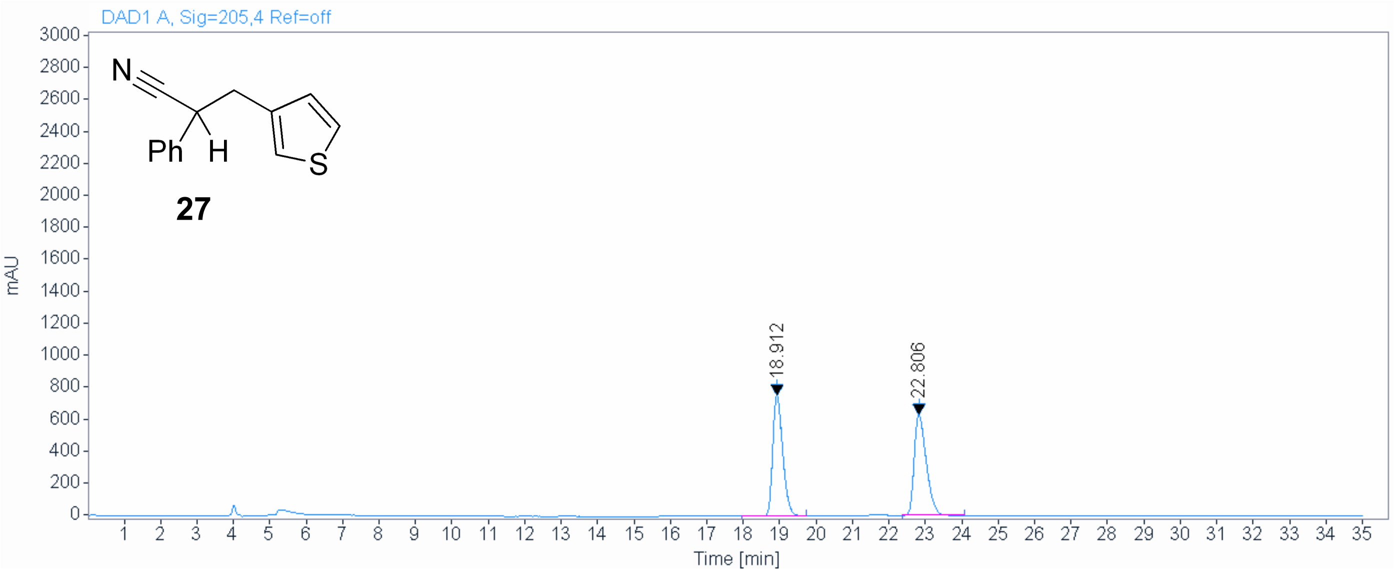

Signal: DAD1 A, Sig=205,4 Ref=off

RT [min]	Width [min]	Height	Area	Area%
18.912	0.2907	753.4446	14454.7842	49.7898
22.806	0.3561	629.6818	14576.8096	50.2102
		Sum	29031.5938	100.0000

zwf-8-034H
Nov 14 2023
SOLVENT: CDCl3
NA = 4
F1 = 400.140015 MHz
F2 = 1.000000 MHz

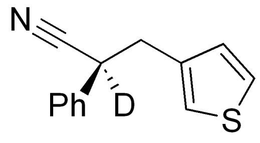

27-d

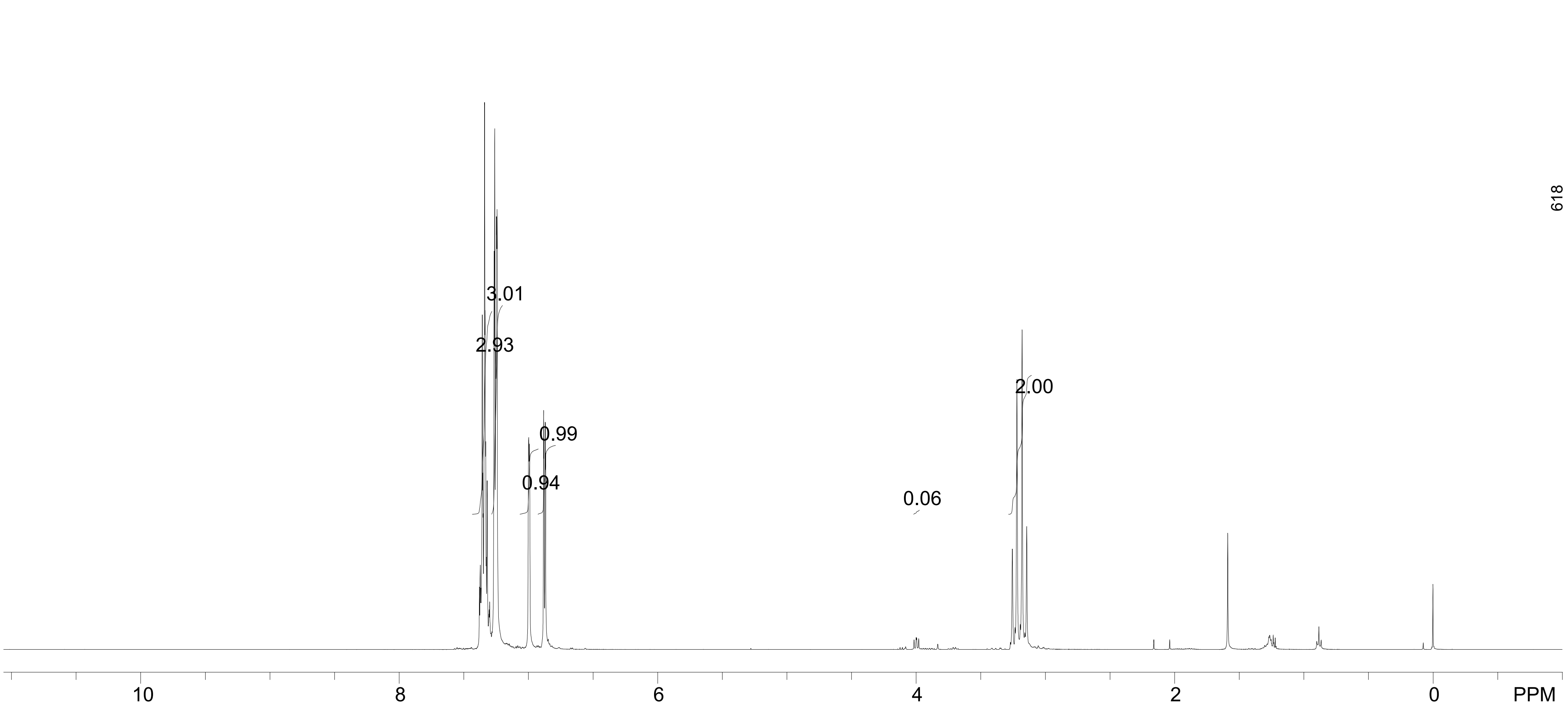

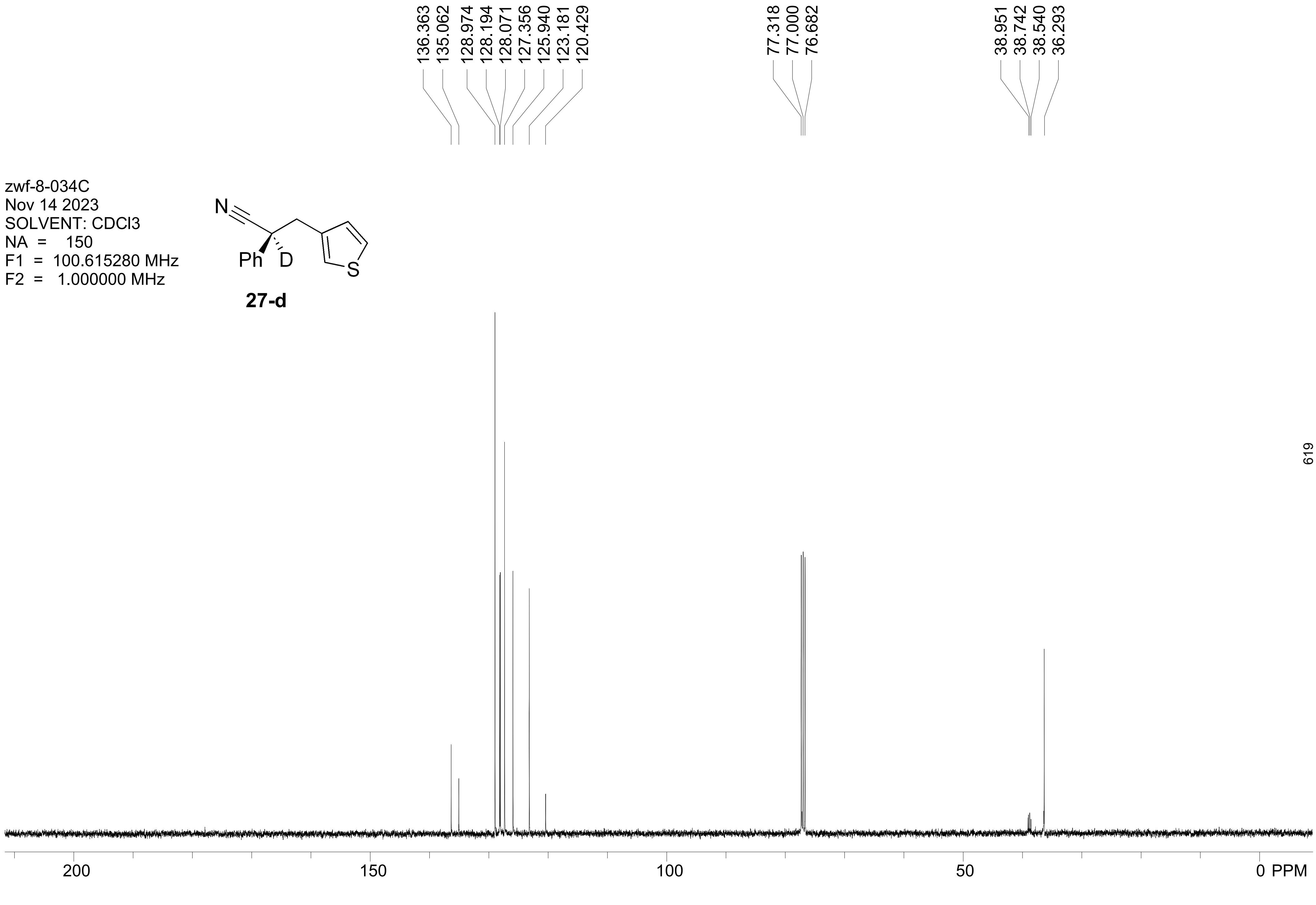
zwf-8-034C
Nov 14 2023
SOLVENT: CDCl3
NA = 150
F1 = 100.615280 MHz
F2 = 1.000000 MHz
N
Ph
D
S
27-d
136.363
135.062
128.974
128.194
128.071
127.356
125.940
123.181
120.429
77.318
77.000
76.682
38.951
38.742
38.540
36.293
200
150
100
50
0 PPM

Area Percent Report

Agilent Technologies

Sample name: zwf-8-034-IE-99-1-0.8-205

Data file: C:\Users\Public\Documents\ChemStation\1\Data\zwf\zwf-S 2023-11-15 14-21-28\013-P1-C7-zwf-8-034.D

Injection Acquired Date 11/15/2023 21:05:16

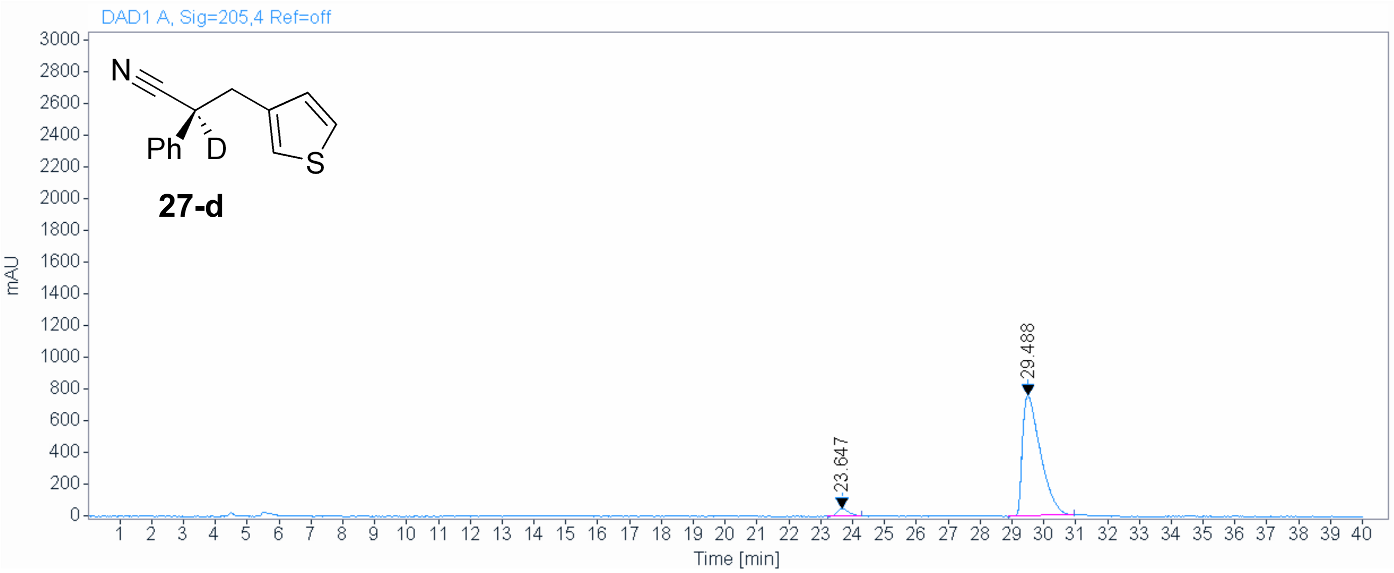

Signal: DAD1 A, Sig=205,4 Ref=off

RT [min]	Width [min]	Height	Area	Area%
23.647	0.3237	48.2951	1247.9852	4.0076
29.488	0.4671	759.4837	29892.4863	95.9924
		Sum	31140.4716	100.0000

Area Percent Report

Agilent Technologies

Sample name: zwf-8-034-rac-IE-99-1-0.8-205

Data file: C:\Users\Public\Documents\ChemStation\1\Data\zwf\zwf-S 2023-11-15 14-21-28\014-P1-C8-zwf-8-034-rac.D

Injection Acquired Date 11/15/2023 21:46:20

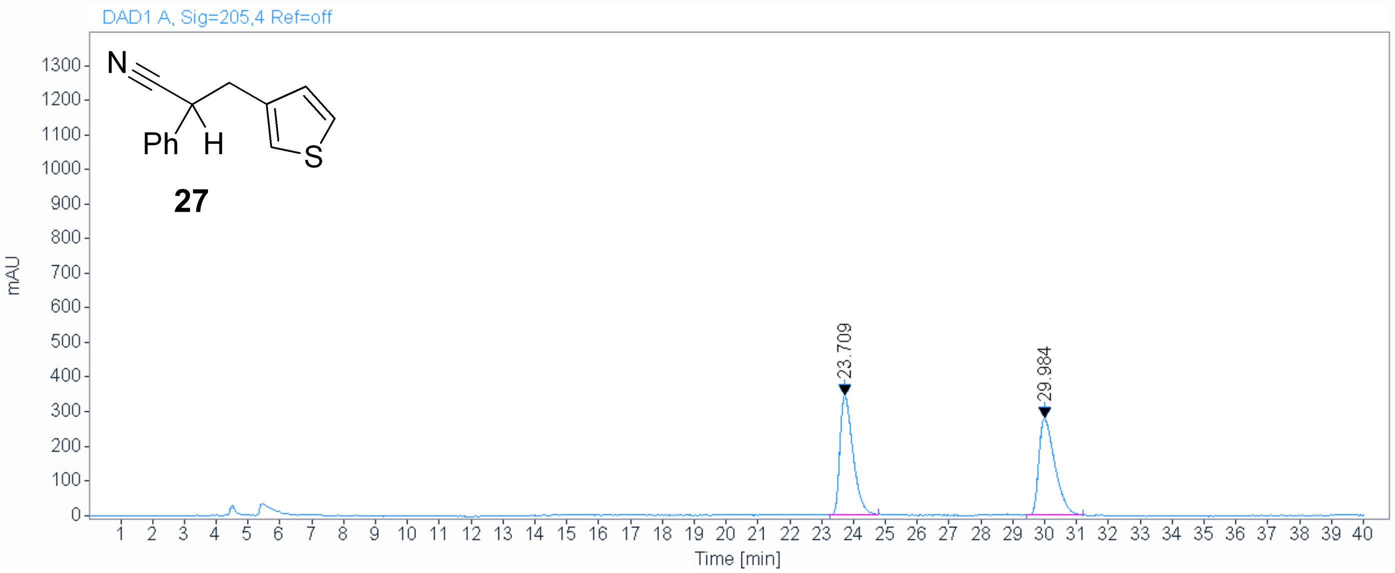

Signal: DAD1 A, Sig=205,4 Ref=off

RT [min]	Width [min]	Height	Area	Area%
23.709	0.3969	344.6831	9785.7500	49.8406
29.984	0.4225	280.6974	9848.3516	50.1594
		Sum	19634.1016	100.0000

zwf-7-073H
Sep 16 2023
SOLVENT: CDCl3
NA = 6
F1 = 400.179993 MHz
F2 = 1.000000 MHz

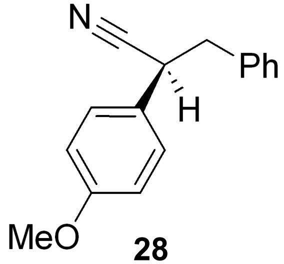

28

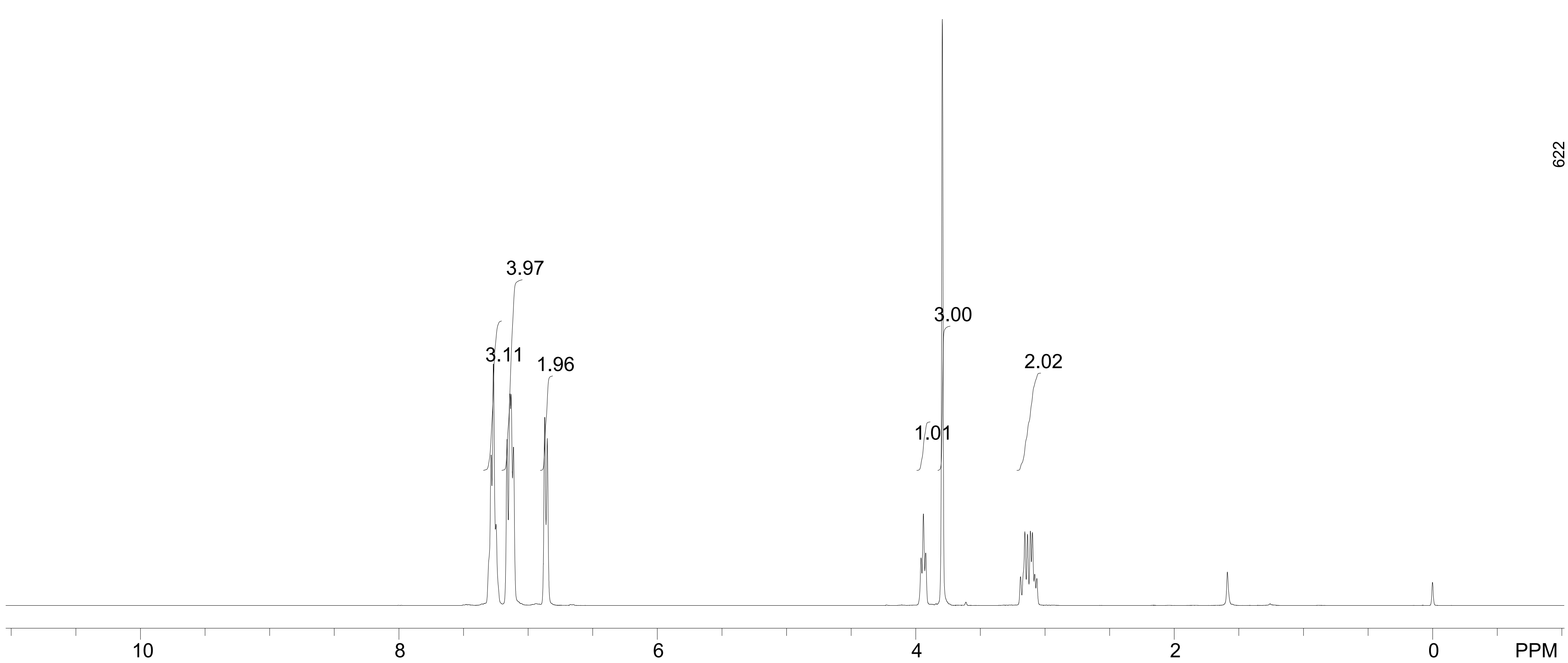

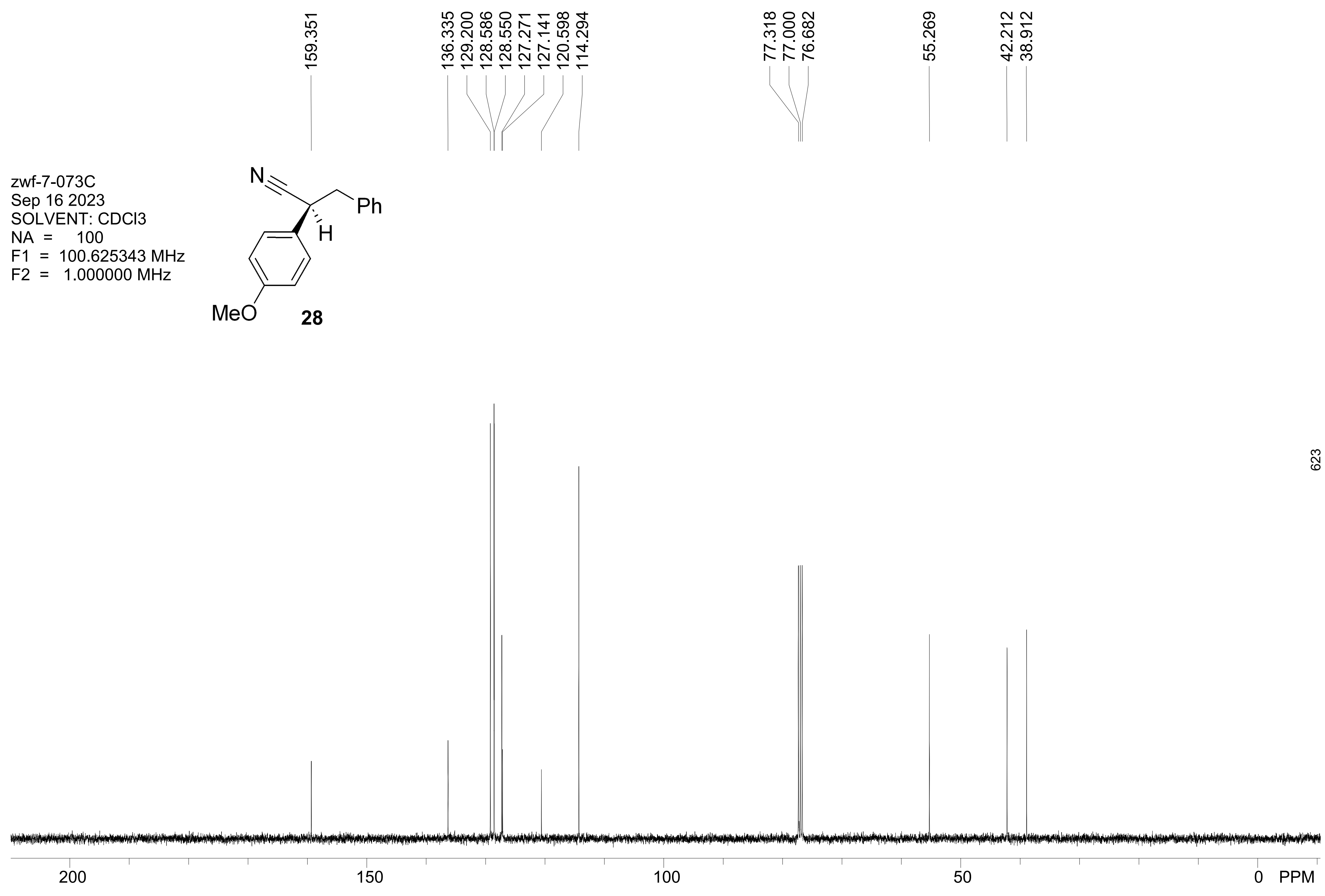
zwf-7-073C
Sep 16 2023
SOLVENT: CDCl3
NA = 100
F1 = 100.625343 MHz
F2 = 1.000000 MHz
N
Ph
H
MeO
28
159.351
136.335
129.200
128.586
128.550
127.271
127.141
120.598
114.294
77.318
77.000
76.682
55.269
42.212
38.912
200
150
100
50
0 PPM

Area Percent Report

Sample name: zwf-7-073-IE-99-1-0.8-205

Data file: C:\Users\Public\Documents\ChemStation\1\Data\zwf\zwf-S 2023-09-21 19-24-40\005-P1-C3-zwf-7-073.D

Injection Acquired Date 09/21/2023 21:23:47

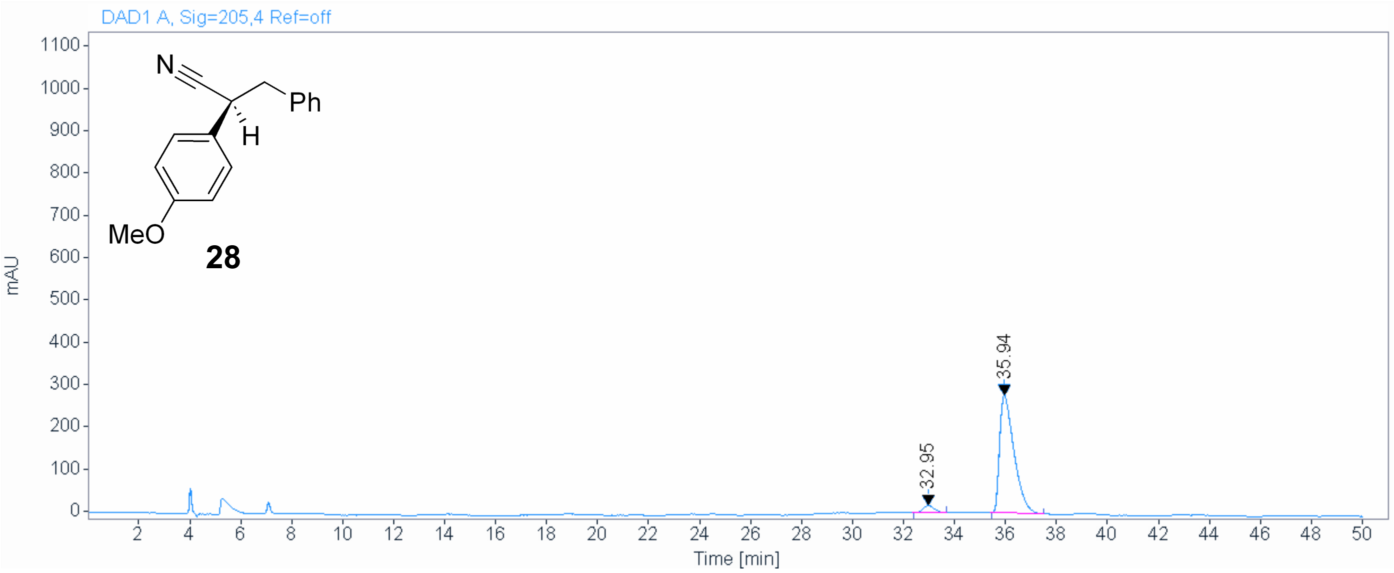

Signal: DAD1 A, Sig=205,4 Ref=off

RT [min]	Width [min]	Height	Area	Area%
32.950	0.3650	16.5893	499.0410	4.3400
35.940	0.5556	278.0309	10999.6885	95.6600
		Sum	11498.7295	100.0000

Area Percent Report

Sample name: zwf-7-073-rac-IE-99-1-0.8-205

Data file: C:\Users\Public\Documents\ChemStation\1\Data\zwf\zwf-S 2023-09-21 19-24-40\006-P1-C4-zwf-7-073-rac.D

Injection Acquired Date 09/21/2023 22:14:51

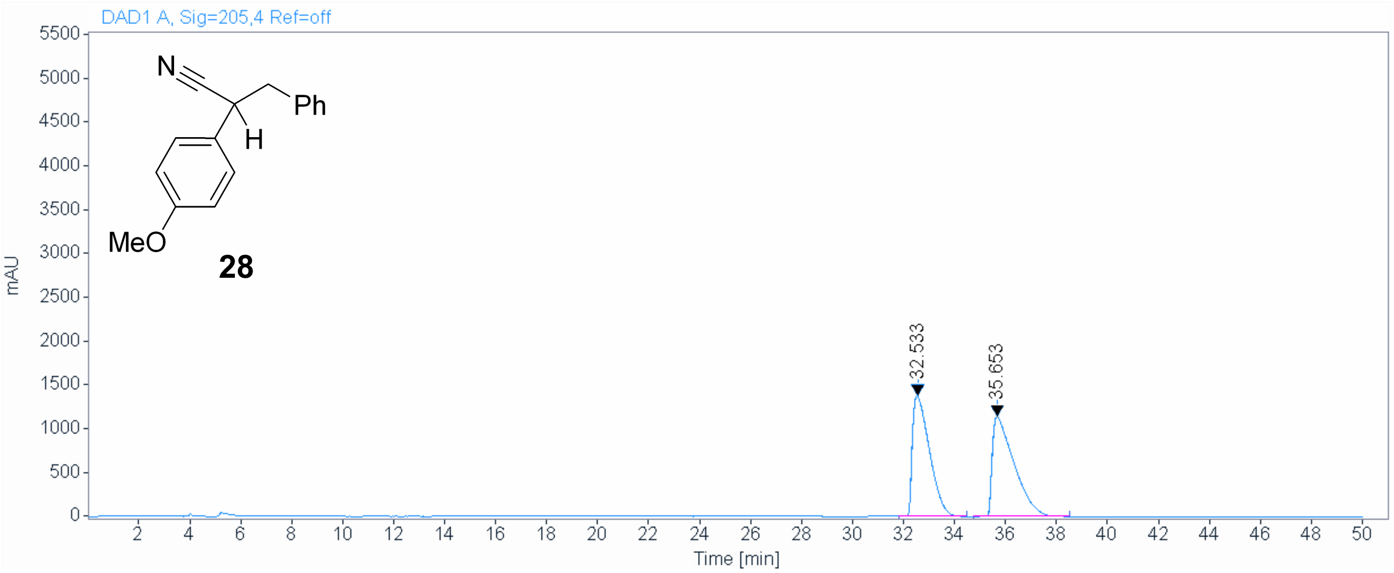

Signal: DAD1 A, Sig=205,4 Ref=off

RT [min]	Width [min]	Height	Area	Area%
32.533	0.5608	1379.3893	64173.3945	48.6207
35.653	0.7704	1145.9283	67814.5078	51.3793
		Sum	131987.9023	100.0000

zwf-8-035H
Nov 14 2023
SOLVENT: CDCl3
NA = 4
F1 = 400.179993 MHz
F2 = 1.000000 MHz

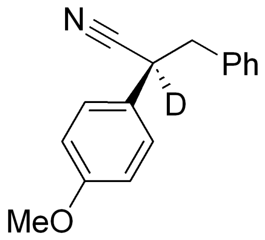

28-d

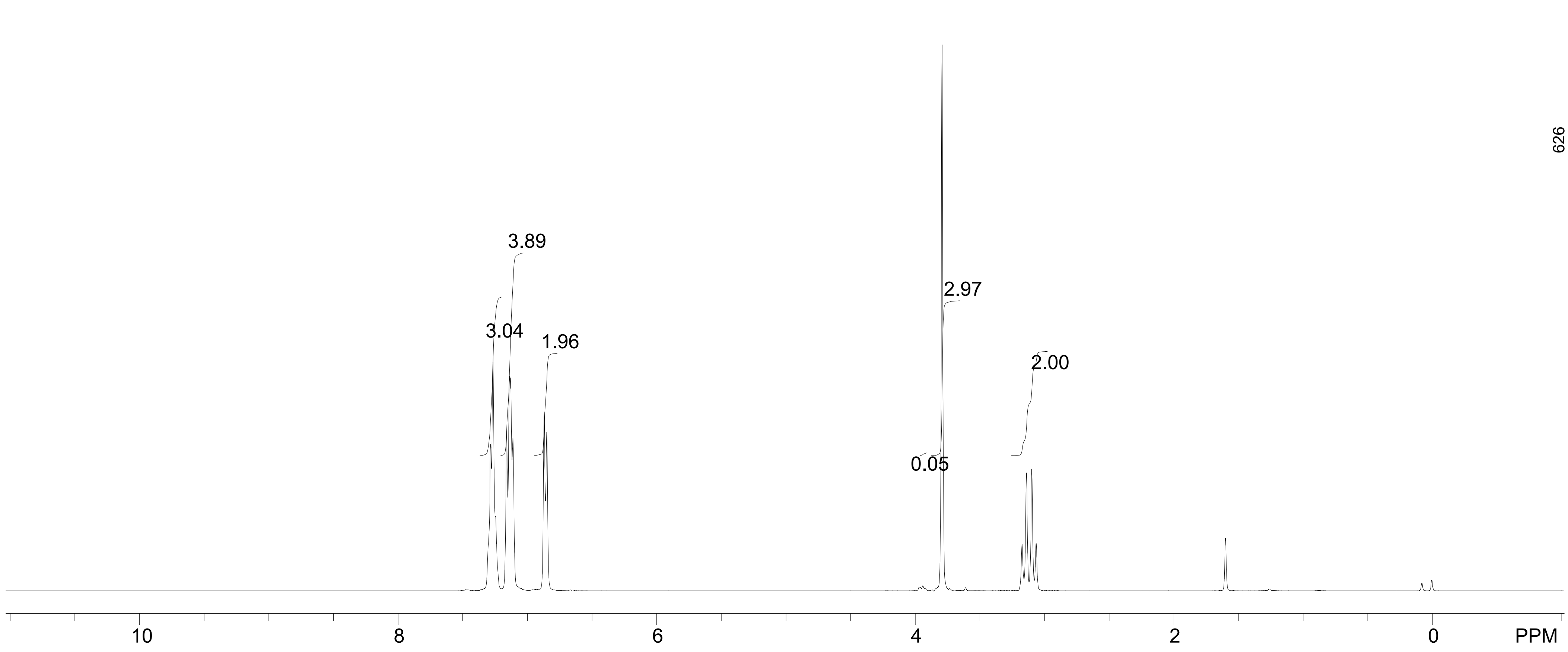

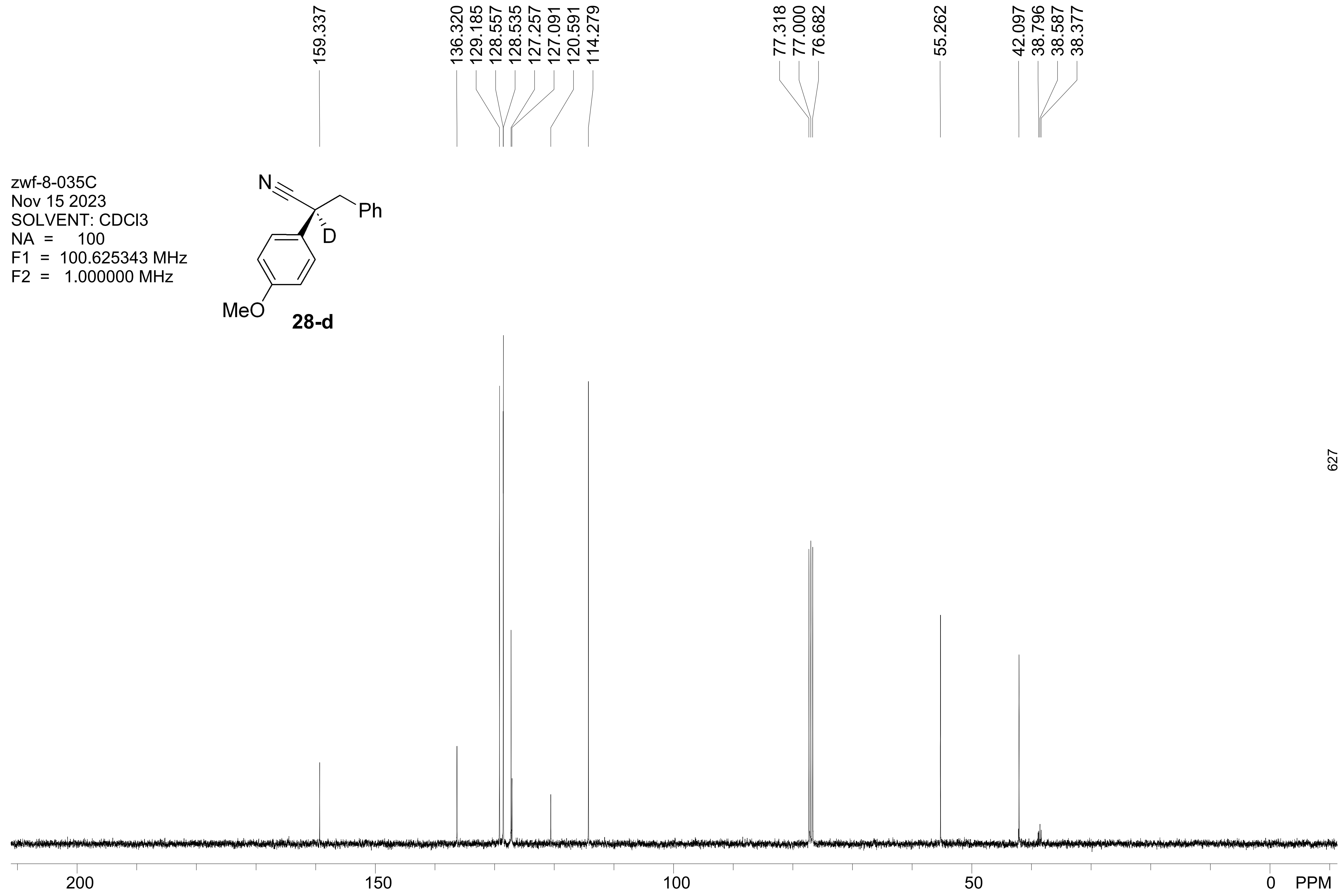

159.337
136.320
129.185
128.557
128.535
127.257
127.091
120.591
114.279
77.318
77.000
76.682
55.262
42.097
38.796
38.587
38.377
zwf-8-035C
Nov 15 2023
SOLVENT: CDCl3
NA = 100
F1 = 100.625343 MHz
F2 = 1.000000 MHz
N
Ph
D
MeO
28-d
200
150
100
50
0
PPM

Area Percent Report

Agilent Technologies

Sample name: zwf-8-035-IE-99-1-0.8-205

Data file: C:\Users\Public\Documents\ChemStation\1\Data\zwf\zwf-S 2023-11-15 14-21-28\015-P1-B1-zwf-8-035.D

Injection Acquired Date 11/15/2023 22:27:28

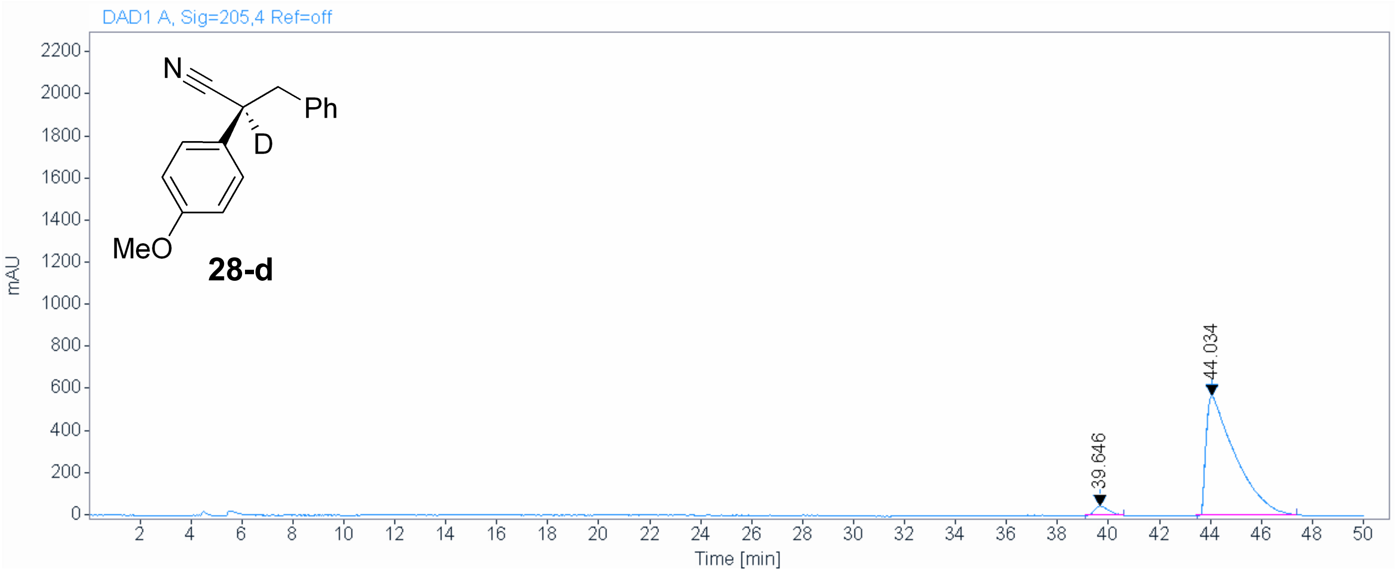

Signal: DAD1 A, Sig=205,4 Ref=off

RT [min]	Width [min]	Height	Area	Area%
39.646	0.6360	43.8552	1673.6031	3.5643
44.034	0.9401	570.4342	45280.8438	96.4357
		Sum	46954.4469	100.0000

Area Percent Report

Agilent Technologies

Sample name: zwf-8-035-rac-IE-99-1-0.8-205

Data file: C:\Users\Public\Documents\ChemStation\1\Data\zwf\zwf-S 2023-11-15 14-21-28\016-P1-B2-zwf-8-035-rac.D

Injection Acquired Date 11/15/2023 23:18:30

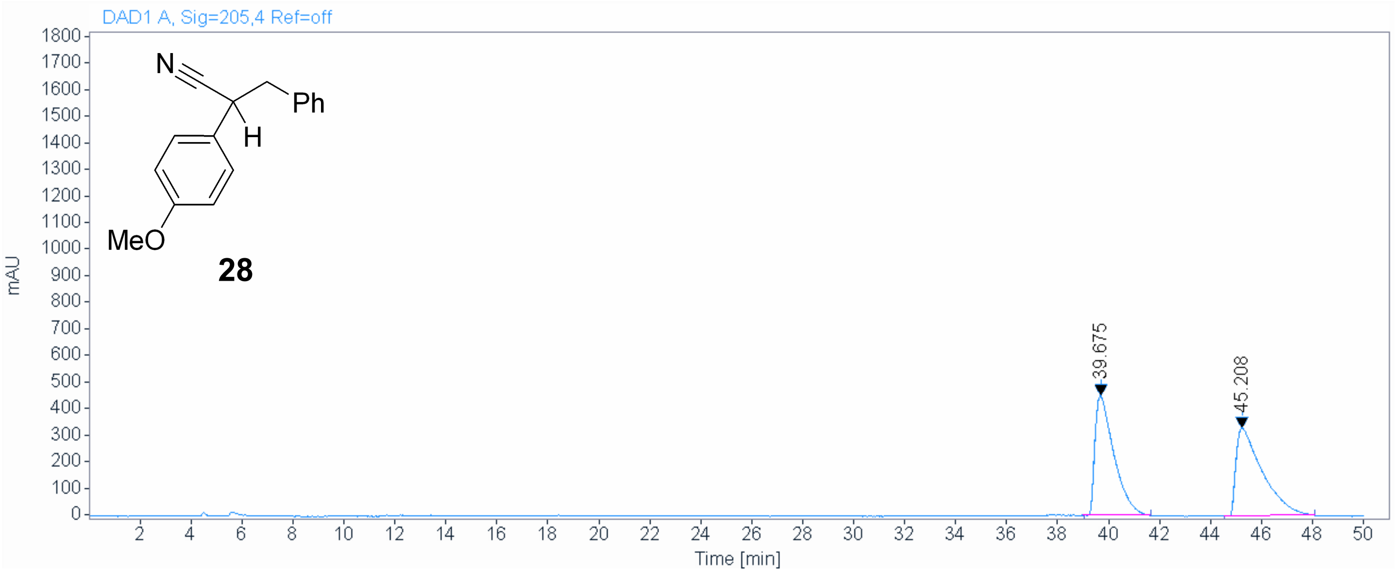

Signal: DAD1 A, Sig=205,4 Ref=off

RT [min]	Width [min]	Height	Area	Area%
39.675	0.6203	451.4163	23394.8594	49.4577
45.208	0.8488	331.2462	23907.8848	50.5423
		Sum	47302.7441	100.0000

zwf-7-116H
Oct 17 2023
SOLVENT: CDCl3
NA = 4
F1 = 400.140015 MHz
F2 = 1.000000 MHz

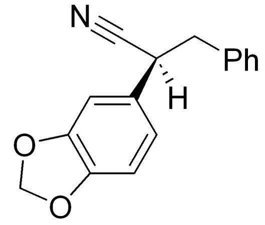

29

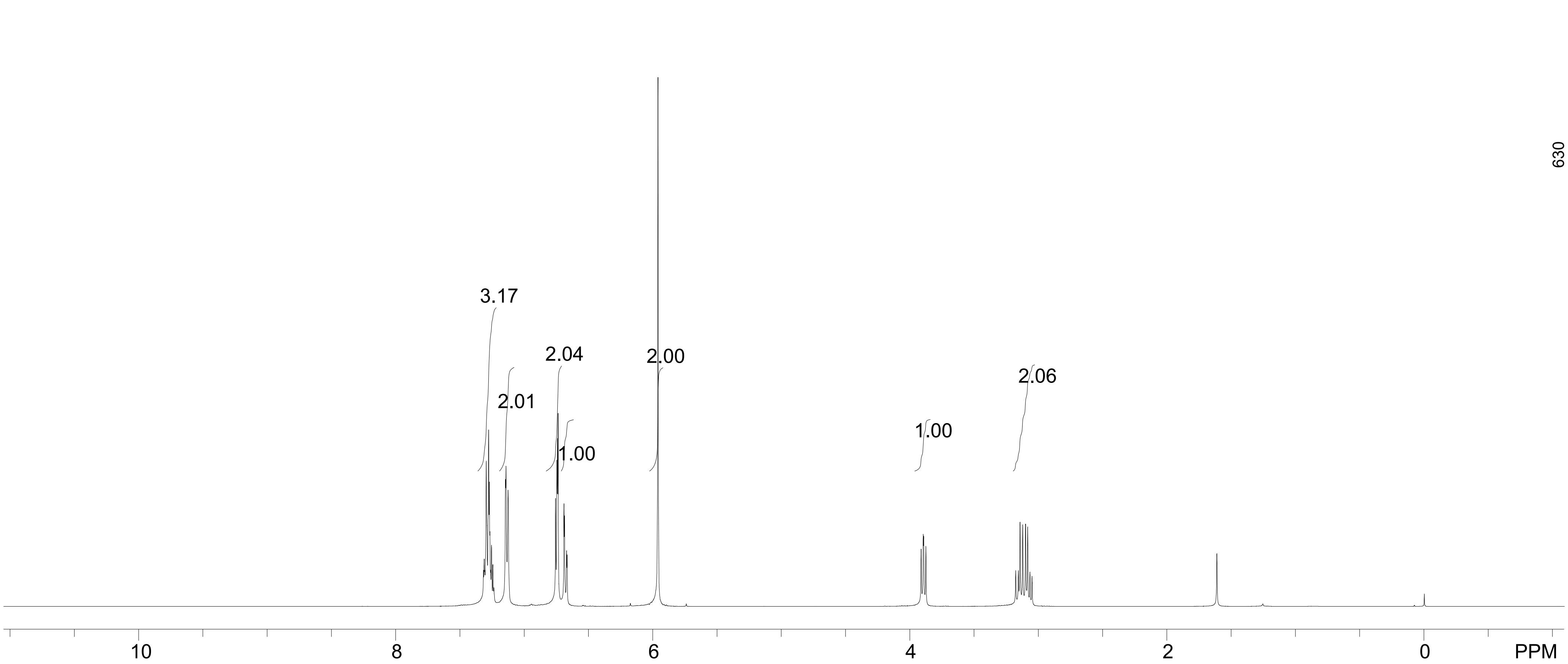

zwf-7-116C
Oct 17 2023
SOLVENT: CDCl3
NA = 100
F1 = 100.615280 MHz
F2 = 1.000000 MHz

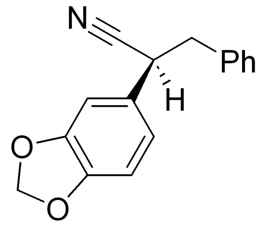

29

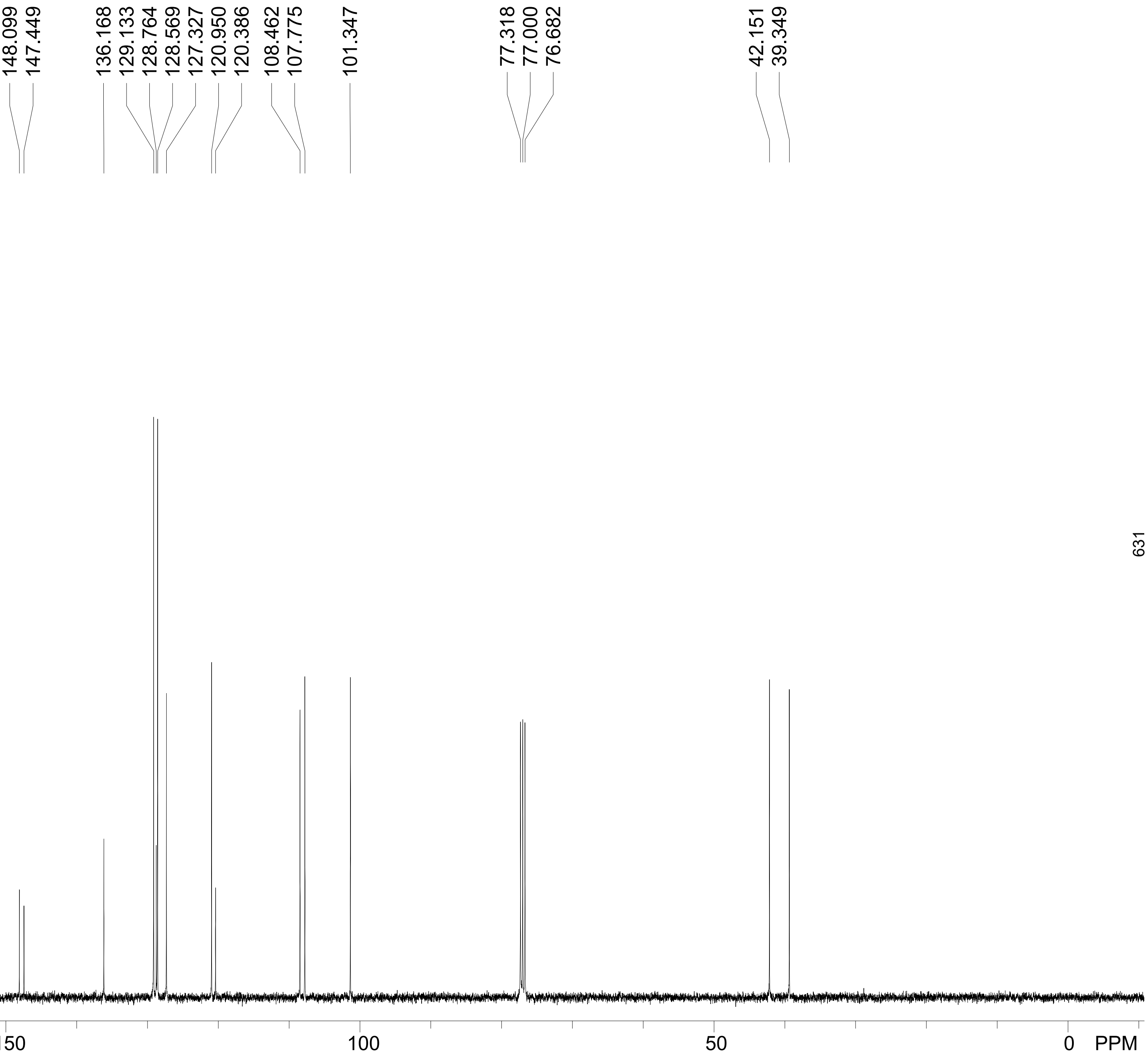

Area Percent Report

Sample name: zwf-7-116-IE-90-10-0.8-205

Data file: C:\Users\Public\Documents\ChemStation\1\Data\zwf\zwf-S 2023-10-16 21-38-05\008-P1-B5-zwf-7-116.D

Injection Acquired Date 10/17/2023 01:03:04

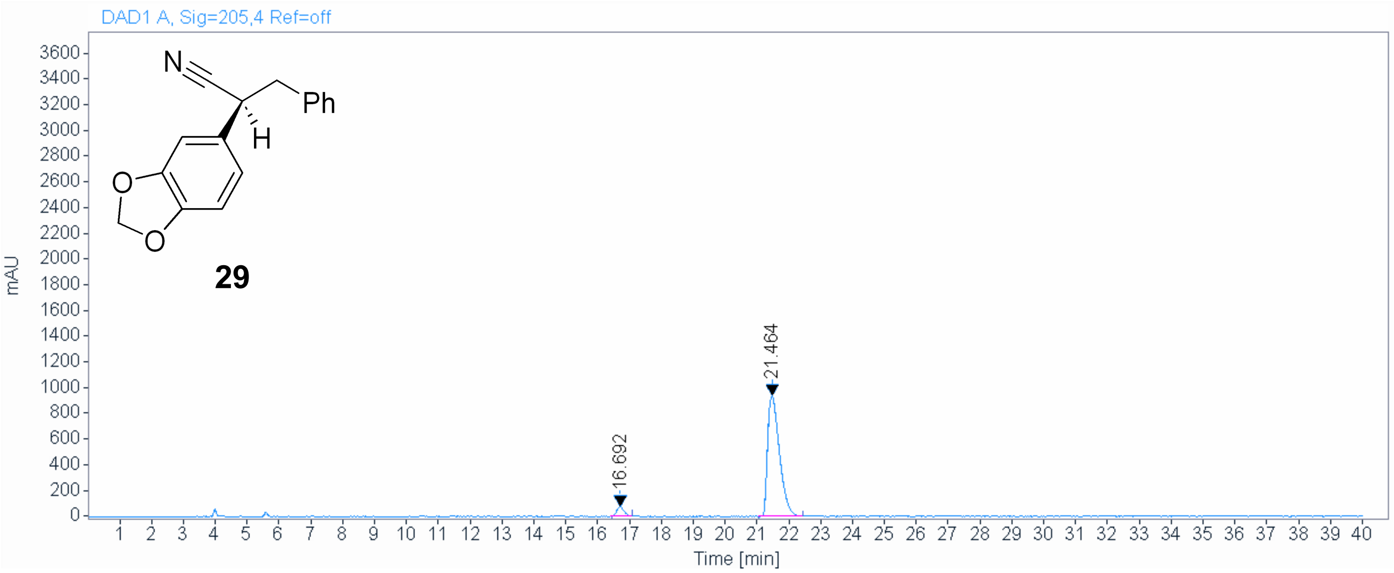

Signal: DAD1 A, Sig=205,4 Ref=off

RT [min]	Width [min]	Height	Area	Area%
16.692	0.2640	75.2886	1192.6740	4.6900
21.464	0.3268	940.0390	24237.3633	95.3100
		Sum	25430.0372	100.0000

Area Percent Report

Agilent Technologies

Sample name: zwf-7-116-rac-IE-90-10-0.8-205

Data file: C:\Users\Public\Documents\ChemStation\1\Data\zwf\zwf-S 2023-10-16 21-38-05\009-P1-B6-zwf-7-116-rac.D

Injection Acquired Date 10/17/2023 01:44:06

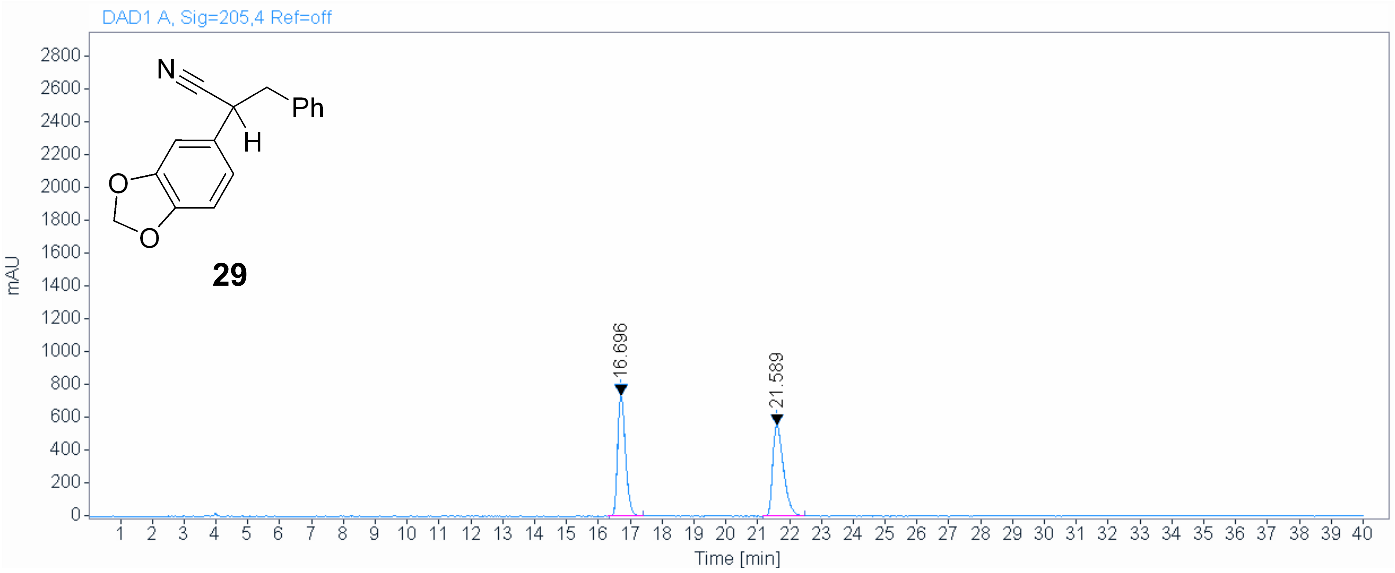

Signal: DAD1 A, Sig=205,4 Ref=off

RT [min]	Width [min]	Height	Area	Area%
16.696	0.2551	735.0172	12677.5420	49.2192
21.589	0.3554	554.1385	13079.7559	50.7808
		Sum	25757.2979	100.0000

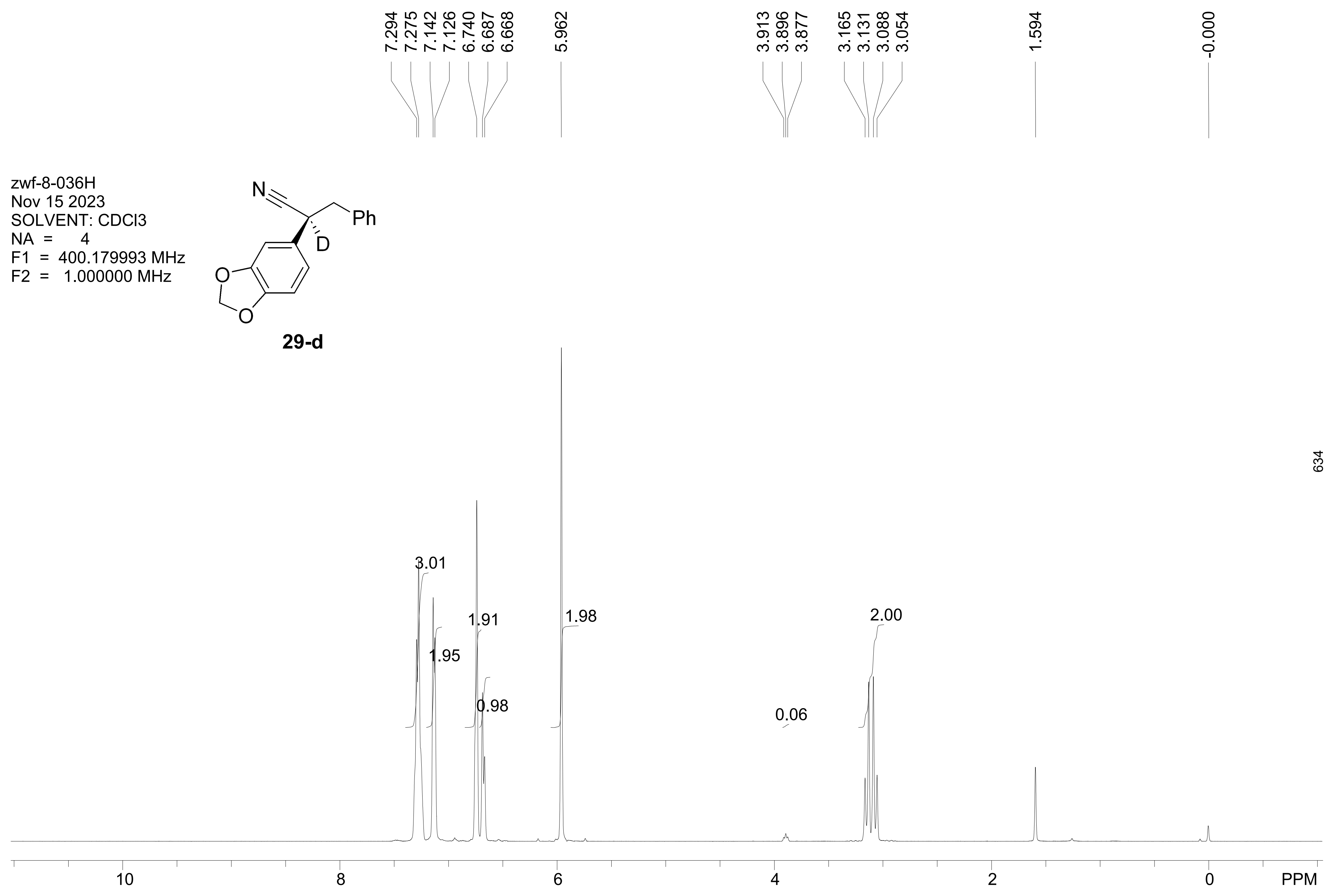

7.294
7.275
7.142
7.126
6.740
6.687
6.668
5.962
3.913
3.896
3.877
3.165
3.131
3.088
3.054
1.594
-0.000
zwf-8-036H
Nov 15 2023
SOLVENT: CDCl3
NA = 4
F1 = 400.179993 MHz
F2 = 1.000000 MHz
N
D
Ph
O
O
29-d
3.01
1.95
1.91
0.98
1.98
0.06
2.00
10
8
6
4
2
0
PPM

zwf-8-036C
Nov 15 2023
SOLVENT: CDCl3
NA = 100
F1 = 100.625343 MHz
F2 = 1.000000 MHz

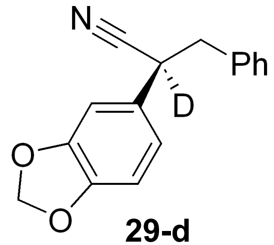

29-d

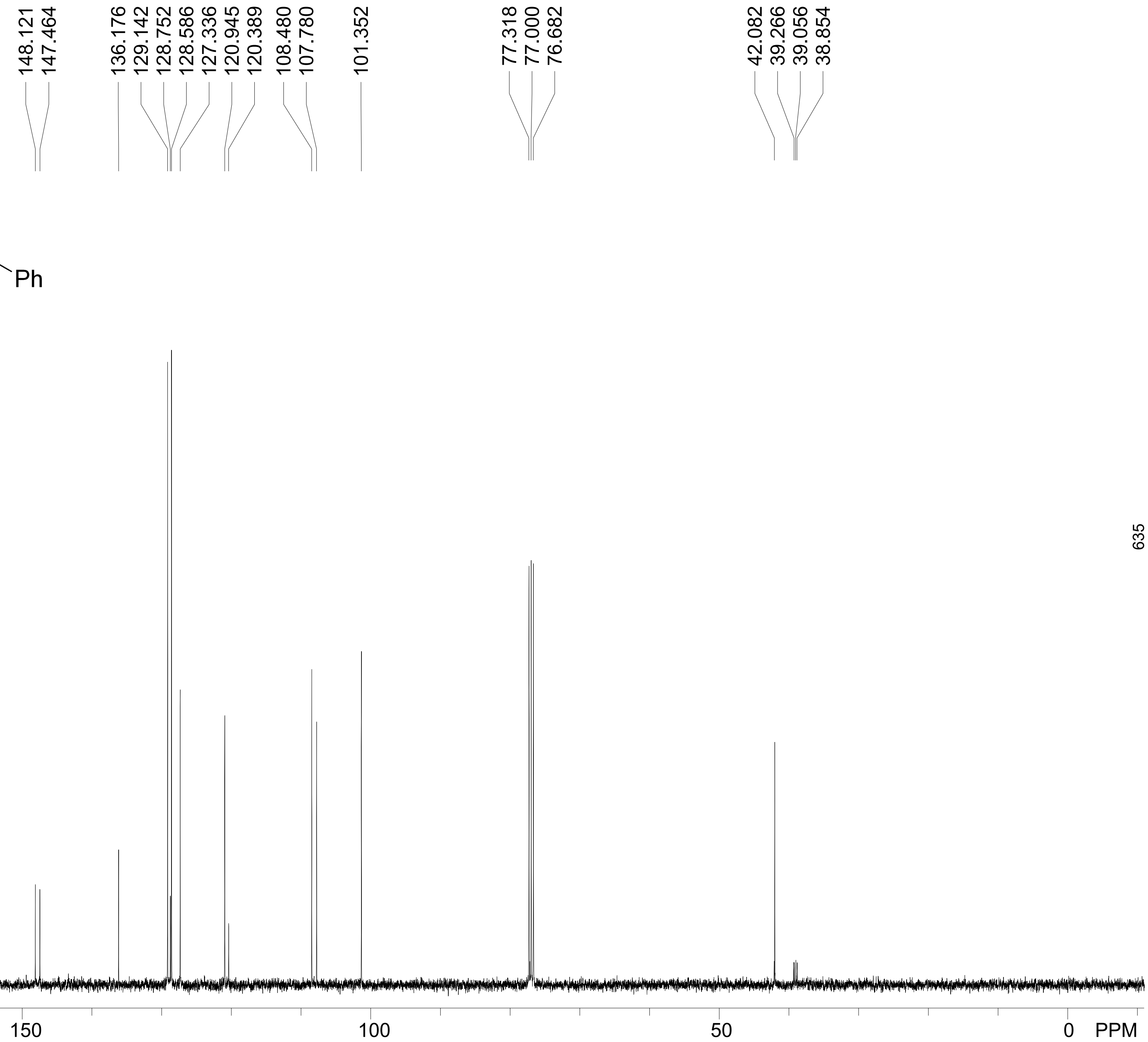

Area Percent Report

Sample name: zwf-8-036-IE-90-10-0.8-205

Data file: C:\Users\Public\Documents\ChemStation\1\Data\zwf\zwf-S 2023-11-15 14-21-28\020-P1-B5-zwf-8-036.D

Injection Acquired Date 11/16/2023 01:37:41

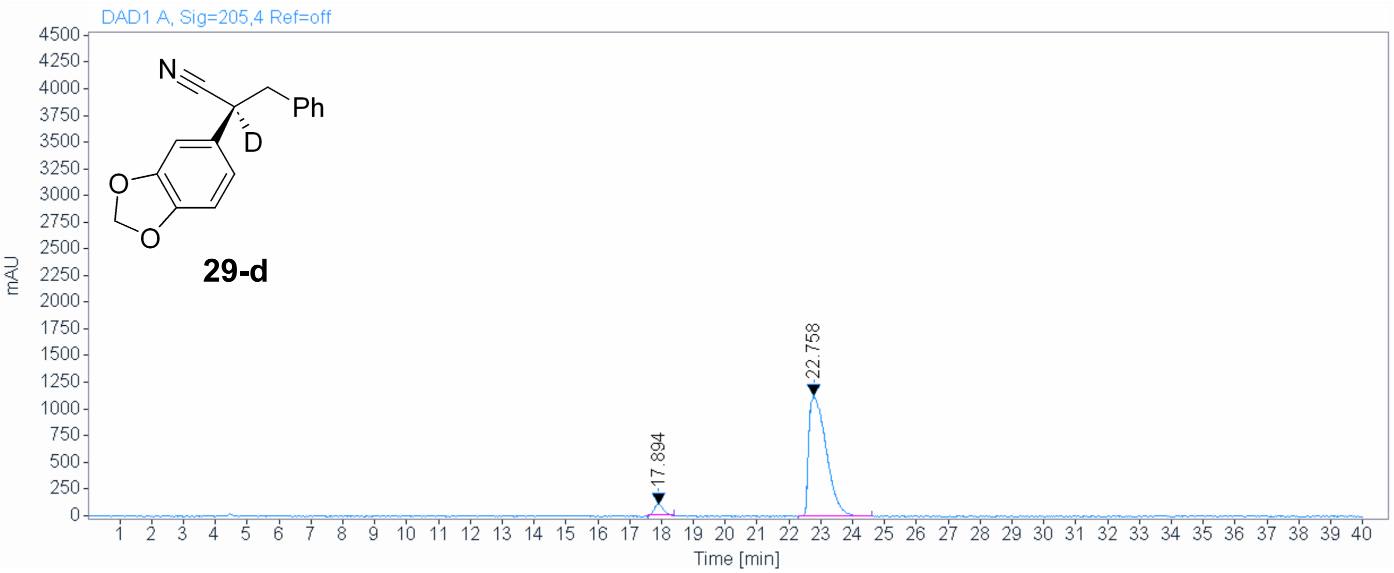

Signal: DAD1 A, Sig=205,4 Ref=off

RT [min]	Width [min]	Height	Area	Area%
17.894	0.3273	103.8875	2040.1187	4.5379
22.758	0.6325	1130.9608	42917.0938	95.4621
		Sum	44957.2124	100.0000

Area Percent Report

Agilent Technologies

Sample name: zwf-8-036-rac-IE-90-10-0.8-205

Data file: C:\Users\Public\Documents\ChemStation\1\Data\zwf\zwf-S 2023-11-15 14-21-28\021-P1-B6-zwf-8-036-rac.D

Injection Acquired Date 11/16/2023 02:18:42

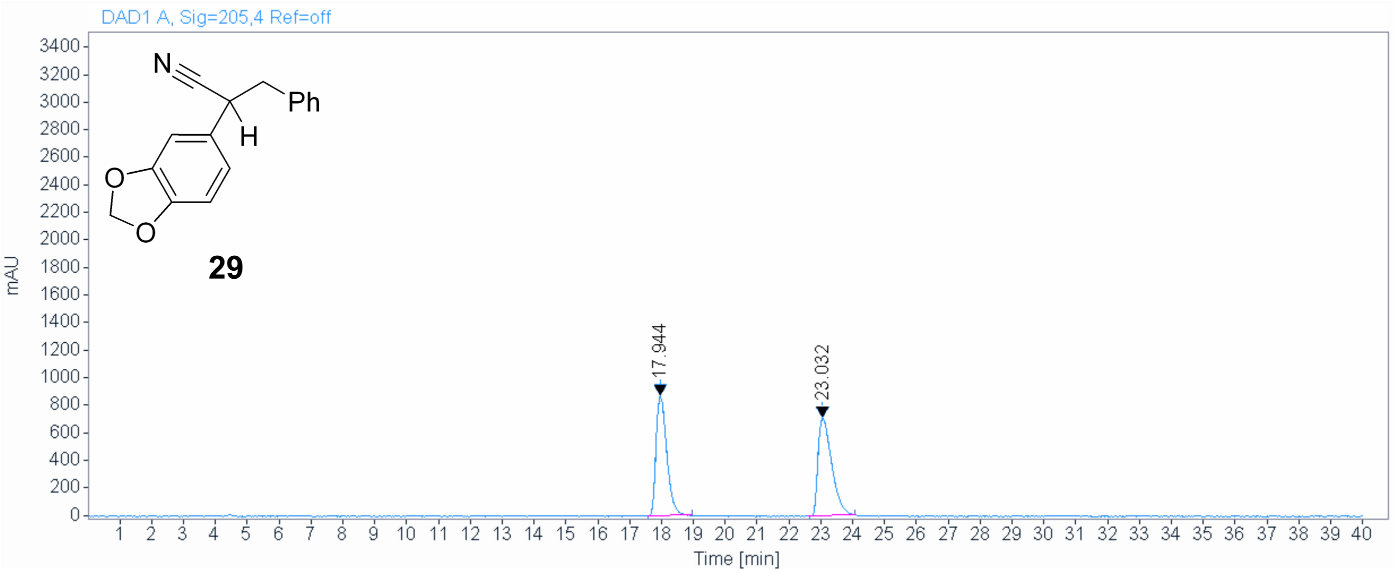

Signal: DAD1 A, Sig=205,4 Ref=off

RT [min]	Width [min]	Height	Area	Area%
17.944	0.2900	874.2382	20535.2676	48.9915
23.032	0.3629	714.8408	21380.6816	51.0085
		Sum	41915.9492	100.0000

zwf-7-114H
Oct 15 2023
SOLVENT: CDCl3
NA = 4
F1 = 400.140015 MHz
F2 = 1.000000 MHz

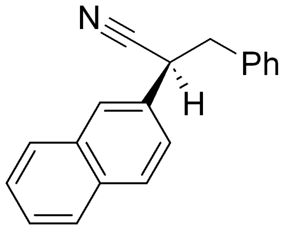

30

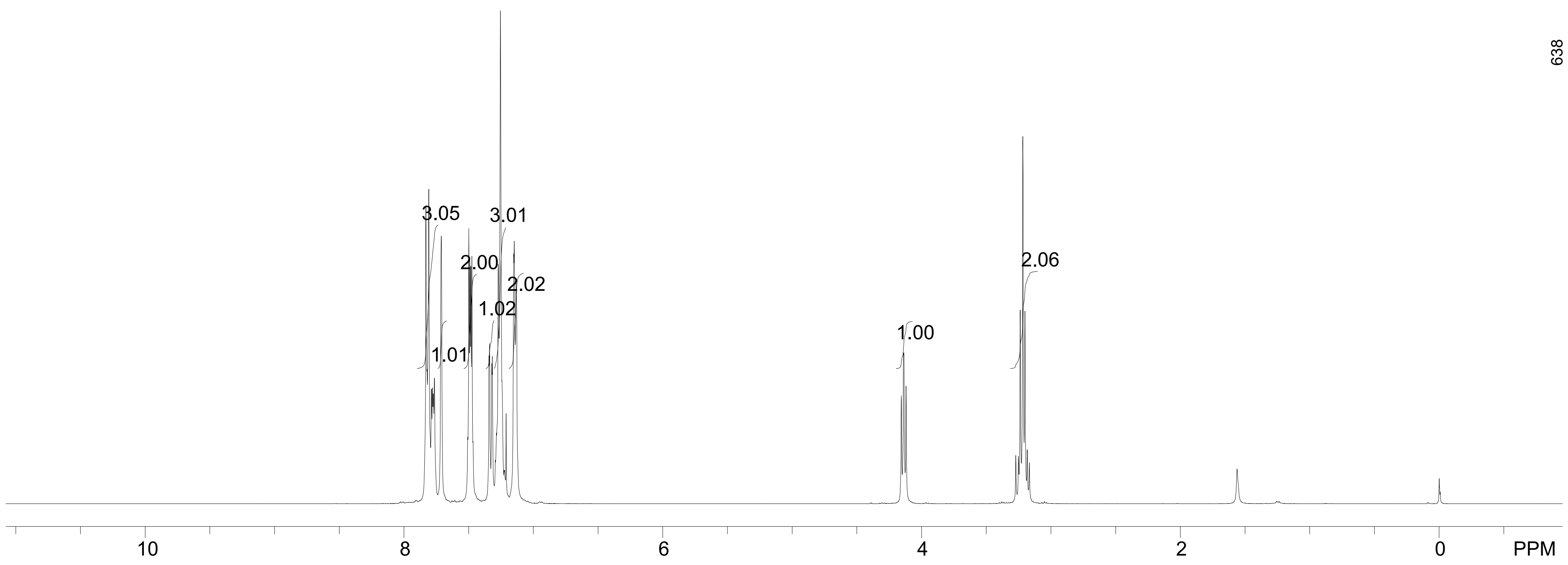

zwf-7-114C
Oct 15 2023
SOLVENT: CDCl3
NA = 100
F1 = 100.615280 MHz
F2 = 1.000000 MHz

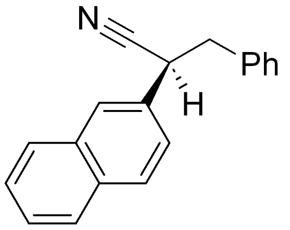

30

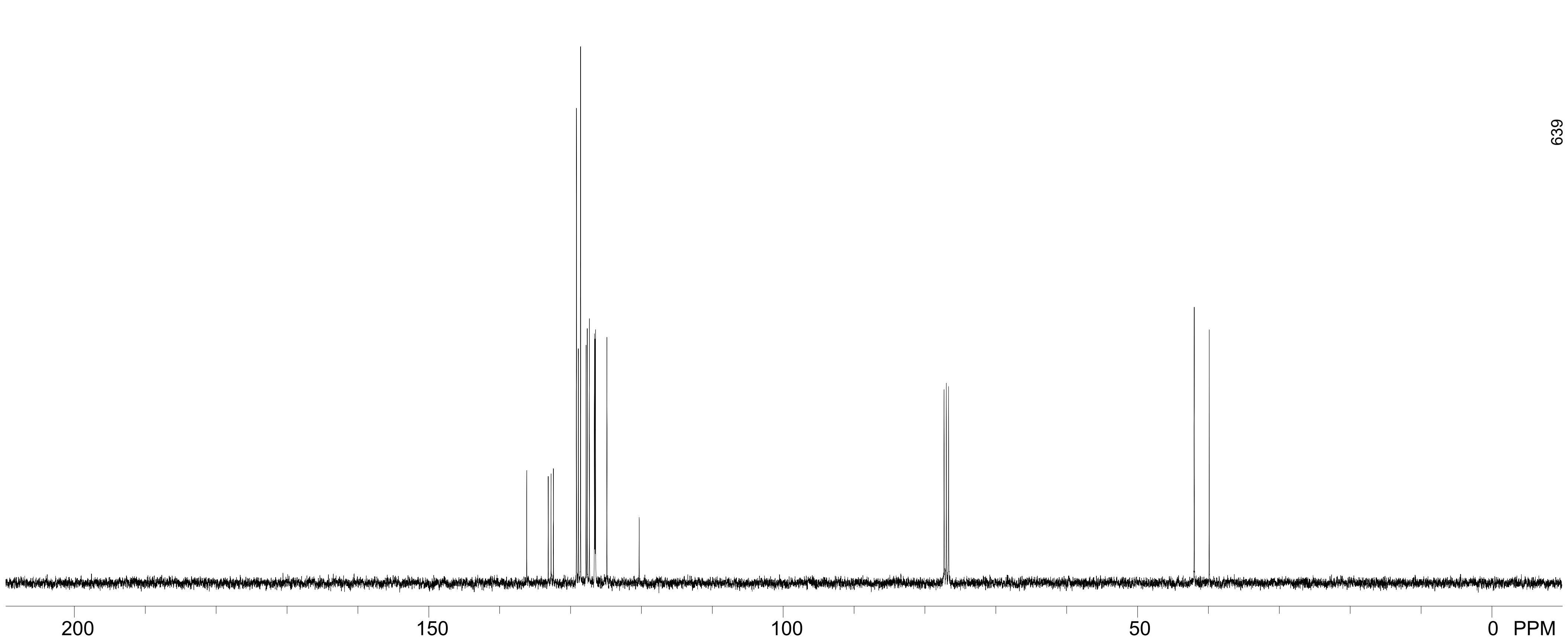

Area Percent Report

Agilent Technologies

Sample name: zwf-7-114-IE-99-1-0.8-205

Data file: C:\Users\Public\Documents\ChemStation\1\Data\zwf\zwf-S 2023-10-15 16-11-49\004-P1-C7-zwf-7-114.D

Injection Acquired Date 10/15/2023 17:41:51

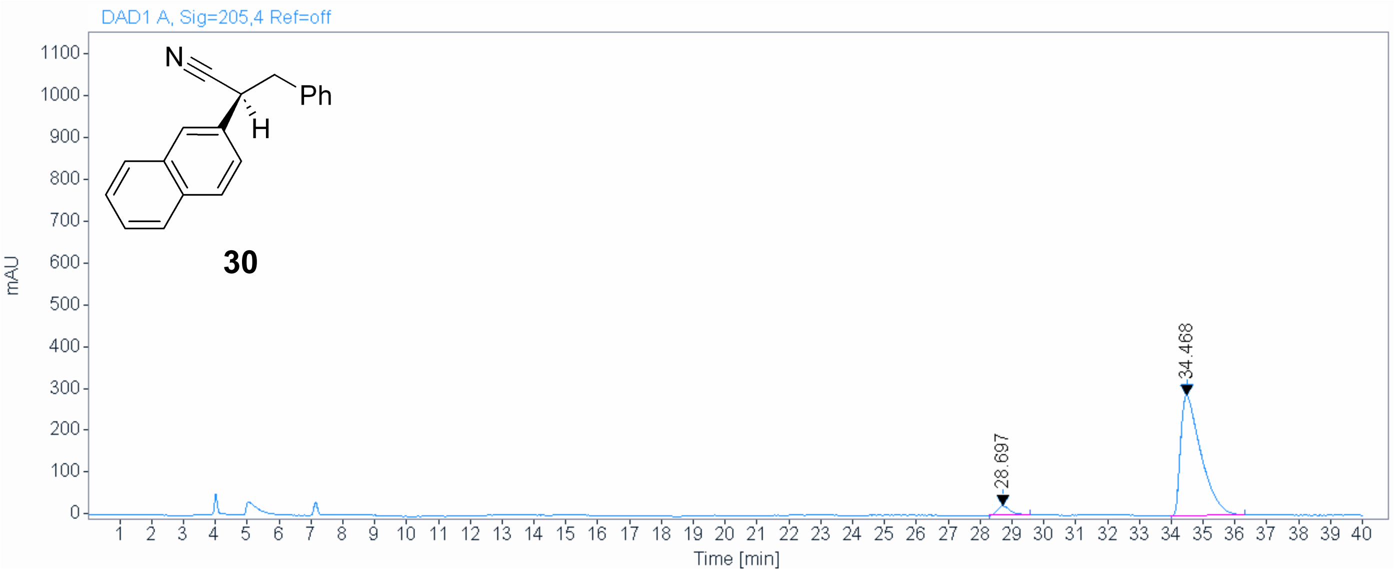

Signal: DAD1 A, Sig=205,4 Ref=off

RT [min]	Width [min]	Height	Area	Area%
28.697	0.4731	22.3803	635.2792	4.6929
34.468	0.6195	287.5031	12901.8311	95.3071
		Sum	13537.1103	100.0000

Area Percent Report

Agilent Technologies

Sample name: zwf-7-114-rac-IE-99-1-0.8-205

Data file: C:\Users\Public\Documents\ChemStation\1\Data\zwf\zwf-S 2023-10-14 09-03-23\023-P1-C8-zwf-7-114-rac.D

Injection Acquired Date 10/15/2023 00:14:47

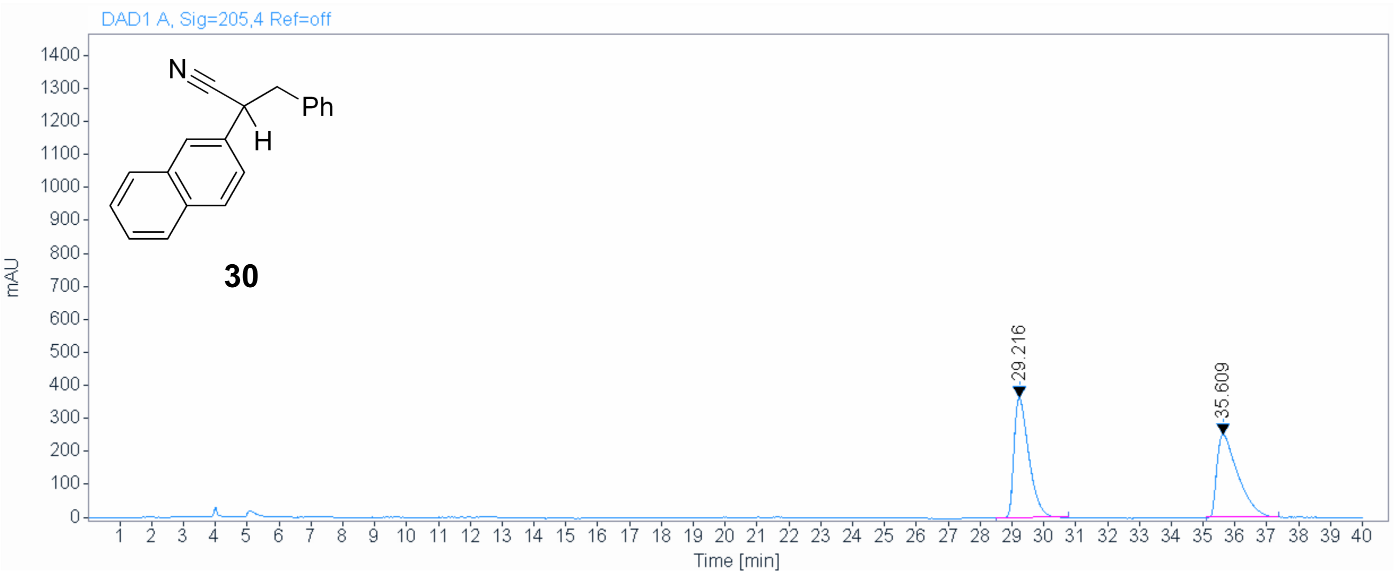

Signal: DAD1 A, Sig=205,4 Ref=off

RT [min]	Width [min]	Height	Area	Area%
29.216	0.4563	364.2504	11809.9063	50.4055
35.609	0.6310	251.5135	11619.8955	49.5945
		Sum	23429.8018	100.0000

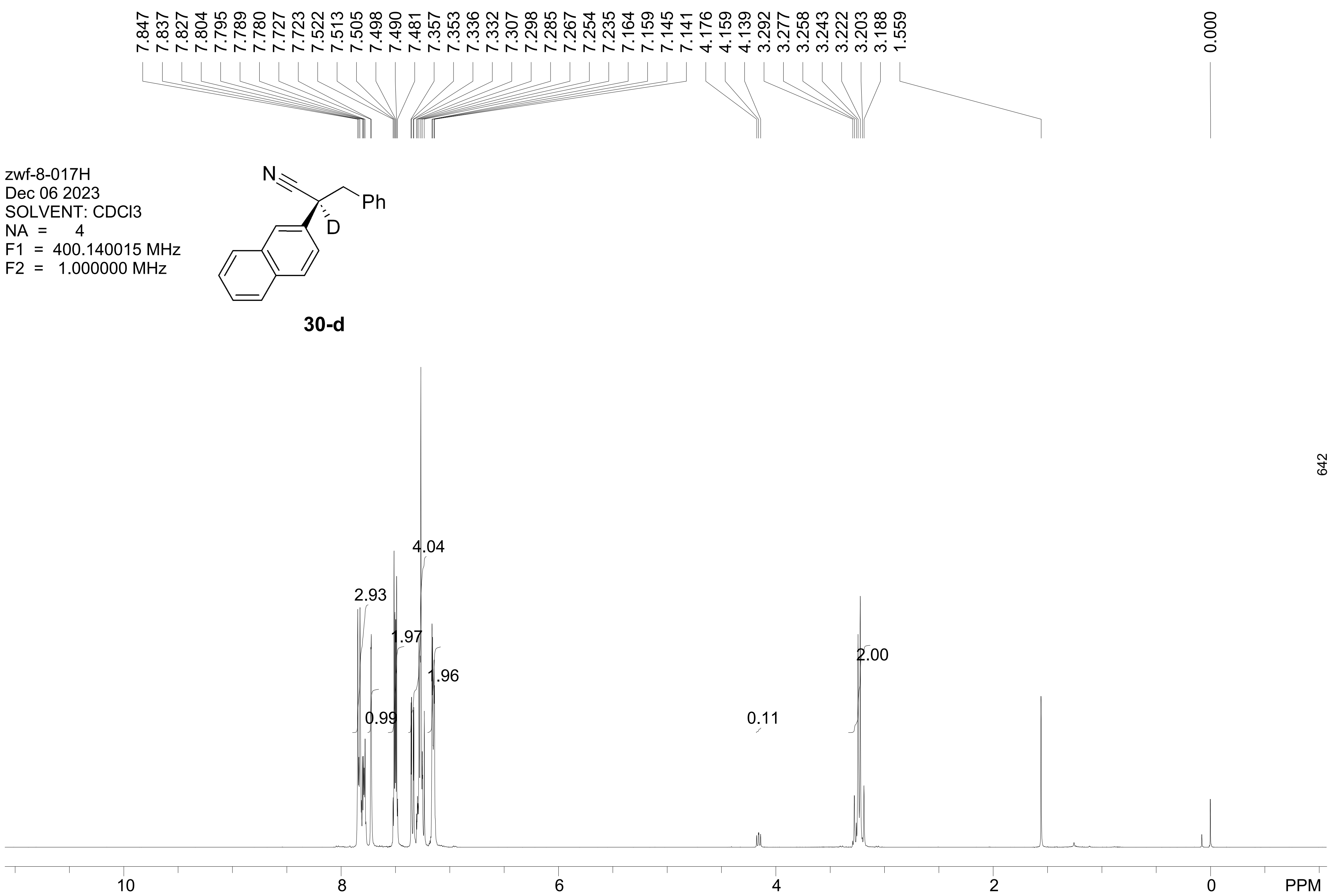
7.847
7.837
7.827
7.804
7.795
7.789
7.780
7.727
7.723
7.522
7.513
7.505
7.498
7.490
7.481
7.357
7.353
7.336
7.332
7.307
7.298
7.285
7.267
7.254
7.235
7.164
7.159
7.145
7.141
4.176
4.159
4.139
3.292
3.277
3.258
3.243
3.222
3.203
3.188
1.559
0.000
zwf-8-017H
Dec 06 2023
SOLVENT: CDCl3
NA = 4
F1 = 400.140015 MHz
F2 = 1.000000 MHz
N
Ph
D
30-d
4.04
2.93
1.97
1.96
0.99
0.11
2.00
10
8
6
4
2
0
PPM

zwf-8-017C
Dec 06 2023
SOLVENT: CDCl3
NA = 100
F1 = 100.615280 MHz
F2 = 1.000000 MHz

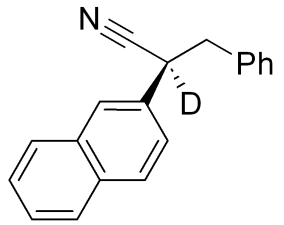

30-d

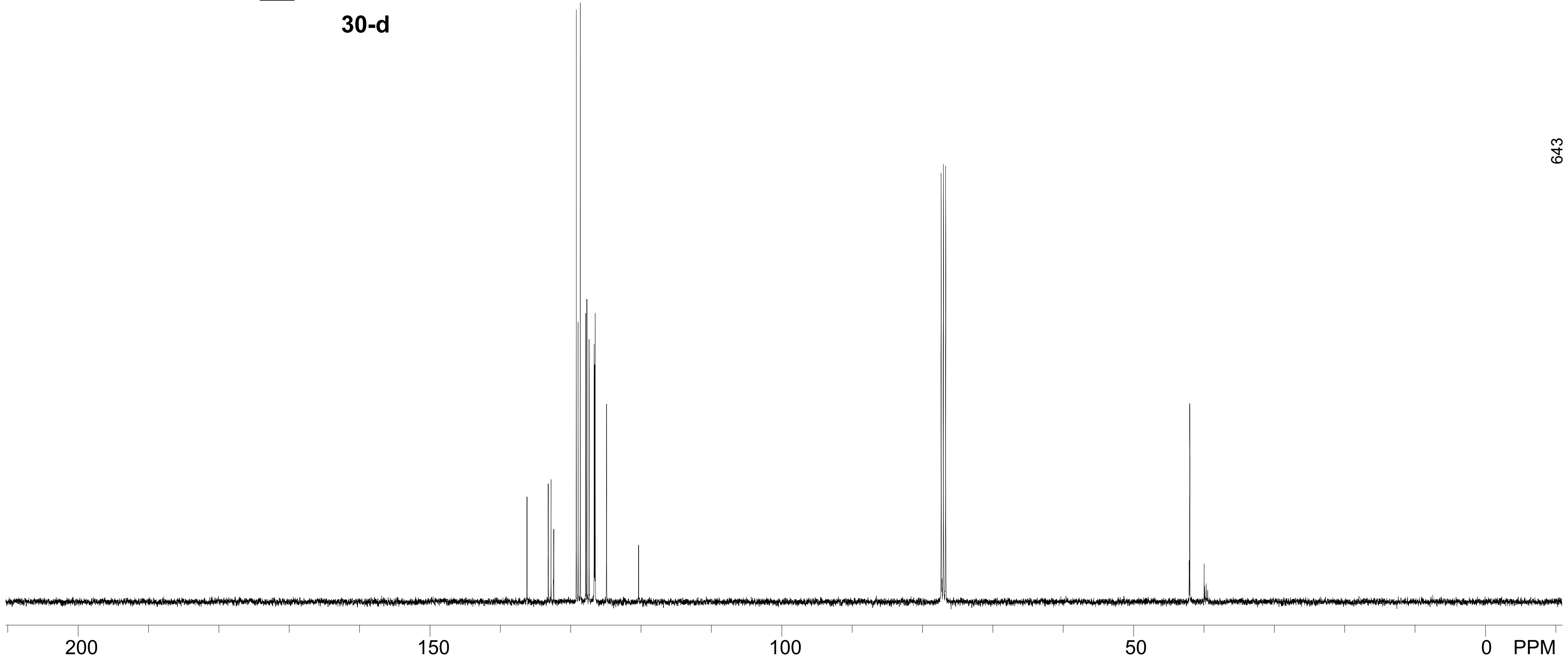

Area Percent Report

Agilent Technologies

Sample name: zwf-8-017-IE-99-1-0.8-205

Data file: C:\Users\Public\Documents\ChemStation\1\Data\zwf\zwf-S 2023-12-06 20-07-35\017-P1-B4-zwf-8-017.D

Injection Acquired Date 12/07/2023 09:12:56

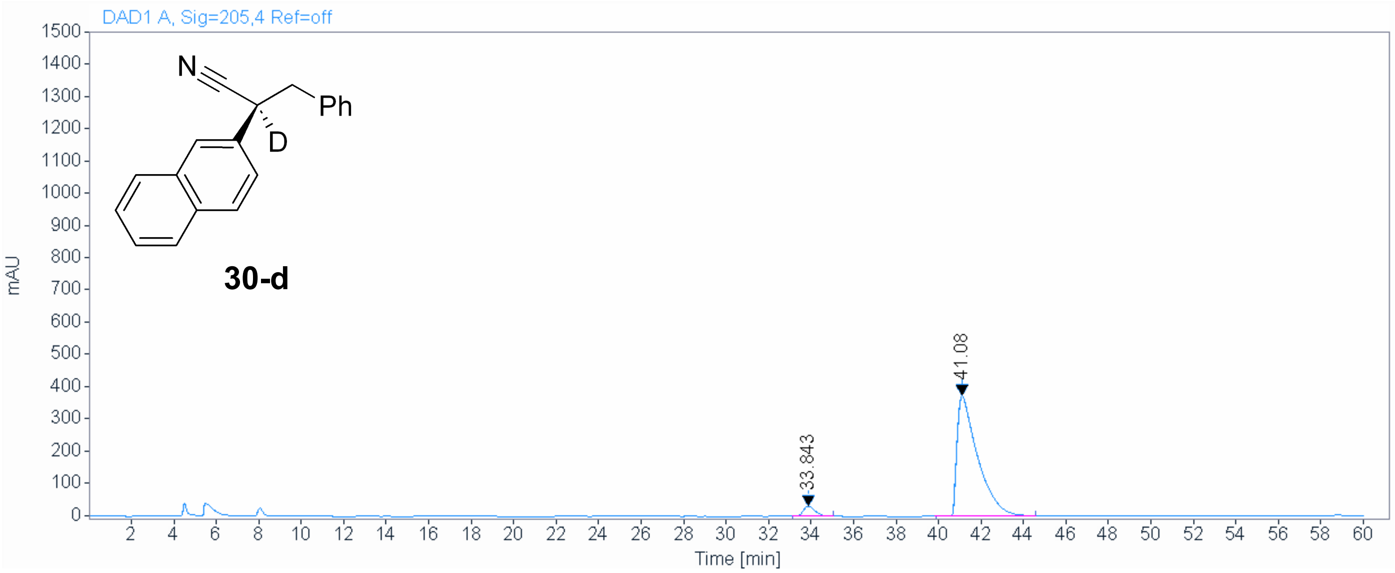

Signal: DAD1 A, Sig=205,4 Ref=off

RT [min]	Width [min]	Height	Area	Area%
33.843	0.5876	31.3340	1212.9625	4.5942
41.080	0.9730	374.3401	25188.8145	95.4058
		Sum	26401.7770	100.0000

Area Percent Report

Agilent Technologies

Sample name: zwf-8-017-rac-IE-99-1-0.8-205

Data file: C:\Users\Public\Documents\ChemStation\1\Data\zwf\zwf-S 2023-12-06 20-07-35\018-P1-B6-zwf-8-017-rac.D

Injection Acquired Date 12/07/2023 10:14:00

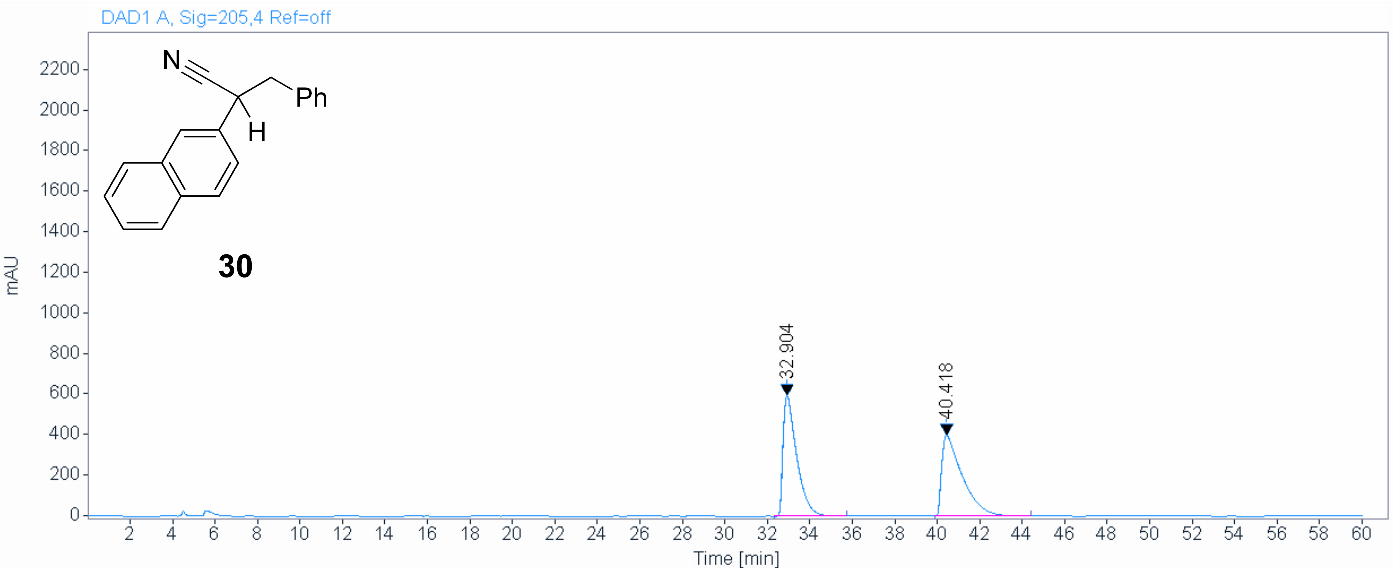

Signal: DAD1 A, Sig=205,4 Ref=off

RT [min]	Width [min]	Height	Area	Area%
32.904	0.6594	595.5720	26616.6309	49.9166
40.418	0.9495	399.7120	26705.5527	50.0834
		Sum	53322.1836	100.0000

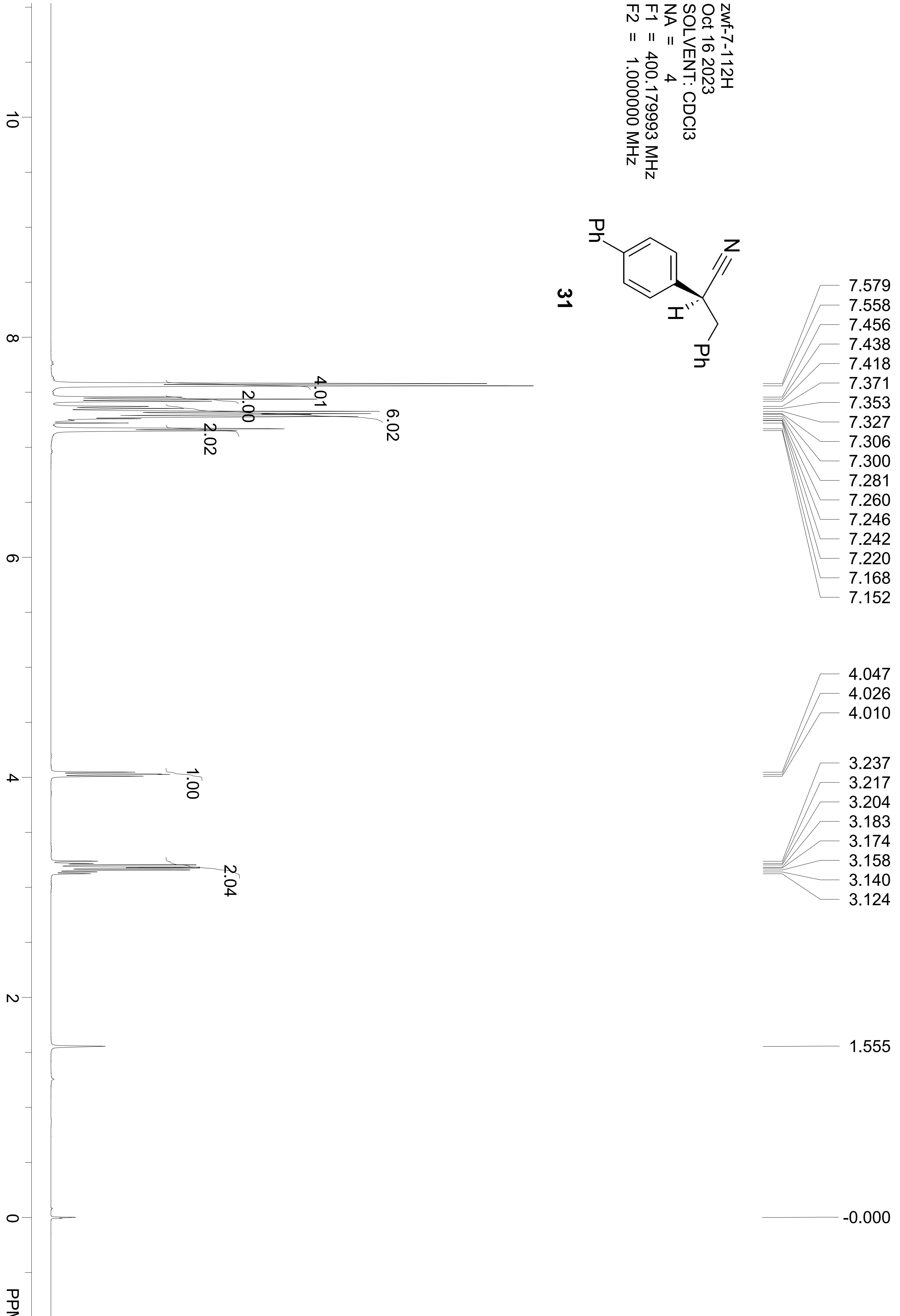
zwf-7-112H
Oct 16 2023
SOLVENT: CDCl3
NA = 4
F1 = 400.179993 MHz
F2 = 1.000000 MHz
Ph
N
H
Ph
31
7.579
7.558
7.456
7.438
7.418
7.371
7.353
7.327
7.306
7.300
7.281
7.260
7.246
7.242
7.220
7.168
7.152
4.047
4.026
4.010
3.237
3.217
3.204
3.183
3.174
3.158
3.140
3.124
1.555
-0.000
4.01
2.00
6.02
2.02
1.00
2.04
10
8
6
4
2
0
PPM

zwf-7-112C
Oct 16 2023
SOLVENT: CDCl3
NA = 100
F1 = 100.625343 MHz
F2 = 1.000000 MHz

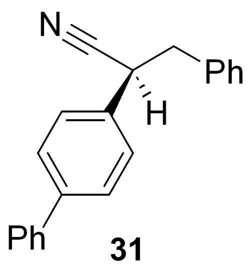

31

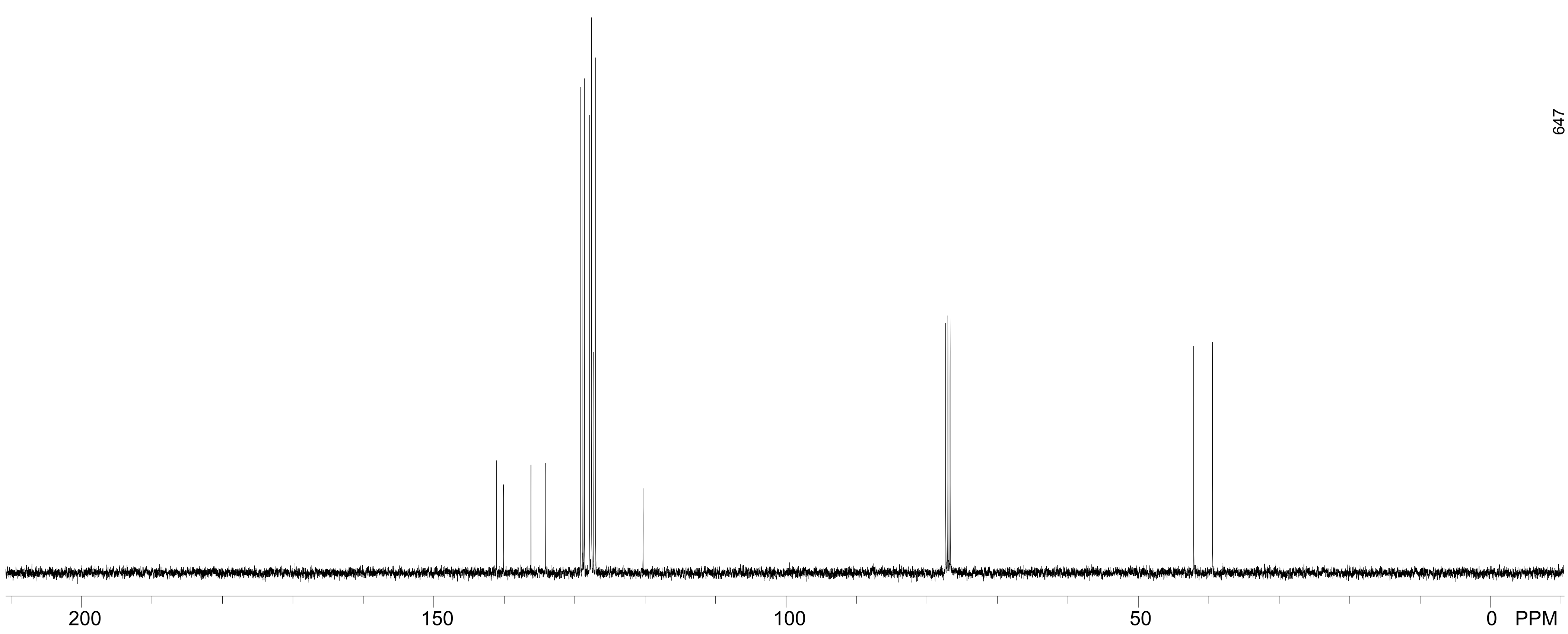

Area Percent Report

Agilent Technologies

Sample name: zwf-7-112-IE-99-1-0.8-205

Data file: C:\Users\Public\Documents\ChemStation\1\Data\zwf\zwf-S 2023-10-17 08-55-39\008-P1-C3-zwf-7-112.D

Injection Acquired Date 10/17/2023 12:15:16

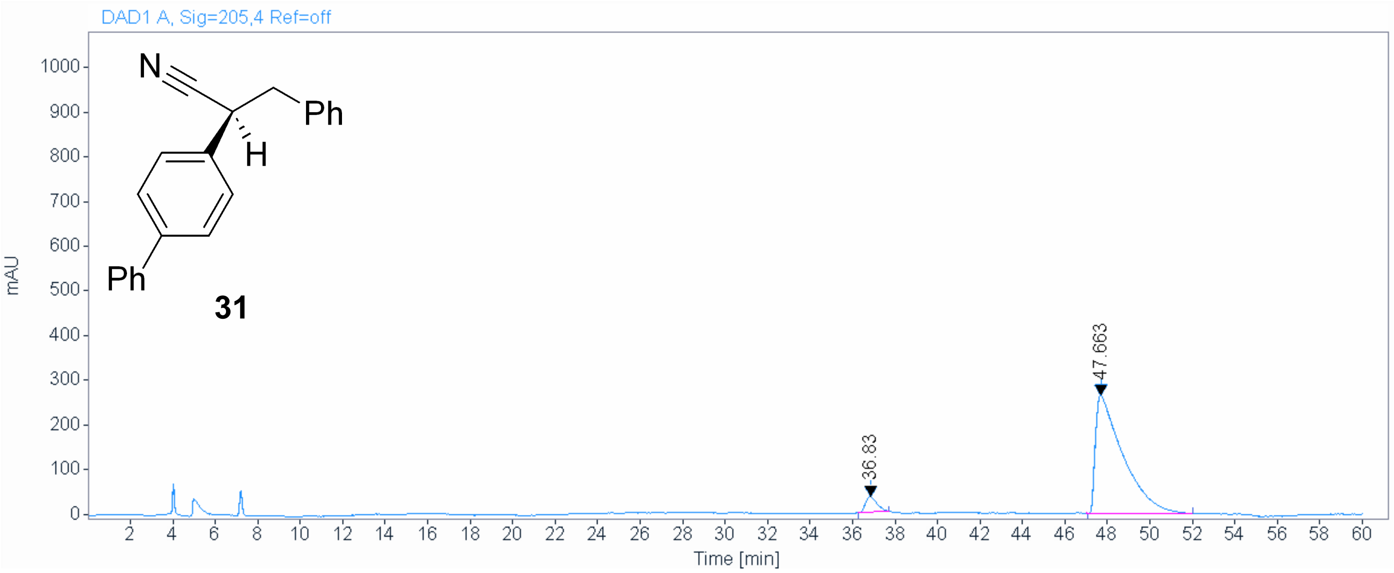

Signal: DAD1 A, Sig=205,4 Ref=off

RT [min]	Width [min]	Height	Area	Area%
36.830	0.6399	34.5335	1325.8190	5.1964
47.663	1.5182	265.5361	24188.1777	94.8036
		Sum	25513.9967	100.0000

Area Percent Report

Agilent Technologies

Sample name: zwf-7-112-rac-IE-99-1-0.8-205

Data file: C:\Users\Public\Documents\ChemStation\1\Data\zwf\zwf-S 2023-10-17 08-55-39\009-P1-C4-zwf-7-112-rac.D

Injection Acquired Date 10/17/2023 13:16:21

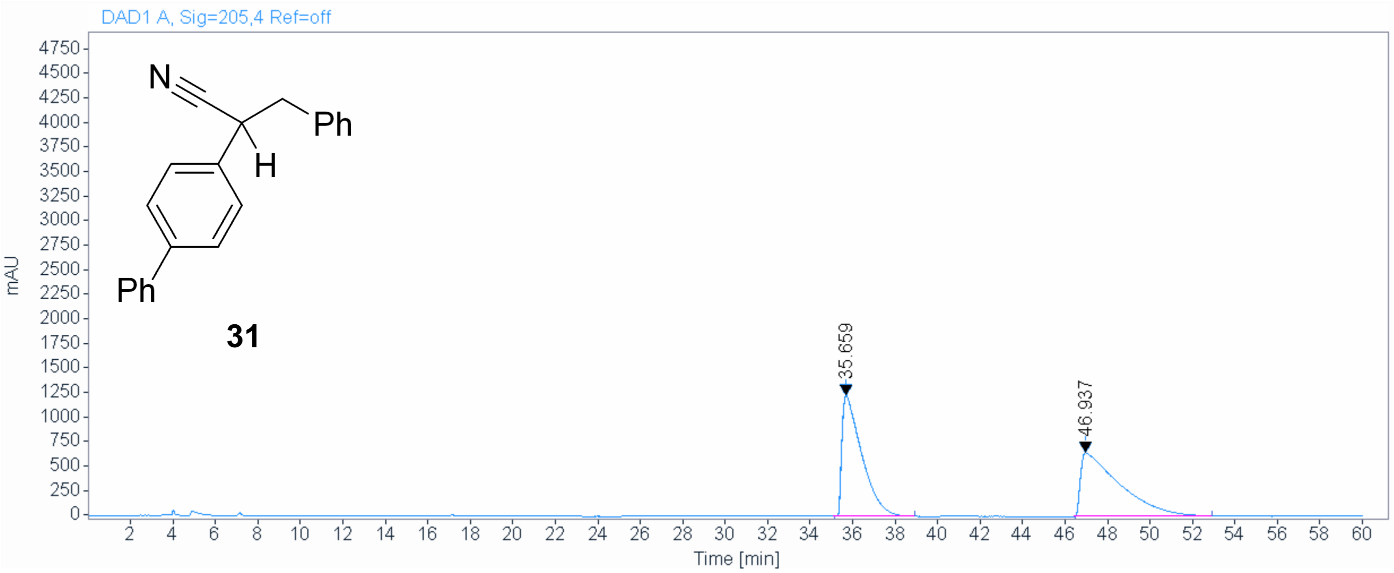

Signal: DAD1 A, Sig=205,4 Ref=off

RT [min]	Width [min]	Height	Area	Area%
35.659	0.8397	1229.2224	80039.8438	49.0649
46.937	1.4991	651.7643	83090.6641	50.9351
		Sum	163130.5078	100.0000

zwf-8-016H
Dec 06 2023
SOLVENT: CDCl3
NA = 4
F1 = 400.140015 MHz
F2 = 1.000000 MHz

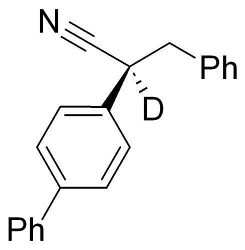

31-d

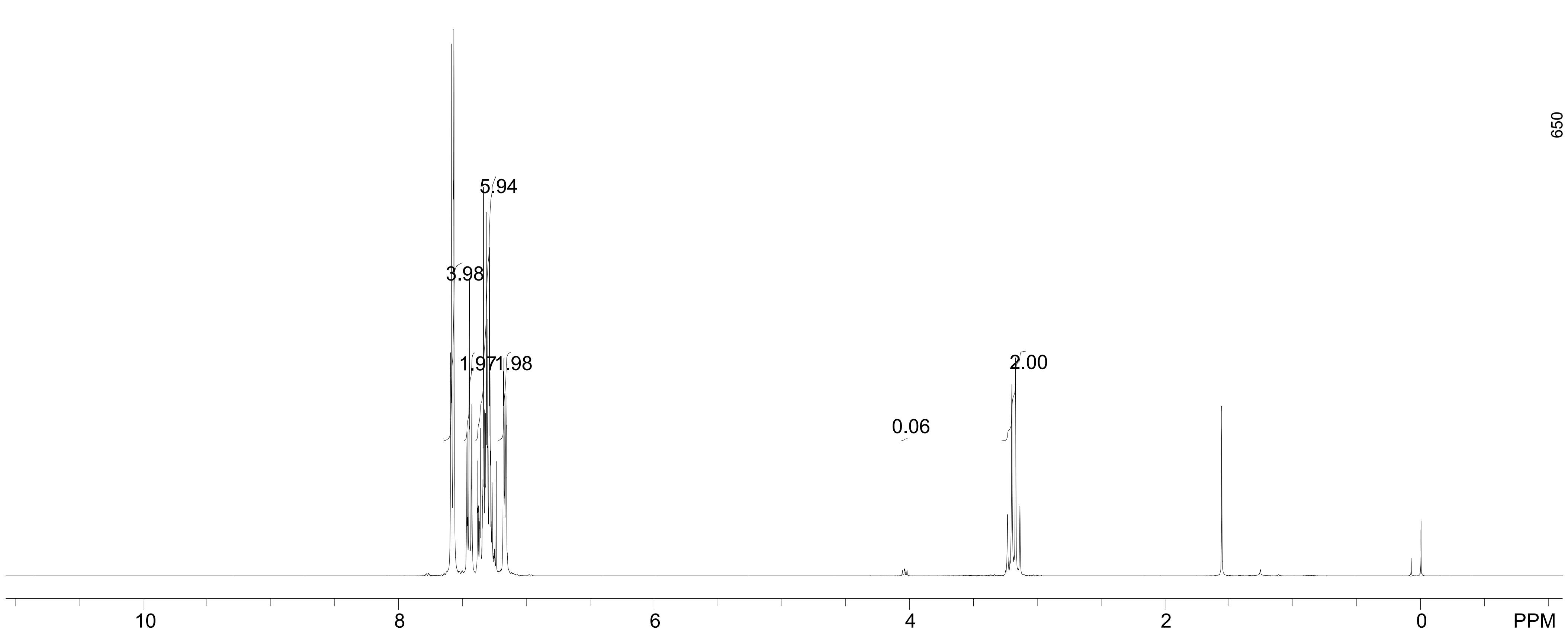

zwf-8-016C
Dec 06 2023
SOLVENT: CDCl3
NA = 100
F1 = 100.615280 MHz
F2 = 1.000000 MHz

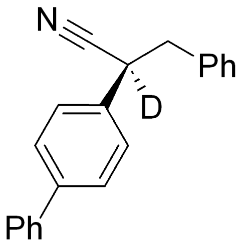

31-d

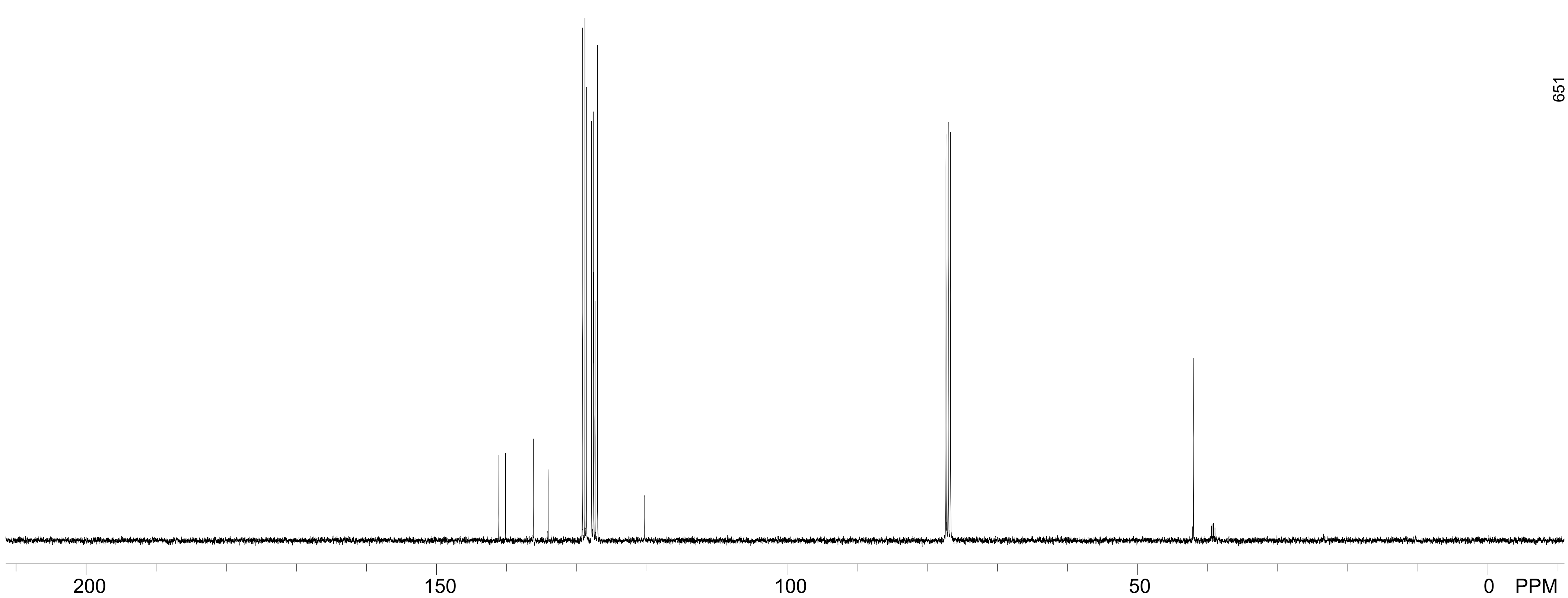

Area Percent Report

Agilent Technologies

Sample name: zwf-8-016-IE-99-1-0.8-205

Data file: C:\Users\Public\Documents\ChemStation\1\Data\zwf\zwf-S 2023-12-06 20-07-35\014-P1-B2-zwf-8-016.D

Injection Acquired Date 12/07/2023 05:49:37

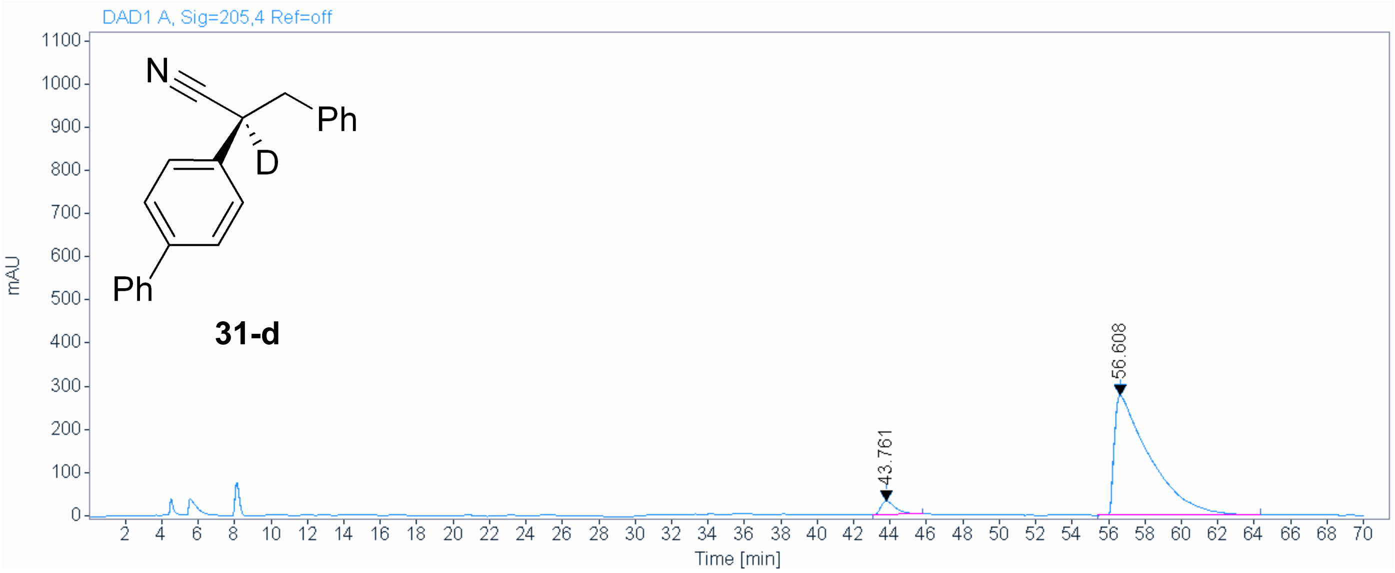

Signal: DAD1 A, Sig=205,4 Ref=off

RT [min]	Width [min]	Height	Area	Area%
43.761	0.8291	31.1808	1799.0400	4.5855
56.608	2.2526	276.9695	37434.6133	95.4145
		Sum	39233.6533	100.0000

Area Percent Report

Agilent Technologies

Sample name: zwf-8-016-rac-IE-99-1-0.8-205

Data file: C:\Users\Public\Documents\ChemStation\1\Data\zwf\zwf-S 2023-12-06 20-07-35\015-P1-B3-zwf-8-016-rac.D

Injection Acquired Date 12/07/2023 07:00:40

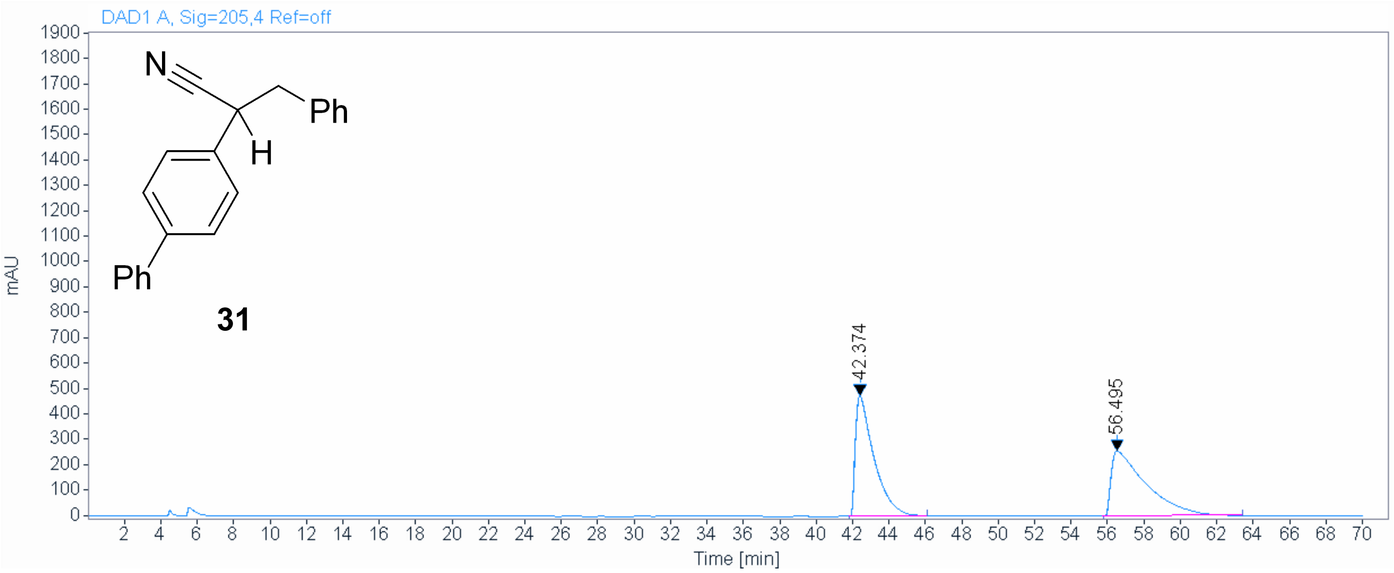

Signal: DAD1 A, Sig=205,4 Ref=off

RT [min]	Width [min]	Height	Area	Area%
42.374	1.0204	474.8948	33640.8477	49.5557
56.495	1.7842	256.4013	34244.1133	50.4443
		Sum	67884.9609	100.0000

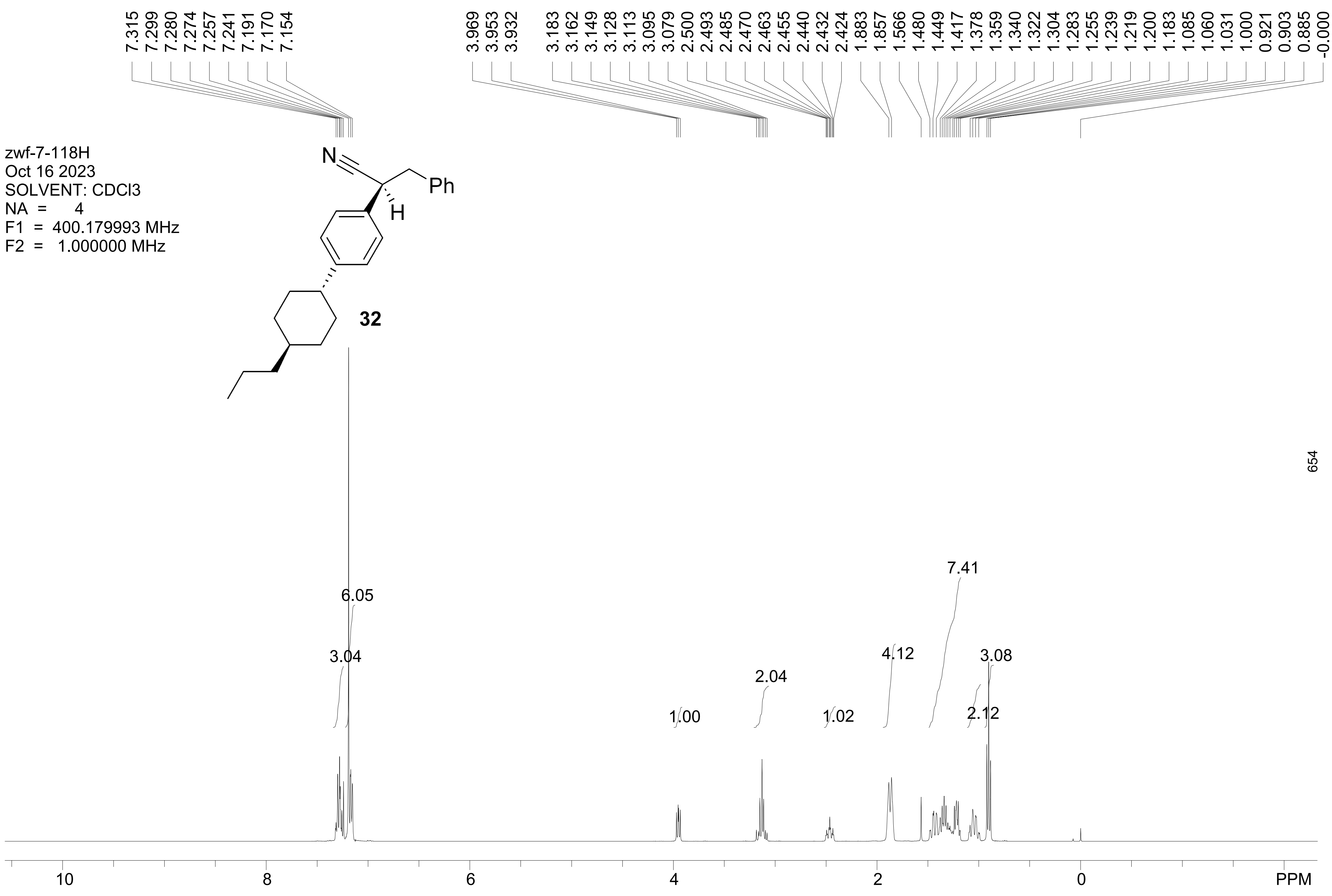
zwf-7-118H
Oct 16 2023
SOLVENT: CDCl3
NA = 4
F1 = 400.179993 MHz
F2 = 1.000000 MHz
32
Ph
H
N
7.315
7.299
7.280
7.274
7.257
7.241
7.191
7.170
7.154
3.969
3.953
3.932
3.183
3.162
3.149
3.128
3.113
3.095
3.079
2.500
2.493
2.485
2.470
2.463
2.455
2.440
2.432
2.424
1.883
1.857
1.566
1.480
1.449
1.417
1.378
1.359
1.340
1.322
1.304
1.283
1.255
1.239
1.219
1.200
1.183
1.085
1.060
1.031
1.000
0.921
0.903
0.885
-0.000
3.04
6.05
1.00
2.04
1.02
4.12
7.41
2.12
3.08
10
8
6
4
2
0
PPM

zwf-7-118C
Oct 16 2023
SOLVENT: CDCl3
NA = 100
F1 = 100.625343 MHz
F2 = 1.000000 MHz

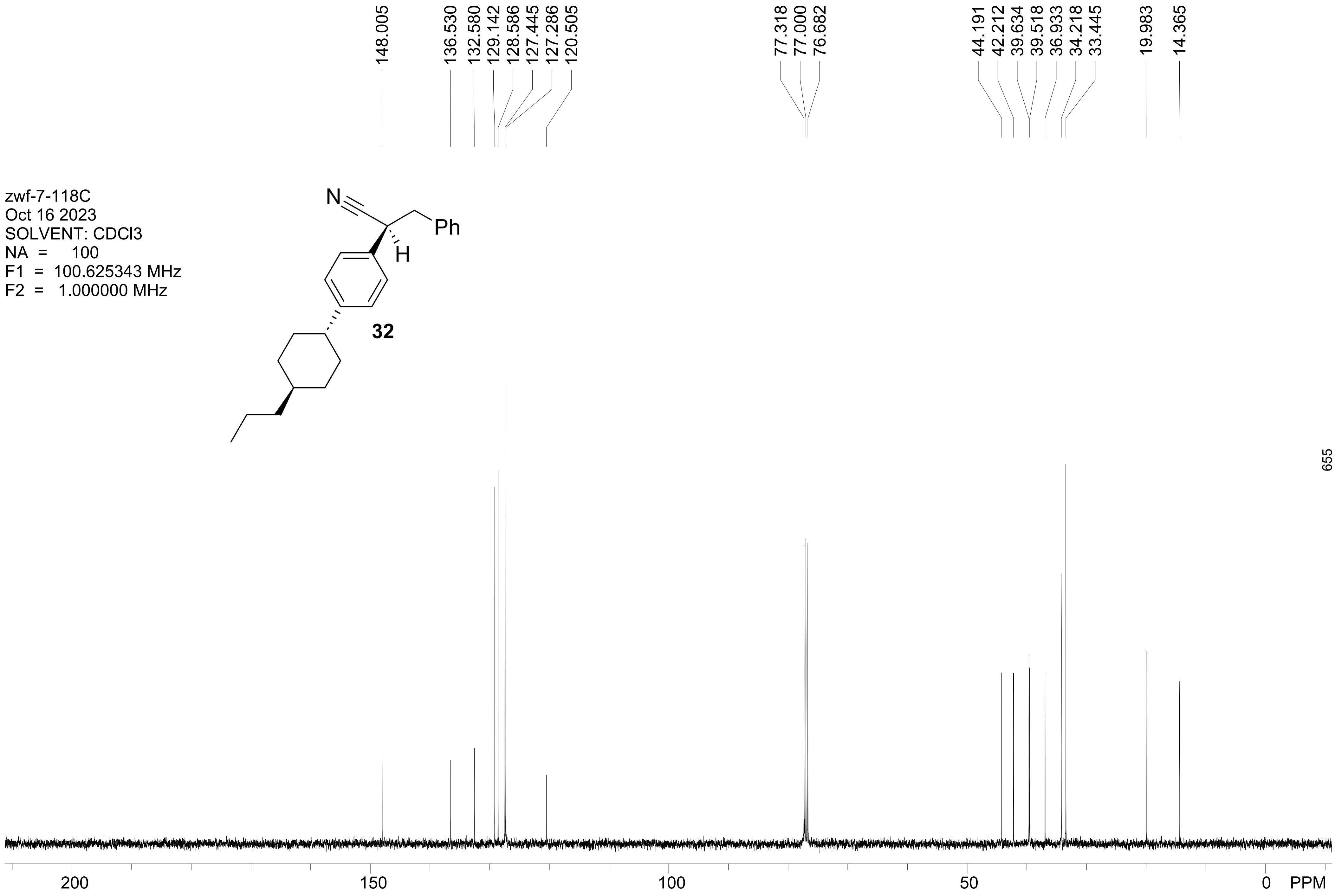

Area Percent Report

Agilent Technologies

Sample name: zwf-7-118-IE-99-1-0.8-205

Data file: C:\Users\Public\Documents\ChemStation\1\Data\zwf\zwf-S 2023-10-16 16-43-27\003-P1-C5-zwf-7-118.D

Injection Acquired Date 10/16/2023 17:37:21

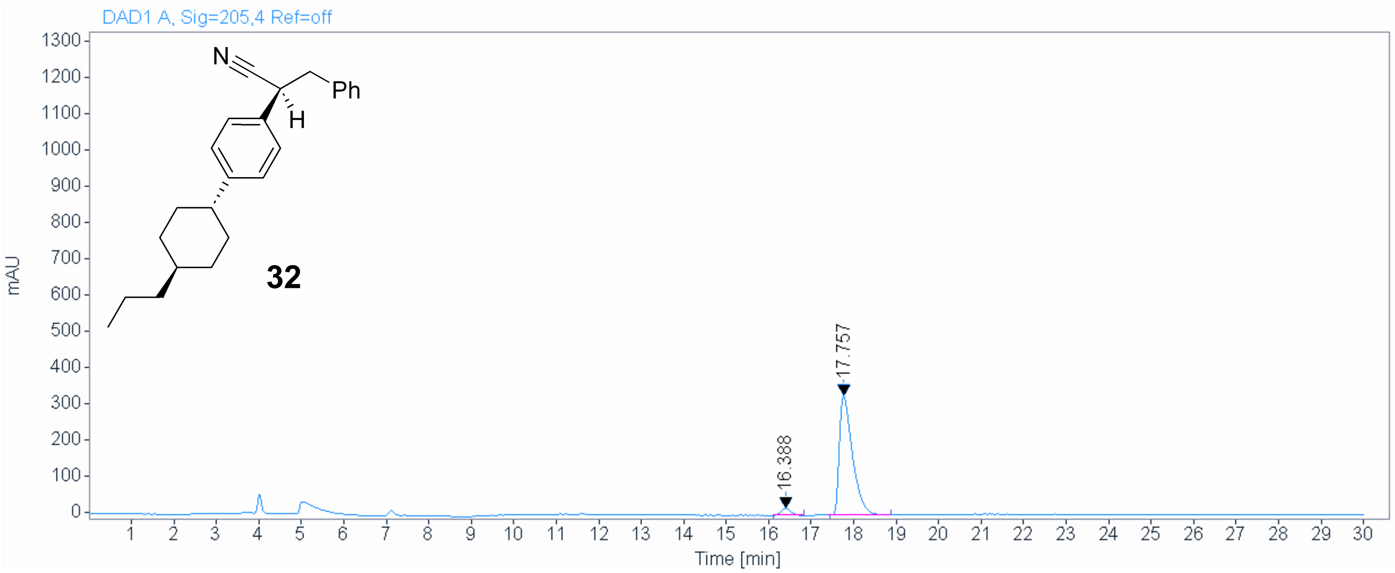

Signal: DAD1 A, Sig=205,4 Ref=off

RT [min]	Width [min]	Height	Area	Area%
16.388	0.2759	19.8966	329.3092	4.4344
17.757	0.3209	330.0141	7096.8804	95.5656
		Sum	7426.1896	100.0000

Area Percent Report

Sample name: zwf-7-118-rac-IE-99-1-0.8-205

Data file: C:\Users\Public\Documents\ChemStation\1\Data\zwf\zwf-S 2023-10-16 16-43-27\004-P1-C6-zwf-7-018-rac.D

Injection Acquired Date 10/16/2023 18:08:25

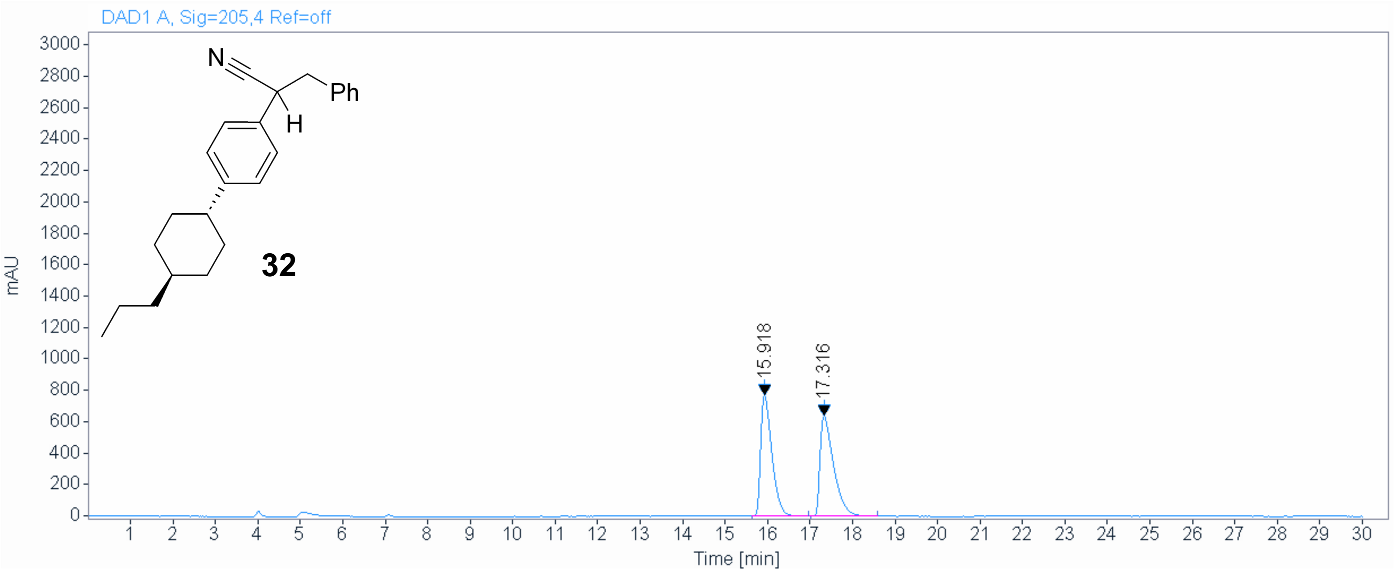

Signal: DAD1 A, Sig=205,4 Ref=off

RT [min]	Width [min]	Height	Area	Area%
15.918	0.2765	769.3799	14143.7715	49.8204
17.316	0.3280	638.9597	14245.7354	50.1796
		Sum	28389.5068	100.0000

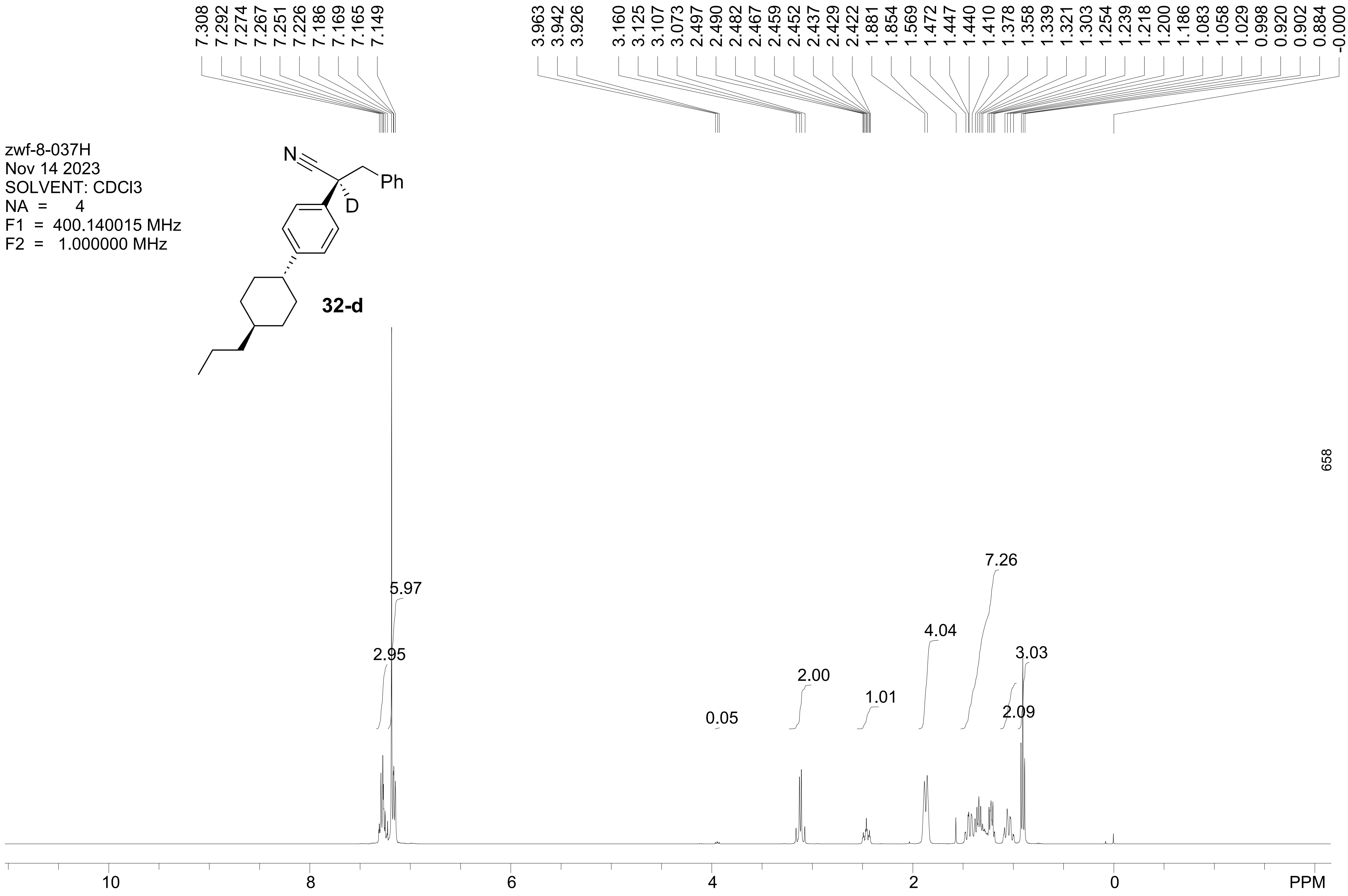
zwf-8-037H
Nov 14 2023
SOLVENT: CDCl3
NA = 4
F1 = 400.140015 MHz
F2 = 1.000000 MHz
N
D
Ph
7.308
7.292
7.274
7.267
7.251
7.226
7.186
7.169
7.165
7.149
3.963
3.942
3.926
3.160
3.125
3.107
3.073
2.497
2.490
2.482
2.467
2.459
2.452
2.437
2.429
2.422
1.881
1.854
1.569
1.472
1.447
1.440
1.410
1.378
1.358
1.339
1.321
1.303
1.254
1.239
1.218
1.200
1.186
1.083
1.058
1.029
0.998
0.920
0.902
0.884
-0.000
2.95
5.97
0.05
2.00
1.01
4.04
7.26
2.09
3.03
10
8
6
4
2
0
PPM

32-d

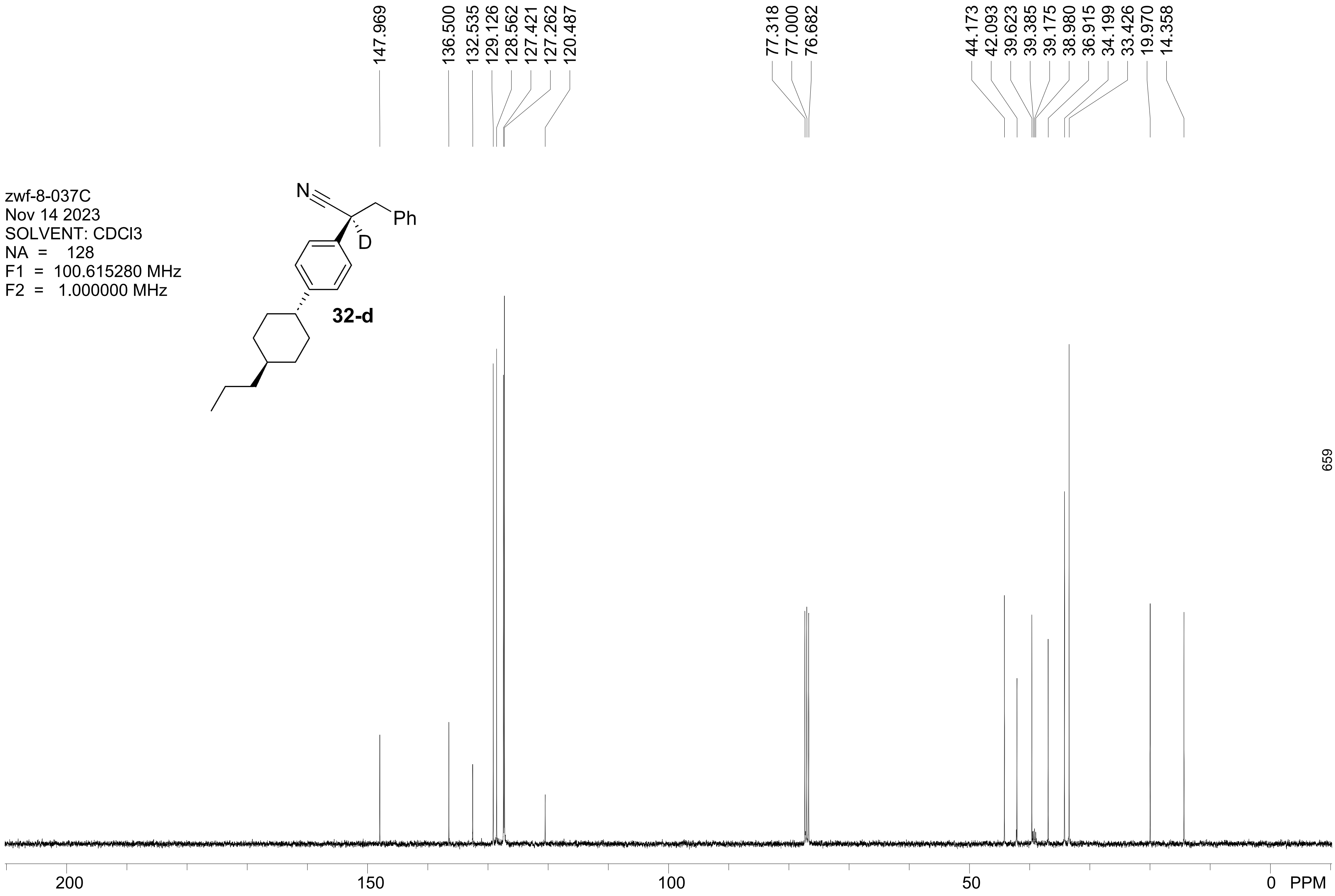
zwf-8-037C
Nov 14 2023
SOLVENT: CDCl3
NA = 128
F1 = 100.615280 MHz
F2 = 1.000000 MHz
N
D
Ph
32-d
147.969
136.500
132.535
129.126
128.562
127.421
127.262
120.487
77.318
77.000
76.682
44.173
42.093
39.623
39.385
39.175
38.980
36.915
34.199
33.426
19.970
14.358
200
150
100
50
0 PPM

Area Percent Report

Sample name: zwf-8-037-IE-99-1-0.8-205

Data file: C:\Users\Public\Documents\ChemStation\1\Data\zwf\zwf-S 2023-11-15 14-21-28\017-P1-B3-zwf-8-037.D

Injection Acquired Date 11/16/2023 00:09:33

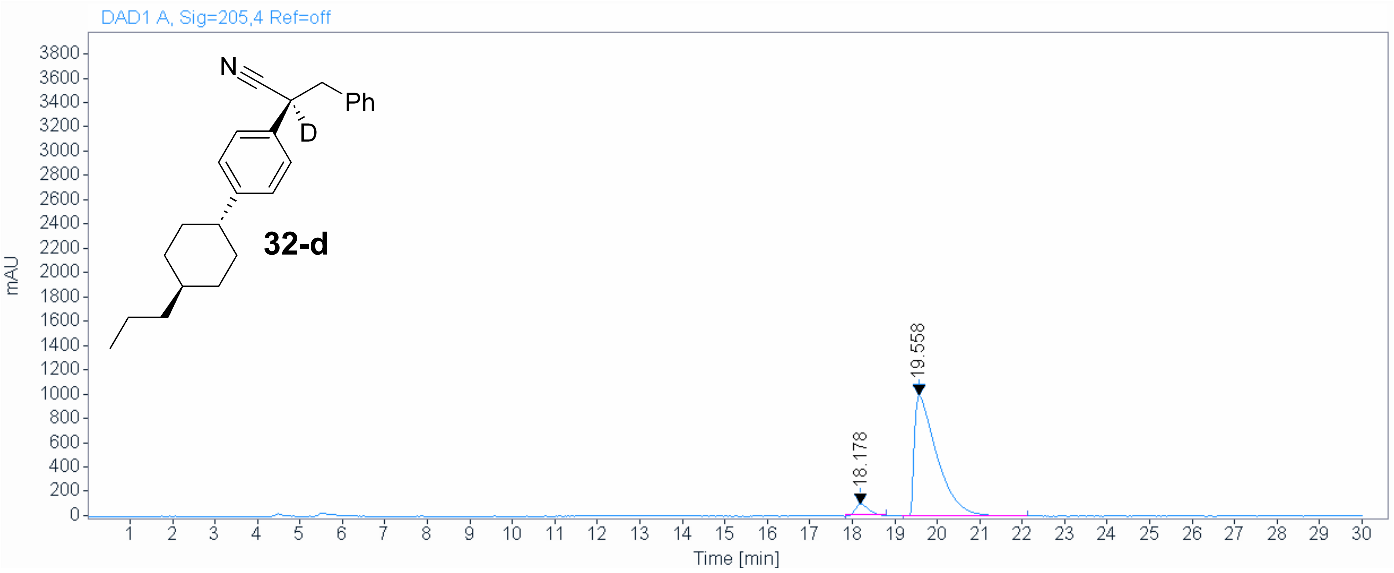

Signal: DAD1 A, Sig=205,4 Ref=off

RT [min]	Width [min]	Height	Area	Area%
18.178	0.3467	87.9855	1830.3241	4.7206
19.558	0.6198	993.4391	36943.1406	95.2794
		Sum	38773.4647	100.0000

Area Percent Report

Agilent Technologies

Sample name: zwf-8-037-rac-IE-99-1-0.8-205

Data file: C:\Users\Public\Documents\ChemStation\1\Data\zwf\zwf-S 2023-11-15 14-21-28\018-P1-B4-zwf-8-037-rac.D

Injection Acquired Date 11/16/2023 00:40:34

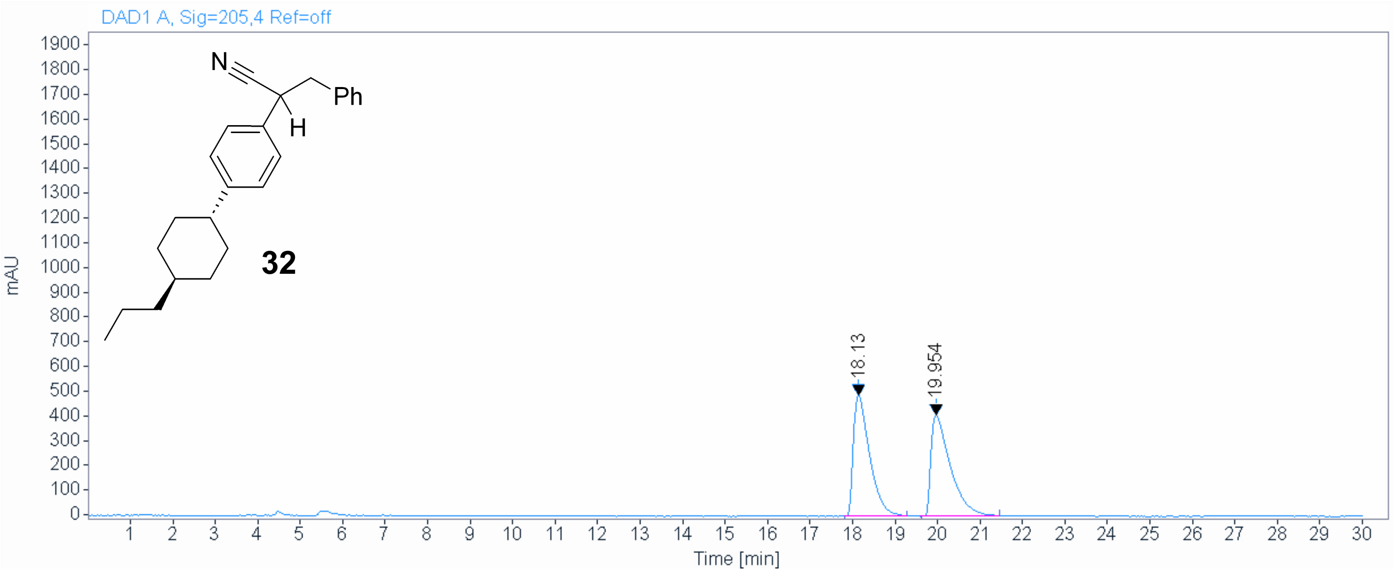

Signal: DAD1 A, Sig=205,4 Ref=off

RT [min]	Width [min]	Height	Area	Area%
18.130	0.3772	487.8553	12937.8613	49.7860
19.954	0.4369	408.2378	13049.1074	50.2140
		Sum	25986.9688	100.0000

zwf-7-117H
Oct 17 2023
SOLVENT: CDCl3
NA = 4
F1 = 400.179993 MHz
F2 = 1.000000 MHz

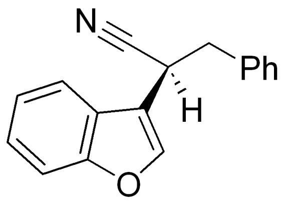

33

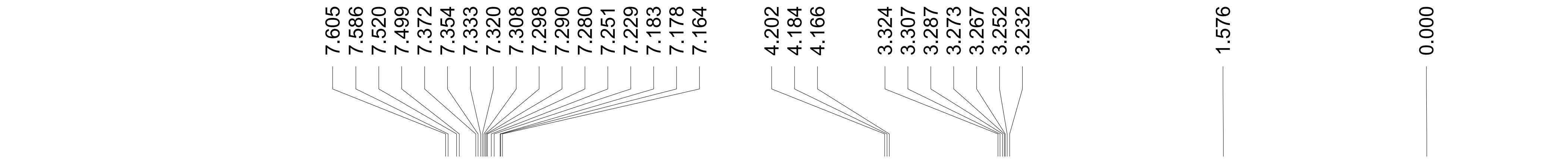

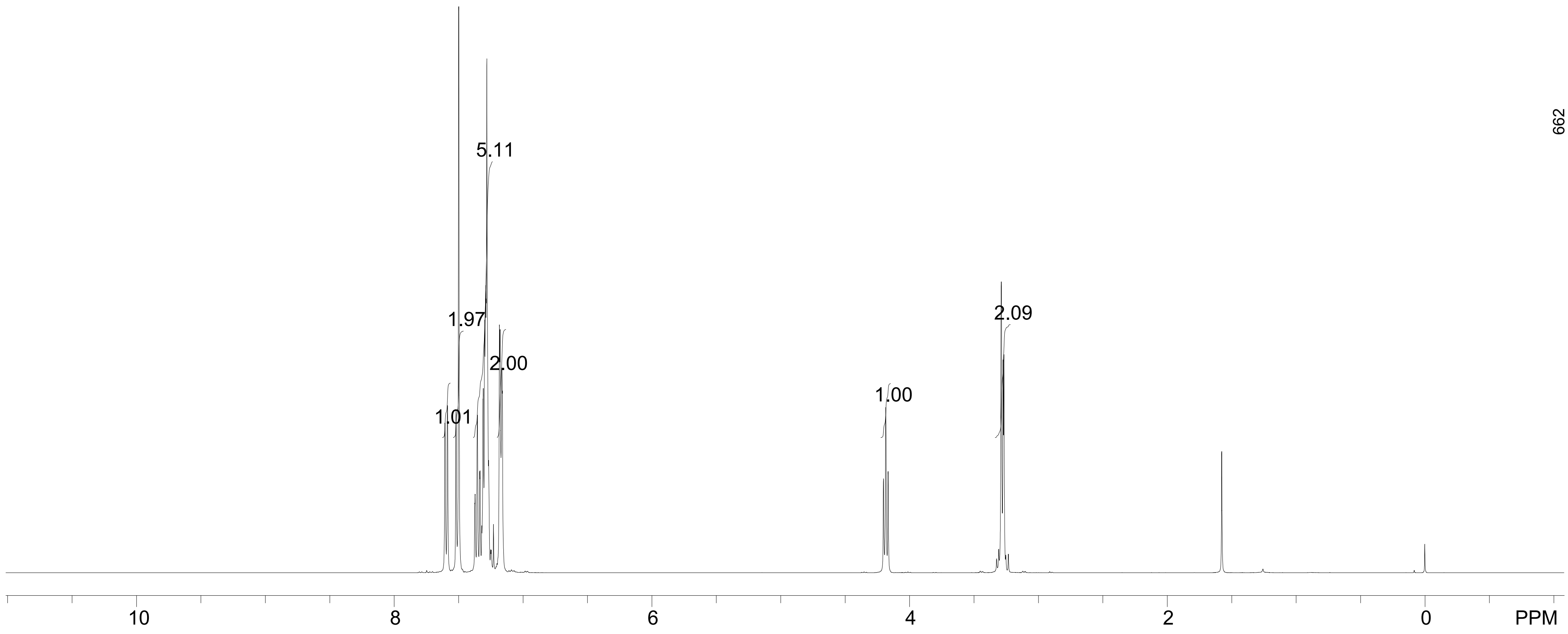

zwf-7-117C
Oct 17 2023
SOLVENT: CDCl3
NA = 100
F1 = 100.625343 MHz
F2 = 1.000000 MHz

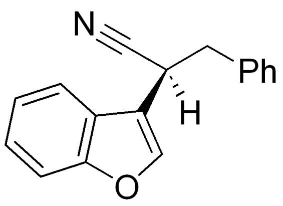

33

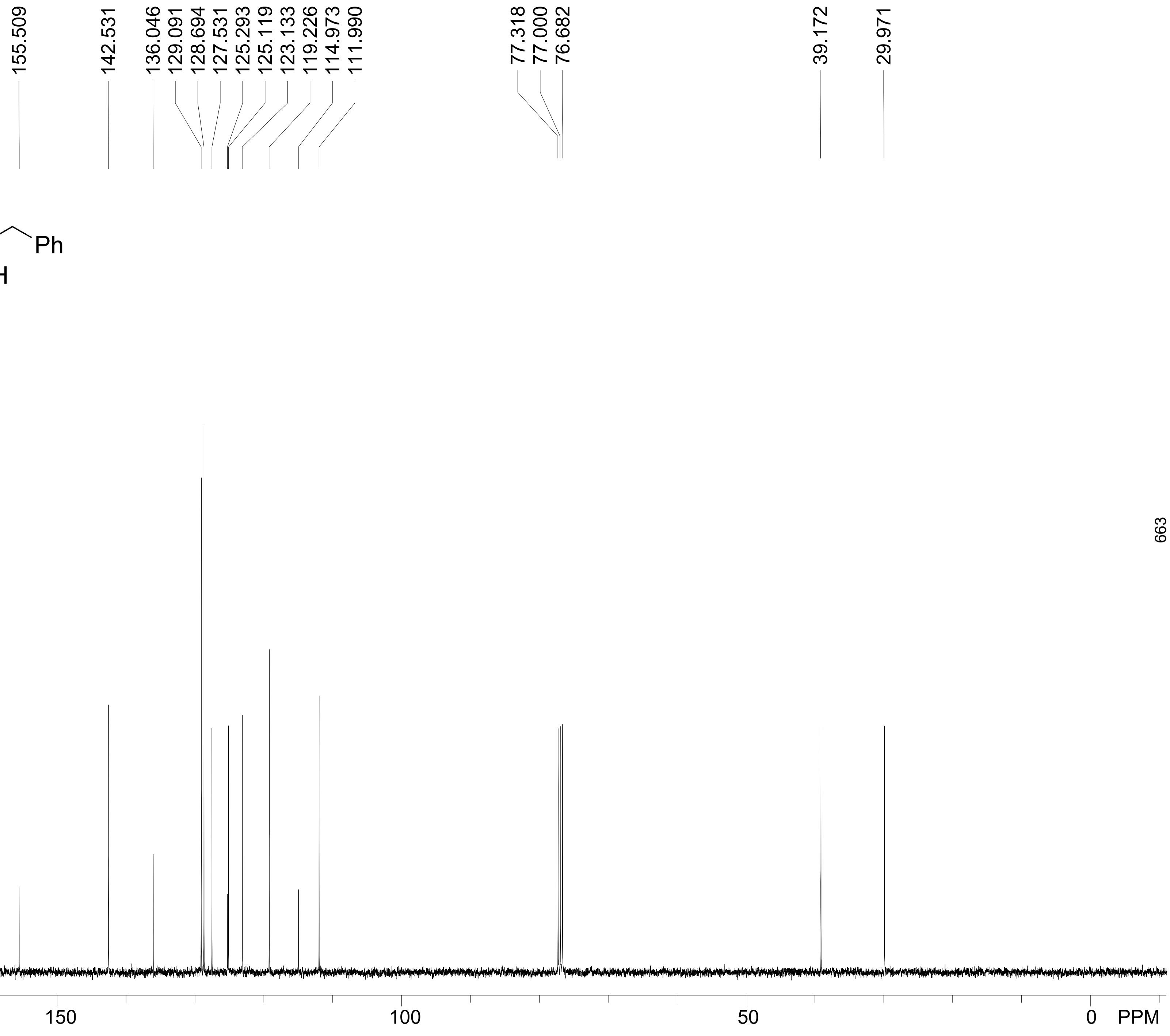

Area Percent Report

Agilent Technologies

Sample name: zwf-7-117-IE-99-1-0.8-205

Data file: C:\Users\Public\Documents\ChemStation\1\Data\zwf\zwf-S 2023-10-17 08-55-39\020-P1-C2-zwf-7-117.D

Injection Acquired Date 10/17/2023 18:38:01

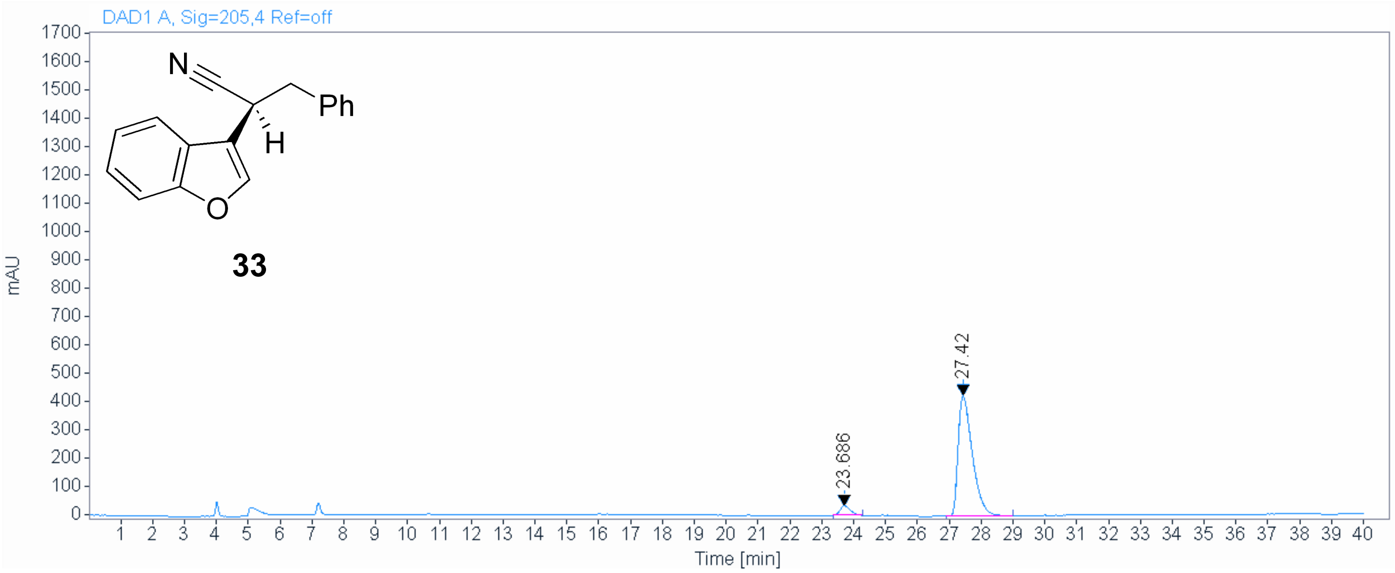

Signal: DAD1 A, Sig=205,4 Ref=off

RT [min]	Width [min]	Height	Area	Area%
23.686	0.3611	32.3386	700.7220	5.0096
27.420	0.5182	427.3054	13286.8896	94.9904
		Sum	13987.6117	100.0000

Area Percent Report

Agilent Technologies

Sample name: zwf-7-117-rac-IE-99-1-0.8-205

Data file: C:\Users\Public\Documents\ChemStation\1\Data\zwf\zwf-S 2023-10-16 21-38-05\003-P1-B2-zwf-7-117-rac.D

Injection Acquired Date 10/16/2023 22:37:48

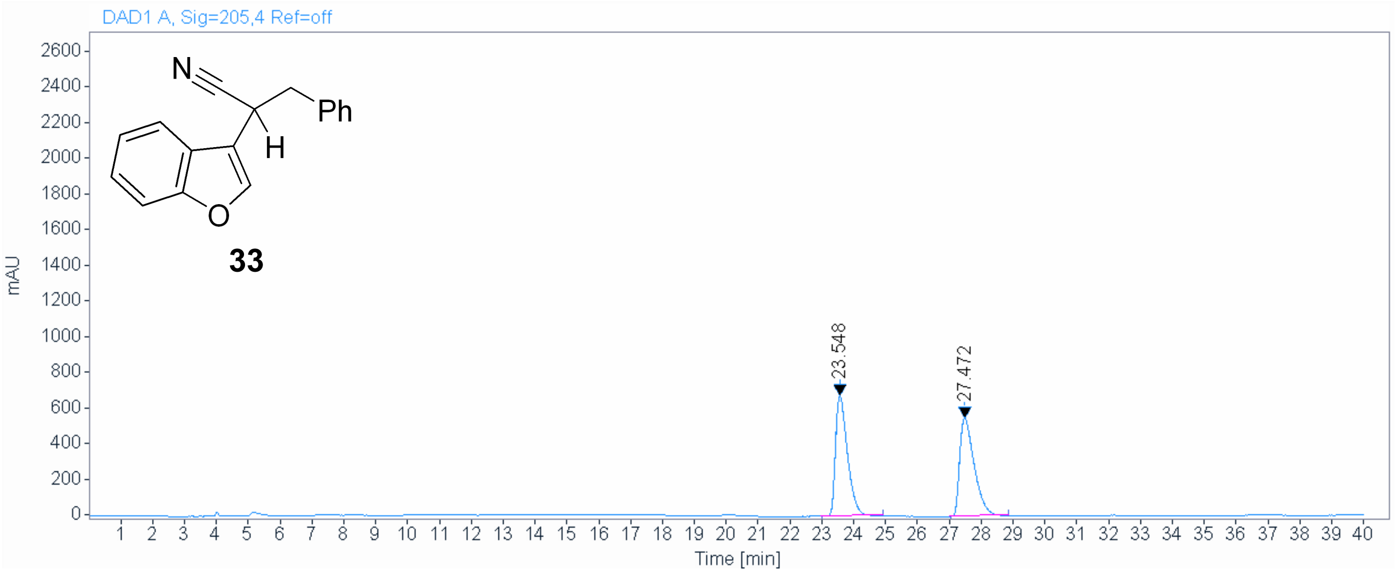

Signal: DAD1 A, Sig=205,4 Ref=off

RT [min]	Width [min]	Height	Area	Area%
23.548	0.3933	675.4202	17557.6074	50.0810
27.472	0.4643	551.4433	17500.8418	49.9190
		Sum	35058.4492	100.0000

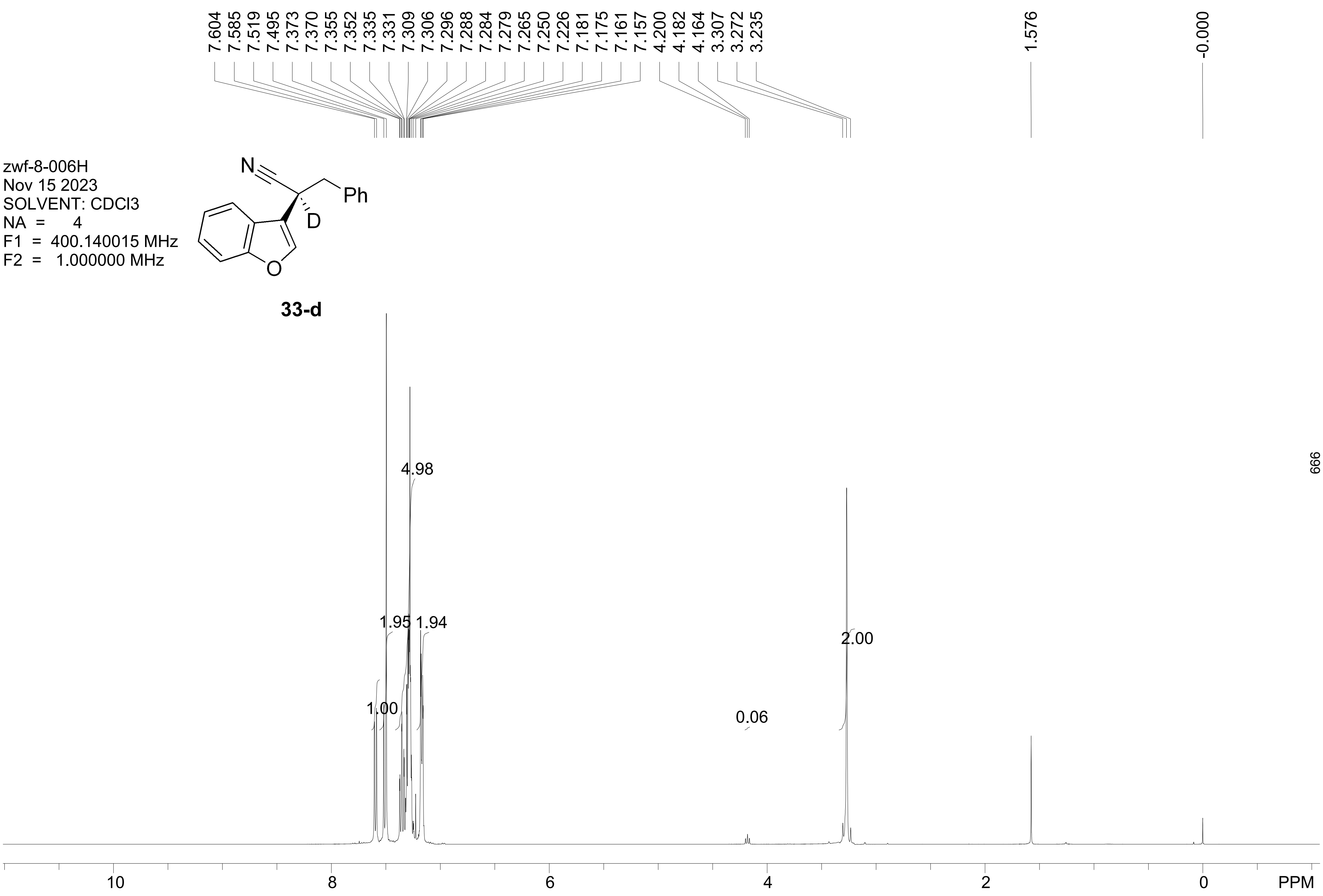
zwf-8-006H
Nov 15 2023
SOLVENT: CDCl3
NA = 4
F1 = 400.140015 MHz
F2 = 1.000000 MHz
N
Ph
D
O
33-d
7.604
7.585
7.519
7.495
7.373
7.370
7.355
7.352
7.335
7.331
7.309
7.306
7.296
7.288
7.284
7.279
7.265
7.250
7.226
7.181
7.175
7.161
7.157
4.200
4.182
4.164
3.307
3.272
3.235
1.576
-0.000
1.00
1.95
4.98
1.94
0.06
2.00
10
8
6
4
2
0
PPM

zwf-8-006C
Nov 15 2023
SOLVENT: CDCl3
NA = 150
F1 = 100.615280 MHz
F2 = 1.000000 MHz

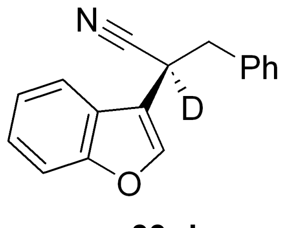

33-d

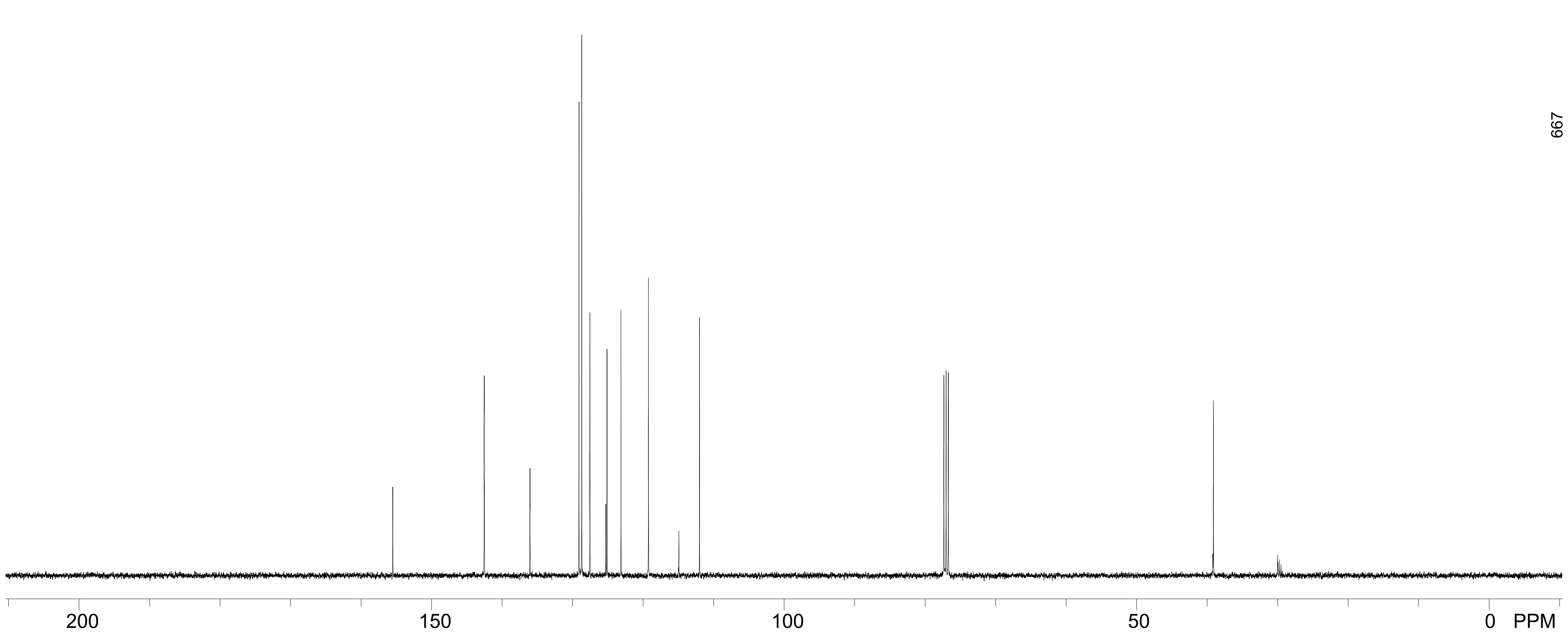

Area Percent Report

Agilent Technologies

Sample name: zwf-8-006-IE-99-1-0.8-205

Data file: C:\Users\Public\Documents\ChemStation\1\Data\zwf\zwf-S 2023-11-17 07-38-48\014-P1-C3-zwf-8-006.D

Injection Acquired Date 11/17/2023 15:03:40

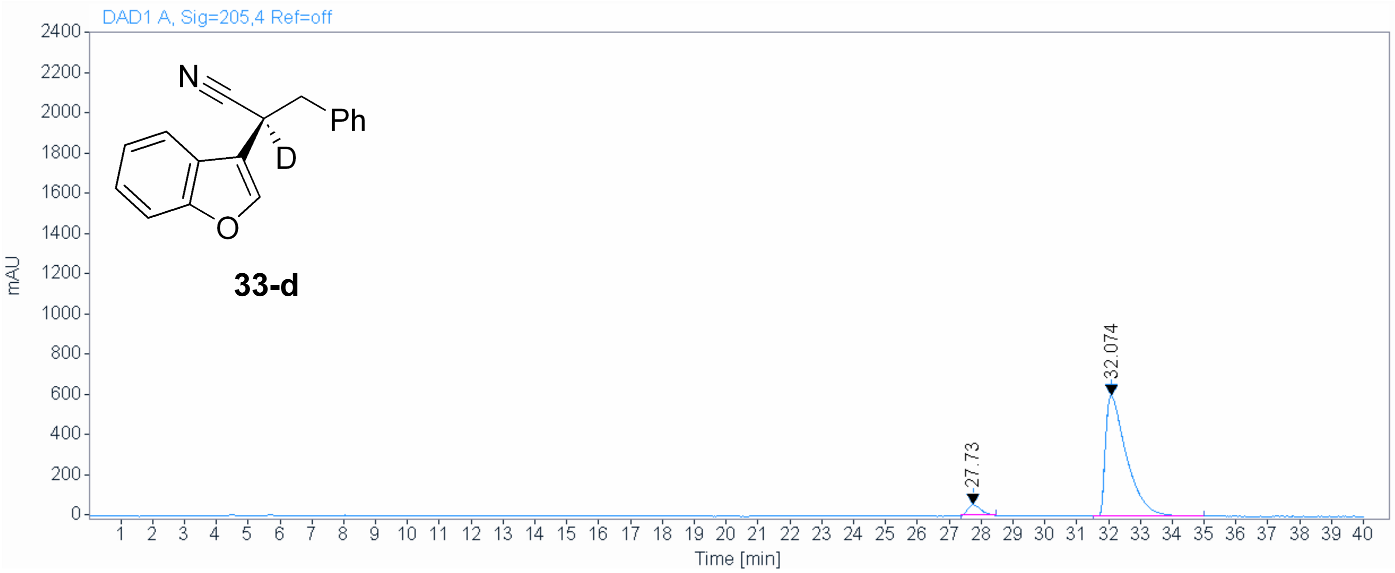

Signal: DAD1 A, Sig=205,4 Ref=off

RT [min]	Width [min]	Height	Area	Area%
27.730	0.4686	53.6067	1507.1616	5.1893
32.074	0.7659	599.1905	27536.3203	94.8107
		Sum	29043.4819	100.0000

Area Percent Report

Sample name: zwf-8-006-rac-IE-99-1-0.8-205

Data file: C:\Users\Public\Documents\ChemStation\1\Data\zwf\zwf-S 2023-11-17 07-38-48\015-P1-C4-zwf-8-006-rac.D

Injection Acquired Date 11/17/2023 15:44:39

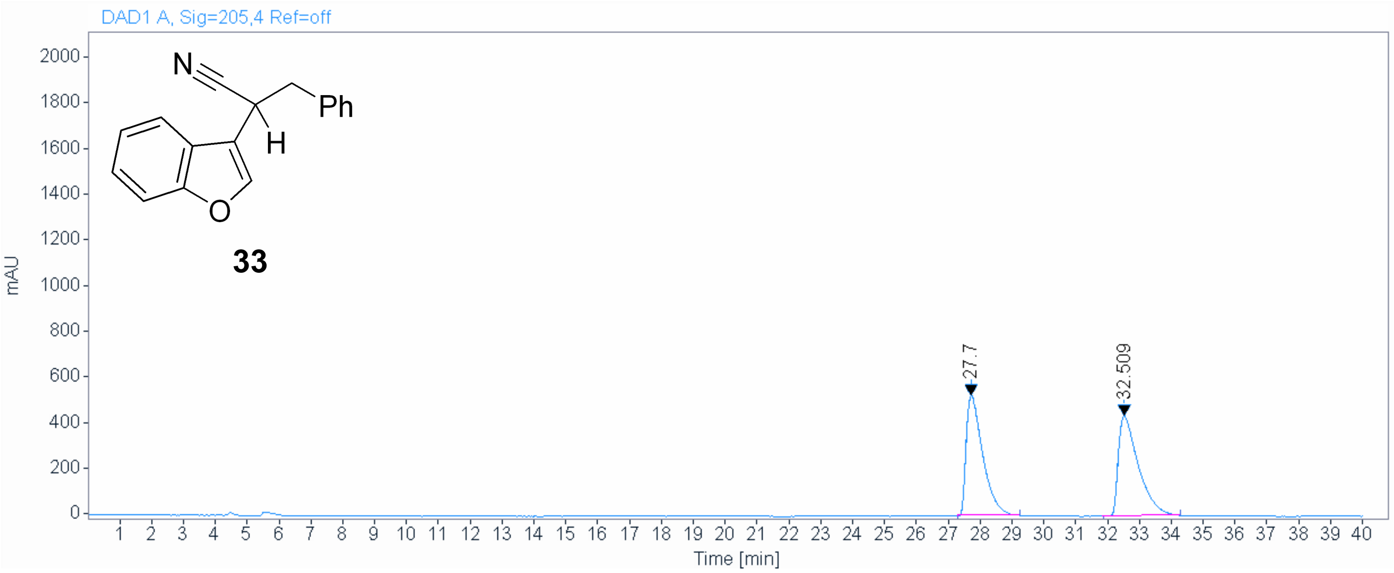

Signal: DAD1 A, Sig=205,4 Ref=off

RT [min]	Width [min]	Height	Area	Area%
27.700	0.6043	525.2626	19044.1445	49.7673
32.509	0.5277	438.0961	19222.2578	50.2327
		Sum	38266.4023	100.0000

zwf-7-113H
Oct 15 2023
SOLVENT: CDCl3
NA = 4
F1 = 400.140015 MHz
F2 = 1.000000 MHz

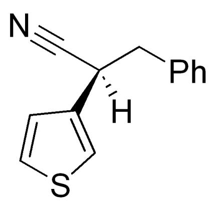

34

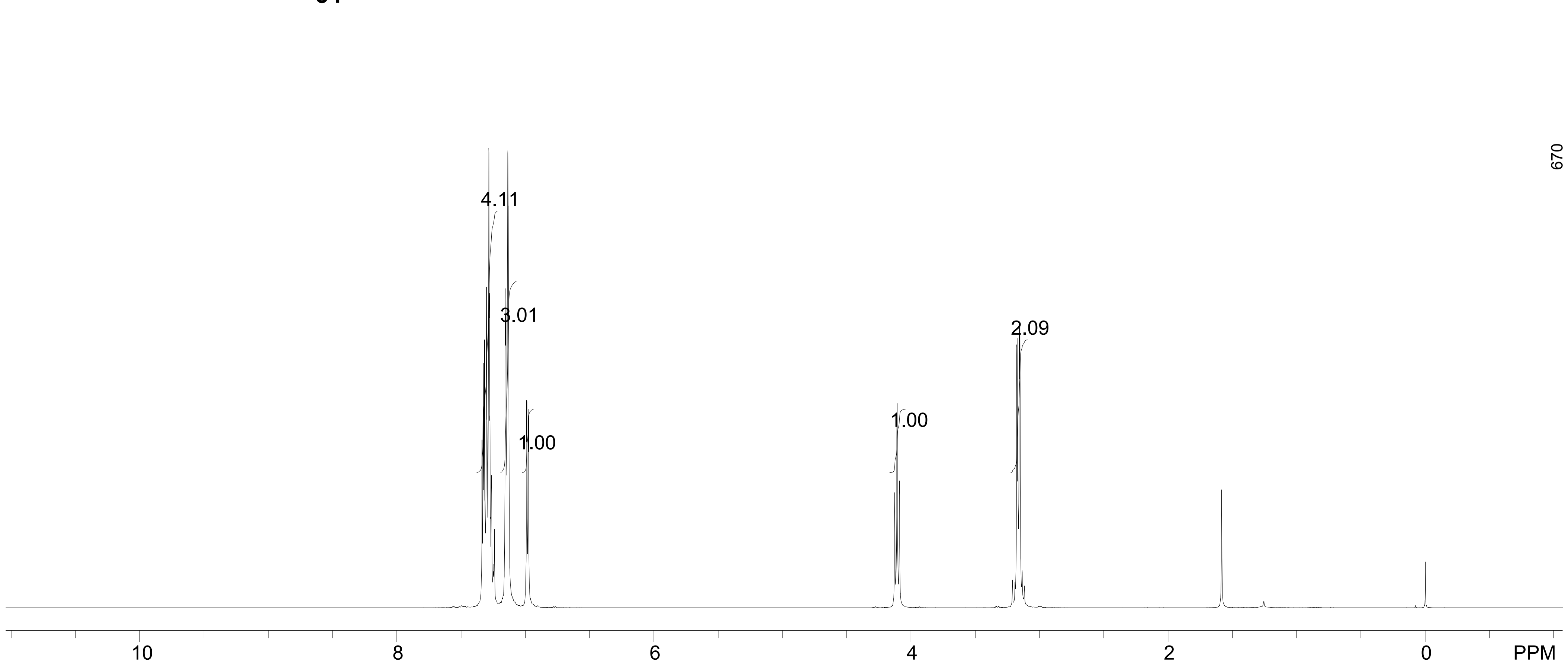

zwf-7-113C
Oct 15 2023
SOLVENT: CDCl3
NA = 100
F1 = 100.615280 MHz
F2 = 1.000000 MHz

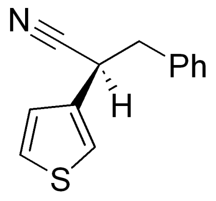

34

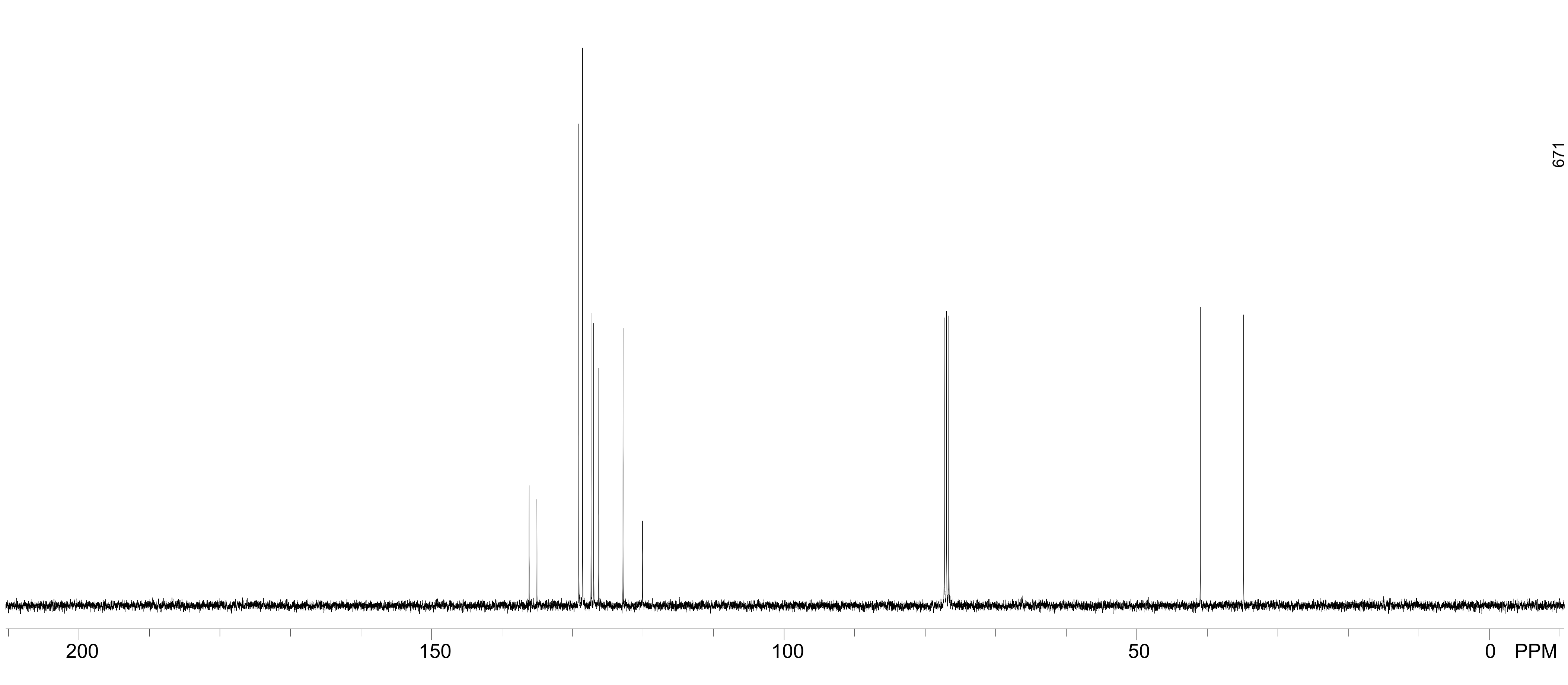

Area Percent Report

Sample name: zwf-7-113-IE-99-1-0.8-205

Data file: C:\Users\Public\Documents\ChemStation\1\Data\zwf\zwf-S 2023-10-15 16-11-49\003-P1-C5-zwf-7-113.D

Injection Acquired Date 10/15/2023 17:00:46

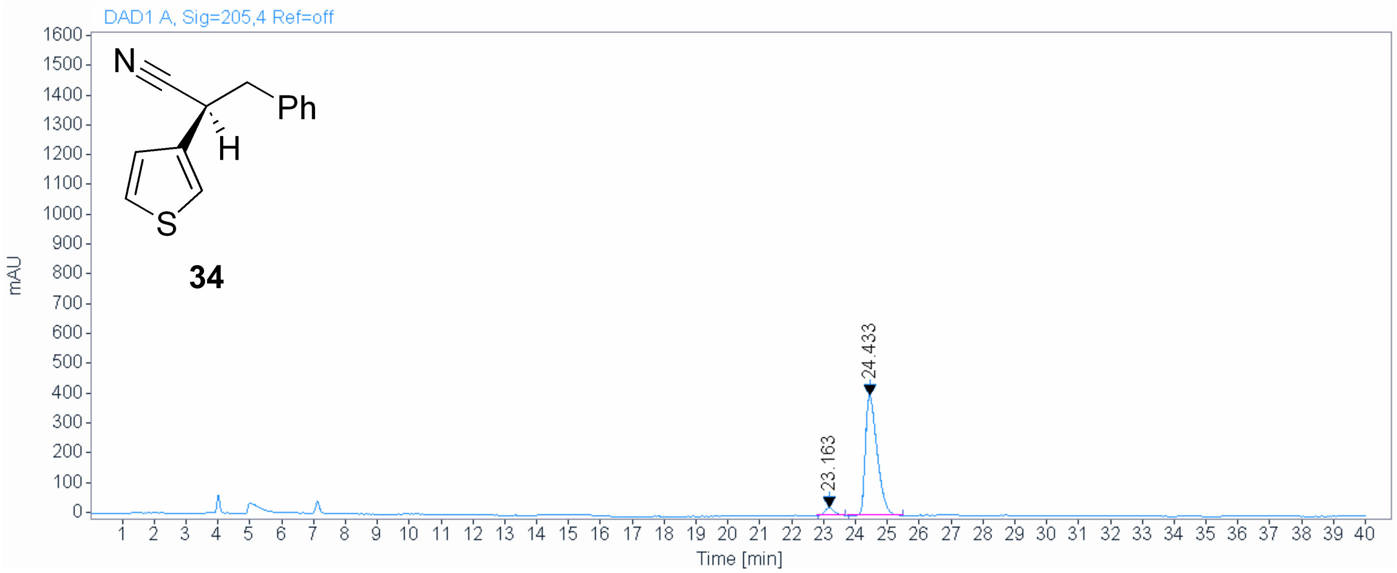

Signal: DAD1 A, Sig=205,4 Ref=off

RT [min]	Width [min]	Height	Area	Area%
23.163	0.3545	26.0545	554.2269	5.0864
24.433	0.3899	402.3197	10342.0537	94.9136
		Sum	10896.2806	100.0000

Area Percent Report

Sample name: zwf-7-113-rac-IE-99-1-0.8-205

Data file: C:\Users\Public\Documents\ChemStation\1\Data\zwf\zwf-S 2023-10-14 09-03-23\021-P1-C6-zwf-7-113-rac.D

Injection Acquired Date 10/14/2023 22:52:45

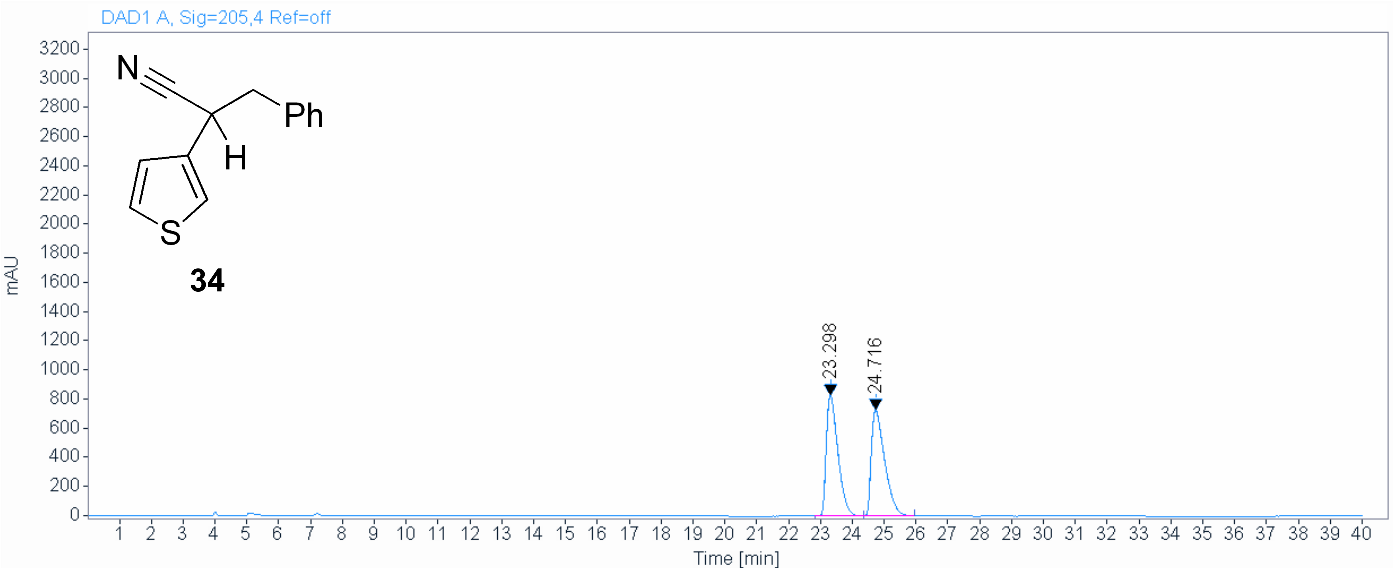

Signal: DAD1 A, Sig=205,4 Ref=off

RT [min]	Width [min]	Height	Area	Area%
23.298	0.3694	827.6647	21101.4629	49.9645
24.716	0.4089	727.4751	21131.4805	50.0355
		Sum	42232.9434	100.0000

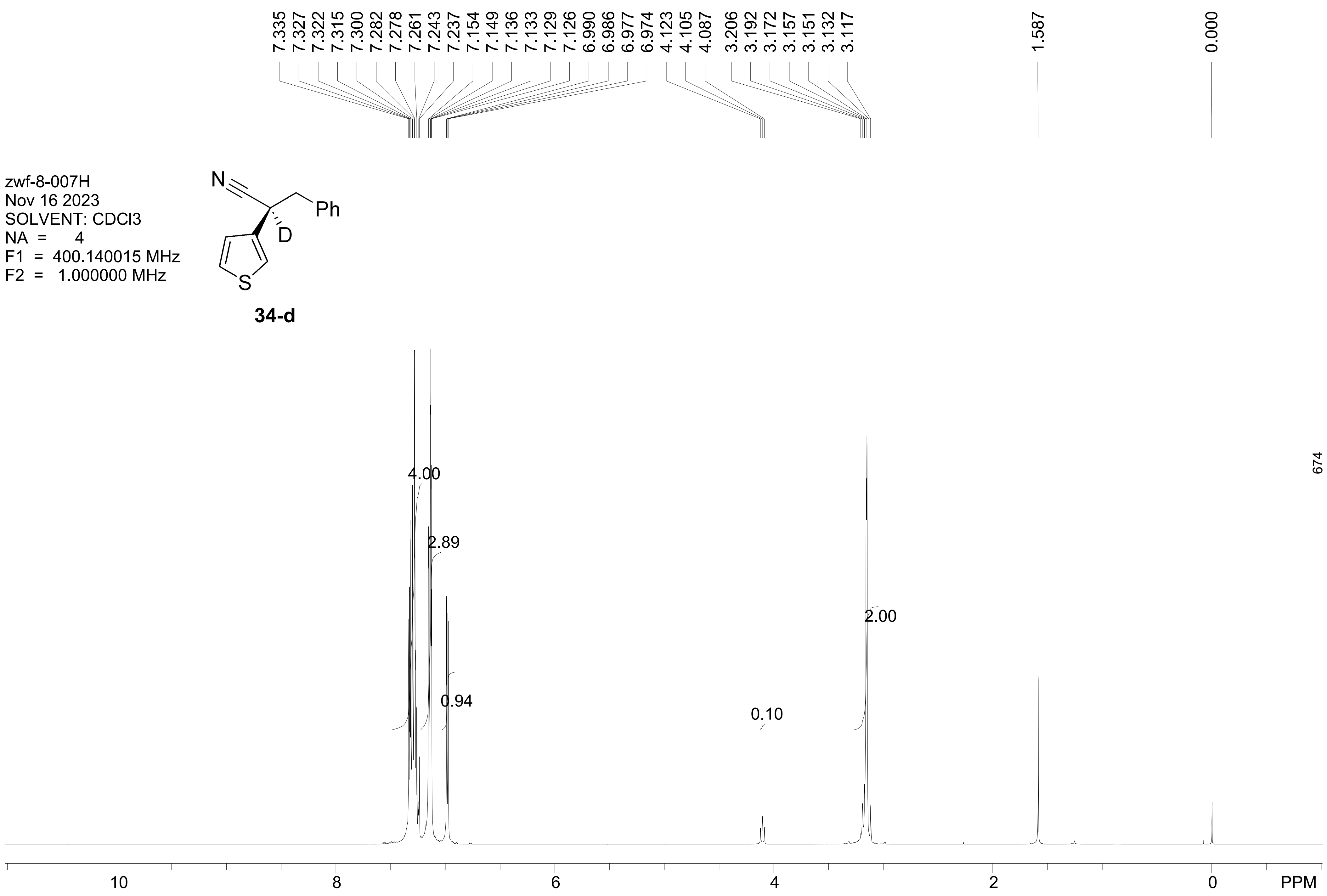
zwf-8-007H
Nov 16 2023
SOLVENT: CDCl3
NA = 4
F1 = 400.140015 MHz
F2 = 1.000000 MHz
N
Ph
D
S
34-d
7.335
7.327
7.322
7.315
7.300
7.282
7.278
7.261
7.243
7.237
7.154
7.149
7.136
7.133
7.129
7.126
6.990
6.986
6.977
6.974
4.123
4.105
4.087
3.206
3.192
3.172
3.157
3.151
3.132
3.117
1.587
0.000
4.00
2.89
0.94
0.10
2.00
10
8
6
4
2
0
PPM

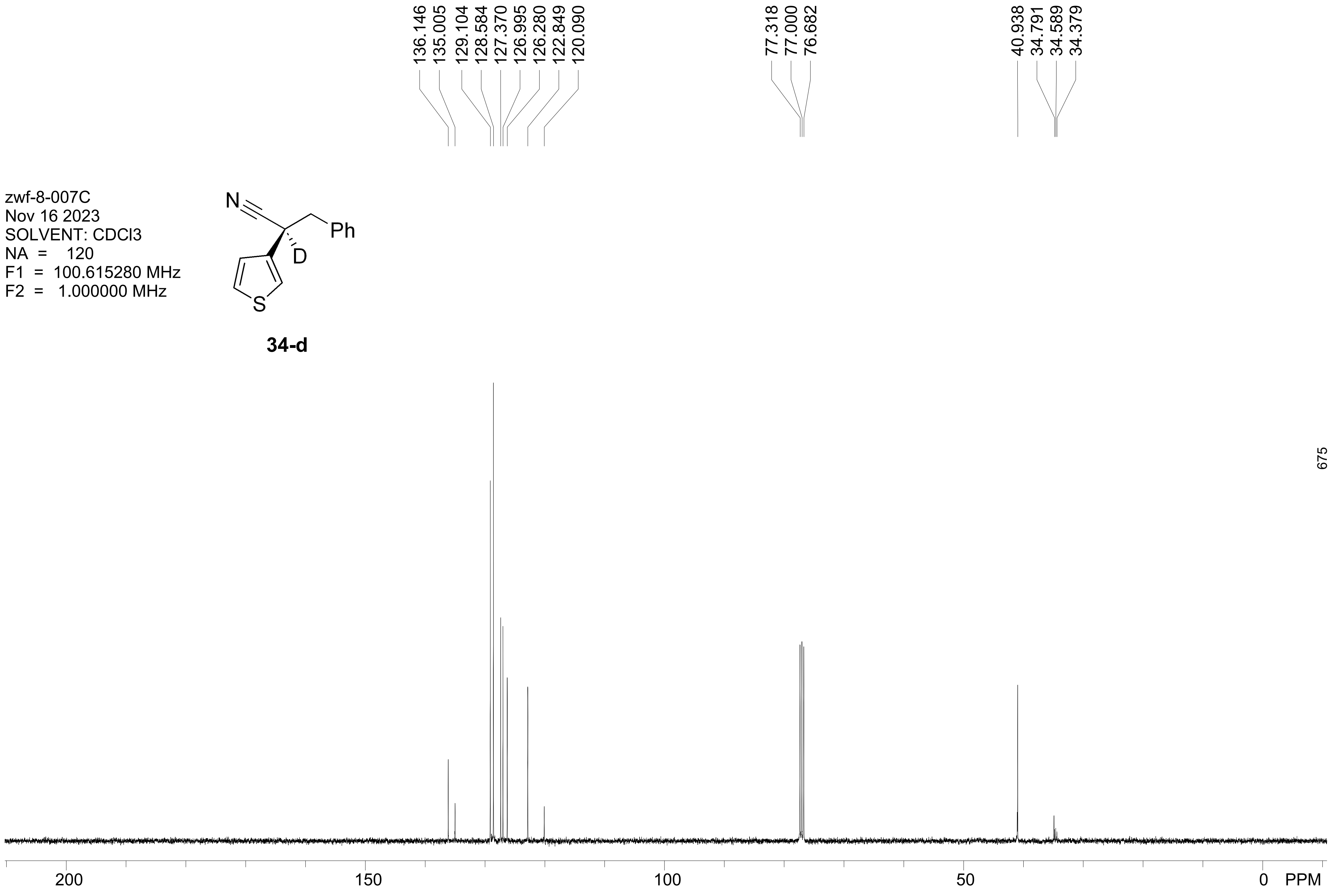
zwf-8-007C
Nov 16 2023
SOLVENT: CDCl3
NA = 120
F1 = 100.615280 MHz
F2 = 1.000000 MHz
N
D
Ph
S
34-d
136.146
135.005
129.104
128.584
127.370
126.995
126.280
122.849
120.090
77.318
77.000
76.682
40.938
34.791
34.589
34.379
200
150
100
50
0 PPM

Area Percent Report

Agilent Technologies

Sample name: zwf-8-007-IE-99-1-0.8-205

Data file: C:\Users\Public\Documents\ChemStation\1\Data\zwf\zwf-S 2023-11-17 07-38-48\031-P1-C5-zwf-8-007.D

Injection Acquired Date 11/17/2023 23:21:48

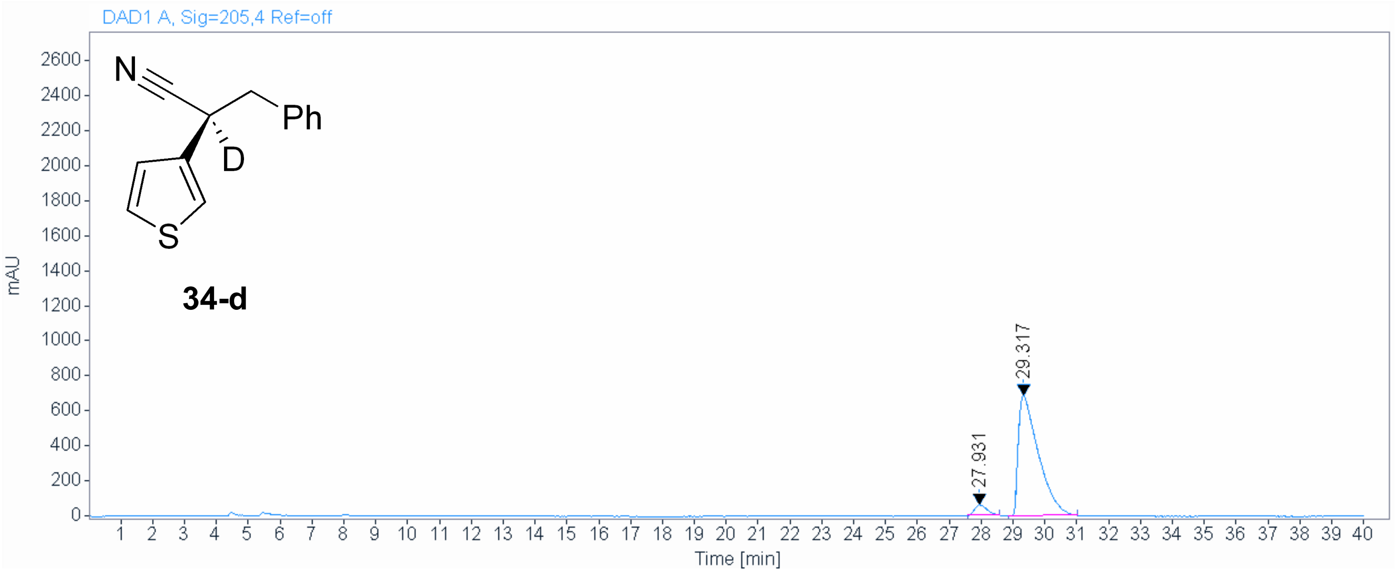

Signal: DAD1 A, Sig=205,4 Ref=off

RT [min]	Width [min]	Height	Area	Area%
27.931	0.4423	58.2739	1546.6301	4.8642
29.317	0.5950	687.7681	30249.5039	95.1358
		Sum	31796.1340	100.0000

Area Percent Report

Agilent Technologies

Sample name: zwf-8-007-rac-IE-99-1-0.8-205

Data file: C:\Users\Public\Documents\ChemStation\1\Data\zwf\zwf-S 2023-11-17 07-38-48\032-P1-C6-zwf-8-007-rac.D

Injection Acquired Date 11/18/2023 00:02:48

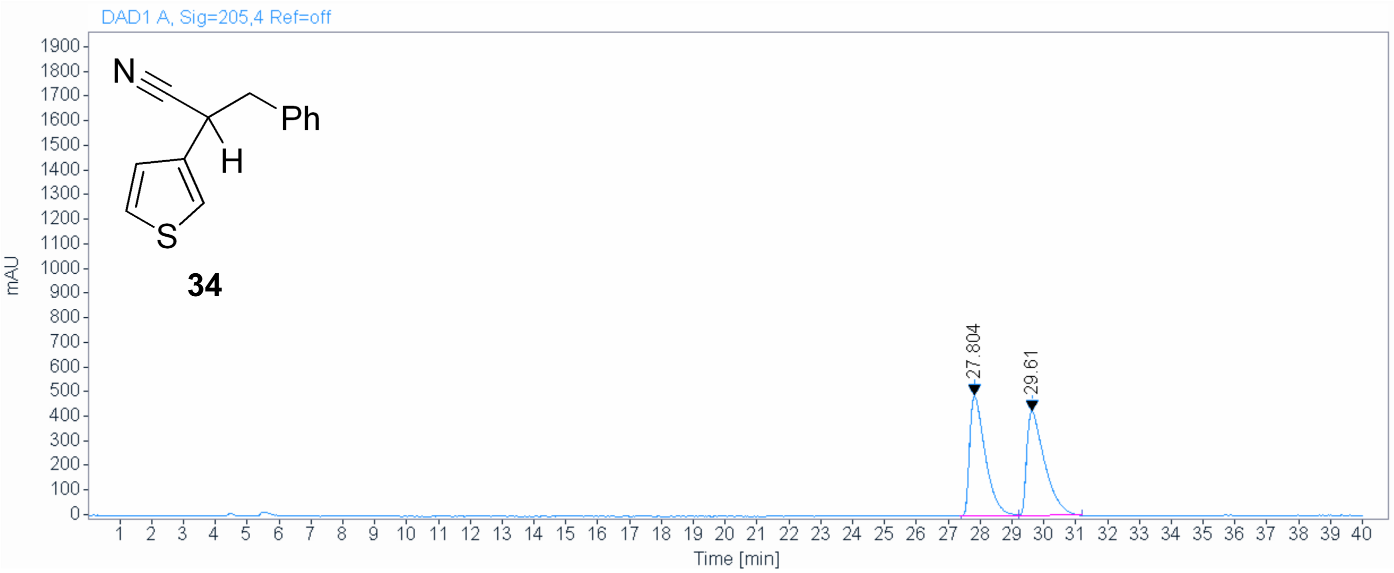

Signal: DAD1 A, Sig=205,4 Ref=off

RT [min]	Width [min]	Height	Area	Area%
27.804	0.4613	488.8792	16741.6094	49.9649
29.610	0.5311	424.1911	16765.1074	50.0351
		Sum	33506.7168	100.0000

zwf-7-143H
Nov 05 2023
SOLVENT: CDCl3
NA = 4
F1 = 400.179993 MHz
F2 = 1.000000 MHz

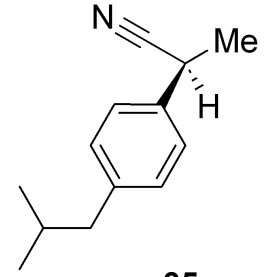

35

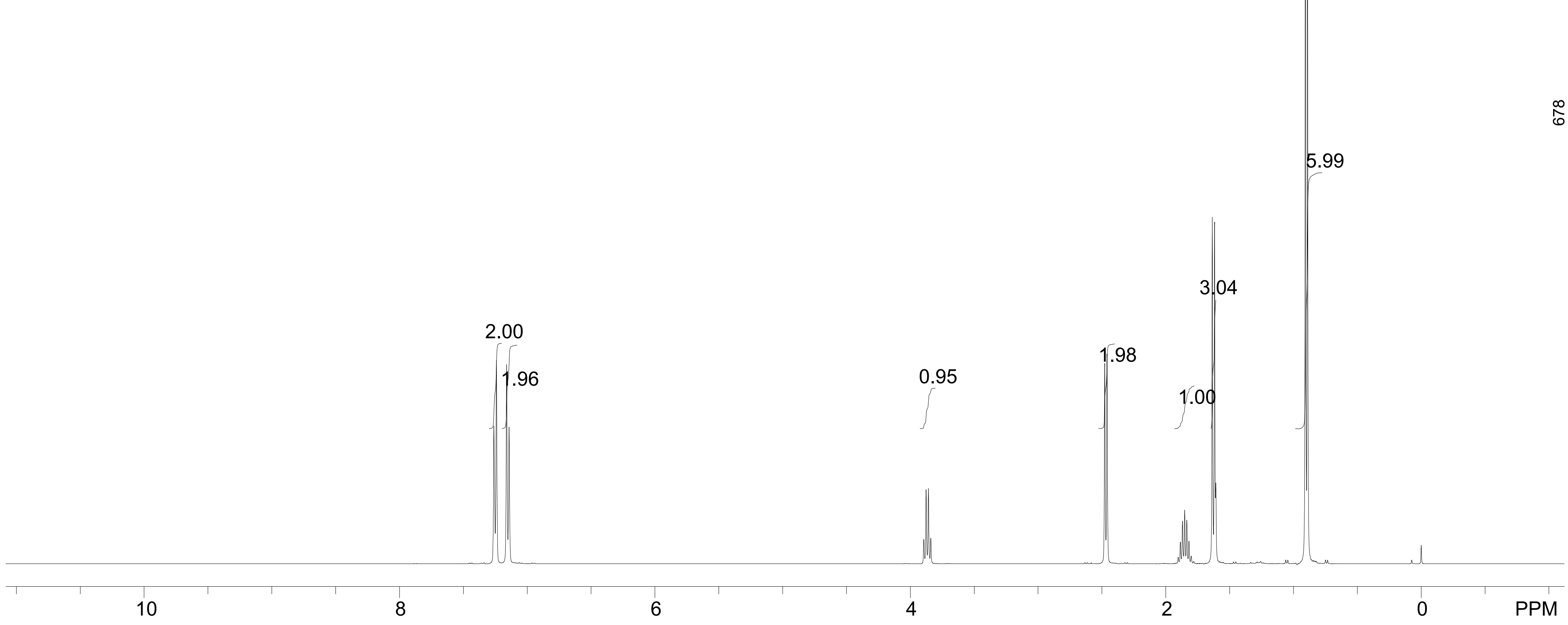

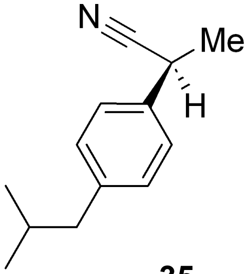

35

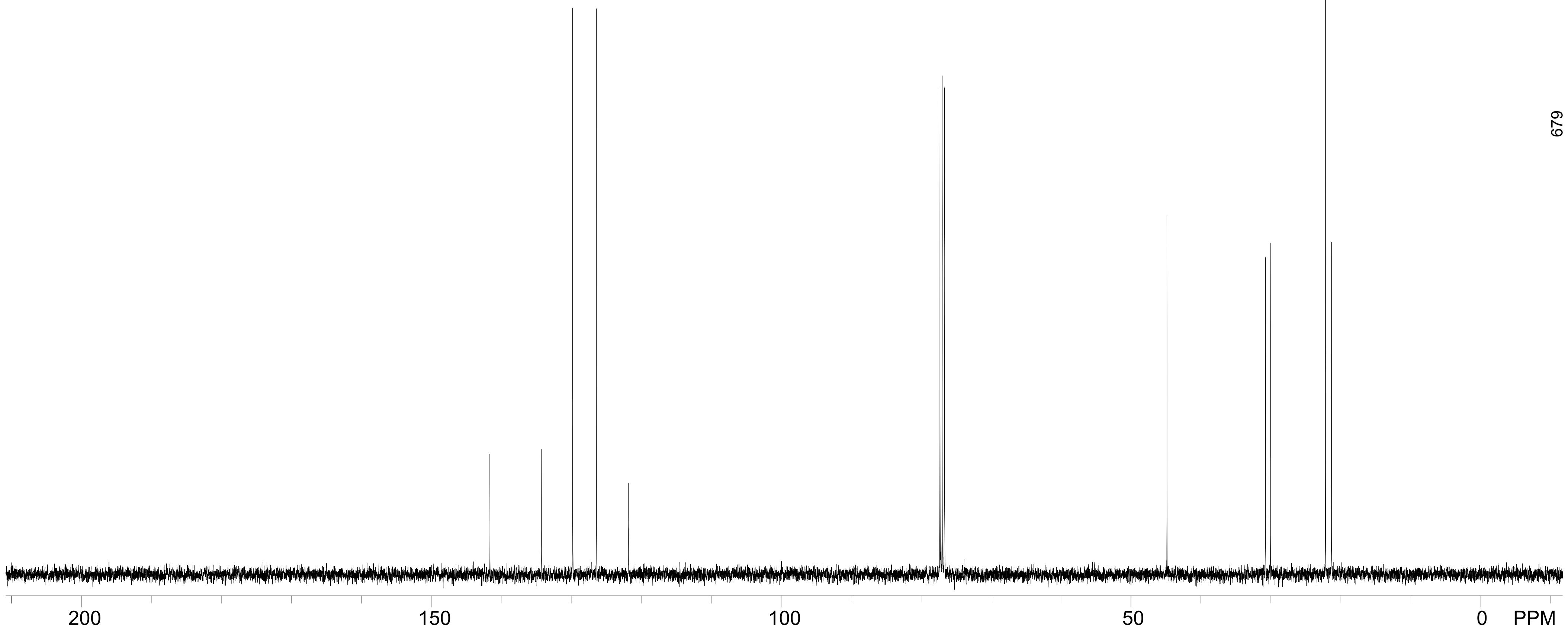

zwf-7-143C
Nov 05 2023
SOLVENT: CDCl3
NA = 100
F1 = 100.625343 MHz
F2 = 1.000000 MHz
141.600
134.255
129.770
126.398
121.783
77.318
77.000
76.682
44.899
30.838
30.130
22.265
21.384
200
150
100
50
0
PPM

Area Percent Report

Agilent Technologies

Sample name: zwf-7-143-IG-99-1-0.8-205

Data file: C:\Users\Public\Documents\ChemStation\1\Data\zwf\zwf-S 2023-11-05 14-11-17\013-P1-C7-zwf-7-143.D

Injection Acquired Date 11/05/2023 18:25:55

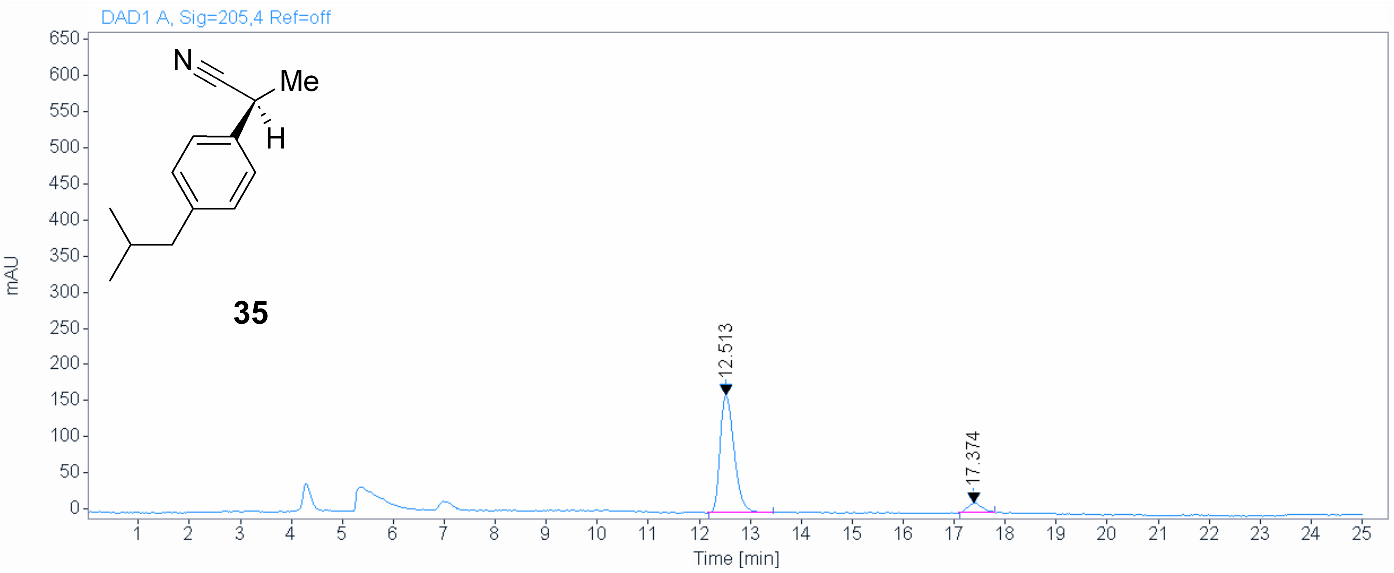

Signal: DAD1 A, Sig=205,4 Ref=off

RT [min]	Width [min]	Height	Area	Area%
12.513	0.2863	162.3186	3128.6145	92.9480
17.374	0.3217	12.2958	237.3701	7.0520
		Sum	3365.9846	100.0000

Area Percent Report

Agilent Technologies

Sample name: zwf-7-143-rac-IG-99-1-0.8-205

Data file: C:\Users\Public\Documents\ChemStation\1\Data\zwf\zwf-S 2023-11-05 14-11-17\014-P1-C8-zwf-7-143-rac.D

Injection Acquired Date 11/05/2023 18:51:58

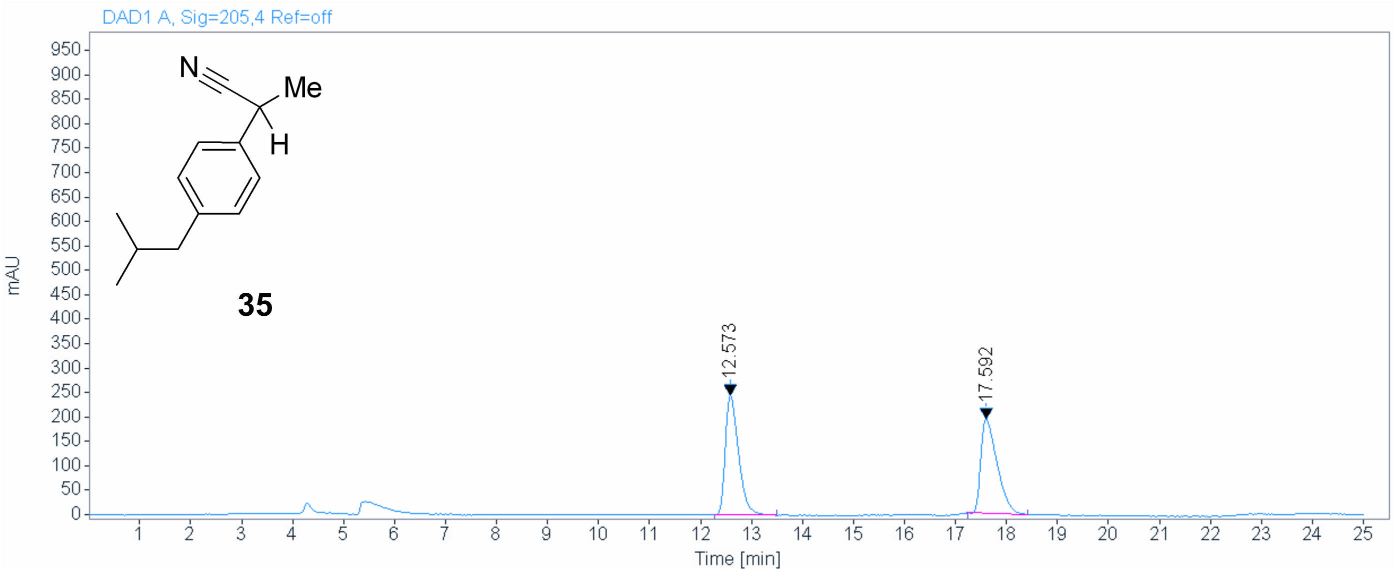

Signal: DAD1 A, Sig=205,4 Ref=off

RT [min]	Width [min]	Height	Area	Area%
12.573	0.2615	244.5728	4433.3882	50.6032
17.592	0.3726	193.5651	4327.6982	49.3968
		Sum	8761.0864	100.0000

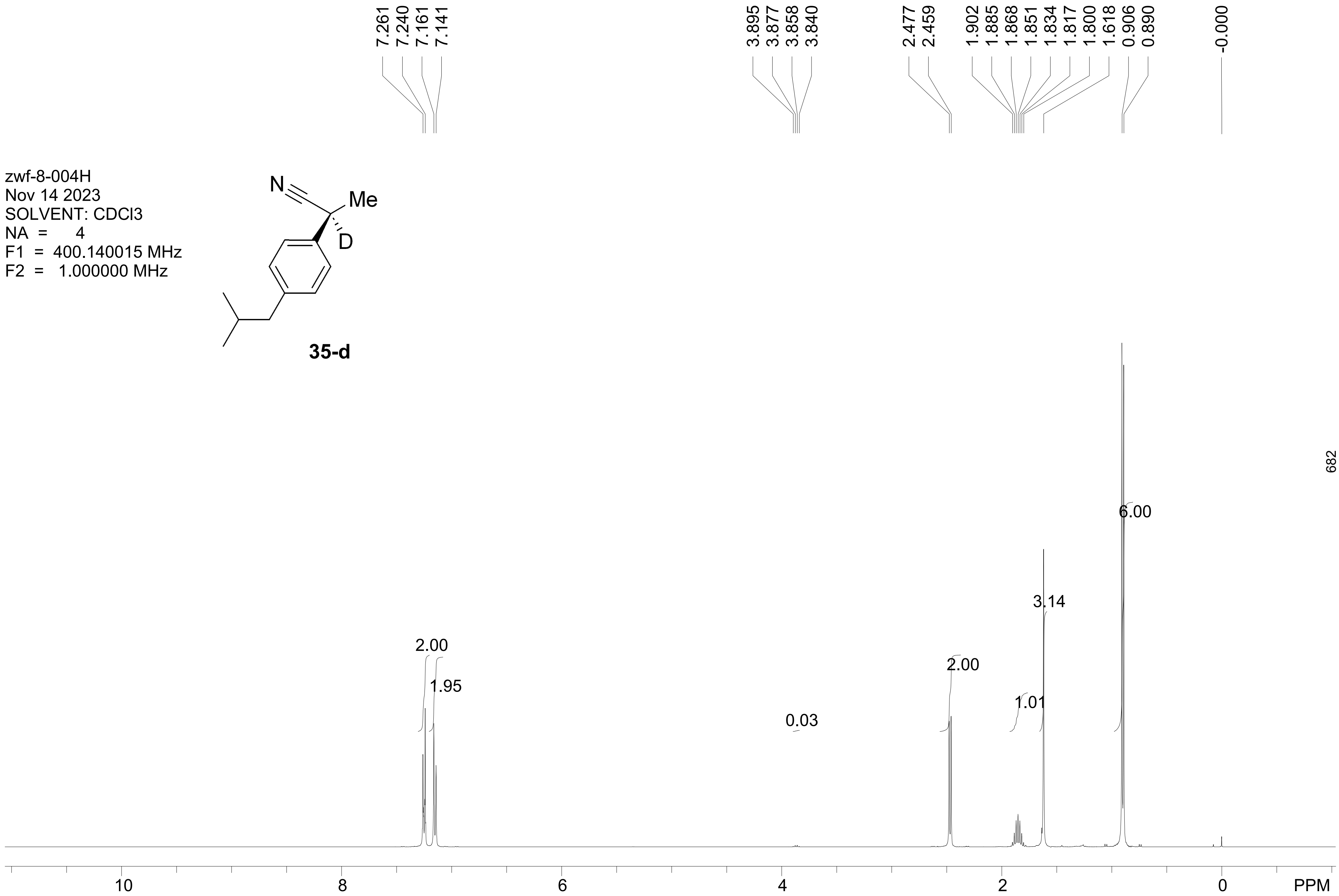
zwf-8-004H
Nov 14 2023
SOLVENT: CDCl3
NA = 4
F1 = 400.140015 MHz
F2 = 1.000000 MHz
N
Me
D
35-d
7.261
7.240
7.161
7.141
3.895
3.877
3.858
3.840
2.477
2.459
1.902
1.885
1.868
1.851
1.834
1.817
1.800
1.618
0.906
0.890
-0.000
2.00
1.95
0.03
2.00
1.01
3.14
6.00
10
8
6
4
2
0
PPM

zwf-8-004C
Nov 14 2023
SOLVENT: CDCl3
NA = 150
F1 = 100.615280 MHz
F2 = 1.000000 MHz

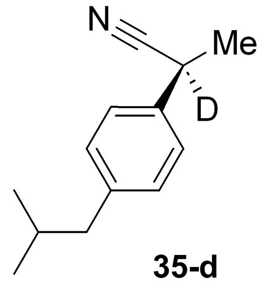

35-d

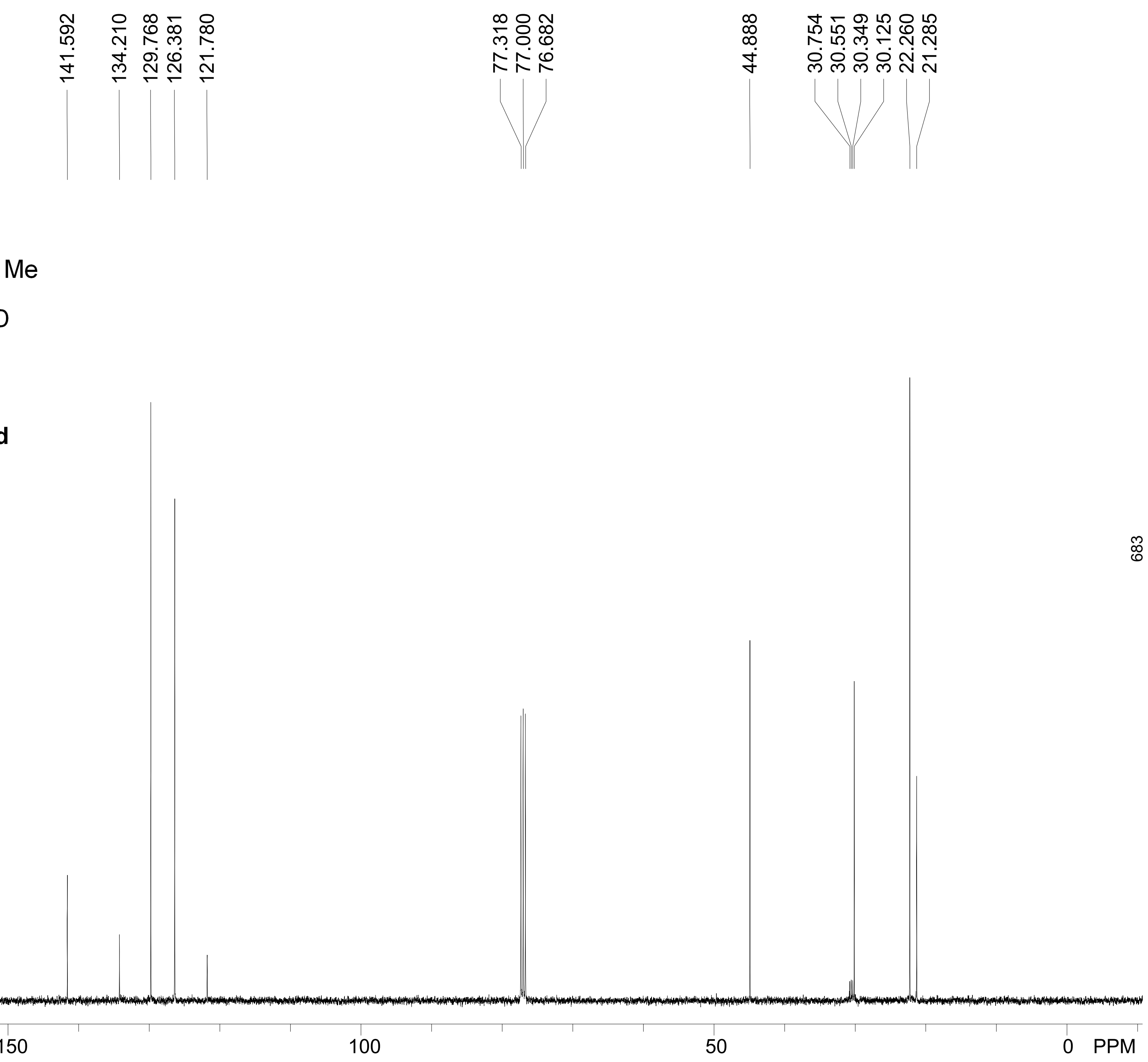

Area Percent Report

Agilent Technologies

Sample name: zwf-8-004-IG-99-1-0.8-205

Data file: C:\Users\Public\Documents\ChemStation\1\Data\zwf\zwf-S 2023-11-15 14-21-28\002-P1-A1-zwf-8-004.D

Injection Acquired Date 11/15/2023 14:48:30

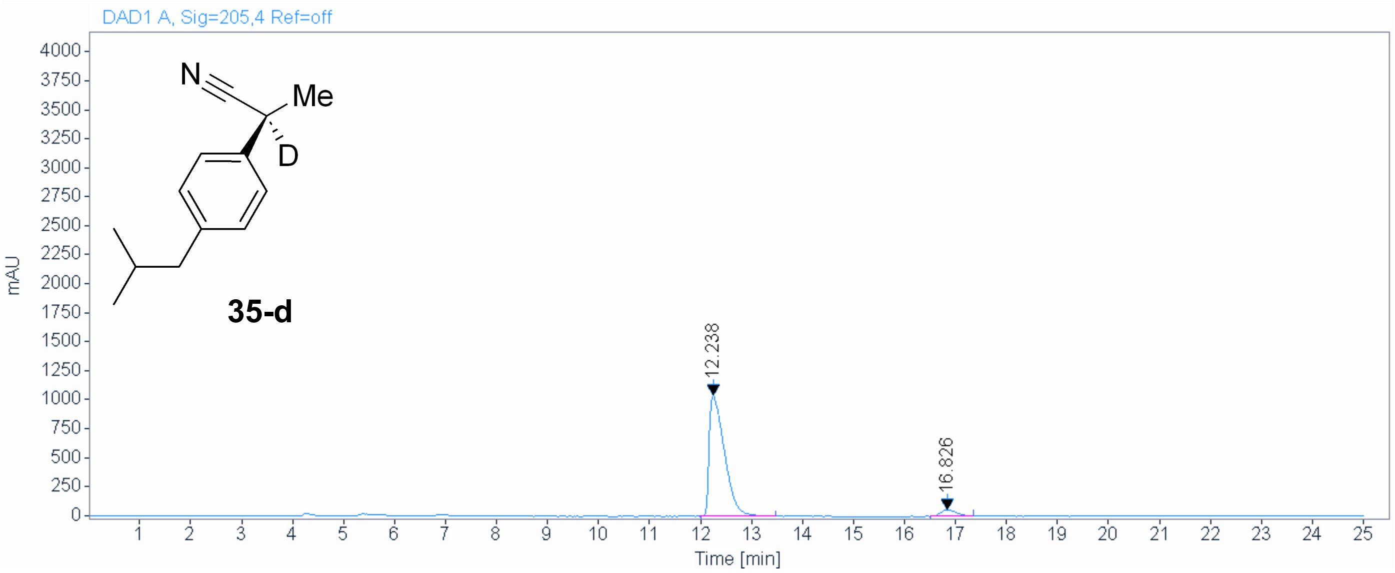

Signal: DAD1 A, Sig=205,4 Ref=off

RT [min]	Width [min]	Height	Area	Area%
12.238	0.2810	1041.8860	21725.4551	94.9805
16.826	0.3553	53.8536	1148.1439	5.0195
		Sum	22873.5990	100.0000

Area Percent Report

Agilent Technologies

Sample name: zwf-8-004-rac-IG-99-1-0.8-205

Data file: C:\Users\Public\Documents\ChemStation\1\Data\zwf\zwf-S 2023-11-15 14-21-28\003-P1-A2-zwf-8-004-rac.D

Injection Acquired Date 11/15/2023 15:14:34

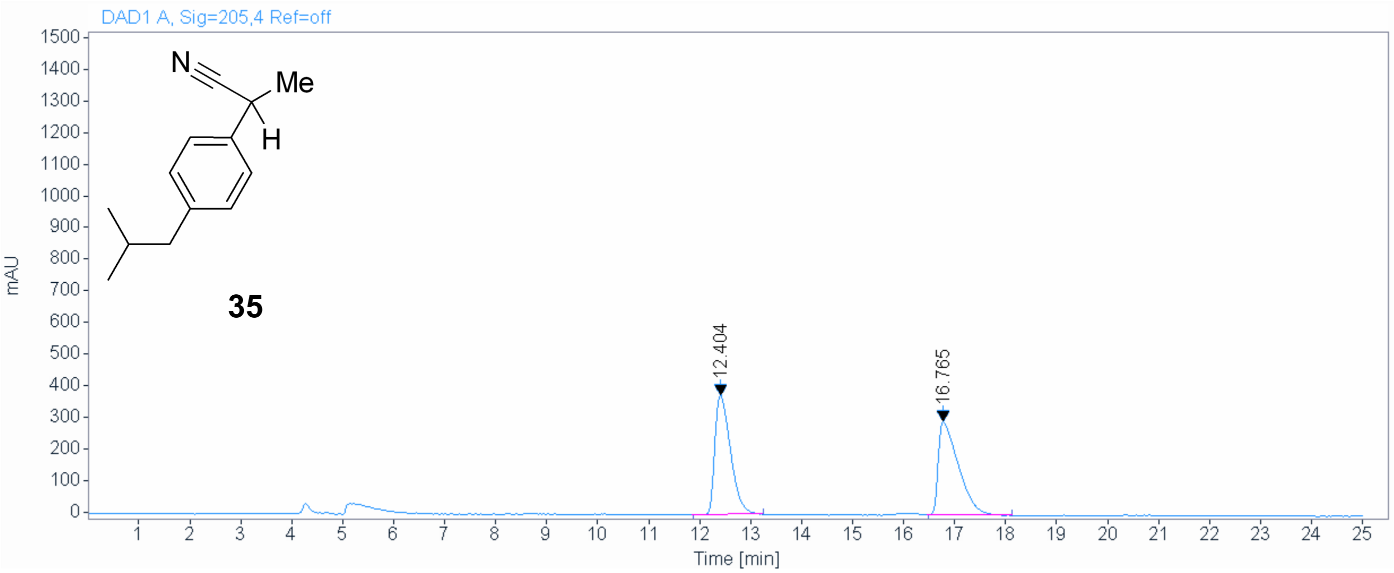

Signal: DAD1 A, Sig=205,4 Ref=off

RT [min]	Width [min]	Height	Area	Area%
12.404	0.3083	376.3254	8084.3867	49.8595
16.765	0.3733	294.5800	8129.9434	50.1405
		Sum	16214.3301	100.0000

zwf-7-142H
Nov 05 2023
SOLVENT: CDCl3
NA = 4
F1 = 400.179993 MHz
F2 = 1.000000 MHz

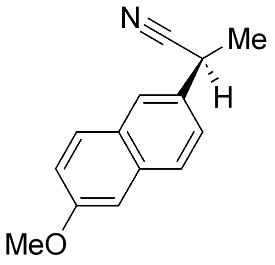

36

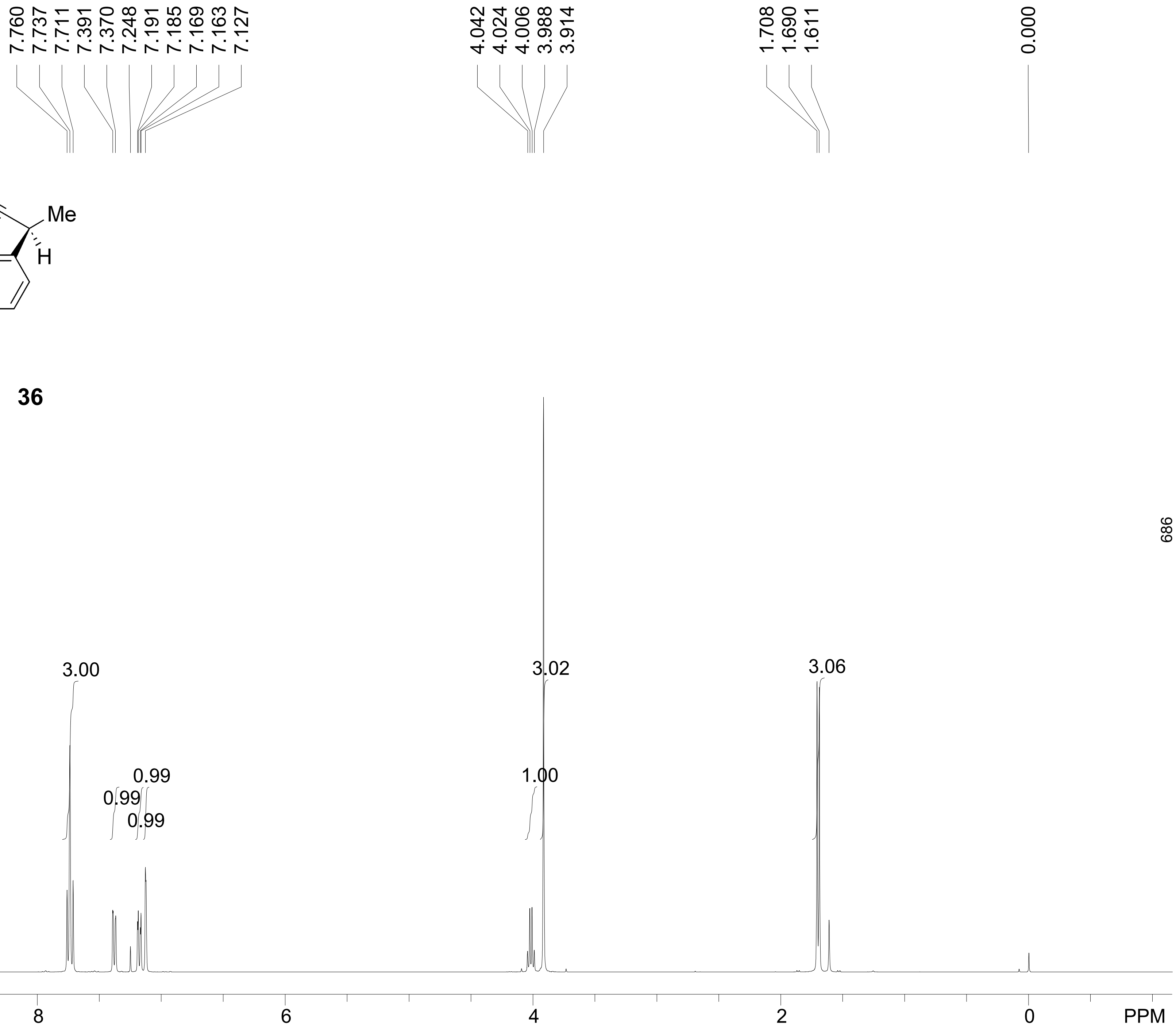

zwf-7-142C
Nov 05 2023
SOLVENT: CDCl3
NA = 100
F1 = 100.625343 MHz
F2 = 1.000000 MHz

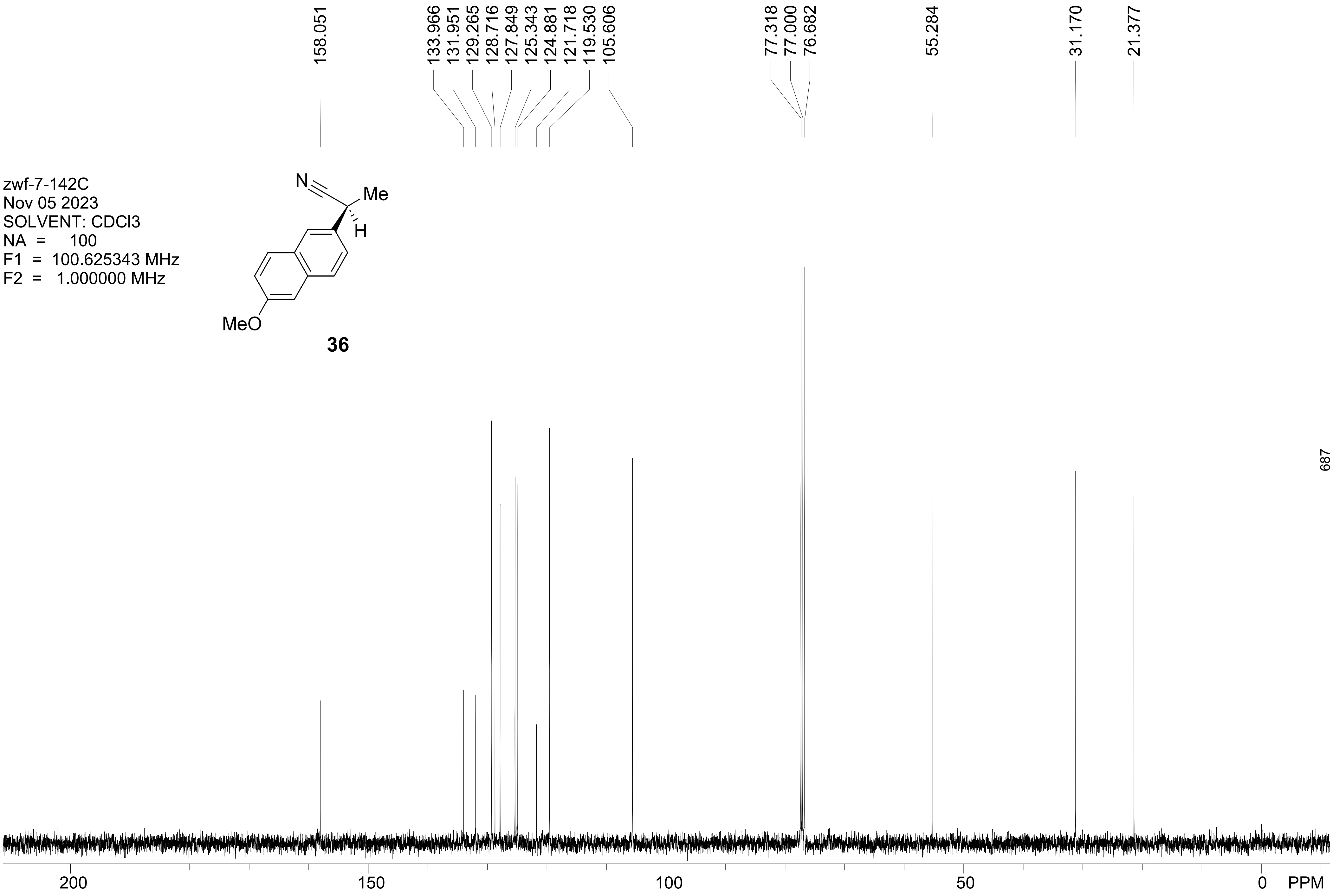

Area Percent Report

Agilent Technologies

Sample name: zwf-7-142-IE-90-10-1.1-205

Data file: C:\Users\Public\Documents\ChemStation\1\Data\zwf\zwf-S 2023-11-05 14-11-17\009-P1-C5-zwf-7-142.D

Injection Acquired Date 11/05/2023 17:06:33

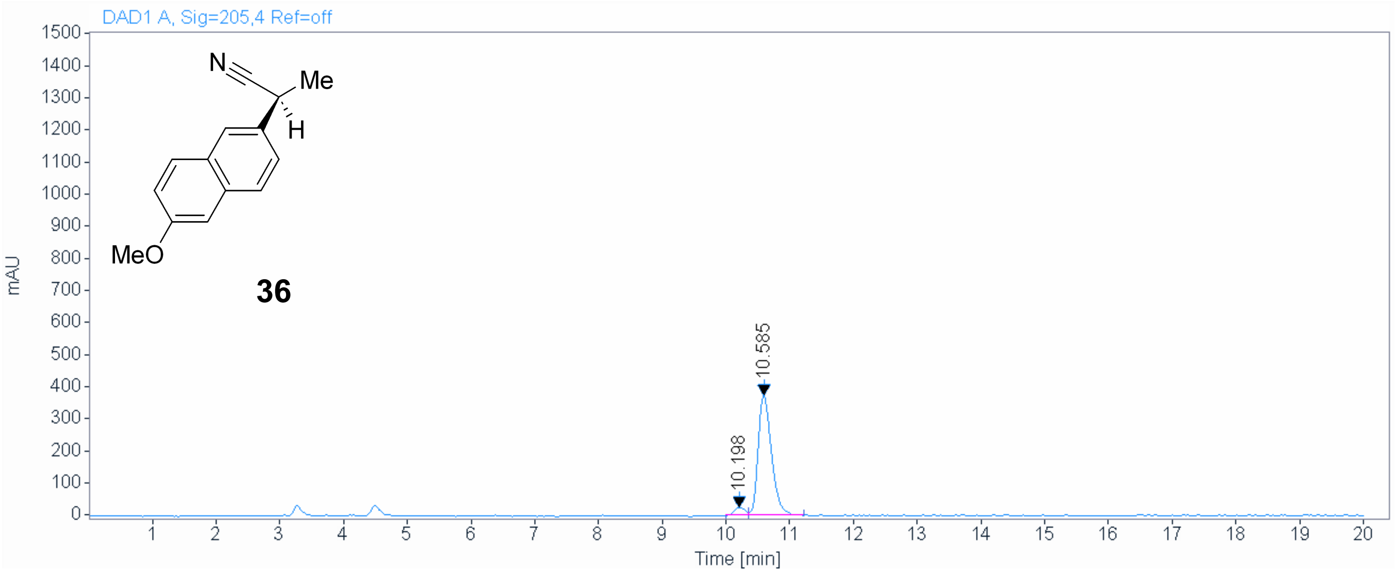

Signal: DAD1 A, Sig=205,4 Ref=off

RT [min]	Width [min]	Height	Area	Area%
10.198	0.1968	23.6317	278.9757	4.8892
10.585	0.2424	373.0768	5426.9785	95.1108
		Sum	5705.9542	100.0000

Area Percent Report

Sample name: zwf-7-142-rac-IE-90-10-1.1-205

Data file: C:\Users\Public\Documents\ChemStation\1\Data\zwf\zwf-S 2023-11-05 14-11-17\010-P1-C6-zwf-7-142-rac.D

Injection Acquired Date 11/05/2023 17:27:36

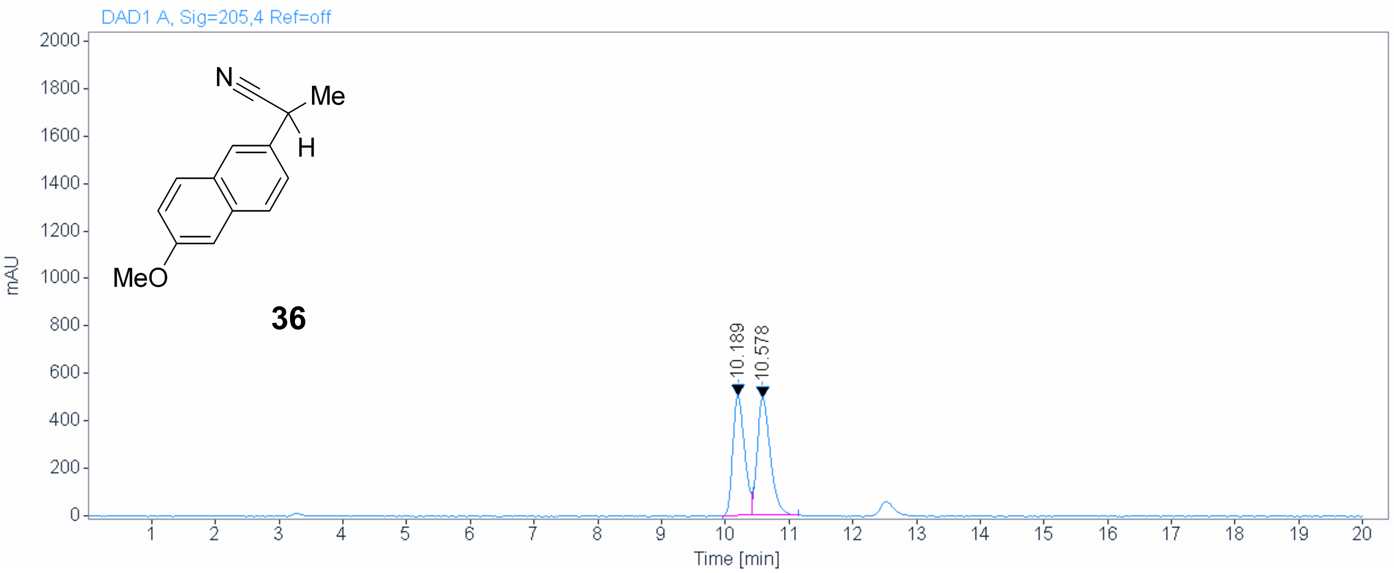

Signal: DAD1 A, Sig=205,4 Ref=off

RT [min]	Width [min]	Height	Area	Area%
10.189	0.2167	506.6721	6586.7407	48.4502
10.578	0.2350	496.9706	7008.1172	51.5498
		Sum	13594.8579	100.0000

zwf-8-003H
Nov 13 2023
SOLVENT: CDCl3
NA = 4
F1 = 400.140015 MHz
F2 = 1.000000 MHz

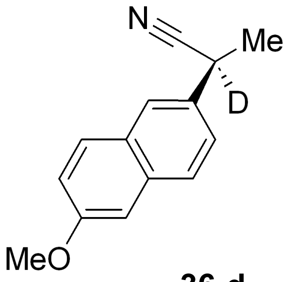

36-d

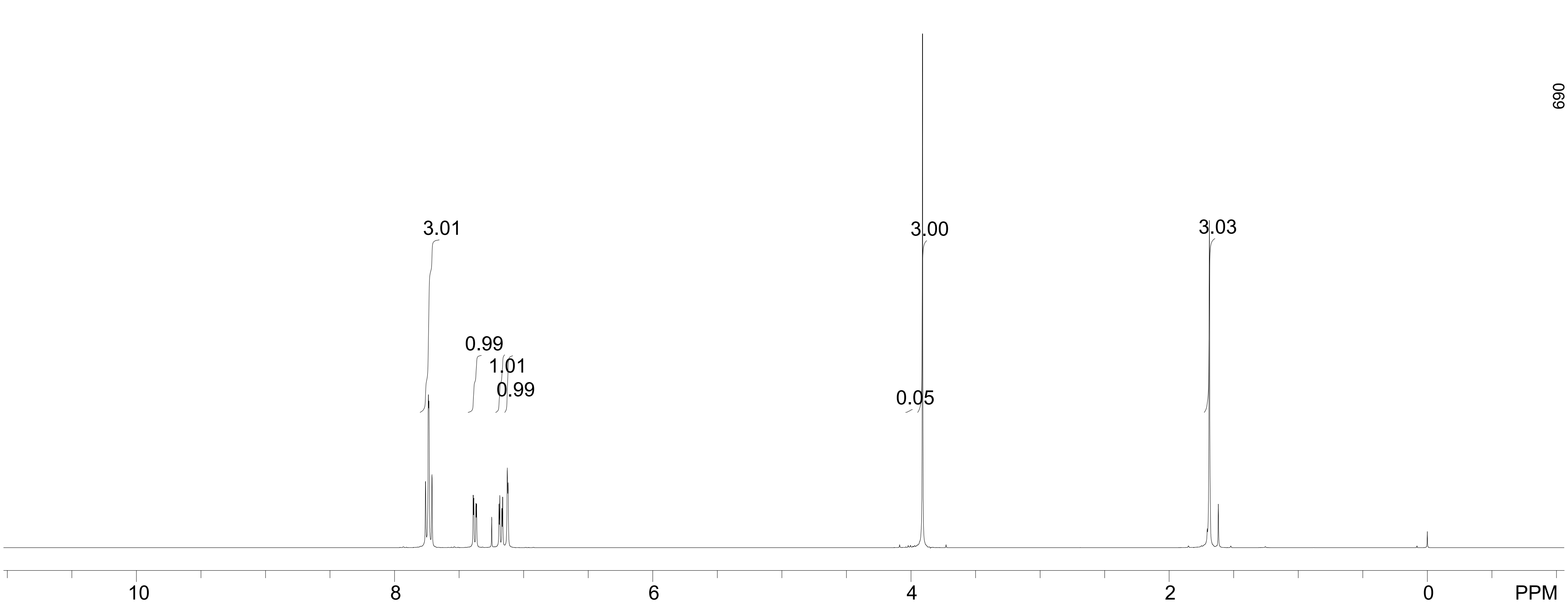

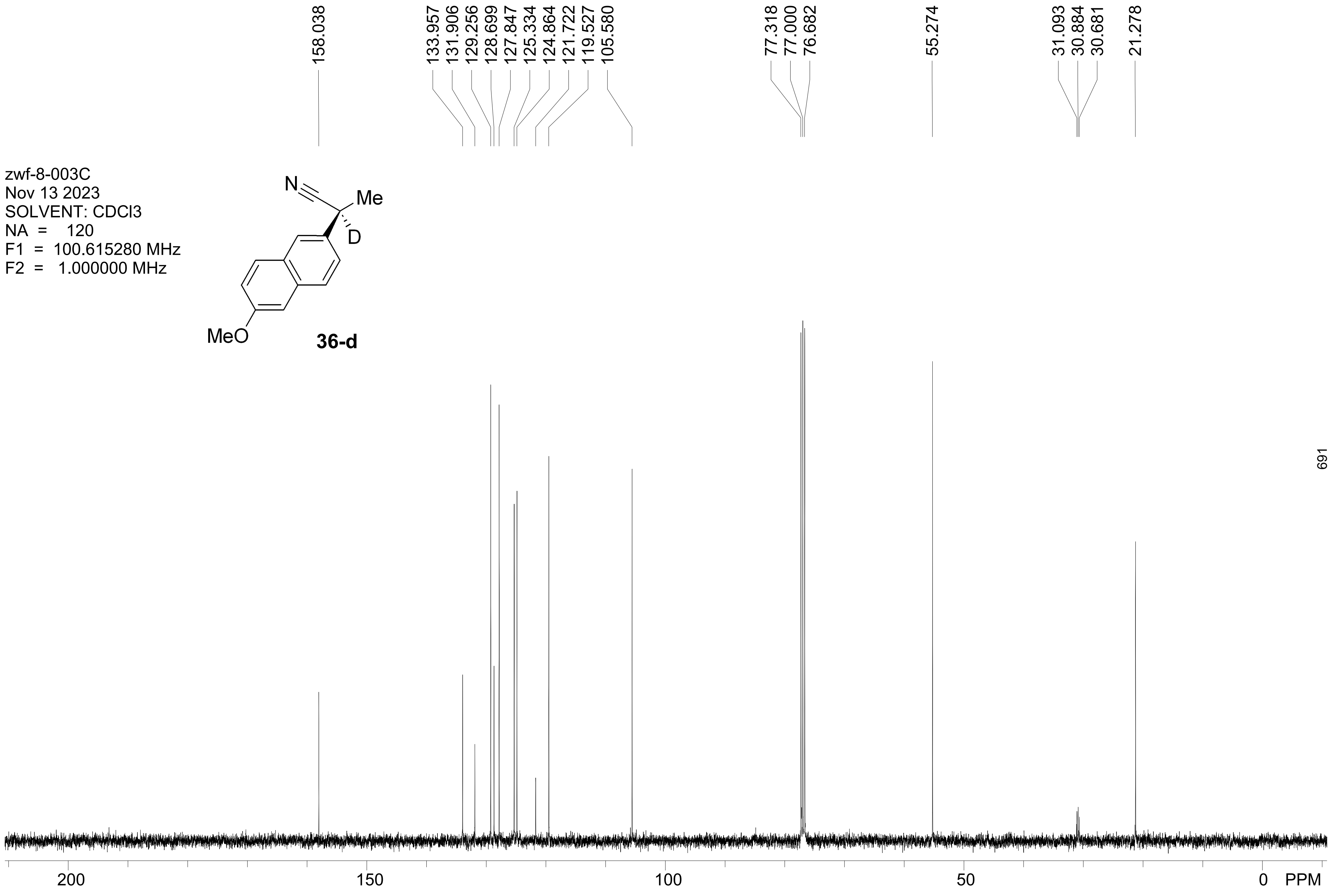
zwf-8-003C
Nov 13 2023
SOLVENT: CDCl3
NA = 120
F1 = 100.615280 MHz
F2 = 1.000000 MHz
MeO
N
Me
D
36-d
158.038
133.957
131.906
129.256
128.699
127.847
125.334
124.864
121.722
119.527
105.580
77.318
77.000
76.682
55.274
31.093
30.884
30.681
21.278
200
150
100
50
0 PPM

Area Percent Report

Agilent Technologies

Sample name: zwf-8-003-IE-90-10-1.1-205

Data file: C:\Users\Public\Documents\ChemStation\1\Data\zwf\zwf-S 2023-11-14 20-02-58\004-P1-C1-zwf-8-003.D

Injection Acquired Date 11/14/2023 21:17:07

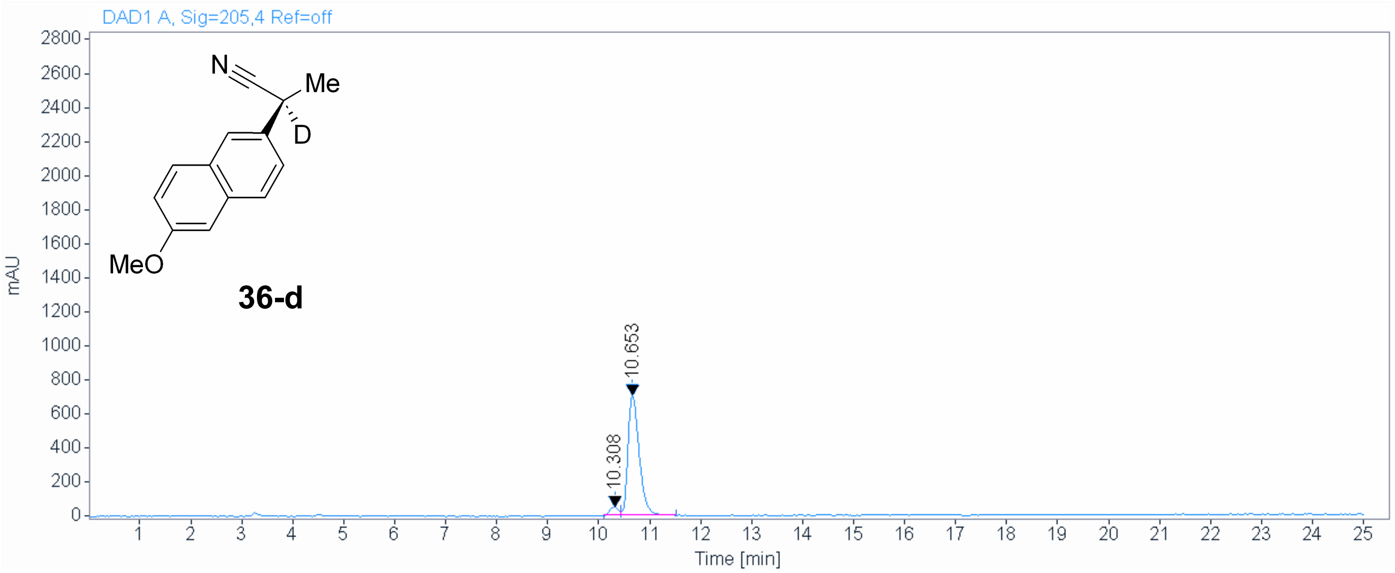

Signal: DAD1 A, Sig=205,4 Ref=off

RT [min]	Width [min]	Height	Area	Area%
10.308	0.1938	47.1185	547.8278	4.8540
10.653	0.2546	702.9714	10738.2217	95.1460
		Sum	11286.0495	100.0000

Area Percent Report

Agilent Technologies

Sample name: zwf-8-003-rac-IE-90-10-1.1-205

Data file: C:\Users\Public\Documents\ChemStation\1\Data\zwf\zwf-S 2023-11-14 20-02-58\005-P1-C2-zwf-8-003-rac.D

Injection Acquired Date 11/14/2023 21:43:08

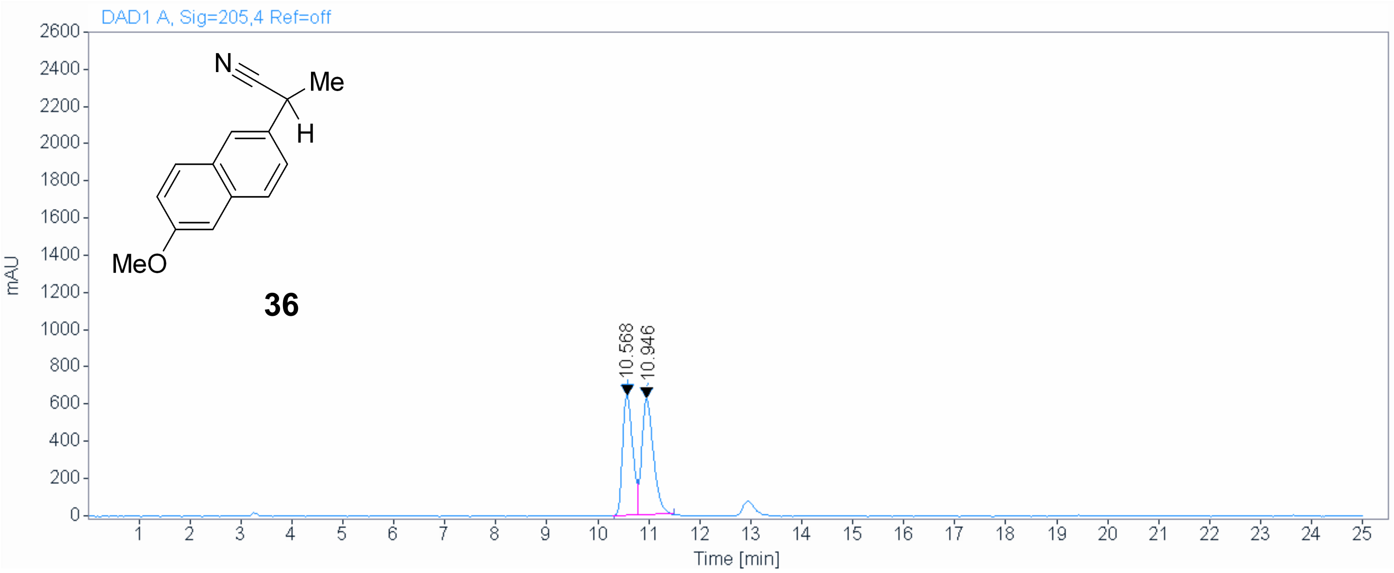

Signal: DAD1 A, Sig=205,4 Ref=off

RT [min]	Width [min]	Height	Area	Area%
10.568	0.2120	645.2787	8962.7227	47.2099
10.946	0.2360	628.5030	10022.1104	52.7901
		Sum	18984.8330	100.0000

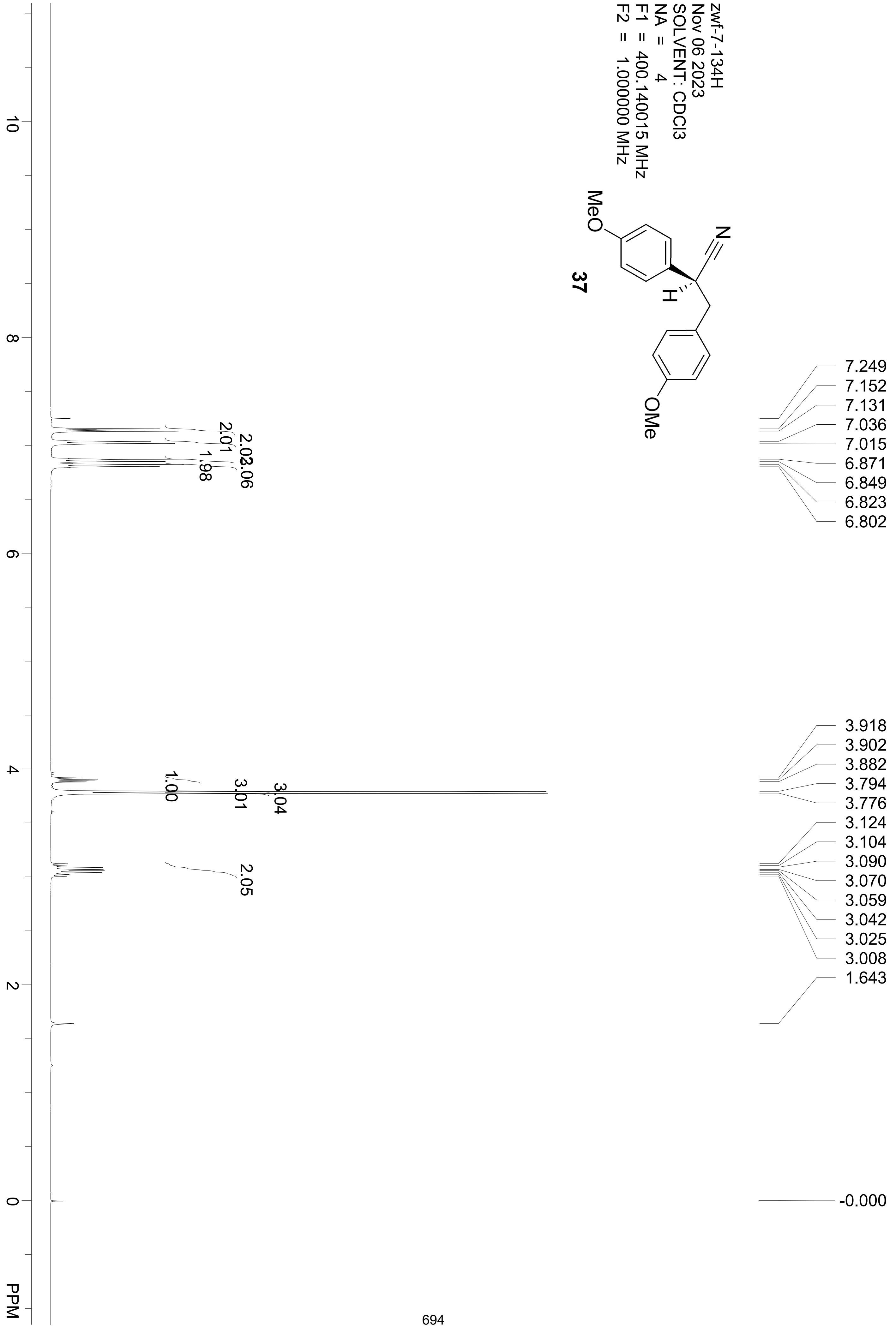
zwf-7-134H
Nov 06 2023
SOLVENT: CDCl3
NA = 4
F1 = 400.140015 MHz
F2 = 1.000000 MHz
MeO
N
H
OMe
37
7.249
7.152
7.131
7.036
7.015
6.871
6.849
6.823
6.802
3.918
3.902
3.882
3.794
3.776
3.124
3.104
3.090
3.070
3.059
3.042
3.025
3.008
1.643
-0.000
2.01
2.03
2.06
1.98
1.00
3.01
3.04
2.05
10
8
6
4
2
0
PPM

zwf-7-134C
Nov 06 2023
SOLVENT: CDCl3
NA = 100
F1 = 100.615280 MHz
F2 = 1.000000 MHz

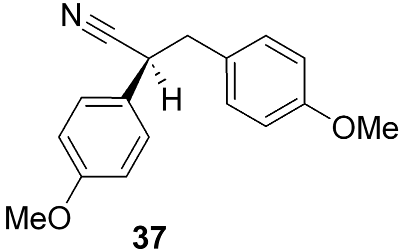

37

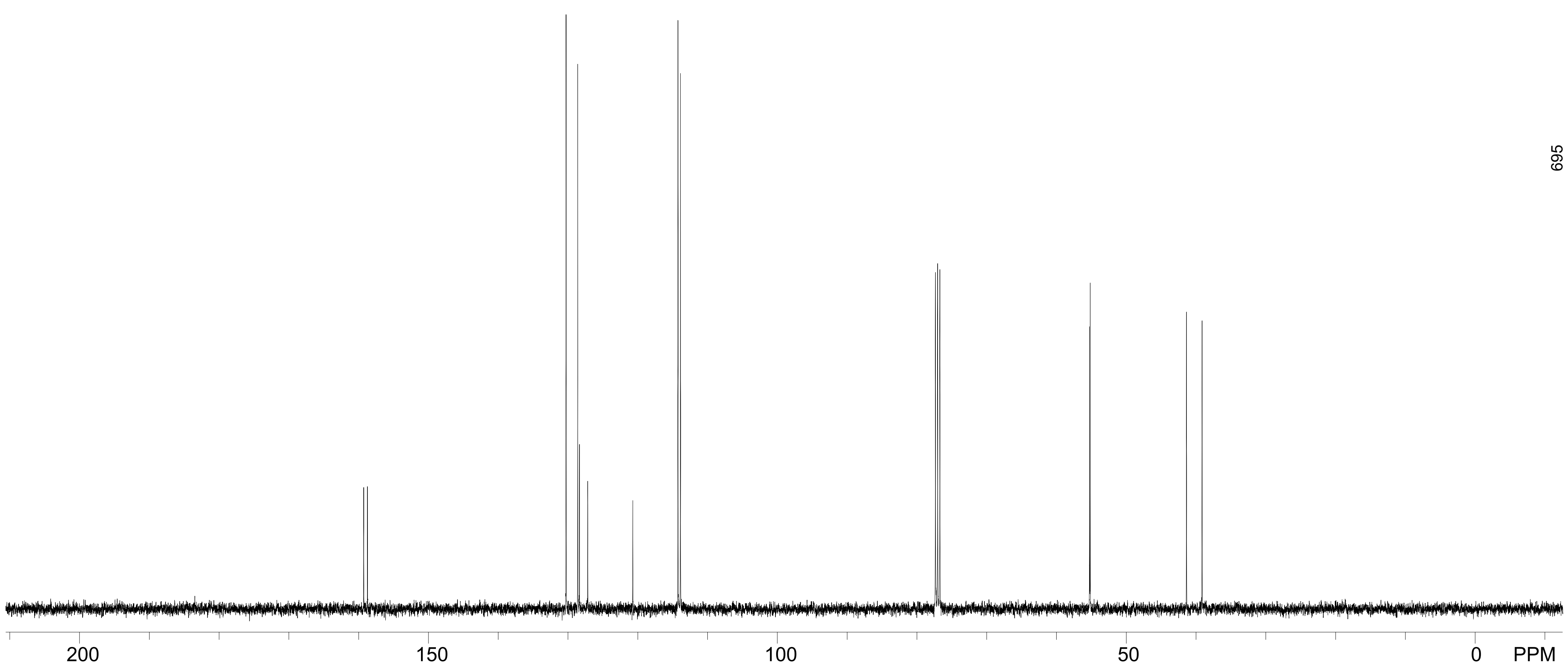

Area Percent Report

Agilent Technologies

Sample name: zwf-7-134-IE-90-10-0.8-205

Data file: C:\Users\Public\Documents\ChemStation\1\Data\zwf\zwf-S 2023-11-06 14-35-09\005-P1-C3-zwf-7-134.D

Injection Acquired Date 11/06/2023 16:36:22

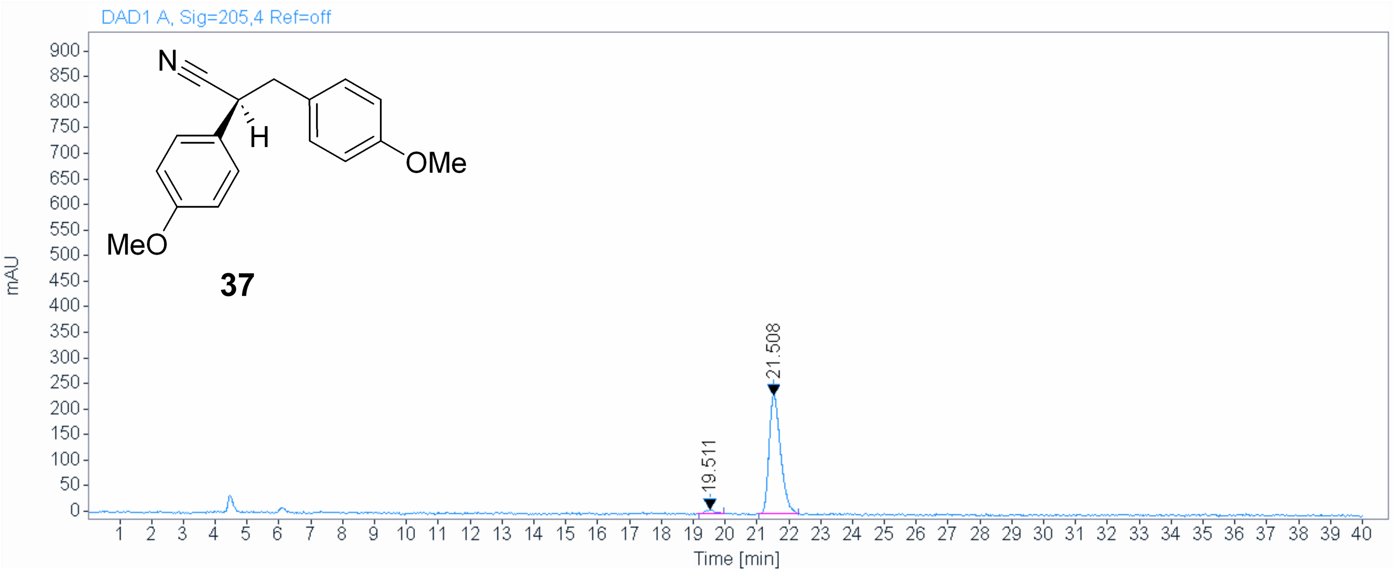

Signal: DAD1 A, Sig=205,4 Ref=off

RT [min]	Width [min]	Height	Area	Area%
19.511	0.3446	8.3128	171.8583	2.8223
21.508	0.3630	232.4727	5917.5093	97.1777
		Sum	6089.3676	100.0000

Area Percent Report

Sample name: zwf-7-134-rac-IE-90-10-0.8-205

Data file: C:\Users\Public\Documents\ChemStation\1\Data\zwf\zwf-S 2023-11-06 14-35-09\006-P1-C4-zwf-7-134-rac.D

Injection Acquired Date 11/06/2023 17:17:32

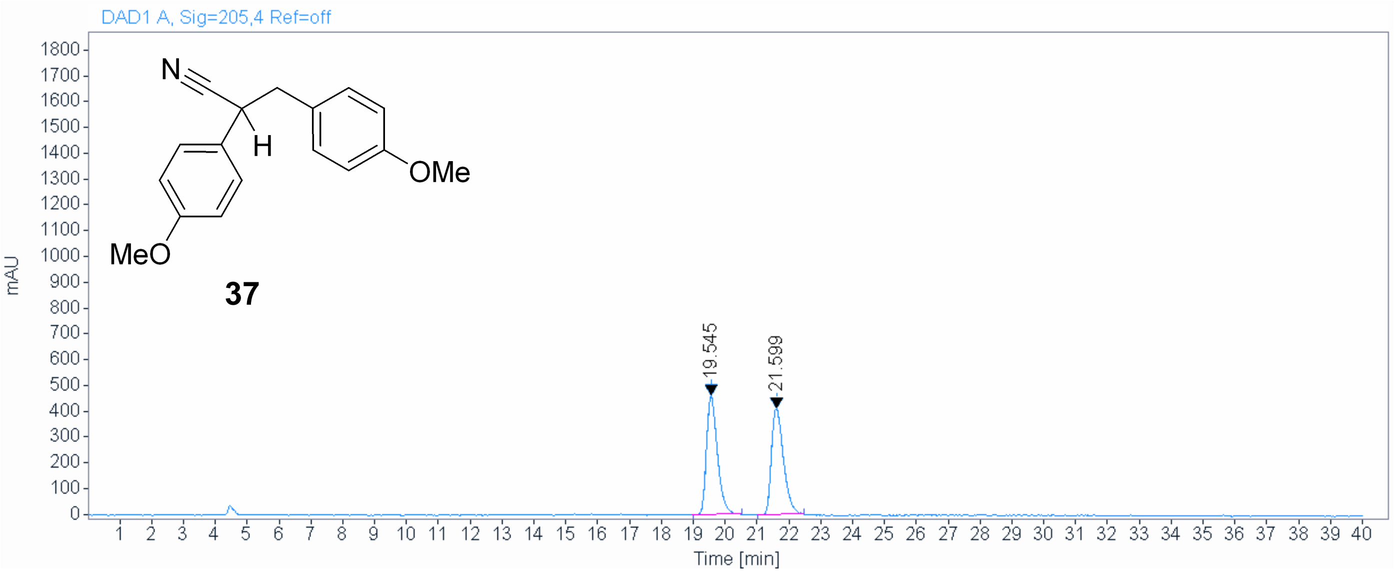

Signal: DAD1 A, Sig=205,4 Ref=off

RT [min]	Width [min]	Height	Area	Area%
19.545	0.3413	462.5063	11083.1855	50.3454
21.599	0.3218	412.9438	10931.0986	49.6546
		Sum	22014.2842	100.0000

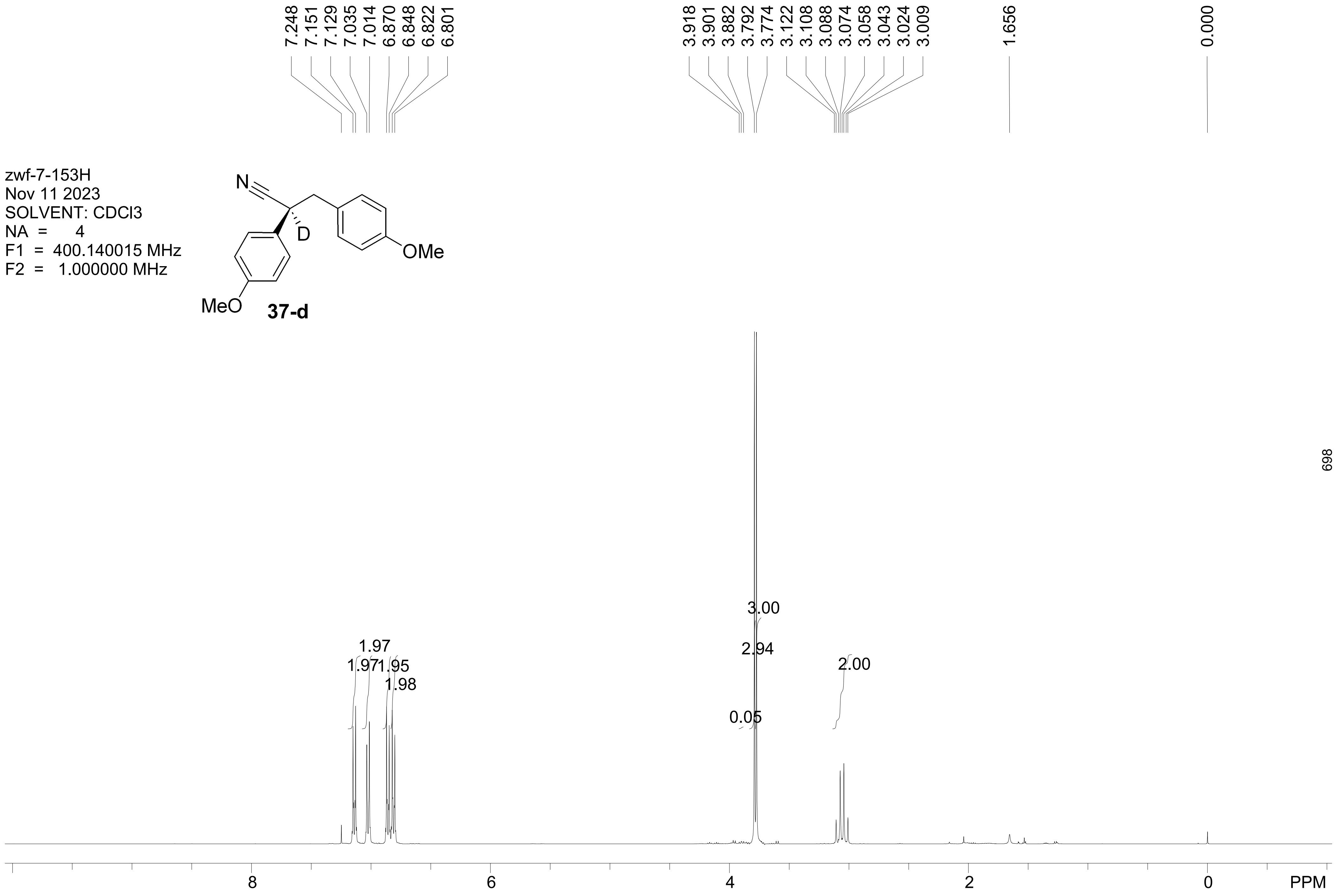
zwf-7-153H
Nov 11 2023
SOLVENT: CDCl3
NA = 4
F1 = 400.140015 MHz
F2 = 1.000000 MHz
N
D
OMe
MeO
37-d
7.248
7.151
7.129
7.035
7.014
6.870
6.848
6.822
6.801
3.918
3.901
3.882
3.792
3.774
3.122
3.108
3.088
3.074
3.058
3.043
3.024
3.009
1.656
0.000
1.97
1.97
1.95
1.98
0.05
2.94
3.00
2.00
8
6
4
2
0
PPM

zwf-7-153C
Nov 11 2023
SOLVENT: CDCl3
NA = 150
F1 = 100.615280 MHz
F2 = 1.000000 MHz

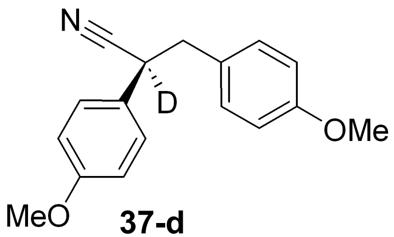

37-d

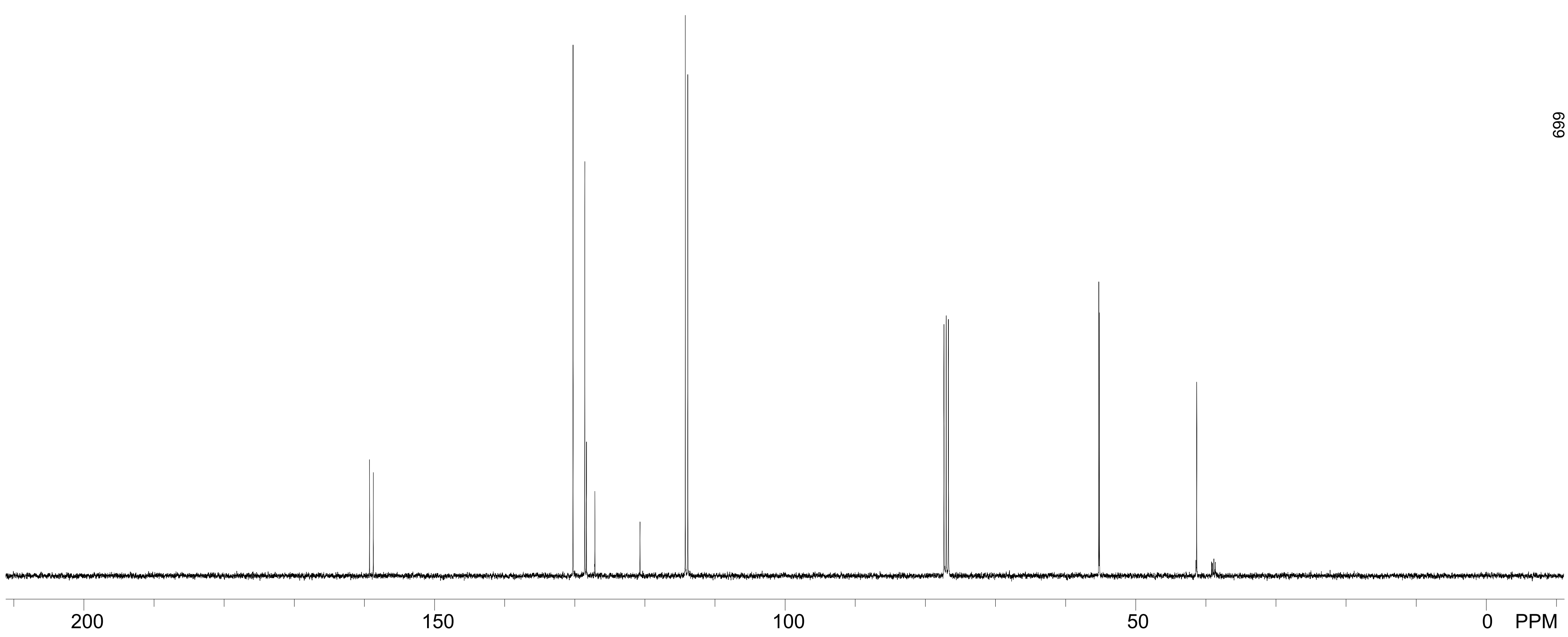

Area Percent Report

Agilent Technologies

Sample name: zwf-7-153-IE-90-10-0.8-205

Data file: C:\Users\Public\Documents\ChemStation\1\Data\zwf\zwf-S 2023-11-12 11-02-17\006-P1-C1-zwf-7-153.D

Injection Acquired Date 11/12/2023 13:47:32

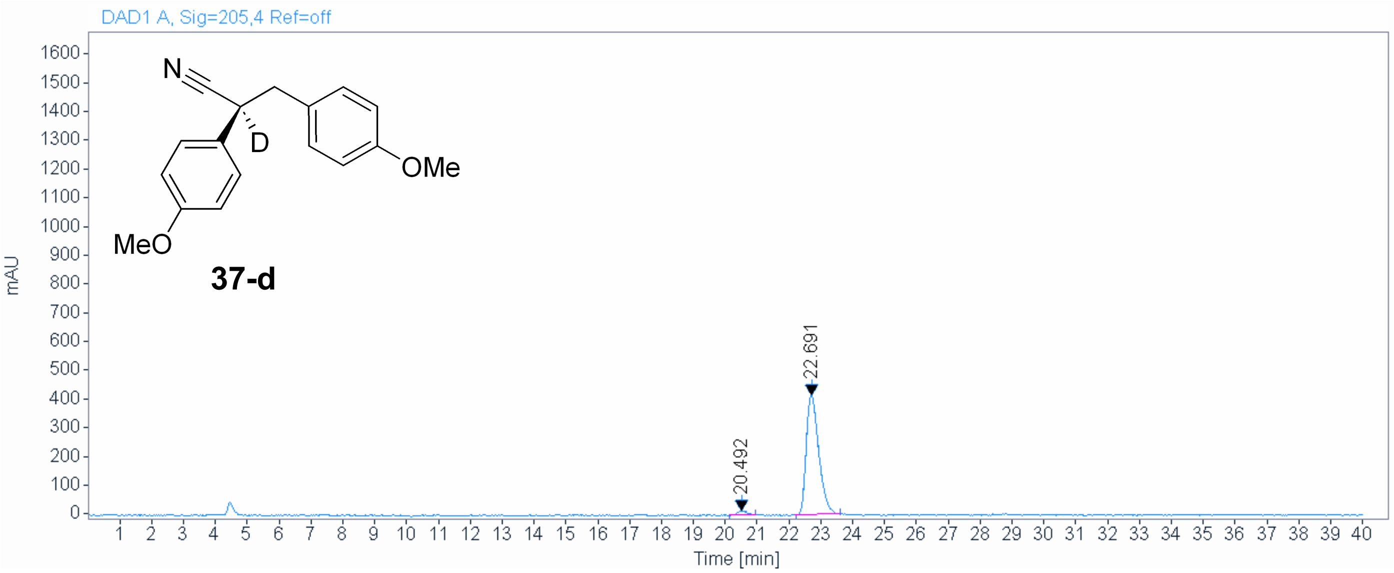

Signal: DAD1 A, Sig=205,4 Ref=off

RT [min]	Width [min]	Height	Area	Area%
20.492	0.3614	13.9564	302.6197	2.5757
22.691	0.3273	415.6847	11446.2578	97.4243
		Sum	11748.8775	100.0000

Area Percent Report

Agilent Technologies

Sample name: zwf-7-153-rac-IE-90-10-0.8-205

Data file: C:\Users\Public\Documents\ChemStation\1\Data\zwf\zwf-S 2023-11-12 11-02-17\007-P1-C2-zwf-7-153-rac.D

Injection Acquired Date 11/12/2023 14:28:40

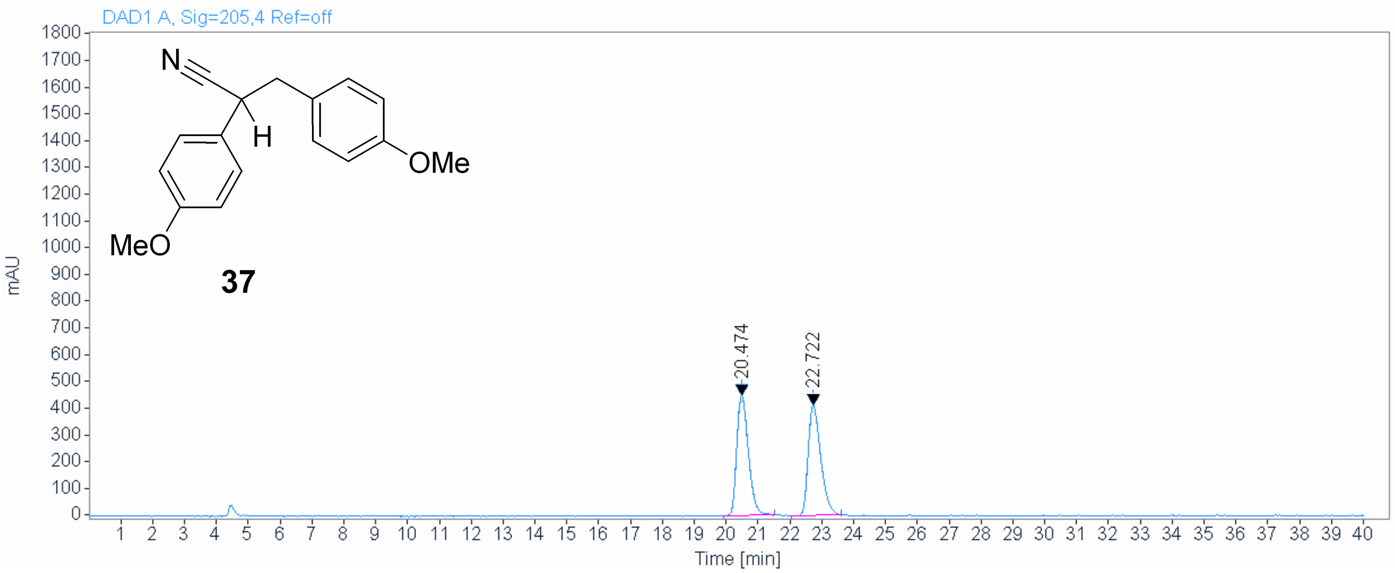

Signal: DAD1 A, Sig=205,4 Ref=off

RT [min]	Width [min]	Height	Area	Area%
20.474	0.4243	448.8350	11426.6113	49.7666
22.722	0.3870	413.6336	11533.7988	50.2334
		Sum	22960.4102	100.0000

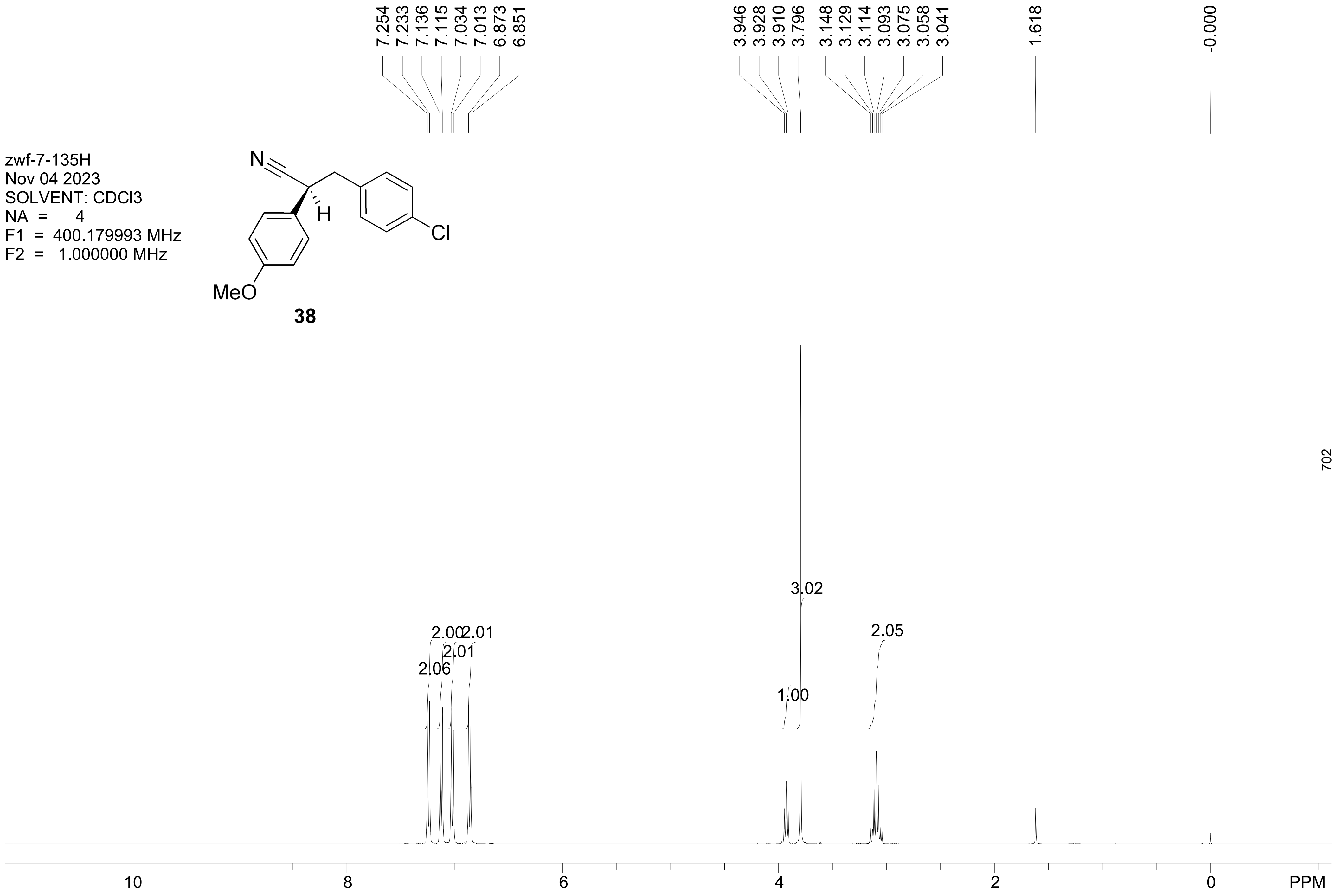
zwf-7-135H
Nov 04 2023
SOLVENT: CDCl3
NA = 4
F1 = 400.179993 MHz
F2 = 1.000000 MHz
N
H
Cl
MeO
38
7.254
7.233
7.136
7.115
7.034
7.013
6.873
6.851
3.946
3.928
3.910
3.796
3.148
3.129
3.114
3.093
3.075
3.058
3.041
1.618
-0.000
2.06
2.00
2.01
2.01
1.00
3.02
2.05
10
8
6
4
2
0
PPM

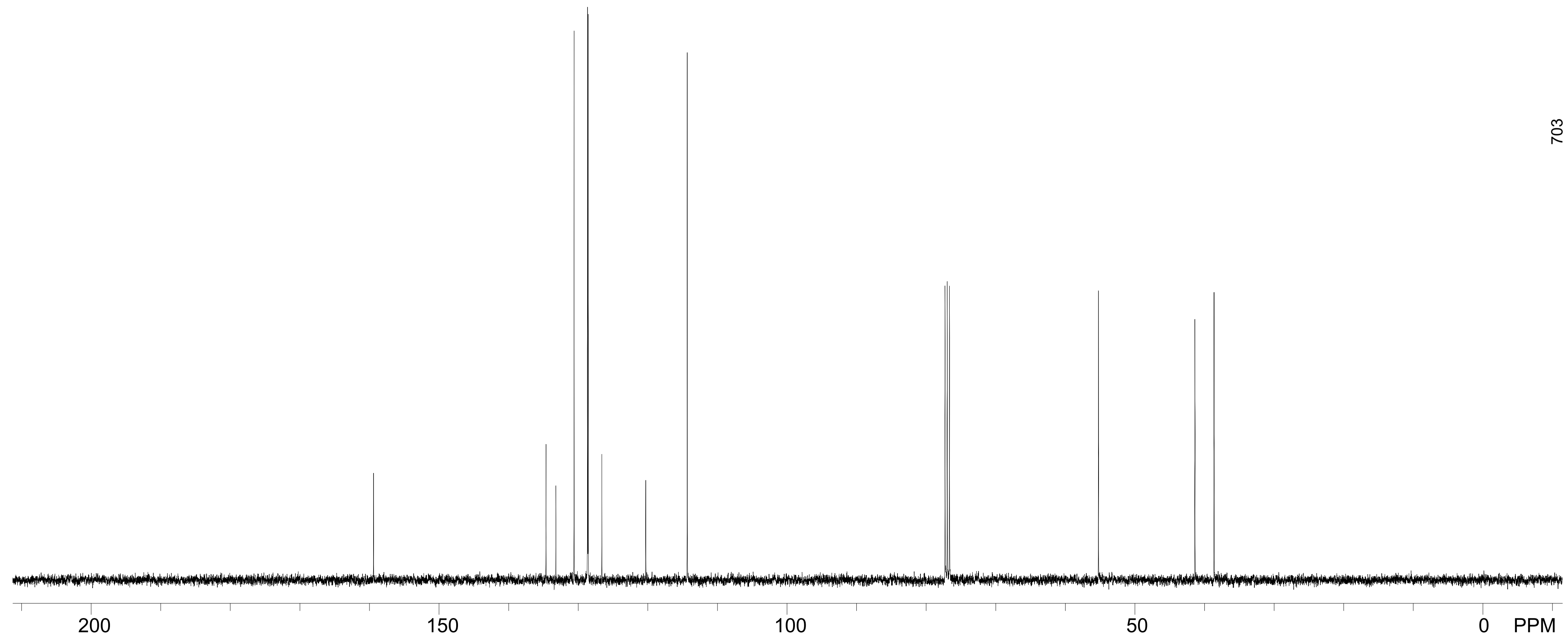
zwf-7-135C
Nov 04 2023
SOLVENT: CDCl3
NA = 100
F1 = 100.625343 MHz
F2 = 1.000000 MHz
159.423
134.638
133.215
130.601
128.665
128.571
126.614
120.302
114.344
77.318
77.000
76.682
55.262
41.389
38.637
200
150
100
50
0
PPM

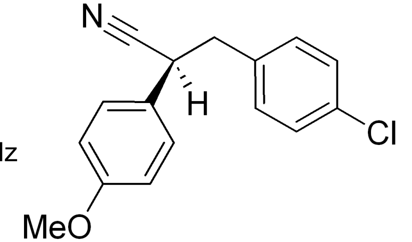
N
H
Cl
MeO

38

Area Percent Report

Agilent Technologies

Sample name: zwf-7-135-IE-99-1-0.8-205

Data file: C:\Users\Public\Documents\ChemStation\1\Data\zwf\zwf-S 2023-11-04 09-48-00\018-P1-C1-zwf-7-135.D

Injection Acquired Date 11/04/2023 16:46:42

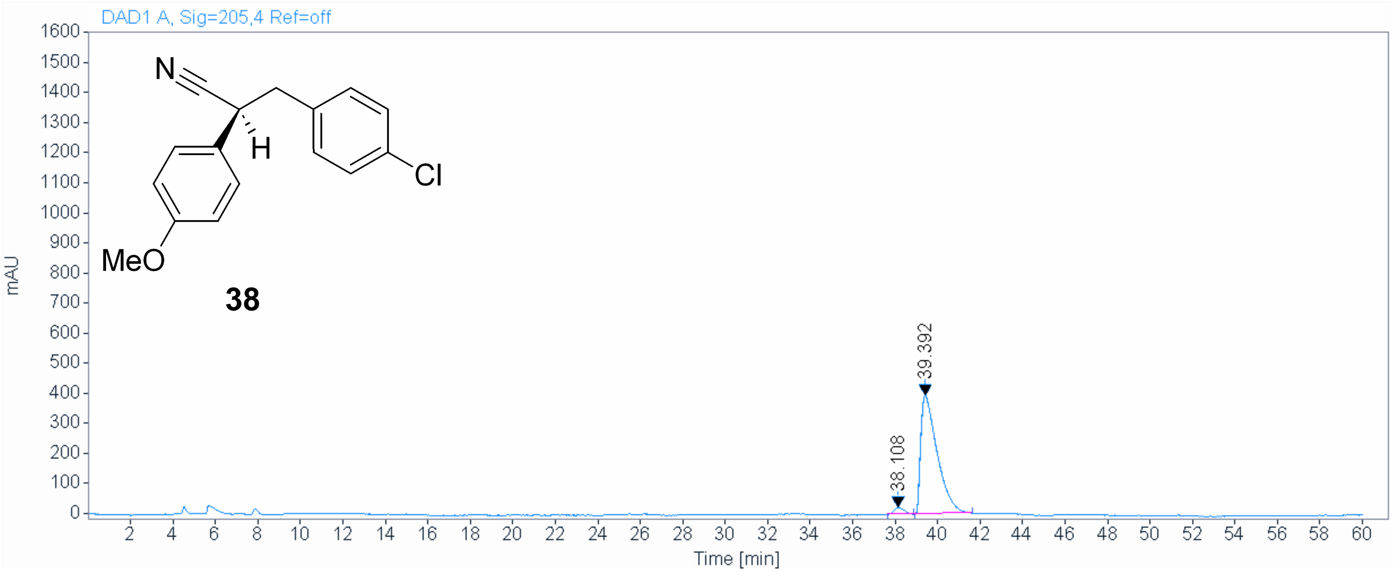

Signal: DAD1 A, Sig=205,4 Ref=off

RT [min]	Width [min]	Height	Area	Area%
38.108	0.5732	23.5984	811.6147	3.6409
39.392	0.6383	395.0918	21479.9648	96.3591
		Sum	22291.5796	100.0000

Area Percent Report

Agilent Technologies

Sample name: zwf-7-135-rac-IE-99-1-0.8-205

Data file: C:\Users\Public\Documents\ChemStation\1\Data\zwf\zwf-S 2023-11-04 09-48-00\019-P1-C2-zwf-7-135-rac.D

Injection Acquired Date 11/04/2023 17:47:43

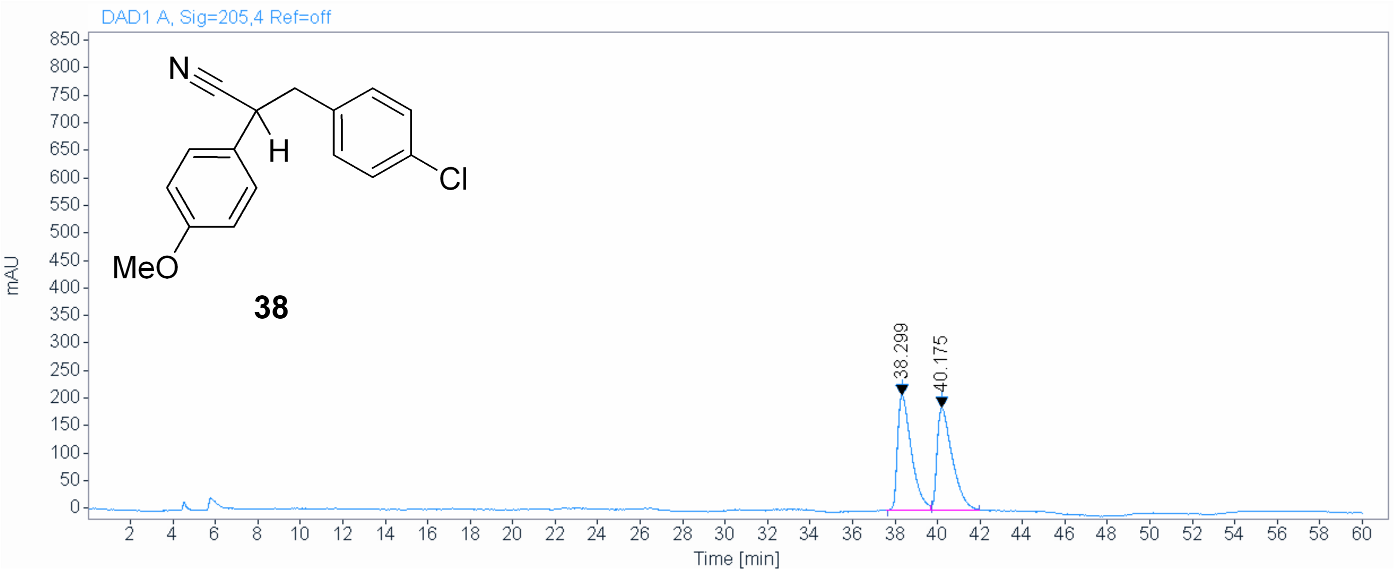

38

Signal: DAD1 A, Sig=205,4 Ref=off

RT [min]	Width [min]	Height	Area	Area%
38.299	0.5883	208.2410	9536.4492	50.6120
40.175	0.6646	186.3660	9305.8350	49.3880
		Sum	18842.2842	100.0000

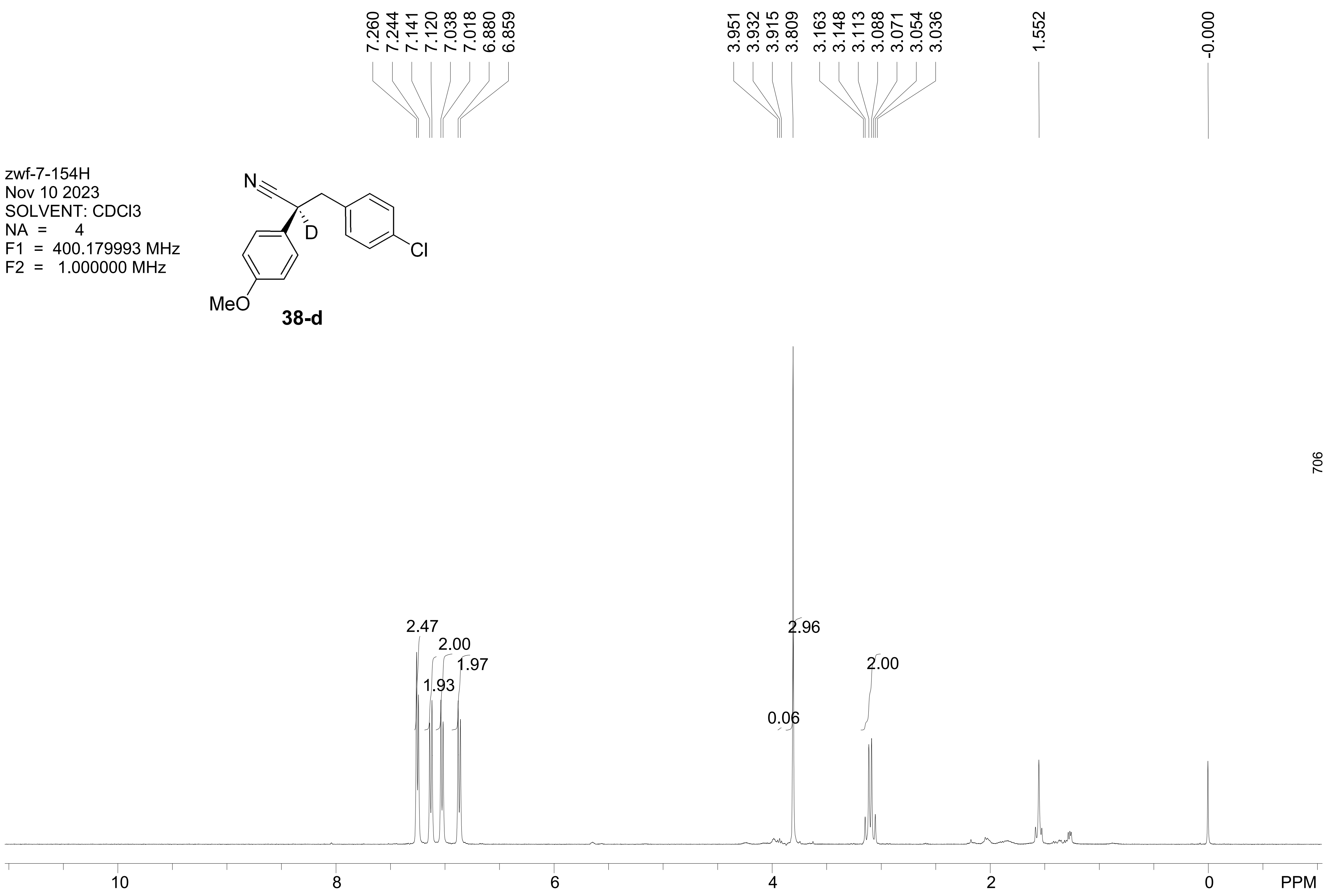
zwf-7-154H
Nov 10 2023
SOLVENT: CDCl3
NA = 4
F1 = 400.179993 MHz
F2 = 1.000000 MHz
N
D
Cl
MeO
38-d
7.260
7.244
7.141
7.120
7.038
7.018
6.880
6.859
3.951
3.932
3.915
3.809
3.163
3.148
3.113
3.088
3.071
3.054
3.036
1.552
-0.000
2.47
2.00
1.97
1.93
2.96
0.06
2.00
10
8
6
4
2
0
PPM

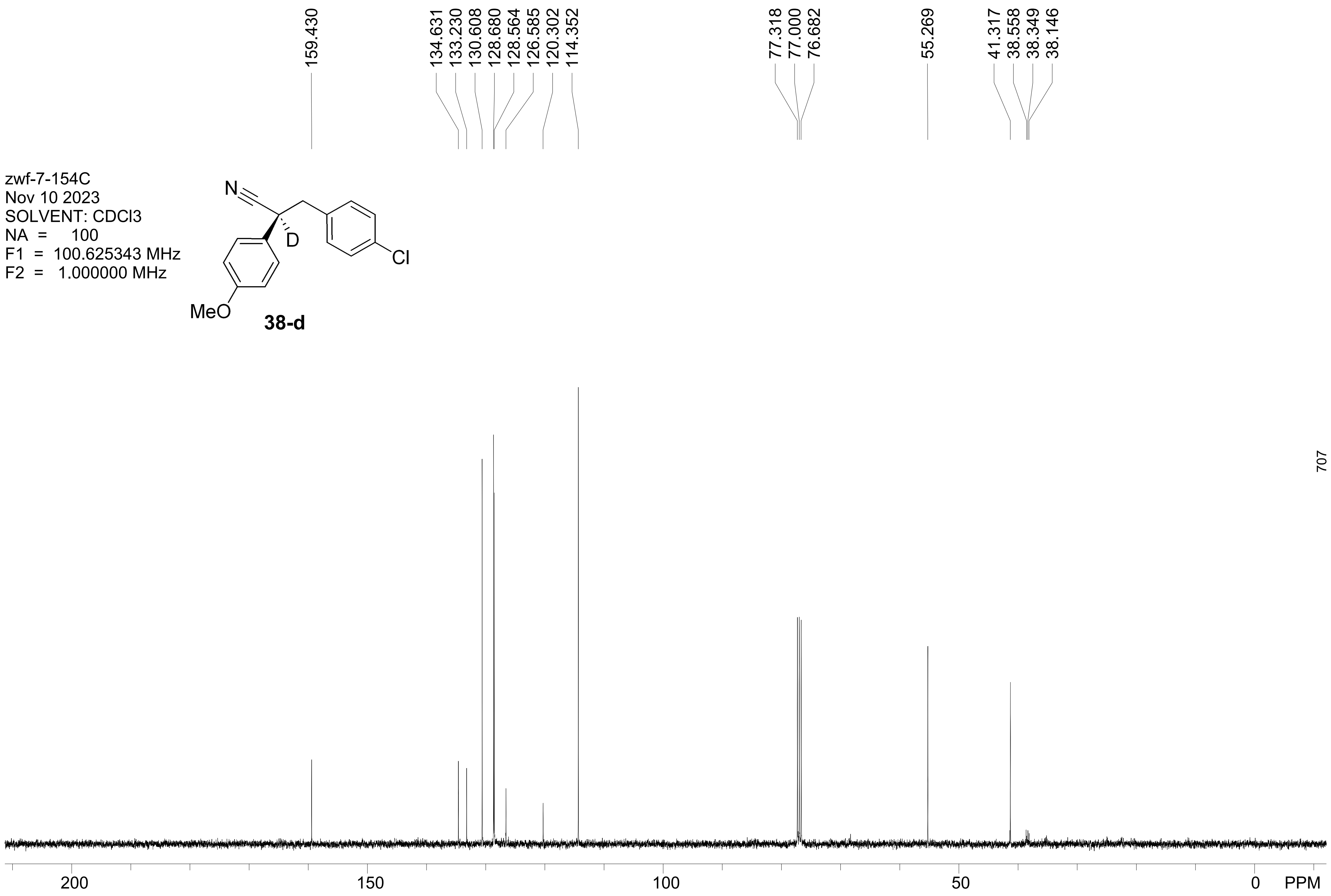
zwf-7-154C
Nov 10 2023
SOLVENT: CDCl3
NA = 100
F1 = 100.625343 MHz
F2 = 1.000000 MHz
N
D
Cl
MeO
38-d
159.430
134.631
133.230
130.608
128.680
128.564
126.585
120.302
114.352
77.318
77.000
76.682
55.269
41.317
38.558
38.349
38.146
200
150
100
50
0
PPM

Area Percent Report

Sample name: zwf-7-154-IE-99-1-0.8-205

Data file: C:\Users\Public\Documents\ChemStation\1\Data\zwf\zwf-S 2023-11-10 18-08-41\004-P1-C5-zwf-7-154.D

Injection Acquired Date 11/10/2023 19:33:30

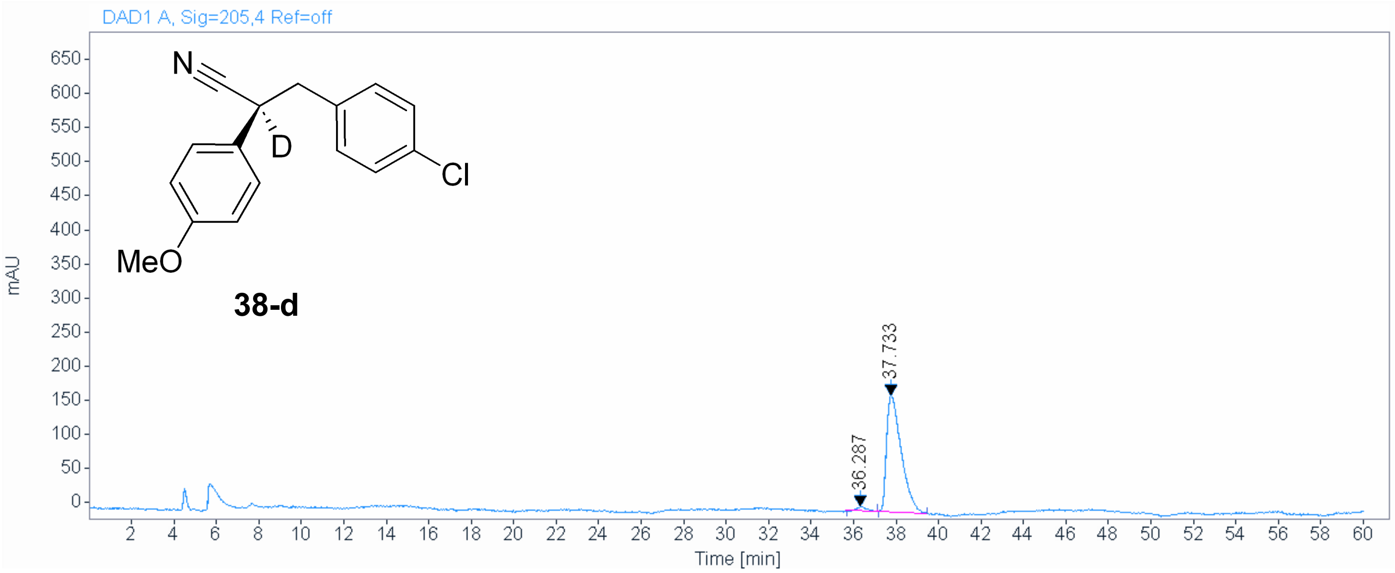

Signal: DAD1 A, Sig=205,4 Ref=off

RT [min]	Width [min]	Height	Area	Area%
36.287	0.6051	6.9859	253.6267	3.0070
37.733	0.5876	170.6758	8181.0078	96.9930
		Sum	8434.6345	100.0000

Area Percent Report

Sample name: zwf-7-154-rac-IE-99-1-0.8-205

Data file: C:\Users\Public\Documents\ChemStation\1\Data\zwf\zwf-S 2023-11-10 18-08-41\005-P1-C6-zwf-7-154-rac.D

Injection Acquired Date 11/10/2023 20:34:37

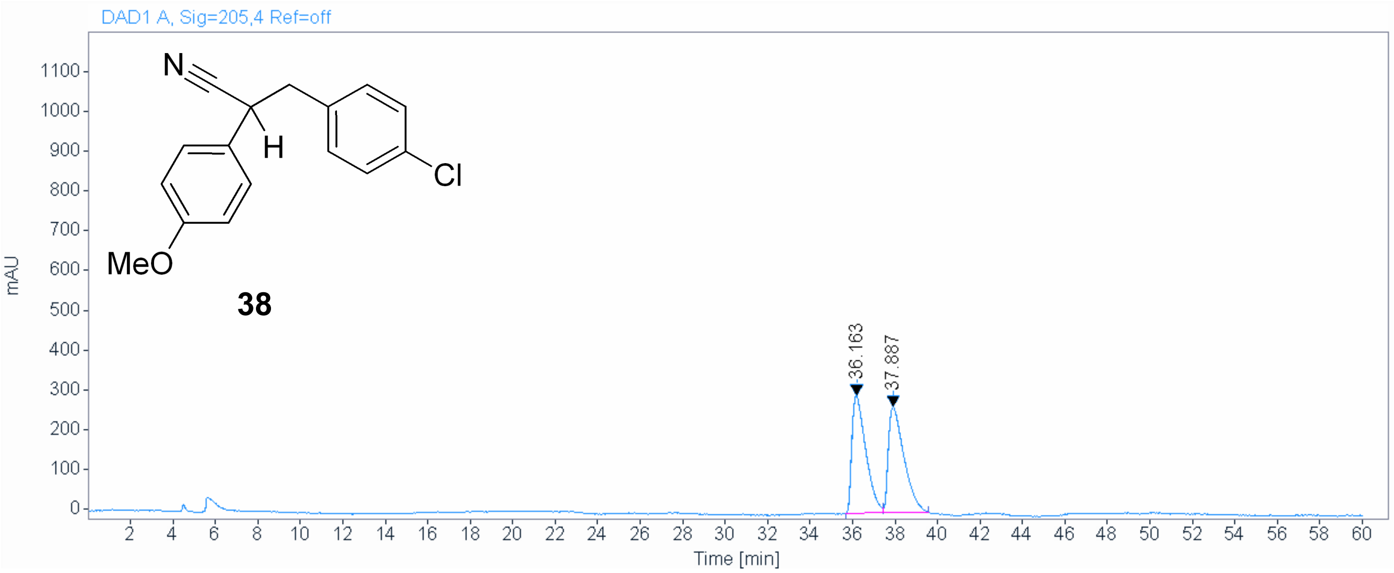

Signal: DAD1 A, Sig=205,4 Ref=off

RT [min]	Width [min]	Height	Area	Area%
36.163	0.5606	296.2097	13628.3584	49.9410
37.887	0.6087	266.9799	13660.5527	50.0590
		Sum	27288.9111	100.0000

zwf-7-138H
Nov 03 2023
SOLVENT: CDCl3
NA = 4
F1 = 400.179993 MHz
F2 = 1.000000 MHz

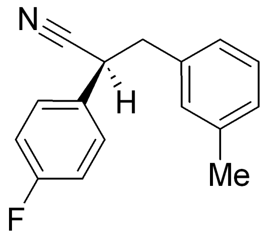

39

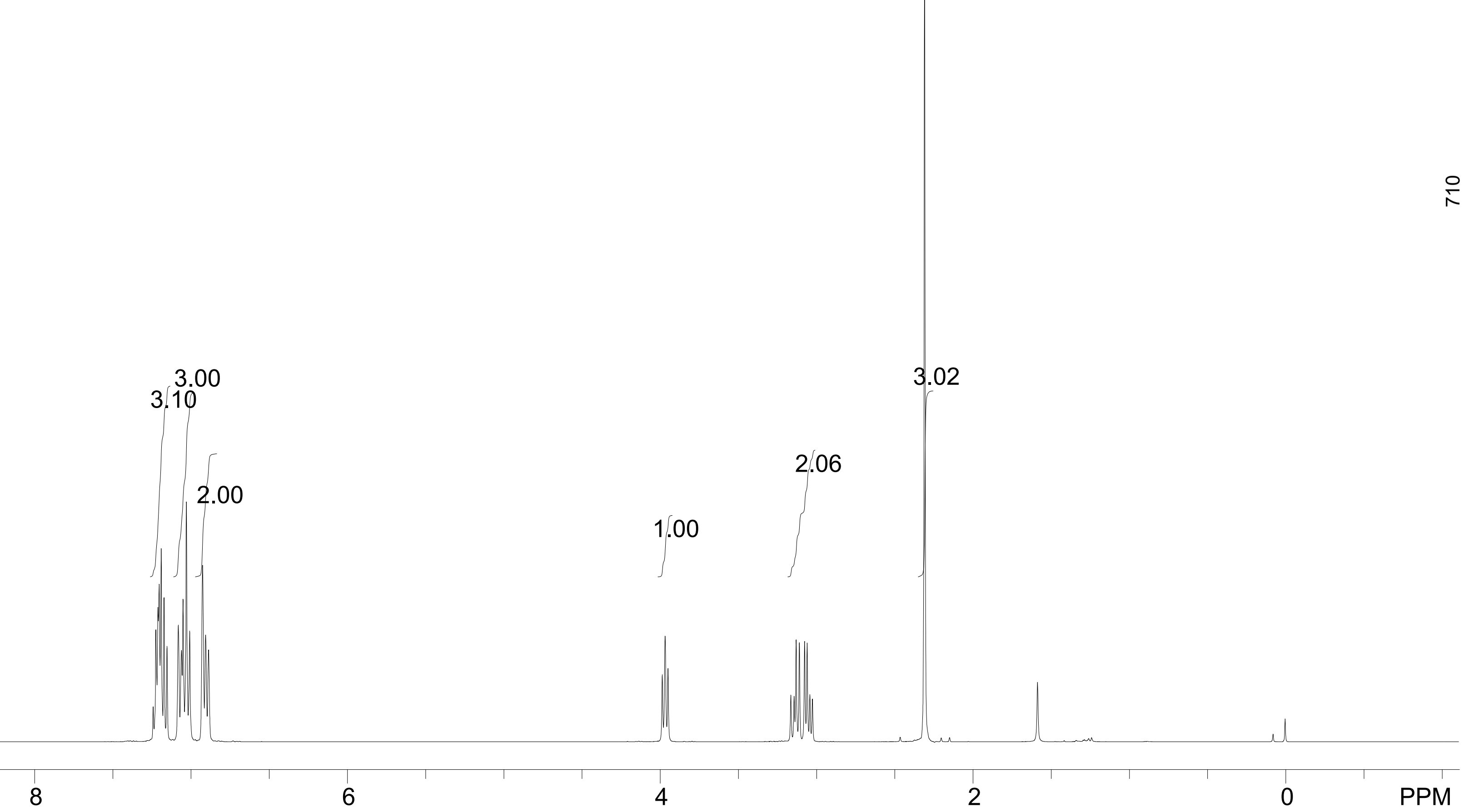

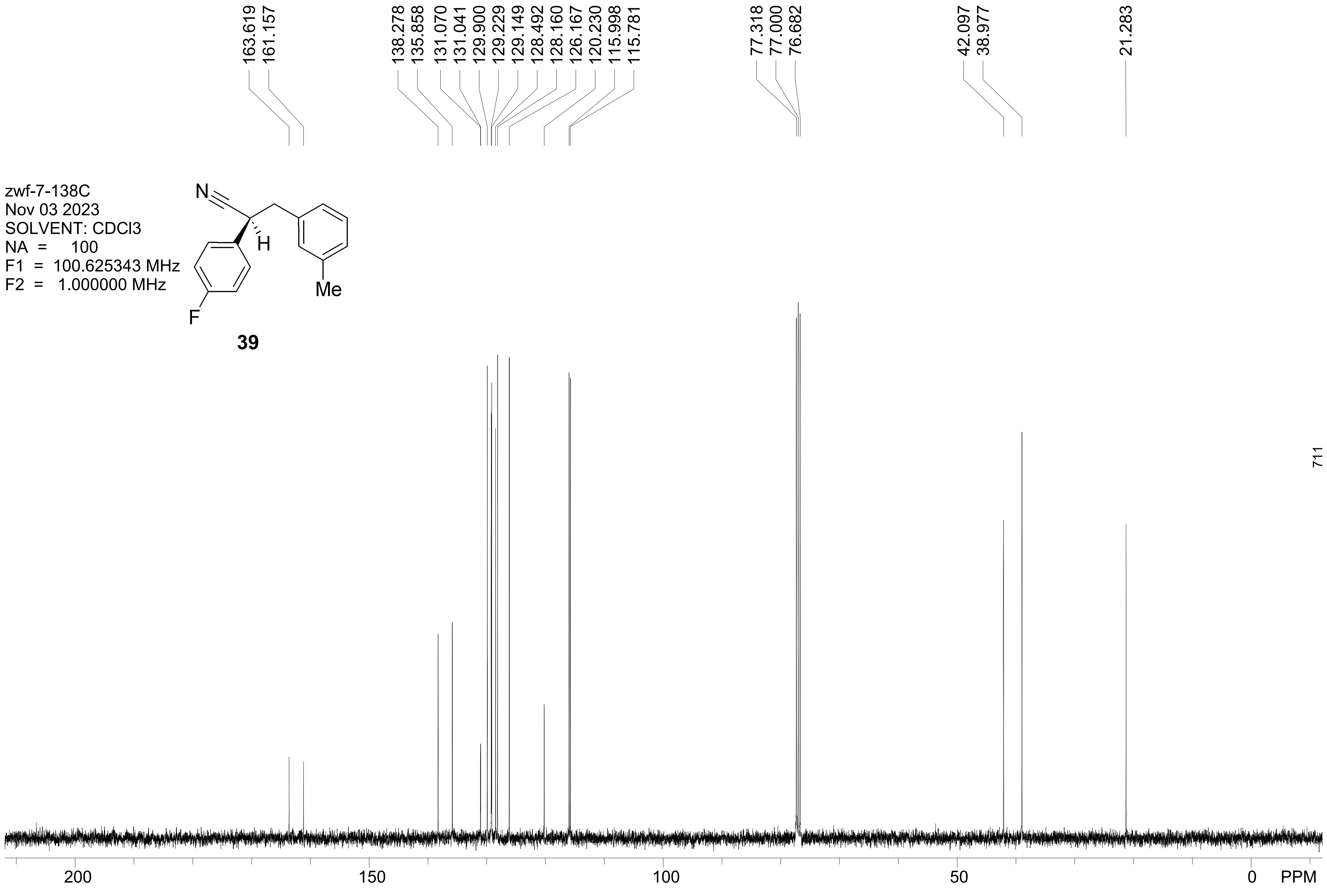
zwf-7-138C
Nov 03 2023
SOLVENT: CDCl3
NA = 100
F1 = 100.625343 MHz
F2 = 1.000000 MHz
N
H
Me
F
39
163.619
161.157
138.278
135.858
131.070
131.041
129.900
129.229
129.149
128.492
128.160
126.167
120.230
115.998
115.781
77.318
77.000
76.682
42.097
38.977
21.283
200
150
100
50
0
PPM

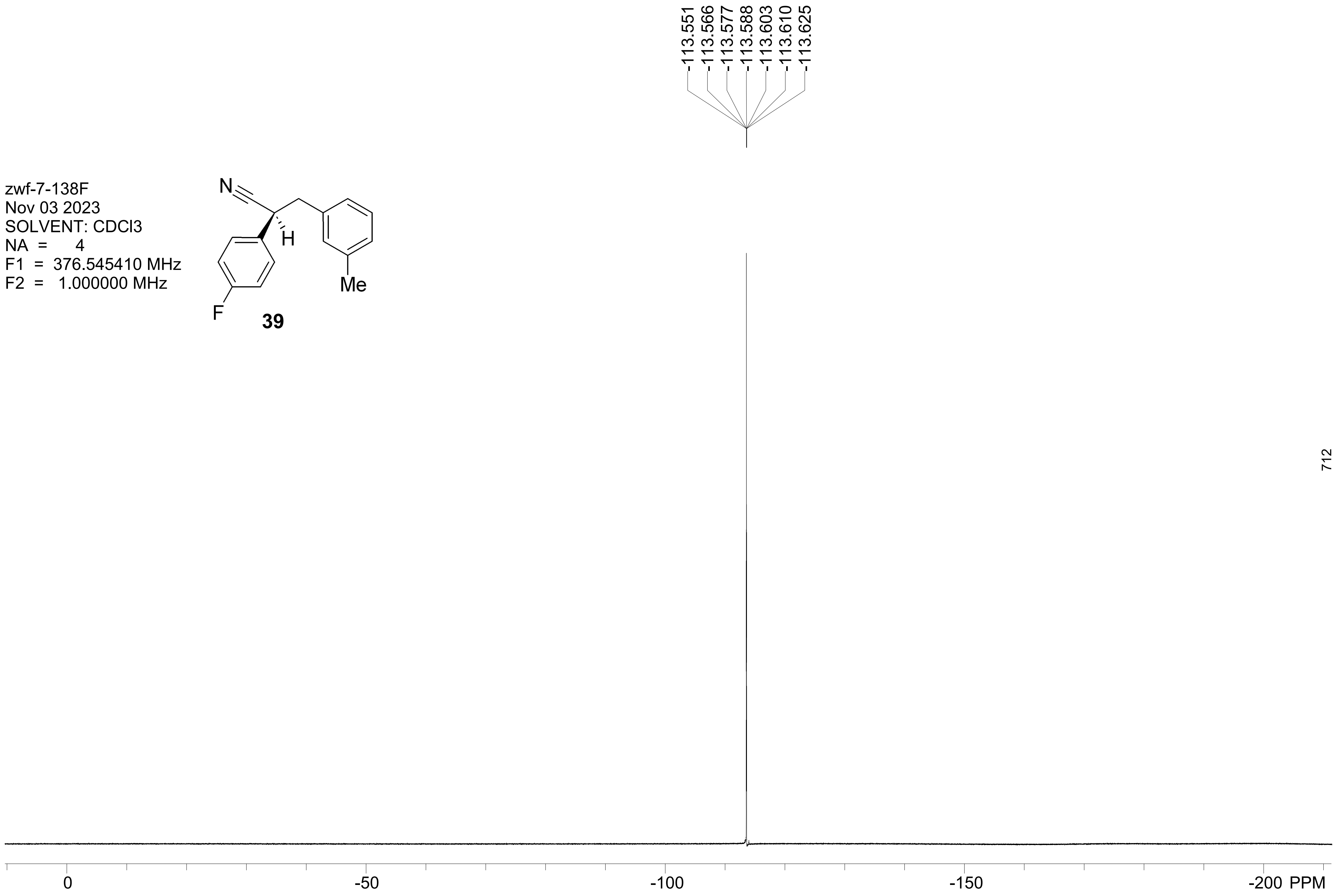
zwf-7-138F
Nov 03 2023
SOLVENT: CDCl3
NA = 4
F1 = 376.545410 MHz
F2 = 1.000000 MHz
N
H
Me
F
39
-113.551
-113.566
-113.577
-113.588
-113.603
-113.610
-113.625
0
-50
-100
-150
-200 PPM

Area Percent Report

Agilent Technologies

Sample name: zwf-7-138-IE-99-1-0.8-205

Data file: C:\Users\Public\Documents\ChemStation\1\Data\zwf\zwf-S 2023-11-04 09-48-00\012-P1-B3-zwf-7-138.D

Injection Acquired Date 11/04/2023 14:10:32

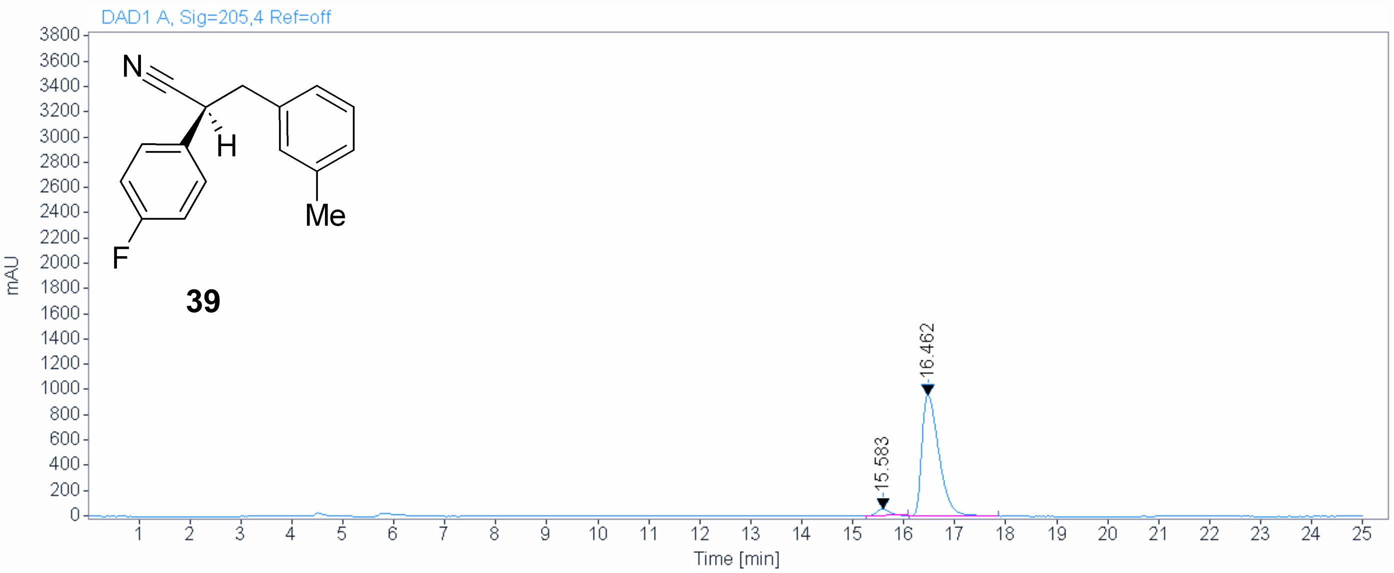

Signal: DAD1 A, Sig=205,4 Ref=off

RT [min]	Width [min]	Height	Area	Area%
15.583	0.3282	52.6617	1036.8624	4.4370
16.462	0.3898	954.8560	22331.7227	95.5630
		Sum	23368.5851	100.0000

Area Percent Report

Agilent Technologies

Sample name: zwf-7-138-rac-IE-99-1-0.8-205

Data file: C:\Users\Public\Documents\ChemStation\1\Data\zwf\zwf-S 2023-11-04 09-48-00\013-P1-B4-zwf-7-138-rac.D

Injection Acquired Date 11/04/2023 14:36:34

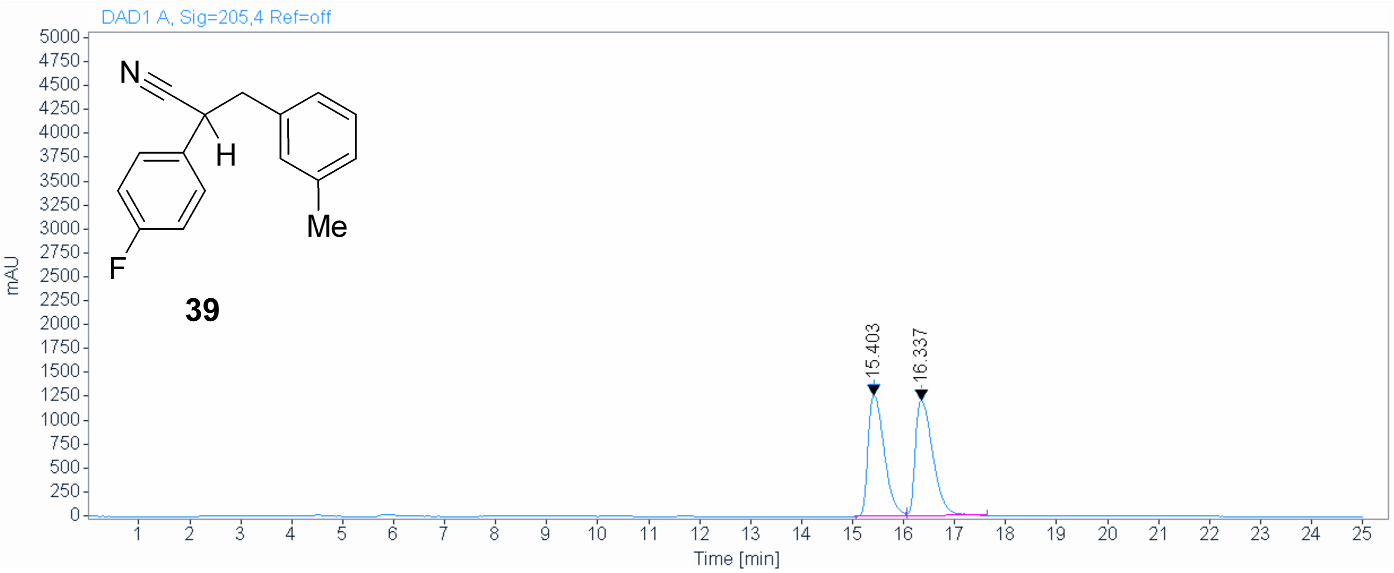

Signal: DAD1 A, Sig=205,4 Ref=off

RT [min]	Width [min]	Height	Area	Area%
15.403	0.3001	1262.7349	27933.1211	49.0409
16.337	0.3582	1206.1570	29025.6816	50.9591
		Sum	56958.8027	100.0000

zwf-7-157H
Nov 10 2023
SOLVENT: CDCl3
NA = 4
F1 = 400.140015 MHz
F2 = 1.000000 MHz

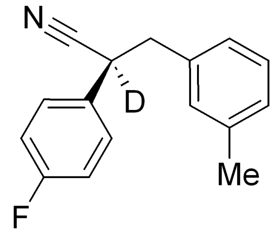

39-d

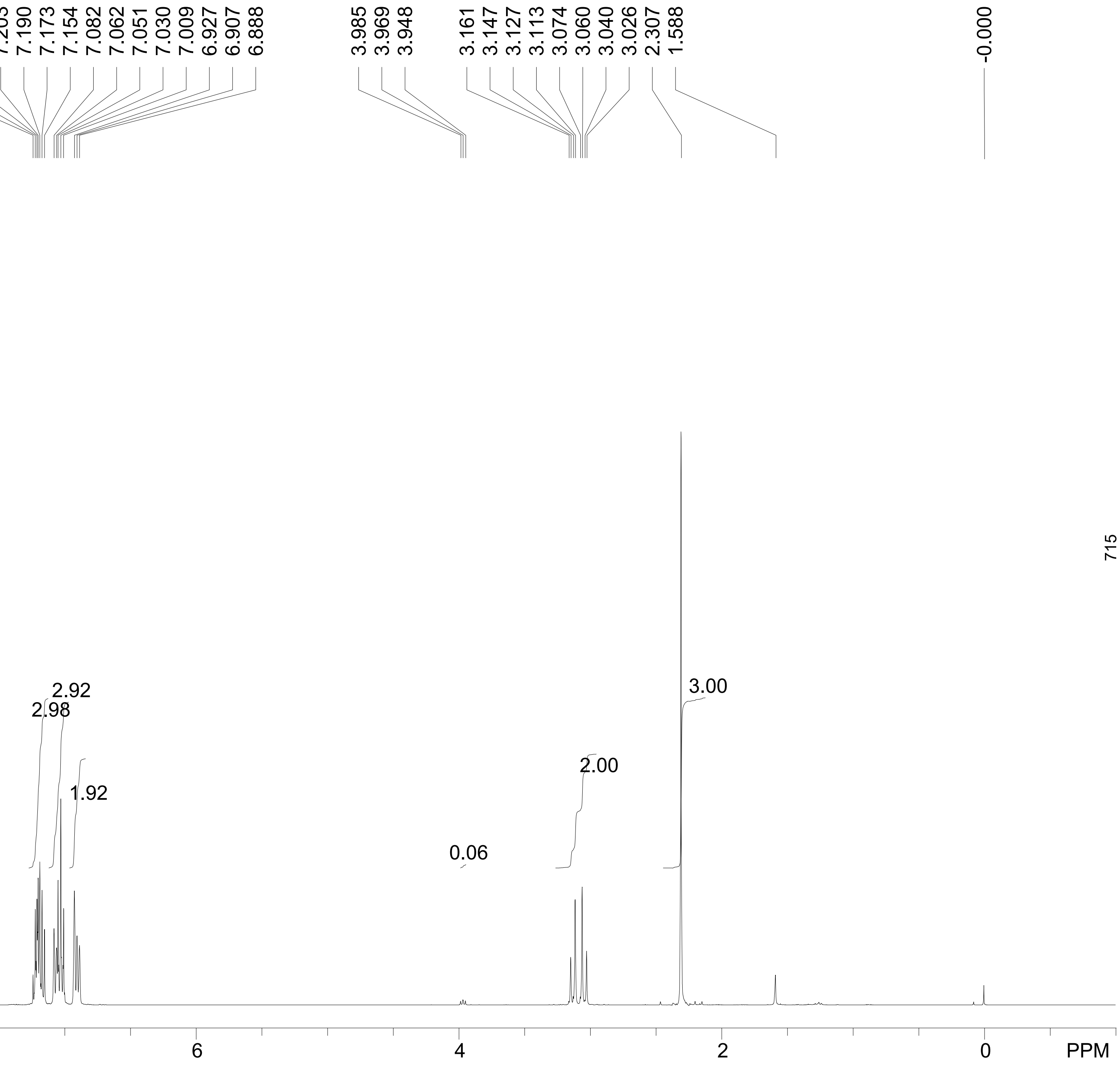

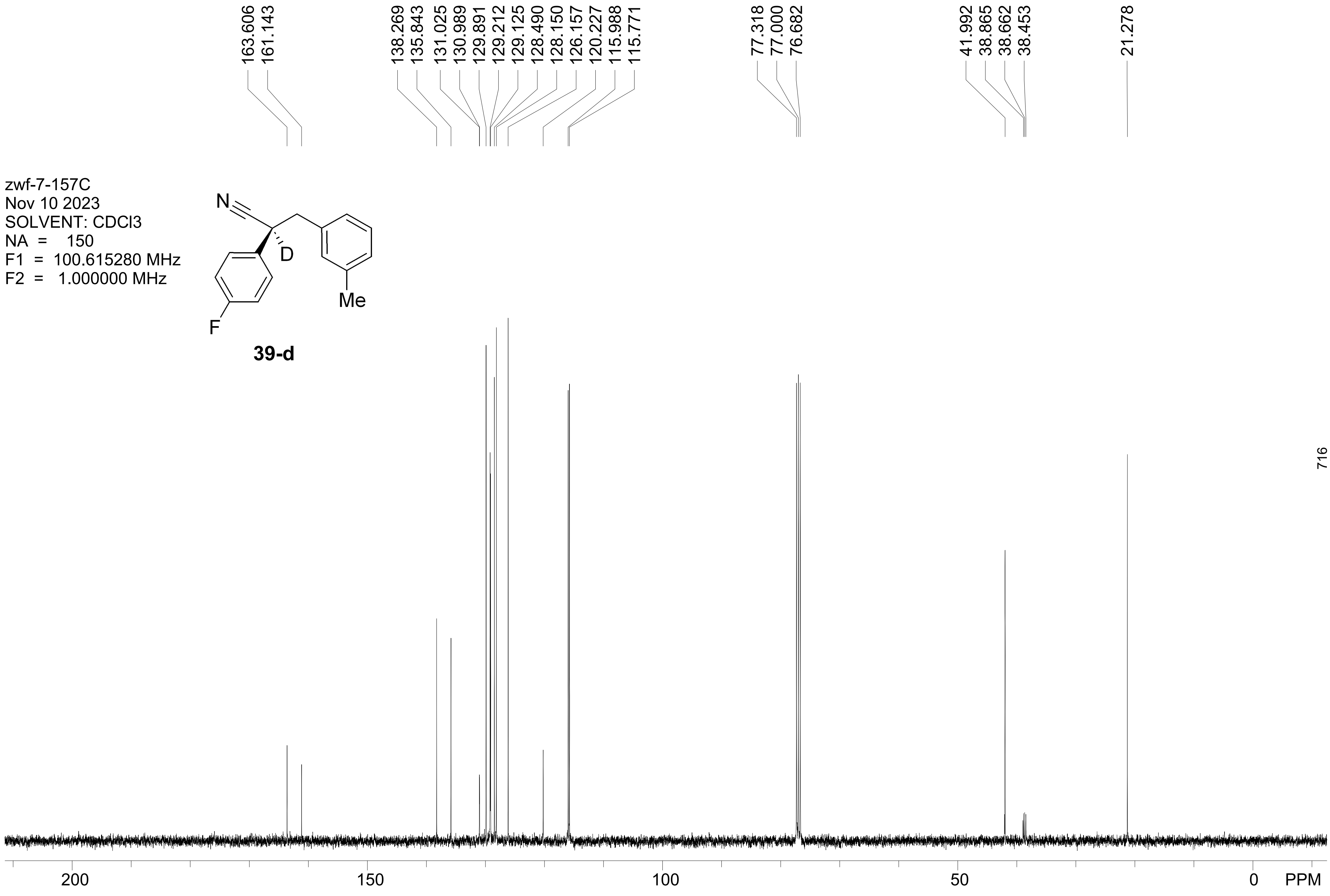
zwf-7-157C
Nov 10 2023
SOLVENT: CDCl3
NA = 150
F1 = 100.615280 MHz
F2 = 1.000000 MHz
N
D
Me
F
39-d
163.606
161.143
138.269
135.843
131.025
130.989
129.891
129.212
129.125
128.490
128.150
126.157
120.227
115.988
115.771
77.318
77.000
76.682
41.992
38.865
38.662
38.453
21.278
200
150
100
50
0
PPM

zwf-7-157F
Nov 10 2023
SOLVENT: CDCl3
NA = 4
F1 = 376.507782 MHz
F2 = 1.000000 MHz

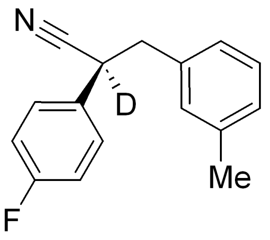

39-d

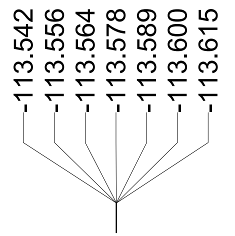

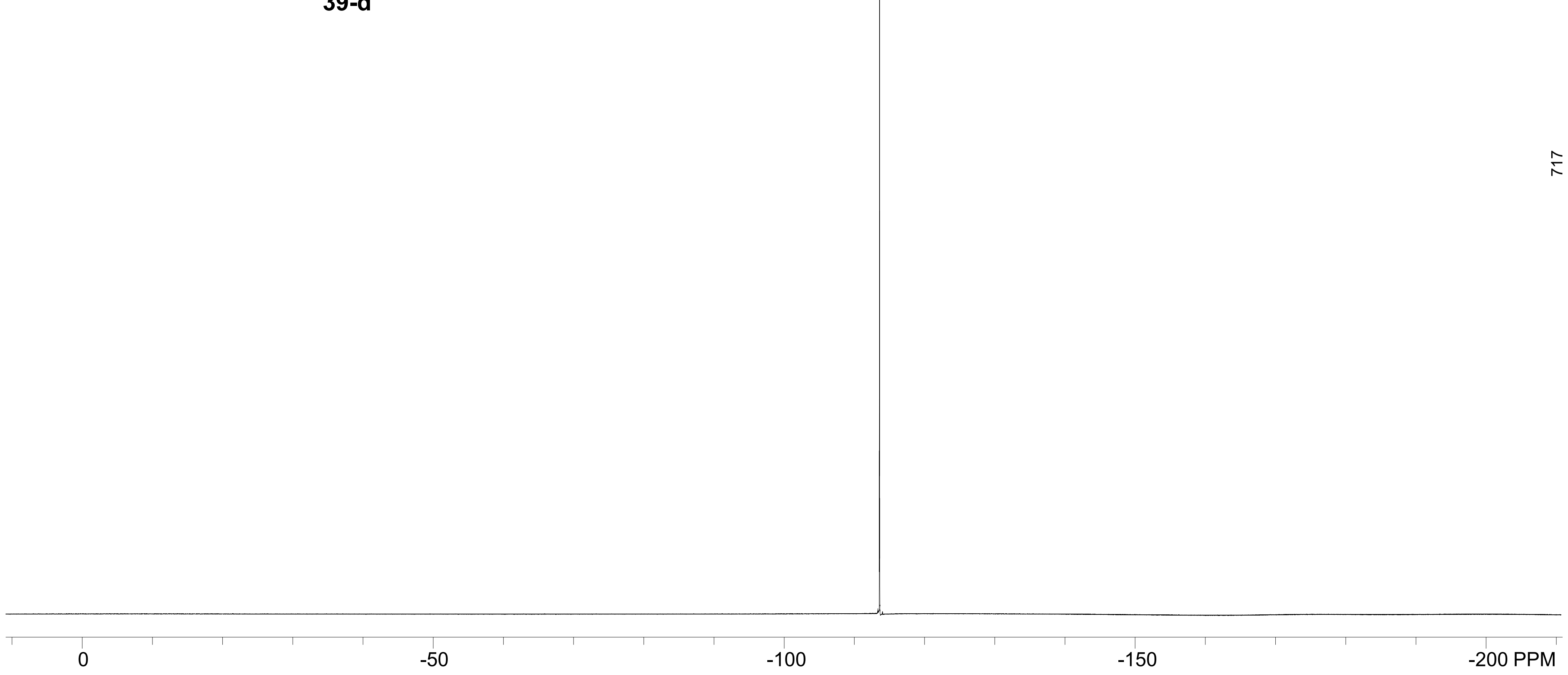

Area Percent Report

Agilent Technologies

Sample name: zwf-7-157-IE-99-1-0.8-205

Data file: C:\Users\Public\Documents\ChemStation\1\Data\zwf\zwf-S 2023-11-11 19-01-34\003-P1-C1-zwf-7-157.D

Injection Acquired Date 11/11/2023 19:51:47

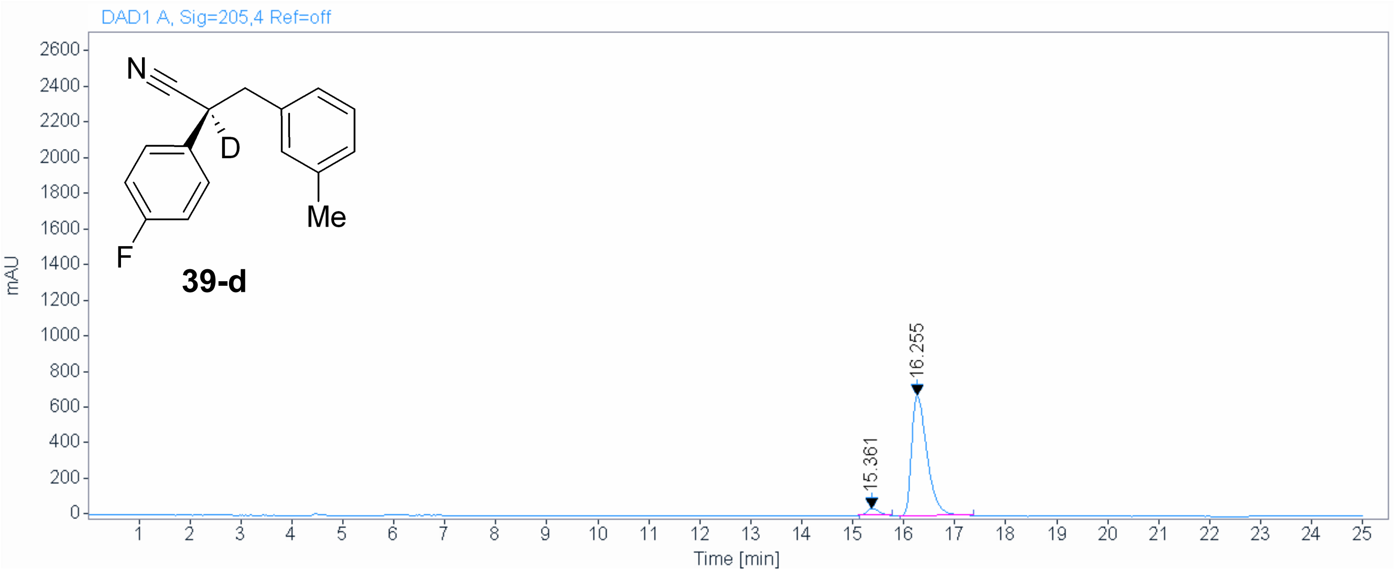

Signal: DAD1 A, Sig=205,4 Ref=off

RT [min]	Width [min]	Height	Area	Area%
15.361	0.2852	36.8670	630.8620	4.2220
16.255	0.3129	674.5451	14311.4570	95.7780
		Sum	14942.3190	100.0000

Area Percent Report

Agilent Technologies

Sample name: zwf-7-157-rac-IE-99-1-0.8-205

Data file: C:\Users\Public\Documents\ChemStation\1\Data\zwf\zwf-S 2023-11-11 19-01-34\004-P1-C2-zwf-7-157-rac.D

Injection Acquired Date 11/11/2023 20:17:44

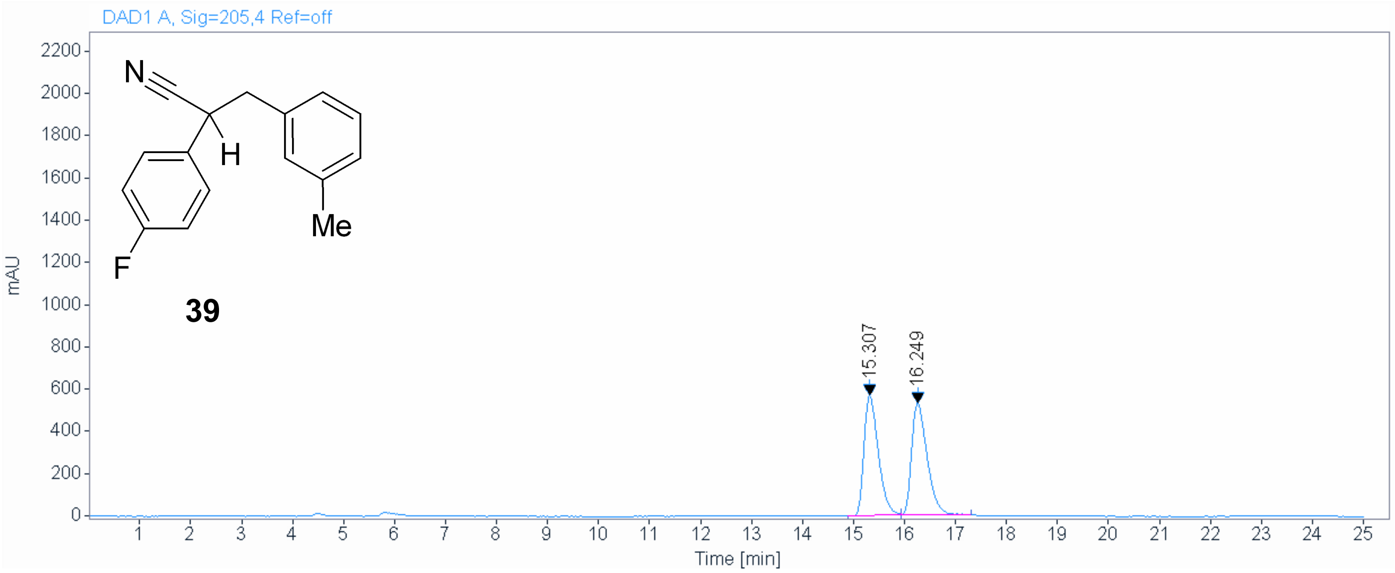

Signal: DAD1 A, Sig=205,4 Ref=off

RT [min]	Width [min]	Height	Area	Area%
15.307	0.2826	568.7192	11144.3887	49.5151
16.249	0.3172	529.8550	11362.6621	50.4849
		Sum	22507.0508	100.0000

zwf-7-139H
Nov 03 2023
SOLVENT: CDCl3
NA = 4
F1 = 400.179993 MHz
F2 = 1.000000 MHz

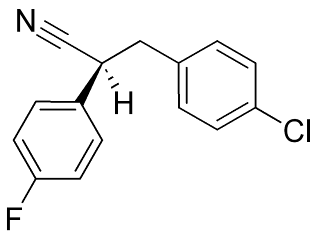

40

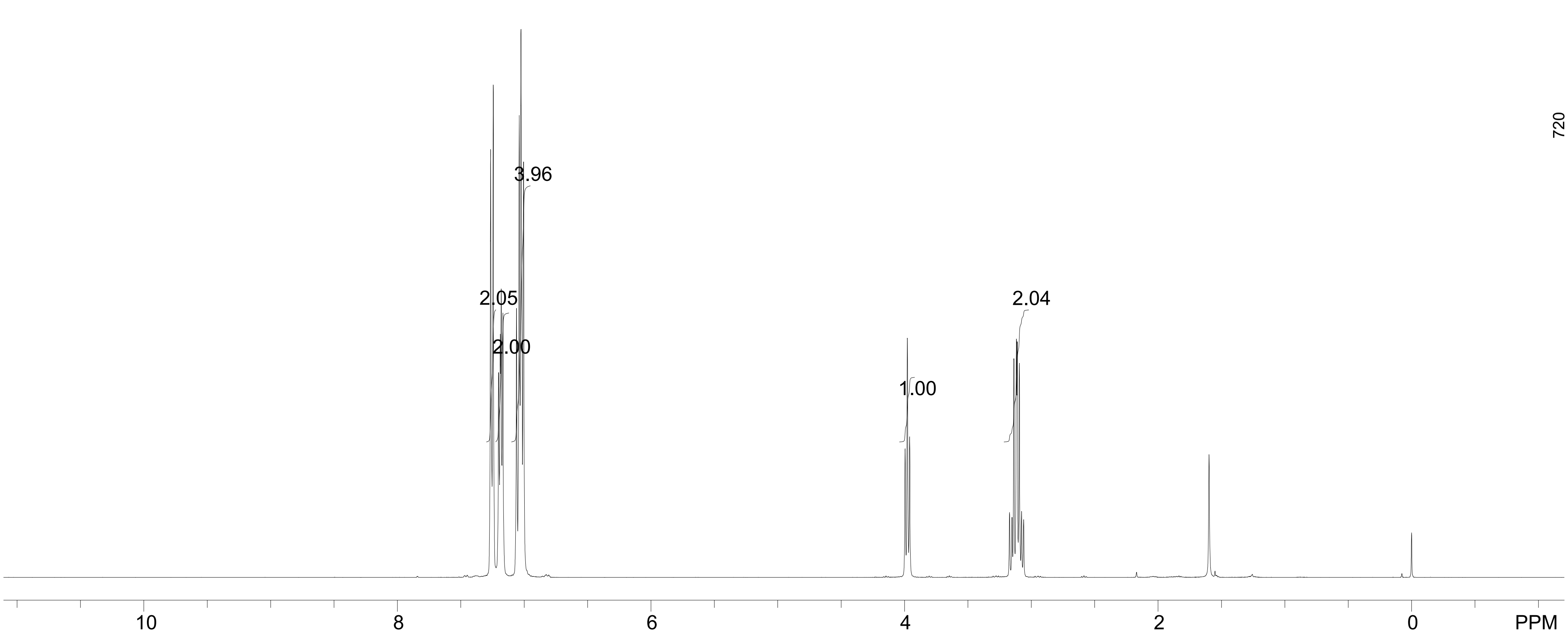

zwf-7-139C
Nov 03 2023
SOLVENT: CDCl3
NA = 100
F1 = 100.625343 MHz
F2 = 1.000000 MHz

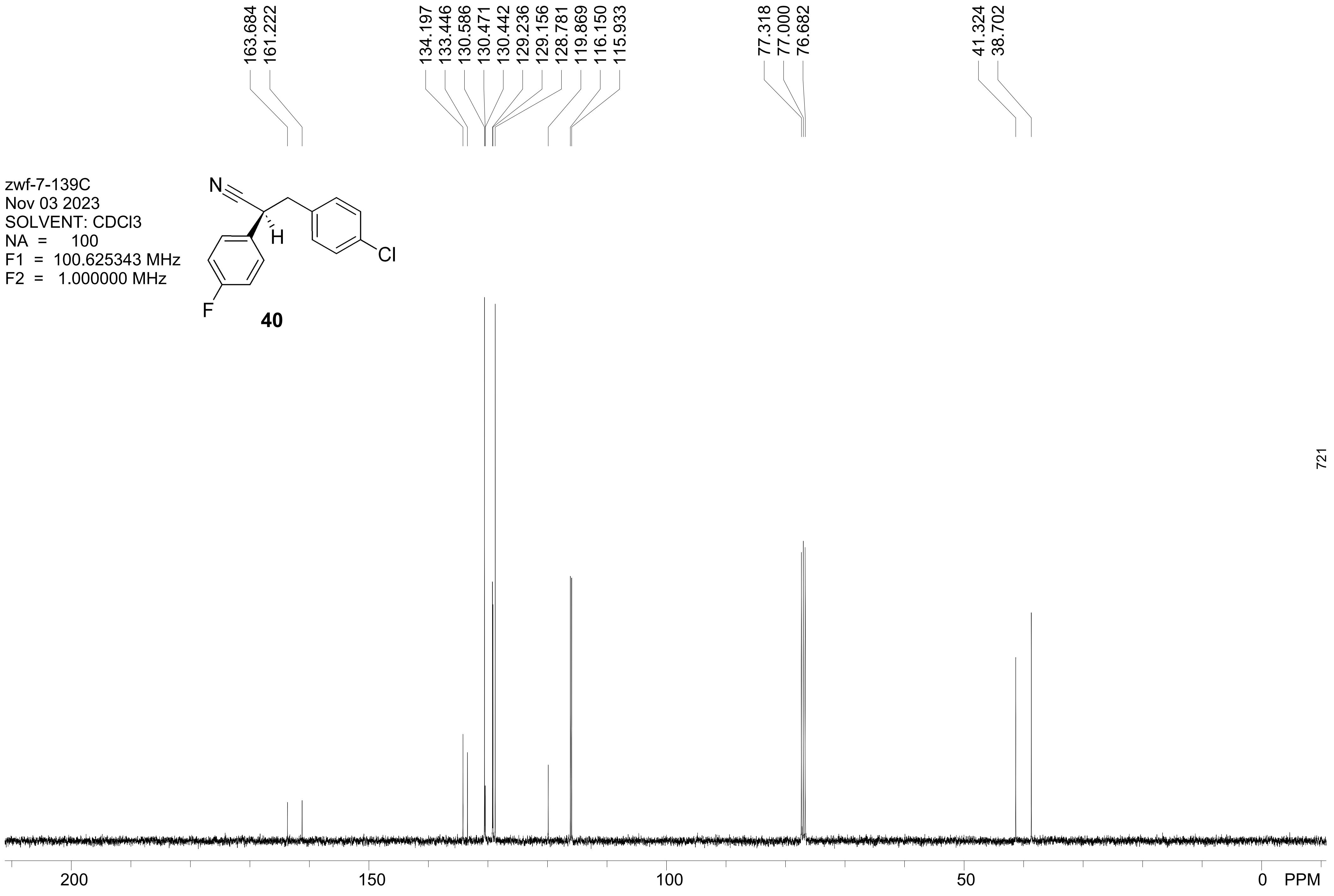

40

zwf-7-139F
Nov 03 2023
SOLVENT: CDCl3
NA = 4
F1 = 376.545410 MHz
F2 = 1.000000 MHz

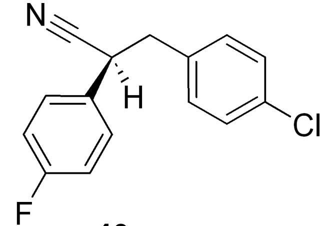

40

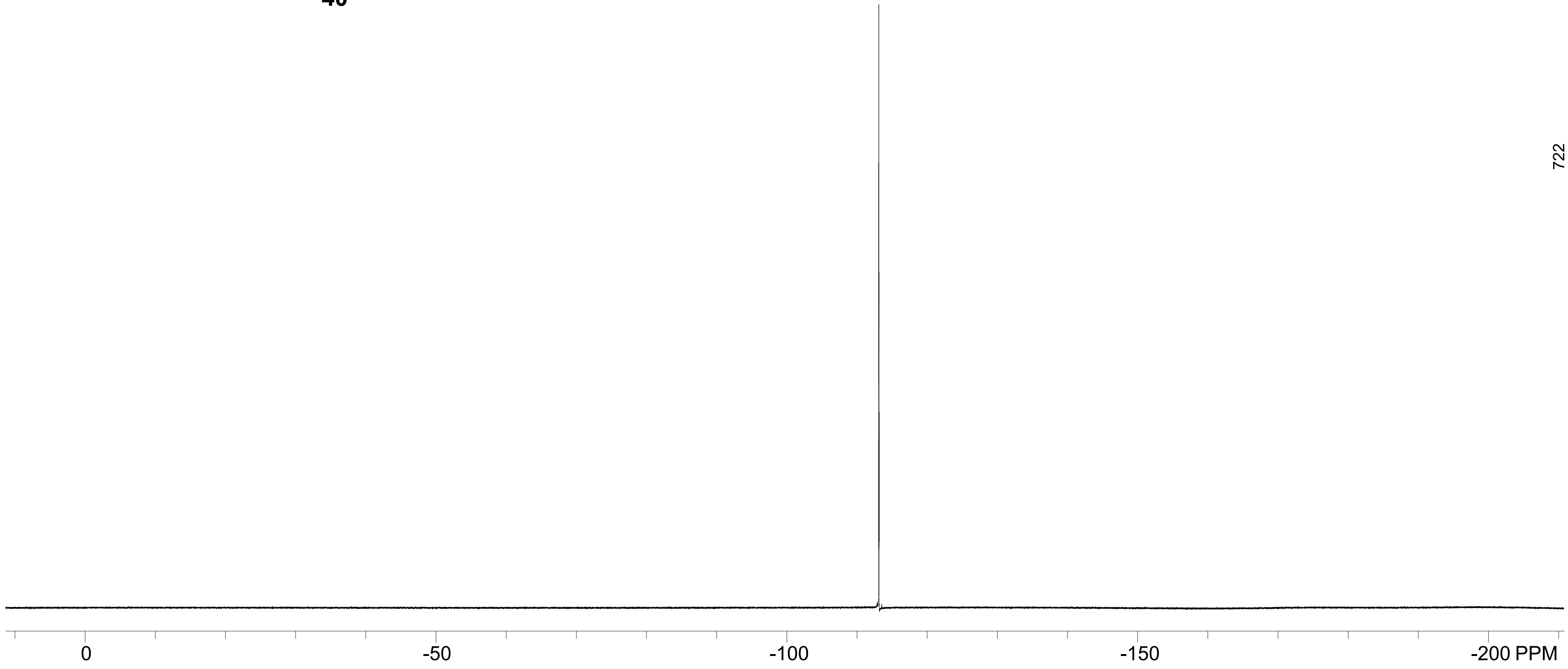

Area Percent Report

Agilent Technologies

Sample name: zwf-7-139-IE-99-1-0.8-205

Data file: C:\Users\Public\Documents\ChemStation\1\Data\zwf\zwf-S 2023-11-04 09-48-00\014-P1-B5-zwf-7-139.D

Injection Acquired Date 11/04/2023 15:02:34

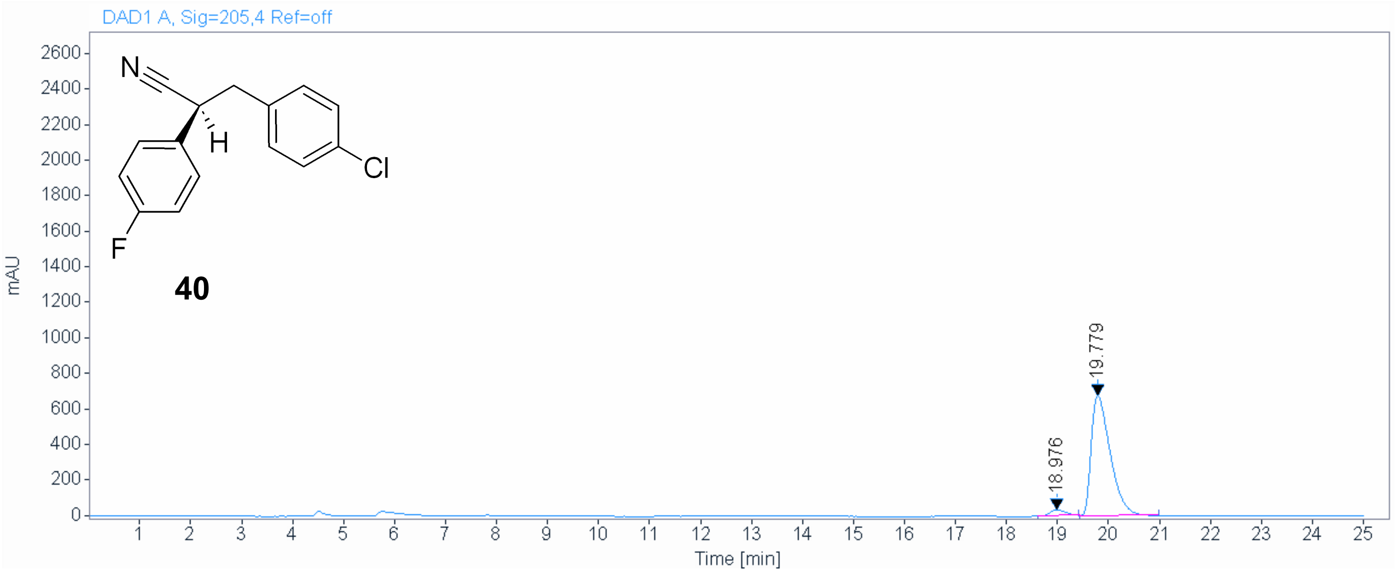

Signal: DAD1 A, Sig=205,4 Ref=off

RT [min]	Width [min]	Height	Area	Area%
18.976	0.3430	34.8041	716.2949	3.9052
19.779	0.4333	677.9386	17625.7246	96.0948
		Sum	18342.0195	100.0000

Area Percent Report

Agilent Technologies

Sample name: zwf-7-139-rac-IE-99-1-0.8-205

Data file: C:\Users\Public\Documents\ChemStation\1\Data\zwf\zwf-S 2023-11-04 09-48-00\015-P1-B6-zwf-7-139-rac.D

Injection Acquired Date 11/04/2023 15:28:36

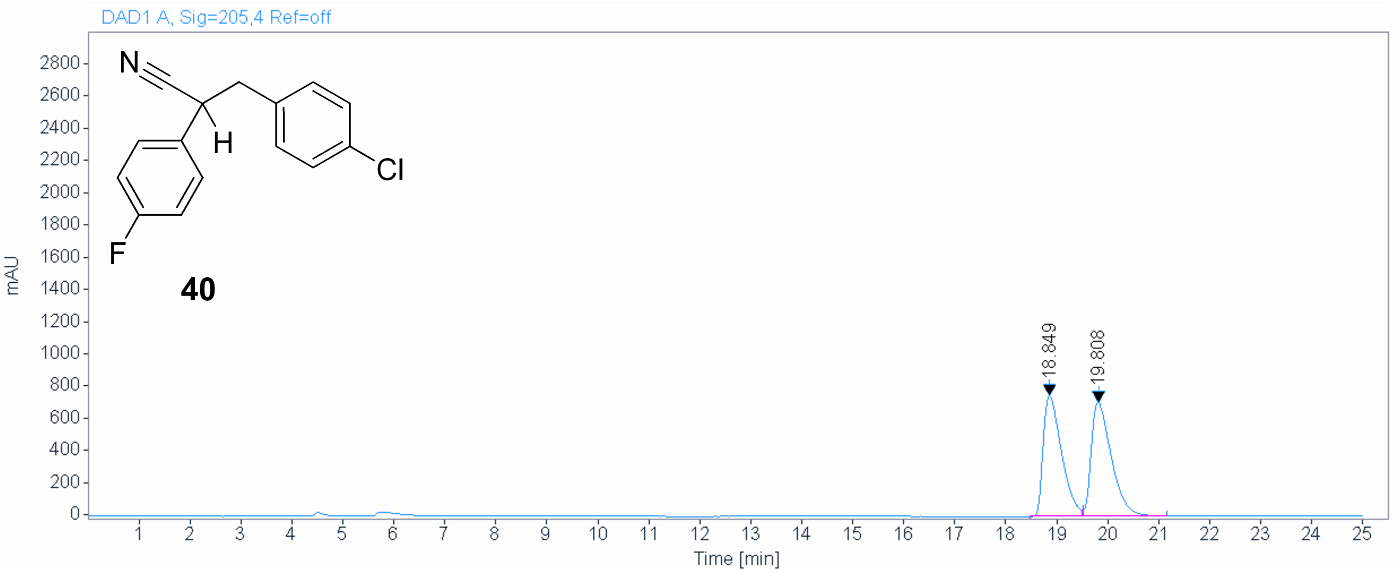

Signal: DAD1 A, Sig=205,4 Ref=off

RT [min]	Width [min]	Height	Area	Area%
18.849	0.3708	749.4243	18810.0879	49.1874
19.808	0.3847	706.9037	19431.5625	50.8126
		Sum	38241.6504	100.0000

zwf-8-001H
Nov 09 2023
SOLVENT: CDCl3
NA = 4
F1 = 400.179993 MHz
F2 = 1.000000 MHz

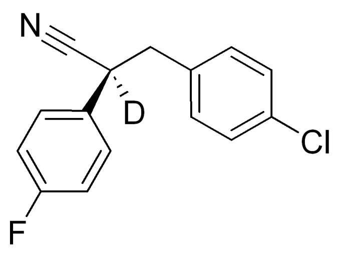

40-d

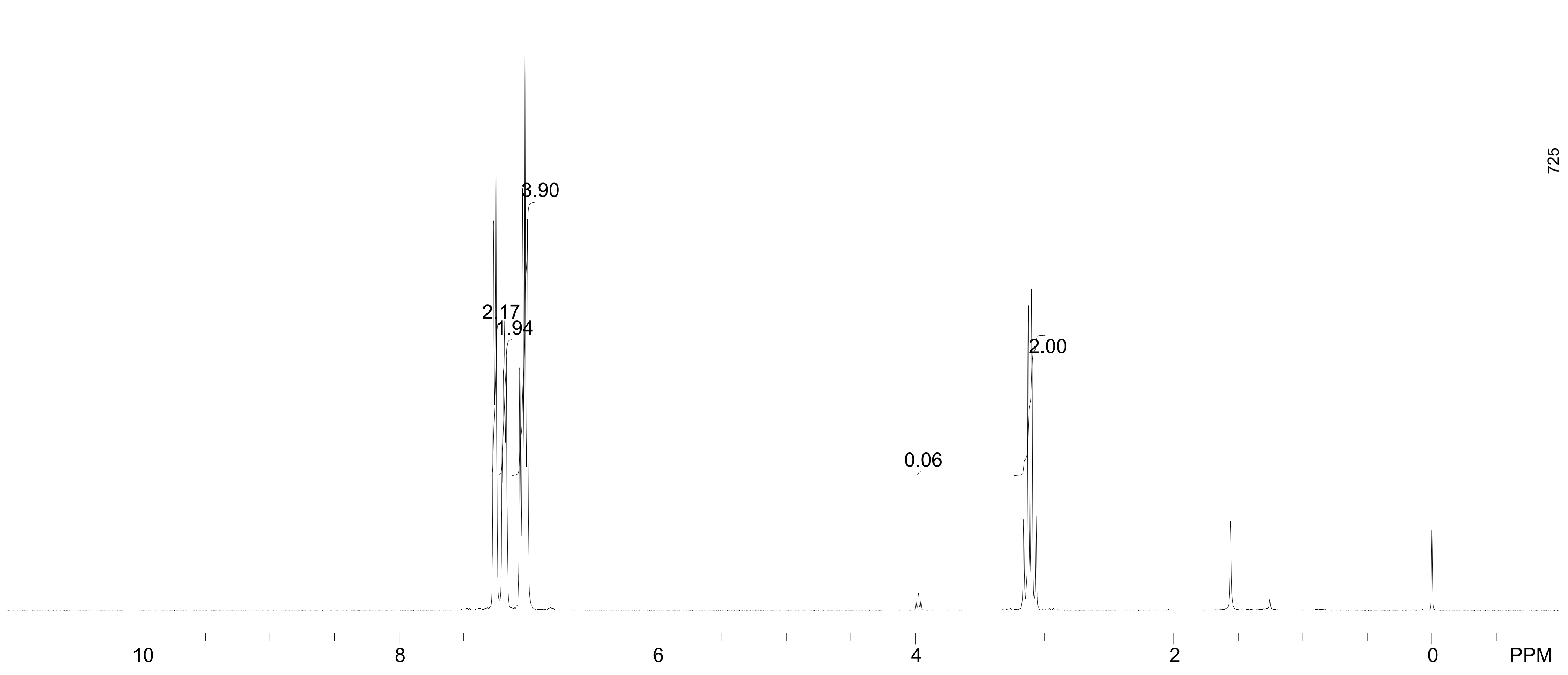

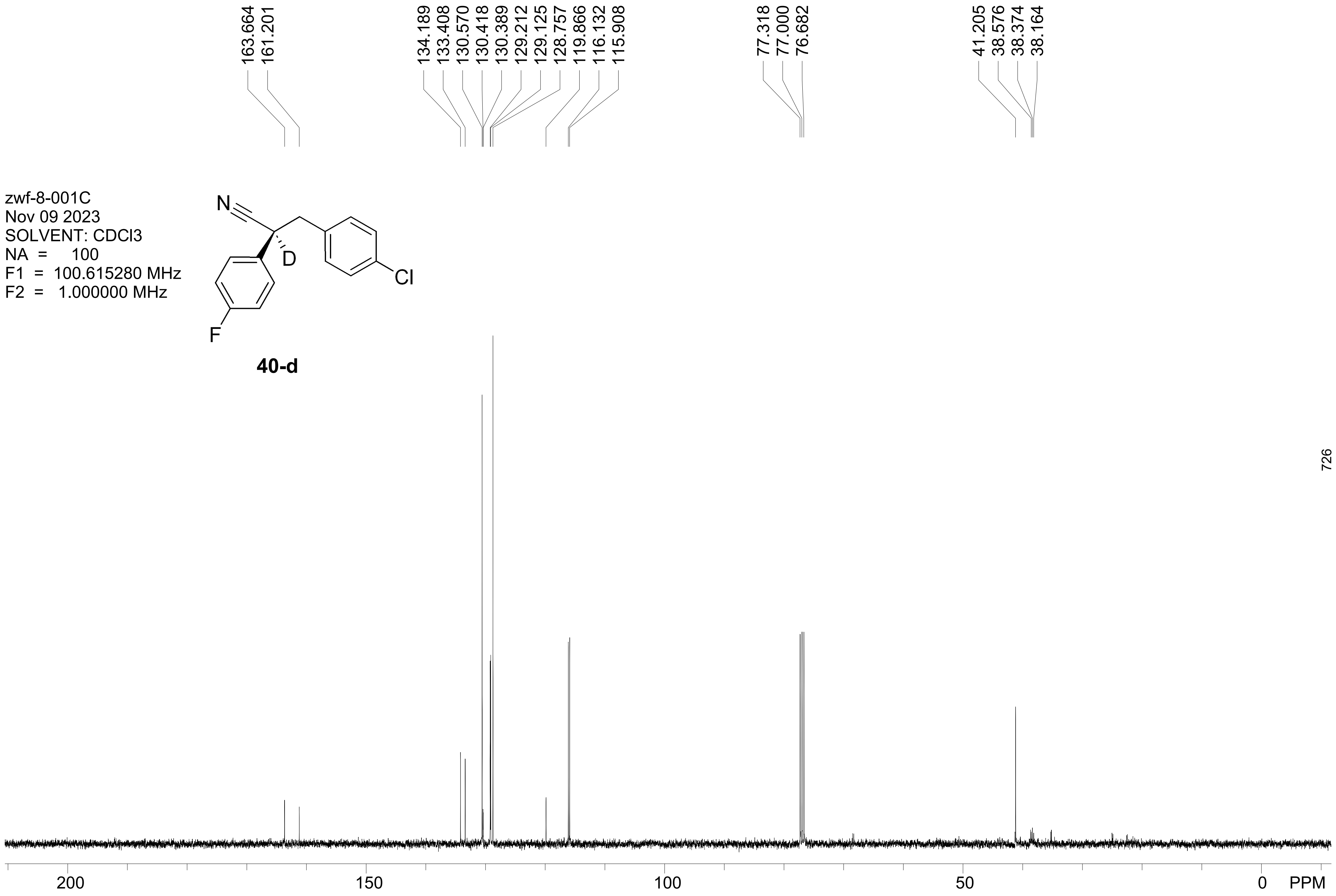
zwf-8-001C
Nov 09 2023
SOLVENT: CDCl3
NA = 100
F1 = 100.615280 MHz
F2 = 1.000000 MHz
N
D
Cl
F
163.664
161.201
134.189
133.408
130.570
130.418
130.389
129.212
129.125
128.757
119.866
116.132
115.908
77.318
77.000
76.682
41.205
38.576
38.374
38.164
200
150
100
50
0
PPM

40-d

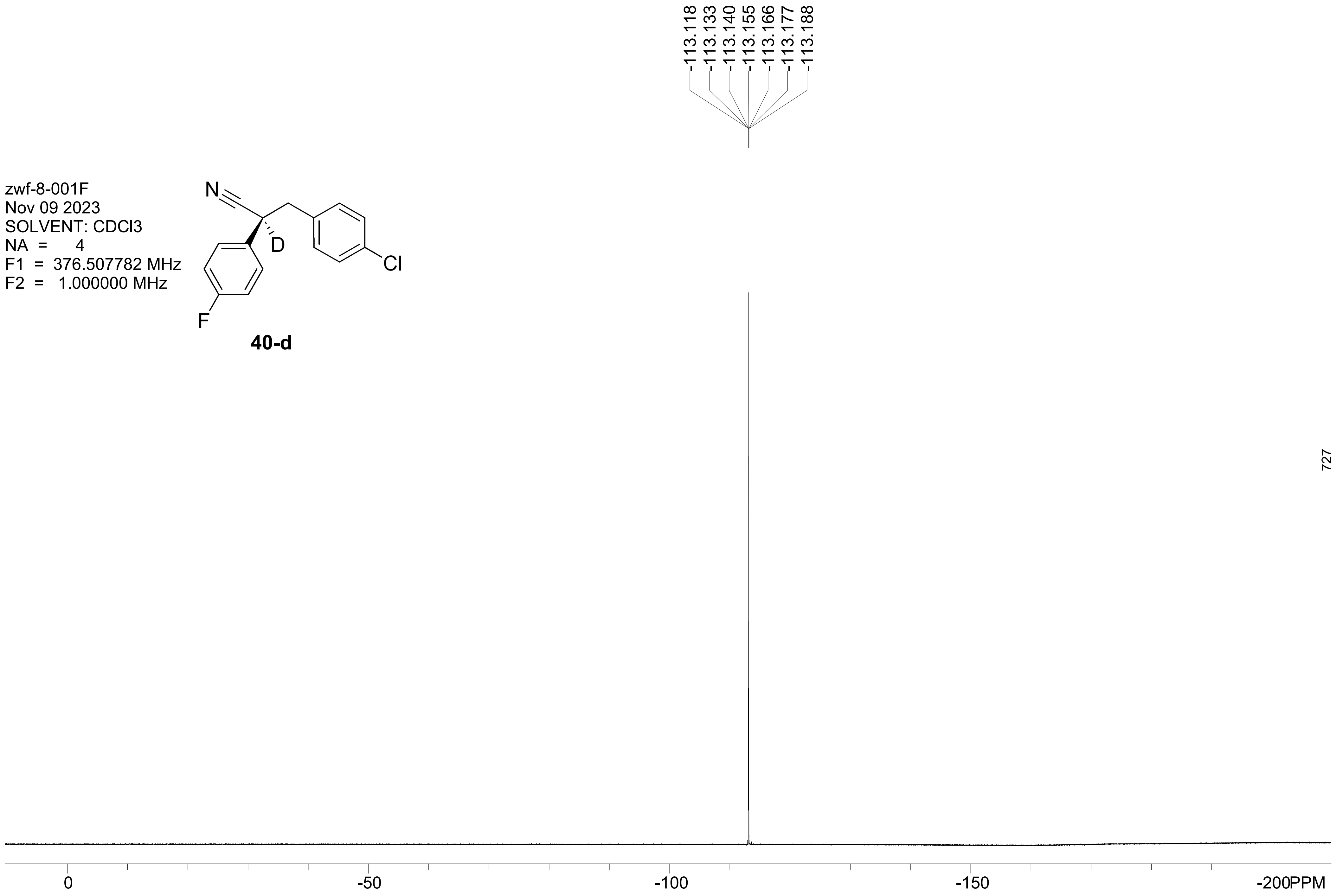
zwf-8-001F
Nov 09 2023
SOLVENT: CDCl3
NA = 4
F1 = 376.507782 MHz
F2 = 1.000000 MHz
-113.118
-113.133
-113.140
-113.155
-113.166
-113.177
-113.188
0
-50
-100
-150
-200PPM

40-d

Area Percent Report

Agilent Technologies

Sample name: zwf-8-001-IE-99-1-0.8-205

Data file: C:\Users\Public\Documents\ChemStation\1\Data\zwf\zwf-S 2023-11-11 19-01-34\005-P1-C3-zwf-8-001.D

Injection Acquired Date 11/11/2023 20:43:42

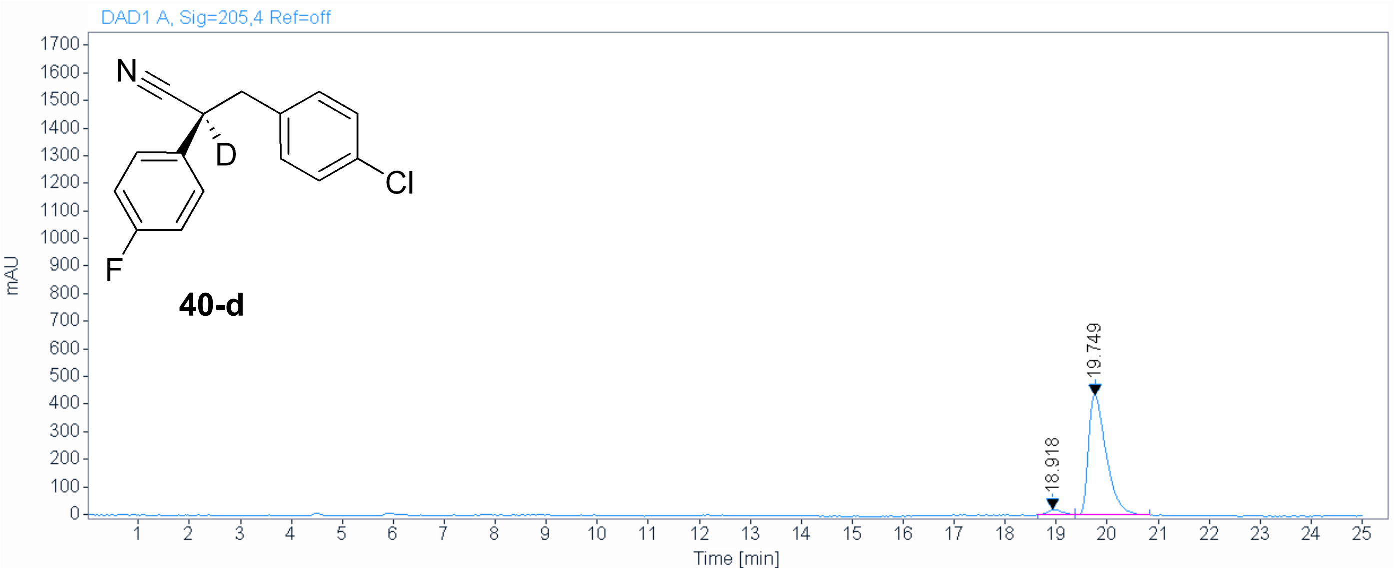

Signal: DAD1 A, Sig=205,4 Ref=off

RT [min]	Width [min]	Height	Area	Area%
18.918	0.3369	19.6671	397.5887	3.6440
19.749	0.3473	432.7063	10513.2461	96.3560
		Sum	10910.8348	100.0000

Area Percent Report

Sample name: zwf-8-001-rac-IE-99-1-0.8-205

Data file: C:\Users\Public\Documents\ChemStation\1\Data\zwf\zwf-S 2023-11-11 19-01-34\006-P1-C4-zwf-8-001-rac.D

Injection Acquired Date 11/11/2023 21:09:43

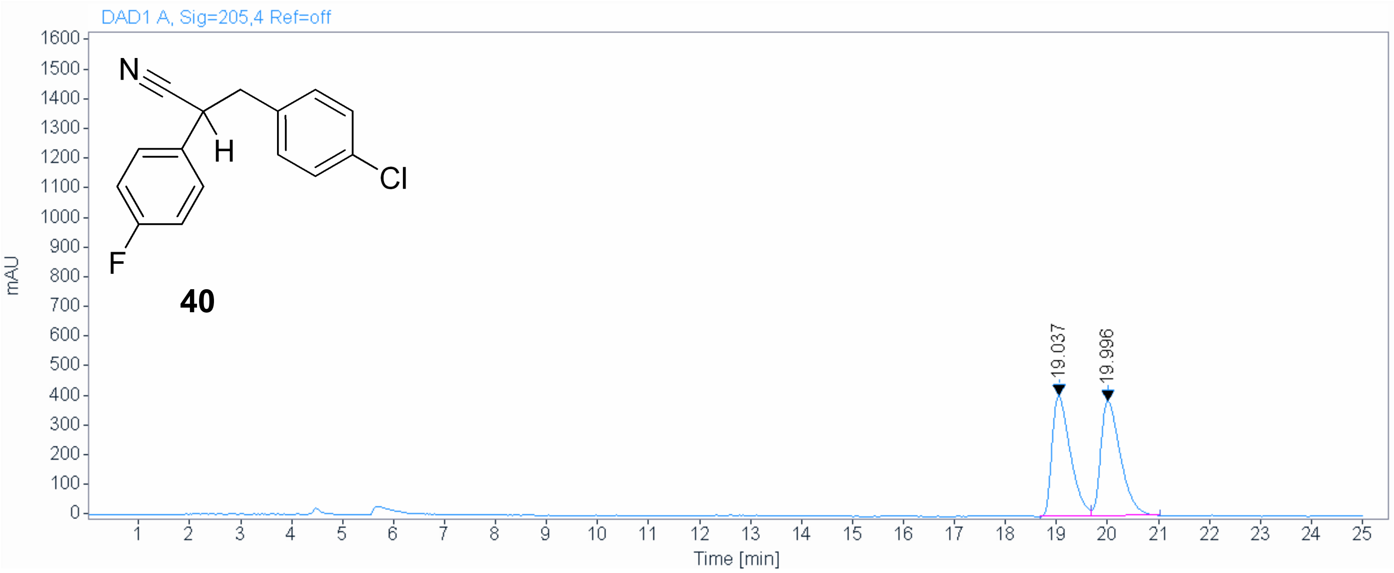

Signal: DAD1 A, Sig=205,4 Ref=off

RT [min]	Width [min]	Height	Area	Area%
19.037	0.3659	407.4520	10196.1729	49.3038
19.996	0.3615	387.4202	10484.1367	50.6962
		Sum	20680.3096	100.0000

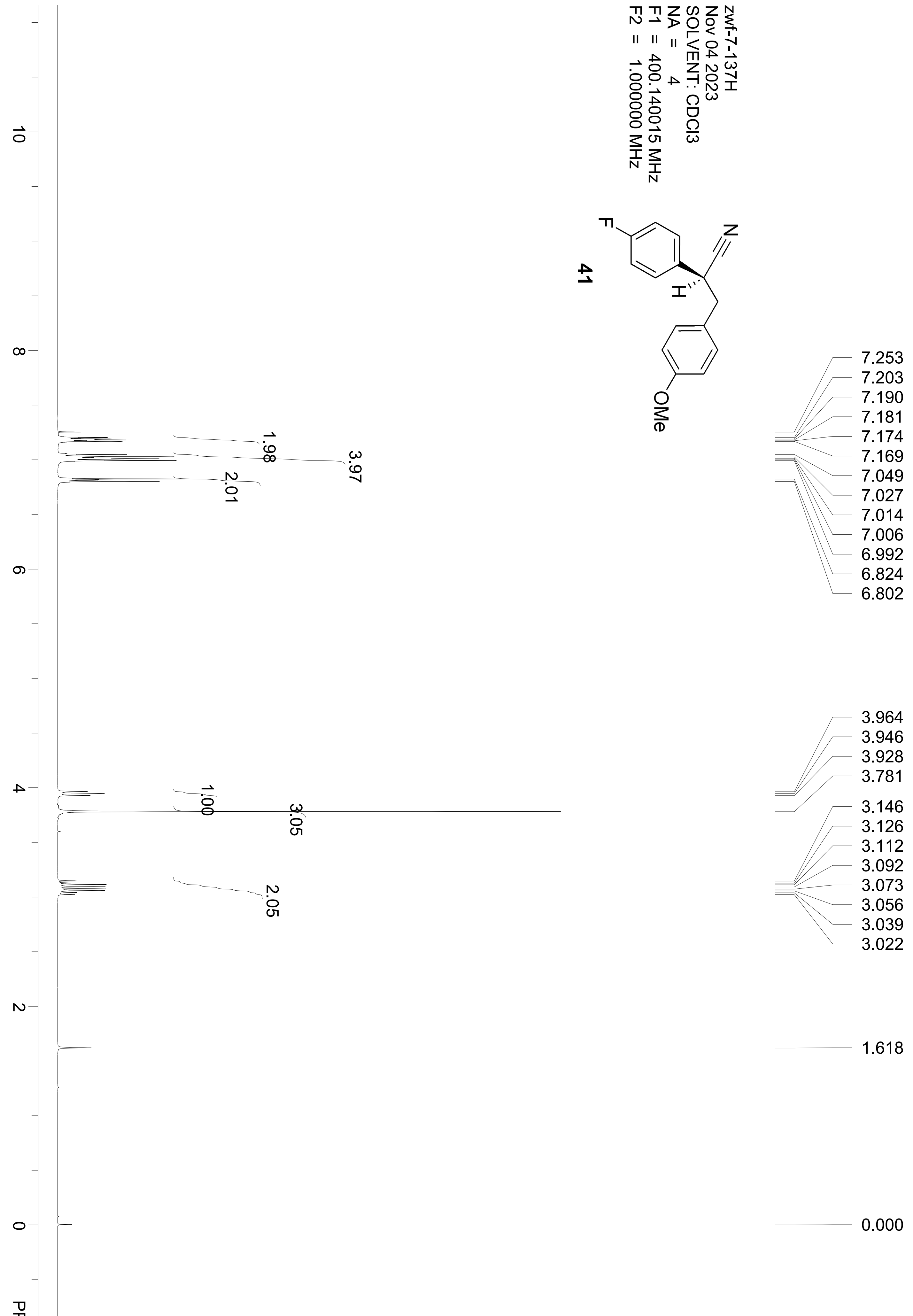
zwf-7-137H
Nov 04 2023
SOLVENT: CDCl3
NA = 4
F1 = 400.140015 MHz
F2 = 1.000000 MHz
N
H
F
OMe
41
7.253
7.203
7.190
7.181
7.174
7.169
7.049
7.027
7.014
7.006
6.992
6.824
6.802
3.964
3.946
3.928
3.781
3.146
3.126
3.112
3.092
3.073
3.056
3.039
3.022
1.618
0.000
1.98
3.97
2.01
1.00
3.05
2.05
10
8
6
4
2
0
PPM

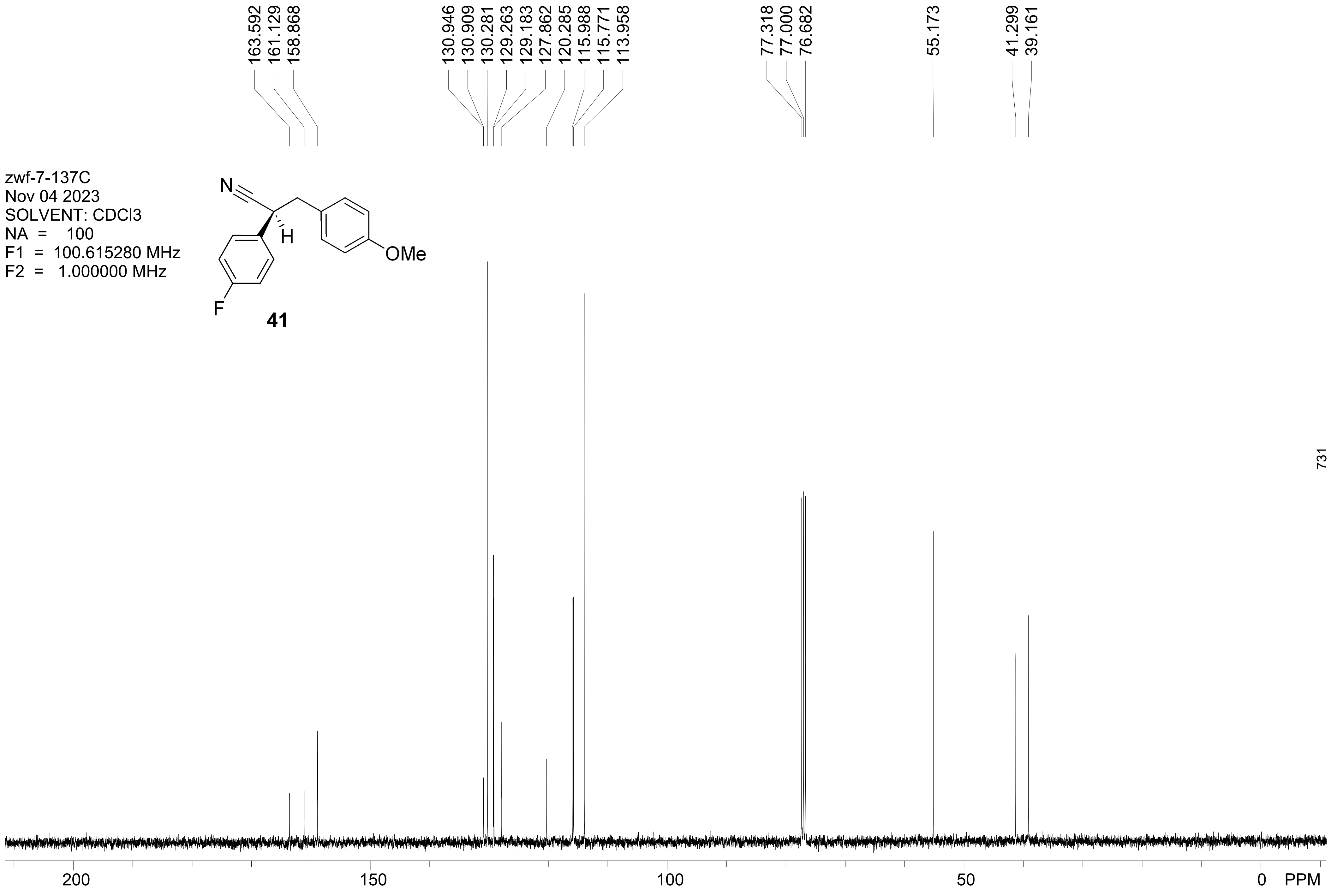
zwf-7-137C
Nov 04 2023
SOLVENT: CDCl3
NA = 100
F1 = 100.615280 MHz
F2 = 1.000000 MHz
N
H
F
OMe
41
163.592
161.129
158.868
130.946
130.909
130.281
129.263
129.183
127.862
120.285
115.988
115.771
113.958
77.318
77.000
76.682
55.173
41.299
39.161
200
150
100
50
0 PPM

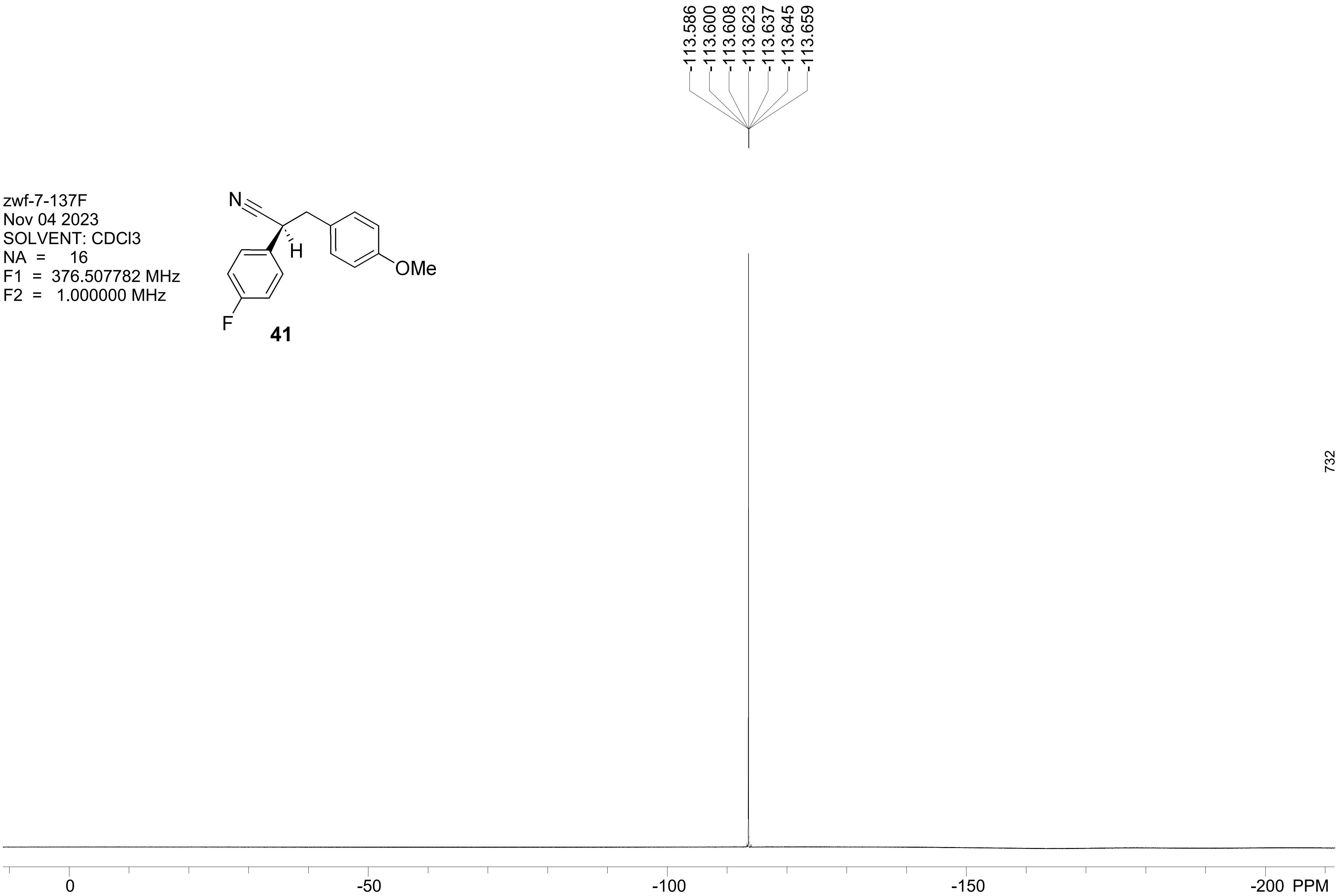
zwf-7-137F
Nov 04 2023
SOLVENT: CDCl3
NA = 16
F1 = 376.507782 MHz
F2 = 1.000000 MHz
41
-113.586
-113.600
-113.608
-113.623
-113.637
-113.645
-113.659
0
-50
-100
-150
-200 PPM

Area Percent Report

Agilent Technologies

Sample name: zwf-7-137-IE-99-1-0.8-205

Data file: C:\Users\Public\Documents\ChemStation\1\Data\zwf\zwf-S 2023-11-04 09-48-00\022-P1-C5-zwf-7-137.D

Injection Acquired Date 11/04/2023 21:10:55

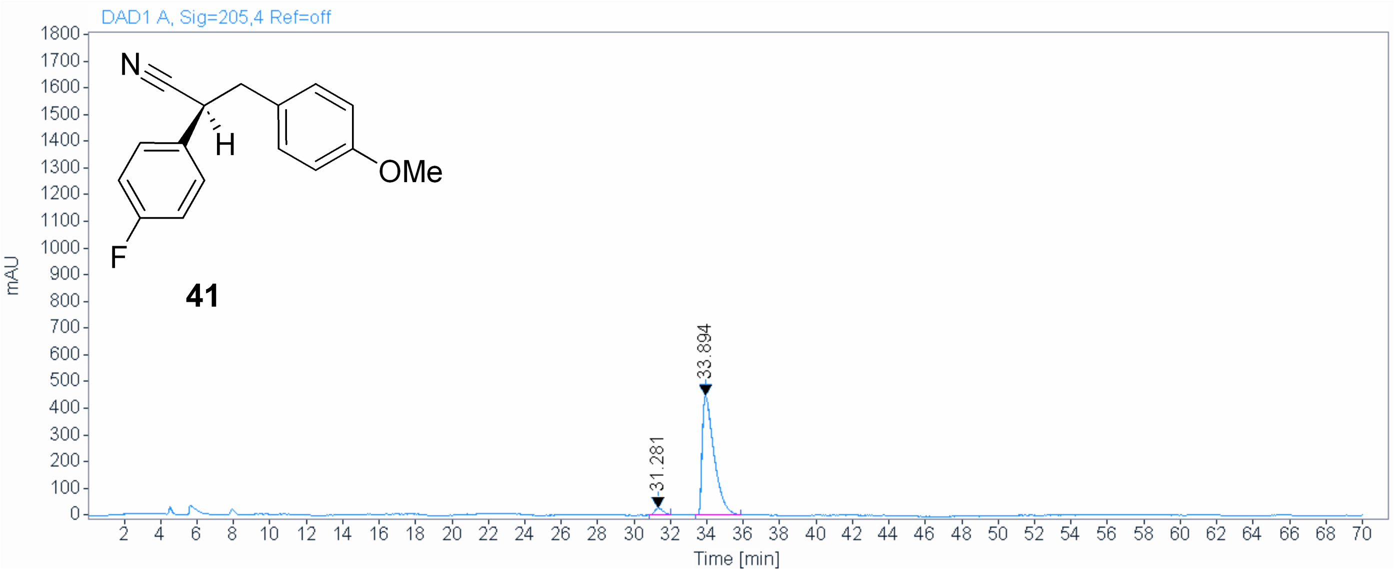

Signal: DAD1 A, Sig=205,4 Ref=off

RT [min]	Width [min]	Height	Area	Area%
31.281	0.5307	25.9764	827.2120	3.9479
33.894	0.7513	446.4782	20125.9316	96.0521
		Sum	20953.1436	100.0000

Area Percent Report

Agilent Technologies

Sample name: zwf-7-137-rac-IE-99-1-0.8-205

Data file: C:\Users\Public\Documents\ChemStation\1\Data\zwf\zwf-S 2023-11-04 09-48-00\023-P1-C6-zwf-7-137-rac.D

Injection Acquired Date 11/04/2023 22:22:00

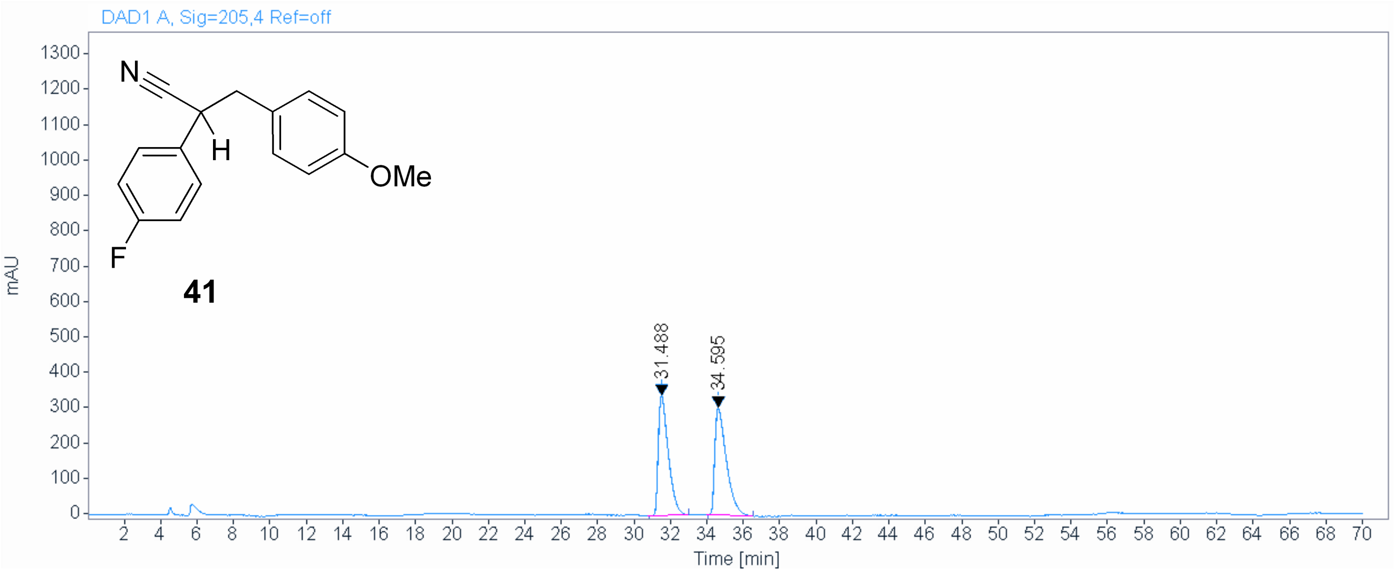

Signal: DAD1 A, Sig=205,4 Ref=off

RT [min]	Width [min]	Height	Area	Area%
31.488	0.5252	339.1407	13007.3623	49.1268
34.595	0.5981	304.4769	13469.7803	50.8732
		Sum	26477.1426	100.0000

zwf-7-156H
Nov 10 2023
SOLVENT: CDCl3
NA = 4
F1 = 400.140015 MHz
F2 = 1.000000 MHz

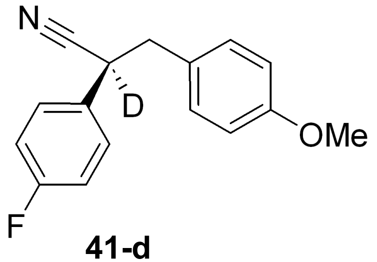

41-d

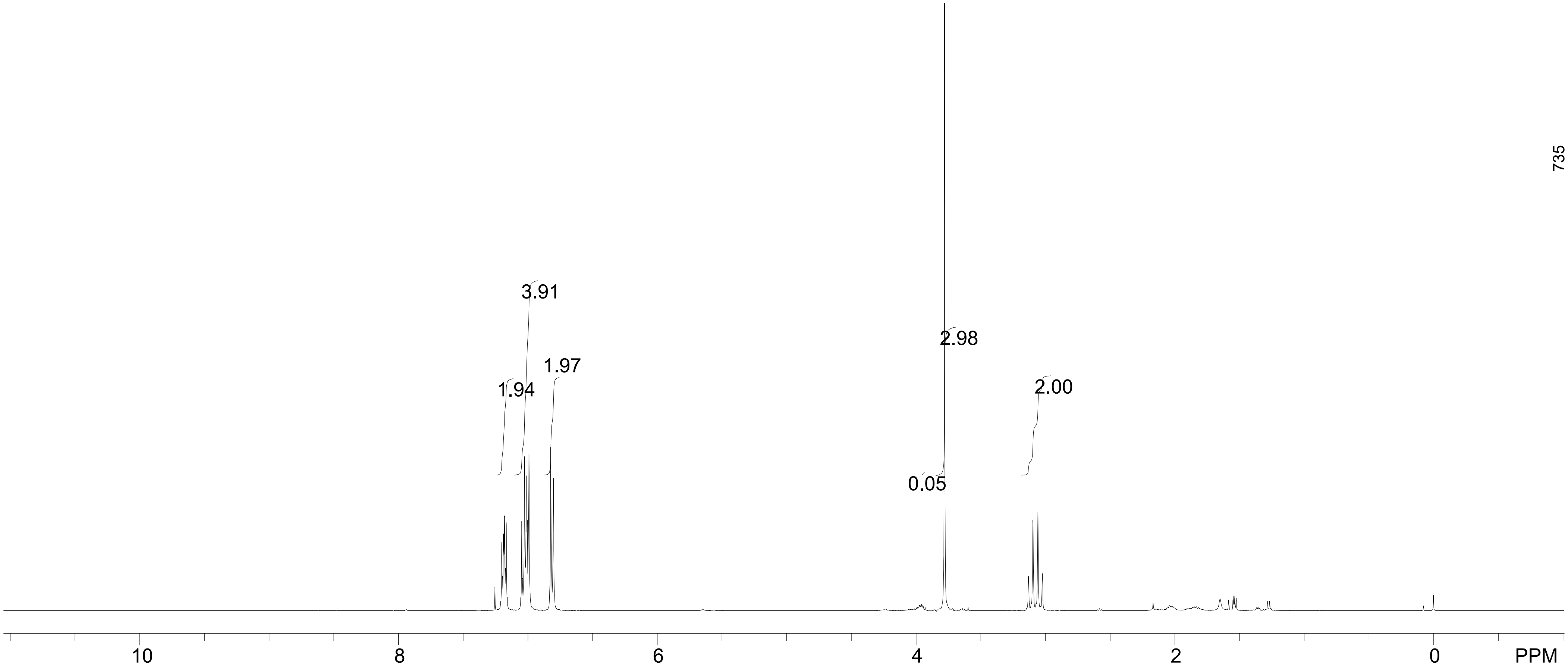

zwf-7-156C
Nov 10 2023
SOLVENT: CDCl3
NA = 150
F1 = 100.615280 MHz
F2 = 1.000000 MHz

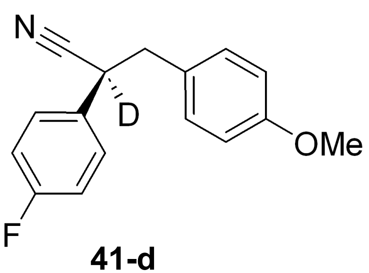

41-d

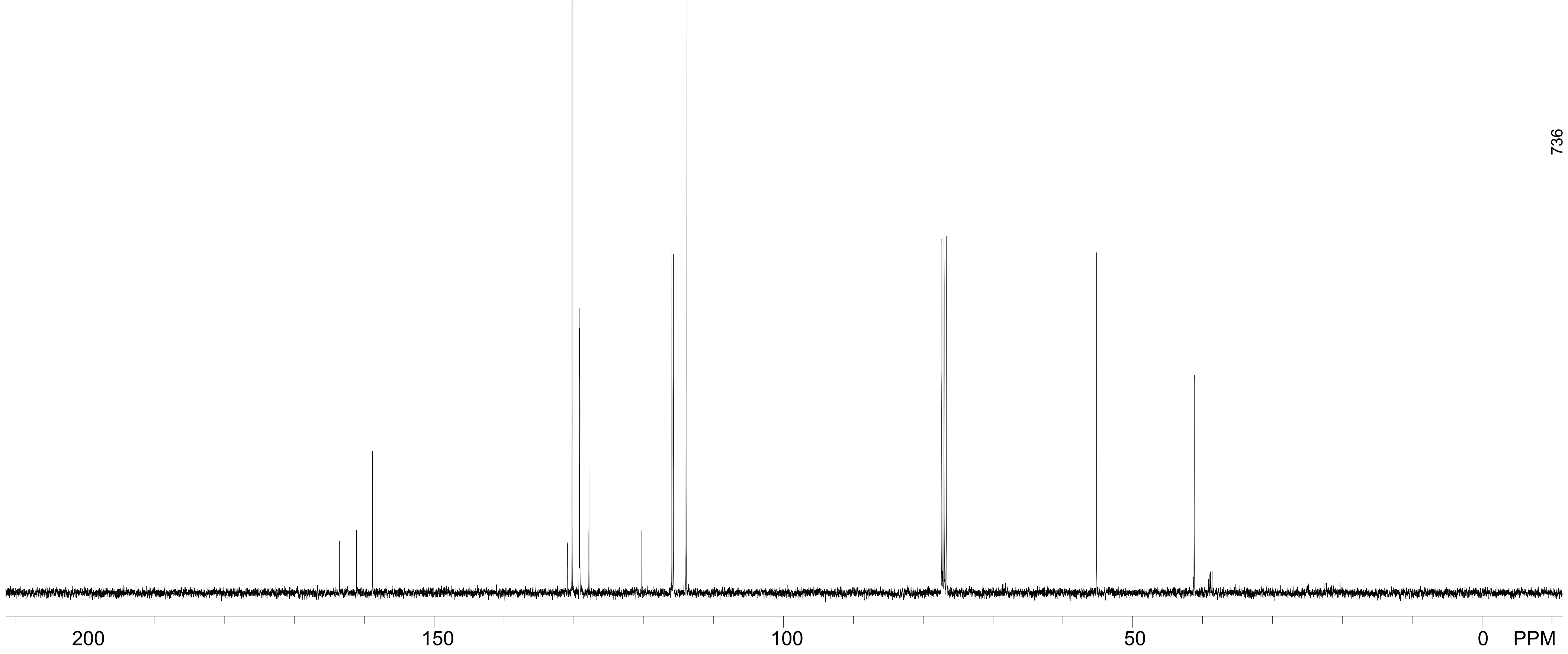

zwf-7-156F
Nov 10 2023
SOLVENT: CDCl3
NA =   4
F1 = 376.507782 MHz
F2 =   1.000000 MHz

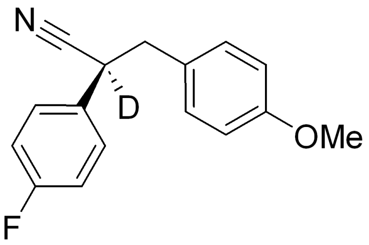

41-d

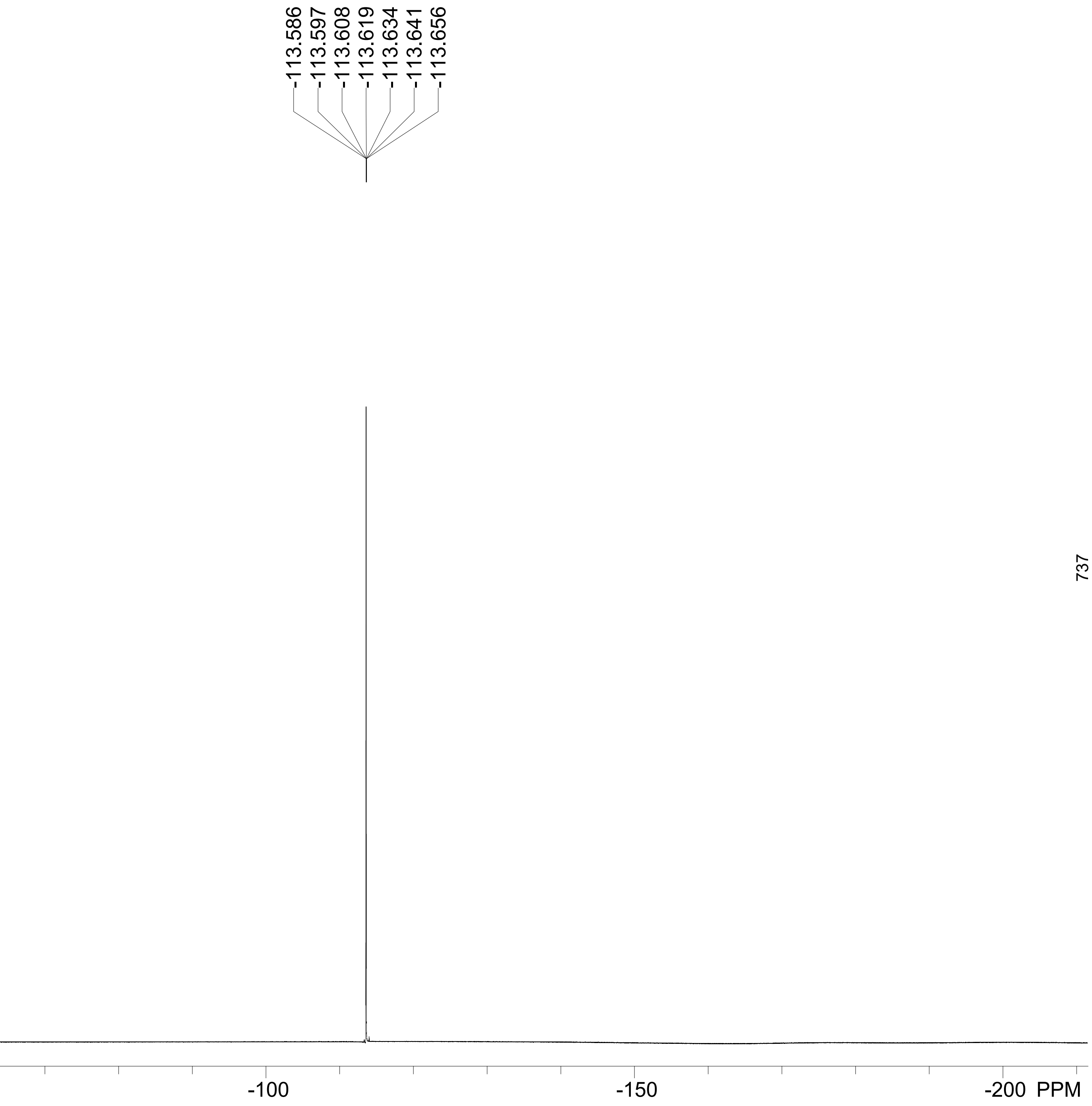

Area Percent Report

Agilent Technologies

Sample name: zwf-7-156-IE-99-1-0.8-205

Data file: C:\Users\Public\Documents\ChemStation\1\Data\zwf\zwf-S 2023-11-12 11-02-17\013-P1-C3-zwf-7-156.D

Injection Acquired Date 11/12/2023 20:20:35

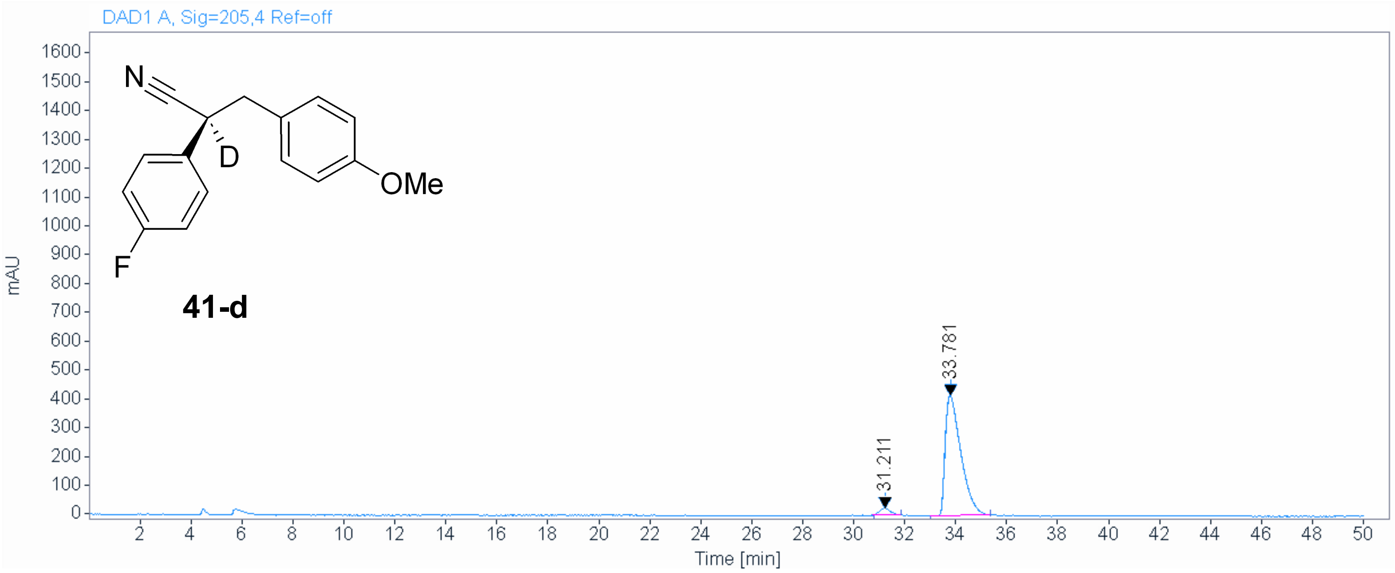

Signal: DAD1 A, Sig=205,4 Ref=off

RT [min]	Width [min]	Height	Area	Area%
31.211	0.4972	23.8854	712.5997	3.8347
33.781	0.5134	416.9475	17870.2969	96.1653
		Sum	18582.8965	100.0000

Area Percent Report

Sample name: zwf-7-156-rac-IE-99-1-0.8-205

Data file: C:\Users\Public\Documents\ChemStation\1\Data\zwf\zwf-S 2023-11-12 11-02-17\014-P1-C4-zwf-7-156-rac.D

Injection Acquired Date 11/12/2023 21:11:38

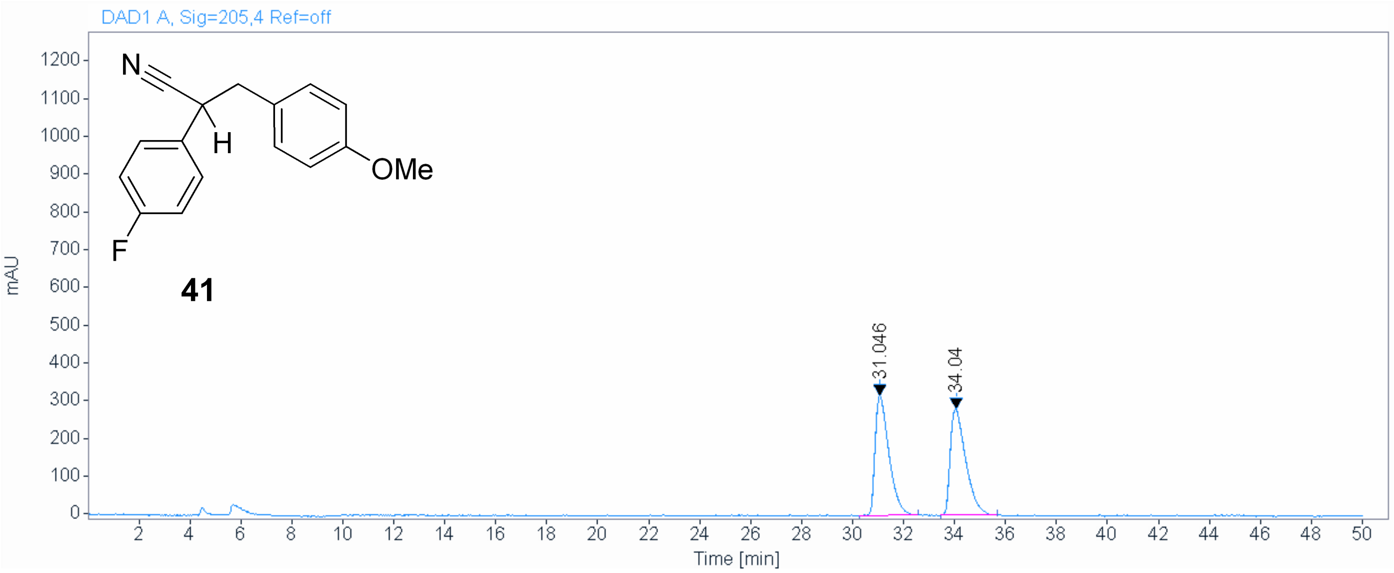

Signal: DAD1 A, Sig=205,4 Ref=off

RT [min]	Width [min]	Height	Area	Area%
31.046	0.4865	318.1661	12087.6719	50.1153
34.040	0.5193	283.0215	12032.0391	49.8847
		Sum	24119.7109	100.0000

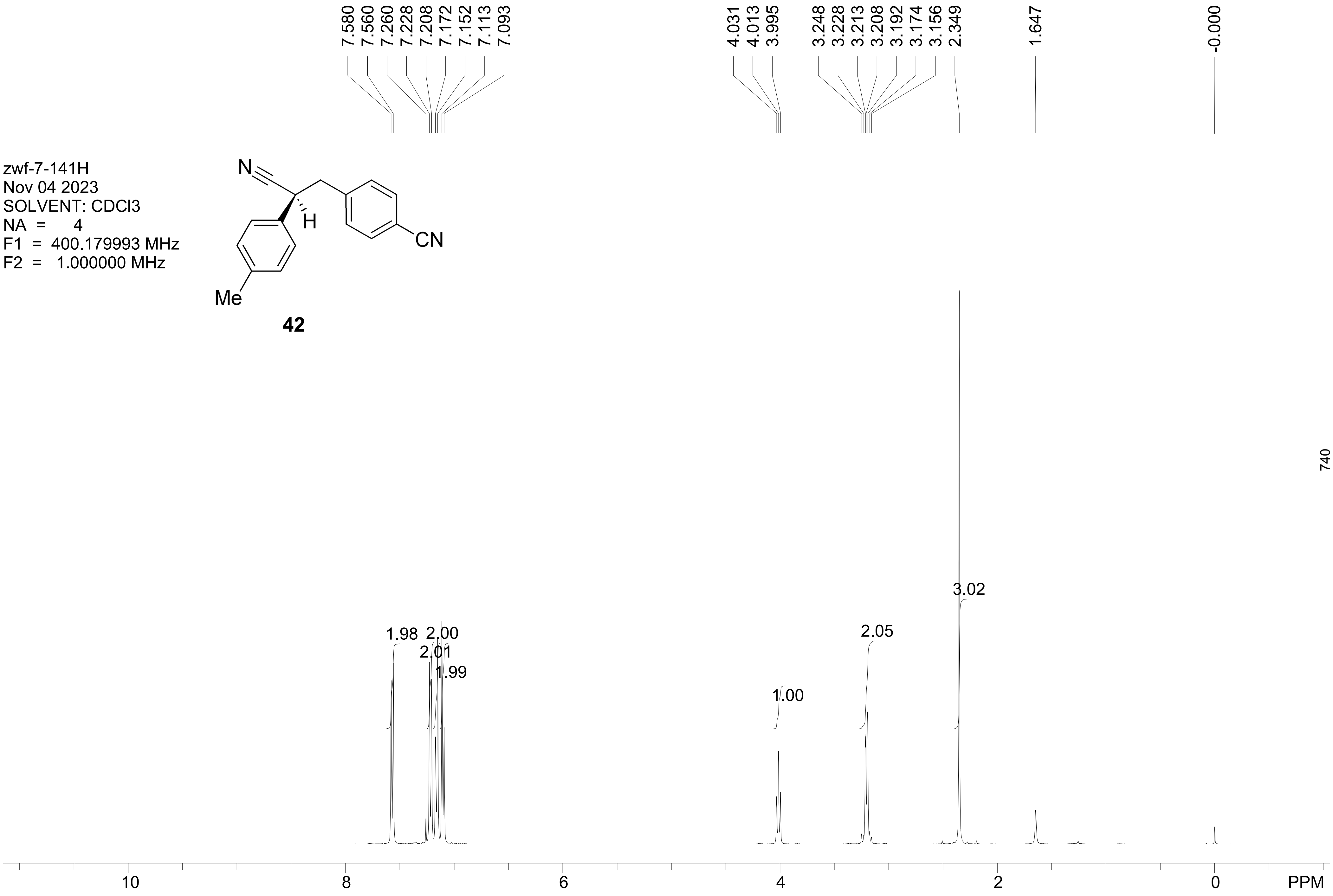
zwf-7-141H
Nov 04 2023
SOLVENT: CDCl3
NA = 4
F1 = 400.179993 MHz
F2 = 1.000000 MHz
N
H
CN
Me
7.580
7.560
7.260
7.228
7.208
7.172
7.152
7.113
7.093
4.031
4.013
3.995
3.248
3.228
3.213
3.208
3.192
3.174
3.156
2.349
1.647
-0.000
1.98
2.00
2.01
1.99
1.00
2.05
3.02
10
8
6
4
2
0
PPM

42

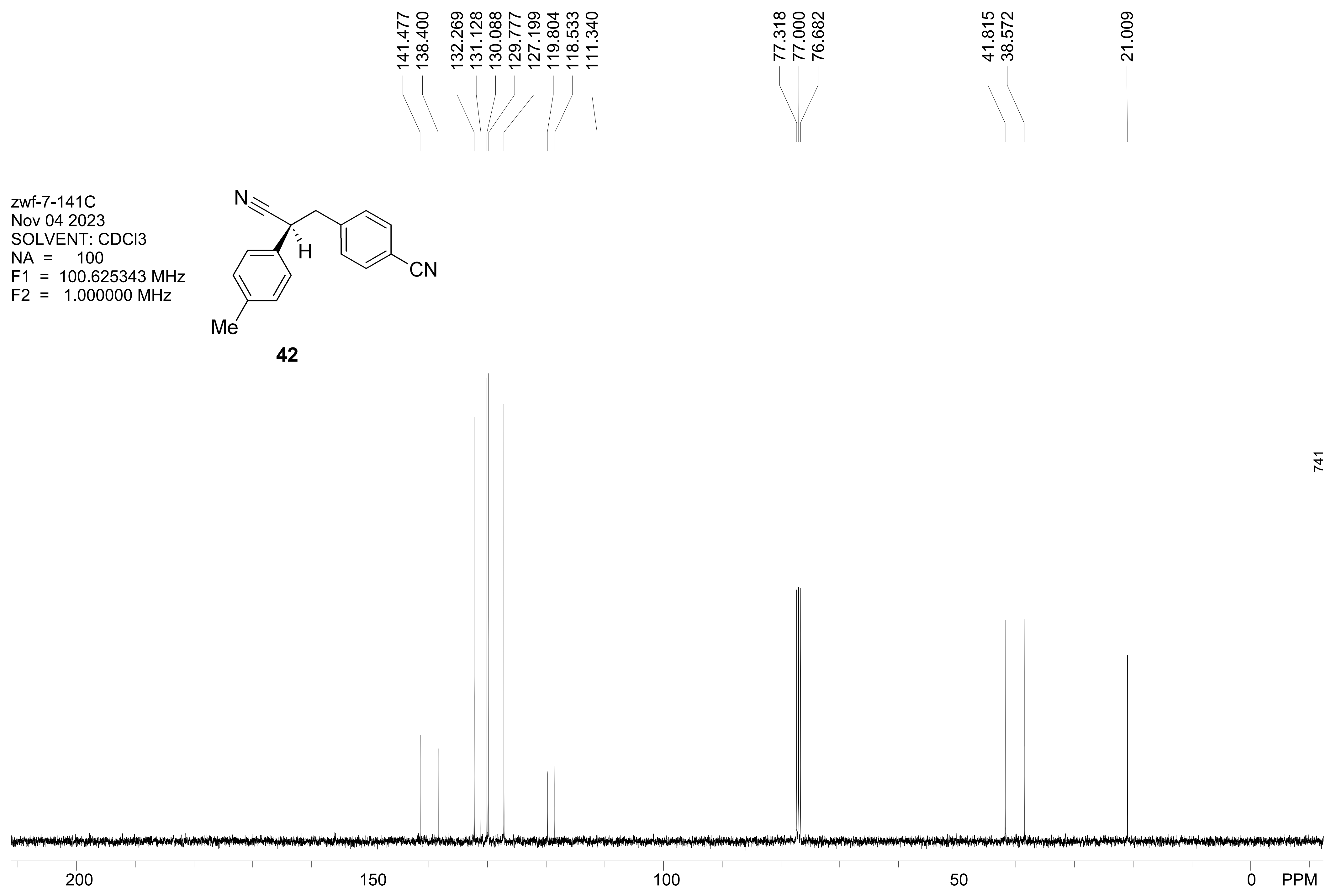
141.477
138.400
132.269
131.128
130.088
129.777
127.199
119.804
118.533
111.340
77.318
77.000
76.682
41.815
38.572
21.009
zwf-7-141C
Nov 04 2023
SOLVENT: CDCl3
NA = 100
F1 = 100.625343 MHz
F2 = 1.000000 MHz
N
H
CN
Me
42
200
150
100
50
0
PPM

Area Percent Report

Sample name: zwf-7-141-IE-90-10-1.1-205

Data file: C:\Users\Public\Documents\ChemStation\1\Data\zwf\zwf-S 2023-11-04 09-48-00\008-P1-B1-zwf-7-141.D

Injection Acquired Date 11/04/2023 12:21:19

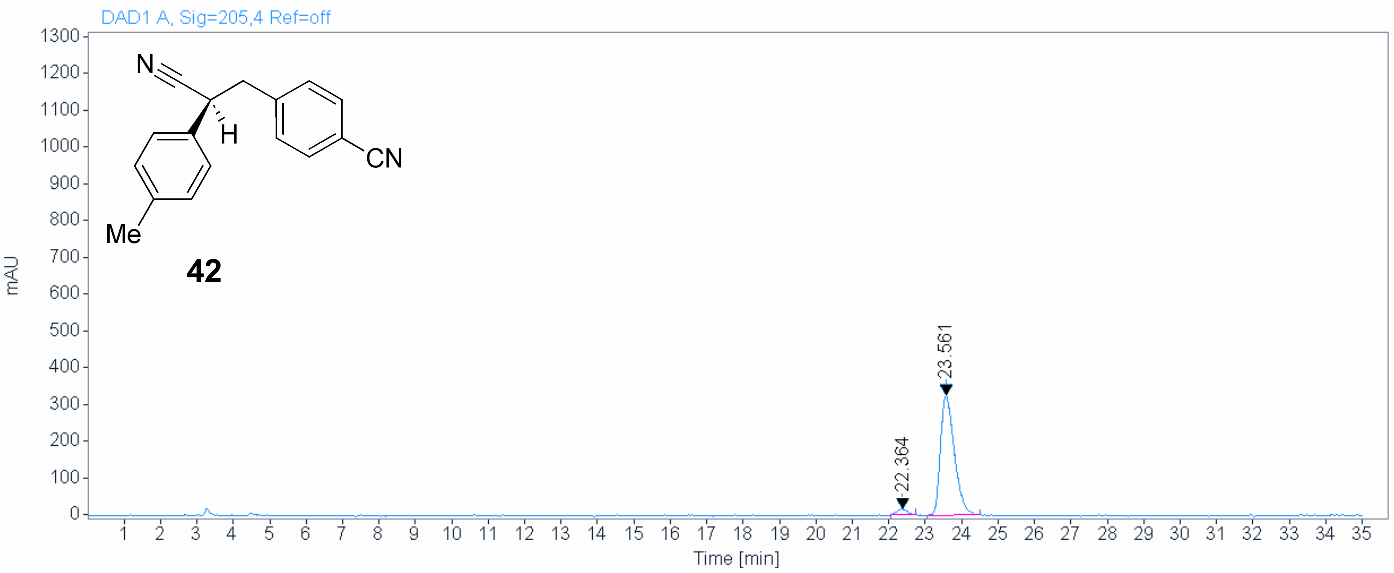

Signal: DAD1 A, Sig=205,4 Ref=off

RT [min]	Width [min]	Height	Area	Area%
22.364	0.3331	15.8476	316.7082	3.5105
23.561	0.3554	326.1116	8705.1123	96.4895
		Sum	9021.8205	100.0000

Area Percent Report

Agilent Technologies

Sample name: zwf-7-141-rac-IE-90-10-1.1-205

Data file: C:\Users\Public\Documents\ChemStation\1\Data\zwf\zwf-S 2023-11-04 09-48-00\009-P1-B2-zwf-7-141-rac.D

Injection Acquired Date 11/04/2023 12:57:23

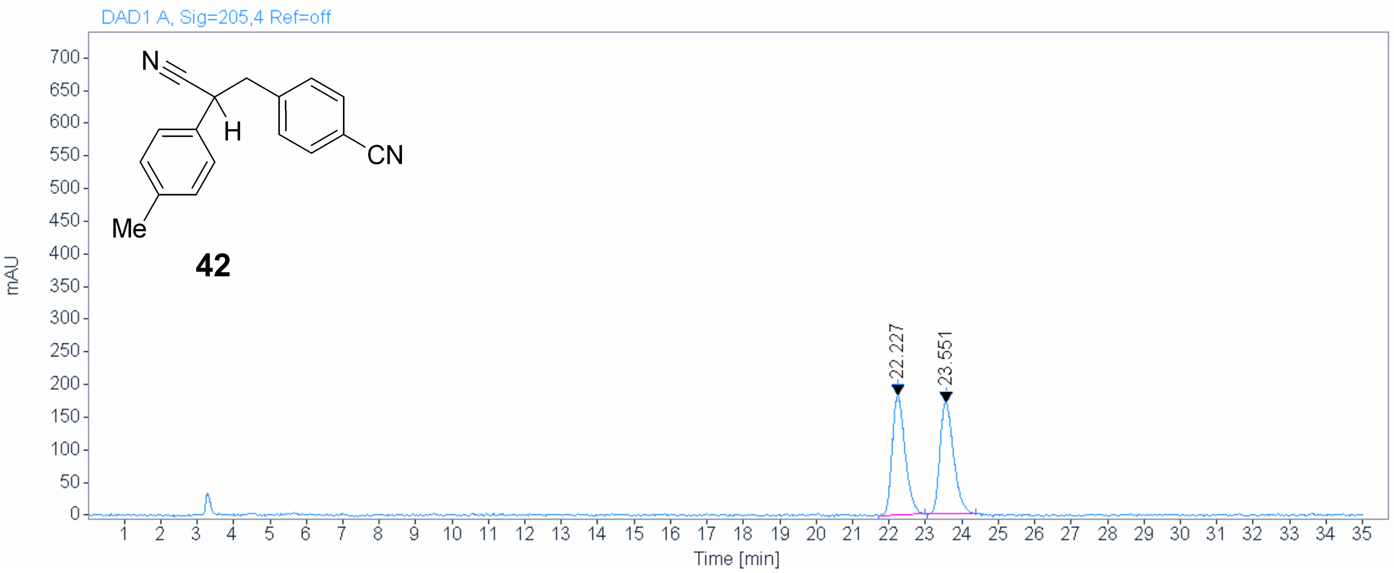

Signal: DAD1 A, Sig=205,4 Ref=off

RT [min]	Width [min]	Height	Area	Area%
22.227	0.3476	183.4779	4574.1641	50.1011
23.551	0.3341	170.9896	4555.7056	49.8989
		Sum	9129.8696	100.0000

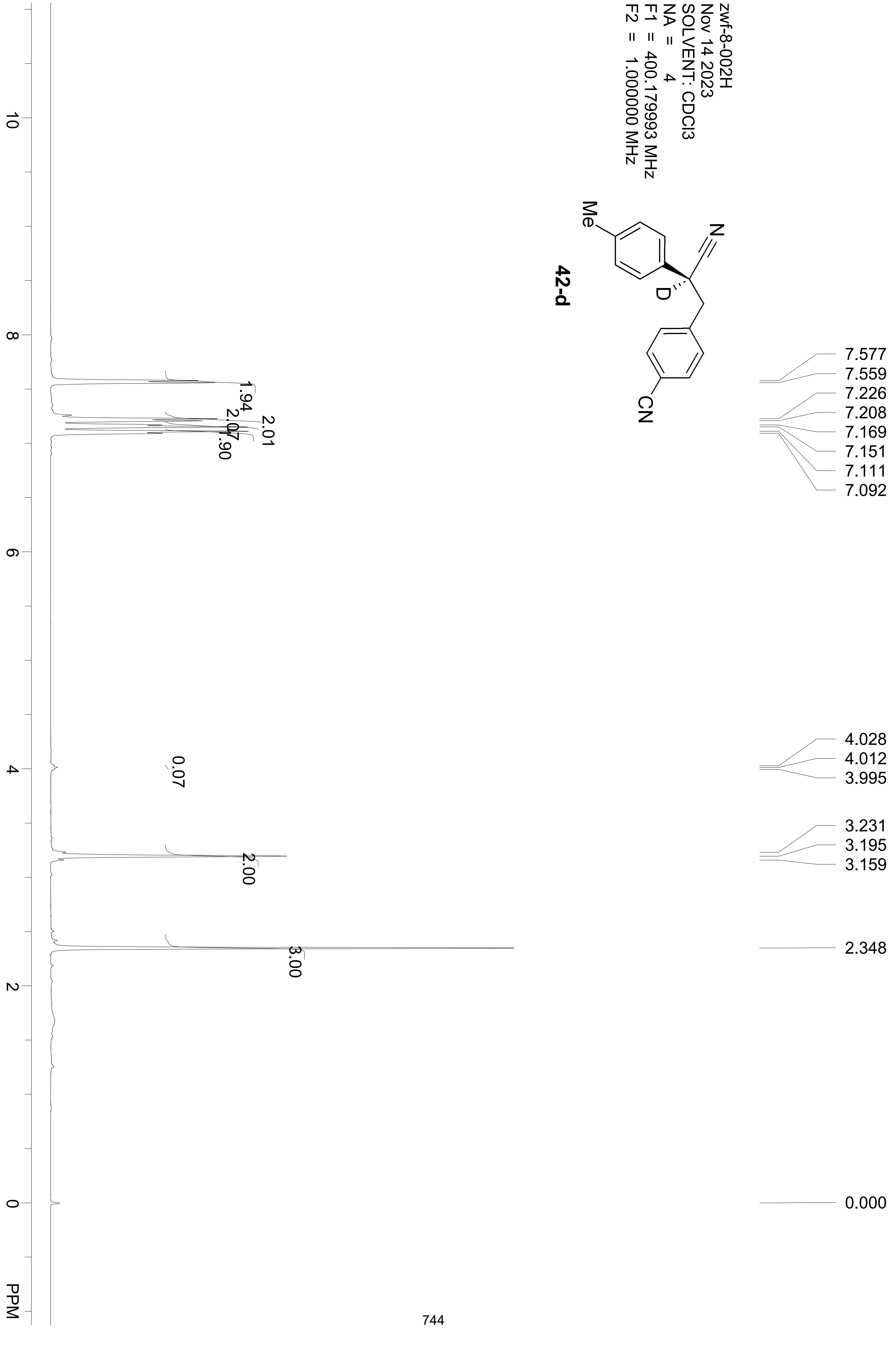
zwf-8-002H
Nov 14 2023
SOLVENT: CDCl3
NA = 4
F1 = 400.179993 MHz
F2 = 1.000000 MHz
42-d
Me
N
D
CN
7.577
7.559
7.226
7.208
7.169
7.151
7.111
7.092
4.028
4.012
3.995
3.231
3.195
3.159
2.348
0.000
1.94
2.01
2.07
1.90
0.07
2.00
3.00
10
8
6
4
2
0
PPM

zwf-8-002C
Nov 14 2023
SOLVENT: CDCl3
NA = 100
F1 = 100.625343 MHz
F2 = 1.000000 MHz

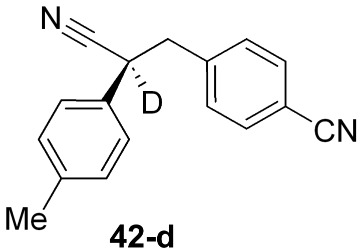

42-d

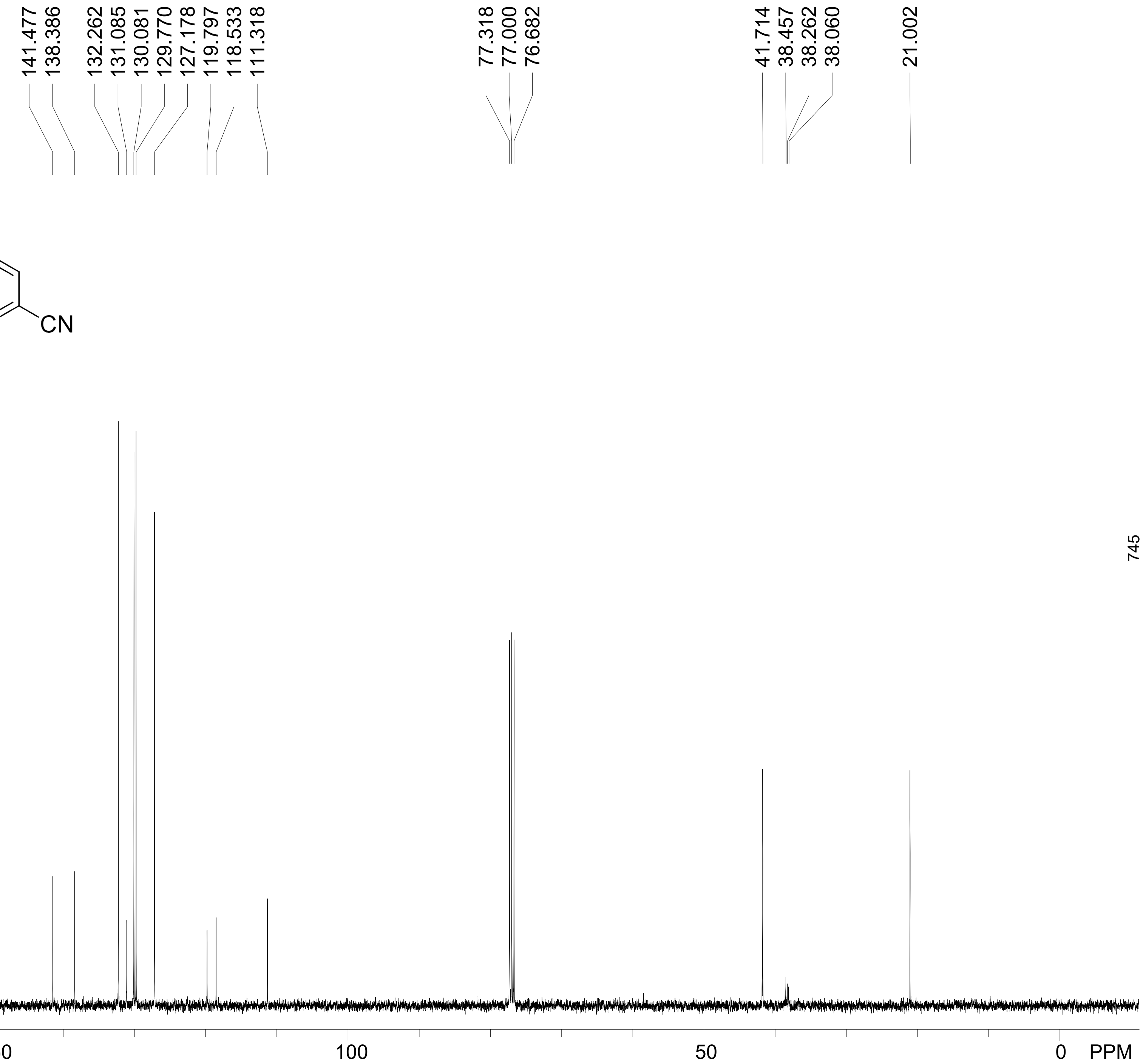

Area Percent Report

Agilent Technologies

Sample name: zwf-8-002-IE-90-10-1.1-205

Data file: C:\Users\Public\Documents\ChemStation\1\Data\zwf\zwf-S 2023-11-14 20-02-58\006-P1-B5-zwf-8-002.D

Injection Acquired Date 11/14/2023 22:09:11

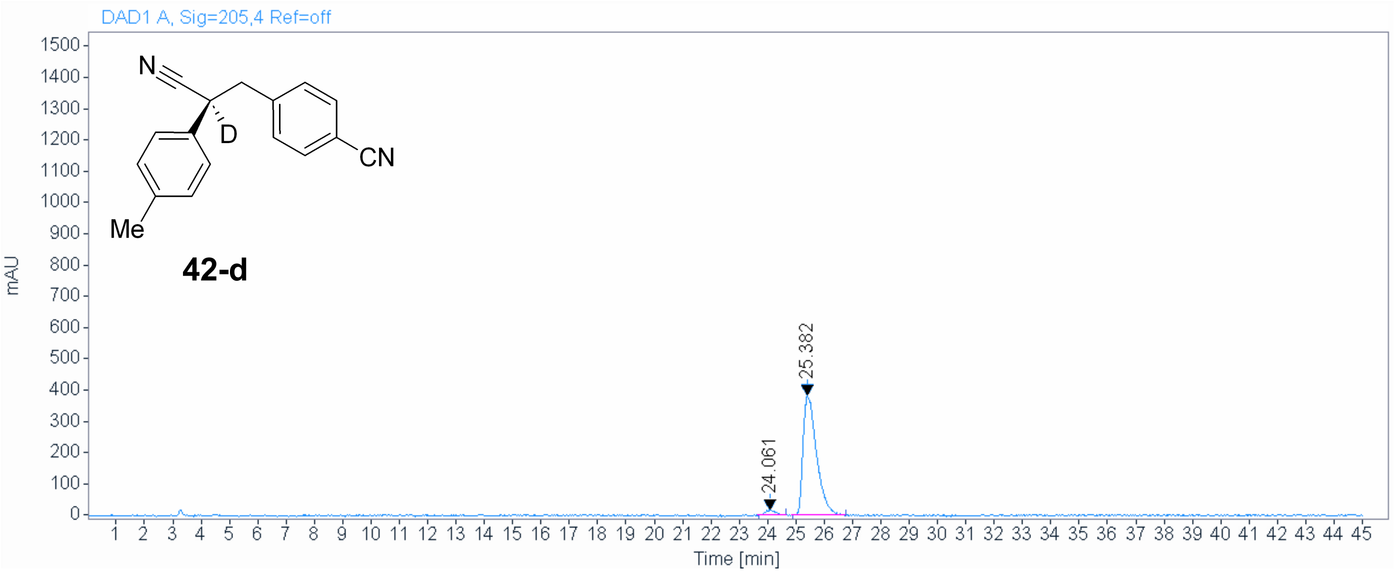

Signal: DAD1 A, Sig=205,4 Ref=off

RT [min]	Width [min]	Height	Area	Area%
24.061	0.4232	16.2230	411.9297	3.1067
25.382	0.5584	383.4370	12847.3438	96.8933
		Sum	13259.2735	100.0000

Area Percent Report

Agilent Technologies

Sample name: zwf-8-002-rac-IE-90-10-1.1-205

Data file: C:\Users\Public\Documents\ChemStation\1\Data\zwf\zwf-S 2023-11-14 20-02-58\007-P1-B6-zwf-8-002-rac.D

Injection Acquired Date 11/14/2023 22:55:18

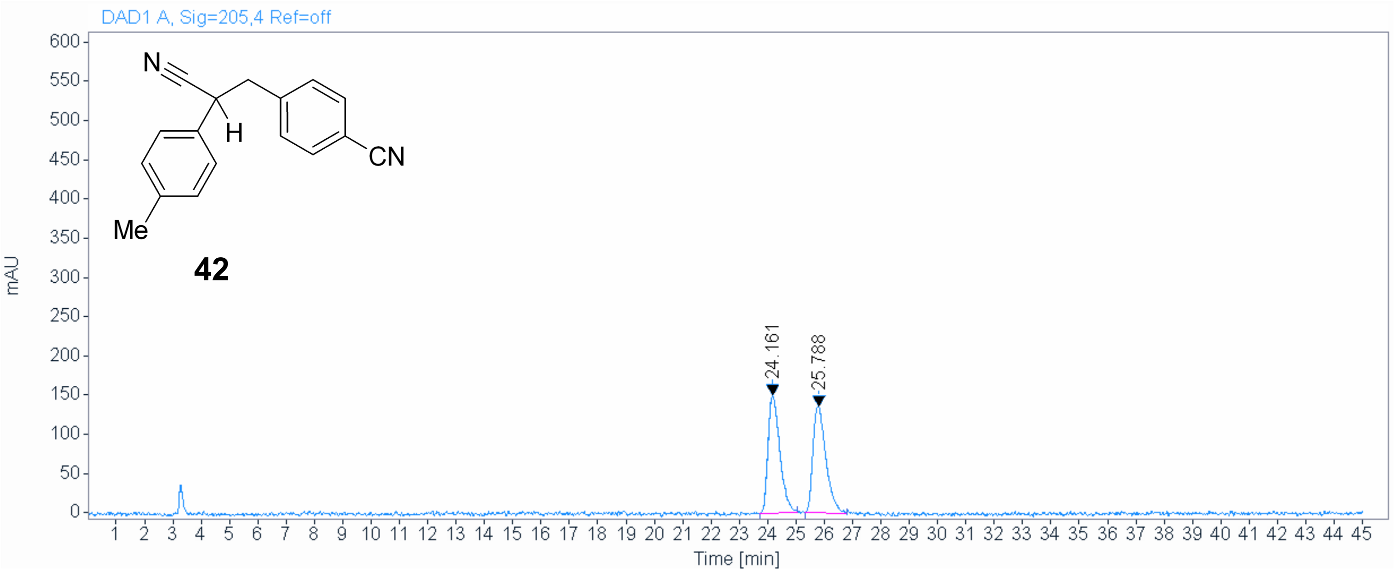

Signal: DAD1 A, Sig=205,4 Ref=off

RT [min]	Width [min]	Height	Area	Area%
24.161	0.3520	150.2414	4391.1470	49.8880
25.788	0.5387	136.4572	4410.8677	50.1120
		Sum	8802.0146	100.0000

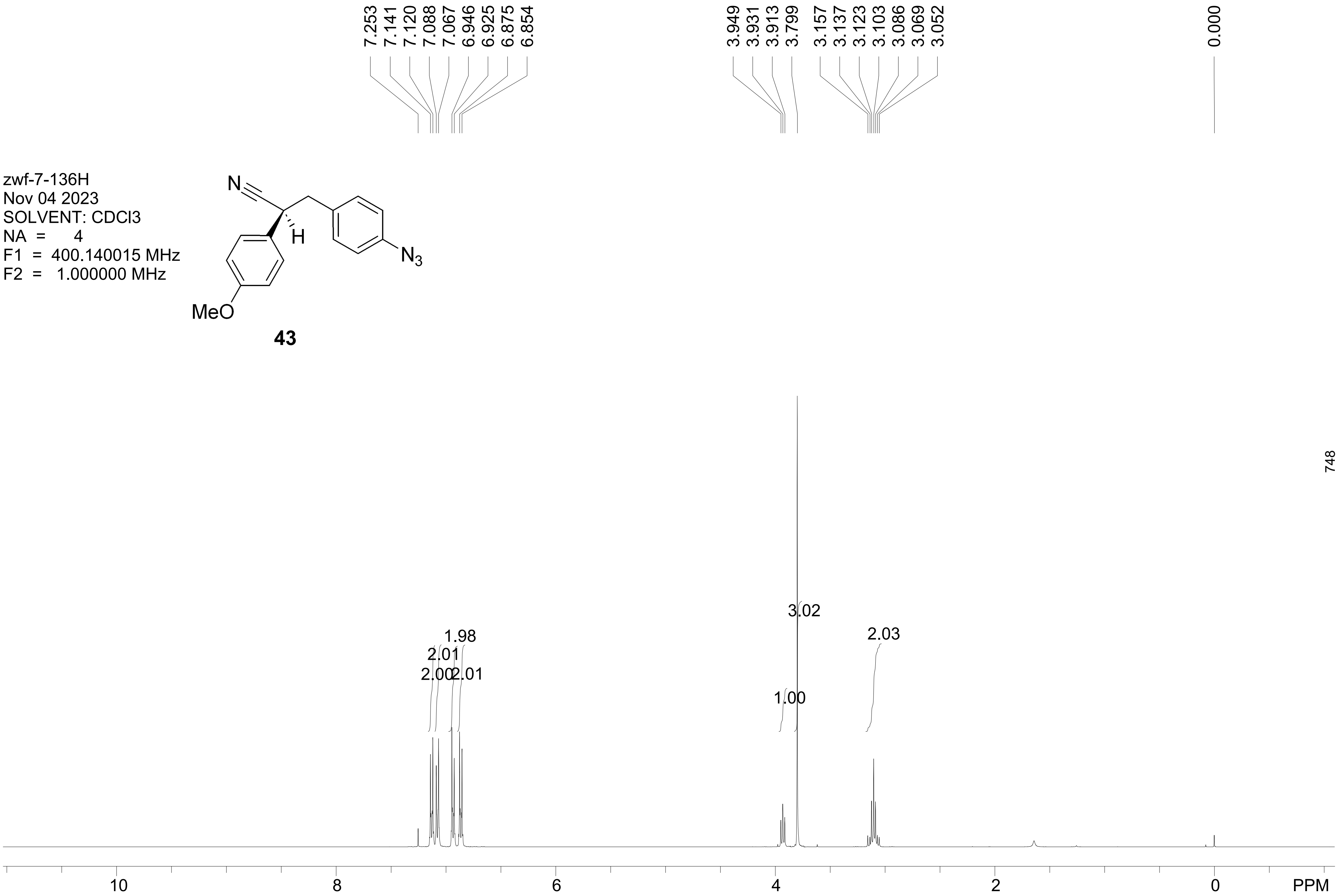
zwf-7-136H
Nov 04 2023
SOLVENT: CDCl3
NA = 4
F1 = 400.140015 MHz
F2 = 1.000000 MHz
N
H
N3
MeO
43
7.253
7.141
7.120
7.088
7.067
6.946
6.925
6.875
6.854
3.949
3.931
3.913
3.799
3.157
3.137
3.123
3.103
3.086
3.069
3.052
0.000
1.98
2.01
2.00
2.01
3.02
1.00
2.03
10
8
6
4
2
0
PPM

zwf-7-136C
Nov 04 2023
SOLVENT: CDCl3
NA =   100
F1 = 100.615280 MHz
F2 =   1.000000 MHz

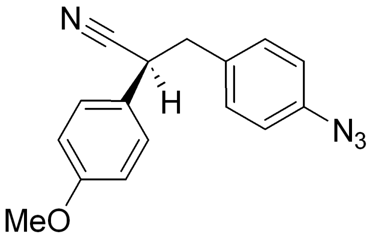

43

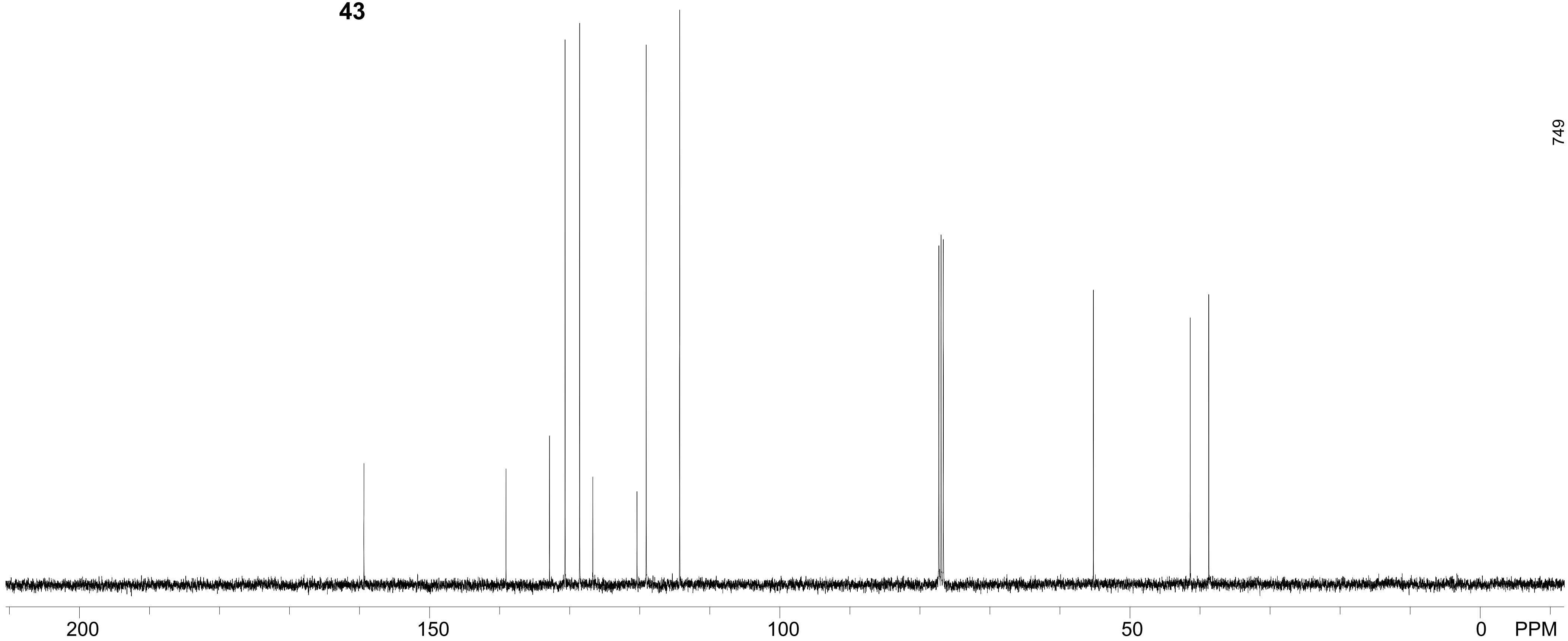

Area Percent Report

Agilent Technologies

Sample name: zwf-7-136-IE-99-1-0.8-205

Data file: C:\Users\Public\Documents\ChemStation\1\Data\zwf\zwf-S 2023-11-04 09-48-00\020-P1-C3-zwf-7-136.D

Injection Acquired Date 11/04/2023 18:48:46

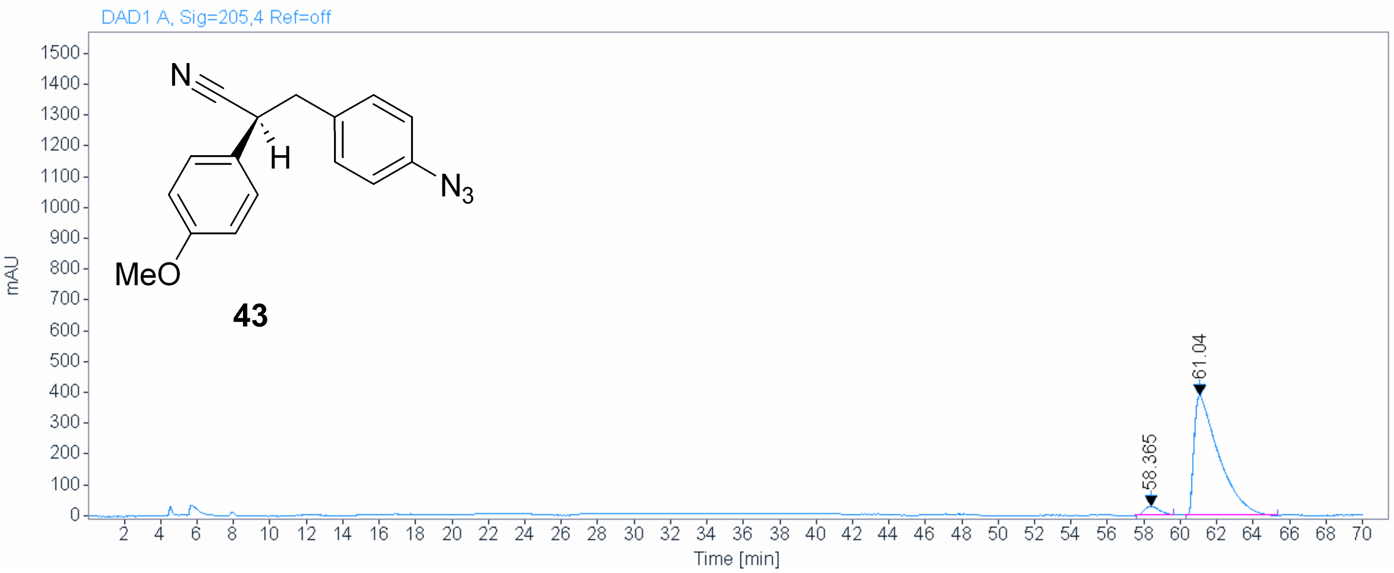

Signal: DAD1 A, Sig=205,4 Ref=off

RT [min]	Width [min]	Height	Area	Area%
58.365	0.9289	28.2356	1573.7017	4.1198
61.040	1.5758	387.3678	36625.0391	95.8802
		Sum	38198.7407	100.0000

Area Percent Report

Sample name: zwf-7-136-rac-IE-99-1-0.8-205

Data file: C:\Users\Public\Documents\ChemStation\1\Data\zwf\zwf-S 2023-11-04 09-48-00\021-P1-C4-zwf-7-136-rac.D

Injection Acquired Date 11/04/2023 19:59:50

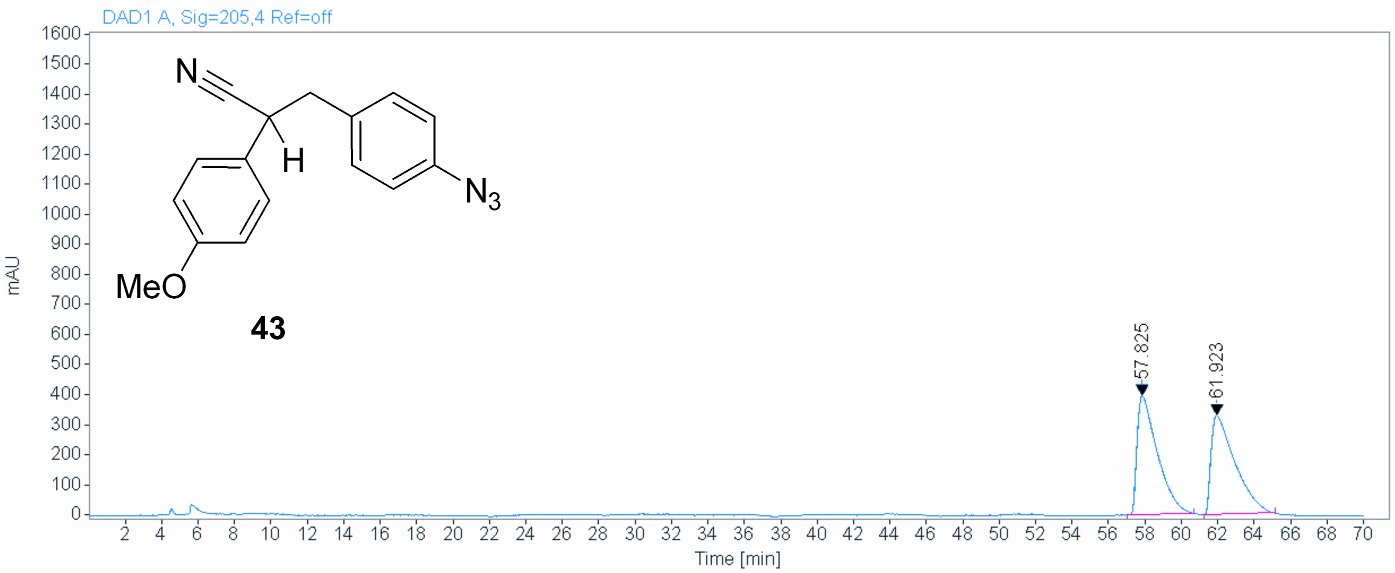

Signal: DAD1 A, Sig=205,4 Ref=off

RT [min]	Width [min]	Height	Area	Area%
57.825	0.9303	397.5055	30956.9512	50.3957
61.923	1.0857	331.5714	30470.7539	49.6043
		Sum	61427.7051	100.0000

zwf-7-155H
Nov 10 2023
SOLVENT: CDCl3
NA = 4
F1 = 400.140015 MHz
F2 = 1.000000 MHz

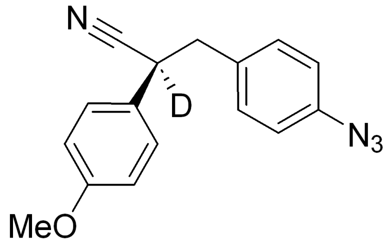

43-d

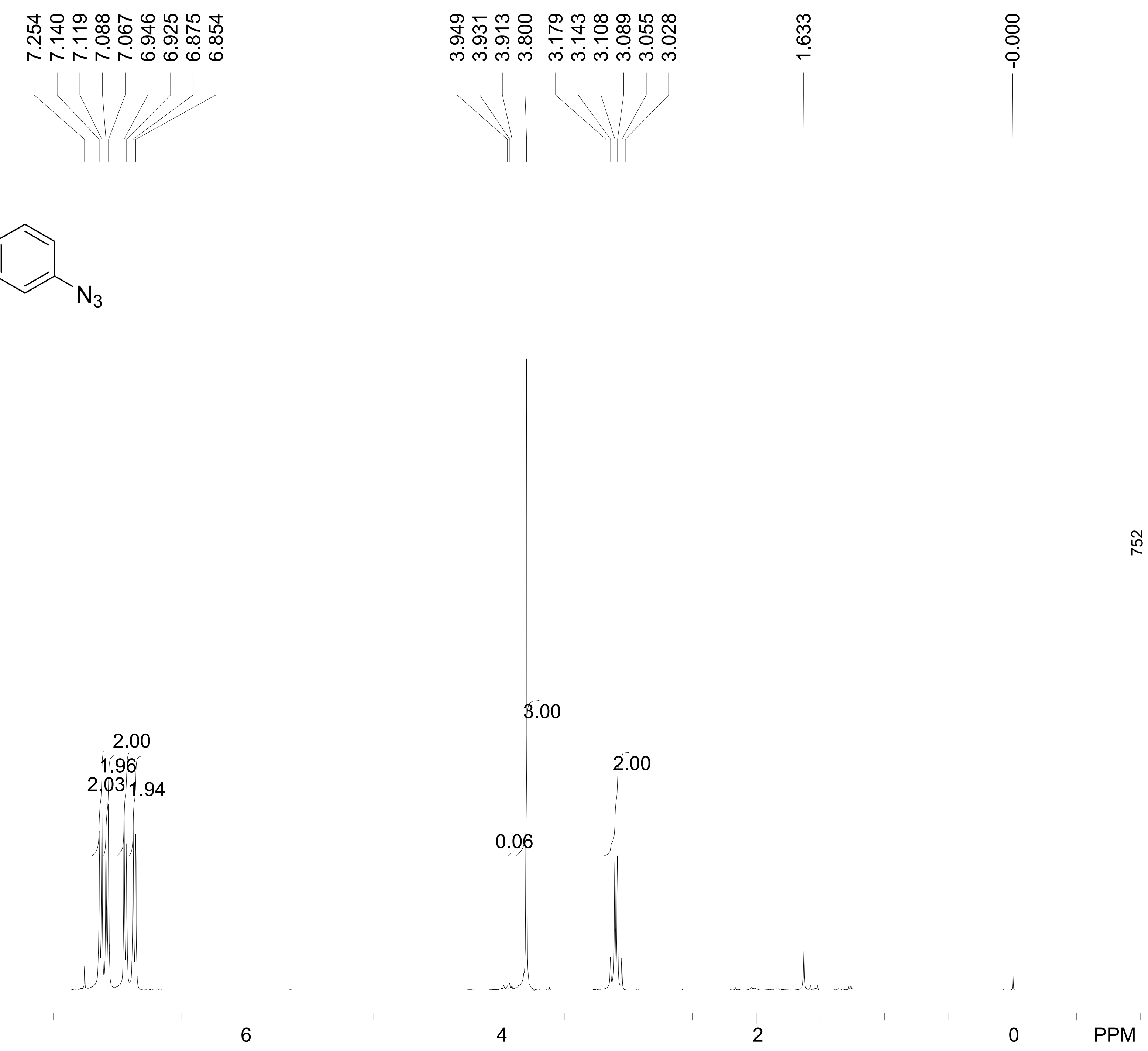

zwf-7-155C
Nov 10 2023
SOLVENT: CDCl3
NA = 150
F1 = 100.615280 MHz
F2 = 1.000000 MHz

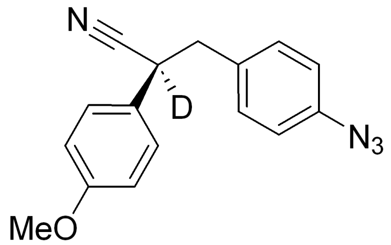

43-d

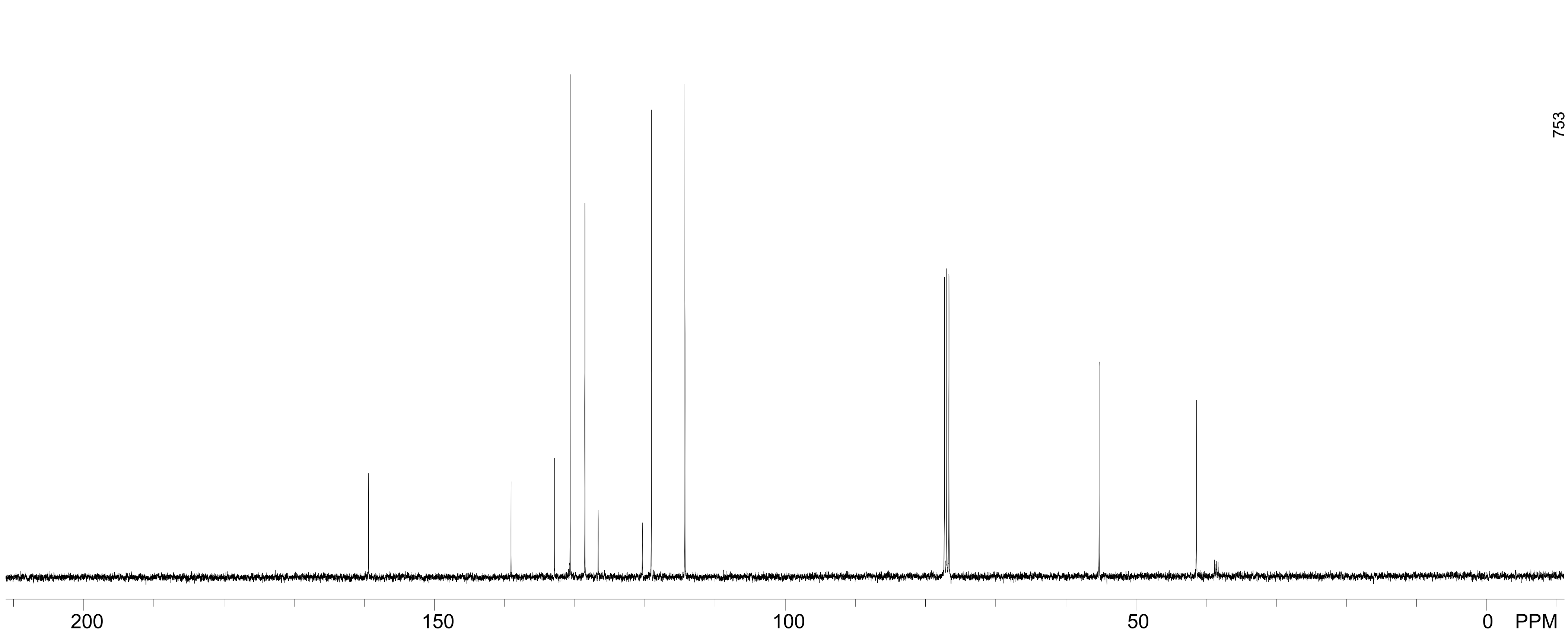

Area Percent Report

Sample name: zwf-7-155-IE-99-1-0.8-205

Data file: C:\Users\Public\Documents\ChemStation\1\Data\zwf\zwf-S 2023-11-10 18-08-41\006-P1-C7-zwf-7-155.D

Injection Acquired Date 11/10/2023 21:35:46

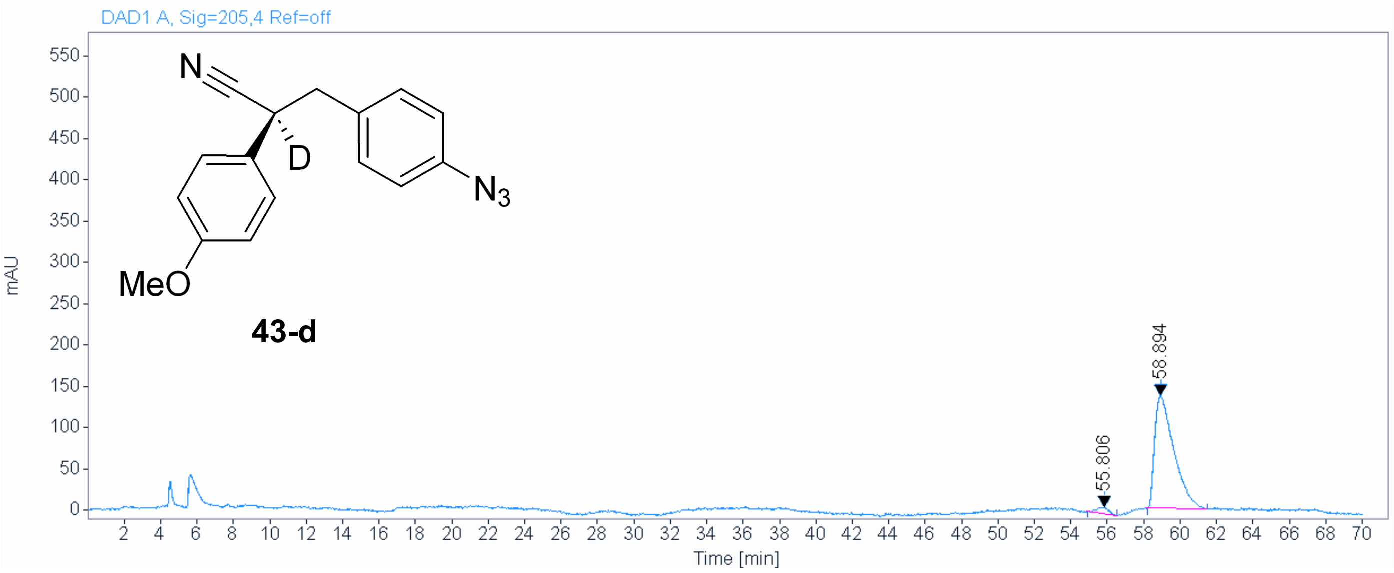

Signal: DAD1 A, Sig=205,4 Ref=off

RT [min]	Width [min]	Height	Area	Area%
55.806	0.7782	8.7221	407.2643	3.8622
58.894	0.8597	137.1922	10137.7451	96.1378
		Sum	10545.0094	100.0000

Area Percent Report

Sample name: zwf-7-155-rac-IE-99-1-0.8-205

Data file: C:\Users\Public\Documents\ChemStation\1\Data\zwf\zwf-S 2023-11-10 18-08-41\007-P1-C8-zwf-7-155-rac.D

Injection Acquired Date 11/10/2023 22:46:53

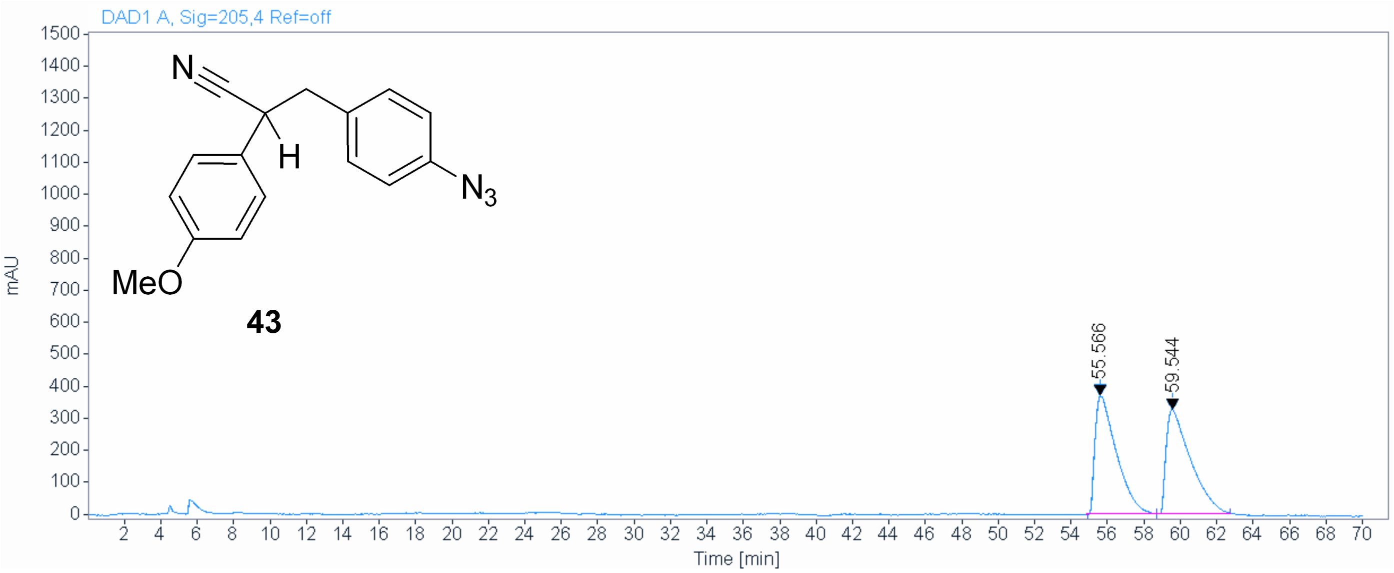

43

Signal: DAD1 A, Sig=205,4 Ref=off

RT [min]	Width [min]	Height	Area	Area%
55.566	1.3580	371.2296	30247.5586	49.7468
59.544	1.0978	326.4747	30555.4824	50.2532
		Sum	60803.0410	100.0000

zwf-7-062H
Sep 14 2023
SOLVENT: CDCl3
NA = 4
F1 = 400.140015 MHz
F2 = 1.000000 MHz

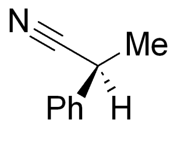

44

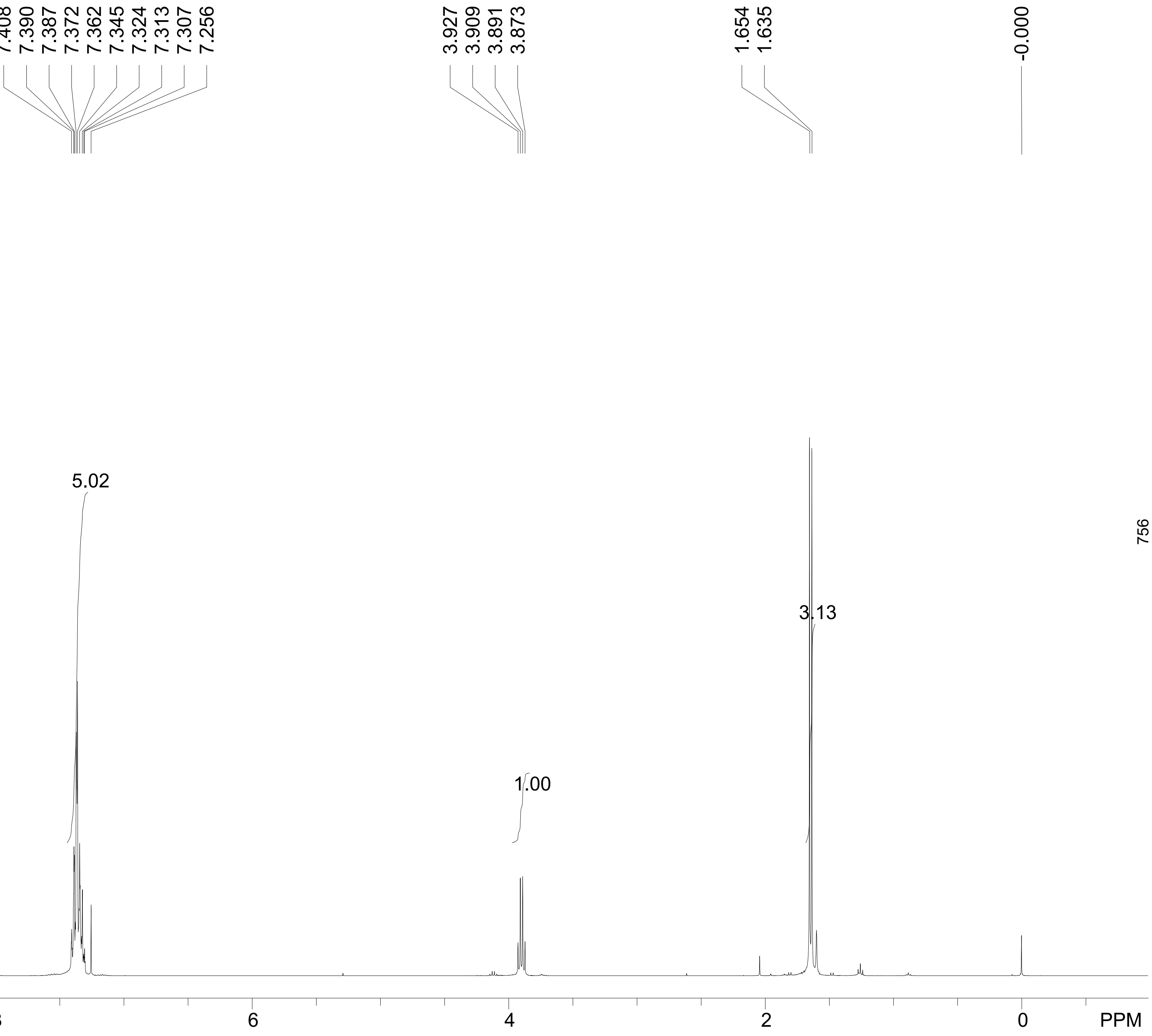

zwf-7-062C
Sep 14 2023
SOLVENT: CDCl3
NA = 100
F1 = 100.615280 MHz
F2 = 1.000000 MHz

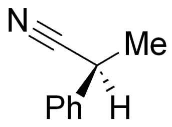

44

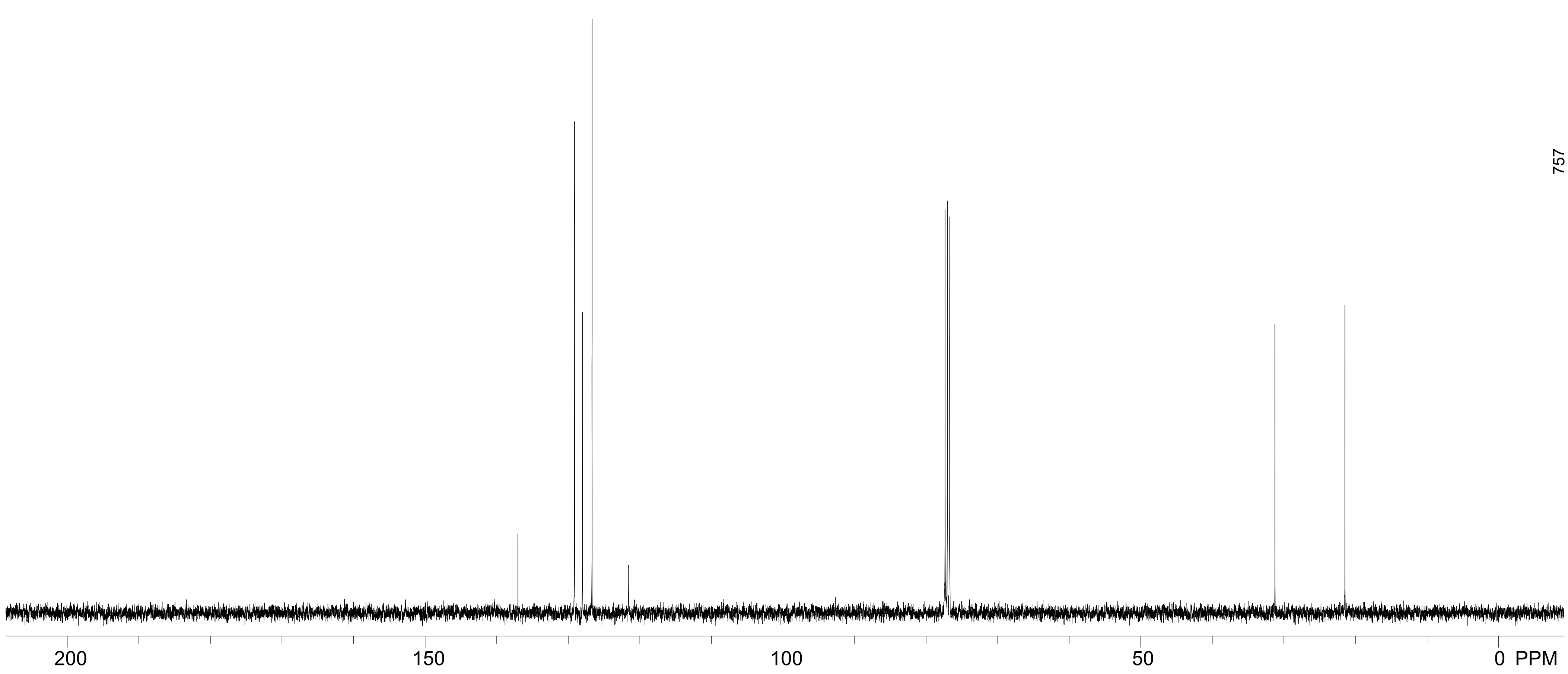

Area Percent Report

Agilent Technologies

Sample name: zwf-7-062-IE-99-1-0.8-205

Data file: C:\Users\Public\Documents\ChemStation\1\Data\zwf\zwf-S 2023-09-24 18-53-06\002-P1-C1-zwf-7-062.D

Injection Acquired Date 09/24/2023 19:15:54

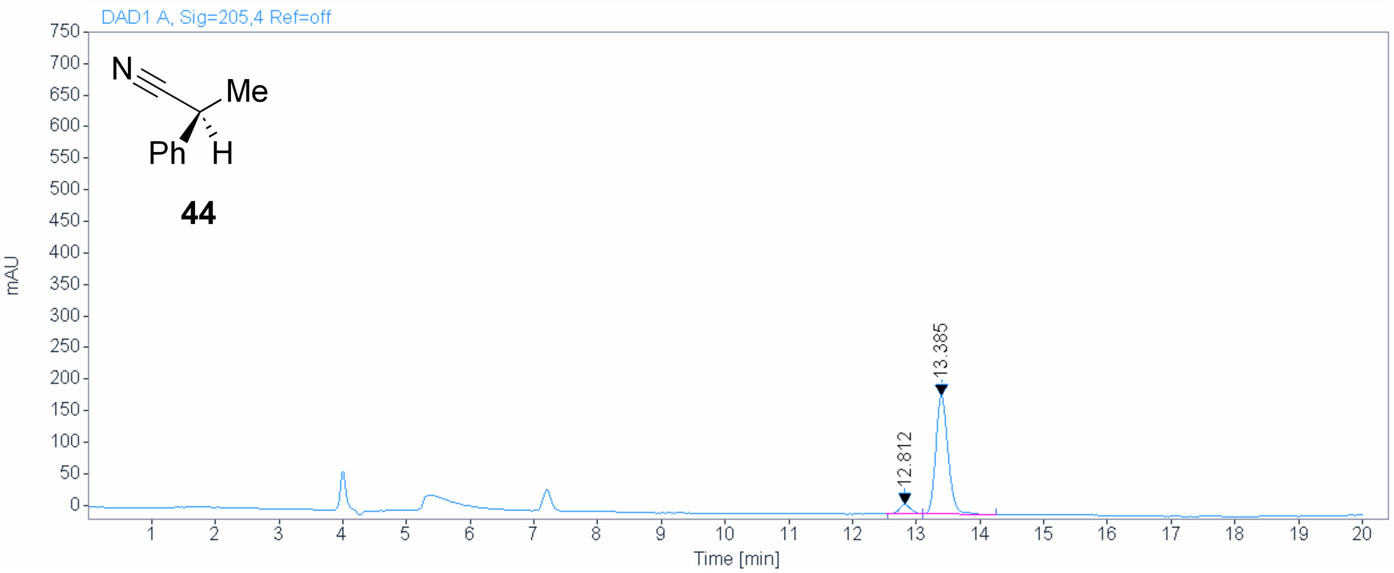

Signal: DAD1 A, Sig=205,4 Ref=off

RT [min]	Width [min]	Height	Area	Area%
12.812	0.1942	15.3816	197.9857	7.1873
13.385	0.2071	188.3624	2556.6926	92.8127
		Sum	2754.6783	100.0000

Area Percent Report

Agilent Technologies

Sample name: zwf-7-062-rac-IE-99-1-0.8-205

Data file: C:\Users\Public\Documents\ChemStation\1\Data\zwf\zwf-S 2023-09-24 18-53-06\003-P1-C2-zwf-7-062-rac.D

Injection Acquired Date 09/24/2023 19:36:56

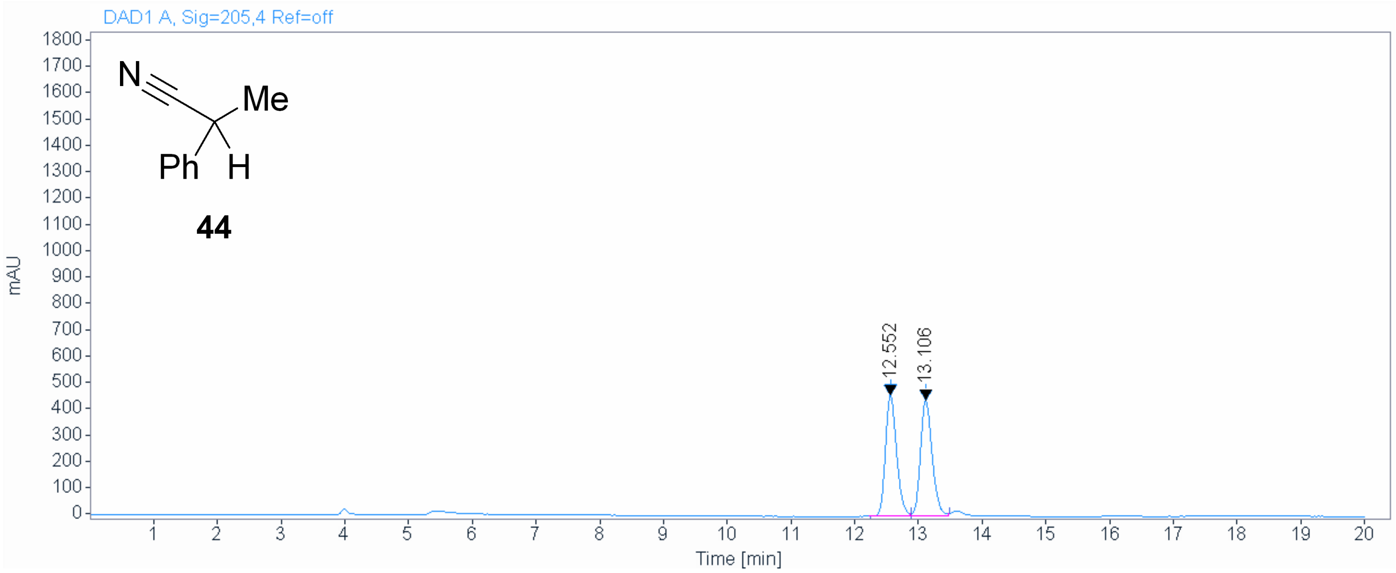

Signal: DAD1 A, Sig=205,4 Ref=off

RT [min]	Width [min]	Height	Area	Area%
12.552	0.1885	458.3181	5599.1963	49.9008
13.106	0.2132	439.3607	5621.4570	50.0992
		Sum	11220.6533	100.0000

zwf-8-009H
Nov 12 2023
SOLVENT: CDCl3
NA = 4
F1 = 400.140015 MHz
F2 = 1.000000 MHz

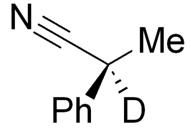

44-d

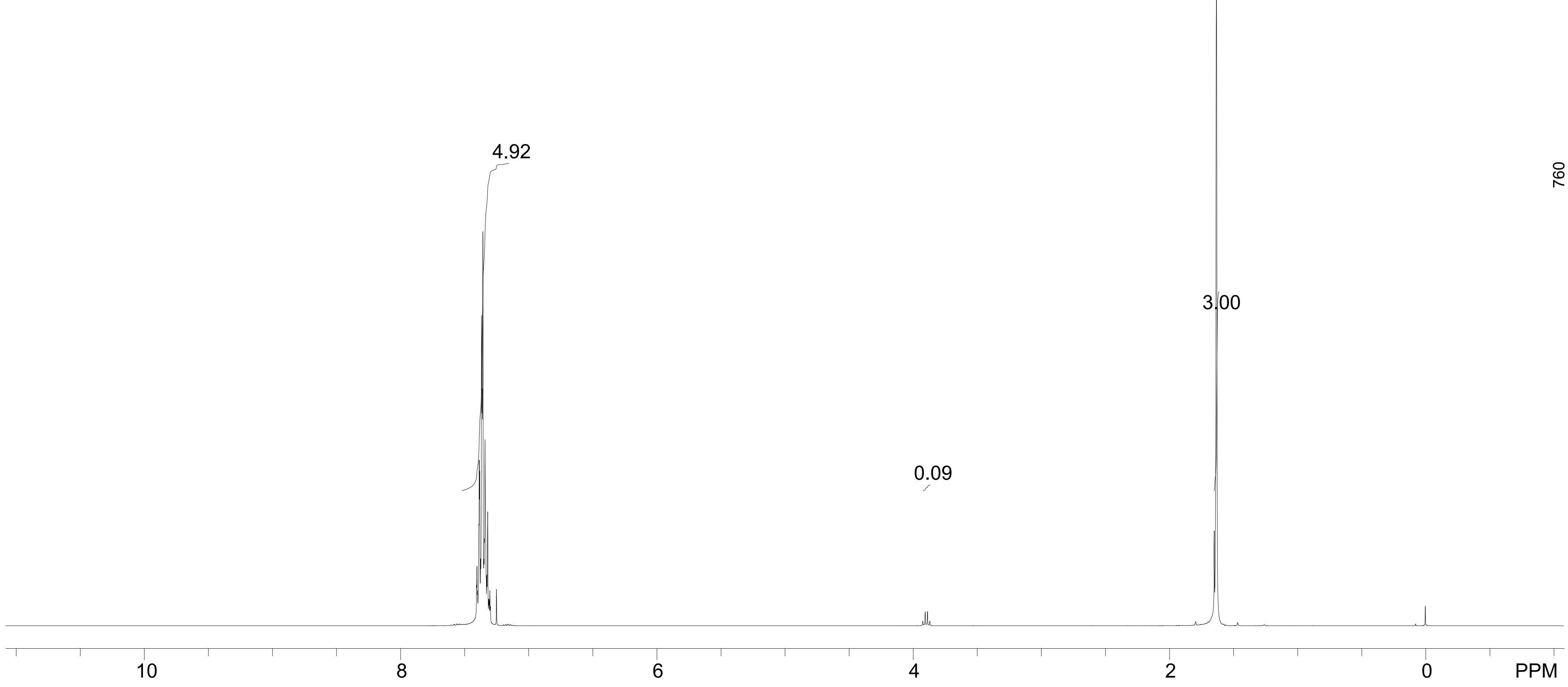

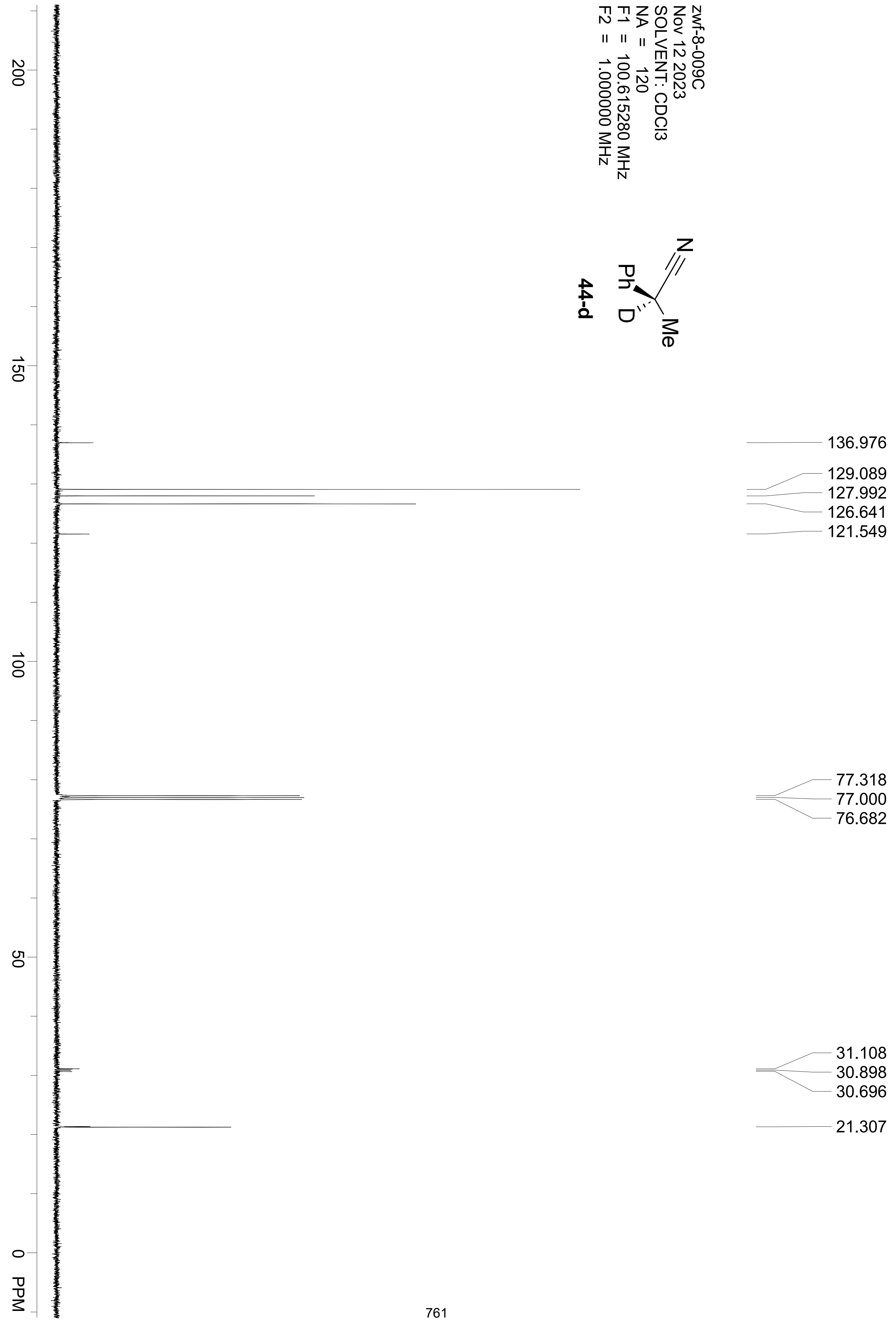

zwf-8-009C
Nov 12 2023
SOLVENT: CDCl3
NA = 120
F1 = 100.615280 MHz
F2 = 1.000000 MHz
N
Me
Ph
D
44-d
136.976
129.089
127.992
126.641
121.549
77.318
77.000
76.682
31.108
30.898
30.696
21.307
200
150
100
50
0
PPM

Area Percent Report

Agilent Technologies

Sample name: zwf-8-009-IE-99-1-0.8-205

Data file: C:\Users\Public\Documents\ChemStation\1\Data\zwf\zwf-S 2023-11-12 11-02-17\026-P1-C9-zwf-8-009.D

Injection Acquired Date 11/13/2023 08:28:34

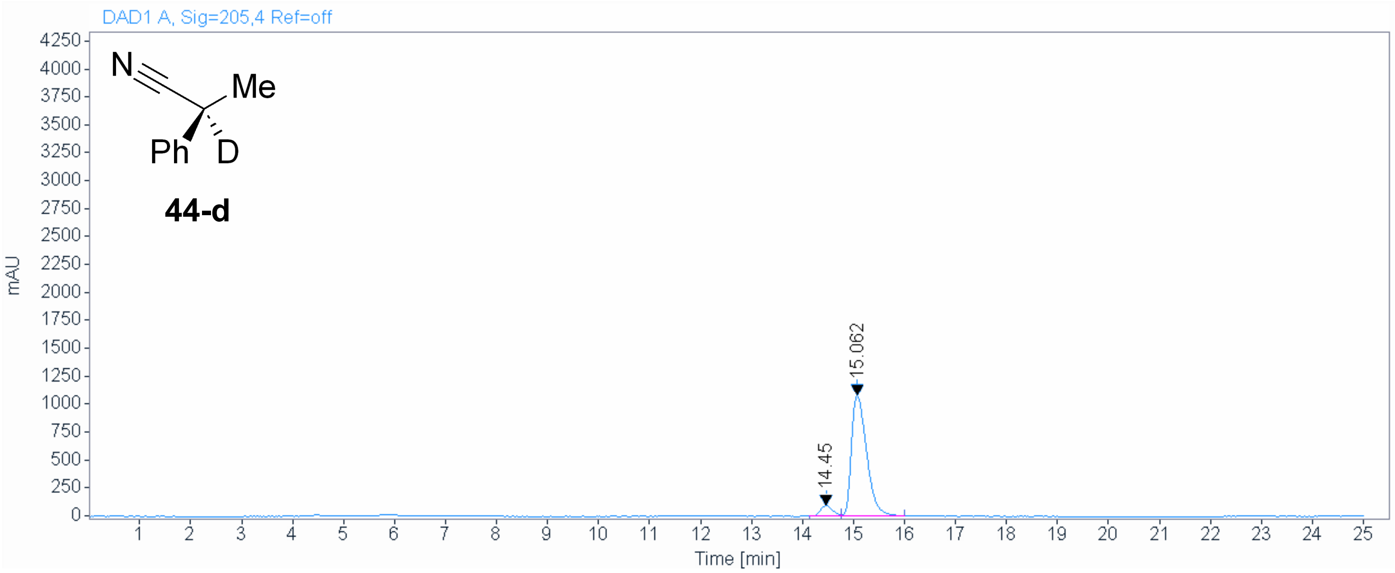

Signal: DAD1 A, Sig=205,4 Ref=off

RT [min]	Width [min]	Height	Area	Area%
14.450	0.2837	94.0585	1600.7911	6.6633
15.062	0.3462	1079.3831	22423.3359	93.3367
		Sum	24024.1271	100.0000

Area Percent Report

Agilent Technologies

Sample name: zwf-8-009-rac-IE-99-1-0.8-205

Data file: C:\Users\Public\Documents\ChemStation\1\Data\zwf\zwf-S 2023-11-12 11-02-17\027-P1-C10-zwf-8-009-rac.D

Injection Acquired Date 11/13/2023 08:54:35

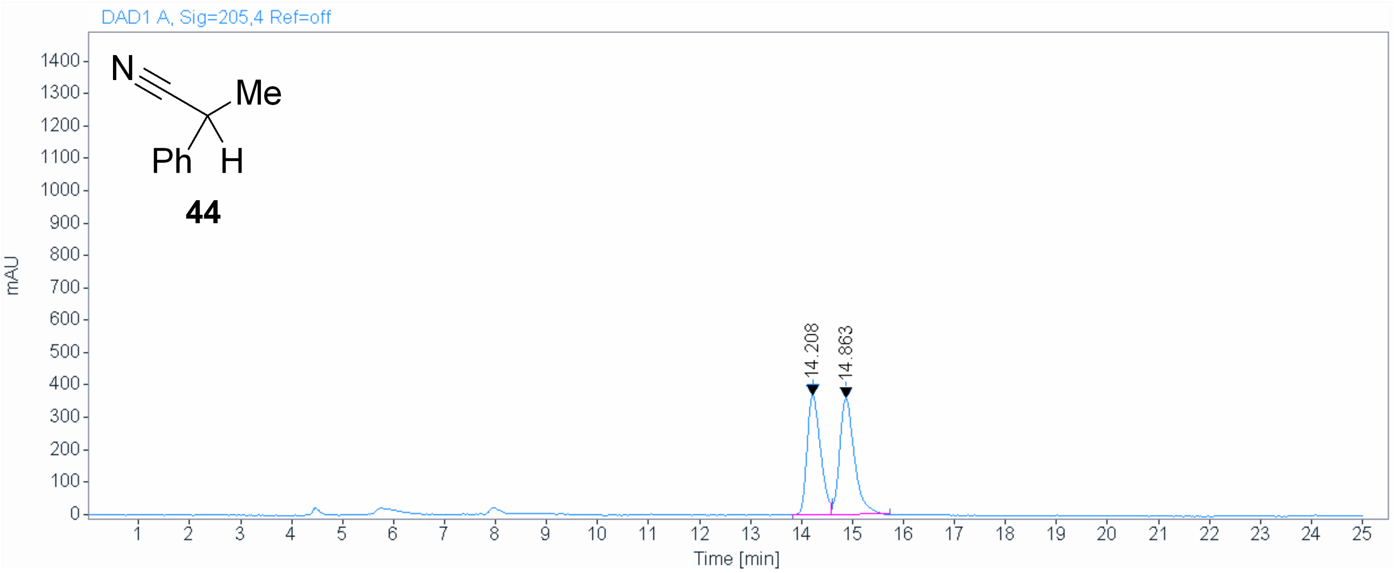

Signal: DAD1 A, Sig=205,4 Ref=off

RT [min]	Width [min]	Height	Area	Area%
14.208	0.2789	368.8295	6794.3110	48.1467
14.863	0.2988	360.5335	7317.3765	51.8533
		Sum	14111.6875	100.0000

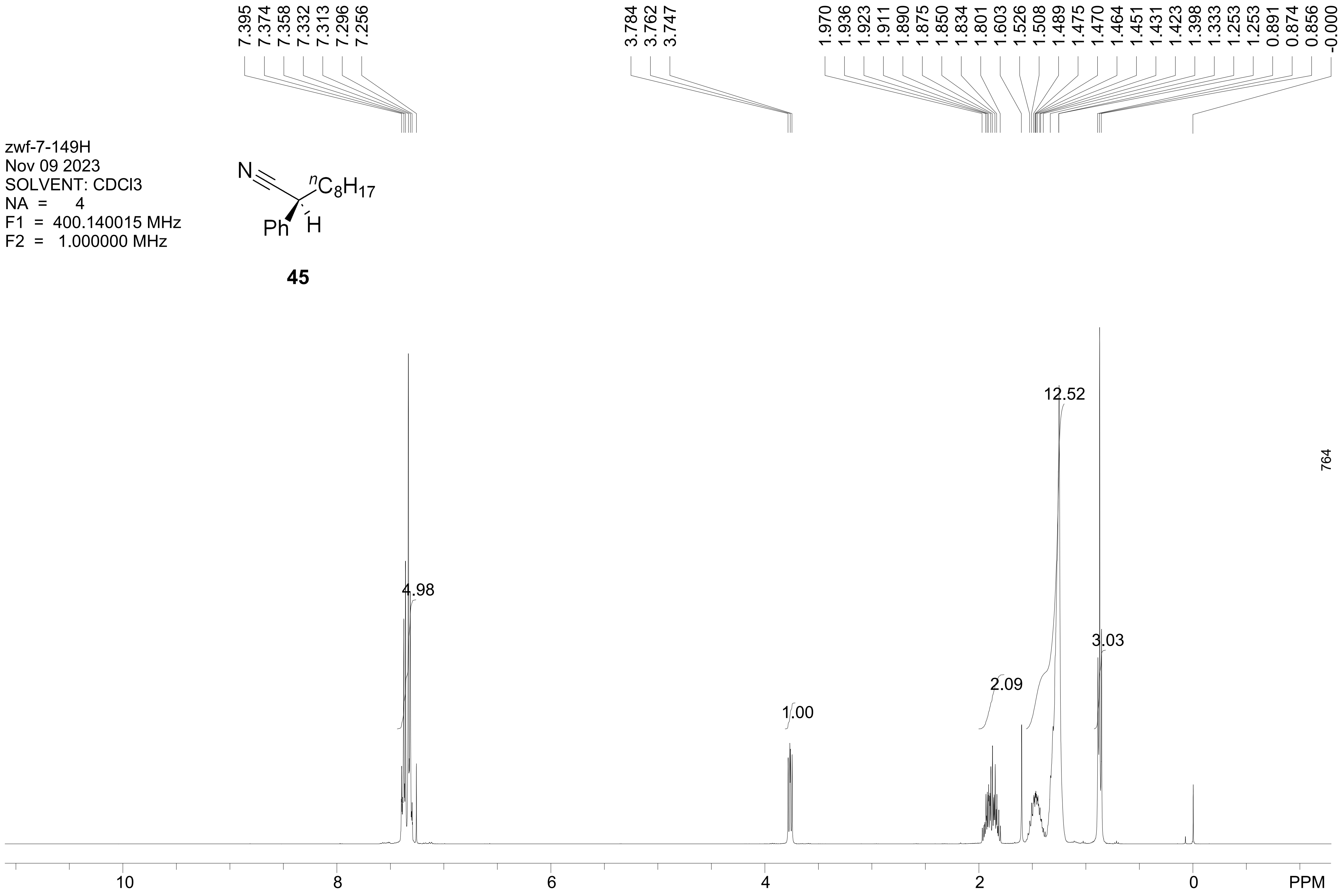
zwf-7-149H
Nov 09 2023
SOLVENT: CDCl3
NA = 4
F1 = 400.140015 MHz
F2 = 1.000000 MHz
45
7.395
7.374
7.358
7.332
7.313
7.296
7.256
3.784
3.762
3.747
1.970
1.936
1.923
1.911
1.890
1.875
1.850
1.834
1.801
1.603
1.526
1.508
1.489
1.475
1.470
1.464
1.451
1.431
1.423
1.398
1.333
1.253
1.253
0.891
0.874
0.856
-0.000
4.98
1.00
2.09
12.52
3.03
10
8
6
4
2
0
PPM

zwf-7-149C
Nov 09 2023
SOLVENT: CDCl3
NA = 100
F1 = 100.615280 MHz
F2 = 1.000000 MHz

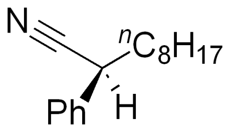

45

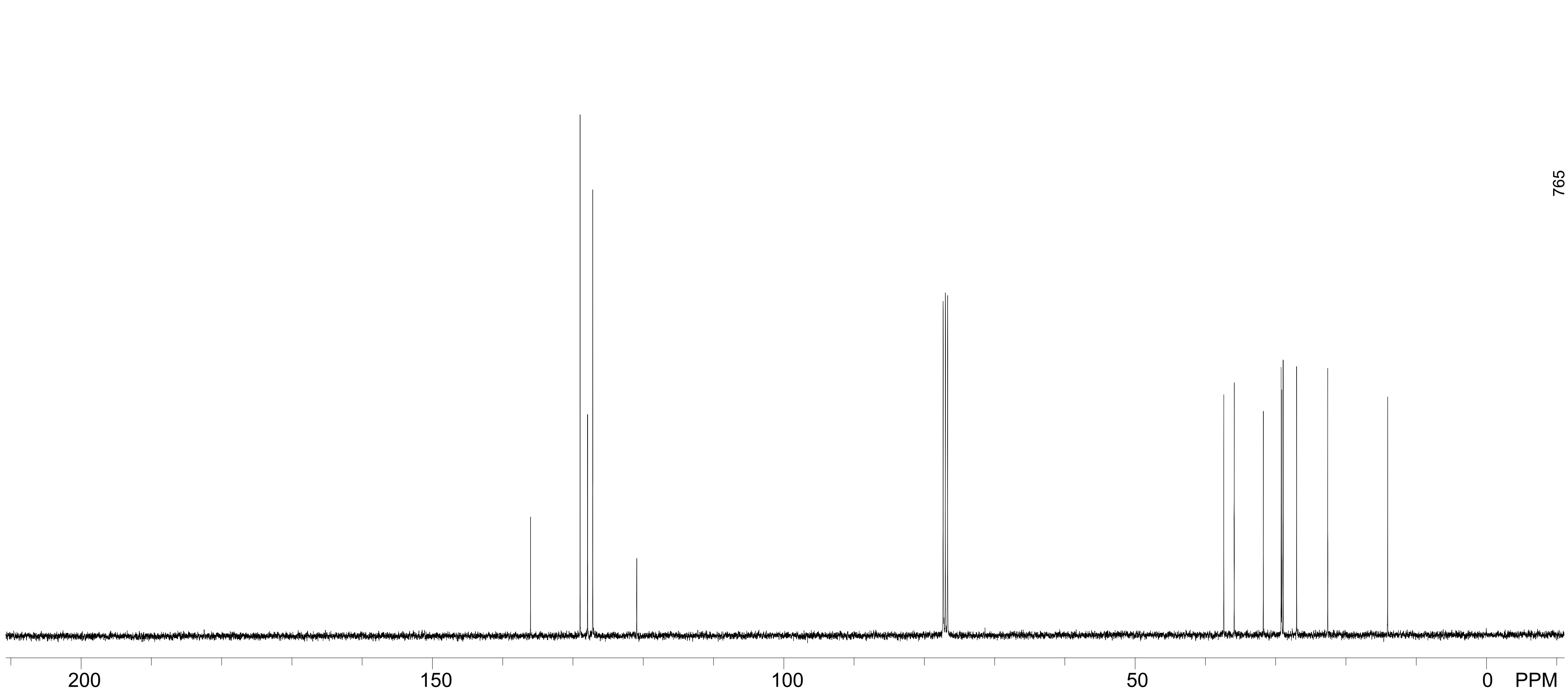

Area Percent Report

Agilent Technologies

Sample name: zwf-7-149-IE-99-1-0.8-205

Data file: C:\Users\Public\Documents\ChemStation\1\Data\zwf\zwf-S 2023-11-09 09-41-30\002-P1-C1-zwf-7-149.D

Injection Acquired Date 11/09/2023 10:04:39

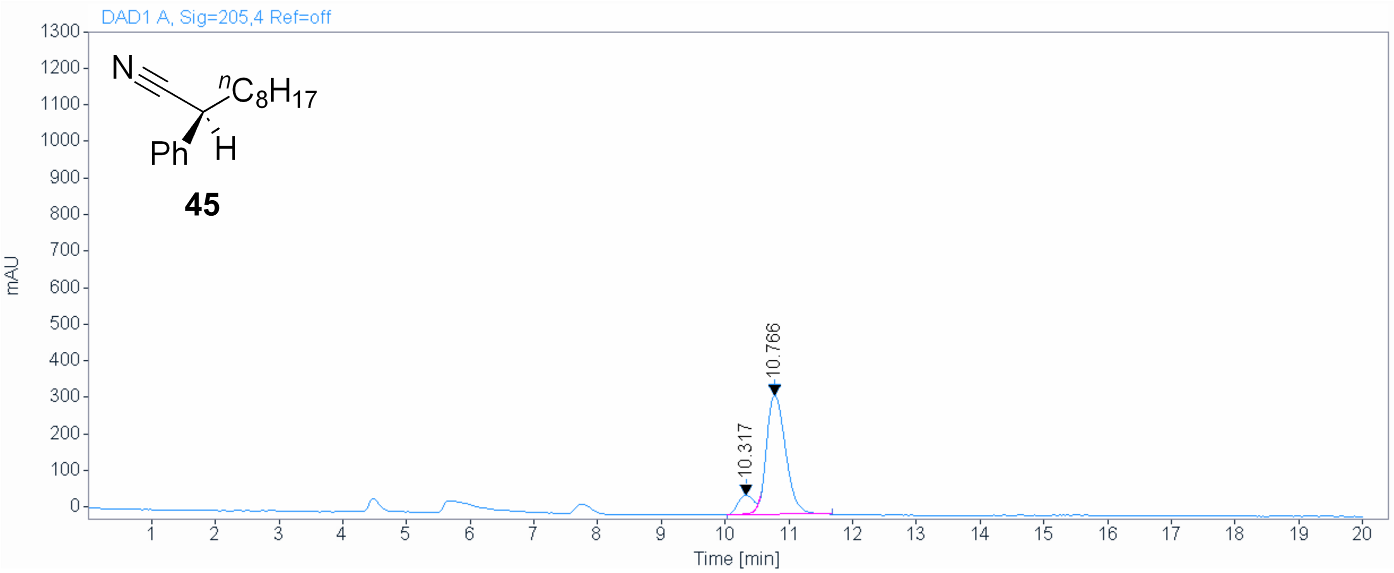

Signal: DAD1 A, Sig=205,4 Ref=off

RT [min]	Width [min]	Height	Area	Area%
10.317	0.2500	52.0103	864.8786	11.0604
10.766	0.3267	325.9630	6954.7339	88.9396
		Sum	7819.6125	100.0000

Area Percent Report

Agilent Technologies

Sample name: zwf-7-149-rac-IE-99-1-0.8-205

Data file: C:\Users\Public\Documents\ChemStation\1\Data\zwf\zwf-S 2023-11-09 09-41-30\003-P1-C2-zwf-7-149-rac.D

Injection Acquired Date 11/09/2023 10:25:41

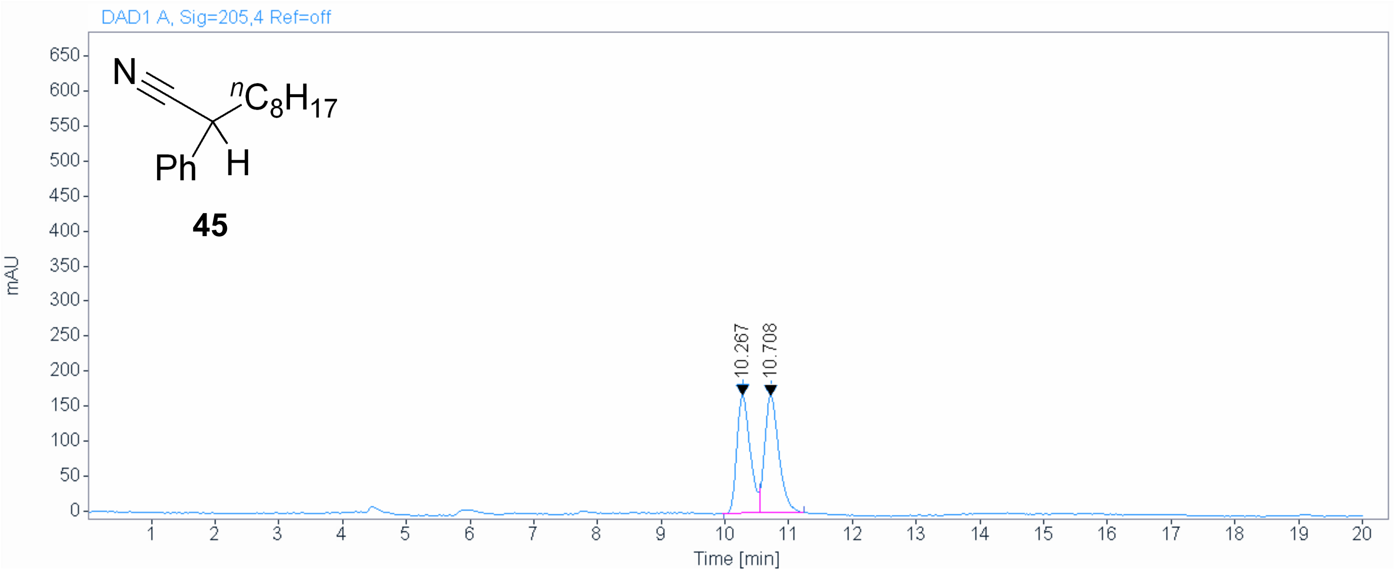

Signal: DAD1 A, Sig=205,4 Ref=off

RT [min]	Width [min]	Height	Area	Area%
10.267	0.2445	168.0040	2464.9304	49.0285
10.708	0.2562	166.7115	2562.6113	50.9715
		Sum	5027.5417	100.0000

zwf-8-010H
Nov 12 2023
SOLVENT: CDCl3
NA = 4
F1 = 400.140015 MHz
F2 = 1.000000 MHz

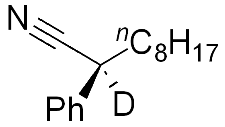

45-d

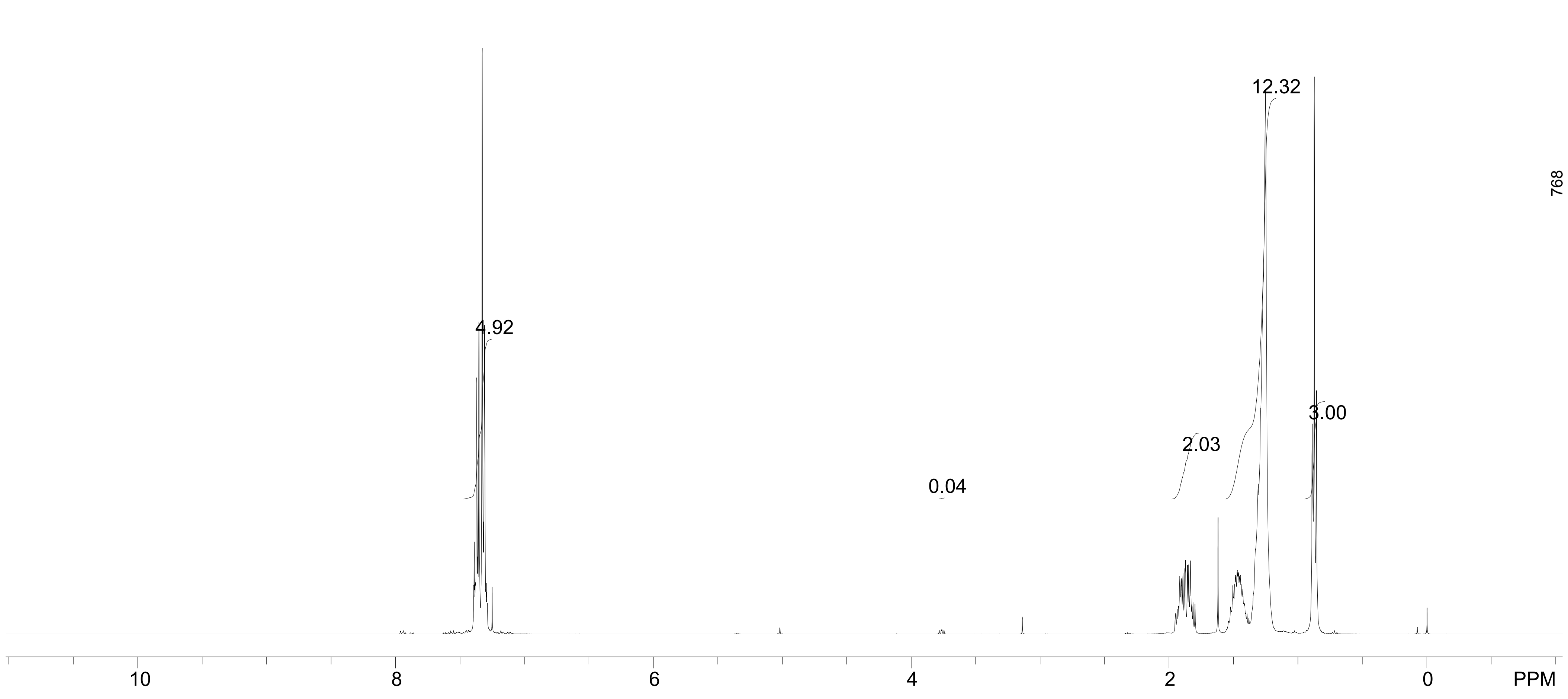

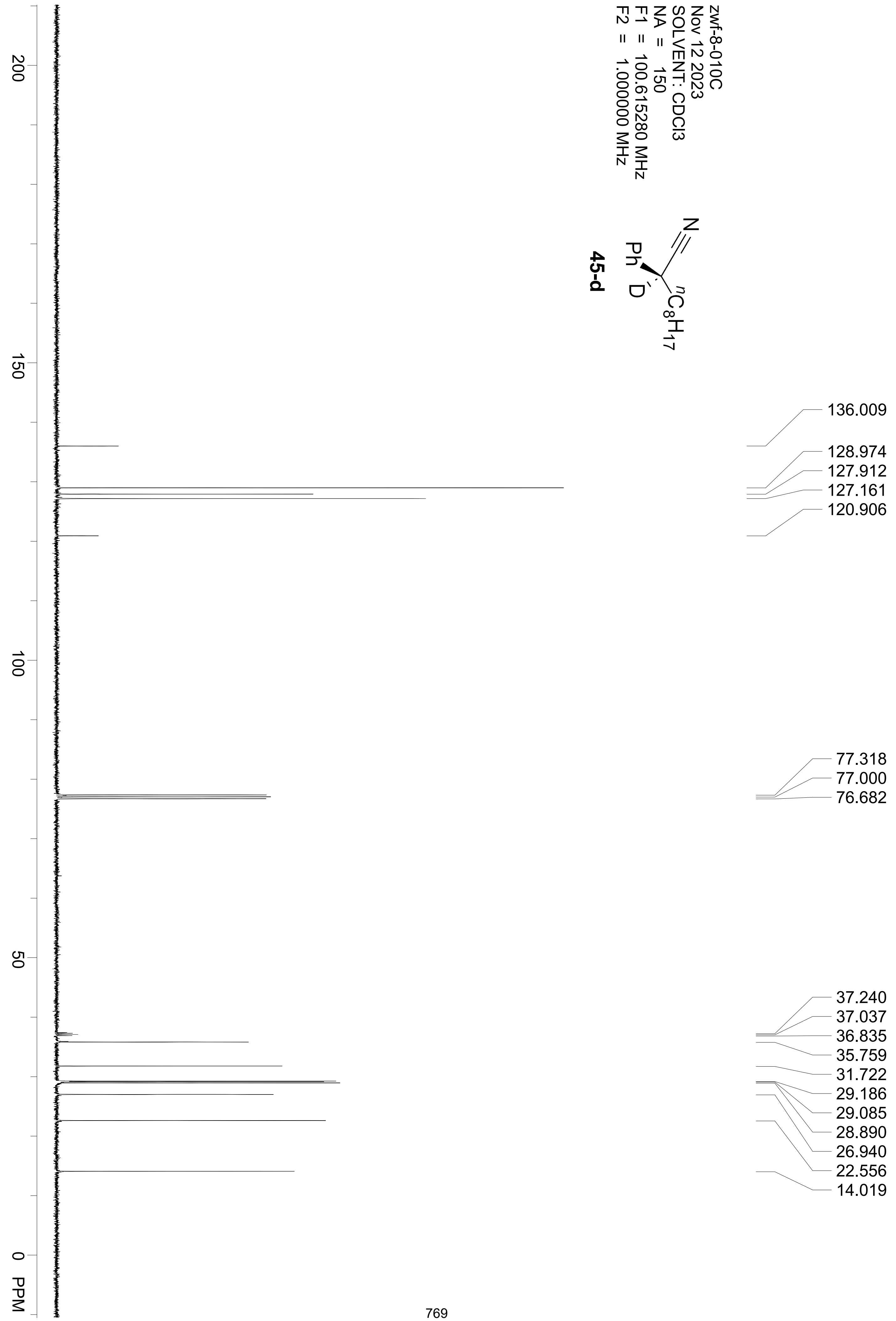
zwf-8-010C
Nov 12 2023
SOLVENT: CDCl3
NA = 150
F1 = 100.615280 MHz
F2 = 1.000000 MHz
45-d
136.009
128.974
127.912
127.161
120.906
77.318
77.000
76.682
37.240
37.037
36.835
35.759
31.722
29.186
29.085
28.890
26.940
22.556
14.019
200
150
100
50
0 PPM

Area Percent Report

Agilent Technologies

Sample name: zwf-8-010-IE-99-1-0.8-205

Data file: C:\Users\Public\Documents\ChemStation\1\Data\zwf\zwf-S 2023-11-12 11-02-17\040-P1-A1-zwf-8-010.D

Injection Acquired Date 11/13/2023 16:03:08

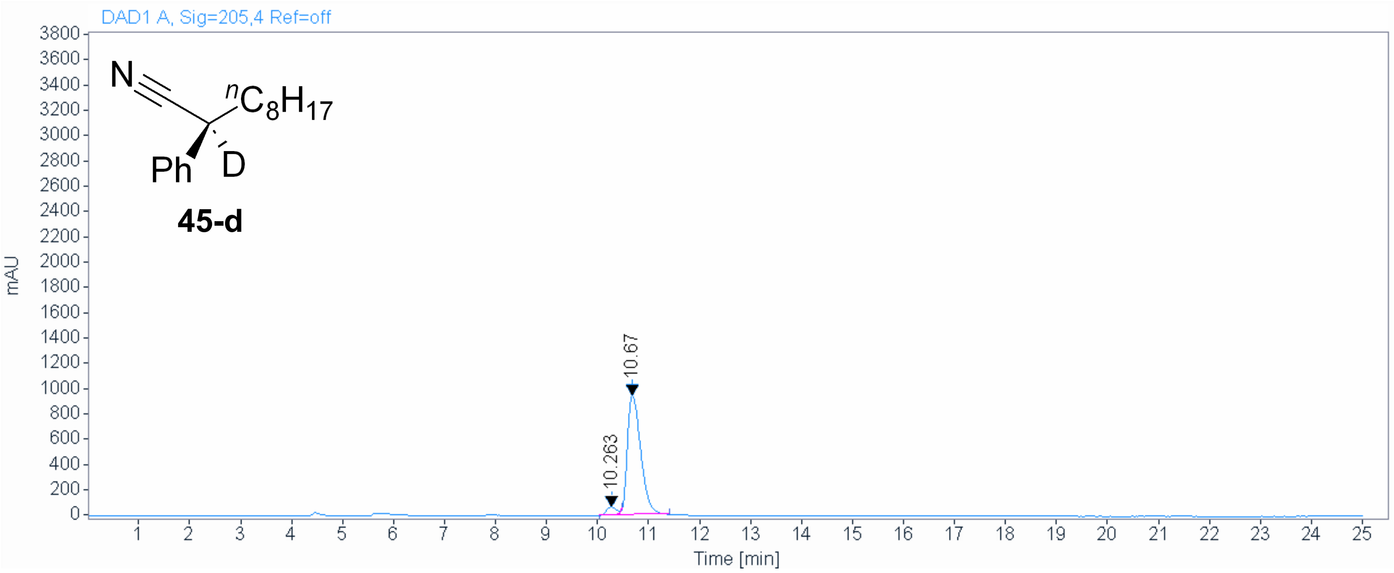

Signal: DAD1 A, Sig=205,4 Ref=off

RT [min]	Width [min]	Height	Area	Area%
10.263	0.1994	67.6815	959.3065	5.2109
10.670	0.2552	945.5165	17450.2910	94.7891
		Sum	18409.5975	100.0000

Area Percent Report

Sample name: zwf-8-010-rac-IE-99-1-0.8-205

Data file: C:\Users\Public\Documents\ChemStation\1\Data\zwf\zwf-S 2023-11-12 11-02-17\041-P1-A2-zwf-8-010-rac.D

Injection Acquired Date 11/13/2023 16:29:10

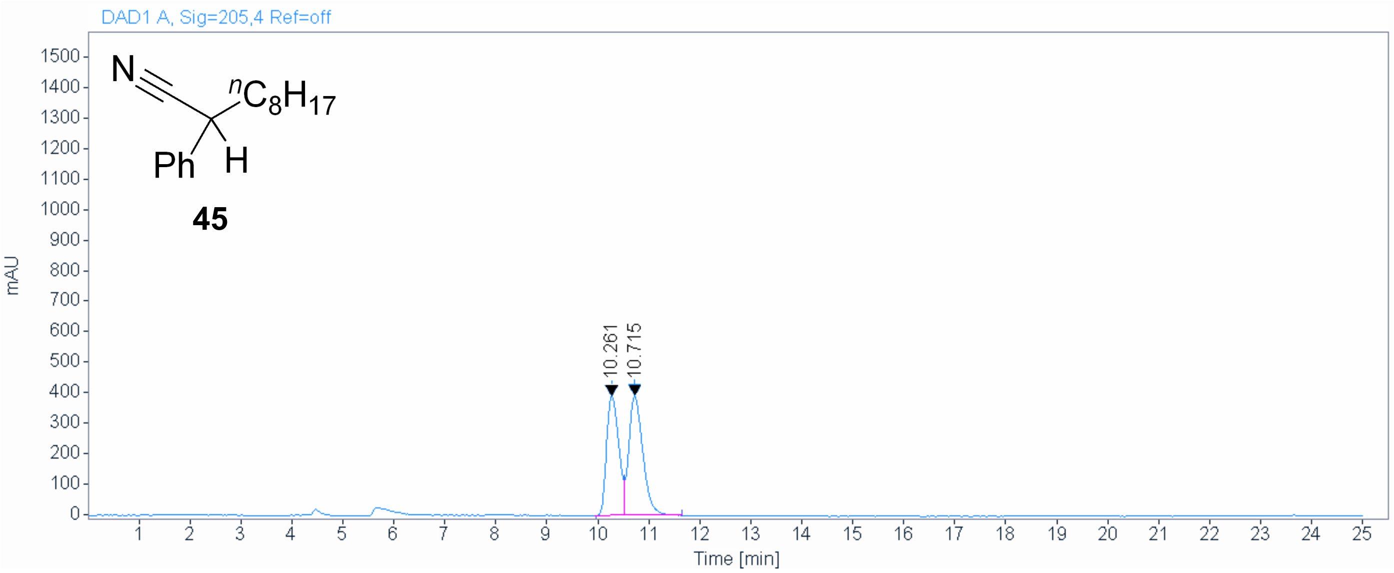

Signal: DAD1 A, Sig=205,4 Ref=off

RT [min]	Width [min]	Height	Area	Area%
10.261	0.2553	392.3394	6568.4009	46.3645
10.715	0.2866	393.7477	7598.4673	53.6355
		Sum	14166.8682	100.0000

zwf-7-144H
Dec 06 2023
SOLVENT: CDCl3
NA = 4
F1 = 400.140015 MHz
F2 = 1.000000 MHz

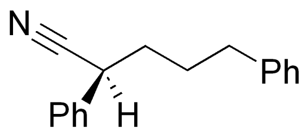

46

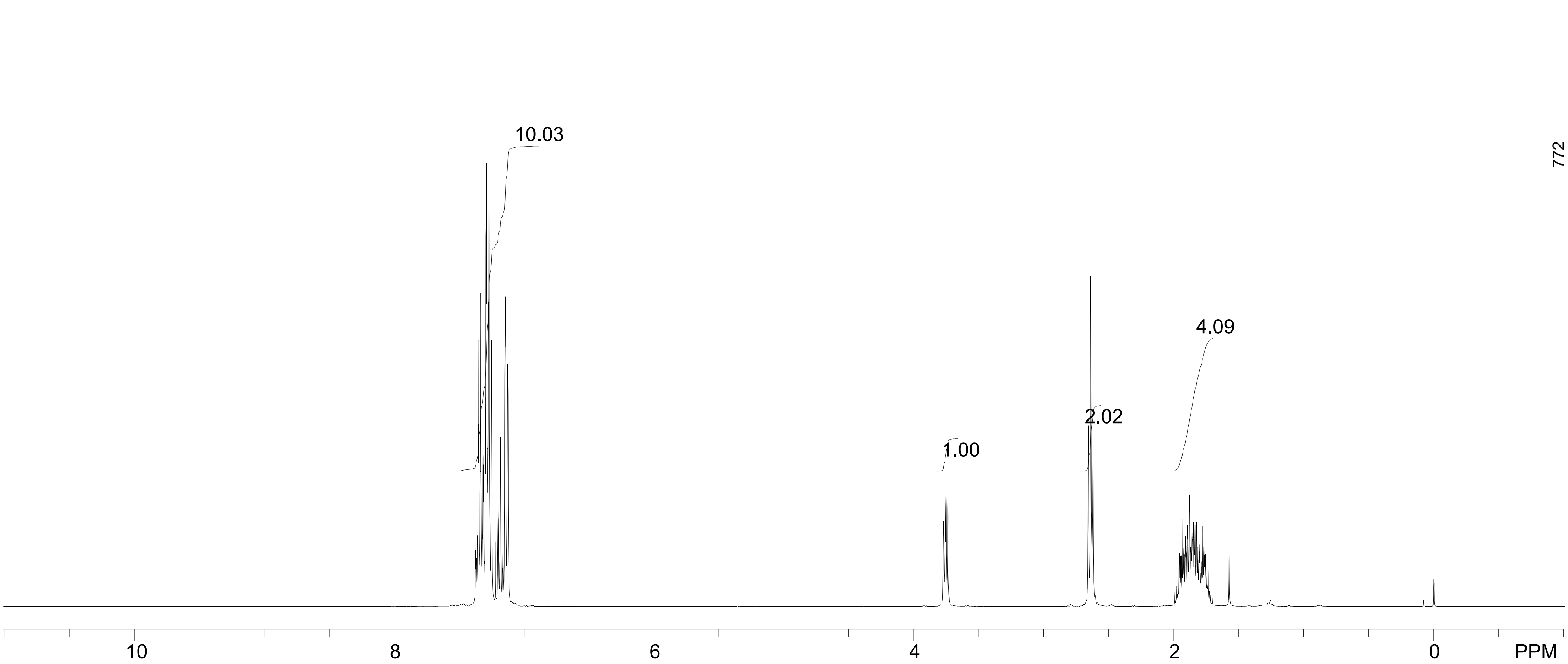

zwf-7-144C
Dec 06 2023
SOLVENT: CDCl3
NA = 100
F1 = 100.615280 MHz
F2 = 1.000000 MHz

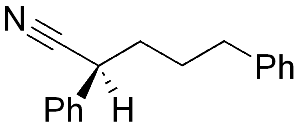

46

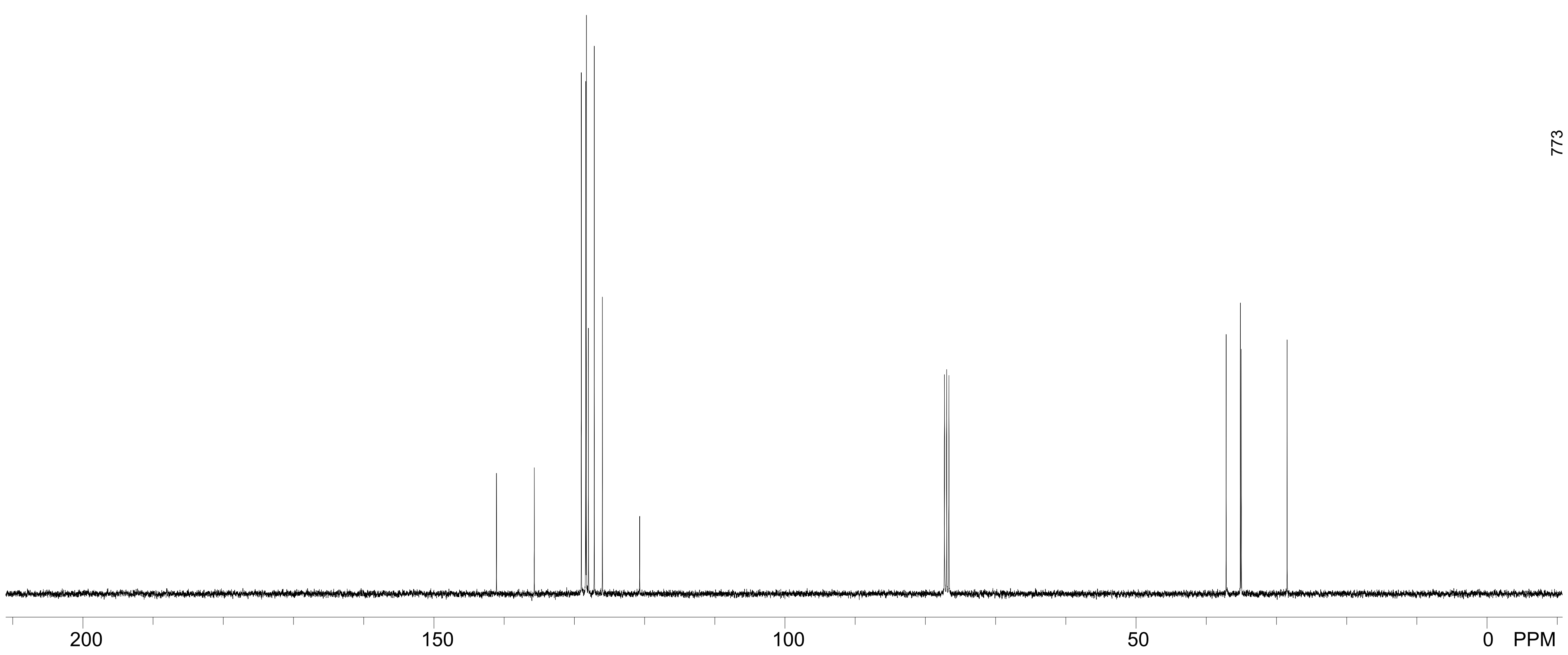

Area Percent Report

Agilent Technologies

Sample name: zwf-7-144-IE-99-1-0.8-205

Data file: C:\Users\Public\Documents\ChemStation\1\Data\zwf\zwf-S 2023-12-06 20-07-35\019-P1-C8-zwf-7-144.D

Injection Acquired Date 12/07/2023 11:15:04

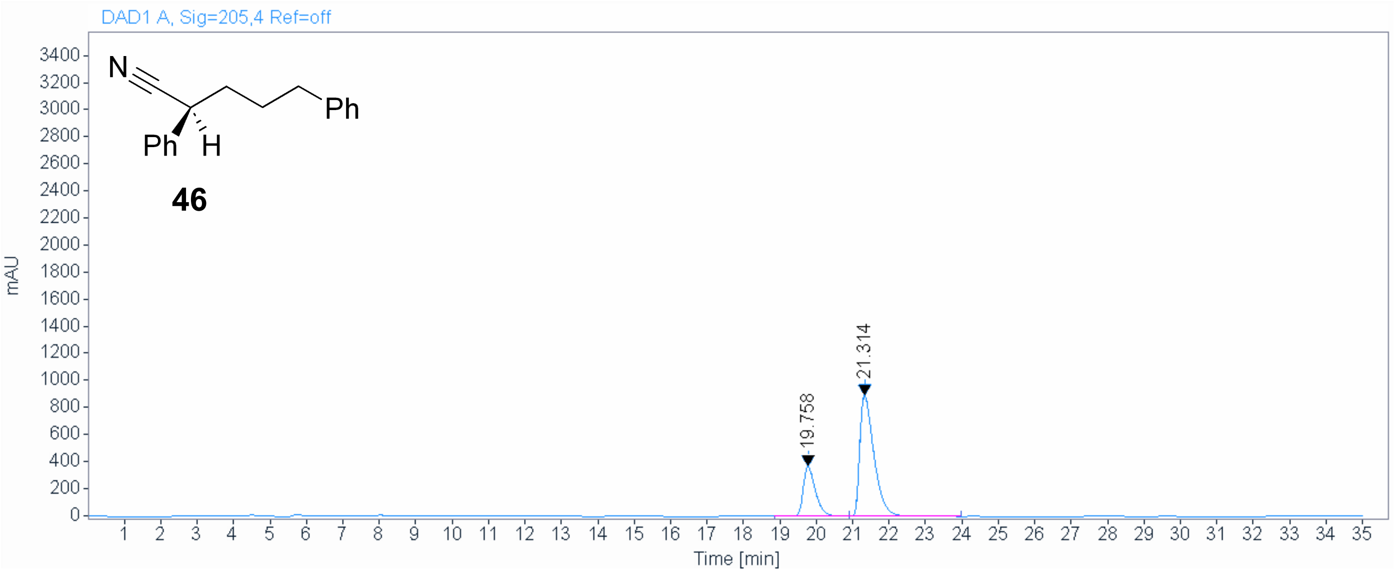

Signal: DAD1 A, Sig=205,4 Ref=off

RT [min]	Width [min]	Height	Area	Area%
19.758	0.3578	368.5594	8650.7998	26.3331
21.314	0.4125	892.0545	24200.6641	73.6669
		Sum	32851.4639	100.0000

Area Percent Report

Agilent Technologies

Sample name: zwf-7-144-rac-IE-99-1-0.8-205

Data file: C:\Users\Public\Documents\ChemStation\1\Data\zwf\zwf-S 2023-12-06 20-07-35\012-P1-C9-zwf-7-144-rac.D

Injection Acquired Date 12/07/2023 04:02:20

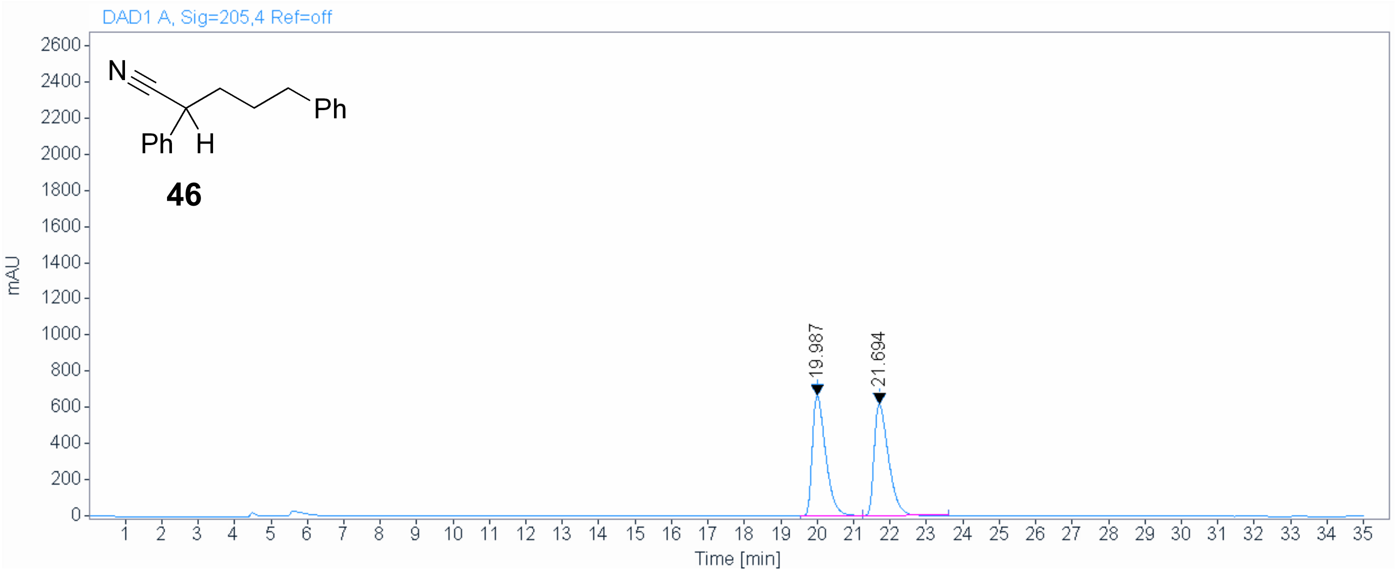

Signal: DAD1 A, Sig=205,4 Ref=off

RT [min]	Width [min]	Height	Area	Area%
19.987	0.3926	666.5826	17061.7617	49.9747
21.694	0.4237	619.3473	17079.0352	50.0253
		Sum	34140.7969	100.0000

zwf-8-043H
Nov 26 2023
SOLVENT: CDCl3
NA = 4
F1 = 400.179993 MHz
F2 = 1.000000 MHz

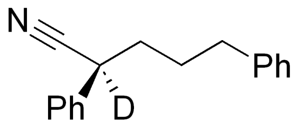

46-d

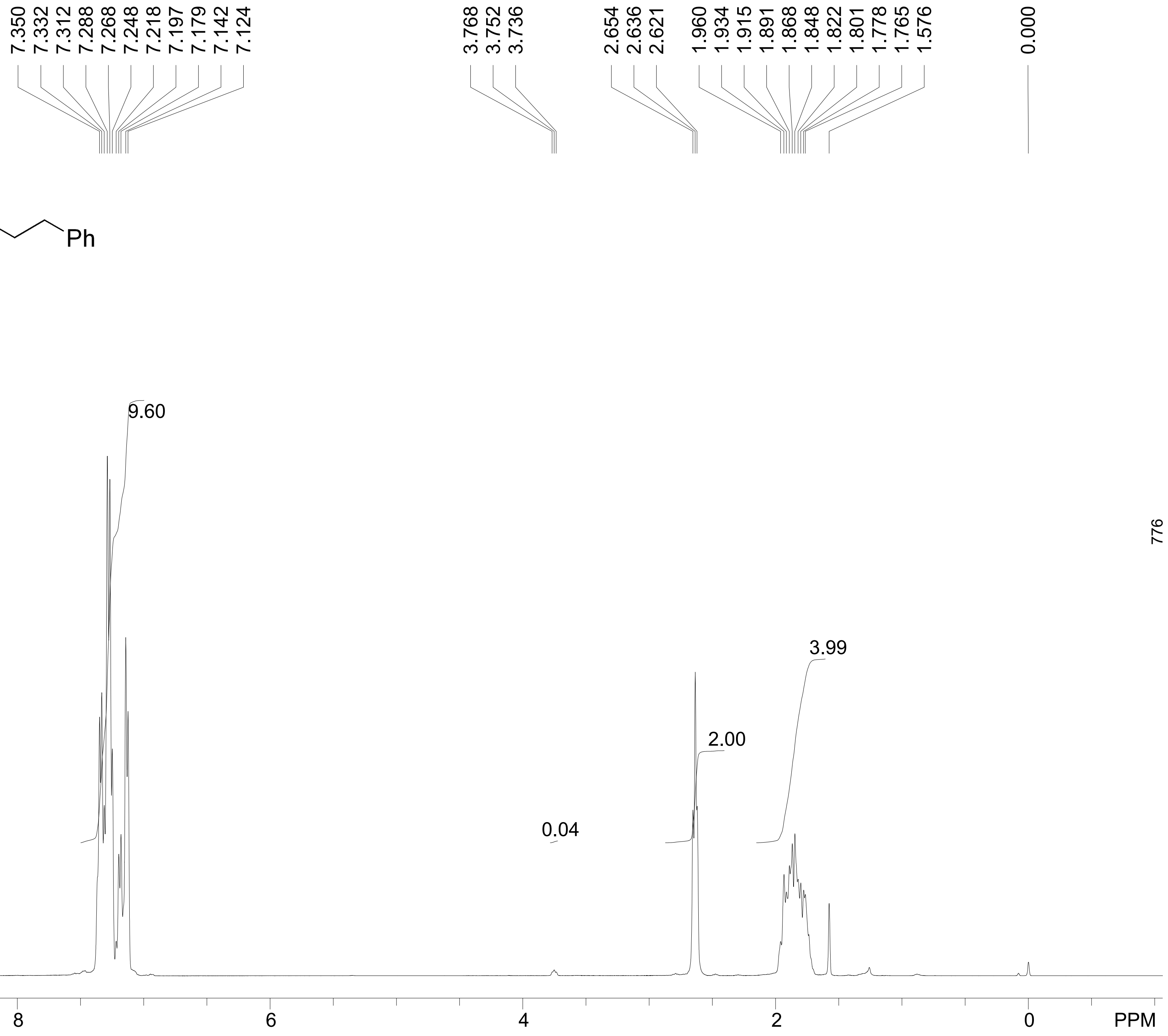

zwf-8-043C
Nov 26 2023
SOLVENT: CDCl3
NA = 100
F1 = 100.625343 MHz
F2 = 1.000000 MHz

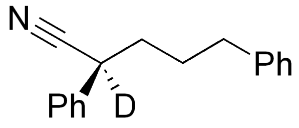

46-d

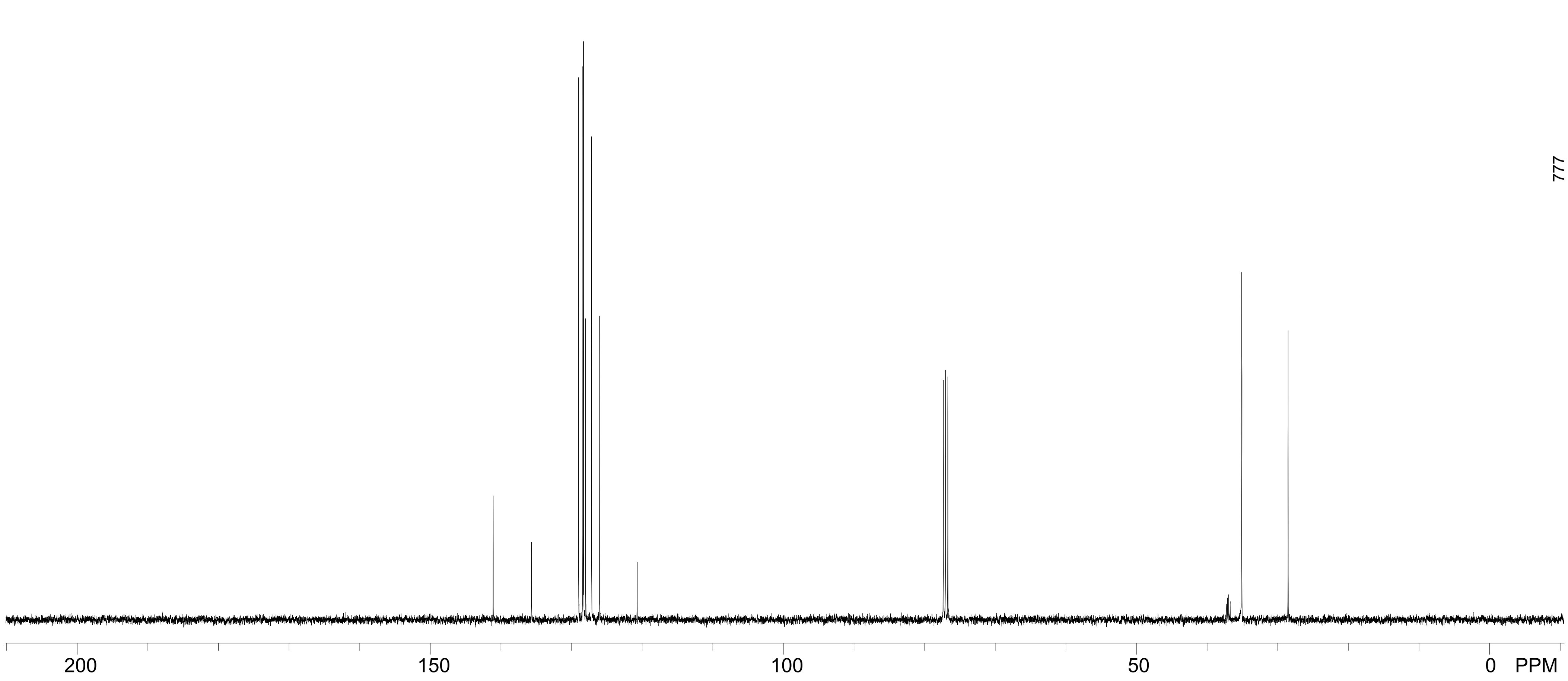

Area Percent Report

Agilent Technologies

Sample name: zwf-8-043-IE-99-1-0.8-205

Data file: C:\Users\Public\Documents\ChemStation\1\Data\zwf\zwf-S 2023-11-26 13-46-16\021-P1-B7-zwf-8-043.D

Injection Acquired Date 11/27/2023 03:43:34

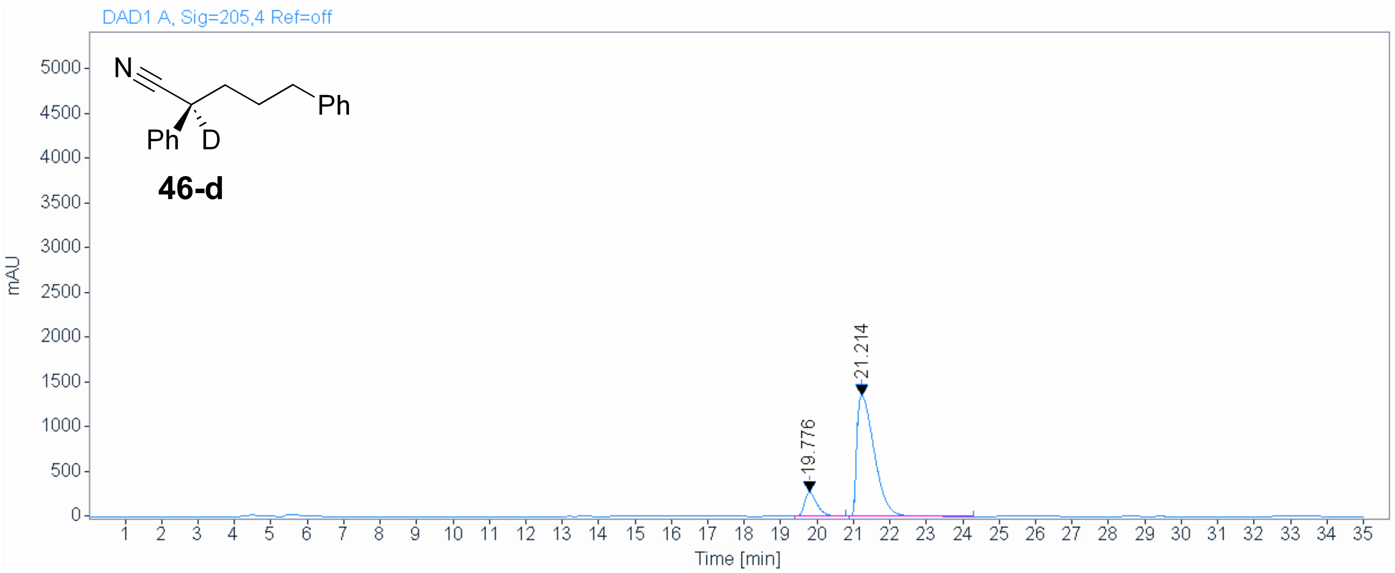

Signal: DAD1 A, Sig=205,4 Ref=off

RT [min]	Width [min]	Height	Area	Area%
19.776	0.3939	269.5325	6370.6279	12.4080
21.214	0.5206	1349.9984	44972.2383	87.5920
		Sum	51342.8662	100.0000

Area Percent Report

Agilent Technologies

Sample name: zwf-8-043-rac-IE-99-1-0.8-205

Data file: C:\Users\Public\Documents\ChemStation\1\Data\zwf\zwf-S 2023-11-26 13-46-16\022-P1-B8-zwf-8-043-arc.D

Injection Acquired Date 11/27/2023 04:19:35

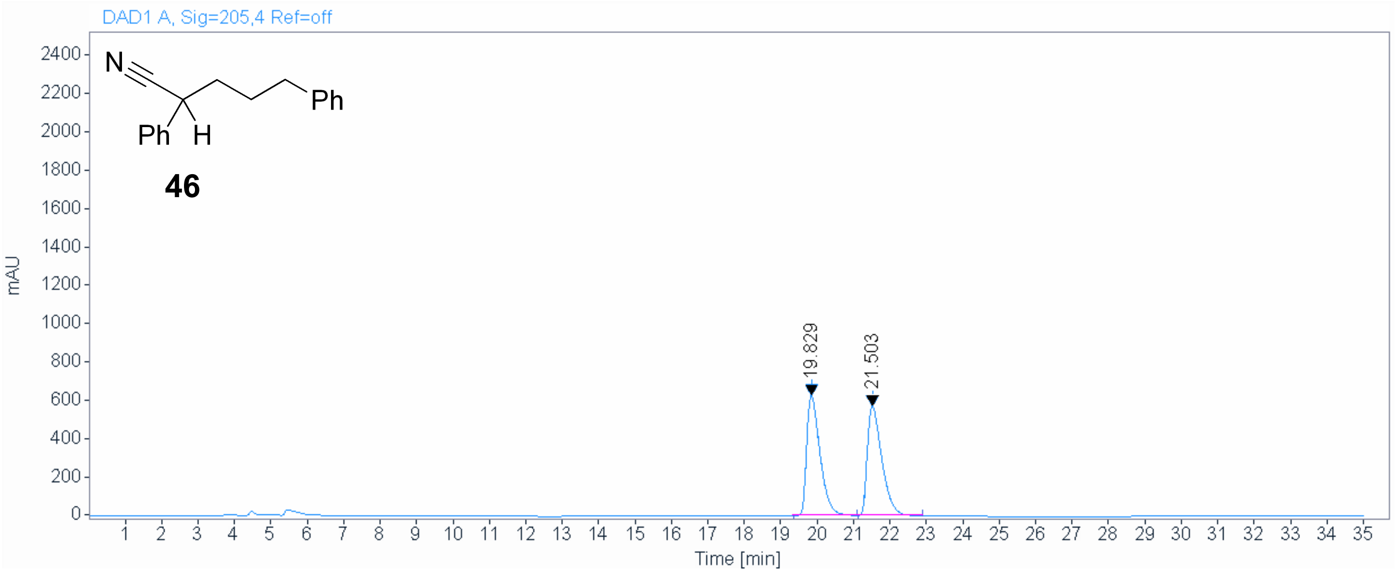

Signal: DAD1 A, Sig=205,4 Ref=off

RT [min]	Width [min]	Height	Area	Area%
19.829	0.3907	626.3342	16034.0625	50.2161
21.503	0.4269	570.8279	15896.0400	49.7839
		Sum	31930.1025	100.0000

zwf-7-150H
Nov 09 2023
SOLVENT: CDCl3
NA = 4
F1 = 400.140015 MHz
F2 = 1.000000 MHz

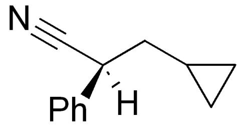

47

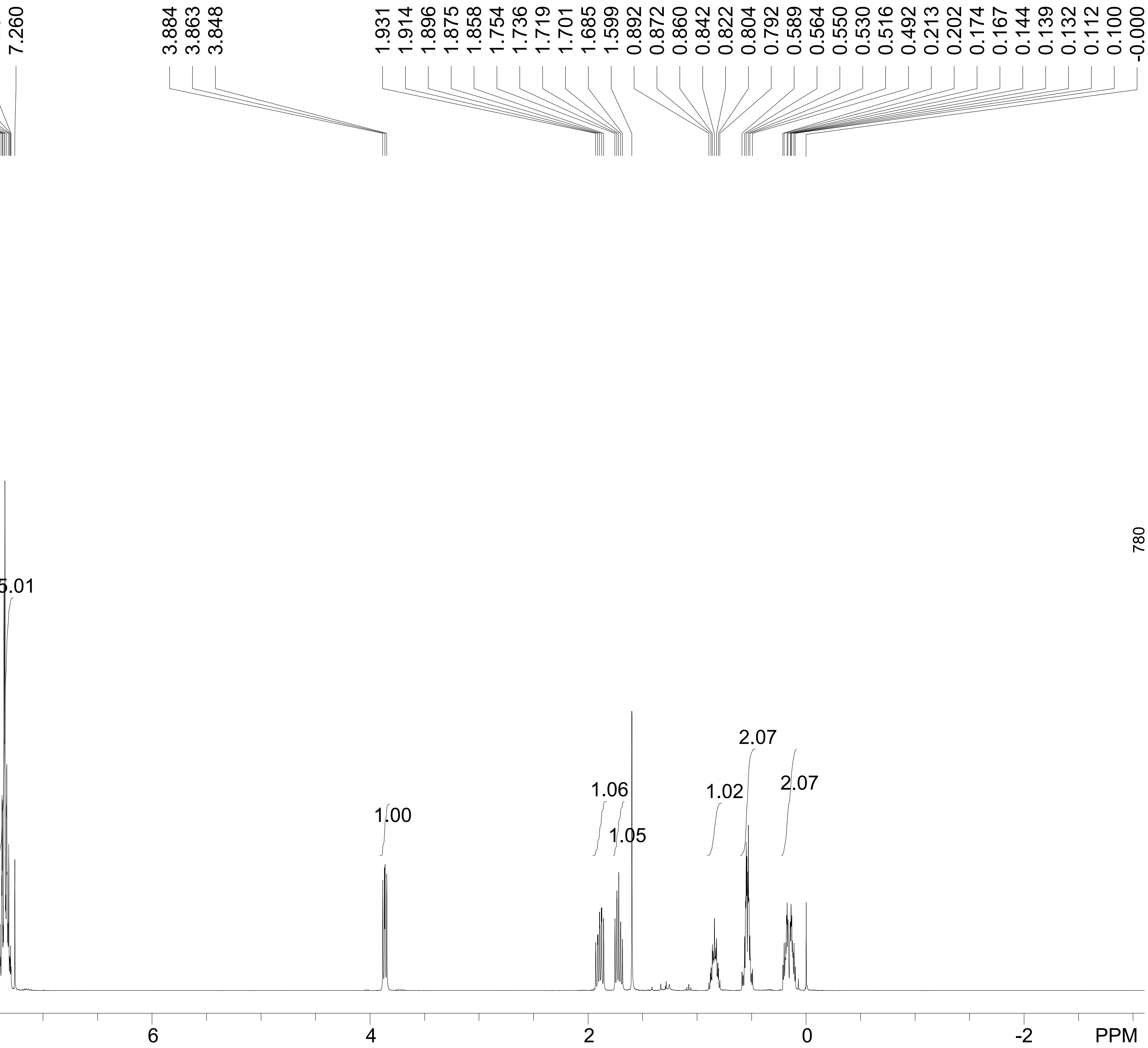

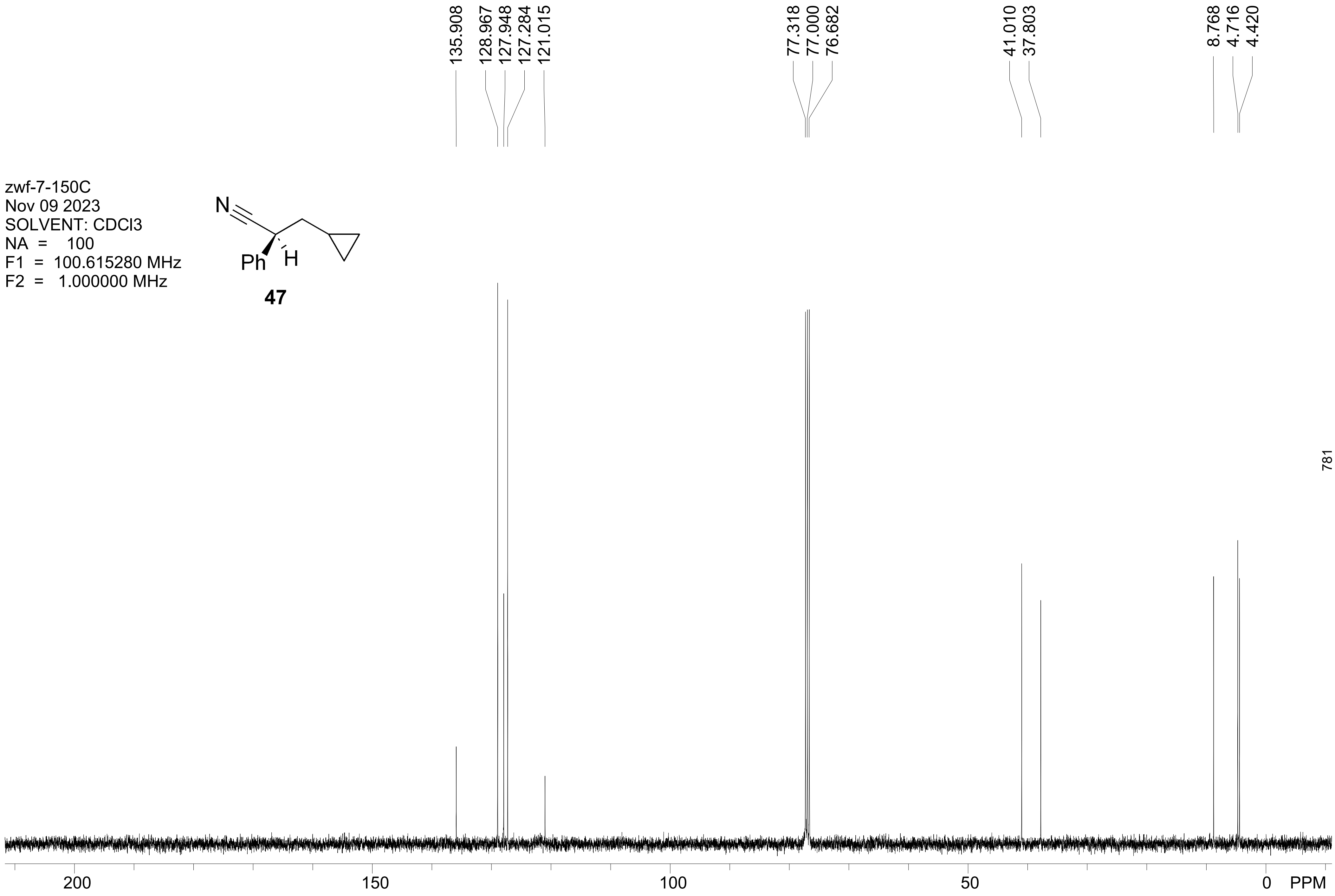
zwf-7-150C
Nov 09 2023
SOLVENT: CDCl3
NA = 100
F1 = 100.615280 MHz
F2 = 1.000000 MHz
135.908
128.967
127.948
127.284
121.015
77.318
77.000
76.682
41.010
37.803
8.768
4.716
4.420
200
150
100
50
0
PPM
N
Ph
H

47

Area Percent Report

Agilent Technologies

Sample name: zwf-7-150-IE-99-1-0.8-205

Data file: C:\Users\Public\Documents\ChemStation\1\Data\zwf\zwf-S 2023-11-09 09-41-30\004-P1-C3-zwf-7-150.D

Injection Acquired Date 11/09/2023 10:46:47

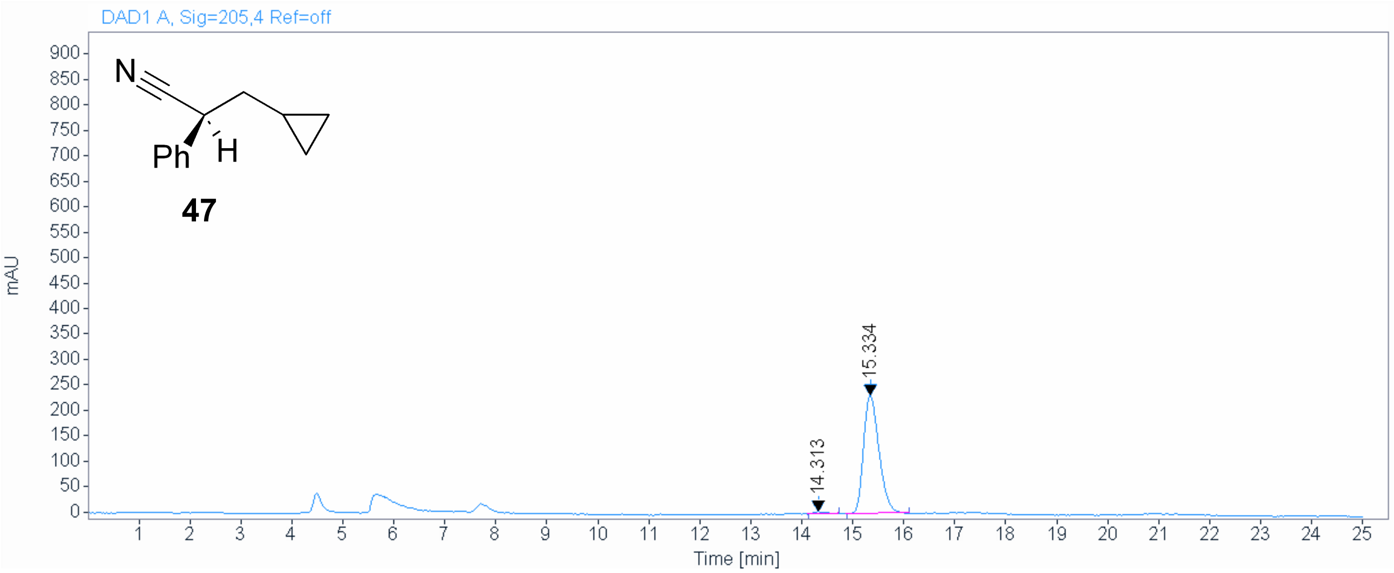

Signal: DAD1 A, Sig=205,4 Ref=off

RT [min]	Width [min]	Height	Area	Area%
14.313	0.3226	4.6642	90.2838	1.8070
15.334	0.3209	231.8116	4906.0991	98.1930
		Sum	4996.3829	100.0000

Area Percent Report

Agilent Technologies

Sample name: zwf-7-150-rac-IE-99-1-0.8-205

Data file: C:\Users\Public\Documents\ChemStation\1\Data\zwf\zwf-S 2023-11-09 09-41-30\005-P1-C4-zwf-7-150-rac.D

Injection Acquired Date 11/09/2023 11:12:53

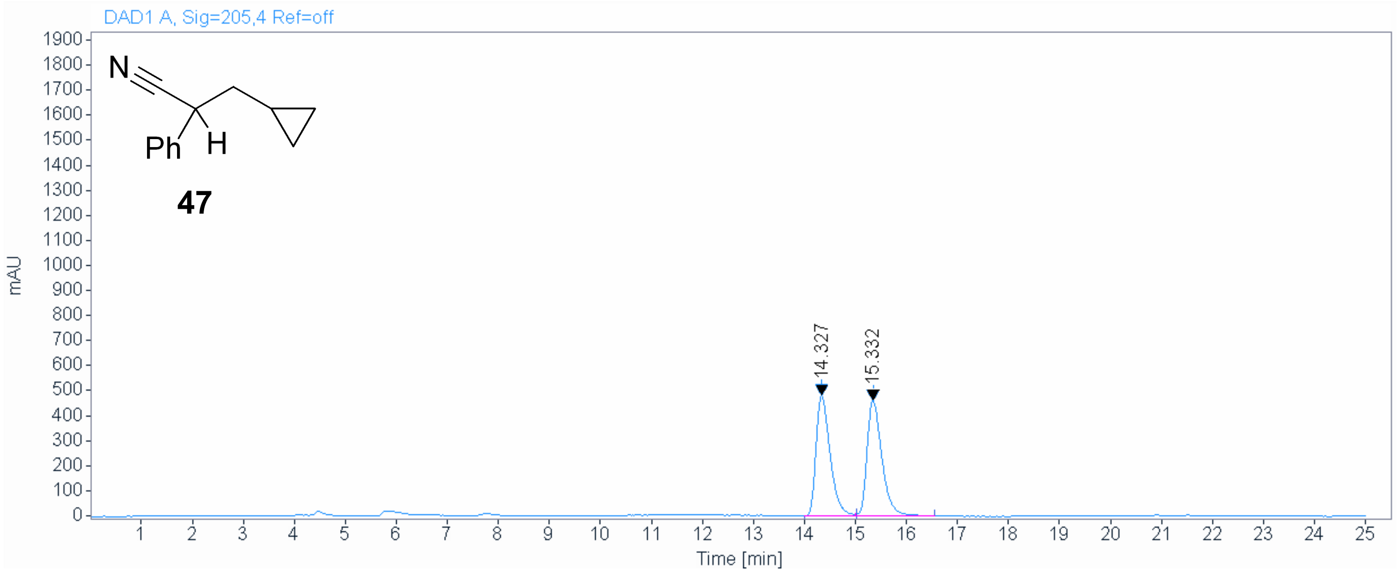

Signal: DAD1 A, Sig=205,4 Ref=off

RT [min]	Width [min]	Height	Area	Area%
14.327	0.2858	480.4202	9216.5127	49.6330
15.332	0.3066	459.5301	9352.8301	50.3670
		Sum	18569.3428	100.0000

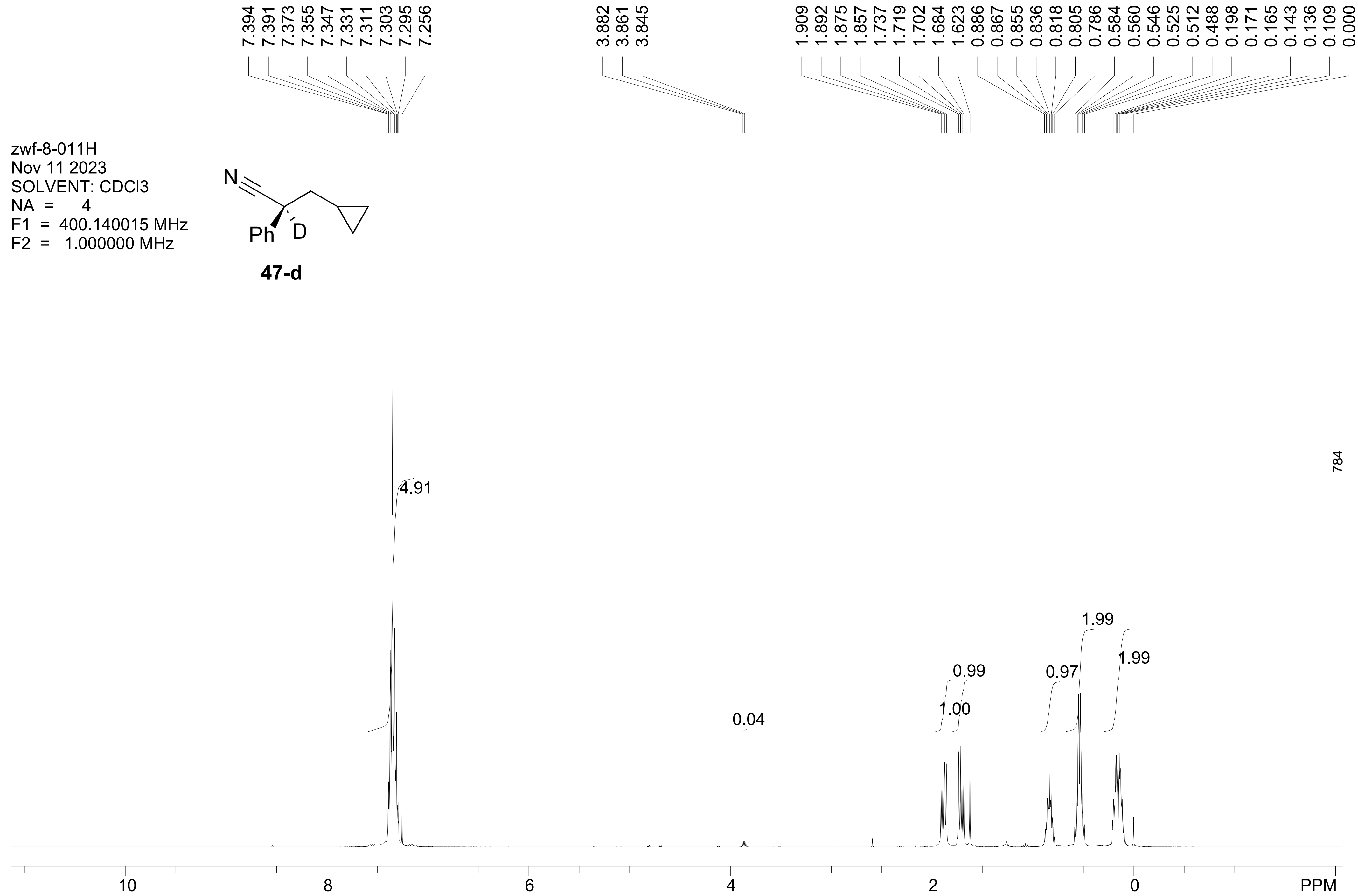
zwf-8-011H
Nov 11 2023
SOLVENT: CDCl3
NA = 4
F1 = 400.140015 MHz
F2 = 1.000000 MHz
N
Ph
D
47-d
7.394
7.391
7.373
7.355
7.347
7.331
7.311
7.303
7.295
7.256
3.882
3.861
3.845
1.909
1.892
1.875
1.857
1.737
1.719
1.702
1.684
1.623
0.886
0.867
0.855
0.836
0.818
0.805
0.786
0.584
0.560
0.546
0.525
0.512
0.488
0.198
0.171
0.165
0.143
0.136
0.109
0.000
4.91
0.04
1.00
0.99
0.97
1.99
1.99
10
8
6
4
2
0
PPM

zwf-8-011C
Nov 13 2023
SOLVENT: CDCl3
NA = 150
F1 = 100.615280 MHz
F2 = 1.000000 MHz

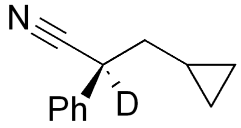

47-d

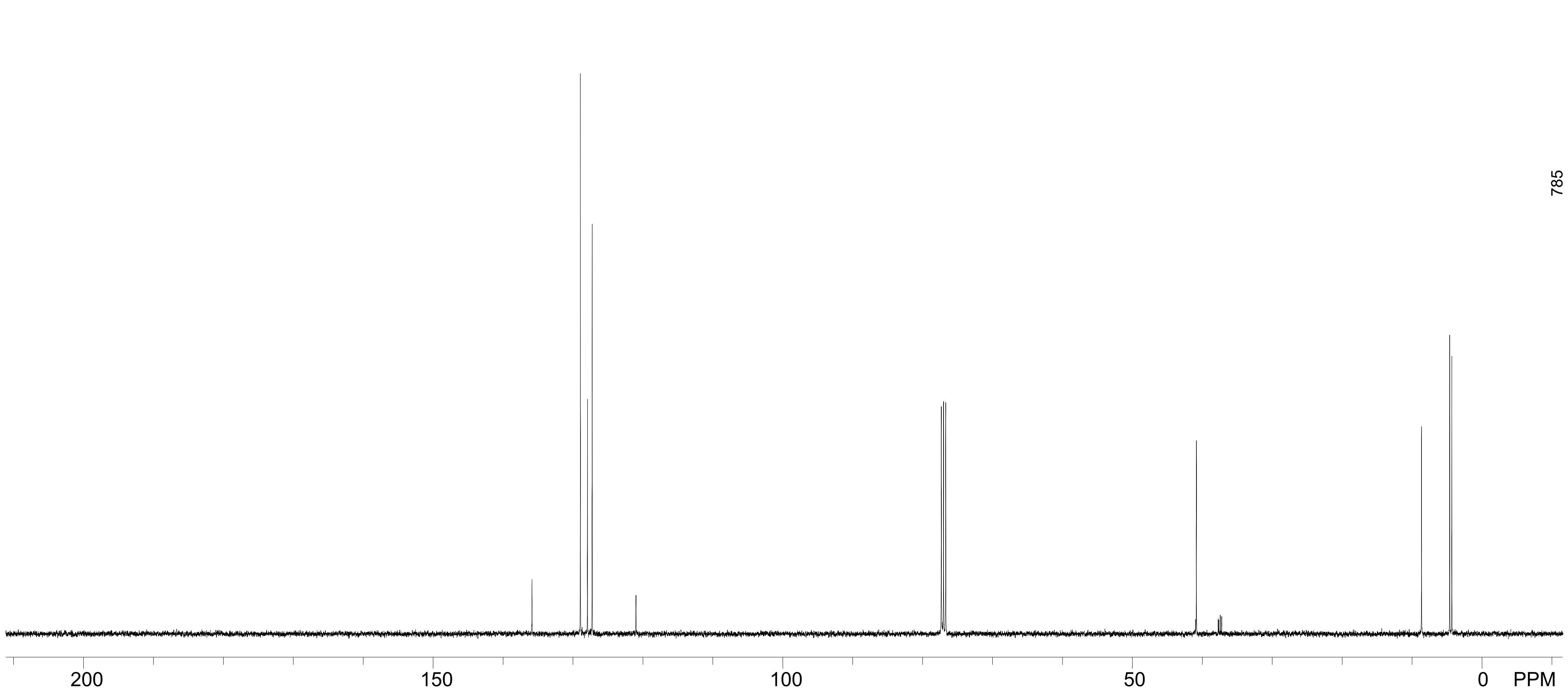

Area Percent Report

Agilent Technologies

Sample name: zwf-8-011-IE-99-1-0.8-205

Data file: C:\Users\Public\Documents\ChemStation\1\Data\zwf\zwf-S 2023-11-12 11-02-17\042-P1-A3-zwf-8-011.D

Injection Acquired Date 11/13/2023 16:55:11

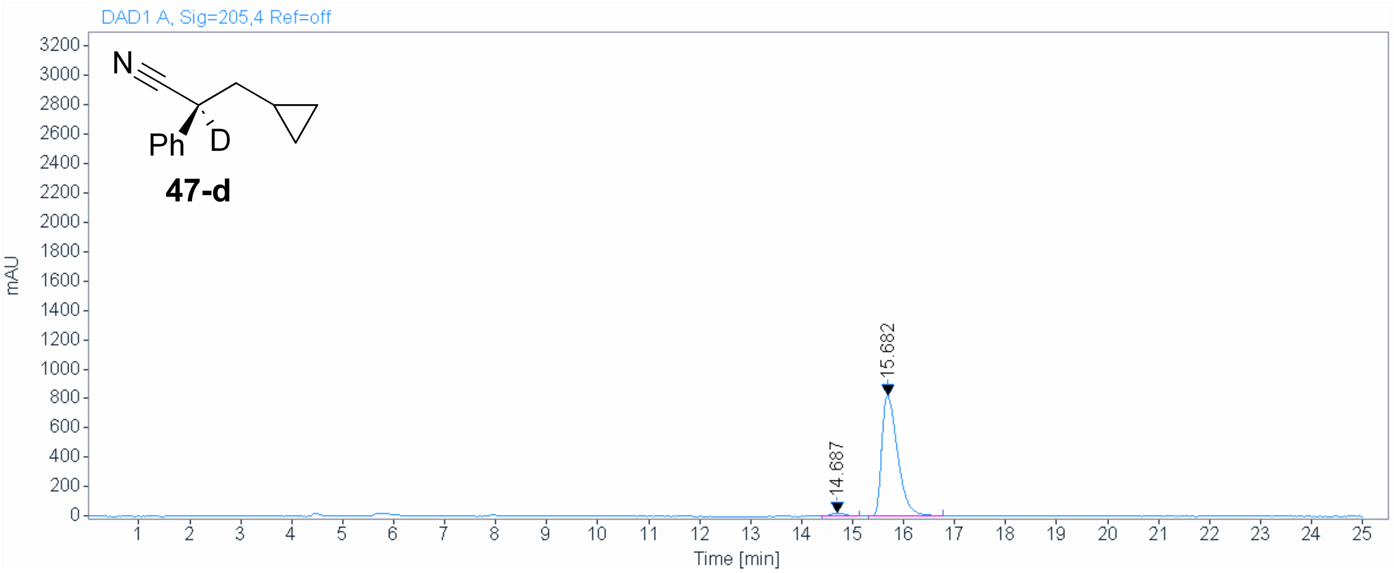

Signal: DAD1 A, Sig=205,4 Ref=off

RT [min]	Width [min]	Height	Area	Area%
14.687	0.2265	21.1574	396.2180	2.1819
15.682	0.2981	821.8474	17763.1816	97.8181
		Sum	18159.3996	100.0000

Area Percent Report

Agilent Technologies

Sample name: zwf-8-011-rac-IE-99-1-0.8-205

Data file: C:\Users\Public\Documents\ChemStation\1\Data\zwf\zwf-S 2023-11-12 11-02-17\043-P1-A4-zwf-8-011-rac.D

Injection Acquired Date 11/13/2023 17:21:12

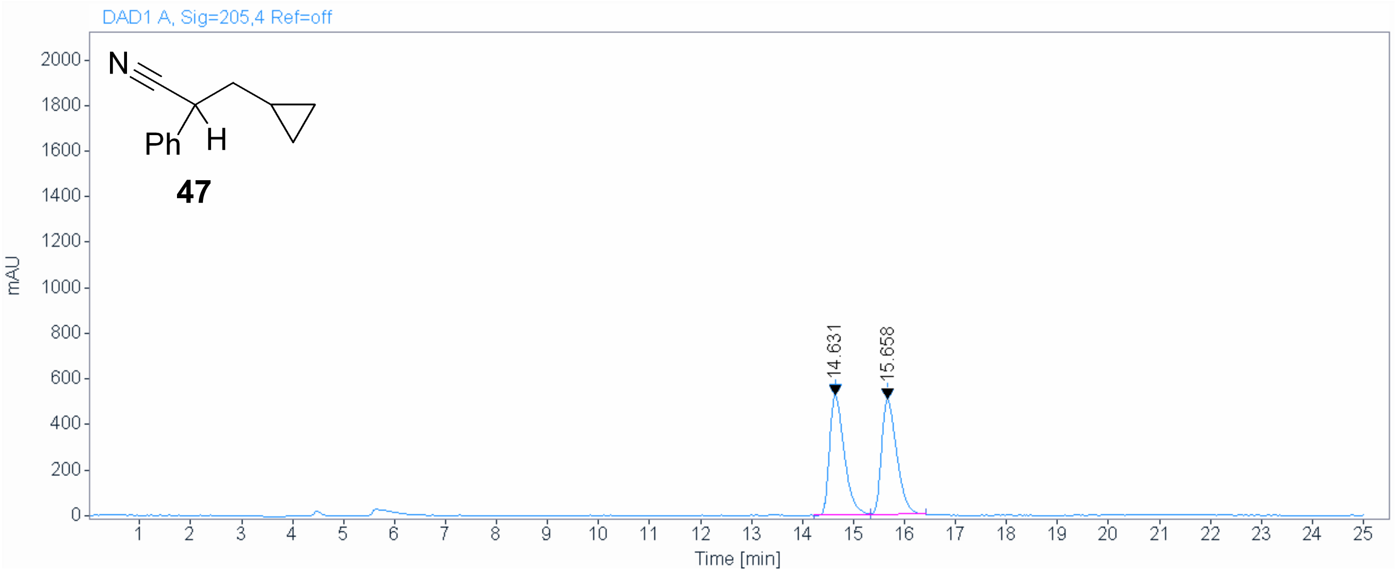

Signal: DAD1 A, Sig=205,4 Ref=off

RT [min]	Width [min]	Height	Area	Area%
14.631	0.3085	525.6851	10657.3281	50.2037
15.658	0.3214	506.5823	10570.8281	49.7963
		Sum	21228.1563	100.0000

zwf-7-089H
Sep 26 2023
SOLVENT: CDCl3
NA = 4
F1 = 400.140015 MHz
F2 = 1.000000 MHz

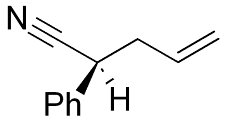

48

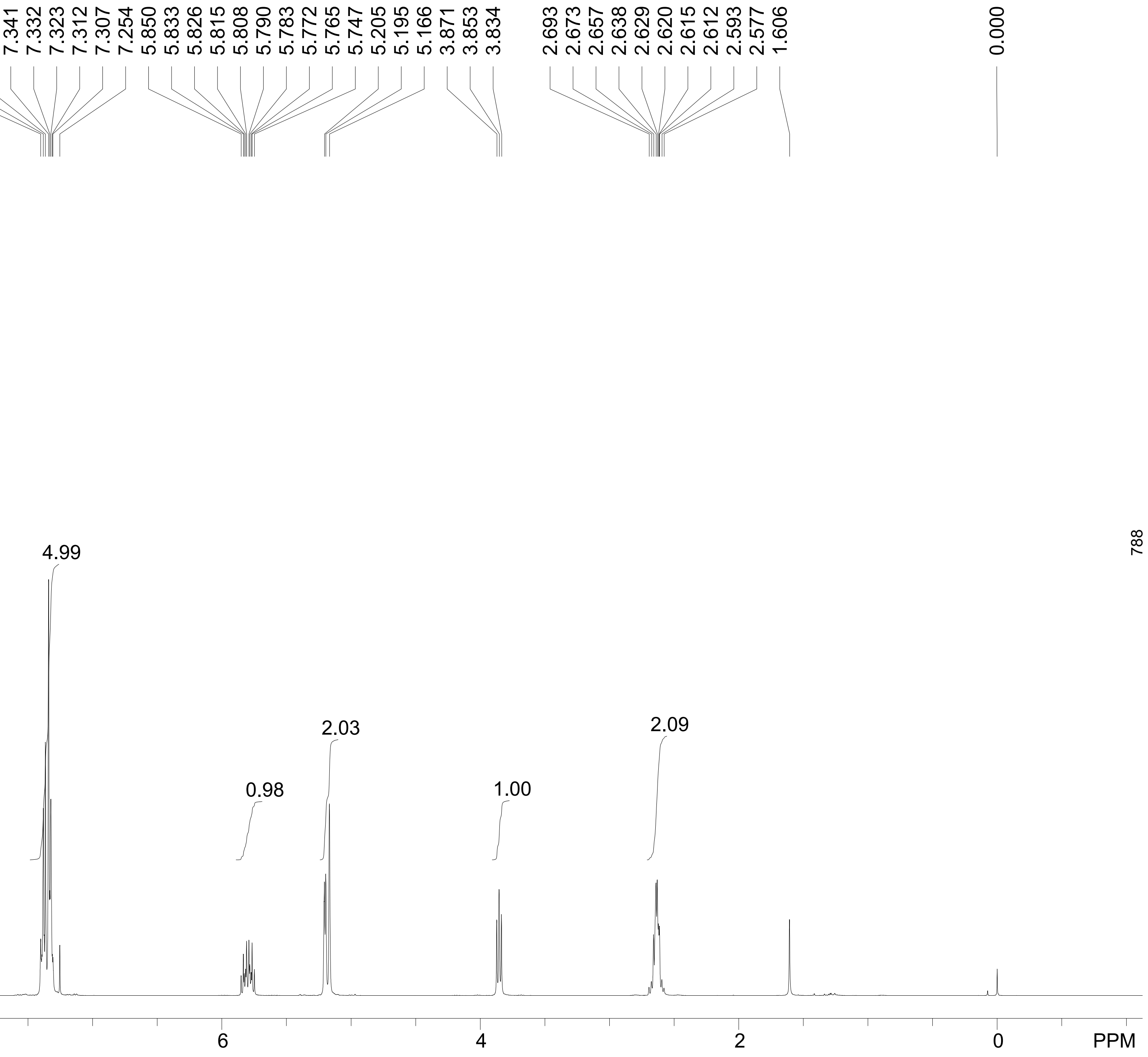

zwf-7-089C
Sep 26 2023
SOLVENT: CDCl3
NA = 150
F1 = 100.615280 MHz
F2 = 1.000000 MHz

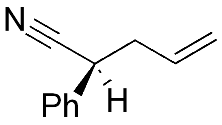

48

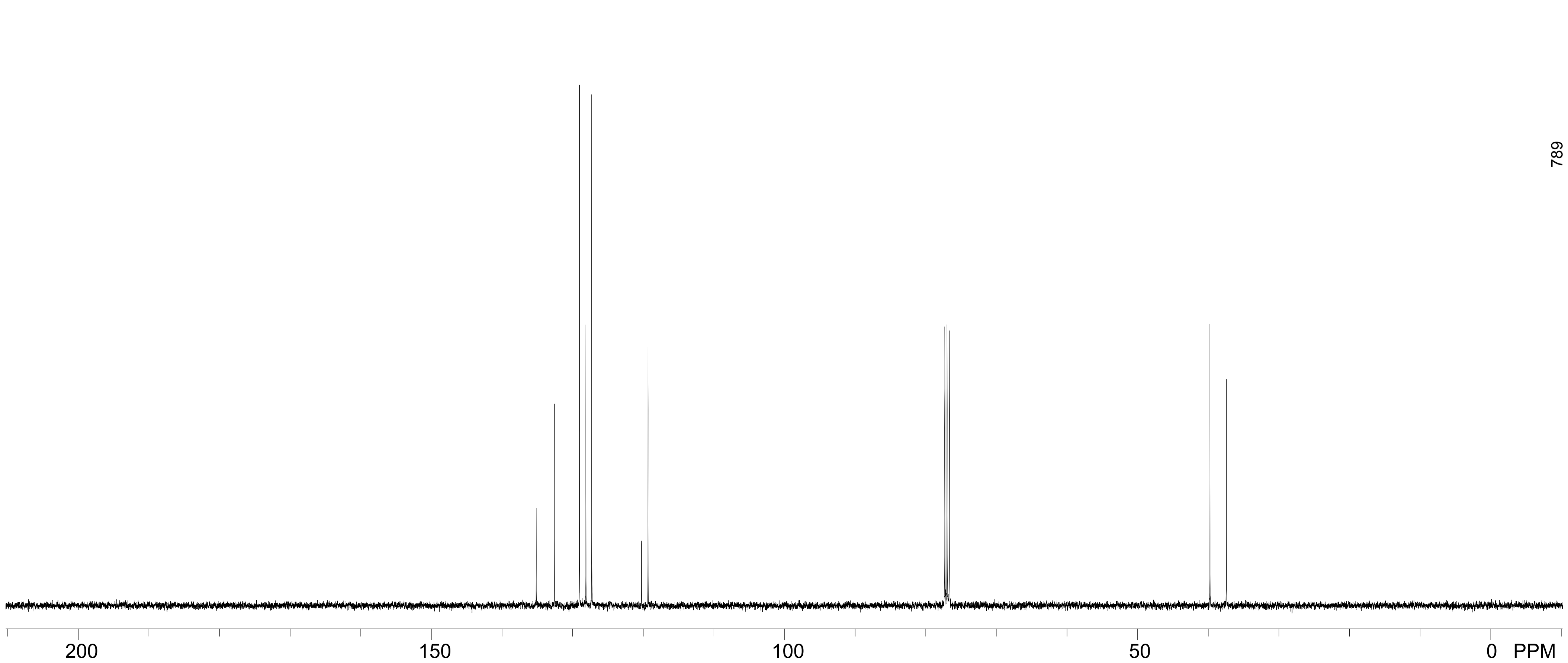

Area Percent Report

Agilent Technologies

Sample name: zwf-7-089-IE-99-1-0.8-205

Data file: C:\Users\Public\Documents\ChemStation\1\Data\zwf\zwf-S 2023-09-26 14-33-49\014-P1-A2-zwf-7-089.D

Injection Acquired Date 09/26/2023 20:16:37

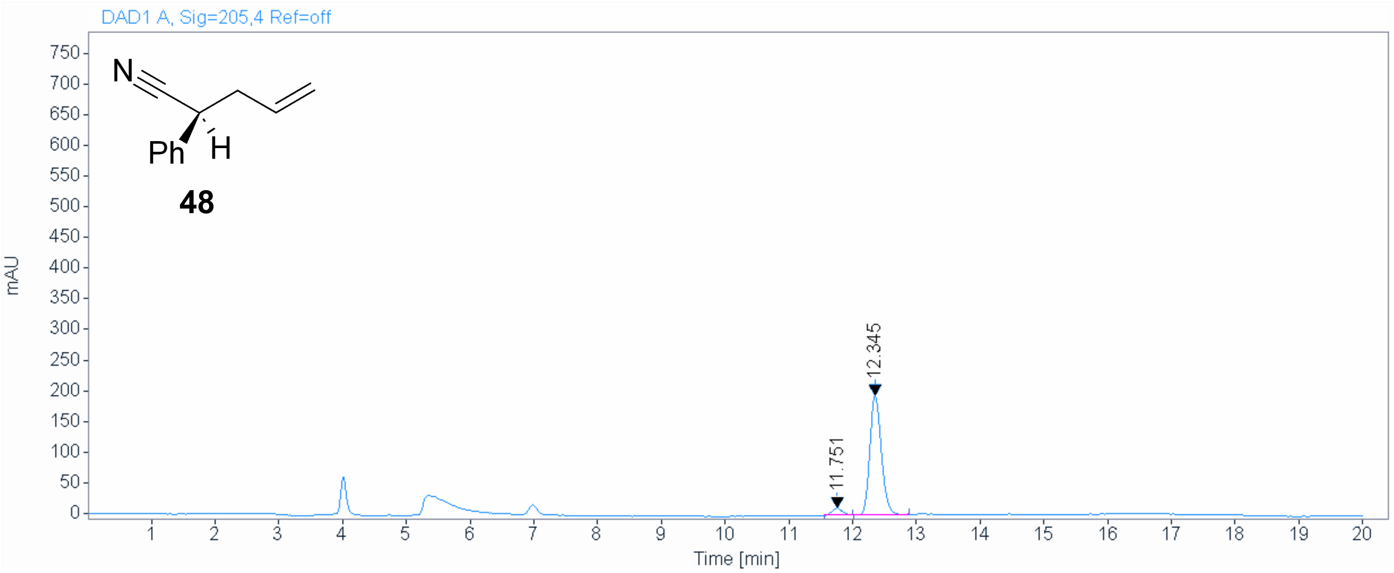

Signal: DAD1 A, Sig=205,4 Ref=off

RT [min]	Width [min]	Height	Area	Area%
11.751	0.1894	11.6737	132.6731	5.1536
12.345	0.2089	194.8030	2441.7175	94.8464
		Sum	2574.3907	100.0000

Area Percent Report

Agilent Technologies

Sample name: zwf-7-089-rac-IE-99-1-0.8-205

Data file: C:\Users\Public\Documents\ChemStation\1\Data\zwf\zwf-S 2023-09-26 14-33-49\013-P1-C4-zwf-7-089-rac.D

Injection Acquired Date 09/26/2023 19:55:32

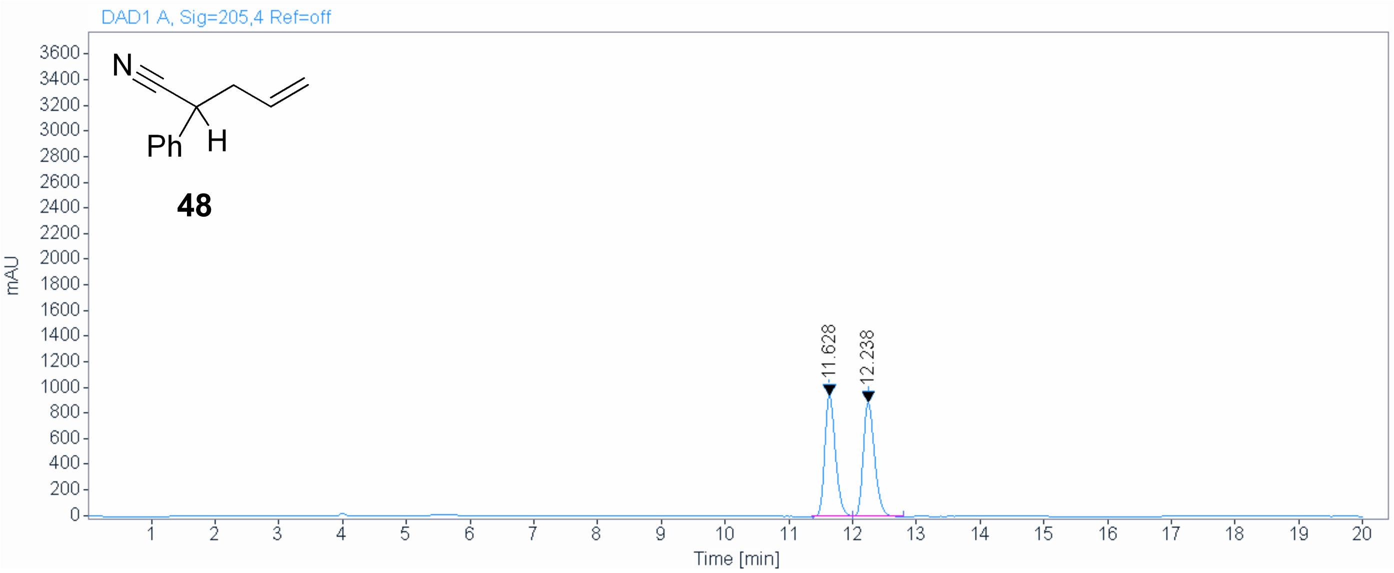

Signal: DAD1 A, Sig=205,4 Ref=off

RT [min]	Width [min]	Height	Area	Area%
11.628	0.1820	943.9556	11005.8330	49.9512
12.238	0.1925	890.1953	11027.3516	50.0488
		Sum	22033.1846	100.0000

zwf-8-012H
Nov 12 2023
SOLVENT: CDCl3
NA = 4
F1 = 400.140015 MHz
F2 = 1.000000 MHz

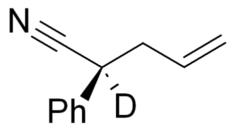

48-d

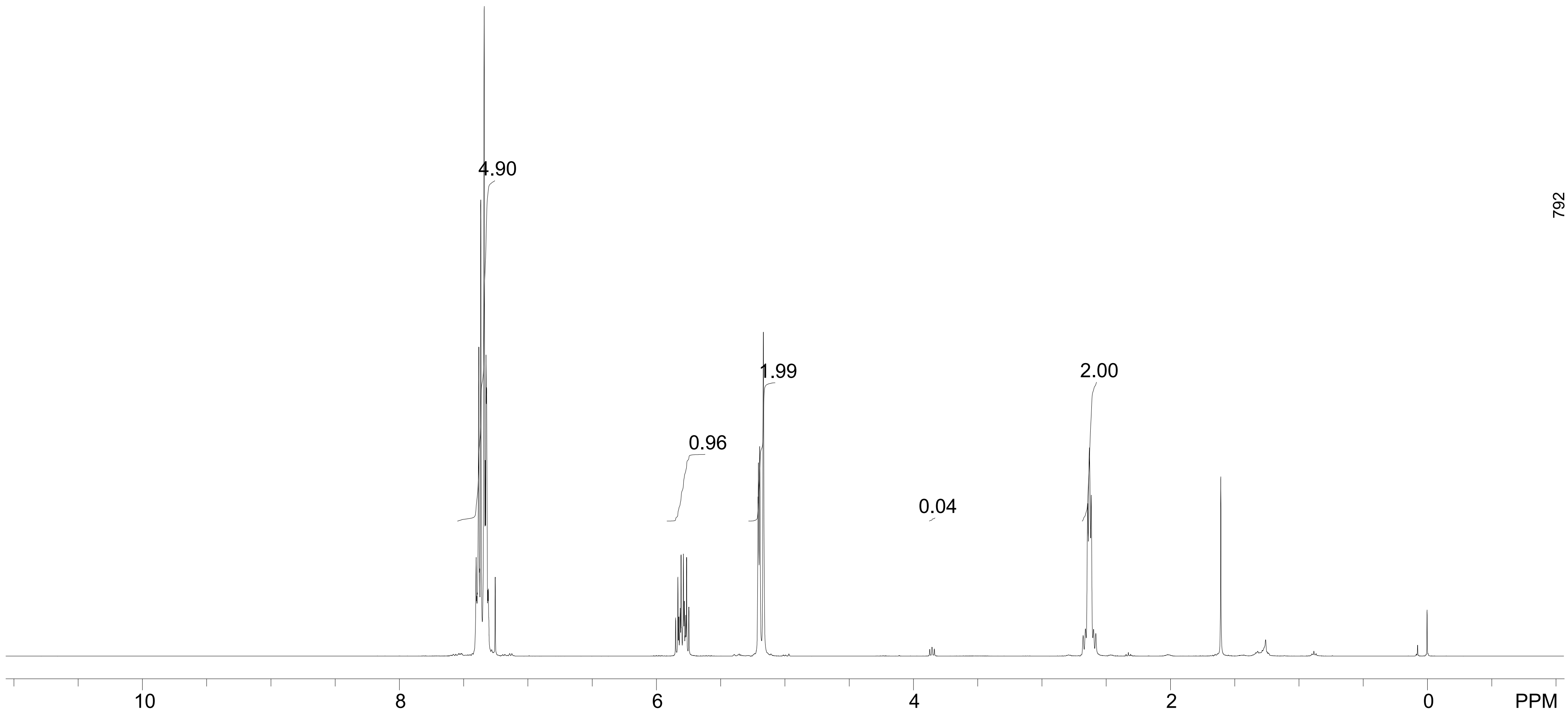

zwf-8-012C
Nov 12 2023
SOLVENT: CDCl3
NA = 150
F1 = 100.615280 MHz
F2 = 1.000000 MHz

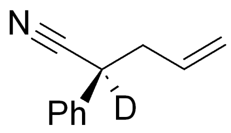

48-d

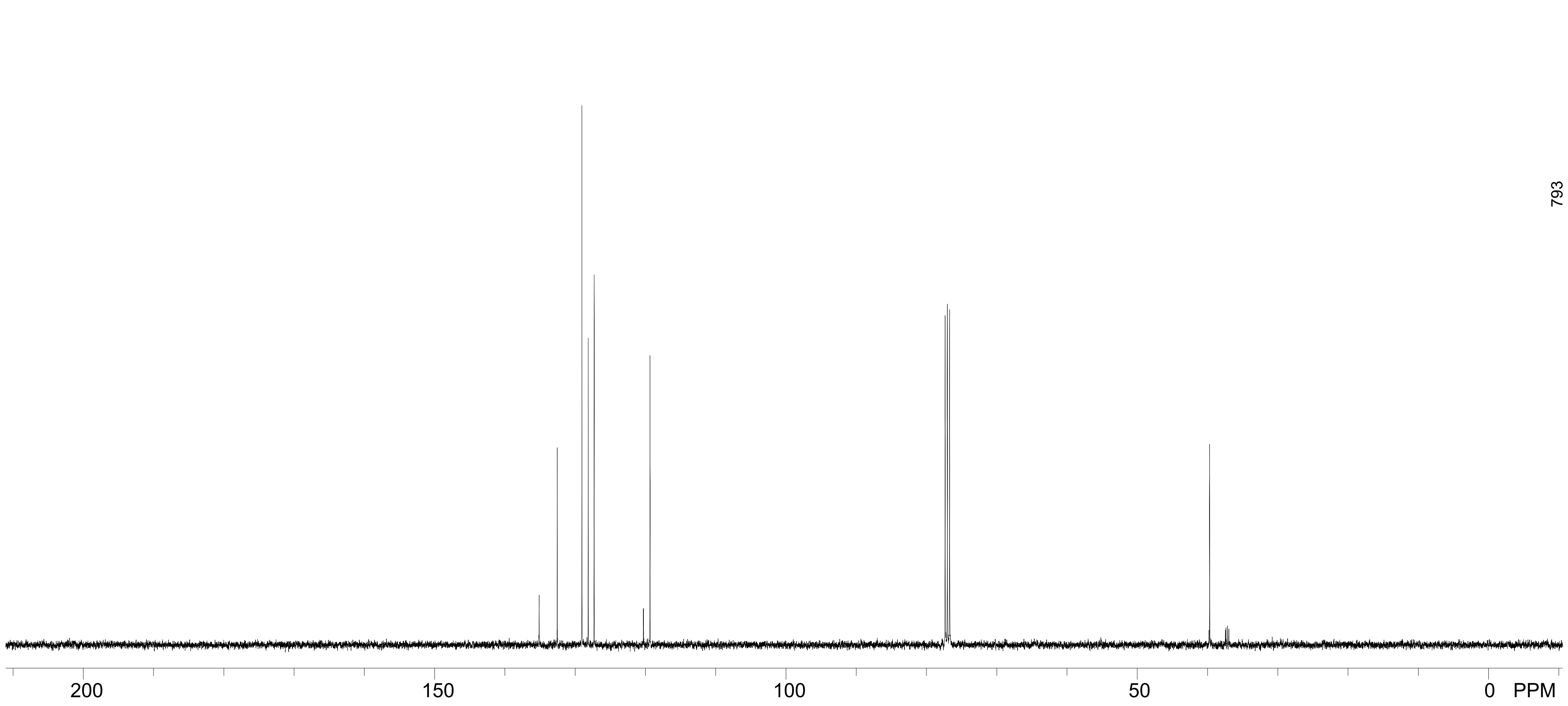

Area Percent Report

Agilent Technologies

Sample name: zwf-8-012-IE-99-1-0.8-205

Data file: C:\Users\Public\Documents\ChemStation\1\Data\zwf\zwf-S 2023-11-12 11-02-17\044-P1-A5-zwf-8-012.D

Injection Acquired Date 11/13/2023 17:47:15

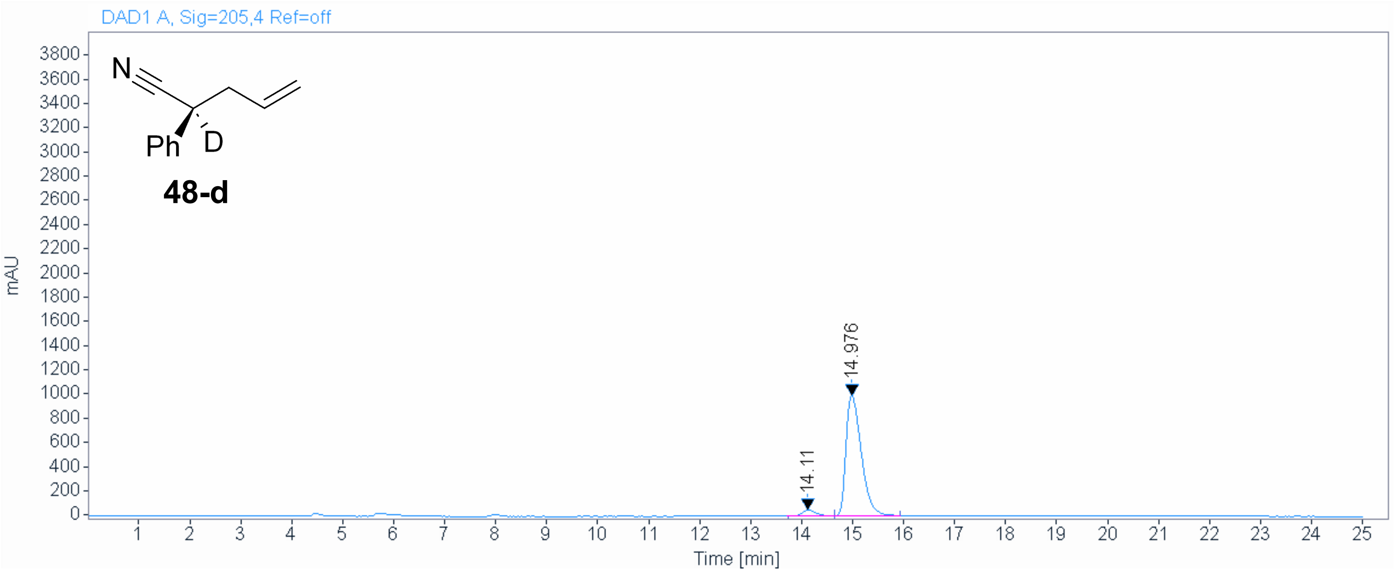

Signal: DAD1 A, Sig=205,4 Ref=off

RT [min]	Width [min]	Height	Area	Area%
14.110	0.2259	51.1805	929.5098	4.2794
14.976	0.3143	992.9155	20790.9922	95.7206
		Sum	21720.5020	100.0000

Area Percent Report

Sample name: zwf-8-012-rac-IE-99-1-0.8-205

Data file: C:\Users\Public\Documents\ChemStation\1\Data\zwf\zwf-S 2023-11-12 11-02-17\048-P1-A6-zwf-8-012-rac.D

Injection Acquired Date 11/13/2023 19:41:23

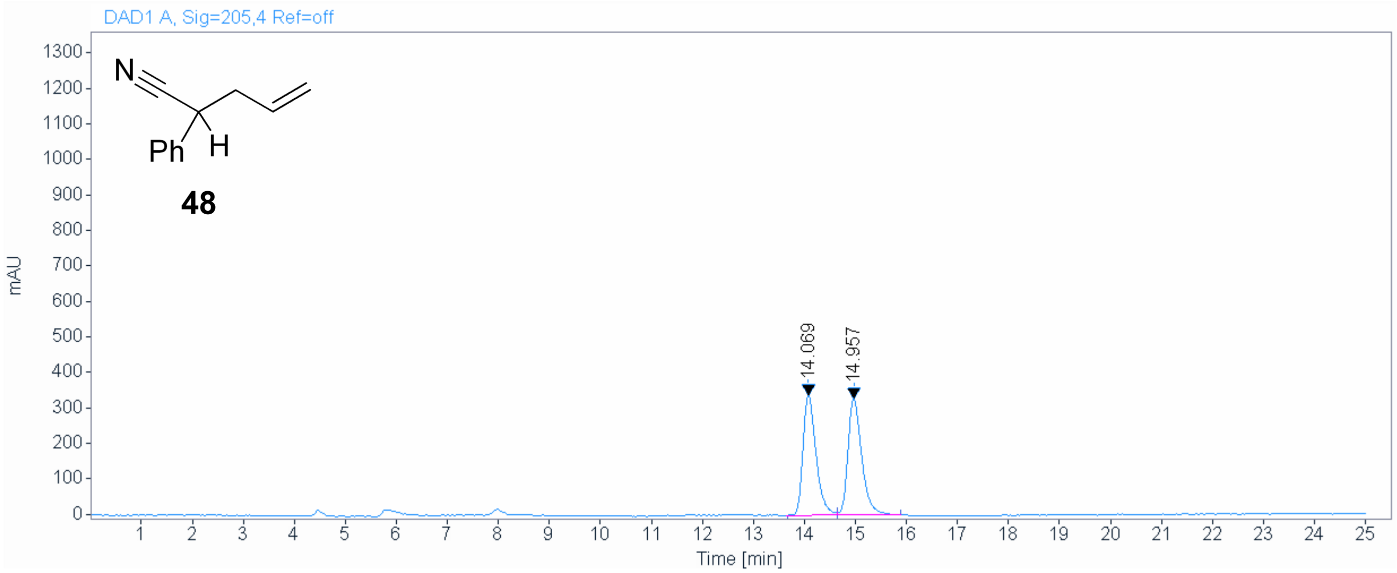

Signal: DAD1 A, Sig=205,4 Ref=off

RT [min]	Width [min]	Height	Area	Area%
14.069	0.2723	338.4803	6101.1787	49.4208
14.957	0.2716	328.1117	6244.1777	50.5792
		Sum	12345.3564	100.0000

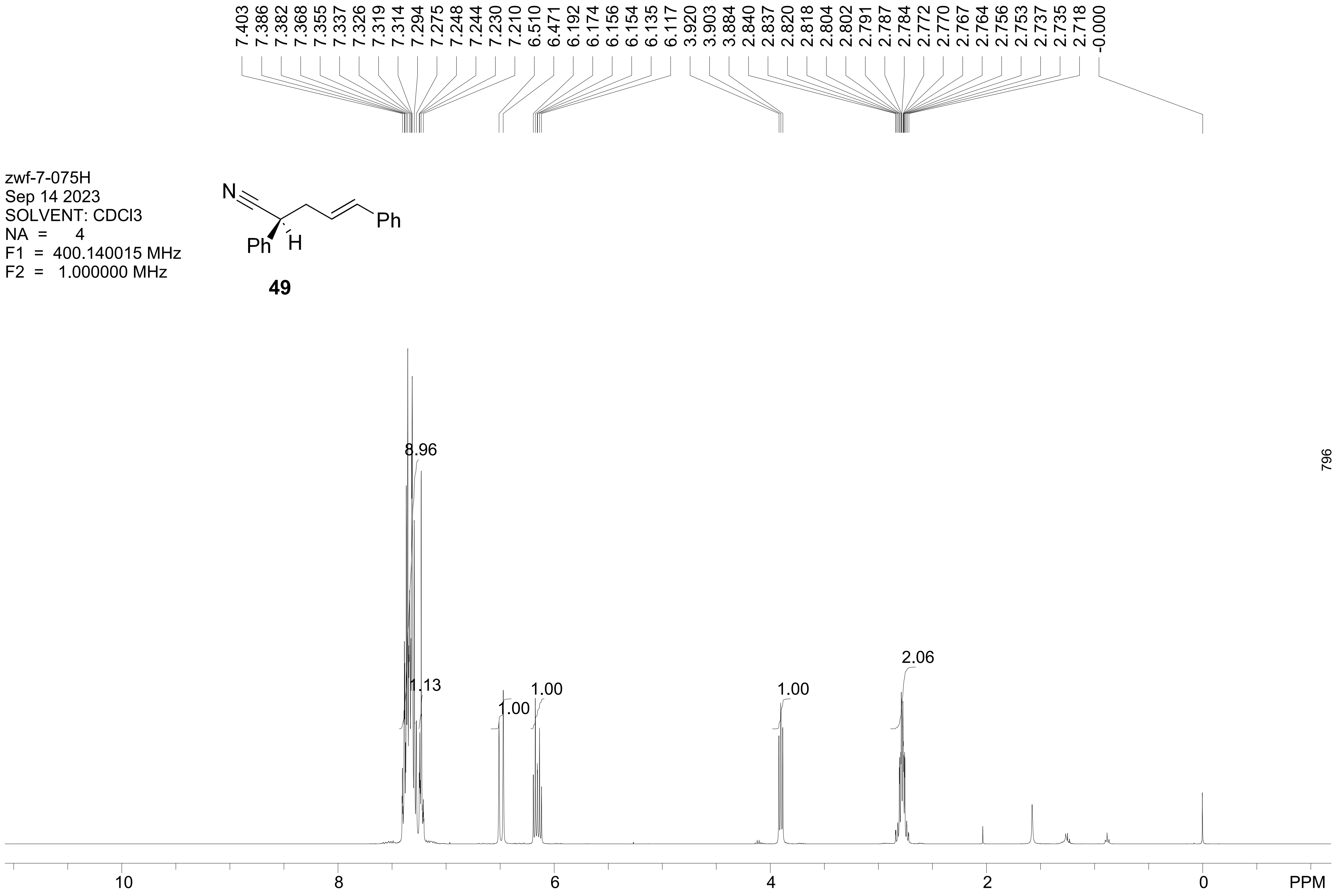
zwf-7-075H
Sep 14 2023
SOLVENT: CDCl3
NA = 4
F1 = 400.140015 MHz
F2 = 1.000000 MHz
N
Ph
Ph
H
49
7.403
7.386
7.382
7.368
7.355
7.337
7.326
7.319
7.314
7.294
7.275
7.248
7.244
7.230
7.210
6.510
6.471
6.192
6.174
6.156
6.154
6.135
6.117
3.920
3.903
3.884
2.840
2.837
2.820
2.818
2.804
2.802
2.791
2.787
2.784
2.772
2.770
2.767
2.764
2.756
2.753
2.737
2.735
2.718
-0.000
8.96
1.13
1.00
1.00
1.00
2.06
10
8
6
4
2
0
PPM

zwf-7-075C
Sep 14 2023
SOLVENT: CDCl3
NA = 100
F1 = 100.615280 MHz
F2 = 1.000000 MHz

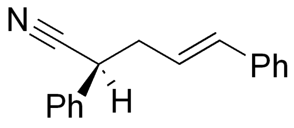

49

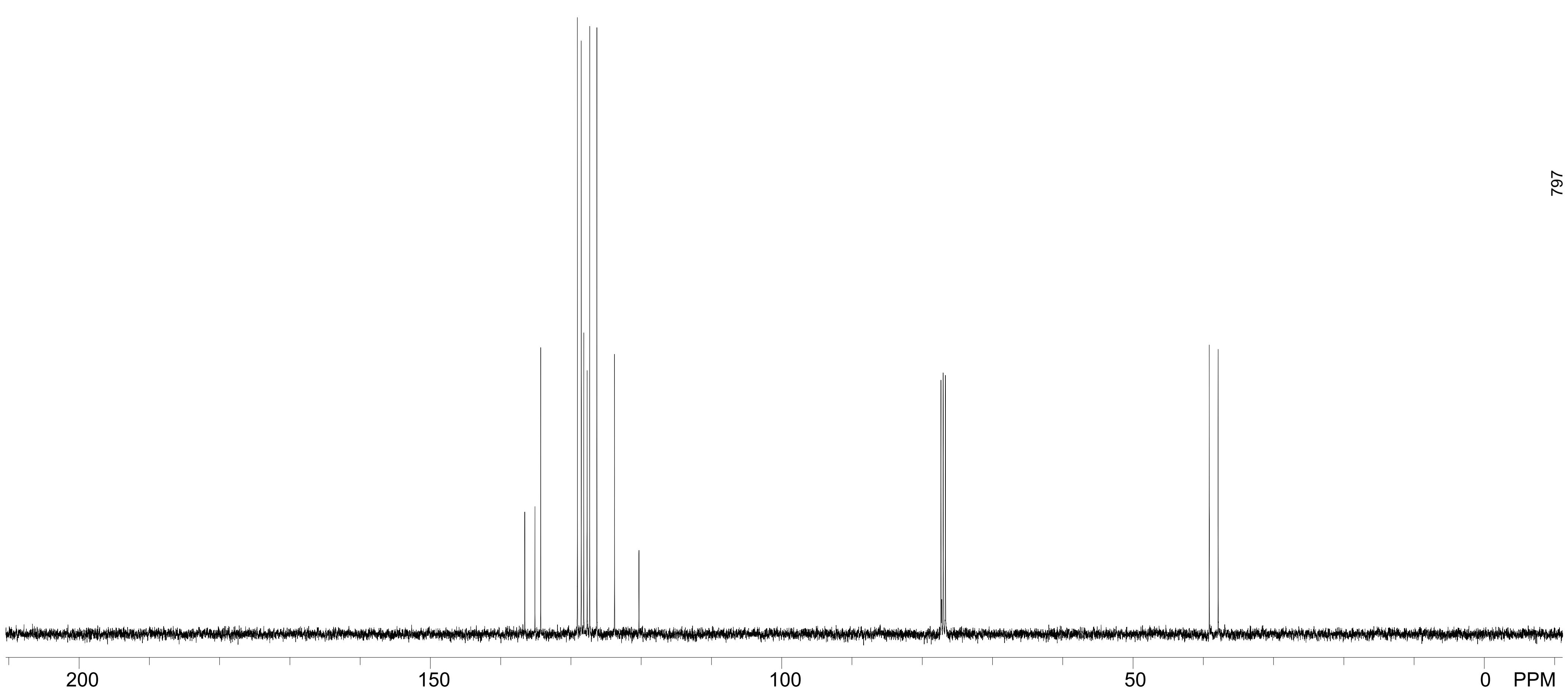

Area Percent Report

Agilent Technologies

Sample name: zwf-7-075-IE-99-1-0.8-205

Data file: C:\Users\Public\Documents\ChemStation\1\Data\zwf\zwf-S 2023-09-23 15-02-57\004-P1-C1-zwf-7-075.D

Injection Acquired Date 09/23/2023 16:32:51

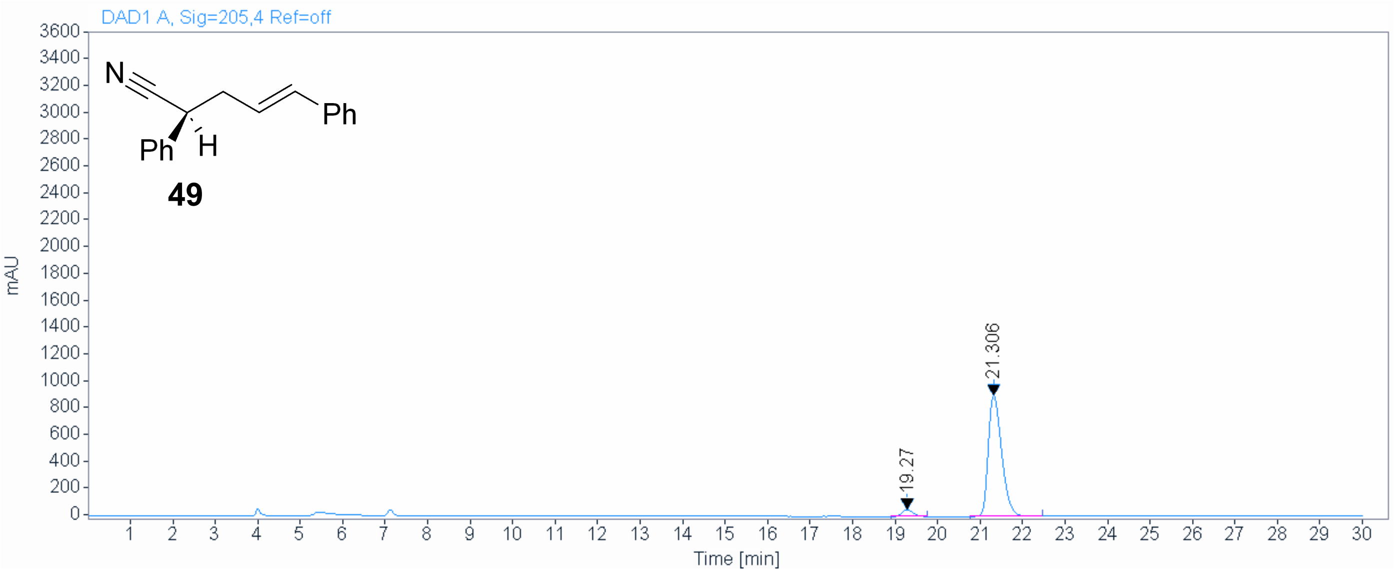

Signal: DAD1 A, Sig=205,4 Ref=off

RT [min]	Width [min]	Height	Area	Area%
19.270	0.2849	52.9759	984.8961	4.7351
21.306	0.3669	900.1759	19815.1055	95.2649
		Sum	20800.0015	100.0000

Area Percent Report

Agilent Technologies

Sample name: zwf-7-075-rac-IE-99-1-0.8-205

Data file: C:\Users\Public\Documents\ChemStation\1\Data\zwf\zwf-S 2023-09-23 15-02-57\003-P1-C2-zwf-7-075-rac.D

Injection Acquired Date 09/23/2023 16:01:48

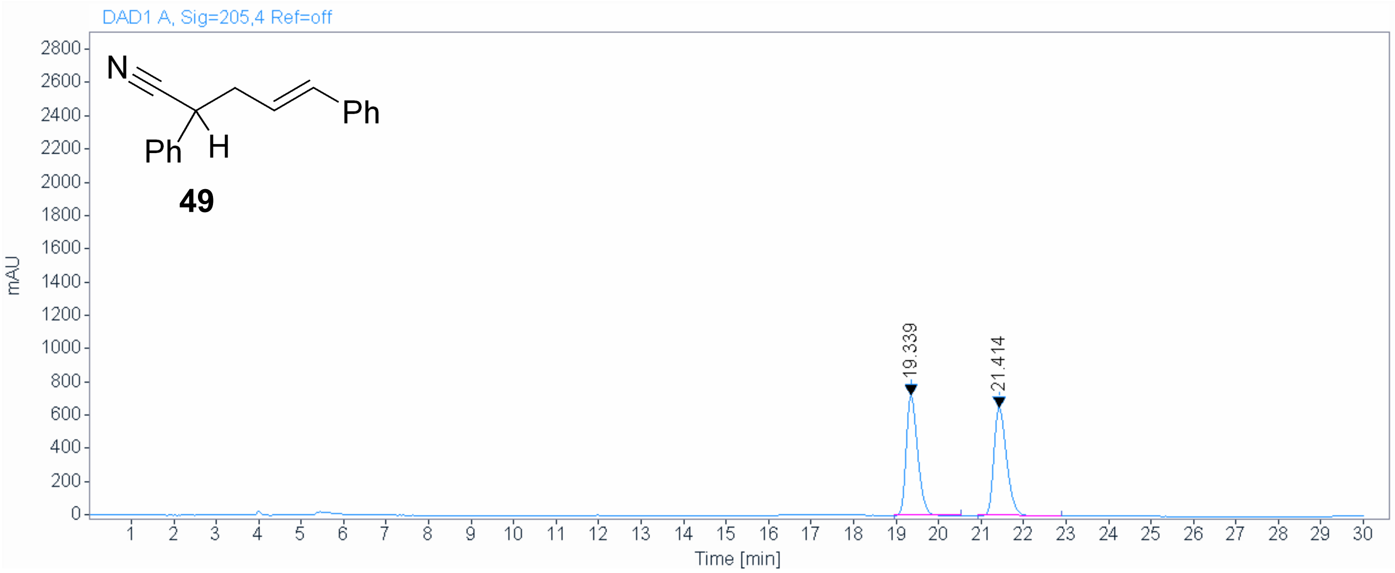

Signal: DAD1 A, Sig=205,4 Ref=off

RT [min]	Width [min]	Height	Area	Area%
19.339	0.2890	721.0330	13537.8018	49.7804
21.414	0.3245	650.3003	13657.2656	50.2196
		Sum	27195.0674	100.0000

zwf-8-014H
Nov 16 2023
SOLVENT: CDCl3
NA = 4
F1 = 400.140015 MHz
F2 = 1.000000 MHz

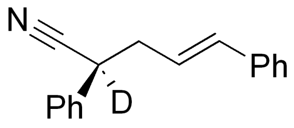

49-d

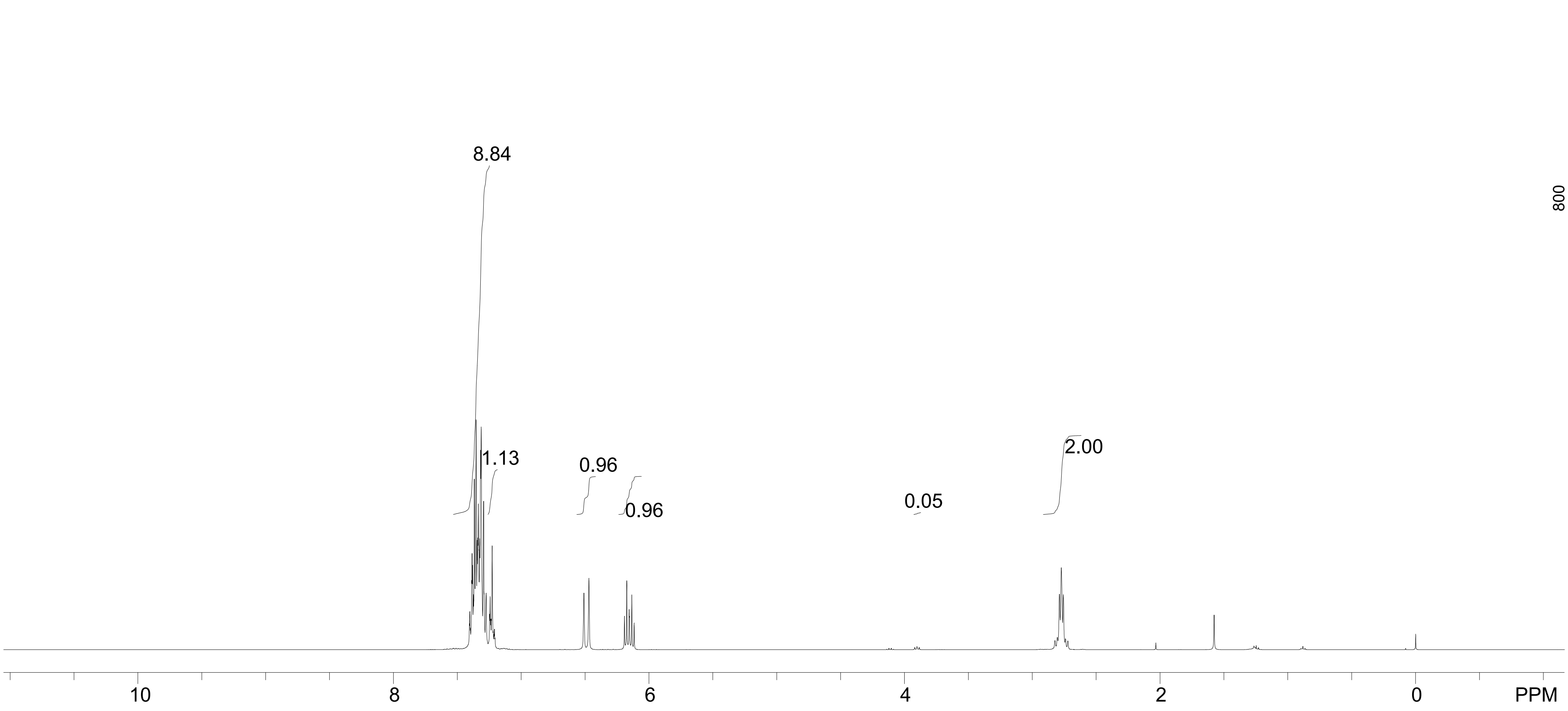

zwf-8-014C
Nov 16 2023
SOLVENT: CDCl3
NA = 150
F1 = 100.615280 MHz
F2 = 1.000000 MHz

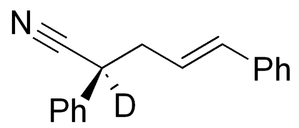

49-d

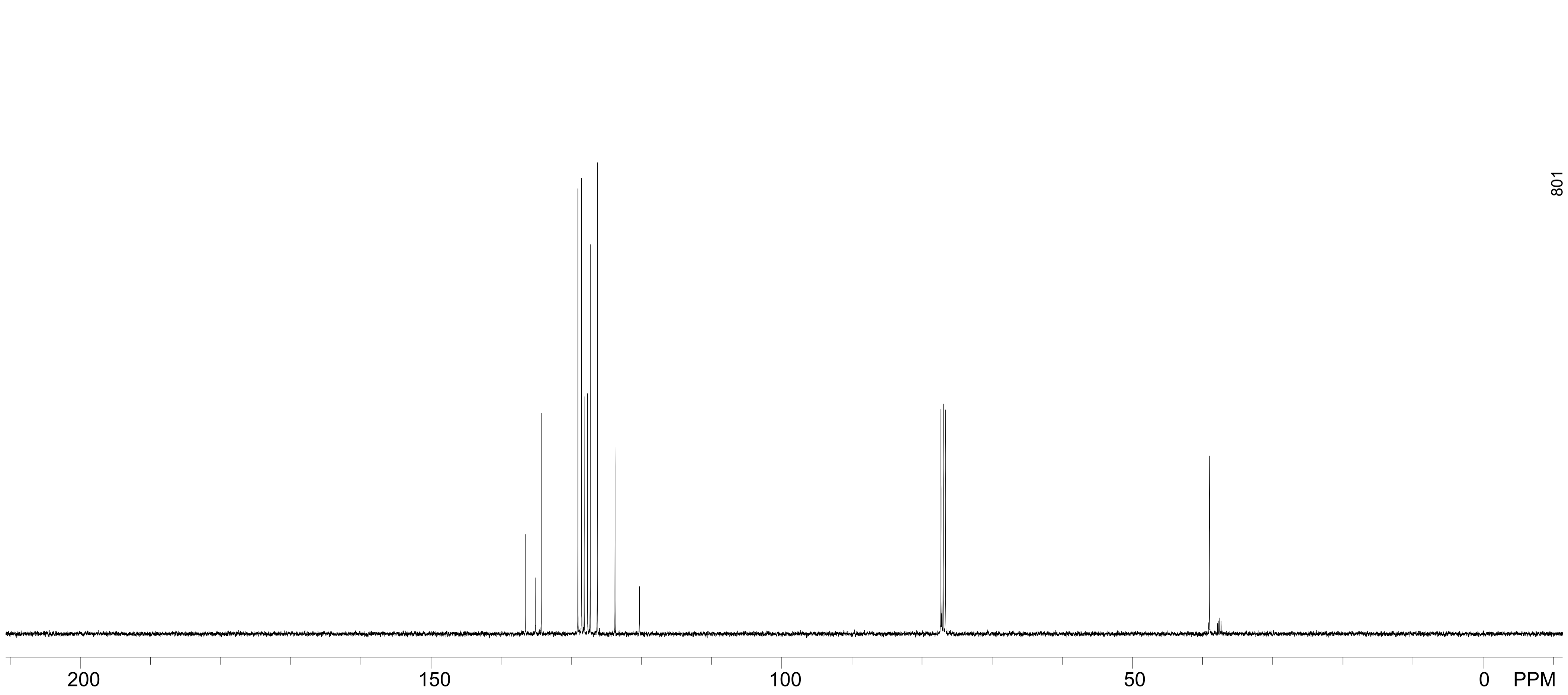

Area Percent Report

Agilent Technologies

Sample name: zwf-8-014-IE-99-1-0.8-205

Data file: C:\Users\Public\Documents\ChemStation\1\Data\zwf\zwf-S 2023-11-17 07-38-48\009-P1-B3-zwf-8-014.D

Injection Acquired Date 11/17/2023 11:48:42

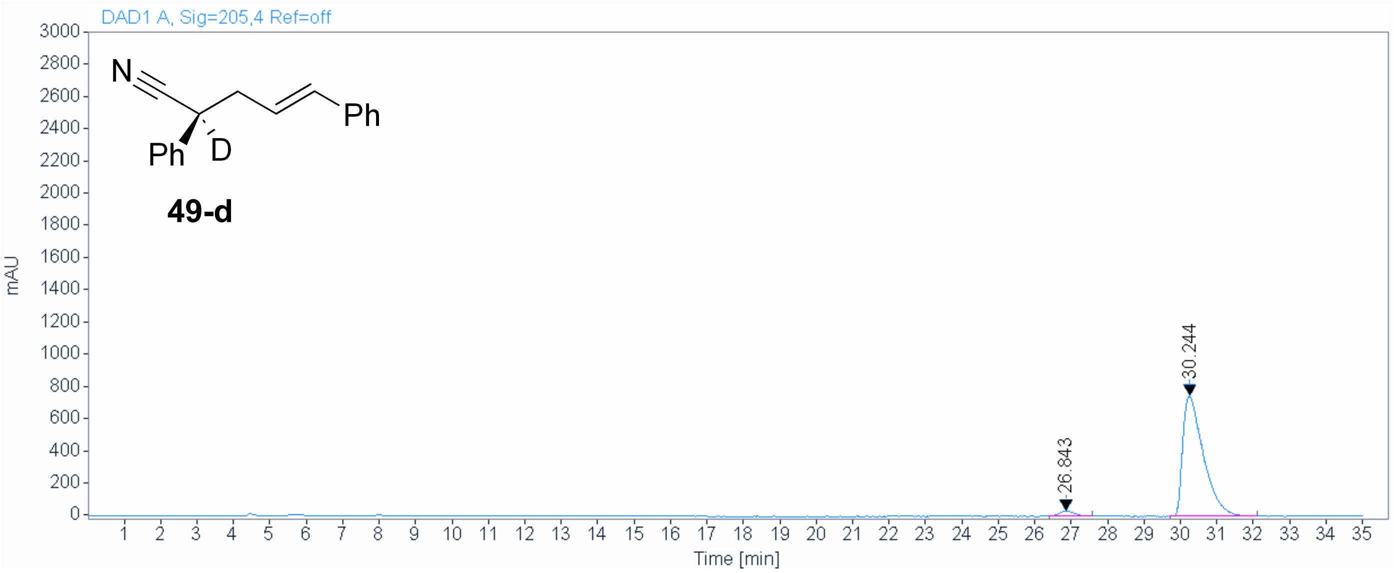

Signal: DAD1 A, Sig=205,4 Ref=off

RT [min]	Width [min]	Height	Area	Area%
26.843	0.4903	35.4591	1043.1909	3.4150
30.244	0.4710	749.8715	29503.7520	96.5850
		Sum	30546.9429	100.0000

Area Percent Report

Sample name: zwf-8-014-rac-IE-99-1-0.8-205

Data file: C:\Users\Public\Documents\ChemStation\1\Data\zwf\zwf-S 2023-11-17 07-38-48\010-P1-B4-zwf-8-014-rac.D

Injection Acquired Date 11/17/2023 12:24:41

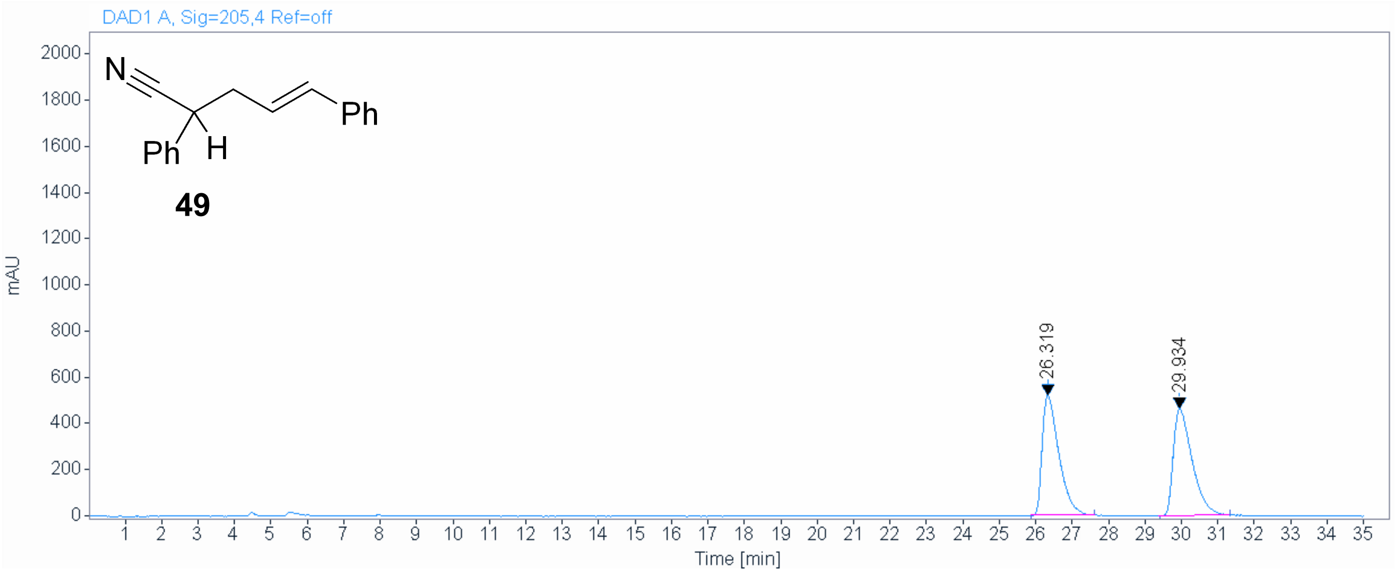

Signal: DAD1 A, Sig=205,4 Ref=off

RT [min]	Width [min]	Height	Area	Area%
26.319	0.4593	520.5095	16652.6074	49.8923
29.934	0.4943	464.0563	16724.5176	50.1077
		Sum	33377.1250	100.0000

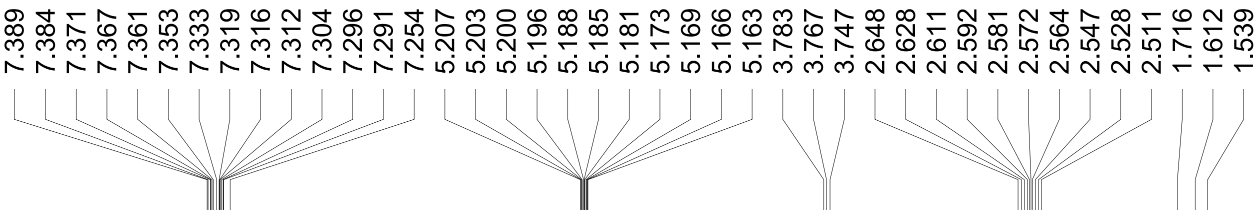

zwf-7-121H
Oct 20 2023
SOLVENT: CDCl3
NA = 4
F1 = 400.140015 MHz
F2 = 1.000000 MHz

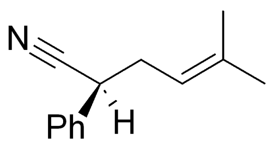

50

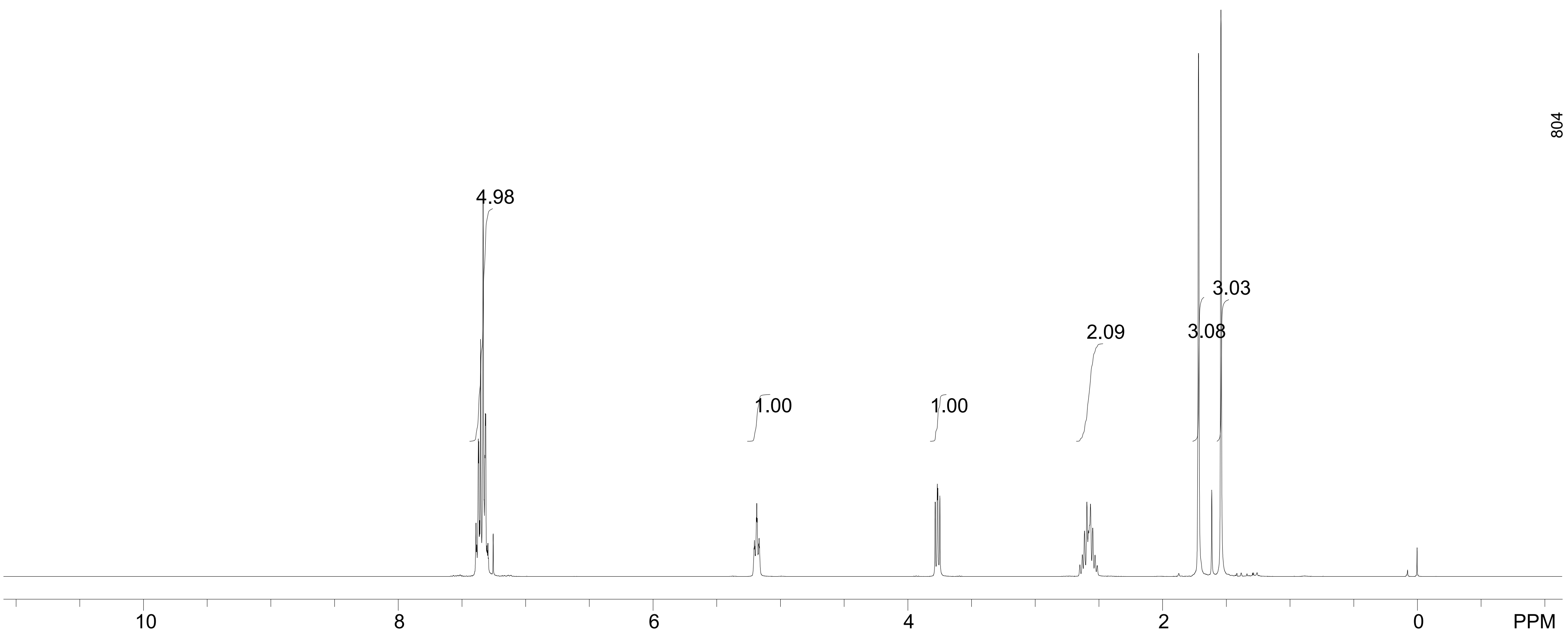

zwf-7-121C
Oct 20 2023
SOLVENT: CDCl3
NA = 100
F1 = 100.615280 MHz
F2 = 1.000000 MHz

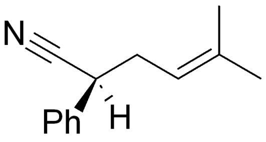

50

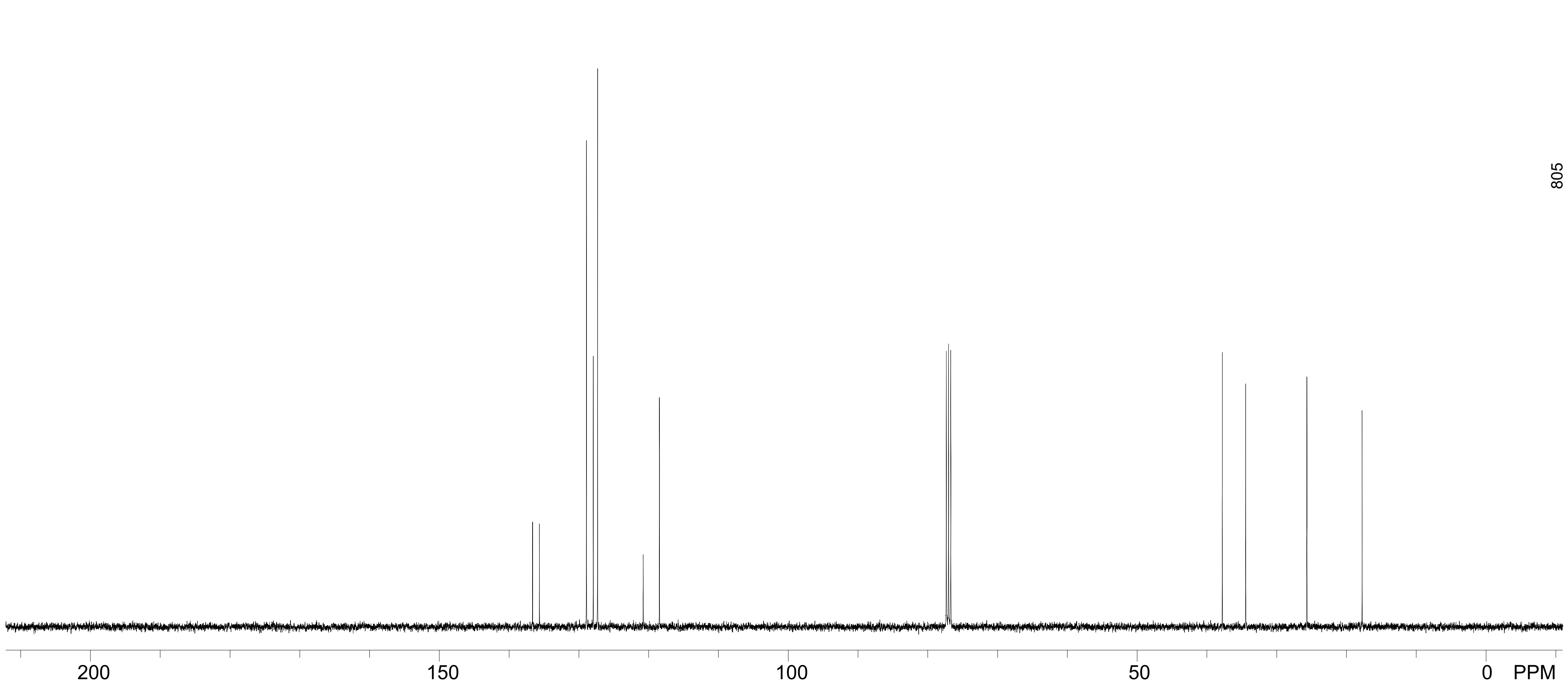

Area Percent Report

Sample name: zwf-7-121-IE-99-1-0.8-205

Data file: C:\Users\Public\Documents\ChemStation\1\Data\zwf\zwf-S 2023-10-21 17-25-18\002-P1-C4-zwf-7-121.D

Injection Acquired Date 10/21/2023 17:52:25

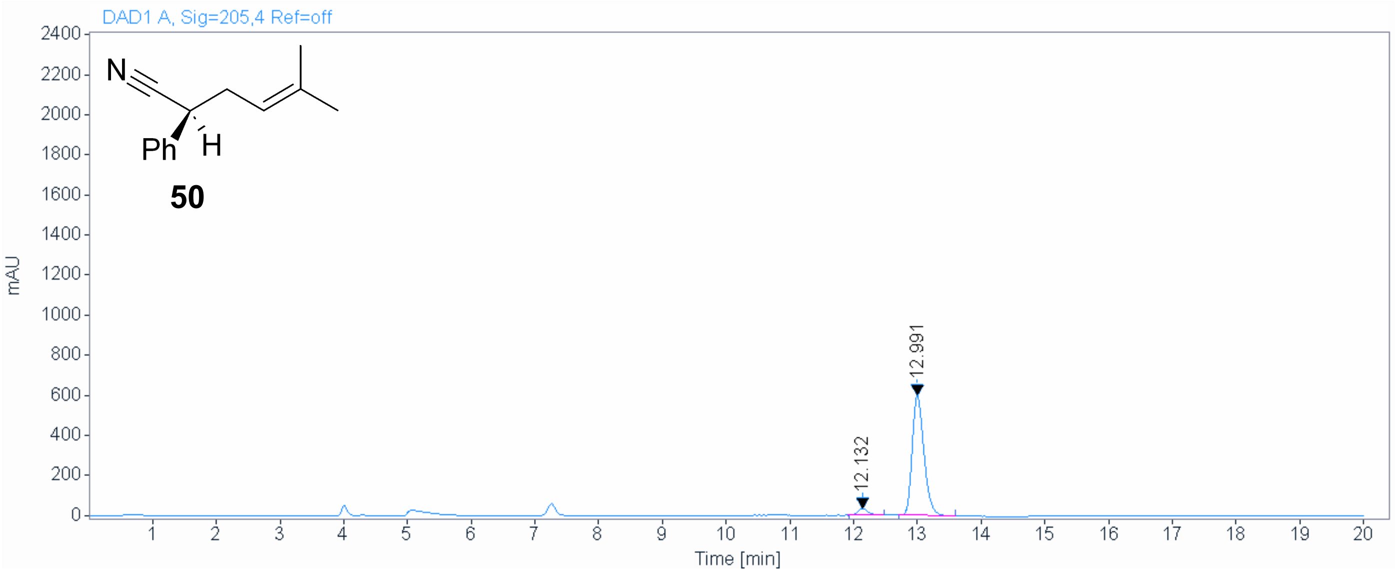

Signal: DAD1 A, Sig=205,4 Ref=off

RT [min]	Width [min]	Height	Area	Area%
12.132	0.1946	31.7814	371.0226	4.5616
12.991	0.1989	599.9845	7762.5464	95.4384
		Sum	8133.5690	100.0000

Area Percent Report

Sample name: zwf-7-121-rac-IE-99-1-0.8-205

Data file: C:\Users\Public\Documents\ChemStation\1\Data\zwf\zwf-S 2023-10-21 12-48-40\002-P1-C1-zwf-7-121-rac.D

Injection Acquired Date 10/21/2023 13:15:44

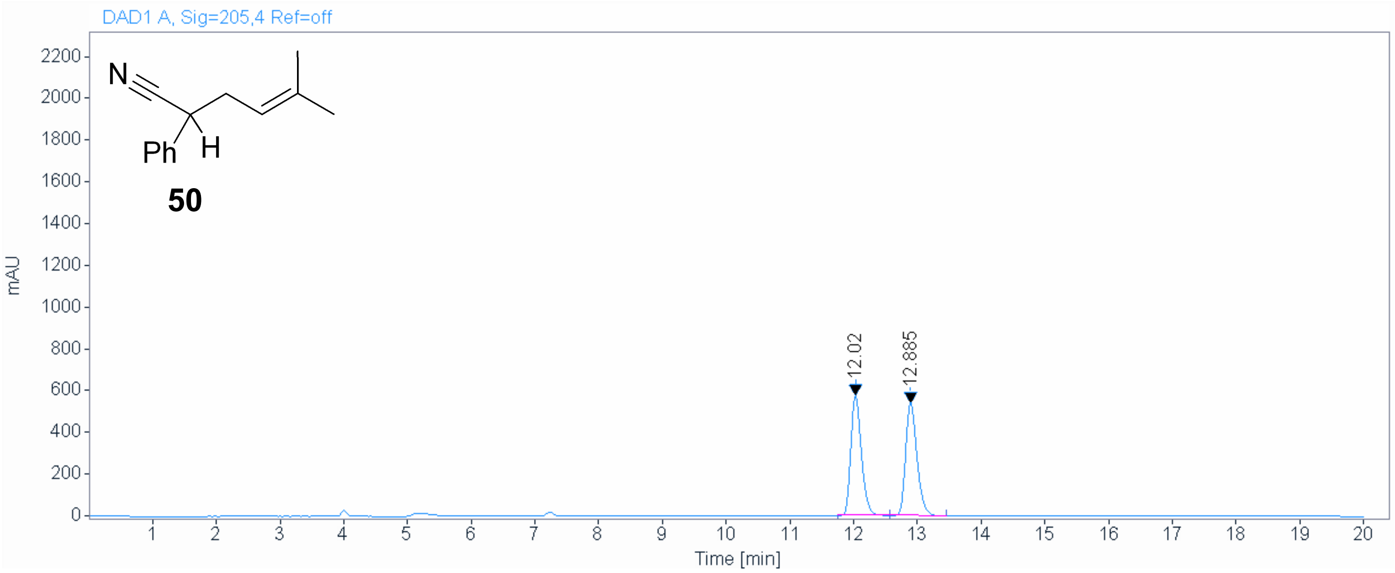

Signal: DAD1 A, Sig=205,4 Ref=off

RT [min]	Width [min]	Height	Area	Area%
12.020	0.1796	575.2828	6688.3140	49.9278
12.885	0.1891	539.1556	6707.6538	50.0722
		Sum	13395.9678	100.0000

zwf-8-013H
Nov 16 2023
SOLVENT: CDCl3
NA = 4
F1 = 400.140015 MHz
F2 = 1.000000 MHz

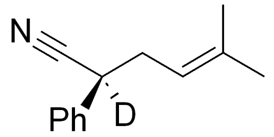

50-d

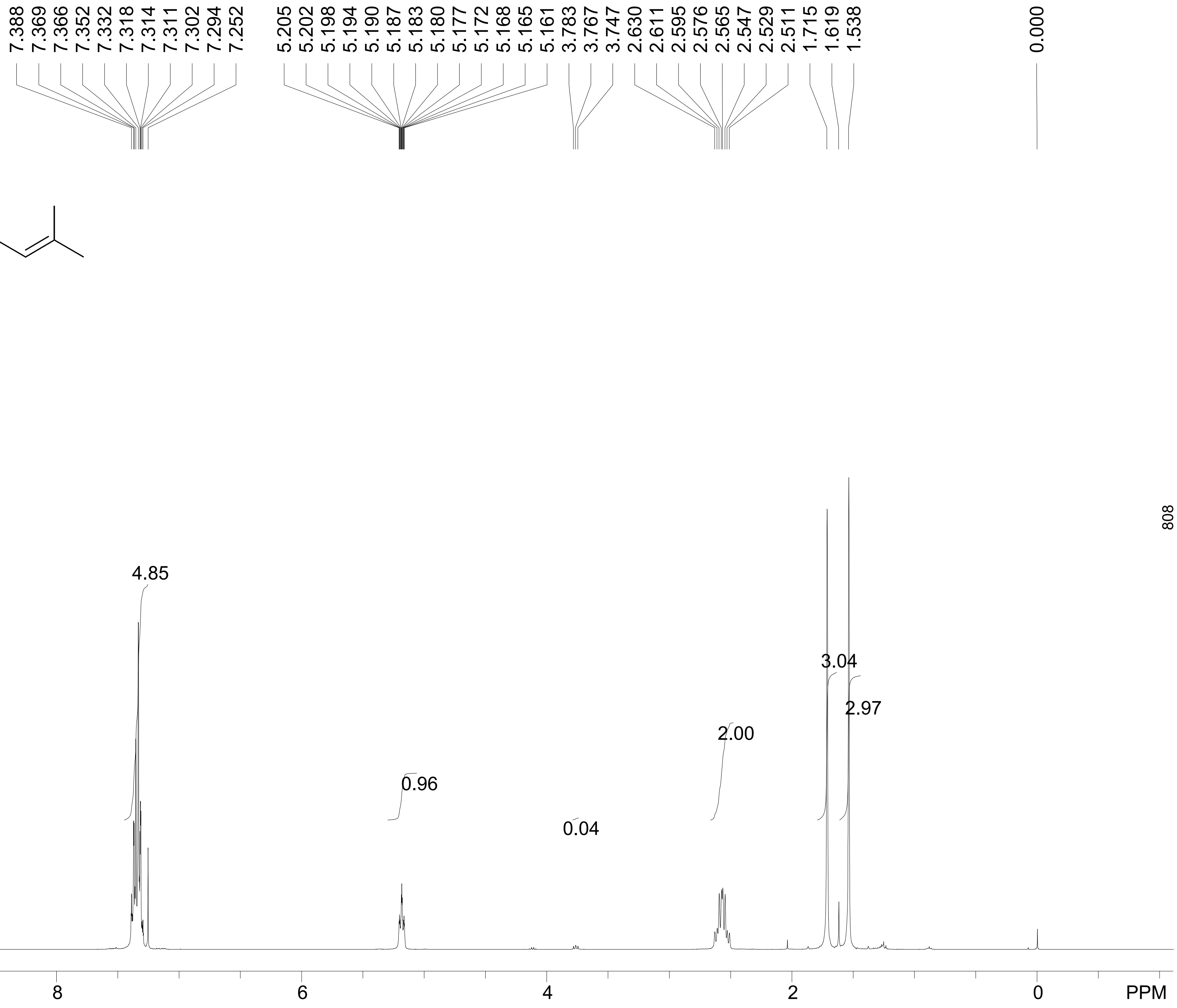

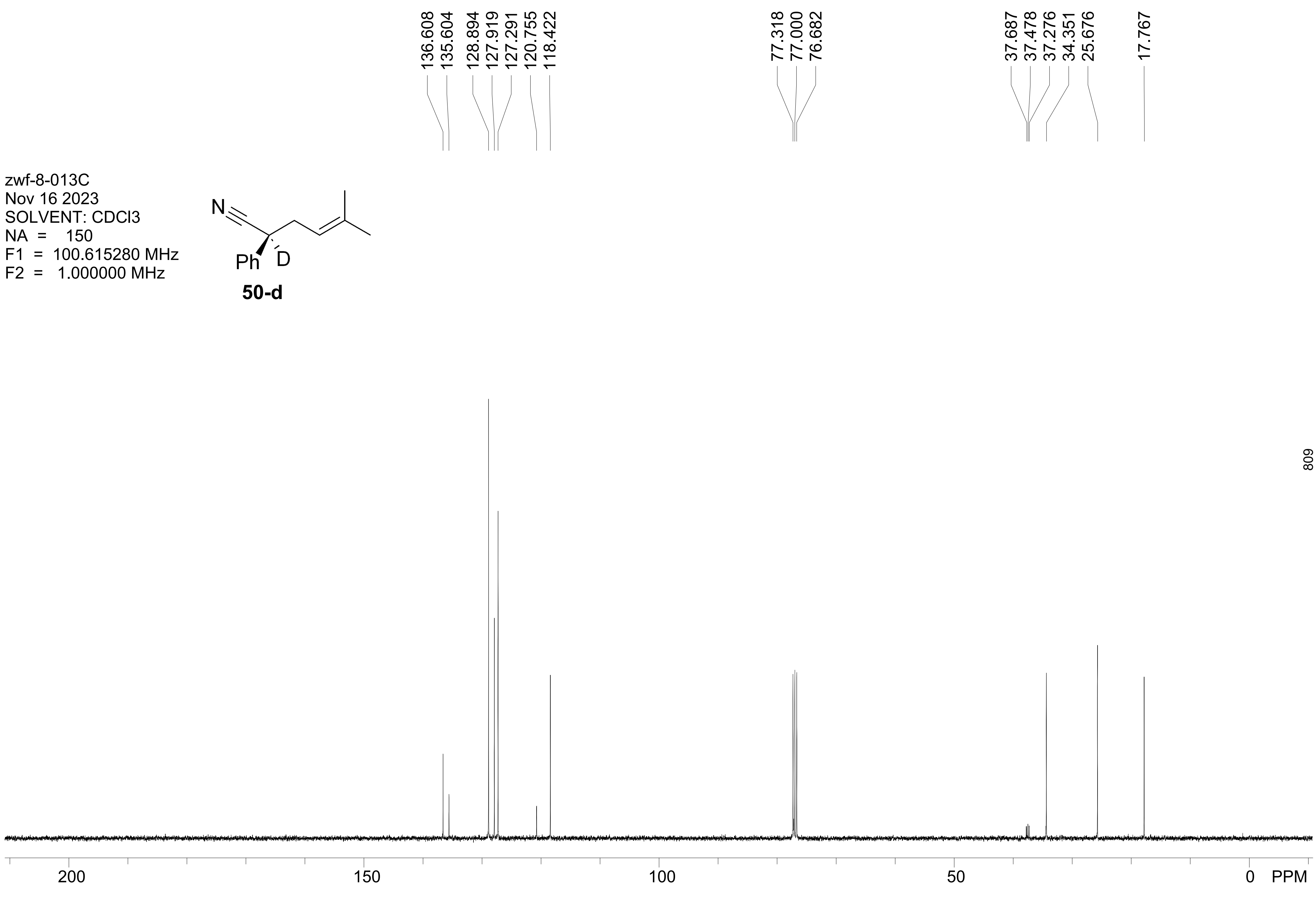
zwf-8-013C
Nov 16 2023
SOLVENT: CDCl3
NA = 150
F1 = 100.615280 MHz
F2 = 1.000000 MHz
N
Ph
D
136.608
135.604
128.894
127.919
127.291
120.755
118.422
77.318
77.000
76.682
37.687
37.478
37.276
34.351
25.676
17.767
200
150
100
50
0
PPM

50-d

Area Percent Report

Agilent Technologies

Sample name: zwf-8-013-IE-99-1-0.8-205

Data file: C:\Users\Public\Documents\ChemStation\1\Data\zwf\zwf-S 2023-11-17 07-38-48\006-P1-B1-zwf-8-013.D

Injection Acquired Date 11/17/2023 10:15:43

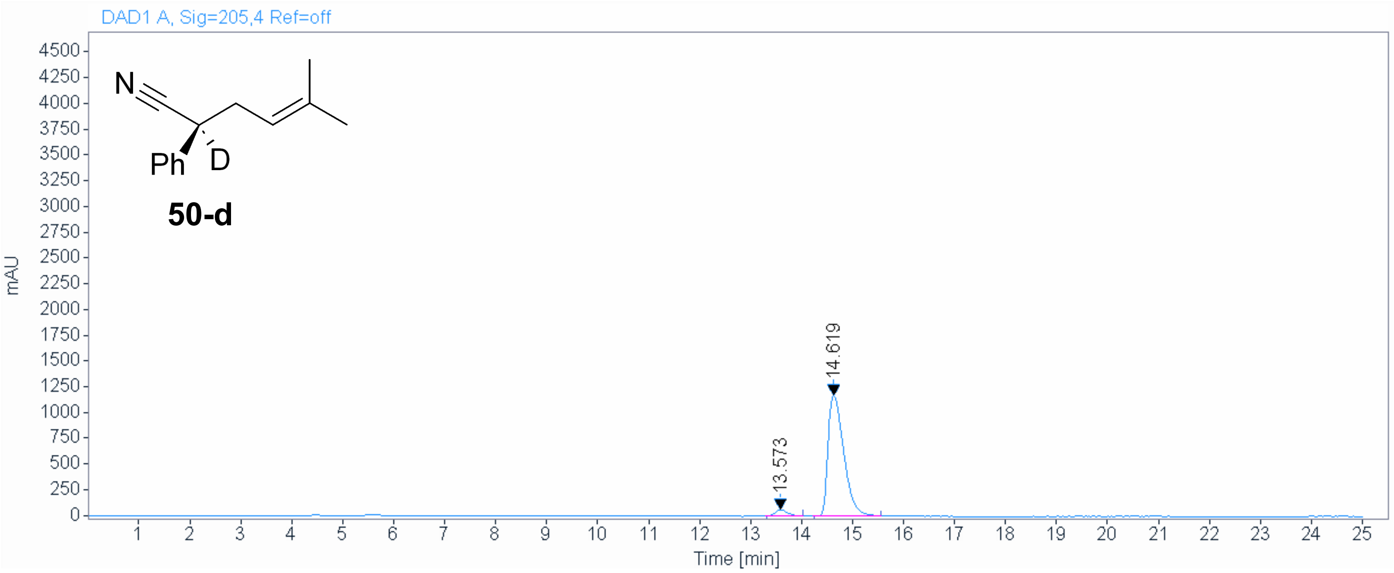

Signal: DAD1 A, Sig=205,4 Ref=off

RT [min]	Width [min]	Height	Area	Area%
13.573	0.2767	61.5018	1021.1705	3.8974
14.619	0.3230	1170.3214	25180.2266	96.1026
		Sum	26201.3971	100.0000

Area Percent Report

Agilent Technologies

Sample name: zwf-8-013-rac-IE-99-1-0.8-205

Data file: C:\Users\Public\Documents\ChemStation\1\Data\zwf\zwf-S 2023-11-17 07-38-48\005-P1-B2-zwf-8-013-rac.D

Injection Acquired Date 11/17/2023 09:49:42

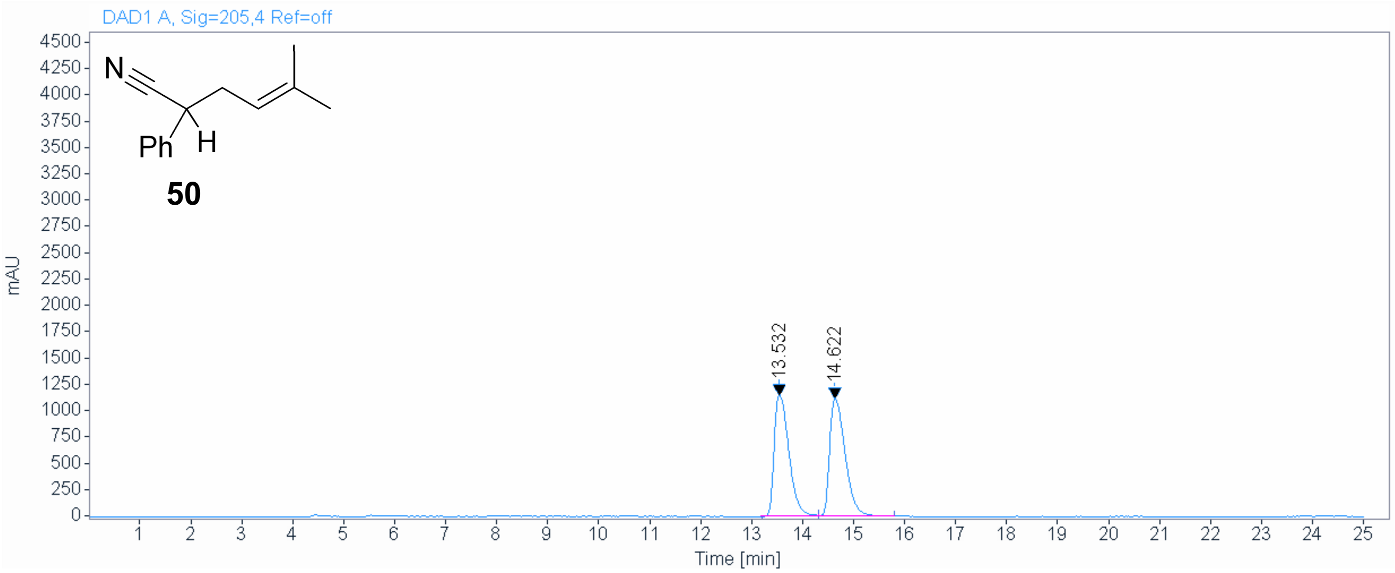

Signal: DAD1 A, Sig=205,4 Ref=off

RT [min]	Width [min]	Height	Area	Area%
13.532	0.3083	1148.7616	23268.6699	49.5029
14.622	0.2839	1116.9513	23736.0293	50.4971
		Sum	47004.6992	100.0000

zwf-7-090H
Sep 26 2023
SOLVENT: CDCl3
NA = 4
F1 = 400.140015 MHz
F2 = 1.000000 MHz

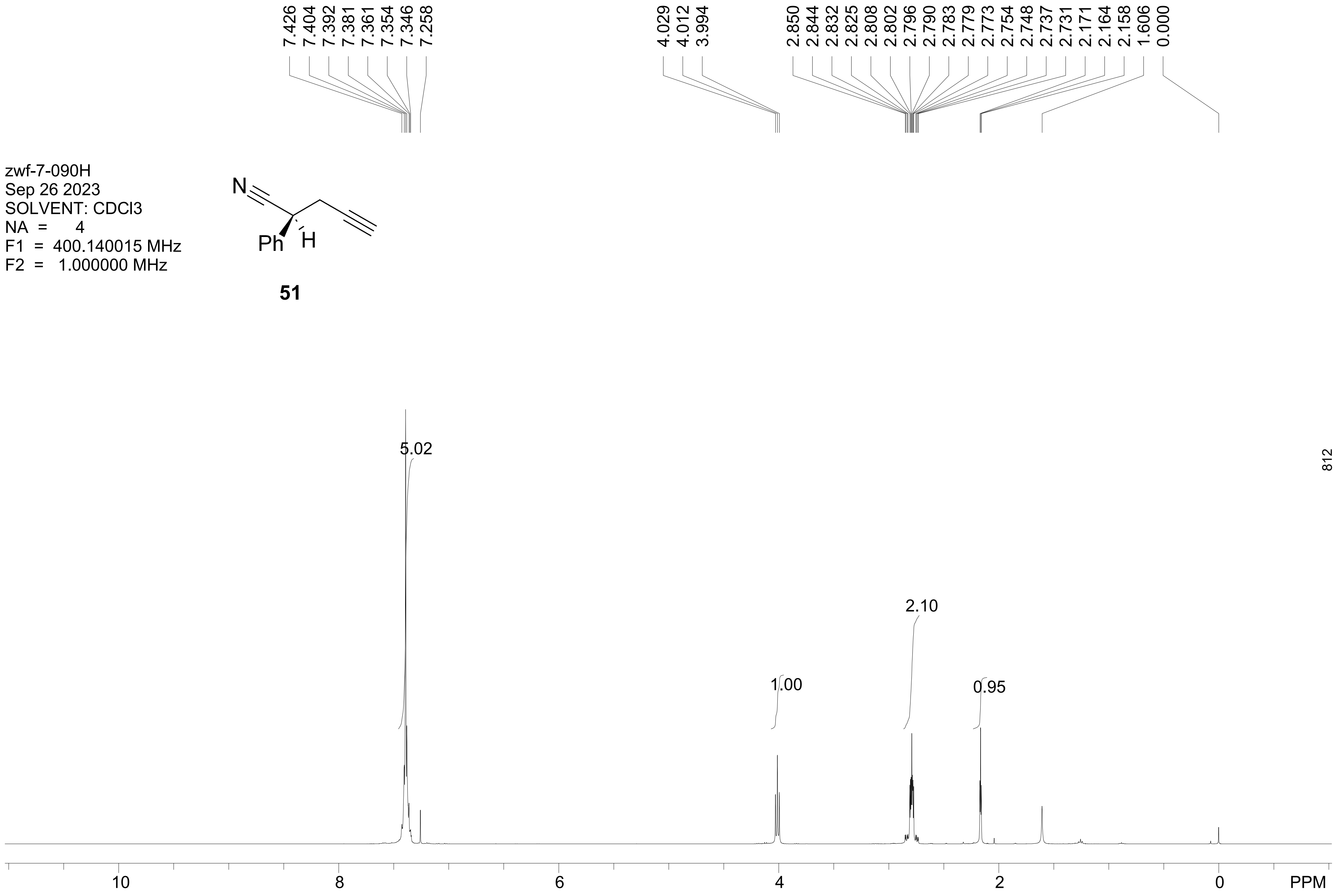

51

zwf-7-090C
Sep 26 2023
SOLVENT: CDCl3
NA = 150
F1 = 100.615280 MHz
F2 = 1.000000 MHz

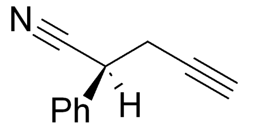

51

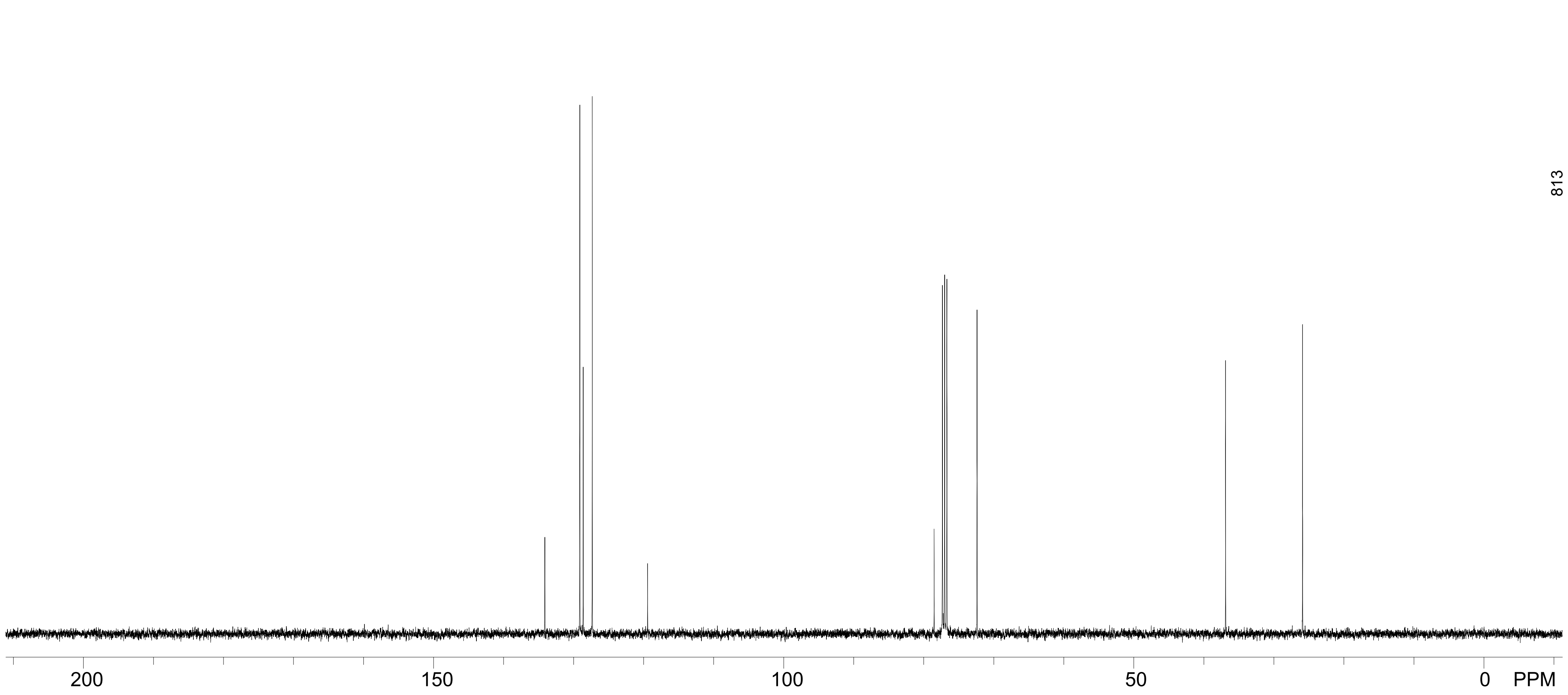

Area Percent Report

Agilent Technologies

Sample name: zwf-7-090-IE-99-1-0.8-205

Data file: C:\Users\Public\Documents\ChemStation\1\Data\zwf\zwf-S 2023-09-26 14-33-49\015-P1-C5-zwf-7-090.D

Injection Acquired Date 09/26/2023 20:37:47

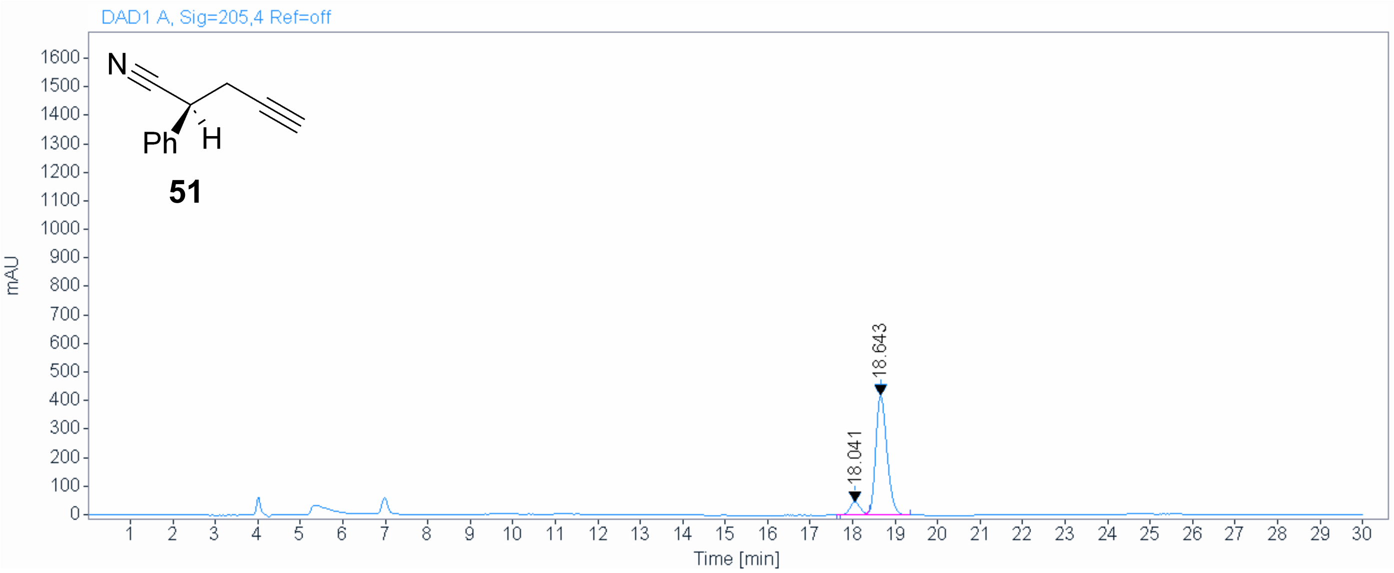

Signal: DAD1 A, Sig=205,4 Ref=off

RT [min]	Width [min]	Height	Area	Area%
18.041	0.2600	44.7873	759.9614	8.9106
18.643	0.2845	418.6144	7768.8018	91.0894
		Sum	8528.7631	100.0000

Area Percent Report

Agilent Technologies

Sample name: zwf-7-090-rac-IE-99-1-0.8-205

Data file: C:\Users\Public\Documents\ChemStation\1\Data\zwf\zwf-S 2023-09-26 14-33-49\016-P1-C6-zwf-7-090-rac.D

Injection Acquired Date 09/26/2023 21:08:55

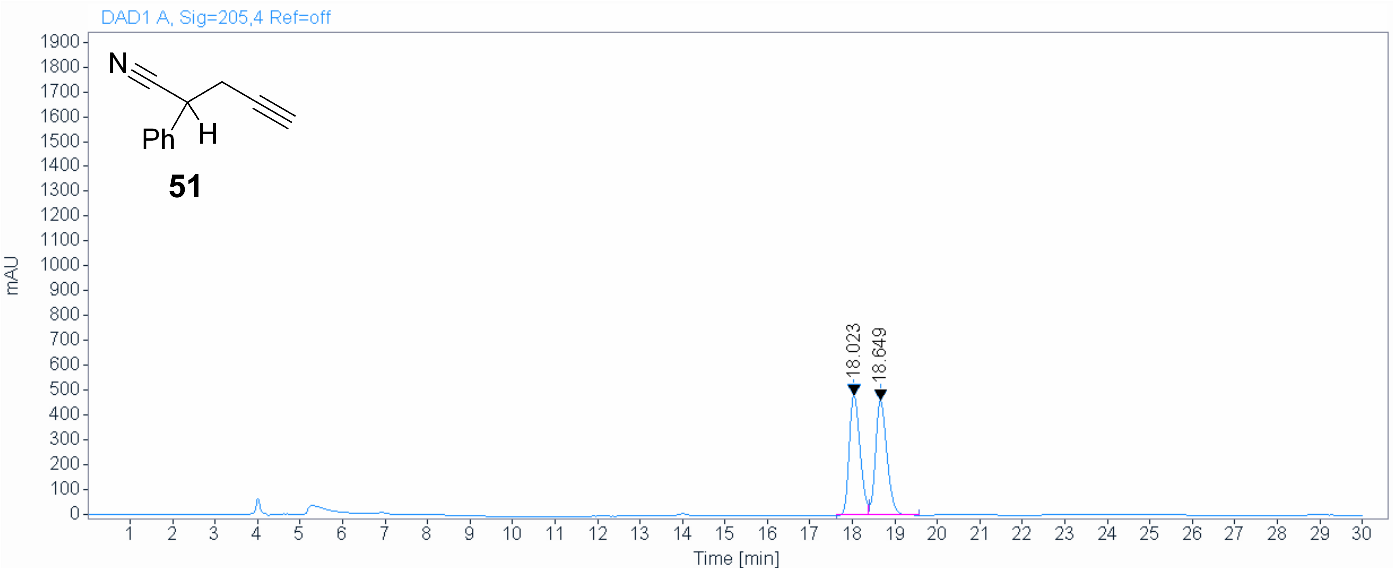

Signal: DAD1 A, Sig=205,4 Ref=off

RT [min]	Width [min]	Height	Area	Area%
18.023	0.2712	482.2716	8480.7422	49.3739
18.649	0.2896	462.0265	8695.8174	50.6261
		Sum	17176.5596	100.0000

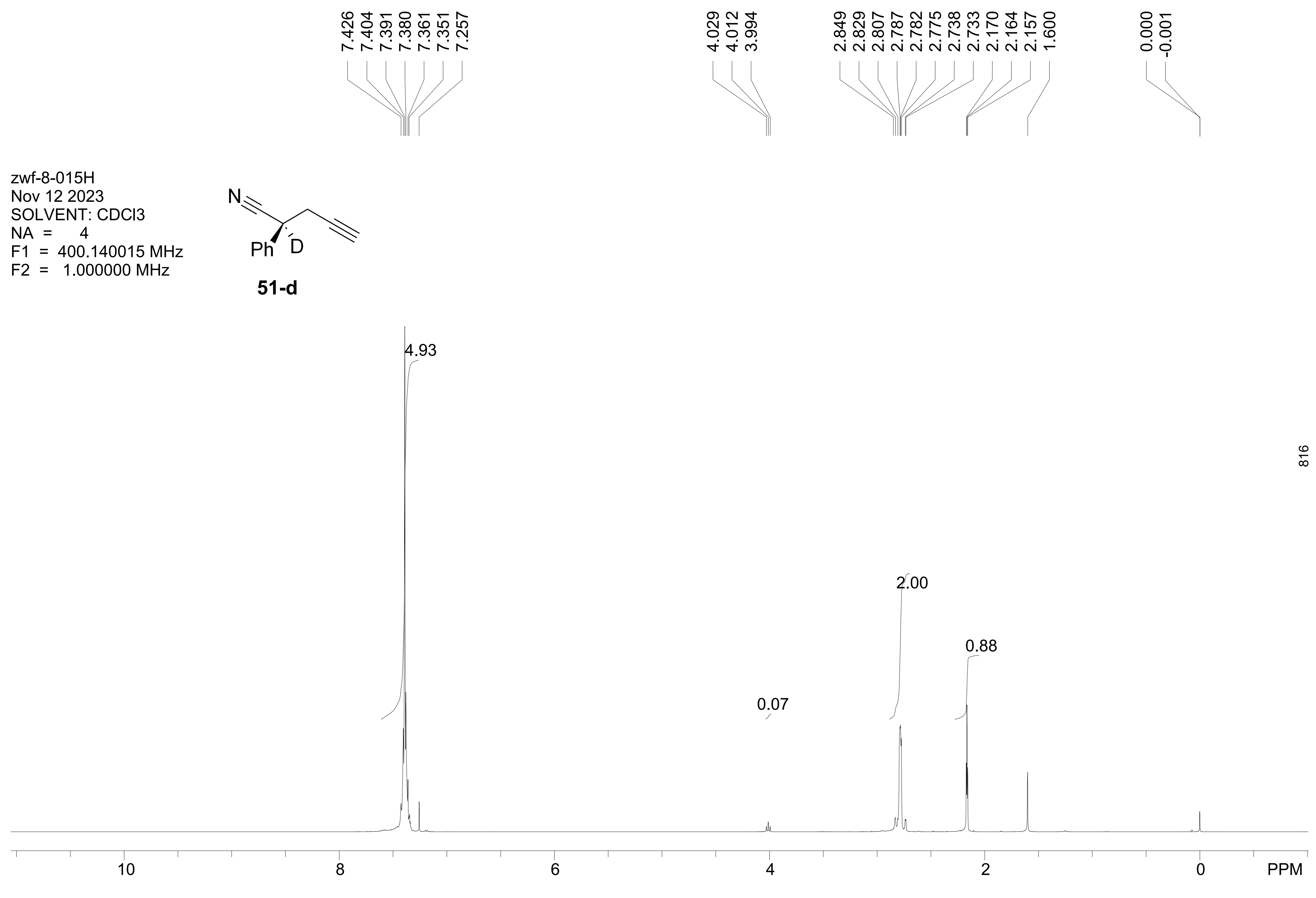
zwf-8-015H
Nov 12 2023
SOLVENT: CDCl3
NA = 4
F1 = 400.140015 MHz
F2 = 1.000000 MHz
N
Ph
D
51-d
7.426
7.404
7.391
7.380
7.361
7.351
7.257
4.029
4.012
3.994
2.849
2.829
2.807
2.787
2.782
2.775
2.738
2.733
2.170
2.164
2.157
1.600
0.000
-0.001
4.93
0.07
2.00
0.88
10
8
6
4
2
0
PPM

zwf-8-015C
Nov 12 2023
SOLVENT: CDCl3
NA = 150
F1 = 100.615280 MHz
F2 = 1.000000 MHz

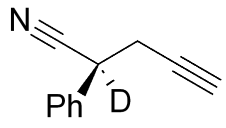

51-d

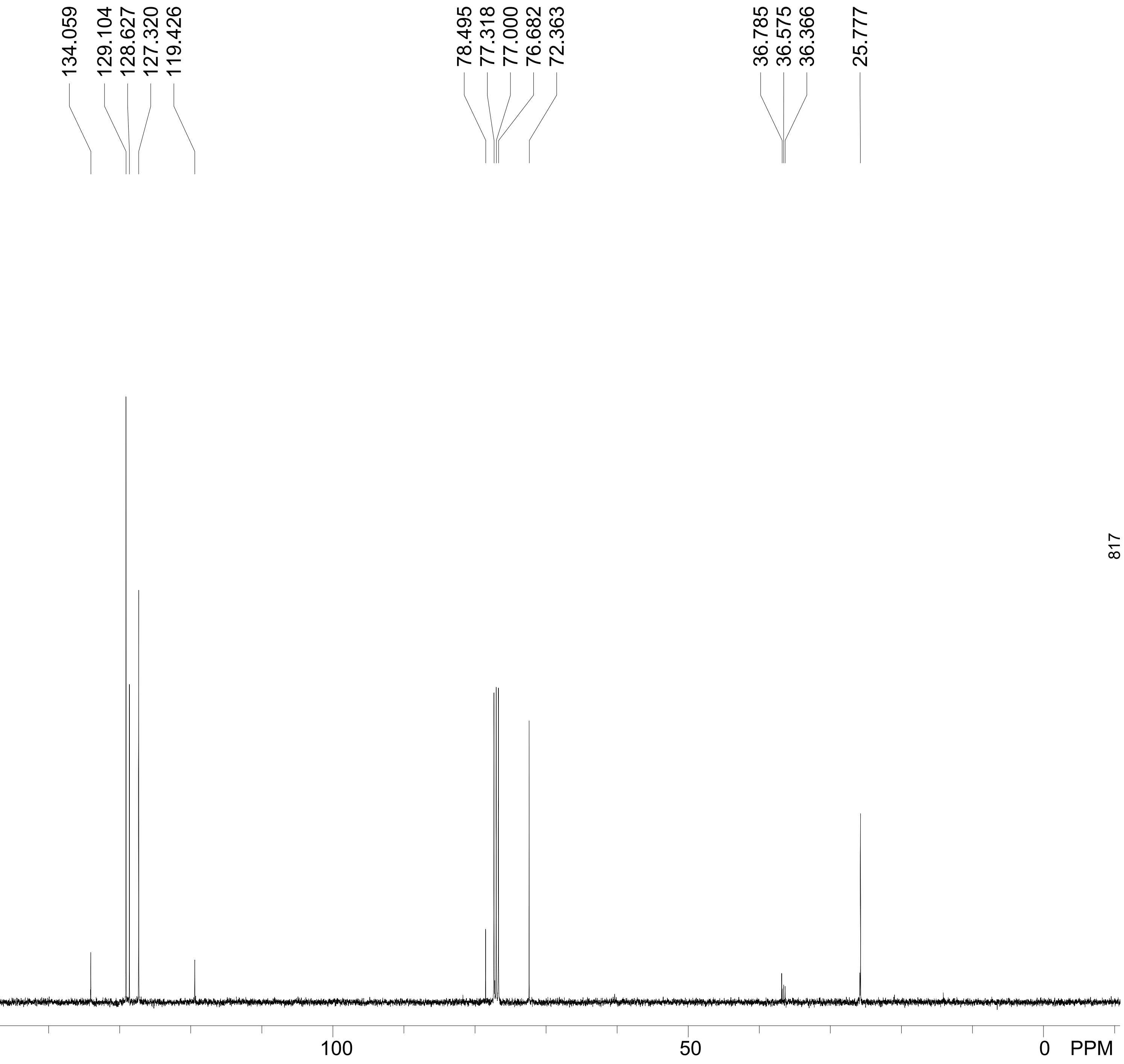

Area Percent Report

Agilent Technologies

Sample name: zwf-8-015-IE-99-1-0.8-205

Data file: C:\Users\Public\Documents\ChemStation\1\Data\zwf\zwf-S 2023-11-12 11-02-17\046-P1-A7-zwf-8-015.D

Injection Acquired Date 11/13/2023 18:39:16

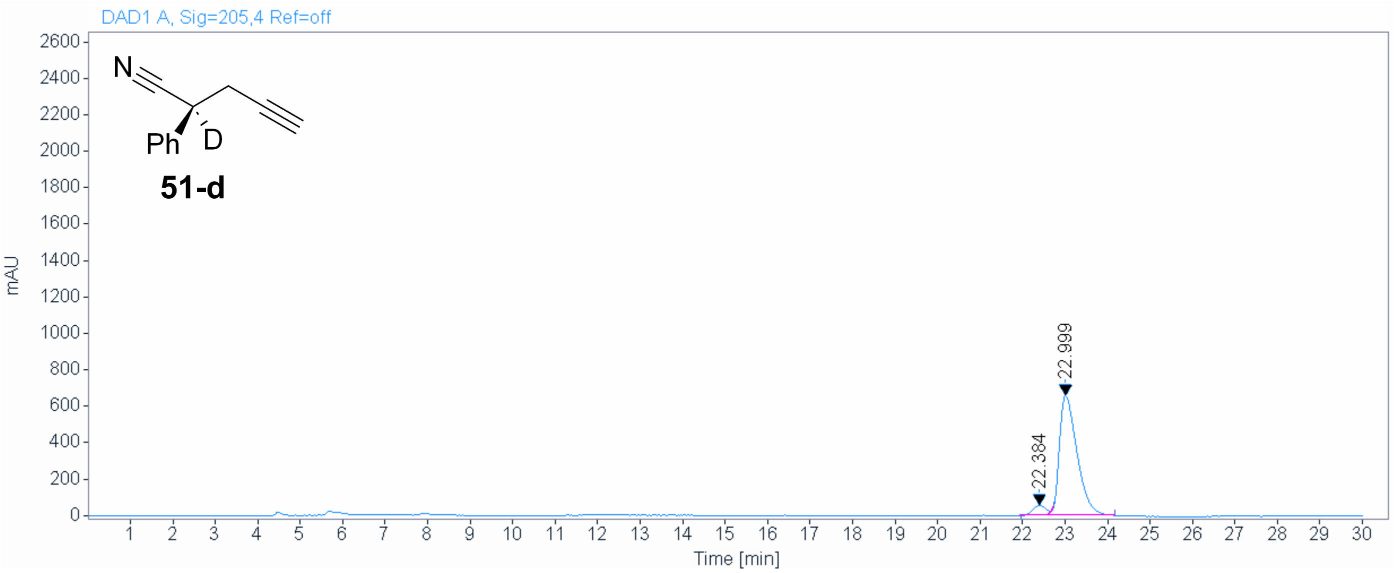

Signal: DAD1 A, Sig=205,4 Ref=off

RT [min]	Width [min]	Height	Area	Area%
22.384	0.2583	55.8367	1207.4501	5.9385
22.999	0.3529	658.4080	19125.2129	94.0615
		Sum	20332.6630	100.0000

Area Percent Report

Agilent Technologies

Sample name: zwf-8-015-rac-IE-99-1-0.8-205

Data file: C:\Users\Public\Documents\ChemStation\1\Data\zwf\zwf-S 2023-11-12 11-02-17\047-P1-A8-zwf-8-015-rac.D

Injection Acquired Date 11/13/2023 19:10:21

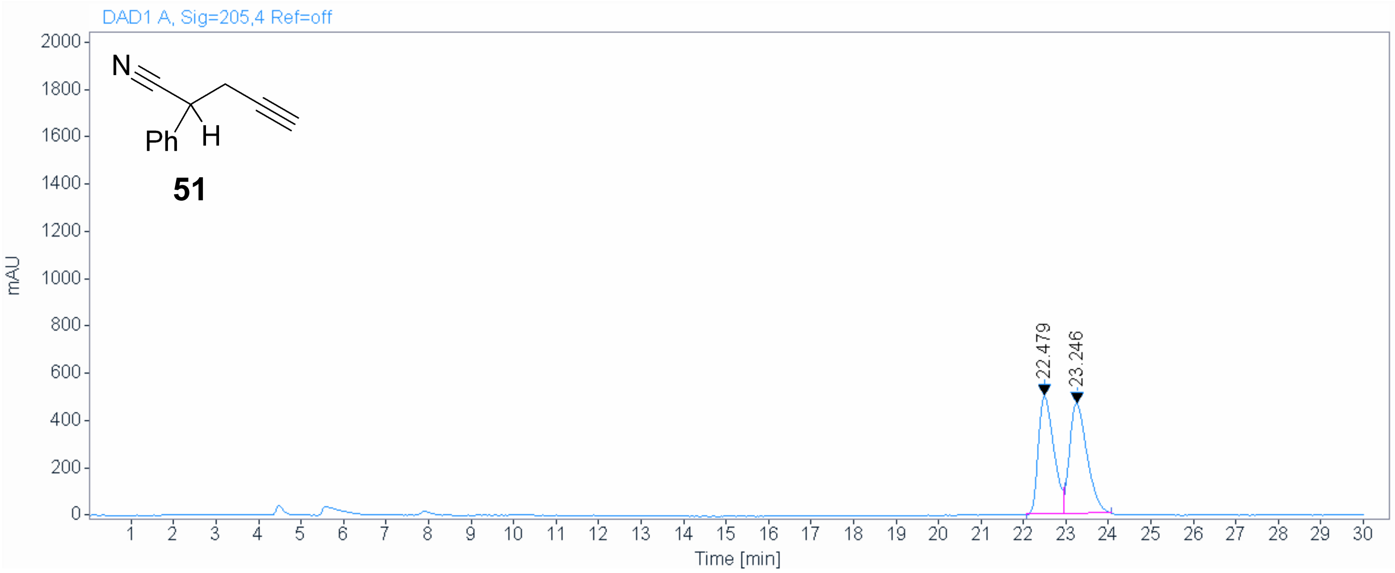

Signal: DAD1 A, Sig=205,4 Ref=off

RT [min]	Width [min]	Height	Area	Area%
22.479	0.4252	501.9209	12803.6318	49.0195
23.246	0.4752	466.9882	13315.8252	50.9805
		Sum	26119.4570	100.0000

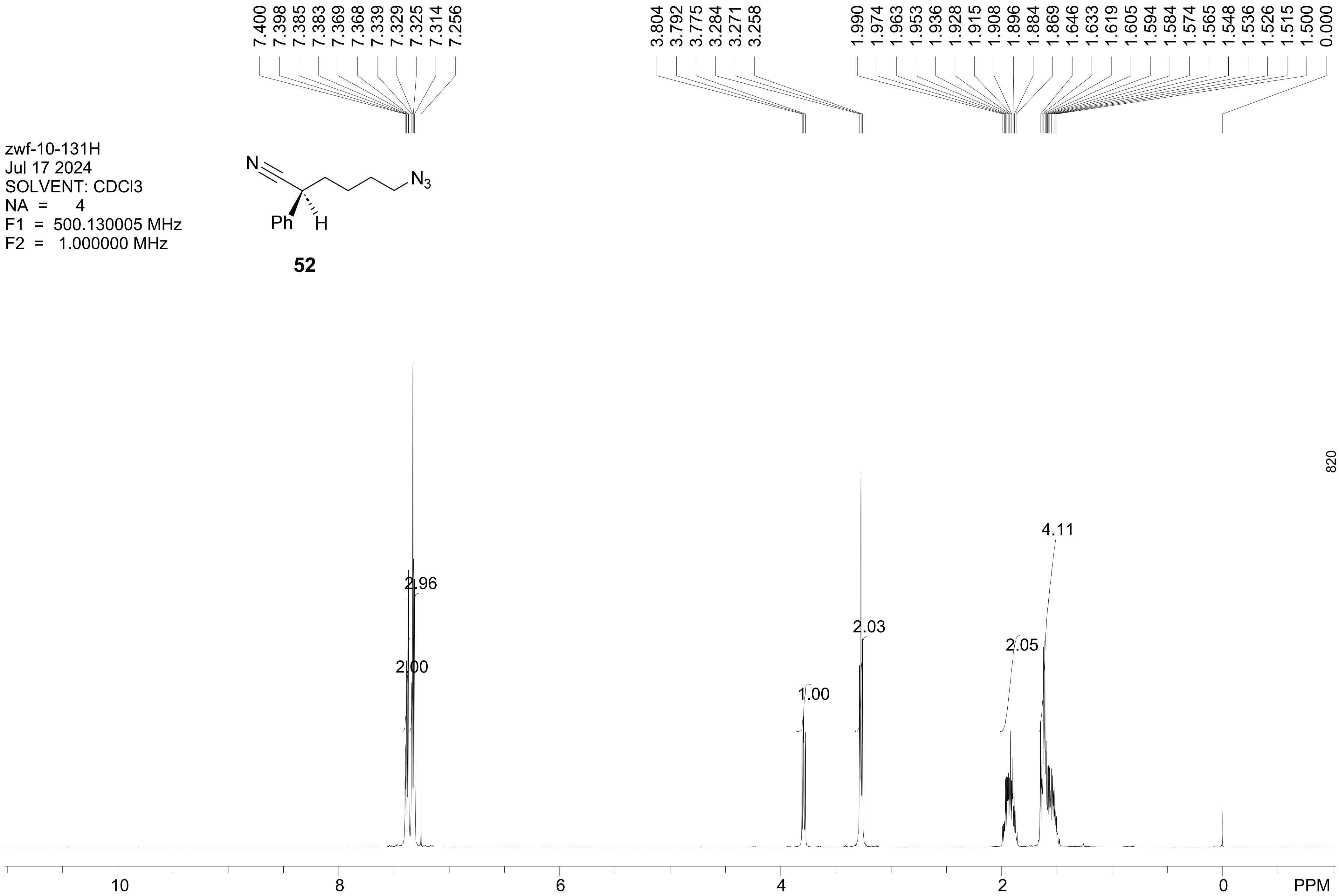
zwf-10-131H
Jul 17 2024
SOLVENT: CDCl3
NA = 4
F1 = 500.130005 MHz
F2 = 1.000000 MHz
N
N_3
Ph
H
7.400
7.398
7.385
7.383
7.369
7.368
7.339
7.329
7.325
7.314
7.256
3.804
3.792
3.775
3.284
3.271
3.258
1.990
1.974
1.963
1.953
1.936
1.928
1.915
1.908
1.896
1.884
1.869
1.646
1.633
1.619
1.605
1.594
1.584
1.574
1.565
1.548
1.536
1.526
1.515
1.500
0.000
2.96
2.00
1.00
2.03
2.05
4.11
10
8
6
4
2
0
PPM

52

zwf-10-131C
Jul 17 2024
SOLVENT: CDCl3
NA = 100
F1 = 125.757790 MHz
F2 = 1.000000 MHz

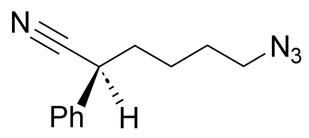

52

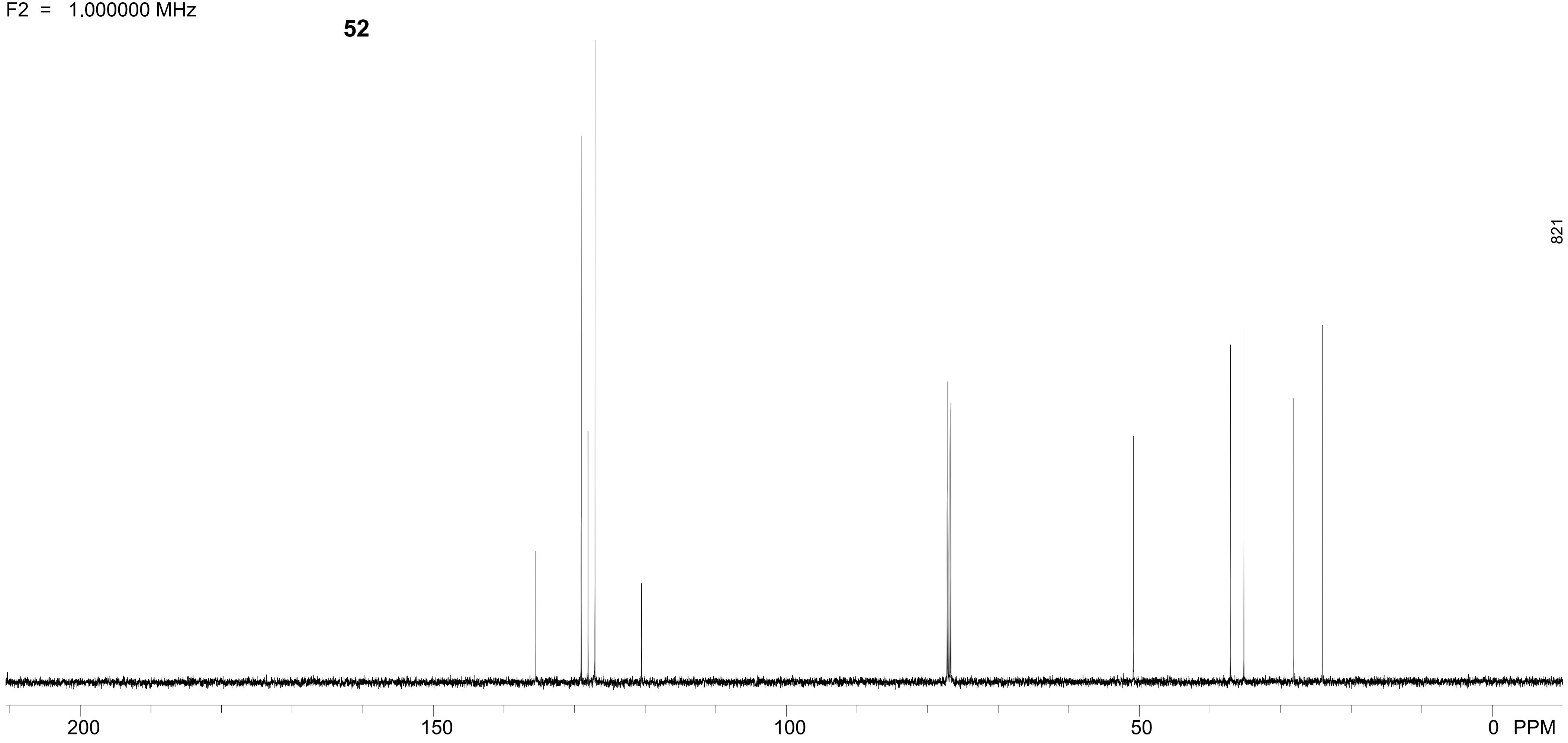

Area Percent Report

Agilent Technologies

Sample name: zwf-10-131-IE-99-1-0.8-205

Data file: C:\Users\Public\Documents\ChemStation\1\Data\zwf\zwf 2024-07-17 16-57-58\004-P1-C3-zwf-10-131.D

Injection Acquired Date 07/17/2024 18:08:07

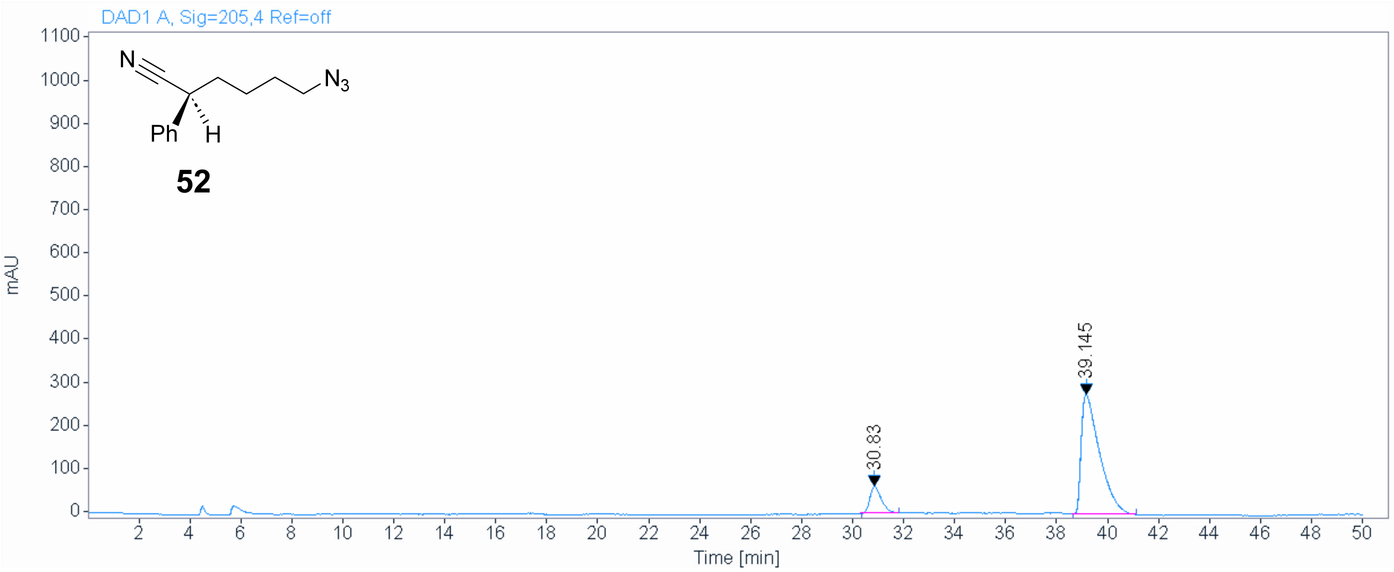

Signal: DAD1 A, Sig=205,4 Ref=off

RT [min]	Width [min]	Height	Area	Area%
30.830	0.4011	62.4242	1985.7385	12.5298
39.145	0.6877	276.1122	13862.3643	87.4702
		Sum	15848.1028	100.0000

Area Percent Report

Agilent Technologies

Sample name: zwf-10-133-rac-IE-99-1-0.8-205

Data file: C:\Users\Public\Documents\ChemStation\1\Data\zwf\zwf 2024-07-17 16-57-58\006-P1-C5-zwf-10-133-rac.D

Injection Acquired Date 07/17/2024 19:50:12

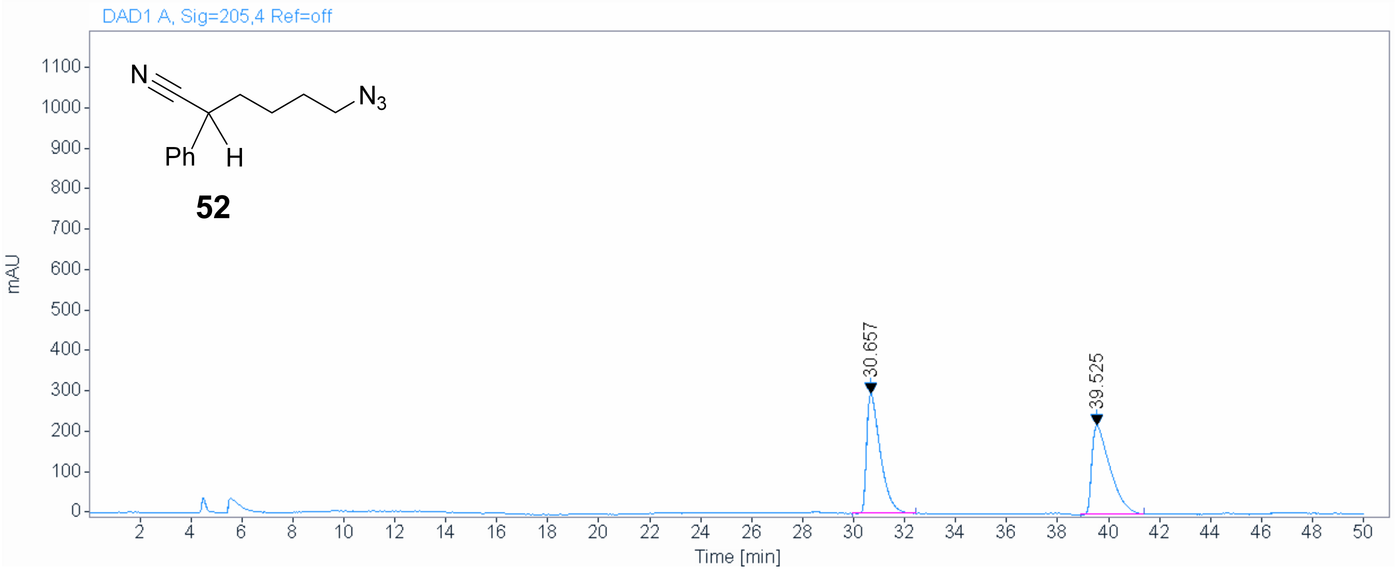

Signal: DAD1 A, Sig=205,4 Ref=off

RT [min]	Width [min]	Height	Area	Area%
30.657	0.5319	295.7906	11102.5430	50.3102
39.525	0.6636	219.9734	10965.6338	49.6898
		Sum	22068.1768	100.0000

zwf-10-133H
Jul 17 2024
SOLVENT: CDCl3
NA = 4
F1 = 500.130005 MHz
F2 = 1.000000 MHz

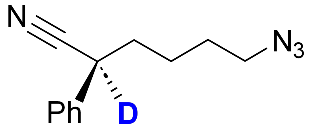

52-d

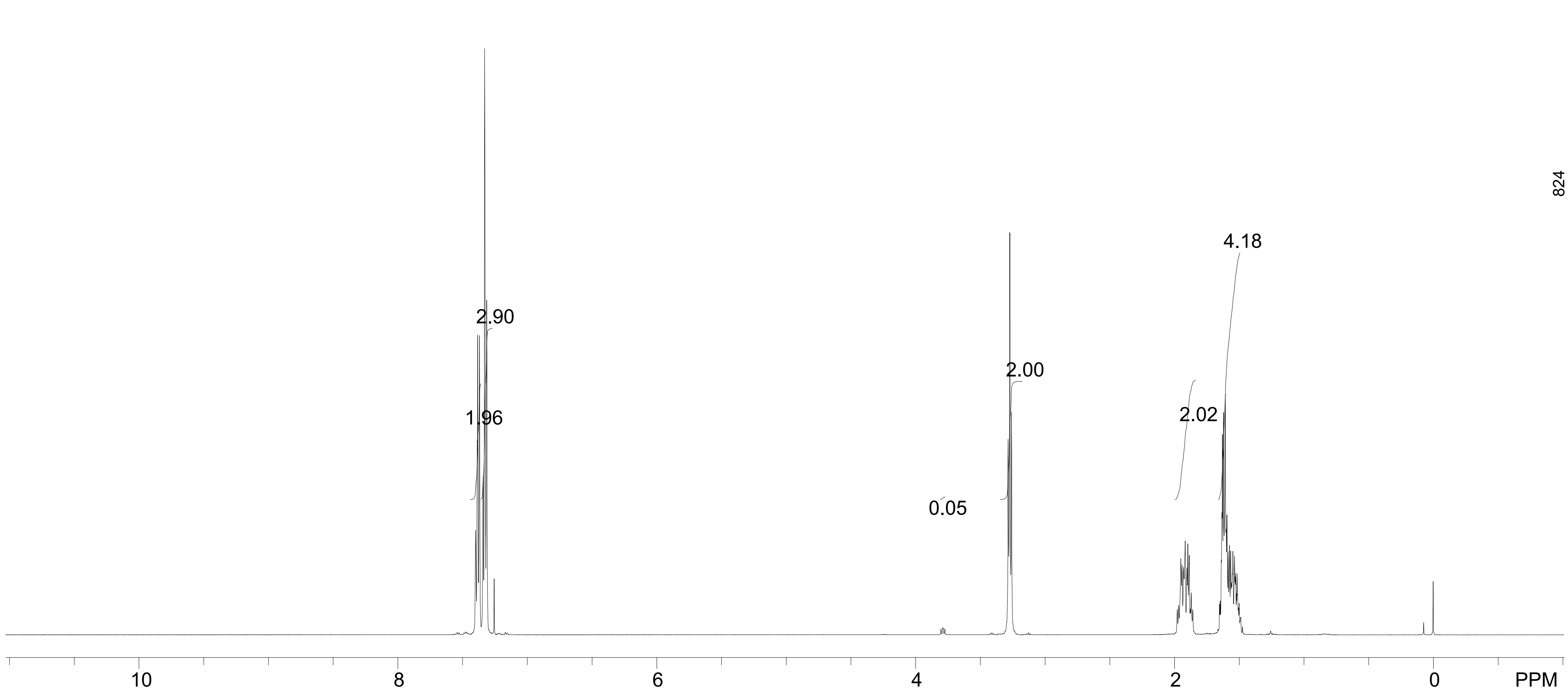

zwf-10-133C
Jul 17 2024
SOLVENT: CDCl3
NA = 100
F1 = 125.757790 MHz
F2 = 1.000000 MHz

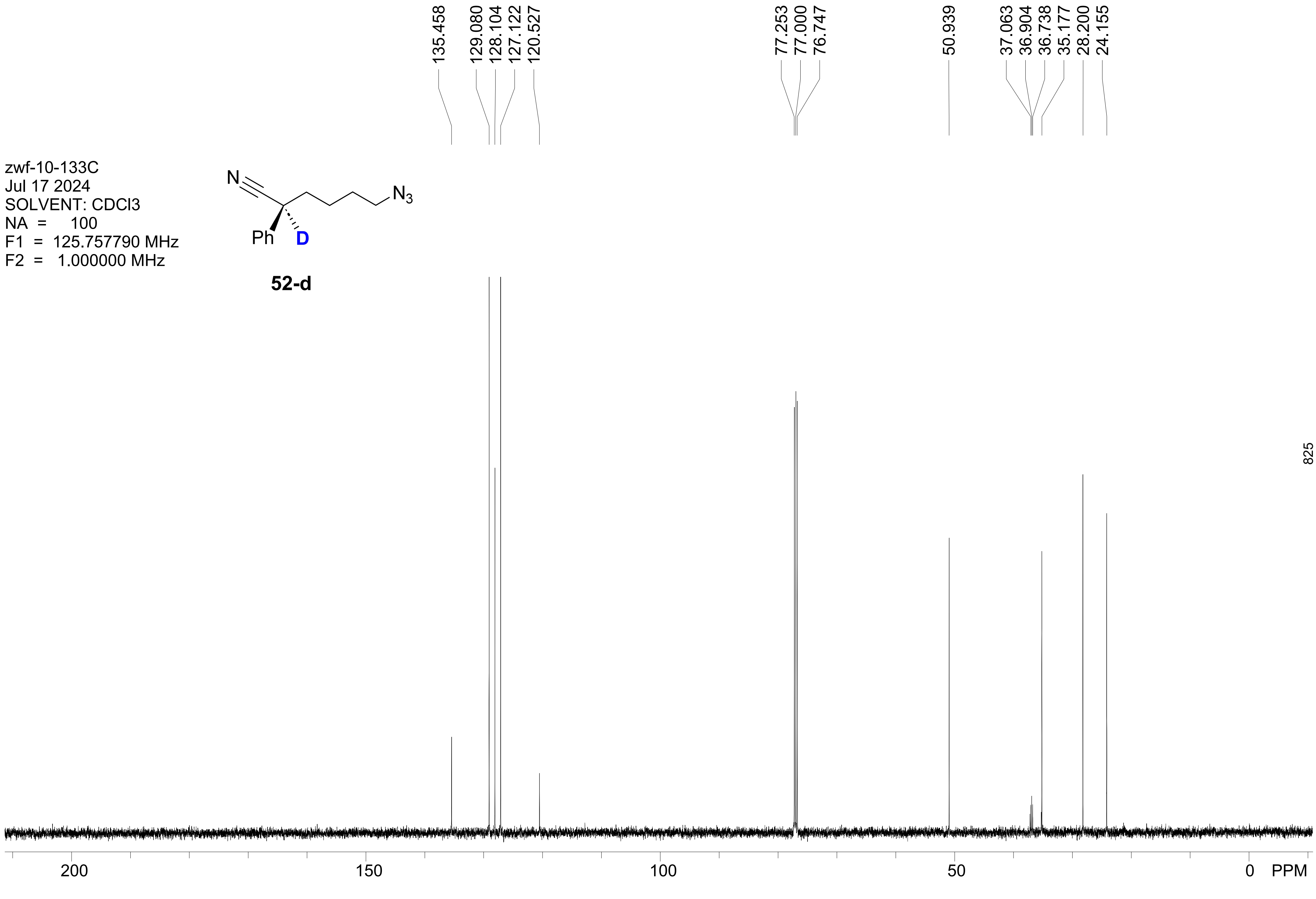

52-d

Area Percent Report

Agilent Technologies

Sample name: zwf-10-133-IE-99-1-0.8-205

Data file: C:\Users\Public\Documents\ChemStation\1\Data\zwf\zwf 2024-07-17 16-57-58\005-P1-C4-zwf-10-133.D

Injection Acquired Date 07/17/2024 18:59:07

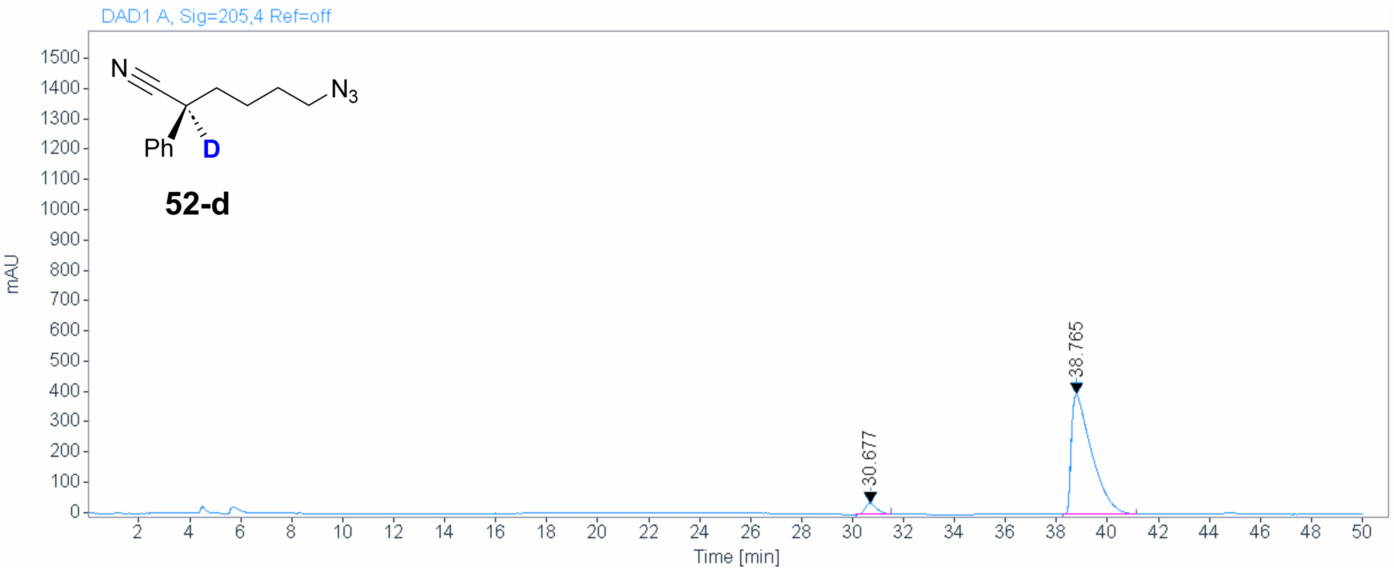

Signal: DAD1 A, Sig=205,4 Ref=off

RT [min]	Width [min]	Height	Area	Area%
30.677	0.3755	37.2141	1165.7572	5.0630
38.765	0.6563	397.0303	21859.4395	94.9370
		Sum	23025.1967	100.0000

Area Percent Report

Sample name: zwf-10-133-rac-IE-99-1-0.8-205

Data file: C:\Users\Public\Documents\ChemStation\1\Data\zwf\zwf 2024-07-17 16-57-58\006-P1-C5-zwf-10-133-rac.D

Injection Acquired Date 07/17/2024 19:50:12

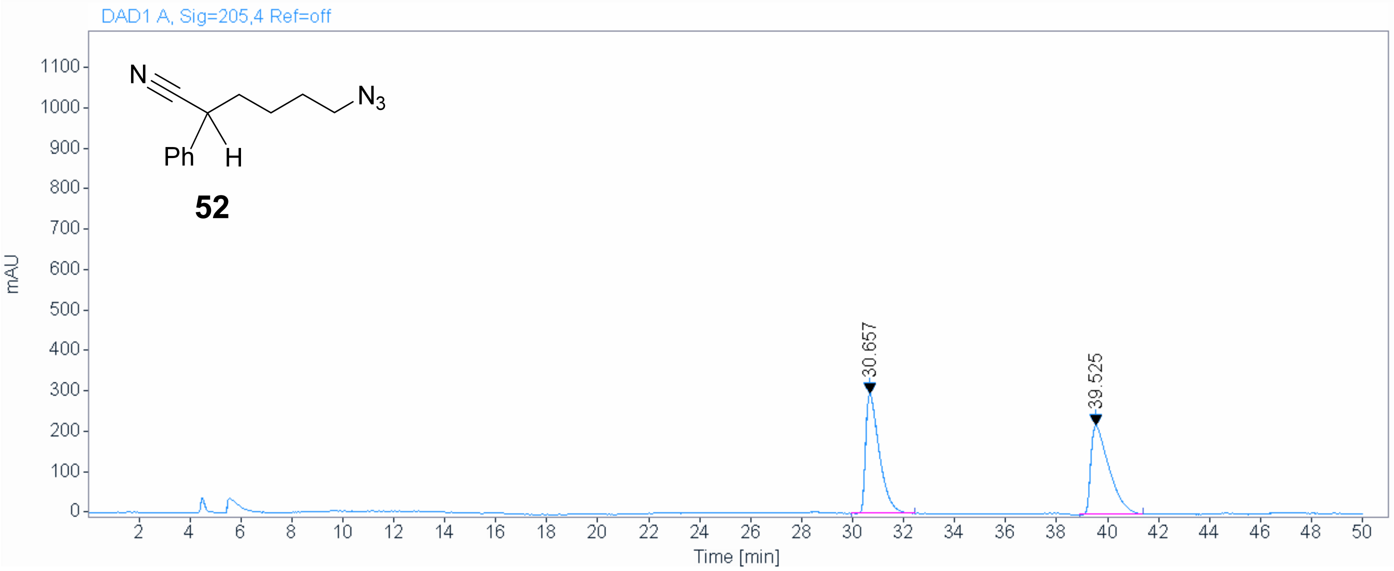

Signal: DAD1 A, Sig=205,4 Ref=off

RT [min]	Width [min]	Height	Area	Area%
30.657	0.5319	295.7906	11102.5430	50.3102
39.525	0.6636	219.9734	10965.6338	49.6898
		Sum	22068.1768	100.0000

zwf-7-146H
Nov 09 2023
SOLVENT: CDCl3
NA = 4
F1 = 400.140015 MHz
F2 = 1.000000 MHz

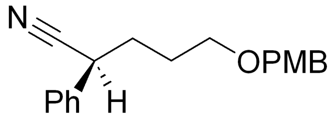

54

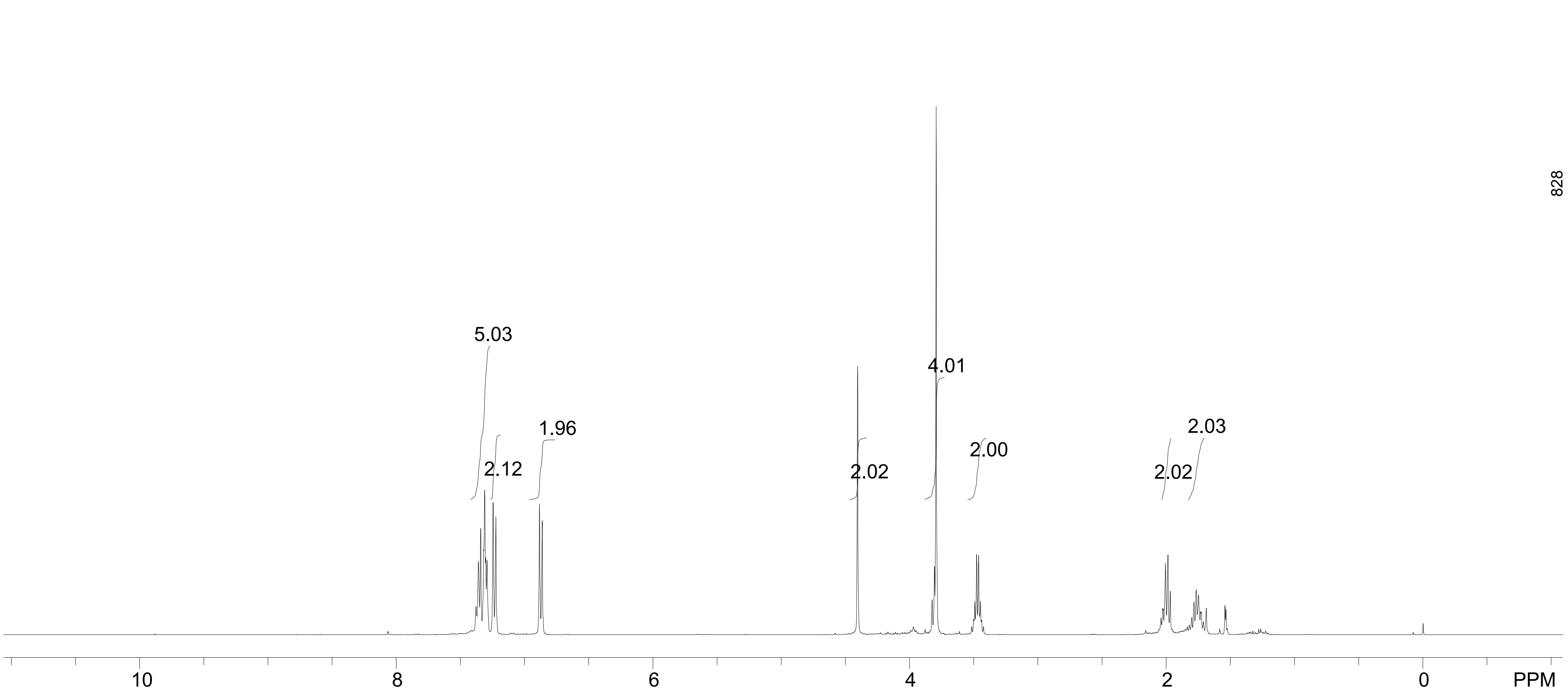

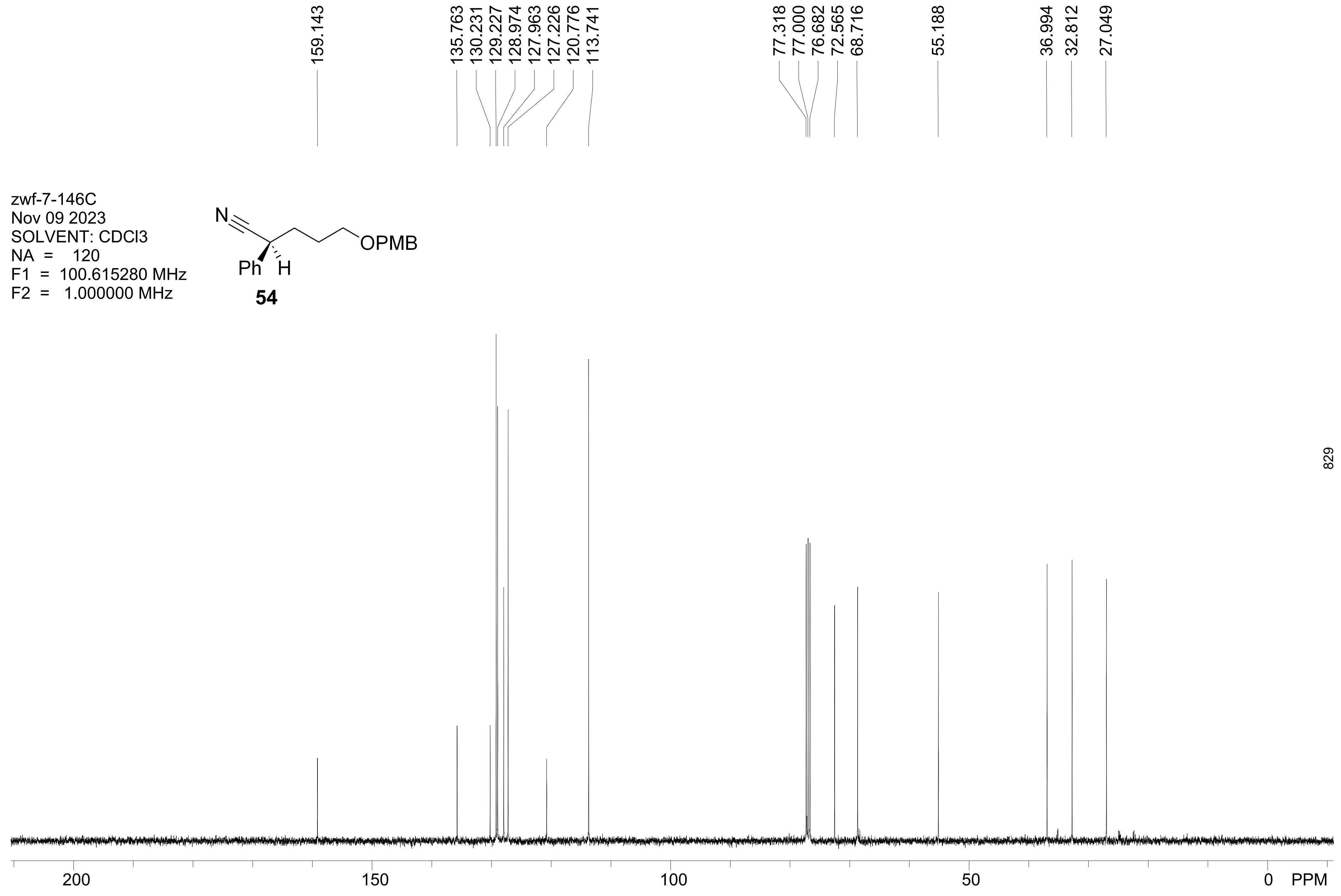
159.143
135.763
130.231
129.227
128.974
127.963
127.226
120.776
113.741
77.318
77.000
76.682
72.565
68.716
55.188
36.994
32.812
27.049
zwf-7-146C
Nov 09 2023
SOLVENT: CDCl3
NA = 120
F1 = 100.615280 MHz
F2 = 1.000000 MHz
N
OPMB
Ph
H
54
200
150
100
50
0
PPM

Area Percent Report

Agilent Technologies

Sample name: zwf-7-146-IE-90-10-0.8-205

Data file: C:\Users\Public\Documents\ChemStation\1\Data\zwf\zwf-S 2023-11-09 14-19-21\010-P1-C5-zwf-7-146.D

Injection Acquired Date 11/09/2023 21:55:47

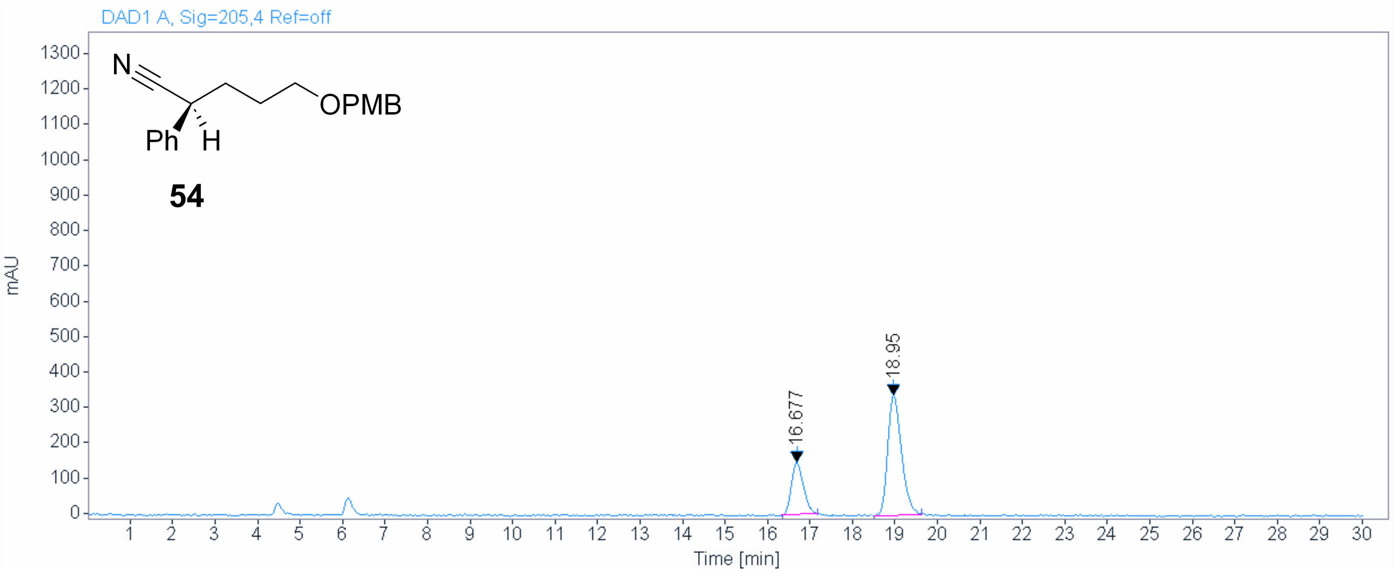

Signal: DAD1 A, Sig=205,4 Ref=off

RT [min]	Width [min]	Height	Area	Area%
16.677	0.3325	147.7875	2948.0315	27.6360
18.950	0.2811	338.8845	7719.3125	72.3640
		Sum	10667.3440	100.0000

Area Percent Report

Agilent Technologies

Sample name: zwf-7-146-rac-IE-90-10-0.8-205

Data file: C:\Users\Public\Documents\ChemStation\1\Data\zwf\zwf-S 2023-11-09 14-19-21\011-P1-C6-zwf-7-146-rac.D

Injection Acquired Date 11/09/2023 22:26:54

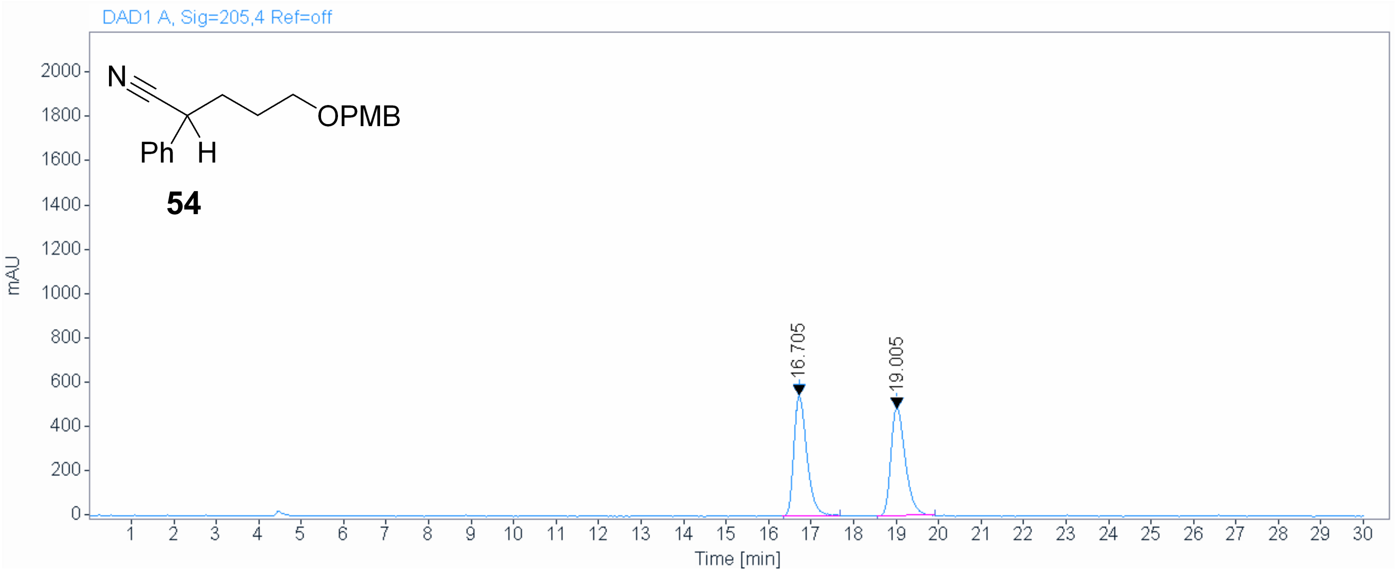

Signal: DAD1 A, Sig=205,4 Ref=off

RT [min]	Width [min]	Height	Area	Area%
16.705	0.2769	544.5705	11676.3506	50.3053
19.005	0.3565	483.1382	11534.6074	49.6947
		Sum	23210.9580	100.0000

zwf-8-044H
Nov 28 2023
SOLVENT: CDCl3
NA = 4
F1 = 400.140015 MHz
F2 = 1.000000 MHz

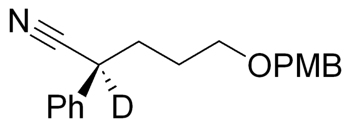

54-d

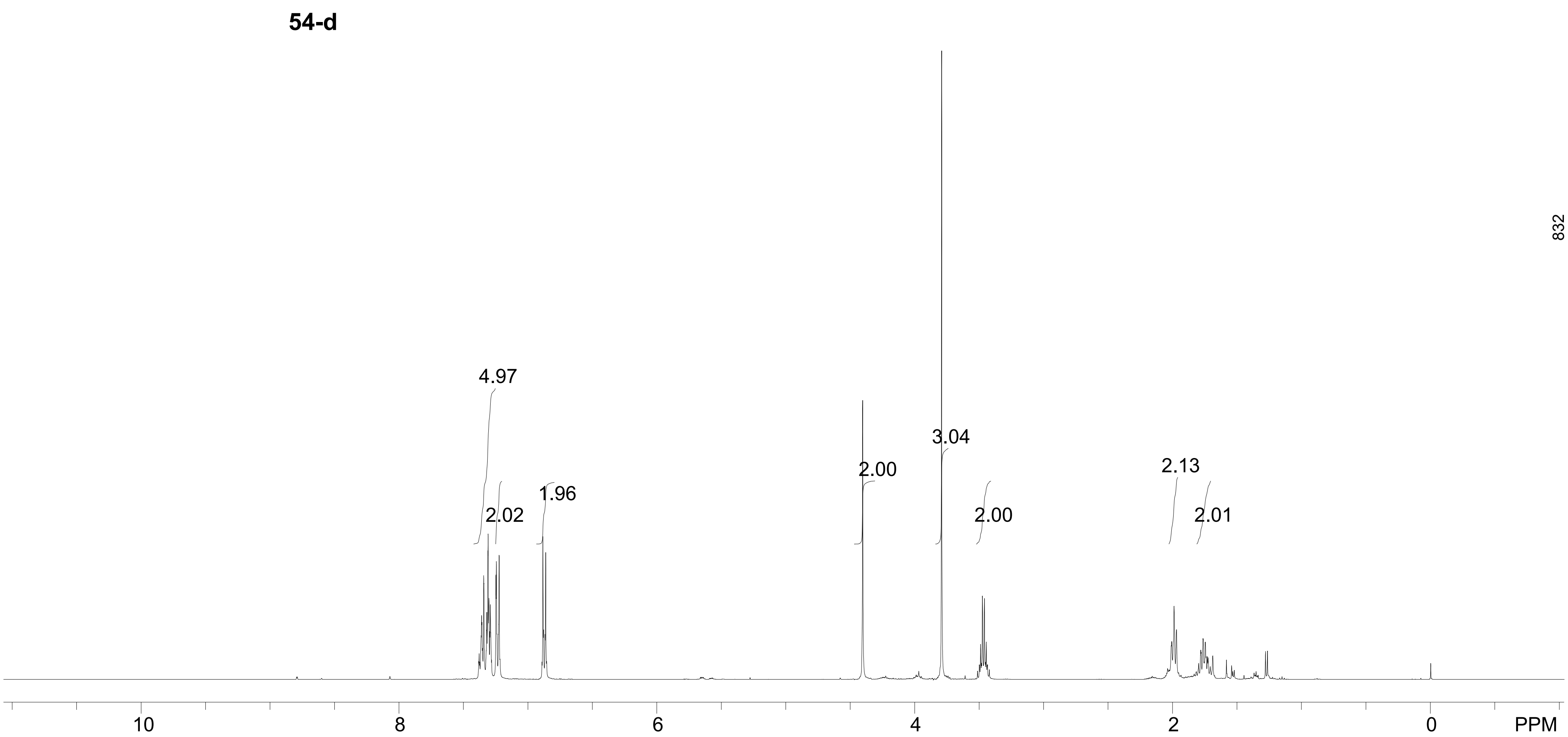

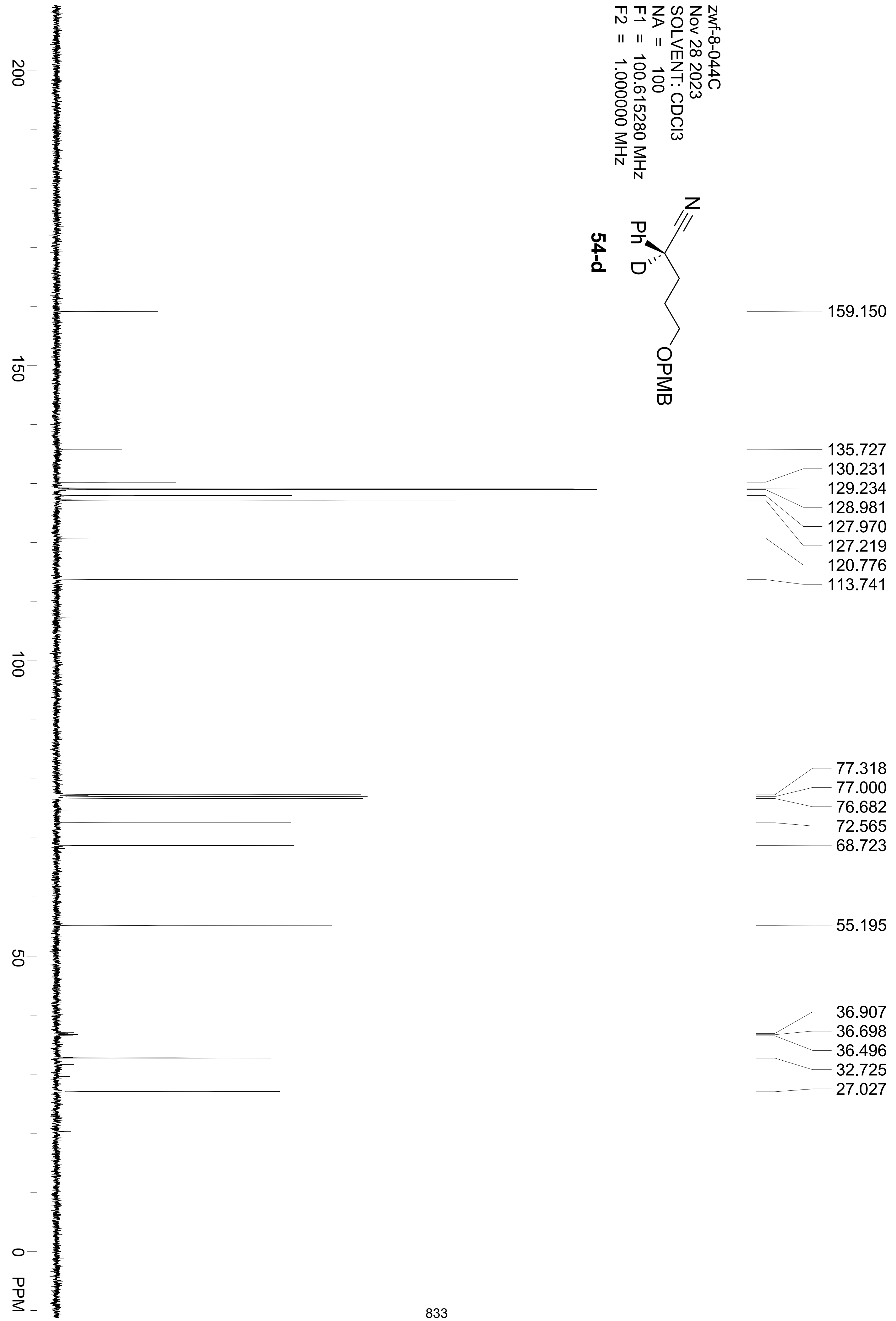
zwf-8-044C
Nov 28 2023
SOLVENT: CDCl3
NA = 100
F1 = 100.615280 MHz
F2 = 1.000000 MHz
N
Ph
D
OPMB
54-d
159.150
135.727
130.231
129.234
128.981
127.970
127.219
120.776
113.741
77.318
77.000
76.682
72.565
68.723
55.195
36.907
36.698
36.496
32.725
27.027
200
150
100
50
0
PPM

Area Percent Report

Agilent Technologies

Sample name: zwf-8-044-IE-90-10-0.8-205

Data file: C:\Users\Public\Documents\ChemStation\1\Data\zwf\zwf-S 2023-11-29 11-54-07\006-P1-C3-zwf-8-044.D

Injection Acquired Date 11/29/2023 14:15:54

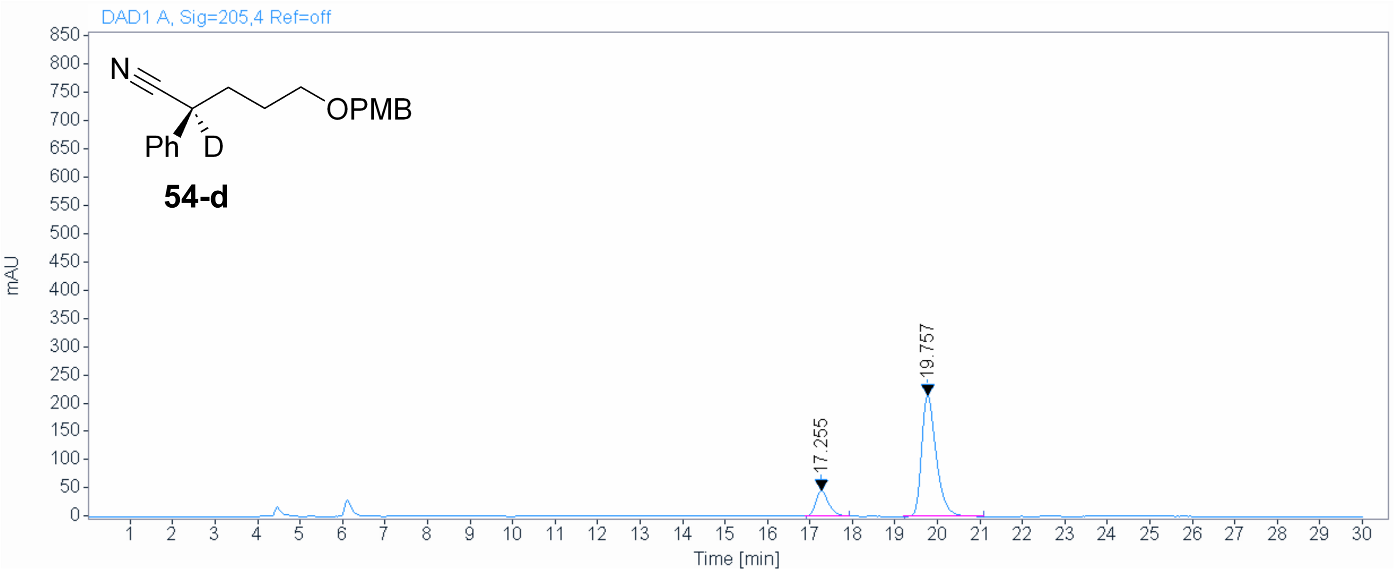

Signal: DAD1 A, Sig=205,4 Ref=off

RT [min]	Width [min]	Height	Area	Area%
17.255	0.3458	44.4983	923.3362	15.2351
19.757	0.3640	214.0580	5137.2446	84.7649
		Sum	6060.5808	100.0000

Area Percent Report

Agilent Technologies

Sample name: zwf-8-044-rac-IE-90-10-0.8-205

Data file: C:\Users\Public\Documents\ChemStation\1\Data\zwf\zwf-S 2023-11-29 11-54-07\007-P1-C4-zwf-8-044-rac.D

Injection Acquired Date 11/29/2023 14:46:55

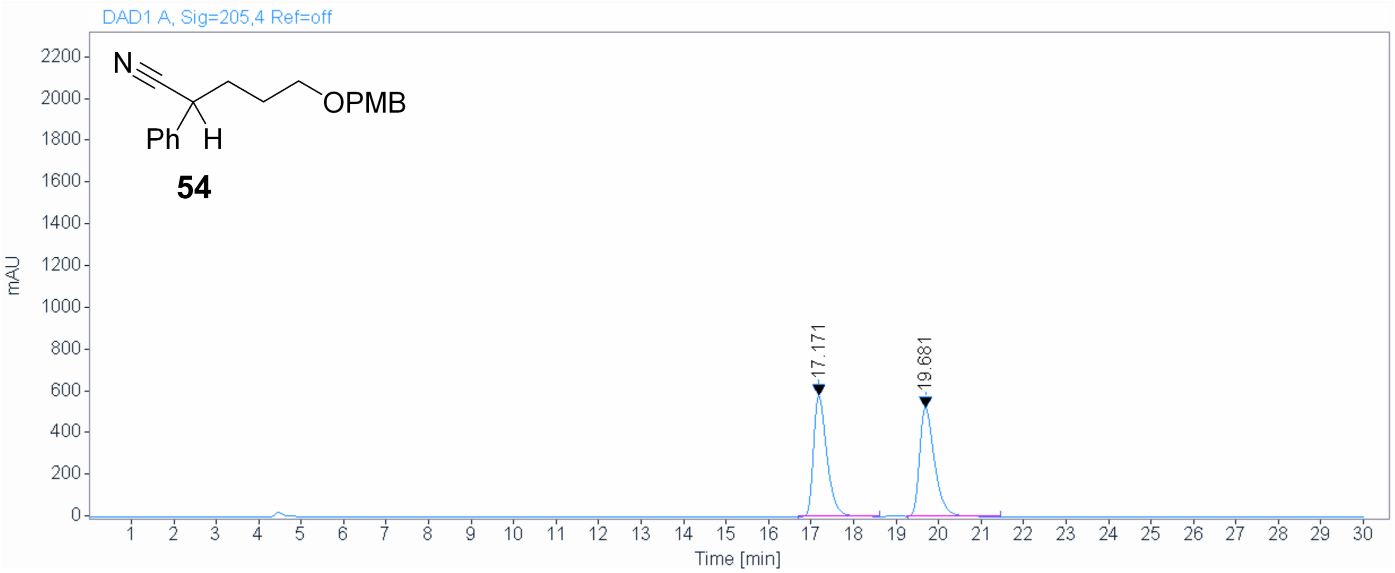

Signal: DAD1 A, Sig=205,4 Ref=off

RT [min]	Width [min]	Height	Area	Area%
17.171	0.3371	579.2752	12765.1123	49.9462
19.681	0.4100	520.0203	12792.6123	50.0538
		Sum	25557.7246	100.0000

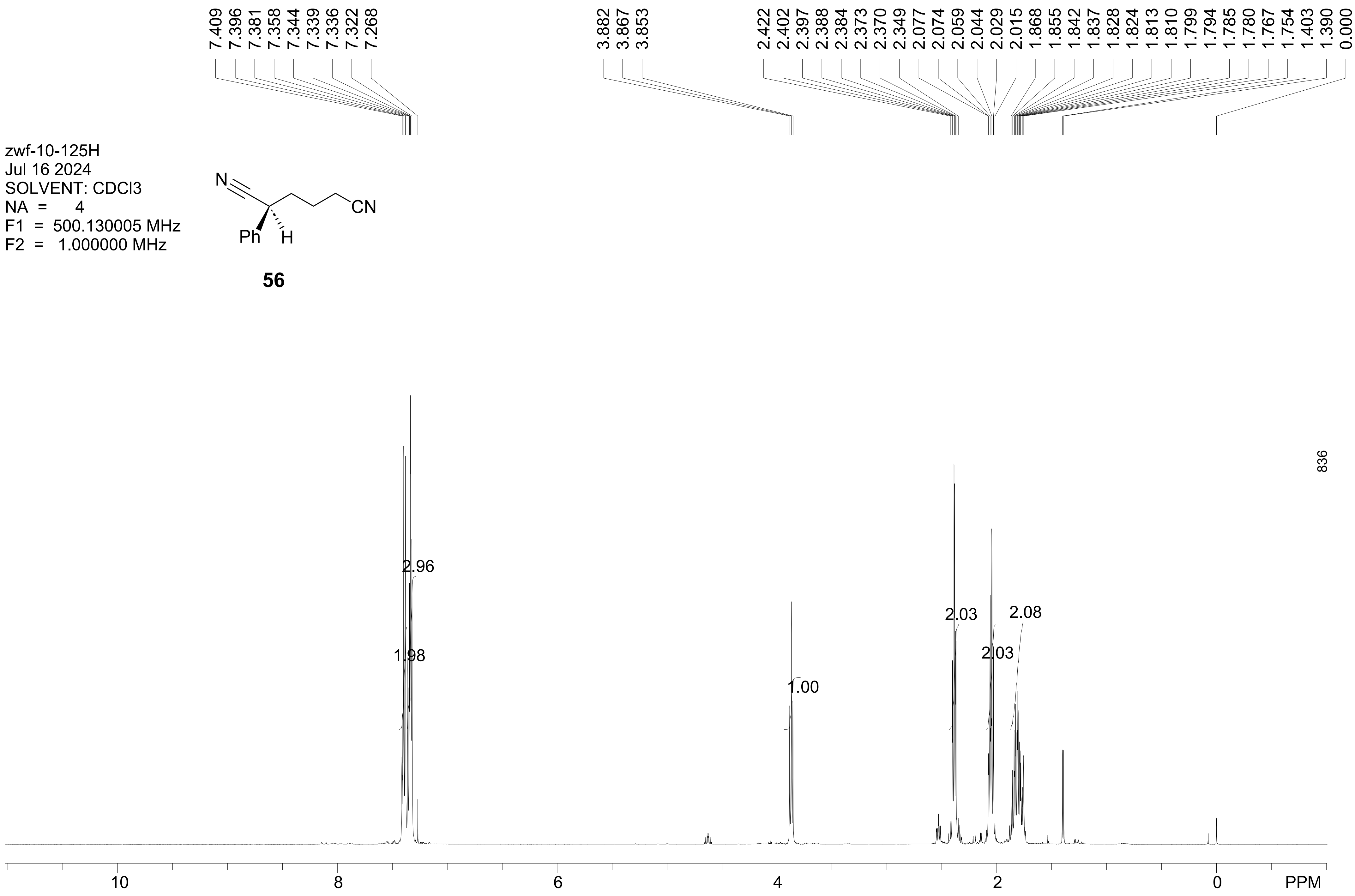
zwf-10-125H
Jul 16 2024
SOLVENT: CDCl3
NA = 4
F1 = 500.130005 MHz
F2 = 1.000000 MHz
N
CN
Ph
H
56
7.409
7.396
7.381
7.358
7.344
7.339
7.336
7.322
7.268
3.882
3.867
3.853
2.422
2.402
2.397
2.388
2.384
2.373
2.370
2.349
2.077
2.074
2.059
2.044
2.029
2.015
1.868
1.855
1.842
1.837
1.828
1.824
1.813
1.810
1.799
1.794
1.785
1.780
1.767
1.754
1.403
1.390
0.000
2.96
1.98
1.00
2.03
2.03
2.08
10
8
6
4
2
0
PPM

zwf-10-125C
Jul 16 2024
SOLVENT: CDCl3
NA = 100
F1 = 125.757790 MHz
F2 = 1.000000 MHz

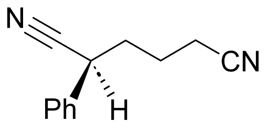

56

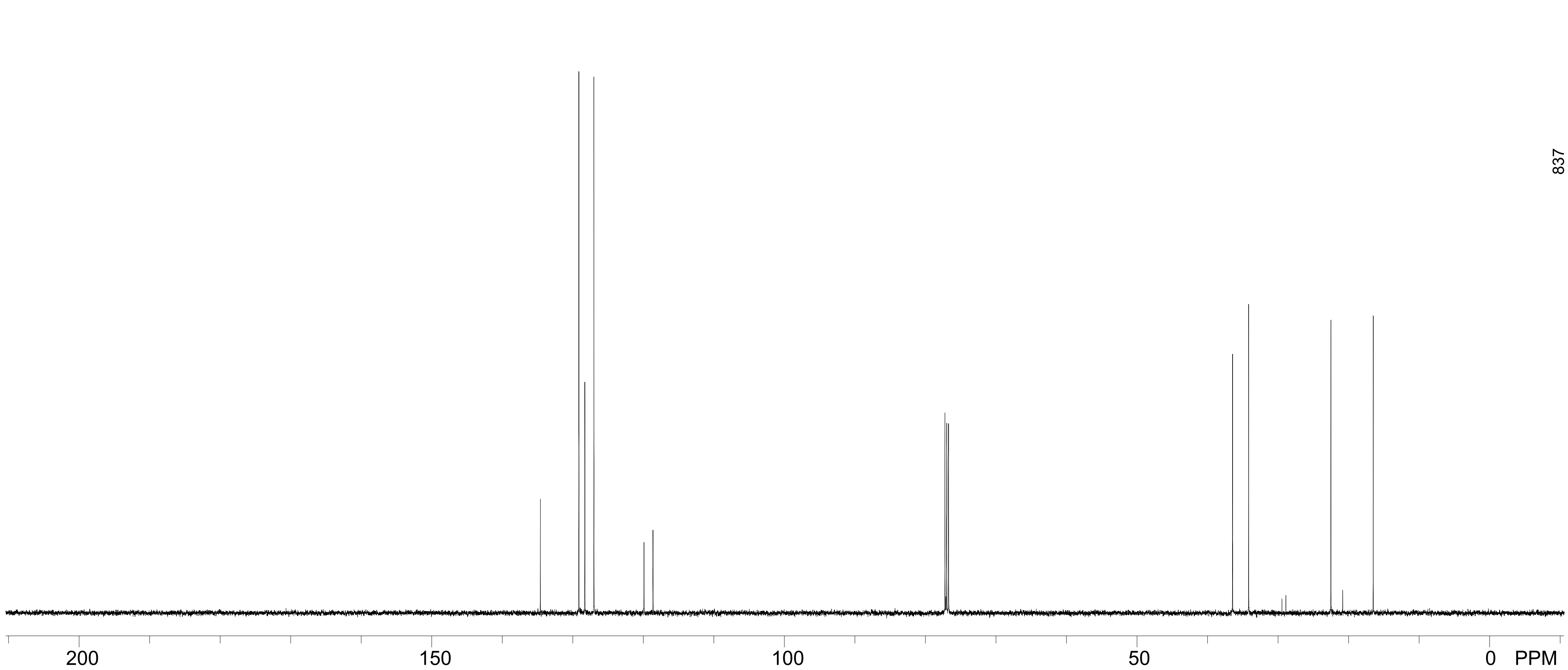

Area Percent Report

Agilent Technologies

Sample name: zwf-10-125-IE-99-1-0.8-205

Data file: C:\Users\Public\Documents\ChemStation\1\Data\zwf\zwf 2024-07-16 14-15-56\008-P1-C4-zwf-10-125.D

Injection Acquired Date 07/16/2024 18:08:48

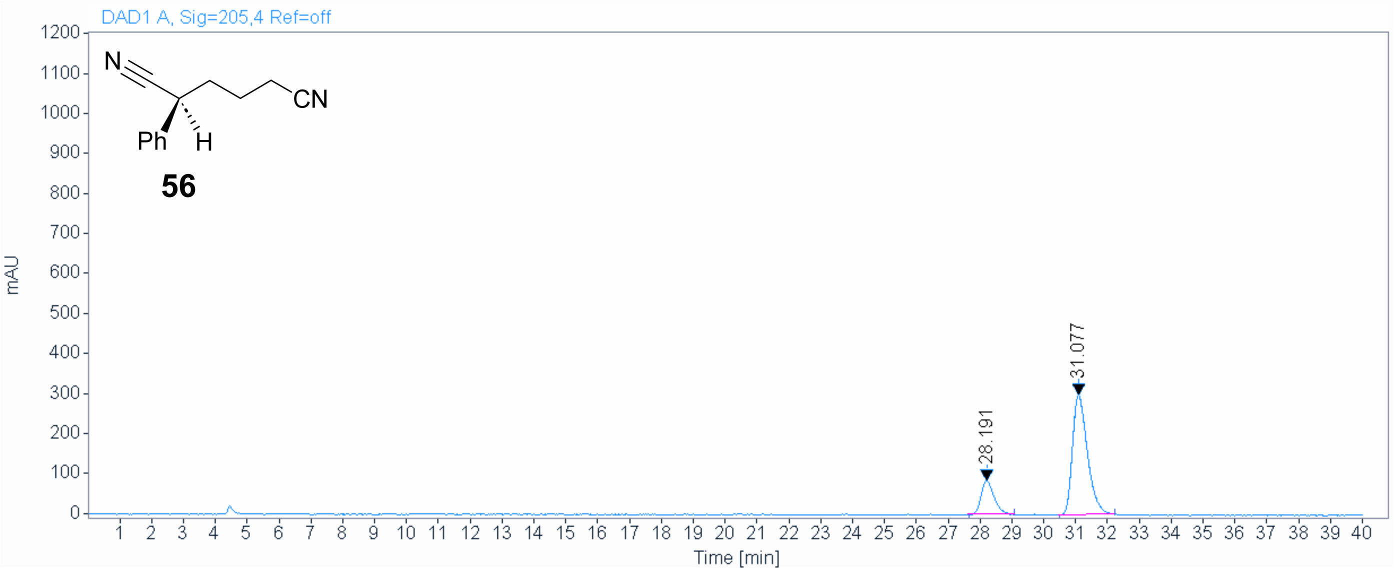

Signal: DAD1 A, Sig=205,4 Ref=off

RT [min]	Width [min]	Height	Area	Area%
28.191	0.3541	85.4259	2461.1755	20.0174
31.077	0.4661	299.7962	9834.0273	79.9826
		Sum	12295.2029	100.0000

Area Percent Report

Agilent Technologies

Sample name: zwf-10-127-rac-IE-99-1-0.8-205

Data file: C:\Users\Public\Documents\ChemStation\1\Data\zwf\zwf 2024-07-16 14-15-56\010-P1-C6-zwf-10-127-rac.D

Injection Acquired Date 07/16/2024 19:30:56

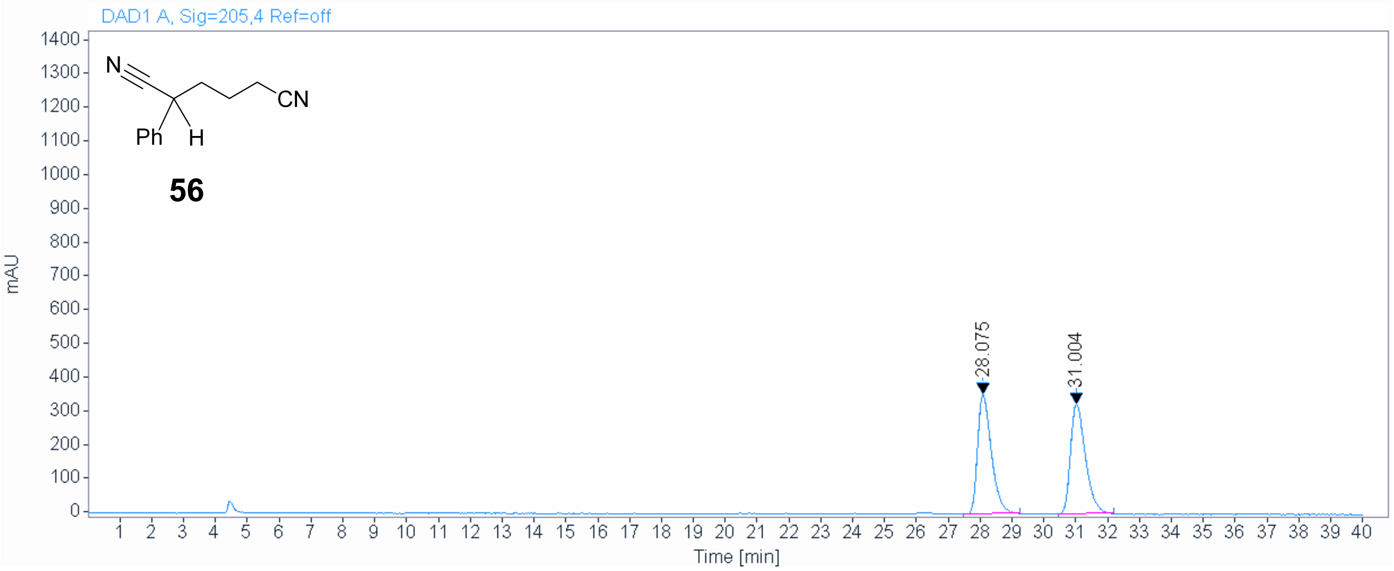

Signal: DAD1 A, Sig=205,4 Ref=off

RT [min]	Width [min]	Height	Area	Area%
28.075	0.4130	356.6580	10762.3125	50.1001
31.004	0.4489	326.5891	10719.3154	49.8999
		Sum	21481.6279	100.0000

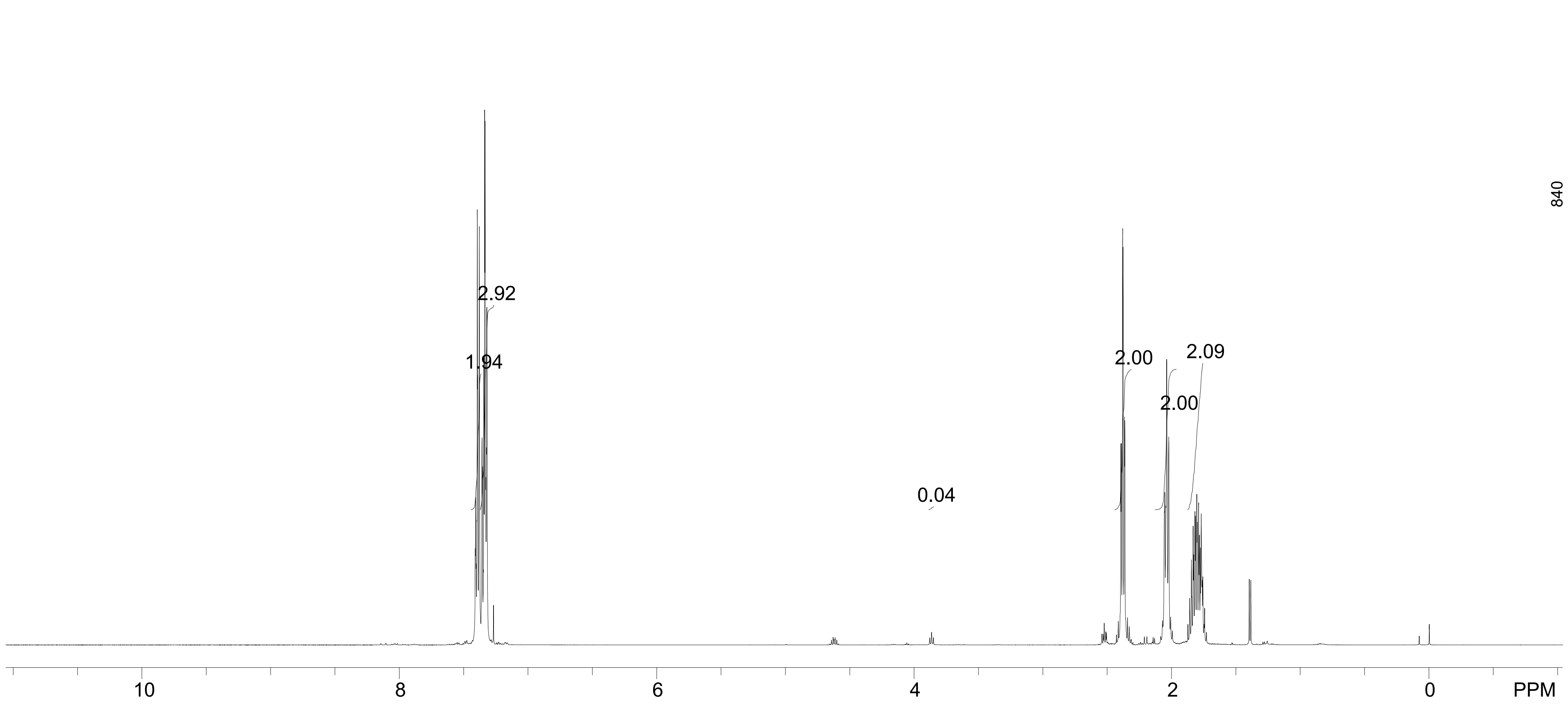
zwf-10-127H
Jul 16 2024
SOLVENT: CDCl3
NA = 4
F1 = 500.130005 MHz
F2 = 1.000000 MHz
7.410
7.407
7.404
7.397
7.394
7.391
7.379
7.356
7.354
7.347
7.342
7.336
7.333
7.328
7.323
7.319
7.268
3.880
3.866
3.851
2.416
2.396
2.392
2.382
2.378
2.368
2.365
2.344
2.072
2.055
2.040
2.023
2.009
1.875
1.861
1.848
1.834
1.821
1.816
1.806
1.802
1.792
1.772
1.759
1.745
0.000
2.92
1.94
0.04
2.00
2.09
2.00
10
8
6
4
2
0
PPM

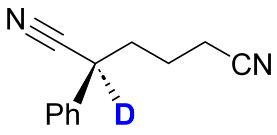
N
CN
Ph
D

56-d

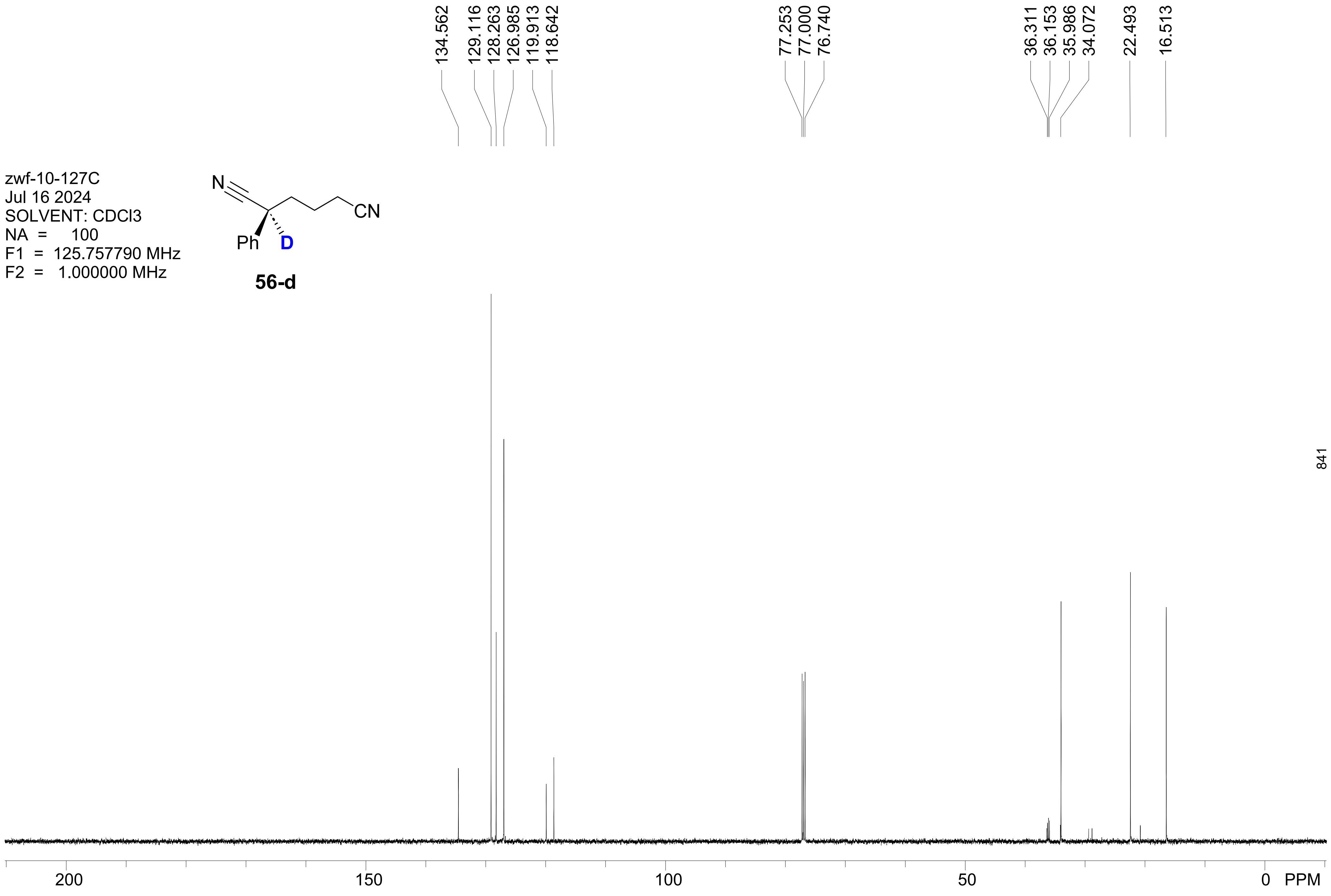
zwf-10-127C
Jul 16 2024
SOLVENT: CDCl3
NA = 100
F1 = 125.757790 MHz
F2 = 1.000000 MHz
N
CN
Ph
D
56-d
134.562
129.116
128.263
126.985
119.913
118.642
77.253
77.000
76.740
36.311
36.153
35.986
34.072
22.493
16.513
200
150
100
50
0 PPM

Area Percent Report

Agilent Technologies

Sample name: zwf-10-127-IE-99-1-0.8-205

Data file: C:\Users\Public\Documents\ChemStation\1\Data\zwf\zwf 2024-07-16 14-15-56\009-P1-C5-zwf-10-127.D

Injection Acquired Date 07/16/2024 18:49:50

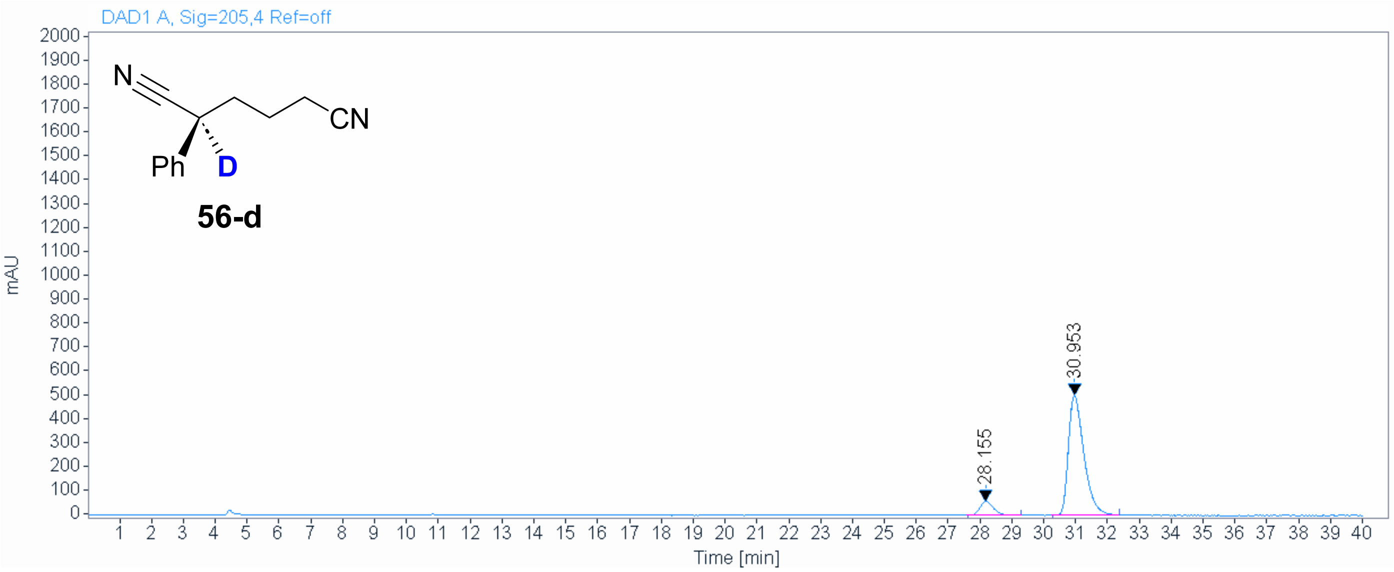

Signal: DAD1 A, Sig=205,4 Ref=off

RT [min]	Width [min]	Height	Area	Area%
28.155	0.3668	59.1334	1779.3966	9.4724
30.953	0.4636	504.1083	17005.7500	90.5276
		Sum	18785.1466	100.0000

Area Percent Report

Sample name: zwf-10-127-rac-IE-99-1-0.8-205

Data file: C:\Users\Public\Documents\ChemStation\1\Data\zwf\zwf 2024-07-16 14-15-56\010-P1-C6-zwf-10-127-rac.D

Injection Acquired Date 07/16/2024 19:30:56

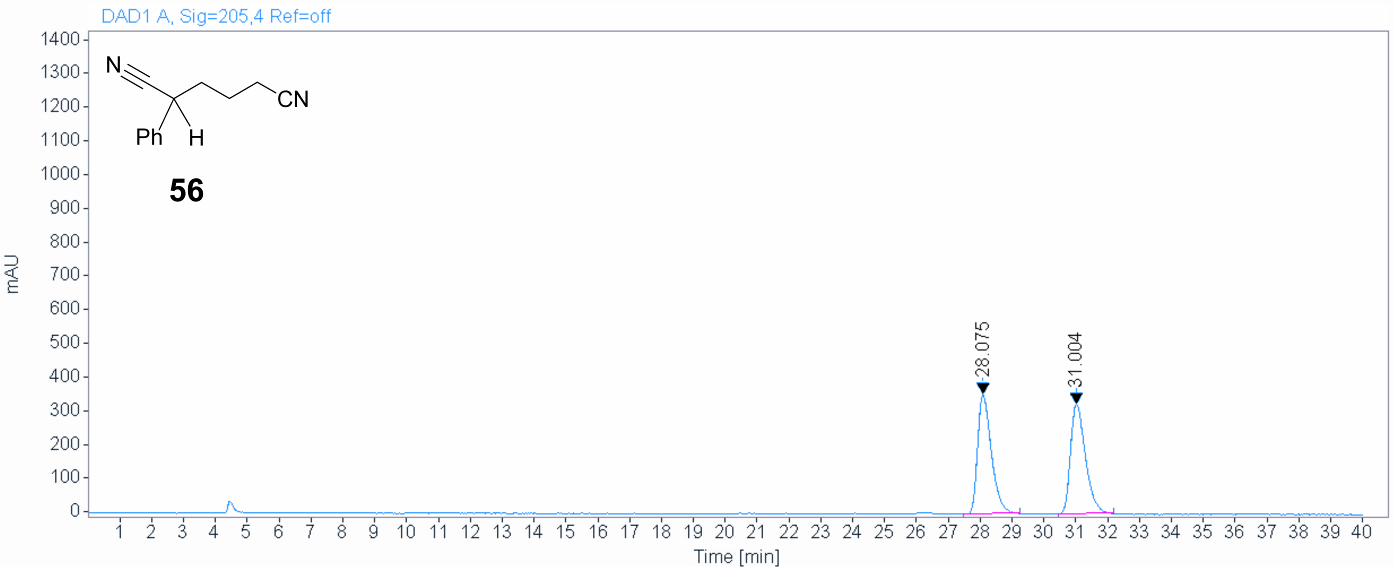

Signal: DAD1 A, Sig=205,4 Ref=off

RT [min]	Width [min]	Height	Area	Area%
28.075	0.4130	356.6580	10762.3125	50.1001
31.004	0.4489	326.5891	10719.3154	49.8999
		Sum	21481.6279	100.0000

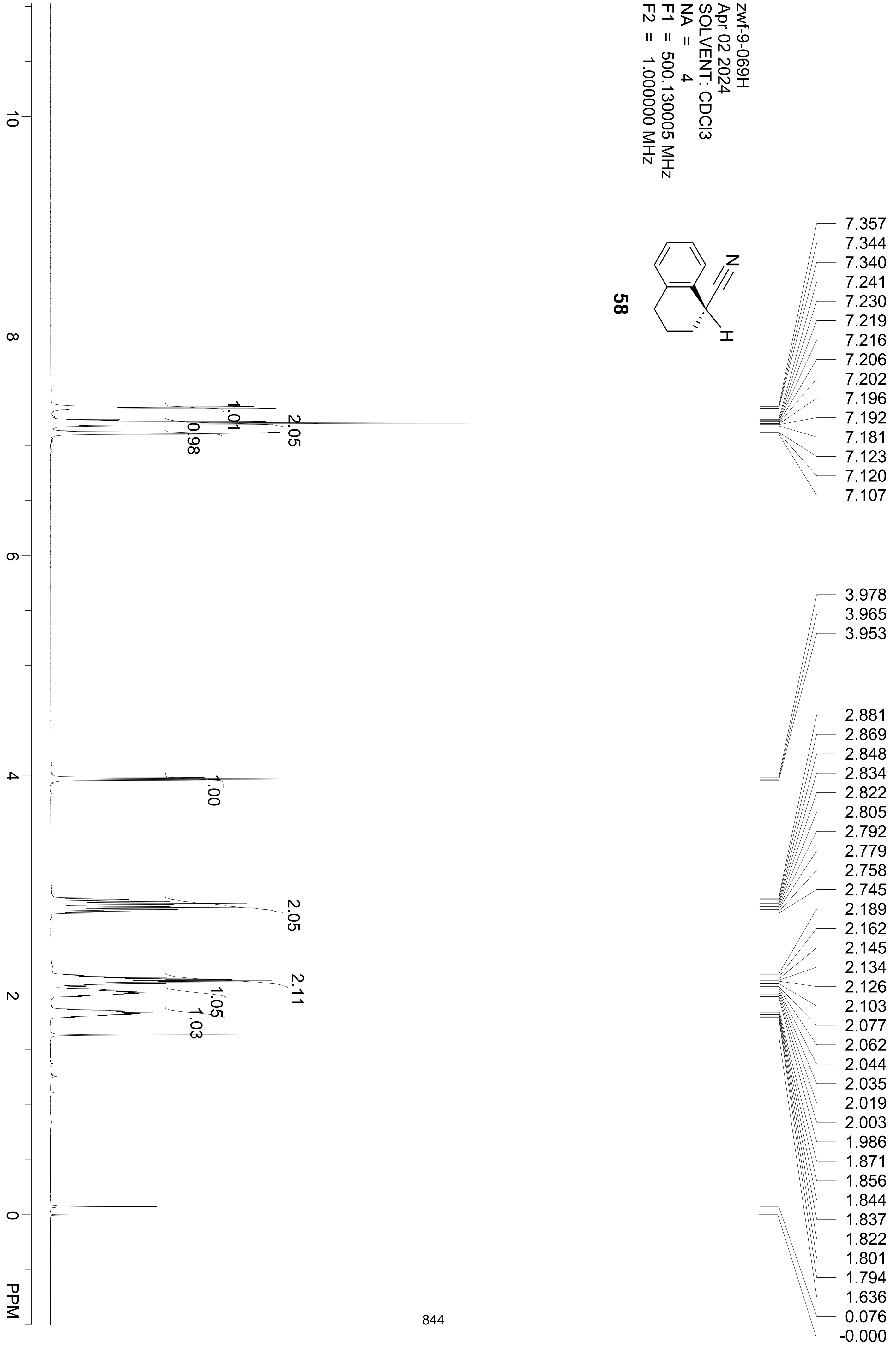
zwf-9-069H
Apr 02 2024
SOLVENT: CDCl3
NA = 4
F1 = 500.130005 MHz
F2 = 1.000000 MHz
58
7.357
7.344
7.340
7.241
7.230
7.219
7.216
7.206
7.202
7.196
7.192
7.181
7.123
7.120
7.107
3.978
3.965
3.953
2.881
2.869
2.848
2.834
2.822
2.805
2.792
2.779
2.758
2.745
2.189
2.162
2.145
2.134
2.126
2.103
2.077
2.062
2.044
2.035
2.019
2.003
1.986
1.871
1.856
1.844
1.837
1.822
1.801
1.794
1.636
0.076
-0.000
1.01
2.05
0.98
1.00
2.05
2.11
1.05
1.03
10
8
6
4
2
0
PPM

zwf-9-069C
Apr 0 2024
SOLVENT: CDCl3
NA = 100
F1 = 125.757790 MHz
F2 = 1.000000 MHz

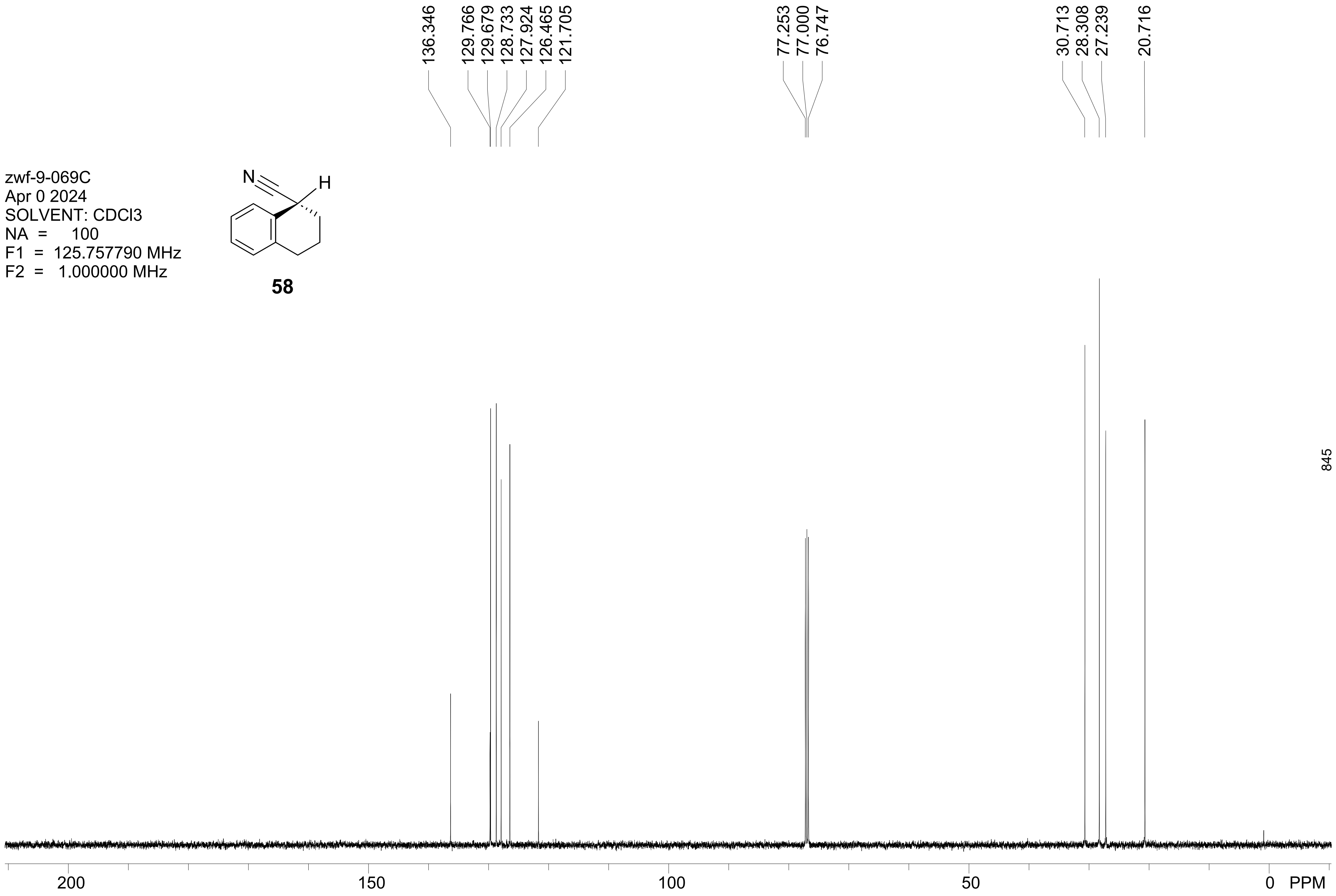

58

Area Percent Report

Agilent Technologies

Sample name: zwf-9-069-IG-99-1-0.8-205

Data file: C:\Users\Public\Documents\ChemStation\1\Data\zwf\zwf-S 2024-04-02 08-29-47\004-P1-C3-zwf-9-0-69.D

Injection Acquired Date 04/02/2024 09:59:33

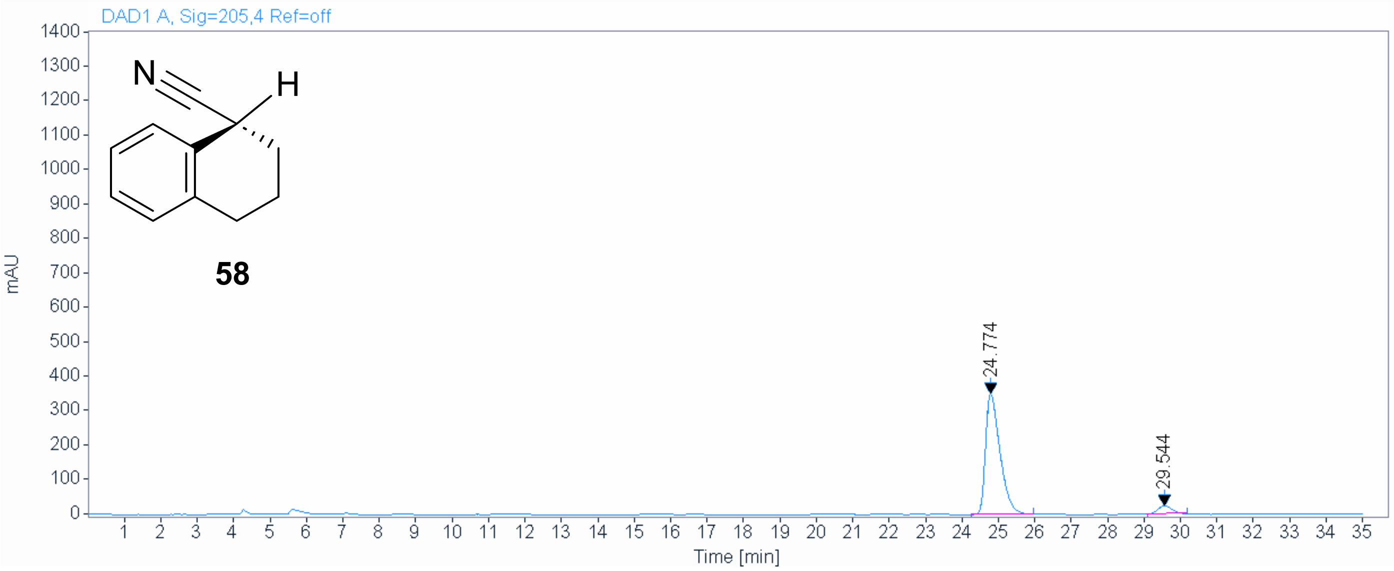

Signal: DAD1 A, Sig=205,4 Ref=off

RT [min]	Width [min]	Height	Area	Area%
24.774	0.4194	349.8931	9639.9619	93.7455
29.544	0.4630	23.1502	643.1541	6.2545
		Sum	10283.1160	100.0000

Area Percent Report

Agilent Technologies

Sample name: zwf-9-069-rac-IG-99-1-0.8-205

Data file: C:\Users\Public\Documents\ChemStation\1\Data\zwf\zwf-S 2024-04-02 08-29-47\005-P1-C2-zwf-9-069-rac.D

Injection Acquired Date 04/02/2024 10:35:32

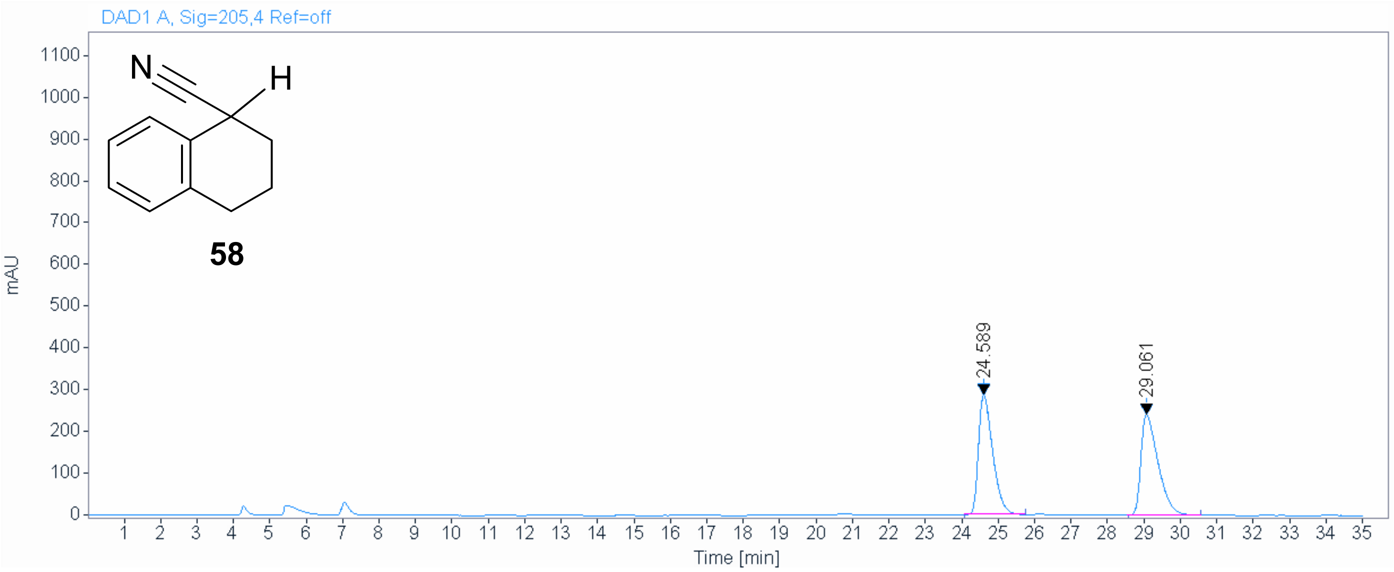

Signal: DAD1 A, Sig=205,4 Ref=off

RT [min]	Width [min]	Height	Area	Area%
24.589	0.4197	287.2987	7824.2192	49.9576
29.061	0.4799	240.6737	7837.5078	50.0424
		Sum	15661.7271	100.0000

zwf-9-070H
Apr 02 2024
SOLVENT: CDCl3
NA = 4
F1 = 400.140015 MHz
F2 = 1.000000 MHz

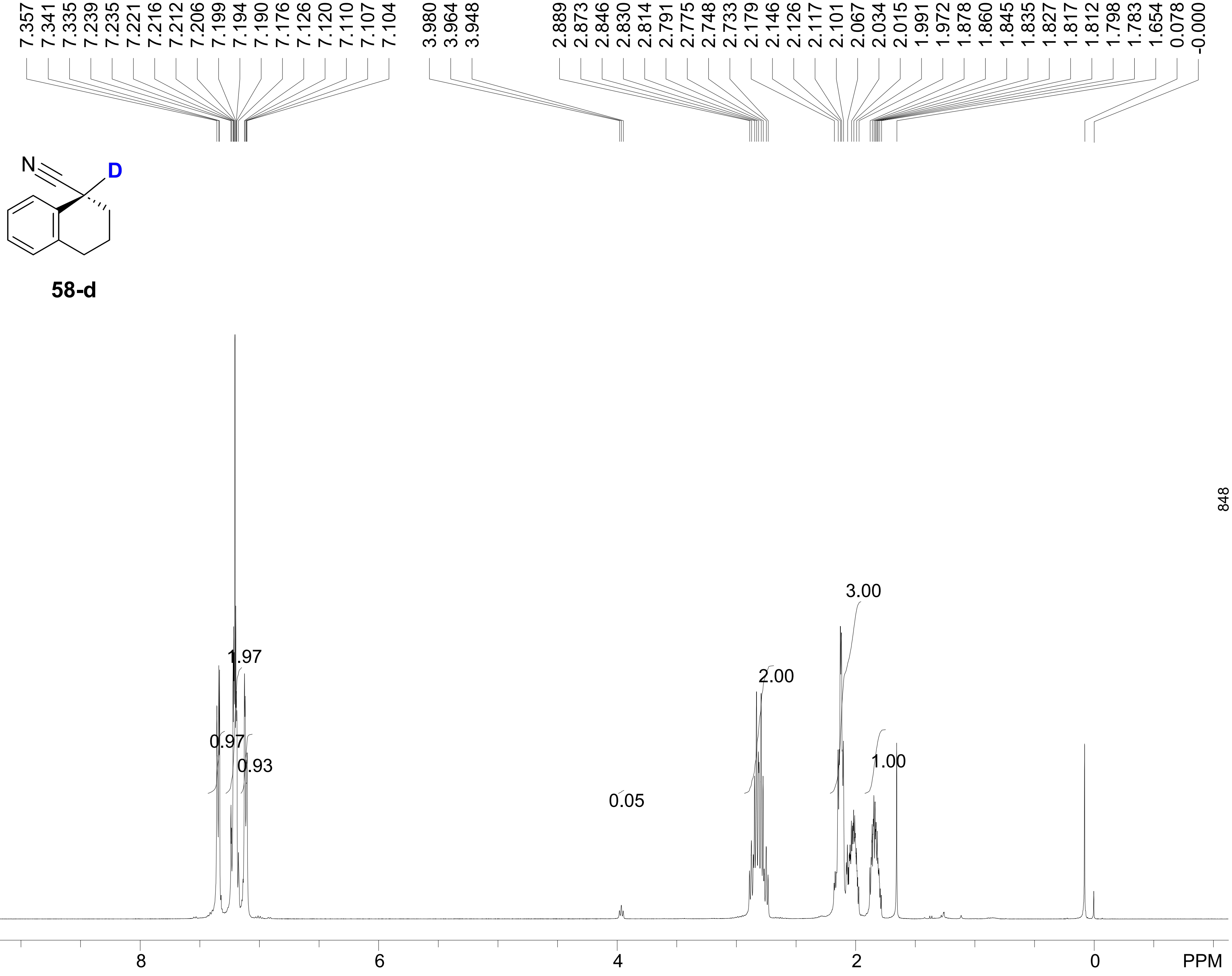

58-d

zwf-9-070C
Apr 02 2024
SOLVENT: CDCl3
NA = 100
F1 = 100.615280 MHz
F2 = 1.000000 MHz

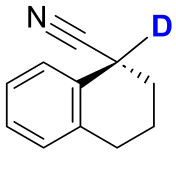

58-d

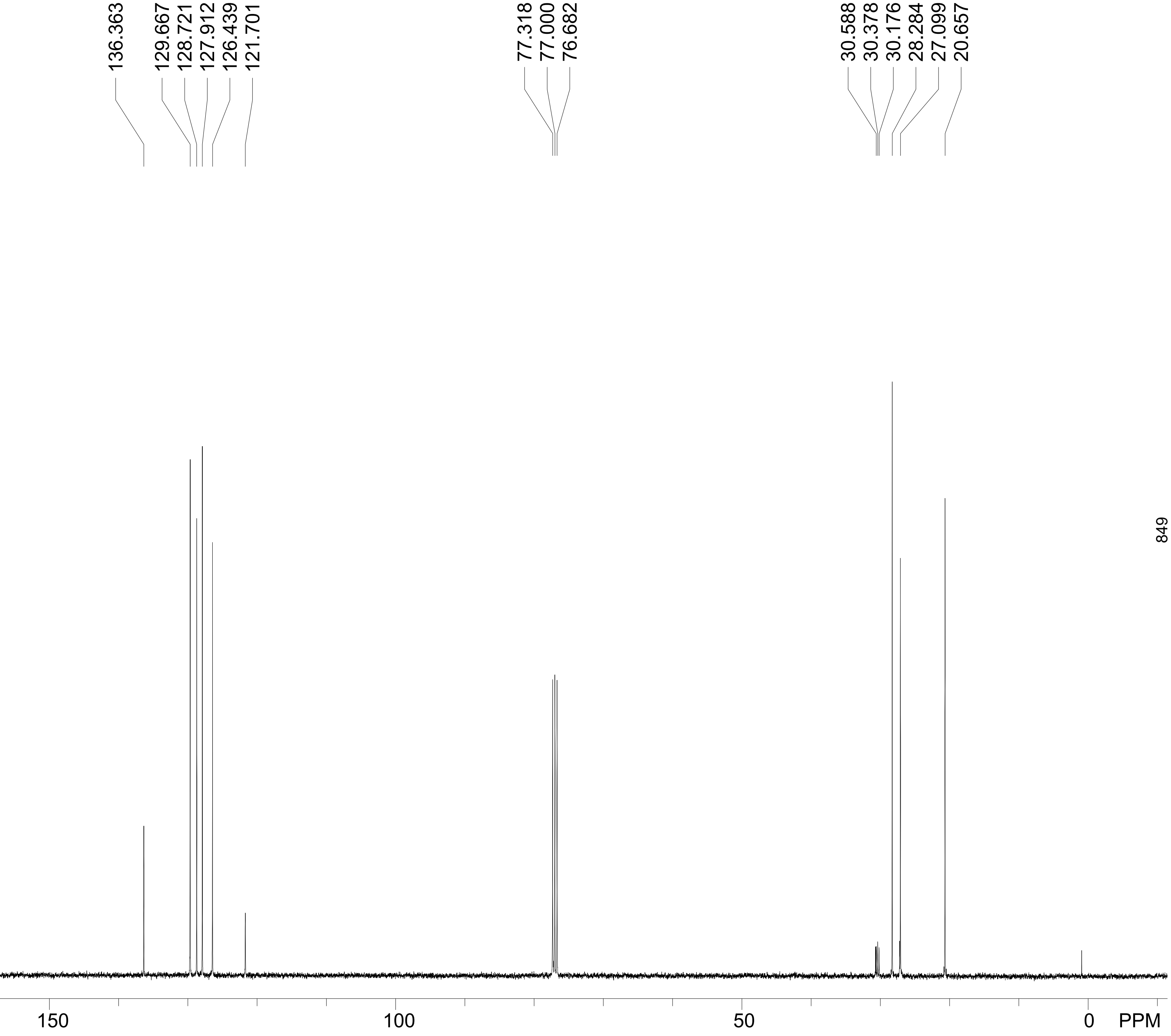

Area Percent Report

Agilent Technologies

Sample name: zwf-9-070-IG-99-1-0.8-205

Data file: C:\Users\Public\Documents\ChemStation\1\Data\zwf\zwf-S 2024-04-02 08-29-47\003-P1-C4-zwf-9-070.D

Injection Acquired Date 04/02/2024 09:23:35

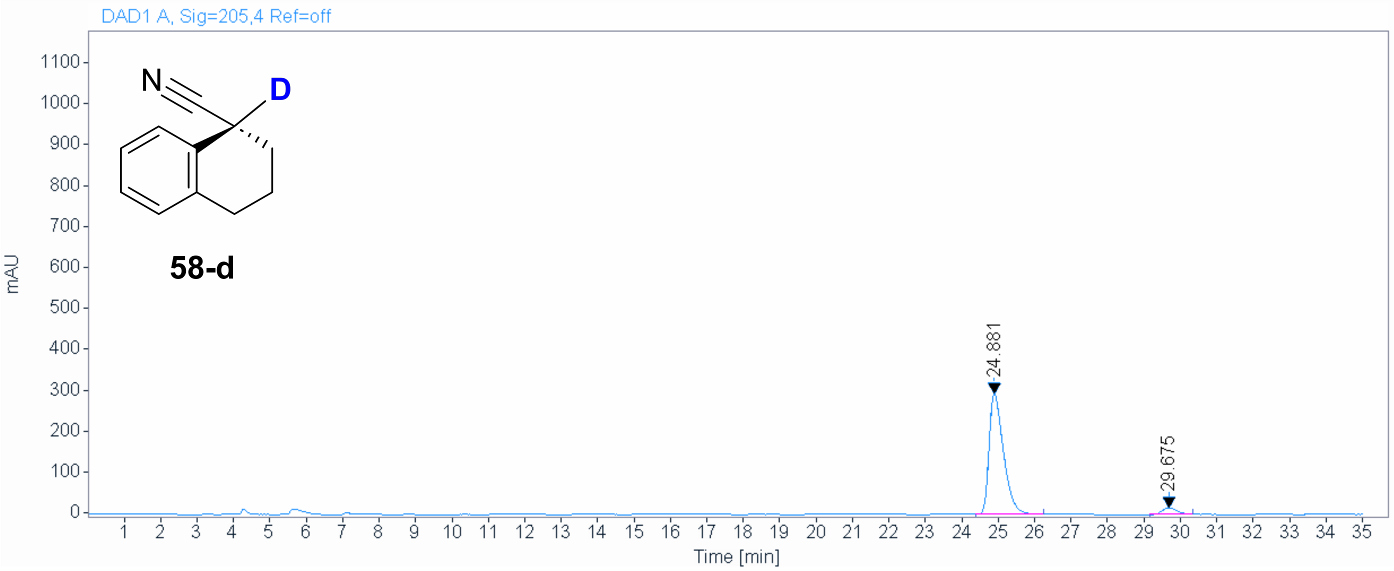

Signal: DAD1 A, Sig=205,4 Ref=off

RT [min]	Width [min]	Height	Area	Area%
24.881	0.4136	294.2918	8112.8291	95.0335
29.675	0.4583	15.4183	423.9797	4.9665
		Sum	8536.8088	100.0000

Area Percent Report

Agilent Technologies

Sample name: zwf-9-069-rac-IG-99-1-0.8-205

Data file: C:\Users\Public\Documents\ChemStation\1\Data\zwf\zwf-S 2024-04-02 08-29-47\005-P1-C2-zwf-9-069-rac.D

Injection Acquired Date 04/02/2024 10:35:32

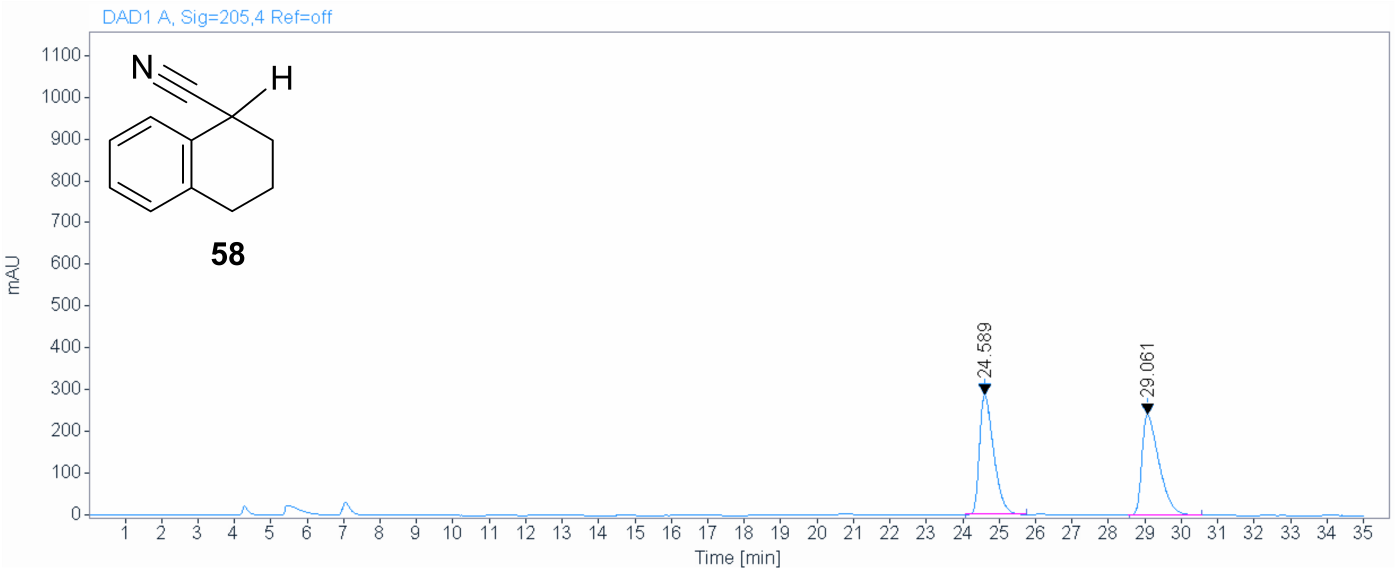

Signal: DAD1 A, Sig=205,4 Ref=off

RT [min]	Width [min]	Height	Area	Area%
24.589	0.4197	287.2987	7824.2192	49.9576
29.061	0.4799	240.6737	7837.5078	50.0424
		Sum	15661.7271	100.0000

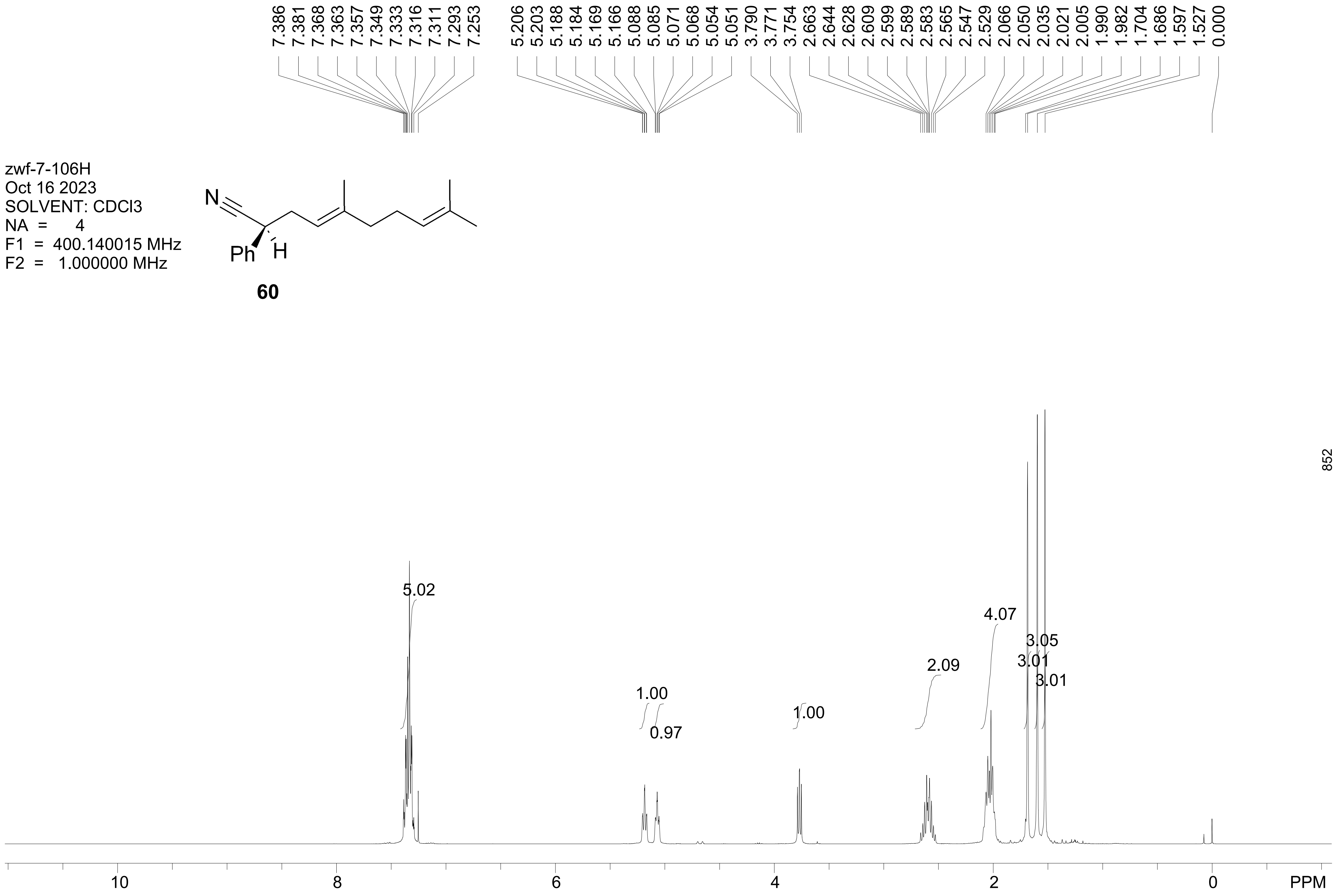
zwf-7-106H
Oct 16 2023
SOLVENT: CDCl3
NA = 4
F1 = 400.140015 MHz
F2 = 1.000000 MHz
N
Ph
H
60
7.386
7.381
7.368
7.363
7.357
7.349
7.333
7.316
7.311
7.293
7.253
5.206
5.203
5.188
5.184
5.169
5.166
5.088
5.085
5.071
5.068
5.054
5.051
3.790
3.771
3.754
2.663
2.644
2.628
2.609
2.599
2.589
2.583
2.565
2.547
2.529
2.066
2.050
2.035
2.021
2.005
1.990
1.982
1.704
1.686
1.597
1.527
0.000
5.02
1.00
0.97
1.00
2.09
4.07
3.05
3.01
3.01
10
8
6
4
2
0
PPM

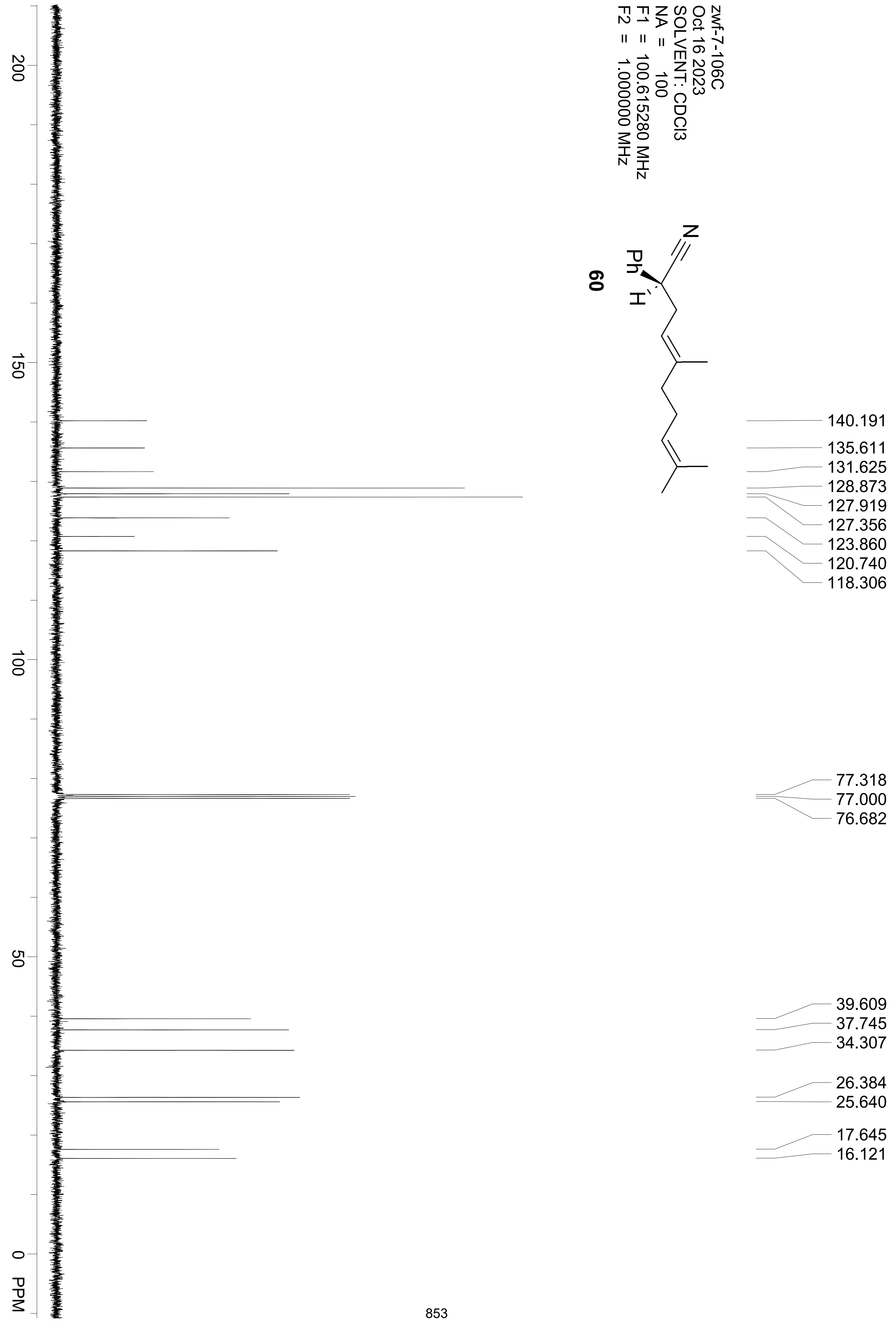
zwf-7-106C
Oct 16 2023
SOLVENT: CDCl3
NA = 100
F1 = 100.615280 MHz
F2 = 1.000000 MHz
N
Ph
H
60
140.191
135.611
131.625
128.873
127.919
127.356
123.860
120.740
118.306
77.318
77.000
76.682
39.609
37.745
34.307
26.384
25.640
17.645
16.121
200
150
100
50
0
PPM

Area Percent Report

Agilent Technologies

Sample name: zwf-7-106-IE-99-1-0.8-205

Data file: C:\Users\Public\Documents\ChemStation\1\Data\zwf\zwf-S 2023-10-17 08-55-39\007-P1-C5-zwf-7-106.D

Injection Acquired Date 10/17/2023 11:54:08

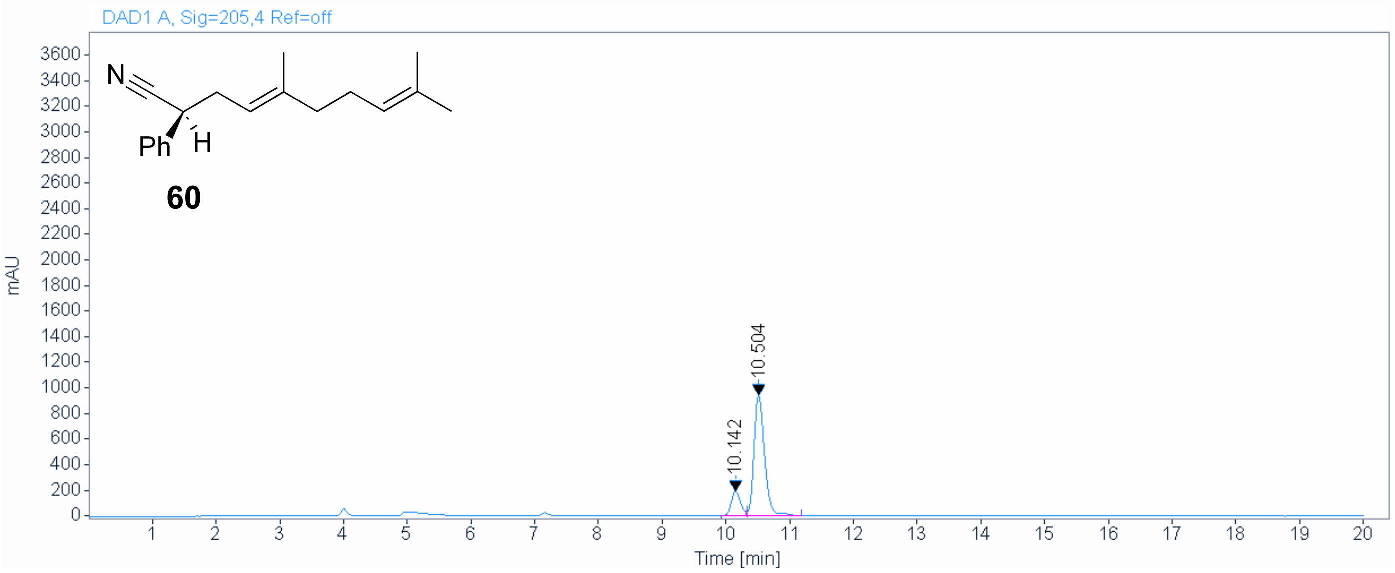

Signal: DAD1 A, Sig=205,4 Ref=off

RT [min]	Width [min]	Height	Area	Area%
10.142	0.1566	190.2760	1934.5532	15.1545
10.504	0.1760	939.6860	10831.0176	84.8455
		Sum	12765.5708	100.0000

Area Percent Report

Agilent Technologies

Sample name: zwf-7-106-rac-IE-99-1-0.8-205

Data file: C:\Users\Public\Documents\ChemStation\1\Data\zwf\zwf-S 2023-10-17 08-55-39\006-P1-C6-zwf-7-106-rac.D

Injection Acquired Date 10/17/2023 11:33:07

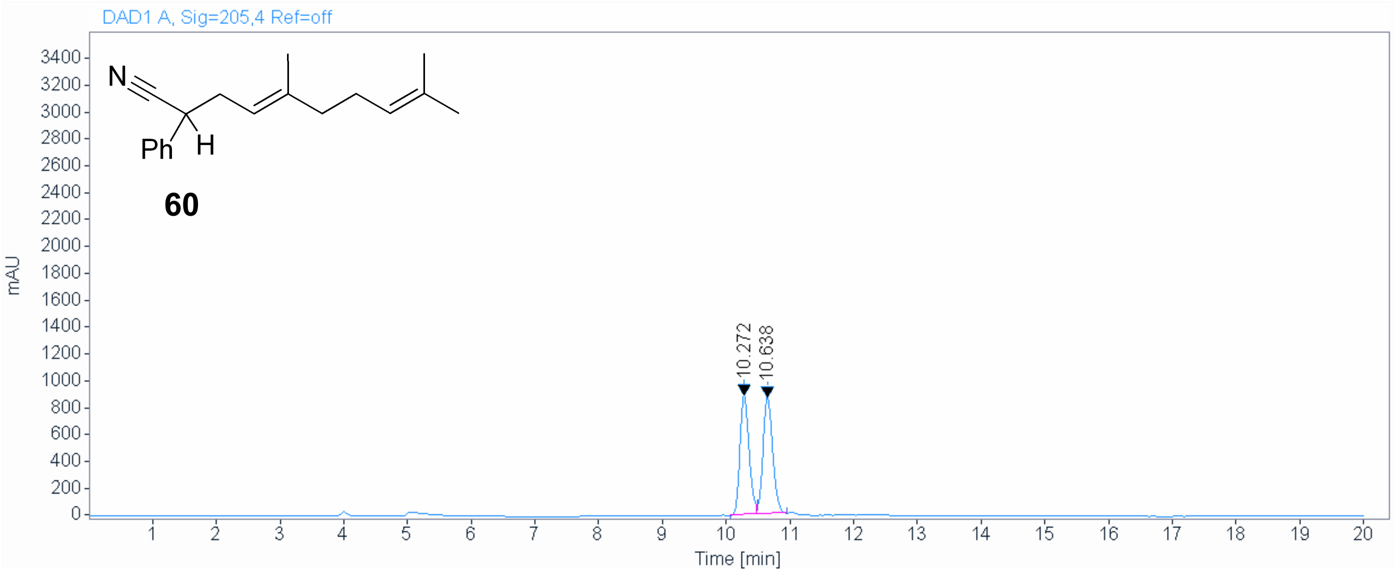

Signal: DAD1 A, Sig=205,4 Ref=off

RT [min]	Width [min]	Height	Area	Area%
10.272	0.1573	887.8339	8925.7930	49.3916
10.638	0.1631	866.6233	9145.6855	50.6084
		Sum	18071.4785	100.0000

zwf-8-040H
Nov 15 2023
SOLVENT: CDCl3
NA = 4
F1 = 400.140015 MHz
F2 = 1.000000 MHz

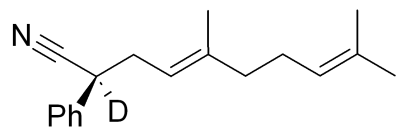

60-d

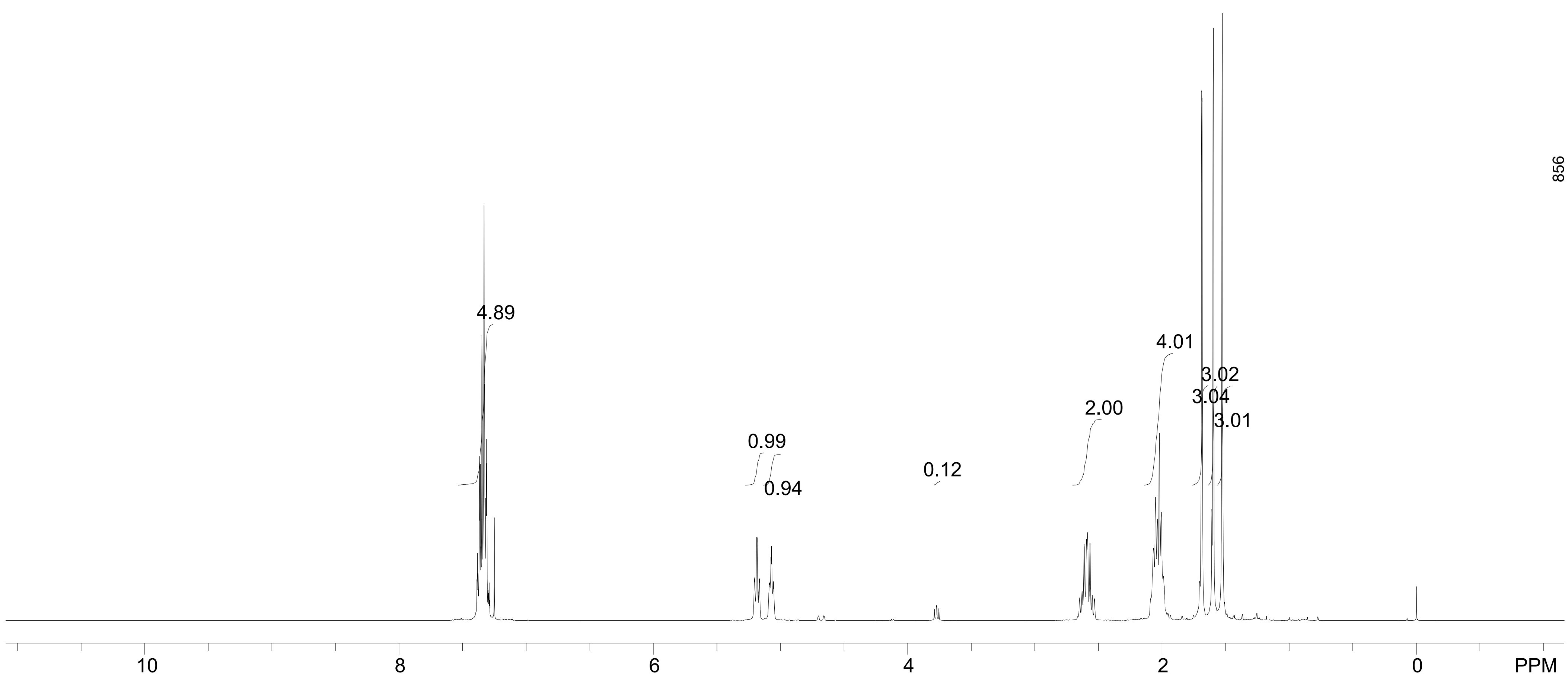

zwf-8-040C
Nov 15 2023
SOLVENT: CDCl3
NA = 150
F1 = 100.615280 MHz
F2 = 1.000000 MHz

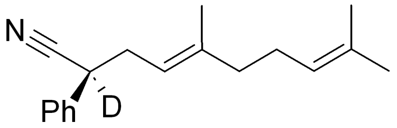

60-d

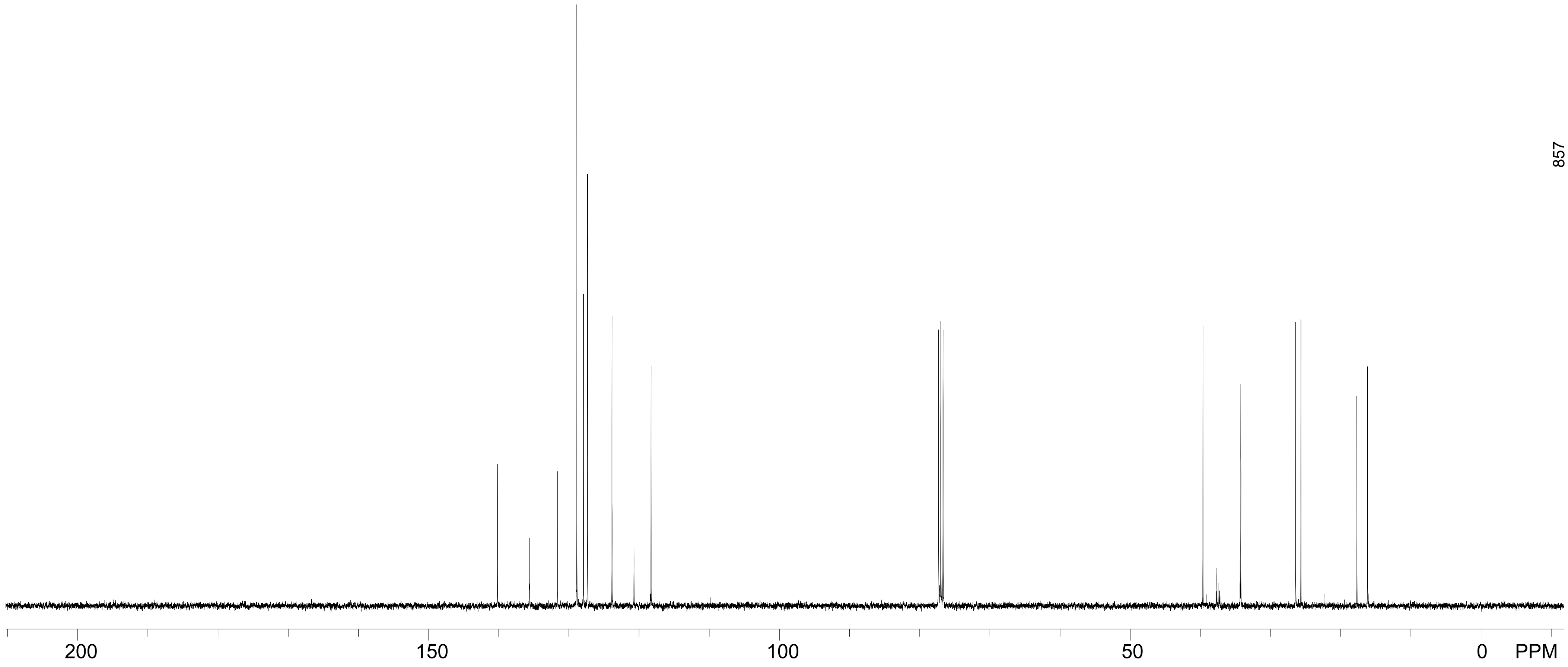

Area Percent Report

Agilent Technologies

Sample name: zwf-8-040-IE-99-1-0.8-205

Data file: C:\Users\Public\Documents\ChemStation\1\Data\zwf\zwf-S 2023-11-26 13-46-16\010-P1-C2-zwf-8-040.D

Injection Acquired Date 11/26/2023 18:42:11

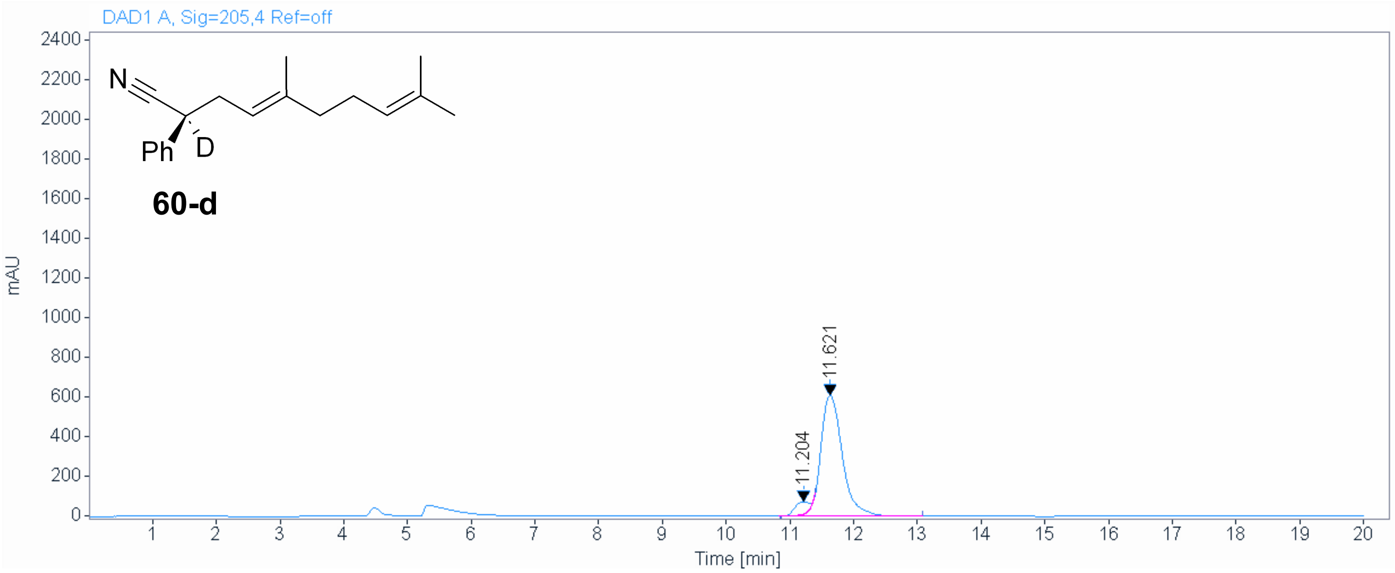

Signal: DAD1 A, Sig=205,4 Ref=off

RT [min]	Width [min]	Height	Area	Area%
11.204	0.2513	65.8029	1044.9337	6.7818
11.621	0.3691	609.1367	14362.9971	93.2182
		Sum	15407.9308	100.0000

Area Percent Report

Agilent Technologies

Sample name: zwf-8-040-rac-IE-99-1-0.8-205

Data file: C:\Users\Public\Documents\ChemStation\1\Data\zwf\zwf-S 2023-11-26 13-46-16\012-P1-C3-zwf-8-040-rac.D

Injection Acquired Date 11/26/2023 19:24:13

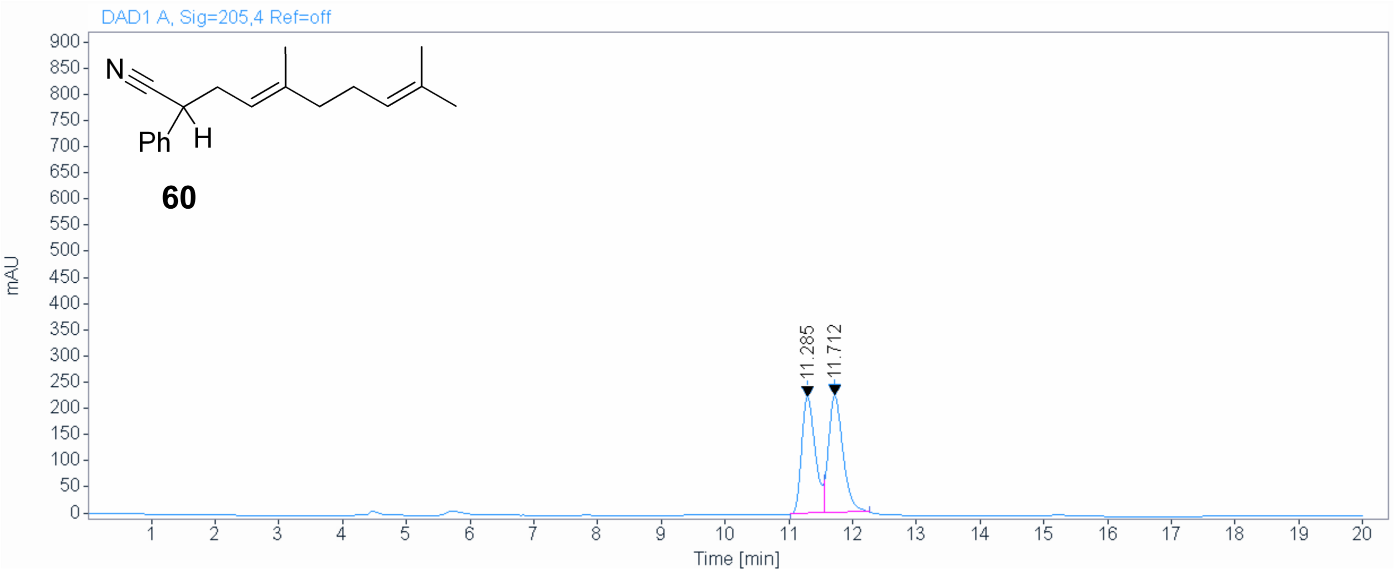

Signal: DAD1 A, Sig=205,4 Ref=off

RT [min]	Width [min]	Height	Area	Area%
11.285	0.2519	223.0674	3371.1340	48.8518
11.712	0.2623	224.2821	3529.5962	51.1482
		Sum	6900.7302	100.0000

zwf-10-136H
Jul 18 2024
SOLVENT: CDCl3
NA = 4
F1 = 500.130005 MHz
F2 = 1.000000 MHz

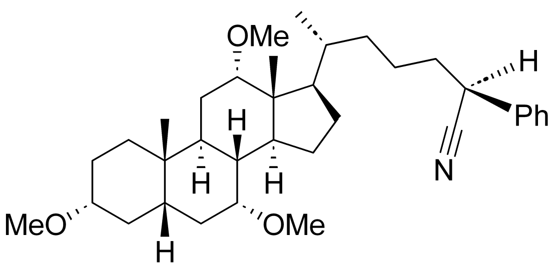

61

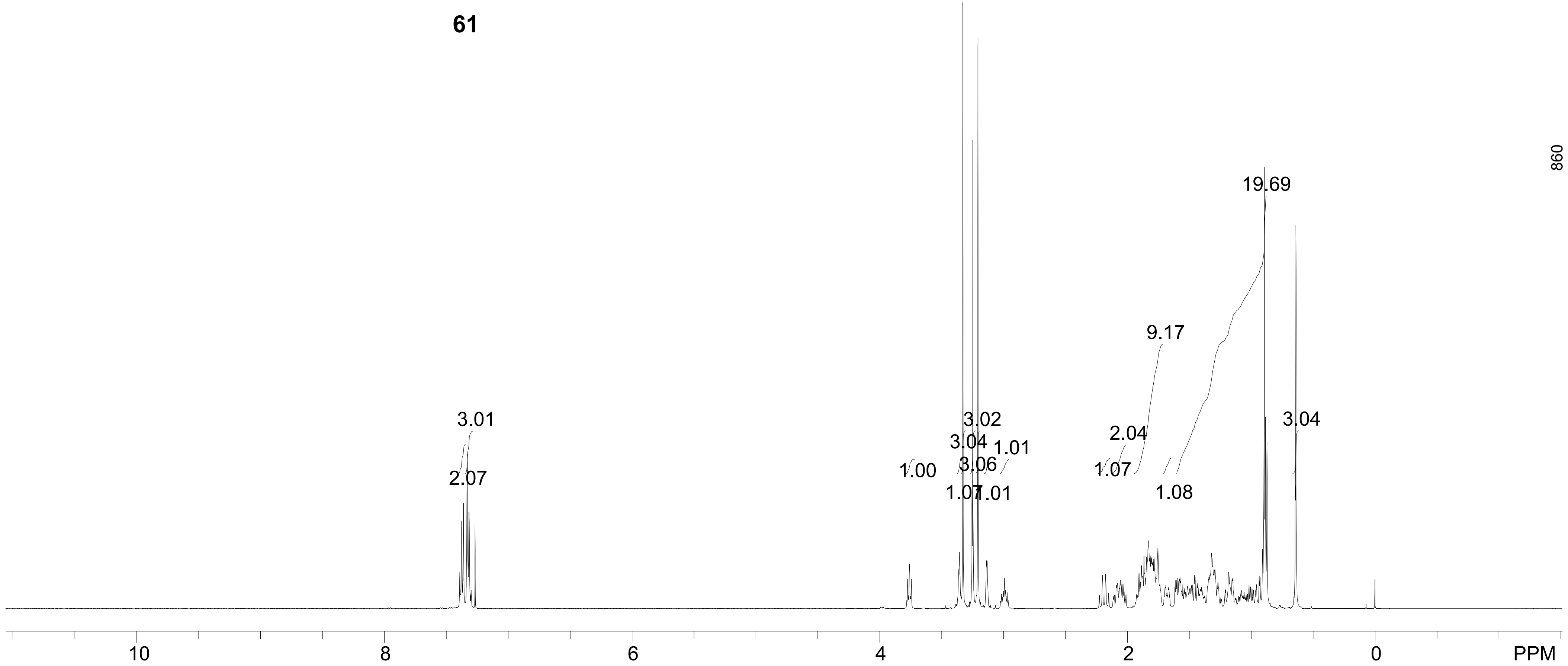

zwf-10-136C
Jul 18 2024
SOLVENT: CDCl3
NA = 100
F1 = 125.757797 MHz
F2 = 1.000000 MHz

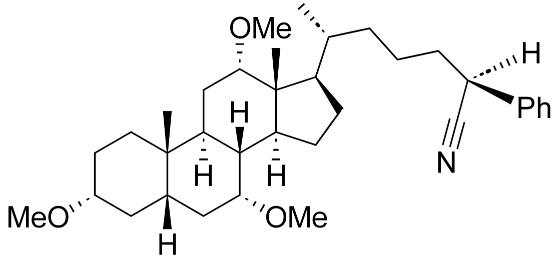

61

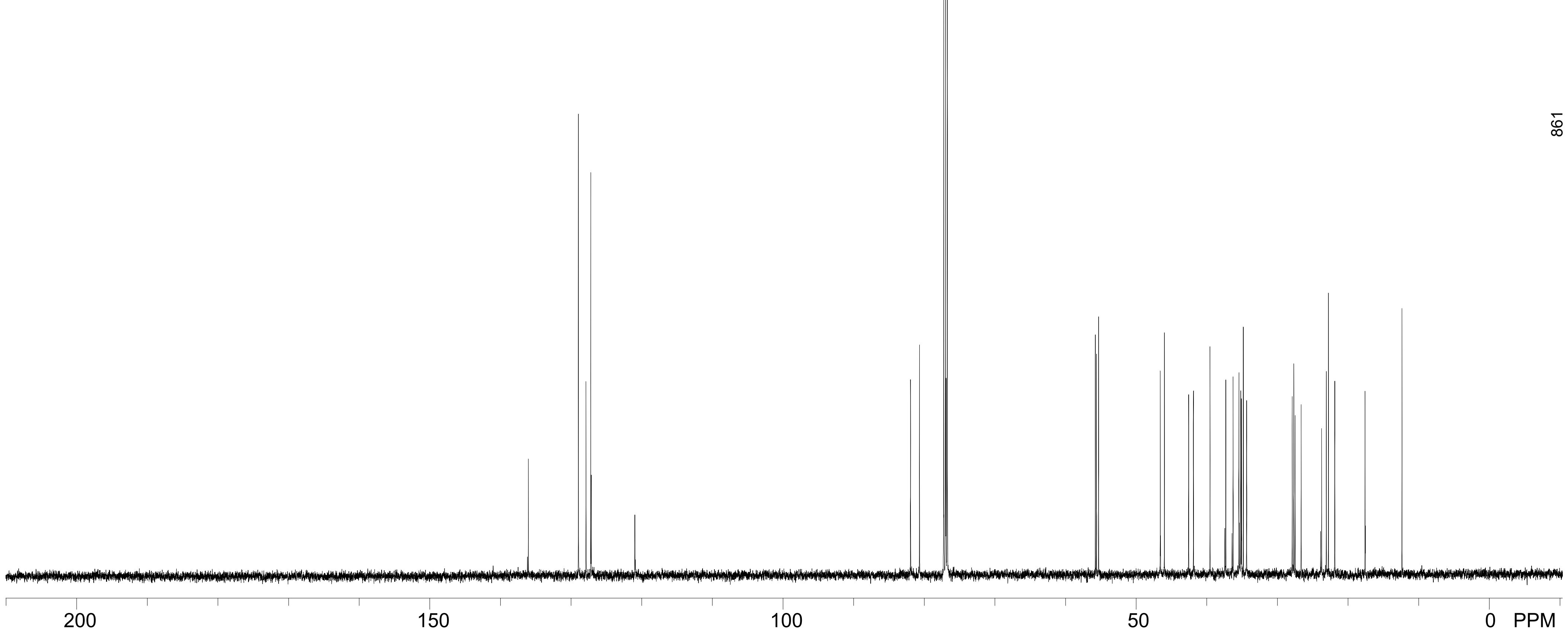

Area Percent Report

Agilent Technologies

Sample name: zwf-10-136-IE-90-10-0.8-205

Data file: C:\Users\Public\Documents\ChemStation\1\Data\zwf\zwf 2024-07-18 14-42-04\007-P1-C4-zwf-10-136.D

Injection Acquired Date 07/18/2024 21:38:36

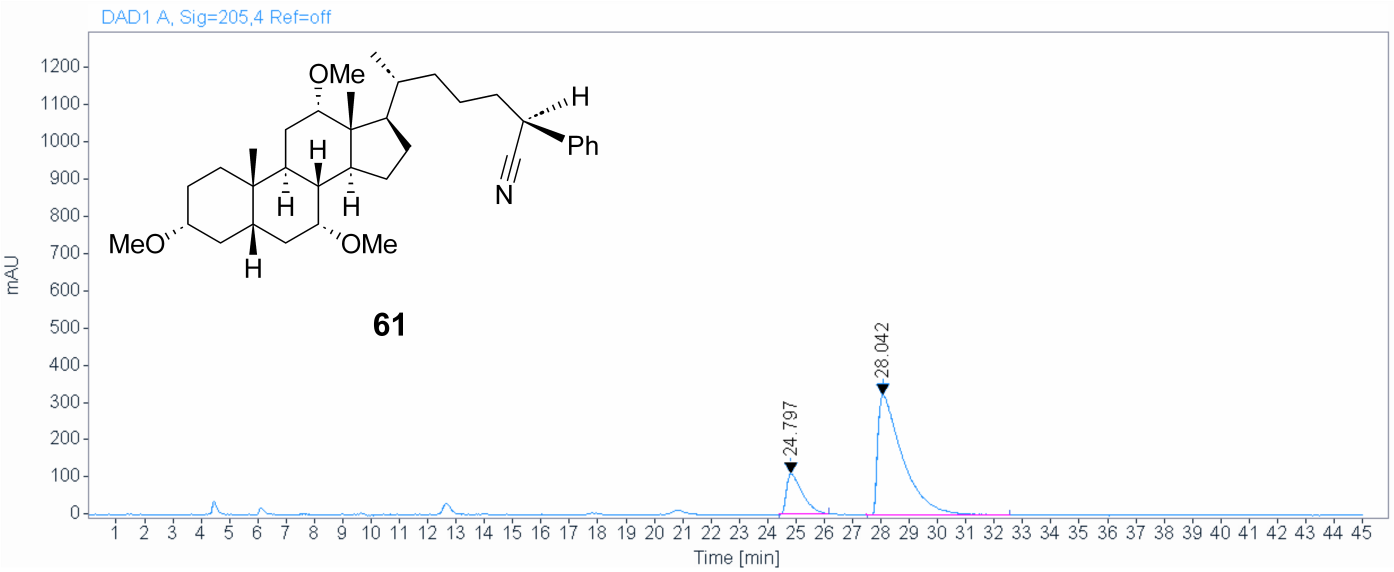

Signal: DAD1 A, Sig=205,4 Ref=off

RT [min]	Width [min]	Height	Area	Area%
24.797	0.6412	109.4468	4210.3203	17.4432
28.042	1.0259	323.7363	19927.0273	82.5568
		Sum	24137.3477	100.0000

Area Percent Report

Sample name: zwf-10-138-rac-IE-90-10-0.8-205

Data file: C:\Users\Public\Documents\ChemStation\1\Data\zwf\zwf 2024-07-18 14-42-04\009-P1-C7-zwf-10-138-rac.D

Injection Acquired Date 07/18/2024 23:10:53

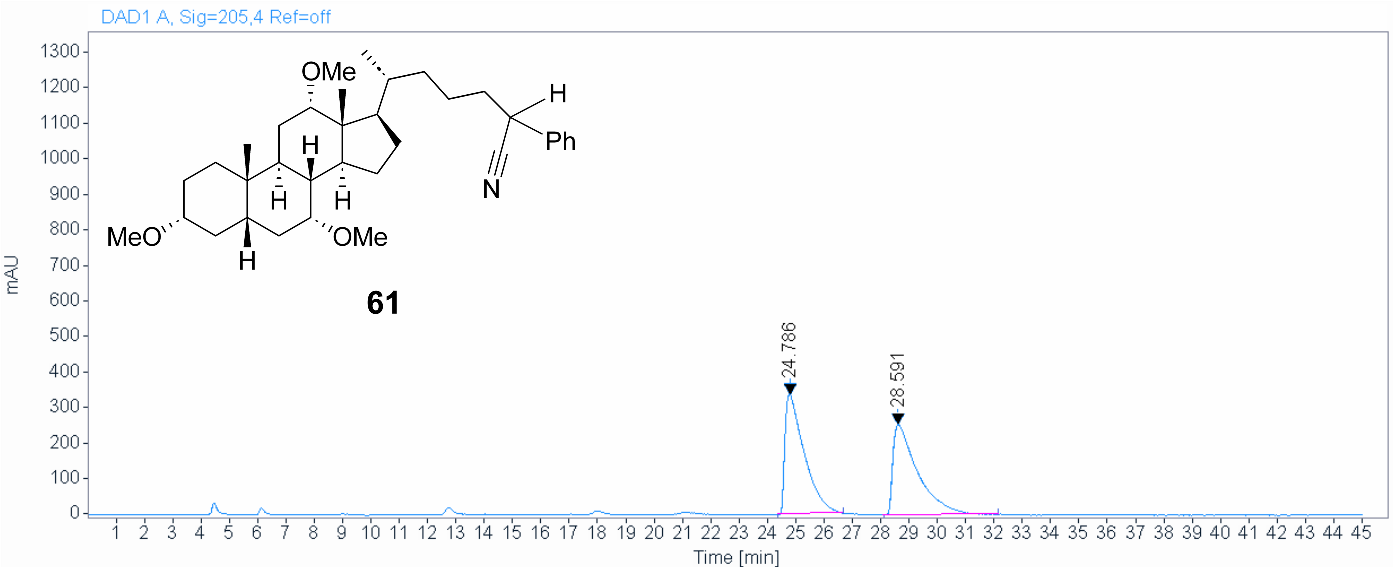

Signal: DAD1 A, Sig=205,4 Ref=off

RT [min]	Width [min]	Height	Area	Area%
24.786	0.7926	336.2056	15989.0996	50.7110
28.591	1.0170	254.6939	15540.7480	49.2890
		Sum	31529.8477	100.0000

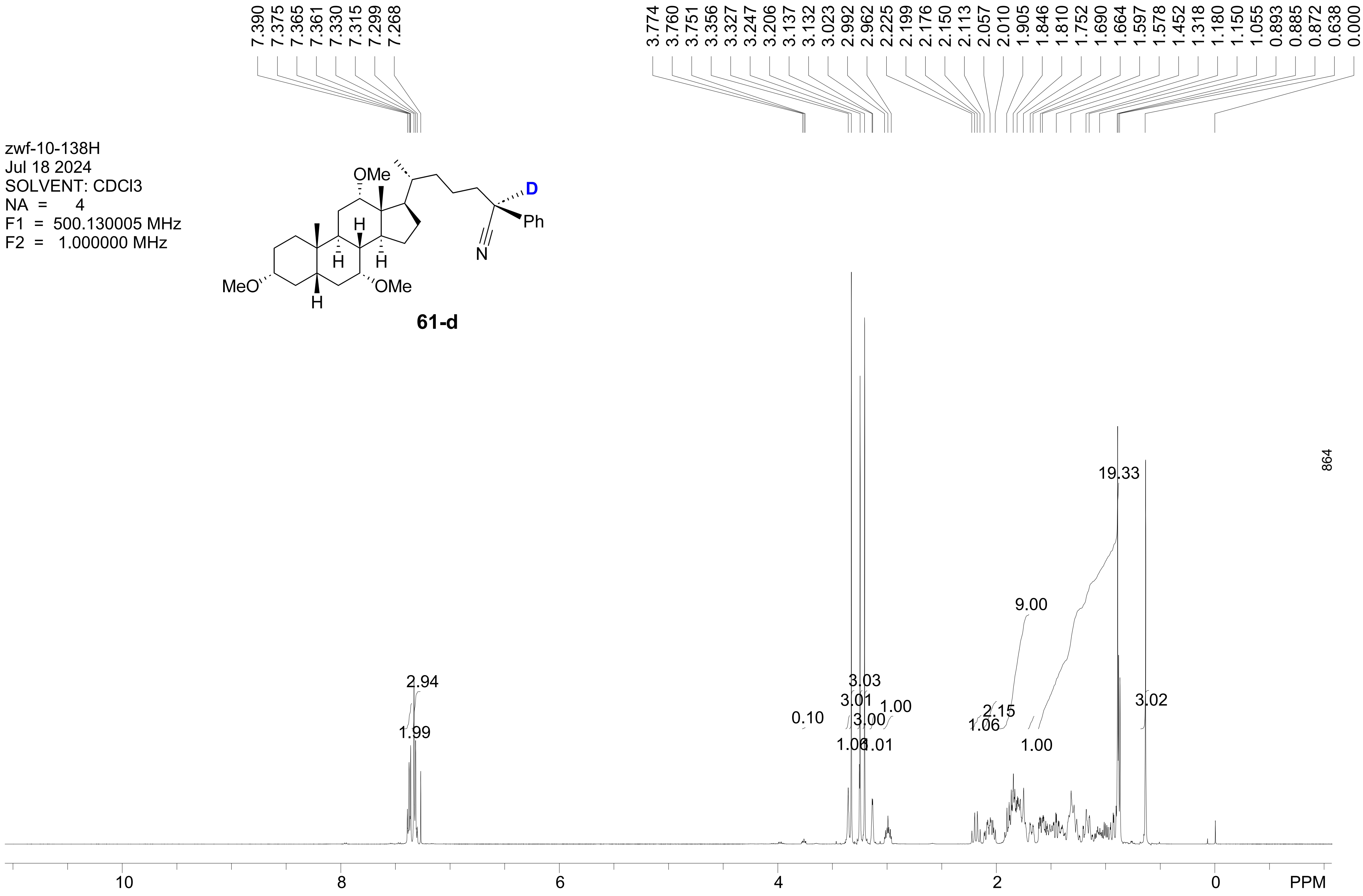
zwf-10-138H
Jul 18 2024
SOLVENT: CDCl3
NA = 4
F1 = 500.130005 MHz
F2 = 1.000000 MHz
OMe
MeO
OMe
H
H
H
H
D
Ph
N
7.390
7.375
7.365
7.361
7.330
7.315
7.299
7.268
3.774
3.760
3.751
3.356
3.327
3.247
3.206
3.137
3.132
3.023
2.992
2.962
2.225
2.199
2.176
2.150
2.113
2.057
2.010
1.905
1.846
1.810
1.752
1.690
1.664
1.597
1.578
1.452
1.318
1.180
1.150
1.055
0.893
0.885
0.872
0.638
0.000
2.94
1.99
0.10
3.03
3.01
3.00
1.00
1.01
2.15
1.06
9.00
1.00
19.33
3.02
10
8
6
4
2
0
PPM

61-d

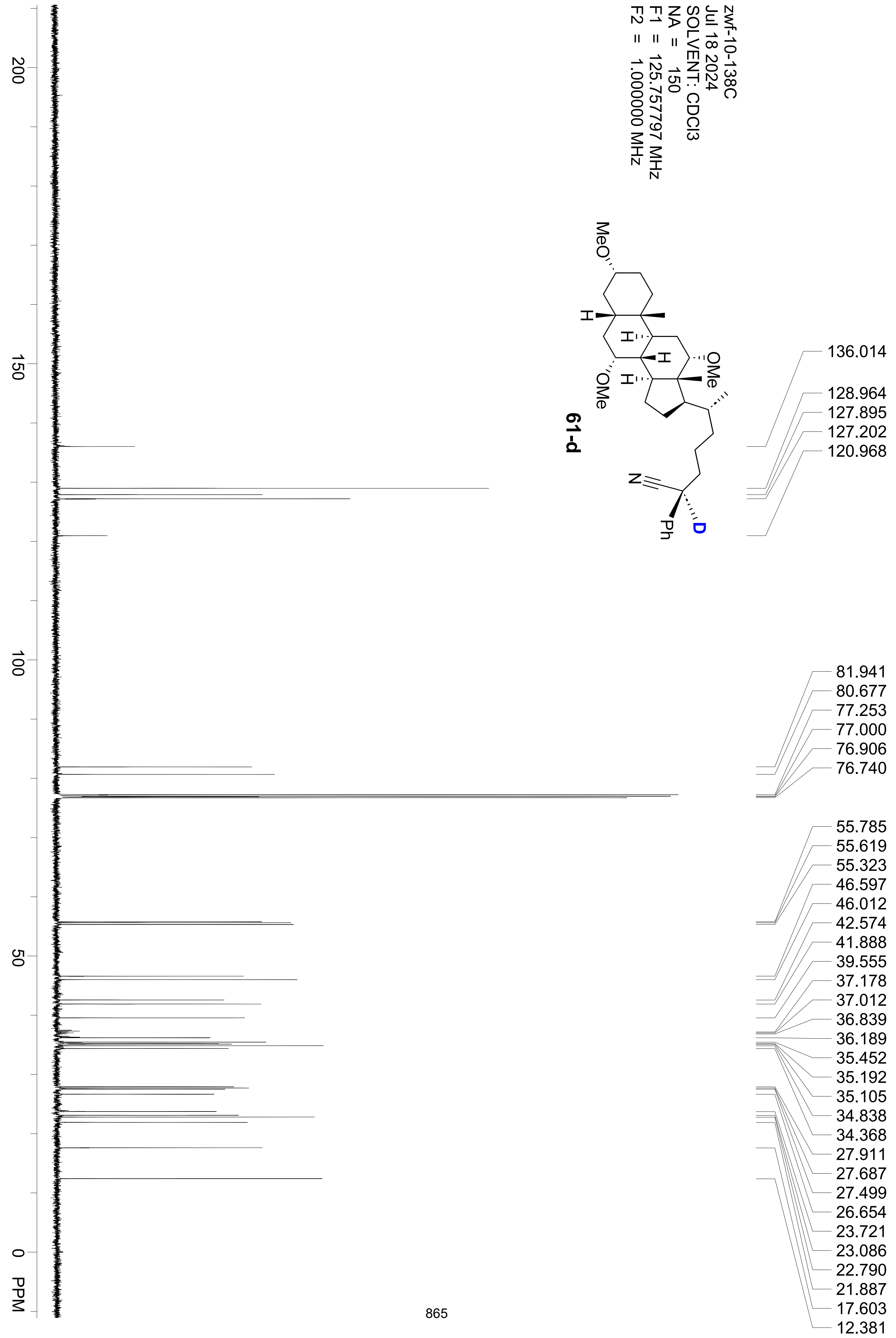
zwf-10-138C
Jul 18 2024
SOLVENT: CDCl3
NA = 150
F1 = 125.757797 MHz
F2 = 1.000000 MHz
MeO
OMe
OMe
H
H
H
H
N
D
Ph
61-d
136.014
128.964
127.895
127.202
120.968
81.941
80.677
77.253
77.000
76.906
76.740
55.785
55.619
55.323
46.597
46.012
42.574
41.888
39.555
37.178
37.012
36.839
36.189
35.452
35.192
35.105
34.838
34.368
27.911
27.687
27.499
26.654
23.721
23.086
22.790
21.887
17.603
12.381
200
150
100
50
0
PPM

Area Percent Report

Agilent Technologies

Sample name: zwf-10-138-IE-90-10-0.8-205

Data file: C:\Users\Public\Documents\ChemStation\1\Data\zwf\zwf 2024-07-18 14-42-04\008-P1-C5-zwf-10-138.D

Injection Acquired Date 07/18/2024 22:24:45

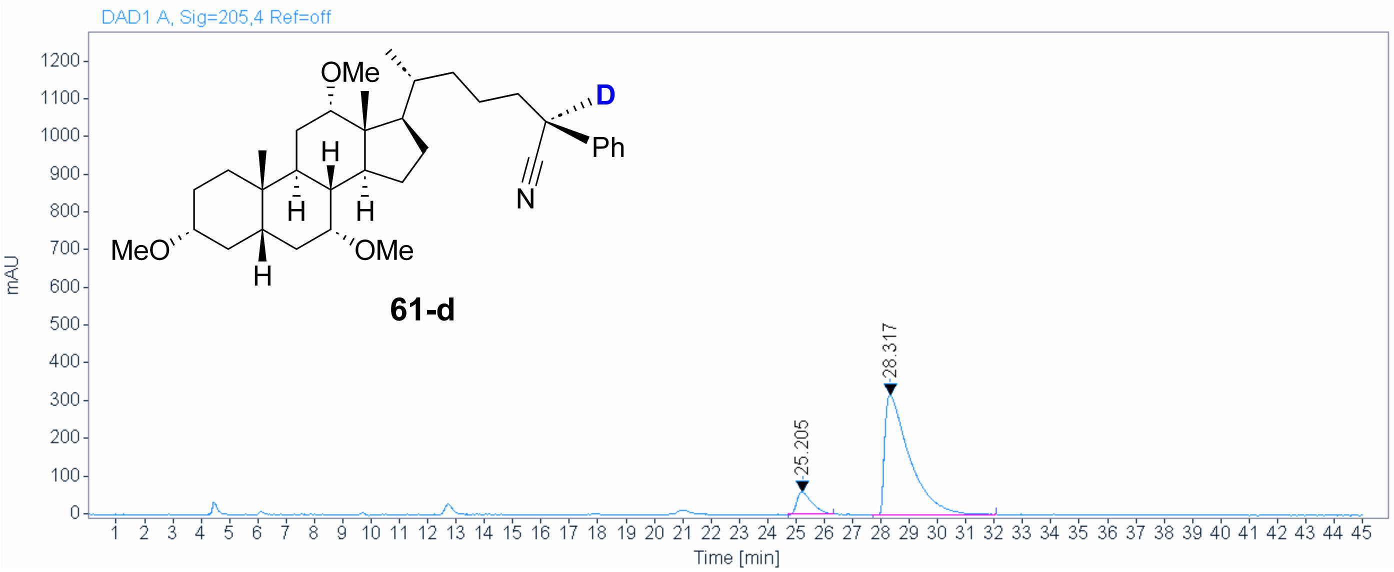

Signal: DAD1 A, Sig=205,4 Ref=off

RT [min]	Width [min]	Height	Area	Area%
25.205	0.6172	58.8633	2179.9783	9.9234
28.317	1.0335	319.1266	19788.1270	90.0766
		Sum	21968.1052	100.0000

Area Percent Report

Agilent Technologies

Sample name: zwf-10-138-rac-IE-90-10-0.8-205

Data file: C:\Users\Public\Documents\ChemStation\1\Data\zwf\zwf 2024-07-18 14-42-04\009-P1-C7-zwf-10-138-rac.D

Injection Acquired Date 07/18/2024 23:10:53

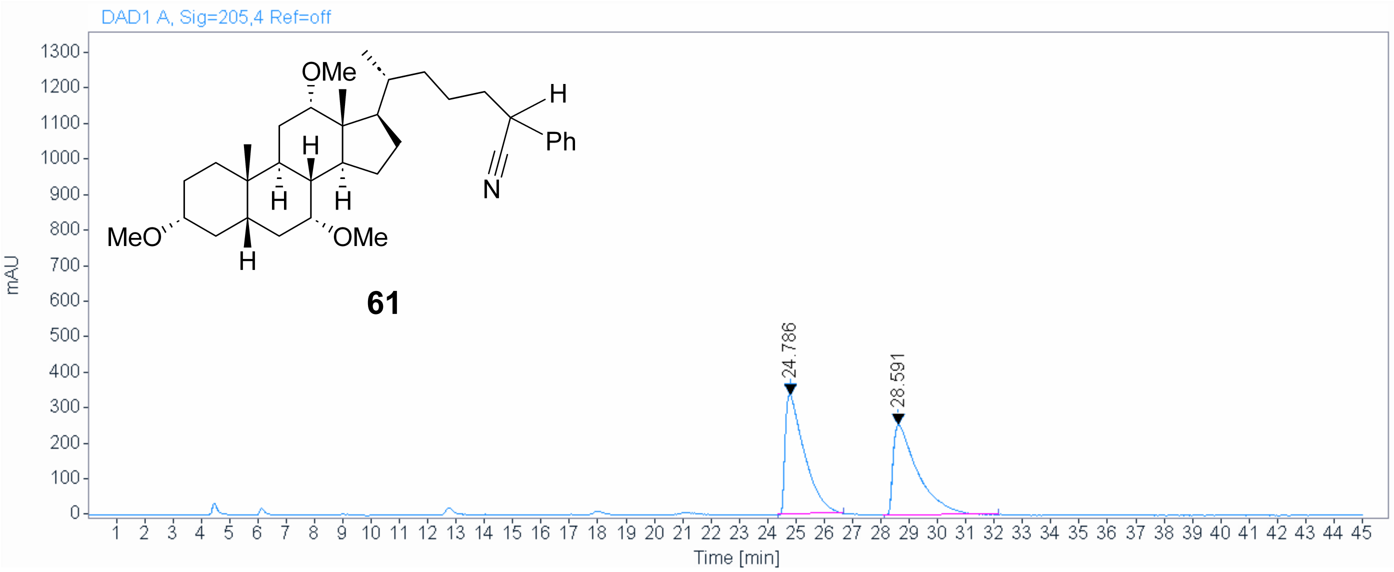

Signal: DAD1 A, Sig=205,4 Ref=off

RT [min]	Width [min]	Height	Area	Area%
24.786	0.7926	336.2056	15989.0996	50.7110
28.591	1.0170	254.6939	15540.7480	49.2890
		Sum	31529.8477	100.0000

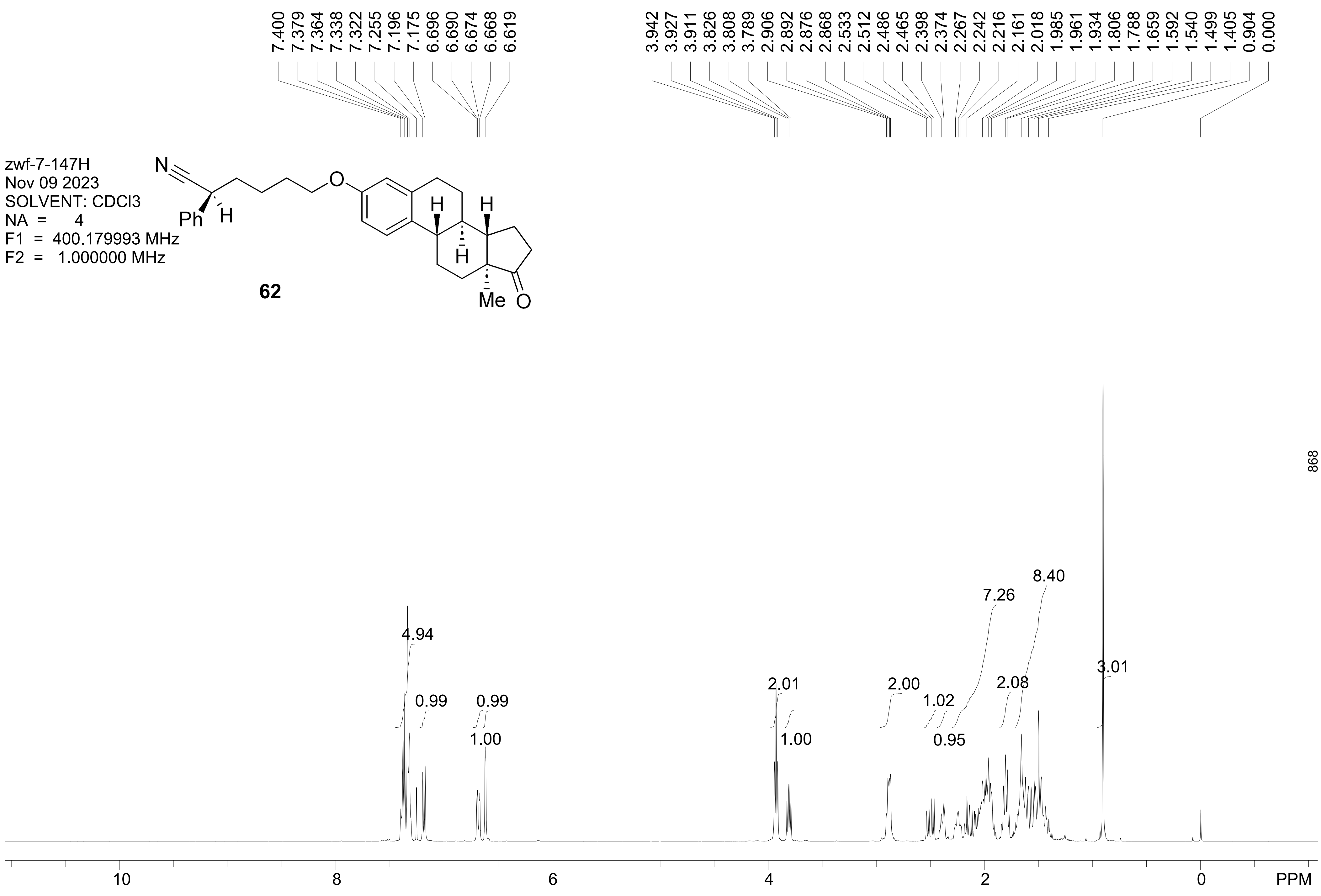
zwf-7-147H
Nov 09 2023
SOLVENT: CDCl3
NA = 4
F1 = 400.179993 MHz
F2 = 1.000000 MHz
62
Ph
H
Me
7.400 7.379 7.364 7.338 7.322 7.255 7.196 7.175 6.696 6.690 6.674 6.668 6.619
3.942 3.927 3.911 3.826 3.808 3.789 2.906 2.892 2.876 2.868 2.533 2.512 2.486 2.465 2.398 2.374 2.267 2.242 2.216 2.161 2.018 1.985 1.961 1.934 1.806 1.788 1.659 1.592 1.540 1.499 1.405 0.904 0.000
4.94 0.99 0.99 1.00 2.01 1.00 2.00 1.02 0.95 7.26 2.08 8.40 3.01
10 8 6 4 2 0 PPM

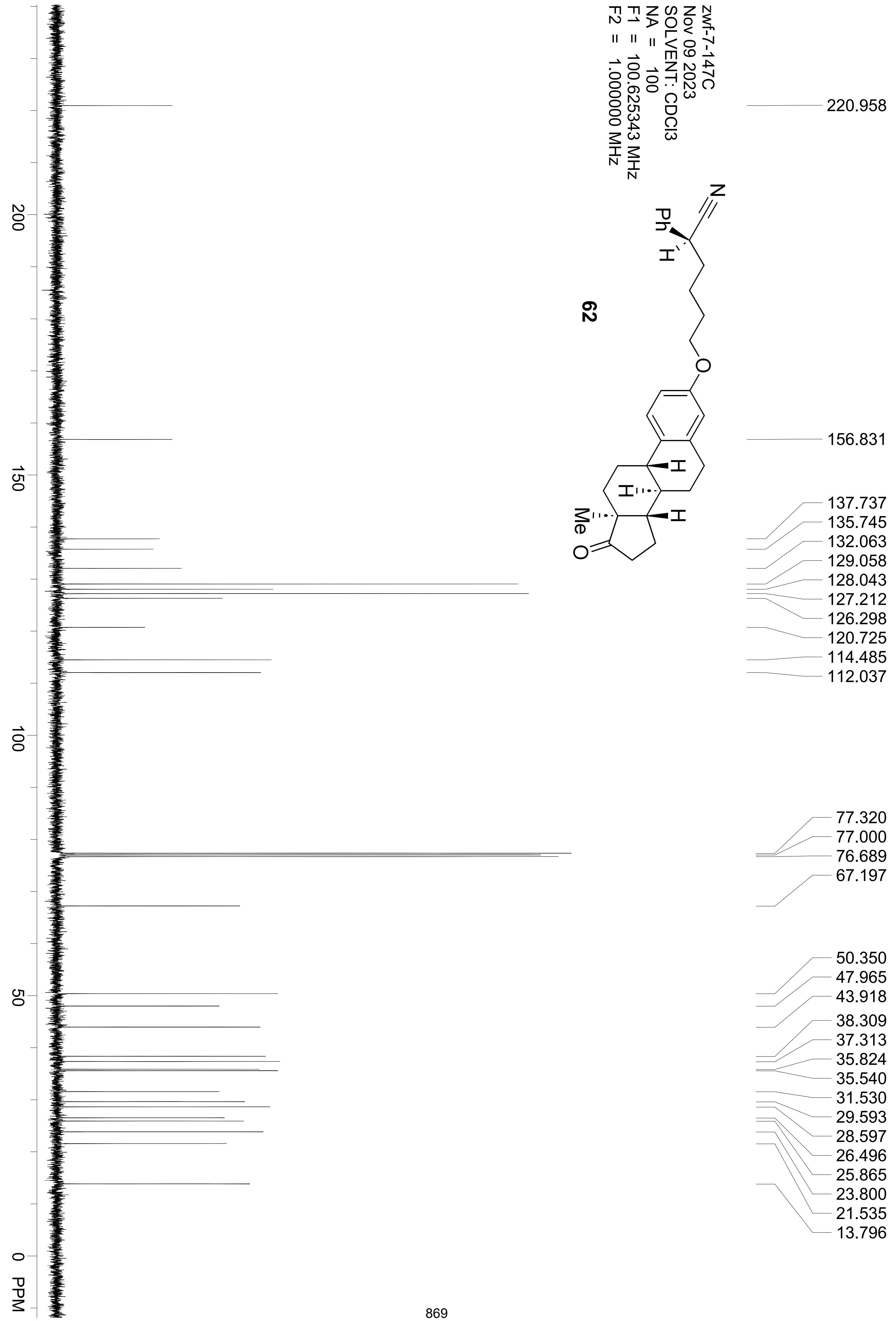

zwf-7-147C
Nov 09 2023
SOLVENT: CDCl3
NA = 100
F1 = 100.625343 MHz
F2 = 1.000000 MHz
62
N
Ph
H
O
H
H
H
Me
O
220.958
156.831
137.737
135.745
132.063
129.058
128.043
127.212
126.298
120.725
114.485
112.037
77.320
77.000
76.689
67.197
50.350
47.965
43.918
38.309
37.313
35.824
35.540
31.530
29.593
28.597
26.496
25.865
23.800
21.535
13.796
200
150
100
50
0
PPM

Area Percent Report

Agilent Technologies

Sample name: zwf-7-147-IE-80-20-0.8-205

Data file: C:\Users\Public\Documents\ChemStation\1\Data\zwf\zwf-S 2023-11-09 14-19-21\005-P1-C2-zwf-7-147 80-20-0.8.D

Injection Acquired Date 11/09/2023 18:40:02

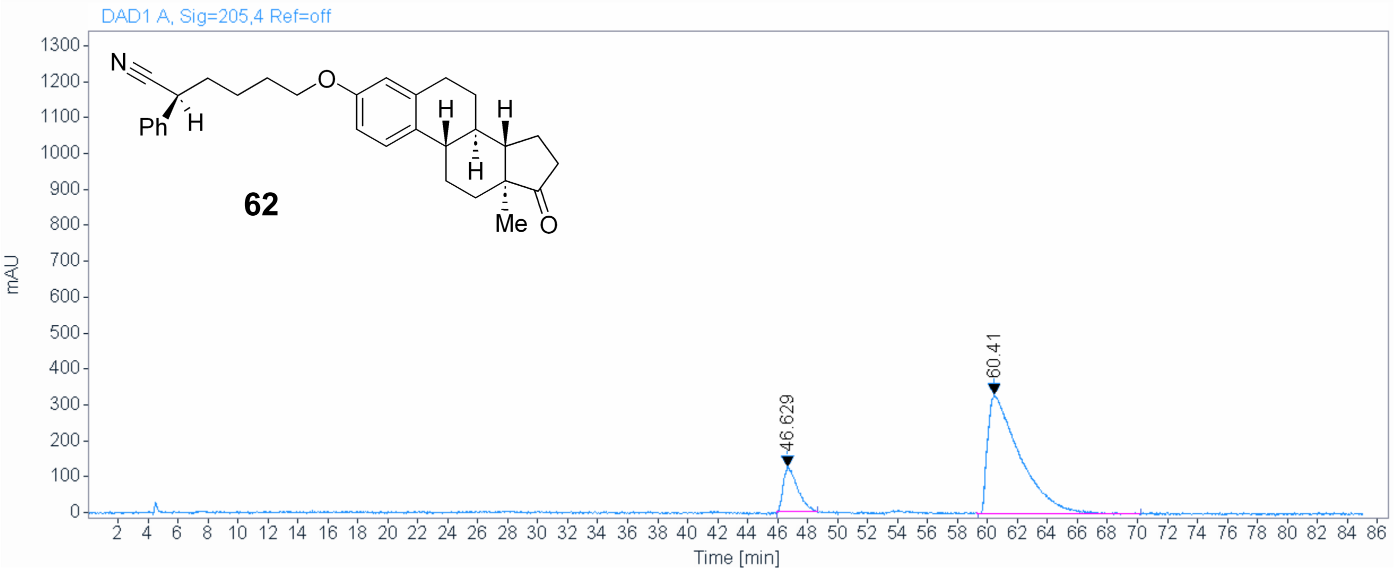

Signal: DAD1 A, Sig=205,4 Ref=off

RT [min]	Width [min]	Height	Area	Area%
46.629	1.1075	126.5271	8407.6836	14.2196
60.410	2.5315	333.9254	50719.7148	85.7804
		Sum	59127.3984	100.0000

Area Percent Report

Sample name: zwf-7-147-rac-IE-80-20-0.8-205

Data file: C:\Users\Public\Documents\ChemStation\1\Data\zwf\zwf-S 2023-11-09 14-19-21\004-P1-C1-zwf-7-147-rac 80-20-0.8.D

Injection Acquired Date 11/09/2023 17:13:48

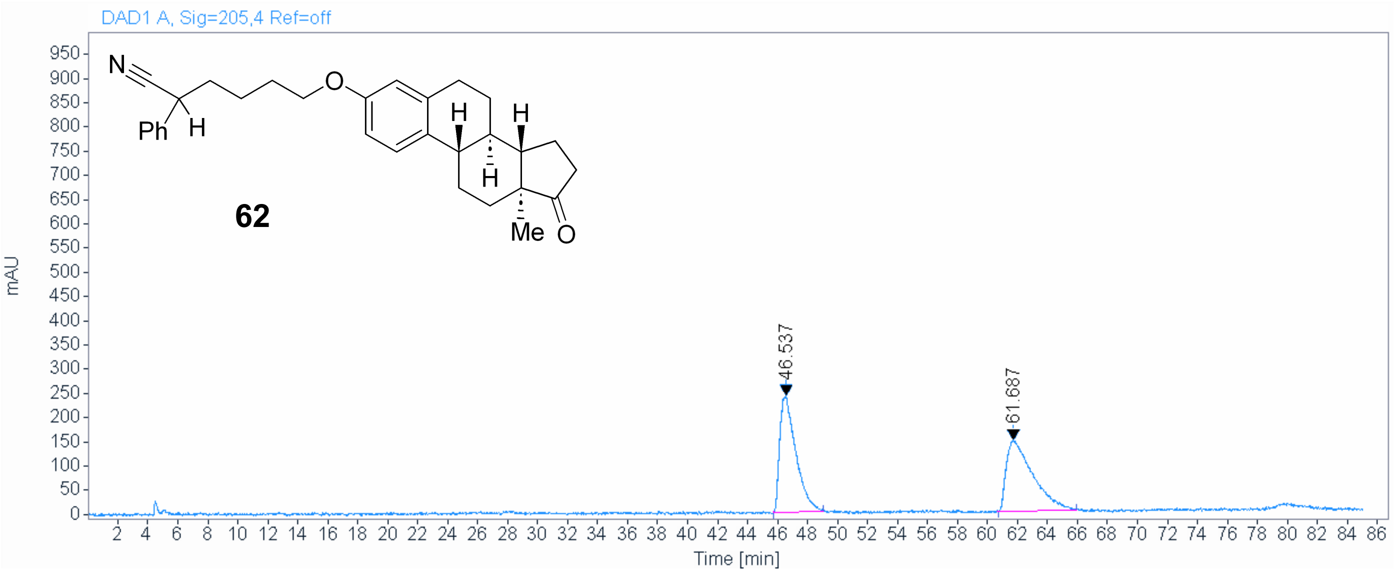

Signal: DAD1 A, Sig=205,4 Ref=off

RT [min]	Width [min]	Height	Area	Area%
46.537	1.2558	242.2372	18251.4629	49.9964
61.687	2.0631	147.4665	18254.0801	50.0036
		Sum	36505.5430	100.0000

zwf-8-008H
Nov 11 2023
SOLVENT: CDCl3
NA = 4
F1 = 400.140015 MHz
F2 = 1.000000 MHz

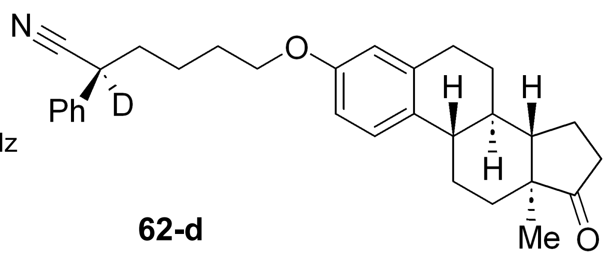

62-d

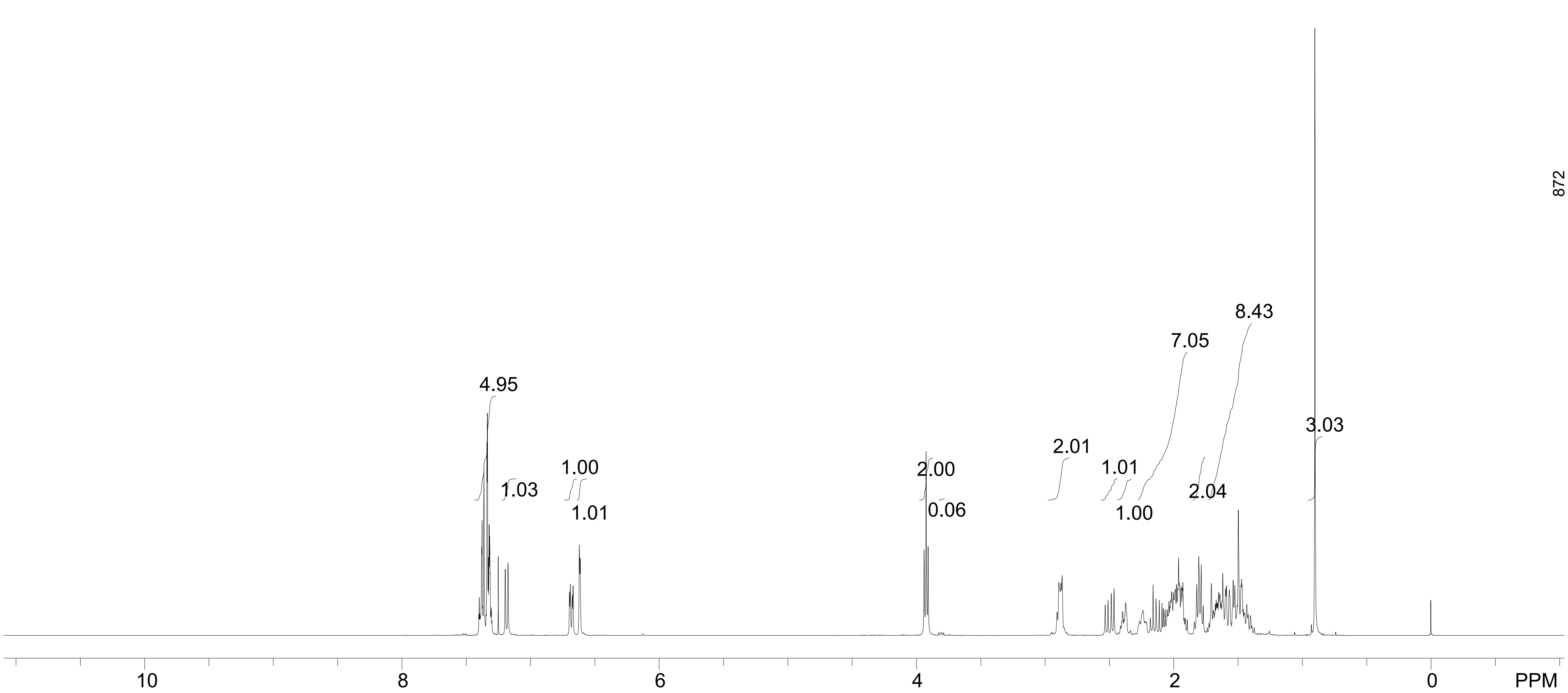

zwf-8-008C
Nov 11 2023
SOLVENT: CDCl3
NA = 150
F1 = 100.615280 MHz
F2 = 1.000000 MHz

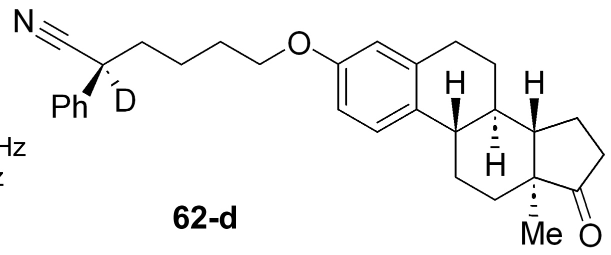

62-d

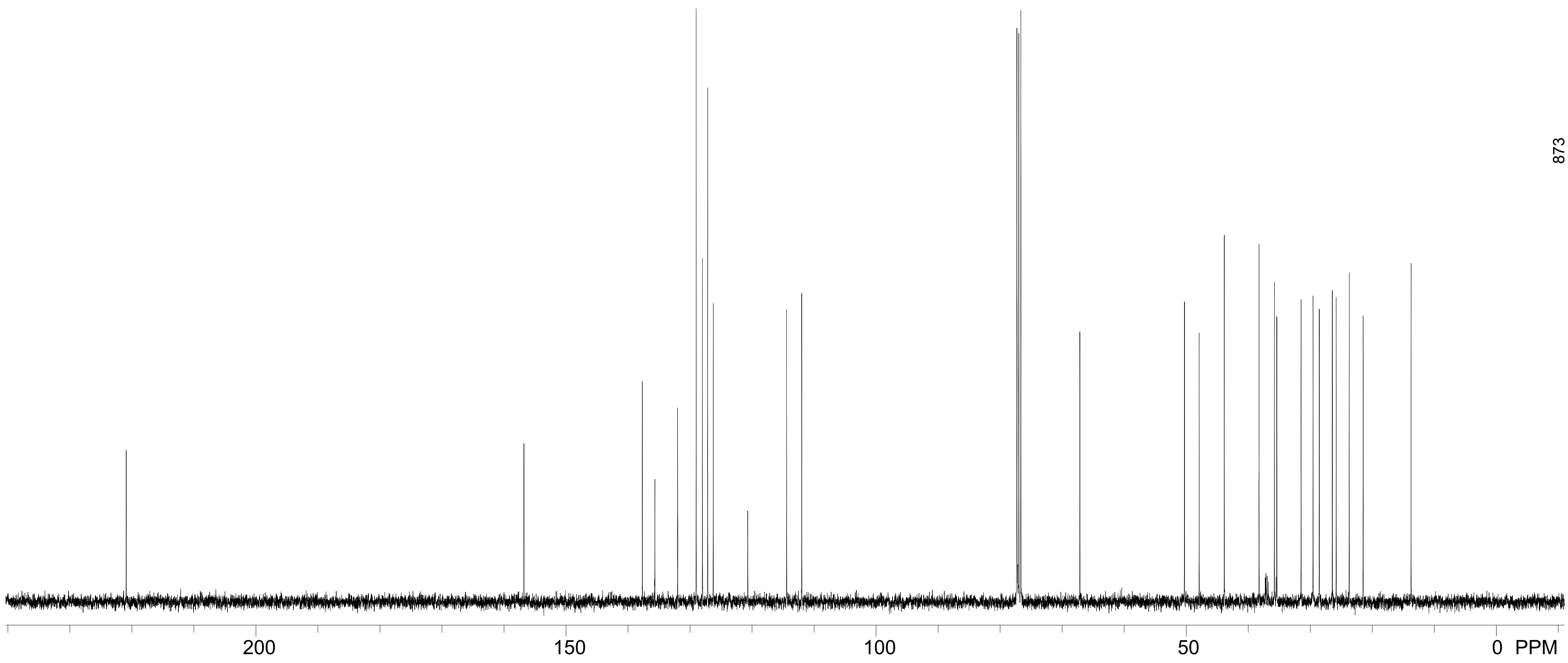

Area Percent Report

Agilent Technologies

Sample name: zwf-8-008-IE-80-20-0.8-205

Data file: C:\Users\Public\Documents\ChemStation\1\Data\zwf\zwf-S 2023-11-12 11-02-17\009-P1-B1-zwf-8-008 80-20-0.8.D

Injection Acquired Date 11/12/2023 15:35:52

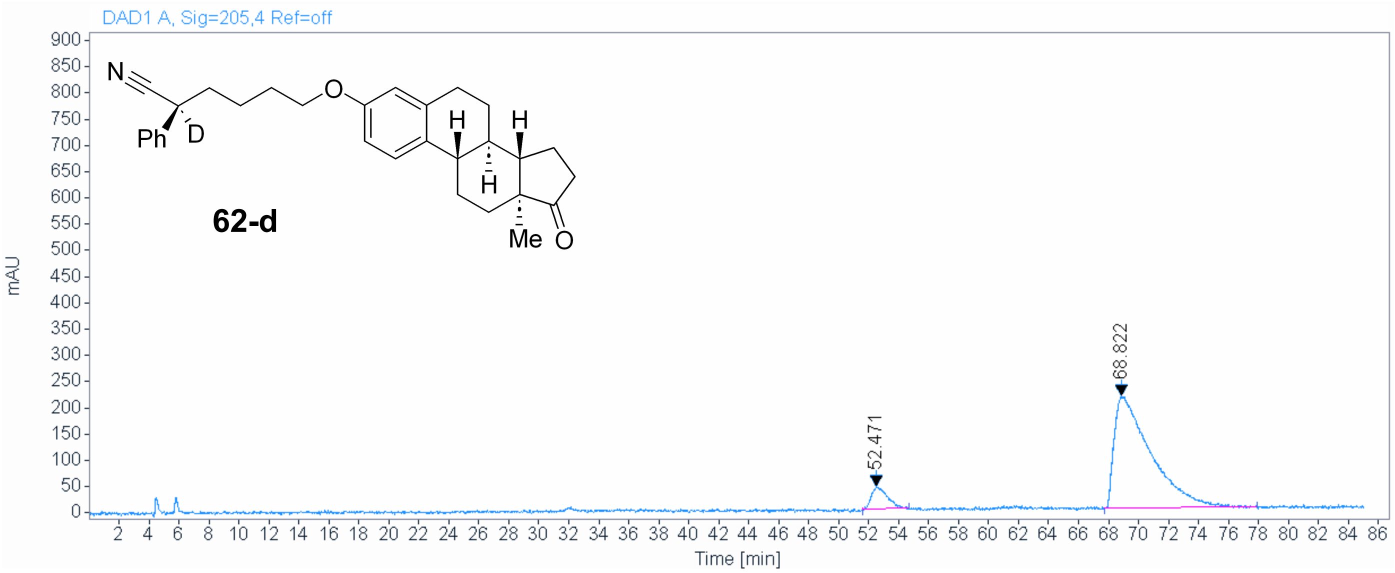

Signal: DAD1 A, Sig=205,4 Ref=off

RT [min]	Width [min]	Height	Area	Area%
52.471	1.2396	43.8066	3258.2441	8.2017
68.822	2.8327	214.5691	36468.3828	91.7983
		Sum	39726.6270	100.0000

Area Percent Report

Sample name: zwf-8-008-rac-IE-80-20-0.8-205

Data file: C:\Users\Public\Documents\ChemStation\1\Data\zwf\zwf-S 2023-11-12 11-02-17\011-P1-B2-zwf-8-008-rac 80-20-0.8.D

Injection Acquired Date 11/12/2023 18:28:19

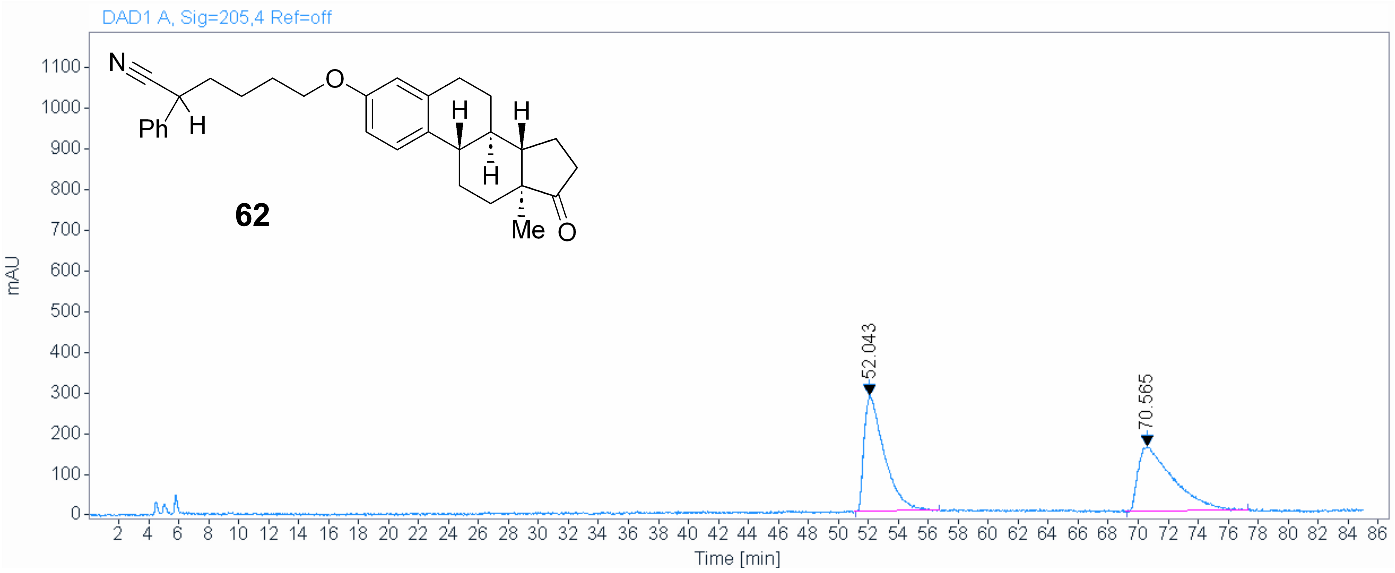

Signal: DAD1 A, Sig=205,4 Ref=off

RT [min]	Width [min]	Height	Area	Area%
52.043	1.5767	285.5154	27010.7578	50.6920
70.565	2.7233	160.7919	26273.3184	49.3080
		Sum	53284.0762	100.0000

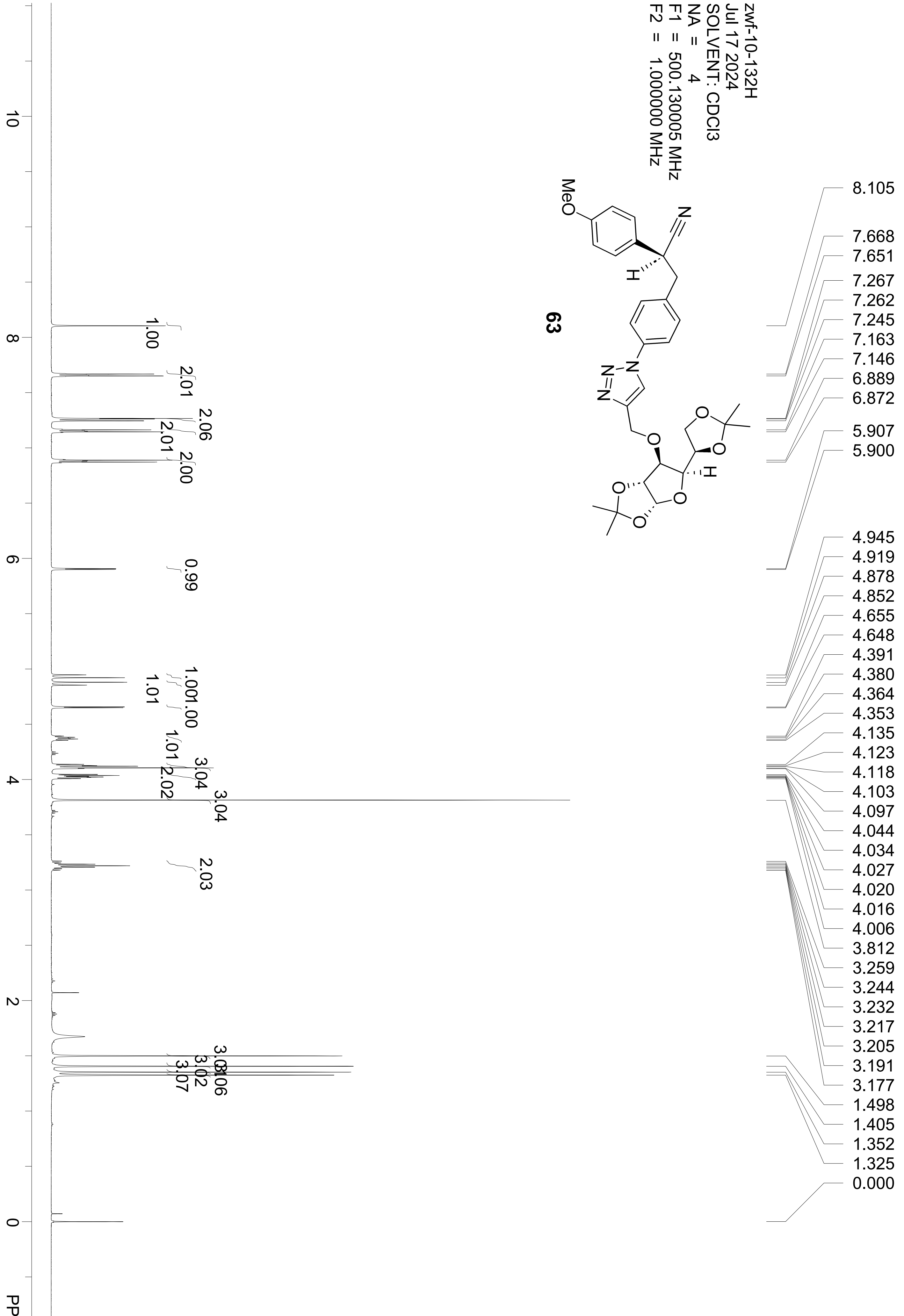
zwf-10-132H
Jul 17 2024
SOLVENT: CDCl3
NA = 4
F1 = 500.130005 MHz
F2 = 1.000000 MHz
63
MeO
N
H
8.105
7.668
7.651
7.267
7.262
7.245
7.163
7.146
6.889
6.872
5.907
5.900
4.945
4.919
4.878
4.852
4.655
4.648
4.391
4.380
4.364
4.353
4.135
4.123
4.118
4.103
4.097
4.044
4.034
4.027
4.020
4.016
4.006
3.812
3.259
3.244
3.232
3.217
3.205
3.191
3.177
1.498
1.405
1.352
1.325
0.000
PPM

zwf-10-132C
Jul 17 2024
SOLVENT: CDCl3
NA = 600
F1 = 125.757790 MHz
F2 = 1.000000 MHz

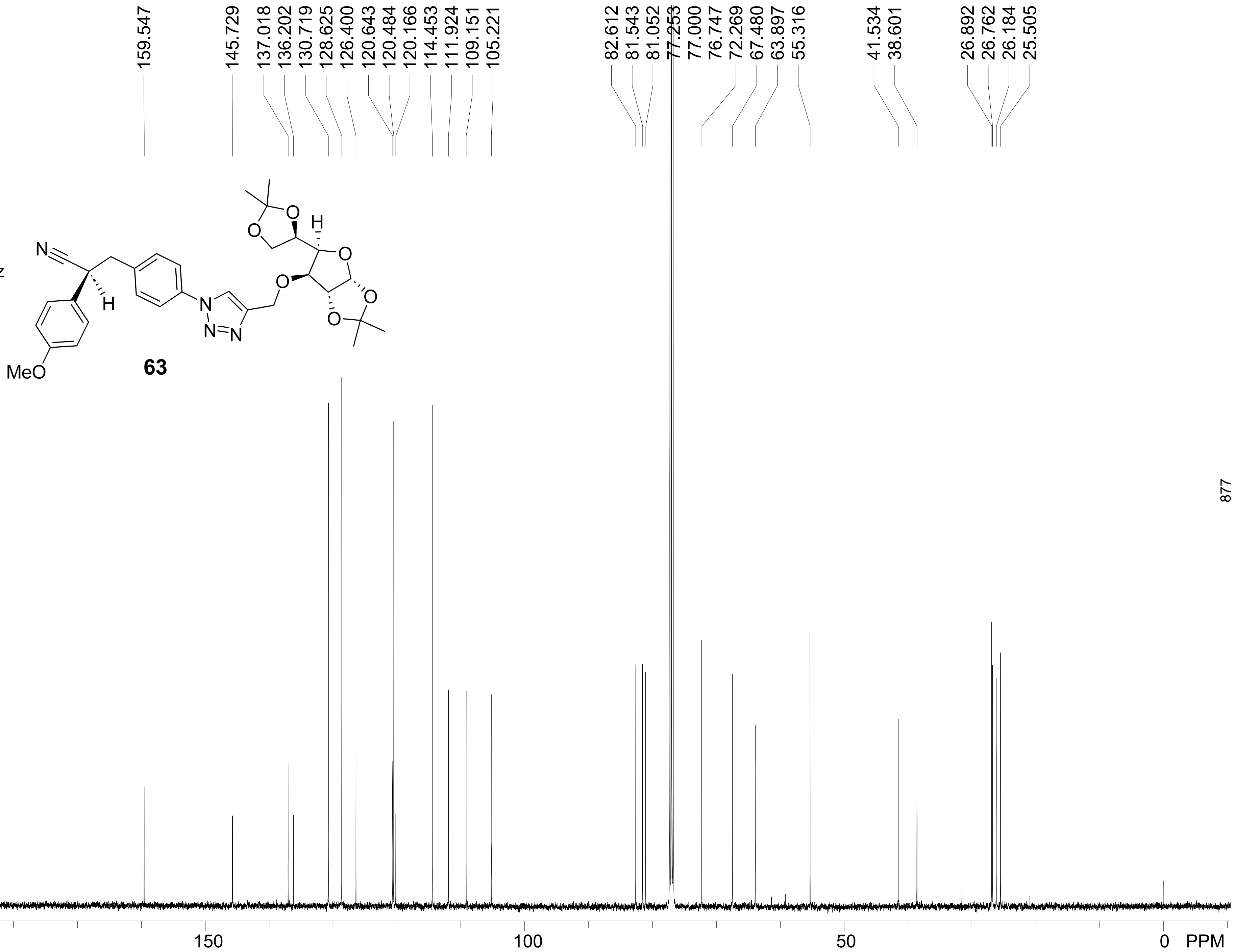

63

Area Percent Report

Agilent Technologies

Sample name: zwf-10-132-IE-70-30-0.8-205

Data file: C:\Users\Public\Documents\ChemStation\1\Data\zwf\zwf 2024-07-17 16-57-58\008-P1-C6-zwf-10-132.D

Injection Acquired Date 07/17/2024 21:02:22

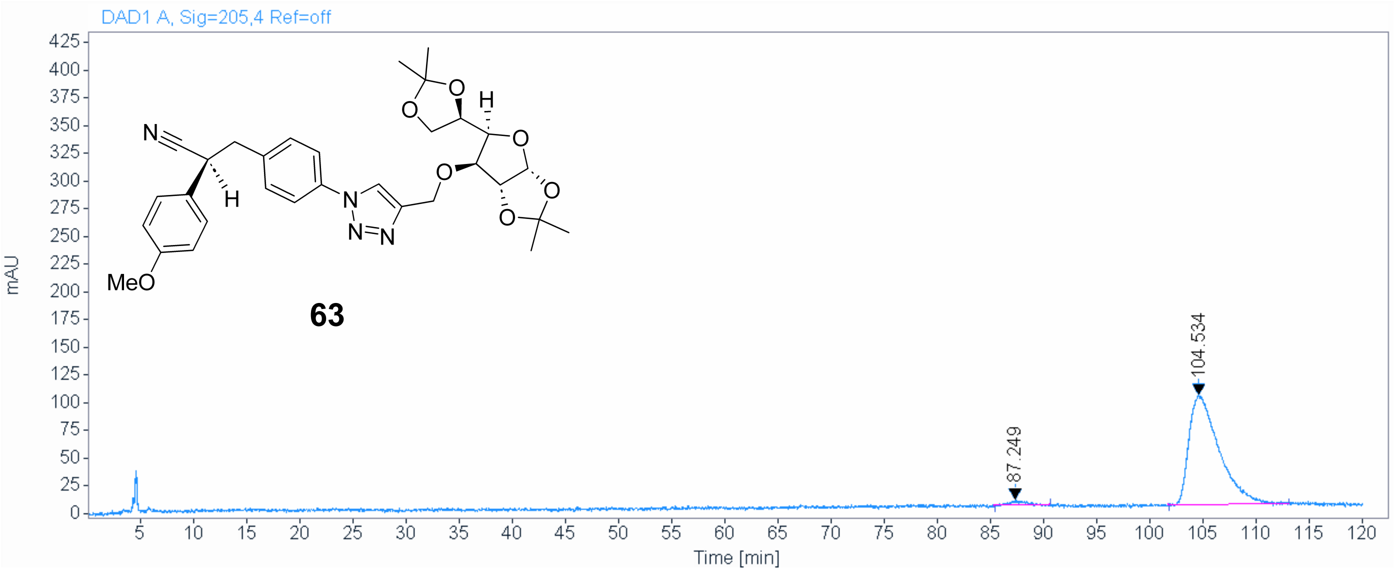

Signal: DAD1 A, Sig=205,4 Ref=off

RT [min]	Width [min]	Height	Area	Area%
87.249	1.6994	5.5956	570.5417	2.8096
104.534	3.3059	99.5032	19736.6250	97.1904
		Sum	20307.1667	100.0000

Area Percent Report

Agilent Technologies

Sample name: zwf-10-134-rac-IE-70-30-0.8-205

Data file: C:\Users\Public\Documents\ChemStation\1\Data\zwf\zwf 2024-07-17 16-57-58\010-P1-C8-zwf-10-134-rac.D

Injection Acquired Date 07/18/2024 01:04:49

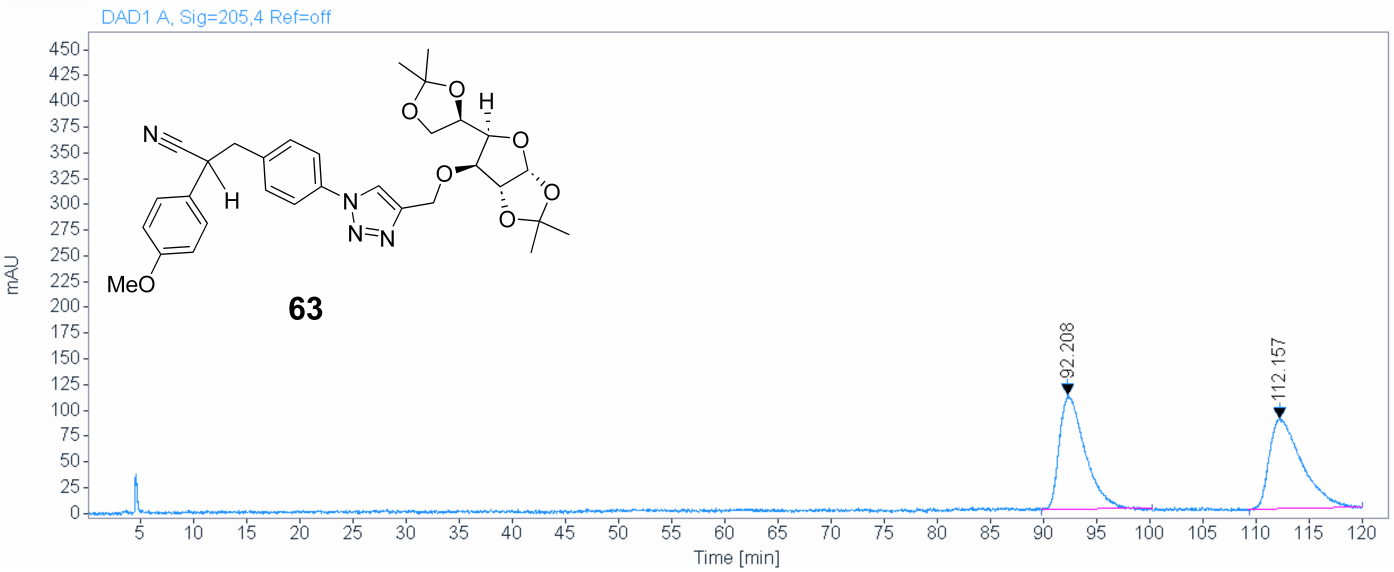

Signal: DAD1 A, Sig=205,4 Ref=off

RT [min]	Width [min]	Height	Area	Area%
92.208	2.8230	111.3638	18862.6719	49.8370
112.157	3.5765	88.4752	18986.0273	50.1630
		Sum	37848.6992	100.0000

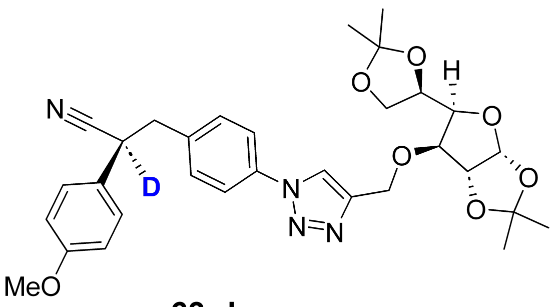

63-d

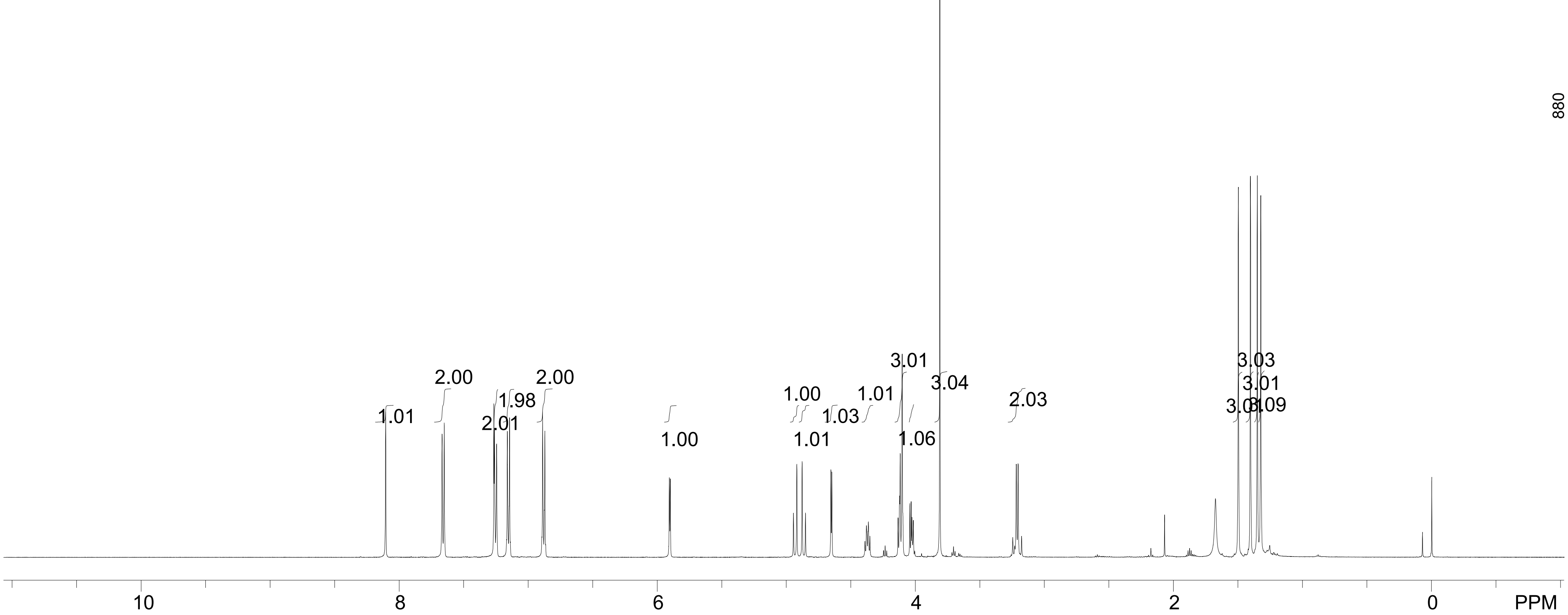
zwf-10-134H
Jul 17 2024
SOLVENT: CDCl3
NA = 4
F1 = 500.130005 MHz
F2 = 1.000000 MHz
8.105
7.668
7.651
7.267
7.262
7.245
7.162
7.145
6.889
6.872
5.907
5.900
4.945
4.919
4.878
4.852
4.655
4.648
4.391
4.380
4.365
4.353
4.135
4.123
4.118
4.103
4.044
4.033
4.027
4.016
3.811
3.246
3.219
3.205
3.178
1.498
1.405
1.352
1.325
0.000
1.01
2.00
2.01
1.98
2.00
1.00
1.00
1.01
1.03
1.01
3.01
1.06
3.04
2.03
3.03
3.01
3.09
10
8
6
4
2
0
PPM

zwf-10-134C
Jul 17
SOLVENT: CDCl3
NA = 800
F1 = 125.757790 MHz
F2 = 1.000000 MHz

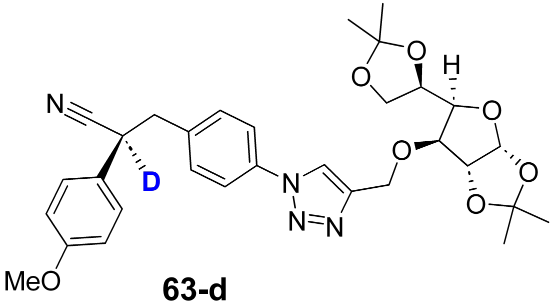

63-d

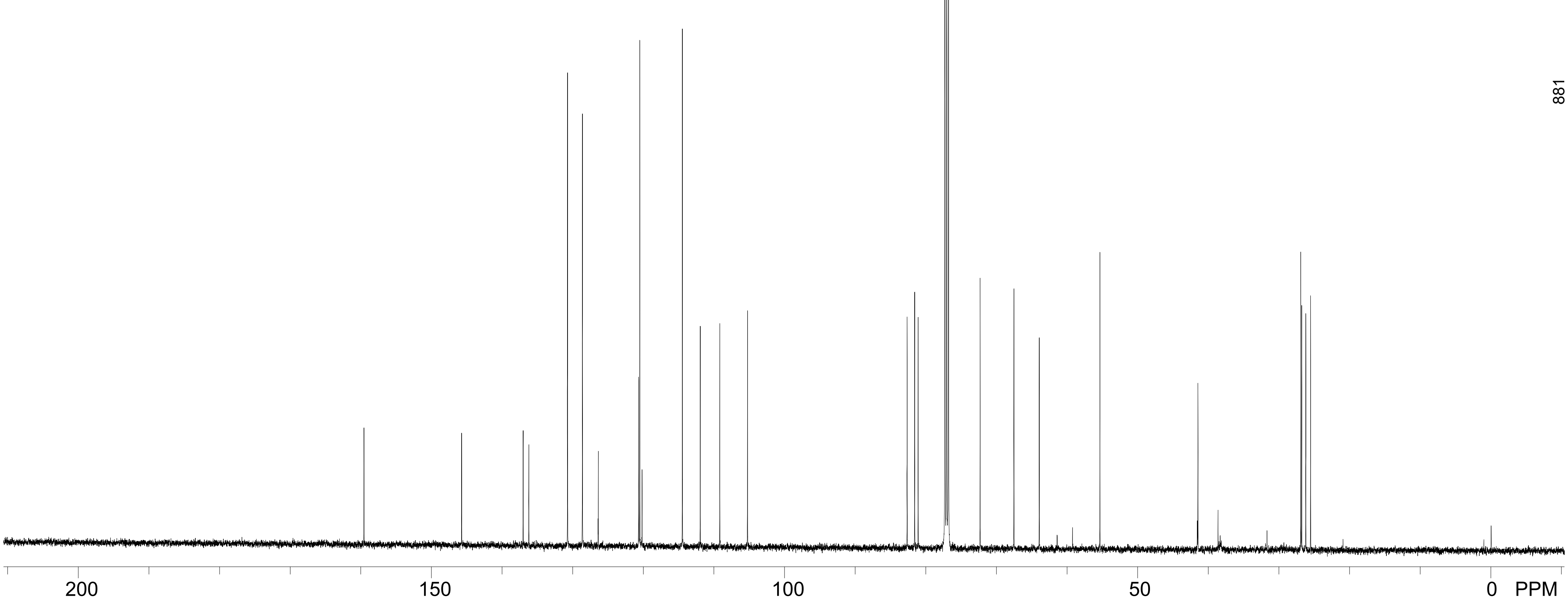

Area Percent Report

Agilent Technologies

Sample name: zwf-10-134-IE-70-30-0.8-205

Data file: C:\Users\Public\Documents\ChemStation\1\Data\zwf\zwf 2024-07-17 16-57-58\009-P1-C7-zwf-10-134.D

Injection Acquired Date 07/17/2024 23:03:37

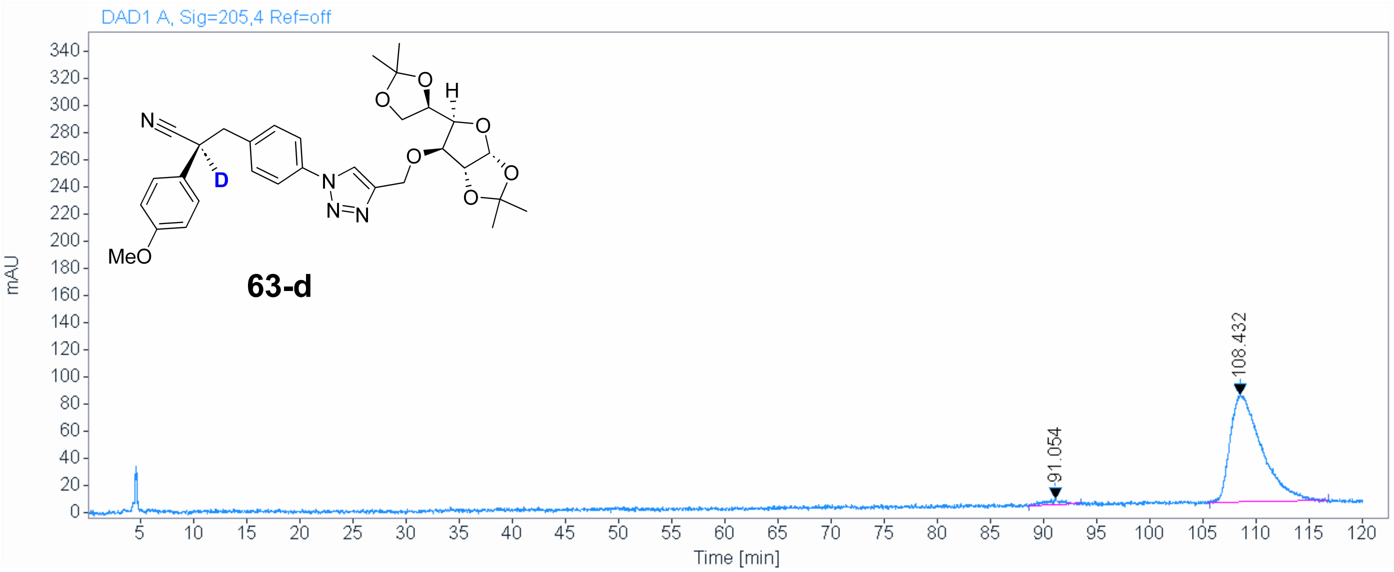

Signal: DAD1 A, Sig=205,4 Ref=off

RT [min]	Width [min]	Height	Area	Area%
91.054	1.7481	4.8160	505.1218	2.9663
108.432	3.4828	79.0732	16523.7441	97.0337
		Sum	17028.8659	100.0000

Area Percent Report

Agilent Technologies

Sample name: zwf-10-134-rac-IE-70-30-0.8-205

Data file: C:\Users\Public\Documents\ChemStation\1\Data\zwf\zwf 2024-07-17 16-57-58\010-P1-C8-zwf-10-134-rac.D

Injection Acquired Date 07/18/2024 01:04:49

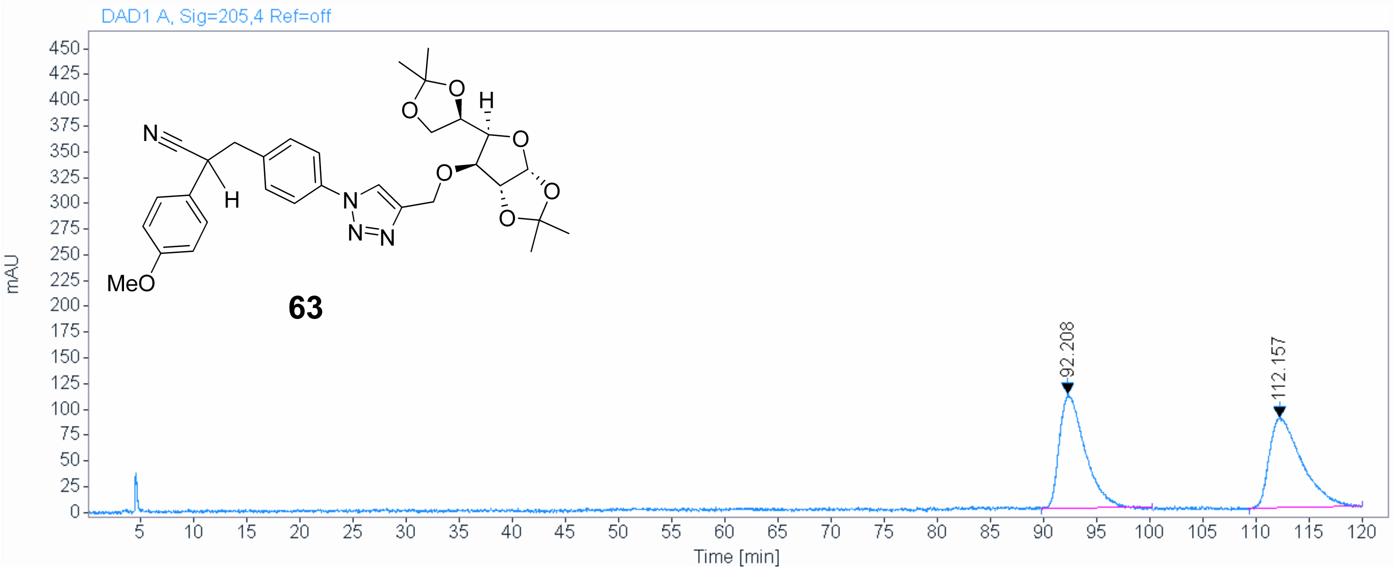

Signal: DAD1 A, Sig=205,4 Ref=off

RT [min]	Width [min]	Height	Area	Area%
92.208	2.8230	111.3638	18862.6719	49.8370
112.157	3.5765	88.4752	18986.0273	50.1630
		Sum	37848.6992	100.0000

zwf-10-137H
Jul 17 2024
SOLVENT: CDCl3
NA = 4
F1 = 500.130005 MHz
F2 = 1.000000 MHz

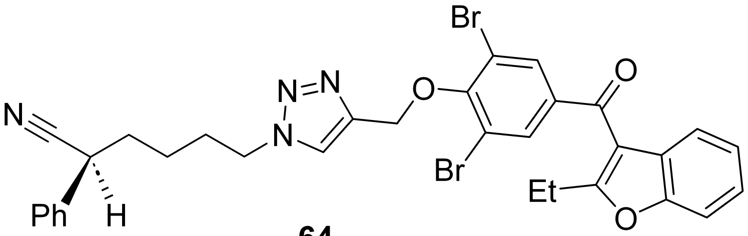

64

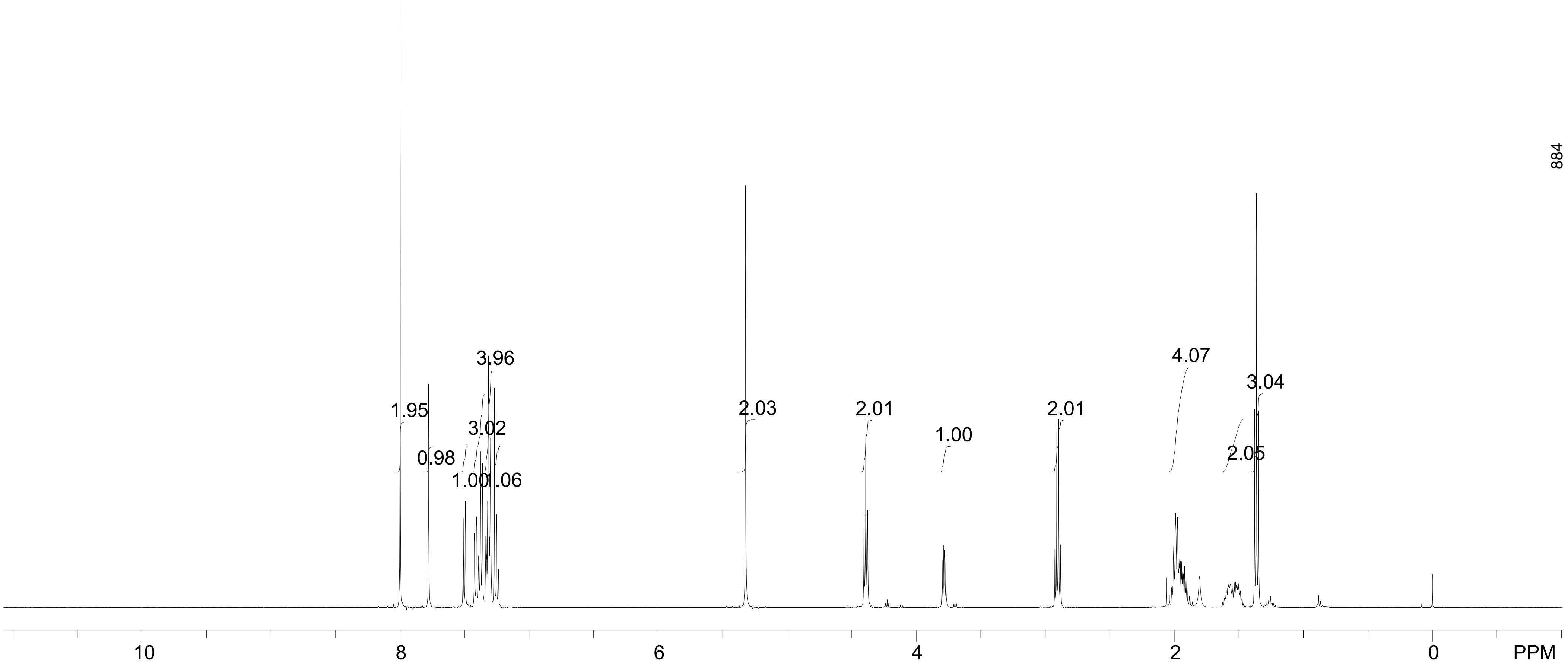

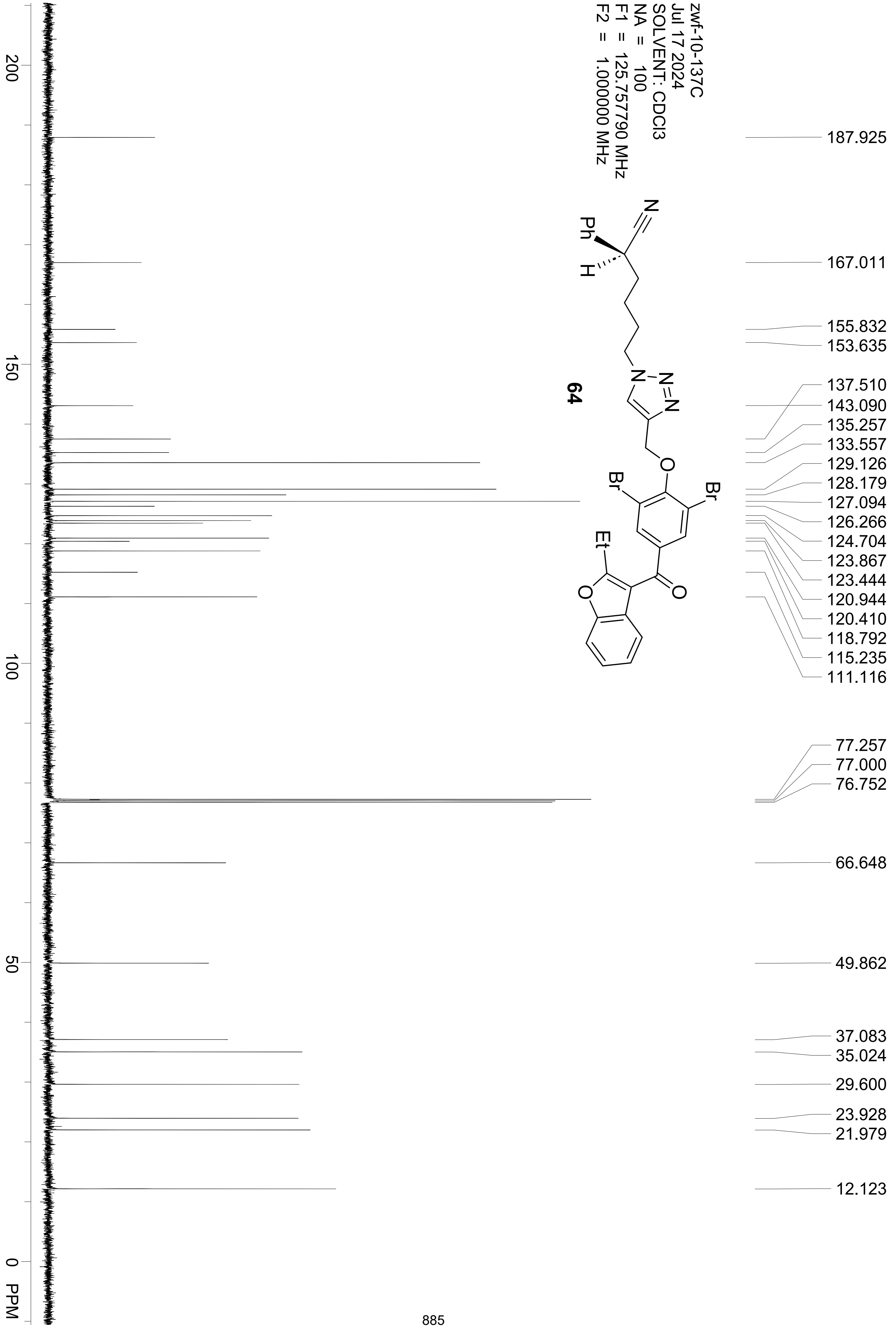
zwf-10-137C
Jul 17 2024
SOLVENT: CDCl3
NA = 100
F1 = 125.757790 MHz
F2 = 1.000000 MHz
64
Ph
H
N
Br
Br
Et
O
187.925
167.011
155.832
153.635
137.510
143.090
135.257
133.557
129.126
128.179
127.094
126.266
124.704
123.867
123.444
120.944
120.410
118.792
115.235
111.116
77.257
77.000
76.752
66.648
49.862
37.083
35.024
29.600
23.928
21.979
12.123
200
150
100
50
0
PPM

Area Percent Report

Sample name: zwf-10-137-IE-70-30-0.8-205

Data file: C:\Users\Public\Documents\ChemStation\1\Data\zwf\zwf 2024-07-18 14-42-04\002-P1-C1-zwf-10-137.D

Injection Acquired Date 07/18/2024 15:17:53

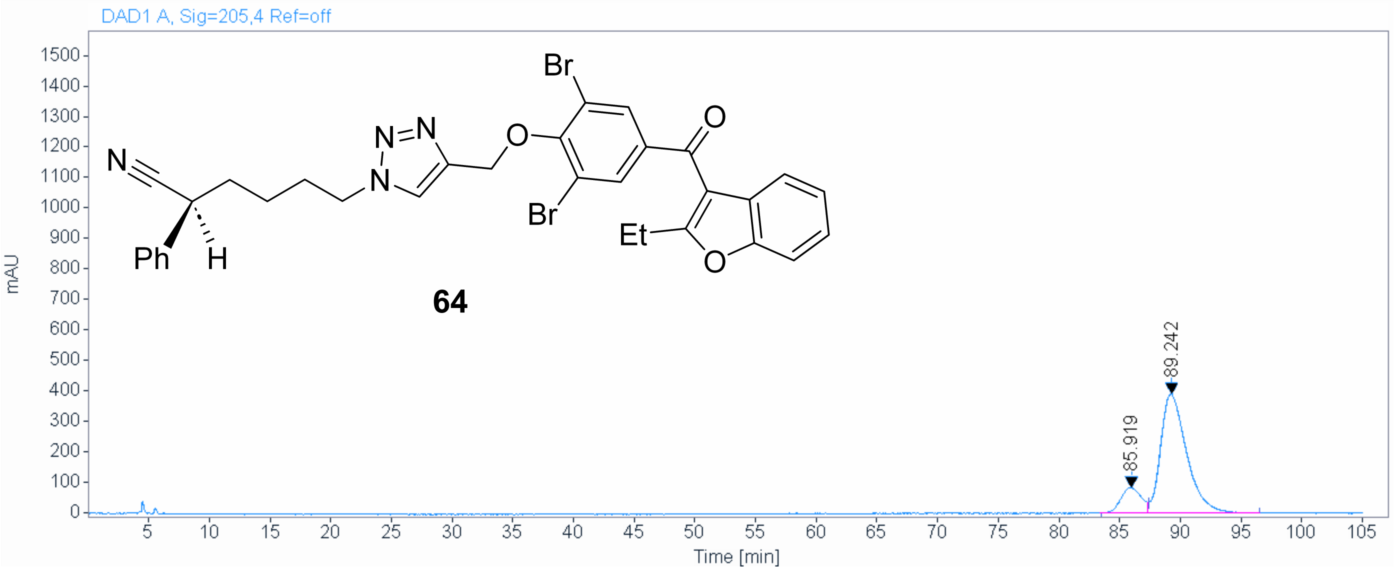

Signal: DAD1 A, Sig=205,4 Ref=off

RT [min]	Width [min]	Height	Area	Area%
85.919	1.9564	83.2283	9769.7178	14.4164
89.242	2.4735	390.7993	57998.3984	85.5836
		Sum	67768.1162	100.0000

Area Percent Report

Agilent Technologies

Sample name: zwf-10-139-rac-IE-70-30-0.8-205

Data file: C:\Users\Public\Documents\ChemStation\1\Data\zwf\zwf 2024-07-18 14-42-04\003-P1-C3-zwf-10-139-rac.D

Injection Acquired Date 07/18/2024 17:04:04

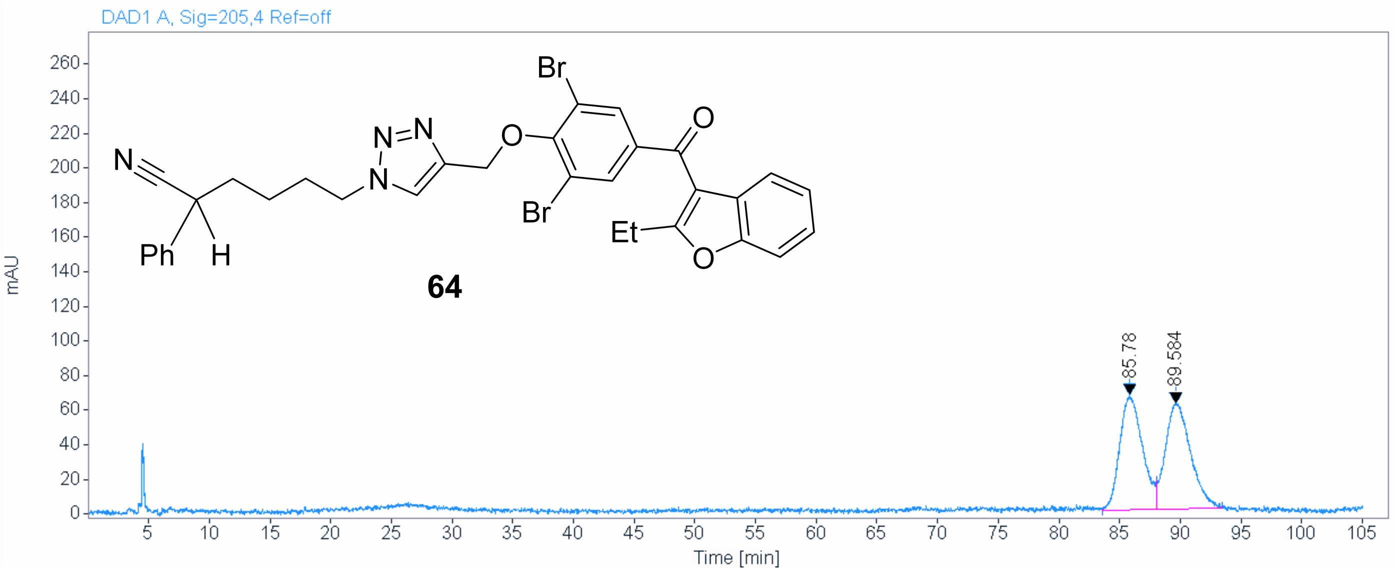

Signal: DAD1 A, Sig=205,4 Ref=off

RT [min]	Width [min]	Height	Area	Area%
85.780	2.1950	66.9039	8811.4287	49.6814
89.584	2.4209	61.4412	8924.4482	50.3186
		Sum	17735.8770	100.0000

zwf-10-139H
Jul 17 2024
SOLVENT: CDCl3
NA = 4
F1 = 500.130005 MHz
F2 = 1.000000 MHz

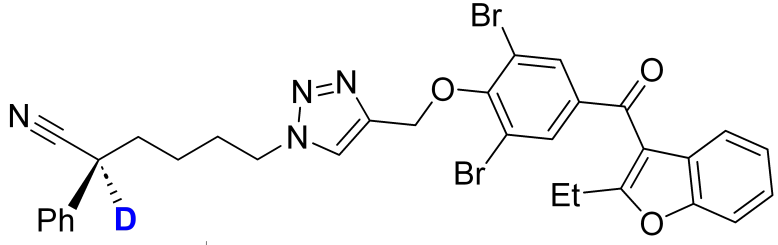

64-d

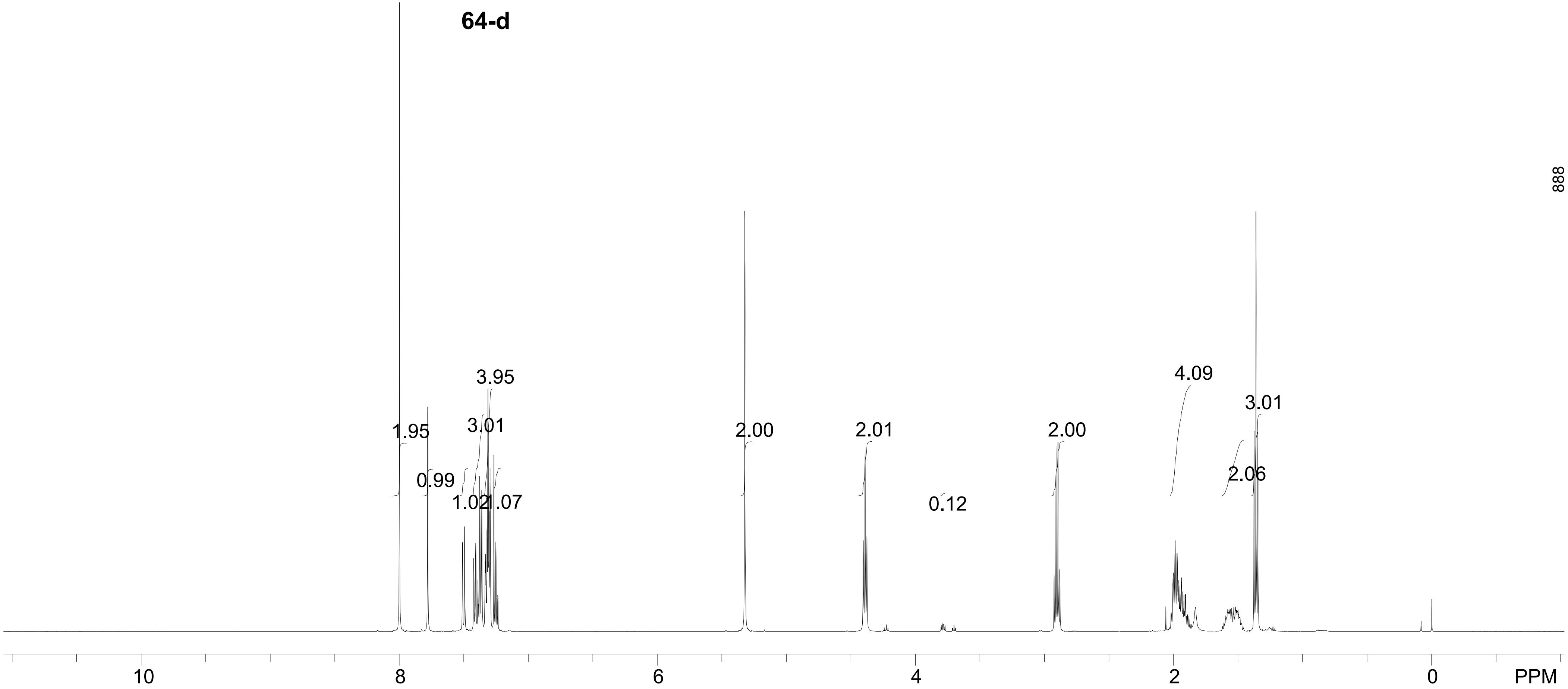

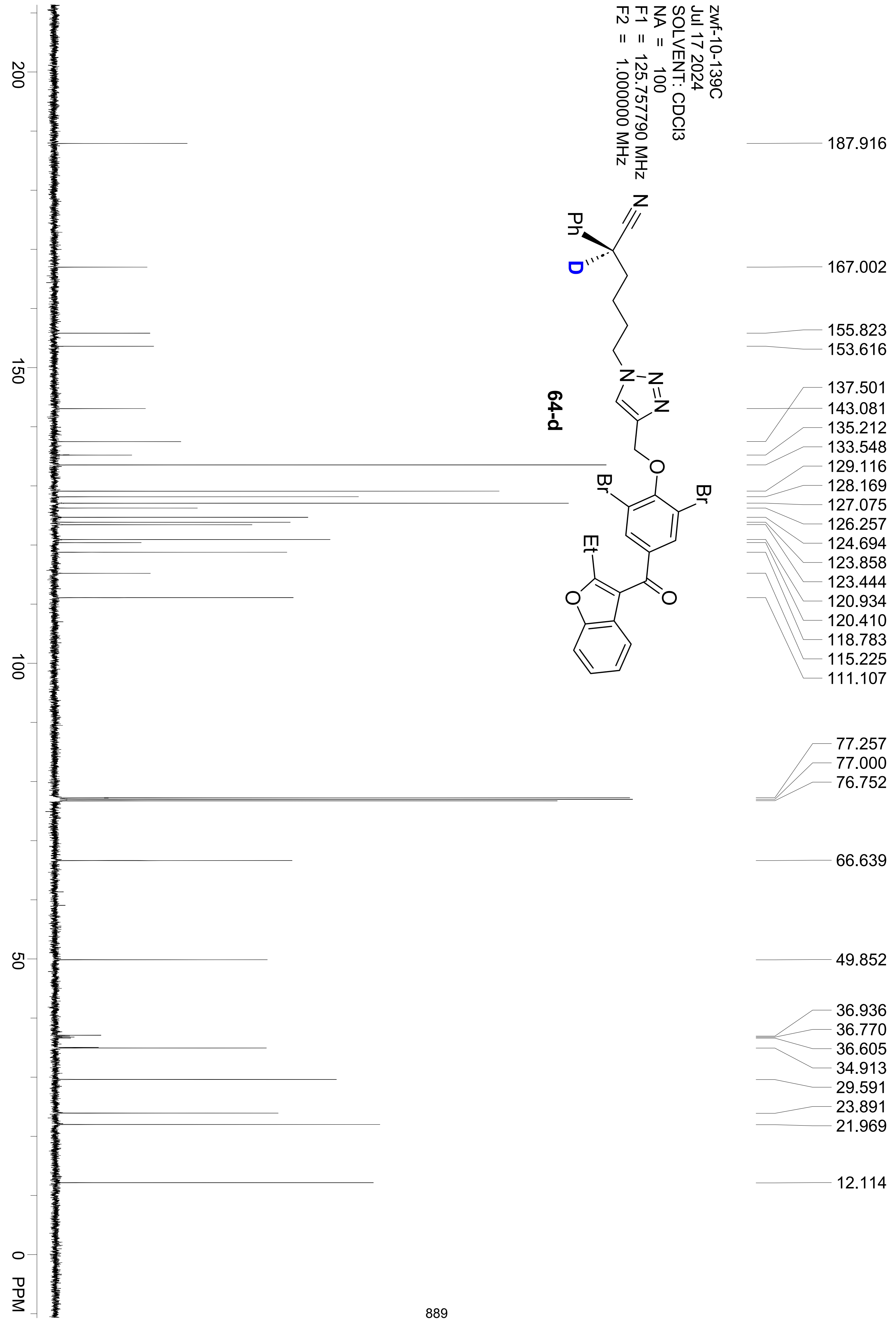
zwf-10-139C
Jul 17 2024
SOLVENT: CDCl3
NA = 100
F1 = 125.757790 MHz
F2 = 1.000000 MHz
64-d
187.916
167.002
155.823
153.616
137.501
143.081
135.212
133.548
129.116
128.169
127.075
126.257
124.694
123.858
123.444
120.934
120.410
118.783
115.225
111.107
77.257
77.000
76.752
66.639
49.852
36.936
36.770
36.605
34.913
29.591
23.891
21.969
12.114
200
150
100
50
0 PPM

Area Percent Report

Agilent Technologies

Sample name: zwf-10-139-IE-70-30-0.8-205

Data file: C:\Users\Public\Documents\ChemStation\1\Data\zwf\zwf 2024-07-18 14-42-04\004-P1-C2-zwf-10-139.D

Injection Acquired Date 07/18/2024 18:50:15

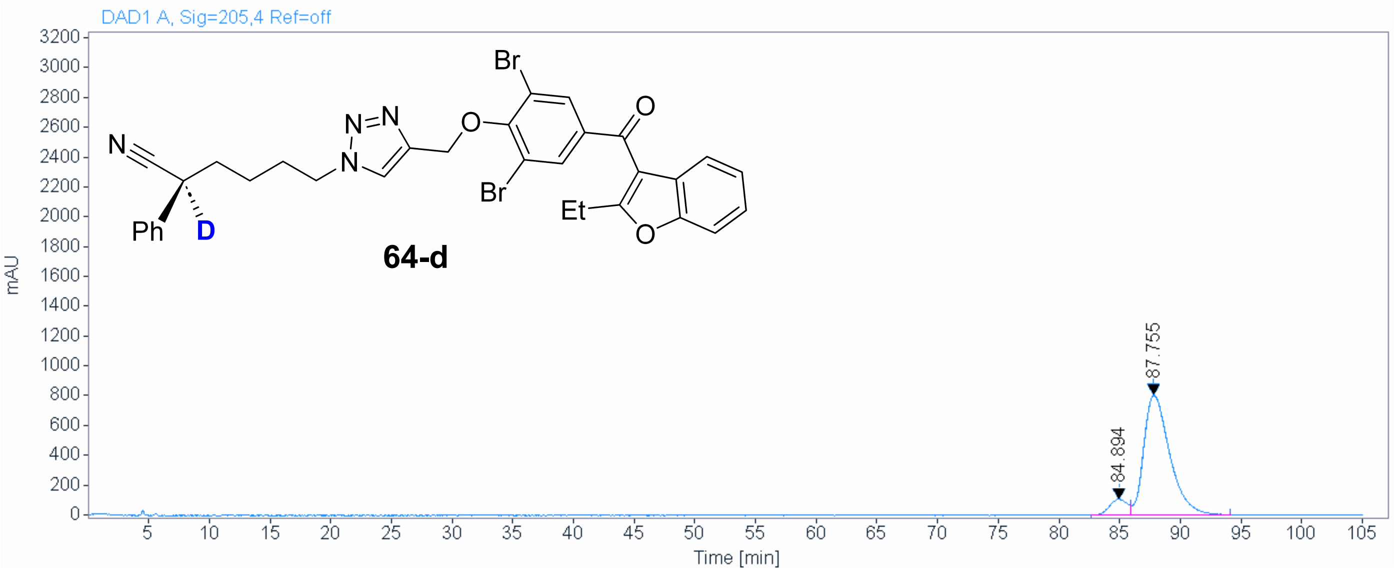

Signal: DAD1 A, Sig=205,4 Ref=off

RT [min]	Width [min]	Height	Area	Area%
84.894	1.7123	105.5991	10849.2090	8.3173
87.755	2.4746	805.4762	119592.1719	91.6827
		Sum	130441.3809	100.0000

Area Percent Report

Sample name: zwf-10-139-rac-IE-70-30-0.8-205

Data file: C:\Users\Public\Documents\ChemStation\1\Data\zwf\zwf 2024-07-18 14-42-04\003-P1-C3-zwf-10-139-rac.D

Injection Acquired Date 07/18/2024 17:04:04

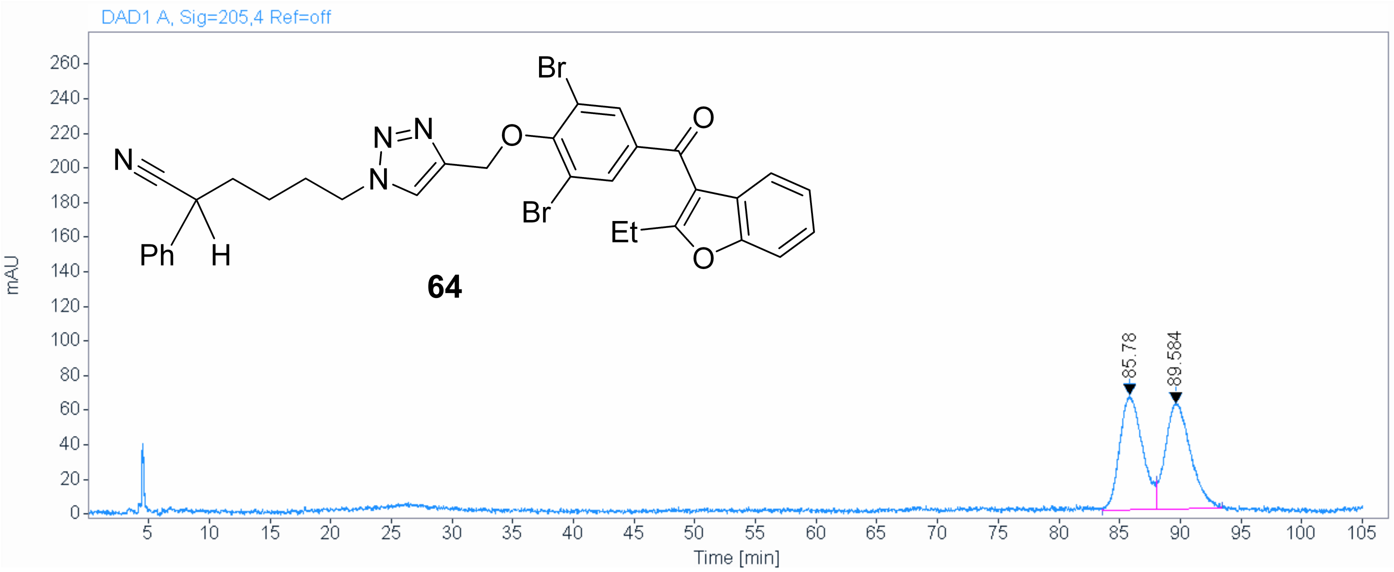

Signal: DAD1 A, Sig=205,4 Ref=off

RT [min]	Width [min]	Height	Area	Area%
85.780	2.1950	66.9039	8811.4287	49.6814
89.584	2.4209	61.4412	8924.4482	50.3186
		Sum	17735.8770	100.0000

zwf-7-063H
Sep 27 2023
SOLVENT: CDCl3
NA = 2
F1 = 400.140015 MHz
F2 = 1.000000 MHz

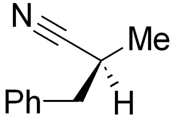

65

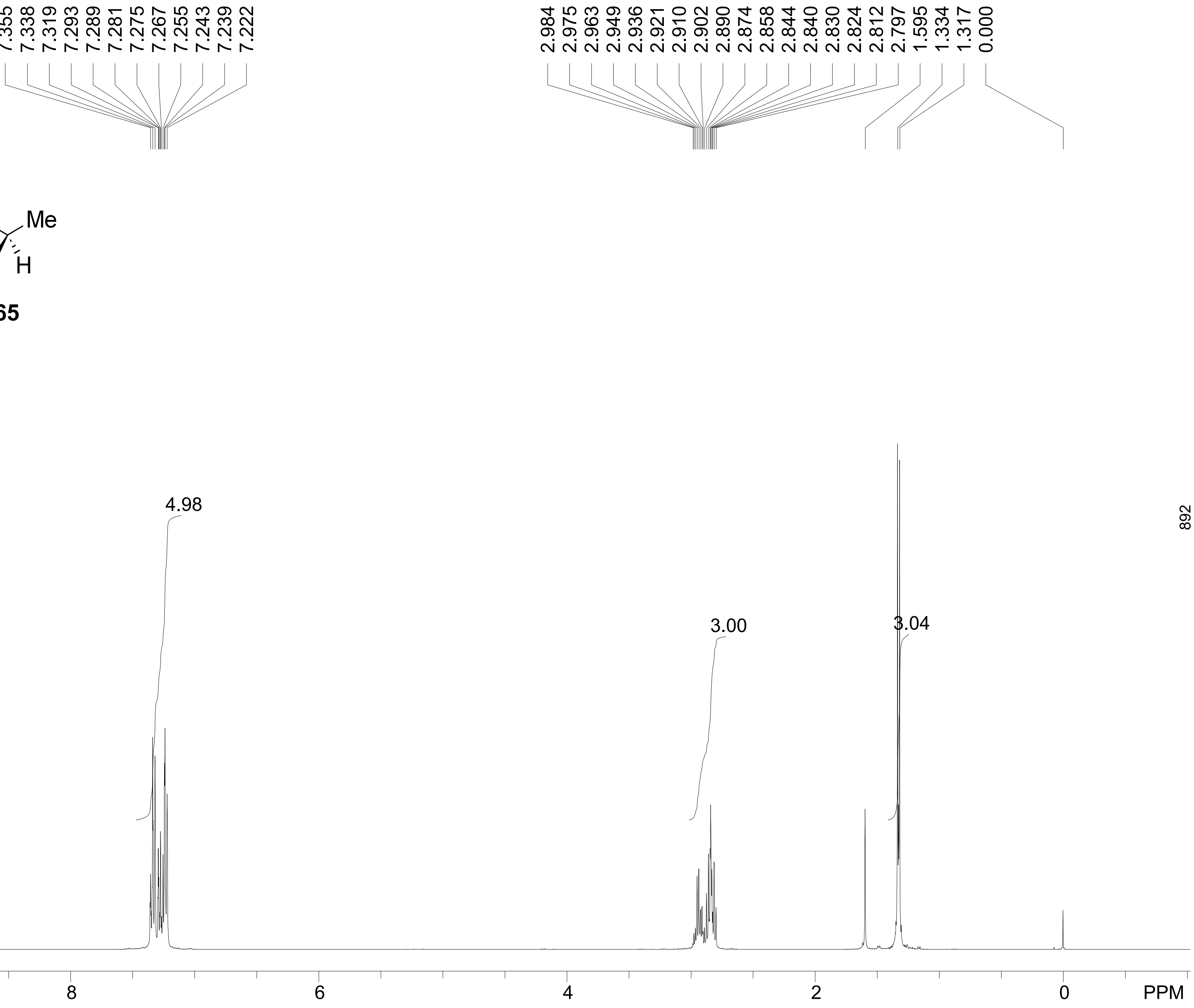

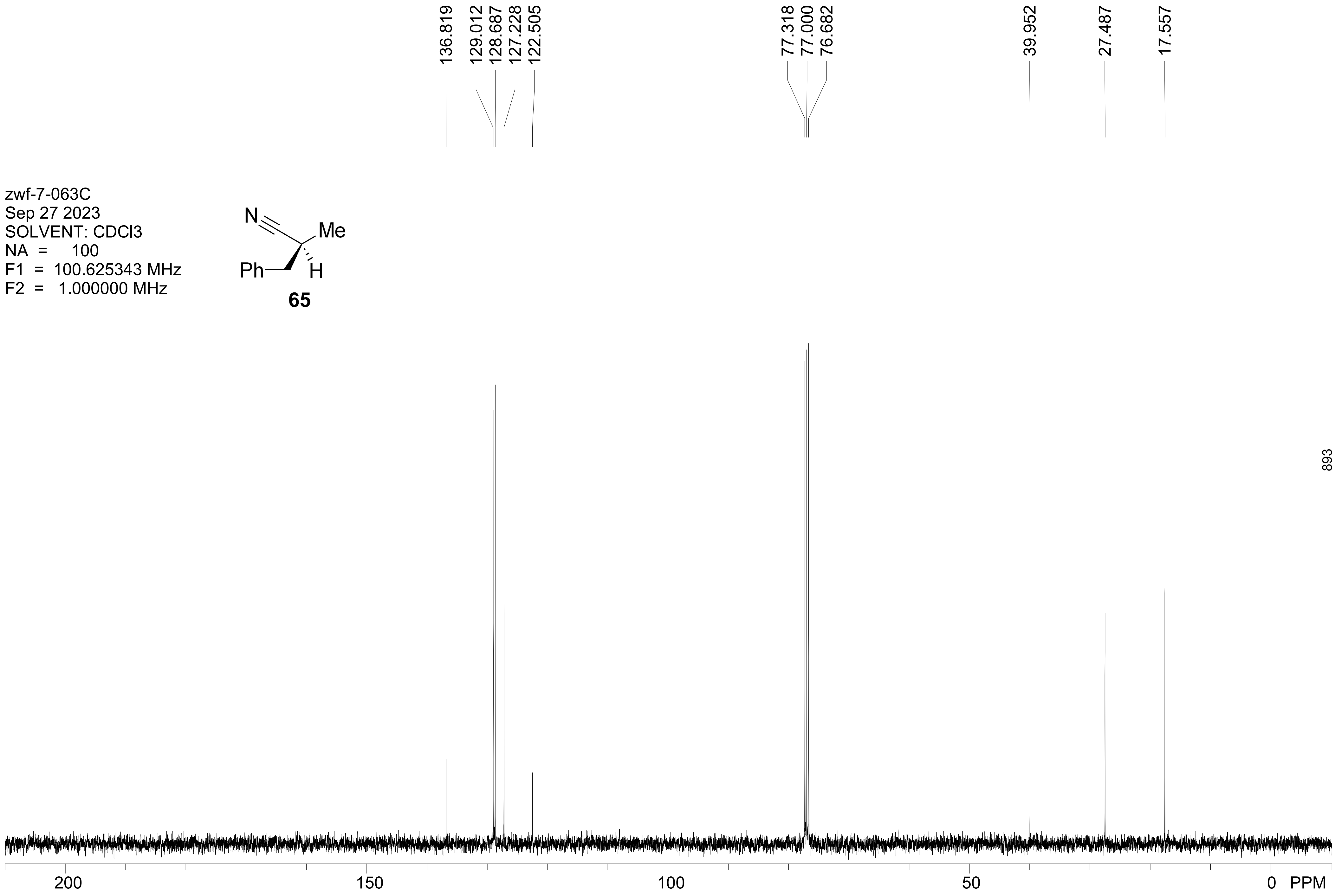
136.819
129.012
128.687
127.228
122.505
77.318
77.000
76.682
39.952
27.487
17.557
zwf-7-063C
Sep 27 2023
SOLVENT: CDCl3
NA = 100
F1 = 100.625343 MHz
F2 = 1.000000 MHz
N
Me
Ph
H
65
200
150
100
50
0 PPM

Area Percent Report

Sample name: zwf-7-063-IG-99-1-0.8-205

Data file: C:\Users\Public\Documents\ChemStation\1\Data\zwf\zwf-S 2023-09-27 14-32-25\002-P1-C4-zwf-7-063.D

Injection Acquired Date 09/27/2023 14:59:19

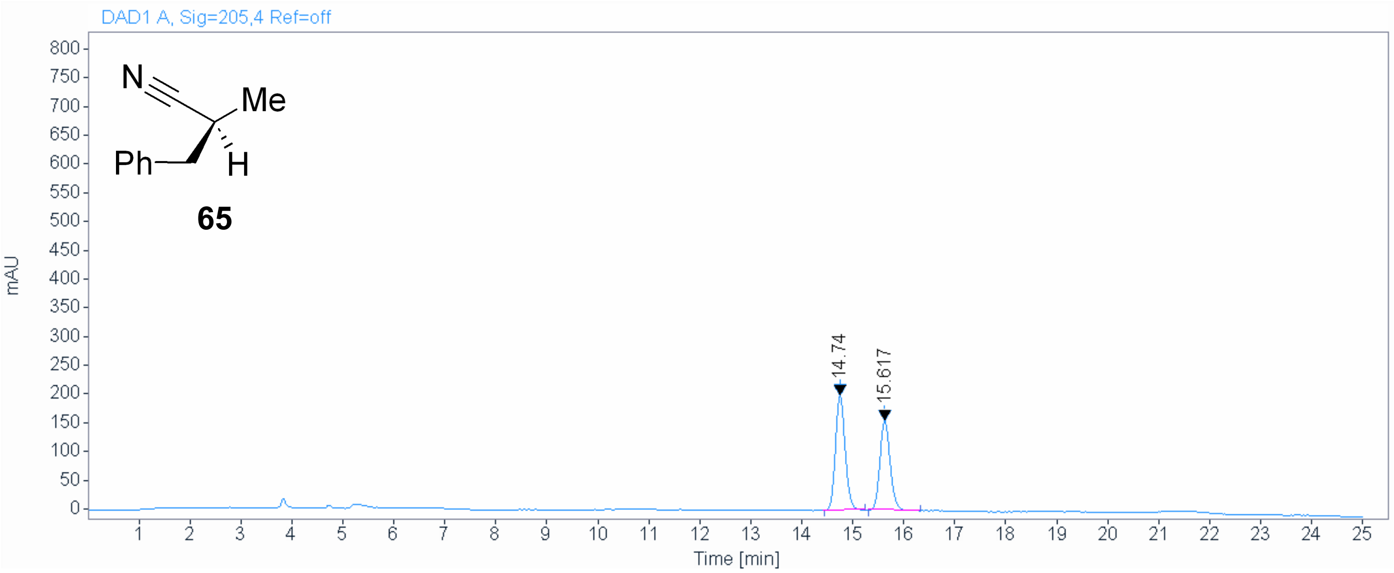

Signal: DAD1 A, Sig=205,4 Ref=off

RT [min]	Width [min]	Height	Area	Area%
14.740	0.2062	199.5608	2637.8279	54.6739
15.617	0.2181	155.4505	2186.8291	45.3261
		Sum	4824.6570	100.0000

Area Percent Report

Agilent Technologies

Sample name: zwf-7-063-rac-IG-99-1-0.8-205

Data file: C:\Users\Public\Documents\ChemStation\1\Data\zwf\zwf-S 2023-09-27 14-32-25\001-P1-C3-zwf-7-063-rac.D

Injection Acquired Date 09/27/2023 14:33:21

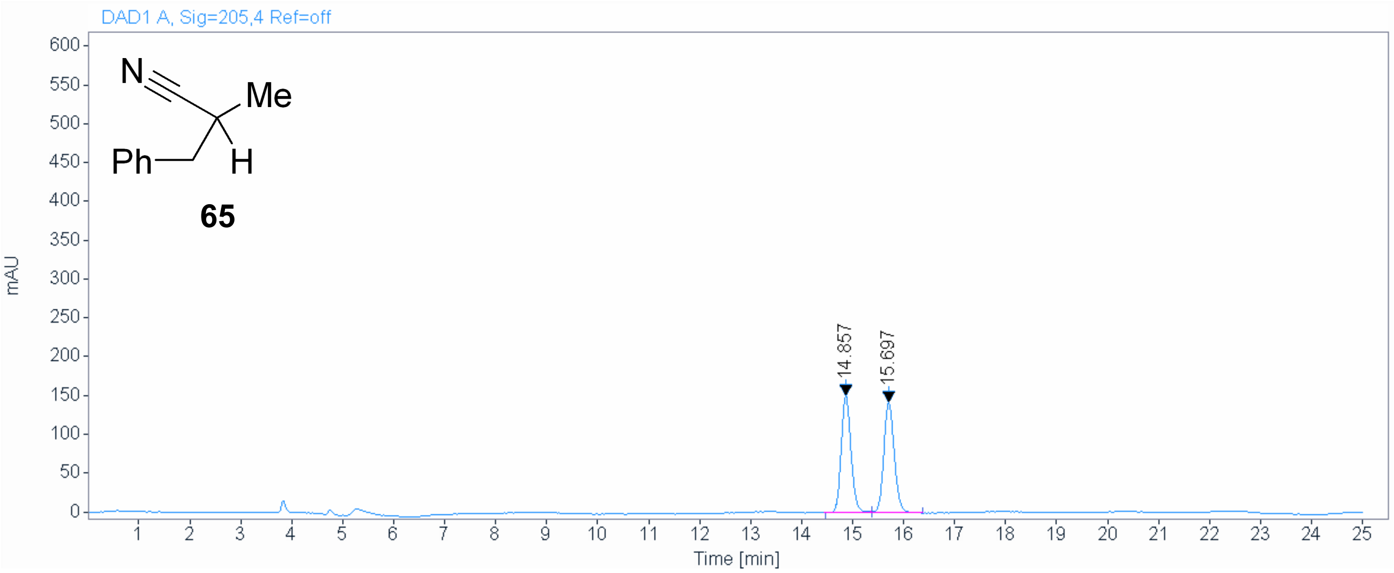

Signal: DAD1 A, Sig=205,4 Ref=off

RT [min]	Width [min]	Height	Area	Area%
14.857	0.2071	150.7261	2030.0676	49.7802
15.697	0.2237	142.4696	2047.9928	50.2198
		Sum	4078.0604	100.0000

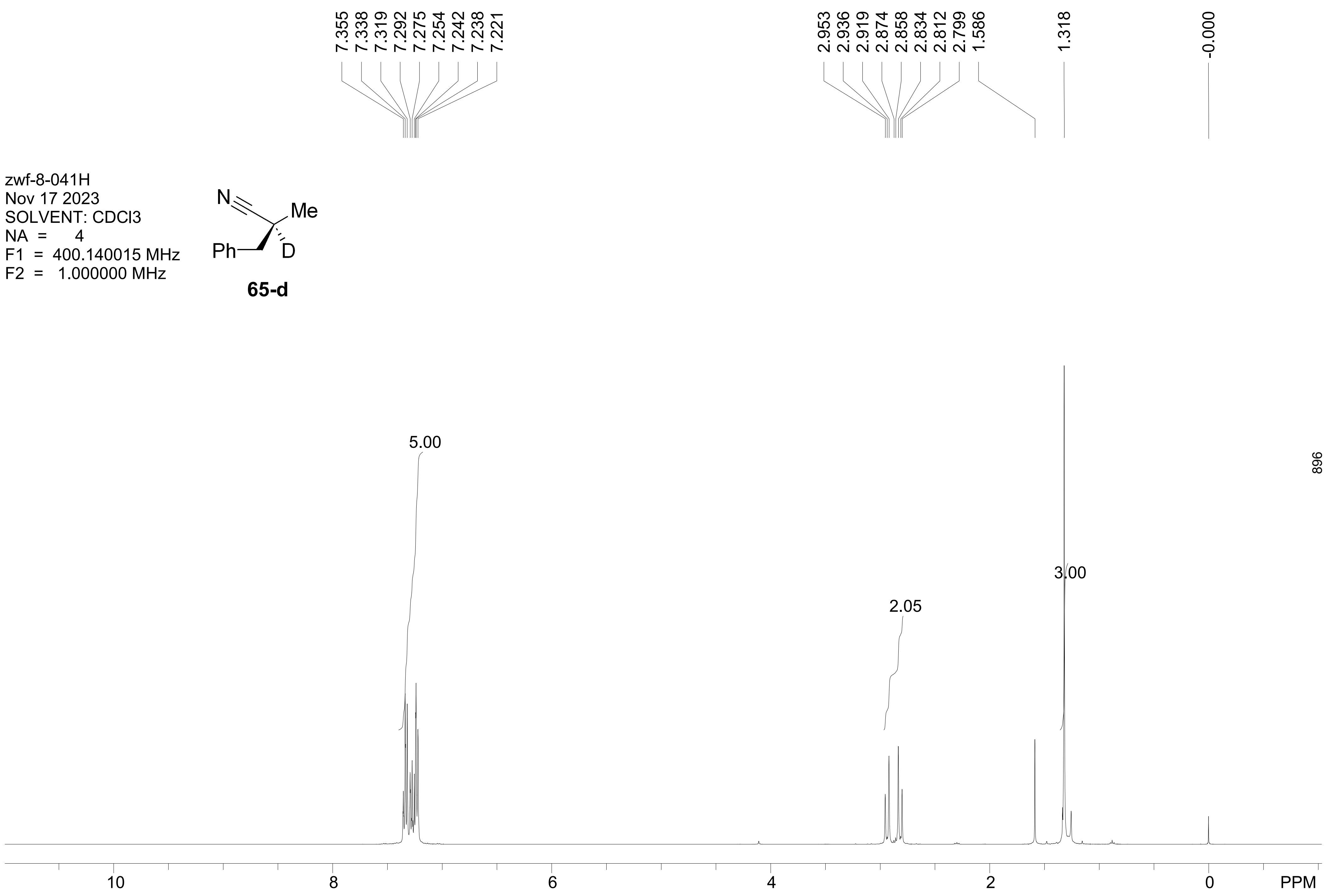
zwf-8-041H
Nov 17 2023
SOLVENT: CDCl3
NA = 4
F1 = 400.140015 MHz
F2 = 1.000000 MHz
N
Me
Ph
D
65-d
7.355
7.338
7.319
7.292
7.275
7.254
7.242
7.238
7.221
2.953
2.936
2.919
2.874
2.858
2.834
2.812
2.799
1.586
1.318
-0.000
5.00
2.05
3.00
10
8
6
4
2
0
PPM

zwf-8-041C
Nov 17 2023
SOLVENT: CDCl3
NA = 300
F1 = 100.615280 MHz
F2 = 1.000000 MHz

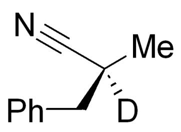

65-d

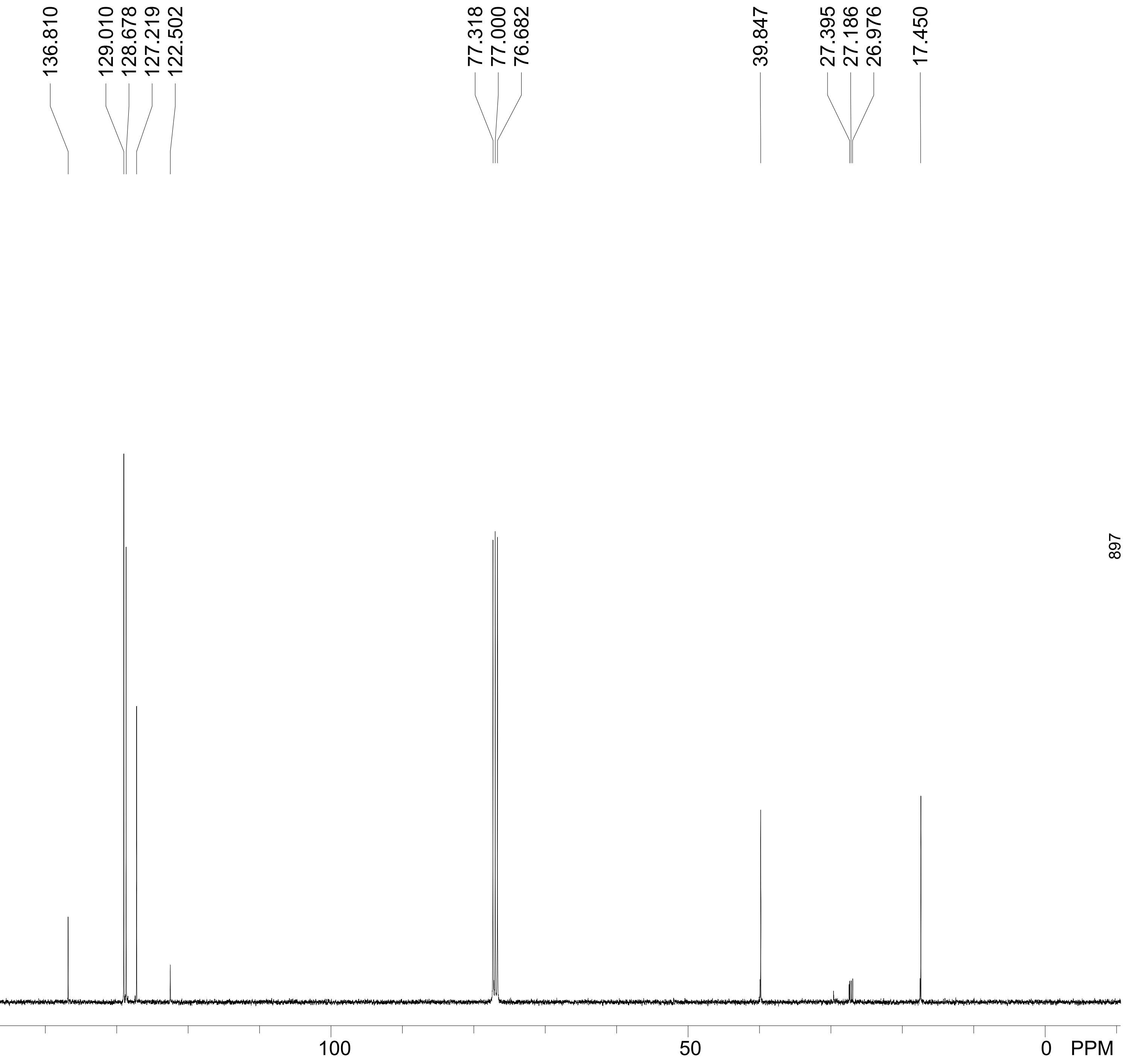

Area Percent Report

Sample name: zwf-8-041-IG-99-1-0.8-205

Data file: C:\Users\Public\Documents\ChemStation\1\Data\zwf\zwf-S 2023-11-17 07-38-48\024-P1-C1-zwf-8-041.D

Injection Acquired Date 11/17/2023 19:39:20

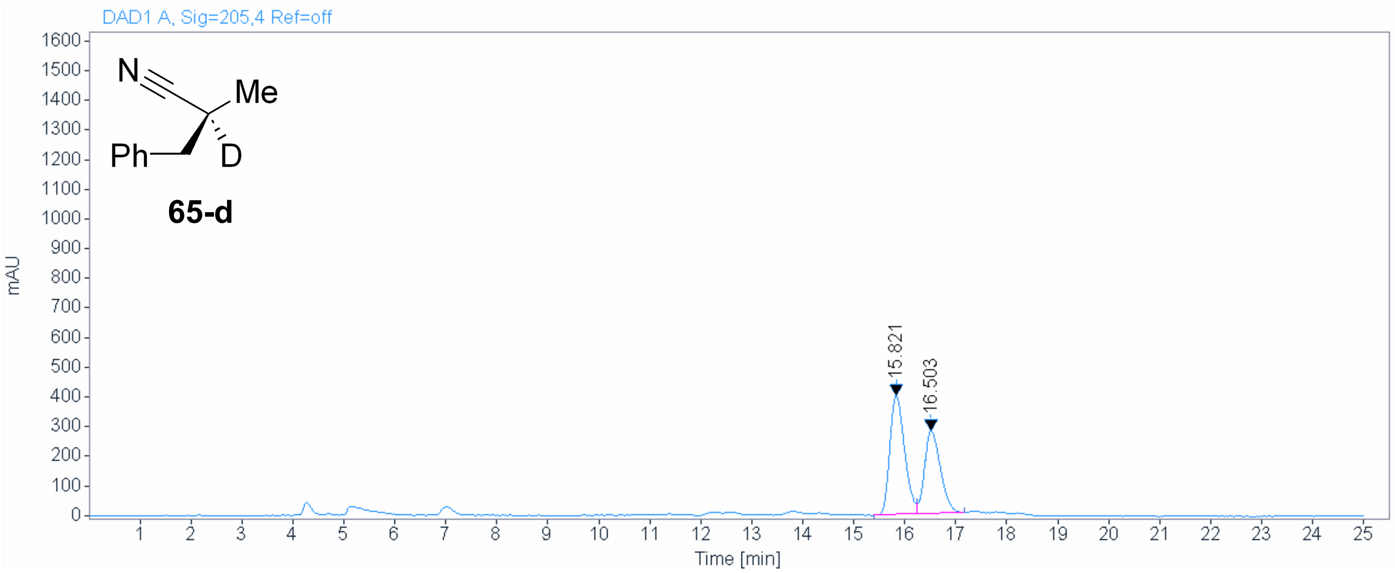

Signal: DAD1 A, Sig=205,4 Ref=off

RT [min]	Width [min]	Height	Area	Area%
15.821	0.3053	401.6018	8101.1035	57.0365
16.503	0.3305	279.7824	6102.2705	42.9635
		Sum	14203.3740	100.0000

Area Percent Report

Agilent Technologies

Sample name: zwf-8-041-rac-IG-99-1-0.8-205

Data file: C:\Users\Public\Documents\ChemStation\1\Data\zwf\zwf-S 2023-11-17 07-38-48\021-P1-C2-zwf-8-041-rac.D

Injection Acquired Date 11/17/2023 18:45:46

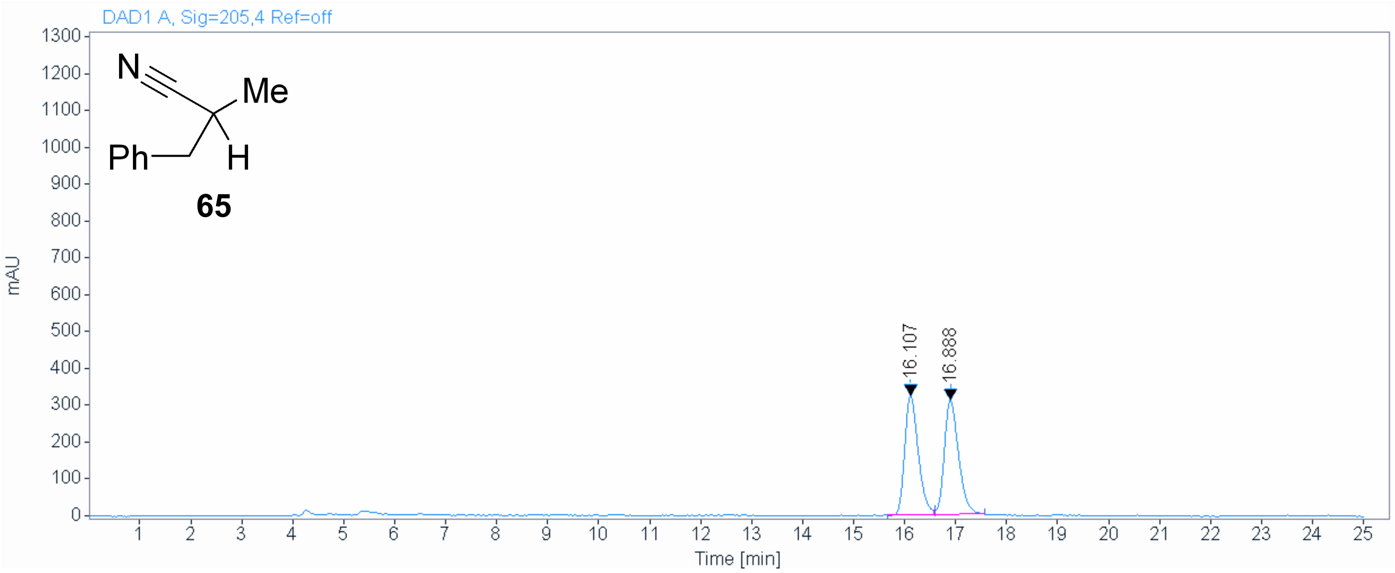

Signal: DAD1 A, Sig=205,4 Ref=off

RT [min]	Width [min]	Height	Area	Area%
16.107	0.2778	325.0391	6010.9058	49.6648
16.888	0.2916	311.5104	6092.0366	50.3352
		Sum	12102.9424	100.0000

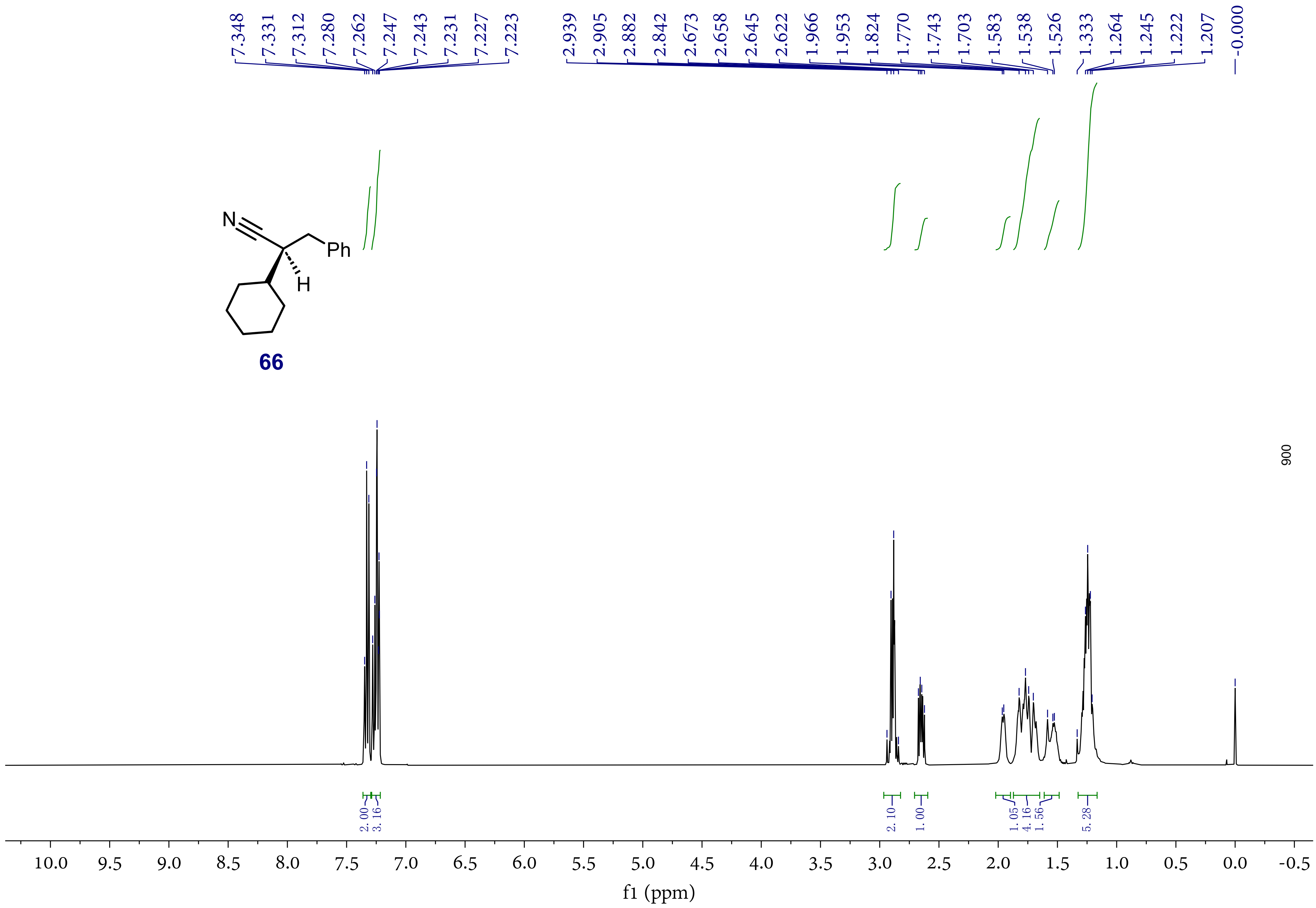
7.348
7.331
7.312
7.280
7.262
7.247
7.243
7.231
7.227
7.223
2.939
2.905
2.882
2.842
2.673
2.658
2.645
2.622
1.966
1.953
1.824
1.770
1.743
1.703
1.583
1.538
1.526
1.333
1.264
1.245
1.222
1.207
-0.000
N
Ph
H
66
2.00
3.16
2.10
1.00
1.05
4.16
1.56
5.28
10.0 9.5 9.0 8.5 8.0 7.5 7.0 6.5 6.0 5.5 5.0 4.5 4.0 3.5 3.0 2.5 2.0 1.5 1.0 0.5 0.0 -0.5
f1 (ppm)

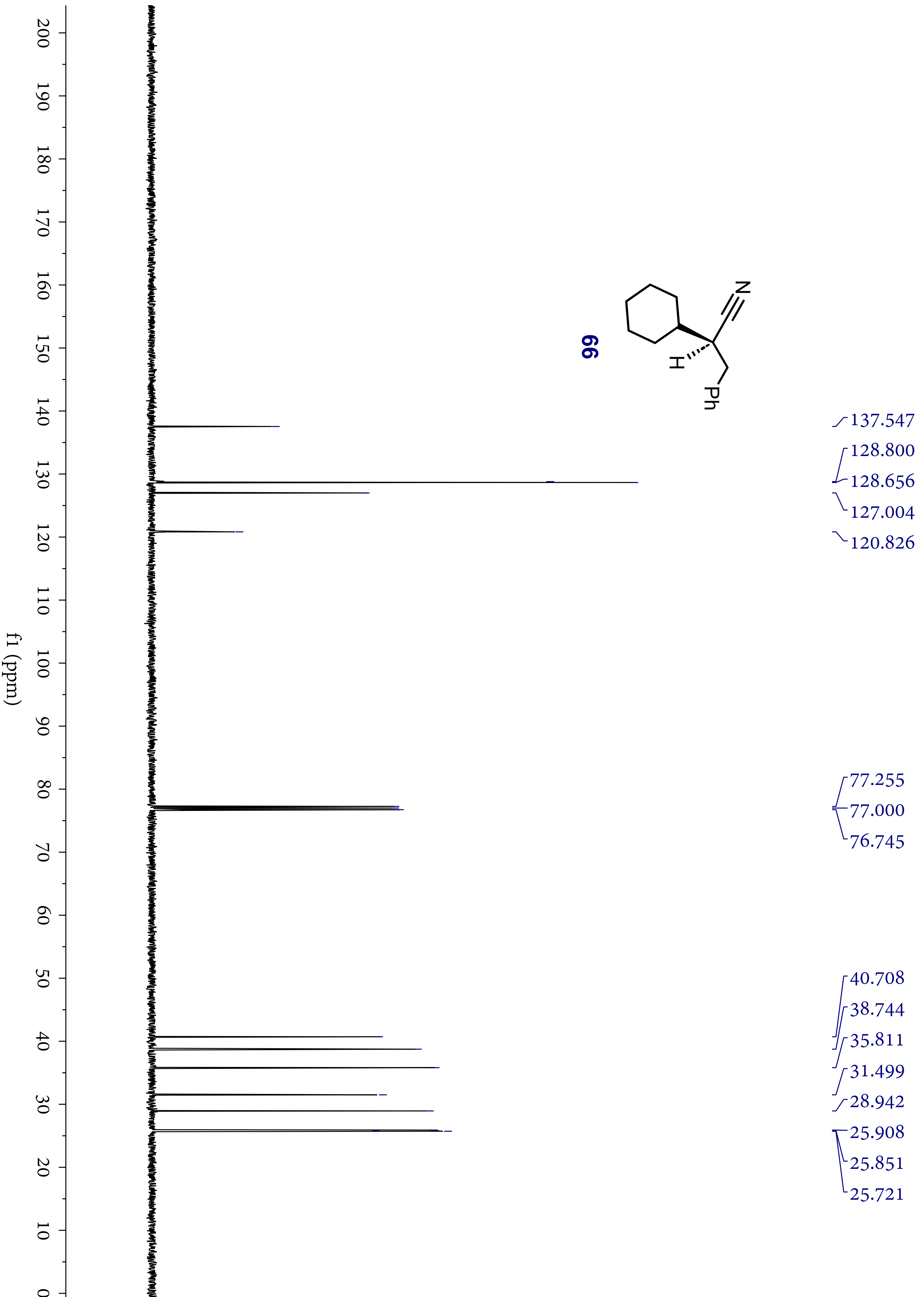

f1 (ppm)
N
H
Ph
137.547
128.800
128.656
127.004
120.826
77.255
77.000
76.745
40.708
38.744
35.811
31.499
28.942
25.908
25.851
25.721

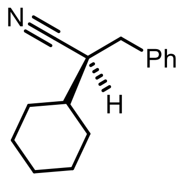

(-)-**66**

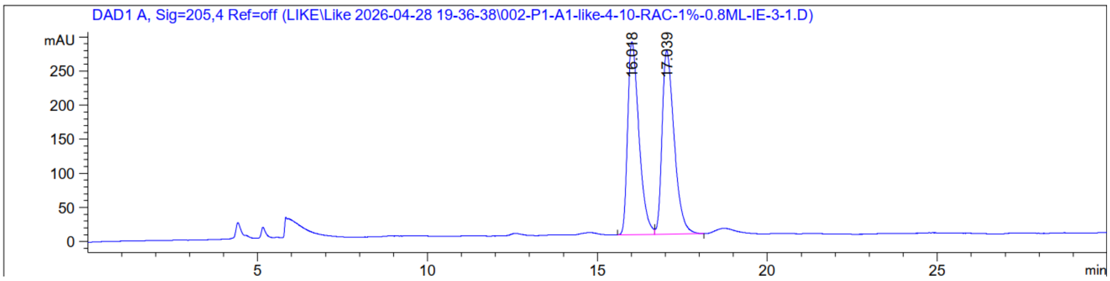

Signal 1: DAD1 A, Sig=205,4 Ref=off

Peak #	RetTime [min]	Type	Width [min]	Area [mAU*s]	Height [mAU]	Area %
1	16.018	BV	0.3560	6547.45605	282.88535	49.5543
2	17.039	VB	0.3803	6665.23340	269.70755	50.4457

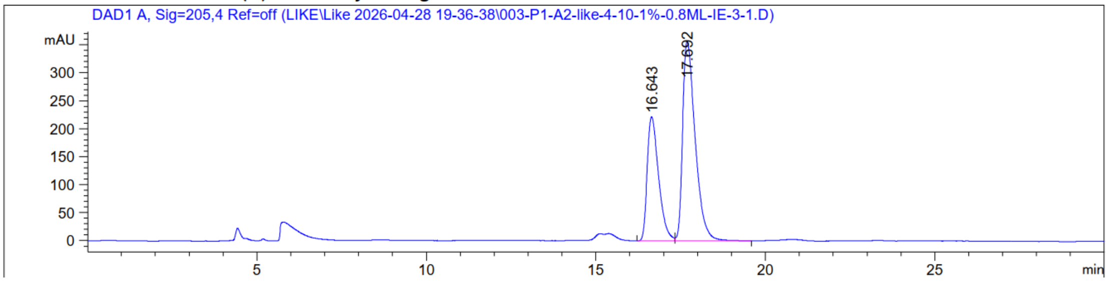

Signal 1: DAD1 A, Sig=205,4 Ref=off

Peak #	RetTime [min]	Type	Width [min]	Area [mAU*s]	Height [mAU]	Area %
1	16.643	BV	0.3555	5203.07275	221.91504	36.1167
2	17.692	VB	0.3910	9203.22363	356.74011	63.8833

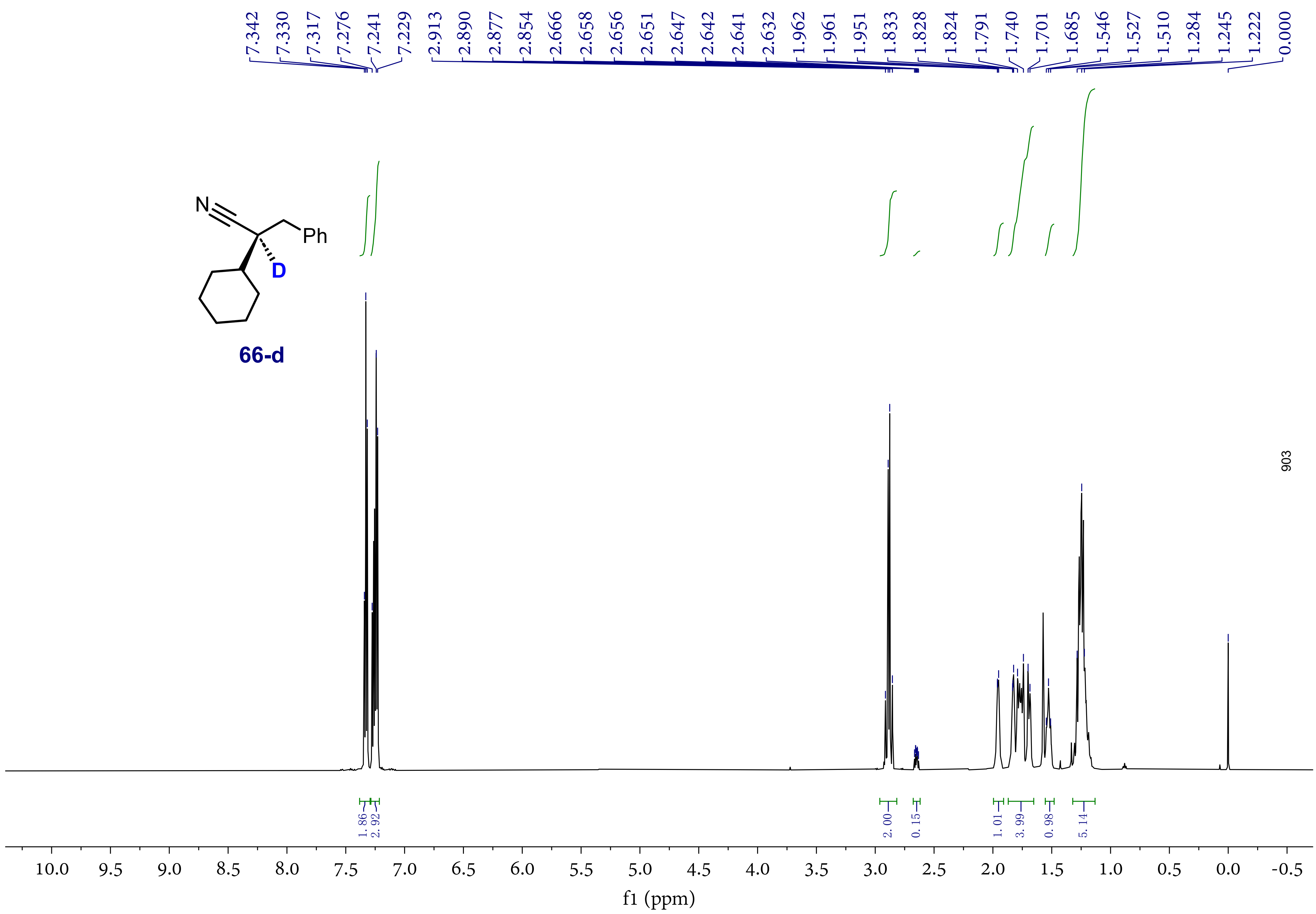
7.342
7.330
7.317
7.276
7.241
7.229
2.913
2.890
2.877
2.854
2.666
2.658
2.656
2.651
2.647
2.642
2.641
2.632
1.962
1.961
1.951
1.833
1.828
1.824
1.791
1.740
1.701
1.685
1.546
1.527
1.510
1.284
1.245
1.222
0.000
N
Ph
D
66-d
1.86
2.92
2.00
0.15
1.01
3.99
0.98
5.14
10.0 9.5 9.0 8.5 8.0 7.5 7.0 6.5 6.0 5.5 5.0 4.5 4.0 3.5 3.0 2.5 2.0 1.5 1.0 0.5 0.0 -0.5
f1 (ppm)

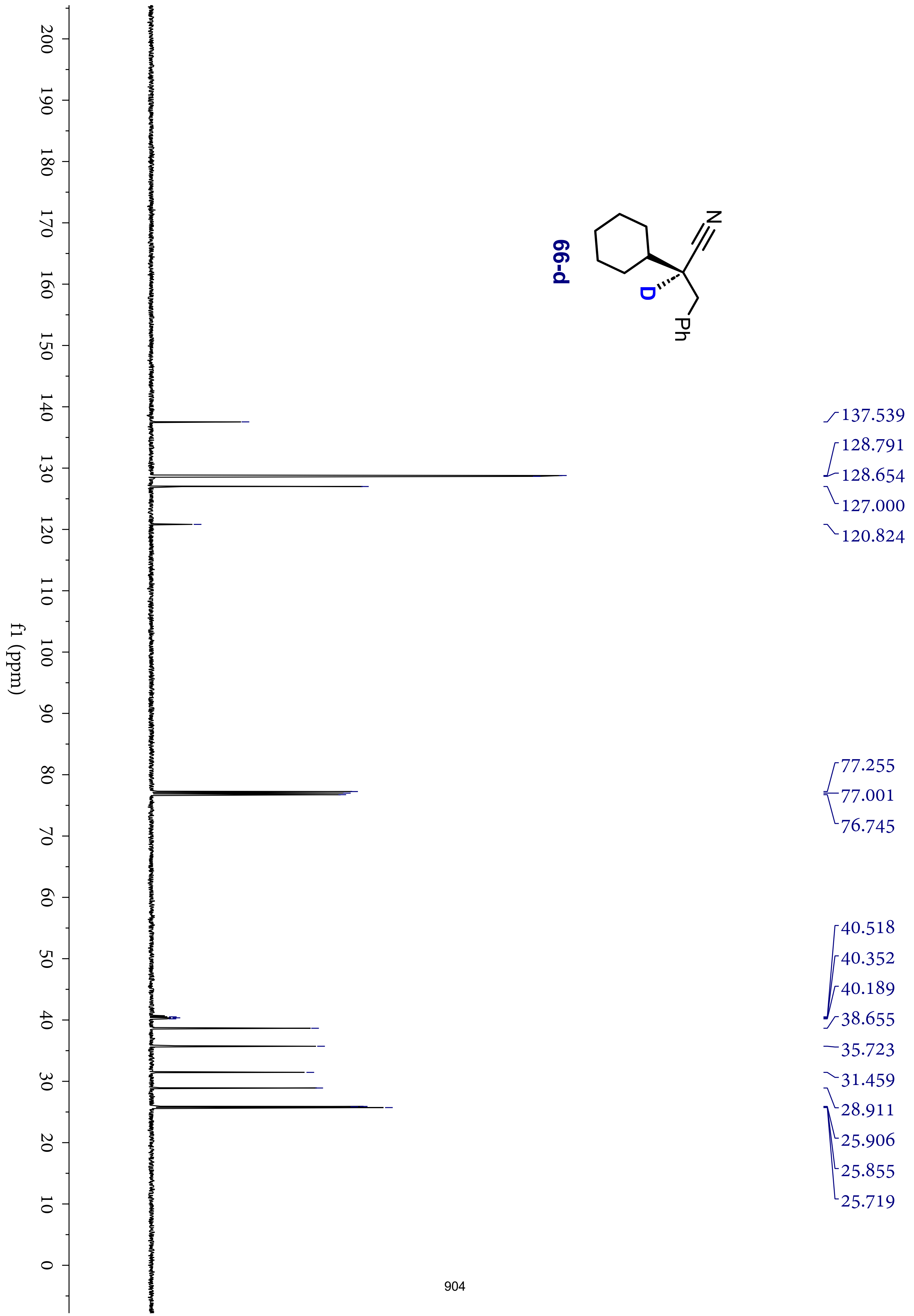
66-d
D
Ph
N
137.539
128.791
128.654
127.000
120.824
77.255
77.001
76.745
40.518
40.352
40.189
38.655
35.723
31.459
28.911
25.906
25.855
25.719
f1 (ppm)
200
190
180
170
160
150
140
130
120
110
100
90
80
70
60
50
40
30
20
10
0

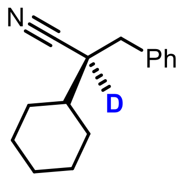

(-)-**66**-d

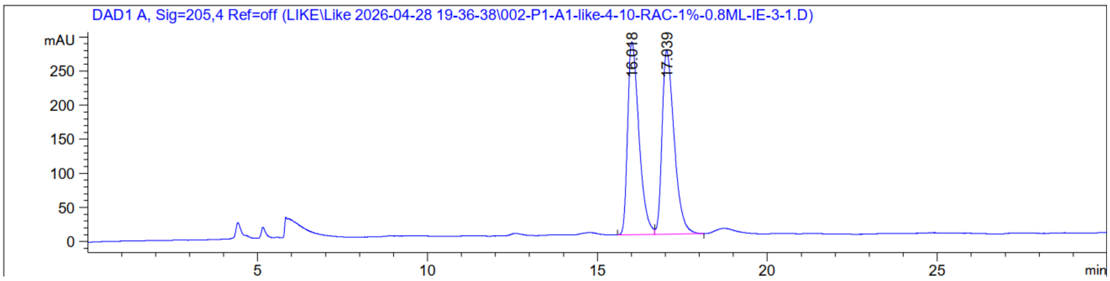

Signal 1: DAD1 A, Sig=205,4 Ref=off

Peak #	RetTime [min]	Type	Width [min]	Area [mAU*s]	Height [mAU]	Area %
1	16.018	BV	0.3560	6547.45605	282.88535	49.5543
2	17.039	VB	0.3803	6665.23340	269.70755	50.4457

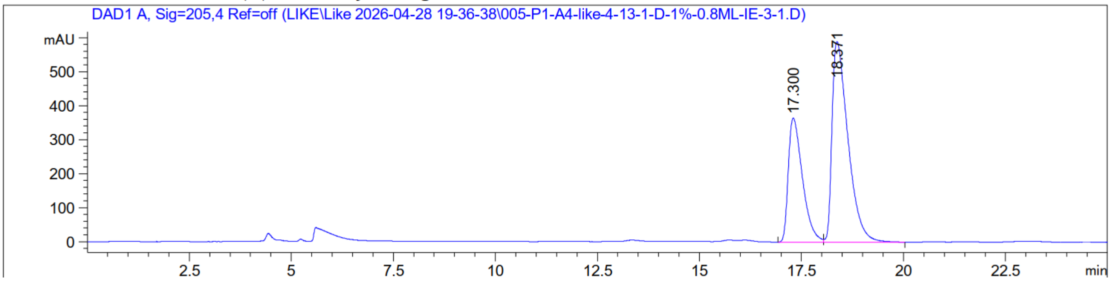

Signal 1: DAD1 A, Sig=205,4 Ref=off

Peak #	RetTime [min]	Type	Width [min]	Area [mAU*s]	Height [mAU]	Area %
1	17.300	BV	0.3689	8798.21094	365.42517	35.4239
2	18.371	VB	0.4030	1.60387e4	590.20221	64.5761

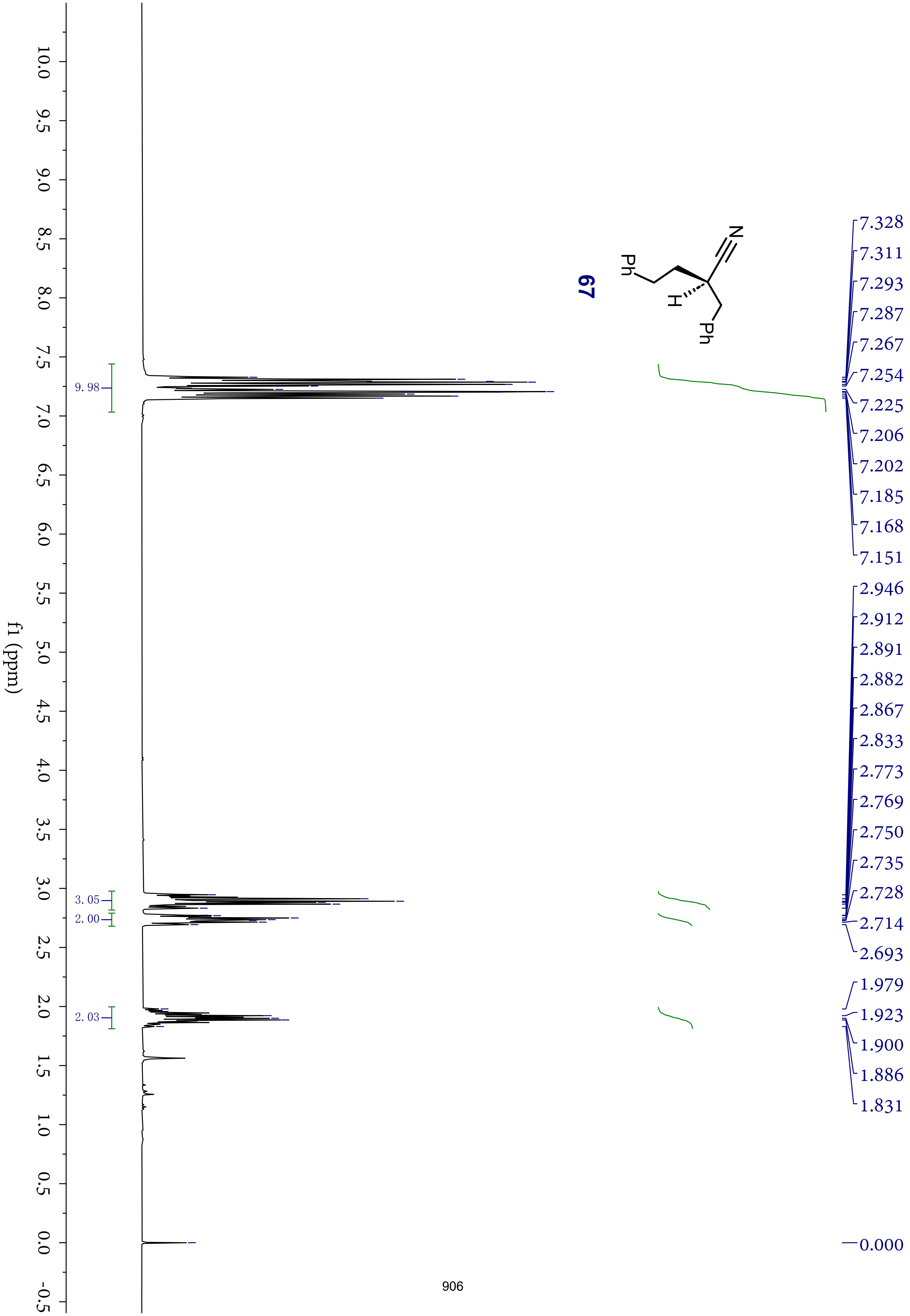
67
Ph
H
N
Ph
7.328
7.311
7.293
7.287
7.267
7.254
7.225
7.206
7.202
7.185
7.168
7.151
2.946
2.912
2.891
2.882
2.867
2.833
2.773
2.769
2.750
2.735
2.728
2.714
2.693
1.979
1.923
1.900
1.886
1.831
0.000
9.98
3.05
2.00
2.03
f1 (ppm)

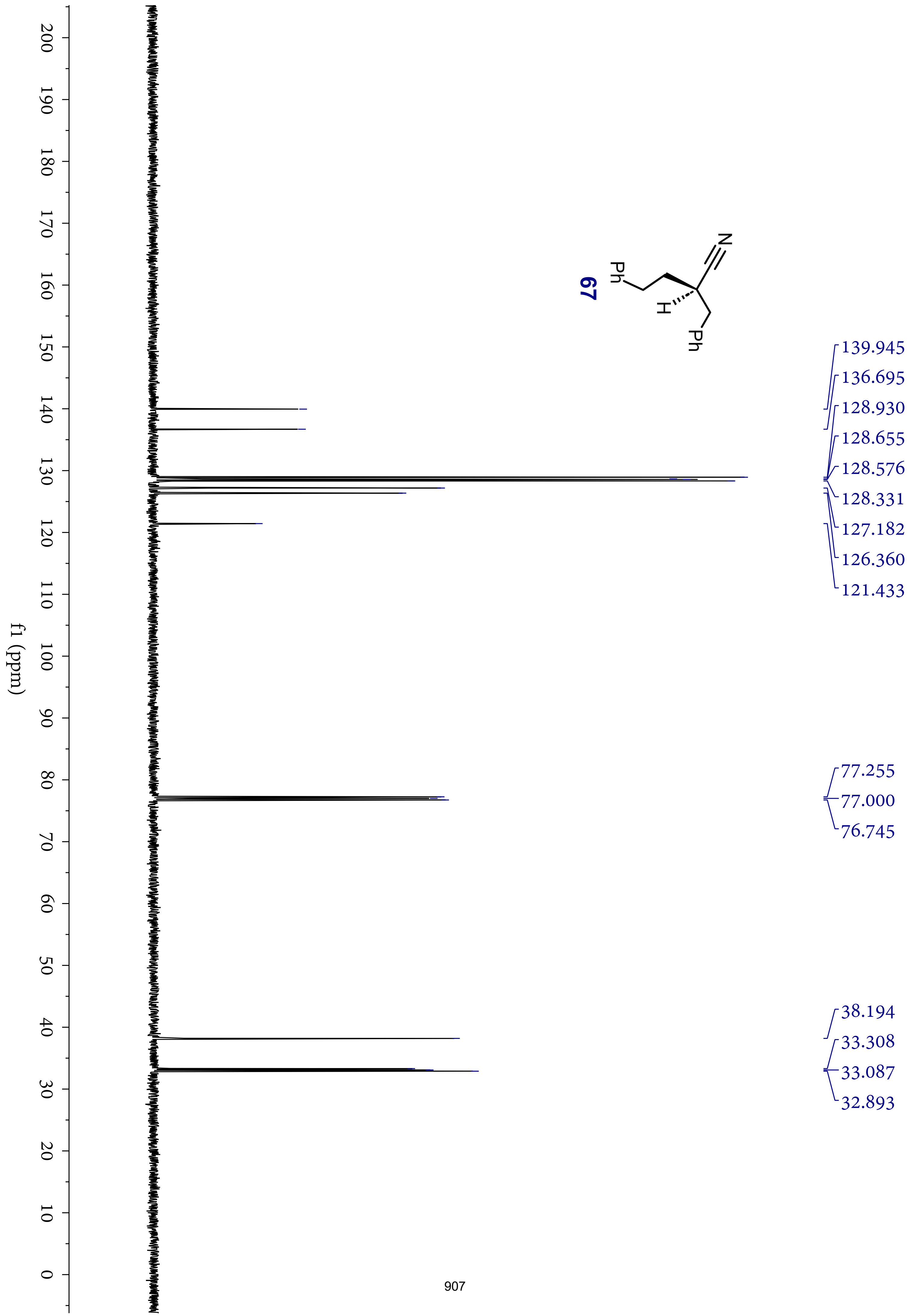
67
Ph
H
Ph
N
139.945
136.695
128.930
128.655
128.576
128.331
127.182
126.360
121.433
77.255
77.000
76.745
38.194
33.308
33.087
32.893
f1 (ppm)
200
190
180
170
160
150
140
130
120
110
100
90
80
70
60
50
40
30
20
10
0

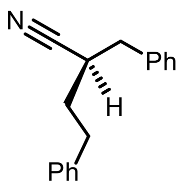

(+)-**67**

1ml/min

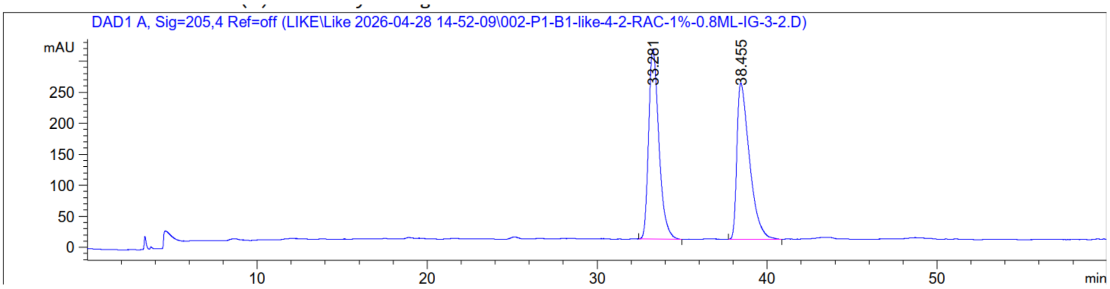

Signal 1: DAD1 A, Sig=205,4 Ref=off

Peak #	RetTime [min]	Type	Width [min]	Area [mAU*s]	Height [mAU]	Area %
1	33.281	BB	0.6323	1.28782e4	306.40787	49.8439
2	38.455	BB	0.7421	1.29589e4	251.95070	50.1561

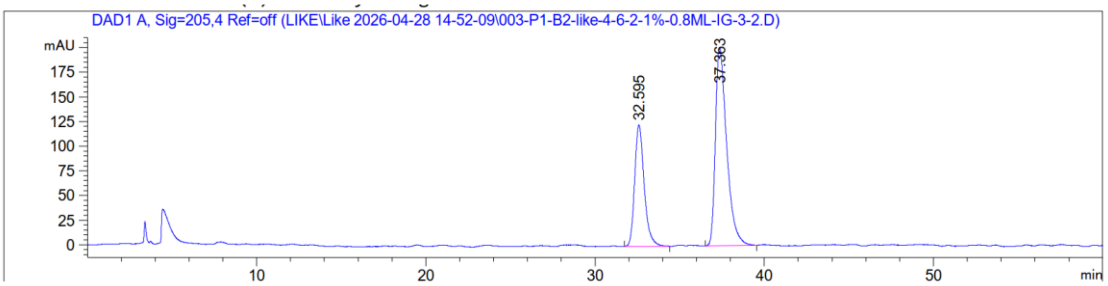

Signal 1: DAD1 A, Sig=205,4 Ref=off

Peak #	RetTime [min]	Type	Width [min]	Area [mAU*s]	Height [mAU]	Area %
1	32.595	BB	0.5869	4814.67041	122.95235	34.1664
2	37.363	BB	0.6842	9277.16699	201.09042	65.8336

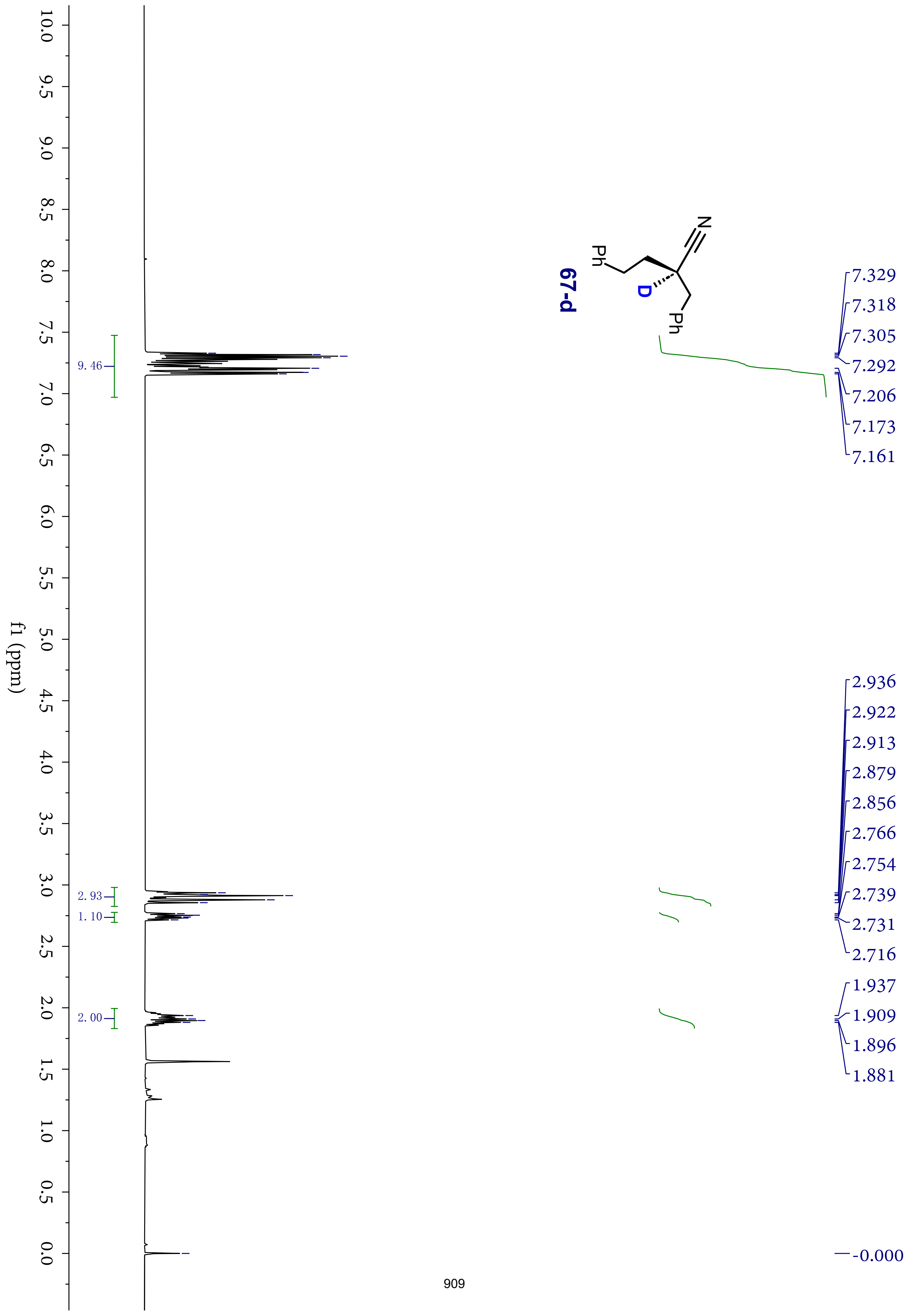
67-d
D
Ph
Ph
N
7.329
7.318
7.305
7.292
7.206
7.173
7.161
2.936
2.922
2.913
2.879
2.856
2.766
2.754
2.739
2.731
2.716
1.937
1.909
1.896
1.881
-0.000
9.46
2.93
1.10
2.00
f1 (ppm)
10.0
9.5
9.0
8.5
8.0
7.5
7.0
6.5
6.0
5.5
5.0
4.5
4.0
3.5
3.0
2.5
2.0
1.5
1.0
0.5
0.0

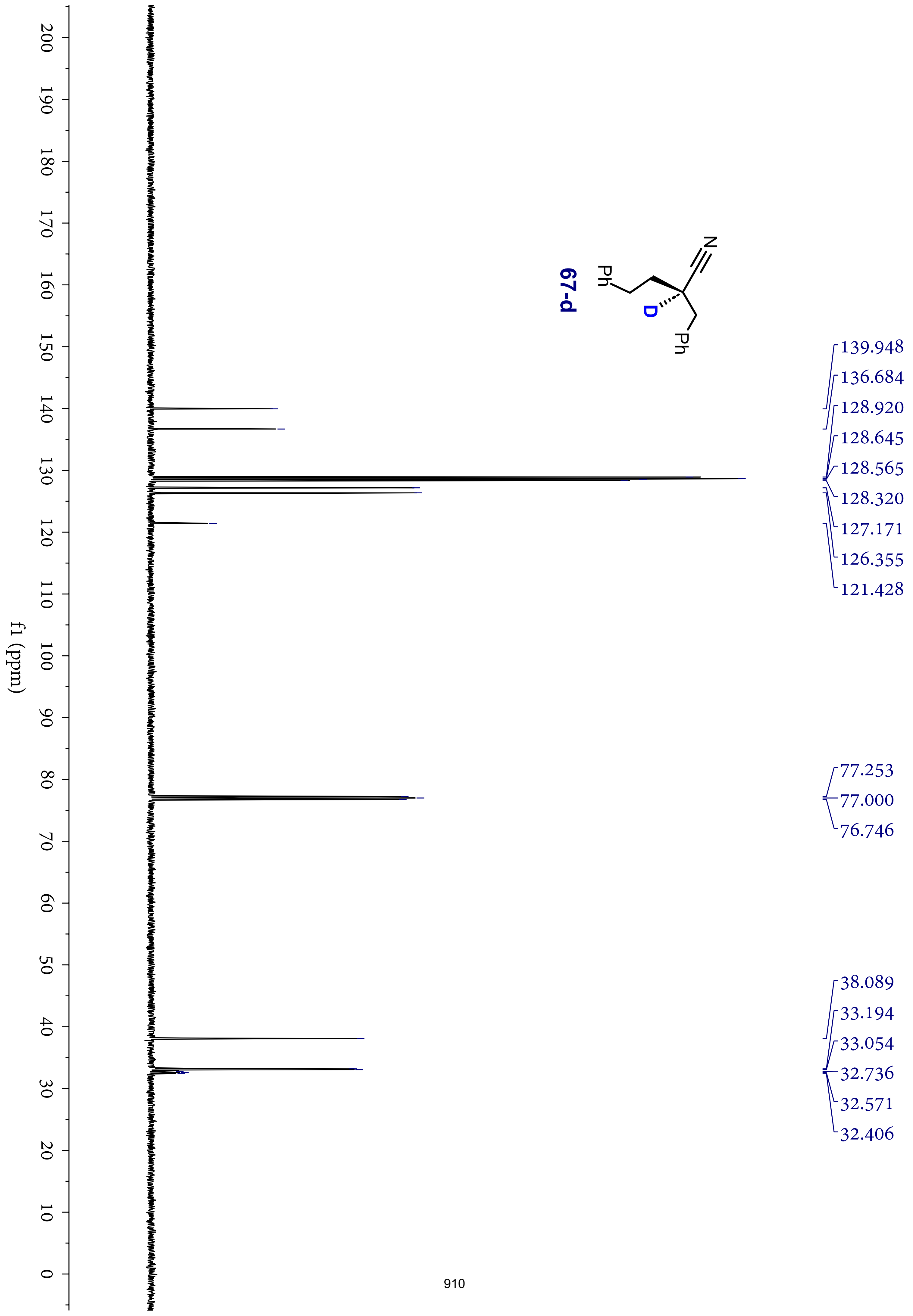

N
D
Ph
Ph
67-d
139.948
136.684
128.920
128.645
128.565
128.320
127.171
126.355
121.428
77.253
77.000
76.746
38.089
33.194
33.054
32.736
32.571
32.406
f1 (ppm)

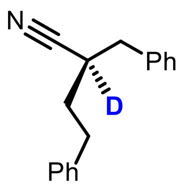

(+)-**67**-d

0.8ml/min

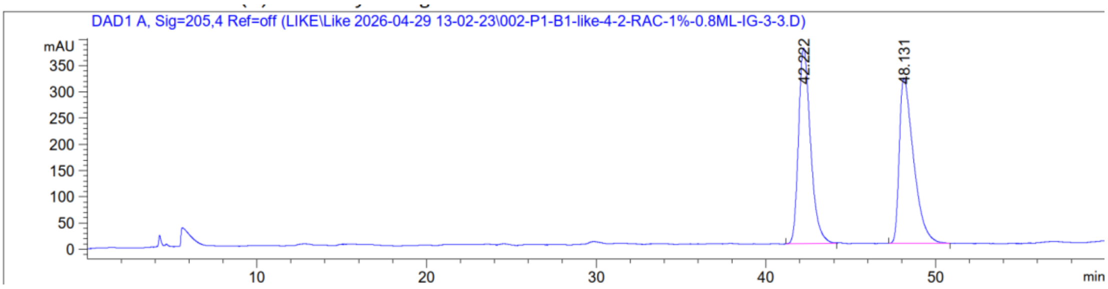

Signal 1: DAD1 A, Sig=205,4 Ref=off

Peak #	RetTime [min]	Type	Width [min]	Area [mAU*s]	Height [mAU]	Area %
1	42.222	BB	0.7406	1.83575e4	372.70892	49.8836
2	48.131	BB	0.8459	1.84432e4	315.55502	50.1164

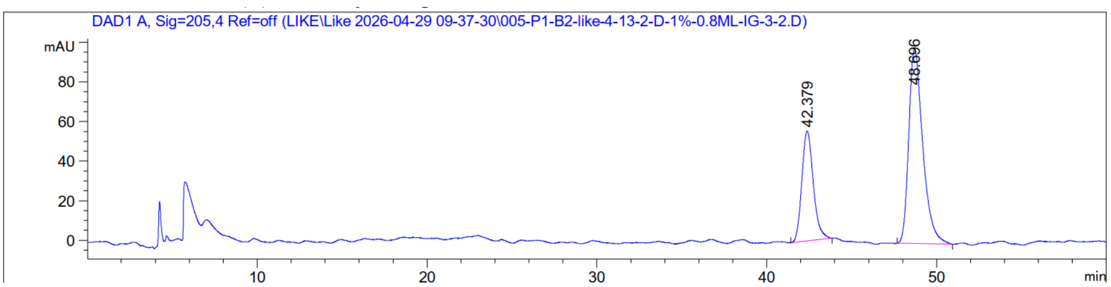

Signal 1: DAD1 A, Sig=205,4 Ref=off

Peak #	RetTime [min]	Type	Width [min]	Area [mAU*s]	Height [mAU]	Area %
1	42.379	BB	0.6702	2603.53442	55.40428	31.6007
2	48.696	BB	0.8152	5635.31543	98.53439	68.3993

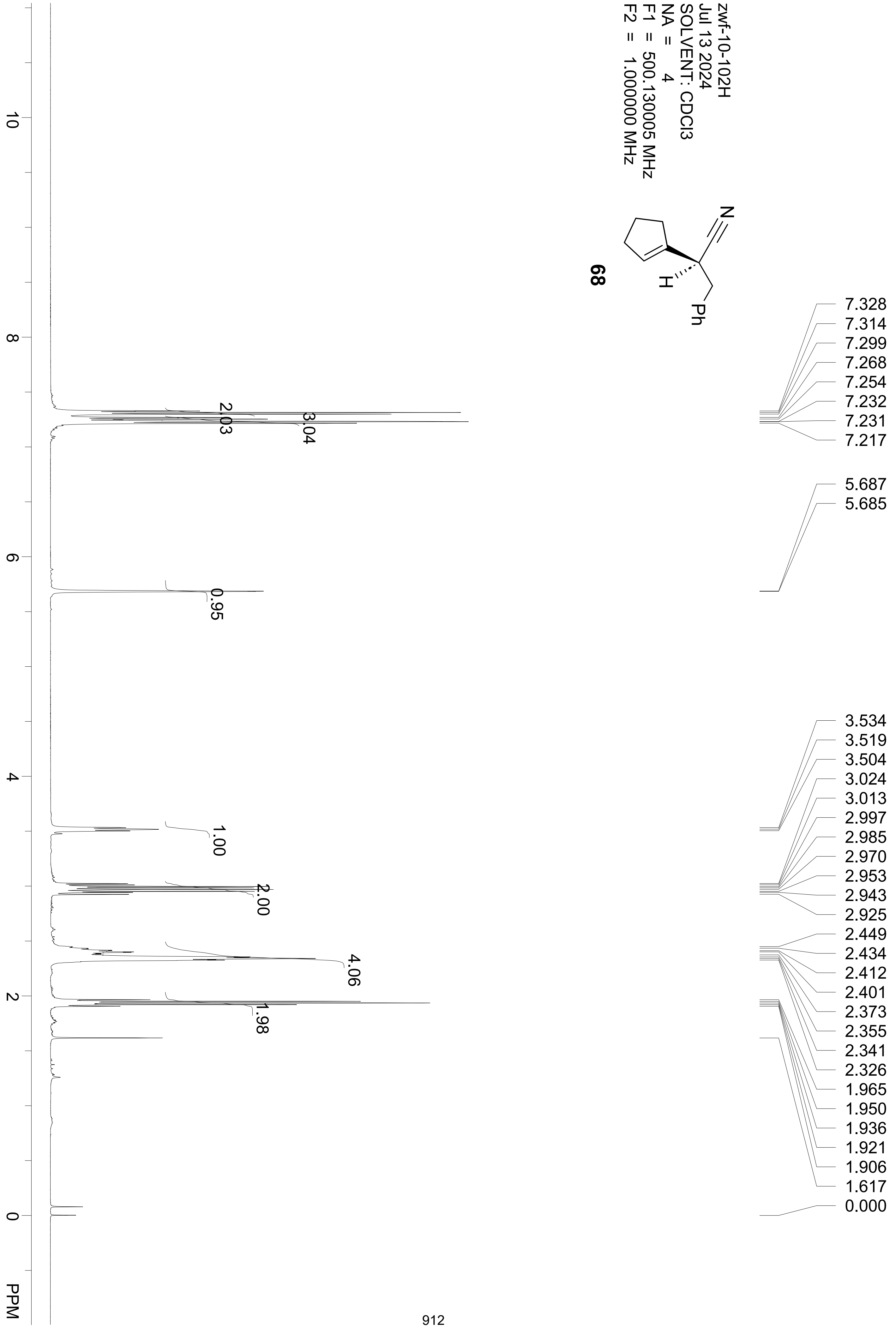
zwf-10-102H
Jul 13 2024
SOLVENT: CDCl3
NA = 4
F1 = 500.130005 MHz
F2 = 1.000000 MHz
N
H
Ph
68
7.328
7.314
7.299
7.268
7.254
7.232
7.231
7.217
5.687
5.685
3.534
3.519
3.504
3.024
3.013
2.997
2.985
2.970
2.953
2.943
2.925
2.449
2.434
2.412
2.401
2.373
2.355
2.341
2.326
1.965
1.950
1.936
1.921
1.906
1.617
0.000
2.03
3.04
0.95
1.00
2.00
4.06
1.98
10
8
6
4
2
0
PPM

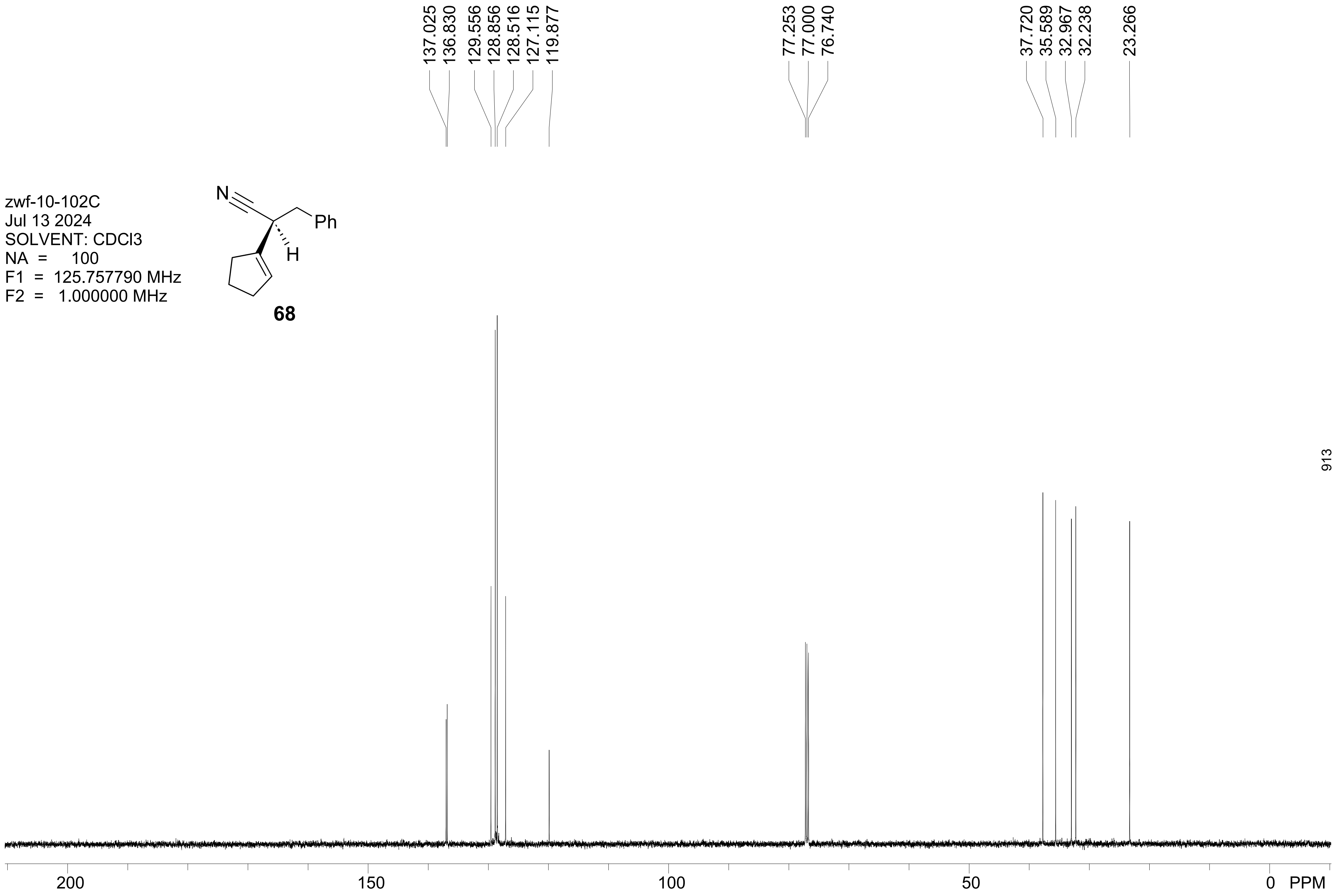
zwf-10-102C
Jul 13 2024
SOLVENT: CDCl3
NA = 100
F1 = 125.757790 MHz
F2 = 1.000000 MHz
N
Ph
H
68
137.025
136.830
129.556
128.856
128.516
127.115
119.877
77.253
77.000
76.740
37.720
35.589
32.967
32.238
23.266
200
150
100
50
0 PPM

Area Percent Report

Agilent Technologies

Sample name: zwf-10-102-IE-99-1-0.8-205

Data file: C:\Users\Public\Documents\ChemStation\1\Data\zwf\zwf-S 2024-07-13 12-02-28\003-P1-C1-zwf-10-102.D

Injection Acquired Date 07/13/2024 13:00:33

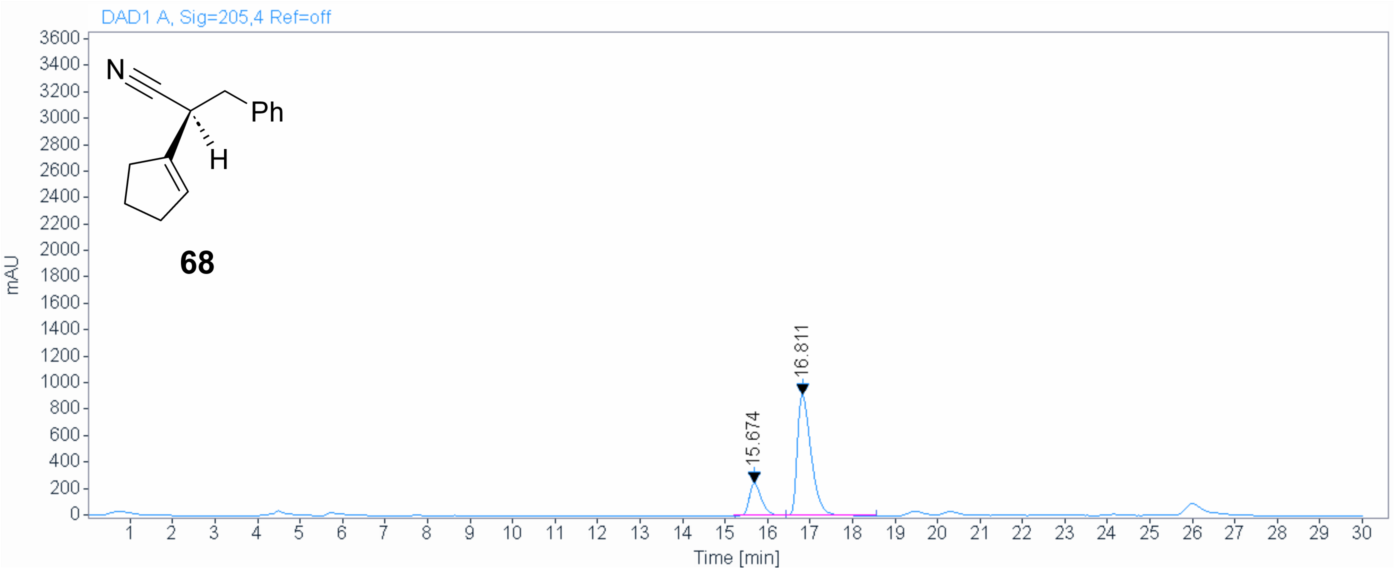

Signal: DAD1 A, Sig=205,4 Ref=off

RT [min]	Width [min]	Height	Area	Area%
15.674	0.3060	245.6491	4928.0146	19.2493
16.811	0.3482	912.9289	20673.0586	80.7507
		Sum	25601.0732	100.0000

Area Percent Report

Sample name: zwf-10-104-rac-IE-99-1-0.8-205

Data file: C:\Users\Public\Documents\ChemStation\1\Data\zwf\zwf-S 2024-07-13 15-16-36\004-P1-C3-zwf-10-104-rac.D

Injection Acquired Date 07/13/2024 16:36:52

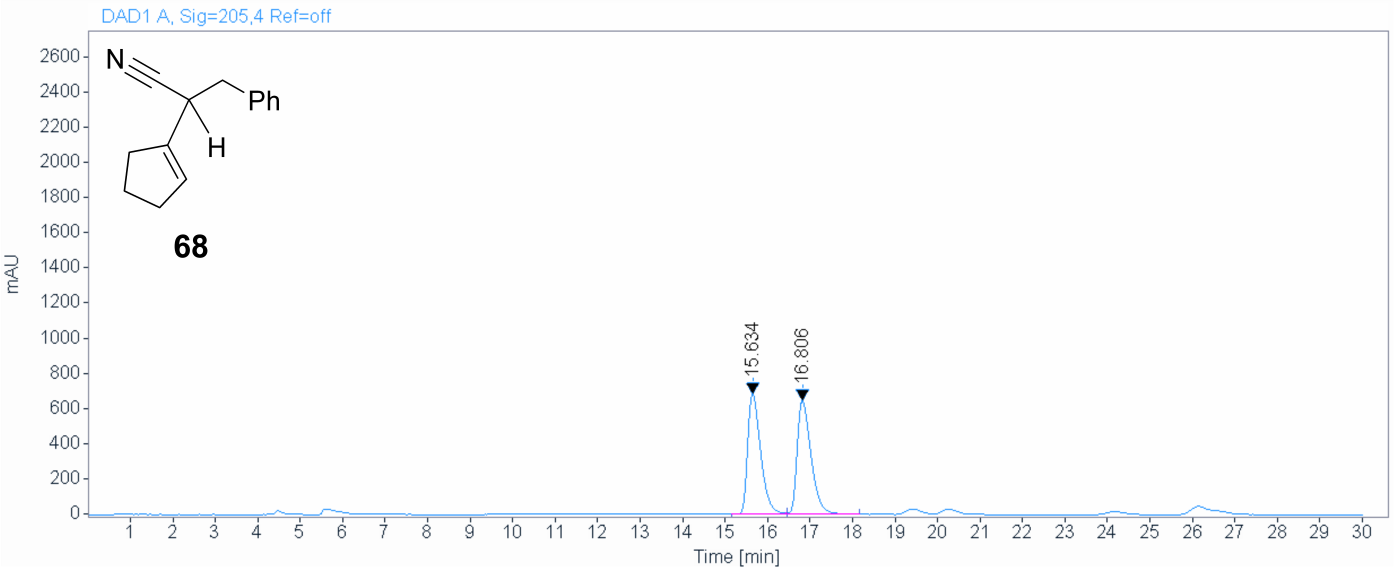

Signal: DAD1 A, Sig=205,4 Ref=off

RT [min]	Width [min]	Height	Area	Area%
15.634	0.3298	684.3140	14653.5107	49.7218
16.806	0.3493	646.6847	14817.4883	50.2782
		Sum	29470.9990	100.0000

zwf-10-104H
Jul 13 2024
SOLVENT: CDCl3
NA = 4
F1 = 500.130005 MHz
F2 = 1.000000 MHz

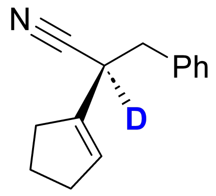

68-d

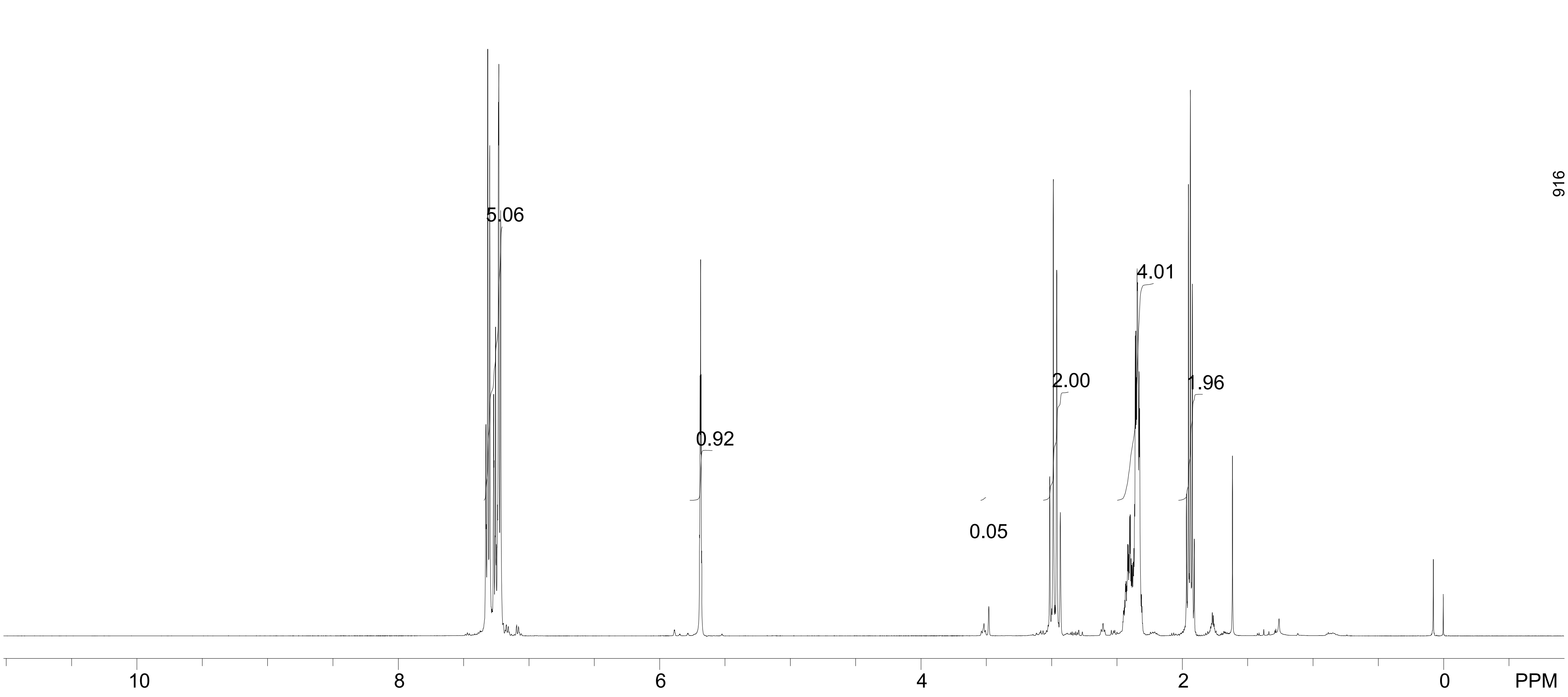

zwf-10-104C
Jul 13 2024
SOLVENT: CDCl3
NA = 100
F1 = 125.757790 MHz
F2 = 1.000000 MHz

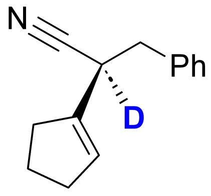

68-d

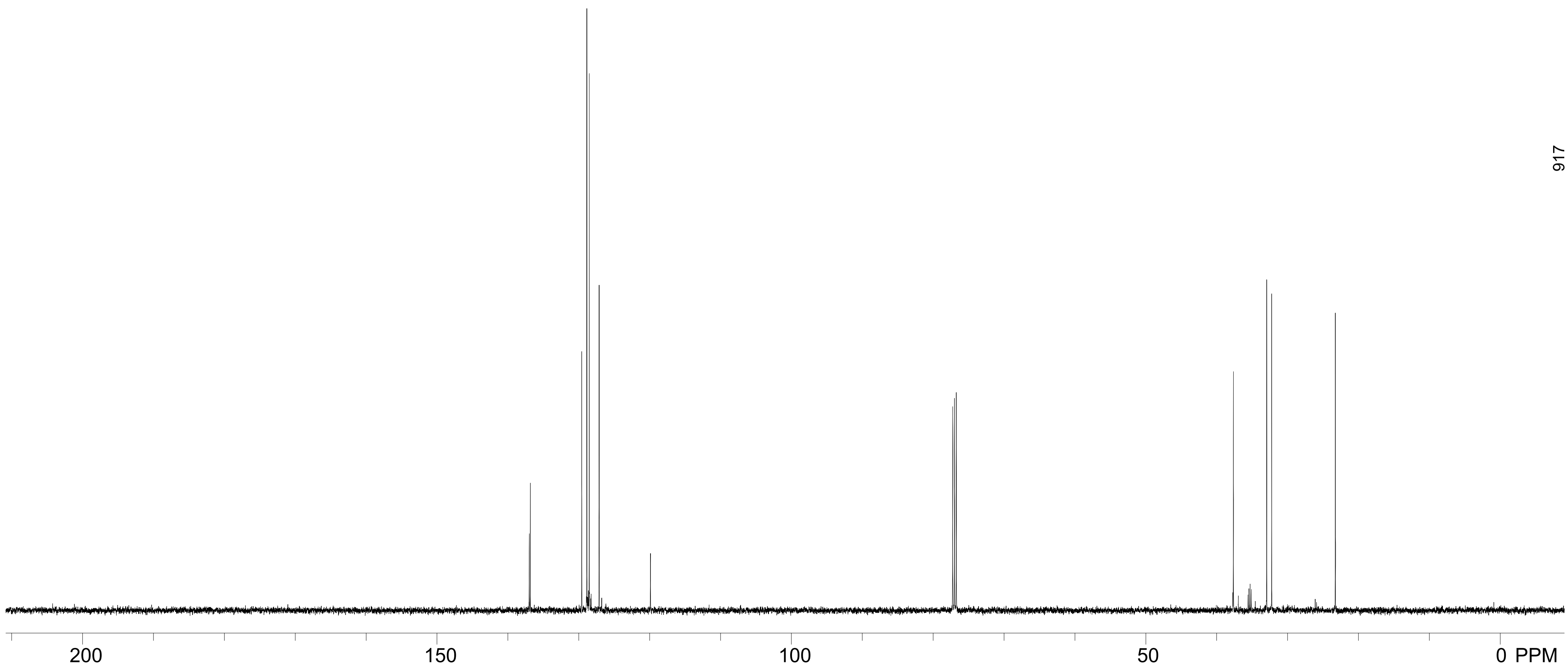

Area Percent Report

Agilent Technologies

Sample name: zwf-10-104-IE-99-1-0.8-205

Data file: C:\Users\Public\Documents\ChemStation\1\Data\zwf\zwf-S 2024-07-13 12-02-28\004-P1-C2-zwf-10-104.D

Injection Acquired Date 07/13/2024 13:31:34

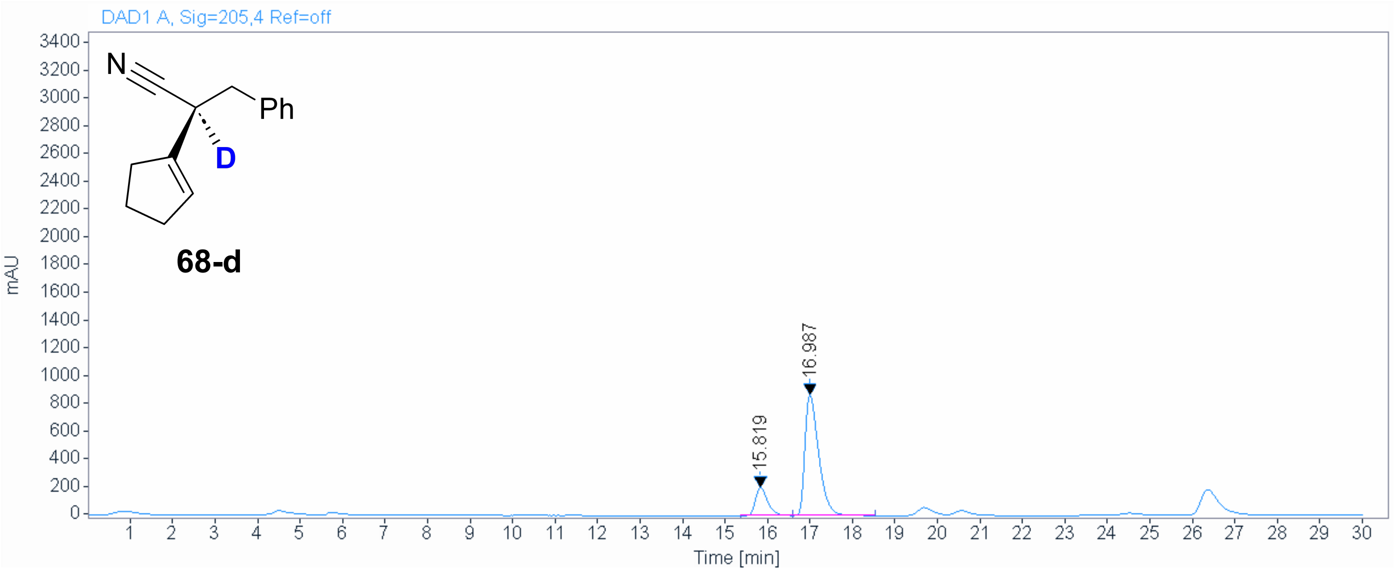

Signal: DAD1 A, Sig=205,4 Ref=off

RT [min]	Width [min]	Height	Area	Area%
15.819	0.3092	202.7239	4086.1482	17.1327
16.987	0.3368	870.8082	19763.7852	82.8673
		Sum	23849.9333	100.0000

Area Percent Report

Agilent Technologies

Sample name: zwf-10-104-rac-IE-99-1-0.8-205

Data file: C:\Users\Public\Documents\ChemStation\1\Data\zwf\zwf-S 2024-07-13 15-16-36\004-P1-C3-zwf-10-104-rac.D

Injection Acquired Date 07/13/2024 16:36:52

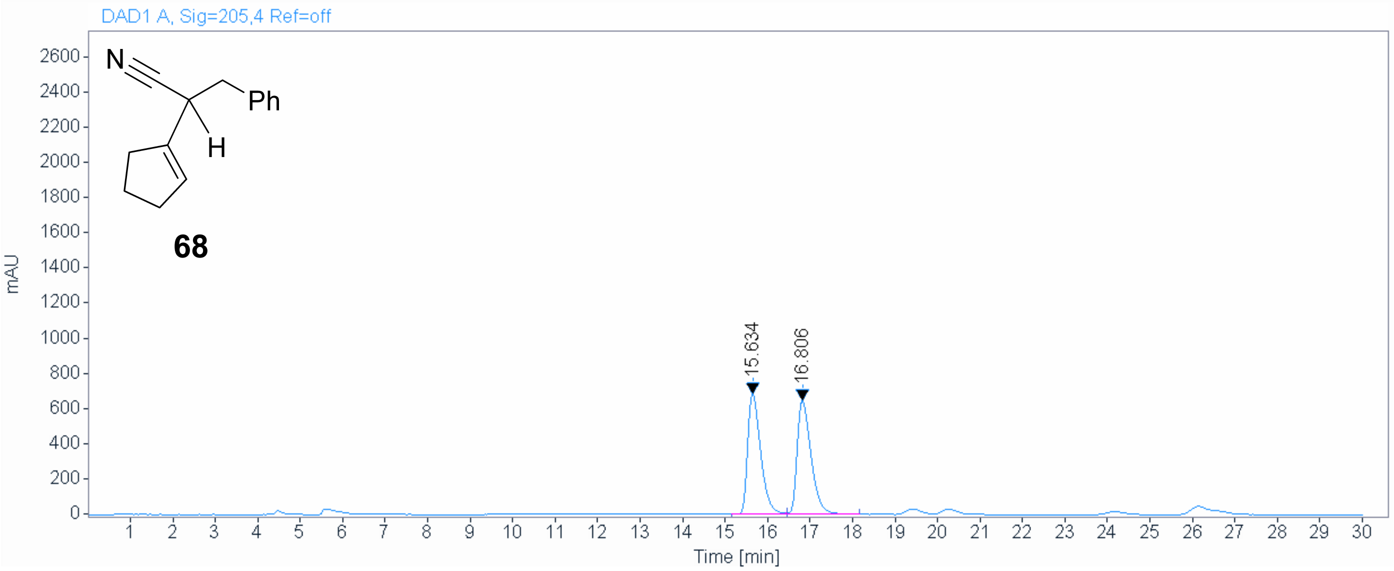

Signal: DAD1 A, Sig=205,4 Ref=off

RT [min]	Width [min]	Height	Area	Area%
15.634	0.3298	684.3140	14653.5107	49.7218
16.806	0.3493	646.6847	14817.4883	50.2782
		Sum	29470.9990	100.0000

zwf-10-070H
Jun 24 2024
SOLVENT: CDCl3
NA = 4
F1 = 500.130005 MHz
F2 = 1.000000 MHz

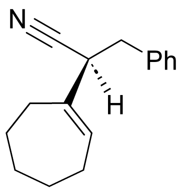

69

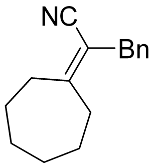

69'

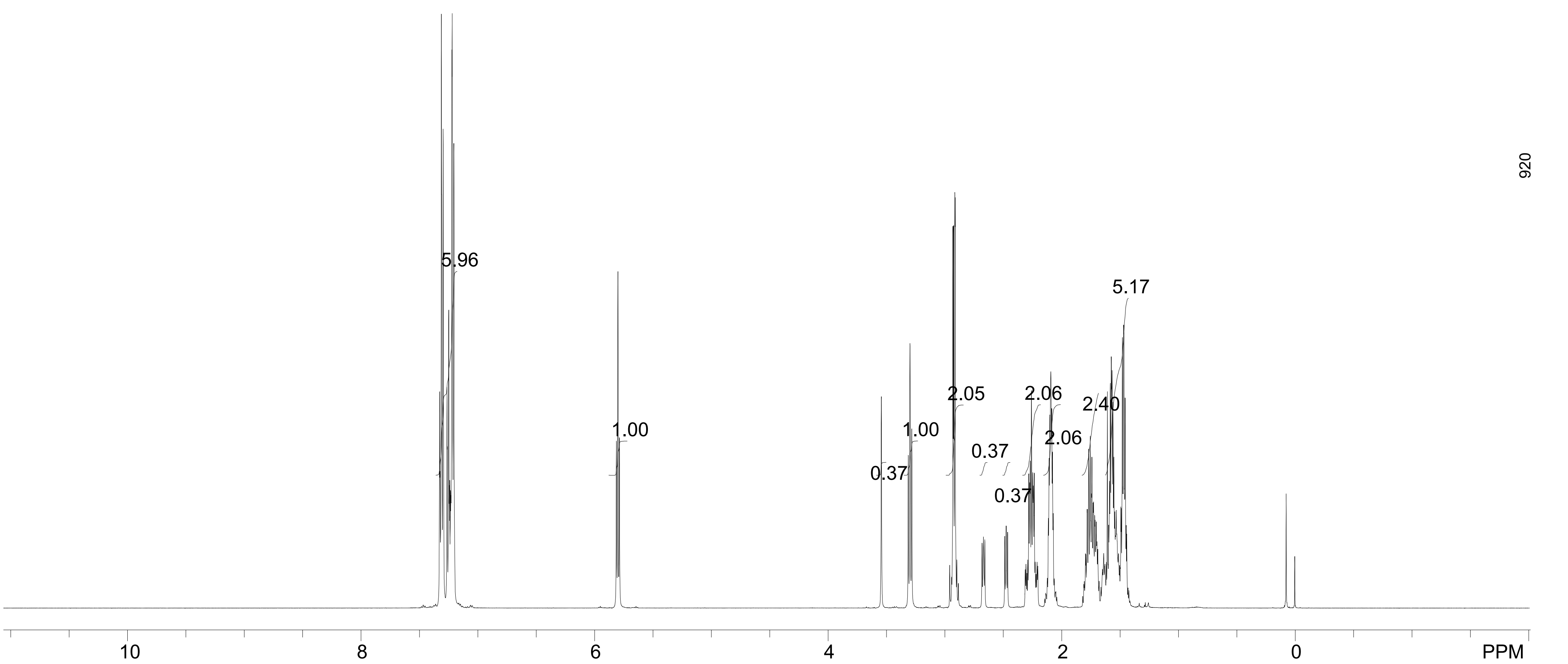

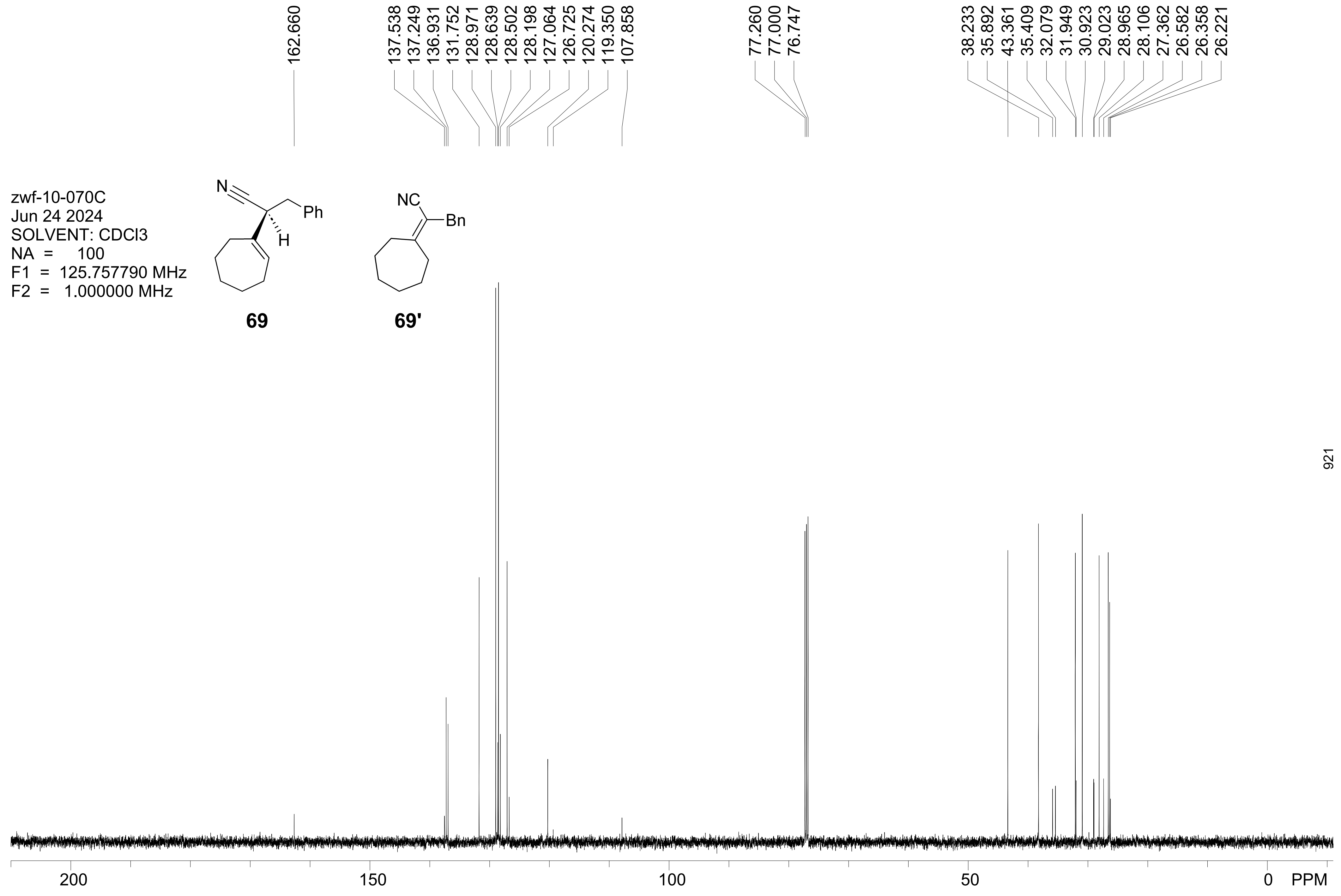
zwf-10-070C
Jun 24 2024
SOLVENT: CDCl3
NA = 100
F1 = 125.757790 MHz
F2 = 1.000000 MHz
N
Ph
H
69
NC
Bn
69'
162.660
137.538
137.249
136.931
131.752
128.971
128.639
128.502
128.198
127.064
126.725
120.274
119.350
107.858
77.260
77.000
76.747
38.233
35.892
43.361
35.409
32.079
31.949
30.923
29.023
28.965
28.106
27.362
26.582
26.358
26.221
200
150
100
50
0 PPM

Area Percent Report

Agilent Technologies

Sample name: zwf-10-070-IE-99-1-0.8-205

Data file: C:\Users\Public\Documents\ChemStation\1\Data\zwf\zwf-S 2024-06-24 12-10-25\006-P1-C2-zwf-10-070.D

Injection Acquired Date 06/24/2024 14:22:31

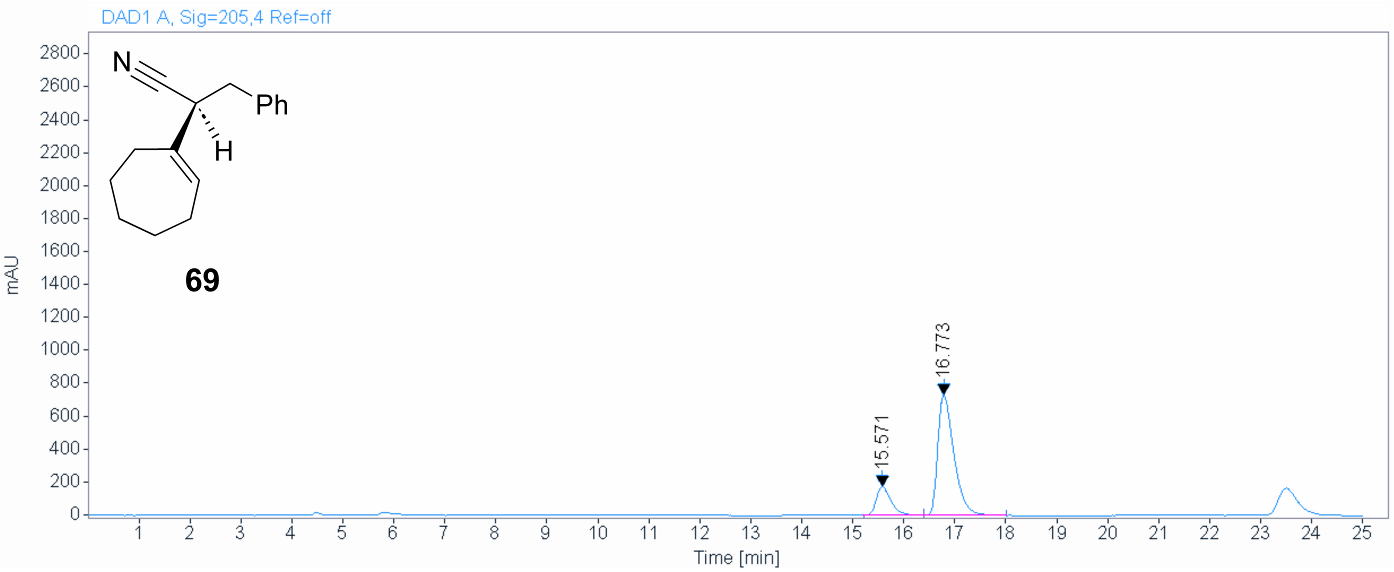

Signal: DAD1 A, Sig=205,4 Ref=off

RT [min]	Width [min]	Height	Area	Area%
15.571	0.3077	173.8230	3482.5217	17.7211
16.773	0.3358	731.7194	16169.2773	82.2789
		Sum	19651.7991	100.0000

Area Percent Report

Agilent Technologies

Sample name: zwf-10-066-rac-IE-99-1-0.8-205

Data file: C:\Users\Public\Documents\ChemStation\1\Data\zwf\zwf-S 2024-06-24 12-10-25\004-P1-C3-zwf-10-066-rac.D

Injection Acquired Date 06/24/2024 13:30:30

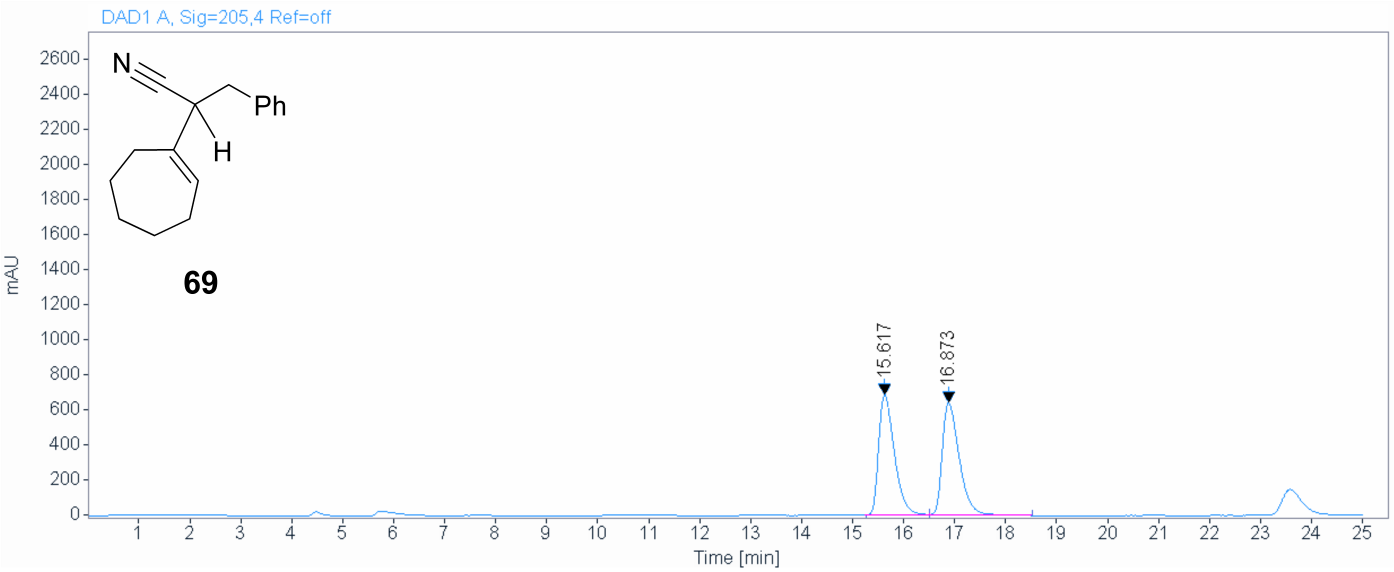

Signal: DAD1 A, Sig=205,4 Ref=off

RT [min]	Width [min]	Height	Area	Area%
15.617	0.3295	685.4927	14780.1338	49.7108
16.873	0.3554	642.6280	14952.0811	50.2892
		Sum	29732.2148	100.0000

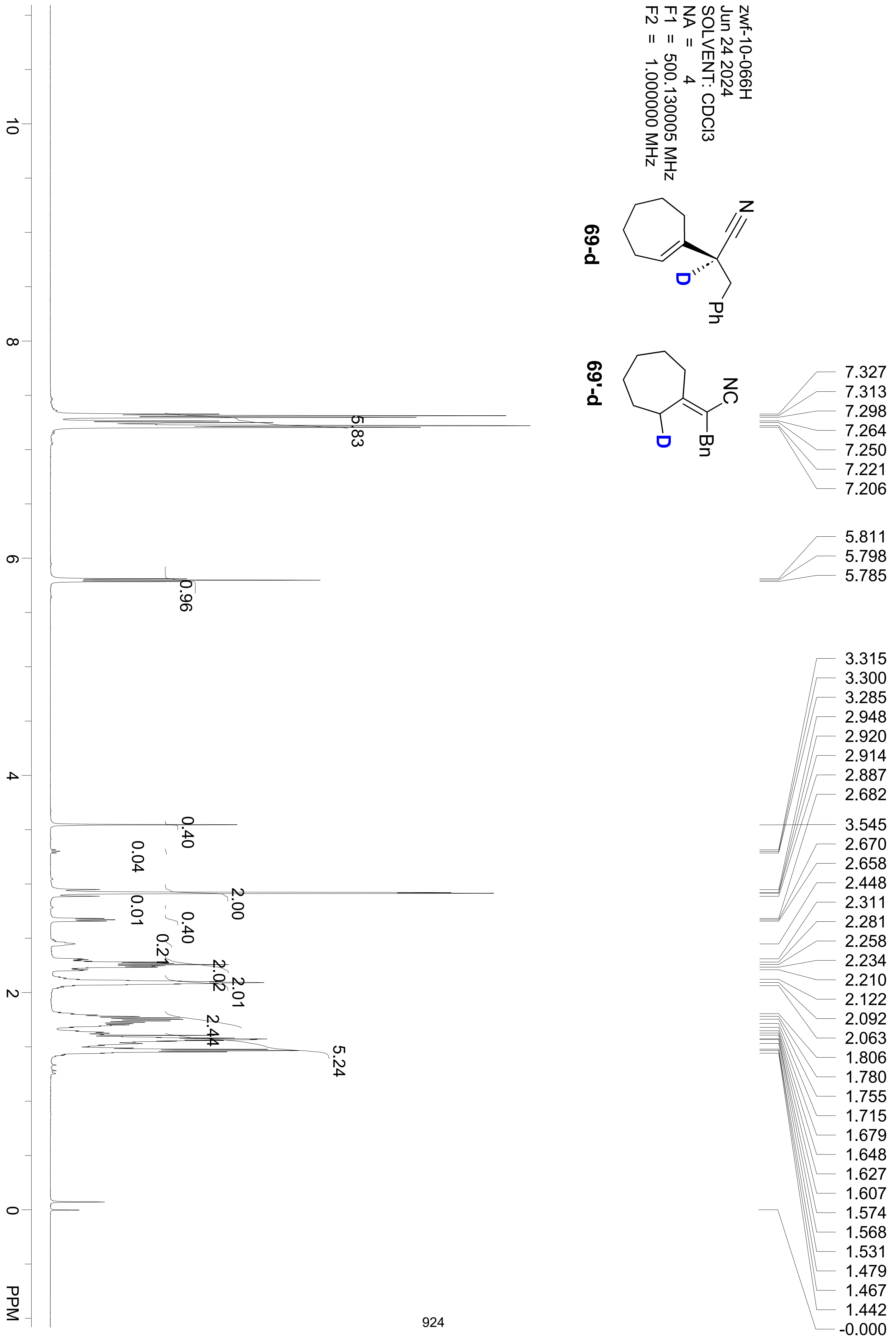
zwf-10-066H
Jun 24 2024
SOLVENT: CDCl3
NA = 4
F1 = 500.130005 MHz
F2 = 1.000000 MHz
N
Ph
D
69-d
NC
Bn
D
69'-d
7.327
7.313
7.298
7.264
7.250
7.221
7.206
5.811
5.798
5.785
3.315
3.300
3.285
2.948
2.920
2.914
2.887
2.682
3.545
2.670
2.658
2.448
2.311
2.281
2.258
2.234
2.210
2.122
2.092
2.063
1.806
1.780
1.755
1.715
1.679
1.648
1.627
1.607
1.574
1.568
1.531
1.479
1.467
1.442
-0.000
5.83
0.96
0.40
0.04
2.00
0.01
0.40
0.21
2.02
2.01
2.44
5.24
10
8
6
4
2
0
PPM

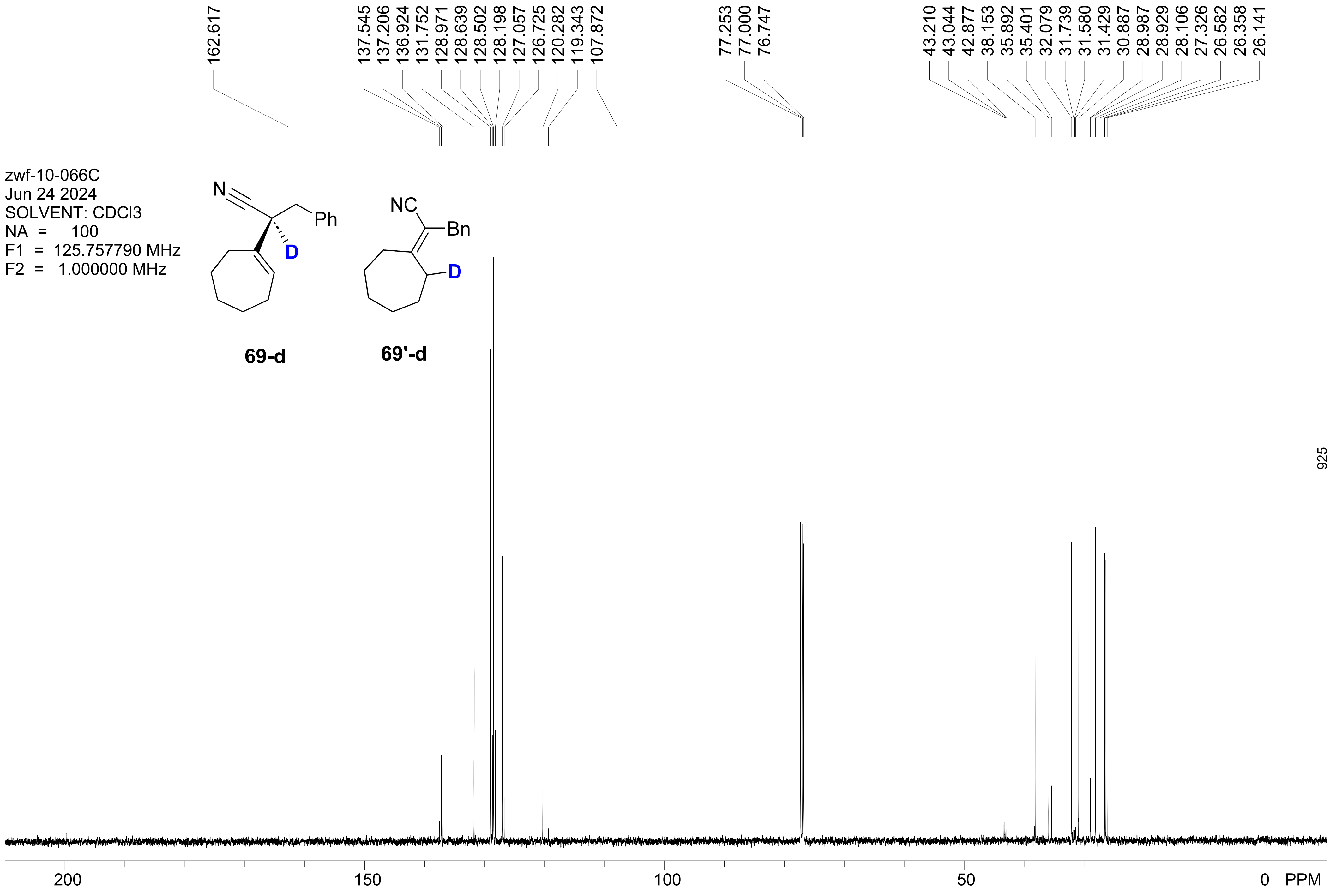
zwf-10-066C
Jun 24 2024
SOLVENT: CDCl3
NA = 100
F1 = 125.757790 MHz
F2 = 1.000000 MHz
N
Ph
D
69-d
NC
Bn
D
69'-d
162.617
137.545
137.206
136.924
131.752
128.971
128.639
128.502
128.198
127.057
126.725
120.282
119.343
107.872
77.253
77.000
76.747
43.210
43.044
42.877
38.153
35.892
35.401
32.079
31.739
31.580
31.429
30.887
28.987
28.929
28.106
27.326
26.582
26.358
26.141
200
150
100
50
0 PPM

Area Percent Report

Agilent Technologies

Sample name: zwf-10-066-IE-99-1-0.8-205

Data file: C:\Users\Public\Documents\ChemStation\1\Data\zwf\zwf-S 2024-06-24 12-10-25\005-P1-C1-zwf-10-066.D

Injection Acquired Date 06/24/2024 13:56:31

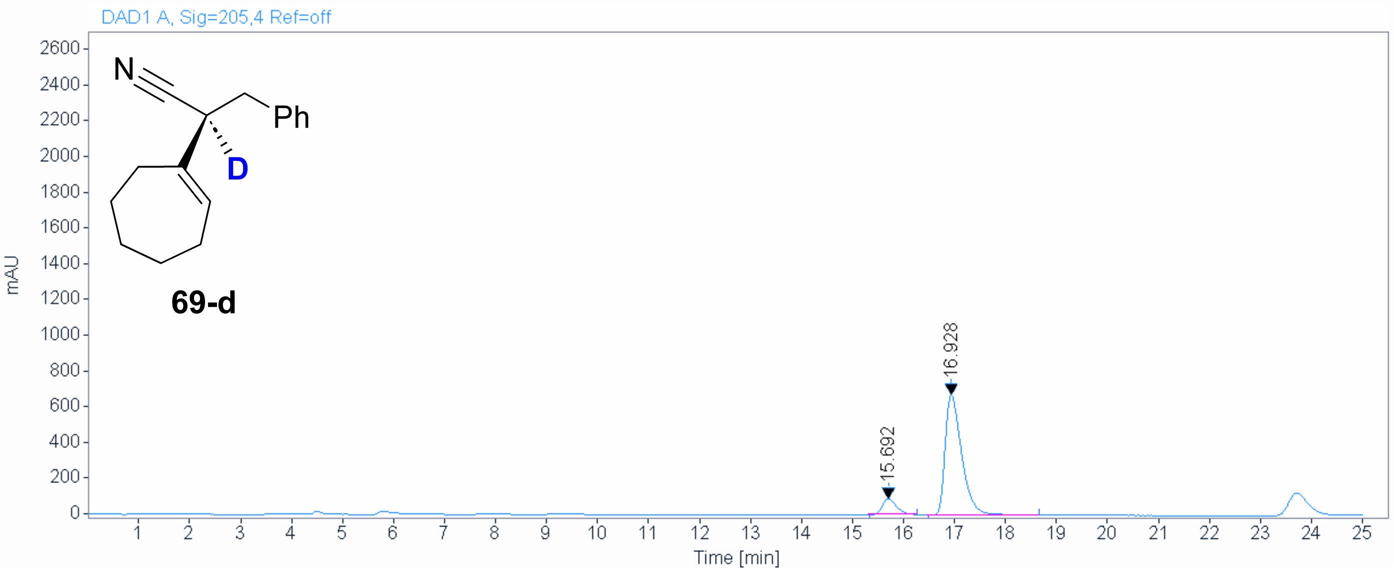

Signal: DAD1 A, Sig=205,4 Ref=off

RT [min]	Width [min]	Height	Area	Area%
15.692	0.3215	88.9129	1715.3192	10.1606
16.928	0.3758	672.6162	15166.7656	89.8394
		Sum	16882.0848	100.0000

Area Percent Report

Agilent Technologies

Sample name: zwf-10-066-rac-IE-99-1-0.8-205

Data file: C:\Users\Public\Documents\ChemStation\1\Data\zwf\zwf-S 2024-06-24 12-10-25\004-P1-C3-zwf-10-066-rac.D

Injection Acquired Date 06/24/2024 13:30:30

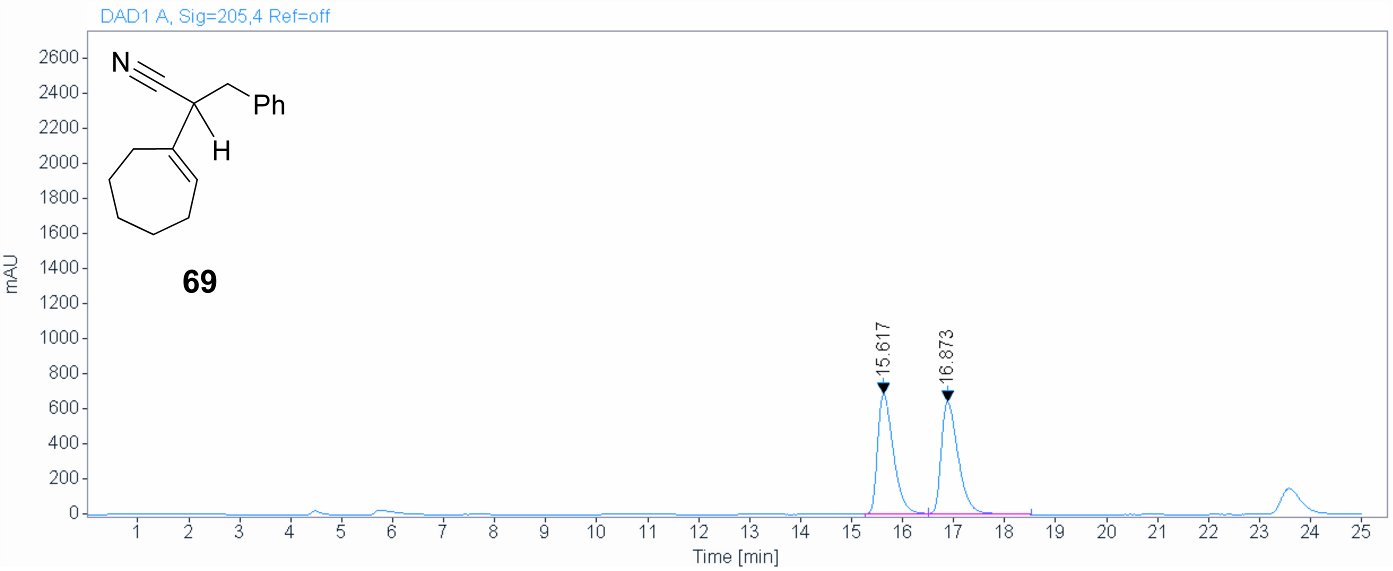

Signal: DAD1 A, Sig=205,4 Ref=off

RT [min]	Width [min]	Height	Area	Area%
15.617	0.3295	685.4927	14780.1338	49.7108
16.873	0.3554	642.6280	14952.0811	50.2892
		Sum	29732.2148	100.0000

zwf-10-071H
Jun 24 2024
SOLVENT: CDCl3
NA = 4
F1 = 500.130005 MHz
F2 = 1.000000 MHz

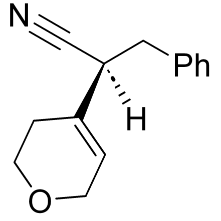

70

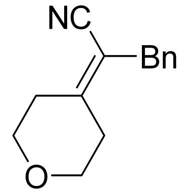

70’

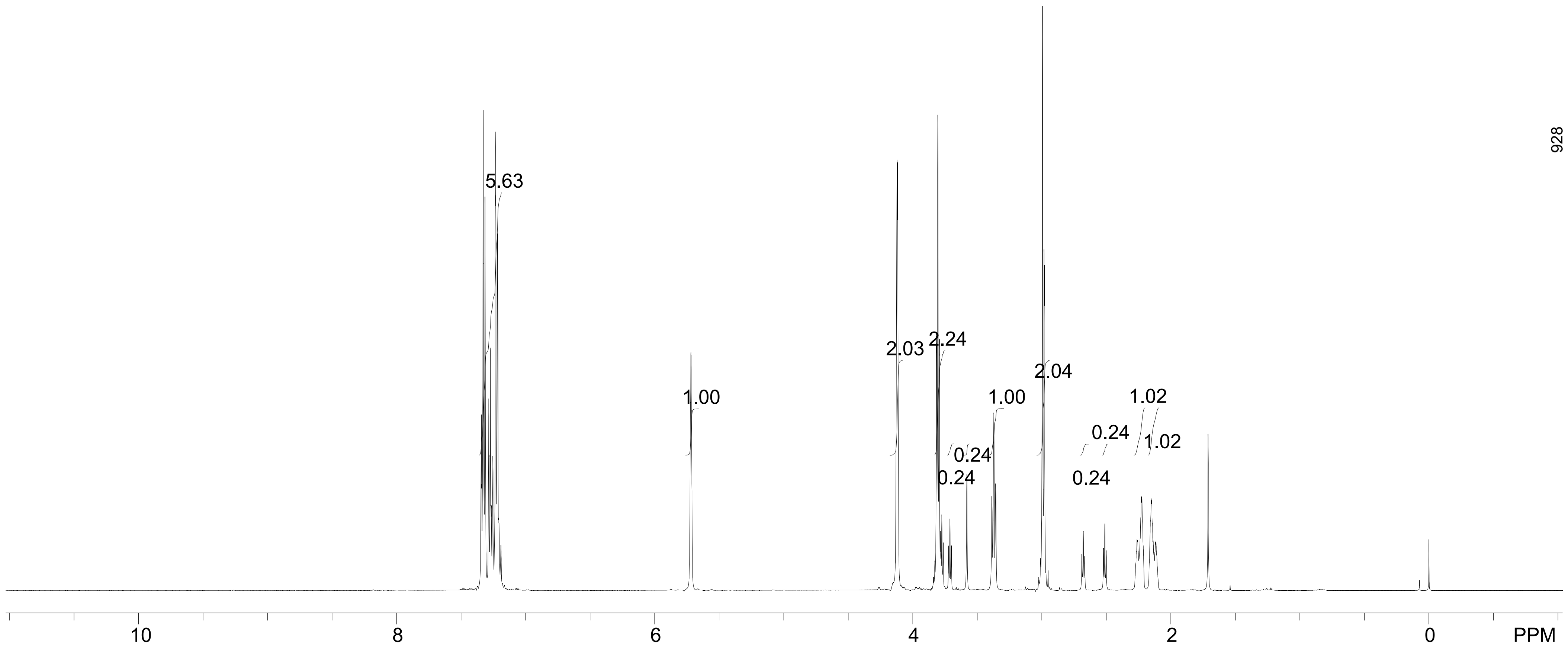

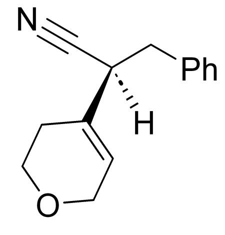
N
Ph
H
O

70

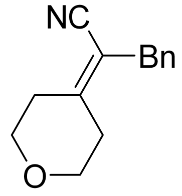
NC
Bn
O

70’

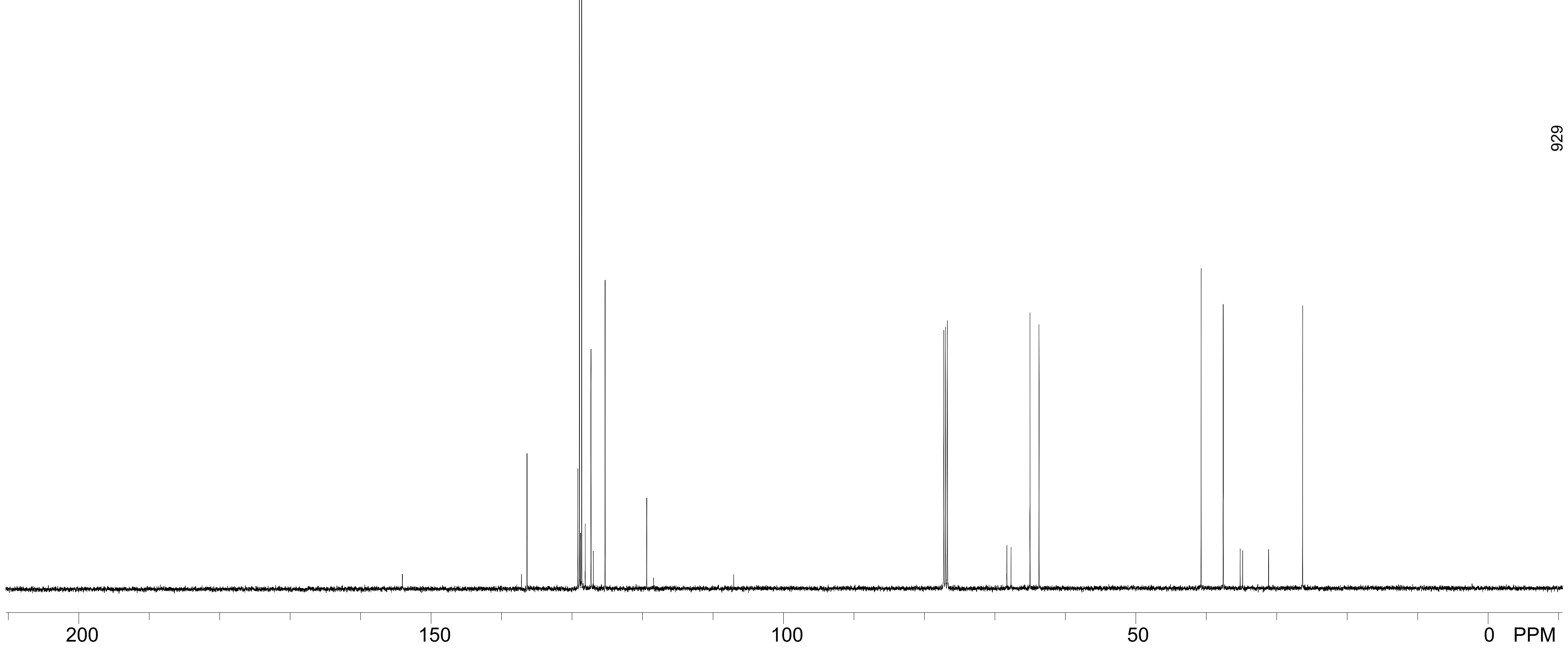
zwf-10-071C
Jun 24 2024
SOLVENT: CDCl3
NA = 100
F1 = 125.757790 MHz
F2 = 1.000000 MHz
154.079
137.177
136.397
129.188
128.964
128.805
128.639
128.141
127.317
126.985
125.324
119.415
118.440
107.078
77.260
77.000
76.747
68.310
67.725
65.031
63.760
40.754
37.619
35.214
34.860
31.183
26.351
200
150
100
50
0 PPM

Area Percent Report

Agilent Technologies

Sample name: zwf-10-071-IE-98-2-0.8-205

Data file: C:\Users\Public\Documents\ChemStation\1\Data\zwf\zwf-S 2024-06-24 12-10-25\009-P1-C5-zwf-10-071.D

Injection Acquired Date 06/24/2024 15:55:35

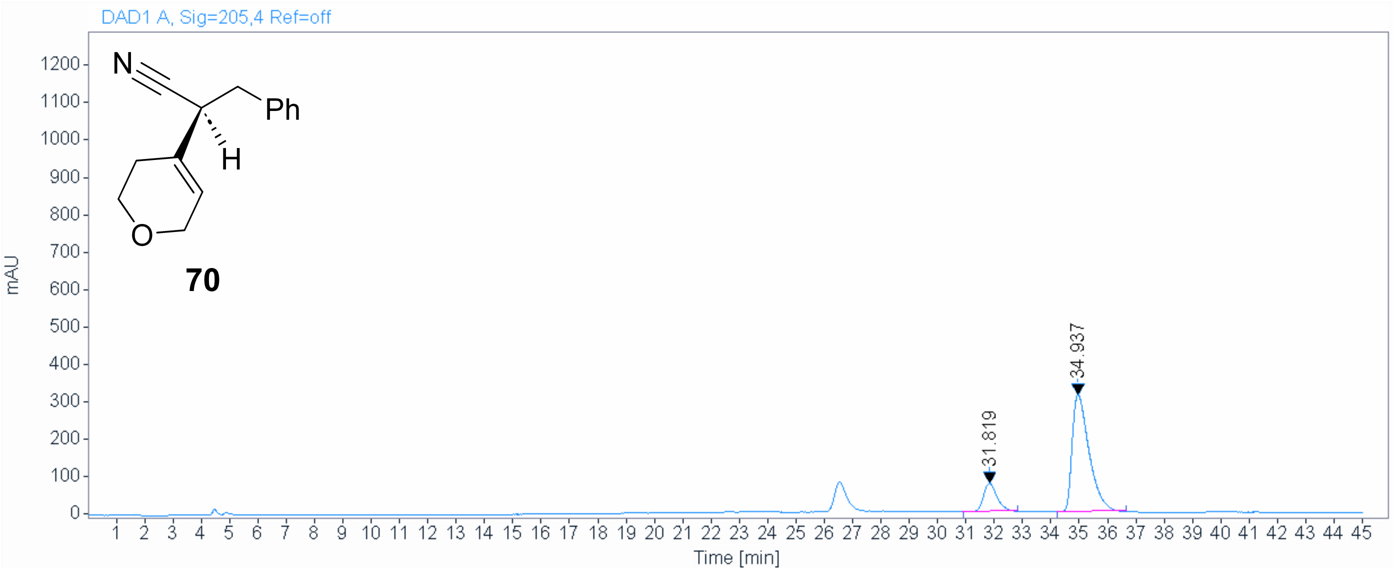

Signal: DAD1 A, Sig=205,4 Ref=off

RT [min]	Width [min]	Height	Area	Area%
31.819	0.4578	76.0814	2581.0134	16.5367
34.937	0.6026	312.3535	13026.7764	83.4633
		Sum	15607.7898	100.0000

Area Percent Report

Agilent Technologies

Sample name: zwf-10-067-rac-IE-98-2-0.8-205

Data file: C:\Users\Public\Documents\ChemStation\1\Data\zwf\zwf-S 2024-06-24 12-10-25\010-P1-C6-zwf-10-067-rac.D

Injection Acquired Date 06/24/2024 16:41:41

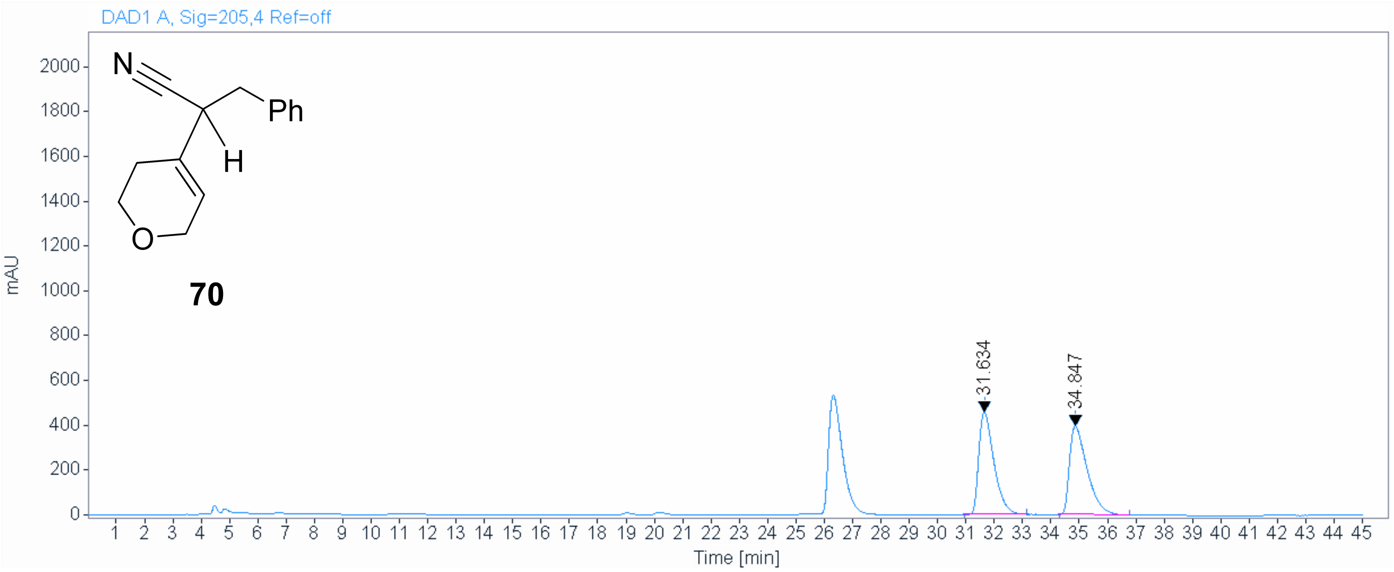

Signal: DAD1 A, Sig=205,4 Ref=off

RT [min]	Width [min]	Height	Area	Area%
31.634	0.5353	457.9002	17086.4160	49.7170
34.847	0.6138	397.0473	17280.9355	50.2830
		Sum	34367.3516	100.0000

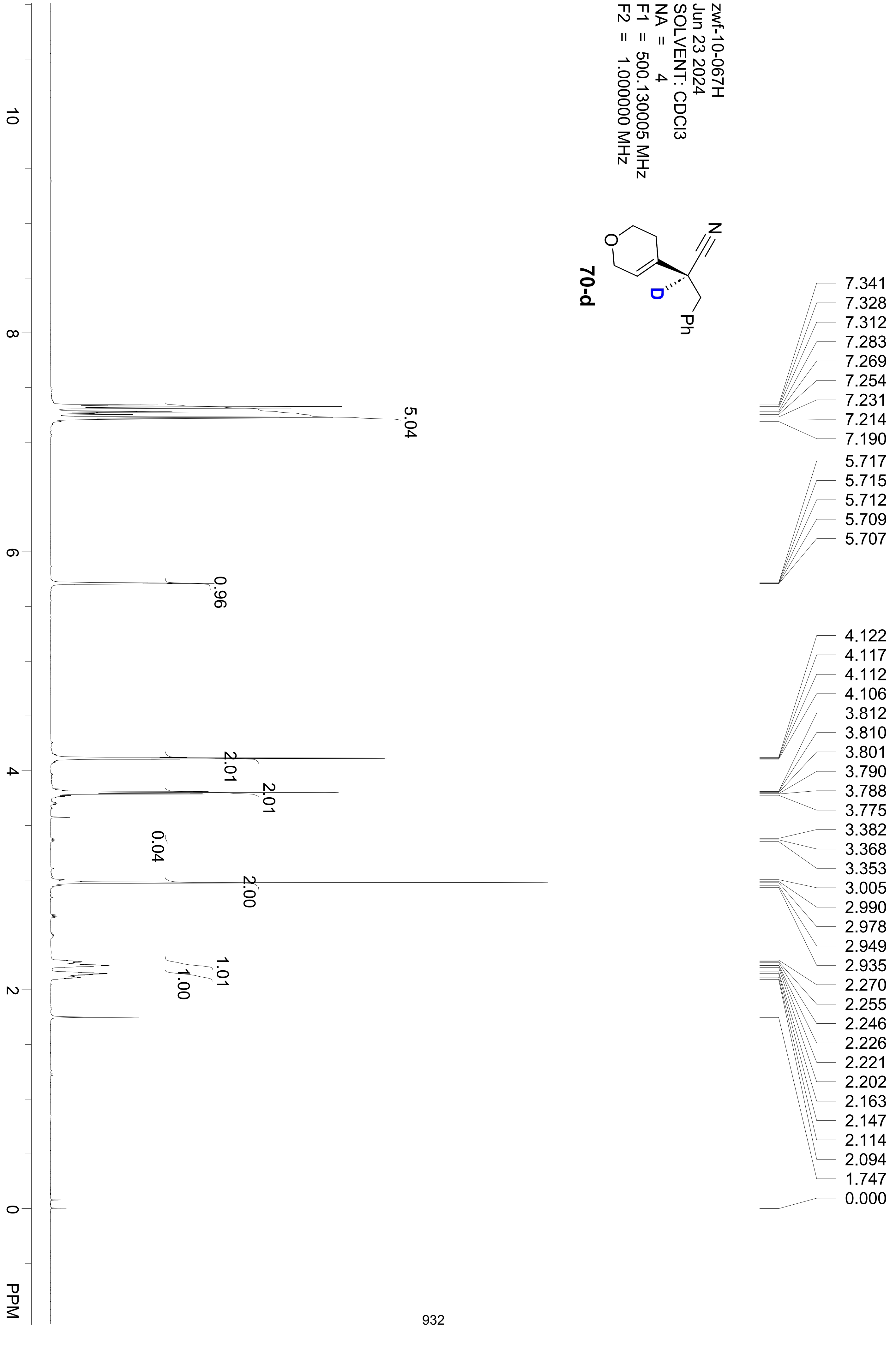
zwf-10-067H
Jun 23 2024
SOLVENT: CDCl3
NA = 4
F1 = 500.130005 MHz
F2 = 1.000000 MHz
N
D
Ph
O
70-d
7.341
7.328
7.312
7.283
7.269
7.254
7.231
7.214
7.190
5.717
5.715
5.712
5.709
5.707
4.122
4.117
4.112
4.106
3.812
3.810
3.801
3.790
3.788
3.775
3.382
3.368
3.353
3.005
2.990
2.978
2.949
2.935
2.270
2.255
2.246
2.226
2.221
2.202
2.163
2.147
2.114
2.094
1.747
0.000
5.04
0.96
2.01
2.01
0.04
2.00
1.01
1.00
10
8
6
4
2
0
PPM

zwf-10-067C
Jun 23 2024
SOLVENT: CDCl3
NA = 100
F1 = 125.757790 MHz
F2 = 1.000000 MHz

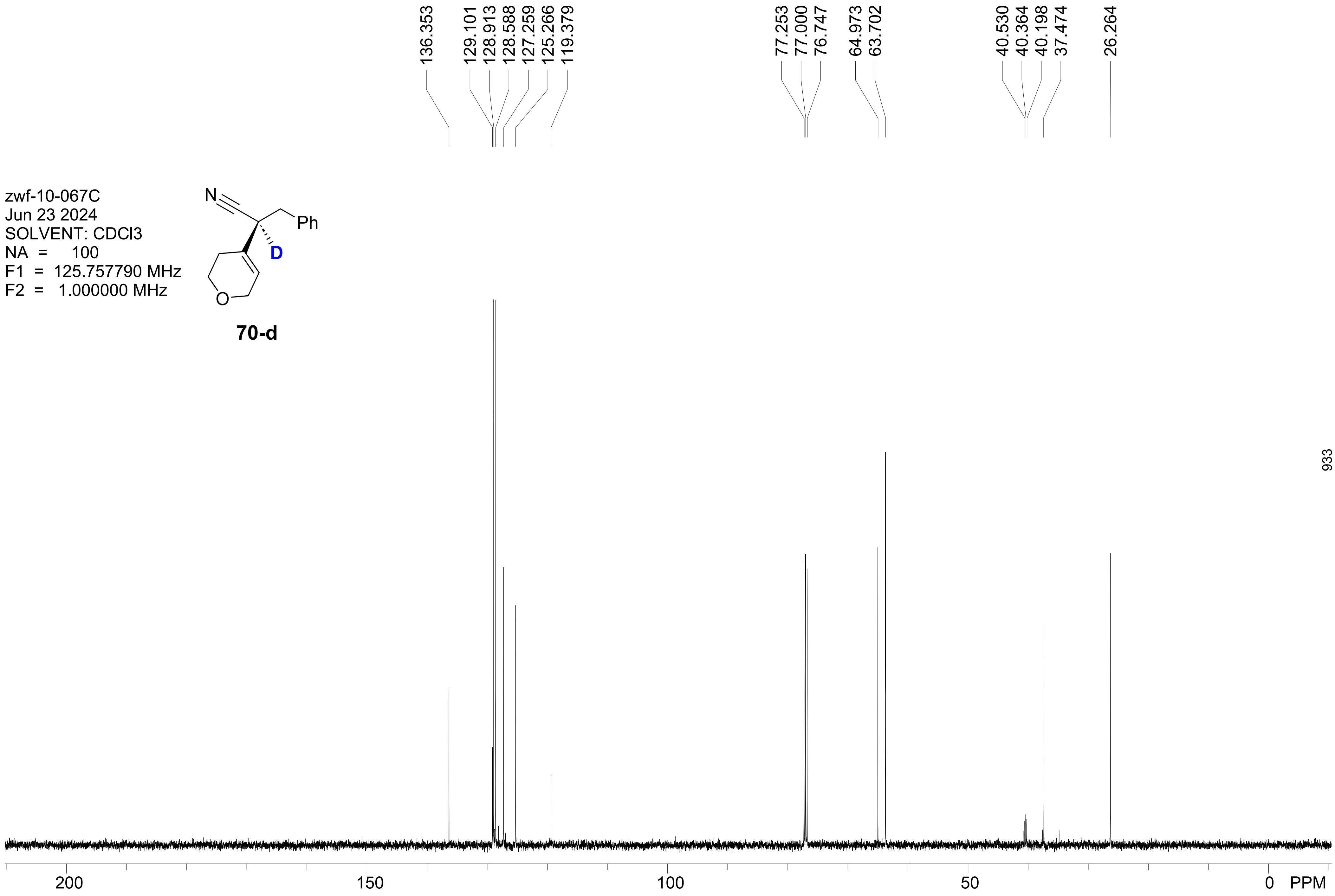

Area Percent Report

Agilent Technologies

Sample name: zwf-10-067-IE-98-2-0.8-205

Data file: C:\Users\Public\Documents\ChemStation\1\Data\zwf\zwf-S 2024-06-24 12-10-25\008-P1-C4-zwf-10-067.D

Injection Acquired Date 06/24/2024 15:09:34

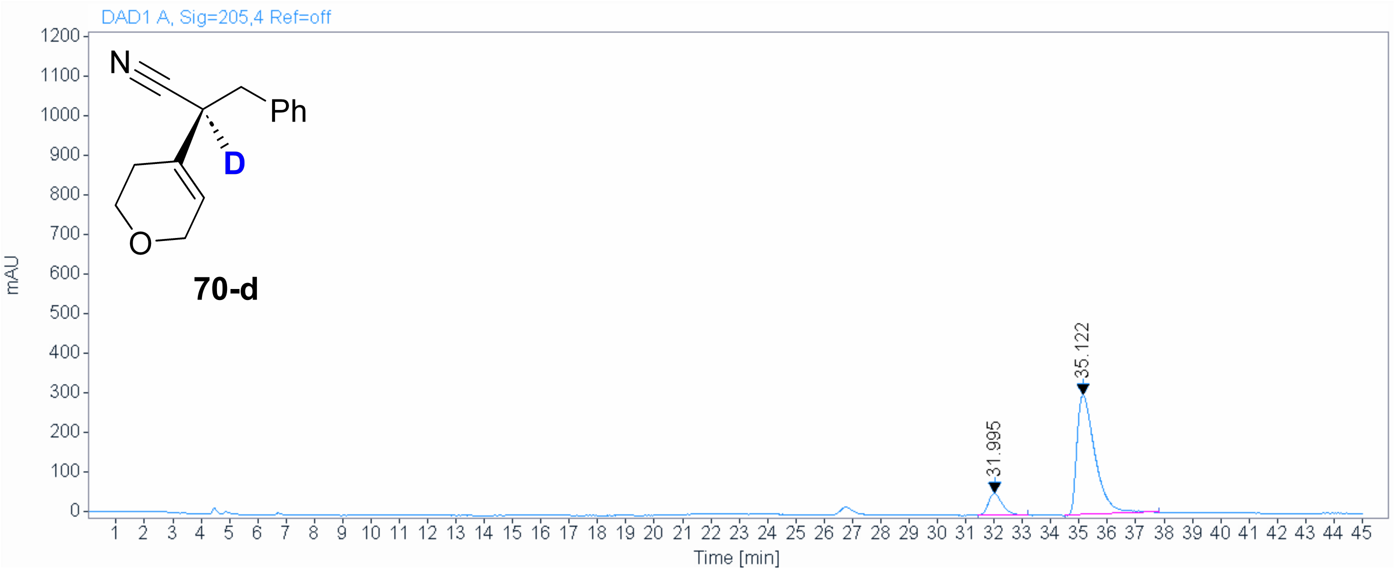

Signal: DAD1 A, Sig=205,4 Ref=off

RT [min]	Width [min]	Height	Area	Area%
31.995	0.4298	54.9103	1881.2496	12.0595
35.122	0.7523	303.9154	13718.4590	87.9405
		Sum	15599.7086	100.0000

Area Percent Report

Agilent Technologies

Sample name: zwf-10-067-rac-IE-98-2-0.8-205

Data file: C:\Users\Public\Documents\ChemStation\1\Data\zwf\zwf-S 2024-06-24 12-10-25\010-P1-C6-zwf-10-067-rac.D

Injection Acquired Date 06/24/2024 16:41:41

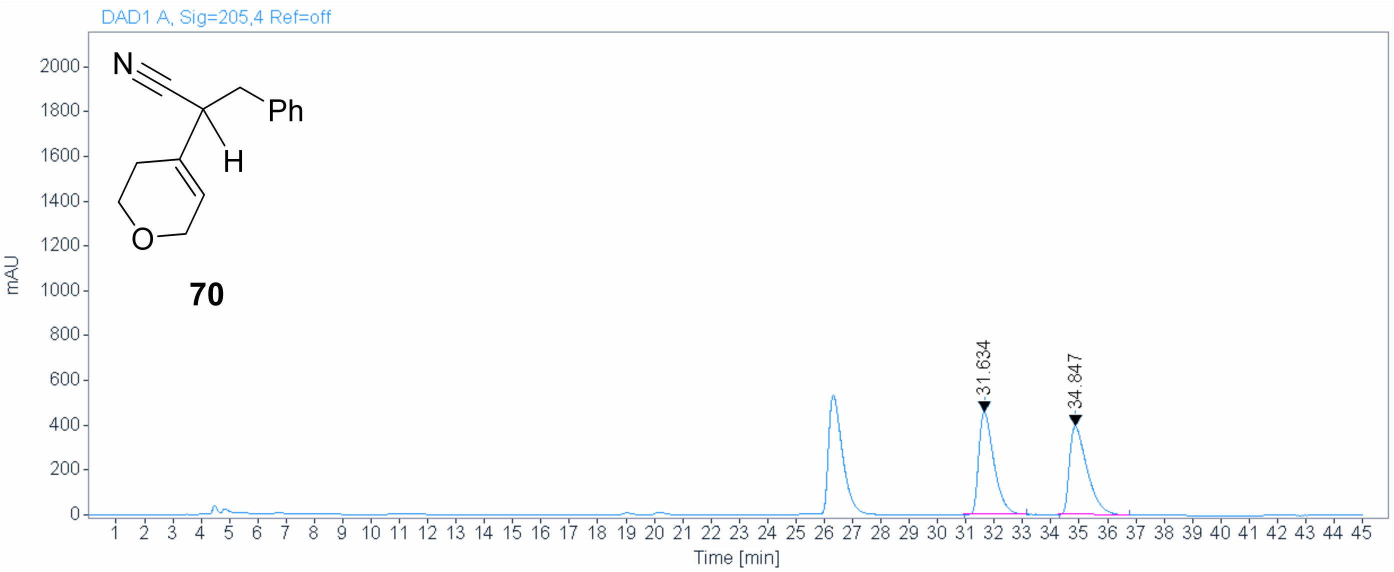

Signal: DAD1 A, Sig=205,4 Ref=off

RT [min]	Width [min]	Height	Area	Area%
31.634	0.5353	457.9002	17086.4160	49.7170
34.847	0.6138	397.0473	17280.9355	50.2830
		Sum	34367.3516	100.0000

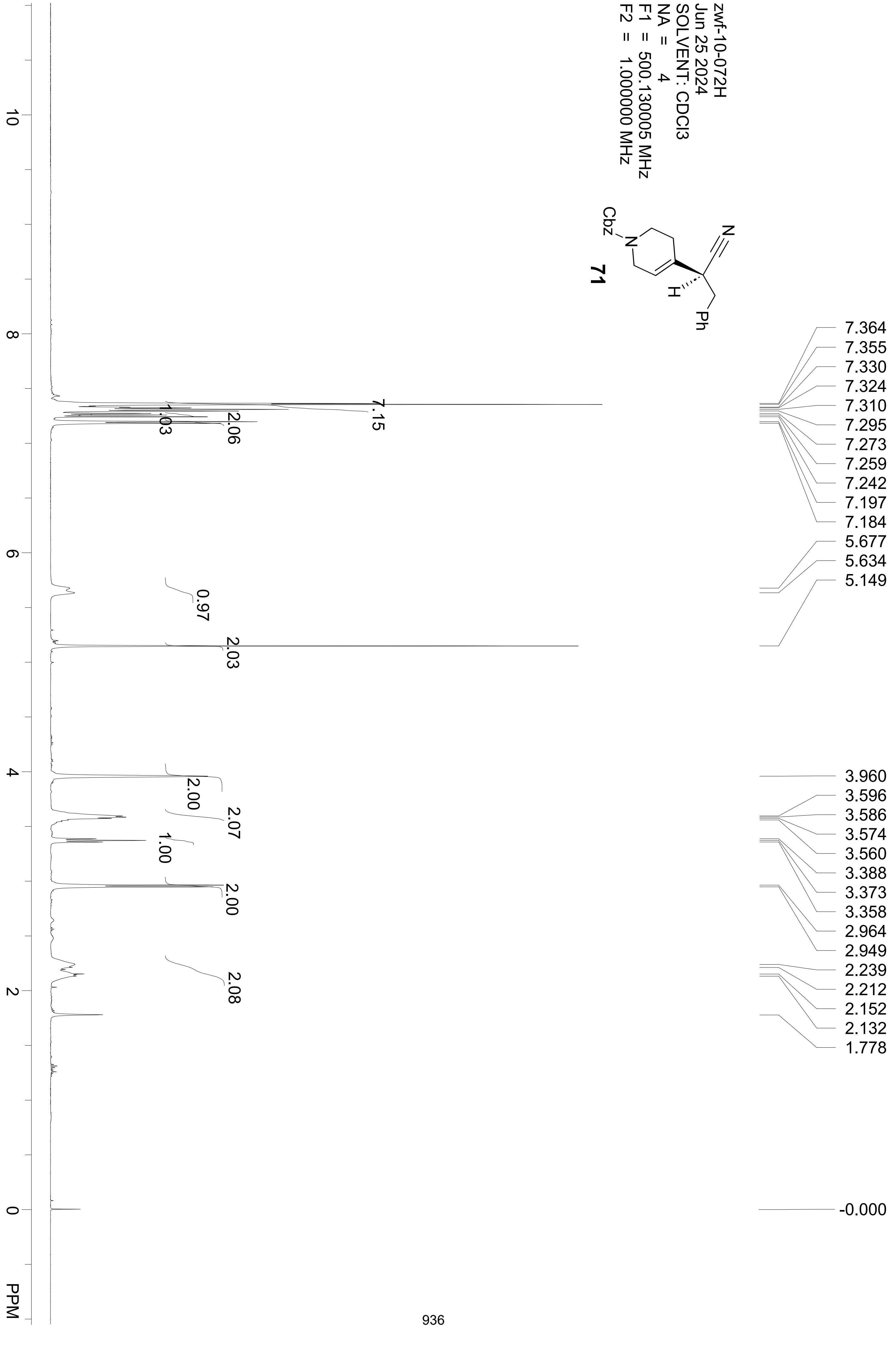
zwf-10-072H
Jun 25 2024
SOLVENT: CDCl3
NA = 4
F1 = 500.130005 MHz
F2 = 1.000000 MHz
Cbz
N
H
Ph
71
7.364
7.355
7.330
7.324
7.310
7.295
7.273
7.259
7.242
7.197
7.184
5.677
5.634
5.149
3.960
3.596
3.586
3.574
3.560
3.388
3.373
3.358
2.964
2.949
2.239
2.212
2.152
2.132
1.778
-0.000
7.15
1.03
2.06
0.97
2.03
2.00
2.07
1.00
2.00
2.08
10
8
6
4
2
0
PPM

zwf-10-072C
Jun 25 2024
SOLVENT: CDCl3
NA = 150
F1 = 125.757790 MHz
F2 = 1.000000 MHz

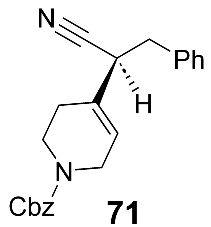

71

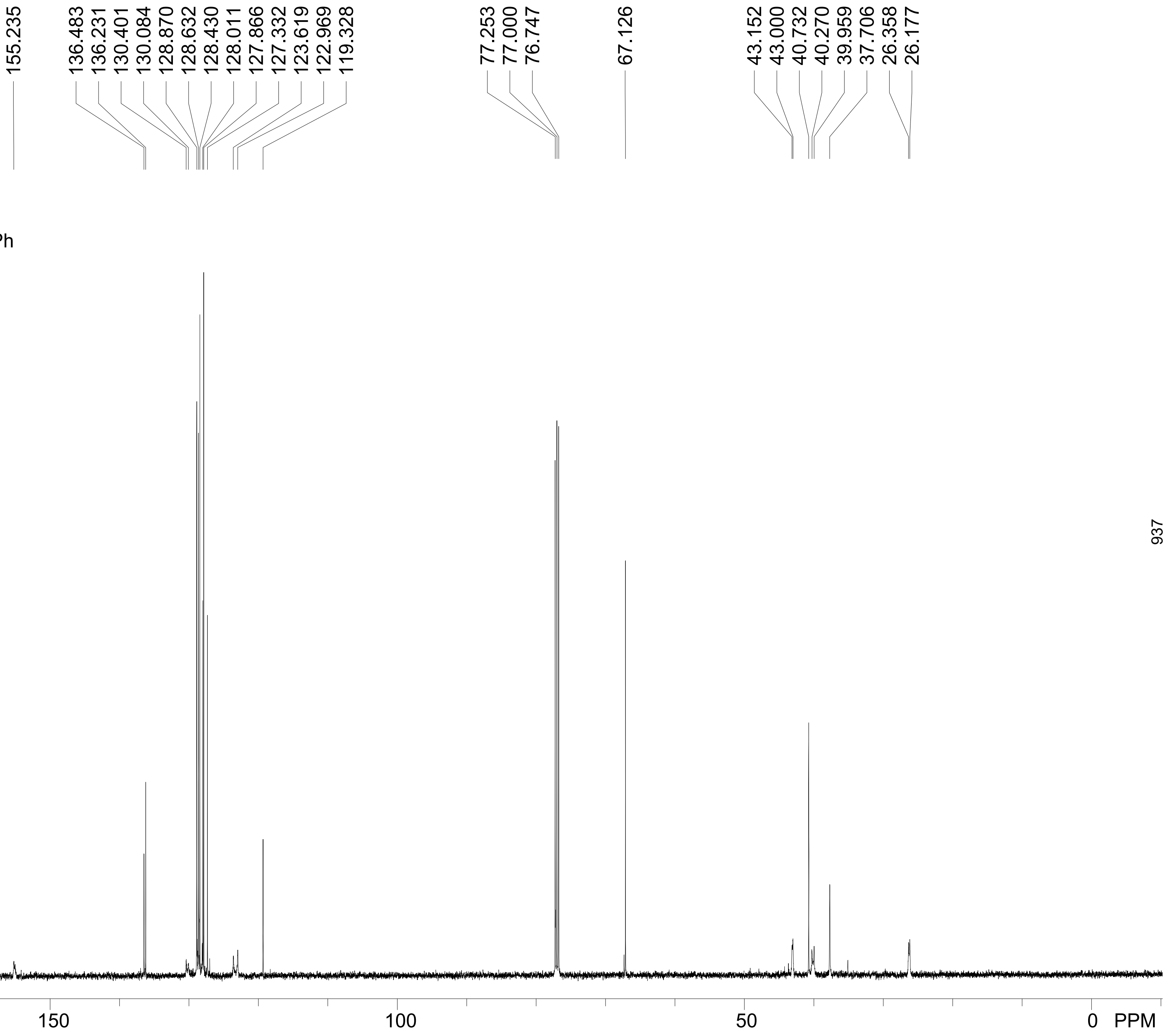

Area Percent Report

Agilent Technologies

Sample name: zwf-10-072-IE-80-20-0.8-205

Data file: C:\Users\Public\Documents\ChemStation\1\Data\zwf\zwf-S 2024-06-25 11-52-53\009-P1-C6-zwf-10-072.D

Injection Acquired Date 06/25/2024 15:38:19

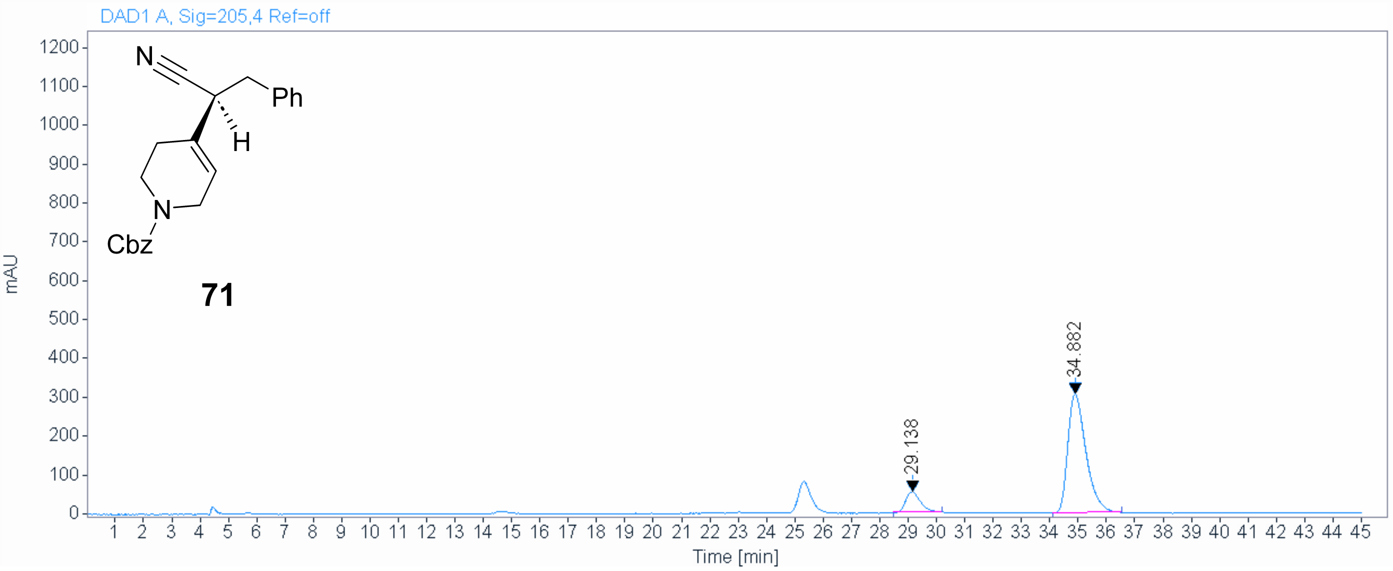

Signal: DAD1 A, Sig=205,4 Ref=off

RT [min]	Width [min]	Height	Area	Area%
29.138	0.6098	53.5459	1959.2574	12.5350
34.882	0.5401	306.2732	13671.0088	87.4650
		Sum	15630.2662	100.0000

Area Percent Report

Agilent Technologies

Sample name: zwf-10-068-rac-IE-80-20-0.8-205

Data file: C:\Users\Public\Documents\ChemStation\1\Data\zwf\zwf-S 2024-06-25 11-52-53\010-P1-C7-zwf-10-068-rac.D

Injection Acquired Date 06/25/2024 16:24:25

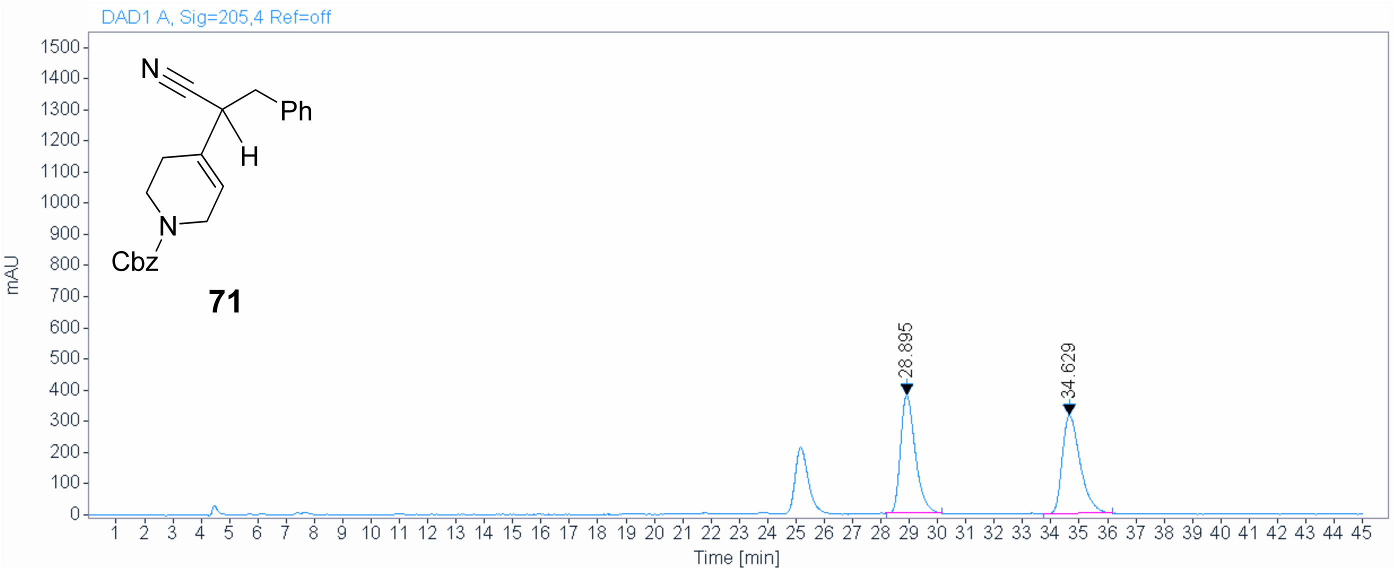

Signal: DAD1 A, Sig=205,4 Ref=off

RT [min]	Width [min]	Height	Area	Area%
28.895	0.4915	380.4985	13876.2881	49.7930
34.629	0.5498	316.4758	13991.6494	50.2070
		Sum	27867.9375	100.0000

zwf-10-068H
Jun 25 2024
SOLVENT: CDCl3
NA = 4
F1 = 500.130005 MHz
F2 = 1.000000 MHz

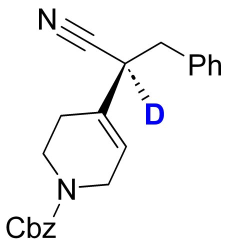

71-d

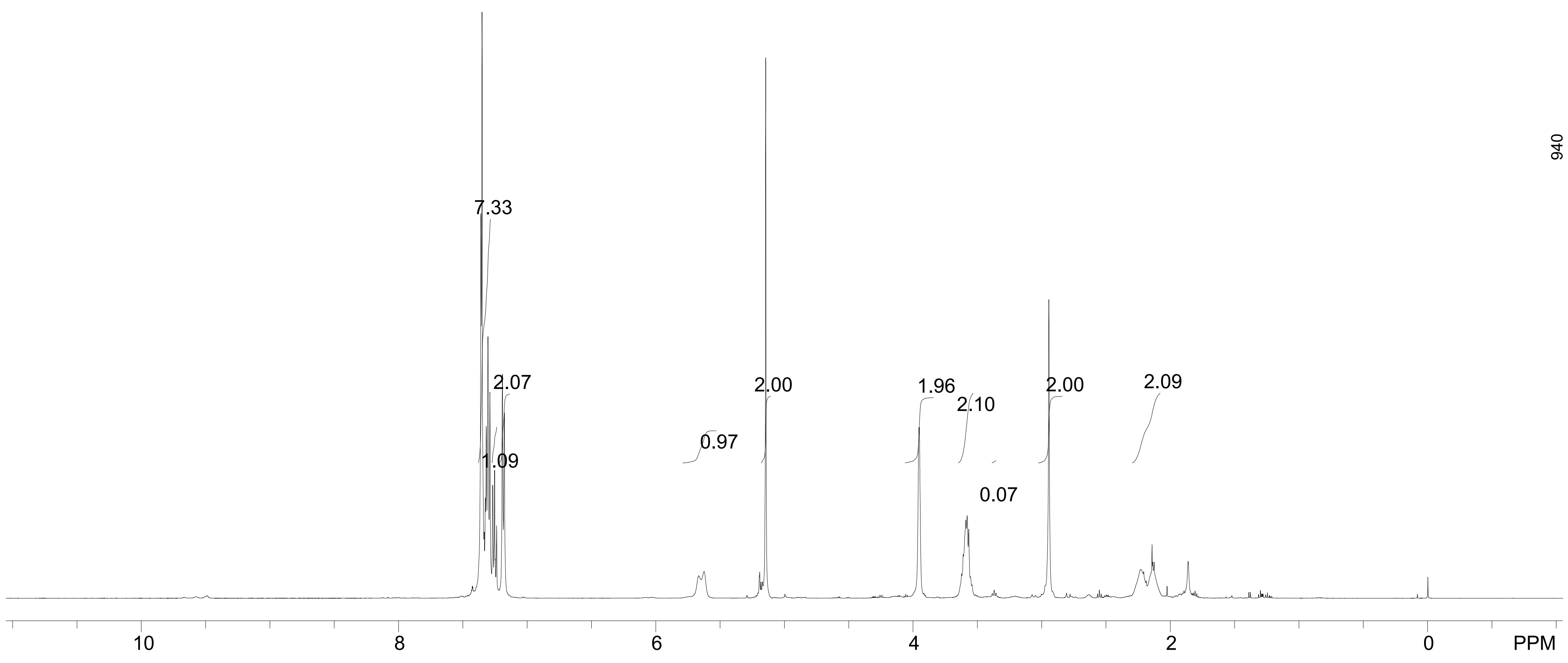

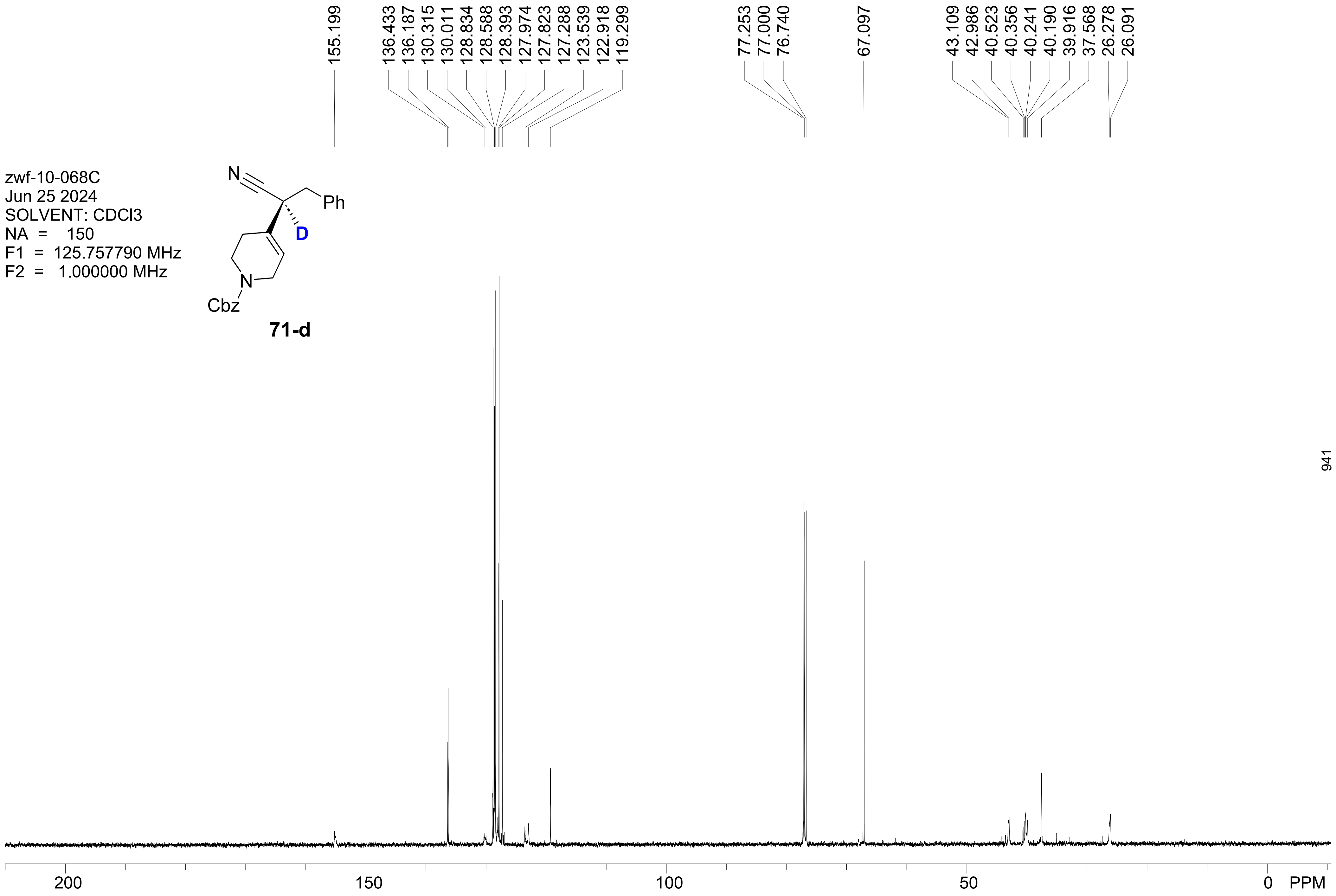

zwf-10-068C
Jun 25 2024
SOLVENT: CDCl3
NA = 150
F1 = 125.757790 MHz
F2 = 1.000000 MHz
N
Ph
D
N
Cbz
71-d
155.199
136.433
136.187
130.315
130.011
128.834
128.588
128.393
127.974
127.823
127.288
123.539
122.918
119.299
77.253
77.000
76.740
67.097
43.109
42.986
40.523
40.356
40.241
40.190
39.916
37.568
26.278
26.091
200
150
100
50
0
PPM

Area Percent Report

Sample name: zwf-10-068-IE-80-20-0.8-205

Data file: C:\Users\Public\Documents\ChemStation\1\Data\zwf\zwf-S 2024-06-25 11-52-53\008-P1-C5-zwf-10-068.D

Injection Acquired Date 06/25/2024 14:52:17

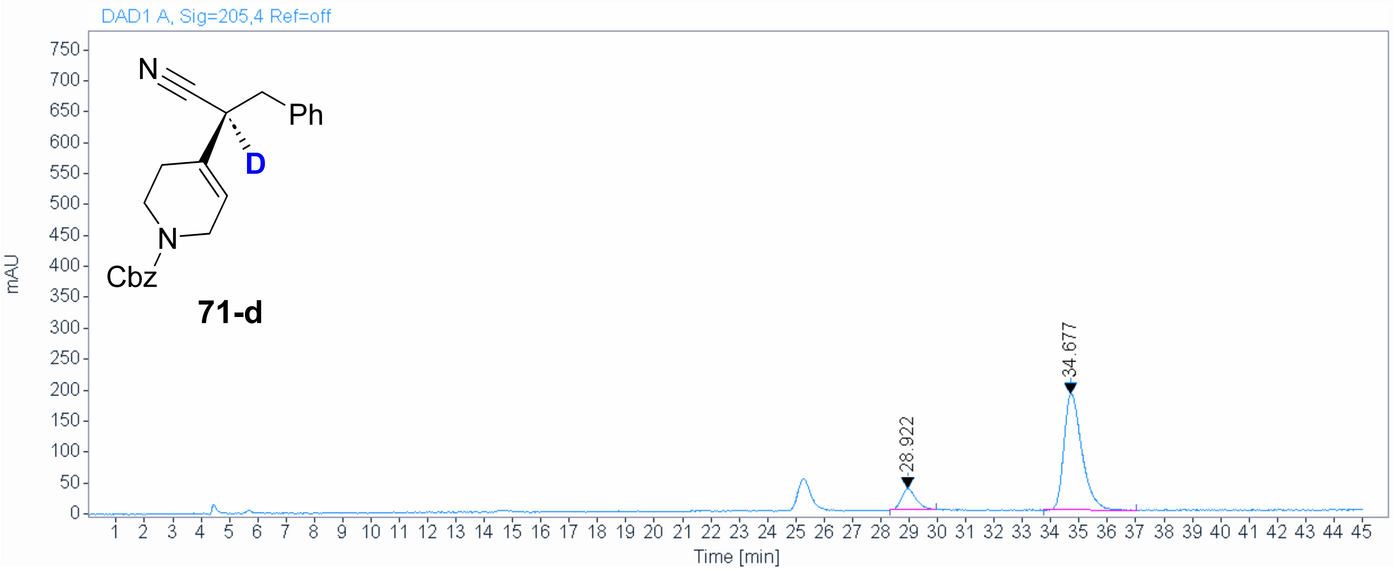

Signal: DAD1 A, Sig=205,4 Ref=off

RT [min]	Width [min]	Height	Area	Area%
28.922	0.5899	34.3766	1216.6731	12.5699
34.677	0.7477	188.6274	8462.5791	87.4301
		Sum	9679.2522	100.0000

Area Percent Report

Agilent Technologies

Sample name: zwf-10-068-rac-IE-80-20-0.8-205

Data file: C:\Users\Public\Documents\ChemStation\1\Data\zwf\zwf-S 2024-06-25 11-52-53\010-P1-C7-zwf-10-068-rac.D

Injection Acquired Date 06/25/2024 16:24:25

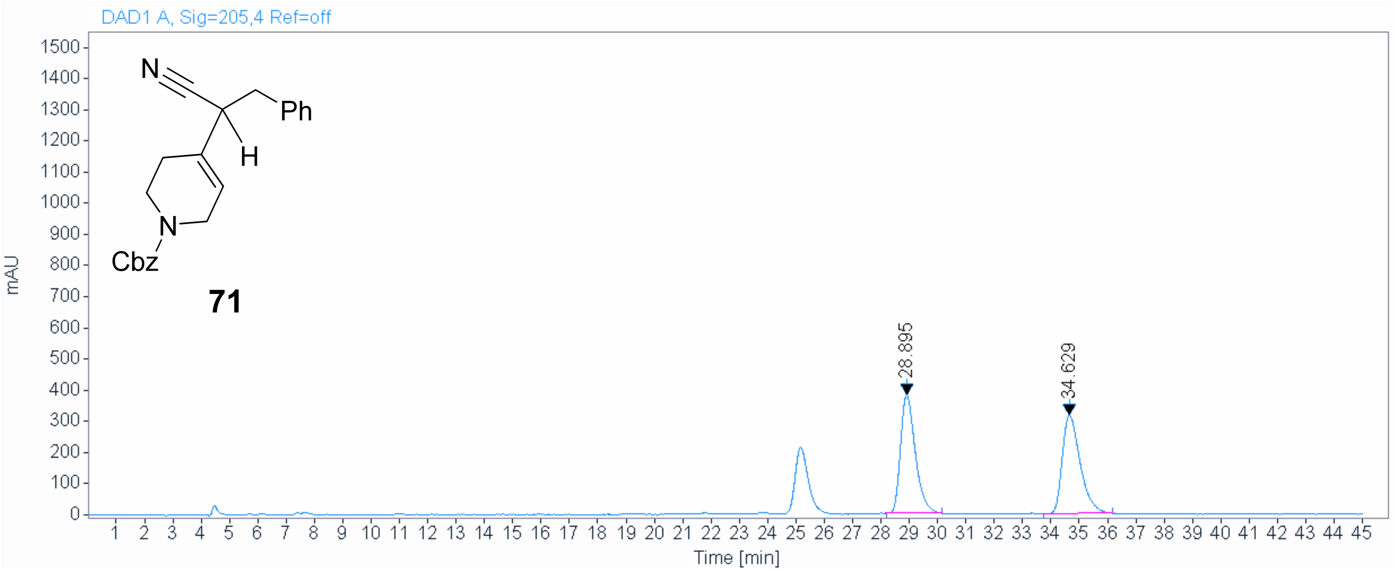

Signal: DAD1 A, Sig=205,4 Ref=off

RT [min]	Width [min]	Height	Area	Area%
28.895	0.4915	380.4985	13876.2881	49.7930
34.629	0.5498	316.4758	13991.6494	50.2070
		Sum	27867.9375	100.0000

zwf-10-022H
Jun 14 2024
SOLVENT: CDCl3
NA = 4
F1 = 400.140015 MHz
F2 = 1.000000 MHz

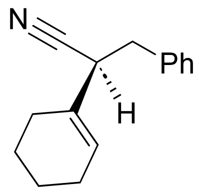

72

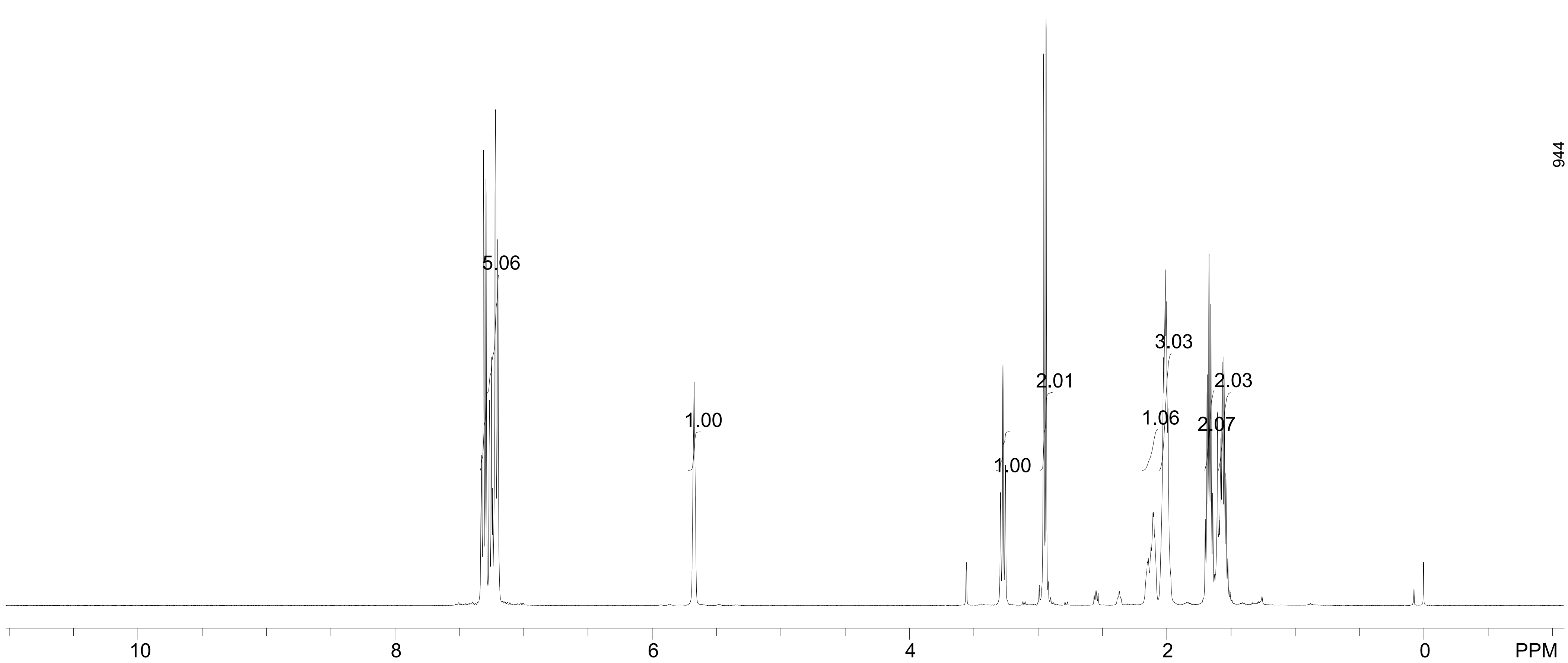

zwf-10-022C
Jun 14 2024
SOLVENT: CDCl3
NA = 100
F1 = 100.615280 MHz
F2 = 1.000000 MHz

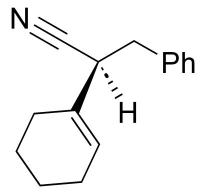

72

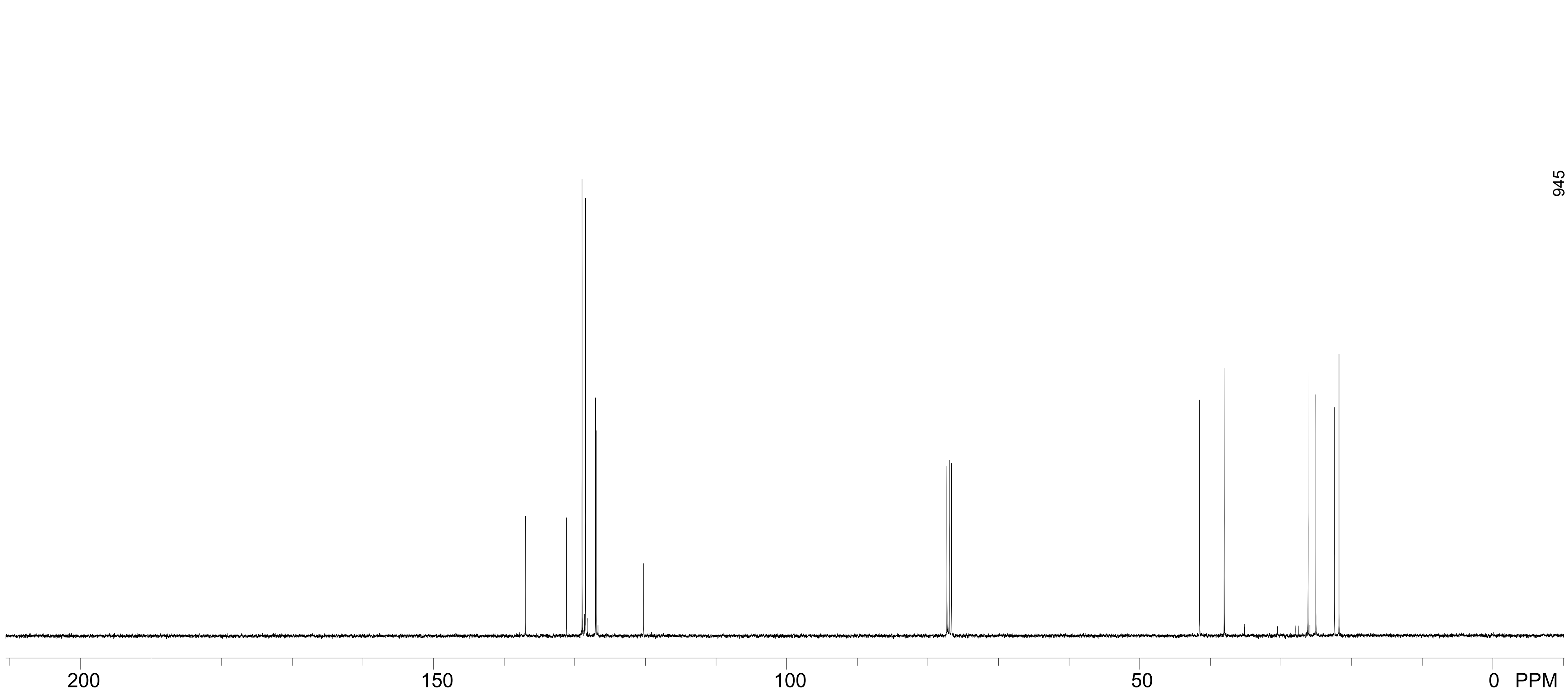

Area Percent Report

Agilent Technologies

Sample name: zwf-10-022-IE-99-1-0.8-205

Data file: C:\Users\Public\Documents\ChemStation\1\Data\zwf\zwf-S 2024-06-10 16-45-36\004-P1-C1-zwf-10-022.D

Injection Acquired Date 06/10/2024 17:42:48

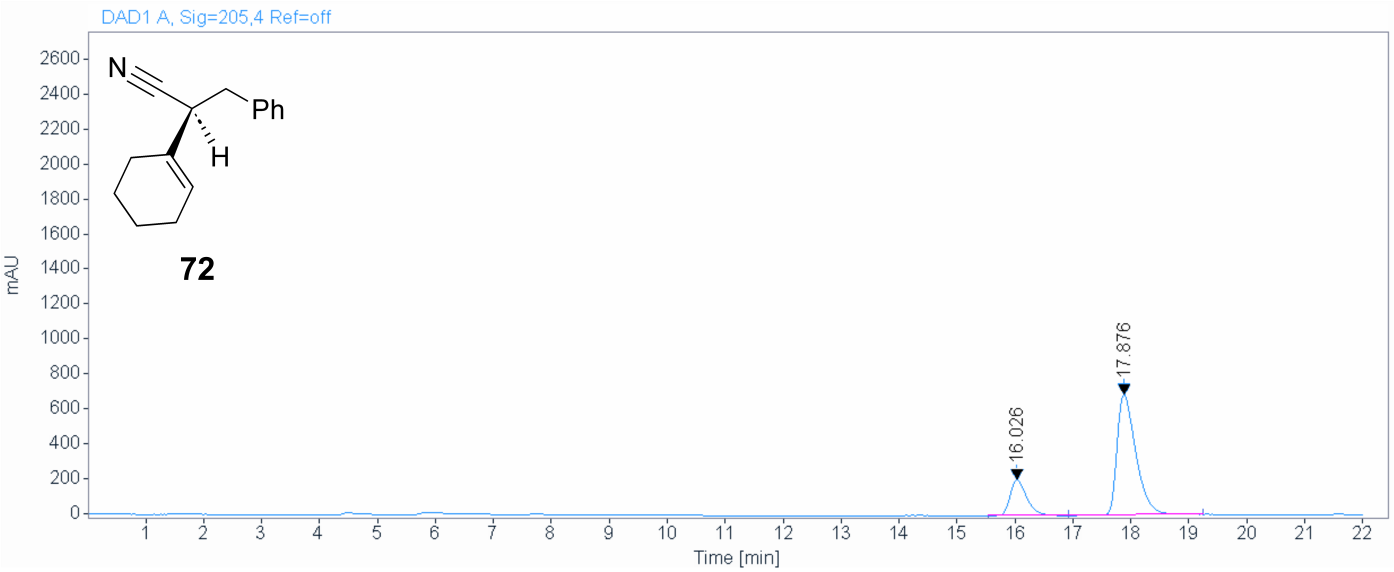

Signal: DAD1 A, Sig=205,4 Ref=off

RT [min]	Width [min]	Height	Area	Area%
16.026	0.3046	204.0758	4069.1790	20.6552
17.876	0.3512	687.6362	15631.3291	79.3448
		Sum	19700.5081	100.0000

Area Percent Report

Agilent Technologies

Sample name: zwf-10-022-rac-IE-99-1-0.8-205

Data file: C:\Users\Public\Documents\ChemStation\1\Data\zwf\zwf-S 2024-06-10 16-45-36\002-P1-C3-zwf-10-022-rac.D

Injection Acquired Date 06/10/2024 17:03:45

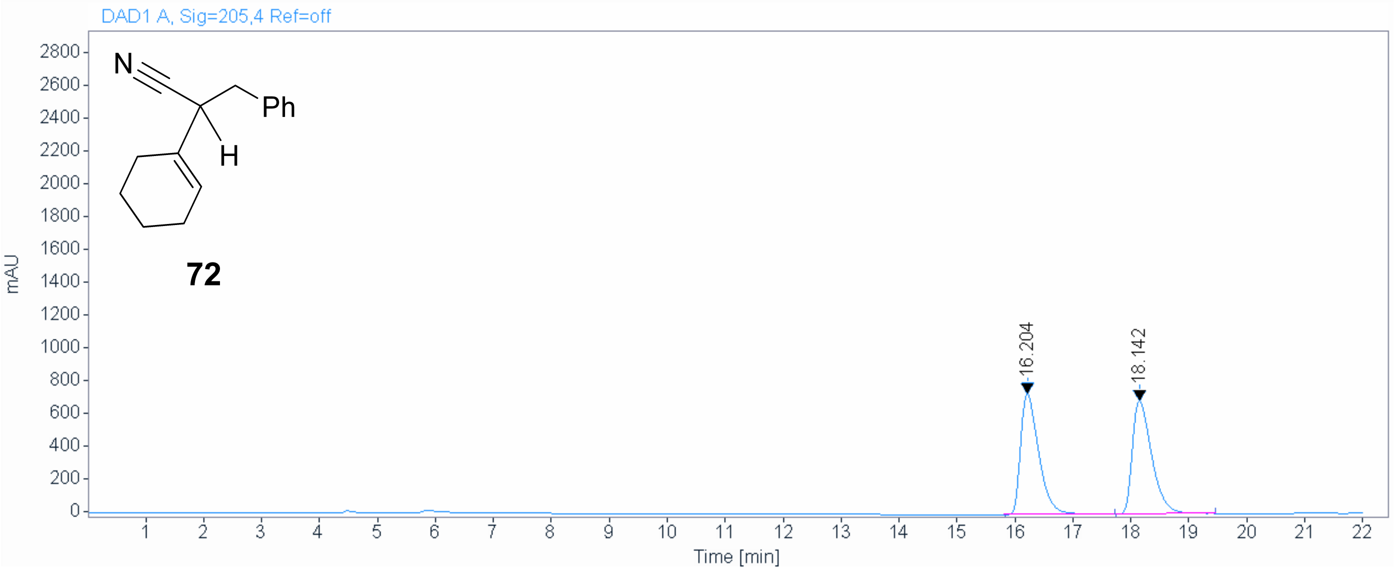

Signal: DAD1 A, Sig=205,4 Ref=off

RT [min]	Width [min]	Height	Area	Area%
16.204	0.3363	736.2170	16047.0342	49.9783
18.142	0.3560	688.8250	16060.9863	50.0217
		Sum	32108.0205	100.0000

zwf-10-023H
Jun 14 2024
SOLVENT: CDCl3
NA = 4
F1 = 500.130005 MHz
F2 = 1.000000 MHz

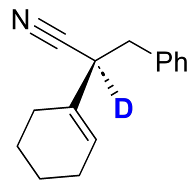

72-d

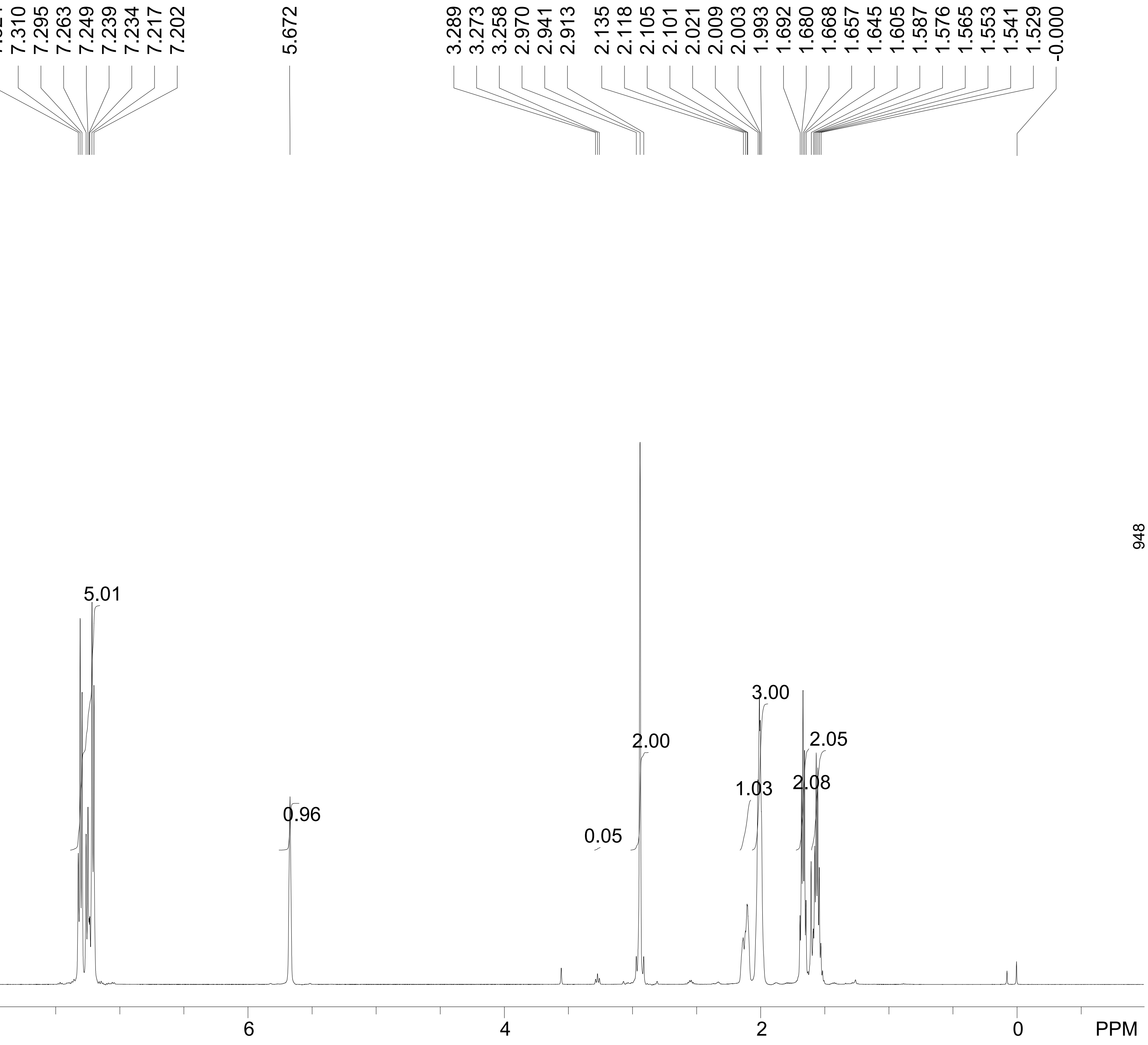

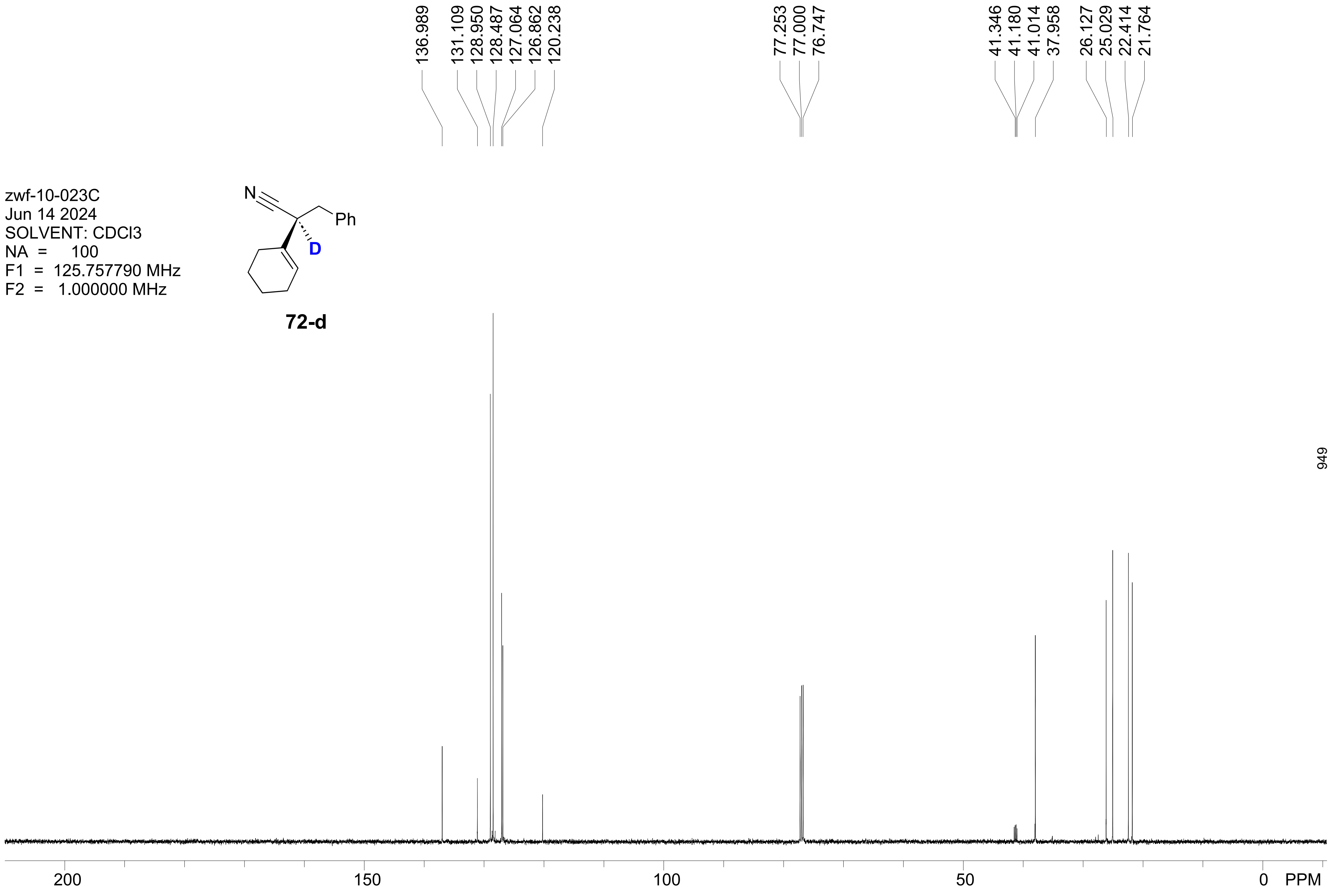

zwf-10-023C
Jun 14 2024
SOLVENT: CDCl3
NA = 100
F1 = 125.757790 MHz
F2 = 1.000000 MHz
136.989
131.109
128.950
128.487
127.064
126.862
120.238
77.253
77.000
76.747
41.346
41.180
41.014
37.958
26.127
25.029
22.414
21.764
200
150
100
50
0 PPM
N
Ph
D

72-d

Area Percent Report

Agilent Technologies

Sample name: zwf-10-023-IE-99-1-0.8-205

Data file: C:\Users\Public\Documents\ChemStation\1\Data\zwf\zwf-S 2024-06-10 16-45-36\006-P1-C2-zwf-10-023.D

Injection Acquired Date 06/10/2024 18:21:49

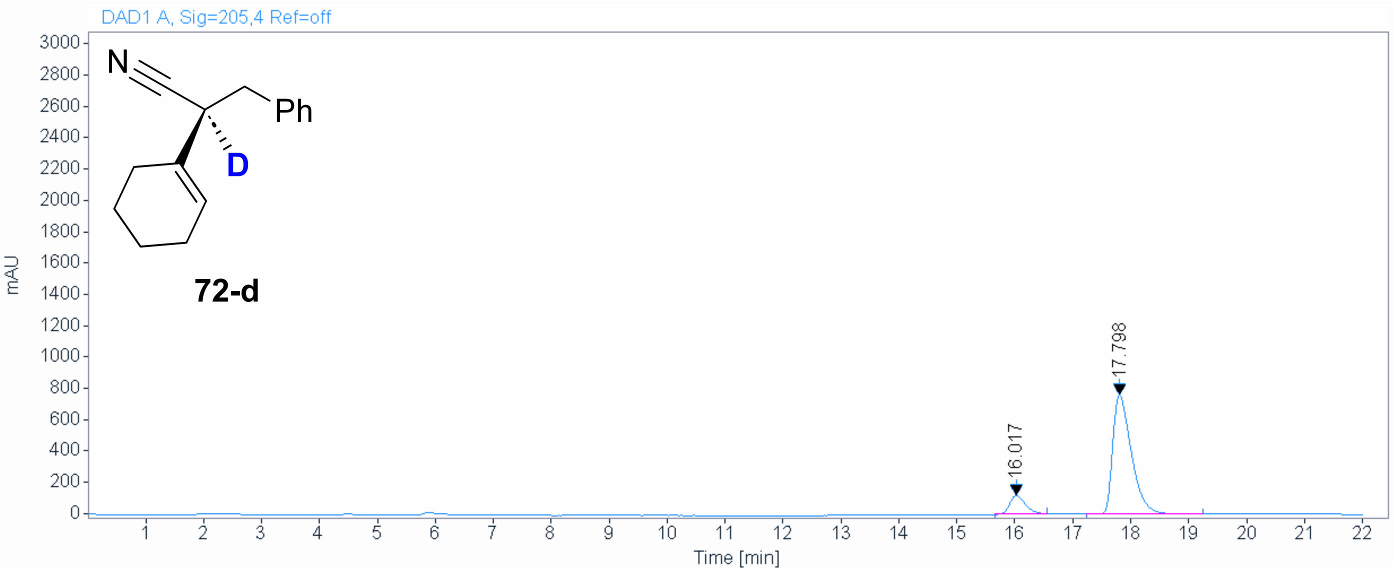

Signal: DAD1 A, Sig=205,4 Ref=off

RT [min]	Width [min]	Height	Area	Area%
16.017	0.3111	118.6335	2214.5508	11.4776
17.798	0.3397	761.3806	17080.0156	88.5224
		Sum	19294.5664	100.0000

Area Percent Report

Agilent Technologies

Sample name: zwf-10-022-rac-IE-99-1-0.8-205

Data file: C:\Users\Public\Documents\ChemStation\1\Data\zwf\zwf-S 2024-06-10 16-45-36\002-P1-C3-zwf-10-022-rac.D

Injection Acquired Date 06/10/2024 17:03:45

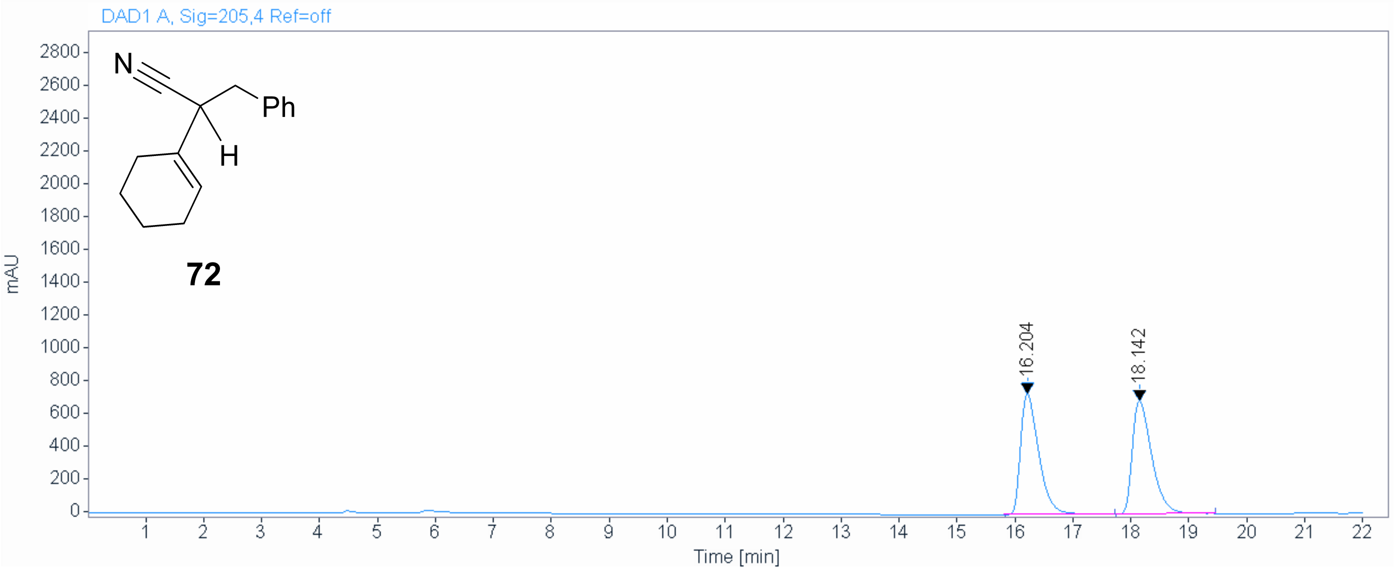

Signal: DAD1 A, Sig=205,4 Ref=off

RT [min]	Width [min]	Height	Area	Area%
16.204	0.3363	736.2170	16047.0342	49.9783
18.142	0.3560	688.8250	16060.9863	50.0217
		Sum	32108.0205	100.0000

7.337
7.323
7.308
7.270
7.255
7.251
7.240
7.238
7.224

2.921
2.902
2.894
2.880
2.875
2.868
2.853
2.841
2.661
2.650
2.643
2.639
2.632
2.621
1.958
1.954
1.947
1.828
1.818
1.785
1.763
1.735
1.697
1.678
1.607
1.530
1.521
1.514
1.282
1.259
1.240
1.222
1.202
-0.000

zwf-10-144H
Jul 23 2024
SOLVENT: CDCl3
NA = 4
F1 = 500.130005 MHz
F2 = 1.000000 MHz

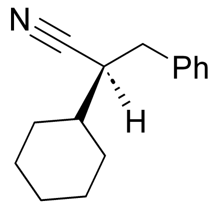

66

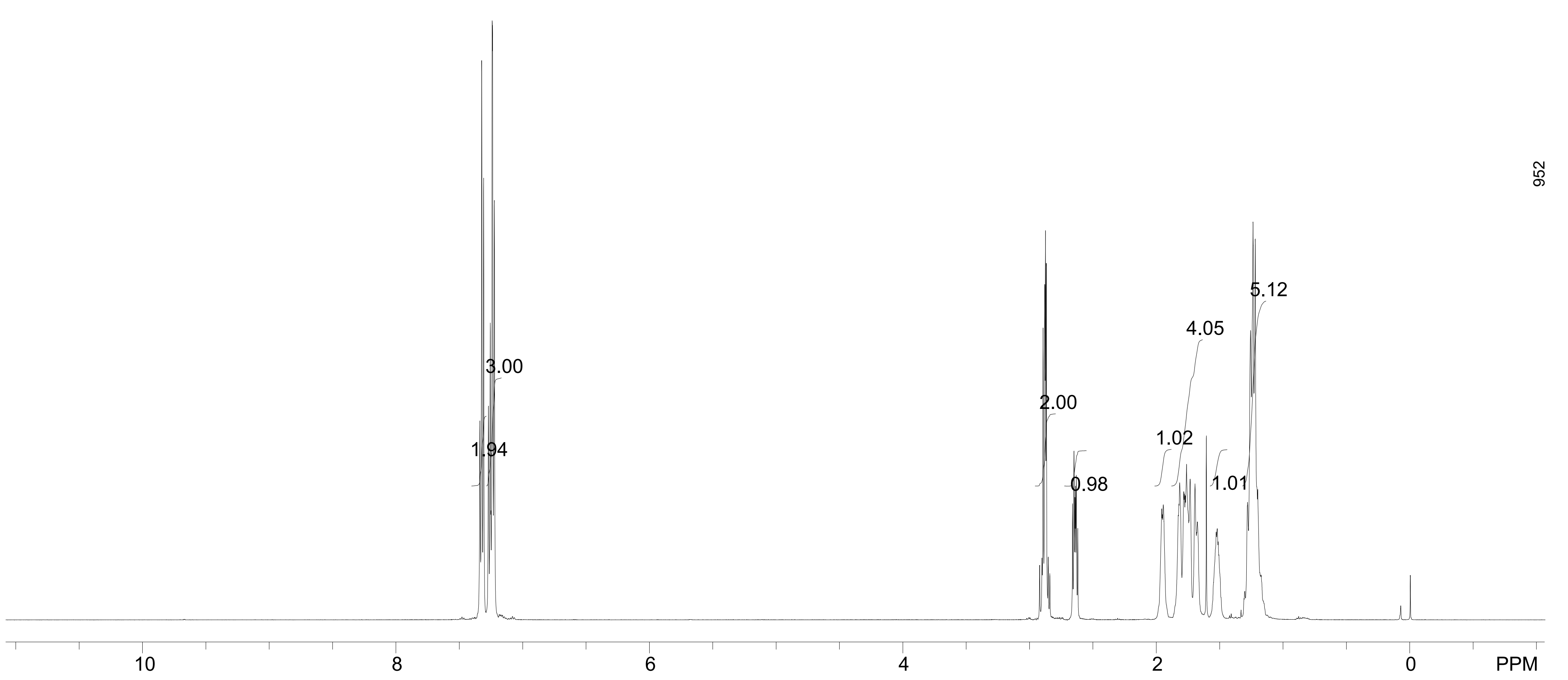

Area Percent Report

Sample name: zwf-10-144-IE-99-1-0.8-205

Data file: C:\Users\Public\Documents\ChemStation\1\Data\zwf\zwf 2024-07-23 17-22-19\002-P1-C1-zwf-10-144.D

Injection Acquired Date 07/23/2024 17:50:29

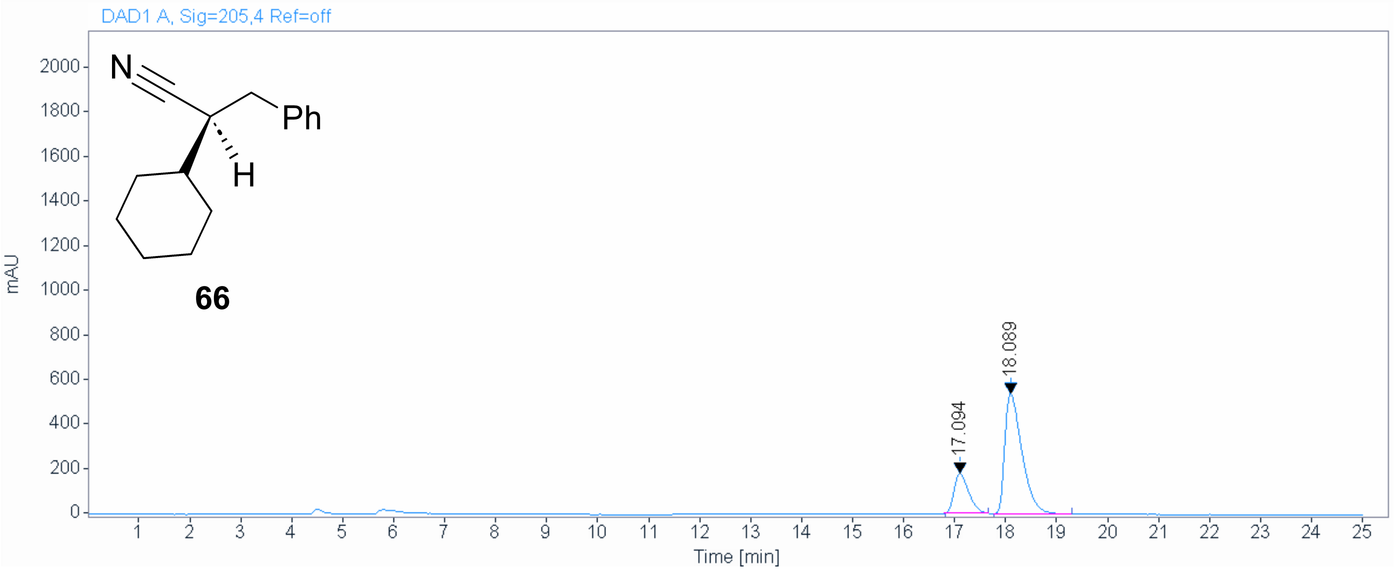

Signal: DAD1 A, Sig=205,4 Ref=off

RT [min]	Width [min]	Height	Area	Area%
17.094	0.3290	177.4075	3502.5283	21.6897
18.089	0.3501	538.2884	12645.8193	78.3103
		Sum	16148.3477	100.0000

Area Percent Report

Agilent Technologies

Sample name: zwf-10-145-rac-IE-99-1-0.8-205

Data file: C:\Users\Public\Documents\ChemStation\1\Data\zwf\zwf 2024-07-23 17-22-19\004-P1-C3-zwf-10-145-rac.D

Injection Acquired Date 07/23/2024 18:42:30

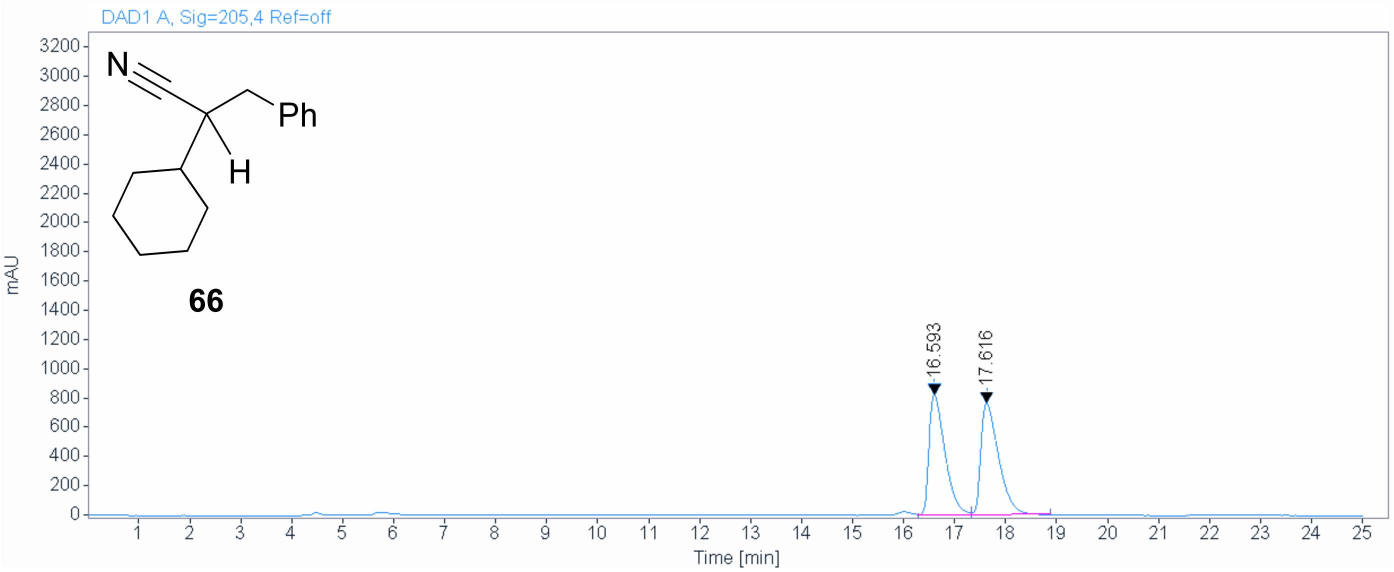

66

Signal: DAD1 A, Sig=205,4 Ref=off

RT [min]	Width [min]	Height	Area	Area%
16.593	0.3639	821.5510	17937.7402	49.1207
17.616	0.4050	764.5584	18579.9375	50.8793
		Sum	36517.6777	100.0000

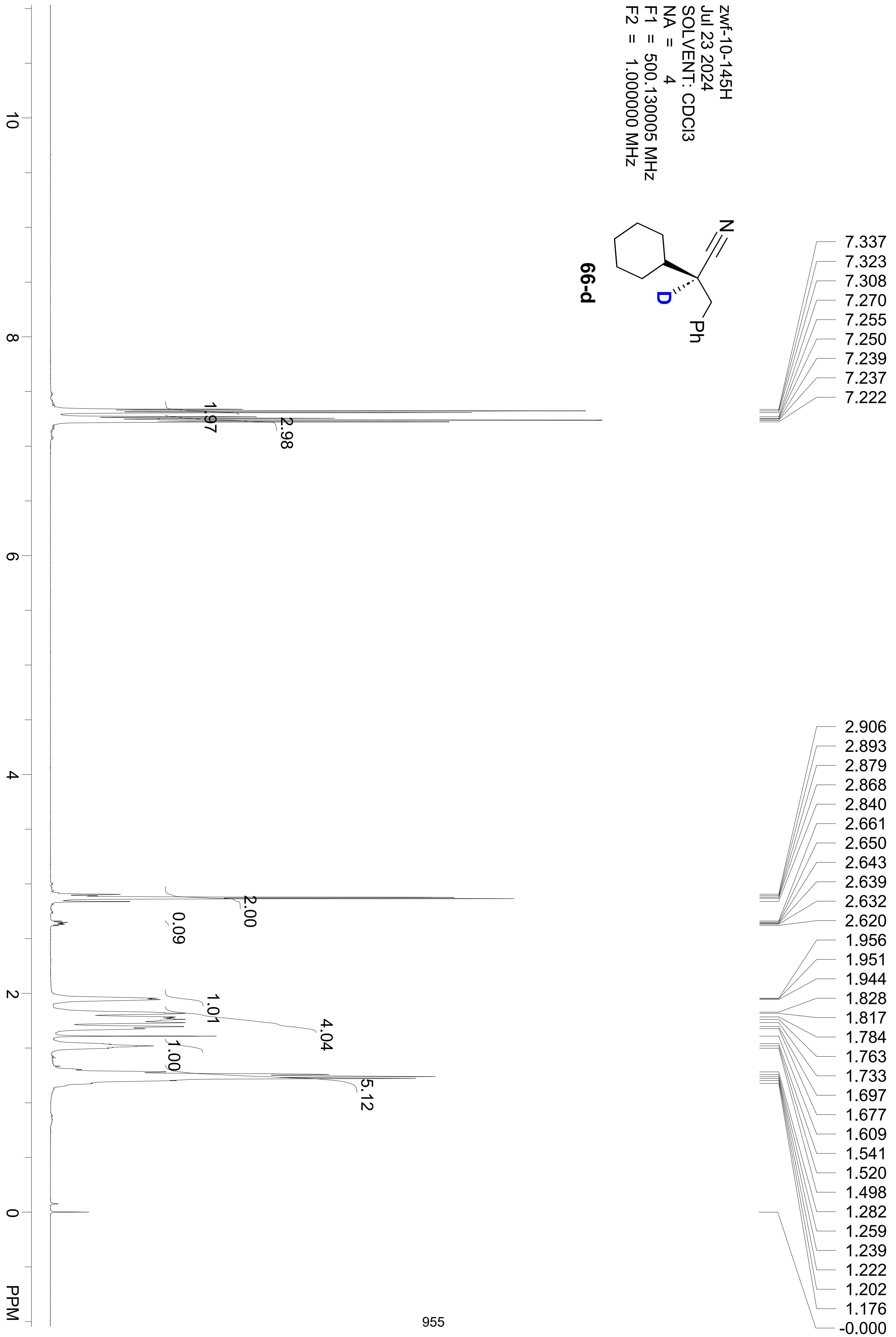
zwf-10-145H
Jul 23 2024
SOLVENT: CDCl3
NA = 4
F1 = 500.130005 MHz
F2 = 1.000000 MHz
N
Ph
D
66-d
7.337
7.323
7.308
7.270
7.255
7.250
7.239
7.237
7.222
2.906
2.893
2.879
2.868
2.840
2.661
2.650
2.643
2.639
2.632
2.620
1.956
1.951
1.944
1.828
1.817
1.784
1.763
1.733
1.697
1.677
1.609
1.541
1.520
1.498
1.282
1.259
1.239
1.222
1.202
1.176
-0.000
1.97
2.98
2.00
0.09
1.01
4.04
1.00
5.12
10
8
6
4
2
0
PPM

Area Percent Report

Agilent Technologies

Sample name: zwf-10-145-IE-99-1-0.8-205

Data file: C:\Users\Public\Documents\ChemStation\1\Data\zwf\zwf 2024-07-23 17-22-19\003-P1-C2-zwf-10-145.D

Injection Acquired Date 07/23/2024 18:16:28

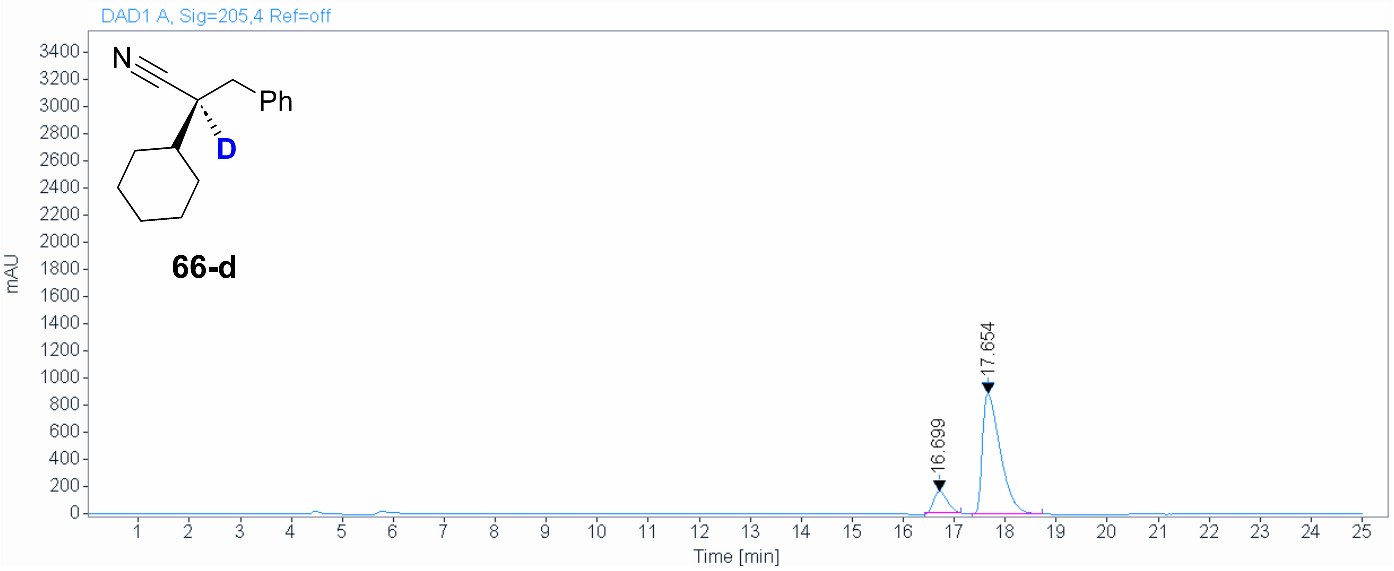

Signal: DAD1 A, Sig=205,4 Ref=off

RT [min]	Width [min]	Height	Area	Area%
16.699	0.3149	162.5947	3071.8855	12.4139
17.654	0.3644	888.9844	21673.7109	87.5861
		Sum	24745.5964	100.0000

Area Percent Report

Agilent Technologies

Sample name: zwf-10-145-rac-IE-99-1-0.8-205

Data file: C:\Users\Public\Documents\ChemStation\1\Data\zwf\zwf 2024-07-23 17-22-19\004-P1-C3-zwf-10-145-rac.D

Injection Acquired Date 07/23/2024 18:42:30

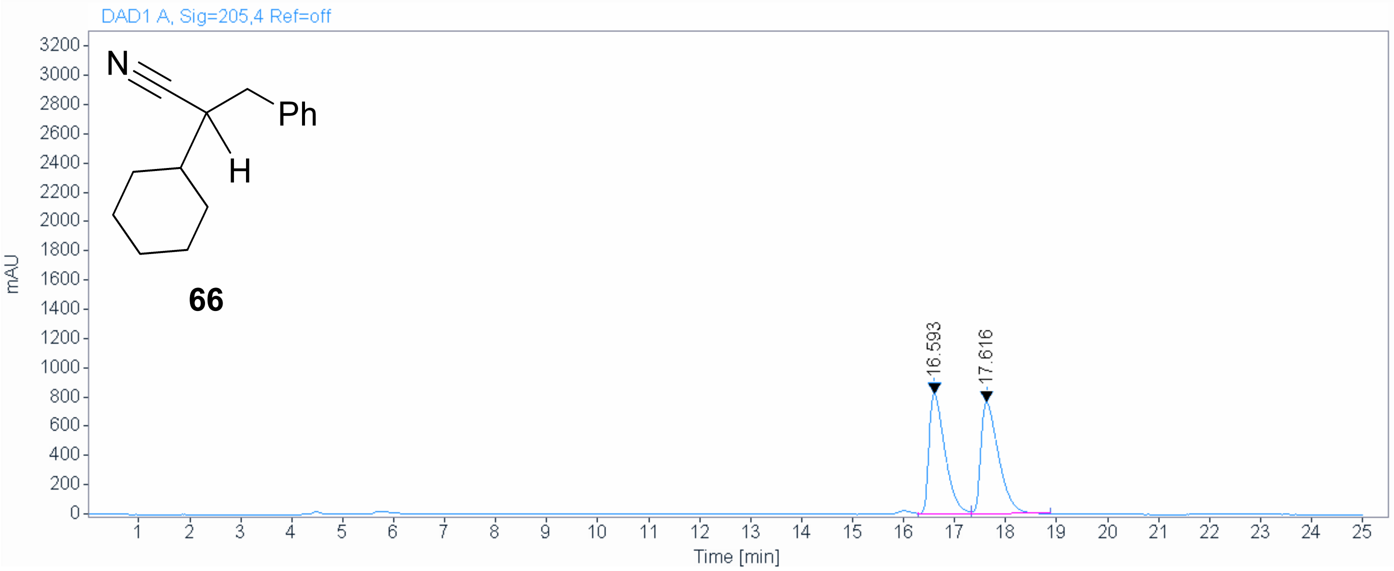

Signal: DAD1 A, Sig=205,4 Ref=off

RT [min]	Width [min]	Height	Area	Area%
16.593	0.3639	821.5510	17937.7402	49.1207
17.616	0.4050	764.5584	18579.9375	50.8793
		Sum	36517.6777	100.0000

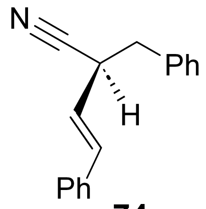

74

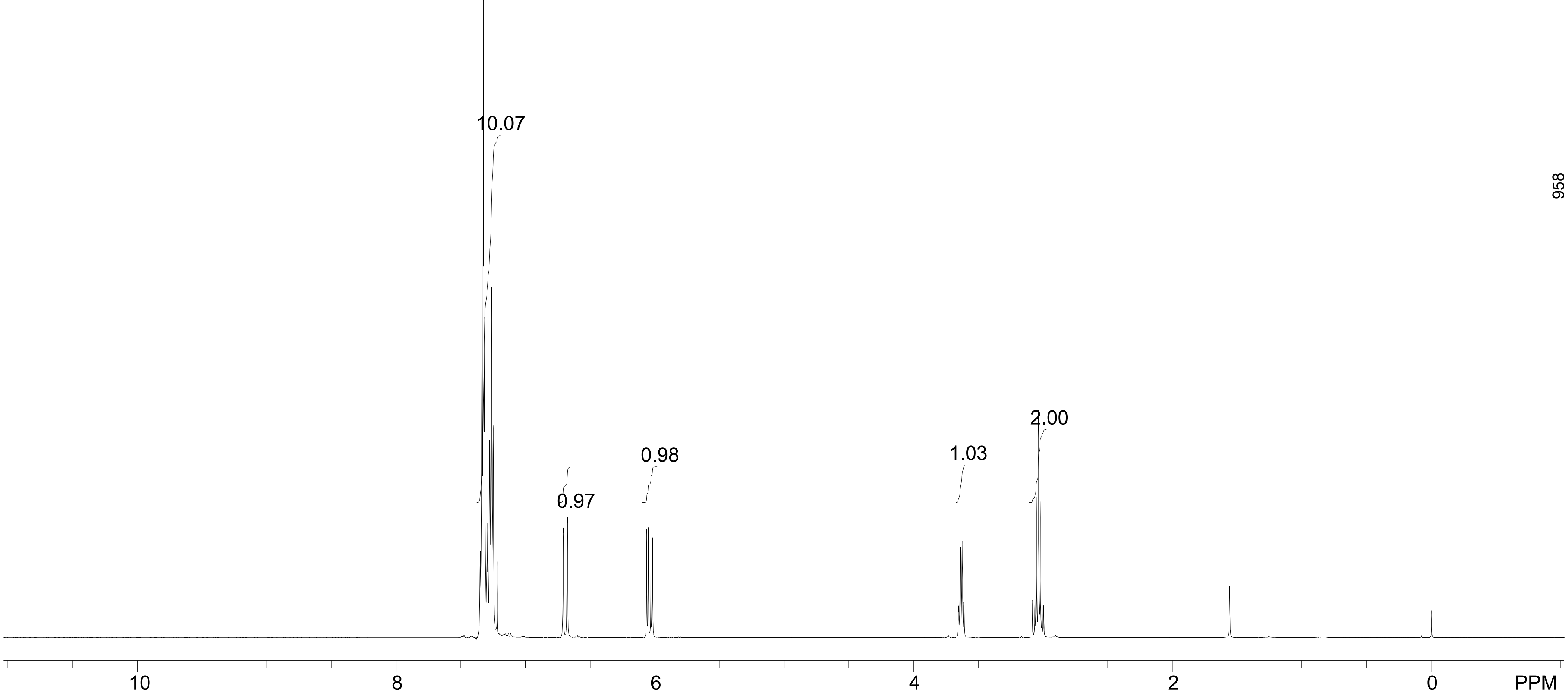
zwf-11-062H
Aug 22 2024
SOLVENT: CDCl3
NA = 4
F1 = 500.130035 MHz
F2 = 1.000000 MHz
7.350
7.336
7.326
7.322
7.314
7.298
7.291
7.276
7.263
7.250
7.218
6.709
6.707
6.677
6.675
6.062
6.050
6.031
6.018
3.656
3.653
3.642
3.640
3.627
3.614
3.611
3.081
3.065
3.054
3.037
3.023
3.009
2.996
1.561
-0.000
10.07
0.97
0.98
1.03
2.00
10
8
6
4
2
0
PPM

zwf-11-062C
Aug 22 2024
SOLVENT: CDCl3
NA = 100
F1 = 125.757790 MHz
F2 = 1.000000 MHz

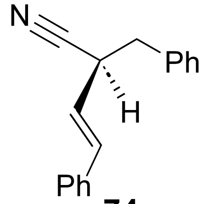

74

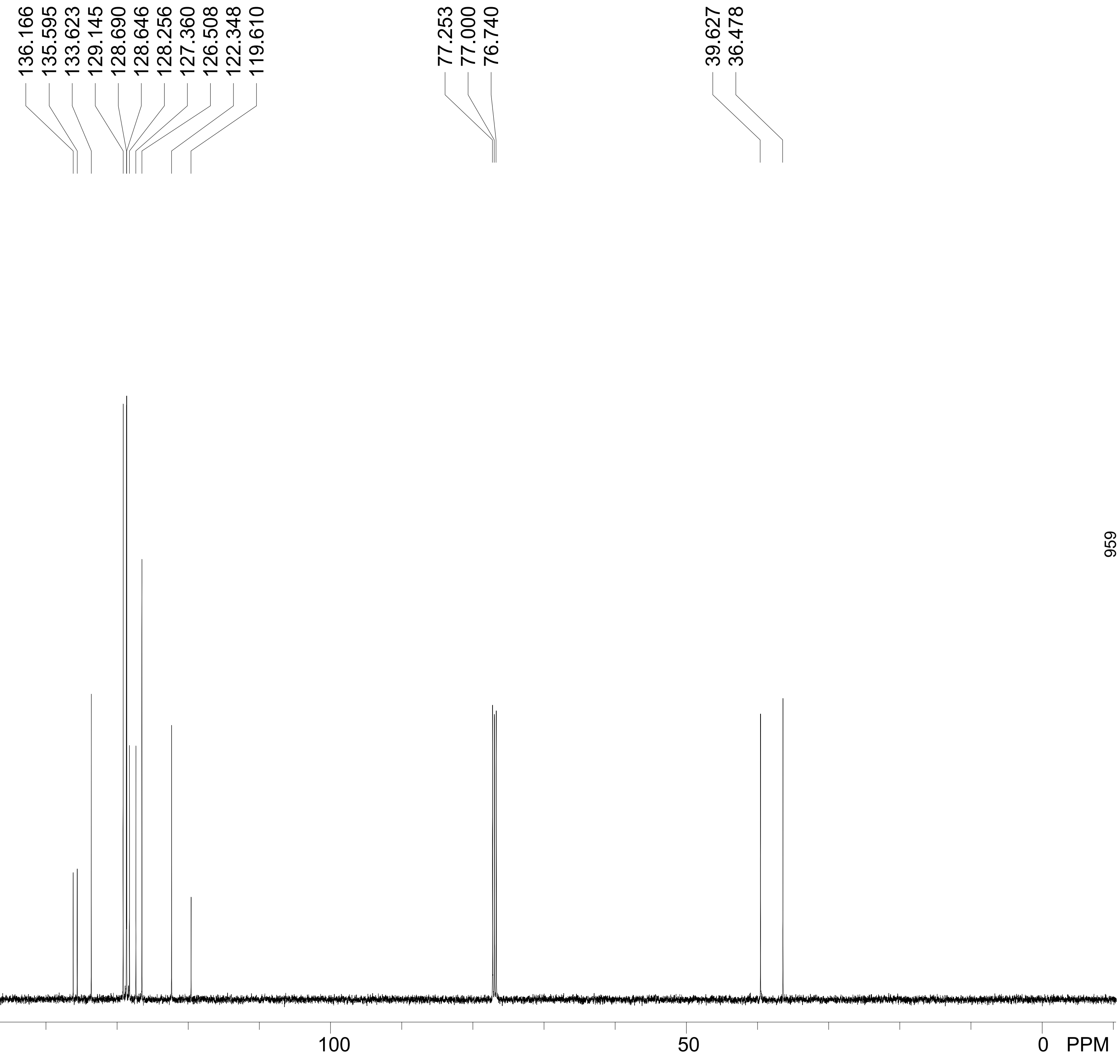

Area Percent Report

Sample name: zwf-11-062-IE-99-1-0.8-205

Data file: C:\Users\Public\Documents\ChemStation\1\Data\zwf\zwf 2024-08-22 20-21-38\003-P1-C3-zwf-11-062.D

Injection Acquired Date 08/22/2024 21:08:45

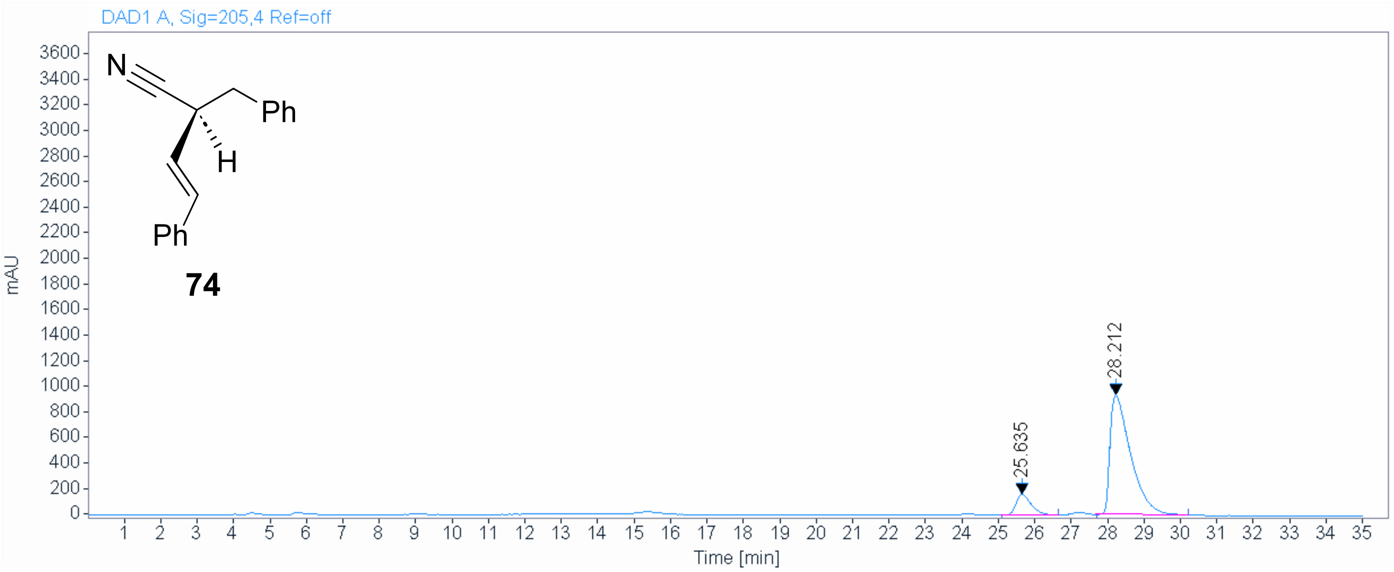

Signal: DAD1 A, Sig=205,4 Ref=off

RT [min]	Width [min]	Height	Area	Area%
25.635	0.3790	164.1307	4768.8799	11.4914
28.212	0.5178	935.9283	36730.7188	88.5086
		Sum	41499.5986	100.0000

Area Percent Report

Agilent Technologies

Sample name: zwf-11-062-rac-IE-99-1-0.8-205

Data file: C:\Users\Public\Documents\ChemStation\1\Data\zwf\zwf 2024-08-22 20-21-38\002-P1-C6-zwf-11-062-rac.D

Injection Acquired Date 08/22/2024 20:32:45

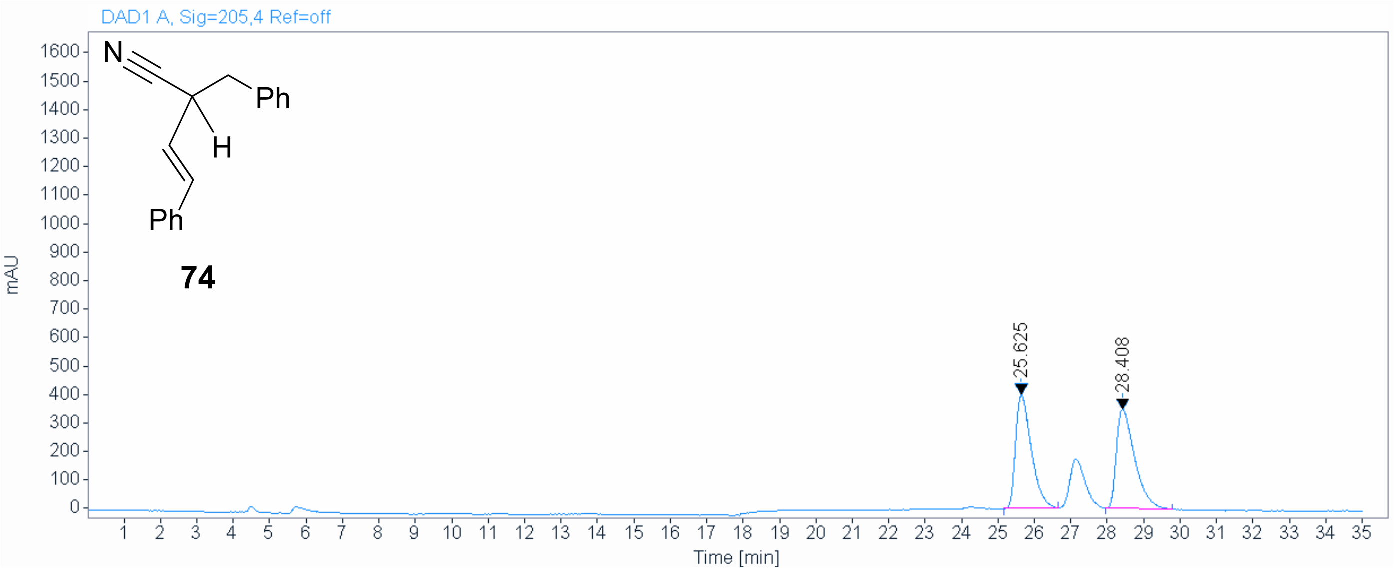

Signal: DAD1 A, Sig=205,4 Ref=off

RT [min]	Width [min]	Height	Area	Area%
25.625	0.4395	400.5646	12135.9375	49.7932
28.408	0.4246	347.6837	12236.7217	50.2068
		Sum	24372.6592	100.0000

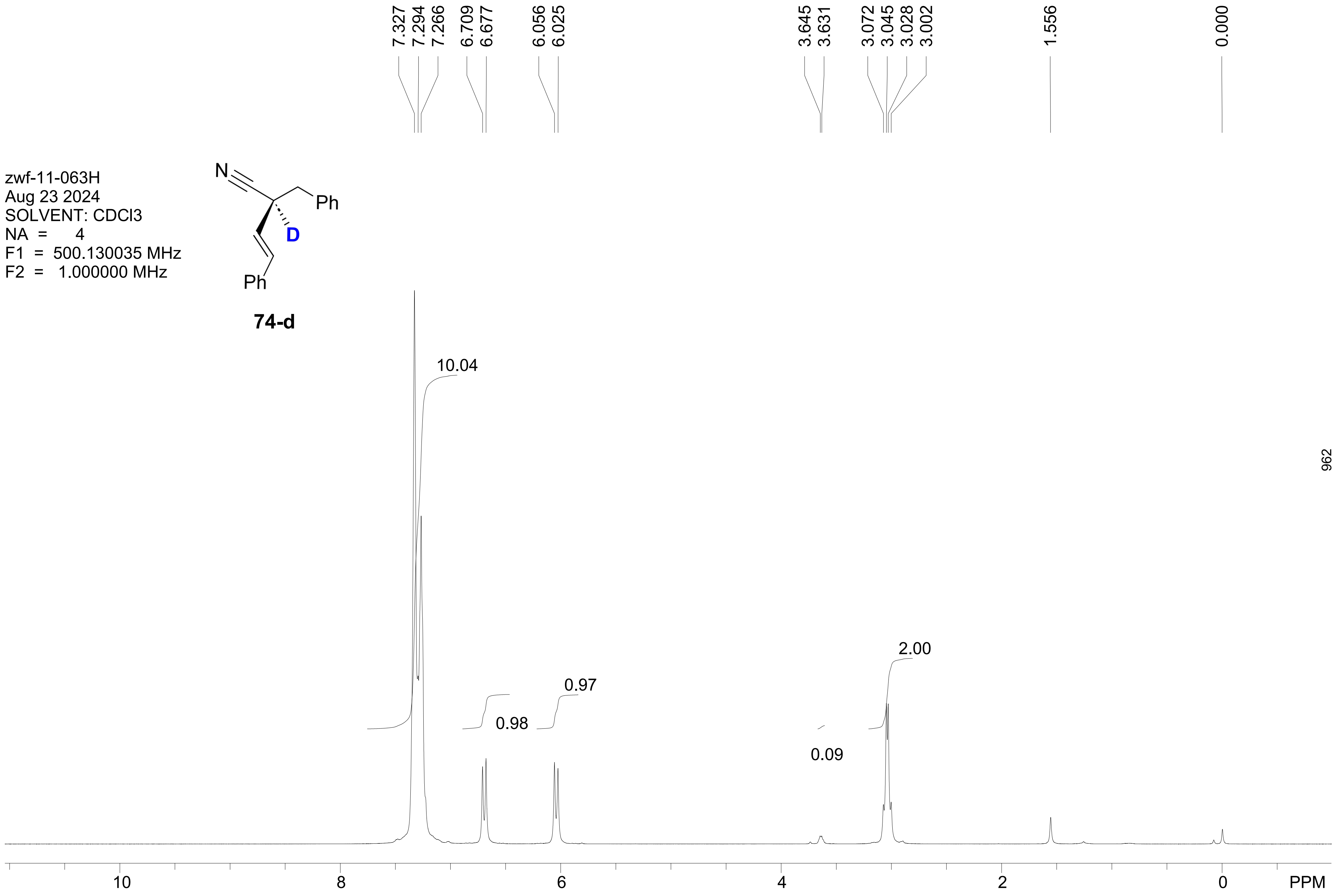

zwf-11-063H
Aug 23 2024
SOLVENT: CDCl3
NA = 4
F1 = 500.130035 MHz
F2 = 1.000000 MHz
N
Ph
D
Ph
74-d
7.327
7.294
7.266
6.709
6.677
6.056
6.025
3.645
3.631
3.072
3.045
3.028
3.002
1.556
0.000
10.04
0.98
0.97
0.09
2.00
10
8
6
4
2
0
PPM

zwf-11-063C
Aug 23 2024
SOLVENT: CDCl3
NA = 100
F1 = 125.757790 MHz
F2 = 1.000000 MHz

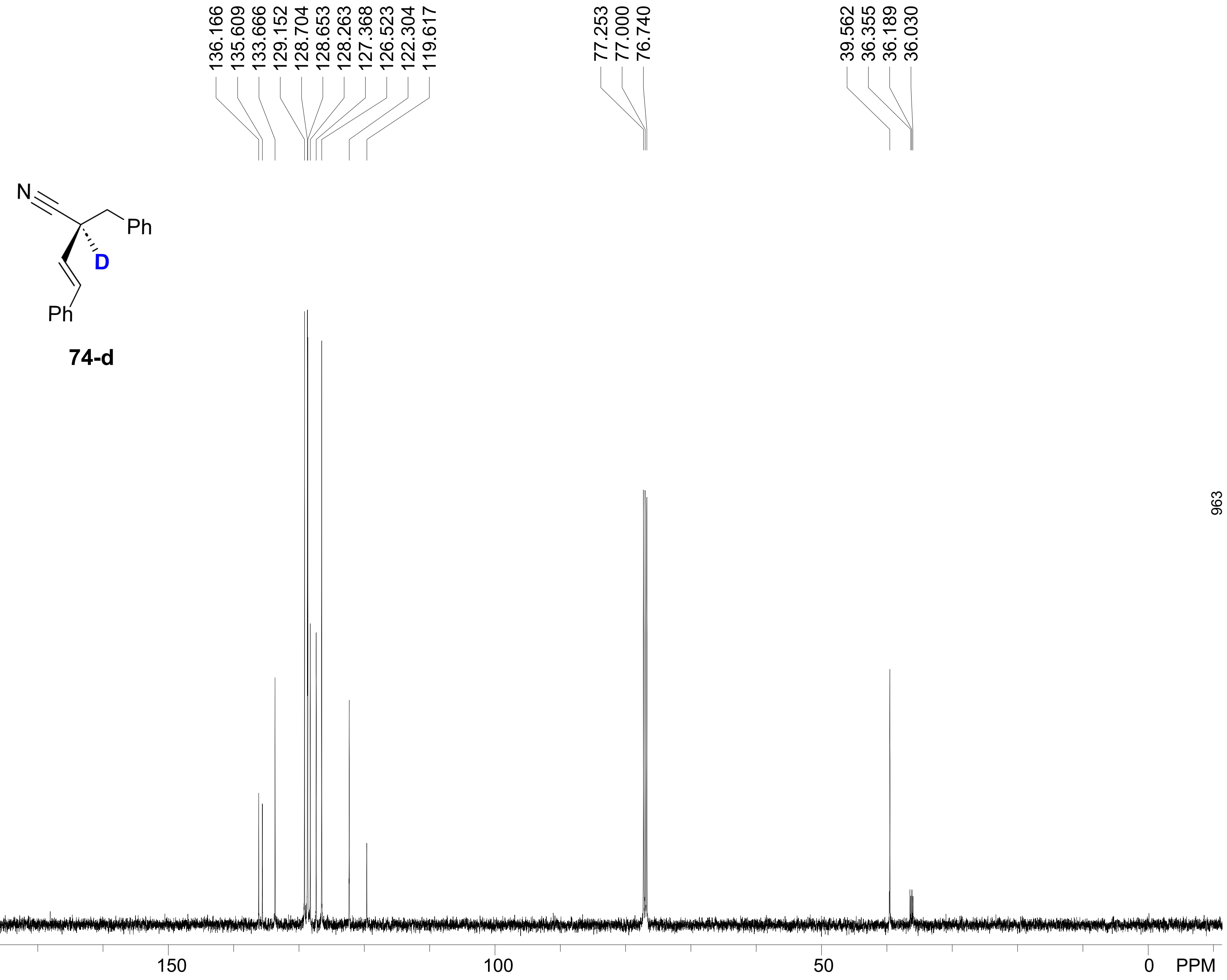

Area Percent Report

Agilent Technologies

Sample name: zwf-11-063-IE-99-1-0.8-205

Data file: C:\Users\Public\Documents\ChemStation\1\Data\zwf\zwf 2024-08-23 08-52-32\005-P1-C3-zwf-11-063.D

Injection Acquired Date 08/23/2024 10:53:00

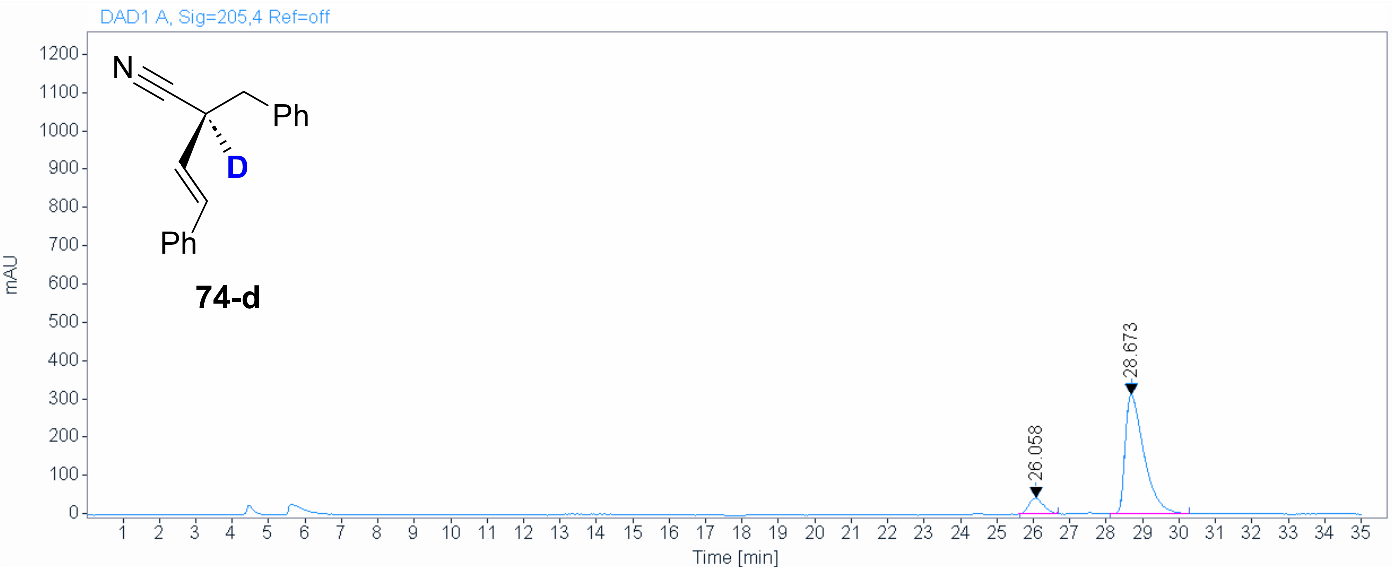

Signal: DAD1 A, Sig=205,4 Ref=off

RT [min]	Width [min]	Height	Area	Area%
26.058	0.4560	41.1518	1125.9441	9.1680
28.673	0.4781	311.3035	11155.2900	90.8320
		Sum	12281.2341	100.0000

Area Percent Report

Sample name: zwf-11-063-rac-IE-99-1-0.8-205

Data file: C:\Users\Public\Documents\ChemStation\1\Data\zwf\zwf 2024-08-23 08-52-32\007-P1-C4-zwf-11-063-rac.D

Injection Acquired Date 08/23/2024 12:05:04

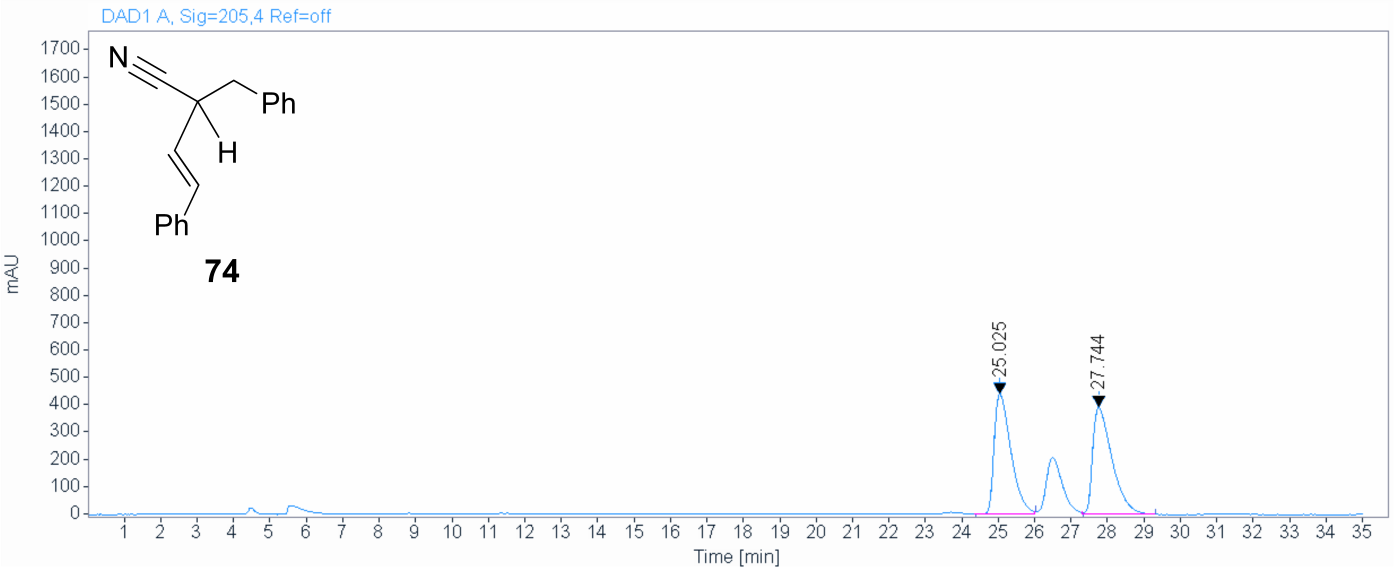

Signal: DAD1 A, Sig=205,4 Ref=off

RT [min]	Width [min]	Height	Area	Area%
25.025	0.4702	439.9899	14111.0674	49.3898
27.744	0.4955	392.7797	14459.7432	50.6102
		Sum	28570.8105	100.0000

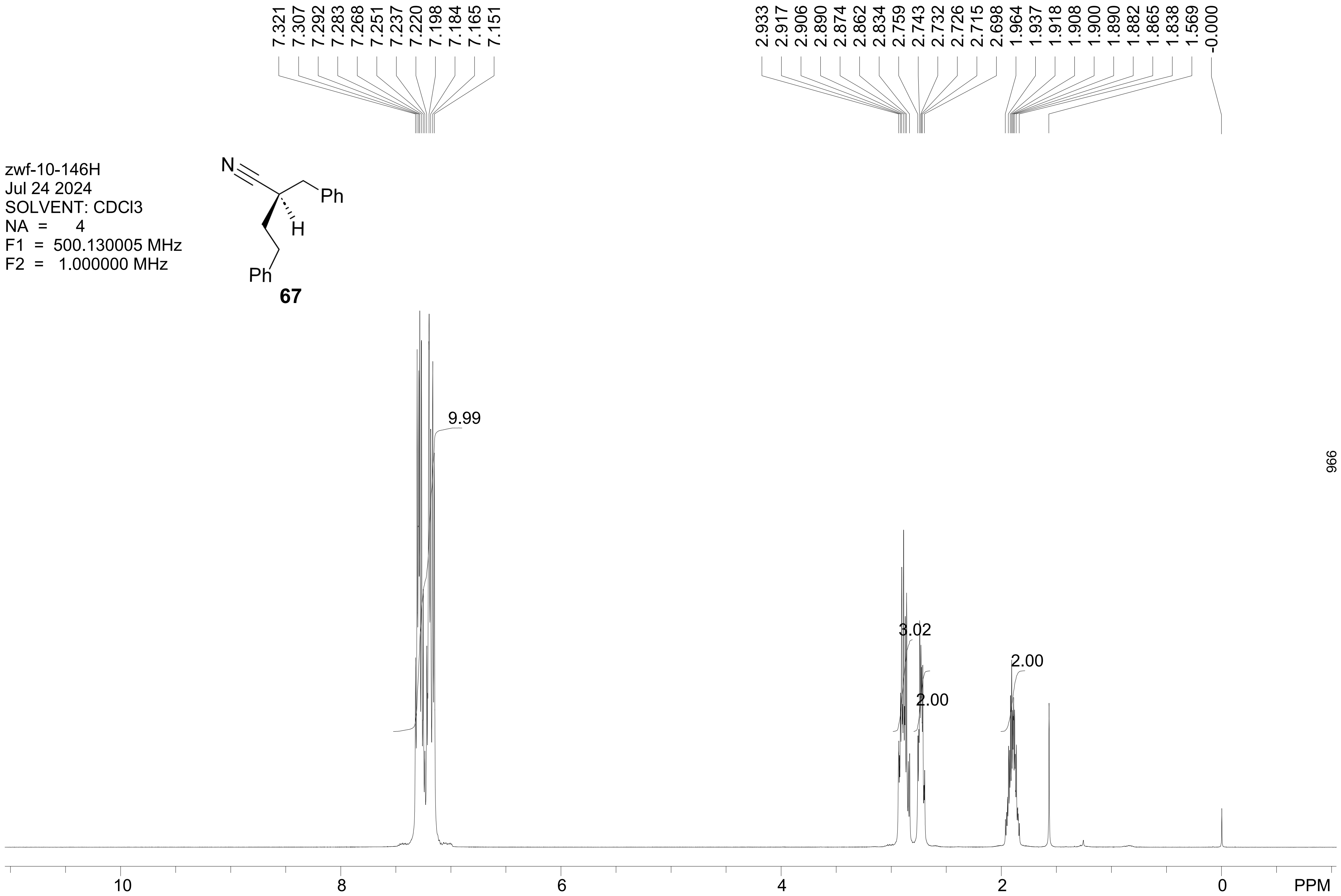
zwf-10-146H
Jul 24 2024
SOLVENT: CDCl3
NA = 4
F1 = 500.130005 MHz
F2 = 1.000000 MHz
7.321
7.307
7.292
7.283
7.268
7.251
7.237
7.220
7.198
7.184
7.165
7.151
2.933
2.917
2.906
2.890
2.874
2.862
2.834
2.759
2.743
2.732
2.726
2.715
2.698
1.964
1.937
1.918
1.908
1.900
1.890
1.882
1.865
1.838
1.569
-0.000
9.99
3.02
2.00
2.00
10
8
6
4
2
0
PPM
N
Ph
H
Ph

67

Area Percent Report

Agilent Technologies

Sample name: zwf-10-146-IG-99-1-0.8-205

Data file: C:\Users\Public\Documents\ChemStation\1\Data\zwf\zwf 2024-07-25 18-31-54\002-P1-C4-zwf-10-146.D

Injection Acquired Date 07/25/2024 18:48:57

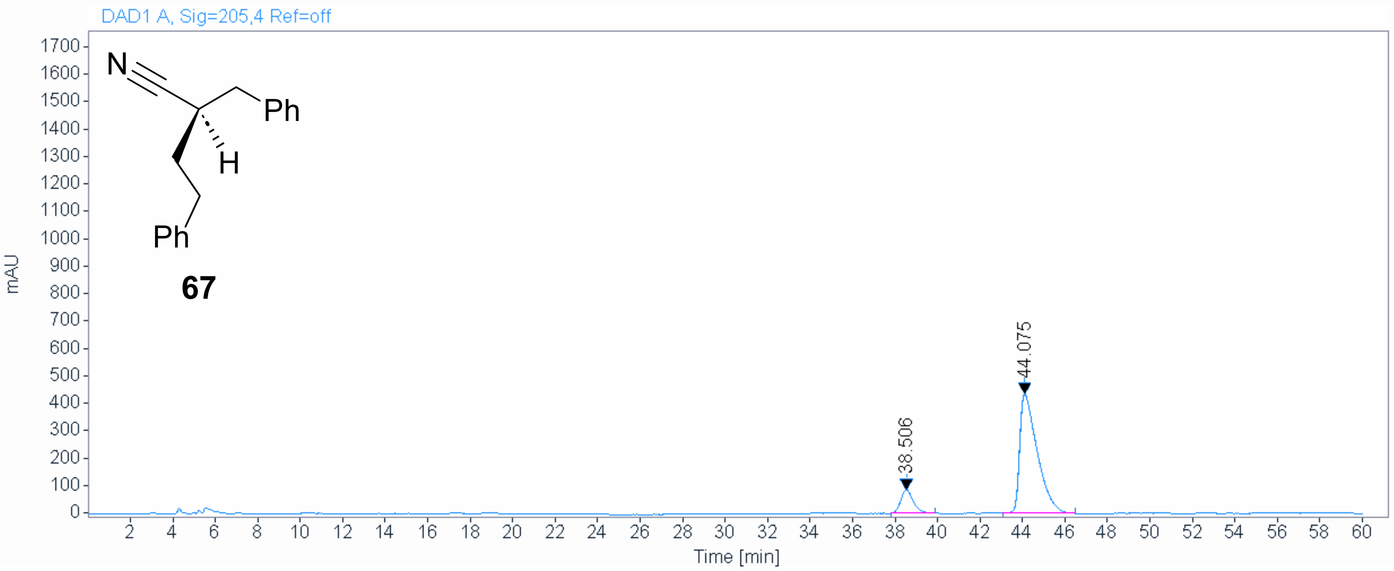

Signal: DAD1 A, Sig=205,4 Ref=off

RT [min]	Width [min]	Height	Area	Area%
38.506	0.6848	84.6273	3477.3933	12.2525
44.075	0.7722	434.7078	24903.7949	87.7475
		Sum	28381.1882	100.0000

Area Percent Report

Agilent Technologies

Sample name: zwf-10-146-rac-IG-99-1-0.8-205

Data file: C:\Users\Public\Documents\ChemStation\1\Data\zwf\zwf 2024-07-25 13-25-56\009-P1-C5-zwf-10-146-rac.D

Injection Acquired Date 07/25/2024 16:48:31

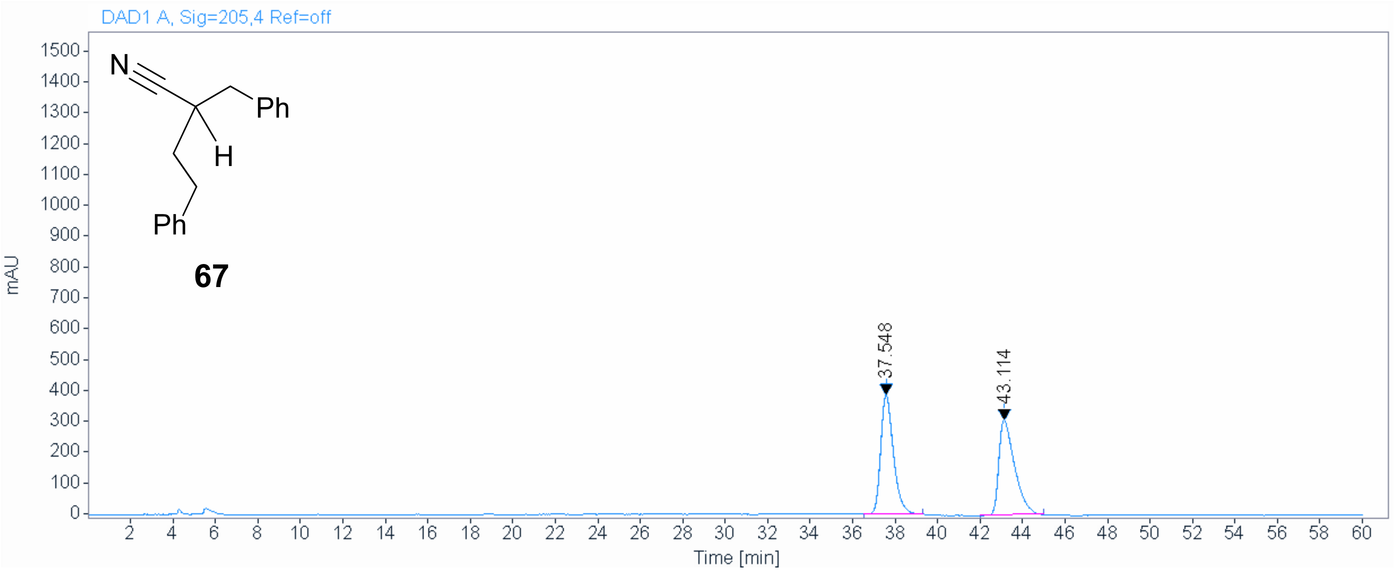

Signal: DAD1 A, Sig=205,4 Ref=off

RT [min]	Width [min]	Height	Area	Area%
37.548	0.5988	385.8750	16036.2197	49.9920
43.114	0.7038	308.9370	16041.3213	50.0080
		Sum	32077.5410	100.0000

zwf-10-147H
Jul 24 2024
SOLVENT: CDCl3
NA = 4
F1 = 500.130005 MHz
F2 = 1.000000 MHz

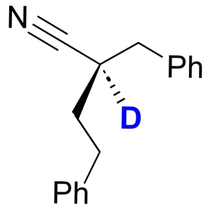

67-d

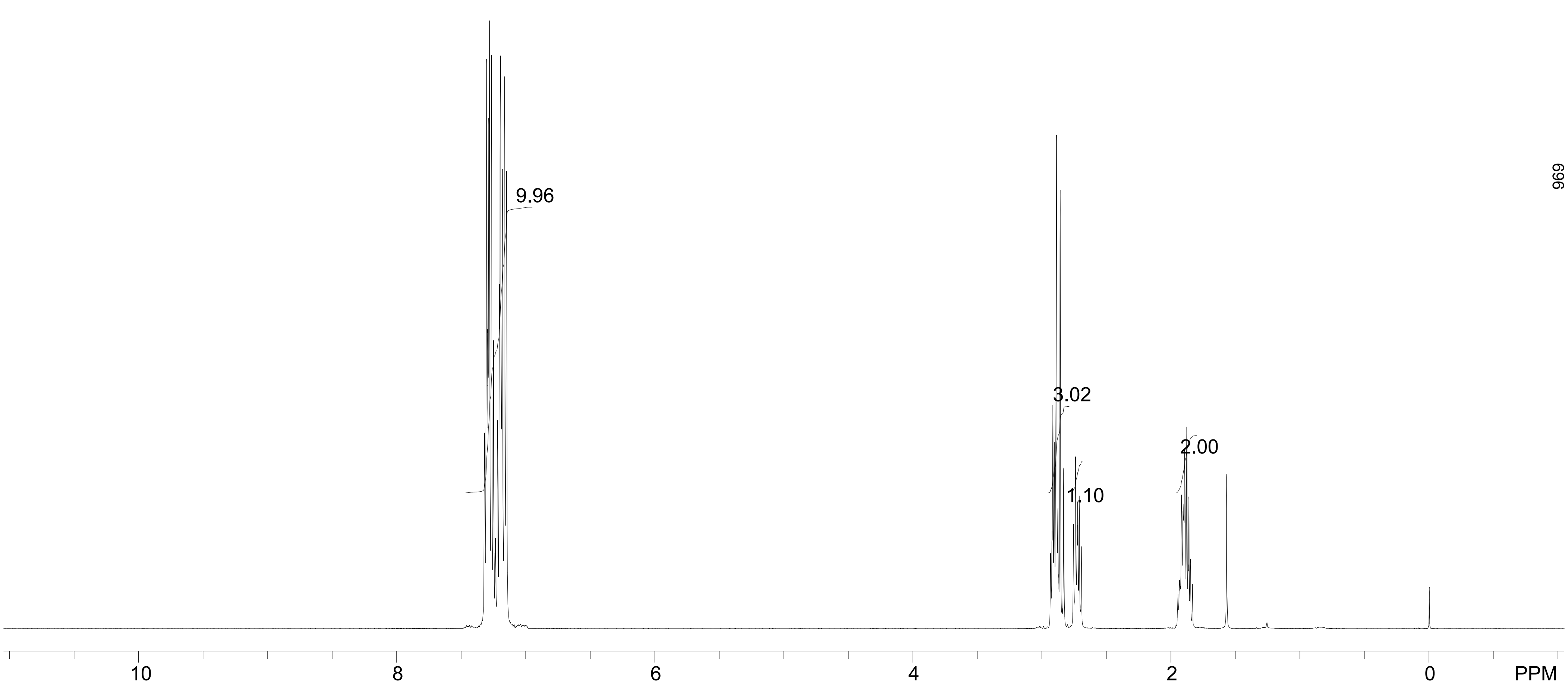

Area Percent Report

Agilent Technologies

Sample name: zwf-10-147-IG-99-1-0.8-205

Data file: C:\Users\Public\Documents\ChemStation\1\Data\zwf\zwf 2024-07-25 01-13-01\009-P1-C4-zwf-10-147.D

Injection Acquired Date 07/25/2024 05:03:08

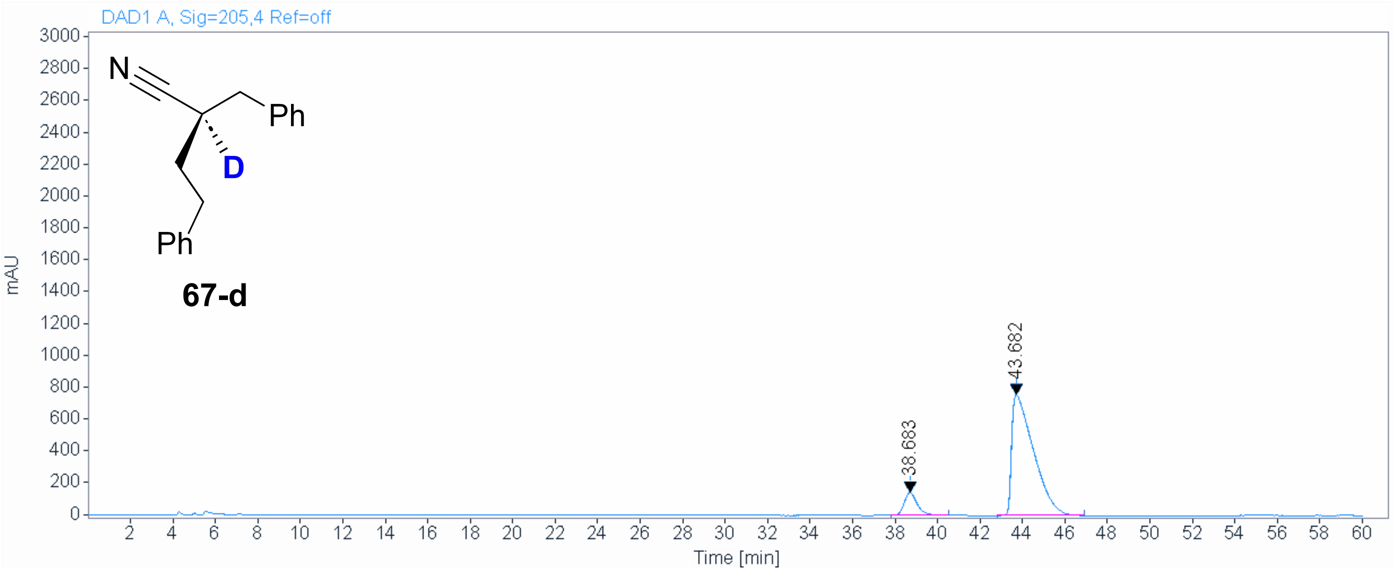

Signal: DAD1 A, Sig=205,4 Ref=off

RT [min]	Width [min]	Height	Area	Area%
38.683	0.5232	143.6461	6145.8237	10.1862
43.682	0.9098	758.2382	54188.7734	89.8138
		Sum	60334.5972	100.0000

Area Percent Report

Agilent Technologies

Sample name: zwf-10-147-rac-IG-99-1-0.8-205

Data file: C:\Users\Public\Documents\ChemStation\1\Data\zwf\zwf 2024-07-25 01-13-01\010-P1-C5-zwf-10-147-rac.D

Injection Acquired Date 07/25/2024 06:04:09

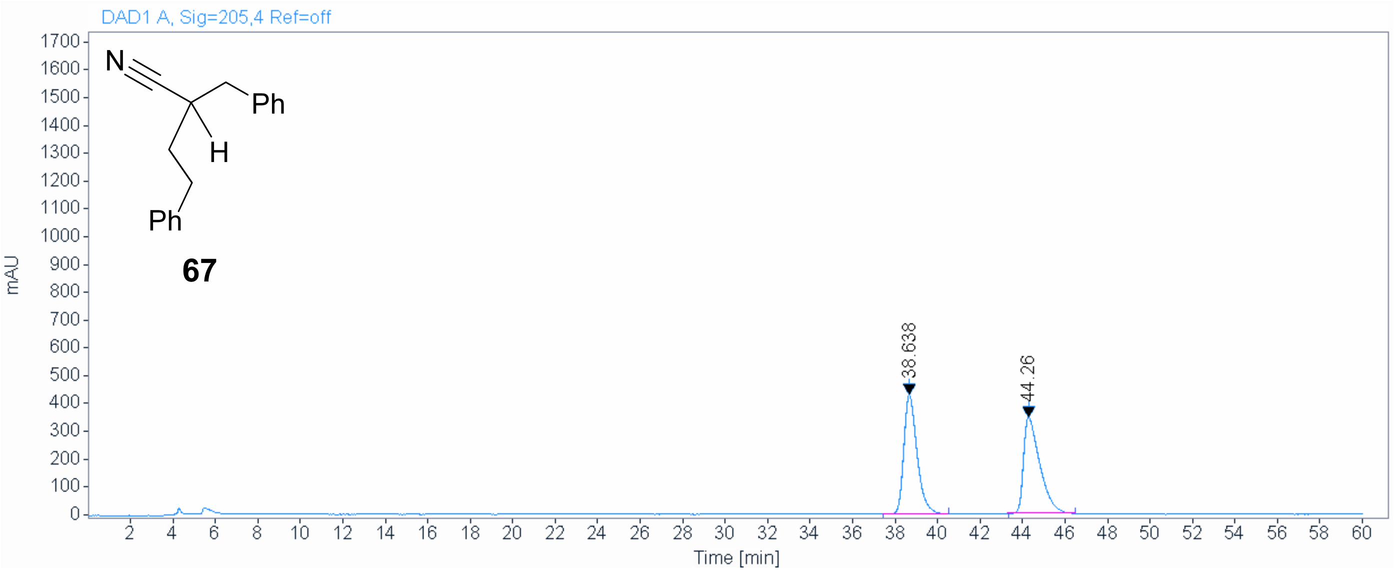

Signal: DAD1 A, Sig=205,4 Ref=off

RT [min]	Width [min]	Height	Area	Area%
38.638	0.5828	428.2350	18601.1348	50.1026
44.260	0.6687	345.9920	18524.9375	49.8974
		Sum	37126.0723	100.0000

zwf-11-047H
Aug 19 2024
SOLVENT: CDCl3
NA = 4
F1 = 400.140015 MHz
F2 = 1.000000 MHz

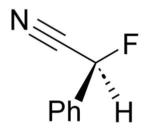

76

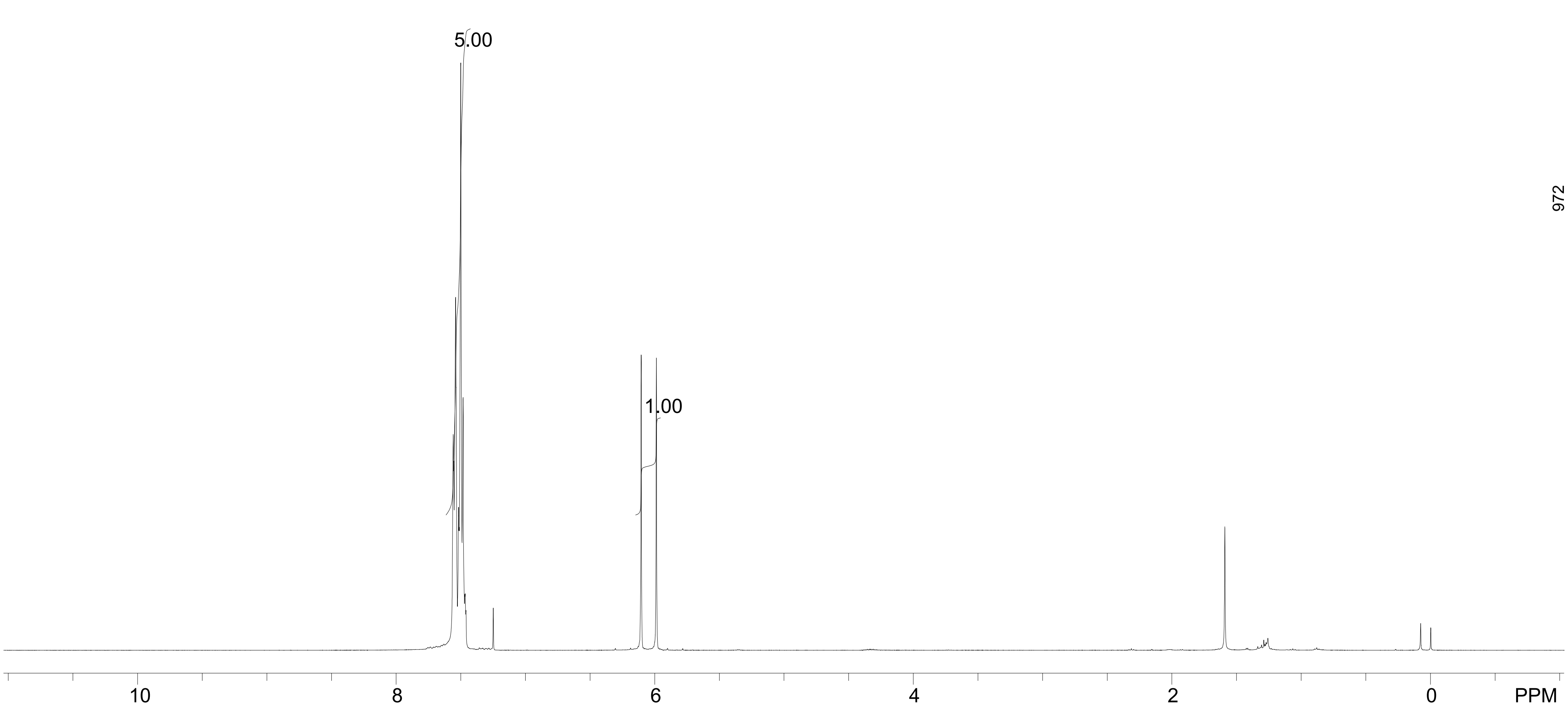

zwf-11-047C
Aug 19 2024
SOLVENT: CDCl3
NA = 100
F1 = 100.615280 MHz
F2 = 1.000000 MHz

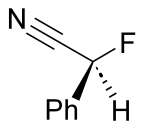

76

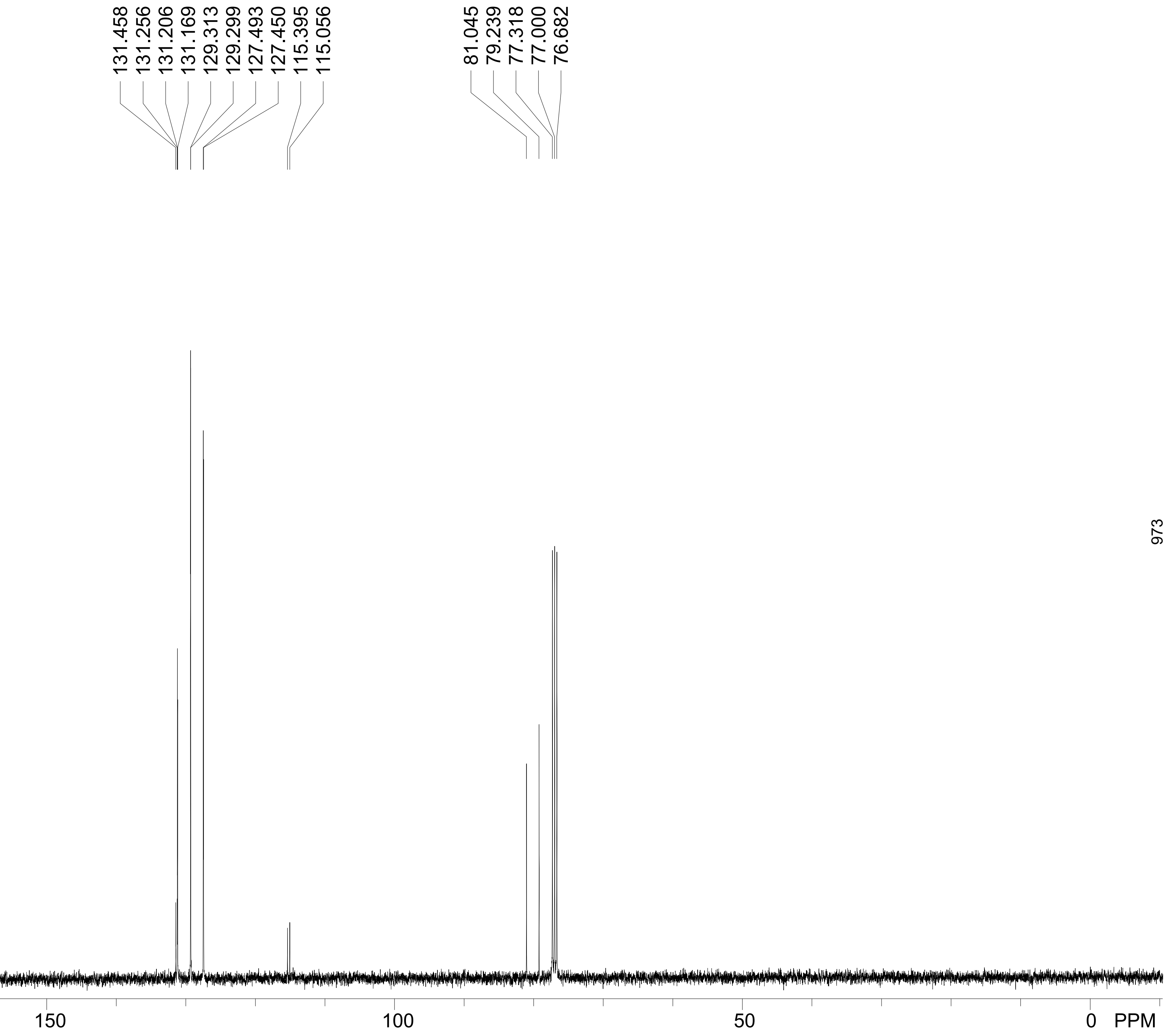

zwf-11-047F
Aug 19 2024
SOLVENT: CDCl3
NA = 16
F1 = 376.507782 MHz
F2 = 1.000000 MHz

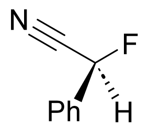

76

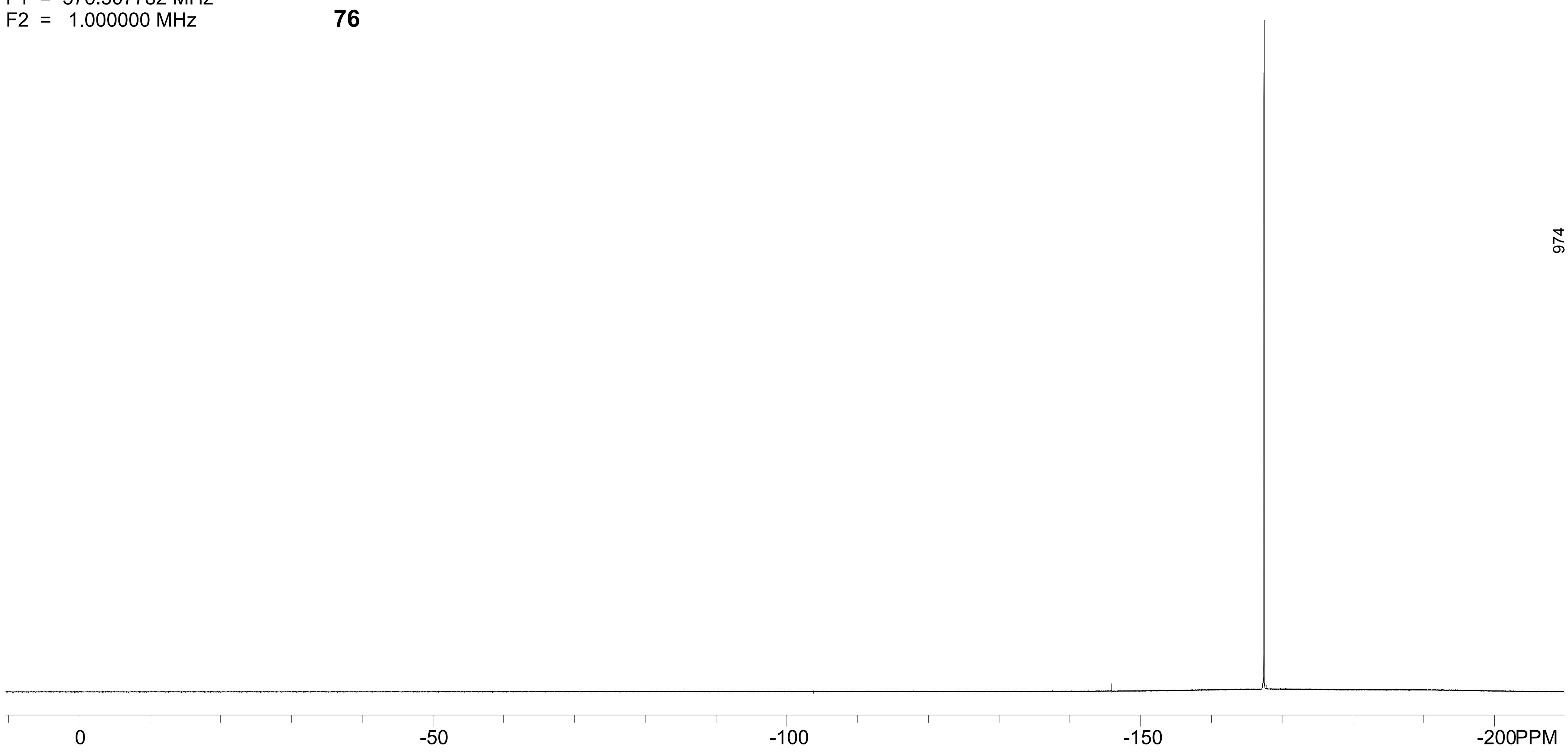

Area Percent Report

Agilent Technologies

Sample name: zwf-11-047-IG-99-1-0.8-205

Data file: C:\Users\Public\Documents\ChemStation\1\Data\zwf\zwf 2024-08-19 05-16-29\027-P1-C7-zwf-11-047.D

Injection Acquired Date 08/19/2024 16:24:16

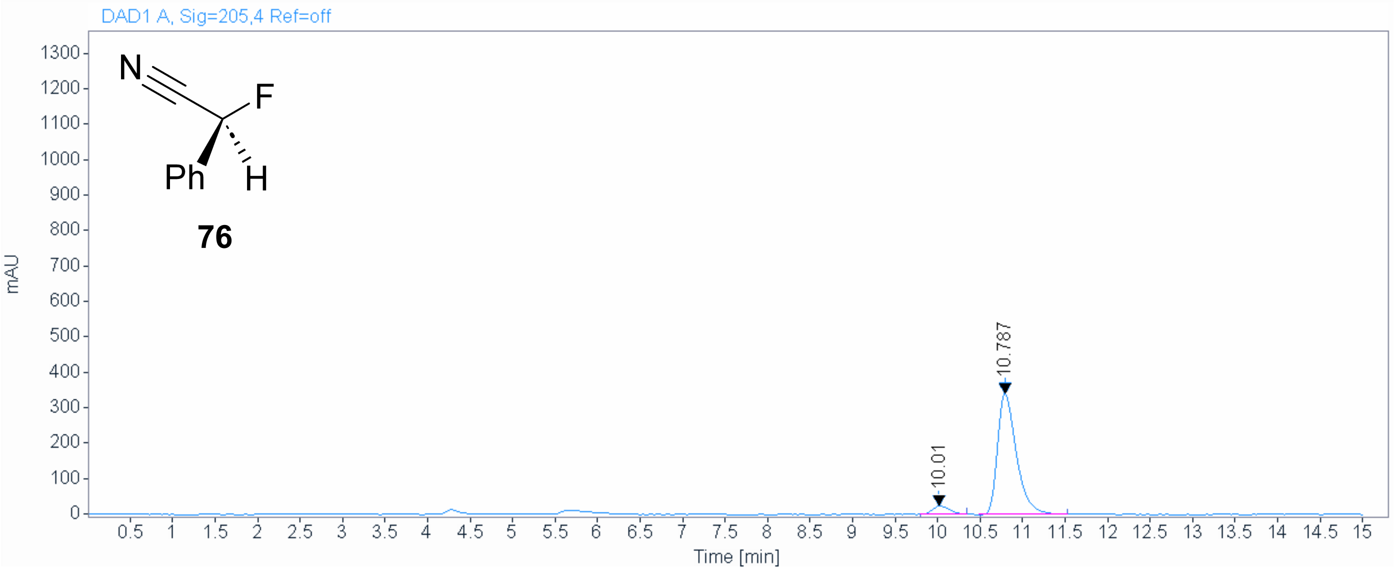

Signal: DAD1 A, Sig=205,4 Ref=off

RT [min]	Width [min]	Height	Area	Area%
10.010	0.2260	22.4413	304.3638	5.5165
10.787	0.2322	340.4283	5212.9438	94.4835
		Sum	5517.3077	100.0000

Area Percent Report

Sample name: zwf-11-047-rac-IG-99-1-0.8-205

Data file: C:\Users\Public\Documents\ChemStation\1\Data\zwf\zwf 2024-08-19 05-16-29\024-P1-C9-zwf-11-047-rac.D

Injection Acquired Date 08/19/2024 15:31:16

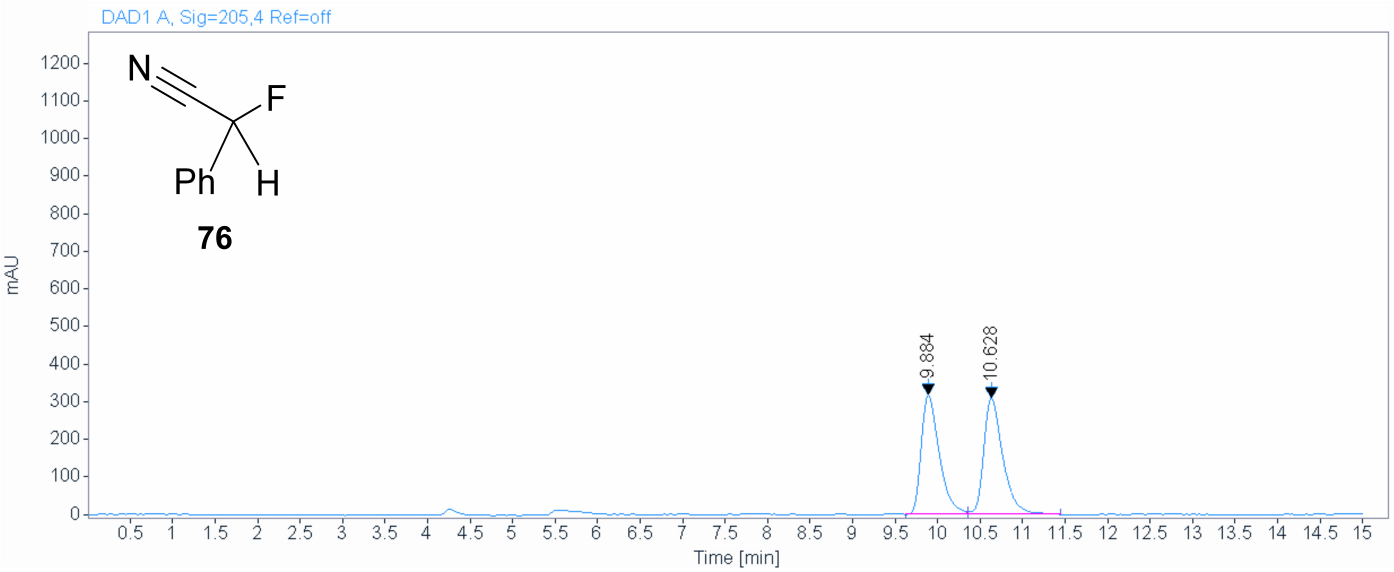

Signal: DAD1 A, Sig=205,4 Ref=off

RT [min]	Width [min]	Height	Area	Area%
9.884	0.2257	318.2497	4737.8438	49.2293
10.628	0.2378	310.2276	4886.1895	50.7707
		Sum	9624.0332	100.0000

zwf-11-048H
Aug 18 2024
SOLVENT: CDCl3
NA = 4
F1 = 600.169983 MHz
F2 = 1.000000 MHz

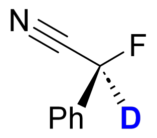

76-d

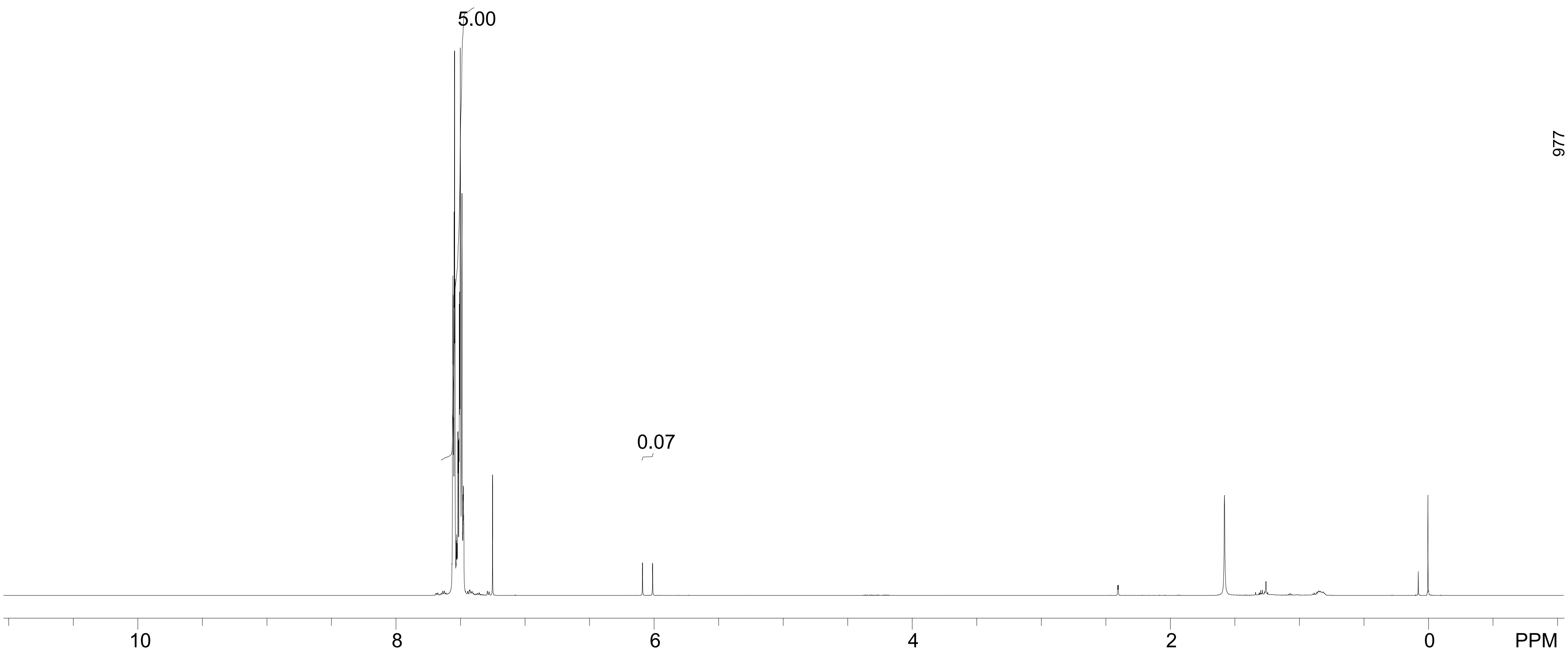

zwf-11-048C
Aug 18 2024
SOLVENT: CDCl3
NA = 400
F1 = 125.757790 MHz
F2 = 1.000000 MHz

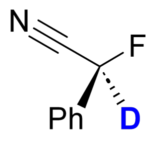

76-d

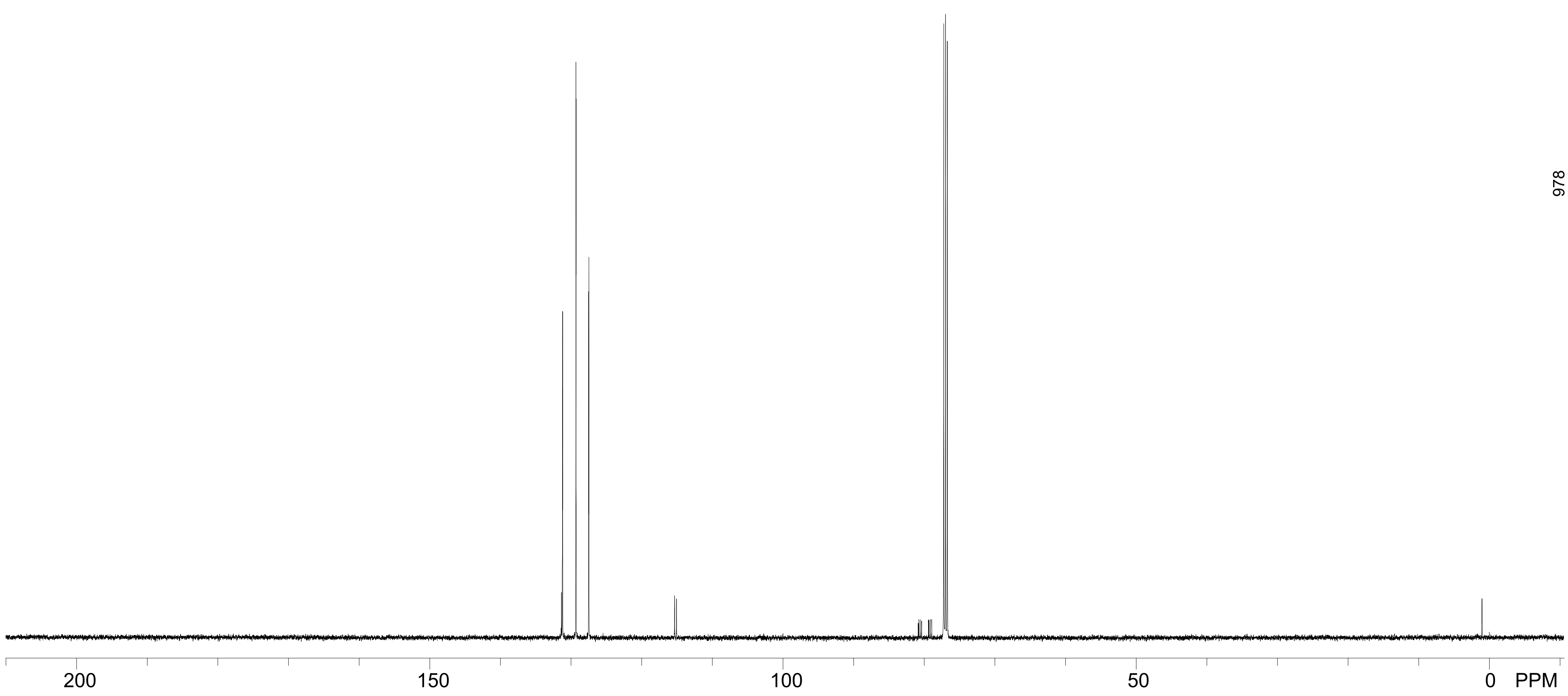

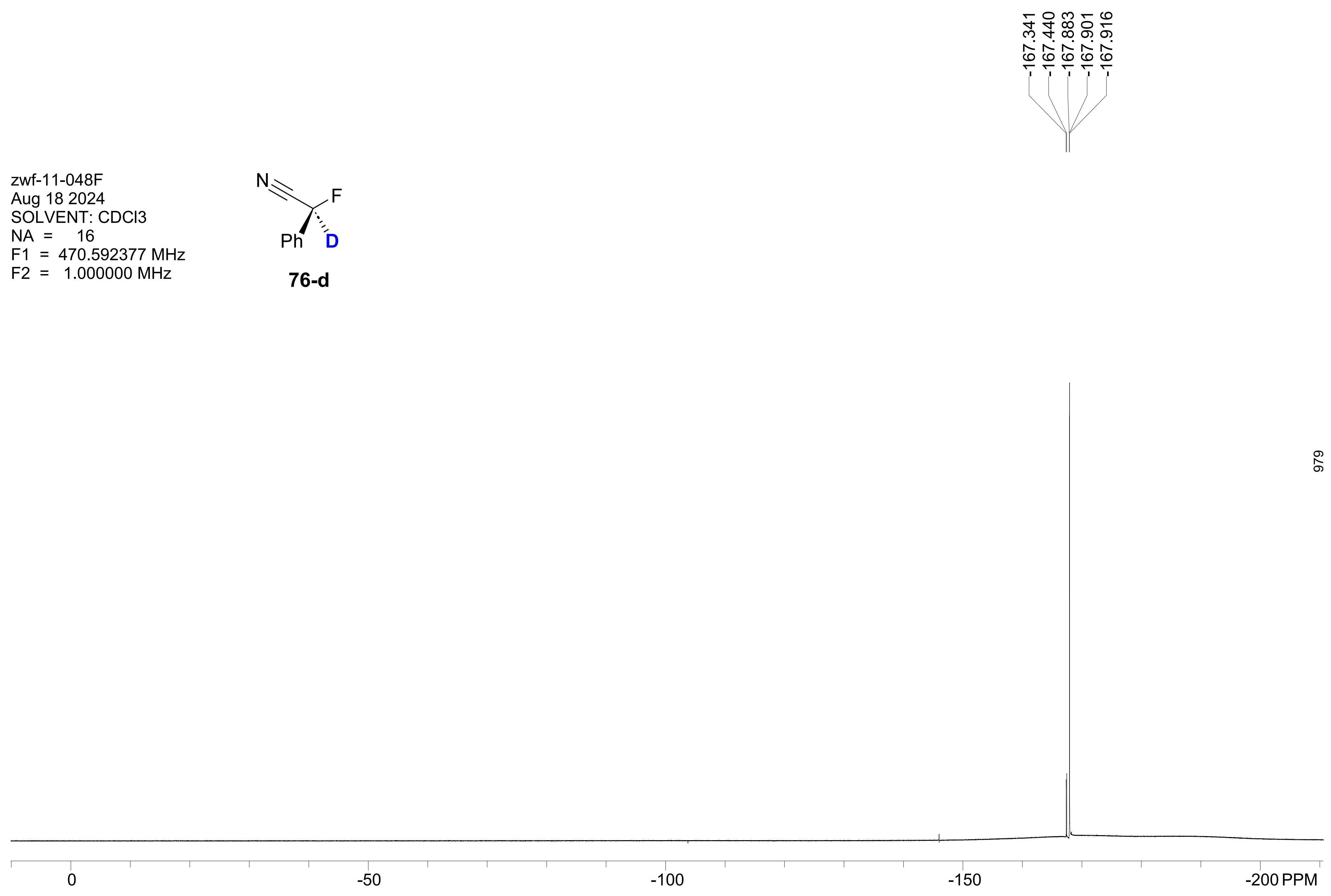
zwf-11-048F
Aug 18 2024
SOLVENT: CDCl3
NA = 16
F1 = 470.592377 MHz
F2 = 1.000000 MHz
N
F
Ph
D
76-d
-167.341
-167.440
-167.883
-167.901
-167.916
0
-50
-100
-150
-200 PPM

Area Percent Report

Agilent Technologies

Sample name: zwf-11-048-IG-99-1-0.8-205

Data file: C:\Users\Public\Documents\ChemStation\1\Data\zwf\zwf 2024-08-19 05-16-29\025-P1-C8-zwf-11-048.D

Injection Acquired Date 08/19/2024 15:47:15

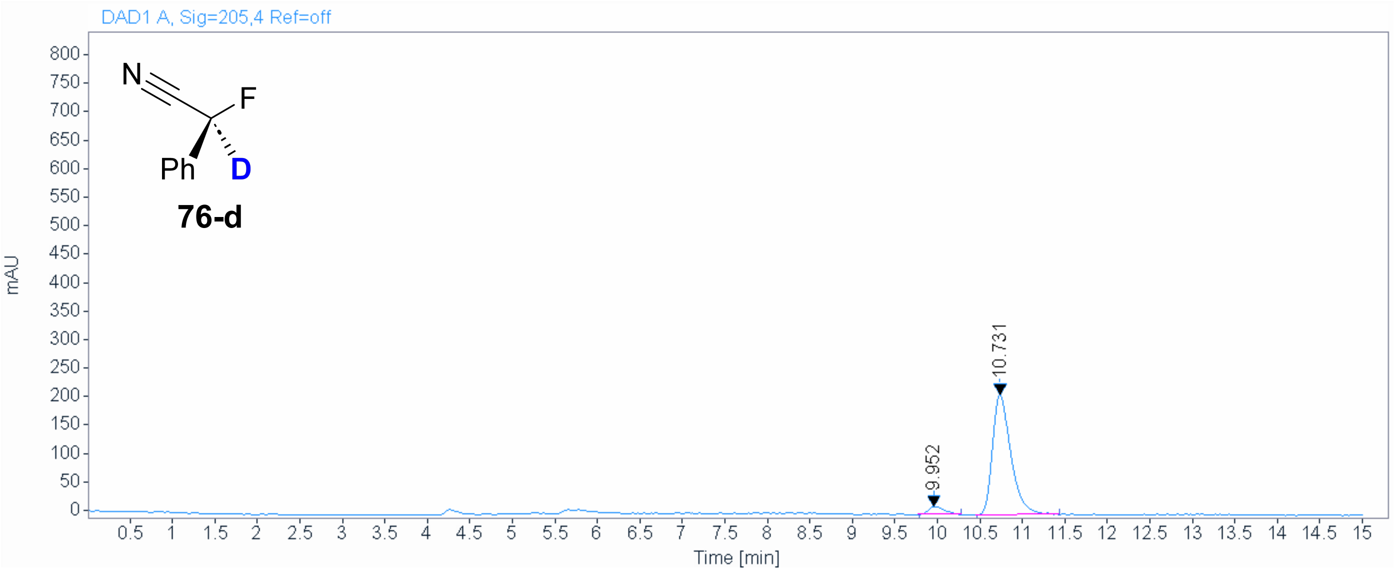

Signal: DAD1 A, Sig=205,4 Ref=off

RT [min]	Width [min]	Height	Area	Area%
9.952	0.2251	13.8189	186.6456	5.5196
10.731	0.2518	211.4583	3194.8315	94.4804
		Sum	3381.4772	100.0000

Area Percent Report

Agilent Technologies

Sample name: zwf-11-047-rac-IG-99-1-0.8-205

Data file: C:\Users\Public\Documents\ChemStation\1\Data\zwf\zwf 2024-08-19 05-16-29\024-P1-C9-zwf-11-047-rac.D

Injection Acquired Date 08/19/2024 15:31:16

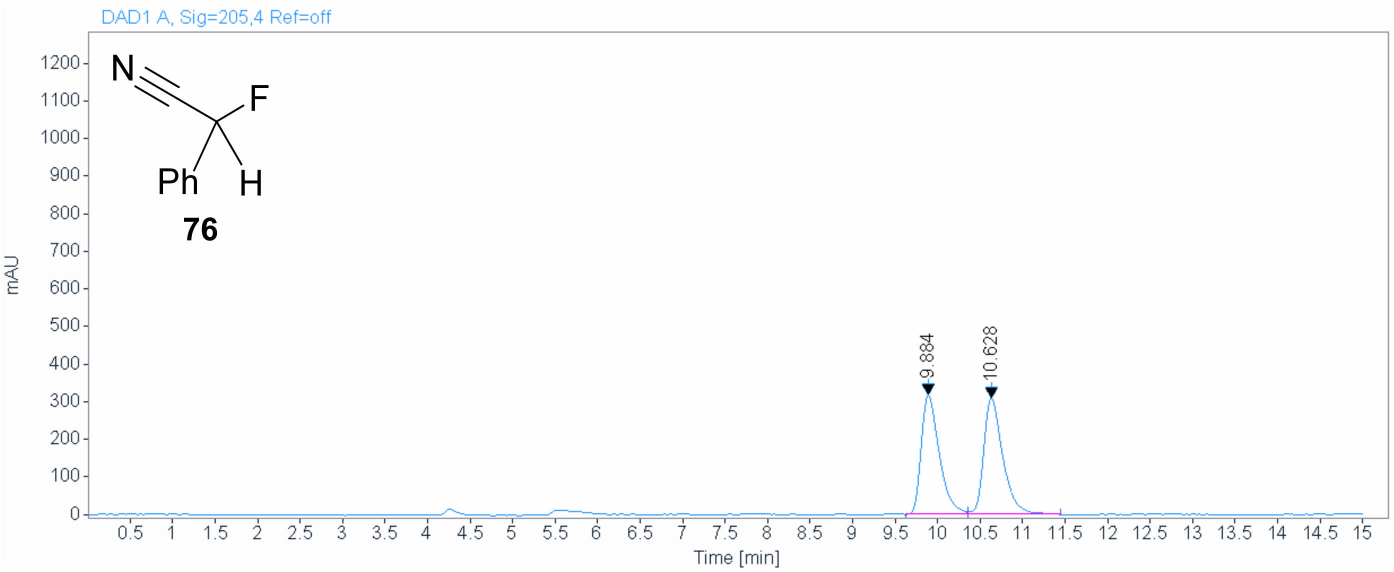

Signal: DAD1 A, Sig=205,4 Ref=off

RT [min]	Width [min]	Height	Area	Area%
9.884	0.2257	318.2497	4737.8438	49.2293
10.628	0.2378	310.2276	4886.1895	50.7707
		Sum	9624.0332	100.0000

zwf-11-040H
Aug 19 2024
SOLVENT: CDCl3
NA = 4
F1 = 500.130005 MHz
F2 = 1.000000 MHz

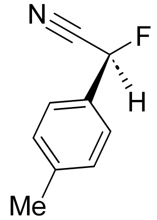

77

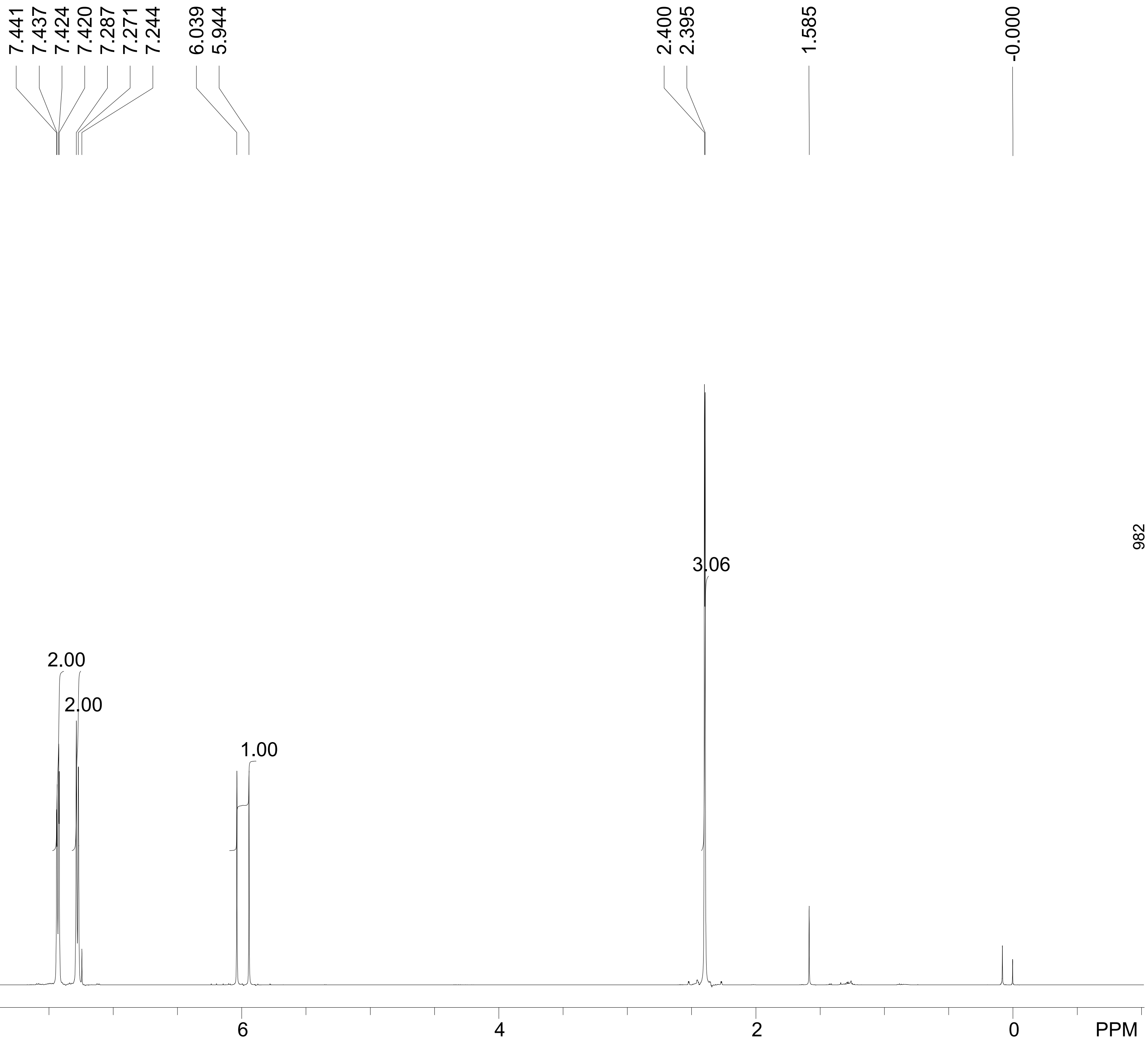

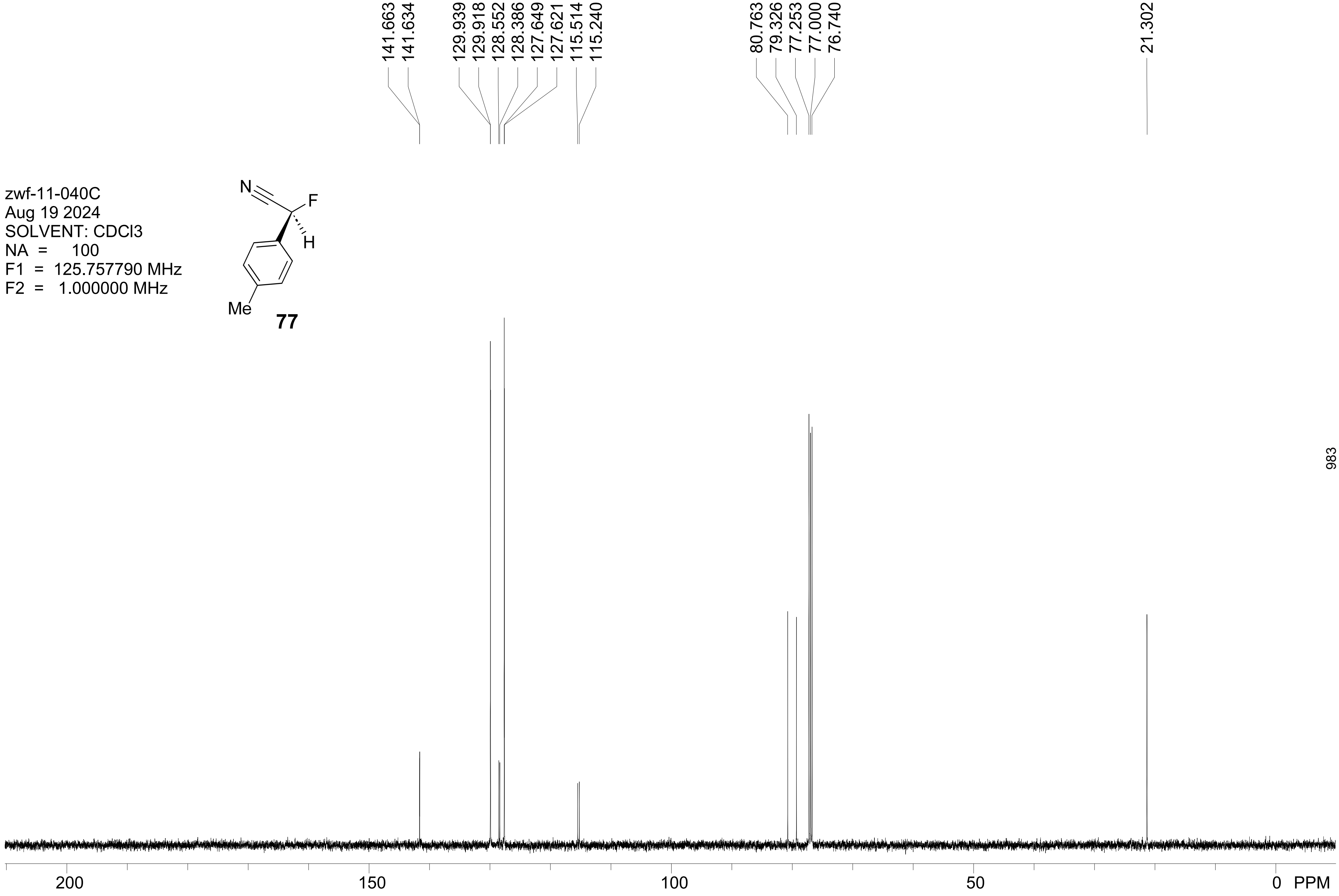
zwf-11-040C
Aug 19 2024
SOLVENT: CDCl3
NA = 100
F1 = 125.757790 MHz
F2 = 1.000000 MHz
141.663
141.634
129.939
129.918
128.552
128.386
127.649
127.621
115.514
115.240
80.763
79.326
77.253
77.000
76.740
21.302
200
150
100
50
0 PPM
N
F
H
Me

77

zwf-11-040F
Aug 19 2024
SOLVENT: CDCl3
NA = 16
F1 = 470.592377 MHz
F2 = 1.000000 MHz

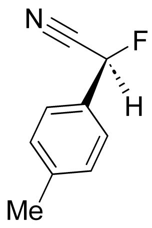

77

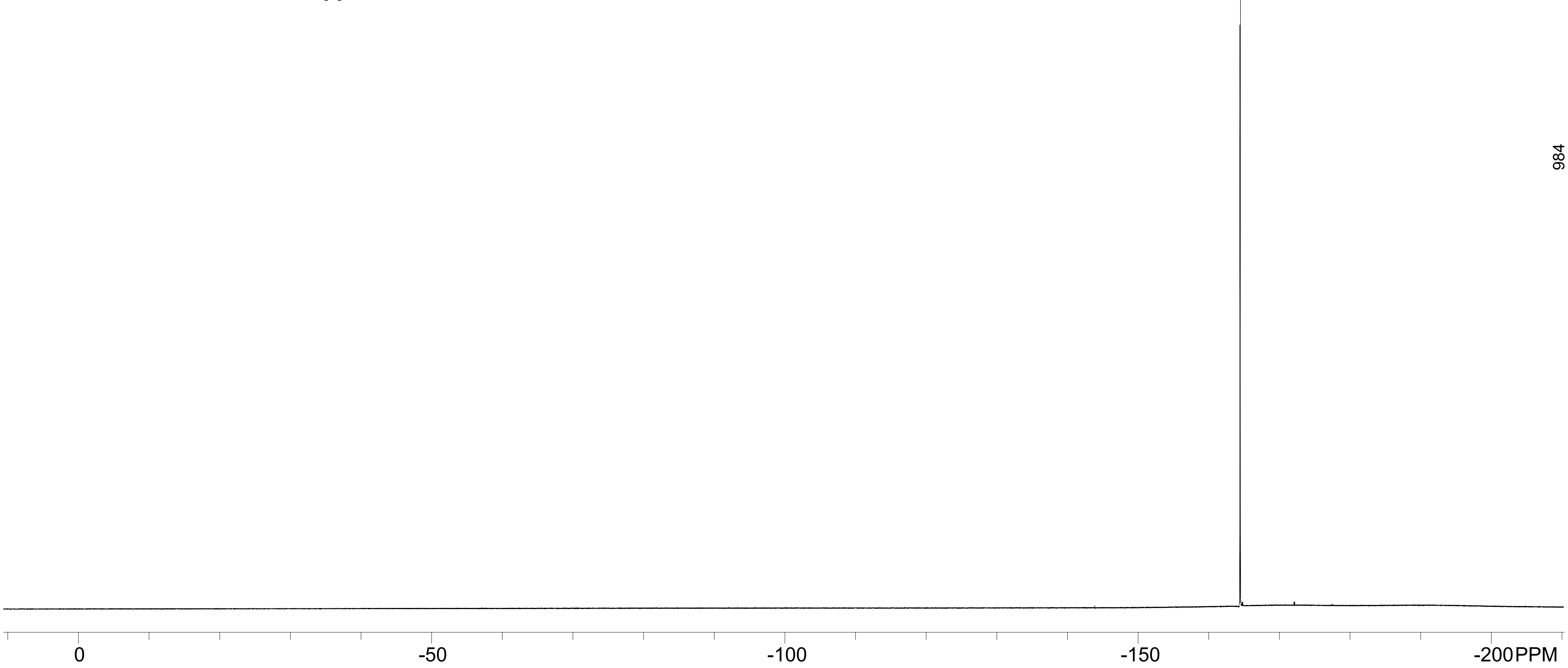

Area Percent Report

Agilent Technologies

Sample name: zwf-11-040-IG-99-1-0.8-205

Data file: C:\Users\Public\Documents\ChemStation\1\Data\zwf\zwf 2024-08-19 05-16-29\021-P1-C4-zwf-11-040.D

Injection Acquired Date 08/19/2024 14:43:21

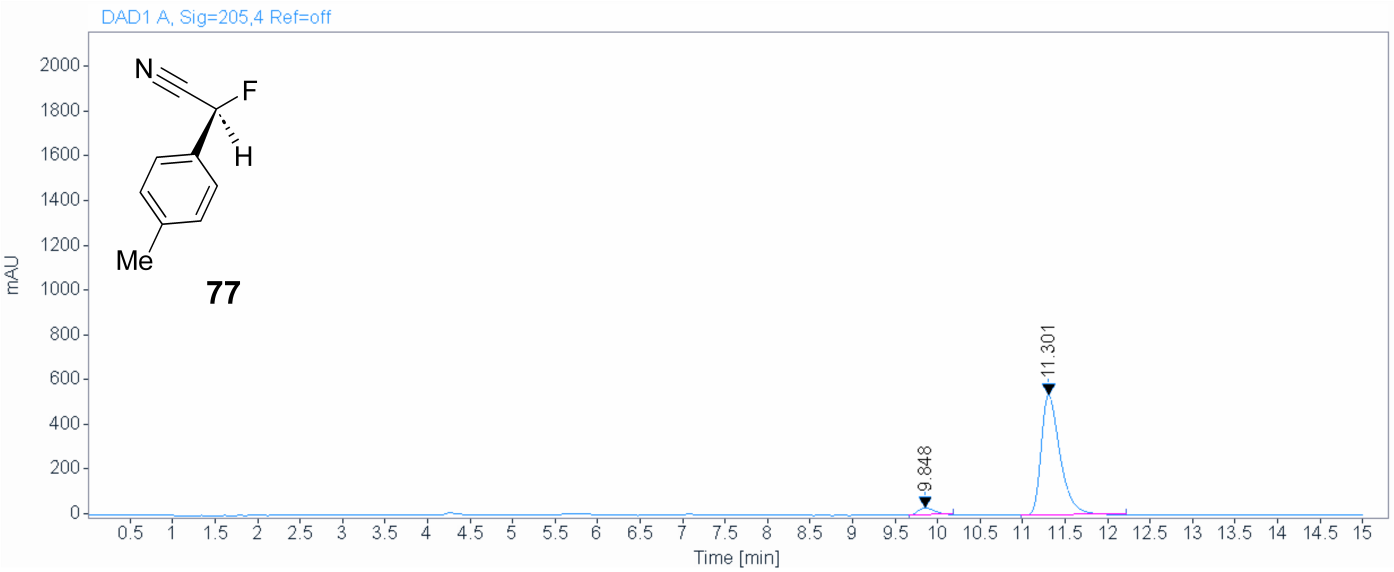

Signal: DAD1 A, Sig=205,4 Ref=off

RT [min]	Width [min]	Height	Area	Area%
9.848	0.2285	32.0085	438.7619	4.9910
11.301	0.2373	537.3704	8352.3135	95.0090
		Sum	8791.0754	100.0000

Area Percent Report

Agilent Technologies

Sample name: zwf-11-040-rac-IG-99-1-0.8-205

Data file: C:\Users\Public\Documents\ChemStation\1\Data\zwf\zwf 2024-08-19 05-16-29\020-P1-C6-zwf-11-040-rac.D

Injection Acquired Date 08/19/2024 14:27:20

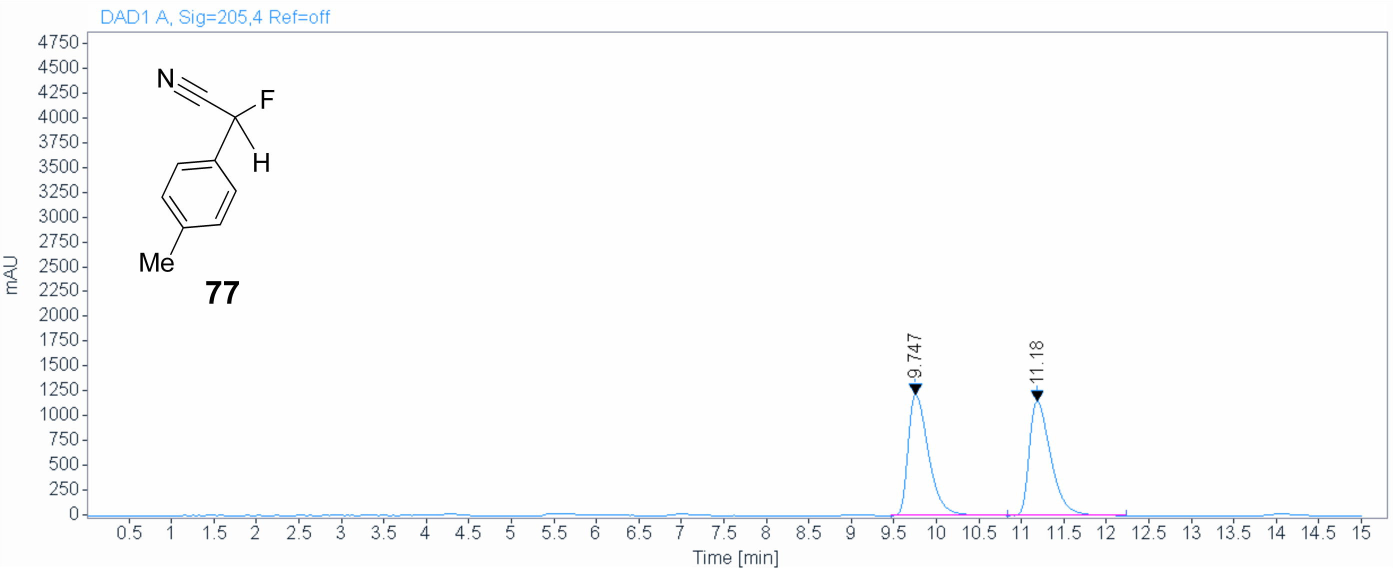

Signal: DAD1 A, Sig=205,4 Ref=off

RT [min]	Width [min]	Height	Area	Area%
9.747	0.2582	1216.0194	20501.4121	50.2739
11.180	0.2661	1147.8894	20278.0039	49.7261
		Sum	40779.4160	100.0000

zwf-10-118H
Jul 18 2024
SOLVENT: CDCl3
NA = 4
F1 = 500.130005 MHz
F2 = 1.000000 MHz

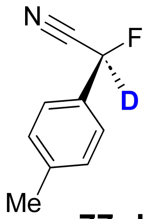

77-d

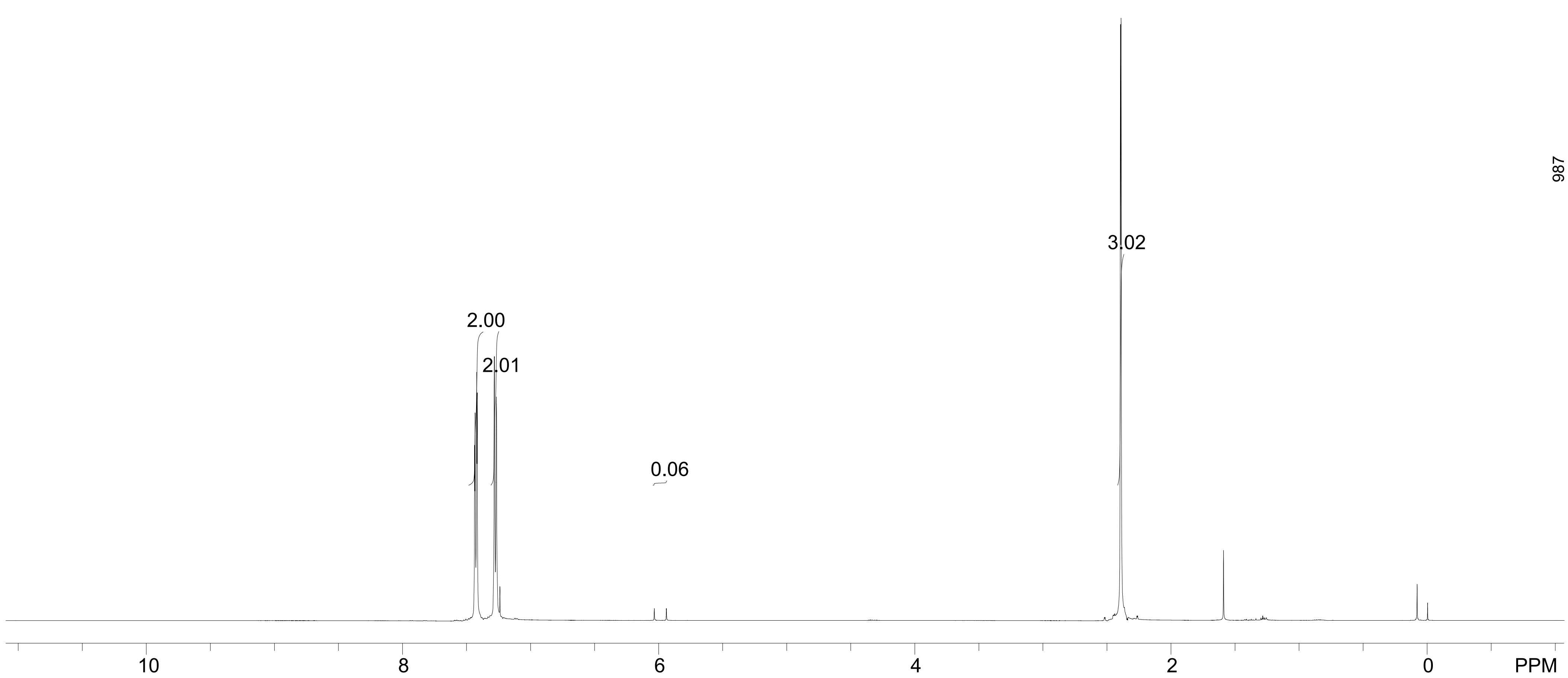

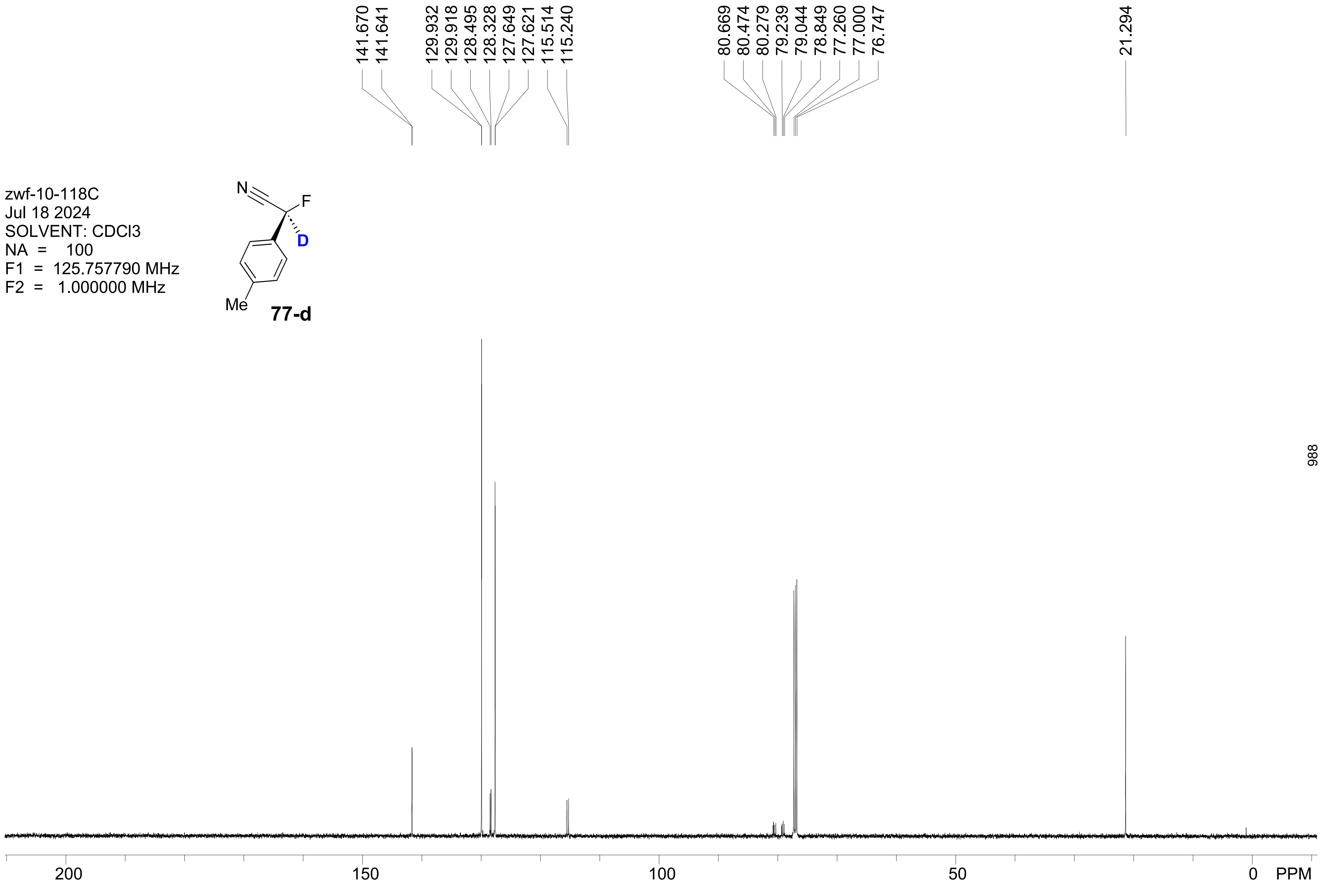
zwf-10-118C
Jul 18 2024
SOLVENT: CDCl3
NA = 100
F1 = 125.757790 MHz
F2 = 1.000000 MHz
N
F
D
Me
77-d
141.670
141.641
129.932
129.918
128.495
128.328
127.649
127.621
115.514
115.240
80.669
80.474
80.279
79.239
79.044
78.849
77.260
77.000
76.747
21.294
200
150
100
50
0
PPM

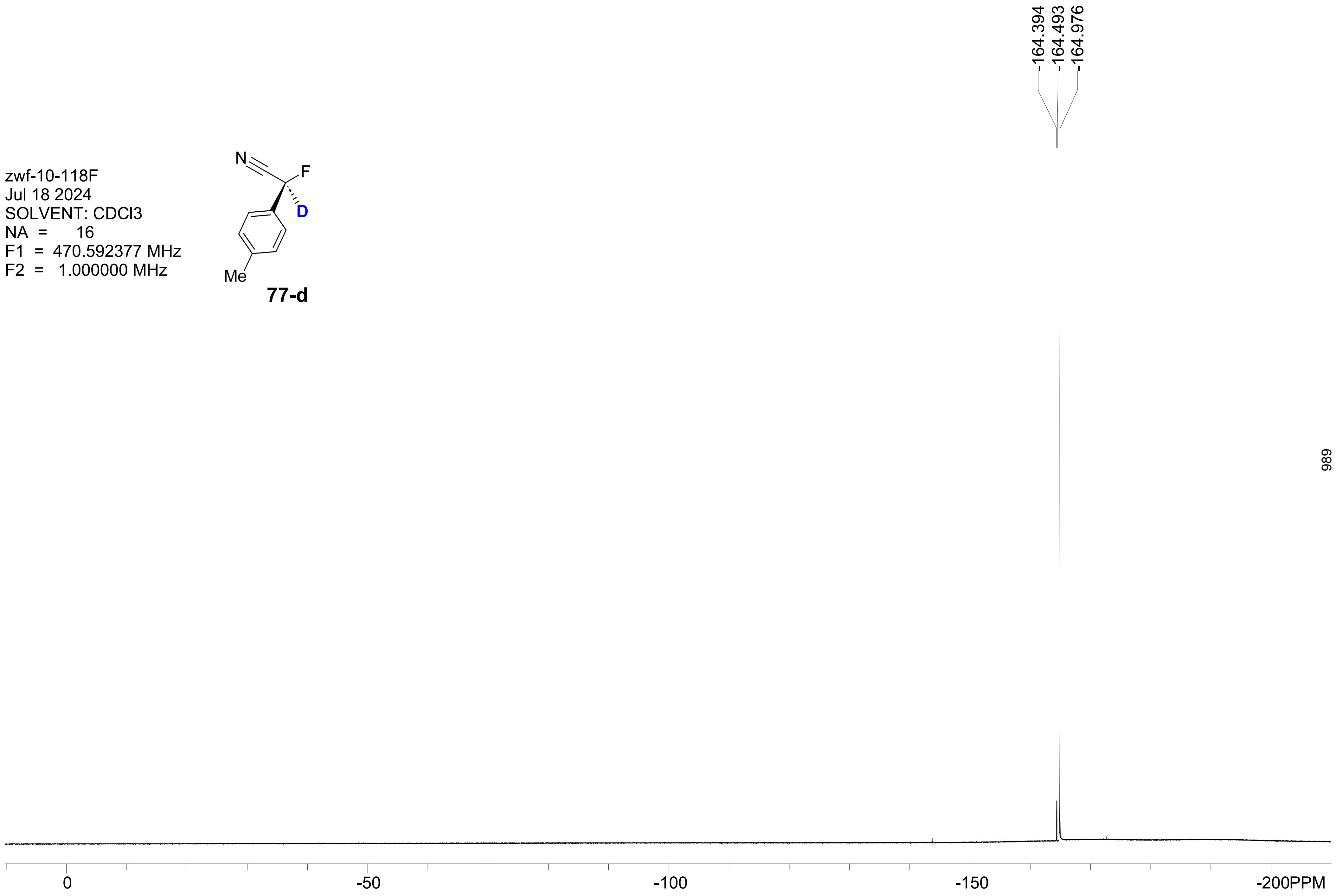
zwf-10-118F
Jul 18 2024
SOLVENT: CDCl3
NA = 16
F1 = 470.592377 MHz
F2 = 1.000000 MHz
N
F
D
Me
77-d
-164.394
-164.493
-164.976
0
-50
-100
-150
-200PPM

Area Percent Report

Agilent Technologies

Sample name: zwf-10-118-IE-99-1-0.8-205

Data file: C:\Users\Public\Documents\ChemStation\1\Data\zwf\zwf 2024-07-19 12-07-32\010-P1-C6-zwf-10-118.D

Injection Acquired Date 07/19/2024 18:49:08

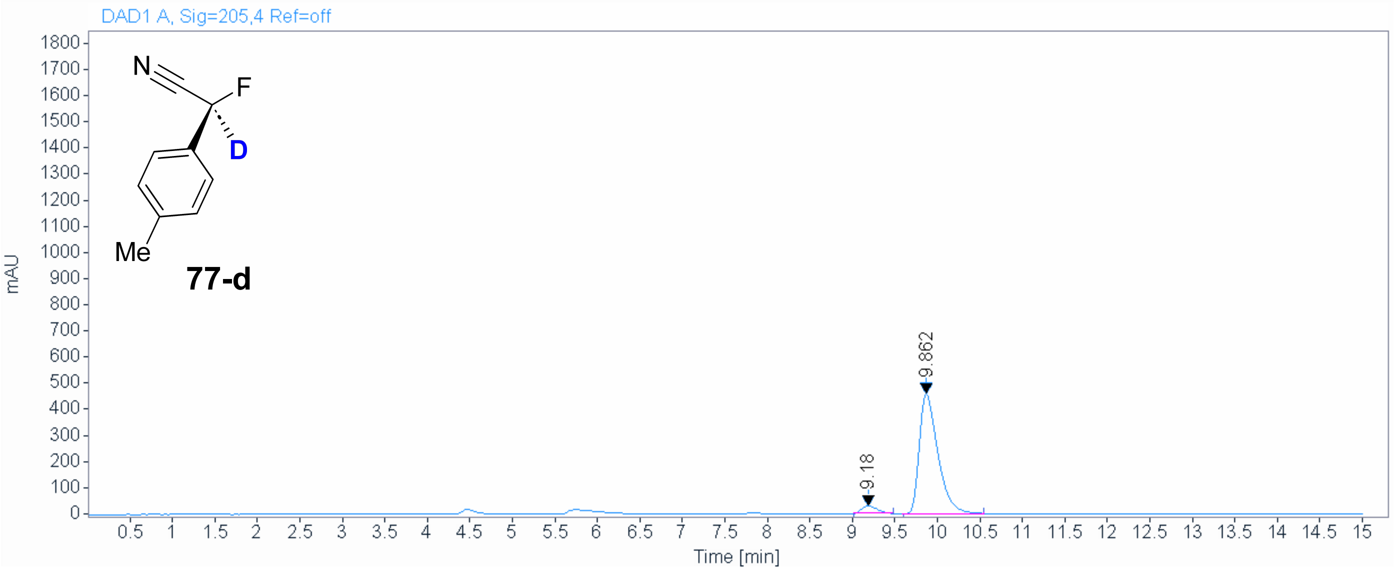

Signal: DAD1 A, Sig=205,4 Ref=off

RT [min]	Width [min]	Height	Area	Area%
9.180	0.2135	28.7952	368.8416	4.9064
9.862	0.2577	462.2761	7148.7783	95.0936
		Sum	7517.6199	100.0000

Area Percent Report

Agilent Technologies

Sample name: zwf-10-116-rac-IE-99-1-0.8-205

Data file: C:\Users\Public\Documents\ChemStation\1\Data\zwf\zwf 2024-07-19 12-07-32\008-P1-C4-zwf-10-116-rac.D

Injection Acquired Date 07/19/2024 18:17:04

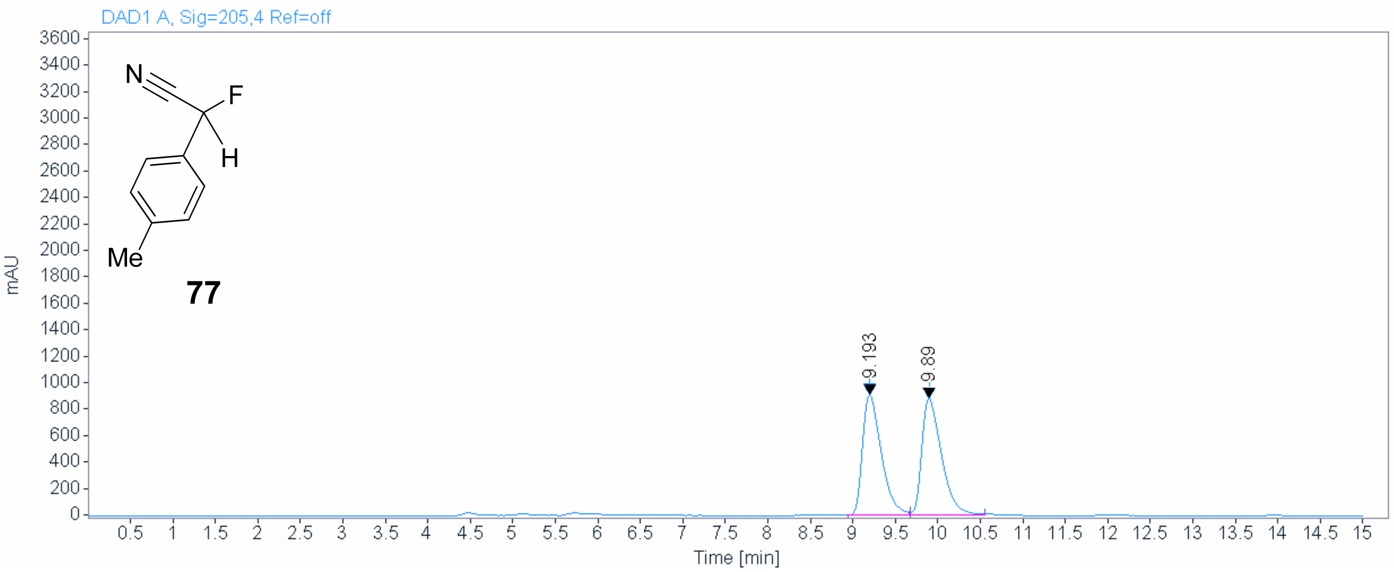

Signal: DAD1 A, Sig=205,4 Ref=off

RT [min]	Width [min]	Height	Area	Area%
9.193	0.2317	908.0942	13988.0010	49.3155
9.890	0.2713	883.1600	14376.3340	50.6845
		Sum	28364.3350	100.0000

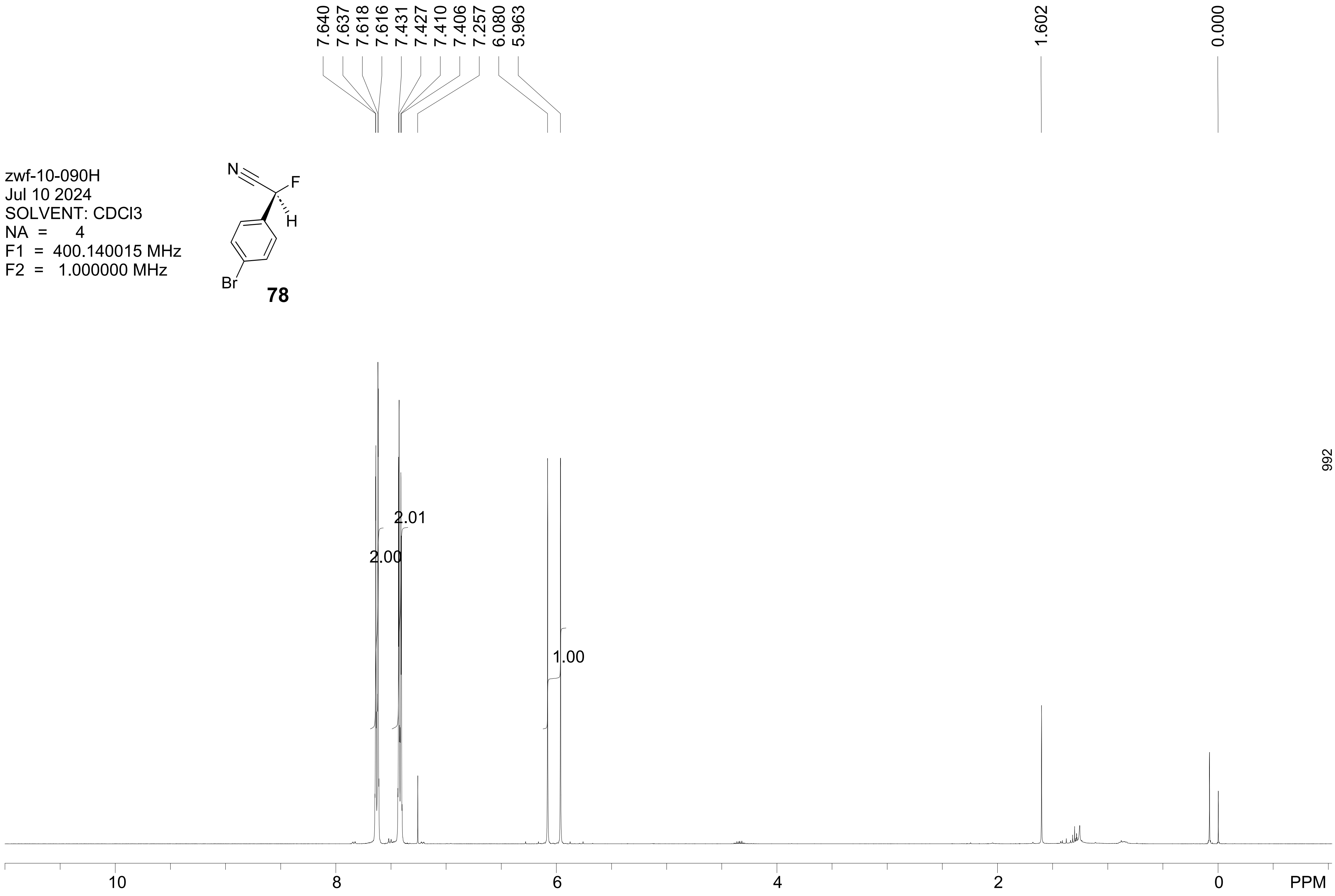

zwf-10-090H
Jul 10 2024
SOLVENT: CDCl3
NA = 4
F1 = 400.140015 MHz
F2 = 1.000000 MHz
N
F
H
Br
78
7.640
7.637
7.618
7.616
7.431
7.427
7.410
7.406
7.257
6.080
5.963
1.602
0.000
2.00
2.01
1.00
10
8
6
4
2
0
PPM

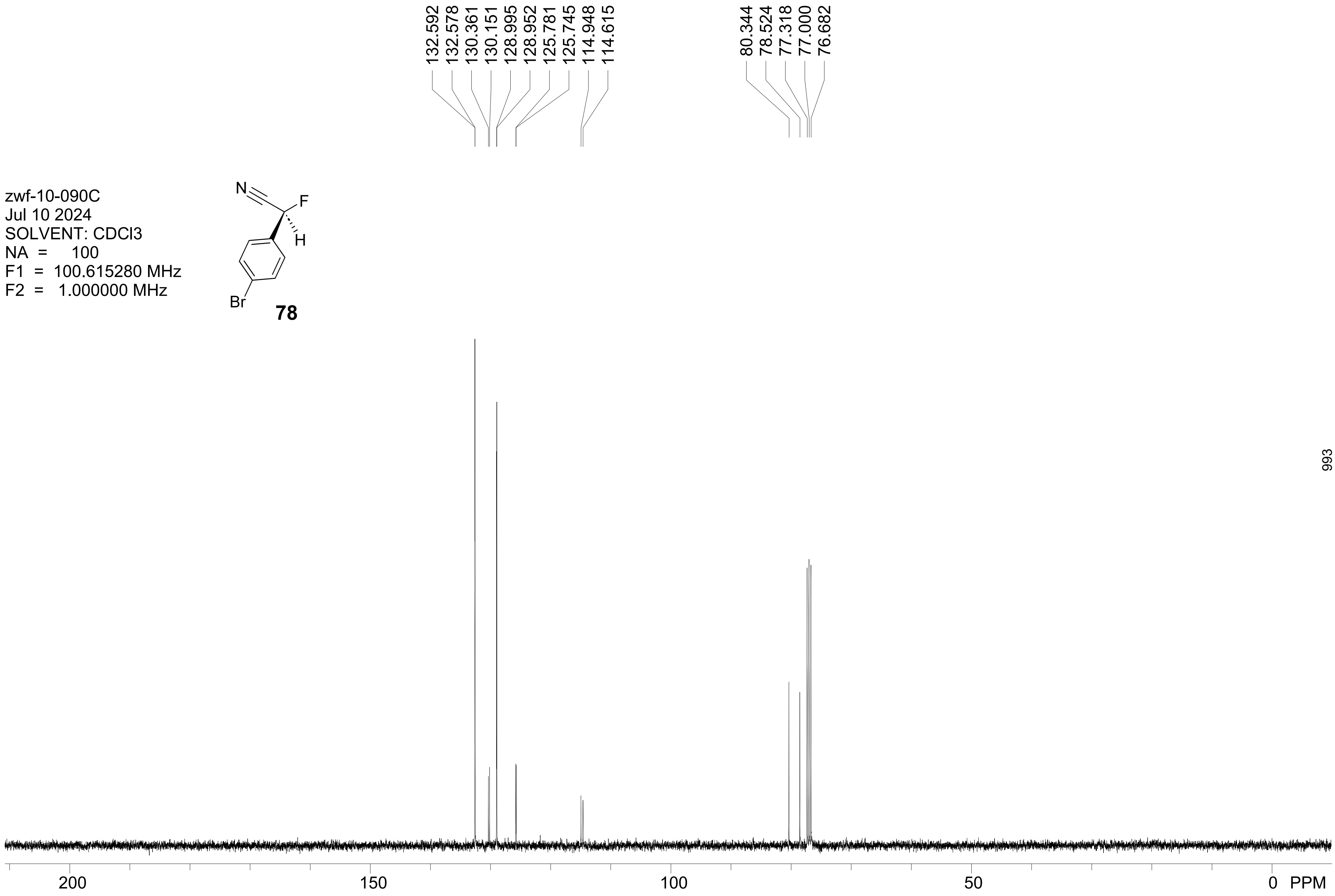
zwf-10-090C
Jul 10 2024
SOLVENT: CDCl3
NA = 100
F1 = 100.615280 MHz
F2 = 1.000000 MHz
N
F
H
Br
78
132.592
132.578
130.361
130.151
128.995
128.952
125.781
125.745
114.948
114.615
80.344
78.524
77.318
77.000
76.682
200
150
100
50
0 PPM

zwf-10-090F
Jul 10 2024
SOLVENT: CDCl3
NA = 16
F1 = 376.507782 MHz
F2 = 1.000000 MHz

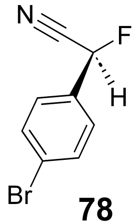

78

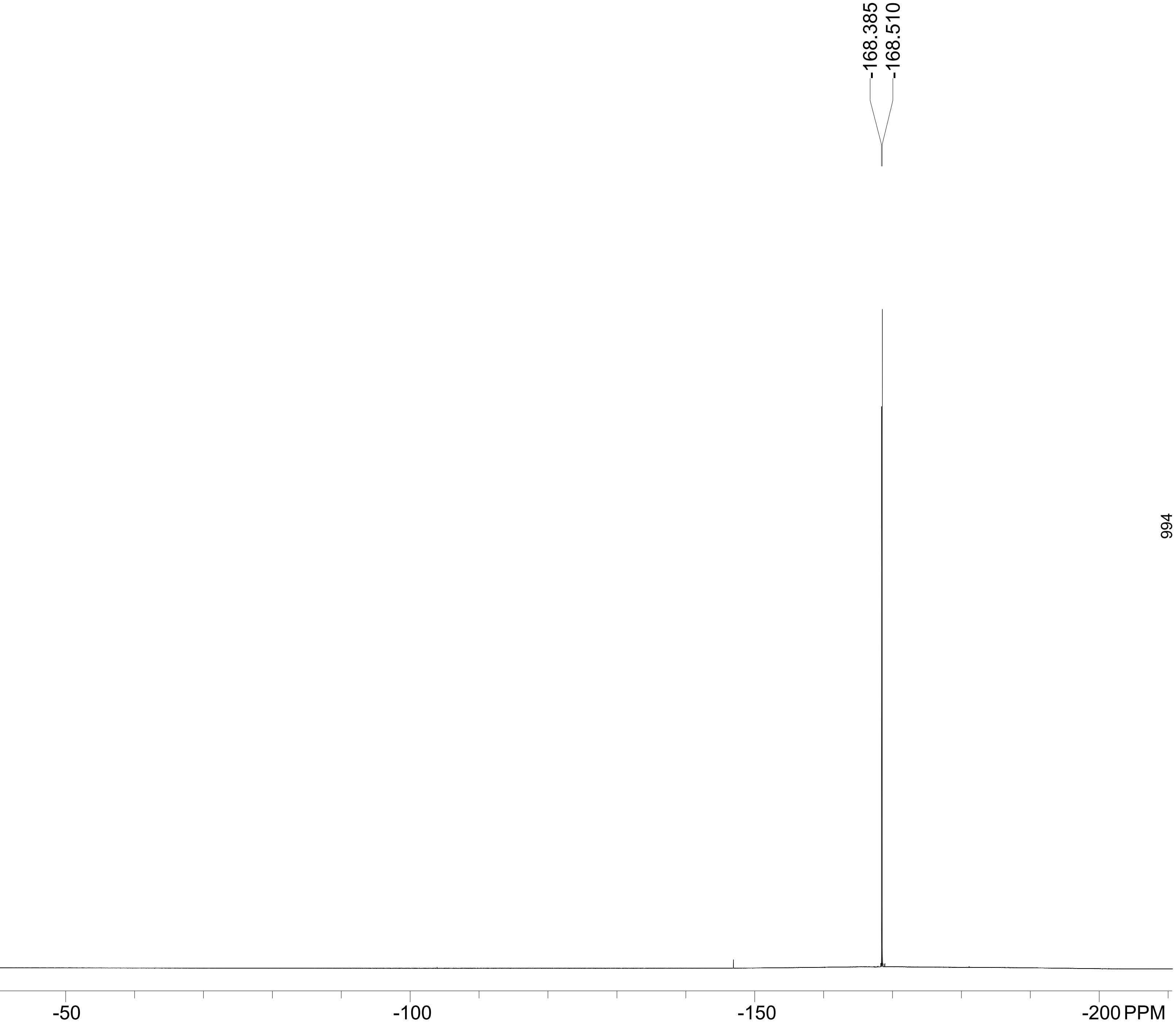

Area Percent Report

Sample name: zwf-10-090-IG-99-1-0.8-205

Data file: C:\Users\Public\Documents\ChemStation\1\Data\zwf\zwf 2024-07-10 11-54-33\008-P1-C5-zwf-10-090.D

Injection Acquired Date 07/10/2024 14:57:54

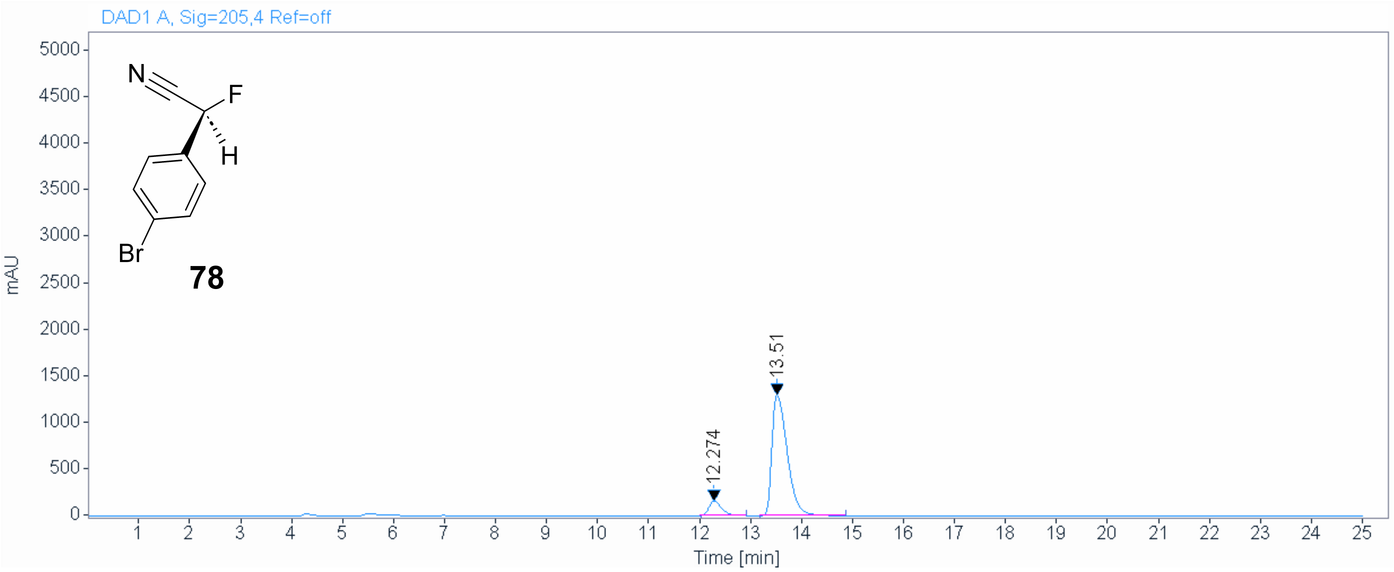

Signal: DAD1 A, Sig=205,4 Ref=off

RT [min]	Width [min]	Height	Area	Area%
12.274	0.2842	158.0926	2696.2383	9.0818
13.510	0.3106	1298.3055	26992.0547	90.9182
		Sum	29688.2930	100.0000

Area Percent Report

Agilent Technologies

Sample name: zwf-10-092-rac-IG-99-1-0.8-205

Data file: C:\Users\Public\Documents\ChemStation\1\Data\zwf\zwf 2024-07-10 11-54-33\007-P1-C6-zwf-10-092-rac.D

Injection Acquired Date 07/10/2024 14:31:50

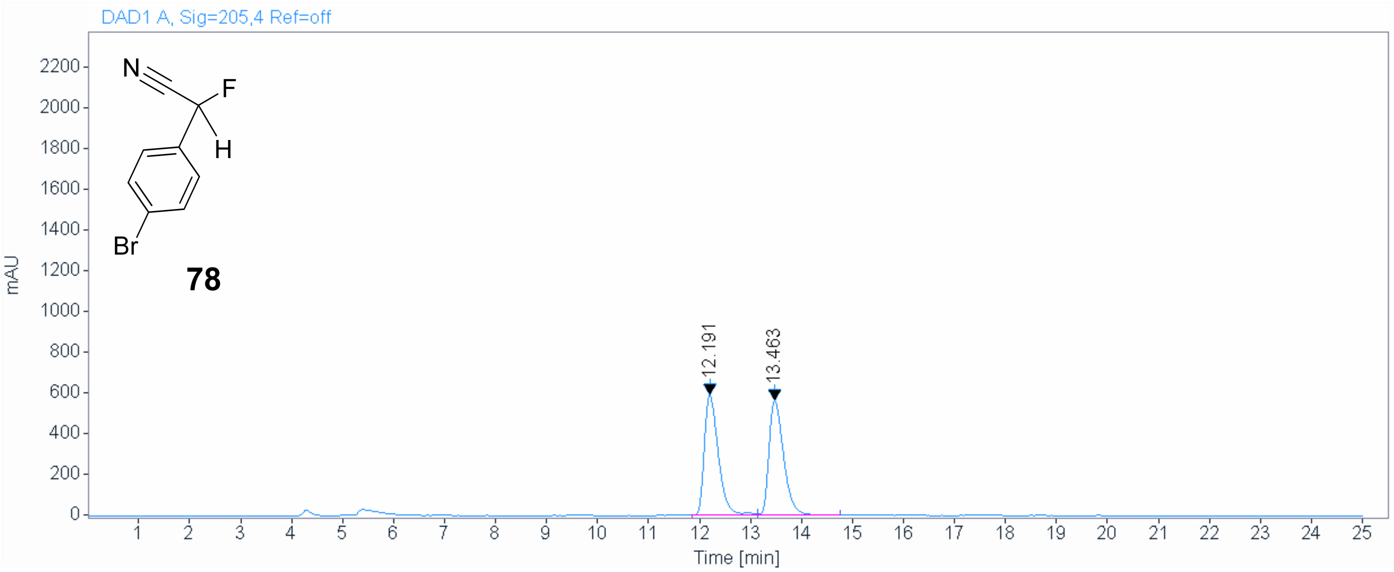

Signal: DAD1 A, Sig=205,4 Ref=off

RT [min]	Width [min]	Height	Area	Area%
12.191	0.2929	591.9988	11377.0381	50.2523
13.463	0.2989	564.1960	11262.8174	49.7477
		Sum	22639.8555	100.0000

zwf-10-092H
Jul 10 2024
SOLVENT: CDCl3
NA = 4
F1 = 400.140015 MHz
F2 = 1.000000 MHz

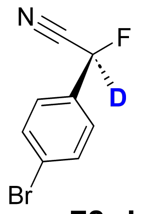

78-d

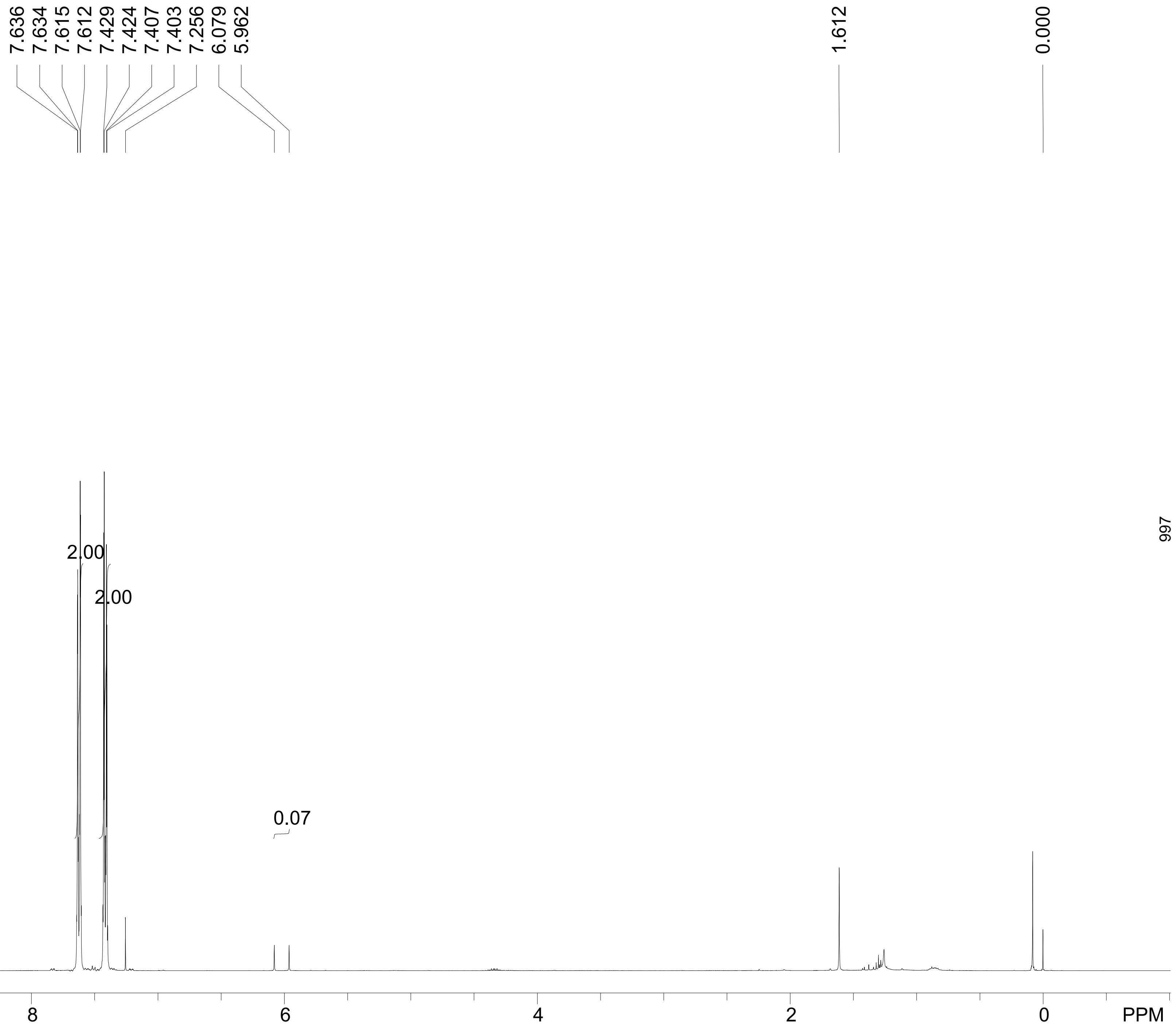

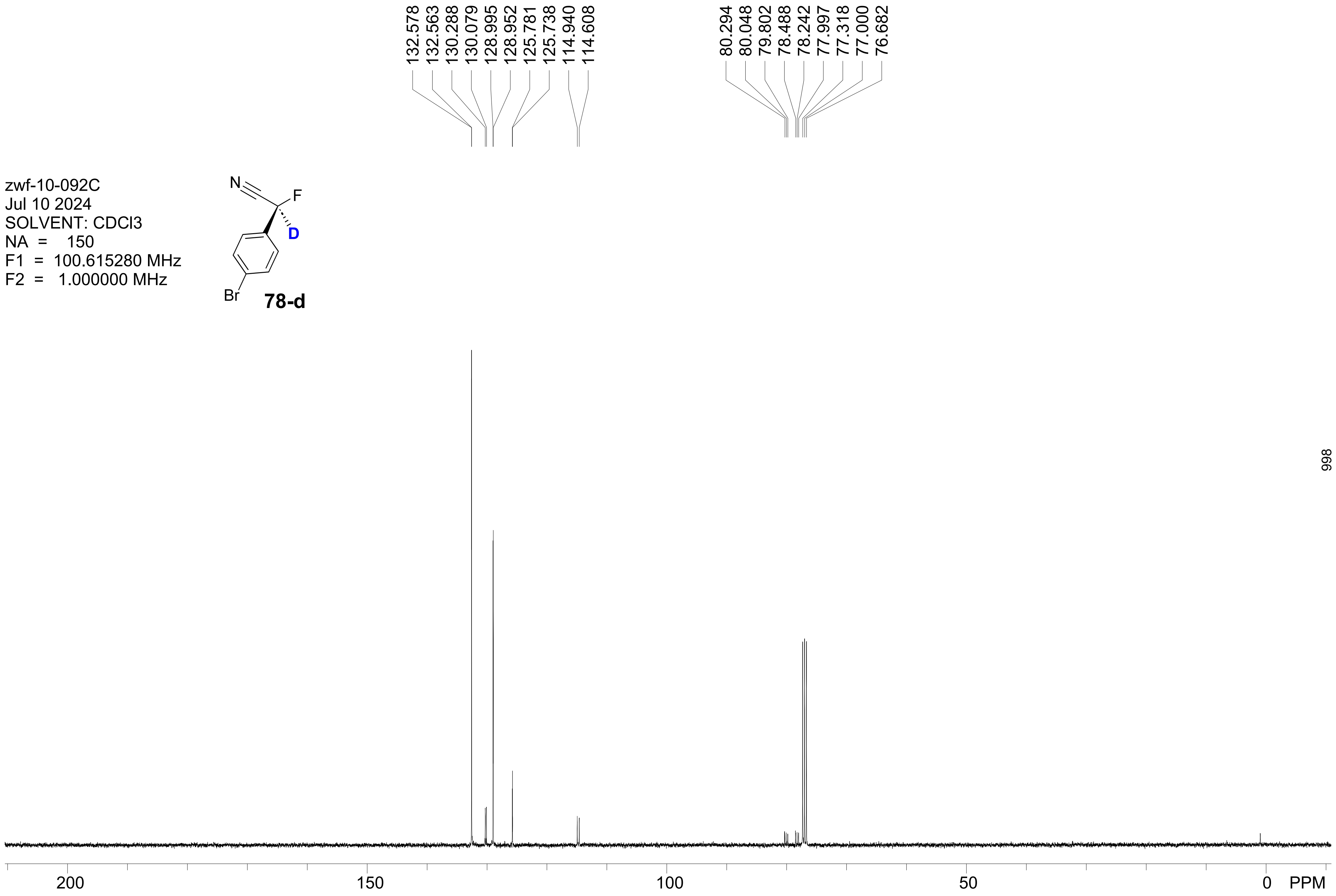
zwf-10-092C
Jul 10 2024
SOLVENT: CDCl3
NA = 150
F1 = 100.615280 MHz
F2 = 1.000000 MHz
N
F
D
Br
78-d
132.578
132.563
130.288
130.079
128.995
128.952
125.781
125.738
114.940
114.608
80.294
80.048
79.802
78.488
78.242
77.997
77.318
77.000
76.682
200
150
100
50
0
PPM

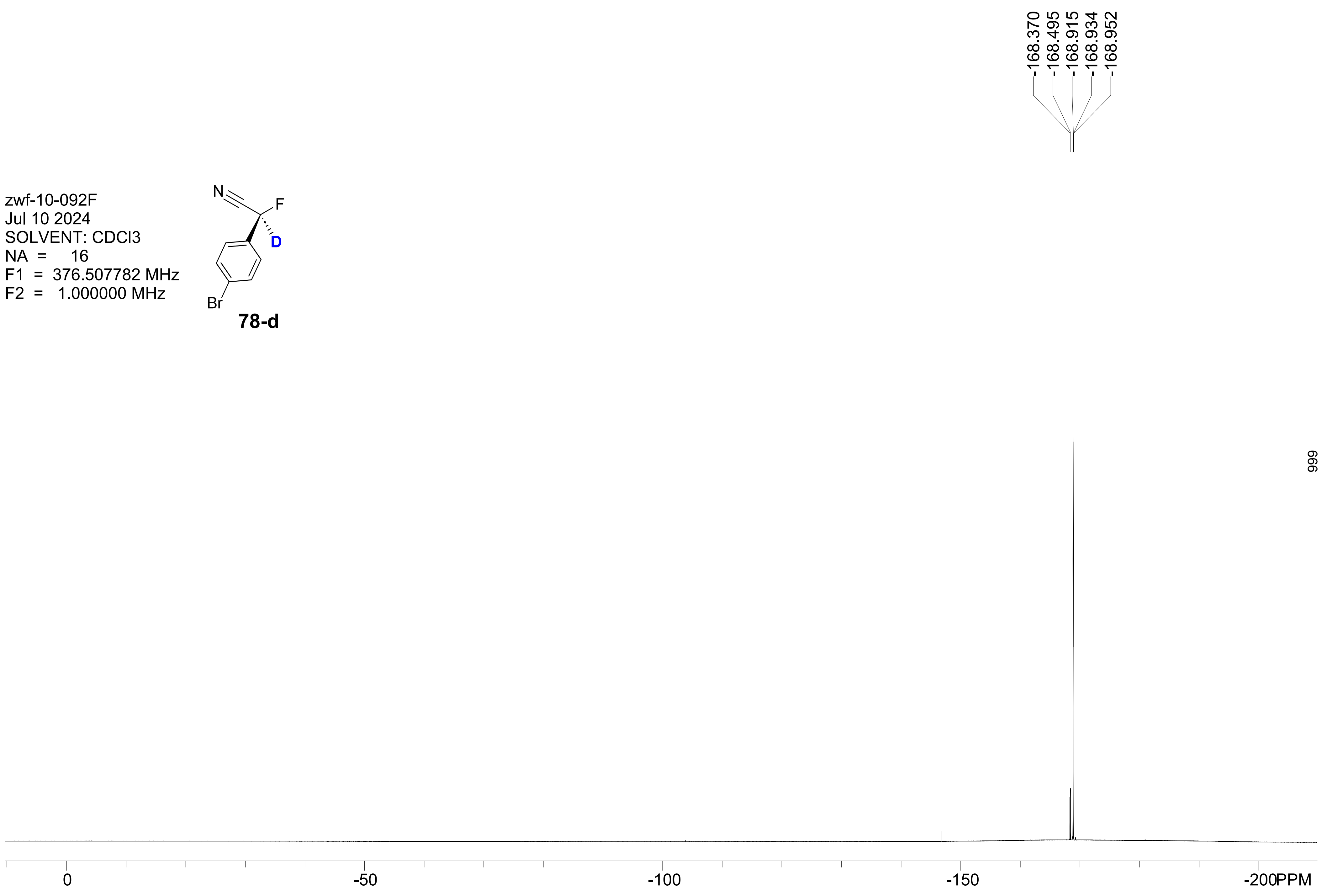

zwf-10-092F
Jul 10 2024
SOLVENT: CDCl3
NA = 16
F1 = 376.507782 MHz
F2 = 1.000000 MHz
N
F
D
Br
78-d
-168.370
-168.495
-168.915
-168.934
-168.952
0
-50
-100
-150
-200PPM

Area Percent Report

Agilent Technologies

Sample name: zwf-10-092-IG-99-1-0.8-205

Data file: C:\Users\Public\Documents\ChemStation\1\Data\zwf\zwf 2024-07-10 11-54-33\009-P1-C4-zwf-10-092.D

Injection Acquired Date 07/10/2024 15:23:56

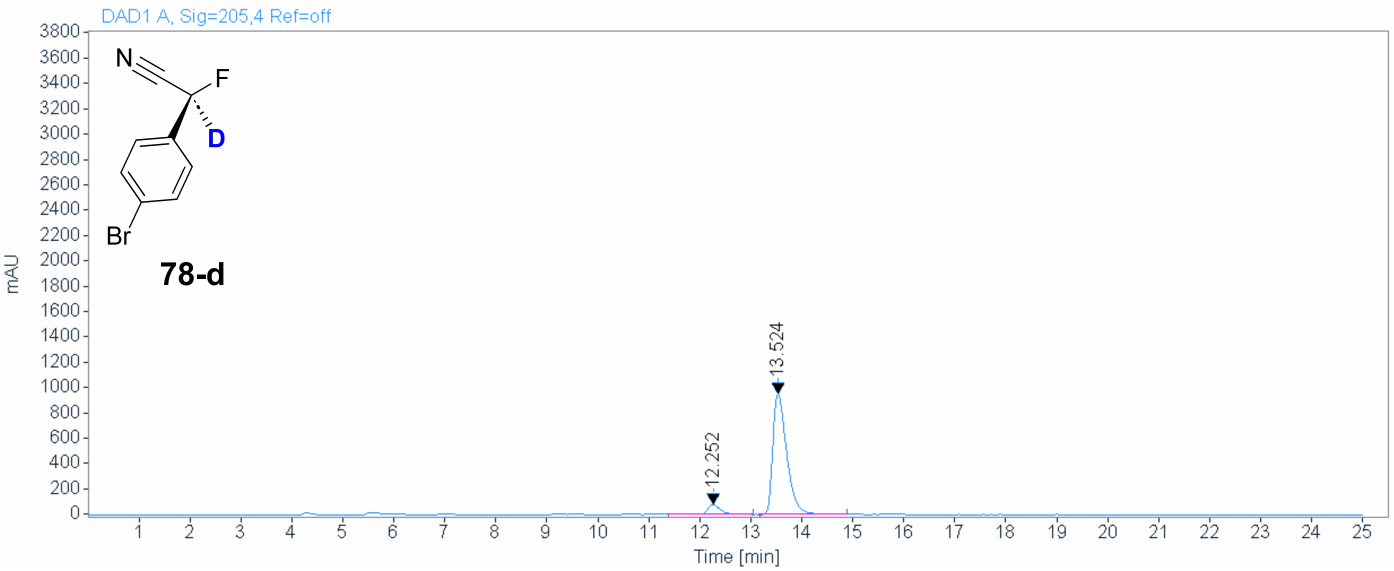

78-d

Signal: DAD1 A, Sig=205,4 Ref=off

RT [min]	Width [min]	Height	Area	Area%
12.252	0.2511	79.1137	1368.3033	7.0255
13.524	0.2879	952.1479	18107.8711	92.9745
		Sum	19476.1744	100.0000

Area Percent Report

Sample name: zwf-10-092-rac-IG-99-1-0.8-205

Data file: C:\Users\Public\Documents\ChemStation\1\Data\zwf\zwf 2024-07-10 11-54-33\007-P1-C6-zwf-10-092-rac.D

Injection Acquired Date 07/10/2024 14:31:50

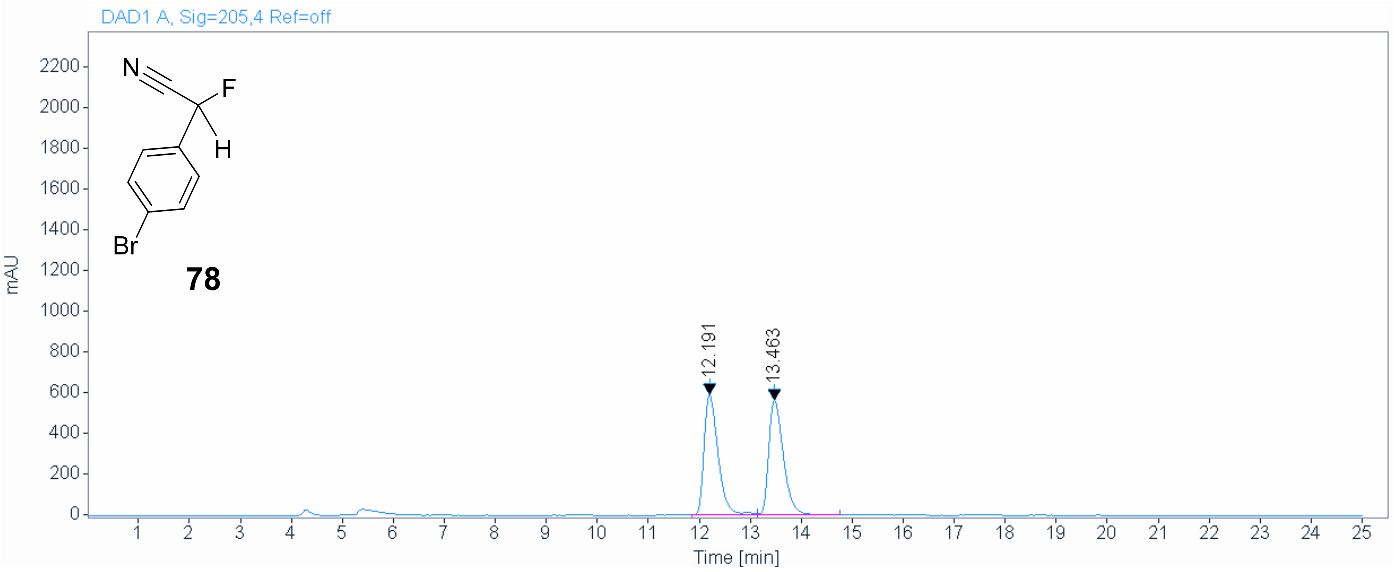

Signal: DAD1 A, Sig=205,4 Ref=off

RT [min]	Width [min]	Height	Area	Area%
12.191	0.2929	591.9988	11377.0381	50.2523
13.463	0.2989	564.1960	11262.8174	49.7477
		Sum	22639.8555	100.0000

HM-I-96-3H
Dec 31 2024
SOLVENT: CDCl3
NA = 16
F1 = 500.130005 MHz
F2 = 1.000000 MHz

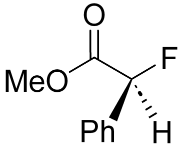

83

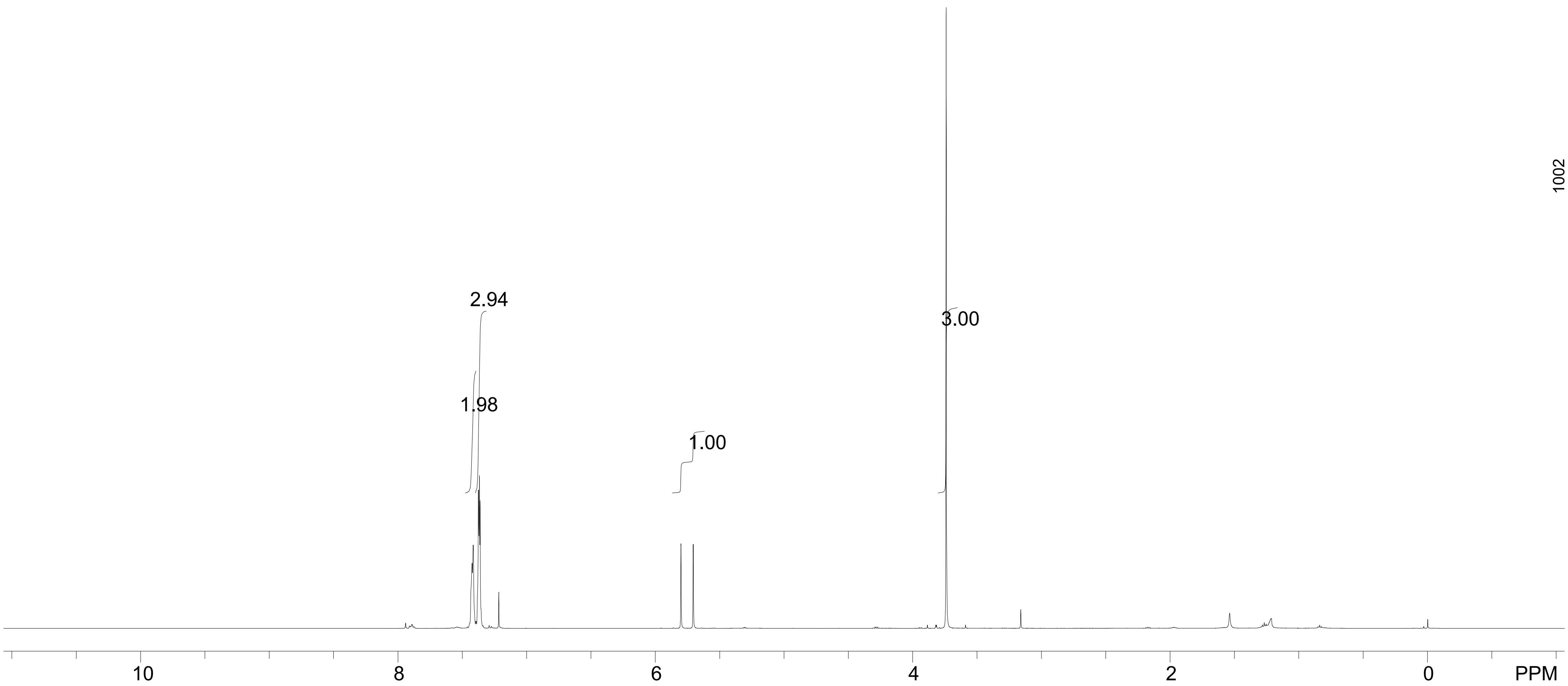

HM-I-96-3C
Dec 31 2024
SOLVENT: CDCl3
NA = 360
F1 = 125.757790 MHz
F2 = 1.000000 MHz

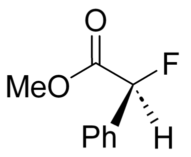

83

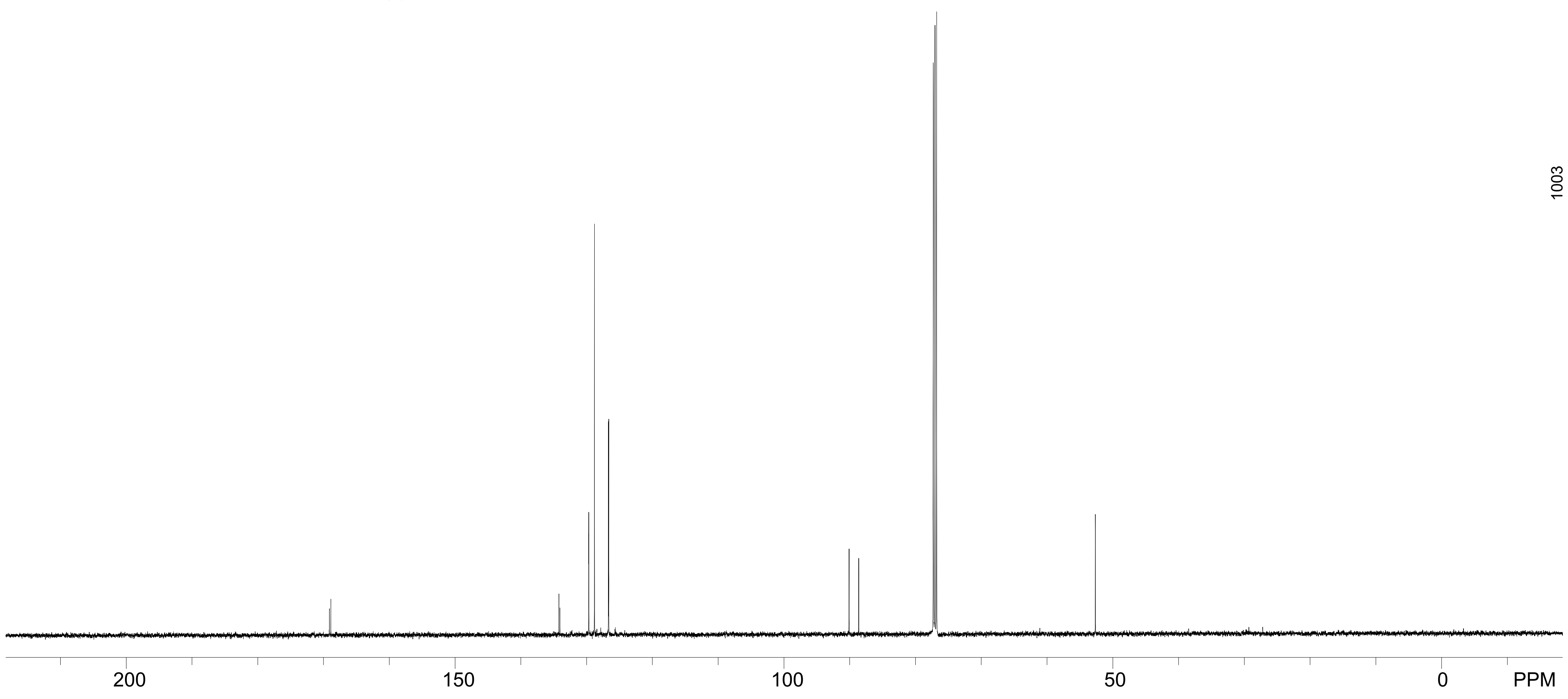

HM-I-96-3F
Dec 31 2024
SOLVENT: CDCl3
NA = 16
F1 = 470.592377 MHz
F2 = 1.000000 MHz

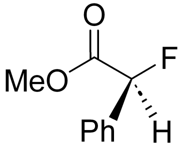

83

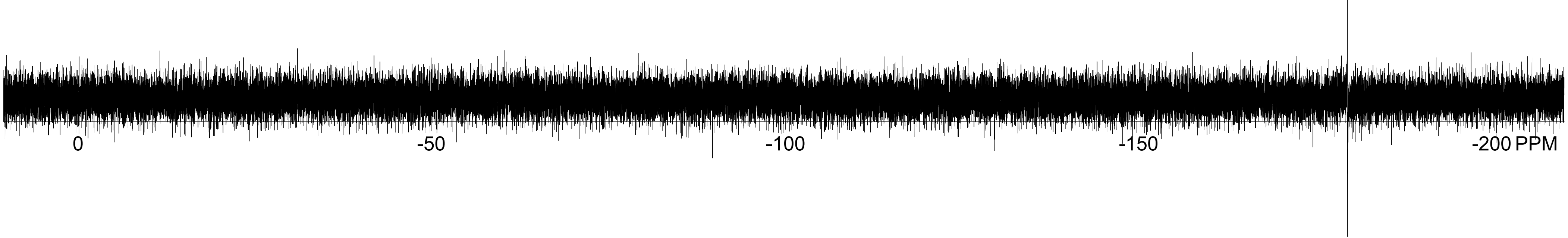

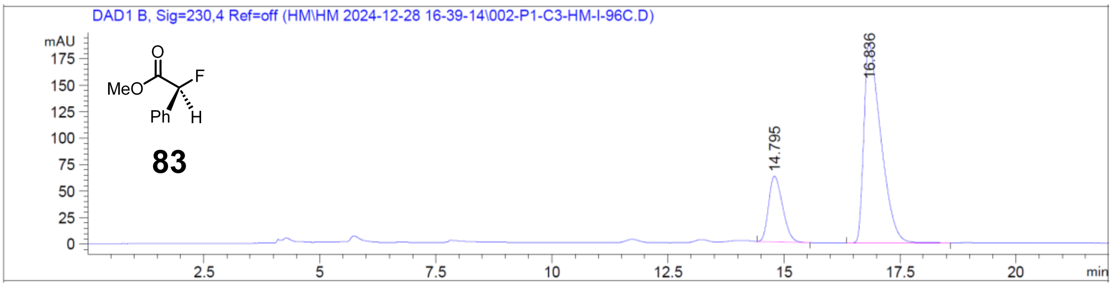

Signal 2: DAD1 B, Sig=230,4 Ref=off

Peak #	RetTime [min]	Type	Width [min]	Area [mAU*s]	Height [mAU]	Area %
1	14.795	BB	0.3271	1292.13977	62.02084	21.2119
2	16.836	BB	0.3763	4799.44727	188.99962	78.7881
Totals :				6091.58704	251.02045	

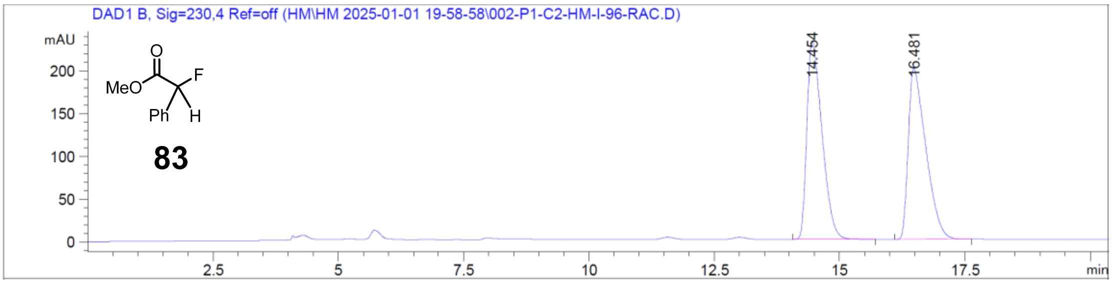

Signal 2: DAD1 B, Sig=230,4 Ref=off

Peak #	RetTime [min]	Type	Width [min]	Area [mAU*s]	Height [mAU]	Area %
1	14.454	BB	0.3235	4870.47412	231.50133	50.0689
2	16.481	BB	0.3654	4857.07422	199.96335	49.9311
Totals :				9727.54834	431.46468	

HM-I-96-2H
Dec 31 2024
SOLVENT: CDCl3
NA = 16
F1 = 500.130005 MHz
F2 = 1.000000 MHz

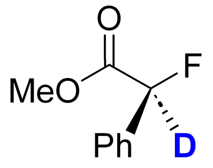

83-d

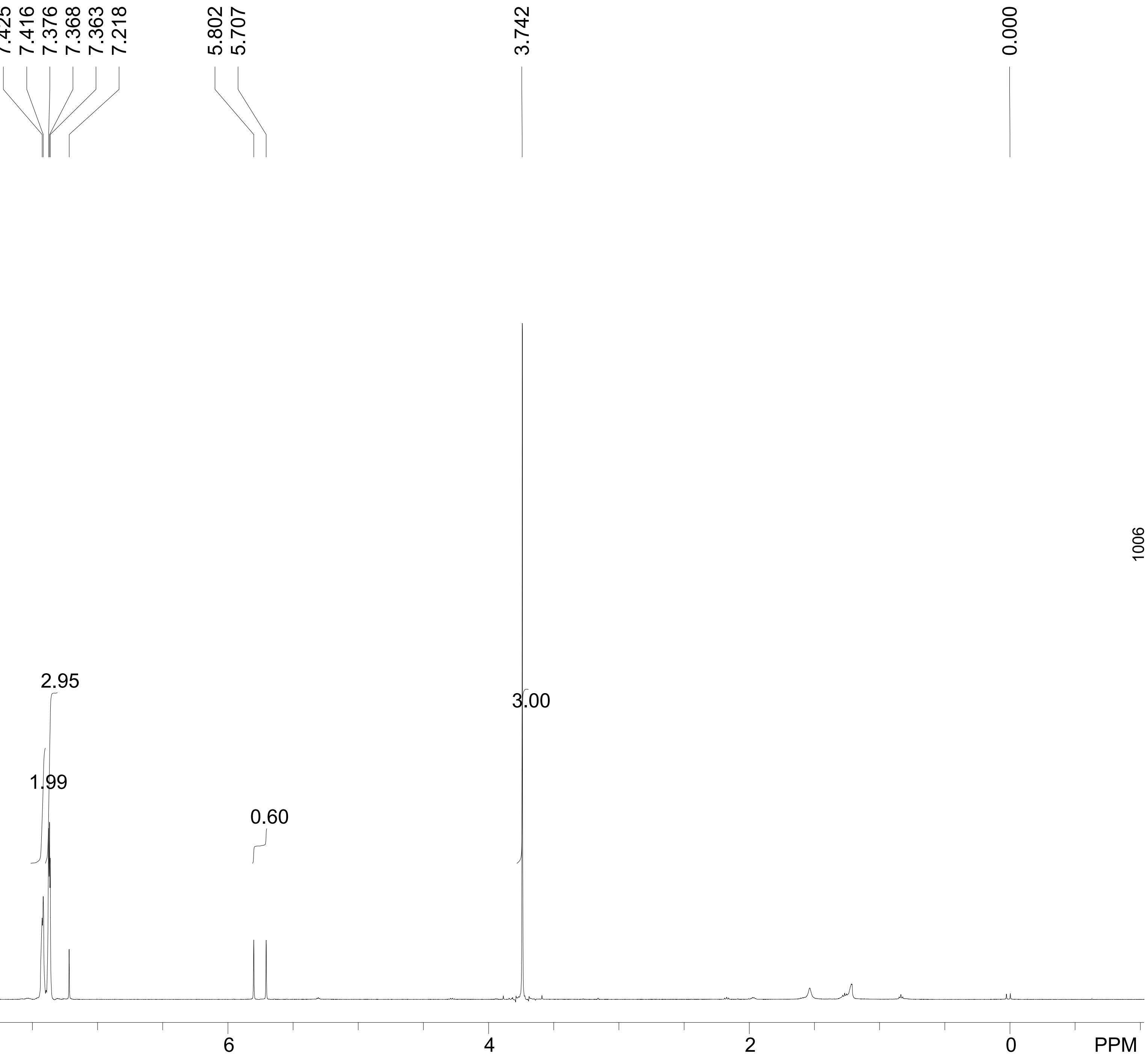

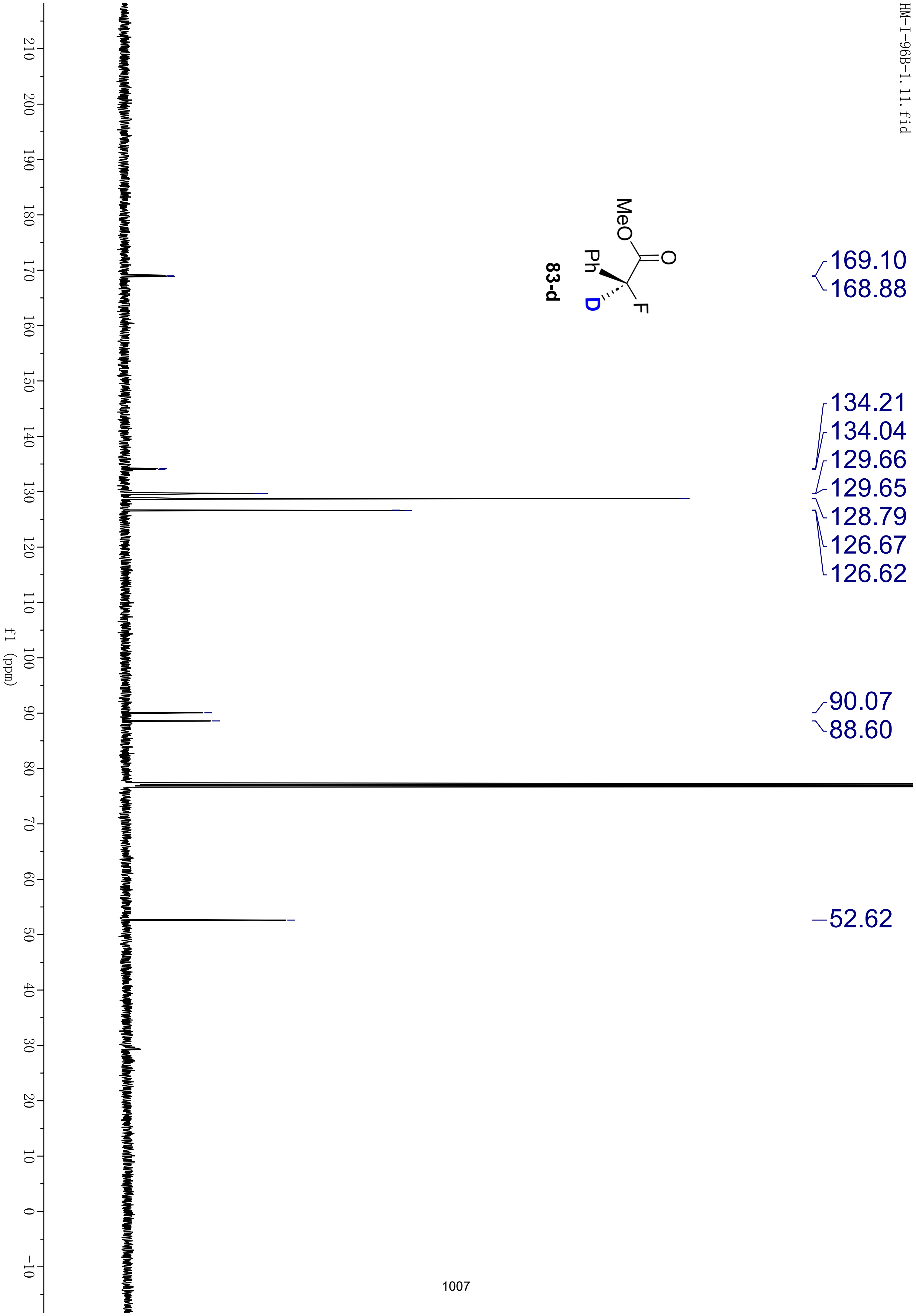
HM-I-96B-1.11.fid
MeO
O
Ph
D
F
83-d
169.10
168.88
134.21
134.04
129.66
129.65
128.79
126.67
126.62
90.07
88.60
52.62
f1 (ppm)

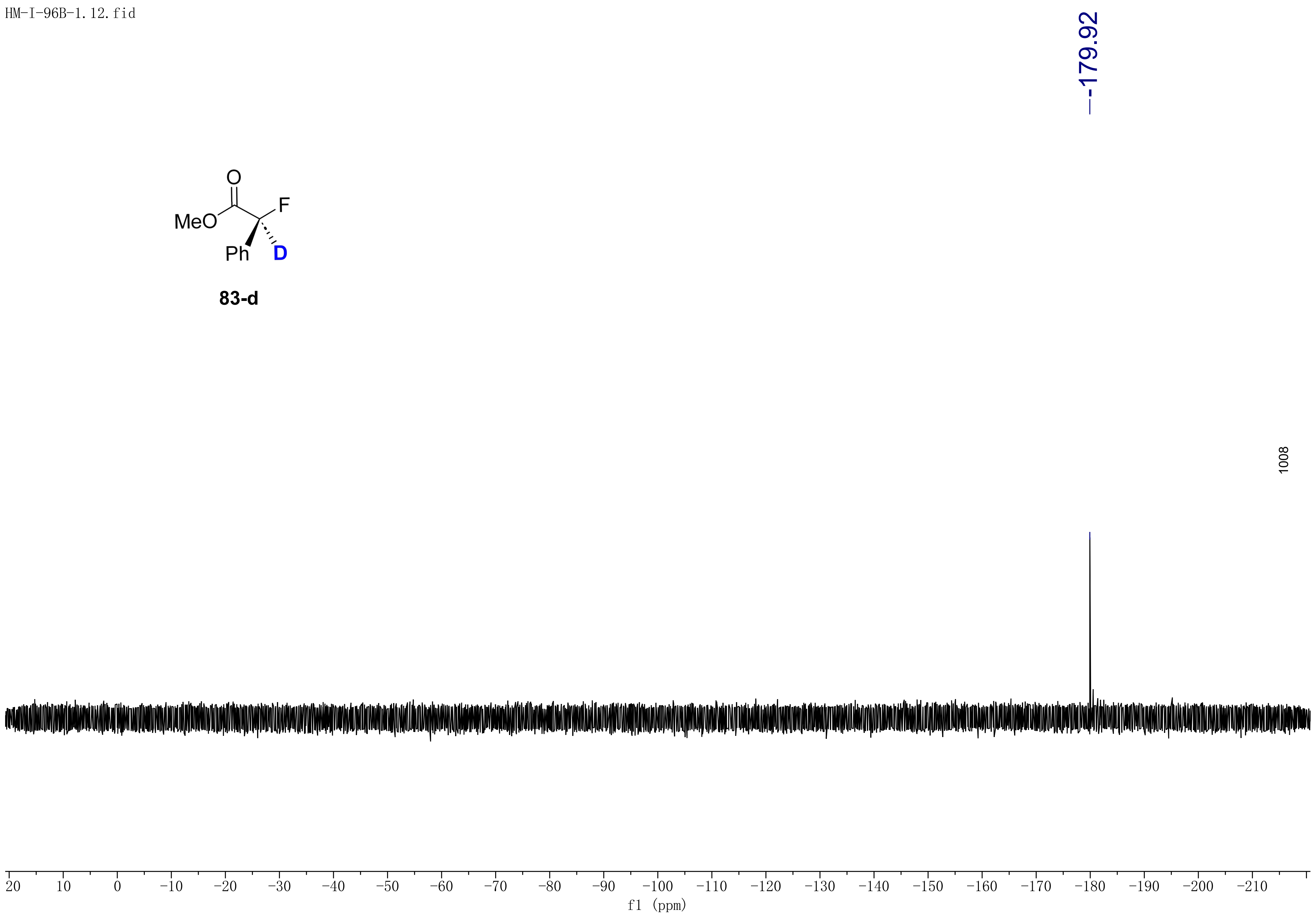
HM-I-96B-1.12.fid
-179.92
MeO
O
F
Ph
D
83-d
20
10
0
−10
−20
−30
−40
−50
−60
−70
−80
−90
−100
−110
−120
−130
−140
−150
−160
−170
−180
−190
−200
−210
f1 (ppm)

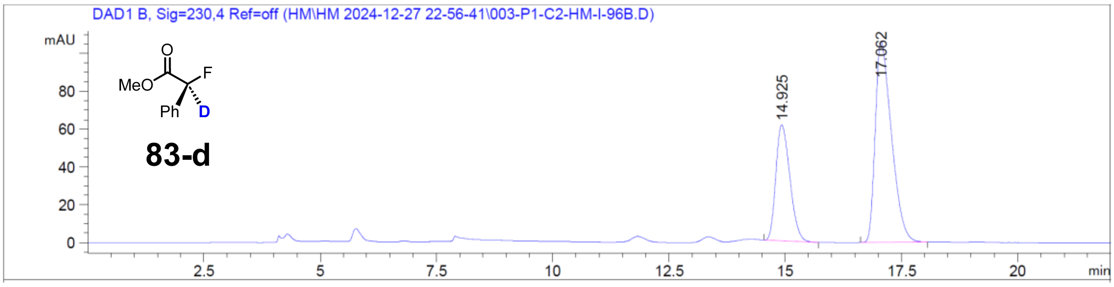

Signal 2: DAD1 B, Sig=230,4 Ref=off

Peak #	RetTime [min]	Type	Width [min]	Area [mAU*s]	Height [mAU]	Area %
1	14.925	BB	0.3274	1291.21375	61.38041	33.4023
2	17.062	BB	0.3704	2574.43359	106.33870	66.5977
Totals :				3865.64734	167.71911	

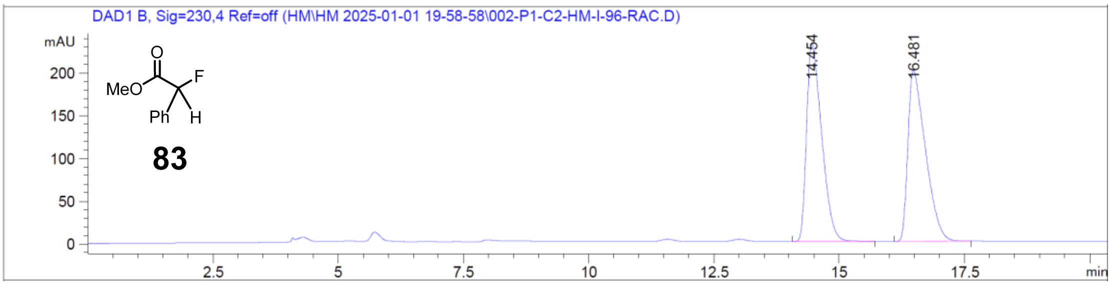

Signal 2: DAD1 B, Sig=230,4 Ref=off

Peak #	RetTime [min]	Type	Width [min]	Area [mAU*s]	Height [mAU]	Area %
1	14.454	BB	0.3235	4870.47412	231.50133	50.0689
2	16.481	BB	0.3654	4857.07422	199.96335	49.9311
Totals :				9727.54834	431.46468	

zwf-8-056H
Dec 14 2023
SOLVENT: CDCl3
NA =   4
F1 = 400.140015 MHz
F2 =   1.000000 MHz

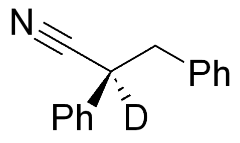

1-d

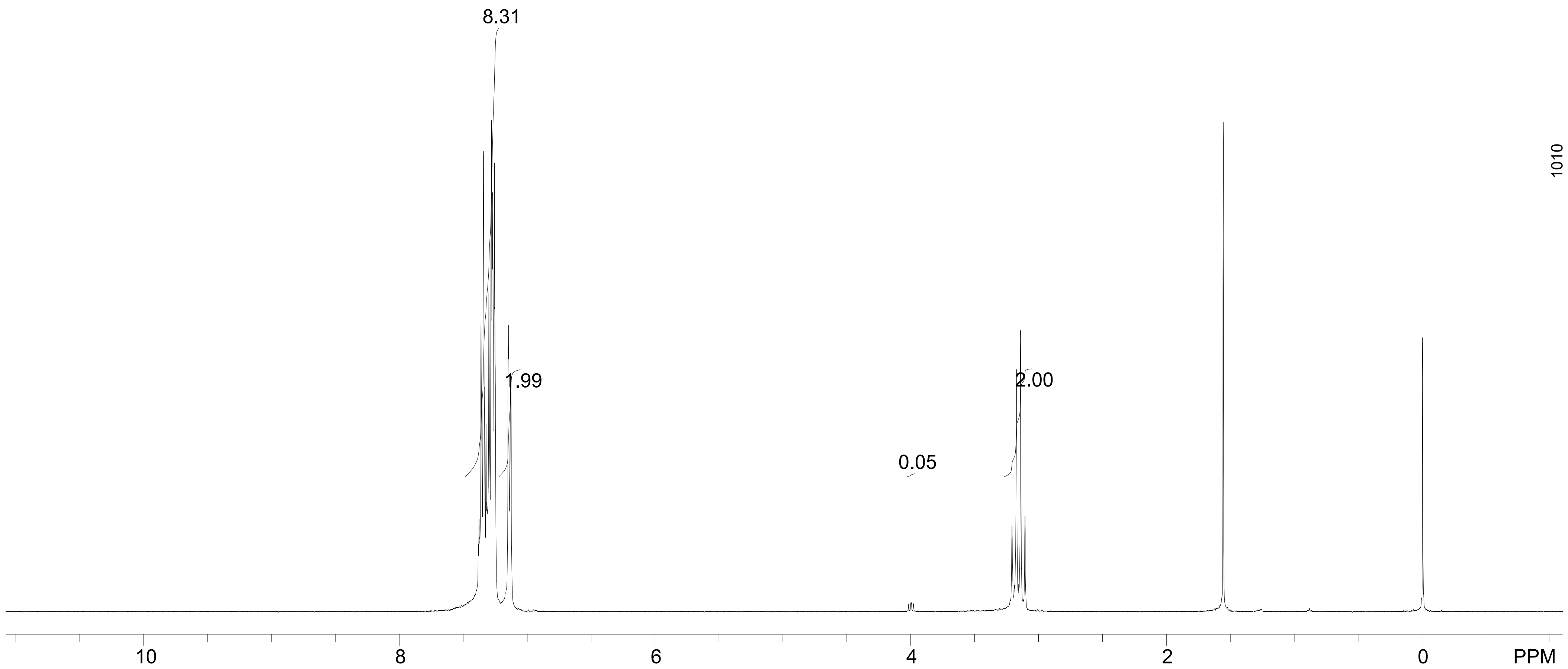

Area Percent Report

Agilent Technologies

Sample name: zwf-8-056-IE-99-1-0.8-205

Data file: C:\Users\Public\Documents\ChemStation\1\Data\zwf\zwf-S 2023-12-14 18-43-09\002-P1-C1-zwf-8-056.D

Injection Acquired Date 12/14/2023 19:05:59

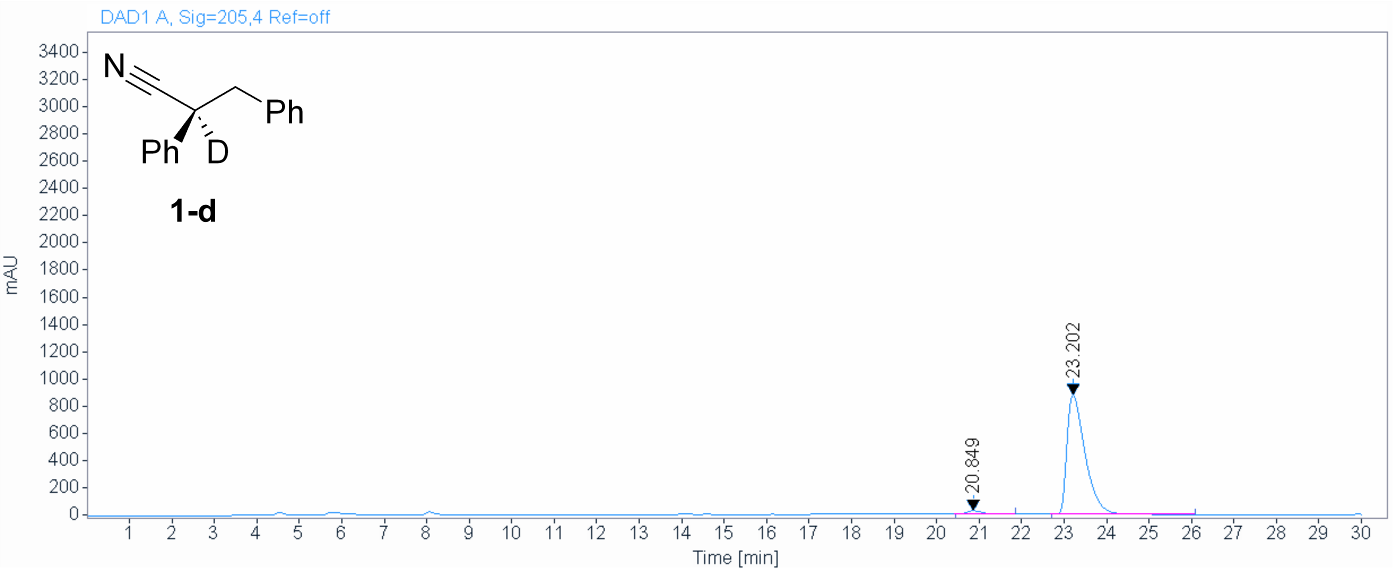

Signal: DAD1 A, Sig=205,4 Ref=off

RT [min]	Width [min]	Height	Area	Area%
20.849	0.3640	24.3397	575.7212	2.1689
23.202	0.4502	875.1977	25968.5332	97.8311
		Sum	26544.2544	100.0000

Area Percent Report

Agilent Technologies

Sample name: zwf-8-056-rac-IE-99-1-0.8-205

Data file: C:\Users\Public\Documents\ChemStation\1\Data\zwf\zwf-S 2023-12-14 18-43-09\003-P1-C2-zwf-8-056-rac.D

Injection Acquired Date 12/14/2023 19:36:59

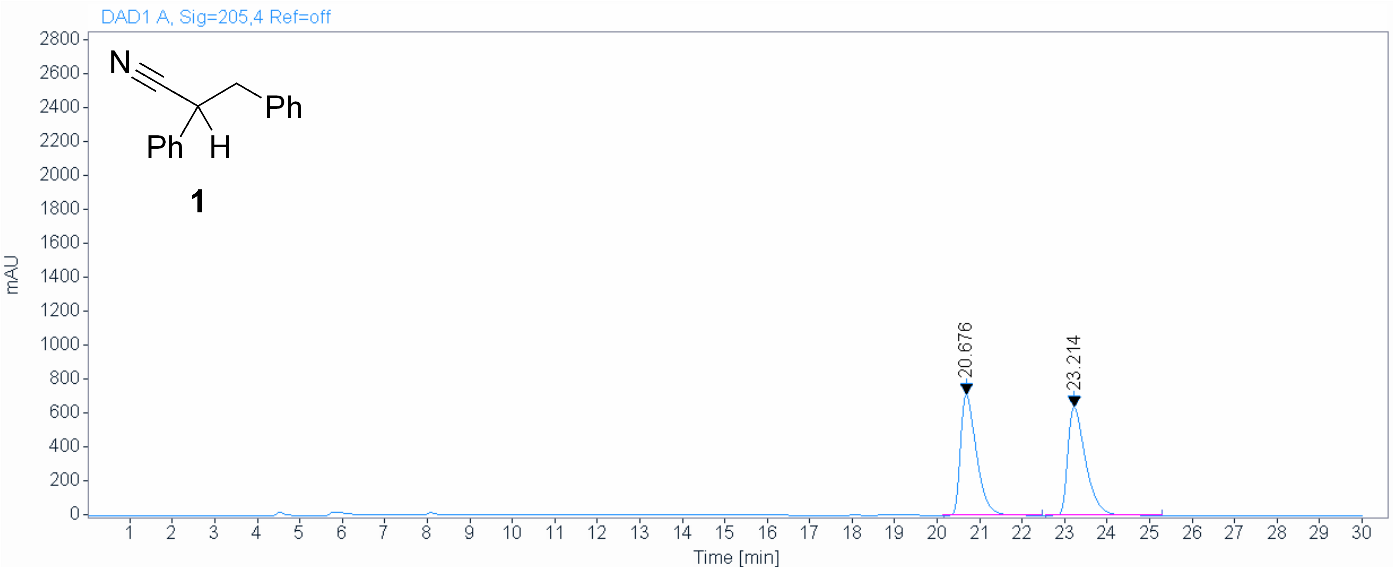

Signal: DAD1 A, Sig=205,4 Ref=off

RT [min]	Width [min]	Height	Area	Area%
20.676	0.3972	712.4696	18514.5332	50.0377
23.214	0.4424	637.4114	18486.6680	49.9623
		Sum	37001.2012	100.0000

7.292 7.274 7.255 7.237 7.215 7.197 7.183 7.166 7.131 7.113 7.030 7.012 4.356 3.576 3.558 3.544 3.526 3.257 3.233 3.225 3.075 3.062 3.041 2.953 2.919 2.882 2.848 1.694 1.377 -0.000

zwf-8-060H
Dec 17 2023
SOLVENT: CDCl3
NA = 4
F1 = 400.179993 MHz
F2 = 1.000000 MHz

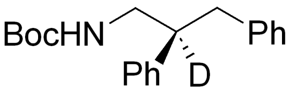

84-d

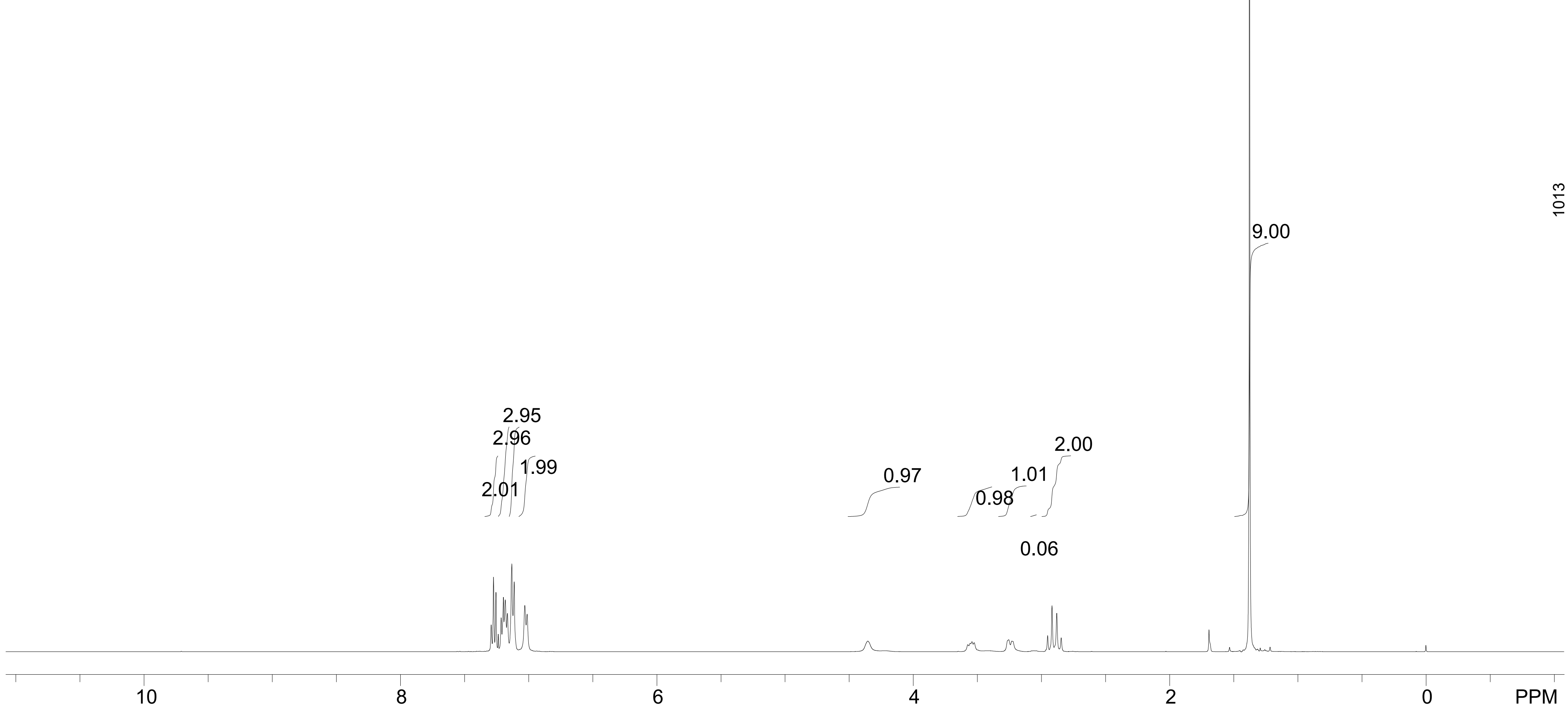

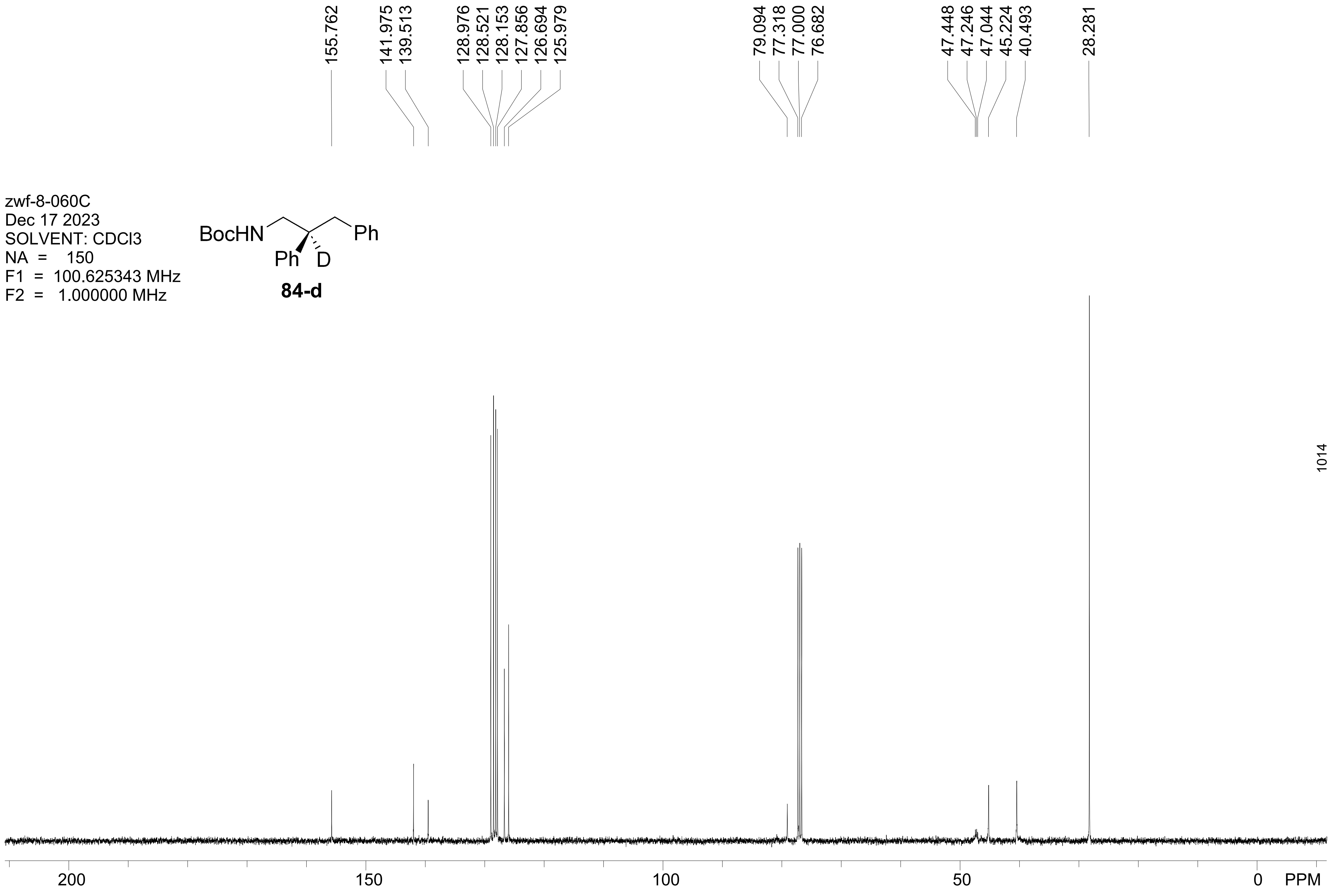
155.762
141.975
139.513
128.976
128.521
128.153
127.856
126.694
125.979
79.094
77.318
77.000
76.682
47.448
47.246
47.044
45.224
40.493
28.281
zwf-8-060C
Dec 17 2023
SOLVENT: CDCl3
NA = 150
F1 = 100.625343 MHz
F2 = 1.000000 MHz
BocHN
Ph
Ph
D
84-d
200
150
100
50
0
PPM

Area Percent Report

Sample name: zwf-8-060-IA-98-2-1.0-205

Data file: C:\Users\Public\Documents\ChemStation\1\Data\zwf\zwf-S 2023-12-17 10-43-39\003-P1-C3-zwf-8-060.D

Injection Acquired Date 12/17/2023 11:31:36

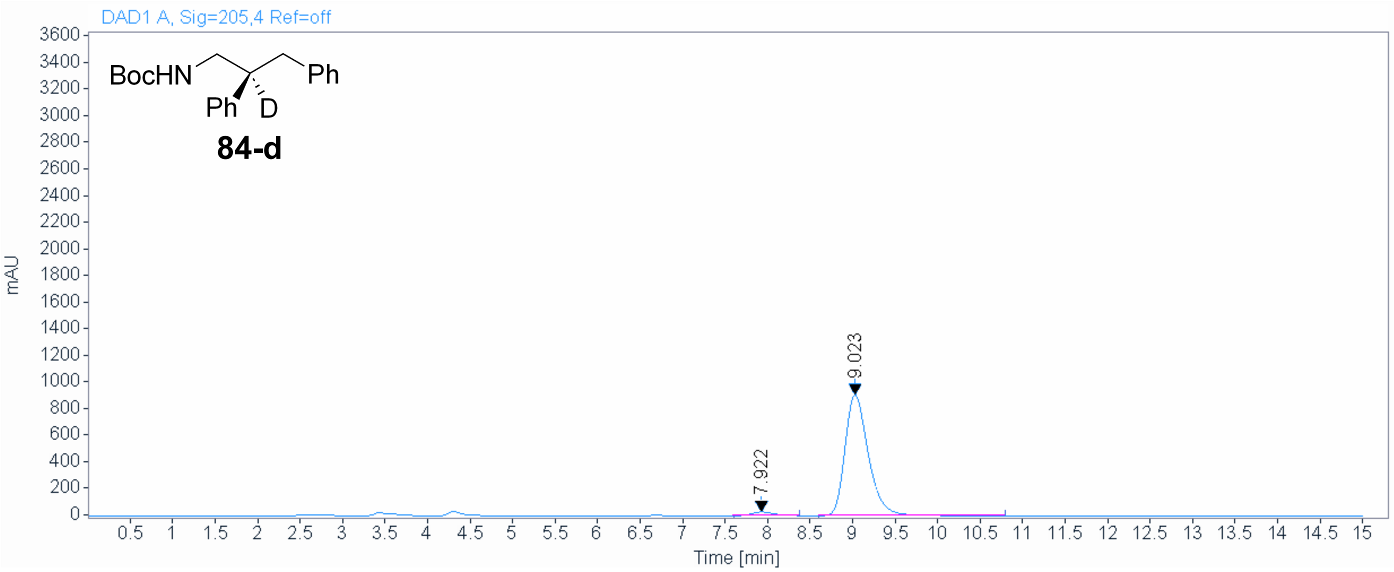

Signal: DAD1 A, Sig=205,4 Ref=off

RT [min]	Width [min]	Height	Area	Area%
7.922	0.2517	25.3407	382.6199	2.1509
9.023	0.2938	907.4096	17406.1895	97.8491
		Sum	17788.8094	100.0000

Area Percent Report

Agilent Technologies

Sample name: zwf-8-060-rac-IA-98-2-1.0-205

Data file: C:\Users\Public\Documents\ChemStation\1\Data\zwf\zwf-S 2023-12-17 10-43-39\004-P1-C4-zwf-8-060-rac.D

Injection Acquired Date 12/17/2023 11:47:39

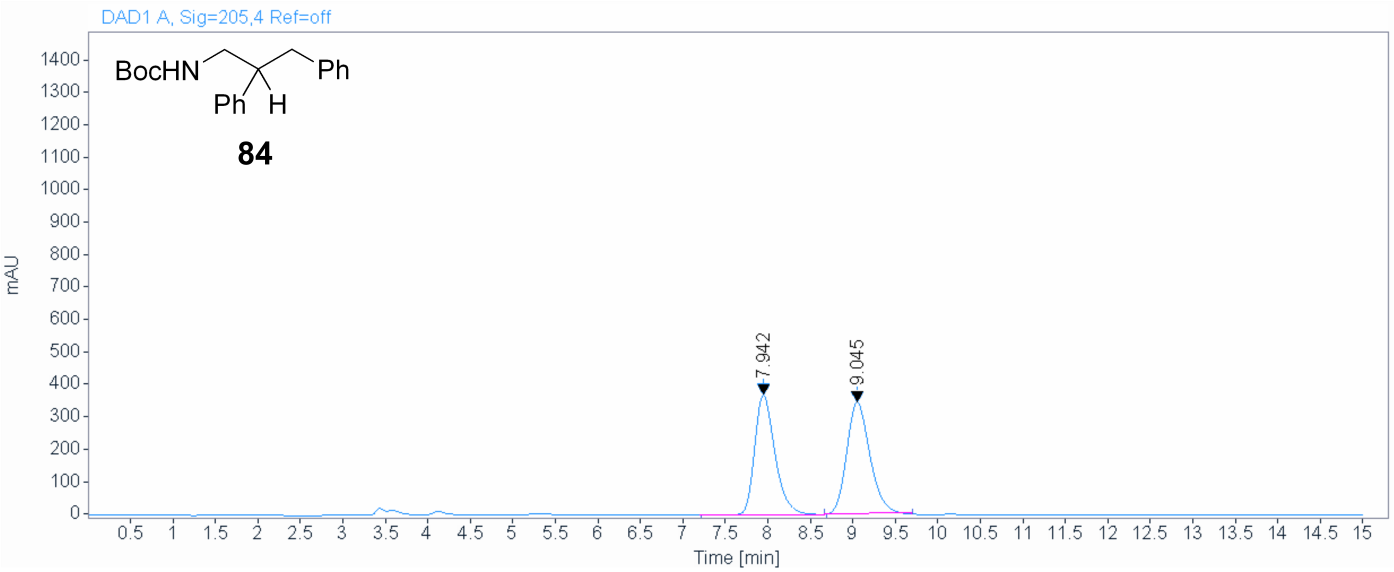

Signal: DAD1 A, Sig=205,4 Ref=off

RT [min]	Width [min]	Height	Area	Area%
7.942	0.2821	371.0627	6280.6567	49.0297
9.045	0.3162	344.1467	6529.2368	50.9703
		Sum	12809.8936	100.0000

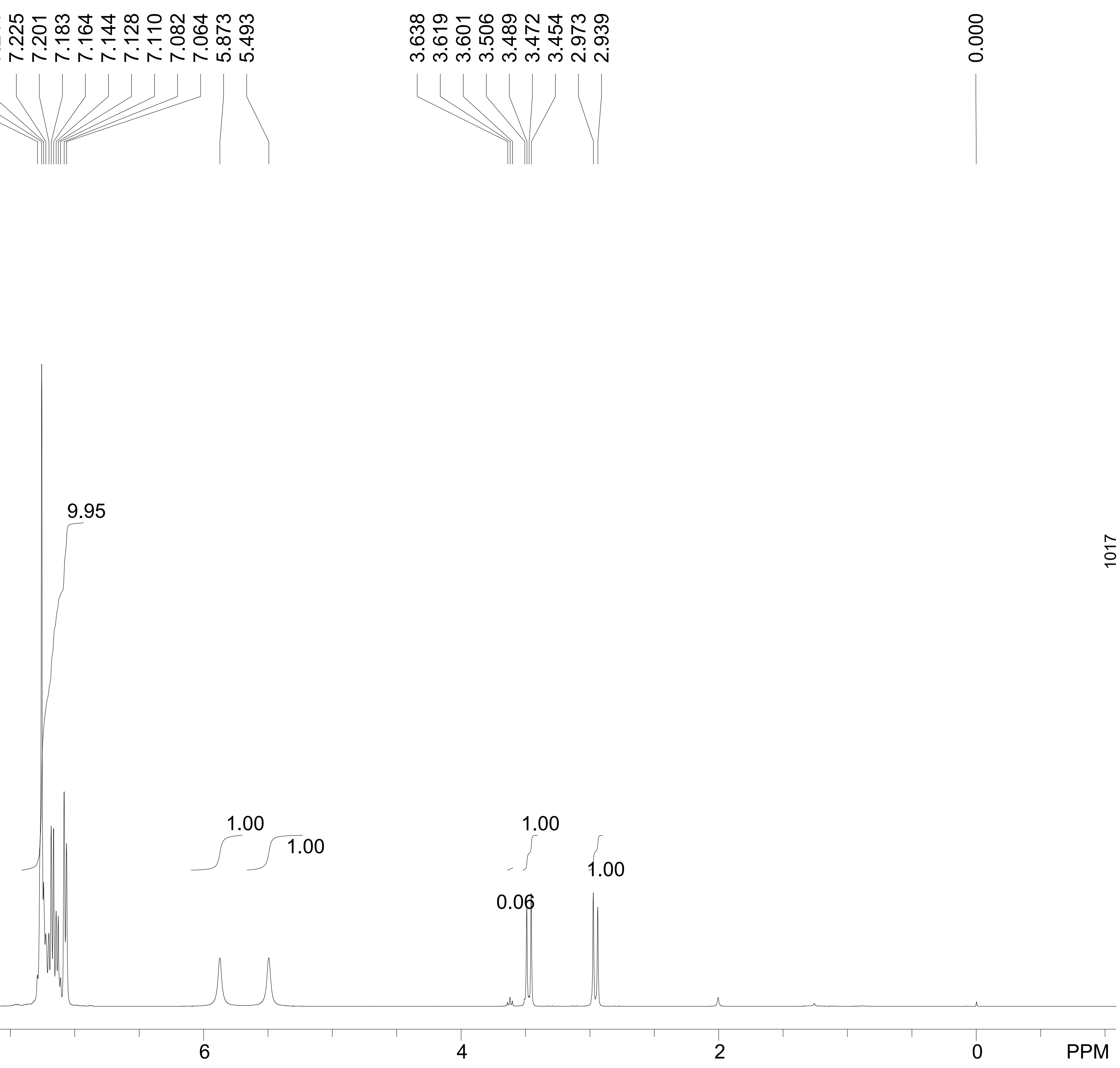
zwf-8-059H
Dec 17 2023
SOLVENT: CDCl3
NA = 4
F1 = 400.179993 MHz
F2 = 1.000000 MHz
7.290
7.256
7.241
7.225
7.201
7.183
7.164
7.144
7.128
7.110
7.082
7.064
5.873
5.493
3.638
3.619
3.601
3.506
3.489
3.472
3.454
2.973
2.939
0.000
9.95
1.00
1.00
1.00
0.06
1.00
10
8
6
4
2
0
PPM

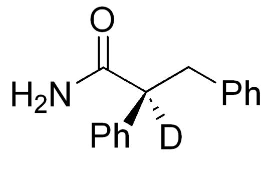

85-d

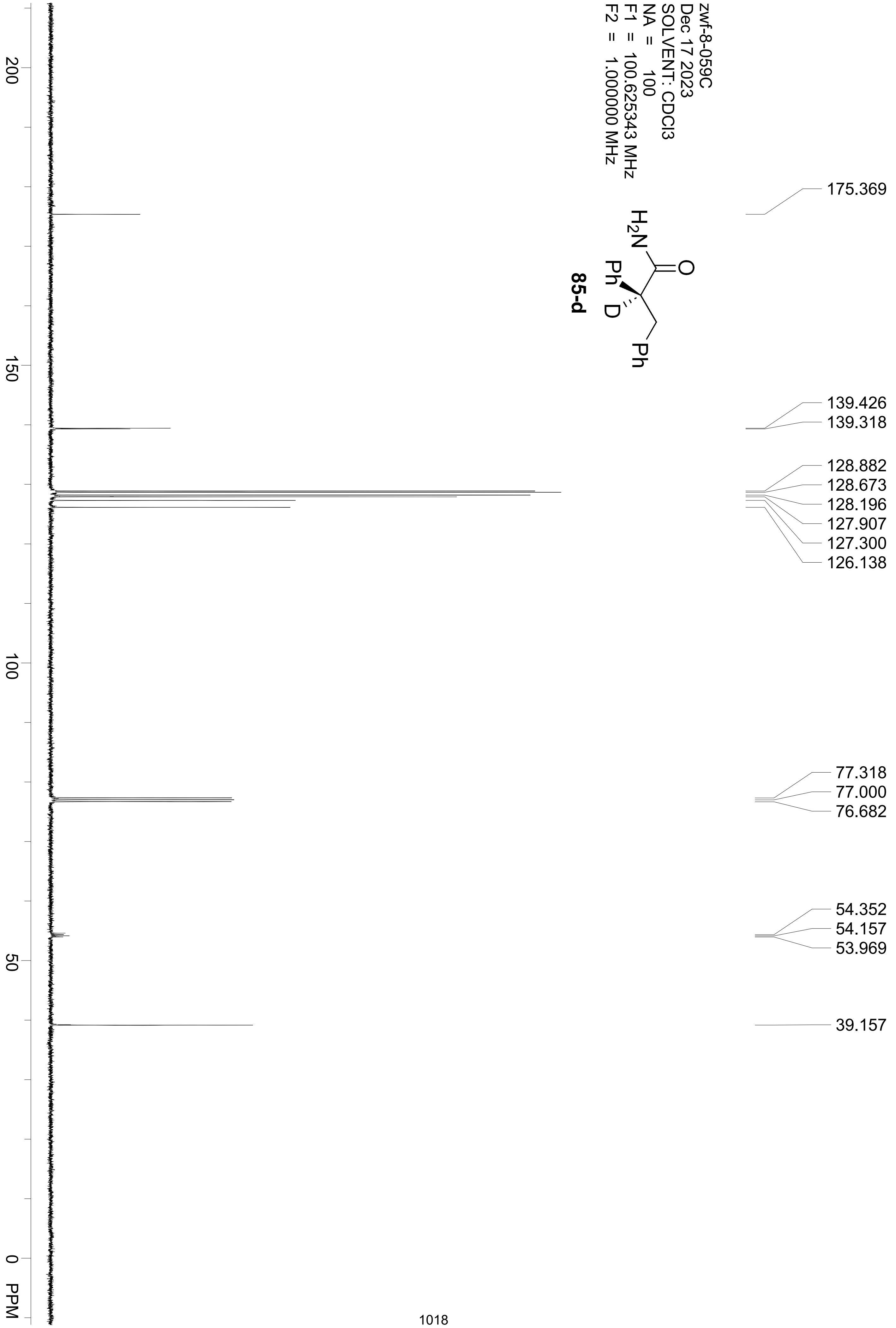
zwf-8-059C
Dec 17 2023
SOLVENT: CDCl3
NA = 100
F1 = 100.625343 MHz
F2 = 1.000000 MHz
H2N
O
Ph
D
Ph
85-d
175.369
139.426
139.318
128.882
128.673
128.196
127.907
127.300
126.138
77.318
77.000
76.682
54.352
54.157
53.969
39.157
200
150
100
50
0
PPM

Area Percent Report

Sample name: zwf-8-059-IA-98-2-1.0-205

Data file: C:\Users\Public\Documents\ChemStation\1\Data\zwf\zwf-S 2023-12-17 10-43-39\007-P1-C1-zwf-8-059.D

Injection Acquired Date 12/17/2023 13:45:46

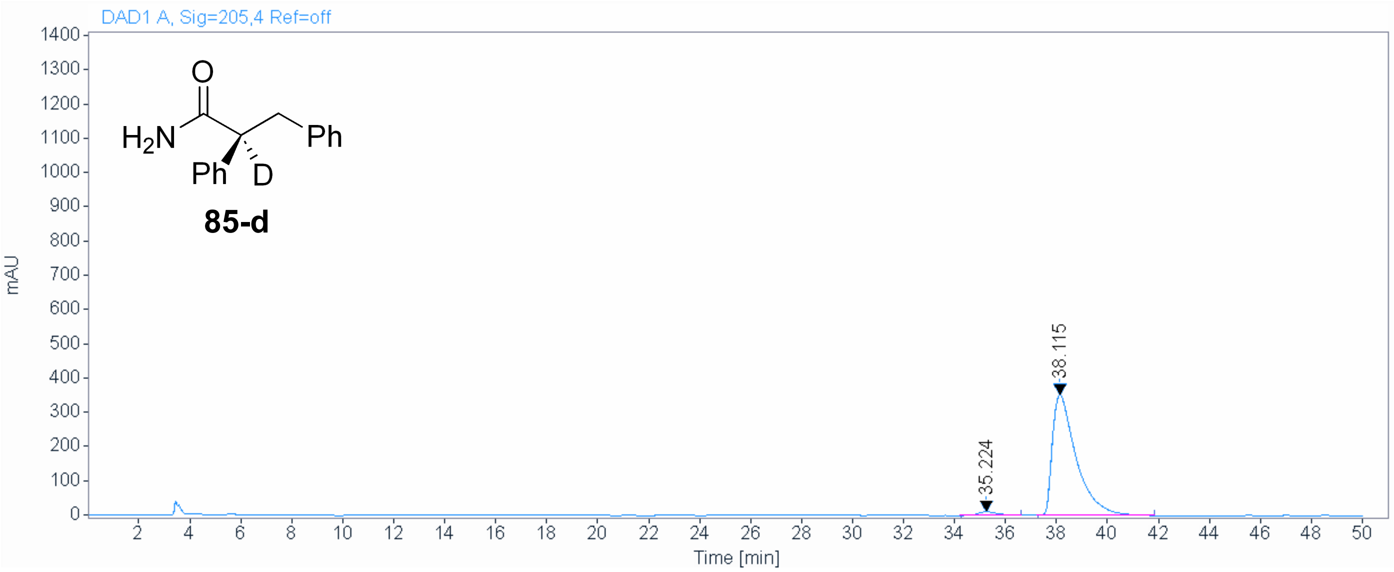

Signal: DAD1 A, Sig=205,4 Ref=off

RT [min]	Width [min]	Height	Area	Area%
35.224	0.6360	10.3328	519.0241	2.2325
38.115	0.9517	350.9091	22730.0469	97.7675
		Sum	23249.0710	100.0000

Area Percent Report

Sample name: zwf-8-059-rac-IA-98-2-1.0-205

Data file: C:\Users\Public\Documents\ChemStation\1\Data\zwf\zwf-S 2023-12-17 10-43-39\006-P1-C2-zwf-8-059-rac.D

Injection Acquired Date 12/17/2023 12:54:40

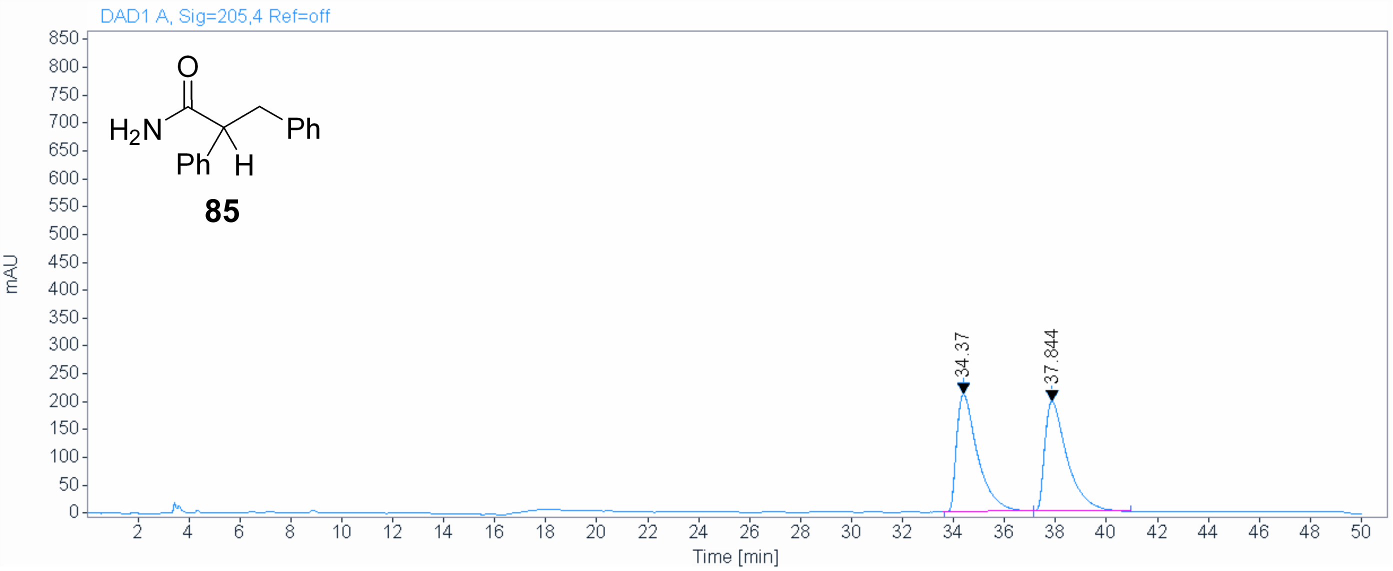

Signal: DAD1 A, Sig=205,4 Ref=off

RT [min]	Width [min]	Height	Area	Area%
34.370	0.8581	211.5761	12116.8438	50.0670
37.844	0.9174	197.0663	12084.4043	49.9330
		Sum	24201.2480	100.0000

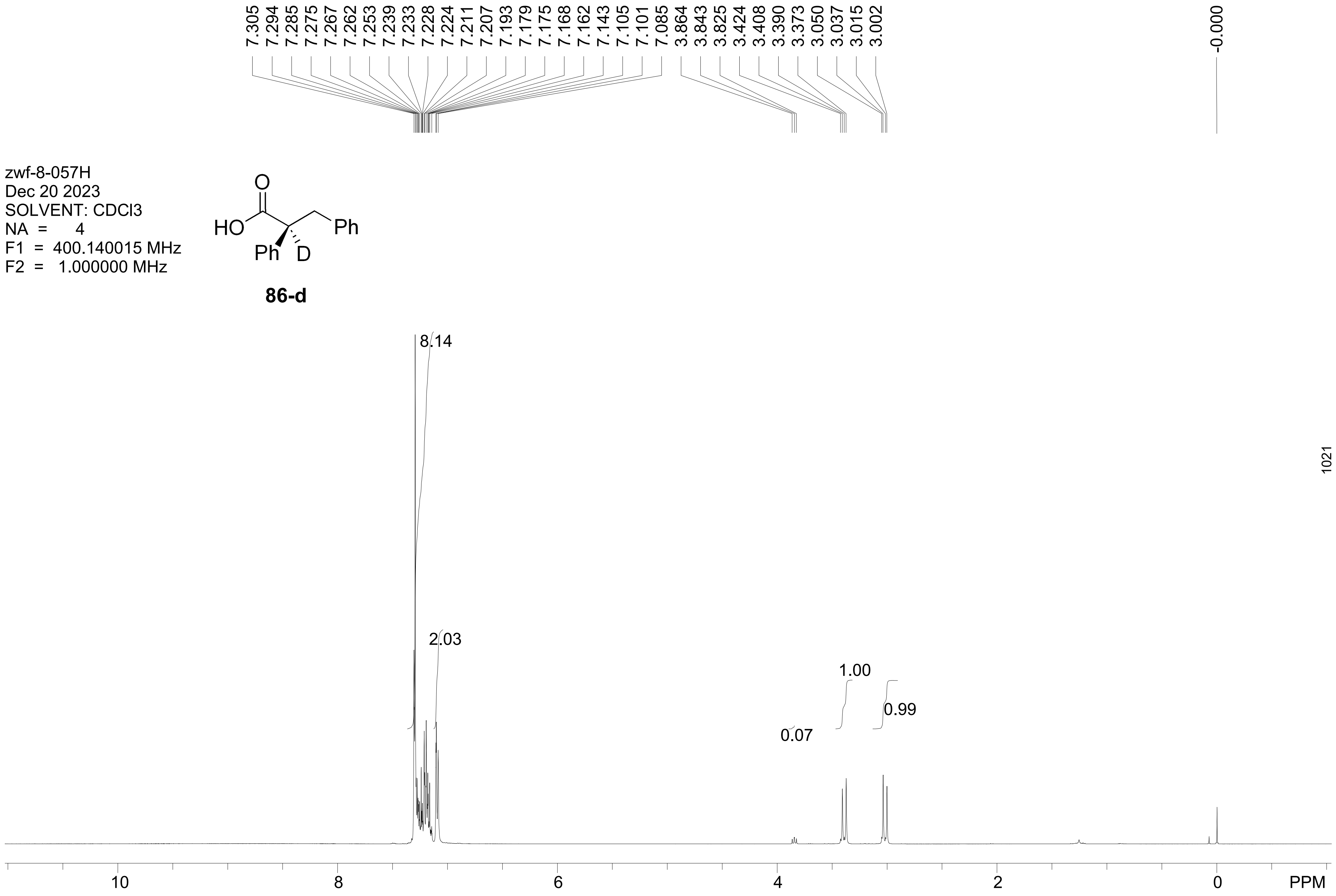
zwf-8-057H
Dec 20 2023
SOLVENT: CDCl3
NA = 4
F1 = 400.140015 MHz
F2 = 1.000000 MHz
HO
O
Ph
Ph
D
86-d
7.305
7.294
7.285
7.275
7.267
7.262
7.253
7.239
7.233
7.228
7.224
7.211
7.207
7.193
7.179
7.175
7.168
7.162
7.143
7.105
7.101
7.085
3.864
3.843
3.825
3.424
3.408
3.390
3.373
3.050
3.037
3.015
3.002
-0.000
8.14
2.03
1.00
0.99
0.07
10
8
6
4
2
0
PPM

zwf-8-057C
Dec 20 2023
SOLVENT: CDCl3
NA = 200
F1 = 100.615280 MHz
F2 = 1.000000 MHz

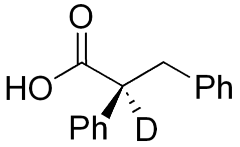

86-d

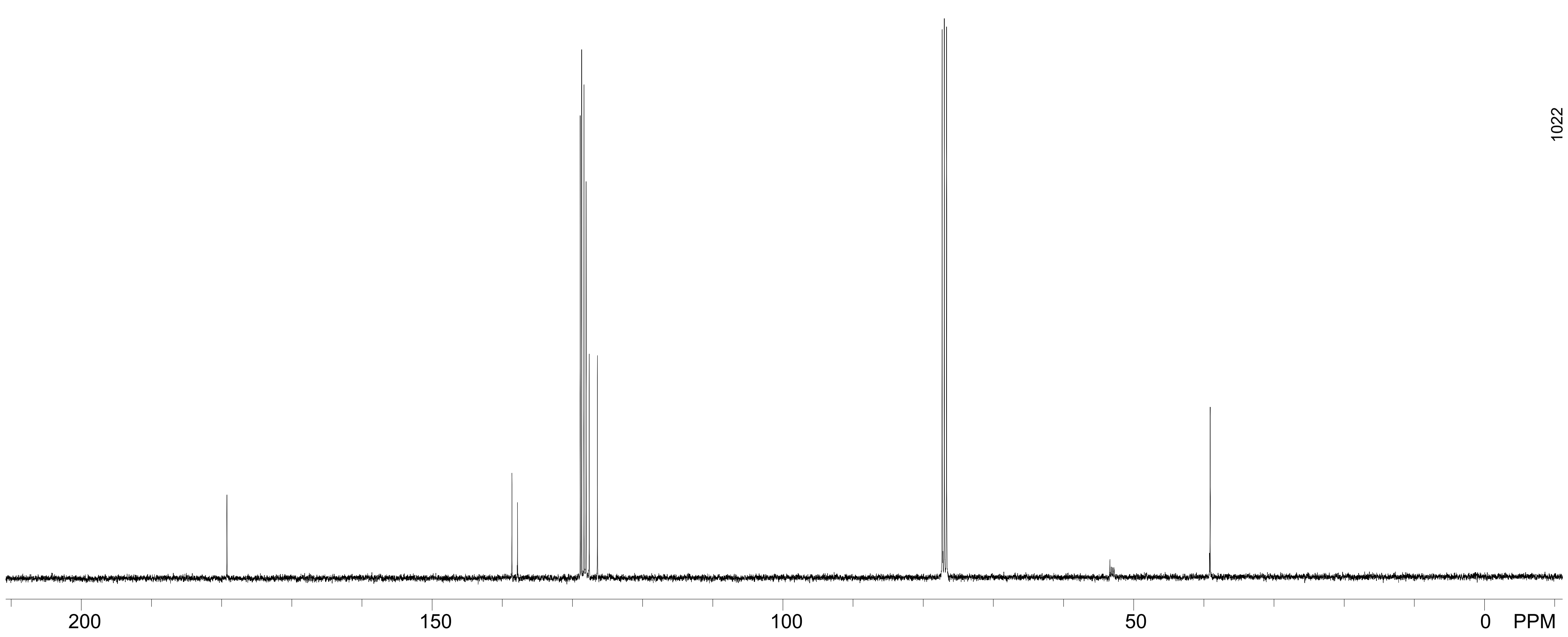

Area Percent Report

Agilent Technologies

Sample name: zwf-8-057-IE-99-1-0.8-205

Data file: C:\Users\Public\Documents\ChemStation\1\Data\zwf\zwf-S 2023-12-21 13-17-55\007-P1-C5-zwf-8-057.D

Injection Acquired Date 12/21/2023 17:06:53

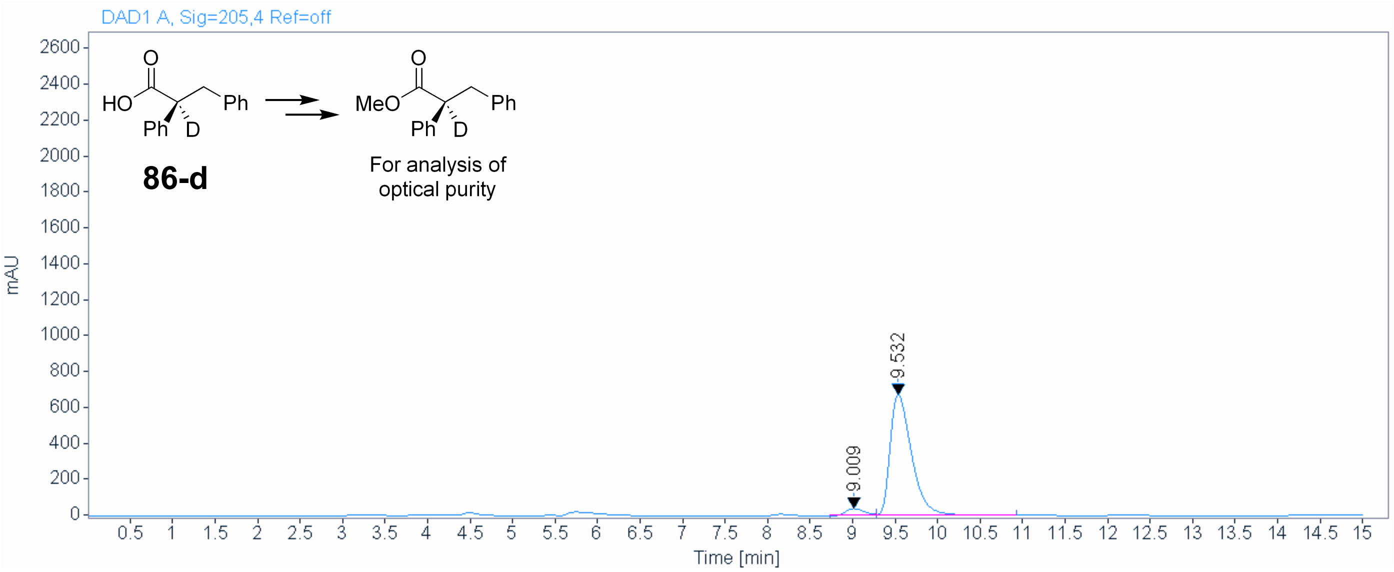

Signal: DAD1 A, Sig=205,4 Ref=off

RT [min]	Width [min]	Height	Area	Area%
9.009	0.2599	39.5972	617.5510	4.9691
9.532	0.2924	673.1405	11810.3799	95.0309
		Sum	12427.9309	100.0000

Area Percent Report

Sample name: zwf-8-058-rac-IE-99-1-0.8-205

Data file: C:\Users\Public\Documents\ChemStation\1\Data\zwf\zwf-S 2023-12-21 13-17-55\009-P1-C3-zwf-8-058-rac.D

Injection Acquired Date 12/21/2023 17:38:54

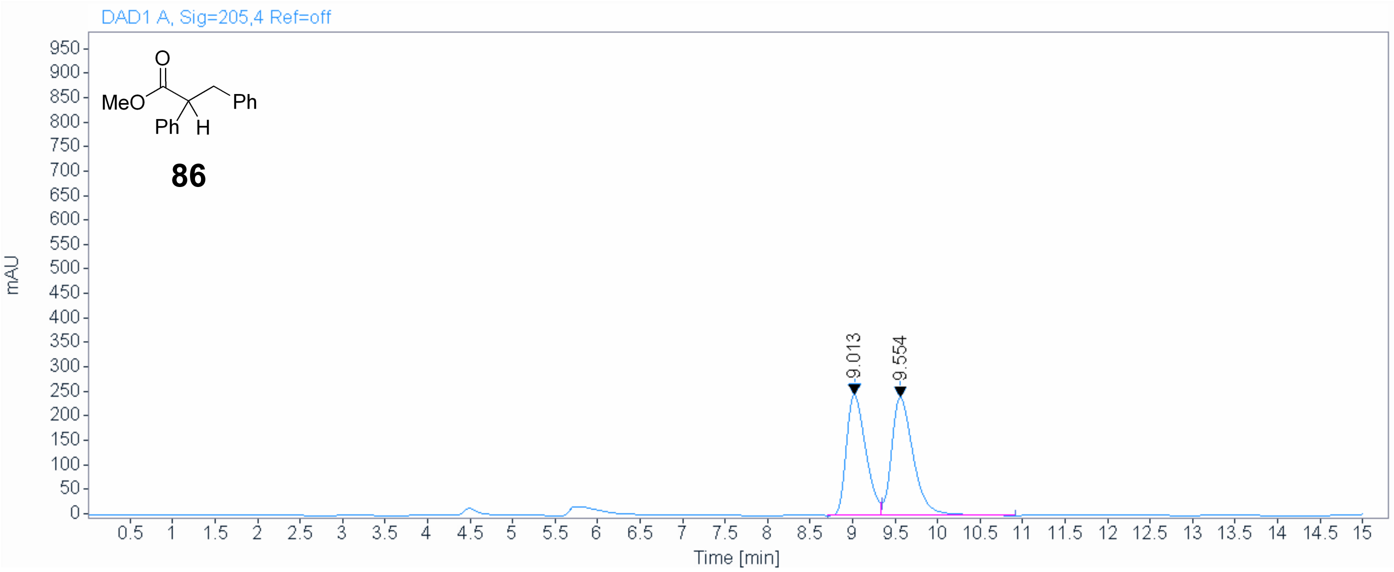

Signal: DAD1 A, Sig=205,4 Ref=off

RT [min]	Width [min]	Height	Area	Area%
9.013	0.2477	246.3666	3921.0508	47.8456
9.554	0.2705	241.4558	4274.1738	52.1544
		Sum	8195.2246	100.0000

zwf-11-079H
Aug 26 2024
SOLVENT: CDCl3
NA = 4
F1 = 600.169983 MHz
F2 = 1.000000 MHz

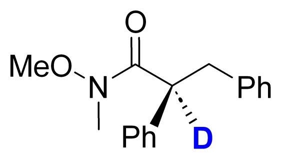

87-d

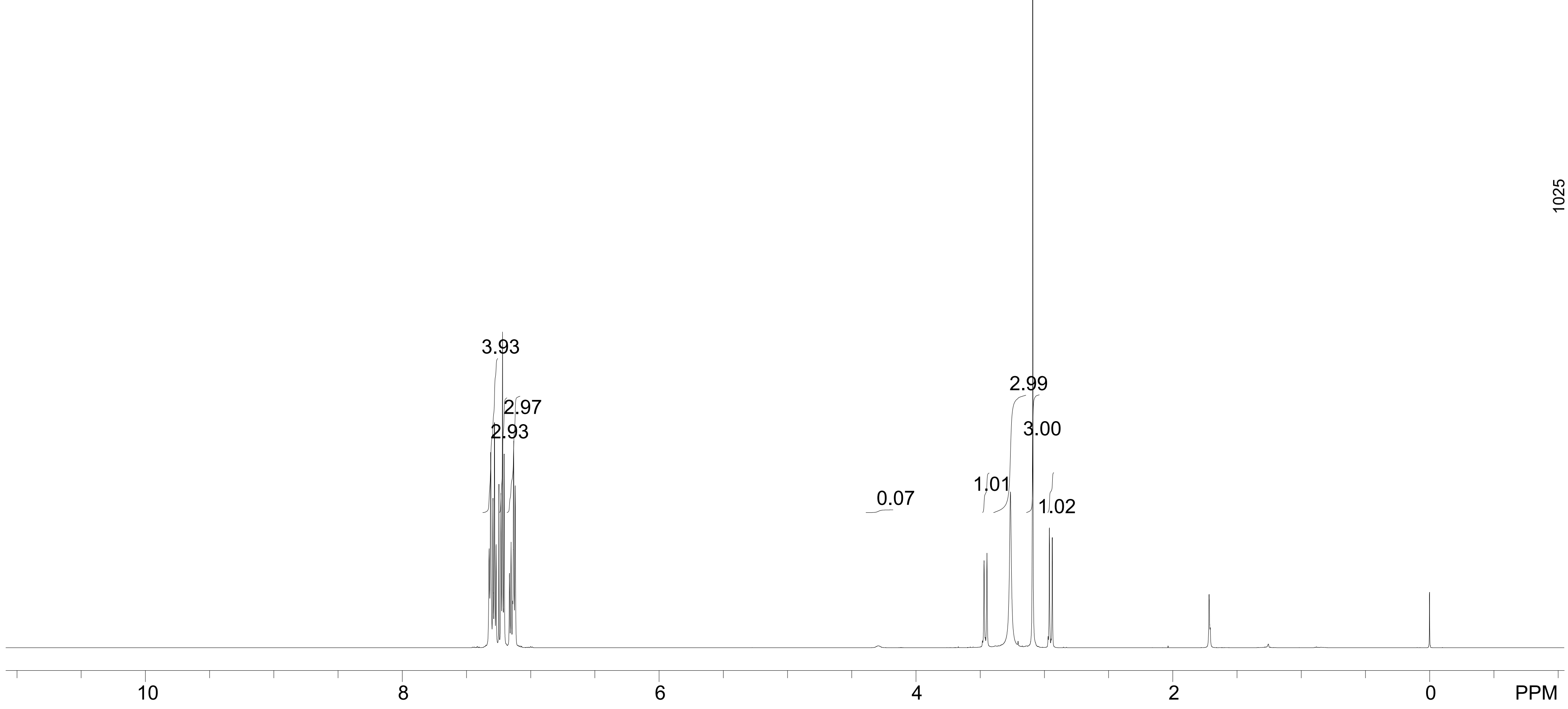

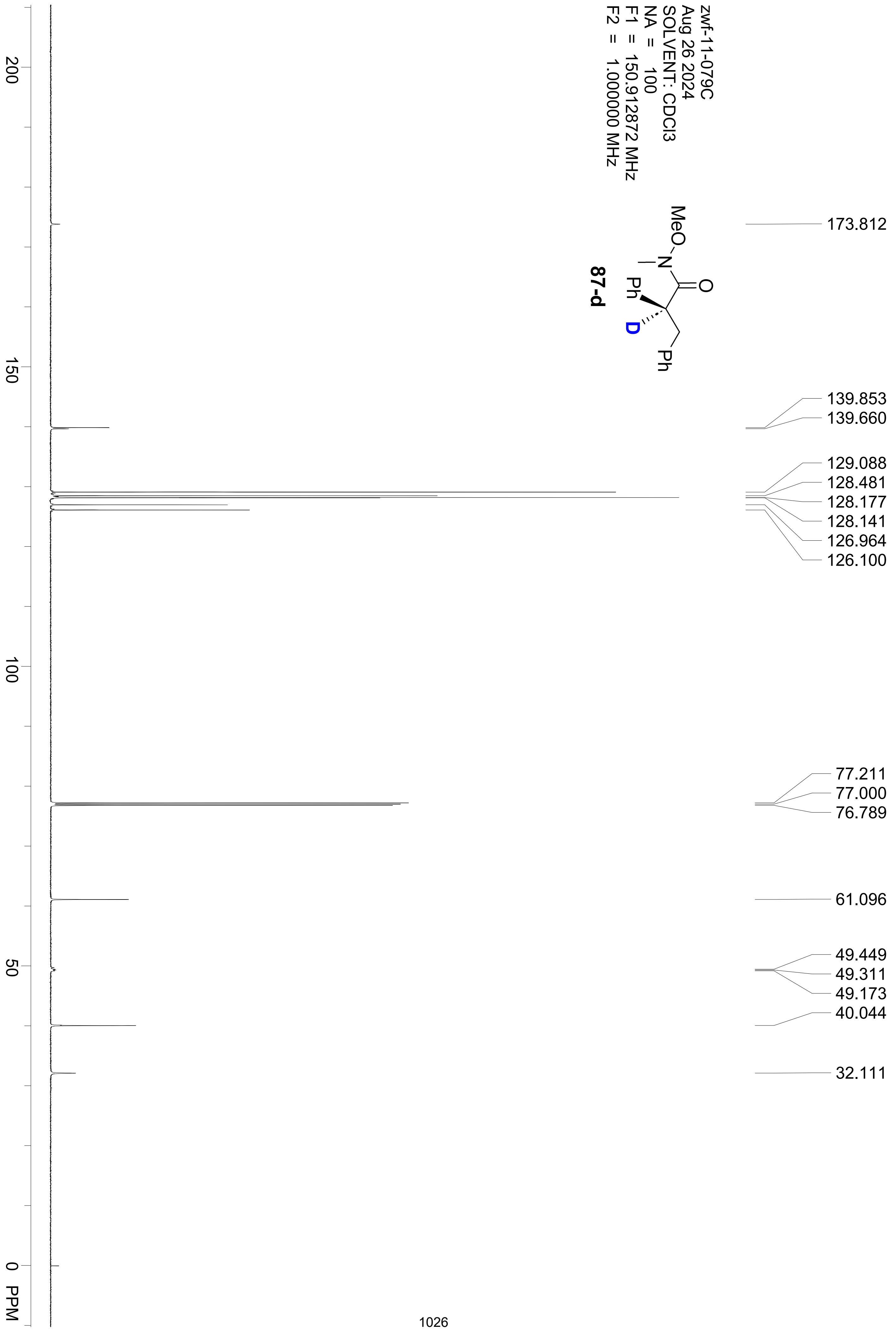
zwf-11-079C
Aug 26 2024
SOLVENT: CDCl3
NA = 100
F1 = 150.912872 MHz
F2 = 1.000000 MHz
MeO
N
O
Ph
D
Ph
87-d
173.812
139.853
139.660
129.088
128.481
128.177
128.141
126.964
126.100
77.211
77.000
76.789
61.096
49.449
49.311
49.173
40.044
32.111
200
150
100
50
0
PPM

Area Percent Report

Agilent Technologies

Sample name: zwf-11-079-IC-95-5-1.0-205

Data file: C:\Users\Public\Documents\ChemStation\1\Data\zwf\zwf 2024-08-27 12-13-37\012-P1-C4-zwf-11-079.D

Injection Acquired Date 08/27/2024 15:11:32

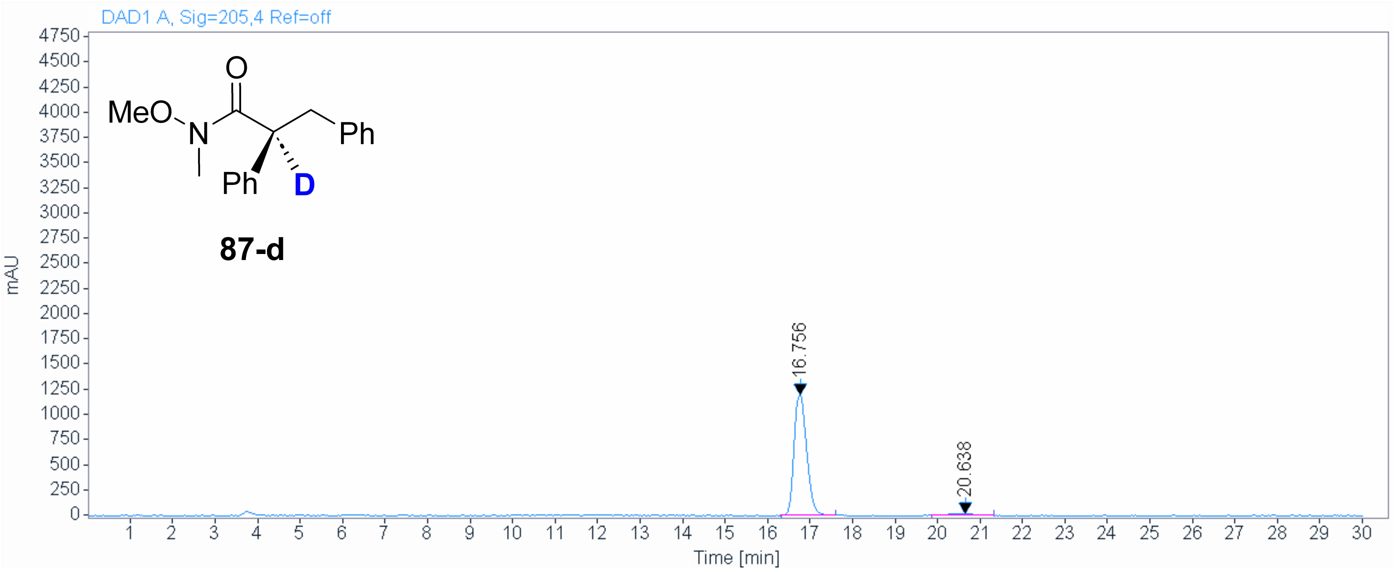

Signal: DAD1 A, Sig=205,4 Ref=off

RT [min]	Width [min]	Height	Area	Area%
16.756	0.2481	1198.3929	24873.7461	97.0038
20.638	0.7275	17.6019	768.2906	2.9962
		Sum	25642.0367	100.0000

Area Percent Report

Sample name: zwf-11-079-rac-IC-95-5-1.0-205

Data file: C:\Users\Public\Documents\ChemStation\1\Data\zwf\zwf 2024-08-27 12-13-37\013-P1-C5-zwf-11-079-rac.D

Injection Acquired Date 08/27/2024 15:42:34

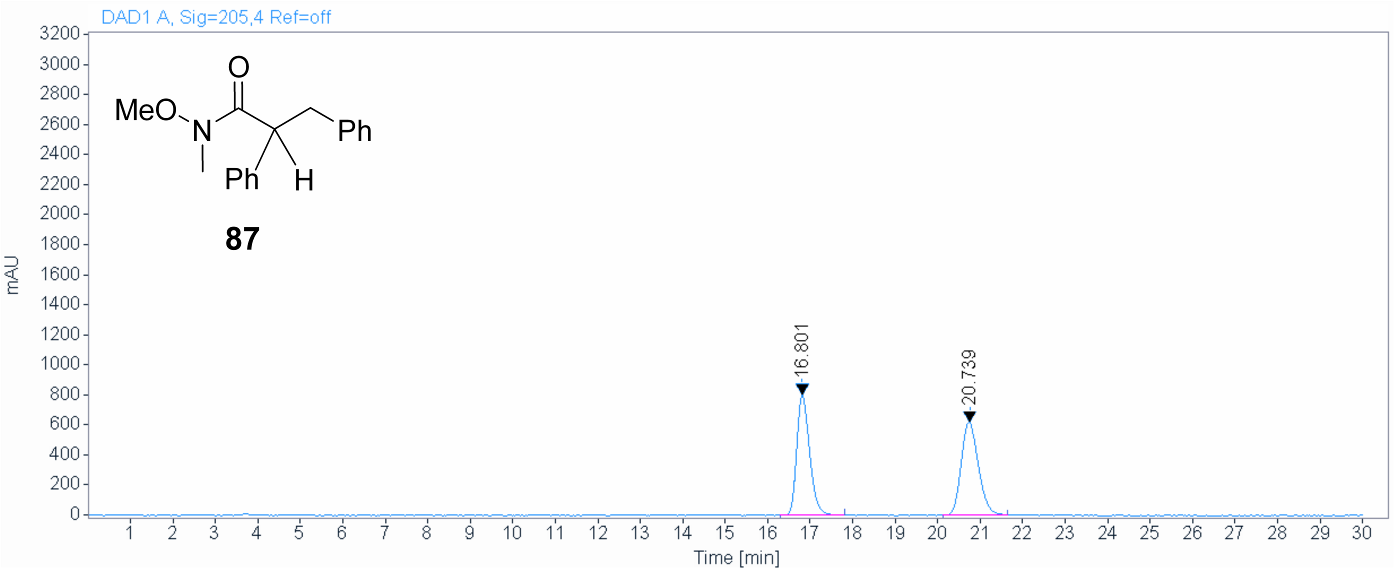

Signal: DAD1 A, Sig=205,4 Ref=off

RT [min]	Width [min]	Height	Area	Area%
16.801	0.2868	802.0137	16974.6035	49.4366
20.739	0.3670	619.4366	17361.5020	50.5634
		Sum	34336.1055	100.0000

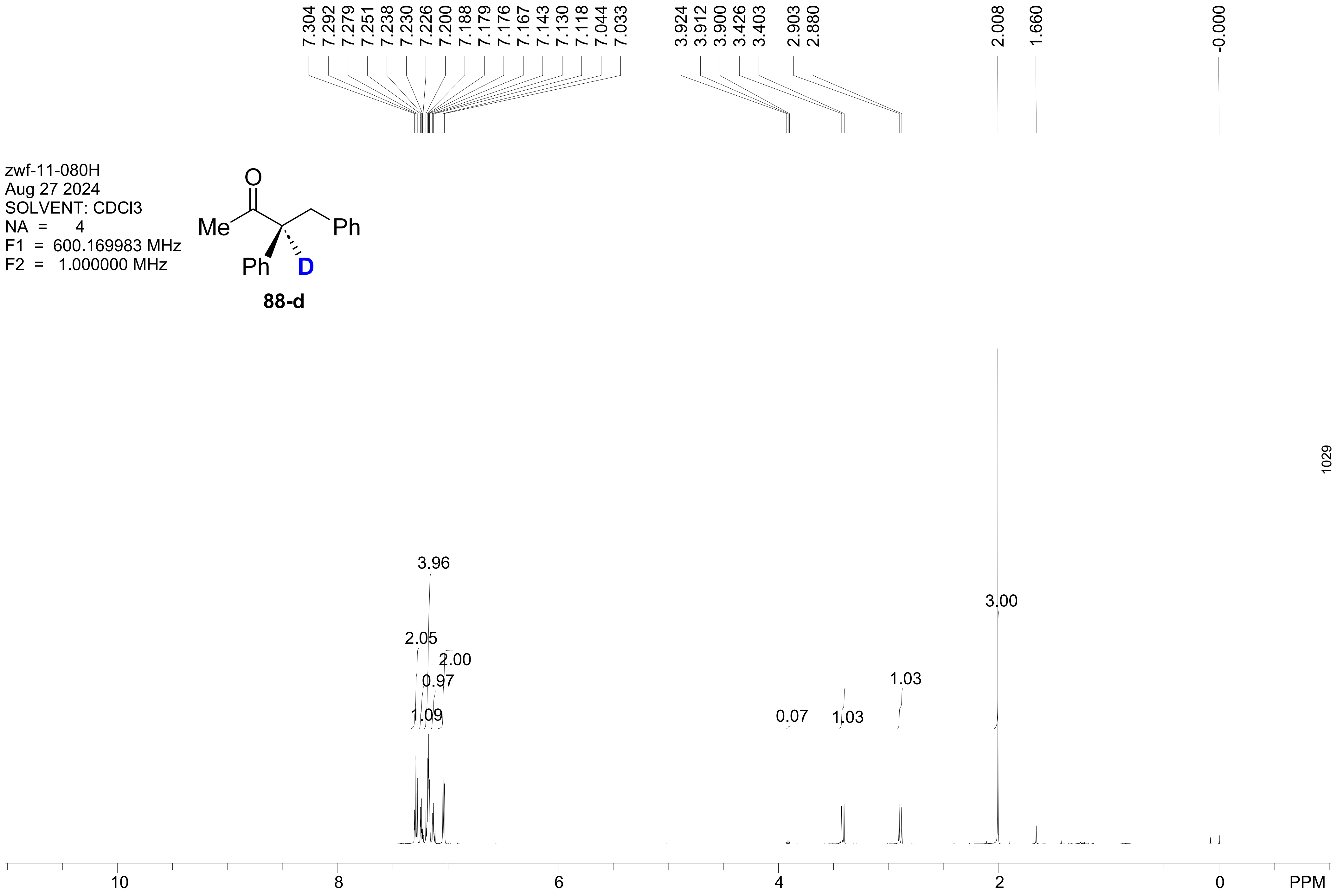
zwf-11-080H
Aug 27 2024
SOLVENT: CDCl3
NA = 4
F1 = 600.169983 MHz
F2 = 1.000000 MHz
O
Me
Ph
Ph
D
88-d
7.304
7.292
7.279
7.251
7.238
7.230
7.226
7.200
7.188
7.179
7.176
7.167
7.143
7.130
7.118
7.044
7.033
3.924
3.912
3.900
3.426
3.403
2.903
2.880
2.008
1.660
-0.000
3.96
2.05
2.00
0.97
1.09
0.07
1.03
1.03
3.00
10
8
6
4
2
0
PPM

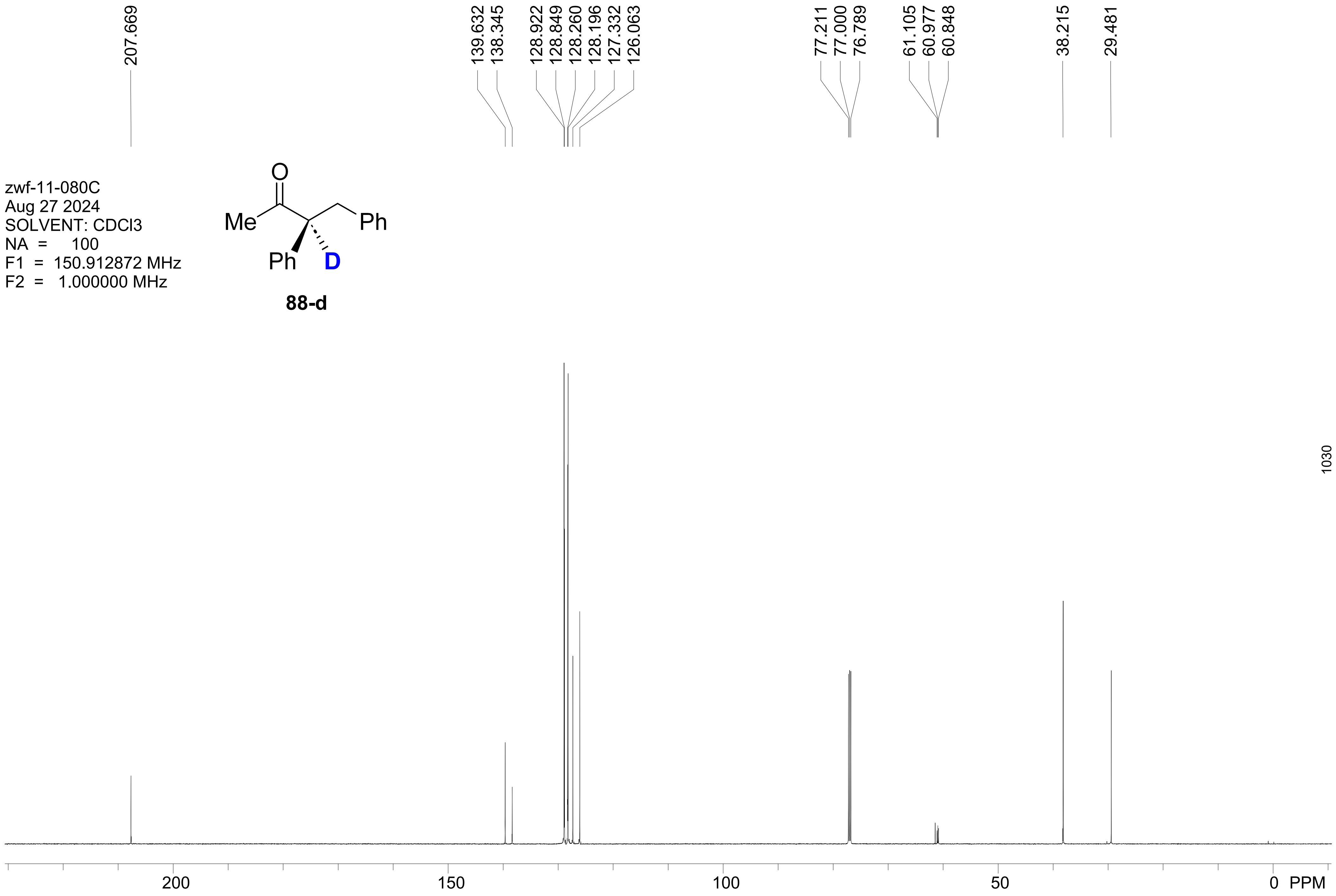
zwf-11-080C
Aug 27 2024
SOLVENT: CDCl3
NA = 100
F1 = 150.912872 MHz
F2 = 1.000000 MHz
Me
O
Ph
Ph
D
207.669
139.632
138.345
128.922
128.849
128.260
128.196
127.332
126.063
77.211
77.000
76.789
61.105
60.977
60.848
38.215
29.481
200
150
100
50
0 PPM

88-d

Area Percent Report

Agilent Technologies

Sample name: zwf-11-080-IC-99-1-0.8-205

Data file: C:\Users\Public\Documents\ChemStation\1\Data\zwf\zwf 2024-08-27 12-13-37\016-P1-C1-zwf-11-080.D

Injection Acquired Date 08/27/2024 16:31:08

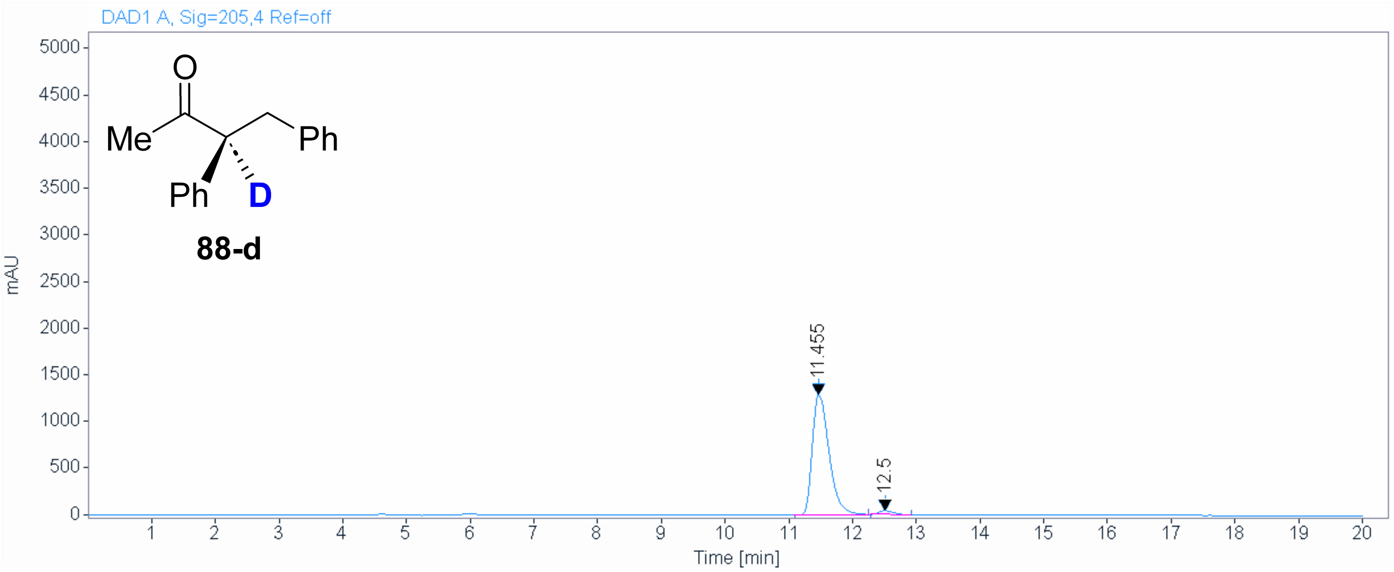

Signal: DAD1 A, Sig=205,4 Ref=off

RT [min]	Width [min]	Height	Area	Area%
11.455	0.3090	1290.0421	23920.8574	96.9714
12.500	0.2788	44.6586	747.0829	3.0286
		Sum	24667.9404	100.0000

Area Percent Report

Sample name: zwf-11-080-rac-IC-99-1-0.8-205

Data file: C:\Users\Public\Documents\ChemStation\1\Data\zwf\zwf 2024-08-27 12-13-37\017-P1-C2-zwf-11-080-rac.D

Injection Acquired Date 08/27/2024 16:52:07

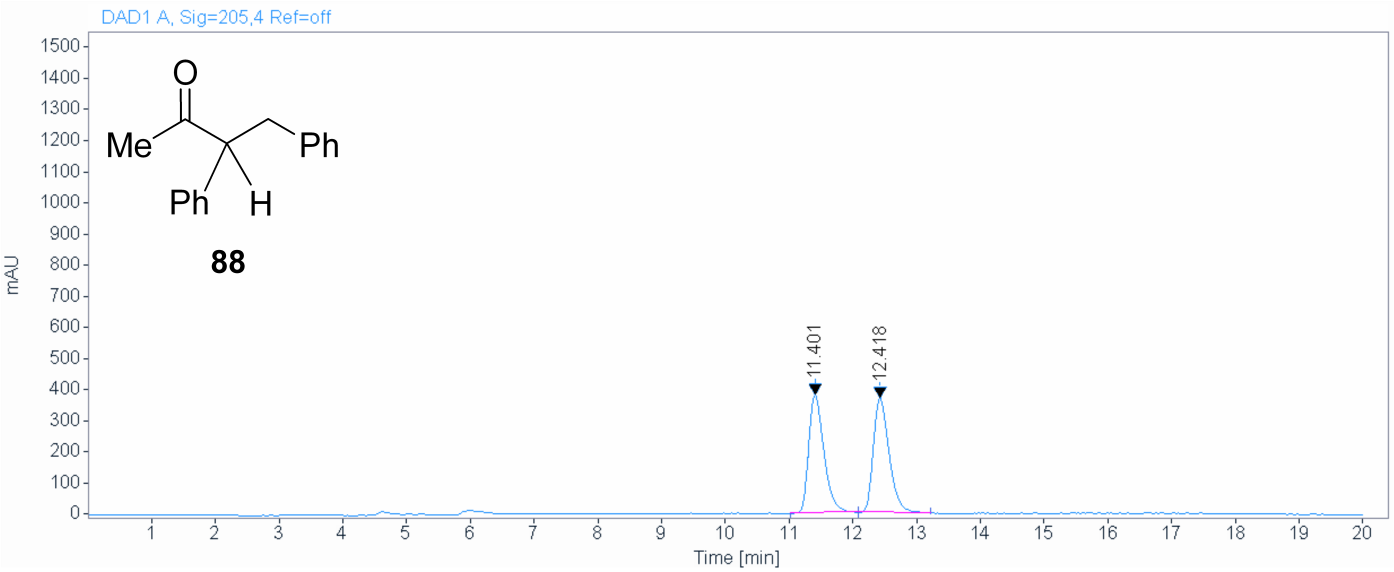

Signal: DAD1 A, Sig=205,4 Ref=off

RT [min]	Width [min]	Height	Area	Area%
11.401	0.2525	379.8796	6334.1099	49.8731
12.418	0.2647	368.5030	6366.3330	50.1269
		Sum	12700.4429	100.0000

zwf-8-058H
Dec 17 2023
SOLVENT: CDCl3
NA = 4
F1 = 400.140015 MHz
F2 = 1.000000 MHz

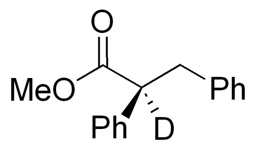

89-d

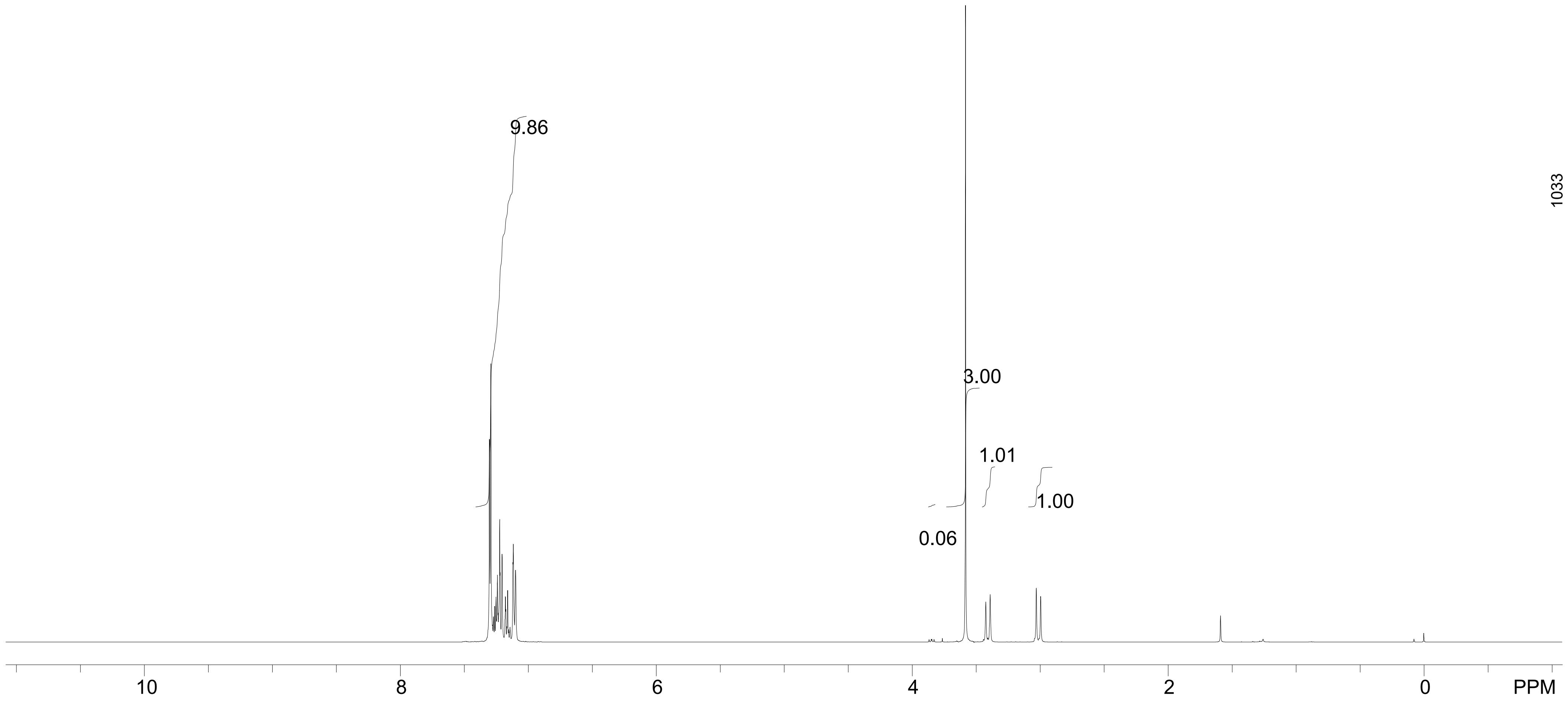

zwf-8-058C
Dec 17 2023
SOLVENT: CDCl3
NA = 100
F1 = 100.615280 MHz
F2 = 1.000000 MHz

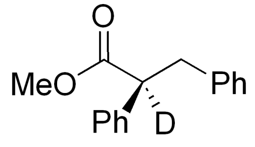

89-d

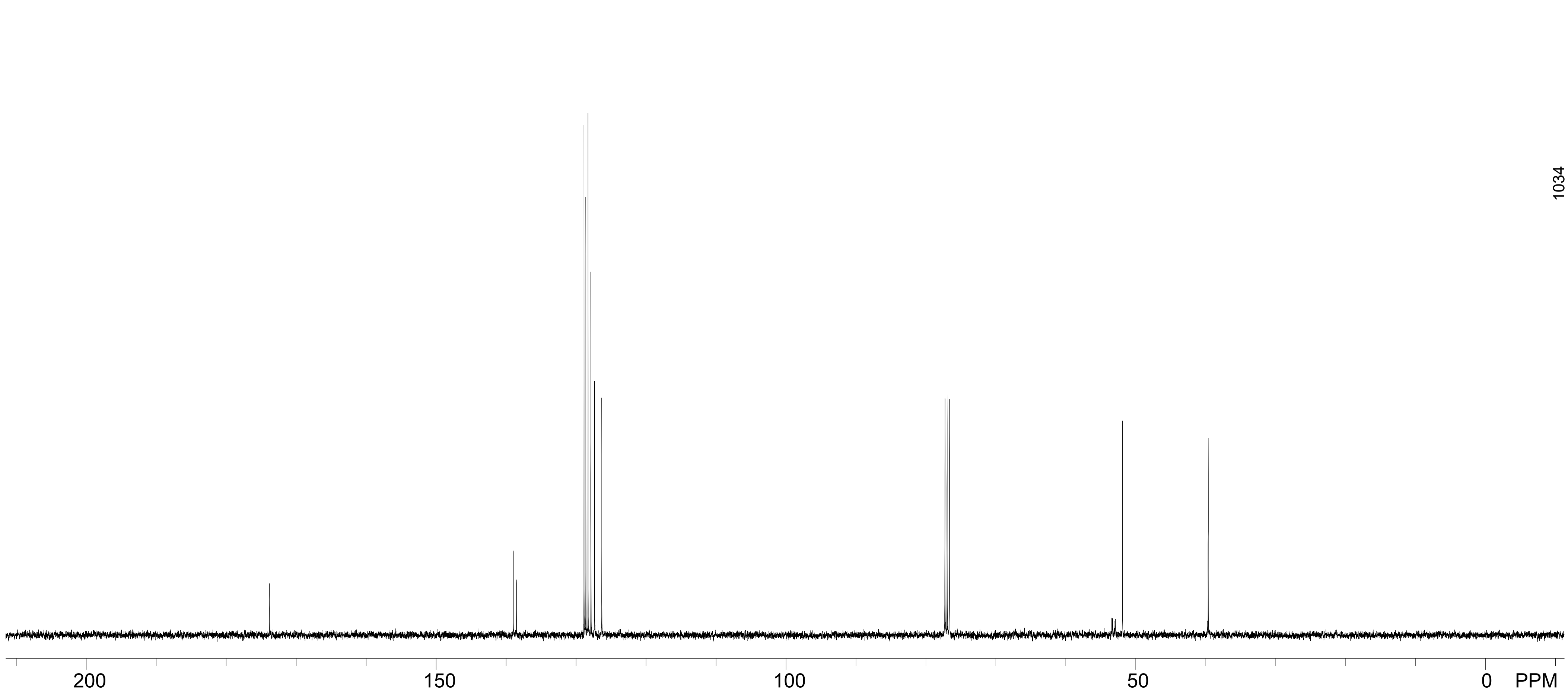

Area Percent Report

Agilent Technologies

Sample name: zwf-8-058-IE-99-1-0.8-205

Data file: C:\Users\Public\Documents\ChemStation\1\Data\zwf\zwf-S 2023-12-21 13-17-55\006-P1-C4-zwf-8-058.D

Injection Acquired Date 12/21/2023 16:50:51

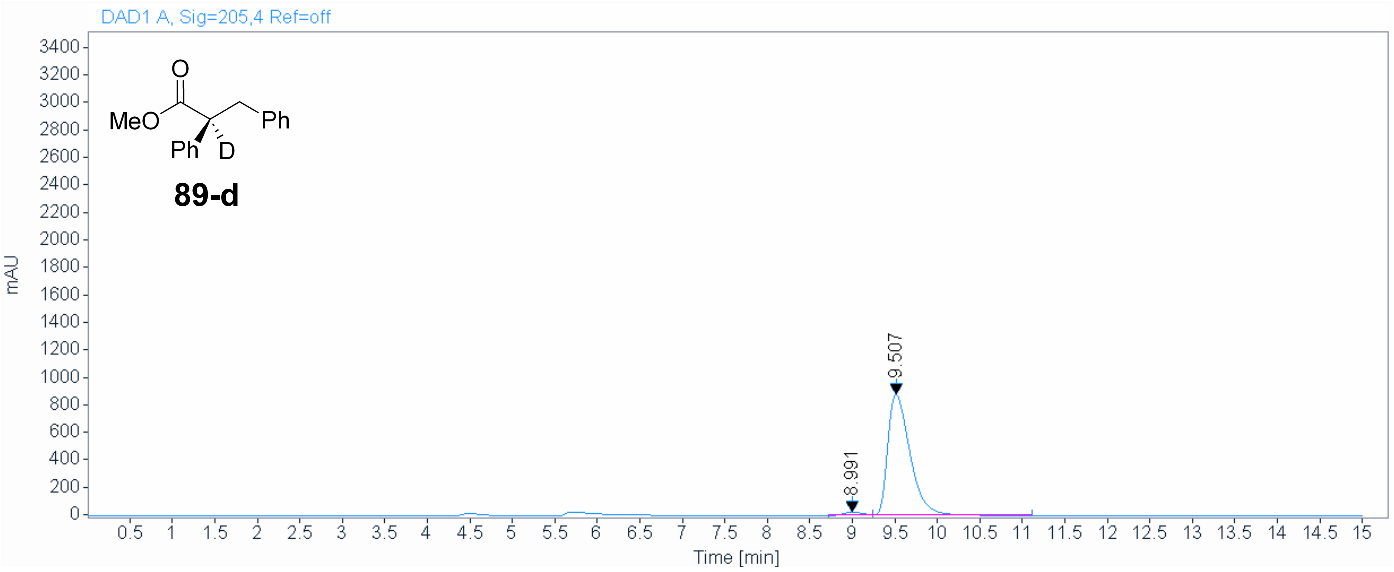

Signal: DAD1 A, Sig=205,4 Ref=off

RT [min]	Width [min]	Height	Area	Area%
8.991	0.2615	24.0719	377.6251	2.3106
9.507	0.3026	879.4771	15965.2266	97.6894
		Sum	16342.8517	100.0000

Area Percent Report

Agilent Technologies

Sample name: zwf-8-058-rac-IE-99-1-0.8-205

Data file: C:\Users\Public\Documents\ChemStation\1\Data\zwf\zwf-S 2023-12-21 13-17-55\009-P1-C3-zwf-8-058-rac.D

Injection Acquired Date 12/21/2023 17:38:54

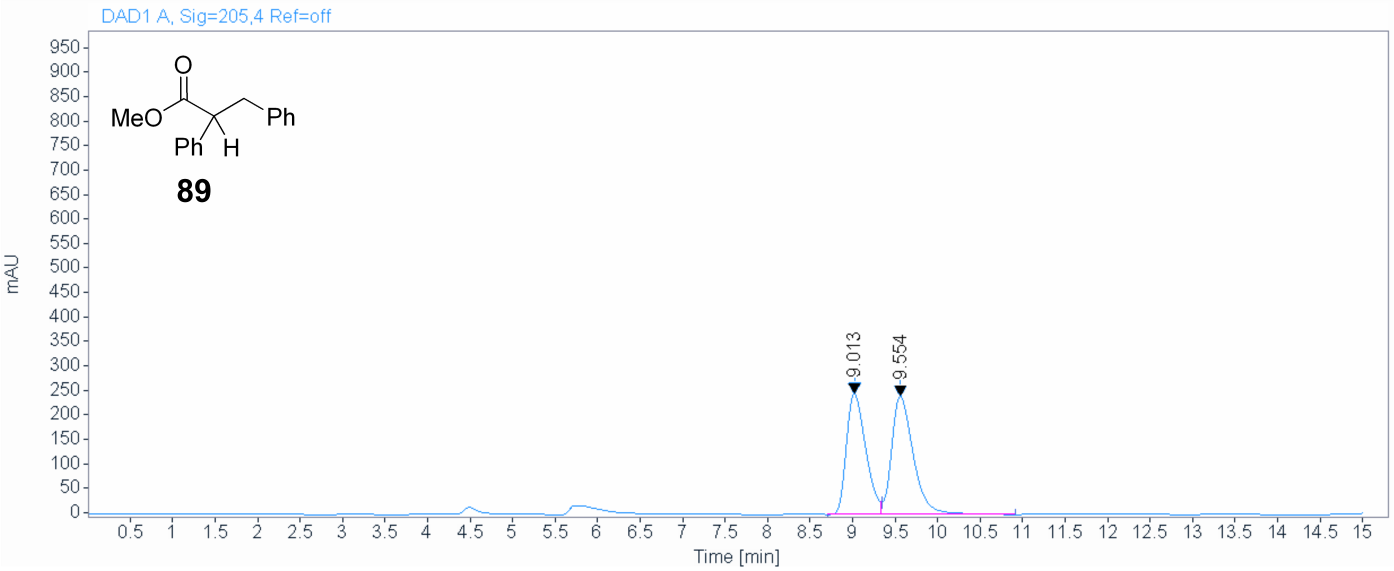

Signal: DAD1 A, Sig=205,4 Ref=off

RT [min]	Width [min]	Height	Area	Area%
9.013	0.2477	246.3666	3921.0508	47.8456
9.554	0.2705	241.4558	4274.1738	52.1544
		Sum	8195.2246	100.0000

zwf-8-072H
Dec 21 2023
SOLVENT: CDCl3
NA =   4
F1 = 400.179993 MHz
F2 =   1.000000 MHz

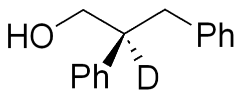

90-d

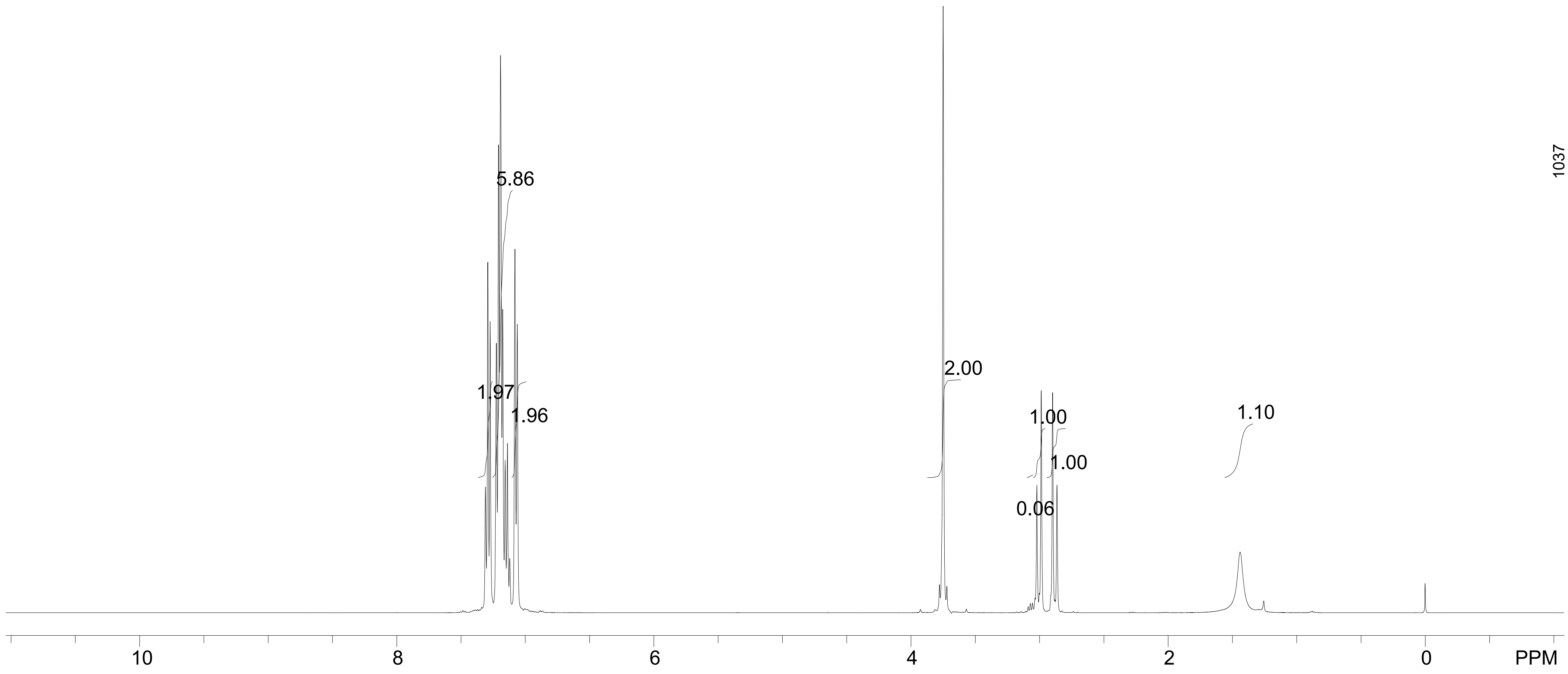

zwf-8-072C
Dec 21 2023
SOLVENT: CDCl3
NA = 100
F1 = 100.625343 MHz
F2 = 1.000000 MHz

90-d

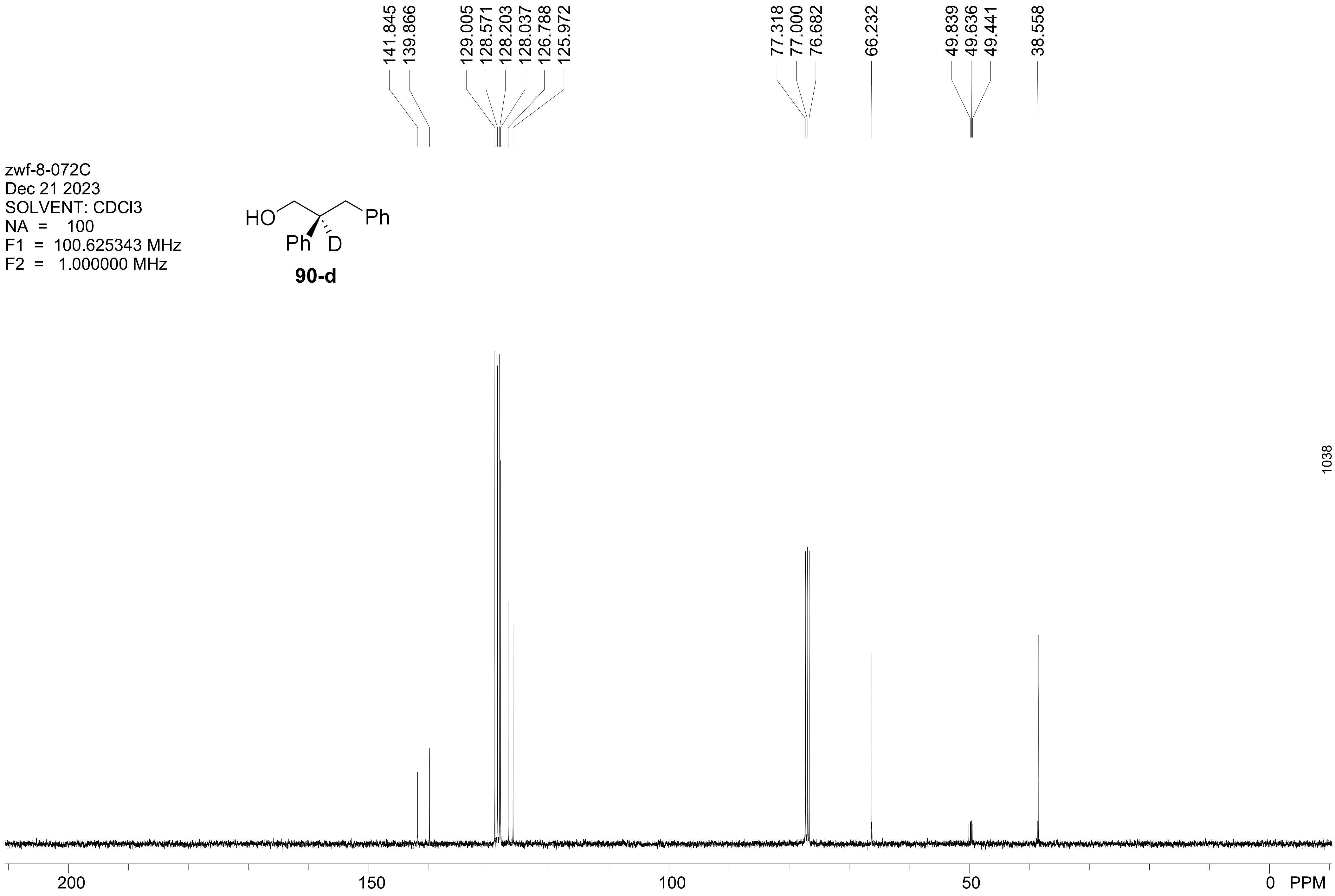

Area Percent Report

Sample name: zwf-8-072-IE-99-1-0.8-205

Data file: C:\Users\Public\Documents\ChemStation\1\Data\zwf\zwf-S 2023-12-21 13-17-55\004-P1-C2-zwf-8-072.D

Injection Acquired Date 12/21/2023 15:28:45

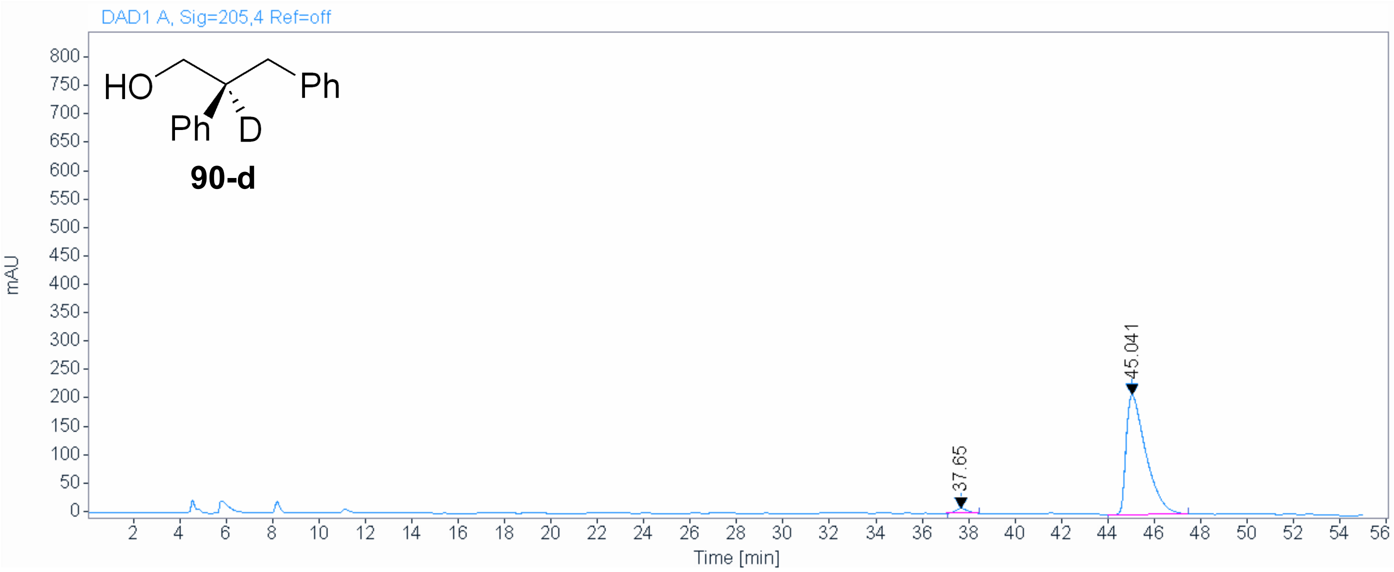

Signal: DAD1 A, Sig=205,4 Ref=off

RT [min]	Width [min]	Height	Area	Area%
37.650	0.6292	8.6126	325.1590	2.5341
45.041	0.8684	210.7322	12506.2842	97.4659
		Sum	12831.4432	100.0000

Area Percent Report

Sample name: zwf-8-072-rac-IE-99-1-0.8-205

Data file: C:\Users\Public\Documents\ChemStation\1\Data\zwf\zwf-S 2023-12-21 13-17-55\002-P1-C1-zwf-8-071-rac.D

Injection Acquired Date 12/21/2023 13:36:40

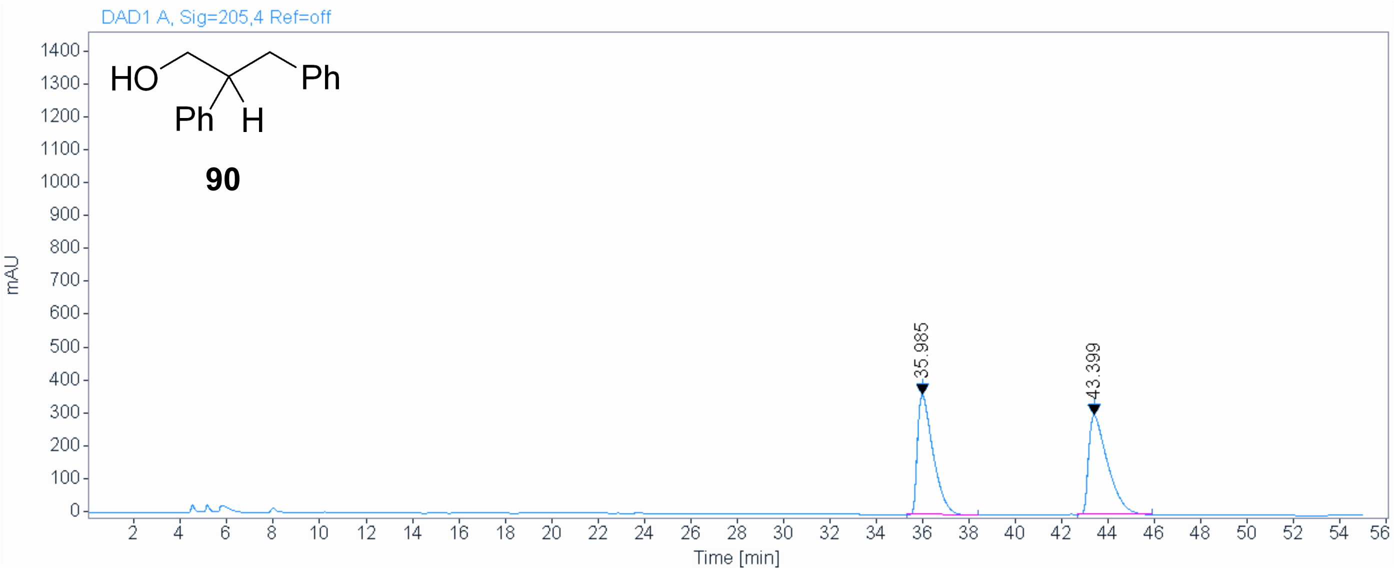

Signal: DAD1 A, Sig=205,4 Ref=off

RT [min]	Width [min]	Height	Area	Area%
35.985	0.7152	364.6537	17229.5215	50.0029
43.399	0.8478	300.9660	17227.5508	49.9971
		Sum	34457.0723	100.0000

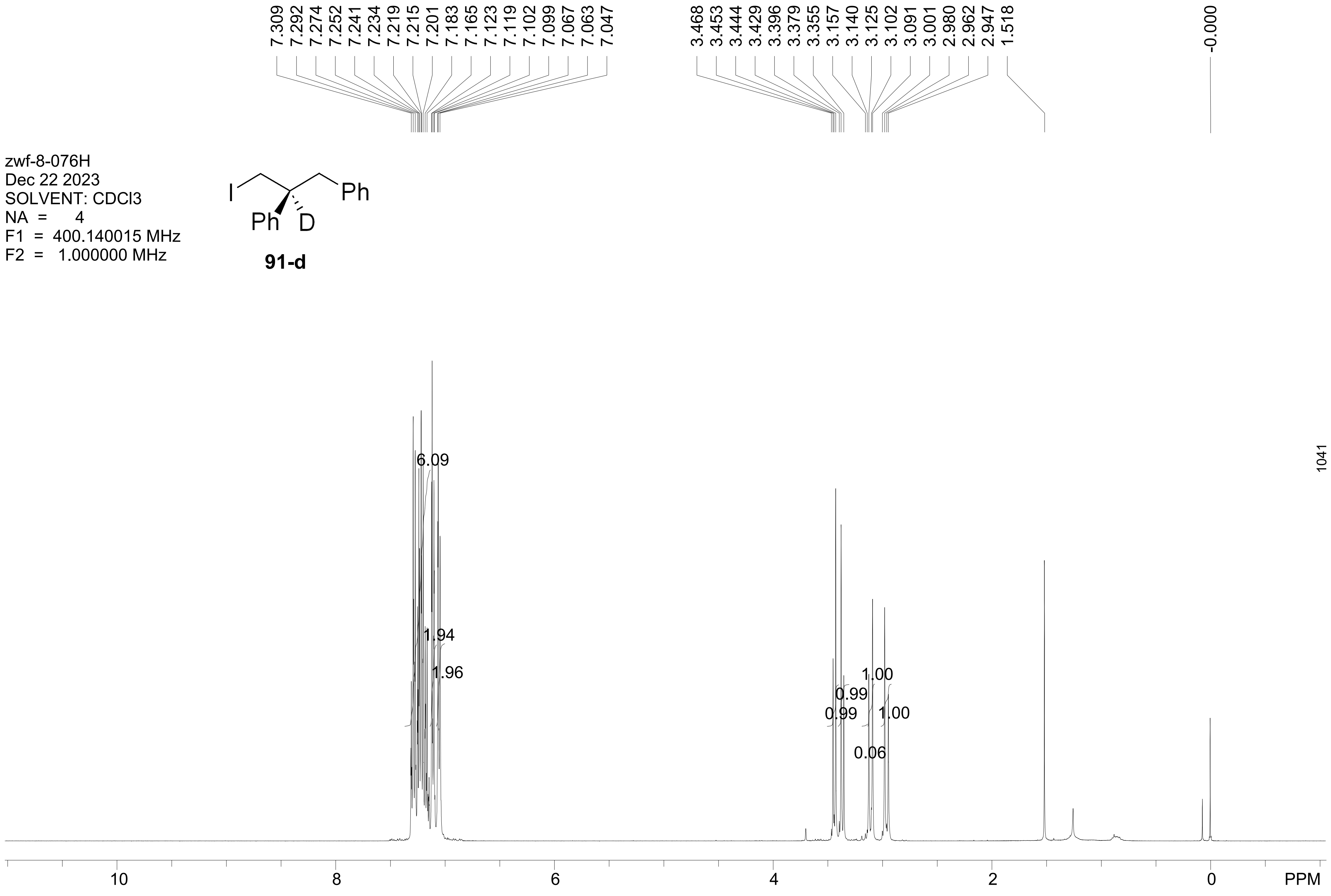
zwf-8-076H
Dec 22 2023
SOLVENT: CDCl3
NA = 4
F1 = 400.140015 MHz
F2 = 1.000000 MHz
I
Ph
Ph
D
91-d
7.309 7.292 7.274 7.252 7.241 7.234 7.219 7.215 7.201 7.183 7.165 7.123 7.119 7.102 7.099 7.067 7.063 7.047
3.468 3.453 3.444 3.429 3.396 3.379 3.355 3.157 3.140 3.125 3.102 3.091 3.001 2.980 2.962 2.947 1.518
-0.000
6.09
1.94
1.96
1.00
0.99
0.99
1.00
0.06
10
8
6
4
2
0
PPM

zwf-8-076C
Dec 22 2023
SOLVENT: CDCl3
NA = 120
F1 = 100.615280 MHz
F2 = 1.000000 MHz

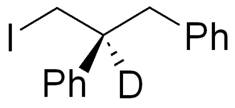

91-d

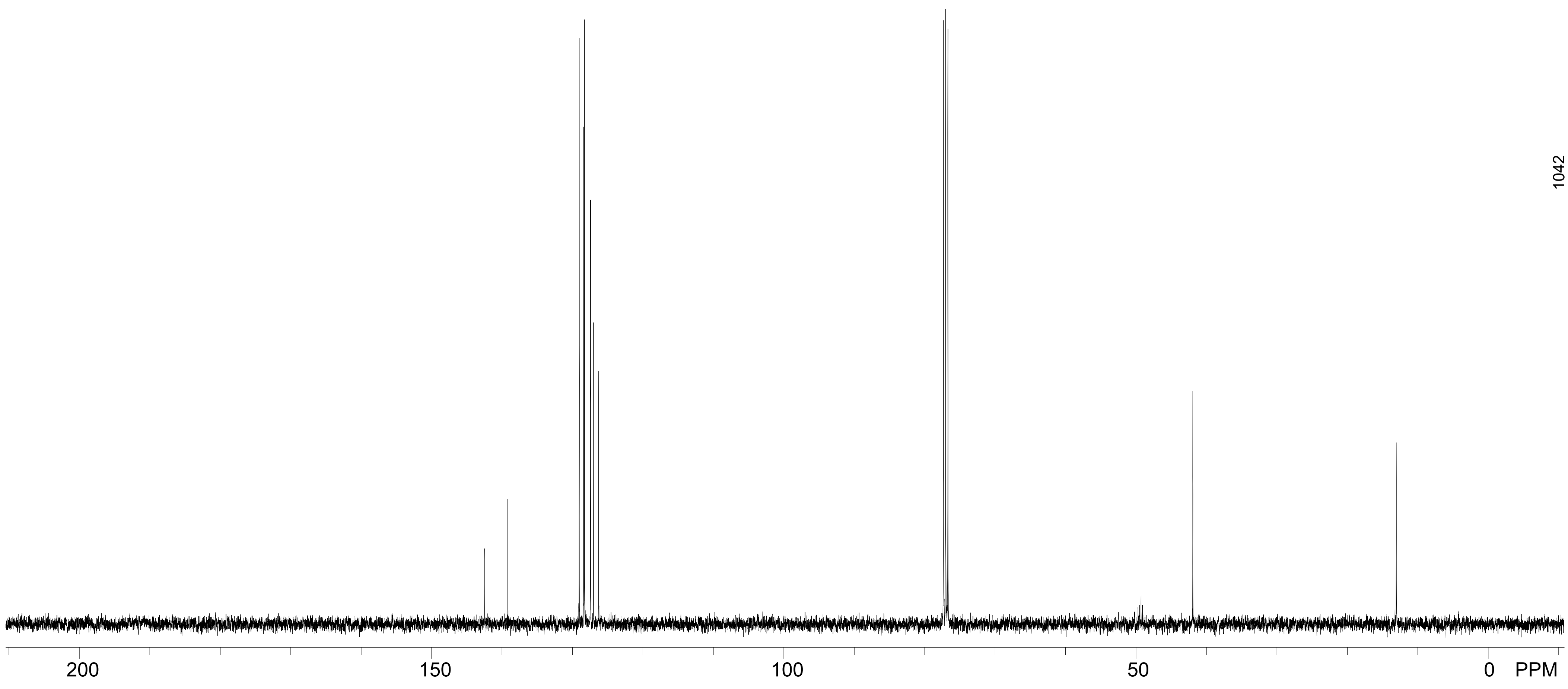

Area Percent Report

Agilent Technologies

Sample name: zwf-8-076-IE-100-0-0.8-205

Data file: C:\Users\Public\Documents\ChemStation\1\Data\zwf\zwf-S 2023-12-23 14-54-51\013-P1-C3-zwf-8-076 IE-100-0-1.0.D

Injection Acquired Date 12/23/2023 20:00:36

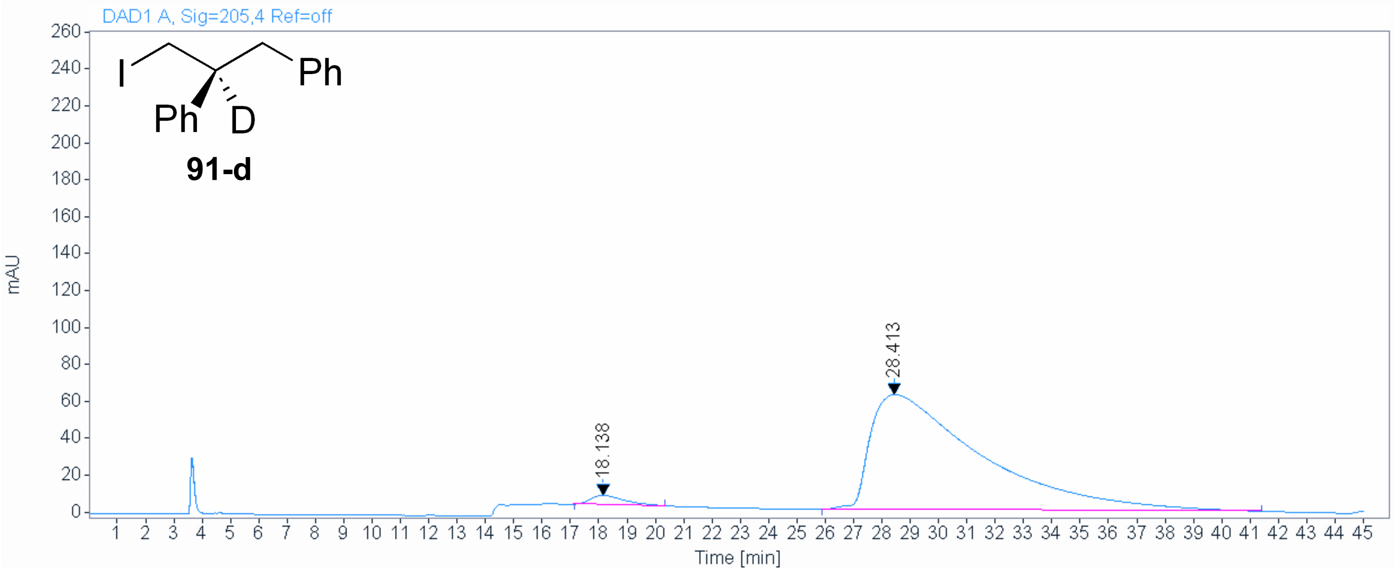

Signal: DAD1 A, Sig=205,4 Ref=off

RT [min]	Width [min]	Height	Area	Area%
18.138	1.3821	4.9708	412.2004	2.3642
28.413	3.1997	62.3424	17022.9805	97.6358
		Sum	17435.1809	100.0000

Area Percent Report

Agilent Technologies

Sample name: zwf-8-076-rac-IE-100-0-0.8-205

Data file: C:\Users\Public\Documents\ChemStation\1\Data\zwf\zwf-S 2023-12-23 14-54-51\012-P1-C4-zwf-8-076-rac IE-100-0-0.8.D

Injection Acquired Date 12/23/2023 19:14:31

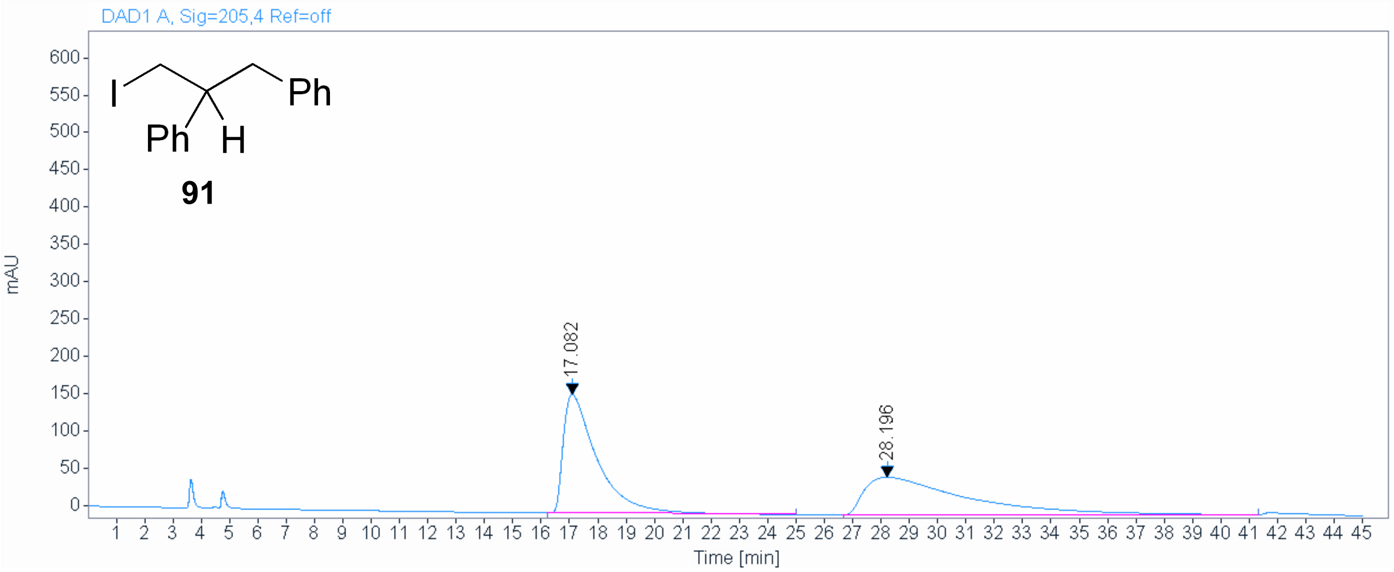

91

Signal: DAD1 A, Sig=205,4 Ref=off

RT [min]	Width [min]	Height	Area	Area%
17.082	1.1883	158.9727	13303.3584	50.4511
28.196	4.3079	50.5490	13065.4590	49.5489
		Sum	26368.8174	100.0000

zwf-8-064-1H
Dec 18 2023
SOLVENT: CDCl3
NA = 4
F1 = 400.179993 MHz
F2 = 1.000000 MHz

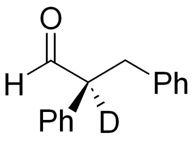

92-d

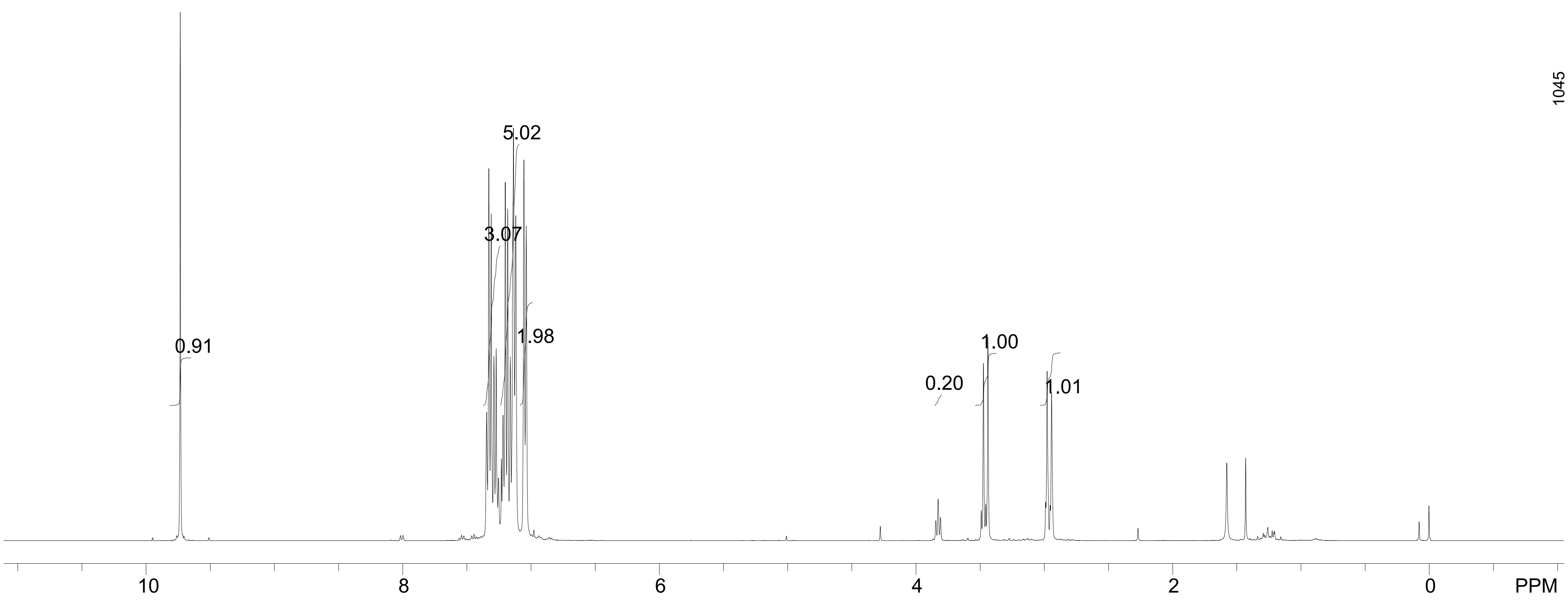

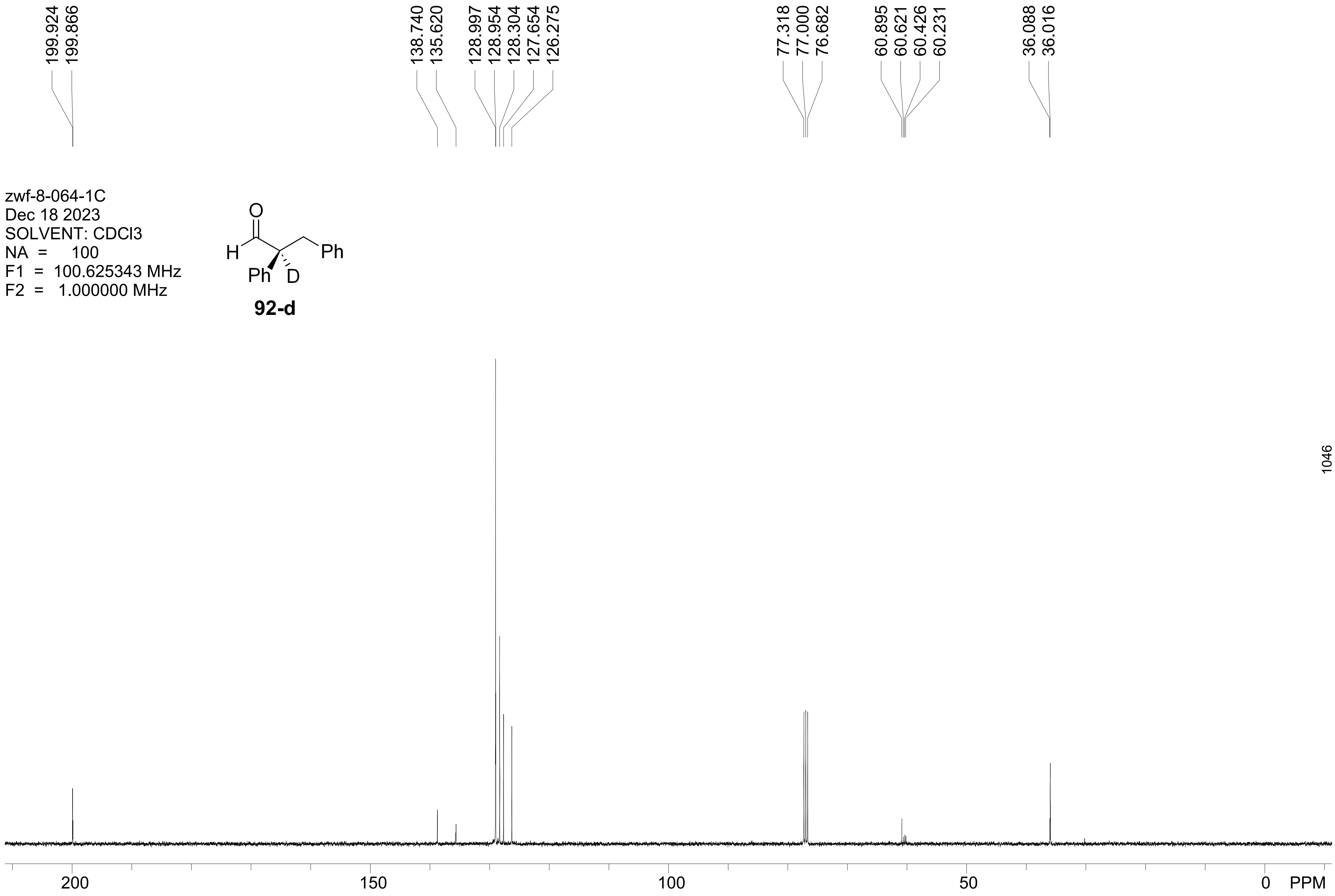
199.924
199.866
138.740
135.620
128.997
128.954
128.304
127.654
126.275
77.318
77.000
76.682
60.895
60.621
60.426
60.231
36.088
36.016
zwf-8-064-1C
Dec 18 2023
SOLVENT: CDCl3
NA = 100
F1 = 100.625343 MHz
F2 = 1.000000 MHz
O
H
Ph
Ph
D
92-d
200
150
100
50
0
PPM

Area Percent Report

Agilent Technologies

Sample name: zwf-8-064-1-IE-99-1-0.8-205

Data file: C:\Users\Public\Documents\ChemStation\1\Data\zwf\zwf-S 2023-12-18 14-32-51\017-P1-C7-zwf-8-064-1.D

Injection Acquired Date 12/18/2023 19:28:32

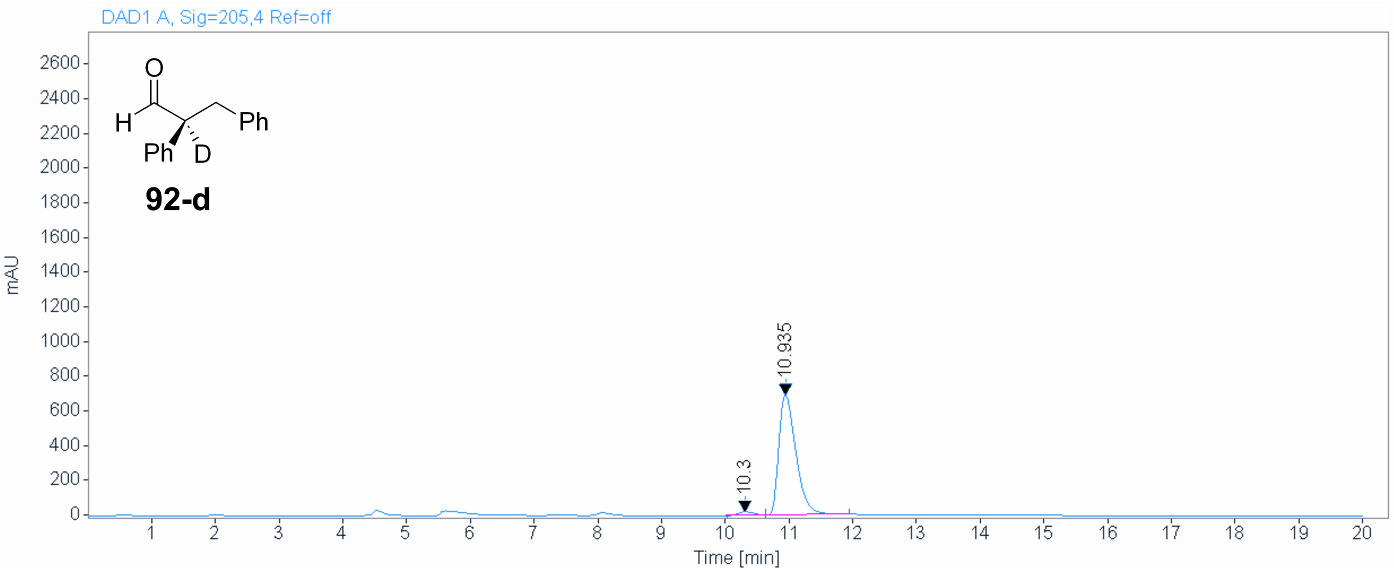

Signal: DAD1 A, Sig=205,4 Ref=off

RT [min]	Width [min]	Height	Area	Area%
10.300	0.2721	20.7768	339.1954	2.5447
10.935	0.2908	692.5563	12990.1924	97.4553
		Sum	13329.3878	100.0000

Area Percent Report

Agilent Technologies

Sample name: zwf-8-064-1-rac-IE-99-1-0.8-205

Data file: C:\Users\Public\Documents\ChemStation\1\Data\zwf\zwf-S 2023-12-18 14-32-51\009-P1-C2-zwf-8-061-rac.D

Injection Acquired Date 12/18/2023 16:40:21

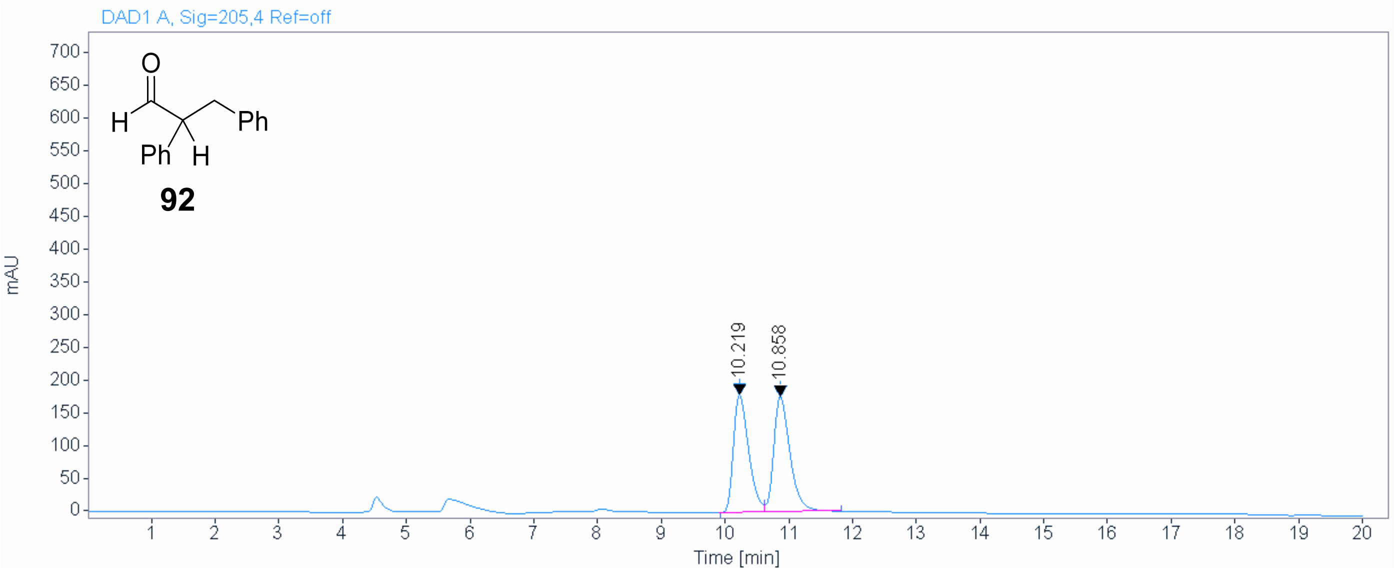

Signal: DAD1 A, Sig=205,4 Ref=off

RT [min]	Width [min]	Height	Area	Area%
10.219	0.2564	179.6582	2993.4497	48.7773
10.858	0.2723	176.0778	3143.5171	51.2227
		Sum	6136.9668	100.0000

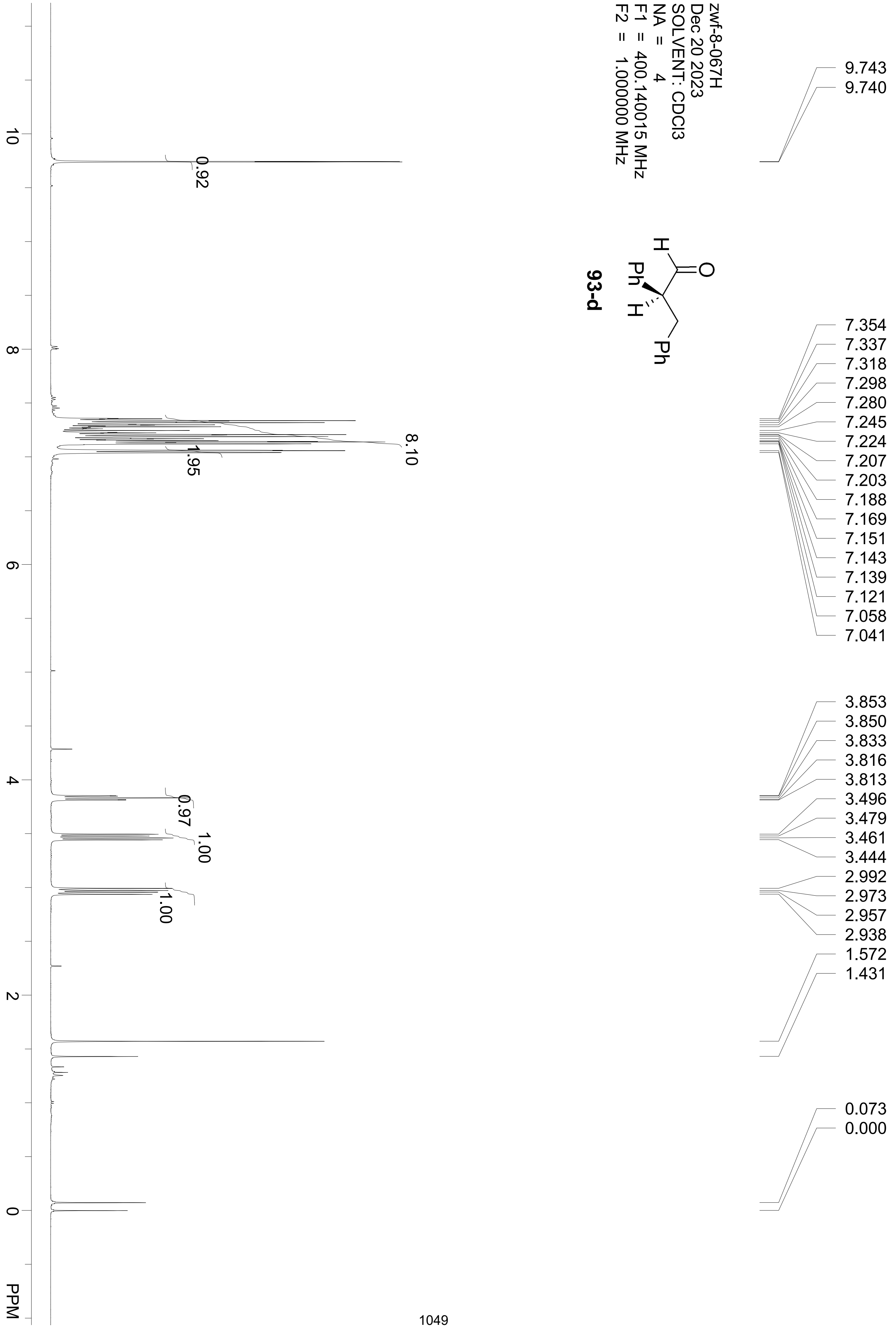
zwf-8-067H
Dec 20 2023
SOLVENT: CDCl3
NA = 4
F1 = 400.140015 MHz
F2 = 1.000000 MHz
93-d
9.743
9.740
7.354
7.337
7.318
7.298
7.280
7.245
7.224
7.207
7.203
7.188
7.169
7.151
7.143
7.139
7.121
7.058
7.041
3.853
3.850
3.833
3.816
3.813
3.496
3.479
3.461
3.444
2.992
2.973
2.957
2.938
1.572
1.431
0.073
0.000
0.92
8.10
1.95
0.97
1.00
1.00
PPM

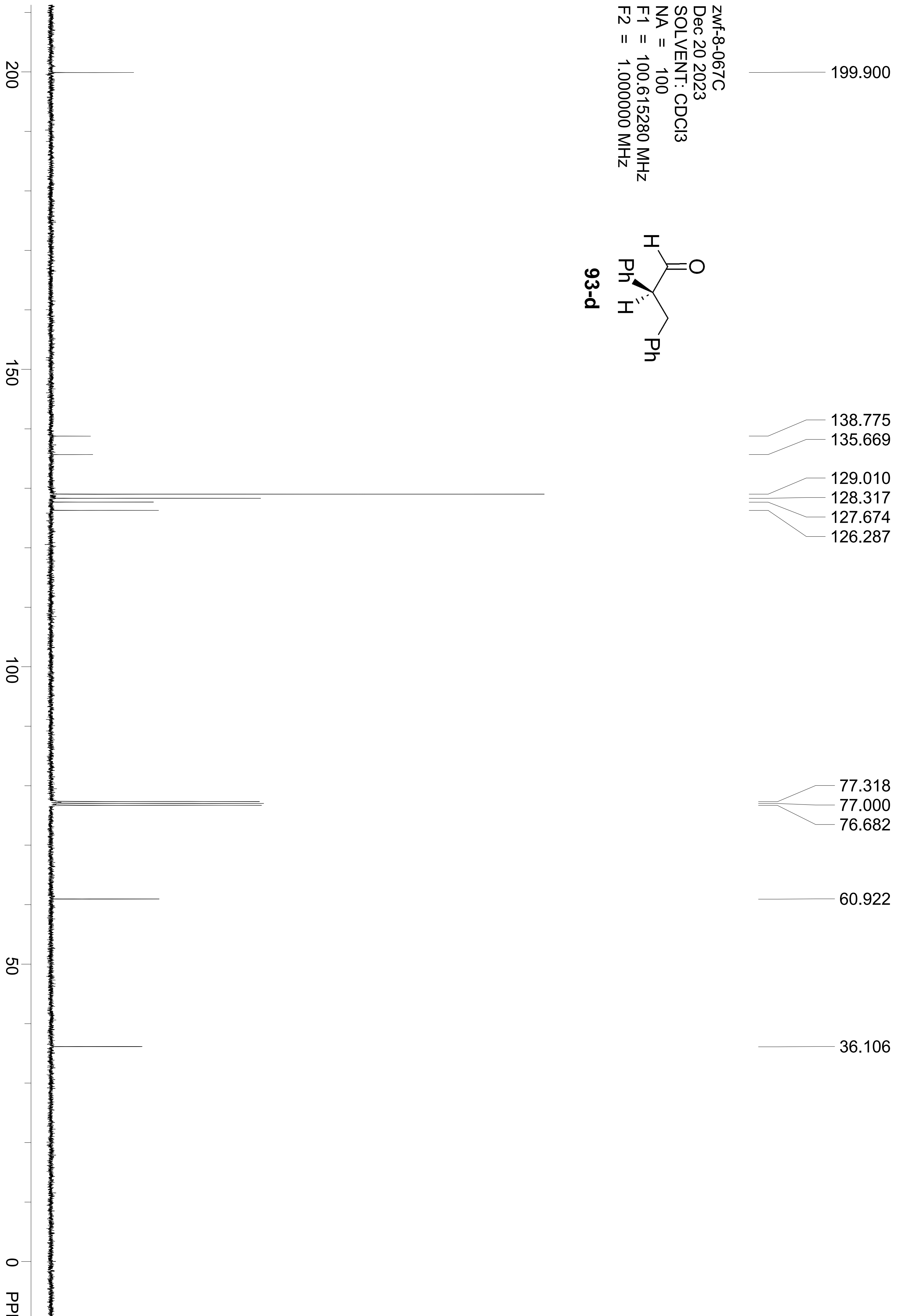
zwf-8-067C
Dec 20 2023
SOLVENT: CDCl3
NA = 100
F1 = 100.615280 MHz
F2 = 1.000000 MHz
93-d
199.900
138.775
135.669
129.010
128.317
127.674
126.287
77.318
77.000
76.682
60.922
36.106
200
150
100
50
0
PPM

Area Percent Report

Agilent Technologies

Sample name: zwf-8-067-IE-99-1-0.8-205

Data file: C:\Users\Public\Documents\ChemStation\1\Data\zwf\zwf-S 2023-12-20 15-51-10\002-P1-C1-zwf-8-067.D

Injection Acquired Date 12/20/2023 16:13:12

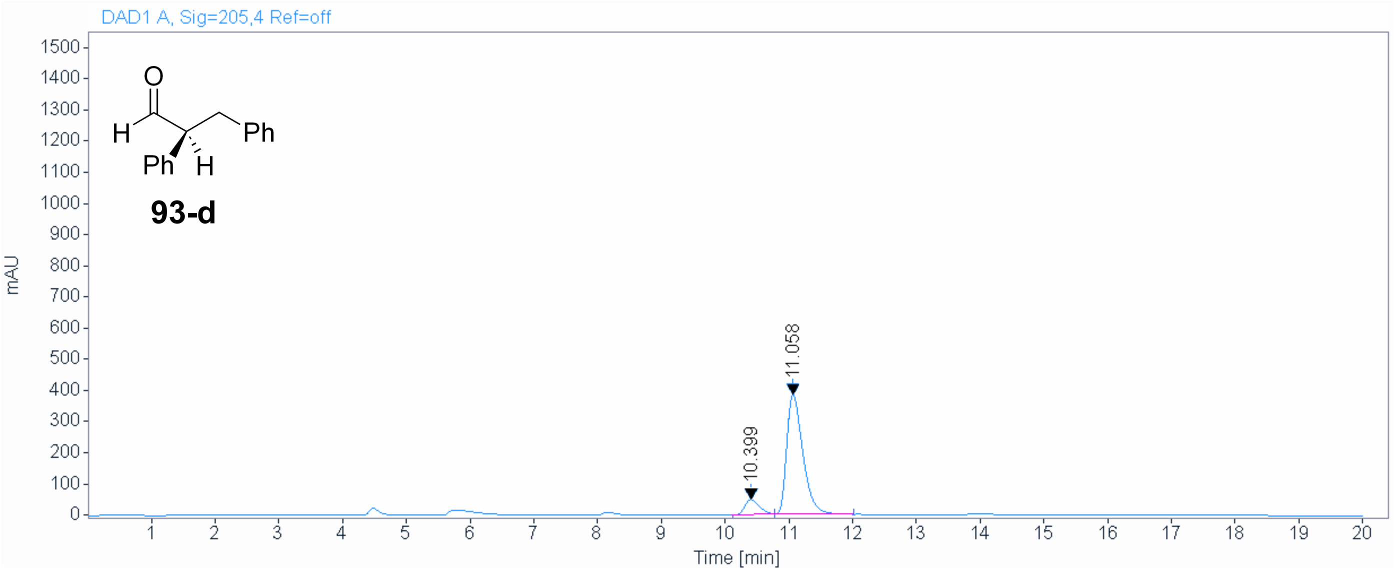

Signal: DAD1 A, Sig=205,4 Ref=off

RT [min]	Width [min]	Height	Area	Area%
10.399	0.2758	49.1411	813.1101	10.6217
11.058	0.2966	384.4664	6842.0981	89.3783
		Sum	7655.2083	100.0000

Area Percent Report

Agilent Technologies

Sample name: zwf-8-067-rac-IE-99-1-0.8-205

Data file: C:\Users\Public\Documents\ChemStation\1\Data\zwf\zwf-S 2023-12-20 15-51-10\007-P1-C4-zwf-8-067-rac.D

Injection Acquired Date 12/20/2023 17:58:21

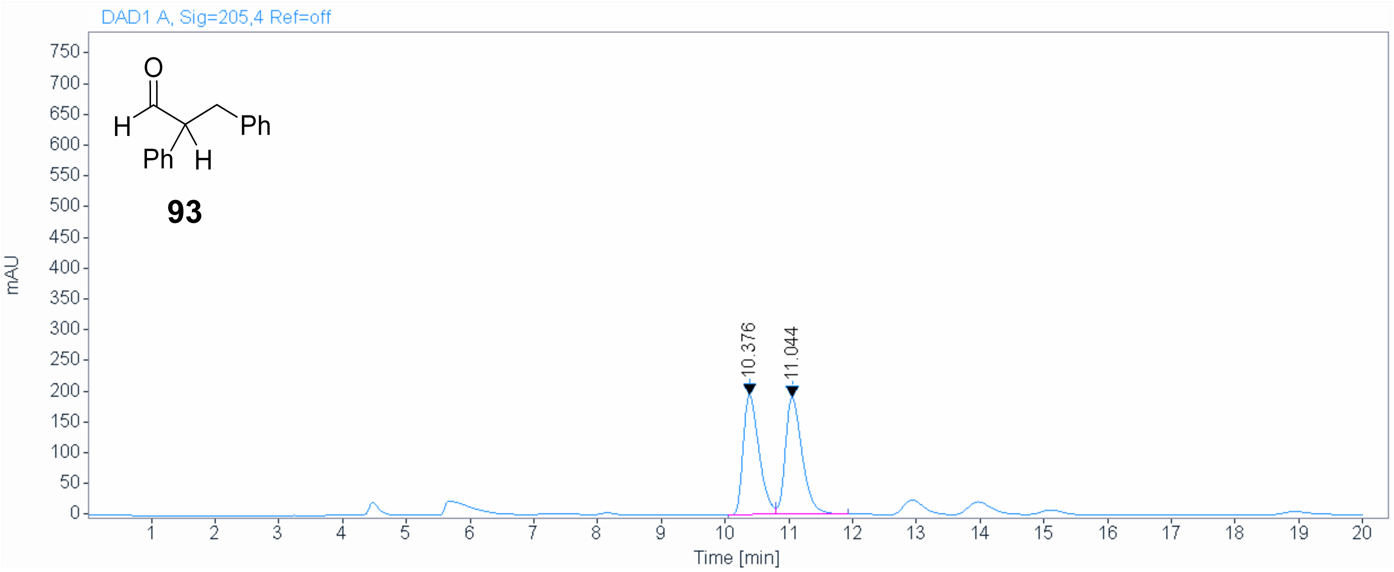

Signal: DAD1 A, Sig=205,4 Ref=off

RT [min]	Width [min]	Height	Area	Area%
10.376	0.2708	195.3129	3394.4314	49.1018
11.044	0.2828	191.0905	3518.6162	50.8982
		Sum	6913.0476	100.0000

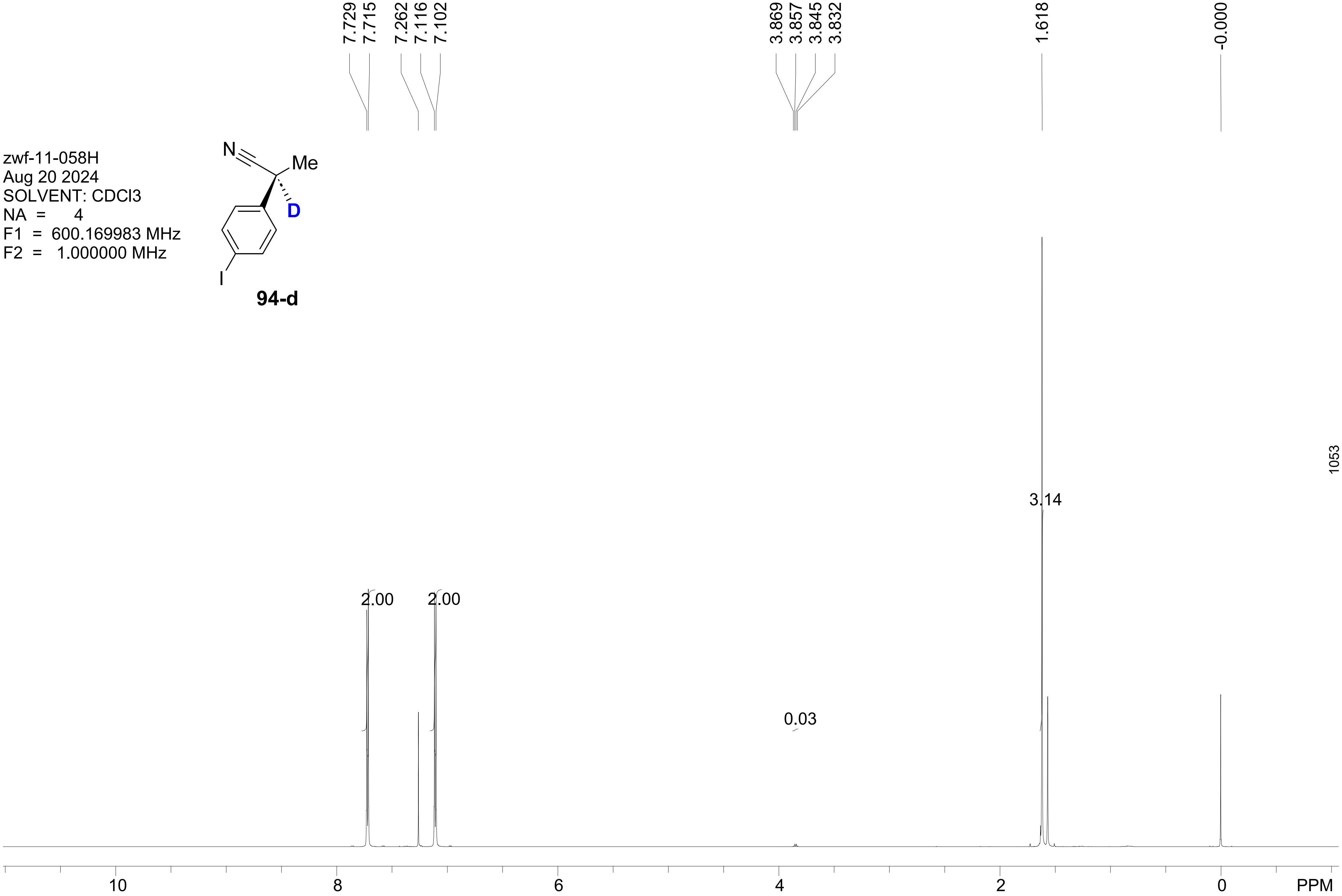
zwf-11-058H
Aug 20 2024
SOLVENT: CDCl3
NA = 4
F1 = 600.169983 MHz
F2 = 1.000000 MHz
N
Me
D
I
94-d
7.729
7.715
7.262
7.116
7.102
3.869
3.857
3.845
3.832
1.618
-0.000
2.00
2.00
0.03
3.14
10
8
6
4
2
0
PPM

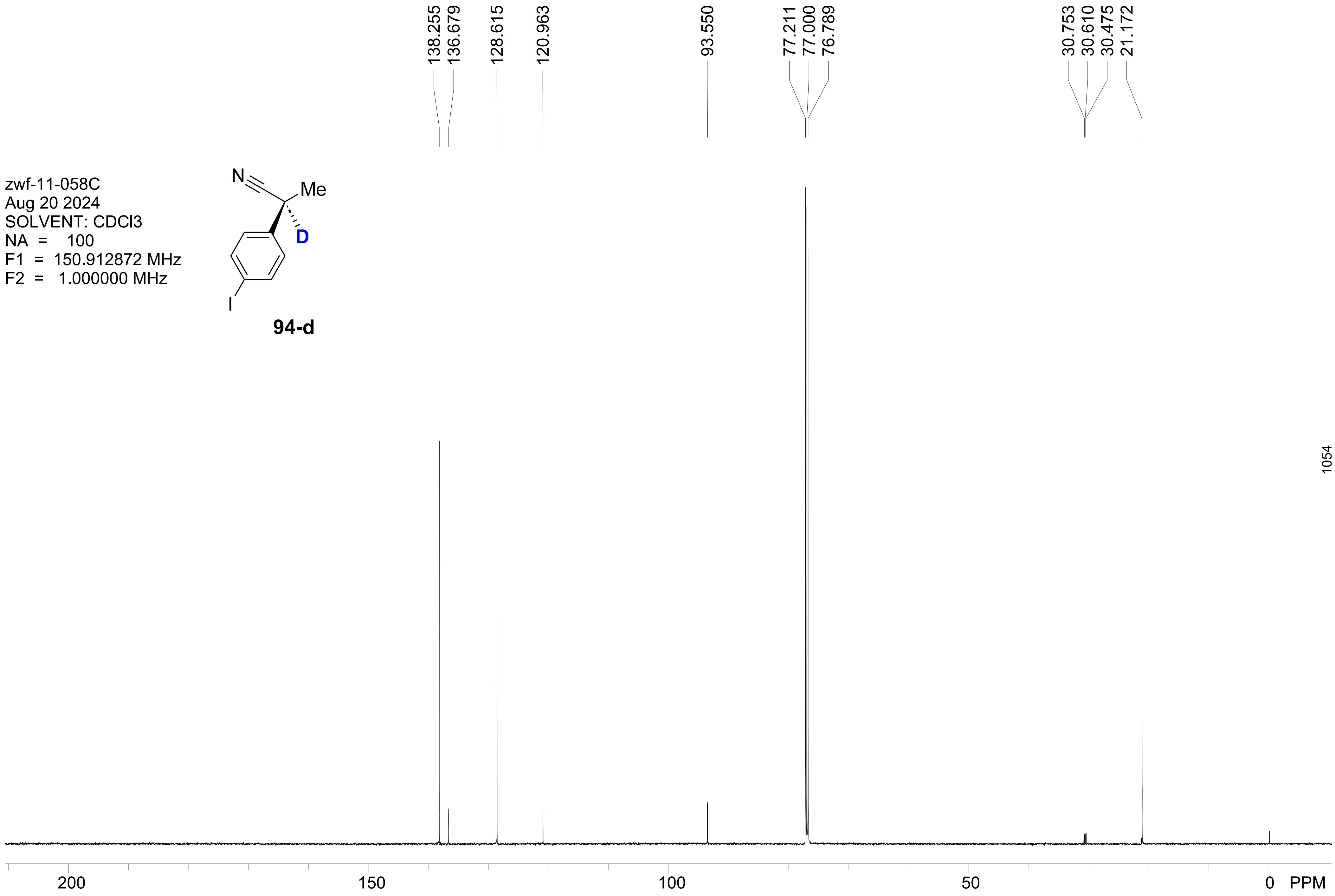
zwf-11-058C
Aug 20 2024
SOLVENT: CDCl3
NA = 100
F1 = 150.912872 MHz
F2 = 1.000000 MHz
N
Me
D
I
138.255
136.679
128.615
120.963
93.550
77.211
77.000
76.789
30.753
30.610
30.475
21.172
200
150
100
50
0 PPM

94-d

Area Percent Report

Agilent Technologies

Sample name: zwf-11-058-IE-99-1-0.8-205

Data file: C:\Users\Public\Documents\ChemStation\1\Data\zwf\zwf 2024-08-20 13-51-56\004-P1-C1-zwf-11-058.D

Injection Acquired Date 08/20/2024 15:12:02

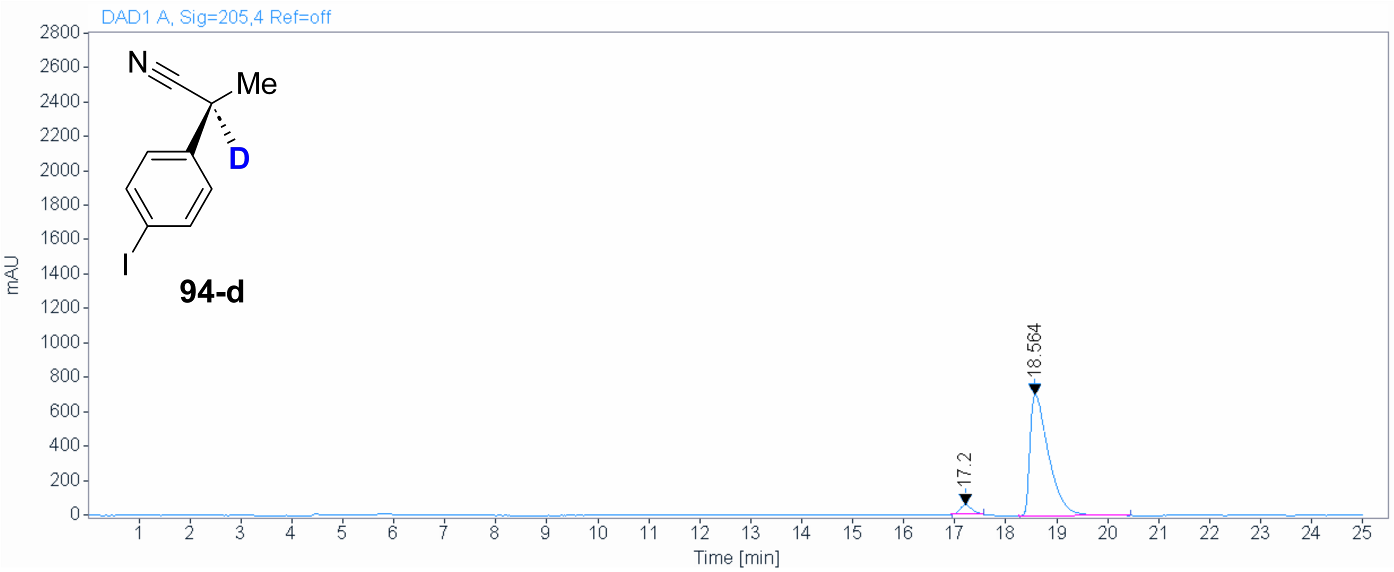

Signal: DAD1 A, Sig=205,4 Ref=off

RT [min]	Width [min]	Height	Area	Area%
17.200	0.2843	54.9860	937.9191	4.9198
18.564	0.4291	703.9789	18126.1113	95.0802
		Sum	19064.0305	100.0000

Area Percent Report

Agilent Technologies

Sample name: zwf-11-058-rac-IE-99-1-0.8-205

Data file: C:\Users\Public\Documents\ChemStation\1\Data\zwf\zwf 2024-08-20 13-51-56\003-P1-C2-zwf-11-058-rac.D

Injection Acquired Date 08/20/2024 14:46:01

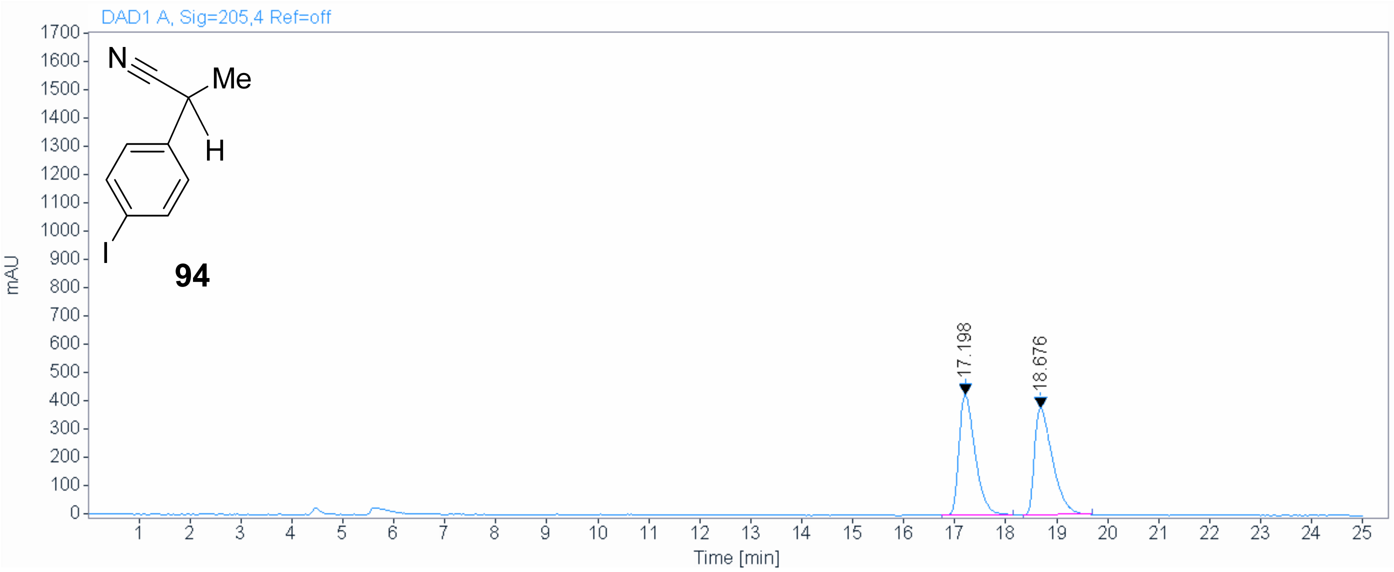

Signal: DAD1 A, Sig=205,4 Ref=off

RT [min]	Width [min]	Height	Area	Area%
17.198	0.3188	426.5037	9386.4883	50.1676
18.676	0.3716	378.1796	9323.7627	49.8324
		Sum	18710.2510	100.0000

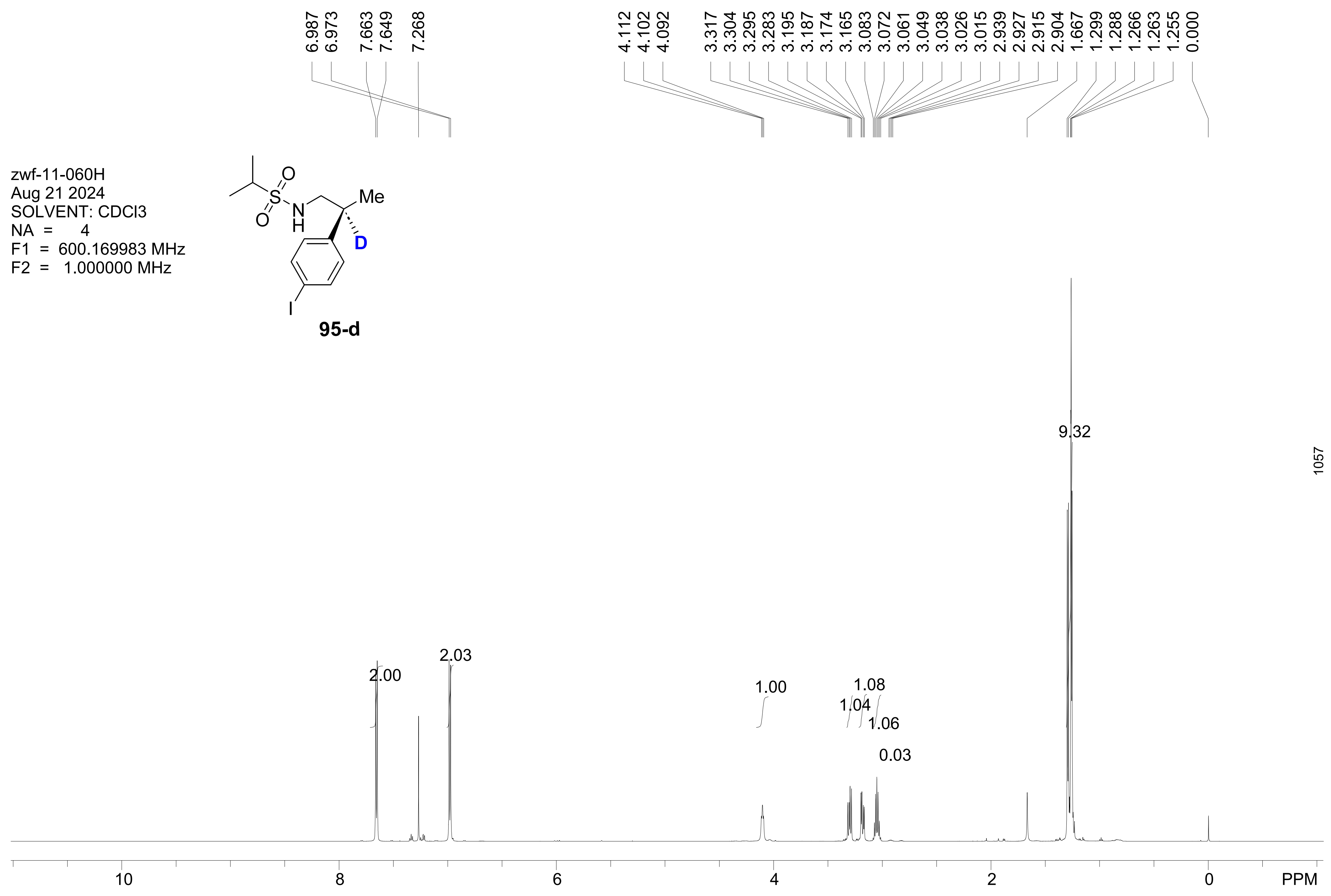
zwf-11-060H
Aug 21 2024
SOLVENT: CDCl3
NA = 4
F1 = 600.169983 MHz
F2 = 1.000000 MHz
Me
D
95-d
6.987
6.973
7.663
7.649
7.268
4.112
4.102
4.092
3.317
3.304
3.295
3.283
3.195
3.187
3.174
3.165
3.083
3.072
3.061
3.049
3.038
3.026
3.015
2.939
2.927
2.915
2.904
1.667
1.299
1.288
1.266
1.263
1.255
0.000
2.00
2.03
1.00
1.04
1.08
1.06
0.03
9.32
10
8
6
4
2
0
PPM

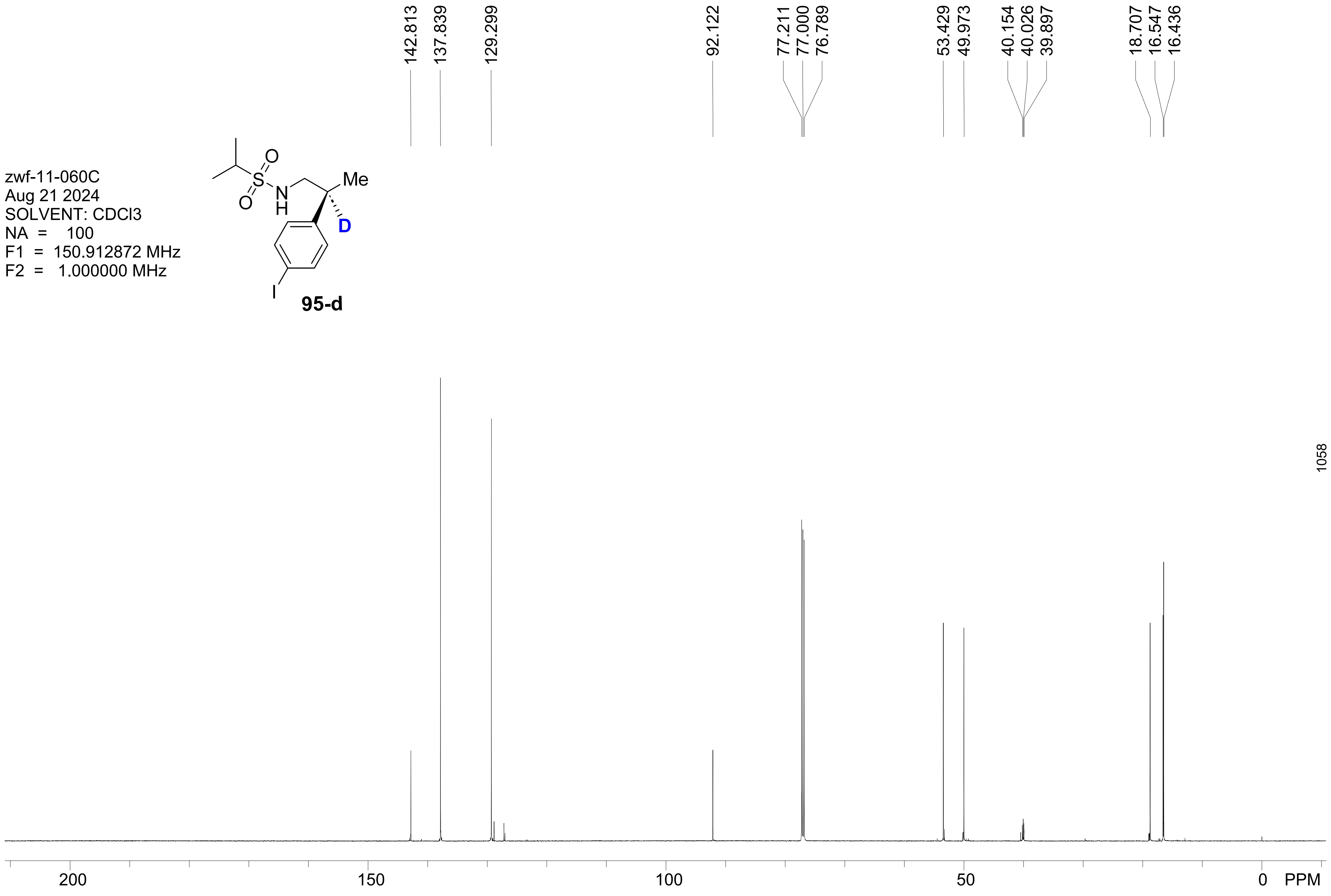
zwf-11-060C
Aug 21 2024
SOLVENT: CDCl3
NA = 100
F1 = 150.912872 MHz
F2 = 1.000000 MHz
O
S
O
N
H
Me
D
I
95-d
142.813
137.839
129.299
92.122
77.211
77.000
76.789
53.429
49.973
40.154
40.026
39.897
18.707
16.547
16.436
200
150
100
50
0
PPM

Area Percent Report

Agilent Technologies

Sample name: zwf-11-060-IE-90-10-0.8-205

Data file: C:\Users\Public\Documents\ChemStation\1\Data\zwf\zwf 2024-08-21 16-39-43\005-P1-C1-zwf-11-060.D

Injection Acquired Date 08/21/2024 19:15:11

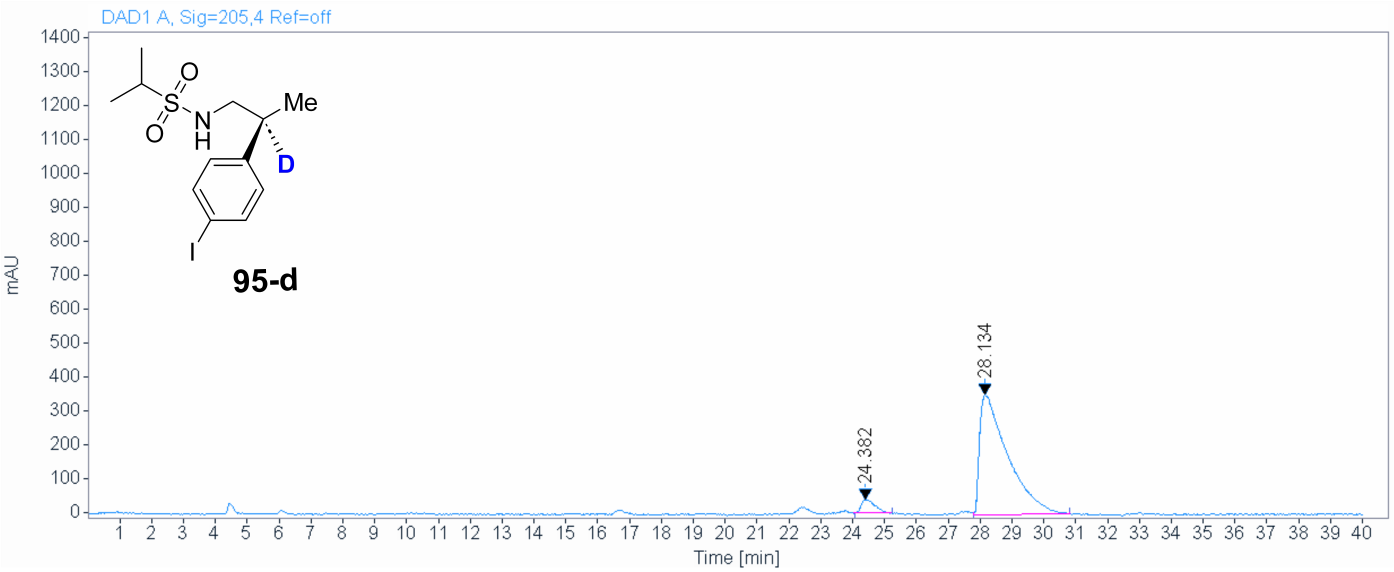

Signal: DAD1 A, Sig=205,4 Ref=off

RT [min]	Width [min]	Height	Area	Area%
24.382	0.4983	39.4062	1178.0537	5.1144
28.134	1.0264	354.8825	21856.1152	94.8856
		Sum	23034.1689	100.0000

Area Percent Report

Sample name: zwf-11-060-rac-IE-90-10-0.8-205

Data file: C:\Users\Public\Documents\ChemStation\1\Data\zwf\zwf 2024-08-21 16-39-43\004-P1-C2-zwf-11-060-rac.D

Injection Acquired Date 08/21/2024 18:34:02

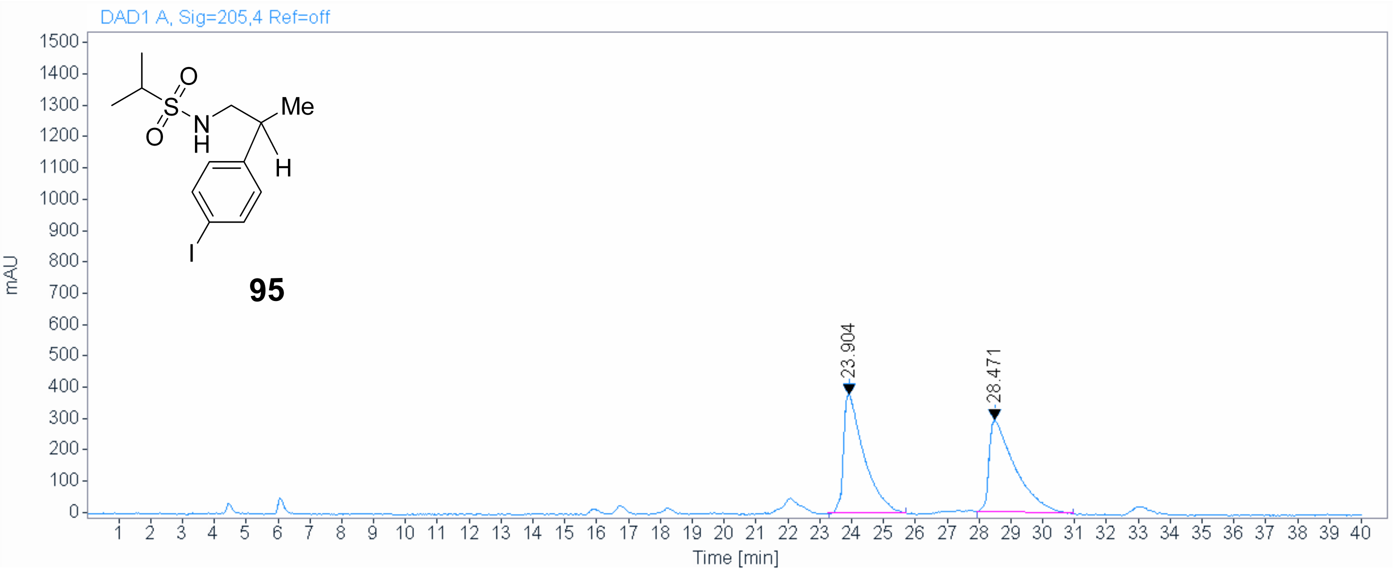

95

Signal: DAD1 A, Sig=205,4 Ref=off

RT [min]	Width [min]	Height	Area	Area%
23.904	0.7518	377.8478	17044.8574	50.2844
28.471	0.9523	294.9423	16852.0625	49.7156
		Sum	33896.9199	100.0000

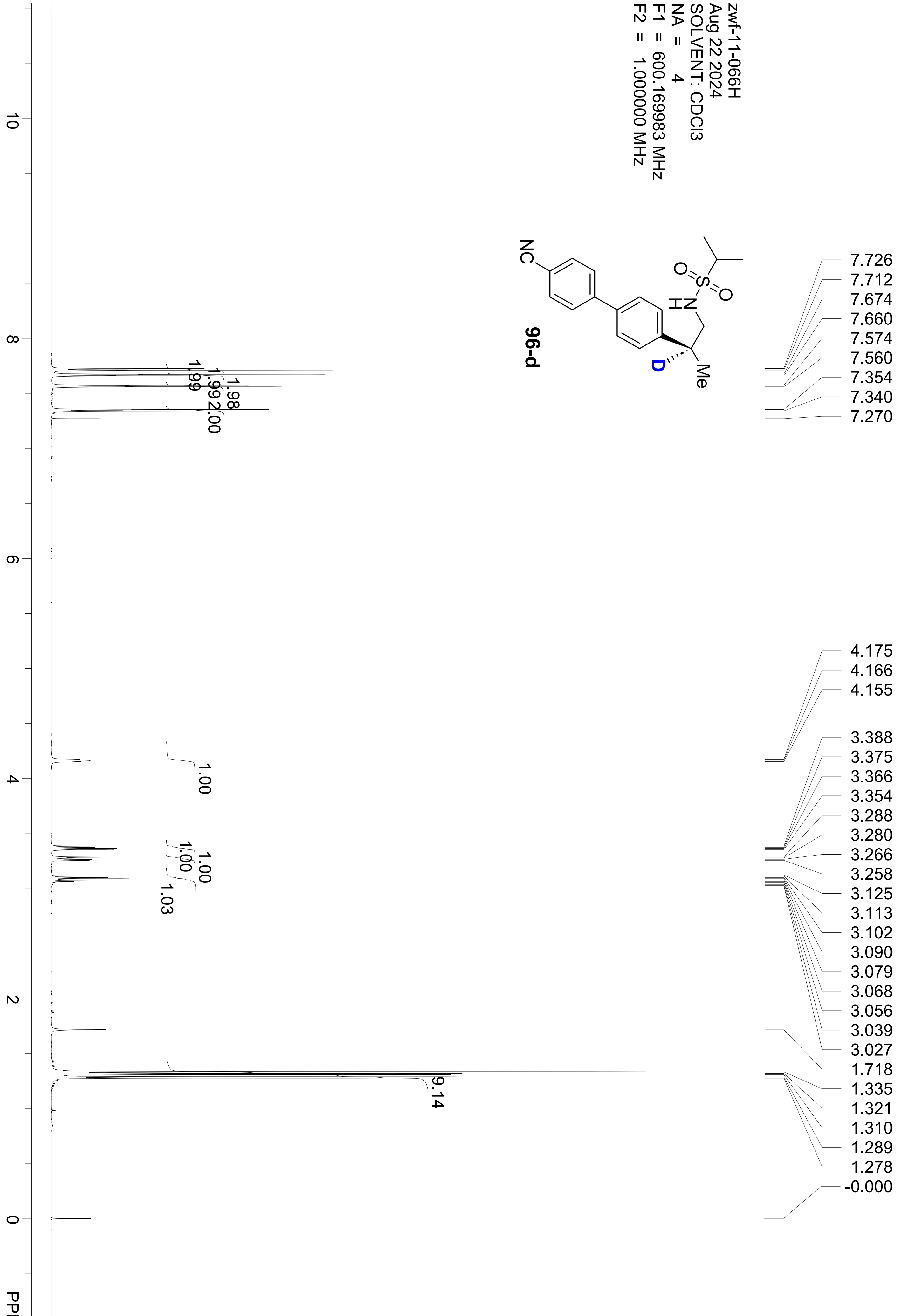
zwf-11-066H
Aug 22 2024
SOLVENT: CDCl3
NA = 4
F1 = 600.169983 MHz
F2 = 1.000000 MHz
NC
O
S
O
N
H
Me
D
96-d
7.726
7.712
7.674
7.660
7.574
7.560
7.354
7.340
7.270
4.175
4.166
4.155
3.388
3.375
3.366
3.354
3.288
3.280
3.266
3.258
3.125
3.113
3.102
3.090
3.079
3.068
3.056
3.039
3.027
1.718
1.335
1.321
1.310
1.289
1.278
-0.000
1.99
1.99
1.98
2.00
1.00
1.00
1.00
1.03
9.14
10
8
6
4
2
0
PPM

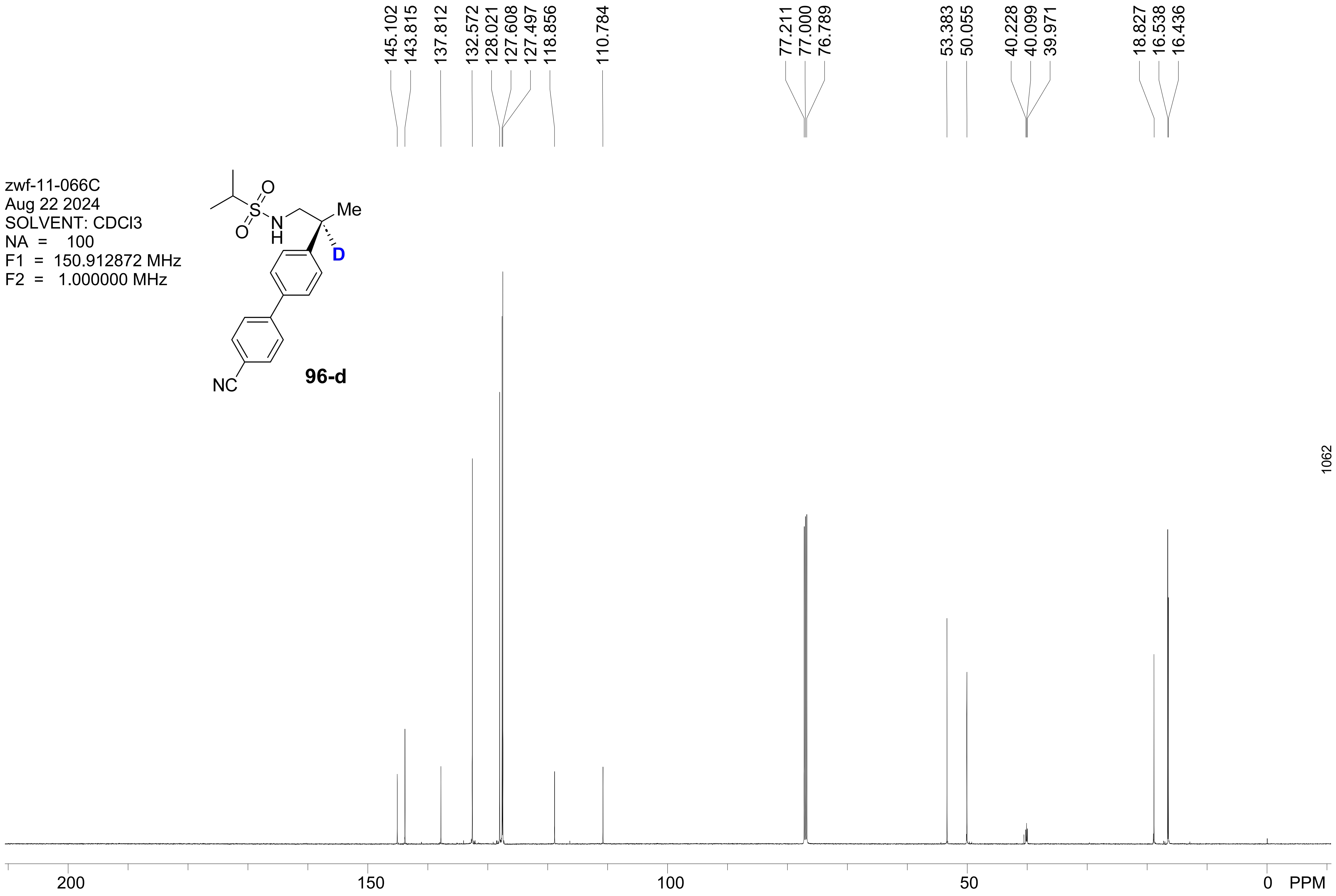

145.102
143.815
137.812
132.572
128.021
127.608
127.497
118.856
110.784
77.211
77.000
76.789
53.383
50.055
40.228
40.099
39.971
18.827
16.538
16.436
zwf-11-066C
Aug 22 2024
SOLVENT: CDCl3
NA = 100
F1 = 150.912872 MHz
F2 = 1.000000 MHz
Me
D
NC
96-d
200
150
100
50
0 PPM

Area Percent Report

Agilent Technologies

Sample name: zwf-11-066-IB-70-30-0.8-254

Data file: C:\Users\Public\Documents\ChemStation\1\Data\zwf\zwf 2024-08-22 18-25-08\003-P1-C2-zwf-11-066.D

Injection Acquired Date 08/22/2024 19:21:32

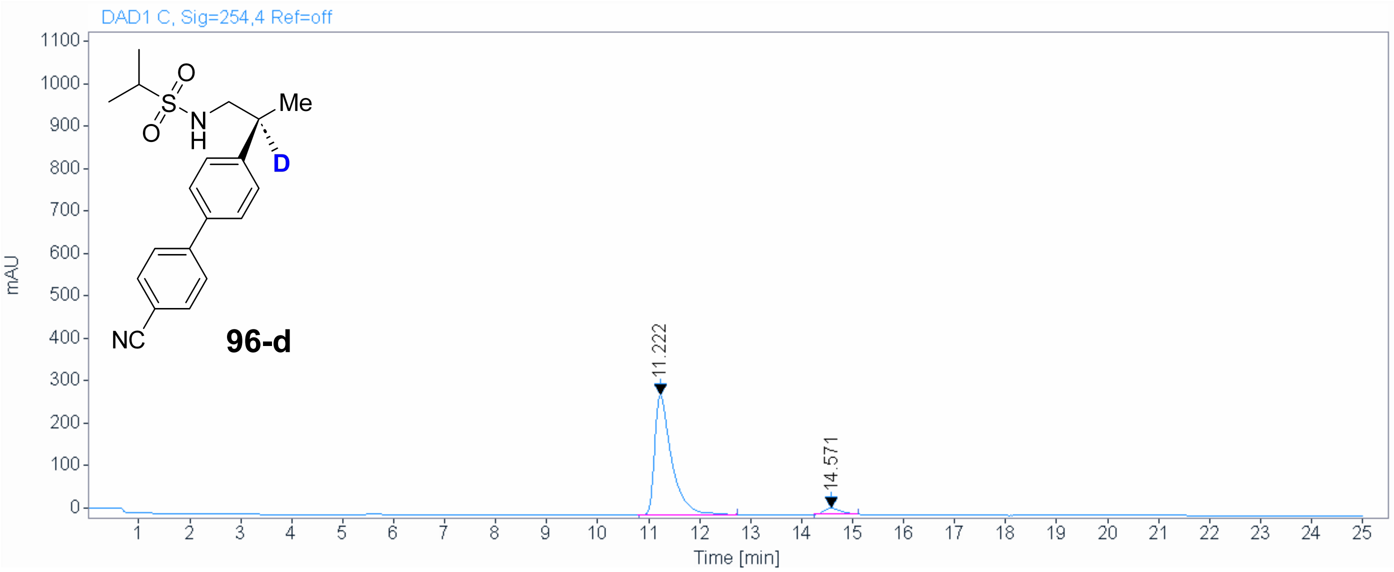

Signal: DAD1 C, Sig=254,4 Ref=off

RT [min]	Width [min]	Height	Area	Area%
11.222	0.3867	283.6993	6582.4878	95.0008
14.571	0.3777	15.2867	346.3884	4.9992
		Sum	6928.8762	100.0000

Area Percent Report

Sample name: zwf-11-066-rac-IB-70-30-0.8-254

Data file: C:\Users\Public\Documents\ChemStation\1\Data\zwf\zwf 2024-08-22 18-25-08\002-P1-C1-zwf-11-066-rac.D

Injection Acquired Date 08/22/2024 18:55:29

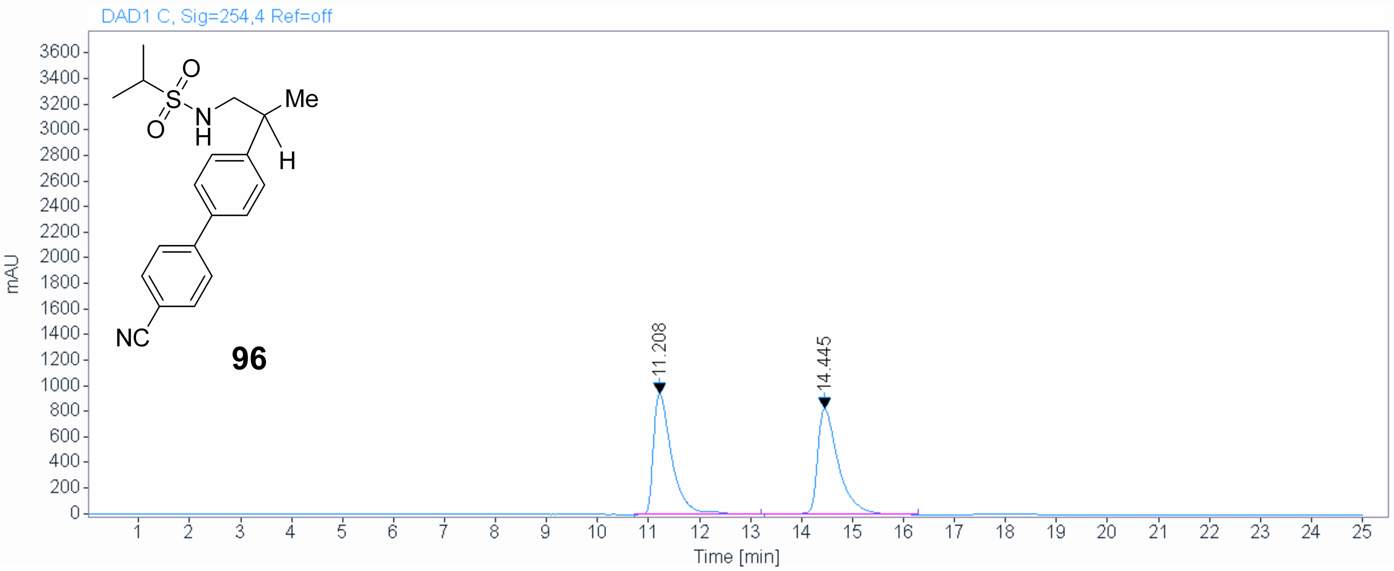

Signal: DAD1 C, Sig=254,4 Ref=off

RT [min]	Width [min]	Height	Area	Area%
11.208	0.3793	942.5358	23693.4355	50.2800
14.445	0.4199	827.3516	23429.5215	49.7200
		Sum	47122.9570	100.0000

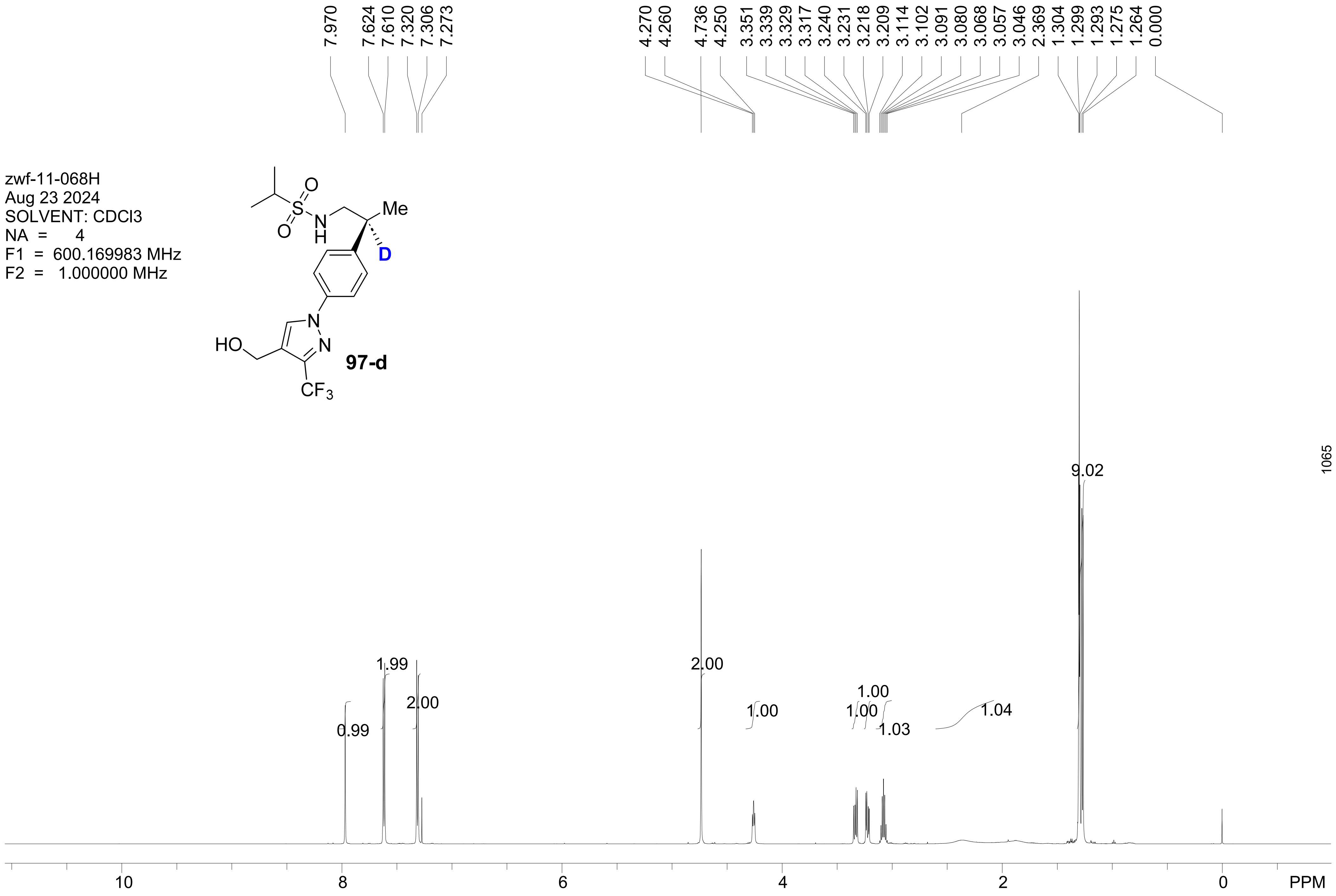
zwf-11-068H
Aug 23 2024
SOLVENT: CDCl3
NA = 4
F1 = 600.169983 MHz
F2 = 1.000000 MHz
97-d
Me
D
HO
CF3
7.970
7.624
7.610
7.320
7.306
7.273
4.270
4.260
4.736
4.250
3.351
3.339
3.329
3.317
3.240
3.231
3.218
3.209
3.114
3.102
3.091
3.080
3.068
3.057
3.046
2.369
1.304
1.299
1.293
1.275
1.264
0.000
0.99
1.99
2.00
2.00
1.00
1.00
1.00
1.03
1.04
9.02
10
8
6
4
2
0
PPM

zwf-11-068C
Aug 23 2024
SOLVENT: CDCl3
NA = 100
F1 = 150.912872 MHz
F2 = 1.000000 MHz

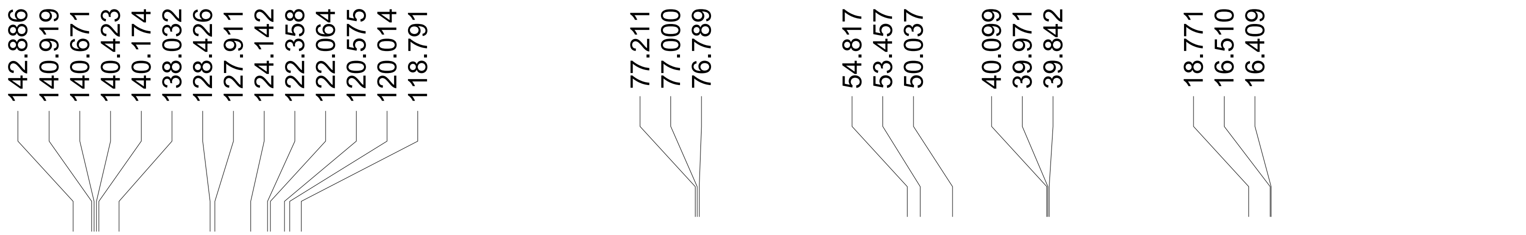

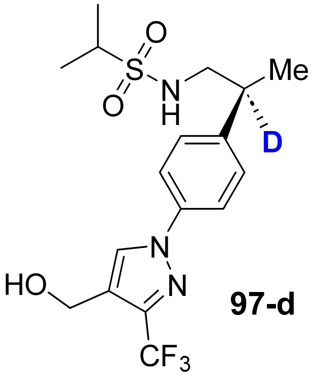

97-d

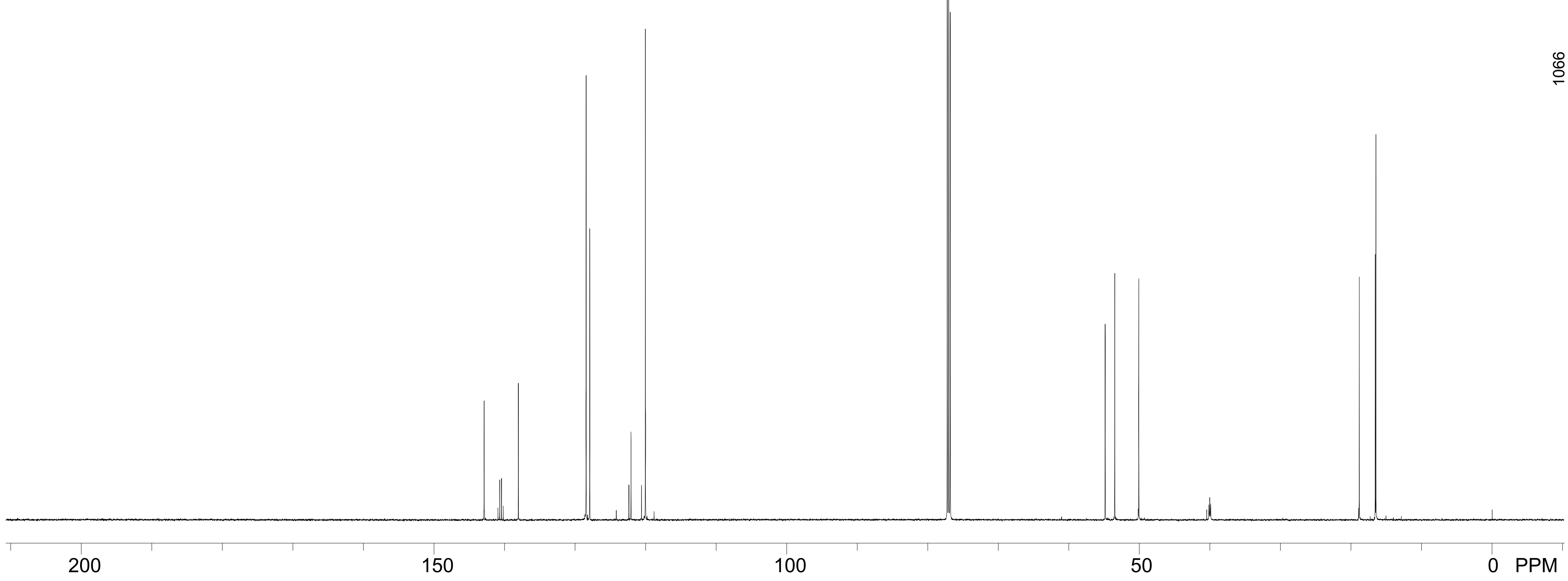

zwf-11-068F
Aug 23 2024
SOLVENT: CDCl3
NA = 16
F1 = 564.723999 MHz
F2 = 1.000000 MHz

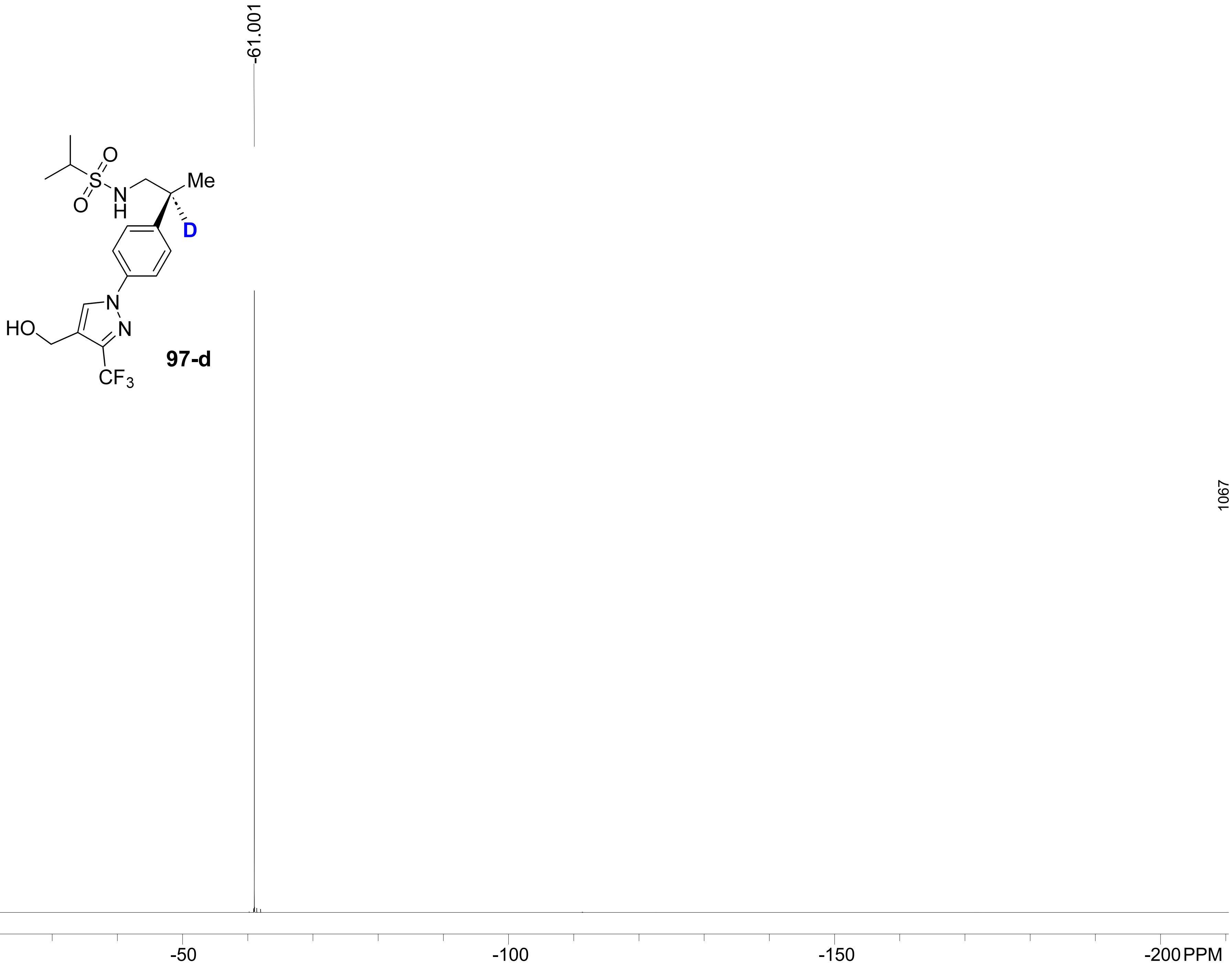

97-d

Area Percent Report

Agilent Technologies

Sample name: zwf-11-068-IB-80-20-0.8-205

Data file: C:\Users\Public\Documents\ChemStation\1\Data\zwf\zwf 2024-08-23 08-52-32\016-P1-C8-zwf-11-068.D

Injection Acquired Date 08/23/2024 16:01:39

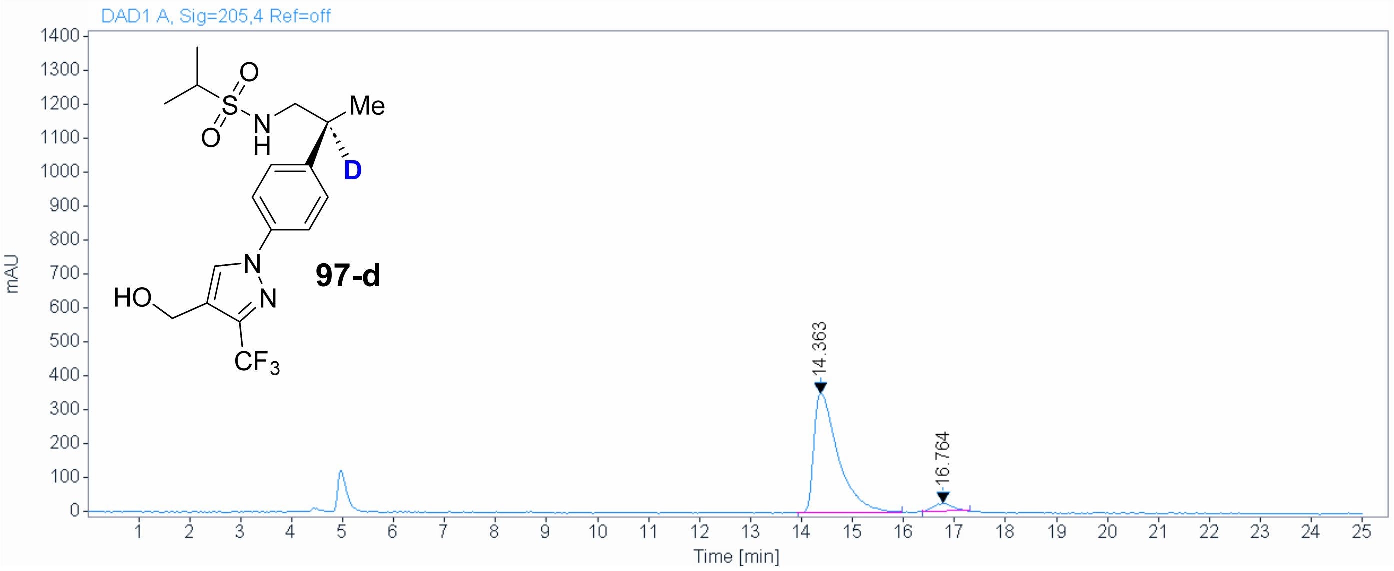

Signal: DAD1 A, Sig=205,4 Ref=off

RT [min]	Width [min]	Height	Area	Area%
14.363	0.5557	352.0674	11738.3271	94.9628
16.764	0.4218	24.6024	622.6525	5.0372
		Sum	12360.9797	100.0000

Area Percent Report

Sample name: zwf-11-068-rac-IB-80-20-0.8-205

Data file: C:\Users\Public\Documents\ChemStation\1\Data\zwf\zwf 2024-08-23 08-52-32\014-P1-C7-zwf-11-068-rac.D

Injection Acquired Date 08/23/2024 15:07:10

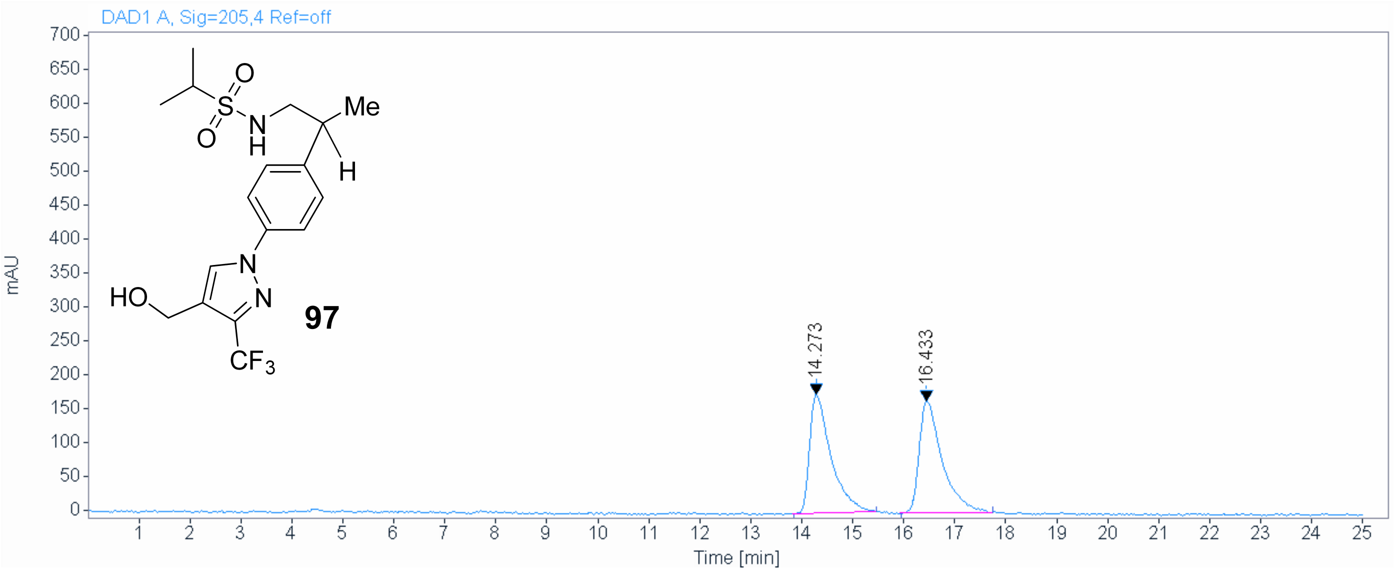

Signal: DAD1 A, Sig=205,4 Ref=off

RT [min]	Width [min]	Height	Area	Area%
14.273	0.4918	175.0458	5165.6152	49.5822
16.433	0.5301	165.1541	5252.6719	50.4178
		Sum	10418.2871	100.0000

zwf-10-121H
Jul 15 2024
SOLVENT: CDCl3
NA = 4
F1 = 500.130005 MHz
F2 = 1.000000 MHz

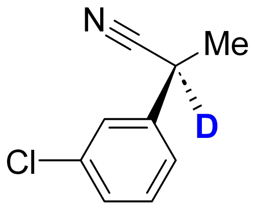

98-d

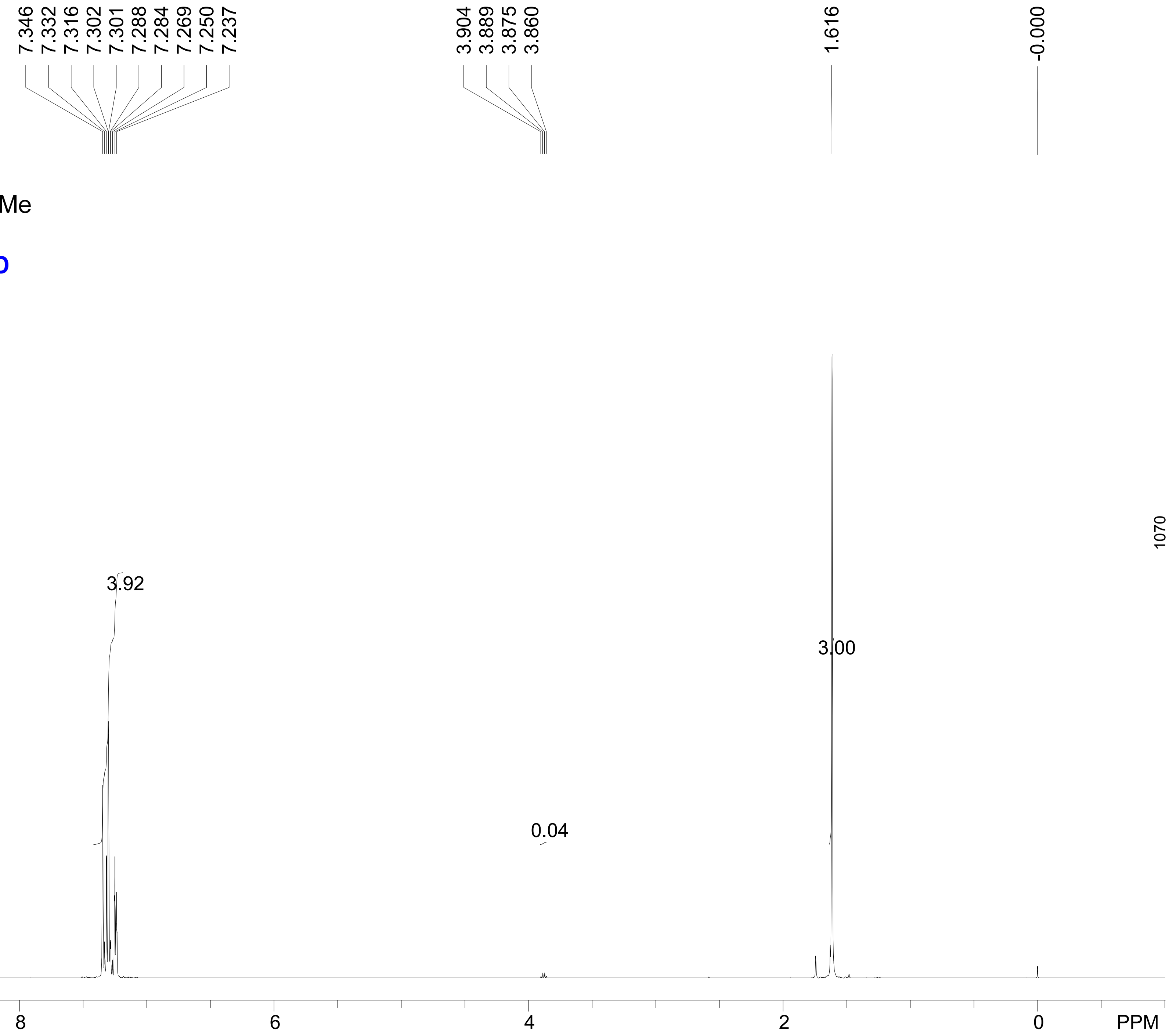

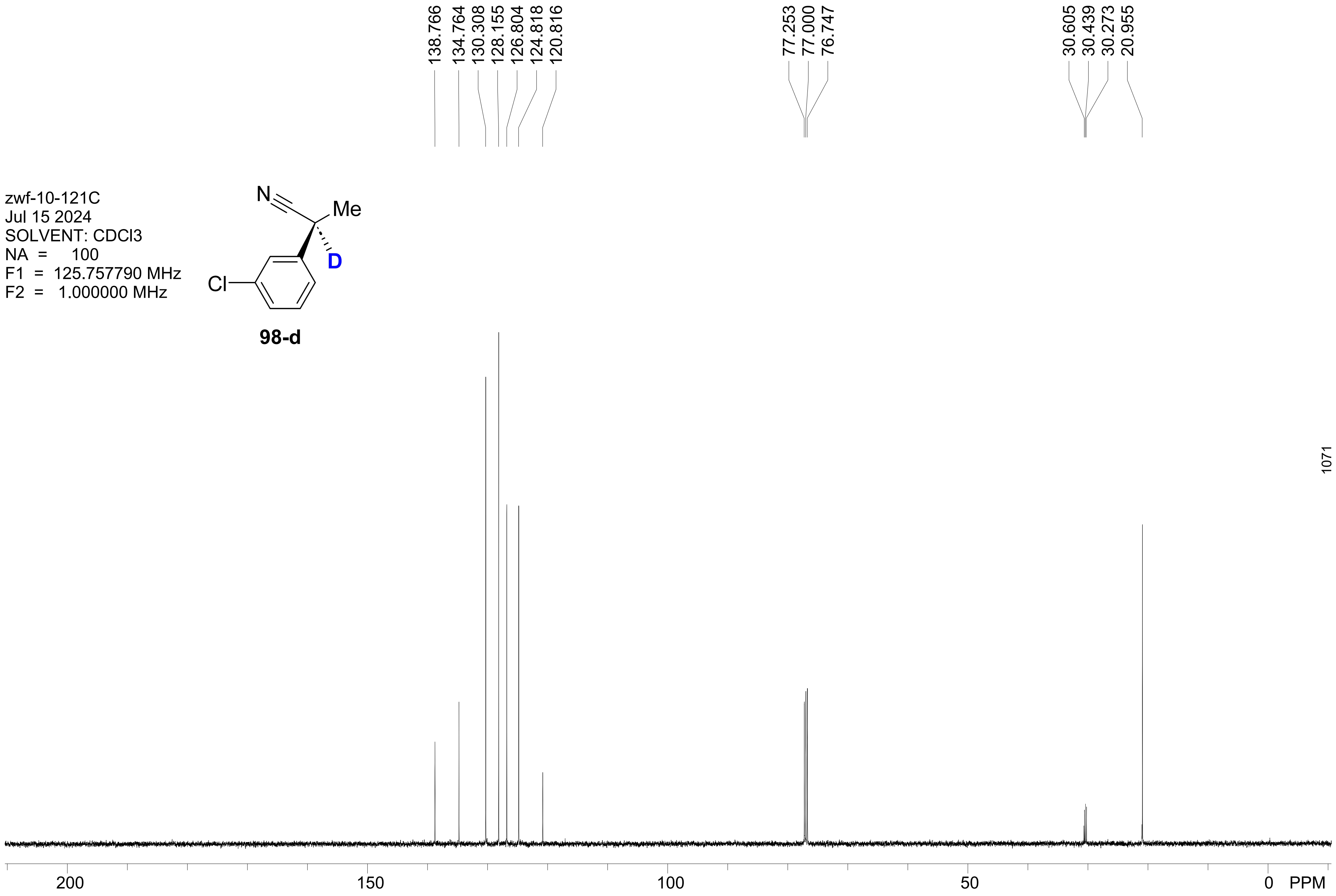

138.766
134.764
130.308
128.155
126.804
124.818
120.816
77.253
77.000
76.747
30.605
30.439
30.273
20.955
zwf-10-121C
Jul 15 2024
SOLVENT: CDCl3
NA = 100
F1 = 125.757790 MHz
F2 = 1.000000 MHz
N
Me
D
Cl
200
150
100
50
0 PPM

98-d

Area Percent Report

Agilent Technologies

Sample name: zwf-10-121-IE-99-1-0.8-205

Data file: C:\Users\Public\Documents\ChemStation\1\Data\zwf\zwf 2024-07-15 15-20-39\002-P1-C1-zwf-10-121.D

Injection Acquired Date 07/15/2024 15:48:33

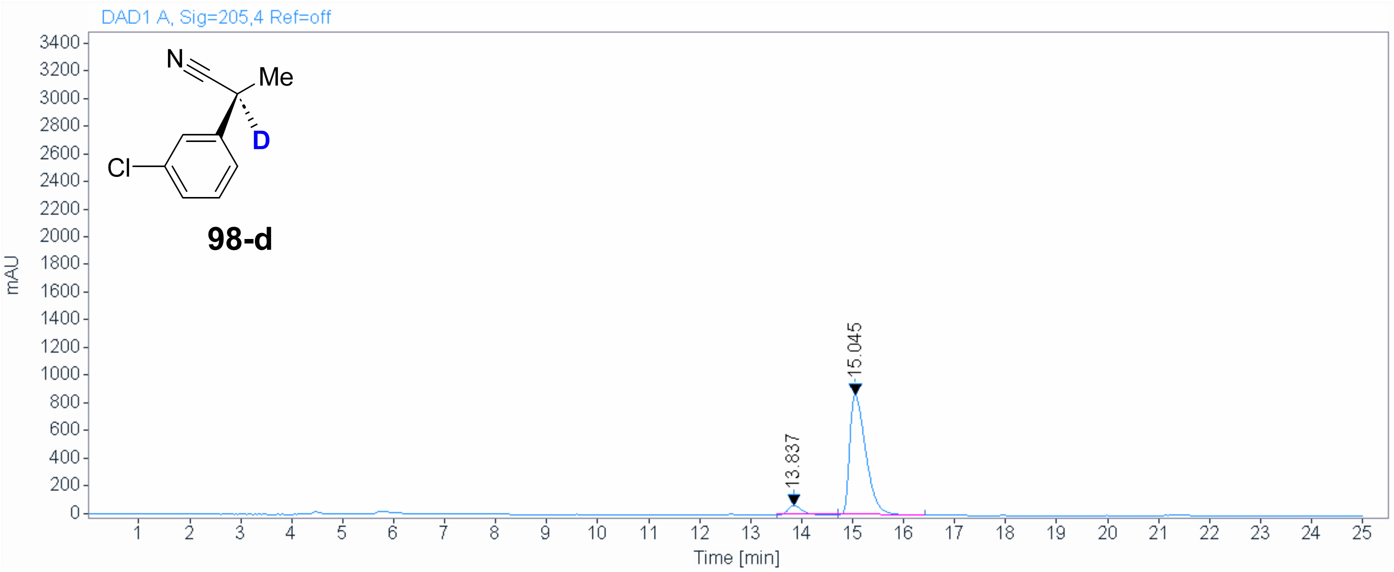

Signal: DAD1 A, Sig=205,4 Ref=off

RT [min]	Width [min]	Height	Area	Area%
13.837	0.2762	66.3219	1228.9027	6.0565
15.045	0.3245	867.0716	19061.6172	93.9435
		Sum	20290.5199	100.0000

Area Percent Report

Agilent Technologies

Sample name: zwf-10-121-rac-IE-99-1-0.8-205

Data file: C:\Users\Public\Documents\ChemStation\1\Data\zwf\zwf 2024-07-15 15-20-39\003-P1-C2-zwf-10-121-rac.D

Injection Acquired Date 07/15/2024 16:14:36

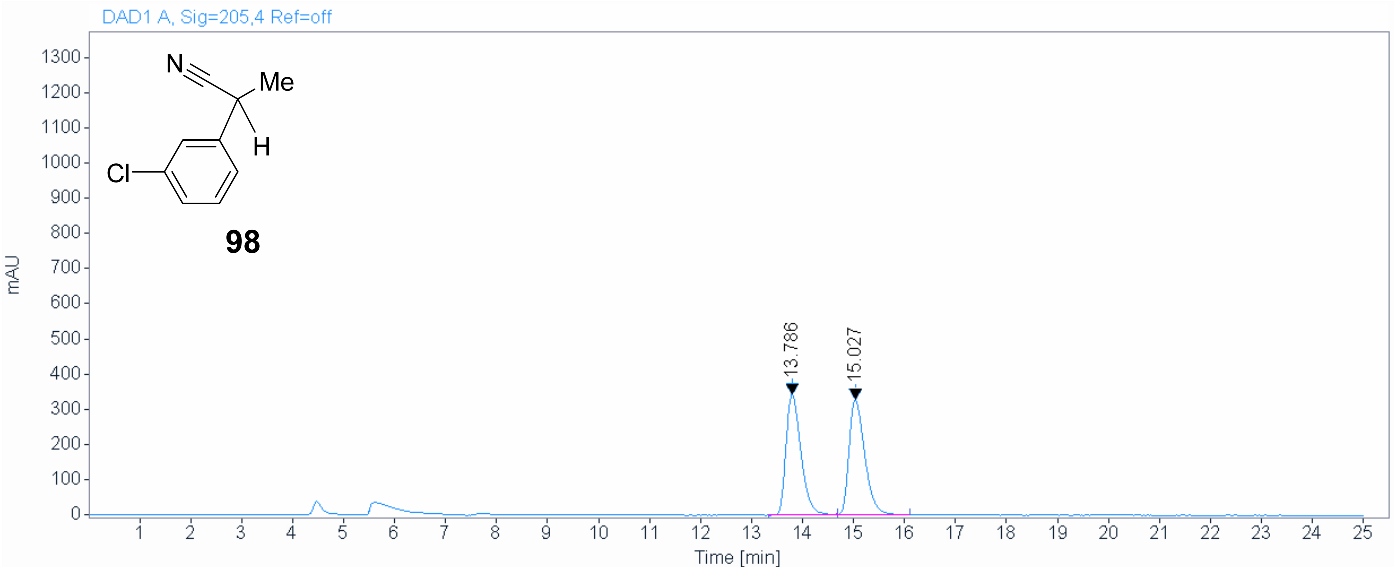

Signal: DAD1 A, Sig=205,4 Ref=off

RT [min]	Width [min]	Height	Area	Area%
13.786	0.3194	342.6283	7031.5522	49.8694
15.027	0.3395	327.9128	7068.3716	50.1306
		Sum	14099.9238	100.0000

zwf-10-152H
Jul 24 2024
SOLVENT: CDCl3
NA = 4
F1 = 500.130005 MHz
F2 = 1.000000 MHz

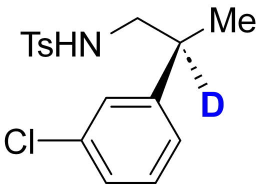

99-d

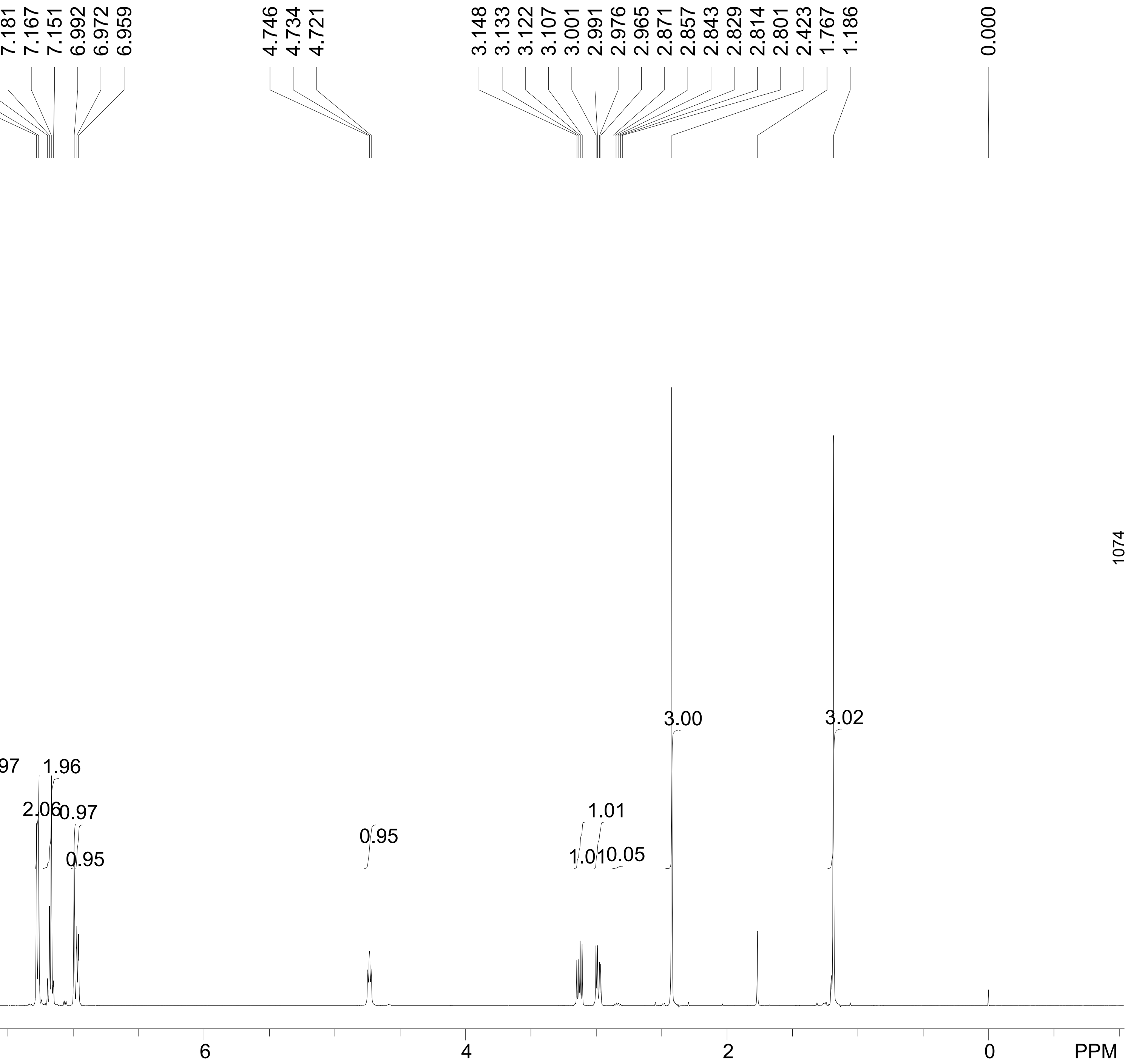

zwf-10-152C
Jul 24 2024
SOLVENT: CDCl3
NA = 100
F1 = 125.757790 MHz
F2 = 1.000000 MHz

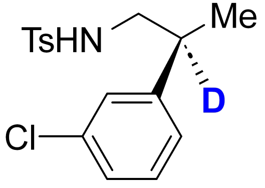

99-d

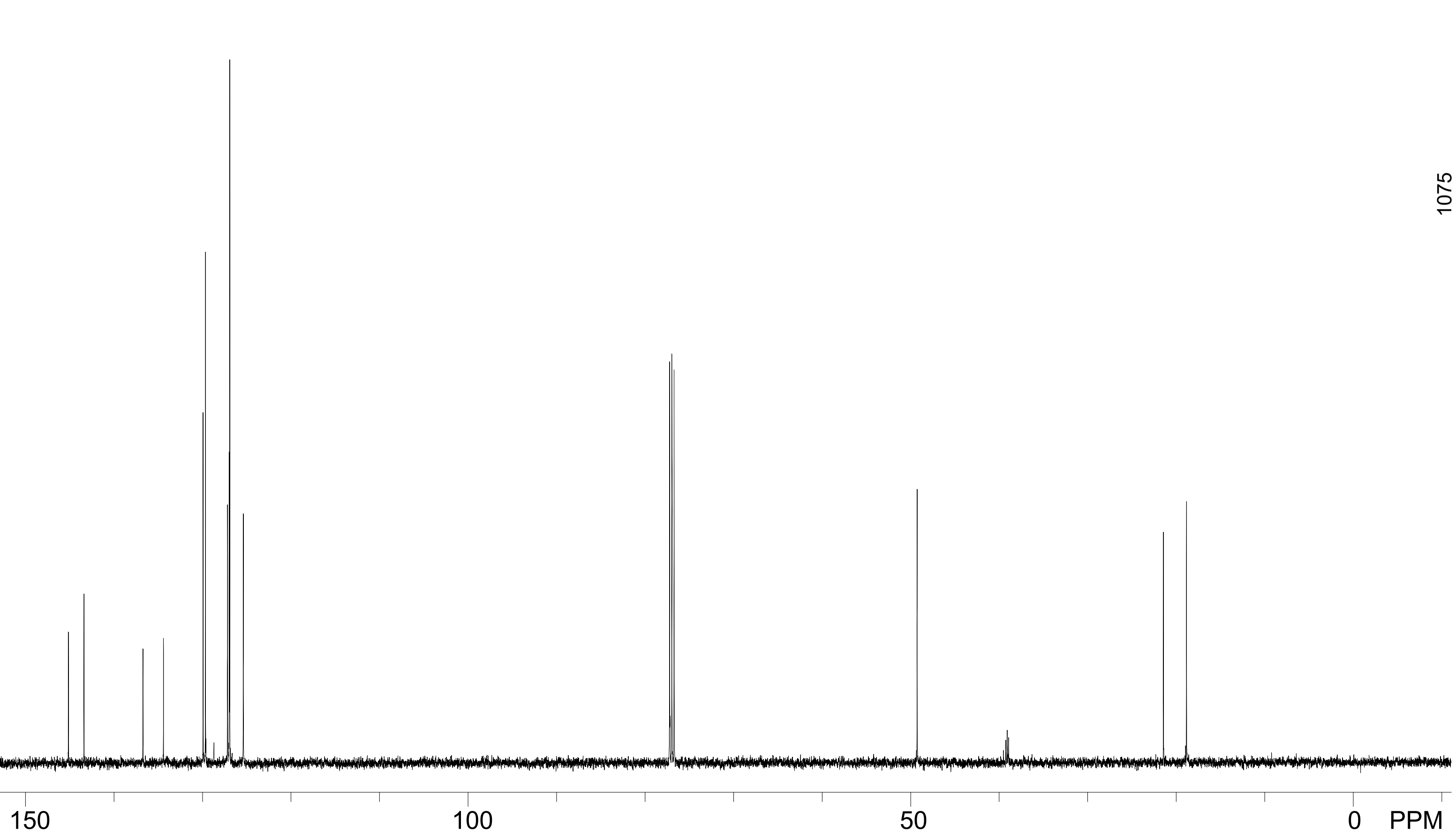

Area Percent Report

Agilent Technologies

Sample name: zwf-10-152-IB-98-2-1.0-205

Data file: C:\Users\Public\Documents\ChemStation\1\Data\zwf\zwf 2024-07-25 01-13-01\003-P1-C1-zwf-10-152.D

Injection Acquired Date 07/25/2024 01:47:04

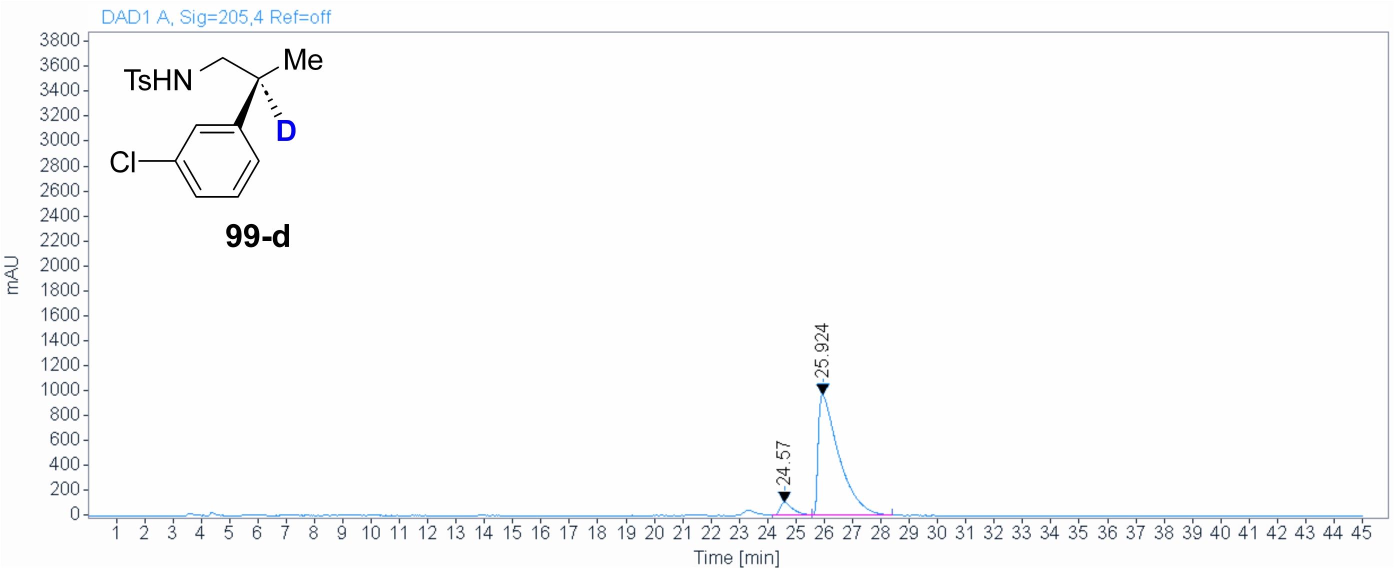

Signal: DAD1 A, Sig=205,4 Ref=off

RT [min]	Width [min]	Height	Area	Area%
24.570	0.4237	101.2038	3171.9192	6.0825
25.924	0.6044	964.5555	48976.1016	93.9175
		Sum	52148.0208	100.0000

Area Percent Report

Agilent Technologies

Sample name: zwf-10-152-rac-IB-98-2-1.0-205

Data file: C:\Users\Public\Documents\ChemStation\1\Data\zwf\zwf 2024-07-25 01-13-01\004-P1-C2-zwf-10-152-rac.D

Injection Acquired Date 07/25/2024 02:33:04

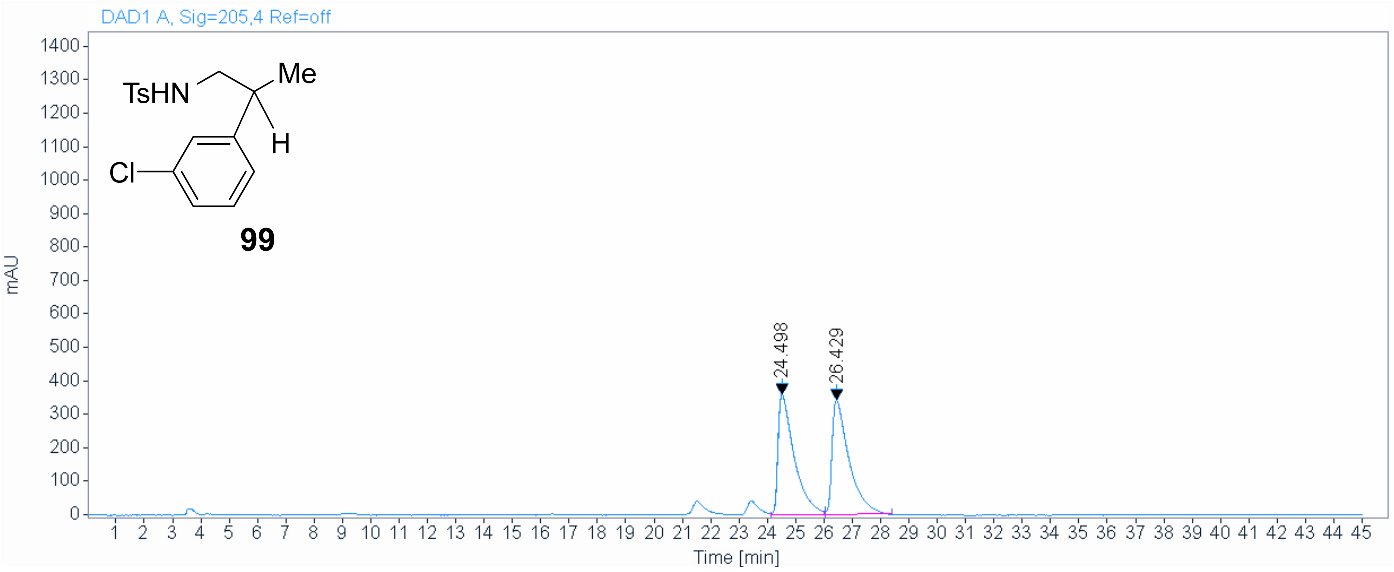

Signal: DAD1 A, Sig=205,4 Ref=off

RT [min]	Width [min]	Height	Area	Area%
24.498	0.5245	359.5455	14375.2100	50.0161
26.429	0.5605	342.6126	14365.9834	49.9839
		Sum	28741.1934	100.0000

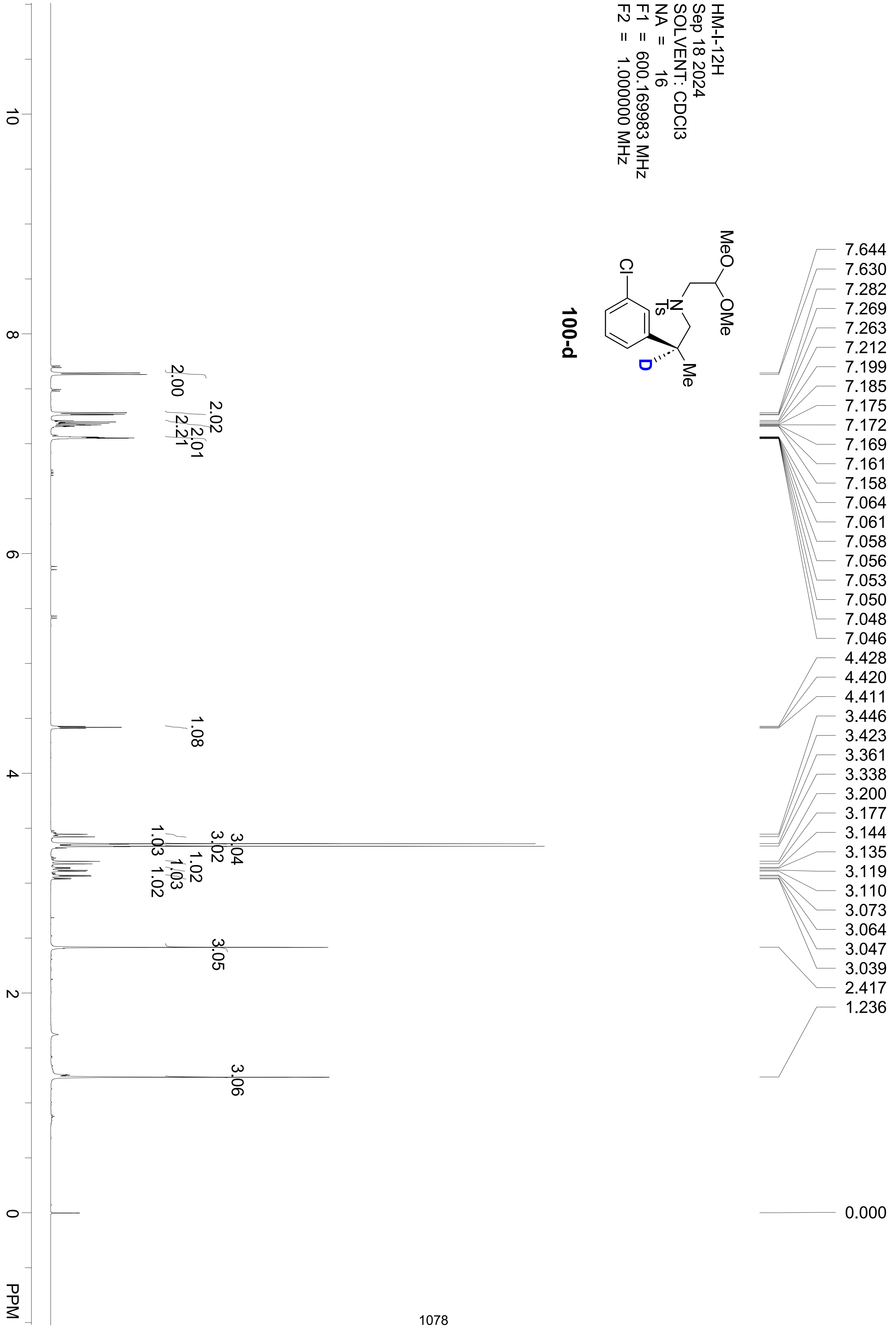
HM-I-12H
Sep 18 2024
SOLVENT: CDCl3
NA = 16
F1 = 600.169983 MHz
F2 = 1.000000 MHz
MeO
OMe
N
Ts
Cl
Me
D
100-d
7.644
7.630
7.282
7.269
7.263
7.212
7.199
7.185
7.175
7.172
7.169
7.161
7.158
7.064
7.061
7.058
7.056
7.053
7.050
7.048
7.046
4.428
4.420
4.411
3.446
3.423
3.361
3.338
3.200
3.177
3.144
3.135
3.119
3.110
3.073
3.064
3.047
3.039
2.417
1.236
0.000
2.00
2.02
2.21
2.01
1.08
3.04
3.02
1.02
1.03
1.03
1.02
3.05
3.06
PPM

HM-I-12C
Sep 18 2024
SOLVENT: CDCl3
NA = 520
F1 = 150.912872 MHz
F2 = 1.000000 MHz

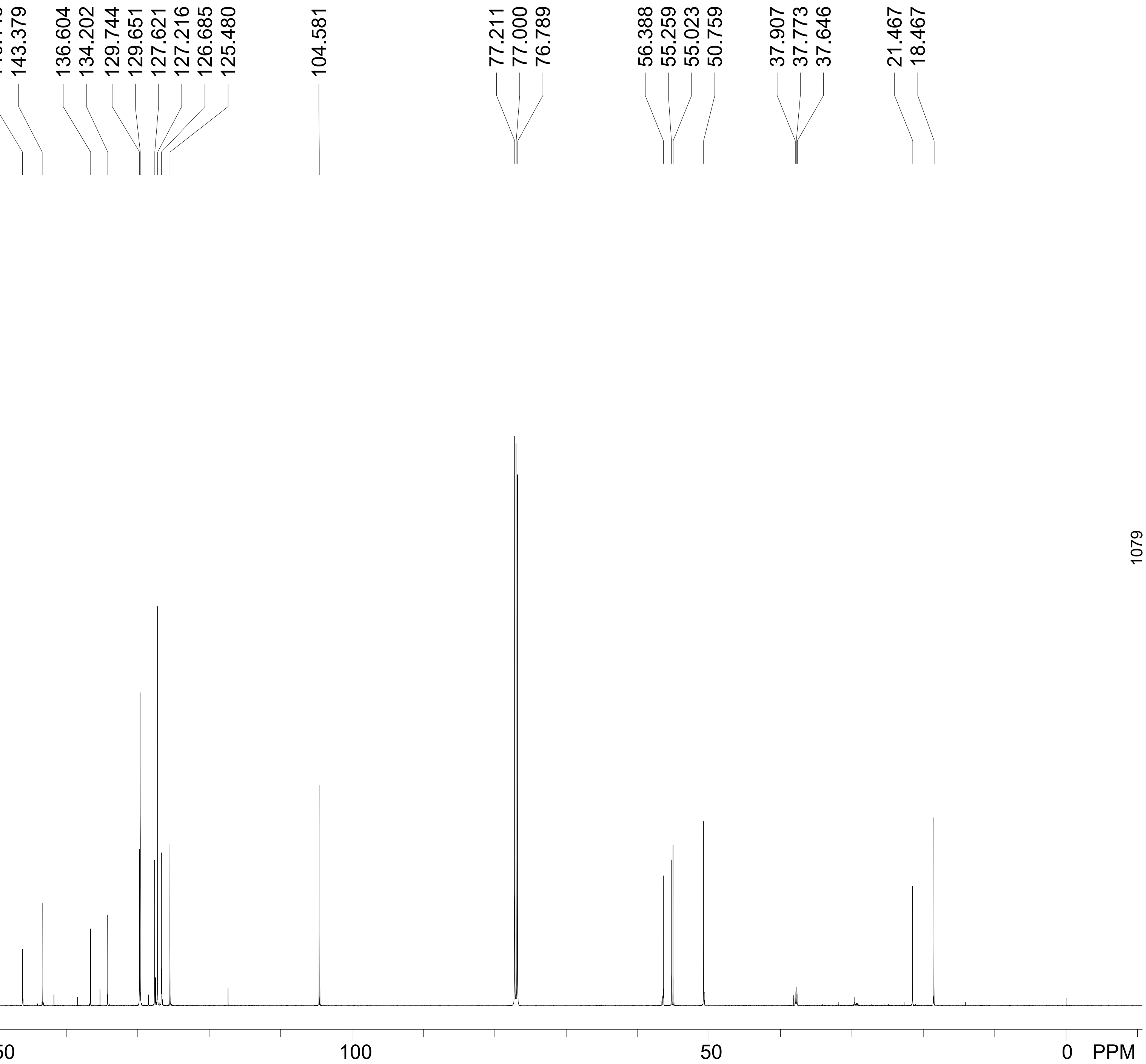

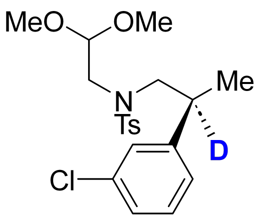

100-d

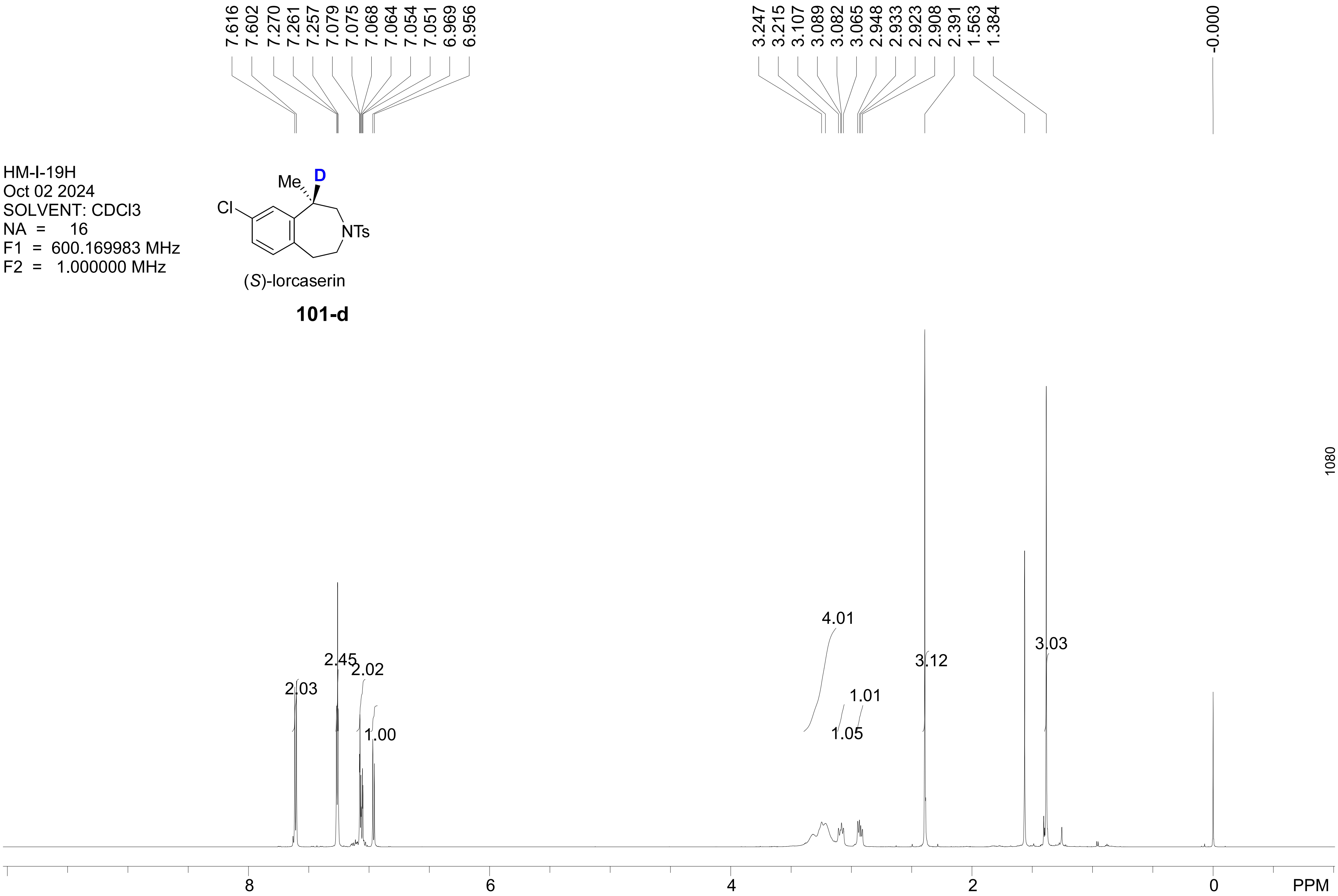

(*S*)-lorcaserin

101-d

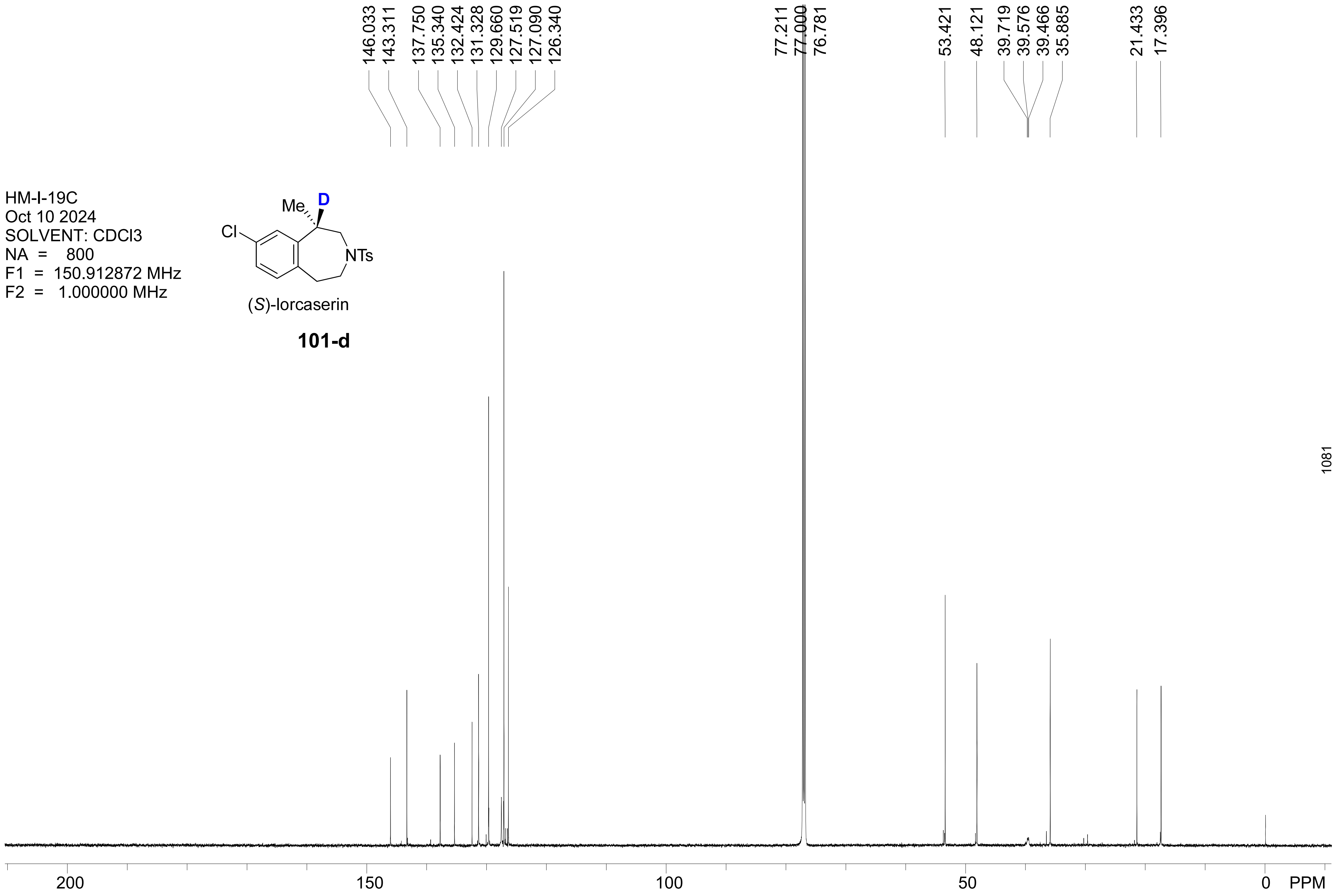
HM-I-19C
Oct 10 2024
SOLVENT: CDCl3
NA = 800
F1 = 150.912872 MHz
F2 = 1.000000 MHz
Cl
Me
D
NTs
(S)-lorcaserin
101-d
146.033
143.311
137.750
135.340
132.424
131.328
129.660
127.519
127.090
126.340
77.211
77.000
76.781
53.421
48.121
39.719
39.576
39.466
35.885
21.433
17.396
200
150
100
50
0
PPM

Area Percent Report

Agilent Technologies

Sample name: HM-I-19-1-IF-95-5-1.0-210

Data file: C:\Users\Public\Documents\ChemStation\1\Data\HM\HM 2024-10-06 14-14-51\003-P1-C2-HM-I-19-IF.D

Injection Acquired Date 10/06/2024 15:08:44

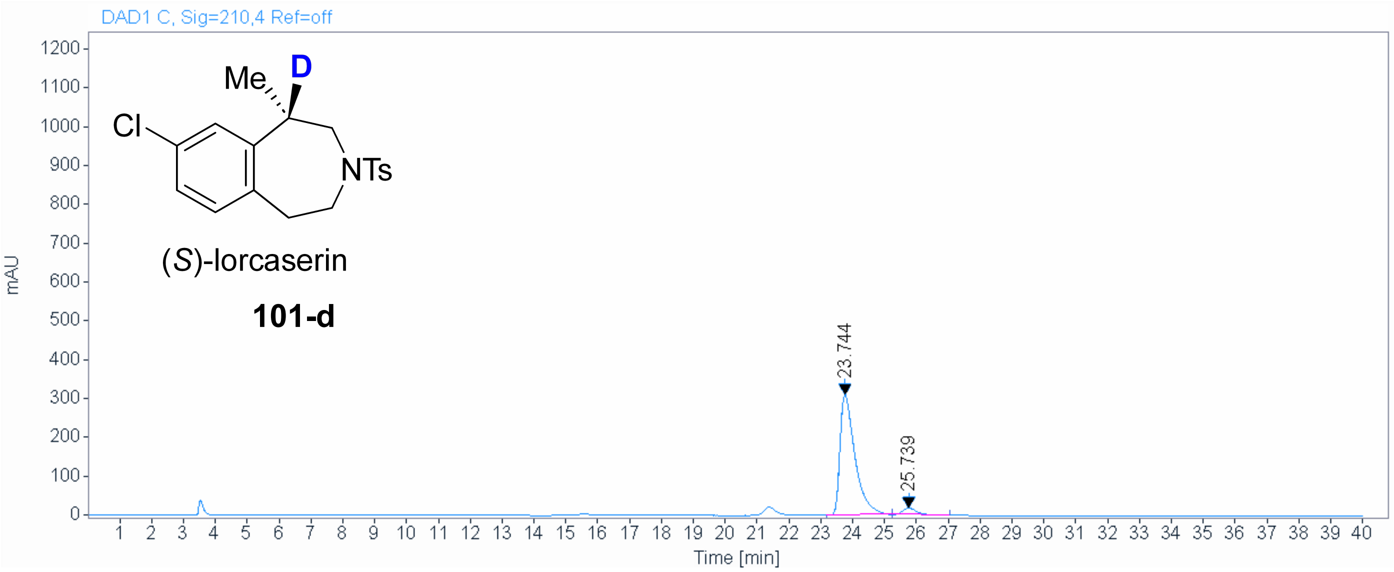

Signal: DAD1 C, Sig=210,4 Ref=off

RT [min]	Width [min]	Height	Area	Area%
23.744	0.4901	309.7108	10143.8154	94.8698
25.739	0.4778	17.3982	548.5378	5.1302
		Sum	10692.3533	100.0000

Area Percent Report

Agilent Technologies

Sample name: HM-I-19-RAC-IF-95-5-1.0-210

Data file: C:\Users\Public\Documents\ChemStation\1\Data\HM\HM 2024-10-06 14-14-51\002-P1-C1-HM-I-13-rac-IF.D

Injection Acquired Date 10/06/2024 14:27:37

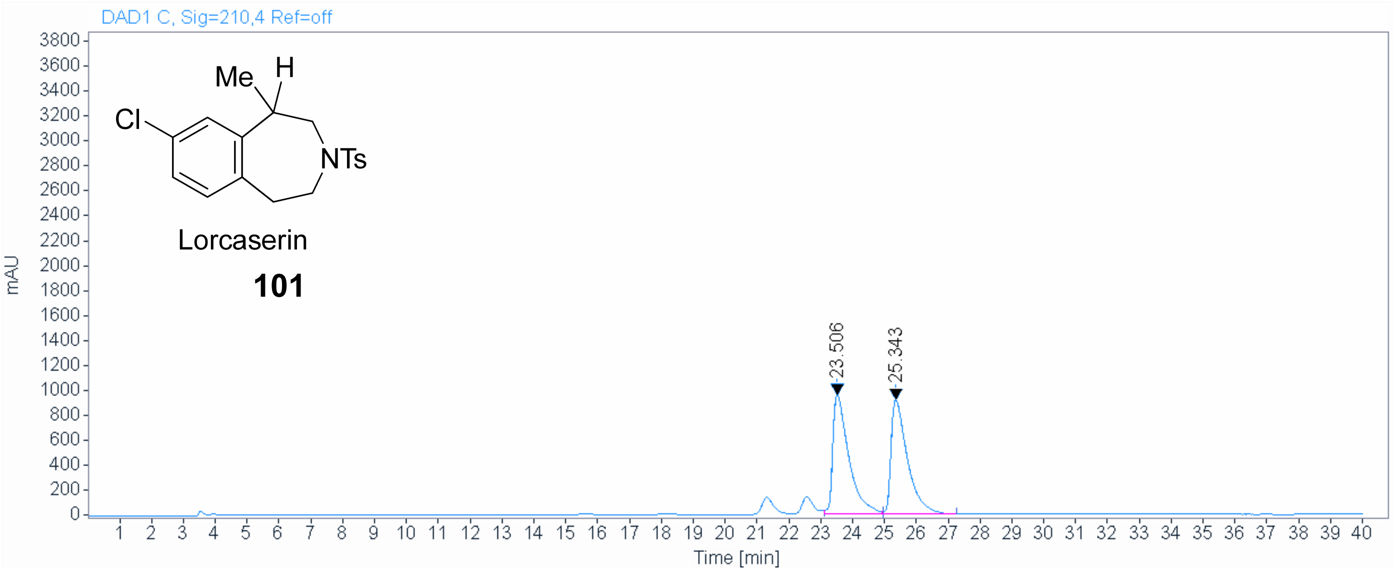

Signal: DAD1 C, Sig=210,4 Ref=off

RT [min]	Width [min]	Height	Area	Area%
23.506	0.5842	953.6111	33427.6016	50.5173
25.343	0.5299	914.1365	32743.0098	49.4827
		Sum	66170.6113	100.0000

zwf-11-064H
Aug 21 2024
SOLVENT: CDCl3
NA = 4
F1 = 600.169983 MHz
F2 = 1.000000 MHz

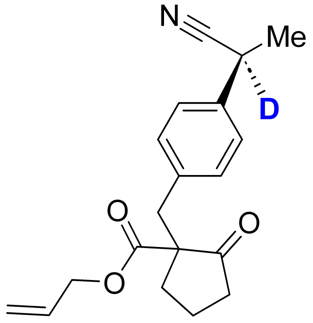

103-d

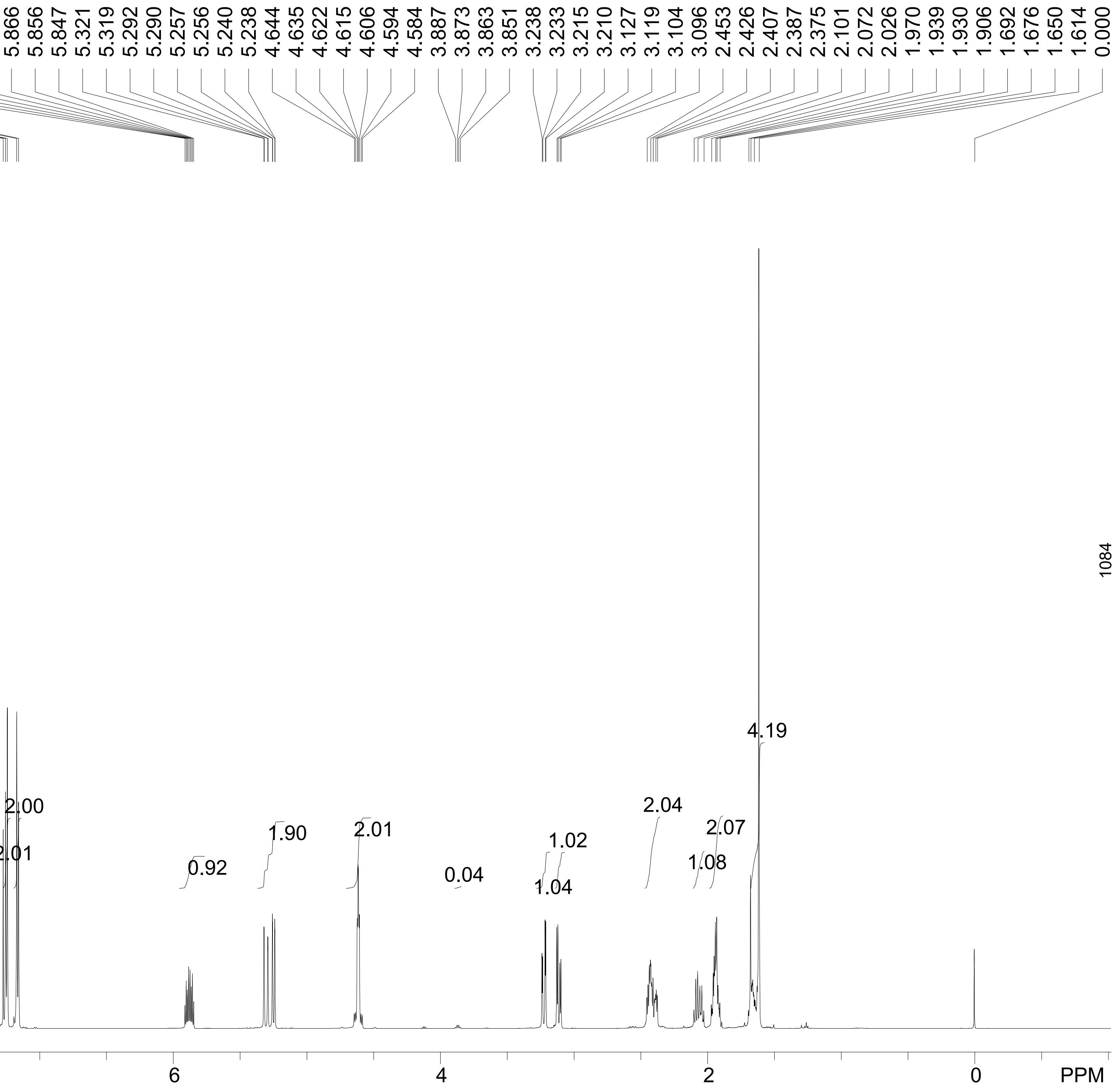

zwf-11-064C
Aug 21 2024
SOLVENT: CDCl3
NA = 100
F1 = 150.912872 MHz
F2 = 1.000000 MHz

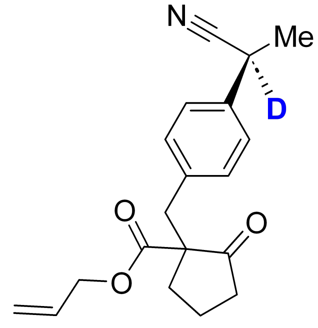

103-d

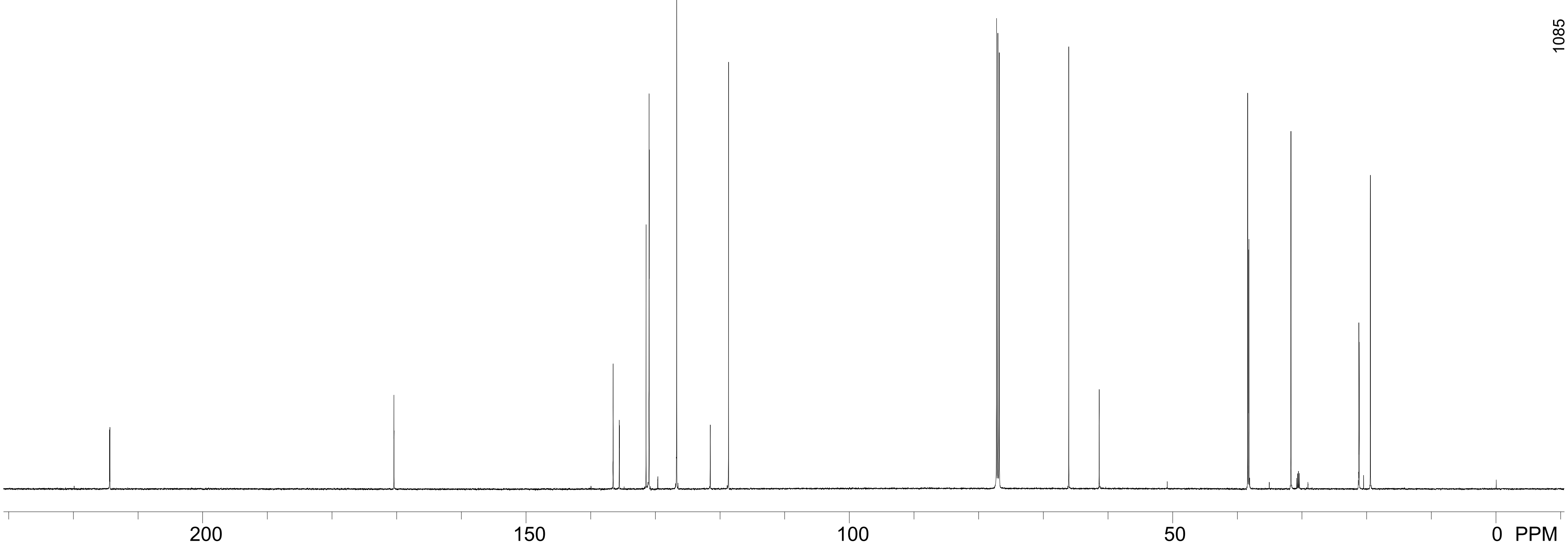

Area Percent Report

Agilent Technologies

Sample name: zwf-11-064-IH-98-2-1.1-205

Data file: C:\Users\Public\Documents\ChemStation\1\Data\zwf\zwf 2024-08-21 16-39-43\009-P1-C3-zwf-11-064 IH-98-2-1.1.D

Injection Acquired Date 08/21/2024 21:44:38

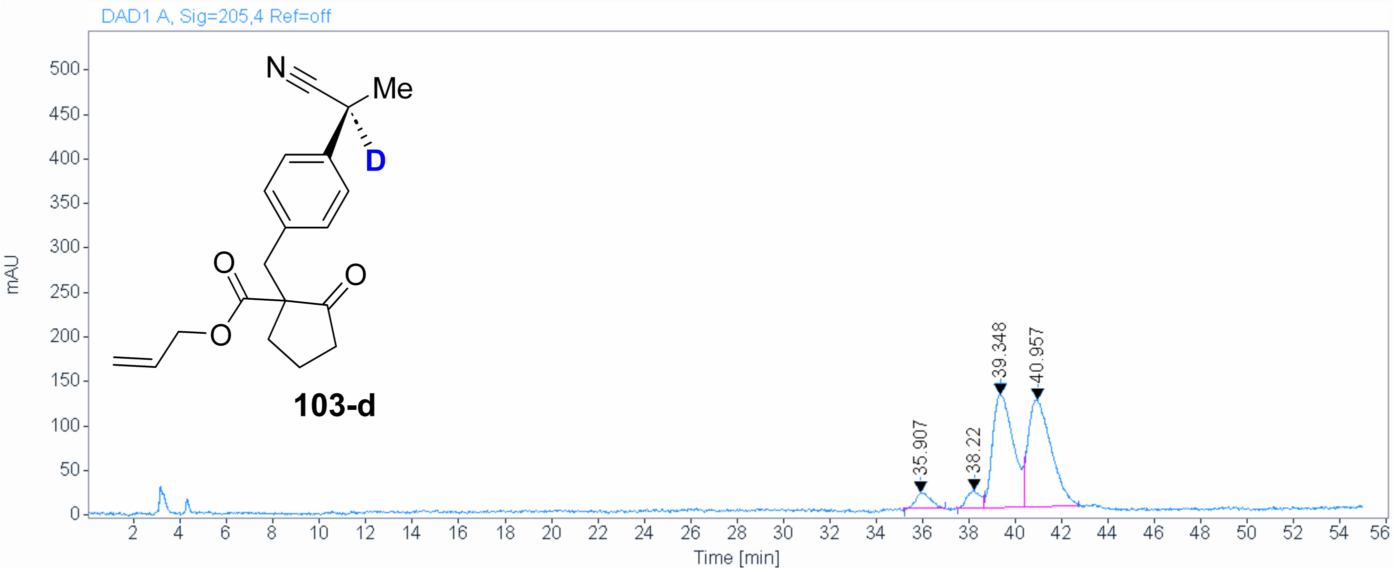

Signal: DAD1 A, Sig=205,4 Ref=off

RT [min]	Width [min]	Height	Area	Area%
35.907	0.7585	18.4117	837.9229	4.6464
38.220	0.6830	20.4822	839.3813	4.6545
39.348	1.0674	127.4759	8164.4463	45.2730
40.957	1.1277	121.0697	8192.0527	45.4261
		Sum	18033.8032	100.0000

Area Percent Report

Agilent Technologies

Sample name: zwf-11-064-rac-IH-98-2-1.1-205

Data file: C:\Users\Public\Documents\ChemStation\1\Data\zwf\zwf 2024-08-21 16-39-43\010-P1-C4-zwf-11-064-rac IH-98-2-1.1.D

Injection Acquired Date 08/21/2024 22:40:48

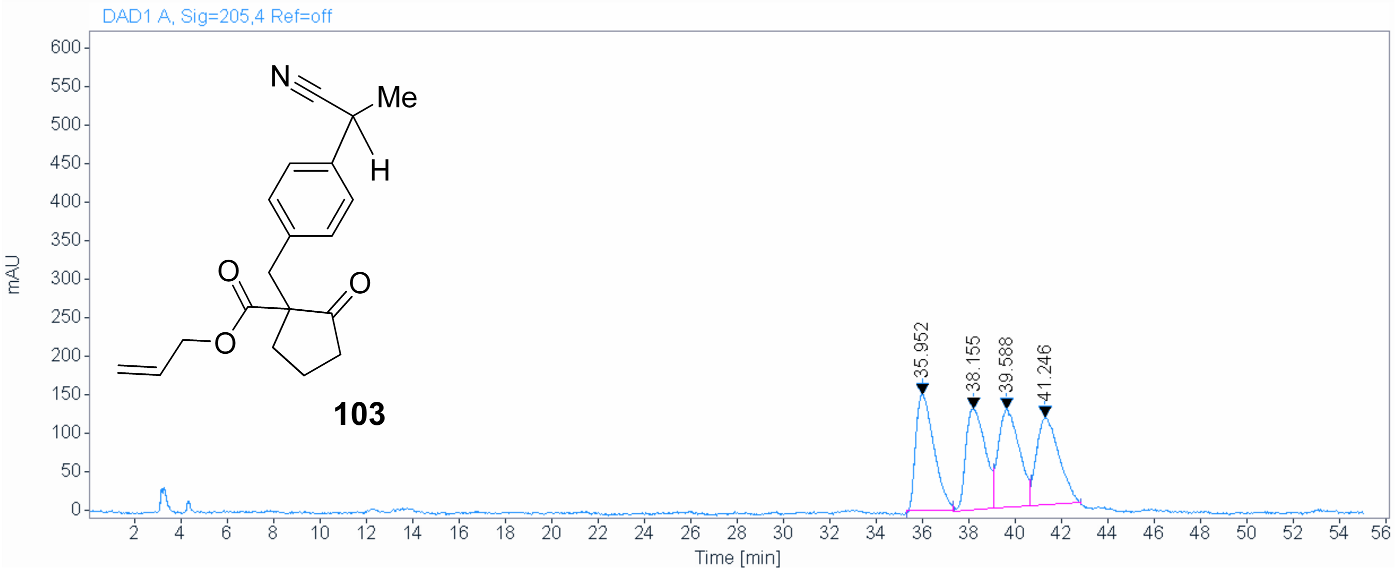

Signal: DAD1 A, Sig=205,4 Ref=off

RT [min]	Width [min]	Height	Area	Area%
35.952	0.9142	150.7081	8266.8965	25.8760
38.155	0.9860	131.9402	7805.6807	24.4324
39.588	1.0491	128.7260	8102.5361	25.3616
41.246	1.1268	114.9722	7772.9824	24.3300
		Sum	31948.0957	100.0000

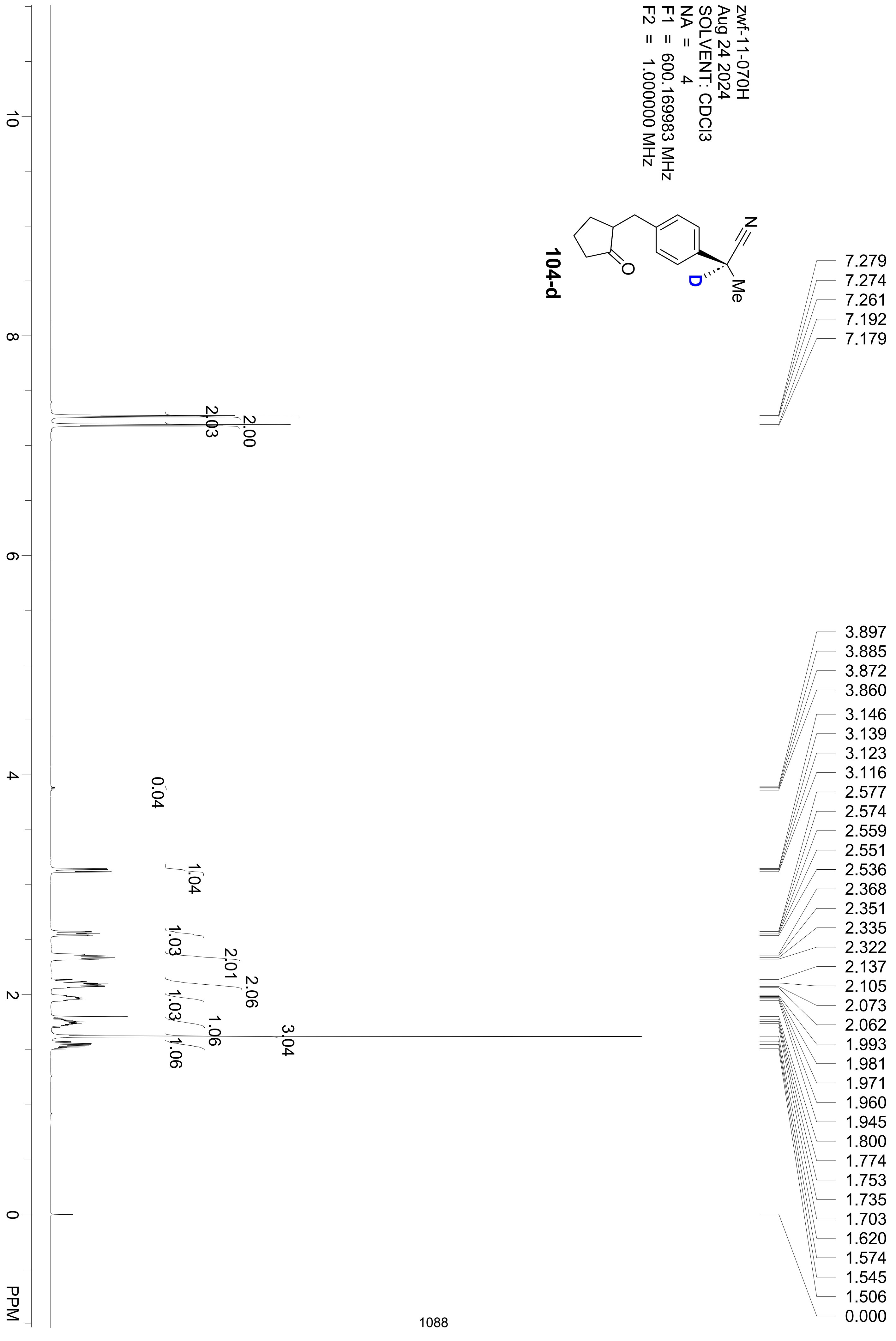
zwf-11-070H
Aug 24 2024
SOLVENT: CDCl3
NA = 4
F1 = 600.169983 MHz
F2 = 1.000000 MHz
N
D
Me
O
104-d
7.279
7.274
7.261
7.192
7.179
3.897
3.885
3.872
3.860
3.146
3.139
3.123
3.116
2.577
2.574
2.559
2.551
2.536
2.368
2.351
2.335
2.322
2.137
2.105
2.073
2.062
1.993
1.981
1.971
1.960
1.945
1.800
1.774
1.753
1.735
1.703
1.620
1.574
1.545
1.506
0.000
2.03
2.00
0.04
1.04
1.03
2.01
2.06
1.03
1.06
3.04
1.06
10
8
6
4
2
0
PPM

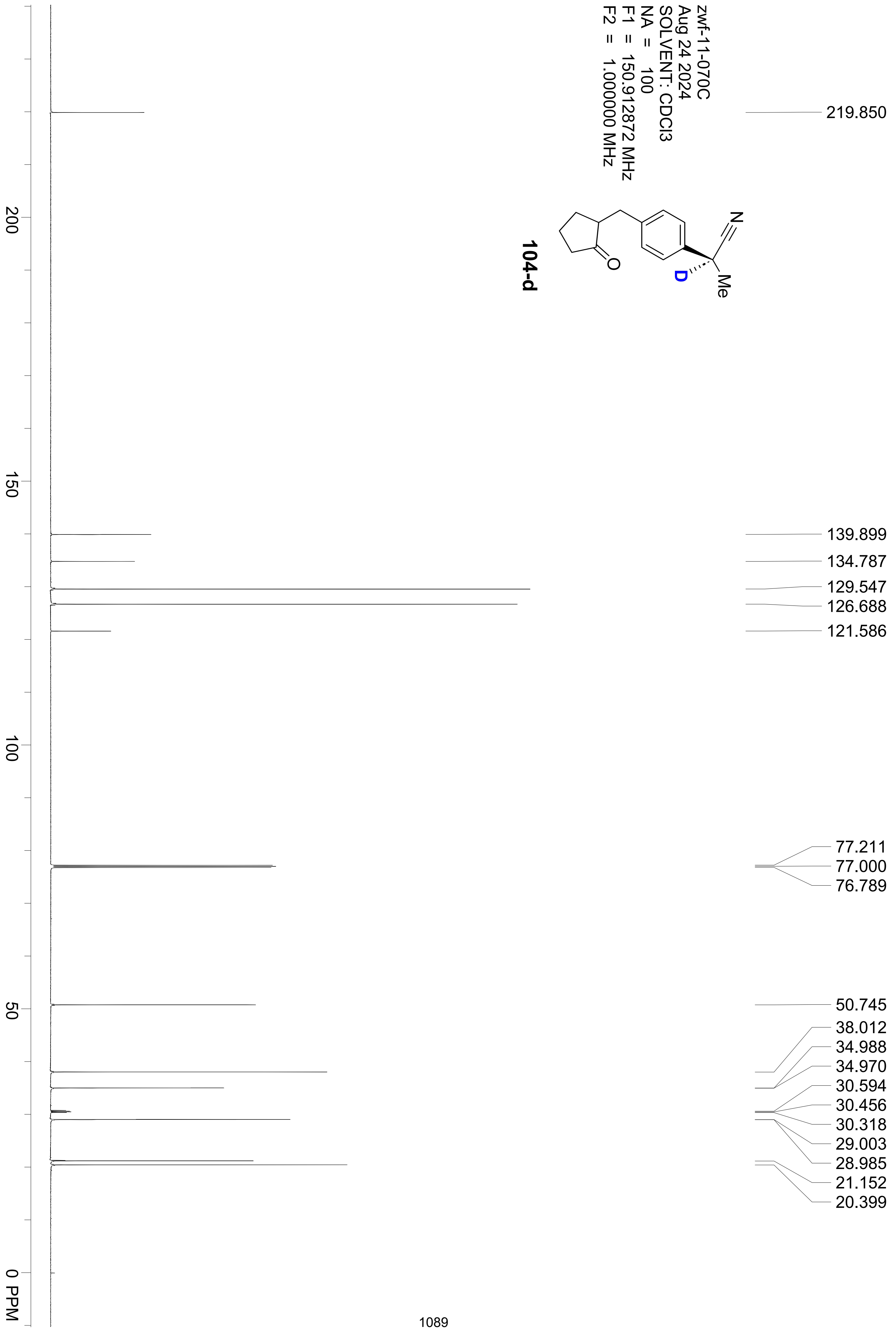
zwf-11-070C
Aug 24 2024
SOLVENT: CDCl3
NA = 100
F1 = 150.912872 MHz
F2 = 1.000000 MHz
104-d
N
Me
D
O
219.850
139.899
134.787
129.547
126.688
121.586
77.211
77.000
76.789
50.745
38.012
34.988
34.970
30.594
30.456
30.318
29.003
28.985
21.152
20.399
200
150
100
50
0 PPM

Area Percent Report

Agilent Technologies

Sample name: zwf-11-070-IH-98-2-1.1-205

Data file: C:\Users\Public\Documents\ChemStation\1\Data\zwf\zwf 2024-08-24 14-20-07\003-P1-C1-zwf-11-070.D

Injection Acquired Date 08/24/2024 14:46:24

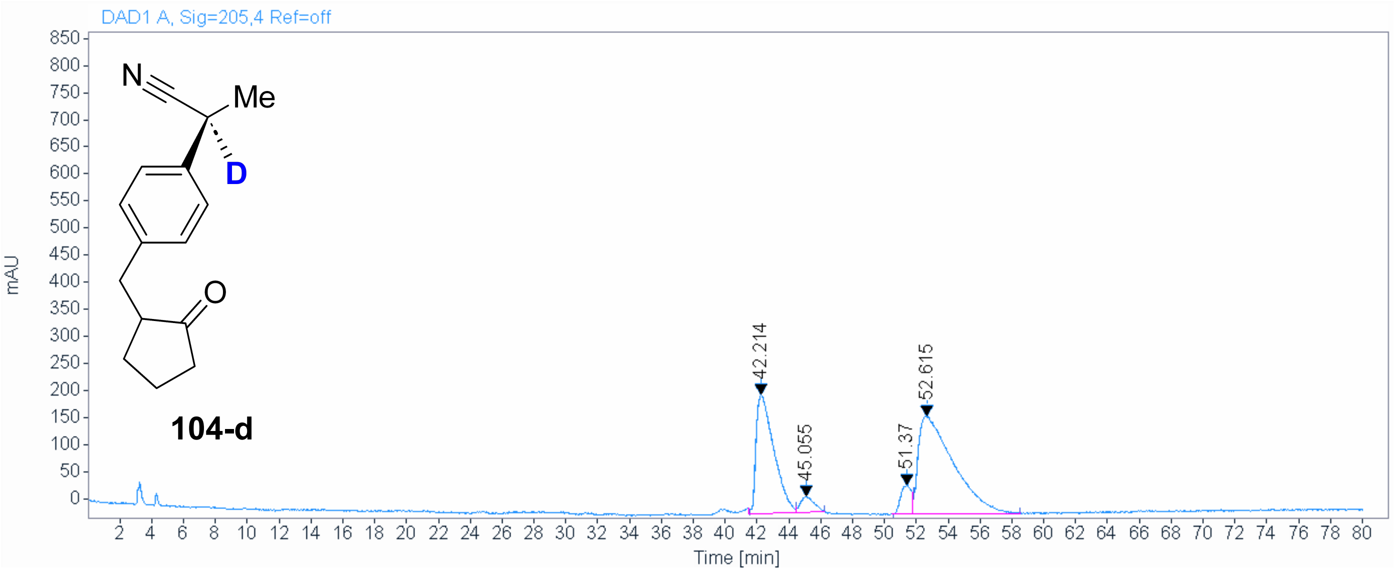

Signal: DAD1 A, Sig=205,4 Ref=off

RT [min]	Width [min]	Height	Area	Area%
42.214	1.3067	219.4572	17206.1250	35.0485
45.055	0.9720	29.3545	1712.0143	3.4873
51.370	0.8101	52.8021	2566.6270	5.2282
52.615	2.5558	180.0326	27607.5742	56.2360
		Sum	49092.3405	100.0000

Area Percent Report

Agilent Technologies

Sample name: zwf-11-070-rac-IH-98-2-1.1-205

Data file: C:\Users\Public\Documents\ChemStation\1\Data\zwf\zwf 2024-08-23 08-52-32\024-P1-C5-zwf-11-070-rac.D

Injection Acquired Date 08/23/2024 21:38:45

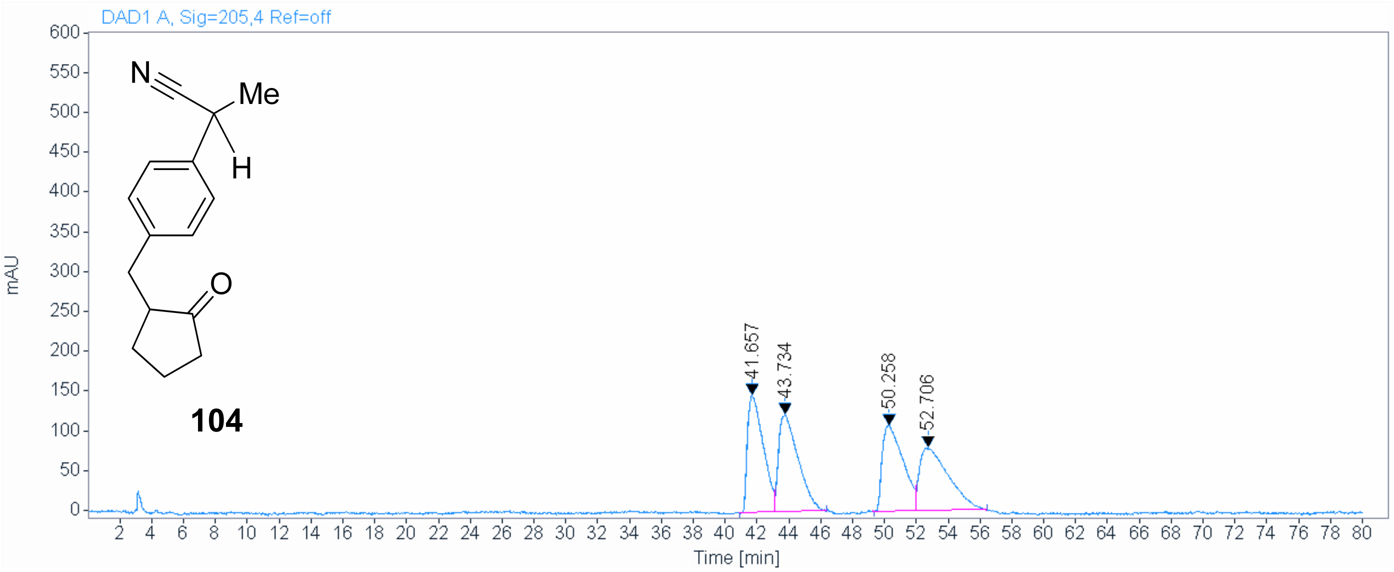

Signal: DAD1 A, Sig=205,4 Ref=off

RT [min]	Width [min]	Height	Area	Area%
41.657	1.1553	148.5073	10294.1689	25.1589
43.734	1.4212	123.5092	10532.1816	25.7406
50.258	1.5286	108.8806	9986.1982	24.4062
52.706	2.0931	80.4572	10104.1152	24.6944
		Sum	40916.6641	100.0000

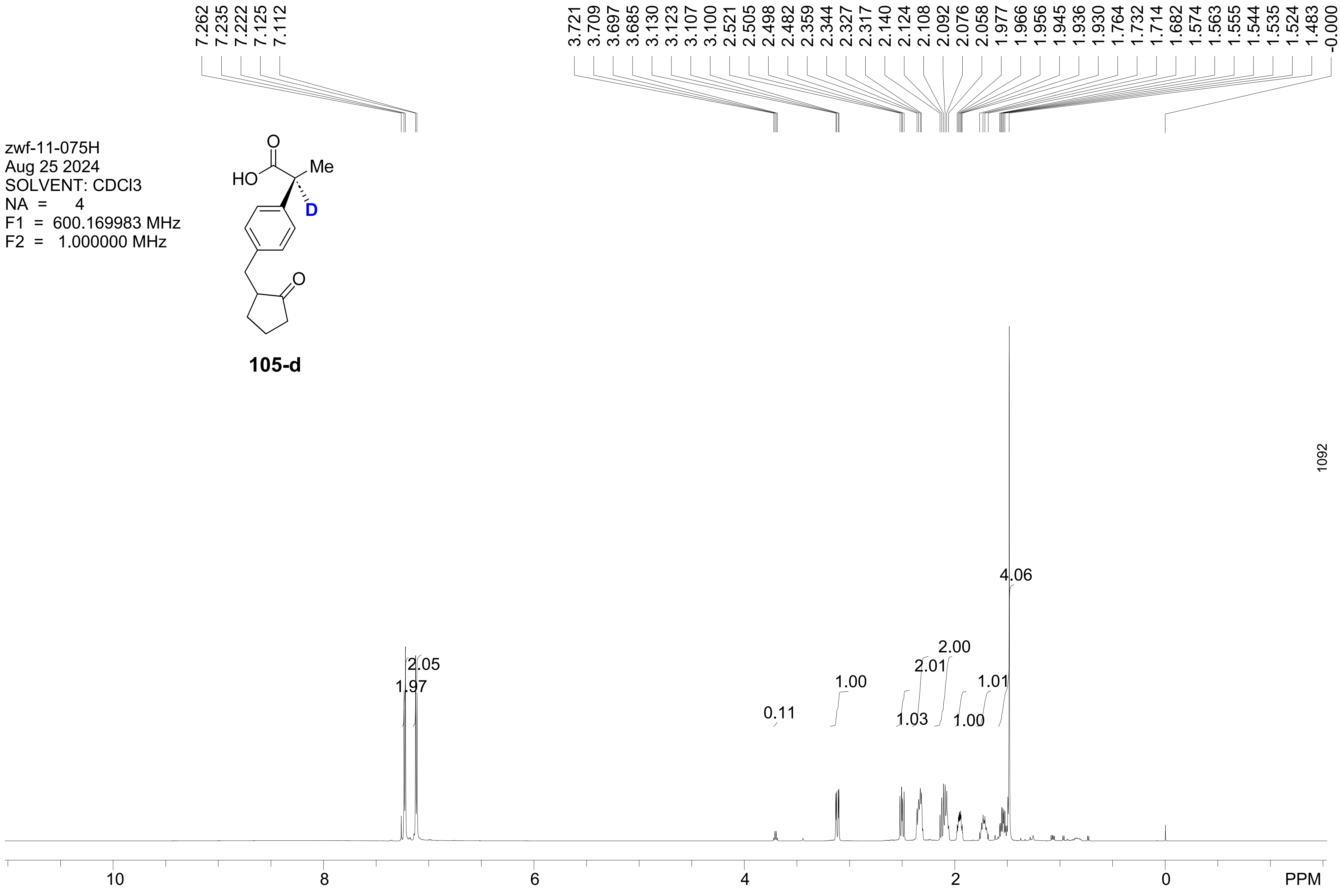
7.262
7.235
7.222
7.125
7.112
3.721
3.709
3.697
3.685
3.130
3.123
3.107
3.100
2.521
2.505
2.498
2.482
2.359
2.344
2.327
2.317
2.140
2.124
2.108
2.092
2.076
2.058
1.977
1.966
1.956
1.945
1.936
1.930
1.764
1.732
1.714
1.682
1.574
1.563
1.555
1.544
1.535
1.524
1.483
-0.000
zwf-11-075H
Aug 25 2024
SOLVENT: CDCl3
NA = 4
F1 = 600.169983 MHz
F2 = 1.000000 MHz
HO
O
Me
D
O
105-d
2.05
1.97
0.11
1.00
1.03
2.01
2.00
1.00
1.01
4.06
10
8
6
4
2
0
PPM

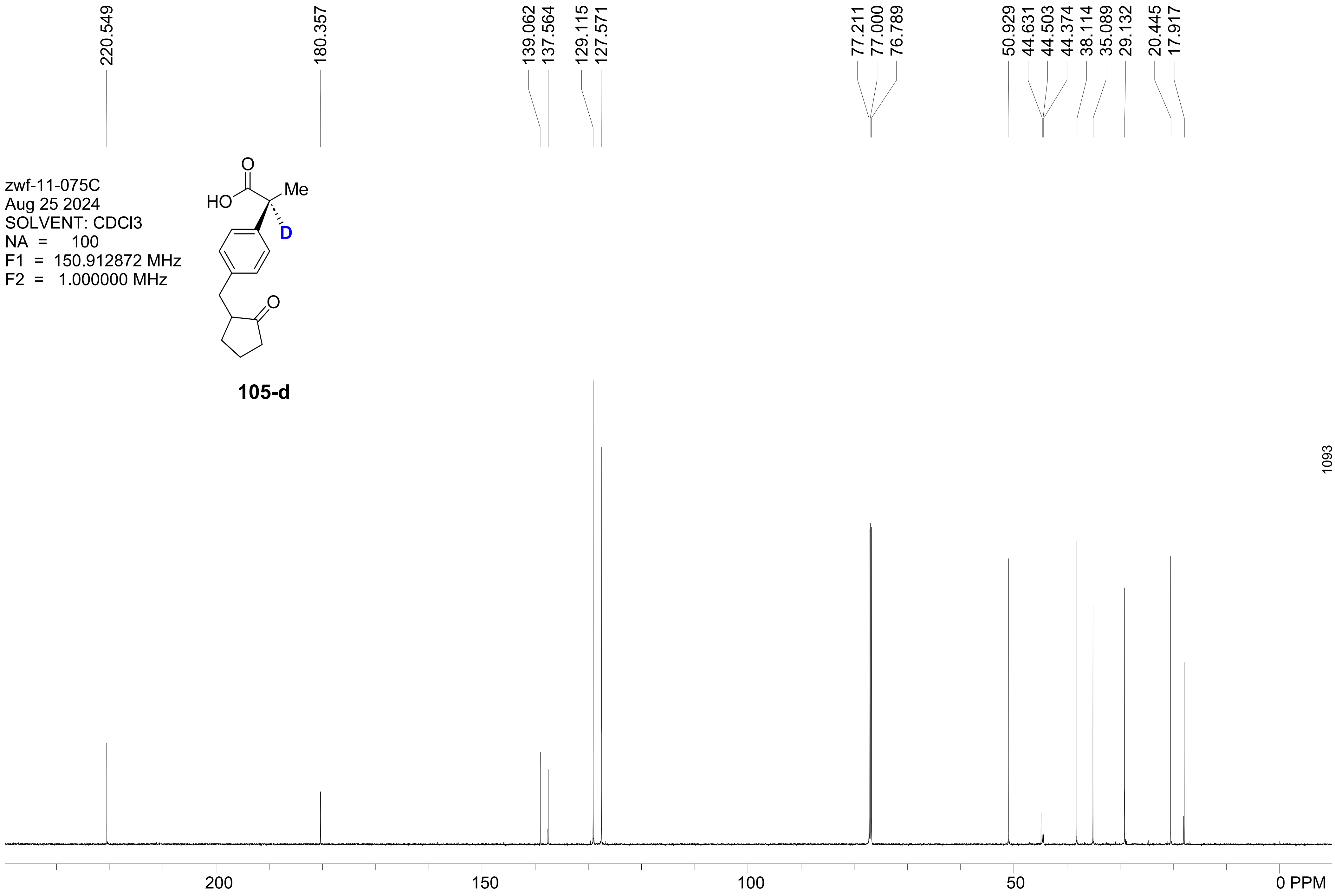
220.549
180.357
139.062
137.564
129.115
127.571
77.211
77.000
76.789
50.929
44.631
44.503
44.374
38.114
35.089
29.132
20.445
17.917
zwf-11-075C
Aug 25 2024
SOLVENT: CDCl3
NA = 100
F1 = 150.912872 MHz
F2 = 1.000000 MHz
HO
O
Me
D
O
105-d
200
150
100
50
0 PPM

Area Percent Report

Agilent Technologies

Sample name: zwf-11-075-rac-IG-98.5-1.5-1.3-205

Data file: C:\Users\Public\Documents\ChemStation\1\Data\zwf\zwf 2024-08-26 08-35-31\011-P1-C2-zwf-11-075.D

Injection Acquired Date 08/26/2024 12:04:45

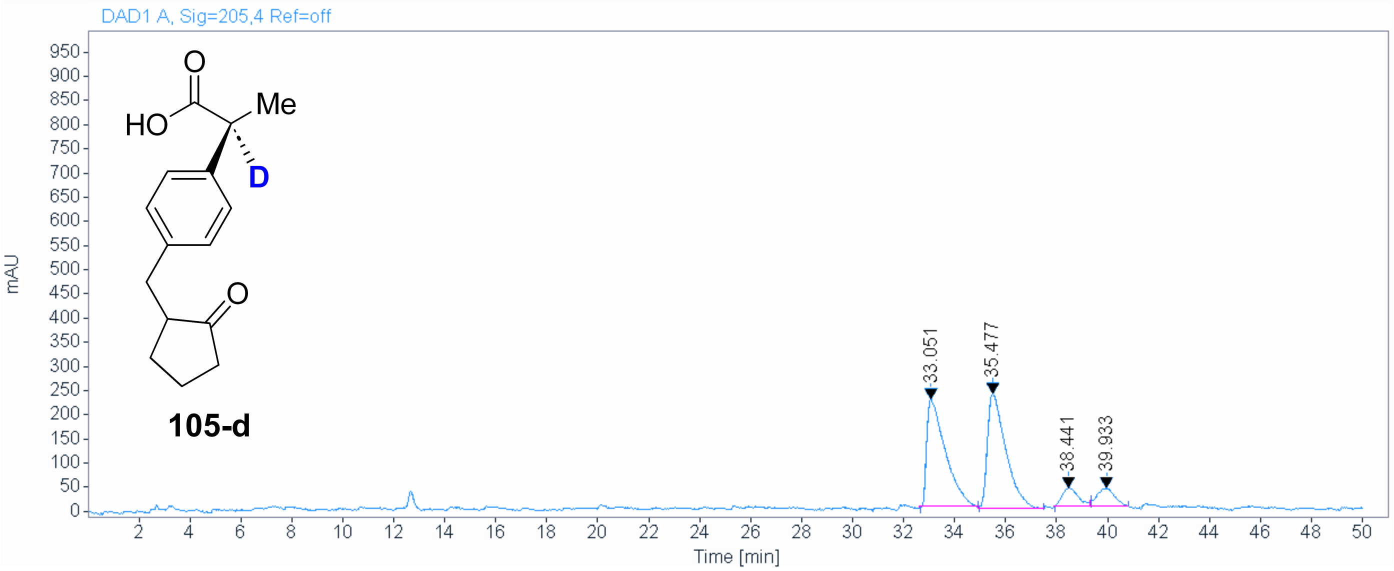

Signal: DAD1 A, Sig=205,4 Ref=off

RT [min]	Width [min]	Height	Area	Area%
33.051	0.8535	222.4371	11390.7119	42.2995
35.477	0.8448	236.9735	12012.0977	44.6071
38.441	0.7465	38.0592	1704.5966	6.3300
39.933	0.8020	37.8505	1821.2853	6.7634
		Sum	26928.6914	100.0000

Area Percent Report

Agilent Technologies

Sample name: zwf-11-075-rac-IG-98.5-1.5-1.3-205

Data file: C:\Users\Public\Documents\ChemStation\1\Data\zwf\zwf 2024-08-26 08-35-31\012-P1-C1-zwf-11-075-rac.D

Injection Acquired Date 08/26/2024 12:55:44

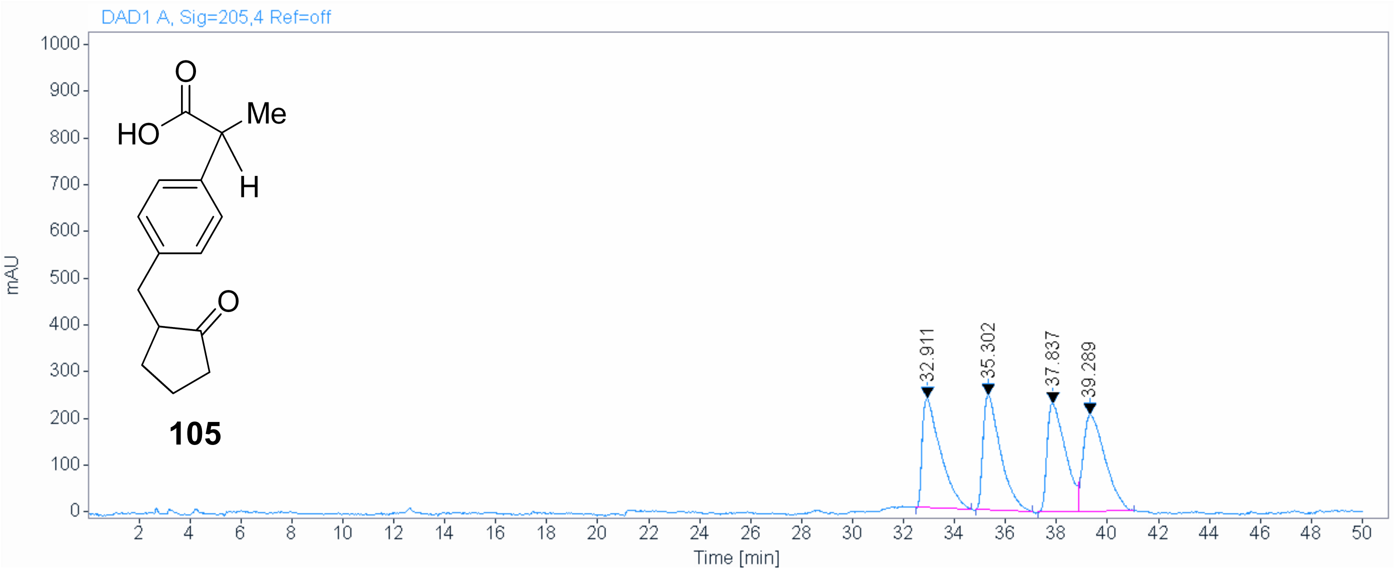

Signal: DAD1 A, Sig=205,4 Ref=off

RT [min]	Width [min]	Height	Area	Area%
32.911	0.8310	235.3448	11734.1650	24.6477
35.302	0.7986	246.4183	11808.0020	24.8028
37.837	0.8423	232.7720	11763.5459	24.7094
39.289	0.9854	208.0648	12301.8906	25.8402
		Sum	47607.6035	100.0000

zwf-10-088H
Aug 20 2024
SOLVENT: CDCl3
NA = 4
F1 = 600.169983 MHz
F2 = 1.000000 MHz

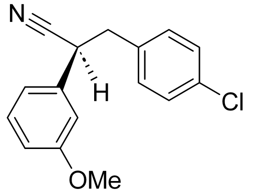

108

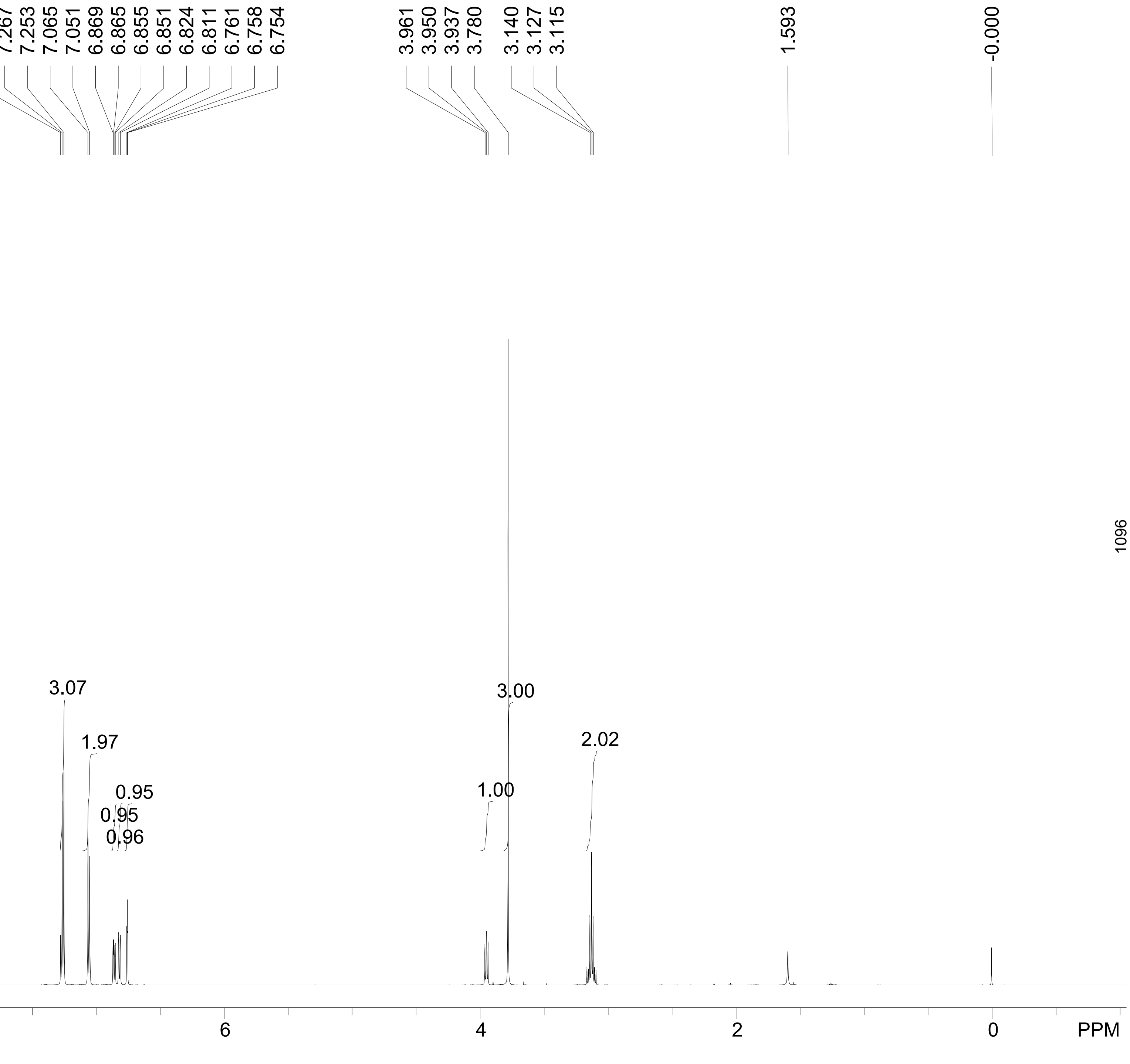

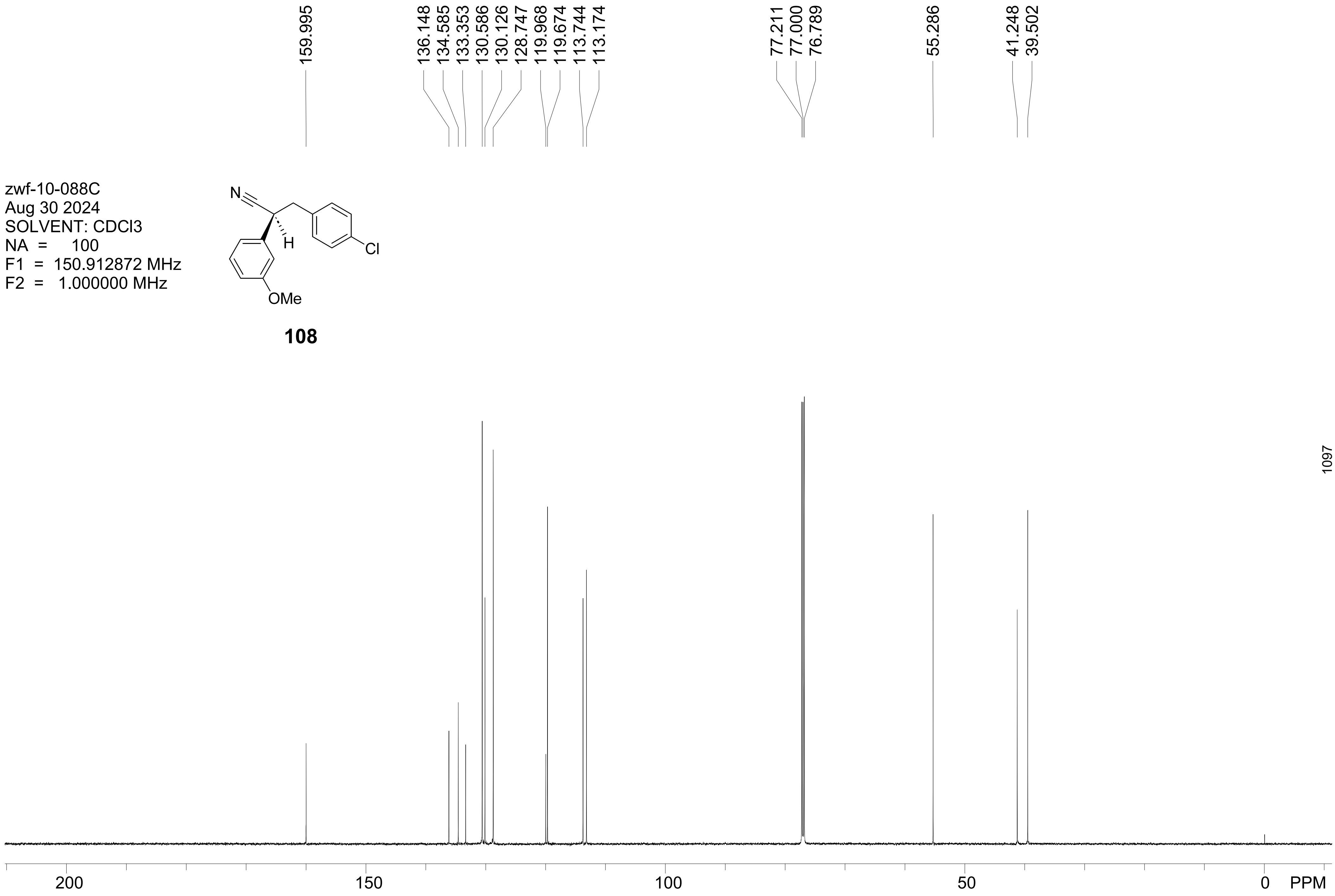
zwf-10-088C
Aug 30 2024
SOLVENT: CDCl3
NA = 100
F1 = 150.912872 MHz
F2 = 1.000000 MHz
N
H
Cl
OMe
108
159.995
136.148
134.585
133.353
130.586
130.126
128.747
119.968
119.674
113.744
113.174
77.211
77.000
76.789
55.286
41.248
39.502
200
150
100
50
0
PPM

Area Percent Report

Agilent Technologies

Sample name: zwf-11-088-IE-99-1-0.8-205

Data file: C:\Users\Public\Documents\ChemStation\1\Data\zwf\zwf 2024-08-30 10-18-54\005-P1-C2-zwf-11-088.D

Injection Acquired Date 08/30/2024 13:27:02

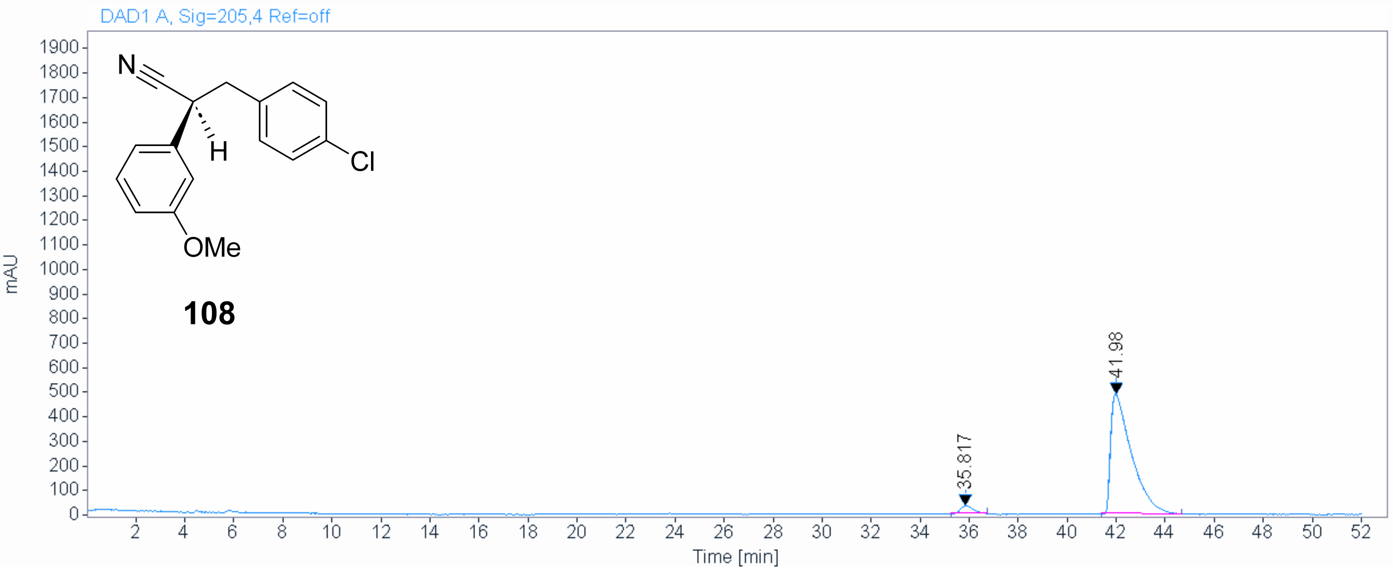

Signal: DAD1 A, Sig=205,4 Ref=off

RT [min]	Width [min]	Height	Area	Area%
35.817	0.6050	33.9583	1232.7849	4.0693
41.980	0.9907	488.9308	29062.0918	95.9307
		Sum	30294.8767	100.0000

Area Percent Report

Agilent Technologies

Sample name: zwf-11-088-rac-IE-99-1-0.8-205

Data file: C:\Users\Public\Documents\ChemStation\1\Data\zwf\zwf 2024-08-30 10-18-54\006-P1-C1-zwf-11-088-rac.D

Injection Acquired Date 08/30/2024 14:20:02

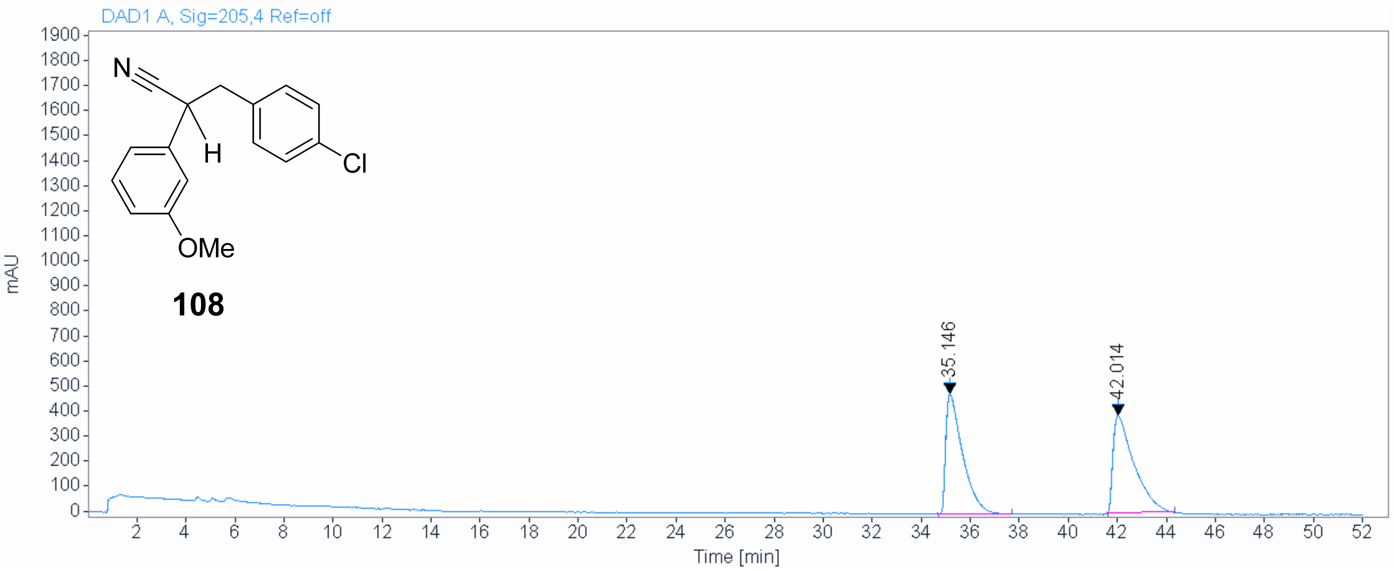

Signal: DAD1 A, Sig=205,4 Ref=off

RT [min]	Width [min]	Height	Area	Area%
35.146	0.8015	481.7709	23169.1523	49.7563
42.014	0.9975	390.9091	23396.1211	50.2437
		Sum	46565.2734	100.0000

zwf-11-102H
Sep 04 2024
SOLVENT: CDCl3
NA = 4
F1 = 600.130005 MHz
F2 = 1.000000 MHz

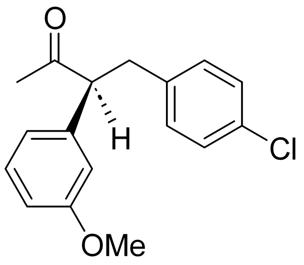

109

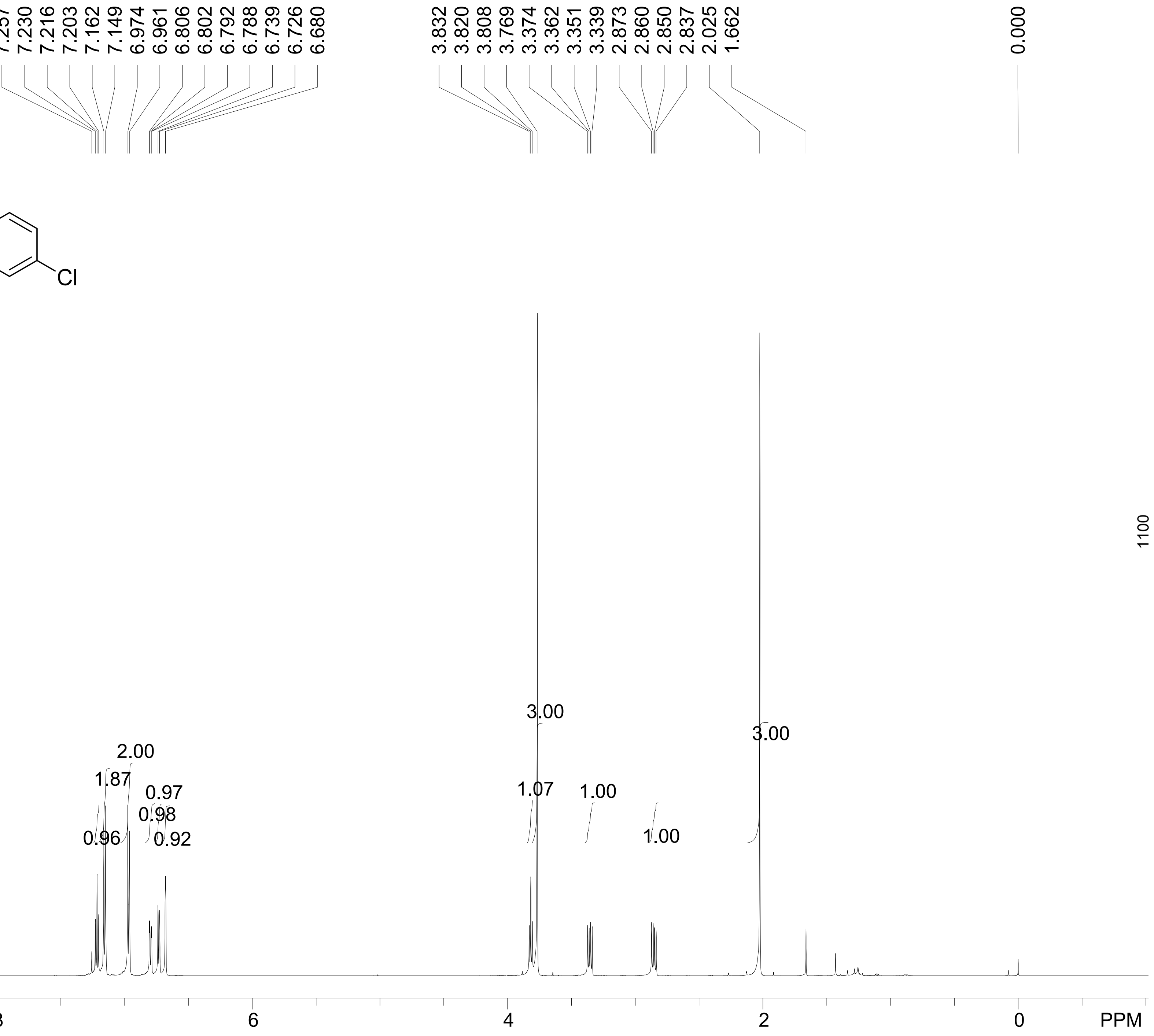

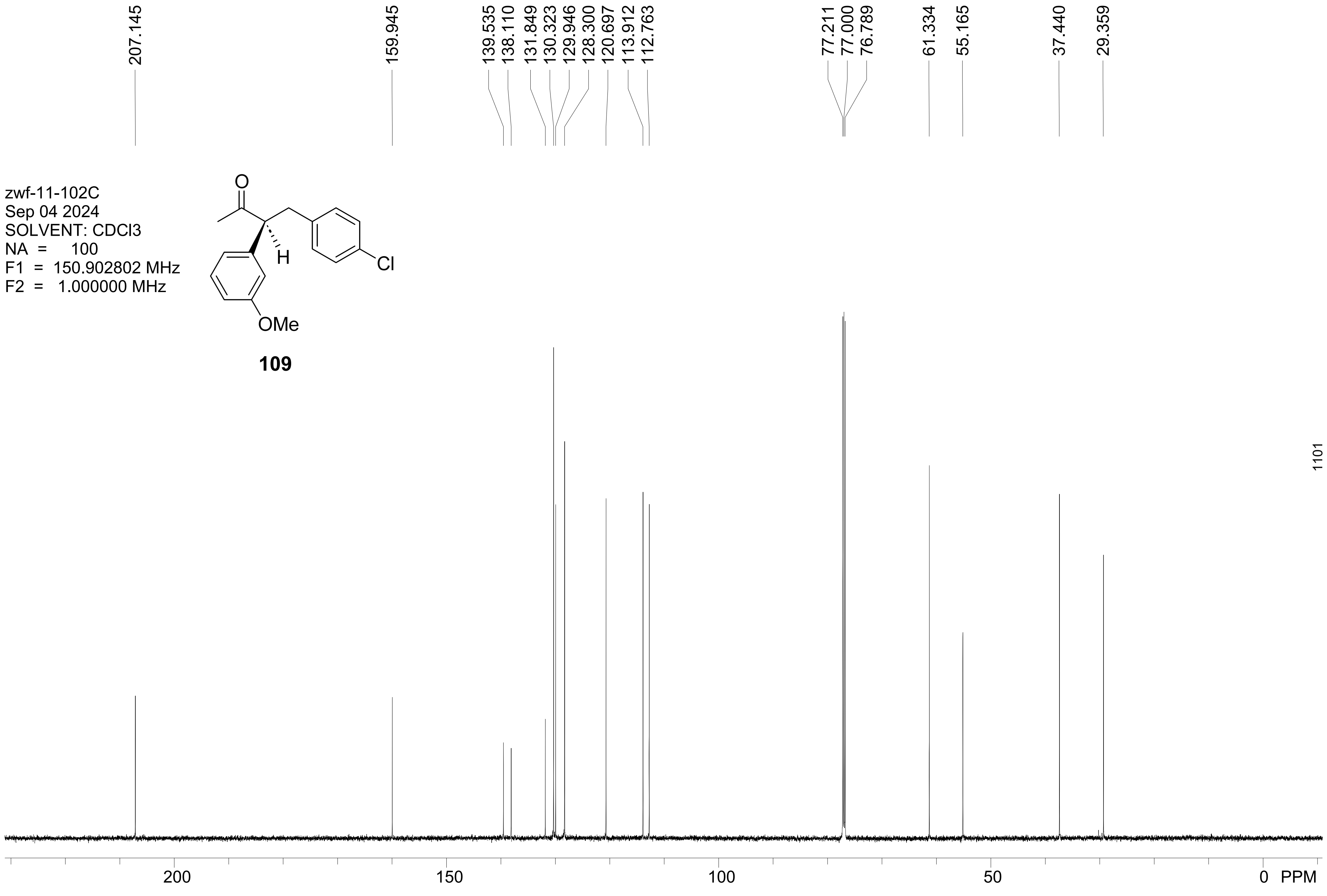
zwf-11-102C
Sep 04 2024
SOLVENT: CDCl3
NA = 100
F1 = 150.902802 MHz
F2 = 1.000000 MHz
O
H
Cl
OMe
109
207.145
159.945
139.535
138.110
131.849
130.323
129.946
128.300
120.697
113.912
112.763
77.211
77.000
76.789
61.334
55.165
37.440
29.359
200
150
100
50
0 PPM

Area Percent Report

Agilent Technologies

Sample name: zwf-11-102-IG-90-10-0.8-205

Data file: C:\Users\Public\Documents\ChemStation\1\Data\zwf\zwf 2024-09-04 08-51-35\003-P1-C2-zwf-11-102.D

Injection Acquired Date 09/04/2024 09:28:54

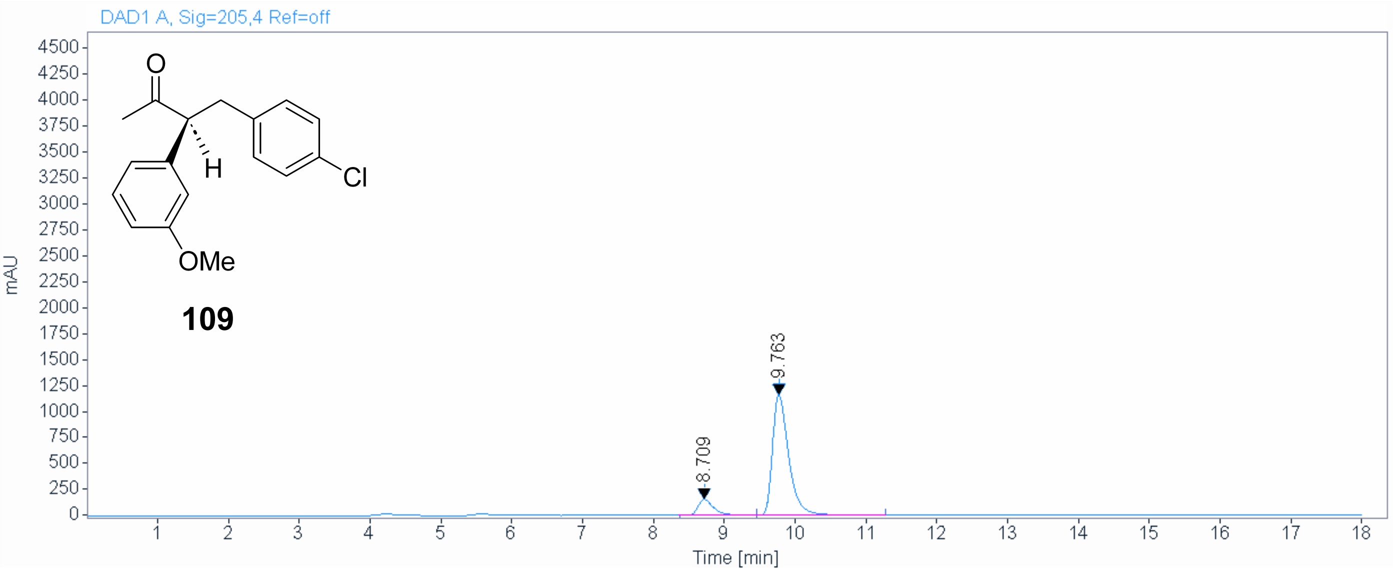

Signal: DAD1 A, Sig=205,4 Ref=off

RT [min]	Width [min]	Height	Area	Area%
8.709	0.2539	152.2012	2319.0837	11.1867
9.763	0.2415	1157.6984	18411.6172	88.8133
		Sum	20730.7009	100.0000

Area Percent Report

Agilent Technologies

Sample name: zwf-11-102-rac-IG-90-10-0.8-205

Data file: C:\Users\Public\Documents\ChemStation\1\Data\zwf\zwf 2024-09-04 08-51-35\002-P1-C1-zwf-11-102-rac.D

Injection Acquired Date 09/04/2024 09:09:56

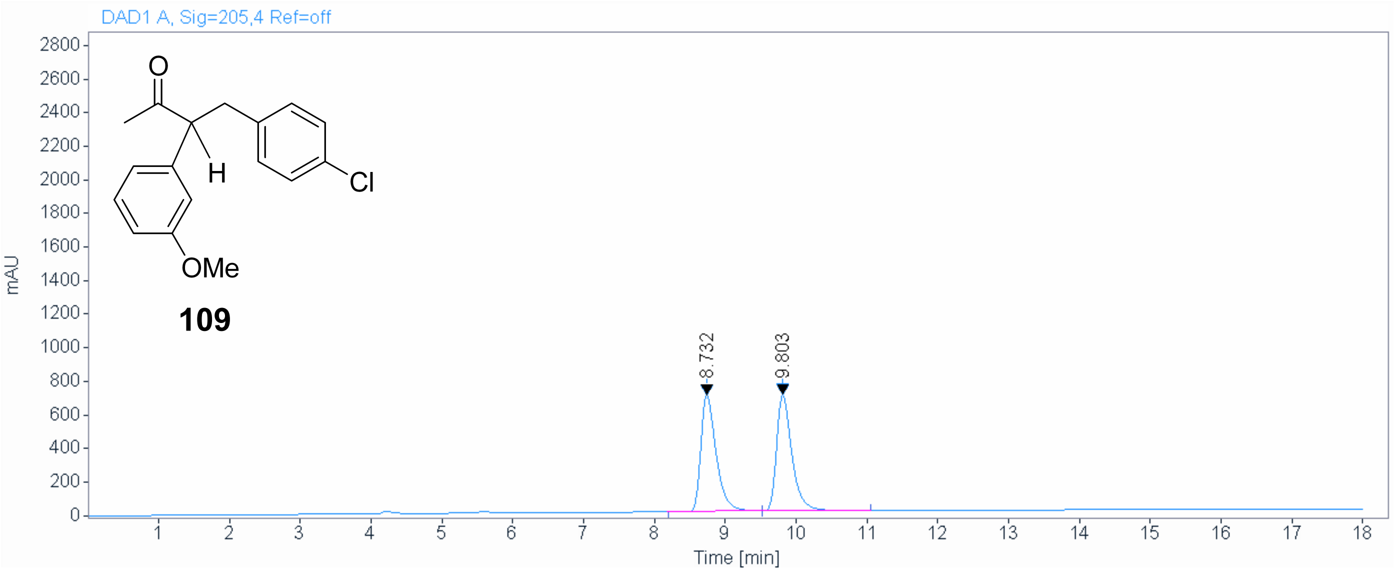

Signal: DAD1 A, Sig=205,4 Ref=off

RT [min]	Width [min]	Height	Area	Area%
8.732	0.2286	693.1561	10492.0459	49.9260
9.803	0.2293	692.5268	10523.1396	50.0740
		Sum	21015.1855	100.0000

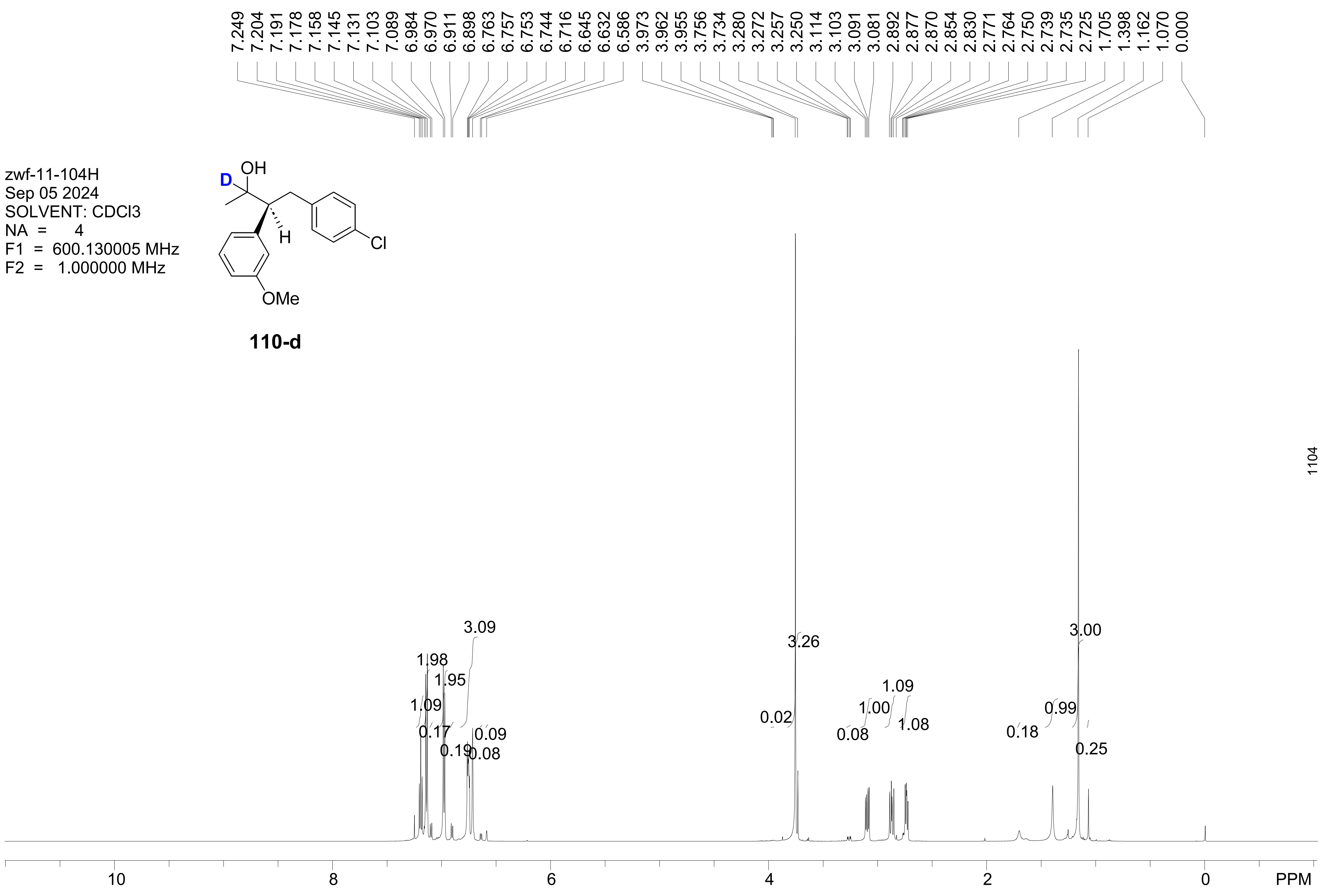
zwf-11-104H
Sep 05 2024
SOLVENT: CDCl3
NA = 4
F1 = 600.130005 MHz
F2 = 1.000000 MHz
D
OH
H
Cl
OMe
7.249
7.204
7.191
7.178
7.158
7.145
7.131
7.103
7.089
6.984
6.970
6.911
6.898
6.763
6.757
6.753
6.744
6.716
6.645
6.632
6.586
3.973
3.962
3.955
3.756
3.734
3.280
3.272
3.257
3.250
3.114
3.103
3.091
3.081
2.892
2.877
2.870
2.854
2.830
2.771
2.764
2.750
2.739
2.735
2.725
1.705
1.398
1.162
1.070
0.000
3.09
1.98
1.95
1.09
0.17
0.19
0.09
0.08
3.26
0.02
1.09
1.00
1.08
0.08
3.00
0.99
0.18
0.25
10
8
6
4
2
0
PPM

110-d

zwf-11-104C
Sep 05 2024
SOLVENT: CDCl3
NA = 100
F1 = 150.902802 MHz
F2 = 1.000000 MHz

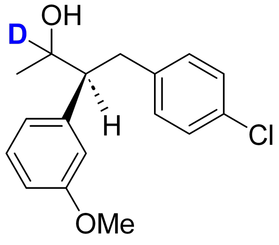

110-d

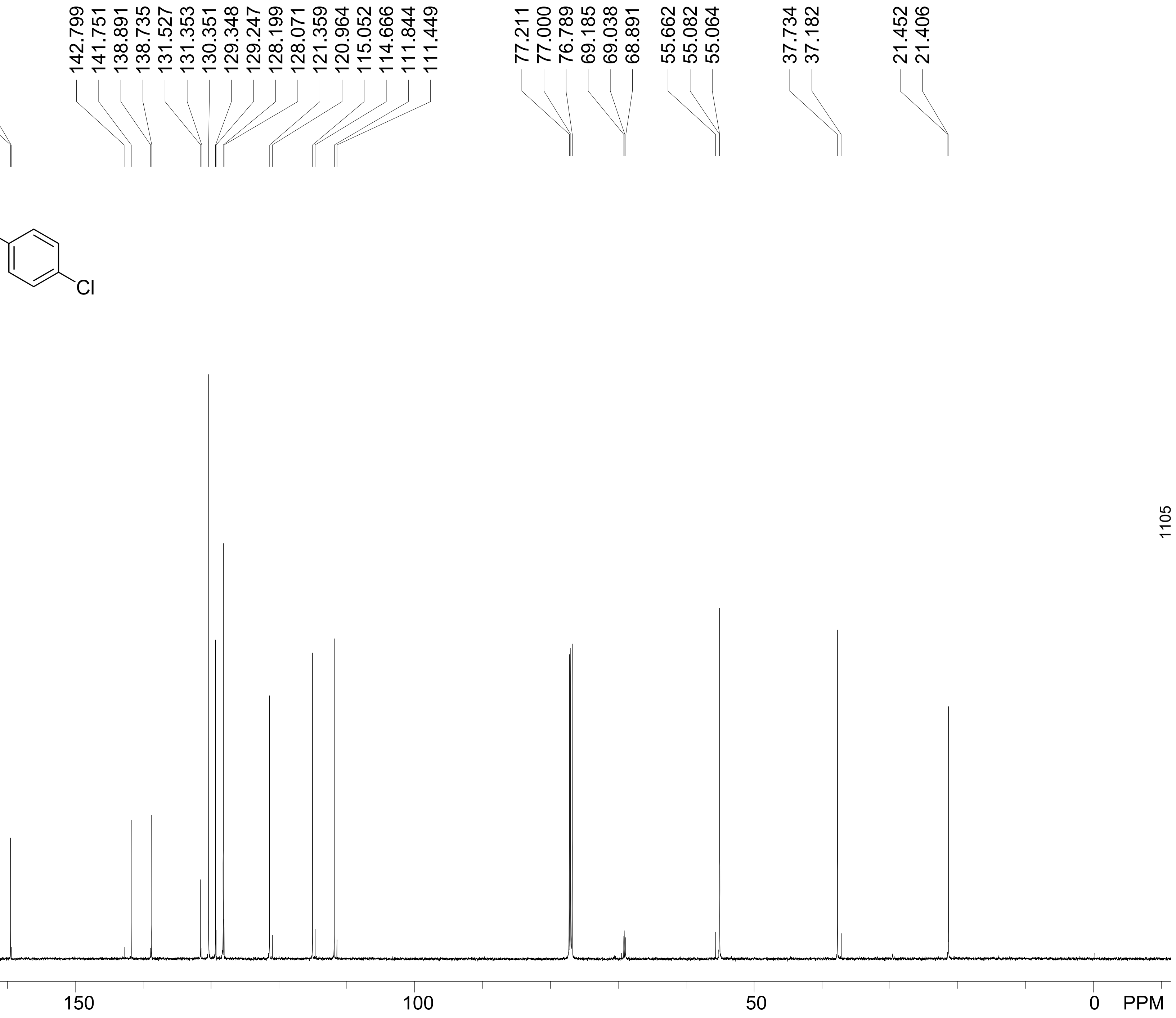

Area Percent Report

Agilent Technologies

Sample name: zwf-11-104-IG-90-10-0.8-205

Data file: C:\Users\Public\Documents\ChemStation\1\Data\zwf\zwf 2024-09-05 15-14-44\007-P1-C2-zwf-11-104 IG-90-10-0.8.D

Injection Acquired Date 09/05/2024 17:19:27

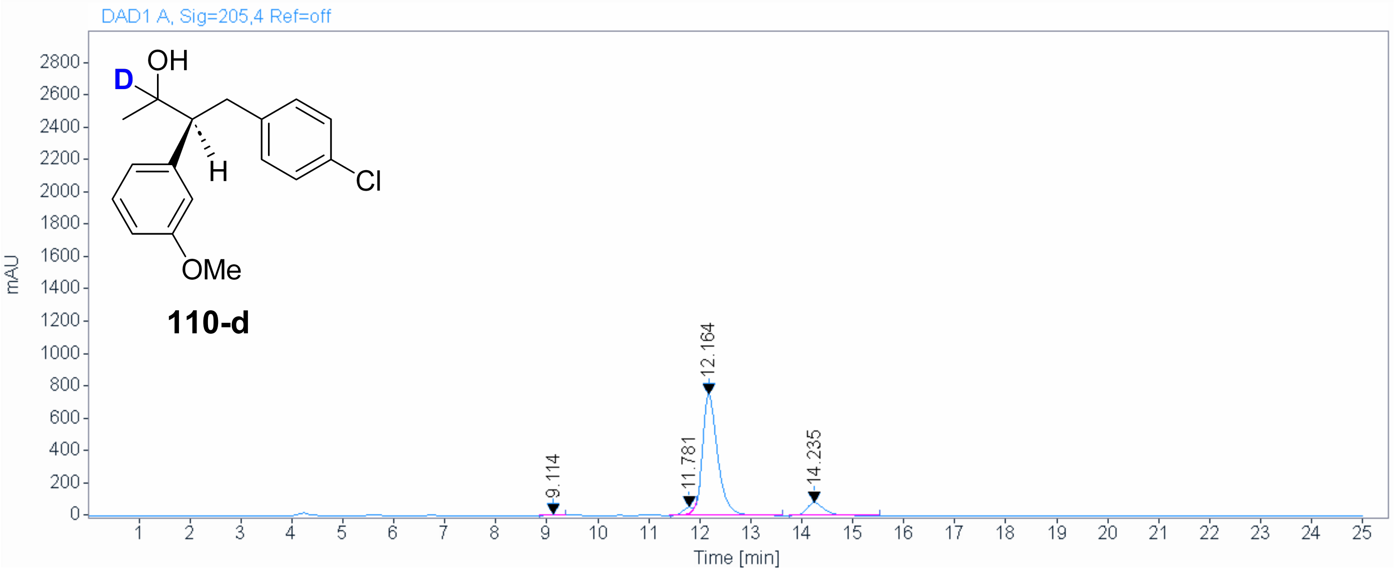

Signal: DAD1 A, Sig=205,4 Ref=off

RT [min]	Width [min]	Height	Area	Area%
9.114	0.2544	6.1423	106.5786	0.5836
11.781	0.2115	42.3186	593.1035	3.2478
12.164	0.3186	748.2784	15691.2734	85.9243
14.235	0.3587	79.4458	1870.7952	10.2443
		Sum	18261.7507	100.0000

Area Percent Report

Agilent Technologies

Sample name: zwf-11-104-rac-IG-90-10-0.8-205

Data file: C:\Users\Public\Documents\ChemStation\1\Data\zwf\zwf 2024-09-05 15-14-44\006-P1-C1-zwf-11-104-rac IG-90-10-0.8.D

Injection Acquired Date 09/05/2024 16:53:30

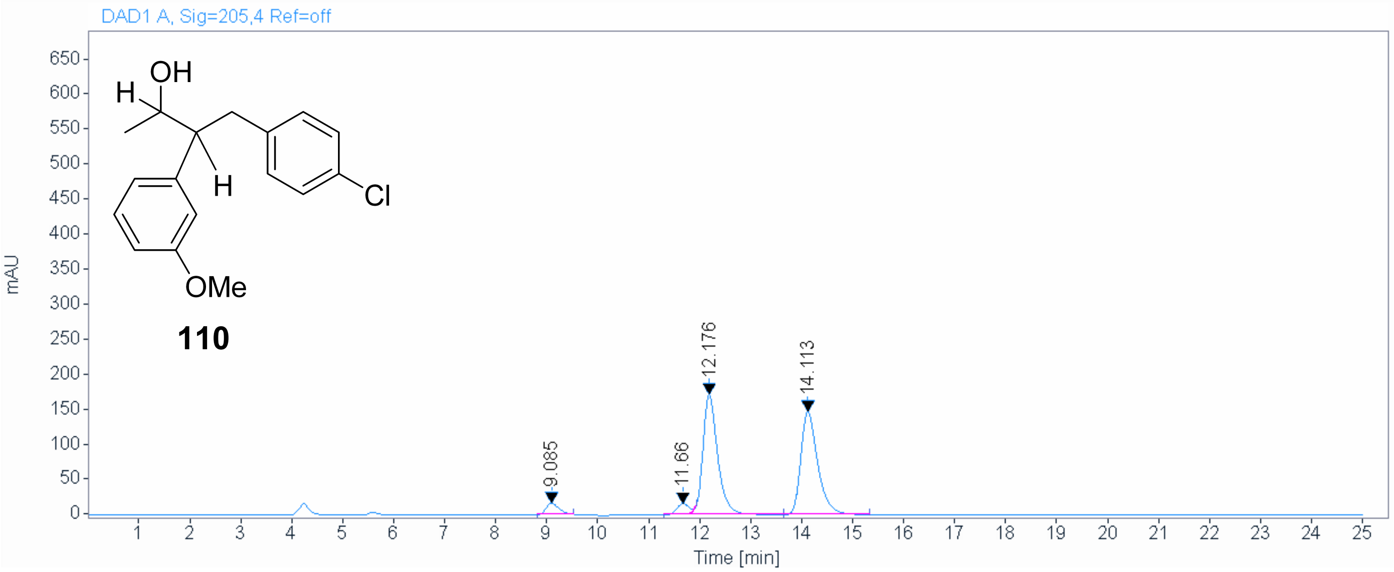

110

Signal: DAD1 A, Sig=205,4 Ref=off

RT [min]	Width [min]	Height	Area	Area%
9.085	0.2837	15.8647	270.0044	3.6355
11.660	0.2621	15.5724	261.7020	3.5237
12.176	0.3048	171.5358	3481.3228	46.8741
14.113	0.3548	147.0839	3413.9341	45.9668
		Sum	7426.9632	100.0000

zwf-10-089H
Aug 30 2024
SOLVENT: CDCl3
NA = 4
F1 = 600.169983 MHz
F2 = 1.000000 MHz

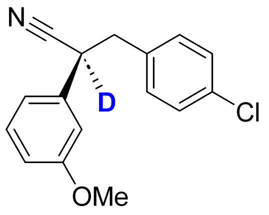

111-d

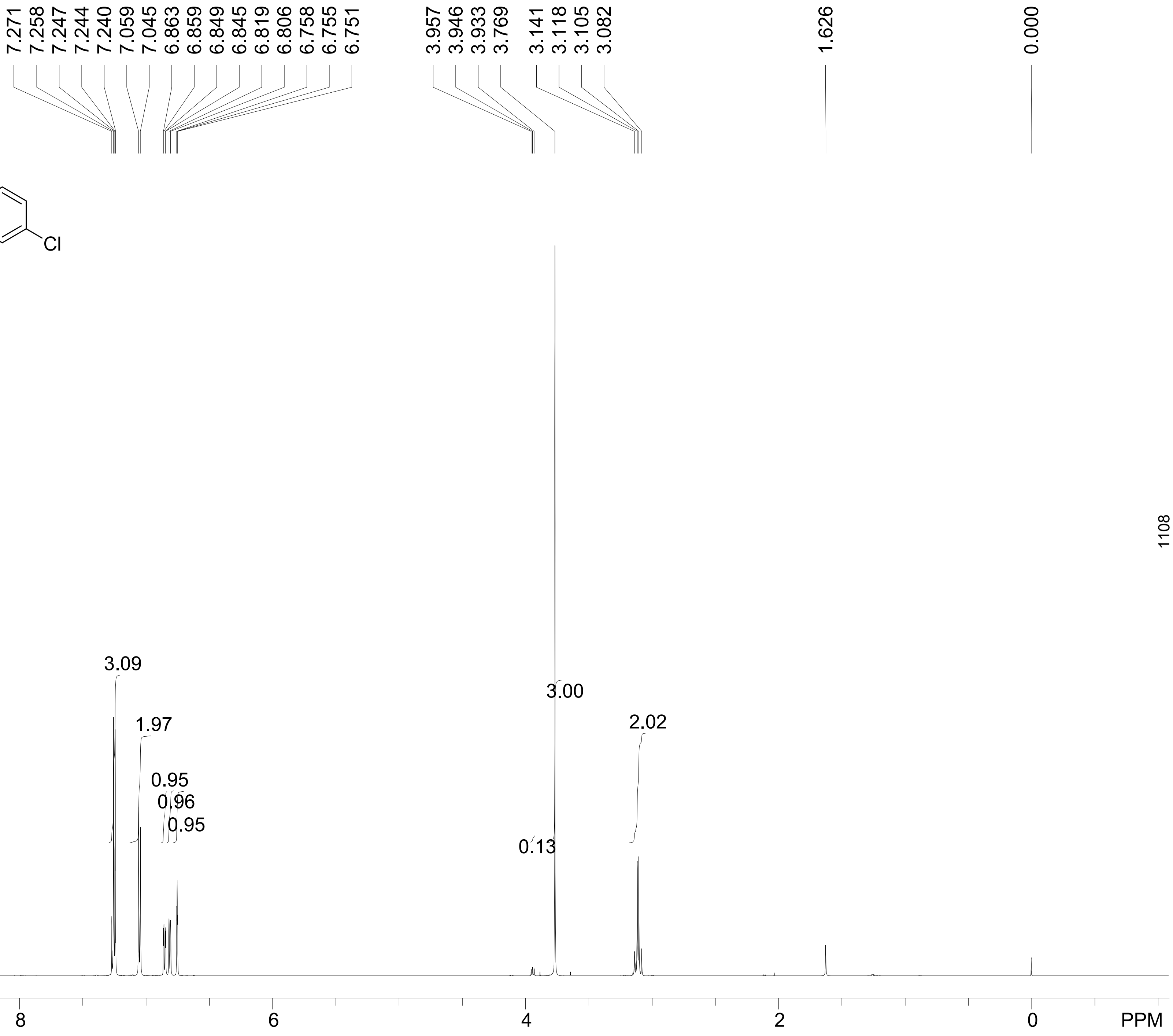

zwf-10-089C
Aug 30 2024
SOLVENT: CDCl3
NA = 100
F1 = 150.912872 MHz
F2 = 1.000000 MHz

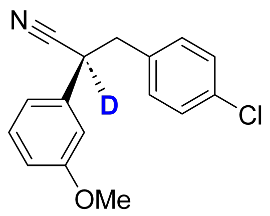

111-d

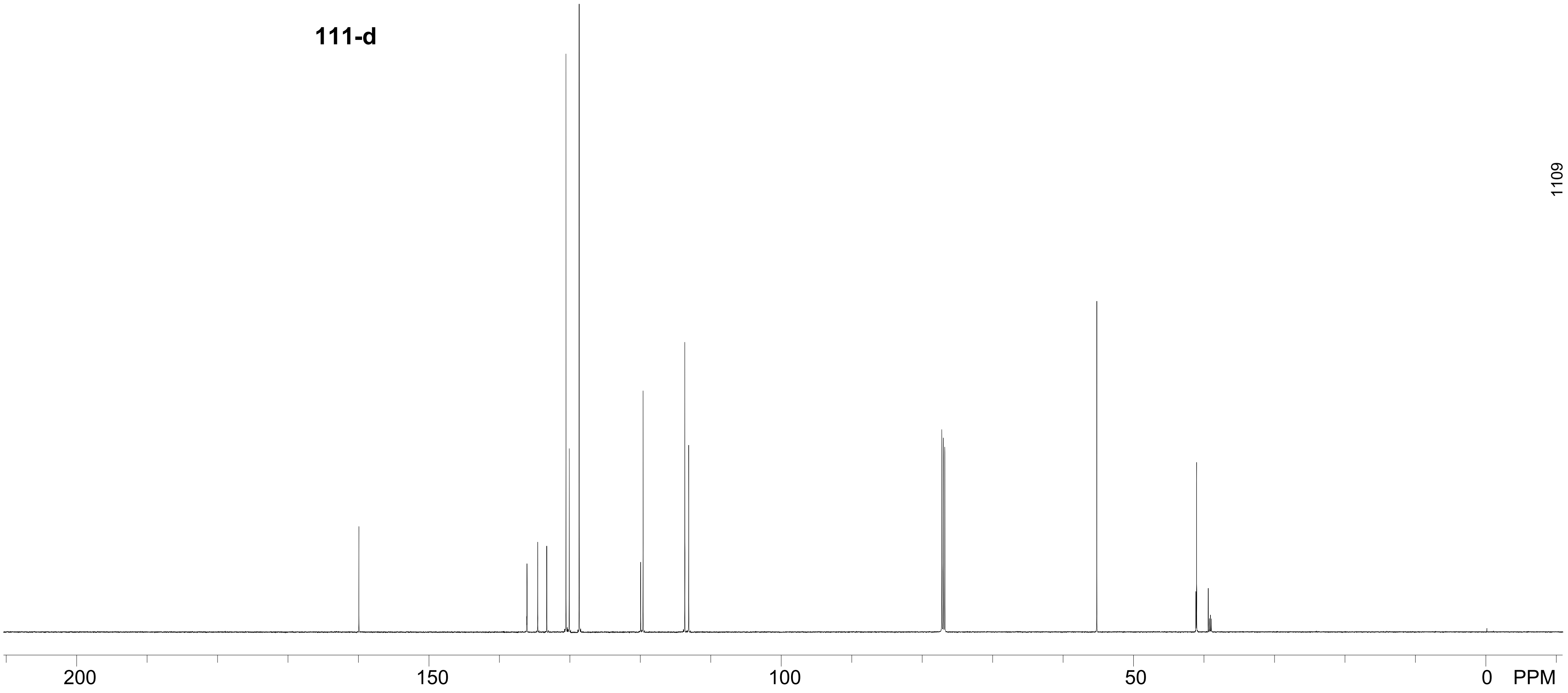

Area Percent Report

Agilent Technologies

Sample name: zwf-11-089-IE-99-1-0.8-205

Data file: C:\Users\Public\Documents\ChemStation\1\Data\zwf\zwf 2024-08-30 10-18-54\007-P1-C3-zwf-11-089.D

Injection Acquired Date 08/30/2024 15:13:02

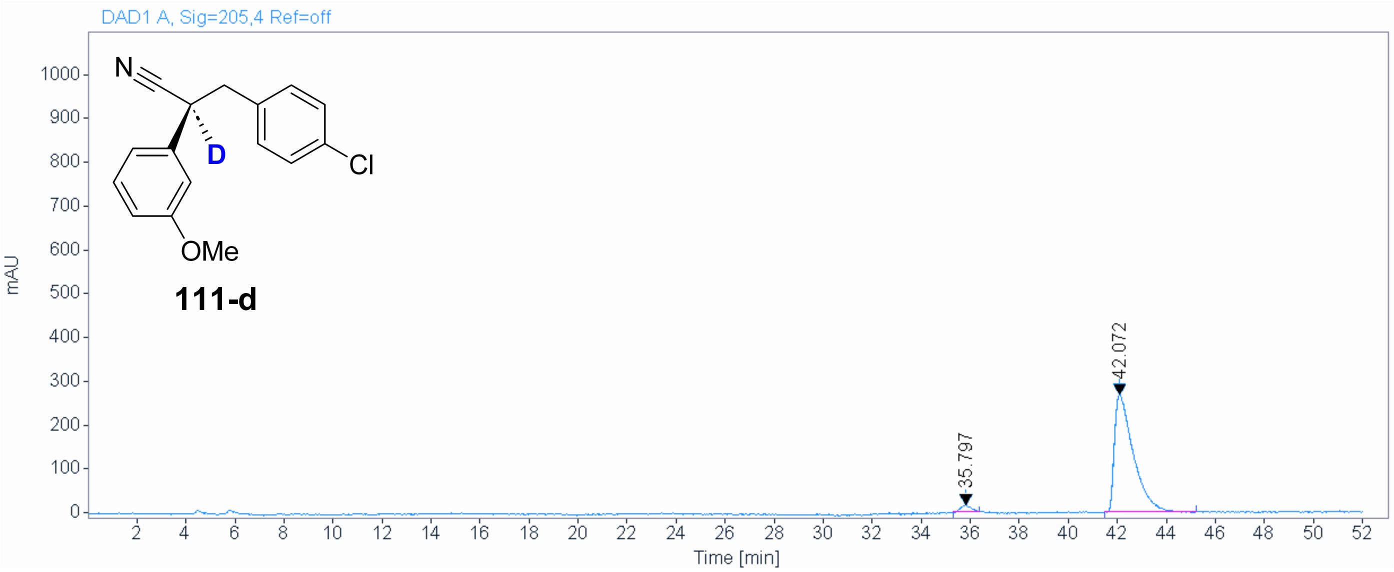

Signal: DAD1 A, Sig=205,4 Ref=off

RT [min]	Width [min]	Height	Area	Area%
35.797	0.5120	15.4672	475.1774	3.2069
42.072	0.8937	267.4693	14342.3740	96.7931
		Sum	14817.5514	100.0000

Area Percent Report

Sample name: zwf-11-088-rac-IE-99-1-0.8-205

Data file: C:\Users\Public\Documents\ChemStation\1\Data\zwf\zwf 2024-08-30 10-18-54\006-P1-C1-zwf-11-088-rac.D

Injection Acquired Date 08/30/2024 14:20:02

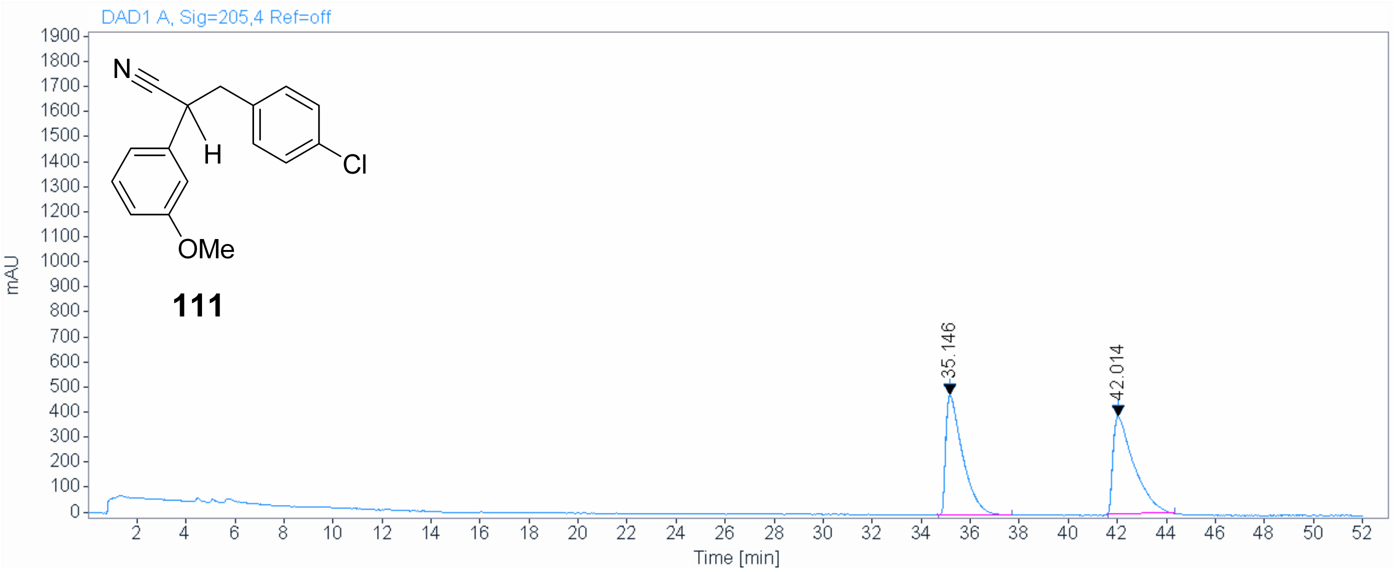

Signal: DAD1 A, Sig=205,4 Ref=off

RT [min]	Width [min]	Height	Area	Area%
35.146	0.8015	481.7709	23169.1523	49.7563
42.014	0.9975	390.9091	23396.1211	50.2437
		Sum	46565.2734	100.0000

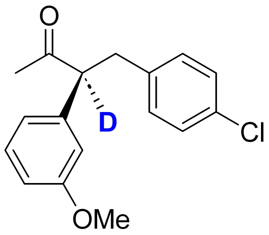

112-d

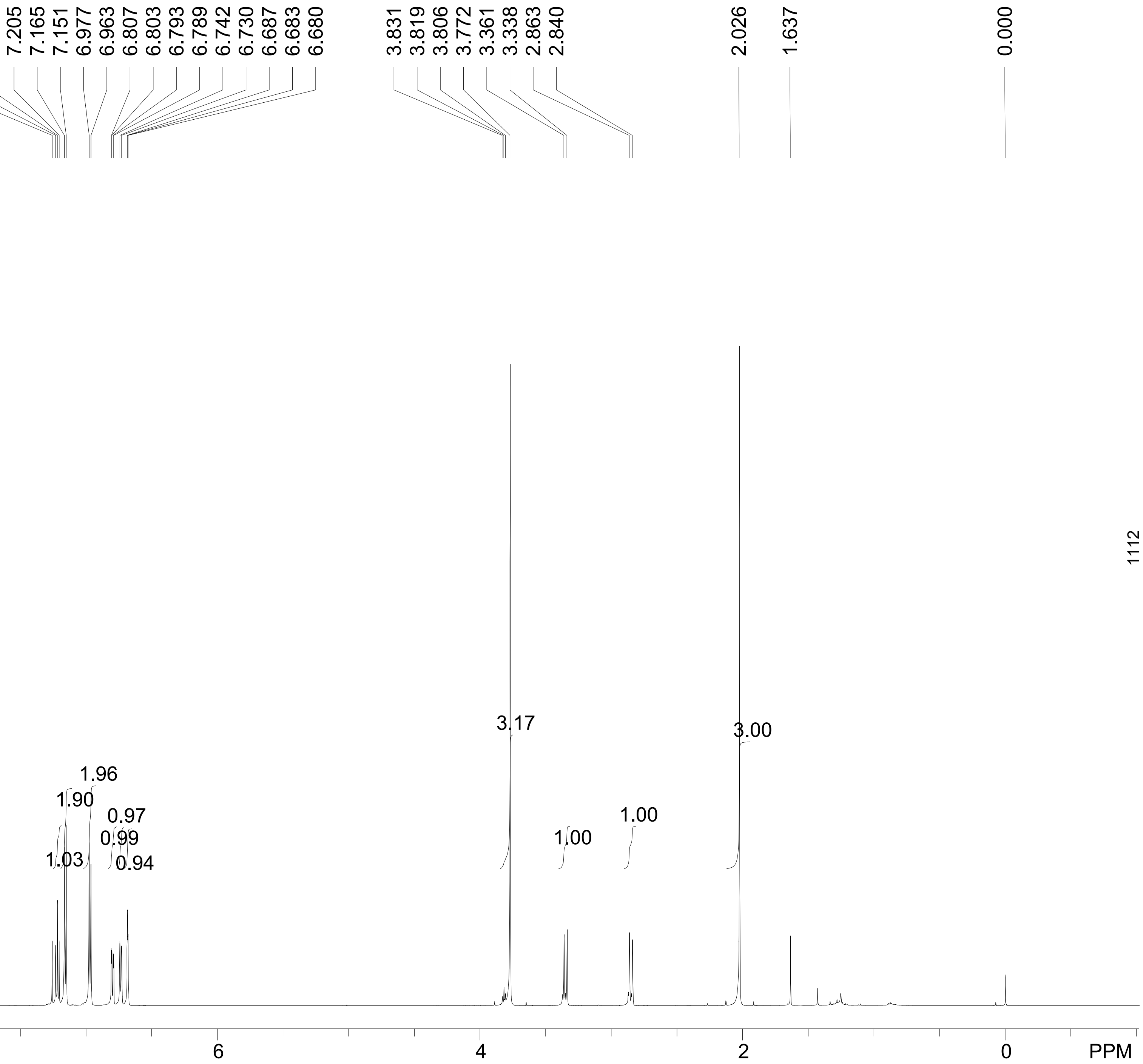
zwf-11-103H
Sep 04 2024
SOLVENT: CDCl3
NA = 4
F1 = 600.130005 MHz
F2 = 1.000000 MHz
7.258
7.231
7.218
7.205
7.165
7.151
6.977
6.963
6.807
6.803
6.793
6.789
6.742
6.730
6.687
6.683
6.680
3.831
3.819
3.806
3.772
3.361
3.338
2.863
2.840
2.026
1.637
0.000
1.96
1.90
1.03
0.97
0.99
0.94
3.17
1.00
1.00
3.00
10
8
6
4
2
0
PPM

zwf-11-103C
Sep 04 2024
SOLVENT: CDCl3
NA = 100
F1 = 150.902802 MHz
F2 = 1.000000 MHz

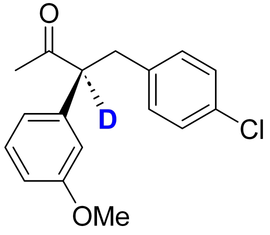

112-d

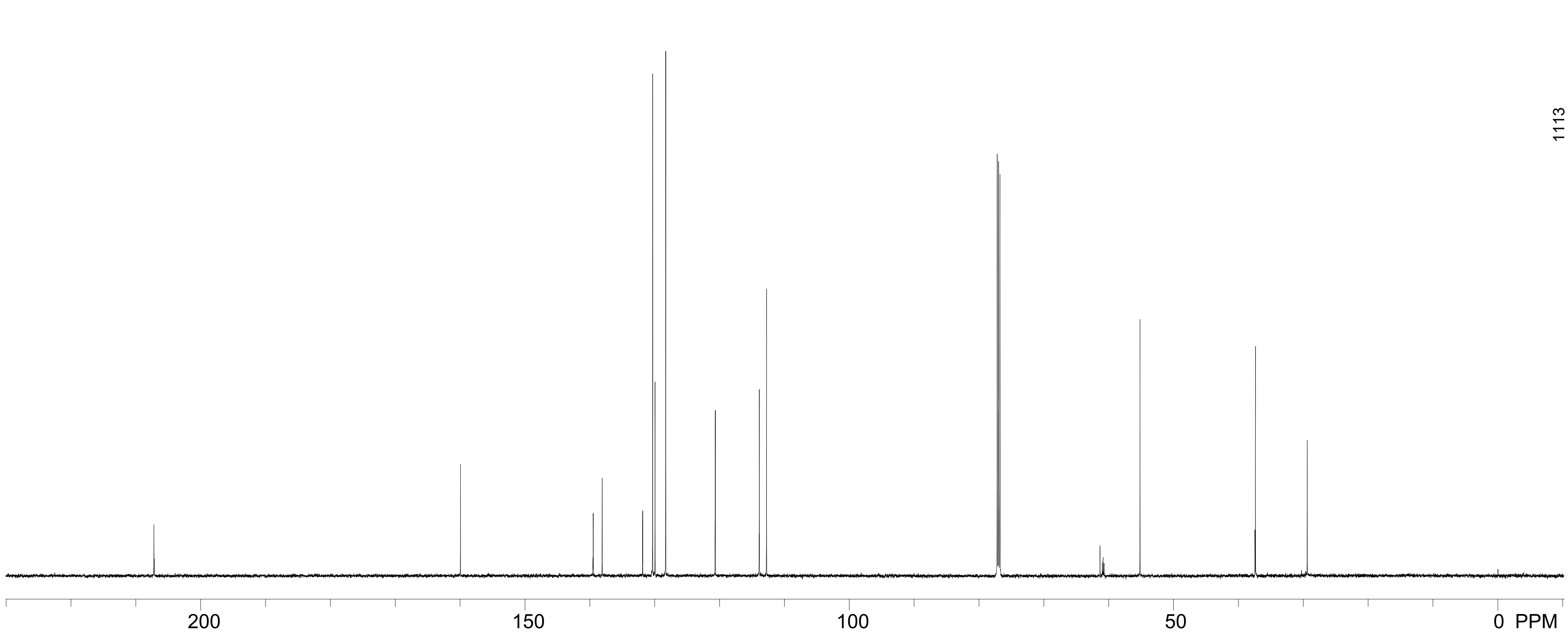

Area Percent Report

Agilent Technologies

Sample name: zwf-11-103-IG-90-10-0.8-205

Data file: C:\Users\Public\Documents\ChemStation\1\Data\zwf\zwf 2024-09-04 08-51-35\006-P1-C3-zwf-11-103.D

Injection Acquired Date 09/04/2024 10:25:49

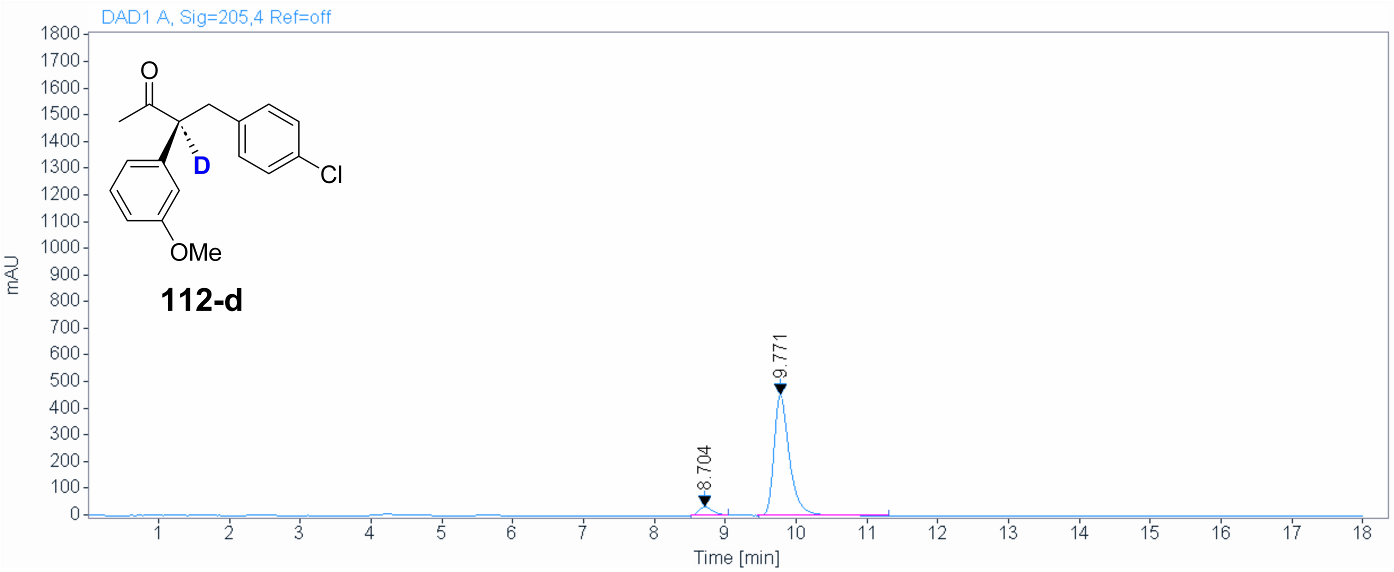

Signal: DAD1 A, Sig=205,4 Ref=off

RT [min]	Width [min]	Height	Area	Area%
8.704	0.2269	31.7913	432.7992	5.9105
9.771	0.2275	452.7563	6889.7759	94.0895
		Sum	7322.5750	100.0000

Area Percent Report

Agilent Technologies

Sample name: zwf-11-102-rac-IG-90-10-0.8-205

Data file: C:\Users\Public\Documents\ChemStation\1\Data\zwf\zwf 2024-09-04 08-51-35\002-P1-C1-zwf-11-102-rac.D

Injection Acquired Date 09/04/2024 09:09:56

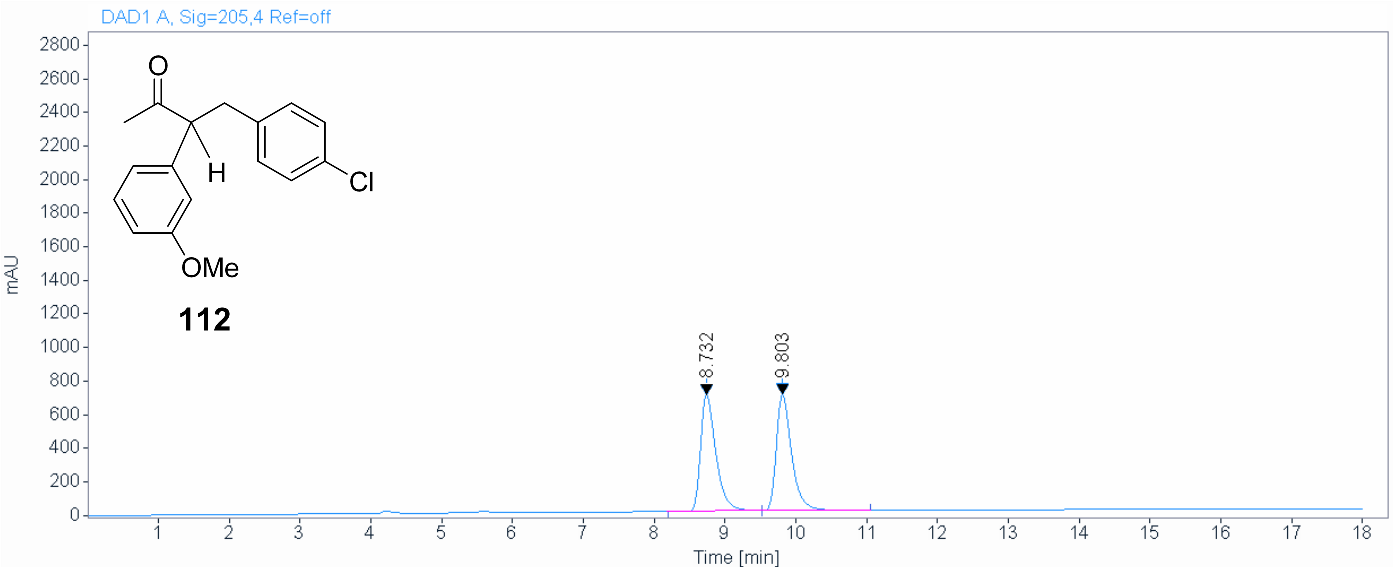

Signal: DAD1 A, Sig=205,4 Ref=off

RT [min]	Width [min]	Height	Area	Area%
8.732	0.2286	693.1561	10492.0459	49.9260
9.803	0.2293	692.5268	10523.1396	50.0740
		Sum	21015.1855	100.0000

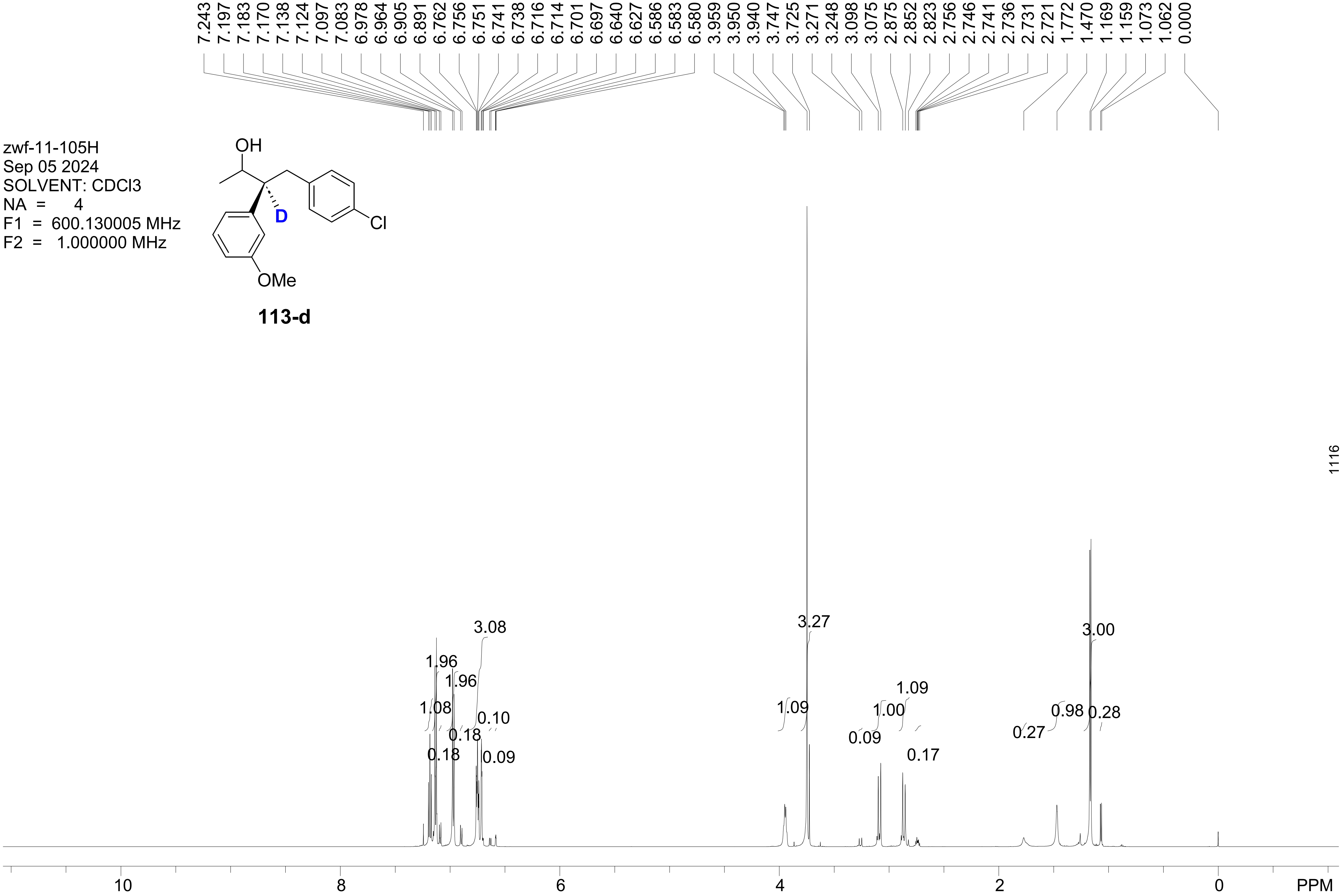
zwf-11-105H
Sep 05 2024
SOLVENT: CDCl3
NA = 4
F1 = 600.130005 MHz
F2 = 1.000000 MHz
OH
D
Cl
OMe
113-d
7.243 7.197 7.183 7.170 7.138 7.124 7.097 7.083 6.978 6.964 6.905 6.891 6.762 6.756 6.751 6.741 6.738 6.716 6.714 6.701 6.697 6.640 6.627 6.586 6.583 6.580 3.959 3.950 3.940 3.747 3.725 3.271 3.248 3.098 3.075 2.875 2.852 2.823 2.756 2.746 2.741 2.736 2.731 2.721 1.772 1.470 1.169 1.159 1.073 1.062 0.000
3.08 1.96 1.96 1.08 0.10 0.18 0.18 0.09
3.27 1.09 1.09 1.00 0.09 0.17
3.00 0.98 0.28 0.27
10 8 6 4 2 0 PPM

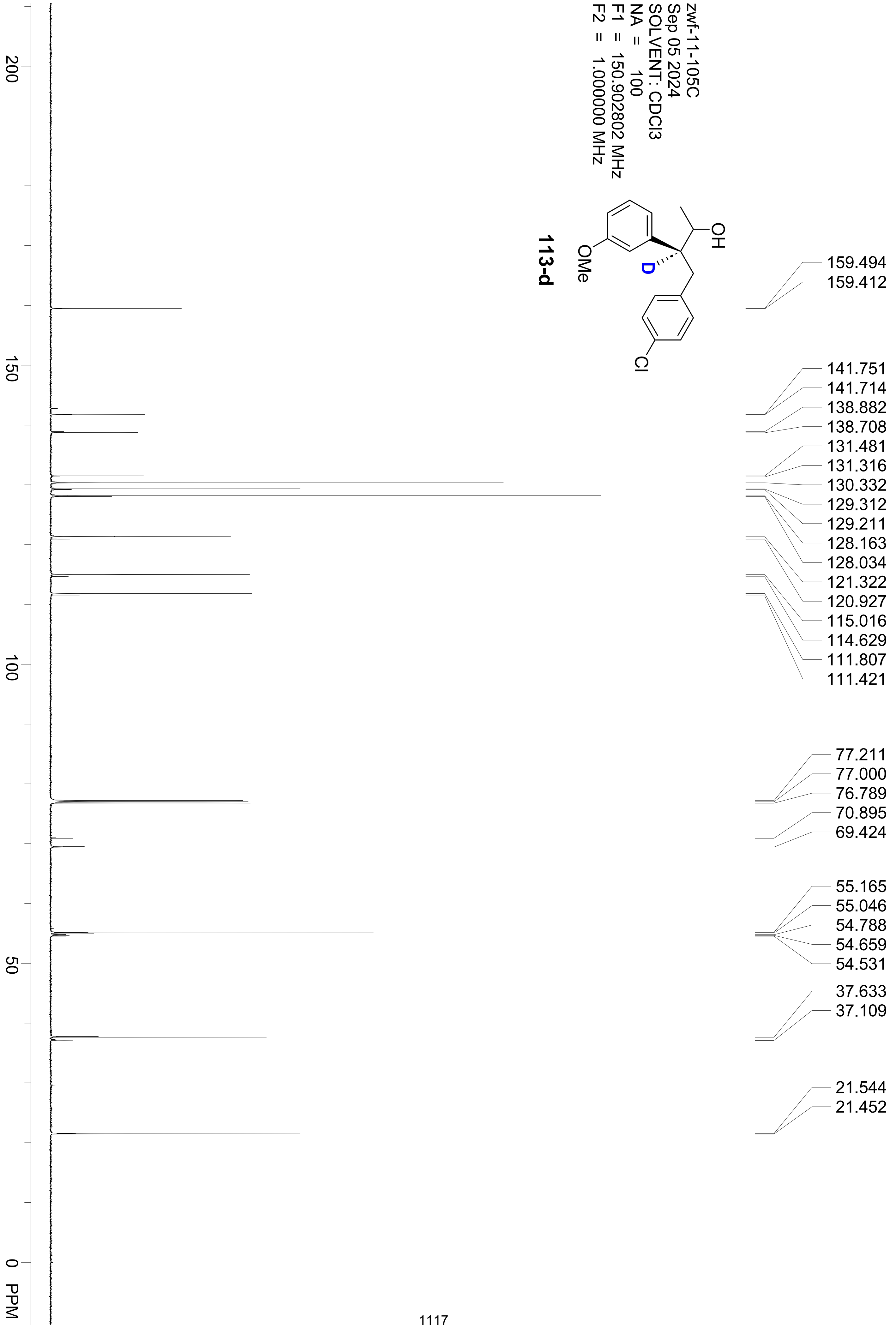

zwf-11-105C
Sep 05 2024
SOLVENT: CDCl3
NA = 100
F1 = 150.902802 MHz
F2 = 1.000000 MHz
OH
D
OMe
Cl
113-d
159.494
159.412
141.751
141.714
138.882
138.708
131.481
131.316
130.332
129.312
129.211
128.163
128.034
121.322
120.927
115.016
114.629
111.807
111.421
77.211
77.000
76.789
70.895
69.424
55.165
55.046
54.788
54.659
54.531
37.633
37.109
21.544
21.452
200
150
100
50
0
PPM

Area Percent Report

Sample name: zwf-11-105-IG-90-10-0.8-205

Data file: C:\Users\Public\Documents\ChemStation\1\Data\zwf\zwf 2024-09-05 15-14-44\008-P1-C3-zwf-11-105 IG-90-10-0.8.D

Injection Acquired Date 09/05/2024 17:45:25

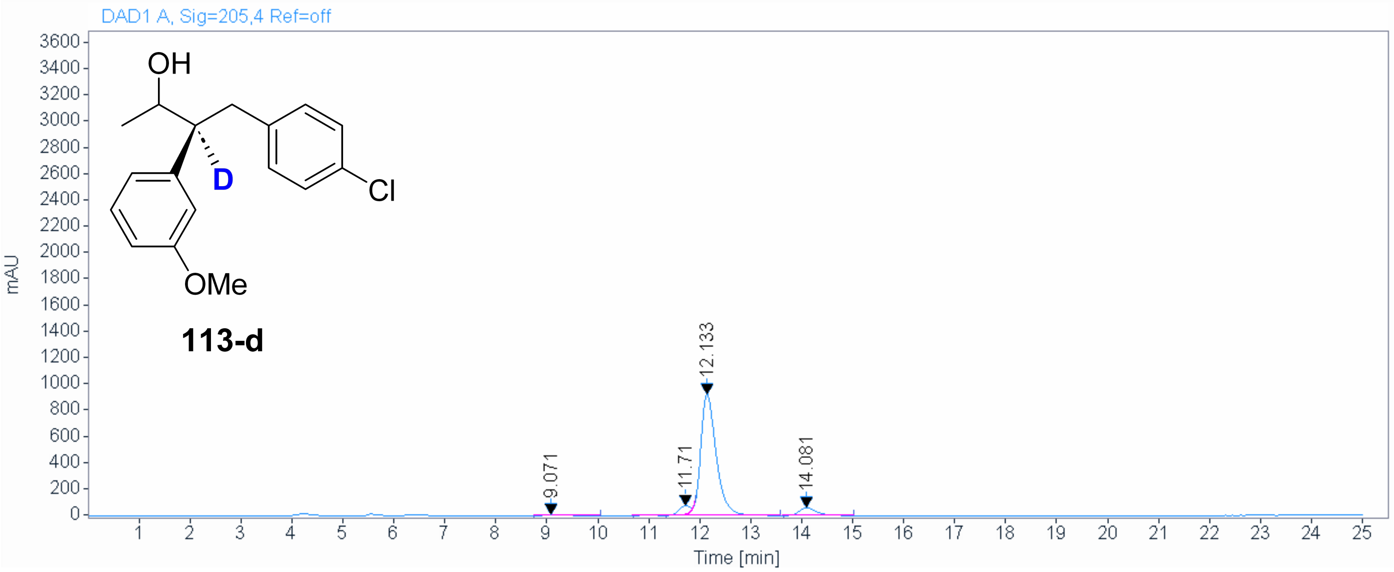

Signal: DAD1 A, Sig=205,4 Ref=off

RT [min]	Width [min]	Height	Area	Area%
9.071	0.3813	6.2550	179.7819	0.8061
11.710	0.2366	69.2161	1059.7025	4.7517
12.133	0.3248	920.8713	19703.4766	88.3508
14.081	0.3578	58.7318	1358.4425	6.0913
		Sum	22301.4035	100.0000

Area Percent Report

Agilent Technologies

Sample name: zwf-11-104-rac-IG-90-10-0.8-205

Data file: C:\Users\Public\Documents\ChemStation\1\Data\zwf\zwf 2024-09-05 15-14-44\006-P1-C1-zwf-11-104-rac IG-90-10-0.8.D

Injection Acquired Date 09/05/2024 16:53:30

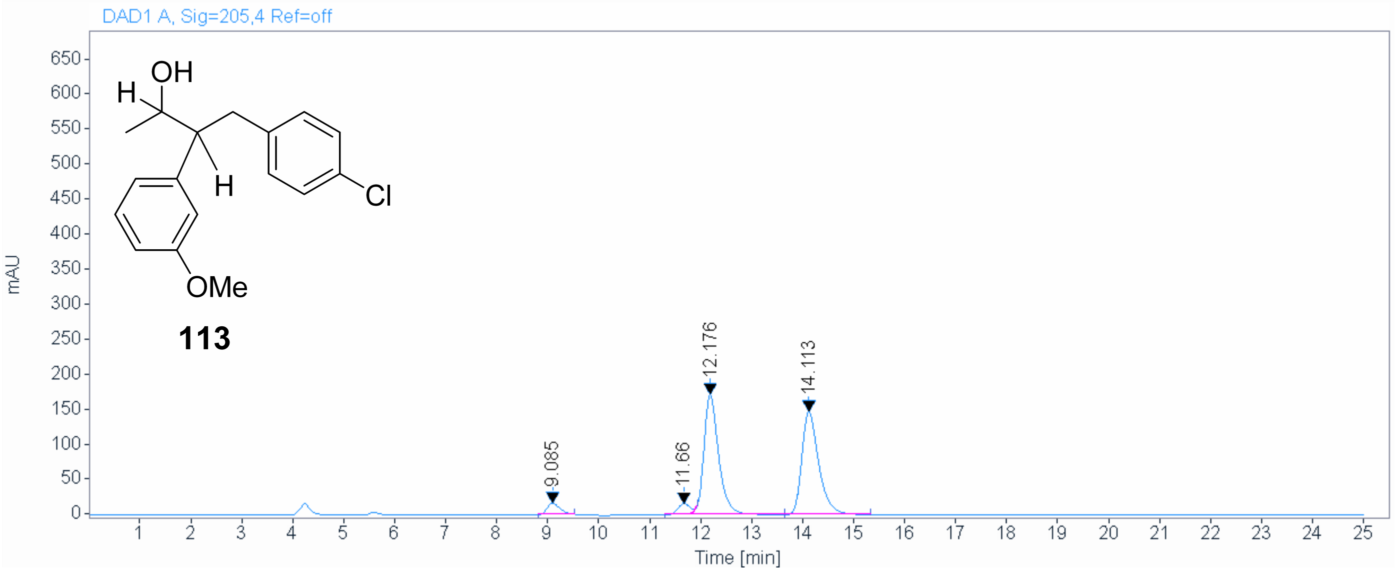

Signal: DAD1 A, Sig=205,4 Ref=off

RT [min]	Width [min]	Height	Area	Area%
9.085	0.2837	15.8647	270.0044	3.6355
11.660	0.2621	15.5724	261.7020	3.5237
12.176	0.3048	171.5358	3481.3228	46.8741
14.113	0.3548	147.0839	3413.9341	45.9668
		Sum	7426.9632	100.0000
